# Global burden of 288 causes of death and life expectancy decomposition in 204 countries and territories and 811 subnational locations, 1990–2021: a systematic analysis for the Global Burden of Disease Study 2021

**DOI:** 10.1016/S0140-6736(24)00367-2

**Published:** 2024-05-18

**Authors:** GBD 2021 Causes of Death Collaborators, Mohsen Naghavi, Kanyin Liane Ong, Amirali Aali, Hazim S Ababneh, Yohannes Habtegiorgis Abate, Cristiana Abbafati, Rouzbeh Abbasgholizadeh, Mohammadreza Abbasian, Mohsen Abbasi-Kangevari, Hedayat Abbastabar, Samar Abd ElHafeez, Michael Abdelmasseh, Sherief Abd-Elsalam, Ahmed Abdelwahab, Mohammad Abdollahi, Mohammad-Amin Abdollahifar, Meriem Abdoun, Deldar Morad Abdulah, Auwal Abdullahi, Mesfin Abebe, Samrawit Shawel Abebe, Aidin Abedi, Kedir Hussein Abegaz, E S Abhilash, Hassan Abidi, Olumide Abiodun, Richard Gyan Aboagye, Hassan Abolhassani, Meysam Abolmaali, Mohamed Abouzid, Girma Beressa Aboye, Lucas Guimarães Abreu, Woldu Aberhe Abrha, Dariush Abtahi, Samir Abu Rumeileh, Hasan Abualruz, Bilyaminu Abubakar, Eman Abu-Gharbieh, Niveen ME Abu-Rmeileh, Salahdein Aburuz, Ahmed Abu-Zaid, Manfred Mario Kokou Accrombessi, Tadele Girum Adal, Abdu A Adamu, Isaac Yeboah Addo, Giovanni Addolorato, Akindele Olupelumi Adebiyi, Victor Adekanmbi, Abiola Victor Adepoju, Charles Oluwaseun Adetunji, Juliana Bunmi Adetunji, Temitayo Esther Adeyeoluwa, Daniel Adedayo Adeyinka, Olorunsola Israel Adeyomoye, Biruk Adie Adie Admass, Qorinah Estiningtyas Sakilah Adnani, Saryia Adra, Aanuoluwapo Adeyimika Afolabi, Muhammad Sohail Afzal, Saira Afzal, Suneth Buddhika Agampodi, Pradyumna Agasthi, Manik Aggarwal, Shahin Aghamiri, Feleke Doyore Agide, Antonella Agodi, Anurag Agrawal, Williams Agyemang-Duah, Bright Opoku Ahinkorah, Aqeel Ahmad, Danish Ahmad, Firdos Ahmad, Muayyad M Ahmad, Sajjad Ahmad, Shahzaib Ahmad, Tauseef Ahmad, Keivan Ahmadi, Amir Mahmoud Ahmadzade, Ali Ahmed, Ayman Ahmed, Haroon Ahmed, Luai A Ahmed, Mehrunnisha Sharif Ahmed, Meqdad Saleh Ahmed, Muktar Beshir Ahmed, Syed Anees Ahmed, Marjan Ajami, Budi Aji, Essona Matatom Akara, Hossein Akbarialiabad, Karolina Akinosoglou, Tomi Akinyemiju, Mohammed Ahmed Akkaif, Samuel Akyirem, Hanadi Al Hamad, Syed Mahfuz Al Hasan, Fares Alahdab, Samer O Alalalmeh, Tariq A Alalwan, Ziyad Al-Aly, Khurshid Alam, Manjurul Alam, Noore Alam, Rasmieh Mustafa Al-amer, Fahad Mashhour Alanezi, Turki M Alanzi, Sayer Al-Azzam, Almaza Albakri, Mohammed Albashtawy, Mohammad T AlBataineh, Jacqueline Elizabeth Alcalde-Rabanal, Khalifah A Aldawsari, Wafa A Aldhaleei, Robert W Aldridge, Haileselasie Berhane Alema, Mulubirhan Assefa Alemayohu, Sharifullah Alemi, Yihun Mulugeta Alemu, Adel Ali Saeed Al-Gheethi, Khalid F Alhabib, Fadwa Alhalaiqa Naji Alhalaiqa, Mohammed Khaled Al-Hanawi, Abid Ali, Amjad Ali, Liaqat Ali, Mohammed Usman Ali, Rafat Ali, Shahid Ali, Syed Shujait Shujait Ali, Gianfranco Alicandro, Sheikh Mohammad Alif, Reyhaneh Alikhani, Yousef Alimohamadi, Ahmednur Adem Aliyi, Mohammad A M Aljasir, Syed Mohamed Aljunid, François Alla, Peter Allebeck, Sabah Al-Marwani, Sadeq Ali Ali Al-Maweri, Joseph Uy Almazan, Hesham M Al-Mekhlafi, Louay Almidani, Omar Almidani, Mahmoud A Alomari, Basem Al-Omari, Jordi Alonso, Jaber S Alqahtani, Shehabaldin Alqalyoobi, Ahmed Yaseen Alqutaibi, Salman Khalifah Al-Sabah, Zaid Altaany, Awais Altaf, Jaffar A Al-Tawfiq, Khalid A Altirkawi, Deborah Oyine Aluh, Nelson Alvis-Guzman, Hassan Alwafi, Yaser Mohammed Al-Worafi, Hany Aly, Safwat Aly, Karem H Alzoubi, Reza Amani, Azmeraw T Amare, Prince M Amegbor, Edward Kwabena Ameyaw, Tarek Tawfik Amin, Alireza Amindarolzarbi, Sohrab Amiri, Mohammad Hosein Amirzade-Iranaq, Hubert Amu, Dickson A Amugsi, Ganiyu Adeniyi Amusa, Robert Ancuceanu, Deanna Anderlini, David B Anderson, Pedro Prata Andrade, Catalina Liliana Andrei, Tudorel Andrei, Colin Angus, Abhishek Anil, Sneha Anil, Amir Anoushiravani, Hossein Ansari, Ansariadi Ansariadi, Alireza Ansari-Moghaddam, Catherine M Antony, Ernoiz Antriyandarti, Davood Anvari, Saeid Anvari, Saleha Anwar, Sumadi Lukman Anwar, Razique Anwer, Anayochukwu Edward Anyasodor, Muhammad Aqeel, Juan Pablo Arab, Jalal Arabloo, Mosab Arafat, Aleksandr Y Aravkin, Demelash Areda, Abdulfatai Aremu, Olatunde Aremu, Hany Ariffin, Mesay Arkew, Benedetta Armocida, Michael Benjamin Arndt, Johan Ärnlöv, Mahwish Arooj, Anton A Artamonov, Judie Arulappan, Raphael Taiwo Aruleba, Ashokan Arumugam, Malke Asaad, Mohsen Asadi-Lari, Akeza Awealom Asgedom, Mona Asghariahmadabad, Mohammad Asghari-Jafarabadi, Muhammad Ashraf, Armin Aslani, Thomas Astell-Burt, Mohammad Athar, Seyyed Shamsadin Athari, Bantalem Tilaye Tilaye Atinafu, Habtamu Wondmagegn Atlaw, Prince Atorkey, Maha Moh'd Wahbi Atout, Alok Atreya, Avinash Aujayeb, Marcel Ausloos, Abolfazl Avan, Atalel Fentahun Awedew, Amlaku Mulat Aweke, Beatriz Paulina Ayala Quintanilla, Haleh Ayatollahi, Jose L Ayuso-Mateos, Seyed Mohammad Ayyoubzadeh, Sina Azadnajafabad, Rui M S Azevedo, Ahmed Y Azzam, Darshan B B, Abraham Samuel Babu, Muhammad Badar, Ashish D Badiye, Soroush Baghdadi, Nasser Bagheri, Sara Bagherieh, Sulaiman Bah, Saeed Bahadorikhalili, Najmeh Bahmanziari, Ruhai Bai, Atif Amin Baig, Jennifer L Baker, Abdulaziz T Bako, Ravleen Kaur Bakshi, Senthilkumar Balakrishnan, Madhan Balasubramanian, Ovidiu Constantin Baltatu, Kiran Bam, Maciej Banach, Soham Bandyopadhyay, Palash Chandra Banik, Hansi Bansal, Kannu Bansal, Franca Barbic, Martina Barchitta, Mainak Bardhan, Erfan Bardideh, Suzanne Lyn Barker-Collo, Till Winfried Bärnighausen, Francesco Barone-Adesi, Hiba Jawdat Barqawi, Lope H Barrero, Amadou Barrow, Sandra Barteit, Lingkan Barua, Zarrin Basharat, Azadeh Bashiri, Afisu Basiru, Pritish Baskaran, Buddha Basnyat, Quique Bassat, João Diogo Basso, Ann V L Basting, Sanjay Basu, Kavita Batra, Bernhard T Baune, Mohsen Bayati, Nebiyou Simegnew Bayileyegn, Thomas Beaney, Neeraj Bedi, Massimiliano Beghi, Emad Behboudi, Priyamadhaba Behera, Amir Hossein Behnoush, Masoud Behzadifar, Maryam Beiranvand, Diana Fernanda Bejarano Ramirez, Yannick Béjot, Sefealem Assefa Belay, Chalie Mulu Belete, Michelle L Bell, Muhammad Bashir Bello, Olorunjuwon Omolaja Bello, Luis Belo, Apostolos Beloukas, Rose Grace Bender, Isabela M Bensenor, Azizullah Beran, Zombor Berezvai, Alemshet Yirga Berhie, Betyna N Berice, Robert S Bernstein, Gregory J Bertolacci, Paulo J G Bettencourt, Kebede A Beyene, Devidas S Bhagat, Akshaya Srikanth Bhagavathula, Neeraj Bhala, Ashish Bhalla, Dinesh Bhandari, Kayleigh Bhangdia, Nikha Bhardwaj, Pankaj Bhardwaj, Prarthna V Bhardwaj, Ashish Bhargava, Sonu Bhaskar, Vivek Bhat, Gurjit Kaur Bhatti, Jasvinder Singh Bhatti, Manpreet S Bhatti, Rajbir Bhatti, Zulfiqar A Bhutta, Boris Bikbov, Jessica Devin Bishai, Catherine Bisignano, Francesca Bisulli, Atanu Biswas, Bijit Biswas, Saeid Bitaraf, Bikes Destaw Bitew, Veera R Bitra, Tone Bjørge, Micheal Kofi Boachie, Mary Sefa Boampong, Anca Vasilica Bobirca, Virginia Bodolica, Aadam Olalekan Bodunrin, Eyob Ketema Bogale, Kassawmar Angaw Bogale, Somayeh Bohlouli, Obasanjo Afolabi Bolarinwa, Archith Boloor, Milad Bonakdar Hashemi, Aime Bonny, Kaustubh Bora, Berrak Bora Basara, Hamed Borhany, Arturo Borzutzky, Souad Bouaoud, Antoine Boustany, Christopher Boxe, Edward J Boyko, Oliver J Brady, Dejana Braithwaite, Luisa C Brant, Michael Brauer, Alexandra Brazinova, Javier Brazo-Sayavera, Nicholas J K Breitborde, Susanne Breitner, Hermann Brenner, Andrey Nikolaevich Briko, Nikolay Ivanovich Briko, Gabrielle Britton, Julie Brown, Traolach Brugha, Norma B Bulamu, Lemma N Bulto, Danilo Buonsenso, Richard A Burns, Reinhard Busse, Yasser Bustanji, Nadeem Shafique Butt, Zahid A Butt, Florentino Luciano Caetano dos Santos, Daniela Calina, Luis Alberto Cámera, Luciana Aparecida Campos, Ismael R Campos-Nonato, Chao Cao, Yin Cao, Angelo Capodici, Rosario Cárdenas, Sinclair Carr, Giulia Carreras, Juan J Carrero, Andrea Carugno, Cristina G Carvalheiro, Felix Carvalho, Márcia Carvalho, Joao Mauricio Castaldelli-Maia, Carlos A Castañeda-Orjuela, Giulio Castelpietra, Ferrán Catalá-López, Alberico L Catapano, Maria Sofia Cattaruzza, Christopher R Cederroth, Luca Cegolon, Francieli Cembranel, Muthia Cenderadewi, Kelly M Cercy, Ester Cerin, Muge Cevik, Joshua Chadwick, Yaacoub Chahine, Chiranjib Chakraborty, Promit Ananyo Chakraborty, Jeffrey Shi Kai Chan, Raymond N C Chan, Rama Mohan Chandika, Eeshwar K Chandrasekar, Chin-Kuo Chang, Jung-Chen Chang, Gashaw Sisay Chanie, Periklis Charalampous, Vijay Kumar Chattu, Pankaj Chaturvedi, Victoria Chatzimavridou-Grigoriadou, Akhilanand Chaurasia, Angela W Chen, An-Tian Chen, Catherine S Chen, Haowei Chen, Meng Xuan Chen, Simiao Chen, Ching-Yu Cheng, Esther T W Cheng, Nicolas Cherbuin, Wondimye Ashenafi Cheru, Ju-Huei Chien, Odgerel Chimed-Ochir, Ritesh Chimoriya, Patrick R Ching, Jesus Lorenzo Chirinos-Caceres, Abdulaal Chitheer, William C S Cho, Bryan Chong, Hitesh Chopra, Sonali Gajanan Choudhari, Rajiv Chowdhury, Devasahayam J Christopher, Isaac Sunday Chukwu, Eric Chung, Erin Chung, Eunice Chung, Sheng-Chia Chung, Muhammad Chutiyami, Zinhle Cindi, Iolanda Cioffi, Mareli M Claassens, Rafael M Claro, Kaleb Coberly, Rebecca M Cogen, Alyssa Columbus, Haley Comfort, Joao Conde, Samuele Cortese, Paolo Angelo Cortesi, Vera Marisa Costa, Simona Costanzo, Ewerton Cousin, Rosa A S Couto, Richard G Cowden, Kenneth Michael Cramer, Michael H Criqui, Natália Cruz-Martins, Silvia Magali Cuadra-Hernández, Garland T Culbreth, Patricia Cullen, Matthew Cunningham, Maria paula Curado, Sriharsha Dadana, Omid Dadras, Siyu Dai, Xiaochen Dai, Zhaoli Dai, Lachlan L Dalli, Giovanni Damiani, Jiregna Darega Gela, Jai K Das, Saswati Das, Subasish Das, Ana Maria Dascalu, Nihar Ranjan Dash, Mohsen Dashti, Anna Dastiridou, Gail Davey, Claudio Alberto Dávila-Cervantes, Nicole Davis Weaver, Kairat Davletov, Diego De Leo, Katie de Luca, Aklilu Tamire Debele, Shayom Debopadhaya, Louisa Degenhardt, Azizallah Dehghan, Lee Deitesfeld, Cristian Del Bo', Ivan Delgado-Enciso, Berecha Hundessa Demessa, Andreas K Demetriades, Ke Deng, Xinlei Deng, Edgar Denova-Gutiérrez, Niloofar Deravi, Nebiyu Dereje, Nikolaos Dervenis, Emina Dervišević, Don C Des Jarlais, Hardik Dineshbhai Desai, Rupak Desai, Vinoth Gnana Chellaiyan Devanbu, Syed Masudur Rahman Dewan, Arkadeep Dhali, Kuldeep Dhama, Meghnath Dhimal, Sameer Dhingra, Vishal R Dhulipala, Diana Dias da Silva, Daniel Diaz, Michael J Diaz, Adriana Dima, Delaney D Ding, Huanghe Ding, Ricardo Jorge Dinis-Oliveira, M Ashworth Dirac, Shirin Djalalinia, Thao Huynh Phuong Do, Camila Bruneli do Prado, Saeid Doaei, Masoud Dodangeh, Milad Dodangeh, Sushil Dohare, Klara Georgieva Dokova, Christiane Dolecek, Regina-Mae Villanueva Dominguez, Wanyue Dong, Deepa Dongarwar, Mario D'Oria, Fariba Dorostkar, E Ray Dorsey, Wendel Mombaque dos Santos, Rajkumar Doshi, Leila Doshmangir, Robert Kokou Dowou, Tim Robert Driscoll, Haneil Larson Dsouza, Viola Dsouza, Mi Du, John Dube, Bruce B Duncan, Andre Rodrigues Duraes, Senbagam Duraisamy, Oyewole Christopher Durojaiye, Laura Dwyer-Lindgren, Paulina Agnieszka Dzianach, Arkadiusz Marian Dziedzic, Abdel Rahman E'mar, Ejemai Eboreime, Alireza Ebrahimi, Chidiebere Peter Echieh, Hisham Atan Edinur, David Edvardsson, Kristina Edvardsson, Defi Efendi, Ferry Efendi, Diyan Ermawan Effendi, Terje Andreas Eikemo, Ebrahim Eini, Michael Ekholuenetale, Temitope Cyrus Ekundayo, Iman El Sayed, Iffat Elbarazi, Teshome Bekele Elema, Noha Mousaad Elemam, Frank J Elgar, Islam Y Elgendy, Ghada Metwally Tawfik ElGohary, Hala Rashad Elhabashy, Muhammed Elhadi, Waseem El-Huneidi, Legesse Tesfaye Elilo, Omar Abdelsadek Abdou Elmeligy, Mohamed A Elmonem, Mohammed Elshaer, Ibrahim Elsohaby, Theophilus I Emeto, Luchuo Engelbert Bain, Ryenchindorj Erkhembayar, Christopher Imokhuede Esezobor, Babak Eshrati, Sharareh Eskandarieh, Juan Espinosa-Montero, Habtamu Esubalew, Farshid Etaee, Natalia Fabin, Adewale Oluwaseun Fadaka, Adeniyi Francis Fagbamigbe, Ayesha Fahim, Saman Fahimi, Aliasghar Fakhri-Demeshghieh, Luca Falzone, Mohammad Fareed, Carla Sofia e Sá Farinha, MoezAlIslam Ezzat Mahmoud Faris, Pawan Sirwan Faris, Andre Faro, Abidemi Omolara Fasanmi, Ali Fatehizadeh, Hamed Fattahi, Nelsensius Klau Fauk, Pooria Fazeli, Valery L Feigin, Alireza Feizkhah, Ginenus Fekadu, Xiaoru Feng, Seyed-Mohammad Fereshtehnejad, Abdullah Hamid Feroze, Daniela Ferrante, Alize J Ferrari, Nuno Ferreira, Getahun Fetensa, Bikila Regassa Feyisa, Irina Filip, Florian Fischer, Joanne Flavel, David Flood, Bobirca Teodor Florin, Nataliya A Foigt, Morenike Oluwatoyin Folayan, Artem Alekseevich Fomenkov, Behzad Foroutan, Masoud Foroutan, Ingeborg Forthun, Daniela Fortuna, Matteo Foschi, Kayode Raphael Fowobaje, Kate Louise Francis, Richard Charles Franklin, Alberto Freitas, Joseph Friedman, Sara D Friedman, Takeshi Fukumoto, John E Fuller, Blima Fux, Peter Andras Gaal, Muktar A Gadanya, Abhay Motiramji Gaidhane, Santosh Gaihre, Emmanuela Gakidou, Yaseen Galali, Natalie C Galles, Silvano Gallus, Mandukhai Ganbat, Aravind P Gandhi, Balasankar Ganesan, Mohammad Arfat Ganiyani, MA Garcia-Gordillo, William M Gardner, Jalaj Garg, Naval Garg, Rupesh K Gautam, Semiu Olatunde Gbadamosi, Tilaye Gebru Gebi, Miglas W Gebregergis, Mesfin Gebrehiwot, Teferi Gebru Gebremeskel, Simona Roxana Georgescu, Tamirat Getachew, Peter W Gething, Molla Getie, Keyghobad Ghadiri, Sulmaz Ghahramani, Khalid Yaser Ghailan, Mohammad-Reza Ghasemi, Ghazal Ghasempour Dabaghi, Afsaneh Ghasemzadeh, Ahmad Ghashghaee, Fariba Ghassemi, Ramy Mohamed Ghazy, Ajnish Ghimire, Sama Ghoba, Maryam Gholamalizadeh, Asadollah Gholamian, Ali Gholamrezanezhad, Nasim Gholizadeh, Mahsa Ghorbani, Pooyan Ghorbani Vajargah, Aloke Gopal Ghoshal, Paramjit Singh Gill, Tiffany K Gill, Richard F Gillum, Themba G Ginindza, Alem Girmay, James C Glasbey, Elena V Gnedovskaya, Laszlo Göbölös, Myron Anthony Godinho, Amit Goel, Ali Golchin, Mohamad Goldust, Mahaveer Golechha, Pouya Goleij, Nelson G M Gomes, Philimon N Gona, Sameer Vali Gopalani, Giuseppe Gorini, Houman Goudarzi, Alessandra C Goulart, Bárbara Niegia Garcia Goulart, Anmol Goyal, Ayman Grada, Simon Matthew Graham, Michal Grivna, Giuseppe Grosso, Shi-Yang Guan, Giovanni Guarducci, Mohammed Ibrahim Mohialdeen Gubari, Mesay Dechasa Gudeta, Avirup Guha, Stefano Guicciardi, Rafael Alves Guimarães, Snigdha Gulati, Damitha Asanga Gunawardane, Sasidhar Gunturu, Cui Guo, Anish Kumar Gupta, Bhawna Gupta, Manoj Kumar Gupta, Mohak Gupta, Rajat Das Gupta, Rajeev Gupta, Sapna Gupta, Veer Bala Gupta, Vijai Kumar Gupta, Vivek Kumar Gupta, Lami Gurmessa, Reyna Alma Gutiérrez, Farrokh Habibzadeh, Parham Habibzadeh, Rasool Haddadi, Mostafa Hadei, Najah R Hadi, Nils Haep, Nima Hafezi-Nejad, Alemayehu Hailu, Arvin Haj-Mirzaian, Esam S Halboub, Brian J Hall, Sebastian Haller, Rabih Halwani, Randah R Hamadeh, Sajid Hameed, Samer Hamidi, Erin B Hamilton, Chieh Han, Qiuxia Han, Asif Hanif, Nasrin Hanifi, Graeme J Hankey, Fahad Hanna, Md Abdul Hannan, Md Nuruzzaman Haque, Harapan Harapan, Arief Hargono, Josep Maria Haro, Ahmed I Hasaballah, Ikramul Hasan, M Tasdik Hasan, Hamidreza Hasani, Mohammad Hasanian, Abdiwahab Hashi, Md Saquib Hasnain, Ikrama Hassan, Soheil Hassanipour, Hadi Hassankhani, Johannes Haubold, Rasmus J Havmoeller, Simon I Hay, Jiawei He, Jeffrey J Hebert, Omar E Hegazi, Golnaz Heidari, Mohammad Heidari, Mahsa Heidari-Foroozan, Bartosz Helfer, Delia Hendrie, Brenda Yuliana Herrera-Serna, Claudiu Herteliu, Hamed Hesami, Kamal Hezam, Catherine L Hill, Yuta Hiraike, Ramesh Holla, Nobuyuki Horita, Md Mahbub Hossain, Sahadat Hossain, Mohammad-Salar Hosseini, Hassan Hosseinzadeh, Mehdi Hosseinzadeh, Ahmad Hosseinzadeh Adli, Mihaela Hostiuc, Sorin Hostiuc, Mohamed Hsairi, Vivian Chia-rong Hsieh, Rebecca L Hsu, Chengxi Hu, Junjie Huang, Michael Hultström, Ayesha Humayun, Tsegaye Gebreyes Hundie, Javid Hussain, M Azhar Hussain, Nawfal R Hussein, Foziya Mohammed Hussien, Hong-Han Huynh, Bing-Fang Hwang, Segun Emmanuel Ibitoye, Khalid S Ibrahim, Pulwasha Maria Iftikhar, Desta Ijo, Adalia I Ikiroma, Kevin S Ikuta, Paul Chukwudi Ikwegbue, Olayinka Stephen Ilesanmi, Irena M Ilic, Milena D Ilic, Mohammad Tarique Imam, Mustapha Immurana, Sumant Inamdar, Endang Indriasih, Muhammad Iqhrammullah, Arnaud Iradukunda, Kenneth Chukwuemeka Iregbu, Md Rabiul Islam, Sheikh Mohammed Shariful Islam, Farhad Islami, Faisal Ismail, Nahlah Elkudssiah Ismail, Hiroyasu Iso, Gaetano Isola, Masao Iwagami, Chidozie C D Iwu, Ihoghosa Osamuyi Iyamu, Mahalaxmi Iyer, Linda Merin J, Jalil Jaafari, Louis Jacob, Kathryn H Jacobsen, Farhad Jadidi-Niaragh, Morteza Jafarinia, Abdollah Jafarzadeh, Khushleen Jaggi, Kasra Jahankhani, Nader Jahanmehr, Haitham Jahrami, Nityanand Jain, Ammar Abdulrahman Jairoun, Abhishek Jaiswal, Elham Jamshidi, Mark M Janko, Abubakar Ibrahim Jatau, Sabzali Javadov, Tahereh Javaheri, Sathish Kumar Jayapal, Shubha Jayaram, Rime Jebai, Sun Ha Jee, Jayakumar Jeganathan, Anil K Jha, Ravi Prakash Jha, Heng Jiang, Yingzhao Jin, Olatunji Johnson, Mohammad Jokar, Jost B Jonas, Tamas Joo, Abel Joseph, Nitin Joseph, Charity Ehimwenma Joshua, Grace Joshy, Jacek Jerzy Jozwiak, Mikk Jürisson, Vaishali K, Billingsley Kaambwa, Ali Kabir, Zubair Kabir, Vidya Kadashetti, Dler Hussein Kadir, Rizwan Kalani, Laleh R Kalankesh, Leila R Kalankesh, Feroze Kaliyadan, Sanjay Kalra, Vineet Kumar Kamal, Sivesh Kathir Kamarajah, Rajesh Kamath, Zahra Kamiab, Naser Kamyari, Thanigaivelan Kanagasabai, Tanuj Kanchan, Himal Kandel, Arun R Kanmanthareddy, Edmund Wedam Kanmiki, Kehinde Kazeem Kanmodi, Suthanthira Kannan S, Sushil Kumar Kansal, Rami S Kantar, Neeti Kapoor, Mehrdad Karajizadeh, Shama D Karanth, Reema A Karasneh, Ibraheem M Karaye, André Karch, Asima Karim, Salah Eddin Karimi, Arman Karimi Behnagh, Faizan Zaffar Kashoo, Qalandar Hussein Abdulkarim Kasnazani, Hengameh Kasraei, Nicholas J Kassebaum, Molly B Kassel, Joonas H Kauppila, Navjot Kaur, Norito Kawakami, Gbenga A Kayode, Foad Kazemi, Sina Kazemian, Tahseen Haider Kazmi, Getu Mosisa Kebebew, Adera Debella Kebede, Fassikaw Kebede, Tibebeselassie S Keflie, Peter Njenga Keiyoro, Cathleen Keller, Jaimon Terence Kelly, John H Kempen, Jessica A Kerr, Emmanuelle Kesse-Guyot, Himanshu Khajuria, Amirmohammad Khalaji, Nauman Khalid, Anees Ahmed Khalil, Alireza Khalilian, Faham Khamesipour, Ajmal Khan, Asaduzzaman Khan, Gulfaraz Khan, Ikramullah Khan, Imteyaz A Khan, M Nuruzzaman Khan, Maseer Khan, Mohammad Jobair Khan, Moien AB Khan, Zeeshan Ali Khan, Mahammed Ziauddin Khan suheb, Shaghayegh Khanmohammadi, Khaled Khatab, Fatemeh Khatami, Haitham Khatatbeh, Moawiah Mohammad Khatatbeh, Armin Khavandegar, Hamid Reza Khayat Kashani, Feriha Fatima Khidri, Elaheh Khodadoust, Mohammad Khorgamphar, Moein Khormali, Zahra Khorrami, Ahmad Khosravi, Mohammad Ali Khosravi, Zemene Demelash Kifle, Grace Kim, Jihee Kim, Kwanghyun Kim, Min Seo Kim, Yun Jin Kim, Ruth W Kimokoti, Kasey E Kinzel, Adnan Kisa, Sezer Kisa, Desmond Klu, Ann Kristin Skrindo Knudsen, Jonathan M Kocarnik, Sonali Kochhar, Timea Kocsis, David S Q Koh, Ali-Asghar Kolahi, Kairi Kolves, Farzad Kompani, Gerbrand Koren, Soewarta Kosen, Karel Kostev, Parvaiz A Koul, Sindhura Lakshmi Koulmane Laxminarayana, Kewal Krishan, Hare Krishna, Varun Krishna, Vijay Krishnamoorthy, Yuvaraj Krishnamoorthy, Kris J Krohn, Barthelemy Kuate Defo, Burcu Kucuk Bicer, Md Abdul Kuddus, Mohammed Kuddus, Ilari Kuitunen, Mukhtar Kulimbet, Vishnutheertha Kulkarni, Akshay Kumar, Ashish Kumar, Harish Kumar, Manasi Kumar, Rakesh Kumar, Madhulata Kumari, Fantahun Tarekegn Kumie, Satyajit Kundu, Om P Kurmi, Asep Kusnali, Dian Kusuma, Alexander Kwarteng, Ilias Kyriopoulos, Hmwe Hmwe Kyu, Carlo La Vecchia, Ben Lacey, Muhammad Awwal Ladan, Lucie Laflamme, Abraham K Lagat, Anton C J Lager, Abdelilah Lahmar, Daphne Teck Ching Lai, Dharmesh Kumar Lal, Ratilal Lalloo, Tea Lallukka, Hilton Lam, Judit Lám, Kelsey R Landrum, Francesco Lanfranchi, Justin J Lang, Berthold Langguth, Van Charles Lansingh, Ariane Laplante-Lévesque, Bagher Larijani, Anders O Larsson, Savita Lasrado, Zohra S Lassi, Kamaluddin Latief, Kaveh Latifinaibin, Paolo Lauriola, Nhi Huu Hanh Le, Thao Thi Thu Le, Trang Diep Thanh Le, Caterina Ledda, Jorge R Ledesma, Munjae Lee, Paul H Lee, Seung Won Lee, Shaun Wen Huey Lee, Wei-Chen Lee, Yo Han Lee, Kate E LeGrand, James Leigh, Elvynna Leong, Temesgen L Lerango, Ming-Chieh Li, Wei Li, Xiaopan Li, Yichong Li, Zhihui Li, Virendra S Ligade, Andrew Tiyamike Makhiringa Likaka, Lee-Ling Lim, Stephen S Lim, Megan Lindstrom, Christine Linehan, Chaojie Liu, Gang Liu, Jue Liu, Runben Liu, Shiwei Liu, Xiaofeng Liu, Xuefeng Liu, Erand Llanaj, Michael J Loftus, Rubén López-Bueno, Platon D Lopukhov, Arianna Maever Loreche, Stefan Lorkowski, Paulo A Lotufo, Rafael Lozano, Jailos Lubinda, Giancarlo Lucchetti, Alessandra Lugo, Raimundas Lunevicius, Zheng Feei Ma, Kelsey Lynn Maass, Nikolaos Machairas, Monika Machoy, Farzan Madadizadeh, Christian Madsen, Áurea M Madureira-Carvalho, Azzam A Maghazachi, Sandeep B Maharaj, Soleiman Mahjoub, Mansour Adam Mahmoud, Alireza Mahmoudi, Elham Mahmoudi, Razzagh Mahmoudi, Azeem Majeed, Irsa Fatima Makhdoom, Elaheh Malakan Rad, Venkatesh Maled, Reza Malekzadeh, Armaan K Malhotra, Kashish Malhotra, Ahmad Azam Malik, Iram Malik, Deborah Carvalho Malta, Abdullah A Mamun, Pejman Mansouri, Mohammad Ali Mansournia, Lorenzo Giovanni Mantovani, Sajid Maqsood, Bishnu P Marasini, Hamid Reza Marateb, Joemer C Maravilla, Agustina M Marconi, Parham Mardi, Mirko Marino, Abdoljalal Marjani, Gabriel Martinez, Bernardo Alfonso Martinez-Guerra, Ramon Martinez-Piedra, Daniela Martini, Santi Martini, Francisco Rogerlândio Martins-Melo, Miquel Martorell, Wolfgang Marx, Sharmeen Maryam, Roy Rillera Marzo, Anthony Masaka, Awoke Masrie, Stephanie Mathieson, Alexander G Mathioudakis, Manu Raj Mathur, Jishanth Mattumpuram, Richard Matzopoulos, Richard James Maude, Andrea Maugeri, Pallab K Maulik, Mahsa Mayeli, Maryam Mazaheri, Mohsen Mazidi, John J McGrath, Martin McKee, Anna Laura W McKowen, Susan A McLaughlin, Steven M McPhail, Enkeleint A Mechili, John Robert Carabeo Medina, Rishi P Mediratta, Jitendra Kumar Meena, Rahul Mehra, Kamran Mehrabani-Zeinabad, Entezar Mehrabi Nasab, Tesfahun Mekene Meto, Gebrekiros Gebremichael Meles, Max Alberto Mendez Mendez-Lopez, Walter Mendoza, Ritesh G Menezes, Belayneh Mengist, Alexios-Fotios A Mentis, Sultan Ayoub Meo, Haftu Asmerom Meresa, Atte Meretoja, Tuomo J Meretoja, Abera M Mersha, Bezawit Afework Mesfin, Tomislav Mestrovic, Kukulege Chamila Dinushi Mettananda, Sachith Mettananda, Peter Meylakhs, Adquate Mhlanga, Laurette Mhlanga, Tianyue Mi, Tomasz Miazgowski, Georgia Micha, Irmina Maria Michalek, Ted R Miller, Edward J Mills, Le Huu Nhat Minh, GK Mini, Pouya Mir Mohammad Sadeghi, Andreea Mirica, Antonio Mirijello, Erkin M Mirrakhimov, Mizan Kiros Mirutse, Maryam Mirzaei, Awoke Misganaw, Ashim Mishra, Sanjeev Misra, Philip B Mitchell, Prasanna Mithra, Chaitanya Mittal, Mohammadreza Mobayen, Madeline E Moberg, Ashraf Mohamadkhani, Jama Mohamed, Mouhand F H Mohamed, Nouh Saad Mohamed, Sakineh Mohammad-Alizadeh-Charandabi, Soheil Mohammadi, Abdollah Mohammadian-Hafshejani, Noushin Mohammadifard, Hassen Mohammed, Hussen Mohammed, Mustapha Mohammed, Salahuddin Mohammed, Shafiu Mohammed, Viswanathan Mohan, Hoda Mojiri-Forushani, Amin Mokari, Ali H Mokdad, Sabrina Molinaro, Mariam Molokhia, Sara Momtazmanesh, Lorenzo Monasta, Stefania Mondello, Mohammad Ali Moni, AmirAli Moodi Ghalibaf, Maryam Moradi, Yousef Moradi, Maziar Moradi-Lakeh, Maliheh Moradzadeh, Paula Moraga, Lidia Morawska, Rafael Silveira Moreira, Negar Morovatdar, Shane Douglas Morrison, Jakub Morze, Jonathan F Mosser, Rohith Motappa, Vincent Mougin, Simin Mouodi, Parsa Mousavi, Seyed Ehsan Mousavi, Amin Mousavi Khaneghah, Emmanuel A Mpolya, Matías Mrejen, Sumaira Mubarik, Lorenzo Muccioli, Ulrich Otto Mueller, Faraz Mughal, Sumoni Mukherjee, Francesk Mulita, Kavita Munjal, Efrén Murillo-Zamora, Fungai Musaigwa, Khaled M Musallam, Ahmad Mustafa, Ghulam Mustafa, Saravanan Muthupandian, Raman Muthusamy, Muhammad Muzaffar, Woojae Myung, Ahamarshan Jayaraman Nagarajan, Gabriele Nagel, Pirouz Naghavi, Aliya Naheed, Ganesh R Naik, Gurudatta Naik, Firzan Nainu, Sanjeev Nair, Hastyar Hama Rashid Najmuldeen, Noureddin Nakhostin Ansari, Vinay Nangia, Atta Abbas Naqvi, Sreenivas Narasimha Swamy, Aparna Ichalangod Narayana, Shumaila Nargus, Bruno Ramos Nascimento, Gustavo G Nascimento, Samar Nasehi, Abdulqadir J Nashwan, Zuhair S Natto, Javaid Nauman, Muhammad Naveed, Biswa Prakash Nayak, Vinod C Nayak, Athare Nazri-Panjaki, Rawlance Ndejjo, Sabina Onyinye Nduaguba, Hadush Negash, Ionut Negoi, Ruxandra Irina Negoi, Serban Mircea Negru, Seyed Aria Nejadghaderi, Chakib Nejjari, Evangelia Nena, Samata Nepal, Marie Ng, Haruna Asura Nggada, Georges Nguefack-Tsague, Josephine W Ngunjiri, Anh Hoang Nguyen, Dang H Nguyen, Hau Thi Hien Nguyen, Phat Tuan Nguyen, Van Thanh Nguyen, Robina Khan Niazi, Katie R Nielsen, Yeshambel T Nigatu, Taxiarchis Konstantinos Nikolouzakis, Ali Nikoobar, Fatemeh Nikoomanesh, Amin Reza Nikpoor, Dina Nur Anggraini Ningrum, Chukwudi A Nnaji, Lawrence Achilles Nnyanzi, Efaq Ali Noman, Shuhei Nomura, Mamoona Noreen, Nafise Noroozi, Bo Norrving, Jean Jacques Noubiap, Amanda Novotney, Chisom Adaobi Nri-Ezedi, George Ntaios, Mpiko Ntsekhe, Virginia Nuñez-Samudio, Dieta Nurrika, Jerry John Nutor, Bogdan Oancea, Kehinde O Obamiro, Mary Aigbiremo Oboh, Ismail A Odetokun, Nkechi Martina Odogwu, Martin James O'Donnell, Michael Safo Oduro, Akinyemi O D Ofakunrin, Abiola Ogunkoya, Ayodipupo Sikiru Oguntade, In-Hwan Oh, Hassan Okati-Aliabad, Sylvester Reuben Okeke, Akinkunmi Paul Okekunle, Osaretin Christabel Okonji, Andrew T Olagunju, Muideen Tunbosun Olaiya, Matthew Idowu Olatubi, Gláucia Maria Moraes Oliveira, Isaac Iyinoluwa Olufadewa, Bolajoko Olubukunola Olusanya, Jacob Olusegun Olusanya, Yinka Doris Oluwafemi, Hany A Omar, Ahmed Omar Bali, Goran Latif Omer, Maureene Auma Ondayo, Sokking Ong, Obinna E Onwujekwe, Kenneth Ikenna Onyedibe, Michal Ordak, Orish Ebere Orisakwe, Verner N Orish, Doris V Ortega-Altamirano, Alberto Ortiz, Wael M S Osman, Samuel M Ostroff, Uchechukwu Levi Osuagwu, Adrian Otoiu, Nikita Otstavnov, Stanislav S Otstavnov, Amel Ouyahia, Guoqing Ouyang, Mayowa O Owolabi, Yaz Ozten, Mahesh Padukudru P A, Alicia Padron-Monedero, Jagadish Rao Padubidri, Pramod Kumar Pal, Tamás Palicz, Claudia Palladino, Raffaele Palladino, Raul Felipe Palma-Alvarez, Feng Pan, Hai-Feng Pan, Adrian Pana, Paramjot Panda, Songhomitra Panda-Jonas, Seithikurippu R Pandi-Perumal, Helena Ullyartha Pangaribuan, Georgios D Panos, Leonidas D Panos, Ioannis Pantazopoulos, Anca Mihaela Pantea Stoian, Paraskevi Papadopoulou, Romil R Parikh, Seoyeon Park, Ashwaghosha Parthasarathi, Ava Pashaei, Maja Pasovic, Roberto Passera, Deepak Kumar Pasupula, Hemal M Patel, Jay Patel, Sangram Kishor Patel, Shankargouda Patil, Dimitrios Patoulias, Venkata Suresh Patthipati, Uttam Paudel, Hamidreza Pazoki Toroudi, Spencer A Pease, Amy E Peden, Paolo Pedersini, Umberto Pensato, Veincent Christian Filipino Pepito, Emmanuel K Peprah, Prince Peprah, João Perdigão, Marcos Pereira, Mario F P Peres, Arokiasamy Perianayagam, Norberto Perico, Richard G Pestell, Konrad Pesudovs, Fanny Emily Petermann-Rocha, William A Petri, Hoang Tran Pham, Anil K Philip, Michael R Phillips, Daniela Pierannunzio, Manon Pigeolet, David M Pigott, Thomas Pilgrim, Zahra Zahid Piracha, Michael A Piradov, Saeed Pirouzpanah, Nishad Plakkal, Evgenii Plotnikov, Vivek Podder, Dimitri Poddighe, Suzanne Polinder, Kevan R Polkinghorne, Ramesh Poluru, Ville T Ponkilainen, Fabio Porru, Maarten J Postma, Govinda Raj Poudel, Akram Pourshams, Naeimeh Pourtaheri, Sergio I Prada, Pranil Man Singh Pradhan, Thejeswar N Prakasham, Manya Prasad, Akila Prashant, Elton Junio Sady Prates, Daniel Prieto Alhambra, TINA PRISCILLA, Natalie Pritchett, Bharathi M Purohit, Jagadeesh Puvvula, Nameer Hashim Qasim, Ibrahim Qattea, Asma Saleem Qazi, Gangzhen Qian, Suli Qiu, Maryam Faiz Qureshi, Mehrdad Rabiee Rad, Amir Radfar, Raghu Anekal Radhakrishnan, Venkatraman Radhakrishnan, Hadi Raeisi Shahraki, Quinn Rafferty, Alberto Raggi, Pankaja Raghav Raghav, Nasiru Raheem, Fakher Rahim, Md Jillur Rahim, Vafa Rahimi-Movaghar, Md Mosfequr Rahman, Mohammad Hifz Ur Rahman, Mosiur Rahman, Muhammad Aziz Rahman, Amir Masoud Rahmani, Shayan Rahmani, Vahid Rahmanian, Sathish Rajaa, Prashant Rajput, Ivo Rakovac, Shakthi Kumaran Ramasamy, Sheena Ramazanu, Kritika Rana, Chhabi Lal Ranabhat, Nemanja Rancic, Amey Rane, Chythra R Rao, Indu Ramachandra Rao, Mithun Rao, Sowmya J Rao, Drona Prakash Rasali, Davide Rasella, Sina Rashedi, Vahid Rashedi, Mohammad-Mahdi Rashidi, Ashkan Rasouli-Saravani, Azad Rasul, Giridhara Rathnaiah Babu, Santosh Kumar Rauniyar, Ramin Ravangard, Nakul Ravikumar, David Laith Rawaf, Salman Rawaf, Lal Rawal, Reza Rawassizadeh, Bharat Rawlley, Rabail Zehra Raza, Christian Razo, Elrashdy Moustafa Mohamed Redwan, Faizan Ur Rehman, Lennart Reifels, Robert C Reiner Jr, Giuseppe Remuzzi, Luis Felipe Reyes, Maryam Rezaei, Nazila Rezaei, Negar Rezaei, Mohsen Rezaeian, Taeho Gregory Rhee, Mavra A Riaz, Antonio Luiz P Ribeiro, Jennifer Rickard, Hannah R Riva, Hannah Elizabeth Robinson-Oden, Célia Fortuna Rodrigues, Mónica Rodrigues, Leonardo Roever, Emma Lynn Best Rogowski, Peter Rohloff, Debby Syahru Romadlon, Esperanza Romero-Rodríguez, Michele Romoli, Luca Ronfani, Gholamreza Roshandel, Gregory A Roth, Himanshu Sekhar Rout, Nitai Roy, Priyanka Roy, Enrico Rubagotti, Guilherme de Andrade Ruela, Susan Fred Rumisha, Tilleye Runghien, Godfrey M Rwegerera, Andrzej Rynkiewicz, Chandan S N, Aly M A Saad, Zahra Saadatian, Korosh Saber, Maha Mohamed Saber-Ayad, Morteza SaberiKamarposhti, Siamak Sabour, Simona Sacco, Perminder S Sachdev, Rajesh Sachdeva, Basema Saddik, Adam Saddler, Bashdar Abuzed Sadee, Ehsan Sadeghi, Erfan Sadeghi, Farideh Sadeghian, Mohammad Reza Saeb, Umar Saeed, Fahimeh Safaeinejad, Sher Zaman Safi, Rajesh Sagar, Amene Saghazadeh, Dominic Sagoe, Fatemeh Saheb Sharif-Askari, Narjes Saheb Sharif-Askari, Amirhossein Sahebkar, Soumya Swaroop Sahoo, Umakanta Sahoo, Monalisha Sahu, Zahra Saif, Mirza Rizwan Sajid, Joseph W Sakshaug, Nasir Salam, Payman Salamati, Afeez Abolarinwa Salami, Luciane B Salaroli, Mohamed A Saleh, Sana Salehi, Marwa Rashad Salem, Mohammed Z Y Salem, Sohrab Salimi, Hossein Samadi Kafil, Sara Samadzadeh, Saad Samargandy, Yoseph Leonardo Samodra, Abdallah M Samy, Juan Sanabria, Francesca Sanna, Damian Francesco Santomauro, Itamar S Santos, Milena M Santric-Milicevic, Bruno Piassi Sao Jose, Made Ary Sarasmita, Sivan Yegnanarayana Iyer Saraswathy, Aswini Saravanan, Babak Saravi, Yaser Sarikhani, Tanmay Sarkar, Rodrigo Sarmiento-Suárez, Gargi Sachin Sarode, Sachin C Sarode, Arash Sarveazad, Brijesh Sathian, Thirunavukkarasu Sathish, Maheswar Satpathy, Abu Sayeed, Md Abu Sayeed, Mete Saylan, Mehdi Sayyah, Nikolaos Scarmeas, Benedikt Michael Schaarschmidt, Markus P Schlaich, Winfried Schlee, Maria Inês Schmidt, Ione Jayce Ceola Schneider, Art Schuermans, Austin E Schumacher, Aletta Elisabeth Schutte, Michaël Schwarzinger, David C Schwebel, Falk Schwendicke, Mario Šekerija, Siddharthan Selvaraj, Sabyasachi Senapati, Subramanian Senthilkumaran, Sadaf G Sepanlou, Dragos Serban, Yashendra Sethi, Feng Sha, Maryam Shabany, Amir Shafaat, Mahan Shafie, Nilay S Shah, Pritik A Shah, Syed Mahboob Shah, Saeed Shahabi, Ataollah Shahbandi, Izza Shahid, Samiah Shahid, Wajeehah Shahid, Hamid R Shahsavari, Moyad Jamal Shahwan, Ahmed Shaikh, Masood Ali Shaikh, Alireza Shakeri, Ali S Shalash, Sunder Sham, Muhammad Aaqib Shamim, Mehran Shams-Beyranvand, Hina Shamshad, Mohammad Anas Shamsi, Mohd Shanawaz, Abhishek Shankar, Sadaf Sharfaei, Amin Sharifan, Javad Sharifi-Rad, Rajesh Sharma, Saurab Sharma, Ujjawal Sharma, Vishal Sharma, Rajesh P Shastry, Amin Shavandi, Maryam Shayan, Amr Mohamed Elsayed Shehabeldine, Aziz Sheikh, Rahim Ali Sheikhi, Jiabin Shen, Adithi Shetty, B Suresh Kumar Shetty, Pavanchand H Shetty, Peilin Shi, Kenji Shibuya, Desalegn Shiferaw, Mika Shigematsu, Min-Jeong Shin, Youn Ho Shin, Rahman Shiri, Reza Shirkoohi, Nebiyu Aniley Shitaye, Aminu Shittu, Ivy Shiue, K M Shivakumar, Velizar Shivarov, Farhad Shokraneh, Azad Shokri, Sina Shool, Seyed Afshin Shorofi, Sunil Shrestha, Kerem Shuval, Emmanuel Edwar Siddig, João Pedro Silva, Luís Manuel Lopes Rodrigues Silva, Soraia Silva, Colin R Simpson, Anjali Singal, Abhinav Singh, Balbir Bagicha Singh, Garima Singh, Jasbir Singh, Narinder Pal Singh, Paramdeep Singh, Surjit Singh, Dhirendra Narain Sinha, Robert Sinto, Md Shahjahan Siraj, Sarah Brooke Sirota, Freddy Sitas, Shravan Sivakumar, Valentin Yurievich Skryabin, Anna Aleksandrovna Skryabina, David A Sleet, Bogdan Socea, Anton Sokhan, Ranjan Solanki, Shipra Solanki, Hamidreza Soleimani, Sameh S M Soliman, Suhang Song, Yimeng Song, Reed J D Sorensen, Joan B Soriano, Ireneous N Soyiri, Michael Spartalis, Sandra Spearman, Chandrashekhar T Sreeramareddy, Vijay Kumar Srivastava, Jeffrey D Stanaway, Muhammad Haroon Stanikzai, Benjamin A Stark, Joseph R Starnes, Antonina V Starodubova, Caroline Stein, Dan J Stein, Fridolin Steinbeis, Caitlyn Steiner, Jaimie D Steinmetz, Paschalis Steiropoulos, Aleksandar Stevanović, Leo Stockfelt, Mark A Stokes, Stefan Stortecky, Vetriselvan Subramaniyan, Muhammad Suleman, Rizwan Suliankatchi Abdulkader, Abida Sultana, Haitong Zhe Sun, Jing Sun, Johan Sundström, David Sunkersing, Katharina S Sunnerhagen, Chandan Kumar Swain, Lukasz Szarpak, Mindy D Szeto, Miklós Szócska, Payam Tabaee Damavandi, Rafael Tabarés-Seisdedos, Seyyed Mohammad Tabatabaei, Ozra Tabatabaei Malazy, Seyed-Amir Tabatabaeizadeh, Shima Tabatabai, Mohammad Tabish, JYOTHI TADAKAMADLA, Santosh Kumar Tadakamadla, Yasaman Taheri Abkenar, Moslem Taheri Soodejani, Jabeen Taiba, Ken Takahashi, Iman M Talaat, Ashis Talukder, Mircea Tampa, Jacques Lukenze Tamuzi, Ker-Kan Tan, Sarmila Tandukar, Haosu Tang, Hong K Tang, Ingan Ukur Tarigan, Mengistie Kassahun Tariku, Md Tariqujjaman, Elvis Enowbeyang Tarkang, Razieh Tavakoli Oliaee, Seyed Mohammad Tavangar, Nuno Taveira, Yibekal Manaye Tefera, Mohamad-Hani Temsah, Reem Mohamad Hani Temsah, Masayuki Teramoto, Riki Tesler, Enoch Teye-Kwadjo, Rishu Thakur, Pugazhenthan Thangaraju, Kavumpurathu Raman Thankappan, Samar Tharwat, Rasiah Thayakaran, Nihal Thomas, Nikhil Kenny Thomas, Azalea M Thomson, Amanda G Thrift, Chern Choong Chern Thum, Lau Caspar Thygesen, Jing Tian, Ales Tichopad, Jansje Henny Vera Ticoalu, Tala Tillawi, Tenaw Yimer Tiruye, Mariya Vladimirovna Titova, Marcello Tonelli, Roman Topor-Madry, Adetunji T Toriola, Anna E Torre, Mathilde Touvier, Marcos Roberto Tovani-Palone, Jasmine T Tran, Nghia Minh Tran, Domenico Trico, Samuel Joseph Tromans, Thien Tan Tri Tai Truyen, Aristidis Tsatsakis, Guesh Mebrahtom Tsegay, Evangelia Eirini Tsermpini, Munkhtuya Tumurkhuu, Kang Tung, Stefanos Tyrovolas, Sayed Mohammad Nazim Uddin, Aniefiok John Udoakang, Arit Udoh, Atta Ullah, Irfan Ullah, Saeed Ullah, Sana Ullah, Srikanth Umakanthan, Chukwuma David Umeokonkwo, Brigid Unim, Bhaskaran Unnikrishnan, Carolyn Anne Unsworth, Era Upadhyay, Daniele Urso, Jibrin Sammani Usman, Seyed Mohammad Vahabi, Asokan Govindaraj Vaithinathan, Rohollah Valizadeh, Sarah M Van de Velde, Jef Van den Eynde, Orsolya Varga, Priya Vart, Shoban Babu Varthya, Tommi Juhani Vasankari, Milena Vasic, Siavash Vaziri, Balachandar Vellingiri, Narayanaswamy Venketasubramanian, Nicholas Alexander Verghese, Madhur Verma, Massimiliano Veroux, Georgios-Ioannis Verras, Dominique Vervoort, Jorge Hugo Villafañe, Gabriela Ines Villanueva, Manish Vinayak, Francesco S Violante, Maria Viskadourou, Sergey Konstantinovitch Vladimirov, Vasily Vlassov, Bay Vo, Stein Emil Vollset, Avina Vongpradith, Theo Vos, Isidora S Vujcic, Rade Vukovic, Hatem A Wafa, Yasir Waheed, Richard G Wamai, Cong Wang, Ning Wang, Shu Wang, Song Wang, Yanzhong Wang, Yuan-Pang Wang, Muhammad Waqas, Paul Ward, Emebet Gashaw Wassie, Stefanie Watson, Stephanie Louise Watson Watson, Kosala Gayan Weerakoon, Melissa Y Wei, Robert G Weintraub, Daniel J Weiss, Ronny Westerman, Joanna L Whisnant, Taweewat Wiangkham, Dakshitha Praneeth Wickramasinghe, Nuwan Darshana Wickramasinghe, Angga Wilandika, Caroline Wilkerson, Peter Willeit, Shadrach Wilson, Marcin W Wojewodzic, Demewoz H Woldegebreal, Axel Walter Wolf, Charles D A Wolfe, Yohannes Addisu Wondimagegene, Yen Jun Wong, Utoomporn Wongsin, Ai-Min Wu, Chenkai Wu, Felicia Wu, Xinsheng Wu, Zenghong Wu, Juan Xia, Hong Xiao, Yang Xie, Suowen Xu, Wang-Dong Xu, Xiaoyue Xu, Yvonne Yiru Xu, Ali Yadollahpour, Kazumasa Yamagishi, Danting Yang, Lin Yang, Yuichiro Yano, Yao Yao, Habib Yaribeygi, Pengpeng Ye, Sisay Shewasinad Yehualashet, Metin Yesiltepe, Subah Abderehim Yesuf, Saber Yezli, Siyan Yi, Amanuel Yigezu, Arzu Yiğit, Vahit Yiğit, Paul Yip, Malede Berihun Yismaw, Yazachew Yismaw, Dong Keon Yon, Naohiro Yonemoto, Seok-Jun Yoon, Yuyi You, Mustafa Z Younis, Zabihollah Yousefi, Chuanhua Yu, Yong Yu, Faith H Yuh, Siddhesh Zadey, Vesna Zadnik, Nima Zafari, Fathiah Zakham, Nazar Zaki, Sojib Bin Zaman, Nelson Zamora, Ramin Zand, Moein Zangiabadian, Heather J Zar, Iman Zare, Armin Zarrintan, Mohammed G M Zeariya, Zahra Zeinali, Haijun Zhang, Jianrong Zhang, Jingya Zhang, Liqun Zhang, Yunquan Zhang, Zhi-Jiang Zhang, Hanqing Zhao, Chenwen Zhong, Juexiao Zhou, Bin Zhu, Lei Zhu, Makan Ziafati, Magdalena Zielińska, Osama A Zitoun, Mohammad Zoladl, Zhiyong Zou, Liesl J Zuhlke, Alimuddin Zumla, Elric Zweck, Samer H Zyoud, Eve E Wool, Christopher J L Murray

## Abstract

**Background:**

Regular, detailed reporting on population health by underlying cause of death is fundamental for public health decision making. Cause-specific estimates of mortality and the subsequent effects on life expectancy worldwide are valuable metrics to gauge progress in reducing mortality rates. These estimates are particularly important following large-scale mortality spikes, such as the COVID-19 pandemic. When systematically analysed, mortality rates and life expectancy allow comparisons of the consequences of causes of death globally and over time, providing a nuanced understanding of the effect of these causes on global populations.

**Methods:**

The Global Burden of Diseases, Injuries, and Risk Factors Study (GBD) 2021 cause-of-death analysis estimated mortality and years of life lost (YLLs) from 288 causes of death by age-sex-location-year in 204 countries and territories and 811 subnational locations for each year from 1990 until 2021. The analysis used 56 604 data sources, including data from vital registration and verbal autopsy as well as surveys, censuses, surveillance systems, and cancer registries, among others. As with previous GBD rounds, cause-specific death rates for most causes were estimated using the Cause of Death Ensemble model—a modelling tool developed for GBD to assess the out-of-sample predictive validity of different statistical models and covariate permutations and combine those results to produce cause-specific mortality estimates—with alternative strategies adapted to model causes with insufficient data, substantial changes in reporting over the study period, or unusual epidemiology. YLLs were computed as the product of the number of deaths for each cause-age-sex-location-year and the standard life expectancy at each age. As part of the modelling process, uncertainty intervals (UIs) were generated using the 2·5th and 97·5th percentiles from a 1000-draw distribution for each metric. We decomposed life expectancy by cause of death, location, and year to show cause-specific effects on life expectancy from 1990 to 2021. We also used the coefficient of variation and the fraction of population affected by 90% of deaths to highlight concentrations of mortality. Findings are reported in counts and age-standardised rates. Methodological improvements for cause-of-death estimates in GBD 2021 include the expansion of under-5-years age group to include four new age groups, enhanced methods to account for stochastic variation of sparse data, and the inclusion of COVID-19 and other pandemic-related mortality—which includes excess mortality associated with the pandemic, excluding COVID-19, lower respiratory infections, measles, malaria, and pertussis. For this analysis, 199 new country-years of vital registration cause-of-death data, 5 country-years of surveillance data, 21 country-years of verbal autopsy data, and 94 country-years of other data types were added to those used in previous GBD rounds.

**Findings:**

The leading causes of age-standardised deaths globally were the same in 2019 as they were in 1990; in descending order, these were, ischaemic heart disease, stroke, chronic obstructive pulmonary disease, and lower respiratory infections. In 2021, however, COVID-19 replaced stroke as the second-leading age-standardised cause of death, with 94·0 deaths (95% UI 89·2–100·0) per 100 000 population. The COVID-19 pandemic shifted the rankings of the leading five causes, lowering stroke to the third-leading and chronic obstructive pulmonary disease to the fourth-leading position. In 2021, the highest age-standardised death rates from COVID-19 occurred in sub-Saharan Africa (271·0 deaths [250·1–290·7] per 100 000 population) and Latin America and the Caribbean (195·4 deaths [182·1–211·4] per 100 000 population). The lowest age-standardised death rates from COVID-19 were in the high-income super-region (48·1 deaths [47·4–48·8] per 100 000 population) and southeast Asia, east Asia, and Oceania (23·2 deaths [16·3–37·2] per 100 000 population). Globally, life expectancy steadily improved between 1990 and 2019 for 18 of the 22 investigated causes. Decomposition of global and regional life expectancy showed the positive effect that reductions in deaths from enteric infections, lower respiratory infections, stroke, and neonatal deaths, among others have contributed to improved survival over the study period. However, a net reduction of 1·6 years occurred in global life expectancy between 2019 and 2021, primarily due to increased death rates from COVID-19 and other pandemic-related mortality. Life expectancy was highly variable between super-regions over the study period, with southeast Asia, east Asia, and Oceania gaining 8·3 years (6·7–9·9) overall, while having the smallest reduction in life expectancy due to COVID-19 (0·4 years). The largest reduction in life expectancy due to COVID-19 occurred in Latin America and the Caribbean (3·6 years). Additionally, 53 of the 288 causes of death were highly concentrated in locations with less than 50% of the global population as of 2021, and these causes of death became progressively more concentrated since 1990, when only 44 causes showed this pattern. The concentration phenomenon is discussed heuristically with respect to enteric and lower respiratory infections, malaria, HIV/AIDS, neonatal disorders, tuberculosis, and measles.

**Interpretation:**

Long-standing gains in life expectancy and reductions in many of the leading causes of death have been disrupted by the COVID-19 pandemic, the adverse effects of which were spread unevenly among populations. Despite the pandemic, there has been continued progress in combatting several notable causes of death, leading to improved global life expectancy over the study period. Each of the seven GBD super-regions showed an overall improvement from 1990 and 2021, obscuring the negative effect in the years of the pandemic. Additionally, our findings regarding regional variation in causes of death driving increases in life expectancy hold clear policy utility. Analyses of shifting mortality trends reveal that several causes, once widespread globally, are now increasingly concentrated geographically. These changes in mortality concentration, alongside further investigation of changing risks, interventions, and relevant policy, present an important opportunity to deepen our understanding of mortality-reduction strategies. Examining patterns in mortality concentration might reveal areas where successful public health interventions have been implemented. Translating these successes to locations where certain causes of death remain entrenched can inform policies that work to improve life expectancy for people everywhere.

**Funding:**

Bill & Melinda Gates Foundation.

## Introduction

For more than three decades, the Global Burden of Diseases, Injuries, and Risk Factors Study (GBD) has been systematically and comprehensively recording and analysing causes of human death stratified by age, sex, and time across the world.^1^, ^2^ This information has been used to guide policy solutions, reduce modifiable risk factors, monitor and evaluate national and sub-national health interventions, and ultimately improve health recommendations at both regional and local levels.^1^ Assessing trends in cause-specific mortality is essential to inform health policy that must continuously evolve to account for rapid changes to the global health landscape, such as the COVID-19 pandemic.^3^ Comprehensive updates to levels and trends in causes of death give insight into emerging global health challenges and can facilitate benchmarking in the case of a new pandemic or other events that can lead to a staggering loss of life. Therefore, documenting novel changes to mortality, such as an emerging pandemic, in real time, is important.

Causes of death are not uniformly distributed between populations; rather, large variability in the leading causes often reflects important social and geographical differences.^4^ These differences can include access to and quality of health care, timeliness of health system responsiveness, and exposure to causes that are endemic to specific geographical locations.^4^ Mortality patterns continually evolve, as some areas become successful in their reduction efforts, whereas other causes persist within specific locations. The past 30 years have seen improvements among many causes of mortality, some of which have considerably narrowed in geographical range and are now concentrated within smaller areas worldwide. This change enables us to identify the resulting areas of concentrated mortality—areas where deaths from that cause are occurring within a limited subset of the global population. Our analysis provides an opportunity to answer important epidemiological questions that have been at the forefront of global and public health discourse—eg, which causes have contributed to the largest increase or decrease in life expectancy, which locations are experiencing greater concentrations of preventable causes of death, and how has COVID-19 and other pandemic-related mortality (OPRM) affected life expectancy and the overall fatal burden of diseases? Regional variation in many of the leading causes of death remains evident in these most recent estimates, representing important opportunities for creating tailored health policy to improve disparities and alleviate concentrations of mortality.

GBD 2021 provides an updated, comprehensive set of the fatal burden of disease summarised with cause-specific mortality metrics and years-of-life-lost (YLLs) metrics for 288 causes by age and sex across 204 countries and territories from 1990 to 2021, an update from the previously published estimates covering 1990–2019. In this study, we present mortality concentrations and a decomposition analysis of life expectancy due to different causes of death and illustrate the impact of causes of death on global, regional, and country-specific life expectancy, as well as highlighting locations that are most affected by concentrated geographical mortality burden. As with previous iterations of GBD, this cycle incorporates newly available data sources and improved methodological approaches to re-estimate the entire time series, providing updated estimates that supersede all previous GBD cause-of-death publications. GBD 2021 includes an estimation of several different models for disease and injury outcomes. This manuscript was produced as part of the GBD Collaborator Network and in accordance with the GBD Protocol.^5^

**Research in context**

**Evidence before this study**

The Global Burden of Diseases, Injuries, and Risk Factors Study (GBD) has provided regular updates on the complex patterns and trends in population health around the world since the first GBD publication in 1993. With each subsequent iteration, there have been important methodological updates, new datasets included, and an expanded list of causes, risk factors, and locations for which estimates of the burden of disease are produced. In 1993, mortality and years of life lost (YLLs) were reported for 107 categories of diseases that covered all possible causes of death, for eight regions. In the last GBD cycle—GBD 2019—estimates of mortality and YLLs were produced for 286 causes of death in 204 countries and territories, including all WHO member states, and for subnational locations in 21 countries and territories, for every year from 1990 to 2019. Although many groups have reported on national-level, cause-specific mortality and other population-health metrics, including the WHO World Health Statistics reports, GBD is the most detailed and transparent research effort to date. Further, estimates of COVID-19-related deaths in 2020 and 2021 have been reported by several sources, including GBD studies that have quantified excess mortality due to the pandemic within a subset of GBD locations. However, no previous publications have quantified the effect of COVID-19 on life expectancy, while considering the full spectrum of disease mortality over the past three decades, across all countries and territories. This study presents, for the first time, 288 causes of death from 1990 to 2021, complementary to the all-cause mortality findings presented in the GBD 2021 Demographics analysis. Combined, these studies provide a comprehensive view of all-cause and cause-specific mortality from 1990 to 2021.

**Added value of this study**

Alongside the all-cause mortality and life-expectancy assessments in companion publications for GBD 2021, this analysis delineates cause-specific mortality and its effect on life expectancy. This study includes a comprehensive decomposition analysis elucidating the primary cause of death influencing life expectancy on a global, regional, and national level. Additionally, we present causes of death and YLLs for all countries and territories, providing policy makers with valuable insights into variations in cause-specific mortality. This study is also the first of its kind to publish 2021 estimates of COVID-19-related deaths and YLLs for 204 countries and territories in the context of the global burden of disease. Although other publications have estimated deaths due to COVID-19, those deaths have not previously been compared with deaths from other causes. By modelling COVID-19 deaths within a hierarchy of mutually exclusive and collectively exhaustive causes of death, this study provides policy makers with information that is essential for setting health priorities around the world. To obtain more comprehensive insights from life expectancy, it is necessary to break it down into age-specific mortality, which is influenced by cause-specific mortality rates. We examined the effect of COVID-19 and other causes of death on life expectancy by decomposing death counts into different cause-specific mortality rates across various dimensions, including country or territory, region, super-region, and five distinct time periods: 1990–2000, 2000–2010, 2010–2019, 2019–2021, and 1990–2021. We could therefore systematically calibrate the COVID-19 pandemic against other causes of mortality over the period 1990–2021. Finally, our study identified several causes of death that exhibited increased geographical concentration over time—ie, causes with a disproportionate impact within a specific geographical area compared with the rest of the global observations. This analysis provides policy makers important information on regional variation and inequalities in cause-specific mortality. Also new to GBD 2021, we report on 12 additional causes of death: COVID-19 and other pandemic-related mortality, pulmonary arterial hypertension, and nine cancer types—hepatoblastoma, Burkitt lymphoma, other non-Hodgkin lymphoma, eye cancer, retinoblastoma, other eye cancers, soft tissue and other extraosseous sarcomas, malignant neoplasm of bone and articular cartilage, and neuroblastoma and other peripheral nervous-cell tumours. Granularity of the estimation of deaths in children younger than 5 years was enhanced by the addition of four new age groups: 1–5 months, 6–11 months, 12–23 months, and 2–4 years.

**Implications of all the available evidence**

Our study provides a full analysis of causes of death worldwide and across time, alongside the changing patterns in life expectancy precipitated by those causes. Increasing geographical concentration of mortality was observed for many causes of death, highlighting disparities between regions and substantial differences in cause-specific contributions to life expectancy. On a global scale, this information provides an opportunity to examine whether reductions in mortality were resilient to the onset of a novel pandemic. On a regional level, the estimates generated by our study provide important detail on the evolving impact of causes of death among countries, allowing crucial insight into differential success by geography, time, and cause. The comprehensive nature of GBD 2021 cause-of-death estimation provides valuable opportunities to learn from mortality gains and losses, helping to accelerate progress in reducing mortality.

## Methods

### Overview

In GBD 2021, we produced estimates for each epidemiological quantity of interest for 288 causes of death by age-sex-location-year for 25 age groups from birth to 95 years and older; for males, females, and both sexes combined; in 204 countries and territories grouped into 21 regions and seven super-regions; and for every year from 1990 to 2021. GBD 2021 also includes subnational analyses for 21 countries and territories (appendix 1 section 2.1). An international network of collaborators provides, reviews, and analyses the available data to generate these metrics; GBD 2021 drew on the expertise of more than 11 000 collaborators from more than 160 countries and territories.

The methods used to generate these estimates closely followed those for GBD 2019.^6^ These methods have been extensively peer reviewed over previous rounds of the GBD study^4^, ^6^, ^7^, ^8^, ^9^ and as part of the peer-review process for GBD 2021. Here, we provide an overview of the methods with an emphasis on the main methodology changes since GBD 2019; a comprehensive description of the analytical methods for GBD 2021 is provided in appendix 1. Detailed descriptions of analytical methods and models for each cause of death are also available in a searchable online tool.

The GBD 2021 cause-of-death estimates described here include cause-specific mortality and the premature death metric (YLLs). We calculated YLLs as the number of deaths for each cause-age-sex-location-year multiplied by the standard life expectancy at each age (appendix 1 section 6.3). Standard life expectancy is calculated from the lowest age-specific mortality rate between countries.^10^ Briefly, we estimated cause-specific death rates for 209 causes using the Cause of Death Ensemble model (CODEm), and we used alternative strategies to model causes with little data, substantial changes in reporting over the study period, or unusual epidemiology. The modelling strategy used for all causes of death can be found in appendix 1 (table S10). CODEm is a modelling tool developed specifically for GBD that assesses the out-of-sample predictive validity of different statistical models and covariate permutations and then combines the results from those assessments to produce cause-specific estimates of the burden of mortality. Methodological improvements for cause-of-death estimates in the present round of estimation focused on several key areas. First, cause-of-death data were updated to include age data for the following age groups younger than 5 years: 1–5 months, 6–11 months, 12–23 months, and 2–4 years. Second, we implemented enhanced methods to account for stochastic variation in cause-of-death data and improve the estimation of small cause fractions present in less common causes of death. Third, we added 199 new country-years of vital registration cause-of-death data, 5 country-years of surveillance data, 21 country-years of verbal autopsy data, and 94 country-years of other data types. Lastly, we incorporated COVID-19 and OPRM, which includes excess mortality associated with the COVID-19 pandemic, excluding deaths from COVID-19, lower respiratory infections, measles, malaria, and pertussis.

### The GBD disease and injury hierarchy

GBD classifies diseases and injuries into a hierarchy with four levels that include both fatal and non-fatal causes. Level 1 causes include three broad aggregate categories (communicable, maternal, neonatal, and nutritional [CMNN] diseases; non-communicable diseases [NCDs]; and injuries) and Level 2 disaggregates those categories into 22 clusters of causes, which are further disaggregated into Level 3 and Level 4 causes. At the most detailed level, 288 fatal causes are estimated. For a full list of causes of death by level, see appendix 1 (table S2). For GBD 2021, we separately report on 12 causes of death for the first time: COVID-19, OPRM, pulmonary arterial hypertension, and nine cancer types: hepatoblastoma, Burkitt lymphoma, other non-Hodgkin lymphoma, eye cancer, retinoblastoma, other eye cancers, soft tissue and other extraosseous sarcomas, malignant neoplasm of bone and articular cartilage, and neuroblastoma and other peripheral nervous cell tumours.

### Data sources, processing, and assessing for completeness

The GBD 2021 cause-of-death database included data sources identified in previous rounds of estimation in addition to 9248 new sources (appendix 1 table S5). We included multiple data types to capture the widest array of information, including vital registration and verbal autopsy for all 288 causes as well as survey, census, surveillance, cancer registry, police records, open-source databases, and minimally invasive tissue sampling. To standardise these data so that they can be compared by cause, age, sex, location, and time, we applied a set of data processing corrections. First, deaths with insufficient age data to estimate the GBD age groups or missing age and sex data underwent age and sex splitting to assign GBD age groups as well as sex (appendix 1 section 3.5). Additionally, garbage codes, which are non-specific, implausible, or intermediate, rather than underlying cause of death codes from the International Classification of Diseases, were redistributed to appropriate targets to assign the underlying cause of death.^11^ We excluded data sources with more than 50% of all deaths assigned to major garbage codes (class 1 or class 2 garbage codes) in a given year for a specific location (location-year) to mitigate the potential for bias from these sources (appendix 1 section 3.7). For GBD 2021, we established a buffer system so that location-years that were included in the previous GBD cycle would not be dropped from the current cycle as long as less than 55% of all deaths were assigned to major garbage codes. This 5% buffer ensured greater consistency in data source inclusion from one cycle to the next.

Assessing data completeness illustrates the coverage from a data source on overall mortality for the country. Vital registration and verbal autopsy data completeness—a source-specific estimate of the percentage of total cause-specific deaths that are reported in a given location and year—was assessed by location-year, and sources with less than 50% completeness were excluded. We excluded 142 country-years of data because of completeness. As with garbage codes, we used a 5% buffer so that sources included in the previous GBD cycle would not be excluded from the current cycle if they had at least 45% completeness, allowing us to retain 24 country-years that had previously been dropped. We then multiplied the estimated all-cause mortality for each age-sex-location-year by the cause fraction for the corresponding age-sex-location-year to adjust all included sources to 100% completeness. Verbal autopsy and vital registration data availability, completeness, and quality rating for each location-year are available in appendix 1 (section 3), as well as full details on all data processing corrections.

### Improvements in GBD 2021 to cause of death data processing and estimation

#### Adjustments for stochastic variation

In GBD 2021, we made two primary improvements to the methods used to reduce stochastic variation, most affecting causes of death with small sample sizes. First, we updated the Bayesian algorithm used in the noise reduction of these data to improve the preservation of real trends in data with large sample sizes, and imparted additional information from regional trends for data with small sample sizes. Second, the non-zero floor, a method that addresses distorted data shapes and nonsensical trends caused by small numbers when transformed to log space, was updated to be time-invariant and independent of demographic inputs. The full details of these two key improvements, as well as other improvements that address stochastic variation, can be found in appendix 1 (section 3.14).

#### COVID-19 and OPRM estimation

We derived COVID-19 and OPRM estimates from an analysis of the overall excess mortality due to the COVID-19 pandemic from January 1, 2020, to December 31, 2021. Full details of the estimation of excess mortality, COVID-19 deaths, and OPRM are provided in appendix 1 (section 5). To estimate excess mortality, we first developed a database of all-cause mortality by week and month after accounting for reporting lags, anomalies such as heat waves, and under-registration of deaths. Next, we developed an ensemble model to predict expected deaths in the absence of the COVID-19 pandemic for the years 2020 and 2021. In location and time combinations with data used for these models, we estimated excess mortality as observed mortality minus expected mortality. To estimate excess mortality for location-years without data, we developed a statistical model to directly predict the excess mortality due to COVID-19, using covariates that pertained to both the COVID-19 pandemic and background population-health-related metrics at the population level before SARS-CoV-2 emerged. Uncertainty was propagated through each step of this estimation procedure.^12^

To produce the final estimates of COVID-19 deaths used in GBD 2021, we used a counterfactual approach. The counterfactual estimates the number of deaths if infection detection rates were at the highest observed value for each location-year. Using the ratio of counterfactual over estimated excess deaths and the ratio of reported COVID-19 deaths over excess deaths, we calculated the ratio of total COVID-19 deaths over reported COVID-19 deaths and multiplied this figure by the number of reported COVID-19 deaths for our final estimates of COVID-19 deaths.^12^

To account for increases in excess mortality in 2020 and 2021 that could not be attributed to particular causes, we introduced a residual cause, OPRM. We identified four causes of death—lower respiratory infections, measles, malaria, and pertussis—as related to the COVID-19 pandemic and having reliable enough estimates to not contribute to OPRM. Thus, we calculated OPRM as the difference between excess mortality and the sum of deaths due to COVID-19 and these four causes.^12^

### Presentation of cause-specific mortality estimates

Cause-specific mortality estimates for 2021 are given in death counts and age-standardised rates per 100 000 population, calculated using the GBD standard-population structure.^10^ For changes over time, we present percentage changes over the period 1990–2021, and annualised rates of change as the difference in the natural log of the values at the start and end of the time interval divided by the number of years in the interval. We computed uncertainty intervals (UIs) for all metrics using the mean estimate across 1000 draws (appendix 1 sections 2–3), and 95% UIs are given as the 2·5th and 97·5th percentiles of that distribution.

### Life-expectancy decomposition

The objective of life-expectancy decomposition is to analyse the difference in life expectancy by age and location, quantifying contributions from specific causes (appendix 1 section 7). We examined temporal trends in causes over continuous time periods across different locations. We aimed to identify the effect of causes of death on life expectancy by using three main decomposition steps. For this study, we investigated the top-20 Level 2 and Level 3 GBD causes contributing to change in life expectancy. The remaining causes were then combined as “other communicable and maternal disorders” or “other NCDs”. The first step involved decomposing the difference in life expectancy by age. We calculated age-specific contributions to understand the variation in life expectancy across different age groups. In the second step, each age-specific contribution was further decomposed into cause-age-specific contributions. This analysis allowed for the identification of the specific causes of death that contributed to the differences in life expectancy within each age group. Finally, we aggregated the cause-age-specific contributions across age groups to produce cause-specific contributions to the overall difference in life expectancy. This aggregation provided a comprehensive understanding of how different causes of death contributed to the observed variations in life expectancy. By applying this decomposition approach, we gain insights into the relative effect of different causes of death on changes in life expectancy by age and location.

### Calculation of mortality concentration

Concentrated causes in GBD refer to causes that exhibit a disproportionate impact in a specific geographical subset of the data compared with the rest of the global observations. In GBD 2021, we used two different methods to identify these concentrated causes: coefficient of variation and mortality concentration.

#### Coefficient of variation

For each GBD cause, we calculated a coefficient of variation using standard statistical methods. This measure assesses the variability of a population relative to its mean.^13^ The observations considered for this calculation were national, age-standardised, both-sex mortality rates, using the mean mortality rate between 2019 and 2021. Causes with larger coefficients of variation have data that are less centred around the mean and indicate a greater likelihood of a concentrated cause.

#### Mortality concentration

To identify concentrations of mortality—geographical locations or groups of locations with populations that are disproportionately affected by a particular cause—we first calculated the total number of all-age, both-sex deaths in 2021 by cause in each of the 811 subnational locations and sorted these locations by number of deaths in descending order. We then calculated the cumulative percentage of deaths by dividing location-specific cumulative deaths by the number of global deaths for each cause. When the cumulative percentage reached or exceeded 90% for a given cause, we divided the population of the geographical subset included in that cumulative percentage by the total global population in 2021, using population estimates from the GBD population model described in previous publications.^10^, ^12^ This identification of geographical subsets that contain at least 90% of deaths from a given cause but represent a comparatively small share of the global population was used to identify potential inequalities in the incidence of mortality between locations and populations. In addition to identifying these concentrations of mortality in 2021, we repeated this same analysis for 1990. By comparing the respective proportions of affected global population in these two years, we were able to differentiate causes that showed increased, decreased, or unchanged concentrations of mortality. The causes highlighted in this study were those characterised by an age-standardised mortality rate greater than 0·5 per 100 000 population. The purpose of presenting mortality concentrations is to illustrate causes that are disproportionately affecting specific populations, when previously that cause affected large swaths of the population. Thus, we did not calculate the mortality concentration for causes that are endemic to certain regions, as the mortality rate is already known to be concentrated among specific parts of the global population. We excluded two endemic causes, Ebola virus disease and Chagas disease, from this calculation.

### GBD research and reporting practices

This research is compliant with the Guidelines for Accurate and Transparent Health Estimates Reporting recommendations (GATHER; appendix 1 table S4).^14^ Software packages used in the cause-of-death analysis for GBD 2021 were Python (version 3.10.4), Stata (version 13.1), and R (version 4.2.1). Statistical code used for GBD estimation is publicly available online.

### Role of the funding source

The funder of this study had no role in study design, data collection, data analysis, data interpretation, or the writing of the report.

## Results

Estimates described in the Article are viewable in appendix 2. Detailed results for each cause of death in the analysis are available in downloadable form through the GBD Results tool and via visual exploration through the online tool GBD Compare. Summaries of results for each cause of death included in the analysis are available online.

### Global causes of death

Estimates described in the Article are viewable in appendix 2. Detailed results for each cause of death in the analysis are available in downloadable form through the GBD Results tool and via visual exploration through the online tool GBD Compare. Summaries of results for each cause of death included in the analysis are available online.

**Figure 1:**
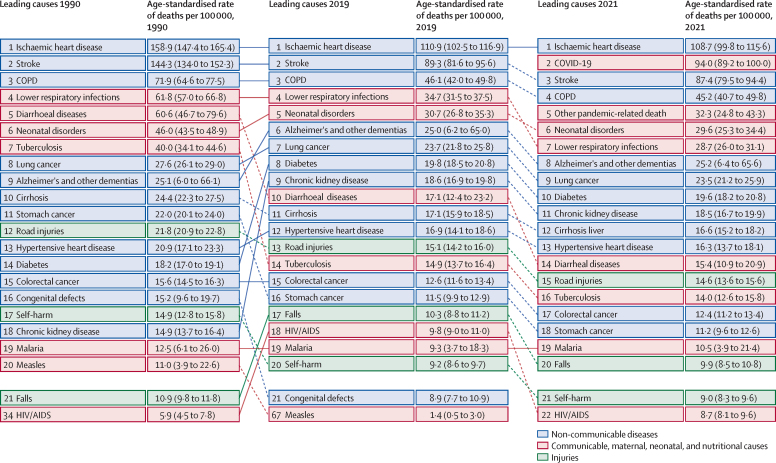
Leading Level 3 causes of global deaths and age-standardised death rate per 100 000 population for males and females combined, 1990, 2019, and 2021 Figure shows the 20 leading causes of death in descending order. Causes are connected by lines between time periods; solid lines represent an increase or lateral shift in ranking and dashed lines are decreases in rank. COPD=chronic obstructive pulmonary disease. Lung cancer=tracheal, bronchus, and lung cancer.

**Figure 2:**
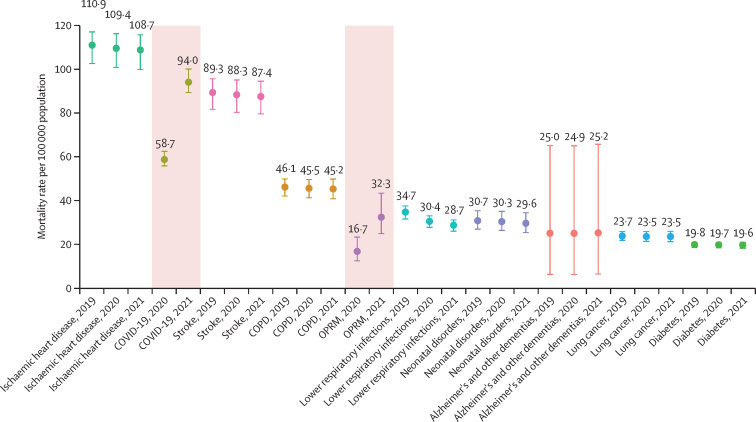
Age-standardised mortality rate per 100 000 population for the ten leading Level 3 causes of death globally, 2019–21 Whisker plot in which the y-axis represents the age-standardised mortality rate and the x-axis represents a selected cause-year. Causes are arranged from highest to lowest age-standardised mortality rate, with each cause assigned a distinct colour for identification. The whiskers represent the 95% uncertainty interval. COPD=chronic obstructive pulmonary disease. OPRM=other pandemic-related mortality.

From 1990 to 2019, the annual rate of change in global deaths from all causes ranged from –0·9% (95% UI –2·7 to 0·8) to 2·4% (0·1 to 4·7; appendix 2 figure S1). The corresponding annual rates of change in the global age-standardised mortality rate ranged from –3·3% (–5·0 to –1·6) to 0·4% (–1·9 to 2·5). In 2020, however, the total number of deaths worldwide increased by 10·8% (6·4 to 15·4) compared with 2019, from 57·0 million deaths (54·9 to 59·5) in 2019 to 63·1 million deaths (60·6 to 65·9) in 2020. This trend persisted in 2021, with an increase of 7·5% (3·1 to 12·4) relative to 2020, to 67·9 million (65·0 to 70·8) deaths. The age-standardised mortality rate followed a similar pattern, increasing by 8·1% (3·9 to 12·4) in 2020 and an additional 5·2% (1·0 to 9·7) in 2021. In 2020 and 2021, deaths from COVID-19 and OPRM changed the pattern of mortality for the leading causes of age-standardised death (Figure 1, Figure 2; table 1). At Level 3 of the GBD cause-classification hierarchy, the rankings of the four causes of death with the highest age-standardised mortality rates were the same in 2019 as they were in 1990, with each showing a steady decline in its age-standardised death rate (figure 1). These causes were, in descending order, ischaemic heart disease, stroke, chronic obstructive pulmonary disease, and lower respiratory infections. In 2021, however, COVID-19 replaced stroke as the second leading cause of age-standardised death globally (with 94·0 deaths [95% UI 89·2 to 100·0] per 100 000 population), with stroke becoming the third leading cause. Additionally, OPRM—which includes excess mortality associated with the pandemic, excluding COVID-19, lower respiratory infections, measles, and pertussis causes—emerged as the fifth leading cause of age-standardised deaths in 2021; lower respiratory infections decreased from the fourth to the seventh leading cause. The effect of COVID-19 on age-standardised mortality was similar to that of chronic obstructive pulmonary disease in 2020 but increased by 60·2% (53·1 to 67·6) in 2021, becoming similar to that of stroke and ischaemic heart disease (figure 2; table 1).

**Table 1:** Number of deaths and age-standardised mortality rates for ten leading Level 3 causes of death in 2020 and 2021, globally and by super-region, for all ages and males and females combined

		**Global**	**Central Europe, eastern Europe, and central Asia**	**High income**	**Latin America and Caribbean**	**North Africa and Middle East**	**South Asia**	**Southeast Asia, east Asia, and Oceania**	**Sub-Saharan Africa**
**2020**									
1									
	Cause	Ischaemic heart disease	Ischaemic heart disease	Ischaemic heart disease	COVID-19	Ischaemic heart disease	Ischaemic heart disease	Stroke	COVID-19
	Age-standardised rate (per 100 000 population)	109·4 (100·7–116·1)	215·3 (199·2–225·7)	51·4 (45·1–54·6)	133.7 (121·5–145·3)	205·2 (182·7–225·6)	150·3 (139·7–162·2)	142·8 (123·9–159·8)	158·9 (148·5–170·0)
	Number	8 840 000 (8 180 000–9 360 000)	1 410 000 (1 310 000–1 480 000)	1 290 000 (1 110 000–1 390 000)	799 000 (725 000–869 000)	760 000 (681 000–838 000)	1 960 000 (1 820 000–2 110 000)	3 460 000 (3 030 000–3 880 000)	659 000 (615 000–706 000)
2									
	Cause	Stroke	Stroke	COVID-19	Ischaemic heart disease	COVID-19	Chronic obstructive pulmonary disease	Ischaemic heart disease	Stroke
	Age-standardised rate (per 100 000 population)	88·3 (80·2–95·0)	110·7 (102·7–115·6)	41·8 (40·8–42·8)	84·3 (77·2–89·4)	123·9 (106·8–137·1)	104·1 (92·3–117·0)	110·8 (97·3–124·6)	126·2 (113·4–140·4)
	Number	7 140 000 (6 500 000–7 680 000)	726 000 (675 000–758 000)	930 000 (908 000–952 000)	496 000 (454 000–525 000)	483 000 (415 000–537 000)	1 230 000 (1 090 000–1 370 000)	2 570 000 (2 260 000–2 880 000)	481 000 (432 000–538 000)
3									
	Cause	COVID-19	COVID-19	Stroke	Stroke	Stroke	COVID-19	Chronic obstructive pulmonary disease	Ischaemic heart disease
	Age-standardised rate (per 100 000 population)	58·7 (55·8–62·4)	72·9 (64·1–81·7)	29·0 (24·7–31·2)	47·5 (43·4–50·5)	103·8 (92·0–115·6)	101·8 (95·0–108·5)	66·9 (57·4–77·0)	92·9 (83·1–103·0)
	Number	4 800 000 (4 560 000–5 110 000)	467 000 (411 000–523 000)	764 000 (636 000–830 000)	278 000 (255 000–296 000)	370 000 (329 000–414 000)	1 320 000 (1 230 000–1 400 000)	1 500 000 (1 290 000–1 730 000)	346 000 (309 000–388 000)
4									
	Cause	Chronic obstructive pulmonary disease	Other COVID-19 pandemic-related outcomes	Alzheimer's disease and other dementias	Diabetes mellitus	Hypertensive heart disease	Stroke	Tracheal, bronchus, and lung cancer	Lower respiratory infections
	Age-standardised rate (per 100 000 population)	45·5 (41·2–49·6)	41·0 (32·9–51·9)	26·5 (6·74–65·1)	36·5 (33·9–38·9)	40·2 (32·0–46·7)	83·3 (75·7–90·4)	34·8 (29·0–41·0)	88·5 (77·8–98·2)
	Number	3 650 000 (3 320 000–3 970 000)	264 000 (212 000–333 000)	774 000 (198 000–1 900 000)	217 000 (202 000–231 000)	138 000 (110 000–160 000)	1 060 000 (969 000–1 150 000)	938 000 (783 000–1 110 000)	588 000 (494 000–686 000)
5									
	Cause	Lower respiratory infections	Tracheal, bronchus, and lung cancer	Tracheal, bronchus, and lung cancer	Lower respiratory infections	Chronic kidney disease	Diarrhoeal diseases	Alzheimer's disease and other dementias	Malaria
	Age-standardised rate (per 100 000 population)	30·4 (27·7–32·9)	25·5 (24·4–26·5)	25·9 (23·8–27·0)	32·8 (29·6–35·1)	37·9 (33·3–42·4)	50·2 (32·0–79·4)	27·9 (6·76–74·8)	67·9 (22·6–145·0)
	Number	2 280 000 (2 080 000–2 460 000)	168 000 (161 000–174 000)	581 000 (526 000–610 000)	187 000 (169 000–200 000)	142 000 (125 000–159 000)	591 000 (381 000–940 000)	562 000 (136 000–1 490 000)	713 000 (251 000–1 480 000)
6									
	Cause	Neonatal disorders	Cirrhosis and other chronic liver diseases	Chronic obstructive pulmonary disease	Chronic kidney disease	Other COVID-19 pandemic-related outcomes	Neonatal disorders	Lower respiratory infections	Tuberculosis
	Age-standardised rate (per 100 000 population)	30·3 (26·3–35·0)	22·5 (21·7–23·3)	19·2 (16·9–20·3)	30·9 (28·3–33·1)	30·4 (11·4–52·0)	43·8 (37·2–51·6)	21·2 (18·9–23·6)	67·3 (56·7–77·8)
	Number	1 910 000 (1 650 000–2 200 000)	131 000 (127 000–136 000)	490 000 (424 000–522 000)	184 000 (169 000–197 000)	121 000 (46 500–207 000)	672 000 (571 000–792 000)	424 000 (378 000–469 000)	378 000 (313 000–442 000)
7									
	Cause	Alzheimer's disease and other dementias	Alzheimer's disease and other dementias	Colon and rectum cancer	Chronic obstructive pulmonary disease	Diabetes mellitus	Lower respiratory infections	Hypertensive heart disease	HIV/AIDS
	Age-standardised rate (per 100 000 population)	24·9 (6·16–65·0)	20·8 (4·88–55·3)	14·7 (13·2–15·6)	25·0 (22·5–26·5)	29·4 (26·4–32·3)	40·0 (35·8–44·7)	20·1 (14·1–24·8)	65·8 (59·9–73·2)
	Number	1 890 000 (470 000–4 940 000)	136 000 (32 100–362 000)	344 000 (300 000–367 000)	144 000 (130 000–152 000)	113 000 (101 000–124 000)	522 000 (465 000–582 000)	459 000 (320 000–562 000)	539 000 (487 000–612 000)
8									
	Cause	Tracheal, bronchus, and lung cancer	Lower respiratory infections	Chronic kidney disease	Interpersonal violence	Chronic obstructive pulmonary disease	Tuberculosis	Stomach cancer	Diarrhoeal diseases
	Age-standardised rate (per 100 000 population)	23·5 (21·3–25·8)	19·5 (18·3–20·8)	14·0 (12·1–15·3)	23·5 (22·4–24·8)	26·9 (23·9–29·7)	34·2 (30·1–40·1)	18·4 (14·2–22·0)	57·0 (36·2–79·4)
	Number	1 970 000 (1 780 000–2 160 000)	96 200 (91 200–101 000)	364 000 (307 000–399 000)	147 000 (140 000–155 000)	92 400 (82 500–102 000)	509 000 (450 000–597 000)	491 000 (380 000–589 000)	452 000 (324 000–588 000)
9									
	Cause	Diabetes mellitus	Cardiomyopathy and myocarditis	Lower respiratory infections	Other COVID-19 pandemic-related outcomes	Alzheimer's disease and other dementias	Diabetes mellitus	Road injuries	Other COVID-19 pandemic-related outcomes
	Age-standardised rate (per 100 000 population)	19·7 (18·4–20·9)	19·2 (17·9–20·4)	13·6 (11·8–14·6)	20·9 (10·3–33·3)	25·7 (6·30–67·6)	33·1 (29·8–36·0)	15·7 (13·9–17·6)	50·5 (31·3–70·8)
	Number	1 630 000 (1 520 000–1 720 000)	113 000 (105 000–121 000)	361 000 (306 000–390 000)	125 000 (59 600–199 000)	73 600 (17 900–198 000)	419 000 (378 000–457 000)	380 000 (335 000–429 000)	245 000 (159 000–339 000)
10									
	Cause	Chronic kidney disease	Colon and rectum cancer	Self-harm	Alzheimer's disease and other dementias	Lower respiratory infections	Other COVID-19 pandemic-related outcomes	Chronic kidney disease	Neonatal disorders
	Age-standardised rate (per 100 000 population)	18·6 (16·9–19·9)	18·6 (17·6–19·4)	10·9 (10·5–11·2)	20·8 (5·14–53·8)	25·4 (22·4–28·5)	28·2 (18·5–39·5)	15·3 (13·4–17·0)	50·0 (42·1–59·2)
	Number	1 500 000 (1 360 000–1 610 000)	122 000 (115 000–127 000)	149 000 (142 000–153 000)	119 000 (29 200–308 000)	103 000 (91 000–116 000)	370 000 (246 000–514 000)	376 000 (333 000–420 000)	889 000 (749 000–1 050 000)
**2021**									
1									
	Cause	Ischaemic heart disease	Ischaemic heart disease	Ischaemic heart disease	COVID-19	Ischaemic heart disease	COVID-19	Stroke	COVID-19
	Age-standardised rate (per 100 000 population)	108·7 (99·8–115·6)	213·6 (196·1–229·1)	51·0 (44·9–54·2)	195·4 (182·1–211·4)	202·8 (179·7–225·9)	156·5 (150·4–164·4)	141·1 (123·2–159·7)	271·0 (250·1–290·7)
	Number	8 990 000 (8 290 000–9 550 000)	1 410 000 (1 290 000–1 510 000)	1 310 000 (1 120 000–1 400 000)	1 200 000 (1 110 000–1 290 000)	769 000 (679 000–863 000)	2 060 000 (1 980 000–2 170 000)	3 550 000 (3 100 000–4 020 000)	1 150 000 (1 060 000–1 240 000)
2									
	Cause	COVID-19	COVID-19	COVID-19	Ischaemic heart disease	COVID-19	Ischaemic heart disease	Ischaemic heart disease	Stroke
	Age-standardised rate (per 100 000 population)	94·0 (89·2–100·0)	168·8 (150·6–186·1)	48·1 (47·4–48·8)	83·8 (75·9–90·6)	172·4 (150·3–191·5)	149·1 (136·4–161·8)	110·4 (94·9–124·6)	124·7 (111·8–138·6)
	Number	7 890 000 (7 490 000–8 400 000)	1 100 000 (982 000–1 210 000)	1 070 000 (1 060 000–1 090 000)	504 000 (457 000–545 000)	698 000 (608 000–777 000)	1 990 000 (1 820 000–2 160 000)	2 660 000 (2 290 000–3 000 000)	484 000 (432 000–544 000)
3									
	Cause	Stroke	Stroke	Stroke	Stroke	Stroke	Chronic obstructive pulmonary disease	Chronic obstructive pulmonary disease	Other COVID-19 pandemic-related outcomes
	Age-standardised rate (per 100 000 population)	87·4 (79·5–94·4)	109·8 (101·6–116·6)	28·8 (24·5–30·9)	46·7 (42·3–50·2)	101·9 (89·2–114·4)	101·6 (90·3–114·2)	66·6 (56·2–77·7)	123·9 (87·7–159.5)
	Number	7 250 000 (6 600 000–7 820 000)	725 000 (671 000–770 000)	771 000 (641 000–838 000)	279 000 (254 000–301 000)	372 000 (325 000–421 000)	1 230 000 (1 100 000–1 380 000)	1 560 000 (1 310 000–1 820 000)	584 000 (418 000–757 000)
4									
	Cause	Chronic obstructive pulmonary disease	Other COVID-19 pandemic-related outcomes	Alzheimer's disease and other dementias	Other COVID-19 pandemic-related outcomes	Other COVID-19 pandemic-related outcomes	Stroke	Tracheal, bronchus, and lung cancer	Ischaemic heart disease
	Age-standardised rate (per 100 000 population)	45·2 (40·7–49·8)	50·0 (34·8–68·7)	26·5 (6·74–64·8)	39·0 (22·5–58·4)	64·5 (34·4–100·6)	81·8 (74·2–89·6)	34·8 (28·8–41·1)	92·8 (83·3–103·5)
	Number	3 720 000 (3 360 000–4 090 000)	321 000 (223 000–438 000)	792 000 (203 000–1 940 000)	236 000 (135 000–355 000)	265 000 (139 000–414 000)	1 070 000 (968 000–1 170 000)	970 000 (800 000–1 150 000)	352 000 (316 000–396 000)
5									
	Cause	Other COVID-19 pandemic-related outcomes	Tracheal, bronchus, and lung cancer	Tracheal, bronchus, and lung cancer	Diabetes mellitus	Hypertensive heart disease	Other COVID-19 pandemic-related outcomes	Alzheimer's disease and other dementias	Lower respiratory infections
	Age-standardised rate (per 100 000 population)	32·3 (24·8–43·3)	25·1 (23·7–26·6)	25·9 (23·8–27·0)	36·3 (33·2–39·3)	39·5 (31·3–46·3)	63·3 (50·4–77·2)	28·9 (7·41–78·6)	85·4 (75·3–95·0)
	Number	2 690 000 (2 060 000–3 610 000)	167 000 (157 000–176 000)	591 000 (537 000–620 000)	221 000 (202 000–239 000)	138 000 (109 000–162 000)	838 000 (674 000–1 020 000)	608 000 (155 000–1 670 000)	563 000 (472 000–655 000)
6									
	Cause	Neonatal disorders	Cirrhosis and other chronic liver diseases	Chronic obstructive pulmonary disease	Chronic kidney disease	Chronic kidney disease	Diarrhoeal diseases	COVID-19	Malaria
	Age-standardised rate (per 100 000 population)	29·6 (25·3–34·4)	22·3 (21·0–23·5)	19·1 (16·8–20·2)	30·7 (27·8–33·5)	37·7 (32·7–42·8)	47·8 (30·2–75·7)	23·2 (16·3–37·2)	65·9 (23·6–136·7)
	Number	1 830 000 (1 570 000–2 130 000)	131 000 (123 000–138 000)	495 000 (428 000–527 000)	187 000 (170 000–204 000)	145 000 (126 000–164 000)	573 000 (372 000–908 000)	606 000 (425 000–974 000)	704 000 (265 000–1 400 000)
7									
	Cause	Lower respiratory infections	Alzheimer's disease and other dementias	Colon and rectum cancer	Lower respiratory infections	Diabetes mellitus	Neonatal disorders	Lower respiratory infections	Tuberculosis
	Age-standardised rate (per 100 000 population)	28·7 (26·0–31·1)	20·8 (4·94–55·6)	14·7 (13·1–15·5)	30·4 (27·0–33·3)	29·3 (25·9–32·5)	42·0 (35·6–50·2)	20·9 (18·6–23·4)	65·8 (56·1–76·9)
	Number	2 180 000 (1 980 000–2 360 000)	137 000 (32 500–370 000)	348 000 (304 000–372 000)	177 000 (157 000–194 000)	116 000 (102 000–129 000)	636 000 (538 000–760 000)	431 000 (384 000–482 000)	373 000 (313 000–439 000)
8									
	Cause	Alzheimer's disease and other dementias	Cardiomyopathy and myocarditis	Chronic kidney disease	Chronic obstructive pulmonary disease	Chronic obstructive pulmonary disease	Lower respiratory infections	Hypertensive heart disease	HIV/AIDS
	Age-standardised rate (per 100 000 population)	25·2 (6·36–65·6)	19·1 (17·5–20·7)	13·9 (12·0–15·1)	24·7 (22·1–26·4)	26·4 (23·2–29·6)	39·2 (34·2–44·6)	19·8 (14·0–24·3)	61·4 (55·8–68·5)
	Number	1 960 000 (499 000–5 120 000)	112 000 (103 000–122 000)	368 000 (310 000–402 000)	145 000 (130 000–156 000)	92 700 (82 000–104 000)	516 000 (451 000–584 000)	470 000 (333 000–575 000)	515 000 (467 000–583 000)
9									
	Cause	Tracheal, bronchus, and lung cancer	Colon and rectum cancer	Lower respiratory infections	Interpersonal violence	Alzheimer's disease and other dementias	Tuberculosis	Stomach cancer	Diarrhoeal diseases
	Age-standardised rate (per 100 000 population)	23·5 (21·2–25·9)	18·5 (17·4–19·6)	11·9 (10·2–12·7)	23·3 (21·7–24·8)	25·7 (6·22–66·8)	33·1 (29·0–39·1)	18·1 (14·4–21·8)	54·4 (33·9–76·7)
	Number	2 020 000 (1 820 000–2 220 000)	122 000 (115 000–129 000)	321 000 (267 000–348 000)	147 000 (137 000–156 000)	73 900 (18 000–198 000)	501 000 (441 000–587 000)	500 000 (397 000–605 000)	434 000 (310 000–570 000)
10									
	Cause	Diabetes mellitus	Lower respiratory infections	Self-harm	Alzheimer's disease and other dementias	Cirrhosis and other chronic liver diseases	Diabetes mellitus	Road injuries	Neonatal disorders
	Age-standardised rate (per 100 000 population)	19·6 (18·2–20·8)	16·5 (15·4–17·7)	10·8 (10·4–11·0)	20·8 (5·18–54·3)	23·2 (20·2–26·8)	32·8 (29·5–36·1)	15·5 (13·6–17·5)	48·6 (40·3–58·1)
	Number	1 660 000 (1 540 000–1 760 000)	82 800 (77 800–87 500)	148 000 (141 000–152 000)	121 000 (30 300–317 000)	99 600 (86 100–116 000)	426 000 (383 000–468 000)	379 000 (331 000–430 000)	873 000 (724 000–1 040 000)

### COVID-19 and OPRM

Our estimates show that 4·80 million (95% UI 4·56–5·11) deaths due to COVID-19 occurred globally in 2020, and 7·89 million (7·49–8·40) in 2021. Age-standardised rates of death due to COVID-19 were highly variable among GBD super-regions (table 1). In 2021, the rankings from highest to lowest were sub-Saharan Africa (271·0 deaths [250·1–290·7] per 100 000 population); Latin America and the Caribbean (195·4 deaths [182·1–211·4] per 100 000 population); north Africa and the Middle East (172·4 deaths [150·3–191·5] per 100 000 population); central Europe, eastern Europe, and central Asia (168·8 deaths [150·6–186·1] per 100 000 population); south Asia (156·5 deaths [150·4–164·4] per 100 000 population); high income (48·1 deaths [47·4–48·8] per 100 000 population); and southeast Asia, east Asia, and Oceania (23·2 deaths [16·3–37·2] per 100 000 population; table 1).

Deaths from both COVID-19 and OPRM also varied substantially by age, with older ages being disproportionately affected (table 2). Individuals aged 70–74 years had the highest number of deaths from both COVID-19 and OPRM in 2020 and again in 2021. The highest percentage of total deaths from COVID-19 was found in those aged 40–44 years, whereas the highest mortality rate occurred in those aged 95 years and older. Death rates from OPRM were high among older age groups and among the youngest ages, with a rate of 141·2 deaths (95% UI 58·0–277·5) per 100 000 population for infants aged 0–6 days, and 77·3 deaths (44·0–118·0) per 100 000 population in infants aged 7–27 days. At a global scale, COVID-19 deaths and OPRM were slightly higher for males than for females in most age groups in 2021 (appendix 2 figure S5). Exceptions to this trend include those aged 90–94 years and those aged 95 years and older (appendix 2 figure S5).

**Table 2:** Number of deaths, age-standardised mortality rates, and percentage of total deaths due to COVID-19 and other pandemic-related mortality by age, globally

	**Deaths**				**Deaths per 100 000 population**				**Percentage of total deaths**			
	COVID-19 2020	COVID-19 2021	Other COVID-19 pandemic-related outcomes 2020	Other COVID-19 pandemic-related outcomes 2021	COVID-19 2020	COVID-19 2021	Other COVID-19 pandemic-related outcomes 2020	Other COVID-19 pandemic-related outcomes 2021	COVID-19 2020	COVID-19 2021	Other COVID-19 pandemic-related outcomes 2020	Other COVID-19 pandemic-related outcomes 2021
Early neonatal	0	1	3518	3462	0·0	<0·1	141·4	141·2	0·0%	<0·1%	0·2%	0·2%
Late neonatal	3	5	5069	5641	<0·1	0·1	68·5	77·3	<0·1%	<0·1%	1·1%	1·3%
1–5 months	170	287	24269	26 647	0·3	0·5	44·4	49·6	<0·1%	<0·1%	3·1%	3·6%
6–11 months	234	394	20 478	30 883	0·4	0·6	31·7	48·9	<0·1%	0·1%	3·5%	5·5%
12–23 months	998	1644	19 042	30 550	0·8	1·3	14·5	23·8	0·2%	0·3%	3·7%	6·2%
2–4 years	8500	14 386	14 730	23 574	2·1	3·6	3·6	5·8	1·2%	2·1%	2·0%	3·4%
5–9 years	7052	11 393	5377	8196	1·0	1·7	0·8	1·2	1·9%	3·2%	1·5%	2·3%
10–14 years	8553	14 405	1588	2715	1·3	2·2	0·2	0·4	2·8%	4·8%	0·5%	0·9%
15–19 years	17 032	26 852	5932	12 576	2·8	4·3	1·0	2·0	3·1%	4·8%	1·1%	2·2%
20–24 years	25 528	40 743	8219	17 453	4·3	6·8	1·4	2·9	3·6%	5·5%	1·2%	2·4%
25–29 years	47 857	78 496	12581	28 816	8·1	13·3	2·1	4·9	5·9%	9·2%	1·6%	3·4%
30–34 years	81 232	137 979	21 625	49 808	13·4	22·8	3·6	8·2	7·9%	12·3%	2·1%	4·5%
35–39 years	112 228	195 380	29 877	69 402	20·5	34·8	5·5	12·4	9·0%	14·1%	2·4%	5·0%
40–44 years	165 337	287 099	44 391	102 041	33·5	57·4	9·0	20·4	10·3%	16·0%	2·8%	5·7%
45–49 years	207 940	355 388	55 989	124 899	44·0	75·1	11·8	26·4	10·1%	15·7%	2·7%	5·5%
50–54 years	253 491	426 785	67 629	147 651	57·7	95·9	15·4	33·2	9·1%	14·0%	2·4%	4·8%
55–59 years	336 162	564 508	90 815	191 441	87·5	142·7	23·6	48·4	9·0%	13·8%	2·4%	4·7%
60–64 years	460 769	774 879	125 433	262 008	146·1	242·1	39·8	81·9	9·8%	15·0%	2·7%	5·1%
65–69 years	564 371	957 557	155 431	321 301	209·4	347·1	57·7	116·5	9·4%	14·5%	2·6%	4·9%
70–74 years	585 549	989 888	156 931	325 295	298·7	480·9	80·1	158·0	8·8%	13·2%	2·4%	4·3%
75–79 years	539 515	861 796	135 849	276 402	417·1	653·4	105·0	209·6	7·9%	11·8%	2·0%	3·8%
80–84 years	551 014	888 813	146 084	277786	638·9	1014·8	169·4	317·2	7·5%	11·3%	2·0%	3·5%
85–89 years	427 770	658 875	106 842	191 824	959·3	1441·1	239·6	419·5	6·9%	10·0%	1·7%	2·9%
90–94 years	280 605	426 185	67 297	114 449	1608·9	2382·3	385·9	639·8	7·5%	10·8%	1·8%	2·9%
≥95 years	120 173	174 390	24 074	42 104	2298·6	3199·6	460·5	772·5	7·8%	10·7%	1·6%	2·6%

### Leading causes of global YLLs

The causes of death with the highest age-standardised YLL rates show shifting epidemiological trends from CMNN diseases to NCDs at Level 3 of the cause hierarchy (appendix 2 figure S2). Globally, the leading three causes of age-standardised YLLs in 1990 were all CMNN diseases. Ranked in descending order, these causes were neonatal disorders, lower respiratory infections, and diarrhoeal diseases. In 2019, neonatal disorders remained the leading cause of age-standardised YLLs, but the second and third leading causes were replaced by NCDs: ischaemic heart disease (ranked second) and stroke (ranked third). In 2021, COVID-19 was the second-leading cause of global age-standardised YLLs, making the leading two causes CMNN diseases (with neonatal disorders ranked first), with ischaemic heart disease ranked third. Among the leading causes of age-standardised YLLs, malaria was the only cause to show an increase in age-standardised YLL rates between 2019 and 2021 (ranking ninth in 2019 and seventh in 2021).

### Decomposition of global life expectancy

**Figure 3:**
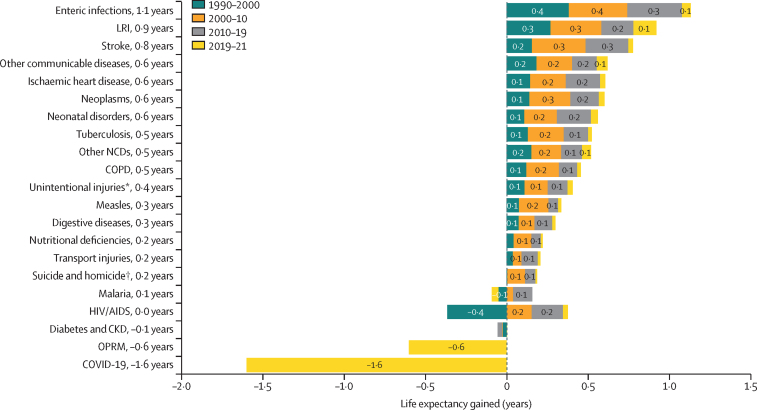
Change in life expectancy attributable to leading causes of death for males and females combined, 1990–2000, 2000–10, 2010–19, and 2019–21, globally Each row represents the change in global life expectancy from 1990 to 2021 for a given cause. The total change in life expectancy is further broken down by different colours to represent changes over time periods. A bar to the right of 0 represents an increase in life expectancy due to changes in the given time period, and a bar to the left of 0 represents a decrease in life expectancy due to a given time period. For readability, labels indicating a change in life expectancy of less than 0·05 years are not shown. CKD=chronic kidney disease. COPD=chronic obstructive pulmonary disease. LRI=lower respiratory infection. NCD=non-communicable disease. OPRM=other pandemic-related mortality. *Does not include natural disasters. †Does not include war and terrorism.

We found long-standing positive trends in global life expectancy since the early 1990s, with steady increases occurring across each decade between 1990 and 2019 (appendix 2 table S4). Altogether, the global increase in life expectancy from 1990 to 2019 totalled 7·8 years (95% UI 7·1–8·5). In 2019–21, however, we found a global decline in life expectancy of 2·2 years due to deaths from COVID-19 and OPRM combined. This decrease was partly offset by reductions in other diseases, for a net reduction in global life expectancy of 1·6 years. Despite this notable reduction, we observed an overall increase in life expectancy of 6·2 years (5·4–7·0) across the entire study period. This decomposition analysis provides insights into the specific causes that influenced changes in life expectancy over the defined time periods. Among the various contributing factors to a change in life expectancy, the cause with the greatest effect on the increase in life expectancy worldwide was the reduction in deaths caused by enteric infections (figure 3). This category includes diarrhoeal, typhoid, and paratyphoid diseases. A reduction in deaths from these diseases is responsible for a substantial increase in life expectancy of 1·1 years during 1990–2021, but this increase was most pronounced between 1990 and 2000 compared with other time periods. The second-largest effect on increasing life expectancy is attributed to the reduction in deaths from lower respiratory infection, contributing 0·9 years of gained life expectancy from 1990 to 2021. Other leading factors include reduced mortality from stroke, CMNN diseases, neonatal deaths, ischaemic heart disease, and neoplasms, each of which increased global life expectancy by 0·6–0·8 years over the study period. Changing rates of HIV/AIDS and malaria mortality both contributed positively to the overall global life expectancy in some years but negatively affected life expectancy in others. Beginning in 2000, reductions in HIV/AIDS-related mortality were evident following substantial negative effects in earlier years. Reductions in deaths from malaria, however, were less sustained, increasing life expectancy by 0·1 years from 2010 to 2019 but having no effect from 2019 to 2021. Across all causes, the largest effect on the change in global life expectancy was from COVID-19, which resulted in a decline of 1·6 years between 2019 and 2021.

### Decomposition of super-region, regional, and country-level life expectancy

**Figure 4:**
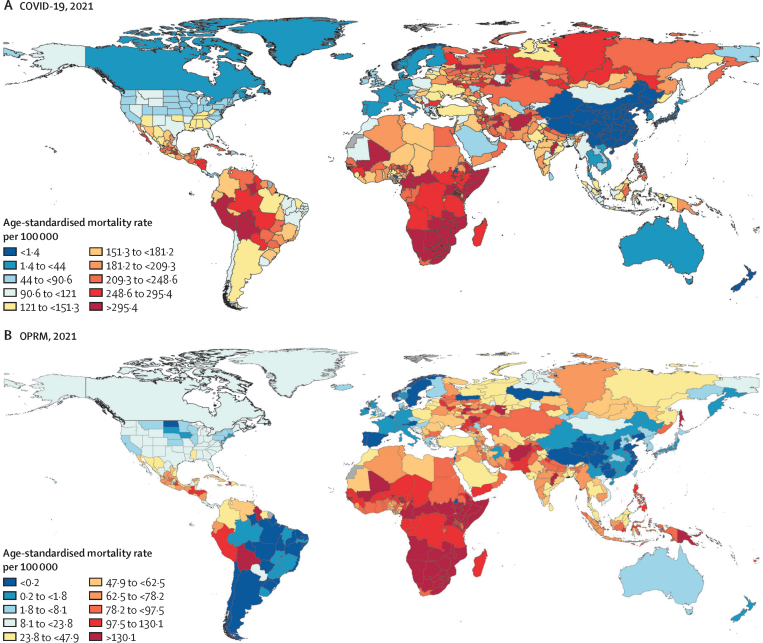
Age-standardised mortality rate of COVID-19 and OPRM, 2021 Global choropleth maps of COVID-19 (A) and OPRM (B) for 2021 that show sub-national detail where available. OPRM=other pandemic-related mortality.

**Figure 5:**
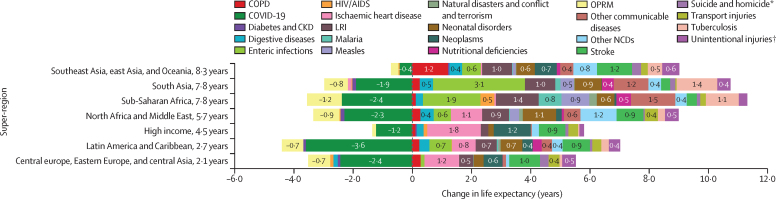
Change in life expectancy attributable to leading causes of death among super-regions, 1990–2021 Each row represents the change in life expectancy from 1990 to 2021 for a given super-region. A bar to the right of 0 represents an increase in life expectancy due to changes in the given cause, and a bar to the left of 0 represents a decrease in life expectancy for a given cause. For readability, labels indicating a change in life expectancy of less than 0·3 years are not shown. CKD=chronic kidney disease. COPD=chronic obstructive pulmonary disease. LRI=lower respiratory infection. NCD=non-communicable disease. OPRM=other pandemic-related mortality. *Does not include natural disasters. †Does not include war and terrorism.

Each of the seven super-regions experienced an overall increase in life expectancy between 1990 and 2021, despite progress in each being differentially affected by COVID-19 (Figure 4, Figure 5). Southeast Asia, east Asia, and Oceania showed the highest gain, with a net improvement of 8·3 years (95% UI 6·7–9·9), while also being the least affected by COVID-19, which contributed a loss in life expectancy of just 0·4 years. The overall increase in life expectancy in southeast Asia, east Asia, and Oceania can largely be attributed to reduced mortality from chronic respiratory diseases, contributing to a gain of 1·2 years, whereas reduced mortality from stroke, lower respiratory infections, and neoplasms were among other causes that contributed to the 8·3-year (6·7–9·9) increase. The second-largest gain occurred in south Asia, where life expectancy increased by 7·8 years (6·7–8·9), which can be largely attributed to reduced mortality from enteric infectious diseases, contributing a substantial gain of 3·1 years in life expectancy. The largest reduction in overall life expectancy due to COVID-19 occurred in the super-region of Latin America and the Caribbean, which experienced a loss of 3·6 years. Reductions in deaths due to malaria throughout sub-Saharan Africa led to an increase in life expectancy of 0·8 years for the super-region.

**Figure 6:**
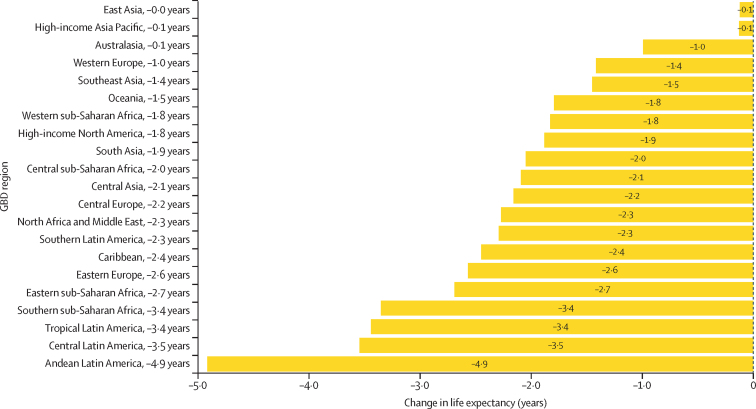
Effect of COVID-19 on life expectancy by GBD region, 2019–21 For readability, labels indicating a change in life expectancy of less than 0·05 years are not shown. GBD=Global Burden of Diseases, Injuries, and Risk Factors Study.

The differential effect of COVID-19 on reduced life expectancy was observed across GBD regions (figure 6). Although most regions experienced overall improvements in life expectancy between 1990 and 2021, a reduction occurred in southern sub-Saharan Africa, which faced the greatest impact of HIV and was also heavily affected by COVID-19. The overall decrease in life expectancy of 4·3 years (95% UI 3·0–5·8) included a reduction of 2·4 years due to HIV/AIDS and 3·4 years due to COVID-19, which were only partly offset by reductions in mortality due to other causes. Notably, COVID-19 reduced life expectancy in Andean Latin America by 4·9 years, although the region had an overall gain of 2·6 years (1·0–4·1) between 1990 and 2021. The effect of COVID-19 in eastern sub-Saharan Africa, which resulted in a reduction in life expectancy of 2·7 years, was offset by steady improvements across many different causes, which resulted in the highest overall increase in life expectancy among GBD regions (10·7 years [9·0–12·2]). Control of enteric infections in this region contributed to an increase in life expectancy of 1·9 years, along with reductions in lower respiratory infections and tuberculosis, each of which contributed to an additional 1·6 years' increase in life expectancy. Each region in sub-Saharan Africa experienced reductions in the number of enteric infections, which improved life expectancy in those regions between 0·8 and 2·4 years.

HIV/AIDS had a substantial negative effect on life-expectancy trends in southern sub-Saharan Africa from 1990 to 2021 (appendix 2 figure S27). Despite improvements in each of the time periods 2000–2010, 2010–2019, and 2019–2021, this region was unable to recover the 9·0 years lost during 1990–2000. Although we found a net decline in deaths due to HIV/AIDS between 2000 and 2019, improvements slowed substantially from 2019 to 2021, when only 0·2 years in life expectancy were gained as a result of reduced HIV/AIDS mortality. Conversely, eastern sub-Saharan Africa had the highest level of recovery to their life expectancy among the regions, gaining 1·5 years of life expectancy over the entire study period.

In 1990, malaria-related deaths had almost no effect on life expectancy in eight of the 21 GBD regions (appendix 2 figure S13). By 2021, however, 90% of malaria deaths across all age groups occurred in locations with only 12% of the global population. Efforts to control malaria in various regions of sub-Saharan Africa have yielded modest gains in life expectancy. Central sub-Saharan Africa gained 0·7 years in life expectancy between 2000 and 2010, western sub-Saharan Africa gained 0·9 years during 2010–19, and eastern sub-Saharan Africa gained 0·7 years in 2000–10. Despite these advancements, many regions with malaria transmission experienced a decline in life expectancy from 2019 to 2021. The most noticeable reductions were in eastern sub-Saharan Africa, with a decrease of 0·2 years, followed by western sub-Saharan Africa, which lost 0·1 years in life expectancy over the same period.

At the national level, some of the highest gains in life expectancy between 1990 and 2021 occurred in the eastern region of sub-Saharan Africa (appendix 2 figure S12). Life expectancy in Ethiopia increased by 18·2 years (95% UI 16·3–19·8) as a result of reductions in deaths from many causes, most notably other communicable and maternal disorders (3·2 years), tuberculosis (3·1 years), and enteric infectious diseases (2·4 year). The largest reduction in life expectancy occurred in Lesotho, at 12·9 years (10·1–15·7), largely attributed to increased deaths from HIV/AIDS, which resulted in a reduction of 7·3 years (appendix 2 figures S12, S27, table S4).

### Effect of CMNN diseases on life expectancy and trends in mortality concentration

**Figure 7:**
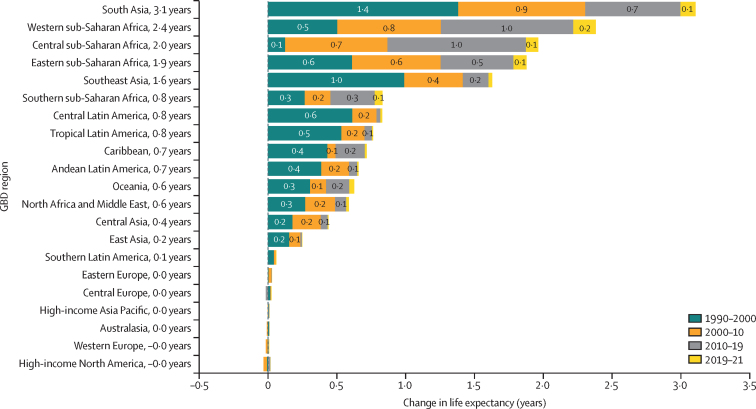
Effect of enteric infectious diseases on life expectancy by time period and GBD region, 1990–2021 For readability, labels indicating a change in life expectancy of less than 0·05 years are not shown. GBD=Global Burden of Diseases, Injuries, and Risk Factors Study.

**Figure 8:**
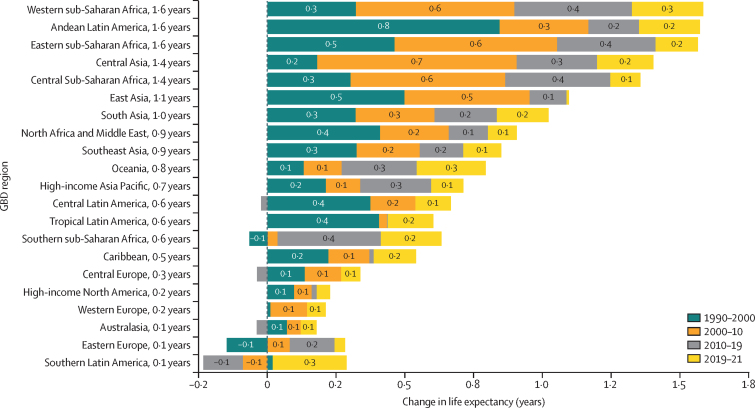
Effect of lower respiratory infections on life expectancy by time period and GBD region, 1990–2021 For readability, labels indicating a change in life expectancy of less than 0·05 years are not shown. GBD=Global Burden of Diseases, Injuries, and Risk Factors Study.

**Figure 9:**
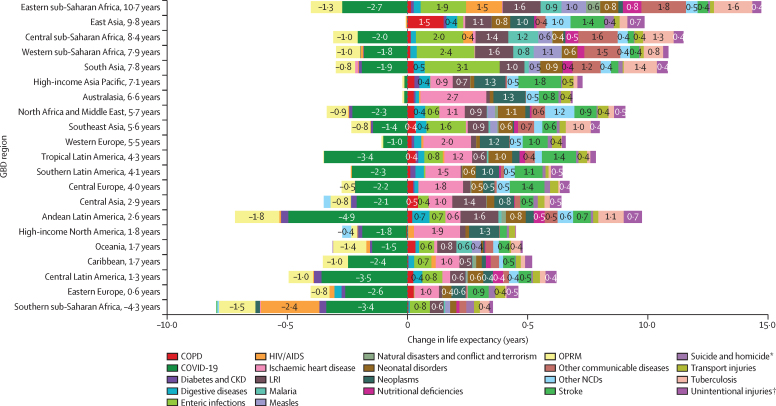
Change in life expectancy attributable to leading causes of death among GBD regions, 1990–2021 Each row represents the change in life expectancy from 1990 to 2021 for a given GBD region. A bar to the right of 0 represents an increase in life expectancy due to changes in the given cause, and a bar to the left of 0 represents a decrease in life expectancy for a given cause. For readability, labels indicating a change in life expectancy of less than 0·3 years are not shown. CKD=chronic kidney disease. COPD=chronic obstructive pulmonary disease. GBD=Global Burden of Diseases, Injuries, and Risk Factors Study. LRI=lower respiratory infection. NCD=non-communicable disease. OPRM=other pandemic-related mortality. *Does not include war and terrorism. †Does not include natural disasters.

Among CMNN causes, several key trends emerged in their effect on global life expectancy and the localisation of deaths over time. First, the reduction of deaths due to enteric disease had a substantial impact on global life expectancy, with notable regional variations (figure 7). As 160 countries and territories made progress in reducing CMNN disease-related mortality, mortality concentration emerged. Deaths became more concentrated into certain countries or regions, persisting alongside advancements made in other parts of the world. An illustrative example is the shift in deaths due to enteric diseases in children younger than 5 years, with 90% of deaths occurring in locations containing 63% of the population of children younger than 5 years in 1990, decreasing to locations containing 51% of the population by 2021 (appendix 2 figure S28). Second, the reduction in the number of lower respiratory infections yielded positive effects on life expectancy in some regions. Regions such as Andean Latin America and western and eastern sub-Saharan Africa had gains of 1·6 years in life expectancy due to reduced deaths from lower respiratory infections. This progress is further underscored by the transformation from 90% of deaths from lower respiratory infections in children younger than 5 years occurring in locations with 71% of the population of the under-5 population in 1990 to 90% occurring in locations with 58% of the under-5 population by 2021, signalling substantial improvements in some regions and increased concentration of this cause in others (figure 8; appendix 2 figure S29). Third, HIV/AIDS had a substantial impact on life-expectancy trends, particularly in southern sub-Saharan Africa, and with 90% of deaths concentrated in locations containing 46% of the entire population and 39% of the under-5 population in 2021 (appendix 2 figures S27, S30). However, HIV/AIDS was less concentrated in 2021 than in 1990. Fourth, efforts to control malaria in sub-Saharan Africa resulted in modest gains in life expectancy. Similarly, 90% of malaria-related deaths in 2021 occurred in locations containing only 12% of the entire population and 20% of the under-5 population, showing mortality concentration (figure 5; appendix 2 figures S13, 31). Fifth, reductions in tuberculosis-related deaths had a positive effect on life expectancy across all regions, and changes in mortality rates indicated mortality concentration, with 90% of deaths occurring in locations containing 66% of the entire population in 1990, decreasing to 62% by 2021 (figure 9; appendix 2 figure S14). Lastly, although measles had a relatively small global effect on life expectancy, this cause showed high mortality concentration. The disease remained contained globally, with 90% of deaths concentrated in locations containing only 15% of the entire population and 24% of the under-5 population in 2021 (figure 3; appendix 2 figure S15).

Reductions in neonatal deaths contributed to a 0·6-year increase in global life expectancy. Also, 90% of neonatal deaths were concentrated in locations containing 71% of the population in 1990, decreasing to 51% by 2021 (appendix 2 figures S16, S34). Finally, nutritional deficiencies had a relatively small global impact on life expectancy but substantial effects on specific regions—eastern sub-Saharan Africa, central sub-Saharan Africa, and south Asia saw notable increases. We found a shift towards mortality concentration, with 90% of nutritional deficiency-related deaths in children younger than 5 years concentrated in locations containing 49% of the population in this age group by 2021, compared with 59% in 1990 (appendix 2 figures S18, S35). Overall, CMNN diseases showed a large degree of mortality concentration.

### Effect of NCDs on life expectancy and trends in mortality concentration

Among NCDs, several findings reflect their effect on global life expectancy and death concentration. Reductions in stroke led to a notable gain in life expectancy of 0·8 years, but stroke deaths were not concentrated, with 90% occurring in locations containing 84% of the global population (appendix 2 figures S23, S36). Similarly, ischaemic heart disease had a substantial effect on improvement to life expectancy, contributing 0·6 years to global life expectancy; yet, as with stroke, ischaemic heart disease showed little mortality concentration, with 90% of deaths concentrated in locations containing 84% of the population in 2021 (appendix 2 figures S17, S37). Neoplasms added 0·6 years to life expectancy, with high-income regions greatly benefiting; as with other NCDs, 90% of neoplasms deaths occurred in locations containing 86% of the population in 2021, indicating a consistent risk of dying from cancer regardless of geography (appendix 2 figures S19, S38). Chronic respiratory diseases contributed an increase of 0·5 years to life expectancy, with east Asia contributing the most to this increase through substantial improvements in mortality in China. Chronic respiratory diseases also showed little mortality concentration, with 90% of deaths occurring in locations containing 79% of the population (appendix 2 figures S20, S39). Digestive diseases and cirrhosis had a substantial negative effect on life expectancy, with little improvement from 2010 to 2019, and showed little mortality concentration (appendix 2 figures S21, S40). Diabetes and kidney diseases had a negative effect on life expectancy, resulting in a global loss of 0·1 years in life expectancy. This cause also had little mortality concentration, with 90% of deaths occurring in locations representing 89% of the population (appendix 2 figures S22, S41). Overall, NCDs largely did not show concentration, meaning that we did not observe mortality from these causes moving towards more restricted geographical areas (appendix 2 figure S42).

### Effect of injuries on life expectancy and trends in mortality concentration

The reduction in transport injuries had a positive effect on life expectancy, contributing to a gain of 0·2 years. However, as with NCDs, transport injury-related mortality was not concentrated, with 90% of deaths concentrated in locations containing 88% of the population in 1990, decreasing slightly to 84% of the population by 2021 (appendix 2 figures S24, S43). Unintentional injuries also showed little mortality concentration, with 90% of deaths occurring in locations containing 88% of the population in 2021 (appendix 2 figures S26, S44). Lastly, the overall reduction in mortality rates from self-harm and interpersonal violence contributed to a 0·2-year increase in life expectancy with variable mortality concentration, showing concentration in central and tropical Latin America and South Africa, but not exclusively in these locations (appendix 2 figures S25, S45).

## Discussion

### Main findings

The COVID-19 pandemic has emerged as one of the most defining global health events of recent history. Our latest comprehensive estimates of cause-specific mortality give insight into the global landscape of disease before and during the first 2 years of the pandemic, revealing the important changes in disease-burden patterns that followed. After more than three decades of consistent improvements in global life expectancy and declining age-standardised death rates, COVID-19 reversed long-standing progress and disrupted trends in the epidemiological transition. As the second leading cause of age-standardised deaths in 2021, COVID-19 had a pronounced influence on the reduction in global life expectancy that occurred. The heterogeneous influence of the disease across the globe provides important insights for improving future pandemic preparedness and ensuring that nations are equitably equipped to respond to new outbreaks. Additionally, our analysis of geographical and temporal trends in mortality enables us to observe the changing patterns in causes of death worldwide. Many causes have exhibited a reduced geographical reach—a reflection of dedicated and persistent mitigation efforts to reduce the burden of certain causes, as well as potential changes to risk-factor exposure.^15^ This study offers an opportunity to apply the lessons learned from these successes to further reduce deaths from causes that are now present within smaller, more concentrated areas throughout the world.

### The COVID-19 pandemic

The emergence and spread of COVID-19 follows a similar pattern of regional heterogeneity that is common among many leading communicable causes of death, with higher rates of infection and increased fatalities occurring in lower-resource settings.^6^, ^16^, ^17^ Although heterogeneity in COVID-19 outcomes in 2020 and 2021 varied by the income status of a country or territory, outcomes were also directly related to age, government actions to close borders, and the implementation of transmission-reduction policies.^18^ This general pattern did not always hold true at the national level, however, where estimates from some high-income countries showed a much greater burden than would have been expected, indicating important opportunities for improved pandemic preparedness and response in these nations.^19^ The varying effects across locations emphasises the complexity of the pandemic. Diverse social, economic, and political influences contributed to the variations in death rates observed between locations. In general, areas with advanced health-care systems and robust medical facilities were better able to manage abrupt increases in the number of COVID-19 cases. By contrast, locations with poorer health-care infrastructure were less equipped to handle the surge in infections that occurred,^20^ although strong health-care systems did not singularly influence the outcome of the pandemic.^19^ Improving preparedness for future pandemics should also include engagement strategies to enhance the trust that individuals place in public health recommendations.^19^ Additionally, identifying methods to enhance death-reporting systems^3^ and overcome political obstacles to ensure accurate reporting will be crucial steps for monitoring COVID-19 and future pandemic occurrences.^21^, ^22^

Our study shows that COVID-19 was one of the leading global causes of death during the first 2 years of the pandemic and provides an opportunity to delineate between the disease's direct and indirect mortality effects as well as its effect on life expectancy. As previously predicted,^3^ COVID-19 shifted baseline patterns of mortality for diseases and injuries that were affected by physical-distancing measures and other government-mandated restrictions. Deferred care-seeking during the height of the pandemic also probably contributed to shifts in patterns of mortality for some diseases and injuries and might also have contributed to the emergence of pandemic-related deaths not attributable directly to COVID-19, lower respiratory infections, measles, malaria, or pertussis (OPRM). Deferred care-seeking might also have been a contributing factor in the notable divergence in the age distribution in deaths between COVID-19 and OPRM, whereby COVID-19 deaths were substantially higher in older ages, whereas the highest rate of OPRM was seen in older ages as well as in children younger than 23 months. Mortality might have increased in the youngest ages because caregivers might have hesitated to seek medical care during the peak of the virus's spread. Understanding these disparities is imperative for shaping future health policies and preparedness efforts.

### Important trends in life expectancy

Advancements over the past three decades in the prevention and control of infectious diseases have contributed to increases in life expectancy in many locations, increasing the need to support populations living with NCDs.^23^ The global decline in life expectancy that occurred in 2020 and 2021 confounds the longer-term trend of increase.^10^ Our decomposition analysis suggests that this decline was predominantly a result of the pandemic (combined COVID-19 and OPRM), but the degree of severity varied greatly by location. Although large improvements in many causes—including HIV/AIDS and lower respiratory and enteric infections—somewhat counterbalanced the decline, the decrease in life expectancy was also compounded by increasing rates of mortality from other causes, such as diabetes and kidney diseases.

The effect of COVID-19 on life expectancy showed varying degrees of severity, ranging from a large loss of 4·9 years in Andean Latin America to almost no change in east Asia. From 1990 to 2021, reductions in many of the leading causes of death resulted in overall life-expectancy increases across most regions, despite heavy setbacks for many because of the COVID-19 pandemic. We found that despite Andean Latin America having the largest regional reduction in life expectancy due to the pandemic, overall life-expectancy reductions across the region were tempered by improvements in other causes, with reductions in rates of death from lower respiratory infections and neonatal disorders responsible for an increase in life expectancy of 2·6 years overall between 1990 and 2021. The impressive reductions in neonatal disorders throughout many countries in Andean Latin America have been attributed to the improvements made in implementing effective maternal and neonatal health intervention strategies.^24^

The reduction in life expectancy in southern sub-Saharan Africa also exceeded the global average by a substantial margin, with a reduction of 3·4 years due to COVID-19. Although life expectancy in the region was substantially affected by the COVID-19 pandemic, the reduction was also attributable to high mortality rates from HIV/AIDS. Some nations with high pandemic-related death tolls were among those already burdened by high rates of other infectious diseases. Several countries in southern sub-Saharan Africa navigated the challenges of the pandemic, alongside long histories of combatting some of the highest HIV/AIDS prevalence rates in the world.^25^, ^26^ A subset of countries were faced with a triple burden of COVID-19, HIV/AIDS, and tuberculosis.^27^ The combined burden of these causes across southern sub-Saharan Africa was not offset by sufficient improvements in mortality from other causes, leading to an overall reduction in the region's life expectancy of more than 4 years over the entire study period.

### Cause-specific patterns of mortality concentration

Estimates of mortality concentration reflect shifting patterns of disease over time, from diseases that have a widespread presence moving to more geographically reduced subsets of the global population. These changes highlight differences between populations and their progress towards reducing mortality due to diseases and injuries. These findings also provide an important opportunity to improve how best public health practices are applied to further disease reduction. Broadly, widespread declines in many communicable diseases resulted in mortality from these causes exhibiting more concentrated geographical distributions in 2021 relative to patterns seen in 1990. The degree of mortality concentration estimated by this study for enteric and lower respiratory infections, malaria, HIV/AIDS, neonatal disorders, and tuberculosis reflects substantial global progress in reducing mortality from these causes over the study period, underscoring the success of several public health campaigns, global commitments, and improvements in communicable-disease programmes.^28^, ^29^, ^30^ Estimates of mortality concentration can be used to examine where disease mitigation strategies have been successful, where they can be further implemented to reduce inequality, and where more research might be needed to develop effective treatment and intervention strategies.

Notably, our estimates support previous findings^31^ that show deaths from malaria are becoming increasingly concentrated and are now particularly concentrated within western sub-Saharan Africa, with an additional corridor running through central Africa and into Mozambique. Countries in western sub-Saharan Africa with the highest under-5 death rates from malaria in 2021 included Burkina Faso, Sierra Leone, and Niger. This concentration of malaria mortality reflects both differential rates of population growth across Africa, as well as the varying rates of progress in reducing transmission, most notably by malaria nets treated with long-lasting insecticide and in strengthening case management.^32^ At a time of growing threats to progress against malaria, including emerging parasite and vector resistance and budgetary pressures, but also amid promising new tools such as second vaccine for malaria, it is more important than ever that changing patterns of mortality are quantified and understood.^33^, ^34^

Enteric infections showed large disease concentration. Under-5 deaths from enteric infections were largely concentrated within sub-Saharan Africa and south Asia. Countries in sub-Saharan Africa and south Asia with the highest under-5 death rates from enteric infections in 2021 included Chad, South Sudan, and the Central African Republic. There are many contributing factors that should be considered when examining how to reduce enteric infections in the remaining concentrated locations. Alongside the provision of oral rehydration solution and rotavirus vaccines, critical public health improvements such as in water, sanitation, and hygiene might have contributed to decreases in enteric deaths.^35^, ^36^ Childhood growth failure, also a leading risk factor for deaths from lower respiratory infections, malaria, and measles, must be addressed through interventions to improve women's health including anaemia, promotion of early exclusive breastfeeding, and management of acute malnutrition, among others.^37^, ^38^ Countries with the highest burden of infectious disease mortality in children younger than 5 years tend to be geographically clustered, suggesting multisectoral approaches are necessary to continue reducing mortality in the countries with the highest rates.^39^

A broad and recurring theme from this study is that reductions in enteric infections contributed to improved life expectancies over the past several decades. The reductions in childhood mortality associated with diarrhoeal diseases that have occurred across many parts of Africa^35^, ^40^, ^41^, ^42^ can also be partly explained by many combined local efforts in improved immunisation;^43^ access to water, sanitation, and hygiene facilities;^12^, ^44^ breastfeeding;^45^ oral rehydration therapy;^46^ and zinc supplementation,^15^ alongside global initiatives such as the Global Action Plan for the Prevention and Control of Pneumonia and Diarrhea.^47^ Given that enteric disease-related mortality and specifically diarrhoeal disease-related mortality continued to decline during the COVID-19 pandemic, the post-pandemic period might offer opportunities to accelerate progress on prevention and treatment. Diarrhoeal diseases are particularly amenable to public health intervention, and given this cause's high burden among children, we must continue to direct resources towards its prevention.^47^, ^48^ Several locations still do not have the necessary financing, governance, and political commitment to reduce rates of enteric infections.^49^ To accelerate progress in reducing enteric disease-related mortality, routine and catch-up immunisation programmes must be strengthened and expanded, including building on the global success of the rotavirus roll-out^50^ and countering disruptions in childhood immunisation during the pandemic.^51^ Additionally, efforts should focus on advancing candidate vaccines against enterotoxigenic *Escherichia coli*, norovirus, and shigella.^51^, ^52^, ^53^, ^54^, ^55^

Our study also found that some vaccine-preventable diseases, such as measles, have shown widespread reductions in mortality rates and were geographically concentrated. Under-5 deaths from measles were concentrated within western and eastern sub-Saharan Africa. Although multiple factors contribute to decreases in infectious disease burden, improvements in measles mortality have largely been attributable to the global availability of a safe and effective vaccine against measles, producing life-long immunity, with two-dose efficacy exceeding 95%.^56^ Measles incidence has decreased dramatically where vaccination efforts have been successful, including North America, South America, Europe, and Australia;^57^, ^58^, ^59^, ^60^, ^61^ although, since 2016, endemic measles transmission has been re-established in ten countries that previously had achieved measles elimination.^61^ We found that, as of 2021, measles mortality was concentrated in countries and regions with insufficient access to the measles vaccine, particularly in sub-Saharan Africa. Although valuable insights can be drawn from countries that have achieved measles control through effective vaccination programmes and surveillance systems, interventions still must be tailored to the affected communities and countries for successful reductions in mortality.^62^

Some infectious diseases, such as HIV/AIDS, also showed mortality concentration. Deaths from HIV/AIDS were largely concentrated within sub-Saharan Africa, most notably southern sub-Saharan Africa. Countries in sub-Saharan Africa with the highest age-standardised mortality rate in 2021 included Lesotho, Eswatini, and Botswana. Countries in sub-Saharan Africa with the highest under-5 death rates from HIV in 2021 included Lesotho, Equatorial Guinea, and Guinea-Bissau. This concentration highlights how HIV-control campaigns, preventative measures,^63^, ^64^ improved treatment with the emergence of antiretroviral therapy,^65^ access to testing and health care,^66^ and research advancements might have contributed to the reduced global mortality of HIV. Despite these successes, substantial barriers remain to reducing HIV mortality, such as stigma discouraging people from accessing treatment and care,^67^, ^68^ insufficient health-care infrastructure,^69^ access to testing,^70^ coverage of antiretroviral therapy,^71^ and complications due to co-occurring diseases such as tuberculosis and HIV.^72^ Preventative measures are particularly important for the reduction of HIV mortality because HIV prevalence is the primary contributor to high mortality rates. Although countries can learn from successful HIV campaigns and strategies, global support is needed to ensure HIV treatment and preventative measures are accessible to all populations at risk.^70^, ^73^, ^74^

In many high-income nations, the overall rate of neonatal deaths decreased between 1990 and 2021, becoming more concentrated over time. Deaths from neonatal disorders in 2021 were concentrated within sub-Saharan Africa and south Asia.^75^ Countries in these regions with the highest under-5 death rates from neonatal disorders in 2021 included Mali, South Sudan, and Sierra Leone. However, the disparity in mortality between high-income and low-income countries and regions highlights inequality in progress. Newborn care that can reduce mortality includes resuscitation, prevention of hypothermia and infection, in-facility delivery, and exclusive breastfeeding.^76^, ^77^ Neonatal mortality might be reduced globally if policy makers examine the strategies that led to successes elsewhere.^78^

Conversely, although the burden of many NCDs has also been reducing, these causes have typically not followed the same pattern of mortality concentration seen in CMNN diseases. These trends emphasise a key distinction in the spatial dynamics of NCDs compared with many communicable diseases. Although non-communicable causes might not exhibit the same degree of concentration as communicable causes, the mortality burden has changed in distribution, reducing over time in high-income countries and regions, while persisting in low-income countries and regions. Age-standardised mortality rates due to NCDs decreased in most locations within the high-income; Latin America and the Caribbean; north Africa and the Middle east; and central Europe, eastern Europe, and central Asia super-regions between 1990 and 2021. However, NCDs in the south Asia; sub-Saharan Africa; and southeast Asia, east Asia, and Oceania super-regions have either increased or decreased at notably lower levels in 2021 compared with in 1990. Examples of this trend include ischaemic heart disease, neoplasms, and stroke, all of which largely declined over the study period—although their reductions have been widely dispersed and not as targeted as the CMNN causes. These findings show that NCDs do not appear to be moving towards more condensed geographical locations over time in the same way that many CMNN diseases are, which could make interventions and policies more complex to implement.

Ultimately, the extent of mortality concentration reflects both the progress achieved in health-care advancements and the shortcomings that persist in their equitable implementation. Disease concentration is evidence that there are effective interventions and policies that have successfully reduced disease burden in many locations, but these innovations have not been equitably distributed throughout the world or have been ineffective at addressing the specific challenges certain populations face. There remains a global need to improve access to new interventions and vaccines, to invest in the implementation of validated public health policies, and to strategise with geographical sources of disease in mind. Future efforts should continue the ongoing mitigation of communicable diseases, focusing on locations where these causes have become more geographically concentrated, while also initiating efforts to combat chronic causes within low-resourced settings. Additionally, patterns of high geographical concentration among infectious causes and low geographical concentration among chronic causes reflect the global epidemiological transition, wherein mortality rates of infectious deaths declined throughout most years of our study. The increased concentration of a cause of death, particularly communicable diseases, illustrates success in mitigation that can be adapted within the countries and regions with mortality concentration identified in our study, with the potential to greatly reduce mortality from those causes of death.

### Limitations

Methodological advancements have enabled GBD 2021 to produce cause-specific estimates of mortality more easily than in previous iterations; however, as with any study of this scope, there are several important limitations to acknowledge. Cause-specific limitations for every cause of death in GBD are detailed in appendix 1 (section 3). Here, we describe cross-cutting limitations with applicability across many causes. First, sparsity of data or unreliability of data from specific regions, time periods, or age groups can influence the accuracy of our estimates, particularly poor data quality and coverage from western, eastern, southern, and central sub-Saharan Africa and south Asia. Second, the quality of cause-of-death and verbal-autopsy data rely on accurately coded death certificates to the international standards set by the International Classification of Diseases and are subject to the practice of the doctor completing the death certificate, who may or may not have received training to facilitate comparability of reporting underlying causes of death. This process is further complicated by comorbidities at the time of death, which might affect the accuracy of both vital-registration and verbal-autopsy data sources. A key data-processing method for GBD is the re-allocation of incorrectly or vaguely assigned deaths—referred to as garbage codes^11^—to a more accurate, plausible underlying cause of death. This step helps to create comparable cause-specific estimates of mortality by underlying cause. Third, GBD assesses quality of cause-of-death data partly by examining levels of completeness, which indicate the accuracy with which the vital registration can capture deaths that occur in a location-year, irrespective of the percentage of garbage coding. Data completeness depends on the percentage of well-certified data, which is not necessarily indicative of low garbage coding. Fourth, some sources of uncertainty, including the covariates used in models, are not captured in our estimation process. Fifth, we used a negative binomial modelling approach to improve our estimation of deaths for some causes with over-dispersed data, but do not have a standardised empirical approach for selecting causes to which we apply this method. Sixth, to provide estimates for locations with low levels of completeness, as well as to address the lags in data reporting that occur, our estimates for the most recent years depend more heavily on the modelling process. For causes where data are limited, providing estimates with appropriate uncertainty is preferable to providing no information. Seventh, in the calculation of life expectancy decomposition, there is instability when the difference in all-cause deaths is too small. In this case, we use the reduced Das Gupta equation (appendix 1 section 7). Additionally, to avoid assigning positive life-expectancy contributions to COVID-19-related causes, if the signs for the change in life expectancy and all-cause deaths were the same, we used the same reduced Das Gupta formula, except in the case that the cause in question was COVID-19-related (either COVID-19 or OPRM), when a modified version was used. When viewing life expectancy decomposition, it is important to understand the effects of fatal discontinuity events, such as earthquakes or conflict. If life-expectancy decomposition is calculated for 2 consecutive years, we can see the effect of unique, stochastic events, but for the longer time periods, the interpretation of the effect of these events will be misleading. This method works well with causes that have continuous time trends, and not for causes that have mortality spikes in select years and locations. This type of event confounds true health trends within a time period because the absence or presence of a disaster is seen as a change in life expectancy. Finally, this cycle of GBD contains additional limitations that pertain to modelling deaths and related mortality from the COVID-19 pandemic. The limitations of the methods used to calculate COVID-19 have been fully outlined in previous publications,^12^ but it is important to reiterate that COVID-19 estimates are limited by data-source availability. The methods to estimate COVID-19-related deaths were especially limited in certain regions, such as sub-Saharan Africa, which means our estimates in these areas are solely driven by relationships with covariates. Future development of these data sources is crucial because estimates improve as the quality of the underlying data sources improves. Subsequent GBD cycles will provide revised estimates after additional data for recent years become available.

### Future directions

In the next iteration of GBD, we will include over 100 location-years of vital registration and other data types that have been reported since GBD 2021 estimates were produced. Additionally, we will continue to expand the estimation of causes of death by disaggregating broad categories of causes of death into more detailed causes where available. These improvements aim to enhance precision and timeliness of estimates of COVID-19-related deaths and other cause of death. We also plan to simplify our approach to estimating COVID-19-related deaths. In lieu of the residual OPRM category reported in GBD 2021, we will use all available location-years of cause-of-death data to attribute mortality to specific causes, removing this residual category. We anticipate that this method will facilitate more timely and actionable insights for public health planning and policy making, especially as we expect to observe more regular and modellable mortality patterns in the post-pandemic years. Through these advancements, we will improve the utility and accuracy of the GBD study as a tool for effective public strategies.

### Conclusion

Findings from GBD 2021 provide a comprehensive overview of long-term mortality trends along with important insights into the COVID-19 pandemic years. The COVID-19 pandemic fundamentally changed the landscape of global health and mortality. As a leading cause of death, COVID-19 reduced life expectancy in 2 years nearly as much as reductions in communicable and NCDs have improved it over decades. The changes in mortality caused by the pandemic were not predictable through the standard GBD estimation methods and required the development and application of novel estimation methods as the pandemic emerged in real time. These timely updates on causes of death are essential for monitoring progress, identifying prevailing health concerns, guiding targeted interventions, and optimising resource allocation. GBD 2021 shows that better life expectancy outcomes might be achieved by leveraging past successes in mortality reduction. If future policy efforts are guided by the successes made in countries and regions with effective disease-mitigation programmes, such achievements might be replicated in locations where high mortality persists. While COVID-19 and other health challenges continue, GBD 2021 can offer valuable guidance for public health investment and policy making.

For the **GBD data sources** see http://ghdx.healthdata.org/gbd-2021

For the **statistical code** see http://ghdx.healthdata.org/gbd-2021/code

## Data sharing

To download the data used in these analyses, please visit the Global health Data Exchange GBD 2021 website (https://ghdx.healthdata.org/gbd-2021/sources).

**This online publication has been corrected. The corrected version first appeared at thelancet.com on April 19, 2024**

## Declaration of interests

S Afzal reports support for the present manuscript from King Edward Medical University including study material, research articles, valid data sources and authentic real time information for this manuscript; payment or honoraria for lectures, presentations, speakers bureaus, manuscript writing or educational events from King Edward Medical University and collaborative partners including University of Johns Hopkins, University of California, University of Massachusetts, KEMCAANA, KEMCA-UK Scientific Conferences and Webinars; support for attending meetings and/or travel from King Edward Medical University to attend meetings; participation on a Data Safety Monitoring Board or Advisory Board with National Bioethics Committee Pakistan, King Edward Medical University Ethical Review Board, as well as Ethical Review Board Fatima Jinnah Medical University and Sir Ganga Ram Hospital; leadership or fiduciary roles in board, society, committee or advocacy groups, paid or unpaid with Pakistan Association of Medical Editors, Fellow of Faculty of Public Health Royal Colleges UK (FFPH), and Society of Prevention, Advocacy And Research, King Edward Medical University (SPARK); and other support as Dean of Public Health and Preventive Medicine at King Edward Medical University, as the Chief Editor Annals of King Edward Medical University, as the Director of Quality Enhancement Cell King Edward Medical University, as an international-level Fellow of Faculty of Public Health United Kingdom, as an Advisory Board Member and Chair Scientific Session KEMCA-UK, as a Chairperson of KEMCAANA (the International Scientific Conference), as a national-level member on the Research and Publications Higher Education Commission (HEC Pakistan), as a member of the Research and Journals Committee (Pakistan) the Medical and Dental Council (Pakistan), the National Bioethics Committee (Pakistan), the Corona Experts Advisory Group (Punjab), the Chair of the Dengue Experts Advisory Group, and a member of the Punjab Residency Program Research Committee; all outside the submitted work. R Ancuceanu reports consulting fees from Abbvie; payment or honoraria for lectures, presentations, speakers' bureaus, manuscript writing or educational events from Abbvie, Sandoz, B. Braun, Laropharm, and MagnaPharm; all outside the submitted work. J Ärnlöv reports payment or honoraria for lectures, presentations, speakers' bureaus, manuscript writing or educational events from AstraZeneca and Novartis for lecture fees; participation on a Data Safety Monitoring Board or Advisory Board with AstraZeneca, Astella, Boehringer Ingelheim; all outside the submitted work. O C Baltatu reports support for the present manuscript from National Council for Scientific and Technological Development (CNPq, 304224/2022-7) and Anima Institute (AI research professor fellowship); Leadership or fiduciary role in other board, society, committee or advocacy group, paid or unpaid, as Founding Member of the Health and Biotechnology Advisory Board at Technology Park São José dos Campos–Center for Innovation in Health Technologies (CITS), outside the submitted work. T W Bärnighausen reports grants or contracts from National Institutes of Health, Alexander von Humboldt Foundation, German National Research Foundation (DFG), European Union, German Ministry of Education and Research, German Ministry of the Environment, Wellcome, and KfW, all as payments to their institution; participation on a Data Safety Monitoring Board or Advisory Board on two Scientific Advisory Boards for NIH-funded research projects in Africa on Climate Change and Health; stock or stock options in CHEERS, an SME focusing on approaches to measure climate change and health-related variables in population cohorts; all outside the submitted work. S Barteit reports grants from Carl-Zeiss Foundation and the German research foundation (DFG); stock or stock options in CHEERS, a for-profit company focusing on climate change and health evaluation and response systems; all outside the submitted work. M Beghi reports consulting fees from Lundbeck and Angelini, all outside the submitted work. Y Bejot reports consulting fees from Medtronic and Novartis; payment or honoraria for lectures, presentations, speakers bureaus, manuscript writing or educational events from BMS, Pfizer, Medtronic, Amgen, NovoNordisk, and Servier; support for attending meetings and/or travel from Medtronic; leadership or fiduciary role in other board, society, committee or advocacy group, unpaid, with the French Neurovascular Society; all outside the submitted work. M Bell reports grants or contracts from US EPA, NIH, High Tide Foundation, Health Effects Institute, Yale Women Faculty Forum, Environmental Defense Fund, Yale Climate Change and Health Center, Wellcome Trust Foundation, Robert Wood Johnson Foundation, and the Hutchinson Postdoctoral Fellowship (all paid to their institution); Consulting fees from Clinique; Payment or honoraria for lectures, presentations, speakers bureaus, manuscript writing or education events from Colorado School of Public Health, Duke University, University of Texas, Data4Justice, Korea University, Organization of Teratology Information Specialists, University of Pennsylvania, Boston University, IOP Publishing, NIH, Health Canada, PAC-10, UKRI, AXA Research Fund Fellowship, Harvard University and the University of Montana; Support for attending meeting and/or travel from Colorado School of Public Health, University of Texas, Duke University, Boston University, University of Pennsylvania, Harvard University, American Journal of Public Health, Columbia University and Harvard University; Leadership or fiduciary role in other board, society, committee or advocacy group, unpaid with Fifth National Climate Assessment and Lancet Countdown, Johns Hopkins Advisory Board, Harvard external advisory committee for training grant, WHO Global Air Pollution and Health Technical Advisory group and the National Academies Panels and Committee; and paid with the US EPA Clean Air Scientific Advisory Committee (CASAC); outside the submitted work. L Belo reports other financial or non-financial interests with UCIBIO – FFUP through support from FCT in the scope of the project UIDP/04378/2020 and UIDB/04378/2020 of UCIBIO and the project LA/P/0140/2020 of i4HB, all outside the submitted work. R S Bernstein reports other financial or non-financial support as a full-time Medical Consultant employee of the California Department of Public Health in the Center for Heal Care Quality; outside the submitted work. P J G Bettencourt reports patents planned, issued, or pending (WO2020229805A1, BR112021022592A2, EP3965809A1, OA1202100511, US2023173050A1, EP4265271A2, and EP4275700A2); other financial or non-financial interests with the Botnar Foundation as project reviewer, outside the submitted work. S Bhaskar reports grants or contracts from the Japan Society for the Promotion of Science (JSPS), Japanese Ministry of Education, Culture, Sports, Science and Technology (MEXT) and JSPS and the Australian Academy of Science; Leadership or fiduciary role in other board, society, committee or advocacy group, paid or unpaid with Rotary District 9675 as the district chair, Global Health & Migration Hub Community as Chair and Manager (Berlin, Germany), PLOS One, BMC Neurology, Frontiers in Neurology, Frontiers in Stroke, Frontiers in Public Health and the BMC Medical Research Methodology as an Editorial Board Member, and as a Member of the College of Reviewers (Canadian Institutes of Health Research, Canada); outside the submitted work. B Bikbov reports grants or contracts from the European Commission and The University of Rome; Support for attending meetings/travel from the European Renal Association; Leadership or fiduciary role in other board, society, committee or advocacy group, unpaid in an advocacy group with the International Society of Nephrology and unpaid on the Western Europe Regional Board of the International Society of Nephrology; Other financial or non-financial support from Scientific-Tools.org; outside the submitted work. A Biswas reports consulting fees from INTAS Pharmaceuticals Ltd, India, Lupin Pharmaceuticals, Ltd, India, and Alkem Laboratories, India as personal payments; payment or honoraria for lectures, presentations, speakers' bureaus, manuscript writing or educational events from Roche Diagnostics, India, as personal payments; all outside the submitted work. E J Boyko reports payment or Honoria for lectures, presentations, speakers bureaus, manuscript writing or education events from the Korean Diabetes Association, Diabetes Association of the R.O.C (Taiwan), the American Diabetes Association, and the International Society for the Diabetic Foot; Support for attending meetings and/or travel from the Korean Diabetes Association; Diabetes Association of the R.O.C (Taiwan), International Society for the Diabetic Foot; outside the submitted work. M Carvalho reports other financial or non-financial interests from LAQV/REQUIMTE, University of Porto (Porto, Portugal) and acknowledges the support from FCT under the scope of the project UIDP/50006/2020; outside the submitted work. E Chung reports support for the present manuscript from the National Institute of Health NICHD T32HD007233. J Conde reports grants or contracts form the European Research Council Starting Grant ERC-StG-2019-848325 (funding 1.5M Euro), outside the submitted work. S Cortese reports grants or contracts from National Institute for Health and Care Research (NIHR)and the European Research Executive Agency; payment or honoraria for lectures, presentations, speakers bureaus, manuscript writing or educational events from the Association of Child and Adolescent Mental Health, British Association of Psychopharmacology, Medice, and Canadian ADHD Resource Alliance; support for attending meetings and/or travel the Association of Child and Adolescent Mental Health, British Association of Psychopharmacology, Medice, and Canadian ADHD Resource Alliance; leadership or fiduciary role in other board, society, committee or advocacy group, unpaid, with the European ADHD Guidelines Group and the European Network for Hyperkinetic Disorders; all outside the submitted work. Sa Das reports leadership or fiduciary role in other board, society, committee or advocacy group, unpaid, with the Association for Diagnostics and Laboratory Medicine as program chair, and the Women in Global Health India Chapter, outside the submitted work. A Dastiridou reports support for attending meetings and/or travel from THEA and ABBVIE, outside the submitted work. L Degenhardt reports untied educational grants from Indivior and Seqirus to examine new opioid medications in Australia, outside the submitted work. A K Demetriades reports leadership or fiduciary role in other board, society, committee or advocacy group, unpaid, with the AO Knowledge Forum Degenerative Steering Committee, Global Neuro Foundation Board, and the European Association of Neurological Societies Board of Officers, all outside the submitted work. A Faro reports support for the present manuscript from National Council for Scientific and Technological Development, CNPq, Brazil as CNPq Researcher (scholarship). I Filip reports support for the present manuscript from Avicenna Medical and Clinical Research Institute. D Flood reports grants or contracts from NHLBI (award number K23HL161271), the University of Michigan Claude D. Pepper Older Americans Independence Center (award number 5P30AG024824), and the University of Michigan Caswell Diabetes Institute Clinical Translational Research Scholars Program; consulting fees from the World Health Organization as payments to their institution; leadership or fiduciary role in other board, society, committee or advocacy group, unpaid, as Staff Physician for Maya Health Alliance, a non-governmental health organization in Guatemala; all outside the submitted work. A A Fomenkov reports support for the present manuscript from Development of effective biotechnologies based on cell cultures, tissues and organs of higher plants, microalgae and cyanobacteria. The research carried out within the state assignment of Ministry of Science and Higher Education of the Russian Federation (theme No. 122042600086-7). M Foschi reports consulting fees from Roche and Novartis; support for attending meetings and/or travel from Biogen, Roche, Novartis, Sanofi, Bristol, and Merck; leadership or fiduciary role in other board, society, committee or advocacy group, unpaid, with MBase Foundation; all outside the submitted work. R Franklin reports grants or contracts from Heatwaves (Queensland Government, Queensland, Australia) and Arc Flash (Human Factors, Queensland Government, Queensland, Australia), and Mobile Plant Safety (Agrifutures); Support for attending meetings and/or travel from ACTM Tropical Medicine and Travel Medicine Conference 2022 and 2023, and ISTM Travel Medicine Conference in Basel 2023; leadership or fiduciary role in other board, society, committee or advocacy group, paid or unpaid as the president/director of Kidsafe, the director of Auschem, a member of the governance committee of ISASH, the director of Farmsafe, the Vice President of ACTM, and as a PHAA Injury Prevention SIG Convenor; outside the submitted work. P S Gill reports support for the present manuscript from the National Institute for Health and Care Research (NIHR) as Senior Investigator with payments to their institution; the views expressed in this publication are those of the author(s) and not necessarily those of the NIHR or the UK Department of Health and Social Care. A Guha reports grants or contracts from the American Heart Association and Department of Defense; consulting fees from Pfizer, Novartis, and Myovant; leadership or fiduciary role in other board, society, committee or advocacy group, paid or unpaid, with ZERO Prostate Cancer Health Equity Task Force and Doctopedia as a founding medical partner; all outside the submitted work. C Herteliu reports grants or contracts from the Romanian Ministry of Research Innovation and Digitalization, MCID, project number ID-585-CTR-42-PFE-2021; grant of the European Commission Horizon 4P-CAN (Personalised Cancer Primary Prevention Research through Citizen Participation and Digitally Enabled Social Innovation); Project “Societal and Economic Resilience within multi-hazards environment in Romania” funded by European Union – NextgenerationEU and Romanian Government, under National Recovery and Resilience Plan for Romania, contract no.760050/ 23.05.2023, cod PNRR-C9-I8-CF 267/ 29.11.2022, through the Romanian Ministry of Research, Innovation and Digitalization, within Component 9, Investment I8; Project “A better understanding of socio-economic systems using quantitative methods from Physics” funded by European Union–NextgenerationEU and Romanian Government, under National Recovery and Resilience Plan for Romania, contract number 760034/ 23.05.2023, cod PNRR-C9-I8-CF 255/ 29.11.2022, through the Romanian Ministry of Research, Innovation and Digitalization, within Component 9, Investment I8; outside the submitted work. M Hultström reports support for the present manuscript from Knut och Alice Wallenberg Foundation and the Swedish Heart-Lung Foundation, all as payments to their institution; Support for attending meetings and/or travel from the American Physiological Society and the Swedish Society for Anaesthesiology and Intensive Care; leadership or fiduciary role in other board, society, committee or advocacy group, paid or unpaid, with the American Physiological Society, Water and Electrolyte Section; all outside the submitted work. I Ilic and M Ilic report support for the present manuscript from Ministry of Science, Technological Development and Innovation of the Republic of Serbia. S M Islam reports support for the present manuscript from NHMRC and Heart Foundation, N E Ismail reports leadership or fiduciary role in other board, society, committee or advocacy group, unpaid, as Bursar (Council Member) of the Malaysian Academy of Pharmacy, outside the submitted work. T Joo reports support for the present manuscript from National Research, Development, and Innovation Office in Hungary (RRF-2.3.1-21-2022-00006), Data-Driven Health Division of National Laboratory for Health Security. G Joshy reports grants or contracts from the Department of Health and Aged Care 2023 (Understanding the fatal burden of COVID-19 in residential aged care facilities); support for attending meetings and/or travel from the Statistical Society of Australia Grant 2023 supporting conference registration; participation on a Data Safety Monitoring Board with the Australian Mathematical Sciences Institute (AMSI) and the Statistical Society of Australia (SSA) for the project Community-led nutrition and Lifestyle program for weight loss and metabolic Health: a randomised Controlled trial (ELCHO), 2022; all outside the submitted work. J Jozwiak reports payment or honoraria for lectures, presentations, speakers bureaus, manuscript writing or educational events from Novartis, Adamed, and Amgen; outside the submitted work. N Kawakami reports grants or contracts from the Junpukai Foundation and the Department of Digital Mental Health is an endowment department, supported with an unrestricted grant from 15 enterprises (https://dmh.m.u-tokyo.ac.jp/c); consulting fees from Riken Institute, JAXA, Sekisui Chemicals, and SB@WORK; leadership or fiduciary role in other board, society, committee or advocacy group, paid or unpaid, with the Japan Society for Occupational Health; all outside the submitted work. J H Kempen reports grants or contracts from the Massachusetts Eye and Ear Surgery Program and Sight for Souls through payments to their institution; leadership or fiduciary role in other board, society, committee or advocacy group, paid or unpaid, as President of Sight for Souls; stock or stock options with Betaliq and Tarsier; all outside the submitted work. T Kocsis reports grants or contracts from Novartis Magyarország Ltd through payment for market access activities, outside the submitted work. K Krishan reports other non-financial interests from the UGC Centre of Advanced Study, CAS II, awarded to the Department of Anthropology, Panjab University (Chandigarh, India); outside the submitted work. B Lacey reports support for the present manuscript from the UK Biobank, funded largely by the UK Medical Research Council and Wellcome, through their employment at the University of Oxford. M Lee reports support for the present manuscript from the Ministry of Education of the Republic of Korea and the National Research Foundation of Korea (NRF-2021R1I1A4A01057428) and Bio-convergence Technology Education Program through the Korea Institute for Advancement Technology (KIAT) funded by the Ministry of Trade, Industry and Energy (No. P0017805). M-C Li reports grants or contracts from The National Science and Technology Council in Taiwan (NSTC 112-2410-H-003-031; leadership or fiduciary role in other board, society, committee or advocacy group, paid or unpaid, as the technical editor of the Journal of the American Heart Association; outside the submitted work. J Liu reports support for the present manuscript from the National Natural Science Foundation of China (grant number: 72122001; 72211540398). S Lorkowski reports grants or contracts from Akcea Therapeutics Germany through payments to their institution; consulting fees from Danone, Novartis Pharma, Swedish Orphan Biovitrum (SOBI), and Upfield; payment or honoraria for lectures, presentations, speakers bureaus, manuscript writing or educational events from Akcea Therapeutics Germany, AMARIN Germany, Amedes Holding, AMGEN, Berlin-Chemie, Boehringer Ingelheim Pharma, Daiichi Sankyo Deutschland, Danone, Hubert Burda Media Holding, Janssen-Cilag, Lilly Deutschland, Novartis Pharma, Novo Nordisk Pharma, Roche Pharma, Sanofi-Aventis, and SYNLAB Holding Deutschland & SYNLAB Akademie; support for attending meetings and/or travel from AMGEN; participation on a Data Safety Monitoring Board or Advisory Board with Akcea Therapeutics Germany, AMGEN, Daiichi Sankyo Deutschland, Novartis Pharma, and Sanofi-Aventis; all outside the submitted work. M A Mahmoud reports grant or contract funding from the Deputyship for Research and Innovation, Ministry of Education in Saudi Arabia (project number 445-5-748). L G Mantovani reports support for the present manuscript from the Italian Ministry of Health. H R Marateb reports support for the present manuscript from The Beatriu de Pinós post-doctoral programme from the Office of the Secretary of Universities and Research from the Ministry of Business and Knowledge of the Government of Catalonia programme: 2020 BP 00261. R Matzopoulos reports consulting fees from New York University and DG Murray Trust; Support for attending meetings/travel paid by SA MRC and University of Cape Town; leadership or fiduciary role in other board, society, committee or advocacy group, unpaid, as a Board member of Gun Free South Africa; Stock or Stock options with Sanlam; outside the submitted work. R J Maude reports support for the present manuscript from Wellcome Trust [Grant number 220211] as it provides core funding for Mahidol Oxford Tropical Medicine Research and contributes to his salary. A-F A Mentis reports grants or contract funding from ‘MilkSafe: A novel pipeline to enrich formula milk using omics technologies’, a research co financed by the European Regional Development Fund of the European Union and Greek national funds through the Operational Program Competitiveness, Entrepreneurship and Innovation, under the call RESEARCH - CREATE - INNOVATE (project code: T2EDK-02222), as well as from ELIDEK (Hellenic Foundation for Research and Innovation, MIMS-860) (both outside of the present manuscript); payment for expert testimony from serving as external peer-reviewer for FONDAZIONE CARIPLO, ITALY; participation in a Data Safety Monitoring or Advisory Board as Editorial Board Member for “Systematic Reviews”, for “Annals of Epidemiology”, and as Associate Editor for “Translational Psychiatry”; stock or stock options from a family winery; and other financial interests as the current scientific officer for BGI Group; outside the submitted work. S A Meo reports support from King Saud University, Riyadh, Saudi Arabia (RSP-2024 R47). T R Miller reports grants or contracts from AB InBev Foundation, National Institute for Mental Health (Santa Clara County, CA), and Everytown for Gun Safety; payment for expert testimony in the states of Michigan, Nevada & New Mexico Mobile County Board of Health; outside the submitted work. P B Mitchell reports payment or honoraria for lectures, presentations, speakers' bureaus, manuscript writing or educational events, from Janssen (Australia), outside the submitted work. L Monasta reports support for the present manuscript from the Ministry of Health (Ricerca Corrente 34/2017) through payments made to the Institute for Maternal and Child Health IRCCS Burlo Garofolo. R S Moreira reports grants or contracts from CNPq Research Productivity Scholarship (National Council for Scientific and Technological Development) scholarship registration number 316607/2021-5; outside the submitted work. J Mosser reports support for the present manuscript from the Bill and Melinda Gates Foundation; grants or contractions from Gavi; Support for attending meetings and/or travel from the Bill and Melinda Gates Foundation; outside the submitted work. S Nomura reports support for the present manuscript from Ministry of Education, Culture, Sports, Science and Technology of Japan (21H03203) and Precursory Research for Embryonic Science and Technology from the Japan Science and Technology Agency (JPMJPR22R8). B Norrving reports participation on a Data Safety Monitoring Board or Advisory Board with Simbec Orion, outside the submitted work. A P Okekunle reports support for the present manuscript from the National Research Foundation of Korea funded by the Ministry of Science and ICT (2020H1D3A1A04081265). A Ortiz reports grants or contracts from Sanofi as payments to their institution; consulting fees, speaker fees or support for travel from, Advicciene, Astellas, Astrazeneca, Amicus, Amgen, Boehringer Ingelheim, Fresenius Medical, Care, GSK, Bayer, Sanofi-Genzyme, Menarini, Mundipharma, Kyowa Kirin, Lilly, Alexion, Freeline, Idorsia, Chiesi, Otsuka, Novo-Nordisk, Sysmex and Vifor Fresenius Medical Care Renal, Pharma and is Director of the Catedra, Mundipharma-UAM of diabetic kidney disease, and the Catedra Astrazeneca-UAM of chronic, kidney disease and electrolytes; Leadership or fiduciary role in other board, society, committee or advocacy group, paid or unpaid, with the European Renal Association; stock or stock options with Telara Farma; all outside the submitted work. P K Pal reports grants or contracts paid to their institution from the Indian Council of Medical Research (ICMR), the Department of Science & Technology (DST)-Science and Engineering Research Board, the Department of Biotechnology (DBT), DST-Cognitive Science Research Initiative, Wellcome Trust UK-India Alliance DBT, PACE scheme of BIRAC, Michael J. Fox Foundation, and SKAN (Scientific Knowledge for Ageing and Neurological ailments)-Research Trust; Payment and honoraria for lectures, presentations, speakers bureaus, manuscript writing or educational events as Faculty/Speaker/Author from the International Parkinson and Movement Disorder Society, and Movement Disorder Societies of Korea, Taiwan and Bangladesh; support for attending meetings and/or travel from the National Institute of Mental Health and Neurosciences (NIMHANS), International Parkinson and Movement Disorder Society, and Movement Disorder Societies of Korea, Taiwan and Bangladesh; Leadership or fiduciary role in other board, society, committee or advocacy group, unpaid, as the Past President of Indian Academy of Neurology, Past Secretary of Asian and Oceanian subsection of International Parkinson and Movement Disorder Society (MDS-AOS), Editor-in-Chief of Annals of Movement Disorders, Chair of the Education Committee of International Parkinson and Movement Disorder Society (IPMDS), President of the Parkinson Society of Karnataka, Chair of Infection Related Movement Disorders Study Group of MDS, Member of Rare Movement Disorders Study Group of International Parkinson and Movement Disorder Society (IPMDS), Member of Education Committee of IAPRD, Member of Rating Scales Education and Training Program Committee of IPMDS, Member of Neurophysiology Task Force of IPMDS, Member of Movement Disorders in Asia Study Group, Member of Post-Stroke Movement Disorders, Member of Ataxia Study Group of IPMDS, and as a Member of Ataxia Global Initiative; all outside the submitted work. C Palladino reports grants or contracts from FCT – Fundação para a Ciência e a Tecnologia, I.P. (national funding), under a contract-programme as defined by DL No. 57/2016 and Law No. 57/2017 (DL57/2016/CP1376/CT0004). DOI 10.54499/DL57/2016/CP1376/CT0004 (https://doi.org/10.54499/DL57/2016/CP1376/CT0004). R F Palma-Alvarez reports payment or honoraria for lectures, presentations, speakers bureaus, manuscript writing or educational events from Angelini, Lundbeck, Casen Recordati and Takeda; support for attending meetings and/or travel from Janssen and Lundbeck; all outside the submitted work. A M Pantea Stoian reports consulting fees from AstraZeneca, Boehringer Ingelheim, Eli Lilly, Novo Nordisk, Novartis, Sandoz, and Sanofi; payment or honoraria for lectures, presentations, speakers bureaus, manuscript writing or educational events from Astra Zeneca, Boehringer Ingelheim, Eli Lilly, Novo Nordisk, Novartis, Sandoz, Medochemie, Servier, and Sanofi; support for attending meetings and/or travel from Sanofi, Novo Nordisk, and Medochemie; Participation on a Data Safety Monitoring Board or Advisory Board with Astra Zeneca, Eli Lilly, Novo Nordisk, and Sanofi; Leadership or fiduciary role in other board, society, committee or advocacy group, unpaid, with the Central European Diabetes Association and the Association for Renal-Metabolic & Nutritional Studies(ASRMN); outside the submitted work. R Passera reports Participation on a Data Safety Monitoring Board or Advisory Board with the non-profit clinical trial “Consolidation with ADCT-402 (loncastuximab tesirine) after immunochemotherapy: a phase II study in BTKi-treated/ineligible Relapse/Refractory Mantle Cell Lymphoma (MCL) patients” - sponsor FIL, Fondazione Italiana Linfomi, Alessandria-I (unpaid role); leadership or fiduciary role in other board, society, committee or advocacy group, paid or unpaid, Member of the Statistical Committee of the EBMT – European Society for Bone and Marrow Transplantation, Paris-F (unpaid role); outside the submitted work. A E Peden reports support for the present manuscript from the [Australian] National Health and Medical Research Council (Grant Number: APP2009306). V C F Pepito reports grants or contracts from Sanofi Consumer Healthcare and the International Initiative for Impact Evaluation; outside the submitted work. M Pigeolet reports a grant from the Belgian Kids' Fund for Pediatric Research, outside the submitted work. T Pilgrim reports grants paid to the institution without personal remuneration from Biotronik, Edwards Lifesciences, and ATSens; Payment or honoraria for lectures, presentations, speakers bureaus, manuscript writing or educational events from Biotronik, Boston Scientific, Edwards Lifesciences, Abbott, Medtronic, Biosensors, and Highlife; Participation on a Data Safety Monitoring Board or Advisory Board for EMPIRE study sponsored by Biosensors; and receipt of equipment (AT-Patches) from ATSens; outside the submitted work. D Prieto-Alhambra reports support for the present manuscript from European Medicines Agency and Innovative Medicines Initiative, through their institution; grants or contracts from Amgen, Chiesi-Taylor, Lilly, Janssen, Novartis, and UCB Biopharma through their institution; consulting fees from Astra Zeneca and UCB Biopharma; other financial or non-financial interest in Amgen, Astellas, Janssen, Synapse Management Partners and UCB Biopharma for supported training programmes; outside the submitted work. A Radfar reports support for the present manuscript from Avicenna Medical and Clinical Research Institute. A Rane reports stock or stock options as a full-time employee at Agios Pharmaceuticals; outside the submitted work. L F Reyes reports grants or contracts form MSD and Pfizer; consulting fees from GSK, MSD, and Pfizer; Payment or honoraria for lectures, presentations, speakers' bureaus, manuscript writing or educational events from GSK and Pfizer; payment for expert testimony from GSK and MSD; support for attending meetings and/or travel from GSK; outside the submitted work. T G Rhee reports grants or contracts from the NIH (R21AG070666; R21DA057540; R21AG078972; R01MH131528; R01AG080647); outside the submitted work. S Sacco reports grants or contracts from Novartis and Uriach; consulting fees from Novartis, Allergan-Abbvie, Teva, Lilly, Lundbeck, Pfizer, Novo Nordisk, Abbott, AstraZeneca; Payment or honoraria for lectures, presentations, speakers bureaus, manuscript writing or educational events from Novartis, Allergan-Abbvie, Teva, Lilly, Lundbeck, Pfizer, Novo Nordisk, Abbott, AstraZeneca; support for attending meetings and/or travel from Lilly, Novartis, Teva, Lundbeck; leadership or fiduciary role in other board, society, committee or advocacy group, paid or unpaid, as the President elect of the European Stroke Organization, and the Second vice-president of the European Headache Federation; receipt of equipment, materials, drugs, medical writing, gifts or other services from Allergan-Abbvie, Novo Nordisk; all outside the submitted work. P Sachdev reports grants or contracts from national Health and Medical Research Council of Australia and the US National Institutes of Health; Payment or honoraria for lectures from Alkem Labs for the Frontiers of Psychiatry June 2023 Seminar, Mumbai, India; Participation on a Data Safety Monitoring Board or Advisory Board with Biogen Australia and Roche Australia; leadership or fiduciary role in other board, society, committee or advocacy group, unpaid, with the VASCOG Society and the World Psychiatric Association; all outside the submitted work. Y L Samodra reports grants or contracts from Taipei Medical University; leadership or fiduciary role in other board, society, committee or advocacy group, paid or unpaid, with the Benang Merah Research Center; all outside the submitted work. J Sanabria reports support for attending meetings and/or travel from the Department of Surgery, Marshall University School of Medicine; three patents pending; participation in quality assessment and assurance for surgeries of the Department of Surgery; leadership or fiduciary role in other board, society, committee or advocacy group, paid or unpaid with SSAT, ASTS, AHPBA, IHPBA, and AASLD; all outside the submitted work. N Scarmeas reports grants or contracts with Novo Nordisk as the Local PI of recruiting site for multinational, multicenter industry sponsored phase III treatment trial for Alzheimer's disease with funding paid to the institution; Participation on a Data Safety Monitoring Board or Advisory Board with Albert Einstein College of Medicine (NIH funded study) as the Chair of Data Safety Monitoring Board; all outside the submitted work. A E Schutte reports Speaker Honoraria from Servier, Novartis, Sanofi, Medtronic, Abbott, Omron, Aktiia; Support for attending meetings and/or travel from Servier, Medtronic, and Abbott; Participation on a Data Safety Monitoring Board or Advisory Board with Abbott Pharmaceuticals Advisory Board, Skylabs devices Advisory Board; Leadership or fiduciary role in other board, society, committee or advocacy group, paid or unpaid, with Co-Chair: National Hypertension Taskforce of Australia, Board Member: Hypertension Australia, Company Secretary: Australian Cardiovascular Alliance; all outside the submitted work. B M Schaarschmidt reports research grants from Else Kröner-Fresenius Foundatuin, DFG, and PharmaCept; Payment or honoraria for lectures, presentations, speakers bureaus, manuscript writing or educational events from AstraZeneca; support for attending meetings and/or travel from Bayer AG; all outside the submitted work. M Šekerija reports consulting fees from Roche; Payment or Honoraria for lectures, presentations, speakers bureaus, manuscript writing or educational events from Johnson and Johnson, and Astellas; outside the submitted work. A Sharifan reports leadership or fiduciary role in other board, society, committee or advocacy group, unpaid with Cochrane as a steering member of the Cochrane Early Career Professionals Network; and receipt of thirty days of complimentary access to ScienceDirect, Scopus, Reaxys, and Geofacets after reviewing manuscripts for two journals published by Elsevier; outside the submitted work. S Sharma reports support for the present manuscript from the John J. Bonica Postdoctoral Fellowship from the International Association for the Study of Pain (IASP; 2021-2023); Payment or honoraria for lectures, presentations, speakers bureaus, manuscript writing or educational events and a travel grant for delivering a talk on “Technologies for pain education in developing countries” conducted by the Pain Education SIG of the IASP at the World Pain Congress in Toronto (2022); outside the submitted work. V Sharma reports other financial or non-financial support from DFSS (MHA)'s research project (DFSS28(1)2019/EMR/6) at Institute of Forensic Science & Criminology, Panjab University, Chandigarh, India, outside the submitted work. K Shibuya reports support for the present manuscript from Tokyo Foundation for Policy Research. V Shivarov reports one patent and one utility model with the Bulgarian Patent Office; stock or stock options from ICONplc (RSUs); and other financial interests from an ICONplc salary; all outside the submitted work. S Shrestha reports other financial interests from the Graduate Research Merit Scholarship from the School of Pharmacy at Monash University Malaysia, outside the submitted work. J P Silva reports support for the present manuscript from the Portuguese Foundation for Science and Technology through payment of their salary (contract with reference 2021.01789.CEECIND/CP1662/CT0014). L M L R Silva reports grants or contracts from CENTRO-04-3559-FSE-000162, Fundo Social Europeu (FSE), outside the submitted work. C R Simpson reports grants or contracts from MBIE (NZ), HRC (NZ), Ministry of Health (NZ), MRC (UK), and CSO (UK); Leadership or fiduciary role in other board, society, committee or advocacy group, paid or unpaid with the New Zealand Government Data Ethics Advisory Group as the Chair; outside the submitted work. D J Stein reports consulting fees from Discovery Vitality, Johnson & Johnson, Kanna, L'Oreal, Lundbeck, Orion, Sanofi, Servier, Takeda, and Vistagen, outside the submitted work. K Stibrant Sunnerhagen reports Leadership or fiduciary role in other board, society, committee or advocacy group, paid or unpaid as the head of the scientific committee of the Sweidhs Stroke Foundation; outside the submitted work. S Stortecky reports grants or contracts paid to their institution from Edwards Lifesciences, Medtronic, Abbott, and Boston Scientific; consulting fees from Teleflex; Payment or honoraria for lectures, presentations, speakers bureaus, manuscript writing or educational events from Boston Scientific/BTG; outside the submitted work. A G Thrift reports grants or contracts paid to their institution from the National Health & Medical Research Council (Australia) (grant numbers 1171966, 1182071), Heart Foundation (Aus) and the Stroke Foundation (Australia); outside the submitted work. J H V Ticoalu reports Leadership or fiduciary role in other board, society, committee or advocacy group, paid or unpaid, with Benang Merah Research Center as co-founder, outside the submitted work. M V Titova reports support for the present manuscript from the Ministry of Science and Higher Education of the Russian Federation (theme No. 122042600086-7). S J Tromans reports grants or contracts from the 2023 Adult Psychiatric Morbidity Survey team, collecting epidemiological data on community-based adults living in England. This is a contracted study from NHS Digital, via the Department of Health and Social Care; outside the submitted work. P Willeit reports consulting fees from Novartis; outside the submitted work. M Zielińska reports other financial interest as an AstraZeneca employee, outside the submitted work. A Zumla reports grants or contracts from The Pan-African Network on Emerging and Re-Emerging Infections (PANDORA-ID-NET, CANTAM-3, and EACCR-3) funded by the European and Developing Countries Clinical Trials Partnership, the EU Horizon 2020 Framework Programme, UK National Institute for Health and Care Research Senior Investigator, and Mahathir Science Award and EU-EDCTP Pascoal Mocumbi Prize Laureate; Participation on a Data Safety Monitoring Board or Advisory Board member of the WHO Mass Gatherings Expert Group and WHO Health Emergencies Programme in Geneva, a member of the EU-EDCTP3-Global Health (Brussels) Scientific Committee; all outside the submitted work.
