
JOURNAL INFORMATION
==============================
NLM Title Abbreviation: Lancet
Journal ID: Lancet
NLM Journal ID: 2985213R

Lancet (London, England)

ISSN: 0140-6736
EISSN: 1474-547X

ARTICLE INFORMATION
==============================
PMCID: PMC11120180
PMID: 38648809
DOI: 10.1016/S0140-6736(24)00824-9
Article ID: S0140-6736(24)00824-9
Article version: 1
Subjects: Department of Error

Department of Error

Print publication date: 2024 May 18
Volume: 403
Issue: 10440
First page: 1988
Last page: 1988
Copyright: © 2024 The Author(s). Published by Elsevier Ltd.
Copyright year: 2024
Copyright holder: Elsevier Ltd
License: This is an open access article under the CC BY license (http://creativecommons.org/licenses/by/4.0/).
License URL: https://creativecommons.org/licenses/by/4.0/

Erratum for: Global burden of 288 causes of death and life expectancy decomposition in 204 countries and territories and 811 subnational locations, 1990–2021: a systematic analysis for the Global Burden of Disease Study 2021 403 10440 18 5 2024 2100 2132 Lancet (London, England) 10.1016/S0140-6736(24)00367-2 PMC11126520 38582094

==============================
GBD 2021 Causes of Death Collaborators. Global burden of 288 causes of death and life expectancy decomposition in 204 countries and territories and 811 subnational locations, 1990–2021: a systematic analysis for the Global Burden of Disease Study 2021. Lancet 2024; published online April 3. https://doi.org/10.1016/S0140-6736(24)00367-2—In this Article, the key in figure 1 has been corrected, the URL for the GBD data sources has been corrected, and the appendix has been updated. These changes have been made to the online version as of April 19, 2024 and the printed version is correct.

