# Supplementary material for: Global burden of 288 causes of death and life expectancy decomposition in 204 countries and territories and 811 subnational locations, 1990–2021: a systematic analysis for the Global Burden of Disease Study 2021
Source: Lancet. 2024 May 18;403(10440):2100–32. doi: 10.1016/S0140-6736(24)00367-2 (PMC11126520; doi:10.1016/S0140-6736(24)00367-2)
Supplement: Supplementary appendix 1 [file mmc1.pdf]

THE LANCET

Supplementary appendix

This appendix formed part of the original submission and has been peer reviewed. We post it as supplied by the authors.

This online publication has been corrected. The corrected version first appeared at thelancet.com on April 19, 2024

Supplement to: GBD 2021 Causes of Death Collaborators. Global burden of 288 causes of death and life expectancy decomposition in 204 countries and territories and 811 subnational locations, 1990–2021: a systematic analysis for the Global Burden of Disease Study 2021. *Lancet* 2024; published online April 3. https://doi.org/10.1016/S0140-6736(24)00367-2.

Appendix 1: methods appendix to "Global burden of 288 causes of death and life expectancy decomposition in 204 countries and territories and 811 subnational locations, 1990–2021: a systematic analysis for the Global Burden of Disease Study 2021"

This appendix provides further methodological detail for "Global burden of 288 causes of death and life expectancy decomposition in 204 countries and territories and 811 subnational locations, 1990–2021: a systematic analysis for the Global Burden of Disease Study 2021."

Preamble

This appendix provides further methodological detail for "Global burden of 288 causes of death and life expectancy decomposition in 204 countries and territories and 811 subnational locations, 1990–2021: a systematic analysis for the Global Burden of Disease Study 2021." This study complies with the Guidelines for Accurate and Transparent Health Estimates Reporting (GATHER) recommendations.[1] It includes detailed tables and information on data in an effort to maximise transparency in our estimation processes and provide a comprehensive description of analytical steps. We intend this appendix to be a living document, to be updated with each iteration of the Global Burden of Disease Study.

Portions of this appendix have been reproduced or adapted from appendices for GBD 2017 Causes of Death Collaborators,[2] and GBD 2019 Demographics Collaborators.[3] References are provided for reproduced or adapted sections.

Table of Contents

Preamble 2

Section 1: List of appendix figures and tables 5

Section 2: GBD overview 6
- Section 2.1: Geographical locations of the analysis 6
- Section 2.2: Time period of the analysis 6
- Section 2.3: GBD cause list 6
- Section 2.4: Statement of GATHER compliance 7
- Section 2.5: Data input sources overview 7
- Section 2.6: Funding sources 7
- Section 2.7: Abbreviations 7

Section 3: GBD 2021 causes of death database 8
- Section 3.1: CoD data identification 8
- Section 3.2: Verbal autopsy[2] 10
- Section 3.3: Standardise input data (step 1)[2] 13
- Section 3.4: Map to GBD cause list (step 2)[2] 15
- Section 3.5: Age-sex splitting (step 3)[2] 16
- Section 3.6: Correction for miscoding of Alzheimer's and other dementias, Parkinson's disease, and atrial fibrillation and flutter (step 4)[3] 18
- Section 3.7: Redistribute (Step 5)[2] 19
- Section 3.8: HIV/AIDS misclassification correction (step 6)[2] 24
- Section 3.9: Scale strata to province (step 7)[2] 25
- Section 3.10: Restrictions post-redistribution (step 8)[2] 26
- Section 3.11: Drop VR country-years or mark as non-representative (step 9)[2] 26
- Section 3.12: Cause aggregation (step 10)[2] 26
- Section 3.13: Remove shocks and HIV/AIDS maternal adjustments (step 11)[2] 26
- Section 3.14: Noise reduction (step 12)[2] 30
- Section 3.15: Cause of death database and outlier identification (step 13)[2] 33
- Section 3.16: Causes of death data star-rating calculation[2] 33

Section 4: Causes of death modelling methods 35
- Section 4.1: CODEm[2] 35
- Section 4.2: Causes modelled outside of CODEm[2] 37

Section 5: COVID-19 and OPRM estimation 41
- Section 5.1: Excess mortality due to the COVID-19 pandemic 41
- Section 5.2: Excess mortality age-sex splitting 42
- Section 5.3: Total COVID-19 mortality estimation 43

Section 5.4: Balancing equation to calculate OPRM ... 43
Section 5.5: COVID-19, OPRM, and excess mortality harmonisation ... 43
Section 6: Central computation[2] ... 43
Section 6.1: Imported cases ... 43
Section 6.2: CoDCorrect ... 44
Section 6.3: Years of life lost calculation ... 45
Section 6.4: GBD world population age standard ... 45
Section 7: Life expectancy decomposition ... 45
Section 8: References ... 48
Section 9: Authors' Contributions ... 52
Managing the overall research enterprise ... 52
Writing the first draft of the manuscript ... 52
Primary responsibility for applying analytical methods to produce estimates ... 52
Primary responsibility for seeking, cataloguing, extracting, or cleaning data; designing or coding figures and tables ... 52
Providing data or critical feedback on data sources ... 52
Developing methods or computational machinery ... 56
Providing critical feedback on methods or results ... 56
Drafting the work or revising it critically for important intellectual content ... 64
Managing the estimation or publications process ... 70
Section 10: Tables and figures ... 71
Section 11: CoD cause-specific modelling descriptions ... 316

Section 1: List of appendix figures and tables

See section 10 for all the appendix figures and tables listed here.

Appendix figures:

Appendix Figure S1. Analytical flowchart for the development of the GBD 2021 cause of death database (A) and different strategies used to model different causes (B) and ultimately combine them into a consistent set of cause-specific deaths for each location, age, sex, and year

Appendix Figure S2. GBD 2021 causes of death estimation flowchart by modelling group

Appendix Figure S3. Vital registration and verbal autopsy data availability by country, 1980−2021

Appendix Figure S4. Percentage of vital registration deaths assigned to major garbage codes for all ages and sexes by country, 1980−2021

Appendix Figure S5a. Classification of national time series of vital registration and verbal autopsy data, 1980−2021

Appendix Figure S5b. Classification of national time series of vital registration and verbal autopsy data, 2010−2021

Appendix Figure S6: Out-of-sample model performance for CODEm models for GBD 2021 and age-standardised cause-specific mortality rate by Level 2 causes

Appendix tables:

Appendix Table S1. GBD location hierarchy with levels

Appendix Table S2. GBD cause hierarchy with levels

Appendix Table S3. CoDCorrect cause hierarchy with levels

Appendix Table S4. GATHER checklist

Appendix Table S5. Total number of site-years by cause and source type for GBD 2021

Appendix Table S6. List of International Classification of Diseases (ICD) codes mapped to the GBD cause list for causes of death

Appendix Table S7. Restrictions on age and sex by cause for GBD 2021

Appendix Table S8. HIV/AIDS-related garbage code redistribution packages

Appendix Table S9. Underlying indicators for percentage well-certified for data source with maximum percentage well-certified in each five-year time interval for 204 countries, 1980−2020

Appendix Table S10. Modelling strategy for individual cause of death models in GBD 2021

Appendix Table S11. Percentage change before and after CoDCorrect by cause for all ages, both sexes, global, 2021

Appendix Table S12. CODEm covariates used, level of covariate, and expected direction of covariate by cause, sex, and age

Appendix Table S13. CODEm predictive validity results by cause, model type, sex, and age

Appendix Table S14: Comparison of GBD 2019 and GBD 2021 covariates and level of covariates used in cause of death modelling

Appendix Table S15: Theoretical minimum risk life table, which is a life table composed of the minimum mortality rate observed across GBD national locations with population at least 5 million, from 2010 to 2021.

Section 2: GBD overview

Section 2.1: Geographical locations of the analysis

We produced estimates for 204 countries and territories that were grouped into 21 regions and seven super-regions (table S1). The seven super-regions are central Europe, eastern Europe, and central Asia; high income; Latin America and the Caribbean; north Africa and the Middle East; south Asia; southeast Asia, east Asia, and Oceania; and sub-Saharan Africa. In GBD 2021 we continue to analyse at subnational levels countries that were added in previous cycles including Brazil, China, Ethiopia, India, Indonesia, Iran, Italy, Japan, Kenya, Mexico, New Zealand, Nigeria, Norway, Pakistan, Russia, the Philippines, Poland, South Africa, Sweden, the UK, and the USA. All analyses are at the first level of administrative organisation within each country except for New Zealand (by Māori ethnicity), Sweden (by Stockholm and non-Stockholm), the UK (by local government authorities), and the Philippines (by provinces). To meet data use requirements, in this publication we present subnational estimates for Brazil, Ethiopia, Indonesia, Iran, Japan, Kenya, Mexico, Norway, Pakistan, South Africa, Sweden, the UK, and the USA; given space constraints, these results are presented in Appendix 2 instead of the main text. Additionally, subnational estimates for China, India, Nigeria, and Russia are included in maps but are not reported in appendix tables. Subnational estimates for other countries will be released in separate publications.

At the most detailed spatial resolution, we generated estimates for 983 unique locations. As was done in GBD 2019, in GBD 2021 we continue to use the set of locations defined as standard locations and non-standard locations. Standard GBD locations are defined as the set of all subnationals belonging to countries where data quality is high and with populations over 200 million, in addition to all other countries. Standard locations include the subnationals for China, India, the USA, and Brazil, but not Indonesia; data for China, India, the USA, and Brazil are also included at the country level. All other countries with subnational estimates are defined as non-standard locations.

Section 2.2: Time period of the analysis

We estimated numbers and rates of incidence, prevalence, years lived with disability (YLDs), and disability-adjusted life-years (DALYs) for the years 1990–2021; we estimated deaths and years of life lost (YLLs) for 1980–2021.

Section 2.3: GBD cause list

The GBD cause and sequelae list for causes of death is organised hierarchically (see table S2) to accommodate different purposes and needs of various users.

The first two levels aggregate causes into general groupings. At Level 1 there are three cause groups: communicable, maternal, neonatal, and nutritional diseases (Group 1 diseases); non-communicable diseases (Group 2); and injuries (Group 3). These Level 1 aggregates are subdivided at Level 2 of the hierarchy into 22 cause groupings (eg, neonatal disorders, neurological disorders, and transport injuries). The disaggregation into Levels 3 and 4 contains the finest level of detail for causes captured in GBD 2021. The greatest detail available for some causes, such as anxiety disorders or rheumatoid arthritis, is at Level 3 of the hierarchy, while other specific causes are at Level 4 of the hierarchy with an aggregate category at Level 3 (for example, depressive disorders at Level 3, which encompasses major depressive disorders and dysthymia at Level 4). Sequelae of diseases and injuries are organised at Levels 5 and 6 of the hierarchy. In GBD, sequelae are defined as distinct, mutually exclusive categories of health consequences that can be directly attributed to a cause. For example, both neuropathy and blindness due to diabetic retinopathy are sequelae of diabetes; stroke and ischaemic heart disease are not, as these consequences cannot be categorically ascribed to diabetes in an individual despite good evidence for increased risk of these outcomes. The finest detail for all sequelae estimated in GBD is at Level 6 and is aggregated into summary sequelae categories (Level 5) for causes with large numbers of sequelae. Examples include the grouping of the infectious disease episodes and long-term sequelae of meningitis. For GBD 2021 there are 3499 mutually exclusive and collectively exhaustive sequelae, 2089 cause sequelae, and 1410 injuries sequelae, and thus our YLD estimates at

each level of the hierarchy sum to the total of the level above. Prevalence and incidence aggregation is estimated at the level of individuals who may have more than one sequela or disease and therefore are not additive.

The GBD cause list continues to evolve to reflect the policy relevance and public health and medical care importance of the causes of major losses of health. The cause and sequelae list expanded based on input from the Scientific Council and GBD Collaborator Network. For GBD 2021, the causes of death cause list has increased to 288 causes, from the 286 causes in GBD 2019. The non-fatal cause list has expanded from 364 causes in GBD 2019 to 365 causes in GBD 2021. The total number of fatal and non-fatal causes combined for GBD 2021 is 371. As in GBD 2019, we made no estimates for YLDs for just five causes, either because no disability is possible (as is the case with sudden infant death syndrome); because disability may occur rarely but at levels too low for accurate estimation given the data (as for aortic aneurysm); or because the disability is captured by the complicating causes that led to that cause of death (as for indirect maternal deaths, late maternal deaths, and maternal deaths aggravated by HIV/AIDS).

Section 2.4: Statement of GATHER compliance

This study complies with the Guidelines for Accurate and Transparent Health Estimates Reporting (GATHER) recommendations.[1] We have documented the steps in our analytical procedures and detailed the data sources used. See appendix table S4 for the GATHER checklist. The GATHER recommendations can be found at the GATHER website under GATHER Statement.

Section 2.5: Data input sources overview

GBD 2021 synthesises a large and growing number of data input sources including surveys, censuses, vital statistics, and other health-related data sources. The data from these sources are used to estimate morbidity; illness, and injury; and attributable risk for 204 countries and territories from 1990 to 2021; cause-specific mortality is estimated from 1980 to 2021. The input sources are accessible through an interactive citation tool available in the GHDx.

Citations for specific GBD components, causes and risks, and locations can be found through the GBD Sources Tool in the GHDx: http://ghdx.healthdata.org/gbd-2021/sources. This tool allows users to view and access GHDx records for input sources and export a comma-separated value (CSV) file that includes metadata, citations, and information about where the data were used in GBD, as data use agreements allow. As required by GATHER, additional metadata for input sources are available through the citation tool as well.

Section 2.6: Funding sources

This publication and the research it presents was funded by the Bill & Melinda Gates Foundation; Queensland Department of Health, Australia; the National Health and Medical Research Council, Australia; Public Health England; the Norwegian Institute of Public Health; St. Jude Children's Research Hospital; the Cardiovascular Medical Research and Education Fund; the National Institute on Aging of the National Institutes of Health (award P30AG047845); and the National Institute of Mental Health of the National Institutes of Health (award R01MH110163). The funders of the study had no role in study design, data collection, data analysis, data interpretation, or writing of the report. All authors had full access to all data in the study and had final responsibility for the decision to submit for publication.

Section 2.7: Abbreviations

ART	antiretroviral therapy
BTL	basic tabulation list
CDC	United States Centers for Disease Control & Prevention
CHAMPS	Child Health and Mortality Prevention Surveillance
CoD	causes of death
CODEm	Cause of Death Ensemble modelling
CRVS	civil registration and vital statistics system
DALYs	disability-adjusted life-years

DHS	Demographic and Health Survey
DisMod-MR	disease model-Bayesian meta-regression
DSP	disease surveillance points
EMR	excess mortality rate
EUREG	European Registry
GATHER	Guidelines for Accurate and Transparent Health Estimates Reporting
GBD	Global Burden of Diseases, Injuries, and Risk Factors Study
GHDx	Global Health Data Exchange
GPR	Gaussian process regression
HDSS	Hierarchical Data Storage System
ICD	International Classification of Diseases
IHME	Institute for Health Metrics and Evaluation
INDEPTH	International Network for the Demographic Evaluation of Populations and their Health
InterVA	Interpreting Verbal Autopsy
LMER	linear mixed effects regression
MCCD	Medical Certification of Causes of Death
MITS	minimally invasive tissue sample
MMR	maternal mortality ratio
NORDCAN	database of cancer statistics for the Nordic countries
OPRM	other pandemic-related mortality
PAF	population attributable fraction
PCVA	Physician-Certified Verbal Autopsy
PHMRC	Population Health Metrics Research Consortium
PWC	piecewise continuous
RSME	root mean square error
SD	standard deviation
SRS	Sample Registration System
ST-GPR	spatiotemporal Gaussian process regression
UI	uncertainty interval
UNAIDS	Joint United Nations Programme on HIV and AIDS
UN	United Nations
USA	United States of America
USSR	Union of Soviet Socialist Republics
VA	verbal autopsy
VR	vital registration
WHO	World Health Organization
YLDs	years lived with disability
YLLs	years of life lost

Section 3: GBD 2021 causes of death database

All available data on causes of death (CoD) are standardised and pooled into a single database used to generate cause-specific mortality estimates by age, sex, year, and geography. Appendix figures 1 and 2 show the high-level view of data inputs, analytical steps, and outputs of the CoD analysis framework. Section 3 of this appendix provides details on each step in the development of the CoD database as illustrated in appendix figure 1.

Section 3.1: CoD data identification[2]

Section 3.1.1: Overview of data types

The CoD database contains eight types of data sources (table S5): vital registration (VR), verbal autopsy (VA), cancer registry, police records, sibling history, surveillance, survey/census, and minimally invasive tissue sample (MITS)

diagnoses. In countries with VR systems with high completeness and low garbage coding, vital registration is often the primary source of data for causes of death, but police data, surveillance data, and open-source databases are also used for select causes, such as injuries, violence, and maternal causes of death. Less than half of the world's population has deaths captured in a VR system; therefore, in countries with incomplete VR systems, vital statistics for causes of death may be supplemented with other data types (appendix figure 3).

Section 3.1.2: ICD detail

A majority of the CoD data are VR data obtained from the World Health Organization (WHO) Mortality Database, a compilation of data submitted to WHO by individual countries. VR is also obtained from country-specific mortality databases operated by official offices. Each cause is coded directly to the three- or four-digit ICD-coded cause of death when possible, whereas cause codes in data tabulated by International Classification of Disease (ICD) are coded to aggregated cause groups. The CoD database contains 2608 country-years of detailed data from 1980 to 2018, which includes underlying CoD coded with three- to four-digit codes by country, year, sex, and age groups. Detailed causes are coded to one of the following ICD-detail coding systems: ICD-8, ICD-9, or ICD-10 (table S6). Each coding system has a similar cause hierarchy and cause list that has continually developed over time. ICD-10 is the current standard and the most exhaustive cause list. Within the cause lists, five-digit codes, where available, are truncated to four-digit codes to condense the lists. Updates to ICD-detail occur biannually as WHO releases new versions or as country collaborators provide additional data. Updates to data from WHO increasingly include ICD-10 CoD data as it is the most current classification of CoD, while updates to ICD-8 and ICD-9 detailed lists are less common. In the case of overlapping data, preference is given to data from pre-determined country collaborations, which are updated annually.

Section 3.1.3: ICD tabulations list

The ICD tabulation lists include the ICD-8 List A (ICD-8A and ICD-8B), ICD-9 Basic Tabulation List (BTL), ICD-10 Mortality Tabulation, Russia Tabulation; the former Union of Soviet Socialist Republics (USSR) Tabulation, Russia Tabulation from 1989-2001, Russia Tabulation after 2001, and India Medical Certification of Cause of Death (MCCD) and China Disease Surveillance Points (DSP) ICD-9 and ICD-10. These data sources make up 1096 country-years from 1980 to 2017 in the CoD database. All are condensed versions of the ICD-8, ICD-9, and ICD-10 detail lists with some differences in the format of cause lists depending on the data source. ICD-8A, ICD-9 BTL, and ICD-10 Mortality Tabulation CoD are assigned to subtotal groups (referred to as chapters) and cause groups respective to ICD-detail groups. Additionally, ICD-9 BTL includes ICD-9 detail codes for some cancers and a custom tabulation scheme for the former USSR countries. The Russia Tabulation lists and India MCCD cause lists each have custom nomenclatures based on ICD-detail cause codes.

Two of the drawbacks in using tabulation lists are discrepancies in the accuracy of death counts and lack of detail due to aggregated cause groups. There are instances where the sum of deaths in chapter subtotals are not equal to the sum of cause groups within the chapter. To account for any missing or duplicate deaths reported within the cause groupings, death counts are systematically adjusted by calculating the differences between subtotals and sub-causes within the cause groups. Any differences are assigned to a remainder cause group. To account for the lack of cause code detail, select cause groups are disaggregated (Step 1.1) to create a complete cause list. Updates to ICD tabulation lists obtained from WHO occur less frequently compared to ICD-detailed lists as more countries are reporting deaths in ICD-detail. In instances of overlapping data, preference is given first to detailed collaborator data, followed by detailed WHO data, then tabulated collaborator data, and finally tabulated WHO data.

Section 3.1.4: China Disease Surveillance Points /China Center for Disease Control and Prevention

The two primary sources of data for China are surveillance data from the China Disease Surveillance Points (DSP) system and VR data collected by the Chinese Center for Disease Control and Prevention (CDC). In the China DSP data, deaths were reported across 145 disease surveillance points used from 1991 to 2003, 161 disease surveillance points from 2004 to 2012, and 605 disease surveillance points from 2013 to 2017. While China DSP with ICD-10 coding is considered sample VR data, it provides national coverage and cause detail. Thus, it receives similar processing and treatment to the China CDC VR from 2008 to 2016. From 2008 to 2017, all of the deaths and CoD information from the DSP system and other system points throughout China were collected and reported via the Mortality Registration and Reporting System, an online reporting system of the Chinese CDC. We process DSP using

a country-specific cause map. For DSP ICD-9, which contains aggregated causes, we use a tabulated cause map. For DSP ICD-10, we disaggregate causes using the ICD code detail in the CDC data. The deaths in these data are reported at the strata level, a metric that is specific to China. Counties are stratified by urban and rural classification, but definitions of urbanity vary across counties. In Step 7, we use a method developed to scale up deaths from strata level to the province level.

Section 3.1.5: Sample registration system

Sample registration systems are expanding in several countries and are key sources of data in Indonesia and India. The Sample Registration System (SRS) is a dual-record system wherein a resident part-time enumerator continuously records births and deaths in each household within the sample unit every month. A full-time SRS supervisor thereafter independently collects the vital events along with other related details for each of the preceding six-month periods during the calendar year. Cause of death in SRS systems is recorded using either the detailed ICD-10 coding list, or the INDEPTH ICD-10 short list.[4]

Section 3.1.6: India Medical Certification of Cause of Death

The India MCCD has data for the urban parts of the majority of the states and union territories beginning in 1980. Deaths reported in this data source have been medically certified and are considered VR data. The CoD are reported in a tabulation list with a unique numbering scheme that conforms to ICD-9 and ICD-10 detail codes, which must be disaggregated. MCCD is state-split to fill in data gaps (Step 1.2 state splitting) prior to age-sex splitting. Because SRS is widely considered a more credible assessment of CoD in India, we chose to use MCCD data only in certain cases for modelling with Cause of Death Ensemble modelling (CODEm). We preserved MCCD data in the database for two primary reasons. First, where the three midpoint years of SRS data resulted in the loss of a clear time trend, as was the case for maternal mortality, we chose to preserve MCCD in addition to SRS. Second, MCCD has an advantage over SRS in cases where VA is not a valid instrument for ascertaining CoD, like encephalitis and dengue fever. In these cases, we kept MCCD over SRS.

Section 3.2: Verbal autopsy[2]

Section 3.2.1: Verbal autopsy coded to ICD-10 and other lists

In countries without VR systems, VA studies are a viable data source to inform CoD. Data are obtained by trained interviewers who use a standardised questionnaire to ask relatives about the signs, symptoms, and demographic characteristics of family members deceased within a year. CoD is assigned based on the answers to the questionnaires using a variety of methods, explained in more detailed in section 3.2.2.

VA data are highly heterogeneous: studies use different instruments, different cause lists (from single causes to full ICD cause lists), different methods for assigning CoD, different recall periods, and different age groups. Cultural differences may also affect the interpretation of specific questions. VAs are likely accurate in assigning CoD to road injury or homicide but less accurate for causes requiring medical certification, such as cardiovascular causes. Studies may also occur as standalone assessments or as part of an extended network, such as the International Network for the Demographic Evaluation of Populations and their Health (INDEPTH) Network[4]— a continuous surveillance source with several Demographic Surveillance Systems sites that collect data coded to ICD-detail causes.

Section 3.2.2: Methods of ascertaining cause of death from VA questionnaires

Verbal autopsy (VA) is one of the most common tools used to obtain data on causes of death in countries that lack a functional civil registration and vital statistics (CRVS) system. As such, a wide variety of methods have been developed for ascertaining cause of death based on a VA interview. The historical standard and still one of the most popular methods is Physician-Certified Verbal Autopsy (PCVA), in which two physicians review the interview responses and formulate the most likely cause of death independently. A third physician may arbitrate the result if there is disagreement. This method has some important advantages, including incorporating the knowledge and expertise of local physicians, but is often expensive and slow, includes the variability inherent to human judgement, and has a high opportunity cost in terms of physicians' time spent delivering health care.

In response to these challenges, a variety of automated CoD ascertainment methods have been developed based on algorithmic or probabilistic approaches.[5,6] Two popular automated methods are Tariff 2.0/SmartVA and Interpreting Verbal Autopsy (InterVA). The Tariff method is an algorithm designed and validated by the Population Health Metrics Research Consortium (PHMRC) and IHME. It is based on the association between individual signs and symptoms and causes of death and is trained on the PHMRC gold standard validation study database, a database of VAs collected for deaths that are linked to hospital records in India, the Philippines, Mexico, and Tanzania, where the hospital records provide the true cause of death.[7] The InterVA (Interpreting Verbal Autopsy) method is a Bayesian method, based on the probability of answering yes to a given line item conditional on the true cause of death. These conditional probabilities are determined using a mix of expert opinion and reference data.[8]

A 2014 study by the PHMRC and IHME found that InterVA showed markedly lower performance in predicting causes of death in the PHMRC gold standard validation database when compared to three other automated CoD ascertainment methods, including Tariff.[9] As a result, we exclude all InterVA-modelled VAs from our analysis, except for InterVA data on injuries and maternal causes, which are easier for most VA CoD ascertainment methods to diagnose.

Each GBD round, a systematic review of verbal autopsy literature data is conducted to ascertain and include new VA studies. We perform two searches, one using PubMed and one through Google Scholar. The PubMed search typically returns fewer articles, while Google Scholar returns more articles but tends to be less precise for capturing raw VA data.

The search strings for the literature search are below.

Google Scholar search strings:

"verbal autopsy" AND ("cause of death" OR "causes of death" OR "reason for death" OR "reasons for death" OR "cause-specific mortality"), 2019-2019, 2020-2020

PubMed search strings:

("verbal autopsy") AND ("2019/04/24"[Date - Publication] : "3000"[Date - Publication])

Inclusion criteria:

- Mentions verbal autopsy
- Mentions causes of death or cause fractions
- References data collection or raw/crude data
- Data must provide a viable, non-biased CoD distribution at an acceptable GBD location level: for example, we do not use global or regional data.

Exclusion criteria:

- Studies already included in previous rounds of GBD
- Non-representative demographics, aka "sub-populations" (eg, HIV-positive people, ICU/NICU patients)
    - This does not include non-representative locations, eg, certain villages, cities, provinces, states, etc. within a GBD location
- Hospital-based sampling
- Methods/theory papers
- Review papers
    - We never extract from review papers, but instead trace the underlying studies and extract the dataset from those

- Papers published by the GBD
- Protocols
- Papers that only report on stillbirths
- Papers that cover exclusively pre-1980
- VA that uses the InterVA assignment method (except for injury and maternal-related deaths)
- Small sample sizes (<50 deaths)
    - Some exceptions have been made for studies from data-sparse countries
- Deaths for one cause without a denominator, for example, only cancer deaths without total deaths
- National level where we model subnationally (eg, South Africa, India, Indonesia)
- Locations reported that are higher than country level (eg, results for several countries together cannot be used)
- Adjusted/modelled estimates (ie, not raw counts) – if the numbers have uncertainty intervals, this is a good sign that they are actually modelled estimates
- Studies where the denominator is not "all deaths due to all causes," eg, datasets where the denominator is all tuberculosis deaths

Figure A. PRISMA diagram for systematic review of verbal autopsy data included in GBD 2021

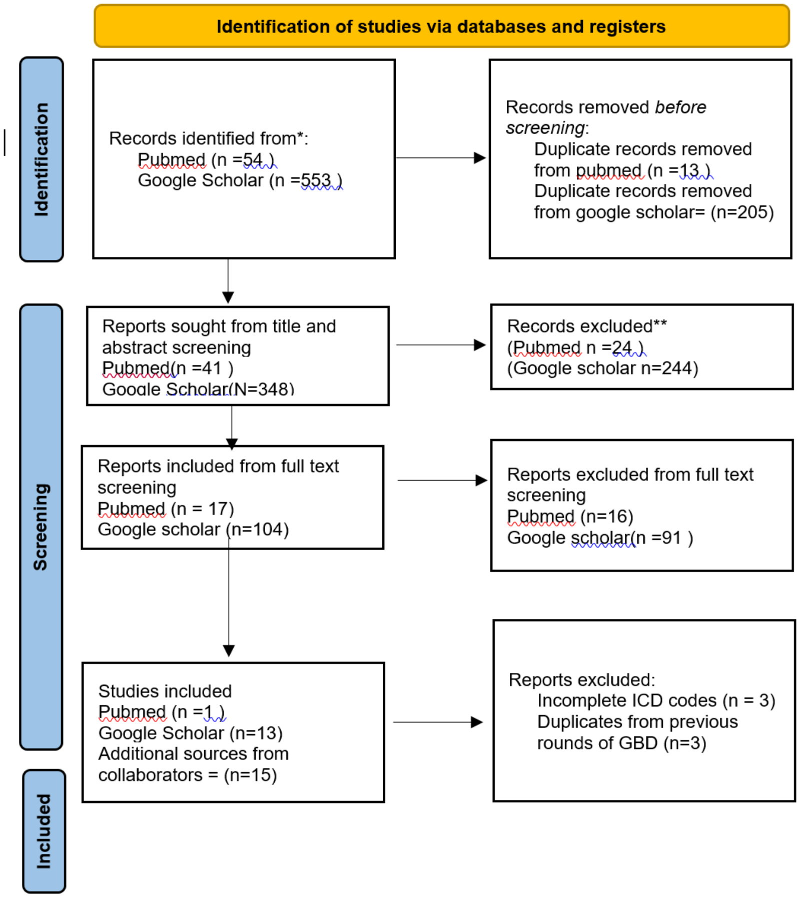

Section 3.2.3: Other data types

Section: 3.2.3.1: Maternal mortality data

In locations with low-quality or no VR, maternal mortality metrics can be found in surveillance, surveys, census, and sibling history data sources. The best data have death counts due to maternal causes and the total number of deaths for

women within the reproductive ages of 10–54 by year. If a data source is missing these components, creating a complete cause list is necessary by using livebirths and all-cause mortality deaths.[3] The livebirths are used to scale down the envelope to match the coverage of the raw data. Though death counts are the preferred metric, maternal mortality is often measured by using the maternal mortality ratio (MMR), which is easily converted to deaths by using livebirths. The China Maternal and Child Surveillance data are adjusted by scaling data from the strata to the province level (Step 7).

Section: 3.2.3.2: Surveys and censuses reporting fraction of deaths due to selected injuries

Surveys and censuses are often used in countries with less-developed VR systems; in countries with adequate VR, surveys and censuses are supplementary. Much like VAs, the CoD validity is a concern because of lack of medical certification at the time of death. For these data sources, we keep only causes related to maternal mortality and injuries. The remaining causes are accounted for as a remainder of total deaths in the sample size. One example of these surveys is DHS sibling history. In these surveys, females are interviewed regarding their sisters and survival status. Further questions are asked regarding the deceased siblings such as whether the sibling was pregnant or was within six weeks post-partum at the time of death. From these results, we can discern maternal mortality after applying correction factors including the Gakidou-King weights[10] and removing incidental HIV deaths.

Section 3.2.4: Police records

In most countries, police and crime reports are an important source of information for some types of injury deaths, notably road injuries and interpersonal violence. Our police data come from reports on road traffic and crime trends. The police reports used in this analysis were obtained from published studies, national agencies, and institutional surveys such as the United Nations (UN) Crime Trends survey and the UN Office on Drugs and Crime Global Study on Homicides. We assessed whether police reports were likely to be complete and to cover the entire country by comparing police trends with those seen in VR. Data are excluded in instances where police data for road traffic injuries are significantly lower than the VR. Inclusion methods for police data are based on data quality and completeness. For countries with high-quality data, with a star rating of 4 or 5, we utilise police records if the death counts provided are higher than those in the VR. For countries with lower-quality data, with a star rating of 0–3 stars, we always include police records. Police data that meet our inclusion criteria and provide complete coverage are uploaded to the database for use in road injuries and interpersonal violence deaths estimation.

Section 3.2.5: Population-based cancer registries

Section 3.2.5.1: Cancer registries with incidence

Data on cancer incidence were sought from population-based cancer registries as well as from databases that include multiple registries, including Cancer Incidence in Five Continents, NORDCAN, and EUREG. Cancer registries were identified through the membership list of the International Association of Cancer Registries, through the GBD Collaborator Network, or through publications. Registries were excluded if they were not representative of the coverage population, if the data were limited to years prior to 1980, if the source did not provide details on the population covered, or if the list of cancer types included was not comprehensive for the age group covered. Beginning in GBD 2019, childhood cancer-specific population-based cancer registry data were sought and included.

Section 3.2.5.2: Cancer registries with incidence and high-quality mortality data

In addition to incidence, some high-quality cancer registries also report cancer mortality data. These data were also extracted and used as inputs to the mortality-to-incidence model.

Section 3.3: Standardise input data (step 1)[2]

The input data to the CoD database are received in various formats and must be standardised to run through central CoD machinery to then upload to the database. Raw data inputs come from data sources such as mortality databases, literature reviews, or reports. Usable data sources must have a clear sample size of the number of deaths in the population and exhaustive cause lists. The complexity of the data cleaning process varies drastically across data sources. For VR microdata with the location, age, sex, year, and ICD-coded cause of every death, very little effort is necessary to standardise it into a consistent structure. Other sources such as VA, sibling history, and surveillance data

may require careful review to accurately extract scans of hardcover CoD reports into spreadsheets that can be transformed and standardised based on the standard CoD process described below.

At this point, data are assigned source identifiers so that they can be linked to the GHDx and cited appropriately. Any aggregate age and sex categories are flagged for age-sex splitting. The methods of cause-of-death assignment and data collection are reviewed to determine which source type to assign; for example, we distinguish sibling history data from surveys with a VA module. Only data at the most detailed level of the GBD location hierarchy are used. Documentation from the source is reviewed to determine if the population is representative of the location or only a subset of the population in that location. Data sources representing a subset of the population are flagged as non-representative; this flag is used by CODEm to increase the variance associated with such datapoints.

Finally, diagnostics are reviewed at this stage to avoid sending cleaning errors downstream. We review cause-specific deaths for each demographic group to ensure the data are reasonable. For example, it is unlikely that male breast cancer deaths are higher than female breast cancer deaths or deaths from neonatal causes occur in age groups over 1 year. All death totals are compared with the sum of cause-specific deaths to ensure the observed deaths are accounted for and sample size is complete.

Section 3.3.1: Disaggregation (step 1.1)

Causes of death in tabulated code systems are specified using tabulated codes, which consist of ranges of detailed codes from the corresponding detailed code systems. These tabulated codes represent groups of related causes, some of which are too general to be mapped directly to estimated GBD causes.

To correct for this, we use a set of age-, sex-, and super-region-specific proportions during data preparation to split tabulated codes into GBD causes and garbage codes that can then be mapped to a GBD cause. These proportions were calculated in GBD 2016 based on detailed VR from code systems ICD-10, ICD-9 detail, and ICD-8 detail. The target causes and garbage codes for a given tabulated code were determined based on the detailed codes that were missing from the tabulated cause list. For any cause and demographic group where we lacked ICD detail, global proportions were used.

As an example, the ICD-10 tabulated list includes a generic tabulated code for "Other intestinal infectious diseases." In order to make this cause coding compatible with standard GBD cause mapping, it requires splitting onto the detailed causes it represents. To do this, we first define the list of target causes for which the tabulated code will be split. This includes diarrhoea, typhoid fever, and paratyphoid fever in this example. Next, detailed ICD-10 data are used to generate the proportions by which the tabulated code will be split across the target causes. Proportions are generated using all years of available detailed ICD-10 data by super-region, age, and sex. For example, in ICD-10 detail data, in Latin America and the Caribbean, for all-age males, 94.5% of deaths within "Other intestinal infectious diseases" were coded to diarrhoea. Thus, in ICD-10 tabulation data, 94.5% of the "Other intestinal infectious diseases" code were assigned to diarrhoea for countries within this super-region and demographic group.

Section 3.3.2: State splitting (step 1.2)

Two sources for CoD estimation in India are the MCCD report, which reports medically certified deaths from health facilities in mostly urban areas,[11] and the SRS, which collects information via VA about one-half of 1% of the total population in India, including both urban and rural areas, from 8853 sampling units as of 2014.[12] For MCCD, missing data impedes estimation of trends at the state level. We used a first-order, log-linear model of the four-way contingency table of deaths by sex, age, state, and year to estimate the missing state-years. We fit the model to all available data for MCCD separately for each cause, including state-specific all-age measurements and age-specific national measurements. From this, we produced estimates for each combination of sex, age, state, and year. We then used these estimates wherever the raw data did not include sex-specific, age-specific, and state-specific death counts.

For MCCD, the model was fit separately for ICD-10-based and ICD-9-based reports by using the tabulated cause list present in the data.

Section 3.3.3: Calculate non-maternal deaths (step 1.3)

In cases when maternal mortality metrics do not include both deaths due to maternal causes and deaths due to non-maternal causes for women of reproductive age, livebirths and all-cause mortality estimates can be used to calculate deaths, which is the same method used in sibling history data. Many studies report maternal deaths as the MMR. MMR is the number of maternal deaths per 100 000 livebirths and can be used to calculate deaths when it has been derived from primary data and not estimated. Maternal deaths were calculated by using MMR and livebirths; if livebirths were missing, we substituted livebirth estimates and used the following equation:

$$Maternal\ deaths\ = \frac{MMR}{100{,}000} \times Live\ births$$

If a study was non-representative, we extracted sample size and livebirths from that study. After maternal deaths were calculated, we used the difference from all-cause mortality estimates to determine non-maternal deaths.

A more accurate and data-inclusive method of calculating maternal and non-maternal deaths incorporates coverage and splits deaths for a range of years into individual years. If there were livebirths in the study, we adjusted the coverage.

$$Coverage = \frac{Live\ births}{GBD\ estimated\ live\ births}$$

After coverage was calculated, totals deaths were scaled to be more representative. This gives a more accurate death count since the envelope assumes representative coverage. We then calculated non-maternal deaths by using all-cause mortality as an all-cause total.

$$Maternal\ envelope\ with\ coverage\ = Maternal\ envelope \times Coverage$$

An additional adjustment can be applied to maternal data spanning over a range of consecutive years, which allows for more data inclusion. The years within specified year ranges are separated into individual years, and total deaths within the year range were split between each individual year by using the fixed proportions of maternal deaths from VR in that particular country. We used only VR data to inform the proportions because it was both high-quality and representative.

Section 3.4: Map to GBD cause list (step 2)[2]

In GBD 2021, we used 439 maps to translate causes found in the input data to the GBD 2021 cause list (table S2). This included 31 maps for VR data, 314 for VA data sources, and 98 for other data types. The largest and most universal maps used were those for ICD-9 and ICD-10 VR data. The input data causes varied from three- to four-digit ICD codes to custom cause lists with cause names such as “cholera” or “hepatitis”. Our mapping process enabled us to compare these various data sources across demographic groups, by mapping all ICD codes to the mutually exclusive and collectively exhaustive list of GBD causes.

A crucial aspect of enhancing the comparability of data for cause of death is to deal with uninformative, so-called garbage codes. Garbage codes are codes to which deaths were assigned that cannot or should not be considered as the underlying cause of death, for example: heart failure, ill-defined cancer site, senility, ill-defined external causes of injuries, and septicaemia. Additionally, any codes not specific enough to assign to a detailed cause of death is considered a garbage code, such as unspecified infectious disease or unspecified injury. In GBD 2019, we developed additional maps to translate ICD codes found in the input data that are non-underlying causes to appropriate target codes based on the levels of the GBD cause list. These garbage codes were mapped to Class 1–4 of the GBD cause list according to the following criteria:

Class 1 includes all garbage codes for which a Level 1 GBD causes (communicable, maternal, neonatal, and nutritional diseases; injuries; and non-communicable diseases) cannot be directly assigned. For example, the underlying causes of "sepsis" or "peritonitis", if not specified in the data, could be an injury, a non-communicable disease, or a type of communicable disease. In these cases, deaths will be redistributed across all three of these Level 1 causes. In addition, deaths coded to impossible or ill-defined causes of death (including "senility" and "unspecified causes") fall into this category, as they will be redistributed onto all causes.

Class 2 includes all garbage codes that can be assigned to one Level 1 cause in the GBD cause list. This would include deaths coded to "unspecified injuries" (X59), which are redistributed onto all injuries.

Class 3 includes all garbage codes for which we know the Level 2 CoD and can redistribute onto one Level 3 cause. This includes deaths coded to causes such as "unspecified cardiovascular disease", which falls within the Level 2 cause "cardiovascular diseases", as well as those coded to "unspecified cancer site", which falls within the Level 2 cause "neoplasms".

Class 4 includes all garbage codes for underlying causes of death that can be redistributed within a Level 4 cause. This includes garbage codes such as "unspecified stroke" or "unspecified road injuries."

Section 3.5: Age-sex splitting (step 3)[2]

Different sources, particularly VA studies, report deaths for a wide range of age groups with varying intervals. For the analysis of CoD, we mapped these different age intervals to the GBD standard set of age groups. While the proportions informing this mapping were updated in GBD 2021, the approach to undertake this mapping was the same as in the prior GBD studies (GBD 2019, GBD 2017, GBD 2016, GBD 2015, GBD 2013, and GBD 2010).

In the process of assembling a consolidated demographic database, we found that the aggregation of age groups is perhaps the strongest source of inconsistency. By convention, such data are reported in broad age groupings such as 0–4, 5–14, and 15–49, or with both sexes together. The issue of comparability between age-sex groups arose when assembling the GBD CoD database. We developed a tool called age-sex splitting that takes aggregated age groupings and the "both sexes combined" grouping and divides them into what their constituent age groups would likely have been if respective cause-specific and country-specific age distributions had been used. The analytical framework for GBD includes six age categories for infants and children under 5: early neonatal (0–6 days), late neonatal (7–27 days), 1–5 months, 6–11 months, 12–23 months, and 2–4 years, and 19 age categories for those over 5: 5–9 years, 10–14 years, and so forth, proceeding in five-year age groups until the terminal age group of 95 years and older. We treat unknown ages and sexes in the same manner we treated the "all ages combined" age category and "both sexes combined" sex group. Through this process, we were able to directly compare all data sources on even terms.

The approach to age splitting is based on the following formula. The key assumption underlying this formula is that the relative risk of death by age group compared to a reference age group is invariant across populations. Although this assumption is likely violated in specific cases, a strong biologically based pattern of the relative risk of death for a cause by age is observed for most causes. The basic formula is as follows:

$$D_a = R_a N_a \left(\frac{D_a^{a+x}}{\sum_a^{a+x}(R_a N_a)}\right)$$

Where:

D_a= the number of deaths from a cause in age group *a*

R_a= global cause-specific mortality rate of age group *a*

N_a= the country-year-sex-specific population in age group *a*

D_a^{a+x}= the number of deaths in the age group *a* to *a*+*x*

With the assumption of invariant relative risks of death by age with respect to a reference age group, this equation can be used, along with population distribution by age, to split an aggregate number of deaths for the age groups a to $a+x$ into specific deaths for each age group within the aggregate interval.

$$D_{as} = R_{as}N_{as}\left(\frac{D_{as}^{a+x,s}}{\sum_{a}^{a+x}(R_{as}N_{as})}\right)$$

Where:

D_{as}= the number of deaths from a cause in age group $a,$ sex s

R_{as}= global cause-specific mortality rate of age group $a,$ sex s

N_{as}= the country-year-sex-specific population in age group a for sex s

$D_{a,s}^{a+x,s}$= the number of deaths in the age group a to $a+x$ for sex s

In some cases, deaths are reported for an aggregate age group for both sexes combined. The task in this case is more complicated, but the same principle can be applied. In this case, we assumed that the relative risks of death by age and sex are constant.

This equation can be used to split data aggregated by age and sex. The assumption, however, of invariant relative risks across age and sex is a stronger assumption. Fortunately, data pooled across sexes are less common in the published or unpublished CoD data.

The relative risk of death in a particular age group for a given sex is derived from the global distribution of cause-specific mortality rates found in available VR data. Location-years from the following code systems are used, provided they report the requisite age detail and sex detail: ICD-7, ICD-8, ICD-9 BTL, ICD-10 tabulated, ICD-9, and ICD-10. Upon compiling these data, we mapped them to GBD causes and retained any observation coded to either cause Level 3 or a most detailed cause. Cause aggregation was then performed to ensure accurate death totals at all levels of the cause hierarchy. Thus, age distributions were generated for most detailed causes, as well as all Level 1, 2, and 3 causes. In the event that a cause does not have a distribution, it will be split using the distribution of its nearest parent cause.

We next adjusted separately for estimated adult and child VR completeness. Location-year-age-sex-cause-specific deaths and population were then aggregated across all location-years to produce cause-specific mortality rates by age and sex. These were used to determine the risk of death at any age relative to any reference age group, as shown in the above equations.

Section 3.5.1: Remap unmodelled age-sex combinations

Occasionally, data sources include deaths by a cause for which consensus exists that death is rare for the sex and/or age. For example, some number of deaths may be attributed to cervical cancer in males, or to maternal causes in children younger than 10 years. We have constructed a list of age-sex-cause combinations we do not model. Some sex/cause combinations cannot be modelled because the input data for causes of death is limited to binary male and female sex groups and thus constrains the combinations of cause and gender that can be modelled for the GBD. Because of these limitations to our input data, when we encounter a death in an unmodelled age-sex-cause combination, cause/sex combinations occur, we reassign them to a related cause or garbage package. For example, IBD may be remapped to other digestive disorders. All unmodelled combinations are included in table S7.

Section 3.6: Correction for miscoding of Alzheimer's and other dementias, Parkinson's disease, and atrial fibrillation and flutter (step 4)[3]

Section 3.6.1: Objective

For certain causes of death, mortality rates reported in VR systems are impossible to reconcile with observed trends in disease prevalence and excess mortality. For dementia,[13–21] Parkinson's disease,[22,23] and atrial fibrillation and flutter, these disparities can largely be attributed to death certification practices which lead to under-coding or over-coding in many country-years. We sought to address the known bias in CoD data by first identifying the proportion of all deaths that should be assigned to these causes and next determining the GBD causes and garbage groups to which these deaths are being incorrectly assigned.

In past GBD iterations, we estimated Alzheimer's disease and other dementias, Parkinson's disease, and atrial fibrillation and flutter on the basis of longitudinal prevalence and excess mortality data to help account for changing patterns in death certification and corresponding implausible time trends in many VR sources. This method was first implemented for Alzheimer's disease and other dementias in GBD 2013. We added atrial fibrillation and flutter to the causes modelled in GBD 2015 and Parkinson's disease to the causes modelled in GBD 2016 by using this strategy. All of these causes were processed in CoDCorrect in a manner that was agnostic to the likely targets of misclassification, which inappropriately led to changes in mortality estimates for causes unrelated to these three in GBD 2015. For GBD 2016, we improved this process by completing a literature review to identify the causes of death most closely associated with Parkinson's and Alzheimer's diseases[3,11,12,18] and limiting the CoDCorrect adjustments to include only those causes. For GBD 2017, we refined this approach further by using multiple CoD data to determine the GBD causes and garbage codes from which we move deaths as well as the pattern of misclassification.

Section 3.6.2: Correction process

Changes in coding practices for Alzheimer's diseases and other dementias, Parkinson's disease, and atrial fibrillation and flutter result in spatiotemporal mortality trends that are incompatible with prevalence and case-fatality trends.[24] These changes in coding practices are believed to be the result of shifting consensus in cause of death certification, meaning there is a bias in vital registration (VR) data that needs correction. For Parkinson's disease and atrial fibrillation and flutter, we first estimated excess mortality from prevalence and CoD data in countries with the highest ratio of cause-specific mortality to prevalence, which represents the greatest willingness to code to an under-coded cause. Then, using DisMod-MR 2.1 (see Section 4.2.3), we derived estimates of cause-specific mortality rates from available prevalence surveys as well as the estimates of excess mortality rate, applied across all countries and over time. We divide this value by the all-cause mortality rate to determine the fraction of overall mortality to attribute to each under-coded cause. For dementia, the modelling process was redesigned in 2019 to no longer depend on vital registration data from the highest dementia mortality locations. Instead, we used relative risk data from cohort studies to calculate total number of excess deaths due to dementia, and end-stage disease proportions from linked hospital to death records to subset these deaths to the proportion of excess deaths with end-stage conditions, which we attributed to dementia. Finally, we used log-linear interpolation to interpolate final estimates of death due to dementia for the entire time series and saved as a custom CoD model.

To ascertain the causes from which we would move deaths to under-coded causes, we leveraged multiple CoD data from the USA—by looking to the combinations of intermediate and immediate causes (ie, chain causes) present on death certificates with an under-coded cause listed as underlying and identifying other death certificates with similar or identical chain causes, we can determine the expected pattern of miscoded deaths.

The first stage in this process is to parse out country-years of data where we believe coding practices to be relatively stable. For dementia, this "gold standard" dataset features USA 2010–2015; for Parkinson's, USA 2005–2015; and for atrial fibrillation and flutter, USA 2014–2015. We then collect all deaths in those years with the under-coded cause listed as underlying and remove any mention of the under-coded cause from the death certificate. Next, for each unique chain identified in this manner, we search the entire time series of USA data (1980–2015) to identify the distribution of underlying causes that share that chain. The premise here is that if the diagnosis of dementia, Parkinson's, or atrial fibrillation and flutter were missed, the other causes listed on the death certificate would have

been the basis for certification. We then reallocate the under-coded cause deaths in the gold standard years by chain based on that alternative underlying cause distributions from the full time series.

Upon iterating through all unique chains, we are left with a counterfactual dataset excluding under-coded causes of death. Each remaining cause can be subdivided into correctly coded deaths and deaths that have been recoded from an under-coded cause by the process described (although not all causes are necessarily targeted by the recoding algorithm). We then calculate the ratio of recoded deaths to total deaths by cause, age, and sex in our counterfactual dataset. This ratio represents the proportion of each cause that we believe to be miscoded Parkinson's disease, Alzheimer's disease and other dementia, or atrial fibrillation and flutter in a counterfactual scenario of 100% under-reporting for these causes.

We multiply the ratios derived from the multiple cause data by the cause-specific deaths in each VR dataset to determine the local pattern of miscoding. In this way, the method is sensitive to the observed epidemiology of a given country and year. Then, we calculate the deficit in under-coded cause mortality for each location, year, age, and sex by taking the difference in the expected cause fraction for a given under-coded cause based on prevalence and excess mortality compared to the proportion of deaths actually certified by the VR system. Finally, we scale the cause-specific miscoded deaths to match the deficit and then move them accordingly. We assumed that misclassification of actual dementia and Parkinson's deaths in past years occurred only for reported causes of deaths that were plausibly the direct result of dementia or resulted from misdiagnosis of other organic brain diseases based on clinical expert judgement. A similar assumption is used for atrial fibrillation and flutter, for which only cardiovascular causes and ill-defined garbage codes are considered. Finally, while we assumed that Parkinson's disease and atrial fibrillation and flutter are strictly under-coded in the VR, we assumed that Alzheimer's disease and other dementias can be under- or over-coded. Anywhere where our expected cause fraction for Alzheimer's disease and other dementia was lower than the observed, we removed deaths from this cause and redistributed them as non-specific garbage.

Because the deaths being reallocated vary by location-year, we need a mechanism to ensure plausible limits to how many deaths are extracted from each GBD cause and garbage code. To achieve this, we first run the above-mentioned algorithm on all 5-star VR data (see Section 3.16 for an explanation of the star data quality rating system). Then, we determine the 95th percentile of the proportion of deaths moved to an under-coded cause for each GBD cause and garbage code group by age and sex across location-years among these data. Those values are subsequently stored and applied as the limits for deaths moved by this process for all other VR data.

Section 3.7: Redistribute (Step 5)[2]

A crucial aspect of enhancing the comparability of data for CoD is to deal with uninformative, so-called garbage codes. Garbage codes, ie, codes that are not specific enough, are an immediate or intermediate CoD, or impossible CoD, should not be considered as the underlying cause of death—for example: heart failure, ill-defined cancer site, senility, ill-defined external causes of injuries, and septicaemia. The methods for redistributing these garbage-coded deaths are outlined in detail in Johnson et al,[25] and the primary algorithm for redistributing deaths assigned to these codes has not changed since GBD 2013.

Section 3.7.1: Redistribute HIV-related garbage codes (step 5.1)

Because of the disparate nature of HIV/AIDS mortality across space and time, dynamic redistribution of HIV/AIDS-related garbage codes was needed (table S8). To inform this redistribution, we generated target proportions for each garbage group by age band (under 1 month, 1–59 months, 5–19 years, 20–49 years, 50–59 years, 60–69 years, 70–79 years, and 80 years and older), five-year time interval, and sex. The garbage groups either target HIV or a remainder target. The allotment of deaths to either of these is based on the regional increase in the mortality rate of all codes in the group relative to the rates seen from 1980 to 1984—an increase greater than 5% is assumed to be HIV/AIDS-related, and the proportion of those deaths exceeding 5% are redistributed to HIV/AIDS. Any increase less than or equal to 5% is then assigned to the remainder target.

Section 3.7.2: Regress garbage codes versus non-garbage codes (step 5.2)

For each redistribution package, we defined the "universe" of data as all deaths coded to either the package's garbage codes or the package's redistribution targets for each country, year, age, and sex. We then ran a regression based on the following equation separately for each target group and sex:

$$TG_{crt} = \alpha + \beta_1 Gar_{crt} + \beta_2 Age_{crt} Gar_{crt} + \theta_r Gar_{crt} + \gamma_r + \varepsilon_{ct}$$

Where:

TG_{crt} = percentage of deaths within the given garbage code's universe that were coded to a given target group, by country

Gar_{crt} = percentage of deaths within the given garbage code's universe that were coded to a given set of garbage codes

Age_{crt} = age interaction term for the fixed effect on the interaction of garbage and age

α = constant

β_1 = slope coefficient describing the association between Gar_{crt} and TG_{crt}

β_2 = slope coefficient describing the association between the interaction $Age_{crt}Gar_{crt}$ and G_{crt}

γ_r = region-specific random intercept (or super-region if the random effect on region is not significant)

θ_r = region-specific random slope (or super-region if the random effect on region is not significant)

ε_{ct} = standard error, normally distributed and calculated by bootstrapping

This regression was adjusted from GBD 2013 to include fixed effects on the interaction of garbage and age to ensure smooth age patterns. We made this decision after investigating diagnostic visualisations that showed unlikely gaps between proportions assigned to different age groups.

Once proportions were produced for each country, sex, age, and target group, certain adjustments were made to conform our packages to the best medical evidence available. In some cases, we implemented restrictions on the proportions that the regressions could yield. For example, we did not allow any redistribution onto "Chagas disease" outside of Latin America and the Caribbean or "self-harm" under the age of 15 years. In other cases, we capped the proportion for some targets to the level that would be produced from proportional redistribution; for example, "haemoglobinopathy" and "haemolytic anaemia" were restricted to the level of proportional redistribution in the redistribution of "left heart failure". Occasionally, further adjustments were made on a case-by-case basis per country, age, sex, and target group to suppress the impact of outliers based on existing epidemiological evidence and expert judgment.

In GBD 2019, we updated the regressions for stroke and diabetes. We dropped the proportion of garbage from the regression formula and ran regression on high-quality, low proportion garbage data (4/5 stars, <50% GC). We also included all covariates included in the CODEm models for both stroke and diabetes.

Section 3.7.3: Development of an algorithm for redistribution of garbage codes based on multiple CoD data

Multiple CoD data are a form of individual record causes of death data that include an underlying CoD along with other causes in the death chain, including intermediate and immediate causes. By analysing this type of data, we can sometimes find the true underlying CoD in other CoD data where the underlying cause is a garbage code or a mis-

assigned CoD.

As of GBD 2019, this method has been expanded and used in redistribution of the following intermediate causes: sepsis, embolism (pulmonary and arterial), heart failure (left, right, and unspecified), acute kidney injury, hepatic failure, acute respiratory failure, pneumonitis, and unspecified central nervous system disorders. Using multiple CoD records for the USA, Mexico, Brazil, Taiwan (province of China), Italy, Canada, New Zealand, Austria, South Africa, and Colombia, we identified the fraction of deaths where the underlying cause of death and the intermediate cause was in the causal chain. Using a mixed effect linear regression, we estimated the fraction of intermediate-cause-related deaths by underlying GBD cause. These fractions were multiplied by the GBD 2021 CoDCorrect result to calculate the number of deaths intermediate cause-related deaths for each GBD cause. Lastly, we calculated the "intermediate cause fraction", with total intermediate-cause-related deaths as the denominator, by age, sex, location, year, and GBD cause. These fractions were used to redistribute the intermediate-cause-related deaths to a GBD cause. An example is given below for sepsis, where a, s, l, y, c denotes a given age group, sex, location, year, and underlying cause of death:

1. $sepsis\ fraction = \beta_{HAQ\ Index} + \beta_{age\ group} + \beta_{sex} + Y_{cause} + \varepsilon$

2. $sepsis\ deaths_{a,s,l,y,c} = sepsis\ fraction_{a,s,l,y,c} * GBD\ deaths_{a,s,l,y,c}$

3. $total\ sepsis\ deaths_{a,s,l,y} = \sum_c sepsis\ deaths_{a,s,l,y,c}$

4. $fraction\ of\ sepsis\ to\ redistribute_{a,s,l,y} = \frac{sepsis\ deaths_{a,s,l,y,c}}{total\ sepsis\ deaths_{a,s,l,y}}$

To redistribute X59 and Y34 (unspecified injuries) deaths, we used a multi-step approach that utilised the pattern of nature of injury codes in the causal chain in the multiple CoD data. First, we looked at deaths where X59, Y34, and GBD injuries causes were the underlying cause of death and got the pattern of nature of injury codes in the chain. We then derived a cause-specific redistribution proportion based on the probability of a given pattern being coded to X59/Y34 or a GBD injuries cause and summing up these proportions for all patterns. An example below is given for X59:

5. $P_{(pattern_j|UCoD\ X59)} = \frac{\#\ of\ pattern_j\ deaths\ |\ UCoD\ X59}{\sum_{j=0}^{m}(\#\ of\ pattern_j\ deaths\ |UCoD\ X59)}$

6. $P_{(GBD\ injuries\ cause_i|pattern_j)} = \frac{\#\ of\ UCoD\ GBD\ injuries\ cause_i\ deaths\ |\ pattern_j}{\sum_{i=0}^{n}(\#\ of\ UCoD\ GBD\ injuries\ cause_i\ deaths\ |pattern_j)}$

7. $redistribution\ proportion_{GBD\ injuries\ cause_i} = \sum_{j=0}^{m}\left(P\left(pattern_j \middle| UCoDX59\right) * P(GBD\ injuries\ cause_i|pattern_j)\right)$

Where:

$pattern_j$ = a given nature of injury code pattern in the chain of the multiple CoD data

UCoD X59 = a death with X59 coded as the underlying cause of death (UCoD)

UCoD GBD injuries $cause_i$ = a death with a GBD injuries causes coded as the UCoD

We applied these cause-specific redistribution proportions on the data where X59/Y34 were the underlying cause of death to get the number of X59/Y34 deaths "attributable" to each GBD injuries cause. Then, for each GBD injuries cause in the multiple CoD data, we calculated the fraction of redistributed X59/Y34 deaths over the fraction of total

injuries death for that cause and modelled this intermediate cause fraction using a mixed effects linear regression similar to the one mentioned above. Like mentioned above, these fractions were then multiplied by GBD 2021 CoDCorrect results, and the cause fractions for X59 and Y34 were calculated by age, sex, location, year, and GBD injuries cause, and then used to redistribute X59 and Y34 deaths to GBD injuries causes.

Additionally, multiple CoD data were used in the correction of the mis-assignment of deaths due to drug overdoses to unintentional other poisoning. More than 90% of these types of poisonings are due to exposure to narcotics, psychodysleptics, and other drugs, specified or unspecified. More than 97% of these poisonings by substance or drug occurred in ages 15–65 years. These are clearly not cases of accidental ingestion of substances but rather deliberate ingestion and unintentional poisoning. Using multiple CoD records from the USA, Mexico, Brazil, Taiwan (province of China), Italy, Colombia, Australia, and various European countries from 1980 to 2017, we selected all deaths with underlying causes coded to X40–X44 (Table A below). Table B shows the combination of other potential causes that can be found in the multiple CoD data for these underlying causes, and table A shows the ICD-10 codes corresponding to these causes. On the basis of Table B, we proportionally redistributed mis-assigned unintentional poisoning deaths to one of these causes. The main assumption behind this algorithm is the predominance of the fatality of some substances when a combination of drugs is considered. Given the combination of different drugs and substances in these codes, opium is the main cause of fatality.[26,27] Other substances, like cocaine, methamphetamine, and alcohol in combination with cannabis are less likely to be dominant in fatality.[28]

For example, if the multiple CoD data show that 40% of deaths include opioid use disorders as an intermediate cause where the underlying cause is X40–X44, the redistribution proportion for opioid use disorders will be exactly 40% due to the dominance of the fatality of opioid use disorders compared to other drugs in the above table. Additionally, in our final results, cannabis and psychoactive and psychedelic drug use disorder deaths were mapped to other drug use disorders.

Table A. ICD-10 codes for substances or drugs used to assign deaths coded to an underlying cause of unintentional poisoning by using multiple CoD data

Accidental poisoning codes	All X40, X41, X42, X43, X44 codes
Opioid codes	T40.0, T40.1, T40.2, T40.3, T40.4, T40.6, F11.0, F11.1, F11.2, F11.3, F11.4, F11.5, F11.6, F11.7, F11.8, F11.9
Amphetamine codes	T43.6, F15.0, F15.1, F15.2, F15.3, F15.4, F15.5, F15.6, F15.7, F15.8, F15.9
Cocaine codes	T40.5, F14.0, F14.1, F14.2, F14.3, F14.4, F14.5, F14.6, F14.7, F14.8, F14.9
Psychoactive and psychedelic drug codes	T40.8, T40.9, F16.0, F16.1, F16.2, F16.3, F16.4, F16.5, F16.6, F16.7, F16.8, F16.9
Alcohol codes	T51.0, T51.1, T51.2, T51.3, T51.8, T51.9, F10.0, F10.1, F10.2, F10.3, F10.4, F10.5, F10.6, F10.7, F10.8, F10.9
Cannabis codes	T40.7, F12.0, F12.1, F12.2, F12.3, F12.4, F12.5, F12.6, F12.7, F12.8, F12.9

Table B. Multiple cause of death selection algorithm used for redistributing unintentional poisoning causes of death to substance or drug use cause of death

Selection algorithm						
	Opioids	Cannabis	Cocaine	Amphetamines	Alcohol	Psychoactive and psychedelic drugs
Opioids	Opioids	Opioids	Opioids	Opioids	Opioids	Opioids

Cannabis	Opioids	Cannabis	Cocaine	Amphetamines	Alcohol	Psychoactive and psychedelic drugs
Cocaine	Opioids	Cocaine	Cocaine	Amphetamines + cocaine	Cocaine + alcohol	Cocaine
Amphetamines	Opioids	Amphetamines	Amphetamines + cocaine	Amphetamines	Amphetamines + alcohol	Amphetamines
Alcohol	Opioids	Alcohol	Cocaine + alcohol	Amphetamines + alcohol	Alcohol	Psychoactive and psychedelic drugs
Psychoactive and psychedelic drugs	Opioids	Psychoactive and psychedelic drugs	Cocaine	Amphetamines	Psychoactive and psychedelic drugs	Psychoactive and psychedelic drugs

Multiple CoD data were only available to us for the USA, Mexico, Brazil, Taiwan (province of China), Italy, Colombia, Australia, and various European countries. Because of this limited sample, we applied the result from the multiple CoD analysis from each country to its respective super-region and used global proportions for sub-Saharan Africa. We hope for increased availability of multiple CoD data in future analyses to achieve a more precise distribution for more locations.

Section 3.7.4: Verbal autopsy anaemia adjustment (step 5.3)

To compensate for the over-representative cause fractions from anaemia found in VA studies, we redistributed these deaths based on the causal attribution of severe anaemia from GBD 2015. The proportions were country-year-age-sex-specific.

Section 3.7.5: Calculate redistribution uncertainty (step 5.4)

We categorised garbage codes into four levels in order of increasing specificity (see Section 3.4). Some garbage codes are redistributed on all causes (eg, unspecified causes of death) and others are only redistributed onto specific causes (eg, unspecified cancer). Major garbage refers to garbage codes in Levels 1 or 2. Because of the variation in redistribution, estimating uncertainty from garbage redistribution for CODEm modelling was an important goal for GBD 2019.

We assigned redistribution variance to each datapoint in the CoD database by calculating residual variance from a regression predicting the percentage of garbage-coded deaths redistributed to a cause, given the proportion of garbage codes we observed for that location, year, age, sex, cause, and the age-standardised relative rate of major garbage codes across all causes. If there is a cause that has greater residual variance, we assume greater redistribution uncertainty.

The two model inputs are the observed percentage of Levels 1, 2, and 3 garbage codes (by cause, age, sex, location, and year) in redistributed CoD data and the percentage of garbage codes in the raw data (calculated as the age-standardised mortality rate ratio of major garbage-coded deaths to all deaths in the raw data by location, year, and sex). Level 4 garbage codes were excluded from the model to avoid overestimating uncertainty in countries with high percentages of major garbage codes. Additionally, the classification of Level 4 garbage codes is not stable between successive GBD rounds—for example, "unspecified diabetes" was not a garbage code in GBD 2016, and in GBD 2017 was reclassified as a Level 4 garbage code to permit estimation of diabetes by type. These deaths are still taken into account later in the uncertainty estimation process. The model predicts the percentage of garbage-coded deaths redistributed to a cause, given the proportion of garbage codes we observed for that location, year, age, sex, cause, and the age-standardised relative rate of major garbage codes across all causes. From this model, we calculate residual variance. It is important to note that the variance here is a measurement of uncertainty of redistribution, not of the level of miscoding in the raw CoD data for a given demographic.

To calculate variance, a dataset was generated that contained percentage garbage by location, year, age, sex, and cause, where percentage garbage is determined by the equation:

$$pct_{garbage} = \frac{deaths_{redistributed} - deaths_{raw}}{deaths_{redistributed}}$$

A mixed-effect linear regression model was then fit to predict the logit percentage of deaths from redistribution by age-standardised relative rate of major garbage codes.

$$logit\left(pct_{garbage_{ij}}\right) = \beta_0 + \beta_1 * \log\left(ASR_{majorgarbage_{ij}}\right) + \beta_2 * 15yearage_{ij} + \gamma_{1j} * \log\left(ASR_{majorgarbage_{ij}}\right) + u_j + e_{ij},\ \ \theta_{\{i\}} \sim N(0, \sigma^2)$$

Where:

i indexes dataset-location-year-age-sex-cause datapoints nested within j groups by GBD region

$ASR_{majorgarbage_{ij}}$ is age-standardised relative rate of major garbage

Residual variance, as estimated by the mean absolute deviation, was calculated for each cause, sex, and age.

The next step was to use the residual variance to calculate uncertainty around each datapoint in the CoD database. First, we calculated the percentage garbage of each datapoint by treating all deaths that could not be directly mapped to a GBD cause as garbage, including Level 4 garbage codes. Percentage garbage was calculated as

$$pct_{garbage} = \frac{deaths_{redistributed} - deaths_{corrected}}{deaths_{corrected}}$$

Where:

$deaths_{corrected}$: deaths post misdiagnosis correction (Section 3.6)

$deaths_{redistributed}$: deaths post redistribution (Section 3.7)

Residual variance was matched to each datapoint, and 100 draws were sampled from a normal distribution by using the cause-age-sex-specific residual variance and mean of 0. The logit transformed percentage garbage was added to each value in the distribution. Each draw was then transformed out of logit space, and the post-redistribution deaths were calculated as:

$$deaths = \frac{deaths_{corrected}}{1 - pct\_garbage}$$

Draws of deaths were processed through noise reduction before calculating the final redistribution variance passed to CODEm, which was added to the total data variance. The mean of the draws was not used as the final estimate because it was found that the logit transformation biased the distribution of cause fractions higher. Instead, only point estimates were used.

Section 3.8: HIV/AIDS misclassification correction (step 6)[2]

In many location-years, certain causes of death known to be comorbid with HIV/AIDS (eg, tuberculosis, other infectious diseases) are seen to have age patterns that diverge from those observed in location-years without widespread HIV epidemics and are in fact more reflective of HIV mortality trends. To identify these instances, a

global relative age pattern is generated by using all VR deaths in countries with observed HIV prevalence less than 1% in 2010 by using the following equation:

$$RR_{asc} = \frac{R_{asc}}{\bar{x}(R_{65sc}, R_{70sc}, R_{75sc})}$$

Where:

RR_{asc} is the relative death rate for age group a, sex s, and cause c;

R_{asc} is the rate for that age group

$\bar{x}(R_{65sc}, R_{70sc}, R_{75sc})$ is the mean of the rates in ages 65–69, 60–74, and 75–79 for that sex and cause. This is preferable to comparing mortality rates because we are able to isolate divergence in age pattern while accounting for varying levels of overall mortality by fixing death rates to age groups that are unlikely to be confounded by the presence of HIV. Expected deaths for an identified cause were then determined by the equation:

$$ED_{lyasc} = \bar{x}\left(R_{ly65sc}, R_{ly70sc}, R_{ly75sc}\right) \times p_{lyas} \times RR_{asc}$$

Where:

ED_{lyasc} are deaths for location l, year y, age group a, sex s, and cause c;

$\bar{x}(R_{l65sc}, R_{l70sc}, R_{l75sc})$ is the mean of the rates for ages 65–69, 60–74, and 75–79 for that location-year-sex-cause;

p_{lyas} is the population for that location-year-age-sex

RR_{asc} is the global standard relative rate determined in the previous step for that age-sex-cause. The expected deaths remain attributed to that particular cause, while the difference between observed and expected are reallocated to HIV/AIDS.

Section 3.9: Scale strata to province (step 7)[2]

Over time, a higher proportion of deaths have been registered in China through the expansion of the DSP system and provincial/county efforts to increase CoD registration. With the expansion of coverage, it is possible that province aggregates do not accurately represent the population distribution between urban and rural areas in each year. For this reason, we stratified the data preparation by urban and rural status for each county within each province. Stratification was based on the median level of urbanisation across counties within each province as recorded in the 2010 China census. In the provinces of Tibet and Hainan, all counties were placed into one stratum based on largely homogeneous urbanisation levels within each province. This yielded a total of 62 analytical province-strata. Macao and Hong Kong were not included in this stratification system as the VR systems there are independent from that on the mainland; no weighting scheme needs to be carried out in these complete VR systems with quality CoD data.

Within each province-strata, a larger proportion of deaths in-hospital might be reported than that of deaths outside of hospital because of the internet hospital reporting system. To avoid bias, we reweighted in-hospital and out-of-hospital deaths based on the age-sex-province-specific fraction of deaths in and out of hospital in the DSP system. DSP data have been used to establish these percentages because in these communities, there is a concerted effort to identify all out-of-hospital deaths. Province-strata death rates are combined to produce overall province death rates by weighting each stratum by population in each age-sex-year group. Province death rates are rescaled so that all-cause mortality equals the estimated death rate in each age-sex-year estimated in the life-table analysis. The Bayesian noise reduction algorithm was used to deal with zero counts and small number issues for rare causes.[29]

Section 3.10: Restrictions post-redistribution (step 8)[2]

Some causes of death can only be reliably assigned through an autopsy by a trained physician. For example, a VA would be unlikely to reliably distinguish between ischaemic and haemorrhagic stroke.

This step ensures that the detail of the cause list at this point in the data prep process is reasonable given the detail of the original data source and the methods by which the CoD was assigned. A "bridge map" is applied over a certain set of sources to ensure that these sources do not contain causes that could not reliably be determined by the methods used. These causes, identified to be too detailed, are then aggregated to their parent cause. This correction is applied to ICD-9 detail, ICD-9 BTL, ICD-10 tabulated, ICD-8 detail, ICD-8 A, China DSP (tabulated ICD-9), India MCCD, India SRS, USSR tabulated ICD-9, the Philippine Vital Statistics Reports, Iran ICD-10 VR from the Ministry of Health and Medical Education, and all VA. An example of this would be the aggregation of all sub-types of lower respiratory infection to lower respiratory infection in ICD-9 BTL.

Section 3.11: Drop VR country-years or mark as non-representative (step 9)[2]

Lozano and colleagues[30] describe the negative impact that low-completeness VR data could have on CoD modelling for GBD 2010. In particular, in settings where a data source does not capture all deaths in a population, the cause composition of deaths captured might be different from those that are not. However, a completeness sensitivity test found that low-completeness VR data had little impact on the cause-specific mortality trends at the global level.

For GBD 2019, we investigated the impact of these data at the country and subnational levels and determined that these data produced unlikely trends in the models affected. Despite the minimal impact on global trends, better models were produced by eliminating or marking as non-representative data with extremely low completeness. VR completeness was estimated as the number of deaths registered divided by the number of deaths estimated in the GBD mortality envelope.

For this round, VR location-years with completeness less than 50% were dropped, while location-years with completeness between 50% and 69% were marked as non-representative.

In addition, any country-year with a number of deaths registered to major garbage codes greater than 50% of the deaths registered was dropped. Major garbage coding refers to garbage codes redistributed across Levels 1 and 2 of the cause hierarchy. When we redistribute garbage codes across Levels 1 and 2 of the cause hierarchy, this is because we do not have enough information to distribute them to more detailed levels [3 and 4].

Section 3.12: Cause aggregation (step 10)[2]

The cause list is organised in a top-down hierarchical format containing four levels. The first group, or Level 1, sums all causes. Following all-cause mortality are Level 2 causes, which include three broad groupings of causes of deaths: "communicable, maternal, neonatal, and nutritional diseases"; "non-communicable diseases"; and "injuries". Within those Level 2 groupings are finer levels used for modelling. Level 3, or parent causes, are aggregated; the mortality estimate for a parent cause in the hierarchy represents the sum of the causes under that rubric. Sub-causes within Level 3 causes—Level 4—are more detailed. For example, the parent cause "intestinal infectious diseases" contains the three sub-causes: "typhoid fever", "paratyphoid fever", and "other intestinal infectious diseases". Included in the parent cause estimate are deaths mapped directly to the parent and any Level 4 sub-causes. In data where there was not enough information to assign a Level 4 cause, we aggregated to the Level 3 parent cause. Exceptions to aggregating the Level 4 sub-causes to the parent are instances when certain sub-causes are not present. The United Nations Crime Trends police data only identify homicides, and aggregating homicides to injuries would not accurately represent all injuries.

Section 3.13: Remove shocks and HIV/AIDS maternal adjustments (step 11)[2]

For GBD 2021, CODEm models use an HIV/AIDS- and shock-free envelope. To be comparable, cause fractions must also be HIV/AIDS- and shock-free. Cause fractions were uploaded to the CoD database as the number of deaths

due to the cause over an adjusted sample in which the number of deaths due to "HIV/AIDS", "conflict and terrorism", "police conflict and executions", and "exposure to forces of nature" were removed.

Section 3.13.1: Remove HIV/AIDS and shocks from denominator where cause list includes HIV/AIDS (step 11.1)

The first step to generate HIV- and shock-free cause fractions was to remove any deaths from the sample that were directly coded to "HIV/AIDS", "collective violence and legal intervention", or "exposure to forces of nature". The cause fraction uploaded to the database can be calculated by a simple equation:

$$CF_{l,t,a,x,c} = \frac{D_{l,t,a,x,c}}{D_{l,t,a,x} - D_{l,t,a,x,hiv} - D_{l,t,a,x,war} - D_{l,t,a,x,disaster}}$$

Where:

$CF_{l,t,a,x,c}$ is the cause fraction for a location *l*, year *t*, age *a*, sex *x*, and cause *c*

$D_{l,t,a,x,c}$ is the number of deaths observed for cause *c* in location *l*, year *t*, age *a*, and sex *x*

$D_{l,t,a,x}$ is the total number of deaths due to all causes observed in location *l*, year *t*, age *a*, and sex *x*

$D_{l,t,a,x,hiv}$, $D_{l,t,a,x,war}$, and $D_{l,t,a,x,disaster}$ are the numbers of deaths observed in location *l*, year *t*, age *a*, and sex *x* for causes "HIV/AIDS", "collective violence and legal intervention", and "exposure to forces of nature", respectively

Cause fractions for HIV/AIDS and shock causes were also uploaded to the database for use in separate estimation processes described by Wang et al.[31] In this case, cause fractions followed the standard equation, with variables following the same explanation.

$$CF_{l,t,a,x,c} = \frac{D_{l,t,a,x,c}}{D_{l,t,a,x}}$$

Section 3.13.2: Remove HIV/AIDS deaths from maternal mortality sources (step 11.2)

HIV-free cause fractions were also uploaded for sources on mortality due to maternal causes. In these cases, the sample of all deaths observed in the study is likely to contain some number of deaths due to HIV/AIDS and shocks, but the sample only includes cause information on maternal deaths. To account for the presence of HIV/AIDS and shocks in the entire sample, we assumed the same proportion of total deaths due to HIV/AIDS by location, age, sex, and year as provided from the estimation of HIV/AIDS and all-cause mortality described by Wang et al.[31]

Maternal mortality studies were only corrected for HIV/AIDS if the sample of total deaths was provided in the data source. Where sources provided only the MMR, we applied the rate to the HIV- and shock-free envelope produced by the analysis described in Wang et al.[31] and thus did not need to adjust cause fractions at this point in the process.

Where a correction was applied, we used the following equation:

$$CF_{l,t,a,x,mat} = \frac{D_{l,t,a,x,maternal}}{D_{l,t,a,x,maternal} + \frac{E[D_{l,t,a,x,hiv\_shock\_free}]}{E[D_{l,t,a,x}]} D_{l,t,a,x,non-maternal}}$$

Where:

$CF_{l,t,a,x,mat}$ is the resulting cause fraction due to maternal causes for the location (l), year (t), age (a), sex (x);

$D_{l,t,a,x,mat}$ is the number of observed deaths in the sample due to maternal causes

$D_{l,t,a,x,non-maternal}$ is the number of observed deaths in the sample due to non-maternal causes

$E[D_{l,t,a,x}]$ is the GBD estimate of all-cause mortality in the location, year, age, and sex

$E[D_{l,t,a,x,hiv\_shock\_free}]$ is the GBD estimate of HIV- and shock-free mortality in the location, year, age, and sex

Section 3.13.3: HIV/AIDS correction of sibling history, census, and survey data (step 11.3)

As described in our analysis from GBD 2013, many studies have failed to find increased mortality in HIV+ pregnant mothers, but those who have advanced HIV are known to have increased baseline mortality. Prior to GBD 2013, we did not distinguish between deaths in HIV+ women that were caused by pregnancy and those for whom the pregnancy was incidental to their death. To more explicitly quantify the contribution of pregnancy to death in HIV+ women, and therefore more accurately estimate the maternal death count, we completed two additional analyses for GBD 2013 and all subsequent GBD analyses. First, we determined the population attributable fraction (PAF) of HIV/AIDS to pregnancy-related death. Second, we determined the proportion of pregnancy-related deaths in HIV+ pregnant mothers that are aggravated by pregnancy and are therefore by definition maternal deaths.

$$PAF = \frac{P(RR-1)}{1+P(RR-1)}$$

Where:

PAF is the population attributable fraction

P denotes the prevalence of HIV in pregnancy

RR is relative risk of mortality in HIV+ vs HIV- pregnant mothers.

To recap our analysis for GBD 2013, we used the paper published by Calvert and Ronsmans[32] to identify sources that could inform Step 1 of our HIV-correction analysis. We independently reviewed each of the component studies in Calvert and Ronsmans' review and extracted data directly, not from the systematic review paper. We identified only one additional study that was not used in Calvert and Ronsmans' analysis. We have, however, not used all the studies included in that review. Specific details are as follows:

1) Figueroa-Damian et al.[33] was excluded for not including any postpartum deaths at all.

2) In the case of Ryder et al.[34] and Zvandasara et al.,[35] we excluded those deaths that occurred more than 12 months after delivery.

3) We excluded the results from Chilongozi et al.[36] from the site that did not include any HIV- patients.

4) Leroy et al.[37] was not in the bibliography. We could not locate it for review, so it was excluded.

5) Kourtis et al.[38] was extracted with adjustment of the denominator based on the average number of hospitalisations per delivery in each group.

6) Ticconi et al.[39] was excluded for being both non-representative and including subgroup data from mothers with malaria infection.

A total of 21 sources were included in our analysis of the increased mortality risk of HIV+ versus HIV- women in pregnancy.[40] We performed DerSimonian-Laird random effects meta-analysis to derive a pooled estimate of RR of death during pregnancy given HIV positivity.[41] The pooled effect size was 6·40 (95% uncertainty interval [UI] 3·98–10·29), which was then used to calculate an HIV PAF for each country, age group, and year. To determine the proportion of those HIV-related deaths that were attributable to maternal causes, we performed a second systematic literature review. This time we sought evidence for the excess mortality risk of pregnancy in those women who are already HIV+. Most studies have failed to find such an effect, but most also did not stratify their study population by stage of HIV or ART (antiretroviral therapy) status. Only two studies did this stratification, with a pooled effect size of 1·13 (95% UI 0·73−1·77).[42,43]

An updated literature review to inform the relative risk of mortality in pregnancy in HIV+ versus HIV- women had 14 non-usable sources. We completed this search on May 10, 2019, using the following search strings:

((HIV[Title/Abstract] OR "Acquired Immunodeficiency Syndrome"[Title/Abstract] OR AIDS[Title/Abstract]) AND (“pregnant”[Title/Abstract] OR “pregnancy”[Title/Abstract] OR “postpartum”[Title/Abstract] OR "post partum"[Title/Abstract]) AND (“mortality”[Title/Abstract] OR “death”[Title/Abstract]) NOT "case report" NOT (animals[MeSH] NOT humans[MeSH])

AND (2016/08/15[PDat] : 3000/12/31[PDat]))

Prevalence of HIV in pregnant women was calculated by using the Joint United Nations Programme on HIV and AIDS (UNAIDS) Spectrum model,[44] a compartmental HIV progression model used to generate age-specific incidence, prevalence, and death rates from pre-calculated incidence curves and assumptions about intervention scale-up and local variation in epidemiology. For each location, we used UNAIDS’ age-specific ratios of fertility in women living with HIV to fertility in women not living with HIV. In most locations, this ratio is assumed to be greater than one in women aged 15–24 years and less than one and decreasing as age increases beyond 24 years. Since Spectrum assumes fertile ages of 15–49 years, we used the ratio of HIV prevalence in pregnant women to HIV prevalence in the general population at either end of that range to extend estimates to age bands 10–14 years and 50–54 years.

Unlike GBD 2013, when we applied the PAF correction to the envelope of maternal deaths predicted by CODEm, we instead applied country-year-age-group-specific PAF to maternal mortality input data prior to modelling in CODEm. This ensured that both the numerator and denominator of all CF data were internally consistent in their exclusion of background HIV/AIDS mortality. The cause fractions for maternal deaths in sibling history, survey, and census data were therefore adjusted as follows:

$$CF_{l,t,a,x,mat_{adj}} = CF_{l,t,a,x,mat} \times \left(1 - ProP_{hiv_{l,t,a,x}}\right)$$

$$ProP_{hiv_{l,t,a,x}} = PAF_{l,t,a,x,hivpos} \times (1 - rr_{mat})$$

$$CF_{l,t,a,x,mat_{hiv}} = CF_{l,t,a,x,mat} \times ProP_{maternalhiv_{l,t,a,x}}$$

$$ProP_{maternalhiv_{l,t,a,x}} = PAF_{l,t,a,x,hivpos} \times rr_{mat}$$

Where:

$CF_{l,t,a,x,mat}$ = The proportion of deaths due to all maternal causes before HIV/AIDS correction for the location, year, age, and sex.

$CF_{l,t,a,x,mat_{adj}}$= The proportion of deaths due to maternal causes after the adjustment for the location, year, age, and sex.

$CF_{l,t,a,x,mat_{hiv}}$ = The proportion of deaths due to maternal deaths aggravated by HIV/AIDS after the adjustment for the location, year, age, and sex.

$PAF_{l,t,a,x,hivpos}$ = The PAF that describes the percentage of all maternal deaths that were HIV-related for the location, year, age, and sex.

$ProP_{hiv_{l,t,a,x}}$ = The proportion of deaths in pregnancy for the location, year, age, and sex that are estimated to be incidental deaths due to HIV/AIDS and therefore not a maternal CoD.

$ProP_{maternalhiv_{l,t,a,x}}$ =The proportion of deaths in pregnancy for the location, year, age, and sex that are estimated to be HIV+ and maternal deaths that are aggravated by HIV/AIDS.

rr_{mat} = 0.13/1.13 = The proportion of HIV/AIDS deaths during pregnancy that were exacerbated by the pregnancy.

Section 3.13.4: HIV/AIDS correction of other maternal mortality data (step 11.4)

Although a specific subset of codes in ICD-10 corresponds to HIV/AIDS deaths aggravated by pregnancy, these codes are sparsely used and unreliable. We therefore adapted the method described to also correct VR and VA sources for the systematic exclusion of HIV-related maternal deaths. This correction was calculated in the same manner, by using the same input data as above, with the only difference being that HIV correction of VR and VA sources resulted in a net increase in the maternal correction factor maternal deaths aggravated by HIV/AIDS are calculated in the following way:

$$CF_{l,t,a,x,mat_{hiv}} = CF_{l,t,a,x,mat} \times ProP_{maternalhiv_{l,t,a,x}}$$

$$ProP_{maternalhiv_{l,t,a,x}} = \frac{PAF_{l,t,a,x,hivpos} \times rr_{mat}}{1 - PAF_{l,t,a,x,hivpos} \times rr_{mat}}$$

Section 3.14: Noise reduction (step 12)[2]

To deal with problems of zero counts and stochastic variation in VR, VA, MITS diagnosed data, and maternal surveys, censuses, surveillance, and sibling histories, we use a Bayesian noise-reduction algorithm. We estimate a prior for a given series of data by running a Poisson regression to estimate the number of deaths due to each respective cause and sex with dummy variables for country, age, and year. With several notable exceptions (detailed below), these regressions are sex-, cause-, and GBD region-specific, so borrowing strength over age, year, and country is only within a given data type, GBD region, cause, and sex. The posterior estimate for each data is a weighted average between the prior and data:

$$posterior\ cause\ fraction = X \times weight + \mu \times (1 - weight)$$

Where:

X is the mean of the data in units of cause fractions

μ is the mean of the prior in units of cause fractions

The weight is determined by a function of the mean deaths per year in a given location-age-sex-cause for vital registration data:

$$VR\ weight_{l,a,s,c} = 1 - \frac{a}{b \times mean_y\left(deaths_{l,y,a,s,c}\right) + 1}$$

In this equation, a and b are hyper-parameters set to 0.99 and 0.1, respectively. This value of a was chosen to give the data only 1% weight when data are extremely sparse and therefore average approximately zero deaths per year for a given location, age, sex, and cause. This value of b was chosen based on a grid search to determine the optimal value of b that gives as much weight to the data as possible while still resulting in optimal smoothing of small numbers.

For non-VR, the weight is instead based on the variance of the prior, τ^2, and the variance of the data, σ^2. τ^2 is estimated from the Poisson regression, taking into account the variance-covariance matrix of the regression coefficients. For the data variance, σ^2, we use the Wilson approximation which provides an estimate of σ^2 even in cases with a zero count of cause-specific deaths. The weight used for non-VR is:

$$non - VR\ weight = \frac{\tau^2}{\tau^2 + \sigma^2}$$

This approach to noise reduction adjusts zero counts to non-zero values, mitigating the problem that zero counts in a log rate model or a logit cause fraction CODEm must be dropped from the regression, leading to upward bias in the estimates. This is particularly important in three settings: small high-income countries with low death counts overall; rare causes with small numbers of cause-specific deaths; and the analysis of sibling history, verbal autopsy, and survey data with small samples. For example, in sibling history data, for any given age group in any given year the number of deaths reported in the survey that are pregnancy-related or the number of deaths from all causes in that age group may be small.

There are several important exceptions to the data pooling and regression specifications outlined above. First, for country-series of VR from subnationally estimated countries, regressions are run on data from only that country with a fixed effect on subnational unit. Third, for VA data, all VA data for a given super-region are pooled together and a random effect on study-location is added to the regressions, allowing for different studies and surveillance sites to borrow strength from one another within a super-region. Unless the data are part of a time series (ie, the Matlab Health and Demographic Surveillance System and India Sample Registration System), the regression has no year component. Fourth, for VA data on malaria specifically, data are pooled into regional groups defined by the endemicity of malaria, as defined by the *Plasmodium falciparum* parasitaemia rate (PFPR). We include groups for hyper- (PFPR ≥0.4), meso- (PFPR 0.05 – <0.04), and hypo- (PFPR <0.05) endemic regions, separately for India; sub-Saharan Africa not including South Africa, Cabo Verde, and Mauritius, and including Yemen; and all remaining locations. Fifth, we run a separate regression for each cause in MITS diagnosed data from the Child Health and Mortality Prevention Surveillance (CHAMPS) study with fixed effects on age group and sex and a random effect on study-location. We include data from both sexes in one model and borrow strength across sex in order to combat data sparsity, and because CHAMPS includes only children under 5, an age demographic where sex differences are less extreme. Sixth, several individual VA studies are excepted from the above data pooling rules and regressed in their own separate models due to sample sizes that are too large and therefore dominate super-region-level models: Indonesia Sample Registration System (SRS), India verbal autopsy studies, and the Nepal Burden of Disease VA Study. Seventh, several VA study groups are excepted from the above regression specifications and run with individualised specifications: malaria-specific VA data from SSA are regressed with fixed effects on age group and study-location-year; VA data from the southeast Asia, east Asia, and Oceania super-region are regressed with fixed effects on age group and study-location; Matlab HDSS data are regressed with fixed effects on age group and year; and India SRS data are regressed with fixed effects on age group, year, and subnational location.

We also employ several strategies to combat data sparsity and failure to converge in noise reduction models. First, for models run on GBD regions of VR data, if a given year has only one country of data in that region, we bin this year with the preceding year in the Poisson regression. Second, for VA data, if a given cause/super-region/sex group has six or fewer observations or zero non-zero observations, we include all VA globally available for that cause/sex in a regression with fixed effects on age group and study-location and use the predictions from this global model as the prior for that cause/super-region/sex group. Third, for VA and CHAMPS data, if a model that includes random effects fails to converge, we rerun the model with the corresponding fixed effects. Fourth, after this, for all noise

reduction models, if the model fails to converge, we use a weighted average of the observed cause fractions within age groups in the model input data as the prior in noise reduction. This is essentially pooling information across study, location, and year within a grouping of data with similar data type and geography.

During noise reduction, all datapoints with cause fractions of zero are raised to a non-zero value through the Bayesian average with a non-zero prior. However, some of these values can be extremely small in magnitude (eg, on the order of 10^{-50} or smaller), to the point that they would be unobservable in the raw data. Cause fractions of this magnitude would become extremely large in absolute value when log- or logit-transformed during the CODEm process, leading to poor model fit in other ranges of the input data. However, these datapoints must remain non-zero in order to include them in a log- or logit-transformed model at all and avoid upwardly biasing estimates. To address this problem, we enforce a set of non-zero cause-, age- and sex-specific minimums, known as the "non-zero floor," on all cause fractions in the cause of death database. These floor values are chosen to be high enough to be relatively close to the bulk of the remaining data in log- or logit-space, but low enough to avoid significantly upwardly biasing the distribution of the data.

Floor values are determined based on VR data for cause/age/sex groups where we have enough data to do so accurately. We first consider VR data from all countries with greater than 50 million person-years represented in the database, after cause mapping and age-sex splitting but before any further processing. We calculate all-year cause fractions for each country, cause, age, and sex in this dataset. If the minimum cause fraction across country for a given cause/age/sex is non-zero, then we consider this cause/age/sex group to have sufficient data to determine a data-driven floor value. Otherwise, we consider this cause/age/sex to be too rare to have sufficient data to determine a minimum observable non-zero rate.

For cause/age/sex groups that have sufficient data, we set floor values based on all national-level noise-reduced VR datapoints in the CoD database that are non-zero before noise reduction. The floor value for a given cause/age/sex (c, a, s) is the minimum cause fraction across country-years (l, y) in this non-zero subset:

$$floor_{c,a,s} = \min_{l,y}\left(post\ NR\ VR\ cause\ fraction_{c,a,s,l,y}\right),$$

$$\text{where } cause\ fraction_{c,a,s,ly} \neq 0\ before\ NR$$

For all other cause/age/sex groups, which have insufficient data to inform the above calculation, we set a $floor_{c,a,s}$ value such that: (a) all floor values for a given cause add up to one death globally per year and (b) the age/sex distribution of the floor values in each cause follows the age/sex distribution of the global cause-specific mortality rates calculated in Section 2.6 for the purposes of age/sex splitting:

$$floor_{c,a,s} \times mortality\ envelope_{a,s} = \frac{R_{c,a,s} \times pop_{a,s}}{\sum_{a,s} R_{c,a,s} \times pop_{a,s}}$$

$floor_{c,a,s}$= floor value for cause c, age group a, sex s

$mortality\ envelope_{a,s}$= total estimated global deaths in 2018 for age group a, sex s

$pop_{a,s}$= total estimated global population in 2018 for age group a, sex s

$R_{c,a,s}$= global cause-specific mortality rate of cause c, age group a, sex s

This ensures that we add at most one death in the world per cause per year for causes where we have insufficient data to determine a plausible minimum observable cause fraction from the data.

Section 3.15: Cause of death database and outlier identification (step 13)[2]

Death rates for different causes of death generally have a stable age pattern. In large populations, these patterns will not change very rapidly over time. We can assume a relatively stable pattern in death rates for all causes except for some epidemic diseases and specific types of injuries. Rare causes in large populations and prevalent causes in small populations usually have stochastic patterns. To correct for these stochastic patterns, we implemented a noise-reduction process, explained in Step 12.

In VR data, we infrequently find one or more datapoints for specific geography/age/sex/year combinations that lie very far from the stable pattern of death rates. In these situations, the model usually ignores the datapoint(s). If the model fails to ignore these data, dramatic jumps or drops can occur in the death rates. When no logical explanation exists for variation in the death rates to this degree, we regard the datapoint(s) as outlier(s). The selection of datapoints to regard as outliers occurs after data have been prepped for modelling, as well as during preliminary reviews of the models.

In non-VR sources, data-collection methods and data quality can vary widely from source to source. Where datapoints in each age-sex-geography-year are very sparse, extreme datapoints can have a bad effect on regional estimation. In these situations, we investigate the study's methods and consider lower-quality datapoints as outliers.

Identifying outliers in the CoD data occurs prior to finalisation of models for each cause. We do not automate the selection of outliers but investigate the source of the offending data as well as reviewing other data sources for the same cause, geography, and year. Ultimately, outliers are identified based on the judgement of the modeller and senior faculty. Outlier decisions are reversible and may be revisited.

Section 3.16: Causes of death data star-rating calculation[2]

GBD estimates are most accurate when computed with a full time series of complete VR with a low percentage of garbage codes. For GBD 2016, we developed a simple star-rating system from 0 to 5 to give a picture of the quality of data available in a given country over the full time series used in GBD estimates. Countries improve in the star rating as they increase availability, completeness, and detail of their mortality data and reduce the percentage of deaths coded to ill-defined garbage codes or highly aggregated causes (figures 5a and 5b). Underlying indicators for the percentage well-certified calculation are listed in table S9.

We assign star ratings to rate the quality of data for any given location year. Two dimensions determine this star rating: (I) the percentage of total deaths determined to be major garbage (such as ill-defined). Causes such as "injuries" or "cancer" will also be included in major garbage percentage because this percentage includes use of highly aggregated causes; and (II) the level of completeness of death registration (percentage of total deaths captured by the death registration). These two values were used to create a "percentage well-certified" value between 0 and 1, determined as:

$$pct_{wellcertified} = Completeness \times (1 - pct_{majgarbage})$$

The mapping of percentage well certified to star rating is as followed:

0 star: $0\% = pct_{wellcertified}$

1 star: $0\% < pct_{wellcertified} < 10\%$

2 star: $10\% <= pct_{wellcertified} < 35\%$

3 star: $35\% <= pct_{wellcertified} < 65\%$

4 star: $65\% <= pct_{wellcertified} < 85\%$

5 star: $pct_{wellcertified} \geq 85\%$

While stars are calculated for each five-year time interval as well as the full time series from 1980 to 2020, stars in the main text are presented for the full time series only.

In the case of VA, all garbage codes are considered ill-defined because redistribution for VA is highly imprecise.

For each VA data source, percentage well-certified is:

$$pct_{wellcertified} = VerbalAutopsyAdjustment \times (1 - pct_{majgarbage})$$

Where:

$$VerbalAutopsyAdjustment = SubAdj \times RegAdj \times AgeSexCoverage$$

SubAdj is 10% for subnationally representative studies; 100% for nationally representative studies. This adjustment, while arbitrary in its specific value, reflects the bias that can be associated with studies that only cover a potentially non-representative sample of a country's population.

RegAdj is 64% for all VA data sources. This accounts for the inaccuracy of VA in assigning CoD compared to medically verified VR. The specific multiplier 0·64 is based on the chance-corrected concordance of Physician Certified Verbal Autopsy (PCVA) versus medical certification by the Population Health Metrics Research Consortium.[45]

Age-sex coverage is the number of deaths estimated in the GBD mortality envelope for the ages and sexes in the study for the country and year divided by the number of deaths estimated in the GBD mortality envelope for the country and year. Studies that only cover children under 5 years or maternal mortality, for example, will be highly discounted by this multiplier.

Once percentage well-certified is calculated for each location-year of VR and each VA study-year, we then combine these into one measurement for each five-year time interval and the full time series 1980–2020. For each five-year time interval, we take the maximum percentage well-certified. Then for 1980–2020, we take the average of the maximum percentages well-certified for the seven five-year time intervals. Any five-year time interval in which no data were available were given a percentage well-certified value of zero.

Prior to GBD 2019, the causes of death team used an all-ages, both-sex cause fraction to estimate the percentage of garbage-coded deaths in a given location-year. Thus, the percentage of garbage for a given location year was determined as:

$$CF_G = \frac{D_G}{D}$$

Where:

CF_G represents the cause fraction of percentage garbage

D_G represents total garbage-coded deaths

D represents the total deaths in a given location/year.

In GBD 2019, we moved to calculating the percentage of garbage-coded deaths using an age-standardised cause fraction. The steps for creating these age-standardised cause fractions, in the case of garbage, are as follows:

1. Create both-sex, age-specific cause fractions of garbage for each age group.

2. Scale these cause fractions by a set of both-sex age weights, determined by global mortality estimates from 2010 to present. That is, weights for each GBD age group were determined as:

$$W_a = \frac{D_a}{D}$$

Where:

W_a is the weight for given age group "a"

D_a is the total both-sex, global deaths from 2010 to present in age group "a"

D is the total both-sex, global deaths from 2010 to present across all ages.

3. Sum these weighted cause fractions across all age groups to produce the age-standardised cause fraction.

In the case of percentage garbage for a given location-year, the formula to calculate percentage garbage would be given as the sum of the weighted age-specific cause fractions across all age groups "a":

$$CF_G = \Sigma_a(\frac{G_a}{D_a} \times W_a)$$

Where:

G_a represents the total both-sex garbage deaths in age group "a"

D_a represents the total both-sex deaths in age group "a"

W_a represents the weight generated from mortality estimates for age group "a"

ICD-10 and ICD-9 codes assigned to Level 1 or 2 garbage can be found in table S6.

In GBD 2021, a buffer system was implemented to prevent frequent star rating changes due to small fluctuations in completeness and percentage of garbage round over round. If a location-year decreases in star rating, as long as its PWC value remains within 3% of the cut-off for its former star rating, it will retain the former star rating.

Section 4: Causes of death modelling methods

Section 4.1: CODEm[2]

Section 4.1.1: Overview of methods

Cause of death ensemble modelling (CODEm) is the framework used to model most cause-specific death rates in the GBD.[46] It relies on four key components:

First, all available data are identified and gathered to be used in the modelling process. Although the data may vary in quality, they all contain some signal of the true epidemiological process.

Second, a diverse set of plausible models are developed to capture well-documented associations in the estimates. Using a wide variety of individual models to create an ensemble predictive model has been shown to outperform techniques using only a single model both in CoD estimation[46] and in more general prediction applications.[47,48]

Third, the out-of-sample predictive validity is assessed for all individual models, which are then ranked for use in the ensemble modelling stage.

Finally, differently weighted combinations of individual models are evaluated to select the ensemble model with the highest out-of-sample predictive validity.

For some causes (eg, lower respiratory infections), evidence exists that the relationship between covariates and death rates might differ between children and adults. Separate models are therefore run for different age ranges, when applicable. Additionally, separate models are developed for countries with extensive, complete, and representative VR for every cause to ensure that uncertainty can better reflect the more complete data in these locations.

In order to ensure the addition of subnational locations is not driving changes in estimates, in GBD 2021, we run a global model that excludes data from non-standard locations; the resulting covariate betas are then used as priors for the true global model.

In addition to CoD modelling, we also estimate fatal discontinuities. Fatal discontinuities are events that are stochastic in nature, that cannot be modelled because they do not have a predictable time trend. The fatal discontinuities by cause are aggregated by age and sex and added to the estimated number of deaths in CoD modelling for those causes during CoDCorrect. Details on their methods can be found in Section 3.4.

Section 4.1.2: Model pool development

Because many factors may co-vary with any given CoD, a range of plausible statistical models are developed for each cause. In the CODEm framework, four families of statistical models are used: linear mixed effects regression (LMER) models of the natural log of the cause-specific death rate, LMER models of the logit of the cause fraction, spatiotemporal Gaussian process regression (ST-GPR) models of the natural logarithm of the cause-specific death rate, and ST-GPR models of the logit of the cause fraction (see the 2x2 table in Foreman et al).[46] For more on ST-GPR, see section 4.3.3. For each family of models, all plausible relationships between covariates and the response variable are identified. Because all possible combinations of selected covariates are considered for each family of models, multi-collinearity between covariates may produce implausible signs on coefficients or unstable coefficients. Each combination is therefore tested for statistical significance (covariate coefficients must have a coefficient with p-value $<0{\cdot}05$) and plausibility (the coefficients must have the directions expected on the basis of the literature). Only covariate combinations meeting these criteria are retained. This selection process is run for both cause fractions and death rates, then ST-GPR and LMER-only models are created for each set of covariates. For a detailed explanation of the covariate selection algorithm, see Foreman et al.[46]

Section 4.1.3: Data variance estimation

The families of models that go through ST-GPR described in Section 4.1.2 incorporate information about data variance. The main inputs for a Gaussian process regression (GPR) are a mean function, a covariance function, and data variance for each datapoint. These inputs are described in detail in Foreman et al.[46] For GBD 2019, we updated this calculation to incorporate garbage code redistribution uncertainty.

Three components of data variance are now used in CODEm: sampling variance, non-sampling variance, and garbage code redistribution variance. The computation of sampling variance and non-sampling variance has not changed since previous iterations of the GBD and is also described in Foreman et al.[46] Garbage code redistribution variance is computed in the CoD database process described in Section 2.7 of this appendix. Since variance is additive, we calculate total data variance as the sum of sampling variance, non-sampling variance, and redistribution variance. Increased data variance in GPR results in the GPR draws not following the datapoint as closely.

Section 4.1.4: Testing model pool on 15% sample

The performance of all models (individual and ensemble) is evaluated by means of out-of-sample predictive validity tests. 30% of the data are randomly excluded from the initial model fits. These individual model fits are evaluated and ranked by using half of the excluded data (15% of the total), then used to construct the ensembles on the basis of their performance. Data are held out from the analysis on the basis of the cause-specific missingness patterns for ages and years across locations. Out-of-sample predictive validity testing is repeated 20 times for each model, which has been shown to produce stable results.[46] These performance tests include the root mean square error (RMSE) for

the log of the cause-specific death rate, the direction of the predicted versus actual trend in the data, and the coverage of the predicted 95% UI.

Section 4.1.5: Ensemble development and testing

The component models are weighted on the basis of their predictive validity rank to determine their contribution to the ensemble estimate. The relative weights are determined both by the model ranks and by a parameter ψ, whose value determines how quickly the weights taper off as rank decreases. The distribution of ψ is described in more detail in Foreman et al.[46] A set of ensemble models is then created by using the weights constructed from the combinations of ranks and ψ values. These ensembles are tested by using the predictive validity metrics described in Section 4.1.4 on the remaining 15% of the data, and the ensemble with the best performance in out-of-sample trend and RMSE is chosen as the final model.

Section 4.1.6 Final estimation

Once a weighting scheme has been chosen, 1000 draws are created for the final ensemble, and the number of draws contributed by each model is proportional to its weight. The mean of the draws is used as the final estimate for the CODEm process, and a 95% UI is created from the 0·025 and 0·975 quantiles of the draws. The validity of the UI can be checked via its coverage of the out-of-sample data; ideally, the 95% UI would capture 95% of these data. Higher coverage suggests that the UIs are too large, and lower coverage suggests overfitting.

Section 4.1.7: Selection of causes for which CODEm is used

CODEm is used to model 193 causes, described in detail in Section 3.3. However, it is unsuitable for use in modelling certain causes, including those with very low death counts, those where cause-specific death record availability is inadequate, or those for which there are marked biases or variability for CoD certification over time that cannot be fully accounted for with the current garbage code redistribution algorithms. Criteria for causes where CODEm is not used are discussed in further detail in Section 3.2.

Section 4.1.8: Model-specific covariates

Modellers select covariates to be used in CODEm, but those covariates may not be significant or in the direction specified during the covariate selection step of CODEm and will therefore not be used in the model. These covariates are listed with a '—' for number of draws. Additionally, covariates may be selected by CODEm but only exist in submodels that perform poorly and may end up with zero draws included in the final ensemble. Finally, all other covariates are listed with the number of draws in the final ensemble from submodels that had the covariate.

Section 4.2: Causes modelled outside of CODEm[2]

Section 4.2.1: Overview

A number of causes required alternative modelling strategies to those used for CODEm because they were not compatible with CODEm estimation infrastructure and processes. Such unsuitability included having very low death counts; inadequate availability of cause-specific death records; and marked biases or variability for CoD certification over time that could not be fully accounted for with current garbage code redistribution algorithms. The inclusion of these causes in CODEm often renders its out-of-sample predictive validity testing unstable, but the validity of this type of testing is a key advantage of using CODEm for CoD estimation. Alternately, CODEm simply fails to generate plausible mortality rates in the absence of enough VR or VA data when these causes are included. Because of increased data availability and redistribution algorithm refinements, we were able to incorporate several new causes, which were modelled separately for GBD 2013, into CODEm for this iteration of the GBD study; with each annual update of GBD, we aim to add more causes within the CODEm estimation space. For GBD 2021, we used alternative modelling approaches for these causes, including negative binomial models, natural history models, sub-cause proportion models, and prevalence-based models (table S10).

Section 4.2.2: Negative binomial models

For eight rare causes of death, too few observed deaths were included in the CoD database to produce stable estimates. For these causes, we ran negative binomial regression models, with either a constant or a constant multiplied by the mean assumption for the dispersion parameter, by using reverse step-wise model building. We selected one of the two model dispersion assumptions based on best fit to the data by using the same method as

GBD 2013. Beginning in GBD 2015, we also tested zero-inflated Poisson models for these rare causes of death but rejected them after finding that they did not substantially affect the mean predictions but instead produced unrealistically large UIs. Descriptions of the modelling process for each of these causes follows in the next sections.

Section 4.2.3: DisMod-MR 2.1

Until GBD 2010, non-fatal estimates were based on a single data source on prevalence, incidence, remission, or a mortality risk selected by the researcher as most relevant to a particular location and time. For GBD 2010, we set a more ambitious goal: to evaluate all available information on a disease that passes a minimum quality standard. That required a different analytical tool that would be able to pool disparate information presented in varying age groupings and from data sources by using different methods. The DisMod-MR 1.0 tool used in GBD 2010 evaluated and pooled all available data, adjusted data for systematic bias associated with methods that varied from the reference and produced estimates with UIs by world regions. For GBD 2013, the improved DisMod-MR 2.0 had increased computational speed, allowing computations that were consistent between all disease parameters at the country rather than the region level. The hundred-fold increase in speed of DisMod-MR 2.0 was partly due to a more efficient rewrite of the code in C++ but also to changing to a model specification using log rates rather than a negative binomial model used in DisMod-MR 1.0. In cross-validation tests, the log rates specification worked as well as or better than the negative binomial specification.[49] For GBD 2015, the computational engine (DisMod-MR 2.1) remained substantively unchanged, but we rewrote the wrapper code that organised the flow of data and settings at each level of the analytical cascade. The sequence of estimation occurred at five levels: global, super-region, region, country, and, where applicable, subnational locations (see flow diagram of DisMod-MR 2.1 cascade that follows). The super-region priors were generated at the global level with mixed-effects, non-linear regression by using all available data; the super-region fit, in turn, informed the region fit, and so on down the cascade. The wrapper gave analysts the choice to branch the cascade in terms of time and sex at different levels depending on data density. The default used in most models was to branch by sex after the global fit but to retain all years of data until the lowest level in the cascade. For GBD 2015, we generated fits for the years 1990, 1995, 2000, 2005, 2010, and 2015.

In updating the wrapper, we consolidated the code base into a single language, Python, to make the code more transparent and efficient and to better deal with subnational estimation. The computational engine is limited to three levels of random effects; we differentiated estimates at the super-region, region, and country levels. In GBD 2013, the subnational units of China, Mexico, and the UK were treated as countries, such that a random effect was estimated for every location with contributing data. However, the lack of a hierarchy between country and subnational units meant that the fit to country data contributed as much to the estimation of a subnational unit as the fits for all other countries in the region. We found inconsistency between the country fit and the aggregation of subnational estimates when the country's epidemiology varied from the average of the region. Adding an additional level of random effects required a prohibitively comprehensive rewrite of the underlying DisMod-MR engine. Instead, we added a fifth layer to the cascade, with subnational estimation informed by the country fit and country covariates, plus an adjustment based on the average of the residuals between the subnational unit's available data and its prior. This procedure mimicked the impact of a random effect on estimates between subnationals.

For GBD 2015, we improved how country covariates differentiate non-fatal estimates for diseases with sparse data. The coefficients for country covariates were re-estimated at each level of the cascade. For a given location, country coefficients were calculated by using both data and prior information available for that location. In the absence of data, the coefficient of its parent location was chosen to utilise the predictive power of our covariates in data-sparse situations.

For GBD 2017, the DisMod-MR 2.1 tool was used. Updates included estimation of new age groups through the GBD 2017 terminal age group of 95 years and older in addition to the new locations added for the GBD 2017 cycle.

Section 4.2.4: DisMod-MR 2.1 likelihood estimation

Analysts have the choice of using a Gaussian, log-Gaussian, Laplace, or log-Laplace likelihood function in DisMod-MR 2.1. The default log-Gaussian equation for the data likelihood is as follows:

$$-log\left[p\left(y_j|\Phi\right)\right] = \log\left(\sqrt{2\pi}\right) + \log\left(\delta_j + s_j\right) + \frac{1}{2}\left(\frac{\log\left(a_j + \eta_j\right) - \log\left(m_j + \eta_j\right)}{\delta_j + s_j}\right)^2$$

Where:

y_j is a measurement value (ie, datapoint)

Φ denotes all model random variables

η_j is the offset value, *eta*, for a particular integrand (prevalence, incidence, remission, excess mortality rate, with-condition mortality rate, cause-specific mortality rate, relative risk, or standardised mortality ratio)

a_j is the adjusted measurement for datapoint j, defined by:

$$a_j = e^{(-u_j - c_j)} y_j$$

Where:

u_j is the total area effect (ie, the sum of the random effects at three levels of the cascade: super-region, region, and country)

c_j is the total covariate effect (ie, the mean combined fixed effects for sex, study-level, and country-level covariates), defined by:

$$c_j = \sum_{k=0}^{K[I(j)]-1} \beta_{I(j),k} \hat{X}_{k,j}$$

with standard deviation (SD)

$$s_j = \sum_{l=0}^{L[I(j)]-1} \zeta_{I(j),l} \hat{Z}_{k,j}$$

Where:

k denotes the mean value of each datapoint in relation to a covariate (also called x-covariate)

$I(j)$ denotes a datapoint for a particular integrand, j

$\beta_{I(j),k}$ is the multiplier of the kth x-covariate for the ith integrand

$\hat{X}_{k,j}$ is the covariate value corresponding to the datapoint j for covariate k

l denotes the SD of each datapoint in relation to a covariate (also called z-covariate)

$\zeta_{I(j),k}$ is the multiplier of the lth z-covariate for the ith integrand

δ_j is the SD for adjusted measurement j, defined by

$$\delta_j = log\left[y_j + e^{(-u_j - c_j)}\eta_j + c_j\right] - log\left[y_j + e^{(-u_j - c_j)}\eta_j\right]$$

Where mj denotes the model for the jth measurement, not counting effects or measurement noise and defined by:

$$m_j = \frac{1}{B(j) - A(j)} \int_{A(j)}^{B(j)} I_j(\mathrm{a})\, \mathrm{da}$$

Where:

$A(j)$ is the lower bound of the age range for a datapoint j

$B(j)$ is the upper bound of the age range for a datapoint j

$I(j)$ denotes the function of age corresponding to the integrand for datapoint j

The source code for DisMod-MR 2.1 as well as the wrapper code is available at https://github.com/ihmeuw/ihme-modelling/tree/master/gbd_2017/shared_code/central_comp/nonfatal/dismod.

Section 4.2.5: Natural history models

For some causes for which CoD data may be systematically biased either owing to misclassification or because the disease exists in focal communities without VR or VA studies, we have developed natural history models. In natural history models, incidence and case-fatality rates are modelled separately and then combined to produce estimates of cause-specific mortality.

Section 4.2.6: Prevalence-based models

The modelling strategies for atrial fibrillation and flutter are distinct from those used for other causes modelled as natural history models. These models use prevalence estimates and excess mortality rates (EMR) generated through DisMod-MR 2.1 rather than incidence and case-fatality rates.

Section 4.2.7: Sub-cause proportion models

For certain sub-causes for which accurate diagnoses are known to be very difficult, we first modelled the parent cause in the GBD hierarchy with CODEm and then allocated deaths to specific causes by using proportions of the parent cause for each age-sex-location-year for each sub-cause. For these causes, we identified no significant predictors in negative binomial regressions. This approach was taken because the available data on these specific causes may come from sources other than VR, such as end-stage renal disease registries, or may come from too few places to model the death rates directly. Details for each cluster of causes analysed in this way follow.

Section 5: COVID-19 and OPRM estimation

Section 5.1: Excess mortality due to the COVID-19 pandemic

We used the methodology described in a previous publication to estimate excess mortality due to the COVID-19 pandemic[50] with some key updates. Our analysis of excess mortality due to the COVID-19 pandemic from January 1, 2020, to December 31, 2021, followed four key steps. First, we developed a database of all-cause mortality by week and month after accounting for reporting lags, anomalies such as heat waves, and under-registration of death. Second, we developed an ensemble model to predict expected deaths in the absence of the COVID-19 pandemic for years 2020 and 2021. In location and time combinations with data meeting our inclusion criteria, excess mortality was estimated as observed mortality minus expected mortality. Third, to estimate excess mortality due to COVID-19 over the entire study period for all countries and territories in the analysis, and especially for locations without weekly or monthly all-cause mortality data, we developed a statistical model to directly predict the excess mortality due to COVID-19, using covariates that pertained to both the COVID-19 pandemic and background population health-related metrics at the population level before SARS-CoV-2 emerged. Fourth, we estimated excess mortality due to COVID-19, propagating uncertainty from each step.

To estimate expected mortality, we developed six models, each fit separately by location. The first four models were based on first estimating the weekly (or monthly) seasonal pattern of mortality and then estimating the time trend in weekly or monthly mortality not explained by seasonality. We used a Bayesian spline to estimate the weekly seasonal pattern for each location using data from 2010, or the earliest year after 2010 when such data first became available, until around February 2020, when the COVID-19 pandemic started for each location. Second, using the same Bayesian spline, we estimated the time trend in the residuals. By combining the seasonal and secular trends, we generated predictions of the expected level of mortality in 2020 and 2021.

The specification of the spline can have a sizeable impact on the estimated expected mortality for a particular location. To make the results more robust to model specification, we included in our ensemble four variants according to where the second to last knot in the spline was placed: 6 months, 12 months, 18 months, and 24 months before the end of the period for the input data before the COVID-19 pandemic started for each location. We also included in the ensemble a Poisson model with fixed effects on week and year, and a model that assumed that expected mortality for 2020 and 2021 was the same as the corresponding weekly mortality observed in 2019. To derive weights for the different models in the ensemble, we assessed how each model performed in an out-of-sample predictive validity test. We fit the model to all data prior to March 1, 2019, and then evaluated how each model performed in predicting mortality between March 2019, and February 2020, compared with observed mortality in the same time period. We then weighted component models in the ensemble using 1 over the root mean squared error (RMSE) of the predictions for each component to down-weight component models with larger RMSE (and thus less accurate predictions) in the ensemble. A global weighting scheme was used for all locations. Expected mortality from the ensemble model was subtracted from observed mortality in 2020 and 2021 to estimate excess mortality due to the COVID-19 pandemic.

Based on our estimation of expected mortality, and after applying the exclusions that account for late registration and other anomalies as previously described, we generated excess mortality estimates for each location where we had input data. In addition, we added excess mortality estimates from two countries where the ensemble model could not be applied due to data constraints: South Africa (only the provinces) and India. We obtained national and province-level excess mortality rate estimates for South Africa which are regularly updated by the Medical Research Council of South Africa, and excess mortality estimates for select periods during the first and second waves of the COVID-19 epidemic for 12 states in India. Using these empirical excess mortality estimates, we developed a statistical model to predict excess mortality for all 191 national and 252 subnational locations in our analysis for the uniform period of January 1, 2020, to December 31, 2021. This model was crucial for directly estimating excess mortality in countries where reported all-cause mortality data have not been available during the pandemic.

Various studies have examined the associations between particular underlying medical conditions and increased risk of severe COVID-19. We examined all available and relevant covariates on the basis of a meta-analysis conducted by the US Centers for Disease Control and Prevention, as well as covariates directly related to the COVID-19

pandemic, including seroprevalence (lagged by 25 days), mobility (lagged by 19 days), infection-detection ratio (IDR; lagged by 19 days), and reported crude death rate due to COVID-19. To help identify covariates that have sensible direction of effect on excess mortality rate, we used ROVER, a method developed at IHME based on Bayesian model averaging (BMA). ROVER is conceptually similar to the BMA method, which is widely used to explore the parameter space and aggregate estimates across candidate models based on performance metrics.[51] The main difference is that while BMA uses marginal likelihood, ROVER focuses on out-of-sample performance. The implementation of ROVER used for this process can be found at https://github.com/ihmeuw-msca/modrover. Based on the outputs of ROVER, 11 covariates were included in our final log-linear model, where the dependent variable was excess mortality in logarithmic scale: lagged cumulative infection rate (seroprevalence) in log space, COVID-19 mortality rate in log space, crude death rate in log space, lagged IDR, annual inpatient admissions per capita, diabetes mortality rate in log space, HIV mortality rate in log space, average absolute latitude, smoking prevalence, Healthcare Access and Quality (HAQ) Index, and proportion of the population aged 75 years or older. To reduce model volatility, after fitting the initial model, datapoints with residuals in the top and bottom 2.5th percentiles were excluded and the model was refit. Altogether, these covariates explained 86.9% of the variation in the input data to this regression. We also calculated in-sample residuals for the locations that had directly observed excess mortality rates used in the regression (ie, India and South Africa). Regional and super-regional residuals, per the GBD regional classification system, were calculated as the mean residuals from locations included in each regional aggregate. We also calculated a country-level residual for India using the residual from the 12 states to recognise the dispersed periods and geographical regions covered by the civil registration data. To validate our modelling process, we conducted out-of-sample predictive validity testing. Given the sparsity of input data on empirical excess mortality, we validated our model by repeatedly leaving one location out of the input data, then re-estimated the model and made predictions for the left-out location. Our analysis showed that the mean relative error of predicted excess mortality rate is 10.2% and the root mean squared error of predicted excess mortality rate is 0.00066, indicating a precise prediction model that has low bias.

With this model, we predicted estimates of excess mortality for the periods of 2020, 2021, and the combined time period of January 1, 2020, to December 21, 2021. Given the broader availability of data covering the entire time period, the yearly estimates were scaled to the entire time period for locations without direct estimates of excess mortality from the first stage model. Ratios of estimated excess mortality to reported COVID-19 deaths were computed using the modelled excess mortality and the reported COVID-19 counts for each location.

As noted previously, policy makers and researchers have proposed many other causes of death that might have been affected by lockdown restrictions, unemployment, and increased poverty. To date, insufficient data are available to widely test for increases in deaths due to pandemic-related elements, such as deferred care, or for reductions in deaths, such as decreases in injury-related deaths due to reduced mobility.

Section 5.2: Excess mortality age-sex splitting

In order to reconcile all-ages, all-sexes excess mortality estimates with age-sex-specific all-cause mortality estimates, age-sex distributions of excess mortality were created to split the aggregate estimate. Distributions of excess were calculated by taking the difference between two all-cause envelope versions for location with VR in 2020–2021: 1) an envelope with pandemic years VR data, 2) a counterfactual envelope without pandemic years VR data. The difference between these envelope versions was the implied excess mortality and provided age- and sex-specific rates of excess. Beyond location-specific distributions of excess mortality, global distributions of positive and negative excess were created by summing the implied excess of all countries with positive and negative excess, respectively. The global positive distribution was calculated separately for 2020 and 2021, while the global negative distribution was calculated using all location-years with implied negative excess.

These distributions of excess were applied to the total COVID-19 excess mortality values and scaled such that the total excess mortality was conserved, and the implied under-5 excess remained constant. Location-years with VR largely used a location-year-specific age-sex distribution while locations without VR used either the positive or negative global distribution of excess depending on the value of excess mortality. Location-years for 2021 could also use the distribution from 2020 if VR was present in 2020, but not 2021. Other locations with VR could also be forced to use the global distribution if estimated excess mortality was below a threshold (50 000) or if the implied

distribution was implausible or differed in sign from the estimated total excess mortality. All subnational locations used the same age-sex distribution as their parent locations if the sign agreed, or the corresponding global distribution if it did not.

Section 5.3: Total COVID-19 mortality estimation

To estimate total COVID deaths, we use a counterfactual approach based on the COVID-19 excess mortality regression model. The counterfactual estimate sets lagged infection-detection rate to the maximum observed value and recalculates COVID-19 excess mortality. The ratio of counterfactual excess deaths over regular excess deaths gives us an estimate of "true COVID as a proportion of excess". Using this value and the ratio of COVID over excess deaths, we then calculate the "ratio of true over reported COVID" as:

$$\frac{\max(\mathit{true\ COVID\ as\ a\ proportion\ of\ excess},\ \mathit{ratio\ of\ reported\ COVID\ over\ excess})}{\mathit{ratio\ of\ reported\ COVID\ over\ excess}}$$

Finally, this ratio is multiplied by reported COVID deaths to give an estimate of total COVID deaths. We also have checks throughout this process to handle cases of negative excess mortality, zero reported COVID, or a ratio of COVID over excess greater than 1—in these cases, we set the ratio of true over reported COVID to 1.

Section 5.4: Balancing equation to calculate OPRM

The balancing equation formally reconciled deaths associated with COVID-19 excess mortality and cause-specific pandemic-related causes. This was done by creating a measure of other pandemic-related mortality (OPRM) which is equal to the difference between excess mortality and the sum of COVID-19-specific deaths, measles, lower respiratory infection (LRI), and pertussis. Measles, LRI, and pertussis were referred to as indirect COVID causes as mortality from these diseases was affected by the pandemic and was deemed to be measured reliably enough to account for components of excess mortality. OPRM captures all deaths due to the pandemic which were not specifically caused by COVID-19 or the indirect COVID causes. It can be positive or negative, though as we assert that GBD Level 1 causes cannot be negative, negative OPRM is removed in the next process.

Section 5.5: COVID-19, OPRM, and excess mortality harmonisation

To create consensus between COVID-19-specific mortality, OPRM, and all-cause mortality, we reconciled the difference between estimated and implied excess mortality, using estimates from the model life table system, First, any negative OPRM was added to the all-cause envelope and set to zero. For locations with VR data in either 2020 or 2021, the difference between implied and separately estimated excess mortality was split between the all-cause envelope, COVID-19 deaths, and other pandemic-related causes via weighted proportional allocation with weights chosen by visual inspection of graphs to minimise discontinuities in expected non-pandemic mortality time trends. Steps were taken such that values were positive and that final COVID-19 deaths were never less than the reported COVID-19 deaths from each location. Additionally, we adjusted mortality for locations that had VR data in 2020, but not 2021, by multiplying the envelope by the ratio of the adjusted envelope in 2020 over the unadjusted envelope. Locations with no VR in either 2020 or 2021 instead use a ratio of expected deaths from the COVID EM estimation pipeline and the estimate from model life tables. This, in addition to distributing negative OPRM among the all-cause envelope and COVID mortality, allows us to have consistency between our with- and without-pandemic data mortality results, COVID mortality estimation, and COVID excess mortality estimation. Subnational estimates for these indicators were scaled to the national level.

Section 6: Central computation[2]

Section 6.1: Imported cases

Imported cases are fatalities that occur in a geographic area where a particular CoD is known to be eradicated in a specific time period or where infection cannot occur. We apply space-time restrictions to these causes in the

modelling strategy for that location and time period. However, in some rare cases, deaths from these causes occur outside of restricted locations and time periods. These deaths are referred to as imported cases.

Illustrating this concept, some diseases are transmitted only in certain regions of the world. For those diseases, we restricted our models to only endemic locations, excluding non-endemic countries from the analysis. In some cases, however, deaths for these geographically restricted diseases may occur in non-endemic countries. For example, individuals may become infected with a disease while travelling or residing in an endemic location. Through travel or migration, however, they may move to a non-endemic location prior to dying from that disease. Imported cases account for these kinds of deaths.

To calculate these imported cases, we find all cases from the VRs of data-rich countries for any CoD that is otherwise geographically or temporally restricted. We then create a beta distribution from that datapoint by using the sample size of the VR for that datapoint and upload these draws as a custom CoD model. This model is then used as an input to CoDCorrect.

Section 6.2: CoDCorrect

Section 6.2.1: Objective of CoDCorrect

As mentioned in the main text, the CoD models are cause-specific. As such, there is no guarantee that the sum of these models will equal the results of the all-cause mortality estimates or that model results of child causes add up to the parent model results. The CoDCorrect process is used to make the CoD and all-cause mortality estimates internally consistent by using a very simple algorithm.

Section 6.2.2: Algorithm and levels

The core algorithm remains the same as it did in GBD 2013. The equation can be written as follows:

$$CD_{lyasjd} = D_{lyasjd}\left(\frac{PD_{lyasjd}}{\sum_{j=1}^{j=k} D_{lyasjd}}\right)$$

Where:

CD_{lyasjd} is the corrected number of deaths for a location l, year y, age a, sex s, cause j, and draw d

PD_{lyasjd} is the parent CoD for a location l, year y, age a, sex s, cause j, and draw d

D_{lyasjd} is the uncorrected number of deaths estimated from a cause-specific model for a l, year y, age a, sex s, cause j, and draw d

The CoDCorrect process starts by rescaling the Level 1 causes to match the all-cause mortality estimates (used for PD_{lyasjd} in the previous equation). Level 2 causes are then rescaled to their corrected parent causes. This process continues until all levels of the hierarchy have been rescaled. Causes and their levels within the CoDCorrect hierarchy can be found in table S3.

Since GBD 2017, HIV has not been included in the CoDCorrect process. To account for this change, Level 1 CoDCorrect causes are rescaled to HIV-deleted mortality estimates that are produced as part of the mortality and HIV estimation process. Results from the GBD version of Spectrum are added to the post-CoDCorrect death estimates with fatal discontinuities and imported cases to generate the full set of death estimates.

Section 6.2.3: Diagnostic results of CoDCorrect by cause and location

For more detail on diagnostic results of CoDCorrect by cause see table S11.

Section 6.3: Years of life lost calculation

Years of life lost (YLLs) owing to premature mortality were computed for 1082 locations and 39 years. First, we used the lowest observed age-specific mortality rates by location and sex across all estimation years from locations with total populations greater than 5 million in 2016 to establish a theoretical minimum risk reference life table.

The YLL is a metric that is computed by multiplying the number of estimated deaths by the predicted life expectancy by age, sex, location, and year. The metric therefore highlights premature deaths by applying a larger weight to deaths that occur in younger age groups. We propagated uncertainty from CoDCorrected deaths for all demographics. The predicted life expectancy is calculated with two main components, a global, sex-agnostic, all-time theoretical "best" life expectancy (assuming that males and females in all countries in the world could theoretically have this life expectancy) and the average age of death from with-shock life tables for each location, sex, age group, and year.[3]

Section 6.4: GBD world population age standard

Age-standardised populations in the GBD were calculated by using the GBD world population age standard. For GBD 2013, GBD 2015, and GBD 2016, the age-specific proportional distributions of all national locations from the UN Population Division World Population Prospects 2012 revision for all years from 2010 to 2035 were used to generate a standard population age structure by using the non-weighted mean across all the aforementioned country-years. For GBD 2017, we used the non-weighted mean of 2017 age-specific proportional distributions from the GBD 2017 population estimates for all national locations with a population greater than 5 million people in 2017 to generate an updated standard population age structure.[52] For GBD 2021, we have continued to use this method using GBD 2021 population estimates.[3]

Section 7: Life expectancy decomposition

LE decomposition has two main steps:

1. Decompose the change in life expectancy from a start and end point by age.

2. Decompose life expectancy contributions for each cause and age group.

Once we have life expectancy contributions by cause and age group, the sum across age groups is taken as the cause's impact on at-birth life expectancy from the start point to the end point.

Before an at-birth life expectancy decomposition by cause can be computed for a start and end time period, we must first compute a decomposition by age for the population over the same time period.

Because age is a function of time and we are measuring the difference in life expectancy for a given time period, we cannot consider time to be an independent variable in our calculations without first separating the contribution to the difference which is due to the age structure of the given population.

The two input life table parameters are l_x, the proportion of the cohort surviving to age x, and e_x, the life expectancy at age x or the mean number of years lived after x-th birthday by those surviving to age x.

The method used is described in detail in Andreev's technique for age-specific contributions to life expectancy.[53]

In brief, for each age group pair x, $x + 1$ we can calculate the life expectancy difference for age group x from time 1 to time 2, δ_x^{2-1}, as:

$$\delta_x^{2-1} = l_x^2\,(e_x^2 - e_x^1) \; - \; l_{x+1}^2\,(e_{x+1}^2 - e_{x+1}^1)$$

where l_x^2 is lx for age group x at time 2, e_x^2 is ex for age group x at time 2, e_x^1 is ex for age group x at time 1, l_{x+1}^2 is lx for age group $x + 1$ at time 2, e_{x+1}^2 is ex for age group $x + 1$ at time 2, and e_{x+1}^1 is ex for age group $x + 1$ at time 1.

Age group pairs are immediately adjacent. For example, if x is 5 - 9, then $x + 1$ is 10 - 14. For the terminal age group, there is no subsequent age group, so the subtraction term is 0.

This function is asymmetrical depending on which year is considered "start" and which is "end", so we take the average of decompositions where the two are interchanged. It's expected that the sum of the decomposed life expectancy by age is the same as the difference between the at-birth life expectancies for the year pair.

Once age-specific decompositions, δ_a, have been calculated, we can perform decomposition by cause for every age group using mean deaths for every cause in rate space, mx_c as well as all-cause mortality, mx. Cause-specific contributions are calculated for each age group and then the sum over age groups is taken as the cause's effect on at-birth life expectancy.

The original version of the Das Gupta method is described in Das Gupta decomposition of additive functions<das_gupta>. In GBD 2021, the modification (3) bolded below was added to improve stability in the results, particularly for COVID-related causes.

The modified Das Gupta method, calculates cause-specific contributions for cause c to differences in life expectancy by age a from time 1 to time 2, δ_{ac}^{2-1}, in the following way:

1) If the differences between all-cause deaths for the start year and the end year are not too small (>10^{-5}) AND the signs for the change in life expectancy, δ_a, and all-cause deaths are different, use the normal Das Gupta equation:

$$\delta_{ac}^{2-1} = \delta_a^{2-1} * \frac{mx_{ac}^2 - mx_{ac}^1}{mx_a^2 - mx_a^1}$$

2) If the all-cause deaths difference is too small (<10^{-5}), use the reduced Das Gupta equation to avoid instability from a denominator that approaches 0.

$$\delta_{ac}^{2-1} = \delta_a^{2-1} * \frac{mx_{ac}^1}{mx_a^1}$$

3) **MODIFICATION:** If the signs for the change in life expectancy and all-cause deaths are the same, use the reduced Das Gupta formula (2) EXCEPT in the case that the cause in question is COVID-related (either COVID-19 or other pandemic-related mortality) where a modified version is used:

$$\delta_{ac}^{2-1} = \delta_a^{2-1} * \frac{mx_{ac}^2}{mx_a^2}$$

where δ_a^{2-1} is the age-specific life expectancy change from time 1 to time 2, mx_{ac}^2 is the age- and cause-specific mortality rate in time 2, mx_{ac}^1 is the age- and cause-specific mortality rate in time 1, mx_a^2 is the age-specific all-cause mortality rate in time 2, and mx_a^1 is the age-specific all-cause mortality rate in time 1.

Once δ_{ac}^{2-1} has been calculated for all age groups a for cause c, the sum across age groups is taken to calculate the cause's contribution to the change in at-birth life expectancy, δ_c^{2-1}:

$$\delta_c^{2-1} = \sum_a \delta_{ac}^{2-1}$$

Using the original Das Gupta decomposition, for a mutually exclusive and collectively exhaustive set of causes, the sum of δ_c^{2-1} adds up to the overall change in life expectancy e_x^{2-1}. However, because of the added modification, this is no longer always true. Typically, the differences are fairly small (–2.4214694 vs. –2.4208824) but do not meet the validation criteria for consistency that existed prior to the modification.

With GBD 2021 came our first attempt at producing estimates for COVID and the impacts of the resulting pandemic. It presented new challenges surrounding how to estimate its effects on mortality, population, and life tables, which are crucial inputs to LE decomposition.

Preliminary attempts showed unrealistic results for COVID-related causes, in particular, when the sign for the change in life expectancy between two years and the change in all-cause deaths are the same (ie, life expectancy increases AND all-cause deaths increase). The modification (3) can help minimise the issue.

There were also concerns that when the all-cause deaths difference is too small and the simplified formula (2) is used, contributions from COVID-related causes would drop to zero as any year start of interest, eg, 2019, would not include COVID deaths as the disease did not yet exist. Since the impact of COVID-related causes on overall life expectancy was an important focus, we added special handling in such cases.

Section 8: References

1 Stevens GA, Alkema L, Black RE, *et al.* Guidelines for Accurate and Transparent Health Estimates Reporting: the GATHER statement. *Lancet* 2016; **388**: e19–23.

2 GBD 2017 Causes of Death Collaborators. Global, regional, and national age-sex-specific mortality for 282 causes of death in 195 countries and territories, 1980-2017: a systematic analysis for the Global Burden of Disease Study 2017. *Lancet* 2018; **392**: 1736–88.

3 GBD 2019 Demographics Collaborators. Global age-sex-specific fertility, mortality, healthy life expectancy (HALE), and population estimates in 204 countries and territories, 1950-2019: a comprehensive demographic analysis for the Global Burden of Disease Study 2019. *Lancet* 2020; **396**: 1160–203.

4 Sankoh O, Byass P. The INDEPTH Network: filling vital gaps in global epidemiology. *Int J Epidemiol* 2012; **41**: 579–88.

5 Uneke CJ, Uro-Chukwu HC, Chukwu OE. Validation of verbal autopsy methods for assessment of child mortality in sub-Saharan Africa and the policy implication: a rapid review. *Pan Afr Med J* 2019; **33**: 318.

6 Thomas L-M, D'Ambruoso L, Balabanova D. Verbal autopsy in health policy and systems: a literature review. *BMJ Glob Health* 2018; **3**: e000639.

7 Serina P, Riley I, Stewart A, *et al.* Improving performance of the Tariff Method for assigning causes of death to verbal autopsies. *BMC Med* 2015; **13**: 291.

8 Byass P, Hussain-Alkhateeb L, D'Ambruoso L, *et al.* An integrated approach to processing WHO-2016 verbal autopsy data: the InterVA-5 model. *BMC Med* 2019; **17**: 102.

9 Murray CJL, Lozano R, Flaxman AD, *et al.* Using verbal autopsy to measure causes of death: the comparative performance of existing methods. *BMC Med* 2014; **12**: 5.

10 Gakidou E, King G. Death by survey: estimating adult mortality without selection bias from sibling survival data. *Demography* 2006; **43**: 569–85.

11 Government of India. Medical certification of cause of death. 2023; published online Aug 11. https://censusindia.gov.in/census.website/node/381 (accessed Sept 29, 2023).

12 Government of India. ORGI SRS. 2023; published online Aug 11. https://censusindia.gov.in/census.website/node/294 (accessed Oct 16, 2023).

13 Ganguli M, Rodriguez EG. Reporting of dementia on death certificates: a community study. *J Am Geriatr Soc* 1999; **47**: 842–9.

14 Wachterman M, Kiely DK, Mitchell SL. Reporting dementia on the death certificates of nursing home residents dying with end-stage dementia. *JAMA* 2008; **300**: 2608–10.

15 Macera CA, Sun RK, Yeager KK, Brandes DA. Sensitivity and specificity of death certificate diagnoses for dementing illnesses, 1988-1990. *J Am Geriatr Soc* 1992; **40**: 479–81.

16 Olichney JM, Hofstetter CR, Galasko D, Thal LJ, Katzman R. Death certificate reporting of dementia and mortality in an Alzheimer's disease research center cohort. *J Am Geriatr Soc* 1995; **43**: 890–3.

17 GBD 2019 Collaborators. Global mortality from dementia: application of a new method and results from the Global Burden of Disease Study 2019. *Alzheimers Dement N Y N* 2021; **7**: e12200.

18 Todd S, Barr S, Passmore AP. Cause of death in Alzheimer's disease: a cohort study. *QJM* 2013; **106**: 747–53.

19 Brunnström HR, Englund EM. Cause of death in patients with dementia disorders. *Eur J Neurol* 2009; **16**: 488–92.

20 Gao L, Calloway R, Zhao E, Brayne C, Matthews FE, Medical Research Council Cognitive Function and Ageing Collaboration. Accuracy of death certification of dementia in population-based samples of older people: analysis over time. *Age Ageing* 2018; **47**: 589–94.

21 Romero JP, Benito-León J, Mitchell AJ, Trincado R, Bermejo-Pareja F. Under reporting of dementia deaths on death certificates using data from a population-based study (NEDICES). *J Alzheimers Dis JAD* 2014; **39**: 741–8.

22 Moscovich M, Boschetti G, Moro A, Teive HAG, Hassan A, Munhoz RP. Death certificate data and causes of death in patients with parkinsonism. *Parkinsonism Relat Disord* 2017; **41**: 99–103.

23 Pressley JC, Tang M-X, Marder K, Cote LJ, Mayeux R. Disparities in the recording of Parkinson's disease on death certificates. *Mov Disord* 2005; **20**: 315–21.

24 Duncan ME, Pitcher A, Goldacre MJ. Atrial fibrillation as a cause of death increased steeply in England between 1995 and 2010. *EP Eur* 2014; **16**: 797–802.

25 Johnson SC, Cunningham M, Dippenaar IN, *et al.* Public health utility of cause of death data: applying empirical algorithms to improve data quality. *BMC Med Inform Decis Mak* 2021; **21**: 175.

26 Barker B, Degenhardt L. Accidental drug-induced deaths in Australia 1997 - 2001. Sydney: National Drug and Alcohol Research Centre, 2003.

27 Roxburgh, A., Burns, L. Accidental drug-induced deaths due to opioids in Australia, 2013. Sydney: National Drug and Alcohol Research Centre, 2013.

28 Roxburgh A, Burns L. Cocaine and methamphetamine related drug-induced deaths in Australia, 2011. Sydney: National Drug and Alcohol Research Centre, 2015.

29 GBD 2013 Mortality and Causes of Death Collaborators. Global, regional, and national age–sex specific all-cause and cause-specific mortality for 240 causes of death, 1990–2013: a systematic analysis for the Global Burden of Disease Study 2013. *Lancet* 2015; **385**: 117–71.

30 Lozano R, Naghavi M, Foreman K, *et al.* Global and regional mortality from 235 causes of death for 20 age groups in 1990 and 2010: a systematic analysis for the Global Burden of Disease Study 2010. *Lancet* 2012; **380**: 2095–128.

31 Wang H, Abajobir AA, Abate KH, *et al.* Global, regional, and national under-5 mortality, adult mortality, age-specific mortality, and life expectancy, 1970–2016: a systematic analysis for the Global Burden of Disease Study 2016. *Lancet* 2017; **390**: 1084–150.

32 Calvert C, Ronsmans C. The contribution of HIV to pregnancy-related mortality: a systematic review and meta-analysis. *AIDS Lond Engl* 2013; **27**: 1631–9.

33 Figueroa-Damián R. Pregnancy outcome in women infected with the human immunodeficiency virus. *Salud Publica Mex* 1999; **41**: 362–7.

34 Ryder RW, Nsuami M, Nsa W, *et al.* Mortality in HIV-1-seropositive women, their spouses and their newly born children during 36 months of follow-up in Kinshasa, Zaïre. *AIDS* 1994; **8**: 667–72.

35 Zvandasara P, Saungweme G, Mlambo JT, Moyo J. Post Caesarean section infective morbidity in HIV-positive women at a tertiary training hospital in Zimbabwe. *Cent Afr J Med* 2007; **53**: 43–7.

36 Chilongozi D, Wang L, Brown L, *et al.* Morbidity and mortality among a cohort of human immunodeficiency virus type 1-infected and uninfected pregnant women and their infants from Malawi, Zambia, and Tanzania. *Pediatr Infect Dis J* 2008; **27**: 808–14.

37 Leroy V, Ladner J, Nyiraziraje M, *et al.* Effect of HIV-1 infection on pregnancy outcome in women in Kigali, Rwanda, 1992-1994. Pregnancy and HIV Study Group. *AIDS* 1998; **12**: 643–50.

38 Kourtis AP, Bansil P, McPheeters M, Meikle SF, Posner SF, Jamieson DJ. Hospitalizations of pregnant HIV-infected women in the USA prior to and during the era of HAART, 1994-2003. *AIDS* 2006; **20**: 1823–31.

39 Ticconi C, Mapfumo M, Dorrucci M, *et al.* Effect of maternal HIV and malaria infection on pregnancy and perinatal outcome in Zimbabwe. *J Acquir Immune Defic Syndr 1999* 2003; **34**: 289–94.

40 Brown T, Peerapatanapokin W. The Asian Epidemic Model: a process model for exploring HIV policy and programme alternatives in Asia. *Sex Transm Infect* 2004; **80**: i19–24.

41 DerSimonian R, Laird N. Meta-analysis in clinical trials. *Control Clin Trials* 1986; **7**: 177–88.

42 Matthews LT, Kaida A, Kanters S, *et al.* HIV-infected women on antiretroviral treatment have increased mortality during pregnant and postpartum periods. *AIDS* 2013; **27 Suppl 1**: S105-112.

43 Westreich D, Maskew M, Evans D, Firnhaber C, Majuba P, Sanne I. Incident pregnancy and time to death or AIDS among HIV-positive women receiving antiretroviral therapy. *PLOS ONE* 2013; **8**: e58117.

44 Stover J. AIM: A computer program for making HIV/AIDS projections and examining the social and economic impact of AIDS. Glastonbury, CT: USAID, 2005.

45 Lozano R, Lopez AD, Atkinson C, Naghavi M, Flaxman AD, Murray CJL. Performance of physician-certified verbal autopsies: multisite validation study using clinical diagnostic gold standards. *Popul Health Metr* 2011; **9**: 32.

46 Foreman KJ, Lozano R, Lopez AD, Murray CJL. Modeling causes of death: an integrated approach using CODEm. *Popul Health Metr* 2012; **10**: 1.

47 Bell RM, Koren Y. Lessons from the Netflix prize challenge. *ACM SIGKDD Explor Newsl* 2007; **9**: 75–9.

48 Bell RM, Koren Y, Volinsky C. All together now: A perspective on the NETFLIX PRIZE. *CHANCE* 2010; **23**: 24–24.

49 Flaxman AD, Vos T, Murray CJL. An integrative metaregression framework for descriptive epidemiology. Univ. Wash. Press. 2015; published online Oct. https://uwapress.uw.edu/book/9780295991849/an-integrative-metaregression-framework-for-descriptive-epidemiology (accessed July 7, 2023).

50 COVID-19 Excess Mortality Collaborators. Estimating excess mortality due to the COVID-19 pandemic: a systematic analysis of COVID-19-related mortality, 2020-21. *Lancet* 2022; **399**: 1513–36.

51 Fragoso TM, Bertoli W, Louzada F. Bayesian model averaging: a systematic review and conceptual classification. *Int Stat Rev* 2018; **86**: 1–28.

52 GBD 2017 Population and Fertility Collaborators. Population and fertility by age and sex for 195 countries and territories, 1950-2017: a systematic analysis for the Global Burden of Disease Study 2017. *Lancet* 2018; **392**: 1995–2051.

53 Jdanov DA, Shkolnikov VM, van Raalte AA, Andreev EM. Decomposing current mortality differences into initial differences and differences in trends: the contour decomposition method. *Demography* 2017; **54**: 1579–602.

Section 9: Authors' Contributions

Managing the overall research enterprise

Nicole Davis Weaver, Simon I Hay, Christopher J L Murray, Mohsen Naghavi, Kanyin Liane Ong, and Eve E Wool.

Writing the first draft of the manuscript

Matthew Cunningham, Nicole Davis Weaver, Mohsen Naghavi, and Eve E Wool.

Primary responsibility for applying analytical methods to produce estimates

Gregory J Bertolacci, Matthew Cunningham, Chieh Han, Rebecca Hsu, and Vincent Mougin.

Primary responsibility for seeking, cataloguing, extracting, or cleaning data; designing or coding figures and tables

Gregory J Bertolacci, Matthew Cunningham, Sama Ghoba, Rebecca Hsu, and Vincent Mougin.

Providing data or critical feedback on data sources

Yohannes Habtegiorgis Abate, Cristiana Abbafati, Hedayat Abbastabar, Samar Abd ElHafeez, Michael Abdelmasseh, Ahmed Abdelwahab, Meriem Abdoun, Auwal Abdullahi, Samrawit Shawel Abebe, Aidin Abedi, E S Abhilash, Hassan Abidi, Richard Gyan Aboagye, Hassan Abolhassani, Lucas Guimarães Abreu, Dariush Abtahi, Niveen ME Abu-Rmeileh, Salahdein Aburuz, Ahmed Abu-Zaid, Akindele Olupelumi Adebiyi, Victor Adekanmbi, Abiola Victor Adepoju, Temitayo Esther Adeyeoluwa, Qorinah Estiningtyas Sakilah Adnani, Muhammad Sohail Afzal, Saira Afzal, Feleke Doyore Agide, Antonella Agodi, Bright Opoku Ahinkorah, Danish Ahmad, Muayyad M Ahmad, Sajjad Ahmad, Ali Ahmed, Ayman Ahmed, Haroon Ahmed, Muktar Beshir Ahmed, Budi Aji, Hossein Akbarialiabad, Hanadi Al Hamad, Fares Alahdab, Tariq A Alalwan, Fahad Mashhour Alanezi, Turki M Alanzi, Almaza Albakri, Mohammed Albashtawy, Mohammad T AlBataineh, Jacqueline Elizabeth Alcalde-Rabanal, Robert W Aldridge, Mulubirhan Assefa Alemayohu, Yihun Mulugeta Alemu, Khalid F Alhabib, Abid Ali, Amjad Ali, Liaqat Ali, Syed Shujait Shujait Ali, Reyhaneh Alikhani, Mohammad A M Aljasir, Syed Mohamed Aljunid, François Alla, Peter Allebeck, Joseph Uy Almazan, Louay Almidani, Omar Almidani, Mahmoud A Alomari, Awais Altaf, Nelson Alvis-Guzman, Hassan Alwafi, Hany Aly, Azmeraw T Amare, Edward Kwabena Ameyaw, Hubert Amu, Ganiyu Adeniyi Amusa, Deanna Anderlini, David B Anderson, Pedro Prata Andrade, Hossein Ansari, Davood Anvari, Saeid Anvari, Saleha Anwar, Sumadi Lukman Anwar, Razique Anwer, Muhammad Aqeel, Jalal Arabloo, Olatunde Aremu, Hany Ariffin, Benedetta Armocida, Johan Ärnlöv, Anton A Artamonov, Judie Arulappan, Mohsen Asadi-Lari, Akeza Awealom Asgedom, Seyyed Shamsadin Athari, Bantalem Tilaye Tilaye Atinafu, Prince Atorkey, Alok Atreya, Beatriz Paulina Ayala Quintanilla, Ahmed Y Azzam, Ashish D Badiye, Soroush Baghdadi, Sara Bagherieh, Atif Amin Baig, Abdulaziz T Bako, Senthilkumar Balakrishnan, Ovidiu Constantin Baltatu, Maciej Banach, Palash Chandra Banik, Martina Barchitta, Mainak Bardhan, Till Winfried Bärnighausen, Hiba Jawdat Barqawi, Lope H Barrero, Amadou Barrow, Sandra Barteit, Zarrin Basharat, Sanjay Basu, Nebiyou Simegnew Bayileyegn, Emad Behboudi, Priyamadhaba Behera, Masoud Behzadifar, Diana Fernanda Bejarano Ramirez, Yannick Béjot, Sefealem Assefa Belay, Chalie Mulu Belete, Michelle L Bell, Olorunjuwon Omolaja Bello, Apostolos Beloukas, Azizullah Beran, Betyna N Berice, Akshaya Srikanth Bhagavathula, Dinesh Bhandari, Kayleigh Bhangdia, Sonu Bhaskar, Vivek Bhat, Gurjit Kaur Bhatti, Jasvinder Singh Bhatti, Zulfiqar A Bhutta, Boris Bikbov, Jessica Devin Bishai, Anca Vasilica Bobirca, Virginia Bodolica, Obasanjo Afolabi Bolarinwa, Archith Boloor, Berrak Bora Basara, Hamed Borhany, Souad Bouaoud, Antoine Boustany, Christopher Boxe, Dejana Braithwaite, Luisa C Brant, Michael Brauer, Traolach Brugha, Lemma N Bulto, Danilo Buonsenso, Florentino Luciano Caetano dos Santos, Luciana Aparecida Campos, Chao Cao,

Juan J Carrero, Márcia Carvalho, Joao Mauricio Castaldelli-Maia, Carlos A Castañeda-Orjuela, Ferrán Catalá-López, Alberico L Catapano, Christopher R Cederroth, Francieli Cembranel, Muthia Cenderadewi, Joshua Chadwick, Gashaw Sisay Chanie, Periklis Charalampous, Vijay Kumar Chattu, Akhilanand Chaurasia, Angela W Chen, Ching-Yu Cheng, Abdulaal Chitheer, William C S Cho, Bryan Chong, Hitesh Chopra, Rajiv Chowdhury, Eric Chung, Eunice Chung, Zinhle Cindi, Rafael M Claro, Alyssa Columbus, Haley Comfort, Joao Conde, Samuele Cortese, Paolo Angelo Cortesi, Ewerton Cousin, Rosa A S Couto, Michael H Criqui, Natália Cruz-Martins, Matthew Cunningham, Siyu Dai, Xiaochen Dai, Giovanni Damiani, Saswati Das, Ana Maria Dascalu, Claudio Alberto Dávila-Cervantes, Aklilu Tamire Debele, Shayom Debopadhaya, Louisa Degenhardt, Lee Deitesfeld, Ivan Delgado-Enciso, Berecha Hundessa Demessa, Andreas K Demetriades, Niloofar Deravi, Nebiyu Dereje, Nikolaos Dervenis, Emina Dervišević, Hardik Dineshbhai Desai, Rupak Desai, Vinoth Gnana Chellaiyan Devanbu, Meghnath Dhimal, Sameer Dhingra, Vishal R Dhulipala, Diana Dias da Silva, Daniel Diaz, Michael J Diaz, Huanghe Ding, Ricardo Jorge Dinis-Oliveira, M Ashworth Dirac, Thao Huynh Phuong Do, Masoud Dodangeh, Milad Dodangeh, Klara Georgieva Dokova, Christiane Dolecek, Regina-Mae Villanueva Dominguez, Wendel Mombaque dos Santos, Rajkumar Doshi, Leila Doshmangir, Robert Kokou Dowou, Tim Robert Driscoll, Haneil Larson Dsouza, Viola Dsouza, Bruce B Duncan, Andre Rodrigues Duraes, Senbagam Duraisamy, Paulina Agnieszka Dzianach, Chidiebere Peter Echieh, Diyan Ermawan Effendi, Ebrahim Eini, Michael Ekholuenetale, Temitope Cyrus Ekundayo, Teshome Bekele Elema, Legesse Tesfaye Elilo, Ryenchindorj Erkhembayar, Sharareh Eskandarieh, Habtamu Esubalew, Adewale Oluwaseun Fadaka, Adeniyi Francis Fagbamigbe, Mohammad Fareed, Carla Sofia e Sá Farinha, Andre Faro, Abidemi Omolara Fasanmi, Ali Fatehizadeh, Pooria Fazeli, Alireza Feizkhah, Seyed-Mohammad Fereshtehnejad, David Flood, Artem Alekseevich Fomenkov, Richard Charles Franklin, Joseph Friedman, Takeshi Fukumoto, John E Fuller, Peter Andras Gaal, Muktar A Gadanya, Santosh Gaihre, Yaseen Galali, Silvano Gallus, Mandukhai Ganbat, William M Gardner, Tilaye Gebru Gebi, Teferi Gebru Gebremeskel, Simona Roxana Georgescu, Peter W Gething, Molla Getie, Ahmad Ghashghaee, Ajnish Ghimire, Sama Ghoba, Alem Girmay, Laszlo Göbölös, Ali Golchin, Mahaveer Golechha, Pouya Goleij, Nelson G M Gomes, Houman Goudarzi, Bárbara Niegia Garcia Goulart, Ayman Grada, Giuseppe Grosso, Avirup Guha, Rafael Alves Guimarães, Snigdha Gulati, Anish Kumar Gupta, Manoj Kumar Gupta, Rajat Das Gupta, Rajeev Gupta, Sapna Gupta, Vijai Kumar Gupta, Najah R Hadi, Nils Haep, Nima Hafezi-Nejad, Alemayehu Hailu, Arvin Haj-Mirzaian, Brian J Hall, Rabih Halwani, Erin B Hamilton, Chieh Han, Arief Hargono, Josep Maria Haro, M Tasdik Hasan, Soheil Hassanipour, Hadi Hassankhani, Johannes Haubold, Simon I Hay, Jeffrey J Hebert, Mahsa Heidari-Foroozan, Claudiu Herteliu, Nobuyuki Horita, Md Mahbub Hossain, Mehdi Hosseinzadeh, Chengxi Hu, Michael Hultström, Javid Hussain, Nawfal R Hussein, Hong-Han Huynh, Segun Emmanuel Ibitoye, Desta Ijo, Kevin S Ikuta, Olayinka Stephen Ilesanmi, Arnaud Iradukunda, Sheikh Mohammed Shariful Islam, Nahlah Elkudssiah Ismail, Gaetano Isola, Mahalaxmi Iyer, Linda Merin J, Jalil Jaafari, Kathryn H Jacobsen, Morteza Jafarinia, Khushleen Jaggi, Nader Jahanmehr, Haitham Jahrami, Nityanand Jain, Tahereh Javaheri, Sathish Kumar Jayapal, Shubha Jayaram, Yingzhao Jin, Jost B Jonas, Tamas Joo, Abel Joseph, Charity Ehimwenma Joshua, Jacek Jerzy Jozwiak, Mikk Jürisson, Vaishali K, Billingsley Kaambwa, Zubair Kabir, Vidya Kadashetti, Dler Hussein Kadir, Leila R Kalankesh, Sivesh Kathir Kamarajah, Naser Kamyari, Himal Kandel, Arun R Kanmanthareddy, Rami S Kantar, Neeti Kapoor, André Karch, Salah Eddin Karimi, Faizan Zaffar Kashoo, Nicholas J Kassebaum, Norito Kawakami, Gbenga A Kayode, Tahseen Haider Kazmi, Fassikaw Kebede, Peter Njenga Keiyoro, Cathleen Keller, Emmanuelle Kesse-Guyot, Himanshu Khajuria, Nauman Khalid, Faham Khamesipour, Ikramullah Khan, M Nuruzzaman Khan, Maseer Khan, Moien AB Khan, Mahammed Ziauddin Khan suheb, Khaled Khatab, Fatemeh Khatami, Feriha Fatima Khidri, Mohammad Khorgamphar, Grace Kim, Jihee Kim, Min Seo Kim,

Yun Jin Kim, Adnan Kisa, Sezer Kisa, Desmond Klu, Ann Kristin Skrindo Knudsen, Jonathan M Kocarnik, Sonali Kochhar, Timea Kocsis, Gerbrand Koren, Soewarta Kosen, Sindhura Lakshmi Koulmane Laxminarayana, Kewal Krishan, Hare Krishna, Vijay Krishnamoorthy, Yuvaraj Krishnamoorthy, Barthelemy Kuate Defo, Burcu Kucuk Bicer, Akshay Kumar, Ashish Kumar, Dian Kusuma, Alexander Kwarteng, Hmwe Hmwe Kyu, Anton C J Lager, Dharmesh Kumar Lal, Savita Lasrado, Kamaluddin Latief, Kaveh Latifinaibin, Nhi Huu Hanh Le, Thao Thi Thu Le, Trang Diep Thanh Le, Caterina Ledda, Munjae Lee, Seung Won Lee, Shaun Wen Huey Lee, James Leigh, Yichong Li, Andrew Tiyamike Makhiringa Likaka, Lee-Ling Lim, Stephen S Lim, Megan Lindstrom, Gang Liu, Jue Liu, Shiwei Liu, Xuefeng Liu, Erand Llanaj, Michael J Loftus, Rubén López-Bueno, Stefan Lorkowski, Paulo A Lotufo, Rafael Lozano, Jailos Lubinda, Zheng Feei Ma, Christian Madsen, Azzam A Maghazachi, Razzagh Mahmoudi, Azeem Majeed, Elaheh Malakan Rad, Venkatesh Maled, Kashish Malhotra, Deborah Carvalho Malta, Abdullah A Mamun, Mohammad Ali Mansournia, Sajid Maqsood, Joemer C Maravilla, Agustina M Marconi, Parham Mardi, Abdoljalal Marjani, Bernardo Alfonso Martinez-Guerra, Francisco Rogerlândio Martins-Melo, Sharmeen Maryam, Roy Rillera Marzo, Awoke Masrie, Alexander G Mathioudakis, Jishanth Mattumpuram, Richard Matzopoulos, Andrea Maugeri, Mohsen Mazidi, Anna Laura W McKowen, Steven M McPhail, Enkeleint A Mechili, John Robert Carabeo Medina, Jitendra Kumar Meena, Entezar Mehrabi Nasab, Tesfahun Mekene Meto, Gebrekiros Gebremichael Meles, Walter Mendoza, Ritesh G Menezes, Haftu Asmerom Meresa, Atte Meretoja, Kukulege Chamila Dinushi Mettananda, Sachith Mettananda, Georgia Micha, Irmina Maria Michalek, Ted R Miller, Edward J Mills, Le Huu Nhat Minh, Erkin M Mirrakhimov, Awoke Misganaw, Mohammadreza Mobayen, Madeline E Moberg, Soheil Mohammadi, Abdollah Mohammadian-Hafshejani, Mustapha Mohammed, Salahuddin Mohammed, Shafiu Mohammed, Viswanathan Mohan, Ali H Mokdad, Mariam Molokhia, Sara Momtazmanesh, Lorenzo Monasta, Mohammad Ali Moni, Yousef Moradi, Maziar Moradi-Lakeh, Shane Douglas Morrison, Jakub Morze, Jonathan F Mosser, Vincent Mougin, Emmanuel A Mpolya, Sumaira Mubarik, Ulrich Otto Mueller, Faraz Mughal, Francesk Mulita, Christopher J L Murray, Fungai Musaigwa, Ghulam Mustafa, Saravanan Muthupandian, Raman Muthusamy, Ahamarshan Jayaraman Nagarajan, Gabriele Nagel, Mohsen Naghavi, Ganesh R Naik, Sanjeev Nair, Sreenivas Narasimha Swamy, Aparna Ichalangod Narayana, Shumaila Nargus, Bruno Ramos Nascimento, Zuhair S Natto, Biswa Prakash Nayak, Vinod C Nayak, Ionut Negoi, Ruxandra Irina Negoi, Haruna Asura Nggada, Josephine W Ngunjiri, Anh Hoang Nguyen, Dang H Nguyen, Hau Thi Hien Nguyen, Phat Tuan Nguyen, Van Thanh Nguyen, Robina Khan Niazi, Taxiarchis Konstantinos Nikolouzakis, Chukwudi A Nnaji, Lawrence Achilles Nnyanzi, Shuhei Nomura, Bo Norrving, Jean Jacques Noubiap, Mpiko Ntsekhe, Dieta Nurrika, Jerry John Nutor, Bogdan Oancea, Kehinde O Obamiro, Mary Aigbiremo Oboh, Ismail A Odetokun, Nkechi Martina Odogwu, Ayodipupo Sikiru Oguntade, Osaretin Christabel Okonji, Andrew T Olagunju, Matthew Idowu Olatubi, Bolajoko Olubukunola Olusanya, Jacob Olusegun Olusanya, Yinka Doris Oluwafemi, Hany A Omar, Ahmed Omar Bali, Maureene Auma Ondayo, Kanyin Liane Ong, Sokking Ong, Obinna E Onwujekwe, Kenneth Ikenna Onyedibe, Alberto Ortiz, Samuel M Ostroff, Adrian Otoiu, Stanislav S Otstavnov, Amel Ouyahia, Mayowa O Owolabi, Yaz Ozten, Mahesh Padukudru P A, Jagadish Rao Padubidri, Raffaele Palladino, Adrian Pana, Songhomitra Panda-Jonas, Seithikurippu R Pandi-Perumal, Helena Ullyartha Pangaribuan, Leonidas D Panos, Anca Mihaela Pantea Stoian, Paraskevi Papadopoulou, Romil R Parikh, Seoyeon Park, Ashwaghosha Parthasarathi, Maja Pasovic, Hemal M Patel, Jay Patel, Sangram Kishor Patel, Shankargouda Patil, Uttam Paudel, Spencer A Pease, Veincent Christian Filipino Pepito, Prince Peprah, Mario F P Peres, Arokiasamy Perianayagam, Konrad Pesudovs, William A Petri, Hoang Tran Pham, Anil K Philip, Daniela Pierannunzio, David M Pigott, Zahra Zahid Piracha, Vivek Podder, Kevan R Polkinghorne, Ramesh Poluru, Maarten J Postma, Naeimeh Pourtaheri, Sergio I Prada, Elton Junio Sady Prates, TINA PRISCILLA, Natalie

Pritchett, Bharathi M Purohit, Jagadeesh Puvvula, Asma Saleem Qazi, Raghu Anekal Radhakrishnan, Venkatraman Radhakrishnan, Hadi Raeisi Shahraki, Quinn Rafferty, Nasiru Raheem, Fakher Rahim, Md Jillur Rahim, Vafa Rahimi-Movaghar, Mohammad Hifz Ur Rahman, Amir Masoud Rahmani, Shakthi Kumaran Ramasamy, Sheena Ramazanu, Chhabi Lal Ranabhat, Nemanja Rancic, Chythra R Rao, Mithun Rao, Sowmya J Rao, Sina Rashedi, Vahid Rashedi, Santosh Kumar Rauniyar, Ramin Ravangard, Nakul Ravikumar, Salman Rawaf, Lal Rawal, Reza Rawassizadeh, Christian Razo, Elrashdy Moustafa Mohamed Redwan, Lennart Reifels, Luis Felipe Reyes, Maryam Rezaei, Taeho Gregory Rhee, Mavra A Riaz, Antonio Luiz P Ribeiro, Hannah R Riva, Hannah Elizabeth Robinson-Oden, Célia Fortuna Rodrigues, Mónica Rodrigues, Leonardo Roever, Emma Lynn Best Rogowski, Peter Rohloff, Debby Syahru Romadlon, Michele Romoli, Luca Ronfani, Gholamreza Roshandel, Himanshu Sekhar Rout, Enrico Rubagotti, Susan Fred Rumisha, Tilleye Runghien, Godfrey M Rwegerera, Andrzej Rynkiewicz, Aly M A Saad, Zahra Saadatian, Siamak Sabour, Basema Saddik, Adam Saddler, Bashdar Abuzed Sadee, Ehsan Sadeghi, Umar Saeed, Sher Zaman Safi, Rajesh Sagar, Narjes Saheb Sharif-Askari, Zahra Saif, Mirza Rizwan Sajid, Afeez Abolarinwa Salami, Marwa Rashad Salem, Sara Samadzadeh, Abdallah M Samy, Juan Sanabria, Francesca Sanna, Damian Francesco Santomauro, Itamar S Santos, Milena M Santric-Milicevic, Babak Saravi, Sachin C Sarode, Brijesh Sathian, Maheswar Satpathy, Md Abu Sayeed, Mete Saylan, Mehdi Sayyah, Markus P Schlaich, Maria Inês Schmidt, David C Schwebel, Mario Šekerija, Subramanian Senthilkumaran, Dragos Serban, Yashendra Sethi, Amir Shafaat, Pritik A Shah, Syed Mahboob Shah, Masood Ali Shaikh, Sunder Sham, Muhammad Aaqib Shamim, Mehran Shams-Beyranvand, Abhishek Shankar, Javad Sharifi-Rad, Vishal Sharma, Rajesh P Shastry, Maryam Shayan, Jiabin Shen, Adithi Shetty, B Suresh Kumar Shetty, Peilin Shi, Kenji Shibuya, Aminu Shittu, K M Shivakumar, Farhad Shokraneh, Sina Shool, Luís Manuel Lopes Rodrigues Silva, Abhinav Singh, Narinder Pal Singh, Paramdeep Singh, Valentin Yurievich Skryabin, Anna Aleksandrovna Skryabina, Sameh S M Soliman, Reed J D Sorensen, Joan B Soriano, Ireneous N Soyiri, Michael Spartalis, Chandrashekhar T Sreeramareddy, Jeffrey D Stanaway, Muhammad Haroon Stanikzai, Benjamin A Stark, Caroline Stein, Fridolin Steinbeis, Caitlyn Steiner, Jaimie D Steinmetz, Aleksandar Stevanović, Mark A Stokes, Vetriselvan Subramaniyan, Muhammad Suleman, Rizwan Suliankatchi Abdulkader, Abida Sultana, Haitong Zhe Sun, Johan Sundström, Chandan Kumar Swain, Mindy D Szeto, Miklós Szócska, Rafael Tabarés-Seisdedos, Seyyed Mohammad Tabatabaei, Shima Tabatabai, Mircea Tampa, Ker-Kan Tan, Elvis Enowbeyang Tarkang, Nuno Taveira, Yibekal Manaye Tefera, Pugazhenthan Thangaraju, Nihal Thomas, Nikhil Kenny Thomas, Azalea M Thomson, Chern Choong Chern Thum, Tala Tillawi, Roman Topor-Madry, Mathilde Touvier, Marcos Roberto Tovani-Palone, Jasmine T Tran, Domenico Trico, Guesh Mebrahtom Tsegay, Munkhtuya Tumurkhuu, Irfan Ullah, Sana Ullah, Srikanth Umakanthan, Chukwuma David Umeokonkwo, Bhaskaran Unnikrishnan, Era Upadhyay, Jibrin Sammani Usman, Jef Van den Eynde, Shoban Babu Varthya, Tommi Juhani Vasankari, Balachandar Vellingiri, Narayanaswamy Venketasubramanian, Nicholas Alexander Verghese, Georgios-Ioannis Verras, Maria Viskadourou, Sergey Konstantinovitch Vladimirov, Vasily Vlassov, Bay Vo, Avina Vongpradith, Theo Vos, Yasir Waheed, Shu Wang, Stefanie Watson, Daniel J Weiss, Ronny Westerman, Joanna L Whisnant, Taweewat Wiangkham, Dakshitha Praneeth Wickramasinghe, Angga Wilandika, Peter Willeit, Demewoz H Woldegebreal, Charles D A Wolfe, Yen Jun Wong, Utoomporn Wongsin, Eve E Wool, Juan Xia, Suowen Xu, Yvonne Yiru Xu, Ali Yadollahpour, Kazumasa Yamagishi, Yao Yao, Habib Yaribeygi, Pengpeng Ye, Sisay Shewasinad Yehualashet, Siyan Yi, Amanuel Yigezu, Dong Keon Yon, Naohiro Yonemoto, Mustafa Z Younis, Chuanhua Yu, Yong Yu, Faith H Yuh, Siddhesh Zadey, Vesna Zadnik, Iman Zare, Mohammed G M Zeariya, Zhi-Jiang Zhang, Magdalena Zielińska, Mohammad Zoladl, Zhiyong Zou, Liesl J Zuhlke, Alimuddin Zumla, Elric Zweck.

Developing methods or computational machinery

Cristiana Abbafati, Samrawit Shawel Abebe, Dariush Abtahi, Qorinah Estiningtyas Sakilah Adnani, Saira Afzal, Muktar Beshir Ahmed, Hossein Akbarialiabad, Mohammed Albashtawy, Yihun Mulugeta Alemu, Hubert Amu, Hossein Ansari, Davood Anvari, Aleksandr Y Aravkin, Michael Benjamin Arndt, Judie Arulappan, Ahmed Y Azzam, Gregory J Bertolacci, Akshaya Srikanth Bhagavathula, Jessica Devin Bishai, Somayeh Bohlouli, Milad Bonakdar Hashemi, Hamed Borhany, Kelly M Cercy, Catherine S Chen, Kaleb Coberly, Rebecca M Cogen, Haley Comfort, Rosa A S Couto, Garland T Culbreth, Matthew Cunningham, Xiaochen Dai, Hardik Dineshbhai Desai, Paulina Agnieszka Dzianach, Michael Ekholuenetale, Ali Fatehizadeh, Natalie C Galles, MA Garcia-Gordillo, William M Gardner, Tilaye Gebru Gebi, Peter W Gething, Sama Ghoba, Alem Girmay, Shi-Yang Guan, Rafael Alves Guimarães, Manoj Kumar Gupta, Chieh Han, Mohammad Hasanian, Simon I Hay, Hamed Hesami, Mehdi Hosseinzadeh, Rebecca L Hsu, Hong-Han Huynh, Kevin S Ikuta, Arnaud Iradukunda, Gaetano Isola, Linda Merin J, Morteza Jafarinia, Khushleen Jaggi, Haitham Jahrami, Abubakar Ibrahim Jatau, Tahereh Javaheri, Sathish Kumar Jayapal, Olatunji Johnson, Charity Ehimwenma Joshua, Dler Hussein Kadir, Sivesh Kathir Kamarajah, Faizan Zaffar Kashoo, Nicholas J Kassebaum, Peter Njenga Keiyoro, Cathleen Keller, Alireza Khalilian, M Nuruzzaman Khan, Zahra Khorrami, Adnan Kisa, Sezer Kisa, Jonathan M Kocarnik, Alexander Kwarteng, Nhi Huu Hanh Le, Thao Thi Thu Le, James Leigh, Megan Lindstrom, Runben Liu, Erand Llanaj, Kelsey Lynn Maass, Razzagh Mahmoudi, Awoke Masrie, Le Huu Nhat Minh, Madeline E Moberg, Abdollah Mohammadian-Hafshejani, Hoda Mojiri-Forushani, Ali H Mokdad, Mohammad Ali Moni, Yousef Moradi, Vincent Mougin, Francesk Mulita, Christopher J L Murray, Mohsen Naghavi, Pirouz Naghavi, Josephine W Ngunjiri, Phat Tuan Nguyen, Van Thanh Nguyen, Osaretin Christabel Okonji, Bolajoko Olubukunola Olusanya, Jacob Olusegun Olusanya, Kanyin Liane Ong, Michal Ordak, Adrian Pana, Maja Pasovic, Spencer A Pease, Hoang Tran Pham, Saeed Pirouzpanah, Hadi Raeisi Shahraki, Quinn Rafferty, Mohammad Hifz Ur Rahman, Amir Masoud Rahmani, Chhabi Lal Ranabhat, Giridhara Rathnaiah Babu, Ramin Ravangard, Reza Rawassizadeh, Christian Razo, Robert C Reiner Jr, Maryam Rezaei, Mónica Rodrigues, Emma Lynn Best Rogowski, Gregory A Roth, Himanshu Sekhar Rout, Enrico Rubagotti, Susan Fred Rumisha, Adam Saddler, Umar Saeed, Zahra Saif, Abdallah M Samy, Francesca Sanna, Maheswar Satpathy, Mehdi Sayyah, Austin E Schumacher, Javad Sharifi-Rad, Kenji Shibuya, Youn Ho Shin, Sarah Brooke Sirota, Reed J D Sorensen, Michael Spartalis, Sandra Spearman, Jeffrey D Stanaway, Caitlyn Steiner, Jaimie D Steinmetz, Muhammad Suleman, Chandan Kumar Swain, Yibekal Manaye Tefera, Pugazhenthan Thangaraju, Chern Choong Chern Thum, Tala Tillawi, Era Upadhyay, Bay Vo, Theo Vos, Daniel J Weiss, Ronny Westerman, Joanna L Whisnant, Shadrach Wilson, Demewoz H Woldegebreal, Yen Jun Wong, Eve E Wool, Zenghong Wu, Wang-Dong Xu, Yvonne Yiru Xu, Amanuel Yigezu, Mohammed G M Zeariya, Jingya Zhang.

Providing critical feedback on methods or results

Amirali Aali, Hazim S Ababneh, Yohannes Habtegiorgis Abate, Cristiana Abbafati, Rouzbeh Abbasgholizadeh, Hedayat Abbastabar, Samar Abd ElHafeez, Michael Abdelmasseh, Sherief Abd-Elsalam, Mohammad Abdollahi, Meriem Abdoun, Deldar Morad Abdulah, Auwal Abdullahi, Mesfin Abebe, Samrawit Shawel Abebe, Kedir Hussein Abegaz, E S Abhilash, Hassan Abidi, Richard Gyan Aboagye, Hassan Abolhassani, Meysam Abolmaali, Mohamed Abouzid, Girma Beressa Aboye, Lucas Guimarães Abreu, Woldu Aberhe Abrha, Dariush Abtahi, Samir Abu Rumeileh, Hasan Abualruz, Bilyaminu Abubakar, Eman Abu-Gharbieh, Salahdein Aburuz, Ahmed Abu-Zaid, Manfred Mario Kokou Accrombessi, Tadele Girum Adal, Abdu A Adamu, Isaac Yeboah Addo, Akindele Olupelumi Adebiyi, Victor Adekanmbi, Abiola Victor Adepoju, Charles Oluwaseun Adetunji, Juliana Bunmi Adetunji, Temitayo Esther Adeyeoluwa, Daniel Adedayo Adeyinka, Olorunsola Israel Adeyomoye, Biruk Adie Adie Admass, Qorinah Estiningtyas Sakilah

Adnani, Saryia Adra, Aanuoluwapo Adeyimika Afolabi, Muhammad Sohail Afzal, Saira Afzal, Suneth Buddhika Agampodi, Pradyumna Agasthi, Manik Aggarwal, Shahin Aghamiri, Feleke Doyore Agide, Antonella Agodi, Anurag Agrawal, Williams Agyemang-Duah, Bright Opoku Ahinkorah, Aqeel Ahmad, Danish Ahmad, Firdos Ahmad, Muayyad M Ahmad, Sajjad Ahmad, Shahzaib Ahmad, Tauseef Ahmad, Keivan Ahmadi, Amir Mahmoud Ahmadzade, Ali Ahmed, Ayman Ahmed, Haroon Ahmed, Luai A Ahmed, Mehrunnisha Sharif Ahmed, Meqdad Saleh Ahmed, Muktar Beshir Ahmed, Syed Anees Ahmed, Budi Aji, Essona Matatom Akara, Hossein Akbarialiabad, Karolina Akinosoglou, Tomi Akinyemiju, Samuel Akyirem, Hanadi Al Hamad, Syed Mahfuz Al Hasan, Fares Alahdab, Samer O Alalalmeh, Ziyad Al-Aly, Khurshid Alam, Manjurul Alam, Noore Alam, Rasmieh Mustafa Al-amer, Fahad Mashhour Alanezi, Turki M Alanzi, Sayer Al-Azzam, Almaza Albakri, Mohammed Albashtawy, Mohammad T AlBataineh, Jacqueline Elizabeth Alcalde-Rabanal, Khalifah A Aldawsari, Robert W Aldridge, Haileselasie Berhane Alema, Mulubirhan Assefa Alemayohu, Sharifullah Alemi, Yihun Mulugeta Alemu, Adel Ali Saeed Al-Gheethi, Khalid F Alhabib, Mohammed Khaled Al-Hanawi, Abid Ali, Amjad Ali, Liaqat Ali, Mohammed Usman Ali, Rafat Ali, Shahid Ali, Syed Shujait Shujait Ali, Gianfranco Alicandro, Sheikh Mohammad Alif, Reyhaneh Alikhani, Yousef Alimohamadi, Ahmednur Adem Aliyi, Mohammad A M Aljasir, Syed Mohamed Aljunid, Peter Allebeck, Sabah Al-Marwani, Sadeq Ali Ali Al-Maweri, Joseph Uy Almazan, Hesham M Al-Mekhlafi, Louay Almidani, Omar Almidani, Mahmoud A Alomari, Basem Al-Omari, Jaber S Alqahtani, Shehabaldin Alqalyoobi, Ahmed Yaseen Alqutaibi, Salman Khalifah Al-Sabah, Zaid Altaany, Awais Altaf, Jaffar A Al-Tawfiq, Khalid A Altirkawi, Nelson Alvis-Guzman, Hassan Alwafi, Yaser Mohammed Al-Worafi, Hany Aly, Safwat Aly, Karem H Alzoubi, Azmeraw T Amare, Prince M Amegbor, Edward Kwabena Ameyaw, Tarek Tawfik Amin, Alireza Amindarolzarbi, Sohrab Amiri, Hubert Amu, Dickson A Amugsi, Ganiyu Adeniyi Amusa, Robert Ancuceanu, Deanna Anderlini, David B Anderson, Pedro Prata Andrade, Catalina Liliana Andrei, Tudorel Andrei, Colin Angus, Amir Anoushiravani, Ansariadi Ansariadi, Alireza Ansari-Moghaddam, Ernoiz Antriyandarti, Davood Anvari, Saeid Anvari, Saleha Anwar, Sumadi Lukman Anwar, Razique Anwer, Anayochukwu Edward Anyasodor, Juan Pablo Arab, Jalal Arabloo, Mosab Arafat, Demelash Areda, Abdulfatai Aremu, Olatunde Aremu, Hany Ariffin, Mesay Arkew, Benedetta Armocida, Mahwish Arooj, Anton A Artamonov, Judie Arulappan, Raphael Taiwo Aruleba, Ashokan Arumugam, Akeza Awealom Asgedom, Mohammad Asghari-Jafarabadi, Muhammad Ashraf, Armin Aslani, Thomas Astell-Burt, Mohammad Athar, Seyyed Shamsadin Athari, Bantalem Tilaye Tilaye Atinafu, Habtamu Wondmagegn Atlaw, Prince Atorkey, Maha Moh'd Wahbi Atout, Alok Atreya, Avinash Aujayeb, Marcel Ausloos, Abolfazl Avan, Atalel Fentahun Awedew, Amlaku Mulat Aweke, Beatriz Paulina Ayala Quintanilla, Haleh Ayatollahi, Jose L Ayuso-Mateos, Seyed Mohammad Ayyoubzadeh, Sina Azadnajafabad, Rui M S Azevedo, Ahmed Y Azzam, Darshan B B, Abraham Samuel Babu, Muhammad Badar, Ashish D Badiye, Soroush Baghdadi, Nasser Bagheri, Sara Bagherieh, Sulaiman Bah, Saeed Bahadorikhalili, Najmeh Bahmanziari, Ruhai Bai, Atif Amin Baig, Jennifer L Baker, Abdulaziz T Bako, Ravleen Kaur Bakshi, Senthilkumar Balakrishnan, Ovidiu Constantin Baltatu, Maciej Banach, Palash Chandra Banik, Hansi Bansal, Kannu Bansal, Franca Barbic, Martina Barchitta, Mainak Bardhan, Erfan Bardideh, Till Winfried Bärnighausen, Hiba Jawdat Barqawi, Lope H Barrero, Amadou Barrow, Sandra Barteit, Lingkan Barua, Zarrin Basharat, Pritish Baskaran, Buddha Basnyat, Quique Bassat, João Diogo Basso, Ann V L Basting, Sanjay Basu, Kavita Batra, Bernhard T Baune, Mohsen Bayati, Nebiyou Simegnew Bayileyegn, Thomas Beaney, Massimiliano Beghi, Emad Behboudi, Priyamadhaba Behera, Amir Hossein Behnoush, Masoud Behzadifar, Sefealem Assefa Belay, Chalie Mulu Belete, Michelle L Bell, Olorunjuwon Omolaja Bello, Luis Belo, Apostolos Beloukas, Rose Grace Bender, Isabela M Bensenor, Alemshet Yirga Berhie, Betyna N Berice, Robert S Bernstein, Gregory J Bertolacci, Devidas S Bhagat, Akshaya Srikanth Bhagavathula, Neeraj Bhala, Ashish Bhalla, Dinesh Bhandari, Nikha Bhardwaj, Pankaj

Bhardwaj, Prarthna V Bhardwaj, Ashish Bhargava, Sonu Bhaskar, Vivek Bhat, Gurjit Kaur Bhatti, Jasvinder Singh Bhatti, Manpreet S Bhatti, Rajbir Bhatti, Zulfiqar A Bhutta, Boris Bikbov, Jessica Devin Bishai, Francesca Bisulli, Atanu Biswas, Bijit Biswas, Bikes Destaw Bitew, Veera R Bitra, Micheal Kofi Boachie, Virginia Bodolica, Aadam Olalekan Bodunrin, Eyob Ketema Bogale, Kassawmar Angaw Bogale, Archith Boloor, Milad Bonakdar Hashemi, Aime Bonny, Kaustubh Bora, Hamed Borhany, Arturo Borzutzky, Souad Bouaoud, Antoine Boustany, Christopher Boxe, Edward J Boyko, Oliver J Brady, Dejana Braithwaite, Luisa C Brant, Alexandra Brazinova, Javier Brazo-Sayavera, Nicholas J K Breitborde, Hermann Brenner, Gabrielle Britton, Julie Brown, Traolach Brugha, Norma B Bulamu, Lemma N Bulto, Danilo Buonsenso, Richard A Burns, Reinhard Busse, Yasser Bustanji, Nadeem Shafique Butt, Zahid A Butt, Florentino Luciano Caetano dos Santos, Luis Alberto Cámera, Luciana Aparecida Campos, Ismael R Campos-Nonato, Chao Cao, Angelo Capodici, Rosario Cárdenas, Cristina G Carvalheiro, Márcia Carvalho, Joao Mauricio Castaldelli-Maia, Carlos A Castañeda-Orjuela, Giulio Castelpietra, Ferrán Catalá-López, Alberico L Catapano, Christopher R Cederroth, Luca Cegolon, Francieli Cembranel, Muthia Cenderadewi, Ester Cerin, Muge Cevik, Joshua Chadwick, Yaacoub Chahine, Chiranjib Chakraborty, Promit Ananyo Chakraborty, Raymond N C Chan, Eeshwar K Chandrasekar, Chin-Kuo Chang, Jung-Chen Chang, Periklis Charalampous, Vijay Kumar Chattu, Pankaj Chaturvedi, Victoria Chatzimavridou-Grigoriadou, Akhilanand Chaurasia, An-Tian Chen, Haowei Chen, Meng Xuan Chen, Ching-Yu Cheng, Esther T W Cheng, Nicolas Cherbuin, Wondimye Ashenafi Cheru, Odgerel Chimed-Ochir, Ritesh Chimoriya, Jesus Lorenzo Chirinos-Caceres, William C S Cho, Bryan Chong, Hitesh Chopra, Sonali Gajanan Choudhari, Rajiv Chowdhury, Devasahayam J Christopher, Isaac Sunday Chukwu, Eric Chung, Erin Chung, Eunice Chung, Sheng-Chia Chung, Muhammad Chutiyami, Zinhle Cindi, Iolanda Cioffi, Mareli M Claassens, Alyssa Columbus, Joao Conde, Samuele Cortese, Paolo Angelo Cortesi, Vera Marisa Costa, Simona Costanzo, Ewerton Cousin, Rosa A S Couto, Richard G Cowden, Kenneth Michael Cramer, Michael H Criqui, Natália Cruz-Martins, Silvia Magali Cuadra-Hernández, Garland T Culbreth, Patricia Cullen, Matthew Cunningham, Omid Dadras, Siyu Dai, Xiaochen Dai, Zhaoli Dai, Lachlan L Dalli, Giovanni Damiani, Jiregna Darega Gela, Jai K Das, Saswati Das, Subasish Das, Ana Maria Dascalu, Nihar Ranjan Dash, Mohsen Dashti, Anna Dastiridou, Gail Davey, Claudio Alberto Dávila-Cervantes, Nicole Davis Weaver, Kairat Davletov, Diego De Leo, Katie de Luca, Aklilu Tamire Debele, Shayom Debopadhaya, Louisa Degenhardt, Azizallah Dehghan, Cristian Del Bo', Ivan Delgado-Enciso, Berecha Hundessa Demessa, Andreas K Demetriades, Ke Deng, Xinlei Deng, Niloofar Deravi, Nebiyu Dereje, Nikolaos Dervenis, Emina Dervišević, Don C Des Jarlais, Hardik Dineshbhai Desai, Rupak Desai, Vinoth Gnana Chellaiyan Devanbu, Syed Masudur Rahman Dewan, Arkadeep Dhali, Kuldeep Dhama, Meghnath Dhimal, Sameer Dhingra, Vishal R Dhulipala, Diana Dias da Silva, Daniel Diaz, Michael J Diaz, Adriana Dima, Delaney D Ding, Huanghe Ding, Ricardo Jorge Dinis-Oliveira, M Ashworth Dirac, Shirin Djalalinia, Thao Huynh Phuong Do, Camila Bruneli do Prado, Saeid Doaei, Masoud Dodangeh, Milad Dodangeh, Klara Georgieva Dokova, Christiane Dolecek, Deepa Dongarwar, Mario D'Oria, Fariba Dorostkar, E Ray Dorsey, Wendel Mombaque dos Santos, Rajkumar Doshi, Leila Doshmangir, Robert Kokou Dowou, Haneil Larson Dsouza, Viola Dsouza, Mi Du, John Dube, Senbagam Duraisamy, Oyewole Christopher Durojaiye, Laura Dwyer-Lindgren, Paulina Agnieszka Dzianach, Arkadiusz Marian Dziedzic, Abdel Rahman E'mar, Ejemai Eboreime, Alireza Ebrahimi, Chidiebere Peter Echieh, Hisham Atan Edinur, David Edvardsson, Kristina Edvardsson, Defi Efendi, Ferry Efendi, Diyan Ermawan Effendi, Terje Andreas Eikemo, Ebrahim Eini, Michael Ekholuenetale, Temitope Cyrus Ekundayo, Iman El Sayed, Noha Mousaad Elemam, Frank J Elgar, Islam Y Elgendy, Ghada Metwally Tawfik ElGohary, Hala Rashad Elhabashy, Muhammed Elhadi, Waseem El-Huneidi, Legesse Tesfaye Elilo, Omar Abdelsadek Abdou Elmeligy, Mohamed A Elmonem, Ibrahim Elsohaby, Theophilus I Emeto, Luchuo Engelbert Bain, Babak Eshrati, Sharareh Eskandarieh, Juan Espinosa-Montero, Habtamu Esubalew, Farshid

Etaee, Natalia Fabin, Adewale Oluwaseun Fadaka, Adeniyi Francis Fagbamigbe, Ayesha Fahim, Saman Fahimi, Aliasghar Fakhri-Demeshghieh, Luca Falzone, Mohammad Fareed, Carla Sofia e Sá Farinha, Pawan Sirwan Faris, Andre Faro, Abidemi Omolara Fasanmi, Ali Fatehizadeh, Hamed Fattahi, Pooria Fazeli, Valery L Feigin, Ginenus Fekadu, Xiaoru Feng, Seyed-Mohammad Fereshtehnejad, Daniela Ferrante, Alize J Ferrari, Getahun Fetensa, Bikila Regassa Feyisa, Irina Filip, Florian Fischer, Joanne Flavel, David Flood, Bobirca Teodor Florin, Nataliya A Foigt, Morenike Oluwatoyin Folayan, Artem Alekseevich Fomenkov, Masoud Foroutan, Ingeborg Forthun, Daniela Fortuna, Matteo Foschi, Kayode Raphael Fowobaje, Kate Louise Francis, Richard Charles Franklin, Alberto Freitas, Joseph Friedman, Sara D Friedman, Takeshi Fukumoto, Blima Fux, Peter Andras Gaal, Muktar A Gadanya, Abhay Motiramji Gaidhane, Santosh Gaihre, Emmanuela Gakidou, Yaseen Galali, Natalie C Galles, Mandukhai Ganbat, Aravind P Gandhi, Balasankar Ganesan, Mohammad Arfat Ganiyani, William M Gardner, Jalaj Garg, Naval Garg, Rupesh K Gautam, Semiu Olatunde Gbadamosi, Tilaye Gebru Gebi, Miglas W Gebregergis, Mesfin Gebrehiwot, Teferi Gebru Gebremeskel, Simona Roxana Georgescu, Tamirat Getachew, Molla Getie, Keyghobad Ghadiri, Sulmaz Ghahramani, Khalid Yaser Ghailan, Mohammad-Reza Ghasemi, Ghazal Ghasempour Dabaghi, Afsaneh Ghasemzadeh, Ahmad Ghashghaee, Fariba Ghassemi, Ramy Mohamed Ghazy, Ajnish Ghimire, Sama Ghoba, Maryam Gholamalizadeh, Asadollah Gholamian, Ali Gholamrezanezhad, Pooyan Ghorbani Vajargah, Aloke Gopal Ghoshal, Paramjit Singh Gill, Tiffany K Gill, Themba G Ginindza, Alem Girmay, James C Glasbey, Elena V Gnedovskaya, Laszlo Göbölös, Myron Anthony Godinho, Amit Goel, Mohamad Goldust, Mahaveer Golechha, Nelson G M Gomes, Philimon N Gona, Sameer Vali Gopalani, Alessandra C Goulart, Bárbara Niegia Garcia Goulart, Anmol Goyal, Ayman Grada, Simon Matthew Graham, Michal Grivna, Shi-Yang Guan, Mohammed Ibrahim Mohialdeen Gubari, Mesay Dechasa Gudeta, Avirup Guha, Stefano Guicciardi, Rafael Alves Guimarães, Snigdha Gulati, Damitha Asanga Gunawardane, Sasidhar Gunturu, Cui Guo, Anish Kumar Gupta, Bhawna Gupta, Manoj Kumar Gupta, Mohak Gupta, Rajat Das Gupta, Rajeev Gupta, Sapna Gupta, Veer Bala Gupta, Vijai Kumar Gupta, Vivek Kumar Gupta, Lami Gurmessa, Reyna Alma Gutiérrez, Farrokh Habibzadeh, Parham Habibzadeh, Rasool Haddadi, Mostafa Hadei, Najah R Hadi, Nils Haep, Nima Hafezi-Nejad, Alemayehu Hailu, Arvin Haj-Mirzaian, Esam S Halboub, Brian J Hall, Sebastian Haller, Rabih Halwani, Randah R Hamadeh, Samer Hamidi, Erin B Hamilton, Chieh Han, Qiuxia Han, Asif Hanif, Fahad Hanna, Md Nuruzzaman Haque, Harapan Harapan, Ahmed I Hasaballah, Ikramul Hasan, M Tasdik Hasan, Hamidreza Hasani, Abdiwahab Hashi, Md Saquib Hasnain, Ikrama Hassan, Soheil Hassanipour, Hadi Hassankhani, Johannes Haubold, Rasmus J Havmoeller, Simon I Hay, Jiawei He, Omar E Hegazi, Golnaz Heidari, Mohammad Heidari, Mahsa Heidari-Foroozan, Bartosz Helfer, Delia Hendrie, Brenda Yuliana Herrera-Serna, Claudiu Herteliu, Hamed Hesami, Kamal Hezam, Catherine L Hill, Yuta Hiraike, Ramesh Holla, Nobuyuki Horita, Md Mahbub Hossain, Sahadat Hossain, Mohammad-Salar Hosseini, Hassan Hosseinzadeh, Mehdi Hosseinzadeh, Ahmad Hosseinzadeh Adli, Mihaela Hostiuc, Mohamed Hsairi, Vivian Chia-rong Hsieh, Rebecca L Hsu, Chengxi Hu, Michael Hultström, Ayesha Humayun, Tsegaye Gebreyes Hundie, Javid Hussain, M Azhar Hussain, Nawfal R Hussein, Foziya Mohammed Hussien, Hong-Han Huynh, Bing-Fang Hwang, Segun Emmanuel Ibitoye, Khalid S Ibrahim, Pulwasha Maria Iftikhar, Desta Ijo, Kevin S Ikuta, Paul Chukwudi Ikwegbue, Olayinka Stephen Ilesanmi, Irena M Ilic, Milena D Ilic, Mohammad Tarique Imam, Mustapha Immurana, Sumant Inamdar, Muhammad Iqhrammullah, Arnaud Iradukunda, Kenneth Chukwuemeka Iregbu, Md Rabiul Islam, Sheikh Mohammed Shariful Islam, Farhad Islami, Faisal Ismail, Nahlah Elkudssiah Ismail, Gaetano Isola, Masao Iwagami, Chidozie C D Iwu, Mahalaxmi Iyer, Linda Merin J, Jalil Jaafari, Louis Jacob, Kathryn H Jacobsen, Farhad Jadidi-Niaragh, Morteza Jafarinia, Abdollah Jafarzadeh, Khushleen Jaggi, Nader Jahanmehr, Haitham Jahrami, Nityanand Jain, Ammar Abdulrahman Jairoun, Abhishek Jaiswal, Elham Jamshidi, Mark M Janko,

Abubakar Ibrahim Jatau, Sabzali Javadov, Tahereh Javaheri, Sathish Kumar Jayapal, Shubha Jayaram, Rime Jebai, Sun Ha Jee, Jayakumar Jeganathan, Anil K Jha, Ravi Prakash Jha, Heng Jiang, Yingzhao Jin, Olatunji Johnson, Mohammad Jokar, Jost B Jonas, Tamas Joo, Abel Joseph, Nitin Joseph, Charity Ehimwenma Joshua, Grace Joshy, Jacek Jerzy Jozwiak, Mikk Jürisson, Vaishali K, Billingsley Kaambwa, Ali Kabir, Zubair Kabir, Vidya Kadashetti, Dler Hussein Kadir, Rizwan Kalani, Laleh R Kalankesh, Leila R Kalankesh, Feroze Kaliyadan, Sanjay Kalra, Vineet Kumar Kamal, Sivesh Kathir Kamarajah, Rajesh Kamath, Zahra Kamiab, Naser Kamyari, Thanigaivelan Kanagasabai, Tanuj Kanchan, Himal Kandel, Arun R Kanmanthareddy, Edmund Wedam Kanmiki, Kehinde Kazeem Kanmodi, Suthanthira Kannan S, Sushil Kumar Kansal, Rami S Kantar, Neeti Kapoor, Mehrdad Karajizadeh, Ibraheem M Karaye, André Karch, Asima Karim, Salah Eddin Karimi, Arman Karimi Behnagh, Faizan Zaffar Kashoo, Qalandar Hussein Abdulkarim Kasnazani, Nicholas J Kassebaum, Joonas H Kauppila, Navjot Kaur, Gbenga A Kayode, Foad Kazemi, Sina Kazemian, Tahseen Haider Kazmi, Getu Mosisa Kebebew, Adera Debella Kebede, Fassikaw Kebede, Peter Njenga Keiyoro, Jaimon Terence Kelly, John H Kempen, Jessica A Kerr, Himanshu Khajuria, Amirmohammad Khalaji, Nauman Khalid, Anees Ahmed Khalil, Alireza Khalilian, Faham Khamesipour, Ajmal Khan, Asaduzzaman Khan, Ikramullah Khan, Imteyaz A Khan, M Nuruzzaman Khan, Maseer Khan, Mohammad Jobair Khan, Moien AB Khan, Zeeshan Ali Khan, Mahammed Ziauddin Khan suheb, Shaghayegh Khanmohammadi, Khaled Khatab, Haitham Khatatbeh, Moawiah Mohammad Khatatbeh, Armin Khavandegar, Feriha Fatima Khidri, Mohammad Khorgamphar, Moein Khormali, Zahra Khorrami, Ahmad Khosravi, Mohammad Ali Khosravi, Zemene Demelash Kifle, Grace Kim, Jihee Kim, Kwanghyun Kim, Min Seo Kim, Yun Jin Kim, Ruth W Kimokoti, Adnan Kisa, Sezer Kisa, Desmond Klu, Ann Kristin Skrindo Knudsen, Jonathan M Kocarnik, Sonali Kochhar, Timea Kocsis, David S Q Koh, Ali-Asghar Kolahi, Kairi Kolves, Farzad Kompani, Gerbrand Koren, Karel Kostev, Parvaiz A Koul, Sindhura Lakshmi Koulmane Laxminarayana, Kewal Krishan, Hare Krishna, Varun Krishna, Vijay Krishnamoorthy, Yuvaraj Krishnamoorthy, Barthelemy Kuate Defo, Md Abdul Kuddus, Mohammed Kuddus, Ilari Kuitunen, Vishnutheertha Kulkarni, Akshay Kumar, Ashish Kumar, Harish Kumar, Manasi Kumar, Fantahun Tarekegn Kumie, Satyajit Kundu, Om P Kurmi, Asep Kusnali, Dian Kusuma, Alexander Kwarteng, Ilias Kyriopoulos, Hmwe Hmwe Kyu, Carlo La Vecchia, Muhammad Awwal Ladan, Abraham K Lagat, Daphne Teck Ching Lai, Dharmesh Kumar Lal, Tea Lallukka, Hilton Lam, Judit Lám, Francesco Lanfranchi, Justin J Lang, Van Charles Lansingh, Savita Lasrado, Zohra S Lassi, Kamaluddin Latief, Kaveh Latifinaibin, Paolo Lauriola, Nhi Huu Hanh Le, Thao Thi Thu Le, Trang Diep Thanh Le, Caterina Ledda, Jorge R Ledesma, Munjae Lee, Seung Won Lee, Shaun Wen Huey Lee, Wei-Chen Lee, Yo Han Lee, Kate E LeGrand, James Leigh, Elvynna Leong, Temesgen L Lerango, Ming-Chieh Li, Wei Li, Xiaopan Li, Zhihui Li, Virendra S Ligade, Andrew Tiyamike Makhiringa Likaka, Lee-Ling Lim, Stephen S Lim, Chaojie Liu, Gang Liu, Jue Liu, Runben Liu, Shiwei Liu, Xiaofeng Liu, Xuefeng Liu, Erand Llanaj, Rubén López-Bueno, Arianna Maever Loreche, Stefan Lorkowski, Paulo A Lotufo, Jailos Lubinda, Giancarlo Lucchetti, Alessandra Lugo, Zheng Feei Ma, Monika Machoy, Farzan Madadizadeh, Christian Madsen, Áurea M Madureira-Carvalho, Azzam A Maghazachi, Sandeep B Maharaj, Soleiman Mahjoub, Mansour Adam Mahmoud, Alireza Mahmoudi, Elham Mahmoudi, Razzagh Mahmoudi, Azeem Majeed, Elaheh Malakan Rad, Venkatesh Maled, Reza Malekzadeh, Armaan K Malhotra, Kashish Malhotra, Ahmad Azam Malik, Iram Malik, Deborah Carvalho Malta, Abdullah A Mamun, Pejman Mansouri, Mohammad Ali Mansournia, Lorenzo Giovanni Mantovani, Sajid Maqsood, Hamid Reza Marateb, Joemer C Maravilla, Agustina M Marconi, Parham Mardi, Mirko Marino, Abdoljalal Marjani, Gabriel Martinez, Bernardo Alfonso Martinez-Guerra, Ramon Martinez-Piedra, Daniela Martini, Santi Martini, Francisco Rogerlândio Martins-Melo, Miquel Martorell, Wolfgang Marx, Sharmeen Maryam, Roy Rillera Marzo, Anthony Masaka, Awoke Masrie, Alexander G Mathioudakis, Manu Raj Mathur, Jishanth Mattumpuram, Richard Matzopoulos,

Richard James Maude, Andrea Maugeri, Mahsa Mayeli, Maryam Mazaheri, Mohsen Mazidi, John J McGrath, Martin McKee, Anna Laura W McKowen, Steven M McPhail, Enkeleint A Mechili, John Robert Carabeo Medina, Rishi P Mediratta, Jitendra Kumar Meena, Rahul Mehra, Kamran Mehrabani-Zeinabad, Entezar Mehrabi Nasab, Tesfahun Mekene Meto, Gebrekiros Gebremichael Meles, Max Alberto Mendez Mendez-Lopez, Walter Mendoza, Ritesh G Menezes, Belayneh Mengist, Alexios-Fotios A Mentis, Sultan Ayoub Meo, Haftu Asmerom Meresa, Atte Meretoja, Tuomo J Meretoja, Abera M Mersha, Bezawit Afework Mesfin, Tomislav Mestrovic, Kukulege Chamila Dinushi Mettananda, Sachith Mettananda, Peter Meylakhs, Adquate Mhlanga, Laurette Mhlanga, Tianyue Mi, Tomasz Miazgowski, Georgia Micha, Irmina Maria Michalek, Ted R Miller, Edward J Mills, Le Huu Nhat Minh, GK Mini, Andreea Mirica, Erkin M Mirrakhimov, Mizan Kiros Mirutse, Maryam Mirzaei, Awoke Misganaw, Sanjeev Misra, Prasanna Mithra, Chaitanya Mittal, Madeline E Moberg, Ashraf Mohamadkhani, Jama Mohamed, Mouhand F H Mohamed, Nouh Saad Mohamed, Sakineh Mohammad-Alizadeh-Charandabi, Soheil Mohammadi, Abdollah Mohammadian-Hafshejani, Noushin Mohammadifard, Hassen Mohammed, Mustapha Mohammed, Salahuddin Mohammed, Shafiu Mohammed, Hoda Mojiri-Forushani, Amin Mokari, Ali H Mokdad, Sabrina Molinaro, Mariam Molokhia, Sara Momtazmanesh, Lorenzo Monasta, Stefania Mondello, Mohammad Ali Moni, AmirAli Moodi Ghalibaf, Maryam Moradi, Yousef Moradi, Maziar Moradi-Lakeh, Maliheh Moradzadeh, Paula Moraga, Lidia Morawska, Rafael Silveira Moreira, Negar Morovatdar, Jonathan F Mosser, Rohith Motappa, Vincent Mougin, Simin Mouodi, Seyed Ehsan Mousavi, Amin Mousavi Khaneghah, Emmanuel A Mpolya, Sumaira Mubarik, Lorenzo Muccioli, Ulrich Otto Mueller, Faraz Mughal, Sumoni Mukherjee, Francesk Mulita, Kavita Munjal, Efrén Murillo-Zamora, Christopher J L Murray, Fungai Musaigwa, Khaled M Musallam, Ghulam Mustafa, Saravanan Muthupandian, Raman Muthusamy, Muhammad Muzaffar, Woojae Myung, Ahamarshan Jayaraman Nagarajan, Mohsen Naghavi, Pirouz Naghavi, Aliya Naheed, Ganesh R Naik, Gurudatta Naik, Firzan Nainu, Sanjeev Nair, Hastyar Hama Rashid Najmuldeen, Vinay Nangia, Atta Abbas Naqvi, Sreenivas Narasimha Swamy, Aparna Ichalangod Narayana, Shumaila Nargus, Bruno Ramos Nascimento, Abdulqadir J Nashwan, Zuhair S Natto, Javaid Nauman, Muhammad Naveed, Biswa Prakash Nayak, Vinod C Nayak, Athare Nazri-Panjaki, Rawlance Ndejjo, Hadush Negash, Ionut Negoi, Ruxandra Irina Negoi, Seyed Aria Nejadghaderi, Chakib Nejjari, Evangelia Nena, Samata Nepal, Marie Ng, Haruna Asura Nggada, Georges Nguefack-Tsague, Josephine W Ngunjiri, Anh Hoang Nguyen, Dang H Nguyen, Phat Tuan Nguyen, Van Thanh Nguyen, Robina Khan Niazi, Katie R Nielsen, Yeshambel T Nigatu, Ali Nikoobar, Amin Reza Nikpoor, Dina Nur Anggraini Ningrum, Chukwudi A Nnaji, Lawrence Achilles Nnyanzi, Efaq Ali Noman, Shuhei Nomura, Mamoona Noreen, Nafise Noroozi, Bo Norrving, Jean Jacques Noubiap, Chisom Adaobi Nri-Ezedi, George Ntaios, Mpiko Ntsekhe, Dieta Nurrika, Jerry John Nutor, Bogdan Oancea, Kehinde O Obamiro, Mary Aigbiremo Oboh, Ismail A Odetokun, Nkechi Martina Odogwu, Martin James O'Donnell, Michael Safo Oduro, Akinyemi O D Ofakunrin, Ayodipupo Sikiru Oguntade, Hassan Okati-Aliabad, Akinkunmi Paul Okekunle, Osaretin Christabel Okonji, Andrew T Olagunju, Muideen Tunbosun Olaiya, Matthew Idowu Olatubi, Gláucia Maria Moraes Oliveira, Isaac Iyinoluwa Olufadewa, Bolajoko Olubukunola Olusanya, Jacob Olusegun Olusanya, Yinka Doris Oluwafemi, Hany A Omar, Ahmed Omar Bali, Goran Latif Omer, Maureene Auma Ondayo, Kanyin Liane Ong, Obinna E Onwujekwe, Kenneth Ikenna Onyedibe, Michal Ordak, Doris V Ortega-Altamirano, Alberto Ortiz, Wael M S Osman, Samuel M Ostroff, Uchechukwu Levi Osuagwu, Adrian Otoiu, Nikita Otstavnov, Stanislav S Otstavnov, Amel Ouyahia, Mayowa O Owolabi, Mahesh Padukudru P A, Jagadish Rao Padubidri, Pramod Kumar Pal, Tamás Palicz, Claudia Palladino, Raffaele Palladino, Feng Pan, Hai-Feng Pan, Adrian Pana, Paramjot Panda, Songhomitra Panda-Jonas, Seithikurippu R Pandi-Perumal, Helena Ullyartha Pangaribuan, Georgios D Panos, Leonidas D Panos, Ioannis Pantazopoulos, Anca Mihaela Pantea Stoian,

Paraskevi Papadopoulou, Romil R Parikh, Seoyeon Park, Ashwaghosha Parthasarathi, Ava Pashaei, Maja Pasovic, Roberto Passera, Deepak Kumar Pasupula, Hemal M Patel, Jay Patel, Sangram Kishor Patel, Shankargouda Patil, Dimitrios Patoulias, Uttam Paudel, Amy E Peden, Paolo Pedersini, Veincent Christian Filipino Pepito, Emmanuel K Peprah, Prince Peprah, João Perdigão, Marcos Pereira, Mario F P Peres, Arokiasamy Perianayagam, Richard G Pestell, Konrad Pesudovs, Fanny Emily Petermann-Rocha, William A Petri, Hoang Tran Pham, Anil K Philip, Michael R Phillips, Daniela Pierannunzio, Manon Pigeolet, David M Pigott, Zahra Zahid Piracha, Michael A Piradov, Saeed Pirouzpanah, Nishad Plakkal, Evgenii Plotnikov, Vivek Podder, Dimitri Poddighe, Kevan R Polkinghorne, Ramesh Poluru, Ville T Ponkilainen, Fabio Porru, Maarten J Postma, Govinda Raj Poudel, Akram Pourshams, Naeimeh Pourtaheri, Sergio I Prada, Pranil Man Singh Pradhan, Manya Prasad, Akila Prashant, Elton Junio Sady Prates, TINA PRISCILLA, Natalie Pritchett, Bharathi M Purohit, Jagadeesh Puvvula, Nameer Hashim Qasim, Ibrahim Qattea, Asma Saleem Qazi, Gangzhen Qian, Suli Qiu, Maryam Faiz Qureshi, Amir Radfar, Raghu Anekal Radhakrishnan, Venkatraman Radhakrishnan, Hadi Raeisi Shahraki, Quinn Rafferty, Alberto Raggi, Pankaja Raghav Raghav, Fakher Rahim, Md Jillur Rahim, Vafa Rahimi-Movaghar, Md Mosfequr Rahman, Mohammad Hifz Ur Rahman, Mosiur Rahman, Muhammad Aziz Rahman, Amir Masoud Rahmani, Shayan Rahmani, Vahid Rahmanian, Sathish Rajaa, Prashant Rajput, Ivo Rakovac, Shakthi Kumaran Ramasamy, Kritika Rana, Chhabi Lal Ranabhat, Nemanja Rancic, Amey Rane, Chythra R Rao, Indu Ramachandra Rao, Mithun Rao, Sowmya J Rao, Drona Prakash Rasali, Sina Rashedi, Vahid Rashedi, Mohammad-Mahdi Rashidi, Azad Rasul, Giridhara Rathnaiah Babu, Santosh Kumar Rauniyar, Ramin Ravangard, Nakul Ravikumar, David Laith Rawaf, Salman Rawaf, Lal Rawal, Reza Rawassizadeh, Bharat Rawlley, Rabail Zehra Raza, Christian Razo, Elrashdy Moustafa Mohamed Redwan, Faizan Ur Rehman, Lennart Reifels, Robert C Reiner Jr, Luis Felipe Reyes, Maryam Rezaei, Nazila Rezaei, Negar Rezaei, Mohsen Rezaeian, Taeho Gregory Rhee, Mavra A Riaz, Antonio Luiz P Ribeiro, Jennifer Rickard, Hannah R Riva, Célia Fortuna Rodrigues, Mónica Rodrigues, Leonardo Roever, Emma Lynn Best Rogowski, Peter Rohloff, Debby Syahru Romadlon, Esperanza Romero-Rodríguez, Gholamreza Roshandel, Himanshu Sekhar Rout, Nitai Roy, Priyanka Roy, Enrico Rubagotti, Godfrey M Rwegerera, Andrzej Rynkiewicz, Chandan S N, Aly M A Saad, Zahra Saadatian, Korosh Saber, Maha Mohamed Saber-Ayad, Morteza SaberiKamarposhti, Siamak Sabour, Perminder S Sachdev, Basema Saddik, Bashdar Abuzed Sadee, Ehsan Sadeghi, Erfan Sadeghi, Mohammad Reza Saeb, Umar Saeed, Sher Zaman Safi, Rajesh Sagar, Amene Saghazadeh, Fatemeh Saheb Sharif-Askari, Narjes Saheb Sharif-Askari, Soumya Swaroop Sahoo, Umakanta Sahoo, Monalisha Sahu, Zahra Saif, Mirza Rizwan Sajid, Joseph W Sakshaug, Payman Salamati, Afeez Abolarinwa Salami, Mohamed A Saleh, Sana Salehi, Marwa Rashad Salem, Hossein Samadi Kafil, Sara Samadzadeh, Saad Samargandy, Yoseph Leonardo Samodra, Abdallah M Samy, Juan Sanabria, Itamar S Santos, Milena M Santric-Milicevic, Bruno Piassi Sao Jose, Sivan Yegnanarayana Iyer Saraswathy, Babak Saravi, Yaser Sarikhani, Tanmay Sarkar, Rodrigo Sarmiento-Suárez, Gargi Sachin Sarode, Sachin C Sarode, Arash Sarveazad, Brijesh Sathian, Thirunavukkarasu Sathish, Maheswar Satpathy, Abu Sayeed, Md Abu Sayeed, Mete Saylan, Mehdi Sayyah, Nikolaos Scarmeas, Benedikt Michael Schaarschmidt, Markus P Schlaich, Winfried Schlee, Ione Jayce Ceola Schneider, Art Schuermans, Austin E Schumacher, Aletta Elisabeth Schutte, Michaël Schwarzinger, David C Schwebel, Falk Schwendicke, Mario Šekerija, Sabyasachi Senapati, Subramanian Senthilkumaran, Sadaf G Sepanlou, Dragos Serban, Yashendra Sethi, Feng Sha, Amir Shafaat, Mahan Shafie, Nilay S Shah, Pritik A Shah, Syed Mahboob Shah, Saeed Shahabi, Ataollah Shahbandi, Izza Shahid, Samiah Shahid, Wajeehah Shahid, Hamid R Shahsavari, Moyad Jamal Shahwan, Masood Ali Shaikh, Alireza Shakeri, Ali S Shalash, Muhammad Aaqib Shamim, Mehran Shams-Beyranvand, Hina Shamshad, Mohammad Anas Shamsi, Mohd Shanawaz, Abhishek Shankar, Sadaf Sharfaei, Amin Sharifan, Javad Sharifi-Rad, Rajesh Sharma, Saurab Sharma, Vishal

Sharma, Rajesh P Shastry, Amin Shavandi, Maryam Shayan, Aziz Sheikh, Rahim Ali Sheikhi, Jiabin Shen, Pavanchand H Shetty, Kenji Shibuya, Desalegn Shiferaw, Mika Shigematsu, Min-Jeong Shin, Youn Ho Shin, Rahman Shiri, Nebiyu Aniley Shitaye, Aminu Shittu, Ivy Shiue, K M Shivakumar, Velizar Shivarov, Farhad Shokraneh, Azad Shokri, Sina Shool, Seyed Afshin Shorofi, Sunil Shrestha, Kerem Shuval, Emmanuel Edwar Siddig, João Pedro Silva, Luís Manuel Lopes Rodrigues Silva, Soraia Silva, Anjali Singal, Abhinav Singh, Balbir Bagicha Singh, Garima Singh, Jasbir Singh, Narinder Pal Singh, Paramdeep Singh, Dhirendra Narain Sinha, Robert Sinto, Md Shahjahan Siraj, Freddy Sitas, Shravan Sivakumar, Valentin Yurievich Skryabin, Anna Aleksandrovna Skryabina, David A Sleet, Hamidreza Soleimani, Sameh S M Soliman, Suhang Song, Yimeng Song, Reed J D Sorensen, Ireneous N Soyiri, Michael Spartalis, Chandrashekhar T Sreeramareddy, Jeffrey D Stanaway, Muhammad Haroon Stanikzai, Benjamin A Stark, Joseph R Starnes, Antonina V Starodubova, Fridolin Steinbeis, Caitlyn Steiner, Jaimie D Steinmetz, Paschalis Steiropoulos, Aleksandar Stevanović, Leo Stockfelt, Mark A Stokes, Stefan Stortecky, Vetriselvan Subramaniyan, Muhammad Suleman, Rizwan Suliankatchi Abdulkader, Abida Sultana, Haitong Zhe Sun, Jing Sun, David Sunkersing, Chandan Kumar Swain, Lukasz Szarpak, Mindy D Szeto, Miklós Szócska, Payam Tabaee Damavandi, Rafael Tabarés-Seisdedos, Shima Tabatabai, Mohammad Tabish, JYOTHI TADAKAMADLA, Santosh Kumar Tadakamadla, Yasaman Taheri Abkenar, Moslem Taheri Soodejani, Jabeen Taiba, Ken Takahashi, Iman M Talaat, Ashis Talukder, Mircea Tampa, Jacques Lukenze Tamuzi, Ker-Kan Tan, Sarmila Tandukar, Haosu Tang, Hong K Tang, Ingan Ukur Tarigan, Mengistie Kassahun Tariku, Md Tariqujjaman, Elvis Enowbeyang Tarkang, Razieh Tavakoli Oliaee, Seyed Mohammad Tavangar, Yibekal Manaye Tefera, Mohamad-Hani Temsah, Reem Mohamad Hani Temsah, Masayuki Teramoto, Enoch Teye-Kwadjo, Rishu Thakur, Pugazhenthan Thangaraju, Kavumpurathu Raman Thankappan, Rasiah Thayakaran, Nihal Thomas, Nikhil Kenny Thomas, Azalea M Thomson, Amanda G Thrift, Chern Choong Chern Thum, Lau Caspar Thygesen, Jing Tian, Ales Tichopad, Jansje Henny Vera Ticoalu, Tala Tillawi, Tenaw Yimer Tiruye, Marcello Tonelli, Roman Topor-Madry, Adetunji T Toriola, Mathilde Touvier, Marcos Roberto Tovani-Palone, Jasmine T Tran, Nghia Minh Tran, Domenico Trico, Samuel Joseph Tromans, Guesh Mebrahtom Tsegay, Evangelia Eirini Tsermpini, Munkhtuya Tumurkhuu, Kang Tung, Stefanos Tyrovolas, Sayed Mohammad Nazim Uddin, Aniefiok John Udoakang, Arit Udoh, Atta Ullah, Irfan Ullah, Saeed Ullah, Sana Ullah, Srikanth Umakanthan, Chukwuma David Umeokonkwo, Bhaskaran Unnikrishnan, Carolyn Anne Unsworth, Era Upadhyay, Daniele Urso, Jibrin Sammani Usman, Seyed Mohammad Vahabi, Asokan Govindaraj Vaithinathan, Rohollah Valizadeh, Sarah M Van de Velde, Jef Van den Eynde, Orsolya Varga, Priya Vart, Shoban Babu Varthya, Milena Vasic, Siavash Vaziri, Balachandar Vellingiri, Narayanaswamy Venketasubramanian, Madhur Verma, Massimiliano Veroux, Georgios-Ioannis Verras, Dominique Vervoort, Jorge Hugo Villafañe, Gabriela Ines Villanueva, Manish Vinayak, Francesco S Violante, Maria Viskadourou, Bay Vo, Stein Emil Vollset, Theo Vos, Isidora S Vujcic, Hatem A Wafa, Yasir Waheed, Richard G Wamai, Cong Wang, Shu Wang, Song Wang, Yanzhong Wang, Yuan-Pang Wang, Muhammad Waqas, Paul Ward, Emebet Gashaw Wassie, Stephanie Louise Watson Watson, Kosala Gayan Weerakoon, Melissa Y Wei, Ronny Westerman, Joanna L Whisnant, Taweewat Wiangkham, Dakshitha Praneeth Wickramasinghe, Nuwan Darshana Wickramasinghe, Angga Wilandika, Caroline Wilkerson, Peter Willeit, Shadrach Wilson, Demewoz H Woldegebreal, Axel Walter Wolf, Yohannes Addisu Wondimagegene, Yen Jun Wong, Utoomporn Wongsin, Eve E Wool, Ai-Min Wu, Chenkai Wu, Felicia Wu, Zenghong Wu, Juan Xia, Hong Xiao, Yang Xie, Wang-Dong Xu, Xiaoyue Xu, Yvonne Yiru Xu, Ali Yadollahpour, Kazumasa Yamagishi, Danting Yang, Lin Yang, Yuichiro Yano, Yao Yao, Habib Yaribeygi, Pengpeng Ye, Sisay Shewasinad Yehualashet, Metin Yesiltepe, Subah Abderehim Yesuf, Saber Yezli, Siyan Yi, Amanuel Yigezu, Arzu Yiğit, Vahit Yiğit, Paul Yip, Malede Berihun Yismaw, Yazachew Yismaw, Dong Keon Yon, Naohiro Yonemoto, Seok-Jun Yoon, Yuyi You,

Mustafa Z Younis, Zabihollah Yousefi, Chuanhua Yu, Yong Yu, Faith H Yuh, Siddhesh Zadey, Vesna Zadnik, Nima Zafari, Fathiah Zakham, Nazar Zaki, Sojib Bin Zaman, Nelson Zamora, Moein Zangiabadian, Armin Zarrintan, Mohammed G M Zeariya, Haijun Zhang, Jianrong Zhang, Jingya Zhang, Liqun Zhang, Yunquan Zhang, Zhi-Jiang Zhang, Hanqing Zhao, Chenwen Zhong, Juexiao Zhou, Bin Zhu, Lei Zhu, Magdalena Zielińska, Osama A Zitoun, Mohammad Zoladl, Liesl J Zuhlke, Alimuddin Zumla, Elric Zweck, Samer H Zyoud.

Drafting the work or revising it critically for important intellectual content

Amirali Aali, Hazim S Ababneh, Yohannes Habtegiorgis Abate, Cristiana Abbafati, Mohammadreza Abbasian, Mohsen Abbasi-Kangevari, Samar Abd ElHafeez, Michael Abdelmasseh, Sherief Abd-Elsalam, Ahmed Abdelwahab, Mohammad-Amin Abdollahifar, Auwal Abdullahi, Mesfin Abebe, Samrawit Shawel Abebe, Aidin Abedi, Kedir Hussein Abegaz, Hassan Abidi, Olumide Abiodun, Hassan Abolhassani, Meysam Abolmaali, Mohamed Abouzid, Girma Beressa Aboye, Lucas Guimarães Abreu, Dariush Abtahi, Samir Abu Rumeileh, Hasan Abualruz, Bilyaminu Abubakar, Eman Abu-Gharbieh, Niveen ME Abu-Rmeileh, Salahdein Aburuz, Ahmed Abu-Zaid, Tadele Girum Adal, Abdu A Adamu, Isaac Yeboah Addo, Giovanni Addolorato, Akindele Olupelumi Adebiyi, Victor Adekanmbi, Abiola Victor Adepoju, Charles Oluwaseun Adetunji, Juliana Bunmi Adetunji, Daniel Adedayo Adeyinka, Olorunsola Israel Adeyomoye, Biruk Adie Adie Admass, Qorinah Estiningtyas Sakilah Adnani, Saryia Adra, Aanuoluwapo Adeyimika Afolabi, Muhammad Sohail Afzal, Saira Afzal, Pradyumna Agasthi, Manik Aggarwal, Feleke Doyore Agide, Antonella Agodi, Bright Opoku Ahinkorah, Danish Ahmad, Firdos Ahmad, Muayyad M Ahmad, Ali Ahmed, Ayman Ahmed, Haroon Ahmed, Luai A Ahmed, Muktar Beshir Ahmed, Syed Anees Ahmed, Marjan Ajami, Essona Matatom Akara, Hossein Akbarialiabad, Mohammed Ahmed Akkaif, Samuel Akyirem, Fares Alahdab, Samer O Alalalmeh, Tariq A Alalwan, Khurshid Alam, Rasmieh Mustafa Al-amer, Sayer Al-Azzam, Almaza Albakri, Mohammed Albashtawy, Mohammad T AlBataineh, Khalifah A Aldawsari, Wafa A Aldhaleei, Mulubirhan Assefa Alemayohu, Yihun Mulugeta Alemu, Khalid F Alhabib, Fadwa Alhalaiqa Naji Alhalaiqa, Mohammed Khaled Al-Hanawi, Abid Ali, Amjad Ali, Liaqat Ali, Mohammed Usman Ali, Rafat Ali, Shahid Ali, Syed Shujait Shujait Ali, Gianfranco Alicandro, Ahmednur Adem Aliyi, Peter Allebeck, Sadeq Ali Ali Al-Maweri, Louay Almidani, Mahmoud A Alomari, Jordi Alonso, Jaber S Alqahtani, Shehabaldin Alqalyoobi, Ahmed Yaseen Alqutaibi, Awais Altaf, Jaffar A Al-Tawfiq, Deborah Oyine Aluh, Nelson Alvis-Guzman, Hassan Alwafi, Yaser Mohammed Al-Worafi, Hany Aly, Safwat Aly, Karem H Alzoubi, Reza Amani, Azmeraw T Amare, Prince M Amegbor, Tarek Tawfik Amin, Alireza Amindarolzarbi, Sohrab Amiri, Mohammad Hosein Amirzade-Iranaq, Hubert Amu, Dickson A Amugsi, Ganiyu Adeniyi Amusa, Robert Ancuceanu, Deanna Anderlini, David B Anderson, Pedro Prata Andrade, Catalina Liliana Andrei, Tudorel Andrei, Colin Angus, Abhishek Anil, Sneha Anil, Amir Anoushiravani, Hossein Ansari, Catherine M Antony, Ernoiz Antriyandarti, Saeid Anvari, Saleha Anwar, Anayochukwu Edward Anyasodor, Muhammad Aqeel, Juan Pablo Arab, Jalal Arabloo, Mosab Arafat, Abdulfatai Aremu, Olatunde Aremu, Hany Ariffin, Mesay Arkew, Benedetta Armocida, Johan Ärnlöv, Mahwish Arooj, Judie Arulappan, Raphael Taiwo Aruleba, Ashokan Arumugam, Malke Asaad, Akeza Awealom Asgedom, Mona Asghariahmadabad, Muhammad Ashraf, Armin Aslani, Seyyed Shamsadin Athari, Bantalem Tilaye Tilaye Atinafu, Habtamu Wondmagegn Atlaw, Prince Atorkey, Maha Moh'd Wahbi Atout, Alok Atreya, Avinash Aujayeb, Marcel Ausloos, Abolfazl Avan, Amlaku Mulat Aweke, Beatriz Paulina Ayala Quintanilla, Sina Azadnajafabad, Rui M S Azevedo, Ahmed Y Azzam, Abraham Samuel Babu, Muhammad Badar, Ashish D Badiye, Soroush Baghdadi, Sara Bagherieh, Sulaiman Bah, Ruhai Bai, Atif Amin Baig, Jennifer L Baker, Abdulaziz T Bako, Senthilkumar Balakrishnan, Madhan Balasubramanian, Ovidiu Constantin Baltatu, Kiran Bam, Maciej Banach, Soham Bandyopadhyay, Palash Chandra Banik, Hansi Bansal, Kannu Bansal, Martina Barchitta, Mainak Bardhan, Erfan Bardideh, Suzanne

Lyn Barker-Collo, Till Winfried Bärnighausen, Francesco Barone-Adesi, Hiba Jawdat Barqawi, Amadou Barrow, Azadeh Bashiri, Afisu Basiru, Pritish Baskaran, Buddha Basnyat, Quique Bassat, João Diogo Basso, Sanjay Basu, Bernhard T Baune, Thomas Beaney, Neeraj Bedi, Massimiliano Beghi, Priyamadhaba Behera, Amir Hossein Behnoush, Masoud Behzadifar, Maryam Beiranvand, Diana Fernanda Bejarano Ramirez, Yannick Béjot, Sefealem Assefa Belay, Chalie Mulu Belete, Michelle L Bell, Muhammad Bashir Bello, Olorunjuwon Omolaja Bello, Luis Belo, Apostolos Beloukas, Isabela M Bensenor, Zombor Berezvai, Gregory J Bertolacci, Paulo J G Bettencourt, Kebede A Beyene, Akshaya Srikanth Bhagavathula, Neeraj Bhala, Dinesh Bhandari, Prarthna V Bhardwaj, Ashish Bhargava, Sonu Bhaskar, Vivek Bhat, Gurjit Kaur Bhatti, Jasvinder Singh Bhatti, Manpreet S Bhatti, Rajbir Bhatti, Boris Bikbov, Catherine Bisignano, Atanu Biswas, Saeid Bitaraf, Veera R Bitra, Tone Bjørge, Mary Sefa Boampong, Anca Vasilica Bobirca, Virginia Bodolica, Aadam Olalekan Bodunrin, Milad Bonakdar Hashemi, Aime Bonny, Kaustubh Bora, Hamed Borhany, Arturo Borzutzky, Souad Bouaoud, Christopher Boxe, Edward J Boyko, Oliver J Brady, Dejana Braithwaite, Luisa C Brant, Javier Brazo-Sayavera, Nicholas J K Breitborde, Susanne Breitner, Hermann Brenner, Andrey Nikolaevich Briko, Nikolay Ivanovich Briko, Gabrielle Britton, Julie Brown, Traolach Brugha, Norma B Bulamu, Danilo Buonsenso, Yasser Bustanji, Florentino Luciano Caetano dos Santos, Daniela Calina, Luciana Aparecida Campos, Ismael R Campos-Nonato, Yin Cao, Angelo Capodici, Sinclair Carr, Giulia Carreras, Juan J Carrero, Andrea Carugno, Felix Carvalho, Joao Mauricio Castaldelli-Maia, Carlos A Castañeda-Orjuela, Giulio Castelpietra, Ferrán Catalá-López, Alberico L Catapano, Maria Sofia Cattaruzza, Christopher R Cederroth, Luca Cegolon, Francieli Cembranel, Muthia Cenderadewi, Ester Cerin, Muge Cevik, Joshua Chadwick, Yaacoub Chahine, Chiranjib Chakraborty, Jeffrey Shi Kai Chan, Rama Mohan Chandika, Eeshwar K Chandrasekar, Chin-Kuo Chang, Vijay Kumar Chattu, Pankaj Chaturvedi, Victoria Chatzimavridou-Grigoriadou, Akhilanand Chaurasia, An-Tian Chen, Haowei Chen, Meng Xuan Chen, Simiao Chen, Nicolas Cherbuin, Wondimye Ashenafi Cheru, Ju-Huei Chien, Ritesh Chimoriya, Patrick R Ching, Jesus Lorenzo Chirinos-Caceres, William C S Cho, Bryan Chong, Hitesh Chopra, Rajiv Chowdhury, Devasahayam J Christopher, Eric Chung, Muhammad Chutiyami, Iolanda Cioffi, Mareli M Claassens, Rafael M Claro, Alyssa Columbus, Joao Conde, Samuele Cortese, Paolo Angelo Cortesi, Vera Marisa Costa, Ewerton Cousin, Rosa A S Couto, Richard G Cowden, Michael H Criqui, Natália Cruz-Martins, Silvia Magali Cuadra-Hernández, Patricia Cullen, Matthew Cunningham, Sriharsha Dadana, Siyu Dai, Zhaoli Dai, Giovanni Damiani, Saswati Das, Subasish Das, Ana Maria Dascalu, Nihar Ranjan Dash, Mohsen Dashti, Gail Davey, Claudio Alberto Dávila-Cervantes, Nicole Davis Weaver, Diego De Leo, Katie de Luca, Shayom Debopadhaya, Louisa Degenhardt, Cristian Del Bo', Ivan Delgado-Enciso, Andreas K Demetriades, Edgar Denova-Gutiérrez, Niloofar Deravi, Nebiyu Dereje, Nikolaos Dervenis, Don C Des Jarlais, Hardik Dineshbhai Desai, Rupak Desai, Syed Masudur Rahman Dewan, Arkadeep Dhali, Meghnath Dhimal, Sameer Dhingra, Vishal R Dhulipala, Diana Dias da Silva, Daniel Diaz, Michael J Diaz, Adriana Dima, Delaney D Ding, M Ashworth Dirac, Camila Bruneli do Prado, Masoud Dodangeh, Milad Dodangeh, Sushil Dohare, Wanyue Dong, Deepa Dongarwar, Mario D'Oria, E Ray Dorsey, Wendel Mombaque dos Santos, Rajkumar Doshi, Leila Doshmangir, Robert Kokou Dowou, Tim Robert Driscoll, Haneil Larson Dsouza, Mi Du, John Dube, Bruce B Duncan, Senbagam Duraisamy, Oyewole Christopher Durojaiye, Arkadiusz Marian Dziedzic, Abdel Rahman E’mar, Ejemai Eboreime, Chidiebere Peter Echieh, David Edvardsson, Diyan Ermawan Effendi, Ebrahim Eini, Michael Ekholuenetale, Iman El Sayed, Iffat Elbarazi, Teshome Bekele Elema, Noha Mousaad Elemam, Frank J Elgar, Islam Y Elgendy, Ghada Metwally Tawfik ElGohary, Muhammed Elhadi, Omar Abdelsadek Abdou Elmeligy, Mohamed A Elmonem, Mohammed Elshaer, Ibrahim Elsohaby, Theophilus I Emeto, Ryenchindorj Erkhembayar, Christopher Imokhuede Esezobor, Sharareh Eskandarieh, Juan Espinosa-Montero, Farshid Etaee, Natalia Fabin, Adeniyi Francis Fagbamigbe, Ayesha Fahim, Saman

Fahimi, Aliasghar Fakhri-Demeshghieh, Luca Falzone, Mohammad Fareed, MoezAlIslam Ezzat Mahmoud Faris, Andre Faro, Ali Fatehizadeh, Nelsensius Klau Fauk, Pooria Fazeli, Valery L Feigin, Seyed-Mohammad Fereshtehnejad, Abdullah Hamid Feroze, Nuno Ferreira, Getahun Fetensa, Irina Filip, Florian Fischer, Joanne Flavel, David Flood, Nataliya A Foigt, Morenike Oluwatoyin Folayan, Behzad Foroutan, Masoud Foroutan, Ingeborg Forthun, Matteo Foschi, Kate Louise Francis, Richard Charles Franklin, Alberto Freitas, Joseph Friedman, Takeshi Fukumoto, Peter Andras Gaal, Muktar A Gadanya, Santosh Gaihre, Yaseen Galali, Silvano Gallus, Aravind P Gandhi, Balasankar Ganesan, Mohammad Arfat Ganiyani, MA Garcia-Gordillo, Jalaj Garg, Semiu Olatunde Gbadamosi, Tilaye Gebru Gebi, Miglas W Gebregergis, Simona Roxana Georgescu, Tamirat Getachew, Sulmaz Ghahramani, Mohammad-Reza Ghasemi, Ghazal Ghasempour Dabaghi, Afsaneh Ghasemzadeh, Fariba Ghassemi, Ramy Mohamed Ghazy, Ajnish Ghimire, Sama Ghoba, Nasim Gholizadeh, Mahsa Ghorbani, Paramjit Singh Gill, Tiffany K Gill, Richard F Gillum, Alem Girmay, James C Glasbey, Elena V Gnedovskaya, Laszlo Göbölös, Myron Anthony Godinho, Mohamad Goldust, Nelson G M Gomes, Philimon N Gona, Sameer Vali Gopalani, Giuseppe Gorini, Alessandra C Goulart, Bárbara Niegia Garcia Goulart, Anmol Goyal, Ayman Grada, Michal Grivna, Shi-Yang Guan, Giovanni Guarducci, Mesay Dechasa Gudeta, Avirup Guha, Stefano Guicciardi, Snigdha Gulati, Damitha Asanga Gunawardane, Sasidhar Gunturu, Cui Guo, Bhawna Gupta, Manoj Kumar Gupta, Mohak Gupta, Rajat Das Gupta, Rajeev Gupta, Sapna Gupta, Veer Bala Gupta, Vivek Kumar Gupta, Lami Gurmessa, Reyna Alma Gutiérrez, Farrokh Habibzadeh, Parham Habibzadeh, Mostafa Hadei, Najah R Hadi, Nils Haep, Nima Hafezi-Nejad, Alemayehu Hailu, Arvin Haj-Mirzaian, Esam S Halboub, Brian J Hall, Rabih Halwani, Randah R Hamadeh, Sajid Hameed, Nasrin Hanifi, Graeme J Hankey, Fahad Hanna, Md Abdul Hannan, Harapan Harapan, Josep Maria Haro, Ahmed I Hasaballah, M Tasdik Hasan, Hamidreza Hasani, Abdiwahab Hashi, Md Saquib Hasnain, Johannes Haubold, Rasmus J Havmoeller, Simon I Hay, Jeffrey J Hebert, Omar E Hegazi, Golnaz Heidari, Mohammad Heidari, Bartosz Helfer, Claudiu Herteliu, Hamed Hesami, Kamal Hezam, Yuta Hiraike, Ramesh Holla, Nobuyuki Horita, Md Mahbub Hossain, Sahadat Hossain, Mohammad-Salar Hosseini, Ahmad Hosseinzadeh Adli, Sorin Hostiuc, Vivian Chia-rong Hsieh, Rebecca L Hsu, Junjie Huang, Michael Hultström, Tsegaye Gebreyes Hundie, Javid Hussain, M Azhar Hussain, Foziya Mohammed Hussien, Hong-Han Huynh, Segun Emmanuel Ibitoye, Pulwasha Maria Iftikhar, Desta Ijo, Adalia I Ikiroma, Paul Chukwudi Ikwegbue, Olayinka Stephen Ilesanmi, Irena M Ilic, Milena D Ilic, Mustapha Immurana, Sumant Inamdar, Endang Indriasih, Muhammad Iqhrammullah, Arnaud Iradukunda, Kenneth Chukwuemeka Iregbu, Md Rabiul Islam, Sheikh Mohammed Shariful Islam, Farhad Islami, Faisal Ismail, Nahlah Elkudssiah Ismail, Hiroyasu Iso, Gaetano Isola, Chidozie C D Iwu, Ihoghosa Osamuyi Iyamu, Mahalaxmi Iyer, Linda Merin J, Louis Jacob, Kathryn H Jacobsen, Morteza Jafarinia, Abdollah Jafarzadeh, Khushleen Jaggi, Kasra Jahankhani, Nader Jahanmehr, Haitham Jahrami, Abhishek Jaiswal, Mark M Janko, Sathish Kumar Jayapal, Shubha Jayaram, Rime Jebai, Ravi Prakash Jha, Jost B Jonas, Tamas Joo, Abel Joseph, Nitin Joseph, Charity Ehimwenma Joshua, Jacek Jerzy Jozwiak, Mikk Jürisson, Ali Kabir, Vidya Kadashetti, Rizwan Kalani, Laleh R Kalankesh, Feroze Kaliyadan, Sanjay Kalra, Sivesh Kathir Kamarajah, Rajesh Kamath, Thanigaivelan Kanagasabai, Himal Kandel, Arun R Kanmanthareddy, Edmund Wedam Kanmiki, Kehinde Kazeem Kanmodi, Suthanthira Kannan S, Rami S Kantar, Neeti Kapoor, Shama D Karanth, Reema A Karasneh, André Karch, Asima Karim, Faizan Zaffar Kashoo, Hengameh Kasraei, Nicholas J Kassebaum, Joonas H Kauppila, Navjot Kaur, Norito Kawakami, Gbenga A Kayode, Foad Kazemi, Tahseen Haider Kazmi, Getu Mosisa Kebebew, Fassikaw Kebede, Tibebeselassie S Keflie, Peter Njenga Keiyoro, Jaimon Terence Kelly, John H Kempen, Jessica A Kerr, Emmanuelle Kesse-Guyot, Himanshu Khajuria, Amirmohammad Khalaji, Nauman Khalid, Anees Ahmed Khalil, Ajmal Khan, Gulfaraz Khan, Imteyaz A Khan, M Nuruzzaman Khan, Maseer Khan, Mohammad Jobair

Khan, Moien AB Khan, Zeeshan Ali Khan, Mahammed Ziauddin Khan suheb, Shaghayegh Khanmohammadi, Khaled Khatab, Haitham Khatatbeh, Moawiah Mohammad Khatatbeh, Armin Khavandegar, Hamid Reza Khayat Kashani, Feriha Fatima Khidri, Elaheh Khodadoust, Mohammad Khorgamphar, Ahmad Khosravi, Grace Kim, Jihee Kim, Kwanghyun Kim, Min Seo Kim, Yun Jin Kim, Kasey E Kinzel, Adnan Kisa, Desmond Klu, Ann Kristin Skrindo Knudsen, Jonathan M Kocarnik, Sonali Kochhar, Timea Kocsis, Kairi Kolves, Farzad Kompani, Parvaiz A Koul, Sindhura Lakshmi Koulmane Laxminarayana, Kewal Krishan, Varun Krishna, Kris J Krohn, Barthelemy Kuate Defo, Burcu Kucuk Bicer, Md Abdul Kuddus, Mohammed Kuddus, Ilari Kuitunen, Mukhtar Kulimbet, Vishnutheertha Kulkarni, Akshay Kumar, Manasi Kumar, Rakesh Kumar, Madhulata Kumari, Fantahun Tarekegn Kumie, Om P Kurmi, Asep Kusnali, Dian Kusuma, Alexander Kwarteng, Hmwe Hmwe Kyu, Carlo La Vecchia, Ben Lacey, Muhammad Awwal Ladan, Lucie Laflamme, Anton C J Lager, Abdelilah Lahmar, Ratilal Lalloo, Tea Lallukka, Hilton Lam, Judit Lám, Kelsey R Landrum, Justin J Lang, Berthold Langguth, Van Charles Lansingh, Ariane Laplante-Lévesque, Bagher Larijani, Anders O Larsson, Savita Lasrado, Kamaluddin Latief, Kaveh Latifinaibin, Nhi Huu Hanh Le, Thao Thi Thu Le, Caterina Ledda, Munjae Lee, Paul H Lee, Kate E LeGrand, Elvynna Leong, Wei Li, Zhihui Li, Lee-Ling Lim, Christine Linehan, Chaojie Liu, Jue Liu, Runben Liu, Xiaofeng Liu, Erand Llanaj, Michael J Loftus, Rubén López-Bueno, Platon D Lopukhov, Paulo A Lotufo, Giancarlo Lucchetti, Alessandra Lugo, Raimundas Lunevicius, Zheng Feei Ma, Nikolaos Machairas, Monika Machoy, Farzan Madadizadeh, Áurea M Madureira-Carvalho, Soleiman Mahjoub, Mansour Adam Mahmoud, Elham Mahmoudi, Razzagh Mahmoudi, Irsa Fatima Makhdoom, Elaheh Malakan Rad, Venkatesh Maled, Reza Malekzadeh, Armaan K Malhotra, Kashish Malhotra, Ahmad Azam Malik, Deborah Carvalho Malta, Abdullah A Mamun, Lorenzo Giovanni Mantovani, Sajid Maqsood, Bishnu P Marasini, Hamid Reza Marateb, Joemer C Maravilla, Agustina M Marconi, Parham Mardi, Mirko Marino, Bernardo Alfonso Martinez-Guerra, Ramon Martinez-Piedra, Daniela Martini, Francisco Rogerlândio Martins-Melo, Miquel Martorell, Wolfgang Marx, Sharmeen Maryam, Roy Rillera Marzo, Awoke Masrie, Stephanie Mathieson, Alexander G Mathioudakis, Jishanth Mattumpuram, Andrea Maugeri, Pallab K Maulik, Mahsa Mayeli, Anna Laura W McKowen, Susan A McLaughlin, Steven M McPhail, Enkeleint A Mechili, Rishi P Mediratta, Jitendra Kumar Meena, Kamran Mehrabani-Zeinabad, Entezar Mehrabi Nasab, Max Alberto Mendez Mendez-Lopez, Walter Mendoza, Ritesh G Menezes, Alexios-Fotios A Mentis, Sultan Ayoub Meo, Haftu Asmerom Meresa, Atte Meretoja, Tuomo J Meretoja, Tomislav Mestrovic, Kukulege Chamila Dinushi Mettananda, Sachith Mettananda, Tomasz Miazgowski, Georgia Micha, Irmina Maria Michalek, Ted R Miller, Edward J Mills, Le Huu Nhat Minh, GK Mini, Pouya Mir Mohammad Sadeghi, Antonio Mirijello, Awoke Misganaw, Ashim Mishra, Philip B Mitchell, Prasanna Mithra, Chaitanya Mittal, Mouhand F H Mohamed, Nouh Saad Mohamed, Sakineh Mohammad-Alizadeh-Charandabi, Soheil Mohammadi, Abdollah Mohammadian-Hafshejani, Hussen Mohammed, Mustapha Mohammed, Salahuddin Mohammed, Shafiu Mohammed, Viswanathan Mohan, Amin Mokari, Ali H Mokdad, Sabrina Molinaro, Mariam Molokhia, Sara Momtazmanesh, Lorenzo Monasta, Stefania Mondello, Mohammad Ali Moni, AmirAli Moodi Ghalibaf, Maryam Moradi, Yousef Moradi, Maziar Moradi-Lakeh, Maliheh Moradzadeh, Paula Moraga, Rafael Silveira Moreira, Shane Douglas Morrison, Jakub Morze, Jonathan F Mosser, Rohith Motappa, Vincent Mougin, Simin Mouodi, Parsa Mousavi, Seyed Ehsan Mousavi, Amin Mousavi Khaneghah, Emmanuel A Mpolya, Matías Mrejen, Ulrich Otto Mueller, Faraz Mughal, Francesk Mulita, Efrén Murillo-Zamora, Christopher J L Murray, Fungai Musaigwa, Khaled M Musallam, Ahmad Mustafa, Ghulam Mustafa, Saravanan Muthupandian, Muhammad Muzaffar, Ahamarshan Jayaraman Nagarajan, Gabriele Nagel, Mohsen Naghavi, Sanjeev Nair, Noureddin Nakhostin Ansari, Sreenivas Narasimha Swamy, Shumaila Nargus, Bruno Ramos Nascimento, Gustavo G Nascimento, Samar Nasehi, Abdulqadir J Nashwan, Zuhair S Natto, Javaid Nauman, Biswa

Prakash Nayak, Vinod C Nayak, Sabina Onyinye Nduaguba, Hadush Negash, Ionut Negoi, Ruxandra Irina Negoi, Serban Mircea Negru, Seyed Aria Nejadghaderi, Samata Nepal, Georges Nguefack-Tsague, Josephine W Ngunjiri, Dang H Nguyen, Phat Tuan Nguyen, Van Thanh Nguyen, Robina Khan Niazi, Katie R Nielsen, Yeshambel T Nigatu, Taxiarchis Konstantinos Nikolouzakis, Fatemeh Nikoomanesh, Amin Reza Nikpoor, Lawrence Achilles Nnyanzi, Mamoona Noreen, Bo Norrving, Jean Jacques Noubiap, Chisom Adaobi Nri-Ezedi, George Ntaios, Mpiko Ntsekhe, Virginia Nuñez-Samudio, Dieta Nurrika, Bogdan Oancea, Kehinde O Obamiro, Ismail A Odetokun, Nkechi Martina Odogwu, Martin James O'Donnell, Akinyemi O D Ofakunrin, Abiola Ogunkoya, Ayodipupo Sikiru Oguntade, In-Hwan Oh, Sylvester Reuben Okeke, Akinkunmi Paul Okekunle, Osaretin Christabel Okonji, Andrew T Olagunju, Matthew Idowu Olatubi, Bolajoko Olubukunola Olusanya, Jacob Olusegun Olusanya, Hany A Omar, Maureene Auma Ondayo, Obinna E Onwujekwe, Kenneth Ikenna Onyedibe, Michal Ordak, Orish Ebere Orisakwe, Verner N Orish, Doris V Ortega-Altamirano, Alberto Ortiz, Wael M S Osman, Samuel M Ostroff, Uchechukwu Levi Osuagwu, Adrian Otoiu, Nikita Otstavnov, Stanislav S Otstavnov, Amel Ouyahia, Guoqing Ouyang, Mayowa O Owolabi, Mahesh Padukudru P A, Alicia Padron-Monedero, Jagadish Rao Padubidri, Tamás Palicz, Raul Felipe Palma-Alvarez, Feng Pan, Paramjot Panda, Songhomitra Panda-Jonas, Helena Ullyartha Pangaribuan, Leonidas D Panos, Ioannis Pantazopoulos, Anca Mihaela Pantea Stoian, Romil R Parikh, Ashwaghosha Parthasarathi, Maja Pasovic, Roberto Passera, Jay Patel, Shankargouda Patil, Dimitrios Patoulias, Venkata Suresh Patthipati, Uttam Paudel, Hamidreza Pazoki Toroudi, Amy E Peden, Paolo Pedersini, Umberto Pensato, Veincent Christian Filipino Pepito, João Perdigão, Marcos Pereira, Mario F P Peres, Norberto Perico, Richard G Pestell, Konrad Pesudovs, Fanny Emily Petermann-Rocha, Hoang Tran Pham, Anil K Philip, Michael R Phillips, Daniela Pierannunzio, Manon Pigeolet, Thomas Pilgrim, Zahra Zahid Piracha, Michael A Piradov, Saeed Pirouzpanah, Nishad Plakkal, Vivek Podder, Dimitri Poddighe, Suzanne Polinder, Ville T Ponkilainen, Fabio Porru, Maarten J Postma, Govinda Raj Poudel, Sergio I Prada, Pranil Man Singh Pradhan, Thejeswar N Prakasham, Manya Prasad, Elton Junio Sady Prates, TINA PRISCILLA, Nameer Hashim Qasim, Ibrahim Qattea, Asma Saleem Qazi, Suli Qiu, Mehrdad Rabiee Rad, Amir Radfar, Raghu Anekal Radhakrishnan, Venkatraman Radhakrishnan, Hadi Raeisi Shahraki, Alberto Raggi, Pankaja Raghav Raghav, Fakher Rahim, Vafa Rahimi-Movaghar, Mohammad Hifz Ur Rahman, Shayan Rahmani, Sathish Rajaa, Prashant Rajput, Ivo Rakovac, Shakthi Kumaran Ramasamy, Kritika Rana, Chhabi Lal Ranabhat, Nemanja Rancic, Chythra R Rao, Indu Ramachandra Rao, Mithun Rao, Sowmya J Rao, Davide Rasella, Vahid Rashedi, Ashkan Rasouli-Saravani, Giridhara Rathnaiah Babu, Nakul Ravikumar, David Laith Rawaf, Salman Rawaf, Lal Rawal, Bharat Rawlley, Rabail Zehra Raza, Christian Razo, Elrashdy Moustafa Mohamed Redwan, Faizan Ur Rehman, Lennart Reifels, Giuseppe Remuzzi, Luis Felipe Reyes, Maryam Rezaei, Nazila Rezaei, Taeho Gregory Rhee, Mavra A Riaz, Antonio Luiz P Ribeiro, Jennifer Rickard, Hannah R Riva, Mónica Rodrigues, Leonardo Roever, Emma Lynn Best Rogowski, Peter Rohloff, Debby Syahru Romadlon, Esperanza Romero-Rodríguez, Michele Romoli, Luca Ronfani, Himanshu Sekhar Rout, Nitai Roy, Priyanka Roy, Enrico Rubagotti, Guilherme de Andrade Ruela, Susan Fred Rumisha, Godfrey M Rwegerera, Chandan S N, Aly M A Saad, Zahra Saadatian, Korosh Saber, Maha Mohamed Saber-Ayad, Morteza SaberiKamarposhti, Siamak Sabour, Simona Sacco, Perminder S Sachdev, Rajesh Sachdeva, Basema Saddik, Bashdar Abuzed Sadee, Farideh Sadeghian, Umar Saeed, Fahimeh Safaeinejad, Rajesh Sagar, Dominic Sagoe, Fatemeh Saheb Sharif-Askari, Narjes Saheb Sharif-Askari, Amirhossein Sahebkar, Soumya Swaroop Sahoo, Monalisha Sahu, Zahra Saif, Mirza Rizwan Sajid, Joseph W Sakshaug, Nasir Salam, Afeez Abolarinwa Salami, Luciane B Salaroli, Marwa Rashad Salem, Mohammed Z Y Salem, Sohrab Salimi, Hossein Samadi Kafil, Sara Samadzadeh, Saad Samargandy, Abdallah M Samy, Juan Sanabria, Itamar S Santos, Milena M Santric-Milicevic, Bruno Piassi Sao Jose, Made Ary Sarasmita, Aswini Saravanan, Babak

Saravi, Yaser Sarikhani, Tanmay Sarkar, Rodrigo Sarmiento-Suárez, Gargi Sachin Sarode, Sachin C Sarode, Arash Sarveazad, Thirunavukkarasu Sathish, Maheswar Satpathy, Abu Sayeed, Md Abu Sayeed, Mete Saylan, Mehdi Sayyah, Nikolaos Scarmeas, Benedikt Michael Schaarschmidt, Maria Inês Schmidt, Ione Jayce Ceola Schneider, Art Schuermans, Aletta Elisabeth Schutte, David C Schwebel, Falk Schwendicke, Mario Šekerija, Siddharthan Selvaraj, Sabyasachi Senapati, Sadaf G Sepanlou, Dragos Serban, Yashendra Sethi, Maryam Shabany, Mahan Shafie, Nilay S Shah, Pritik A Shah, Syed Mahboob Shah, Saeed Shahabi, Izza Shahid, Samiah Shahid, Moyad Jamal Shahwan, Ahmed Shaikh, Alireza Shakeri, Ali S Shalash, Muhammad Aaqib Shamim, Mehran Shams-Beyranvand, Mohammad Anas Shamsi, Mohd Shanawaz, Abhishek Shankar, Amin Sharifan, Javad Sharifi-Rad, Rajesh Sharma, Saurab Sharma, Ujjawal Sharma, Vishal Sharma, Rajesh P Shastry, Amr Mohamed Elsayed Shehabeldine, Jiabin Shen, Pavanchand H Shetty, Kenji Shibuya, Mika Shigematsu, Youn Ho Shin, Reza Shirkoohi, Nebiyu Aniley Shitaye, Aminu Shittu, K M Shivakumar, Sina Shool, Seyed Afshin Shorofi, Sunil Shrestha, Kerem Shuval, Emmanuel Edwar Siddig, João Pedro Silva, Luís Manuel Lopes Rodrigues Silva, Soraia Silva, Colin R Simpson, Abhinav Singh, Balbir Bagicha Singh, Garima Singh, Jasbir Singh, Narinder Pal Singh, Paramdeep Singh, Surjit Singh, Robert Sinto, Shravan Sivakumar, Valentin Yurievich Skryabin, Anna Aleksandrovna Skryabina, Bogdan Socea, Anton Sokhan, Ranjan Solanki, Shipra Solanki, Hamidreza Soleimani, Sameh S M Soliman, Suhang Song, Joan B Soriano, Ireneous N Soyiri, Michael Spartalis, Chandrashekhar T Sreeramareddy, Jeffrey D Stanaway, Muhammad Haroon Stanikzai, Joseph R Starnes, Antonina V Starodubova, Dan J Stein, Fridolin Steinbeis, Paschalis Steiropoulos, Leo Stockfelt, Stefan Stortecky, Vetriselvan Subramaniyan, Muhammad Suleman, Abida Sultana, Haitong Zhe Sun, Johan Sundström, David Sunkersing, Katharina S Sunnerhagen, Chandan Kumar Swain, Lukasz Szarpak, Miklós Szócska, Payam Tabaee Damavandi, Rafael Tabarés-Seisdedos, Ozra Tabatabaei Malazy, Seyed-Amir Tabatabaeizadeh, Shima Tabatabai, Mohammad Tabish, JYOTHI TADAKAMADLA, Santosh Kumar Tadakamadla, Yasaman Taheri Abkenar, Iman M Talaat, Mircea Tampa, Jacques Lukenze Tamuzi, Ker-Kan Tan, Elvis Enowbeyang Tarkang, Razieh Tavakoli Oliaee, Seyed Mohammad Tavangar, Nuno Taveira, Yibekal Manaye Tefera, Mohamad-Hani Temsah, Reem Mohamad Hani Temsah, Masayuki Teramoto, Riki Tesler, Pugazhenthan Thangaraju, Samar Tharwat, Nihal Thomas, Amanda G Thrift, Chern Choong Chern Thum, Jing Tian, Ales Tichopad, Tala Tillawi, Tenaw Yimer Tiruye, Mariya Vladimirovna Titova, Marcello Tonelli, Roman Topor-Madry, Adetunji T Toriola, Mathilde Touvier, Marcos Roberto Tovani-Palone, Jasmine T Tran, Nghia Minh Tran, Domenico Trico, Samuel Joseph Tromans, Thien Tan Tri Tai Truyen, Aristidis Tsatsakis, Evangelia Eirini Tsermpini, Kang Tung, Stefanos Tyrovolas, Aniefiok John Udoakang, Arit Udoh, Atta Ullah, Irfan Ullah, Saeed Ullah, Srikanth Umakanthan, Brigid Unim, Bhaskaran Unnikrishnan, Carolyn Anne Unsworth, Era Upadhyay, Daniele Urso, Jibrin Sammani Usman, Asokan Govindaraj Vaithinathan, Jef Van den Eynde, Orsolya Varga, Shoban Babu Varthya, Tommi Juhani Vasankari, Balachandar Vellingiri, Narayanaswamy Venketasubramanian, Madhur Verma, Massimiliano Veroux, Georgios-Ioannis Verras, Dominique Vervoort, Jorge Hugo Villafañe, Gabriela Ines Villanueva, Francesco S Violante, Maria Viskadourou, Vasily Vlassov, Stein Emil Vollset, Rade Vukovic, Hatem A Wafa, Yasir Waheed, Richard G Wamai, Cong Wang, Ning Wang, Shu Wang, Song Wang, Yanzhong Wang, Yuan-Pang Wang, Paul Ward, Emebet Gashaw Wassie, Stephanie Louise Watson Watson, Melissa Y Wei, Robert G Weintraub, Ronny Westerman, Taweewat Wiangkham, Dakshitha Praneeth Wickramasinghe, Nuwan Darshana Wickramasinghe, Caroline Wilkerson, Peter Willeit, Shadrach Wilson, Marcin W Wojewodzic, Axel Walter Wolf, Yen Jun Wong, Eve E Wool, Ai-Min Wu, Xinsheng Wu, Juan Xia, Hong Xiao, Kazumasa Yamagishi, Lin Yang, Yao Yao, Sisay Shewasinad Yehualashet, Metin Yesiltepe, Saber Yezli, Amanuel Yigezu, Arzu Yiğit, Vahit Yiğit, Dong Keon Yon, Naohiro Yonemoto, Nima Zafari, Sojib Bin Zaman, Ramin Zand, Heather J Zar, Iman Zare, Armin Zarrintan, Mohammed G M Zeariya,

Zahra Zeinali, Haijun Zhang, Jianrong Zhang, Chenwen Zhong, Makan Ziafati, Magdalena Zielińska, Osama A Zitoun, Mohammad Zoladl, Liesl J Zuhlke, Alimuddin Zumla, Samer H Zyoud.

Managing the estimation or publications process

Robert W Aldridge, Catherine M Antony, Catherine S Chen, Matthew Cunningham, Nicole Davis Weaver, Alize J Ferrari, Erin B Hamilton, Simon I Hay, Nicholas J Kassebaum, Molly B Kassel, Jonathan M Kocarnik, Kris J Krohn, Kate E LeGrand, Anna Laura W McKowen, Madeline E Moberg, Ali H Mokdad, Christopher J L Murray, Mohsen Naghavi, Amanda Novotney, Kanyin Liane Ong, Maja Pasovic, Caitlyn Steiner, Jaimie D Steinmetz, Anna E Torre, Eve E Wool.

Section 10: Tables and figures

Appendix Figure S1: Analytical flowchart for the development of the GBD 2021 cause of death database (A) and different strategies used to model different causes (B) and ultimately combine them into a consistent set of cause-specific deaths for each location, age, sex, and year.

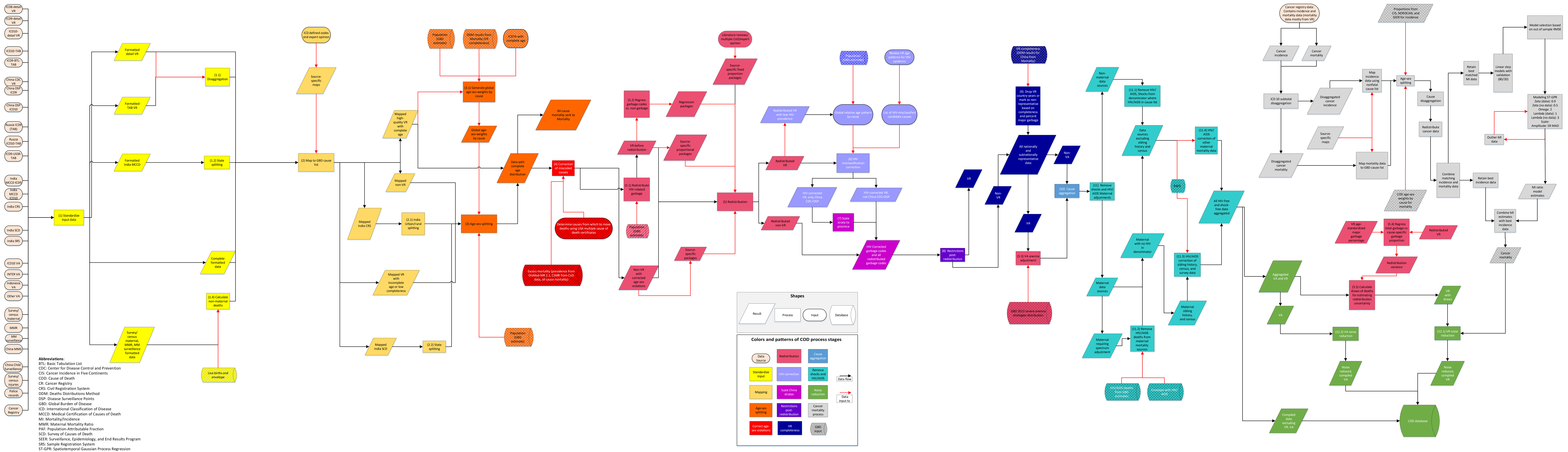

Abbreviations:
BTL: Basic Tabulation List
CDC: Center for Disease Control and Prevention
CI5: Cancer Incidence in Five Continents
COD: Cause of Death
CR: Cancer Registry
CRS: Civil Registration System
DDM: Deaths Distributions Method
DSP: Disease Surveillance Points
GBD: Global Burden of Disease
ICD: International Classification of Disease
MCCD: Medical Certification of Causes of Death
MI: Mortality/Incidence
MMR: Maternal Mortality Ratio
PAF: Population-Attributable Fraction
SCD: Survey of Causes of Death
SEER: Surveillance, Epidemiology, and End Results Program
SRS: Sample Registration System
ST-GPR: Spatiotemporal Gaussian Process Regression
TAB: Tabulation List
VA: Verbal Autopsy
VR: Vital Registration

Appendix Figure S2: GBD 2019 causes of death estimation flowchart by modeling group

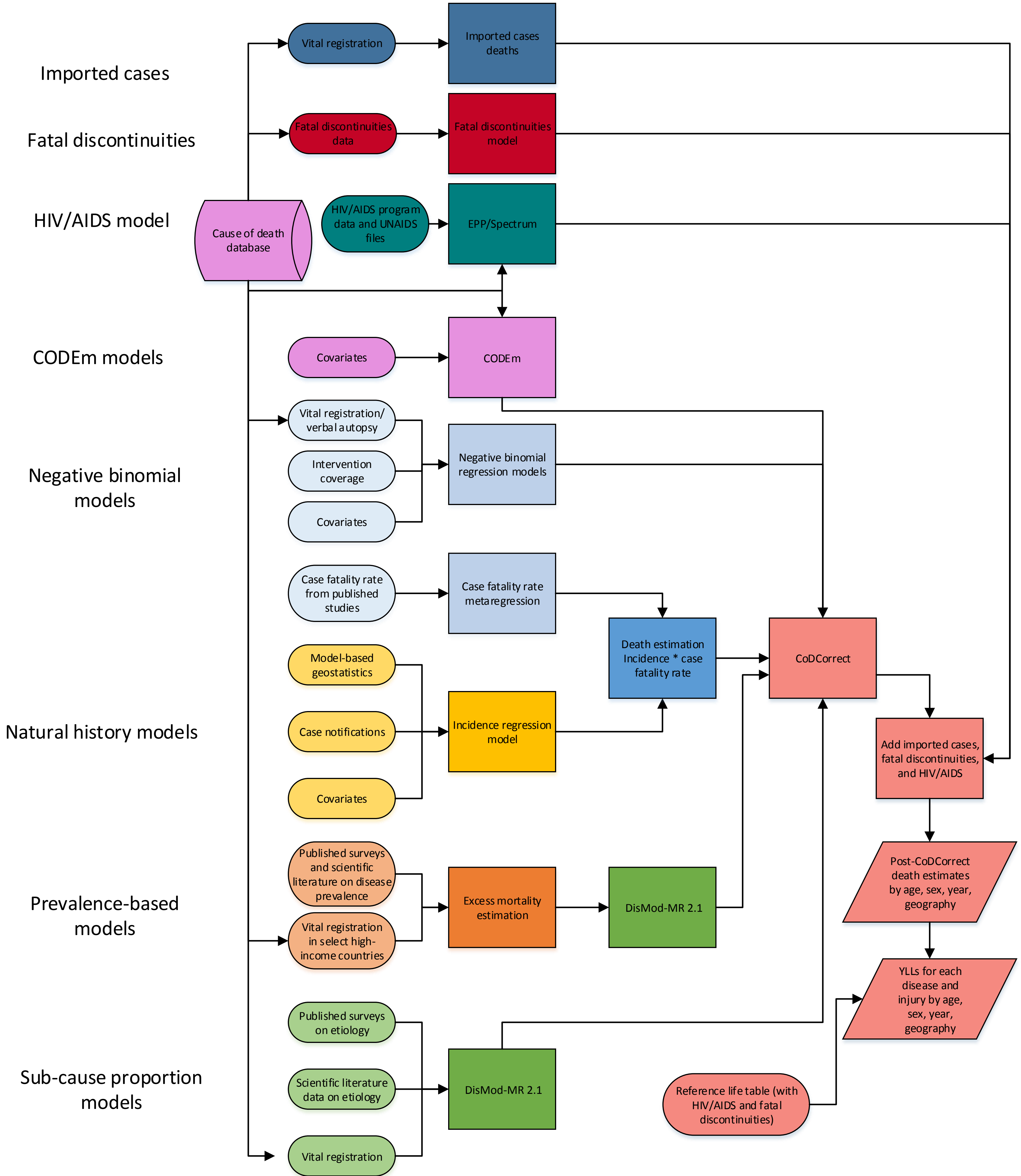

Abbreviations:
CODEm: Cause of death ensemble model
GBD: Global Burden of Disease
YLL: years of life lost

Appendix Figure S3: Vital Registration and Verbal Autopsy data availability by country, 1980−2021

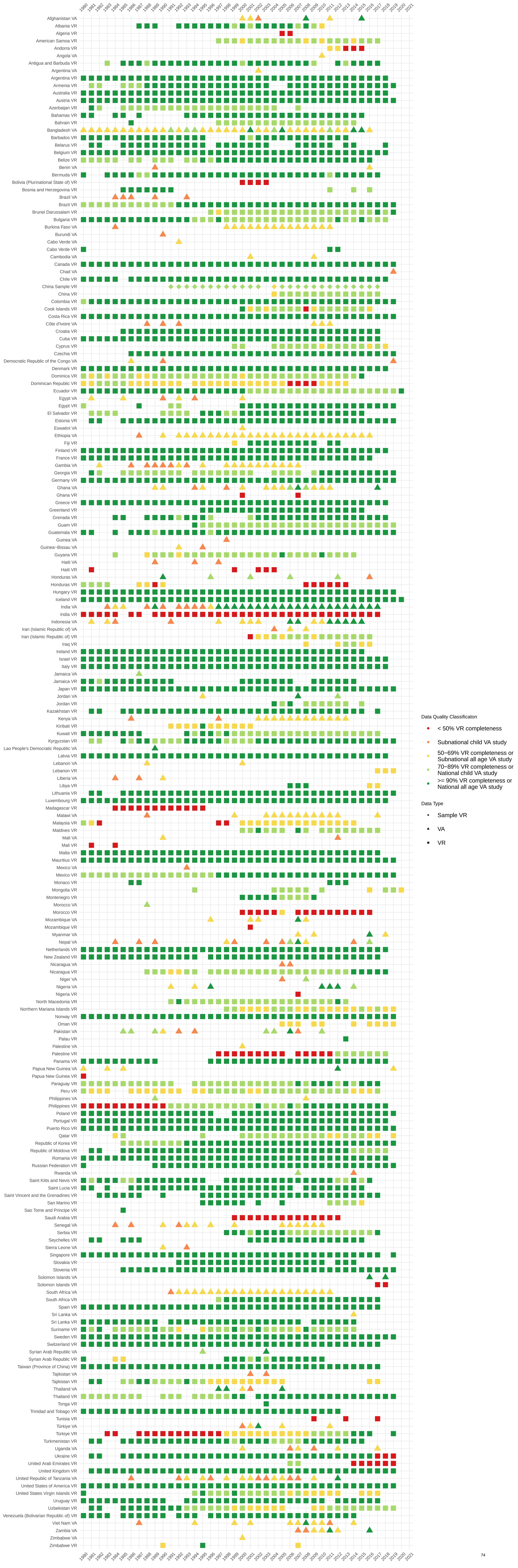

Appendix Figure S4: Percent of vital registration deaths assigned to major garbage codes for all ages and sexes by country, 1980-2021

Year

Country	1980	1981	1982	1983	1984	1985	1986	1987	1988	1989	1990	1991	1992	1993	1994	1995	1996	1997	1998	1999	2000
Albania								38	36	35			36	33	36	30	29	26	28	25	27
Algeria																					
American Samoa																		26	25	20	17
Andorra																					
Antigua and Barbuda				29		38	40	38	33	30	26	24	25	24	24	28	20	26	22	21	19
Argentina	25	24	27	29	30	30	29	29	29	29	30	30	31	31	30	31	30	32	32	32	32
Armenia		11	13			13	12	12	32	14	13	13	14	17	19	16	15	14	13	13	13
Australia	7	7	7	7	7	7	6	7	6	7	7	6	6	7	6	8	7	7	8	8	8
Austria	9	9	9	15	8	7	7	7	7	7	7	7	8	8	8	8	8	8	8	9	9
Azerbaijan		12	12			12	11	12	12	14	13	12	11	11	17	14	14	14	14	15	15
Bahrain							25											33	43	42	39
Barbados	24	25	24	24	24	25	22	22	23	23	22	23	20	22	22	20					23
Belarus		14	14			13	12	12	13	14	20	23	23	23	22	23		22	20	19	20
Belgium	21	20	20	21	21	22	21	19	21	21	20	21	18	16	16	16	14	14	14	15	14
Belize	33	26	36	39	32		29	25		32	40	37		36	38	33	38	31	25	25	30
Bermuda	10			20	8	10	20	10	11	9	11	10	10	12	16	20	5	5	7	10	8
Bolivia																					74
Bosnia and Herzegovina						41	40	36	35	36	34	35									
Brazil	38	38	38	38	39	37	37	36	36	35	34	34	34	34	33	31	30	30	30	29	28
Brunei																	29	20	16	20	19
Bulgaria	21	21	20	21	19	19	19	21	21	21	20	19	20	19	20	22	24	26	27	26	27
Canada	11	9	11	11	11	12	9	10	10	10	11	10	10	10	11	11	11	13	11	10	10
Cape Verde	50																				
Chile	27	26	26	25	26		24	25	24	24	21	21	20	20	17	16	15	15	15	15	12
China												23	21	18	14	16	16	17	15	15	17
Colombia	29	30	27	25	24	24	24	23	22	22	22	22	21	19	18	19	18	17	14	12	11
Cook Islands																					40
Costa Rica	24	25	21	19	18	18	18	17	17	17	21	20	17	17	18	18	17	11	10	9	8
Croatia						18	18	18	17	15	14	15	17	18	18	18	18	19	17	15	14
Cuba	15	15	15	14	14	14	13	15	13	13	13	14	14	15	15	16	15	10	10	10	11
Cyprus																				63	65
Czechia							9	10	10	10	10	10	11	11	12	14	16	18	16	15	13
Denmark	17	16	17	17	17	18	18	19	19	20	21	22	23	22	14	14	17	14	13	12	12
Dominica	33	27	31	29	29	39	37	37	32	36	34	40	36	34	43	37	40	38	36	32	32
Dominican Republic	51	47	44	40	37	36	35	34	34	35	34	35	34		33	33	32	30	28	28	26
Ecuador	37	35	35	34	34	33	33	33	32	32	31	30	30	29	34	34	30	34	35	35	34
Egypt	69							65				59	58								60
El Salvador		43	46	49	48						40	38	38	38		36	32	36	36	32	31
Estonia		9	9			9	9	9	9	12	15	15	19	19	6	7	7	7	7	7	8
Fiji																				47	
Finland	17	19	18	18	18	19	19	8	9	10	10	10	9	9	9	9	5	5	4	4	4
France	24	24	23	23	22	21	21	21	20	20	19	19	19	18	18	18	19	19	18	18	18
Georgia		10	12			11	12	12	9	8	8	9	10		10	10	12	15	14	14	13
Germany	20	20	20	20	19	19	20	19	19	17	17	15	14	14	14	14	14	14	14	14	15
Ghana																					34
Greece	19	19	18	17	16	16	19	24	24	24	24	23	23	22	22	21	21	21	22	22	22
Greenland																16	19	15	15	19	14
Grenada					26	42		36	34	36	37	50	35	34	35	30					
Guam															8	9	10	10	7	8	10
Guatemala	33	36			31		34	32	33	34	35	36	38	38	34	32	33	32	31	30	33
Guyana					37				23	25	25	27	25	24	23	23	24	23	26	22	17
Haiti		70																		61	
Honduras	56	54	64	77				38	36	43	38										
Hungary	9	9	10	10	11	11	11	10	10	10	10	10	10	10	10	9	9	8	9	9	9
Iceland	15	10	7	8	8	7	8	9	7	7	7	6	6	4	6	6	5	6	7	6	8
India	31	36	40	39	35		29	38		44	41	42	42	40	41	42	41	42	41	42	42
Iran																					
Iraq																					
Ireland	10	9	9	9	9	8	9	8	8	8	8	8	8	7	8	8	8	8	8	9	8

Country	2001	2002	2003	2004	2005	2006	2007	2008	2009	2010	2011	2012	2013	2014	2015	2016	2017	2018	2019	2020	2021
Albania	28	27	27	27	29	28	25	25	25	23											
Algeria					51	50															
American Samoa	16	19	16	24	16	30	25	14	20	18	24	22	22	18	15	13	18				
Andorra											23	19	17	20	17						
Antigua and Barbuda	23	22	17	19	27	26	18	21	20			29	27	22	25	24	23				
Argentina	32	33	32	31	31	31	32	32	32	32	32	31	31	29	27	27	26	28			
Armenia	13	14	15			9	11	9	10	10	11	11	11	11	10	10	15	20	22		
Australia	7	8	9	9	9	8	9	9	9	9	9	9	9	9	9	10	10	10			
Austria	8	6	5	6	6	7	7	7	8	9	9	10	9	9	11	11	11	12	12		
Azerbaijan	7	8	8	10			45														
Bahrain	40	42	39	42	39	40	38	40	43	47	44	41	38	34							
Barbados	22	21	25	28	27	18	18	20	18	18	18	20	20								
Belarus	20	18	18				18	19	17	16	19		18	16				11			
Belgium	14	14	15	14	14	14	15	14	15	17	17	18	17	17	18	18	19	18			
Belize	24	24	27	25	22	20	21	16	13	13	12	13	13	13	13	15					
Bermuda	12	7	8	12	12	14	11	11	20	14	14	11	9	7	10	10	10				
Bolivia	72	70	67																		
Bosnia and Herzegovina											34			27		32					
Brazil	28	27	26	25	23	20	20	19	19	19	18	18	17	17	18	18	17	17	17		
Brunei	19	19	20	20	19	19	19	19	24	24	17	14	15	16	17	18	15	16	14		
Bulgaria	27	27	28	28	25	27	26	26	30	31	34	28	30	29	31	32	33	33			
Canada	10	10	10	10	10	11	10	10	10	10	10	10	10	10	11	12	12	15	14		
Cape Verde											30	30									
Chile	11	10	9	9	9	9	10	10	10	10	10	10	10	10	10	10	11	12			
China	26	25		6	5	9	8	14	13	13	12	11	10	8	8	8	7				
Colombia	11	10	11	11	11	11	11	11	11	11	12	11	10	9	9	9	9	9	9		
Cook Islands	27	36	30	37	38	30	42	33	24	23	30	25	25	26	21	22					
Costa Rica	8	9	7	8	8	10	9	10	10	9	9	9	10	9	8	10	8	8	8		
Croatia	14	15	14	12	12	12	12	11	10	8	8	8	7	7	7	6	8				
Cuba	10	9	9	9	9	8	8	8	8	8	8	8	8	8	8	8	8				
Cyprus				28	23	22	19	20	20	19	15	16	14	15	14	13	13	13			
Czechia	14	15	15	16	15	15	13	15	15	15	14	14	12	12	13	14	15	14	15		
Denmark	13	15	15	14	13	13	17	16	18	17	17	15	16	16	16	13	14	18			
Dominica	34	35	35	30	13	11	19	18	18	17	15	21	17	29	21						
Dominican Republic	25	24	23	23	21	19	20	18	16	17	19	22	19								
Ecuador	33	35	34	31	30	27	27	28	25	25	25	25	22	20	17	15	16	16	16	15	
Egypt	62	60	59	60	59	60	61	61	60	60	56	54	59	54	57	56	57	58	61		
El Salvador	32	33	34	32	31	31	33	33	33	34	33	33	36	37	40						
Estonia	8	9	9	9	9	8	10	7	7	8	8	9	9	9	11	8	7	8			
Fiji	57	48	47	47	45	44	43	42	41		35	35									
Finland	5	5	5	5	5	6	6	6	6	5	6	6	6	6	5	5	6	6			
France	18	18	19	17	18	18	18	18	18	19	19	20	21	19	20	20					
Georgia	12			23	35	40	40		47	69	54	48	49	38	40	43	39	29	26		
Germany	15	15	15	14	15	14	15	15	15	15	14	14	15	15	15	15	15	15	16		
Ghana							36														
Greece	20	21	20	20	20	20	16	21	15	17	17	17	16	18	18	16	17	16			
Greenland	12	16	15	15	14	19	16	16	14	18	24	23	22	35	41						
Grenada	24	20	26	22	20	24	23	26	18	15	13	14	13	18	18	14	15	12			
Guam	9	10	9	7	9	8	6	6	8	11	10	11	8	11	10	11	12	11	14		
Guatemala	33	30	31	31	31	30	30	28	25	24	23	22	23	22	24	26	26	28	28		
Guyana	21	19	16	17	16	17	18	18	21	19	19	21	20	20							
Haiti		47	48	52																	
Honduras								13	13	13	13	10	11								
Hungary	8	8	7	7	7	6	6	6	6	6	6	6	6	6	6	7	7	7	6		
Iceland	7	6	8	6	8	9	8	7	9	8	9	9	11	9	11	10	11	14	13	14	
India	41	40	38	40	41	41	37	43	45	44	38	37	54	43	45	45	44				
Iran	43	38	35	26	23	20	19	18	17	18	17	16	18	17	19	14					
Iraq								46				38	40	40	40	39					
Ireland	9	9	9	8	8	11	7	7	7	8	8	7	8	7	7						

Israel	21	21	20	19	21	19	18	18	19	17	19	19	21	17	17	17	16	20	19	19	18	18	18	18	19	19	19	19	19	19	18	19	20	20	20	21	22	22	21		
Italy	11	11	11	11	11	12	12	11	11	11	11	10	10	10	10	11	11	11	11	11	11	11	11	10	10	10	10	10	10	10	10	10	11	11	11	12	11	12	12		
Jamaica	34	35	31	28	29	28	27	26	25	26	30	28									21	27	30	22	16	20	13			12	13	11	12	12	12						
Japan	18	17	17	18	18	18	18	18	19	19	19	19	19	19	17	11	12	12	12	12	12	12	12	12	12	13	13	13	14	14	15	15	15	16	16	16	17	17	18	18	
Jordan																									23	21	19		19	18	18	20	21	22		22					
Kazakhstan		19	18			18	16	16	16	15	15	11	12	11	11	12	12	13	13	16	14	15	15	15	17	16	17	17	18	23	28	34	35	18	18	17		20			
Kiribati												53	48	46	51	45	55	43	35	48	45	53																			
Kuwait	27	26	31	29	27	25	28	22						23	27	31	24	28	22	29	25	21	24	23	20	19	20	19	22	18	16	16	18	22	18	19	15	17			
Kyrgyzstan		19	18			17	15	15	14	15	18	20	20	22	24	24	23	21	20	19	11	9	8	8	8	8	6	7	7	6	6	6	6	6	6	7	7	7	7	7	
Latvia	10	10	10	9	9	8	9	9	9	9	11	15	16	16	18	19	10	9	8	8	9	10	11	11	13	13	13	11	12	11	11	9	8	7	7	6	6	7	7		
Lebanon																																						44	42	40	
Libya																											87	91	89								51	52			
Lithuania		10	9			8	8	8	8	7	7	12	13	10	8	7	6	5	7	7	8	7	7	7	7	7	7	8	7	7	7	8	7	6	7	8	8	7	9	7	
Luxembourg	16	16	15	14	13	13	13	13	15	14	14	18	13	14	13	12	15	12	17	17	15	15	17	18	17	18	19	18	17	19	17	19	15	15	14	14	13	14	20		
Madagascar					54	51	53	50	54	53	49	52	52	53	52	50																									
Malaysia	41	40	27															33	33		37	40	38	39	40	39	38	36	36	27	27	26	26	26	26						
Maldives																					66	60	56	60	61	81		53	51		47	38	46	42	43	47	48	43			
Mali		71			74																																				
Malta	19	20	18	18	17	19	25	25	20	12	12	11	10	10	11	10	9	10	9	12	10	9	9	12	8	8	9	8	8	6	6	8	11	7	7	7	6	7			
Mauritius	39	34	28	28	27	26	26	24	23	23	25	23	26	28	26	28	31	30	23	23	21	18	18	18	18	16	17	15	16	16	16	15	16	15	13	14	14	15	15	15	
Mexico	30	29	27	25	25	23	23	22	20	19	18	18	17	17	16	15	15	14	14	13	13	13	13	12	12	12	12	13	13	13	13	13	12	13	12	12	11	11	11	11	
Moldova		10	10			9	9	9	10	11	20	21	23	24	23	20	13	10	10	9	7	5	4	4	3	3	3	3	3	3	3	3	3	3	3	4	3	4	4		
Monaco							24	25																								30	24	19							
Mongolia															24										76	77	77	74	74		8						7		9	8	10
Montenegro																					30	35	34	33	32	24	30	25	28	31											
Morocco																					57	58	60	61	57	58		58	58	56	57	60	53	51	51	51	50				
Mozambique																						37																			
Netherlands	12	11	13	14	13	13	13	12	12	13	14	14	14	15	14	15	14	14	15	15	16	16	16	16	15	15	15	14	15	14	14	14	14	15	15	14	15	14	15		
New Zealand	5	5	5	5	5	5	5	5	5	5	5	5	5	5	4		4	4	3	3	3	4	3	4	3	3	4	4	4	4	4	4	4	4	4	4	5	5			
Nicaragua									27	28	27	26	25	26	26		24	19	17	16	17	14	14	13	13	13	14	14	14	12	11	10	10	10	10	12	9	10	10		
Nigeria																												97													
North Macedonia												21	21	21	19	20	20	18	18	18	17	17	19	17	28	29	21	21	21	20	21	23	22	23							
Northern Mariana Islands																			21	35	26	17	31	20	20	29	15	14	24	19	24	32	21	15	13	11	11	15	15	20	
Norway	10	10	10	9	9	9	9	9	9	9	9	10	9	10	10	10	9	9	10	11	11	12	12	13	13	14	14	15	15	15	15	15	15	16	15	15	14	14	14	15	
Oman																										18	17	18		46	59				56		49	62	74	70	
Palau																																		51							
Palestine																		43	36	34	35	38	39	39	34	34		34	35	31	31	30	31	31	27	33	32	29	11		
Panama	26	26	25	26	25	24	23	22	25	24							26	25	18	17	15	15	13	15	16	14	17	14	14	14	16	17	16	17	15	16	18	17	15		
Papua New Guinea	30																																								
Paraguay	37	37	39	39	41	39	36	37	36	34	33	31			26	27	27	34	33	34	34	31	33	34	28	28	25	26	25	24	22	21	22	21	21	19	17	16			
Peru	27	26	27	28			47	50	46	46	48	50	45		48	40	38	41	38	35	31	33	32	29	29	61	64	23	25	27	25	25	25	24	23	19	20	28			
Philippines	99	99	99	99	99	99	99	99	99	99	99	82	82	83	83	83	83	84	84	52	53	52	47	51	47	47	16	16	16	16	16	16	18	17	17	16	15	14	14		
Poland	38	37	37	37	36	37	36	37	37	37	37	37	37	37	36	36	35			27	25	25	24	25	25	24	24	25	25	26	26	24	25	26	26	27	26	25	25	24	
Portugal	24	23	23	24	23	22	23	23	24	23	23	23	22	23	23	22	23	24	24	24	25	21	18	19	18	24	26	22	21	21	21	19	20	19	14	15	14	14	14		
Puerto Rico	24	22	26	24	24	24	23	23	23	23	23	21	22	22	16	16	16	16	17	16	17	16	17	17	15	17	17	17	16	17	16	16	16	15	17	17	16	17	18	17	
Qatar					82	80										27					100	36	100	100	41	34	34	34	40	31	36	38	41	37	41	36	29	29		29	
Romania	23	23	24	24	24	23	22	22	22	21	22	22	23	17	17	17	17	16	16	14	14	14	14	13	13	13	13	13	13	13	13	13	14	13	15	15	15	15	15		
Russia	7									8	10	11	12	13	14	14	14	13	13	13	13	13	13	13	13	12	11	11	11	11	11	12	12	13	15	15	15	15	15	15	
Saint Kitts and Nevis	26	29	31	28	32	28	32	32	33	37	42	37	28	33	27	19			19	31	28	39	22	30	34	21	15	12	14	14	11	16	10	11	11	9	8				
Saint Lucia	43	38		31			36	32	33	37	31	28	27	27	26	28	30	27	28	32	24	18	23	23	20	29	26		27	16	18	17	14	17	14	17					
Saint Vincent and the Grenadines			36	31	47	35	31	40			37					20	23	18	14	16	16	17	14	18	16	15	12	12	20	29	20	18	20	15	12	17	29	16			
San Marino																25	31	25	29	30	26		23			23						15	19	20	19	17					
Saudi Arabia																				46	47	46	49	50	49	46	46	50	52	51	53	54	56								
Serbia																			22	23	23	23	23	22	24	19	18	18	18	18	18	20	19	20	21	22	21	21			
Seychelles		29	30			36	35	29														23	23	24	23	23	24	23	21	25	24	21	22	22	23	22					
Singapore	13	12	9	9	10	8	13	8	8	10	8	6	6	6	5	5	5	4	5	5	6	5	5	5	5	5	5	6	6	6	7	6	4	3	3	4	4	4		5	
Slovakia													18	17	16	18	18	18	18	16	15	15	15	15	15	16	26	22	11	11	11		8	8	9						
Slovenia						7	9	10	9	12	11	7	6	7	7	8	8	9	10	10	11	11	12	11	11	13	12	11	10	10	10	10	10	12	11	12	12	13	11	13	
Solomon Islands																																						13	16		
South Africa																		39	38	34	33	33	33	32	32	32	33	32	32	32	32	32	32	31	31	31	33	32			
South Korea						38	37	38	38	35	32	33	28	25	26	27	28	28	27	26	23	20	16	17	18	19	19	17	18	18	17	17	17	17	16	16	17	17	18	19	
Spain	22	22	22	22	21	21	20	19	19	19	18	17	17	16	15	14	14	14	13	14	14	14	14	14	14	14	14	15	15	14	13	13	13	12	12	13	12	12			
Sri Lanka	57	57	62	58	58	58	57	58	56	57		59	59	59	60	57	58	49	49	48	43	42	41	39	46	42	38	37		36	35	37	37	37	37						

Suriname	48	44	37		36	34	32	30	33	33	30	32	31			32	34	33	31	31	28	25	24	23	25	25	20	21	23	21	21	22	23	24	24					
Sweden	8	9	9	9	10	10	9	9	9	10	10	10	10	10	11	11	11	10	11	11	11	11	11	11	11	11	12	13	13	13	16	15	14	14	13	15	14	13	14	14
Switzerland	25	25	25	24	24	24	24	24	24	25	25	26	26	26	26	14	13	13	13	14	13	13	14	14	14	13	13	12	13	13	13	13	13	13	13	14	14	14		
Syria	71				77	78													54	47	47	49	48	42	34	42	38	33	33	26	42									
São Tomé and Príncipe						41																																		
Taiwan (province of China)	19	21	21	21	21	21	21	21	21	21	22	22	22	23	22	20	19	18	18	17	16	16	16	15	15	15	15	15	18	17	17	17	15	15	14	15	15	15		
Tajikistan		16	18			24	23	24	21	19	21	21	27	33	32	31	35	37	40	36	38	35	32	33	34	35											32	33		
Thailand	72	72	72	72	72	72	72	70			67	66	66		65	66	65	67	66	63	62		56	52	55	55	56	55	54	54	52	48	47	43	40	38	36	36	35	32
The Bahamas	26	26			21	24		18						24	18	18	25	16	16	12	13	12	24	15	15	16	14	15	14	13	14	12	15	14	15	15				
Tonga																								47																
Trinidad and Tobago	21	19	19	17	18	18	16	15	16	15	16	15	15	14	14	15	14	14	14	11	10	11	10	10	10	11	11	12	13	12	12	10	10							
Tunisia																														26				35				35		
Turkey				64	65			61	60	60	61	60	59	58	60	60	60	60	57	59	56	54	55	55	62	59	60	60	60	21	21	21	22	15	16	16	17			24
Turkmenistan		11	12			12	12	16	13	16	18	13	12	11	20	20	22	27	29	9	12	10	12	11	11	13	15	16	16	16	17	19	19	21	22	23				
UK		8	7	7	6	6	6	6	6	7	6	6	6	7	7	8	8	8	9	9	9	8	8	8	8	8	8	9	9	9	9	8	8	9	8	9	9	9	9	9
USA	10	10	10	10	11	11	12	12	12	10	10	10	10	10	10	10	11	11	11	11	11	12	12	12	12	12	12	12	13	13	13	13	13	13	13	14	14	14	14	15
Ukraine		12	12			11	12	13	15	17	21	22	22	20	18	18	19	17	17	16	16	16	17	18	18	12	12	12	11	10	10	9	9	8	9	9	9	6	9	7
United Arab Emirates																											49	46							34	31	35	38	39	61
Uruguay	23	23	22	23	24	23	24	23	23	22	22			23	22	23	23	18	20	21	19	19	20	20	21	19	20	21	21	22	22		23	22	21	20	20	20		
Uzbekistan		14	13			13	14	14	14	13	13	14	14	15	16	16	18	19	18	18	17	18	22	24	13	12				10	10	11	11	12	14	14	14	16	16	17
Venezuela	31	30	30	21		32	32	28	27	28	18		18	18	17		13	13	13	12	10	9	9	10	9	9	9	9	9	10	9	9	9	8	8					
Virgin Islands	19														10	11	12	10	12	10	11	11	13	12	9	13	11	11	10	11	15	13	17			19	12	17		
Zimbabwe											36					42												26												

Color Scale: 0 | 10 | 20 | 30 | 40 | 50 | 60 | 70 | 80 | 90 | 100

Cells are colour coded by percent of data redistributed in a given country-year from garbage coding to a likely underlying cause of death. Blank white cells indicate lack of VR for that year. High percentages of vaguely-coded deaths highlight the importance of improvements in accurate reporting of mortality data to avoid redistribution of deaths to broad level causes. Countries without VR for 1980-2020 are not listed. VR=vital registration.

Appendix Figure S5: Classification of national time series of vital registration and verbal autopsy data 1980−2021

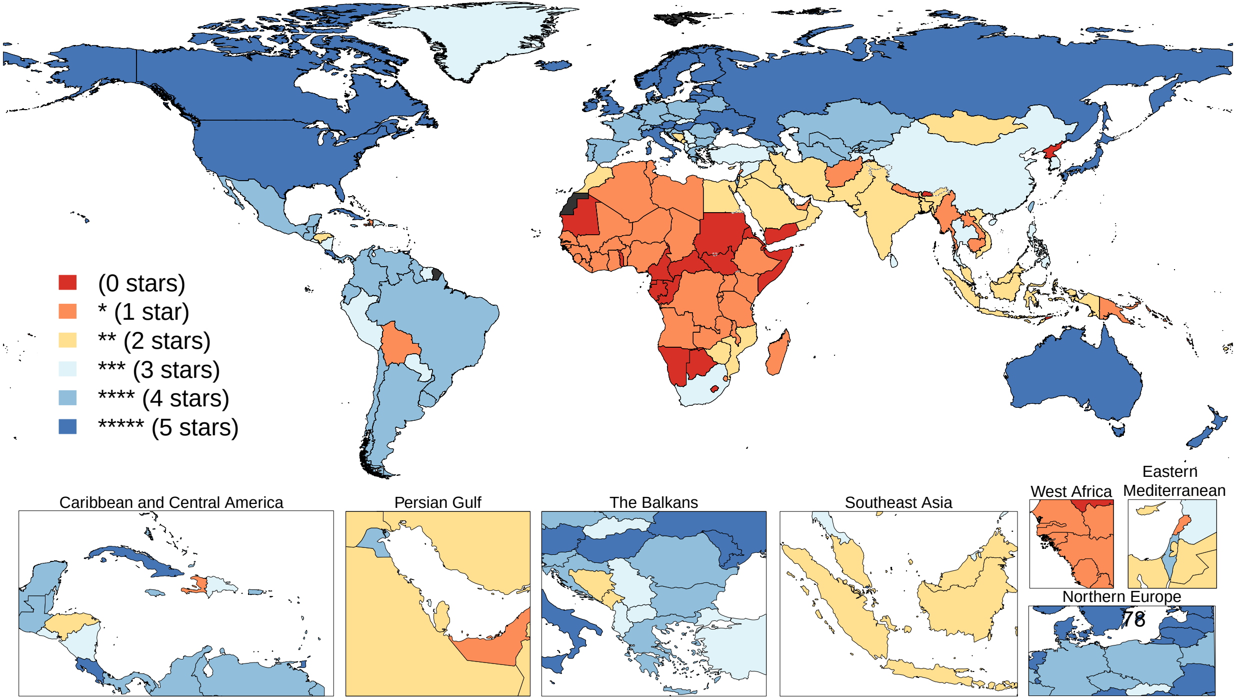

Appendix Figure S5b: Classification of national time series of vital registration and verbal autopsy data 2010−2021

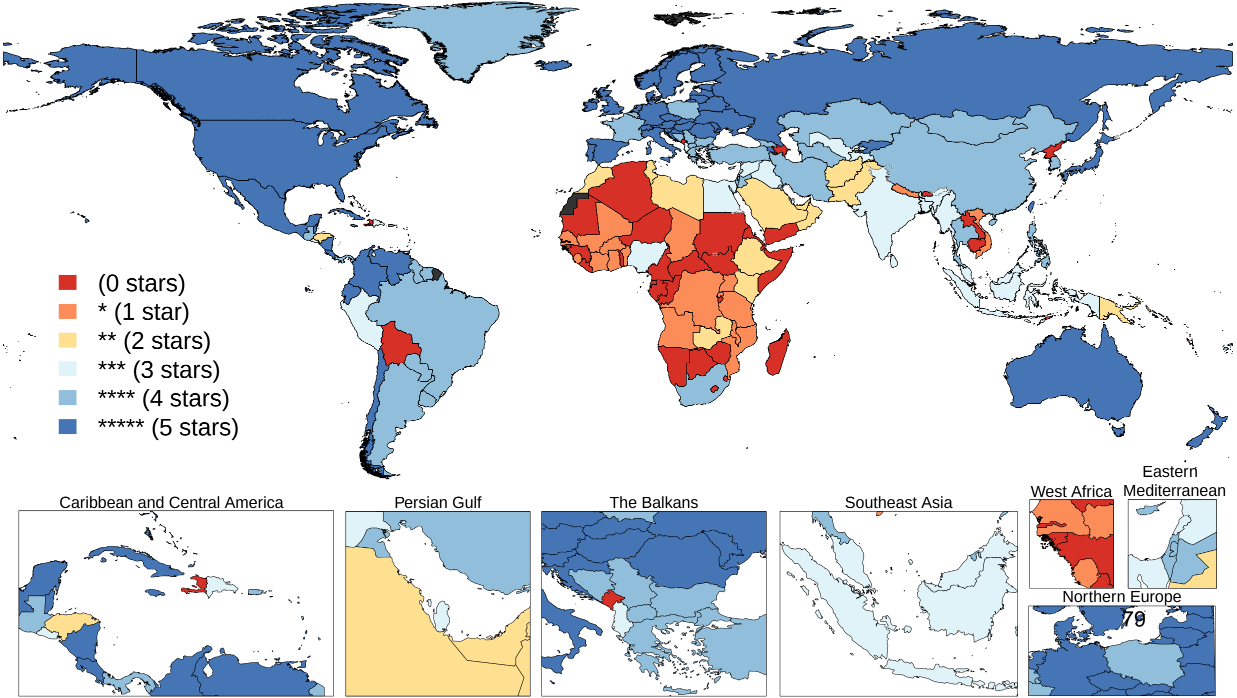

Appendix Figure S6: Out-of-sample model performance for CODEm models for GBD 2021 and age-standardized cause-specific mortality rate by level 2 causes

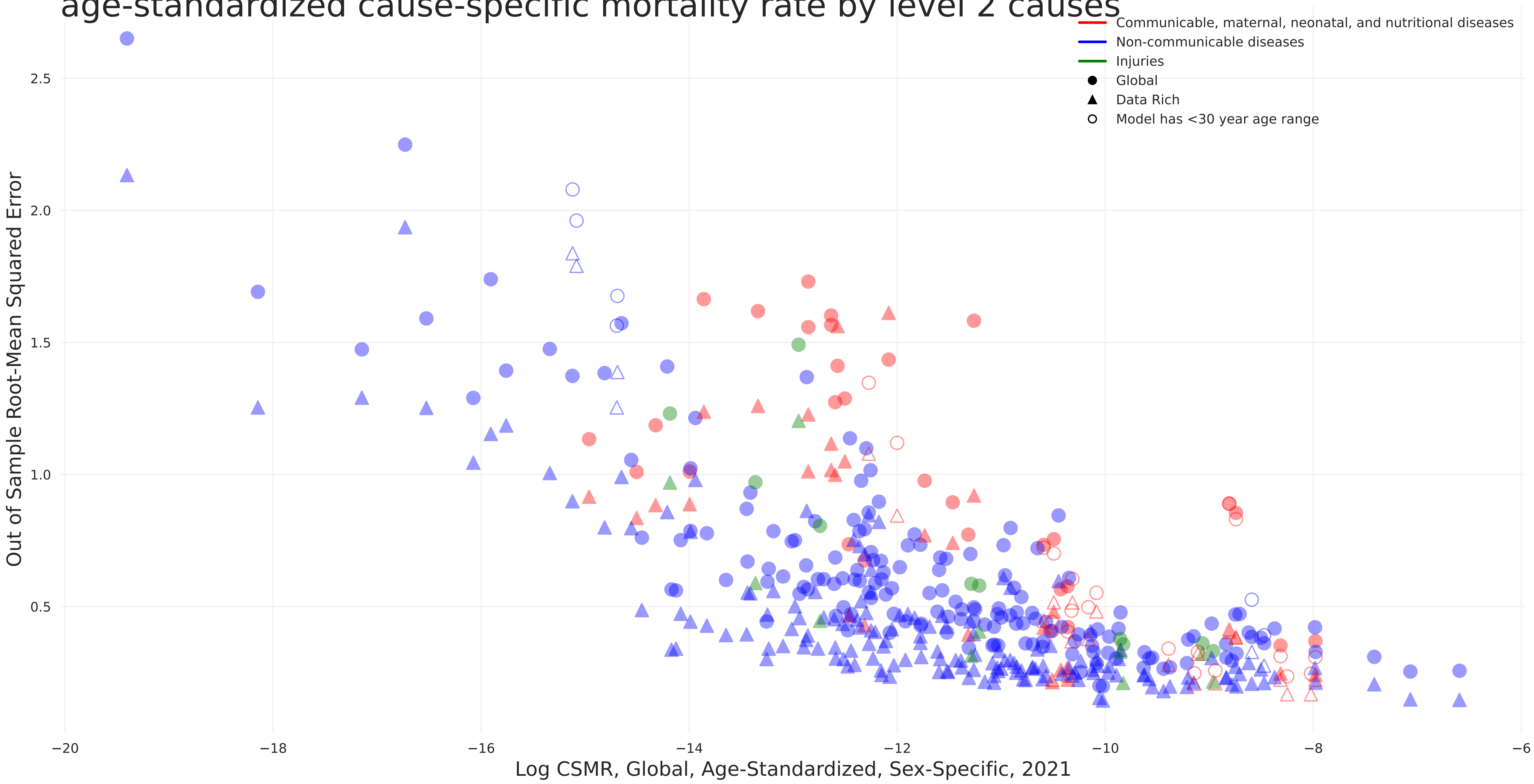

Appendix Table S1: GBD location hierarchy with levels	
**Geography**	**level**
Global	0
Central Europe, eastern Europe, and central Asia	1
Central Asia	2
Armenia	3
Azerbaijan	3
Georgia	3
Kazakhstan	3
Kyrgyzstan	3
Mongolia	3
Tajikistan	3
Turkmenistan	3
Uzbekistan	3
Central Europe	2
Albania	3
Bosnia and Herzegovina	3
Bulgaria	3
Croatia	3
Czechia	3
Hungary	3
Montenegro	3
North Macedonia	3
Poland	3
Romania	3
Serbia	3
Slovakia	3
Slovenia	3
Eastern Europe	2
Belarus	3
Estonia	3
Latvia	3
Lithuania	3
Moldova	3
Russia	3
Ukraine	3
High income	1
Australasia	2
Australia	3
New Zealand	3
High-income Asia Pacific	2
Brunei	3
Japan	3
Aichi	4
Akita	4
Aomori	4

Chiba	4
Ehime	4
Fukui	4
Fukuoka	4
Fukushima	4
Gifu	4
Gunma	4
Hiroshima	4
Hokkaidō	4
Hyōgo	4
Ibaraki	4
Ishikawa	4
Iwate	4
Kagawa	4
Kagoshima	4
Kanagawa	4
Kōchi	4
Kumamoto	4
Kyōto	4
Mie	4
Miyagi	4
Miyazaki	4
Nagano	4
Nagasaki	4
Nara	4
Niigata	4
Ōita	4
Okayama	4
Okinawa	4
Ōsaka	4
Saga	4
Saitama	4
Shiga	4
Shimane	4
Shizuoka	4
Tochigi	4
Tokushima	4
Tōkyō	4
Tottori	4
Toyama	4
Wakayama	4
Yamagata	4
Yamaguchi	4
Yamanashi	4
South Korea	3
Singapore	3

High-income North America	2
Canada	3
Greenland	3
USA	3
Alabama	4
Alaska	4
Arizona	4
Arkansas	4
California	4
Colorado	4
Connecticut	4
Delaware	4
Washington, DC	4
Florida	4
Georgia	4
Hawaii	4
Idaho	4
Illinois	4
Indiana	4
Iowa	4
Kansas	4
Kentucky	4
Louisiana	4
Maine	4
Maryland	4
Massachusetts	4
Michigan	4
Minnesota	4
Mississippi	4
Missouri	4
Montana	4
Nebraska	4
Nevada	4
New Hampshire	4
New Jersey	4
New Mexico	4
New York	4
North Carolina	4
North Dakota	4
Ohio	4
Oklahoma	4
Oregon	4
Pennsylvania	4
Rhode Island	4
South Carolina	4
South Dakota	4

Tennessee	4
Texas	4
Utah	4
Vermont	4
Virginia	4
Washington	4
West Virginia	4
Wisconsin	4
Wyoming	4
Southern Latin America	2
Argentina	3
Chile	3
Uruguay	3
Western Europe	2
Andorra	3
Austria	3
Belgium	3
Cyprus	3
Denmark	3
Finland	3
France	3
Germany	3
Greece	3
Iceland	3
Ireland	3
Israel	3
Italy	3
Abruzzo	4
Basilicata	4
Calabria	4
Campania	4
Emilia-Romagna	4
Friuli-Venezia Giulia	4
Lazio	4
Liguria	4
Lombardia	4
Marche	4
Molise	4
Piemonte	4
Provincia autonoma di Bolzano	4
Provincia autonoma di Trento	4
Puglia	4
Sardegna	4
Sicilia	4
Toscana	4
Umbria	4

Valle d'Aosta	4
Veneto	4
Luxembourg	3
Malta	3
Monaco	3
Netherlands	3
Norway	3
Agder	4
Innlandet	4
Møre og Romsdal	4
Nordland	4
Oslo	4
Rogaland	4
Troms og Finnmark	4
Trøndelag	4
Vestfold og Telemark	4
Vestland	4
Viken	4
Portugal	3
San Marino	3
Spain	3
Sweden	3
Stockholm	4
Sweden except Stockholm	4
Switzerland	3
UK	3
England	4
East Midlands	5
Derby	6
Derbyshire	6
Leicester	6
Leicestershire	6
Lincolnshire	6
Northamptonshire	6
Nottingham	6
Nottinghamshire	6
Rutland	6
East of England	5
Bedford	6
Cambridgeshire	6
Central Bedfordshire	6
Essex	6
Hertfordshire	6
Luton	6
Norfolk	6
Peterborough	6

Southend-on-Sea	6
Suffolk	6
Thurrock	6
Greater London	5
Barking and Dagenham	6
Barnet	6
Bexley	6
Brent	6
Bromley	6
Camden	6
Croydon	6
Ealing	6
Enfield	6
Greenwich	6
Hackney	6
Hammersmith and Fulham	6
Haringey	6
Harrow	6
Havering	6
Hillingdon	6
Hounslow	6
Islington	6
Kensington and Chelsea	6
Kingston upon Thames	6
Lambeth	6
Lewisham	6
Merton	6
Newham	6
Redbridge	6
Richmond upon Thames	6
Southwark	6
Sutton	6
Tower Hamlets	6
Waltham Forest	6
Wandsworth	6
Westminster	6
North East England	5
County Durham	6
Darlington	6
Gateshead	6
Hartlepool	6
Middlesbrough	6
Newcastle upon Tyne	6
North Tyneside	6
Northumberland	6
Redcar and Cleveland	6

South Tyneside	6
Stockton-on-Tees	6
Sunderland	6
North West England	5
Blackburn with Darwen	6
Blackpool	6
Bolton	6
Bury	6
Cheshire East	6
Cheshire West and Chester	6
Cumbria	6
Halton	6
Knowsley	6
Lancashire	6
Liverpool	6
Manchester	6
Oldham	6
Rochdale	6
Salford	6
Sefton	6
St Helens	6
Stockport	6
Tameside	6
Trafford	6
Warrington	6
Wigan	6
Wirral	6
South East England	5
Bracknell Forest	6
Brighton and Hove	6
Buckinghamshire	6
East Sussex	6
Hampshire	6
Isle of Wight	6
Kent	6
Medway	6
Milton Keynes	6
Oxfordshire	6
Portsmouth	6
Reading	6
Slough	6
Southampton	6
Surrey	6
West Berkshire	6
West Sussex	6
Windsor and Maidenhead	6

Wokingham	6
South West England	5
Bath and North East Somerset	6
Bournemouth	6
Bristol, City of	6
Cornwall	6
Devon	6
Dorset	6
Gloucestershire	6
North Somerset	6
Plymouth	6
Poole	6
Somerset	6
South Gloucestershire	6
Swindon	6
Torbay	6
Wiltshire	6
West Midlands	5
Birmingham	6
Coventry	6
Dudley	6
Herefordshire, County of	6
Sandwell	6
Shropshire	6
Solihull	6
Staffordshire	6
Stoke-on-Trent	6
Telford and Wrekin	6
Walsall	6
Warwickshire	6
Wolverhampton	6
Worcestershire	6
Yorkshire and the Humber	5
Barnsley	6
Bradford	6
Calderdale	6
Doncaster	6
East Riding of Yorkshire	6
Kingston upon Hull, City of	6
Kirklees	6
Leeds	6
North East Lincolnshire	6
North Lincolnshire	6
North Yorkshire	6
Rotherham	6
Sheffield	6

Wakefield	6
York	6
Northern Ireland	4
Scotland	4
Wales	4
Latin America and Caribbean	1
Andean Latin America	2
Bolivia	3
Ecuador	3
Peru	3
Caribbean	2
Antigua and Barbuda	3
The Bahamas	3
Barbados	3
Belize	3
Bermuda	3
Cuba	3
Dominica	3
Dominican Republic	3
Grenada	3
Guyana	3
Haiti	3
Jamaica	3
Puerto Rico	3
Saint Kitts and Nevis	3
Saint Lucia	3
Saint Vincent and the Grenadines	3
Suriname	3
Trinidad and Tobago	3
Virgin Islands	3
Central Latin America	2
Colombia	3
Costa Rica	3
El Salvador	3
Guatemala	3
Honduras	3
Mexico	3
Aguascalientes	4
Baja California	4
Baja California Sur	4
Campeche	4
Chiapas	4
Chihuahua	4
Coahuila	4
Colima	4
Durango	4

Guanajuato	4
Guerrero	4
Hidalgo	4
Jalisco	4
México	4
Mexico City	4
Michoacán de Ocampo	4
Morelos	4
Nayarit	4
Nuevo León	4
Oaxaca	4
Puebla	4
Querétaro	4
Quintana Roo	4
San Luis Potosí	4
Sinaloa	4
Sonora	4
Tabasco	4
Tamaulipas	4
Tlaxcala	4
Veracruz de Ignacio de la Llave	4
Yucatán	4
Zacatecas	4
Nicaragua	3
Panama	3
Venezuela	3
Tropical Latin America	2
Brazil	3
Acre	4
Alagoas	4
Amapá	4
Amazonas	4
Bahia	4
Ceará	4
Distrito Federal	4
Espírito Santo	4
Goiás	4
Maranhão	4
Mato Grosso	4
Mato Grosso do Sul	4
Minas Gerais	4
Pará	4
Paraíba	4
Paraná	4
Pernambuco	4
Piauí	4

Rio de Janeiro	4
Rio Grande do Norte	4
Rio Grande do Sul	4
Rondônia	4
Roraima	4
Santa Catarina	4
São Paulo	4
Sergipe	4
Tocantins	4
Paraguay	3
North Africa and Middle East	1
North Africa and Middle East	2
Afghanistan	3
Algeria	3
Bahrain	3
Egypt	3
Iran	3
Alborz	4
Ardebil	4
Bushehr	4
Chahar Mahaal and Bakhtiari	4
East Azarbayejan	4
Fars	4
Gilan	4
Golestan	4
Hamadan	4
Hormozgan	4
Ilam	4
Isfahan	4
Kerman	4
Kermanshah	4
Khorasan-e-Razavi	4
Khuzestan	4
Kohgiluyeh and Boyer-Ahmad	4
Kurdistan	4
Lorestan	4
Markazi	4
Mazandaran	4
North Khorasan	4
Qazvin	4
Qom	4
Semnan	4
Sistan and Baluchistan	4
South Khorasan	4
Tehran	4
West Azarbayejan	4

Yazd	4
Zanjan	4
Iraq	3
Jordan	3
Kuwait	3
Lebanon	3
Libya	3
Morocco	3
Oman	3
Palestine	3
Qatar	3
Saudi Arabia	3
Sudan	3
Syria	3
Tunisia	3
Türkiye	3
United Arab Emirates	3
Yemen	3
South Asia	1
South Asia	2
Bangladesh	3
Bhutan	3
India	3
Nepal	3
Pakistan	3
Azad Jammu & Kashmir	4
Balochistan	4
Gilgit-Baltistan	4
Islamabad Capital Territory	4
Khyber Pakhtunkhwa	4
Punjab	4
Sindh	4
Southeast Asia, east Asia, and Oceania	1
East Asia	2
China	3
North Korea	3
Taiwan (province of China)	3
Oceania	2
American Samoa	3
Cook Islands	3
Fiji	3
Guam	3
Kiribati	3
Marshall Islands	3
Federated States of Micronesia	3
Nauru	3

Niue	3
Northern Mariana Islands	3
Palau	3
Papua New Guinea	3
Samoa	3
Solomon Islands	3
Tokelau	3
Tonga	3
Tuvalu	3
Vanuatu	3
Southeast Asia	2
Cambodia	3
Indonesia	3
Aceh	4
Bali	4
Bangka-Belitung Islands	4
Banten	4
Bengkulu	4
Gorontalo	4
Jakarta	4
Jambi	4
West Java	4
Central Java	4
East Java	4
West Kalimantan	4
South Kalimantan	4
Central Kalimantan	4
East Kalimantan	4
North Kalimantan	4
Riau Islands	4
Lampung	4
Maluku	4
North Maluku	4
West Nusa Tenggara	4
East Nusa Tenggara	4
Papua	4
West Papua	4
Riau	4
West Sulawesi	4
South Sulawesi	4
Central Sulawesi	4
Southeast Sulawesi	4
North Sulawesi	4
West Sumatra	4
South Sumatra	4
North Sumatra	4

Yogyakarta	4
Laos	3
Malaysia	3
Maldives	3
Mauritius	3
Myanmar	3
Philippines	3
Abra	4
Agusan Del Norte	4
Agusan Del Sur	4
Aklan	4
Albay	4
Antique	4
Apayao	4
Aurora	4
Basilan	4
Bataan	4
Batanes	4
Batangas	4
Benguet	4
Biliran	4
Bohol	4
Bukidnon	4
Bulacan	4
Cagayan	4
Camarines Norte	4
Camarines Sur	4
Camiguin	4
Capiz	4
Catanduanes	4
Cavite	4
Cebu	4
Cotabato (North Cotabato)	4
Davao de Oro	4
Davao Del Norte	4
Davao Del Sur	4
Davao Occidental	4
Davao Oriental	4
Dinagat Islands	4
Eastern Samar	4
Guimaras	4
Ifugao	4
Ilocos Norte	4
Ilocos Sur	4
Iloilo	4
Isabela	4

Kalinga	4
La Union	4
Laguna	4
Lanao Del Norte	4
Lanao Del Sur	4
Leyte	4
Maguindanao	4
Marinduque	4
Masbate	4
Misamis Occidental	4
Misamis Oriental	4
Mountain Province	4
National Capital Region	4
Negros Occidental	4
Negros Oriental	4
Northern Samar	4
Nueva Ecija	4
Nueva Vizcaya	4
Occidental Mindoro	4
Oriental Mindoro	4
Palawan	4
Pampanga	4
Pangasinan	4
Quezon	4
Quirino	4
Rizal	4
Romblon	4
Samar (Western Samar)	4
Sarangani	4
Siquijor	4
Sorsogon	4
South Cotabato	4
Southern Leyte	4
Sultan Kudarat	4
Sulu	4
Surigao Del Norte	4
Surigao Del Sur	4
Tarlac	4
Tawi-Tawi	4
Zambales	4
Zamboanga Del Norte	4
Zamboanga Del Sur	4
Zamboanga Sibugay	4
Seychelles	3
Sri Lanka	3
Thailand	3

Timor-Leste	3
Viet Nam	3
Sub-Saharan Africa	1
Central sub-Saharan Africa	2
Angola	3
Central African Republic	3
Congo (Brazzaville)	3
DR Congo	3
Equatorial Guinea	3
Gabon	3
Eastern sub-Saharan Africa	2
Burundi	3
Comoros	3
Djibouti	3
Eritrea	3
Ethiopia	3
Addis Ababa	4
Afar	4
Amhara	4
Benishangul-Gumuz	4
Dire Dawa	4
Gambella	4
Harari	4
Oromia	4
Somali	4
Southern Nations, Nationalities, and Peoples	4
Tigray	4
Kenya	3
Baringo	4
Bomet	4
Bungoma	4
Busia	4
Elgeyo Marakwet	4
Embu	4
Garissa	4
Homa Bay	4
Isiolo	4
Kajiado	4
Kakamega	4
Kericho	4
Kiambu	4
Kilifi	4
Kirinyaga	4
Kisii	4
Kisumu	4
Kitui	4

Kwale	4
Laikipia	4
Lamu	4
Machakos	4
Makueni	4
Mandera	4
Marsabit	4
Meru	4
Migori	4
Mombasa	4
Murang'a	4
Nairobi	4
Nakuru	4
Nandi	4
Narok	4
Nyamira	4
Nyandarua	4
Nyeri	4
Samburu	4
Siaya	4
Taita Taveta	4
Tana River	4
Tharaka Nithi	4
Trans Nzoia	4
Turkana	4
Uasin Gishu	4
Vihiga	4
Wajir	4
West Pokot	4
Madagascar	3
Malawi	3
Mozambique	3
Rwanda	3
Somalia	3
South Sudan	3
Uganda	3
Tanzania	3
Zambia	3
Southern sub-Saharan Africa	2
Botswana	3
Eswatini	3
Lesotho	3
Namibia	3
South Africa	3
Eastern Cape	4
Free State	4

Gauteng	4
KwaZulu-Natal	4
Limpopo	4
Mpumalanga	4
North West	4
Northern Cape	4
Western Cape	4
Zimbabwe	3
Western sub-Saharan Africa	2
Benin	3
Burkina Faso	3
Cabo Verde	3
Cameroon	3
Chad	3
Côte d'Ivoire	3
The Gambia	3
Ghana	3
Guinea	3
Guinea-Bissau	3
Liberia	3
Mali	3
Mauritania	3
Niger	3
Nigeria	3
São Tomé and Príncipe	3
Senegal	3
Sierra Leone	3
Togo	3

Appendix Table S2: GBD cause hierarchy with levels	
All causes	level
Communicable, maternal, neonatal, and nutritional diseases	0
	1
HIV/AIDS and sexually transmitted infections	2
HIV/AIDS	3
HIV/AIDS - Drug-susceptible Tuberculosis	4
extensive drug resistance	4
HIV/AIDS - Extensively drug-resistant Tuberculosis	4
HIV/AIDS resulting in other diseases	4
Sexually transmitted infections excluding HIV	3
Syphilis	4
Chlamydial infection	4
Gonococcal infection	4
Trichomoniasis	4
Genital herpes	4
Other sexually transmitted infections	4
Respiratory infections and tuberculosis	2
Tuberculosis	3
Latent tuberculosis infection	4
Drug-susceptible tuberculosis	4
resistance	4
Extensively drug-resistant tuberculosis	4
Lower respiratory infections	3
Upper respiratory infections	3
Otitis media	3
COVID-19	3
Enteric infections	2
Diarrheal diseases	3
Typhoid and paratyphoid	3
Typhoid fever	4
Paratyphoid fever	4
Invasive Non-typhoidal Salmonella (iNTS)	3
Other intestinal infectious diseases	3
Neglected tropical diseases and malaria	2
Malaria	3
Chagas disease	3
Leishmaniasis	3
Visceral leishmaniasis	4
Cutaneous and mucocutaneous leishmaniasis	4
African trypanosomiasis	3
Schistosomiasis	3
Cysticercosis	3
Cystic echinococcosis	3
Lymphatic filariasis	3
Onchocerciasis	3

Trachoma	3
Dengue	3
Yellow fever	3
Rabies	3
Intestinal nematode infections	3
Ascariasis	4
Trichuriasis	4
Hookworm disease	4
Food-borne trematodiases	3
Leprosy	3
Ebola	3
Zika virus	3
Guinea worm disease	3
Other neglected tropical diseases	3
Other infectious diseases	2
Meningitis	3
Encephalitis	3
Diphtheria	3
Pertussis	3
Tetanus	3
Measles	3
Varicella and herpes zoster	3
Acute hepatitis	3
Acute hepatitis A	4
Acute hepatitis B	4
Acute hepatitis C	4
Acute hepatitis E	4
Other unspecified infectious diseases	3
Maternal and neonatal disorders	2
Maternal disorders	3
Maternal hemorrhage	4
Maternal sepsis and other maternal infections	4
Maternal hypertensive disorders	4
Maternal obstructed labor and uterine rupture	4
Maternal abortion and miscarriage	4
Ectopic pregnancy	4
Indirect maternal deaths	4
Late maternal deaths	4
Maternal deaths aggravated by HIV/AIDS	4
Other direct maternal disorders	4
Neonatal disorders	3
Neonatal preterm birth	4
Neonatal encephalopathy due to birth asphyxia and trauma	4
Neonatal sepsis and other neonatal infections	4
Hemolytic disease and other neonatal jaundice	4
Other neonatal disorders	4

Nutritional deficiencies	2
Protein-energy malnutrition	3
Iodine deficiency	3
Vitamin A deficiency	3
Dietary iron deficiency	3
Other nutritional deficiencies	3
Non-communicable diseases	1
Neoplasms	2
Lip and oral cavity cancer	3
Nasopharynx cancer	3
Other pharynx cancer	3
Esophageal cancer	3
Stomach cancer	3
Colon and rectum cancer	3
Liver cancer	3
Liver cancer due to hepatitis B	4
Liver cancer due to hepatitis C	4
Liver cancer due to alcohol use	4
Liver cancer due to NASH	4
Hepatoblastoma	4
Liver cancer due to other causes	4
Gallbladder and biliary tract cancer	3
Pancreatic cancer	3
Larynx cancer	3
Tracheal, bronchus, and lung cancer	3
Malignant skin melanoma	3
Non-melanoma skin cancer	3
Non-melanoma skin cancer (squamous-cell carcinoma)	4
Non-melanoma skin cancer (basal-cell carcinoma)	4
Soft tissue and other extraosseous sarcomas	3
Malignant neoplasm of bone and articular cartilage	3
Breast cancer	3
Cervical cancer	3
Uterine cancer	3
Ovarian cancer	3
Prostate cancer	3
Testicular cancer	3
Kidney cancer	3
Bladder cancer	3
Brain and central nervous system cancer	3
Eye cancer	3
Retinoblastoma	4
Other eye cancers	4
Neuroblastoma and other peripheral nervous cell tumors	3
Thyroid cancer	3
Mesothelioma	3

Hodgkin lymphoma	3
Non-Hodgkin lymphoma	3
Burkitt lymphoma	4
Other non-Hodgkin lymphoma	4
Multiple myeloma	3
Leukemia	3
Acute lymphoid leukemia	4
Chronic lymphoid leukemia	4
Acute myeloid leukemia	4
Chronic myeloid leukemia	4
Other leukemia	4
Other malignant neoplasms	3
Other neoplasms	3
neoplasms	4
Benign and in situ intestinal neoplasms	4
Benign and in situ cervical and uterine neoplasms	4
Other benign and in situ neoplasms	4
Cardiovascular diseases	2
Rheumatic heart disease	3
Ischemic heart disease	3
Stroke	3
Ischemic stroke	4
Intracerebral hemorrhage	4
Subarachnoid hemorrhage	4
Hypertensive heart disease	3
Non-rheumatic valvular heart disease	3
Non-rheumatic calcific aortic valve disease	4
Non-rheumatic degenerative mitral valve disease	4
Other non-rheumatic valve diseases	4
Cardiomyopathy and myocarditis	3
Myocarditis	4
Alcoholic cardiomyopathy	4
Other cardiomyopathy	4
Pulmonary Arterial Hypertension	3
Atrial fibrillation and flutter	3
Aortic aneurysm	3
Lower extremity peripheral arterial disease	3
Endocarditis	3
Other cardiovascular and circulatory diseases	3
Chronic respiratory diseases	2
Chronic obstructive pulmonary disease	3
Pneumoconiosis	3
Silicosis	4
Asbestosis	4
Coal workers pneumoconiosis	4
Other pneumoconiosis	4

Asthma	3
Interstitial lung disease and pulmonary sarcoidosis	3
Other chronic respiratory diseases	3
Digestive diseases	2
Cirrhosis and other chronic liver diseases	3
Chronic hepatitis B including cirrhosis	4
Chronic hepatitis C including cirrhosis	4
Cirrhosis due to alcohol	4
Nonalcoholic fatty liver disease including cirrhosis	4
Cirrhosis due to other causes	4
Upper digestive system diseases	3
Peptic ulcer disease	4
Gastritis and duodenitis	4
Gastroesophageal reflux disease	4
Appendicitis	3
Paralytic ileus and intestinal obstruction	3
Inguinal, femoral, and abdominal hernia	3
Inflammatory bowel disease	3
Vascular intestinal disorders	3
Gallbladder and biliary diseases	3
Pancreatitis	3
Other digestive diseases	3
Neurological disorders	2
Alzheimer's disease and other dementias	3
Parkinson's disease	3
Idiopathic epilepsy	3
Multiple sclerosis	3
Motor neuron disease	3
Headache disorders	3
Migraine	4
Tension-type headache	4
Other neurological disorders	3
Mental disorders	2
Schizophrenia	3
Depressive disorders	3
Major depressive disorder	4
Dysthymia	4
Bipolar disorder	3
Anxiety disorders	3
Eating disorders	3
Anorexia nervosa	4
Bulimia nervosa	4
Autism spectrum disorders	3
Attention-deficit/hyperactivity disorder	3
Conduct disorder	3
Idiopathic developmental intellectual disability	3

Other mental disorders	3
Substance use disorders	2
Alcohol use disorders	3
Drug use disorders	3
Opioid use disorders	4
Cocaine use disorders	4
Amphetamine use disorders	4
Cannabis use disorders	4
Other drug use disorders	4
Diabetes and kidney diseases	2
Diabetes mellitus	3
Diabetes mellitus type 1	4
Diabetes mellitus type 2	4
Chronic kidney disease	3
Chronic kidney disease due to diabetes mellitus type 1	4
Chronic kidney disease due to diabetes mellitus type 2	4
Chronic kidney disease due to hypertension	4
Chronic kidney disease due to glomerulonephritis	4
Chronic kidney disease due to other and unspecified causes	4
Acute glomerulonephritis	3
Skin and subcutaneous diseases	2
Dermatitis	3
Atopic dermatitis	4
Contact dermatitis	4
Seborrhoeic dermatitis	4
Psoriasis	3
Bacterial skin diseases	3
Cellulitis	4
Pyoderma	4
Scabies	3
Fungal skin diseases	3
Viral skin diseases	3
Acne vulgaris	3
Alopecia areata	3
Pruritus	3
Urticaria	3
Decubitus ulcer	3
Other skin and subcutaneous diseases	3
Sense organ diseases	2
Blindness and vision loss	3
Glaucoma	4
Cataract	4
Age-related macular degeneration	4
Refraction disorders	4
Near vision loss	4
Other vision loss	4

Age-related and other hearing loss	3
Other sense organ diseases	3
Musculoskeletal disorders	2
Rheumatoid arthritis	3
Osteoarthritis	3
Osteoarthritis hip	4
Osteoarthritis knee	4
Osteoarthritis hand	4
Osteoarthritis other	4
Low back pain	3
Neck pain	3
Gout	3
Other musculoskeletal disorders	3
Other non-communicable diseases	2
Congenital birth defects	3
Neural tube defects	4
Congenital heart anomalies	4
Orofacial clefts	4
Down syndrome	4
Turner syndrome	4
Klinefelter syndrome	4
Other chromosomal abnormalities	4
Congenital musculoskeletal and limb anomalies	4
Urogenital congenital anomalies	4
Digestive congenital anomalies	4
Other congenital birth defects	4
Urinary diseases and male infertility	3
Urinary tract infections and interstitial nephritis	4
Urolithiasis	4
Benign prostatic hyperplasia	4
Male infertility	4
Other urinary diseases	4
Gynecological diseases	3
Uterine fibroids	4
Polycystic ovarian syndrome	4
Female infertility	4
Endometriosis	4
Genital prolapse	4
Premenstrual syndrome	4
Other gynecological diseases	4
Hemoglobinopathies and hemolytic anemias	3
Thalassemias	4
Thalassemias trait	4
Sickle cell disorders	4
Sickle cell trait	4
G6PD deficiency	4

G6PD trait	4
Other hemoglobinopathies and hemolytic anemias	4
Endocrine, metabolic, blood, and immune disorders	3
Oral disorders	3
Caries of deciduous teeth	4
Caries of permanent teeth	4
Periodontal diseases	4
Edentulism	4
Other oral disorders	4
Sudden infant death syndrome	3
Injuries	1
Transport injuries	2
Road injuries	3
Pedestrian road injuries	4
Cyclist road injuries	4
Motorcyclist road injuries	4
Motor vehicle road injuries	4
Other road injuries	4
Other transport injuries	3
Unintentional injuries	2
Falls	3
Drowning	3
Fire, heat, and hot substances	3
Poisonings	3
Poisoning by carbon monoxide	4
Poisoning by other means	4
Exposure to mechanical forces	3
Unintentional firearm injuries	4
Other exposure to mechanical forces	4
Adverse effects of medical treatment	3
Animal contact	3
Venomous animal contact	4
Non-venomous animal contact	4
Foreign body	3
Pulmonary aspiration and foreign body in airway	4
Foreign body in eyes	4
Foreign body in other body part	4
Environmental heat and cold exposure	3
Exposure to forces of nature	3
Other unintentional injuries	3
Self-harm and interpersonal violence	2
Self-harm	3
Self-harm by firearm	4
Self-harm by other specified means	4
Interpersonal violence	3
Physical violence by firearm	4

Physical violence by sharp object	4
Sexual violence	4
Physical violence by other means	4
Conflict and terrorism	3
Police conflict and executions	3
Other COVID-19 pandemic-related outcomes	1
Total cancers	1
Total burden related to hepatitis B	1
Total burden related to hepatitis C	1
Total burden related to Non-alcoholic fatty liver disease (NAFLD)	1
Total Cancers excluding Non-melanoma skin cancer	1

Appendix Table S3: CodCorrect cause hierarchy with levels

Cause Name	CodCorrect Level
All causes	0
Sexually transmitted infections excluding HIV	1
Syphilis	2
Chlamydial infection	2
Gonococcal infection	2
Other sexually transmitted infections	2
Tuberculosis	1
Drug-susceptible tuberculosis	2
Multidrug-resistant tuberculosis without extensive drug resistance	2
Extensively drug-resistant tuberculosis	2
Lower respiratory infections	1
Upper respiratory infections	1
Otitis media	1
Diarrheal diseases	1
Typhoid fever	1
Paratyphoid fever	1
Invasive Non-typhoidal Salmonella (iNTS)	1
Other intestinal infectious diseases	1
Malaria	1
Chagas disease	1
Visceral leishmaniasis	1
African trypanosomiasis	1
Schistosomiasis	1
Cysticercosis	1
Cystic echinococcosis	1
Dengue	1
Yellow fever	1
Rabies	1
Ascariasis	1
Zika virus	1
Other neglected tropical diseases	1
Meningitis	1
Encephalitis	1
Diphtheria	1
Pertussis	1
Tetanus	1
Measles	1
Varicella and herpes zoster	1
Acute hepatitis	1
Acute hepatitis A	2
Acute hepatitis B	2
Acute hepatitis C	2
Acute hepatitis E	2
Other unspecified infectious diseases	1

Appendix Table S3: CodCorrect cause hierarchy with levels

Cause Name	CodCorrect Level
Maternal disorders	1
Maternal hemorrhage	2
Maternal sepsis and other maternal infections	2
Maternal hypertensive disorders	2
Maternal obstructed labor and uterine rupture	2
Maternal abortion and miscarriage	2
Ectopic pregnancy	2
Indirect maternal deaths	2
Late maternal deaths	2
Maternal deaths aggravated by HIV/AIDS	2
Other direct maternal disorders	2
Neonatal disorders	1
Neonatal preterm birth	2
Neonatal encephalopathy due to birth asphyxia and trauma	2
Neonatal sepsis and other neonatal infections	2
Hemolytic disease and other neonatal jaundice	2
Other neonatal disorders	2
Nutritional deficiencies	1
Protein-energy malnutrition	2
Other nutritional deficiencies	2
Lip and oral cavity cancer	1
Nasopharynx cancer	1
Other pharynx cancer	1
Esophageal cancer	1
Stomach cancer	1
Colon and rectum cancer	1
Liver cancer due to hepatitis B	1
Liver cancer due to hepatitis C	1
Liver cancer due to alcohol use	1
Liver cancer due to NASH	1
Hepatoblastoma	1
Liver cancer due to other causes (internal)	1
Gallbladder and biliary tract cancer	1
Pancreatic cancer	1
Larynx cancer	1
Tracheal, bronchus, and lung cancer	1
Malignant skin melanoma	1
Non-melanoma skin cancer (squamous-cell carcinoma)	1
Soft tissue and other extraosseous sarcomas	1
Malignant neoplasm of bone and articular cartilage	1
Breast cancer	1
Cervical cancer	1
Uterine cancer	1
Ovarian cancer	1

Appendix Table S3: CodCorrect cause hierarchy with levels

Cause Name	CodCorrect Level
Prostate cancer	1
Testicular cancer	1
Kidney cancer	1
Bladder cancer	1
Brain and central nervous system cancer	1
Retinoblastoma	1
Other eye cancers	1
Neuroblastoma and other peripheral nervous cell tumors	1
Thyroid cancer	1
Mesothelioma	1
Hodgkin lymphoma	1
Non-Hodgkin lymphoma	1
Burkitt lymphoma	2
Other non-Hodgkin lymphoma	2
Multiple myeloma	1
Leukemia	1
Acute lymphoid leukemia	2
Chronic lymphoid leukemia	2
Acute myeloid leukemia	2
Chronic myeloid leukemia	2
Other leukemia	2
Other malignant neoplasms (internal)	1
Myelodysplastic, myeloproliferative, and other hematopoietic neoplasms	1
Other benign and in situ neoplasms	1
Cardiovascular diseases	1
Rheumatic heart disease	2
Ischemic heart disease	2
Stroke	2
Ischemic stroke	3
Intracerebral hemorrhage	3
Subarachnoid hemorrhage	3
Hypertensive heart disease	2
Non-rheumatic valvular heart disease	2
Non-rheumatic calcific aortic valve disease	3
Non-rheumatic degenerative mitral valve disease	3
Other non-rheumatic valve diseases	3
Cardiomyopathy and myocarditis	2
Myocarditis	3
Alcoholic cardiomyopathy	3
Other cardiomyopathy	3
Pulmonary Arterial Hypertension	2
Atrial fibrillation and flutter	2
Aortic aneurysm	2

Appendix Table S3: CodCorrect cause hierarchy with levels

Cause Name	CodCorrect Level
Lower extremity peripheral arterial disease	2
Endocarditis	2
Other cardiovascular and circulatory diseases (internal)	2
Chronic respiratory diseases	1
Chronic obstructive pulmonary disease	2
Pneumoconiosis	2
Silicosis	3
Asbestosis	3
Coal workers pneumoconiosis	3
Other pneumoconiosis	3
Asthma	2
Interstitial lung disease and pulmonary sarcoidosis	2
Other chronic respiratory diseases	2
Digestive diseases	1
Cirrhosis and other chronic liver diseases	2
Cirrhosis and other chronic liver diseases due to hepatitis B	3
Cirrhosis and other chronic liver diseases due to hepatitis C	3
Cirrhosis and other chronic liver diseases due to alcohol use	3
Cirrhosis and other chronic liver diseases due to NAFLD	3
Cirrhosis and other chronic liver diseases due to other causes	3
Upper digestive system diseases	2
Peptic ulcer disease	3
Gastritis and duodenitis	3
Appendicitis	2
Paralytic ileus and intestinal obstruction	2
Inguinal, femoral, and abdominal hernia	2
Inflammatory bowel disease	2
Ulcerative colitis	3
Crohn's disease	3
Vascular intestinal disorders	2
Gallbladder and biliary diseases	2
Pancreatitis	2
Other digestive diseases	2
Alzheimer's disease and other dementias	1
Parkinson's disease	1
Idiopathic epilepsy	1
Multiple sclerosis	1
Motor neuron disease	1
Other neurological disorders	1
Anorexia nervosa	1
Alcohol use disorders	1

Appendix Table S3: CodCorrect cause hierarchy with levels

Cause Name	CodCorrect Level
Drug use disorders	1
Opioid use disorders	2
Cocaine use disorders	2
Amphetamine use disorders	2
Other drug use disorders	2
Diabetes mellitus	1
Diabetes mellitus type 1	2
Diabetes mellitus type 2	2
Chronic kidney disease	1
Chronic kidney disease due to diabetes mellitus type 1	2
Chronic kidney disease due to diabetes mellitus type 2	2
Chronic kidney disease due to hypertension	2
Chronic kidney disease due to glomerulonephritis	2
Chronic kidney disease due to other and unspecified causes	2
Acute glomerulonephritis	1
Skin and subcutaneous diseases	1
Bacterial skin diseases	2
Cellulitis	3
Pyoderma	3
Decubitus ulcer	2
Other skin and subcutaneous diseases	2
Musculoskeletal disorders	1
Rheumatoid arthritis	2
Other musculoskeletal disorders	2
Congenital birth defects	1
Neural tube defects	2
Congenital heart anomalies	2
Orofacial clefts	2
Down syndrome	2
Other chromosomal abnormalities	2
Congenital musculoskeletal and limb anomalies	2
Urogenital congenital anomalies	2
Digestive congenital anomalies	2
Other congenital birth defects	2
Urinary diseases and male infertility	1
Urinary tract infections and interstitial nephritis	2
Urolithiasis	2
Other urinary diseases	2
Gynecological diseases	1
Uterine fibroids	2
Endometriosis	2
Genital prolapse	2
Other gynecological diseases	2

Appendix Table S3: CodCorrect cause hierarchy with levels

Cause Name	CodCorrect Level
Hemoglobinopathies and hemolytic anemias	1
Thalassemias	2
Sickle cell disorders	2
G6PD deficiency	2
Other hemoglobinopathies and hemolytic anemias	2
Endocrine, metabolic, blood, and immune disorders	1
Thyroid diseases	2
Other endocrine, metabolic, blood, and immune disorders	2
Sudden infant death syndrome	1
Transport injuries	1
Road injuries	2
Pedestrian road injuries	3
Cyclist road injuries	3
Motorcyclist road injuries	3
Motor vehicle road injuries	3
Other road injuries	3
Other transport injuries	2
Falls	1
Drowning	1
Fire, heat, and hot substances	1
Poisonings	1
Poisoning by carbon monoxide	2
Poisoning by other means	2
Exposure to mechanical forces	1
Unintentional firearm injuries	2
Other exposure to mechanical forces	2
Adverse effects of medical treatment	1
Animal contact	1
Venomous animal contact	2
Non-venomous animal contact	2
Foreign body	1
Pulmonary aspiration and foreign body in airway	2
Foreign body in other body part	2
Electrocution	1
Environmental heat and cold exposure	1
Other unintentional injuries (internal)	1
Self-harm	1
Self-harm by firearm	2
Self-harm by other specified means	2
Interpersonal violence	1
Physical violence by firearm	2
Physical violence by sharp object	2
Physical violence by other means	2
Police conflict and executions	1

Appendix Table S4: GATHER checklist of information that should be included in reports of global health estimates, with description of compliance and location of information for "Global burden of 288 causes of death and life-expectancy decomposition in 204 countries and territories and 811 subnational locations, 1990-2021: a systematic analysis for the Global Burden of Disease Study 2021"			
**#**	**GATHER checklist item**	**Description of compliance**	**Reference**
**Objectives and funding**			
1	Define the indicators, populations, and time periods for which estimates were made.	Narrative provided in paper and methods appendix describing indicators, definitions, and populations	Manuscript (Methods) and methods appendix section 1
2	List the funding sources for the work.	Funding sources listed in paper	Manuscript (Funding) and appendix section 1
**Data Inputs**			
For all data inputs from multiple sources that are synthesized as part of the study:			
3	Describe how the data were identified and how the data were accessed.	Narrative description of data seeking methods provided	Manuscript (Methods) and methods appendix Section 2
4	Specify the inclusion and exclusion criteria. Identify all ad-hoc exclusions.	Narrative about inclusion and exclusion criteria by data type provided; ad-hoc exclusions in cause-specific write ups	Methods appendix Section 2
5	Provide information on all included data sources and their main characteristics. For each data source used, report reference information or contact name/institution, population represented, data collection method, year(s) of data collection, sex and age range, diagnostic criteria or measurement method, and sample size, as relevant.	An interactive, online data source tool that provides metadata for data sources by component, geography, cause, risk, or impairment has been developed	Online data citation tool https://ghdx.healthdata.org/gbd-2021/sources
6	Identify and describe any categories of input data that have potentially important biases (e.g., based on characteristics listed in item 5).	Summary of known biases by cause included in methods appendix	Methods appendix Section 2 and in each cause's methods write up
For data inputs that contribute to the analysis but were not synthesized as part of the study:			
7	Describe and give sources for any other data inputs.	Included in online data source tool	Input data can be accessed:https://ghdx.healthdata.org/gbd-2021/sources
For all data inputs:			

8	Provide all data inputs in a file format from which data can be efficiently extracted (e.g., a spreadsheet as opposed to a PDF), including all relevant meta-data listed in item 5. For any data inputs that cannot be shared due to ethical or legal reasons, such as third-party ownership, provide a contact name or the name of the institution that retains the right to the data.	Downloads of input data are available through an online data repository; input data not available in this form will be made available upon request.	Data inputs are accesible on the Global Health Data Exchange https://ghdx.healthdata.org/gbd-2021/sources
**Data analysis**			
9	Provide a conceptual overview of the data analysis method. A diagram may be helpful.	Flow diagrams of the overall methodological processes, as well as cause-specific modeling processes, have been provided	Manuscript (Methods) and methods appendix (appendix Figure 1 & 2)
10	Provide a detailed description of all steps of the analysis, including mathematical formulae. This description should cover, as relevant, data cleaning, data pre-processing, data adjustments and weighting of data sources, and mathematical or statistical model(s).	Flow diagrams and corresponding methodological write- ups for each cause, as well as the databases and modeling processes, have been provided	Manuscript (Methods) and methods appendix (Appendix Figure 1, Appendix Section 2 and 3)
11	Describe how candidate models were evaluated and how the final model(s) were selected.	Provided in the methodological write-ups	Appendix section 3
12	Provide the results of an evaluation of model performance, if done, as well as the results of any relevant sensitivity analysis.	Provided in the methodological write-ups	Appendix Section 3
13	Describe methods for calculating uncertainty of the estimates. State which sources of uncertainty were, and were not, accounted for in the uncertainty analysis.	Provided in the methods appendix and in the methodological write ups	Appendix section 2 and section 3
14	State how analytic or statistical source code used to generate estimates can be accessed.	Access statement provided	Code is provided at https://ghdx.healthdata.org/gbd-2021/code
**Results and Discussion**			

15	Provide published estimates in a file format from which data can be efficiently extracted.	GBD 2021 results are available through an online repository on the Global Health Data Exchange.	Manuscript, supplementary results, are available at; https://ghdx.healthdata.org/record/ihme-data/gbd-2021-cause-specific-mortality-1990-2021
16	Report a quantitative measure of the uncertainty of the estimates (e.g. uncertainty intervals).	Uncertainty intervals are provided with all results.	Uncertainty is available at: https://ghdx.healthdata.org/record/ihme-data/gbd-2021-cause-specific-mortality-1990-2021
17	Interpret results in light of existing evidence. If updating a previous set of estimates, describe the reasons for changes in estimates.	Discussion of methodological changes between GBD rounds provided in the narrative of the manuscript and methods appendix	Manuscript (Research in Context, Methods and Discussion) and methods appendix section 3
18	Discuss limitations of the estimates. Include a discussion of any modeling assumptions or data limitations that affect interpretation of the estimates.	Discussion of limitations provided in the narrative of the manuscript, as well as in the methodological write-ups in the methods appendix	Manuscript (Limitations) and methods appendix

Appendix Table S5: Total number of site years by cause and source type for GBD2021

Cause	Level	Vital Registration	Vital Registration - Sample	Verbal Autopsy	Surveillance	Survey/Census	Sibling History	MITS diagnosed	Cancer Registry	Police Records
All causes	0	25094	825	3157	4549	1605	5904	10	5278	1617
Communicable, maternal, neonatal, and nutritional diseases	1	25094	825	2915	3393	1538	5904	10		
HIV/AIDS and sexually transmitted infections	2	24169	825	562	411			9		
HIV/AIDS	3	24169	825	546	411			9		
HIV/AIDS - Drug-susceptible Tuberculosis	4	13272	448		11			9		
HIV/AIDS resulting in other diseases	4	20475	448		58			9		
Sexually transmitted infections excluding HIV	3	23663	825	366	239			4		
Syphilis	4	23171	825		239			4		
Chlamydial infection	4	21419	825							
Gonococcal infection	4	22866	825							
Other sexually transmitted infections	4	22614	825							
Respiratory infections and tuberculosis	2	24181	825	1825	681			9		
Tuberculosis	3	24181	825	1763	680			9		
Drug-susceptible tuberculosis	4	13837	448		679			9		
Multidrug-resistant tuberculosis without extensive drug resistance	4	13268	448					9		
Lower respiratory infections	3	24181	825	1794	681			9		
Influenza	4	20477	448					9		
Pneumococcal pneumonia	4	20470	448					9		
H influenzae type B pneumonia	4	20470	448					9		
Respiratory syncytial virus pneumonia	4	20470	448					9		
Other lower respiratory infections	4	20470	448					9		
Upper respiratory infections	3	21453	825					2		
Otitis media	3	21557	825					2		
COVID-19	3	2								
Enteric infections	2	24181	825	1790	575			9		
Diarrheal diseases	3	24181	825	1785	575			9		
Typhoid and paratyphoid	3	24132	825	409				4		
Typhoid fever	4	21331	448					4		
Paratyphoid fever	4	20566	448							
Invasive Non-typhoidal Salmonella (iNTS)	3	21088	825					8		
Other intestinal infectious diseases	3	21056	448							
Neglected tropical diseases and malaria	2	24185	825	1554	310			9		
Malaria	3	22694	825	1461	1			9		
Chagas disease	3	3258								
Leishmaniasis	3	22618	825	187				4		
Visceral leishmaniasis	4	22557	825	187				4		
African trypanosomiasis	3	20345	448							
Schistosomiasis	3	21450	448					1		
Cysticercosis	3	21645	825							
Cystic echinococcosis	3	21962	825							
Dengue	3	23556	825	342	1					
Yellow fever	3	21256	448	126				1		
Rabies	3	23824	825	924	278			1		
Intestinal nematode infections	3	22360	825	47						
Ascariasis	4	20888	825							
Ebola	3	370	56		30					
Zika virus	3	2128	799							
Other neglected tropical diseases	3	21473	825					5		
Other infectious diseases	2	24181	825	1718	912			10		
Meningitis	3	23669	825	1499	611			9		
Encephalitis	3	23375	825	375				5		
Diphtheria	3	23505	825							
Pertussis	3	23653	825	589				9		
Tetanus	3	23675	825	1361	396			6		
Measles	3	23650	825	1157	585			5		
Varicella and herpes zoster	3	22556	825	291						
Acute hepatitis	3	23057	825	1176				1		
Acute hepatitis A	4	20477	448					1		
Acute hepatitis B	4	20477	448					1		
Acute hepatitis C	4	13268	448					1		
Acute hepatitis E	4	20470	448					1		
Other unspecified infectious diseases	3	24177	825	1080	890			6		
Maternal and neonatal disorders	2	25087	825	2725	3014	1538	5904	10		
Maternal disorders	3	25085	825	2249	2332	1537	5904			
Maternal hemorrhage	4	22627	825	1134	948	9				
Maternal sepsis and other maternal infections	4	22626	825	840	556	7				
Maternal hypertensive disorders	4	22634	825	1006	909	9				
Maternal obstructed labor and uterine rupture	4	22627	825	862	667	8				
Maternal abortion and miscarriage	4	22646	825	363	441	7				
Ectopic pregnancy	4	22062	825		629					
Indirect maternal deaths	4	22627	825	931	1036	9				
Late maternal deaths	4	14144	825		152					
Maternal deaths aggravated by HIV/AIDS	4	25085	825	2249	2332	1537	5904			
Other direct maternal disorders	4	22627	825	330	888	4				
Neonatal disorders	3	23622	823	1695	682	1		10		
Neonatal preterm birth	4	22019	823	600	681			10		
Neonatal encephalopathy due to birth asphyxia and trauma	4	22020	823	590	681			10		
Neonatal sepsis and other neonatal infections	4	22007	823	168	634			8		
Hemolytic disease and other neonatal jaundice	4	22002	823	371				6		
Other neonatal disorders	4	22006	823	175	664			6		
Nutritional deficiencies	2	23630	825	1487	578			10		
Protein-energy malnutrition	3	22559	825	1421				10		
Dietary iron deficiency	3			741						
Other nutritional deficiencies	3	22012	825							
Non-communicable diseases	1	24191	825	1786	756			10	5278	64
Neoplasms	2	23615	825	1430	723			7	5278	
Lip and oral cavity cancer	3	22825	825	394					4636	
Nasopharynx cancer	3	22825	825						5154	
Other pharynx cancer	3	22825	825						5113	
Esophageal cancer	3	23111	825	420					5206	
Stomach cancer	3	23115	825						5247	
Colon and rectum cancer	3	23115	825	424					5247	
Liver cancer	3	22826	825					3	5206	
Liver cancer due to hepatitis B	4	20470	448							
Liver cancer due to hepatitis C	4	20470	448							
Liver cancer due to alcohol use	4	20470	448							
Liver cancer due to NASH	4	20470	448							
Hepatoblastoma	4	22798	824					3	947	
Liver cancer due to other causes	4	20470	448							
Gallbladder and biliary tract cancer	3	22262	825						5145	
Pancreatic cancer	3	22826	825						5166	
Larynx cancer	3	23111	825						5214	
Tracheal, bronchus, and lung cancer	3	23615	825	413					5225	
Malignant skin melanoma	3	22825	825						5119	
Non-melanoma skin cancer	3	22825	825							
Non-melanoma skin cancer (squamous-cell carcinoma)	4	22263	825							
Soft tissue and other extraosseous sarcomas	3	20470	448					2	4601	
Malignant neoplasm of bone and articular cartilage	3	22255	825						5159	
Breast cancer	3	23615	825	434	1				5244	
Cervical cancer	3	23115	825						4955	
Uterine cancer	3	23112	825						4956	
Ovarian cancer	3	22825	825						4977	
Prostate cancer	3	23112	825						4923	
Testicular cancer	3	22111	825						4838	
Kidney cancer	3	22825	825						5052	
Bladder cancer	3	22825	825						5025	
Brain and central nervous system cancer	3	22826	825						5189	
Eye cancer	3	20570	448						4171	
Retinoblastoma	4	20412	448						4011	
Other eye cancers	4	20470	448						4046	
Neuroblastoma and other peripheral nervous cell tumors	3	20470	448					2	4362	
Thyroid cancer	3	22824	825						5219	
Mesothelioma	3	14144	448						2485	
Hodgkin lymphoma	3	22824	825						5149	
Non-Hodgkin lymphoma	3	22826	825						5188	
Burkitt lymphoma	4	20470	448						5168	
Other non-Hodgkin lymphoma	4	20470	448						5187	
Multiple myeloma	3	22207	825						5145	
Leukemia	3	23614	825		650			4	5197	
Acute lymphoid leukemia	4	20154	448						3081	
Chronic lymphoid leukemia	4	19877	434						2968	
Acute myeloid leukemia	4	20154	448						3059	
Chronic myeloid leukemia	4	19977	448						2971	
Other leukemia	4	20048	448						2519	
Other malignant neoplasms	3	22828	825		72			3	5235	
Other neoplasms	3	20607	448							
Myelodysplastic, myeloproliferative, and other hematopoietic neoplasms	4	20468	448							

Appendix Table S5: Total number of site years by cause and source type for GBD2020

Cause	Level	Vital Registration	Vital Registration - Sample	Verbal Autopsy	Surveillance	Survey/Census	Sibling History	MITS diagnosed	Cancer Registry	Police Records
Other benign and in situ neoplasms	4	19304	448							
Cardiovascular diseases	2	24191	825	1588	2			4		
Rheumatic heart disease	3	22831	825	187						
Ischemic heart disease	3	23129	825	1474						
Stroke	3	23629	825	1322	1					
Ischemic stroke	4	21353	825							
Intracerebral hemorrhage	4	21346	825							
Subarachnoid hemorrhage	4	21346	825							
Hypertensive heart disease	3	22224	825							
Non-rheumatic valvular heart disease	3	21498	825							
Non-rheumatic calcific aortic valve disease	4	21333	448							
Non-rheumatic degenerative mitral valve disease	4	21333	448							
Other non-rheumatic valve diseases	4	21158	448							
Cardiomyopathy and myocarditis	3	22225	825					4		
Myocarditis	4	21498	448					4		
Alcoholic cardiomyopathy	4	21407	448							
Other cardiomyopathy	4	21498	448					3		
Pulmonary Arterial Hypertension	3	20570	448							
Atrial fibrillation and flutter	3	20570	448							
Aortic aneurysm	3	21454	448							
Lower extremity peripheral arterial disease	3	20566	448							
Endocarditis	3	21447	825					3		
Other cardiovascular and circulatory diseases	3	22226	825					1		
Chronic respiratory diseases	2	23630	825	1532	549			5		
Chronic obstructive pulmonary disease	3	21453	825							
Pneumoconiosis	3	21231	448							
Silicosis	4	21075	448							
Asbestosis	4	21105	448							
Coal workers pneumoconiosis	4	21077	448							
Other pneumoconiosis	4	21095	448							
Asthma	3	21446	448					5		
Interstitial lung disease and pulmonary sarcoidosis	3	21446	448					2		
Other chronic respiratory diseases	3	21453	825					2		
Digestive diseases	2	23117	825	1388	602			7		
Cirrhosis and other chronic liver diseases	3	23117	825	1251				5		
Cirrhosis and other chronic liver diseases due to hepatitis B	4	7202								
Cirrhosis and other chronic liver diseases due to hepatitis C	4	7202								
Cirrhosis and other chronic liver diseases due to alcohol use	4	7202								
Cirrhosis and other chronic liver diseases due to NAFLD	4	7202								
Cirrhosis and other chronic liver diseases due to other causes	4	7202								
Upper digestive system diseases	3	23116	825	350				1		
Peptic ulcer disease	4	21747	448					1		
Gastritis and duodenitis	4	21734	448					1		
Appendicitis	3	23113	825	186				1		
Paralytic ileus and intestinal obstruction	3	22826	825	383				7		
Inguinal, femoral, and abdominal hernia	3	22626	448	689				3		
Inflammatory bowel disease	3	21453	448					1		
Ulcerative colitis	4	20470	448					1		
Crohn's disease	4	20470	448					1		
Vascular intestinal disorders	3	21346	448					1		
Gallbladder and biliary diseases	3	22919	825					1		
Pancreatitis	3	21741	448					1		
Other digestive diseases	3	22786	825					4		
Neurological disorders	2	23112	825	953				5		
Alzheimer's disease and other dementias	3	22824	825							
Parkinson's disease	3	22071	825							
Idiopathic epilepsy	3	22921	825	953				5		
Multiple sclerosis	3	22070	774							
Motor neuron disease	3	20993	825					2		
Other neurological disorders	3	21453	825					5		
Mental disorders	2	20499	825							
Eating disorders	3	19918	392							
Anorexia nervosa	4	18866	392							
Substance use disorders	2	23065	825	313						64
Alcohol use disorders	3	23065	825	306						
Drug use disorders	3	23040	825	264						64
Opioid use disorders	4	21307	448							
Cocaine use disorders	4	21304	448							
Amphetamine use disorders	4	21304	448							
Other drug use disorders	4	21322	448							
Diabetes and kidney diseases	2	23617	825	1454				6		
Diabetes mellitus	3	23117	825	1244				4		
Diabetes mellitus type 1	4	21379	448					4		
Diabetes mellitus type 2	4	21379	448							
Chronic kidney disease	3	23617	825	1372				4		
Chronic kidney disease due to diabetes mellitus type 1	4	13268	448					3		
Chronic kidney disease due to diabetes mellitus type 2	4	13268	448							
Chronic kidney disease due to hypertension	4	20470	448							
Chronic kidney disease due to glomerulonephritis	4	20470	448					3		
Chronic kidney disease due to other and unspecified causes	4	20470	448					4		
Acute glomerulonephritis	3	21365	825							
Skin and subcutaneous diseases	2	23103	825	382				4		
Bacterial skin diseases	3	23103	825	352				4		
Cellulitis	4	20241	448					2		
Pyoderma	4	20470	448					4		
Decubitus ulcer	3	21482	825	150						
Other skin and subcutaneous diseases	3	20558	825							
Musculoskeletal disorders	2	23111	825	359						
Rheumatoid arthritis	3	21327	825							
Other musculoskeletal disorders	3	21454	825							
Other non-communicable diseases	2	23640	825	1461	683			10		
Congenital birth defects	3	23640	825	1404	683			10		
Neural tube defects	4	22301	825	111	590			10		
Congenital heart anomalies	4	22301	825	451	680			8		
Orofacial clefts	4	21189	448					2		
Down syndrome	4	21353	825		593			8		
Other chromosomal abnormalities	4	21342	448					5		
Congenital musculoskeletal and limb anomalies	4	21340	448					3		
Urogenital congenital anomalies	4	21344	448		500			2		
Digestive congenital anomalies	4	21346	448					6		
Other congenital birth defects	4	23600	825					6		
Urinary diseases and male infertility	3	23111	825	389						
Urinary tract infections and interstitial nephritis	4	22492	825	152						
Urolithiasis	4	21441	825	139						
Other urinary diseases	4	20570	825							
Gynecological diseases	3	22677	825	651						
Uterine fibroids	4	20470	448	90						
Endometriosis	4	19635	406							
Genital prolapse	4	20078	448							
Other gynecological diseases	4	20470	448							
Hemoglobinopathies and hemolytic anemias	3	23110	825	994				5		
Thalassemias	4	21741	825	137				1		
Sickle cell disorders	4	21453	825	328				5		
G6PD deficiency	4	16580	392							
Other hemoglobinopathies and hemolytic anemias	4	21453	825					1		
Endocrine, metabolic, blood, and immune disorders	3	23112	825	701				3		
Thyroid diseases	4	20477	448					1		
Other endocrine, metabolic, blood, and immune disorders	4	20477	448					3		
Sudden infant death syndrome	3	17726	28							
Injuries	1	23617	825	1708	1837	295		6		1617
Transport injuries	2	23617	825	1486	667	264		5		60
Road injuries	3	22515	825	188		12		5		60
Pedestrian road injuries	4	20570	448	188				5		55
Cyclist road injuries	4	20570	448	184				4		33
Motorcyclist road injuries	4	20570	448	186		2		5		35
Motor vehicle road injuries	4	20570	448	186		8		5		46
Other road injuries	4	20570	448	183				2		34
Other transport injuries	3	21340	448	186				5		
Unintentional injuries	2	23615	825	1596	678	12		6		223
Falls	3	23115	825	1219	654	3		5		
Drowning	3	23614	825	1386	647	3		5		223
Fire, heat, and hot substances	3	23114	825	1339		1		5		191
Poisonings	3	23114	825	457	582			6		188
Poisoning by carbon monoxide	4	22299	825	95						188
Poisoning by other means	4	20360	825					6		
Exposure to mechanical forces	3	22825	825	381	669	8		5		
Unintentional firearm injuries	4	21132	825	273		1		2		

Appendix Table S5: Total number of site years by cause and source type for GBD2020

Cause	Level	Vital Registration	Vital Registration - Sample	Verbal Autopsy	Surveillance	Survey/Census	Sibling History	MITS diagnosed	Cancer Registry	Police Records
Other exposure to mechanical forces	4	20570	825	368	669	4		5		
Adverse effects of medical treatment	3	22826	825	187				6		
Animal contact	3	22550	825	1182		1		5		
Venomous animal contact	4	20863	825	450				5		
Non-venomous animal contact	4	20569	825					5		
Foreign body	3	22263	825	369				6		
Pulmonary aspiration and foreign body in airway	4	20571	825	180				6		
Foreign body in other body part	4	20570	825					5		
Electrocution	3	20470	448	7						
Environmental heat and cold exposure	3	23090	825	286				5		
Exposure to forces of nature	3	22769	825	262		5		2		
Other unintentional injuries (internal)	3	22826	825	373		1		5		
Self-harm and interpersonal violence	2	23617	825	1567	1157	12		5		1334
Self-harm	3	22873	825	1522	12	3				
Self-harm by firearm	4	20116	448							
Self-harm by other specified means	4	20116	448							
Interpersonal violence	3	23371	825	1358		7		5		1334
Physical violence by firearm	4	20326	448	351		3		5		33
Physical violence by sharp object	4	20326	448	367						33
Physical violence by other means	4	20326	448	370				5		33
Conflict and terrorism	3	21975	825	84		3				
Police conflict and executions	3	21410	825	84	1144					

Appendix Table S6: List of International Classification of Diseases (ICD) codes mapped to the Global Burden of Diseases cause list for causes of death		
**Cause**	**ICD10**	**ICD9**
Communicable, maternal, neonatal, and nutritional diseases	A00-A00.9, A01.0-A14, A15-A28.9, A32-A39.9, A48.1-A48.2, A48.4-A48.5, A50-A58, A60-A60.9, A63-A63.8, A65-A65.0, A68-A70, A74, A74.8-A75.9, A77-A96.9, A98-A98.8, B00-B06.9, B10-B10.8, B15-B16.2, B17.0, B17.2, B19.1, B20-B27.9, B29.4, B33-B33.1, B33.3-B33.8, B34.2, B47-B48.8, B50-B53.8, B55.0, B56-B57.5, B60-B60.8, B63, B65-B67.9, B69-B72.0, B74.3-B75, B77-B77.9, B83-B83.8, B90-B91, B94.1, B95-B95.5, B97.2, B97.4-B97.6, C58-C58.0, D50.1-D50.8, D51-D52.0, D52.8-D53.9, D70.3, D89.3, E00-E02, E40-E46.9, E51-E61.9, E63-E64.0, E64.2-E64.9, F02.1, F02.4, F07.1, G00.0-G00.8, G03-G03.8, G04-G05.8, G14-G14.6, G21.3, H70-H70.9, I00, I02, I02.9, I98.0-I98.1, J00-J02.8, J03-J03.8, J04-J04.2, J05-J05.1, J06.0-J06.8, J09-J15.8, J16-J16.9, J20-J21.9, J36-J36.0, J91.0, K52.1-K52.3, K67.0-K67.8, K75.3, K76.3, K77.0, K93.0-K93.1, M03.1, M12.1, M49.0-M49.1, M73.0-M73.1, M89.6, N74.1, N96, N98-N98.9, O00-O07.9, O09-O16.9, O20-O26.9, O28-O36.9, O40-O48.1, O60-O77.9, O80-O92.7, O96-O98.6, O98.8-P04.2, P04.5-P05.9, P07-P15.9, P19-P22.9, P23.0-P23.4, P24-P29.9, P35-P37.2, P37.5-P39.9, P50-P61.9, P70-P70.1, P70.3-P72.9, P74-P78.9, P80-P81.9, P83-P84, P90-P92.9, P94-P94.9, P96, P96.3-P96.4, P96.8, R19.7, U04-U04.9, U06-U07.2, U82-U89, Z16-Z16.3	001-001.9, 002.0-029, 032-034.9, 036-037.9, 040, 040.1-041.0, 042-066.9, 070.0-070.2, 071-075.9, 078.3-078.7, 079-079.7, 080-084.9, 085.0, 086-088, 088.8-088.9, 090-101.6, 104-104.9, 120-124.9, 125.4-125.9, 127-127.1, 128-129.0, 136-136.2, 137-139.0, 181-181.9, 244.2, 260-263.9, 265-269.9, 281.0-281.9, 320.0-320.8, 321-323.9, 381-383.9, 390-390.9, 392, 392.9, 425.6, 460-464.4, 464.8-464.9, 465.0-465.8, 466-469, 470.0, 475-475.9, 476.9, 480-482.8, 483.0-483.9, 484.0-484.7, 487-489, 558.2-558.9, 630-636.9, 638-638.9, 640-679.1, 716.0, 730.4-730.6, 760-760.6, 760.8-768, 768.2-770, 770.1-775.0, 775.4-779.3, 779.6-779.8, V09-V09.9
HIV/AIDS and sexually transmitted infections	A50-A58, A60-A60.9, A63-A63.8, B20-B24.9, B63, F02.4, I98.0, K67.0-K67.2, M03.1, M73.0-M73.1	042-044.9, 054.1, 090-099.9
HIV/AIDS	B20-B24.9, F02.4	042-044.9
HIV/AIDS - Drug-susceptible Tuberculosis	B20.0	
HIV/AIDS - Multidrug-resistant Tuberculosis without extensive drug resistance		
HIV/AIDS - Extensively drug-resistant Tuberculosis		
HIV/AIDS resulting in other diseases	B20, B20.1-B24.9, F02.4	042-044.9
Sexually transmitted infections excluding HIV	A50-A58, A60-A60.9, A63-A63.8, B63, I98.0, K67.0-K67.2, M03.1, M73.0-M73.1	054.1, 090-099.9
Syphilis	A50-A53.9, I98.0, K67.2, M03.1, M73.1	090-097.9
Chlamydial infection	A55-A56.8, K67.0	
Gonococcal infection	A54-A54.9, K67.1, M73.0	098-098.9
Other sexually transmitted infections	A57-A58, A63-A63.8, B63	099-099.9
Respiratory infections and tuberculosis	A10-A14, A15-A19.9, A48.1, A70, B34.2, B90-B90.9, B97.2, B97.4-B97.6, H70-H70.9, J00-J02.8, J03-J03.8, J04-J04.2, J05-J05.1, J06.0-J06.8, J09-J15.8, J16-J16.9, J20-J21.9, J36-J36.0, J91.0, K67.3, K93.0, M49.0, N74.1, P23.0-P23.4, P37.0, U04-U04.9, U07-U07.2, U84.3	010-019.9, 034.0, 079.6, 137-137.9, 138.0-138.9, 320.4, 381-383.9, 460-464.4, 464.8-464.9, 465.0-465.8, 466-469, 470.0, 475-475.9, 476.9, 480-482.8, 483.0-483.9, 484.1-484.2, 484.6-484.7, 487-489, 730.4-730.6
Tuberculosis	A10-A14, A15-A19.9, B90-B90.9, K67.3, K93.0, M49.0, N74.1, P37.0, U84.3	010-019.9, 137-137.9, 138.0-138.9, 320.4, 730.4-730.6
Drug-susceptible tuberculosis	A10-A14, A15-A19.9, B90-B90.9, K67.3, K93.0, M49.0, N74.1, P37.0	010-019.9, 137-137.9, 138.0-138.9, 320.4, 730.4-730.6
Multidrug-resistant tuberculosis without extensive drug resistance	U84.3	
Extensively drug-resistant tuberculosis		
Lower respiratory infections	A48.1, A70, B34.2, B97.2, B97.4-B97.6, J09-J15.8, J16-J16.9, J20-J21.9, J91.0, P23.0-P23.4, U04-U04.9	079.6, 466-469, 470.0, 480-482.8, 483.0-483.9, 484.1-484.2, 484.6-484.7, 487-489
Influenza	J09-J11.8, U04-U04.9	487-489
Pneumococcal pneumonia	J13-J13.9, J15.3-J15.4, J15.6	481-481.9
H influenzae type B pneumonia	J14-J14.0	482.2
Respiratory syncytial virus pneumonia	B97.4-B97.6, J12.1	079.6, 480.1
Other lower respiratory infections	A48.1, A70, B34.2, B97.2, J12-J12.0, J12.2-J12.9, J15-J15.2, J15.5, J15.7-J15.8, J16-J16.9, J20-J21.9, J91.0, P23.0-P23.4	466-469, 470.0, 480-480.0, 480.2-480.9, 482-482.1, 482.3-482.8, 483.0-483.9, 484.1-484.2, 484.6-484.7
Upper respiratory infections	J00-J02.8, J03-J03.8, J04-J04.2, J05-J05.1, J06.0-J06.8, J36-J36.0	034.0, 460-464.4, 464.8-464.9, 465.0-465.8, 475-475.9, 476.9
Otitis media	H70-H70.9	381-383.9
COVID-19	U07-U07.2	
Enteric infections	A00-A00.9, A01.0-A09.9, A80-A80.9, K52.1-K52.3, R19.7	001-001.9, 002.0-009.9, 045-045.9, 138, 558.2-558.9
Diarrheal diseases	A00-A00.9, A02-A02.0, A02.8-A07, A07.2-A07.4, A08-A09.9, K52.1-K52.3, R19.7	001-001.9, 003.8-006.9, 007.4-007.8, 008.2-009.9, 558.2-558.9
Leprosy	A30-A30.9	030-030.9
Typhoid and paratyphoid	A01.0-A01.4	002.0-002.9
Typhoid fever	A01.0	002.0
Paratyphoid fever	A01.1-A01.4	002.1-002.9
Invasive Non-typhoidal Salmonella (iNTS)	A02.1-A02.2	003-003.7
Other intestinal infectious diseases	A07.0-A07.1, A07.8-A07.9, A80-A80.9	007-007.3, 007.9-008.1, 045-045.9, 138
Neglected tropical diseases and malaria	A68-A68.9, A69.2-A69.9, A75-A75.9, A77-A79.9, A82-A82.9, A90-A96.9, A98-A98.8, B33.0-B33.1, B50-B53.8, B55.0, B56-B57.5, B60-B60.8, B65-B67.9, B69-B72.0, B74.3-B75, B77-B77.9, B83-B83.8, K93.1, P37.1, U06-U06.9	060-061.8, 065-066.9, 071-071.9, 080-084.9, 085.0, 086-088, 088.8-088.9, 120-124.9, 125.4-125.9, 127-127.1, 128-129.0, 425.6
Malaria	B50-B53.8	084-084.9
Chagas disease	B57-B57.5, K93.1	086-086.2, 086.9, 425.6
Leishmaniasis	B55.0	085.0
Visceral leishmaniasis	B55.0	085.0
African trypanosomiasis	B56-B56.9	086.3-086.5
Schistosomiasis	B65-B65.9	120-120.9
Cysticercosis	B69-B69.9	123.1
Cystic echinococcosis	B67-B67.4, B67.8-B67.9	122-122.4, 122.8-122.9
Dengue	A90-A91.9	061-061.8
Yellow fever	A95-A95.9	060-060.9
Rabies	A82-A82.9	071-071.9
Intestinal nematode infections	B77-B77.9	127.0
Ascariasis	B77-B77.9	127.0

Appendix Table S6: List of International Classification of Diseases (ICD) codes mapped to the Global Burden of Diseases cause list for causes of death		
**Cause**	**ICD10**	**ICD9**
Ebola	A98.4	
Zika virus	U06-U06.9	
Other neglected tropical diseases	A68-A68.9, A69.2-A69.9, A75-A75.9, A77-A79.9, A92-A94.0, A96-A96.9, A98-A98.3, A98.5-A98.8, B33.0-B33.1, B60-B60.8, B67.5-B67.7, B70-B71.9, B74.3-B75, B83-B83.8, P37.1	065-066.9, 080-083.9, 087-088, 088.8-088.9, 122.5-122.7, 123-123.0, 123.2-124.9, 125.4-125.6, 125.9, 127, 127.1, 128-129.0
Other infectious diseases	A20-A28.9, A32-A39.9, A48.2, A48.4-A48.5, A65-A65.0, A69-A69.1, A74, A74.8-A74.9, A81-A81.9, A83-A89.9, B00-B06.9, B10-B10.8, B15-B16.2, B17.0, B17.2, B19.1, B25-B27.9, B29.4, B33, B33.3-B33.8, B47-B48.8, B91, B94.1, B95-B95.5, D70.3, D89.3, F02.1, F07.1, G00.0-G00.8, G03-G03.8, G04-G05.8, G14-G14.6, G21.3, I00, I02, I02.9, I98.1, K67.8, K75.3, K76.3, K77.0, M49.1, M89.6, P35-P35.9, P37, P37.2, P37.5-P37.9, U82-U84, U85-U89, Z16-Z16.3	020-029, 032-034, 034.1-034.9, 036-037.9, 040, 040.1-041.0, 046-054.0, 054.2-059.9, 062-064.9, 070.0-070.2, 072-075.9, 078.3-078.7, 079-079.5, 079.7, 100-101.6, 104-104.9, 136-136.2, 139-139.0, 320.0-320.3, 320.5-320.8, 321-323.9, 390-390.9, 392, 392.9, 484.0, 484.3-484.5, 771.0-771.3, V09-V09.9
Meningitis	A39-A39.9, A87-A87.9, G00.0-G00.8, G03-G03.8	036-036.9, 047-049.9, 320.0-320.3, 320.5-320.8, 321-322.9
Encephalitis	A83-A86.4, B94.1, F07.1, G04-G05.8, G21.3	062-064.9, 139.0, 323, 323.4-323.9
Diphtheria	A36-A36.9	032-032.9
Pertussis	A37-A37.9	033-033.9, 484.3
Tetanus	A33-A35.0	037-037.9, 771.3
Measles	B05-B05.9	055-055.9, 484.0
Varicella and herpes zoster	B01-B02.9, P35.8	052-053.9
Acute hepatitis	B15-B16.2, B17.0, B17.2, B19.1, P35.3	070.0-070.2
Acute hepatitis A	B15-B15.9	070.0-070.1
Acute hepatitis B	B16-B16.2, B17.0, B19.1, P35.3	070.2
Acute hepatitis C		
Acute hepatitis E	B17.2	
Other unspecified infectious diseases	A20-A28.9, A32-A32.9, A38-A38.9, A48.2, A48.4-A48.5, A65-A65.0, A69-A69.1, A74, A74.8-A74.9, A81-A81.9, A88-A89.9, B00-B00.9, B03-B04, B06-B06.9, B10-B10.8, B25-B27.9, B29.4, B33, B33.3-B33.8, B47-B48.8, B91, B95-B95.5, D70.3, D89.3, F02.1, G14-G14.6, I00, I02, I02.9, I98.1, K67.8, K75.3, K76.3, K77.0, M49.1, M89.6, P35-P35.2, P35.9, P37, P37.2, P37.5-P37.9, U82-U84, U85-U89, Z16-Z16.3	020-029, 034, 034.1-034.9, 040, 040.1-041.0, 046-046.9, 050-051.9, 054-054.0, 054.2-054.9, 056-059.9, 072-075.9, 078.3-078.7, 079-079.5, 079.7, 100-101.6, 104-104.9, 136-136.2, 139, 323.0-323.3, 390-390.9, 392, 392.9, 484.4-484.5, 771.0-771.2, V09-V09.9
Maternal and neonatal disorders	C58-C58.0, N96, N98-N98.9, O00-O07.9, O09-O16.9, O20-O26.9, O28-O36.9, O40-O48.1, O60-O77.9, O80-O92.7, O96-O98.6, O98.8-P04.2, P04.5-P05.9, P07-P15.9, P19-P22.9, P24-P29.9, P36-P36.9, P38-P39.9, P50-P61.9, P70-P70.1, P70.3-P72.9, P74-P78.9, P80-P81.9, P83-P84, P90-P92.9, P94-P94.9, P96, P96.3-P96.4, P96.8	181-181.9, 630-636.9, 638-638.9, 640-679.1, 760-760.6, 760.8-768, 768.2-770, 770.1-771, 771.4-775.0, 775.4-779.3, 779.6-779.8
Maternal disorders	C58-C58.0, N96, N98-N98.9, O00-O07.9, O09-O16.9, O20-O26.9, O28-O36.9, O40-O48.1, O60-O77.9, O80-O92.7, O96-O98.6, O98.8-O99.9	181-181.9, 630-636.9, 638-638.9, 640-679.1
Maternal hemorrhage	O20-O20.9, O43.2, O44-O46.9, O62-O62.9, O67-O67.9, O70, O72-O72.3	640-641.9, 661-661.9, 665, 666-666.9
Maternal sepsis and other maternal infections	O23-O23.9, O85-O86.8, O91-O91.2	659.3, 670-670.9
Maternal hypertensive disorders	O10-O16.9	642-642.9
Maternal obstructed labor and uterine rupture	O32-O33.9, O64-O66.9, O71-O71.9	652-653.9, 660-660.9, 665.0-665.3
Maternal abortion and miscarriage	N96, O01-O07.9	630-632.9, 634-636.9, 638-638.9, 646.3
Ectopic pregnancy	O00-O00.9	633-633.9
Indirect maternal deaths	O24-O25.3, O98-O98.6, O98.8-O99.9	646-646.2, 646.4-649.9
Late maternal deaths	O96-O97.9	
Maternal deaths aggravated by HIV/AIDS		
Other direct maternal disorders	C58-C58.0, N98-N98.9, O09-O09.9, O21-O22.9, O26-O26.9, O28-O31.8, O34-O36.9, O40-O43.1, O43.8-O43.9, O47-O48.1, O60-O61.9, O63-O63.9, O68-O69.9, O70.0-O70.9, O73-O77.9, O80-O84.9, O87-O90.9, O92-O92.7	181-181.9, 643-645.2, 650-651.9, 654-659.2, 659.4-659.9, 662-664.9, 665.4-665.9, 667-669.9, 671-679.1
Neonatal disorders	P00-P04.2, P04.5-P05.9, P07-P15.9, P19-P22.9, P24-P29.9, P36-P36.9, P38-P39.9, P50-P61.9, P70-P70.1, P70.3-P72.9, P74-P78.9, P80-P81.9, P83-P84, P90-P92.9, P94-P94.9, P96, P96.3-P96.4, P96.8	760-760.6, 760.8-768, 768.2-770, 770.1-771, 771.4-775.0, 775.4-779.3, 779.6-779.8
Neonatal preterm birth	P01.0-P01.1, P05-P05.9, P07-P07.3, P22-P22.9, P25-P28.9, P52-P52.9, P61.2, P77-P77.9, P78.0-P78.9	761.0-761.1, 764-765.9, 769-769.9, 770.2-770.9, 772.1-772.9, 776.6, 777.0-777.9
Neonatal encephalopathy due to birth asphyxia and trauma	P01.7, P02-P03.9, P10-P15.9, P20-P21.9, P24-P24.9, P90-P91.9	761.7-763.9, 767-768, 768.2-768.9, 770.1, 779.0-779.2
Neonatal sepsis and other neonatal infections	P36-P36.9, P38-P39.9	771.4-771.9
Hemolytic disease and other neonatal jaundice	P55-P59.9	773-774.9
Other neonatal disorders	P00-P01, P01.2-P01.6, P01.8-P01.9, P04-P04.2, P04.5-P04.9, P08-P09, P19-P19.9, P29-P29.9, P50-P51.9, P53-P54.9, P60-P61.1, P61.3-P61.9, P70-P70.1, P70.3-P72.9, P74-P76.9, P78, P80-P81.9, P83-P84, P92-P92.9, P94-P94.9, P96, P96.3-P96.4, P96.8	760-760.6, 760.8-761, 761.2-761.6, 766-766.9, 770, 771, 772-772.0, 775-775.0, 775.4-776.5, 776.7-777, 778-779, 779.3, 779.6-779.8
Nutritional deficiencies	D50.1-D50.8, D51-D52.0, D52.8-D53.9, E00-E02, E40-E46.9, E51-E61.9, E63-E64.0, E64.2-E64.9, M12.1	244.2, 260-263.9, 265-269.9, 281.0-281.9, 716.0
Protein-energy malnutrition	E40-E46.9, E64.0	260-263.9
Other nutritional deficiencies	D51-D52.0, D52.8-D53.9, E00-E02, E51-E61.9, E63-E64, E64.2-E64.9, M12.1	244.2, 265-269.9, 281.0-281.9, 716.0

Appendix Table S6: List of International Classification of Diseases (ICD) codes mapped to the Global Burden of Diseases cause list for causes of death

Cause	ICD10	ICD9
Non-communicable diseases	A46-A46.0, A66-A67.9, B18-B18.9, B33.2, B86, C00-C13.9, C15-C22.8, C23-C25.9, C30-C34.9, C37-C38.8, C40-C41.9, C43-C45.9, C47-C54.9, C56-C57.8, C60-C63.8, C64-C67.9, C68.0-C68.8, C69.0-C69.8, C70-C73.9, C75-C75.8, C81-C82.9, C83.0-C83.8, C84-C85.0, C85.2-C85.8, C86-C86.6, C88-C91.0, C91.2-C91.3, C91.6, C92-C92.6, C93-C93.1, C93.3, C93.8, C94-C94.5, C94.7-C96.9, D00.1-D00.2, D01.0-D01.3, D02.0-D02.3, D03-D06.9, D07.0-D07.2, D07.4-D07.5, D09.0, D09.2-D09.3, D09.8, D10.0-D10.7, D11-D12.9, D13.0-D13.7, D14.0-D14.3, D15-D16.9, D22-D27.9, D28.0-D28.7, D29.0-D29.8, D30.0-D30.8, D31-D36, D36.1-D36.7, D37.1-D37.5, D38.0-D38.5, D39.1-D39.2, D39.8, D40.0-D40.8, D41.0-D41.8, D42-D43.9, D44.0-D44.8, D45-D47.9, D48.0-D48.6, D49.2-D49.4, D49.6, D55-D58.9, D59.1, D59.3, D59.5, D60-D61.9, D63.1, D64.0, D66-D67, D68.0-D69.4, D69.6-D69.8, D70-D70.0, D70.4-D75.8, D76-D77, D86-D86.9, D89-D89.2, E03-E03.1, E03.3-E06.3, E06.5-E07.1, E10-E11.9, E16.1-E16.9, E20-E23.0, E23.2-E24.1, E24.3-E27.2, E27.4-E34, E34.1-E34.8, E65-E66.0, E66.2-E68, E70-E85.2, E88-E88.2, E88.4-E88.9, F00-F02.0, F02.2-F02.3, F02.8-F03.9, F10-F16.9, F18-F18.9, F24, F50.0-F50.5, G10-G13.8, G20-G20.9, G23-G24, G24.1-G25.0, G25.2-G25.3, G25.5, G25.8-G26.0, G30-G31.9, G35-G37.9, G40-G41.9, G45-G46.8, G47.3, G61-G61.9, G62.1, G70-G72, G72.1-G73.7, G90-G90.9, G95-G95.9, H05.0-H05.1, I01-I01.9, I02.0, I05-I09.9, I11-I13.9, I20-I25.9, I27.0-I27.2, I28-I28.9, I30-I31.1, I31.8-I37.8, I38-I41.9, I42.1-I42.8, I43-I43.9, I47-I48.9, I51.0-I51.4, I60-I63.9, I65-I66.9, I67.0-I67.3, I67.5-I67.7, I68.0-I68.2, I69.0-I69.3, I70.2-I70.8, I71-I73.9, I77-I89.9, I98, I98.2, J30-J35.9, J37-J39.9, J41-J46.9, J60-J63.8, J66-J68.9, J70, J70.8-J70.9, J82, J84-J84.9, J91, J91.8-J92.9, K20-K20.9, K22-K22.6, K22.8-K29.9, K31-K31.8, K35-K38.9, K40-K42.9, K44-K46.9, K50-K52, K52.8-K52.9, K55-K62.6, K62.8-K62.9, K63.5, K64-K64.9, K66.8, K67, K68, K70-K70.3, K71.7, K73-K75, K75.1-K75.2, K75.4-K76.2, K76.4-K77, K77.8, K80-K83.9, K85-K86.9, K90-K90.9, K92.8, K93.8, L00-L05.9, L08-L08.9, L10-L14.0, L51-L51.9, L88-L89.9, L93-L93.2, L97-L98.4, M00-M03.0, M03.2-M03.6, M05-M09.8, M30-M36.8, M40-M43.1, M65-M65.0, M71.0-M71.1, M72.5-M72.6, M80-M82.8, M86.3-M86.4, M87-M87.0, M88-M89.0, M89.5, M89.7-M89.9, N00-N08.8, N10-N12.9, N13.6, N15-N16.8, N18-N18.9, N20-N23.0, N25-N28.1, N29-N30.3, N30.8-N32.0, N32.3-N32.4, N34-N34.3, N36-N36.9, N39-N39.2, N41-N41.9, N44-N44.0, N45-N45.9, N49-N49.9, N60-N60.9, N72-N72.0, N75-N77.8, N80-N81.9, N83-N83.9, N84.0-N84.1, N87-N87.9, P04.3-P04.4, P70.2, P96.0-P96.1, Q00-Q07.9, Q10.4-Q18.9, Q20-Q28.9, Q30-Q36, Q37-Q45.9, Q50-Q87.8, Q89-Q89.8, Q90-Q93.9, Q95-Q99.8, R78.0-R78.5, R95-R95.9, X45-X45.9, X65-X65.9, Y15-Y15.9	035-035.9, 102-103.9, 133-133.6, 135-135.9, 140-148.9, 150-155.1, 155.3-158.9, 160-164.9, 170-175.9, 180-180.9, 182-183.8, 184.0-184.4, 184.8, 185-186.9, 187.1-187.8, 188-188.9, 189.0-189.8, 190-190.8, 191-193.9, 194.1-194.8, 200-202.8, 203-204.0, 204.2, 205-205.3, 206-206.1, 207-208.9, 209.0-209.1, 209.4-209.5, 210.0-210.9, 211.0-211.8, 212.0-212.8, 213-213.9, 217-220.9, 221.0-221.8, 222.0-222.8, 223.0-223.8, 224-228.9, 229.0, 229.8, 230.1-230.8, 231.0-231.2, 232-232.9, 233.0-233.2, 233.4-233.5, 233.7, 234.0-234.8, 235.0, 235.4, 235.6-235.8, 236.0-236.2, 236.4-236.5, 236.7, 237-237.3, 237.5-237.9, 238.0-238.9, 239.2-239.4, 239.6, 240-243.9, 245-246.9, 251-251.2, 251.4-253.6, 253.8-259.1, 259.3-259.9, 270-273.9, 275-276, 277-277.2, 277.4-277.9, 278.0-278.8, 282-284.9, 286-286.5, 286.7-289.0, 289.4-289.7, 290-292.9, 294.1-294.9, 303-303.9, 304.0-304.8, 305.0, 305.2-305.8, 307.1, 327.2-327.8, 330-331.2, 331.5-332.0, 333-337.9, 340-341.9, 345-345.9, 349, 349.2-349.8, 353.8-353.9, 356-356.9, 357.0-357.1, 357.3-357.5, 357.7, 358-359.9, 376.0-376.1, 391-391.9, 392.0, 393-398.9, 402-404.9, 410-414.9, 416.0-416.1, 417-417.9, 420-423, 423.1-423.9, 424.0-424.3, 424.8, 425.0-425.5, 425.7-425.8, 427.0-427.3, 427.6-427.8, 429.0, 430-435.9, 437.0-437.2, 437.4-437.8, 440.2, 440.4, 441-443.9, 446-457, 457.1-457.9, 459, 459.1-459.3, 470, 470.9-474.9, 476-476.1, 477-479, 491-493.9, 495-504.9, 506-506.9, 508-509, 515, 516-517.8, 518.6, 518.9, 519.1-519.4, 530-530.0, 530.2-530.6, 531-536.1, 537-537.6, 537.8, 538, 540-543.9, 550-551.1, 551.3-552.1, 552.3-553.1, 553.3-558.0, 560-560.3, 560.8-560.9, 562-562.1, 564-564.1, 564.5-564.7, 565-566.9, 569.0-569.5, 569.7, 571-571.9, 572.2-573.0, 573.4-577.9, 579-579.2, 579.4-583.9, 585-585.9, 588-590.9, 592-593.8, 594-598.1, 598.8-599.6, 599.8, 601-602.9, 604-604.9, 608.2, 610-610.9, 617-618.9, 620-620.9, 621.4-621.9, 622.1-622.7, 629-629.8, 680-689, 694-695.5, 707-707.9, 710-711.9, 714-714.3, 714.8-714.9, 730.1, 732-732.9, 733.0-733.1, 740-749.0, 749.2-758.9, 759.0-759.8, 760.7, 775.1-775.3, 788.0, 790.3, 798-798.0, E850, E860
Neoplasms	C00-C13.9, C15-C22.8, C23-C25.9, C30-C34.9, C37-C38.8, C40-C41.9, C43-C45.9, C47-C54.9, C56-C57.8, C60-C63.8, C64-C67.9, C68.0-C68.8, C69.0-C69.8, C70-C73.9, C75-C75.8, C81-C82.9, C83.0-C83.8, C84-C85.0, C85.2-C85.8, C86-C86.6, C88-C91.0, C91.2-C91.3, C91.6, C92-C92.6, C93-C93.1, C93.3, C93.8, C94-C94.5, C94.7-C96.9, D00.1-D00.2, D01.0-D01.3, D02.0-D02.3, D03-D06.9, D07.0-D07.2, D07.4-D07.5, D09.0, D09.2-D09.3, D09.8, D10.0-D10.7, D11-D12.9, D13.0-D13.7, D14.0-D14.3, D15-D16.9, D22-D24.9, D26.0-D27.9, D28.0-D28.1, D28.7, D29.0-D29.8, D30.0-D30.8, D31-D36, D36.1-D36.7, D37.1-D37.5, D38.0-D38.5, D39.1-D39.2, D39.8, D40.0-D40.8, D41.0-D41.8, D42-D43.9, D44.0-D44.8, D45-D47.9, D48.0-D48.6, D49.2-D49.4, D49.6, K62.0-K62.1, K63.5, N60-N60.9, N84.0-N84.1, N87-N87.9	140-148.9, 150-155.1, 155.3-158.9, 160-164.9, 170-175.9, 180-180.9, 182-183.8, 184.0-184.4, 184.8, 185-186.9, 187.1-187.8, 188-188.9, 189.0-189.8, 190-190.8, 191-193.9, 194.1-194.8, 200-202.8, 203-204.0, 204.2, 205-205.3, 206-206.1, 207-208.9, 209.0-209.1, 209.4-209.5, 210.0-210.9, 211.0-211.8, 212.0-212.8, 213-213.9, 217-217.8, 219.0, 220-220.9, 221.0-221.8, 222.0-222.8, 223.0-223.8, 224-228.9, 229.0, 229.8, 230.1-230.8, 231.0-231.2, 232-232.9, 233.0-233.2, 233.4-233.5, 233.7, 234.0-234.8, 235.0, 235.4, 235.6-235.8, 236.1-236.2, 236.4-236.5, 236.7, 237-237.3, 237.5-237.9, 238.0-238.9, 239.2-239.4, 239.6, 569.0, 610-610.9, 622.1-622.2, 622.7
Lip and oral cavity cancer	C00-C08.9, D10.0-D10.5, D11-D11.9	140-145.9, 210.0-210.6, 235.0
Nasopharynx cancer	C11-C11.9, D10.6	147-147.9, 210.7-210.9
Other pharynx cancer	C09-C10.9, C12-C13.9, D10.7	146-146.9, 148-148.9
Esophageal cancer	C15-C15.9, D00.1, D13.0	150-150.9, 211.0, 230.1
Stomach cancer	C16-C16.9, D00.2, D13.1, D37.1	151-151.9, 211.1, 230.2
Colon and rectum cancer	C18-C21.9, D01.0-D01.3, D12-D12.9, D37.3-D37.5	153-154.9, 209.1, 209.5, 211.3-211.4, 230.3-230.6, 569.0
Liver cancer	C22-C22.8, D13.4	155-155.1, 155.3-155.9, 211.5
Liver cancer due to hepatitis B		
Liver cancer due to hepatitis C		
Liver cancer due to alcohol use		
Liver cancer due to NASH		
Hepatoblastoma	C22.2	
Liver cancer due to other causes		
Gallbladder and biliary tract cancer	C23-C24.9, D13.5	156-156.9
Pancreatic cancer	C25-C25.9, D13.6-D13.7	157-157.9, 211.6-211.7
Larynx cancer	C32-C32.9, D02.0, D14.1, D38.0	161-161.9, 212.1, 231.0, 235.6
Tracheal, bronchus, and lung cancer	C33-C34.9, D02.1-D02.3, D14.2-D14.3, D38.1	162-162.9, 212.2-212.3, 231.1-231.2, 235.7
Malignant skin melanoma	C43-C43.9, D03-D03.9, D22-D23.9, D48.5	172-172.9
Non-melanoma skin cancer	C44-C44.9, D04-D04.9, D49.2	173-173.9, 222.4, 232-232.9, 238.2
Non-melanoma skin cancer (squamous-cell carcinoma)	C44-C44.9, D04-D04.9, D49.2	173-173.9, 222.4, 232-232.9, 238.2
Soft tissue and other extraosseous sarcomas	C49-C49.9	171-171.9
Malignant neoplasm of bone and articular cartilage	C40-C41.9	170-170.9
Breast cancer	C50-C50.9, D05-D05.9, D24-D24.9, D48.6, D49.3	174-175.9, 217-217.8, 233.0, 238.3, 239.3, 610-610.9
Cervical cancer	C53-C53.9, D06-D06.9, D26.0	180-180.9, 219.0, 233.1, 622.1-622.2, 622.7
Uterine cancer	C54-C54.9, D07.0-D07.2, D26.1-D26.9	182-182.9, 233.2
Ovarian cancer	C56-C56.9, D27-D27.9, D39.1	183-183.0, 220-220.9, 236.2
Prostate cancer	C61-C61.9, D07.5, D29.1, D40.0	185-185.9, 222.2, 236.5
Testicular cancer	C62-C62.9, D29.2-D29.8, D40.1-D40.8	186-186.9, 222.0, 222.3, 236.4
Kidney cancer	C64-C65.9, D30.0-D30.1, D41.0-D41.1	189.0-189.1, 189.5-189.6, 223.0-223.1
Bladder cancer	C67-C67.9, D09.0, D30.3, D41.4-D41.8, D49.4	188-188.9, 223.3, 233.7, 236.7, 239.4
Brain and central nervous system cancer	C70-C72.9, C75.1-C75.3	191-192.9, 194.3-194.4
Eye cancer	C69.0-C69.8	190-190.8

Appendix Table S6: List of International Classification of Diseases (ICD) codes mapped to the Global Burden of Diseases cause list for causes of death

Cause	ICD10	ICD9
Retinoblastoma	C69.2	190.5
Other eye cancers	C69.0-C69.1, C69.3-C69.8	190-190.4, 190.6-190.8
Neuroblastoma and other peripheral nervous cell tumors	C47-C47.9	
Thyroid cancer	C73-C73.9, D09.3, D09.8, D34-D34.9, D44.0	193-193.9, 226-226.9
Mesothelioma	C45-C45.9	
Hodgkin lymphoma	C81-C81.9	201-201.9
Non-Hodgkin lymphoma	C82-C82.9, C83.0-C83.8, C84-C85.0, C85.2-C85.8, C86-C86.6, C96-C96.9	200-200.9, 202-202.8
Burkitt lymphoma	C83.7	200.2
Other non-Hodgkin lymphoma	C82-C82.9, C83.0-C83.6, C83.8, C84-C85.0, C85.2-C85.8, C86-C86.6, C96-C96.9	200-200.1, 200.3-200.9, 202-202.8
Multiple myeloma	C88-C90.9	203-203.9
Leukemia	C91-C91.0, C91.2-C91.3, C91.6, C92-C92.6, C93-C93.1, C93.3, C93.8, C94-C94.5, C94.7-C95.9	204-204.0, 204.2, 205-205.3, 206-206.1, 207-208.9
Acute lymphoid leukemia	C91.0, C91.2-C91.3, C91.6	204.0, 204.2
Chronic lymphoid leukemia		
Acute myeloid leukemia	C92.0, C92.3-C92.6, C93.0, C94.0, C94.2, C94.4-C94.5	205.0, 205.2-205.3, 206.0, 207.0, 207.2-207.8
Chronic myeloid leukemia	C92.1-C92.2	205.1
Other leukemia	C93.1, C93.3, C93.8, C94.1, C94.3, C94.7-C94.8	206.1, 207.1, 207.9
Other malignant neoplasms	C17-C17.9, C30-C31.9, C37-C38.8, C48-C48.9, C4A, C51-C52.9, C57-C57.8, C60-C60.9, C63-C63.8, C66-C66.9, C68.0-C68.8, C75-C75.0, C75.4-C75.8, D07.4, D09.2, D13.2-D13.3, D14.0, D15-D16.9, D28.0-D28.1, D28.7, D29.0, D30.2, D30.4-D30.8, D31-D31.9, D35-D35.2, D35.5-D36, D36.1-D36.7, D37.2, D38.2-D38.5, D39.2, D39.8, D41.2-D41.3, D44.1-D44.8, D48.0-D48.4	152-152.9, 158-158.9, 160-160.9, 163-164.9, 183.2-183.8, 184.0-184.4, 184.8, 187.1-187.8, 189.2-189.4, 189.8, 194.1, 194.5-194.8, 209.0, 209.4, 211.2, 211.8, 212.0, 212.4-212.8, 213-213.9, 221.0-221.8, 222.1, 222.8, 223.2, 223.8, 224-224.9, 227-228.9, 229.0, 229.8, 230.7-230.8, 233.4-233.5, 234.0-234.8, 235.4, 235.8, 236.1, 238.0-238.1, 239.2
Other neoplasms	D32-D33.9, D35.3-D35.4, D42-D43.9, D45-D47.9, D49.6, K62.0-K62.1, K63.5, N60-N60.9, N84.0-N84.1, N87-N87.9	225-225.9, 237-237.3, 237.5-237.9, 238.4-238.9, 239.6
Myelodysplastic, myeloproliferative, and other hematopoietic neoplasms	D45-D47.9	238.4-238.9
Other benign and in situ neoplasms	N60-N60.9	
Cardiovascular diseases	B33.2, G45-G46.8, I01-I01.9, I02.0, I05-I09.9, I11-I11.9, I20-I25.9, I27.0, I27.2, I28-I28.9, I30-I31.1, I31.8-I37.8, I38-I41.9, I42.1-I42.8, I43-I43.9, I47-I48.9, I51.0-I51.4, I60-I63.9, I65-I66.9, I67.0-I67.3, I67.5-I67.6, I68.0-I68.2, I69.0-I69.3, I70.2-I70.8, I71-I73.9, I77-I83.9, I86-I89.0, I89.9, I98, K75.1	391-391.9, 392.0, 393-398.9, 402-402.9, 410-414.9, 416.0, 417-417.9, 420-423, 423.1-423.9, 424.0-424.3, 424.8, 425.0-425.5, 425.7-425.8, 427.0-427.3, 427.6-427.8, 429.0, 430-435.9, 437.0-437.2, 437.5-437.8, 440.2, 440.4, 441-443.9, 447-454.9, 456, 456.3-457, 457.1, 457.8-457.9, 459, 459.1-459.3
Rheumatic heart disease	I01-I01.9, I02.0, I05-I09.9	391-391.9, 392.0, 393-398.9
Ischemic heart disease	I20-I25.9	410-414.9
Stroke	G45-G46.8, I60-I63.9, I65-I66.9, I67.0-I67.3, I67.5-I67.6, I68.1-I68.2, I69.0-I69.3	430-435.9, 437.0-437.2, 437.5-437.8
Ischemic stroke	G45-G46.8, I63-I63.9, I65-I66.9, I67.2-I67.3, I67.5-I67.6, I69.3	433-435.9, 437.0-437.1, 437.5-437.8
Intracerebral hemorrhage	I61-I62, I62.1-I62.9, I68.1-I68.2, I69.1-I69.2	431-432.9, 437.2
Subarachnoid hemorrhage	I60-I60.9, I62.0, I67.0-I67.1, I69.0	430-430.9
Hypertensive heart disease	I11-I11.9	402-402.9
Non-rheumatic valvular heart disease	I34-I37.8	424.0-424.3, 424.8
Non-rheumatic calcific aortic valve disease	I35-I35.9	424.1
Non-rheumatic degenerative mitral valve disease	I34-I34.9	424.0
Other non-rheumatic valve diseases	I36-I37.8	424.2-424.3, 424.8
Cardiomyopathy and myocarditis	B33.2, I40-I41.9, I42.1-I42.8, I43-I43.9, I51.4	422-422.9, 425.0-425.5, 425.7-425.8, 429.0
Myocarditis	B33.2, I40-I41.9, I51.4	422-422.9
Alcoholic cardiomyopathy	I42.6	425.5
Other cardiomyopathy	I42.1-I42.5, I42.7-I42.8, I43-I43.9	425.0-425.4, 425.7-425.8, 429.0
Pulmonary Arterial Hypertension	I27.0	416.0
Atrial fibrillation and flutter	I48-I48.9	427.3
Aortic aneurysm	I71-I71.9	441-441.9
Lower extremity peripheral arterial disease	I70.2-I70.8, I73-I73.9	440.2, 440.4, 443.0-443.9
Endocarditis	I33-I33.9, I38-I39.9	421-421.9
Other cardiovascular and circulatory diseases	I27.2, I28-I28.9, I30-I31.1, I31.8-I32.8, I47-I47.9, I51.0-I51.3, I68.0, I72-I72.9, I77-I83.9, I86-I89.0, I89.9, I98, K75.1	417-417.9, 420-420.9, 423, 423.1-423.9, 427.0-427.2, 427.6-427.8, 442-443, 447-454.9, 456, 456.3-457, 457.1, 457.8-457.9, 459, 459.1-459.3
Chronic respiratory diseases	D86-D86.2, D86.9, G47.3, J30-J35.9, J37-J39.9, J41-J46.9, J60-J63.8, J66-J68.9, J70, J70.8-J70.9, J82, J84-J84.9, J91, J91.8-J92.9	135-135.9, 327.2-327.8, 470, 470.9-474.9, 476-476.1, 477-479, 491-493.9, 495-504.9, 506-506.9, 508-509, 515, 516-517.8, 518.6, 518.9, 519.1-519.4
Chronic obstructive pulmonary disease	J41-J44.9	491-492.9, 496-499
Pneumoconiosis	J60-J63.8, J92.0	500-504.9
Silicosis	J62-J62.9	502-502.9, 503.0, 503.9
Asbestosis	J61-J61.0, J92.0	501
Coal workers pneumoconiosis	J60-J60.0	500-500.9, 501.0-501.9
Other pneumoconiosis	J63-J63.8	503, 503.1, 504-504.9
Asthma	J45-J46.9	493-493.9
Interstitial lung disease and pulmonary sarcoidosis	D86-D86.2, D86.9, J84-J84.9	135-135.9, 515, 516-516.9
Other chronic respiratory diseases	G47.3, J30-J35.9, J37-J39.9, J66-J68.9, J70, J70.8-J70.9, J82, J91, J91.8-J92, J92.9	327.2-327.8, 470, 470.9-474.9, 476-476.1, 477-479, 495-495.9, 506-506.9, 508-509, 517-517.8, 518.6, 518.9, 519.1-519.4
Digestive diseases	B18-B18.9, I84-I85.9, I98.2, K20-K20.9, K22-K22.6, K22.8-K29.9, K31-K31.8, K35-K38.9, K40-K42.9, K44-K46.9, K50-K52, K52.8-K52.9, K55-K62, K62.2-K62.6, K62.8-K62.9, K64-K64.9, K66.8, K67, K68, K70-K70.3, K71.7, K73-K75, K75.2, K75.4-K76.2, K76.4-K77, K77.8, K80-K83.9, K85-K86.9, K90-K90.9, K92.8, K93.8, M09.1	455-455.9, 456.0-456.2, 530-530.0, 530.2-530.6, 531-536.1, 537-537.6, 537.8, 538, 540-543.9, 550-551.1, 551.3-552.1, 552.3-553.1, 553.3-558.0, 560-560.3, 560.8-560.9, 562-562.1, 564-564.1, 564.5-564.7, 565-566.9, 569.1-569.5, 569.7, 571-571.9, 572.2-573.0, 573.4-577.9, 579-579.2, 579.4-579.9
Cirrhosis and other chronic liver diseases	B18-B18.9, I85-I85.9, I98.2, K70-K70.3, K71.7, K73-K75, K75.2, K75.4-K76.2, K76.4-K76.9, K77.8	456.0-456.2, 571-571.9, 572.2-573.0, 573.4-573.9
Cirrhosis and other chronic liver diseases due to hepatitis B		
Cirrhosis and other chronic liver diseases due to hepatitis C		

Appendix Table S6: List of International Classification of Diseases (ICD) codes mapped to the Global Burden of Diseases cause list for causes of death		
**Cause**	**ICD10**	**ICD9**
Cirrhosis and other chronic liver diseases due to alcohol use		
Cirrhosis and other chronic liver diseases due to NAFLD		
Cirrhosis and other chronic liver diseases due to other causes		
Upper digestive system diseases	K25-K29.9	531-535.9
Peptic ulcer disease	K25-K28.9	531-534.9
Gastritis and duodenitis	K29-K29.9	535-535.9
Appendicitis	K35-K37.9, K38.3-K38.9	540-542.9
Paralytic ileus and intestinal obstruction	K56-K56.9	560-560.3, 560.8-560.9
Inguinal, femoral, and abdominal hernia	K40-K42.9, K44-K46.9	550-551.1, 551.3-552.1, 552.3-553.1, 553.3-553.9
Inflammatory bowel disease	K50-K52, K52.8-K52.9, M09.1	555-556.9, 558-558.0, 569.5
Ulcerative colitis	K51-K52, K52.8-K52.9	556-556.9, 558.0
Crohn's disease	K50-K50.9, M09.1	555-555.9
Vascular intestinal disorders	K55-K55.9	557-557.9
Gallbladder and biliary diseases	K80-K83.9	574-576.9
Pancreatitis	K85-K86.9	577-577.9, 579.4
Other digestive diseases	I84-I84.9, K20-K20.9, K22-K22.6, K22.8-K24, K31-K31.8, K38-K38.2, K57-K62, K62.2-K62.6, K62.8-K62.9, K64-K64.9, K66.8, K67, K68, K77, K90-K90.9, K92.8, K93.8	455-455.9, 530-530.0, 530.2-530.6, 536-536.1, 537-537.6, 537.8, 538, 543-543.9, 562-562.1, 564-564.1, 564.5-564.7, 565-566.9, 569.1-569.4, 569.7, 579-579.2, 579.8-579.9
Neurological disorders	F00-F02.0, F02.2-F02.3, F02.8-F03.9, G10-G13.8, G20-G20.9, G23-G24, G24.1-G25.0, G25.2-G25.3, G25.5, G25.8-G26.0, G30-G31.1, G31.8-G31.9, G35-G37.9, G40-G41.9, G61-G61.9, G70-G71.1, G71.3-G72, G72.2-G73.7, G90-G90.9, G95-G95.9, M33-M33.9	290-290.9, 294.1-294.9, 330-331.2, 331.5-332.0, 333-337.9, 340-341.9, 345-345.9, 349, 349.2-349.8, 353.8-353.9, 356-356.9, 357.0-357.1, 357.3-357.4, 357.7, 358-359.9, 775.2
Alzheimer's disease and other dementias	F00-F02.0, F02.8-F03.9, G30-G31.1, G31.8-G31.9	290-290.9, 294.1-294.9, 331-331.2
Parkinson's disease	F02.3, G20-G20.9	332-332.0
Idiopathic epilepsy	G40-G41.9	345-345.9
Multiple sclerosis	G35-G35.9	340-340.9
Motor neuron disease	G12.2-G12.9	335-335.2, 335.8-335.9
Other neurological disorders	F02.2, G10-G12.1, G13-G13.8, G23-G24, G24.1-G25.0, G25.2-G25.3, G25.5, G25.8-G26.0, G36-G37.9, G61-G61.9, G70-G71.1, G71.3-G72, G72.2-G73.7, G90-G90.9, G95-G95.9, M33-M33.9	330-330.9, 331.5-331.9, 333-334.9, 335.3, 336-337.9, 341-341.9, 349, 349.2-349.8, 353.8-353.9, 356-356.9, 357.0-357.1, 357.3-357.4, 357.7, 358-359.9, 775.2
Mental disorders	F24, F50.0-F50.5	307.1
Eating disorders	F50.0-F50.5	307.1
Anorexia nervosa	F50.0-F50.5	307.1
Substance use disorders	E24.4, F10-F16.9, F18-F18.9, G31.2, G62.1, G72.1, P04.3-P04.4, P96.1, Q86.0, R78.0-R78.5, X45-X45.9, X65-X65.9, Y15-Y15.9	291-292.9, 303-303.9, 304.0-304.8, 305.0, 305.2-305.8, 357.5, 760.7, 790.3, E850, E860
Alcohol use disorders	E24.4, F10-F10.9, G31.2, G62.1, G72.1, P04.3, Q86.0, R78.0, X45-X45.9, X65-X65.9, Y15-Y15.9	291-291.9, 303-303.9, 305.0, 357.5, 790.3, E860
Drug use disorders	F11-F16.9, F18-F18.9, P04.4, P96.1, R78.1-R78.5	292-292.9, 304.0-304.8, 305.2-305.8, 760.7, E850
Opioid use disorders	F11-F11.9, P96.1, R78.1	304.0, 305.5
Cocaine use disorders	F14-F14.9, R78.2	304.2, 305.6
Amphetamine use disorders	F15-F15.9	304.4, 305.7
Other drug use disorders	F13-F13.9, F16-F16.9, F18-F18.9, P04.4, R78.3-R78.5	292-292.9, 304.1, 304.5-304.8, 305.3-305.4, 305.8, 760.7
Diabetes and kidney diseases	D63.1, E10-E11.9, I12-I13.9, N00-N08.8, N15.0, N18-N18.9, P70.2, Q61-Q62.8	403-404.9, 580-583.9, 585-585.9, 589-589.9, 753-753.3, 775.1
Diabetes mellitus	E10-E10.1, E10.3-E11.1, E11.3-E11.9, P70.2	775.1
Diabetes mellitus type 1	E10-E10.1, E10.3-E10.9, P70.2	775.1
Diabetes mellitus type 2	E11-E11.1, E11.3-E11.9	
Chronic kidney disease	D63.1, E10.2, E11.2, I12-I13.9, N02-N08.8, N15.0, N18-N18.9, Q61-Q62.8	403-404.9, 581-583.9, 585-585.9, 589-589.9, 753-753.3
Chronic kidney disease due to diabetes mellitus type 1	E10.2	
Chronic kidney disease due to diabetes mellitus type 2	E11.2	
Chronic kidney disease due to hypertension	I12-I13.9	403-404.9
Chronic kidney disease due to glomerulonephritis	N03-N06.9	581-583.9
Chronic kidney disease due to other and unspecified causes	N02-N02.9, N07-N08.8, N15.0, Q61-Q62.8	589-589.9, 753-753.3
Acute glomerulonephritis	N00-N01.9	580-580.9
Skin and subcutaneous diseases	A46-A46.0, A66-A67.9, B86, D86.3, H05.0-H05.1, I89.1-I89.8, L00-L05.9, L08-L08.9, L10-L14.0, L51-L51.9, L88-L89.9, L97-L98.4, M72.5-M72.6	035-035.9, 102-103.9, 133-133.6, 376.0-376.1, 457.2-457.3, 680-689, 694-695.3, 707-707.9
Bacterial skin diseases	A46-A46.0, A66-A67.9, H05.0-H05.1, I89.1-I89.8, L00-L05.9, L08-L08.9, L88, L97-L98.4, M72.5-M72.6	035-035.9, 102-103.9, 376.0-376.1, 457.2-457.3, 680-689
Cellulitis	H05.0, L03-L03.9, M72.5-M72.6	681-682.9
Pyoderma	A46-A46.0, A66-A67.9, H05.1, I89.1-I89.8, L00-L02.9, L04-L05.9, L08-L08.9, L88, L97-L98.4	035-035.9, 102-103.9, 376.0-376.1, 457.2-457.3, 680-680.9, 683-689
Decubitus ulcer	L89-L89.9	707-707.9
Other skin and subcutaneous diseases	D86.3, L10-L14.0, L51-L51.9	694-695.3
Musculoskeletal disorders	I27.1, I67.7, L93-L93.2, M00-M03.0, M03.2-M03.6, M05-M09.0, M09.2-M09.8, M30-M32.9, M34-M36.8, M40-M43.1, M65-M65.0, M71.0-M71.1, M80-M82.8, M86.3-M86.4, M87-M87.0, M88-M89.0, M89.5, M89.7-M89.9	416.1, 437.4, 446-446.9, 695.4-695.5, 710-711.9, 714-714.3, 714.8-714.9, 730.1, 732-732.9, 733.0-733.1
Rheumatoid arthritis	M05-M06.9, M08.0-M08.8	714-714.3, 714.8-714.9
Other musculoskeletal disorders	I27.1, I67.7, L93-L93.2, M00-M03.0, M03.2-M03.6, M07-M08, M08.9-M09.0, M09.2-M09.8, M30-M32.9, M34-M36.8, M40-M43.1, M65-M65.0, M71.0-M71.1, M80-M82.8, M86.3-M86.4, M87-M87.0, M88-M89.0, M89.5, M89.7-M89.9	416.1, 437.4, 446-446.9, 695.4-695.5, 710-711.9, 730.1, 732-732.9, 733.0-733.1

Appendix Table S6: List of International Classification of Diseases (ICD) codes mapped to the Global Burden of Diseases cause list for causes of death		
Cause	ICD10	ICD9
Other non-communicable diseases	D25-D26, D28.2, D55-D58.9, D59.1, D59.3, D59.5, D60-D61.9, D64.0, D66-D67, D68.0-D69.4, D69.6-D69.8, D70-D70.0, D70.4-D75.8, D76-D77, D86.8, D89-D89.2, E03-E03.1, E03.3-E06.3, E06.5-E07.1, E16.1-E16.9, E20-E23.0, E23.2-E24.1, E24.3, E24.8-E27.2, E27.4-E34, E34.1-E34.8, E65-E66.0, E66.2-E68, E70-E85.2, E88-E88.2, E88.4-E88.9, G71.2, N10-N12.9, N13.6, N15, N15.1-N16.8, N20-N23.0, N25-N28.1, N29-N30.3, N30.8-N32.0, N32.3-N32.4, N34-N34.3, N36-N36.9, N39-N39.2, N41-N41.9, N44-N44.0, N45-N45.9, N49-N49.9, N72-N72.0, N75-N77.8, N80-N81.9, N83-N83.9, P96.0, Q00-Q07.9, Q10.4-Q18.9, Q20-Q28.9, Q30-Q36, Q37-Q45.9, Q50-Q60.6, Q63-Q86, Q86.1-Q87.8, Q89-Q89.8, Q90-Q93.9, Q95-Q99.8, R95-R95.9	218-219, 219.1-219.9, 236.0, 240-243.9, 245-246.9, 251-251.2, 251.4-253.6, 253.8-259.1, 259.3-259.9, 270-273.9, 275-276, 277-277.2, 277.4-277.9, 278.0-278.8, 282-284.9, 286-286.5, 286.7-289.0, 289.4-289.7, 588-588.9, 590-590.9, 592-593.8, 594-598.1, 598.8-599.6, 599.8, 601-602.9, 604-604.9, 608.2, 617-618.9, 620-620.9, 621.4-621.9, 622.3-622.6, 629-629.8, 740-749.0, 749.2-752.9, 753.4-758.9, 759.0-759.8, 775.3, 788.0, 798-798.0
Congenital birth defects	G71.2, P96.0, Q00-Q07.9, Q10.4-Q18.9, Q20-Q28.9, Q30-Q36, Q37-Q45.9, Q50-Q60.6, Q63-Q86, Q86.1-Q87.8, Q89-Q89.8, Q90-Q93.9, Q95-Q99.8	740-749.0, 749.2-752.9, 753.4-758.9, 759.0-759.8
Neural tube defects	Q00-Q01.9, Q05-Q05.9	740-741.9, 742.0
Congenital heart anomalies	Q20-Q28.9	745-747.9
Orofacial clefts	Q35-Q36, Q37-Q37.9	749-749.0, 749.2-749.9
Down syndrome	Q90-Q90.9	758.0
Other chromosomal abnormalities	Q87-Q87.8, Q91-Q93.9, Q95-Q95.9, Q97-Q97.9, Q99-Q99.8	758, 758.1-758.6, 758.8-758.9
Congenital musculoskeletal and limb anomalies	Q65-Q79, Q79.6-Q79.9	742.5, 754-756.5, 756.8-756.9
Urogenital congenital anomalies	P96.0, Q50-Q60.6, Q63-Q64.9	752-752.9, 753.4-753.9
Digestive congenital anomalies	Q38-Q45.9, Q79.0-Q79.5	750-751.9, 756.6-756.7
Other congenital birth defects	G71.2, Q02-Q04.9, Q06-Q07.9, Q10.4-Q18.9, Q30-Q34.9, Q80-Q86, Q86.1-Q86.8, Q89-Q89.8	742, 742.1-742.4, 742.8-744.9, 748-748.9, 757-757.9, 759.0-759.8
Urinary diseases and male infertility	N10-N12.9, N13.6, N15, N15.1-N16.8, N20-N23.0, N25-N28.1, N29-N30.3, N30.8-N32.0, N32.3-N32.4, N34-N34.3, N36-N36.9, N39-N39.2, N41-N41.9, N44-N44.0, N45-N45.9, N49-N49.9	588-588.9, 590-590.9, 592-593.8, 594-598.1, 598.8-599.6, 599.8, 601-602.9, 604-604.9, 608.2, 788.0
Urinary tract infections and interstitial nephritis	N10-N12.9, N13.6, N15, N15.1-N16.8, N30-N30.3, N30.8-N30.9, N34-N34.3, N39.0-N39.2	590-590.9, 595-595.9, 597-597.9, 599.0
Urolithiasis	N20-N23.0	592-592.9, 594-594.9, 788.0
Other urinary diseases	N25-N28.1, N29-N29.8, N31-N32.0, N32.3-N32.4, N36-N36.9, N39, N41-N41.9, N44-N44.0, N45-N45.9, N49-N49.9	588-588.9, 593-593.8, 596-596.9, 598-598.1, 598.8-599, 599.1-599.6, 599.8, 601-602.9, 604-604.9, 608.2
Gynecological diseases	D25-D26, D28.2, E28.2, N72-N72.0, N75-N77.8, N80-N81.9, N83-N83.9	218-219, 219.1-219.9, 236.0, 256.4, 617-618.9, 620-620.9, 621.4-621.9, 622.3-622.6, 629-629.8
Uterine fibroids	D25-D26, D28.2	218-219, 219.1-219.9, 236.0
Endometriosis	N80-N80.9	617-617.9
Genital prolapse	N81-N81.9	618-618.9
Other gynecological diseases	N72-N72.0, N75-N77.8, N83-N83.9	620-620.9, 621.4-621.9, 622.3-622.6, 629-629.8
Hemoglobinopathies and hemolytic anemias	D55-D58.9, D59.1, D59.3, D59.5, D60-D61.9, D64.0	282-284.9
Thalassemias	D56-D56.9	282.4-282.5
Sickle cell disorders	D57-D57.8	282.6
G6PD deficiency	D55-D55.2	282.2-282.3
Other hemoglobinopathies and hemolytic anemias	D55.3-D55.9, D58-D58.9, D59.1, D59.3, D59.5, D60-D61.9, D64.0	282-282.1, 282.7-284.9
Endocrine, metabolic, blood, and immune disorders	D66-D67, D68.0-D69.4, D69.6-D69.8, D70-D70.0, D70.4-D75.8, D76-D77, D86.8, D89-D89.2, E03-E03.1, E03.3-E06.3, E06.5-E07.1, E16.1-E16.9, E20-E23.0, E23.2-E24.1, E24.3, E24.8-E27.2, E27.4-E28.1, E28.3-E34, E34.1-E34.8, E65-E66.0, E66.2-E68, E70-E85.2, E88-E88.2, E88.4-E88.9	240-243.9, 245-246.9, 251-251.2, 251.4-253.6, 253.8-256.3, 256.8-259.1, 259.3-259.9, 270-273.9, 275-276, 277-277.2, 277.4-277.9, 278.0-278.8, 286-286.5, 286.7-289.0, 289.4-289.7, 775.3
Thyroid diseases	E03-E03.1, E03.3-E06.3, E06.5-E07, E07.1	240-243.9, 245-245.9, 775.3
Other endocrine, metabolic, blood, and immune disorders	D66-D67, D68.0-D69.4, D69.6-D69.8, D70-D70.0, D70.4-D75.8, D76-D77, D86.8, D89-D89.2, E07.0, E16.1-E16.9, E20-E23.0, E23.2-E24.1, E24.3, E24.8-E27.2, E27.4-E28.1, E28.3-E34, E34.1-E34.8, E67-E68, E70-E77.9, E79-E83.9, E85-E85.2, E88-E88.2, E88.4-E88.9	246-246.9, 251-251.2, 251.4-253.6, 253.8-256.3, 256.8-259.1, 259.3-259.9, 270-271.9, 273-273.9, 275-276, 277, 277.1-277.2, 277.4-277.9, 278.2-278.8, 286-286.5, 286.7-289.0, 289.4-289.7
Sudden infant death syndrome	R95-R95.9	798-798.0
Injuries	D52.1, D59.0, D59.2, D59.6, D69.5, D70.1-D70.2, D78-D78.8, E03.2, E06.4, E09-E09.9, E16.0, E23.1, E24.2, E27.3, E36-E36.8, E66.1, E88.3, E89-E89.9, G21.0-G21.1, G24.0, G25.1, G25.4, G25.6-G25.7, G72.0, G93.7, G97-G97.9, I95.2-I95.3, I97-I97.9, I98.9, J70.0-J70.5, J95-J95.9, K43-K43.9, K52.0, K62.7, K91-K91.9, K94-K95.8, L55-L55.9, L56.3, L56.8-L56.9, L58-L58.9, M87.1, N14-N14.4, N30.4, N65-N65.1, N99-N99.9, P93-P93.8, P96.2, P96.5, R50.2, U00-U03, V00-V86.9, V87.2-V87.3, V88.2-V88.3, V90-V98.8, W00-W46.2, W49-W62.9, W64-W70.9, W73-W75.9, W77-W81.9, W83-W94.9, W97.9, W99-X06.9, X08-X39.9, X47-X48.9, X50-X54.9, X57-X58.9, X60-X64.9, X66-X83.9, X85-Y08.9, Y35-Y84.9, Y87.0-Y87.1, Y88-Y88.3, Y89.0-Y89.1	244.0-244.1, 244.3-244.8, 251.3, 253.7, 349.0-349.1, 357.6, 457.0, 518.7, 519.0, 536.4, 539-539.9, 551.2, 552.2, 553.2, 558.1, 564.2-564.4, 569.6, 579.3, 598.2, 779.4-779.5, E800-E807, E830-E838, E840-E849, E856-E857, E861-E865, E867-E869, E870-E876, E878-E879, E880-E886, E888-E928, E930-E979, E990-E999
Transport injuries	V00-V86.9, V87.2-V87.3, V88.2-V88.3, V90-V98.8	E800-E807, E830-E838, E840-E849
Road injuries	V01-V04.9, V06-V80.9, V82-V82.9, V87.2-V87.3	
Pedestrian road injuries	V01-V04.9, V06-V09.9	
Cyclist road injuries	V10-V19.9	
Motorcyclist road injuries	V20-V29.9	
Motor vehicle road injuries	V30-V79.9, V87.2-V87.3	
Other road injuries	V80-V80.9, V82-V82.9	
Other transport injuries	V00-V00.8, V05-V05.9, V81-V81.9, V83-V86.9, V88.2-V88.3, V90-V98.8	E800-E807, E830-E838, E840-E849

Appendix Table S6: List of International Classification of Diseases (ICD) codes mapped to the Global Burden of Diseases cause list for causes of death		
**Cause**	**ICD10**	**ICD9**
Unintentional injuries	D52.1, D59.0, D59.2, D59.6, D69.5, D70.1-D70.2, D78-D78.8, E03.2, E06.4, E09-E09.9, E16.0, E23.1, E24.2, E27.3, E36-E36.8, E66.1, E88.3, E89-E89.9, G21.0-G21.1, G24.0, G25.1, G25.4, G25.6-G25.7, G72.0, G93.7, G97-G97.9, I95.2-I95.3, I97-I97.9, I98.9, J70.0-J70.5, J95-J95.9, K43-K43.9, K52.0, K62.7, K91-K91.9, K94-K95.8, L55-L55.9, L56.3, L56.8-L56.9, L58-L58.9, M87.1, N14-N14.4, N30.4, N65-N65.1, N99-N99.9, P93-P93.8, P96.2, P96.5, R50.2, W00-W46.2, W49-W62.9, W64-W70.9, W73-W75.9, W77-W81.9, W83-W94.9, W97.9, W99-X06.9, X08-X39.9, X47-X48.9, X50-X54.9, X57-X58.9, Y40-Y84.9, Y88-Y88.3	244.0-244.1, 244.3-244.8, 251.3, 253.7, 349.0-349.1, 357.6, 457.0, 518.7, 519.0, 536.4, 539-539.9, 551.2, 552.2, 553.2, 558.1, 564.2-564.4, 569.6, 579.3, 598.2, 779.4-779.5, E856-E857, E861-E865, E867-E869, E870-E876, E878-E879, E880-E886, E888-E928, E930-E949
Falls	W00-W19.9	E880-E886, E888
Drowning	W65-W70.9, W73-W74.9	E910
Fire, heat, and hot substances	X00-X06.9, X08-X19.9	E890-E899, E924
Poisonings	X47-X48.9	E856-E857, E861-E865, E867-E869
Poisoning by carbon monoxide	X47-X47.9	E862, E868-E869
Poisoning by other means	X48-X48.9	E856-E857, E861, E863-E865, E867
Exposure to mechanical forces	W20-W38.9, W40-W43.9, W45.0-W45.2, W46-W46.2, W49-W52	E916-E922
Unintentional firearm injuries	W32-W34.9	E922
Other exposure to mechanical forces	W20-W31.9, W35-W38.9, W40-W43.9, W45.0-W45.2, W46-W46.2, W49-W52	E916-E921
Still Born	P95-P95.9	768.0-768.1
Adverse effects of medical treatment	D52.1, D59.0, D59.2, D59.6, D69.5, D70.1-D70.2, D78-D78.8, E03.2, E06.4, E09-E09.9, E16.0, E23.1, E24.2, E27.3, E36-E36.8, E66.1, E88.3, E89-E89.9, G21.0-G21.1, G24.0, G25.1, G25.4, G25.6-G25.7, G72.0, G93.7, G97-G97.9, I95.2-I95.3, I97-I97.9, I98.9, J70.0-J70.5, J95-J95.9, K43-K43.9, K52.0, K62.7, K91-K91.9, K94-K95.8, M87.1, N14-N14.4, N30.4, N65-N65.1, N99-N99.9, P93-P93.8, P96.2, P96.5, R50.2, Y40-Y84.9, Y88-Y88.3	244.0-244.1, 244.3-244.8, 251.3, 253.7, 349.0-349.1, 357.6, 457.0, 518.7, 519.0, 536.4, 539-539.9, 551.2, 552.2, 553.2, 558.1, 564.2-564.4, 569.6, 579.3, 598.2, 779.4-779.5, E870-E876, E878-E879, E930-E949
Animal contact	W52.0-W62.9, W64-W64.9, X20-X29.9	E905-E906
Venomous animal contact	X20-X29.9	E905
Non-venomous animal contact	W52.0-W62.9, W64-W64.9	E906
Foreign body	W44-W45, W45.3-W45.9, W75-W75.9, W78-W80.9, W83-W84.9	E911-E915
Pulmonary aspiration and foreign body in airway	W75-W75.9, W78-W80.9, W83-W84.9	E911-E913
Foreign body in other body part	W44-W45, W45.3-W45.9	E914-E915
Electrocution	W85-W87.9	E925
Environmental heat and cold exposure	L55-L55.9, L56.3, L56.8-L56.9, L58-L58.9, W88-W94.9, W97.9, W99-W99.9, X30-X32.9, X39-X39.9	E900-E902, E926
Exposure to forces of nature	X33-X38.9	E907-E909
Garbage Code (GBD Level 1)	A40-A41.9, A48.0, A48.3, A49.0-A49.1, A59-A59.9, A71-A71.9, A74.0, B07-B07.9, B30-B30.9, B35-B36.9, B85-B85.4, B87-B88.9, B94.0, D50-D50.0, D50.9, D62-D63.0, D63.8-D64, D64.1-D65.9, D68, D69.9, E15-E16, E50-E50.9, E64.1, E85.3-E87.6, E87.8-E87.9, F06.2-F06.4, F07.2, F09-F09.9, F19-F23.9, F25-F49, F51-F99.0, G06-G08.0, G32-G32.8, G43-G44.2, G44.4-G44.8, G47-G47.2, G47.4-G47.9, G50-G60.9, G62-G62.0, G62.2-G65.2, G80-G83.9, G89-G89.4, G91-G91.2, G91.4-G93, G93.1-G93.2, G93.4-G93.6, G94.0-G94.8, G99-H05, H05.2-H69.9, H71-H99, I26-I26.9, I31.2-I31.4, I46-I46.9, I50.0-I50.4, I76, I95-I95.1, I95.8-I95.9, J69-J69.9, J80-J80.9, J81.0, J85-J85.3, J86-J86.9, J90-J90.0, J93-J93.1, J93.8-J94.9, J96-J96.9, J98.1-J98.3, K00-K19, K30, K65-K66.1, K66.9, K68.1-K68.9, K71-K71.6, K71.8-K72.9, K75.0, L20-L30.9, L40-L50.9, L52-L54.8, L56-L56.2, L56.4-L56.5, L57-L57.9, L59-L68.9, L70-L76.8, L80-L87.9, L90-L92.9, L94-L96, L98.5-L99.8, M04, M10-M12.0, M12.2-M29, M37-M39, M43.2-M49, M49.2-M64, M65.1-M71, M71.2-M72.4, M72.8-M73, M73.8-M79.9, M83-M86.2, M86.5-M86.9, M87.2-M87.9, M89.1-M89.4, M90-M99.9, N17-N17.9, N19-N19.9, N32.1-N32.2, N32.8-N33.8, N35-N35.9, N37-N37.8, N39.3-N39.8, N42-N43.4, N44.1-N44.8, N46-N48.9, N50-N53.9, N61-N64.9, N82-N82.9, N91-N91.5, N95, N95.1-N95.9, N97-N97.9, R02-R02.9, R03.1-R04, R04.1-R04.9, R07.0, R08-R12.0, R14-R19.6, R19.8-R23, R23.1-R30.9, R32-R50.1, R50.8-R57.9, R58.0-R72.9, R74-R78, R78.6-R94.8, R96-R99.9, U05, U08-U81, U89.9-U99, X40-X44.9, X46-X46.9, X49-X49.9, Y10-Y14.9, Y16-Y19.9, Z00-Z15.8, Z17-unsp.	038-038.9, 040.0, 041.1, 076-078.2, 110-111.9, 125-125.3, 126-126.9, 127.2-127.9, 131-132.9, 133.8-134.9, 136.6, 139.1, 139.9, 247-248, 264-264.9, 274-274.9, 276.0-276.9, 277.3, 280-281, 285-285.9, 286.6, 289.1-289.3, 293, 294-294.0, 295-302.9, 305, 305.9-307.0, 307.2-307.4, 307.6-319.9, 324-327.1, 328-329, 338-339.1, 339.3-339.8, 342-344.9, 346-346.9, 350-353.6, 354-355.9, 360-362, 362.1-376, 376.2-380.9, 384-389.9, 415-415.9, 423.0, 424, 424.4-424.5, 424.9, 427.5, 427.9-428.9, 437.3, 458-458.9, 459.0, 507-507.9, 510-513.9, 518.1-518.3, 520-529.9, 536.3, 536.8-536.9, 537.7, 537.9, 564.8-564.9, 567-568.9, 570-570.9, 572-572.1, 573.1-573.3, 584-584.9, 586-587.9, 603-603.9, 605-608.1, 608.3-609, 611-612.1, 615-616.9, 619-619.9, 621-621.3, 622-622.0, 622.8-623.6, 623.8-624.5, 624.8-628.9, 629.9, 690-693.9, 695.8-706.9, 708-709.9, 712-713.8, 715-716, 716.2-721.6, 721.8-730.0, 730.2-730.3, 730.7-731.9, 733, 733.2-734.2, 737-738, 738.2-739.9, 780-782.4, 782.6-784.6, 784.9, 785.4-786, 786.6, 786.8, 787, 787.3-788, 788.3-790.1, 790.4-796.1, 796.3-797.9, 798.1-799.9, E851-E855, E858, E866, E980-E982, V01-V08, V10-uns
Garbage Code (GBD Level 2)	A14.9, A29-A30.9, A45-A45.9, A47-A48, A48.8-A49, A49.3-A49.9, A61-A62, A72-A73, A76, A97, B08-B09, B11-B14, B28-B29, B31-B32.4, B34-B34.1, B34.3-B34.9, B61-B62, B68-B68.9, B73-B74.2, B76-B76.9, B78-B81.8, B84, B92-B94, B94.8-B94.9, B95.6-B97.1, B97.3, B97.7-B99.9, D59, D59.4, D59.8-D59.9, F17-F17.9, G44.3, G91.3, G93.0, G93.3, I10-I10.9, I15-I15.9, I27, I27.8-I27.9, I50, I50.8-I50.9, I67.4, I70-I70.1, I70.9, I74-I75.8, J81, J81.1, K92.0-K92.2, N70-N71.9, N73-N74.0, N74.2-N74.8, R03-R03.0, R04.0, R05-R06.9, R13-R13.9, R23.0, R58, S00-T98.3, W47-W48, W63, W71-W72, W76-W76.9, W82, W95-W97, W98, X07, X55-X56, X59-X59.9, Y20-Y34.9, Y86-Y87, Y87.2, Y89, Y89.9-Y99.9	000-000.9, 030-030.9, 041.2-041.9, 067-069, 078.8-078.9, 079.8-079.9, 089-089.9, 105-109.9, 119, 136.8-136.9, 139.8, 304, 304.9, 305.1, 339.2, 401-401.9, 405-405.9, 416, 416.2-416.9, 440-440.1, 440.3, 440.8-440.9, 444-445.8, 490-490.9, 494-494.9, 514-514.9, 515.0-515.9, 518-518.0, 518.4-518.5, 518.8, 536.2, 578-578.9, 599.7, 613-614.9, 714.4, 716.1, 721.7, 735-736.9, 738.0-738.1, 784.7-784.8, 786.3, 787.0-787.2, 796.2, 800-E80, E83, E839, E85, E859, E87, E877, E88, E887, E929, E983-E985, E988-E989

Appendix Table S6: List of International Classification of Diseases (ICD) codes mapped to the Global Burden of Diseases cause list for causes of death		
**Cause**	**ICD10**	**ICD9**
Garbage Code (GBD Level 3)	A01, A31-A31.9, A42-A44.9, A49.2, A64-A64.0, A99-A99.0, B17, B17.1, B17.8-B17.9, B19-B19.0, B19.2-B19.9, B37-B46.9, B49-B49.9, B55, B55.1-B55.9, B58-B59.9, B89, B94.2, C14-C14.9, C22.9, C26-C29, C35-C36, C39-C39.9, C42, C46-C46.9, C55-C55.9, C57.9, C59, C63.9, C68, C68.9, C74-C74.9, C75.9-C80.9, C83, C83.9, C85.1, C85.9, C87, C94.6, C97-D00.0, D01, D01.4-D02, D02.4-D02.9, D07, D07.3, D07.6-D09, D09.1, D09.7, D09.9-D10, D10.9, D13, D13.9-D14, D14.4, D17-D21.9, D28, D28.9-D29, D29.9-D30, D30.9, D36.0, D36.9-D37.0, D37.6-D38, D38.6-D39.0, D39.7, D39.9-D40, D40.9-D41, D41.9, D44, D44.9, D48, D48.7-D49.1, D49.5, D49.7-D49.9, D54, D75.9, D79-D85, D87-D88, D89.8-D99, E07.8-E08.9, E17-E19, E34.0, E34.9-E35.8, E37-E39, E47-E49., E62, E69, E87.7, E90-E998, F04-F06.1, F06.5-F07.0, F07.8-F08, F50, F50.8-F50.9, G09-G09.9, G15-G19, G21, G21.2, G21.4-G22.0, G27-G29, G33-G34, G38-G39., G42, G48-G49, G66-G69, G74-G79, G84-G88, G93.8-G94, G96-G96.9, G98-G98.9, I00.0, I03-I04., I14-I14., I16-I19, I29-I29.9, I44-I45.9, I49-I49.9, I51, I51.6-I59, I90-I94, I96-I96.9, I98.4-I98.8, I99-ID5.9, J02.9, J03.9, J04.3, J06, J06.9, J40-J40.9, J47-J59, J65-J65.0, J71-J79, J81.9, J83, J85.9, J87-J89, J90.9, J93.6, J97-J98.0, J98.4-J99.8, K21-K21.9, K22.7, K31.9-K34, K39, K47-K49, K53-K54, K63-K63.4, K63.8-K63.9, K69, K70.4-K70.9, K78-K79, K84, K87-K89, K92, K92.9-K93, K96-K99, L06-L07, L09, L15-L19, L31-L39, L69, L77-L79, N09, N13-N13.5, N13.7-N13.9, N24, N28.8-N28.9, N38, N39.9-N40.9, N54-N59, N66-N69, N78-N79, N84, N84.2-N86, N88-N90.9, N92-N94.9, N95.0, O08-O08.9, O17-O19, O27, O37-O39, O49-O59, O78-O79, O93-O95.9, P06, P16-P18, P30-P34.2, P40-P49, P62-P69, P73, P79, P82, P85-P89, P96.9-P99.9, Q08-Q10.3, Q19, Q29-Q29., Q36.0-Q36.9, Q46-Q49, Q88, Q89.9, Q94, Q99.9-R01.2, R07, R07.1-R07.9, R31-R31.9	002, 031-031.9, 039-039.9, 070, 070.4-070.9, 085, 085.1-085.9, 088.0-088.7, 112-118.9, 130-130.9, 136.3-136.5, 149-149.9, 155.2, 159-159.9, 165-169, 176-179.9, 183.9-184, 184.5, 184.9, 187, 187.9, 189, 189.9, 190.9, 195-199.9, 202.9, 209, 209.2-209.3, 209.6-210, 211, 211.9-212, 212.9, 214-216.9, 221, 221.9-222, 222.9-223, 223.9, 229, 229.1, 229.9-230.0, 230.9-231, 231.8-231.9, 233, 233.3, 233.6, 233.9-234, 234.9-235, 235.1-235.3, 235.5, 235.9-236, 236.3, 236.6, 236.9, 237.4, 239-239.1, 239.5, 239.7-239.9, 249-249.9, 259.2, 278, 279-279.9, 293.0-293.9, 331.3-331.4, 332.1-332.9, 347-348.9, 349.9, 357, 357.8-357.9, 399-400.0, 406-409.4, 418-419.9, 426-427, 427.4, 429, 429.2-429.9, 459.5-459.9, 464.5, 465, 465.9, 505-505.9, 519, 519.8-519.9, 530.1, 530.7-530.9, 544-549, 559-559.0, 560.4-560.7, 561, 562.2-563, 569, 569.8-569.9, 591-591.9, 593.9, 599.9-600.9, 623.7, 624.6, 637-637.9, 639-639.9, 749.1, 759, 759.9, 779.9, 782.5, 785-785.3, 786.0-786.2, 786.4-786.5, 786.7, 786.9, 788.1-788.2, E986-E987
Garbage Code (GBD Level 4)	B16.9, B54-B54.0, B64, B82-B82.9, B83.9, C69, C69.9, C91.1, C91.4-C91.5, C91.7-C91.9, C92.7-C92.9, C93.2, C93.5-C93.7, C93.9, E12-E14.9, G00, G00.9-G02.8, G03.9, I37.9, I42-I42.0, I42.9, I51.5, I64-I64.9, I67, I67.8-I68, I68.8-I69, I69.4-I69.9, J07-J08, J15.9, J17-J19.6, J22-J29, J64-J64.9, P23, P23.5-P23.9, P37.3-P37.4, R73-R73.9, V87-V87.1, V87.4-V88.1, V88.4-V89.9, V99-V99.0, X84-X84.9, Y09-Y09.9, Y85-Y85.9	070.3, 194-194.0, 194.9, 204.1, 204.5-204.9, 205.8-205.9, 206.2-206.9, 238, 244, 244.9, 250-250.9, 289.8-289.9, 307.5, 320, 320.9, 357.2, 362.0, 425, 425.9, 429.1, 436-437, 437.9-439.6, 482.9-483, 484, 484.8-486.9, 770.0, 790.2, E808-E829
Other unintentional injuries (internal)	W39-W39.9, W77-W77.9, W81-W81.9, X50-X54.9, X57-X58.9	E903-E904, E923, E927-E928
Self-harm and interpersonal violence	U00-U03, X60-X64.9, X66-X83.9, X85-Y08.9, Y35-Y38.9, Y87.0-Y87.1, Y89.0-Y89.1	E950-E979, E990-E999
Self-harm	X60-X64.9, X66-X83.9, Y87.0	E950-E959
Self-harm by firearm	X72-X74.9	E955
Self-harm by other specified means	X60-X64.9, X66-X71.9, X75-X83.9, Y87.0	E950-E954, E956-E959
Interpersonal violence	X85-Y08.9, Y87.1	E960-E969
Physical violence by firearm	X93-X95.9	E965
Physical violence by sharp object	X99-X99.9	E966
Physical violence by other means	X85-X92.9, X96-X98.9, Y00-Y04.9, Y06-Y08.9, Y87.1	E961-E964, E967-E969
Conflict and terrorism	U00-U03, Y36-Y38.9, Y89.1	E979, E990-E999
Police conflict and executions	Y35-Y35.9, Y89.0	E970-E978
None		

Appendix Table S7: Restrictions on age and sex by cause for GBD 2021			
**Cause**	**Minimum Age**	**Maximum Age**	**Sex Restrictions**
HIV/AIDS and sexually transmitted infections			
HIV/AIDS	1 month		
HIV/AIDS - Drug-susceptible Tuberculosis	1 month		
HIV/AIDS - Multidrug-resistant Tuberculosis without extensive drug resistance	1 month		
HIV/AIDS - Extensively drug-resistant Tuberculosis	1 month		
HIV/AIDS resulting in other diseases	1 month		
Sexually transmitted infections excluding HIV			
Syphilis			
Chlamydial infection	10		
Gonococcal infection	10		
Other sexually transmitted infections	10		
Respiratory infections and tuberculosis			
Tuberculosis	1 month		
Drug-susceptible tuberculosis	1 month		
Multidrug-resistant tuberculosis without extensive drug resistance	1 month		
Extensively drug-resistant tuberculosis	1 month		
Lower respiratory infections			
Influenza			
Pneumococcal pneumonia			
H influenzae type B pneumonia			
Group B streptococcus lri			
Respiratory syncytial virus pneumonia			
Other lower respiratory infections			
Upper respiratory infections			
Otitis media			
COVID-19			
Enteric infections			
Diarrhoeal diseases			
Cholera			
Other salmonella infections			
Shigellosis			
Enteropathogenic E coli infection			
Enterotoxigenic E coli infection			
Campylobacter enteritis			
Amoebiasis	1 month		
Cryptosporidiosis			
Rotaviral enteritis			
Aeromonas			
Clostridium difficile			
Norovirus			
Adenovirus			
Other bacterial foodborne diarrhea			
Other diarrheal diseases			
Typhoid and paratyphoid	7 days		
Typhoid fever	7 days		
Paratyphoid fever	7 days		
iNTS	7 days		
Polio	7 days		
Other intestinal infectious diseases	7 days		
Neglected tropical diseases and malaria			
Malaria			
Malaria falciparum			
Malaria vivax			
Other type and mix form of malaria			
Chagas disease	1 month		
Leishmaniasis	1 month		
Visceral leishmaniasis	1 month		
African trypanosomiasis	2		
Schistosomiasis	6 months		
Cysticercosis	2		
Cystic echinococcosis	2		
Dengue	7 days		
Yellow fever	7 days		
Rabies	1 month		
Intestinal nematode infections	1 month		
Ascariasis	1 month		
Ebola virus disease			
Zika virus disease			
Other neglected tropical diseases			
Other infectious diseases			
Meningitis			
Pneumococcal meningitis			
H influenzae type B meningitis			
Group B streptococcus meningitis			
Meningococcal infection			
Viral meningitis			
Other meningitis			
Encephalitis			

Appendix Table S7: Restrictions on age and sex by cause for GBD 2020			
Cause	Minimum Age	Maximum Age	Sex Restrictions
Diphtheria	1 month	59	
Pertussis	1 month	59	
Tetanus			
Measles	6 months	64	
Varicella and herpes zoster			
Rubella			
Mumps			
Acute hepatitis	1 month		
Acute hepatitis A	1 month		
Acute hepatitis B	1 month		
Acute hepatitis C	1 month		
Acute hepatitis E	1 month		
Zoonotic bacterial diseases	1 month		
Non-genital herpes infection	1 month		
Other unspecified infectious diseases			
Other drug-resistant infectious diseases			
Maternal and neonatal disorders		54	
Maternal disorders	10	54	Females Only
Maternal haemorrhage	10	54	Females Only
Maternal sepsis and other pregnancy related infections	10	54	Females Only
Maternal hypertensive disorders	10	54	Females Only
Maternal obstructed labour and uterine rupture	10	54	Females Only
Maternal abortive outcome	10	54	Females Only
Ectopic pregnancy	10	54	Females Only
Indirect maternal deaths	10	54	Females Only
Late maternal deaths	10	54	Females Only
Maternal deaths aggravated by HIV/AIDS	10	54	Females Only
Other maternal disorders	10	54	Females Only
Neonatal disorders		4	
Neonatal preterm birth		4	
Neonatal encephalopathy due to birth asphyxia and trauma		4	
Neonatal sepsis and other neonatal infections		4	
Hemolytic disease and other neonatal jaundice		4	
Other neonatal disorders		4	
Nutritional deficiencies	1 month		
Protein-energy malnutrition	1 month		
Other nutritional deficiencies	1 month		
Neoplasms			
Lip and oral cavity cancer	15		
Nasopharynx cancer	5		
Other pharynx cancer	20		
Oesophageal cancer	20		
Stomach cancer	15		
Colon and rectum cancer	15		
Liver cancer			
Liver cancer due to hepatitis B	10		
Liver cancer due to hepatitis C	10		
Liver cancer due to alcohol use	15		
Liver cancer due to NASH	15		
Hepatoblastoma		9	
Liver cancer due to other causes	10		
Gallbladder and biliary tract cancer	20		
Pancreatic cancer	15		
Larynx cancer	20		
Tracheal, bronchus, and lung cancer	15		
Malignant skin melanoma	15		
Non-melanoma skin cancer	20		
Non-melanoma skin cancer (squamous-cell carcinoma)	20		
Soft tissue and other extraosseous sarcomas			
Malignant neoplasm of bone and articular cartilage	12 months		
Breast cancer	15		
Cervical cancer	15		Females Only
Uterine cancer	20		Females Only
Ovarian cancer	15		Females Only
Prostate cancer	20		Males Only
Testicular cancer	15		Males Only
Kidney cancer			
Bladder cancer	15		
Brain and nervous system cancer			
Eye cancer			
Retinoblastoma		9	
Other eye cancers	10		
Neuroblastoma and other peripheral nervous cell tumors			
Thyroid cancer	5		
Mesothelioma	20		
Hodgkin lymphoma	2		
Non-Hodgkin's lymphoma	12 months		
Burkitt lymphoma	12 months		

Appendix Table S7: Restrictions on age and sex by cause for GBD 2020			
**Cause**	**Minimum Age**	**Maximum Age**	**Sex Restrictions**
Other non-Hodgkin lymphoma	12 months		
Multiple myeloma	20		
Leukaemia			
Acute lymphoid leukaemia			
Chronic lymphoid leukaemia	20		
Acute myeloid leukaemia			
Chronic myeloid leukaemia			
Other leukaemia			
Other malignant cancers			
Other neoplasms			
Myelodysplastic, myeloproliferative, and other hematopoietic neoplasms			
Benign and in situ CNS neoplasms	1 month		
Other benign and in situ neoplasms	1 month		
Cardiovascular diseases			
Rheumatic heart disease	12 months		
Ischaemic heart disease	15		
Angina	15		
Acute myocardial infarction	15		
Chronic ischemic heart disease	15		
Stroke			
Ischaemic stroke			
Intracerebral hemorrhage			
Subarachnoid hemorrhage			
Hypertensive heart disease	15		
Non-rheumatic valvular heart disease	15		
Non-rheumatic calcific aortic valve disease	15		
Non-rheumatic degenerative mitral valve disease	15		
Other non-rheumatic valve diseases	15		
Cardiomyopathy and myocarditis			
Myocarditis			
Alcoholic cardiomyopathy	15		
Other cardiomyopathy			
Pulmonary Arterial Hypertension			
Atrial fibrillation and flutter	30		
Aortic aneurysm	15		
Peripheral vascular disease	40		
Endocarditis			
Phlebitis and thrombophlebitis			
Varicose veins	15		
Other cardiovascular and circulatory diseases			
Chronic respiratory diseases			
Chronic obstructive pulmonary disease	15		
Pneumoconiosis	15		
Silicosis	15		
Asbestosis	15		
Coal workers pneumoconiosis	15		
Other pneumoconiosis	15		
Asthma	12 months		
Interstitial lung disease and pulmonary sarcoidosis	2		
Chronic diseases of upper respiratory system	12 months		
Allergic disorders of upper respiratory system	12 months		
Sleep apnea	15		
Other chronic respiratory diseases			
Digestive diseases			
Cirrhosis and other chronic liver diseases	12 months		
Cirrhosis and other chronic liver diseases due to hepatitis B	12 months		
Cirrhosis and other chronic liver diseases due to hepatitis C	12 months		
Cirrhosis and other chronic liver diseases due to alcohol use	15		
Cirrhosis due to NASH	15		
Cirrhosis and other chronic liver diseases due to other causes	12 months		
Upper digestive system diseases	6 months		
Peptic ulcer disease	2		
Gastritis and duodenitis	2		
Appendicitis	12 months		
Paralytic ileus and intestinal obstruction			
Inguinal, femoral, and abdominal hernia			
Inguinal hernia			
Femoral hernia			
Diaphragmatic hernia			
Abdominal and other hernia			
Inflammatory bowel disease	2		
Ulcerative colitis	2		
Crohn's disease	2		
Vascular intestinal disorders	2		
Gallbladder and biliary diseases	2		
Pancreatitis	2		
Diverticular disease of intestines	2		

Appendix Table S7: Restrictions on age and sex by cause for GBD 2020			
**Cause**	**Minimum Age**	**Maximum Age**	**Sex Restrictions**
Hemorrhoids, anal fissure, anal abscess, and fistula	2		
Celiac disease	12 months		
Esophageal diseases	2		
Other digestive diseases	12 months		
Neurological disorders			
Alzheimer's disease and other dementias	40		
Parkinson's disease	20		
Idiopathic epilepsy			
Multiple sclerosis	5		
Motor neuron disease			
Other neurological disorders			
Fibromyalgia and other neuropathies			
Muscular dystrophy			
Mental disorders	5		
Eating disorders	5	49	
Anorexia nervosa	5	49	
Substance use disorders			
Alcohol use disorders	10		
Drug use disorders			
Opioid use disorders			
Cocaine use disorders	10		
Amphetamine use disorders	10		
Other drug use disorders	10		
Diabetes and kidney diseases			
Diabetes mellitus	1 month		
Diabetes mellitus type 1	1 month		
Diabetes mellitus type 2	15		
Chronic kidney disease			
Chronic kidney disease due to diabetes mellitus type 1			
Chronic kidney disease due to diabetes mellitus type 2	15		
Chronic kidney disease due to hypertension	15		
Chronic kidney disease due to glomerulonephritis			
Chronic kidney disease due to other and unspecified causes			
Acute glomerulonephritis			
Skin and subcutaneous diseases			
Bacterial skin diseases			
Cellulitis			
Pyoderma			
Decubitus ulcer	2		
Other skin and subcutaneous diseases			
Musculoskeletal disorders	1 month		
Rheumatoid arthritis	5		
Osteoporosis and related pathological fracture	1 month		
Pyogenic and reactive arthritis	1 month		
Systemic and discoid lupus erythematous	1 month		
Shoulder lesions	1 month		
Osteomyelitis non-traumatic	1 month		
Other myositis	1 month		
Other musculoskeletal disorders	1 month		
Other non-communicable diseases			
Congenital anomalies		69	
Neural tube defects		69	
Congenital heart anomalies		69	
Orofacial clefts		4	
Down's syndrome		69	
Other chromosomal abnormalities		69	
Congenital musculoskeletal and limb anomalies		69	
Urogenital congenital anomalies		69	
Digestive congenital anomalies		69	
Other congenital anomalies		69	
Urinary diseases and male infertility			
Urinary tract infections and interstitial nephritis			
Urolithiasis	2		
Other urinary diseases			
Gynecological diseases	10		Females Only
Uterine fibroids	10		Females Only
Endometriosis	10	54	Females Only
Genital prolapse	10		Females Only
Other gynecological diseases	10		Females Only
Hemoglobinopathies and hemolytic anaemias			
Thalassemias			
Sickle cell disorders			
G6PD deficiency			
Other hemoglobinopathies and hemolytic anaemias			
Endocrine, metabolic, blood, and immune disorders			
Thyroid diseases			
Obesity			
Lipoprotein metabolism and other lipidaemias disorders			

Appendix Table S7: Restrictions on age and sex by cause for GBD 2020			
**Cause**	**Minimum Age**	**Maximum Age**	**Sex Restrictions**
Cystic fibrosis			
Other endocrine, metabolic, blood, and immune disorders			
Sudden infant death syndrome	7 days	23 months	
Transport injuries			
Road injuries			
Pedestrian road injuries			
Cyclist road injuries	12 months	94	
Motorcyclist road injuries		94	
Motor vehicle road injuries			
Other road injuries			
Other transport injuries			
Unintentional injuries			
Falls			
Drowning			
Fire, heat, and hot substances			
Poisonings			
Poisoning by carbon monoxide			
Poisoning by other means			
Exposure to mechanical forces			
Unintentional firearm injuries			
Other exposure to mechanical forces			
Adverse effects of medical treatment			
Post procedural or drug treatment disorders			
Animal contact			
Venomous animal contact			
Non-venomous animal contact			
Foreign body			
Pulmonary aspiration and foreign body in airway			
Foreign body in other body part			
Electrocution			
Environmental heat and cold exposure			
Exposure to forces of nature			
Victim of lightning			
Earthquake			
Volcanic eruption			
Avalanche, landslide and other earth movements			
Cataclysmic storm			
Flood			
Other forms of forces of nature			
Other unintentional injuries			
Self-harm and interpersonal violence			
Self-harm	10		
Self-harm by firearm	10		
Self-harm by other specified means	10		
Interpersonal violence			
Assault by firearm			
Assault by sharp object			
Assault by other means			
Conflict and terrorism			
Terrorism			
Military operations			
Executions and police conflict			

Appendix Table S8. HIV/AIDS-related garbage code redistribution packages

Package Name	ICD10 codes	ICD9 codes
Actinomycosis	A42-A42.9	039-039.9, 113-113.6
Bartonellosis	A44-A44.9	088.0-088.7
Urogenital Candidiasis	B37.3-B37.4	112.1-112.2
Candidiasis	B37-B37.2, B37.5-B37.9	112-112.0, 112.3-112.9
Coccidioidomycosis	B38-B38.9	114-114.9
Histoplasmosis	B39-B39.9	115-115.9
Blastomycosis	B40-B40.9	116-116.0, 116.2-116.9
Paracoccidioidomycosis	B41-B41.9	116.1
Sporotrichosis and Chromomycosis	B42-B43.9	117.1
Zygomycosis	B46-B46.9	117.3
Aspergillosis	B44-B44.9	117.7
Toxoplasmosis	B58-B58.9	130-130.9
Pneumocystosis	B59-B59.9	136.3-136.5
Cryptococcosis	B45-B45.9	117.5
Nocardiosis	A43-A43.9	117.2
Unspecified mycosis	B49-B49.9	117-117.0, 117.4, 117.6, 117.8-118.9
Cutaneous leishmaniasis	B55, B55.1-B55.9	085.1-085.5
Mycobacterial skin infection	A31.1-A31.2	031.1
Other Mycobacterial infection	A31-A31.0, A31.8-A31.9	031-031.0, 031.2-031.9
Immunodeficiency cell	D81-D82.9	279.2-279.4
Immunodeficiency antibody	D80-D80.9	279.0-279.1
Immunodeficiency other	D83-D84.9, D89.8-D89.9	279, 279.5-279.9
Kaposi's sarcoma	C46-C46.9	176-176.9

Appendix Table S9: Underlying indicators for percent well-certified for data source with maximum percent well certified in each 5-year time interval for 204 countries, 1980-2020.

Location	Time Window	Stars	Percent Well-Certified [PWC] (%)	Max PWC Data Year	Max PWC Data Source	Completeness (%)	Percent Major Garbage (%)	Verbal Autopsy Adjustment (None for VR) (%)
Afghanistan	1980-1984	0						
Afghanistan	1985-1989	0						
Afghanistan	1990-1994	0						
Afghanistan	1995-1999	0						
Afghanistan	2000-2004	1	3.4	2001	Afghanistan - Badghis Nutrition and Health Survey 2002		47.3	6.4
Afghanistan	2005-2009	2	28.3	2008	Afghanistan Special Demographic and Health Survey 2010		55.8	64.0
Afghanistan	2010-2020	2	28.0	2015	Afghanistan Health Survey 2015		8.0	30.5
Albania	1980-1984	0						
Albania	1985-1989	4	65.4	1989	Vital Registration	100.0	34.6	
Albania	1990-1994	3	64.7	1993	Vital Registration	96.3	32.8	
Albania	1995-1999	4	68.8	1996	Vital Registration	97.2	29.2	
Albania	2000-2004	4	70.8	2003	Vital Registration	97.2	27.1	
Albania	2005-2009	4	68.9	2006	Vital Registration	95.6	27.9	
Albania	2010-2020	3	50.5	2010	Vital Registration	65.3	22.7	
Algeria	1980-1984	0						
Algeria	1985-1989	0						
Algeria	1990-1994	0						
Algeria	1995-1999	0						
Algeria	2000-2004	0						
Algeria	2005-2009	2	15.6	2006	Vital Registration	31.0	49.6	
Algeria	2010-2020	0						
American Samoa	1980-1984	0						
American Samoa	1985-1989	0						
American Samoa	1990-1994	0						
American Samoa	1995-1999	3	61.2	1997	Vital Registration	83.0	26.3	
American Samoa	2000-2004	4	68.1	2002	Vital Registration	83.6	18.5	
American Samoa	2005-2009	4	68.3	2009	Vital Registration	85.6	20.2	
American Samoa	2010-2020	4	67.1	2015	Vital Registration	79.1	15.2	
Andorra	1980-1984	0						
Andorra	1985-1989	0						
Andorra	1990-1994	0						
Andorra	1995-1999	0						
Andorra	2000-2004	0						
Andorra	2005-2009	0						
Andorra	2010-2020	3	45.6	2012	Vital Registration	56.6	19.4	
Angola	1980-1984	0						
Angola	1985-1989	0						
Angola	1990-1994	0						
Angola	1995-1999	0						
Angola	2000-2004	0						
Angola	2005-2009	0						
Angola	2010-2020	1	2.7	2010	Angola - Dande Health and Demographic Surveillance System		57.8	6.4
Antigua and Barbuda	1980-1984	3	58.0	1983	Vital Registration	81.9	29.2	
Antigua and Barbuda	1985-1989	4	70.0	1989	Vital Registration	100.0	30.0	
Antigua and Barbuda	1990-1994	4	73.1	1993	Vital Registration	96.7	24.4	
Antigua and Barbuda	1995-1999	4	79.1	1999	Vital Registration	100.0	20.9	
Antigua and Barbuda	2000-2004	4	82.6	2003	Vital Registration	99.3	16.8	
Antigua and Barbuda	2005-2009	4	78.8	2008	Vital Registration	100.0	21.2	
Antigua and Barbuda	2010-2020	4	78.4	2014	Vital Registration	100.0	21.6	
Argentina	1980-1984	4	75.9	1981	Vital Registration	100.0	24.1	
Argentina	1985-1989	4	71.2	1988	Vital Registration	100.0	28.8	
Argentina	1990-1994	4	70.4	1990	Vital Registration	100.0	29.6	
Argentina	1995-1999	4	69.7	1996	Vital Registration	100.0	30.3	
Argentina	2000-2004	4	68.8	2004	Vital Registration	100.0	31.2	
Argentina	2005-2009	4	68.9	2005	Vital Registration	100.0	31.1	
Argentina	2010-2020	4	73.6	2017	Vital Registration	100.0	26.4	
Armenia	1980-1984	4	73.2	1982	Vital Registration	84.1	12.9	
Armenia	1985-1989	4	78.3	1986	Vital Registration	89.0	12.0	
Armenia	1990-1994	4	81.1	1992	Vital Registration	94.1	13.8	
Armenia	1995-1999	4	84.5	1999	Vital Registration	97.3	13.2	
Armenia	2000-2004	5	86.0	2002	Vital Registration	100.0	14.0	
Armenia	2005-2009	5	89.9	2006	Vital Registration	99.1	9.3	
Armenia	2010-2020	5	87.7	2010	Vital Registration	97.9	10.5	
Australia	1980-1984	5	93.3	1983	Vital Registration	100.0	6.7	
Australia	1985-1989	5	93.7	1986	Vital Registration	100.0	6.3	
Australia	1990-1994	5	93.6	1991	Vital Registration	100.0	6.4	
Australia	1995-1999	5	93.5	1996	Vital Registration	100.0	6.5	
Australia	2000-2004	5	92.5	2001	Vital Registration	100.0	7.5	
Australia	2005-2009	5	91.9	2006	Vital Registration	100.0	8.1	
Australia	2010-2020	5	91.2	2013	Vital Registration	100.0	8.8	
Austria	1980-1984	5	92.2	1984	Vital Registration	100.0	7.8	
Austria	1985-1989	5	93.1	1987	Vital Registration	100.0	6.9	
Austria	1990-1994	5	92.8	1990	Vital Registration	100.0	7.2	
Austria	1995-1999	5	92.3	1996	Vital Registration	100.0	7.7	
Austria	2000-2004	5	94.7	2003	Vital Registration	100.0	5.3	
Austria	2005-2009	5	93.6	2005	Vital Registration	100.0	6.4	
Austria	2010-2020	5	91.1	2010	Vital Registration	100.0	8.9	
Azerbaijan	1980-1984	4	79.7	1981	Vital Registration	91.0	12.4	
Azerbaijan	1985-1989	4	76.0	1985	Vital Registration	86.8	12.4	
Azerbaijan	1990-1994	4	70.5	1992	Vital Registration	79.3	11.1	
Azerbaijan	1995-1999	4	65.3	1995	Vital Registration	76.0	14.1	
Azerbaijan	2000-2004	4	68.6	2003	Vital Registration	74.6	8.0	
Azerbaijan	2005-2009	3	39.9	2007	Vital Registration	72.6	45.0	
Azerbaijan	2010-2020	0						
Bahrain	1980-1984	0						
Bahrain	1985-1989	4	75.1	1986	Vital Registration	100.0	24.9	
Bahrain	1990-1994	0						
Bahrain	1995-1999	3	53.4	1997	Vital Registration	79.9	33.2	
Bahrain	2000-2004	3	51.7	2000	Vital Registration	84.3	38.7	
Bahrain	2005-2009	3	47.9	2006	Vital Registration	79.2	39.5	
Bahrain	2010-2020	3	50.2	2014	Vital Registration	76.2	34.1	
Bangladesh	1980-1984	1	2.2	1983	Bangladesh - Matlab Health and Demographic Surveillance System		66.2	6.4
Bangladesh	1985-1989	1	3.3	1989	Bangladesh - Matlab Health and Demographic Surveillance System		48.8	6.4
Bangladesh	1990-1994	2	28.9	1991	Causes of childhood deaths in Bangladesh: an update		1.8	29.4
Bangladesh	1995-1999	1	3.9	1998	Bangladesh - Matlab Health and Demographic Surveillance System		39.4	6.4
Bangladesh	2000-2004	2	19.0	2004	Bangladesh Demographic and Health Survey 2004		4.4	19.9
Bangladesh	2005-2009	2	22.6	2005	Bangladesh Child Health and Injury Survey 2005		1.7	23.0
Bangladesh	2010-2020	3	53.2	2015	Verbal Autopsy		1.7	54.1
Barbados	1980-1984	4	75.8	1983	Vital Registration	100.0	24.2	
Barbados	1985-1989	4	78.3	1986	Vital Registration	100.0	21.7	
Barbados	1990-1994	4	80.0	1992	Vital Registration	100.0	20.0	
Barbados	1995-1999	4	79.8	1995	Vital Registration	100.0	20.2	
Barbados	2000-2004	4	77.3	2000	Vital Registration	100.0	22.7	
Barbados	2005-2009	4	80.6	2009	Vital Registration	98.7	18.3	
Barbados	2010-2020	4	80.1	2011	Vital Registration	97.8	18.1	
Belarus	1980-1984	5	85.7	1982	Vital Registration	100.0	14.3	
Belarus	1985-1989	5	88.0	1986	Vital Registration	100.0	12.0	
Belarus	1990-1994	4	79.9	1990	Vital Registration	100.0	20.1	
Belarus	1995-1999	4	80.6	1999	Vital Registration	100.0	19.4	
Belarus	2000-2004	4	82.4	2002	Vital Registration	100.0	17.6	
Belarus	2005-2009	4	83.2	2009	Vital Registration	100.0	16.8	
Belarus	2010-2020	5	85.4	2018	Vital Registration	95.5	10.6	
Belgium	1980-1984	4	80.0	1981	Vital Registration	100.0	20.0	
Belgium	1985-1989	4	80.6	1987	Vital Registration	100.0	19.4	
Belgium	1990-1994	4	84.1	1994	Vital Registration	100.0	15.9	
Belgium	1995-1999	5	86.2	1998	Vital Registration	100.0	13.8	
Belgium	2000-2004	5	86.1	2000	Vital Registration	100.0	13.9	
Belgium	2005-2009	5	86.3	2008	Vital Registration	100.0	13.7	
Belgium	2010-2020	4	82.7	2011	Vital Registration	99.5	16.9	
Belize	1980-1984	3	59.9	1980	Vital Registration	89.1	32.7	
Belize	1985-1989	3	62.9	1987	Vital Registration	83.8	25.0	
Belize	1990-1994	3	57.7	1993	Vital Registration	89.5	35.5	
Belize	1995-1999	4	74.7	1998	Vital Registration	100.0	25.3	
Belize	2000-2004	4	75.8	2001	Vital Registration	100.0	24.2	
Belize	2005-2009	5	87.2	2009	Vital Registration	100.0	12.8	
Belize	2010-2020	5	87.5	2011	Vital Registration	100.0	12.5	
Benin	1980-1984	0						
Benin	1985-1989	1	2.1	1989	Incidence de décès de 0 à 1 an dans une cohorte de 802 enfants en milieu rural au sud du Bénin		6.0	2.2
Benin	1990-1994	0						
Benin	1995-1999	0						
Benin	2000-2004	0						
Benin	2005-2009	0						
Benin	2010-2020	1	1.2	2016	A population-based cardiovascular cohort in Subsaharian Africa: The pilot project Tanve Health Study (Tahes) in Benin		54.4	2.7
Bermuda	1980-1984	5	89.7	1980	Vital Registration	100.0	10.3	
Bermuda	1985-1989	5	90.7	1989	Vital Registration	100.0	9.3	
Bermuda	1990-1994	5	90.3	1992	Vital Registration	100.0	9.7	
Bermuda	1995-1999	5	94.9	1996	Vital Registration	100.0	5.1	
Bermuda	2000-2004	5	91.8	2002	Vital Registration	98.6	6.9	
Bermuda	2005-2009	5	88.6	2007	Vital Registration	100.0	11.4	
Bermuda	2010-2020	5	92.8	2014	Vital Registration	99.9	7.0	
Bhutan	1980-1984	0						
Bhutan	1985-1989	0						
Bhutan	1990-1994	0						
Bhutan	1995-1999	0						
Bhutan	2000-2004	0						
Bhutan	2005-2009	0						
Bhutan	2010-2020	0						
Bolivia	1980-1984	0						
Bolivia	1985-1989	0						
Bolivia	1990-1994	0						
Bolivia	1995-1999	0						
Bolivia	2000-2004	2	12.7	2003	Vital Registration	38.9	67.3	
Bolivia	2005-2009	0						
Bolivia	2010-2020	0						
Bosnia and Herzegovina	1980-1984	0						
Bosnia and Herzegovina	1985-1989	3	64.6	1988	Vital Registration	100.0	35.4	
Bosnia and Herzegovina	1990-1994	4	65.9	1990	Vital Registration	100.0	34.1	
Bosnia and Herzegovina	1995-1999	0						
Bosnia and Herzegovina	2000-2004	0						
Bosnia and Herzegovina	2005-2009	0						
Bosnia and Herzegovina	2010-2020	4	65.1	2014	Vital Registration	89.6	27.3	
Botswana	1980-1984	0						
Botswana	1985-1989	0						
Botswana	1990-1994	0						
Botswana	1995-1999	0						
Botswana	2000-2004	0						
Botswana	2005-2009	0						
Botswana	2010-2020	0						
Brazil	1980-1984	3	52.5	1984	Vital Registration	85.6	38.7	
Brazil	1985-1989	3	57.1	1989	Vital Registration	87.9	35.0	
Brazil	1990-1994	3	64.3	1994	Vital Registration	95.8	32.9	
Brazil	1995-1999	4	67.9	1999	Vital Registration	96.2	29.4	
Brazil	2000-2004	4	72.9	2004	Vital Registration	97.3	25.1	
Brazil	2005-2009	4	78.7	2009	Vital Registration	97.1	19.0	
Brazil	2010-2020	4	83.3	2017	Vital Registration	100.0	16.7	
Brunei	1980-1984	0						
Brunei	1985-1989	0						
Brunei	1990-1994	0						
Brunei	1995-1999	3	61.2	1998	Vital Registration	72.5	15.5	
Brunei	2000-2004	4	65.7	2002	Vital Registration	81.3	19.2	
Brunei	2005-2009	4	70.0	2007	Vital Registration	86.5	19.1	
Brunei	2010-2020	4	79.2	2019	Vital Registration	92.3	14.1	
Bulgaria	1980-1984	4	79.6	1982	Vital Registration	100.0	20.4	
Bulgaria	1985-1989	4	78.2	1985	Vital Registration	96.8	19.2	
Bulgaria	1990-1994	4	77.6	1991	Vital Registration	96.3	19.4	
Bulgaria	1995-1999	4	70.1	1995	Vital Registration	89.6	21.7	
Bulgaria	2000-2004	4	65.8	2002	Vital Registration	89.6	26.6	
Bulgaria	2005-2009	4	66.8	2005	Vital Registration	89.6	25.4	
Bulgaria	2010-2020	4	65.1	2012	Vital Registration	90.2	27.9	
Burkina Faso	1980-1984	1	3.7	1984	The burden of malaria mortality among African children in the year 2000		10.1	4.1
Burkina Faso	1985-1989	0						
Burkina Faso	1990-1994	0						
Burkina Faso	1995-1999	1	4.6	1998	Measuring the local burden of disease. A study of years of life lost in sub-Saharan Africa		27.4	6.4

Appendix Table S9: Underlying indicators for percent well-certified for data source with maximum percent well certified in each 5-year time interval for 204 countries, 1980-2020.

Location	Time Window	Stars	Percent Well-Certified [PWC] (%)	Max PWC Data Year	Max PWC Data Source	Completeness (%)	Percent Major Garbage (%)	Verbal Autopsy Adjustment (None for VR) (%)
Burkina Faso	2000-2004	1	6.3	2000	Burkina Faso - Nouna Health and Demographic Surveillance System		0.9	6.4
Burkina Faso	2005-2009	1	2.3	2009	An improved method for physician-certified verbal autopsy reduces the rate of discrepancy: experiences in the Nouna Health and Demographic Surveillance Site (NHDSS), Burkina Faso		63.7	6.4
Burkina Faso	2010-2020	1	0.3	2010	Africa, Asia, Oceania - INDEPTH Network Cause-Specific Mortality - Release 2014		95.6	6.4
Burundi	1980-1984	0						
Burundi	1985-1989	0						
Burundi	1990-1994	1	3.2	1990	Mortality and morbidity at young ages in a stable hyperendemic malaria region, community Nyanza-Lac, Imbo South, Burundi		4.3	3.3
Burundi	1995-1999	0						
Burundi	2000-2004	0						
Burundi	2005-2009	0						
Burundi	2010-2020	0						
Cambodia	1980-1984	0						
Cambodia	1985-1989	0						
Cambodia	1990-1994	0						
Cambodia	1995-1999	0						
Cambodia	2000-2004	1	0.9	2001	Community-based surveillance: a pilot study from rural Cambodia		86.6	6.4
Cambodia	2005-2009	1	4.2	2009	Mortality in Cambodia An 18-Month Prospective Community-based Surveillance of All-age Deaths Using Verbal Autopsies		35.0	6.4
Cambodia	2010-2020	0						
Cameroon	1980-1984	0						
Cameroon	1985-1989	0						
Cameroon	1990-1994	0						
Cameroon	1995-1999	0						
Cameroon	2000-2004	0						
Cameroon	2005-2009	0						
Cameroon	2010-2020	0						
Canada	1980-1984	5	90.5	1981	Vital Registration	100.0	9.5	
Canada	1985-1989	5	90.7	1986	Vital Registration	100.0	9.3	
Canada	1990-1994	5	89.9	1992	Vital Registration	100.0	10.1	
Canada	1995-1999	5	89.5	1999	Vital Registration	100.0	10.5	
Canada	2000-2004	5	90.0	2001	Vital Registration	100.0	10.0	
Canada	2005-2009	5	90.4	2009	Vital Registration	100.0	9.6	
Canada	2010-2020	5	90.3	2013	Vital Registration	100.0	9.7	
Cape Verde	1980-1984	3	47.6	1980	Vital Registration	95.3	50.0	
Cape Verde	1985-1989	0						
Cape Verde	1990-1994	1	0.3	1992	Deaths among women of reproductive age in Cape Verde: causes and avoidability		6.2	0.3
Cape Verde	1995-1999	0						
Cape Verde	2000-2004	0						
Cape Verde	2005-2009	0						
Cape Verde	2010-2020	4	68.3	2012	Vital Registration	98.0	30.3	
Central African Republic	1980-1984	0						
Central African Republic	1985-1989	0						
Central African Republic	1990-1994	0						
Central African Republic	1995-1999	0						
Central African Republic	2000-2004	0						
Central African Republic	2005-2009	0						
Central African Republic	2010-2020	0						
Chad	1980-1984	0						
Chad	1985-1989	0						
Chad	1990-1994	0						
Chad	1995-1999	0						
Chad	2000-2004	0						
Chad	2005-2009	0						
Chad	2010-2020	1	1.1	2019	Validation of elimination of maternal and neonatal tetanus in Chad - Weekly Epidemiological Record 2019		0.4	1.1
Chile	1980-1984	4	73.8	1983	Vital Registration	98.0	24.6	
Chile	1985-1989	4	75.4	1988	Vital Registration	99.4	24.1	
Chile	1990-1994	4	82.0	1994	Vital Registration	99.1	17.2	
Chile	1995-1999	5	85.2	1999	Vital Registration	100.0	14.8	
Chile	2000-2004	5	90.8	2004	Vital Registration	99.6	8.8	
Chile	2005-2009	5	90.5	2005	Vital Registration	99.2	8.7	
Chile	2010-2020	5	90.4	2012	Vital Registration	100.0	9.6	
China	1980-1984	0						
China	1985-1989	1	0.9	1986	Infant mortality among various nationalities in the middle part of Guizhou, China		1.5	0.9
China	1990-1994	4	69.9	1994	Vital Registration - Sample	81.5	14.2	
China	1995-1999	4	73.6	1998	Vital Registration - Sample	86.4	14.9	
China	2000-2004	4	66.6	2000	Vital Registration - Sample	80.0	16.7	
China	2005-2009	4	72.1	2009	Vital Registration - Sample	76.9	6.3	
China	2010-2020	4	71.8	2010	Vital Registration - Sample	76.6	6.3	
Colombia	1980-1984	4	69.3	1983	Vital Registration	92.6	25.3	
Colombia	1985-1989	4	72.4	1988	Vital Registration	93.0	22.2	
Colombia	1990-1994	4	76.3	1994	Vital Registration	92.9	17.9	
Colombia	1995-1999	5	88.2	1999	Vital Registration	100.0	11.8	
Colombia	2000-2004	5	89.7	2002	Vital Registration	100.0	10.3	
Colombia	2005-2009	5	89.1	2008	Vital Registration	100.0	10.8	
Colombia	2010-2020	5	91.4	2018	Vital Registration	100.0	8.6	
Comoros	1980-1984	0						
Comoros	1985-1989	0						
Comoros	1990-1994	0						
Comoros	1995-1999	0						
Comoros	2000-2004	0						
Comoros	2005-2009	0						
Comoros	2010-2020	0						
Congo (Brazzaville)	1980-1984	0						
Congo (Brazzaville)	1985-1989	0						
Congo (Brazzaville)	1990-1994	0						
Congo (Brazzaville)	1995-1999	0						
Congo (Brazzaville)	2000-2004	0						
Congo (Brazzaville)	2005-2009	0						
Congo (Brazzaville)	2010-2020	0						
Cook Islands	1980-1984	0						
Cook Islands	1985-1989	0						
Cook Islands	1990-1994	0						
Cook Islands	1995-1999	0						
Cook Islands	2000-2004	3	60.1	2000	Vital Registration	100.0	39.9	
Cook Islands	2005-2009	3	50.2	2006	Vital Registration	71.7	30.0	
Cook Islands	2010-2020	4	66.1	2014	Vital Registration	89.6	26.2	
Costa Rica	1980-1984	4	81.5	1984	Vital Registration	100.0	18.5	
Costa Rica	1985-1989	4	83.1	1989	Vital Registration	100.0	16.9	
Costa Rica	1990-1994	4	83.2	1993	Vital Registration	100.0	16.8	
Costa Rica	1995-1999	5	91.0	1999	Vital Registration	99.6	8.7	
Costa Rica	2000-2004	5	92.7	2003	Vital Registration	100.0	7.3	
Costa Rica	2005-2009	5	91.6	2005	Vital Registration	100.0	8.4	
Costa Rica	2010-2020	5	91.9	2018	Vital Registration	99.8	7.9	
Croatia	1980-1984	0						
Croatia	1985-1989	4	84.7	1989	Vital Registration	100.0	15.3	
Croatia	1990-1994	5	85.7	1990	Vital Registration	100.0	14.3	
Croatia	1995-1999	4	84.7	1999	Vital Registration	100.0	15.3	
Croatia	2000-2004	5	86.3	2004	Vital Registration	98.1	12.0	
Croatia	2005-2009	5	89.0	2009	Vital Registration	99.1	10.3	
Croatia	2010-2020	5	92.6	2015	Vital Registration	99.4	6.8	
Cuba	1980-1984	5	85.9	1984	Vital Registration	99.9	14.0	
Cuba	1985-1989	5	87.1	1989	Vital Registration	100.0	12.9	
Cuba	1990-1994	5	86.6	1990	Vital Registration	100.0	13.4	
Cuba	1995-1999	5	89.8	1997	Vital Registration	100.0	10.2	
Cuba	2000-2004	5	91.3	2004	Vital Registration	100.0	8.7	
Cuba	2005-2009	5	92.1	2009	Vital Registration	100.0	7.9	
Cuba	2010-2020	5	92.3	2012	Vital Registration	100.0	7.7	
Cyprus	1980-1984	0						
Cyprus	1985-1989	0						
Cyprus	1990-1994	0						
Cyprus	1995-1999	2	27.6	1999	Vital Registration	74.4	62.9	
Cyprus	2000-2004	3	55.9	2004	Vital Registration	78.1	28.4	
Cyprus	2005-2009	3	60.2	2005	Vital Registration	78.7	23.4	
Cyprus	2010-2020	4	65.0	2015	Vital Registration	75.8	14.2	
Czechia	1980-1984	0						
Czechia	1985-1989	5	91.2	1986	Vital Registration	100.0	8.8	
Czechia	1990-1994	5	90.4	1990	Vital Registration	100.0	9.6	
Czechia	1995-1999	5	85.8	1995	Vital Registration	100.0	14.2	
Czechia	2000-2004	5	86.5	2000	Vital Registration	100.0	13.5	
Czechia	2005-2009	5	86.5	2007	Vital Registration	100.0	13.5	
Czechia	2010-2020	5	87.9	2013	Vital Registration	100.0	12.1	
Côte d'Ivoire	1980-1984	0						
Côte d'Ivoire	1985-1989	1	3.6	1988	Effet de l'observance des d'approvisionnement en eau et de la therapie par voie orale sur les diarrhees chez les enfants de moins de 5 de la Cote d'Ivoire		8.0	3.9
Côte d'Ivoire	1990-1994	1	3.5	1990	Effet de l'observance des d'approvisionnement en eau et de la therapie par voie orale sur les diarrhees chez les enfants de moins de 5 de la Cote d'Ivoire		8.4	3.9
Côte d'Ivoire	1995-1999	0						
Côte d'Ivoire	2000-2004	0						
Côte d'Ivoire	2005-2009	1	0.2	2009	Africa, Asia, Oceania - INDEPTH Network Cause-Specific Mortality - Release 2014		97.3	6.4
Côte d'Ivoire	2010-2020	1	0.1	2010	Africa, Asia, Oceania - INDEPTH Network Cause-Specific Mortality - Release 2014		98.1	6.4
DR Congo	1980-1984	0						
DR Congo	1985-1989	1	0.8	1986	Etude de la mortalité globale et de la mortalité liée au paludisme dans le Kivu montagneux, Zaire		86.9	6.4
DR Congo	1990-1994	1	3.6	1990	Influence of nutritional status on child mortality in rural Zaire		2.0	3.7
DR Congo	1995-1999	0						
DR Congo	2000-2004	0						
DR Congo	2005-2009	0						
DR Congo	2010-2020	1	0.7	2019	Validation of maternal and neonatal tetanus elimination in the Democratic Republic of the Congo - Weekly Epidemiological Record 2019		0.6	0.7
Denmark	1980-1984	4	83.6	1981	Vital Registration	100.0	16.4	
Denmark	1985-1989	4	82.3	1985	Vital Registration	100.0	17.7	
Denmark	1990-1994	5	85.7	1994	Vital Registration	100.0	14.3	
Denmark	1995-1999	5	88.1	1999	Vital Registration	100.0	11.9	
Denmark	2000-2004	5	87.7	2000	Vital Registration	100.0	12.3	
Denmark	2005-2009	5	87.0	2005	Vital Registration	100.0	13.0	
Denmark	2010-2020	5	87.1	2016	Vital Registration	99.8	12.7	
Djibouti	1980-1984	0						
Djibouti	1985-1989	0						
Djibouti	1990-1994	0						
Djibouti	1995-1999	0						
Djibouti	2000-2004	0						
Djibouti	2005-2009	0						
Djibouti	2010-2020	0						
Dominica	1980-1984	3	53.3	1984	Vital Registration	75.1	29.1	
Dominica	1985-1989	3	48.1	1986	Vital Registration	75.8	36.5	
Dominica	1990-1994	3	52.5	1993	Vital Registration	80.2	34.4	
Dominica	1995-1999	3	60.5	1999	Vital Registration	88.4	31.6	
Dominica	2000-2004	3	55.9	2004	Vital Registration	80.0	30.1	
Dominica	2005-2009	4	69.3	2006	Vital Registration	78.1	11.2	
Dominica	2010-2020	4	71.8	2015	Vital Registration	90.7	20.8	
Dominican Republic	1980-1984	3	46.5	1984	Vital Registration	74.3	37.5	
Dominican Republic	1985-1989	3	46.7	1985	Vital Registration	72.9	35.9	
Dominican Republic	1990-1994	3	41.4	1990	Vital Registration	63.1	34.3	
Dominican Republic	1995-1999	3	47.7	1999	Vital Registration	66.1	27.8	
Dominican Republic	2000-2004	3	48.3	2004	Vital Registration	63.1	23.4	
Dominican Republic	2005-2009	3	46.7	2005	Vital Registration	59.4	21.5	
Dominican Republic	2010-2020	3	48.7	2013	Vital Registration	60.3	19.3	
Ecuador	1980-1984	4	65.6	1984	Vital Registration	100.0	34.4	
Ecuador	1985-1989	4	68.3	1988	Vital Registration	100.0	31.7	
Ecuador	1990-1994	4	70.3	1992	Vital Registration	100.0	29.7	
Ecuador	1995-1999	4	66.1	1996	Vital Registration	94.8	30.2	
Ecuador	2000-2004	3	61.3	2000	Vital Registration	93.3	34.3	
Ecuador	2005-2009	3	56.3	2009	Vital Registration	75.1	25.0	
Ecuador	2010-2020	5	85.5	2020	Vital Registration	100.0	14.5	
Egypt	1980-1984	2	24.4	1980	Vital Registration	77.6	68.5	
Egypt	1985-1989	2	31.9	1987	Vital Registration	90.3	64.7	
Egypt	1990-1994	3	36.3	1992	Vital Registration	87.4	58.5	
Egypt	1995-1999	0						
Egypt	2000-2004	3	38.6	2003	Vital Registration	95.3	59.5	
Egypt	2005-2009	3	38.9	2009	Vital Registration	98.2	60.4	
Egypt	2010-2020	3	46.3	2014	Vital Registration	100.0	53.7	

Appendix Table S9: Underlying indicators for percent well-certified for data source with maximum percent well certified in each 5-year time interval for 204 countries, 1980-2020.

Location	Time Window	Stars	Percent Well-Certified [PWC] (%)	Max PWC Data Year	Max PWC Data Source	Completeness (%)	Percent Major Garbage (%)	Verbal Autopsy Adjustment (None for VR) (%)
El Salvador	1980-1984	3	50.1	1981	Vital Registration	88.4	43.4	
El Salvador	1985-1989	0						
El Salvador	1990-1994	3	55.6	1993	Vital Registration	89.2	37.6	
El Salvador	1995-1999	3	62.0	1996	Vital Registration	91.5	32.2	
El Salvador	2000-2004	4	65.3	2001	Vital Registration	96.1	32.0	
El Salvador	2005-2009	4	64.7	2005	Vital Registration	93.7	31.0	
El Salvador	2010-2020	3	64.6	2012	Vital Registration	96.0	32.8	
Equatorial Guinea	1980-1984	0						
Equatorial Guinea	1985-1989	0						
Equatorial Guinea	1990-1994	0						
Equatorial Guinea	1995-1999	0						
Equatorial Guinea	2000-2004	0						
Equatorial Guinea	2005-2009	0						
Equatorial Guinea	2010-2020	0						
Eritrea	1980-1984	0						
Eritrea	1985-1989	0						
Eritrea	1990-1994	0						
Eritrea	1995-1999	0						
Eritrea	2000-2004	0						
Eritrea	2005-2009	0						
Eritrea	2010-2020	0						
Estonia	1980-1984	5	91.1	1982	Vital Registration	100.0	8.9	
Estonia	1985-1989	5	91.4	1986	Vital Registration	100.0	8.6	
Estonia	1990-1994	5	93.8	1994	Vital Registration	100.0	6.2	
Estonia	1995-1999	5	93.2	1995	Vital Registration	100.0	6.8	
Estonia	2000-2004	5	92.3	2000	Vital Registration	100.0	7.7	
Estonia	2005-2009	5	92.9	2008	Vital Registration	100.0	7.1	
Estonia	2010-2020	5	93.3	2018	Vital Registration	100.0	6.7	
Eswatini	1980-1984	0						
Eswatini	1985-1989	0						
Eswatini	1990-1994	0						
Eswatini	1995-1999	0						
Eswatini	2000-2004	1	0.5	2000	Effect of HIV infection on pregnancy-related mortality in sub-Saharan Africa: secondary analyses of pooled community-based data from the network for Analysing Longitudinal Population-based HIV/AIDS data on Africa (ALPHA)		12.2	0.6
Eswatini	2005-2009	0						
Eswatini	2010-2020	0						
Ethiopia	1980-1984	0						
Ethiopia	1985-1989	1	3.2	1987	The Butajira rural health project in Ethiopia: mortality pattern of the under fives		7.0	3.5
Ethiopia	1990-1994	1	3.4	1992	Patterns of childhood mortality in three districts of north Gondar Administrative Zone. A community based study using the verbal autopsy method		2.0	3.4
Ethiopia	1995-1999	1	0.9	1997	The use of simplified verbal autopsy in identifying causes of adult death in a predominantly rural population in Ethiopia		6.9	1.0
Ethiopia	2000-2004	1	3.2	2001	HIV/AIDS-Related Mortality In Addis Ababa City Administration		49.8	6.4
Ethiopia	2005-2009	1	1.8	2006	Ethiopia - Addis Ababa Mortality Surveillance Program 2006		41.0	3.1
Ethiopia	2010-2020	2	27.7	2019	Child Health and Mortality Prevention Surveillance Network Program		0.6	27.8
Federated States of Micronesia	1980-1984	0						
Federated States of Micronesia	1985-1989	0						
Federated States of Micronesia	1990-1994	0						
Federated States of Micronesia	1995-1999	0						
Federated States of Micronesia	2000-2004	0						
Federated States of Micronesia	2005-2009	0						
Federated States of Micronesia	2010-2020	0						
Fiji	1980-1984	0						
Fiji	1985-1989	0						
Fiji	1990-1994	0						
Fiji	1995-1999	2	32.6	1999	Vital Registration	61.8	47.2	
Fiji	2000-2004	3	53.0	2003	Vital Registration	100.0	47.0	
Fiji	2005-2009	3	59.3	2009	Vital Registration	100.0	40.7	
Fiji	2010-2020	4	65.5	2012	Vital Registration	100.0	34.5	
Finland	1980-1984	4	82.7	1980	Vital Registration	100.0	17.3	
Finland	1985-1989	5	91.6	1987	Vital Registration	100.0	8.4	
Finland	1990-1994	5	91.4	1992	Vital Registration	100.0	8.6	
Finland	1995-1999	5	96.2	1998	Vital Registration	100.0	3.8	
Finland	2000-2004	5	95.4	2000	Vital Registration	99.7	4.3	
Finland	2005-2009	5	94.4	2008	Vital Registration	99.9	5.6	
Finland	2010-2020	5	94.7	2016	Vital Registration	100.0	5.3	
France	1980-1984	4	77.8	1984	Vital Registration	100.0	22.2	
France	1985-1989	4	80.2	1989	Vital Registration	100.0	19.8	
France	1990-1994	4	81.8	1993	Vital Registration	100.0	18.2	
France	1995-1999	4	81.8	1999	Vital Registration	100.0	18.2	
France	2000-2004	4	82.6	2004	Vital Registration	100.0	17.4	
France	2005-2009	4	82.5	2006	Vital Registration	100.0	17.5	
France	2010-2020	4	80.9	2011	Vital Registration	100.0	19.1	
Gabon	1980-1984	0						
Gabon	1985-1989	0						
Gabon	1990-1994	0						
Gabon	1995-1999	0						
Gabon	2000-2004	0						
Gabon	2005-2009	0						
Gabon	2010-2020	0						
Georgia	1980-1984	4	80.9	1981	Vital Registration	90.3	10.4	
Georgia	1985-1989	4	77.2	1985	Vital Registration	87.1	11.3	
Georgia	1990-1994	4	72.3	1990	Vital Registration	78.5	7.8	
Georgia	1995-1999	4	66.4	1998	Vital Registration	76.9	13.6	
Georgia	2000-2004	4	67.7	2000	Vital Registration	77.5	12.7	
Georgia	2005-2009	3	47.0	2009	Vital Registration	88.0	46.6	
Georgia	2010-2020	4	74.5	2019	Vital Registration	100.0	25.5	
Germany	1980-1984	4	80.5	1984	Vital Registration	100.0	19.5	
Germany	1985-1989	4	83.1	1989	Vital Registration	100.0	16.9	
Germany	1990-1994	5	86.0	1994	Vital Registration	100.0	14.0	
Germany	1995-1999	5	86.1	1995	Vital Registration	100.0	13.9	
Germany	2000-2004	5	85.4	2002	Vital Registration	100.0	14.6	
Germany	2005-2009	5	85.7	2006	Vital Registration	100.0	14.3	
Germany	2010-2020	5	85.8	2011	Vital Registration	100.0	14.2	
Ghana	1980-1984	0						
Ghana	1985-1989	1	0.4	1989	Maternal mortality among the Kassena-Nankana of northern Ghana		10.1	0.4
Ghana	1990-1994	1	2.2	1990	Vitamin A supplementation in northern Ghana: effects on clinic attendances, hospital admissions, and child mortality		1.8	2.3
Ghana	1995-1999	1	0.9	1998	Trend and causes of neonatal mortality in the Kassena-Nankana district of northern Ghana, 1995-2002		0.3	0.9
Ghana	2000-2004	1	8.7	2000	Vital Registration	13.3	34.1	
Ghana	2005-2009	2	21.9	2006	Ghana Child Verbal Autopsy Study 2008		0.8	22.1
Ghana	2010-2020	1	5.4	2017	Ghana Special Demographic and Health Survey 2017		0.6	5.4
Greece	1980-1984	4	83.8	1984	Vital Registration	100.0	16.2	
Greece	1985-1989	4	84.1	1985	Vital Registration	100.0	15.9	
Greece	1990-1994	4	78.4	1994	Vital Registration	100.0	21.6	
Greece	1995-1999	4	79.1	1995	Vital Registration	100.0	20.9	
Greece	2000-2004	4	79.9	2004	Vital Registration	100.0	20.1	
Greece	2005-2009	4	84.7	2009	Vital Registration	100.0	15.3	
Greece	2010-2020	4	84.2	2018	Vital Registration	100.0	15.8	
Greenland	1980-1984	0						
Greenland	1985-1989	0						
Greenland	1990-1994	0						
Greenland	1995-1999	4	84.9	1997	Vital Registration	100.0	15.1	
Greenland	2000-2004	5	87.9	2001	Vital Registration	100.0	12.1	
Greenland	2005-2009	5	86.5	2005	Vital Registration	100.0	13.5	
Greenland	2010-2020	4	82.3	2010	Vital Registration	100.0	17.7	
Grenada	1980-1984	4	70.9	1984	Vital Registration	95.9	26.0	
Grenada	1985-1989	3	63.7	1989	Vital Registration	95.8	33.6	
Grenada	1990-1994	4	65.7	1994	Vital Registration	100.0	34.3	
Grenada	1995-1999	3	64.2	1995	Vital Registration	98.8	35.1	
Grenada	2000-2004	4	80.1	2002	Vital Registration	100.0	19.9	
Grenada	2005-2009	4	82.2	2009	Vital Registration	100.0	17.8	
Grenada	2010-2020	5	87.7	2018	Vital Registration	100.0	12.3	
Guam	1980-1984	0						
Guam	1985-1989	0						
Guam	1990-1994	4	83.5	1994	Vital Registration	90.4	7.6	
Guam	1995-1999	4	77.3	1999	Vital Registration	84.0	7.9	
Guam	2000-2004	4	70.0	2004	Vital Registration	75.4	7.1	
Guam	2005-2009	4	73.5	2007	Vital Registration	78.5	6.5	
Guam	2010-2020	4	69.8	2010	Vital Registration	78.5	11.0	
Guatemala	1980-1984	4	66.8	1984	Vital Registration	96.3	30.7	
Guatemala	1985-1989	3	64.0	1987	Vital Registration	94.3	32.1	
Guatemala	1990-1994	4	65.9	1994	Vital Registration	100.0	34.1	
Guatemala	1995-1999	4	69.1	1998	Vital Registration	100.0	30.9	
Guatemala	2000-2004	4	65.2	2002	Vital Registration	93.6	30.3	
Guatemala	2005-2009	4	70.4	2009	Vital Registration	93.9	25.1	
Guatemala	2010-2020	4	73.1	2014	Vital Registration	93.4	21.7	
Guinea	1980-1984	0						
Guinea	1985-1989	0						
Guinea	1990-1994	0						
Guinea	1995-1999	1	3.5	1998	Guinea - Mandiana Mortality Study 1998-1999		0.6	3.5
Guinea	2000-2004	0						
Guinea	2005-2009	0						
Guinea	2010-2020	0						
Guinea-Bissau	1980-1984	0						
Guinea-Bissau	1985-1989	0						
Guinea-Bissau	1990-1994	1	0.4	1992	Maternal mortality in Guinea-Bissau: the use of verbal autopsy in a multi-ethnic population		10.7	0.4
Guinea-Bissau	1995-1999	1	2.4	1995	BCG vaccination scar associated with better childhood survival in Guinea-Bissau		2.6	2.4
Guinea-Bissau	2000-2004	0						
Guinea-Bissau	2005-2009	0						
Guinea-Bissau	2010-2020	0						
Guyana	1980-1984	3	46.3	1984	Vital Registration	73.0	36.6	
Guyana	1985-1989	3	60.7	1989	Vital Registration	81.2	25.3	
Guyana	1990-1994	3	56.4	1990	Vital Registration	74.8	24.6	
Guyana	1995-1999	4	66.9	1997	Vital Registration	86.9	23.0	
Guyana	2000-2004	4	72.9	2004	Vital Registration	88.0	17.1	
Guyana	2005-2009	4	76.0	2005	Vital Registration	90.2	15.7	
Guyana	2010-2020	4	73.5	2010	Vital Registration	90.5	18.8	
Haiti	1980-1984	2	15.1	1981	Vital Registration	49.6	69.6	
Haiti	1985-1989	1	1.9	1989	The utility of verbal autopsies for identifying HIV-1-related deaths in Haitian children		2.9	2.0
Haiti	1990-1994	1	1.7	1994	Survey on Infant mortality in Mirebalais, Haiti		4.2	1.8
Haiti	1995-1999	2	11.9	1999	Vital Registration	30.1	60.5	
Haiti	2000-2004	1	5.4	2003	Vital Registration	10.3	47.8	
Haiti	2005-2009	0						
Haiti	2010-2020	0						
Honduras	1980-1984	2	33.1	1980	Vital Registration	74.5	55.5	
Honduras	1985-1989	3	41.7	1988	Vital Registration	65.5	36.4	
Honduras	1990-1994	3	38.4	1990	Vital Registration	61.9	38.0	
Honduras	1995-1999	2	16.6	1996	Honduras Demographic and Health Survey 2005-2006		3.4	17.1
Honduras	2000-2004	2	12.9	2001	Honduras Demographic and Health Survey 2005-2006		3.4	13.4
Honduras	2005-2009	2	12.4	2008	Vital Registration	14.3	13.4	
Honduras	2010-2020	2	12.6	2013	Vital Registration	14.2	11.4	
Hungary	1980-1984	5	91.1	1981	Vital Registration	100.0	8.9	
Hungary	1985-1989	5	90.2	1988	Vital Registration	100.0	9.8	
Hungary	1990-1994	5	90.3	1994	Vital Registration	100.0	9.7	
Hungary	1995-1999	5	91.5	1997	Vital Registration	100.0	8.5	
Hungary	2000-2004	5	93.2	2004	Vital Registration	100.0	6.8	
Hungary	2005-2009	5	93.9	2008	Vital Registration	100.0	6.1	
Hungary	2010-2020	5	94.2	2014	Vital Registration	100.0	5.8	
Iceland	1980-1984	5	92.8	1982	Vital Registration	100.0	7.2	
Iceland	1985-1989	5	93.3	1988	Vital Registration	100.0	6.7	
Iceland	1990-1994	5	95.5	1993	Vital Registration	99.5	4.0	
Iceland	1995-1999	5	94.9	1996	Vital Registration	100.0	5.1	
Iceland	2000-2004	5	94.2	2004	Vital Registration	100.0	5.8	
Iceland	2005-2009	5	92.5	2008	Vital Registration	100.0	7.5	
Iceland	2010-2020	5	91.5	2010	Vital Registration	100.0	8.5	
India	1980-1984	1	2.7	1983	Morbidity and mortality in diarrhea in rural Haryana Indian		1.6	2.7
India	1985-1989	1	4.6	1989	Maternal mortality in seven districts of Uttar Pradesh - an ICMR task force study		10.4	5.1
India	1990-1994	1	2.7	1994	Vital Registration	4.6	41.1	
India	1995-1999	1	5.0	1999	Vital Registration	8.8	42.4	
India	2000-2004	1	6.3	2003	India Study on Causes of Death by Verbal Autopsy 2003		1.8	6.4

Appendix Table S9: Underlying indicators for percent well-certified for data source with maximum percent well certified in each 5-year time interval for 204 countries, 1980-2020.

Location	Time Window	Stars	Percent Well-Certified [PWC] (%)	Max PWC Data Year	Max PWC Data Source	Completeness (%)	Percent Major Garbage (%)	Verbal Autopsy Adjustment (None for VR) (%)
India	2005-2009	3	49.5	2005	Verbal Autopsy		22.6	64.0
India	2010-2020	3	45.7	2012	Verbal Autopsy		28.6	64.0
Indonesia	1980-1984	1	1.7	1984	The pattern of the causes of death in children in rural swampy area of South Sumatra, Indonesia		0.8	1.7
Indonesia	1985-1989	0						
Indonesia	1990-1994	1	1.7	1991	Care-seeking for fatal illnesses in young children in Indramayu, West Java, Indonesia		3.0	1.8
Indonesia	1995-1999	1	0.6	1997	Age- and cause-specific childhood mortality in Lombok, Indonesia, as a factor for determining the appropriateness of introducing Haemophilus influenzae type b and pneumococcal vaccines		1.8	0.6
Indonesia	2000-2004	1	0.3	2001	Determining the Cause of Death: Mortality Surveillance Using Verbal Autopsy in Indonesia		96.0	6.4
Indonesia	2005-2009	3	53.7	2006	Indonesia Basic Health Research 2007-2008		2.1	54.9
Indonesia	2010-2020	3	63.4	2012	Indonesia Sample Registration System - Deaths 2012		1.0	64.0
Iran	1980-1984	0						
Iran	1985-1989	0						
Iran	1990-1994	0						
Iran	1995-1999	0						
Iran	2000-2004	3	53.4	2004	Vital Registration	72.6	26.4	
Iran	2005-2009	3	63.5	2007	Vital Registration	78.0	18.6	
Iran	2010-2020	4	77.0	2016	Vital Registration	89.3	13.8	
Iraq	1980-1984	0						
Iraq	1985-1989	0						
Iraq	1990-1994	0						
Iraq	1995-1999	0						
Iraq	2000-2004	0						
Iraq	2005-2009	3	37.6	2008	Vital Registration	69.5	45.9	
Iraq	2010-2020	3	44.6	2014	Vital Registration	73.8	39.6	
Ireland	1980-1984	5	91.3	1984	Vital Registration	100.0	8.7	
Ireland	1985-1989	5	92.2	1988	Vital Registration	100.0	7.8	
Ireland	1990-1994	5	92.7	1993	Vital Registration	100.0	7.3	
Ireland	1995-1999	5	92.0	1997	Vital Registration	100.0	8.0	
Ireland	2000-2004	5	91.7	2000	Vital Registration	100.0	8.3	
Ireland	2005-2009	5	92.8	2007	Vital Registration	100.0	7.2	
Ireland	2010-2020	5	92.8	2015	Vital Registration	100.0	7.2	
Israel	1980-1984	4	80.7	1983	Vital Registration	100.0	19.3	
Israel	1985-1989	4	82.5	1989	Vital Registration	100.0	17.5	
Israel	1990-1994	4	83.4	1994	Vital Registration	100.0	16.6	
Israel	1995-1999	4	84.0	1996	Vital Registration	100.0	16.0	
Israel	2000-2004	4	82.3	2003	Vital Registration	100.0	17.7	
Israel	2005-2009	4	81.3	2009	Vital Registration	100.0	18.7	
Israel	2010-2020	4	82.3	2010	Vital Registration	100.0	17.7	
Italy	1980-1984	5	89.5	1980	Vital Registration	100.0	10.5	
Italy	1985-1989	5	89.5	1989	Vital Registration	100.0	10.5	
Italy	1990-1994	5	90.0	1994	Vital Registration	100.0	10.0	
Italy	1995-1999	5	89.2	1995	Vital Registration	100.0	10.8	
Italy	2000-2004	5	89.7	2003	Vital Registration	100.0	10.3	
Italy	2005-2009	5	90.2	2008	Vital Registration	100.0	9.8	
Italy	2010-2020	5	89.8	2010	Vital Registration	100.0	10.2	
Jamaica	1980-1984	4	71.8	1983	Vital Registration	100.0	28.2	
Jamaica	1985-1989	4	72.3	1985	Vital Registration	100.0	27.7	
Jamaica	1990-1994	4	65.9	1991	Vital Registration	91.7	28.1	
Jamaica	1995-1999	0						
Jamaica	2000-2004	4	82.6	2004	Vital Registration	98.6	16.3	
Jamaica	2005-2009	4	84.0	2006	Vital Registration	96.1	12.6	
Jamaica	2010-2020	5	88.1	2014	Vital Registration	100.0	11.9	
Japan	1980-1984	4	82.7	1982	Vital Registration	100.0	17.3	
Japan	1985-1989	4	81.9	1985	Vital Registration	100.0	18.1	
Japan	1990-1994	4	83.4	1994	Vital Registration	100.0	16.6	
Japan	1995-1999	5	89.1	1995	Vital Registration	100.0	10.9	
Japan	2000-2004	5	88.0	2001	Vital Registration	100.0	12.0	
Japan	2005-2009	5	87.0	2005	Vital Registration	100.0	13.0	
Japan	2010-2020	5	85.5	2011	Vital Registration	100.0	14.5	
Jordan	1980-1984	0						
Jordan	1985-1989	0						
Jordan	1990-1994	0						
Jordan	1995-1999	1	1.7	1995	Mortality and causes of death in Jordan 1995-96: assessment by verbal autopsy		73.9	6.4
Jordan	2000-2004	4	73.2	2004	Vital Registration	94.5	22.5	
Jordan	2005-2009	4	78.2	2006	Vital Registration	96.4	18.8	
Jordan	2010-2020	4	66.5	2011	Vital Registration	83.0	19.8	
Kazakhstan	1980-1984	4	82.5	1982	Vital Registration	100.0	17.5	
Kazakhstan	1985-1989	4	83.8	1987	Vital Registration	100.0	16.2	
Kazakhstan	1990-1994	5	88.9	1991	Vital Registration	99.7	10.8	
Kazakhstan	1995-1999	5	87.9	1995	Vital Registration	99.7	11.9	
Kazakhstan	2000-2004	4	81.3	2000	Vital Registration	95.0	14.4	
Kazakhstan	2005-2009	4	78.8	2008	Vital Registration	96.3	18.1	
Kazakhstan	2010-2020	4	79.0	2015	Vital Registration	94.9	16.7	
Kenya	1980-1984	0						
Kenya	1985-1989	1	3.4	1986	Mortality patterns in a rural Kenyan community		1.3	3.5
Kenya	1990-1994	0						
Kenya	1995-1999	1	2.7	1997	The burden of malaria mortality among African children in the year 2000		9.0	2.9
Kenya	2000-2004	1	4.3	2003	Kenya - Nairobi Urban Health and Demographic Surveillance System		30.7	6.2
Kenya	2005-2009	1	5.1	2006	Kenya - Nairobi Urban Health and Demographic Surveillance System		20.6	6.4
Kenya	2010-2020	2	22.2	2018	Child Health and Mortality Prevention Surveillance Network Program		1.2	22.5
Kiribati	1980-1984	0						
Kiribati	1985-1989	0						
Kiribati	1990-1994	3	36.4	1993	Vital Registration	67.8	46.3	
Kiribati	1995-1999	3	55.0	1995	Vital Registration	100.0	45.0	
Kiribati	2000-2004	2	33.5	2000	Vital Registration	61.2	45.2	
Kiribati	2005-2009	0						
Kiribati	2010-2020	0						
Kuwait	1980-1984	4	74.4	1981	Vital Registration	100.0	25.6	
Kuwait	1985-1989	4	77.6	1987	Vital Registration	99.0	21.7	
Kuwait	1990-1994	4	74.9	1993	Vital Registration	97.9	23.4	
Kuwait	1995-1999	4	72.2	1998	Vital Registration	92.1	21.7	
Kuwait	2000-2004	4	70.5	2001	Vital Registration	89.4	21.1	
Kuwait	2005-2009	4	66.5	2009	Vital Registration	81.1	17.9	
Kuwait	2010-2020	4	65.2	2010	Vital Registration	77.8	16.2	
Kyrgyzstan	1980-1984	4	71.1	1982	Vital Registration	86.2	17.5	
Kyrgyzstan	1985-1989	4	78.5	1988	Vital Registration	91.0	13.8	
Kyrgyzstan	1990-1994	4	71.2	1994	Vital Registration	93.6	24.0	
Kyrgyzstan	1995-1999	4	72.0	1997	Vital Registration	90.9	20.8	
Kyrgyzstan	2000-2004	5	85.2	2004	Vital Registration	92.3	7.6	
Kyrgyzstan	2005-2009	5	91.4	2006	Vital Registration	97.4	6.2	
Kyrgyzstan	2010-2020	5	93.3	2014	Vital Registration	99.3	6.1	
Laos	1980-1984	0						
Laos	1985-1989	1	4.1	1989	The Lao People's Democratic Republic: maternal mortality and female mortality: determining causes of deaths		9.6	4.5
Laos	1990-1994	0						
Laos	1995-1999	0						
Laos	2000-2004	0						
Laos	2005-2009	0						
Laos	2010-2020	0						
Latvia	1980-1984	5	91.4	1983	Vital Registration	100.0	8.6	
Latvia	1985-1989	5	91.5	1985	Vital Registration	100.0	8.5	
Latvia	1990-1994	5	88.8	1990	Vital Registration	100.0	11.2	
Latvia	1995-1999	5	91.9	1999	Vital Registration	100.0	8.1	
Latvia	2000-2004	5	91.3	2000	Vital Registration	100.0	8.7	
Latvia	2005-2009	5	89.2	2007	Vital Registration	100.0	10.8	
Latvia	2010-2020	5	94.1	2016	Vital Registration	100.0	5.9	
Lebanon	1980-1984	0						
Lebanon	1985-1989	1	3.8	1988	Non-communicable disease mortality rates using the verbal autopsy in a cohort of middle aged and older populations in Beirut during wartime, 1983-93		13.3	4.4
Lebanon	1990-1994	0						
Lebanon	1995-1999	0						
Lebanon	2000-2004	1	0.2	2000	Facility-based audit of maternal mortality in Lebanon: A feasibility study		12.1	0.3
Lebanon	2005-2009	0						
Lebanon	2010-2020	3	37.5	2019	Vital Registration	63.0	40.5	
Lesotho	1980-1984	0						
Lesotho	1985-1989	0						
Lesotho	1990-1994	0						
Lesotho	1995-1999	0						
Lesotho	2000-2004	0						
Lesotho	2005-2009	0						
Lesotho	2010-2020	0						
Liberia	1980-1984	1	4.0	1984	Infant and child mortality in two counties of Liberia: results of a survey in 1988 and trends since 1984		5.6	4.2
Liberia	1985-1989	1	3.9	1987	Infant and child mortality in two counties of Liberia: results of a survey in 1988 and trends since 1984		6.1	4.2
Liberia	1990-1994	1	4.2	1990	Application of the verbal autopsy during a clinical trial		9.6	4.7
Liberia	1995-1999	0						
Liberia	2000-2004	0						
Liberia	2005-2009	0						
Liberia	2010-2020	0						
Libya	1980-1984	0						
Libya	1985-1989	0						
Libya	1990-1994	0						
Libya	1995-1999	0						
Libya	2000-2004	0						
Libya	2005-2009	2	12.7	2006	Vital Registration	100.0	87.3	
Libya	2010-2020	2	28.5	2016	Vital Registration	58.6	51.3	
Lithuania	1980-1984	5	91.0	1982	Vital Registration	99.9	9.0	
Lithuania	1985-1989	5	92.6	1989	Vital Registration	99.9	7.3	
Lithuania	1990-1994	5	92.8	1990	Vital Registration	100.0	7.2	
Lithuania	1995-1999	5	94.8	1997	Vital Registration	100.0	5.2	
Lithuania	2000-2004	5	93.2	2003	Vital Registration	100.0	6.8	
Lithuania	2005-2009	5	92.8	2006	Vital Registration	100.0	7.2	
Lithuania	2010-2020	5	93.6	2013	Vital Registration	100.0	6.4	
Luxembourg	1980-1984	5	86.8	1984	Vital Registration	100.0	13.2	
Luxembourg	1985-1989	5	87.2	1986	Vital Registration	100.0	12.8	
Luxembourg	1990-1994	5	87.1	1992	Vital Registration	100.0	12.9	
Luxembourg	1995-1999	5	87.7	1997	Vital Registration	99.9	12.2	
Luxembourg	2000-2004	4	83.0	2001	Vital Registration	97.5	14.9	
Luxembourg	2005-2009	4	81.9	2007	Vital Registration	100.0	18.1	
Luxembourg	2010-2020	4	83.9	2012	Vital Registration	98.5	14.8	
Madagascar	1980-1984	1	2.3	1984	Vital Registration	5.0	53.9	
Madagascar	1985-1989	1	2.9	1986	Vital Registration	6.1	52.7	
Madagascar	1990-1994	1	2.1	1990	Vital Registration	4.2	49.4	
Madagascar	1995-1999	1	2.2	1995	Vital Registration	4.4	49.7	
Madagascar	2000-2004	0						
Madagascar	2005-2009	0						
Madagascar	2010-2020	0						
Malawi	1980-1984	0						
Malawi	1985-1989	1	3.9	1988	Infant and second-year mortality in rural Malawi: causes and descriptive epidemiology		4.7	4.1
Malawi	1990-1994	0						
Malawi	1995-1999	1	0.7	1999	Estimation of AIDS adult mortality by verbal autopsy in rural Malawi		2.9	0.7
Malawi	2000-2004	1	3.3	2004	Declining child mortality in northern Malawi despite high rates of infection with HIV		3.4	3.4
Malawi	2005-2009	1	3.3	2008	Rates and causes of death in Chiradzulu District, Malawi, 2008: a key informant study		48.9	6.4
Malawi	2010-2020	1	4.1	2017	Can a village headman use an electronic village register and a simplified community-based verbal autopsy tool to record numbers and causes of death in rural Malawi? [Unpublished data]		33.8	6.2
Malaysia	1980-1984	3	43.4	1980	Vital Registration	73.9	41.3	
Malaysia	1985-1989	0						
Malaysia	1990-1994	0						
Malaysia	1995-1999	2	31.2	1997	Vital Registration	46.5	32.8	
Malaysia	2000-2004	3	35.8	2004	Vital Registration	59.3	39.6	
Malaysia	2005-2009	3	40.2	2008	Vital Registration	62.5	35.7	
Malaysia	2010-2020	3	40.3	2014	Vital Registration	54.1	25.5	
Maldives	1980-1984	0						
Maldives	1985-1989	0						
Maldives	1990-1994	0						
Maldives	1995-1999	0						
Maldives	2000-2004	3	39.8	2002	Vital Registration	91.2	56.4	
Maldives	2005-2009	3	43.6	2007	Vital Registration	93.3	53.2	
Maldives	2010-2020	3	53.8	2011	Vital Registration	87.3	38.4	
Mali	1980-1984	1	2.2	1981	Vital Registration	7.8	71.3	
Mali	1985-1989	0						

Appendix Table S9. Underlying indicators for percent well-certified for data source with maximum percent well certified in each 5-year time interval for 204 countries, 1980-2020.

Location	Time Window	Stars	Percent Well-Certified [PWC] (%)	Max PWC Data Year	Max PWC Data Source	Completeness (%)	Percent Major Garbage (%)	Verbal Autopsy Adjustment (None for VR) (%)
Mali	1990-1994	1	0.3	1990	Assessment of maternal mortality and late maternal mortality among a cohort of pregnant women in Bamako, Mali		10.7	0.3
Mali	1995-1999	0						
Mali	2000-2004	0						
Mali	2005-2009	0						
Mali	2010-2020	1	5.5	2018	Child Health and Mortality Prevention Surveillance Network Program		2.2	5.7
Malta	1980-1984	4	82.7	1984	Vital Registration	100.0	17.3	
Malta	1985-1989	5	85.6	1989	Vital Registration	97.7	12.4	
Malta	1990-1994	5	89.7	1992	Vital Registration	100.0	10.3	
Malta	1995-1999	5	90.5	1998	Vital Registration	100.0	9.5	
Malta	2000-2004	5	90.6	2002	Vital Registration	99.1	8.6	
Malta	2005-2009	5	94.4	2009	Vital Registration	100.0	5.6	
Malta	2010-2020	5	93.2	2015	Vital Registration	99.8	6.6	
Marshall Islands	1980-1984	0						
Marshall Islands	1985-1989	0						
Marshall Islands	1990-1994	0						
Marshall Islands	1995-1999	0						
Marshall Islands	2000-2004	0						
Marshall Islands	2005-2009	0						
Marshall Islands	2010-2020	0						
Mauritania	1980-1984	0						
Mauritania	1985-1989	0						
Mauritania	1990-1994	0						
Mauritania	1995-1999	0						
Mauritania	2000-2004	0						
Mauritania	2005-2009	0						
Mauritania	2010-2020	0						
Mauritius	1980-1984	4	73.2	1984	Vital Registration	100.0	26.8	
Mauritius	1985-1989	4	77.4	1988	Vital Registration	100.0	22.6	
Mauritius	1990-1994	4	76.5	1991	Vital Registration	100.0	23.5	
Mauritius	1995-1999	4	77.0	1999	Vital Registration	100.0	23.0	
Mauritius	2000-2004	4	82.3	2002	Vital Registration	100.0	17.7	
Mauritius	2005-2009	5	85.2	2007	Vital Registration	100.0	14.8	
Mauritius	2010-2020	5	87.1	2014	Vital Registration	100.0	12.9	
Mexico	1980-1984	3	63.5	1984	Vital Registration	84.2	24.6	
Mexico	1985-1989	4	72.0	1989	Vital Registration	88.5	18.7	
Mexico	1990-1994	4	73.5	1994	Vital Registration	87.5	16.0	
Mexico	1995-1999	4	79.2	1999	Vital Registration	91.3	13.2	
Mexico	2000-2004	4	81.4	2004	Vital Registration	92.7	12.1	
Mexico	2005-2009	4	82.5	2005	Vital Registration	93.7	12.0	
Mexico	2010-2020	5	89.3	2016	Vital Registration	100.0	10.7	
Moldova	1980-1984	5	89.7	1981	Vital Registration	100.0	10.3	
Moldova	1985-1989	5	91.2	1985	Vital Registration	100.0	8.8	
Moldova	1990-1994	4	79.2	1990	Vital Registration	99.4	20.3	
Moldova	1995-1999	5	86.7	1996	Vital Registration	100.0	13.3	
Moldova	2000-2004	5	89.2	2003	Vital Registration	92.9	4.0	
Moldova	2005-2009	5	89.3	2005	Vital Registration	92.5	3.4	
Moldova	2010-2020	5	88.8	2012	Vital Registration	91.5	3.0	
Monaco	1980-1984	0						
Monaco	1985-1989	4	75.9	1986	Vital Registration	100.0	24.1	
Monaco	1990-1994	0						
Monaco	1995-1999	0						
Monaco	2000-2004	0						
Monaco	2005-2009	0						
Monaco	2010-2020	4	80.5	2013	Vital Registration	100.0	19.5	
Mongolia	1980-1984	0						
Mongolia	1985-1989	0						
Mongolia	1990-1994	3	53.9	1994	Vital Registration	71.3	24.4	
Mongolia	1995-1999	0						
Mongolia	2000-2004	2	17.8	2004	Vital Registration	74.0	76.0	
Mongolia	2005-2009	2	19.4	2007	Vital Registration	75.8	74.4	
Mongolia	2010-2020	4	73.2	2010	Vital Registration	79.4	7.9	
Montenegro	1980-1984	0						
Montenegro	1985-1989	0						
Montenegro	1990-1994	0						
Montenegro	1995-1999	0						
Montenegro	2000-2004	4	68.4	2000	Vital Registration	98.2	30.3	
Montenegro	2005-2009	3	63.0	2009	Vital Registration	90.6	30.5	
Montenegro	2010-2020	0						
Morocco	1980-1984	0						
Morocco	1985-1989	2	23.7	1988	Morocco National Survey on Causes and Circumstances of Infant and Child Deaths 1988-1989		2.7	24.4
Morocco	1990-1994	0						
Morocco	1995-1999	0						
Morocco	2000-2004	2	10.4	2004	Vital Registration	24.5	57.4	
Morocco	2005-2009	2	28.7	2005	Vital Registration	68.5	58.1	
Morocco	2010-2020	2	11.8	2014	Vital Registration	24.1	51.1	
Mozambique	1980-1984	0						
Mozambique	1985-1989	0						
Mozambique	1990-1994	0						
Mozambique	1995-1999	1	0.3	1996	Quality of registration of maternal deaths in Mozambique: a community-based study in rural and urban areas		10.8	0.3
Mozambique	2000-2004	2	13.5	2001	Vital Registration	21.6	37.3	
Mozambique	2005-2009	3	63.3	2007	Mozambique National Survey on the Causes of Death 2007-2008		1.0	64.0
Mozambique	2010-2020	1	3.6	2017	Child Health and Mortality Prevention Surveillance Network Program		0.5	3.7
Myanmar	1980-1984	0						
Myanmar	1985-1989	0						
Myanmar	1990-1994	0						
Myanmar	1995-1999	0						
Myanmar	2000-2004	0						
Myanmar	2005-2009	1	2.8	2007	Cause of Death Verification Study in Myanmar		55.1	6.2
Myanmar	2010-2020	3	49.1	2016	Myanmar National Mortality Survey 2016		23.3	64.0
Namibia	1980-1984	0						
Namibia	1985-1989	0						
Namibia	1990-1994	0						
Namibia	1995-1999	0						
Namibia	2000-2004	0						
Namibia	2005-2009	0						
Namibia	2010-2020	0						
Nauru	1980-1984	0						
Nauru	1985-1989	0						
Nauru	1990-1994	0						
Nauru	1995-1999	0						
Nauru	2000-2004	0						
Nauru	2005-2009	0						
Nauru	2010-2020	0						
Nepal	1980-1984	1	3.4	1984	Impact of a pilot acute respiratory infection (ARI) control programme in a rural community of the hill region of Nepal		2.7	3.5
Nepal	1985-1989	1	3.3	1987	Reduction in total under-five mortality in western Nepal through community-based antimicrobial treatment of pneumonia		1.9	3.3
Nepal	1990-1994	0						
Nepal	1995-1999	1	1.2	1999	Evaluation of neonatal verbal autopsy using physician review versus algorithm-based cause-of-death assignment in rural Nepal		2.1	1.2
Nepal	2000-2004	1	1.0	2003	Effect of daily zinc supplementation on child mortality in southern Nepal: a community-based, cluster randomised, placebo-controlled trial		1.9	1.0
Nepal	2005-2009	3	38.0	2007	Nepal Assessment of Burden of Disease Verbal Autopsy Data 2005-2007		40.6	64.0
Nepal	2010-2020	1	6.1	2016	Nepal Demographic and Health Survey 2016-2017		0.0	6.1
Netherlands	1980-1984	5	88.9	1981	Vital Registration	100.0	11.1	
Netherlands	1985-1989	5	88.1	1987	Vital Registration	100.0	11.9	
Netherlands	1990-1994	5	86.4	1991	Vital Registration	100.0	13.6	
Netherlands	1995-1999	5	85.9	1996	Vital Registration	100.0	14.1	
Netherlands	2000-2004	4	84.6	2004	Vital Registration	100.0	15.4	
Netherlands	2005-2009	5	85.8	2009	Vital Registration	100.0	14.2	
Netherlands	2010-2020	5	86.2	2012	Vital Registration	100.0	13.8	
New Zealand	1980-1984	5	95.1	1980	Vital Registration	100.0	4.9	
New Zealand	1985-1989	5	95.1	1987	Vital Registration	100.0	4.9	
New Zealand	1990-1994	5	95.3	1994	Vital Registration	99.7	4.4	
New Zealand	1995-1999	5	96.8	1999	Vital Registration	100.0	3.2	
New Zealand	2000-2004	5	97.0	2004	Vital Registration	100.0	3.0	
New Zealand	2005-2009	5	96.5	2006	Vital Registration	100.0	3.5	
New Zealand	2010-2020	5	96.1	2012	Vital Registration	100.0	3.9	
Nicaragua	1980-1984	0						
Nicaragua	1985-1989	3	51.5	1988	Vital Registration	70.5	27.0	
Nicaragua	1990-1994	3	54.6	1994	Vital Registration	73.8	26.0	
Nicaragua	1995-1999	4	71.2	1998	Vital Registration	85.8	17.0	
Nicaragua	2000-2004	4	72.1	2003	Vital Registration	82.7	12.8	
Nicaragua	2005-2009	4	73.7	2008	Vital Registration	85.4	13.7	
Nicaragua	2010-2020	5	85.2	2016	Vital Registration	93.7	9.0	
Niger	1980-1984	0						
Niger	1985-1989	0						
Niger	1990-1994	0						
Niger	1995-1999	0						
Niger	2000-2004	0						
Niger	2005-2009	3	42.0	2008	Direct estimates of national neonatal and child cause-specific mortality proportions in Niger by expert algorithm and physician-coded analysis of verbal autopsy interviews		0.9	42.4
Niger	2010-2020	0						
Nigeria	1980-1984	0						
Nigeria	1985-1989	0						
Nigeria	1990-1994	1	4.0	1991	Community-based surveillance of paediatric deaths in Cross River State, Nigeria		2.2	4.1
Nigeria	1995-1999	1	3.4	1996	Using verbal autopsy to identify and proportionally assign cause of death in Ibadan, southwest Nigeria		84.9	22.8
Nigeria	2000-2004	0						
Nigeria	2005-2009	1	0.0	2007	Vital Registration	1.5	96.6	
Nigeria	2010-2020	3	36.4	2014	Direct estimates of cause-specific mortality fractions and rates of under-five deaths in the northern and southern regions of Nigeria by verbal autopsy interview		0.7	36.7
Niue	1980-1984	0						
Niue	1985-1989	0						
Niue	1990-1994	0						
Niue	1995-1999	0						
Niue	2000-2004	0						
Niue	2005-2009	0						
Niue	2010-2020	0						
North Korea	1980-1984	0						
North Korea	1985-1989	0						
North Korea	1990-1994	0						
North Korea	1995-1999	0						
North Korea	2000-2004	0						
North Korea	2005-2009	0						
North Korea	2010-2020	0						
North Macedonia	1980-1984	0						
North Macedonia	1985-1989	0						
North Macedonia	1990-1994	4	72.1	1992	Vital Registration	91.1	20.8	
North Macedonia	1995-1999	4	71.9	1998	Vital Registration	87.3	17.6	
North Macedonia	2000-2004	4	73.7	2000	Vital Registration	88.6	16.9	
North Macedonia	2005-2009	4	70.8	2007	Vital Registration	89.8	21.1	
North Macedonia	2010-2020	4	70.2	2012	Vital Registration	90.4	22.3	
Northern Mariana Islands	1980-1984	0						
Northern Mariana Islands	1985-1989	0						
Northern Mariana Islands	1990-1994	0						
Northern Mariana Islands	1995-1999	3	62.5	1998	Vital Registration	79.2	21.2	
Northern Mariana Islands	2000-2004	3	56.8	2004	Vital Registration	71.2	20.3	
Northern Mariana Islands	2005-2009	3	60.7	2009	Vital Registration	75.3	19.3	
Northern Mariana Islands	2010-2020	3	64.1	2015	Vital Registration	71.8	10.8	
Norway	1980-1984	5	90.9	1984	Vital Registration	100.0	9.1	
Norway	1985-1989	5	91.4	1988	Vital Registration	100.0	8.6	
Norway	1990-1994	5	90.9	1990	Vital Registration	100.0	9.1	
Norway	1995-1999	5	90.9	1996	Vital Registration	100.0	9.0	
Norway	2000-2004	5	89.0	2000	Vital Registration	100.0	11.0	
Norway	2005-2009	5	86.3	2005	Vital Registration	100.0	13.7	
Norway	2010-2020	5	86.0	2017	Vital Registration	100.0	14.0	
Oman	1980-1984	0						
Oman	1985-1989	0						
Oman	1990-1994	0						
Oman	1995-1999	0						
Oman	2000-2004	0						
Oman	2005-2009	3	53.2	2007	Vital Registration	65.1	18.3	
Oman	2010-2020	2	28.2	2016	Vital Registration	55.3	49.1	

Appendix Table S9. Underlying indicators for percent well-certified for data source with maximum percent well certified in each 5-year time interval for 204 countries, 1980-2020.

Location	Time Window	Stars	Percent Well-Certified [PWC] (%)	Max PWC Data Year	Max PWC Data Source	Completeness (%)	Percent Major Garbage (%)	Verbal Autopsy Adjustment (None for VR) (%)
Pakistan	1980-1984	0						
Pakistan	1985-1989	2	30.9	1986	Acute respiratory infections in children: a case management intervention in Abbottabad District, Pakistan		1.8	31.5
Pakistan	1990-1994	1	1.9	1990	Time to focus child survival programmes on the newborn: assessment of levels and causes of infant mortality in rural Pakistan		2.4	2.0
Pakistan	1995-1999	0						
Pakistan	2000-2004	2	12.8	2003	Impact of a community-based perinatal and newborn preventive care package on perinatal and neonatal mortality in a remote mountainous district in Northern Pakistan		0.0	12.8
Pakistan	2005-2009	2	21.3	2006	Pakistan Demographic and Health Survey 2006-2007		3.5	22.1
Pakistan	2010-2020	2	20.2	2010	Cause of Death in under 5 Children in a Demographic Surveillance Site in Pakistan		2.7	20.7
Palau	1980-1984	0						
Palau	1985-1989	0						
Palau	1990-1994	0						
Palau	1995-1999	0						
Palau	2000-2004	0						
Palau	2005-2009	0						
Palau	2010-2020	3	48.6	2013	Vital Registration	100.0	51.4	
Palestine	1980-1984	0						
Palestine	1985-1989	0						
Palestine	1990-1994	0						
Palestine	1995-1999	2	27.7	1999	Vital Registration	41.9	33.8	
Palestine	2000-2004	2	27.8	2000	Vital Registration	42.5	34.5	
Palestine	2005-2009	2	29.6	2009	Vital Registration	43.2	31.4	
Palestine	2010-2020	4	70.5	2018	Vital Registration	79.0	10.7	
Panama	1980-1984	4	74.2	1983	Vital Registration	99.7	25.5	
Panama	1985-1989	4	77.8	1987	Vital Registration	100.0	22.2	
Panama	1990-1994	0						
Panama	1995-1999	4	83.3	1999	Vital Registration	100.0	16.7	
Panama	2000-2004	5	86.7	2002	Vital Registration	100.0	13.3	
Panama	2005-2009	5	86.5	2007	Vital Registration	100.0	13.5	
Panama	2010-2020	4	84.8	2018	Vital Registration	100.0	15.2	
Papua New Guinea	1980-1984	1	8.7	1980	Vital Registration	12.4	29.9	
Papua New Guinea	1985-1989	1	3.6	1985	Mortality rates and the utilization of health services during terminal illness in the Asaro Valley, Eastern Highlands Province, Papua New Guinea		43.2	6.4
Papua New Guinea	1990-1994	0						
Papua New Guinea	1995-1999	0						
Papua New Guinea	2000-2004	0						
Papua New Guinea	2005-2009	0						
Papua New Guinea	2010-2020	2	26.2	2012	Epidemiological transition in Papua New Guinea: new evidence from verbal autopsy studies		39.0	42.9
Paraguay	1980-1984	3	54.7	1980	Vital Registration	86.8	36.9	
Paraguay	1985-1989	3	54.1	1989	Vital Registration	81.6	33.7	
Paraguay	1990-1994	3	62.5	1994	Vital Registration	84.5	26.1	
Paraguay	1995-1999	3	63.1	1996	Vital Registration	86.6	27.2	
Paraguay	2000-2004	3	64.4	2004	Vital Registration	90.0	28.4	
Paraguay	2005-2009	4	69.2	2009	Vital Registration	91.7	24.5	
Paraguay	2010-2020	4	79.3	2016	Vital Registration	95.6	17.0	
Peru	1980-1984	3	52.6	1980	Vital Registration	72.0	26.9	
Peru	1985-1989	2	34.5	1989	Vital Registration	64.4	46.5	
Peru	1990-1994	3	35.0	1992	Vital Registration	63.2	44.6	
Peru	1995-1999	3	46.4	1999	Vital Registration	71.8	35.3	
Peru	2000-2004	3	56.8	2004	Vital Registration	80.5	29.4	
Peru	2005-2009	3	58.7	2008	Vital Registration	77.8	24.5	
Peru	2010-2020	3	55.7	2015	Vital Registration	69.0	19.2	
Philippines	1980-1984	1	0.3	1980	Vital Registration	36.0	99.1	
Philippines	1985-1989	2	12.6	1989	Effect of not breastfeeding on the risk of diarrheal and respiratory mortality in children under 2 years of age in Metro Cebu, The Philippines		5.7	13.3
Philippines	1990-1994	2	16.2	1992	Vital Registration	87.8	81.6	
Philippines	1995-1999	3	41.4	1999	Vital Registration	86.9	52.4	
Philippines	2000-2004	3	47.7	2002	Vital Registration	90.1	47.1	
Philippines	2005-2009	4	77.5	2009	Vital Registration	92.1	15.9	
Philippines	2010-2020	4	82.6	2018	Vital Registration	95.6	13.6	
Poland	1980-1984	3	63.7	1984	Vital Registration	100.0	36.3	
Poland	1985-1989	3	63.8	1986	Vital Registration	100.0	36.2	
Poland	1990-1994	3	63.6	1994	Vital Registration	100.0	36.4	
Poland	1995-1999	4	73.2	1999	Vital Registration	100.0	26.8	
Poland	2000-2004	4	75.6	2002	Vital Registration	100.0	24.4	
Poland	2005-2009	4	75.7	2006	Vital Registration	100.0	24.3	
Poland	2010-2020	4	75.7	2011	Vital Registration	100.0	24.3	
Portugal	1980-1984	4	77.4	1984	Vital Registration	100.0	22.6	
Portugal	1985-1989	4	77.7	1985	Vital Registration	100.0	22.3	
Portugal	1990-1994	4	77.6	1992	Vital Registration	100.0	22.4	
Portugal	1995-1999	4	77.7	1995	Vital Registration	100.0	22.3	
Portugal	2000-2004	4	82.2	2002	Vital Registration	100.0	17.8	
Portugal	2005-2009	4	79.2	2009	Vital Registration	100.0	20.8	
Portugal	2010-2020	5	85.8	2014	Vital Registration	99.9	14.0	
Puerto Rico	1980-1984	4	78.0	1981	Vital Registration	100.0	22.0	
Puerto Rico	1985-1989	4	77.2	1989	Vital Registration	100.0	22.8	
Puerto Rico	1990-1994	4	84.1	1994	Vital Registration	100.0	15.9	
Puerto Rico	1995-1999	4	83.8	1997	Vital Registration	100.0	16.2	
Puerto Rico	2000-2004	4	84.5	2004	Vital Registration	100.0	15.5	
Puerto Rico	2005-2009	4	83.6	2008	Vital Registration	100.0	16.4	
Puerto Rico	2010-2020	4	84.4	2012	Vital Registration	100.0	15.6	
Qatar	1980-1984	2	11.6	1984	Vital Registration	63.9	81.9	
Qatar	1985-1989	2	15.1	1985	Vital Registration	74.8	79.8	
Qatar	1990-1994	0						
Qatar	1995-1999	3	53.2	1995	Vital Registration	72.6	26.6	
Qatar	2000-2004	3	47.8	2001	Vital Registration	75.3	36.5	
Qatar	2005-2009	3	54.9	2006	Vital Registration	82.6	33.6	
Qatar	2010-2020	3	49.2	2016	Vital Registration	69.7	29.5	
Romania	1980-1984	4	76.9	1980	Vital Registration	100.0	23.1	
Romania	1985-1989	4	78.6	1989	Vital Registration	100.0	21.4	
Romania	1990-1994	4	83.4	1994	Vital Registration	100.0	16.6	
Romania	1995-1999	5	85.8	1999	Vital Registration	100.0	14.2	
Romania	2000-2004	5	87.0	2004	Vital Registration	100.0	13.0	
Romania	2005-2009	5	87.4	2007	Vital Registration	100.0	12.6	
Romania	2010-2020	5	87.0	2013	Vital Registration	100.0	13.0	
Russia	1980-1984	5	92.5	1980	Vital Registration	100.0	7.5	
Russia	1985-1989	5	86.6	1989	Vital Registration	94.4	8.2	
Russia	1990-1994	5	90.1	1990	Vital Registration	100.0	9.9	
Russia	1995-1999	5	86.9	1999	Vital Registration	100.0	13.1	
Russia	2000-2004	5	87.4	2004	Vital Registration	100.0	12.6	
Russia	2005-2009	5	88.9	2009	Vital Registration	100.0	11.1	
Russia	2010-2020	5	88.5	2010	Vital Registration	100.0	11.5	
Rwanda	1980-1984	0						
Rwanda	1985-1989	0						
Rwanda	1990-1994	0						
Rwanda	1995-1999	0						
Rwanda	2000-2004	0						
Rwanda	2005-2009	2	27.1	2007	Rwanda Child Verbal Autopsy Study 2008		1.1	27.4
Rwanda	2010-2020	1	0.6	2014	Four delays of child mortality in Rwanda: a mixed methods analysis of verbal social autopsies		2.7	0.6
Saint Kitts and Nevis	1980-1984	4	67.1	1982	Vital Registration	97.0	30.9	
Saint Kitts and Nevis	1985-1989	3	64.5	1987	Vital Registration	94.2	31.6	
Saint Kitts and Nevis	1990-1994	4	69.7	1994	Vital Registration	95.3	26.9	
Saint Kitts and Nevis	1995-1999	4	80.7	1998	Vital Registration	99.2	18.7	
Saint Kitts and Nevis	2000-2004	4	71.3	2002	Vital Registration	91.8	22.3	
Saint Kitts and Nevis	2005-2009	5	85.6	2008	Vital Registration	100.0	14.4	
Saint Kitts and Nevis	2010-2020	5	89.1	2014	Vital Registration	100.0	10.9	
Saint Lucia	1980-1984	4	67.1	1983	Vital Registration	97.3	31.0	
Saint Lucia	1985-1989	4	67.7	1987	Vital Registration	100.0	32.3	
Saint Lucia	1990-1994	4	73.6	1994	Vital Registration	100.0	26.4	
Saint Lucia	1995-1999	4	73.3	1997	Vital Registration	100.0	26.7	
Saint Lucia	2000-2004	4	81.6	2001	Vital Registration	100.0	18.4	
Saint Lucia	2005-2009	4	84.2	2009	Vital Registration	99.9	15.7	
Saint Lucia	2010-2020	5	86.4	2014	Vital Registration	100.0	13.6	
Saint Vincent and the Grenadines	1980-1984	4	69.3	1983	Vital Registration	100.0	30.7	
Saint Vincent and the Grenadines	1985-1989	3	62.3	1986	Vital Registration	90.9	31.4	
Saint Vincent and the Grenadines	1990-1994	3	60.3	1990	Vital Registration	95.4	36.7	
Saint Vincent and the Grenadines	1995-1999	5	85.6	1998	Vital Registration	100.0	14.4	
Saint Vincent and the Grenadines	2000-2004	5	85.6	2002	Vital Registration	100.0	14.4	
Saint Vincent and the Grenadines	2005-2009	5	88.0	2006	Vital Registration	100.0	12.0	
Saint Vincent and the Grenadines	2010-2020	5	88.0	2014	Vital Registration	100.0	12.0	
Samoa	1980-1984	0						
Samoa	1985-1989	0						
Samoa	1990-1994	0						
Samoa	1995-1999	0						
Samoa	2000-2004	0						
Samoa	2005-2009	0						
Samoa	2010-2020	0						
San Marino	1980-1984	0						
San Marino	1985-1989	0						
San Marino	1990-1994	0						
San Marino	1995-1999	4	75.3	1995	Vital Registration	100.0	24.7	
San Marino	2000-2004	4	76.8	2002	Vital Registration	100.0	23.2	
San Marino	2005-2009	4	76.2	2005	Vital Registration	99.2	23.2	
San Marino	2010-2020	4	67.1	2012	Vital Registration	83.2	19.4	
Saudi Arabia	1980-1984	0						
Saudi Arabia	1985-1989	0						
Saudi Arabia	1990-1994	0						
Saudi Arabia	1995-1999	2	17.7	1999	Vital Registration	32.8	46.0	
Saudi Arabia	2000-2004	2	20.8	2004	Vital Registration	40.9	49.3	
Saudi Arabia	2005-2009	2	22.2	2005	Vital Registration	41.3	46.2	
Saudi Arabia	2010-2020	2	20.5	2010	Vital Registration	43.8	53.1	
Senegal	1980-1984	1	2.8	1984	Senegal - Risk of Death Associated with Different Nutritional States in Children of Preschool age: Study Conducted in Niakhar (Senegal) 1983-1986		1.7	2.8
Senegal	1985-1989	1	3.5	1986	International differences in clinical patterns of diarrhoeal deaths: a comparison of children from Brazil, Senegal, Bangladesh, and India		5.2	3.7
Senegal	1990-1994	1	3.6	1990	Childhood mortality and probable causes of death using verbal autopsy in Niakhar, Senegal, 1989-2000		3.8	3.7
Senegal	1995-1999	1	3.4	1996	Childhood mortality and probable causes of death using verbal autopsy in Niakhar, Senegal, 1989-2000		2.6	3.5
Senegal	2000-2004	0						
Senegal	2005-2009	1	0.0	2008	Africa, Asia, Oceania - INDEPTH Network Cause-Specific Mortality - Release 2014		99.8	6.4
Senegal	2010-2020	1	0.0	2010	Africa, Asia, Oceania - INDEPTH Network Cause-Specific Mortality - Release 2014		100.0	6.4
Serbia	1980-1984	0						
Serbia	1985-1989	0						
Serbia	1990-1994	0						
Serbia	1995-1999	4	71.6	1999	Vital Registration	93.0	22.9	
Serbia	2000-2004	4	72.2	2003	Vital Registration	92.8	22.2	
Serbia	2005-2009	4	73.5	2005	Vital Registration	90.6	18.8	
Serbia	2010-2020	4	72.5	2010	Vital Registration	88.0	17.6	
Seychelles	1980-1984	4	71.0	1981	Vital Registration	100.0	29.0	
Seychelles	1985-1989	4	71.3	1987	Vital Registration	100.0	28.7	
Seychelles	1990-1994	0						
Seychelles	1995-1999	0						
Seychelles	2000-2004	4	77.1	2002	Vital Registration	100.0	22.9	
Seychelles	2005-2009	4	78.6	2008	Vital Registration	100.0	21.4	
Seychelles	2010-2020	4	79.1	2011	Vital Registration	100.0	20.9	
Sierra Leone	1980-1984	0						
Sierra Leone	1985-1989	0						
Sierra Leone	1990-1994	1	4.0	1990	Malaria in a rural area of Sierra Leone. I. Initial results		1.1	4.1
Sierra Leone	1995-1999	0						
Sierra Leone	2000-2004	0						
Sierra Leone	2005-2009	0						
Sierra Leone	2010-2020	1	2.9	2019	Child Health and Mortality Prevention Surveillance Network Program		2.0	3.0
Singapore	1980-1984	5	91.1	1983	Vital Registration	100.0	8.9	
Singapore	1985-1989	5	92.1	1988	Vital Registration	100.0	7.9	
Singapore	1990-1994	5	94.5	1994	Vital Registration	100.0	5.5	
Singapore	1995-1999	5	95.6	1997	Vital Registration	100.0	4.4	
Singapore	2000-2004	5	95.2	2003	Vital Registration	100.0	4.8	
Singapore	2005-2009	5	95.1	2005	Vital Registration	100.0	4.9	
Singapore	2010-2020	5	96.8	2014	Vital Registration	100.0	3.2	
Slovakia	1980-1984	0						
Slovakia	1985-1989	0						
Slovakia	1990-1994	4	83.7	1994	Vital Registration	100.0	16.3	
Slovakia	1995-1999	4	83.4	1999	Vital Registration	99.9	16.5	

Appendix Table S9: Underlying indicators for percent well-certified for data source with maximum percent well certified in each 5-year time interval for 204 countries, 1980-2020.

Location	Time Window	Stars	Percent Well-Certified [PWC] (%)	Max PWC Data Year	Max PWC Data Source	Completeness (%)	Percent Major Garbage (%)	Verbal Autopsy Adjustment (None for VR) (%)
Slovakia	2000-2004	5	85.4	2003	Vital Registration	100.0	14.6	
Slovakia	2005-2009	5	88.6	2008	Vital Registration	99.2	10.6	
Slovakia	2010-2020	5	91.6	2012	Vital Registration	99.5	7.9	
Slovenia	1980-1984	0						
Slovenia	1985-1989	5	92.5	1985	Vital Registration	100.0	7.5	
Slovenia	1990-1994	5	93.6	1992	Vital Registration	99.7	6.2	
Slovenia	1995-1999	5	91.8	1996	Vital Registration	100.0	8.2	
Slovenia	2000-2004	5	89.4	2000	Vital Registration	100.0	10.6	
Slovenia	2005-2009	5	89.7	2008	Vital Registration	100.0	10.3	
Slovenia	2010-2020	5	90.2	2011	Vital Registration	100.0	9.8	
Solomon Islands	1980-1984	0						
Solomon Islands	1985-1989	0						
Solomon Islands	1990-1994	0						
Solomon Islands	1995-1999	0						
Solomon Islands	2000-2004	0						
Solomon Islands	2005-2009	0						
Solomon Islands	2010-2020	3	42.5	2018	Verbal Autopsy		33.6	64.0
Somalia	1980-1984	0						
Somalia	1985-1989	0						
Somalia	1990-1994	0						
Somalia	1995-1999	0						
Somalia	2000-2004	0						
Somalia	2005-2009	0						
Somalia	2010-2020	0						
South Africa	1980-1984	0						
South Africa	1985-1989	0						
South Africa	1990-1994	1	1.2	1991	'A bothersome death' -- narrative accounts of infant mortality in Cape Town, South Africa		1.9	1.3
South Africa	1995-1999	4	66.0	1999	Vital Registration	100.0	34.0	
South Africa	2000-2004	4	67.9	2004	Vital Registration	100.0	32.1	
South Africa	2005-2009	4	67.7	2005	Vital Registration	100.0	32.3	
South Africa	2010-2020	4	69.0	2014	Vital Registration	100.0	31.0	
South Korea	1980-1984	0						
South Korea	1985-1989	3	55.1	1985	Vital Registration	89.2	38.2	
South Korea	1990-1994	4	72.2	1994	Vital Registration	97.2	25.7	
South Korea	1995-1999	4	73.8	1999	Vital Registration	100.0	26.2	
South Korea	2000-2004	4	83.6	2002	Vital Registration	100.0	16.4	
South Korea	2005-2009	4	82.5	2007	Vital Registration	100.0	17.5	
South Korea	2010-2020	4	83.8	2015	Vital Registration	100.0	16.2	
South Sudan	1980-1984	0						
South Sudan	1985-1989	0						
South Sudan	1990-1994	0						
South Sudan	1995-1999	0						
South Sudan	2000-2004	0						
South Sudan	2005-2009	0						
South Sudan	2010-2020	0						
Spain	1980-1984	4	79.2	1984	Vital Registration	100.0	20.8	
Spain	1985-1989	4	81.5	1989	Vital Registration	100.0	18.5	
Spain	1990-1994	5	85.2	1994	Vital Registration	100.0	14.8	
Spain	1995-1999	5	86.6	1998	Vital Registration	100.0	13.4	
Spain	2000-2004	5	86.2	2004	Vital Registration	100.0	13.8	
Spain	2005-2009	5	86.2	2009	Vital Registration	100.0	13.8	
Spain	2010-2020	5	87.9	2014	Vital Registration	100.0	12.1	
Sri Lanka	1980-1984	3	43.3	1980	Vital Registration	100.0	56.7	
Sri Lanka	1985-1989	3	43.6	1988	Vital Registration	100.0	56.4	
Sri Lanka	1990-1994	3	41.4	1992	Vital Registration	100.0	58.6	
Sri Lanka	1995-1999	3	52.2	1999	Vital Registration	100.0	47.8	
Sri Lanka	2000-2004	3	61.0	2003	Vital Registration	100.0	39.0	
Sri Lanka	2005-2009	4	63.5	2009	Vital Registration	100.0	36.5	
Sri Lanka	2010-2020	3	64.7	2010	Vital Registration	100.0	35.3	
Sudan	1980-1984	0						
Sudan	1985-1989	0						
Sudan	1990-1994	0						
Sudan	1995-1999	0						
Sudan	2000-2004	0						
Sudan	2005-2009	0						
Sudan	2010-2020	0						
Suriname	1980-1984	3	57.1	1984	Vital Registration	89.1	35.9	
Suriname	1985-1989	3	61.6	1989	Vital Registration	92.7	33.5	
Suriname	1990-1994	3	56.2	1991	Vital Registration	82.5	31.9	
Suriname	1995-1999	3	62.1	1999	Vital Registration	90.2	31.1	
Suriname	2000-2004	4	69.0	2002	Vital Registration	90.4	23.7	
Suriname	2005-2009	4	69.7	2008	Vital Registration	91.0	23.4	
Suriname	2010-2020	4	70.7	2010	Vital Registration	89.8	21.2	
Sweden	1980-1984	5	92.3	1980	Vital Registration	100.0	7.7	
Sweden	1985-1989	5	90.7	1987	Vital Registration	100.0	9.3	
Sweden	1990-1994	5	90.2	1990	Vital Registration	100.0	9.8	
Sweden	1995-1999	5	89.7	1997	Vital Registration	100.0	10.3	
Sweden	2000-2004	5	89.3	2004	Vital Registration	100.0	10.7	
Sweden	2005-2009	5	88.6	2005	Vital Registration	100.0	11.4	
Sweden	2010-2020	5	86.7	2014	Vital Registration	99.9	13.2	
Switzerland	1980-1984	4	76.0	1983	Vital Registration	100.0	24.0	
Switzerland	1985-1989	4	75.9	1986	Vital Registration	100.0	24.1	
Switzerland	1990-1994	4	75.2	1990	Vital Registration	100.0	24.8	
Switzerland	1995-1999	5	87.1	1997	Vital Registration	100.0	12.9	
Switzerland	2000-2004	5	86.5	2000	Vital Registration	100.0	13.5	
Switzerland	2005-2009	5	87.5	2009	Vital Registration	100.0	12.5	
Switzerland	2010-2020	5	87.4	2012	Vital Registration	100.0	12.6	
Syria	1980-1984	2	29.3	1980	Vital Registration	100.0	70.7	
Syria	1985-1989	2	13.5	1985	Vital Registration	60.9	77.9	
Syria	1990-1994	0						
Syria	1995-1999	3	50.7	1999	Vital Registration	95.8	47.0	
Syria	2000-2004	3	60.5	2004	Vital Registration	91.5	33.9	
Syria	2005-2009	4	73.2	2009	Vital Registration	98.7	25.9	
Syria	2010-2020	3	57.9	2010	Vital Registration	100.0	42.1	
São Tomé and Príncipe	1980-1984	0						
São Tomé and Príncipe	1985-1989	3	58.7	1985	Vital Registration	100.0	41.3	
São Tomé and Príncipe	1990-1994	0						
São Tomé and Príncipe	1995-1999	0						
São Tomé and Príncipe	2000-2004	0						
São Tomé and Príncipe	2005-2009	0						
São Tomé and Príncipe	2010-2020	0						
Taiwan (province of China)	1980-1984	4	80.7	1980	Vital Registration	100.0	19.3	
Taiwan (province of China)	1985-1989	4	79.4	1987	Vital Registration	100.0	20.6	
Taiwan (province of China)	1990-1994	4	78.2	1994	Vital Registration	100.0	21.8	
Taiwan (province of China)	1995-1999	4	83.3	1999	Vital Registration	100.0	16.7	
Taiwan (province of China)	2000-2004	4	84.8	2004	Vital Registration	100.0	15.2	
Taiwan (province of China)	2005-2009	5	85.1	2005	Vital Registration	100.0	14.9	
Taiwan (province of China)	2010-2020	5	85.6	2014	Vital Registration	100.0	14.4	
Tajikistan	1980-1984	4	78.6	1981	Vital Registration	94.0	16.3	
Tajikistan	1985-1989	4	73.4	1988	Vital Registration	93.1	21.2	
Tajikistan	1990-1994	4	66.7	1990	Vital Registration	84.7	21.2	
Tajikistan	1995-1999	3	51.3	1995	Vital Registration	74.9	31.4	
Tajikistan	2000-2004	3	45.2	2002	Vital Registration	66.1	31.6	
Tajikistan	2005-2009	3	43.9	2005	Vital Registration	67.3	34.7	
Tajikistan	2010-2020	3	45.9	2016	Vital Registration	67.2	31.7	
Tanzania	1980-1984	0						
Tanzania	1985-1989	1	3.8	1986	Risk factors for deaths in children under 5 years old in Bagamoyo district, Tanzania		2.6	3.9
Tanzania	1990-1994	1	3.3	1993	Community based studies on childhood mortality in a malaria holoendemic area on the Tanzanian coast		6.7	3.6
Tanzania	1995-1999	1	2.3	1995	The Policy Implications of Tanzania's Mortality Burden		64.3	6.4
Tanzania	2000-2004	1	6.3	2000	Tanzania - Rufiji Health and Demographic Surveillance System		0.9	6.4
Tanzania	2005-2009	1	3.1	2005	The contribution of reduction in malaria as a cause of rapid decline of under-five mortality: evidence from the Rufiji Health and Demographic Surveillance System (HDSS) in rural Tanzania		0.8	3.1
Tanzania	2010-2020	1	2.9	2012	Mortality of neurological disorders in Tanzania: analysis of baseline data from sample vital registration with verbal autopsy (SAVVY)		95.5	64.0
Thailand	1980-1984	2	23.6	1983	Vital Registration	84.5	72.0	
Thailand	1985-1989	2	24.3	1987	Vital Registration	82.2	70.5	
Thailand	1990-1994	2	29.6	1994	Vital Registration	85.3	65.3	
Thailand	1995-1999	3	62.6	1998	Thailand Burden of Disease and Injuries 1998-1999		2.2	64.0
Thailand	2000-2004	3	47.1	2003	Vital Registration	98.1	52.0	
Thailand	2005-2009	3	61.6	2005	Thailand Verbal Autopsy Study 2005		3.7	64.0
Thailand	2010-2020	4	67.6	2019	Vital Registration	100.0	32.4	
The Bahamas	1980-1984	4	75.0	1984	Vital Registration	95.1	21.2	
The Bahamas	1985-1989	4	82.3	1987	Vital Registration	100.0	17.7	
The Bahamas	1990-1994	4	82.1	1994	Vital Registration	100.0	17.9	
The Bahamas	1995-1999	5	86.0	1999	Vital Registration	97.2	11.5	
The Bahamas	2000-2004	5	87.7	2001	Vital Registration	100.0	12.3	
The Bahamas	2005-2009	5	86.8	2009	Vital Registration	100.0	13.2	
The Bahamas	2010-2020	5	88.0	2011	Vital Registration	100.0	12.0	
The Gambia	1980-1984	1	3.8	1982	Deaths in infancy and early childhood in a well-vaccinated, rural, West African population		1.7	3.8
The Gambia	1985-1989	1	3.3	1989	Changes in the pattern of infant and childhood mortality in upper river division, The Gambia, from 1989 to 1993		3.8	3.5
The Gambia	1990-1994	1	3.3	1990	Changes in the pattern of infant and childhood mortality in upper river division, The Gambia, from 1989 to 1993		4.8	3.4
The Gambia	1995-1999	1	2.7	1999	Reaching millennium development goal 4 - the Gambia		7.4	3.0
The Gambia	2000-2004	1	2.5	2002	Reaching millennium development goal 4 - the Gambia		8.0	2.7
The Gambia	2005-2009	1	2.2	2006	Preventive measures in infancy to reduce under-five mortality: a case-control study in The Gambia		6.3	2.4
The Gambia	2010-2020	0						
Timor-Leste	1980-1984	0						
Timor-Leste	1985-1989	0						
Timor-Leste	1990-1994	0						
Timor-Leste	1995-1999	0						
Timor-Leste	2000-2004	0						
Timor-Leste	2005-2009	0						
Timor-Leste	2010-2020	0						
Togo	1980-1984	0						
Togo	1985-1989	0						
Togo	1990-1994	0						
Togo	1995-1999	0						
Togo	2000-2004	0						
Togo	2005-2009	0						
Togo	2010-2020	0						
Tokelau	1980-1984	0						
Tokelau	1985-1989	0						
Tokelau	1990-1994	0						
Tokelau	1995-1999	0						
Tokelau	2000-2004	0						
Tokelau	2005-2009	0						
Tokelau	2010-2020	0						
Tonga	1980-1984	0						
Tonga	1985-1989	0						
Tonga	1990-1994	0						
Tonga	1995-1999	0						
Tonga	2000-2004	3	52.9	2003	Vital Registration	100.0	47.1	
Tonga	2005-2009	0						
Tonga	2010-2020	0						
Trinidad and Tobago	1980-1984	4	80.2	1983	Vital Registration	96.9	17.3	
Trinidad and Tobago	1985-1989	4	83.6	1989	Vital Registration	98.2	14.9	
Trinidad and Tobago	1990-1994	5	85.8	1994	Vital Registration	100.0	14.2	
Trinidad and Tobago	1995-1999	5	89.3	1999	Vital Registration	100.0	10.7	
Trinidad and Tobago	2000-2004	5	90.3	2002	Vital Registration	100.0	9.7	
Trinidad and Tobago	2005-2009	5	88.8	2005	Vital Registration	100.0	11.2	
Trinidad and Tobago	2010-2020	5	89.9	2012	Vital Registration	100.0	10.1	
Tunisia	1980-1984	0						
Tunisia	1985-1989	0						
Tunisia	1990-1994	0						
Tunisia	1995-1999	0						
Tunisia	2000-2004	0						
Tunisia	2005-2009	2	27.8	2009	Vital Registration	37.5	25.8	
Tunisia	2010-2020	2	28.8	2017	Vital Registration	44.2	34.8	
Turkey	1980-1984	2	12.7	1983	Vital Registration	35.2	64.0	
Turkey	1985-1989	2	15.9	1989	Vital Registration	39.8	60.0	
Turkey	1990-1994	2	17.9	1993	Vital Registration	42.9	58.2	
Turkey	1995-1999	2	21.8	1999	Vital Registration	53.4	59.3	

Appendix Table S9: Underlying indicators for percent well-certified for data source with maximum percent well certified in each 5-year time interval for 204 countries, 1980-2020.

Location	Time Window	Stars	Percent Well-Certified [PWC] (%)	Max PWC Data Year	Max PWC Data Source	Completeness (%)	Percent Major Garbage (%)	Verbal Autopsy Adjustment (None for VR) (%)
Turkey	2000-2004	3	59.1	2002	Turkey Verbal Autopsy Survey 2003		7.7	64.0
Turkey	2005-2009	3	61.0	2009	Vital Registration	77.1	20.9	
Turkey	2010-2020	4	80.4	2016	Vital Registration	96.6	16.7	
Turkmenistan	1980-1984	5	88.5	1981	Vital Registration	100.0	11.5	
Turkmenistan	1985-1989	5	88.0	1986	Vital Registration	100.0	12.0	
Turkmenistan	1990-1994	5	88.5	1993	Vital Registration	100.0	11.5	
Turkmenistan	1995-1999	4	81.9	1999	Vital Registration	89.6	8.6	
Turkmenistan	2000-2004	4	82.5	2002	Vital Registration	93.5	11.7	
Turkmenistan	2005-2009	4	78.7	2009	Vital Registration	93.8	16.1	
Turkmenistan	2010-2020	4	81.5	2010	Vital Registration	97.7	16.6	
Tuvalu	1980-1984	0						
Tuvalu	1985-1989	0						
Tuvalu	1990-1994	0						
Tuvalu	1995-1999	0						
Tuvalu	2000-2004	0						
Tuvalu	2005-2009	0						
Tuvalu	2010-2020	0						
UK	1980-1984	5	93.7	1984	Vital Registration	100.0	6.3	
UK	1985-1989	5	93.7	1985	Vital Registration	100.0	6.3	
UK	1990-1994	5	93.6	1991	Vital Registration	100.0	6.4	
UK	1995-1999	5	92.2	1995	Vital Registration	100.0	7.8	
UK	2000-2004	5	91.7	2002	Vital Registration	100.0	8.3	
UK	2005-2009	5	91.6	2006	Vital Registration	100.0	8.4	
UK	2010-2020	5	91.7	2014	Vital Registration	100.0	8.3	
USA	1980-1984	5	90.5	1980	Vital Registration	100.0	9.5	
USA	1985-1989	5	89.6	1989	Vital Registration	100.0	10.4	
USA	1990-1994	5	90.1	1990	Vital Registration	100.0	9.9	
USA	1995-1999	5	89.6	1995	Vital Registration	100.0	10.4	
USA	2000-2004	5	88.7	2000	Vital Registration	100.0	11.3	
USA	2005-2009	5	87.8	2005	Vital Registration	100.0	12.2	
USA	2010-2020	5	87.4	2010	Vital Registration	100.0	12.6	
Uganda	1980-1984	0						
Uganda	1985-1989	0						
Uganda	1990-1994	0						
Uganda	1995-1999	0						
Uganda	2000-2004	1	0.3	2000	Effect of HIV infection on pregnancy-related mortality in sub-Saharan Africa: secondary analyses of pooled community-based data from the network for Analysing Longitudinal Population-based HIV/AIDS data on Africa (ALPHA)		12.3	0.3
Uganda	2005-2009	1	3.4	2006	Uganda Child Verbal Autopsy Study 2007		1.2	3.5
Uganda	2010-2020	1	0.4	2017	Community health workers trained to conduct verbal autopsies provide better mortality measures than existing surveillance: Results from a cross-sectional study in rural western Uganda		94.1	6.4
Ukraine	1980-1984	5	88.0	1982	Vital Registration	100.0	12.0	
Ukraine	1985-1989	5	88.7	1985	Vital Registration	100.0	11.3	
Ukraine	1990-1994	4	81.6	1994	Vital Registration	100.0	18.4	
Ukraine	1995-1999	4	83.6	1999	Vital Registration	100.0	16.4	
Ukraine	2000-2004	4	84.1	2000	Vital Registration	100.0	15.9	
Ukraine	2005-2009	5	89.8	2009	Vital Registration	100.0	10.2	
Ukraine	2010-2020	5	91.9	2013	Vital Registration	100.0	8.1	
United Arab Emirates	1980-1984	0						
United Arab Emirates	1985-1989	0						
United Arab Emirates	1990-1994	0						
United Arab Emirates	1995-1999	0						
United Arab Emirates	2000-2004	0						
United Arab Emirates	2005-2009	3	41.7	2007	Vital Registration	77.9	46.4	
United Arab Emirates	2010-2020	2	16.1	2019	Vital Registration	40.9	60.5	
Uruguay	1980-1984	4	77.7	1982	Vital Registration	100.0	22.3	
Uruguay	1985-1989	4	77.8	1989	Vital Registration	100.0	22.2	
Uruguay	1990-1994	4	77.7	1994	Vital Registration	99.7	22.1	
Uruguay	1995-1999	4	80.9	1997	Vital Registration	99.3	18.5	
Uruguay	2000-2004	4	80.8	2001	Vital Registration	100.0	19.2	
Uruguay	2005-2009	4	80.5	2005	Vital Registration	100.0	19.5	
Uruguay	2010-2020	4	80.3	2015	Vital Registration	100.0	19.7	
Uzbekistan	1980-1984	5	86.5	1982	Vital Registration	100.0	13.4	
Uzbekistan	1985-1989	5	87.0	1985	Vital Registration	100.0	13.0	
Uzbekistan	1990-1994	4	80.6	1990	Vital Registration	92.9	13.2	
Uzbekistan	1995-1999	4	68.4	1995	Vital Registration	81.4	16.0	
Uzbekistan	2000-2004	3	58.2	2000	Vital Registration	70.3	17.2	
Uzbekistan	2005-2009	3	59.5	2009	Vital Registration	65.9	9.7	
Uzbekistan	2010-2020	3	64.6	2016	Vital Registration	75.1	14.1	
Vanuatu	1980-1984	0						
Vanuatu	1985-1989	0						
Vanuatu	1990-1994	0						
Vanuatu	1995-1999	0						
Vanuatu	2000-2004	0						
Vanuatu	2005-2009	0						
Vanuatu	2010-2020	0						
Venezuela	1980-1984	4	78.8	1983	Vital Registration	100.0	21.2	
Venezuela	1985-1989	4	73.3	1988	Vital Registration	100.0	26.7	
Venezuela	1990-1994	4	82.1	1992	Vital Registration	100.0	17.9	
Venezuela	1995-1999	5	88.4	1999	Vital Registration	100.0	11.6	
Venezuela	2000-2004	5	90.6	2001	Vital Registration	100.0	9.3	
Venezuela	2005-2009	5	89.2	2007	Vital Registration	97.9	8.9	
Venezuela	2010-2020	5	91.6	2014	Vital Registration	100.0	8.4	
Vietnam	1980-1984	0						
Vietnam	1985-1989	1	1.3	1987	Are there social inequities in child morbidity and mortality in rural Vietnam		4.6	1.4
Vietnam	1990-1994	1	0.8	1994	Maternal mortality in Vietnam in 1994-95		9.8	0.9
Vietnam	1995-1999	1	1.2	1999	Applying verbal autopsy to determine cause of death in rural Vietnam		81.2	6.4
Vietnam	2000-2004	1	1.4	2001	Socio-economic status inequality and major causes of death in adults: a 5-year follow-up study in rural Vietnam		74.1	5.6
Vietnam	2005-2009	3	62.0	2008	Mortality measures from sample-based surveillance: evidence of the epidemiological transition in Viet Nam, Unpublished data		3.1	64.0
Vietnam	2010-2020	1	4.8	2010	The causes of deaths in Chililab between 2008-2010 based on verbal autopsy method		25.1	6.4
Virgin Islands	1980-1984	4	81.2	1980	Vital Registration	100.0	18.8	
Virgin Islands	1985-1989	0						
Virgin Islands	1990-1994	4	81.1	1994	Vital Registration	89.8	9.7	
Virgin Islands	1995-1999	5	85.2	1995	Vital Registration	95.8	11.1	
Virgin Islands	2000-2004	4	77.0	2000	Vital Registration	86.8	11.4	
Virgin Islands	2005-2009	4	67.4	2005	Vital Registration	77.2	12.7	
Virgin Islands	2010-2020	3	56.6	2010	Vital Registration	66.9	15.3	
Yemen	1980-1984	0						
Yemen	1985-1989	0						
Yemen	1990-1994	0						
Yemen	1995-1999	0						
Yemen	2000-2004	0						
Yemen	2005-2009	0						
Yemen	2010-2020	0						
Zambia	1980-1984	0						
Zambia	1985-1989	0						
Zambia	1990-1994	0						
Zambia	1995-1999	0						
Zambia	2000-2004	0						
Zambia	2005-2009	1	6.4	2009	Zambia Sample Vital Registration with Verbal Autopsy (SAVVY) Data 2010		0.7	6.4
Zambia	2010-2020	2	32.6	2016	Zambia Sample Vital Registration with Verbal Autopsy (SAVVY) 2015-2016		49.0	64.0
Zimbabwe	1980-1984	0						
Zimbabwe	1985-1989	0						
Zimbabwe	1990-1994	3	35.3	1990	Vital Registration	54.9	35.7	
Zimbabwe	1995-1999	3	58.1	1995	Vital Registration	100.0	41.9	
Zimbabwe	2000-2004	1	0.4	2000	Effect of HIV infection on pregnancy-related mortality in sub-Saharan Africa: secondary analyses of pooled community-based data from the network for Analysing Longitudinal Population-based HIV/AIDS data on Africa (ALPHA)		12.4	0.4
Zimbabwe	2005-2009	3	42.6	2007	Vital Registration	57.2	25.6	
Zimbabwe	2010-2020	0						

Appendix Table S10: Modeling strategy for individual cause of death models in GBD 2021		
**Cause Name**	**Level**	**Model type**
Communicable, maternal, neonatal, and nutritional diseases	Aggregate	
HIV/AIDS and sexually transmitted infections	Aggregate	
HIV/AIDS	3	EPP-ASM, Spectrum
HIV-AIDS - Drug-susceptible Tuberculosis	4	Data proportion
HIV/AIDS - Multidrug-resistant Tuberculosis without extensive drug resistance	4	Data proportion
HIV/AIDS - Extensively drug-resistant Tuberculosis	4	Data proportion
HIV/AIDS resulting in other diseases	4	Data proportion
Sexually transmitted infections excluding HIV	3	CODEm; natural history model (congenital syphilis)
Syphilis	4	Data proportion (age/sex-specific VR); natural history model (congenital syphilis)
Chlamydial infection	4	Data proportion (age/sex-specific VR)
Gonococcal infection	4	Data proportion (age/sex-specific VR)
Other sexually transmitted diseases	4	Data proportion (age/sex-specific VR)
Respiratory infections and tuberculosis	Aggregate	
Tuberculosis	3	CODEm
Drug-susceptible tuberculosis	4	Spatio-temporal Gaussian process regression proportion
Multidrug-resistant tuberculosis without extensive drug resistance	4	Spatio-temporal Gaussian process regression proportion
Extensively drug-resistant tuberculosis	4	Spatio-temporal Gaussian process regression proportion
Lower respiratory infections	3	CODEm
Upper respiratory infections	3	CODEm
Otitis media	3	CODEm
Enteric infections	Aggregate	
Diarrheal diseases	3	CODEm; Fatal Discontinuity
Typhoid and paratyphoid	Aggregate	
Typhoid fever	4	CODEm (data rich countries); natural history model (non-data rich countries)
Paratyphoid fever	4	CODEm (data rich countries); natural history model (non-data rich countries)
Invasive Non-typhoidal Salmonella (iNTS)	3	CODEm (data rich countries); natural history model (non-data rich countries)
Other intestinal infectious diseases	3	Negative binomial regression
Neglected tropical diseases and malaria	Aggregate	
Malaria	3	CODEm (P. falciparum outside of Africa); natural history model (P. falciparum within Africa); negative binomial regression (P. vivax)
Chagas disease	3	CODEm
Leishmaniasis	Aggregate	
Visceral leishmaniasis	4	Natural history model
African trypanosomiasis	3	Natural history model
Schistosomiasis	3	Negative binomial regression
Cysticercosis	3	Negative binomial regression
Cystic echinococcosis	3	Negative binomial regression
Dengue	3	CODEm, Fatal Discontinuity
Yellow fever	3	Natural history model
Rabies	3	CODEm
Intestinal nematode infections	Aggregate	
Ascariasis	4	Negative binomial regression
Ebola	3	Fatal Discontinuity
Zika virus	3	Natural history model
Other neglected tropical diseases	3	CODEm
Other infectious diseases	Aggregate	
Meningitis	3	CODEm, Fatal Discontinuity
Encephalitis	3	CODEm
Diphtheria	3	CODEm (data rich countries); negative binomial regression (non-data rich countries)
Whooping cough	3	CODEm (data rich countries); natural history model (non-data rich countries)
Tetanus	3	CODEm
Measles	3	CODEm (data rich countries); natural history model (non-data rich countries), Fatal Discontinuity
Varicella and herpes zoster	3	CODEm (data rich countries); negative binomial regression (non-data rich countries)
Acute hepatitis	3	CODEm
Acute hepatitis A	4	CODEm

Appendix Table S10: Modeling strategy for individual cause of death models in GBD 2021		
**Cause Name**	**Level**	**Model type**
Acute hepatitis B	4	CODEm
Acute hepatitis C	4	CODEm
Acute hepatitis E	4	CODEm
Other unspecified infectious diseases	3	CODEm, Fatal Discontinuity
Maternal and neonatal disorders	Aggregate	
Maternal disorders	3	CODEm
Maternal hemorrhage	4	Spatio-temporal Gaussian process regression
Maternal sepsis and other maternal infections	4	Spatio-temporal Gaussian process regression
Maternal hypertensive disorders	4	Spatio-temporal Gaussian process regression
Maternal obstructed labor and uterine rupture	4	Spatio-temporal Gaussian process regression
Maternal abortion and miscarriage	4	Spatio-temporal Gaussian process regression
Ectopic pregnancy	4	Spatio-temporal Gaussian process regression proportion
Indirect maternal deaths	4	Spatio-temporal Gaussian process regression
Late maternal deaths	4	DisMod MR-2.1 proportion model
Maternal deaths aggravated by HIV/AIDS	4	Spatio-temporal Gaussian process regression
Other maternal disorders	4	Spatio-temporal Gaussian process regression
Neonatal disorders	3	CODEm
Neonatal preterm birth	4	CODEm
Neonatal encephalopathy due to birth asphyxia and trauma	4	CODEm
Neonatal sepsis and other neonatal infections	4	CODEm
Hemolytic disease and other neonatal jaundice	4	CODEm
Other neonatal disorders	4	CODEm
Nutritional deficiencies	2	CODEm
Protein-energy malnutrition	3	CODEm; Fatal Discontinuity
Other nutritional deficiencies	3	CODEm
Non-communicable diseases	Aggregate	
Neoplasms	Aggregate	
Lip and oral cavity cancer	3	CODEm
Nasopharynx cancer	3	CODEm
Other pharynx cancer	3	CODEm
Esophageal cancer	3	CODEm
Stomach cancer	3	CODEm
Colon and rectum cancer	3	CODEm
Liver cancer	3	CODEm
Liver cancer due to hepatitis B	4	DisMod MR-2.1 proportion model
Liver cancer due to hepatitis C	4	DisMod MR-2.1 proportion model
Liver cancer due to alcohol use	4	DisMod MR-2.1 proportion model
Liver cancer due to NASH	4	DisMod MR-2.1 proportion model
Liver cancer due to other causes	4	DisMod MR-2.1 proportion model
Gallbladder and biliary tract cancer	3	CODEm
Pancreatic cancer	3	CODEm
Larynx cancer	3	CODEm
Tracheal, bronchus, and lung cancer	3	CODEm
Malignant skin melanoma	3	CODEm
Non-melanoma skin cancer	Aggregate	
Non-melanoma skin cancer (squamous-cell carcinoma)	4	CODEm
Breast cancer	3	CODEm
Cervical cancer	3	CODEm
Uterine cancer	3	CODEm
Ovarian cancer	3	CODEm
Prostate cancer	3	CODEm
Testicular cancer	3	CODEm
Kidney cancer	3	CODEm
Bladder cancer	3	CODEm
Brain and nervous system cancer	3	CODEm
Thyroid cancer	3	CODEm
Mesothelioma	3	CODEm
Hodgkin lymphoma	3	CODEm
Non-Hodgkin lymphoma	3	CODEm
Multiple myeloma	3	CODEm
Leukemia	3	CODEm
Acute lymphoid leukemia	4	CODEm
Chronic lymphoid leukemia	4	CODEm
Acute myeloid leukemia	4	CODEm
Chronic myeloid leukemia	4	CODEm
Other leukemia	4	CODEm
Other malignant neoplasms	3	CODEm
Other neoplasms	Aggregate	
Myelodysplastic, myeloproliferative, and other hematopoietic neoplasms	4	CODEm

Appendix Table S10: Modeling strategy for individual cause of death models in GBD 2021		
**Cause Name**	**Level**	**Model type**
Other benign and in situ neoplasms	4	CODEm
Cardiovascular diseases	2	CODEm
Rheumatic heart disease	3	CODEm
Ischemic heart disease	3	CODEm
Stroke	3	CODEm
Ischemic stroke	4	CODEm
Intracerebral hemorrhage	4	CODEm
Subarachnoid hemorrhage	4	CODEm
Hypertensive heart disease	3	CODEm
Non-rheumatic valvular heart disease	3	CODEm
Non-rheumatic calcific aortic valve disease	4	CODEm
Non-rheumatic degenerative mitral valve disease	4	CODEm
Other non-rheumatic valve diseases	4	CODEm
Cardiomyopathy and myocarditis	3	CODEm
Myocarditis	4	CODEm
Alcoholic cardiomyopathy	4	CODEm
Other cardiomyopathy	4	CODEm
Atrial fibrillation and flutter	3	CODEm
Aortic aneurysm	3	CODEm
Peripheral artery disease	3	CODEm
Endocarditis	3	CODEm
Other cardiovascular and circulatory diseases	3	CODEm
Chronic respiratory diseases	2	CODEm
Chronic obstructive pulmonary disease	3	CODEm
Pneumoconiosis	3	CODEm
Silicosis	4	CODEm
Asbestosis	4	CODEm
Coal workers pneumoconiosis	4	CODEm
Other pneumoconiosis	4	CODEm
Asthma	3	CODEm
Interstitial lung disease and pulmonary sarcoidosis	3	CODEm
Other chronic respiratory diseases	3	CODEm
Digestive diseases	2	CODEm
Cirrhosis and other chronic liver diseases	3	CODEm
Chronic hepatitis B including cirrhosis	4	DisMod MR-2.1 proportion model
Chronic hepatitis C including cirrhosis	4	DisMod MR-2.1 proportion model
Cirrhosis due to alcohol use	4	DisMod MR-2.1 proportion model
Nonalcoholic fatty liver disease including cirrhosis	4	DisMod MR-2.1 proportion model
Cirrhosis due to other causes	4	DisMod MR-2.1 proportion model
Upper digestive system diseases	3	CODEm
Peptic ulcer disease	4	CODEm
Gastritis and duodenitis	4	CODEm
Appendicitis	3	CODEm
Paralytic ileus and intestinal obstruction	3	CODEm
Inguinal, femoral, and abdominal hernia	3	CODEm
Inflammatory bowel disease	3	CODEm
Crohn's disease	4	CODEm
Ulcerative colitis	4	CODEm
Vascular intestinal disorders	3	CODEm
Gallbladder and biliary diseases	3	CODEm
Pancreatitis	3	CODEm
Other digestive diseases	3	CODEm
Neurological disorders	Aggregate	
Alzheimer's disease and other dementias	3	DisMod MR-2.1; custom excess mortality analysis
Parkinson's disease	3	CODEm
Epilepsy	3	CODEm
Multiple sclerosis	3	CODEm
Motor neuron disease	3	CODEm
Other neurological disorders	3	CODEm
Mental disorders	Aggregate	
Eating disorders	3	CODEm
Anorexia nervosa	4	CODEm
Substance use disorders	Aggregate	
Alcohol use disorders	3	CODEm, imported cases
Drug use disorders	3	CODEm
Opioid use disorders	4	CODEm, imported cases
Cocaine use disorders	4	CODEm
Amphetamine use disorders	4	CODEm
Other drug use disorders	4	CODEm

Appendix Table S10: Modeling strategy for individual cause of death models in GBD 2021		
Cause Name	Level	Model type
Diabetes and kidney diseases	Aggregate	
Diabetes mellitus	3	CODEm
Diabetes mellitus type 1	4	CODEm
Diabetes mellitus type 2	4	CODEm
Chronic kidney disease	3	CODEm
Chronic kidney disease due to diabetes mellitus type 1	4	DisMod MR-2.1 proportion model
Chronic kidney disease due to diabetes mellitus type 2	4	DisMod MR-2.1 proportion model
Chronic kidney disease due to hypertension	4	DisMod MR-2.1 proportion model
Chronic kidney disease due to glomerulonephritis	4	DisMod MR-2.1 proportion model
Chronic kidney disease due to other causes	4	DisMod MR-2.1 proportion model
Acute glomerulonephritis	3	CODEm
Skin and subcutaneous diseases	2	CODEm
Bacterial skin diseases	3	CODEm
Cellulitis	4	CODEm
Pyoderma	4	CODEm
Decubitus ulcer	3	CODEm
Other skin and subcutaneous diseases	3	CODEm
Musculoskeletal disorders	2	CODEm
Rheumatoid arthritis	3	CODEm
Other musculoskeletal disorders	3	CODEm
Other non-communicable diseases	Aggregate	
Congenital birth defects	3	CODEm
Neural tube defects	4	CODEm
Congenital heart anomalies	4	CODEm
Orofacial clefts	4	CODEm
Down syndrome	4	CODEm
Other chromosomal abnormalities	4	CODEm
Congenital musculoskeletal and limb anomalies	4	CODEm
Urogenital congenital anomalies	4	CODEm
Digestive congenital anomalies	4	CODEm
Other congenital birth defects	4	CODEm
Urinary diseases and male infertility	3	CODEm
Urinary tract infections	4	CODEm
Urolithiasis	4	CODEm
Other urinary diseases	4	CODEm
Gynecological diseases	3	CODEm
Uterine fibroids	4	CODEm
Endometriosis	4	CODEm
Genital prolapse	4	CODEm
Other gynecological diseases	4	CODEm
Hemoglobinopathies and hemolytic anemias	3	CODEm
Thalassemias	4	DisMod MR-2.1 cause-specific mortality model
Sickle cell disorders	4	DisMod MR-2.1 cause-specific mortality model
G6PD deficiency	4	DisMod MR-2.1 cause-specific mortality model
Other hemoglobinopathies and hemolytic anemias	4	Data proportion
Endocrine, metabolic, blood, and immune disorders	3	CODEm
Thyroid disorders	4	CODEm
Other endocrine, metabolic, blood, and immune disorders	4	CODEm
Sudden infant death syndrome	3	CODEm
Injuries	Aggregate	
Transport injuries	2	CODEm
Road injuries	3	CODEm
Pedestrian road injuries	4	CODEm
Cyclist road injuries	4	CODEm
Motorcyclist road injuries	4	CODEm
Motor vehicle road injuries	4	CODEm
Other road injuries	4	CODEm
Other transport injuries	3	CODEm; Fatal Discontinuity
Unintentional injuries	Aggregate	
Falls	3	CODEm
Drowning	3	CODEm
Fire, heat, and hot substances	3	CODEm; Fatal Discontinuity
Poisonings	3	CODEm
Poisoning by carbon monoxide	4	CODEm
Poisoning by other means	4	CODEm, Fatal Discontinuity
Exposure to mechanical forces	Aggregate	
Unintentional firearm injuries	4	CODEm
Other exposure to mechanical forces	4	CODEm; Fatal Discontinuity
Adverse effects of medical treatment	3	CODEm

Appendix Table S10: Modeling strategy for individual cause of death models in GBD 2021		
**Cause Name**	**Level**	**Model type**
Animal contact	3	CODEm
Venomous animal contact	4	CODEm
Non-venomous animal contact	4	CODEm, Fatal Discontinuity
Foreign body	Aggregate	
Pulmonary aspiration and foreign body in airway	4	CODEm
Foreign body in other body part	4	CODEm
Environmental heat and cold exposure	3	CODEm; Fatal Discontinuity
Exposure to forces of nature	3	Fatal Discontinuity
Other unintentional injuries	3	CODEm
Self-harm and interpersonal violence	Aggregate	
Self-harm	3	CODEm
Self-harm by firearm	4	CODEm
Self-harm by other specified means	4	CODEm
Interpersonal violence	3	CODEm
Physical violence by firearm	4	CODEm, Fatal Discontinuity
Physical violence by sharp object	4	CODEm, Fatal Discontinuity
Physical violence by other means	4	CODEm, Fatal Discontinuity
Conflict and terrorism	3	Fatal Discontinuity
Executions and police conflict	3	CODEm, Fatal Discontinuity
Pulmonary arterial hypertension	3	CODEm
Hepatoblastoma	4	CODEm (age split)
Burkitt lymphoma	4	CODEm
Other non-Hodgkin lymphoma	4	CODEm
Eye cancer	Aggregate	
Retinoblastoma	4	CODEm
Other eye cancers	4	CODEm
Soft tissue and other extraosseous sarcomas	3	CODEm
Malignant neoplasm of bone and articular cartilage	3	CODEm
Neuroblastoma and other peripheral nervous cell tumors	3	CODEm

Appendix Table S11: Percent change before and after CoDCorrect by cause for all ages, both sexes, global, 2021		
Cause name	CoDCorrect level	Percent change
All causes	0	19.01<br>11.06 to 23.28
Communicable, maternal, neonatal, and nutritional disorders	1	71.06<br>55.64 to 83.44
Tuberculosis	3	15.4<br>11.67 to 18.1
Diarrhoeal diseases	3	12.12<br>5.28 to 17.98
Typhoid fever	4	10.0<br>1.41 to 19.61
Paratyphoid fever	4	10.18<br>0.66 to 20.98
Other intestinal infectious diseases	3	14.57<br>0.29 to 27.23
Lower respiratory infections	3	-0.61<br>-6.34 to 3.66
Upper respiratory infections	3	11.89<br>0.39 to 19.94
Otitis media	3	11.18<br>0.54 to 22.15
Meningitis	3	15.67<br>4.73 to 23.76
Encephalitis	3	12.41<br>8.21 to 16.23
Diphtheria	3	18.44<br>-8.87 to 50.12
Pertussis	3	-44.61<br>-58.48 to -28.87
Tetanus	3	5.79<br>-4.63 to 12.05
Measles	3	-18.02<br>-50.79 to 81.48
Varicella and herpes zoster	3	12.16<br>4.8 to 17.65
Neglected tropical diseases and malaria	2	12.38<br>-9.83 to 29.24
Malaria	3	12.46<br>-10.54 to 35.57
Chagas disease	3	1.85<br>-5.04 to 5.37
Leishmaniasis	3	1.51<br>-5.43 to 17.24
Visceral leishmaniasis	4	1.51<br>-5.43 to 17.24
African trypanosomiasis	3	16.13<br>4.42 to 24.02
Schistosomiasis	3	17.36<br>10.58 to 21.36
Cysticercosis	3	15.24<br>8.83 to 19.62
Cystic echinococcosis	3	14.61<br>10.48 to 18.44
Dengue	3	8.5<br>3.53 to 14.13
Yellow fever	3	15.15<br>-0.29 to 25.8
Rabies	3	15.41<br>10.82 to 19.54
Intestinal nematode infections	3	16.33<br>-11.42 to 39.68

Appendix Table S11: Percent change before and after CoDCorrect by cause for all ages, both sexes, global, 2021

Cause name	CoDCorrect level	Percent change
Ascariasis	4	16.33<br>-11.42 to 39.68
Other neglected tropical diseases	3	14.92<br>-3.7 to 27.65
Maternal disorders	3	2.4<br>1.55 to 3.17
Maternal haemorrhage	4	2.23<br>1.62 to 2.81
Maternal sepsis and other pregnancy related infections	4	2.01<br>1.4 to 2.62
Maternal hypertensive disorders	4	2.15<br>1.49 to 2.88
Maternal obstructed labour and uterine rupture	4	2.42<br>1.65 to 3.2
Ectopic pregnancy	4	4.77<br>-4.07 to 11.51
Indirect maternal deaths	4	2.94<br>2.06 to 3.78
Late maternal deaths	4	2.2<br>1.49 to 2.99
Other maternal disorders	4	2.82<br>1.96 to 3.69
Neonatal disorders	3	-0.68<br>-4.95 to 5.86
Neonatal preterm birth	4	16.98<br>5.8 to 23.69
Neonatal encephalopathy due to birth asphyxia and trauma	4	19.67<br>9.78 to 25.82
Neonatal sepsis and other neonatal infections	4	20.44<br>10.35 to 27.25
Hemolytic disease and other neonatal jaundice	4	33.15<br>15.46 to 44.66
Other neonatal disorders	4	20.18<br>9.48 to 27.13
Nutritional deficiencies	2	9.9<br>1.78 to 15.96
Protein-energy malnutrition	3	13.92<br>-4.38 to 27.91
Other nutritional deficiencies	3	-1.27<br>-13.92 to 10.96
Sexually transmitted infections excluding HIV	3	2.63<br>-3.04 to 9.53
Syphilis	4	1.99<br>-3.45 to 9.09
Chlamydial infection	4	13.69<br>7.58 to 18.03
Gonococcal infection	4	13.27<br>7.79 to 16.94
Other sexually transmitted infections	4	13.0<br>7.19 to 17.23
Acute hepatitis	3	14.98<br>10.29 to 18.9
Acute hepatitis A	4	617.28<br>339.56 to 954.06
Acute hepatitis B	4	252.9<br>141.02 to 355.9
Acute hepatitis C	4	710.64<br>301.19 to 1058.32
Acute hepatitis E	4	566.84<br>375.27 to 787.18

Appendix Table S11: Percent change before and after CoDCorrect by cause for all ages, both sexes, global, 2021		
**Cause name**	**CoDCorrect level**	**Percent change**
Other unspecified infectious diseases	3	9.08<br>4.6 to 12.35
Non-communicable diseases	1	5.67<br>-2.48 to 9.61
Neoplasms	2	4.26<br>-1.91 to 7.53
Oesophageal cancer	3	4.28<br>-0.82 to 7.49
Stomach cancer	3	4.12<br>-1.54 to 7.42
Liver cancer	3	4.94<br>-0.31 to 7.91
Liver cancer due to hepatitis B	4	5.43<br>2.09 to 8.14
Liver cancer due to hepatitis C	4	4.04<br>-4.4 to 8.34
Liver cancer due to alcohol use	4	4.78<br>0.24 to 7.27
Liver cancer due to other causes	4	4.83<br>-0.29 to 7.69
Larynx cancer	3	6.53<br>2.83 to 8.9
Tracheal, bronchus, and lung cancer	3	2.62<br>-3.24 to 5.83
Breast cancer	3	5.69<br>-0.7 to 9.17
Cervical cancer	3	9.16<br>4.92 to 12.05
Uterine cancer	3	4.08<br>-3.69 to 8.1
Prostate cancer	3	3.88<br>-3.22 to 7.3
Colon and rectum cancer	3	3.43<br>-4.42 to 7.33
Lip and oral cavity cancer	3	9.01<br>4.64 to 11.71
Nasopharynx cancer	3	7.43<br>4.25 to 10.08
Other pharynx cancer	3	8.19<br>5.06 to 10.47
Gallbladder and biliary tract cancer	3	4.95<br>-3.5 to 9.5
Pancreatic cancer	3	2.25<br>-5.58 to 6.21
Malignant skin melanoma	3	2.59<br>-4.14 to 5.95
Non-melanoma skin cancer	3	3.82<br>-5.14 to 8.32
Ovarian cancer	3	4.97<br>-1.67 to 8.6
Testicular cancer	3	8.32<br>5.79 to 10.67
Kidney cancer	3	2.63<br>-4.1 to 5.96
Bladder cancer	3	3.4<br>-4.91 to 7.6
Brain and nervous system cancer	3	3.86<br>-0.22 to 6.3
Thyroid cancer	3	6.91<br>0.39 to 10.52

Appendix Table S11: Percent change before and after CoDCorrect by cause for all ages, both sexes, global, 2021		
Cause name	CoDCorrect level	Percent change
Mesothelioma	3	3.07<br>-3.65 to 6.41
Hodgkin lymphoma	3	10.73<br>7.18 to 13.47
Non-Hodgkin's lymphoma	3	5.62<br>-0.82 to 9.06
Multiple myeloma	3	3.24<br>-4.72 to 7.32
Leukaemia	3	5.2<br>-0.48 to 8.38
Other malignant cancers	3	6.19<br>0.2 to 9.61
Other neoplasms	3	1.71<br>-10.25 to 7.74
Cardiovascular diseases	2	6.03<br>-1.71 to 9.93
Rheumatic heart disease	3	5.86<br>-0.52 to 11.28
Ischaemic heart disease	3	6.18<br>-1.25 to 10.48
Stroke	3	9.14<br>1.56 to 13.66
Ischaemic stroke	4	5.14<br>-5.06 to 12.07
Intracerebral hemorrhage	4	9.5<br>2.2 to 16.67
Subarachnoid hemorrhage	4	5.19<br>-1.74 to 11.76
Hypertensive heart disease	3	8.89<br>-0.11 to 14.57
Cardiomyopathy and myocarditis	3	6.16<br>-0.12 to 9.99
Atrial fibrillation and flutter	3	5.83<br>-9.06 to 13.52
Aortic aneurysm	3	4.18<br>-5.26 to 9.14
Lower extremity peripheral arterial disease	3	3.91<br>-8.17 to 9.99
Endocarditis	3	5.64<br>-3.53 to 11.04
Non-rheumatic valvular heart disease	3	3.37<br>-11.91 to 11.39
Other cardiovascular and circulatory diseases	3	8.1<br>1.28 to 12.38
Chronic respiratory diseases	2	7.88<br>0.31 to 11.96
Chronic obstructive pulmonary disease	3	37.35<br>25.52 to 47.5
Pneumoconiosis	3	11.2<br>0.64 to 17.9
Silicosis	4	31.97<br>-3.49 to 107.45
Asbestosis	4	18.5<br>2.1 to 32.8
Coal workers pneumoconiosis	4	41.13<br>4.83 to 113.53
Other pneumoconiosis	4	74.64<br>24.83 to 145.21
Asthma	3	75.92<br>48.88 to 113.45

Appendix Table S11: Percent change before and after CoDCorrect by cause for all ages, both sexes, global, 2021		
**Cause name**	**CoDCorrect level**	**Percent change**
Interstitial lung disease and pulmonary sarcoidosis	3	29.48<br>15.04 to 42.63
Other chronic respiratory diseases	3	48.49<br>30.05 to 70.27
Cirrhosis and other chronic liver diseases	3	11.76<br>4.38 to 20.93
Chronic hepatitis B including cirrhosis	4	10.49<br>2.46 to 21.93
Chronic hepatitis C including cirrhosis	4	12.25<br>6.27 to 20.22
Cirrhosis due to alcohol	4	6.94<br>-0.88 to 16.4
Cirrhosis due to other causes	4	10.73<br>2.19 to 20.12
Digestive diseases	2	7.92<br>2.59 to 10.98
Peptic ulcer disease	4	12.21<br>-4.31 to 30.28
Gastritis and duodenitis	4	16.44<br>2.28 to 33.54
Appendicitis	3	15.01<br>-2.96 to 31.33
Paralytic ileus and intestinal obstruction	3	13.99<br>2.61 to 24.3
Inguinal, femoral, and abdominal hernia	3	16.63<br>5.68 to 26.29
Inflammatory bowel disease	3	6.13<br>-5.5 to 13.75
Vascular intestinal disorders	3	3.62<br>-8.35 to 10.68
Gallbladder and biliary diseases	3	10.01<br>-3.07 to 18.55
Pancreatitis	3	9.62<br>1.37 to 20.42
Other digestive diseases	3	9.56<br>-2.02 to 17.46
Neurological disorders	2	0.83<br>-11.15 to 11.06
Alzheimer's disease and other dementias	3	-0.77<br>-12.09 to 12.73
Parkinson's disease	3	4.99<br>-5.38 to 10.1
Idiopathic epilepsy	3	12.14<br>8.72 to 15.12
Multiple sclerosis	3	0.69<br>-4.3 to 3.28
Motor neuron disease	3	0.52<br>-6.16 to 3.98
Other neurological disorders	3	2.65<br>-3.53 to 5.95
Mental disorders	2	-2.21<br>-3.5 to -0.85
Alcohol use disorders	3	4.25<br>2.27 to 5.57
Drug use disorders	3	1.58<br>-0.29 to 3.2
Opioid use disorders	4	2.28<br>-2.3 to 10.3
Cocaine use disorders	4	6.76<br>0.5 to 14.25

Appendix Table S11: Percent change before and after CoDCorrect by cause for all ages, both sexes, global, 2021		
**Cause name**	**CoDCorrect level**	**Percent change**
Amphetamine use disorders	4	5.53<br>0.07 to 14.34
Other drug use disorders	4	26.1<br>21.36 to 32.53
Eating disorders	3	-2.21<br>-3.5 to -0.85
Anorexia nervosa	4	-2.21<br>-3.5 to -0.85
Diabetes mellitus	3	8.08<br>2.16 to 11.24
Acute glomerulonephritis	3	4.7<br>-1.11 to 7.9
Chronic kidney disease	3	6.6<br>-1.41 to 10.76
Chronic kidney disease due to hypertension	4	7.4<br>-1.35 to 11.85
Chronic kidney disease due to glomerulonephritis	4	11.86<br>6.4 to 15.05
Chronic kidney disease due to other and unspecified causes	4	8.86<br>-0.66 to 13.75
Urinary diseases and male infertility	3	7.75<br>-1.1 to 12.54
Urinary tract infections and interstitial nephritis	4	26.45<br>13.57 to 38.35
Urolithiasis	4	33.6<br>15.61 to 54.2
Other urinary diseases	4	67.91<br>43.46 to 105.39
Gynecological diseases	3	11.87<br>6.41 to 15.89
Uterine fibroids	4	42.97<br>1.46 to 70.62
Endometriosis	4	55.04<br>22.96 to 106.09
Genital prolapse	4	95.64<br>53.18 to 137.74
Other gynecological diseases	4	112.71<br>60.68 to 162.21
Hemoglobinopathies and hemolytic anaemias	3	8.54<br>-3.81 to 14.47
Thalassemias	4	12.11<br>5.41 to 16.81
Sickle cell disorders	4	-15.93<br>-32.97 to -4.73
G6PD deficiency	4	14.05<br>9.02 to 17.39
Other hemoglobinopathies and hemolytic anaemias	4	1.41<br>-5.59 to 5.56
Endocrine, metabolic, blood, and immune disorders	3	3.6<br>-3.96 to 7.59
Musculoskeletal disorders	2	6.73<br>-1.45 to 11.07
Rheumatoid arthritis	3	26.91<br>6.47 to 40.22
Other musculoskeletal disorders	3	17.7<br>4.22 to 28.93
Other non-communicable diseases	2	5.81<br>0.29 to 9.29
Congenital anomalies	3	4.76<br>-1.8 to 8.95

Appendix Table S11: Percent change before and after CoDCorrect by cause for all ages, both sexes, global, 2021		
**Cause name**	**CoDCorrect level**	**Percent change**
Neural tube defects	4	25.28<br>7.61 to 59.03
Congenital heart anomalies	4	8.09<br>-5.3 to 25.82
Orofacial clefts	4	24.29<br>1.99 to 50.63
Down's syndrome	4	18.54<br>6.86 to 47.93
Other chromosomal abnormalities	4	12.27<br>3.12 to 35.97
Congenital musculoskeletal and limb anomalies	4	18.43<br>4.09 to 43.56
Urogenital congenital anomalies	4	12.66<br>-0.65 to 38.42
Digestive congenital anomalies	4	19.99<br>4.7 to 42.91
Other congenital anomalies	4	10.19<br>-8.0 to 32.4
Skin and subcutaneous diseases	2	6.45<br>-2.04 to 10.9
Cellulitis	4	46.66<br>28.77 to 64.76
Pyoderma	4	36.76<br>17.74 to 50.85
Decubitus ulcer	3	39.44<br>7.21 to 85.87
Other skin and subcutaneous diseases	3	63.9<br>26.34 to 197.29
Sudden infant death syndrome	3	6.28<br>-1.89 to 14.45
Injuries	1	12.21<br>9.17 to 14.5
Transport injuries	2	9.91<br>7.89 to 11.64
Road injuries	3	3.27<br>-1.14 to 9.36
Pedestrian road injuries	4	14.66<br>4.88 to 24.97
Cyclist road injuries	4	16.79<br>5.23 to 32.96
Motorcyclist road injuries	4	17.82<br>8.56 to 29.33
Motor vehicle road injuries	4	15.31<br>6.65 to 24.26
Other road injuries	4	16.5<br>9.04 to 24.49
Other transport injuries	3	10.44<br>5.49 to 18.0
Unintentional injuries	2	10.62<br>5.79 to 13.84
Falls	3	8.74<br>0.65 to 13.35
Drowning	3	9.87<br>6.5 to 12.76
Fire, heat, and hot substances	3	13.39<br>9.03 to 16.68
Poisonings	3	7.63<br>4.67 to 9.55
Poisoning by carbon monoxide	4	7.2<br>-13.91 to 39.9

Appendix Table S11: Percent change before and after CoDCorrect by cause for all ages, both sexes, global, 2021		
**Cause name**	**CoDCorrect level**	**Percent change**
Poisoning by other means	4	39.28<br>13.77 to 86.47
Exposure to mechanical forces	3	10.49<br>8.11 to 12.47
Unintentional firearm injuries	4	7.19<br>-16.29 to 29.69
Other exposure to mechanical forces	4	13.51<br>-3.21 to 40.09
Adverse effects of medical treatment	3	11.74<br>6.63 to 15.53
Animal contact	3	16.95<br>12.84 to 20.96
Venomous animal contact	4	39.88<br>8.61 to 60.31
Non-venomous animal contact	4	-2.91<br>-25.05 to 29.8
Foreign body	3	4.85<br>-1.13 to 8.38
Pulmonary aspiration and foreign body in airway	4	-0.42<br>-19.02 to 14.88
Foreign body in other body part	4	-11.25<br>-34.77 to 30.33
Self-harm and interpersonal violence	2	17.21<br>14.89 to 19.32
Self-harm	3	8.03<br>5.47 to 10.06
Self-harm by firearm	4	10.54<br>1.35 to 20.55
Self-harm by other specified means	4	9.27<br>-3.04 to 19.24
Interpersonal violence	3	9.02<br>7.4 to 10.45
Assault by firearm	4	0.4<br>-1.82 to 3.15
Assault by sharp object	4	0.72<br>-3.16 to 4.76
Assault by other means	4	-4.89<br>-9.87 to 0.18
Maternal deaths aggravated by HIV/AIDS	4	3.03<br>1.33 to 4.67
Environmental heat and cold exposure	3	24.95<br>19.67 to 31.82
Acute lymphoid leukaemia	4	59.03<br>24.96 to 92.6
Chronic lymphoid leukaemia	4	45.91<br>28.19 to 64.96
Acute myeloid leukaemia	4	50.56<br>33.52 to 69.72
Chronic myeloid leukaemia	4	58.56<br>36.12 to 86.4
Non-melanoma skin cancer (squamous-cell carcinoma)	4	3.82<br>-5.14 to 8.32
Executions and police conflict	3	114.31<br>77.97 to 139.95
Drug-susceptible tuberculosis	4	15.31<br>11.6 to 17.98
Zika virus disease	3	1.46<br>-0.66 to 3.35
Alcoholic cardiomyopathy	4	8.26<br>2.56 to 13.37

Appendix Table S11: Percent change before and after CoDCorrect by cause for all ages, both sexes, global, 2021		
**Cause name**	**CoDCorrect level**	**Percent change**
Myocarditis	4	21.06<br>9.54 to 36.18
Other leukaemia	4	48.34<br>26.39 to 79.76
Other cardiomyopathy	4	8.95<br>-2.49 to 21.68
Multidrug-resistant tuberculosis without extensive drug resistance	4	16.63<br>12.06 to 20.2
Extensively drug-resistant tuberculosis	4	10.97<br>6.8 to 14.63
HIV/AIDS and sexually transmitted infections	2	1030.3<br>509.76 to 2379.83
Respiratory infections and tuberculosis	2	148.42<br>134.02 to 162.48
Enteric infections	2	12.12<br>5.1 to 18.16
Typhoid and paratyphoid	3	10.03<br>1.59 to 19.83
iNTS	3	16.04<br>-9.44 to 36.41
Other infectious diseases	2	-1.98<br>-17.1 to 11.46
Maternal and neonatal disorders	2	-0.4<br>-4.37 to 5.51
Myelodysplastic, myeloproliferative, and other hematopoietic neoplasms	4	1.71<br>-10.26 to 7.74
Other benign and in situ neoplasms	4	3.82<br>-1.87 to 8.16
Non-rheumatic calcific aortic valve disease	4	6.95<br>-10.33 to 16.73
Non-rheumatic degenerative mitral valve disease	4	15.86<br>0.87 to 29.57
Other non-rheumatic valve diseases	4	42.03<br>18.4 to 62.23
Nonalcoholic fatty liver disease including cirrhosis	4	5.07<br>-1.54 to 11.25
Substance use disorders	2	3.02<br>1.32 to 4.33
Diabetes and kidney diseases	2	7.35<br>0.35 to 10.99
Diabetes mellitus type 1	4	19.01<br>9.25 to 32.88
Diabetes mellitus type 2	4	12.23<br>2.3 to 22.97
Bacterial skin diseases	3	4.27<br>-9.79 to 40.0
Upper digestive system diseases	3	11.6<br>-0.7 to 24.18
Maternal abortive outcome	4	1.79<br>1.23 to 2.39
Liver cancer due to NASH	4	6.09<br>0.11 to 9.36
Chronic kidney disease due to diabetes mellitus type 1	4	9.17<br>7.15 to 10.93
Chronic kidney disease due to diabetes mellitus type 2	4	7.18<br>-1.25 to 11.52
Pulmonary Arterial Hypertension	3	8.82<br>1.11 to 13.6
Hepatoblastoma	4	10.02<br>0.26 to 17.09

Appendix Table S11: Percent change before and after CoDCorrect by cause for all ages, both sexes, global, 2021		
**Cause name**	**CoDCorrect level**	**Percent change**
Burkitt lymphoma	4	-1.02<br>-40.71 to 33.45
Other non-Hodgkin lymphoma	4	12.27<br>4.12 to 22.52
Eye cancer	3	9.63<br>3.17 to 13.69
Retinoblastoma	4	11.94<br>-5.88 to 25.82
Other eye cancers	4	8.78<br>3.1 to 12.54
Soft tissue and other extraosseous sarcomas	3	6.75<br>1.66 to 9.73
Malignant neoplasm of bone and articular cartilage	3	7.74<br>3.63 to 10.71
Neuroblastoma and other peripheral nervous cell tumours	3	6.81<br>3.7 to 9.4
Total burden related to hepatitis B	1	13.41<br>5.89 to 21.55
Total burden related to hepatitis C	1	10.85<br>4.83 to 16.71
Total burden related to Non-alcoholic fatty liver disease (NAFLD)	1	5.37<br>-0.97 to 10.48
Total cancers	1	5.5<br>-0.76 to 8.84
Other unintentional injuries	3	10.92<br>8.54 to 13.05
Total Cancers excluding Non-melanoma skin cancer	1	5.51<br>-0.72 to 8.85

Appendix Table S12: CODEm covariates used, level of covariate, and expected direction of covariate by cause, sex, and age

Cause	Sex	Age Start	Age End	Model Version Type	Direction	Level	Covariate Name	Number of Draws
Sexually transmitted infections excluding HIV	Female	10-14 years	95+ years	Data Rich	1	1	Syphilis prevalence (proportion)	--
Sexually transmitted infections excluding HIV	Female	10-14 years	95+ years	Data Rich	-1	2	Maternal care and immunization	83
Sexually transmitted infections excluding HIV	Female	10-14 years	95+ years	Data Rich	-1	2	Legality of Abortion	122
Sexually transmitted infections excluding HIV	Female	10-14 years	95+ years	Data Rich	-1	2	Education (years per capita)	228
Sexually transmitted infections excluding HIV	Female	10-14 years	95+ years	Data Rich	-1	2	Healthcare access and quality index	315
Sexually transmitted infections excluding HIV	Female	10-14 years	95+ years	Data Rich	1	2	Age-Specific Fertility Rate	--
Sexually transmitted infections excluding HIV	Female	10-14 years	95+ years	Data Rich	1	2	Total Fertility Rate	--
Sexually transmitted infections excluding HIV	Female	10-14 years	95+ years	Data Rich	-1	3	Antenatal Care (4 visits) Coverage (proportion)	21
Sexually transmitted infections excluding HIV	Female	10-14 years	95+ years	Data Rich	-1	3	Antenatal Care (1 visit) Coverage (proportion)	28
Sexually transmitted infections excluding HIV	Female	10-14 years	95+ years	Data Rich	-1	3	LDI (I$ per capita)	--
Sexually transmitted infections excluding HIV	Female	10-14 years	95+ years	Global	1	1	Syphilis prevalence (proportion)	--
Sexually transmitted infections excluding HIV	Female	10-14 years	95+ years	Global	-1	2	Maternal care and immunization	99
Sexually transmitted infections excluding HIV	Female	10-14 years	95+ years	Global	-1	2	Healthcare access and quality index	145
Sexually transmitted infections excluding HIV	Female	10-14 years	95+ years	Global	-1	2	Education (years per capita)	355
Sexually transmitted infections excluding HIV	Female	10-14 years	95+ years	Global	-1	2	Legality of Abortion	--
Sexually transmitted infections excluding HIV	Female	10-14 years	95+ years	Global	1	2	Age-Specific Fertility Rate	--
Sexually transmitted infections excluding HIV	Female	10-14 years	95+ years	Global	1	2	Total Fertility Rate	--
Sexually transmitted infections excluding HIV	Female	10-14 years	95+ years	Global	-1	3	Antenatal Care (1 visit) Coverage (proportion)	247
Sexually transmitted infections excluding HIV	Female	10-14 years	95+ years	Global	-1	3	Antenatal Care (4 visits) Coverage (proportion)	247
Sexually transmitted infections excluding HIV	Female	10-14 years	95+ years	Global	-1	3	LDI (I$ per capita)	--
Sexually transmitted infections excluding HIV	Male	10-14 years	95+ years	Data Rich	1	1	Syphilis prevalence (proportion)	--
Sexually transmitted infections excluding HIV	Male	10-14 years	95+ years	Data Rich	-1	2	Maternal care and immunization	331
Sexually transmitted infections excluding HIV	Male	10-14 years	95+ years	Data Rich	-1	2	Legality of Abortion	380
Sexually transmitted infections excluding HIV	Male	10-14 years	95+ years	Data Rich	-1	2	Healthcare access and quality index	444
Sexually transmitted infections excluding HIV	Male	10-14 years	95+ years	Data Rich	-1	2	Education (years per capita)	648
Sexually transmitted infections excluding HIV	Male	10-14 years	95+ years	Data Rich	1	2	Age-Specific Fertility Rate	--
Sexually transmitted infections excluding HIV	Male	10-14 years	95+ years	Data Rich	1	2	Total Fertility Rate	--
Sexually transmitted infections excluding HIV	Male	10-14 years	95+ years	Data Rich	-1	3	Antenatal Care (4 visits) Coverage (proportion)	64
Sexually transmitted infections excluding HIV	Male	10-14 years	95+ years	Data Rich	-1	3	Antenatal Care (1 visit) Coverage (proportion)	84
Sexually transmitted infections excluding HIV	Male	10-14 years	95+ years	Data Rich	-1	3	LDI (I$ per capita)	--
Sexually transmitted infections excluding HIV	Male	10-14 years	95+ years	Global	1	1	Syphilis prevalence (proportion)	--
Sexually transmitted infections excluding HIV	Male	10-14 years	95+ years	Global	-1	2	Healthcare access and quality index	261
Sexually transmitted infections excluding HIV	Male	10-14 years	95+ years	Global	-1	2	Maternal care and immunization	532
Sexually transmitted infections excluding HIV	Male	10-14 years	95+ years	Global	-1	2	Education (years per capita)	616
Sexually transmitted infections excluding HIV	Male	10-14 years	95+ years	Global	-1	2	Legality of Abortion	633
Sexually transmitted infections excluding HIV	Male	10-14 years	95+ years	Global	1	2	Age-Specific Fertility Rate	--
Sexually transmitted infections excluding HIV	Male	10-14 years	95+ years	Global	1	2	Total Fertility Rate	--
Sexually transmitted infections excluding HIV	Male	10-14 years	95+ years	Global	-1	3	Antenatal Care (4 visits) Coverage (proportion)	118
Sexually transmitted infections excluding HIV	Male	10-14 years	95+ years	Global	-1	3	Antenatal Care (1 visit) Coverage (proportion)	359
Sexually transmitted infections excluding HIV	Male	10-14 years	95+ years	Global	-1	3	LDI (I$ per capita)	--
Lower respiratory infections	Female	0-6 days	2-4 years	Data Rich	-1	1	Hib3 lagged five year coverage (proportion)	1
Lower respiratory infections	Female	0-6 days	2-4 years	Data Rich	-1	1	PCV3 lagged five year coverage (proportion)	107
Lower respiratory infections	Female	0-6 days	2-4 years	Data Rich	-1	1	Antibiotics for LRI	805
Lower respiratory infections	Female	0-6 days	2-4 years	Data Rich	1	1	Log-transformed SEV scalar: LRI	9
Lower respiratory infections	Female	0-6 days	2-4 years	Data Rich	1	1	Age- and sex-specific SEV for Child wasting	28
Lower respiratory infections	Female	0-6 days	2-4 years	Data Rich	1	1	Indoor Air Pollution (All Cooking Fuels)	130
Lower respiratory infections	Female	0-6 days	2-4 years	Data Rich	1	1	Age- and sex-specific SEV for Child underweight	154
Lower respiratory infections	Female	0-6 days	2-4 years	Data Rich	1	1	Age- and sex-specific SEV for Child stunting	458
Lower respiratory infections	Female	0-6 days	2-4 years	Data Rich	-1	2	DTP3 lagged five year coverage (proportion)	252
Lower respiratory infections	Female	0-6 days	2-4 years	Data Rich	-1	2	Healthcare access and quality index	--
Lower respiratory infections	Female	0-6 days	2-4 years	Data Rich	1	2	Age- and sex-specific SEV for Household air pollution	0
Lower respiratory infections	Female	0-6 days	2-4 years	Data Rich	1	2	Vitamin A Deficiency Prevalence (age-standardized)	43
Lower respiratory infections	Female	0-6 days	2-4 years	Data Rich	1	2	Age-standardized SEV for Handwashing	52
Lower respiratory infections	Female	0-6 days	2-4 years	Data Rich	1	2	Age- and sex-specific SEV for Ambient particulate matter	160
Lower respiratory infections	Female	0-6 days	2-4 years	Data Rich	1	2	Zinc deficiency	362
Lower respiratory infections	Female	0-6 days	2-4 years	Data Rich	1	2	Outdoor Air Pollution (PM2.5)	465
Lower respiratory infections	Female	0-6 days	2-4 years	Data Rich	1	2	Secondhand smoke	956
Lower respiratory infections	Female	0-6 days	2-4 years	Data Rich	-1	3	Socio-demographic Index	212
Lower respiratory infections	Female	0-6 days	2-4 years	Data Rich	-1	3	Maternal Education (years per capita)	490
Lower respiratory infections	Female	0-6 days	2-4 years	Data Rich	1	3	Population Density (under 150 ppl/sqkm, proportion)	0
Lower respiratory infections	Female	0-6 days	2-4 years	Data Rich	1	3	Age- and sex-specific SEV for Unsafe sanitation	325
Lower respiratory infections	Female	0-6 days	2-4 years	Data Rich	1	3	Population Density (over 1000 ppl/sqkm, proportion)	647
Lower respiratory infections	Female	0-6 days	2-4 years	Global	-1	1	Hib3 lagged five year coverage (proportion)	320
Lower respiratory infections	Female	0-6 days	2-4 years	Global	-1	1	Antibiotics for LRI	499
Lower respiratory infections	Female	0-6 days	2-4 years	Global	-1	1	PCV3 lagged five year coverage (proportion)	692
Lower respiratory infections	Female	0-6 days	2-4 years	Global	1	1	Age- and sex-specific SEV for Child underweight	2
Lower respiratory infections	Female	0-6 days	2-4 years	Global	1	1	Age- and sex-specific SEV for Child wasting	5
Lower respiratory infections	Female	0-6 days	2-4 years	Global	1	1	Log-transformed SEV scalar: LRI	7
Lower respiratory infections	Female	0-6 days	2-4 years	Global	1	1	Age- and sex-specific SEV for Child stunting	248
Lower respiratory infections	Female	0-6 days	2-4 years	Global	1	1	Indoor Air Pollution (All Cooking Fuels)	385
Lower respiratory infections	Female	0-6 days	2-4 years	Global	-1	2	DTP3 lagged five year coverage (proportion)	222
Lower respiratory infections	Female	0-6 days	2-4 years	Global	-1	2	Healthcare access and quality index	--
Lower respiratory infections	Female	0-6 days	2-4 years	Global	1	2	Outdoor Air Pollution (PM2.5)	0
Lower respiratory infections	Female	0-6 days	2-4 years	Global	1	2	Zinc deficiency	0
Lower respiratory infections	Female	0-6 days	2-4 years	Global	1	2	Age-standardized SEV for Handwashing	16
Lower respiratory infections	Female	0-6 days	2-4 years	Global	1	2	Age- and sex-specific SEV for Household air pollution	172
Lower respiratory infections	Female	0-6 days	2-4 years	Global	1	2	Secondhand smoke	558
Lower respiratory infections	Female	0-6 days	2-4 years	Global	1	2	Age- and sex-specific SEV for Ambient particulate matter	--
Lower respiratory infections	Female	0-6 days	2-4 years	Global	-1	3	Socio-demographic Index	420
Lower respiratory infections	Female	0-6 days	2-4 years	Global	-1	3	Maternal Education (years per capita)	441
Lower respiratory infections	Female	0-6 days	2-4 years	Global	1	3	Age- and sex-specific SEV for Unsafe sanitation	26
Lower respiratory infections	Female	0-6 days	2-4 years	Global	1	3	Population Density (over 1000 ppl/sqkm, proportion)	96
Lower respiratory infections	Female	5-9 years	95+ years	Data Rich	1	1	Indoor Air Pollution (All Cooking Fuels)	0
Lower respiratory infections	Female	5-9 years	95+ years	Data Rich	1	1	Log-transformed SEV scalar: LRI	255
Lower respiratory infections	Female	5-9 years	95+ years	Data Rich	1	1	Smoking Prevalence	295
Lower respiratory infections	Female	5-9 years	95+ years	Data Rich	1	1	Outdoor Air Pollution (PM2.5)	556
Lower respiratory infections	Female	5-9 years	95+ years	Data Rich	1	1	Secondhand smoke	591
Lower respiratory infections	Female	5-9 years	95+ years	Data Rich	-1	2	PCV3 lagged five year coverage (proportion)	143
Lower respiratory infections	Female	5-9 years	95+ years	Data Rich	-1	2	DTP3 lagged five year coverage (proportion)	247
Lower respiratory infections	Female	5-9 years	95+ years	Data Rich	-1	2	Healthcare access and quality index	--
Lower respiratory infections	Female	5-9 years	95+ years	Data Rich	1	2	No access to handwashing facility	1
Lower respiratory infections	Female	5-9 years	95+ years	Data Rich	1	2	Age-standardized proportion adult underweight	453
Lower respiratory infections	Female	5-9 years	95+ years	Data Rich	-1	3	Socio-demographic Index	77
Lower respiratory infections	Female	5-9 years	95+ years	Data Rich	-1	3	Education (years per capita)	284
Lower respiratory infections	Female	5-9 years	95+ years	Data Rich	-1	3	LDI (I$ per capita)	--
Lower respiratory infections	Female	5-9 years	95+ years	Data Rich	1	3	Age- and sex-specific SEV for Unsafe sanitation	60
Lower respiratory infections	Female	5-9 years	95+ years	Global	1	1	Smoking Prevalence	14
Lower respiratory infections	Female	5-9 years	95+ years	Global	1	1	Indoor Air Pollution (All Cooking Fuels)	464
Lower respiratory infections	Female	5-9 years	95+ years	Global	1	1	Secondhand smoke	475
Lower respiratory infections	Female	5-9 years	95+ years	Global	1	1	Log-transformed SEV scalar: LRI	534
Lower respiratory infections	Female	5-9 years	95+ years	Global	1	1	Outdoor Air Pollution (PM2.5)	--
Lower respiratory infections	Female	5-9 years	95+ years	Global	-1	2	PCV3 lagged five year coverage (proportion)	0
Lower respiratory infections	Female	5-9 years	95+ years	Global	-1	2	DTP3 lagged five year coverage (proportion)	53
Lower respiratory infections	Female	5-9 years	95+ years	Global	-1	2	Healthcare access and quality index	--
Lower respiratory infections	Female	5-9 years	95+ years	Global	1	2	No access to handwashing facility	150
Lower respiratory infections	Female	5-9 years	95+ years	Global	1	2	Age-standardized proportion adult underweight	380
Lower respiratory infections	Female	5-9 years	95+ years	Global	-1	3	Socio-demographic Index	16
Lower respiratory infections	Female	5-9 years	95+ years	Global	-1	3	Education (years per capita)	120
Lower respiratory infections	Female	5-9 years	95+ years	Global	-1	3	LDI (I$ per capita)	--
Lower respiratory infections	Female	5-9 years	95+ years	Global	1	3	Age- and sex-specific SEV for Unsafe sanitation	3
Lower respiratory infections	Male	0-6 days	2-4 years	Data Rich	-1	1	Hib3 lagged five year coverage (proportion)	0
Lower respiratory infections	Male	0-6 days	2-4 years	Data Rich	-1	1	Antibiotics for LRI	3

CODEm covariates used, level of covariate, and expected direction of covariate by cause, sex, and age

Cause	Sex	Age Start	Age End	Model Version Type	Direction	Level	Covariate Name	Number of Draws
Lower respiratory infections	Male	0-6 days	2-4 years	Data Rich	-1	1	PCV3 lagged five year coverage (proportion)	24
Lower respiratory infections	Male	0-6 days	2-4 years	Data Rich	1	1	Log-transformed SEV scalar: LRI	15
Lower respiratory infections	Male	0-6 days	2-4 years	Data Rich	1	1	Age- and sex-specific SEV for Child underweight	35
Lower respiratory infections	Male	0-6 days	2-4 years	Data Rich	1	1	Age- and sex-specific SEV for Child wasting	52
Lower respiratory infections	Male	0-6 days	2-4 years	Data Rich	1	1	Indoor Air Pollution (All Cooking Fuels)	202
Lower respiratory infections	Male	0-6 days	2-4 years	Data Rich	1	1	Age- and sex-specific SEV for Child stunting	869
Lower respiratory infections	Male	0-6 days	2-4 years	Data Rich	-1	2	DTP3 lagged five year coverage (proportion)	887
Lower respiratory infections	Male	0-6 days	2-4 years	Data Rich	-1	2	Healthcare access and quality index	--
Lower respiratory infections	Male	0-6 days	2-4 years	Data Rich	1	2	Age- and sex-specific SEV for Household air pollution	44
Lower respiratory infections	Male	0-6 days	2-4 years	Data Rich	1	2	Age- and sex-specific SEV for Ambient particulate matter	67
Lower respiratory infections	Male	0-6 days	2-4 years	Data Rich	1	2	Age-standardized SEV for Handwashing	281
Lower respiratory infections	Male	0-6 days	2-4 years	Data Rich	1	2	Zinc deficiency	661
Lower respiratory infections	Male	0-6 days	2-4 years	Data Rich	1	2	Outdoor Air Pollution (PM2.5)	918
Lower respiratory infections	Male	0-6 days	2-4 years	Data Rich	1	2	Secondhand smoke	994
Lower respiratory infections	Male	0-6 days	2-4 years	Data Rich	-1	3	Socio-demographic Index	22
Lower respiratory infections	Male	0-6 days	2-4 years	Data Rich	-1	3	Maternal Education (years per capita)	346
Lower respiratory infections	Male	0-6 days	2-4 years	Data Rich	1	3	Age- and sex-specific SEV for Unsafe sanitation	407
Lower respiratory infections	Male	0-6 days	2-4 years	Data Rich	1	3	Population Density (over 1000 ppl/sqkm, proportion)	701
Lower respiratory infections	Male	0-6 days	2-4 years	Global	-1	1	Hib3 lagged five year coverage (proportion)	21
Lower respiratory infections	Male	0-6 days	2-4 years	Global	-1	1	PCV3 lagged five year coverage (proportion)	334
Lower respiratory infections	Male	0-6 days	2-4 years	Global	-1	1	Antibiotics for LRI	990
Lower respiratory infections	Male	0-6 days	2-4 years	Global	1	1	Age- and sex-specific SEV for Child underweight	0
Lower respiratory infections	Male	0-6 days	2-4 years	Global	1	1	Age- and sex-specific SEV for Child wasting	0
Lower respiratory infections	Male	0-6 days	2-4 years	Global	1	1	Log-transformed SEV scalar: LRI	0
Lower respiratory infections	Male	0-6 days	2-4 years	Global	1	1	Indoor Air Pollution (All Cooking Fuels)	216
Lower respiratory infections	Male	0-6 days	2-4 years	Global	1	1	Age- and sex-specific SEV for Child stunting	376
Lower respiratory infections	Male	0-6 days	2-4 years	Global	-1	2	DTP3 lagged five year coverage (proportion)	291
Lower respiratory infections	Male	0-6 days	2-4 years	Global	-1	2	Healthcare access and quality index	--
Lower respiratory infections	Male	0-6 days	2-4 years	Global	1	2	Age- and sex-specific SEV for Ambient particulate matter	0
Lower respiratory infections	Male	0-6 days	2-4 years	Global	1	2	Age-standardized SEV for Handwashing	0
Lower respiratory infections	Male	0-6 days	2-4 years	Global	1	2	Outdoor Air Pollution (PM2.5)	0
Lower respiratory infections	Male	0-6 days	2-4 years	Global	1	2	Zinc deficiency	0
Lower respiratory infections	Male	0-6 days	2-4 years	Global	1	2	Age- and sex-specific SEV for Household air pollution	364
Lower respiratory infections	Male	0-6 days	2-4 years	Global	1	2	Secondhand smoke	976
Lower respiratory infections	Male	0-6 days	2-4 years	Global	-1	3	Socio-demographic Index	32
Lower respiratory infections	Male	0-6 days	2-4 years	Global	-1	3	Maternal Education (years per capita)	147
Lower respiratory infections	Male	0-6 days	2-4 years	Global	1	3	Population Density (over 1000 ppl/sqkm, proportion)	149
Lower respiratory infections	Male	0-6 days	2-4 years	Global	1	3	Age- and sex-specific SEV for Unsafe sanitation	156
Lower respiratory infections	Male	5-9 years	95+ years	Data Rich	1	1	Outdoor Air Pollution (PM2.5)	71
Lower respiratory infections	Male	5-9 years	95+ years	Data Rich	1	1	Smoking Prevalence	439
Lower respiratory infections	Male	5-9 years	95+ years	Data Rich	1	1	Log-transformed SEV scalar: LRI	448
Lower respiratory infections	Male	5-9 years	95+ years	Data Rich	1	1	Secondhand smoke	735
Lower respiratory infections	Male	5-9 years	95+ years	Data Rich	1	1	Indoor Air Pollution (All Cooking Fuels)	--
Lower respiratory infections	Male	5-9 years	95+ years	Data Rich	-1	2	PCV3 lagged five year coverage (proportion)	33
Lower respiratory infections	Male	5-9 years	95+ years	Data Rich	-1	2	DTP3 lagged five year coverage (proportion)	--
Lower respiratory infections	Male	5-9 years	95+ years	Data Rich	-1	2	Healthcare access and quality index	--
Lower respiratory infections	Male	5-9 years	95+ years	Data Rich	1	2	Age-standardized proportion adult underweight	510
Lower respiratory infections	Male	5-9 years	95+ years	Data Rich	1	2	No access to handwashing facility	--
Lower respiratory infections	Male	5-9 years	95+ years	Data Rich	-1	3	Education (years per capita)	--
Lower respiratory infections	Male	5-9 years	95+ years	Data Rich	-1	3	LDI (I$ per capita)	--
Lower respiratory infections	Male	5-9 years	95+ years	Data Rich	-1	3	Socio-demographic Index	--
Lower respiratory infections	Male	5-9 years	95+ years	Data Rich	1	3	Age- and sex-specific SEV for Unsafe sanitation	9
Lower respiratory infections	Male	5-9 years	95+ years	Global	1	1	Log-transformed SEV scalar: LRI	257
Lower respiratory infections	Male	5-9 years	95+ years	Global	1	1	Indoor Air Pollution (All Cooking Fuels)	294
Lower respiratory infections	Male	5-9 years	95+ years	Global	1	1	Secondhand smoke	347
Lower respiratory infections	Male	5-9 years	95+ years	Global	1	1	Smoking Prevalence	408
Lower respiratory infections	Male	5-9 years	95+ years	Global	1	1	Outdoor Air Pollution (PM2.5)	--
Lower respiratory infections	Male	5-9 years	95+ years	Global	-1	2	DTP3 lagged five year coverage (proportion)	18
Lower respiratory infections	Male	5-9 years	95+ years	Global	-1	2	PCV3 lagged five year coverage (proportion)	303
Lower respiratory infections	Male	5-9 years	95+ years	Global	-1	2	Healthcare access and quality index	--
Lower respiratory infections	Male	5-9 years	95+ years	Global	1	2	No access to handwashing facility	102
Lower respiratory infections	Male	5-9 years	95+ years	Global	1	2	Age-standardized proportion adult underweight	450
Lower respiratory infections	Male	5-9 years	95+ years	Global	-1	3	Socio-demographic Index	0
Lower respiratory infections	Male	5-9 years	95+ years	Global	-1	3	Education (years per capita)	10
Lower respiratory infections	Male	5-9 years	95+ years	Global	-1	3	LDI (I$ per capita)	--
Lower respiratory infections	Male	5-9 years	95+ years	Global	1	3	Age- and sex-specific SEV for Unsafe sanitation	18
Upper respiratory infections	Female	0-6 days	95+ years	Data Rich	1	1	Smoking Prevalence	1000
Upper respiratory infections	Female	0-6 days	95+ years	Data Rich	-1	2	Healthcare access and quality index	583
Upper respiratory infections	Female	0-6 days	95+ years	Data Rich	1	2	Indoor Air Pollution (All Cooking Fuels)	172
Upper respiratory infections	Female	0-6 days	95+ years	Data Rich	1	2	Outdoor Air Pollution (PM2.5)	257
Upper respiratory infections	Female	0-6 days	95+ years	Data Rich	-1	3	Socio-demographic Index	337
Upper respiratory infections	Female	0-6 days	95+ years	Data Rich	-1	3	Education (years per capita)	440
Upper respiratory infections	Female	0-6 days	95+ years	Data Rich	-1	3	LDI (I$ per capita)	--
Upper respiratory infections	Female	0-6 days	95+ years	Global	1	1	Smoking Prevalence	1000
Upper respiratory infections	Female	0-6 days	95+ years	Global	-1	2	Healthcare access and quality index	236
Upper respiratory infections	Female	0-6 days	95+ years	Global	1	2	Indoor Air Pollution (All Cooking Fuels)	336
Upper respiratory infections	Female	0-6 days	95+ years	Global	1	2	Outdoor Air Pollution (PM2.5)	--
Upper respiratory infections	Female	0-6 days	95+ years	Global	-1	3	Education (years per capita)	133
Upper respiratory infections	Female	0-6 days	95+ years	Global	-1	3	Socio-demographic Index	160
Upper respiratory infections	Female	0-6 days	95+ years	Global	-1	3	LDI (I$ per capita)	--
Upper respiratory infections	Male	0-6 days	95+ years	Data Rich	1	1	Smoking Prevalence	221
Upper respiratory infections	Male	0-6 days	95+ years	Data Rich	-1	2	Healthcare access and quality index	--
Upper respiratory infections	Male	0-6 days	95+ years	Data Rich	1	2	Indoor Air Pollution (All Cooking Fuels)	72
Upper respiratory infections	Male	0-6 days	95+ years	Data Rich	1	2	Outdoor Air Pollution (PM2.5)	477
Upper respiratory infections	Male	0-6 days	95+ years	Data Rich	-1	3	Education (years per capita)	588
Upper respiratory infections	Male	0-6 days	95+ years	Data Rich	-1	3	LDI (I$ per capita)	--
Upper respiratory infections	Male	0-6 days	95+ years	Data Rich	-1	3	Socio-demographic Index	--
Upper respiratory infections	Male	0-6 days	95+ years	Global	1	1	Smoking Prevalence	1000
Upper respiratory infections	Male	0-6 days	95+ years	Global	-1	2	Healthcare access and quality index	160
Upper respiratory infections	Male	0-6 days	95+ years	Global	1	2	Indoor Air Pollution (All Cooking Fuels)	370
Upper respiratory infections	Male	0-6 days	95+ years	Global	1	2	Outdoor Air Pollution (PM2.5)	--
Upper respiratory infections	Male	0-6 days	95+ years	Global	-1	3	Socio-demographic Index	146
Upper respiratory infections	Male	0-6 days	95+ years	Global	-1	3	Education (years per capita)	179
Upper respiratory infections	Male	0-6 days	95+ years	Global	-1	3	LDI (I$ per capita)	--
Otitis media	Female	0-6 days	95+ years	Data Rich	1	1	Log-transformed SEV scalar: Otitis	475
Otitis media	Female	0-6 days	95+ years	Data Rich	1	1	Smoking Prevalence	719
Otitis media	Female	0-6 days	95+ years	Data Rich	-1	2	Healthcare access and quality index	249
Otitis media	Female	0-6 days	95+ years	Data Rich	1	2	Indoor Air Pollution (All Cooking Fuels)	247
Otitis media	Female	0-6 days	95+ years	Data Rich	1	2	Outdoor Air Pollution (PM2.5)	636
Otitis media	Female	0-6 days	95+ years	Data Rich	-1	3	Socio-demographic Index	42
Otitis media	Female	0-6 days	95+ years	Data Rich	-1	3	Education (years per capita)	157
Otitis media	Female	0-6 days	95+ years	Data Rich	-1	3	LDI (I$ per capita)	--
Otitis media	Female	0-6 days	95+ years	Global	1	1	Smoking Prevalence	653
Otitis media	Female	0-6 days	95+ years	Global	1	1	Log-transformed SEV scalar: Otitis	935
Otitis media	Female	0-6 days	95+ years	Global	-1	2	Healthcare access and quality index	495
Otitis media	Female	0-6 days	95+ years	Global	1	2	Indoor Air Pollution (All Cooking Fuels)	147
Otitis media	Female	0-6 days	95+ years	Global	1	2	Outdoor Air Pollution (PM2.5)	315
Otitis media	Female	0-6 days	95+ years	Global	-1	3	Socio-demographic Index	358
Otitis media	Female	0-6 days	95+ years	Global	-1	3	Education (years per capita)	442
Otitis media	Female	0-6 days	95+ years	Global	-1	3	LDI (I$ per capita)	--
Otitis media	Male	0-6 days	95+ years	Data Rich	1	1	Smoking Prevalence	554
Otitis media	Male	0-6 days	95+ years	Data Rich	1	1	Log-transformed SEV scalar: Otitis	810
Otitis media	Male	0-6 days	95+ years	Data Rich	-1	2	Healthcare access and quality index	671
Otitis media	Male	0-6 days	95+ years	Data Rich	1	2	Indoor Air Pollution (All Cooking Fuels)	224
Otitis media	Male	0-6 days	95+ years	Data Rich	1	2	Outdoor Air Pollution (PM2.5)	770
Otitis media	Male	0-6 days	95+ years	Data Rich	-1	3	Education (years per capita)	111

CODEm covariates used, level of covariate, and expected direction of covariate by cause, sex, and age

Cause	Sex	Age Start	Age End	Model Version Type	Direction	Level	Covariate Name	Number of Draws
Otitis media	Male	0-6 days	95+ years	Data Rich	-1	3	Socio-demographic Index	113
Otitis media	Male	0-6 days	95+ years	Data Rich	-1	3	LDI (I$ per capita)	--
Otitis media	Male	0-6 days	95+ years	Global	1	1	Log-transformed SEV scalar: Otitis	628
Otitis media	Male	0-6 days	95+ years	Global	1	1	Smoking Prevalence	823
Otitis media	Male	0-6 days	95+ years	Global	-1	2	Healthcare access and quality index	118
Otitis media	Male	0-6 days	95+ years	Global	1	2	Indoor Air Pollution (All Cooking Fuels)	515
Otitis media	Male	0-6 days	95+ years	Global	1	2	Outdoor Air Pollution (PM2.5)	633
Otitis media	Male	0-6 days	95+ years	Global	-1	3	Socio-demographic Index	255
Otitis media	Male	0-6 days	95+ years	Global	-1	3	Education (years per capita)	348
Otitis media	Male	0-6 days	95+ years	Global	-1	3	LDI (I$ per capita)	--
Diarrhoeal diseases	Female	0-6 days	2-4 years	Data Rich	-1	1	Improved Water Source (proportion with access)	196
Diarrhoeal diseases	Female	0-6 days	2-4 years	Data Rich	-1	1	Rotavirus coverage (proportion)	215
Diarrhoeal diseases	Female	0-6 days	2-4 years	Data Rich	-1	1	ORS (oral rehydration)	228
Diarrhoeal diseases	Female	0-6 days	2-4 years	Data Rich	-1	1	Sanitation (proportion with access)	549
Diarrhoeal diseases	Female	0-6 days	2-4 years	Data Rich	-1	2	Zinc treatment for diarrhea	0
Diarrhoeal diseases	Female	0-6 days	2-4 years	Data Rich	-1	2	Healthcare access and quality index	--
Diarrhoeal diseases	Female	0-6 days	2-4 years	Data Rich	1	2	Zinc deficiency	183
Diarrhoeal diseases	Female	0-6 days	2-4 years	Data Rich	-1	3	Maternal Education (years per capita)	106
Diarrhoeal diseases	Female	0-6 days	2-4 years	Data Rich	-1	3	Socio-demographic Index	167
Diarrhoeal diseases	Female	0-6 days	2-4 years	Data Rich	-1	3	LDI (I$ per capita)	--
Diarrhoeal diseases	Female	0-6 days	2-4 years	Data Rich	1	3	No access to handwashing facility	131
Diarrhoeal diseases	Female	0-6 days	2-4 years	Global	-1	1	ORS (oral rehydration)	254
Diarrhoeal diseases	Female	0-6 days	2-4 years	Global	-1	1	Improved Water Source (proportion with access)	560
Diarrhoeal diseases	Female	0-6 days	2-4 years	Global	-1	1	Rotavirus coverage (proportion)	818
Diarrhoeal diseases	Female	0-6 days	2-4 years	Global	-1	1	Sanitation (proportion with access)	999
Diarrhoeal diseases	Female	0-6 days	2-4 years	Global	-1	2	Healthcare access and quality index	0
Diarrhoeal diseases	Female	0-6 days	2-4 years	Global	-1	2	Zinc treatment for diarrhea	312
Diarrhoeal diseases	Female	0-6 days	2-4 years	Global	1	2	Zinc deficiency	384
Diarrhoeal diseases	Female	0-6 days	2-4 years	Global	-1	3	LDI (I$ per capita)	0
Diarrhoeal diseases	Female	0-6 days	2-4 years	Global	-1	3	Maternal Education (years per capita)	0
Diarrhoeal diseases	Female	0-6 days	2-4 years	Global	-1	3	Socio-demographic Index	6
Diarrhoeal diseases	Female	0-6 days	2-4 years	Global	1	3	No access to handwashing facility	152
Diarrhoeal diseases	Female	5-9 years	95+ years	Data Rich	-1	1	Sanitation (proportion with access)	460
Diarrhoeal diseases	Female	5-9 years	95+ years	Data Rich	-1	1	Improved Water Source (proportion with access)	632
Diarrhoeal diseases	Female	5-9 years	95+ years	Data Rich	-1	2	Healthcare access and quality index	--
Diarrhoeal diseases	Female	5-9 years	95+ years	Data Rich	-1	2	ORS (oral rehydration)	--
Diarrhoeal diseases	Female	5-9 years	95+ years	Data Rich	-1	2	Rotavirus coverage (proportion)	--
Diarrhoeal diseases	Female	5-9 years	95+ years	Data Rich	-1	3	Education (years per capita)	--
Diarrhoeal diseases	Female	5-9 years	95+ years	Data Rich	-1	3	LDI (I$ per capita)	--
Diarrhoeal diseases	Female	5-9 years	95+ years	Data Rich	-1	3	Socio-demographic Index	--
Diarrhoeal diseases	Female	5-9 years	95+ years	Data Rich	1	3	No access to handwashing facility	303
Diarrhoeal diseases	Female	5-9 years	95+ years	Data Rich	1	3	Age-standardized proportion adult underweight	--
Diarrhoeal diseases	Female	5-9 years	95+ years	Global	-1	1	Sanitation (proportion with access)	587
Diarrhoeal diseases	Female	5-9 years	95+ years	Global	-1	1	Improved Water Source (proportion with access)	677
Diarrhoeal diseases	Female	5-9 years	95+ years	Global	-1	2	Healthcare access and quality index	208
Diarrhoeal diseases	Female	5-9 years	95+ years	Global	-1	2	ORS (oral rehydration)	--
Diarrhoeal diseases	Female	5-9 years	95+ years	Global	-1	2	Rotavirus coverage (proportion)	--
Diarrhoeal diseases	Female	5-9 years	95+ years	Global	-1	3	Education (years per capita)	--
Diarrhoeal diseases	Female	5-9 years	95+ years	Global	-1	3	LDI (I$ per capita)	--
Diarrhoeal diseases	Female	5-9 years	95+ years	Global	-1	3	Socio-demographic Index	--
Diarrhoeal diseases	Female	5-9 years	95+ years	Global	1	3	Age-standardized proportion adult underweight	69
Diarrhoeal diseases	Female	5-9 years	95+ years	Global	1	3	No access to handwashing facility	313
Diarrhoeal diseases	Male	0-6 days	2-4 years	Data Rich	-1	1	Improved Water Source (proportion with access)	225
Diarrhoeal diseases	Male	0-6 days	2-4 years	Data Rich	-1	1	ORS (oral rehydration)	230
Diarrhoeal diseases	Male	0-6 days	2-4 years	Data Rich	-1	1	Rotavirus coverage (proportion)	246
Diarrhoeal diseases	Male	0-6 days	2-4 years	Data Rich	-1	1	Sanitation (proportion with access)	716
Diarrhoeal diseases	Male	0-6 days	2-4 years	Data Rich	-1	2	Zinc treatment for diarrhea	0
Diarrhoeal diseases	Male	0-6 days	2-4 years	Data Rich	-1	2	Healthcare access and quality index	--
Diarrhoeal diseases	Male	0-6 days	2-4 years	Data Rich	1	2	Zinc deficiency	123
Diarrhoeal diseases	Male	0-6 days	2-4 years	Data Rich	-1	3	Socio-demographic Index	81
Diarrhoeal diseases	Male	0-6 days	2-4 years	Data Rich	-1	3	Maternal Education (years per capita)	154
Diarrhoeal diseases	Male	0-6 days	2-4 years	Data Rich	-1	3	LDI (I$ per capita)	--
Diarrhoeal diseases	Male	0-6 days	2-4 years	Data Rich	1	3	No access to handwashing facility	72
Diarrhoeal diseases	Male	0-6 days	2-4 years	Global	-1	1	ORS (oral rehydration)	153
Diarrhoeal diseases	Male	0-6 days	2-4 years	Global	-1	1	Rotavirus coverage (proportion)	377
Diarrhoeal diseases	Male	0-6 days	2-4 years	Global	-1	1	Improved Water Source (proportion with access)	527
Diarrhoeal diseases	Male	0-6 days	2-4 years	Global	-1	1	Sanitation (proportion with access)	870
Diarrhoeal diseases	Male	0-6 days	2-4 years	Global	-1	2	Healthcare access and quality index	0
Diarrhoeal diseases	Male	0-6 days	2-4 years	Global	-1	2	Zinc treatment for diarrhea	1
Diarrhoeal diseases	Male	0-6 days	2-4 years	Global	1	2	Zinc deficiency	95
Diarrhoeal diseases	Male	0-6 days	2-4 years	Global	-1	3	LDI (I$ per capita)	0
Diarrhoeal diseases	Male	0-6 days	2-4 years	Global	-1	3	Maternal Education (years per capita)	35
Diarrhoeal diseases	Male	0-6 days	2-4 years	Global	-1	3	Socio-demographic Index	56
Diarrhoeal diseases	Male	0-6 days	2-4 years	Global	1	3	No access to handwashing facility	151
Diarrhoeal diseases	Male	5-9 years	95+ years	Data Rich	-1	1	Sanitation (proportion with access)	316
Diarrhoeal diseases	Male	5-9 years	95+ years	Data Rich	-1	1	Improved Water Source (proportion with access)	771
Diarrhoeal diseases	Male	5-9 years	95+ years	Data Rich	-1	2	Healthcare access and quality index	--
Diarrhoeal diseases	Male	5-9 years	95+ years	Data Rich	-1	2	ORS (oral rehydration)	--
Diarrhoeal diseases	Male	5-9 years	95+ years	Data Rich	-1	2	Rotavirus coverage (proportion)	--
Diarrhoeal diseases	Male	5-9 years	95+ years	Data Rich	-1	3	Education (years per capita)	--
Diarrhoeal diseases	Male	5-9 years	95+ years	Data Rich	-1	3	LDI (I$ per capita)	--
Diarrhoeal diseases	Male	5-9 years	95+ years	Data Rich	-1	3	Socio-demographic Index	--
Diarrhoeal diseases	Male	5-9 years	95+ years	Data Rich	1	3	No access to handwashing facility	240
Diarrhoeal diseases	Male	5-9 years	95+ years	Data Rich	1	3	Age-standardized proportion adult underweight	386
Diarrhoeal diseases	Male	5-9 years	95+ years	Global	-1	1	Improved Water Source (proportion with access)	441
Diarrhoeal diseases	Male	5-9 years	95+ years	Global	-1	1	Sanitation (proportion with access)	859
Diarrhoeal diseases	Male	5-9 years	95+ years	Global	-1	2	Healthcare access and quality index	188
Diarrhoeal diseases	Male	5-9 years	95+ years	Global	-1	2	ORS (oral rehydration)	223
Diarrhoeal diseases	Male	5-9 years	95+ years	Global	-1	2	Rotavirus coverage (proportion)	--
Diarrhoeal diseases	Male	5-9 years	95+ years	Global	-1	3	Education (years per capita)	--
Diarrhoeal diseases	Male	5-9 years	95+ years	Global	-1	3	LDI (I$ per capita)	--
Diarrhoeal diseases	Male	5-9 years	95+ years	Global	-1	3	Socio-demographic Index	--
Diarrhoeal diseases	Male	5-9 years	95+ years	Global	1	3	Age-standardized proportion adult underweight	408
Diarrhoeal diseases	Male	5-9 years	95+ years	Global	1	3	No access to handwashing facility	488
Rabies	Female	1-5 months	95+ years	Data Rich	-1	1	Socio-demographic Index	374
Rabies	Female	1-5 months	95+ years	Data Rich	-1	1	Antenatal Care (4 visits) Coverage (proportion)	687
Rabies	Female	1-5 months	95+ years	Data Rich	-1	1	In-Facility Delivery (proportion)	912
Rabies	Female	1-5 months	95+ years	Data Rich	-1	2	Skilled Birth Attendance (proportion)	360
Rabies	Female	1-5 months	95+ years	Data Rich	-1	2	Maternal care and immunization	627
Rabies	Female	1-5 months	95+ years	Data Rich	-1	2	Healthcare access and quality index	861
Rabies	Female	1-5 months	95+ years	Data Rich	1	3	Population Density (500-1000 ppl/sqkm, proportion)	--
Rabies	Female	1-5 months	95+ years	Data Rich	1	3	Population Density (under 150 ppl/sqkm, proportion)	--
Rabies	Female	1-5 months	95+ years	Global	-1	1	Antenatal Care (4 visits) Coverage (proportion)	143
Rabies	Female	1-5 months	95+ years	Global	-1	1	In-Facility Delivery (proportion)	205
Rabies	Female	1-5 months	95+ years	Global	-1	1	Socio-demographic Index	674
Rabies	Female	1-5 months	95+ years	Global	-1	2	Healthcare access and quality index	211
Rabies	Female	1-5 months	95+ years	Global	-1	2	Skilled Birth Attendance (proportion)	377
Rabies	Female	1-5 months	95+ years	Global	1	3	Population Density (500-1000 ppl/sqkm, proportion)	--
Rabies	Female	1-5 months	95+ years	Global	1	3	Population Density (under 150 ppl/sqkm, proportion)	--
Rabies	Male	1-5 months	95+ years	Data Rich	-1	1	In-Facility Delivery (proportion)	266
Rabies	Male	1-5 months	95+ years	Data Rich	-1	1	Antenatal Care (4 visits) Coverage (proportion)	608
Rabies	Male	1-5 months	95+ years	Data Rich	-1	1	Socio-demographic Index	930
Rabies	Male	1-5 months	95+ years	Data Rich	-1	2	Maternal care and immunization	0
Rabies	Male	1-5 months	95+ years	Data Rich	-1	2	Healthcare access and quality index	12
Rabies	Male	1-5 months	95+ years	Data Rich	-1	2	Skilled Birth Attendance (proportion)	812

CODEm covariates used, level of covariate, and expected direction of covariate by cause, sex, and age								
Cause	Sex	Age Start	Age End	Model Version Type	Direction	Level	Covariate Name	Number of Draws
Rabies	Male	1-5 months	95+ years	Data Rich	1	3	Population Density (500-1000 ppl/sqkm, proportion)	--
Rabies	Male	1-5 months	95+ years	Data Rich	1	3	Population Density (under 150 ppl/sqkm, proportion)	--
Rabies	Male	1-5 months	95+ years	Global	-1	1	In-Facility Delivery (proportion)	307
Rabies	Male	1-5 months	95+ years	Global	-1	1	Antenatal Care (4 visits) Coverage (proportion)	327
Rabies	Male	1-5 months	95+ years	Global	-1	1	Socio-demographic Index	366
Rabies	Male	1-5 months	95+ years	Global	-1	2	Skilled Birth Attendance (proportion)	194
Rabies	Male	1-5 months	95+ years	Global	-1	2	Healthcare access and quality index	258
Rabies	Male	1-5 months	95+ years	Global	1	3	Population Density (500-1000 ppl/sqkm, proportion)	--
Rabies	Male	1-5 months	95+ years	Global	1	3	Population Density (under 150 ppl/sqkm, proportion)	--
Other neglected tropical diseases	Female	0-6 days	95+ years	Data Rich	-1	1	Healthcare access and quality index	637
Other neglected tropical diseases	Female	0-6 days	95+ years	Data Rich	1	1	Latitude Under 15 (proportion)	474
Other neglected tropical diseases	Female	0-6 days	95+ years	Data Rich	-1	2	Sanitation (proportion with access)	178
Other neglected tropical diseases	Female	0-6 days	95+ years	Data Rich	1	2	Rainfall Quintile 5 (proportion)	--
Other neglected tropical diseases	Female	0-6 days	95+ years	Data Rich	-1	3	Socio-demographic Index	145
Other neglected tropical diseases	Female	0-6 days	95+ years	Data Rich	-1	3	Education (years per capita)	--
Other neglected tropical diseases	Female	0-6 days	95+ years	Data Rich	-1	3	LDI (I$ per capita)	--
Other neglected tropical diseases	Female	0-6 days	95+ years	Global	-1	1	Healthcare access and quality index	654
Other neglected tropical diseases	Female	0-6 days	95+ years	Global	1	1	Latitude Under 15 (proportion)	572
Other neglected tropical diseases	Female	0-6 days	95+ years	Global	-1	2	Sanitation (proportion with access)	253
Other neglected tropical diseases	Female	0-6 days	95+ years	Global	1	2	Rainfall Quintile 5 (proportion)	--
Other neglected tropical diseases	Female	0-6 days	95+ years	Global	-1	3	Socio-demographic Index	42
Other neglected tropical diseases	Female	0-6 days	95+ years	Global	-1	3	Education (years per capita)	353
Other neglected tropical diseases	Female	0-6 days	95+ years	Global	-1	3	LDI (I$ per capita)	--
Other neglected tropical diseases	Male	0-6 days	95+ years	Data Rich	-1	1	Healthcare access and quality index	616
Other neglected tropical diseases	Male	0-6 days	95+ years	Data Rich	1	1	Latitude Under 15 (proportion)	503
Other neglected tropical diseases	Male	0-6 days	95+ years	Data Rich	-1	2	Sanitation (proportion with access)	182
Other neglected tropical diseases	Male	0-6 days	95+ years	Data Rich	1	2	Rainfall Quintile 5 (proportion)	--
Other neglected tropical diseases	Male	0-6 days	95+ years	Data Rich	-1	3	Socio-demographic Index	69
Other neglected tropical diseases	Male	0-6 days	95+ years	Data Rich	-1	3	Education (years per capita)	--
Other neglected tropical diseases	Male	0-6 days	95+ years	Data Rich	-1	3	LDI (I$ per capita)	--
Other neglected tropical diseases	Male	0-6 days	95+ years	Global	-1	1	Healthcare access and quality index	670
Other neglected tropical diseases	Male	0-6 days	95+ years	Global	1	1	Latitude Under 15 (proportion)	697
Other neglected tropical diseases	Male	0-6 days	95+ years	Global	-1	2	Sanitation (proportion with access)	197
Other neglected tropical diseases	Male	0-6 days	95+ years	Global	1	2	Rainfall Quintile 5 (proportion)	--
Other neglected tropical diseases	Male	0-6 days	95+ years	Global	-1	3	Socio-demographic Index	118
Other neglected tropical diseases	Male	0-6 days	95+ years	Global	-1	3	Education (years per capita)	316
Other neglected tropical diseases	Male	0-6 days	95+ years	Global	-1	3	LDI (I$ per capita)	--
Meningitis	Female	0-6 days	2-4 years	Data Rich	-1	1	Hib3 transformed: population-level coverage, including indirect effects (proportion)	259
Meningitis	Female	0-6 days	2-4 years	Data Rich	-1	1	PCV3 lagged five year coverage (proportion)	871
Meningitis	Female	0-6 days	2-4 years	Data Rich	-1	1	Proportion of total population covered by menafrivac initiative (meningitis meningococcal type A vaccine)	--
Meningitis	Female	0-6 days	2-4 years	Data Rich	1	1	meningitis belt (proportion)	--
Meningitis	Female	0-6 days	2-4 years	Data Rich	-1	2	Maternal care and immunization	52
Meningitis	Female	0-6 days	2-4 years	Data Rich	-1	2	Healthcare access and quality index	256
Meningitis	Female	0-6 days	2-4 years	Data Rich	-1	2	Improved Water Source (proportion with access)	--
Meningitis	Female	0-6 days	2-4 years	Data Rich	1	2	Age- and sex-specific SEV for Child underweight	41
Meningitis	Female	0-6 days	2-4 years	Data Rich	-1	3	Maternal Education (years per capita)	115
Meningitis	Female	0-6 days	2-4 years	Data Rich	-1	3	Sanitation (proportion with access)	119
Meningitis	Female	0-6 days	2-4 years	Data Rich	-1	3	Socio-demographic Index	251
Meningitis	Female	0-6 days	2-4 years	Data Rich	-1	3	LDI (I$ per capita)	--
Meningitis	Female	0-6 days	2-4 years	Global	-1	1	Proportion of total population covered by menafrivac initiative (meningitis meningococcal type A vaccine)	0
Meningitis	Female	0-6 days	2-4 years	Global	-1	1	Hib3 transformed: population-level coverage, including indirect effects (proportion)	391
Meningitis	Female	0-6 days	2-4 years	Global	-1	1	PCV3 lagged five year coverage (proportion)	869
Meningitis	Female	0-6 days	2-4 years	Global	1	1	meningitis belt (proportion)	0
Meningitis	Female	0-6 days	2-4 years	Global	-1	2	Maternal care and immunization	186
Meningitis	Female	0-6 days	2-4 years	Global	-1	2	Healthcare access and quality index	260
Meningitis	Female	0-6 days	2-4 years	Global	-1	2	Improved Water Source (proportion with access)	--
Meningitis	Female	0-6 days	2-4 years	Global	1	2	Age- and sex-specific SEV for Child underweight	72
Meningitis	Female	0-6 days	2-4 years	Global	-1	3	Maternal Education (years per capita)	92
Meningitis	Female	0-6 days	2-4 years	Global	-1	3	Sanitation (proportion with access)	112
Meningitis	Female	0-6 days	2-4 years	Global	-1	3	Socio-demographic Index	171
Meningitis	Female	0-6 days	2-4 years	Global	-1	3	LDI (I$ per capita)	--
Meningitis	Female	5-9 years	95+ years	Data Rich	-1	1	Hib3 transformed: population-level coverage, including indirect effects (proportion)	49
Meningitis	Female	5-9 years	95+ years	Data Rich	-1	1	PCV3 lagged five year coverage (proportion)	539
Meningitis	Female	5-9 years	95+ years	Data Rich	-1	1	Proportion of total population covered by menafrivac initiative (meningitis meningococcal type A vaccine)	627
Meningitis	Female	5-9 years	95+ years	Data Rich	1	1	meningitis belt (proportion)	--
Meningitis	Female	5-9 years	95+ years	Data Rich	-1	2	Maternal care and immunization	0
Meningitis	Female	5-9 years	95+ years	Data Rich	-1	2	Healthcare access and quality index	34
Meningitis	Female	5-9 years	95+ years	Data Rich	-1	2	Improved Water Source (proportion with access)	--
Meningitis	Female	5-9 years	95+ years	Data Rich	1	2	Age-standardized SEV for Child underweight	279
Meningitis	Female	5-9 years	95+ years	Data Rich	-1	3	Maternal Education (years per capita)	34
Meningitis	Female	5-9 years	95+ years	Data Rich	-1	3	Socio-demographic Index	153
Meningitis	Female	5-9 years	95+ years	Data Rich	-1	3	Sanitation (proportion with access)	303
Meningitis	Female	5-9 years	95+ years	Data Rich	-1	3	LDI (I$ per capita)	--
Meningitis	Female	5-9 years	95+ years	Global	-1	1	Proportion of total population covered by menafrivac initiative (meningitis meningococcal type A vaccine)	0
Meningitis	Female	5-9 years	95+ years	Global	-1	1	Hib3 transformed: population-level coverage, including indirect effects (proportion)	541
Meningitis	Female	5-9 years	95+ years	Global	-1	1	PCV3 lagged five year coverage (proportion)	786
Meningitis	Female	5-9 years	95+ years	Global	1	1	meningitis belt (proportion)	--
Meningitis	Female	5-9 years	95+ years	Global	-1	2	Maternal care and immunization	8
Meningitis	Female	5-9 years	95+ years	Global	-1	2	Healthcare access and quality index	268
Meningitis	Female	5-9 years	95+ years	Global	-1	2	Improved Water Source (proportion with access)	--
Meningitis	Female	5-9 years	95+ years	Global	1	2	Age-standardized SEV for Child underweight	104
Meningitis	Female	5-9 years	95+ years	Global	-1	3	Maternal Education (years per capita)	63
Meningitis	Female	5-9 years	95+ years	Global	-1	3	Socio-demographic Index	200
Meningitis	Female	5-9 years	95+ years	Global	-1	3	Sanitation (proportion with access)	275
Meningitis	Female	5-9 years	95+ years	Global	-1	3	LDI (I$ per capita)	--
Meningitis	Male	0-6 days	2-4 years	Data Rich	-1	1	Hib3 transformed: population-level coverage, including indirect effects (proportion)	349
Meningitis	Male	0-6 days	2-4 years	Data Rich	-1	1	PCV3 lagged five year coverage (proportion)	825
Meningitis	Male	0-6 days	2-4 years	Data Rich	-1	1	Proportion of total population covered by menafrivac initiative (meningitis meningococcal type A vaccine)	--
Meningitis	Male	0-6 days	2-4 years	Data Rich	1	1	meningitis belt (proportion)	--
Meningitis	Male	0-6 days	2-4 years	Data Rich	-1	2	Maternal care and immunization	46
Meningitis	Male	0-6 days	2-4 years	Data Rich	-1	2	Healthcare access and quality index	495
Meningitis	Male	0-6 days	2-4 years	Data Rich	-1	2	Improved Water Source (proportion with access)	--
Meningitis	Male	0-6 days	2-4 years	Data Rich	1	2	Age- and sex-specific SEV for Child underweight	22
Meningitis	Male	0-6 days	2-4 years	Data Rich	-1	3	Socio-demographic Index	68
Meningitis	Male	0-6 days	2-4 years	Data Rich	-1	3	Sanitation (proportion with access)	117
Meningitis	Male	0-6 days	2-4 years	Data Rich	-1	3	Maternal Education (years per capita)	285
Meningitis	Male	0-6 days	2-4 years	Data Rich	-1	3	LDI (I$ per capita)	--
Meningitis	Male	0-6 days	2-4 years	Global	-1	1	Proportion of total population covered by menafrivac initiative (meningitis meningococcal type A vaccine)	175
Meningitis	Male	0-6 days	2-4 years	Global	-1	1	PCV3 lagged five year coverage (proportion)	618
Meningitis	Male	0-6 days	2-4 years	Global	-1	1	Hib3 transformed: population-level coverage, including indirect effects (proportion)	641
Meningitis	Male	0-6 days	2-4 years	Global	1	1	meningitis belt (proportion)	171
Meningitis	Male	0-6 days	2-4 years	Global	-1	2	Maternal care and immunization	213
Meningitis	Male	0-6 days	2-4 years	Global	-1	2	Healthcare access and quality index	238
Meningitis	Male	0-6 days	2-4 years	Global	-1	2	Improved Water Source (proportion with access)	--

CODEm covariates used, level of covariate, and expected direction of covariate by cause, sex, and age

Cause	Sex	Age Start	Age End	Model Version Type	Direction	Level	Covariate Name	Number of Draws
Meningitis	Male	0-6 days	2-4 years	Global	1	2	Age- and sex-specific SEV for Child underweight	116
Meningitis	Male	0-6 days	2-4 years	Global	-1	3	Sanitation (proportion with access)	46
Meningitis	Male	0-6 days	2-4 years	Global	-1	3	Maternal Education (years per capita)	67
Meningitis	Male	0-6 days	2-4 years	Global	-1	3	Socio-demographic Index	139
Meningitis	Male	0-6 days	2-4 years	Global	-1	3	LDI (I$ per capita)	--
Meningitis	Male	5-9 years	95+ years	Data Rich	-1	1	Hib3 transformed: population-level coverage, including indirect effects (proportion)	15
Meningitis	Male	5-9 years	95+ years	Data Rich	-1	1	Proportion of total population covered by menafrivac initiative (meningitis meningococcal type A vaccine)	636
Meningitis	Male	5-9 years	95+ years	Data Rich	-1	1	PCV3 lagged five year coverage (proportion)	707
Meningitis	Male	5-9 years	95+ years	Data Rich	1	1	meningitis belt (proportion)	--
Meningitis	Male	5-9 years	95+ years	Data Rich	-1	2	Maternal care and immunization	0
Meningitis	Male	5-9 years	95+ years	Data Rich	-1	2	Healthcare access and quality index	123
Meningitis	Male	5-9 years	95+ years	Data Rich	-1	2	Improved Water Source (proportion with access)	--
Meningitis	Male	5-9 years	95+ years	Data Rich	1	2	Age-standardized SEV for Child underweight	211
Meningitis	Male	5-9 years	95+ years	Data Rich	-1	3	Maternal Education (years per capita)	50
Meningitis	Male	5-9 years	95+ years	Data Rich	-1	3	Socio-demographic Index	168
Meningitis	Male	5-9 years	95+ years	Data Rich	-1	3	Sanitation (proportion with access)	420
Meningitis	Male	5-9 years	95+ years	Data Rich	-1	3	LDI (I$ per capita)	--
Meningitis	Male	5-9 years	95+ years	Global	-1	1	Proportion of total population covered by menafrivac initiative (meningitis meningococcal type A vaccine)	5
Meningitis	Male	5-9 years	95+ years	Global	-1	1	PCV3 lagged five year coverage (proportion)	453
Meningitis	Male	5-9 years	95+ years	Global	-1	1	Hib3 transformed: population-level coverage, including indirect effects (proportion)	910
Meningitis	Male	5-9 years	95+ years	Global	1	1	meningitis belt (proportion)	63
Meningitis	Male	5-9 years	95+ years	Global	-1	2	Maternal care and immunization	195
Meningitis	Male	5-9 years	95+ years	Global	-1	2	Healthcare access and quality index	419
Meningitis	Male	5-9 years	95+ years	Global	-1	2	Improved Water Source (proportion with access)	--
Meningitis	Male	5-9 years	95+ years	Global	1	2	Age-standardized SEV for Child underweight	101
Meningitis	Male	5-9 years	95+ years	Global	-1	3	Sanitation (proportion with access)	97
Meningitis	Male	5-9 years	95+ years	Global	-1	3	Maternal Education (years per capita)	98
Meningitis	Male	5-9 years	95+ years	Global	-1	3	Socio-demographic Index	136
Meningitis	Male	5-9 years	95+ years	Global	-1	3	LDI (I$ per capita)	--
Encephalitis	Female	0-6 days	95+ years	Data Rich	1	1	Age- and sex-specific SEV for Child underweight	331
Encephalitis	Female	0-6 days	95+ years	Data Rich	1	1	Japanese encelphalitis endemic area (binary)	669
Encephalitis	Female	0-6 days	95+ years	Data Rich	-1	2	Healthcare access and quality index	28
Encephalitis	Female	0-6 days	95+ years	Data Rich	-1	2	Maternal care and immunization	102
Encephalitis	Female	0-6 days	95+ years	Data Rich	-1	2	LDI (I$ per capita)	--
Encephalitis	Female	0-6 days	95+ years	Data Rich	-1	3	DTP3 Coverage (proportion)	16
Encephalitis	Female	0-6 days	95+ years	Data Rich	-1	3	Sanitation (proportion with access)	145
Encephalitis	Female	0-6 days	95+ years	Data Rich	-1	3	Socio-demographic Index	159
Encephalitis	Female	0-6 days	95+ years	Data Rich	-1	3	Improved Water Source (proportion with access)	256
Encephalitis	Female	0-6 days	95+ years	Data Rich	-1	3	Maternal Education (years per capita)	467
Encephalitis	Female	0-6 days	95+ years	Data Rich	-1	3	In-Facility Delivery (proportion)	--
Encephalitis	Female	0-6 days	95+ years	Global	1	1	Japanese encelphalitis endemic area (binary)	423
Encephalitis	Female	0-6 days	95+ years	Global	1	1	Age- and sex-specific SEV for Child underweight	875
Encephalitis	Female	0-6 days	95+ years	Global	-1	2	Maternal care and immunization	40
Encephalitis	Female	0-6 days	95+ years	Global	-1	2	Healthcare access and quality index	161
Encephalitis	Female	0-6 days	95+ years	Global	-1	2	LDI (I$ per capita)	--
Encephalitis	Female	0-6 days	95+ years	Global	-1	3	DTP3 Coverage (proportion)	121
Encephalitis	Female	0-6 days	95+ years	Global	-1	3	Maternal Education (years per capita)	123
Encephalitis	Female	0-6 days	95+ years	Global	-1	3	Socio-demographic Index	205
Encephalitis	Female	0-6 days	95+ years	Global	-1	3	Improved Water Source (proportion with access)	--
Encephalitis	Female	0-6 days	95+ years	Global	-1	3	In-Facility Delivery (proportion)	--
Encephalitis	Female	0-6 days	95+ years	Global	-1	3	Sanitation (proportion with access)	--
Encephalitis	Male	0-6 days	95+ years	Data Rich	1	1	Age- and sex-specific SEV for Child underweight	388
Encephalitis	Male	0-6 days	95+ years	Data Rich	1	1	Japanese encelphalitis endemic area (binary)	612
Encephalitis	Male	0-6 days	95+ years	Data Rich	-1	2	Healthcare access and quality index	145
Encephalitis	Male	0-6 days	95+ years	Data Rich	-1	2	Maternal care and immunization	340
Encephalitis	Male	0-6 days	95+ years	Data Rich	-1	2	LDI (I$ per capita)	--
Encephalitis	Male	0-6 days	95+ years	Data Rich	-1	3	DTP3 Coverage (proportion)	21
Encephalitis	Male	0-6 days	95+ years	Data Rich	-1	3	Sanitation (proportion with access)	28
Encephalitis	Male	0-6 days	95+ years	Data Rich	-1	3	Socio-demographic Index	121
Encephalitis	Male	0-6 days	95+ years	Data Rich	-1	3	Improved Water Source (proportion with access)	256
Encephalitis	Male	0-6 days	95+ years	Data Rich	-1	3	Maternal Education (years per capita)	556
Encephalitis	Male	0-6 days	95+ years	Data Rich	-1	3	In-Facility Delivery (proportion)	--
Encephalitis	Male	0-6 days	95+ years	Global	1	1	Japanese encelphalitis endemic area (binary)	1000
Encephalitis	Male	0-6 days	95+ years	Global	1	1	Age- and sex-specific SEV for Child underweight	--
Encephalitis	Male	0-6 days	95+ years	Global	-1	2	Healthcare access and quality index	215
Encephalitis	Male	0-6 days	95+ years	Global	-1	2	LDI (I$ per capita)	--
Encephalitis	Male	0-6 days	95+ years	Global	-1	2	Maternal care and immunization	--
Encephalitis	Male	0-6 days	95+ years	Global	-1	3	Socio-demographic Index	222
Encephalitis	Male	0-6 days	95+ years	Global	-1	3	DTP3 Coverage (proportion)	278
Encephalitis	Male	0-6 days	95+ years	Global	-1	3	Improved Water Source (proportion with access)	--
Encephalitis	Male	0-6 days	95+ years	Global	-1	3	In-Facility Delivery (proportion)	--
Encephalitis	Male	0-6 days	95+ years	Global	-1	3	Maternal Education (years per capita)	--
Encephalitis	Male	0-6 days	95+ years	Global	-1	3	Sanitation (proportion with access)	--
Tetanus	Female	0-6 days	6-11 months	Data Rich	-1	1	DTP3 lagged five year coverage (proportion)	217
Tetanus	Female	0-6 days	6-11 months	Data Rich	-1	1	tetanus toxoid maternal protection at birth	988
Tetanus	Female	0-6 days	6-11 months	Data Rich	-1	2	In-Facility Delivery (proportion)	14
Tetanus	Female	0-6 days	6-11 months	Data Rich	-1	2	Skilled Birth Attendance (proportion)	712
Tetanus	Female	0-6 days	6-11 months	Data Rich	-1	2	Healthcare access and quality index	907
Tetanus	Female	0-6 days	6-11 months	Data Rich	-1	3	Education (years per capita)	235
Tetanus	Female	0-6 days	6-11 months	Data Rich	-1	3	Socio-demographic Index	331
Tetanus	Female	0-6 days	6-11 months	Data Rich	-1	3	LDI (I$ per capita)	--
Tetanus	Female	0-6 days	6-11 months	Global	-1	1	DTP3 lagged five year coverage (proportion)	684
Tetanus	Female	0-6 days	6-11 months	Global	-1	1	tetanus toxoid maternal protection at birth	696
Tetanus	Female	0-6 days	6-11 months	Global	-1	2	In-Facility Delivery (proportion)	90
Tetanus	Female	0-6 days	6-11 months	Global	-1	2	Healthcare access and quality index	589
Tetanus	Female	0-6 days	6-11 months	Global	-1	2	Skilled Birth Attendance (proportion)	589
Tetanus	Female	0-6 days	6-11 months	Global	-1	3	Education (years per capita)	318
Tetanus	Female	0-6 days	6-11 months	Global	-1	3	Socio-demographic Index	402
Tetanus	Female	0-6 days	6-11 months	Global	-1	3	LDI (I$ per capita)	--
Tetanus	Female	12-23 months	95+ years	Data Rich	-1	1	DTP3 lagged five year coverage (proportion)	1000
Tetanus	Female	12-23 months	95+ years	Data Rich	-1	2	Healthcare access and quality index	578
Tetanus	Female	12-23 months	95+ years	Data Rich	-1	3	Socio-demographic Index	379
Tetanus	Female	12-23 months	95+ years	Data Rich	-1	3	Sanitation (proportion with access)	669
Tetanus	Female	12-23 months	95+ years	Data Rich	-1	3	Education (years per capita)	866
Tetanus	Female	12-23 months	95+ years	Data Rich	-1	3	LDI (I$ per capita)	--
Tetanus	Female	12-23 months	95+ years	Global	-1	1	DTP3 lagged five year coverage (proportion)	1000
Tetanus	Female	12-23 months	95+ years	Global	-1	2	Healthcare access and quality index	566
Tetanus	Female	12-23 months	95+ years	Global	-1	3	Sanitation (proportion with access)	233
Tetanus	Female	12-23 months	95+ years	Global	-1	3	Education (years per capita)	304
Tetanus	Female	12-23 months	95+ years	Global	-1	3	Socio-demographic Index	387
Tetanus	Female	12-23 months	95+ years	Global	-1	3	LDI (I$ per capita)	--
Tetanus	Male	0-6 days	6-11 months	Data Rich	-1	1	tetanus toxoid maternal protection at birth	304
Tetanus	Male	0-6 days	6-11 months	Data Rich	-1	1	DTP3 lagged five year coverage (proportion)	950
Tetanus	Male	0-6 days	6-11 months	Data Rich	-1	2	Skilled Birth Attendance (proportion)	271
Tetanus	Male	0-6 days	6-11 months	Data Rich	-1	2	In-Facility Delivery (proportion)	536
Tetanus	Male	0-6 days	6-11 months	Data Rich	-1	2	Healthcare access and quality index	625
Tetanus	Male	0-6 days	6-11 months	Data Rich	-1	3	Socio-demographic Index	359
Tetanus	Male	0-6 days	6-11 months	Data Rich	-1	3	Education (years per capita)	813
Tetanus	Male	0-6 days	6-11 months	Data Rich	-1	3	LDI (I$ per capita)	--
Tetanus	Male	0-6 days	6-11 months	Global	-1	1	tetanus toxoid maternal protection at birth	597
Tetanus	Male	0-6 days	6-11 months	Global	-1	1	DTP3 lagged five year coverage (proportion)	641
Tetanus	Male	0-6 days	6-11 months	Global	-1	2	Skilled Birth Attendance (proportion)	242
Tetanus	Male	0-6 days	6-11 months	Global	-1	2	In-Facility Delivery (proportion)	350
Tetanus	Male	0-6 days	6-11 months	Global	-1	2	Healthcare access and quality index	587

CODEm covariates used, level of covariate, and expected direction of covariate by cause, sex, and age								
**Cause**	**Sex**	**Age Start**	**Age End**	**Model Version Type**	**Direction**	**Level**	**Covariate Name**	**Number of Draws**
Tetanus	Male	0-6 days	6-11 months	Global	-1	3	Socio-demographic Index	225
Tetanus	Male	0-6 days	6-11 months	Global	-1	3	Education (years per capita)	426
Tetanus	Male	0-6 days	6-11 months	Global	-1	3	LDI (I$ per capita)	--
Tetanus	Male	12-23 months	95+ years	Data Rich	-1	1	DTP3 lagged five year coverage (proportion)	436
Tetanus	Male	12-23 months	95+ years	Data Rich	-1	2	Healthcare access and quality index	721
Tetanus	Male	12-23 months	95+ years	Data Rich	-1	3	Sanitation (proportion with access)	178
Tetanus	Male	12-23 months	95+ years	Data Rich	-1	3	Socio-demographic Index	349
Tetanus	Male	12-23 months	95+ years	Data Rich	-1	3	Education (years per capita)	519
Tetanus	Male	12-23 months	95+ years	Data Rich	-1	3	LDI (I$ per capita)	--
Tetanus	Male	12-23 months	95+ years	Global	-1	1	DTP3 lagged five year coverage (proportion)	1000
Tetanus	Male	12-23 months	95+ years	Global	-1	2	Healthcare access and quality index	486
Tetanus	Male	12-23 months	95+ years	Global	-1	3	Education (years per capita)	140
Tetanus	Male	12-23 months	95+ years	Global	-1	3	Sanitation (proportion with access)	201
Tetanus	Male	12-23 months	95+ years	Global	-1	3	Socio-demographic Index	344
Tetanus	Male	12-23 months	95+ years	Global	-1	3	LDI (I$ per capita)	--
Acute hepatitis	Female	1-5 months	95+ years	Data Rich	1	1	Hepatitis A Seroprevalence (anti-HAV) age standardized	129
Acute hepatitis	Female	1-5 months	95+ years	Data Rich	1	1	Hepatitis E Seroprevalence (anti-HEV) age standardized	159
Acute hepatitis	Female	1-5 months	95+ years	Data Rich	1	1	Vaccine adjusted HbSAg serporevalence age standardized	203
Acute hepatitis	Female	1-5 months	95+ years	Data Rich	1	1	Log-transformed SEV scalar: Hep	500
Acute hepatitis	Female	1-5 months	95+ years	Data Rich	1	1	Hepatitis C Seroprevalence (anti-HCV) age standardized	833
Acute hepatitis	Female	1-5 months	95+ years	Data Rich	-1	2	Healthcare access and quality index	0
Acute hepatitis	Female	1-5 months	95+ years	Data Rich	-1	2	Socio-demographic Index	0
Acute hepatitis	Female	1-5 months	95+ years	Data Rich	-1	2	Hepatitis B vaccine coverage (proportion), aged through time	53
Acute hepatitis	Female	1-5 months	95+ years	Data Rich	1	2	Age- and sex-specific SEV for Unsafe sanitation	13
Acute hepatitis	Female	1-5 months	95+ years	Data Rich	1	2	Age- and sex-specific SEV for Unsafe water	53
Acute hepatitis	Female	1-5 months	95+ years	Data Rich	1	2	Intravenous drug use (proportion by age)	--
Acute hepatitis	Female	1-5 months	95+ years	Data Rich	-1	3	Education (years per capita)	0
Acute hepatitis	Female	1-5 months	95+ years	Data Rich	-1	3	LDI (I$ per capita)	--
Acute hepatitis	Female	1-5 months	95+ years	Global	1	1	Log-transformed SEV scalar: Hep	144
Acute hepatitis	Female	1-5 months	95+ years	Global	1	1	Hepatitis A Seroprevalence (anti-HAV) age standardized	271
Acute hepatitis	Female	1-5 months	95+ years	Global	1	1	Hepatitis C Seroprevalence (anti-HCV) age standardized	463
Acute hepatitis	Female	1-5 months	95+ years	Global	1	1	Hepatitis E Seroprevalence (anti-HEV) age standardized	539
Acute hepatitis	Female	1-5 months	95+ years	Global	1	1	Vaccine adjusted HbSAg serporevalence age standardized	981
Acute hepatitis	Female	1-5 months	95+ years	Global	-1	2	Socio-demographic Index	0
Acute hepatitis	Female	1-5 months	95+ years	Global	-1	2	Healthcare access and quality index	2
Acute hepatitis	Female	1-5 months	95+ years	Global	-1	2	Hepatitis B vaccine coverage (proportion), aged through time	160
Acute hepatitis	Female	1-5 months	95+ years	Global	1	2	Age- and sex-specific SEV for Unsafe water	5
Acute hepatitis	Female	1-5 months	95+ years	Global	1	2	Age- and sex-specific SEV for Unsafe sanitation	493
Acute hepatitis	Female	1-5 months	95+ years	Global	1	2	Intravenous drug use (proportion by age)	--
Acute hepatitis	Female	1-5 months	95+ years	Global	-1	3	Education (years per capita)	0
Acute hepatitis	Female	1-5 months	95+ years	Global	-1	3	LDI (I$ per capita)	--
Acute hepatitis	Male	1-5 months	95+ years	Data Rich	1	1	Log-transformed SEV scalar: Hep	117
Acute hepatitis	Male	1-5 months	95+ years	Data Rich	1	1	Hepatitis E Seroprevalence (anti-HEV) age standardized	271
Acute hepatitis	Male	1-5 months	95+ years	Data Rich	1	1	Hepatitis A Seroprevalence (anti-HAV) age standardized	417
Acute hepatitis	Male	1-5 months	95+ years	Data Rich	1	1	Vaccine adjusted HbSAg serporevalence age standardized	483
Acute hepatitis	Male	1-5 months	95+ years	Data Rich	1	1	Hepatitis C Seroprevalence (anti-HCV) age standardized	910
Acute hepatitis	Male	1-5 months	95+ years	Data Rich	-1	2	Healthcare access and quality index	0
Acute hepatitis	Male	1-5 months	95+ years	Data Rich	-1	2	Socio-demographic Index	0
Acute hepatitis	Male	1-5 months	95+ years	Data Rich	-1	2	Hepatitis B vaccine coverage (proportion), aged through time	147
Acute hepatitis	Male	1-5 months	95+ years	Data Rich	1	2	Age- and sex-specific SEV for Unsafe water	114
Acute hepatitis	Male	1-5 months	95+ years	Data Rich	1	2	Age- and sex-specific SEV for Unsafe sanitation	188
Acute hepatitis	Male	1-5 months	95+ years	Data Rich	1	2	Intravenous drug use (proportion by age)	--
Acute hepatitis	Male	1-5 months	95+ years	Data Rich	-1	3	Education (years per capita)	1
Acute hepatitis	Male	1-5 months	95+ years	Data Rich	-1	3	LDI (I$ per capita)	--
Acute hepatitis	Male	1-5 months	95+ years	Global	1	1	Log-transformed SEV scalar: Hep	245
Acute hepatitis	Male	1-5 months	95+ years	Global	1	1	Hepatitis A Seroprevalence (anti-HAV) age standardized	496
Acute hepatitis	Male	1-5 months	95+ years	Global	1	1	Hepatitis E Seroprevalence (anti-HEV) age standardized	629
Acute hepatitis	Male	1-5 months	95+ years	Global	1	1	Vaccine adjusted HbSAg serporevalence age standardized	926
Acute hepatitis	Male	1-5 months	95+ years	Global	1	1	Hepatitis C Seroprevalence (anti-HCV) age standardized	946
Acute hepatitis	Male	1-5 months	95+ years	Global	-1	2	Healthcare access and quality index	3
Acute hepatitis	Male	1-5 months	95+ years	Global	-1	2	Socio-demographic Index	5
Acute hepatitis	Male	1-5 months	95+ years	Global	-1	2	Hepatitis B vaccine coverage (proportion), aged through time	12
Acute hepatitis	Male	1-5 months	95+ years	Global	1	2	Age- and sex-specific SEV for Unsafe water	444
Acute hepatitis	Male	1-5 months	95+ years	Global	1	2	Age- and sex-specific SEV for Unsafe sanitation	549
Acute hepatitis	Male	1-5 months	95+ years	Global	1	2	Intravenous drug use (proportion by age)	--
Acute hepatitis	Male	1-5 months	95+ years	Global	-1	3	Education (years per capita)	11
Acute hepatitis	Male	1-5 months	95+ years	Global	-1	3	LDI (I$ per capita)	--
Acute hepatitis A	Female	1-5 months	95+ years	Data Rich	1	1	Log-transformed SEV scalar: Hep	854
Acute hepatitis A	Female	1-5 months	95+ years	Data Rich	1	1	Hepatitis A Seroprevalence (anti-HAV) age standardized	892
Acute hepatitis A	Female	1-5 months	95+ years	Data Rich	-1	2	Healthcare access and quality index	117
Acute hepatitis A	Female	1-5 months	95+ years	Data Rich	-1	2	Socio-demographic Index	218
Acute hepatitis A	Female	1-5 months	95+ years	Data Rich	1	2	Age- and sex-specific SEV for Unsafe sanitation	117
Acute hepatitis A	Female	1-5 months	95+ years	Data Rich	1	2	Age- and sex-specific SEV for Unsafe water	390
Acute hepatitis A	Female	1-5 months	95+ years	Data Rich	-1	3	Education (years per capita)	288
Acute hepatitis A	Female	1-5 months	95+ years	Data Rich	-1	3	LDI (I$ per capita)	--
Acute hepatitis A	Female	1-5 months	95+ years	Global	1	1	Hepatitis A Seroprevalence (anti-HAV) age standardized	474
Acute hepatitis A	Female	1-5 months	95+ years	Global	1	1	Log-transformed SEV scalar: Hep	955
Acute hepatitis A	Female	1-5 months	95+ years	Global	-1	2	Socio-demographic Index	71
Acute hepatitis A	Female	1-5 months	95+ years	Global	-1	2	Healthcare access and quality index	305
Acute hepatitis A	Female	1-5 months	95+ years	Global	1	2	Age- and sex-specific SEV for Unsafe water	480
Acute hepatitis A	Female	1-5 months	95+ years	Global	1	2	Age- and sex-specific SEV for Unsafe sanitation	639
Acute hepatitis A	Female	1-5 months	95+ years	Global	-1	3	Education (years per capita)	262
Acute hepatitis A	Female	1-5 months	95+ years	Global	-1	3	LDI (I$ per capita)	--
Acute hepatitis A	Male	1-5 months	95+ years	Data Rich	1	1	Log-transformed SEV scalar: Hep	444
Acute hepatitis A	Male	1-5 months	95+ years	Data Rich	1	1	Hepatitis A Seroprevalence (anti-HAV) age standardized	718
Acute hepatitis A	Male	1-5 months	95+ years	Data Rich	-1	2	Healthcare access and quality index	134
Acute hepatitis A	Male	1-5 months	95+ years	Data Rich	-1	2	Socio-demographic Index	278
Acute hepatitis A	Male	1-5 months	95+ years	Data Rich	1	2	Age- and sex-specific SEV for Unsafe sanitation	122
Acute hepatitis A	Male	1-5 months	95+ years	Data Rich	1	2	Age- and sex-specific SEV for Unsafe water	424
Acute hepatitis A	Male	1-5 months	95+ years	Data Rich	-1	3	Education (years per capita)	411
Acute hepatitis A	Male	1-5 months	95+ years	Data Rich	-1	3	LDI (I$ per capita)	--
Acute hepatitis A	Male	1-5 months	95+ years	Global	1	1	Log-transformed SEV scalar: Hep	431
Acute hepatitis A	Male	1-5 months	95+ years	Global	1	1	Hepatitis A Seroprevalence (anti-HAV) age standardized	1000
Acute hepatitis A	Male	1-5 months	95+ years	Global	-1	2	Socio-demographic Index	51
Acute hepatitis A	Male	1-5 months	95+ years	Global	-1	2	Healthcare access and quality index	326
Acute hepatitis A	Male	1-5 months	95+ years	Global	1	2	Age- and sex-specific SEV for Unsafe water	237
Acute hepatitis A	Male	1-5 months	95+ years	Global	1	2	Age- and sex-specific SEV for Unsafe sanitation	578
Acute hepatitis A	Male	1-5 months	95+ years	Global	-1	3	Education (years per capita)	195
Acute hepatitis A	Male	1-5 months	95+ years	Global	-1	3	LDI (I$ per capita)	--
Acute hepatitis B	Female	1-5 months	95+ years	Data Rich	-1	1	Hepatitis B vaccine coverage (proportion), aged through time	416
Acute hepatitis B	Female	1-5 months	95+ years	Data Rich	1	1	Vaccine adjusted HbSAg serporevalence age standardized	736
Acute hepatitis B	Female	1-5 months	95+ years	Data Rich	1	1	Intravenous drug use (proportion by age)	--
Acute hepatitis B	Female	1-5 months	95+ years	Data Rich	-1	2	Socio-demographic Index	55
Acute hepatitis B	Female	1-5 months	95+ years	Data Rich	-1	2	Healthcare access and quality index	--
Acute hepatitis B	Female	1-5 months	95+ years	Data Rich	1	2	Log-transformed SEV scalar: Hep	130
Acute hepatitis B	Female	1-5 months	95+ years	Data Rich	-1	3	Education (years per capita)	313
Acute hepatitis B	Female	1-5 months	95+ years	Data Rich	-1	3	LDI (I$ per capita)	--
Acute hepatitis B	Female	1-5 months	95+ years	Global	-1	1	Hepatitis B vaccine coverage (proportion), aged through time	546

CODEm covariates used, level of covariate, and expected direction of covariate by cause, sex, and age

Cause	Sex	Age Start	Age End	Model Version Type	Direction	Level	Covariate Name	Number of Draws
Acute hepatitis B	Female	1-5 months	95+ years	Global	1	1	Vaccine adjusted HbSAg serporevalence age standardized	848
Acute hepatitis B	Female	1-5 months	95+ years	Global	1	1	Intravenous drug use (proportion by age)	--
Acute hepatitis B	Female	1-5 months	95+ years	Global	-1	2	Socio-demographic Index	6
Acute hepatitis B	Female	1-5 months	95+ years	Global	-1	2	Healthcare access and quality index	79
Acute hepatitis B	Female	1-5 months	95+ years	Global	1	2	Log-transformed SEV scalar: Hep	350
Acute hepatitis B	Female	1-5 months	95+ years	Global	-1	3	Education (years per capita)	105
Acute hepatitis B	Female	1-5 months	95+ years	Global	-1	3	LDI (I$ per capita)	--
Acute hepatitis B	Male	1-5 months	95+ years	Data Rich	-1	1	Hepatitis B vaccine coverage (proportion), aged through time	1000
Acute hepatitis B	Male	1-5 months	95+ years	Data Rich	1	1	Vaccine adjusted HbSAg serporevalence age standardized	514
Acute hepatitis B	Male	1-5 months	95+ years	Data Rich	1	1	Intravenous drug use (proportion by age)	--
Acute hepatitis B	Male	1-5 months	95+ years	Data Rich	-1	2	Socio-demographic Index	112
Acute hepatitis B	Male	1-5 months	95+ years	Data Rich	-1	2	Healthcare access and quality index	262
Acute hepatitis B	Male	1-5 months	95+ years	Data Rich	1	2	Log-transformed SEV scalar: Hep	336
Acute hepatitis B	Male	1-5 months	95+ years	Data Rich	-1	3	Education (years per capita)	181
Acute hepatitis B	Male	1-5 months	95+ years	Data Rich	-1	3	LDI (I$ per capita)	--
Acute hepatitis B	Male	1-5 months	95+ years	Global	-1	1	Hepatitis B vaccine coverage (proportion), aged through time	679
Acute hepatitis B	Male	1-5 months	95+ years	Global	1	1	Vaccine adjusted HbSAg serporevalence age standardized	716
Acute hepatitis B	Male	1-5 months	95+ years	Global	1	1	Intravenous drug use (proportion by age)	--
Acute hepatitis B	Male	1-5 months	95+ years	Global	-1	2	Socio-demographic Index	35
Acute hepatitis B	Male	1-5 months	95+ years	Global	-1	2	Healthcare access and quality index	125
Acute hepatitis B	Male	1-5 months	95+ years	Global	1	2	Log-transformed SEV scalar: Hep	222
Acute hepatitis B	Male	1-5 months	95+ years	Global	-1	3	Education (years per capita)	121
Acute hepatitis B	Male	1-5 months	95+ years	Global	-1	3	LDI (I$ per capita)	--
Acute hepatitis C	Female	1-5 months	95+ years	Data Rich	1	1	Hepatitis C Seroprevalence (anti-HCV) age standardized	603
Acute hepatitis C	Female	1-5 months	95+ years	Data Rich	1	1	Log-transformed SEV scalar: Hep	926
Acute hepatitis C	Female	1-5 months	95+ years	Data Rich	-1	2	Socio-demographic Index	80
Acute hepatitis C	Female	1-5 months	95+ years	Data Rich	-1	2	Healthcare access and quality index	672
Acute hepatitis C	Female	1-5 months	95+ years	Data Rich	1	2	Intravenous drug use (proportion by age)	--
Acute hepatitis C	Female	1-5 months	95+ years	Data Rich	-1	3	Education (years per capita)	719
Acute hepatitis C	Female	1-5 months	95+ years	Data Rich	-1	3	LDI (I$ per capita)	--
Acute hepatitis C	Female	1-5 months	95+ years	Global	1	1	Hepatitis C Seroprevalence (anti-HCV) age standardized	226
Acute hepatitis C	Female	1-5 months	95+ years	Global	1	1	Log-transformed SEV scalar: Hep	598
Acute hepatitis C	Female	1-5 months	95+ years	Global	-1	2	Healthcare access and quality index	348
Acute hepatitis C	Female	1-5 months	95+ years	Global	-1	2	Socio-demographic Index	518
Acute hepatitis C	Female	1-5 months	95+ years	Global	1	2	Intravenous drug use (proportion by age)	--
Acute hepatitis C	Female	1-5 months	95+ years	Global	-1	3	Education (years per capita)	632
Acute hepatitis C	Female	1-5 months	95+ years	Global	-1	3	LDI (I$ per capita)	--
Acute hepatitis C	Male	1-5 months	95+ years	Data Rich	1	1	Hepatitis C Seroprevalence (anti-HCV) age standardized	681
Acute hepatitis C	Male	1-5 months	95+ years	Data Rich	1	1	Intravenous drug use (proportion by age)	--
Acute hepatitis C	Male	1-5 months	95+ years	Data Rich	-1	2	Socio-demographic Index	174
Acute hepatitis C	Male	1-5 months	95+ years	Data Rich	-1	2	Healthcare access and quality index	635
Acute hepatitis C	Male	1-5 months	95+ years	Data Rich	1	2	Log-transformed SEV scalar: Hep	635
Acute hepatitis C	Male	1-5 months	95+ years	Data Rich	-1	3	Education (years per capita)	518
Acute hepatitis C	Male	1-5 months	95+ years	Data Rich	-1	3	LDI (I$ per capita)	--
Acute hepatitis C	Male	1-5 months	95+ years	Global	1	1	Hepatitis C Seroprevalence (anti-HCV) age standardized	1000
Acute hepatitis C	Male	1-5 months	95+ years	Global	1	1	Intravenous drug use (proportion by age)	--
Acute hepatitis C	Male	1-5 months	95+ years	Global	-1	2	Healthcare access and quality index	352
Acute hepatitis C	Male	1-5 months	95+ years	Global	-1	2	Socio-demographic Index	588
Acute hepatitis C	Male	1-5 months	95+ years	Global	1	2	Log-transformed SEV scalar: Hep	588
Acute hepatitis C	Male	1-5 months	95+ years	Global	-1	3	Education (years per capita)	395
Acute hepatitis C	Male	1-5 months	95+ years	Global	-1	3	LDI (I$ per capita)	--
Acute hepatitis E	Female	1-5 months	95+ years	Data Rich	1	1	Proportion of the population living in the classic monsoon region	460
Acute hepatitis E	Female	1-5 months	95+ years	Data Rich	1	1	Hepatitis E Seroprevalence (anti-HEV) age standardized	747
Acute hepatitis E	Female	1-5 months	95+ years	Data Rich	1	1	Log-transformed SEV scalar: Hep	--
Acute hepatitis E	Female	1-5 months	95+ years	Data Rich	-1	2	Healthcare access and quality index	174
Acute hepatitis E	Female	1-5 months	95+ years	Data Rich	-1	2	Socio-demographic Index	--
Acute hepatitis E	Female	1-5 months	95+ years	Data Rich	1	2	Age- and sex-specific SEV for Unsafe sanitation	217
Acute hepatitis E	Female	1-5 months	95+ years	Data Rich	1	2	Age- and sex-specific SEV for Unsafe water	377
Acute hepatitis E	Female	1-5 months	95+ years	Data Rich	-1	3	Education (years per capita)	66
Acute hepatitis E	Female	1-5 months	95+ years	Data Rich	-1	3	LDI (I$ per capita)	--
Acute hepatitis E	Female	1-5 months	95+ years	Global	1	1	Hepatitis E Seroprevalence (anti-HEV) age standardized	579
Acute hepatitis E	Female	1-5 months	95+ years	Global	1	1	Log-transformed SEV scalar: Hep	--
Acute hepatitis E	Female	1-5 months	95+ years	Global	-1	2	Socio-demographic Index	167
Acute hepatitis E	Female	1-5 months	95+ years	Global	-1	2	Healthcare access and quality index	--
Acute hepatitis E	Female	1-5 months	95+ years	Global	1	2	Age- and sex-specific SEV for Unsafe water	200
Acute hepatitis E	Female	1-5 months	95+ years	Global	1	2	Age- and sex-specific SEV for Unsafe sanitation	221
Acute hepatitis E	Female	1-5 months	95+ years	Global	1	2	Proportion of the population living in the classic monsoon region	417
Acute hepatitis E	Female	1-5 months	95+ years	Global	-1	3	Education (years per capita)	244
Acute hepatitis E	Female	1-5 months	95+ years	Global	-1	3	LDI (I$ per capita)	--
Acute hepatitis E	Male	1-5 months	95+ years	Data Rich	1	1	Hepatitis E Seroprevalence (anti-HEV) age standardized	1000
Acute hepatitis E	Male	1-5 months	95+ years	Data Rich	1	1	Log-transformed SEV scalar: Hep	--
Acute hepatitis E	Male	1-5 months	95+ years	Data Rich	-1	2	Socio-demographic Index	174
Acute hepatitis E	Male	1-5 months	95+ years	Data Rich	-1	2	Healthcare access and quality index	208
Acute hepatitis E	Male	1-5 months	95+ years	Data Rich	1	2	Proportion of the population living in the classic monsoon region	174
Acute hepatitis E	Male	1-5 months	95+ years	Data Rich	1	2	Age- and sex-specific SEV for Unsafe sanitation	--
Acute hepatitis E	Male	1-5 months	95+ years	Data Rich	1	2	Age- and sex-specific SEV for Unsafe water	--
Acute hepatitis E	Male	1-5 months	95+ years	Data Rich	-1	3	Education (years per capita)	145
Acute hepatitis E	Male	1-5 months	95+ years	Data Rich	-1	3	LDI (I$ per capita)	--
Acute hepatitis E	Male	1-5 months	95+ years	Global	1	1	Hepatitis E Seroprevalence (anti-HEV) age standardized	--
Acute hepatitis E	Male	1-5 months	95+ years	Global	1	1	Log-transformed SEV scalar: Hep	--
Acute hepatitis E	Male	1-5 months	95+ years	Global	-1	2	Socio-demographic Index	89
Acute hepatitis E	Male	1-5 months	95+ years	Global	-1	2	Healthcare access and quality index	203
Acute hepatitis E	Male	1-5 months	95+ years	Global	1	2	Age- and sex-specific SEV for Unsafe sanitation	228
Acute hepatitis E	Male	1-5 months	95+ years	Global	1	2	Age- and sex-specific SEV for Unsafe water	295
Acute hepatitis E	Male	1-5 months	95+ years	Global	1	2	Proportion of the population living in the classic monsoon region	587
Acute hepatitis E	Male	1-5 months	95+ years	Global	-1	3	Education (years per capita)	111
Acute hepatitis E	Male	1-5 months	95+ years	Global	-1	3	LDI (I$ per capita)	--
Other unspecified infectious diseases	Female	0-6 days	95+ years	Data Rich	-1	1	DTP3 Coverage (proportion)	--
Other unspecified infectious diseases	Female	0-6 days	95+ years	Data Rich	-1	2	Sanitation (proportion with access)	207
Other unspecified infectious diseases	Female	0-6 days	95+ years	Data Rich	-1	2	Improved Water Source (proportion with access)	325
Other unspecified infectious diseases	Female	0-6 days	95+ years	Data Rich	-1	2	Healthcare access and quality index	532
Other unspecified infectious diseases	Female	0-6 days	95+ years	Data Rich	-1	3	Socio-demographic Index	171
Other unspecified infectious diseases	Female	0-6 days	95+ years	Data Rich	-1	3	Antenatal Care (1 visit) Coverage (proportion)	653
Other unspecified infectious diseases	Female	0-6 days	95+ years	Global	-1	1	DTP3 Coverage (proportion)	454
Other unspecified infectious diseases	Female	0-6 days	95+ years	Global	-1	2	Healthcare access and quality index	--
Other unspecified infectious diseases	Female	0-6 days	95+ years	Global	-1	2	Improved Water Source (proportion with access)	--
Other unspecified infectious diseases	Female	0-6 days	95+ years	Global	-1	2	Sanitation (proportion with access)	--
Other unspecified infectious diseases	Female	0-6 days	95+ years	Global	-1	3	Antenatal Care (1 visit) Coverage (proportion)	546
Other unspecified infectious diseases	Female	0-6 days	95+ years	Global	-1	3	Socio-demographic Index	--
Other unspecified infectious diseases	Male	0-6 days	95+ years	Data Rich	-1	1	DTP3 Coverage (proportion)	--
Other unspecified infectious diseases	Male	0-6 days	95+ years	Data Rich	-1	2	Healthcare access and quality index	307
Other unspecified infectious diseases	Male	0-6 days	95+ years	Data Rich	-1	2	Improved Water Source (proportion with access)	653
Other unspecified infectious diseases	Male	0-6 days	95+ years	Data Rich	-1	2	Sanitation (proportion with access)	698
Other unspecified infectious diseases	Male	0-6 days	95+ years	Data Rich	-1	3	Socio-demographic Index	171
Other unspecified infectious diseases	Male	0-6 days	95+ years	Data Rich	-1	3	Antenatal Care (1 visit) Coverage (proportion)	544
Other unspecified infectious diseases	Male	0-6 days	95+ years	Global	-1	1	DTP3 Coverage (proportion)	133
Other unspecified infectious diseases	Male	0-6 days	95+ years	Global	-1	2	Healthcare access and quality index	211
Other unspecified infectious diseases	Male	0-6 days	95+ years	Global	-1	2	Improved Water Source (proportion with access)	258
Other unspecified infectious diseases	Male	0-6 days	95+ years	Global	-1	2	Sanitation (proportion with access)	398
Other unspecified infectious diseases	Male	0-6 days	95+ years	Global	-1	3	Antenatal Care (1 visit) Coverage (proportion)	194
Other unspecified infectious diseases	Male	0-6 days	95+ years	Global	-1	3	Socio-demographic Index	--

CODEm covariates used, level of covariate, and expected direction of covariate by cause, sex, and age

Cause	Sex	Age Start	Age End	Model Version Type	Direction	Level	Covariate Name	Number of Draws
Neonatal disorders	Female	0-6 days	2-4 years	Data Rich	-1	1	Maternal care and immunization	0
Neonatal disorders	Female	0-6 days	2-4 years	Data Rich	1	1	Age-standardized SEV for Household air pollution	0
Neonatal disorders	Female	0-6 days	2-4 years	Data Rich	1	1	Age-standardized SEV for Smoking	0
Neonatal disorders	Female	0-6 days	2-4 years	Data Rich	1	1	Age-standardized SEV for Low birth weight	309
Neonatal disorders	Female	0-6 days	2-4 years	Data Rich	1	1	Age-standardized SEV for Short gestation	491
Neonatal disorders	Female	0-6 days	2-4 years	Data Rich	1	1	Age-standardized SEV for Ambient particulate matter	661
Neonatal disorders	Female	0-6 days	2-4 years	Data Rich	-1	2	Healthcare access and quality index	0
Neonatal disorders	Female	0-6 days	2-4 years	Data Rich	-1	2	Proportion of the population with at least 12 years of education, maternal	0
Neonatal disorders	Female	0-6 days	2-4 years	Data Rich	-1	2	Proportion of the population with at least 6 years of education, maternal	0
Neonatal disorders	Female	0-6 days	2-4 years	Data Rich	-1	2	Socio-demographic Index	0
Neonatal disorders	Female	0-6 days	2-4 years	Data Rich	1	2	Live Births 35+ (proportion)	427
Neonatal disorders	Female	0-6 days	2-4 years	Data Rich	-1	3	Antenatal Care (1 visit) Coverage (proportion)	0
Neonatal disorders	Female	0-6 days	2-4 years	Data Rich	-1	3	Antenatal Care (4 visits) Coverage (proportion)	0
Neonatal disorders	Female	0-6 days	2-4 years	Data Rich	-1	3	In-Facility Delivery (proportion)	0
Neonatal disorders	Female	0-6 days	2-4 years	Data Rich	-1	3	Skilled Birth Attendance (proportion)	0
Neonatal disorders	Female	0-6 days	2-4 years	Data Rich	-1	3	LDI (I$ per capita)	--
Neonatal disorders	Female	0-6 days	2-4 years	Data Rich	1	3	Total Fertility Rate	--
Neonatal disorders	Female	0-6 days	2-4 years	Global	-1	1	Maternal care and immunization	0
Neonatal disorders	Female	0-6 days	2-4 years	Global	1	1	Age-standardized SEV for Smoking	0
Neonatal disorders	Female	0-6 days	2-4 years	Global	1	1	Age-standardized SEV for Household air pollution	22
Neonatal disorders	Female	0-6 days	2-4 years	Global	1	1	Age-standardized SEV for Low birth weight	426
Neonatal disorders	Female	0-6 days	2-4 years	Global	1	1	Age-standardized SEV for Short gestation	427
Neonatal disorders	Female	0-6 days	2-4 years	Global	1	1	Age-standardized SEV for Ambient particulate matter	579
Neonatal disorders	Female	0-6 days	2-4 years	Global	-1	2	Proportion of the population with at least 6 years of education, maternal	21
Neonatal disorders	Female	0-6 days	2-4 years	Global	-1	2	Socio-demographic Index	28
Neonatal disorders	Female	0-6 days	2-4 years	Global	-1	2	Proportion of the population with at least 12 years of education, maternal	37
Neonatal disorders	Female	0-6 days	2-4 years	Global	-1	2	Healthcare access and quality index	68
Neonatal disorders	Female	0-6 days	2-4 years	Global	1	2	Live Births 35+ (proportion)	608
Neonatal disorders	Female	0-6 days	2-4 years	Global	-1	3	In-Facility Delivery (proportion)	0
Neonatal disorders	Female	0-6 days	2-4 years	Global	-1	3	Skilled Birth Attendance (proportion)	0
Neonatal disorders	Female	0-6 days	2-4 years	Global	-1	3	Antenatal Care (1 visit) Coverage (proportion)	5
Neonatal disorders	Female	0-6 days	2-4 years	Global	-1	3	Antenatal Care (4 visits) Coverage (proportion)	12
Neonatal disorders	Female	0-6 days	2-4 years	Global	-1	3	LDI (I$ per capita)	--
Neonatal disorders	Female	0-6 days	2-4 years	Global	1	3	Total Fertility Rate	--
Neonatal disorders	Male	0-6 days	2-4 years	Data Rich	-1	1	Maternal care and immunization	0
Neonatal disorders	Male	0-6 days	2-4 years	Data Rich	1	1	Age-standardized SEV for Household air pollution	0
Neonatal disorders	Male	0-6 days	2-4 years	Data Rich	1	1	Age-standardized SEV for Smoking	0
Neonatal disorders	Male	0-6 days	2-4 years	Data Rich	1	1	Age-standardized SEV for Low birth weight	360
Neonatal disorders	Male	0-6 days	2-4 years	Data Rich	1	1	Age-standardized SEV for Ambient particulate matter	508
Neonatal disorders	Male	0-6 days	2-4 years	Data Rich	1	1	Age-standardized SEV for Short gestation	640
Neonatal disorders	Male	0-6 days	2-4 years	Data Rich	-1	2	Proportion of the population with at least 6 years of education, maternal	0
Neonatal disorders	Male	0-6 days	2-4 years	Data Rich	-1	2	Proportion of the population with at least 12 years of education, maternal	5
Neonatal disorders	Male	0-6 days	2-4 years	Data Rich	-1	2	Socio-demographic Index	6
Neonatal disorders	Male	0-6 days	2-4 years	Data Rich	-1	2	Healthcare access and quality index	11
Neonatal disorders	Male	0-6 days	2-4 years	Data Rich	1	2	Live Births 35+ (proportion)	828
Neonatal disorders	Male	0-6 days	2-4 years	Data Rich	-1	3	Skilled Birth Attendance (proportion)	0
Neonatal disorders	Male	0-6 days	2-4 years	Data Rich	-1	3	Antenatal Care (1 visit) Coverage (proportion)	1
Neonatal disorders	Male	0-6 days	2-4 years	Data Rich	-1	3	Antenatal Care (4 visits) Coverage (proportion)	3
Neonatal disorders	Male	0-6 days	2-4 years	Data Rich	-1	3	In-Facility Delivery (proportion)	3
Neonatal disorders	Male	0-6 days	2-4 years	Data Rich	-1	3	LDI (I$ per capita)	--
Neonatal disorders	Male	0-6 days	2-4 years	Data Rich	1	3	Total Fertility Rate	--
Neonatal disorders	Male	0-6 days	2-4 years	Global	-1	1	Maternal care and immunization	14
Neonatal disorders	Male	0-6 days	2-4 years	Global	1	1	Age-standardized SEV for Smoking	9
Neonatal disorders	Male	0-6 days	2-4 years	Global	1	1	Age-standardized SEV for Household air pollution	55
Neonatal disorders	Male	0-6 days	2-4 years	Global	1	1	Age-standardized SEV for Low birth weight	346
Neonatal disorders	Male	0-6 days	2-4 years	Global	1	1	Age-standardized SEV for Short gestation	481
Neonatal disorders	Male	0-6 days	2-4 years	Global	1	1	Age-standardized SEV for Ambient particulate matter	574
Neonatal disorders	Male	0-6 days	2-4 years	Global	-1	2	Proportion of the population with at least 6 years of education, maternal	17
Neonatal disorders	Male	0-6 days	2-4 years	Global	-1	2	Socio-demographic Index	32
Neonatal disorders	Male	0-6 days	2-4 years	Global	-1	2	Proportion of the population with at least 12 years of education, maternal	72
Neonatal disorders	Male	0-6 days	2-4 years	Global	-1	2	Healthcare access and quality index	85
Neonatal disorders	Male	0-6 days	2-4 years	Global	1	2	Live Births 35+ (proportion)	370
Neonatal disorders	Male	0-6 days	2-4 years	Global	-1	3	Antenatal Care (1 visit) Coverage (proportion)	0
Neonatal disorders	Male	0-6 days	2-4 years	Global	-1	3	Skilled Birth Attendance (proportion)	0
Neonatal disorders	Male	0-6 days	2-4 years	Global	-1	3	In-Facility Delivery (proportion)	2
Neonatal disorders	Male	0-6 days	2-4 years	Global	-1	3	Antenatal Care (4 visits) Coverage (proportion)	3
Neonatal disorders	Male	0-6 days	2-4 years	Global	-1	3	LDI (I$ per capita)	--
Neonatal disorders	Male	0-6 days	2-4 years	Global	1	3	Total Fertility Rate	--
Neonatal preterm birth	Female	0-6 days	2-4 years	Data Rich	-1	1	Maternal care and immunization	116
Neonatal preterm birth	Female	0-6 days	2-4 years	Data Rich	1	1	Age-standardized SEV for Household air pollution	92
Neonatal preterm birth	Female	0-6 days	2-4 years	Data Rich	1	1	Age-standardized SEV for Ambient particulate matter	244
Neonatal preterm birth	Female	0-6 days	2-4 years	Data Rich	1	1	Age-standardized SEV for Smoking	338
Neonatal preterm birth	Female	0-6 days	2-4 years	Data Rich	1	1	Age-standardized SEV for Low birth weight	658
Neonatal preterm birth	Female	0-6 days	2-4 years	Data Rich	1	1	Age-standardized SEV for Short gestation	936
Neonatal preterm birth	Female	0-6 days	2-4 years	Data Rich	-1	2	Healthcare access and quality index	0
Neonatal preterm birth	Female	0-6 days	2-4 years	Data Rich	-1	2	Proportion of the population with at least 6 years of education, maternal	1
Neonatal preterm birth	Female	0-6 days	2-4 years	Data Rich	-1	2	Socio-demographic Index	69
Neonatal preterm birth	Female	0-6 days	2-4 years	Data Rich	-1	2	Proportion of the population with at least 12 years of education, maternal	83
Neonatal preterm birth	Female	0-6 days	2-4 years	Data Rich	1	2	Live Births 35+ (proportion)	0
Neonatal preterm birth	Female	0-6 days	2-4 years	Data Rich	-1	3	Antenatal Care (1 visit) Coverage (proportion)	9
Neonatal preterm birth	Female	0-6 days	2-4 years	Data Rich	-1	3	Antenatal Care (4 visits) Coverage (proportion)	52
Neonatal preterm birth	Female	0-6 days	2-4 years	Data Rich	-1	3	Skilled Birth Attendance (proportion)	56
Neonatal preterm birth	Female	0-6 days	2-4 years	Data Rich	-1	3	In-Facility Delivery (proportion)	103
Neonatal preterm birth	Female	0-6 days	2-4 years	Data Rich	-1	3	LDI (I$ per capita)	--
Neonatal preterm birth	Female	0-6 days	2-4 years	Data Rich	1	3	Total Fertility Rate	--
Neonatal preterm birth	Female	0-6 days	2-4 years	Global	-1	1	Maternal care and immunization	0
Neonatal preterm birth	Female	0-6 days	2-4 years	Global	1	1	Age-standardized SEV for Household air pollution	0
Neonatal preterm birth	Female	0-6 days	2-4 years	Global	1	1	Age-standardized SEV for Smoking	0
Neonatal preterm birth	Female	0-6 days	2-4 years	Global	1	1	Age-standardized SEV for Low birth weight	517
Neonatal preterm birth	Female	0-6 days	2-4 years	Global	1	1	Age-standardized SEV for Ambient particulate matter	726
Neonatal preterm birth	Female	0-6 days	2-4 years	Global	1	1	Age-standardized SEV for Short gestation	808
Neonatal preterm birth	Female	0-6 days	2-4 years	Global	-1	2	Healthcare access and quality index	0
Neonatal preterm birth	Female	0-6 days	2-4 years	Global	-1	2	Proportion of the population with at least 12 years of education, maternal	0
Neonatal preterm birth	Female	0-6 days	2-4 years	Global	-1	2	Socio-demographic Index	0
Neonatal preterm birth	Female	0-6 days	2-4 years	Global	-1	2	Proportion of the population with at least 6 years of education, maternal	58
Neonatal preterm birth	Female	0-6 days	2-4 years	Global	1	2	Live Births 35+ (proportion)	152
Neonatal preterm birth	Female	0-6 days	2-4 years	Global	-1	3	Antenatal Care (1 visit) Coverage (proportion)	0
Neonatal preterm birth	Female	0-6 days	2-4 years	Global	-1	3	Antenatal Care (4 visits) Coverage (proportion)	0
Neonatal preterm birth	Female	0-6 days	2-4 years	Global	-1	3	In-Facility Delivery (proportion)	0
Neonatal preterm birth	Female	0-6 days	2-4 years	Global	-1	3	Skilled Birth Attendance (proportion)	0
Neonatal preterm birth	Female	0-6 days	2-4 years	Global	-1	3	LDI (I$ per capita)	--
Neonatal preterm birth	Female	0-6 days	2-4 years	Global	1	3	Total Fertility Rate	--
Neonatal preterm birth	Male	0-6 days	2-4 years	Data Rich	-1	1	Maternal care and immunization	0
Neonatal preterm birth	Male	0-6 days	2-4 years	Data Rich	1	1	Age-standardized SEV for Household air pollution	55
Neonatal preterm birth	Male	0-6 days	2-4 years	Data Rich	1	1	Age-standardized SEV for Smoking	205
Neonatal preterm birth	Male	0-6 days	2-4 years	Data Rich	1	1	Age-standardized SEV for Low birth weight	571
Neonatal preterm birth	Male	0-6 days	2-4 years	Data Rich	1	1	Age-standardized SEV for Ambient particulate matter	592

CODEm covariates used, level of covariate, and expected direction of covariate by cause, sex, and age

Cause	Sex	Age Start	Age End	Model Version Type	Direction	Level	Covariate Name	Number of Draws
Neonatal preterm birth	Male	0-6 days	2-4 years	Data Rich	1	1	Age-standardized SEV for Short gestation	1000
Neonatal preterm birth	Male	0-6 days	2-4 years	Data Rich	-1	2	Healthcare access and quality index	0
Neonatal preterm birth	Male	0-6 days	2-4 years	Data Rich	-1	2	Proportion of the population with at least 12 years of education, maternal	0
Neonatal preterm birth	Male	0-6 days	2-4 years	Data Rich	-1	2	Proportion of the population with at least 6 years of education, maternal	0
Neonatal preterm birth	Male	0-6 days	2-4 years	Data Rich	-1	2	Socio-demographic Index	0
Neonatal preterm birth	Male	0-6 days	2-4 years	Data Rich	1	2	Live Births 35+ (proportion)	351
Neonatal preterm birth	Male	0-6 days	2-4 years	Data Rich	-1	3	In-Facility Delivery (proportion)	0
Neonatal preterm birth	Male	0-6 days	2-4 years	Data Rich	-1	3	Antenatal Care (1 visit) Coverage (proportion)	5
Neonatal preterm birth	Male	0-6 days	2-4 years	Data Rich	-1	3	Antenatal Care (4 visits) Coverage (proportion)	46
Neonatal preterm birth	Male	0-6 days	2-4 years	Data Rich	-1	3	Skilled Birth Attendance (proportion)	76
Neonatal preterm birth	Male	0-6 days	2-4 years	Data Rich	-1	3	LDI (I$ per capita)	--
Neonatal preterm birth	Male	0-6 days	2-4 years	Data Rich	1	3	Total Fertility Rate	--
Neonatal preterm birth	Male	0-6 days	2-4 years	Global	-1	1	Maternal care and immunization	0
Neonatal preterm birth	Male	0-6 days	2-4 years	Global	1	1	Age-standardized SEV for Smoking	10
Neonatal preterm birth	Male	0-6 days	2-4 years	Global	1	1	Age-standardized SEV for Household air pollution	14
Neonatal preterm birth	Male	0-6 days	2-4 years	Global	1	1	Age-standardized SEV for Low birth weight	199
Neonatal preterm birth	Male	0-6 days	2-4 years	Global	1	1	Age-standardized SEV for Ambient particulate matter	719
Neonatal preterm birth	Male	0-6 days	2-4 years	Global	1	1	Age-standardized SEV for Short gestation	720
Neonatal preterm birth	Male	0-6 days	2-4 years	Global	-1	2	Socio-demographic Index	4
Neonatal preterm birth	Male	0-6 days	2-4 years	Global	-1	2	Proportion of the population with at least 12 years of education, maternal	14
Neonatal preterm birth	Male	0-6 days	2-4 years	Global	-1	2	Healthcare access and quality index	18
Neonatal preterm birth	Male	0-6 days	2-4 years	Global	-1	2	Proportion of the population with at least 6 years of education, maternal	43
Neonatal preterm birth	Male	0-6 days	2-4 years	Global	1	2	Live Births 35+ (proportion)	568
Neonatal preterm birth	Male	0-6 days	2-4 years	Global	-1	3	Antenatal Care (1 visit) Coverage (proportion)	0
Neonatal preterm birth	Male	0-6 days	2-4 years	Global	-1	3	Antenatal Care (4 visits) Coverage (proportion)	0
Neonatal preterm birth	Male	0-6 days	2-4 years	Global	-1	3	In-Facility Delivery (proportion)	0
Neonatal preterm birth	Male	0-6 days	2-4 years	Global	-1	3	Skilled Birth Attendance (proportion)	0
Neonatal preterm birth	Male	0-6 days	2-4 years	Global	-1	3	LDI (I$ per capita)	--
Neonatal preterm birth	Male	0-6 days	2-4 years	Global	1	3	Total Fertility Rate	--
Neonatal encephalopathy due to birth asphyxia and trauma	Female	0-6 days	2-4 years	Data Rich	-1	1	Maternal care and immunization	355
Neonatal encephalopathy due to birth asphyxia and trauma	Female	0-6 days	2-4 years	Data Rich	1	1	Age-standardized SEV for Low birth weight	17
Neonatal encephalopathy due to birth asphyxia and trauma	Female	0-6 days	2-4 years	Data Rich	1	1	Age-standardized SEV for Short gestation	24
Neonatal encephalopathy due to birth asphyxia and trauma	Female	0-6 days	2-4 years	Data Rich	1	1	Age-standardized SEV for Smoking	315
Neonatal encephalopathy due to birth asphyxia and trauma	Female	0-6 days	2-4 years	Data Rich	1	1	Age-standardized SEV for Household air pollution	382
Neonatal encephalopathy due to birth asphyxia and trauma	Female	0-6 days	2-4 years	Data Rich	1	1	Age-standardized SEV for Ambient particulate matter	487
Neonatal encephalopathy due to birth asphyxia and trauma	Female	0-6 days	2-4 years	Data Rich	-1	2	Socio-demographic Index	14
Neonatal encephalopathy due to birth asphyxia and trauma	Female	0-6 days	2-4 years	Data Rich	-1	2	Proportion of the population with at least 6 years of education, maternal	373
Neonatal encephalopathy due to birth asphyxia and trauma	Female	0-6 days	2-4 years	Data Rich	-1	2	Proportion of the population with at least 12 years of education, maternal	791
Neonatal encephalopathy due to birth asphyxia and trauma	Female	0-6 days	2-4 years	Data Rich	-1	2	Healthcare access and quality index	--
Neonatal encephalopathy due to birth asphyxia and trauma	Female	0-6 days	2-4 years	Data Rich	1	2	Live Births 35+ (proportion)	209
Neonatal encephalopathy due to birth asphyxia and trauma	Female	0-6 days	2-4 years	Data Rich	-1	3	Antenatal Care (4 visits) Coverage (proportion)	29
Neonatal encephalopathy due to birth asphyxia and trauma	Female	0-6 days	2-4 years	Data Rich	-1	3	Antenatal Care (1 visit) Coverage (proportion)	76
Neonatal encephalopathy due to birth asphyxia and trauma	Female	0-6 days	2-4 years	Data Rich	-1	3	Skilled Birth Attendance (proportion)	157
Neonatal encephalopathy due to birth asphyxia and trauma	Female	0-6 days	2-4 years	Data Rich	-1	3	In-Facility Delivery (proportion)	277
Neonatal encephalopathy due to birth asphyxia and trauma	Female	0-6 days	2-4 years	Data Rich	-1	3	LDI (I$ per capita)	--
Neonatal encephalopathy due to birth asphyxia and trauma	Female	0-6 days	2-4 years	Data Rich	1	3	Total Fertility Rate	--
Neonatal encephalopathy due to birth asphyxia and trauma	Female	0-6 days	2-4 years	Global	-1	1	Antenatal Care (1 visit) Coverage (proportion)	67
Neonatal encephalopathy due to birth asphyxia and trauma	Female	0-6 days	2-4 years	Global	-1	1	Maternal care and immunization	372
Neonatal encephalopathy due to birth asphyxia and trauma	Female	0-6 days	2-4 years	Global	1	1	Age-standardized SEV for Ambient particulate matter	26
Neonatal encephalopathy due to birth asphyxia and trauma	Female	0-6 days	2-4 years	Global	1	1	Age-standardized SEV for Household air pollution	127
Neonatal encephalopathy due to birth asphyxia and trauma	Female	0-6 days	2-4 years	Global	1	1	Age-standardized SEV for Smoking	184
Neonatal encephalopathy due to birth asphyxia and trauma	Female	0-6 days	2-4 years	Global	1	1	Age-standardized SEV for Low birth weight	--
Neonatal encephalopathy due to birth asphyxia and trauma	Female	0-6 days	2-4 years	Global	1	1	Age-standardized SEV for Short gestation	--
Neonatal encephalopathy due to birth asphyxia and trauma	Female	0-6 days	2-4 years	Global	-1	2	Proportion of the population with at least 6 years of education, maternal	76
Neonatal encephalopathy due to birth asphyxia and trauma	Female	0-6 days	2-4 years	Global	-1	2	Socio-demographic Index	121
Neonatal encephalopathy due to birth asphyxia and trauma	Female	0-6 days	2-4 years	Global	-1	2	Proportion of the population with at least 12 years of education, maternal	689
Neonatal encephalopathy due to birth asphyxia and trauma	Female	0-6 days	2-4 years	Global	-1	2	Healthcare access and quality index	--
Neonatal encephalopathy due to birth asphyxia and trauma	Female	0-6 days	2-4 years	Global	1	2	Live Births 35+ (proportion)	--
Neonatal encephalopathy due to birth asphyxia and trauma	Female	0-6 days	2-4 years	Global	-1	3	Antenatal Care (4 visits) Coverage (proportion)	12
Neonatal encephalopathy due to birth asphyxia and trauma	Female	0-6 days	2-4 years	Global	-1	3	Skilled Birth Attendance (proportion)	37
Neonatal encephalopathy due to birth asphyxia and trauma	Female	0-6 days	2-4 years	Global	-1	3	In-Facility Delivery (proportion)	154
Neonatal encephalopathy due to birth asphyxia and trauma	Female	0-6 days	2-4 years	Global	-1	3	LDI (I$ per capita)	--
Neonatal encephalopathy due to birth asphyxia and trauma	Female	0-6 days	2-4 years	Global	1	3	Total Fertility Rate	--
Neonatal encephalopathy due to birth asphyxia and trauma	Male	0-6 days	2-4 years	Data Rich	-1	1	Maternal care and immunization	169
Neonatal encephalopathy due to birth asphyxia and trauma	Male	0-6 days	2-4 years	Data Rich	1	1	Age-standardized SEV for Short gestation	238
Neonatal encephalopathy due to birth asphyxia and trauma	Male	0-6 days	2-4 years	Data Rich	1	1	Age-standardized SEV for Low birth weight	254
Neonatal encephalopathy due to birth asphyxia and trauma	Male	0-6 days	2-4 years	Data Rich	1	1	Age-standardized SEV for Household air pollution	311
Neonatal encephalopathy due to birth asphyxia and trauma	Male	0-6 days	2-4 years	Data Rich	1	1	Age-standardized SEV for Smoking	526
Neonatal encephalopathy due to birth asphyxia and trauma	Male	0-6 days	2-4 years	Data Rich	1	1	Age-standardized SEV for Ambient particulate matter	624
Neonatal encephalopathy due to birth asphyxia and trauma	Male	0-6 days	2-4 years	Data Rich	-1	2	Proportion of the population with at least 6 years of education, maternal	0
Neonatal encephalopathy due to birth asphyxia and trauma	Male	0-6 days	2-4 years	Data Rich	-1	2	Socio-demographic Index	16
Neonatal encephalopathy due to birth asphyxia and trauma	Male	0-6 days	2-4 years	Data Rich	-1	2	Proportion of the population with at least 12 years of education, maternal	978
Neonatal encephalopathy due to birth asphyxia and trauma	Male	0-6 days	2-4 years	Data Rich	-1	2	Healthcare access and quality index	--
Neonatal encephalopathy due to birth asphyxia and trauma	Male	0-6 days	2-4 years	Data Rich	1	2	Live Births 35+ (proportion)	--
Neonatal encephalopathy due to birth asphyxia and trauma	Male	0-6 days	2-4 years	Data Rich	-1	3	Antenatal Care (1 visit) Coverage (proportion)	168
Neonatal encephalopathy due to birth asphyxia and trauma	Male	0-6 days	2-4 years	Data Rich	-1	3	Antenatal Care (4 visits) Coverage (proportion)	249
Neonatal encephalopathy due to birth asphyxia and trauma	Male	0-6 days	2-4 years	Data Rich	-1	3	In-Facility Delivery (proportion)	280
Neonatal encephalopathy due to birth asphyxia and trauma	Male	0-6 days	2-4 years	Data Rich	-1	3	Skilled Birth Attendance (proportion)	322
Neonatal encephalopathy due to birth asphyxia and trauma	Male	0-6 days	2-4 years	Data Rich	-1	3	LDI (I$ per capita)	--
Neonatal encephalopathy due to birth asphyxia and trauma	Male	0-6 days	2-4 years	Data Rich	1	3	Total Fertility Rate	--
Neonatal encephalopathy due to birth asphyxia and trauma	Male	0-6 days	2-4 years	Global	-1	1	Antenatal Care (1 visit) Coverage (proportion)	26
Neonatal encephalopathy due to birth asphyxia and trauma	Male	0-6 days	2-4 years	Global	-1	1	Maternal care and immunization	264
Neonatal encephalopathy due to birth asphyxia and trauma	Male	0-6 days	2-4 years	Global	1	1	Age-standardized SEV for Ambient particulate matter	45
Neonatal encephalopathy due to birth asphyxia and trauma	Male	0-6 days	2-4 years	Global	1	1	Age-standardized SEV for Household air pollution	184
Neonatal encephalopathy due to birth asphyxia and trauma	Male	0-6 days	2-4 years	Global	1	1	Age-standardized SEV for Smoking	208
Neonatal encephalopathy due to birth asphyxia and trauma	Male	0-6 days	2-4 years	Global	1	1	Age-standardized SEV for Low birth weight	--
Neonatal encephalopathy due to birth asphyxia and trauma	Male	0-6 days	2-4 years	Global	1	1	Age-standardized SEV for Short gestation	--
Neonatal encephalopathy due to birth asphyxia and trauma	Male	0-6 days	2-4 years	Global	-1	2	Proportion of the population with at least 6 years of education, maternal	15
Neonatal encephalopathy due to birth asphyxia and trauma	Male	0-6 days	2-4 years	Global	-1	2	Socio-demographic Index	95
Neonatal encephalopathy due to birth asphyxia and trauma	Male	0-6 days	2-4 years	Global	-1	2	Healthcare access and quality index	218
Neonatal encephalopathy due to birth asphyxia and trauma	Male	0-6 days	2-4 years	Global	-1	2	Proportion of the population with at least 12 years of education, maternal	535
Neonatal encephalopathy due to birth asphyxia and trauma	Male	0-6 days	2-4 years	Global	1	2	Live Births 35+ (proportion)	--

CODEm covariates used, level of covariate, and expected direction of covariate by cause, sex, and age

Cause	Sex	Age Start	Age End	Model Version Type	Direction	Level	Covariate Name	Number of Draws
Neonatal encephalopathy due to birth asphyxia and trauma	Male	0-6 days	2-4 years	Global	-1	3	Antenatal Care (4 visits) Coverage (proportion)	9
Neonatal encephalopathy due to birth asphyxia and trauma	Male	0-6 days	2-4 years	Global	-1	3	Skilled Birth Attendance (proportion)	19
Neonatal encephalopathy due to birth asphyxia and trauma	Male	0-6 days	2-4 years	Global	-1	3	In-Facility Delivery (proportion)	251
Neonatal encephalopathy due to birth asphyxia and trauma	Male	0-6 days	2-4 years	Global	-1	3	LDI (I$ per capita)	--
Neonatal encephalopathy due to birth asphyxia and trauma	Male	0-6 days	2-4 years	Global	1	3	Total Fertility Rate	--
Neonatal sepsis and other neonatal infections	Female	0-6 days	2-4 years	Data Rich	-1	1	Maternal care and immunization	479
Neonatal sepsis and other neonatal infections	Female	0-6 days	2-4 years	Data Rich	1	1	Age-standardized SEV for Household air pollution	19
Neonatal sepsis and other neonatal infections	Female	0-6 days	2-4 years	Data Rich	1	1	Age-standardized SEV for Smoking	25
Neonatal sepsis and other neonatal infections	Female	0-6 days	2-4 years	Data Rich	1	1	Age- and sex-specific SEV for Short gestation	187
Neonatal sepsis and other neonatal infections	Female	0-6 days	2-4 years	Data Rich	1	1	Age- and sex-specific SEV for Low birth weight	441
Neonatal sepsis and other neonatal infections	Female	0-6 days	2-4 years	Data Rich	1	1	Age-standardized SEV for Ambient particulate matter	776
Neonatal sepsis and other neonatal infections	Female	0-6 days	2-4 years	Data Rich	-1	2	Healthcare access and quality index	9
Neonatal sepsis and other neonatal infections	Female	0-6 days	2-4 years	Data Rich	-1	2	Socio-demographic Index	58
Neonatal sepsis and other neonatal infections	Female	0-6 days	2-4 years	Data Rich	-1	2	Proportion of the population with at least 12 years of education, maternal	80
Neonatal sepsis and other neonatal infections	Female	0-6 days	2-4 years	Data Rich	-1	2	Proportion of the population with at least 6 years of education, maternal	219
Neonatal sepsis and other neonatal infections	Female	0-6 days	2-4 years	Data Rich	1	2	Live Births 35+ (proportion)	176
Neonatal sepsis and other neonatal infections	Female	0-6 days	2-4 years	Data Rich	-1	3	Antenatal Care (1 visit) Coverage (proportion)	67
Neonatal sepsis and other neonatal infections	Female	0-6 days	2-4 years	Data Rich	-1	3	Antenatal Care (4 visits) Coverage (proportion)	100
Neonatal sepsis and other neonatal infections	Female	0-6 days	2-4 years	Data Rich	-1	3	In-Facility Delivery (proportion)	123
Neonatal sepsis and other neonatal infections	Female	0-6 days	2-4 years	Data Rich	-1	3	Skilled Birth Attendance (proportion)	319
Neonatal sepsis and other neonatal infections	Female	0-6 days	2-4 years	Data Rich	-1	3	LDI (I$ per capita)	--
Neonatal sepsis and other neonatal infections	Female	0-6 days	2-4 years	Data Rich	1	3	Total Fertility Rate	--
Neonatal sepsis and other neonatal infections	Female	0-6 days	2-4 years	Global	-1	1	Maternal care and immunization	27
Neonatal sepsis and other neonatal infections	Female	0-6 days	2-4 years	Global	1	1	Age-standardized SEV for Smoking	0
Neonatal sepsis and other neonatal infections	Female	0-6 days	2-4 years	Global	1	1	Age- and sex-specific SEV for Low birth weight	27
Neonatal sepsis and other neonatal infections	Female	0-6 days	2-4 years	Global	1	1	Age- and sex-specific SEV for Short gestation	178
Neonatal sepsis and other neonatal infections	Female	0-6 days	2-4 years	Global	1	1	Age-standardized SEV for Household air pollution	506
Neonatal sepsis and other neonatal infections	Female	0-6 days	2-4 years	Global	1	1	Age-standardized SEV for Ambient particulate matter	803
Neonatal sepsis and other neonatal infections	Female	0-6 days	2-4 years	Global	-1	2	Proportion of the population with at least 12 years of education, maternal	26
Neonatal sepsis and other neonatal infections	Female	0-6 days	2-4 years	Global	-1	2	Proportion of the population with at least 6 years of education, maternal	164
Neonatal sepsis and other neonatal infections	Female	0-6 days	2-4 years	Global	-1	2	Healthcare access and quality index	576
Neonatal sepsis and other neonatal infections	Female	0-6 days	2-4 years	Global	-1	2	Socio-demographic Index	--
Neonatal sepsis and other neonatal infections	Female	0-6 days	2-4 years	Global	1	2	Live Births 35+ (proportion)	186
Neonatal sepsis and other neonatal infections	Female	0-6 days	2-4 years	Global	-1	3	Antenatal Care (1 visit) Coverage (proportion)	0
Neonatal sepsis and other neonatal infections	Female	0-6 days	2-4 years	Global	-1	3	Antenatal Care (4 visits) Coverage (proportion)	145
Neonatal sepsis and other neonatal infections	Female	0-6 days	2-4 years	Global	-1	3	Skilled Birth Attendance (proportion)	159
Neonatal sepsis and other neonatal infections	Female	0-6 days	2-4 years	Global	-1	3	In-Facility Delivery (proportion)	176
Neonatal sepsis and other neonatal infections	Female	0-6 days	2-4 years	Global	-1	3	LDI (I$ per capita)	--
Neonatal sepsis and other neonatal infections	Female	0-6 days	2-4 years	Global	1	3	Total Fertility Rate	--
Neonatal sepsis and other neonatal infections	Male	0-6 days	2-4 years	Data Rich	-1	1	Maternal care and immunization	119
Neonatal sepsis and other neonatal infections	Male	0-6 days	2-4 years	Data Rich	1	1	Age-standardized SEV for Smoking	18
Neonatal sepsis and other neonatal infections	Male	0-6 days	2-4 years	Data Rich	1	1	Age-standardized SEV for Low birth weight	27
Neonatal sepsis and other neonatal infections	Male	0-6 days	2-4 years	Data Rich	1	1	Age-standardized SEV for Short gestation	41
Neonatal sepsis and other neonatal infections	Male	0-6 days	2-4 years	Data Rich	1	1	Age-standardized SEV for Household air pollution	86
Neonatal sepsis and other neonatal infections	Male	0-6 days	2-4 years	Data Rich	1	1	Age-standardized SEV for Ambient particulate matter	914
Neonatal sepsis and other neonatal infections	Male	0-6 days	2-4 years	Data Rich	-1	2	Proportion of the population with at least 12 years of education, maternal	73
Neonatal sepsis and other neonatal infections	Male	0-6 days	2-4 years	Data Rich	-1	2	Proportion of the population with at least 6 years of education, maternal	225
Neonatal sepsis and other neonatal infections	Male	0-6 days	2-4 years	Data Rich	-1	2	Socio-demographic Index	397
Neonatal sepsis and other neonatal infections	Male	0-6 days	2-4 years	Data Rich	-1	2	Healthcare access and quality index	584
Neonatal sepsis and other neonatal infections	Male	0-6 days	2-4 years	Data Rich	1	2	Live Births 35+ (proportion)	40
Neonatal sepsis and other neonatal infections	Male	0-6 days	2-4 years	Data Rich	-1	3	Skilled Birth Attendance (proportion)	5
Neonatal sepsis and other neonatal infections	Male	0-6 days	2-4 years	Data Rich	-1	3	Antenatal Care (1 visit) Coverage (proportion)	189
Neonatal sepsis and other neonatal infections	Male	0-6 days	2-4 years	Data Rich	-1	3	In-Facility Delivery (proportion)	194
Neonatal sepsis and other neonatal infections	Male	0-6 days	2-4 years	Data Rich	-1	3	Antenatal Care (4 visits) Coverage (proportion)	262
Neonatal sepsis and other neonatal infections	Male	0-6 days	2-4 years	Data Rich	-1	3	LDI (I$ per capita)	--
Neonatal sepsis and other neonatal infections	Male	0-6 days	2-4 years	Data Rich	1	3	Total Fertility Rate	--
Neonatal sepsis and other neonatal infections	Male	0-6 days	2-4 years	Global	-1	1	Maternal care and immunization	71
Neonatal sepsis and other neonatal infections	Male	0-6 days	2-4 years	Global	1	1	Age-standardized SEV for Smoking	0
Neonatal sepsis and other neonatal infections	Male	0-6 days	2-4 years	Global	1	1	Age-standardized SEV for Low birth weight	66
Neonatal sepsis and other neonatal infections	Male	0-6 days	2-4 years	Global	1	1	Age-standardized SEV for Short gestation	178
Neonatal sepsis and other neonatal infections	Male	0-6 days	2-4 years	Global	1	1	Age-standardized SEV for Household air pollution	630
Neonatal sepsis and other neonatal infections	Male	0-6 days	2-4 years	Global	1	1	Age-standardized SEV for Ambient particulate matter	787
Neonatal sepsis and other neonatal infections	Male	0-6 days	2-4 years	Global	-1	2	Socio-demographic Index	0
Neonatal sepsis and other neonatal infections	Male	0-6 days	2-4 years	Global	-1	2	Proportion of the population with at least 12 years of education, maternal	157
Neonatal sepsis and other neonatal infections	Male	0-6 days	2-4 years	Global	-1	2	Proportion of the population with at least 6 years of education, maternal	256
Neonatal sepsis and other neonatal infections	Male	0-6 days	2-4 years	Global	-1	2	Healthcare access and quality index	578
Neonatal sepsis and other neonatal infections	Male	0-6 days	2-4 years	Global	1	2	Live Births 35+ (proportion)	186
Neonatal sepsis and other neonatal infections	Male	0-6 days	2-4 years	Global	-1	3	Antenatal Care (1 visit) Coverage (proportion)	43
Neonatal sepsis and other neonatal infections	Male	0-6 days	2-4 years	Global	-1	3	Antenatal Care (4 visits) Coverage (proportion)	57
Neonatal sepsis and other neonatal infections	Male	0-6 days	2-4 years	Global	-1	3	Skilled Birth Attendance (proportion)	63
Neonatal sepsis and other neonatal infections	Male	0-6 days	2-4 years	Global	-1	3	In-Facility Delivery (proportion)	100
Neonatal sepsis and other neonatal infections	Male	0-6 days	2-4 years	Global	-1	3	LDI (I$ per capita)	--
Neonatal sepsis and other neonatal infections	Male	0-6 days	2-4 years	Global	1	3	Total Fertility Rate	--
Hemolytic disease and other neonatal jaundice	Female	0-6 days	2-4 years	Data Rich	-1	1	Maternal care and immunization	666
Hemolytic disease and other neonatal jaundice	Female	0-6 days	2-4 years	Data Rich	1	1	Age-standardized SEV for Low birth weight	0
Hemolytic disease and other neonatal jaundice	Female	0-6 days	2-4 years	Data Rich	1	1	Age-standardized SEV for Short gestation	43
Hemolytic disease and other neonatal jaundice	Female	0-6 days	2-4 years	Data Rich	1	1	Age-standardized SEV for Ambient particulate matter	129
Hemolytic disease and other neonatal jaundice	Female	0-6 days	2-4 years	Data Rich	1	1	Age-standardized SEV for Household air pollution	467
Hemolytic disease and other neonatal jaundice	Female	0-6 days	2-4 years	Data Rich	1	1	Age-standardized SEV for Smoking	644
Hemolytic disease and other neonatal jaundice	Female	0-6 days	2-4 years	Data Rich	-1	2	Socio-demographic Index	82
Hemolytic disease and other neonatal jaundice	Female	0-6 days	2-4 years	Data Rich	-1	2	Proportion of the population with at least 6 years of education, maternal	131
Hemolytic disease and other neonatal jaundice	Female	0-6 days	2-4 years	Data Rich	-1	2	Healthcare access and quality index	496
Hemolytic disease and other neonatal jaundice	Female	0-6 days	2-4 years	Data Rich	-1	2	Proportion of the population with at least 12 years of education, maternal	928
Hemolytic disease and other neonatal jaundice	Female	0-6 days	2-4 years	Data Rich	1	2	Live Births 35+ (proportion)	--
Hemolytic disease and other neonatal jaundice	Female	0-6 days	2-4 years	Data Rich	-1	3	Antenatal Care (4 visits) Coverage (proportion)	13
Hemolytic disease and other neonatal jaundice	Female	0-6 days	2-4 years	Data Rich	-1	3	Antenatal Care (1 visit) Coverage (proportion)	70
Hemolytic disease and other neonatal jaundice	Female	0-6 days	2-4 years	Data Rich	-1	3	Skilled Birth Attendance (proportion)	209
Hemolytic disease and other neonatal jaundice	Female	0-6 days	2-4 years	Data Rich	-1	3	In-Facility Delivery (proportion)	234
Hemolytic disease and other neonatal jaundice	Female	0-6 days	2-4 years	Data Rich	-1	3	LDI (I$ per capita)	--
Hemolytic disease and other neonatal jaundice	Female	0-6 days	2-4 years	Data Rich	1	3	Total Fertility Rate	--
Hemolytic disease and other neonatal jaundice	Female	0-6 days	2-4 years	Global	-1	1	Maternal care and immunization	335
Hemolytic disease and other neonatal jaundice	Female	0-6 days	2-4 years	Global	1	1	Age-standardized SEV for Short gestation	2
Hemolytic disease and other neonatal jaundice	Female	0-6 days	2-4 years	Global	1	1	Age-standardized SEV for Ambient particulate matter	96
Hemolytic disease and other neonatal jaundice	Female	0-6 days	2-4 years	Global	1	1	Age-standardized SEV for Household air pollution	243
Hemolytic disease and other neonatal jaundice	Female	0-6 days	2-4 years	Global	1	1	Age-standardized SEV for Smoking	850
Hemolytic disease and other neonatal jaundice	Female	0-6 days	2-4 years	Global	1	1	Age-standardized SEV for Low birth weight	--
Hemolytic disease and other neonatal jaundice	Female	0-6 days	2-4 years	Global	-1	2	Socio-demographic Index	170
Hemolytic disease and other neonatal jaundice	Female	0-6 days	2-4 years	Global	-1	2	Proportion of the population with at least 6 years of education, maternal	230
Hemolytic disease and other neonatal jaundice	Female	0-6 days	2-4 years	Global	-1	2	Healthcare access and quality index	334
Hemolytic disease and other neonatal jaundice	Female	0-6 days	2-4 years	Global	-1	2	Proportion of the population with at least 12 years of education, maternal	385
Hemolytic disease and other neonatal jaundice	Female	0-6 days	2-4 years	Global	1	2	Live Births 35+ (proportion)	--
Hemolytic disease and other neonatal jaundice	Female	0-6 days	2-4 years	Global	-1	3	Skilled Birth Attendance (proportion)	29
Hemolytic disease and other neonatal jaundice	Female	0-6 days	2-4 years	Global	-1	3	Antenatal Care (4 visits) Coverage (proportion)	55
Hemolytic disease and other neonatal jaundice	Female	0-6 days	2-4 years	Global	-1	3	Antenatal Care (1 visit) Coverage (proportion)	75

CODEm covariates used, level of covariate, and expected direction of covariate by cause, sex, and age								
Cause	Sex	Age Start	Age End	Model Version Type	Direction	Level	Covariate Name	Number of Draws
Hemolytic disease and other neonatal jaundice	Female	0-6 days	2-4 years	Global	-1	3	In-Facility Delivery (proportion)	78
Hemolytic disease and other neonatal jaundice	Female	0-6 days	2-4 years	Global	-1	3	LDI (I$ per capita)	--
Hemolytic disease and other neonatal jaundice	Female	0-6 days	2-4 years	Global	1	3	Total Fertility Rate	--
Hemolytic disease and other neonatal jaundice	Male	0-6 days	2-4 years	Data Rich	-1	1	Maternal care and immunization	527
Hemolytic disease and other neonatal jaundice	Male	0-6 days	2-4 years	Data Rich	1	1	Age-standardized SEV for Short gestation	0
Hemolytic disease and other neonatal jaundice	Male	0-6 days	2-4 years	Data Rich	1	1	Age-standardized SEV for Low birth weight	1
Hemolytic disease and other neonatal jaundice	Male	0-6 days	2-4 years	Data Rich	1	1	Age-standardized SEV for Ambient particulate matter	65
Hemolytic disease and other neonatal jaundice	Male	0-6 days	2-4 years	Data Rich	1	1	Age-standardized SEV for Household air pollution	439
Hemolytic disease and other neonatal jaundice	Male	0-6 days	2-4 years	Data Rich	1	1	Age-standardized SEV for Smoking	719
Hemolytic disease and other neonatal jaundice	Male	0-6 days	2-4 years	Data Rich	-1	2	Proportion of the population with at least 6 years of education, maternal	95
Hemolytic disease and other neonatal jaundice	Male	0-6 days	2-4 years	Data Rich	-1	2	Socio-demographic Index	130
Hemolytic disease and other neonatal jaundice	Male	0-6 days	2-4 years	Data Rich	-1	2	Healthcare access and quality index	222
Hemolytic disease and other neonatal jaundice	Male	0-6 days	2-4 years	Data Rich	-1	2	Proportion of the population with at least 12 years of education, maternal	619
Hemolytic disease and other neonatal jaundice	Male	0-6 days	2-4 years	Data Rich	1	2	Live Births 35+ (proportion)	--
Hemolytic disease and other neonatal jaundice	Male	0-6 days	2-4 years	Data Rich	-1	3	Antenatal Care (1 visit) Coverage (proportion)	14
Hemolytic disease and other neonatal jaundice	Male	0-6 days	2-4 years	Data Rich	-1	3	Antenatal Care (4 visits) Coverage (proportion)	30
Hemolytic disease and other neonatal jaundice	Male	0-6 days	2-4 years	Data Rich	-1	3	Skilled Birth Attendance (proportion)	221
Hemolytic disease and other neonatal jaundice	Male	0-6 days	2-4 years	Data Rich	-1	3	In-Facility Delivery (proportion)	235
Hemolytic disease and other neonatal jaundice	Male	0-6 days	2-4 years	Data Rich	-1	3	LDI (I$ per capita)	--
Hemolytic disease and other neonatal jaundice	Male	0-6 days	2-4 years	Data Rich	1	3	Total Fertility Rate	--
Hemolytic disease and other neonatal jaundice	Male	0-6 days	2-4 years	Global	-1	1	Maternal care and immunization	487
Hemolytic disease and other neonatal jaundice	Male	0-6 days	2-4 years	Global	1	1	Age-standardized SEV for Short gestation	29
Hemolytic disease and other neonatal jaundice	Male	0-6 days	2-4 years	Global	1	1	Age-standardized SEV for Ambient particulate matter	31
Hemolytic disease and other neonatal jaundice	Male	0-6 days	2-4 years	Global	1	1	Age-standardized SEV for Low birth weight	39
Hemolytic disease and other neonatal jaundice	Male	0-6 days	2-4 years	Global	1	1	Age-standardized SEV for Household air pollution	326
Hemolytic disease and other neonatal jaundice	Male	0-6 days	2-4 years	Global	1	1	Age-standardized SEV for Smoking	816
Hemolytic disease and other neonatal jaundice	Male	0-6 days	2-4 years	Global	-1	2	Healthcare access and quality index	106
Hemolytic disease and other neonatal jaundice	Male	0-6 days	2-4 years	Global	-1	2	Socio-demographic Index	119
Hemolytic disease and other neonatal jaundice	Male	0-6 days	2-4 years	Global	-1	2	Proportion of the population with at least 6 years of education, maternal	276
Hemolytic disease and other neonatal jaundice	Male	0-6 days	2-4 years	Global	-1	2	Proportion of the population with at least 12 years of education, maternal	366
Hemolytic disease and other neonatal jaundice	Male	0-6 days	2-4 years	Global	1	2	Live Births 35+ (proportion)	--
Hemolytic disease and other neonatal jaundice	Male	0-6 days	2-4 years	Global	-1	3	Antenatal Care (4 visits) Coverage (proportion)	91
Hemolytic disease and other neonatal jaundice	Male	0-6 days	2-4 years	Global	-1	3	Antenatal Care (1 visit) Coverage (proportion)	93
Hemolytic disease and other neonatal jaundice	Male	0-6 days	2-4 years	Global	-1	3	Skilled Birth Attendance (proportion)	93
Hemolytic disease and other neonatal jaundice	Male	0-6 days	2-4 years	Global	-1	3	In-Facility Delivery (proportion)	191
Hemolytic disease and other neonatal jaundice	Male	0-6 days	2-4 years	Global	-1	3	LDI (I$ per capita)	--
Hemolytic disease and other neonatal jaundice	Male	0-6 days	2-4 years	Global	1	3	Total Fertility Rate	--
Other neonatal disorders	Female	0-6 days	2-4 years	Data Rich	-1	1	Maternal care and immunization	1
Other neonatal disorders	Female	0-6 days	2-4 years	Data Rich	1	1	Age-standardized SEV for Smoking	0
Other neonatal disorders	Female	0-6 days	2-4 years	Data Rich	1	1	Age-standardized SEV for Household air pollution	1
Other neonatal disorders	Female	0-6 days	2-4 years	Data Rich	1	1	Age-standardized SEV for Low birth weight	180
Other neonatal disorders	Female	0-6 days	2-4 years	Data Rich	1	1	Age-standardized SEV for Short gestation	195
Other neonatal disorders	Female	0-6 days	2-4 years	Data Rich	1	1	Age-standardized SEV for Ambient particulate matter	968
Other neonatal disorders	Female	0-6 days	2-4 years	Data Rich	-1	2	Proportion of the population with at least 6 years of education, maternal	1
Other neonatal disorders	Female	0-6 days	2-4 years	Data Rich	-1	2	Proportion of the population with at least 12 years of education, maternal	139
Other neonatal disorders	Female	0-6 days	2-4 years	Data Rich	-1	2	Healthcare access and quality index	211
Other neonatal disorders	Female	0-6 days	2-4 years	Data Rich	-1	2	Socio-demographic Index	439
Other neonatal disorders	Female	0-6 days	2-4 years	Data Rich	1	2	Live Births 35+ (proportion)	138
Other neonatal disorders	Female	0-6 days	2-4 years	Data Rich	-1	3	Skilled Birth Attendance (proportion)	3
Other neonatal disorders	Female	0-6 days	2-4 years	Data Rich	-1	3	Antenatal Care (1 visit) Coverage (proportion)	47
Other neonatal disorders	Female	0-6 days	2-4 years	Data Rich	-1	3	Antenatal Care (4 visits) Coverage (proportion)	60
Other neonatal disorders	Female	0-6 days	2-4 years	Data Rich	-1	3	In-Facility Delivery (proportion)	322
Other neonatal disorders	Female	0-6 days	2-4 years	Data Rich	-1	3	LDI (I$ per capita)	--
Other neonatal disorders	Female	0-6 days	2-4 years	Data Rich	1	3	Total Fertility Rate	--
Other neonatal disorders	Female	0-6 days	2-4 years	Global	-1	1	Maternal care and immunization	9
Other neonatal disorders	Female	0-6 days	2-4 years	Global	1	1	Age-standardized SEV for Smoking	0
Other neonatal disorders	Female	0-6 days	2-4 years	Global	1	1	Age-standardized SEV for Short gestation	179
Other neonatal disorders	Female	0-6 days	2-4 years	Global	1	1	Age-standardized SEV for Household air pollution	287
Other neonatal disorders	Female	0-6 days	2-4 years	Global	1	1	Age-standardized SEV for Ambient particulate matter	692
Other neonatal disorders	Female	0-6 days	2-4 years	Global	1	1	Age-standardized SEV for Low birth weight	--
Other neonatal disorders	Female	0-6 days	2-4 years	Global	-1	2	Proportion of the population with at least 6 years of education, maternal	0
Other neonatal disorders	Female	0-6 days	2-4 years	Global	-1	2	Socio-demographic Index	169
Other neonatal disorders	Female	0-6 days	2-4 years	Global	-1	2	Proportion of the population with at least 12 years of education, maternal	430
Other neonatal disorders	Female	0-6 days	2-4 years	Global	-1	2	Healthcare access and quality index	--
Other neonatal disorders	Female	0-6 days	2-4 years	Global	1	2	Live Births 35+ (proportion)	234
Other neonatal disorders	Female	0-6 days	2-4 years	Global	-1	3	Skilled Birth Attendance (proportion)	2
Other neonatal disorders	Female	0-6 days	2-4 years	Global	-1	3	In-Facility Delivery (proportion)	88
Other neonatal disorders	Female	0-6 days	2-4 years	Global	-1	3	Antenatal Care (1 visit) Coverage (proportion)	121
Other neonatal disorders	Female	0-6 days	2-4 years	Global	-1	3	Antenatal Care (4 visits) Coverage (proportion)	243
Other neonatal disorders	Female	0-6 days	2-4 years	Global	-1	3	LDI (I$ per capita)	--
Other neonatal disorders	Female	0-6 days	2-4 years	Global	1	3	Total Fertility Rate	--
Other neonatal disorders	Male	0-6 days	2-4 years	Data Rich	-1	1	Maternal care and immunization	0
Other neonatal disorders	Male	0-6 days	2-4 years	Data Rich	1	1	Age-standardized SEV for Household air pollution	1
Other neonatal disorders	Male	0-6 days	2-4 years	Data Rich	1	1	Age-standardized SEV for Smoking	2
Other neonatal disorders	Male	0-6 days	2-4 years	Data Rich	1	1	Age-standardized SEV for Low birth weight	137
Other neonatal disorders	Male	0-6 days	2-4 years	Data Rich	1	1	Age-standardized SEV for Short gestation	214
Other neonatal disorders	Male	0-6 days	2-4 years	Data Rich	1	1	Age-standardized SEV for Ambient particulate matter	871
Other neonatal disorders	Male	0-6 days	2-4 years	Data Rich	-1	2	Proportion of the population with at least 6 years of education, maternal	0
Other neonatal disorders	Male	0-6 days	2-4 years	Data Rich	-1	2	Healthcare access and quality index	115
Other neonatal disorders	Male	0-6 days	2-4 years	Data Rich	-1	2	Proportion of the population with at least 12 years of education, maternal	191
Other neonatal disorders	Male	0-6 days	2-4 years	Data Rich	-1	2	Socio-demographic Index	404
Other neonatal disorders	Male	0-6 days	2-4 years	Data Rich	1	2	Live Births 35+ (proportion)	138
Other neonatal disorders	Male	0-6 days	2-4 years	Data Rich	-1	3	Skilled Birth Attendance (proportion)	0
Other neonatal disorders	Male	0-6 days	2-4 years	Data Rich	-1	3	Antenatal Care (1 visit) Coverage (proportion)	8
Other neonatal disorders	Male	0-6 days	2-4 years	Data Rich	-1	3	Antenatal Care (4 visits) Coverage (proportion)	34
Other neonatal disorders	Male	0-6 days	2-4 years	Data Rich	-1	3	In-Facility Delivery (proportion)	384
Other neonatal disorders	Male	0-6 days	2-4 years	Data Rich	-1	3	LDI (I$ per capita)	--
Other neonatal disorders	Male	0-6 days	2-4 years	Data Rich	1	3	Total Fertility Rate	--
Other neonatal disorders	Male	0-6 days	2-4 years	Global	-1	1	Maternal care and immunization	14
Other neonatal disorders	Male	0-6 days	2-4 years	Global	1	1	Age-standardized SEV for Smoking	0
Other neonatal disorders	Male	0-6 days	2-4 years	Global	1	1	Age-standardized SEV for Short gestation	178
Other neonatal disorders	Male	0-6 days	2-4 years	Global	1	1	Age-standardized SEV for Household air pollution	277
Other neonatal disorders	Male	0-6 days	2-4 years	Global	1	1	Age-standardized SEV for Ambient particulate matter	700
Other neonatal disorders	Male	0-6 days	2-4 years	Global	1	1	Age-standardized SEV for Low birth weight	--
Other neonatal disorders	Male	0-6 days	2-4 years	Global	-1	2	Proportion of the population with at least 6 years of education, maternal	0
Other neonatal disorders	Male	0-6 days	2-4 years	Global	-1	2	Socio-demographic Index	174
Other neonatal disorders	Male	0-6 days	2-4 years	Global	-1	2	Proportion of the population with at least 12 years of education, maternal	455
Other neonatal disorders	Male	0-6 days	2-4 years	Global	-1	2	Healthcare access and quality index	--
Other neonatal disorders	Male	0-6 days	2-4 years	Global	1	2	Live Births 35+ (proportion)	234
Other neonatal disorders	Male	0-6 days	2-4 years	Global	-1	3	Skilled Birth Attendance (proportion)	50
Other neonatal disorders	Male	0-6 days	2-4 years	Global	-1	3	In-Facility Delivery (proportion)	84
Other neonatal disorders	Male	0-6 days	2-4 years	Global	-1	3	Antenatal Care (1 visit) Coverage (proportion)	87
Other neonatal disorders	Male	0-6 days	2-4 years	Global	-1	3	Antenatal Care (4 visits) Coverage (proportion)	160
Other neonatal disorders	Male	0-6 days	2-4 years	Global	-1	3	LDI (I$ per capita)	--
Other neonatal disorders	Male	0-6 days	2-4 years	Global	1	3	Total Fertility Rate	--
Nutritional deficiencies	Female	1-5 months	95+ years	Data Rich	-1	1	Proportion of households using iodized salt (adjusted)	137
Nutritional deficiencies	Female	1-5 months	95+ years	Data Rich	-1	1	energy unadjusted(kcal)	249

CODEm covariates used, level of covariate, and expected direction of covariate by cause, sex, and age

Cause	Sex	Age Start	Age End	Model Version Type	Direction	Level	Covariate Name	Number of Draws
Nutritional deficiencies	Female	1-5 months	95+ years	Data Rich	1	1	Age-Standardize Prevalence of Severe Anemia	0
Nutritional deficiencies	Female	1-5 months	95+ years	Data Rich	1	1	Log-transformed SEV scalar: Diarrhea	22
Nutritional deficiencies	Female	1-5 months	95+ years	Data Rich	1	1	Age-standardized SEV for Child underweight	147
Nutritional deficiencies	Female	1-5 months	95+ years	Data Rich	1	1	Age-standardized SEV for Child wasting	547
Nutritional deficiencies	Female	1-5 months	95+ years	Data Rich	-1	2	Healthcare access and quality index	0
Nutritional deficiencies	Female	1-5 months	95+ years	Data Rich	-1	2	Improved Water Source (proportion with access)	0
Nutritional deficiencies	Female	1-5 months	95+ years	Data Rich	-1	2	Maternal care and immunization	0
Nutritional deficiencies	Female	1-5 months	95+ years	Data Rich	-1	2	Sanitation (proportion with access)	7
Nutritional deficiencies	Female	1-5 months	95+ years	Data Rich	-1	2	Rainfall Quintile 1 (proportion)	--
Nutritional deficiencies	Female	1-5 months	95+ years	Data Rich	1	2	Rainfall Quintile 2 (proportion)	0
Nutritional deficiencies	Female	1-5 months	95+ years	Data Rich	1	2	Mortality Rate Due to Death Shocks in Last 10 Years (per 1 person)	--
Nutritional deficiencies	Female	1-5 months	95+ years	Data Rich	-1	3	Maternal Education (years per capita)	4
Nutritional deficiencies	Female	1-5 months	95+ years	Data Rich	-1	3	Education (years per capita)	61
Nutritional deficiencies	Female	1-5 months	95+ years	Data Rich	-1	3	Socio-demographic Index	188
Nutritional deficiencies	Female	1-5 months	95+ years	Data Rich	-1	3	LDI (I$ per capita)	--
Nutritional deficiencies	Female	1-5 months	95+ years	Data Rich	1	3	Age- and sex-specific SEV for Alcohol use	--
Nutritional deficiencies	Female	1-5 months	95+ years	Global	-1	1	energy unadjusted(kcal)	133
Nutritional deficiencies	Female	1-5 months	95+ years	Global	-1	1	Proportion of households using iodized salt (adjusted)	293
Nutritional deficiencies	Female	1-5 months	95+ years	Global	1	1	Age-standardized SEV for Child wasting	107
Nutritional deficiencies	Female	1-5 months	95+ years	Global	1	1	Log-transformed SEV scalar: Diarrhea	519
Nutritional deficiencies	Female	1-5 months	95+ years	Global	1	1	Age-Standardize Prevalence of Severe Anemia	621
Nutritional deficiencies	Female	1-5 months	95+ years	Global	1	1	Age-standardized SEV for Child underweight	985
Nutritional deficiencies	Female	1-5 months	95+ years	Global	-1	2	Healthcare access and quality index	0
Nutritional deficiencies	Female	1-5 months	95+ years	Global	-1	2	Socio-demographic Index	8
Nutritional deficiencies	Female	1-5 months	95+ years	Global	-1	2	Maternal care and immunization	30
Nutritional deficiencies	Female	1-5 months	95+ years	Global	1	2	Age- and sex-specific SEV for Unsafe sanitation	39
Nutritional deficiencies	Female	1-5 months	95+ years	Global	1	2	Rainfall Quintile 1 (proportion)	52
Nutritional deficiencies	Female	1-5 months	95+ years	Global	1	2	Age- and sex-specific SEV for Unsafe water	79
Nutritional deficiencies	Female	1-5 months	95+ years	Global	1	2	Rainfall Quintile 2 (proportion)	156
Nutritional deficiencies	Female	1-5 months	95+ years	Global	1	2	Mortality Rate Due to Death Shocks in Last 10 Years (per 1 person)	--
Nutritional deficiencies	Female	1-5 months	95+ years	Global	-1	3	Maternal Education (years per capita)	0
Nutritional deficiencies	Female	1-5 months	95+ years	Global	-1	3	Education (years per capita)	37
Nutritional deficiencies	Female	1-5 months	95+ years	Global	-1	3	LDI (I$ per capita)	--
Nutritional deficiencies	Female	1-5 months	95+ years	Global	1	3	Age- and sex-specific SEV for Alcohol use	1
Nutritional deficiencies	Male	1-5 months	95+ years	Data Rich	-1	1	Proportion of households using iodized salt (adjusted)	218
Nutritional deficiencies	Male	1-5 months	95+ years	Data Rich	-1	1	energy unadjusted(kcal)	586
Nutritional deficiencies	Male	1-5 months	95+ years	Data Rich	1	1	Age-Standardize Prevalence of Severe Anemia	0
Nutritional deficiencies	Male	1-5 months	95+ years	Data Rich	1	1	Age-standardized SEV for Child underweight	77
Nutritional deficiencies	Male	1-5 months	95+ years	Data Rich	1	1	Log-transformed SEV scalar: Diarrhea	96
Nutritional deficiencies	Male	1-5 months	95+ years	Data Rich	1	1	Age-standardized SEV for Child wasting	402
Nutritional deficiencies	Male	1-5 months	95+ years	Data Rich	-1	2	Maternal care and immunization	0
Nutritional deficiencies	Male	1-5 months	95+ years	Data Rich	-1	2	Sanitation (proportion with access)	27
Nutritional deficiencies	Male	1-5 months	95+ years	Data Rich	-1	2	Healthcare access and quality index	36
Nutritional deficiencies	Male	1-5 months	95+ years	Data Rich	-1	2	Improved Water Source (proportion with access)	105
Nutritional deficiencies	Male	1-5 months	95+ years	Data Rich	-1	2	Rainfall Quintile 1 (proportion)	--
Nutritional deficiencies	Male	1-5 months	95+ years	Data Rich	1	2	Rainfall Quintile 2 (proportion)	45
Nutritional deficiencies	Male	1-5 months	95+ years	Data Rich	1	2	Mortality Rate Due to Death Shocks in Last 10 Years (per 1 person)	--
Nutritional deficiencies	Male	1-5 months	95+ years	Data Rich	-1	3	Maternal Education (years per capita)	0
Nutritional deficiencies	Male	1-5 months	95+ years	Data Rich	-1	3	Education (years per capita)	60
Nutritional deficiencies	Male	1-5 months	95+ years	Data Rich	-1	3	Socio-demographic Index	61
Nutritional deficiencies	Male	1-5 months	95+ years	Data Rich	-1	3	LDI (I$ per capita)	--
Nutritional deficiencies	Male	1-5 months	95+ years	Data Rich	1	3	Age- and sex-specific SEV for Alcohol use	--
Nutritional deficiencies	Male	1-5 months	95+ years	Global	-1	1	Proportion of households using iodized salt (adjusted)	2
Nutritional deficiencies	Male	1-5 months	95+ years	Global	-1	1	energy unadjusted(kcal)	203
Nutritional deficiencies	Male	1-5 months	95+ years	Global	1	1	Age-standardized SEV for Child wasting	214
Nutritional deficiencies	Male	1-5 months	95+ years	Global	1	1	Log-transformed SEV scalar: Diarrhea	223
Nutritional deficiencies	Male	1-5 months	95+ years	Global	1	1	Age-Standardize Prevalence of Severe Anemia	485
Nutritional deficiencies	Male	1-5 months	95+ years	Global	1	1	Age-standardized SEV for Child underweight	980
Nutritional deficiencies	Male	1-5 months	95+ years	Global	-1	2	Healthcare access and quality index	0
Nutritional deficiencies	Male	1-5 months	95+ years	Global	-1	2	Socio-demographic Index	0
Nutritional deficiencies	Male	1-5 months	95+ years	Global	-1	2	Maternal care and immunization	15
Nutritional deficiencies	Male	1-5 months	95+ years	Global	1	2	Rainfall Quintile 2 (proportion)	0
Nutritional deficiencies	Male	1-5 months	95+ years	Global	1	2	Age- and sex-specific SEV for Unsafe sanitation	15
Nutritional deficiencies	Male	1-5 months	95+ years	Global	1	2	Rainfall Quintile 1 (proportion)	180
Nutritional deficiencies	Male	1-5 months	95+ years	Global	1	2	Age- and sex-specific SEV for Unsafe water	373
Nutritional deficiencies	Male	1-5 months	95+ years	Global	1	2	Mortality Rate Due to Death Shocks in Last 10 Years (per 1 person)	--
Nutritional deficiencies	Male	1-5 months	95+ years	Global	-1	3	Maternal Education (years per capita)	0
Nutritional deficiencies	Male	1-5 months	95+ years	Global	-1	3	Education (years per capita)	29
Nutritional deficiencies	Male	1-5 months	95+ years	Global	-1	3	LDI (I$ per capita)	--
Nutritional deficiencies	Male	1-5 months	95+ years	Global	1	3	Age- and sex-specific SEV for Alcohol use	--
Protein-energy malnutrition	Female	1-5 months	2-4 years	Data Rich	-1	1	energy unadjusted(kcal)	--
Protein-energy malnutrition	Female	1-5 months	2-4 years	Data Rich	1	1	Age-Standardize Prevalence of Severe Anemia	80
Protein-energy malnutrition	Female	1-5 months	2-4 years	Data Rich	1	1	Log-transformed SEV scalar: Diarrhea	241
Protein-energy malnutrition	Female	1-5 months	2-4 years	Data Rich	1	1	Age- and sex-specific SEV for Child wasting	262
Protein-energy malnutrition	Female	1-5 months	2-4 years	Data Rich	-1	2	Healthcare access and quality index	19
Protein-energy malnutrition	Female	1-5 months	2-4 years	Data Rich	-1	2	Maternal care and immunization	39
Protein-energy malnutrition	Female	1-5 months	2-4 years	Data Rich	-1	2	Socio-demographic Index	70
Protein-energy malnutrition	Female	1-5 months	2-4 years	Data Rich	1	2	Age- and sex-specific SEV for Unsafe sanitation	2
Protein-energy malnutrition	Female	1-5 months	2-4 years	Data Rich	1	2	Age- and sex-specific SEV for Unsafe water	84
Protein-energy malnutrition	Female	1-5 months	2-4 years	Data Rich	1	2	Rainfall Quintile 1 (proportion)	220
Protein-energy malnutrition	Female	1-5 months	2-4 years	Data Rich	1	2	Rainfall Quintile 2 (proportion)	243
Protein-energy malnutrition	Female	1-5 months	2-4 years	Data Rich	1	2	Mortality Rate Due to Death Shocks in Last 10 Years (per 1 person)	--
Protein-energy malnutrition	Female	1-5 months	2-4 years	Data Rich	-1	3	Education (years per capita)	151
Protein-energy malnutrition	Female	1-5 months	2-4 years	Data Rich	-1	3	LDI (I$ per capita)	--
Protein-energy malnutrition	Female	1-5 months	2-4 years	Data Rich	1	3	Age- and sex-specific SEV for Alcohol use	--
Protein-energy malnutrition	Female	1-5 months	2-4 years	Global	-1	1	energy unadjusted(kcal)	--
Protein-energy malnutrition	Female	1-5 months	2-4 years	Global	1	1	Age-Standardize Prevalence of Severe Anemia	228
Protein-energy malnutrition	Female	1-5 months	2-4 years	Global	1	1	Age- and sex-specific SEV for Child wasting	348
Protein-energy malnutrition	Female	1-5 months	2-4 years	Global	1	1	Log-transformed SEV scalar: Diarrhea	569
Protein-energy malnutrition	Female	1-5 months	2-4 years	Global	-1	2	Maternal care and immunization	2
Protein-energy malnutrition	Female	1-5 months	2-4 years	Global	-1	2	Socio-demographic Index	41
Protein-energy malnutrition	Female	1-5 months	2-4 years	Global	-1	2	Healthcare access and quality index	218
Protein-energy malnutrition	Female	1-5 months	2-4 years	Global	1	2	Age- and sex-specific SEV for Unsafe water	41
Protein-energy malnutrition	Female	1-5 months	2-4 years	Global	1	2	Rainfall Quintile 2 (proportion)	150
Protein-energy malnutrition	Female	1-5 months	2-4 years	Global	1	2	Age- and sex-specific SEV for Unsafe sanitation	155
Protein-energy malnutrition	Female	1-5 months	2-4 years	Global	1	2	Rainfall Quintile 1 (proportion)	215
Protein-energy malnutrition	Female	1-5 months	2-4 years	Global	1	2	Mortality Rate Due to Death Shocks in Last 10 Years (per 1 person)	--
Protein-energy malnutrition	Female	1-5 months	2-4 years	Global	-1	3	Education (years per capita)	22
Protein-energy malnutrition	Female	1-5 months	2-4 years	Global	-1	3	LDI (I$ per capita)	--
Protein-energy malnutrition	Female	1-5 months	2-4 years	Global	1	3	Age- and sex-specific SEV for Alcohol use	--
Protein-energy malnutrition	Female	5-9 years	95+ years	Data Rich	-1	1	energy unadjusted(kcal)	--
Protein-energy malnutrition	Female	5-9 years	95+ years	Data Rich	1	1	Age-standardized SEV for Child wasting	104
Protein-energy malnutrition	Female	5-9 years	95+ years	Data Rich	1	1	Log-transformed SEV scalar: Diarrhea	221
Protein-energy malnutrition	Female	5-9 years	95+ years	Data Rich	1	1	Age-Standardize Prevalence of Severe Anemia	488
Protein-energy malnutrition	Female	5-9 years	95+ years	Data Rich	1	1	Malnutrition Shock mortality rate	--
Protein-energy malnutrition	Female	5-9 years	95+ years	Data Rich	-1	2	Healthcare access and quality index	11
Protein-energy malnutrition	Female	5-9 years	95+ years	Data Rich	-1	2	Maternal care and immunization	145
Protein-energy malnutrition	Female	5-9 years	95+ years	Data Rich	-1	2	Rainfall Quintile 1 (proportion)	--
Protein-energy malnutrition	Female	5-9 years	95+ years	Data Rich	1	2	Age- and sex-specific SEV for Unsafe water	174
Protein-energy malnutrition	Female	5-9 years	95+ years	Data Rich	1	2	Rainfall Quintile 2 (proportion)	217
Protein-energy malnutrition	Female	5-9 years	95+ years	Data Rich	1	2	Age- and sex-specific SEV for Unsafe sanitation	--
Protein-energy malnutrition	Female	5-9 years	95+ years	Data Rich	1	2	Mortality Rate Due to Death Shocks in Last 10 Years (per 1 person)	--

CODEm covariates used, level of covariate, and expected direction of covariate by cause, sex, and age								
Cause	Sex	Age Start	Age End	Model Version Type	Direction	Level	Covariate Name	Number of Draws
Protein-energy malnutrition	Female	5-9 years	95+ years	Data Rich	-1	3	Socio-demographic Index	19
Protein-energy malnutrition	Female	5-9 years	95+ years	Data Rich	-1	3	Education (years per capita)	34
Protein-energy malnutrition	Female	5-9 years	95+ years	Data Rich	-1	3	Maternal Education (years per capita)	42
Protein-energy malnutrition	Female	5-9 years	95+ years	Data Rich	-1	3	LDI (I$ per capita)	--
Protein-energy malnutrition	Female	5-9 years	95+ years	Data Rich	1	3	Age- and sex-specific SEV for Alcohol use	--
Protein-energy malnutrition	Female	5-9 years	95+ years	Global	-1	1	energy unadjusted(kcal)	--
Protein-energy malnutrition	Female	5-9 years	95+ years	Global	1	1	Log-transformed SEV scalar: Diarrhea	667
Protein-energy malnutrition	Female	5-9 years	95+ years	Global	1	1	Age-Standardize Prevalence of Severe Anemia	749
Protein-energy malnutrition	Female	5-9 years	95+ years	Global	-1	2	Socio-demographic Index	0
Protein-energy malnutrition	Female	5-9 years	95+ years	Global	-1	2	Healthcare access and quality index	1
Protein-energy malnutrition	Female	5-9 years	95+ years	Global	-1	2	Maternal care and immunization	4
Protein-energy malnutrition	Female	5-9 years	95+ years	Global	1	2	Mortality Rate Due to Death Shocks in Last 10 Years (per 1 person)	14
Protein-energy malnutrition	Female	5-9 years	95+ years	Global	1	2	Age- and sex-specific SEV for Unsafe water	94
Protein-energy malnutrition	Female	5-9 years	95+ years	Global	1	2	Age- and sex-specific SEV for Unsafe sanitation	96
Protein-energy malnutrition	Female	5-9 years	95+ years	Global	1	2	Rainfall Quintile 2 (proportion)	114
Protein-energy malnutrition	Female	5-9 years	95+ years	Global	1	2	Rainfall Quintile 1 (proportion)	169
Protein-energy malnutrition	Female	5-9 years	95+ years	Global	-1	3	Maternal Education (years per capita)	23
Protein-energy malnutrition	Female	5-9 years	95+ years	Global	-1	3	Education (years per capita)	28
Protein-energy malnutrition	Female	5-9 years	95+ years	Global	-1	3	LDI (I$ per capita)	--
Protein-energy malnutrition	Female	5-9 years	95+ years	Global	1	3	Age- and sex-specific SEV for Alcohol use	5
Protein-energy malnutrition	Male	1-5 months	2-4 years	Data Rich	-1	1	energy unadjusted(kcal)	--
Protein-energy malnutrition	Male	1-5 months	2-4 years	Data Rich	1	1	Age-Standardize Prevalence of Severe Anemia	197
Protein-energy malnutrition	Male	1-5 months	2-4 years	Data Rich	1	1	Age- and sex-specific SEV for Child wasting	398
Protein-energy malnutrition	Male	1-5 months	2-4 years	Data Rich	1	1	Log-transformed SEV scalar: Diarrhea	435
Protein-energy malnutrition	Male	1-5 months	2-4 years	Data Rich	-1	2	Healthcare access and quality index	20
Protein-energy malnutrition	Male	1-5 months	2-4 years	Data Rich	-1	2	Socio-demographic Index	70
Protein-energy malnutrition	Male	1-5 months	2-4 years	Data Rich	-1	2	Maternal care and immunization	121
Protein-energy malnutrition	Male	1-5 months	2-4 years	Data Rich	1	2	Age- and sex-specific SEV for Unsafe water	49
Protein-energy malnutrition	Male	1-5 months	2-4 years	Data Rich	1	2	Rainfall Quintile 1 (proportion)	57
Protein-energy malnutrition	Male	1-5 months	2-4 years	Data Rich	1	2	Age- and sex-specific SEV for Unsafe sanitation	111
Protein-energy malnutrition	Male	1-5 months	2-4 years	Data Rich	1	2	Rainfall Quintile 2 (proportion)	208
Protein-energy malnutrition	Male	1-5 months	2-4 years	Data Rich	1	2	Mortality Rate Due to Death Shocks in Last 10 Years (per 1 person)	--
Protein-energy malnutrition	Male	1-5 months	2-4 years	Data Rich	-1	3	Education (years per capita)	46
Protein-energy malnutrition	Male	1-5 months	2-4 years	Data Rich	-1	3	LDI (I$ per capita)	--
Protein-energy malnutrition	Male	1-5 months	2-4 years	Data Rich	1	3	Age- and sex-specific SEV for Alcohol use	--
Protein-energy malnutrition	Male	1-5 months	2-4 years	Global	-1	1	energy unadjusted(kcal)	--
Protein-energy malnutrition	Male	1-5 months	2-4 years	Global	1	1	Age- and sex-specific SEV for Child wasting	323
Protein-energy malnutrition	Male	1-5 months	2-4 years	Global	1	1	Age-Standardize Prevalence of Severe Anemia	570
Protein-energy malnutrition	Male	1-5 months	2-4 years	Global	1	1	Log-transformed SEV scalar: Diarrhea	916
Protein-energy malnutrition	Male	1-5 months	2-4 years	Global	-1	2	Socio-demographic Index	2
Protein-energy malnutrition	Male	1-5 months	2-4 years	Global	-1	2	Healthcare access and quality index	55
Protein-energy malnutrition	Male	1-5 months	2-4 years	Global	-1	2	Maternal care and immunization	85
Protein-energy malnutrition	Male	1-5 months	2-4 years	Global	1	2	Age- and sex-specific SEV for Unsafe sanitation	4
Protein-energy malnutrition	Male	1-5 months	2-4 years	Global	1	2	Age- and sex-specific SEV for Unsafe water	6
Protein-energy malnutrition	Male	1-5 months	2-4 years	Global	1	2	Rainfall Quintile 1 (proportion)	143
Protein-energy malnutrition	Male	1-5 months	2-4 years	Global	1	2	Rainfall Quintile 2 (proportion)	173
Protein-energy malnutrition	Male	1-5 months	2-4 years	Global	1	2	Mortality Rate Due to Death Shocks in Last 10 Years (per 1 person)	--
Protein-energy malnutrition	Male	1-5 months	2-4 years	Global	-1	3	Education (years per capita)	33
Protein-energy malnutrition	Male	1-5 months	2-4 years	Global	-1	3	LDI (I$ per capita)	--
Protein-energy malnutrition	Male	1-5 months	2-4 years	Global	1	3	Age- and sex-specific SEV for Alcohol use	--
Protein-energy malnutrition	Male	5-9 years	95+ years	Data Rich	-1	1	energy unadjusted(kcal)	--
Protein-energy malnutrition	Male	5-9 years	95+ years	Data Rich	1	1	Age-Standardize Prevalence of Severe Anemia	0
Protein-energy malnutrition	Male	5-9 years	95+ years	Data Rich	1	1	Log-transformed SEV scalar: Diarrhea	247
Protein-energy malnutrition	Male	5-9 years	95+ years	Data Rich	1	1	Age-standardized SEV for Child wasting	764
Protein-energy malnutrition	Male	5-9 years	95+ years	Data Rich	1	1	Malnutrition Shock mortality rate	--
Protein-energy malnutrition	Male	5-9 years	95+ years	Data Rich	-1	2	Healthcare access and quality index	0
Protein-energy malnutrition	Male	5-9 years	95+ years	Data Rich	-1	2	Maternal care and immunization	0
Protein-energy malnutrition	Male	5-9 years	95+ years	Data Rich	-1	2	Rainfall Quintile 1 (proportion)	--
Protein-energy malnutrition	Male	5-9 years	95+ years	Data Rich	1	2	Rainfall Quintile 2 (proportion)	75
Protein-energy malnutrition	Male	5-9 years	95+ years	Data Rich	1	2	Age- and sex-specific SEV for Unsafe sanitation	99
Protein-energy malnutrition	Male	5-9 years	95+ years	Data Rich	1	2	Age- and sex-specific SEV for Unsafe water	138
Protein-energy malnutrition	Male	5-9 years	95+ years	Data Rich	1	2	Mortality Rate Due to Death Shocks in Last 10 Years (per 1 person)	--
Protein-energy malnutrition	Male	5-9 years	95+ years	Data Rich	-1	3	Maternal Education (years per capita)	0
Protein-energy malnutrition	Male	5-9 years	95+ years	Data Rich	-1	3	Socio-demographic Index	0
Protein-energy malnutrition	Male	5-9 years	95+ years	Data Rich	-1	3	Education (years per capita)	31
Protein-energy malnutrition	Male	5-9 years	95+ years	Data Rich	-1	3	LDI (I$ per capita)	--
Protein-energy malnutrition	Male	5-9 years	95+ years	Data Rich	1	3	Age- and sex-specific SEV for Alcohol use	--
Protein-energy malnutrition	Male	5-9 years	95+ years	Global	-1	1	energy unadjusted(kcal)	--
Protein-energy malnutrition	Male	5-9 years	95+ years	Global	1	1	Log-transformed SEV scalar: Diarrhea	664
Protein-energy malnutrition	Male	5-9 years	95+ years	Global	1	1	Age-Standardize Prevalence of Severe Anemia	753
Protein-energy malnutrition	Male	5-9 years	95+ years	Global	-1	2	Socio-demographic Index	33
Protein-energy malnutrition	Male	5-9 years	95+ years	Global	-1	2	Healthcare access and quality index	59
Protein-energy malnutrition	Male	5-9 years	95+ years	Global	-1	2	Maternal care and immunization	186
Protein-energy malnutrition	Male	5-9 years	95+ years	Global	1	2	Rainfall Quintile 1 (proportion)	38
Protein-energy malnutrition	Male	5-9 years	95+ years	Global	1	2	Age- and sex-specific SEV for Unsafe sanitation	53
Protein-energy malnutrition	Male	5-9 years	95+ years	Global	1	2	Age- and sex-specific SEV for Unsafe water	120
Protein-energy malnutrition	Male	5-9 years	95+ years	Global	1	2	Mortality Rate Due to Death Shocks in Last 10 Years (per 1 person)	162
Protein-energy malnutrition	Male	5-9 years	95+ years	Global	1	2	Rainfall Quintile 2 (proportion)	--
Protein-energy malnutrition	Male	5-9 years	95+ years	Global	-1	3	Maternal Education (years per capita)	48
Protein-energy malnutrition	Male	5-9 years	95+ years	Global	-1	3	Education (years per capita)	146
Protein-energy malnutrition	Male	5-9 years	95+ years	Global	-1	3	LDI (I$ per capita)	--
Protein-energy malnutrition	Male	5-9 years	95+ years	Global	1	3	Age- and sex-specific SEV for Alcohol use	--
Other nutritional deficiencies	Female	1-5 months	95+ years	Data Rich	1	1	Log-transformed SEV scalar: Diarrhea	321
Other nutritional deficiencies	Female	1-5 months	95+ years	Data Rich	1	1	Age-standardized SEV for Child underweight	679
Other nutritional deficiencies	Female	1-5 months	95+ years	Data Rich	1	1	Age-Standardize Prevalence of Severe Anemia	808
Other nutritional deficiencies	Female	1-5 months	95+ years	Data Rich	-1	2	Healthcare access and quality index	211
Other nutritional deficiencies	Female	1-5 months	95+ years	Data Rich	-1	2	Maternal care and immunization	429
Other nutritional deficiencies	Female	1-5 months	95+ years	Data Rich	-1	2	energy unadjusted(kcal)	505
Other nutritional deficiencies	Female	1-5 months	95+ years	Data Rich	-1	2	Rainfall Quintile 1 (proportion)	506
Other nutritional deficiencies	Female	1-5 months	95+ years	Data Rich	-1	2	Socio-demographic Index	--
Other nutritional deficiencies	Female	1-5 months	95+ years	Data Rich	1	2	Age- and sex-specific SEV for Unsafe sanitation	0
Other nutritional deficiencies	Female	1-5 months	95+ years	Data Rich	1	2	Age- and sex-specific SEV for Unsafe water	16
Other nutritional deficiencies	Female	1-5 months	95+ years	Data Rich	1	2	Rainfall Quintile 2 (proportion)	374
Other nutritional deficiencies	Female	1-5 months	95+ years	Data Rich	1	2	Mortality Rate Due to Death Shocks in Last 10 Years (per 1 person)	--
Other nutritional deficiencies	Female	1-5 months	95+ years	Data Rich	-1	3	Education (years per capita)	483
Other nutritional deficiencies	Female	1-5 months	95+ years	Data Rich	-1	3	LDI (I$ per capita)	--
Other nutritional deficiencies	Female	1-5 months	95+ years	Data Rich	1	3	Age- and sex-specific SEV for Alcohol use	--
Other nutritional deficiencies	Female	1-5 months	95+ years	Data Rich	1	3	Maternal Education (years per capita)	--
Other nutritional deficiencies	Female	1-5 months	95+ years	Global	-1	1	energy unadjusted(kcal)	546
Other nutritional deficiencies	Female	1-5 months	95+ years	Global	1	1	Log-transformed SEV scalar: Diarrhea	18
Other nutritional deficiencies	Female	1-5 months	95+ years	Global	1	1	Age-Standardize Prevalence of Severe Anemia	92
Other nutritional deficiencies	Female	1-5 months	95+ years	Global	1	1	Age-standardized SEV for Child underweight	790
Other nutritional deficiencies	Female	1-5 months	95+ years	Global	-1	2	Socio-demographic Index	0
Other nutritional deficiencies	Female	1-5 months	95+ years	Global	-1	2	Maternal care and immunization	9
Other nutritional deficiencies	Female	1-5 months	95+ years	Global	-1	2	Healthcare access and quality index	264
Other nutritional deficiencies	Female	1-5 months	95+ years	Global	1	2	Age- and sex-specific SEV for Unsafe sanitation	0
Other nutritional deficiencies	Female	1-5 months	95+ years	Global	1	2	Age- and sex-specific SEV for Unsafe water	0
Other nutritional deficiencies	Female	1-5 months	95+ years	Global	1	2	Rainfall Quintile 2 (proportion)	137
Other nutritional deficiencies	Female	1-5 months	95+ years	Global	1	2	Rainfall Quintile 1 (proportion)	384
Other nutritional deficiencies	Female	1-5 months	95+ years	Global	1	2	Mortality Rate Due to Death Shocks in Last 10 Years (per 1 person)	--
Other nutritional deficiencies	Female	1-5 months	95+ years	Global	-1	3	Maternal Education (years per capita)	187
Other nutritional deficiencies	Female	1-5 months	95+ years	Global	-1	3	Education (years per capita)	481
Other nutritional deficiencies	Female	1-5 months	95+ years	Global	-1	3	LDI (I$ per capita)	--
Other nutritional deficiencies	Female	1-5 months	95+ years	Global	1	3	Age- and sex-specific SEV for Alcohol use	--

CODEm covariates used, level of covariate, and expected direction of covariate by cause, sex, and age

Cause	Sex	Age Start	Age End	Model Version Type	Direction	Level	Covariate Name	Number of Draws
Other nutritional deficiencies	Male	1-5 months	95+ years	Data Rich	1	1	Log-transformed SEV scalar: Diarrhea	23
Other nutritional deficiencies	Male	1-5 months	95+ years	Data Rich	1	1	Age-standardized SEV for Child underweight	447
Other nutritional deficiencies	Male	1-5 months	95+ years	Data Rich	1	1	Age-Standardize Prevalence of Severe Anemia	562
Other nutritional deficiencies	Male	1-5 months	95+ years	Data Rich	-1	2	Socio-demographic Index	103
Other nutritional deficiencies	Male	1-5 months	95+ years	Data Rich	-1	2	Healthcare access and quality index	259
Other nutritional deficiencies	Male	1-5 months	95+ years	Data Rich	-1	2	energy unadjusted(kcal)	333
Other nutritional deficiencies	Male	1-5 months	95+ years	Data Rich	-1	2	Rainfall Quintile 1 (proportion)	732
Other nutritional deficiencies	Male	1-5 months	95+ years	Data Rich	-1	2	Maternal care and immunization	972
Other nutritional deficiencies	Male	1-5 months	95+ years	Data Rich	1	2	Age- and sex-specific SEV for Unsafe water	32
Other nutritional deficiencies	Male	1-5 months	95+ years	Data Rich	1	2	Age- and sex-specific SEV for Unsafe sanitation	325
Other nutritional deficiencies	Male	1-5 months	95+ years	Data Rich	1	2	Rainfall Quintile 2 (proportion)	935
Other nutritional deficiencies	Male	1-5 months	95+ years	Data Rich	1	2	Mortality Rate Due to Death Shocks in Last 10 Years (per 1 person)	--
Other nutritional deficiencies	Male	1-5 months	95+ years	Data Rich	-1	3	Education (years per capita)	293
Other nutritional deficiencies	Male	1-5 months	95+ years	Data Rich	-1	3	LDI (I$ per capita)	--
Other nutritional deficiencies	Male	1-5 months	95+ years	Data Rich	1	3	Maternal Education (years per capita)	38
Other nutritional deficiencies	Male	1-5 months	95+ years	Data Rich	1	3	Age- and sex-specific SEV for Alcohol use	--
Other nutritional deficiencies	Male	1-5 months	95+ years	Global	-1	1	energy unadjusted(kcal)	--
Other nutritional deficiencies	Male	1-5 months	95+ years	Global	1	1	Age-standardized SEV for Child underweight	193
Other nutritional deficiencies	Male	1-5 months	95+ years	Global	1	1	Age-Standardize Prevalence of Severe Anemia	279
Other nutritional deficiencies	Male	1-5 months	95+ years	Global	1	1	Log-transformed SEV scalar: Diarrhea	287
Other nutritional deficiencies	Male	1-5 months	95+ years	Global	-1	2	Maternal care and immunization	7
Other nutritional deficiencies	Male	1-5 months	95+ years	Global	-1	2	Healthcare access and quality index	201
Other nutritional deficiencies	Male	1-5 months	95+ years	Global	-1	2	Socio-demographic Index	--
Other nutritional deficiencies	Male	1-5 months	95+ years	Global	1	2	Age- and sex-specific SEV for Unsafe sanitation	0
Other nutritional deficiencies	Male	1-5 months	95+ years	Global	1	2	Rainfall Quintile 2 (proportion)	40
Other nutritional deficiencies	Male	1-5 months	95+ years	Global	1	2	Rainfall Quintile 1 (proportion)	320
Other nutritional deficiencies	Male	1-5 months	95+ years	Global	1	2	Age- and sex-specific SEV for Unsafe water	--
Other nutritional deficiencies	Male	1-5 months	95+ years	Global	1	2	Mortality Rate Due to Death Shocks in Last 10 Years (per 1 person)	--
Other nutritional deficiencies	Male	1-5 months	95+ years	Global	-1	3	Maternal Education (years per capita)	300
Other nutritional deficiencies	Male	1-5 months	95+ years	Global	-1	3	Education (years per capita)	403
Other nutritional deficiencies	Male	1-5 months	95+ years	Global	-1	3	LDI (I$ per capita)	--
Other nutritional deficiencies	Male	1-5 months	95+ years	Global	1	3	Age- and sex-specific SEV for Alcohol use	--
Lip and oral cavity cancer	Female	15-19 years	95+ years	Data Rich	1	1	Cumulative Cigarettes (20 Years)	236
Lip and oral cavity cancer	Female	15-19 years	95+ years	Data Rich	1	1	Liters of alcohol consumed per capita	252
Lip and oral cavity cancer	Female	15-19 years	95+ years	Data Rich	1	1	Cumulative Cigarettes (10 Years)	421
Lip and oral cavity cancer	Female	15-19 years	95+ years	Data Rich	1	1	Tobacco (cigarettes per capita)	579
Lip and oral cavity cancer	Female	15-19 years	95+ years	Data Rich	1	1	Log-transformed SEV scalar: Lip Oral C	--
Lip and oral cavity cancer	Female	15-19 years	95+ years	Data Rich	-1	2	Healthcare access and quality index	70
Lip and oral cavity cancer	Female	15-19 years	95+ years	Data Rich	1	2	Age- and sex-specific SEV for High red meat	60
Lip and oral cavity cancer	Female	15-19 years	95+ years	Data Rich	1	2	Age- and sex-specific SEV for Low fruit	155
Lip and oral cavity cancer	Female	15-19 years	95+ years	Data Rich	1	2	Age- and sex-specific SEV for Low vegetables	--
Lip and oral cavity cancer	Female	15-19 years	95+ years	Data Rich	-1	3	Education (years per capita)	65
Lip and oral cavity cancer	Female	15-19 years	95+ years	Data Rich	1	3	LDI (I$ per capita)	--
Lip and oral cavity cancer	Female	15-19 years	95+ years	Data Rich	1	3	Socio-demographic Index	--
Lip and oral cavity cancer	Female	15-19 years	95+ years	Global	1	1	Log-transformed SEV scalar: Lip Oral C	28
Lip and oral cavity cancer	Female	15-19 years	95+ years	Global	1	1	Cumulative Cigarettes (20 Years)	281
Lip and oral cavity cancer	Female	15-19 years	95+ years	Global	1	1	Tobacco (cigarettes per capita)	300
Lip and oral cavity cancer	Female	15-19 years	95+ years	Global	1	1	Cumulative Cigarettes (10 Years)	318
Lip and oral cavity cancer	Female	15-19 years	95+ years	Global	1	1	Liters of alcohol consumed per capita	553
Lip and oral cavity cancer	Female	15-19 years	95+ years	Global	-1	2	Healthcare access and quality index	387
Lip and oral cavity cancer	Female	15-19 years	95+ years	Global	1	2	Age- and sex-specific SEV for Low vegetables	26
Lip and oral cavity cancer	Female	15-19 years	95+ years	Global	1	2	Age- and sex-specific SEV for Low fruit	192
Lip and oral cavity cancer	Female	15-19 years	95+ years	Global	1	2	Age- and sex-specific SEV for High red meat	454
Lip and oral cavity cancer	Female	15-19 years	95+ years	Global	-1	3	Education (years per capita)	1
Lip and oral cavity cancer	Female	15-19 years	95+ years	Global	1	3	Socio-demographic Index	546
Lip and oral cavity cancer	Female	15-19 years	95+ years	Global	1	3	LDI (I$ per capita)	--
Lip and oral cavity cancer	Male	15-19 years	95+ years	Data Rich	1	1	Cumulative Cigarettes (10 Years)	0
Lip and oral cavity cancer	Male	15-19 years	95+ years	Data Rich	1	1	Cumulative Cigarettes (20 Years)	13
Lip and oral cavity cancer	Male	15-19 years	95+ years	Data Rich	1	1	Tobacco (cigarettes per capita)	28
Lip and oral cavity cancer	Male	15-19 years	95+ years	Data Rich	1	1	Liters of alcohol consumed per capita	494
Lip and oral cavity cancer	Male	15-19 years	95+ years	Data Rich	1	1	Log-transformed SEV scalar: Lip Oral C	763
Lip and oral cavity cancer	Male	15-19 years	95+ years	Data Rich	-1	2	Healthcare access and quality index	1
Lip and oral cavity cancer	Male	15-19 years	95+ years	Data Rich	1	2	Age- and sex-specific SEV for Low vegetables	0
Lip and oral cavity cancer	Male	15-19 years	95+ years	Data Rich	1	2	Age- and sex-specific SEV for Low fruit	9
Lip and oral cavity cancer	Male	15-19 years	95+ years	Data Rich	1	2	Age- and sex-specific SEV for High red meat	258
Lip and oral cavity cancer	Male	15-19 years	95+ years	Data Rich	-1	3	Education (years per capita)	11
Lip and oral cavity cancer	Male	15-19 years	95+ years	Data Rich	1	3	Socio-demographic Index	564
Lip and oral cavity cancer	Male	15-19 years	95+ years	Data Rich	1	3	LDI (I$ per capita)	--
Lip and oral cavity cancer	Male	15-19 years	95+ years	Global	1	1	Liters of alcohol consumed per capita	0
Lip and oral cavity cancer	Male	15-19 years	95+ years	Global	1	1	Tobacco (cigarettes per capita)	40
Lip and oral cavity cancer	Male	15-19 years	95+ years	Global	1	1	Cumulative Cigarettes (10 Years)	234
Lip and oral cavity cancer	Male	15-19 years	95+ years	Global	1	1	Cumulative Cigarettes (20 Years)	337
Lip and oral cavity cancer	Male	15-19 years	95+ years	Global	1	1	Log-transformed SEV scalar: Lip Oral C	981
Lip and oral cavity cancer	Male	15-19 years	95+ years	Global	-1	2	Healthcare access and quality index	48
Lip and oral cavity cancer	Male	15-19 years	95+ years	Global	1	2	Age- and sex-specific SEV for High red meat	0
Lip and oral cavity cancer	Male	15-19 years	95+ years	Global	1	2	Age- and sex-specific SEV for Low fruit	0
Lip and oral cavity cancer	Male	15-19 years	95+ years	Global	1	2	Age- and sex-specific SEV for Low vegetables	186
Lip and oral cavity cancer	Male	15-19 years	95+ years	Global	-1	3	Education (years per capita)	56
Lip and oral cavity cancer	Male	15-19 years	95+ years	Global	1	3	Socio-demographic Index	213
Lip and oral cavity cancer	Male	15-19 years	95+ years	Global	1	3	LDI (I$ per capita)	--
Nasopharynx cancer	Female	5-9 years	95+ years	Data Rich	1	1	Tobacco (cigarettes per capita)	135
Nasopharynx cancer	Female	5-9 years	95+ years	Data Rich	1	1	Log-transformed SEV scalar: Nasoph C	218
Nasopharynx cancer	Female	5-9 years	95+ years	Data Rich	1	1	Liters of alcohol consumed per capita	274
Nasopharynx cancer	Female	5-9 years	95+ years	Data Rich	1	1	Cumulative Cigarettes (10 Years)	401
Nasopharynx cancer	Female	5-9 years	95+ years	Data Rich	1	1	Cumulative Cigarettes (20 Years)	--
Nasopharynx cancer	Female	5-9 years	95+ years	Data Rich	-1	2	Healthcare access and quality index	--
Nasopharynx cancer	Female	5-9 years	95+ years	Data Rich	1	2	Age- and sex-specific SEV for Low vegetables	21
Nasopharynx cancer	Female	5-9 years	95+ years	Data Rich	1	2	Population Density (over 1000 ppl/sqkm, proportion)	276
Nasopharynx cancer	Female	5-9 years	95+ years	Data Rich	-1	3	Education (years per capita)	64
Nasopharynx cancer	Female	5-9 years	95+ years	Data Rich	-1	3	LDI (I$ per capita)	--
Nasopharynx cancer	Female	5-9 years	95+ years	Data Rich	1	3	Age- and sex-specific SEV for Low fruit	64
Nasopharynx cancer	Female	5-9 years	95+ years	Data Rich	1	3	Socio-demographic Index	--
Nasopharynx cancer	Female	5-9 years	95+ years	Global	1	1	Cumulative Cigarettes (10 Years)	19
Nasopharynx cancer	Female	5-9 years	95+ years	Global	1	1	Cumulative Cigarettes (20 Years)	27
Nasopharynx cancer	Female	5-9 years	95+ years	Global	1	1	Tobacco (cigarettes per capita)	159
Nasopharynx cancer	Female	5-9 years	95+ years	Global	1	1	Liters of alcohol consumed per capita	343
Nasopharynx cancer	Female	5-9 years	95+ years	Global	1	1	Log-transformed SEV scalar: Nasoph C	556
Nasopharynx cancer	Female	5-9 years	95+ years	Global	-1	2	Healthcare access and quality index	72
Nasopharynx cancer	Female	5-9 years	95+ years	Global	1	2	Population Density (over 1000 ppl/sqkm, proportion)	154
Nasopharynx cancer	Female	5-9 years	95+ years	Global	1	2	Age- and sex-specific SEV for Low vegetables	487
Nasopharynx cancer	Female	5-9 years	95+ years	Global	-1	3	Education (years per capita)	195
Nasopharynx cancer	Female	5-9 years	95+ years	Global	-1	3	LDI (I$ per capita)	--
Nasopharynx cancer	Female	5-9 years	95+ years	Global	1	3	Age- and sex-specific SEV for Low fruit	209
Nasopharynx cancer	Female	5-9 years	95+ years	Global	1	3	Socio-demographic Index	331
Nasopharynx cancer	Male	5-9 years	95+ years	Data Rich	1	1	Cumulative Cigarettes (10 Years)	83
Nasopharynx cancer	Male	5-9 years	95+ years	Data Rich	1	1	Tobacco (cigarettes per capita)	104
Nasopharynx cancer	Male	5-9 years	95+ years	Data Rich	1	1	Cumulative Cigarettes (20 Years)	112
Nasopharynx cancer	Male	5-9 years	95+ years	Data Rich	1	1	Liters of alcohol consumed per capita	530
Nasopharynx cancer	Male	5-9 years	95+ years	Data Rich	1	1	Log-transformed SEV scalar: Nasoph C	694
Nasopharynx cancer	Male	5-9 years	95+ years	Data Rich	-1	2	Healthcare access and quality index	0
Nasopharynx cancer	Male	5-9 years	95+ years	Data Rich	1	2	Population Density (over 1000 ppl/sqkm, proportion)	2
Nasopharynx cancer	Male	5-9 years	95+ years	Data Rich	1	2	Age- and sex-specific SEV for Low vegetables	58
Nasopharynx cancer	Male	5-9 years	95+ years	Data Rich	-1	3	Education (years per capita)	19
Nasopharynx cancer	Male	5-9 years	95+ years	Data Rich	-1	3	LDI (I$ per capita)	--

CODEm covariates used, level of covariate, and expected direction of covariate by cause, sex, and age

Cause	Sex	Age Start	Age End	Model Version Type	Direction	Level	Covariate Name	Number of Draws
Nasopharynx cancer	Male	5-9 years	95+ years	Data Rich	1	3	Socio-demographic Index	566
Nasopharynx cancer	Male	5-9 years	95+ years	Data Rich	1	3	Age- and sex-specific SEV for Low fruit	--
Nasopharynx cancer	Male	5-9 years	95+ years	Global	1	1	Cumulative Cigarettes (20 Years)	128
Nasopharynx cancer	Male	5-9 years	95+ years	Global	1	1	Cumulative Cigarettes (10 Years)	234
Nasopharynx cancer	Male	5-9 years	95+ years	Global	1	1	Log-transformed SEV scalar: Nasoph C	453
Nasopharynx cancer	Male	5-9 years	95+ years	Global	1	1	Tobacco (cigarettes per capita)	457
Nasopharynx cancer	Male	5-9 years	95+ years	Global	1	1	Liters of alcohol consumed per capita	459
Nasopharynx cancer	Male	5-9 years	95+ years	Global	-1	2	Healthcare access and quality index	373
Nasopharynx cancer	Male	5-9 years	95+ years	Global	1	2	Population Density (over 1000 ppl/sqkm, proportion)	5
Nasopharynx cancer	Male	5-9 years	95+ years	Global	1	2	Age- and sex-specific SEV for Low vegetables	55
Nasopharynx cancer	Male	5-9 years	95+ years	Global	-1	3	Education (years per capita)	137
Nasopharynx cancer	Male	5-9 years	95+ years	Global	-1	3	LDI (I$ per capita)	--
Nasopharynx cancer	Male	5-9 years	95+ years	Global	1	3	Age- and sex-specific SEV for Low fruit	137
Nasopharynx cancer	Male	5-9 years	95+ years	Global	1	3	Socio-demographic Index	298
Other pharynx cancer	Female	20-24 years	95+ years	Data Rich	1	1	Smoking Prevalence	174
Other pharynx cancer	Female	20-24 years	95+ years	Data Rich	1	1	Liters of alcohol consumed per capita	528
Other pharynx cancer	Female	20-24 years	95+ years	Data Rich	1	1	Log-transformed SEV scalar: Oth Phar C	714
Other pharynx cancer	Female	20-24 years	95+ years	Data Rich	-1	2	Healthcare access and quality index	17
Other pharynx cancer	Female	20-24 years	95+ years	Data Rich	1	2	Age- and sex-specific SEV for Low vegetables	19
Other pharynx cancer	Female	20-24 years	95+ years	Data Rich	1	2	Cumulative Cigarettes (5 Years)	27
Other pharynx cancer	Female	20-24 years	95+ years	Data Rich	1	2	Age- and sex-specific SEV for Low fruit	51
Other pharynx cancer	Female	20-24 years	95+ years	Data Rich	1	2	Population Density (over 1000 ppl/sqkm, proportion)	195
Other pharynx cancer	Female	20-24 years	95+ years	Data Rich	1	2	Population Density (under 150 ppl/sqkm, proportion)	--
Other pharynx cancer	Female	20-24 years	95+ years	Data Rich	-1	3	Education (years per capita)	51
Other pharynx cancer	Female	20-24 years	95+ years	Data Rich	1	3	Socio-demographic Index	190
Other pharynx cancer	Female	20-24 years	95+ years	Data Rich	1	3	LDI (I$ per capita)	--
Other pharynx cancer	Female	20-24 years	95+ years	Global	1	1	Log-transformed SEV scalar: Oth Phar C	509
Other pharynx cancer	Female	20-24 years	95+ years	Global	1	1	Smoking Prevalence	793
Other pharynx cancer	Female	20-24 years	95+ years	Global	1	1	Liters of alcohol consumed per capita	--
Other pharynx cancer	Female	20-24 years	95+ years	Global	-1	2	Healthcare access and quality index	416
Other pharynx cancer	Female	20-24 years	95+ years	Global	1	2	Age- and sex-specific SEV for Low fruit	52
Other pharynx cancer	Female	20-24 years	95+ years	Global	1	2	Population Density (under 150 ppl/sqkm, proportion)	81
Other pharynx cancer	Female	20-24 years	95+ years	Global	1	2	Age- and sex-specific SEV for Low vegetables	--
Other pharynx cancer	Female	20-24 years	95+ years	Global	1	2	Cumulative Cigarettes (5 Years)	--
Other pharynx cancer	Female	20-24 years	95+ years	Global	1	2	Population Density (over 1000 ppl/sqkm, proportion)	--
Other pharynx cancer	Female	20-24 years	95+ years	Global	-1	3	Education (years per capita)	250
Other pharynx cancer	Female	20-24 years	95+ years	Global	1	3	LDI (I$ per capita)	--
Other pharynx cancer	Female	20-24 years	95+ years	Global	1	3	Socio-demographic Index	--
Other pharynx cancer	Male	20-24 years	95+ years	Data Rich	1	1	Smoking Prevalence	36
Other pharynx cancer	Male	20-24 years	95+ years	Data Rich	1	1	Log-transformed SEV scalar: Oth Phar C	429
Other pharynx cancer	Male	20-24 years	95+ years	Data Rich	1	1	Liters of alcohol consumed per capita	610
Other pharynx cancer	Male	20-24 years	95+ years	Data Rich	-1	2	Healthcare access and quality index	8
Other pharynx cancer	Male	20-24 years	95+ years	Data Rich	1	2	Age- and sex-specific SEV for Low vegetables	5
Other pharynx cancer	Male	20-24 years	95+ years	Data Rich	1	2	Cumulative Cigarettes (5 Years)	7
Other pharynx cancer	Male	20-24 years	95+ years	Data Rich	1	2	Population Density (over 1000 ppl/sqkm, proportion)	333
Other pharynx cancer	Male	20-24 years	95+ years	Data Rich	1	2	Age- and sex-specific SEV for Low fruit	--
Other pharynx cancer	Male	20-24 years	95+ years	Data Rich	1	2	Population Density (under 150 ppl/sqkm, proportion)	--
Other pharynx cancer	Male	20-24 years	95+ years	Data Rich	-1	3	Education (years per capita)	54
Other pharynx cancer	Male	20-24 years	95+ years	Data Rich	1	3	Socio-demographic Index	599
Other pharynx cancer	Male	20-24 years	95+ years	Data Rich	1	3	LDI (I$ per capita)	--
Other pharynx cancer	Male	20-24 years	95+ years	Global	1	1	Smoking Prevalence	116
Other pharynx cancer	Male	20-24 years	95+ years	Global	1	1	Liters of alcohol consumed per capita	395
Other pharynx cancer	Male	20-24 years	95+ years	Global	1	1	Log-transformed SEV scalar: Oth Phar C	513
Other pharynx cancer	Male	20-24 years	95+ years	Global	-1	2	Healthcare access and quality index	76
Other pharynx cancer	Male	20-24 years	95+ years	Global	1	2	Age- and sex-specific SEV for Low fruit	27
Other pharynx cancer	Male	20-24 years	95+ years	Global	1	2	Cumulative Cigarettes (5 Years)	74
Other pharynx cancer	Male	20-24 years	95+ years	Global	1	2	Population Density (over 1000 ppl/sqkm, proportion)	200
Other pharynx cancer	Male	20-24 years	95+ years	Global	1	2	Age- and sex-specific SEV for Low vegetables	340
Other pharynx cancer	Male	20-24 years	95+ years	Global	1	2	Population Density (under 150 ppl/sqkm, proportion)	--
Other pharynx cancer	Male	20-24 years	95+ years	Global	-1	3	Education (years per capita)	0
Other pharynx cancer	Male	20-24 years	95+ years	Global	1	3	Socio-demographic Index	247
Other pharynx cancer	Male	20-24 years	95+ years	Global	1	3	LDI (I$ per capita)	--
Oesophageal cancer	Female	20-24 years	95+ years	Data Rich	1	1	Smoking Prevalence	341
Oesophageal cancer	Female	20-24 years	95+ years	Data Rich	1	1	Log-transformed age-standardized SEV scalar: Esophag C	390
Oesophageal cancer	Female	20-24 years	95+ years	Data Rich	1	1	Mean BMI	400
Oesophageal cancer	Female	20-24 years	95+ years	Data Rich	1	1	Liters of alcohol consumed per capita	444
Oesophageal cancer	Female	20-24 years	95+ years	Data Rich	-1	2	Healthcare access and quality index	33
Oesophageal cancer	Female	20-24 years	95+ years	Data Rich	1	2	Age- and sex-specific SEV for Low fruit	0
Oesophageal cancer	Female	20-24 years	95+ years	Data Rich	1	2	Tobacco (cigarettes per capita)	64
Oesophageal cancer	Female	20-24 years	95+ years	Data Rich	1	2	Indoor Air Pollution (All Cooking Fuels)	121
Oesophageal cancer	Female	20-24 years	95+ years	Data Rich	1	2	Age- and sex-specific SEV for Low vegetables	203
Oesophageal cancer	Female	20-24 years	95+ years	Data Rich	-1	3	Sanitation (proportion with access)	28
Oesophageal cancer	Female	20-24 years	95+ years	Data Rich	-1	3	Improved Water Source (proportion with access)	40
Oesophageal cancer	Female	20-24 years	95+ years	Data Rich	-1	3	Education (years per capita)	72
Oesophageal cancer	Female	20-24 years	95+ years	Data Rich	1	3	Socio-demographic Index	312
Oesophageal cancer	Female	20-24 years	95+ years	Data Rich	1	3	LDI (I$ per capita)	--
Oesophageal cancer	Female	20-24 years	95+ years	Global	1	1	Smoking Prevalence	59
Oesophageal cancer	Female	20-24 years	95+ years	Global	1	1	Log-transformed age-standardized SEV scalar: Esophag C	497
Oesophageal cancer	Female	20-24 years	95+ years	Global	1	1	Mean BMI	503
Oesophageal cancer	Female	20-24 years	95+ years	Global	1	1	Liters of alcohol consumed per capita	--
Oesophageal cancer	Female	20-24 years	95+ years	Global	-1	2	Healthcare access and quality index	62
Oesophageal cancer	Female	20-24 years	95+ years	Global	1	2	Tobacco (cigarettes per capita)	29
Oesophageal cancer	Female	20-24 years	95+ years	Global	1	2	Age- and sex-specific SEV for Low fruit	423
Oesophageal cancer	Female	20-24 years	95+ years	Global	1	2	Age- and sex-specific SEV for Low vegetables	444
Oesophageal cancer	Female	20-24 years	95+ years	Global	1	2	Indoor Air Pollution (All Cooking Fuels)	457
Oesophageal cancer	Female	20-24 years	95+ years	Global	-1	3	Improved Water Source (proportion with access)	163
Oesophageal cancer	Female	20-24 years	95+ years	Global	-1	3	Education (years per capita)	318
Oesophageal cancer	Female	20-24 years	95+ years	Global	-1	3	Sanitation (proportion with access)	486
Oesophageal cancer	Female	20-24 years	95+ years	Global	1	3	Socio-demographic Index	82
Oesophageal cancer	Female	20-24 years	95+ years	Global	1	3	LDI (I$ per capita)	--
Oesophageal cancer	Male	20-24 years	95+ years	Data Rich	1	1	Liters of alcohol consumed per capita	79
Oesophageal cancer	Male	20-24 years	95+ years	Data Rich	1	1	Smoking Prevalence	117
Oesophageal cancer	Male	20-24 years	95+ years	Data Rich	1	1	Log-transformed age-standardized SEV scalar: Esophag C	373
Oesophageal cancer	Male	20-24 years	95+ years	Data Rich	1	1	Mean BMI	679
Oesophageal cancer	Male	20-24 years	95+ years	Data Rich	-1	2	Healthcare access and quality index	--
Oesophageal cancer	Male	20-24 years	95+ years	Data Rich	1	2	Indoor Air Pollution (All Cooking Fuels)	318
Oesophageal cancer	Male	20-24 years	95+ years	Data Rich	1	2	Age- and sex-specific SEV for Low vegetables	623
Oesophageal cancer	Male	20-24 years	95+ years	Data Rich	1	2	Age- and sex-specific SEV for Low fruit	--
Oesophageal cancer	Male	20-24 years	95+ years	Data Rich	1	2	Tobacco (cigarettes per capita)	--
Oesophageal cancer	Male	20-24 years	95+ years	Data Rich	-1	3	Improved Water Source (proportion with access)	464
Oesophageal cancer	Male	20-24 years	95+ years	Data Rich	-1	3	Education (years per capita)	--
Oesophageal cancer	Male	20-24 years	95+ years	Data Rich	-1	3	Sanitation (proportion with access)	--
Oesophageal cancer	Male	20-24 years	95+ years	Data Rich	1	3	Socio-demographic Index	570
Oesophageal cancer	Male	20-24 years	95+ years	Data Rich	1	3	LDI (I$ per capita)	--
Oesophageal cancer	Male	20-24 years	95+ years	Global	1	1	Liters of alcohol consumed per capita	13
Oesophageal cancer	Male	20-24 years	95+ years	Global	1	1	Log-transformed age-standardized SEV scalar: Esophag C	92
Oesophageal cancer	Male	20-24 years	95+ years	Global	1	1	Smoking Prevalence	281
Oesophageal cancer	Male	20-24 years	95+ years	Global	1	1	Mean BMI	959
Oesophageal cancer	Male	20-24 years	95+ years	Global	-1	2	Healthcare access and quality index	16
Oesophageal cancer	Male	20-24 years	95+ years	Global	1	2	Indoor Air Pollution (All Cooking Fuels)	95
Oesophageal cancer	Male	20-24 years	95+ years	Global	1	2	Age- and sex-specific SEV for Low fruit	352
Oesophageal cancer	Male	20-24 years	95+ years	Global	1	2	Age- and sex-specific SEV for Low vegetables	398
Oesophageal cancer	Male	20-24 years	95+ years	Global	1	2	Tobacco (cigarettes per capita)	--

CODEm covariates used, level of covariate, and expected direction of covariate by cause, sex, and age								
**Cause**	**Sex**	**Age Start**	**Age End**	**Model Version Type**	**Direction**	**Level**	**Covariate Name**	**Number of Draws**
Oesophageal cancer	Male	20-24 years	95+ years	Global	-1	3	Education (years per capita)	0
Oesophageal cancer	Male	20-24 years	95+ years	Global	-1	3	Improved Water Source (proportion with access)	204
Oesophageal cancer	Male	20-24 years	95+ years	Global	-1	3	Sanitation (proportion with access)	468
Oesophageal cancer	Male	20-24 years	95+ years	Global	1	3	Socio-demographic Index	619
Oesophageal cancer	Male	20-24 years	95+ years	Global	1	3	LDI (I$ per capita)	--
Stomach cancer	Female	15-19 years	95+ years	Data Rich	1	1	Log-transformed SEV scalar: Stomach C	269
Stomach cancer	Female	15-19 years	95+ years	Data Rich	1	1	Tobacco (cigarettes per capita)	322
Stomach cancer	Female	15-19 years	95+ years	Data Rich	1	1	Diet high in sodium	678
Stomach cancer	Female	15-19 years	95+ years	Data Rich	-1	2	Sanitation (proportion with access)	53
Stomach cancer	Female	15-19 years	95+ years	Data Rich	-1	2	Improved Water Source (proportion with access)	145
Stomach cancer	Female	15-19 years	95+ years	Data Rich	-1	2	Healthcare access and quality index	813
Stomach cancer	Female	15-19 years	95+ years	Data Rich	1	2	Cumulative Cigarettes (20 Years)	0
Stomach cancer	Female	15-19 years	95+ years	Data Rich	1	2	Age- and sex-specific SEV for Unsafe sanitation	47
Stomach cancer	Female	15-19 years	95+ years	Data Rich	1	2	Age- and sex-specific SEV for Unsafe water	568
Stomach cancer	Female	15-19 years	95+ years	Data Rich	1	2	Mean BMI	--
Stomach cancer	Female	15-19 years	95+ years	Data Rich	-1	3	Socio-demographic Index	234
Stomach cancer	Female	15-19 years	95+ years	Data Rich	-1	3	Education (years per capita)	893
Stomach cancer	Female	15-19 years	95+ years	Data Rich	1	3	Age- and sex-specific SEV for Low fruit	0
Stomach cancer	Female	15-19 years	95+ years	Data Rich	1	3	Age- and sex-specific SEV for Low vegetables	0
Stomach cancer	Female	15-19 years	95+ years	Data Rich	1	3	LDI (I$ per capita)	--
Stomach cancer	Female	15-19 years	95+ years	Global	1	1	Tobacco (cigarettes per capita)	345
Stomach cancer	Female	15-19 years	95+ years	Global	1	1	Log-transformed SEV scalar: Stomach C	431
Stomach cancer	Female	15-19 years	95+ years	Global	1	1	Diet high in sodium	493
Stomach cancer	Female	15-19 years	95+ years	Global	-1	2	Sanitation (proportion with access)	101
Stomach cancer	Female	15-19 years	95+ years	Global	-1	2	Improved Water Source (proportion with access)	117
Stomach cancer	Female	15-19 years	95+ years	Global	-1	2	Healthcare access and quality index	369
Stomach cancer	Female	15-19 years	95+ years	Global	1	2	Age- and sex-specific SEV for Unsafe water	147
Stomach cancer	Female	15-19 years	95+ years	Global	1	2	Cumulative Cigarettes (20 Years)	198
Stomach cancer	Female	15-19 years	95+ years	Global	1	2	Age- and sex-specific SEV for Unsafe sanitation	302
Stomach cancer	Female	15-19 years	95+ years	Global	1	2	Mean BMI	--
Stomach cancer	Female	15-19 years	95+ years	Global	-1	3	Socio-demographic Index	258
Stomach cancer	Female	15-19 years	95+ years	Global	-1	3	Education (years per capita)	276
Stomach cancer	Female	15-19 years	95+ years	Global	1	3	Age- and sex-specific SEV for Low vegetables	45
Stomach cancer	Female	15-19 years	95+ years	Global	1	3	Age- and sex-specific SEV for Low fruit	78
Stomach cancer	Female	15-19 years	95+ years	Global	1	3	LDI (I$ per capita)	--
Stomach cancer	Male	15-19 years	95+ years	Data Rich	1	1	Diet high in sodium	77
Stomach cancer	Male	15-19 years	95+ years	Data Rich	1	1	Log-transformed SEV scalar: Stomach C	112
Stomach cancer	Male	15-19 years	95+ years	Data Rich	1	1	Tobacco (cigarettes per capita)	1000
Stomach cancer	Male	15-19 years	95+ years	Data Rich	-1	2	Improved Water Source (proportion with access)	0
Stomach cancer	Male	15-19 years	95+ years	Data Rich	-1	2	Sanitation (proportion with access)	138
Stomach cancer	Male	15-19 years	95+ years	Data Rich	-1	2	Healthcare access and quality index	447
Stomach cancer	Male	15-19 years	95+ years	Data Rich	1	2	Age- and sex-specific SEV for Unsafe water	29
Stomach cancer	Male	15-19 years	95+ years	Data Rich	1	2	Age- and sex-specific SEV for Unsafe sanitation	342
Stomach cancer	Male	15-19 years	95+ years	Data Rich	1	2	Cumulative Cigarettes (20 Years)	438
Stomach cancer	Male	15-19 years	95+ years	Data Rich	1	2	Mean BMI	--
Stomach cancer	Male	15-19 years	95+ years	Data Rich	-1	3	Socio-demographic Index	296
Stomach cancer	Male	15-19 years	95+ years	Data Rich	-1	3	Education (years per capita)	551
Stomach cancer	Male	15-19 years	95+ years	Data Rich	1	3	Age- and sex-specific SEV for Low vegetables	30
Stomach cancer	Male	15-19 years	95+ years	Data Rich	1	3	Age- and sex-specific SEV for Low fruit	130
Stomach cancer	Male	15-19 years	95+ years	Data Rich	1	3	LDI (I$ per capita)	--
Stomach cancer	Male	15-19 years	95+ years	Global	1	1	Log-transformed SEV scalar: Stomach C	513
Stomach cancer	Male	15-19 years	95+ years	Global	1	1	Diet high in sodium	728
Stomach cancer	Male	15-19 years	95+ years	Global	1	1	Tobacco (cigarettes per capita)	903
Stomach cancer	Male	15-19 years	95+ years	Global	-1	2	Healthcare access and quality index	140
Stomach cancer	Male	15-19 years	95+ years	Global	-1	2	Improved Water Source (proportion with access)	315
Stomach cancer	Male	15-19 years	95+ years	Global	-1	2	Sanitation (proportion with access)	459
Stomach cancer	Male	15-19 years	95+ years	Global	1	2	Age- and sex-specific SEV for Unsafe sanitation	51
Stomach cancer	Male	15-19 years	95+ years	Global	1	2	Cumulative Cigarettes (20 Years)	174
Stomach cancer	Male	15-19 years	95+ years	Global	1	2	Age- and sex-specific SEV for Unsafe water	223
Stomach cancer	Male	15-19 years	95+ years	Global	1	2	Mean BMI	--
Stomach cancer	Male	15-19 years	95+ years	Global	-1	3	Education (years per capita)	284
Stomach cancer	Male	15-19 years	95+ years	Global	-1	3	Socio-demographic Index	426
Stomach cancer	Male	15-19 years	95+ years	Global	1	3	Age- and sex-specific SEV for Low fruit	47
Stomach cancer	Male	15-19 years	95+ years	Global	1	3	Age- and sex-specific SEV for Low vegetables	48
Stomach cancer	Male	15-19 years	95+ years	Global	1	3	LDI (I$ per capita)	--
Colon and rectum cancer	Female	15-19 years	95+ years	Data Rich	-1	1	Total Physical Activity (MET-min/week), Age-specific	86
Colon and rectum cancer	Female	15-19 years	95+ years	Data Rich	1	1	Mean BMI	472
Colon and rectum cancer	Female	15-19 years	95+ years	Data Rich	1	1	Age- and sex-specific SEV for High red meat	511
Colon and rectum cancer	Female	15-19 years	95+ years	Data Rich	1	1	Tobacco (cigarettes per capita)	675
Colon and rectum cancer	Female	15-19 years	95+ years	Data Rich	1	1	Log-transformed SEV scalar: Colorect C	--
Colon and rectum cancer	Female	15-19 years	95+ years	Data Rich	-1	2	pufa adjusted(percent)	--
Colon and rectum cancer	Female	15-19 years	95+ years	Data Rich	1	2	Liters of alcohol consumed per capita	33
Colon and rectum cancer	Female	15-19 years	95+ years	Data Rich	1	2	Cumulative Cigarettes (5 Years)	99
Colon and rectum cancer	Female	15-19 years	95+ years	Data Rich	1	2	Diabetes Fasting Plasma Glucose (mmol/L), age-standardized 25+	180
Colon and rectum cancer	Female	15-19 years	95+ years	Data Rich	1	2	Age- and sex-specific SEV for Low calcium	--
Colon and rectum cancer	Female	15-19 years	95+ years	Data Rich	1	2	Age- and sex-specific SEV for Low fiber	--
Colon and rectum cancer	Female	15-19 years	95+ years	Data Rich	1	2	Age- and sex-specific SEV for Low vegetables	--
Colon and rectum cancer	Female	15-19 years	95+ years	Data Rich	-1	3	Education (years per capita)	--
Colon and rectum cancer	Female	15-19 years	95+ years	Data Rich	-1	3	Healthcare access and quality index	--
Colon and rectum cancer	Female	15-19 years	95+ years	Data Rich	1	3	Socio-demographic Index	248
Colon and rectum cancer	Female	15-19 years	95+ years	Data Rich	1	3	Age- and sex-specific SEV for Low fruit	--
Colon and rectum cancer	Female	15-19 years	95+ years	Data Rich	1	3	Age- and sex-specific SEV for Low milk	--
Colon and rectum cancer	Female	15-19 years	95+ years	Data Rich	1	3	Age- and sex-specific SEV for Low nuts and seeds	--
Colon and rectum cancer	Female	15-19 years	95+ years	Data Rich	1	3	LDI (I$ per capita)	--
Colon and rectum cancer	Female	15-19 years	95+ years	Global	-1	1	Total Physical Activity (MET-min/week), Age-specific	--
Colon and rectum cancer	Female	15-19 years	95+ years	Global	1	1	Log-transformed SEV scalar: Colorect C	134
Colon and rectum cancer	Female	15-19 years	95+ years	Global	1	1	Tobacco (cigarettes per capita)	364
Colon and rectum cancer	Female	15-19 years	95+ years	Global	1	1	Mean BMI	571
Colon and rectum cancer	Female	15-19 years	95+ years	Global	1	1	Age- and sex-specific SEV for High red meat	608
Colon and rectum cancer	Female	15-19 years	95+ years	Global	-1	2	pufa adjusted(percent)	--
Colon and rectum cancer	Female	15-19 years	95+ years	Global	1	2	Age- and sex-specific SEV for Low vegetables	58
Colon and rectum cancer	Female	15-19 years	95+ years	Global	1	2	Age- and sex-specific SEV for Low fiber	77
Colon and rectum cancer	Female	15-19 years	95+ years	Global	1	2	Cumulative Cigarettes (5 Years)	127
Colon and rectum cancer	Female	15-19 years	95+ years	Global	1	2	Liters of alcohol consumed per capita	144
Colon and rectum cancer	Female	15-19 years	95+ years	Global	1	2	Diabetes Fasting Plasma Glucose (mmol/L), age-standardized 25+	195
Colon and rectum cancer	Female	15-19 years	95+ years	Global	1	2	Age- and sex-specific SEV for Low calcium	--
Colon and rectum cancer	Female	15-19 years	95+ years	Global	-1	3	Healthcare access and quality index	37
Colon and rectum cancer	Female	15-19 years	95+ years	Global	-1	3	Education (years per capita)	61
Colon and rectum cancer	Female	15-19 years	95+ years	Global	1	3	Age- and sex-specific SEV for Low nuts and seeds	0
Colon and rectum cancer	Female	15-19 years	95+ years	Global	1	3	Age- and sex-specific SEV for Low fruit	7
Colon and rectum cancer	Female	15-19 years	95+ years	Global	1	3	Socio-demographic Index	37
Colon and rectum cancer	Female	15-19 years	95+ years	Global	1	3	Age- and sex-specific SEV for Low milk	243
Colon and rectum cancer	Female	15-19 years	95+ years	Global	1	3	LDI (I$ per capita)	--
Colon and rectum cancer	Male	15-19 years	95+ years	Data Rich	-1	1	Total Physical Activity (MET-min/week), Age-specific	3
Colon and rectum cancer	Male	15-19 years	95+ years	Data Rich	1	1	Tobacco (cigarettes per capita)	107
Colon and rectum cancer	Male	15-19 years	95+ years	Data Rich	1	1	Log-transformed SEV scalar: Colorect C	141
Colon and rectum cancer	Male	15-19 years	95+ years	Data Rich	1	1	Age- and sex-specific SEV for High red meat	325
Colon and rectum cancer	Male	15-19 years	95+ years	Data Rich	1	1	Mean BMI	609
Colon and rectum cancer	Male	15-19 years	95+ years	Data Rich	-1	2	pufa adjusted(percent)	--
Colon and rectum cancer	Male	15-19 years	95+ years	Data Rich	1	2	Cumulative Cigarettes (5 Years)	19
Colon and rectum cancer	Male	15-19 years	95+ years	Data Rich	1	2	Diabetes Fasting Plasma Glucose (mmol/L), age-standardized 25+	83
Colon and rectum cancer	Male	15-19 years	95+ years	Data Rich	1	2	Age- and sex-specific SEV for Low fiber	84
Colon and rectum cancer	Male	15-19 years	95+ years	Data Rich	1	2	Liters of alcohol consumed per capita	152

CODEm covariates used, level of covariate, and expected direction of covariate by cause, sex, and age

Cause	Sex	Age Start	Age End	Model Version Type	Direction	Level	Covariate Name	Number of Draws
Colon and rectum cancer	Male	15-19 years	95+ years	Data Rich	1	2	Age- and sex-specific SEV for Low calcium	--
Colon and rectum cancer	Male	15-19 years	95+ years	Data Rich	1	2	Age- and sex-specific SEV for Low vegetables	--
Colon and rectum cancer	Male	15-19 years	95+ years	Data Rich	-1	3	Education (years per capita)	0
Colon and rectum cancer	Male	15-19 years	95+ years	Data Rich	-1	3	Healthcare access and quality index	0
Colon and rectum cancer	Male	15-19 years	95+ years	Data Rich	1	3	Age- and sex-specific SEV for Low fruit	0
Colon and rectum cancer	Male	15-19 years	95+ years	Data Rich	1	3	Age- and sex-specific SEV for Low nuts and seeds	0
Colon and rectum cancer	Male	15-19 years	95+ years	Data Rich	1	3	Age- and sex-specific SEV for Low milk	47
Colon and rectum cancer	Male	15-19 years	95+ years	Data Rich	1	3	Socio-demographic Index	249
Colon and rectum cancer	Male	15-19 years	95+ years	Data Rich	1	3	LDI (I$ per capita)	--
Colon and rectum cancer	Male	15-19 years	95+ years	Global	-1	1	Total Physical Activity (MET-min/week), Age-specific	--
Colon and rectum cancer	Male	15-19 years	95+ years	Global	1	1	Tobacco (cigarettes per capita)	183
Colon and rectum cancer	Male	15-19 years	95+ years	Global	1	1	Log-transformed SEV scalar: Colorect C	347
Colon and rectum cancer	Male	15-19 years	95+ years	Global	1	1	Mean BMI	549
Colon and rectum cancer	Male	15-19 years	95+ years	Global	1	1	Age- and sex-specific SEV for High red meat	688
Colon and rectum cancer	Male	15-19 years	95+ years	Global	-1	2	pufa adjusted(percent)	--
Colon and rectum cancer	Male	15-19 years	95+ years	Global	1	2	Age- and sex-specific SEV for Low vegetables	15
Colon and rectum cancer	Male	15-19 years	95+ years	Global	1	2	Liters of alcohol consumed per capita	108
Colon and rectum cancer	Male	15-19 years	95+ years	Global	1	2	Cumulative Cigarettes (20 Years)	113
Colon and rectum cancer	Male	15-19 years	95+ years	Global	1	2	Diabetes Fasting Plasma Glucose (mmol/L), age-standardized 25+	238
Colon and rectum cancer	Male	15-19 years	95+ years	Global	1	2	Age- and sex-specific SEV for Low fiber	280
Colon and rectum cancer	Male	15-19 years	95+ years	Global	1	2	Age- and sex-specific SEV for Low calcium	--
Colon and rectum cancer	Male	15-19 years	95+ years	Global	-1	3	Healthcare access and quality index	17
Colon and rectum cancer	Male	15-19 years	95+ years	Global	-1	3	Education (years per capita)	--
Colon and rectum cancer	Male	15-19 years	95+ years	Global	1	3	Age- and sex-specific SEV for Low fruit	16
Colon and rectum cancer	Male	15-19 years	95+ years	Global	1	3	Age- and sex-specific SEV for Low milk	138
Colon and rectum cancer	Male	15-19 years	95+ years	Global	1	3	Socio-demographic Index	175
Colon and rectum cancer	Male	15-19 years	95+ years	Global	1	3	Age- and sex-specific SEV for Low nuts and seeds	--
Colon and rectum cancer	Male	15-19 years	95+ years	Global	1	3	LDI (I$ per capita)	--
Gallbladder and biliary tract cancer	Female	20-24 years	95+ years	Data Rich	1	1	Log-transformed SEV scalar: Gallblad C	--
Gallbladder and biliary tract cancer	Female	20-24 years	95+ years	Data Rich	1	1	Mean BMI	--
Gallbladder and biliary tract cancer	Female	20-24 years	95+ years	Data Rich	-1	2	Healthcare access and quality index	383
Gallbladder and biliary tract cancer	Female	20-24 years	95+ years	Data Rich	1	2	Age- and sex-specific SEV for Low vegetables	56
Gallbladder and biliary tract cancer	Female	20-24 years	95+ years	Data Rich	1	2	Tobacco (cigarettes per capita)	159
Gallbladder and biliary tract cancer	Female	20-24 years	95+ years	Data Rich	1	2	Liters of alcohol consumed per capita	188
Gallbladder and biliary tract cancer	Female	20-24 years	95+ years	Data Rich	1	2	Smoking Prevalence	376
Gallbladder and biliary tract cancer	Female	20-24 years	95+ years	Data Rich	1	2	Cumulative Cigarettes (5 Years)	495
Gallbladder and biliary tract cancer	Female	20-24 years	95+ years	Data Rich	1	2	Age- and sex-specific SEV for Low fruit	820
Gallbladder and biliary tract cancer	Female	20-24 years	95+ years	Data Rich	1	2	Cumulative Cigarettes (10 Years)	--
Gallbladder and biliary tract cancer	Female	20-24 years	95+ years	Data Rich	1	2	Diabetes Age-Standardized Prevalence (proportion)	--
Gallbladder and biliary tract cancer	Female	20-24 years	95+ years	Data Rich	-1	3	Socio-demographic Index	37
Gallbladder and biliary tract cancer	Female	20-24 years	95+ years	Data Rich	-1	3	Education (years per capita)	100
Gallbladder and biliary tract cancer	Female	20-24 years	95+ years	Data Rich	1	3	LDI (I$ per capita)	--
Gallbladder and biliary tract cancer	Female	20-24 years	95+ years	Global	1	1	Log-transformed SEV scalar: Gallblad C	--
Gallbladder and biliary tract cancer	Female	20-24 years	95+ years	Global	1	1	Mean BMI	--
Gallbladder and biliary tract cancer	Female	20-24 years	95+ years	Global	-1	2	Healthcare access and quality index	150
Gallbladder and biliary tract cancer	Female	20-24 years	95+ years	Global	1	2	Age- and sex-specific SEV for Low fruit	49
Gallbladder and biliary tract cancer	Female	20-24 years	95+ years	Global	1	2	Smoking Prevalence	298
Gallbladder and biliary tract cancer	Female	20-24 years	95+ years	Global	1	2	Liters of alcohol consumed per capita	361
Gallbladder and biliary tract cancer	Female	20-24 years	95+ years	Global	1	2	Tobacco (cigarettes per capita)	457
Gallbladder and biliary tract cancer	Female	20-24 years	95+ years	Global	1	2	Cumulative Cigarettes (10 Years)	470
Gallbladder and biliary tract cancer	Female	20-24 years	95+ years	Global	1	2	Age- and sex-specific SEV for Low vegetables	--
Gallbladder and biliary tract cancer	Female	20-24 years	95+ years	Global	1	2	Cumulative Cigarettes (5 Years)	--
Gallbladder and biliary tract cancer	Female	20-24 years	95+ years	Global	1	2	Diabetes Age-Standardized Prevalence (proportion)	--
Gallbladder and biliary tract cancer	Female	20-24 years	95+ years	Global	-1	3	Education (years per capita)	55
Gallbladder and biliary tract cancer	Female	20-24 years	95+ years	Global	-1	3	Socio-demographic Index	--
Gallbladder and biliary tract cancer	Female	20-24 years	95+ years	Global	1	3	LDI (I$ per capita)	--
Gallbladder and biliary tract cancer	Male	20-24 years	95+ years	Data Rich	1	1	Log-transformed SEV scalar: Gallblad C	494
Gallbladder and biliary tract cancer	Male	20-24 years	95+ years	Data Rich	1	1	Mean BMI	--
Gallbladder and biliary tract cancer	Male	20-24 years	95+ years	Data Rich	-1	2	Healthcare access and quality index	--
Gallbladder and biliary tract cancer	Male	20-24 years	95+ years	Data Rich	1	2	Age- and sex-specific SEV for Low fruit	94
Gallbladder and biliary tract cancer	Male	20-24 years	95+ years	Data Rich	1	2	Smoking Prevalence	162
Gallbladder and biliary tract cancer	Male	20-24 years	95+ years	Data Rich	1	2	Cumulative Cigarettes (10 Years)	195
Gallbladder and biliary tract cancer	Male	20-24 years	95+ years	Data Rich	1	2	Age- and sex-specific SEV for Low vegetables	451
Gallbladder and biliary tract cancer	Male	20-24 years	95+ years	Data Rich	1	2	Tobacco (cigarettes per capita)	451
Gallbladder and biliary tract cancer	Male	20-24 years	95+ years	Data Rich	1	2	Liters of alcohol consumed per capita	663
Gallbladder and biliary tract cancer	Male	20-24 years	95+ years	Data Rich	1	2	Cumulative Cigarettes (5 Years)	--
Gallbladder and biliary tract cancer	Male	20-24 years	95+ years	Data Rich	1	2	Diabetes Age-Standardized Prevalence (proportion)	--
Gallbladder and biliary tract cancer	Male	20-24 years	95+ years	Data Rich	-1	3	Socio-demographic Index	55
Gallbladder and biliary tract cancer	Male	20-24 years	95+ years	Data Rich	-1	3	Education (years per capita)	127
Gallbladder and biliary tract cancer	Male	20-24 years	95+ years	Data Rich	1	3	LDI (I$ per capita)	--
Gallbladder and biliary tract cancer	Male	20-24 years	95+ years	Global	1	1	Mean BMI	528
Gallbladder and biliary tract cancer	Male	20-24 years	95+ years	Global	1	1	Log-transformed SEV scalar: Gallblad C	--
Gallbladder and biliary tract cancer	Male	20-24 years	95+ years	Global	-1	2	Healthcare access and quality index	220
Gallbladder and biliary tract cancer	Male	20-24 years	95+ years	Global	1	2	Cumulative Cigarettes (10 Years)	199
Gallbladder and biliary tract cancer	Male	20-24 years	95+ years	Global	1	2	Tobacco (cigarettes per capita)	342
Gallbladder and biliary tract cancer	Male	20-24 years	95+ years	Global	1	2	Cumulative Cigarettes (5 Years)	391
Gallbladder and biliary tract cancer	Male	20-24 years	95+ years	Global	1	2	Liters of alcohol consumed per capita	562
Gallbladder and biliary tract cancer	Male	20-24 years	95+ years	Global	1	2	Age- and sex-specific SEV for Low fruit	--
Gallbladder and biliary tract cancer	Male	20-24 years	95+ years	Global	1	2	Age- and sex-specific SEV for Low vegetables	--
Gallbladder and biliary tract cancer	Male	20-24 years	95+ years	Global	1	2	Diabetes Age-Standardized Prevalence (proportion)	--
Gallbladder and biliary tract cancer	Male	20-24 years	95+ years	Global	1	2	Smoking Prevalence	--
Gallbladder and biliary tract cancer	Male	20-24 years	95+ years	Global	-1	3	Education (years per capita)	110
Gallbladder and biliary tract cancer	Male	20-24 years	95+ years	Global	-1	3	Socio-demographic Index	--
Gallbladder and biliary tract cancer	Male	20-24 years	95+ years	Global	1	3	LDI (I$ per capita)	--
Pancreatic cancer	Female	15-19 years	95+ years	Data Rich	1	1	Cumulative Cigarettes (20 Years)	16
Pancreatic cancer	Female	15-19 years	95+ years	Data Rich	1	1	Tobacco (cigarettes per capita)	89
Pancreatic cancer	Female	15-19 years	95+ years	Data Rich	1	1	Cumulative Cigarettes (10 Years)	230
Pancreatic cancer	Female	15-19 years	95+ years	Data Rich	1	1	Log-transformed SEV scalar: Pancreas C	491
Pancreatic cancer	Female	15-19 years	95+ years	Data Rich	1	1	Mean BMI	599
Pancreatic cancer	Female	15-19 years	95+ years	Data Rich	-1	2	Healthcare access and quality index	26
Pancreatic cancer	Female	15-19 years	95+ years	Data Rich	1	2	Diabetes Age-Standardized Prevalence (proportion)	104
Pancreatic cancer	Female	15-19 years	95+ years	Data Rich	1	2	Age- and sex-specific SEV for High red meat	169
Pancreatic cancer	Female	15-19 years	95+ years	Data Rich	1	2	Diabetes Fasting Plasma Glucose (mmol/L), age-standardized 25+	255
Pancreatic cancer	Female	15-19 years	95+ years	Data Rich	1	2	Liters of alcohol consumed per capita	269
Pancreatic cancer	Female	15-19 years	95+ years	Data Rich	1	2	energy unadjusted(kcal)	296
Pancreatic cancer	Female	15-19 years	95+ years	Data Rich	-1	3	Education (years per capita)	--
Pancreatic cancer	Female	15-19 years	95+ years	Data Rich	1	3	Socio-demographic Index	62
Pancreatic cancer	Female	15-19 years	95+ years	Data Rich	1	3	Age- and sex-specific SEV for Low fruit	--
Pancreatic cancer	Female	15-19 years	95+ years	Data Rich	1	3	Age- and sex-specific SEV for Low vegetables	--
Pancreatic cancer	Female	15-19 years	95+ years	Data Rich	1	3	LDI (I$ per capita)	--
Pancreatic cancer	Female	15-19 years	95+ years	Global	1	1	Tobacco (cigarettes per capita)	73
Pancreatic cancer	Female	15-19 years	95+ years	Global	1	1	Cumulative Cigarettes (20 Years)	152
Pancreatic cancer	Female	15-19 years	95+ years	Global	1	1	Cumulative Cigarettes (10 Years)	205
Pancreatic cancer	Female	15-19 years	95+ years	Global	1	1	Log-transformed SEV scalar: Pancreas C	618
Pancreatic cancer	Female	15-19 years	95+ years	Global	1	1	Mean BMI	725
Pancreatic cancer	Female	15-19 years	95+ years	Global	-1	2	Healthcare access and quality index	--
Pancreatic cancer	Female	15-19 years	95+ years	Global	1	2	Diabetes Age-Standardized Prevalence (proportion)	41
Pancreatic cancer	Female	15-19 years	95+ years	Global	1	2	Diabetes Fasting Plasma Glucose (mmol/L), age-standardized 25+	54
Pancreatic cancer	Female	15-19 years	95+ years	Global	1	2	energy unadjusted(kcal)	62
Pancreatic cancer	Female	15-19 years	95+ years	Global	1	2	Liters of alcohol consumed per capita	148
Pancreatic cancer	Female	15-19 years	95+ years	Global	1	2	Age- and sex-specific SEV for High red meat	270
Pancreatic cancer	Female	15-19 years	95+ years	Global	-1	3	Education (years per capita)	0
Pancreatic cancer	Female	15-19 years	95+ years	Global	1	3	Age- and sex-specific SEV for Low vegetables	1
Pancreatic cancer	Female	15-19 years	95+ years	Global	1	3	Socio-demographic Index	281

CODEm covariates used, level of covariate, and expected direction of covariate by cause, sex, and age

Cause	Sex	Age Start	Age End	Model Version Type	Direction	Level	Covariate Name	Number of Draws
Pancreatic cancer	Female	15-19 years	95+ years	Global	1	3	Age- and sex-specific SEV for Low fruit	--
Pancreatic cancer	Female	15-19 years	95+ years	Global	1	3	LDI (I$ per capita)	--
Pancreatic cancer	Male	15-19 years	95+ years	Data Rich	1	1	Log-transformed SEV scalar: Pancreas C	46
Pancreatic cancer	Male	15-19 years	95+ years	Data Rich	1	1	Cumulative Cigarettes (10 Years)	68
Pancreatic cancer	Male	15-19 years	95+ years	Data Rich	1	1	Tobacco (cigarettes per capita)	95
Pancreatic cancer	Male	15-19 years	95+ years	Data Rich	1	1	Cumulative Cigarettes (20 Years)	122
Pancreatic cancer	Male	15-19 years	95+ years	Data Rich	1	1	Mean BMI	817
Pancreatic cancer	Male	15-19 years	95+ years	Data Rich	-1	2	Healthcare access and quality index	0
Pancreatic cancer	Male	15-19 years	95+ years	Data Rich	1	2	Diabetes Fasting Plasma Glucose (mmol/L), age-standardized 25+	35
Pancreatic cancer	Male	15-19 years	95+ years	Data Rich	1	2	Liters of alcohol consumed per capita	80
Pancreatic cancer	Male	15-19 years	95+ years	Data Rich	1	2	Diabetes Age-Standardized Prevalence (proportion)	136
Pancreatic cancer	Male	15-19 years	95+ years	Data Rich	1	2	energy unadjusted(kcal)	171
Pancreatic cancer	Male	15-19 years	95+ years	Data Rich	1	2	Age- and sex-specific SEV for High red meat	216
Pancreatic cancer	Male	15-19 years	95+ years	Data Rich	-1	3	Education (years per capita)	--
Pancreatic cancer	Male	15-19 years	95+ years	Data Rich	1	3	Age- and sex-specific SEV for Low fruit	0
Pancreatic cancer	Male	15-19 years	95+ years	Data Rich	1	3	Age- and sex-specific SEV for Low vegetables	7
Pancreatic cancer	Male	15-19 years	95+ years	Data Rich	1	3	Socio-demographic Index	499
Pancreatic cancer	Male	15-19 years	95+ years	Data Rich	1	3	LDI (I$ per capita)	--
Pancreatic cancer	Male	15-19 years	95+ years	Global	1	1	Tobacco (cigarettes per capita)	115
Pancreatic cancer	Male	15-19 years	95+ years	Global	1	1	Cumulative Cigarettes (20 Years)	130
Pancreatic cancer	Male	15-19 years	95+ years	Global	1	1	Log-transformed SEV scalar: Pancreas C	189
Pancreatic cancer	Male	15-19 years	95+ years	Global	1	1	Cumulative Cigarettes (10 Years)	422
Pancreatic cancer	Male	15-19 years	95+ years	Global	1	1	Mean BMI	967
Pancreatic cancer	Male	15-19 years	95+ years	Global	-1	2	Healthcare access and quality index	--
Pancreatic cancer	Male	15-19 years	95+ years	Global	1	2	Liters of alcohol consumed per capita	1
Pancreatic cancer	Male	15-19 years	95+ years	Global	1	2	energy unadjusted(kcal)	54
Pancreatic cancer	Male	15-19 years	95+ years	Global	1	2	Age- and sex-specific SEV for High red meat	85
Pancreatic cancer	Male	15-19 years	95+ years	Global	1	2	Diabetes Age-Standardized Prevalence (proportion)	92
Pancreatic cancer	Male	15-19 years	95+ years	Global	1	2	Diabetes Fasting Plasma Glucose (mmol/L), age-standardized 25+	95
Pancreatic cancer	Male	15-19 years	95+ years	Global	-1	3	Education (years per capita)	--
Pancreatic cancer	Male	15-19 years	95+ years	Global	1	3	Age- and sex-specific SEV for Low vegetables	4
Pancreatic cancer	Male	15-19 years	95+ years	Global	1	3	Socio-demographic Index	492
Pancreatic cancer	Male	15-19 years	95+ years	Global	1	3	Age- and sex-specific SEV for Low fruit	--
Pancreatic cancer	Male	15-19 years	95+ years	Global	1	3	LDI (I$ per capita)	--
Larynx cancer	Female	20-24 years	95+ years	Data Rich	1	1	Liters of alcohol consumed per capita	492
Larynx cancer	Female	20-24 years	95+ years	Data Rich	1	1	Log-transformed SEV scalar: Larynx C	532
Larynx cancer	Female	20-24 years	95+ years	Data Rich	-1	2	Healthcare access and quality index	138
Larynx cancer	Female	20-24 years	95+ years	Data Rich	1	2	Cumulative Cigarettes (10 Years)	3
Larynx cancer	Female	20-24 years	95+ years	Data Rich	1	2	Smoking Prevalence	81
Larynx cancer	Female	20-24 years	95+ years	Data Rich	1	2	Population Density (over 1000 ppl/sqkm, proportion)	200
Larynx cancer	Female	20-24 years	95+ years	Data Rich	1	2	Cumulative Cigarettes (20 Years)	216
Larynx cancer	Female	20-24 years	95+ years	Data Rich	1	2	Asbestos consumption (metric tons per year per capita)	288
Larynx cancer	Female	20-24 years	95+ years	Data Rich	1	2	Age- and sex-specific SEV for Low fruit	--
Larynx cancer	Female	20-24 years	95+ years	Data Rich	1	3	Socio-demographic Index	65
Larynx cancer	Female	20-24 years	95+ years	Data Rich	1	3	Age- and sex-specific SEV for Low vegetables	619
Larynx cancer	Female	20-24 years	95+ years	Data Rich	1	3	LDI (I$ per capita)	--
Larynx cancer	Female	20-24 years	95+ years	Global	1	1	Liters of alcohol consumed per capita	588
Larynx cancer	Female	20-24 years	95+ years	Global	1	1	Log-transformed SEV scalar: Larynx C	1000
Larynx cancer	Female	20-24 years	95+ years	Global	-1	2	Healthcare access and quality index	--
Larynx cancer	Female	20-24 years	95+ years	Global	1	2	Asbestos consumption (metric tons per year per capita)	160
Larynx cancer	Female	20-24 years	95+ years	Global	1	2	Cumulative Cigarettes (20 Years)	192
Larynx cancer	Female	20-24 years	95+ years	Global	1	2	Cumulative Cigarettes (10 Years)	240
Larynx cancer	Female	20-24 years	95+ years	Global	1	2	Smoking Prevalence	400
Larynx cancer	Female	20-24 years	95+ years	Global	1	2	Age- and sex-specific SEV for Low vegetables	--
Larynx cancer	Female	20-24 years	95+ years	Global	1	2	Population Density (over 1000 ppl/sqkm, proportion)	--
Larynx cancer	Female	20-24 years	95+ years	Global	1	3	Age- and sex-specific SEV for Low fruit	229
Larynx cancer	Female	20-24 years	95+ years	Global	1	3	LDI (I$ per capita)	--
Larynx cancer	Female	20-24 years	95+ years	Global	1	3	Socio-demographic Index	--
Larynx cancer	Male	20-24 years	95+ years	Data Rich	1	1	Liters of alcohol consumed per capita	386
Larynx cancer	Male	20-24 years	95+ years	Data Rich	1	1	Log-transformed SEV scalar: Larynx C	892
Larynx cancer	Male	20-24 years	95+ years	Data Rich	-1	2	Healthcare access and quality index	625
Larynx cancer	Male	20-24 years	95+ years	Data Rich	1	2	Cumulative Cigarettes (10 Years)	7
Larynx cancer	Male	20-24 years	95+ years	Data Rich	1	2	Population Density (over 1000 ppl/sqkm, proportion)	216
Larynx cancer	Male	20-24 years	95+ years	Data Rich	1	2	Age- and sex-specific SEV for Low fruit	240
Larynx cancer	Male	20-24 years	95+ years	Data Rich	1	2	Smoking Prevalence	430
Larynx cancer	Male	20-24 years	95+ years	Data Rich	1	2	Asbestos consumption (metric tons per year per capita)	761
Larynx cancer	Male	20-24 years	95+ years	Data Rich	1	2	Cumulative Cigarettes (20 Years)	--
Larynx cancer	Male	20-24 years	95+ years	Data Rich	1	3	Age- and sex-specific SEV for Low vegetables	100
Larynx cancer	Male	20-24 years	95+ years	Data Rich	1	3	Socio-demographic Index	112
Larynx cancer	Male	20-24 years	95+ years	Data Rich	1	3	LDI (I$ per capita)	--
Larynx cancer	Male	20-24 years	95+ years	Global	1	1	Log-transformed SEV scalar: Larynx C	91
Larynx cancer	Male	20-24 years	95+ years	Global	1	1	Liters of alcohol consumed per capita	246
Larynx cancer	Male	20-24 years	95+ years	Global	-1	2	Healthcare access and quality index	219
Larynx cancer	Male	20-24 years	95+ years	Global	1	2	Cumulative Cigarettes (10 Years)	146
Larynx cancer	Male	20-24 years	95+ years	Global	1	2	Age- and sex-specific SEV for Low vegetables	228
Larynx cancer	Male	20-24 years	95+ years	Global	1	2	Population Density (over 1000 ppl/sqkm, proportion)	287
Larynx cancer	Male	20-24 years	95+ years	Global	1	2	Smoking Prevalence	327
Larynx cancer	Male	20-24 years	95+ years	Global	1	2	Cumulative Cigarettes (20 Years)	443
Larynx cancer	Male	20-24 years	95+ years	Global	1	2	Asbestos consumption (metric tons per year per capita)	721
Larynx cancer	Male	20-24 years	95+ years	Global	1	3	Age- and sex-specific SEV for Low fruit	4
Larynx cancer	Male	20-24 years	95+ years	Global	1	3	Socio-demographic Index	454
Larynx cancer	Male	20-24 years	95+ years	Global	1	3	LDI (I$ per capita)	--
Tracheal, bronchus, and lung cancer	Female	15-19 years	95+ years	Data Rich	1	1	Log-transformed age-standardized SEV scalar: Lung C	122
Tracheal, bronchus, and lung cancer	Female	15-19 years	95+ years	Data Rich	1	1	Log-transformed SEV scalar: Lung C	234
Tracheal, bronchus, and lung cancer	Female	15-19 years	95+ years	Data Rich	1	1	Smoking Prevalence	253
Tracheal, bronchus, and lung cancer	Female	15-19 years	95+ years	Data Rich	1	1	Asbestos consumption (metric tons per year per capita)	993
Tracheal, bronchus, and lung cancer	Female	15-19 years	95+ years	Data Rich	-1	2	Healthcare access and quality index	--
Tracheal, bronchus, and lung cancer	Female	15-19 years	95+ years	Data Rich	1	2	Residential radon	0
Tracheal, bronchus, and lung cancer	Female	15-19 years	95+ years	Data Rich	1	2	Cumulative Cigarettes (10 Years)	27
Tracheal, bronchus, and lung cancer	Female	15-19 years	95+ years	Data Rich	1	2	Cumulative Cigarettes (20 Years)	133
Tracheal, bronchus, and lung cancer	Female	15-19 years	95+ years	Data Rich	1	2	Diabetes Fasting Plasma Glucose (mmol/L), age-standardized 25+	275
Tracheal, bronchus, and lung cancer	Female	15-19 years	95+ years	Data Rich	1	2	Indoor Air Pollution (All Cooking Fuels)	--
Tracheal, bronchus, and lung cancer	Female	15-19 years	95+ years	Data Rich	1	2	Outdoor Air Pollution (PM2.5)	--
Tracheal, bronchus, and lung cancer	Female	15-19 years	95+ years	Data Rich	1	2	Secondhand smoke	--
Tracheal, bronchus, and lung cancer	Female	15-19 years	95+ years	Data Rich	-1	3	Education (years per capita)	--
Tracheal, bronchus, and lung cancer	Female	15-19 years	95+ years	Data Rich	1	3	Socio-demographic Index	254
Tracheal, bronchus, and lung cancer	Female	15-19 years	95+ years	Data Rich	1	3	LDI (I$ per capita)	--
Tracheal, bronchus, and lung cancer	Female	15-19 years	95+ years	Global	1	1	Log-transformed SEV scalar: Lung C	435
Tracheal, bronchus, and lung cancer	Female	15-19 years	95+ years	Global	1	1	Smoking Prevalence	443
Tracheal, bronchus, and lung cancer	Female	15-19 years	95+ years	Global	1	1	Log-transformed age-standardized SEV scalar: Lung C	575
Tracheal, bronchus, and lung cancer	Female	15-19 years	95+ years	Global	1	1	Asbestos consumption (metric tons per year per capita)	636
Tracheal, bronchus, and lung cancer	Female	15-19 years	95+ years	Global	-1	2	Healthcare access and quality index	--
Tracheal, bronchus, and lung cancer	Female	15-19 years	95+ years	Global	1	2	Outdoor Air Pollution (PM2.5)	0
Tracheal, bronchus, and lung cancer	Female	15-19 years	95+ years	Global	1	2	Cumulative Cigarettes (10 Years)	20
Tracheal, bronchus, and lung cancer	Female	15-19 years	95+ years	Global	1	2	Cumulative Cigarettes (20 Years)	166
Tracheal, bronchus, and lung cancer	Female	15-19 years	95+ years	Global	1	2	Diabetes Fasting Plasma Glucose (mmol/L), age-standardized 25+	294
Tracheal, bronchus, and lung cancer	Female	15-19 years	95+ years	Global	1	2	Indoor Air Pollution (All Cooking Fuels)	--
Tracheal, bronchus, and lung cancer	Female	15-19 years	95+ years	Global	1	2	Residential radon	--
Tracheal, bronchus, and lung cancer	Female	15-19 years	95+ years	Global	1	2	Secondhand smoke	--
Tracheal, bronchus, and lung cancer	Female	15-19 years	95+ years	Global	-1	3	Education (years per capita)	--
Tracheal, bronchus, and lung cancer	Female	15-19 years	95+ years	Global	1	3	Socio-demographic Index	545
Tracheal, bronchus, and lung cancer	Female	15-19 years	95+ years	Global	1	3	LDI (I$ per capita)	--
Tracheal, bronchus, and lung cancer	Male	15-19 years	95+ years	Data Rich	1	1	Log-transformed SEV scalar: Lung C	239

CODEm covariates used, level of covariate, and expected direction of covariate by cause, sex, and age

Cause	Sex	Age Start	Age End	Model Version Type	Direction	Level	Covariate Name	Number of Draws
Tracheal, bronchus, and lung cancer	Male	15-19 years	95+ years	Data Rich	1	1	Smoking Prevalence	613
Tracheal, bronchus, and lung cancer	Male	15-19 years	95+ years	Data Rich	1	1	Log-transformed age-standardized SEV scalar: Lung C	855
Tracheal, bronchus, and lung cancer	Male	15-19 years	95+ years	Data Rich	1	1	Asbestos consumption (metric tons per year per capita)	970
Tracheal, bronchus, and lung cancer	Male	15-19 years	95+ years	Data Rich	-1	2	Healthcare access and quality index	0
Tracheal, bronchus, and lung cancer	Male	15-19 years	95+ years	Data Rich	1	2	Cumulative Cigarettes (20 Years)	0
Tracheal, bronchus, and lung cancer	Male	15-19 years	95+ years	Data Rich	1	2	Cumulative Cigarettes (10 Years)	1
Tracheal, bronchus, and lung cancer	Male	15-19 years	95+ years	Data Rich	1	2	Secondhand smoke	84
Tracheal, bronchus, and lung cancer	Male	15-19 years	95+ years	Data Rich	1	2	Outdoor Air Pollution (PM2.5)	358
Tracheal, bronchus, and lung cancer	Male	15-19 years	95+ years	Data Rich	1	2	Diabetes Fasting Plasma Glucose (mmol/L), age-standardized 25+	--
Tracheal, bronchus, and lung cancer	Male	15-19 years	95+ years	Data Rich	1	2	Indoor Air Pollution (All Cooking Fuels)	--
Tracheal, bronchus, and lung cancer	Male	15-19 years	95+ years	Data Rich	1	2	Residential radon	--
Tracheal, bronchus, and lung cancer	Male	15-19 years	95+ years	Data Rich	-1	3	Education (years per capita)	0
Tracheal, bronchus, and lung cancer	Male	15-19 years	95+ years	Data Rich	1	3	Socio-demographic Index	630
Tracheal, bronchus, and lung cancer	Male	15-19 years	95+ years	Data Rich	1	3	LDI (I$ per capita)	--
Tracheal, bronchus, and lung cancer	Male	15-19 years	95+ years	Global	1	1	Smoking Prevalence	317
Tracheal, bronchus, and lung cancer	Male	15-19 years	95+ years	Global	1	1	Log-transformed age-standardized SEV scalar: Lung C	347
Tracheal, bronchus, and lung cancer	Male	15-19 years	95+ years	Global	1	1	Asbestos consumption (metric tons per year per capita)	410
Tracheal, bronchus, and lung cancer	Male	15-19 years	95+ years	Global	1	1	Log-transformed SEV scalar: Lung C	456
Tracheal, bronchus, and lung cancer	Male	15-19 years	95+ years	Global	-1	2	Healthcare access and quality index	0
Tracheal, bronchus, and lung cancer	Male	15-19 years	95+ years	Global	1	2	Secondhand smoke	107
Tracheal, bronchus, and lung cancer	Male	15-19 years	95+ years	Global	1	2	Cumulative Cigarettes (10 Years)	110
Tracheal, bronchus, and lung cancer	Male	15-19 years	95+ years	Global	1	2	Cumulative Cigarettes (20 Years)	199
Tracheal, bronchus, and lung cancer	Male	15-19 years	95+ years	Global	1	2	Diabetes Fasting Plasma Glucose (mmol/L), age-standardized 25+	362
Tracheal, bronchus, and lung cancer	Male	15-19 years	95+ years	Global	1	2	Outdoor Air Pollution (PM2.5)	454
Tracheal, bronchus, and lung cancer	Male	15-19 years	95+ years	Global	1	2	Indoor Air Pollution (All Cooking Fuels)	--
Tracheal, bronchus, and lung cancer	Male	15-19 years	95+ years	Global	1	2	Residential radon	--
Tracheal, bronchus, and lung cancer	Male	15-19 years	95+ years	Global	-1	3	Education (years per capita)	0
Tracheal, bronchus, and lung cancer	Male	15-19 years	95+ years	Global	1	3	Socio-demographic Index	379
Tracheal, bronchus, and lung cancer	Male	15-19 years	95+ years	Global	1	3	LDI (I$ per capita)	--
Malignant skin melanoma	Female	15-19 years	95+ years	Data Rich	1	1	Liters of alcohol consumed per capita	328
Malignant skin melanoma	Female	15-19 years	95+ years	Data Rich	-1	2	Latitude Under 15 (proportion)	186
Malignant skin melanoma	Female	15-19 years	95+ years	Data Rich	-1	2	Latitude 30 to 45 (proportion)	351
Malignant skin melanoma	Female	15-19 years	95+ years	Data Rich	-1	2	Healthcare access and quality index	--
Malignant skin melanoma	Female	15-19 years	95+ years	Data Rich	-1	2	Latitude 15 to 30 (proportion)	--
Malignant skin melanoma	Female	15-19 years	95+ years	Data Rich	-1	2	Latitude Over 45 (proportion)	--
Malignant skin melanoma	Female	15-19 years	95+ years	Data Rich	-1	3	Education (years per capita)	--
Malignant skin melanoma	Female	15-19 years	95+ years	Data Rich	-1	3	LDI (I$ per capita)	--
Malignant skin melanoma	Female	15-19 years	95+ years	Data Rich	1	3	Socio-demographic Index	564
Malignant skin melanoma	Female	15-19 years	95+ years	Global	1	1	Liters of alcohol consumed per capita	1000
Malignant skin melanoma	Female	15-19 years	95+ years	Global	-1	2	Latitude Over 45 (proportion)	100
Malignant skin melanoma	Female	15-19 years	95+ years	Global	-1	2	Latitude Under 15 (proportion)	144
Malignant skin melanoma	Female	15-19 years	95+ years	Global	-1	2	Healthcare access and quality index	173
Malignant skin melanoma	Female	15-19 years	95+ years	Global	-1	2	Latitude 15 to 30 (proportion)	--
Malignant skin melanoma	Female	15-19 years	95+ years	Global	-1	2	Latitude 30 to 45 (proportion)	--
Malignant skin melanoma	Female	15-19 years	95+ years	Global	-1	3	Education (years per capita)	--
Malignant skin melanoma	Female	15-19 years	95+ years	Global	-1	3	LDI (I$ per capita)	--
Malignant skin melanoma	Female	15-19 years	95+ years	Global	1	3	Socio-demographic Index	255
Malignant skin melanoma	Male	15-19 years	95+ years	Data Rich	1	1	Liters of alcohol consumed per capita	1000
Malignant skin melanoma	Male	15-19 years	95+ years	Data Rich	-1	2	Healthcare access and quality index	--
Malignant skin melanoma	Male	15-19 years	95+ years	Data Rich	-1	2	Latitude 15 to 30 (proportion)	--
Malignant skin melanoma	Male	15-19 years	95+ years	Data Rich	-1	2	Latitude 30 to 45 (proportion)	--
Malignant skin melanoma	Male	15-19 years	95+ years	Data Rich	-1	2	Latitude Over 45 (proportion)	--
Malignant skin melanoma	Male	15-19 years	95+ years	Data Rich	-1	2	Latitude Under 15 (proportion)	--
Malignant skin melanoma	Male	15-19 years	95+ years	Data Rich	-1	3	Education (years per capita)	--
Malignant skin melanoma	Male	15-19 years	95+ years	Data Rich	-1	3	LDI (I$ per capita)	--
Malignant skin melanoma	Male	15-19 years	95+ years	Data Rich	1	3	Socio-demographic Index	--
Malignant skin melanoma	Male	15-19 years	95+ years	Global	1	1	Liters of alcohol consumed per capita	1000
Malignant skin melanoma	Male	15-19 years	95+ years	Global	-1	2	Latitude Over 45 (proportion)	185
Malignant skin melanoma	Male	15-19 years	95+ years	Global	-1	2	Latitude Under 15 (proportion)	494
Malignant skin melanoma	Male	15-19 years	95+ years	Global	-1	2	Healthcare access and quality index	--
Malignant skin melanoma	Male	15-19 years	95+ years	Global	-1	2	Latitude 15 to 30 (proportion)	--
Malignant skin melanoma	Male	15-19 years	95+ years	Global	-1	2	Latitude 30 to 45 (proportion)	--
Malignant skin melanoma	Male	15-19 years	95+ years	Global	-1	3	Education (years per capita)	--
Malignant skin melanoma	Male	15-19 years	95+ years	Global	-1	3	LDI (I$ per capita)	--
Malignant skin melanoma	Male	15-19 years	95+ years	Global	1	3	Socio-demographic Index	--
Non-melanoma skin cancer (squamous-cell carcinoma)	Female	20-24 years	95+ years	Data Rich	1	1	Cumulative Cigarettes (10 Years)	165
Non-melanoma skin cancer (squamous-cell carcinoma)	Female	20-24 years	95+ years	Data Rich	1	1	Cumulative Cigarettes (15 Years)	437
Non-melanoma skin cancer (squamous-cell carcinoma)	Female	20-24 years	95+ years	Data Rich	1	1	Cumulative Cigarettes (5 Years)	475
Non-melanoma skin cancer (squamous-cell carcinoma)	Female	20-24 years	95+ years	Data Rich	1	1	Smoking Prevalence	--
Non-melanoma skin cancer (squamous-cell carcinoma)	Female	20-24 years	95+ years	Data Rich	-1	2	Average latitude	165
Non-melanoma skin cancer (squamous-cell carcinoma)	Female	20-24 years	95+ years	Data Rich	-1	2	Healthcare access and quality index	559
Non-melanoma skin cancer (squamous-cell carcinoma)	Female	20-24 years	95+ years	Data Rich	-1	3	Education (years per capita)	32
Non-melanoma skin cancer (squamous-cell carcinoma)	Female	20-24 years	95+ years	Data Rich	-1	3	LDI (I$ per capita)	647
Non-melanoma skin cancer (squamous-cell carcinoma)	Female	20-24 years	95+ years	Data Rich	1	3	Socio-demographic Index	493
Non-melanoma skin cancer (squamous-cell carcinoma)	Female	20-24 years	95+ years	Global	1	1	Smoking Prevalence	22
Non-melanoma skin cancer (squamous-cell carcinoma)	Female	20-24 years	95+ years	Global	1	1	Cumulative Cigarettes (15 Years)	57
Non-melanoma skin cancer (squamous-cell carcinoma)	Female	20-24 years	95+ years	Global	1	1	Cumulative Cigarettes (5 Years)	128
Non-melanoma skin cancer (squamous-cell carcinoma)	Female	20-24 years	95+ years	Global	1	1	Cumulative Cigarettes (10 Years)	338
Non-melanoma skin cancer (squamous-cell carcinoma)	Female	20-24 years	95+ years	Global	-1	2	Healthcare access and quality index	355
Non-melanoma skin cancer (squamous-cell carcinoma)	Female	20-24 years	95+ years	Global	-1	2	Average latitude	419
Non-melanoma skin cancer (squamous-cell carcinoma)	Female	20-24 years	95+ years	Global	-1	3	Education (years per capita)	190
Non-melanoma skin cancer (squamous-cell carcinoma)	Female	20-24 years	95+ years	Global	-1	3	LDI (I$ per capita)	235
Non-melanoma skin cancer (squamous-cell carcinoma)	Female	20-24 years	95+ years	Global	1	3	Socio-demographic Index	335
Non-melanoma skin cancer (squamous-cell carcinoma)	Male	20-24 years	95+ years	Data Rich	1	1	Cumulative Cigarettes (15 Years)	90
Non-melanoma skin cancer (squamous-cell carcinoma)	Male	20-24 years	95+ years	Data Rich	1	1	Smoking Prevalence	173
Non-melanoma skin cancer (squamous-cell carcinoma)	Male	20-24 years	95+ years	Data Rich	1	1	Cumulative Cigarettes (10 Years)	353
Non-melanoma skin cancer (squamous-cell carcinoma)	Male	20-24 years	95+ years	Data Rich	1	1	Cumulative Cigarettes (5 Years)	384
Non-melanoma skin cancer (squamous-cell carcinoma)	Male	20-24 years	95+ years	Data Rich	-1	2	Average latitude	649
Non-melanoma skin cancer (squamous-cell carcinoma)	Male	20-24 years	95+ years	Data Rich	-1	2	Healthcare access and quality index	--
Non-melanoma skin cancer (squamous-cell carcinoma)	Male	20-24 years	95+ years	Data Rich	-1	3	LDI (I$ per capita)	628
Non-melanoma skin cancer (squamous-cell carcinoma)	Male	20-24 years	95+ years	Data Rich	-1	3	Education (years per capita)	--
Non-melanoma skin cancer (squamous-cell carcinoma)	Male	20-24 years	95+ years	Data Rich	1	3	Socio-demographic Index	596
Non-melanoma skin cancer (squamous-cell carcinoma)	Male	20-24 years	95+ years	Global	1	1	Cumulative Cigarettes (15 Years)	148
Non-melanoma skin cancer (squamous-cell carcinoma)	Male	20-24 years	95+ years	Global	1	1	Cumulative Cigarettes (5 Years)	186
Non-melanoma skin cancer (squamous-cell carcinoma)	Male	20-24 years	95+ years	Global	1	1	Smoking Prevalence	277
Non-melanoma skin cancer (squamous-cell carcinoma)	Male	20-24 years	95+ years	Global	1	1	Cumulative Cigarettes (10 Years)	--
Non-melanoma skin cancer (squamous-cell carcinoma)	Male	20-24 years	95+ years	Global	-1	2	Average latitude	408
Non-melanoma skin cancer (squamous-cell carcinoma)	Male	20-24 years	95+ years	Global	-1	2	Healthcare access and quality index	--
Non-melanoma skin cancer (squamous-cell carcinoma)	Male	20-24 years	95+ years	Global	-1	3	LDI (I$ per capita)	197
Non-melanoma skin cancer (squamous-cell carcinoma)	Male	20-24 years	95+ years	Global	-1	3	Education (years per capita)	--
Non-melanoma skin cancer (squamous-cell carcinoma)	Male	20-24 years	95+ years	Global	1	3	Socio-demographic Index	527
Soft tissue and other extraosseous sarcomas	Female	0-6 days	95+ years	Data Rich	-1	2	Universal health coverage	131
Soft tissue and other extraosseous sarcomas	Female	0-6 days	95+ years	Data Rich	-1	2	Healthcare access and quality index	156
Soft tissue and other extraosseous sarcomas	Female	0-6 days	95+ years	Data Rich	-1	2	Socio-demographic Index	212
Soft tissue and other extraosseous sarcomas	Female	0-6 days	95+ years	Data Rich	-1	2	LDI (I$ per capita)	--

CODEm covariates used, level of covariate, and expected direction of covariate by cause, sex, and age								
Cause	Sex	Age Start	Age End	Model Version Type	Direction	Level	Covariate Name	Number of Draws
Soft tissue and other extraosseous sarcomas	Female	0-6 days	95+ years	Data Rich	-1	3	Maternal care and immunization	119
Soft tissue and other extraosseous sarcomas	Female	0-6 days	95+ years	Data Rich	-1	3	Education (years per capita)	--
Soft tissue and other extraosseous sarcomas	Female	0-6 days	95+ years	Data Rich	1	3	Log-transformed age-standardized SEV scalar: HIV	210
Soft tissue and other extraosseous sarcomas	Female	0-6 days	95+ years	Data Rich	1	3	Liters of alcohol consumed per capita	384
Soft tissue and other extraosseous sarcomas	Female	0-6 days	95+ years	Data Rich	1	3	Log-transformed SEV scalar: HIV	--
Soft tissue and other extraosseous sarcomas	Female	0-6 days	95+ years	Global	-1	2	Healthcare access and quality index	185
Soft tissue and other extraosseous sarcomas	Female	0-6 days	95+ years	Global	-1	2	LDI (I$ per capita)	--
Soft tissue and other extraosseous sarcomas	Female	0-6 days	95+ years	Global	-1	2	Socio-demographic Index	--
Soft tissue and other extraosseous sarcomas	Female	0-6 days	95+ years	Global	-1	2	Universal health coverage	--
Soft tissue and other extraosseous sarcomas	Female	0-6 days	95+ years	Global	-1	3	Education (years per capita)	--
Soft tissue and other extraosseous sarcomas	Female	0-6 days	95+ years	Global	-1	3	Maternal care and immunization	--
Soft tissue and other extraosseous sarcomas	Female	0-6 days	95+ years	Global	1	3	Log-transformed SEV scalar: HIV	222
Soft tissue and other extraosseous sarcomas	Female	0-6 days	95+ years	Global	1	3	Liters of alcohol consumed per capita	267
Soft tissue and other extraosseous sarcomas	Female	0-6 days	95+ years	Global	1	3	Log-transformed age-standardized SEV scalar: HIV	326
Soft tissue and other extraosseous sarcomas	Male	0-6 days	95+ years	Data Rich	-1	2	Healthcare access and quality index	210
Soft tissue and other extraosseous sarcomas	Male	0-6 days	95+ years	Data Rich	-1	2	Universal health coverage	252
Soft tissue and other extraosseous sarcomas	Male	0-6 days	95+ years	Data Rich	-1	2	LDI (I$ per capita)	--
Soft tissue and other extraosseous sarcomas	Male	0-6 days	95+ years	Data Rich	-1	2	Socio-demographic Index	--
Soft tissue and other extraosseous sarcomas	Male	0-6 days	95+ years	Data Rich	-1	3	Education (years per capita)	111
Soft tissue and other extraosseous sarcomas	Male	0-6 days	95+ years	Data Rich	-1	3	Maternal care and immunization	111
Soft tissue and other extraosseous sarcomas	Male	0-6 days	95+ years	Data Rich	1	3	Log-transformed age-standardized SEV scalar: HIV	235
Soft tissue and other extraosseous sarcomas	Male	0-6 days	95+ years	Data Rich	1	3	Liters of alcohol consumed per capita	280
Soft tissue and other extraosseous sarcomas	Male	0-6 days	95+ years	Data Rich	1	3	Log-transformed SEV scalar: HIV	--
Soft tissue and other extraosseous sarcomas	Male	0-6 days	95+ years	Global	-1	2	Healthcare access and quality index	--
Soft tissue and other extraosseous sarcomas	Male	0-6 days	95+ years	Global	-1	2	LDI (I$ per capita)	--
Soft tissue and other extraosseous sarcomas	Male	0-6 days	95+ years	Global	-1	2	Socio-demographic Index	--
Soft tissue and other extraosseous sarcomas	Male	0-6 days	95+ years	Global	-1	2	Universal health coverage	--
Soft tissue and other extraosseous sarcomas	Male	0-6 days	95+ years	Global	-1	3	Education (years per capita)	--
Soft tissue and other extraosseous sarcomas	Male	0-6 days	95+ years	Global	-1	3	Maternal care and immunization	--
Soft tissue and other extraosseous sarcomas	Male	0-6 days	95+ years	Global	1	3	Liters of alcohol consumed per capita	1000
Soft tissue and other extraosseous sarcomas	Male	0-6 days	95+ years	Global	1	3	Log-transformed age-standardized SEV scalar: HIV	1000
Soft tissue and other extraosseous sarcomas	Male	0-6 days	95+ years	Global	1	3	Log-transformed SEV scalar: HIV	--
Malignant neoplasm of bone and articular cartilage	Female	12-23 months	95+ years	Data Rich	-1	2	Universal health coverage	115
Malignant neoplasm of bone and articular cartilage	Female	12-23 months	95+ years	Data Rich	-1	2	Healthcare access and quality index	293
Malignant neoplasm of bone and articular cartilage	Female	12-23 months	95+ years	Data Rich	-1	2	Socio-demographic Index	332
Malignant neoplasm of bone and articular cartilage	Female	12-23 months	95+ years	Data Rich	-1	2	LDI (I$ per capita)	--
Malignant neoplasm of bone and articular cartilage	Female	12-23 months	95+ years	Data Rich	-1	3	Maternal care and immunization	268
Malignant neoplasm of bone and articular cartilage	Female	12-23 months	95+ years	Data Rich	-1	3	Health worker density	453
Malignant neoplasm of bone and articular cartilage	Female	12-23 months	95+ years	Data Rich	-1	3	Education (years per capita)	471
Malignant neoplasm of bone and articular cartilage	Female	12-23 months	95+ years	Data Rich	1	3	Age- and sex-specific SEV for Low bone mineral density	175
Malignant neoplasm of bone and articular cartilage	Female	12-23 months	95+ years	Data Rich	1	3	Smoking Prevalence	175
Malignant neoplasm of bone and articular cartilage	Female	12-23 months	95+ years	Data Rich	1	3	Log-transformed age-standardized SEV scalar: Osteoarth	494
Malignant neoplasm of bone and articular cartilage	Female	12-23 months	95+ years	Data Rich	1	3	Log-transformed SEV scalar: Osteoarth	596
Malignant neoplasm of bone and articular cartilage	Female	12-23 months	95+ years	Global	-1	2	Universal health coverage	407
Malignant neoplasm of bone and articular cartilage	Female	12-23 months	95+ years	Global	-1	2	Healthcare access and quality index	--
Malignant neoplasm of bone and articular cartilage	Female	12-23 months	95+ years	Global	-1	2	LDI (I$ per capita)	--
Malignant neoplasm of bone and articular cartilage	Female	12-23 months	95+ years	Global	-1	2	Socio-demographic Index	--
Malignant neoplasm of bone and articular cartilage	Female	12-23 months	95+ years	Global	-1	3	Education (years per capita)	--
Malignant neoplasm of bone and articular cartilage	Female	12-23 months	95+ years	Global	-1	3	Health worker density	--
Malignant neoplasm of bone and articular cartilage	Female	12-23 months	95+ years	Global	-1	3	Maternal care and immunization	--
Malignant neoplasm of bone and articular cartilage	Female	12-23 months	95+ years	Global	1	3	Smoking Prevalence	185
Malignant neoplasm of bone and articular cartilage	Female	12-23 months	95+ years	Global	1	3	Log-transformed age-standardized SEV scalar: Osteoarth	452
Malignant neoplasm of bone and articular cartilage	Female	12-23 months	95+ years	Global	1	3	Log-transformed SEV scalar: Osteoarth	511
Malignant neoplasm of bone and articular cartilage	Female	12-23 months	95+ years	Global	1	3	Age- and sex-specific SEV for Low bone mineral density	--
Malignant neoplasm of bone and articular cartilage	Male	12-23 months	95+ years	Data Rich	-1	2	Universal health coverage	134
Malignant neoplasm of bone and articular cartilage	Male	12-23 months	95+ years	Data Rich	-1	2	Healthcare access and quality index	559
Malignant neoplasm of bone and articular cartilage	Male	12-23 months	95+ years	Data Rich	-1	2	LDI (I$ per capita)	--
Malignant neoplasm of bone and articular cartilage	Male	12-23 months	95+ years	Data Rich	-1	2	Socio-demographic Index	--
Malignant neoplasm of bone and articular cartilage	Male	12-23 months	95+ years	Data Rich	-1	3	Education (years per capita)	--
Malignant neoplasm of bone and articular cartilage	Male	12-23 months	95+ years	Data Rich	-1	3	Health worker density	--
Malignant neoplasm of bone and articular cartilage	Male	12-23 months	95+ years	Data Rich	-1	3	Maternal care and immunization	--
Malignant neoplasm of bone and articular cartilage	Male	12-23 months	95+ years	Data Rich	1	3	Log-transformed age-standardized SEV scalar: Osteoarth	107
Malignant neoplasm of bone and articular cartilage	Male	12-23 months	95+ years	Data Rich	1	3	Smoking Prevalence	185
Malignant neoplasm of bone and articular cartilage	Male	12-23 months	95+ years	Data Rich	1	3	Log-transformed SEV scalar: Osteoarth	464
Malignant neoplasm of bone and articular cartilage	Male	12-23 months	95+ years	Data Rich	1	3	Age- and sex-specific SEV for Low bone mineral density	--
Malignant neoplasm of bone and articular cartilage	Male	12-23 months	95+ years	Global	-1	2	Universal health coverage	1000
Malignant neoplasm of bone and articular cartilage	Male	12-23 months	95+ years	Global	-1	2	Healthcare access and quality index	--
Malignant neoplasm of bone and articular cartilage	Male	12-23 months	95+ years	Global	-1	2	LDI (I$ per capita)	--
Malignant neoplasm of bone and articular cartilage	Male	12-23 months	95+ years	Global	-1	2	Socio-demographic Index	--
Malignant neoplasm of bone and articular cartilage	Male	12-23 months	95+ years	Global	-1	3	Health worker density	491
Malignant neoplasm of bone and articular cartilage	Male	12-23 months	95+ years	Global	-1	3	Education (years per capita)	--
Malignant neoplasm of bone and articular cartilage	Male	12-23 months	95+ years	Global	-1	3	Maternal care and immunization	--
Malignant neoplasm of bone and articular cartilage	Male	12-23 months	95+ years	Global	1	3	Log-transformed age-standardized SEV scalar: Osteoarth	491
Malignant neoplasm of bone and articular cartilage	Male	12-23 months	95+ years	Global	1	3	Smoking Prevalence	491
Malignant neoplasm of bone and articular cartilage	Male	12-23 months	95+ years	Global	1	3	Age- and sex-specific SEV for Low bone mineral density	--
Malignant neoplasm of bone and articular cartilage	Male	12-23 months	95+ years	Global	1	3	Log-transformed SEV scalar: Osteoarth	--
Breast cancer	Female	15-19 years	95+ years	Data Rich	1	1	Liters of alcohol consumed per capita	277
Breast cancer	Female	15-19 years	95+ years	Data Rich	1	1	Mean BMI	397
Breast cancer	Female	15-19 years	95+ years	Data Rich	1	1	Log-transformed SEV scalar: Breast C	578
Breast cancer	Female	15-19 years	95+ years	Data Rich	-1	2	Healthcare access and quality index	0
Breast cancer	Female	15-19 years	95+ years	Data Rich	-1	2	Total Fertility Rate	4
Breast cancer	Female	15-19 years	95+ years	Data Rich	-1	2	Age-Specific Fertility Rate	167
Breast cancer	Female	15-19 years	95+ years	Data Rich	1	2	Age- and sex-specific SEV for Low fruit	0
Breast cancer	Female	15-19 years	95+ years	Data Rich	1	2	Age- and sex-specific SEV for Low vegetables	0
Breast cancer	Female	15-19 years	95+ years	Data Rich	1	2	Cumulative Cigarettes (20 Years)	99
Breast cancer	Female	15-19 years	95+ years	Data Rich	1	2	Smoking Prevalence	140
Breast cancer	Female	15-19 years	95+ years	Data Rich	1	2	Diabetes Fasting Plasma Glucose (mmol/L), age-standardized 25+	172
Breast cancer	Female	15-19 years	95+ years	Data Rich	1	2	Cumulative Cigarettes (10 Years)	330
Breast cancer	Female	15-19 years	95+ years	Data Rich	-1	3	LDI (I$ per capita)	--
Breast cancer	Female	15-19 years	95+ years	Data Rich	1	3	Socio-demographic Index	566
Breast cancer	Female	15-19 years	95+ years	Global	1	1	Mean BMI	6
Breast cancer	Female	15-19 years	95+ years	Global	1	1	Liters of alcohol consumed per capita	409
Breast cancer	Female	15-19 years	95+ years	Global	1	1	Log-transformed SEV scalar: Breast C	832
Breast cancer	Female	15-19 years	95+ years	Global	-1	2	Age-Specific Fertility Rate	0
Breast cancer	Female	15-19 years	95+ years	Global	-1	2	Healthcare access and quality index	169
Breast cancer	Female	15-19 years	95+ years	Global	-1	2	Total Fertility Rate	551
Breast cancer	Female	15-19 years	95+ years	Global	1	2	Diabetes Fasting Plasma Glucose (mmol/L), age-standardized 25+	0
Breast cancer	Female	15-19 years	95+ years	Global	1	2	Smoking Prevalence	30
Breast cancer	Female	15-19 years	95+ years	Global	1	2	Age- and sex-specific SEV for Low vegetables	156
Breast cancer	Female	15-19 years	95+ years	Global	1	2	Cumulative Cigarettes (20 Years)	164
Breast cancer	Female	15-19 years	95+ years	Global	1	2	Cumulative Cigarettes (10 Years)	359
Breast cancer	Female	15-19 years	95+ years	Global	1	2	Age- and sex-specific SEV for Low fruit	394
Breast cancer	Female	15-19 years	95+ years	Global	-1	3	LDI (I$ per capita)	--
Breast cancer	Female	15-19 years	95+ years	Global	1	3	Socio-demographic Index	568
Breast cancer	Male	15-19 years	95+ years	Data Rich	1	1	Liters of alcohol consumed per capita	239
Breast cancer	Male	15-19 years	95+ years	Data Rich	1	1	Mean BMI	458
Breast cancer	Male	15-19 years	95+ years	Data Rich	1	1	Log-transformed SEV scalar: Breast C	689
Breast cancer	Male	15-19 years	95+ years	Data Rich	-1	2	Healthcare access and quality index	--
Breast cancer	Male	15-19 years	95+ years	Data Rich	1	2	Cumulative Cigarettes (20 Years)	20
Breast cancer	Male	15-19 years	95+ years	Data Rich	1	2	Cumulative Cigarettes (10 Years)	46
Breast cancer	Male	15-19 years	95+ years	Data Rich	1	2	Diabetes Fasting Plasma Glucose (mmol/L), age-standardized 25+	220
Breast cancer	Male	15-19 years	95+ years	Data Rich	1	2	Age- and sex-specific SEV for Low fruit	--
Breast cancer	Male	15-19 years	95+ years	Data Rich	1	2	Age- and sex-specific SEV for Low vegetables	--
Breast cancer	Male	15-19 years	95+ years	Data Rich	1	2	Smoking Prevalence	--
Breast cancer	Male	15-19 years	95+ years	Data Rich	-1	3	LDI (I$ per capita)	--
Breast cancer	Male	15-19 years	95+ years	Data Rich	1	3	Socio-demographic Index	39

CODEm covariates used, level of covariate, and expected direction of covariate by cause, sex, and age								
**Cause**	**Sex**	**Age Start**	**Age End**	**Model Version Type**	**Direction**	**Level**	**Covariate Name**	**Number of Draws**
Breast cancer	Male	15-19 years	95+ years	Global	1	1	Liters of alcohol consumed per capita	137
Breast cancer	Male	15-19 years	95+ years	Global	1	1	Log-transformed SEV scalar: Breast C	354
Breast cancer	Male	15-19 years	95+ years	Global	1	1	Mean BMI	718
Breast cancer	Male	15-19 years	95+ years	Global	-1	2	Healthcare access and quality index	44
Breast cancer	Male	15-19 years	95+ years	Global	1	2	Cumulative Cigarettes (10 Years)	44
Breast cancer	Male	15-19 years	95+ years	Global	1	2	Diabetes Fasting Plasma Glucose (mmol/L), age-standardized 25+	121
Breast cancer	Male	15-19 years	95+ years	Global	1	2	Age- and sex-specific SEV for Low fruit	--
Breast cancer	Male	15-19 years	95+ years	Global	1	2	Age- and sex-specific SEV for Low vegetables	--
Breast cancer	Male	15-19 years	95+ years	Global	1	2	Cumulative Cigarettes (20 Years)	--
Breast cancer	Male	15-19 years	95+ years	Global	1	2	Smoking Prevalence	--
Breast cancer	Male	15-19 years	95+ years	Global	-1	3	LDI (I$ per capita)	--
Breast cancer	Male	15-19 years	95+ years	Global	1	3	Socio-demographic Index	207
Cervical cancer	Female	15-19 years	95+ years	Data Rich	1	1	Log-transformed SEV scalar: HIV	476
Cervical cancer	Female	15-19 years	95+ years	Data Rich	1	1	Cumulative Cigarettes (5 Years)	538
Cervical cancer	Female	15-19 years	95+ years	Data Rich	1	1	HIV age-standardized prevalence	938
Cervical cancer	Female	15-19 years	95+ years	Data Rich	1	1	Log-transformed age-standardized SEV scalar: HIV	--
Cervical cancer	Female	15-19 years	95+ years	Data Rich	-1	2	Healthcare access and quality index	612
Cervical cancer	Female	15-19 years	95+ years	Data Rich	1	2	Age-Specific Fertility Rate	67
Cervical cancer	Female	15-19 years	95+ years	Data Rich	1	2	Age- and sex-specific SEV for Low vegetables	106
Cervical cancer	Female	15-19 years	95+ years	Data Rich	1	2	Total Fertility Rate	318
Cervical cancer	Female	15-19 years	95+ years	Data Rich	1	2	Age- and sex-specific SEV for Low fruit	--
Cervical cancer	Female	15-19 years	95+ years	Data Rich	1	2	Smoking Prevalence	--
Cervical cancer	Female	15-19 years	95+ years	Data Rich	-1	3	Socio-demographic Index	80
Cervical cancer	Female	15-19 years	95+ years	Data Rich	-1	3	Education (years per capita)	470
Cervical cancer	Female	15-19 years	95+ years	Data Rich	-1	3	LDI (I$ per capita)	--
Cervical cancer	Female	15-19 years	95+ years	Global	1	1	Cumulative Cigarettes (5 Years)	155
Cervical cancer	Female	15-19 years	95+ years	Global	1	1	Log-transformed SEV scalar: HIV	526
Cervical cancer	Female	15-19 years	95+ years	Global	1	1	HIV age-standardized prevalence	665
Cervical cancer	Female	15-19 years	95+ years	Global	1	1	Log-transformed age-standardized SEV scalar: HIV	--
Cervical cancer	Female	15-19 years	95+ years	Global	-1	2	Healthcare access and quality index	40
Cervical cancer	Female	15-19 years	95+ years	Global	1	2	Age- and sex-specific SEV for Low fruit	4
Cervical cancer	Female	15-19 years	95+ years	Global	1	2	Age- and sex-specific SEV for Low vegetables	4
Cervical cancer	Female	15-19 years	95+ years	Global	1	2	Total Fertility Rate	125
Cervical cancer	Female	15-19 years	95+ years	Global	1	2	Age-Specific Fertility Rate	275
Cervical cancer	Female	15-19 years	95+ years	Global	1	2	Smoking Prevalence	--
Cervical cancer	Female	15-19 years	95+ years	Global	-1	3	Socio-demographic Index	287
Cervical cancer	Female	15-19 years	95+ years	Global	-1	3	Education (years per capita)	420
Cervical cancer	Female	15-19 years	95+ years	Global	-1	3	LDI (I$ per capita)	--
Uterine cancer	Female	20-24 years	95+ years	Data Rich	1	1	Log-transformed SEV scalar: Uterus C	357
Uterine cancer	Female	20-24 years	95+ years	Data Rich	1	1	Mean BMI	763
Uterine cancer	Female	20-24 years	95+ years	Data Rich	-1	2	Healthcare access and quality index	63
Uterine cancer	Female	20-24 years	95+ years	Data Rich	-1	2	Total Fertility Rate	144
Uterine cancer	Female	20-24 years	95+ years	Data Rich	1	2	Tobacco (cigarettes per capita)	21
Uterine cancer	Female	20-24 years	95+ years	Data Rich	1	2	Cumulative Cigarettes (5 Years)	37
Uterine cancer	Female	20-24 years	95+ years	Data Rich	1	2	Smoking Prevalence	63
Uterine cancer	Female	20-24 years	95+ years	Data Rich	1	2	Age- and sex-specific SEV for Low vegetables	84
Uterine cancer	Female	20-24 years	95+ years	Data Rich	1	2	Diabetes Age-Standardized Prevalence (proportion)	285
Uterine cancer	Female	20-24 years	95+ years	Data Rich	1	2	Age- and sex-specific SEV for Low fruit	--
Uterine cancer	Female	20-24 years	95+ years	Data Rich	1	2	Cumulative Cigarettes (10 Years)	--
Uterine cancer	Female	20-24 years	95+ years	Data Rich	-1	3	Education (years per capita)	16
Uterine cancer	Female	20-24 years	95+ years	Data Rich	1	3	Socio-demographic Index	9
Uterine cancer	Female	20-24 years	95+ years	Data Rich	1	3	LDI (I$ per capita)	--
Uterine cancer	Female	20-24 years	95+ years	Global	1	1	Mean BMI	242
Uterine cancer	Female	20-24 years	95+ years	Global	1	1	Log-transformed SEV scalar: Uterus C	672
Uterine cancer	Female	20-24 years	95+ years	Global	-1	2	Healthcare access and quality index	148
Uterine cancer	Female	20-24 years	95+ years	Global	-1	2	Total Fertility Rate	153
Uterine cancer	Female	20-24 years	95+ years	Global	1	2	Smoking Prevalence	44
Uterine cancer	Female	20-24 years	95+ years	Global	1	2	Age- and sex-specific SEV for Low fruit	106
Uterine cancer	Female	20-24 years	95+ years	Global	1	2	Age- and sex-specific SEV for Low vegetables	224
Uterine cancer	Female	20-24 years	95+ years	Global	1	2	Diabetes Age-Standardized Prevalence (proportion)	458
Uterine cancer	Female	20-24 years	95+ years	Global	1	2	Cumulative Cigarettes (10 Years)	--
Uterine cancer	Female	20-24 years	95+ years	Global	1	2	Cumulative Cigarettes (5 Years)	--
Uterine cancer	Female	20-24 years	95+ years	Global	1	2	Tobacco (cigarettes per capita)	--
Uterine cancer	Female	20-24 years	95+ years	Global	-1	3	Education (years per capita)	65
Uterine cancer	Female	20-24 years	95+ years	Global	1	3	Socio-demographic Index	165
Uterine cancer	Female	20-24 years	95+ years	Global	1	3	LDI (I$ per capita)	--
Ovarian cancer	Female	15-19 years	95+ years	Data Rich	1	1	Log-transformed SEV scalar: Ovary C	410
Ovarian cancer	Female	15-19 years	95+ years	Data Rich	1	1	Liters of alcohol consumed per capita	1000
Ovarian cancer	Female	15-19 years	95+ years	Data Rich	-1	2	Contraception (Modern) Prevalence (proportion)	10
Ovarian cancer	Female	15-19 years	95+ years	Data Rich	-1	2	Healthcare access and quality index	202
Ovarian cancer	Female	15-19 years	95+ years	Data Rich	-1	2	Total Fertility Rate	480
Ovarian cancer	Female	15-19 years	95+ years	Data Rich	1	2	Mean BMI	180
Ovarian cancer	Female	15-19 years	95+ years	Data Rich	1	2	Cumulative Cigarettes (10 Years)	349
Ovarian cancer	Female	15-19 years	95+ years	Data Rich	1	2	Cumulative Cigarettes (20 Years)	371
Ovarian cancer	Female	15-19 years	95+ years	Data Rich	1	2	Smoking Prevalence	420
Ovarian cancer	Female	15-19 years	95+ years	Data Rich	1	2	Diabetes Age-Standardized Prevalence (proportion)	429
Ovarian cancer	Female	15-19 years	95+ years	Data Rich	1	2	energy unadjusted(kcal)	429
Ovarian cancer	Female	15-19 years	95+ years	Data Rich	1	2	Asbestos consumption (metric tons per year per capita)	909
Ovarian cancer	Female	15-19 years	95+ years	Data Rich	-1	3	Education (years per capita)	0
Ovarian cancer	Female	15-19 years	95+ years	Data Rich	-1	3	LDI (I$ per capita)	--
Ovarian cancer	Female	15-19 years	95+ years	Data Rich	1	3	Age- and sex-specific SEV for Low fruit	12
Ovarian cancer	Female	15-19 years	95+ years	Data Rich	1	3	Socio-demographic Index	296
Ovarian cancer	Female	15-19 years	95+ years	Data Rich	1	3	Age- and sex-specific SEV for Low vegetables	--
Ovarian cancer	Female	15-19 years	95+ years	Global	1	1	Liters of alcohol consumed per capita	624
Ovarian cancer	Female	15-19 years	95+ years	Global	1	1	Log-transformed SEV scalar: Ovary C	792
Ovarian cancer	Female	15-19 years	95+ years	Global	-1	2	Total Fertility Rate	492
Ovarian cancer	Female	15-19 years	95+ years	Global	-1	2	Contraception (Modern) Prevalence (proportion)	--
Ovarian cancer	Female	15-19 years	95+ years	Global	-1	2	Healthcare access and quality index	--
Ovarian cancer	Female	15-19 years	95+ years	Global	1	2	Cumulative Cigarettes (10 Years)	58
Ovarian cancer	Female	15-19 years	95+ years	Global	1	2	energy unadjusted(kcal)	144
Ovarian cancer	Female	15-19 years	95+ years	Global	1	2	Mean BMI	155
Ovarian cancer	Female	15-19 years	95+ years	Global	1	2	Diabetes Age-Standardized Prevalence (proportion)	230
Ovarian cancer	Female	15-19 years	95+ years	Global	1	2	Smoking Prevalence	288
Ovarian cancer	Female	15-19 years	95+ years	Global	1	2	Cumulative Cigarettes (20 Years)	328
Ovarian cancer	Female	15-19 years	95+ years	Global	1	2	Asbestos consumption (metric tons per year per capita)	555
Ovarian cancer	Female	15-19 years	95+ years	Global	-1	3	Education (years per capita)	122
Ovarian cancer	Female	15-19 years	95+ years	Global	-1	3	LDI (I$ per capita)	--
Ovarian cancer	Female	15-19 years	95+ years	Global	1	3	Age- and sex-specific SEV for Low fruit	192
Ovarian cancer	Female	15-19 years	95+ years	Global	1	3	Socio-demographic Index	229
Ovarian cancer	Female	15-19 years	95+ years	Global	1	3	Age- and sex-specific SEV for Low vegetables	248
Prostate cancer	Male	20-24 years	95+ years	Data Rich	1	1	Log-transformed SEV scalar: Prostate C	578
Prostate cancer	Male	20-24 years	95+ years	Data Rich	-1	2	Healthcare access and quality index	61
Prostate cancer	Male	20-24 years	95+ years	Data Rich	1	2	Smoking Prevalence	61
Prostate cancer	Male	20-24 years	95+ years	Data Rich	-1	3	Education (years per capita)	--
Prostate cancer	Male	20-24 years	95+ years	Data Rich	-1	3	LDI (I$ per capita)	--
Prostate cancer	Male	20-24 years	95+ years	Data Rich	1	3	Socio-demographic Index	681
Prostate cancer	Male	20-24 years	95+ years	Global	1	1	Log-transformed SEV scalar: Prostate C	718
Prostate cancer	Male	20-24 years	95+ years	Global	-1	2	Healthcare access and quality index	294
Prostate cancer	Male	20-24 years	95+ years	Global	1	2	Smoking Prevalence	--
Prostate cancer	Male	20-24 years	95+ years	Global	-1	3	Education (years per capita)	--
Prostate cancer	Male	20-24 years	95+ years	Global	-1	3	LDI (I$ per capita)	--
Prostate cancer	Male	20-24 years	95+ years	Global	1	3	Socio-demographic Index	654
Testicular cancer	Male	15-19 years	95+ years	Data Rich	-1	2	Healthcare access and quality index	1
Testicular cancer	Male	15-19 years	95+ years	Data Rich	1	2	Cumulative Cigarettes (20 Years)	106
Testicular cancer	Male	15-19 years	95+ years	Data Rich	1	2	Cumulative Cigarettes (5 Years)	132
Testicular cancer	Male	15-19 years	95+ years	Data Rich	1	2	Tobacco (cigarettes per capita)	154

CODEm covariates used, level of covariate, and expected direction of covariate by cause, sex, and age								
Cause	Sex	Age Start	Age End	Model Version Type	Direction	Level	Covariate Name	Number of Draws
Testicular cancer	Male	15-19 years	95+ years	Data Rich	1	2	Cumulative Cigarettes (10 Years)	179
Testicular cancer	Male	15-19 years	95+ years	Data Rich	1	2	Cumulative Cigarettes (15 Years)	194
Testicular cancer	Male	15-19 years	95+ years	Data Rich	1	2	Smoking Prevalence	300
Testicular cancer	Male	15-19 years	95+ years	Data Rich	1	2	Age- and sex-specific SEV for Low fruit	--
Testicular cancer	Male	15-19 years	95+ years	Data Rich	1	2	Age- and sex-specific SEV for Low vegetables	--
Testicular cancer	Male	15-19 years	95+ years	Data Rich	-1	3	Education (years per capita)	--
Testicular cancer	Male	15-19 years	95+ years	Data Rich	1	3	Socio-demographic Index	191
Testicular cancer	Male	15-19 years	95+ years	Data Rich	1	3	LDI (I$ per capita)	--
Testicular cancer	Male	15-19 years	95+ years	Global	-1	2	Healthcare access and quality index	380
Testicular cancer	Male	15-19 years	95+ years	Global	1	2	Smoking Prevalence	11
Testicular cancer	Male	15-19 years	95+ years	Global	1	2	Cumulative Cigarettes (20 Years)	60
Testicular cancer	Male	15-19 years	95+ years	Global	1	2	Cumulative Cigarettes (15 Years)	67
Testicular cancer	Male	15-19 years	95+ years	Global	1	2	Cumulative Cigarettes (10 Years)	119
Testicular cancer	Male	15-19 years	95+ years	Global	1	2	Cumulative Cigarettes (5 Years)	122
Testicular cancer	Male	15-19 years	95+ years	Global	1	2	Tobacco (cigarettes per capita)	615
Testicular cancer	Male	15-19 years	95+ years	Global	1	2	Age- and sex-specific SEV for Low vegetables	616
Testicular cancer	Male	15-19 years	95+ years	Global	1	2	Age- and sex-specific SEV for Low fruit	695
Testicular cancer	Male	15-19 years	95+ years	Global	-1	3	Education (years per capita)	--
Testicular cancer	Male	15-19 years	95+ years	Global	1	3	Socio-demographic Index	376
Testicular cancer	Male	15-19 years	95+ years	Global	1	3	LDI (I$ per capita)	--
Kidney cancer	Female	0-6 days	95+ years	Data Rich	1	1	Tobacco (cigarettes per capita)	105
Kidney cancer	Female	0-6 days	95+ years	Data Rich	1	1	Log-transformed SEV scalar: Kidney C	555
Kidney cancer	Female	0-6 days	95+ years	Data Rich	1	1	Cumulative Cigarettes (10 Years)	612
Kidney cancer	Female	0-6 days	95+ years	Data Rich	1	1	Mean BMI	776
Kidney cancer	Female	0-6 days	95+ years	Data Rich	-1	2	Healthcare access and quality index	--
Kidney cancer	Female	0-6 days	95+ years	Data Rich	1	2	Liters of alcohol consumed per capita	152
Kidney cancer	Female	0-6 days	95+ years	Data Rich	1	2	Diabetes Age-Standardized Prevalence (proportion)	--
Kidney cancer	Female	0-6 days	95+ years	Data Rich	1	2	Systolic Blood Pressure (mmHg)	--
Kidney cancer	Female	0-6 days	95+ years	Data Rich	-1	3	Education (years per capita)	--
Kidney cancer	Female	0-6 days	95+ years	Data Rich	1	3	LDI (I$ per capita)	--
Kidney cancer	Female	0-6 days	95+ years	Data Rich	1	3	Socio-demographic Index	--
Kidney cancer	Female	0-6 days	95+ years	Global	1	1	Tobacco (cigarettes per capita)	90
Kidney cancer	Female	0-6 days	95+ years	Global	1	1	Cumulative Cigarettes (10 Years)	242
Kidney cancer	Female	0-6 days	95+ years	Global	1	1	Mean BMI	297
Kidney cancer	Female	0-6 days	95+ years	Global	1	1	Log-transformed SEV scalar: Kidney C	848
Kidney cancer	Female	0-6 days	95+ years	Global	-1	2	Healthcare access and quality index	105
Kidney cancer	Female	0-6 days	95+ years	Global	1	2	Systolic Blood Pressure (mmHg)	48
Kidney cancer	Female	0-6 days	95+ years	Global	1	2	Liters of alcohol consumed per capita	120
Kidney cancer	Female	0-6 days	95+ years	Global	1	2	Diabetes Age-Standardized Prevalence (proportion)	229
Kidney cancer	Female	0-6 days	95+ years	Global	-1	3	Education (years per capita)	62
Kidney cancer	Female	0-6 days	95+ years	Global	1	3	Socio-demographic Index	45
Kidney cancer	Female	0-6 days	95+ years	Global	1	3	LDI (I$ per capita)	--
Kidney cancer	Male	0-6 days	95+ years	Data Rich	1	1	Tobacco (cigarettes per capita)	12
Kidney cancer	Male	0-6 days	95+ years	Data Rich	1	1	Mean BMI	500
Kidney cancer	Male	0-6 days	95+ years	Data Rich	1	1	Log-transformed SEV scalar: Kidney C	753
Kidney cancer	Male	0-6 days	95+ years	Data Rich	1	1	Cumulative Cigarettes (10 Years)	--
Kidney cancer	Male	0-6 days	95+ years	Data Rich	-1	2	Healthcare access and quality index	--
Kidney cancer	Male	0-6 days	95+ years	Data Rich	1	2	Liters of alcohol consumed per capita	9
Kidney cancer	Male	0-6 days	95+ years	Data Rich	1	2	Systolic Blood Pressure (mmHg)	58
Kidney cancer	Male	0-6 days	95+ years	Data Rich	1	2	Diabetes Age-Standardized Prevalence (proportion)	229
Kidney cancer	Male	0-6 days	95+ years	Data Rich	-1	3	Education (years per capita)	--
Kidney cancer	Male	0-6 days	95+ years	Data Rich	1	3	Socio-demographic Index	671
Kidney cancer	Male	0-6 days	95+ years	Data Rich	1	3	LDI (I$ per capita)	--
Kidney cancer	Male	0-6 days	95+ years	Global	1	1	Log-transformed SEV scalar: Kidney C	475
Kidney cancer	Male	0-6 days	95+ years	Global	1	1	Mean BMI	530
Kidney cancer	Male	0-6 days	95+ years	Global	1	1	Tobacco (cigarettes per capita)	561
Kidney cancer	Male	0-6 days	95+ years	Global	1	1	Cumulative Cigarettes (10 Years)	--
Kidney cancer	Male	0-6 days	95+ years	Global	-1	2	Healthcare access and quality index	31
Kidney cancer	Male	0-6 days	95+ years	Global	1	2	Systolic Blood Pressure (mmHg)	52
Kidney cancer	Male	0-6 days	95+ years	Global	1	2	Diabetes Age-Standardized Prevalence (proportion)	81
Kidney cancer	Male	0-6 days	95+ years	Global	1	2	Liters of alcohol consumed per capita	204
Kidney cancer	Male	0-6 days	95+ years	Global	-1	3	Education (years per capita)	--
Kidney cancer	Male	0-6 days	95+ years	Global	1	3	Socio-demographic Index	234
Kidney cancer	Male	0-6 days	95+ years	Global	1	3	LDI (I$ per capita)	--
Bladder cancer	Female	15-19 years	95+ years	Data Rich	1	1	Smoking Prevalence	39
Bladder cancer	Female	15-19 years	95+ years	Data Rich	1	1	Schistosomiasis Prevalence Results	279
Bladder cancer	Female	15-19 years	95+ years	Data Rich	1	1	Log-transformed SEV scalar: Bladder C	369
Bladder cancer	Female	15-19 years	95+ years	Data Rich	-1	2	Healthcare access and quality index	12
Bladder cancer	Female	15-19 years	95+ years	Data Rich	1	2	Liters of alcohol consumed per capita	121
Bladder cancer	Female	15-19 years	95+ years	Data Rich	1	2	Cumulative Cigarettes (10 Years)	127
Bladder cancer	Female	15-19 years	95+ years	Data Rich	1	2	Age- and sex-specific SEV for Low vegetables	152
Bladder cancer	Female	15-19 years	95+ years	Data Rich	1	2	Diabetes Fasting Plasma Glucose (mmol/L), age-standardized 25+	540
Bladder cancer	Female	15-19 years	95+ years	Data Rich	1	3	Age- and sex-specific SEV for Low fruit	20
Bladder cancer	Female	15-19 years	95+ years	Data Rich	1	3	LDI (I$ per capita)	181
Bladder cancer	Female	15-19 years	95+ years	Data Rich	1	3	Socio-demographic Index	449
Bladder cancer	Female	15-19 years	95+ years	Global	1	1	Schistosomiasis Prevalence Results	255
Bladder cancer	Female	15-19 years	95+ years	Global	1	1	Smoking Prevalence	445
Bladder cancer	Female	15-19 years	95+ years	Global	1	1	Log-transformed SEV scalar: Bladder C	910
Bladder cancer	Female	15-19 years	95+ years	Global	-1	2	Healthcare access and quality index	9
Bladder cancer	Female	15-19 years	95+ years	Global	1	2	Cumulative Cigarettes (10 Years)	10
Bladder cancer	Female	15-19 years	95+ years	Global	1	2	Age- and sex-specific SEV for Low vegetables	37
Bladder cancer	Female	15-19 years	95+ years	Global	1	2	Liters of alcohol consumed per capita	90
Bladder cancer	Female	15-19 years	95+ years	Global	1	2	Diabetes Fasting Plasma Glucose (mmol/L), age-standardized 25+	310
Bladder cancer	Female	15-19 years	95+ years	Global	1	3	Age- and sex-specific SEV for Low fruit	183
Bladder cancer	Female	15-19 years	95+ years	Global	1	3	LDI (I$ per capita)	335
Bladder cancer	Female	15-19 years	95+ years	Global	1	3	Socio-demographic Index	--
Bladder cancer	Male	15-19 years	95+ years	Data Rich	1	1	Log-transformed SEV scalar: Bladder C	195
Bladder cancer	Male	15-19 years	95+ years	Data Rich	1	1	Smoking Prevalence	264
Bladder cancer	Male	15-19 years	95+ years	Data Rich	1	1	Schistosomiasis Prevalence Results	867
Bladder cancer	Male	15-19 years	95+ years	Data Rich	-1	2	Healthcare access and quality index	83
Bladder cancer	Male	15-19 years	95+ years	Data Rich	1	2	Age- and sex-specific SEV for Low vegetables	74
Bladder cancer	Male	15-19 years	95+ years	Data Rich	1	2	Diabetes Fasting Plasma Glucose (mmol/L), age-standardized 25+	83
Bladder cancer	Male	15-19 years	95+ years	Data Rich	1	2	Liters of alcohol consumed per capita	326
Bladder cancer	Male	15-19 years	95+ years	Data Rich	1	2	Cumulative Cigarettes (10 Years)	--
Bladder cancer	Male	15-19 years	95+ years	Data Rich	1	3	LDI (I$ per capita)	458
Bladder cancer	Male	15-19 years	95+ years	Data Rich	1	3	Socio-demographic Index	458
Bladder cancer	Male	15-19 years	95+ years	Data Rich	1	3	Age- and sex-specific SEV for Low fruit	--
Bladder cancer	Male	15-19 years	95+ years	Global	1	1	Log-transformed SEV scalar: Bladder C	92
Bladder cancer	Male	15-19 years	95+ years	Global	1	1	Schistosomiasis Prevalence Results	158
Bladder cancer	Male	15-19 years	95+ years	Global	1	1	Smoking Prevalence	185
Bladder cancer	Male	15-19 years	95+ years	Global	-1	2	Healthcare access and quality index	123
Bladder cancer	Male	15-19 years	95+ years	Global	1	2	Age- and sex-specific SEV for Low vegetables	171
Bladder cancer	Male	15-19 years	95+ years	Global	1	2	Cumulative Cigarettes (10 Years)	171
Bladder cancer	Male	15-19 years	95+ years	Global	1	2	Diabetes Fasting Plasma Glucose (mmol/L), age-standardized 25+	588
Bladder cancer	Male	15-19 years	95+ years	Global	1	2	Liters of alcohol consumed per capita	588
Bladder cancer	Male	15-19 years	95+ years	Global	1	3	Age- and sex-specific SEV for Low fruit	4
Bladder cancer	Male	15-19 years	95+ years	Global	1	3	LDI (I$ per capita)	256
Bladder cancer	Male	15-19 years	95+ years	Global	1	3	Socio-demographic Index	454
Brain and nervous system cancer	Female	0-6 days	95+ years	Data Rich	1	1	Smoking Prevalence	145
Brain and nervous system cancer	Female	0-6 days	95+ years	Data Rich	1	1	Cumulative Cigarettes (10 Years)	301
Brain and nervous system cancer	Female	0-6 days	95+ years	Data Rich	1	1	Liters of alcohol consumed per capita	--
Brain and nervous system cancer	Female	0-6 days	95+ years	Data Rich	-1	2	Healthcare access and quality index	--
Brain and nervous system cancer	Female	0-6 days	95+ years	Data Rich	1	2	Systolic Blood Pressure (mmHg)	16
Brain and nervous system cancer	Female	0-6 days	95+ years	Data Rich	1	2	Age- and sex-specific SEV for High red meat	522
Brain and nervous system cancer	Female	0-6 days	95+ years	Data Rich	1	2	Age- and sex-specific SEV for Low fruit	--
Brain and nervous system cancer	Female	0-6 days	95+ years	Data Rich	1	2	Age- and sex-specific SEV for Low vegetables	--
Brain and nervous system cancer	Female	0-6 days	95+ years	Data Rich	1	2	Low-Density Lipoprotein (mmol/L)	--

CODEm covariates used, level of covariate, and expected direction of covariate by cause, sex, and age

Cause	Sex	Age Start	Age End	Model Version Type	Direction	Level	Covariate Name	Number of Draws
Brain and nervous system cancer	Female	0-6 days	95+ years	Data Rich	-1	3	Education (years per capita)	--
Brain and nervous system cancer	Female	0-6 days	95+ years	Data Rich	-1	3	LDI (I$ per capita)	--
Brain and nervous system cancer	Female	0-6 days	95+ years	Data Rich	1	3	Socio-demographic Index	704
Brain and nervous system cancer	Female	0-6 days	95+ years	Global	1	1	Liters of alcohol consumed per capita	36
Brain and nervous system cancer	Female	0-6 days	95+ years	Global	1	1	Cumulative Cigarettes (10 Years)	538
Brain and nervous system cancer	Female	0-6 days	95+ years	Global	1	1	Smoking Prevalence	648
Brain and nervous system cancer	Female	0-6 days	95+ years	Global	-1	2	Healthcare access and quality index	276
Brain and nervous system cancer	Female	0-6 days	95+ years	Global	1	2	Systolic Blood Pressure (mmHg)	155
Brain and nervous system cancer	Female	0-6 days	95+ years	Global	1	2	Age- and sex-specific SEV for High red meat	191
Brain and nervous system cancer	Female	0-6 days	95+ years	Global	1	2	Age- and sex-specific SEV for Low fruit	--
Brain and nervous system cancer	Female	0-6 days	95+ years	Global	1	2	Age- and sex-specific SEV for Low vegetables	--
Brain and nervous system cancer	Female	0-6 days	95+ years	Global	1	2	Low-Density Lipoprotein (mmol/L)	--
Brain and nervous system cancer	Female	0-6 days	95+ years	Global	-1	3	LDI (I$ per capita)	45
Brain and nervous system cancer	Female	0-6 days	95+ years	Global	-1	3	Education (years per capita)	349
Brain and nervous system cancer	Female	0-6 days	95+ years	Global	1	3	Socio-demographic Index	183
Brain and nervous system cancer	Male	0-6 days	95+ years	Data Rich	1	1	Liters of alcohol consumed per capita	640
Brain and nervous system cancer	Male	0-6 days	95+ years	Data Rich	1	1	Cumulative Cigarettes (10 Years)	--
Brain and nervous system cancer	Male	0-6 days	95+ years	Data Rich	1	1	Smoking Prevalence	--
Brain and nervous system cancer	Male	0-6 days	95+ years	Data Rich	-1	2	Healthcare access and quality index	--
Brain and nervous system cancer	Male	0-6 days	95+ years	Data Rich	1	2	Systolic Blood Pressure (mmHg)	159
Brain and nervous system cancer	Male	0-6 days	95+ years	Data Rich	1	2	Age- and sex-specific SEV for High red meat	367
Brain and nervous system cancer	Male	0-6 days	95+ years	Data Rich	1	2	Age- and sex-specific SEV for Low fruit	--
Brain and nervous system cancer	Male	0-6 days	95+ years	Data Rich	1	2	Age- and sex-specific SEV for Low vegetables	--
Brain and nervous system cancer	Male	0-6 days	95+ years	Data Rich	1	2	Low-Density Lipoprotein (mmol/L)	--
Brain and nervous system cancer	Male	0-6 days	95+ years	Data Rich	-1	3	Education (years per capita)	--
Brain and nervous system cancer	Male	0-6 days	95+ years	Data Rich	-1	3	LDI (I$ per capita)	--
Brain and nervous system cancer	Male	0-6 days	95+ years	Data Rich	1	3	Socio-demographic Index	360
Brain and nervous system cancer	Male	0-6 days	95+ years	Global	1	1	Cumulative Cigarettes (10 Years)	585
Brain and nervous system cancer	Male	0-6 days	95+ years	Global	1	1	Liters of alcohol consumed per capita	--
Brain and nervous system cancer	Male	0-6 days	95+ years	Global	1	1	Smoking Prevalence	--
Brain and nervous system cancer	Male	0-6 days	95+ years	Global	-1	2	Healthcare access and quality index	--
Brain and nervous system cancer	Male	0-6 days	95+ years	Global	1	2	Age- and sex-specific SEV for High red meat	487
Brain and nervous system cancer	Male	0-6 days	95+ years	Global	1	2	Age- and sex-specific SEV for Low fruit	--
Brain and nervous system cancer	Male	0-6 days	95+ years	Global	1	2	Age- and sex-specific SEV for Low vegetables	--
Brain and nervous system cancer	Male	0-6 days	95+ years	Global	1	2	Low-Density Lipoprotein (mmol/L)	--
Brain and nervous system cancer	Male	0-6 days	95+ years	Global	1	2	Systolic Blood Pressure (mmHg)	--
Brain and nervous system cancer	Male	0-6 days	95+ years	Global	-1	3	Education (years per capita)	161
Brain and nervous system cancer	Male	0-6 days	95+ years	Global	-1	3	LDI (I$ per capita)	--
Brain and nervous system cancer	Male	0-6 days	95+ years	Global	1	3	Socio-demographic Index	282
Retinoblastoma	Female	0-6 days	5-9 years	Data Rich	-1	2	Healthcare access and quality index	398
Retinoblastoma	Female	0-6 days	5-9 years	Data Rich	-1	2	Universal health coverage	888
Retinoblastoma	Female	0-6 days	5-9 years	Data Rich	-1	3	Socio-demographic Index	206
Retinoblastoma	Female	0-6 days	5-9 years	Data Rich	-1	3	Education (years per capita)	261
Retinoblastoma	Female	0-6 days	5-9 years	Data Rich	-1	3	LDI (I$ per capita)	--
Retinoblastoma	Female	0-6 days	5-9 years	Data Rich	-1	3	Maternal care and immunization	--
Retinoblastoma	Female	0-6 days	5-9 years	Global	-1	2	Healthcare access and quality index	344
Retinoblastoma	Female	0-6 days	5-9 years	Global	-1	2	Universal health coverage	656
Retinoblastoma	Female	0-6 days	5-9 years	Global	-1	3	Socio-demographic Index	211
Retinoblastoma	Female	0-6 days	5-9 years	Global	-1	3	Education (years per capita)	--
Retinoblastoma	Female	0-6 days	5-9 years	Global	-1	3	LDI (I$ per capita)	--
Retinoblastoma	Female	0-6 days	5-9 years	Global	-1	3	Maternal care and immunization	--
Retinoblastoma	Male	0-6 days	5-9 years	Data Rich	-1	2	Healthcare access and quality index	494
Retinoblastoma	Male	0-6 days	5-9 years	Data Rich	-1	2	Universal health coverage	743
Retinoblastoma	Male	0-6 days	5-9 years	Data Rich	-1	3	Socio-demographic Index	164
Retinoblastoma	Male	0-6 days	5-9 years	Data Rich	-1	3	Education (years per capita)	222
Retinoblastoma	Male	0-6 days	5-9 years	Data Rich	-1	3	LDI (I$ per capita)	--
Retinoblastoma	Male	0-6 days	5-9 years	Data Rich	-1	3	Maternal care and immunization	--
Retinoblastoma	Male	0-6 days	5-9 years	Global	-1	2	Healthcare access and quality index	487
Retinoblastoma	Male	0-6 days	5-9 years	Global	-1	2	Universal health coverage	706
Retinoblastoma	Male	0-6 days	5-9 years	Global	-1	3	Socio-demographic Index	443
Retinoblastoma	Male	0-6 days	5-9 years	Global	-1	3	Education (years per capita)	--
Retinoblastoma	Male	0-6 days	5-9 years	Global	-1	3	LDI (I$ per capita)	--
Retinoblastoma	Male	0-6 days	5-9 years	Global	-1	3	Maternal care and immunization	--
Other eye cancers	Female	10-14 years	95+ years	Data Rich	-1	2	Healthcare access and quality index	248
Other eye cancers	Female	10-14 years	95+ years	Data Rich	-1	2	Universal health coverage	273
Other eye cancers	Female	10-14 years	95+ years	Data Rich	1	2	Age-standardized melanoma	895
Other eye cancers	Female	10-14 years	95+ years	Data Rich	-1	3	Education (years per capita)	258
Other eye cancers	Female	10-14 years	95+ years	Data Rich	-1	3	Socio-demographic Index	359
Other eye cancers	Female	10-14 years	95+ years	Data Rich	-1	3	LDI (I$ per capita)	--
Other eye cancers	Female	10-14 years	95+ years	Global	-1	2	Healthcare access and quality index	745
Other eye cancers	Female	10-14 years	95+ years	Global	-1	2	Universal health coverage	--
Other eye cancers	Female	10-14 years	95+ years	Global	1	2	Age-standardized melanoma	614
Other eye cancers	Female	10-14 years	95+ years	Global	-1	3	Education (years per capita)	142
Other eye cancers	Female	10-14 years	95+ years	Global	-1	3	LDI (I$ per capita)	--
Other eye cancers	Female	10-14 years	95+ years	Global	-1	3	Socio-demographic Index	--
Other eye cancers	Male	10-14 years	95+ years	Data Rich	-1	2	Universal health coverage	133
Other eye cancers	Male	10-14 years	95+ years	Data Rich	-1	2	Healthcare access and quality index	573
Other eye cancers	Male	10-14 years	95+ years	Data Rich	1	2	Age-standardized melanoma	427
Other eye cancers	Male	10-14 years	95+ years	Data Rich	-1	3	Education (years per capita)	92
Other eye cancers	Male	10-14 years	95+ years	Data Rich	-1	3	Socio-demographic Index	111
Other eye cancers	Male	10-14 years	95+ years	Data Rich	-1	3	LDI (I$ per capita)	395
Other eye cancers	Male	10-14 years	95+ years	Global	-1	2	Healthcare access and quality index	--
Other eye cancers	Male	10-14 years	95+ years	Global	-1	2	Universal health coverage	--
Other eye cancers	Male	10-14 years	95+ years	Global	1	2	Age-standardized melanoma	546
Other eye cancers	Male	10-14 years	95+ years	Global	-1	3	Education (years per capita)	--
Other eye cancers	Male	10-14 years	95+ years	Global	-1	3	LDI (I$ per capita)	--
Other eye cancers	Male	10-14 years	95+ years	Global	-1	3	Socio-demographic Index	--
Neuroblastoma and other peripheral nervous cell tumours	Female	0-6 days	95+ years	Data Rich	-1	3	Health worker density	268
Neuroblastoma and other peripheral nervous cell tumours	Female	0-6 days	95+ years	Data Rich	-1	3	Education (years per capita)	--
Neuroblastoma and other peripheral nervous cell tumours	Female	0-6 days	95+ years	Data Rich	-1	3	Healthcare access and quality index	--
Neuroblastoma and other peripheral nervous cell tumours	Female	0-6 days	95+ years	Data Rich	-1	3	Maternal care and immunization	--
Neuroblastoma and other peripheral nervous cell tumours	Female	0-6 days	95+ years	Data Rich	-1	3	Universal health coverage	--
Neuroblastoma and other peripheral nervous cell tumours	Female	0-6 days	95+ years	Data Rich	1	3	Socio-demographic Index	814
Neuroblastoma and other peripheral nervous cell tumours	Female	0-6 days	95+ years	Data Rich	1	3	LDI (I$ per capita)	--
Neuroblastoma and other peripheral nervous cell tumours	Female	0-6 days	95+ years	Data Rich	1	3	Smoking Prevalence	--
Neuroblastoma and other peripheral nervous cell tumours	Female	0-6 days	95+ years	Global	-1	3	Health worker density	418
Neuroblastoma and other peripheral nervous cell tumours	Female	0-6 days	95+ years	Global	-1	3	Education (years per capita)	--
Neuroblastoma and other peripheral nervous cell tumours	Female	0-6 days	95+ years	Global	-1	3	Healthcare access and quality index	--
Neuroblastoma and other peripheral nervous cell tumours	Female	0-6 days	95+ years	Global	-1	3	Maternal care and immunization	--
Neuroblastoma and other peripheral nervous cell tumours	Female	0-6 days	95+ years	Global	-1	3	Universal health coverage	--
Neuroblastoma and other peripheral nervous cell tumours	Female	0-6 days	95+ years	Global	1	3	Smoking Prevalence	404
Neuroblastoma and other peripheral nervous cell tumours	Female	0-6 days	95+ years	Global	1	3	Socio-demographic Index	636
Neuroblastoma and other peripheral nervous cell tumours	Female	0-6 days	95+ years	Global	1	3	LDI (I$ per capita)	--
Neuroblastoma and other peripheral nervous cell tumours	Male	0-6 days	95+ years	Data Rich	-1	3	Education (years per capita)	217
Neuroblastoma and other peripheral nervous cell tumours	Male	0-6 days	95+ years	Data Rich	-1	3	Healthcare access and quality index	398
Neuroblastoma and other peripheral nervous cell tumours	Male	0-6 days	95+ years	Data Rich	-1	3	Health worker density	464
Neuroblastoma and other peripheral nervous cell tumours	Male	0-6 days	95+ years	Data Rich	-1	3	Maternal care and immunization	--
Neuroblastoma and other peripheral nervous cell tumours	Male	0-6 days	95+ years	Data Rich	-1	3	Universal health coverage	--
Neuroblastoma and other peripheral nervous cell tumours	Male	0-6 days	95+ years	Data Rich	1	3	Socio-demographic Index	861
Neuroblastoma and other peripheral nervous cell tumours	Male	0-6 days	95+ years	Data Rich	1	3	LDI (I$ per capita)	--
Neuroblastoma and other peripheral nervous cell tumours	Male	0-6 days	95+ years	Data Rich	1	3	Smoking Prevalence	--

CODEm covariates used, level of covariate, and expected direction of covariate by cause, sex, and age								
**Cause**	**Sex**	**Age Start**	**Age End**	**Model Version Type**	**Direction**	**Level**	**Covariate Name**	**Number of Draws**
Neuroblastoma and other peripheral nervous cell tumours	Male	0-6 days	95+ years	Global	-1	3	Healthcare access and quality index	101
Neuroblastoma and other peripheral nervous cell tumours	Male	0-6 days	95+ years	Global	-1	3	Health worker density	277
Neuroblastoma and other peripheral nervous cell tumours	Male	0-6 days	95+ years	Global	-1	3	Universal health coverage	297
Neuroblastoma and other peripheral nervous cell tumours	Male	0-6 days	95+ years	Global	-1	3	Education (years per capita)	429
Neuroblastoma and other peripheral nervous cell tumours	Male	0-6 days	95+ years	Global	-1	3	Maternal care and immunization	--
Neuroblastoma and other peripheral nervous cell tumours	Male	0-6 days	95+ years	Global	1	3	Socio-demographic Index	968
Neuroblastoma and other peripheral nervous cell tumours	Male	0-6 days	95+ years	Global	1	3	LDI (I$ per capita)	--
Neuroblastoma and other peripheral nervous cell tumours	Male	0-6 days	95+ years	Global	1	3	Smoking Prevalence	--
Thyroid cancer	Female	5-9 years	95+ years	Data Rich	1	1	Liters of alcohol consumed per capita	635
Thyroid cancer	Female	5-9 years	95+ years	Data Rich	1	1	Log-transformed SEV scalar: Thyroid C	800
Thyroid cancer	Female	5-9 years	95+ years	Data Rich	-1	2	Healthcare access and quality index	2
Thyroid cancer	Female	5-9 years	95+ years	Data Rich	1	2	Tobacco (cigarettes per capita)	9
Thyroid cancer	Female	5-9 years	95+ years	Data Rich	1	2	Age- and sex-specific SEV for Low vegetables	11
Thyroid cancer	Female	5-9 years	95+ years	Data Rich	1	2	Mean BMI	44
Thyroid cancer	Female	5-9 years	95+ years	Data Rich	1	2	Age- and sex-specific SEV for High red meat	367
Thyroid cancer	Female	5-9 years	95+ years	Data Rich	-1	3	Education (years per capita)	5
Thyroid cancer	Female	5-9 years	95+ years	Data Rich	-1	3	Sanitation (proportion with access)	7
Thyroid cancer	Female	5-9 years	95+ years	Data Rich	-1	3	Improved Water Source (proportion with access)	319
Thyroid cancer	Female	5-9 years	95+ years	Data Rich	1	3	Age- and sex-specific SEV for Low fruit	3
Thyroid cancer	Female	5-9 years	95+ years	Data Rich	1	3	Socio-demographic Index	648
Thyroid cancer	Female	5-9 years	95+ years	Data Rich	1	3	LDI (I$ per capita)	--
Thyroid cancer	Female	5-9 years	95+ years	Global	1	1	Log-transformed SEV scalar: Thyroid C	454
Thyroid cancer	Female	5-9 years	95+ years	Global	1	1	Liters of alcohol consumed per capita	782
Thyroid cancer	Female	5-9 years	95+ years	Global	-1	2	Healthcare access and quality index	--
Thyroid cancer	Female	5-9 years	95+ years	Global	1	2	Age- and sex-specific SEV for Low vegetables	135
Thyroid cancer	Female	5-9 years	95+ years	Global	1	2	Age- and sex-specific SEV for High red meat	330
Thyroid cancer	Female	5-9 years	95+ years	Global	1	2	Mean BMI	--
Thyroid cancer	Female	5-9 years	95+ years	Global	1	2	Tobacco (cigarettes per capita)	--
Thyroid cancer	Female	5-9 years	95+ years	Global	-1	3	Improved Water Source (proportion with access)	150
Thyroid cancer	Female	5-9 years	95+ years	Global	-1	3	Education (years per capita)	158
Thyroid cancer	Female	5-9 years	95+ years	Global	-1	3	Sanitation (proportion with access)	--
Thyroid cancer	Female	5-9 years	95+ years	Global	1	3	Socio-demographic Index	225
Thyroid cancer	Female	5-9 years	95+ years	Global	1	3	Age- and sex-specific SEV for Low fruit	--
Thyroid cancer	Female	5-9 years	95+ years	Global	1	3	LDI (I$ per capita)	--
Thyroid cancer	Male	5-9 years	95+ years	Data Rich	1	1	Liters of alcohol consumed per capita	313
Thyroid cancer	Male	5-9 years	95+ years	Data Rich	1	1	Log-transformed SEV scalar: Thyroid C	796
Thyroid cancer	Male	5-9 years	95+ years	Data Rich	-1	2	Healthcare access and quality index	71
Thyroid cancer	Male	5-9 years	95+ years	Data Rich	1	2	Tobacco (cigarettes per capita)	32
Thyroid cancer	Male	5-9 years	95+ years	Data Rich	1	2	Age- and sex-specific SEV for Low vegetables	38
Thyroid cancer	Male	5-9 years	95+ years	Data Rich	1	2	Mean BMI	160
Thyroid cancer	Male	5-9 years	95+ years	Data Rich	1	2	Age- and sex-specific SEV for High red meat	371
Thyroid cancer	Male	5-9 years	95+ years	Data Rich	-1	3	Education (years per capita)	20
Thyroid cancer	Male	5-9 years	95+ years	Data Rich	-1	3	Sanitation (proportion with access)	52
Thyroid cancer	Male	5-9 years	95+ years	Data Rich	-1	3	Improved Water Source (proportion with access)	60
Thyroid cancer	Male	5-9 years	95+ years	Data Rich	1	3	Age- and sex-specific SEV for Low fruit	7
Thyroid cancer	Male	5-9 years	95+ years	Data Rich	1	3	Socio-demographic Index	237
Thyroid cancer	Male	5-9 years	95+ years	Data Rich	1	3	LDI (I$ per capita)	--
Thyroid cancer	Male	5-9 years	95+ years	Global	1	1	Liters of alcohol consumed per capita	692
Thyroid cancer	Male	5-9 years	95+ years	Global	1	1	Log-transformed SEV scalar: Thyroid C	1000
Thyroid cancer	Male	5-9 years	95+ years	Global	-1	2	Healthcare access and quality index	256
Thyroid cancer	Male	5-9 years	95+ years	Global	1	2	Tobacco (cigarettes per capita)	187
Thyroid cancer	Male	5-9 years	95+ years	Global	1	2	Age- and sex-specific SEV for High red meat	651
Thyroid cancer	Male	5-9 years	95+ years	Global	1	2	Age- and sex-specific SEV for Low vegetables	--
Thyroid cancer	Male	5-9 years	95+ years	Global	1	2	Mean BMI	--
Thyroid cancer	Male	5-9 years	95+ years	Global	-1	3	Education (years per capita)	163
Thyroid cancer	Male	5-9 years	95+ years	Global	-1	3	Improved Water Source (proportion with access)	--
Thyroid cancer	Male	5-9 years	95+ years	Global	-1	3	Sanitation (proportion with access)	--
Thyroid cancer	Male	5-9 years	95+ years	Global	1	3	Age- and sex-specific SEV for Low fruit	--
Thyroid cancer	Male	5-9 years	95+ years	Global	1	3	LDI (I$ per capita)	--
Thyroid cancer	Male	5-9 years	95+ years	Global	1	3	Socio-demographic Index	--
Mesothelioma	Female	20-24 years	95+ years	Data Rich	1	1	Smoking Prevalence	88
Mesothelioma	Female	20-24 years	95+ years	Data Rich	1	1	Age-standardized SEV for Occupational asbestos	862
Mesothelioma	Female	20-24 years	95+ years	Data Rich	1	1	Age- and sex-specific SEV for Occupational asbestos	1000
Mesothelioma	Female	20-24 years	95+ years	Data Rich	1	1	Asbestos consumption (metric tons per year per capita)	--
Mesothelioma	Female	20-24 years	95+ years	Data Rich	-1	2	Healthcare access and quality index	--
Mesothelioma	Female	20-24 years	95+ years	Data Rich	1	2	Cumulative Cigarettes (5 Years)	102
Mesothelioma	Female	20-24 years	95+ years	Data Rich	1	2	Population Density (over 1000 ppl/sqkm, proportion)	243
Mesothelioma	Female	20-24 years	95+ years	Data Rich	1	2	Gold production (binary)	386
Mesothelioma	Female	20-24 years	95+ years	Data Rich	1	2	Indoor Air Pollution (All Cooking Fuels)	--
Mesothelioma	Female	20-24 years	95+ years	Data Rich	-1	3	Education (years per capita)	--
Mesothelioma	Female	20-24 years	95+ years	Data Rich	-1	3	LDI (I$ per capita)	--
Mesothelioma	Female	20-24 years	95+ years	Data Rich	1	3	Socio-demographic Index	771
Mesothelioma	Female	20-24 years	95+ years	Global	1	1	Smoking Prevalence	467
Mesothelioma	Female	20-24 years	95+ years	Global	1	1	Age- and sex-specific SEV for Occupational asbestos	705
Mesothelioma	Female	20-24 years	95+ years	Global	1	1	Age-standardized SEV for Occupational asbestos	730
Mesothelioma	Female	20-24 years	95+ years	Global	1	1	Asbestos consumption (metric tons per year per capita)	--
Mesothelioma	Female	20-24 years	95+ years	Global	-1	2	Healthcare access and quality index	50
Mesothelioma	Female	20-24 years	95+ years	Global	1	2	Population Density (over 1000 ppl/sqkm, proportion)	32
Mesothelioma	Female	20-24 years	95+ years	Global	1	2	Cumulative Cigarettes (5 Years)	271
Mesothelioma	Female	20-24 years	95+ years	Global	1	2	Indoor Air Pollution (All Cooking Fuels)	416
Mesothelioma	Female	20-24 years	95+ years	Global	1	2	Gold production (binary)	542
Mesothelioma	Female	20-24 years	95+ years	Global	-1	3	Education (years per capita)	--
Mesothelioma	Female	20-24 years	95+ years	Global	-1	3	LDI (I$ per capita)	--
Mesothelioma	Female	20-24 years	95+ years	Global	1	3	Socio-demographic Index	468
Mesothelioma	Male	20-24 years	95+ years	Data Rich	1	1	Smoking Prevalence	74
Mesothelioma	Male	20-24 years	95+ years	Data Rich	1	1	Asbestos consumption (metric tons per year per capita)	210
Mesothelioma	Male	20-24 years	95+ years	Data Rich	1	1	Age-standardized SEV for Occupational asbestos	891
Mesothelioma	Male	20-24 years	95+ years	Data Rich	1	1	Age- and sex-specific SEV for Occupational asbestos	985
Mesothelioma	Male	20-24 years	95+ years	Data Rich	-1	2	Healthcare access and quality index	--
Mesothelioma	Male	20-24 years	95+ years	Data Rich	1	2	Population Density (over 1000 ppl/sqkm, proportion)	0
Mesothelioma	Male	20-24 years	95+ years	Data Rich	1	2	Cumulative Cigarettes (5 Years)	1
Mesothelioma	Male	20-24 years	95+ years	Data Rich	1	2	Gold production (binary)	316
Mesothelioma	Male	20-24 years	95+ years	Data Rich	1	2	Indoor Air Pollution (All Cooking Fuels)	--
Mesothelioma	Male	20-24 years	95+ years	Data Rich	-1	3	Education (years per capita)	--
Mesothelioma	Male	20-24 years	95+ years	Data Rich	-1	3	LDI (I$ per capita)	--
Mesothelioma	Male	20-24 years	95+ years	Data Rich	1	3	Socio-demographic Index	822
Mesothelioma	Male	20-24 years	95+ years	Global	1	1	Smoking Prevalence	102
Mesothelioma	Male	20-24 years	95+ years	Global	1	1	Asbestos consumption (metric tons per year per capita)	174
Mesothelioma	Male	20-24 years	95+ years	Global	1	1	Age- and sex-specific SEV for Occupational asbestos	314
Mesothelioma	Male	20-24 years	95+ years	Global	1	1	Age-standardized SEV for Occupational asbestos	570
Mesothelioma	Male	20-24 years	95+ years	Global	-1	2	Healthcare access and quality index	56
Mesothelioma	Male	20-24 years	95+ years	Global	1	2	Population Density (over 1000 ppl/sqkm, proportion)	32
Mesothelioma	Male	20-24 years	95+ years	Global	1	2	Indoor Air Pollution (All Cooking Fuels)	104
Mesothelioma	Male	20-24 years	95+ years	Global	1	2	Gold production (binary)	176
Mesothelioma	Male	20-24 years	95+ years	Global	1	2	Cumulative Cigarettes (5 Years)	271
Mesothelioma	Male	20-24 years	95+ years	Global	-1	3	Education (years per capita)	--
Mesothelioma	Male	20-24 years	95+ years	Global	-1	3	LDI (I$ per capita)	--
Mesothelioma	Male	20-24 years	95+ years	Global	1	3	Socio-demographic Index	235

CODEm covariates used, level of covariate, and expected direction of covariate by cause, sex, and age

Cause	Sex	Age Start	Age End	Model Version Type	Direction	Level	Covariate Name	Number of Draws
Hodgkin lymphoma	Female	2-4 years	95+ years	Data Rich	-1	2	Healthcare access and quality index	551
Hodgkin lymphoma	Female	2-4 years	95+ years	Data Rich	-1	3	Socio-demographic Index	170
Hodgkin lymphoma	Female	2-4 years	95+ years	Data Rich	-1	3	Education (years per capita)	449
Hodgkin lymphoma	Female	2-4 years	95+ years	Data Rich	-1	3	LDI (I$ per capita)	--
Hodgkin lymphoma	Female	2-4 years	95+ years	Global	-1	2	Healthcare access and quality index	--
Hodgkin lymphoma	Female	2-4 years	95+ years	Global	-1	3	Education (years per capita)	274
Hodgkin lymphoma	Female	2-4 years	95+ years	Global	-1	3	Socio-demographic Index	602
Hodgkin lymphoma	Female	2-4 years	95+ years	Global	-1	3	LDI (I$ per capita)	--
Hodgkin lymphoma	Male	2-4 years	95+ years	Data Rich	-1	2	Healthcare access and quality index	841
Hodgkin lymphoma	Male	2-4 years	95+ years	Data Rich	-1	3	Socio-demographic Index	98
Hodgkin lymphoma	Male	2-4 years	95+ years	Data Rich	-1	3	Education (years per capita)	259
Hodgkin lymphoma	Male	2-4 years	95+ years	Data Rich	-1	3	LDI (I$ per capita)	--
Hodgkin lymphoma	Male	2-4 years	95+ years	Global	-1	2	Healthcare access and quality index	1000
Hodgkin lymphoma	Male	2-4 years	95+ years	Global	-1	3	Education (years per capita)	274
Hodgkin lymphoma	Male	2-4 years	95+ years	Global	-1	3	LDI (I$ per capita)	--
Hodgkin lymphoma	Male	2-4 years	95+ years	Global	-1	3	Socio-demographic Index	--
Non-Hodgkin's lymphoma	Female	12-23 months	95+ years	Data Rich	-1	2	Healthcare access and quality index	0
Non-Hodgkin's lymphoma	Female	12-23 months	95+ years	Data Rich	-1	2	Universal health coverage	0
Non-Hodgkin's lymphoma	Female	12-23 months	95+ years	Data Rich	1	2	Cumulative Cigarettes (20 Years)	43
Non-Hodgkin's lymphoma	Female	12-23 months	95+ years	Data Rich	1	2	Cumulative Cigarettes (5 Years)	46
Non-Hodgkin's lymphoma	Female	12-23 months	95+ years	Data Rich	1	2	Cumulative Cigarettes (15 Years)	186
Non-Hodgkin's lymphoma	Female	12-23 months	95+ years	Data Rich	1	2	Cumulative Cigarettes (10 Years)	223
Non-Hodgkin's lymphoma	Female	12-23 months	95+ years	Data Rich	1	2	Liters of alcohol consumed per capita	244
Non-Hodgkin's lymphoma	Female	12-23 months	95+ years	Data Rich	1	2	Mean BMI	910
Non-Hodgkin's lymphoma	Female	12-23 months	95+ years	Data Rich	-1	3	Socio-demographic Index	0
Non-Hodgkin's lymphoma	Female	12-23 months	95+ years	Data Rich	-1	3	Total Fertility Rate	903
Non-Hodgkin's lymphoma	Female	12-23 months	95+ years	Data Rich	-1	3	Education (years per capita)	--
Non-Hodgkin's lymphoma	Female	12-23 months	95+ years	Data Rich	-1	3	LDI (I$ per capita)	--
Non-Hodgkin's lymphoma	Female	12-23 months	95+ years	Data Rich	1	3	Log-transformed SEV scalar: HIV	439
Non-Hodgkin's lymphoma	Female	12-23 months	95+ years	Data Rich	1	3	Log-transformed age-standardized SEV scalar: HIV	619
Non-Hodgkin's lymphoma	Female	12-23 months	95+ years	Global	-1	2	Healthcare access and quality index	12
Non-Hodgkin's lymphoma	Female	12-23 months	95+ years	Global	-1	2	Universal health coverage	18
Non-Hodgkin's lymphoma	Female	12-23 months	95+ years	Global	1	2	Cumulative Cigarettes (10 Years)	119
Non-Hodgkin's lymphoma	Female	12-23 months	95+ years	Global	1	2	Cumulative Cigarettes (15 Years)	150
Non-Hodgkin's lymphoma	Female	12-23 months	95+ years	Global	1	2	Cumulative Cigarettes (20 Years)	235
Non-Hodgkin's lymphoma	Female	12-23 months	95+ years	Global	1	2	Cumulative Cigarettes (5 Years)	255
Non-Hodgkin's lymphoma	Female	12-23 months	95+ years	Global	1	2	Liters of alcohol consumed per capita	453
Non-Hodgkin's lymphoma	Female	12-23 months	95+ years	Global	1	2	Mean BMI	631
Non-Hodgkin's lymphoma	Female	12-23 months	95+ years	Global	-1	3	Total Fertility Rate	501
Non-Hodgkin's lymphoma	Female	12-23 months	95+ years	Global	-1	3	Education (years per capita)	--
Non-Hodgkin's lymphoma	Female	12-23 months	95+ years	Global	-1	3	LDI (I$ per capita)	--
Non-Hodgkin's lymphoma	Female	12-23 months	95+ years	Global	-1	3	Socio-demographic Index	--
Non-Hodgkin's lymphoma	Female	12-23 months	95+ years	Global	1	3	Log-transformed SEV scalar: HIV	395
Non-Hodgkin's lymphoma	Female	12-23 months	95+ years	Global	1	3	Log-transformed age-standardized SEV scalar: HIV	--
Non-Hodgkin's lymphoma	Male	12-23 months	95+ years	Data Rich	-1	2	Universal health coverage	9
Non-Hodgkin's lymphoma	Male	12-23 months	95+ years	Data Rich	-1	2	Healthcare access and quality index	15
Non-Hodgkin's lymphoma	Male	12-23 months	95+ years	Data Rich	1	2	Cumulative Cigarettes (20 Years)	8
Non-Hodgkin's lymphoma	Male	12-23 months	95+ years	Data Rich	1	2	Cumulative Cigarettes (15 Years)	97
Non-Hodgkin's lymphoma	Male	12-23 months	95+ years	Data Rich	1	2	Cumulative Cigarettes (5 Years)	150
Non-Hodgkin's lymphoma	Male	12-23 months	95+ years	Data Rich	1	2	Liters of alcohol consumed per capita	434
Non-Hodgkin's lymphoma	Male	12-23 months	95+ years	Data Rich	1	2	Cumulative Cigarettes (10 Years)	511
Non-Hodgkin's lymphoma	Male	12-23 months	95+ years	Data Rich	1	2	Mean BMI	792
Non-Hodgkin's lymphoma	Male	12-23 months	95+ years	Data Rich	-1	3	Socio-demographic Index	45
Non-Hodgkin's lymphoma	Male	12-23 months	95+ years	Data Rich	-1	3	Education (years per capita)	--
Non-Hodgkin's lymphoma	Male	12-23 months	95+ years	Data Rich	-1	3	LDI (I$ per capita)	--
Non-Hodgkin's lymphoma	Male	12-23 months	95+ years	Data Rich	1	3	Log-transformed SEV scalar: HIV	320
Non-Hodgkin's lymphoma	Male	12-23 months	95+ years	Data Rich	1	3	Log-transformed age-standardized SEV scalar: HIV	--
Non-Hodgkin's lymphoma	Male	12-23 months	95+ years	Global	-1	2	Healthcare access and quality index	64
Non-Hodgkin's lymphoma	Male	12-23 months	95+ years	Global	-1	2	Universal health coverage	--
Non-Hodgkin's lymphoma	Male	12-23 months	95+ years	Global	1	2	Cumulative Cigarettes (5 Years)	64
Non-Hodgkin's lymphoma	Male	12-23 months	95+ years	Global	1	2	Liters of alcohol consumed per capita	472
Non-Hodgkin's lymphoma	Male	12-23 months	95+ years	Global	1	2	Mean BMI	746
Non-Hodgkin's lymphoma	Male	12-23 months	95+ years	Global	1	2	Cumulative Cigarettes (10 Years)	--
Non-Hodgkin's lymphoma	Male	12-23 months	95+ years	Global	1	2	Cumulative Cigarettes (15 Years)	--
Non-Hodgkin's lymphoma	Male	12-23 months	95+ years	Global	1	2	Cumulative Cigarettes (20 Years)	--
Non-Hodgkin's lymphoma	Male	12-23 months	95+ years	Global	-1	3	Education (years per capita)	--
Non-Hodgkin's lymphoma	Male	12-23 months	95+ years	Global	-1	3	LDI (I$ per capita)	--
Non-Hodgkin's lymphoma	Male	12-23 months	95+ years	Global	-1	3	Socio-demographic Index	--
Non-Hodgkin's lymphoma	Male	12-23 months	95+ years	Global	1	3	Log-transformed age-standardized SEV scalar: HIV	372
Non-Hodgkin's lymphoma	Male	12-23 months	95+ years	Global	1	3	Log-transformed SEV scalar: HIV	374
Other non-Hodgkin lymphoma	Female	12-23 months	95+ years	Data Rich	-1	2	Universal health coverage	0
Other non-Hodgkin lymphoma	Female	12-23 months	95+ years	Data Rich	-1	2	Healthcare access and quality index	25
Other non-Hodgkin lymphoma	Female	12-23 months	95+ years	Data Rich	1	2	Mean BMI	0
Other non-Hodgkin lymphoma	Female	12-23 months	95+ years	Data Rich	1	2	Liters of alcohol consumed per capita	201
Other non-Hodgkin lymphoma	Female	12-23 months	95+ years	Data Rich	1	2	Cumulative Cigarettes (10 Years)	227
Other non-Hodgkin lymphoma	Female	12-23 months	95+ years	Data Rich	1	2	Cumulative Cigarettes (20 Years)	256
Other non-Hodgkin lymphoma	Female	12-23 months	95+ years	Data Rich	1	2	Cumulative Cigarettes (5 Years)	306
Other non-Hodgkin lymphoma	Female	12-23 months	95+ years	Data Rich	1	2	Cumulative Cigarettes (15 Years)	336
Other non-Hodgkin lymphoma	Female	12-23 months	95+ years	Data Rich	-1	3	Socio-demographic Index	175
Other non-Hodgkin lymphoma	Female	12-23 months	95+ years	Data Rich	-1	3	Total Fertility Rate	422
Other non-Hodgkin lymphoma	Female	12-23 months	95+ years	Data Rich	-1	3	Education (years per capita)	--
Other non-Hodgkin lymphoma	Female	12-23 months	95+ years	Data Rich	-1	3	LDI (I$ per capita)	--
Other non-Hodgkin lymphoma	Female	12-23 months	95+ years	Data Rich	1	3	Log-transformed age-standardized SEV scalar: HIV	0
Other non-Hodgkin lymphoma	Female	12-23 months	95+ years	Data Rich	1	3	Log-transformed SEV scalar: HIV	712
Other non-Hodgkin lymphoma	Female	12-23 months	95+ years	Global	-1	2	Healthcare access and quality index	--
Other non-Hodgkin lymphoma	Female	12-23 months	95+ years	Global	-1	2	Universal health coverage	--
Other non-Hodgkin lymphoma	Female	12-23 months	95+ years	Global	1	2	Mean BMI	154
Other non-Hodgkin lymphoma	Female	12-23 months	95+ years	Global	1	2	Cumulative Cigarettes (20 Years)	207
Other non-Hodgkin lymphoma	Female	12-23 months	95+ years	Global	1	2	Cumulative Cigarettes (10 Years)	224
Other non-Hodgkin lymphoma	Female	12-23 months	95+ years	Global	1	2	Cumulative Cigarettes (5 Years)	313
Other non-Hodgkin lymphoma	Female	12-23 months	95+ years	Global	1	2	Cumulative Cigarettes (15 Years)	329
Other non-Hodgkin lymphoma	Female	12-23 months	95+ years	Global	1	2	Liters of alcohol consumed per capita	416
Other non-Hodgkin lymphoma	Female	12-23 months	95+ years	Global	-1	3	Total Fertility Rate	441
Other non-Hodgkin lymphoma	Female	12-23 months	95+ years	Global	-1	3	Education (years per capita)	--
Other non-Hodgkin lymphoma	Female	12-23 months	95+ years	Global	-1	3	LDI (I$ per capita)	--
Other non-Hodgkin lymphoma	Female	12-23 months	95+ years	Global	-1	3	Socio-demographic Index	--
Other non-Hodgkin lymphoma	Female	12-23 months	95+ years	Global	1	3	Log-transformed age-standardized SEV scalar: HIV	19
Other non-Hodgkin lymphoma	Female	12-23 months	95+ years	Global	1	3	Log-transformed SEV scalar: HIV	498
Other non-Hodgkin lymphoma	Male	12-23 months	95+ years	Data Rich	-1	2	Healthcare access and quality index	50
Other non-Hodgkin lymphoma	Male	12-23 months	95+ years	Data Rich	-1	2	Universal health coverage	--
Other non-Hodgkin lymphoma	Male	12-23 months	95+ years	Data Rich	1	2	Cumulative Cigarettes (20 Years)	50
Other non-Hodgkin lymphoma	Male	12-23 months	95+ years	Data Rich	1	2	Cumulative Cigarettes (10 Years)	200
Other non-Hodgkin lymphoma	Male	12-23 months	95+ years	Data Rich	1	2	Cumulative Cigarettes (15 Years)	200
Other non-Hodgkin lymphoma	Male	12-23 months	95+ years	Data Rich	1	2	Cumulative Cigarettes (5 Years)	250
Other non-Hodgkin lymphoma	Male	12-23 months	95+ years	Data Rich	1	2	Liters of alcohol consumed per capita	550
Other non-Hodgkin lymphoma	Male	12-23 months	95+ years	Data Rich	1	2	Mean BMI	750
Other non-Hodgkin lymphoma	Male	12-23 months	95+ years	Data Rich	-1	3	Education (years per capita)	--
Other non-Hodgkin lymphoma	Male	12-23 months	95+ years	Data Rich	-1	3	LDI (I$ per capita)	--
Other non-Hodgkin lymphoma	Male	12-23 months	95+ years	Data Rich	-1	3	Socio-demographic Index	--
Other non-Hodgkin lymphoma	Male	12-23 months	95+ years	Data Rich	1	3	Log-transformed age-standardized SEV scalar: HIV	50
Other non-Hodgkin lymphoma	Male	12-23 months	95+ years	Data Rich	1	3	Log-transformed SEV scalar: HIV	350
Other non-Hodgkin lymphoma	Male	12-23 months	95+ years	Global	-1	2	Universal health coverage	56
Other non-Hodgkin lymphoma	Male	12-23 months	95+ years	Global	-1	2	Healthcare access and quality index	136
Other non-Hodgkin lymphoma	Male	12-23 months	95+ years	Global	1	2	Cumulative Cigarettes (20 Years)	4
Other non-Hodgkin lymphoma	Male	12-23 months	95+ years	Global	1	2	Cumulative Cigarettes (15 Years)	6
Other non-Hodgkin lymphoma	Male	12-23 months	95+ years	Global	1	2	Cumulative Cigarettes (5 Years)	168
Other non-Hodgkin lymphoma	Male	12-23 months	95+ years	Global	1	2	Cumulative Cigarettes (10 Years)	200
Other non-Hodgkin lymphoma	Male	12-23 months	95+ years	Global	1	2	Liters of alcohol consumed per capita	363
Other non-Hodgkin lymphoma	Male	12-23 months	95+ years	Global	1	2	Mean BMI	669
Other non-Hodgkin lymphoma	Male	12-23 months	95+ years	Global	-1	3	Socio-demographic Index	27
Other non-Hodgkin lymphoma	Male	12-23 months	95+ years	Global	-1	3	Education (years per capita)	33

CODEm covariates used, level of covariate, and expected direction of covariate by cause, sex, and age

Cause	Sex	Age Start	Age End	Model Version Type	Direction	Level	Covariate Name	Number of Draws
Other non-Hodgkin lymphoma	Male	12-23 months	95+ years	Global	-1	3	LDI (I$ per capita)	--
Other non-Hodgkin lymphoma	Male	12-23 months	95+ years	Global	1	3	Log-transformed age-standardized SEV scalar: HIV	0
Other non-Hodgkin lymphoma	Male	12-23 months	95+ years	Global	1	3	Log-transformed SEV scalar: HIV	553
Multiple myeloma	Female	20-24 years	95+ years	Data Rich	1	1	Liters of alcohol consumed per capita	443
Multiple myeloma	Female	20-24 years	95+ years	Data Rich	1	1	Smoking Prevalence	737
Multiple myeloma	Female	20-24 years	95+ years	Data Rich	1	1	Tobacco (cigarettes per capita)	--
Multiple myeloma	Female	20-24 years	95+ years	Data Rich	-1	2	Healthcare access and quality index	--
Multiple myeloma	Female	20-24 years	95+ years	Data Rich	-1	2	Improved Water Source (proportion with access)	--
Multiple myeloma	Female	20-24 years	95+ years	Data Rich	-1	2	Sanitation (proportion with access)	--
Multiple myeloma	Female	20-24 years	95+ years	Data Rich	1	2	Age- and sex-specific SEV for High red meat	105
Multiple myeloma	Female	20-24 years	95+ years	Data Rich	1	2	Mean BMI	715
Multiple myeloma	Female	20-24 years	95+ years	Data Rich	1	2	Age- and sex-specific SEV for Low fruit	--
Multiple myeloma	Female	20-24 years	95+ years	Data Rich	1	2	Age- and sex-specific SEV for Low vegetables	--
Multiple myeloma	Female	20-24 years	95+ years	Data Rich	-1	3	Education (years per capita)	--
Multiple myeloma	Female	20-24 years	95+ years	Data Rich	1	3	Socio-demographic Index	255
Multiple myeloma	Female	20-24 years	95+ years	Data Rich	1	3	LDI (I$ per capita)	--
Multiple myeloma	Female	20-24 years	95+ years	Global	1	1	Liters of alcohol consumed per capita	265
Multiple myeloma	Female	20-24 years	95+ years	Global	1	1	Smoking Prevalence	--
Multiple myeloma	Female	20-24 years	95+ years	Global	1	1	Tobacco (cigarettes per capita)	--
Multiple myeloma	Female	20-24 years	95+ years	Global	-1	2	Healthcare access and quality index	--
Multiple myeloma	Female	20-24 years	95+ years	Global	-1	2	Improved Water Source (proportion with access)	--
Multiple myeloma	Female	20-24 years	95+ years	Global	-1	2	Sanitation (proportion with access)	--
Multiple myeloma	Female	20-24 years	95+ years	Global	1	2	Mean BMI	354
Multiple myeloma	Female	20-24 years	95+ years	Global	1	2	Age- and sex-specific SEV for High red meat	--
Multiple myeloma	Female	20-24 years	95+ years	Global	1	2	Age- and sex-specific SEV for Low fruit	--
Multiple myeloma	Female	20-24 years	95+ years	Global	1	2	Age- and sex-specific SEV for Low vegetables	--
Multiple myeloma	Female	20-24 years	95+ years	Global	-1	3	Education (years per capita)	--
Multiple myeloma	Female	20-24 years	95+ years	Global	1	3	Socio-demographic Index	298
Multiple myeloma	Female	20-24 years	95+ years	Global	1	3	LDI (I$ per capita)	--
Multiple myeloma	Male	20-24 years	95+ years	Data Rich	1	1	Liters of alcohol consumed per capita	1000
Multiple myeloma	Male	20-24 years	95+ years	Data Rich	1	1	Smoking Prevalence	--
Multiple myeloma	Male	20-24 years	95+ years	Data Rich	1	1	Tobacco (cigarettes per capita)	--
Multiple myeloma	Male	20-24 years	95+ years	Data Rich	-1	2	Healthcare access and quality index	--
Multiple myeloma	Male	20-24 years	95+ years	Data Rich	-1	2	Improved Water Source (proportion with access)	--
Multiple myeloma	Male	20-24 years	95+ years	Data Rich	-1	2	Sanitation (proportion with access)	--
Multiple myeloma	Male	20-24 years	95+ years	Data Rich	1	2	Mean BMI	750
Multiple myeloma	Male	20-24 years	95+ years	Data Rich	1	2	Age- and sex-specific SEV for High red meat	--
Multiple myeloma	Male	20-24 years	95+ years	Data Rich	1	2	Age- and sex-specific SEV for Low fruit	--
Multiple myeloma	Male	20-24 years	95+ years	Data Rich	1	2	Age- and sex-specific SEV for Low vegetables	--
Multiple myeloma	Male	20-24 years	95+ years	Data Rich	-1	3	Education (years per capita)	--
Multiple myeloma	Male	20-24 years	95+ years	Data Rich	1	3	LDI (I$ per capita)	--
Multiple myeloma	Male	20-24 years	95+ years	Data Rich	1	3	Socio-demographic Index	--
Multiple myeloma	Male	20-24 years	95+ years	Global	1	1	Liters of alcohol consumed per capita	346
Multiple myeloma	Male	20-24 years	95+ years	Global	1	1	Smoking Prevalence	--
Multiple myeloma	Male	20-24 years	95+ years	Global	1	1	Tobacco (cigarettes per capita)	--
Multiple myeloma	Male	20-24 years	95+ years	Global	-1	2	Healthcare access and quality index	--
Multiple myeloma	Male	20-24 years	95+ years	Global	-1	2	Improved Water Source (proportion with access)	--
Multiple myeloma	Male	20-24 years	95+ years	Global	-1	2	Sanitation (proportion with access)	--
Multiple myeloma	Male	20-24 years	95+ years	Global	1	2	Age- and sex-specific SEV for High red meat	565
Multiple myeloma	Male	20-24 years	95+ years	Global	1	2	Mean BMI	565
Multiple myeloma	Male	20-24 years	95+ years	Global	1	2	Age- and sex-specific SEV for Low fruit	--
Multiple myeloma	Male	20-24 years	95+ years	Global	1	2	Age- and sex-specific SEV for Low vegetables	--
Multiple myeloma	Male	20-24 years	95+ years	Global	-1	3	Education (years per capita)	--
Multiple myeloma	Male	20-24 years	95+ years	Global	1	3	Socio-demographic Index	282
Multiple myeloma	Male	20-24 years	95+ years	Global	1	3	LDI (I$ per capita)	--
Leukaemia	Female	0-6 days	95+ years	Data Rich	1	1	Log-transformed SEV scalar: Leukemia	437
Leukaemia	Female	0-6 days	95+ years	Data Rich	1	1	Log-transformed age-standardized SEV scalar: Leukemia	796
Leukaemia	Female	0-6 days	95+ years	Data Rich	-1	2	Healthcare access and quality index	182
Leukaemia	Female	0-6 days	95+ years	Data Rich	1	2	Cumulative Cigarettes (20 Years)	67
Leukaemia	Female	0-6 days	95+ years	Data Rich	1	2	Tobacco (cigarettes per capita)	115
Leukaemia	Female	0-6 days	95+ years	Data Rich	1	2	Liters of alcohol consumed per capita	198
Leukaemia	Female	0-6 days	95+ years	Data Rich	1	2	Cumulative Cigarettes (10 Years)	258
Leukaemia	Female	0-6 days	95+ years	Data Rich	1	2	Mean BMI	--
Leukaemia	Female	0-6 days	95+ years	Data Rich	-1	3	Socio-demographic Index	103
Leukaemia	Female	0-6 days	95+ years	Data Rich	-1	3	Education (years per capita)	--
Leukaemia	Female	0-6 days	95+ years	Data Rich	1	3	LDI (I$ per capita)	--
Leukaemia	Female	0-6 days	95+ years	Global	1	1	Log-transformed age-standardized SEV scalar: Leukemia	284
Leukaemia	Female	0-6 days	95+ years	Global	1	1	Log-transformed SEV scalar: Leukemia	716
Leukaemia	Female	0-6 days	95+ years	Global	-1	2	Healthcare access and quality index	255
Leukaemia	Female	0-6 days	95+ years	Global	1	2	Cumulative Cigarettes (10 Years)	74
Leukaemia	Female	0-6 days	95+ years	Global	1	2	Tobacco (cigarettes per capita)	75
Leukaemia	Female	0-6 days	95+ years	Global	1	2	Liters of alcohol consumed per capita	112
Leukaemia	Female	0-6 days	95+ years	Global	1	2	Cumulative Cigarettes (20 Years)	181
Leukaemia	Female	0-6 days	95+ years	Global	1	2	Mean BMI	347
Leukaemia	Female	0-6 days	95+ years	Global	-1	3	Socio-demographic Index	81
Leukaemia	Female	0-6 days	95+ years	Global	-1	3	Education (years per capita)	83
Leukaemia	Female	0-6 days	95+ years	Global	1	3	LDI (I$ per capita)	--
Leukaemia	Male	0-6 days	95+ years	Data Rich	1	1	Log-transformed SEV scalar: Leukemia	478
Leukaemia	Male	0-6 days	95+ years	Data Rich	1	1	Log-transformed age-standardized SEV scalar: Leukemia	522
Leukaemia	Male	0-6 days	95+ years	Data Rich	-1	2	Healthcare access and quality index	231
Leukaemia	Male	0-6 days	95+ years	Data Rich	1	2	Liters of alcohol consumed per capita	456
Leukaemia	Male	0-6 days	95+ years	Data Rich	1	2	Cumulative Cigarettes (10 Years)	--
Leukaemia	Male	0-6 days	95+ years	Data Rich	1	2	Cumulative Cigarettes (20 Years)	--
Leukaemia	Male	0-6 days	95+ years	Data Rich	1	2	Mean BMI	--
Leukaemia	Male	0-6 days	95+ years	Data Rich	1	2	Tobacco (cigarettes per capita)	--
Leukaemia	Male	0-6 days	95+ years	Data Rich	-1	3	Socio-demographic Index	302
Leukaemia	Male	0-6 days	95+ years	Data Rich	-1	3	Education (years per capita)	--
Leukaemia	Male	0-6 days	95+ years	Data Rich	1	3	LDI (I$ per capita)	--
Leukaemia	Male	0-6 days	95+ years	Global	1	1	Log-transformed age-standardized SEV scalar: Leukemia	554
Leukaemia	Male	0-6 days	95+ years	Global	1	1	Log-transformed SEV scalar: Leukemia	--
Leukaemia	Male	0-6 days	95+ years	Global	-1	2	Healthcare access and quality index	--
Leukaemia	Male	0-6 days	95+ years	Global	1	2	Cumulative Cigarettes (20 Years)	45
Leukaemia	Male	0-6 days	95+ years	Global	1	2	Cumulative Cigarettes (10 Years)	125
Leukaemia	Male	0-6 days	95+ years	Global	1	2	Tobacco (cigarettes per capita)	201
Leukaemia	Male	0-6 days	95+ years	Global	1	2	Mean BMI	307
Leukaemia	Male	0-6 days	95+ years	Global	1	2	Liters of alcohol consumed per capita	--
Leukaemia	Male	0-6 days	95+ years	Global	-1	3	Socio-demographic Index	184
Leukaemia	Male	0-6 days	95+ years	Global	-1	3	Education (years per capita)	259
Leukaemia	Male	0-6 days	95+ years	Global	1	3	LDI (I$ per capita)	--
Acute lymphoid leukaemia	Female	0-6 days	95+ years	Data Rich	1	1	Log-transformed age-standardized SEV scalar: Leukemia	325
Acute lymphoid leukaemia	Female	0-6 days	95+ years	Data Rich	1	1	Log-transformed SEV scalar: Leukemia	675
Acute lymphoid leukaemia	Female	0-6 days	95+ years	Data Rich	-1	2	Healthcare access and quality index	--
Acute lymphoid leukaemia	Female	0-6 days	95+ years	Data Rich	1	2	Mean BMI	--
Acute lymphoid leukaemia	Female	0-6 days	95+ years	Data Rich	-1	3	Education (years per capita)	--
Acute lymphoid leukaemia	Female	0-6 days	95+ years	Data Rich	-1	3	LDI (I$ per capita)	--
Acute lymphoid leukaemia	Female	0-6 days	95+ years	Data Rich	1	3	Socio-demographic Index	--
Acute lymphoid leukaemia	Female	0-6 days	95+ years	Global	1	1	Log-transformed age-standardized SEV scalar: Leukemia	230
Acute lymphoid leukaemia	Female	0-6 days	95+ years	Global	1	1	Log-transformed SEV scalar: Leukemia	445
Acute lymphoid leukaemia	Female	0-6 days	95+ years	Global	-1	2	Healthcare access and quality index	269
Acute lymphoid leukaemia	Female	0-6 days	95+ years	Global	1	2	Mean BMI	236
Acute lymphoid leukaemia	Female	0-6 days	95+ years	Global	-1	3	Education (years per capita)	170
Acute lymphoid leukaemia	Female	0-6 days	95+ years	Global	-1	3	LDI (I$ per capita)	--
Acute lymphoid leukaemia	Female	0-6 days	95+ years	Global	1	3	Socio-demographic Index	141
Acute lymphoid leukaemia	Male	0-6 days	95+ years	Data Rich	1	1	Log-transformed age-standardized SEV scalar: Leukemia	1000
Acute lymphoid leukaemia	Male	0-6 days	95+ years	Data Rich	1	1	Log-transformed SEV scalar: Leukemia	--

CODEm covariates used, level of covariate, and expected direction of covariate by cause, sex, and age								
**Cause**	**Sex**	**Age Start**	**Age End**	**Model Version Type**	**Direction**	**Level**	**Covariate Name**	**Number of Draws**
Acute lymphoid leukaemia	Male	0-6 days	95+ years	Data Rich	-1	2	Healthcare access and quality index	328
Acute lymphoid leukaemia	Male	0-6 days	95+ years	Data Rich	1	2	Mean BMI	--
Acute lymphoid leukaemia	Male	0-6 days	95+ years	Data Rich	-1	3	Education (years per capita)	145
Acute lymphoid leukaemia	Male	0-6 days	95+ years	Data Rich	-1	3	LDI (I$ per capita)	461
Acute lymphoid leukaemia	Male	0-6 days	95+ years	Data Rich	1	3	Socio-demographic Index	208
Acute lymphoid leukaemia	Male	0-6 days	95+ years	Global	1	1	Log-transformed SEV scalar: Leukemia	194
Acute lymphoid leukaemia	Male	0-6 days	95+ years	Global	1	1	Log-transformed age-standardized SEV scalar: Leukemia	806
Acute lymphoid leukaemia	Male	0-6 days	95+ years	Global	-1	2	Healthcare access and quality index	231
Acute lymphoid leukaemia	Male	0-6 days	95+ years	Global	1	2	Mean BMI	--
Acute lymphoid leukaemia	Male	0-6 days	95+ years	Global	-1	3	Education (years per capita)	193
Acute lymphoid leukaemia	Male	0-6 days	95+ years	Global	-1	3	LDI (I$ per capita)	241
Acute lymphoid leukaemia	Male	0-6 days	95+ years	Global	1	3	Socio-demographic Index	--
Chronic lymphoid leukaemia	Female	20-24 years	95+ years	Data Rich	1	1	Log-transformed SEV scalar: Leukemia	187
Chronic lymphoid leukaemia	Female	20-24 years	95+ years	Data Rich	1	1	Log-transformed age-standardized SEV scalar: Leukemia	369
Chronic lymphoid leukaemia	Female	20-24 years	95+ years	Data Rich	-1	2	Healthcare access and quality index	--
Chronic lymphoid leukaemia	Female	20-24 years	95+ years	Data Rich	1	2	Cumulative Cigarettes (20 Years)	52
Chronic lymphoid leukaemia	Female	20-24 years	95+ years	Data Rich	1	2	Liters of alcohol consumed per capita	210
Chronic lymphoid leukaemia	Female	20-24 years	95+ years	Data Rich	1	2	Mean BMI	210
Chronic lymphoid leukaemia	Female	20-24 years	95+ years	Data Rich	1	2	Smoking Prevalence	427
Chronic lymphoid leukaemia	Female	20-24 years	95+ years	Data Rich	1	2	Cumulative Cigarettes (10 Years)	467
Chronic lymphoid leukaemia	Female	20-24 years	95+ years	Data Rich	1	2	Cumulative Cigarettes (15 Years)	--
Chronic lymphoid leukaemia	Female	20-24 years	95+ years	Data Rich	1	2	Cumulative Cigarettes (5 Years)	--
Chronic lymphoid leukaemia	Female	20-24 years	95+ years	Data Rich	1	2	Tobacco (cigarettes per capita)	--
Chronic lymphoid leukaemia	Female	20-24 years	95+ years	Data Rich	-1	3	LDI (I$ per capita)	204
Chronic lymphoid leukaemia	Female	20-24 years	95+ years	Data Rich	-1	3	Education (years per capita)	--
Chronic lymphoid leukaemia	Female	20-24 years	95+ years	Data Rich	1	3	Socio-demographic Index	476
Chronic lymphoid leukaemia	Female	20-24 years	95+ years	Global	1	1	Log-transformed age-standardized SEV scalar: Leukemia	402
Chronic lymphoid leukaemia	Female	20-24 years	95+ years	Global	1	1	Log-transformed SEV scalar: Leukemia	710
Chronic lymphoid leukaemia	Female	20-24 years	95+ years	Global	-1	2	Healthcare access and quality index	63
Chronic lymphoid leukaemia	Female	20-24 years	95+ years	Global	1	2	Cumulative Cigarettes (10 Years)	31
Chronic lymphoid leukaemia	Female	20-24 years	95+ years	Global	1	2	Cumulative Cigarettes (15 Years)	63
Chronic lymphoid leukaemia	Female	20-24 years	95+ years	Global	1	2	Cumulative Cigarettes (5 Years)	77
Chronic lymphoid leukaemia	Female	20-24 years	95+ years	Global	1	2	Mean BMI	135
Chronic lymphoid leukaemia	Female	20-24 years	95+ years	Global	1	2	Cumulative Cigarettes (20 Years)	141
Chronic lymphoid leukaemia	Female	20-24 years	95+ years	Global	1	2	Tobacco (cigarettes per capita)	183
Chronic lymphoid leukaemia	Female	20-24 years	95+ years	Global	1	2	Liters of alcohol consumed per capita	246
Chronic lymphoid leukaemia	Female	20-24 years	95+ years	Global	1	2	Smoking Prevalence	--
Chronic lymphoid leukaemia	Female	20-24 years	95+ years	Global	-1	3	LDI (I$ per capita)	207
Chronic lymphoid leukaemia	Female	20-24 years	95+ years	Global	-1	3	Education (years per capita)	--
Chronic lymphoid leukaemia	Female	20-24 years	95+ years	Global	1	3	Socio-demographic Index	182
Chronic lymphoid leukaemia	Male	20-24 years	95+ years	Data Rich	1	1	Log-transformed SEV scalar: Leukemia	293
Chronic lymphoid leukaemia	Male	20-24 years	95+ years	Data Rich	1	1	Log-transformed age-standardized SEV scalar: Leukemia	816
Chronic lymphoid leukaemia	Male	20-24 years	95+ years	Data Rich	-1	2	Healthcare access and quality index	42
Chronic lymphoid leukaemia	Male	20-24 years	95+ years	Data Rich	1	2	Cumulative Cigarettes (10 Years)	1
Chronic lymphoid leukaemia	Male	20-24 years	95+ years	Data Rich	1	2	Liters of alcohol consumed per capita	2
Chronic lymphoid leukaemia	Male	20-24 years	95+ years	Data Rich	1	2	Cumulative Cigarettes (15 Years)	3
Chronic lymphoid leukaemia	Male	20-24 years	95+ years	Data Rich	1	2	Cumulative Cigarettes (20 Years)	23
Chronic lymphoid leukaemia	Male	20-24 years	95+ years	Data Rich	1	2	Tobacco (cigarettes per capita)	23
Chronic lymphoid leukaemia	Male	20-24 years	95+ years	Data Rich	1	2	Cumulative Cigarettes (5 Years)	48
Chronic lymphoid leukaemia	Male	20-24 years	95+ years	Data Rich	1	2	Smoking Prevalence	63
Chronic lymphoid leukaemia	Male	20-24 years	95+ years	Data Rich	1	2	Mean BMI	82
Chronic lymphoid leukaemia	Male	20-24 years	95+ years	Data Rich	-1	3	LDI (I$ per capita)	15
Chronic lymphoid leukaemia	Male	20-24 years	95+ years	Data Rich	-1	3	Education (years per capita)	--
Chronic lymphoid leukaemia	Male	20-24 years	95+ years	Data Rich	1	3	Socio-demographic Index	671
Chronic lymphoid leukaemia	Male	20-24 years	95+ years	Global	1	1	Log-transformed SEV scalar: Leukemia	480
Chronic lymphoid leukaemia	Male	20-24 years	95+ years	Global	1	1	Log-transformed age-standardized SEV scalar: Leukemia	798
Chronic lymphoid leukaemia	Male	20-24 years	95+ years	Global	-1	2	Healthcare access and quality index	265
Chronic lymphoid leukaemia	Male	20-24 years	95+ years	Global	1	2	Smoking Prevalence	116
Chronic lymphoid leukaemia	Male	20-24 years	95+ years	Global	1	2	Cumulative Cigarettes (20 Years)	121
Chronic lymphoid leukaemia	Male	20-24 years	95+ years	Global	1	2	Cumulative Cigarettes (5 Years)	121
Chronic lymphoid leukaemia	Male	20-24 years	95+ years	Global	1	2	Mean BMI	265
Chronic lymphoid leukaemia	Male	20-24 years	95+ years	Global	1	2	Cumulative Cigarettes (15 Years)	314
Chronic lymphoid leukaemia	Male	20-24 years	95+ years	Global	1	2	Cumulative Cigarettes (10 Years)	--
Chronic lymphoid leukaemia	Male	20-24 years	95+ years	Global	1	2	Liters of alcohol consumed per capita	--
Chronic lymphoid leukaemia	Male	20-24 years	95+ years	Global	1	2	Tobacco (cigarettes per capita)	--
Chronic lymphoid leukaemia	Male	20-24 years	95+ years	Global	-1	3	LDI (I$ per capita)	93
Chronic lymphoid leukaemia	Male	20-24 years	95+ years	Global	-1	3	Education (years per capita)	--
Chronic lymphoid leukaemia	Male	20-24 years	95+ years	Global	1	3	Socio-demographic Index	411
Acute myeloid leukaemia	Female	0-6 days	95+ years	Data Rich	1	1	Log-transformed SEV scalar: Leukemia	--
Acute myeloid leukaemia	Female	0-6 days	95+ years	Data Rich	1	1	Log-transformed age-standardized SEV scalar: Leukemia	--
Acute myeloid leukaemia	Female	0-6 days	95+ years	Data Rich	-1	2	Healthcare access and quality index	--
Acute myeloid leukaemia	Female	0-6 days	95+ years	Data Rich	1	2	Cumulative Cigarettes (10 Years)	248
Acute myeloid leukaemia	Female	0-6 days	95+ years	Data Rich	1	2	Liters of alcohol consumed per capita	248
Acute myeloid leukaemia	Female	0-6 days	95+ years	Data Rich	1	2	Cumulative Cigarettes (20 Years)	--
Acute myeloid leukaemia	Female	0-6 days	95+ years	Data Rich	1	2	Mean BMI	--
Acute myeloid leukaemia	Female	0-6 days	95+ years	Data Rich	1	2	Smoking Prevalence	--
Acute myeloid leukaemia	Female	0-6 days	95+ years	Data Rich	-1	3	Education (years per capita)	--
Acute myeloid leukaemia	Female	0-6 days	95+ years	Data Rich	1	3	Socio-demographic Index	752
Acute myeloid leukaemia	Female	0-6 days	95+ years	Data Rich	1	3	LDI (I$ per capita)	--
Acute myeloid leukaemia	Female	0-6 days	95+ years	Global	1	1	Log-transformed age-standardized SEV scalar: Leukemia	474
Acute myeloid leukaemia	Female	0-6 days	95+ years	Global	1	1	Log-transformed SEV scalar: Leukemia	526
Acute myeloid leukaemia	Female	0-6 days	95+ years	Global	-1	2	Healthcare access and quality index	--
Acute myeloid leukaemia	Female	0-6 days	95+ years	Global	1	2	Cumulative Cigarettes (10 Years)	85
Acute myeloid leukaemia	Female	0-6 days	95+ years	Global	1	2	Cumulative Cigarettes (20 Years)	101
Acute myeloid leukaemia	Female	0-6 days	95+ years	Global	1	2	Mean BMI	196
Acute myeloid leukaemia	Female	0-6 days	95+ years	Global	1	2	Liters of alcohol consumed per capita	296
Acute myeloid leukaemia	Female	0-6 days	95+ years	Global	1	2	Smoking Prevalence	--
Acute myeloid leukaemia	Female	0-6 days	95+ years	Global	-1	3	Education (years per capita)	--
Acute myeloid leukaemia	Female	0-6 days	95+ years	Global	1	3	Socio-demographic Index	262
Acute myeloid leukaemia	Female	0-6 days	95+ years	Global	1	3	LDI (I$ per capita)	--
Acute myeloid leukaemia	Male	0-6 days	95+ years	Data Rich	1	1	Log-transformed SEV scalar: Leukemia	--
Acute myeloid leukaemia	Male	0-6 days	95+ years	Data Rich	1	1	Log-transformed age-standardized SEV scalar: Leukemia	--
Acute myeloid leukaemia	Male	0-6 days	95+ years	Data Rich	-1	2	Healthcare access and quality index	--
Acute myeloid leukaemia	Male	0-6 days	95+ years	Data Rich	1	2	Mean BMI	624
Acute myeloid leukaemia	Male	0-6 days	95+ years	Data Rich	1	2	Cumulative Cigarettes (10 Years)	--
Acute myeloid leukaemia	Male	0-6 days	95+ years	Data Rich	1	2	Cumulative Cigarettes (20 Years)	--
Acute myeloid leukaemia	Male	0-6 days	95+ years	Data Rich	1	2	Liters of alcohol consumed per capita	--
Acute myeloid leukaemia	Male	0-6 days	95+ years	Data Rich	1	2	Smoking Prevalence	--
Acute myeloid leukaemia	Male	0-6 days	95+ years	Data Rich	-1	3	Education (years per capita)	--
Acute myeloid leukaemia	Male	0-6 days	95+ years	Data Rich	1	3	Socio-demographic Index	494
Acute myeloid leukaemia	Male	0-6 days	95+ years	Data Rich	1	3	LDI (I$ per capita)	--
Acute myeloid leukaemia	Male	0-6 days	95+ years	Global	1	1	Log-transformed age-standardized SEV scalar: Leukemia	185
Acute myeloid leukaemia	Male	0-6 days	95+ years	Global	1	1	Log-transformed SEV scalar: Leukemia	--
Acute myeloid leukaemia	Male	0-6 days	95+ years	Global	-1	2	Healthcare access and quality index	--
Acute myeloid leukaemia	Male	0-6 days	95+ years	Global	1	2	Liters of alcohol consumed per capita	222
Acute myeloid leukaemia	Male	0-6 days	95+ years	Global	1	2	Cumulative Cigarettes (10 Years)	--
Acute myeloid leukaemia	Male	0-6 days	95+ years	Global	1	2	Cumulative Cigarettes (20 Years)	--
Acute myeloid leukaemia	Male	0-6 days	95+ years	Global	1	2	Mean BMI	--
Acute myeloid leukaemia	Male	0-6 days	95+ years	Global	1	2	Smoking Prevalence	--
Acute myeloid leukaemia	Male	0-6 days	95+ years	Global	-1	3	Education (years per capita)	--
Acute myeloid leukaemia	Male	0-6 days	95+ years	Global	1	3	Socio-demographic Index	326
Acute myeloid leukaemia	Male	0-6 days	95+ years	Global	1	3	LDI (I$ per capita)	--
Chronic myeloid leukaemia	Female	0-6 days	95+ years	Data Rich	1	1	Log-transformed SEV scalar: Leukemia	1000
Chronic myeloid leukaemia	Female	0-6 days	95+ years	Data Rich	1	1	Log-transformed age-standardized SEV scalar: Leukemia	--
Chronic myeloid leukaemia	Female	0-6 days	95+ years	Data Rich	-1	2	Healthcare access and quality index	274
Chronic myeloid leukaemia	Female	0-6 days	95+ years	Data Rich	1	2	Liters of alcohol consumed per capita	274
Chronic myeloid leukaemia	Female	0-6 days	95+ years	Data Rich	1	2	Cumulative Cigarettes (10 Years)	--
Chronic myeloid leukaemia	Female	0-6 days	95+ years	Data Rich	1	2	Cumulative Cigarettes (15 Years)	--
Chronic myeloid leukaemia	Female	0-6 days	95+ years	Data Rich	1	2	Cumulative Cigarettes (20 Years)	--
Chronic myeloid leukaemia	Female	0-6 days	95+ years	Data Rich	1	2	Cumulative Cigarettes (5 Years)	--

CODEm covariates used, level of covariate, and expected direction of covariate by cause, sex, and age

Cause	Sex	Age Start	Age End	Model Version Type	Direction	Level	Covariate Name	Number of Draws
Chronic myeloid leukaemia	Female	0-6 days	95+ years	Data Rich	1	2	Mean BMI	--
Chronic myeloid leukaemia	Female	0-6 days	95+ years	Data Rich	1	2	Smoking Prevalence	--
Chronic myeloid leukaemia	Female	0-6 days	95+ years	Data Rich	1	2	Tobacco (cigarettes per capita)	--
Chronic myeloid leukaemia	Female	0-6 days	95+ years	Data Rich	-1	3	Education (years per capita)	269
Chronic myeloid leukaemia	Female	0-6 days	95+ years	Data Rich	-1	3	Socio-demographic Index	378
Chronic myeloid leukaemia	Female	0-6 days	95+ years	Data Rich	1	3	LDI (I$ per capita)	--
Chronic myeloid leukaemia	Female	0-6 days	95+ years	Global	1	1	Log-transformed SEV scalar: Leukemia	1000
Chronic myeloid leukaemia	Female	0-6 days	95+ years	Global	1	1	Log-transformed age-standardized SEV scalar: Leukemia	--
Chronic myeloid leukaemia	Female	0-6 days	95+ years	Global	-1	2	Healthcare access and quality index	143
Chronic myeloid leukaemia	Female	0-6 days	95+ years	Global	1	2	Liters of alcohol consumed per capita	567
Chronic myeloid leukaemia	Female	0-6 days	95+ years	Global	1	2	Cumulative Cigarettes (10 Years)	--
Chronic myeloid leukaemia	Female	0-6 days	95+ years	Global	1	2	Cumulative Cigarettes (15 Years)	--
Chronic myeloid leukaemia	Female	0-6 days	95+ years	Global	1	2	Cumulative Cigarettes (20 Years)	--
Chronic myeloid leukaemia	Female	0-6 days	95+ years	Global	1	2	Cumulative Cigarettes (5 Years)	--
Chronic myeloid leukaemia	Female	0-6 days	95+ years	Global	1	2	Mean BMI	--
Chronic myeloid leukaemia	Female	0-6 days	95+ years	Global	1	2	Smoking Prevalence	--
Chronic myeloid leukaemia	Female	0-6 days	95+ years	Global	1	2	Tobacco (cigarettes per capita)	--
Chronic myeloid leukaemia	Female	0-6 days	95+ years	Global	-1	3	Socio-demographic Index	70
Chronic myeloid leukaemia	Female	0-6 days	95+ years	Global	-1	3	Education (years per capita)	306
Chronic myeloid leukaemia	Female	0-6 days	95+ years	Global	1	3	LDI (I$ per capita)	--
Chronic myeloid leukaemia	Male	0-6 days	95+ years	Data Rich	1	1	Log-transformed age-standardized SEV scalar: Leukemia	594
Chronic myeloid leukaemia	Male	0-6 days	95+ years	Data Rich	1	1	Log-transformed SEV scalar: Leukemia	926
Chronic myeloid leukaemia	Male	0-6 days	95+ years	Data Rich	-1	2	Healthcare access and quality index	8
Chronic myeloid leukaemia	Male	0-6 days	95+ years	Data Rich	1	2	Cumulative Cigarettes (10 Years)	1
Chronic myeloid leukaemia	Male	0-6 days	95+ years	Data Rich	1	2	Cumulative Cigarettes (15 Years)	1
Chronic myeloid leukaemia	Male	0-6 days	95+ years	Data Rich	1	2	Liters of alcohol consumed per capita	122
Chronic myeloid leukaemia	Male	0-6 days	95+ years	Data Rich	1	2	Cumulative Cigarettes (5 Years)	196
Chronic myeloid leukaemia	Male	0-6 days	95+ years	Data Rich	1	2	Smoking Prevalence	287
Chronic myeloid leukaemia	Male	0-6 days	95+ years	Data Rich	1	2	Cumulative Cigarettes (20 Years)	--
Chronic myeloid leukaemia	Male	0-6 days	95+ years	Data Rich	1	2	Mean BMI	--
Chronic myeloid leukaemia	Male	0-6 days	95+ years	Data Rich	1	2	Tobacco (cigarettes per capita)	--
Chronic myeloid leukaemia	Male	0-6 days	95+ years	Data Rich	-1	3	Socio-demographic Index	60
Chronic myeloid leukaemia	Male	0-6 days	95+ years	Data Rich	-1	3	Education (years per capita)	152
Chronic myeloid leukaemia	Male	0-6 days	95+ years	Data Rich	1	3	LDI (I$ per capita)	--
Chronic myeloid leukaemia	Male	0-6 days	95+ years	Global	1	1	Log-transformed SEV scalar: Leukemia	721
Chronic myeloid leukaemia	Male	0-6 days	95+ years	Global	1	1	Log-transformed age-standardized SEV scalar: Leukemia	839
Chronic myeloid leukaemia	Male	0-6 days	95+ years	Global	-1	2	Healthcare access and quality index	--
Chronic myeloid leukaemia	Male	0-6 days	95+ years	Global	1	2	Cumulative Cigarettes (5 Years)	79
Chronic myeloid leukaemia	Male	0-6 days	95+ years	Global	1	2	Smoking Prevalence	120
Chronic myeloid leukaemia	Male	0-6 days	95+ years	Global	1	2	Cumulative Cigarettes (20 Years)	141
Chronic myeloid leukaemia	Male	0-6 days	95+ years	Global	1	2	Liters of alcohol consumed per capita	226
Chronic myeloid leukaemia	Male	0-6 days	95+ years	Global	1	2	Cumulative Cigarettes (10 Years)	--
Chronic myeloid leukaemia	Male	0-6 days	95+ years	Global	1	2	Cumulative Cigarettes (15 Years)	--
Chronic myeloid leukaemia	Male	0-6 days	95+ years	Global	1	2	Mean BMI	--
Chronic myeloid leukaemia	Male	0-6 days	95+ years	Global	1	2	Tobacco (cigarettes per capita)	--
Chronic myeloid leukaemia	Male	0-6 days	95+ years	Global	-1	3	Education (years per capita)	75
Chronic myeloid leukaemia	Male	0-6 days	95+ years	Global	-1	3	Socio-demographic Index	190
Chronic myeloid leukaemia	Male	0-6 days	95+ years	Global	1	3	LDI (I$ per capita)	--
Other leukaemia	Female	0-6 days	95+ years	Data Rich	1	1	Log-transformed age-standardized SEV scalar: Leukemia	253
Other leukaemia	Female	0-6 days	95+ years	Data Rich	1	1	Log-transformed SEV scalar: Leukemia	747
Other leukaemia	Female	0-6 days	95+ years	Data Rich	1	1	Liters of alcohol consumed per capita	--
Other leukaemia	Female	0-6 days	95+ years	Data Rich	-1	2	Healthcare access and quality index	208
Other leukaemia	Female	0-6 days	95+ years	Data Rich	1	2	Cumulative Cigarettes (10 Years)	120
Other leukaemia	Female	0-6 days	95+ years	Data Rich	1	2	Tobacco (cigarettes per capita)	208
Other leukaemia	Female	0-6 days	95+ years	Data Rich	1	2	Cumulative Cigarettes (20 Years)	--
Other leukaemia	Female	0-6 days	95+ years	Data Rich	1	2	Mean BMI	--
Other leukaemia	Female	0-6 days	95+ years	Data Rich	-1	3	Education (years per capita)	145
Other leukaemia	Female	0-6 days	95+ years	Data Rich	-1	3	Socio-demographic Index	174
Other leukaemia	Female	0-6 days	95+ years	Data Rich	1	3	LDI (I$ per capita)	--
Other leukaemia	Female	0-6 days	95+ years	Global	1	1	Log-transformed age-standardized SEV scalar: Leukemia	428
Other leukaemia	Female	0-6 days	95+ years	Global	1	1	Log-transformed SEV scalar: Leukemia	572
Other leukaemia	Female	0-6 days	95+ years	Global	-1	2	Healthcare access and quality index	--
Other leukaemia	Female	0-6 days	95+ years	Global	1	2	Cumulative Cigarettes (10 Years)	213
Other leukaemia	Female	0-6 days	95+ years	Global	1	2	Cumulative Cigarettes (20 Years)	--
Other leukaemia	Female	0-6 days	95+ years	Global	1	2	Liters of alcohol consumed per capita	--
Other leukaemia	Female	0-6 days	95+ years	Global	1	2	Mean BMI	--
Other leukaemia	Female	0-6 days	95+ years	Global	1	2	Tobacco (cigarettes per capita)	--
Other leukaemia	Female	0-6 days	95+ years	Global	-1	3	Socio-demographic Index	111
Other leukaemia	Female	0-6 days	95+ years	Global	-1	3	Education (years per capita)	168
Other leukaemia	Female	0-6 days	95+ years	Global	1	3	LDI (I$ per capita)	236
Other leukaemia	Male	0-6 days	95+ years	Data Rich	1	1	Log-transformed age-standardized SEV scalar: Leukemia	237
Other leukaemia	Male	0-6 days	95+ years	Data Rich	1	1	Log-transformed SEV scalar: Leukemia	775
Other leukaemia	Male	0-6 days	95+ years	Data Rich	-1	2	Healthcare access and quality index	149
Other leukaemia	Male	0-6 days	95+ years	Data Rich	1	2	Mean BMI	290
Other leukaemia	Male	0-6 days	95+ years	Data Rich	1	2	Tobacco (cigarettes per capita)	308
Other leukaemia	Male	0-6 days	95+ years	Data Rich	1	2	Cumulative Cigarettes (10 Years)	--
Other leukaemia	Male	0-6 days	95+ years	Data Rich	1	2	Cumulative Cigarettes (20 Years)	--
Other leukaemia	Male	0-6 days	95+ years	Data Rich	1	2	Liters of alcohol consumed per capita	--
Other leukaemia	Male	0-6 days	95+ years	Data Rich	-1	3	Education (years per capita)	270
Other leukaemia	Male	0-6 days	95+ years	Data Rich	-1	3	Socio-demographic Index	--
Other leukaemia	Male	0-6 days	95+ years	Data Rich	1	3	LDI (I$ per capita)	159
Other leukaemia	Male	0-6 days	95+ years	Global	1	1	Log-transformed SEV scalar: Leukemia	253
Other leukaemia	Male	0-6 days	95+ years	Global	1	1	Log-transformed age-standardized SEV scalar: Leukemia	343
Other leukaemia	Male	0-6 days	95+ years	Global	-1	2	Healthcare access and quality index	--
Other leukaemia	Male	0-6 days	95+ years	Global	1	2	Cumulative Cigarettes (10 Years)	111
Other leukaemia	Male	0-6 days	95+ years	Global	1	2	Mean BMI	181
Other leukaemia	Male	0-6 days	95+ years	Global	1	2	Cumulative Cigarettes (20 Years)	--
Other leukaemia	Male	0-6 days	95+ years	Global	1	2	Liters of alcohol consumed per capita	--
Other leukaemia	Male	0-6 days	95+ years	Global	1	2	Tobacco (cigarettes per capita)	--
Other leukaemia	Male	0-6 days	95+ years	Global	-1	3	Socio-demographic Index	173
Other leukaemia	Male	0-6 days	95+ years	Global	-1	3	Education (years per capita)	--
Other leukaemia	Male	0-6 days	95+ years	Global	1	3	LDI (I$ per capita)	223
Other malignant cancers	Female	0-6 days	95+ years	Data Rich	1	1	Smoking Prevalence	413
Other malignant cancers	Female	0-6 days	95+ years	Data Rich	1	1	Tobacco (cigarettes per capita)	413
Other malignant cancers	Female	0-6 days	95+ years	Data Rich	-1	2	pufa adjusted(percent)	169
Other malignant cancers	Female	0-6 days	95+ years	Data Rich	-1	2	Healthcare access and quality index	--
Other malignant cancers	Female	0-6 days	95+ years	Data Rich	1	2	Age- and sex-specific SEV for Low nuts and seeds	77
Other malignant cancers	Female	0-6 days	95+ years	Data Rich	1	2	Age- and sex-specific SEV for Low fruit	92
Other malignant cancers	Female	0-6 days	95+ years	Data Rich	1	2	Age- and sex-specific SEV for Low vegetables	429
Other malignant cancers	Female	0-6 days	95+ years	Data Rich	-1	3	Education (years per capita)	133
Other malignant cancers	Female	0-6 days	95+ years	Data Rich	1	3	LDI (I$ per capita)	427
Other malignant cancers	Female	0-6 days	95+ years	Data Rich	1	3	Socio-demographic Index	427
Other malignant cancers	Female	0-6 days	95+ years	Global	1	1	Smoking Prevalence	332
Other malignant cancers	Female	0-6 days	95+ years	Global	1	1	Tobacco (cigarettes per capita)	--
Other malignant cancers	Female	0-6 days	95+ years	Global	-1	2	Healthcare access and quality index	203
Other malignant cancers	Female	0-6 days	95+ years	Global	-1	2	pufa adjusted(percent)	--
Other malignant cancers	Female	0-6 days	95+ years	Global	1	2	Age- and sex-specific SEV for Low nuts and seeds	24
Other malignant cancers	Female	0-6 days	95+ years	Global	1	2	Age- and sex-specific SEV for Low fruit	30
Other malignant cancers	Female	0-6 days	95+ years	Global	1	2	Age- and sex-specific SEV for Low vegetables	486
Other malignant cancers	Female	0-6 days	95+ years	Global	-1	3	Education (years per capita)	--
Other malignant cancers	Female	0-6 days	95+ years	Global	1	3	Socio-demographic Index	553
Other malignant cancers	Female	0-6 days	95+ years	Global	1	3	LDI (I$ per capita)	573
Other malignant cancers	Male	0-6 days	95+ years	Data Rich	1	1	Smoking Prevalence	--
Other malignant cancers	Male	0-6 days	95+ years	Data Rich	1	1	Tobacco (cigarettes per capita)	--
Other malignant cancers	Male	0-6 days	95+ years	Data Rich	-1	2	pufa adjusted(percent)	494
Other malignant cancers	Male	0-6 days	95+ years	Data Rich	-1	2	Healthcare access and quality index	--
Other malignant cancers	Male	0-6 days	95+ years	Data Rich	1	2	Age- and sex-specific SEV for Low nuts and seeds	159
Other malignant cancers	Male	0-6 days	95+ years	Data Rich	1	2	Age- and sex-specific SEV for Low vegetables	159

CODEm covariates used, level of covariate, and expected direction of covariate by cause, sex, and age									
**Cause**	**Sex**	**Age Start**	**Age End**	**Model Version Type**	**Direction**	**Level**	**Covariate Name**	**Number of Draws**
Other malignant cancers	Male	0-6 days	95+ years	Data Rich	1	2	Age- and sex-specific SEV for Low fruit	--
Other malignant cancers	Male	0-6 days	95+ years	Data Rich	-1	3	Education (years per capita)	87
Other malignant cancers	Male	0-6 days	95+ years	Data Rich	1	3	LDI (I$ per capita)	229
Other malignant cancers	Male	0-6 days	95+ years	Data Rich	1	3	Socio-demographic Index	402
Other malignant cancers	Male	0-6 days	95+ years	Global	1	1	Smoking Prevalence	539
Other malignant cancers	Male	0-6 days	95+ years	Global	1	1	Tobacco (cigarettes per capita)	--
Other malignant cancers	Male	0-6 days	95+ years	Global	-1	2	Healthcare access and quality index	--
Other malignant cancers	Male	0-6 days	95+ years	Global	-1	2	pufa adjusted(percent)	--
Other malignant cancers	Male	0-6 days	95+ years	Global	1	2	Age- and sex-specific SEV for Low fruit	294
Other malignant cancers	Male	0-6 days	95+ years	Global	1	2	Age- and sex-specific SEV for Low nuts and seeds	294
Other malignant cancers	Male	0-6 days	95+ years	Global	1	2	Age- and sex-specific SEV for Low vegetables	294
Other malignant cancers	Male	0-6 days	95+ years	Global	-1	3	Education (years per capita)	--
Other malignant cancers	Male	0-6 days	95+ years	Global	1	3	Socio-demographic Index	461
Other malignant cancers	Male	0-6 days	95+ years	Global	1	3	LDI (I$ per capita)	--
Myelodysplastic, myeloproliferative, and other hematopoietic neoplasms	Female	0-6 days	95+ years	Data Rich	1	1	Log-transformed SEV scalar: Leukemia	1000
Myelodysplastic, myeloproliferative, and other hematopoietic neoplasms	Female	0-6 days	95+ years	Data Rich	1	1	Log-transformed age-standardized SEV scalar: Leukemia	--
Myelodysplastic, myeloproliferative, and other hematopoietic neoplasms	Female	0-6 days	95+ years	Data Rich	-1	2	Healthcare access and quality index	--
Myelodysplastic, myeloproliferative, and other hematopoietic neoplasms	Female	0-6 days	95+ years	Data Rich	1	2	Liters of alcohol consumed per capita	134
Myelodysplastic, myeloproliferative, and other hematopoietic neoplasms	Female	0-6 days	95+ years	Data Rich	1	2	Cumulative Cigarettes (10 Years)	--
Myelodysplastic, myeloproliferative, and other hematopoietic neoplasms	Female	0-6 days	95+ years	Data Rich	1	2	Cumulative Cigarettes (15 Years)	--
Myelodysplastic, myeloproliferative, and other hematopoietic neoplasms	Female	0-6 days	95+ years	Data Rich	1	2	Cumulative Cigarettes (20 Years)	--
Myelodysplastic, myeloproliferative, and other hematopoietic neoplasms	Female	0-6 days	95+ years	Data Rich	1	2	Cumulative Cigarettes (5 Years)	--
Myelodysplastic, myeloproliferative, and other hematopoietic neoplasms	Female	0-6 days	95+ years	Data Rich	1	2	Smoking Prevalence	--
Myelodysplastic, myeloproliferative, and other hematopoietic neoplasms	Female	0-6 days	95+ years	Data Rich	1	2	Tobacco (cigarettes per capita)	--
Myelodysplastic, myeloproliferative, and other hematopoietic neoplasms	Female	0-6 days	95+ years	Data Rich	-1	3	Education (years per capita)	--
Myelodysplastic, myeloproliferative, and other hematopoietic neoplasms	Female	0-6 days	95+ years	Data Rich	1	3	Socio-demographic Index	512
Myelodysplastic, myeloproliferative, and other hematopoietic neoplasms	Female	0-6 days	95+ years	Data Rich	1	3	LDI (I$ per capita)	--
Myelodysplastic, myeloproliferative, and other hematopoietic neoplasms	Female	0-6 days	95+ years	Global	1	1	Log-transformed age-standardized SEV scalar: Leukemia	427
Myelodysplastic, myeloproliferative, and other hematopoietic neoplasms	Female	0-6 days	95+ years	Global	1	1	Log-transformed SEV scalar: Leukemia	791
Myelodysplastic, myeloproliferative, and other hematopoietic neoplasms	Female	0-6 days	95+ years	Global	-1	2	Healthcare access and quality index	--
Myelodysplastic, myeloproliferative, and other hematopoietic neoplasms	Female	0-6 days	95+ years	Global	1	2	Liters of alcohol consumed per capita	32
Myelodysplastic, myeloproliferative, and other hematopoietic neoplasms	Female	0-6 days	95+ years	Global	1	2	Cumulative Cigarettes (10 Years)	--
Myelodysplastic, myeloproliferative, and other hematopoietic neoplasms	Female	0-6 days	95+ years	Global	1	2	Cumulative Cigarettes (15 Years)	--
Myelodysplastic, myeloproliferative, and other hematopoietic neoplasms	Female	0-6 days	95+ years	Global	1	2	Cumulative Cigarettes (20 Years)	--
Myelodysplastic, myeloproliferative, and other hematopoietic neoplasms	Female	0-6 days	95+ years	Global	1	2	Cumulative Cigarettes (5 Years)	--
Myelodysplastic, myeloproliferative, and other hematopoietic neoplasms	Female	0-6 days	95+ years	Global	1	2	Smoking Prevalence	--
Myelodysplastic, myeloproliferative, and other hematopoietic neoplasms	Female	0-6 days	95+ years	Global	1	2	Tobacco (cigarettes per capita)	--
Myelodysplastic, myeloproliferative, and other hematopoietic neoplasms	Female	0-6 days	95+ years	Global	-1	3	Education (years per capita)	--
Myelodysplastic, myeloproliferative, and other hematopoietic neoplasms	Female	0-6 days	95+ years	Global	1	3	Socio-demographic Index	332
Myelodysplastic, myeloproliferative, and other hematopoietic neoplasms	Female	0-6 days	95+ years	Global	1	3	LDI (I$ per capita)	--
Myelodysplastic, myeloproliferative, and other hematopoietic neoplasms	Male	0-6 days	95+ years	Data Rich	1	1	Log-transformed age-standardized SEV scalar: Leukemia	1000
Myelodysplastic, myeloproliferative, and other hematopoietic neoplasms	Male	0-6 days	95+ years	Data Rich	1	1	Log-transformed SEV scalar: Leukemia	--
Myelodysplastic, myeloproliferative, and other hematopoietic neoplasms	Male	0-6 days	95+ years	Data Rich	-1	2	Healthcare access and quality index	--
Myelodysplastic, myeloproliferative, and other hematopoietic neoplasms	Male	0-6 days	95+ years	Data Rich	1	2	Liters of alcohol consumed per capita	134
Myelodysplastic, myeloproliferative, and other hematopoietic neoplasms	Male	0-6 days	95+ years	Data Rich	1	2	Cumulative Cigarettes (10 Years)	--
Myelodysplastic, myeloproliferative, and other hematopoietic neoplasms	Male	0-6 days	95+ years	Data Rich	1	2	Cumulative Cigarettes (15 Years)	--
Myelodysplastic, myeloproliferative, and other hematopoietic neoplasms	Male	0-6 days	95+ years	Data Rich	1	2	Cumulative Cigarettes (20 Years)	--
Myelodysplastic, myeloproliferative, and other hematopoietic neoplasms	Male	0-6 days	95+ years	Data Rich	1	2	Cumulative Cigarettes (5 Years)	--
Myelodysplastic, myeloproliferative, and other hematopoietic neoplasms	Male	0-6 days	95+ years	Data Rich	1	2	Smoking Prevalence	--
Myelodysplastic, myeloproliferative, and other hematopoietic neoplasms	Male	0-6 days	95+ years	Data Rich	1	2	Tobacco (cigarettes per capita)	--
Myelodysplastic, myeloproliferative, and other hematopoietic neoplasms	Male	0-6 days	95+ years	Data Rich	-1	3	Education (years per capita)	--
Myelodysplastic, myeloproliferative, and other hematopoietic neoplasms	Male	0-6 days	95+ years	Data Rich	1	3	Socio-demographic Index	512
Myelodysplastic, myeloproliferative, and other hematopoietic neoplasms	Male	0-6 days	95+ years	Data Rich	1	3	LDI (I$ per capita)	--
Myelodysplastic, myeloproliferative, and other hematopoietic neoplasms	Male	0-6 days	95+ years	Global	1	1	Log-transformed age-standardized SEV scalar: Leukemia	1000
Myelodysplastic, myeloproliferative, and other hematopoietic neoplasms	Male	0-6 days	95+ years	Global	1	1	Log-transformed SEV scalar: Leukemia	--
Myelodysplastic, myeloproliferative, and other hematopoietic neoplasms	Male	0-6 days	95+ years	Global	-1	2	Healthcare access and quality index	--
Myelodysplastic, myeloproliferative, and other hematopoietic neoplasms	Male	0-6 days	95+ years	Global	1	2	Cumulative Cigarettes (10 Years)	--
Myelodysplastic, myeloproliferative, and other hematopoietic neoplasms	Male	0-6 days	95+ years	Global	1	2	Cumulative Cigarettes (15 Years)	--
Myelodysplastic, myeloproliferative, and other hematopoietic neoplasms	Male	0-6 days	95+ years	Global	1	2	Cumulative Cigarettes (20 Years)	--
Myelodysplastic, myeloproliferative, and other hematopoietic neoplasms	Male	0-6 days	95+ years	Global	1	2	Cumulative Cigarettes (5 Years)	--
Myelodysplastic, myeloproliferative, and other hematopoietic neoplasms	Male	0-6 days	95+ years	Global	1	2	Liters of alcohol consumed per capita	--
Myelodysplastic, myeloproliferative, and other hematopoietic neoplasms	Male	0-6 days	95+ years	Global	1	2	Smoking Prevalence	--
Myelodysplastic, myeloproliferative, and other hematopoietic neoplasms	Male	0-6 days	95+ years	Global	1	2	Tobacco (cigarettes per capita)	--
Myelodysplastic, myeloproliferative, and other hematopoietic neoplasms	Male	0-6 days	95+ years	Global	-1	3	Education (years per capita)	--
Myelodysplastic, myeloproliferative, and other hematopoietic neoplasms	Male	0-6 days	95+ years	Global	1	3	LDI (I$ per capita)	--
Myelodysplastic, myeloproliferative, and other hematopoietic neoplasms	Male	0-6 days	95+ years	Global	1	3	Socio-demographic Index	--
Other benign and in situ neoplasms	Female	0-6 days	95+ years	Data Rich	-1	2	Healthcare access and quality index	--
Other benign and in situ neoplasms	Female	0-6 days	95+ years	Data Rich	1	2	Cumulative Cigarettes (10 Years)	--
Other benign and in situ neoplasms	Female	0-6 days	95+ years	Data Rich	1	2	Smoking Prevalence	--
Other benign and in situ neoplasms	Female	0-6 days	95+ years	Data Rich	1	2	Tobacco (cigarettes per capita)	--
Other benign and in situ neoplasms	Female	0-6 days	95+ years	Data Rich	-1	3	Education (years per capita)	500
Other benign and in situ neoplasms	Female	0-6 days	95+ years	Data Rich	-1	3	Socio-demographic Index	500
Other benign and in situ neoplasms	Female	0-6 days	95+ years	Data Rich	-1	3	LDI (I$ per capita)	--
Other benign and in situ neoplasms	Female	0-6 days	95+ years	Data Rich	1	3	Liters of alcohol consumed per capita	--
Other benign and in situ neoplasms	Female	0-6 days	95+ years	Global	-1	2	Healthcare access and quality index	1000
Other benign and in situ neoplasms	Female	0-6 days	95+ years	Global	1	2	Cumulative Cigarettes (10 Years)	--
Other benign and in situ neoplasms	Female	0-6 days	95+ years	Global	1	2	Smoking Prevalence	--
Other benign and in situ neoplasms	Female	0-6 days	95+ years	Global	1	2	Tobacco (cigarettes per capita)	--
Other benign and in situ neoplasms	Female	0-6 days	95+ years	Global	-1	3	LDI (I$ per capita)	185
Other benign and in situ neoplasms	Female	0-6 days	95+ years	Global	-1	3	Education (years per capita)	222
Other benign and in situ neoplasms	Female	0-6 days	95+ years	Global	-1	3	Socio-demographic Index	--
Other benign and in situ neoplasms	Female	0-6 days	95+ years	Global	1	3	Liters of alcohol consumed per capita	--
Other benign and in situ neoplasms	Male	0-6 days	95+ years	Data Rich	-1	2	Healthcare access and quality index	677
Other benign and in situ neoplasms	Male	0-6 days	95+ years	Data Rich	1	2	Tobacco (cigarettes per capita)	104
Other benign and in situ neoplasms	Male	0-6 days	95+ years	Data Rich	1	2	Cumulative Cigarettes (10 Years)	132
Other benign and in situ neoplasms	Male	0-6 days	95+ years	Data Rich	1	2	Smoking Prevalence	159
Other benign and in situ neoplasms	Male	0-6 days	95+ years	Data Rich	-1	3	LDI (I$ per capita)	150
Other benign and in situ neoplasms	Male	0-6 days	95+ years	Data Rich	-1	3	Education (years per capita)	372
Other benign and in situ neoplasms	Male	0-6 days	95+ years	Data Rich	-1	3	Socio-demographic Index	--
Other benign and in situ neoplasms	Male	0-6 days	95+ years	Data Rich	1	3	Liters of alcohol consumed per capita	--
Other benign and in situ neoplasms	Male	0-6 days	95+ years	Global	-1	2	Healthcare access and quality index	398
Other benign and in situ neoplasms	Male	0-6 days	95+ years	Global	1	2	Cumulative Cigarettes (10 Years)	274
Other benign and in situ neoplasms	Male	0-6 days	95+ years	Global	1	2	Tobacco (cigarettes per capita)	328
Other benign and in situ neoplasms	Male	0-6 days	95+ years	Global	1	2	Smoking Prevalence	--
Other benign and in situ neoplasms	Male	0-6 days	95+ years	Global	-1	3	Education (years per capita)	--
Other benign and in situ neoplasms	Male	0-6 days	95+ years	Global	-1	3	LDI (I$ per capita)	--
Other benign and in situ neoplasms	Male	0-6 days	95+ years	Global	-1	3	Socio-demographic Index	--
Other benign and in situ neoplasms	Male	0-6 days	95+ years	Global	1	3	Liters of alcohol consumed per capita	--
Cardiovascular diseases	Female	0-6 days	95+ years	Data Rich	1	1	Systolic Blood Pressure (mmHg)	258
Cardiovascular diseases	Female	0-6 days	95+ years	Data Rich	1	1	Smoking Prevalence	969

CODEm covariates used, level of covariate, and expected direction of covariate by cause, sex, and age

Cause	Sex	Age Start	Age End	Model Version Type	Direction	Level	Covariate Name	Number of Draws
Cardiovascular diseases	Female	0-6 days	95+ years	Data Rich	1	1	Low-Density Lipoprotein (mmol/L)	--
Cardiovascular diseases	Female	0-6 days	95+ years	Data Rich	-1	2	Healthcare access and quality index	37
Cardiovascular diseases	Female	0-6 days	95+ years	Data Rich	-1	2	Elevation Over 1500m (proportion)	333
Cardiovascular diseases	Female	0-6 days	95+ years	Data Rich	1	2	Outdoor Air Pollution (PM2.5)	6
Cardiovascular diseases	Female	0-6 days	95+ years	Data Rich	1	2	Indoor Air Pollution (All Cooking Fuels)	301
Cardiovascular diseases	Female	0-6 days	95+ years	Data Rich	1	2	Diabetes Fasting Plasma Glucose (mmol/L), age-standardized 25+	--
Cardiovascular diseases	Female	0-6 days	95+ years	Data Rich	1	2	Mean BMI	--
Cardiovascular diseases	Female	0-6 days	95+ years	Data Rich	-1	3	Pulses legumes unadjusted(g)	807
Cardiovascular diseases	Female	0-6 days	95+ years	Data Rich	-1	3	LDI (I$ per capita)	986
Cardiovascular diseases	Female	0-6 days	95+ years	Data Rich	1	3	Age- and sex-specific SEV for Low nuts and seeds	345
Cardiovascular diseases	Female	0-6 days	95+ years	Data Rich	1	3	Age- and sex-specific SEV for Low vegetables	468
Cardiovascular diseases	Female	0-6 days	95+ years	Data Rich	1	3	Age- and sex-specific SEV for Low omega-3	564
Cardiovascular diseases	Female	0-6 days	95+ years	Data Rich	1	3	Age- and sex-specific SEV for Low PUFA	904
Cardiovascular diseases	Female	0-6 days	95+ years	Data Rich	1	3	Age- and sex-specific SEV for Low fruit	909
Cardiovascular diseases	Female	0-6 days	95+ years	Data Rich	1	3	Liters of alcohol consumed per capita	934
Cardiovascular diseases	Female	0-6 days	95+ years	Data Rich	1	3	Diet high in trans fatty acids	946
Cardiovascular diseases	Female	0-6 days	95+ years	Global	1	1	Systolic Blood Pressure (mmHg)	651
Cardiovascular diseases	Female	0-6 days	95+ years	Global	1	1	Smoking Prevalence	871
Cardiovascular diseases	Female	0-6 days	95+ years	Global	1	1	Low-Density Lipoprotein (mmol/L)	--
Cardiovascular diseases	Female	0-6 days	95+ years	Global	-1	2	Elevation Over 1500m (proportion)	103
Cardiovascular diseases	Female	0-6 days	95+ years	Global	-1	2	Healthcare access and quality index	646
Cardiovascular diseases	Female	0-6 days	95+ years	Global	1	2	Indoor Air Pollution (All Cooking Fuels)	155
Cardiovascular diseases	Female	0-6 days	95+ years	Global	1	2	Outdoor Air Pollution (PM2.5)	801
Cardiovascular diseases	Female	0-6 days	95+ years	Global	1	2	Diabetes Fasting Plasma Glucose (mmol/L), age-standardized 25+	--
Cardiovascular diseases	Female	0-6 days	95+ years	Global	1	2	Mean BMI	--
Cardiovascular diseases	Female	0-6 days	95+ years	Global	-1	3	Pulses legumes unadjusted(g)	83
Cardiovascular diseases	Female	0-6 days	95+ years	Global	-1	3	LDI (I$ per capita)	517
Cardiovascular diseases	Female	0-6 days	95+ years	Global	1	3	Age- and sex-specific SEV for Low vegetables	62
Cardiovascular diseases	Female	0-6 days	95+ years	Global	1	3	Age- and sex-specific SEV for Low omega-3	203
Cardiovascular diseases	Female	0-6 days	95+ years	Global	1	3	Diet high in trans fatty acids	404
Cardiovascular diseases	Female	0-6 days	95+ years	Global	1	3	Liters of alcohol consumed per capita	451
Cardiovascular diseases	Female	0-6 days	95+ years	Global	1	3	Age- and sex-specific SEV for Low fruit	456
Cardiovascular diseases	Female	0-6 days	95+ years	Global	1	3	Age- and sex-specific SEV for Low nuts and seeds	556
Cardiovascular diseases	Female	0-6 days	95+ years	Global	1	3	Age- and sex-specific SEV for Low PUFA	688
Cardiovascular diseases	Male	0-6 days	95+ years	Data Rich	1	1	Systolic Blood Pressure (mmHg)	588
Cardiovascular diseases	Male	0-6 days	95+ years	Data Rich	1	1	Smoking Prevalence	896
Cardiovascular diseases	Male	0-6 days	95+ years	Data Rich	1	1	Low-Density Lipoprotein (mmol/L)	--
Cardiovascular diseases	Male	0-6 days	95+ years	Data Rich	-1	2	Healthcare access and quality index	91
Cardiovascular diseases	Male	0-6 days	95+ years	Data Rich	-1	2	Elevation Over 1500m (proportion)	411
Cardiovascular diseases	Male	0-6 days	95+ years	Data Rich	1	2	Outdoor Air Pollution (PM2.5)	191
Cardiovascular diseases	Male	0-6 days	95+ years	Data Rich	1	2	Indoor Air Pollution (All Cooking Fuels)	333
Cardiovascular diseases	Male	0-6 days	95+ years	Data Rich	1	2	Diabetes Fasting Plasma Glucose (mmol/L), age-standardized 25+	--
Cardiovascular diseases	Male	0-6 days	95+ years	Data Rich	1	2	Mean BMI	--
Cardiovascular diseases	Male	0-6 days	95+ years	Data Rich	-1	3	Pulses legumes unadjusted(g)	909
Cardiovascular diseases	Male	0-6 days	95+ years	Data Rich	-1	3	LDI (I$ per capita)	939
Cardiovascular diseases	Male	0-6 days	95+ years	Data Rich	1	3	Age- and sex-specific SEV for Low omega-3	454
Cardiovascular diseases	Male	0-6 days	95+ years	Data Rich	1	3	Age- and sex-specific SEV for Low vegetables	466
Cardiovascular diseases	Male	0-6 days	95+ years	Data Rich	1	3	Diet high in trans fatty acids	502
Cardiovascular diseases	Male	0-6 days	95+ years	Data Rich	1	3	Age- and sex-specific SEV for Low nuts and seeds	631
Cardiovascular diseases	Male	0-6 days	95+ years	Data Rich	1	3	Age- and sex-specific SEV for Low fruit	738
Cardiovascular diseases	Male	0-6 days	95+ years	Data Rich	1	3	Age- and sex-specific SEV for Low PUFA	914
Cardiovascular diseases	Male	0-6 days	95+ years	Data Rich	1	3	Liters of alcohol consumed per capita	999
Cardiovascular diseases	Male	0-6 days	95+ years	Global	1	1	Smoking Prevalence	632
Cardiovascular diseases	Male	0-6 days	95+ years	Global	1	1	Systolic Blood Pressure (mmHg)	963
Cardiovascular diseases	Male	0-6 days	95+ years	Global	1	1	Low-Density Lipoprotein (mmol/L)	--
Cardiovascular diseases	Male	0-6 days	95+ years	Global	-1	2	Elevation Over 1500m (proportion)	291
Cardiovascular diseases	Male	0-6 days	95+ years	Global	-1	2	Healthcare access and quality index	300
Cardiovascular diseases	Male	0-6 days	95+ years	Global	1	2	Indoor Air Pollution (All Cooking Fuels)	285
Cardiovascular diseases	Male	0-6 days	95+ years	Global	1	2	Outdoor Air Pollution (PM2.5)	342
Cardiovascular diseases	Male	0-6 days	95+ years	Global	1	2	Diabetes Fasting Plasma Glucose (mmol/L), age-standardized 25+	487
Cardiovascular diseases	Male	0-6 days	95+ years	Global	1	2	Mean BMI	--
Cardiovascular diseases	Male	0-6 days	95+ years	Global	-1	3	Pulses legumes unadjusted(g)	0
Cardiovascular diseases	Male	0-6 days	95+ years	Global	-1	3	LDI (I$ per capita)	341
Cardiovascular diseases	Male	0-6 days	95+ years	Global	1	3	Age- and sex-specific SEV for Low omega-3	51
Cardiovascular diseases	Male	0-6 days	95+ years	Global	1	3	Age- and sex-specific SEV for Low vegetables	53
Cardiovascular diseases	Male	0-6 days	95+ years	Global	1	3	Liters of alcohol consumed per capita	217
Cardiovascular diseases	Male	0-6 days	95+ years	Global	1	3	Age- and sex-specific SEV for Low nuts and seeds	316
Cardiovascular diseases	Male	0-6 days	95+ years	Global	1	3	Age- and sex-specific SEV for Low fruit	338
Cardiovascular diseases	Male	0-6 days	95+ years	Global	1	3	Age- and sex-specific SEV for Low PUFA	371
Cardiovascular diseases	Male	0-6 days	95+ years	Global	1	3	Diet high in trans fatty acids	408
Rheumatic heart disease	Female	12-23 months	95+ years	Data Rich	-1	1	Improved Water Source (proportion with access)	314
Rheumatic heart disease	Female	12-23 months	95+ years	Data Rich	-1	1	Sanitation (proportion with access)	372
Rheumatic heart disease	Female	12-23 months	95+ years	Data Rich	1	1	Age- and sex-specific SEV for Child underweight	194
Rheumatic heart disease	Female	12-23 months	95+ years	Data Rich	1	1	Log-transformed SEV scalar: RHD	813
Rheumatic heart disease	Female	12-23 months	95+ years	Data Rich	-1	2	Healthcare access and quality index	105
Rheumatic heart disease	Female	12-23 months	95+ years	Data Rich	-1	3	Education (years per capita)	238
Rheumatic heart disease	Female	12-23 months	95+ years	Data Rich	-1	3	LDI (I$ per capita)	--
Rheumatic heart disease	Female	12-23 months	95+ years	Global	-1	1	Improved Water Source (proportion with access)	312
Rheumatic heart disease	Female	12-23 months	95+ years	Global	-1	1	Sanitation (proportion with access)	477
Rheumatic heart disease	Female	12-23 months	95+ years	Global	1	1	Age- and sex-specific SEV for Child underweight	280
Rheumatic heart disease	Female	12-23 months	95+ years	Global	1	1	Log-transformed SEV scalar: RHD	451
Rheumatic heart disease	Female	12-23 months	95+ years	Global	-1	2	Healthcare access and quality index	98
Rheumatic heart disease	Female	12-23 months	95+ years	Global	-1	3	Education (years per capita)	282
Rheumatic heart disease	Female	12-23 months	95+ years	Global	-1	3	LDI (I$ per capita)	--
Rheumatic heart disease	Male	12-23 months	95+ years	Data Rich	-1	1	Improved Water Source (proportion with access)	355
Rheumatic heart disease	Male	12-23 months	95+ years	Data Rich	-1	1	Sanitation (proportion with access)	364
Rheumatic heart disease	Male	12-23 months	95+ years	Data Rich	1	1	Age- and sex-specific SEV for Child underweight	288
Rheumatic heart disease	Male	12-23 months	95+ years	Data Rich	1	1	Log-transformed SEV scalar: RHD	734
Rheumatic heart disease	Male	12-23 months	95+ years	Data Rich	-1	2	Healthcare access and quality index	96
Rheumatic heart disease	Male	12-23 months	95+ years	Data Rich	-1	3	Education (years per capita)	301
Rheumatic heart disease	Male	12-23 months	95+ years	Data Rich	-1	3	LDI (I$ per capita)	--
Rheumatic heart disease	Male	12-23 months	95+ years	Global	-1	1	Improved Water Source (proportion with access)	435
Rheumatic heart disease	Male	12-23 months	95+ years	Global	-1	1	Sanitation (proportion with access)	546
Rheumatic heart disease	Male	12-23 months	95+ years	Global	1	1	Age- and sex-specific SEV for Child underweight	474
Rheumatic heart disease	Male	12-23 months	95+ years	Global	1	1	Log-transformed SEV scalar: RHD	578
Rheumatic heart disease	Male	12-23 months	95+ years	Global	-1	2	Healthcare access and quality index	207
Rheumatic heart disease	Male	12-23 months	95+ years	Global	-1	3	Socio-demographic Index	57
Rheumatic heart disease	Male	12-23 months	95+ years	Global	-1	3	Education (years per capita)	226
Rheumatic heart disease	Male	12-23 months	95+ years	Global	-1	3	LDI (I$ per capita)	--
Ischaemic heart disease	Female	15-19 years	95+ years	Data Rich	1	1	Log-transformed SEV scalar: IHD	109
Ischaemic heart disease	Female	15-19 years	95+ years	Data Rich	1	1	Smoking Prevalence	463
Ischaemic heart disease	Female	15-19 years	95+ years	Data Rich	1	1	Systolic Blood Pressure (mmHg)	770
Ischaemic heart disease	Female	15-19 years	95+ years	Data Rich	1	1	Low-Density Lipoprotein (mmol/L)	--
Ischaemic heart disease	Female	15-19 years	95+ years	Data Rich	-1	2	Elevation Over 1500m (proportion)	260
Ischaemic heart disease	Female	15-19 years	95+ years	Data Rich	-1	2	Healthcare access and quality index	395
Ischaemic heart disease	Female	15-19 years	95+ years	Data Rich	1	2	Outdoor Air Pollution (PM2.5)	202
Ischaemic heart disease	Female	15-19 years	95+ years	Data Rich	1	2	Indoor Air Pollution (All Cooking Fuels)	210
Ischaemic heart disease	Female	15-19 years	95+ years	Data Rich	1	2	Diabetes Fasting Plasma Glucose (mmol/L), age-standardized 25+	--
Ischaemic heart disease	Female	15-19 years	95+ years	Data Rich	1	2	Mean BMI	--
Ischaemic heart disease	Female	15-19 years	95+ years	Data Rich	-1	3	Pulses legumes unadjusted(g)	729
Ischaemic heart disease	Female	15-19 years	95+ years	Data Rich	-1	3	LDI (I$ per capita)	--
Ischaemic heart disease	Female	15-19 years	95+ years	Data Rich	1	3	Age- and sex-specific SEV for Low PUFA	283
Ischaemic heart disease	Female	15-19 years	95+ years	Data Rich	1	3	Age- and sex-specific SEV for Low nuts and seeds	408

CODEm covariates used, level of covariate, and expected direction of covariate by cause, sex, and age

Cause	Sex	Age Start	Age End	Model Version Type	Direction	Level	Covariate Name	Number of Draws
Ischaemic heart disease	Female	15-19 years	95+ years	Data Rich	1	3	Age- and sex-specific SEV for Low omega-3	472
Ischaemic heart disease	Female	15-19 years	95+ years	Data Rich	1	3	Liters of alcohol consumed per capita	590
Ischaemic heart disease	Female	15-19 years	95+ years	Data Rich	1	3	Age- and sex-specific SEV for Low fruit	780
Ischaemic heart disease	Female	15-19 years	95+ years	Data Rich	1	3	Age- and sex-specific SEV for Low vegetables	846
Ischaemic heart disease	Female	15-19 years	95+ years	Data Rich	1	3	Diet high in trans fatty acids	901
Ischaemic heart disease	Female	15-19 years	95+ years	Global	1	1	Log-transformed SEV scalar: IHD	539
Ischaemic heart disease	Female	15-19 years	95+ years	Global	1	1	Smoking Prevalence	618
Ischaemic heart disease	Female	15-19 years	95+ years	Global	1	1	Systolic Blood Pressure (mmHg)	969
Ischaemic heart disease	Female	15-19 years	95+ years	Global	1	1	Low-Density Lipoprotein (mmol/L)	--
Ischaemic heart disease	Female	15-19 years	95+ years	Global	-1	2	Healthcare access and quality index	155
Ischaemic heart disease	Female	15-19 years	95+ years	Global	-1	2	Elevation Over 1500m (proportion)	549
Ischaemic heart disease	Female	15-19 years	95+ years	Global	1	2	Indoor Air Pollution (All Cooking Fuels)	0
Ischaemic heart disease	Female	15-19 years	95+ years	Global	1	2	Diabetes Fasting Plasma Glucose (mmol/L), age-standardized 25+	243
Ischaemic heart disease	Female	15-19 years	95+ years	Global	1	2	Outdoor Air Pollution (PM2.5)	730
Ischaemic heart disease	Female	15-19 years	95+ years	Global	1	2	Mean BMI	--
Ischaemic heart disease	Female	15-19 years	95+ years	Global	-1	3	Pulses legumes unadjusted(g)	0
Ischaemic heart disease	Female	15-19 years	95+ years	Global	-1	3	LDI (I$ per capita)	--
Ischaemic heart disease	Female	15-19 years	95+ years	Global	1	3	Age- and sex-specific SEV for Low omega-3	1
Ischaemic heart disease	Female	15-19 years	95+ years	Global	1	3	Age- and sex-specific SEV for Low vegetables	100
Ischaemic heart disease	Female	15-19 years	95+ years	Global	1	3	Age- and sex-specific SEV for Low nuts and seeds	142
Ischaemic heart disease	Female	15-19 years	95+ years	Global	1	3	Age- and sex-specific SEV for Low fruit	266
Ischaemic heart disease	Female	15-19 years	95+ years	Global	1	3	Liters of alcohol consumed per capita	333
Ischaemic heart disease	Female	15-19 years	95+ years	Global	1	3	Diet high in trans fatty acids	364
Ischaemic heart disease	Female	15-19 years	95+ years	Global	1	3	Age- and sex-specific SEV for Low PUFA	525
Ischaemic heart disease	Male	15-19 years	95+ years	Data Rich	1	1	Log-transformed SEV scalar: IHD	349
Ischaemic heart disease	Male	15-19 years	95+ years	Data Rich	1	1	Systolic Blood Pressure (mmHg)	858
Ischaemic heart disease	Male	15-19 years	95+ years	Data Rich	1	1	Smoking Prevalence	861
Ischaemic heart disease	Male	15-19 years	95+ years	Data Rich	1	1	Low-Density Lipoprotein (mmol/L)	--
Ischaemic heart disease	Male	15-19 years	95+ years	Data Rich	-1	2	Elevation Over 1500m (proportion)	195
Ischaemic heart disease	Male	15-19 years	95+ years	Data Rich	-1	2	Healthcare access and quality index	652
Ischaemic heart disease	Male	15-19 years	95+ years	Data Rich	1	2	Indoor Air Pollution (All Cooking Fuels)	56
Ischaemic heart disease	Male	15-19 years	95+ years	Data Rich	1	2	Outdoor Air Pollution (PM2.5)	190
Ischaemic heart disease	Male	15-19 years	95+ years	Data Rich	1	2	Diabetes Fasting Plasma Glucose (mmol/L), age-standardized 25+	--
Ischaemic heart disease	Male	15-19 years	95+ years	Data Rich	1	2	Mean BMI	--
Ischaemic heart disease	Male	15-19 years	95+ years	Data Rich	-1	3	Pulses legumes unadjusted(g)	825
Ischaemic heart disease	Male	15-19 years	95+ years	Data Rich	-1	3	LDI (I$ per capita)	--
Ischaemic heart disease	Male	15-19 years	95+ years	Data Rich	1	3	Age- and sex-specific SEV for Low vegetables	55
Ischaemic heart disease	Male	15-19 years	95+ years	Data Rich	1	3	Age- and sex-specific SEV for Low PUFA	413
Ischaemic heart disease	Male	15-19 years	95+ years	Data Rich	1	3	Age- and sex-specific SEV for Low nuts and seeds	435
Ischaemic heart disease	Male	15-19 years	95+ years	Data Rich	1	3	Age- and sex-specific SEV for Low omega-3	472
Ischaemic heart disease	Male	15-19 years	95+ years	Data Rich	1	3	Diet high in trans fatty acids	682
Ischaemic heart disease	Male	15-19 years	95+ years	Data Rich	1	3	Liters of alcohol consumed per capita	874
Ischaemic heart disease	Male	15-19 years	95+ years	Data Rich	1	3	Age- and sex-specific SEV for Low fruit	953
Ischaemic heart disease	Male	15-19 years	95+ years	Global	1	1	Smoking Prevalence	528
Ischaemic heart disease	Male	15-19 years	95+ years	Global	1	1	Log-transformed SEV scalar: IHD	685
Ischaemic heart disease	Male	15-19 years	95+ years	Global	1	1	Systolic Blood Pressure (mmHg)	908
Ischaemic heart disease	Male	15-19 years	95+ years	Global	1	1	Low-Density Lipoprotein (mmol/L)	--
Ischaemic heart disease	Male	15-19 years	95+ years	Global	-1	2	Healthcare access and quality index	0
Ischaemic heart disease	Male	15-19 years	95+ years	Global	-1	2	Elevation Over 1500m (proportion)	748
Ischaemic heart disease	Male	15-19 years	95+ years	Global	1	2	Indoor Air Pollution (All Cooking Fuels)	0
Ischaemic heart disease	Male	15-19 years	95+ years	Global	1	2	Mean BMI	62
Ischaemic heart disease	Male	15-19 years	95+ years	Global	1	2	Outdoor Air Pollution (PM2.5)	585
Ischaemic heart disease	Male	15-19 years	95+ years	Global	1	2	Diabetes Fasting Plasma Glucose (mmol/L), age-standardized 25+	614
Ischaemic heart disease	Male	15-19 years	95+ years	Global	-1	3	Pulses legumes unadjusted(g)	0
Ischaemic heart disease	Male	15-19 years	95+ years	Global	-1	3	LDI (I$ per capita)	--
Ischaemic heart disease	Male	15-19 years	95+ years	Global	1	3	Age- and sex-specific SEV for Low vegetables	0
Ischaemic heart disease	Male	15-19 years	95+ years	Global	1	3	Age- and sex-specific SEV for Low omega-3	8
Ischaemic heart disease	Male	15-19 years	95+ years	Global	1	3	Age- and sex-specific SEV for Low PUFA	86
Ischaemic heart disease	Male	15-19 years	95+ years	Global	1	3	Age- and sex-specific SEV for Low fruit	211
Ischaemic heart disease	Male	15-19 years	95+ years	Global	1	3	Age- and sex-specific SEV for Low nuts and seeds	222
Ischaemic heart disease	Male	15-19 years	95+ years	Global	1	3	Diet high in trans fatty acids	245
Ischaemic heart disease	Male	15-19 years	95+ years	Global	1	3	Liters of alcohol consumed per capita	341
Stroke	Female	0-6 days	95+ years	Data Rich	1	1	Log-transformed SEV scalar: Stroke	0
Stroke	Female	0-6 days	95+ years	Data Rich	1	1	Systolic Blood Pressure (mmHg)	519
Stroke	Female	0-6 days	95+ years	Data Rich	1	1	Smoking Prevalence	638
Stroke	Female	0-6 days	95+ years	Data Rich	1	1	Low-Density Lipoprotein (mmol/L)	--
Stroke	Female	0-6 days	95+ years	Data Rich	-1	2	Elevation Over 1500m (proportion)	141
Stroke	Female	0-6 days	95+ years	Data Rich	-1	2	Healthcare access and quality index	746
Stroke	Female	0-6 days	95+ years	Data Rich	1	2	Indoor Air Pollution (All Cooking Fuels)	75
Stroke	Female	0-6 days	95+ years	Data Rich	1	2	Outdoor Air Pollution (PM2.5)	121
Stroke	Female	0-6 days	95+ years	Data Rich	1	2	Diabetes Fasting Plasma Glucose (mmol/L), age-standardized 25+	--
Stroke	Female	0-6 days	95+ years	Data Rich	1	2	Mean BMI	--
Stroke	Female	0-6 days	95+ years	Data Rich	-1	3	Pulses legumes unadjusted(g)	42
Stroke	Female	0-6 days	95+ years	Data Rich	-1	3	LDI (I$ per capita)	--
Stroke	Female	0-6 days	95+ years	Data Rich	1	3	Age- and sex-specific SEV for Low PUFA	10
Stroke	Female	0-6 days	95+ years	Data Rich	1	3	Diet high in trans fatty acids	24
Stroke	Female	0-6 days	95+ years	Data Rich	1	3	Liters of alcohol consumed per capita	101
Stroke	Female	0-6 days	95+ years	Data Rich	1	3	Age- and sex-specific SEV for Low omega-3	240
Stroke	Female	0-6 days	95+ years	Data Rich	1	3	Age- and sex-specific SEV for Low vegetables	257
Stroke	Female	0-6 days	95+ years	Data Rich	1	3	Age- and sex-specific SEV for Low nuts and seeds	273
Stroke	Female	0-6 days	95+ years	Data Rich	1	3	Age- and sex-specific SEV for Low fruit	440
Stroke	Female	0-6 days	95+ years	Global	1	1	Log-transformed SEV scalar: Stroke	0
Stroke	Female	0-6 days	95+ years	Global	1	1	Smoking Prevalence	982
Stroke	Female	0-6 days	95+ years	Global	1	1	Systolic Blood Pressure (mmHg)	982
Stroke	Female	0-6 days	95+ years	Global	1	1	Low-Density Lipoprotein (mmol/L)	--
Stroke	Female	0-6 days	95+ years	Global	-1	2	Healthcare access and quality index	516
Stroke	Female	0-6 days	95+ years	Global	-1	2	Elevation Over 1500m (proportion)	--
Stroke	Female	0-6 days	95+ years	Global	1	2	Outdoor Air Pollution (PM2.5)	90
Stroke	Female	0-6 days	95+ years	Global	1	2	Indoor Air Pollution (All Cooking Fuels)	178
Stroke	Female	0-6 days	95+ years	Global	1	2	Diabetes Fasting Plasma Glucose (mmol/L), age-standardized 25+	--
Stroke	Female	0-6 days	95+ years	Global	1	2	Mean BMI	--
Stroke	Female	0-6 days	95+ years	Global	-1	3	Pulses legumes unadjusted(g)	27
Stroke	Female	0-6 days	95+ years	Global	-1	3	LDI (I$ per capita)	--
Stroke	Female	0-6 days	95+ years	Global	1	3	Diet high in trans fatty acids	0
Stroke	Female	0-6 days	95+ years	Global	1	3	Age- and sex-specific SEV for Low omega-3	15
Stroke	Female	0-6 days	95+ years	Global	1	3	Age- and sex-specific SEV for Low vegetables	63
Stroke	Female	0-6 days	95+ years	Global	1	3	Age- and sex-specific SEV for Low nuts and seeds	77
Stroke	Female	0-6 days	95+ years	Global	1	3	Age- and sex-specific SEV for Low fruit	138
Stroke	Female	0-6 days	95+ years	Global	1	3	Liters of alcohol consumed per capita	141
Stroke	Female	0-6 days	95+ years	Global	1	3	Age- and sex-specific SEV for Low PUFA	303
Stroke	Male	0-6 days	95+ years	Data Rich	1	1	Log-transformed SEV scalar: Stroke	2
Stroke	Male	0-6 days	95+ years	Data Rich	1	1	Systolic Blood Pressure (mmHg)	323
Stroke	Male	0-6 days	95+ years	Data Rich	1	1	Smoking Prevalence	636
Stroke	Male	0-6 days	95+ years	Data Rich	1	1	Low-Density Lipoprotein (mmol/L)	--
Stroke	Male	0-6 days	95+ years	Data Rich	-1	2	Healthcare access and quality index	286
Stroke	Male	0-6 days	95+ years	Data Rich	-1	2	Elevation Over 1500m (proportion)	293
Stroke	Male	0-6 days	95+ years	Data Rich	1	2	Outdoor Air Pollution (PM2.5)	81
Stroke	Male	0-6 days	95+ years	Data Rich	1	2	Indoor Air Pollution (All Cooking Fuels)	232
Stroke	Male	0-6 days	95+ years	Data Rich	1	2	Diabetes Fasting Plasma Glucose (mmol/L), age-standardized 25+	--
Stroke	Male	0-6 days	95+ years	Data Rich	1	2	Mean BMI	--
Stroke	Male	0-6 days	95+ years	Data Rich	-1	3	Pulses legumes unadjusted(g)	203
Stroke	Male	0-6 days	95+ years	Data Rich	-1	3	LDI (I$ per capita)	--
Stroke	Male	0-6 days	95+ years	Data Rich	1	3	Diet high in trans fatty acids	7
Stroke	Male	0-6 days	95+ years	Data Rich	1	3	Age- and sex-specific SEV for Low PUFA	38

CODEm covariates used, level of covariate, and expected direction of covariate by cause, sex, and age								
Cause	Sex	Age Start	Age End	Model Version Type	Direction	Level	Covariate Name	Number of Draws
Stroke	Male	0-6 days	95+ years	Data Rich	1	3	Age- and sex-specific SEV for Low fruit	40
Stroke	Male	0-6 days	95+ years	Data Rich	1	3	Age- and sex-specific SEV for Low nuts and seeds	67
Stroke	Male	0-6 days	95+ years	Data Rich	1	3	Liters of alcohol consumed per capita	73
Stroke	Male	0-6 days	95+ years	Data Rich	1	3	Age- and sex-specific SEV for Low omega-3	80
Stroke	Male	0-6 days	95+ years	Data Rich	1	3	Age- and sex-specific SEV for Low vegetables	261
Stroke	Male	0-6 days	95+ years	Global	1	1	Systolic Blood Pressure (mmHg)	52
Stroke	Male	0-6 days	95+ years	Global	1	1	Smoking Prevalence	597
Stroke	Male	0-6 days	95+ years	Global	1	1	Log-transformed SEV scalar: Stroke	628
Stroke	Male	0-6 days	95+ years	Global	1	1	Low-Density Lipoprotein (mmol/L)	--
Stroke	Male	0-6 days	95+ years	Global	-1	2	Healthcare access and quality index	723
Stroke	Male	0-6 days	95+ years	Global	-1	2	Elevation Over 1500m (proportion)	--
Stroke	Male	0-6 days	95+ years	Global	1	2	Indoor Air Pollution (All Cooking Fuels)	138
Stroke	Male	0-6 days	95+ years	Global	1	2	Outdoor Air Pollution (PM2.5)	708
Stroke	Male	0-6 days	95+ years	Global	1	2	Diabetes Fasting Plasma Glucose (mmol/L), age-standardized 25+	--
Stroke	Male	0-6 days	95+ years	Global	1	2	Mean BMI	--
Stroke	Male	0-6 days	95+ years	Global	-1	3	Pulses legumes unadjusted(g)	21
Stroke	Male	0-6 days	95+ years	Global	-1	3	LDI (I$ per capita)	--
Stroke	Male	0-6 days	95+ years	Global	1	3	Age- and sex-specific SEV for Low nuts and seeds	137
Stroke	Male	0-6 days	95+ years	Global	1	3	Age- and sex-specific SEV for Low omega-3	186
Stroke	Male	0-6 days	95+ years	Global	1	3	Age- and sex-specific SEV for Low vegetables	209
Stroke	Male	0-6 days	95+ years	Global	1	3	Diet high in trans fatty acids	210
Stroke	Male	0-6 days	95+ years	Global	1	3	Age- and sex-specific SEV for Low fruit	303
Stroke	Male	0-6 days	95+ years	Global	1	3	Age- and sex-specific SEV for Low PUFA	437
Stroke	Male	0-6 days	95+ years	Global	1	3	Liters of alcohol consumed per capita	513
Ischaemic stroke	Female	0-6 days	95+ years	Data Rich	1	1	Log-transformed SEV scalar: Isch Stroke	414
Ischaemic stroke	Female	0-6 days	95+ years	Data Rich	1	1	Smoking Prevalence	481
Ischaemic stroke	Female	0-6 days	95+ years	Data Rich	1	1	Systolic Blood Pressure (mmHg)	795
Ischaemic stroke	Female	0-6 days	95+ years	Data Rich	1	1	Low-Density Lipoprotein (mmol/L)	--
Ischaemic stroke	Female	0-6 days	95+ years	Data Rich	-1	2	Healthcare access and quality index	547
Ischaemic stroke	Female	0-6 days	95+ years	Data Rich	-1	2	Elevation Over 1500m (proportion)	--
Ischaemic stroke	Female	0-6 days	95+ years	Data Rich	1	2	Outdoor Air Pollution (PM2.5)	246
Ischaemic stroke	Female	0-6 days	95+ years	Data Rich	1	2	Indoor Air Pollution (All Cooking Fuels)	504
Ischaemic stroke	Female	0-6 days	95+ years	Data Rich	1	2	Diabetes Fasting Plasma Glucose (mmol/L), age-standardized 25+	--
Ischaemic stroke	Female	0-6 days	95+ years	Data Rich	1	2	Mean BMI	--
Ischaemic stroke	Female	0-6 days	95+ years	Data Rich	-1	3	Pulses legumes unadjusted(g)	135
Ischaemic stroke	Female	0-6 days	95+ years	Data Rich	-1	3	LDI (I$ per capita)	--
Ischaemic stroke	Female	0-6 days	95+ years	Data Rich	1	3	Liters of alcohol consumed per capita	32
Ischaemic stroke	Female	0-6 days	95+ years	Data Rich	1	3	Age- and sex-specific SEV for Low PUFA	184
Ischaemic stroke	Female	0-6 days	95+ years	Data Rich	1	3	Age- and sex-specific SEV for Low fruit	267
Ischaemic stroke	Female	0-6 days	95+ years	Data Rich	1	3	Age- and sex-specific SEV for Low vegetables	299
Ischaemic stroke	Female	0-6 days	95+ years	Data Rich	1	3	Diet high in trans fatty acids	306
Ischaemic stroke	Female	0-6 days	95+ years	Data Rich	1	3	Age- and sex-specific SEV for Low omega-3	427
Ischaemic stroke	Female	0-6 days	95+ years	Data Rich	1	3	Age- and sex-specific SEV for Low nuts and seeds	486
Ischaemic stroke	Female	0-6 days	95+ years	Global	1	1	Log-transformed SEV scalar: Isch Stroke	132
Ischaemic stroke	Female	0-6 days	95+ years	Global	1	1	Systolic Blood Pressure (mmHg)	516
Ischaemic stroke	Female	0-6 days	95+ years	Global	1	1	Smoking Prevalence	686
Ischaemic stroke	Female	0-6 days	95+ years	Global	1	1	Low-Density Lipoprotein (mmol/L)	--
Ischaemic stroke	Female	0-6 days	95+ years	Global	-1	2	Elevation Over 1500m (proportion)	4
Ischaemic stroke	Female	0-6 days	95+ years	Global	-1	2	Healthcare access and quality index	32
Ischaemic stroke	Female	0-6 days	95+ years	Global	1	2	Indoor Air Pollution (All Cooking Fuels)	0
Ischaemic stroke	Female	0-6 days	95+ years	Global	1	2	Outdoor Air Pollution (PM2.5)	301
Ischaemic stroke	Female	0-6 days	95+ years	Global	1	2	Diabetes Fasting Plasma Glucose (mmol/L), age-standardized 25+	--
Ischaemic stroke	Female	0-6 days	95+ years	Global	1	2	Mean BMI	--
Ischaemic stroke	Female	0-6 days	95+ years	Global	-1	3	Pulses legumes unadjusted(g)	0
Ischaemic stroke	Female	0-6 days	95+ years	Global	-1	3	LDI (I$ per capita)	--
Ischaemic stroke	Female	0-6 days	95+ years	Global	1	3	Age- and sex-specific SEV for Low vegetables	0
Ischaemic stroke	Female	0-6 days	95+ years	Global	1	3	Age- and sex-specific SEV for Low fruit	190
Ischaemic stroke	Female	0-6 days	95+ years	Global	1	3	Age- and sex-specific SEV for Low PUFA	247
Ischaemic stroke	Female	0-6 days	95+ years	Global	1	3	Age- and sex-specific SEV for Low nuts and seeds	398
Ischaemic stroke	Female	0-6 days	95+ years	Global	1	3	Liters of alcohol consumed per capita	486
Ischaemic stroke	Female	0-6 days	95+ years	Global	1	3	Diet high in trans fatty acids	638
Ischaemic stroke	Female	0-6 days	95+ years	Global	1	3	Age- and sex-specific SEV for Low omega-3	723
Ischaemic stroke	Male	0-6 days	95+ years	Data Rich	1	1	Log-transformed SEV scalar: Isch Stroke	211
Ischaemic stroke	Male	0-6 days	95+ years	Data Rich	1	1	Systolic Blood Pressure (mmHg)	325
Ischaemic stroke	Male	0-6 days	95+ years	Data Rich	1	1	Smoking Prevalence	465
Ischaemic stroke	Male	0-6 days	95+ years	Data Rich	1	1	Low-Density Lipoprotein (mmol/L)	--
Ischaemic stroke	Male	0-6 days	95+ years	Data Rich	-1	2	Elevation Over 1500m (proportion)	389
Ischaemic stroke	Male	0-6 days	95+ years	Data Rich	-1	2	Healthcare access and quality index	781
Ischaemic stroke	Male	0-6 days	95+ years	Data Rich	1	2	Indoor Air Pollution (All Cooking Fuels)	378
Ischaemic stroke	Male	0-6 days	95+ years	Data Rich	1	2	Diabetes Fasting Plasma Glucose (mmol/L), age-standardized 25+	--
Ischaemic stroke	Male	0-6 days	95+ years	Data Rich	1	2	Mean BMI	--
Ischaemic stroke	Male	0-6 days	95+ years	Data Rich	1	2	Outdoor Air Pollution (PM2.5)	--
Ischaemic stroke	Male	0-6 days	95+ years	Data Rich	-1	3	Pulses legumes unadjusted(g)	46
Ischaemic stroke	Male	0-6 days	95+ years	Data Rich	-1	3	LDI (I$ per capita)	--
Ischaemic stroke	Male	0-6 days	95+ years	Data Rich	1	3	Age- and sex-specific SEV for Low PUFA	11
Ischaemic stroke	Male	0-6 days	95+ years	Data Rich	1	3	Age- and sex-specific SEV for Low vegetables	60
Ischaemic stroke	Male	0-6 days	95+ years	Data Rich	1	3	Age- and sex-specific SEV for Low fruit	242
Ischaemic stroke	Male	0-6 days	95+ years	Data Rich	1	3	Liters of alcohol consumed per capita	255
Ischaemic stroke	Male	0-6 days	95+ years	Data Rich	1	3	Age- and sex-specific SEV for Low nuts and seeds	279
Ischaemic stroke	Male	0-6 days	95+ years	Data Rich	1	3	Age- and sex-specific SEV for Low omega-3	280
Ischaemic stroke	Male	0-6 days	95+ years	Data Rich	1	3	Diet high in trans fatty acids	294
Ischaemic stroke	Male	0-6 days	95+ years	Global	1	1	Log-transformed SEV scalar: Isch Stroke	312
Ischaemic stroke	Male	0-6 days	95+ years	Global	1	1	Systolic Blood Pressure (mmHg)	649
Ischaemic stroke	Male	0-6 days	95+ years	Global	1	1	Smoking Prevalence	968
Ischaemic stroke	Male	0-6 days	95+ years	Global	1	1	Low-Density Lipoprotein (mmol/L)	--
Ischaemic stroke	Male	0-6 days	95+ years	Global	-1	2	Healthcare access and quality index	0
Ischaemic stroke	Male	0-6 days	95+ years	Global	-1	2	Elevation Over 1500m (proportion)	123
Ischaemic stroke	Male	0-6 days	95+ years	Global	1	2	Indoor Air Pollution (All Cooking Fuels)	0
Ischaemic stroke	Male	0-6 days	95+ years	Global	1	2	Outdoor Air Pollution (PM2.5)	123
Ischaemic stroke	Male	0-6 days	95+ years	Global	1	2	Diabetes Fasting Plasma Glucose (mmol/L), age-standardized 25+	219
Ischaemic stroke	Male	0-6 days	95+ years	Global	1	2	Mean BMI	--
Ischaemic stroke	Male	0-6 days	95+ years	Global	-1	3	Pulses legumes unadjusted(g)	0
Ischaemic stroke	Male	0-6 days	95+ years	Global	-1	3	LDI (I$ per capita)	--
Ischaemic stroke	Male	0-6 days	95+ years	Global	1	3	Age- and sex-specific SEV for Low vegetables	105
Ischaemic stroke	Male	0-6 days	95+ years	Global	1	3	Liters of alcohol consumed per capita	132
Ischaemic stroke	Male	0-6 days	95+ years	Global	1	3	Age- and sex-specific SEV for Low fruit	134
Ischaemic stroke	Male	0-6 days	95+ years	Global	1	3	Age- and sex-specific SEV for Low nuts and seeds	189
Ischaemic stroke	Male	0-6 days	95+ years	Global	1	3	Age- and sex-specific SEV for Low omega-3	203
Ischaemic stroke	Male	0-6 days	95+ years	Global	1	3	Age- and sex-specific SEV for Low PUFA	205
Ischaemic stroke	Male	0-6 days	95+ years	Global	1	3	Diet high in trans fatty acids	266
Intracerebral hemorrhage	Female	0-6 days	95+ years	Data Rich	1	1	Smoking Prevalence	0
Intracerebral hemorrhage	Female	0-6 days	95+ years	Data Rich	1	1	Systolic Blood Pressure (mmHg)	1000
Intracerebral hemorrhage	Female	0-6 days	95+ years	Data Rich	1	1	Log-transformed SEV scalar: Intrahem Stroke	--
Intracerebral hemorrhage	Female	0-6 days	95+ years	Data Rich	-1	2	Elevation Over 1500m (proportion)	517
Intracerebral hemorrhage	Female	0-6 days	95+ years	Data Rich	-1	2	Healthcare access and quality index	--
Intracerebral hemorrhage	Female	0-6 days	95+ years	Data Rich	1	2	Indoor Air Pollution (All Cooking Fuels)	204
Intracerebral hemorrhage	Female	0-6 days	95+ years	Data Rich	1	2	Outdoor Air Pollution (PM2.5)	313
Intracerebral hemorrhage	Female	0-6 days	95+ years	Data Rich	1	2	Diabetes Fasting Plasma Glucose (mmol/L), age-standardized 25+	--
Intracerebral hemorrhage	Female	0-6 days	95+ years	Data Rich	1	2	Mean BMI	--
Intracerebral hemorrhage	Female	0-6 days	95+ years	Data Rich	-1	3	Pulses legumes unadjusted(g)	144
Intracerebral hemorrhage	Female	0-6 days	95+ years	Data Rich	-1	3	LDI (I$ per capita)	--
Intracerebral hemorrhage	Female	0-6 days	95+ years	Data Rich	-1	3	Low-Density Lipoprotein (mmol/L)	--
Intracerebral hemorrhage	Female	0-6 days	95+ years	Data Rich	1	3	Age- and sex-specific SEV for Low PUFA	216

CODEm covariates used, level of covariate, and expected direction of covariate by cause, sex, and age								
**Cause**	**Sex**	**Age Start**	**Age End**	**Model Version Type**	**Direction**	**Level**	**Covariate Name**	**Number of Draws**
Intracerebral hemorrhage	Female	0-6 days	95+ years	Data Rich	1	3	Age- and sex-specific SEV for Low fruit	237
Intracerebral hemorrhage	Female	0-6 days	95+ years	Data Rich	1	3	Age- and sex-specific SEV for Low nuts and seeds	268
Intracerebral hemorrhage	Female	0-6 days	95+ years	Data Rich	1	3	Diet high in trans fatty acids	654
Intracerebral hemorrhage	Female	0-6 days	95+ years	Data Rich	1	3	Age- and sex-specific SEV for Low omega-3	768
Intracerebral hemorrhage	Female	0-6 days	95+ years	Data Rich	1	3	Liters of alcohol consumed per capita	--
Intracerebral hemorrhage	Female	0-6 days	95+ years	Global	1	1	Systolic Blood Pressure (mmHg)	124
Intracerebral hemorrhage	Female	0-6 days	95+ years	Global	1	1	Log-transformed SEV scalar: Intrahem Stroke	523
Intracerebral hemorrhage	Female	0-6 days	95+ years	Global	1	1	Smoking Prevalence	623
Intracerebral hemorrhage	Female	0-6 days	95+ years	Global	-1	2	Healthcare access and quality index	810
Intracerebral hemorrhage	Female	0-6 days	95+ years	Global	-1	2	Elevation Over 1500m (proportion)	--
Intracerebral hemorrhage	Female	0-6 days	95+ years	Global	1	2	Indoor Air Pollution (All Cooking Fuels)	558
Intracerebral hemorrhage	Female	0-6 days	95+ years	Global	1	2	Outdoor Air Pollution (PM2.5)	703
Intracerebral hemorrhage	Female	0-6 days	95+ years	Global	1	2	Diabetes Fasting Plasma Glucose (mmol/L), age-standardized 25+	--
Intracerebral hemorrhage	Female	0-6 days	95+ years	Global	1	2	Mean BMI	--
Intracerebral hemorrhage	Female	0-6 days	95+ years	Global	-1	3	Pulses legumes unadjusted(g)	28
Intracerebral hemorrhage	Female	0-6 days	95+ years	Global	-1	3	LDI (I$ per capita)	--
Intracerebral hemorrhage	Female	0-6 days	95+ years	Global	-1	3	Low-Density Lipoprotein (mmol/L)	--
Intracerebral hemorrhage	Female	0-6 days	95+ years	Global	1	3	Age- and sex-specific SEV for Low fruit	40
Intracerebral hemorrhage	Female	0-6 days	95+ years	Global	1	3	Age- and sex-specific SEV for Low vegetables	45
Intracerebral hemorrhage	Female	0-6 days	95+ years	Global	1	3	Diet high in trans fatty acids	156
Intracerebral hemorrhage	Female	0-6 days	95+ years	Global	1	3	Age- and sex-specific SEV for Low nuts and seeds	396
Intracerebral hemorrhage	Female	0-6 days	95+ years	Global	1	3	Age- and sex-specific SEV for Low PUFA	668
Intracerebral hemorrhage	Female	0-6 days	95+ years	Global	1	3	Age- and sex-specific SEV for Low omega-3	764
Intracerebral hemorrhage	Female	0-6 days	95+ years	Global	1	3	Liters of alcohol consumed per capita	--
Intracerebral hemorrhage	Male	0-6 days	95+ years	Data Rich	1	1	Systolic Blood Pressure (mmHg)	10
Intracerebral hemorrhage	Male	0-6 days	95+ years	Data Rich	1	1	Smoking Prevalence	21
Intracerebral hemorrhage	Male	0-6 days	95+ years	Data Rich	1	1	Log-transformed SEV scalar: Intrahem Stroke	--
Intracerebral hemorrhage	Male	0-6 days	95+ years	Data Rich	-1	2	Elevation Over 1500m (proportion)	11
Intracerebral hemorrhage	Male	0-6 days	95+ years	Data Rich	-1	2	Healthcare access and quality index	261
Intracerebral hemorrhage	Male	0-6 days	95+ years	Data Rich	1	2	Outdoor Air Pollution (PM2.5)	9
Intracerebral hemorrhage	Male	0-6 days	95+ years	Data Rich	1	2	Indoor Air Pollution (All Cooking Fuels)	550
Intracerebral hemorrhage	Male	0-6 days	95+ years	Data Rich	1	2	Diabetes Fasting Plasma Glucose (mmol/L), age-standardized 25+	--
Intracerebral hemorrhage	Male	0-6 days	95+ years	Data Rich	1	2	Mean BMI	--
Intracerebral hemorrhage	Male	0-6 days	95+ years	Data Rich	-1	3	Pulses legumes unadjusted(g)	53
Intracerebral hemorrhage	Male	0-6 days	95+ years	Data Rich	-1	3	LDI (I$ per capita)	--
Intracerebral hemorrhage	Male	0-6 days	95+ years	Data Rich	-1	3	Low-Density Lipoprotein (mmol/L)	--
Intracerebral hemorrhage	Male	0-6 days	95+ years	Data Rich	1	3	Age- and sex-specific SEV for Low PUFA	46
Intracerebral hemorrhage	Male	0-6 days	95+ years	Data Rich	1	3	Age- and sex-specific SEV for Low vegetables	396
Intracerebral hemorrhage	Male	0-6 days	95+ years	Data Rich	1	3	Age- and sex-specific SEV for Low fruit	453
Intracerebral hemorrhage	Male	0-6 days	95+ years	Data Rich	1	3	Diet high in trans fatty acids	506
Intracerebral hemorrhage	Male	0-6 days	95+ years	Data Rich	1	3	Age- and sex-specific SEV for Low nuts and seeds	566
Intracerebral hemorrhage	Male	0-6 days	95+ years	Data Rich	1	3	Age- and sex-specific SEV for Low omega-3	705
Intracerebral hemorrhage	Male	0-6 days	95+ years	Data Rich	1	3	Liters of alcohol consumed per capita	--
Intracerebral hemorrhage	Male	0-6 days	95+ years	Global	1	1	Systolic Blood Pressure (mmHg)	225
Intracerebral hemorrhage	Male	0-6 days	95+ years	Global	1	1	Log-transformed SEV scalar: Intrahem Stroke	352
Intracerebral hemorrhage	Male	0-6 days	95+ years	Global	1	1	Smoking Prevalence	551
Intracerebral hemorrhage	Male	0-6 days	95+ years	Global	-1	2	Healthcare access and quality index	638
Intracerebral hemorrhage	Male	0-6 days	95+ years	Global	-1	2	Elevation Over 1500m (proportion)	--
Intracerebral hemorrhage	Male	0-6 days	95+ years	Global	1	2	Outdoor Air Pollution (PM2.5)	66
Intracerebral hemorrhage	Male	0-6 days	95+ years	Global	1	2	Indoor Air Pollution (All Cooking Fuels)	582
Intracerebral hemorrhage	Male	0-6 days	95+ years	Global	1	2	Diabetes Fasting Plasma Glucose (mmol/L), age-standardized 25+	--
Intracerebral hemorrhage	Male	0-6 days	95+ years	Global	1	2	Mean BMI	--
Intracerebral hemorrhage	Male	0-6 days	95+ years	Global	-1	3	Pulses legumes unadjusted(g)	0
Intracerebral hemorrhage	Male	0-6 days	95+ years	Global	-1	3	LDI (I$ per capita)	--
Intracerebral hemorrhage	Male	0-6 days	95+ years	Global	-1	3	Low-Density Lipoprotein (mmol/L)	--
Intracerebral hemorrhage	Male	0-6 days	95+ years	Global	1	3	Diet high in trans fatty acids	3
Intracerebral hemorrhage	Male	0-6 days	95+ years	Global	1	3	Age- and sex-specific SEV for Low fruit	14
Intracerebral hemorrhage	Male	0-6 days	95+ years	Global	1	3	Age- and sex-specific SEV for Low nuts and seeds	74
Intracerebral hemorrhage	Male	0-6 days	95+ years	Global	1	3	Age- and sex-specific SEV for Low PUFA	381
Intracerebral hemorrhage	Male	0-6 days	95+ years	Global	1	3	Age- and sex-specific SEV for Low vegetables	387
Intracerebral hemorrhage	Male	0-6 days	95+ years	Global	1	3	Age- and sex-specific SEV for Low omega-3	616
Intracerebral hemorrhage	Male	0-6 days	95+ years	Global	1	3	Liters of alcohol consumed per capita	--
Subarachnoid hemorrhage	Female	0-6 days	95+ years	Data Rich	1	1	Systolic Blood Pressure (mmHg)	244
Subarachnoid hemorrhage	Female	0-6 days	95+ years	Data Rich	1	1	Log-transformed SEV scalar: Sub Hem	626
Subarachnoid hemorrhage	Female	0-6 days	95+ years	Data Rich	1	1	Smoking Prevalence	744
Subarachnoid hemorrhage	Female	0-6 days	95+ years	Data Rich	-1	2	Healthcare access and quality index	108
Subarachnoid hemorrhage	Female	0-6 days	95+ years	Data Rich	-1	3	LDI (I$ per capita)	--
Subarachnoid hemorrhage	Female	0-6 days	95+ years	Data Rich	1	3	Liters of alcohol consumed per capita	--
Subarachnoid hemorrhage	Female	0-6 days	95+ years	Global	1	1	Log-transformed SEV scalar: Sub Hem	459
Subarachnoid hemorrhage	Female	0-6 days	95+ years	Global	1	1	Smoking Prevalence	541
Subarachnoid hemorrhage	Female	0-6 days	95+ years	Global	1	1	Systolic Blood Pressure (mmHg)	--
Subarachnoid hemorrhage	Female	0-6 days	95+ years	Global	-1	2	Healthcare access and quality index	349
Subarachnoid hemorrhage	Female	0-6 days	95+ years	Global	-1	3	LDI (I$ per capita)	--
Subarachnoid hemorrhage	Female	0-6 days	95+ years	Global	1	3	Liters of alcohol consumed per capita	--
Subarachnoid hemorrhage	Male	0-6 days	95+ years	Data Rich	1	1	Systolic Blood Pressure (mmHg)	301
Subarachnoid hemorrhage	Male	0-6 days	95+ years	Data Rich	1	1	Smoking Prevalence	371
Subarachnoid hemorrhage	Male	0-6 days	95+ years	Data Rich	1	1	Log-transformed SEV scalar: Sub Hem	432
Subarachnoid hemorrhage	Male	0-6 days	95+ years	Data Rich	-1	2	Healthcare access and quality index	222
Subarachnoid hemorrhage	Male	0-6 days	95+ years	Data Rich	-1	3	LDI (I$ per capita)	--
Subarachnoid hemorrhage	Male	0-6 days	95+ years	Data Rich	1	3	Liters of alcohol consumed per capita	--
Subarachnoid hemorrhage	Male	0-6 days	95+ years	Global	1	1	Smoking Prevalence	593
Subarachnoid hemorrhage	Male	0-6 days	95+ years	Global	1	1	Log-transformed SEV scalar: Sub Hem	--
Subarachnoid hemorrhage	Male	0-6 days	95+ years	Global	1	1	Systolic Blood Pressure (mmHg)	--
Subarachnoid hemorrhage	Male	0-6 days	95+ years	Global	-1	2	Healthcare access and quality index	222
Subarachnoid hemorrhage	Male	0-6 days	95+ years	Global	-1	3	LDI (I$ per capita)	--
Subarachnoid hemorrhage	Male	0-6 days	95+ years	Global	1	3	Liters of alcohol consumed per capita	--
Hypertensive heart disease	Female	15-19 years	95+ years	Data Rich	1	1	Systolic Blood Pressure (mmHg)	--
Hypertensive heart disease	Female	15-19 years	95+ years	Data Rich	-1	2	Healthcare access and quality index	392
Hypertensive heart disease	Female	15-19 years	95+ years	Data Rich	1	2	Smoking Prevalence	27
Hypertensive heart disease	Female	15-19 years	95+ years	Data Rich	1	2	Mean BMI	429
Hypertensive heart disease	Female	15-19 years	95+ years	Data Rich	1	2	Low-Density Lipoprotein (mmol/L)	--
Hypertensive heart disease	Female	15-19 years	95+ years	Data Rich	-1	3	Pulses legumes unadjusted(g)	156
Hypertensive heart disease	Female	15-19 years	95+ years	Data Rich	-1	3	LDI (I$ per capita)	--
Hypertensive heart disease	Female	15-19 years	95+ years	Data Rich	1	3	Age- and sex-specific SEV for Low fruit	49
Hypertensive heart disease	Female	15-19 years	95+ years	Data Rich	1	3	Age- and sex-specific SEV for Low nuts and seeds	220
Hypertensive heart disease	Female	15-19 years	95+ years	Data Rich	1	3	Socio-demographic Index	252
Hypertensive heart disease	Female	15-19 years	95+ years	Data Rich	1	3	Age- and sex-specific SEV for Low vegetables	292
Hypertensive heart disease	Female	15-19 years	95+ years	Data Rich	1	3	Age- and sex-specific SEV for Low omega-3	364
Hypertensive heart disease	Female	15-19 years	95+ years	Data Rich	1	3	Age- and sex-specific SEV for Low PUFA	--
Hypertensive heart disease	Female	15-19 years	95+ years	Data Rich	1	3	Diet high in trans fatty acids	--
Hypertensive heart disease	Female	15-19 years	95+ years	Data Rich	1	3	Liters of alcohol consumed per capita	--
Hypertensive heart disease	Female	15-19 years	95+ years	Global	1	1	Systolic Blood Pressure (mmHg)	282
Hypertensive heart disease	Female	15-19 years	95+ years	Global	-1	2	Healthcare access and quality index	364
Hypertensive heart disease	Female	15-19 years	95+ years	Global	1	2	Smoking Prevalence	178
Hypertensive heart disease	Female	15-19 years	95+ years	Global	1	2	Low-Density Lipoprotein (mmol/L)	--
Hypertensive heart disease	Female	15-19 years	95+ years	Global	1	2	Mean BMI	--
Hypertensive heart disease	Female	15-19 years	95+ years	Global	-1	3	Pulses legumes unadjusted(g)	237
Hypertensive heart disease	Female	15-19 years	95+ years	Global	-1	3	LDI (I$ per capita)	--
Hypertensive heart disease	Female	15-19 years	95+ years	Global	1	3	Age- and sex-specific SEV for Low PUFA	83
Hypertensive heart disease	Female	15-19 years	95+ years	Global	1	3	Age- and sex-specific SEV for Low fruit	240
Hypertensive heart disease	Female	15-19 years	95+ years	Global	1	3	Age- and sex-specific SEV for Low omega-3	240
Hypertensive heart disease	Female	15-19 years	95+ years	Global	1	3	Age- and sex-specific SEV for Low nuts and seeds	247
Hypertensive heart disease	Female	15-19 years	95+ years	Global	1	3	Age- and sex-specific SEV for Low vegetables	575
Hypertensive heart disease	Female	15-19 years	95+ years	Global	1	3	Diet high in trans fatty acids	--

CODEm covariates used, level of covariate, and expected direction of covariate by cause, sex, and age								
**Cause**	**Sex**	**Age Start**	**Age End**	**Model Version Type**	**Direction**	**Level**	**Covariate Name**	**Number of Draws**
Hypertensive heart disease	Female	15-19 years	95+ years	Global	1	3	Liters of alcohol consumed per capita	--
Hypertensive heart disease	Female	15-19 years	95+ years	Global	1	3	Socio-demographic Index	--
Hypertensive heart disease	Male	15-19 years	95+ years	Data Rich	1	1	Systolic Blood Pressure (mmHg)	--
Hypertensive heart disease	Male	15-19 years	95+ years	Data Rich	-1	2	Healthcare access and quality index	425
Hypertensive heart disease	Male	15-19 years	95+ years	Data Rich	1	2	Low-Density Lipoprotein (mmol/L)	--
Hypertensive heart disease	Male	15-19 years	95+ years	Data Rich	1	2	Mean BMI	--
Hypertensive heart disease	Male	15-19 years	95+ years	Data Rich	1	2	Smoking Prevalence	--
Hypertensive heart disease	Male	15-19 years	95+ years	Data Rich	-1	3	LDI (I$ per capita)	--
Hypertensive heart disease	Male	15-19 years	95+ years	Data Rich	-1	3	Pulses legumes unadjusted(g)	--
Hypertensive heart disease	Male	15-19 years	95+ years	Data Rich	1	3	Age- and sex-specific SEV for Low fruit	134
Hypertensive heart disease	Male	15-19 years	95+ years	Data Rich	1	3	Age- and sex-specific SEV for Low omega-3	161
Hypertensive heart disease	Male	15-19 years	95+ years	Data Rich	1	3	Age- and sex-specific SEV for Low vegetables	441
Hypertensive heart disease	Male	15-19 years	95+ years	Data Rich	1	3	Socio-demographic Index	473
Hypertensive heart disease	Male	15-19 years	95+ years	Data Rich	1	3	Age- and sex-specific SEV for Low PUFA	--
Hypertensive heart disease	Male	15-19 years	95+ years	Data Rich	1	3	Age- and sex-specific SEV for Low nuts and seeds	--
Hypertensive heart disease	Male	15-19 years	95+ years	Data Rich	1	3	Diet high in trans fatty acids	--
Hypertensive heart disease	Male	15-19 years	95+ years	Data Rich	1	3	Liters of alcohol consumed per capita	--
Hypertensive heart disease	Male	15-19 years	95+ years	Global	1	1	Systolic Blood Pressure (mmHg)	--
Hypertensive heart disease	Male	15-19 years	95+ years	Global	-1	2	Healthcare access and quality index	584
Hypertensive heart disease	Male	15-19 years	95+ years	Global	1	2	Mean BMI	584
Hypertensive heart disease	Male	15-19 years	95+ years	Global	1	2	Low-Density Lipoprotein (mmol/L)	--
Hypertensive heart disease	Male	15-19 years	95+ years	Global	1	2	Smoking Prevalence	--
Hypertensive heart disease	Male	15-19 years	95+ years	Global	-1	3	Pulses legumes unadjusted(g)	406
Hypertensive heart disease	Male	15-19 years	95+ years	Global	-1	3	LDI (I$ per capita)	--
Hypertensive heart disease	Male	15-19 years	95+ years	Global	1	3	Age- and sex-specific SEV for Low PUFA	149
Hypertensive heart disease	Male	15-19 years	95+ years	Global	1	3	Age- and sex-specific SEV for Low omega-3	165
Hypertensive heart disease	Male	15-19 years	95+ years	Global	1	3	Age- and sex-specific SEV for Low vegetables	252
Hypertensive heart disease	Male	15-19 years	95+ years	Global	1	3	Age- and sex-specific SEV for Low fruit	258
Hypertensive heart disease	Male	15-19 years	95+ years	Global	1	3	Age- and sex-specific SEV for Low nuts and seeds	366
Hypertensive heart disease	Male	15-19 years	95+ years	Global	1	3	Diet high in trans fatty acids	--
Hypertensive heart disease	Male	15-19 years	95+ years	Global	1	3	Liters of alcohol consumed per capita	--
Hypertensive heart disease	Male	15-19 years	95+ years	Global	1	3	Socio-demographic Index	--
Non-rheumatic valvular heart disease	Female	15-19 years	95+ years	Data Rich	1	1	Systolic Blood Pressure (mmHg)	267
Non-rheumatic valvular heart disease	Female	15-19 years	95+ years	Data Rich	1	1	Log-transformed SEV scalar: Valvular	495
Non-rheumatic valvular heart disease	Female	15-19 years	95+ years	Data Rich	1	1	Smoking Prevalence	671
Non-rheumatic valvular heart disease	Female	15-19 years	95+ years	Data Rich	-1	2	Healthcare access and quality index	83
Non-rheumatic valvular heart disease	Female	15-19 years	95+ years	Data Rich	1	2	Mean BMI	116
Non-rheumatic valvular heart disease	Female	15-19 years	95+ years	Data Rich	1	2	Low-Density Lipoprotein (mmol/L)	--
Non-rheumatic valvular heart disease	Female	15-19 years	95+ years	Data Rich	-1	3	LDI (I$ per capita)	--
Non-rheumatic valvular heart disease	Female	15-19 years	95+ years	Data Rich	1	3	Liters of alcohol consumed per capita	71
Non-rheumatic valvular heart disease	Female	15-19 years	95+ years	Data Rich	1	3	Socio-demographic Index	445
Non-rheumatic valvular heart disease	Female	15-19 years	95+ years	Global	1	1	Systolic Blood Pressure (mmHg)	99
Non-rheumatic valvular heart disease	Female	15-19 years	95+ years	Global	1	1	Smoking Prevalence	456
Non-rheumatic valvular heart disease	Female	15-19 years	95+ years	Global	1	1	Log-transformed SEV scalar: Valvular	793
Non-rheumatic valvular heart disease	Female	15-19 years	95+ years	Global	-1	2	Healthcare access and quality index	75
Non-rheumatic valvular heart disease	Female	15-19 years	95+ years	Global	1	2	Mean BMI	134
Non-rheumatic valvular heart disease	Female	15-19 years	95+ years	Global	1	2	Low-Density Lipoprotein (mmol/L)	--
Non-rheumatic valvular heart disease	Female	15-19 years	95+ years	Global	-1	3	LDI (I$ per capita)	--
Non-rheumatic valvular heart disease	Female	15-19 years	95+ years	Global	1	3	Liters of alcohol consumed per capita	224
Non-rheumatic valvular heart disease	Female	15-19 years	95+ years	Global	1	3	Socio-demographic Index	302
Non-rheumatic valvular heart disease	Male	15-19 years	95+ years	Data Rich	1	1	Systolic Blood Pressure (mmHg)	243
Non-rheumatic valvular heart disease	Male	15-19 years	95+ years	Data Rich	1	1	Smoking Prevalence	345
Non-rheumatic valvular heart disease	Male	15-19 years	95+ years	Data Rich	1	1	Log-transformed SEV scalar: Valvular	471
Non-rheumatic valvular heart disease	Male	15-19 years	95+ years	Data Rich	-1	2	Healthcare access and quality index	10
Non-rheumatic valvular heart disease	Male	15-19 years	95+ years	Data Rich	1	2	Mean BMI	310
Non-rheumatic valvular heart disease	Male	15-19 years	95+ years	Data Rich	1	2	Low-Density Lipoprotein (mmol/L)	--
Non-rheumatic valvular heart disease	Male	15-19 years	95+ years	Data Rich	-1	3	LDI (I$ per capita)	--
Non-rheumatic valvular heart disease	Male	15-19 years	95+ years	Data Rich	1	3	Liters of alcohol consumed per capita	117
Non-rheumatic valvular heart disease	Male	15-19 years	95+ years	Data Rich	1	3	Socio-demographic Index	441
Non-rheumatic valvular heart disease	Male	15-19 years	95+ years	Global	1	1	Smoking Prevalence	193
Non-rheumatic valvular heart disease	Male	15-19 years	95+ years	Global	1	1	Log-transformed SEV scalar: Valvular	488
Non-rheumatic valvular heart disease	Male	15-19 years	95+ years	Global	1	1	Systolic Blood Pressure (mmHg)	626
Non-rheumatic valvular heart disease	Male	15-19 years	95+ years	Global	-1	2	Healthcare access and quality index	187
Non-rheumatic valvular heart disease	Male	15-19 years	95+ years	Global	1	2	Mean BMI	349
Non-rheumatic valvular heart disease	Male	15-19 years	95+ years	Global	1	2	Low-Density Lipoprotein (mmol/L)	--
Non-rheumatic valvular heart disease	Male	15-19 years	95+ years	Global	-1	3	LDI (I$ per capita)	--
Non-rheumatic valvular heart disease	Male	15-19 years	95+ years	Global	1	3	Liters of alcohol consumed per capita	187
Non-rheumatic valvular heart disease	Male	15-19 years	95+ years	Global	1	3	Socio-demographic Index	359
Non-rheumatic calcific aortic valve disease	Female	15-19 years	95+ years	Data Rich	1	1	Log-transformed SEV scalar: CAVD	176
Non-rheumatic calcific aortic valve disease	Female	15-19 years	95+ years	Data Rich	1	1	Systolic Blood Pressure (mmHg)	416
Non-rheumatic calcific aortic valve disease	Female	15-19 years	95+ years	Data Rich	1	1	Mean BMI	533
Non-rheumatic calcific aortic valve disease	Female	15-19 years	95+ years	Data Rich	1	1	Smoking Prevalence	620
Non-rheumatic calcific aortic valve disease	Female	15-19 years	95+ years	Data Rich	-1	2	Healthcare access and quality index	--
Non-rheumatic calcific aortic valve disease	Female	15-19 years	95+ years	Data Rich	1	2	Diabetes Fasting Plasma Glucose (mmol/L), age-standardized 25+	165
Non-rheumatic calcific aortic valve disease	Female	15-19 years	95+ years	Data Rich	1	2	Low-Density Lipoprotein (mmol/L)	--
Non-rheumatic calcific aortic valve disease	Female	15-19 years	95+ years	Data Rich	-1	3	LDI (I$ per capita)	--
Non-rheumatic calcific aortic valve disease	Female	15-19 years	95+ years	Data Rich	1	3	Socio-demographic Index	234
Non-rheumatic calcific aortic valve disease	Female	15-19 years	95+ years	Data Rich	1	3	Liters of alcohol consumed per capita	--
Non-rheumatic calcific aortic valve disease	Female	15-19 years	95+ years	Global	1	1	Systolic Blood Pressure (mmHg)	174
Non-rheumatic calcific aortic valve disease	Female	15-19 years	95+ years	Global	1	1	Log-transformed SEV scalar: CAVD	402
Non-rheumatic calcific aortic valve disease	Female	15-19 years	95+ years	Global	1	1	Smoking Prevalence	492
Non-rheumatic calcific aortic valve disease	Female	15-19 years	95+ years	Global	1	1	Mean BMI	502
Non-rheumatic calcific aortic valve disease	Female	15-19 years	95+ years	Global	-1	2	Healthcare access and quality index	13
Non-rheumatic calcific aortic valve disease	Female	15-19 years	95+ years	Global	1	2	Diabetes Fasting Plasma Glucose (mmol/L), age-standardized 25+	--
Non-rheumatic calcific aortic valve disease	Female	15-19 years	95+ years	Global	1	2	Low-Density Lipoprotein (mmol/L)	--
Non-rheumatic calcific aortic valve disease	Female	15-19 years	95+ years	Global	-1	3	LDI (I$ per capita)	--
Non-rheumatic calcific aortic valve disease	Female	15-19 years	95+ years	Global	1	3	Socio-demographic Index	322
Non-rheumatic calcific aortic valve disease	Female	15-19 years	95+ years	Global	1	3	Liters of alcohol consumed per capita	--
Non-rheumatic calcific aortic valve disease	Male	15-19 years	95+ years	Data Rich	1	1	Log-transformed SEV scalar: CAVD	61
Non-rheumatic calcific aortic valve disease	Male	15-19 years	95+ years	Data Rich	1	1	Mean BMI	601
Non-rheumatic calcific aortic valve disease	Male	15-19 years	95+ years	Data Rich	1	1	Systolic Blood Pressure (mmHg)	618
Non-rheumatic calcific aortic valve disease	Male	15-19 years	95+ years	Data Rich	1	1	Smoking Prevalence	637
Non-rheumatic calcific aortic valve disease	Male	15-19 years	95+ years	Data Rich	-1	2	Healthcare access and quality index	0
Non-rheumatic calcific aortic valve disease	Male	15-19 years	95+ years	Data Rich	1	2	Diabetes Fasting Plasma Glucose (mmol/L), age-standardized 25+	296
Non-rheumatic calcific aortic valve disease	Male	15-19 years	95+ years	Data Rich	1	2	Low-Density Lipoprotein (mmol/L)	--
Non-rheumatic calcific aortic valve disease	Male	15-19 years	95+ years	Data Rich	-1	3	LDI (I$ per capita)	--
Non-rheumatic calcific aortic valve disease	Male	15-19 years	95+ years	Data Rich	1	3	Liters of alcohol consumed per capita	54
Non-rheumatic calcific aortic valve disease	Male	15-19 years	95+ years	Data Rich	1	3	Socio-demographic Index	333
Non-rheumatic calcific aortic valve disease	Male	15-19 years	95+ years	Global	1	1	Smoking Prevalence	101
Non-rheumatic calcific aortic valve disease	Male	15-19 years	95+ years	Global	1	1	Log-transformed SEV scalar: CAVD	199
Non-rheumatic calcific aortic valve disease	Male	15-19 years	95+ years	Global	1	1	Mean BMI	511
Non-rheumatic calcific aortic valve disease	Male	15-19 years	95+ years	Global	1	1	Systolic Blood Pressure (mmHg)	801
Non-rheumatic calcific aortic valve disease	Male	15-19 years	95+ years	Global	-1	2	Healthcare access and quality index	72
Non-rheumatic calcific aortic valve disease	Male	15-19 years	95+ years	Global	1	2	Diabetes Fasting Plasma Glucose (mmol/L), age-standardized 25+	27
Non-rheumatic calcific aortic valve disease	Male	15-19 years	95+ years	Global	1	2	Low-Density Lipoprotein (mmol/L)	--
Non-rheumatic calcific aortic valve disease	Male	15-19 years	95+ years	Global	-1	3	LDI (I$ per capita)	--
Non-rheumatic calcific aortic valve disease	Male	15-19 years	95+ years	Global	1	3	Socio-demographic Index	283
Non-rheumatic calcific aortic valve disease	Male	15-19 years	95+ years	Global	1	3	Liters of alcohol consumed per capita	298
Non-rheumatic degenerative mitral valve disease	Female	15-19 years	95+ years	Data Rich	-1	1	Healthcare access and quality index	488
Non-rheumatic degenerative mitral valve disease	Female	15-19 years	95+ years	Data Rich	1	1	Socio-demographic Index	473
Non-rheumatic degenerative mitral valve disease	Female	15-19 years	95+ years	Data Rich	1	1	LDI (I$ per capita)	--
Non-rheumatic degenerative mitral valve disease	Female	15-19 years	95+ years	Global	-1	1	Healthcare access and quality index	706
Non-rheumatic degenerative mitral valve disease	Female	15-19 years	95+ years	Global	1	1	Socio-demographic Index	254
Non-rheumatic degenerative mitral valve disease	Female	15-19 years	95+ years	Global	1	1	LDI (I$ per capita)	--
Non-rheumatic degenerative mitral valve disease	Male	15-19 years	95+ years	Data Rich	-1	1	Healthcare access and quality index	603
Non-rheumatic degenerative mitral valve disease	Male	15-19 years	95+ years	Data Rich	1	1	Socio-demographic Index	726
Non-rheumatic degenerative mitral valve disease	Male	15-19 years	95+ years	Data Rich	1	1	LDI (I$ per capita)	--
Non-rheumatic degenerative mitral valve disease	Male	15-19 years	95+ years	Global	-1	1	Healthcare access and quality index	622
Non-rheumatic degenerative mitral valve disease	Male	15-19 years	95+ years	Global	1	1	Socio-demographic Index	511
Non-rheumatic degenerative mitral valve disease	Male	15-19 years	95+ years	Global	1	1	LDI (I$ per capita)	--

CODEm covariates used, level of covariate, and expected direction of covariate by cause, sex, and age

Cause	Sex	Age Start	Age End	Model Version Type	Direction	Level	Covariate Name	Number of Draws
Other non-rheumatic valve diseases	Female	15-19 years	95+ years	Data Rich	1	1	Healthcare access and quality index	--
Other non-rheumatic valve diseases	Female	15-19 years	95+ years	Data Rich	1	1	Log-transformed SEV scalar: Valvular	--
Other non-rheumatic valve diseases	Female	15-19 years	95+ years	Data Rich	1	2	Socio-demographic Index	1000
Other non-rheumatic valve diseases	Female	15-19 years	95+ years	Global	1	1	Healthcare access and quality index	1000
Other non-rheumatic valve diseases	Female	15-19 years	95+ years	Global	1	1	Log-transformed SEV scalar: Valvular	--
Other non-rheumatic valve diseases	Female	15-19 years	95+ years	Global	1	2	Socio-demographic Index	--
Other non-rheumatic valve diseases	Male	15-19 years	95+ years	Data Rich	1	1	Log-transformed SEV scalar: Valvular	1000
Other non-rheumatic valve diseases	Male	15-19 years	95+ years	Data Rich	1	1	Healthcare access and quality index	--
Other non-rheumatic valve diseases	Male	15-19 years	95+ years	Data Rich	1	2	Socio-demographic Index	454
Other non-rheumatic valve diseases	Male	15-19 years	95+ years	Global	1	1	Healthcare access and quality index	--
Other non-rheumatic valve diseases	Male	15-19 years	95+ years	Global	1	1	Log-transformed SEV scalar: Valvular	--
Other non-rheumatic valve diseases	Male	15-19 years	95+ years	Global	1	2	Socio-demographic Index	1000
Cardiomyopathy and myocarditis	Female	0-6 days	95+ years	Data Rich	1	1	Log-transformed SEV scalar: CMP	601
Cardiomyopathy and myocarditis	Female	0-6 days	95+ years	Data Rich	1	1	Smoking Prevalence	636
Cardiomyopathy and myocarditis	Female	0-6 days	95+ years	Data Rich	-1	2	Healthcare access and quality index	89
Cardiomyopathy and myocarditis	Female	0-6 days	95+ years	Data Rich	1	2	Mean BMI	389
Cardiomyopathy and myocarditis	Female	0-6 days	95+ years	Data Rich	1	2	Systolic Blood Pressure (mmHg)	420
Cardiomyopathy and myocarditis	Female	0-6 days	95+ years	Data Rich	-1	3	Socio-demographic Index	146
Cardiomyopathy and myocarditis	Female	0-6 days	95+ years	Data Rich	-1	3	LDI (I$ per capita)	--
Cardiomyopathy and myocarditis	Female	0-6 days	95+ years	Global	1	1	Log-transformed SEV scalar: CMP	422
Cardiomyopathy and myocarditis	Female	0-6 days	95+ years	Global	1	1	Smoking Prevalence	646
Cardiomyopathy and myocarditis	Female	0-6 days	95+ years	Global	-1	2	Healthcare access and quality index	555
Cardiomyopathy and myocarditis	Female	0-6 days	95+ years	Global	1	2	Systolic Blood Pressure (mmHg)	297
Cardiomyopathy and myocarditis	Female	0-6 days	95+ years	Global	1	2	Mean BMI	--
Cardiomyopathy and myocarditis	Female	0-6 days	95+ years	Global	-1	3	Socio-demographic Index	41
Cardiomyopathy and myocarditis	Female	0-6 days	95+ years	Global	-1	3	LDI (I$ per capita)	--
Cardiomyopathy and myocarditis	Male	0-6 days	95+ years	Data Rich	1	1	Smoking Prevalence	225
Cardiomyopathy and myocarditis	Male	0-6 days	95+ years	Data Rich	1	1	Log-transformed SEV scalar: CMP	775
Cardiomyopathy and myocarditis	Male	0-6 days	95+ years	Data Rich	-1	2	Healthcare access and quality index	48
Cardiomyopathy and myocarditis	Male	0-6 days	95+ years	Data Rich	1	2	Systolic Blood Pressure (mmHg)	156
Cardiomyopathy and myocarditis	Male	0-6 days	95+ years	Data Rich	1	2	Mean BMI	210
Cardiomyopathy and myocarditis	Male	0-6 days	95+ years	Data Rich	-1	3	Socio-demographic Index	83
Cardiomyopathy and myocarditis	Male	0-6 days	95+ years	Data Rich	-1	3	LDI (I$ per capita)	--
Cardiomyopathy and myocarditis	Male	0-6 days	95+ years	Global	1	1	Smoking Prevalence	524
Cardiomyopathy and myocarditis	Male	0-6 days	95+ years	Global	1	1	Log-transformed SEV scalar: CMP	705
Cardiomyopathy and myocarditis	Male	0-6 days	95+ years	Global	-1	2	Healthcare access and quality index	--
Cardiomyopathy and myocarditis	Male	0-6 days	95+ years	Global	1	2	Systolic Blood Pressure (mmHg)	141
Cardiomyopathy and myocarditis	Male	0-6 days	95+ years	Global	1	2	Mean BMI	--
Cardiomyopathy and myocarditis	Male	0-6 days	95+ years	Global	-1	3	Socio-demographic Index	138
Cardiomyopathy and myocarditis	Male	0-6 days	95+ years	Global	-1	3	LDI (I$ per capita)	--
Myocarditis	Female	0-6 days	95+ years	Data Rich	1	2	Healthcare access and quality index	1000
Myocarditis	Female	0-6 days	95+ years	Data Rich	1	3	LDI (I$ per capita)	--
Myocarditis	Female	0-6 days	95+ years	Data Rich	1	3	Socio-demographic Index	--
Myocarditis	Female	0-6 days	95+ years	Global	-1	2	Healthcare access and quality index	1000
Myocarditis	Female	0-6 days	95+ years	Global	-1	3	Socio-demographic Index	197
Myocarditis	Female	0-6 days	95+ years	Global	-1	3	LDI (I$ per capita)	--
Myocarditis	Male	0-6 days	95+ years	Data Rich	1	2	Healthcare access and quality index	1000
Myocarditis	Male	0-6 days	95+ years	Data Rich	1	3	LDI (I$ per capita)	--
Myocarditis	Male	0-6 days	95+ years	Data Rich	1	3	Socio-demographic Index	--
Myocarditis	Male	0-6 days	95+ years	Global	-1	2	Healthcare access and quality index	602
Myocarditis	Male	0-6 days	95+ years	Global	-1	3	LDI (I$ per capita)	--
Myocarditis	Male	0-6 days	95+ years	Global	-1	3	Socio-demographic Index	--
Alcoholic cardiomyopathy	Female	15-19 years	95+ years	Data Rich	1	1	Age- and sex-specific SEV for Alcohol use	356
Alcoholic cardiomyopathy	Female	15-19 years	95+ years	Data Rich	1	1	Smoking Prevalence	501
Alcoholic cardiomyopathy	Female	15-19 years	95+ years	Data Rich	1	1	Log-transformed SEV scalar: CMP	826
Alcoholic cardiomyopathy	Female	15-19 years	95+ years	Data Rich	-1	2	Healthcare access and quality index	104
Alcoholic cardiomyopathy	Female	15-19 years	95+ years	Data Rich	-1	3	LDI (I$ per capita)	--
Alcoholic cardiomyopathy	Female	15-19 years	95+ years	Global	1	1	Age- and sex-specific SEV for Alcohol use	408
Alcoholic cardiomyopathy	Female	15-19 years	95+ years	Global	1	1	Smoking Prevalence	469
Alcoholic cardiomyopathy	Female	15-19 years	95+ years	Global	1	1	Log-transformed SEV scalar: CMP	481
Alcoholic cardiomyopathy	Female	15-19 years	95+ years	Global	-1	2	Healthcare access and quality index	112
Alcoholic cardiomyopathy	Female	15-19 years	95+ years	Global	-1	3	LDI (I$ per capita)	--
Alcoholic cardiomyopathy	Male	15-19 years	95+ years	Data Rich	1	1	Smoking Prevalence	273
Alcoholic cardiomyopathy	Male	15-19 years	95+ years	Data Rich	1	1	Age- and sex-specific SEV for Alcohol use	582
Alcoholic cardiomyopathy	Male	15-19 years	95+ years	Data Rich	1	1	Log-transformed SEV scalar: CMP	673
Alcoholic cardiomyopathy	Male	15-19 years	95+ years	Data Rich	-1	2	Healthcare access and quality index	188
Alcoholic cardiomyopathy	Male	15-19 years	95+ years	Data Rich	-1	3	LDI (I$ per capita)	--
Alcoholic cardiomyopathy	Male	15-19 years	95+ years	Global	1	1	Smoking Prevalence	415
Alcoholic cardiomyopathy	Male	15-19 years	95+ years	Global	1	1	Log-transformed SEV scalar: CMP	513
Alcoholic cardiomyopathy	Male	15-19 years	95+ years	Global	1	1	Age- and sex-specific SEV for Alcohol use	--
Alcoholic cardiomyopathy	Male	15-19 years	95+ years	Global	-1	2	Healthcare access and quality index	133
Alcoholic cardiomyopathy	Male	15-19 years	95+ years	Global	-1	3	LDI (I$ per capita)	--
Other cardiomyopathy	Female	0-6 days	95+ years	Data Rich	1	1	Systolic Blood Pressure (mmHg)	251
Other cardiomyopathy	Female	0-6 days	95+ years	Data Rich	1	1	Log-transformed SEV scalar: CMP	434
Other cardiomyopathy	Female	0-6 days	95+ years	Data Rich	1	1	Smoking Prevalence	733
Other cardiomyopathy	Female	0-6 days	95+ years	Data Rich	-1	2	Healthcare access and quality index	152
Other cardiomyopathy	Female	0-6 days	95+ years	Data Rich	1	2	Mean BMI	--
Other cardiomyopathy	Female	0-6 days	95+ years	Data Rich	-1	3	LDI (I$ per capita)	--
Other cardiomyopathy	Female	0-6 days	95+ years	Data Rich	1	3	Socio-demographic Index	83
Other cardiomyopathy	Female	0-6 days	95+ years	Global	1	1	Log-transformed SEV scalar: CMP	349
Other cardiomyopathy	Female	0-6 days	95+ years	Global	1	1	Systolic Blood Pressure (mmHg)	672
Other cardiomyopathy	Female	0-6 days	95+ years	Global	1	1	Smoking Prevalence	814
Other cardiomyopathy	Female	0-6 days	95+ years	Global	-1	2	Healthcare access and quality index	370
Other cardiomyopathy	Female	0-6 days	95+ years	Global	1	2	Mean BMI	--
Other cardiomyopathy	Female	0-6 days	95+ years	Global	-1	3	LDI (I$ per capita)	--
Other cardiomyopathy	Female	0-6 days	95+ years	Global	1	3	Socio-demographic Index	216
Other cardiomyopathy	Male	0-6 days	95+ years	Data Rich	1	1	Systolic Blood Pressure (mmHg)	620
Other cardiomyopathy	Male	0-6 days	95+ years	Data Rich	1	1	Smoking Prevalence	679
Other cardiomyopathy	Male	0-6 days	95+ years	Data Rich	1	1	Log-transformed SEV scalar: CMP	--
Other cardiomyopathy	Male	0-6 days	95+ years	Data Rich	-1	2	Healthcare access and quality index	--
Other cardiomyopathy	Male	0-6 days	95+ years	Data Rich	1	2	Mean BMI	72
Other cardiomyopathy	Male	0-6 days	95+ years	Data Rich	-1	3	LDI (I$ per capita)	--
Other cardiomyopathy	Male	0-6 days	95+ years	Data Rich	1	3	Socio-demographic Index	509
Other cardiomyopathy	Male	0-6 days	95+ years	Global	1	1	Log-transformed SEV scalar: CMP	154
Other cardiomyopathy	Male	0-6 days	95+ years	Global	1	1	Smoking Prevalence	326
Other cardiomyopathy	Male	0-6 days	95+ years	Global	1	1	Systolic Blood Pressure (mmHg)	733
Other cardiomyopathy	Male	0-6 days	95+ years	Global	-1	2	Healthcare access and quality index	228
Other cardiomyopathy	Male	0-6 days	95+ years	Global	1	2	Mean BMI	--
Other cardiomyopathy	Male	0-6 days	95+ years	Global	-1	3	LDI (I$ per capita)	--
Other cardiomyopathy	Male	0-6 days	95+ years	Global	1	3	Socio-demographic Index	367
Pulmonary Arterial Hypertension	Female	0-6 days	95+ years	Data Rich	-1	1	Socio-demographic Index	634
Pulmonary Arterial Hypertension	Female	0-6 days	95+ years	Data Rich	1	1	Healthcare access and quality index	280
Pulmonary Arterial Hypertension	Female	0-6 days	95+ years	Data Rich	1	1	Log-transformed SEV scalar: HIV	559
Pulmonary Arterial Hypertension	Female	0-6 days	95+ years	Data Rich	1	1	Schistosomiasis Prevalence Results	--
Pulmonary Arterial Hypertension	Female	0-6 days	95+ years	Global	-1	1	Socio-demographic Index	423
Pulmonary Arterial Hypertension	Female	0-6 days	95+ years	Global	1	1	Log-transformed SEV scalar: HIV	773
Pulmonary Arterial Hypertension	Female	0-6 days	95+ years	Global	1	1	Healthcare access and quality index	--
Pulmonary Arterial Hypertension	Female	0-6 days	95+ years	Global	1	1	Schistosomiasis Prevalence Results	--
Pulmonary Arterial Hypertension	Male	0-6 days	95+ years	Data Rich	-1	1	Socio-demographic Index	404
Pulmonary Arterial Hypertension	Male	0-6 days	95+ years	Data Rich	1	1	Log-transformed SEV scalar: HIV	605
Pulmonary Arterial Hypertension	Male	0-6 days	95+ years	Data Rich	1	1	Healthcare access and quality index	720
Pulmonary Arterial Hypertension	Male	0-6 days	95+ years	Data Rich	1	1	Schistosomiasis Prevalence Results	--
Pulmonary Arterial Hypertension	Male	0-6 days	95+ years	Global	-1	1	Socio-demographic Index	798
Pulmonary Arterial Hypertension	Male	0-6 days	95+ years	Global	1	1	Schistosomiasis Prevalence Results	216
Pulmonary Arterial Hypertension	Male	0-6 days	95+ years	Global	1	1	Log-transformed SEV scalar: HIV	610
Pulmonary Arterial Hypertension	Male	0-6 days	95+ years	Global	1	1	Healthcare access and quality index	--
Atrial fibrillation and flutter	Female	30-34 years	95+ years	Data Rich	1	1	Log-transformed SEV scalar: A Fib	--
Atrial fibrillation and flutter	Female	30-34 years	95+ years	Data Rich	1	1	Smoking Prevalence	--
Atrial fibrillation and flutter	Female	30-34 years	95+ years	Data Rich	1	1	Systolic Blood Pressure (mmHg)	--
Atrial fibrillation and flutter	Female	30-34 years	95+ years	Data Rich	-1	2	Healthcare access and quality index	--
Atrial fibrillation and flutter	Female	30-34 years	95+ years	Data Rich	1	2	Mean BMI	815
Atrial fibrillation and flutter	Female	30-34 years	95+ years	Data Rich	1	2	Diabetes Fasting Plasma Glucose (mmol/L), age-standardized 25+	--
Atrial fibrillation and flutter	Female	30-34 years	95+ years	Data Rich	1	2	Low-Density Lipoprotein (mmol/L)	--
Atrial fibrillation and flutter	Female	30-34 years	95+ years	Data Rich	-1	3	Pulses legumes unadjusted(g)	219

CODEm covariates used, level of covariate, and expected direction of covariate by cause, sex, and age

Cause	Sex	Age Start	Age End	Model Version Type	Direction	Level	Covariate Name	Number of Draws
Atrial fibrillation and flutter	Female	30-34 years	95+ years	Data Rich	-1	3	LDI (I$ per capita)	--
Atrial fibrillation and flutter	Female	30-34 years	95+ years	Data Rich	1	3	Age- and sex-specific SEV for Low fruit	72
Atrial fibrillation and flutter	Female	30-34 years	95+ years	Data Rich	1	3	Age- and sex-specific SEV for Low nuts and seeds	87
Atrial fibrillation and flutter	Female	30-34 years	95+ years	Data Rich	1	3	Age- and sex-specific SEV for Low PUFA	150
Atrial fibrillation and flutter	Female	30-34 years	95+ years	Data Rich	1	3	Age- and sex-specific SEV for Low omega-3	159
Atrial fibrillation and flutter	Female	30-34 years	95+ years	Data Rich	1	3	Age- and sex-specific SEV for Low vegetables	212
Atrial fibrillation and flutter	Female	30-34 years	95+ years	Data Rich	1	3	Socio-demographic Index	347
Atrial fibrillation and flutter	Female	30-34 years	95+ years	Data Rich	1	3	Diet high in trans fatty acids	--
Atrial fibrillation and flutter	Female	30-34 years	95+ years	Data Rich	1	3	Liters of alcohol consumed per capita	--
Atrial fibrillation and flutter	Female	30-34 years	95+ years	Global	1	1	Log-transformed SEV scalar: A Fib	--
Atrial fibrillation and flutter	Female	30-34 years	95+ years	Global	1	1	Smoking Prevalence	--
Atrial fibrillation and flutter	Female	30-34 years	95+ years	Global	1	1	Systolic Blood Pressure (mmHg)	--
Atrial fibrillation and flutter	Female	30-34 years	95+ years	Global	-1	2	Healthcare access and quality index	--
Atrial fibrillation and flutter	Female	30-34 years	95+ years	Global	1	2	Diabetes Fasting Plasma Glucose (mmol/L), age-standardized 25+	340
Atrial fibrillation and flutter	Female	30-34 years	95+ years	Global	1	2	Mean BMI	401
Atrial fibrillation and flutter	Female	30-34 years	95+ years	Global	1	2	Low-Density Lipoprotein (mmol/L)	--
Atrial fibrillation and flutter	Female	30-34 years	95+ years	Global	-1	3	Pulses legumes unadjusted(g)	311
Atrial fibrillation and flutter	Female	30-34 years	95+ years	Global	-1	3	LDI (I$ per capita)	--
Atrial fibrillation and flutter	Female	30-34 years	95+ years	Global	1	3	Age- and sex-specific SEV for Low PUFA	19
Atrial fibrillation and flutter	Female	30-34 years	95+ years	Global	1	3	Age- and sex-specific SEV for Low fruit	19
Atrial fibrillation and flutter	Female	30-34 years	95+ years	Global	1	3	Age- and sex-specific SEV for Low omega-3	44
Atrial fibrillation and flutter	Female	30-34 years	95+ years	Global	1	3	Age- and sex-specific SEV for Low vegetables	74
Atrial fibrillation and flutter	Female	30-34 years	95+ years	Global	1	3	Age- and sex-specific SEV for Low nuts and seeds	98
Atrial fibrillation and flutter	Female	30-34 years	95+ years	Global	1	3	Liters of alcohol consumed per capita	151
Atrial fibrillation and flutter	Female	30-34 years	95+ years	Global	1	3	Socio-demographic Index	448
Atrial fibrillation and flutter	Female	30-34 years	95+ years	Global	1	3	Diet high in trans fatty acids	--
Atrial fibrillation and flutter	Male	30-34 years	95+ years	Data Rich	1	1	Log-transformed SEV scalar: A Fib	--
Atrial fibrillation and flutter	Male	30-34 years	95+ years	Data Rich	1	1	Smoking Prevalence	--
Atrial fibrillation and flutter	Male	30-34 years	95+ years	Data Rich	1	1	Systolic Blood Pressure (mmHg)	--
Atrial fibrillation and flutter	Male	30-34 years	95+ years	Data Rich	-1	2	Healthcare access and quality index	--
Atrial fibrillation and flutter	Male	30-34 years	95+ years	Data Rich	1	2	Diabetes Fasting Plasma Glucose (mmol/L), age-standardized 25+	--
Atrial fibrillation and flutter	Male	30-34 years	95+ years	Data Rich	1	2	Low-Density Lipoprotein (mmol/L)	--
Atrial fibrillation and flutter	Male	30-34 years	95+ years	Data Rich	1	2	Mean BMI	--
Atrial fibrillation and flutter	Male	30-34 years	95+ years	Data Rich	-1	3	Pulses legumes unadjusted(g)	546
Atrial fibrillation and flutter	Male	30-34 years	95+ years	Data Rich	-1	3	LDI (I$ per capita)	--
Atrial fibrillation and flutter	Male	30-34 years	95+ years	Data Rich	1	3	Age- and sex-specific SEV for Low vegetables	454
Atrial fibrillation and flutter	Male	30-34 years	95+ years	Data Rich	1	3	Socio-demographic Index	546
Atrial fibrillation and flutter	Male	30-34 years	95+ years	Data Rich	1	3	Age- and sex-specific SEV for Low PUFA	--
Atrial fibrillation and flutter	Male	30-34 years	95+ years	Data Rich	1	3	Age- and sex-specific SEV for Low fruit	--
Atrial fibrillation and flutter	Male	30-34 years	95+ years	Data Rich	1	3	Age- and sex-specific SEV for Low nuts and seeds	--
Atrial fibrillation and flutter	Male	30-34 years	95+ years	Data Rich	1	3	Age- and sex-specific SEV for Low omega-3	--
Atrial fibrillation and flutter	Male	30-34 years	95+ years	Data Rich	1	3	Diet high in trans fatty acids	--
Atrial fibrillation and flutter	Male	30-34 years	95+ years	Data Rich	1	3	Liters of alcohol consumed per capita	--
Atrial fibrillation and flutter	Male	30-34 years	95+ years	Global	1	1	Log-transformed SEV scalar: A Fib	--
Atrial fibrillation and flutter	Male	30-34 years	95+ years	Global	1	1	Smoking Prevalence	--
Atrial fibrillation and flutter	Male	30-34 years	95+ years	Global	1	1	Systolic Blood Pressure (mmHg)	--
Atrial fibrillation and flutter	Male	30-34 years	95+ years	Global	-1	2	Healthcare access and quality index	--
Atrial fibrillation and flutter	Male	30-34 years	95+ years	Global	1	2	Mean BMI	188
Atrial fibrillation and flutter	Male	30-34 years	95+ years	Global	1	2	Diabetes Fasting Plasma Glucose (mmol/L), age-standardized 25+	400
Atrial fibrillation and flutter	Male	30-34 years	95+ years	Global	1	2	Low-Density Lipoprotein (mmol/L)	--
Atrial fibrillation and flutter	Male	30-34 years	95+ years	Global	-1	3	Pulses legumes unadjusted(g)	239
Atrial fibrillation and flutter	Male	30-34 years	95+ years	Global	-1	3	LDI (I$ per capita)	--
Atrial fibrillation and flutter	Male	30-34 years	95+ years	Global	1	3	Age- and sex-specific SEV for Low fruit	85
Atrial fibrillation and flutter	Male	30-34 years	95+ years	Global	1	3	Age- and sex-specific SEV for Low PUFA	115
Atrial fibrillation and flutter	Male	30-34 years	95+ years	Global	1	3	Age- and sex-specific SEV for Low omega-3	115
Atrial fibrillation and flutter	Male	30-34 years	95+ years	Global	1	3	Age- and sex-specific SEV for Low vegetables	134
Atrial fibrillation and flutter	Male	30-34 years	95+ years	Global	1	3	Socio-demographic Index	202
Atrial fibrillation and flutter	Male	30-34 years	95+ years	Global	1	3	Liters of alcohol consumed per capita	273
Atrial fibrillation and flutter	Male	30-34 years	95+ years	Global	1	3	Age- and sex-specific SEV for Low nuts and seeds	--
Atrial fibrillation and flutter	Male	30-34 years	95+ years	Global	1	3	Diet high in trans fatty acids	--
Aortic aneurysm	Female	15-19 years	95+ years	Data Rich	1	1	Systolic Blood Pressure (mmHg)	47
Aortic aneurysm	Female	15-19 years	95+ years	Data Rich	1	1	Cumulative Cigarettes (10 Years)	165
Aortic aneurysm	Female	15-19 years	95+ years	Data Rich	1	1	Log-transformed SEV scalar: Aort An	921
Aortic aneurysm	Female	15-19 years	95+ years	Data Rich	1	1	Low-Density Lipoprotein (mmol/L)	--
Aortic aneurysm	Female	15-19 years	95+ years	Data Rich	-1	2	Healthcare access and quality index	--
Aortic aneurysm	Female	15-19 years	95+ years	Data Rich	1	2	Mean BMI	232
Aortic aneurysm	Female	15-19 years	95+ years	Data Rich	-1	3	LDI (I$ per capita)	--
Aortic aneurysm	Female	15-19 years	95+ years	Data Rich	-1	3	Pulses legumes unadjusted(g)	--
Aortic aneurysm	Female	15-19 years	95+ years	Data Rich	1	3	Liters of alcohol consumed per capita	405
Aortic aneurysm	Female	15-19 years	95+ years	Data Rich	1	3	Socio-demographic Index	503
Aortic aneurysm	Female	15-19 years	95+ years	Data Rich	1	3	Age- and sex-specific SEV for Low PUFA	--
Aortic aneurysm	Female	15-19 years	95+ years	Data Rich	1	3	Age- and sex-specific SEV for Low fruit	--
Aortic aneurysm	Female	15-19 years	95+ years	Data Rich	1	3	Age- and sex-specific SEV for Low nuts and seeds	--
Aortic aneurysm	Female	15-19 years	95+ years	Data Rich	1	3	Age- and sex-specific SEV for Low omega-3	--
Aortic aneurysm	Female	15-19 years	95+ years	Data Rich	1	3	Age- and sex-specific SEV for Low vegetables	--
Aortic aneurysm	Female	15-19 years	95+ years	Global	1	1	Systolic Blood Pressure (mmHg)	105
Aortic aneurysm	Female	15-19 years	95+ years	Global	1	1	Cumulative Cigarettes (10 Years)	314
Aortic aneurysm	Female	15-19 years	95+ years	Global	1	1	Log-transformed SEV scalar: Aort An	871
Aortic aneurysm	Female	15-19 years	95+ years	Global	1	1	Low-Density Lipoprotein (mmol/L)	--
Aortic aneurysm	Female	15-19 years	95+ years	Global	-1	2	Healthcare access and quality index	252
Aortic aneurysm	Female	15-19 years	95+ years	Global	1	2	Mean BMI	430
Aortic aneurysm	Female	15-19 years	95+ years	Global	-1	3	LDI (I$ per capita)	--
Aortic aneurysm	Female	15-19 years	95+ years	Global	-1	3	Pulses legumes unadjusted(g)	--
Aortic aneurysm	Female	15-19 years	95+ years	Global	1	3	Age- and sex-specific SEV for Low vegetables	55
Aortic aneurysm	Female	15-19 years	95+ years	Global	1	3	Liters of alcohol consumed per capita	85
Aortic aneurysm	Female	15-19 years	95+ years	Global	1	3	Socio-demographic Index	294
Aortic aneurysm	Female	15-19 years	95+ years	Global	1	3	Age- and sex-specific SEV for Low PUFA	--
Aortic aneurysm	Female	15-19 years	95+ years	Global	1	3	Age- and sex-specific SEV for Low fruit	--
Aortic aneurysm	Female	15-19 years	95+ years	Global	1	3	Age- and sex-specific SEV for Low nuts and seeds	--
Aortic aneurysm	Female	15-19 years	95+ years	Global	1	3	Age- and sex-specific SEV for Low omega-3	--
Aortic aneurysm	Male	15-19 years	95+ years	Data Rich	1	1	Cumulative Cigarettes (10 Years)	0
Aortic aneurysm	Male	15-19 years	95+ years	Data Rich	1	1	Log-transformed SEV scalar: Aort An	359
Aortic aneurysm	Male	15-19 years	95+ years	Data Rich	1	1	Systolic Blood Pressure (mmHg)	423
Aortic aneurysm	Male	15-19 years	95+ years	Data Rich	1	1	Low-Density Lipoprotein (mmol/L)	--
Aortic aneurysm	Male	15-19 years	95+ years	Data Rich	-1	2	Healthcare access and quality index	0
Aortic aneurysm	Male	15-19 years	95+ years	Data Rich	1	2	Mean BMI	415
Aortic aneurysm	Male	15-19 years	95+ years	Data Rich	-1	3	LDI (I$ per capita)	--
Aortic aneurysm	Male	15-19 years	95+ years	Data Rich	-1	3	Pulses legumes unadjusted(g)	--
Aortic aneurysm	Male	15-19 years	95+ years	Data Rich	1	3	Age- and sex-specific SEV for Low PUFA	0
Aortic aneurysm	Male	15-19 years	95+ years	Data Rich	1	3	Age- and sex-specific SEV for Low nuts and seeds	0
Aortic aneurysm	Male	15-19 years	95+ years	Data Rich	1	3	Age- and sex-specific SEV for Low vegetables	0
Aortic aneurysm	Male	15-19 years	95+ years	Data Rich	1	3	Age- and sex-specific SEV for Low fruit	32
Aortic aneurysm	Male	15-19 years	95+ years	Data Rich	1	3	Liters of alcohol consumed per capita	178
Aortic aneurysm	Male	15-19 years	95+ years	Data Rich	1	3	Socio-demographic Index	718
Aortic aneurysm	Male	15-19 years	95+ years	Data Rich	1	3	Age- and sex-specific SEV for Low omega-3	--
Aortic aneurysm	Male	15-19 years	95+ years	Global	1	1	Cumulative Cigarettes (10 Years)	24
Aortic aneurysm	Male	15-19 years	95+ years	Global	1	1	Systolic Blood Pressure (mmHg)	213
Aortic aneurysm	Male	15-19 years	95+ years	Global	1	1	Log-transformed SEV scalar: Aort An	680
Aortic aneurysm	Male	15-19 years	95+ years	Global	1	1	Low-Density Lipoprotein (mmol/L)	--
Aortic aneurysm	Male	15-19 years	95+ years	Global	-1	2	Healthcare access and quality index	--
Aortic aneurysm	Male	15-19 years	95+ years	Global	1	2	Mean BMI	500
Aortic aneurysm	Male	15-19 years	95+ years	Global	-1	3	LDI (I$ per capita)	--
Aortic aneurysm	Male	15-19 years	95+ years	Global	-1	3	Pulses legumes unadjusted(g)	--

CODEm covariates used, level of covariate, and expected direction of covariate by cause, sex, and age								
Cause	Sex	Age Start	Age End	Model Version Type	Direction	Level	Covariate Name	Number of Draws
Aortic aneurysm	Male	15-19 years	95+ years	Global	1	3	Age- and sex-specific SEV for Low vegetables	125
Aortic aneurysm	Male	15-19 years	95+ years	Global	1	3	Liters of alcohol consumed per capita	143
Aortic aneurysm	Male	15-19 years	95+ years	Global	1	3	Socio-demographic Index	308
Aortic aneurysm	Male	15-19 years	95+ years	Global	1	3	Age- and sex-specific SEV for Low PUFA	--
Aortic aneurysm	Male	15-19 years	95+ years	Global	1	3	Age- and sex-specific SEV for Low fruit	--
Aortic aneurysm	Male	15-19 years	95+ years	Global	1	3	Age- and sex-specific SEV for Low nuts and seeds	--
Aortic aneurysm	Male	15-19 years	95+ years	Global	1	3	Age- and sex-specific SEV for Low omega-3	--
Lower extremity peripheral arterial disease	Female	40-44 years	95+ years	Data Rich	1	1	Log-transformed SEV scalar: PAD	230
Lower extremity peripheral arterial disease	Female	40-44 years	95+ years	Data Rich	1	1	Systolic Blood Pressure (mmHg)	230
Lower extremity peripheral arterial disease	Female	40-44 years	95+ years	Data Rich	1	1	Smoking Prevalence	388
Lower extremity peripheral arterial disease	Female	40-44 years	95+ years	Data Rich	1	1	Low-Density Lipoprotein (mmol/L)	--
Lower extremity peripheral arterial disease	Female	40-44 years	95+ years	Data Rich	-1	2	Healthcare access and quality index	57
Lower extremity peripheral arterial disease	Female	40-44 years	95+ years	Data Rich	1	2	Diabetes Fasting Plasma Glucose (mmol/L), age-standardized 25+	382
Lower extremity peripheral arterial disease	Female	40-44 years	95+ years	Data Rich	1	2	Mean BMI	644
Lower extremity peripheral arterial disease	Female	40-44 years	95+ years	Data Rich	-1	3	LDI (I$ per capita)	--
Lower extremity peripheral arterial disease	Female	40-44 years	95+ years	Data Rich	-1	3	Pulses legumes unadjusted(g)	--
Lower extremity peripheral arterial disease	Female	40-44 years	95+ years	Data Rich	1	3	Age- and sex-specific SEV for Low fruit	119
Lower extremity peripheral arterial disease	Female	40-44 years	95+ years	Data Rich	1	3	Age- and sex-specific SEV for Low nuts and seeds	119
Lower extremity peripheral arterial disease	Female	40-44 years	95+ years	Data Rich	1	3	Age- and sex-specific SEV for Low vegetables	218
Lower extremity peripheral arterial disease	Female	40-44 years	95+ years	Data Rich	1	3	Socio-demographic Index	258
Lower extremity peripheral arterial disease	Female	40-44 years	95+ years	Data Rich	1	3	Age- and sex-specific SEV for Low PUFA	--
Lower extremity peripheral arterial disease	Female	40-44 years	95+ years	Data Rich	1	3	Age- and sex-specific SEV for Low omega-3	--
Lower extremity peripheral arterial disease	Female	40-44 years	95+ years	Data Rich	1	3	Liters of alcohol consumed per capita	--
Lower extremity peripheral arterial disease	Female	40-44 years	95+ years	Global	1	1	Smoking Prevalence	125
Lower extremity peripheral arterial disease	Female	40-44 years	95+ years	Global	1	1	Systolic Blood Pressure (mmHg)	212
Lower extremity peripheral arterial disease	Female	40-44 years	95+ years	Global	1	1	Log-transformed SEV scalar: PAD	458
Lower extremity peripheral arterial disease	Female	40-44 years	95+ years	Global	1	1	Low-Density Lipoprotein (mmol/L)	--
Lower extremity peripheral arterial disease	Female	40-44 years	95+ years	Global	-1	2	Healthcare access and quality index	--
Lower extremity peripheral arterial disease	Female	40-44 years	95+ years	Global	1	2	Diabetes Fasting Plasma Glucose (mmol/L), age-standardized 25+	257
Lower extremity peripheral arterial disease	Female	40-44 years	95+ years	Global	1	2	Mean BMI	349
Lower extremity peripheral arterial disease	Female	40-44 years	95+ years	Global	-1	3	Pulses legumes unadjusted(g)	144
Lower extremity peripheral arterial disease	Female	40-44 years	95+ years	Global	-1	3	LDI (I$ per capita)	--
Lower extremity peripheral arterial disease	Female	40-44 years	95+ years	Global	1	3	Age- and sex-specific SEV for Low PUFA	141
Lower extremity peripheral arterial disease	Female	40-44 years	95+ years	Global	1	3	Age- and sex-specific SEV for Low fruit	215
Lower extremity peripheral arterial disease	Female	40-44 years	95+ years	Global	1	3	Age- and sex-specific SEV for Low nuts and seeds	279
Lower extremity peripheral arterial disease	Female	40-44 years	95+ years	Global	1	3	Age- and sex-specific SEV for Low vegetables	348
Lower extremity peripheral arterial disease	Female	40-44 years	95+ years	Global	1	3	Socio-demographic Index	474
Lower extremity peripheral arterial disease	Female	40-44 years	95+ years	Global	1	3	Age- and sex-specific SEV for Low omega-3	--
Lower extremity peripheral arterial disease	Female	40-44 years	95+ years	Global	1	3	Liters of alcohol consumed per capita	--
Lower extremity peripheral arterial disease	Male	40-44 years	95+ years	Data Rich	1	1	Smoking Prevalence	135
Lower extremity peripheral arterial disease	Male	40-44 years	95+ years	Data Rich	1	1	Systolic Blood Pressure (mmHg)	175
Lower extremity peripheral arterial disease	Male	40-44 years	95+ years	Data Rich	1	1	Log-transformed SEV scalar: PAD	749
Lower extremity peripheral arterial disease	Male	40-44 years	95+ years	Data Rich	1	1	Low-Density Lipoprotein (mmol/L)	--
Lower extremity peripheral arterial disease	Male	40-44 years	95+ years	Data Rich	-1	2	Healthcare access and quality index	54
Lower extremity peripheral arterial disease	Male	40-44 years	95+ years	Data Rich	1	2	Mean BMI	238
Lower extremity peripheral arterial disease	Male	40-44 years	95+ years	Data Rich	1	2	Diabetes Fasting Plasma Glucose (mmol/L), age-standardized 25+	457
Lower extremity peripheral arterial disease	Male	40-44 years	95+ years	Data Rich	-1	3	LDI (I$ per capita)	--
Lower extremity peripheral arterial disease	Male	40-44 years	95+ years	Data Rich	-1	3	Pulses legumes unadjusted(g)	--
Lower extremity peripheral arterial disease	Male	40-44 years	95+ years	Data Rich	1	3	Age- and sex-specific SEV for Low nuts and seeds	34
Lower extremity peripheral arterial disease	Male	40-44 years	95+ years	Data Rich	1	3	Liters of alcohol consumed per capita	44
Lower extremity peripheral arterial disease	Male	40-44 years	95+ years	Data Rich	1	3	Age- and sex-specific SEV for Low vegetables	151
Lower extremity peripheral arterial disease	Male	40-44 years	95+ years	Data Rich	1	3	Socio-demographic Index	388
Lower extremity peripheral arterial disease	Male	40-44 years	95+ years	Data Rich	1	3	Age- and sex-specific SEV for Low PUFA	--
Lower extremity peripheral arterial disease	Male	40-44 years	95+ years	Data Rich	1	3	Age- and sex-specific SEV for Low fruit	--
Lower extremity peripheral arterial disease	Male	40-44 years	95+ years	Data Rich	1	3	Age- and sex-specific SEV for Low omega-3	--
Lower extremity peripheral arterial disease	Male	40-44 years	95+ years	Global	1	1	Systolic Blood Pressure (mmHg)	46
Lower extremity peripheral arterial disease	Male	40-44 years	95+ years	Global	1	1	Log-transformed SEV scalar: PAD	193
Lower extremity peripheral arterial disease	Male	40-44 years	95+ years	Global	1	1	Smoking Prevalence	922
Lower extremity peripheral arterial disease	Male	40-44 years	95+ years	Global	1	1	Low-Density Lipoprotein (mmol/L)	--
Lower extremity peripheral arterial disease	Male	40-44 years	95+ years	Global	-1	2	Healthcare access and quality index	--
Lower extremity peripheral arterial disease	Male	40-44 years	95+ years	Global	1	2	Mean BMI	311
Lower extremity peripheral arterial disease	Male	40-44 years	95+ years	Global	1	2	Diabetes Fasting Plasma Glucose (mmol/L), age-standardized 25+	461
Lower extremity peripheral arterial disease	Male	40-44 years	95+ years	Global	-1	3	LDI (I$ per capita)	--
Lower extremity peripheral arterial disease	Male	40-44 years	95+ years	Global	-1	3	Pulses legumes unadjusted(g)	--
Lower extremity peripheral arterial disease	Male	40-44 years	95+ years	Global	1	3	Age- and sex-specific SEV for Low nuts and seeds	10
Lower extremity peripheral arterial disease	Male	40-44 years	95+ years	Global	1	3	Age- and sex-specific SEV for Low omega-3	10
Lower extremity peripheral arterial disease	Male	40-44 years	95+ years	Global	1	3	Age- and sex-specific SEV for Low vegetables	31
Lower extremity peripheral arterial disease	Male	40-44 years	95+ years	Global	1	3	Liters of alcohol consumed per capita	262
Lower extremity peripheral arterial disease	Male	40-44 years	95+ years	Global	1	3	Socio-demographic Index	293
Lower extremity peripheral arterial disease	Male	40-44 years	95+ years	Global	1	3	Age- and sex-specific SEV for Low PUFA	--
Lower extremity peripheral arterial disease	Male	40-44 years	95+ years	Global	1	3	Age- and sex-specific SEV for Low fruit	--
Endocarditis	Female	0-6 days	95+ years	Data Rich	1	1	Sanitation (proportion with access)	265
Endocarditis	Female	0-6 days	95+ years	Data Rich	1	1	Log-transformed SEV scalar: Endocar	427
Endocarditis	Female	0-6 days	95+ years	Data Rich	1	1	Improved Water Source (proportion with access)	681
Endocarditis	Female	0-6 days	95+ years	Data Rich	-1	2	Healthcare access and quality index	--
Endocarditis	Female	0-6 days	95+ years	Data Rich	-1	3	LDI (I$ per capita)	--
Endocarditis	Female	0-6 days	95+ years	Data Rich	-1	3	Socio-demographic Index	--
Endocarditis	Female	0-6 days	95+ years	Global	1	1	Sanitation (proportion with access)	328
Endocarditis	Female	0-6 days	95+ years	Global	1	1	Improved Water Source (proportion with access)	--
Endocarditis	Female	0-6 days	95+ years	Global	1	1	Log-transformed SEV scalar: Endocar	--
Endocarditis	Female	0-6 days	95+ years	Global	-1	2	Healthcare access and quality index	--
Endocarditis	Female	0-6 days	95+ years	Global	-1	3	Socio-demographic Index	398
Endocarditis	Female	0-6 days	95+ years	Global	-1	3	LDI (I$ per capita)	--
Endocarditis	Male	0-6 days	95+ years	Data Rich	1	1	Log-transformed SEV scalar: Endocar	425
Endocarditis	Male	0-6 days	95+ years	Data Rich	1	1	Improved Water Source (proportion with access)	526
Endocarditis	Male	0-6 days	95+ years	Data Rich	1	1	Sanitation (proportion with access)	581
Endocarditis	Male	0-6 days	95+ years	Data Rich	-1	2	Healthcare access and quality index	--
Endocarditis	Male	0-6 days	95+ years	Data Rich	-1	3	LDI (I$ per capita)	--
Endocarditis	Male	0-6 days	95+ years	Data Rich	-1	3	Socio-demographic Index	--
Endocarditis	Male	0-6 days	95+ years	Global	1	1	Improved Water Source (proportion with access)	--
Endocarditis	Male	0-6 days	95+ years	Global	1	1	Log-transformed SEV scalar: Endocar	--
Endocarditis	Male	0-6 days	95+ years	Global	1	1	Sanitation (proportion with access)	--
Endocarditis	Male	0-6 days	95+ years	Global	-1	2	Healthcare access and quality index	454
Endocarditis	Male	0-6 days	95+ years	Global	-1	3	Socio-demographic Index	546
Endocarditis	Male	0-6 days	95+ years	Global	-1	3	LDI (I$ per capita)	--
Other cardiovascular and circulatory diseases	Female	0-6 days	95+ years	Data Rich	1	1	Log-transformed SEV scalar: Oth Cardio	--
Other cardiovascular and circulatory diseases	Female	0-6 days	95+ years	Data Rich	1	1	Low-Density Lipoprotein (mmol/L)	--
Other cardiovascular and circulatory diseases	Female	0-6 days	95+ years	Data Rich	1	1	Smoking Prevalence	--
Other cardiovascular and circulatory diseases	Female	0-6 days	95+ years	Data Rich	1	1	Systolic Blood Pressure (mmHg)	--
Other cardiovascular and circulatory diseases	Female	0-6 days	95+ years	Data Rich	-1	2	Elevation Over 1500m (proportion)	--
Other cardiovascular and circulatory diseases	Female	0-6 days	95+ years	Data Rich	-1	2	Healthcare access and quality index	--
Other cardiovascular and circulatory diseases	Female	0-6 days	95+ years	Data Rich	1	2	Diabetes Fasting Plasma Glucose (mmol/L), age-standardized 25+	160
Other cardiovascular and circulatory diseases	Female	0-6 days	95+ years	Data Rich	1	2	Indoor Air Pollution (All Cooking Fuels)	410
Other cardiovascular and circulatory diseases	Female	0-6 days	95+ years	Data Rich	1	2	Mean BMI	410
Other cardiovascular and circulatory diseases	Female	0-6 days	95+ years	Data Rich	1	2	Outdoor Air Pollution (PM2.5)	--
Other cardiovascular and circulatory diseases	Female	0-6 days	95+ years	Data Rich	-1	3	Socio-demographic Index	244
Other cardiovascular and circulatory diseases	Female	0-6 days	95+ years	Data Rich	-1	3	LDI (I$ per capita)	--
Other cardiovascular and circulatory diseases	Female	0-6 days	95+ years	Data Rich	1	3	Age- and sex-specific SEV for Low fruit	192
Other cardiovascular and circulatory diseases	Female	0-6 days	95+ years	Data Rich	1	3	Liters of alcohol consumed per capita	198
Other cardiovascular and circulatory diseases	Female	0-6 days	95+ years	Data Rich	1	3	Age- and sex-specific SEV for Low legumes	256
Other cardiovascular and circulatory diseases	Female	0-6 days	95+ years	Data Rich	1	3	Age- and sex-specific SEV for Low vegetables	466
Other cardiovascular and circulatory diseases	Female	0-6 days	95+ years	Data Rich	1	3	Age- and sex-specific SEV for Low PUFA	--
Other cardiovascular and circulatory diseases	Female	0-6 days	95+ years	Data Rich	1	3	Age- and sex-specific SEV for Low omega-3	--

CODEm covariates used, level of covariate, and expected direction of covariate by cause, sex, and age

Cause	Sex	Age Start	Age End	Model Version Type	Direction	Level	Covariate Name	Number of Draws
Other cardiovascular and circulatory diseases	Female	0-6 days	95+ years	Global	1	1	Systolic Blood Pressure (mmHg)	157
Other cardiovascular and circulatory diseases	Female	0-6 days	95+ years	Global	1	1	Smoking Prevalence	289
Other cardiovascular and circulatory diseases	Female	0-6 days	95+ years	Global	1	1	Log-transformed SEV scalar: Oth Cardio	--
Other cardiovascular and circulatory diseases	Female	0-6 days	95+ years	Global	1	1	Low-Density Lipoprotein (mmol/L)	--
Other cardiovascular and circulatory diseases	Female	0-6 days	95+ years	Global	-1	2	Elevation Over 1500m (proportion)	--
Other cardiovascular and circulatory diseases	Female	0-6 days	95+ years	Global	-1	2	Healthcare access and quality index	--
Other cardiovascular and circulatory diseases	Female	0-6 days	95+ years	Global	1	2	Diabetes Fasting Plasma Glucose (mmol/L), age-standardized 25+	451
Other cardiovascular and circulatory diseases	Female	0-6 days	95+ years	Global	1	2	Mean BMI	643
Other cardiovascular and circulatory diseases	Female	0-6 days	95+ years	Global	1	2	Indoor Air Pollution (All Cooking Fuels)	--
Other cardiovascular and circulatory diseases	Female	0-6 days	95+ years	Global	1	2	Outdoor Air Pollution (PM2.5)	--
Other cardiovascular and circulatory diseases	Female	0-6 days	95+ years	Global	-1	3	Age- and sex-specific SEV for Low nuts and seeds	137
Other cardiovascular and circulatory diseases	Female	0-6 days	95+ years	Global	-1	3	Socio-demographic Index	338
Other cardiovascular and circulatory diseases	Female	0-6 days	95+ years	Global	-1	3	LDI (I$ per capita)	--
Other cardiovascular and circulatory diseases	Female	0-6 days	95+ years	Global	1	3	Age- and sex-specific SEV for Low legumes	81
Other cardiovascular and circulatory diseases	Female	0-6 days	95+ years	Global	1	3	Liters of alcohol consumed per capita	137
Other cardiovascular and circulatory diseases	Female	0-6 days	95+ years	Global	1	3	Age- and sex-specific SEV for Low fruit	339
Other cardiovascular and circulatory diseases	Female	0-6 days	95+ years	Global	1	3	Age- and sex-specific SEV for Low vegetables	419
Other cardiovascular and circulatory diseases	Female	0-6 days	95+ years	Global	1	3	Age- and sex-specific SEV for Low PUFA	--
Other cardiovascular and circulatory diseases	Female	0-6 days	95+ years	Global	1	3	Age- and sex-specific SEV for Low omega-3	--
Other cardiovascular and circulatory diseases	Male	0-6 days	95+ years	Data Rich	1	1	Log-transformed SEV scalar: Oth Cardio	219
Other cardiovascular and circulatory diseases	Male	0-6 days	95+ years	Data Rich	1	1	Smoking Prevalence	219
Other cardiovascular and circulatory diseases	Male	0-6 days	95+ years	Data Rich	1	1	Systolic Blood Pressure (mmHg)	219
Other cardiovascular and circulatory diseases	Male	0-6 days	95+ years	Data Rich	1	1	Low-Density Lipoprotein (mmol/L)	--
Other cardiovascular and circulatory diseases	Male	0-6 days	95+ years	Data Rich	-1	2	Elevation Over 1500m (proportion)	--
Other cardiovascular and circulatory diseases	Male	0-6 days	95+ years	Data Rich	-1	2	Healthcare access and quality index	--
Other cardiovascular and circulatory diseases	Male	0-6 days	95+ years	Data Rich	1	2	Mean BMI	229
Other cardiovascular and circulatory diseases	Male	0-6 days	95+ years	Data Rich	1	2	Diabetes Fasting Plasma Glucose (mmol/L), age-standardized 25+	330
Other cardiovascular and circulatory diseases	Male	0-6 days	95+ years	Data Rich	1	2	Indoor Air Pollution (All Cooking Fuels)	--
Other cardiovascular and circulatory diseases	Male	0-6 days	95+ years	Data Rich	1	2	Outdoor Air Pollution (PM2.5)	--
Other cardiovascular and circulatory diseases	Male	0-6 days	95+ years	Data Rich	-1	3	LDI (I$ per capita)	--
Other cardiovascular and circulatory diseases	Male	0-6 days	95+ years	Data Rich	-1	3	Socio-demographic Index	--
Other cardiovascular and circulatory diseases	Male	0-6 days	95+ years	Data Rich	1	3	Age- and sex-specific SEV for Low fruit	125
Other cardiovascular and circulatory diseases	Male	0-6 days	95+ years	Data Rich	1	3	Age- and sex-specific SEV for Low legumes	125
Other cardiovascular and circulatory diseases	Male	0-6 days	95+ years	Data Rich	1	3	Age- and sex-specific SEV for Low omega-3	125
Other cardiovascular and circulatory diseases	Male	0-6 days	95+ years	Data Rich	1	3	Liters of alcohol consumed per capita	210
Other cardiovascular and circulatory diseases	Male	0-6 days	95+ years	Data Rich	1	3	Age- and sex-specific SEV for Low vegetables	459
Other cardiovascular and circulatory diseases	Male	0-6 days	95+ years	Data Rich	1	3	Age- and sex-specific SEV for Low PUFA	--
Other cardiovascular and circulatory diseases	Male	0-6 days	95+ years	Global	1	1	Smoking Prevalence	26
Other cardiovascular and circulatory diseases	Male	0-6 days	95+ years	Global	1	1	Systolic Blood Pressure (mmHg)	83
Other cardiovascular and circulatory diseases	Male	0-6 days	95+ years	Global	1	1	Log-transformed SEV scalar: Oth Cardio	401
Other cardiovascular and circulatory diseases	Male	0-6 days	95+ years	Global	1	1	Low-Density Lipoprotein (mmol/L)	--
Other cardiovascular and circulatory diseases	Male	0-6 days	95+ years	Global	-1	2	Healthcare access and quality index	297
Other cardiovascular and circulatory diseases	Male	0-6 days	95+ years	Global	-1	2	Elevation Over 1500m (proportion)	--
Other cardiovascular and circulatory diseases	Male	0-6 days	95+ years	Global	1	2	Indoor Air Pollution (All Cooking Fuels)	50
Other cardiovascular and circulatory diseases	Male	0-6 days	95+ years	Global	1	2	Diabetes Fasting Plasma Glucose (mmol/L), age-standardized 25+	337
Other cardiovascular and circulatory diseases	Male	0-6 days	95+ years	Global	1	2	Mean BMI	--
Other cardiovascular and circulatory diseases	Male	0-6 days	95+ years	Global	1	2	Outdoor Air Pollution (PM2.5)	--
Other cardiovascular and circulatory diseases	Male	0-6 days	95+ years	Global	-1	3	Socio-demographic Index	66
Other cardiovascular and circulatory diseases	Male	0-6 days	95+ years	Global	-1	3	Age- and sex-specific SEV for Low nuts and seeds	150
Other cardiovascular and circulatory diseases	Male	0-6 days	95+ years	Global	-1	3	LDI (I$ per capita)	--
Other cardiovascular and circulatory diseases	Male	0-6 days	95+ years	Global	1	3	Age- and sex-specific SEV for Low fruit	7
Other cardiovascular and circulatory diseases	Male	0-6 days	95+ years	Global	1	3	Age- and sex-specific SEV for Low legumes	22
Other cardiovascular and circulatory diseases	Male	0-6 days	95+ years	Global	1	3	Liters of alcohol consumed per capita	380
Other cardiovascular and circulatory diseases	Male	0-6 days	95+ years	Global	1	3	Age- and sex-specific SEV for Low vegetables	400
Other cardiovascular and circulatory diseases	Male	0-6 days	95+ years	Global	1	3	Age- and sex-specific SEV for Low PUFA	--
Other cardiovascular and circulatory diseases	Male	0-6 days	95+ years	Global	1	3	Age- and sex-specific SEV for Low omega-3	--
Chronic respiratory diseases	Female	0-6 days	95+ years	Data Rich	1	1	Cumulative Cigarettes (10 Years)	927
Chronic respiratory diseases	Female	0-6 days	95+ years	Data Rich	1	1	Cumulative Cigarettes (5 Years)	--
Chronic respiratory diseases	Female	0-6 days	95+ years	Data Rich	-1	2	Healthcare access and quality index	--
Chronic respiratory diseases	Female	0-6 days	95+ years	Data Rich	1	2	Elevation Over 1500m (proportion)	220
Chronic respiratory diseases	Female	0-6 days	95+ years	Data Rich	1	2	Outdoor Air Pollution (PM2.5)	220
Chronic respiratory diseases	Female	0-6 days	95+ years	Data Rich	1	2	Indoor Air Pollution (All Cooking Fuels)	373
Chronic respiratory diseases	Female	0-6 days	95+ years	Data Rich	1	2	Smoking Prevalence	--
Chronic respiratory diseases	Female	0-6 days	95+ years	Data Rich	-1	3	Education (years per capita)	--
Chronic respiratory diseases	Female	0-6 days	95+ years	Data Rich	-1	3	LDI (I$ per capita)	--
Chronic respiratory diseases	Female	0-6 days	95+ years	Data Rich	-1	3	Socio-demographic Index	--
Chronic respiratory diseases	Female	0-6 days	95+ years	Data Rich	1	3	Population Density (over 1000 ppl/sqkm, proportion)	73
Chronic respiratory diseases	Female	0-6 days	95+ years	Data Rich	1	3	Elevation 500 to 1500m (proportion)	314
Chronic respiratory diseases	Female	0-6 days	95+ years	Global	1	1	Cumulative Cigarettes (10 Years)	98
Chronic respiratory diseases	Female	0-6 days	95+ years	Global	1	1	Cumulative Cigarettes (5 Years)	183
Chronic respiratory diseases	Female	0-6 days	95+ years	Global	1	1	Healthcare access and quality index	--
Chronic respiratory diseases	Female	0-6 days	95+ years	Global	1	2	Elevation Over 1500m (proportion)	433
Chronic respiratory diseases	Female	0-6 days	95+ years	Global	1	2	Indoor Air Pollution (All Cooking Fuels)	484
Chronic respiratory diseases	Female	0-6 days	95+ years	Global	1	2	Outdoor Air Pollution (PM2.5)	--
Chronic respiratory diseases	Female	0-6 days	95+ years	Global	1	2	Smoking Prevalence	--
Chronic respiratory diseases	Female	0-6 days	95+ years	Global	-1	3	Socio-demographic Index	102
Chronic respiratory diseases	Female	0-6 days	95+ years	Global	-1	3	Education (years per capita)	156
Chronic respiratory diseases	Female	0-6 days	95+ years	Global	-1	3	LDI (I$ per capita)	--
Chronic respiratory diseases	Female	0-6 days	95+ years	Global	1	3	Population Density (over 1000 ppl/sqkm, proportion)	190
Chronic respiratory diseases	Female	0-6 days	95+ years	Global	1	3	Elevation 500 to 1500m (proportion)	553
Chronic respiratory diseases	Male	0-6 days	95+ years	Data Rich	1	1	Cumulative Cigarettes (5 Years)	105
Chronic respiratory diseases	Male	0-6 days	95+ years	Data Rich	1	1	Cumulative Cigarettes (10 Years)	177
Chronic respiratory diseases	Male	0-6 days	95+ years	Data Rich	-1	2	Healthcare access and quality index	--
Chronic respiratory diseases	Male	0-6 days	95+ years	Data Rich	1	2	Outdoor Air Pollution (PM2.5)	28
Chronic respiratory diseases	Male	0-6 days	95+ years	Data Rich	1	2	Smoking Prevalence	143
Chronic respiratory diseases	Male	0-6 days	95+ years	Data Rich	1	2	Indoor Air Pollution (All Cooking Fuels)	495
Chronic respiratory diseases	Male	0-6 days	95+ years	Data Rich	1	2	Elevation Over 1500m (proportion)	599
Chronic respiratory diseases	Male	0-6 days	95+ years	Data Rich	-1	3	Education (years per capita)	24
Chronic respiratory diseases	Male	0-6 days	95+ years	Data Rich	-1	3	Socio-demographic Index	113
Chronic respiratory diseases	Male	0-6 days	95+ years	Data Rich	-1	3	LDI (I$ per capita)	--
Chronic respiratory diseases	Male	0-6 days	95+ years	Data Rich	1	3	Elevation 500 to 1500m (proportion)	304
Chronic respiratory diseases	Male	0-6 days	95+ years	Data Rich	1	3	Population Density (over 1000 ppl/sqkm, proportion)	--
Chronic respiratory diseases	Male	0-6 days	95+ years	Global	1	1	Cumulative Cigarettes (10 Years)	182
Chronic respiratory diseases	Male	0-6 days	95+ years	Global	1	1	Cumulative Cigarettes (5 Years)	266
Chronic respiratory diseases	Male	0-6 days	95+ years	Global	1	1	Smoking Prevalence	273
Chronic respiratory diseases	Male	0-6 days	95+ years	Global	1	1	Indoor Air Pollution (All Cooking Fuels)	609
Chronic respiratory diseases	Male	0-6 days	95+ years	Global	-1	2	Healthcare access and quality index	193
Chronic respiratory diseases	Male	0-6 days	95+ years	Global	1	2	Outdoor Air Pollution (PM2.5)	19
Chronic respiratory diseases	Male	0-6 days	95+ years	Global	1	2	Elevation Over 1500m (proportion)	138
Chronic respiratory diseases	Male	0-6 days	95+ years	Global	-1	3	Education (years per capita)	209
Chronic respiratory diseases	Male	0-6 days	95+ years	Global	-1	3	Socio-demographic Index	233
Chronic respiratory diseases	Male	0-6 days	95+ years	Global	-1	3	LDI (I$ per capita)	--
Chronic respiratory diseases	Male	0-6 days	95+ years	Global	1	3	Elevation 500 to 1500m (proportion)	450
Chronic respiratory diseases	Male	0-6 days	95+ years	Global	1	3	Population Density (over 1000 ppl/sqkm, proportion)	--
Chronic obstructive pulmonary disease	Female	12-23 months	95+ years	Data Rich	-1	1	Healthcare access and quality index	111
Chronic obstructive pulmonary disease	Female	12-23 months	95+ years	Data Rich	1	1	Cumulative Cigarettes (10 Years)	7
Chronic obstructive pulmonary disease	Female	12-23 months	95+ years	Data Rich	1	1	Elevation Over 1500m (proportion)	544
Chronic obstructive pulmonary disease	Female	12-23 months	95+ years	Data Rich	1	1	Log-transformed SEV scalar: COPD	659
Chronic obstructive pulmonary disease	Female	12-23 months	95+ years	Data Rich	1	1	Cumulative Cigarettes (5 Years)	--
Chronic obstructive pulmonary disease	Female	12-23 months	95+ years	Data Rich	1	1	Outdoor Air Pollution (PM2.5)	--
Chronic obstructive pulmonary disease	Female	12-23 months	95+ years	Data Rich	1	2	Smoking Prevalence	127
Chronic obstructive pulmonary disease	Female	12-23 months	95+ years	Data Rich	1	2	Indoor Air Pollution (All Cooking Fuels)	545
Chronic obstructive pulmonary disease	Female	12-23 months	95+ years	Data Rich	-1	3	Socio-demographic Index	27
Chronic obstructive pulmonary disease	Female	12-23 months	95+ years	Data Rich	-1	3	Education (years per capita)	--
Chronic obstructive pulmonary disease	Female	12-23 months	95+ years	Data Rich	-1	3	LDI (I$ per capita)	--
Chronic obstructive pulmonary disease	Female	12-23 months	95+ years	Global	-1	1	Healthcare access and quality index	172
Chronic obstructive pulmonary disease	Female	12-23 months	95+ years	Global	1	1	Cumulative Cigarettes (10 Years)	0

CODEm covariates used, level of covariate, and expected direction of covariate by cause, sex, and age

Cause	Sex	Age Start	Age End	Model Version Type	Direction	Level	Covariate Name	Number of Draws
Chronic obstructive pulmonary disease	Female	12-23 months	95+ years	Global	1	1	Cumulative Cigarettes (5 Years)	0
Chronic obstructive pulmonary disease	Female	12-23 months	95+ years	Global	1	1	Log-transformed SEV scalar: COPD	485
Chronic obstructive pulmonary disease	Female	12-23 months	95+ years	Global	1	1	Elevation Over 1500m (proportion)	940
Chronic obstructive pulmonary disease	Female	12-23 months	95+ years	Global	1	1	Outdoor Air Pollution (PM2.5)	--
Chronic obstructive pulmonary disease	Female	12-23 months	95+ years	Global	1	2	Indoor Air Pollution (All Cooking Fuels)	364
Chronic obstructive pulmonary disease	Female	12-23 months	95+ years	Global	1	2	Smoking Prevalence	--
Chronic obstructive pulmonary disease	Female	12-23 months	95+ years	Global	-1	3	Education (years per capita)	18
Chronic obstructive pulmonary disease	Female	12-23 months	95+ years	Global	-1	3	Socio-demographic Index	154
Chronic obstructive pulmonary disease	Female	12-23 months	95+ years	Global	-1	3	LDI (I$ per capita)	--
Chronic obstructive pulmonary disease	Male	12-23 months	95+ years	Data Rich	1	1	Outdoor Air Pollution (PM2.5)	8
Chronic obstructive pulmonary disease	Male	12-23 months	95+ years	Data Rich	1	1	Cumulative Cigarettes (20 Years)	13
Chronic obstructive pulmonary disease	Male	12-23 months	95+ years	Data Rich	1	1	Cumulative Cigarettes (10 Years)	16
Chronic obstructive pulmonary disease	Male	12-23 months	95+ years	Data Rich	1	1	Cumulative Cigarettes (5 Years)	27
Chronic obstructive pulmonary disease	Male	12-23 months	95+ years	Data Rich	1	1	Log-transformed SEV scalar: COPD	420
Chronic obstructive pulmonary disease	Male	12-23 months	95+ years	Data Rich	1	1	Elevation Over 1500m (proportion)	771
Chronic obstructive pulmonary disease	Male	12-23 months	95+ years	Data Rich	-1	2	Healthcare access and quality index	118
Chronic obstructive pulmonary disease	Male	12-23 months	95+ years	Data Rich	1	2	Indoor Air Pollution (All Cooking Fuels)	210
Chronic obstructive pulmonary disease	Male	12-23 months	95+ years	Data Rich	1	2	Smoking Prevalence	430
Chronic obstructive pulmonary disease	Male	12-23 months	95+ years	Data Rich	-1	3	Education (years per capita)	12
Chronic obstructive pulmonary disease	Male	12-23 months	95+ years	Data Rich	-1	3	Socio-demographic Index	12
Chronic obstructive pulmonary disease	Male	12-23 months	95+ years	Data Rich	-1	3	LDI (I$ per capita)	--
Chronic obstructive pulmonary disease	Male	12-23 months	95+ years	Global	1	1	Log-transformed SEV scalar: COPD	84
Chronic obstructive pulmonary disease	Male	12-23 months	95+ years	Global	1	1	Cumulative Cigarettes (10 Years)	222
Chronic obstructive pulmonary disease	Male	12-23 months	95+ years	Global	1	1	Cumulative Cigarettes (5 Years)	232
Chronic obstructive pulmonary disease	Male	12-23 months	95+ years	Global	1	1	Cumulative Cigarettes (20 Years)	287
Chronic obstructive pulmonary disease	Male	12-23 months	95+ years	Global	1	1	Elevation Over 1500m (proportion)	386
Chronic obstructive pulmonary disease	Male	12-23 months	95+ years	Global	1	1	Outdoor Air Pollution (PM2.5)	--
Chronic obstructive pulmonary disease	Male	12-23 months	95+ years	Global	-1	2	Healthcare access and quality index	68
Chronic obstructive pulmonary disease	Male	12-23 months	95+ years	Global	1	2	Smoking Prevalence	53
Chronic obstructive pulmonary disease	Male	12-23 months	95+ years	Global	1	2	Indoor Air Pollution (All Cooking Fuels)	124
Chronic obstructive pulmonary disease	Male	12-23 months	95+ years	Global	-1	3	Socio-demographic Index	91
Chronic obstructive pulmonary disease	Male	12-23 months	95+ years	Global	-1	3	Education (years per capita)	462
Chronic obstructive pulmonary disease	Male	12-23 months	95+ years	Global	-1	3	LDI (I$ per capita)	--
Pneumoconiosis	Female	15-19 years	95+ years	Data Rich	1	1	Age- and sex-specific SEV for Occupational asbestos	112
Pneumoconiosis	Female	15-19 years	95+ years	Data Rich	1	1	Asbestos consumption (metric tons per year per capita)	510
Pneumoconiosis	Female	15-19 years	95+ years	Data Rich	1	1	Log-transformed coal production (per capita)	622
Pneumoconiosis	Female	15-19 years	95+ years	Data Rich	1	1	Age- and sex-specific SEV for Occupational beryllium	--
Pneumoconiosis	Female	15-19 years	95+ years	Data Rich	1	1	Age- and sex-specific SEV for Occupational silica	--
Pneumoconiosis	Female	15-19 years	95+ years	Data Rich	1	1	Gold production (kg) per capita, smoothed with 20-year lag	--
Pneumoconiosis	Female	15-19 years	95+ years	Data Rich	-1	2	Healthcare access and quality index	6
Pneumoconiosis	Female	15-19 years	95+ years	Data Rich	1	2	Cumulative Cigarettes (5 Years)	--
Pneumoconiosis	Female	15-19 years	95+ years	Data Rich	1	2	Indoor Air Pollution (All Cooking Fuels)	--
Pneumoconiosis	Female	15-19 years	95+ years	Data Rich	1	2	Smoking Prevalence	--
Pneumoconiosis	Female	15-19 years	95+ years	Data Rich	-1	3	Education (years per capita)	22
Pneumoconiosis	Female	15-19 years	95+ years	Data Rich	-1	3	Socio-demographic Index	33
Pneumoconiosis	Female	15-19 years	95+ years	Data Rich	-1	3	LDI (I$ per capita)	--
Pneumoconiosis	Female	15-19 years	95+ years	Global	1	1	Log-transformed coal production (per capita)	463
Pneumoconiosis	Female	15-19 years	95+ years	Global	1	1	Asbestos consumption (metric tons per year per capita)	1000
Pneumoconiosis	Female	15-19 years	95+ years	Global	1	1	Gold production (kg) per capita, smoothed with 20-year lag	--
Pneumoconiosis	Female	15-19 years	95+ years	Global	-1	2	Healthcare access and quality index	--
Pneumoconiosis	Female	15-19 years	95+ years	Global	1	2	Cumulative Cigarettes (5 Years)	--
Pneumoconiosis	Female	15-19 years	95+ years	Global	1	2	Indoor Air Pollution (All Cooking Fuels)	--
Pneumoconiosis	Female	15-19 years	95+ years	Global	1	2	Smoking Prevalence	--
Pneumoconiosis	Female	15-19 years	95+ years	Global	-1	3	Education (years per capita)	120
Pneumoconiosis	Female	15-19 years	95+ years	Global	-1	3	Socio-demographic Index	144
Pneumoconiosis	Female	15-19 years	95+ years	Global	-1	3	LDI (I$ per capita)	--
Pneumoconiosis	Male	15-19 years	95+ years	Data Rich	1	1	Asbestos consumption (metric tons per year per capita)	0
Pneumoconiosis	Male	15-19 years	95+ years	Data Rich	1	1	Log-transformed coal production (per capita)	281
Pneumoconiosis	Male	15-19 years	95+ years	Data Rich	1	1	Age- and sex-specific SEV for Occupational asbestos	469
Pneumoconiosis	Male	15-19 years	95+ years	Data Rich	1	1	Age- and sex-specific SEV for Occupational silica	803
Pneumoconiosis	Male	15-19 years	95+ years	Data Rich	1	1	Age- and sex-specific SEV for Occupational beryllium	--
Pneumoconiosis	Male	15-19 years	95+ years	Data Rich	1	1	Gold production (kg) per capita, smoothed with 20-year lag	--
Pneumoconiosis	Male	15-19 years	95+ years	Data Rich	-1	2	Healthcare access and quality index	18
Pneumoconiosis	Male	15-19 years	95+ years	Data Rich	1	2	Indoor Air Pollution (All Cooking Fuels)	0
Pneumoconiosis	Male	15-19 years	95+ years	Data Rich	1	2	Smoking Prevalence	19
Pneumoconiosis	Male	15-19 years	95+ years	Data Rich	1	2	Cumulative Cigarettes (5 Years)	981
Pneumoconiosis	Male	15-19 years	95+ years	Data Rich	-1	3	Socio-demographic Index	90
Pneumoconiosis	Male	15-19 years	95+ years	Data Rich	-1	3	Education (years per capita)	186
Pneumoconiosis	Male	15-19 years	95+ years	Data Rich	-1	3	LDI (I$ per capita)	--
Pneumoconiosis	Male	15-19 years	95+ years	Global	1	1	Log-transformed coal production (per capita)	513
Pneumoconiosis	Male	15-19 years	95+ years	Global	1	1	Asbestos consumption (metric tons per year per capita)	567
Pneumoconiosis	Male	15-19 years	95+ years	Global	1	1	Gold production (kg) per capita, smoothed with 20-year lag	--
Pneumoconiosis	Male	15-19 years	95+ years	Global	-1	2	Healthcare access and quality index	6
Pneumoconiosis	Male	15-19 years	95+ years	Global	1	2	Indoor Air Pollution (All Cooking Fuels)	0
Pneumoconiosis	Male	15-19 years	95+ years	Global	1	2	Cumulative Cigarettes (5 Years)	294
Pneumoconiosis	Male	15-19 years	95+ years	Global	1	2	Smoking Prevalence	511
Pneumoconiosis	Male	15-19 years	95+ years	Global	-1	3	Education (years per capita)	4
Pneumoconiosis	Male	15-19 years	95+ years	Global	-1	3	Socio-demographic Index	57
Pneumoconiosis	Male	15-19 years	95+ years	Global	-1	3	LDI (I$ per capita)	--
Silicosis	Female	15-19 years	95+ years	Data Rich	1	1	Age- and sex-specific SEV for Occupational silica	--
Silicosis	Female	15-19 years	95+ years	Data Rich	1	1	Gold production (kg) per capita, smoothed with 20-year lag	--
Silicosis	Female	15-19 years	95+ years	Data Rich	-1	2	Healthcare access and quality index	103
Silicosis	Female	15-19 years	95+ years	Data Rich	1	2	Smoking Prevalence	22
Silicosis	Female	15-19 years	95+ years	Data Rich	1	2	Cumulative Cigarettes (5 Years)	215
Silicosis	Female	15-19 years	95+ years	Data Rich	1	2	Indoor Air Pollution (All Cooking Fuels)	--
Silicosis	Female	15-19 years	95+ years	Data Rich	-1	3	Socio-demographic Index	197
Silicosis	Female	15-19 years	95+ years	Data Rich	-1	3	Education (years per capita)	260
Silicosis	Female	15-19 years	95+ years	Data Rich	-1	3	LDI (I$ per capita)	--
Silicosis	Female	15-19 years	95+ years	Global	1	1	Gold production (kg) per capita, smoothed with 20-year lag	--
Silicosis	Female	15-19 years	95+ years	Global	-1	2	Healthcare access and quality index	70
Silicosis	Female	15-19 years	95+ years	Global	1	2	Cumulative Cigarettes (5 Years)	419
Silicosis	Female	15-19 years	95+ years	Global	1	2	Indoor Air Pollution (All Cooking Fuels)	--
Silicosis	Female	15-19 years	95+ years	Global	1	2	Smoking Prevalence	--
Silicosis	Female	15-19 years	95+ years	Global	-1	3	Education (years per capita)	135
Silicosis	Female	15-19 years	95+ years	Global	-1	3	Socio-demographic Index	218
Silicosis	Female	15-19 years	95+ years	Global	-1	3	LDI (I$ per capita)	--
Silicosis	Male	15-19 years	95+ years	Data Rich	1	1	Age- and sex-specific SEV for Occupational silica	--
Silicosis	Male	15-19 years	95+ years	Data Rich	1	1	Gold production (kg) per capita, smoothed with 20-year lag	--
Silicosis	Male	15-19 years	95+ years	Data Rich	-1	2	Healthcare access and quality index	611
Silicosis	Male	15-19 years	95+ years	Data Rich	1	2	Smoking Prevalence	80
Silicosis	Male	15-19 years	95+ years	Data Rich	1	2	Cumulative Cigarettes (5 Years)	106
Silicosis	Male	15-19 years	95+ years	Data Rich	1	2	Indoor Air Pollution (All Cooking Fuels)	--
Silicosis	Male	15-19 years	95+ years	Data Rich	-1	3	Socio-demographic Index	202
Silicosis	Male	15-19 years	95+ years	Data Rich	-1	3	Education (years per capita)	239
Silicosis	Male	15-19 years	95+ years	Data Rich	-1	3	LDI (I$ per capita)	--
Silicosis	Male	15-19 years	95+ years	Global	1	1	Gold production (kg) per capita, smoothed with 20-year lag	--
Silicosis	Male	15-19 years	95+ years	Global	-1	2	Healthcare access and quality index	439
Silicosis	Male	15-19 years	95+ years	Global	1	2	Smoking Prevalence	145
Silicosis	Male	15-19 years	95+ years	Global	1	2	Cumulative Cigarettes (5 Years)	--
Silicosis	Male	15-19 years	95+ years	Global	1	2	Indoor Air Pollution (All Cooking Fuels)	--
Silicosis	Male	15-19 years	95+ years	Global	-1	3	Socio-demographic Index	216
Silicosis	Male	15-19 years	95+ years	Global	-1	3	Education (years per capita)	283
Silicosis	Male	15-19 years	95+ years	Global	-1	3	LDI (I$ per capita)	--

CODEm covariates used, level of covariate, and expected direction of covariate by cause, sex, and age

Cause	Sex	Age Start	Age End	Model Version Type	Direction	Level	Covariate Name	Number of Draws
Asbestosis	Female	15-19 years	95+ years	Data Rich	1	1	Asbestos consumption (metric tons per year per capita)	1000
Asbestosis	Female	15-19 years	95+ years	Data Rich	-1	2	Healthcare access and quality index	--
Asbestosis	Female	15-19 years	95+ years	Data Rich	1	2	Cumulative Cigarettes (10 Years)	--
Asbestosis	Female	15-19 years	95+ years	Data Rich	1	2	Cumulative Cigarettes (5 Years)	--
Asbestosis	Female	15-19 years	95+ years	Data Rich	1	2	Elevation 500 to 1500m (proportion)	--
Asbestosis	Female	15-19 years	95+ years	Data Rich	1	2	Elevation Over 1500m (proportion)	--
Asbestosis	Female	15-19 years	95+ years	Data Rich	1	2	Indoor Air Pollution (All Cooking Fuels)	--
Asbestosis	Female	15-19 years	95+ years	Data Rich	1	2	Smoking Prevalence	--
Asbestosis	Female	15-19 years	95+ years	Data Rich	-1	3	Education (years per capita)	--
Asbestosis	Female	15-19 years	95+ years	Data Rich	-1	3	LDI (I$ per capita)	--
Asbestosis	Female	15-19 years	95+ years	Data Rich	-1	3	Socio-demographic Index	--
Asbestosis	Female	15-19 years	95+ years	Global	1	1	Asbestos consumption (metric tons per year per capita)	1000
Asbestosis	Female	15-19 years	95+ years	Global	-1	2	Healthcare access and quality index	--
Asbestosis	Female	15-19 years	95+ years	Global	1	2	Cumulative Cigarettes (10 Years)	--
Asbestosis	Female	15-19 years	95+ years	Global	1	2	Cumulative Cigarettes (5 Years)	--
Asbestosis	Female	15-19 years	95+ years	Global	1	2	Elevation 500 to 1500m (proportion)	--
Asbestosis	Female	15-19 years	95+ years	Global	1	2	Elevation Over 1500m (proportion)	--
Asbestosis	Female	15-19 years	95+ years	Global	1	2	Indoor Air Pollution (All Cooking Fuels)	--
Asbestosis	Female	15-19 years	95+ years	Global	1	2	Smoking Prevalence	--
Asbestosis	Female	15-19 years	95+ years	Global	-1	3	Education (years per capita)	--
Asbestosis	Female	15-19 years	95+ years	Global	-1	3	LDI (I$ per capita)	--
Asbestosis	Female	15-19 years	95+ years	Global	-1	3	Socio-demographic Index	--
Asbestosis	Male	15-19 years	95+ years	Data Rich	1	1	Asbestos consumption (metric tons per year per capita)	573
Asbestosis	Male	15-19 years	95+ years	Data Rich	1	1	Age- and sex-specific SEV for Occupational asbestos	--
Asbestosis	Male	15-19 years	95+ years	Data Rich	1	1	Smoking Prevalence	--
Asbestosis	Male	15-19 years	95+ years	Data Rich	-1	2	Healthcare access and quality index	--
Asbestosis	Male	15-19 years	95+ years	Data Rich	1	2	Elevation Over 1500m (proportion)	325
Asbestosis	Male	15-19 years	95+ years	Data Rich	1	2	Elevation 500 to 1500m (proportion)	427
Asbestosis	Male	15-19 years	95+ years	Data Rich	1	2	Cumulative Cigarettes (5 Years)	--
Asbestosis	Male	15-19 years	95+ years	Data Rich	1	2	Indoor Air Pollution (All Cooking Fuels)	--
Asbestosis	Male	15-19 years	95+ years	Data Rich	-1	3	Education (years per capita)	--
Asbestosis	Male	15-19 years	95+ years	Data Rich	-1	3	LDI (I$ per capita)	--
Asbestosis	Male	15-19 years	95+ years	Data Rich	-1	3	Socio-demographic Index	--
Asbestosis	Male	15-19 years	95+ years	Global	1	1	Asbestos consumption (metric tons per year per capita)	602
Asbestosis	Male	15-19 years	95+ years	Global	-1	2	Healthcare access and quality index	--
Asbestosis	Male	15-19 years	95+ years	Global	1	2	Elevation 500 to 1500m (proportion)	398
Asbestosis	Male	15-19 years	95+ years	Global	1	2	Elevation Over 1500m (proportion)	726
Asbestosis	Male	15-19 years	95+ years	Global	1	2	Cumulative Cigarettes (5 Years)	--
Asbestosis	Male	15-19 years	95+ years	Global	1	2	Indoor Air Pollution (All Cooking Fuels)	--
Asbestosis	Male	15-19 years	95+ years	Global	1	2	Smoking Prevalence	--
Asbestosis	Male	15-19 years	95+ years	Global	-1	3	Education (years per capita)	--
Asbestosis	Male	15-19 years	95+ years	Global	-1	3	LDI (I$ per capita)	--
Asbestosis	Male	15-19 years	95+ years	Global	-1	3	Socio-demographic Index	--
Coal workers pneumoconiosis	Female	15-19 years	95+ years	Data Rich	1	1	Log-transformed coal production (per capita)	290
Coal workers pneumoconiosis	Female	15-19 years	95+ years	Data Rich	-1	2	Healthcare access and quality index	394
Coal workers pneumoconiosis	Female	15-19 years	95+ years	Data Rich	1	2	Cumulative Cigarettes (5 Years)	29
Coal workers pneumoconiosis	Female	15-19 years	95+ years	Data Rich	1	2	Indoor Air Pollution (All Cooking Fuels)	--
Coal workers pneumoconiosis	Female	15-19 years	95+ years	Data Rich	1	2	Smoking Prevalence	--
Coal workers pneumoconiosis	Female	15-19 years	95+ years	Data Rich	-1	3	Socio-demographic Index	162
Coal workers pneumoconiosis	Female	15-19 years	95+ years	Data Rich	-1	3	Education (years per capita)	271
Coal workers pneumoconiosis	Female	15-19 years	95+ years	Data Rich	-1	3	LDI (I$ per capita)	--
Coal workers pneumoconiosis	Female	15-19 years	95+ years	Global	1	1	Log-transformed coal production (per capita)	1000
Coal workers pneumoconiosis	Female	15-19 years	95+ years	Global	-1	2	Healthcare access and quality index	529
Coal workers pneumoconiosis	Female	15-19 years	95+ years	Global	1	2	Cumulative Cigarettes (5 Years)	30
Coal workers pneumoconiosis	Female	15-19 years	95+ years	Global	1	2	Indoor Air Pollution (All Cooking Fuels)	30
Coal workers pneumoconiosis	Female	15-19 years	95+ years	Global	1	2	Smoking Prevalence	--
Coal workers pneumoconiosis	Female	15-19 years	95+ years	Global	-1	3	Socio-demographic Index	259
Coal workers pneumoconiosis	Female	15-19 years	95+ years	Global	-1	3	Education (years per capita)	304
Coal workers pneumoconiosis	Female	15-19 years	95+ years	Global	-1	3	LDI (I$ per capita)	--
Coal workers pneumoconiosis	Male	15-19 years	95+ years	Data Rich	1	1	Log-transformed coal production (per capita)	1000
Coal workers pneumoconiosis	Male	15-19 years	95+ years	Data Rich	-1	2	Healthcare access and quality index	830
Coal workers pneumoconiosis	Male	15-19 years	95+ years	Data Rich	1	2	Smoking Prevalence	265
Coal workers pneumoconiosis	Male	15-19 years	95+ years	Data Rich	1	2	Cumulative Cigarettes (5 Years)	642
Coal workers pneumoconiosis	Male	15-19 years	95+ years	Data Rich	1	2	Indoor Air Pollution (All Cooking Fuels)	--
Coal workers pneumoconiosis	Male	15-19 years	95+ years	Data Rich	-1	3	Education (years per capita)	37
Coal workers pneumoconiosis	Male	15-19 years	95+ years	Data Rich	-1	3	Socio-demographic Index	269
Coal workers pneumoconiosis	Male	15-19 years	95+ years	Data Rich	-1	3	LDI (I$ per capita)	--
Coal workers pneumoconiosis	Male	15-19 years	95+ years	Global	1	1	Log-transformed coal production (per capita)	1000
Coal workers pneumoconiosis	Male	15-19 years	95+ years	Global	-1	2	Healthcare access and quality index	347
Coal workers pneumoconiosis	Male	15-19 years	95+ years	Global	1	2	Indoor Air Pollution (All Cooking Fuels)	22
Coal workers pneumoconiosis	Male	15-19 years	95+ years	Global	1	2	Cumulative Cigarettes (5 Years)	279
Coal workers pneumoconiosis	Male	15-19 years	95+ years	Global	1	2	Smoking Prevalence	493
Coal workers pneumoconiosis	Male	15-19 years	95+ years	Global	-1	3	Education (years per capita)	115
Coal workers pneumoconiosis	Male	15-19 years	95+ years	Global	-1	3	Socio-demographic Index	265
Coal workers pneumoconiosis	Male	15-19 years	95+ years	Global	-1	3	LDI (I$ per capita)	--
Other pneumoconiosis	Female	15-19 years	95+ years	Data Rich	-1	1	Occupation Professionals	363
Other pneumoconiosis	Female	15-19 years	95+ years	Data Rich	1	1	Age- and sex-specific SEV for Occupational beryllium	631
Other pneumoconiosis	Female	15-19 years	95+ years	Data Rich	1	1	Tuberculosis prevalence (age-standardized)	844
Other pneumoconiosis	Female	15-19 years	95+ years	Data Rich	-1	2	Healthcare access and quality index	61
Other pneumoconiosis	Female	15-19 years	95+ years	Data Rich	1	2	Cumulative Cigarettes (5 Years)	143
Other pneumoconiosis	Female	15-19 years	95+ years	Data Rich	1	2	Indoor Air Pollution (All Cooking Fuels)	--
Other pneumoconiosis	Female	15-19 years	95+ years	Data Rich	1	2	Smoking Prevalence	--
Other pneumoconiosis	Female	15-19 years	95+ years	Data Rich	-1	3	Education (years per capita)	47
Other pneumoconiosis	Female	15-19 years	95+ years	Data Rich	-1	3	Socio-demographic Index	59
Other pneumoconiosis	Female	15-19 years	95+ years	Data Rich	-1	3	LDI (I$ per capita)	--
Other pneumoconiosis	Female	15-19 years	95+ years	Global	-1	1	Occupation Professionals	556
Other pneumoconiosis	Female	15-19 years	95+ years	Global	1	1	Tuberculosis prevalence (age-standardized)	630
Other pneumoconiosis	Female	15-19 years	95+ years	Global	-1	2	Healthcare access and quality index	52
Other pneumoconiosis	Female	15-19 years	95+ years	Global	1	2	Cumulative Cigarettes (5 Years)	200
Other pneumoconiosis	Female	15-19 years	95+ years	Global	1	2	Indoor Air Pollution (All Cooking Fuels)	--
Other pneumoconiosis	Female	15-19 years	95+ years	Global	1	2	Smoking Prevalence	--
Other pneumoconiosis	Female	15-19 years	95+ years	Global	-1	3	Socio-demographic Index	98
Other pneumoconiosis	Female	15-19 years	95+ years	Global	-1	3	Education (years per capita)	134
Other pneumoconiosis	Female	15-19 years	95+ years	Global	-1	3	LDI (I$ per capita)	--
Other pneumoconiosis	Male	15-19 years	95+ years	Data Rich	-1	1	Occupation Professionals	633
Other pneumoconiosis	Male	15-19 years	95+ years	Data Rich	1	1	Tuberculosis prevalence (age-standardized)	1000
Other pneumoconiosis	Male	15-19 years	95+ years	Data Rich	1	1	Age- and sex-specific SEV for Occupational beryllium	--
Other pneumoconiosis	Male	15-19 years	95+ years	Data Rich	-1	2	Healthcare access and quality index	--
Other pneumoconiosis	Male	15-19 years	95+ years	Data Rich	1	2	Smoking Prevalence	208
Other pneumoconiosis	Male	15-19 years	95+ years	Data Rich	1	2	Cumulative Cigarettes (5 Years)	--
Other pneumoconiosis	Male	15-19 years	95+ years	Data Rich	1	2	Indoor Air Pollution (All Cooking Fuels)	--
Other pneumoconiosis	Male	15-19 years	95+ years	Data Rich	-1	3	Socio-demographic Index	360
Other pneumoconiosis	Male	15-19 years	95+ years	Data Rich	-1	3	Education (years per capita)	--
Other pneumoconiosis	Male	15-19 years	95+ years	Data Rich	-1	3	LDI (I$ per capita)	--
Other pneumoconiosis	Male	15-19 years	95+ years	Global	-1	1	Occupation Professionals	552
Other pneumoconiosis	Male	15-19 years	95+ years	Global	1	1	Tuberculosis prevalence (age-standardized)	614
Other pneumoconiosis	Male	15-19 years	95+ years	Global	-1	2	Healthcare access and quality index	223
Other pneumoconiosis	Male	15-19 years	95+ years	Global	1	2	Cumulative Cigarettes (5 Years)	252
Other pneumoconiosis	Male	15-19 years	95+ years	Global	1	2	Smoking Prevalence	468
Other pneumoconiosis	Male	15-19 years	95+ years	Global	1	2	Indoor Air Pollution (All Cooking Fuels)	--
Other pneumoconiosis	Male	15-19 years	95+ years	Global	-1	3	Education (years per capita)	173
Other pneumoconiosis	Male	15-19 years	95+ years	Global	-1	3	Socio-demographic Index	265
Other pneumoconiosis	Male	15-19 years	95+ years	Global	-1	3	LDI (I$ per capita)	--
Asthma	Female	12-23 months	95+ years	Data Rich	-1	1	Healthcare access and quality index	829
Asthma	Female	12-23 months	95+ years	Data Rich	1	1	Log-transformed SEV scalar: Asthma	14
Asthma	Female	12-23 months	95+ years	Data Rich	1	1	Cumulative Cigarettes (5 Years)	70
Asthma	Female	12-23 months	95+ years	Data Rich	1	1	Cumulative Cigarettes (10 Years)	101
Asthma	Female	12-23 months	95+ years	Data Rich	1	2	Smoking Prevalence	0
Asthma	Female	12-23 months	95+ years	Data Rich	1	2	Outdoor Air Pollution (PM2.5)	46

CODEm covariates used, level of covariate, and expected direction of covariate by cause, sex, and age

Cause	Sex	Age Start	Age End	Model Version Type	Direction	Level	Covariate Name	Number of Draws
Asthma	Female	12-23 months	95+ years	Data Rich	1	2	Indoor Air Pollution (All Cooking Fuels)	499
Asthma	Female	12-23 months	95+ years	Data Rich	-1	3	Socio-demographic Index	253
Asthma	Female	12-23 months	95+ years	Data Rich	-1	3	Education (years per capita)	287
Asthma	Female	12-23 months	95+ years	Data Rich	-1	3	LDI (I$ per capita)	--
Asthma	Female	12-23 months	95+ years	Global	-1	1	Healthcare access and quality index	866
Asthma	Female	12-23 months	95+ years	Global	1	1	Cumulative Cigarettes (5 Years)	127
Asthma	Female	12-23 months	95+ years	Global	1	1	Cumulative Cigarettes (10 Years)	211
Asthma	Female	12-23 months	95+ years	Global	1	1	Log-transformed SEV scalar: Asthma	--
Asthma	Female	12-23 months	95+ years	Global	1	2	Indoor Air Pollution (All Cooking Fuels)	32
Asthma	Female	12-23 months	95+ years	Global	1	2	Outdoor Air Pollution (PM2.5)	45
Asthma	Female	12-23 months	95+ years	Global	1	2	Smoking Prevalence	51
Asthma	Female	12-23 months	95+ years	Global	-1	3	Socio-demographic Index	209
Asthma	Female	12-23 months	95+ years	Global	-1	3	Education (years per capita)	417
Asthma	Female	12-23 months	95+ years	Global	-1	3	LDI (I$ per capita)	--
Asthma	Male	12-23 months	95+ years	Data Rich	-1	1	Healthcare access and quality index	474
Asthma	Male	12-23 months	95+ years	Data Rich	1	1	Cumulative Cigarettes (5 Years)	167
Asthma	Male	12-23 months	95+ years	Data Rich	1	1	Cumulative Cigarettes (10 Years)	406
Asthma	Male	12-23 months	95+ years	Data Rich	1	1	Log-transformed SEV scalar: Asthma	--
Asthma	Male	12-23 months	95+ years	Data Rich	1	2	Smoking Prevalence	61
Asthma	Male	12-23 months	95+ years	Data Rich	1	2	Outdoor Air Pollution (PM2.5)	177
Asthma	Male	12-23 months	95+ years	Data Rich	1	2	Indoor Air Pollution (All Cooking Fuels)	183
Asthma	Male	12-23 months	95+ years	Data Rich	-1	3	Socio-demographic Index	251
Asthma	Male	12-23 months	95+ years	Data Rich	-1	3	Education (years per capita)	314
Asthma	Male	12-23 months	95+ years	Data Rich	-1	3	LDI (I$ per capita)	--
Asthma	Male	12-23 months	95+ years	Global	-1	1	Healthcare access and quality index	701
Asthma	Male	12-23 months	95+ years	Global	1	1	Cumulative Cigarettes (5 Years)	164
Asthma	Male	12-23 months	95+ years	Global	1	1	Cumulative Cigarettes (10 Years)	831
Asthma	Male	12-23 months	95+ years	Global	1	1	Log-transformed SEV scalar: Asthma	--
Asthma	Male	12-23 months	95+ years	Global	1	2	Indoor Air Pollution (All Cooking Fuels)	9
Asthma	Male	12-23 months	95+ years	Global	1	2	Outdoor Air Pollution (PM2.5)	53
Asthma	Male	12-23 months	95+ years	Global	1	2	Smoking Prevalence	444
Asthma	Male	12-23 months	95+ years	Global	-1	3	Socio-demographic Index	223
Asthma	Male	12-23 months	95+ years	Global	-1	3	Education (years per capita)	689
Asthma	Male	12-23 months	95+ years	Global	-1	3	LDI (I$ per capita)	--
Interstitial lung disease and pulmonary sarcoidosis	Female	12-23 months	95+ years	Data Rich	-1	1	Occupation Professionals	--
Interstitial lung disease and pulmonary sarcoidosis	Female	12-23 months	95+ years	Data Rich	1	1	Cumulative Cigarettes (10 Years)	--
Interstitial lung disease and pulmonary sarcoidosis	Female	12-23 months	95+ years	Data Rich	1	1	Cumulative Cigarettes (5 Years)	--
Interstitial lung disease and pulmonary sarcoidosis	Female	12-23 months	95+ years	Data Rich	1	1	Smoking Prevalence	--
Interstitial lung disease and pulmonary sarcoidosis	Female	12-23 months	95+ years	Data Rich	-1	2	Healthcare access and quality index	--
Interstitial lung disease and pulmonary sarcoidosis	Female	12-23 months	95+ years	Data Rich	1	2	Elevation 500 to 1500m (proportion)	1000
Interstitial lung disease and pulmonary sarcoidosis	Female	12-23 months	95+ years	Data Rich	1	2	Elevation Over 1500m (proportion)	--
Interstitial lung disease and pulmonary sarcoidosis	Female	12-23 months	95+ years	Data Rich	1	2	Indoor Air Pollution (All Cooking Fuels)	--
Interstitial lung disease and pulmonary sarcoidosis	Female	12-23 months	95+ years	Data Rich	1	2	Outdoor Air Pollution (PM2.5)	--
Interstitial lung disease and pulmonary sarcoidosis	Female	12-23 months	95+ years	Data Rich	-1	3	Education (years per capita)	--
Interstitial lung disease and pulmonary sarcoidosis	Female	12-23 months	95+ years	Data Rich	-1	3	LDI (I$ per capita)	--
Interstitial lung disease and pulmonary sarcoidosis	Female	12-23 months	95+ years	Data Rich	-1	3	Socio-demographic Index	--
Interstitial lung disease and pulmonary sarcoidosis	Female	12-23 months	95+ years	Global	-1	1	Occupation Professionals	--
Interstitial lung disease and pulmonary sarcoidosis	Female	12-23 months	95+ years	Global	1	1	Outdoor Air Pollution (PM2.5)	454
Interstitial lung disease and pulmonary sarcoidosis	Female	12-23 months	95+ years	Global	1	1	Cumulative Cigarettes (10 Years)	--
Interstitial lung disease and pulmonary sarcoidosis	Female	12-23 months	95+ years	Global	1	1	Cumulative Cigarettes (5 Years)	--
Interstitial lung disease and pulmonary sarcoidosis	Female	12-23 months	95+ years	Global	1	1	Indoor Air Pollution (All Cooking Fuels)	--
Interstitial lung disease and pulmonary sarcoidosis	Female	12-23 months	95+ years	Global	1	1	Smoking Prevalence	--
Interstitial lung disease and pulmonary sarcoidosis	Female	12-23 months	95+ years	Global	-1	2	Healthcare access and quality index	--
Interstitial lung disease and pulmonary sarcoidosis	Female	12-23 months	95+ years	Global	1	2	Elevation 500 to 1500m (proportion)	546
Interstitial lung disease and pulmonary sarcoidosis	Female	12-23 months	95+ years	Global	1	2	Elevation Over 1500m (proportion)	--
Interstitial lung disease and pulmonary sarcoidosis	Female	12-23 months	95+ years	Global	-1	3	Education (years per capita)	--
Interstitial lung disease and pulmonary sarcoidosis	Female	12-23 months	95+ years	Global	-1	3	LDI (I$ per capita)	--
Interstitial lung disease and pulmonary sarcoidosis	Female	12-23 months	95+ years	Global	-1	3	Socio-demographic Index	--
Interstitial lung disease and pulmonary sarcoidosis	Male	12-23 months	95+ years	Data Rich	-1	1	Occupation Professionals	--
Interstitial lung disease and pulmonary sarcoidosis	Male	12-23 months	95+ years	Data Rich	1	1	Cumulative Cigarettes (10 Years)	--
Interstitial lung disease and pulmonary sarcoidosis	Male	12-23 months	95+ years	Data Rich	1	1	Cumulative Cigarettes (5 Years)	--
Interstitial lung disease and pulmonary sarcoidosis	Male	12-23 months	95+ years	Data Rich	1	1	Smoking Prevalence	--
Interstitial lung disease and pulmonary sarcoidosis	Male	12-23 months	95+ years	Data Rich	-1	2	Healthcare access and quality index	--
Interstitial lung disease and pulmonary sarcoidosis	Male	12-23 months	95+ years	Data Rich	1	2	Elevation Over 1500m (proportion)	1000
Interstitial lung disease and pulmonary sarcoidosis	Male	12-23 months	95+ years	Data Rich	1	2	Elevation 500 to 1500m (proportion)	--
Interstitial lung disease and pulmonary sarcoidosis	Male	12-23 months	95+ years	Data Rich	1	2	Indoor Air Pollution (All Cooking Fuels)	--
Interstitial lung disease and pulmonary sarcoidosis	Male	12-23 months	95+ years	Data Rich	1	2	Outdoor Air Pollution (PM2.5)	--
Interstitial lung disease and pulmonary sarcoidosis	Male	12-23 months	95+ years	Data Rich	-1	3	Education (years per capita)	--
Interstitial lung disease and pulmonary sarcoidosis	Male	12-23 months	95+ years	Data Rich	-1	3	LDI (I$ per capita)	--
Interstitial lung disease and pulmonary sarcoidosis	Male	12-23 months	95+ years	Data Rich	-1	3	Socio-demographic Index	--
Interstitial lung disease and pulmonary sarcoidosis	Male	12-23 months	95+ years	Global	-1	1	Occupation Professionals	--
Interstitial lung disease and pulmonary sarcoidosis	Male	12-23 months	95+ years	Global	1	1	Cumulative Cigarettes (10 Years)	--
Interstitial lung disease and pulmonary sarcoidosis	Male	12-23 months	95+ years	Global	1	1	Cumulative Cigarettes (5 Years)	--
Interstitial lung disease and pulmonary sarcoidosis	Male	12-23 months	95+ years	Global	1	1	Smoking Prevalence	--
Interstitial lung disease and pulmonary sarcoidosis	Male	12-23 months	95+ years	Global	-1	2	Healthcare access and quality index	--
Interstitial lung disease and pulmonary sarcoidosis	Male	12-23 months	95+ years	Global	1	2	Outdoor Air Pollution (PM2.5)	454
Interstitial lung disease and pulmonary sarcoidosis	Male	12-23 months	95+ years	Global	1	2	Elevation 500 to 1500m (proportion)	546
Interstitial lung disease and pulmonary sarcoidosis	Male	12-23 months	95+ years	Global	1	2	Elevation Over 1500m (proportion)	546
Interstitial lung disease and pulmonary sarcoidosis	Male	12-23 months	95+ years	Global	1	2	Indoor Air Pollution (All Cooking Fuels)	--
Interstitial lung disease and pulmonary sarcoidosis	Male	12-23 months	95+ years	Global	-1	3	Education (years per capita)	--
Interstitial lung disease and pulmonary sarcoidosis	Male	12-23 months	95+ years	Global	-1	3	LDI (I$ per capita)	--
Interstitial lung disease and pulmonary sarcoidosis	Male	12-23 months	95+ years	Global	-1	3	Socio-demographic Index	--
Other chronic respiratory diseases	Female	0-6 days	95+ years	Data Rich	1	1	Outdoor Air Pollution (PM2.5)	679
Other chronic respiratory diseases	Female	0-6 days	95+ years	Data Rich	1	1	Cumulative Cigarettes (5 Years)	--
Other chronic respiratory diseases	Female	0-6 days	95+ years	Data Rich	1	1	Indoor Air Pollution (All Cooking Fuels)	--
Other chronic respiratory diseases	Female	0-6 days	95+ years	Data Rich	1	1	Smoking Prevalence	--
Other chronic respiratory diseases	Female	0-6 days	95+ years	Data Rich	-1	2	Healthcare access and quality index	--
Other chronic respiratory diseases	Female	0-6 days	95+ years	Data Rich	1	2	Elevation Over 1500m (proportion)	757
Other chronic respiratory diseases	Female	0-6 days	95+ years	Data Rich	1	2	Elevation 500 to 1500m (proportion)	--
Other chronic respiratory diseases	Female	0-6 days	95+ years	Data Rich	1	2	Population Density (over 1000 ppl/sqkm, proportion)	--
Other chronic respiratory diseases	Female	0-6 days	95+ years	Data Rich	-1	3	Education (years per capita)	108
Other chronic respiratory diseases	Female	0-6 days	95+ years	Data Rich	-1	3	Socio-demographic Index	108
Other chronic respiratory diseases	Female	0-6 days	95+ years	Data Rich	-1	3	LDI (I$ per capita)	--
Other chronic respiratory diseases	Female	0-6 days	95+ years	Global	1	1	Indoor Air Pollution (All Cooking Fuels)	775
Other chronic respiratory diseases	Female	0-6 days	95+ years	Global	1	1	Cumulative Cigarettes (5 Years)	--
Other chronic respiratory diseases	Female	0-6 days	95+ years	Global	1	1	Outdoor Air Pollution (PM2.5)	--
Other chronic respiratory diseases	Female	0-6 days	95+ years	Global	1	1	Smoking Prevalence	--
Other chronic respiratory diseases	Female	0-6 days	95+ years	Global	-1	2	Healthcare access and quality index	--
Other chronic respiratory diseases	Female	0-6 days	95+ years	Global	1	2	Elevation 500 to 1500m (proportion)	391
Other chronic respiratory diseases	Female	0-6 days	95+ years	Global	1	2	Elevation Over 1500m (proportion)	616
Other chronic respiratory diseases	Female	0-6 days	95+ years	Global	1	2	Population Density (over 1000 ppl/sqkm, proportion)	--
Other chronic respiratory diseases	Female	0-6 days	95+ years	Global	-1	3	Socio-demographic Index	270
Other chronic respiratory diseases	Female	0-6 days	95+ years	Global	-1	3	Education (years per capita)	287
Other chronic respiratory diseases	Female	0-6 days	95+ years	Global	-1	3	LDI (I$ per capita)	--
Other chronic respiratory diseases	Male	0-6 days	95+ years	Data Rich	1	1	Smoking Prevalence	148
Other chronic respiratory diseases	Male	0-6 days	95+ years	Data Rich	1	1	Outdoor Air Pollution (PM2.5)	406
Other chronic respiratory diseases	Male	0-6 days	95+ years	Data Rich	1	1	Indoor Air Pollution (All Cooking Fuels)	701
Other chronic respiratory diseases	Male	0-6 days	95+ years	Data Rich	1	1	Cumulative Cigarettes (5 Years)	--
Other chronic respiratory diseases	Male	0-6 days	95+ years	Data Rich	-1	2	Healthcare access and quality index	--
Other chronic respiratory diseases	Male	0-6 days	95+ years	Data Rich	1	2	Elevation 500 to 1500m (proportion)	110
Other chronic respiratory diseases	Male	0-6 days	95+ years	Data Rich	1	2	Elevation Over 1500m (proportion)	511
Other chronic respiratory diseases	Male	0-6 days	95+ years	Data Rich	1	2	Population Density (over 1000 ppl/sqkm, proportion)	--
Other chronic respiratory diseases	Male	0-6 days	95+ years	Data Rich	-1	3	Education (years per capita)	49
Other chronic respiratory diseases	Male	0-6 days	95+ years	Data Rich	-1	3	LDI (I$ per capita)	--
Other chronic respiratory diseases	Male	0-6 days	95+ years	Data Rich	-1	3	Socio-demographic Index	--
Other chronic respiratory diseases	Male	0-6 days	95+ years	Global	1	1	Indoor Air Pollution (All Cooking Fuels)	718
Other chronic respiratory diseases	Male	0-6 days	95+ years	Global	1	1	Cumulative Cigarettes (5 Years)	--
Other chronic respiratory diseases	Male	0-6 days	95+ years	Global	1	1	Outdoor Air Pollution (PM2.5)	--
Other chronic respiratory diseases	Male	0-6 days	95+ years	Global	1	1	Smoking Prevalence	--
Other chronic respiratory diseases	Male	0-6 days	95+ years	Global	-1	2	Healthcare access and quality index	--
Other chronic respiratory diseases	Male	0-6 days	95+ years	Global	1	2	Elevation 500 to 1500m (proportion)	326
Other chronic respiratory diseases	Male	0-6 days	95+ years	Global	1	2	Elevation Over 1500m (proportion)	608

CODEm covariates used, level of covariate, and expected direction of covariate by cause, sex, and age

Cause	Sex	Age Start	Age End	Model Version Type	Direction	Level	Covariate Name	Number of Draws
Other chronic respiratory diseases	Male	0-6 days	95+ years	Global	1	2	Population Density (over 1000 ppl/sqkm, proportion)	--
Other chronic respiratory diseases	Male	0-6 days	95+ years	Global	-1	3	Socio-demographic Index	193
Other chronic respiratory diseases	Male	0-6 days	95+ years	Global	-1	3	Education (years per capita)	231
Other chronic respiratory diseases	Male	0-6 days	95+ years	Global	-1	3	LDI (I$ per capita)	--
Digestive diseases	Female	0-6 days	95+ years	Data Rich	-1	1	Sanitation (proportion with access)	1000
Digestive diseases	Female	0-6 days	95+ years	Data Rich	1	1	Cumulative Cigarettes (5 Years)	38
Digestive diseases	Female	0-6 days	95+ years	Data Rich	1	1	Smoking Prevalence	38
Digestive diseases	Female	0-6 days	95+ years	Data Rich	1	1	Cumulative Cigarettes (10 Years)	188
Digestive diseases	Female	0-6 days	95+ years	Data Rich	1	1	Mean BMI	428
Digestive diseases	Female	0-6 days	95+ years	Data Rich	1	1	Liters of alcohol consumed per capita	754
Digestive diseases	Female	0-6 days	95+ years	Data Rich	-1	2	Healthcare access and quality index	105
Digestive diseases	Female	0-6 days	95+ years	Data Rich	1	2	Age- and sex-specific SEV for Low fruit	3
Digestive diseases	Female	0-6 days	95+ years	Data Rich	1	2	Age- and sex-specific SEV for Low vegetables	130
Digestive diseases	Female	0-6 days	95+ years	Data Rich	1	2	Age- and sex-specific SEV for High red meat	180
Digestive diseases	Female	0-6 days	95+ years	Data Rich	-1	3	LDI (I$ per capita)	118
Digestive diseases	Female	0-6 days	95+ years	Data Rich	-1	3	Education (years per capita)	249
Digestive diseases	Female	0-6 days	95+ years	Data Rich	-1	3	Socio-demographic Index	--
Digestive diseases	Female	0-6 days	95+ years	Global	-1	1	Sanitation (proportion with access)	538
Digestive diseases	Female	0-6 days	95+ years	Global	1	1	Cumulative Cigarettes (5 Years)	41
Digestive diseases	Female	0-6 days	95+ years	Global	1	1	Cumulative Cigarettes (10 Years)	44
Digestive diseases	Female	0-6 days	95+ years	Global	1	1	Smoking Prevalence	141
Digestive diseases	Female	0-6 days	95+ years	Global	1	1	Liters of alcohol consumed per capita	--
Digestive diseases	Female	0-6 days	95+ years	Global	-1	2	Healthcare access and quality index	176
Digestive diseases	Female	0-6 days	95+ years	Global	1	2	Age- and sex-specific SEV for Low fruit	25
Digestive diseases	Female	0-6 days	95+ years	Global	1	2	Age- and sex-specific SEV for High red meat	92
Digestive diseases	Female	0-6 days	95+ years	Global	1	2	Age- and sex-specific SEV for Low vegetables	378
Digestive diseases	Female	0-6 days	95+ years	Global	1	2	Mean BMI	--
Digestive diseases	Female	0-6 days	95+ years	Global	-1	3	LDI (I$ per capita)	91
Digestive diseases	Female	0-6 days	95+ years	Global	-1	3	Socio-demographic Index	149
Digestive diseases	Female	0-6 days	95+ years	Global	-1	3	Education (years per capita)	447
Digestive diseases	Male	0-6 days	95+ years	Data Rich	-1	1	Sanitation (proportion with access)	970
Digestive diseases	Male	0-6 days	95+ years	Data Rich	1	1	Smoking Prevalence	33
Digestive diseases	Male	0-6 days	95+ years	Data Rich	1	1	Cumulative Cigarettes (10 Years)	97
Digestive diseases	Male	0-6 days	95+ years	Data Rich	1	1	Cumulative Cigarettes (5 Years)	105
Digestive diseases	Male	0-6 days	95+ years	Data Rich	1	1	Liters of alcohol consumed per capita	796
Digestive diseases	Male	0-6 days	95+ years	Data Rich	-1	2	Healthcare access and quality index	14
Digestive diseases	Male	0-6 days	95+ years	Data Rich	1	2	Age- and sex-specific SEV for Low fruit	0
Digestive diseases	Male	0-6 days	95+ years	Data Rich	1	2	Age- and sex-specific SEV for Low vegetables	56
Digestive diseases	Male	0-6 days	95+ years	Data Rich	1	2	Age- and sex-specific SEV for High red meat	245
Digestive diseases	Male	0-6 days	95+ years	Data Rich	1	2	Mean BMI	249
Digestive diseases	Male	0-6 days	95+ years	Data Rich	-1	3	LDI (I$ per capita)	2
Digestive diseases	Male	0-6 days	95+ years	Data Rich	-1	3	Socio-demographic Index	18
Digestive diseases	Male	0-6 days	95+ years	Data Rich	-1	3	Education (years per capita)	232
Digestive diseases	Male	0-6 days	95+ years	Global	-1	1	Sanitation (proportion with access)	761
Digestive diseases	Male	0-6 days	95+ years	Global	1	1	Cumulative Cigarettes (10 Years)	41
Digestive diseases	Male	0-6 days	95+ years	Global	1	1	Cumulative Cigarettes (5 Years)	72
Digestive diseases	Male	0-6 days	95+ years	Global	1	1	Liters of alcohol consumed per capita	271
Digestive diseases	Male	0-6 days	95+ years	Global	1	1	Smoking Prevalence	337
Digestive diseases	Male	0-6 days	95+ years	Global	-1	2	Healthcare access and quality index	87
Digestive diseases	Male	0-6 days	95+ years	Global	1	2	Age- and sex-specific SEV for Low fruit	3
Digestive diseases	Male	0-6 days	95+ years	Global	1	2	Age- and sex-specific SEV for High red meat	89
Digestive diseases	Male	0-6 days	95+ years	Global	1	2	Age- and sex-specific SEV for Low vegetables	98
Digestive diseases	Male	0-6 days	95+ years	Global	1	2	Mean BMI	--
Digestive diseases	Male	0-6 days	95+ years	Global	-1	3	LDI (I$ per capita)	22
Digestive diseases	Male	0-6 days	95+ years	Global	-1	3	Socio-demographic Index	132
Digestive diseases	Male	0-6 days	95+ years	Global	-1	3	Education (years per capita)	180
Cirrhosis and other chronic liver diseases	Female	12-23 months	95+ years	Data Rich	-1	1	Hepatitis B vaccine coverage (proportion), aged through time	360
Cirrhosis and other chronic liver diseases	Female	12-23 months	95+ years	Data Rich	1	1	Liters of alcohol consumed per capita	190
Cirrhosis and other chronic liver diseases	Female	12-23 months	95+ years	Data Rich	1	1	Vaccine adjusted HbSAg serporevalence age standardized	802
Cirrhosis and other chronic liver diseases	Female	12-23 months	95+ years	Data Rich	1	1	Chronic Hepatitis C age standardized	--
Cirrhosis and other chronic liver diseases	Female	12-23 months	95+ years	Data Rich	-1	2	Healthcare access and quality index	0
Cirrhosis and other chronic liver diseases	Female	12-23 months	95+ years	Data Rich	1	2	Mean BMI	461
Cirrhosis and other chronic liver diseases	Female	12-23 months	95+ years	Data Rich	1	2	Diabetes Age-Standardized Prevalence (proportion)	715
Cirrhosis and other chronic liver diseases	Female	12-23 months	95+ years	Data Rich	1	2	Intravenous drug use (proportion by age)	--
Cirrhosis and other chronic liver diseases	Female	12-23 months	95+ years	Data Rich	-1	3	Education (years per capita)	0
Cirrhosis and other chronic liver diseases	Female	12-23 months	95+ years	Data Rich	-1	3	Socio-demographic Index	0
Cirrhosis and other chronic liver diseases	Female	12-23 months	95+ years	Data Rich	-1	3	LDI (I$ per capita)	--
Cirrhosis and other chronic liver diseases	Female	12-23 months	95+ years	Global	-1	1	Hepatitis B vaccine coverage (proportion), aged through time	507
Cirrhosis and other chronic liver diseases	Female	12-23 months	95+ years	Global	1	1	Vaccine adjusted HbSAg serporevalence age standardized	648
Cirrhosis and other chronic liver diseases	Female	12-23 months	95+ years	Global	1	1	Liters of alcohol consumed per capita	779
Cirrhosis and other chronic liver diseases	Female	12-23 months	95+ years	Global	1	1	Chronic Hepatitis C age standardized	--
Cirrhosis and other chronic liver diseases	Female	12-23 months	95+ years	Global	-1	2	Healthcare access and quality index	114
Cirrhosis and other chronic liver diseases	Female	12-23 months	95+ years	Global	1	2	Mean BMI	168
Cirrhosis and other chronic liver diseases	Female	12-23 months	95+ years	Global	1	2	Diabetes Age-Standardized Prevalence (proportion)	427
Cirrhosis and other chronic liver diseases	Female	12-23 months	95+ years	Global	1	2	Intravenous drug use (proportion by age)	--
Cirrhosis and other chronic liver diseases	Female	12-23 months	95+ years	Global	-1	3	Education (years per capita)	73
Cirrhosis and other chronic liver diseases	Female	12-23 months	95+ years	Global	-1	3	Socio-demographic Index	75
Cirrhosis and other chronic liver diseases	Female	12-23 months	95+ years	Global	-1	3	LDI (I$ per capita)	--
Cirrhosis and other chronic liver diseases	Male	12-23 months	95+ years	Data Rich	-1	1	Hepatitis B vaccine coverage (proportion), aged through time	346
Cirrhosis and other chronic liver diseases	Male	12-23 months	95+ years	Data Rich	1	1	Vaccine adjusted HbSAg serporevalence age standardized	302
Cirrhosis and other chronic liver diseases	Male	12-23 months	95+ years	Data Rich	1	1	Liters of alcohol consumed per capita	588
Cirrhosis and other chronic liver diseases	Male	12-23 months	95+ years	Data Rich	1	1	Chronic Hepatitis C age standardized	--
Cirrhosis and other chronic liver diseases	Male	12-23 months	95+ years	Data Rich	-1	2	Healthcare access and quality index	0
Cirrhosis and other chronic liver diseases	Male	12-23 months	95+ years	Data Rich	1	2	Intravenous drug use (proportion by age)	159
Cirrhosis and other chronic liver diseases	Male	12-23 months	95+ years	Data Rich	1	2	Mean BMI	312
Cirrhosis and other chronic liver diseases	Male	12-23 months	95+ years	Data Rich	1	2	Diabetes Age-Standardized Prevalence (proportion)	509
Cirrhosis and other chronic liver diseases	Male	12-23 months	95+ years	Data Rich	-1	3	Education (years per capita)	0
Cirrhosis and other chronic liver diseases	Male	12-23 months	95+ years	Data Rich	-1	3	Socio-demographic Index	0
Cirrhosis and other chronic liver diseases	Male	12-23 months	95+ years	Data Rich	-1	3	LDI (I$ per capita)	--
Cirrhosis and other chronic liver diseases	Male	12-23 months	95+ years	Global	-1	1	Hepatitis B vaccine coverage (proportion), aged through time	735
Cirrhosis and other chronic liver diseases	Male	12-23 months	95+ years	Global	1	1	Vaccine adjusted HbSAg serporevalence age standardized	265
Cirrhosis and other chronic liver diseases	Male	12-23 months	95+ years	Global	1	1	Liters of alcohol consumed per capita	667
Cirrhosis and other chronic liver diseases	Male	12-23 months	95+ years	Global	1	1	Chronic Hepatitis C age standardized	--
Cirrhosis and other chronic liver diseases	Male	12-23 months	95+ years	Global	-1	2	Healthcare access and quality index	3
Cirrhosis and other chronic liver diseases	Male	12-23 months	95+ years	Global	1	2	Intravenous drug use (proportion by age)	324
Cirrhosis and other chronic liver diseases	Male	12-23 months	95+ years	Global	1	2	Mean BMI	433
Cirrhosis and other chronic liver diseases	Male	12-23 months	95+ years	Global	1	2	Diabetes Age-Standardized Prevalence (proportion)	578
Cirrhosis and other chronic liver diseases	Male	12-23 months	95+ years	Global	-1	3	Education (years per capita)	0
Cirrhosis and other chronic liver diseases	Male	12-23 months	95+ years	Global	-1	3	Socio-demographic Index	0
Cirrhosis and other chronic liver diseases	Male	12-23 months	95+ years	Global	-1	3	LDI (I$ per capita)	--
Upper digestive system diseases	Female	6-11 months	95+ years	Data Rich	-1	1	Sanitation (proportion with access)	92
Upper digestive system diseases	Female	6-11 months	95+ years	Data Rich	1	1	Age- and sex-specific SEV for Unsafe water	10
Upper digestive system diseases	Female	6-11 months	95+ years	Data Rich	1	1	Smoking Prevalence	61
Upper digestive system diseases	Female	6-11 months	95+ years	Data Rich	1	1	Cumulative Cigarettes (5 Years)	262
Upper digestive system diseases	Female	6-11 months	95+ years	Data Rich	1	1	Cumulative Cigarettes (10 Years)	613
Upper digestive system diseases	Female	6-11 months	95+ years	Data Rich	-1	2	vegetables unadjusted(g)	59
Upper digestive system diseases	Female	6-11 months	95+ years	Data Rich	-1	2	Healthcare access and quality index	675
Upper digestive system diseases	Female	6-11 months	95+ years	Data Rich	1	2	Liters of alcohol consumed per capita	111
Upper digestive system diseases	Female	6-11 months	95+ years	Data Rich	-1	3	Education (years per capita)	159
Upper digestive system diseases	Female	6-11 months	95+ years	Data Rich	-1	3	Socio-demographic Index	429
Upper digestive system diseases	Female	6-11 months	95+ years	Data Rich	-1	3	LDI (I$ per capita)	--
Upper digestive system diseases	Female	6-11 months	95+ years	Global	-1	1	Sanitation (proportion with access)	226
Upper digestive system diseases	Female	6-11 months	95+ years	Global	1	1	Age- and sex-specific SEV for Unsafe water	12
Upper digestive system diseases	Female	6-11 months	95+ years	Global	1	1	Smoking Prevalence	21
Upper digestive system diseases	Female	6-11 months	95+ years	Global	1	1	Cumulative Cigarettes (5 Years)	464

CODEm covariates used, level of covariate, and expected direction of covariate by cause, sex, and age

Cause	Sex	Age Start	Age End	Model Version Type	Direction	Level	Covariate Name	Number of Draws
Upper digestive system diseases	Female	6-11 months	95+ years	Global	1	1	Cumulative Cigarettes (10 Years)	483
Upper digestive system diseases	Female	6-11 months	95+ years	Global	-1	2	vegetables unadjusted(g)	260
Upper digestive system diseases	Female	6-11 months	95+ years	Global	-1	2	Healthcare access and quality index	615
Upper digestive system diseases	Female	6-11 months	95+ years	Global	1	2	Liters of alcohol consumed per capita	165
Upper digestive system diseases	Female	6-11 months	95+ years	Global	-1	3	Socio-demographic Index	280
Upper digestive system diseases	Female	6-11 months	95+ years	Global	-1	3	Education (years per capita)	583
Upper digestive system diseases	Female	6-11 months	95+ years	Global	-1	3	LDI (I$ per capita)	--
Upper digestive system diseases	Male	6-11 months	95+ years	Data Rich	-1	1	Sanitation (proportion with access)	235
Upper digestive system diseases	Male	6-11 months	95+ years	Data Rich	1	1	Cumulative Cigarettes (10 Years)	138
Upper digestive system diseases	Male	6-11 months	95+ years	Data Rich	1	1	Smoking Prevalence	146
Upper digestive system diseases	Male	6-11 months	95+ years	Data Rich	1	1	Age- and sex-specific SEV for Unsafe water	212
Upper digestive system diseases	Male	6-11 months	95+ years	Data Rich	1	1	Cumulative Cigarettes (5 Years)	643
Upper digestive system diseases	Male	6-11 months	95+ years	Data Rich	-1	2	vegetables unadjusted(g)	413
Upper digestive system diseases	Male	6-11 months	95+ years	Data Rich	-1	2	Healthcare access and quality index	533
Upper digestive system diseases	Male	6-11 months	95+ years	Data Rich	1	2	Liters of alcohol consumed per capita	42
Upper digestive system diseases	Male	6-11 months	95+ years	Data Rich	-1	3	Socio-demographic Index	260
Upper digestive system diseases	Male	6-11 months	95+ years	Data Rich	-1	3	Education (years per capita)	275
Upper digestive system diseases	Male	6-11 months	95+ years	Data Rich	-1	3	LDI (I$ per capita)	--
Upper digestive system diseases	Male	6-11 months	95+ years	Global	-1	1	Sanitation (proportion with access)	124
Upper digestive system diseases	Male	6-11 months	95+ years	Global	1	1	Age- and sex-specific SEV for Unsafe water	88
Upper digestive system diseases	Male	6-11 months	95+ years	Global	1	1	Cumulative Cigarettes (10 Years)	419
Upper digestive system diseases	Male	6-11 months	95+ years	Global	1	1	Cumulative Cigarettes (5 Years)	472
Upper digestive system diseases	Male	6-11 months	95+ years	Global	1	1	Smoking Prevalence	521
Upper digestive system diseases	Male	6-11 months	95+ years	Global	-1	2	vegetables unadjusted(g)	65
Upper digestive system diseases	Male	6-11 months	95+ years	Global	-1	2	Healthcare access and quality index	635
Upper digestive system diseases	Male	6-11 months	95+ years	Global	1	2	Liters of alcohol consumed per capita	177
Upper digestive system diseases	Male	6-11 months	95+ years	Global	-1	3	Socio-demographic Index	281
Upper digestive system diseases	Male	6-11 months	95+ years	Global	-1	3	Education (years per capita)	684
Upper digestive system diseases	Male	6-11 months	95+ years	Global	-1	3	LDI (I$ per capita)	--
Peptic ulcer disease	Female	6-11 months	95+ years	Data Rich	-1	1	Sanitation (proportion with access)	339
Peptic ulcer disease	Female	6-11 months	95+ years	Data Rich	1	1	Age- and sex-specific SEV for Unsafe water	10
Peptic ulcer disease	Female	6-11 months	95+ years	Data Rich	1	1	Smoking Prevalence	24
Peptic ulcer disease	Female	6-11 months	95+ years	Data Rich	1	1	Cumulative Cigarettes (5 Years)	226
Peptic ulcer disease	Female	6-11 months	95+ years	Data Rich	1	1	Cumulative Cigarettes (10 Years)	745
Peptic ulcer disease	Female	6-11 months	95+ years	Data Rich	-1	2	vegetables unadjusted(g)	86
Peptic ulcer disease	Female	6-11 months	95+ years	Data Rich	-1	2	Healthcare access and quality index	760
Peptic ulcer disease	Female	6-11 months	95+ years	Data Rich	1	2	Liters of alcohol consumed per capita	792
Peptic ulcer disease	Female	6-11 months	95+ years	Data Rich	-1	3	Socio-demographic Index	187
Peptic ulcer disease	Female	6-11 months	95+ years	Data Rich	-1	3	Education (years per capita)	623
Peptic ulcer disease	Female	6-11 months	95+ years	Data Rich	-1	3	LDI (I$ per capita)	--
Peptic ulcer disease	Female	6-11 months	95+ years	Global	-1	1	Sanitation (proportion with access)	214
Peptic ulcer disease	Female	6-11 months	95+ years	Global	1	1	Smoking Prevalence	51
Peptic ulcer disease	Female	6-11 months	95+ years	Global	1	1	Age- and sex-specific SEV for Unsafe water	89
Peptic ulcer disease	Female	6-11 months	95+ years	Global	1	1	Cumulative Cigarettes (10 Years)	424
Peptic ulcer disease	Female	6-11 months	95+ years	Global	1	1	Cumulative Cigarettes (5 Years)	498
Peptic ulcer disease	Female	6-11 months	95+ years	Global	-1	2	Healthcare access and quality index	681
Peptic ulcer disease	Female	6-11 months	95+ years	Global	-1	2	vegetables unadjusted(g)	841
Peptic ulcer disease	Female	6-11 months	95+ years	Global	1	2	Liters of alcohol consumed per capita	933
Peptic ulcer disease	Female	6-11 months	95+ years	Global	-1	3	Socio-demographic Index	156
Peptic ulcer disease	Female	6-11 months	95+ years	Global	-1	3	Education (years per capita)	459
Peptic ulcer disease	Female	6-11 months	95+ years	Global	-1	3	LDI (I$ per capita)	--
Peptic ulcer disease	Male	6-11 months	95+ years	Data Rich	-1	1	Sanitation (proportion with access)	378
Peptic ulcer disease	Male	6-11 months	95+ years	Data Rich	1	1	Age- and sex-specific SEV for Unsafe water	24
Peptic ulcer disease	Male	6-11 months	95+ years	Data Rich	1	1	Cumulative Cigarettes (5 Years)	303
Peptic ulcer disease	Male	6-11 months	95+ years	Data Rich	1	1	Smoking Prevalence	307
Peptic ulcer disease	Male	6-11 months	95+ years	Data Rich	1	1	Cumulative Cigarettes (10 Years)	416
Peptic ulcer disease	Male	6-11 months	95+ years	Data Rich	-1	2	vegetables unadjusted(g)	260
Peptic ulcer disease	Male	6-11 months	95+ years	Data Rich	-1	2	Healthcare access and quality index	340
Peptic ulcer disease	Male	6-11 months	95+ years	Data Rich	1	2	Liters of alcohol consumed per capita	101
Peptic ulcer disease	Male	6-11 months	95+ years	Data Rich	-1	3	Socio-demographic Index	490
Peptic ulcer disease	Male	6-11 months	95+ years	Data Rich	-1	3	Education (years per capita)	860
Peptic ulcer disease	Male	6-11 months	95+ years	Data Rich	-1	3	LDI (I$ per capita)	--
Peptic ulcer disease	Male	6-11 months	95+ years	Global	-1	1	Sanitation (proportion with access)	381
Peptic ulcer disease	Male	6-11 months	95+ years	Global	1	1	Age- and sex-specific SEV for Unsafe water	55
Peptic ulcer disease	Male	6-11 months	95+ years	Global	1	1	Cumulative Cigarettes (5 Years)	204
Peptic ulcer disease	Male	6-11 months	95+ years	Global	1	1	Cumulative Cigarettes (10 Years)	251
Peptic ulcer disease	Male	6-11 months	95+ years	Global	1	1	Smoking Prevalence	543
Peptic ulcer disease	Male	6-11 months	95+ years	Global	-1	2	vegetables unadjusted(g)	748
Peptic ulcer disease	Male	6-11 months	95+ years	Global	-1	2	Healthcare access and quality index	809
Peptic ulcer disease	Male	6-11 months	95+ years	Global	1	2	Liters of alcohol consumed per capita	684
Peptic ulcer disease	Male	6-11 months	95+ years	Global	-1	3	Socio-demographic Index	150
Peptic ulcer disease	Male	6-11 months	95+ years	Global	-1	3	Education (years per capita)	346
Peptic ulcer disease	Male	6-11 months	95+ years	Global	-1	3	LDI (I$ per capita)	--
Gastritis and duodenitis	Female	6-11 months	95+ years	Data Rich	-1	1	Sanitation (proportion with access)	211
Gastritis and duodenitis	Female	6-11 months	95+ years	Data Rich	1	1	Age- and sex-specific SEV for Unsafe water	789
Gastritis and duodenitis	Female	6-11 months	95+ years	Data Rich	-1	2	vegetables unadjusted(g)	9
Gastritis and duodenitis	Female	6-11 months	95+ years	Data Rich	-1	2	Healthcare access and quality index	493
Gastritis and duodenitis	Female	6-11 months	95+ years	Data Rich	1	2	Liters of alcohol consumed per capita	11
Gastritis and duodenitis	Female	6-11 months	95+ years	Data Rich	1	2	Smoking Prevalence	25
Gastritis and duodenitis	Female	6-11 months	95+ years	Data Rich	1	2	Cumulative Cigarettes (10 Years)	246
Gastritis and duodenitis	Female	6-11 months	95+ years	Data Rich	1	2	Cumulative Cigarettes (5 Years)	259
Gastritis and duodenitis	Female	6-11 months	95+ years	Data Rich	-1	3	Socio-demographic Index	62
Gastritis and duodenitis	Female	6-11 months	95+ years	Data Rich	-1	3	Education (years per capita)	300
Gastritis and duodenitis	Female	6-11 months	95+ years	Data Rich	-1	3	LDI (I$ per capita)	--
Gastritis and duodenitis	Female	6-11 months	95+ years	Global	-1	1	Sanitation (proportion with access)	756
Gastritis and duodenitis	Female	6-11 months	95+ years	Global	1	1	Age- and sex-specific SEV for Unsafe water	244
Gastritis and duodenitis	Female	6-11 months	95+ years	Global	-1	2	vegetables unadjusted(g)	53
Gastritis and duodenitis	Female	6-11 months	95+ years	Global	-1	2	Healthcare access and quality index	177
Gastritis and duodenitis	Female	6-11 months	95+ years	Global	1	2	Smoking Prevalence	19
Gastritis and duodenitis	Female	6-11 months	95+ years	Global	1	2	Liters of alcohol consumed per capita	108
Gastritis and duodenitis	Female	6-11 months	95+ years	Global	1	2	Cumulative Cigarettes (5 Years)	159
Gastritis and duodenitis	Female	6-11 months	95+ years	Global	1	2	Cumulative Cigarettes (10 Years)	196
Gastritis and duodenitis	Female	6-11 months	95+ years	Global	-1	3	Socio-demographic Index	26
Gastritis and duodenitis	Female	6-11 months	95+ years	Global	-1	3	Education (years per capita)	188
Gastritis and duodenitis	Female	6-11 months	95+ years	Global	-1	3	LDI (I$ per capita)	--
Gastritis and duodenitis	Male	6-11 months	95+ years	Data Rich	-1	1	Sanitation (proportion with access)	709
Gastritis and duodenitis	Male	6-11 months	95+ years	Data Rich	1	1	Age- and sex-specific SEV for Unsafe water	291
Gastritis and duodenitis	Male	6-11 months	95+ years	Data Rich	-1	2	vegetables unadjusted(g)	53
Gastritis and duodenitis	Male	6-11 months	95+ years	Data Rich	-1	2	Healthcare access and quality index	90
Gastritis and duodenitis	Male	6-11 months	95+ years	Data Rich	1	2	Cumulative Cigarettes (5 Years)	84
Gastritis and duodenitis	Male	6-11 months	95+ years	Data Rich	1	2	Cumulative Cigarettes (10 Years)	122
Gastritis and duodenitis	Male	6-11 months	95+ years	Data Rich	1	2	Liters of alcohol consumed per capita	129
Gastritis and duodenitis	Male	6-11 months	95+ years	Data Rich	1	2	Smoking Prevalence	304
Gastritis and duodenitis	Male	6-11 months	95+ years	Data Rich	-1	3	Socio-demographic Index	72
Gastritis and duodenitis	Male	6-11 months	95+ years	Data Rich	-1	3	Education (years per capita)	140
Gastritis and duodenitis	Male	6-11 months	95+ years	Data Rich	-1	3	LDI (I$ per capita)	--
Gastritis and duodenitis	Male	6-11 months	95+ years	Global	-1	1	Sanitation (proportion with access)	706
Gastritis and duodenitis	Male	6-11 months	95+ years	Global	1	1	Age- and sex-specific SEV for Unsafe water	294
Gastritis and duodenitis	Male	6-11 months	95+ years	Global	-1	2	Healthcare access and quality index	243
Gastritis and duodenitis	Male	6-11 months	95+ years	Global	-1	2	vegetables unadjusted(g)	467
Gastritis and duodenitis	Male	6-11 months	95+ years	Global	1	2	Cumulative Cigarettes (10 Years)	200
Gastritis and duodenitis	Male	6-11 months	95+ years	Global	1	2	Smoking Prevalence	354
Gastritis and duodenitis	Male	6-11 months	95+ years	Global	1	2	Liters of alcohol consumed per capita	554
Gastritis and duodenitis	Male	6-11 months	95+ years	Global	1	2	Cumulative Cigarettes (5 Years)	--
Gastritis and duodenitis	Male	6-11 months	95+ years	Global	-1	3	Education (years per capita)	175
Gastritis and duodenitis	Male	6-11 months	95+ years	Global	-1	3	LDI (I$ per capita)	--
Gastritis and duodenitis	Male	6-11 months	95+ years	Global	-1	3	Socio-demographic Index	--
Appendicitis	Female	12-23 months	95+ years	Data Rich	-1	2	Healthcare access and quality index	--
Appendicitis	Female	12-23 months	95+ years	Data Rich	1	2	Age- and sex-specific SEV for Low vegetables	537
Appendicitis	Female	12-23 months	95+ years	Data Rich	1	2	Age- and sex-specific SEV for Low fruit	649
Appendicitis	Female	12-23 months	95+ years	Data Rich	-1	3	Education (years per capita)	243
Appendicitis	Female	12-23 months	95+ years	Data Rich	-1	3	Socio-demographic Index	243
Appendicitis	Female	12-23 months	95+ years	Data Rich	-1	3	LDI (I$ per capita)	--
Appendicitis	Female	12-23 months	95+ years	Global	-1	2	Healthcare access and quality index	389

CODEm covariates used, level of covariate, and expected direction of covariate by cause, sex, and age								
**Cause**	**Sex**	**Age Start**	**Age End**	**Model Version Type**	**Direction**	**Level**	**Covariate Name**	**Number of Draws**
Appendicitis	Female	12-23 months	95+ years	Global	1	2	Age- and sex-specific SEV for Low fruit	296
Appendicitis	Female	12-23 months	95+ years	Global	1	2	Age- and sex-specific SEV for Low vegetables	389
Appendicitis	Female	12-23 months	95+ years	Global	-1	3	Socio-demographic Index	60
Appendicitis	Female	12-23 months	95+ years	Global	-1	3	Education (years per capita)	220
Appendicitis	Female	12-23 months	95+ years	Global	-1	3	LDI (I$ per capita)	--
Appendicitis	Male	12-23 months	95+ years	Data Rich	-1	2	Healthcare access and quality index	--
Appendicitis	Male	12-23 months	95+ years	Data Rich	1	2	Age- and sex-specific SEV for Low fruit	660
Appendicitis	Male	12-23 months	95+ years	Data Rich	1	2	Age- and sex-specific SEV for Low vegetables	716
Appendicitis	Male	12-23 months	95+ years	Data Rich	-1	3	Education (years per capita)	--
Appendicitis	Male	12-23 months	95+ years	Data Rich	-1	3	LDI (I$ per capita)	--
Appendicitis	Male	12-23 months	95+ years	Data Rich	-1	3	Socio-demographic Index	--
Appendicitis	Male	12-23 months	95+ years	Global	-1	2	Healthcare access and quality index	409
Appendicitis	Male	12-23 months	95+ years	Global	1	2	Age- and sex-specific SEV for Low fruit	326
Appendicitis	Male	12-23 months	95+ years	Global	1	2	Age- and sex-specific SEV for Low vegetables	784
Appendicitis	Male	12-23 months	95+ years	Global	-1	3	Socio-demographic Index	156
Appendicitis	Male	12-23 months	95+ years	Global	-1	3	Education (years per capita)	484
Appendicitis	Male	12-23 months	95+ years	Global	-1	3	LDI (I$ per capita)	--
Paralytic ileus and intestinal obstruction	Female	0-6 days	95+ years	Data Rich	-1	2	Healthcare access and quality index	253
Paralytic ileus and intestinal obstruction	Female	0-6 days	95+ years	Data Rich	-1	2	vegetables unadjusted(g)	473
Paralytic ileus and intestinal obstruction	Female	0-6 days	95+ years	Data Rich	-1	2	fruits unadjusted(g)	747
Paralytic ileus and intestinal obstruction	Female	0-6 days	95+ years	Data Rich	-1	3	Education (years per capita)	382
Paralytic ileus and intestinal obstruction	Female	0-6 days	95+ years	Data Rich	-1	3	LDI (I$ per capita)	--
Paralytic ileus and intestinal obstruction	Female	0-6 days	95+ years	Data Rich	-1	3	Socio-demographic Index	--
Paralytic ileus and intestinal obstruction	Female	0-6 days	95+ years	Global	-1	2	vegetables unadjusted(g)	286
Paralytic ileus and intestinal obstruction	Female	0-6 days	95+ years	Global	-1	2	fruits unadjusted(g)	309
Paralytic ileus and intestinal obstruction	Female	0-6 days	95+ years	Global	-1	2	Healthcare access and quality index	405
Paralytic ileus and intestinal obstruction	Female	0-6 days	95+ years	Global	-1	3	Socio-demographic Index	257
Paralytic ileus and intestinal obstruction	Female	0-6 days	95+ years	Global	-1	3	Education (years per capita)	427
Paralytic ileus and intestinal obstruction	Female	0-6 days	95+ years	Global	-1	3	LDI (I$ per capita)	--
Paralytic ileus and intestinal obstruction	Male	0-6 days	95+ years	Data Rich	-1	2	vegetables unadjusted(g)	244
Paralytic ileus and intestinal obstruction	Male	0-6 days	95+ years	Data Rich	-1	2	Healthcare access and quality index	427
Paralytic ileus and intestinal obstruction	Male	0-6 days	95+ years	Data Rich	-1	2	fruits unadjusted(g)	521
Paralytic ileus and intestinal obstruction	Male	0-6 days	95+ years	Data Rich	-1	3	Education (years per capita)	293
Paralytic ileus and intestinal obstruction	Male	0-6 days	95+ years	Data Rich	-1	3	LDI (I$ per capita)	--
Paralytic ileus and intestinal obstruction	Male	0-6 days	95+ years	Data Rich	-1	3	Socio-demographic Index	--
Paralytic ileus and intestinal obstruction	Male	0-6 days	95+ years	Global	-1	2	fruits unadjusted(g)	282
Paralytic ileus and intestinal obstruction	Male	0-6 days	95+ years	Global	-1	2	vegetables unadjusted(g)	294
Paralytic ileus and intestinal obstruction	Male	0-6 days	95+ years	Global	-1	2	Healthcare access and quality index	424
Paralytic ileus and intestinal obstruction	Male	0-6 days	95+ years	Global	-1	3	Education (years per capita)	193
Paralytic ileus and intestinal obstruction	Male	0-6 days	95+ years	Global	-1	3	LDI (I$ per capita)	--
Paralytic ileus and intestinal obstruction	Male	0-6 days	95+ years	Global	-1	3	Socio-demographic Index	--
Inguinal, femoral, and abdominal hernia	Female	0-6 days	95+ years	Data Rich	-1	1	Mean BMI	403
Inguinal, femoral, and abdominal hernia	Female	0-6 days	95+ years	Data Rich	1	1	Smoking Prevalence	12
Inguinal, femoral, and abdominal hernia	Female	0-6 days	95+ years	Data Rich	1	1	Cumulative Cigarettes (5 Years)	267
Inguinal, femoral, and abdominal hernia	Female	0-6 days	95+ years	Data Rich	1	1	Cumulative Cigarettes (10 Years)	520
Inguinal, femoral, and abdominal hernia	Female	0-6 days	95+ years	Data Rich	-1	2	Healthcare access and quality index	907
Inguinal, femoral, and abdominal hernia	Female	0-6 days	95+ years	Data Rich	-1	3	Education (years per capita)	9
Inguinal, femoral, and abdominal hernia	Female	0-6 days	95+ years	Data Rich	-1	3	LDI (I$ per capita)	--
Inguinal, femoral, and abdominal hernia	Female	0-6 days	95+ years	Data Rich	1	3	Socio-demographic Index	110
Inguinal, femoral, and abdominal hernia	Female	0-6 days	95+ years	Global	-1	1	Mean BMI	155
Inguinal, femoral, and abdominal hernia	Female	0-6 days	95+ years	Global	1	1	Smoking Prevalence	132
Inguinal, femoral, and abdominal hernia	Female	0-6 days	95+ years	Global	1	1	Cumulative Cigarettes (10 Years)	280
Inguinal, femoral, and abdominal hernia	Female	0-6 days	95+ years	Global	1	1	Cumulative Cigarettes (5 Years)	472
Inguinal, femoral, and abdominal hernia	Female	0-6 days	95+ years	Global	-1	2	Healthcare access and quality index	204
Inguinal, femoral, and abdominal hernia	Female	0-6 days	95+ years	Global	-1	3	Education (years per capita)	105
Inguinal, femoral, and abdominal hernia	Female	0-6 days	95+ years	Global	-1	3	LDI (I$ per capita)	--
Inguinal, femoral, and abdominal hernia	Female	0-6 days	95+ years	Global	1	3	Socio-demographic Index	40
Inguinal, femoral, and abdominal hernia	Male	0-6 days	95+ years	Data Rich	-1	1	Mean BMI	299
Inguinal, femoral, and abdominal hernia	Male	0-6 days	95+ years	Data Rich	1	1	Cumulative Cigarettes (10 Years)	48
Inguinal, femoral, and abdominal hernia	Male	0-6 days	95+ years	Data Rich	1	1	Smoking Prevalence	54
Inguinal, femoral, and abdominal hernia	Male	0-6 days	95+ years	Data Rich	1	1	Cumulative Cigarettes (5 Years)	599
Inguinal, femoral, and abdominal hernia	Male	0-6 days	95+ years	Data Rich	-1	2	Healthcare access and quality index	251
Inguinal, femoral, and abdominal hernia	Male	0-6 days	95+ years	Data Rich	-1	3	Education (years per capita)	332
Inguinal, femoral, and abdominal hernia	Male	0-6 days	95+ years	Data Rich	-1	3	LDI (I$ per capita)	--
Inguinal, femoral, and abdominal hernia	Male	0-6 days	95+ years	Data Rich	1	3	Socio-demographic Index	62
Inguinal, femoral, and abdominal hernia	Male	0-6 days	95+ years	Global	-1	1	Mean BMI	718
Inguinal, femoral, and abdominal hernia	Male	0-6 days	95+ years	Global	1	1	Smoking Prevalence	104
Inguinal, femoral, and abdominal hernia	Male	0-6 days	95+ years	Global	1	1	Cumulative Cigarettes (5 Years)	178
Inguinal, femoral, and abdominal hernia	Male	0-6 days	95+ years	Global	1	1	Cumulative Cigarettes (10 Years)	--
Inguinal, femoral, and abdominal hernia	Male	0-6 days	95+ years	Global	-1	2	Healthcare access and quality index	372
Inguinal, femoral, and abdominal hernia	Male	0-6 days	95+ years	Global	-1	3	Education (years per capita)	464
Inguinal, femoral, and abdominal hernia	Male	0-6 days	95+ years	Global	-1	3	LDI (I$ per capita)	--
Inguinal, femoral, and abdominal hernia	Male	0-6 days	95+ years	Global	1	3	Socio-demographic Index	--
Inflammatory bowel disease	Female	2-4 years	95+ years	Data Rich	1	1	Age- and sex-specific SEV for Low vegetables	660
Inflammatory bowel disease	Female	2-4 years	95+ years	Data Rich	1	1	Age- and sex-specific SEV for High red meat	796
Inflammatory bowel disease	Female	2-4 years	95+ years	Data Rich	1	1	Age- and sex-specific SEV for Low PUFA	--
Inflammatory bowel disease	Female	2-4 years	95+ years	Data Rich	1	1	Age- and sex-specific SEV for Low fruit	--
Inflammatory bowel disease	Female	2-4 years	95+ years	Data Rich	-1	2	Healthcare access and quality index	--
Inflammatory bowel disease	Female	2-4 years	95+ years	Data Rich	-1	2	Latitude 15 to 30 (proportion)	--
Inflammatory bowel disease	Female	2-4 years	95+ years	Data Rich	1	2	Latitude 30 to 45 (proportion)	147
Inflammatory bowel disease	Female	2-4 years	95+ years	Data Rich	1	2	Latitude Over 45 (proportion)	347
Inflammatory bowel disease	Female	2-4 years	95+ years	Data Rich	-1	3	Education (years per capita)	--
Inflammatory bowel disease	Female	2-4 years	95+ years	Data Rich	1	3	Socio-demographic Index	67
Inflammatory bowel disease	Female	2-4 years	95+ years	Data Rich	1	3	LDI (I$ per capita)	--
Inflammatory bowel disease	Female	2-4 years	95+ years	Global	1	1	Age- and sex-specific SEV for Low fruit	197
Inflammatory bowel disease	Female	2-4 years	95+ years	Global	1	1	Age- and sex-specific SEV for Low PUFA	210
Inflammatory bowel disease	Female	2-4 years	95+ years	Global	1	1	Age- and sex-specific SEV for Low vegetables	1000
Inflammatory bowel disease	Female	2-4 years	95+ years	Global	1	1	Age- and sex-specific SEV for High red meat	--
Inflammatory bowel disease	Female	2-4 years	95+ years	Global	-1	2	Healthcare access and quality index	--
Inflammatory bowel disease	Female	2-4 years	95+ years	Global	-1	2	Latitude 15 to 30 (proportion)	--
Inflammatory bowel disease	Female	2-4 years	95+ years	Global	1	2	Latitude 30 to 45 (proportion)	--
Inflammatory bowel disease	Female	2-4 years	95+ years	Global	1	2	Latitude Over 45 (proportion)	--
Inflammatory bowel disease	Female	2-4 years	95+ years	Global	-1	3	Education (years per capita)	--
Inflammatory bowel disease	Female	2-4 years	95+ years	Global	1	3	LDI (I$ per capita)	--
Inflammatory bowel disease	Female	2-4 years	95+ years	Global	1	3	Socio-demographic Index	--
Inflammatory bowel disease	Male	2-4 years	95+ years	Data Rich	1	1	Age- and sex-specific SEV for High red meat	253
Inflammatory bowel disease	Male	2-4 years	95+ years	Data Rich	1	1	Age- and sex-specific SEV for Low vegetables	857
Inflammatory bowel disease	Male	2-4 years	95+ years	Data Rich	1	1	Age- and sex-specific SEV for Low PUFA	--
Inflammatory bowel disease	Male	2-4 years	95+ years	Data Rich	1	1	Age- and sex-specific SEV for Low fruit	--
Inflammatory bowel disease	Male	2-4 years	95+ years	Data Rich	-1	2	Healthcare access and quality index	49
Inflammatory bowel disease	Male	2-4 years	95+ years	Data Rich	-1	2	Latitude 15 to 30 (proportion)	--
Inflammatory bowel disease	Male	2-4 years	95+ years	Data Rich	1	2	Latitude 30 to 45 (proportion)	138
Inflammatory bowel disease	Male	2-4 years	95+ years	Data Rich	1	2	Latitude Over 45 (proportion)	399
Inflammatory bowel disease	Male	2-4 years	95+ years	Data Rich	-1	3	Education (years per capita)	--
Inflammatory bowel disease	Male	2-4 years	95+ years	Data Rich	1	3	Socio-demographic Index	19
Inflammatory bowel disease	Male	2-4 years	95+ years	Data Rich	1	3	LDI (I$ per capita)	--
Inflammatory bowel disease	Male	2-4 years	95+ years	Global	1	1	Age- and sex-specific SEV for Low PUFA	294
Inflammatory bowel disease	Male	2-4 years	95+ years	Global	1	1	Age- and sex-specific SEV for Low fruit	315
Inflammatory bowel disease	Male	2-4 years	95+ years	Global	1	1	Age- and sex-specific SEV for Low vegetables	431
Inflammatory bowel disease	Male	2-4 years	95+ years	Global	1	1	Age- and sex-specific SEV for High red meat	--
Inflammatory bowel disease	Male	2-4 years	95+ years	Global	-1	2	Healthcare access and quality index	--
Inflammatory bowel disease	Male	2-4 years	95+ years	Global	-1	2	Latitude 15 to 30 (proportion)	--
Inflammatory bowel disease	Male	2-4 years	95+ years	Global	1	2	Latitude 30 to 45 (proportion)	--
Inflammatory bowel disease	Male	2-4 years	95+ years	Global	1	2	Latitude Over 45 (proportion)	--
Inflammatory bowel disease	Male	2-4 years	95+ years	Global	-1	3	Education (years per capita)	--
Inflammatory bowel disease	Male	2-4 years	95+ years	Global	1	3	Socio-demographic Index	214
Inflammatory bowel disease	Male	2-4 years	95+ years	Global	1	3	LDI (I$ per capita)	--
Ulcerative colitis	Female	2-4 years	95+ years	Data Rich	1	1	Age- and sex-specific SEV for High red meat	13

CODEm covariates used, level of covariate, and expected direction of covariate by cause, sex, and age								
**Cause**	**Sex**	**Age Start**	**Age End**	**Model Version Type**	**Direction**	**Level**	**Covariate Name**	**Number of Draws**
Ulcerative colitis	Female	2-4 years	95+ years	Data Rich	1	1	Age- and sex-specific SEV for Low vegetables	1000
Ulcerative colitis	Female	2-4 years	95+ years	Data Rich	1	1	Age- and sex-specific SEV for Low PUFA	--
Ulcerative colitis	Female	2-4 years	95+ years	Data Rich	1	1	Age- and sex-specific SEV for Low fruit	--
Ulcerative colitis	Female	2-4 years	95+ years	Data Rich	-1	2	Latitude 15 to 30 (proportion)	326
Ulcerative colitis	Female	2-4 years	95+ years	Data Rich	-1	2	Healthcare access and quality index	--
Ulcerative colitis	Female	2-4 years	95+ years	Data Rich	1	2	Latitude Over 45 (proportion)	29
Ulcerative colitis	Female	2-4 years	95+ years	Data Rich	1	2	Latitude 30 to 45 (proportion)	326
Ulcerative colitis	Female	2-4 years	95+ years	Data Rich	-1	3	Education (years per capita)	--
Ulcerative colitis	Female	2-4 years	95+ years	Data Rich	1	3	Socio-demographic Index	801
Ulcerative colitis	Female	2-4 years	95+ years	Data Rich	1	3	LDI (I$ per capita)	--
Ulcerative colitis	Female	2-4 years	95+ years	Global	1	1	Age- and sex-specific SEV for Low vegetables	415
Ulcerative colitis	Female	2-4 years	95+ years	Global	1	1	Age- and sex-specific SEV for High red meat	--
Ulcerative colitis	Female	2-4 years	95+ years	Global	1	1	Age- and sex-specific SEV for Low PUFA	--
Ulcerative colitis	Female	2-4 years	95+ years	Global	1	1	Age- and sex-specific SEV for Low fruit	--
Ulcerative colitis	Female	2-4 years	95+ years	Global	-1	2	Healthcare access and quality index	585
Ulcerative colitis	Female	2-4 years	95+ years	Global	-1	2	Latitude 15 to 30 (proportion)	--
Ulcerative colitis	Female	2-4 years	95+ years	Global	1	2	Latitude 30 to 45 (proportion)	--
Ulcerative colitis	Female	2-4 years	95+ years	Global	1	2	Latitude Over 45 (proportion)	--
Ulcerative colitis	Female	2-4 years	95+ years	Global	-1	3	Education (years per capita)	--
Ulcerative colitis	Female	2-4 years	95+ years	Global	1	3	Socio-demographic Index	443
Ulcerative colitis	Female	2-4 years	95+ years	Global	1	3	LDI (I$ per capita)	--
Ulcerative colitis	Male	2-4 years	95+ years	Data Rich	1	1	Age- and sex-specific SEV for High red meat	61
Ulcerative colitis	Male	2-4 years	95+ years	Data Rich	1	1	Age- and sex-specific SEV for Low vegetables	995
Ulcerative colitis	Male	2-4 years	95+ years	Data Rich	1	1	Age- and sex-specific SEV for Low PUFA	--
Ulcerative colitis	Male	2-4 years	95+ years	Data Rich	1	1	Age- and sex-specific SEV for Low fruit	--
Ulcerative colitis	Male	2-4 years	95+ years	Data Rich	-1	2	Latitude 15 to 30 (proportion)	407
Ulcerative colitis	Male	2-4 years	95+ years	Data Rich	-1	2	Healthcare access and quality index	--
Ulcerative colitis	Male	2-4 years	95+ years	Data Rich	1	2	Latitude Over 45 (proportion)	5
Ulcerative colitis	Male	2-4 years	95+ years	Data Rich	1	2	Latitude 30 to 45 (proportion)	95
Ulcerative colitis	Male	2-4 years	95+ years	Data Rich	-1	3	Education (years per capita)	--
Ulcerative colitis	Male	2-4 years	95+ years	Data Rich	1	3	Socio-demographic Index	487
Ulcerative colitis	Male	2-4 years	95+ years	Data Rich	1	3	LDI (I$ per capita)	--
Ulcerative colitis	Male	2-4 years	95+ years	Global	1	1	Age- and sex-specific SEV for Low vegetables	515
Ulcerative colitis	Male	2-4 years	95+ years	Global	1	1	Age- and sex-specific SEV for High red meat	--
Ulcerative colitis	Male	2-4 years	95+ years	Global	1	1	Age- and sex-specific SEV for Low PUFA	--
Ulcerative colitis	Male	2-4 years	95+ years	Global	1	1	Age- and sex-specific SEV for Low fruit	--
Ulcerative colitis	Male	2-4 years	95+ years	Global	-1	2	Healthcare access and quality index	485
Ulcerative colitis	Male	2-4 years	95+ years	Global	-1	2	Latitude 15 to 30 (proportion)	--
Ulcerative colitis	Male	2-4 years	95+ years	Global	1	2	Latitude 30 to 45 (proportion)	--
Ulcerative colitis	Male	2-4 years	95+ years	Global	1	2	Latitude Over 45 (proportion)	--
Ulcerative colitis	Male	2-4 years	95+ years	Global	-1	3	Education (years per capita)	--
Ulcerative colitis	Male	2-4 years	95+ years	Global	1	3	Socio-demographic Index	463
Ulcerative colitis	Male	2-4 years	95+ years	Global	1	3	LDI (I$ per capita)	--
Crohn's disease	Female	2-4 years	95+ years	Data Rich	1	1	Age- and sex-specific SEV for Low vegetables	647
Crohn's disease	Female	2-4 years	95+ years	Data Rich	1	1	Age- and sex-specific SEV for High red meat	923
Crohn's disease	Female	2-4 years	95+ years	Data Rich	1	1	Age- and sex-specific SEV for Low PUFA	--
Crohn's disease	Female	2-4 years	95+ years	Data Rich	1	1	Age- and sex-specific SEV for Low fruit	--
Crohn's disease	Female	2-4 years	95+ years	Data Rich	-1	2	Healthcare access and quality index	--
Crohn's disease	Female	2-4 years	95+ years	Data Rich	-1	2	Latitude 15 to 30 (proportion)	--
Crohn's disease	Female	2-4 years	95+ years	Data Rich	1	2	Latitude 30 to 45 (proportion)	230
Crohn's disease	Female	2-4 years	95+ years	Data Rich	1	2	Latitude Over 45 (proportion)	491
Crohn's disease	Female	2-4 years	95+ years	Data Rich	-1	3	Education (years per capita)	--
Crohn's disease	Female	2-4 years	95+ years	Data Rich	1	3	Socio-demographic Index	699
Crohn's disease	Female	2-4 years	95+ years	Data Rich	1	3	LDI (I$ per capita)	--
Crohn's disease	Female	2-4 years	95+ years	Global	1	1	Age- and sex-specific SEV for High red meat	575
Crohn's disease	Female	2-4 years	95+ years	Global	1	1	Age- and sex-specific SEV for Low vegetables	802
Crohn's disease	Female	2-4 years	95+ years	Global	1	1	Age- and sex-specific SEV for Low PUFA	--
Crohn's disease	Female	2-4 years	95+ years	Global	1	1	Age- and sex-specific SEV for Low fruit	--
Crohn's disease	Female	2-4 years	95+ years	Global	-1	2	Healthcare access and quality index	--
Crohn's disease	Female	2-4 years	95+ years	Global	-1	2	Latitude 15 to 30 (proportion)	--
Crohn's disease	Female	2-4 years	95+ years	Global	1	2	Latitude Over 45 (proportion)	290
Crohn's disease	Female	2-4 years	95+ years	Global	1	2	Latitude 30 to 45 (proportion)	--
Crohn's disease	Female	2-4 years	95+ years	Global	-1	3	Education (years per capita)	--
Crohn's disease	Female	2-4 years	95+ years	Global	1	3	Socio-demographic Index	304
Crohn's disease	Female	2-4 years	95+ years	Global	1	3	LDI (I$ per capita)	--
Crohn's disease	Male	2-4 years	95+ years	Data Rich	1	1	Age- and sex-specific SEV for High red meat	359
Crohn's disease	Male	2-4 years	95+ years	Data Rich	1	1	Age- and sex-specific SEV for Low vegetables	874
Crohn's disease	Male	2-4 years	95+ years	Data Rich	1	1	Age- and sex-specific SEV for Low PUFA	--
Crohn's disease	Male	2-4 years	95+ years	Data Rich	1	1	Age- and sex-specific SEV for Low fruit	--
Crohn's disease	Male	2-4 years	95+ years	Data Rich	-1	2	Healthcare access and quality index	--
Crohn's disease	Male	2-4 years	95+ years	Data Rich	-1	2	Latitude 15 to 30 (proportion)	--
Crohn's disease	Male	2-4 years	95+ years	Data Rich	1	2	Latitude 30 to 45 (proportion)	400
Crohn's disease	Male	2-4 years	95+ years	Data Rich	1	2	Latitude Over 45 (proportion)	--
Crohn's disease	Male	2-4 years	95+ years	Data Rich	-1	3	Education (years per capita)	--
Crohn's disease	Male	2-4 years	95+ years	Data Rich	1	3	Socio-demographic Index	683
Crohn's disease	Male	2-4 years	95+ years	Data Rich	1	3	LDI (I$ per capita)	--
Crohn's disease	Male	2-4 years	95+ years	Global	1	1	Age- and sex-specific SEV for High red meat	744
Crohn's disease	Male	2-4 years	95+ years	Global	1	1	Age- and sex-specific SEV for Low vegetables	1000
Crohn's disease	Male	2-4 years	95+ years	Global	1	1	Age- and sex-specific SEV for Low PUFA	--
Crohn's disease	Male	2-4 years	95+ years	Global	1	1	Age- and sex-specific SEV for Low fruit	--
Crohn's disease	Male	2-4 years	95+ years	Global	-1	2	Healthcare access and quality index	--
Crohn's disease	Male	2-4 years	95+ years	Global	-1	2	Latitude 15 to 30 (proportion)	--
Crohn's disease	Male	2-4 years	95+ years	Global	1	2	Latitude Over 45 (proportion)	133
Crohn's disease	Male	2-4 years	95+ years	Global	1	2	Latitude 30 to 45 (proportion)	364
Crohn's disease	Male	2-4 years	95+ years	Global	-1	3	Education (years per capita)	--
Crohn's disease	Male	2-4 years	95+ years	Global	1	3	Socio-demographic Index	397
Crohn's disease	Male	2-4 years	95+ years	Global	1	3	LDI (I$ per capita)	--
Vascular intestinal disorders	Female	2-4 years	95+ years	Data Rich	1	1	Diabetes Fasting Plasma Glucose (mmol/L), age-standardized 25+	271
Vascular intestinal disorders	Female	2-4 years	95+ years	Data Rich	1	1	Systolic Blood Pressure (mmHg)	666
Vascular intestinal disorders	Female	2-4 years	95+ years	Data Rich	1	1	Low-Density Lipoprotein (mmol/L)	--
Vascular intestinal disorders	Female	2-4 years	95+ years	Data Rich	-1	2	Healthcare access and quality index	169
Vascular intestinal disorders	Female	2-4 years	95+ years	Data Rich	1	2	Mean BMI	190
Vascular intestinal disorders	Female	2-4 years	95+ years	Data Rich	1	2	Smoking Prevalence	282
Vascular intestinal disorders	Female	2-4 years	95+ years	Data Rich	-1	3	Education (years per capita)	3
Vascular intestinal disorders	Female	2-4 years	95+ years	Data Rich	-1	3	Socio-demographic Index	39
Vascular intestinal disorders	Female	2-4 years	95+ years	Data Rich	-1	3	LDI (I$ per capita)	--
Vascular intestinal disorders	Female	2-4 years	95+ years	Data Rich	-1	3	Pulses legumes unadjusted(g)	--
Vascular intestinal disorders	Female	2-4 years	95+ years	Data Rich	1	3	Total Fertility Rate	5
Vascular intestinal disorders	Female	2-4 years	95+ years	Data Rich	1	3	Age- and sex-specific SEV for Low omega-3	30
Vascular intestinal disorders	Female	2-4 years	95+ years	Data Rich	1	3	Age- and sex-specific SEV for Low nuts and seeds	53
Vascular intestinal disorders	Female	2-4 years	95+ years	Data Rich	1	3	Age- and sex-specific SEV for Low vegetables	74
Vascular intestinal disorders	Female	2-4 years	95+ years	Data Rich	1	3	Age- and sex-specific SEV for Low fruit	108
Vascular intestinal disorders	Female	2-4 years	95+ years	Data Rich	1	3	Age- and sex-specific SEV for Low PUFA	--
Vascular intestinal disorders	Female	2-4 years	95+ years	Data Rich	1	3	Liters of alcohol consumed per capita	--
Vascular intestinal disorders	Female	2-4 years	95+ years	Global	1	1	Diabetes Fasting Plasma Glucose (mmol/L), age-standardized 25+	893
Vascular intestinal disorders	Female	2-4 years	95+ years	Global	1	1	Low-Density Lipoprotein (mmol/L)	--
Vascular intestinal disorders	Female	2-4 years	95+ years	Global	1	1	Systolic Blood Pressure (mmHg)	--
Vascular intestinal disorders	Female	2-4 years	95+ years	Global	-1	2	Healthcare access and quality index	48
Vascular intestinal disorders	Female	2-4 years	95+ years	Global	1	2	Mean BMI	80
Vascular intestinal disorders	Female	2-4 years	95+ years	Global	1	2	Smoking Prevalence	195
Vascular intestinal disorders	Female	2-4 years	95+ years	Global	-1	3	Education (years per capita)	24
Vascular intestinal disorders	Female	2-4 years	95+ years	Global	-1	3	Pulses legumes unadjusted(g)	278
Vascular intestinal disorders	Female	2-4 years	95+ years	Global	-1	3	LDI (I$ per capita)	--
Vascular intestinal disorders	Female	2-4 years	95+ years	Global	1	3	Age- and sex-specific SEV for Low vegetables	173
Vascular intestinal disorders	Female	2-4 years	95+ years	Global	1	3	Liters of alcohol consumed per capita	335

CODEm covariates used, level of covariate, and expected direction of covariate by cause, sex, and age

Cause	Sex	Age Start	Age End	Model Version Type	Direction	Level	Covariate Name	Number of Draws
Vascular intestinal disorders	Female	2-4 years	95+ years	Global	1	3	Age- and sex-specific SEV for Low PUFA	--
Vascular intestinal disorders	Female	2-4 years	95+ years	Global	1	3	Age- and sex-specific SEV for Low fruit	--
Vascular intestinal disorders	Female	2-4 years	95+ years	Global	1	3	Age- and sex-specific SEV for Low nuts and seeds	--
Vascular intestinal disorders	Female	2-4 years	95+ years	Global	1	3	Age- and sex-specific SEV for Low omega-3	--
Vascular intestinal disorders	Female	2-4 years	95+ years	Global	1	3	Total Fertility Rate	--
Vascular intestinal disorders	Male	2-4 years	95+ years	Data Rich	1	1	Systolic Blood Pressure (mmHg)	0
Vascular intestinal disorders	Male	2-4 years	95+ years	Data Rich	1	1	Diabetes Fasting Plasma Glucose (mmol/L), age-standardized 25+	967
Vascular intestinal disorders	Male	2-4 years	95+ years	Data Rich	1	1	Low-Density Lipoprotein (mmol/L)	--
Vascular intestinal disorders	Male	2-4 years	95+ years	Data Rich	-1	2	Healthcare access and quality index	78
Vascular intestinal disorders	Male	2-4 years	95+ years	Data Rich	1	2	Smoking Prevalence	80
Vascular intestinal disorders	Male	2-4 years	95+ years	Data Rich	1	2	Mean BMI	--
Vascular intestinal disorders	Male	2-4 years	95+ years	Data Rich	-1	3	Pulses legumes unadjusted(g)	0
Vascular intestinal disorders	Male	2-4 years	95+ years	Data Rich	-1	3	Socio-demographic Index	114
Vascular intestinal disorders	Male	2-4 years	95+ years	Data Rich	-1	3	Education (years per capita)	152
Vascular intestinal disorders	Male	2-4 years	95+ years	Data Rich	-1	3	LDI (I$ per capita)	--
Vascular intestinal disorders	Male	2-4 years	95+ years	Data Rich	1	3	Age- and sex-specific SEV for Low fruit	1
Vascular intestinal disorders	Male	2-4 years	95+ years	Data Rich	1	3	Age- and sex-specific SEV for Low omega-3	1
Vascular intestinal disorders	Male	2-4 years	95+ years	Data Rich	1	3	Liters of alcohol consumed per capita	12
Vascular intestinal disorders	Male	2-4 years	95+ years	Data Rich	1	3	Total Fertility Rate	13
Vascular intestinal disorders	Male	2-4 years	95+ years	Data Rich	1	3	Age- and sex-specific SEV for Low vegetables	244
Vascular intestinal disorders	Male	2-4 years	95+ years	Data Rich	1	3	Age- and sex-specific SEV for Low nuts and seeds	355
Vascular intestinal disorders	Male	2-4 years	95+ years	Data Rich	1	3	Age- and sex-specific SEV for Low PUFA	--
Vascular intestinal disorders	Male	2-4 years	95+ years	Global	1	1	Diabetes Fasting Plasma Glucose (mmol/L), age-standardized 25+	124
Vascular intestinal disorders	Male	2-4 years	95+ years	Global	1	1	Systolic Blood Pressure (mmHg)	621
Vascular intestinal disorders	Male	2-4 years	95+ years	Global	1	1	Low-Density Lipoprotein (mmol/L)	--
Vascular intestinal disorders	Male	2-4 years	95+ years	Global	-1	2	Healthcare access and quality index	17
Vascular intestinal disorders	Male	2-4 years	95+ years	Global	1	2	Smoking Prevalence	1
Vascular intestinal disorders	Male	2-4 years	95+ years	Global	1	2	Mean BMI	612
Vascular intestinal disorders	Male	2-4 years	95+ years	Global	-1	3	Education (years per capita)	1
Vascular intestinal disorders	Male	2-4 years	95+ years	Global	-1	3	Pulses legumes unadjusted(g)	281
Vascular intestinal disorders	Male	2-4 years	95+ years	Global	-1	3	LDI (I$ per capita)	--
Vascular intestinal disorders	Male	2-4 years	95+ years	Global	1	3	Age- and sex-specific SEV for Low fruit	0
Vascular intestinal disorders	Male	2-4 years	95+ years	Global	1	3	Age- and sex-specific SEV for Low nuts and seeds	0
Vascular intestinal disorders	Male	2-4 years	95+ years	Global	1	3	Liters of alcohol consumed per capita	129
Vascular intestinal disorders	Male	2-4 years	95+ years	Global	1	3	Age- and sex-specific SEV for Low vegetables	317
Vascular intestinal disorders	Male	2-4 years	95+ years	Global	1	3	Age- and sex-specific SEV for Low PUFA	--
Vascular intestinal disorders	Male	2-4 years	95+ years	Global	1	3	Age- and sex-specific SEV for Low omega-3	--
Vascular intestinal disorders	Male	2-4 years	95+ years	Global	1	3	Total Fertility Rate	--
Gallbladder and biliary diseases	Female	2-4 years	95+ years	Data Rich	1	1	Age- and sex-specific SEV for Low PUFA	159
Gallbladder and biliary diseases	Female	2-4 years	95+ years	Data Rich	1	1	Mean BMI	--
Gallbladder and biliary diseases	Female	2-4 years	95+ years	Data Rich	-1	2	Healthcare access and quality index	633
Gallbladder and biliary diseases	Female	2-4 years	95+ years	Data Rich	1	2	Age- and sex-specific SEV for High red meat	--
Gallbladder and biliary diseases	Female	2-4 years	95+ years	Data Rich	1	2	Liters of alcohol consumed per capita	--
Gallbladder and biliary diseases	Female	2-4 years	95+ years	Data Rich	1	2	Population Over 65 (proportion)	--
Gallbladder and biliary diseases	Female	2-4 years	95+ years	Data Rich	-1	3	Socio-demographic Index	360
Gallbladder and biliary diseases	Female	2-4 years	95+ years	Data Rich	-1	3	Education (years per capita)	--
Gallbladder and biliary diseases	Female	2-4 years	95+ years	Data Rich	-1	3	LDI (I$ per capita)	--
Gallbladder and biliary diseases	Female	2-4 years	95+ years	Global	1	1	Age- and sex-specific SEV for Low PUFA	1000
Gallbladder and biliary diseases	Female	2-4 years	95+ years	Global	1	1	Mean BMI	--
Gallbladder and biliary diseases	Female	2-4 years	95+ years	Global	-1	2	Healthcare access and quality index	--
Gallbladder and biliary diseases	Female	2-4 years	95+ years	Global	1	2	Liters of alcohol consumed per capita	328
Gallbladder and biliary diseases	Female	2-4 years	95+ years	Global	1	2	Age- and sex-specific SEV for High red meat	--
Gallbladder and biliary diseases	Female	2-4 years	95+ years	Global	1	2	Population Over 65 (proportion)	--
Gallbladder and biliary diseases	Female	2-4 years	95+ years	Global	-1	3	Education (years per capita)	--
Gallbladder and biliary diseases	Female	2-4 years	95+ years	Global	-1	3	LDI (I$ per capita)	--
Gallbladder and biliary diseases	Female	2-4 years	95+ years	Global	-1	3	Socio-demographic Index	--
Gallbladder and biliary diseases	Male	2-4 years	95+ years	Data Rich	1	1	Age- and sex-specific SEV for Low PUFA	--
Gallbladder and biliary diseases	Male	2-4 years	95+ years	Data Rich	1	1	Mean BMI	--
Gallbladder and biliary diseases	Male	2-4 years	95+ years	Data Rich	-1	2	Healthcare access and quality index	325
Gallbladder and biliary diseases	Male	2-4 years	95+ years	Data Rich	1	2	Liters of alcohol consumed per capita	427
Gallbladder and biliary diseases	Male	2-4 years	95+ years	Data Rich	1	2	Age- and sex-specific SEV for High red meat	752
Gallbladder and biliary diseases	Male	2-4 years	95+ years	Data Rich	1	2	Population Over 65 (proportion)	--
Gallbladder and biliary diseases	Male	2-4 years	95+ years	Data Rich	-1	3	Education (years per capita)	248
Gallbladder and biliary diseases	Male	2-4 years	95+ years	Data Rich	-1	3	LDI (I$ per capita)	--
Gallbladder and biliary diseases	Male	2-4 years	95+ years	Data Rich	-1	3	Socio-demographic Index	--
Gallbladder and biliary diseases	Male	2-4 years	95+ years	Global	1	1	Age- and sex-specific SEV for Low PUFA	1000
Gallbladder and biliary diseases	Male	2-4 years	95+ years	Global	1	1	Mean BMI	--
Gallbladder and biliary diseases	Male	2-4 years	95+ years	Global	-1	2	Healthcare access and quality index	--
Gallbladder and biliary diseases	Male	2-4 years	95+ years	Global	1	2	Age- and sex-specific SEV for High red meat	328
Gallbladder and biliary diseases	Male	2-4 years	95+ years	Global	1	2	Liters of alcohol consumed per capita	--
Gallbladder and biliary diseases	Male	2-4 years	95+ years	Global	1	2	Population Over 65 (proportion)	--
Gallbladder and biliary diseases	Male	2-4 years	95+ years	Global	-1	3	Education (years per capita)	--
Gallbladder and biliary diseases	Male	2-4 years	95+ years	Global	-1	3	LDI (I$ per capita)	--
Gallbladder and biliary diseases	Male	2-4 years	95+ years	Global	-1	3	Socio-demographic Index	--
Pancreatitis	Female	2-4 years	95+ years	Data Rich	1	1	Log-transformed SEV scalar: Pancreatit	374
Pancreatitis	Female	2-4 years	95+ years	Data Rich	1	1	Liters of alcohol consumed per capita	567
Pancreatitis	Female	2-4 years	95+ years	Data Rich	-1	2	Healthcare access and quality index	55
Pancreatitis	Female	2-4 years	95+ years	Data Rich	1	2	Mean BMI	337
Pancreatitis	Female	2-4 years	95+ years	Data Rich	-1	3	Education (years per capita)	94
Pancreatitis	Female	2-4 years	95+ years	Data Rich	-1	3	LDI (I$ per capita)	--
Pancreatitis	Female	2-4 years	95+ years	Data Rich	-1	3	Socio-demographic Index	--
Pancreatitis	Female	2-4 years	95+ years	Global	1	1	Log-transformed SEV scalar: Pancreatit	192
Pancreatitis	Female	2-4 years	95+ years	Global	1	1	Liters of alcohol consumed per capita	396
Pancreatitis	Female	2-4 years	95+ years	Global	-1	2	Healthcare access and quality index	133
Pancreatitis	Female	2-4 years	95+ years	Global	1	2	Mean BMI	236
Pancreatitis	Female	2-4 years	95+ years	Global	-1	3	Socio-demographic Index	96
Pancreatitis	Female	2-4 years	95+ years	Global	-1	3	Education (years per capita)	107
Pancreatitis	Female	2-4 years	95+ years	Global	-1	3	LDI (I$ per capita)	--
Pancreatitis	Male	2-4 years	95+ years	Data Rich	1	1	Log-transformed SEV scalar: Pancreatit	162
Pancreatitis	Male	2-4 years	95+ years	Data Rich	1	1	Liters of alcohol consumed per capita	1000
Pancreatitis	Male	2-4 years	95+ years	Data Rich	-1	2	Healthcare access and quality index	55
Pancreatitis	Male	2-4 years	95+ years	Data Rich	1	2	Mean BMI	282
Pancreatitis	Male	2-4 years	95+ years	Data Rich	-1	3	Socio-demographic Index	123
Pancreatitis	Male	2-4 years	95+ years	Data Rich	-1	3	Education (years per capita)	--
Pancreatitis	Male	2-4 years	95+ years	Data Rich	-1	3	LDI (I$ per capita)	--
Pancreatitis	Male	2-4 years	95+ years	Global	1	1	Liters of alcohol consumed per capita	--
Pancreatitis	Male	2-4 years	95+ years	Global	1	1	Log-transformed SEV scalar: Pancreatit	--
Pancreatitis	Male	2-4 years	95+ years	Global	-1	2	Healthcare access and quality index	--
Pancreatitis	Male	2-4 years	95+ years	Global	1	2	Mean BMI	398
Pancreatitis	Male	2-4 years	95+ years	Global	-1	3	Socio-demographic Index	274
Pancreatitis	Male	2-4 years	95+ years	Global	-1	3	Education (years per capita)	328
Pancreatitis	Male	2-4 years	95+ years	Global	-1	3	LDI (I$ per capita)	--
Other digestive diseases	Female	12-23 months	95+ years	Data Rich	1	1	Cumulative Cigarettes (10 Years)	--
Other digestive diseases	Female	12-23 months	95+ years	Data Rich	1	1	Cumulative Cigarettes (5 Years)	--
Other digestive diseases	Female	12-23 months	95+ years	Data Rich	1	1	Liters of alcohol consumed per capita	--
Other digestive diseases	Female	12-23 months	95+ years	Data Rich	1	1	Smoking Prevalence	--
Other digestive diseases	Female	12-23 months	95+ years	Data Rich	-1	2	Healthcare access and quality index	--
Other digestive diseases	Female	12-23 months	95+ years	Data Rich	-1	2	Improved Water Source (proportion with access)	--
Other digestive diseases	Female	12-23 months	95+ years	Data Rich	-1	2	Sanitation (proportion with access)	--
Other digestive diseases	Female	12-23 months	95+ years	Data Rich	1	2	Diabetes Age-Standardized Prevalence (proportion)	506
Other digestive diseases	Female	12-23 months	95+ years	Data Rich	1	2	Age- and sex-specific SEV for High red meat	513
Other digestive diseases	Female	12-23 months	95+ years	Data Rich	1	2	Mean BMI	797
Other digestive diseases	Female	12-23 months	95+ years	Data Rich	1	2	Age- and sex-specific SEV for Low PUFA	--
Other digestive diseases	Female	12-23 months	95+ years	Data Rich	1	2	Age- and sex-specific SEV for Low fruit	--
Other digestive diseases	Female	12-23 months	95+ years	Data Rich	1	2	Age- and sex-specific SEV for Low vegetables	--
Other digestive diseases	Female	12-23 months	95+ years	Data Rich	-1	3	Education (years per capita)	--

CODEm covariates used, level of covariate, and expected direction of covariate by cause, sex, and age								
Cause	Sex	Age Start	Age End	Model Version Type	Direction	Level	Covariate Name	Number of Draws
Other digestive diseases	Female	12-23 months	95+ years	Data Rich	-1	3	LDI (I$ per capita)	--
Other digestive diseases	Female	12-23 months	95+ years	Data Rich	1	3	Socio-demographic Index	519
Other digestive diseases	Female	12-23 months	95+ years	Global	1	1	Smoking Prevalence	254
Other digestive diseases	Female	12-23 months	95+ years	Global	1	1	Cumulative Cigarettes (10 Years)	--
Other digestive diseases	Female	12-23 months	95+ years	Global	1	1	Cumulative Cigarettes (5 Years)	--
Other digestive diseases	Female	12-23 months	95+ years	Global	1	1	Liters of alcohol consumed per capita	--
Other digestive diseases	Female	12-23 months	95+ years	Global	-1	2	Improved Water Source (proportion with access)	99
Other digestive diseases	Female	12-23 months	95+ years	Global	-1	2	Healthcare access and quality index	361
Other digestive diseases	Female	12-23 months	95+ years	Global	-1	2	Sanitation (proportion with access)	366
Other digestive diseases	Female	12-23 months	95+ years	Global	1	2	Age- and sex-specific SEV for Low PUFA	298
Other digestive diseases	Female	12-23 months	95+ years	Global	1	2	Age- and sex-specific SEV for Low fruit	322
Other digestive diseases	Female	12-23 months	95+ years	Global	1	2	Age- and sex-specific SEV for High red meat	361
Other digestive diseases	Female	12-23 months	95+ years	Global	1	2	Diabetes Age-Standardized Prevalence (proportion)	361
Other digestive diseases	Female	12-23 months	95+ years	Global	1	2	Mean BMI	361
Other digestive diseases	Female	12-23 months	95+ years	Global	1	2	Age- and sex-specific SEV for Low vegetables	545
Other digestive diseases	Female	12-23 months	95+ years	Global	-1	3	Socio-demographic Index	171
Other digestive diseases	Female	12-23 months	95+ years	Global	-1	3	Education (years per capita)	214
Other digestive diseases	Female	12-23 months	95+ years	Global	-1	3	LDI (I$ per capita)	--
Other digestive diseases	Male	12-23 months	95+ years	Data Rich	1	1	Cumulative Cigarettes (10 Years)	--
Other digestive diseases	Male	12-23 months	95+ years	Data Rich	1	1	Cumulative Cigarettes (5 Years)	--
Other digestive diseases	Male	12-23 months	95+ years	Data Rich	1	1	Liters of alcohol consumed per capita	--
Other digestive diseases	Male	12-23 months	95+ years	Data Rich	1	1	Smoking Prevalence	--
Other digestive diseases	Male	12-23 months	95+ years	Data Rich	-1	2	Healthcare access and quality index	--
Other digestive diseases	Male	12-23 months	95+ years	Data Rich	-1	2	Improved Water Source (proportion with access)	--
Other digestive diseases	Male	12-23 months	95+ years	Data Rich	-1	2	Sanitation (proportion with access)	--
Other digestive diseases	Male	12-23 months	95+ years	Data Rich	1	2	Age- and sex-specific SEV for Low vegetables	186
Other digestive diseases	Male	12-23 months	95+ years	Data Rich	1	2	Age- and sex-specific SEV for High red meat	491
Other digestive diseases	Male	12-23 months	95+ years	Data Rich	1	2	Diabetes Age-Standardized Prevalence (proportion)	546
Other digestive diseases	Male	12-23 months	95+ years	Data Rich	1	2	Mean BMI	777
Other digestive diseases	Male	12-23 months	95+ years	Data Rich	1	2	Age- and sex-specific SEV for Low PUFA	--
Other digestive diseases	Male	12-23 months	95+ years	Data Rich	1	2	Age- and sex-specific SEV for Low fruit	--
Other digestive diseases	Male	12-23 months	95+ years	Data Rich	-1	3	Education (years per capita)	--
Other digestive diseases	Male	12-23 months	95+ years	Data Rich	-1	3	LDI (I$ per capita)	--
Other digestive diseases	Male	12-23 months	95+ years	Data Rich	-1	3	Socio-demographic Index	--
Other digestive diseases	Male	12-23 months	95+ years	Global	1	1	Cumulative Cigarettes (10 Years)	14
Other digestive diseases	Male	12-23 months	95+ years	Global	1	1	Cumulative Cigarettes (5 Years)	17
Other digestive diseases	Male	12-23 months	95+ years	Global	1	1	Smoking Prevalence	969
Other digestive diseases	Male	12-23 months	95+ years	Global	-1	2	Sanitation (proportion with access)	277
Other digestive diseases	Male	12-23 months	95+ years	Global	-1	2	Improved Water Source (proportion with access)	625
Other digestive diseases	Male	12-23 months	95+ years	Global	-1	2	Healthcare access and quality index	--
Other digestive diseases	Male	12-23 months	95+ years	Global	1	2	Diabetes Age-Standardized Prevalence (proportion)	343
Other digestive diseases	Male	12-23 months	95+ years	Global	1	2	Mean BMI	537
Other digestive diseases	Male	12-23 months	95+ years	Global	1	2	Age- and sex-specific SEV for Low fruit	601
Other digestive diseases	Male	12-23 months	95+ years	Global	1	2	Age- and sex-specific SEV for Low vegetables	679
Other digestive diseases	Male	12-23 months	95+ years	Global	1	2	Age- and sex-specific SEV for Low PUFA	860
Other digestive diseases	Male	12-23 months	95+ years	Global	1	2	Age- and sex-specific SEV for High red meat	880
Other digestive diseases	Male	12-23 months	95+ years	Global	-1	3	Socio-demographic Index	152
Other digestive diseases	Male	12-23 months	95+ years	Global	-1	3	Education (years per capita)	158
Other digestive diseases	Male	12-23 months	95+ years	Global	-1	3	LDI (I$ per capita)	--
Parkinson's disease	Female	20-24 years	95+ years	Data Rich	-1	1	Age- and sex-specific SEV for Low fruit	710
Parkinson's disease	Female	20-24 years	95+ years	Data Rich	-1	1	Cumulative Cigarettes (10 Years)	804
Parkinson's disease	Female	20-24 years	95+ years	Data Rich	-1	2	Healthcare access and quality index	--
Parkinson's disease	Female	20-24 years	95+ years	Data Rich	1	2	Absolute value of average latitude	45
Parkinson's disease	Female	20-24 years	95+ years	Data Rich	1	2	Improved Water Source (proportion with access)	125
Parkinson's disease	Female	20-24 years	95+ years	Data Rich	1	2	Sanitation (proportion with access)	125
Parkinson's disease	Female	20-24 years	95+ years	Data Rich	1	2	Low-Density Lipoprotein (mmol/L)	--
Parkinson's disease	Female	20-24 years	95+ years	Data Rich	-1	3	Education (years per capita)	--
Parkinson's disease	Female	20-24 years	95+ years	Data Rich	1	3	Socio-demographic Index	440
Parkinson's disease	Female	20-24 years	95+ years	Data Rich	1	3	LDI (I$ per capita)	--
Parkinson's disease	Female	20-24 years	95+ years	Global	-1	1	Cumulative Cigarettes (10 Years)	445
Parkinson's disease	Female	20-24 years	95+ years	Global	-1	1	Age- and sex-specific SEV for Low fruit	555
Parkinson's disease	Female	20-24 years	95+ years	Global	-1	2	Healthcare access and quality index	313
Parkinson's disease	Female	20-24 years	95+ years	Global	1	2	Improved Water Source (proportion with access)	219
Parkinson's disease	Female	20-24 years	95+ years	Global	1	2	Sanitation (proportion with access)	219
Parkinson's disease	Female	20-24 years	95+ years	Global	1	2	Absolute value of average latitude	--
Parkinson's disease	Female	20-24 years	95+ years	Global	1	2	Low-Density Lipoprotein (mmol/L)	--
Parkinson's disease	Female	20-24 years	95+ years	Global	-1	3	Education (years per capita)	60
Parkinson's disease	Female	20-24 years	95+ years	Global	1	3	Socio-demographic Index	124
Parkinson's disease	Female	20-24 years	95+ years	Global	1	3	LDI (I$ per capita)	--
Parkinson's disease	Male	20-24 years	95+ years	Data Rich	-1	1	Age- and sex-specific SEV for Low fruit	572
Parkinson's disease	Male	20-24 years	95+ years	Data Rich	-1	1	Cumulative Cigarettes (10 Years)	870
Parkinson's disease	Male	20-24 years	95+ years	Data Rich	-1	2	Healthcare access and quality index	96
Parkinson's disease	Male	20-24 years	95+ years	Data Rich	1	2	Improved Water Source (proportion with access)	83
Parkinson's disease	Male	20-24 years	95+ years	Data Rich	1	2	Sanitation (proportion with access)	100
Parkinson's disease	Male	20-24 years	95+ years	Data Rich	1	2	Absolute value of average latitude	--
Parkinson's disease	Male	20-24 years	95+ years	Data Rich	1	2	Low-Density Lipoprotein (mmol/L)	--
Parkinson's disease	Male	20-24 years	95+ years	Data Rich	-1	3	Education (years per capita)	--
Parkinson's disease	Male	20-24 years	95+ years	Data Rich	1	3	Socio-demographic Index	366
Parkinson's disease	Male	20-24 years	95+ years	Data Rich	1	3	LDI (I$ per capita)	--
Parkinson's disease	Male	20-24 years	95+ years	Global	-1	1	Age- and sex-specific SEV for Low fruit	533
Parkinson's disease	Male	20-24 years	95+ years	Global	-1	1	Cumulative Cigarettes (10 Years)	882
Parkinson's disease	Male	20-24 years	95+ years	Global	-1	2	Healthcare access and quality index	173
Parkinson's disease	Male	20-24 years	95+ years	Global	1	2	Sanitation (proportion with access)	85
Parkinson's disease	Male	20-24 years	95+ years	Global	1	2	Improved Water Source (proportion with access)	181
Parkinson's disease	Male	20-24 years	95+ years	Global	1	2	Absolute value of average latitude	--
Parkinson's disease	Male	20-24 years	95+ years	Global	1	2	Low-Density Lipoprotein (mmol/L)	--
Parkinson's disease	Male	20-24 years	95+ years	Global	-1	3	Education (years per capita)	17
Parkinson's disease	Male	20-24 years	95+ years	Global	1	3	Socio-demographic Index	88
Parkinson's disease	Male	20-24 years	95+ years	Global	1	3	LDI (I$ per capita)	--
Idiopathic epilepsy	Female	0-6 days	95+ years	Data Rich	1	1	Log-transformed SEV scalar: Idiopathic epilepsy	360
Idiopathic epilepsy	Female	0-6 days	95+ years	Data Rich	1	1	Pigs (per capita)	--
Idiopathic epilepsy	Female	0-6 days	95+ years	Data Rich	1	1	Systolic Blood Pressure (mmHg)	--
Idiopathic epilepsy	Female	0-6 days	95+ years	Data Rich	-1	2	Healthcare access and quality index	--
Idiopathic epilepsy	Female	0-6 days	95+ years	Data Rich	1	2	Low-Density Lipoprotein (mmol/L)	--
Idiopathic epilepsy	Female	0-6 days	95+ years	Data Rich	1	2	Mean BMI	--
Idiopathic epilepsy	Female	0-6 days	95+ years	Data Rich	-1	3	Education (years per capita)	273
Idiopathic epilepsy	Female	0-6 days	95+ years	Data Rich	-1	3	LDI (I$ per capita)	--
Idiopathic epilepsy	Female	0-6 days	95+ years	Data Rich	-1	3	Socio-demographic Index	--
Idiopathic epilepsy	Female	0-6 days	95+ years	Data Rich	1	3	Cumulative Cigarettes (5 Years)	159
Idiopathic epilepsy	Female	0-6 days	95+ years	Data Rich	1	3	Cumulative Cigarettes (10 Years)	--
Idiopathic epilepsy	Female	0-6 days	95+ years	Global	1	1	Log-transformed SEV scalar: Idiopathic epilepsy	428
Idiopathic epilepsy	Female	0-6 days	95+ years	Global	1	1	Pigs (per capita)	--
Idiopathic epilepsy	Female	0-6 days	95+ years	Global	1	1	Systolic Blood Pressure (mmHg)	--
Idiopathic epilepsy	Female	0-6 days	95+ years	Global	-1	2	Healthcare access and quality index	351
Idiopathic epilepsy	Female	0-6 days	95+ years	Global	1	2	Mean BMI	228
Idiopathic epilepsy	Female	0-6 days	95+ years	Global	1	2	Low-Density Lipoprotein (mmol/L)	--
Idiopathic epilepsy	Female	0-6 days	95+ years	Global	-1	3	Socio-demographic Index	246
Idiopathic epilepsy	Female	0-6 days	95+ years	Global	-1	3	Education (years per capita)	332
Idiopathic epilepsy	Female	0-6 days	95+ years	Global	-1	3	LDI (I$ per capita)	--
Idiopathic epilepsy	Female	0-6 days	95+ years	Global	1	3	Cumulative Cigarettes (10 Years)	--
Idiopathic epilepsy	Female	0-6 days	95+ years	Global	1	3	Cumulative Cigarettes (5 Years)	--
Idiopathic epilepsy	Male	0-6 days	95+ years	Data Rich	1	1	Log-transformed SEV scalar: Idiopathic epilepsy	489
Idiopathic epilepsy	Male	0-6 days	95+ years	Data Rich	1	1	Systolic Blood Pressure (mmHg)	738
Idiopathic epilepsy	Male	0-6 days	95+ years	Data Rich	1	1	Pigs (per capita)	--
Idiopathic epilepsy	Male	0-6 days	95+ years	Data Rich	-1	2	Healthcare access and quality index	--
Idiopathic epilepsy	Male	0-6 days	95+ years	Data Rich	1	2	Low-Density Lipoprotein (mmol/L)	--

CODEm covariates used, level of covariate, and expected direction of covariate by cause, sex, and age

Cause	Sex	Age Start	Age End	Model Version Type	Direction	Level	Covariate Name	Number of Draws
Idiopathic epilepsy	Male	0-6 days	95+ years	Data Rich	1	2	Mean BMI	--
Idiopathic epilepsy	Male	0-6 days	95+ years	Data Rich	-1	3	Socio-demographic Index	349
Idiopathic epilepsy	Male	0-6 days	95+ years	Data Rich	-1	3	Education (years per capita)	464
Idiopathic epilepsy	Male	0-6 days	95+ years	Data Rich	-1	3	LDI (I$ per capita)	--
Idiopathic epilepsy	Male	0-6 days	95+ years	Data Rich	1	3	Cumulative Cigarettes (10 Years)	51
Idiopathic epilepsy	Male	0-6 days	95+ years	Data Rich	1	3	Cumulative Cigarettes (5 Years)	67
Idiopathic epilepsy	Male	0-6 days	95+ years	Global	1	1	Log-transformed SEV scalar: Idiopathic epilepsy	--
Idiopathic epilepsy	Male	0-6 days	95+ years	Global	1	1	Pigs (per capita)	--
Idiopathic epilepsy	Male	0-6 days	95+ years	Global	1	1	Systolic Blood Pressure (mmHg)	--
Idiopathic epilepsy	Male	0-6 days	95+ years	Global	-1	2	Healthcare access and quality index	636
Idiopathic epilepsy	Male	0-6 days	95+ years	Global	1	2	Low-Density Lipoprotein (mmol/L)	--
Idiopathic epilepsy	Male	0-6 days	95+ years	Global	1	2	Mean BMI	--
Idiopathic epilepsy	Male	0-6 days	95+ years	Global	-1	3	Education (years per capita)	467
Idiopathic epilepsy	Male	0-6 days	95+ years	Global	-1	3	Socio-demographic Index	467
Idiopathic epilepsy	Male	0-6 days	95+ years	Global	-1	3	LDI (I$ per capita)	--
Idiopathic epilepsy	Male	0-6 days	95+ years	Global	1	3	Cumulative Cigarettes (10 Years)	--
Idiopathic epilepsy	Male	0-6 days	95+ years	Global	1	3	Cumulative Cigarettes (5 Years)	--
Multiple sclerosis	Female	5-9 years	95+ years	Data Rich	1	1	Absolute value of average latitude	1000
Multiple sclerosis	Female	5-9 years	95+ years	Data Rich	-1	2	Healthcare access and quality index	--
Multiple sclerosis	Female	5-9 years	95+ years	Data Rich	1	2	Low-Density Lipoprotein (mmol/L)	--
Multiple sclerosis	Female	5-9 years	95+ years	Data Rich	-1	3	Education (years per capita)	--
Multiple sclerosis	Female	5-9 years	95+ years	Data Rich	-1	3	LDI (I$ per capita)	--
Multiple sclerosis	Female	5-9 years	95+ years	Data Rich	1	3	Socio-demographic Index	568
Multiple sclerosis	Female	5-9 years	95+ years	Data Rich	1	3	Cumulative Cigarettes (10 Years)	--
Multiple sclerosis	Female	5-9 years	95+ years	Data Rich	1	3	Cumulative Cigarettes (5 Years)	--
Multiple sclerosis	Female	5-9 years	95+ years	Data Rich	1	3	Smoking Prevalence	--
Multiple sclerosis	Female	5-9 years	95+ years	Global	1	1	Absolute value of average latitude	674
Multiple sclerosis	Female	5-9 years	95+ years	Global	-1	2	Healthcare access and quality index	422
Multiple sclerosis	Female	5-9 years	95+ years	Global	1	2	Low-Density Lipoprotein (mmol/L)	--
Multiple sclerosis	Female	5-9 years	95+ years	Global	-1	3	Education (years per capita)	--
Multiple sclerosis	Female	5-9 years	95+ years	Global	-1	3	LDI (I$ per capita)	--
Multiple sclerosis	Female	5-9 years	95+ years	Global	1	3	Socio-demographic Index	326
Multiple sclerosis	Female	5-9 years	95+ years	Global	1	3	Cumulative Cigarettes (10 Years)	--
Multiple sclerosis	Female	5-9 years	95+ years	Global	1	3	Cumulative Cigarettes (5 Years)	--
Multiple sclerosis	Female	5-9 years	95+ years	Global	1	3	Smoking Prevalence	--
Multiple sclerosis	Male	5-9 years	95+ years	Data Rich	1	1	Absolute value of average latitude	752
Multiple sclerosis	Male	5-9 years	95+ years	Data Rich	-1	2	Healthcare access and quality index	--
Multiple sclerosis	Male	5-9 years	95+ years	Data Rich	1	2	Low-Density Lipoprotein (mmol/L)	--
Multiple sclerosis	Male	5-9 years	95+ years	Data Rich	-1	3	Education (years per capita)	--
Multiple sclerosis	Male	5-9 years	95+ years	Data Rich	-1	3	LDI (I$ per capita)	--
Multiple sclerosis	Male	5-9 years	95+ years	Data Rich	1	3	Socio-demographic Index	675
Multiple sclerosis	Male	5-9 years	95+ years	Data Rich	1	3	Cumulative Cigarettes (10 Years)	--
Multiple sclerosis	Male	5-9 years	95+ years	Data Rich	1	3	Cumulative Cigarettes (5 Years)	--
Multiple sclerosis	Male	5-9 years	95+ years	Data Rich	1	3	Smoking Prevalence	--
Multiple sclerosis	Male	5-9 years	95+ years	Global	1	1	Absolute value of average latitude	699
Multiple sclerosis	Male	5-9 years	95+ years	Global	-1	2	Healthcare access and quality index	206
Multiple sclerosis	Male	5-9 years	95+ years	Global	1	2	Low-Density Lipoprotein (mmol/L)	--
Multiple sclerosis	Male	5-9 years	95+ years	Global	-1	3	Education (years per capita)	--
Multiple sclerosis	Male	5-9 years	95+ years	Global	-1	3	LDI (I$ per capita)	--
Multiple sclerosis	Male	5-9 years	95+ years	Global	1	3	Smoking Prevalence	96
Multiple sclerosis	Male	5-9 years	95+ years	Global	1	3	Cumulative Cigarettes (10 Years)	115
Multiple sclerosis	Male	5-9 years	95+ years	Global	1	3	Cumulative Cigarettes (5 Years)	249
Multiple sclerosis	Male	5-9 years	95+ years	Global	1	3	Socio-demographic Index	380
Motor neuron disease	Female	0-6 days	95+ years	Data Rich	-1	1	Age- and sex-specific SEV for Low fruit	604
Motor neuron disease	Female	0-6 days	95+ years	Data Rich	-1	1	Low-Density Lipoprotein (mmol/L)	--
Motor neuron disease	Female	0-6 days	95+ years	Data Rich	-1	1	Mean BMI	--
Motor neuron disease	Female	0-6 days	95+ years	Data Rich	1	1	Absolute value of average latitude	209
Motor neuron disease	Female	0-6 days	95+ years	Data Rich	1	1	Diabetes Fasting Plasma Glucose (mmol/L), age-standardized 25+	366
Motor neuron disease	Female	0-6 days	95+ years	Data Rich	1	1	Socio-demographic Index	--
Motor neuron disease	Female	0-6 days	95+ years	Data Rich	-1	2	Population-weighted mean temperature	94
Motor neuron disease	Female	0-6 days	95+ years	Data Rich	-1	2	Healthcare access and quality index	--
Motor neuron disease	Female	0-6 days	95+ years	Data Rich	-1	2	Improved Water Source (proportion with access)	--
Motor neuron disease	Female	0-6 days	95+ years	Data Rich	1	2	Sanitation (proportion with access)	527
Motor neuron disease	Female	0-6 days	95+ years	Data Rich	1	3	Education (years per capita)	285
Motor neuron disease	Female	0-6 days	95+ years	Data Rich	1	3	LDI (I$ per capita)	--
Motor neuron disease	Female	0-6 days	95+ years	Global	-1	1	Age- and sex-specific SEV for Low fruit	9
Motor neuron disease	Female	0-6 days	95+ years	Global	-1	1	Low-Density Lipoprotein (mmol/L)	--
Motor neuron disease	Female	0-6 days	95+ years	Global	-1	1	Mean BMI	--
Motor neuron disease	Female	0-6 days	95+ years	Global	1	1	Absolute value of average latitude	152
Motor neuron disease	Female	0-6 days	95+ years	Global	1	1	Diabetes Fasting Plasma Glucose (mmol/L), age-standardized 25+	527
Motor neuron disease	Female	0-6 days	95+ years	Global	1	1	Socio-demographic Index	986
Motor neuron disease	Female	0-6 days	95+ years	Global	-1	2	Improved Water Source (proportion with access)	17
Motor neuron disease	Female	0-6 days	95+ years	Global	-1	2	Healthcare access and quality index	20
Motor neuron disease	Female	0-6 days	95+ years	Global	-1	2	Population-weighted mean temperature	273
Motor neuron disease	Female	0-6 days	95+ years	Global	1	2	Sanitation (proportion with access)	238
Motor neuron disease	Female	0-6 days	95+ years	Global	1	3	Education (years per capita)	471
Motor neuron disease	Female	0-6 days	95+ years	Global	1	3	LDI (I$ per capita)	--
Motor neuron disease	Male	0-6 days	95+ years	Data Rich	-1	1	Age- and sex-specific SEV for Low fruit	19
Motor neuron disease	Male	0-6 days	95+ years	Data Rich	-1	1	Low-Density Lipoprotein (mmol/L)	--
Motor neuron disease	Male	0-6 days	95+ years	Data Rich	-1	1	Mean BMI	--
Motor neuron disease	Male	0-6 days	95+ years	Data Rich	1	1	Absolute value of average latitude	76
Motor neuron disease	Male	0-6 days	95+ years	Data Rich	1	1	Diabetes Fasting Plasma Glucose (mmol/L), age-standardized 25+	133
Motor neuron disease	Male	0-6 days	95+ years	Data Rich	1	1	Socio-demographic Index	593
Motor neuron disease	Male	0-6 days	95+ years	Data Rich	-1	2	Population-weighted mean temperature	47
Motor neuron disease	Male	0-6 days	95+ years	Data Rich	-1	2	Healthcare access and quality index	--
Motor neuron disease	Male	0-6 days	95+ years	Data Rich	-1	2	Improved Water Source (proportion with access)	--
Motor neuron disease	Male	0-6 days	95+ years	Data Rich	1	2	Sanitation (proportion with access)	495
Motor neuron disease	Male	0-6 days	95+ years	Data Rich	1	3	Education (years per capita)	391
Motor neuron disease	Male	0-6 days	95+ years	Data Rich	1	3	LDI (I$ per capita)	--
Motor neuron disease	Male	0-6 days	95+ years	Global	-1	1	Age- and sex-specific SEV for Low fruit	298
Motor neuron disease	Male	0-6 days	95+ years	Global	-1	1	Low-Density Lipoprotein (mmol/L)	--
Motor neuron disease	Male	0-6 days	95+ years	Global	-1	1	Mean BMI	--
Motor neuron disease	Male	0-6 days	95+ years	Global	1	1	Diabetes Fasting Plasma Glucose (mmol/L), age-standardized 25+	213
Motor neuron disease	Male	0-6 days	95+ years	Global	1	1	Socio-demographic Index	349
Motor neuron disease	Male	0-6 days	95+ years	Global	1	1	Absolute value of average latitude	591
Motor neuron disease	Male	0-6 days	95+ years	Global	-1	2	Improved Water Source (proportion with access)	33
Motor neuron disease	Male	0-6 days	95+ years	Global	-1	2	Healthcare access and quality index	--
Motor neuron disease	Male	0-6 days	95+ years	Global	-1	2	Population-weighted mean temperature	--
Motor neuron disease	Male	0-6 days	95+ years	Global	1	2	Sanitation (proportion with access)	320
Motor neuron disease	Male	0-6 days	95+ years	Global	1	3	Education (years per capita)	113
Motor neuron disease	Male	0-6 days	95+ years	Global	1	3	LDI (I$ per capita)	--
Other neurological disorders	Female	0-6 days	95+ years	Data Rich	1	1	Age- and sex-specific SEV for Child underweight	2
Other neurological disorders	Female	0-6 days	95+ years	Data Rich	1	1	Systolic Blood Pressure (mmHg)	75
Other neurological disorders	Female	0-6 days	95+ years	Data Rich	1	1	Age- and sex-specific SEV for High red meat	253
Other neurological disorders	Female	0-6 days	95+ years	Data Rich	1	1	Pigs (per capita)	431
Other neurological disorders	Female	0-6 days	95+ years	Data Rich	1	1	Mean BMI	751
Other neurological disorders	Female	0-6 days	95+ years	Data Rich	1	1	Low-Density Lipoprotein (mmol/L)	--
Other neurological disorders	Female	0-6 days	95+ years	Data Rich	-1	2	Healthcare access and quality index	21
Other neurological disorders	Female	0-6 days	95+ years	Data Rich	-1	2	Age- and sex-specific SEV for Low fruit	307
Other neurological disorders	Female	0-6 days	95+ years	Data Rich	1	2	Population Density (over 1000 ppl/sqkm, proportion)	--
Other neurological disorders	Female	0-6 days	95+ years	Data Rich	-1	3	Education (years per capita)	--
Other neurological disorders	Female	0-6 days	95+ years	Data Rich	-1	3	LDI (I$ per capita)	--
Other neurological disorders	Female	0-6 days	95+ years	Data Rich	1	3	Smoking Prevalence	0
Other neurological disorders	Female	0-6 days	95+ years	Data Rich	1	3	Socio-demographic Index	353
Other neurological disorders	Female	0-6 days	95+ years	Data Rich	1	3	Cumulative Cigarettes (10 Years)	--
Other neurological disorders	Female	0-6 days	95+ years	Data Rich	1	3	Cumulative Cigarettes (5 Years)	--
Other neurological disorders	Female	0-6 days	95+ years	Global	1	1	Mean BMI	186
Other neurological disorders	Female	0-6 days	95+ years	Global	1	1	Systolic Blood Pressure (mmHg)	310

CODEm covariates used, level of covariate, and expected direction of covariate by cause, sex, and age

Cause	Sex	Age Start	Age End	Model Version Type	Direction	Level	Covariate Name	Number of Draws
Other neurological disorders	Female	0-6 days	95+ years	Global	1	1	Age- and sex-specific SEV for High red meat	587
Other neurological disorders	Female	0-6 days	95+ years	Global	1	1	Pigs (per capita)	802
Other neurological disorders	Female	0-6 days	95+ years	Global	1	1	Low-Density Lipoprotein (mmol/L)	--
Other neurological disorders	Female	0-6 days	95+ years	Global	-1	2	Age- and sex-specific SEV for Low fruit	310
Other neurological disorders	Female	0-6 days	95+ years	Global	-1	2	Healthcare access and quality index	--
Other neurological disorders	Female	0-6 days	95+ years	Global	1	2	Population Density (over 1000 ppl/sqkm, proportion)	104
Other neurological disorders	Female	0-6 days	95+ years	Global	-1	3	Education (years per capita)	--
Other neurological disorders	Female	0-6 days	95+ years	Global	-1	3	LDI (I$ per capita)	--
Other neurological disorders	Female	0-6 days	95+ years	Global	1	3	Smoking Prevalence	5
Other neurological disorders	Female	0-6 days	95+ years	Global	1	3	Cumulative Cigarettes (5 Years)	20
Other neurological disorders	Female	0-6 days	95+ years	Global	1	3	Cumulative Cigarettes (10 Years)	27
Other neurological disorders	Female	0-6 days	95+ years	Global	1	3	Socio-demographic Index	818
Other neurological disorders	Male	0-6 days	95+ years	Data Rich	1	1	Age- and sex-specific SEV for Child underweight	0
Other neurological disorders	Male	0-6 days	95+ years	Data Rich	1	1	Age- and sex-specific SEV for High red meat	43
Other neurological disorders	Male	0-6 days	95+ years	Data Rich	1	1	Systolic Blood Pressure (mmHg)	258
Other neurological disorders	Male	0-6 days	95+ years	Data Rich	1	1	Pigs (per capita)	812
Other neurological disorders	Male	0-6 days	95+ years	Data Rich	1	1	Mean BMI	861
Other neurological disorders	Male	0-6 days	95+ years	Data Rich	1	1	Low-Density Lipoprotein (mmol/L)	--
Other neurological disorders	Male	0-6 days	95+ years	Data Rich	-1	2	Healthcare access and quality index	0
Other neurological disorders	Male	0-6 days	95+ years	Data Rich	-1	2	Age- and sex-specific SEV for Low fruit	212
Other neurological disorders	Male	0-6 days	95+ years	Data Rich	1	2	Population Density (over 1000 ppl/sqkm, proportion)	135
Other neurological disorders	Male	0-6 days	95+ years	Data Rich	-1	3	Education (years per capita)	--
Other neurological disorders	Male	0-6 days	95+ years	Data Rich	-1	3	LDI (I$ per capita)	--
Other neurological disorders	Male	0-6 days	95+ years	Data Rich	1	3	Socio-demographic Index	566
Other neurological disorders	Male	0-6 days	95+ years	Data Rich	1	3	Cumulative Cigarettes (10 Years)	--
Other neurological disorders	Male	0-6 days	95+ years	Data Rich	1	3	Cumulative Cigarettes (5 Years)	--
Other neurological disorders	Male	0-6 days	95+ years	Data Rich	1	3	Smoking Prevalence	--
Other neurological disorders	Male	0-6 days	95+ years	Global	1	1	Age- and sex-specific SEV for Child underweight	16
Other neurological disorders	Male	0-6 days	95+ years	Global	1	1	Systolic Blood Pressure (mmHg)	290
Other neurological disorders	Male	0-6 days	95+ years	Global	1	1	Age- and sex-specific SEV for High red meat	370
Other neurological disorders	Male	0-6 days	95+ years	Global	1	1	Mean BMI	692
Other neurological disorders	Male	0-6 days	95+ years	Global	1	1	Pigs (per capita)	749
Other neurological disorders	Male	0-6 days	95+ years	Global	1	1	Low-Density Lipoprotein (mmol/L)	--
Other neurological disorders	Male	0-6 days	95+ years	Global	-1	2	Age- and sex-specific SEV for Low fruit	84
Other neurological disorders	Male	0-6 days	95+ years	Global	-1	2	Healthcare access and quality index	--
Other neurological disorders	Male	0-6 days	95+ years	Global	1	2	Liters of alcohol consumed per capita	7
Other neurological disorders	Male	0-6 days	95+ years	Global	1	2	Population Density (over 1000 ppl/sqkm, proportion)	66
Other neurological disorders	Male	0-6 days	95+ years	Global	-1	3	Education (years per capita)	--
Other neurological disorders	Male	0-6 days	95+ years	Global	-1	3	LDI (I$ per capita)	--
Other neurological disorders	Male	0-6 days	95+ years	Global	1	3	Cumulative Cigarettes (10 Years)	9
Other neurological disorders	Male	0-6 days	95+ years	Global	1	3	Cumulative Cigarettes (5 Years)	10
Other neurological disorders	Male	0-6 days	95+ years	Global	1	3	Socio-demographic Index	596
Other neurological disorders	Male	0-6 days	95+ years	Global	1	3	Smoking Prevalence	--
Anorexia nervosa	Female	5-9 years	45-49 years	Data Rich	-1	1	Age- and sex-specific SEV for Child underweight	--
Anorexia nervosa	Female	5-9 years	45-49 years	Data Rich	1	1	Maternal Education (years per capita)	407
Anorexia nervosa	Female	5-9 years	45-49 years	Data Rich	1	1	Education (years per capita)	--
Anorexia nervosa	Female	5-9 years	45-49 years	Data Rich	1	1	LDI (I$ per capita)	--
Anorexia nervosa	Female	5-9 years	45-49 years	Data Rich	1	1	Sanitation (proportion with access)	--
Anorexia nervosa	Female	5-9 years	45-49 years	Data Rich	-1	2	Healthcare access and quality index	--
Anorexia nervosa	Female	5-9 years	45-49 years	Data Rich	1	3	Socio-demographic Index	464
Anorexia nervosa	Female	5-9 years	45-49 years	Global	-1	1	Age- and sex-specific SEV for Child underweight	--
Anorexia nervosa	Female	5-9 years	45-49 years	Global	1	1	Maternal Education (years per capita)	562
Anorexia nervosa	Female	5-9 years	45-49 years	Global	1	1	Education (years per capita)	--
Anorexia nervosa	Female	5-9 years	45-49 years	Global	1	1	LDI (I$ per capita)	--
Anorexia nervosa	Female	5-9 years	45-49 years	Global	1	1	Sanitation (proportion with access)	--
Anorexia nervosa	Female	5-9 years	45-49 years	Global	-1	2	Healthcare access and quality index	--
Anorexia nervosa	Female	5-9 years	45-49 years	Global	1	3	Socio-demographic Index	289
Anorexia nervosa	Male	5-9 years	45-49 years	Data Rich	-1	1	Age- and sex-specific SEV for Child underweight	--
Anorexia nervosa	Male	5-9 years	45-49 years	Data Rich	1	1	Maternal Education (years per capita)	189
Anorexia nervosa	Male	5-9 years	45-49 years	Data Rich	1	1	Education (years per capita)	--
Anorexia nervosa	Male	5-9 years	45-49 years	Data Rich	1	1	LDI (I$ per capita)	--
Anorexia nervosa	Male	5-9 years	45-49 years	Data Rich	1	1	Sanitation (proportion with access)	--
Anorexia nervosa	Male	5-9 years	45-49 years	Data Rich	-1	2	Healthcare access and quality index	--
Anorexia nervosa	Male	5-9 years	45-49 years	Data Rich	1	3	Socio-demographic Index	88
Anorexia nervosa	Male	5-9 years	45-49 years	Global	-1	1	Age- and sex-specific SEV for Child underweight	--
Anorexia nervosa	Male	5-9 years	45-49 years	Global	1	1	Maternal Education (years per capita)	171
Anorexia nervosa	Male	5-9 years	45-49 years	Global	1	1	Education (years per capita)	--
Anorexia nervosa	Male	5-9 years	45-49 years	Global	1	1	LDI (I$ per capita)	--
Anorexia nervosa	Male	5-9 years	45-49 years	Global	1	1	Sanitation (proportion with access)	--
Anorexia nervosa	Male	5-9 years	45-49 years	Global	-1	2	Healthcare access and quality index	--
Anorexia nervosa	Male	5-9 years	45-49 years	Global	1	3	Socio-demographic Index	61
Alcohol use disorders	Female	15-19 years	95+ years	Data Rich	1	1	Alcohol drinker proportion, age-standardized	38
Alcohol use disorders	Female	15-19 years	95+ years	Data Rich	1	1	Alcohol consumption, age standardized, in grams per day	166
Alcohol use disorders	Female	15-19 years	95+ years	Data Rich	1	1	Alcohol binge drinker proportion, age-standardized	802
Alcohol use disorders	Female	15-19 years	95+ years	Data Rich	1	1	Liters of alcohol consumed per capita	--
Alcohol use disorders	Female	15-19 years	95+ years	Data Rich	-1	2	Healthcare access and quality index	0
Alcohol use disorders	Female	15-19 years	95+ years	Data Rich	1	2	Cumulative Cigarettes (10 Years)	--
Alcohol use disorders	Female	15-19 years	95+ years	Data Rich	1	2	Smoking Prevalence	--
Alcohol use disorders	Female	15-19 years	95+ years	Data Rich	-1	3	Education (years per capita)	0
Alcohol use disorders	Female	15-19 years	95+ years	Data Rich	-1	3	Socio-demographic Index	0
Alcohol use disorders	Female	15-19 years	95+ years	Data Rich	-1	3	LDI (I$ per capita)	--
Alcohol use disorders	Female	15-19 years	95+ years	Global	1	1	Alcohol binge drinker proportion, age-standardized	579
Alcohol use disorders	Female	15-19 years	95+ years	Global	1	1	Alcohol drinker proportion, age-standardized	986
Alcohol use disorders	Female	15-19 years	95+ years	Global	1	1	Alcohol consumption, age standardized, in grams per day	--
Alcohol use disorders	Female	15-19 years	95+ years	Global	1	1	Liters of alcohol consumed per capita	--
Alcohol use disorders	Female	15-19 years	95+ years	Global	-1	2	Healthcare access and quality index	3
Alcohol use disorders	Female	15-19 years	95+ years	Global	1	2	Cumulative Cigarettes (10 Years)	--
Alcohol use disorders	Female	15-19 years	95+ years	Global	1	2	Smoking Prevalence	--
Alcohol use disorders	Female	15-19 years	95+ years	Global	-1	3	Education (years per capita)	0
Alcohol use disorders	Female	15-19 years	95+ years	Global	-1	3	Socio-demographic Index	58
Alcohol use disorders	Female	15-19 years	95+ years	Global	-1	3	LDI (I$ per capita)	--
Alcohol use disorders	Male	15-19 years	95+ years	Data Rich	1	1	Alcohol drinker proportion, age-standardized	11
Alcohol use disorders	Male	15-19 years	95+ years	Data Rich	1	1	Liters of alcohol consumed per capita	46
Alcohol use disorders	Male	15-19 years	95+ years	Data Rich	1	1	Alcohol consumption, age standardized, in grams per day	187
Alcohol use disorders	Male	15-19 years	95+ years	Data Rich	1	1	Alcohol binge drinker proportion, age-standardized	943
Alcohol use disorders	Male	15-19 years	95+ years	Data Rich	-1	2	Healthcare access and quality index	0
Alcohol use disorders	Male	15-19 years	95+ years	Data Rich	1	2	Cumulative Cigarettes (10 Years)	--
Alcohol use disorders	Male	15-19 years	95+ years	Data Rich	1	2	Smoking Prevalence	--
Alcohol use disorders	Male	15-19 years	95+ years	Data Rich	-1	3	Education (years per capita)	--
Alcohol use disorders	Male	15-19 years	95+ years	Data Rich	-1	3	LDI (I$ per capita)	--
Alcohol use disorders	Male	15-19 years	95+ years	Data Rich	-1	3	Socio-demographic Index	--
Alcohol use disorders	Male	15-19 years	95+ years	Global	1	1	Alcohol drinker proportion, age-standardized	1000
Alcohol use disorders	Male	15-19 years	95+ years	Global	1	1	Alcohol binge drinker proportion, age-standardized	--
Alcohol use disorders	Male	15-19 years	95+ years	Global	1	1	Alcohol consumption, age standardized, in grams per day	--
Alcohol use disorders	Male	15-19 years	95+ years	Global	1	1	Liters of alcohol consumed per capita	--
Alcohol use disorders	Male	15-19 years	95+ years	Global	-1	2	Healthcare access and quality index	--
Alcohol use disorders	Male	15-19 years	95+ years	Global	1	2	Cumulative Cigarettes (10 Years)	--
Alcohol use disorders	Male	15-19 years	95+ years	Global	1	2	Smoking Prevalence	--
Alcohol use disorders	Male	15-19 years	95+ years	Global	-1	3	Socio-demographic Index	70
Alcohol use disorders	Male	15-19 years	95+ years	Global	-1	3	Education (years per capita)	136
Alcohol use disorders	Male	15-19 years	95+ years	Global	-1	3	LDI (I$ per capita)	--
Drug use disorders	Female	15-19 years	95+ years	Data Rich	1	1	Intravenous drug use (proportion by age)	203
Drug use disorders	Female	15-19 years	95+ years	Data Rich	1	1	Opioids per million population per day (10 year lag)	495

CODEm covariates used, level of covariate, and expected direction of covariate by cause, sex, and age								
**Cause**	**Sex**	**Age Start**	**Age End**	**Model Version Type**	**Direction**	**Level**	**Covariate Name**	**Number of Draws**
Drug use disorders	Female	15-19 years	95+ years	Data Rich	1	1	Intravenous drug use (age-standardized proportion)	772
Drug use disorders	Female	15-19 years	95+ years	Data Rich	-1	2	Healthcare access and quality index	--
Drug use disorders	Female	15-19 years	95+ years	Data Rich	1	2	Cumulative Cigarettes (10 Years)	--
Drug use disorders	Female	15-19 years	95+ years	Data Rich	1	2	Cumulative Cigarettes (5 Years)	--
Drug use disorders	Female	15-19 years	95+ years	Data Rich	1	2	Smoking Prevalence	--
Drug use disorders	Female	15-19 years	95+ years	Data Rich	1	3	Education (years per capita)	761
Drug use disorders	Female	15-19 years	95+ years	Data Rich	1	3	Socio-demographic Index	840
Drug use disorders	Female	15-19 years	95+ years	Data Rich	1	3	LDI (I$ per capita)	--
Drug use disorders	Female	15-19 years	95+ years	Global	1	1	Intravenous drug use (proportion by age)	834
Drug use disorders	Female	15-19 years	95+ years	Global	1	1	Intravenous drug use (age-standardized proportion)	967
Drug use disorders	Female	15-19 years	95+ years	Global	1	1	Opioids per million population per day (10 year lag)	994
Drug use disorders	Female	15-19 years	95+ years	Global	-1	2	Healthcare access and quality index	0
Drug use disorders	Female	15-19 years	95+ years	Global	1	2	Cumulative Cigarettes (10 Years)	--
Drug use disorders	Female	15-19 years	95+ years	Global	1	2	Cumulative Cigarettes (5 Years)	--
Drug use disorders	Female	15-19 years	95+ years	Global	1	2	Smoking Prevalence	--
Drug use disorders	Female	15-19 years	95+ years	Global	1	3	Education (years per capita)	807
Drug use disorders	Female	15-19 years	95+ years	Global	1	3	Socio-demographic Index	999
Drug use disorders	Female	15-19 years	95+ years	Global	1	3	LDI (I$ per capita)	--
Drug use disorders	Male	15-19 years	95+ years	Data Rich	1	1	Opioids per million population per day (10 year lag)	409
Drug use disorders	Male	15-19 years	95+ years	Data Rich	1	1	Intravenous drug use (proportion by age)	749
Drug use disorders	Male	15-19 years	95+ years	Data Rich	1	1	Intravenous drug use (age-standardized proportion)	838
Drug use disorders	Male	15-19 years	95+ years	Data Rich	-1	2	Healthcare access and quality index	--
Drug use disorders	Male	15-19 years	95+ years	Data Rich	1	2	Cumulative Cigarettes (10 Years)	--
Drug use disorders	Male	15-19 years	95+ years	Data Rich	1	2	Cumulative Cigarettes (5 Years)	--
Drug use disorders	Male	15-19 years	95+ years	Data Rich	1	2	Smoking Prevalence	--
Drug use disorders	Male	15-19 years	95+ years	Data Rich	1	3	Socio-demographic Index	342
Drug use disorders	Male	15-19 years	95+ years	Data Rich	1	3	Education (years per capita)	658
Drug use disorders	Male	15-19 years	95+ years	Data Rich	1	3	LDI (I$ per capita)	--
Drug use disorders	Male	15-19 years	95+ years	Global	1	1	Intravenous drug use (age-standardized proportion)	196
Drug use disorders	Male	15-19 years	95+ years	Global	1	1	Intravenous drug use (proportion by age)	612
Drug use disorders	Male	15-19 years	95+ years	Global	1	1	Opioids per million population per day (10 year lag)	993
Drug use disorders	Male	15-19 years	95+ years	Global	-1	2	Healthcare access and quality index	5
Drug use disorders	Male	15-19 years	95+ years	Global	1	2	Cumulative Cigarettes (10 Years)	--
Drug use disorders	Male	15-19 years	95+ years	Global	1	2	Cumulative Cigarettes (5 Years)	--
Drug use disorders	Male	15-19 years	95+ years	Global	1	2	Smoking Prevalence	--
Drug use disorders	Male	15-19 years	95+ years	Global	1	3	Education (years per capita)	94
Drug use disorders	Male	15-19 years	95+ years	Global	1	3	Socio-demographic Index	705
Drug use disorders	Male	15-19 years	95+ years	Global	1	3	LDI (I$ per capita)	--
Opioid use disorders	Female	15-19 years	95+ years	Data Rich	1	1	Opioids per million population per day (10 year lag)	418
Opioid use disorders	Female	15-19 years	95+ years	Data Rich	1	1	Intravenous drug use (proportion by age)	463
Opioid use disorders	Female	15-19 years	95+ years	Data Rich	1	1	Intravenous drug use (age-standardized proportion)	654
Opioid use disorders	Female	15-19 years	95+ years	Data Rich	-1	2	Healthcare access and quality index	--
Opioid use disorders	Female	15-19 years	95+ years	Data Rich	1	2	Cumulative Cigarettes (10 Years)	--
Opioid use disorders	Female	15-19 years	95+ years	Data Rich	1	2	Cumulative Cigarettes (5 Years)	--
Opioid use disorders	Female	15-19 years	95+ years	Data Rich	1	2	Smoking Prevalence	--
Opioid use disorders	Female	15-19 years	95+ years	Data Rich	1	3	Socio-demographic Index	56
Opioid use disorders	Female	15-19 years	95+ years	Data Rich	1	3	Education (years per capita)	422
Opioid use disorders	Female	15-19 years	95+ years	Data Rich	1	3	LDI (I$ per capita)	--
Opioid use disorders	Female	15-19 years	95+ years	Global	1	1	Intravenous drug use (proportion by age)	281
Opioid use disorders	Female	15-19 years	95+ years	Global	1	1	Intravenous drug use (age-standardized proportion)	562
Opioid use disorders	Female	15-19 years	95+ years	Global	1	1	Opioids per million population per day (10 year lag)	920
Opioid use disorders	Female	15-19 years	95+ years	Global	-1	2	Healthcare access and quality index	--
Opioid use disorders	Female	15-19 years	95+ years	Global	1	2	Cumulative Cigarettes (10 Years)	--
Opioid use disorders	Female	15-19 years	95+ years	Global	1	2	Cumulative Cigarettes (5 Years)	--
Opioid use disorders	Female	15-19 years	95+ years	Global	1	2	Smoking Prevalence	--
Opioid use disorders	Female	15-19 years	95+ years	Global	1	3	Socio-demographic Index	456
Opioid use disorders	Female	15-19 years	95+ years	Global	1	3	Education (years per capita)	797
Opioid use disorders	Female	15-19 years	95+ years	Global	1	3	LDI (I$ per capita)	--
Opioid use disorders	Male	15-19 years	95+ years	Data Rich	1	1	Intravenous drug use (proportion by age)	462
Opioid use disorders	Male	15-19 years	95+ years	Data Rich	1	1	Intravenous drug use (age-standardized proportion)	769
Opioid use disorders	Male	15-19 years	95+ years	Data Rich	1	1	Opioids per million population per day (10 year lag)	800
Opioid use disorders	Male	15-19 years	95+ years	Data Rich	1	2	Cumulative Cigarettes (10 Years)	--
Opioid use disorders	Male	15-19 years	95+ years	Data Rich	1	2	Cumulative Cigarettes (5 Years)	--
Opioid use disorders	Male	15-19 years	95+ years	Data Rich	1	2	Smoking Prevalence	--
Opioid use disorders	Male	15-19 years	95+ years	Global	1	1	Intravenous drug use (proportion by age)	482
Opioid use disorders	Male	15-19 years	95+ years	Global	1	1	Intravenous drug use (age-standardized proportion)	772
Opioid use disorders	Male	15-19 years	95+ years	Global	1	1	Opioids per million population per day (10 year lag)	870
Opioid use disorders	Male	15-19 years	95+ years	Global	1	2	Cumulative Cigarettes (10 Years)	--
Opioid use disorders	Male	15-19 years	95+ years	Global	1	2	Cumulative Cigarettes (5 Years)	--
Opioid use disorders	Male	15-19 years	95+ years	Global	1	2	Smoking Prevalence	--
Cocaine use disorders	Female	15-19 years	95+ years	Data Rich	1	1	Alcohol drinker proportion, age-standardized	8
Cocaine use disorders	Female	15-19 years	95+ years	Data Rich	1	1	Intravenous drug use (age-standardized proportion)	8
Cocaine use disorders	Female	15-19 years	95+ years	Data Rich	1	1	Intravenous drug use (proportion by age)	992
Cocaine use disorders	Female	15-19 years	95+ years	Data Rich	1	1	Cumulative Cigarettes (10 Years)	--
Cocaine use disorders	Female	15-19 years	95+ years	Data Rich	1	1	Cumulative Cigarettes (5 Years)	--
Cocaine use disorders	Female	15-19 years	95+ years	Data Rich	1	1	Liters of alcohol consumed per capita	--
Cocaine use disorders	Female	15-19 years	95+ years	Data Rich	1	1	Smoking Prevalence	--
Cocaine use disorders	Female	15-19 years	95+ years	Data Rich	1	1	Tobacco (cigarettes per capita)	--
Cocaine use disorders	Female	15-19 years	95+ years	Data Rich	-1	2	Healthcare access and quality index	--
Cocaine use disorders	Female	15-19 years	95+ years	Data Rich	1	3	Socio-demographic Index	320
Cocaine use disorders	Female	15-19 years	95+ years	Data Rich	1	3	Education (years per capita)	768
Cocaine use disorders	Female	15-19 years	95+ years	Data Rich	1	3	LDI (I$ per capita)	--
Cocaine use disorders	Female	15-19 years	95+ years	Global	1	1	Intravenous drug use (proportion by age)	296
Cocaine use disorders	Female	15-19 years	95+ years	Global	1	1	Intravenous drug use (age-standardized proportion)	704
Cocaine use disorders	Female	15-19 years	95+ years	Global	1	1	Alcohol drinker proportion, age-standardized	--
Cocaine use disorders	Female	15-19 years	95+ years	Global	1	1	Cumulative Cigarettes (10 Years)	--
Cocaine use disorders	Female	15-19 years	95+ years	Global	1	1	Cumulative Cigarettes (5 Years)	--
Cocaine use disorders	Female	15-19 years	95+ years	Global	1	1	Liters of alcohol consumed per capita	--
Cocaine use disorders	Female	15-19 years	95+ years	Global	1	1	Smoking Prevalence	--
Cocaine use disorders	Female	15-19 years	95+ years	Global	1	1	Tobacco (cigarettes per capita)	--
Cocaine use disorders	Female	15-19 years	95+ years	Global	-1	2	Healthcare access and quality index	11
Cocaine use disorders	Female	15-19 years	95+ years	Global	1	3	Socio-demographic Index	96
Cocaine use disorders	Female	15-19 years	95+ years	Global	1	3	Education (years per capita)	118
Cocaine use disorders	Female	15-19 years	95+ years	Global	1	3	LDI (I$ per capita)	--
Cocaine use disorders	Male	15-19 years	95+ years	Data Rich	1	1	Liters of alcohol consumed per capita	20
Cocaine use disorders	Male	15-19 years	95+ years	Data Rich	1	1	Intravenous drug use (age-standardized proportion)	279
Cocaine use disorders	Male	15-19 years	95+ years	Data Rich	1	1	Intravenous drug use (proportion by age)	721
Cocaine use disorders	Male	15-19 years	95+ years	Data Rich	1	1	Alcohol drinker proportion, age-standardized	--
Cocaine use disorders	Male	15-19 years	95+ years	Data Rich	1	1	Cumulative Cigarettes (10 Years)	--
Cocaine use disorders	Male	15-19 years	95+ years	Data Rich	1	1	Cumulative Cigarettes (5 Years)	--
Cocaine use disorders	Male	15-19 years	95+ years	Data Rich	1	1	Smoking Prevalence	--
Cocaine use disorders	Male	15-19 years	95+ years	Data Rich	1	1	Tobacco (cigarettes per capita)	--
Cocaine use disorders	Male	15-19 years	95+ years	Data Rich	-1	2	Healthcare access and quality index	--
Cocaine use disorders	Male	15-19 years	95+ years	Data Rich	1	3	Education (years per capita)	58
Cocaine use disorders	Male	15-19 years	95+ years	Data Rich	1	3	LDI (I$ per capita)	--
Cocaine use disorders	Male	15-19 years	95+ years	Data Rich	1	3	Socio-demographic Index	--
Cocaine use disorders	Male	15-19 years	95+ years	Global	1	1	Liters of alcohol consumed per capita	49
Cocaine use disorders	Male	15-19 years	95+ years	Global	1	1	Intravenous drug use (proportion by age)	286
Cocaine use disorders	Male	15-19 years	95+ years	Global	1	1	Intravenous drug use (age-standardized proportion)	714
Cocaine use disorders	Male	15-19 years	95+ years	Global	1	1	Alcohol drinker proportion, age-standardized	--
Cocaine use disorders	Male	15-19 years	95+ years	Global	1	1	Cumulative Cigarettes (10 Years)	--
Cocaine use disorders	Male	15-19 years	95+ years	Global	1	1	Cumulative Cigarettes (5 Years)	--
Cocaine use disorders	Male	15-19 years	95+ years	Global	1	1	Smoking Prevalence	--
Cocaine use disorders	Male	15-19 years	95+ years	Global	1	1	Tobacco (cigarettes per capita)	--

CODEm covariates used, level of covariate, and expected direction of covariate by cause, sex, and age								
**Cause**	**Sex**	**Age Start**	**Age End**	**Model Version Type**	**Direction**	**Level**	**Covariate Name**	**Number of Draws**
Cocaine use disorders	Male	15-19 years	95+ years	Global	-1	2	Healthcare access and quality index	--
Cocaine use disorders	Male	15-19 years	95+ years	Global	1	3	Socio-demographic Index	573
Cocaine use disorders	Male	15-19 years	95+ years	Global	1	3	Education (years per capita)	729
Cocaine use disorders	Male	15-19 years	95+ years	Global	1	3	LDI (I$ per capita)	--
Amphetamine use disorders	Female	15-19 years	95+ years	Data Rich	1	1	Intravenous drug use (age-standardized proportion)	179
Amphetamine use disorders	Female	15-19 years	95+ years	Data Rich	1	1	Intravenous drug use (proportion by age)	821
Amphetamine use disorders	Female	15-19 years	95+ years	Data Rich	1	1	Alcohol drinker proportion, age-standardized	--
Amphetamine use disorders	Female	15-19 years	95+ years	Data Rich	1	1	Cumulative Cigarettes (10 Years)	--
Amphetamine use disorders	Female	15-19 years	95+ years	Data Rich	1	1	Cumulative Cigarettes (5 Years)	--
Amphetamine use disorders	Female	15-19 years	95+ years	Data Rich	1	1	Liters of alcohol consumed per capita	--
Amphetamine use disorders	Female	15-19 years	95+ years	Data Rich	1	1	Smoking Prevalence	--
Amphetamine use disorders	Female	15-19 years	95+ years	Data Rich	1	1	Tobacco (cigarettes per capita)	--
Amphetamine use disorders	Female	15-19 years	95+ years	Data Rich	-1	2	Healthcare access and quality index	--
Amphetamine use disorders	Female	15-19 years	95+ years	Data Rich	1	3	Socio-demographic Index	452
Amphetamine use disorders	Female	15-19 years	95+ years	Data Rich	1	3	Education (years per capita)	614
Amphetamine use disorders	Female	15-19 years	95+ years	Data Rich	1	3	LDI (I$ per capita)	--
Amphetamine use disorders	Female	15-19 years	95+ years	Global	1	1	Intravenous drug use (proportion by age)	445
Amphetamine use disorders	Female	15-19 years	95+ years	Global	1	1	Intravenous drug use (age-standardized proportion)	555
Amphetamine use disorders	Female	15-19 years	95+ years	Global	1	1	Alcohol drinker proportion, age-standardized	--
Amphetamine use disorders	Female	15-19 years	95+ years	Global	1	1	Cumulative Cigarettes (10 Years)	--
Amphetamine use disorders	Female	15-19 years	95+ years	Global	1	1	Cumulative Cigarettes (5 Years)	--
Amphetamine use disorders	Female	15-19 years	95+ years	Global	1	1	Liters of alcohol consumed per capita	--
Amphetamine use disorders	Female	15-19 years	95+ years	Global	1	1	Smoking Prevalence	--
Amphetamine use disorders	Female	15-19 years	95+ years	Global	1	1	Tobacco (cigarettes per capita)	--
Amphetamine use disorders	Female	15-19 years	95+ years	Global	-1	2	Healthcare access and quality index	--
Amphetamine use disorders	Female	15-19 years	95+ years	Global	1	3	Education (years per capita)	506
Amphetamine use disorders	Female	15-19 years	95+ years	Global	1	3	Socio-demographic Index	849
Amphetamine use disorders	Female	15-19 years	95+ years	Global	1	3	LDI (I$ per capita)	--
Amphetamine use disorders	Male	15-19 years	95+ years	Data Rich	1	1	Liters of alcohol consumed per capita	10
Amphetamine use disorders	Male	15-19 years	95+ years	Data Rich	1	1	Alcohol drinker proportion, age-standardized	31
Amphetamine use disorders	Male	15-19 years	95+ years	Data Rich	1	1	Intravenous drug use (proportion by age)	316
Amphetamine use disorders	Male	15-19 years	95+ years	Data Rich	1	1	Intravenous drug use (age-standardized proportion)	684
Amphetamine use disorders	Male	15-19 years	95+ years	Data Rich	1	1	Cumulative Cigarettes (10 Years)	--
Amphetamine use disorders	Male	15-19 years	95+ years	Data Rich	1	1	Cumulative Cigarettes (5 Years)	--
Amphetamine use disorders	Male	15-19 years	95+ years	Data Rich	1	1	Smoking Prevalence	--
Amphetamine use disorders	Male	15-19 years	95+ years	Data Rich	1	1	Tobacco (cigarettes per capita)	--
Amphetamine use disorders	Male	15-19 years	95+ years	Data Rich	-1	2	Healthcare access and quality index	--
Amphetamine use disorders	Male	15-19 years	95+ years	Data Rich	1	3	Education (years per capita)	62
Amphetamine use disorders	Male	15-19 years	95+ years	Data Rich	1	3	Socio-demographic Index	756
Amphetamine use disorders	Male	15-19 years	95+ years	Data Rich	1	3	LDI (I$ per capita)	--
Amphetamine use disorders	Male	15-19 years	95+ years	Global	1	1	Liters of alcohol consumed per capita	1
Amphetamine use disorders	Male	15-19 years	95+ years	Global	1	1	Intravenous drug use (proportion by age)	296
Amphetamine use disorders	Male	15-19 years	95+ years	Global	1	1	Intravenous drug use (age-standardized proportion)	704
Amphetamine use disorders	Male	15-19 years	95+ years	Global	1	1	Alcohol drinker proportion, age-standardized	--
Amphetamine use disorders	Male	15-19 years	95+ years	Global	1	1	Cumulative Cigarettes (10 Years)	--
Amphetamine use disorders	Male	15-19 years	95+ years	Global	1	1	Cumulative Cigarettes (5 Years)	--
Amphetamine use disorders	Male	15-19 years	95+ years	Global	1	1	Smoking Prevalence	--
Amphetamine use disorders	Male	15-19 years	95+ years	Global	1	1	Tobacco (cigarettes per capita)	--
Amphetamine use disorders	Male	15-19 years	95+ years	Global	-1	2	Healthcare access and quality index	--
Amphetamine use disorders	Male	15-19 years	95+ years	Global	1	3	Education (years per capita)	53
Amphetamine use disorders	Male	15-19 years	95+ years	Global	1	3	Socio-demographic Index	787
Amphetamine use disorders	Male	15-19 years	95+ years	Global	1	3	LDI (I$ per capita)	--
Other drug use disorders	Female	15-19 years	95+ years	Data Rich	1	1	Alcohol drinker proportion, age-standardized	170
Other drug use disorders	Female	15-19 years	95+ years	Data Rich	1	1	Liters of alcohol consumed per capita	408
Other drug use disorders	Female	15-19 years	95+ years	Data Rich	1	1	Intravenous drug use (age-standardized proportion)	456
Other drug use disorders	Female	15-19 years	95+ years	Data Rich	1	1	Intravenous drug use (proportion by age)	578
Other drug use disorders	Female	15-19 years	95+ years	Data Rich	1	1	Cumulative Cigarettes (10 Years)	--
Other drug use disorders	Female	15-19 years	95+ years	Data Rich	1	1	Cumulative Cigarettes (5 Years)	--
Other drug use disorders	Female	15-19 years	95+ years	Data Rich	1	1	Smoking Prevalence	--
Other drug use disorders	Female	15-19 years	95+ years	Data Rich	1	1	Tobacco (cigarettes per capita)	--
Other drug use disorders	Female	15-19 years	95+ years	Data Rich	-1	2	Healthcare access and quality index	--
Other drug use disorders	Female	15-19 years	95+ years	Data Rich	1	3	Socio-demographic Index	136
Other drug use disorders	Female	15-19 years	95+ years	Data Rich	1	3	Education (years per capita)	306
Other drug use disorders	Female	15-19 years	95+ years	Data Rich	1	3	LDI (I$ per capita)	--
Other drug use disorders	Female	15-19 years	95+ years	Global	1	1	Alcohol drinker proportion, age-standardized	11
Other drug use disorders	Female	15-19 years	95+ years	Global	1	1	Liters of alcohol consumed per capita	46
Other drug use disorders	Female	15-19 years	95+ years	Global	1	1	Intravenous drug use (age-standardized proportion)	59
Other drug use disorders	Female	15-19 years	95+ years	Global	1	1	Intravenous drug use (proportion by age)	941
Other drug use disorders	Female	15-19 years	95+ years	Global	1	1	Cumulative Cigarettes (10 Years)	--
Other drug use disorders	Female	15-19 years	95+ years	Global	1	1	Cumulative Cigarettes (5 Years)	--
Other drug use disorders	Female	15-19 years	95+ years	Global	1	1	Smoking Prevalence	--
Other drug use disorders	Female	15-19 years	95+ years	Global	1	1	Tobacco (cigarettes per capita)	--
Other drug use disorders	Female	15-19 years	95+ years	Global	-1	2	Healthcare access and quality index	--
Other drug use disorders	Female	15-19 years	95+ years	Global	1	3	Education (years per capita)	187
Other drug use disorders	Female	15-19 years	95+ years	Global	1	3	Socio-demographic Index	754
Other drug use disorders	Female	15-19 years	95+ years	Global	1	3	LDI (I$ per capita)	--
Other drug use disorders	Male	15-19 years	95+ years	Data Rich	1	1	Alcohol drinker proportion, age-standardized	0
Other drug use disorders	Male	15-19 years	95+ years	Data Rich	1	1	Liters of alcohol consumed per capita	2
Other drug use disorders	Male	15-19 years	95+ years	Data Rich	1	1	Intravenous drug use (proportion by age)	295
Other drug use disorders	Male	15-19 years	95+ years	Data Rich	1	1	Intravenous drug use (age-standardized proportion)	962
Other drug use disorders	Male	15-19 years	95+ years	Data Rich	1	1	Cumulative Cigarettes (10 Years)	--
Other drug use disorders	Male	15-19 years	95+ years	Data Rich	1	1	Cumulative Cigarettes (5 Years)	--
Other drug use disorders	Male	15-19 years	95+ years	Data Rich	1	1	Smoking Prevalence	--
Other drug use disorders	Male	15-19 years	95+ years	Data Rich	1	1	Tobacco (cigarettes per capita)	--
Other drug use disorders	Male	15-19 years	95+ years	Data Rich	-1	2	Healthcare access and quality index	--
Other drug use disorders	Male	15-19 years	95+ years	Data Rich	1	3	Socio-demographic Index	374
Other drug use disorders	Male	15-19 years	95+ years	Data Rich	1	3	Education (years per capita)	977
Other drug use disorders	Male	15-19 years	95+ years	Data Rich	1	3	LDI (I$ per capita)	--
Other drug use disorders	Male	15-19 years	95+ years	Global	1	1	Liters of alcohol consumed per capita	31
Other drug use disorders	Male	15-19 years	95+ years	Global	1	1	Intravenous drug use (age-standardized proportion)	484
Other drug use disorders	Male	15-19 years	95+ years	Global	1	1	Intravenous drug use (proportion by age)	704
Other drug use disorders	Male	15-19 years	95+ years	Global	1	1	Alcohol drinker proportion, age-standardized	--
Other drug use disorders	Male	15-19 years	95+ years	Global	1	1	Cumulative Cigarettes (10 Years)	--
Other drug use disorders	Male	15-19 years	95+ years	Global	1	1	Cumulative Cigarettes (5 Years)	--
Other drug use disorders	Male	15-19 years	95+ years	Global	1	1	Smoking Prevalence	--
Other drug use disorders	Male	15-19 years	95+ years	Global	1	1	Tobacco (cigarettes per capita)	--
Other drug use disorders	Male	15-19 years	95+ years	Global	-1	2	Healthcare access and quality index	--
Other drug use disorders	Male	15-19 years	95+ years	Global	1	3	Socio-demographic Index	635
Other drug use disorders	Male	15-19 years	95+ years	Global	1	3	Education (years per capita)	--
Other drug use disorders	Male	15-19 years	95+ years	Global	1	3	LDI (I$ per capita)	--
Diabetes mellitus	Female	0-6 days	10-14 years	Data Rich	-1	1	Healthcare access and quality index	677
Diabetes mellitus	Female	0-6 days	10-14 years	Data Rich	1	2	Live Births 35+ (proportion)	323
Diabetes mellitus	Female	0-6 days	10-14 years	Data Rich	1	2	Absolute value of average latitude	--
Diabetes mellitus	Female	0-6 days	10-14 years	Data Rich	1	2	Live Births 40+ (proportion)	--
Diabetes mellitus	Female	0-6 days	10-14 years	Data Rich	-1	3	Socio-demographic Index	186
Diabetes mellitus	Female	0-6 days	10-14 years	Data Rich	-1	3	Education (years per capita)	223
Diabetes mellitus	Female	0-6 days	10-14 years	Global	-1	1	Healthcare access and quality index	1000
Diabetes mellitus	Female	0-6 days	10-14 years	Global	1	2	Absolute value of average latitude	--
Diabetes mellitus	Female	0-6 days	10-14 years	Global	1	2	Live Births 35+ (proportion)	--
Diabetes mellitus	Female	0-6 days	10-14 years	Global	1	2	Live Births 40+ (proportion)	--
Diabetes mellitus	Female	0-6 days	10-14 years	Global	-1	3	Socio-demographic Index	454
Diabetes mellitus	Female	0-6 days	10-14 years	Global	-1	3	Education (years per capita)	--
Diabetes mellitus	Female	15-19 years	95+ years	Data Rich	1	1	Prevalence of obesity	117
Diabetes mellitus	Female	15-19 years	95+ years	Data Rich	1	1	Diabetes Fasting Plasma Glucose (mmol/L), age-standardized 25+	218
Diabetes mellitus	Female	15-19 years	95+ years	Data Rich	1	1	Diabetes Age-Standardized Prevalence (proportion)	352
Diabetes mellitus	Female	15-19 years	95+ years	Data Rich	1	1	Mean BMI	655

CODEm covariates used, level of covariate, and expected direction of covariate by cause, sex, and age

Cause	Sex	Age Start	Age End	Model Version Type	Direction	Level	Covariate Name	Number of Draws
Diabetes mellitus	Female	15-19 years	95+ years	Data Rich	-1	2	Age- and sex-specific SEV for Low vegetables	371
Diabetes mellitus	Female	15-19 years	95+ years	Data Rich	-1	2	Age- and sex-specific SEV for Low fruit	498
Diabetes mellitus	Female	15-19 years	95+ years	Data Rich	1	2	sugar unadjusted(g)	172
Diabetes mellitus	Female	15-19 years	95+ years	Data Rich	1	2	Systolic Blood Pressure (mmHg)	536
Diabetes mellitus	Female	15-19 years	95+ years	Data Rich	1	2	Age- and sex-specific SEV for Alcohol use	--
Diabetes mellitus	Female	15-19 years	95+ years	Data Rich	1	2	Low-Density Lipoprotein (mmol/L)	--
Diabetes mellitus	Female	15-19 years	95+ years	Data Rich	-1	3	Healthcare access and quality index	111
Diabetes mellitus	Female	15-19 years	95+ years	Data Rich	-1	3	Education (years per capita)	176
Diabetes mellitus	Female	15-19 years	95+ years	Data Rich	1	3	LDI (I$ per capita)	--
Diabetes mellitus	Female	15-19 years	95+ years	Global	1	1	Prevalence of obesity	60
Diabetes mellitus	Female	15-19 years	95+ years	Global	1	1	Mean BMI	73
Diabetes mellitus	Female	15-19 years	95+ years	Global	1	1	Diabetes Fasting Plasma Glucose (mmol/L), age-standardized 25+	582
Diabetes mellitus	Female	15-19 years	95+ years	Global	1	1	Diabetes Age-Standardized Prevalence (proportion)	973
Diabetes mellitus	Female	15-19 years	95+ years	Global	-1	2	Age- and sex-specific SEV for Low vegetables	563
Diabetes mellitus	Female	15-19 years	95+ years	Global	-1	2	Age- and sex-specific SEV for Low fruit	641
Diabetes mellitus	Female	15-19 years	95+ years	Global	1	2	sugar unadjusted(g)	735
Diabetes mellitus	Female	15-19 years	95+ years	Global	1	2	Systolic Blood Pressure (mmHg)	841
Diabetes mellitus	Female	15-19 years	95+ years	Global	1	2	Age- and sex-specific SEV for Alcohol use	--
Diabetes mellitus	Female	15-19 years	95+ years	Global	1	2	Low-Density Lipoprotein (mmol/L)	--
Diabetes mellitus	Female	15-19 years	95+ years	Global	-1	3	Education (years per capita)	0
Diabetes mellitus	Female	15-19 years	95+ years	Global	-1	3	Healthcare access and quality index	0
Diabetes mellitus	Female	15-19 years	95+ years	Global	1	3	LDI (I$ per capita)	--
Diabetes mellitus	Male	0-6 days	10-14 years	Data Rich	-1	1	Healthcare access and quality index	677
Diabetes mellitus	Male	0-6 days	10-14 years	Data Rich	1	2	Live Births 40+ (proportion)	323
Diabetes mellitus	Male	0-6 days	10-14 years	Data Rich	1	2	Absolute value of average latitude	--
Diabetes mellitus	Male	0-6 days	10-14 years	Data Rich	1	2	Live Births 35+ (proportion)	--
Diabetes mellitus	Male	0-6 days	10-14 years	Data Rich	-1	3	Socio-demographic Index	186
Diabetes mellitus	Male	0-6 days	10-14 years	Data Rich	-1	3	Education (years per capita)	223
Diabetes mellitus	Male	0-6 days	10-14 years	Global	-1	1	Healthcare access and quality index	407
Diabetes mellitus	Male	0-6 days	10-14 years	Global	1	2	Live Births 35+ (proportion)	267
Diabetes mellitus	Male	0-6 days	10-14 years	Global	1	2	Live Births 40+ (proportion)	326
Diabetes mellitus	Male	0-6 days	10-14 years	Global	1	2	Absolute value of average latitude	--
Diabetes mellitus	Male	0-6 days	10-14 years	Global	-1	3	Socio-demographic Index	185
Diabetes mellitus	Male	0-6 days	10-14 years	Global	-1	3	Education (years per capita)	--
Diabetes mellitus	Male	15-19 years	95+ years	Data Rich	1	1	Diabetes Fasting Plasma Glucose (mmol/L), age-standardized 25+	295
Diabetes mellitus	Male	15-19 years	95+ years	Data Rich	1	1	Prevalence of obesity	345
Diabetes mellitus	Male	15-19 years	95+ years	Data Rich	1	1	Diabetes Age-Standardized Prevalence (proportion)	705
Diabetes mellitus	Male	15-19 years	95+ years	Data Rich	1	1	Mean BMI	--
Diabetes mellitus	Male	15-19 years	95+ years	Data Rich	-1	2	Age- and sex-specific SEV for Low vegetables	137
Diabetes mellitus	Male	15-19 years	95+ years	Data Rich	-1	2	Age- and sex-specific SEV for Low fruit	373
Diabetes mellitus	Male	15-19 years	95+ years	Data Rich	1	2	sugar unadjusted(g)	295
Diabetes mellitus	Male	15-19 years	95+ years	Data Rich	1	2	Age- and sex-specific SEV for Alcohol use	--
Diabetes mellitus	Male	15-19 years	95+ years	Data Rich	1	2	Low-Density Lipoprotein (mmol/L)	--
Diabetes mellitus	Male	15-19 years	95+ years	Data Rich	1	2	Systolic Blood Pressure (mmHg)	--
Diabetes mellitus	Male	15-19 years	95+ years	Data Rich	-1	3	Healthcare access and quality index	59
Diabetes mellitus	Male	15-19 years	95+ years	Data Rich	-1	3	Education (years per capita)	--
Diabetes mellitus	Male	15-19 years	95+ years	Data Rich	1	3	LDI (I$ per capita)	--
Diabetes mellitus	Male	15-19 years	95+ years	Global	1	1	Diabetes Age-Standardized Prevalence (proportion)	204
Diabetes mellitus	Male	15-19 years	95+ years	Global	1	1	Diabetes Fasting Plasma Glucose (mmol/L), age-standardized 25+	294
Diabetes mellitus	Male	15-19 years	95+ years	Global	1	1	Prevalence of obesity	398
Diabetes mellitus	Male	15-19 years	95+ years	Global	1	1	Mean BMI	469
Diabetes mellitus	Male	15-19 years	95+ years	Global	-1	2	Age- and sex-specific SEV for Low vegetables	80
Diabetes mellitus	Male	15-19 years	95+ years	Global	-1	2	Age- and sex-specific SEV for Low fruit	384
Diabetes mellitus	Male	15-19 years	95+ years	Global	1	2	Age- and sex-specific SEV for Alcohol use	22
Diabetes mellitus	Male	15-19 years	95+ years	Global	1	2	sugar unadjusted(g)	104
Diabetes mellitus	Male	15-19 years	95+ years	Global	1	2	Low-Density Lipoprotein (mmol/L)	--
Diabetes mellitus	Male	15-19 years	95+ years	Global	1	2	Systolic Blood Pressure (mmHg)	--
Diabetes mellitus	Male	15-19 years	95+ years	Global	-1	3	Healthcare access and quality index	6
Diabetes mellitus	Male	15-19 years	95+ years	Global	-1	3	Education (years per capita)	7
Diabetes mellitus	Male	15-19 years	95+ years	Global	1	3	LDI (I$ per capita)	--
Diabetes mellitus type 1	Female	0-6 days	95+ years	Data Rich	-1	1	Healthcare access and quality index	1000
Diabetes mellitus type 1	Female	0-6 days	95+ years	Data Rich	1	2	Absolute value of average latitude	--
Diabetes mellitus type 1	Female	0-6 days	95+ years	Data Rich	1	2	Live Births 35+ (proportion)	--
Diabetes mellitus type 1	Female	0-6 days	95+ years	Data Rich	1	2	Live Births 40+ (proportion)	--
Diabetes mellitus type 1	Female	0-6 days	95+ years	Data Rich	-1	3	Socio-demographic Index	454
Diabetes mellitus type 1	Female	0-6 days	95+ years	Data Rich	-1	3	Education (years per capita)	--
Diabetes mellitus type 1	Female	0-6 days	95+ years	Global	-1	1	Healthcare access and quality index	1000
Diabetes mellitus type 1	Female	0-6 days	95+ years	Global	1	2	Absolute value of average latitude	--
Diabetes mellitus type 1	Female	0-6 days	95+ years	Global	1	2	Live Births 35+ (proportion)	--
Diabetes mellitus type 1	Female	0-6 days	95+ years	Global	1	2	Live Births 40+ (proportion)	--
Diabetes mellitus type 1	Female	0-6 days	95+ years	Global	-1	3	Socio-demographic Index	185
Diabetes mellitus type 1	Female	0-6 days	95+ years	Global	-1	3	Education (years per capita)	252
Diabetes mellitus type 1	Male	0-6 days	95+ years	Data Rich	-1	1	Healthcare access and quality index	274
Diabetes mellitus type 1	Male	0-6 days	95+ years	Data Rich	1	2	Live Births 40+ (proportion)	329
Diabetes mellitus type 1	Male	0-6 days	95+ years	Data Rich	1	2	Live Births 35+ (proportion)	397
Diabetes mellitus type 1	Male	0-6 days	95+ years	Data Rich	1	2	Absolute value of average latitude	--
Diabetes mellitus type 1	Male	0-6 days	95+ years	Data Rich	-1	3	Education (years per capita)	--
Diabetes mellitus type 1	Male	0-6 days	95+ years	Data Rich	-1	3	Socio-demographic Index	--
Diabetes mellitus type 1	Male	0-6 days	95+ years	Global	-1	1	Healthcare access and quality index	602
Diabetes mellitus type 1	Male	0-6 days	95+ years	Global	1	2	Absolute value of average latitude	294
Diabetes mellitus type 1	Male	0-6 days	95+ years	Global	1	2	Live Births 35+ (proportion)	398
Diabetes mellitus type 1	Male	0-6 days	95+ years	Global	1	2	Live Births 40+ (proportion)	--
Diabetes mellitus type 1	Male	0-6 days	95+ years	Global	-1	3	Education (years per capita)	--
Diabetes mellitus type 1	Male	0-6 days	95+ years	Global	-1	3	Socio-demographic Index	--
Diabetes mellitus type 2	Female	15-19 years	95+ years	Data Rich	1	1	Diabetes Fasting Plasma Glucose (mmol/L), age-standardized 25+	135
Diabetes mellitus type 2	Female	15-19 years	95+ years	Data Rich	1	1	Prevalence of obesity	325
Diabetes mellitus type 2	Female	15-19 years	95+ years	Data Rich	1	1	Diabetes Age-Standardized Prevalence (proportion)	534
Diabetes mellitus type 2	Female	15-19 years	95+ years	Data Rich	1	1	Mean BMI	534
Diabetes mellitus type 2	Female	15-19 years	95+ years	Data Rich	-1	2	Age- and sex-specific SEV for Low vegetables	300
Diabetes mellitus type 2	Female	15-19 years	95+ years	Data Rich	-1	2	Age- and sex-specific SEV for Low fruit	562
Diabetes mellitus type 2	Female	15-19 years	95+ years	Data Rich	1	2	Systolic Blood Pressure (mmHg)	589
Diabetes mellitus type 2	Female	15-19 years	95+ years	Data Rich	1	2	Age- and sex-specific SEV for Alcohol use	--
Diabetes mellitus type 2	Female	15-19 years	95+ years	Data Rich	1	2	Low-Density Lipoprotein (mmol/L)	--
Diabetes mellitus type 2	Female	15-19 years	95+ years	Data Rich	1	2	sugar unadjusted(g)	--
Diabetes mellitus type 2	Female	15-19 years	95+ years	Data Rich	-1	3	Healthcare access and quality index	96
Diabetes mellitus type 2	Female	15-19 years	95+ years	Data Rich	-1	3	Education (years per capita)	635
Diabetes mellitus type 2	Female	15-19 years	95+ years	Data Rich	1	3	LDI (I$ per capita)	--
Diabetes mellitus type 2	Female	15-19 years	95+ years	Global	1	1	Diabetes Fasting Plasma Glucose (mmol/L), age-standardized 25+	192
Diabetes mellitus type 2	Female	15-19 years	95+ years	Global	1	1	Mean BMI	597
Diabetes mellitus type 2	Female	15-19 years	95+ years	Global	1	1	Diabetes Age-Standardized Prevalence (proportion)	598
Diabetes mellitus type 2	Female	15-19 years	95+ years	Global	1	1	Prevalence of obesity	--
Diabetes mellitus type 2	Female	15-19 years	95+ years	Global	-1	2	Age- and sex-specific SEV for Low fruit	269
Diabetes mellitus type 2	Female	15-19 years	95+ years	Global	-1	2	Age- and sex-specific SEV for Low vegetables	314
Diabetes mellitus type 2	Female	15-19 years	95+ years	Global	1	2	Systolic Blood Pressure (mmHg)	306
Diabetes mellitus type 2	Female	15-19 years	95+ years	Global	1	2	Age- and sex-specific SEV for Alcohol use	--
Diabetes mellitus type 2	Female	15-19 years	95+ years	Global	1	2	Low-Density Lipoprotein (mmol/L)	--
Diabetes mellitus type 2	Female	15-19 years	95+ years	Global	1	2	sugar unadjusted(g)	--
Diabetes mellitus type 2	Female	15-19 years	95+ years	Global	-1	3	Education (years per capita)	75
Diabetes mellitus type 2	Female	15-19 years	95+ years	Global	-1	3	Healthcare access and quality index	112
Diabetes mellitus type 2	Female	15-19 years	95+ years	Global	1	3	LDI (I$ per capita)	--
Diabetes mellitus type 2	Male	15-19 years	95+ years	Data Rich	1	1	Prevalence of obesity	86
Diabetes mellitus type 2	Male	15-19 years	95+ years	Data Rich	1	1	Diabetes Fasting Plasma Glucose (mmol/L), age-standardized 25+	90
Diabetes mellitus type 2	Male	15-19 years	95+ years	Data Rich	1	1	Diabetes Age-Standardized Prevalence (proportion)	472
Diabetes mellitus type 2	Male	15-19 years	95+ years	Data Rich	1	1	Mean BMI	756
Diabetes mellitus type 2	Male	15-19 years	95+ years	Data Rich	-1	2	Age- and sex-specific SEV for Low vegetables	32
Diabetes mellitus type 2	Male	15-19 years	95+ years	Data Rich	-1	2	Age- and sex-specific SEV for Low fruit	614

CODEm covariates used, level of covariate, and expected direction of covariate by cause, sex, and age

Cause	Sex	Age Start	Age End	Model Version Type	Direction	Level	Covariate Name	Number of Draws
Diabetes mellitus type 2	Male	15-19 years	95+ years	Data Rich	1	2	sugar unadjusted(g)	79
Diabetes mellitus type 2	Male	15-19 years	95+ years	Data Rich	1	2	Age- and sex-specific SEV for Alcohol use	--
Diabetes mellitus type 2	Male	15-19 years	95+ years	Data Rich	1	2	Low-Density Lipoprotein (mmol/L)	--
Diabetes mellitus type 2	Male	15-19 years	95+ years	Data Rich	1	2	Systolic Blood Pressure (mmHg)	--
Diabetes mellitus type 2	Male	15-19 years	95+ years	Data Rich	-1	3	Healthcare access and quality index	32
Diabetes mellitus type 2	Male	15-19 years	95+ years	Data Rich	-1	3	Education (years per capita)	384
Diabetes mellitus type 2	Male	15-19 years	95+ years	Data Rich	1	3	LDI (I$ per capita)	--
Diabetes mellitus type 2	Male	15-19 years	95+ years	Global	1	1	Diabetes Fasting Plasma Glucose (mmol/L), age-standardized 25+	38
Diabetes mellitus type 2	Male	15-19 years	95+ years	Global	1	1	Mean BMI	375
Diabetes mellitus type 2	Male	15-19 years	95+ years	Global	1	1	Prevalence of obesity	426
Diabetes mellitus type 2	Male	15-19 years	95+ years	Global	1	1	Diabetes Age-Standardized Prevalence (proportion)	899
Diabetes mellitus type 2	Male	15-19 years	95+ years	Global	-1	2	Age- and sex-specific SEV for Low fruit	21
Diabetes mellitus type 2	Male	15-19 years	95+ years	Global	-1	2	Age- and sex-specific SEV for Low vegetables	296
Diabetes mellitus type 2	Male	15-19 years	95+ years	Global	1	2	sugar unadjusted(g)	186
Diabetes mellitus type 2	Male	15-19 years	95+ years	Global	1	2	Age- and sex-specific SEV for Alcohol use	--
Diabetes mellitus type 2	Male	15-19 years	95+ years	Global	1	2	Low-Density Lipoprotein (mmol/L)	--
Diabetes mellitus type 2	Male	15-19 years	95+ years	Global	1	2	Systolic Blood Pressure (mmHg)	--
Diabetes mellitus type 2	Male	15-19 years	95+ years	Global	-1	3	Education (years per capita)	25
Diabetes mellitus type 2	Male	15-19 years	95+ years	Global	-1	3	Healthcare access and quality index	46
Diabetes mellitus type 2	Male	15-19 years	95+ years	Global	1	3	LDI (I$ per capita)	--
Chronic kidney disease	Female	0-6 days	95+ years	Data Rich	-1	1	Healthcare access and quality index	286
Chronic kidney disease	Female	0-6 days	95+ years	Data Rich	1	1	Systolic Blood Pressure (mmHg)	8
Chronic kidney disease	Female	0-6 days	95+ years	Data Rich	1	1	Diabetes Age-Standardized Prevalence (proportion)	389
Chronic kidney disease	Female	0-6 days	95+ years	Data Rich	1	1	Mean BMI	425
Chronic kidney disease	Female	0-6 days	95+ years	Data Rich	1	1	Diabetes Fasting Plasma Glucose (mmol/L), age-standardized 25+	537
Chronic kidney disease	Female	0-6 days	95+ years	Data Rich	1	2	energy unadjusted(kcal)	105
Chronic kidney disease	Female	0-6 days	95+ years	Data Rich	1	2	Low-Density Lipoprotein (mmol/L)	--
Chronic kidney disease	Female	0-6 days	95+ years	Data Rich	1	2	red meats unadjusted(g)	--
Chronic kidney disease	Female	0-6 days	95+ years	Data Rich	-1	3	Education (years per capita)	11
Chronic kidney disease	Female	0-6 days	95+ years	Data Rich	-1	3	Socio-demographic Index	309
Chronic kidney disease	Female	0-6 days	95+ years	Data Rich	-1	3	LDI (I$ per capita)	--
Chronic kidney disease	Female	0-6 days	95+ years	Global	-1	1	Healthcare access and quality index	81
Chronic kidney disease	Female	0-6 days	95+ years	Global	1	1	Diabetes Fasting Plasma Glucose (mmol/L), age-standardized 25+	260
Chronic kidney disease	Female	0-6 days	95+ years	Global	1	1	Diabetes Age-Standardized Prevalence (proportion)	500
Chronic kidney disease	Female	0-6 days	95+ years	Global	1	1	Mean BMI	920
Chronic kidney disease	Female	0-6 days	95+ years	Global	1	1	Systolic Blood Pressure (mmHg)	--
Chronic kidney disease	Female	0-6 days	95+ years	Global	1	2	energy unadjusted(kcal)	25
Chronic kidney disease	Female	0-6 days	95+ years	Global	1	2	Low-Density Lipoprotein (mmol/L)	--
Chronic kidney disease	Female	0-6 days	95+ years	Global	1	2	red meats unadjusted(g)	--
Chronic kidney disease	Female	0-6 days	95+ years	Global	-1	3	Socio-demographic Index	86
Chronic kidney disease	Female	0-6 days	95+ years	Global	-1	3	Education (years per capita)	153
Chronic kidney disease	Female	0-6 days	95+ years	Global	-1	3	LDI (I$ per capita)	--
Chronic kidney disease	Male	0-6 days	95+ years	Data Rich	-1	1	Healthcare access and quality index	316
Chronic kidney disease	Male	0-6 days	95+ years	Data Rich	1	1	Diabetes Age-Standardized Prevalence (proportion)	105
Chronic kidney disease	Male	0-6 days	95+ years	Data Rich	1	1	Mean BMI	498
Chronic kidney disease	Male	0-6 days	95+ years	Data Rich	1	1	Diabetes Fasting Plasma Glucose (mmol/L), age-standardized 25+	532
Chronic kidney disease	Male	0-6 days	95+ years	Data Rich	1	1	Systolic Blood Pressure (mmHg)	--
Chronic kidney disease	Male	0-6 days	95+ years	Data Rich	1	2	energy unadjusted(kcal)	106
Chronic kidney disease	Male	0-6 days	95+ years	Data Rich	1	2	Low-Density Lipoprotein (mmol/L)	--
Chronic kidney disease	Male	0-6 days	95+ years	Data Rich	1	2	red meats unadjusted(g)	--
Chronic kidney disease	Male	0-6 days	95+ years	Data Rich	-1	3	Education (years per capita)	16
Chronic kidney disease	Male	0-6 days	95+ years	Data Rich	-1	3	Socio-demographic Index	99
Chronic kidney disease	Male	0-6 days	95+ years	Data Rich	-1	3	LDI (I$ per capita)	--
Chronic kidney disease	Male	0-6 days	95+ years	Global	-1	1	Healthcare access and quality index	217
Chronic kidney disease	Male	0-6 days	95+ years	Global	1	1	Diabetes Age-Standardized Prevalence (proportion)	441
Chronic kidney disease	Male	0-6 days	95+ years	Global	1	1	Mean BMI	801
Chronic kidney disease	Male	0-6 days	95+ years	Global	1	1	Diabetes Fasting Plasma Glucose (mmol/L), age-standardized 25+	--
Chronic kidney disease	Male	0-6 days	95+ years	Global	1	1	Systolic Blood Pressure (mmHg)	--
Chronic kidney disease	Male	0-6 days	95+ years	Global	1	2	energy unadjusted(kcal)	72
Chronic kidney disease	Male	0-6 days	95+ years	Global	1	2	Low-Density Lipoprotein (mmol/L)	--
Chronic kidney disease	Male	0-6 days	95+ years	Global	1	2	red meats unadjusted(g)	--
Chronic kidney disease	Male	0-6 days	95+ years	Global	-1	3	Socio-demographic Index	204
Chronic kidney disease	Male	0-6 days	95+ years	Global	-1	3	Education (years per capita)	265
Chronic kidney disease	Male	0-6 days	95+ years	Global	-1	3	LDI (I$ per capita)	--
Acute glomerulonephritis	Female	0-6 days	95+ years	Data Rich	-1	2	Improved Water Source (proportion with access)	151
Acute glomerulonephritis	Female	0-6 days	95+ years	Data Rich	-1	2	Sanitation (proportion with access)	621
Acute glomerulonephritis	Female	0-6 days	95+ years	Data Rich	-1	2	Healthcare access and quality index	860
Acute glomerulonephritis	Female	0-6 days	95+ years	Data Rich	1	2	Systolic Blood Pressure (mmHg)	398
Acute glomerulonephritis	Female	0-6 days	95+ years	Data Rich	-1	3	Socio-demographic Index	27
Acute glomerulonephritis	Female	0-6 days	95+ years	Data Rich	-1	3	Education (years per capita)	63
Acute glomerulonephritis	Female	0-6 days	95+ years	Data Rich	-1	3	LDI (I$ per capita)	--
Acute glomerulonephritis	Female	0-6 days	95+ years	Global	-1	2	Improved Water Source (proportion with access)	261
Acute glomerulonephritis	Female	0-6 days	95+ years	Global	-1	2	Sanitation (proportion with access)	278
Acute glomerulonephritis	Female	0-6 days	95+ years	Global	-1	2	Healthcare access and quality index	606
Acute glomerulonephritis	Female	0-6 days	95+ years	Global	1	2	Systolic Blood Pressure (mmHg)	406
Acute glomerulonephritis	Female	0-6 days	95+ years	Global	-1	3	Socio-demographic Index	131
Acute glomerulonephritis	Female	0-6 days	95+ years	Global	-1	3	Education (years per capita)	236
Acute glomerulonephritis	Female	0-6 days	95+ years	Global	-1	3	LDI (I$ per capita)	--
Acute glomerulonephritis	Male	0-6 days	95+ years	Data Rich	-1	2	Sanitation (proportion with access)	390
Acute glomerulonephritis	Male	0-6 days	95+ years	Data Rich	-1	2	Improved Water Source (proportion with access)	548
Acute glomerulonephritis	Male	0-6 days	95+ years	Data Rich	-1	2	Healthcare access and quality index	751
Acute glomerulonephritis	Male	0-6 days	95+ years	Data Rich	1	2	Systolic Blood Pressure (mmHg)	402
Acute glomerulonephritis	Male	0-6 days	95+ years	Data Rich	-1	3	Socio-demographic Index	41
Acute glomerulonephritis	Male	0-6 days	95+ years	Data Rich	-1	3	Education (years per capita)	81
Acute glomerulonephritis	Male	0-6 days	95+ years	Data Rich	-1	3	LDI (I$ per capita)	--
Acute glomerulonephritis	Male	0-6 days	95+ years	Global	-1	2	Sanitation (proportion with access)	391
Acute glomerulonephritis	Male	0-6 days	95+ years	Global	-1	2	Improved Water Source (proportion with access)	520
Acute glomerulonephritis	Male	0-6 days	95+ years	Global	-1	2	Healthcare access and quality index	--
Acute glomerulonephritis	Male	0-6 days	95+ years	Global	1	2	Systolic Blood Pressure (mmHg)	171
Acute glomerulonephritis	Male	0-6 days	95+ years	Global	-1	3	Socio-demographic Index	150
Acute glomerulonephritis	Male	0-6 days	95+ years	Global	-1	3	Education (years per capita)	685
Acute glomerulonephritis	Male	0-6 days	95+ years	Global	-1	3	LDI (I$ per capita)	--
Skin and subcutaneous diseases	Female	0-6 days	95+ years	Data Rich	-1	1	Improved Water Source (proportion with access)	69
Skin and subcutaneous diseases	Female	0-6 days	95+ years	Data Rich	-1	1	Healthcare access and quality index	187
Skin and subcutaneous diseases	Female	0-6 days	95+ years	Data Rich	1	1	Age- and sex-specific SEV for Unsafe sanitation	479
Skin and subcutaneous diseases	Female	0-6 days	95+ years	Data Rich	1	1	Diabetes Fasting Plasma Glucose (mmol/L), by age	672
Skin and subcutaneous diseases	Female	0-6 days	95+ years	Data Rich	1	1	Prevalence of overweight and obesity	984
Skin and subcutaneous diseases	Female	0-6 days	95+ years	Data Rich	1	2	Cumulative Cigarettes (10 Years)	--
Skin and subcutaneous diseases	Female	0-6 days	95+ years	Data Rich	1	2	Cumulative Cigarettes (5 Years)	--
Skin and subcutaneous diseases	Female	0-6 days	95+ years	Data Rich	1	2	Liters of alcohol consumed per capita	--
Skin and subcutaneous diseases	Female	0-6 days	95+ years	Data Rich	1	2	Smoking Prevalence	--
Skin and subcutaneous diseases	Female	0-6 days	95+ years	Data Rich	-1	3	Education (years per capita)	83
Skin and subcutaneous diseases	Female	0-6 days	95+ years	Data Rich	-1	3	Socio-demographic Index	245
Skin and subcutaneous diseases	Female	0-6 days	95+ years	Data Rich	-1	3	LDI (I$ per capita)	--
Skin and subcutaneous diseases	Female	0-6 days	95+ years	Global	-1	1	Healthcare access and quality index	208
Skin and subcutaneous diseases	Female	0-6 days	95+ years	Global	-1	1	Improved Water Source (proportion with access)	229
Skin and subcutaneous diseases	Female	0-6 days	95+ years	Global	1	1	Age- and sex-specific SEV for Unsafe sanitation	358
Skin and subcutaneous diseases	Female	0-6 days	95+ years	Global	1	1	Diabetes Fasting Plasma Glucose (mmol/L), by age	506
Skin and subcutaneous diseases	Female	0-6 days	95+ years	Global	1	1	Prevalence of overweight and obesity	850
Skin and subcutaneous diseases	Female	0-6 days	95+ years	Global	1	2	Cumulative Cigarettes (10 Years)	--
Skin and subcutaneous diseases	Female	0-6 days	95+ years	Global	1	2	Cumulative Cigarettes (5 Years)	--
Skin and subcutaneous diseases	Female	0-6 days	95+ years	Global	1	2	Liters of alcohol consumed per capita	--
Skin and subcutaneous diseases	Female	0-6 days	95+ years	Global	1	2	Smoking Prevalence	--
Skin and subcutaneous diseases	Female	0-6 days	95+ years	Global	-1	3	Socio-demographic Index	77
Skin and subcutaneous diseases	Female	0-6 days	95+ years	Global	-1	3	Education (years per capita)	180

CODEm covariates used, level of covariate, and expected direction of covariate by cause, sex, and age

Cause	Sex	Age Start	Age End	Model Version Type	Direction	Level	Covariate Name	Number of Draws
Skin and subcutaneous diseases	Female	0-6 days	95+ years	Global	-1	3	LDI (I$ per capita)	--
Skin and subcutaneous diseases	Male	0-6 days	95+ years	Data Rich	-1	1	Healthcare access and quality index	285
Skin and subcutaneous diseases	Male	0-6 days	95+ years	Data Rich	-1	1	Improved Water Source (proportion with access)	--
Skin and subcutaneous diseases	Male	0-6 days	95+ years	Data Rich	1	1	Diabetes Fasting Plasma Glucose (mmol/L), by age	267
Skin and subcutaneous diseases	Male	0-6 days	95+ years	Data Rich	1	1	Age- and sex-specific SEV for Unsafe sanitation	451
Skin and subcutaneous diseases	Male	0-6 days	95+ years	Data Rich	1	1	Prevalence of overweight and obesity	733
Skin and subcutaneous diseases	Male	0-6 days	95+ years	Data Rich	1	2	Cumulative Cigarettes (10 Years)	--
Skin and subcutaneous diseases	Male	0-6 days	95+ years	Data Rich	1	2	Cumulative Cigarettes (5 Years)	--
Skin and subcutaneous diseases	Male	0-6 days	95+ years	Data Rich	1	2	Liters of alcohol consumed per capita	--
Skin and subcutaneous diseases	Male	0-6 days	95+ years	Data Rich	1	2	Smoking Prevalence	--
Skin and subcutaneous diseases	Male	0-6 days	95+ years	Data Rich	-1	3	Education (years per capita)	--
Skin and subcutaneous diseases	Male	0-6 days	95+ years	Data Rich	-1	3	LDI (I$ per capita)	--
Skin and subcutaneous diseases	Male	0-6 days	95+ years	Data Rich	-1	3	Socio-demographic Index	--
Skin and subcutaneous diseases	Male	0-6 days	95+ years	Global	-1	1	Healthcare access and quality index	95
Skin and subcutaneous diseases	Male	0-6 days	95+ years	Global	-1	1	Improved Water Source (proportion with access)	766
Skin and subcutaneous diseases	Male	0-6 days	95+ years	Global	1	1	Diabetes Fasting Plasma Glucose (mmol/L), by age	169
Skin and subcutaneous diseases	Male	0-6 days	95+ years	Global	1	1	Age- and sex-specific SEV for Unsafe sanitation	529
Skin and subcutaneous diseases	Male	0-6 days	95+ years	Global	1	1	Prevalence of overweight and obesity	997
Skin and subcutaneous diseases	Male	0-6 days	95+ years	Global	1	2	Cumulative Cigarettes (10 Years)	--
Skin and subcutaneous diseases	Male	0-6 days	95+ years	Global	1	2	Cumulative Cigarettes (5 Years)	--
Skin and subcutaneous diseases	Male	0-6 days	95+ years	Global	1	2	Liters of alcohol consumed per capita	--
Skin and subcutaneous diseases	Male	0-6 days	95+ years	Global	1	2	Smoking Prevalence	--
Skin and subcutaneous diseases	Male	0-6 days	95+ years	Global	-1	3	Education (years per capita)	133
Skin and subcutaneous diseases	Male	0-6 days	95+ years	Global	-1	3	Socio-demographic Index	154
Skin and subcutaneous diseases	Male	0-6 days	95+ years	Global	-1	3	LDI (I$ per capita)	--
Bacterial skin diseases	Female	0-6 days	95+ years	Data Rich	-1	1	Improved Water Source (proportion with access)	642
Bacterial skin diseases	Female	0-6 days	95+ years	Data Rich	-1	1	Healthcare access and quality index	--
Bacterial skin diseases	Female	0-6 days	95+ years	Data Rich	1	1	Diabetes Fasting Plasma Glucose (mmol/L), by age	102
Bacterial skin diseases	Female	0-6 days	95+ years	Data Rich	1	1	Prevalence of overweight and obesity	1000
Bacterial skin diseases	Female	0-6 days	95+ years	Data Rich	1	1	Age- and sex-specific SEV for Unsafe sanitation	--
Bacterial skin diseases	Female	0-6 days	95+ years	Data Rich	1	2	Cumulative Cigarettes (10 Years)	--
Bacterial skin diseases	Female	0-6 days	95+ years	Data Rich	1	2	Cumulative Cigarettes (5 Years)	--
Bacterial skin diseases	Female	0-6 days	95+ years	Data Rich	1	2	Liters of alcohol consumed per capita	--
Bacterial skin diseases	Female	0-6 days	95+ years	Data Rich	1	2	Smoking Prevalence	--
Bacterial skin diseases	Female	0-6 days	95+ years	Data Rich	-1	3	Education (years per capita)	--
Bacterial skin diseases	Female	0-6 days	95+ years	Data Rich	-1	3	LDI (I$ per capita)	--
Bacterial skin diseases	Female	0-6 days	95+ years	Data Rich	-1	3	Socio-demographic Index	--
Bacterial skin diseases	Female	0-6 days	95+ years	Global	-1	1	Healthcare access and quality index	238
Bacterial skin diseases	Female	0-6 days	95+ years	Global	-1	1	Improved Water Source (proportion with access)	526
Bacterial skin diseases	Female	0-6 days	95+ years	Global	1	1	Diabetes Fasting Plasma Glucose (mmol/L), by age	700
Bacterial skin diseases	Female	0-6 days	95+ years	Global	1	1	Age- and sex-specific SEV for Unsafe sanitation	726
Bacterial skin diseases	Female	0-6 days	95+ years	Global	1	1	Prevalence of overweight and obesity	821
Bacterial skin diseases	Female	0-6 days	95+ years	Global	1	2	Cumulative Cigarettes (10 Years)	--
Bacterial skin diseases	Female	0-6 days	95+ years	Global	1	2	Cumulative Cigarettes (5 Years)	--
Bacterial skin diseases	Female	0-6 days	95+ years	Global	1	2	Liters of alcohol consumed per capita	--
Bacterial skin diseases	Female	0-6 days	95+ years	Global	1	2	Smoking Prevalence	--
Bacterial skin diseases	Female	0-6 days	95+ years	Global	-1	3	Socio-demographic Index	14
Bacterial skin diseases	Female	0-6 days	95+ years	Global	-1	3	Education (years per capita)	119
Bacterial skin diseases	Female	0-6 days	95+ years	Global	-1	3	LDI (I$ per capita)	--
Bacterial skin diseases	Male	0-6 days	95+ years	Data Rich	-1	1	Healthcare access and quality index	--
Bacterial skin diseases	Male	0-6 days	95+ years	Data Rich	-1	1	Improved Water Source (proportion with access)	--
Bacterial skin diseases	Male	0-6 days	95+ years	Data Rich	1	1	Diabetes Fasting Plasma Glucose (mmol/L), by age	137
Bacterial skin diseases	Male	0-6 days	95+ years	Data Rich	1	1	Age- and sex-specific SEV for Unsafe sanitation	617
Bacterial skin diseases	Male	0-6 days	95+ years	Data Rich	1	1	Prevalence of overweight and obesity	863
Bacterial skin diseases	Male	0-6 days	95+ years	Data Rich	1	2	Cumulative Cigarettes (10 Years)	--
Bacterial skin diseases	Male	0-6 days	95+ years	Data Rich	1	2	Cumulative Cigarettes (5 Years)	--
Bacterial skin diseases	Male	0-6 days	95+ years	Data Rich	1	2	Liters of alcohol consumed per capita	--
Bacterial skin diseases	Male	0-6 days	95+ years	Data Rich	1	2	Smoking Prevalence	--
Bacterial skin diseases	Male	0-6 days	95+ years	Data Rich	-1	3	Education (years per capita)	--
Bacterial skin diseases	Male	0-6 days	95+ years	Data Rich	-1	3	LDI (I$ per capita)	--
Bacterial skin diseases	Male	0-6 days	95+ years	Data Rich	-1	3	Socio-demographic Index	--
Bacterial skin diseases	Male	0-6 days	95+ years	Global	-1	1	Healthcare access and quality index	311
Bacterial skin diseases	Male	0-6 days	95+ years	Global	-1	1	Improved Water Source (proportion with access)	479
Bacterial skin diseases	Male	0-6 days	95+ years	Global	1	1	Diabetes Fasting Plasma Glucose (mmol/L), by age	56
Bacterial skin diseases	Male	0-6 days	95+ years	Global	1	1	Age- and sex-specific SEV for Unsafe sanitation	483
Bacterial skin diseases	Male	0-6 days	95+ years	Global	1	1	Prevalence of overweight and obesity	907
Bacterial skin diseases	Male	0-6 days	95+ years	Global	1	2	Cumulative Cigarettes (10 Years)	--
Bacterial skin diseases	Male	0-6 days	95+ years	Global	1	2	Cumulative Cigarettes (5 Years)	--
Bacterial skin diseases	Male	0-6 days	95+ years	Global	1	2	Liters of alcohol consumed per capita	--
Bacterial skin diseases	Male	0-6 days	95+ years	Global	1	2	Smoking Prevalence	--
Bacterial skin diseases	Male	0-6 days	95+ years	Global	-1	3	Education (years per capita)	56
Bacterial skin diseases	Male	0-6 days	95+ years	Global	-1	3	Socio-demographic Index	123
Bacterial skin diseases	Male	0-6 days	95+ years	Global	-1	3	LDI (I$ per capita)	--
Cellulitis	Female	0-6 days	95+ years	Data Rich	-1	1	Healthcare access and quality index	477
Cellulitis	Female	0-6 days	95+ years	Data Rich	1	1	Diabetes Fasting Plasma Glucose (mmol/L), by age	91
Cellulitis	Female	0-6 days	95+ years	Data Rich	1	1	Prevalence of overweight and obesity	909
Cellulitis	Female	0-6 days	95+ years	Data Rich	-1	2	LDI (I$ per capita)	--
Cellulitis	Female	0-6 days	95+ years	Data Rich	-1	3	Education (years per capita)	--
Cellulitis	Female	0-6 days	95+ years	Global	-1	1	Healthcare access and quality index	284
Cellulitis	Female	0-6 days	95+ years	Global	1	1	Diabetes Fasting Plasma Glucose (mmol/L), by age	465
Cellulitis	Female	0-6 days	95+ years	Global	1	1	Prevalence of overweight and obesity	873
Cellulitis	Female	0-6 days	95+ years	Global	-1	2	LDI (I$ per capita)	--
Cellulitis	Female	0-6 days	95+ years	Global	-1	3	Education (years per capita)	--
Cellulitis	Male	0-6 days	95+ years	Data Rich	-1	1	Healthcare access and quality index	--
Cellulitis	Male	0-6 days	95+ years	Data Rich	1	1	Diabetes Fasting Plasma Glucose (mmol/L), by age	358
Cellulitis	Male	0-6 days	95+ years	Data Rich	1	1	Prevalence of overweight and obesity	642
Cellulitis	Male	0-6 days	95+ years	Data Rich	-1	2	LDI (I$ per capita)	--
Cellulitis	Male	0-6 days	95+ years	Data Rich	-1	3	Education (years per capita)	--
Cellulitis	Male	0-6 days	95+ years	Global	-1	1	Healthcare access and quality index	334
Cellulitis	Male	0-6 days	95+ years	Global	1	1	Diabetes Fasting Plasma Glucose (mmol/L), by age	419
Cellulitis	Male	0-6 days	95+ years	Global	1	1	Prevalence of overweight and obesity	896
Cellulitis	Male	0-6 days	95+ years	Global	-1	2	LDI (I$ per capita)	--
Cellulitis	Male	0-6 days	95+ years	Global	-1	3	Education (years per capita)	--
Pyoderma	Female	0-6 days	95+ years	Data Rich	-1	1	Improved Water Source (proportion with access)	96
Pyoderma	Female	0-6 days	95+ years	Data Rich	-1	1	Healthcare access and quality index	--
Pyoderma	Female	0-6 days	95+ years	Data Rich	1	1	Diabetes Fasting Plasma Glucose (mmol/L), by age	295
Pyoderma	Female	0-6 days	95+ years	Data Rich	1	1	Age- and sex-specific SEV for Unsafe sanitation	851
Pyoderma	Female	0-6 days	95+ years	Data Rich	1	1	Prevalence of overweight and obesity	947
Pyoderma	Female	0-6 days	95+ years	Data Rich	1	2	Cumulative Cigarettes (10 Years)	--
Pyoderma	Female	0-6 days	95+ years	Data Rich	1	2	Cumulative Cigarettes (5 Years)	--
Pyoderma	Female	0-6 days	95+ years	Data Rich	1	2	Liters of alcohol consumed per capita	--
Pyoderma	Female	0-6 days	95+ years	Data Rich	1	2	Smoking Prevalence	--
Pyoderma	Female	0-6 days	95+ years	Data Rich	-1	3	Education (years per capita)	--
Pyoderma	Female	0-6 days	95+ years	Data Rich	-1	3	LDI (I$ per capita)	--
Pyoderma	Female	0-6 days	95+ years	Data Rich	-1	3	Socio-demographic Index	--
Pyoderma	Female	0-6 days	95+ years	Global	-1	1	Healthcare access and quality index	577
Pyoderma	Female	0-6 days	95+ years	Global	-1	1	Improved Water Source (proportion with access)	581
Pyoderma	Female	0-6 days	95+ years	Global	1	1	Diabetes Fasting Plasma Glucose (mmol/L), by age	418
Pyoderma	Female	0-6 days	95+ years	Global	1	1	Age- and sex-specific SEV for Unsafe sanitation	493
Pyoderma	Female	0-6 days	95+ years	Global	1	1	Prevalence of overweight and obesity	913
Pyoderma	Female	0-6 days	95+ years	Global	1	2	Cumulative Cigarettes (10 Years)	--
Pyoderma	Female	0-6 days	95+ years	Global	1	2	Cumulative Cigarettes (5 Years)	--

CODEm covariates used, level of covariate, and expected direction of covariate by cause, sex, and age

Cause	Sex	Age Start	Age End	Model Version Type	Direction	Level	Covariate Name	Number of Draws
Pyoderma	Female	0-6 days	95+ years	Global	1	2	Liters of alcohol consumed per capita	--
Pyoderma	Female	0-6 days	95+ years	Global	1	2	Smoking Prevalence	--
Pyoderma	Female	0-6 days	95+ years	Global	-1	3	Education (years per capita)	--
Pyoderma	Female	0-6 days	95+ years	Global	-1	3	LDI (I$ per capita)	--
Pyoderma	Female	0-6 days	95+ years	Global	-1	3	Socio-demographic Index	--
Pyoderma	Male	0-6 days	95+ years	Data Rich	-1	1	Improved Water Source (proportion with access)	240
Pyoderma	Male	0-6 days	95+ years	Data Rich	-1	1	Healthcare access and quality index	--
Pyoderma	Male	0-6 days	95+ years	Data Rich	1	1	Diabetes Fasting Plasma Glucose (mmol/L), by age	6
Pyoderma	Male	0-6 days	95+ years	Data Rich	1	1	Age- and sex-specific SEV for Unsafe sanitation	643
Pyoderma	Male	0-6 days	95+ years	Data Rich	1	1	Prevalence of overweight and obesity	994
Pyoderma	Male	0-6 days	95+ years	Data Rich	1	2	Cumulative Cigarettes (10 Years)	--
Pyoderma	Male	0-6 days	95+ years	Data Rich	1	2	Cumulative Cigarettes (5 Years)	--
Pyoderma	Male	0-6 days	95+ years	Data Rich	1	2	Liters of alcohol consumed per capita	--
Pyoderma	Male	0-6 days	95+ years	Data Rich	1	2	Smoking Prevalence	--
Pyoderma	Male	0-6 days	95+ years	Data Rich	-1	3	Education (years per capita)	--
Pyoderma	Male	0-6 days	95+ years	Data Rich	-1	3	LDI (I$ per capita)	--
Pyoderma	Male	0-6 days	95+ years	Data Rich	-1	3	Socio-demographic Index	--
Pyoderma	Male	0-6 days	95+ years	Global	-1	1	Healthcare access and quality index	258
Pyoderma	Male	0-6 days	95+ years	Global	-1	1	Improved Water Source (proportion with access)	572
Pyoderma	Male	0-6 days	95+ years	Global	1	1	Diabetes Fasting Plasma Glucose (mmol/L), by age	16
Pyoderma	Male	0-6 days	95+ years	Global	1	1	Age- and sex-specific SEV for Unsafe sanitation	222
Pyoderma	Male	0-6 days	95+ years	Global	1	1	Prevalence of overweight and obesity	984
Pyoderma	Male	0-6 days	95+ years	Global	1	2	Cumulative Cigarettes (10 Years)	--
Pyoderma	Male	0-6 days	95+ years	Global	1	2	Cumulative Cigarettes (5 Years)	--
Pyoderma	Male	0-6 days	95+ years	Global	1	2	Liters of alcohol consumed per capita	--
Pyoderma	Male	0-6 days	95+ years	Global	1	2	Smoking Prevalence	--
Pyoderma	Male	0-6 days	95+ years	Global	-1	3	Education (years per capita)	56
Pyoderma	Male	0-6 days	95+ years	Global	-1	3	Socio-demographic Index	198
Pyoderma	Male	0-6 days	95+ years	Global	-1	3	LDI (I$ per capita)	--
Decubitus ulcer	Female	12-23 months	95+ years	Data Rich	-1	1	Improved Water Source (proportion with access)	--
Decubitus ulcer	Female	12-23 months	95+ years	Data Rich	1	1	Prevalence of obesity	389
Decubitus ulcer	Female	12-23 months	95+ years	Data Rich	1	1	Diabetes Fasting Plasma Glucose (mmol/L), age-standardized 25+	606
Decubitus ulcer	Female	12-23 months	95+ years	Data Rich	-1	2	Healthcare access and quality index	430
Decubitus ulcer	Female	12-23 months	95+ years	Data Rich	1	2	Smoking Prevalence	48
Decubitus ulcer	Female	12-23 months	95+ years	Data Rich	1	2	Cumulative Cigarettes (10 Years)	245
Decubitus ulcer	Female	12-23 months	95+ years	Data Rich	1	2	Cumulative Cigarettes (5 Years)	432
Decubitus ulcer	Female	12-23 months	95+ years	Data Rich	1	2	Liters of alcohol consumed per capita	--
Decubitus ulcer	Female	12-23 months	95+ years	Data Rich	-1	3	Education (years per capita)	23
Decubitus ulcer	Female	12-23 months	95+ years	Data Rich	-1	3	Socio-demographic Index	221
Decubitus ulcer	Female	12-23 months	95+ years	Data Rich	-1	3	LDI (I$ per capita)	--
Decubitus ulcer	Female	12-23 months	95+ years	Data Rich	1	3	Age- and sex-specific SEV for Unsafe sanitation	--
Decubitus ulcer	Female	12-23 months	95+ years	Global	-1	1	Improved Water Source (proportion with access)	--
Decubitus ulcer	Female	12-23 months	95+ years	Global	1	1	Diabetes Fasting Plasma Glucose (mmol/L), age-standardized 25+	231
Decubitus ulcer	Female	12-23 months	95+ years	Global	1	1	Liters of alcohol consumed per capita	231
Decubitus ulcer	Female	12-23 months	95+ years	Global	1	1	Prevalence of obesity	282
Decubitus ulcer	Female	12-23 months	95+ years	Global	-1	2	Healthcare access and quality index	--
Decubitus ulcer	Female	12-23 months	95+ years	Global	1	2	Smoking Prevalence	133
Decubitus ulcer	Female	12-23 months	95+ years	Global	1	2	Cumulative Cigarettes (10 Years)	161
Decubitus ulcer	Female	12-23 months	95+ years	Global	1	2	Cumulative Cigarettes (5 Years)	193
Decubitus ulcer	Female	12-23 months	95+ years	Global	-1	3	Education (years per capita)	--
Decubitus ulcer	Female	12-23 months	95+ years	Global	-1	3	LDI (I$ per capita)	--
Decubitus ulcer	Female	12-23 months	95+ years	Global	-1	3	Socio-demographic Index	--
Decubitus ulcer	Female	12-23 months	95+ years	Global	1	3	Age- and sex-specific SEV for Unsafe sanitation	--
Decubitus ulcer	Male	12-23 months	95+ years	Data Rich	-1	1	Improved Water Source (proportion with access)	--
Decubitus ulcer	Male	12-23 months	95+ years	Data Rich	1	1	Diabetes Fasting Plasma Glucose (mmol/L), age-standardized 25+	--
Decubitus ulcer	Male	12-23 months	95+ years	Data Rich	1	1	Prevalence of obesity	--
Decubitus ulcer	Male	12-23 months	95+ years	Data Rich	-1	2	Healthcare access and quality index	155
Decubitus ulcer	Male	12-23 months	95+ years	Data Rich	1	2	Smoking Prevalence	142
Decubitus ulcer	Male	12-23 months	95+ years	Data Rich	1	2	Liters of alcohol consumed per capita	204
Decubitus ulcer	Male	12-23 months	95+ years	Data Rich	1	2	Cumulative Cigarettes (10 Years)	--
Decubitus ulcer	Male	12-23 months	95+ years	Data Rich	1	2	Cumulative Cigarettes (5 Years)	--
Decubitus ulcer	Male	12-23 months	95+ years	Data Rich	-1	3	Education (years per capita)	187
Decubitus ulcer	Male	12-23 months	95+ years	Data Rich	-1	3	Socio-demographic Index	480
Decubitus ulcer	Male	12-23 months	95+ years	Data Rich	-1	3	LDI (I$ per capita)	--
Decubitus ulcer	Male	12-23 months	95+ years	Data Rich	1	3	Age- and sex-specific SEV for Unsafe sanitation	40
Decubitus ulcer	Male	12-23 months	95+ years	Global	-1	1	Improved Water Source (proportion with access)	--
Decubitus ulcer	Male	12-23 months	95+ years	Global	1	1	Liters of alcohol consumed per capita	317
Decubitus ulcer	Male	12-23 months	95+ years	Global	1	1	Prevalence of obesity	374
Decubitus ulcer	Male	12-23 months	95+ years	Global	1	1	Diabetes Fasting Plasma Glucose (mmol/L), age-standardized 25+	475
Decubitus ulcer	Male	12-23 months	95+ years	Global	-1	2	Healthcare access and quality index	--
Decubitus ulcer	Male	12-23 months	95+ years	Global	1	2	Cumulative Cigarettes (5 Years)	120
Decubitus ulcer	Male	12-23 months	95+ years	Global	1	2	Cumulative Cigarettes (10 Years)	--
Decubitus ulcer	Male	12-23 months	95+ years	Global	1	2	Smoking Prevalence	--
Decubitus ulcer	Male	12-23 months	95+ years	Global	-1	3	Education (years per capita)	--
Decubitus ulcer	Male	12-23 months	95+ years	Global	-1	3	LDI (I$ per capita)	--
Decubitus ulcer	Male	12-23 months	95+ years	Global	-1	3	Socio-demographic Index	--
Decubitus ulcer	Male	12-23 months	95+ years	Global	1	3	Age- and sex-specific SEV for Unsafe sanitation	--
Other skin and subcutaneous diseases	Female	0-6 days	95+ years	Data Rich	-1	1	Improved Water Source (proportion with access)	54
Other skin and subcutaneous diseases	Female	0-6 days	95+ years	Data Rich	-1	1	Healthcare access and quality index	933
Other skin and subcutaneous diseases	Female	0-6 days	95+ years	Data Rich	1	1	Age- and sex-specific SEV for Unsafe sanitation	530
Other skin and subcutaneous diseases	Female	0-6 days	95+ years	Data Rich	1	1	Prevalence of overweight and obesity	784
Other skin and subcutaneous diseases	Female	0-6 days	95+ years	Data Rich	1	1	Diabetes Fasting Plasma Glucose (mmol/L), by age	850
Other skin and subcutaneous diseases	Female	0-6 days	95+ years	Data Rich	1	1	Age-standardized SEV for Child underweight	--
Other skin and subcutaneous diseases	Female	0-6 days	95+ years	Data Rich	1	2	Cumulative Cigarettes (10 Years)	162
Other skin and subcutaneous diseases	Female	0-6 days	95+ years	Data Rich	1	2	Smoking Prevalence	165
Other skin and subcutaneous diseases	Female	0-6 days	95+ years	Data Rich	1	2	Cumulative Cigarettes (5 Years)	337
Other skin and subcutaneous diseases	Female	0-6 days	95+ years	Data Rich	1	2	Liters of alcohol consumed per capita	--
Other skin and subcutaneous diseases	Female	0-6 days	95+ years	Data Rich	-1	3	Education (years per capita)	56
Other skin and subcutaneous diseases	Female	0-6 days	95+ years	Data Rich	-1	3	LDI (I$ per capita)	--
Other skin and subcutaneous diseases	Female	0-6 days	95+ years	Data Rich	-1	3	Socio-demographic Index	--
Other skin and subcutaneous diseases	Female	0-6 days	95+ years	Global	-1	1	Improved Water Source (proportion with access)	11
Other skin and subcutaneous diseases	Female	0-6 days	95+ years	Global	-1	1	Healthcare access and quality index	164
Other skin and subcutaneous diseases	Female	0-6 days	95+ years	Global	1	1	Diabetes Fasting Plasma Glucose (mmol/L), by age	459
Other skin and subcutaneous diseases	Female	0-6 days	95+ years	Global	1	1	Prevalence of overweight and obesity	636
Other skin and subcutaneous diseases	Female	0-6 days	95+ years	Global	1	1	Age- and sex-specific SEV for Unsafe sanitation	--
Other skin and subcutaneous diseases	Female	0-6 days	95+ years	Global	1	1	Age-standardized SEV for Child underweight	--
Other skin and subcutaneous diseases	Female	0-6 days	95+ years	Global	1	2	Cumulative Cigarettes (5 Years)	260
Other skin and subcutaneous diseases	Female	0-6 days	95+ years	Global	1	2	Cumulative Cigarettes (10 Years)	275
Other skin and subcutaneous diseases	Female	0-6 days	95+ years	Global	1	2	Smoking Prevalence	277
Other skin and subcutaneous diseases	Female	0-6 days	95+ years	Global	1	2	Liters of alcohol consumed per capita	383
Other skin and subcutaneous diseases	Female	0-6 days	95+ years	Global	-1	3	Socio-demographic Index	1
Other skin and subcutaneous diseases	Female	0-6 days	95+ years	Global	-1	3	Education (years per capita)	--
Other skin and subcutaneous diseases	Female	0-6 days	95+ years	Global	-1	3	LDI (I$ per capita)	--
Other skin and subcutaneous diseases	Male	0-6 days	95+ years	Data Rich	-1	1	Improved Water Source (proportion with access)	65
Other skin and subcutaneous diseases	Male	0-6 days	95+ years	Data Rich	-1	1	Healthcare access and quality index	384
Other skin and subcutaneous diseases	Male	0-6 days	95+ years	Data Rich	1	1	Age-standardized SEV for Child underweight	118
Other skin and subcutaneous diseases	Male	0-6 days	95+ years	Data Rich	1	1	Prevalence of overweight and obesity	671
Other skin and subcutaneous diseases	Male	0-6 days	95+ years	Data Rich	1	1	Age- and sex-specific SEV for Unsafe sanitation	882
Other skin and subcutaneous diseases	Male	0-6 days	95+ years	Data Rich	1	1	Diabetes Fasting Plasma Glucose (mmol/L), by age	--
Other skin and subcutaneous diseases	Male	0-6 days	95+ years	Data Rich	1	2	Cumulative Cigarettes (10 Years)	58
Other skin and subcutaneous diseases	Male	0-6 days	95+ years	Data Rich	1	2	Smoking Prevalence	96

CODEm covariates used, level of covariate, and expected direction of covariate by cause, sex, and age								
Cause	Sex	Age Start	Age End	Model Version Type	Direction	Level	Covariate Name	Number of Draws
Other skin and subcutaneous diseases	Male	0-6 days	95+ years	Data Rich	1	2	Cumulative Cigarettes (5 Years)	105
Other skin and subcutaneous diseases	Male	0-6 days	95+ years	Data Rich	1	2	Liters of alcohol consumed per capita	302
Other skin and subcutaneous diseases	Male	0-6 days	95+ years	Data Rich	-1	3	Education (years per capita)	25
Other skin and subcutaneous diseases	Male	0-6 days	95+ years	Data Rich	-1	3	Socio-demographic Index	128
Other skin and subcutaneous diseases	Male	0-6 days	95+ years	Data Rich	-1	3	LDI (I$ per capita)	--
Other skin and subcutaneous diseases	Male	0-6 days	95+ years	Global	-1	1	Healthcare access and quality index	216
Other skin and subcutaneous diseases	Male	0-6 days	95+ years	Global	-1	1	Improved Water Source (proportion with access)	387
Other skin and subcutaneous diseases	Male	0-6 days	95+ years	Global	1	1	Age- and sex-specific SEV for Unsafe sanitation	8
Other skin and subcutaneous diseases	Male	0-6 days	95+ years	Global	1	1	Age-standardized SEV for Child underweight	21
Other skin and subcutaneous diseases	Male	0-6 days	95+ years	Global	1	1	Prevalence of overweight and obesity	914
Other skin and subcutaneous diseases	Male	0-6 days	95+ years	Global	1	1	Diabetes Fasting Plasma Glucose (mmol/L), by age	--
Other skin and subcutaneous diseases	Male	0-6 days	95+ years	Global	1	2	Cumulative Cigarettes (10 Years)	67
Other skin and subcutaneous diseases	Male	0-6 days	95+ years	Global	1	2	Smoking Prevalence	150
Other skin and subcutaneous diseases	Male	0-6 days	95+ years	Global	1	2	Cumulative Cigarettes (5 Years)	173
Other skin and subcutaneous diseases	Male	0-6 days	95+ years	Global	1	2	Liters of alcohol consumed per capita	411
Other skin and subcutaneous diseases	Male	0-6 days	95+ years	Global	-1	3	Socio-demographic Index	9
Other skin and subcutaneous diseases	Male	0-6 days	95+ years	Global	-1	3	Education (years per capita)	--
Other skin and subcutaneous diseases	Male	0-6 days	95+ years	Global	-1	3	LDI (I$ per capita)	--
Musculoskeletal disorders	Female	1-5 months	95+ years	Data Rich	1	1	Mean BMI	956
Musculoskeletal disorders	Female	1-5 months	95+ years	Data Rich	1	1	Liters of alcohol consumed per capita	--
Musculoskeletal disorders	Female	1-5 months	95+ years	Data Rich	-1	2	Healthcare access and quality index	--
Musculoskeletal disorders	Female	1-5 months	95+ years	Data Rich	1	2	Education (years per capita)	98
Musculoskeletal disorders	Female	1-5 months	95+ years	Data Rich	1	2	Cumulative Cigarettes (5 Years)	328
Musculoskeletal disorders	Female	1-5 months	95+ years	Data Rich	1	2	Cumulative Cigarettes (10 Years)	466
Musculoskeletal disorders	Female	1-5 months	95+ years	Data Rich	1	2	Low bone mineral density	499
Musculoskeletal disorders	Female	1-5 months	95+ years	Data Rich	1	2	Age-standardized bone mineral density among population age 60+ years	515
Musculoskeletal disorders	Female	1-5 months	95+ years	Data Rich	1	2	LDI (I$ per capita)	--
Musculoskeletal disorders	Female	1-5 months	95+ years	Data Rich	1	2	Low-Density Lipoprotein (mmol/L)	--
Musculoskeletal disorders	Female	1-5 months	95+ years	Data Rich	1	2	Smoking Prevalence	--
Musculoskeletal disorders	Female	1-5 months	95+ years	Data Rich	1	3	Socio-demographic Index	353
Musculoskeletal disorders	Female	1-5 months	95+ years	Global	-1	1	vegetables unadjusted(g)	--
Musculoskeletal disorders	Female	1-5 months	95+ years	Global	1	1	Liters of alcohol consumed per capita	719
Musculoskeletal disorders	Female	1-5 months	95+ years	Global	1	1	Mean BMI	742
Musculoskeletal disorders	Female	1-5 months	95+ years	Global	-1	2	Healthcare access and quality index	235
Musculoskeletal disorders	Female	1-5 months	95+ years	Global	1	2	Cumulative Cigarettes (5 Years)	87
Musculoskeletal disorders	Female	1-5 months	95+ years	Global	1	2	Smoking Prevalence	148
Musculoskeletal disorders	Female	1-5 months	95+ years	Global	1	2	Age-standardized bone mineral density among population age 60+ years	312
Musculoskeletal disorders	Female	1-5 months	95+ years	Global	1	2	Low bone mineral density	382
Musculoskeletal disorders	Female	1-5 months	95+ years	Global	1	2	Cumulative Cigarettes (10 Years)	--
Musculoskeletal disorders	Female	1-5 months	95+ years	Global	1	2	Education (years per capita)	--
Musculoskeletal disorders	Female	1-5 months	95+ years	Global	1	2	LDI (I$ per capita)	--
Musculoskeletal disorders	Female	1-5 months	95+ years	Global	1	2	Low-Density Lipoprotein (mmol/L)	--
Musculoskeletal disorders	Female	1-5 months	95+ years	Global	1	3	Socio-demographic Index	178
Musculoskeletal disorders	Male	1-5 months	95+ years	Data Rich	1	1	vegetables unadjusted(g)	852
Musculoskeletal disorders	Male	1-5 months	95+ years	Data Rich	1	1	Mean BMI	984
Musculoskeletal disorders	Male	1-5 months	95+ years	Data Rich	1	1	Liters of alcohol consumed per capita	--
Musculoskeletal disorders	Male	1-5 months	95+ years	Data Rich	-1	2	Healthcare access and quality index	--
Musculoskeletal disorders	Male	1-5 months	95+ years	Data Rich	1	2	Smoking Prevalence	0
Musculoskeletal disorders	Male	1-5 months	95+ years	Data Rich	1	2	Age-standardized bone mineral density among population age 60+ years	3
Musculoskeletal disorders	Male	1-5 months	95+ years	Data Rich	1	2	Low bone mineral density	285
Musculoskeletal disorders	Male	1-5 months	95+ years	Data Rich	1	2	Cumulative Cigarettes (10 Years)	342
Musculoskeletal disorders	Male	1-5 months	95+ years	Data Rich	1	2	Cumulative Cigarettes (5 Years)	361
Musculoskeletal disorders	Male	1-5 months	95+ years	Data Rich	1	2	Education (years per capita)	571
Musculoskeletal disorders	Male	1-5 months	95+ years	Data Rich	1	2	LDI (I$ per capita)	--
Musculoskeletal disorders	Male	1-5 months	95+ years	Data Rich	1	2	Low-Density Lipoprotein (mmol/L)	--
Musculoskeletal disorders	Male	1-5 months	95+ years	Data Rich	1	3	Socio-demographic Index	85
Musculoskeletal disorders	Male	1-5 months	95+ years	Global	1	1	vegetables unadjusted(g)	334
Musculoskeletal disorders	Male	1-5 months	95+ years	Global	1	1	Mean BMI	868
Musculoskeletal disorders	Male	1-5 months	95+ years	Global	1	1	Liters of alcohol consumed per capita	--
Musculoskeletal disorders	Male	1-5 months	95+ years	Global	-1	2	Healthcare access and quality index	132
Musculoskeletal disorders	Male	1-5 months	95+ years	Global	1	2	Education (years per capita)	138
Musculoskeletal disorders	Male	1-5 months	95+ years	Global	1	2	Smoking Prevalence	450
Musculoskeletal disorders	Male	1-5 months	95+ years	Global	1	2	Cumulative Cigarettes (10 Years)	--
Musculoskeletal disorders	Male	1-5 months	95+ years	Global	1	2	Cumulative Cigarettes (5 Years)	--
Musculoskeletal disorders	Male	1-5 months	95+ years	Global	1	2	LDI (I$ per capita)	--
Musculoskeletal disorders	Male	1-5 months	95+ years	Global	1	2	Low-Density Lipoprotein (mmol/L)	--
Musculoskeletal disorders	Male	1-5 months	95+ years	Global	1	3	Socio-demographic Index	288
Rheumatoid arthritis	Female	5-9 years	95+ years	Data Rich	-1	1	Healthcare access and quality index	249
Rheumatoid arthritis	Female	5-9 years	95+ years	Data Rich	-1	1	milk unadjusted(g)	--
Rheumatoid arthritis	Female	5-9 years	95+ years	Data Rich	1	1	Smoking Prevalence	14
Rheumatoid arthritis	Female	5-9 years	95+ years	Data Rich	1	1	Cumulative Cigarettes (10 Years)	43
Rheumatoid arthritis	Female	5-9 years	95+ years	Data Rich	1	1	Cumulative Cigarettes (5 Years)	75
Rheumatoid arthritis	Female	5-9 years	95+ years	Data Rich	1	1	Liters of alcohol consumed per capita	--
Rheumatoid arthritis	Female	5-9 years	95+ years	Data Rich	1	2	Mean BMI	908
Rheumatoid arthritis	Female	5-9 years	95+ years	Data Rich	1	2	Low-Density Lipoprotein (mmol/L)	--
Rheumatoid arthritis	Female	5-9 years	95+ years	Data Rich	1	3	Socio-demographic Index	141
Rheumatoid arthritis	Female	5-9 years	95+ years	Data Rich	1	3	Education (years per capita)	211
Rheumatoid arthritis	Female	5-9 years	95+ years	Data Rich	1	3	LDI (I$ per capita)	--
Rheumatoid arthritis	Female	5-9 years	95+ years	Global	-1	1	Healthcare access and quality index	617
Rheumatoid arthritis	Female	5-9 years	95+ years	Global	1	1	Cumulative Cigarettes (5 Years)	31
Rheumatoid arthritis	Female	5-9 years	95+ years	Global	1	1	Smoking Prevalence	35
Rheumatoid arthritis	Female	5-9 years	95+ years	Global	1	1	milk unadjusted(g)	652
Rheumatoid arthritis	Female	5-9 years	95+ years	Global	1	1	Cumulative Cigarettes (10 Years)	--
Rheumatoid arthritis	Female	5-9 years	95+ years	Global	1	1	Liters of alcohol consumed per capita	--
Rheumatoid arthritis	Female	5-9 years	95+ years	Global	1	2	Mean BMI	322
Rheumatoid arthritis	Female	5-9 years	95+ years	Global	1	2	Low-Density Lipoprotein (mmol/L)	--
Rheumatoid arthritis	Female	5-9 years	95+ years	Global	1	3	Socio-demographic Index	95
Rheumatoid arthritis	Female	5-9 years	95+ years	Global	1	3	Education (years per capita)	250
Rheumatoid arthritis	Female	5-9 years	95+ years	Global	1	3	LDI (I$ per capita)	--
Rheumatoid arthritis	Male	5-9 years	95+ years	Data Rich	-1	1	Healthcare access and quality index	564
Rheumatoid arthritis	Male	5-9 years	95+ years	Data Rich	-1	1	milk unadjusted(g)	--
Rheumatoid arthritis	Male	5-9 years	95+ years	Data Rich	1	1	Cumulative Cigarettes (5 Years)	128
Rheumatoid arthritis	Male	5-9 years	95+ years	Data Rich	1	1	Smoking Prevalence	311
Rheumatoid arthritis	Male	5-9 years	95+ years	Data Rich	1	1	Cumulative Cigarettes (10 Years)	407
Rheumatoid arthritis	Male	5-9 years	95+ years	Data Rich	1	1	Liters of alcohol consumed per capita	--
Rheumatoid arthritis	Male	5-9 years	95+ years	Data Rich	1	2	Mean BMI	322
Rheumatoid arthritis	Male	5-9 years	95+ years	Data Rich	1	2	Low-Density Lipoprotein (mmol/L)	--
Rheumatoid arthritis	Male	5-9 years	95+ years	Data Rich	1	3	Education (years per capita)	191
Rheumatoid arthritis	Male	5-9 years	95+ years	Data Rich	1	3	Socio-demographic Index	254
Rheumatoid arthritis	Male	5-9 years	95+ years	Data Rich	1	3	LDI (I$ per capita)	--
Rheumatoid arthritis	Male	5-9 years	95+ years	Global	-1	1	Healthcare access and quality index	112
Rheumatoid arthritis	Male	5-9 years	95+ years	Global	1	1	Cumulative Cigarettes (5 Years)	159
Rheumatoid arthritis	Male	5-9 years	95+ years	Global	1	1	Cumulative Cigarettes (10 Years)	184
Rheumatoid arthritis	Male	5-9 years	95+ years	Global	1	1	Smoking Prevalence	594
Rheumatoid arthritis	Male	5-9 years	95+ years	Global	1	1	milk unadjusted(g)	828
Rheumatoid arthritis	Male	5-9 years	95+ years	Global	1	1	Liters of alcohol consumed per capita	--
Rheumatoid arthritis	Male	5-9 years	95+ years	Global	1	2	Mean BMI	207
Rheumatoid arthritis	Male	5-9 years	95+ years	Global	1	2	Low-Density Lipoprotein (mmol/L)	--
Rheumatoid arthritis	Male	5-9 years	95+ years	Global	1	3	Socio-demographic Index	81
Rheumatoid arthritis	Male	5-9 years	95+ years	Global	1	3	Education (years per capita)	301
Rheumatoid arthritis	Male	5-9 years	95+ years	Global	1	3	LDI (I$ per capita)	--
Other musculoskeletal disorders	Female	1-5 months	95+ years	Data Rich	1	1	vegetables unadjusted(g)	678
Other musculoskeletal disorders	Female	1-5 months	95+ years	Data Rich	1	1	Mean BMI	781
Other musculoskeletal disorders	Female	1-5 months	95+ years	Data Rich	1	1	Liters of alcohol consumed per capita	--
Other musculoskeletal disorders	Female	1-5 months	95+ years	Data Rich	-1	2	Healthcare access and quality index	70
Other musculoskeletal disorders	Female	1-5 months	95+ years	Data Rich	1	2	Smoking Prevalence	19
Other musculoskeletal disorders	Female	1-5 months	95+ years	Data Rich	1	2	Cumulative Cigarettes (5 Years)	23
Other musculoskeletal disorders	Female	1-5 months	95+ years	Data Rich	1	2	Cumulative Cigarettes (10 Years)	28
Other musculoskeletal disorders	Female	1-5 months	95+ years	Data Rich	1	2	Education (years per capita)	--
Other musculoskeletal disorders	Female	1-5 months	95+ years	Data Rich	1	2	LDI (I$ per capita)	--
Other musculoskeletal disorders	Female	1-5 months	95+ years	Data Rich	1	2	Low-Density Lipoprotein (mmol/L)	--

CODEm covariates used, level of covariate, and expected direction of covariate by cause, sex, and age								
**Cause**	**Sex**	**Age Start**	**Age End**	**Model Version Type**	**Direction**	**Level**	**Covariate Name**	**Number of Draws**
Other musculoskeletal disorders	Female	1-5 months	95+ years	Data Rich	1	3	Socio-demographic Index	479
Other musculoskeletal disorders	Female	1-5 months	95+ years	Global	1	1	Liters of alcohol consumed per capita	10
Other musculoskeletal disorders	Female	1-5 months	95+ years	Global	1	1	vegetables unadjusted(g)	644
Other musculoskeletal disorders	Female	1-5 months	95+ years	Global	1	1	Mean BMI	990
Other musculoskeletal disorders	Female	1-5 months	95+ years	Global	-1	2	Healthcare access and quality index	--
Other musculoskeletal disorders	Female	1-5 months	95+ years	Global	1	2	Cumulative Cigarettes (10 Years)	80
Other musculoskeletal disorders	Female	1-5 months	95+ years	Global	1	2	Cumulative Cigarettes (5 Years)	90
Other musculoskeletal disorders	Female	1-5 months	95+ years	Global	1	2	Smoking Prevalence	130
Other musculoskeletal disorders	Female	1-5 months	95+ years	Global	1	2	Education (years per capita)	523
Other musculoskeletal disorders	Female	1-5 months	95+ years	Global	1	2	LDI (I$ per capita)	--
Other musculoskeletal disorders	Female	1-5 months	95+ years	Global	1	2	Low-Density Lipoprotein (mmol/L)	--
Other musculoskeletal disorders	Female	1-5 months	95+ years	Global	1	3	Socio-demographic Index	386
Other musculoskeletal disorders	Male	1-5 months	95+ years	Data Rich	-1	1	vegetables unadjusted(g)	148
Other musculoskeletal disorders	Male	1-5 months	95+ years	Data Rich	1	1	Mean BMI	1000
Other musculoskeletal disorders	Male	1-5 months	95+ years	Data Rich	1	1	Liters of alcohol consumed per capita	--
Other musculoskeletal disorders	Male	1-5 months	95+ years	Data Rich	-1	2	Healthcare access and quality index	77
Other musculoskeletal disorders	Male	1-5 months	95+ years	Data Rich	1	2	Cumulative Cigarettes (10 Years)	33
Other musculoskeletal disorders	Male	1-5 months	95+ years	Data Rich	1	2	Smoking Prevalence	37
Other musculoskeletal disorders	Male	1-5 months	95+ years	Data Rich	1	2	Cumulative Cigarettes (5 Years)	92
Other musculoskeletal disorders	Male	1-5 months	95+ years	Data Rich	1	2	Education (years per capita)	252
Other musculoskeletal disorders	Male	1-5 months	95+ years	Data Rich	1	2	LDI (I$ per capita)	--
Other musculoskeletal disorders	Male	1-5 months	95+ years	Data Rich	1	2	Low-Density Lipoprotein (mmol/L)	--
Other musculoskeletal disorders	Male	1-5 months	95+ years	Data Rich	1	3	Socio-demographic Index	237
Other musculoskeletal disorders	Male	1-5 months	95+ years	Global	-1	1	vegetables unadjusted(g)	220
Other musculoskeletal disorders	Male	1-5 months	95+ years	Global	1	1	Liters of alcohol consumed per capita	144
Other musculoskeletal disorders	Male	1-5 months	95+ years	Global	1	1	Mean BMI	856
Other musculoskeletal disorders	Male	1-5 months	95+ years	Global	-1	2	Healthcare access and quality index	--
Other musculoskeletal disorders	Male	1-5 months	95+ years	Global	1	2	Cumulative Cigarettes (10 Years)	115
Other musculoskeletal disorders	Male	1-5 months	95+ years	Global	1	2	Cumulative Cigarettes (5 Years)	--
Other musculoskeletal disorders	Male	1-5 months	95+ years	Global	1	2	Education (years per capita)	--
Other musculoskeletal disorders	Male	1-5 months	95+ years	Global	1	2	LDI (I$ per capita)	--
Other musculoskeletal disorders	Male	1-5 months	95+ years	Global	1	2	Low-Density Lipoprotein (mmol/L)	--
Other musculoskeletal disorders	Male	1-5 months	95+ years	Global	1	2	Smoking Prevalence	--
Other musculoskeletal disorders	Male	1-5 months	95+ years	Global	1	3	Socio-demographic Index	255
Congenital anomalies	Female	0-6 days	65-69 years	Data Rich	-1	1	Folic acid unadjusted (ug)	73
Congenital anomalies	Female	0-6 days	65-69 years	Data Rich	1	1	Birth prevalence of congenital chromosomal anomalies	179
Congenital anomalies	Female	0-6 days	65-69 years	Data Rich	1	1	Maternal alcohol consumption during pregnancy (proportion)	218
Congenital anomalies	Female	0-6 days	65-69 years	Data Rich	1	1	Birth prevalence of CHD	402
Congenital anomalies	Female	0-6 days	65-69 years	Data Rich	1	1	Live Births 35+ (proportion)	551
Congenital anomalies	Female	0-6 days	65-69 years	Data Rich	-1	2	Legality of Abortion	44
Congenital anomalies	Female	0-6 days	65-69 years	Data Rich	-1	2	Antenatal Care (1 visit) Coverage (proportion)	--
Congenital anomalies	Female	0-6 days	65-69 years	Data Rich	-1	2	Antenatal Care (4 visits) Coverage (proportion)	--
Congenital anomalies	Female	0-6 days	65-69 years	Data Rich	-1	2	Composite fortification standard and folic acid inclusion	--
Congenital anomalies	Female	0-6 days	65-69 years	Data Rich	-1	2	Healthcare access and quality index	--
Congenital anomalies	Female	0-6 days	65-69 years	Data Rich	-1	2	In-Facility Delivery (proportion)	--
Congenital anomalies	Female	0-6 days	65-69 years	Data Rich	1	2	Age-standardized SEV for Smoking	48
Congenital anomalies	Female	0-6 days	65-69 years	Data Rich	-1	3	Socio-demographic Index	19
Congenital anomalies	Female	0-6 days	65-69 years	Data Rich	-1	3	Maternal Education (years per capita)	68
Congenital anomalies	Female	0-6 days	65-69 years	Data Rich	1	3	Age-standardized SEV for Low fruit	14
Congenital anomalies	Female	0-6 days	65-69 years	Data Rich	1	3	Age-standardized SEV for Low vegetables	23
Congenital anomalies	Female	0-6 days	65-69 years	Data Rich	1	3	Liters of alcohol consumed per capita	258
Congenital anomalies	Female	0-6 days	65-69 years	Data Rich	1	3	Age-standardized SEV for Ambient particulate matter	446
Congenital anomalies	Female	0-6 days	65-69 years	Data Rich	1	3	Age-standardized SEV for Household air pollution	--
Congenital anomalies	Female	0-6 days	65-69 years	Global	-1	1	Folic acid unadjusted (ug)	162
Congenital anomalies	Female	0-6 days	65-69 years	Global	1	1	Birth prevalence of CHD	162
Congenital anomalies	Female	0-6 days	65-69 years	Global	1	1	Maternal alcohol consumption during pregnancy (proportion)	551
Congenital anomalies	Female	0-6 days	65-69 years	Global	1	1	Live Births 35+ (proportion)	680
Congenital anomalies	Female	0-6 days	65-69 years	Global	1	1	Birth prevalence of congenital chromosomal anomalies	698
Congenital anomalies	Female	0-6 days	65-69 years	Global	-1	2	Legality of Abortion	36
Congenital anomalies	Female	0-6 days	65-69 years	Global	-1	2	Antenatal Care (1 visit) Coverage (proportion)	--
Congenital anomalies	Female	0-6 days	65-69 years	Global	-1	2	Antenatal Care (4 visits) Coverage (proportion)	--
Congenital anomalies	Female	0-6 days	65-69 years	Global	-1	2	Composite fortification standard and folic acid inclusion	--
Congenital anomalies	Female	0-6 days	65-69 years	Global	-1	2	Healthcare access and quality index	--
Congenital anomalies	Female	0-6 days	65-69 years	Global	-1	2	In-Facility Delivery (proportion)	--
Congenital anomalies	Female	0-6 days	65-69 years	Global	1	2	Age-standardized SEV for Smoking	88
Congenital anomalies	Female	0-6 days	65-69 years	Global	-1	3	Maternal Education (years per capita)	--
Congenital anomalies	Female	0-6 days	65-69 years	Global	-1	3	Socio-demographic Index	--
Congenital anomalies	Female	0-6 days	65-69 years	Global	1	3	Age-standardized SEV for Low vegetables	42
Congenital anomalies	Female	0-6 days	65-69 years	Global	1	3	Liters of alcohol consumed per capita	219
Congenital anomalies	Female	0-6 days	65-69 years	Global	1	3	Age-standardized SEV for Ambient particulate matter	300
Congenital anomalies	Female	0-6 days	65-69 years	Global	1	3	Age-standardized SEV for Household air pollution	--
Congenital anomalies	Female	0-6 days	65-69 years	Global	1	3	Age-standardized SEV for Low fruit	--
Congenital anomalies	Male	0-6 days	65-69 years	Data Rich	-1	1	Folic acid unadjusted (ug)	313
Congenital anomalies	Male	0-6 days	65-69 years	Data Rich	1	1	Birth prevalence of CHD	90
Congenital anomalies	Male	0-6 days	65-69 years	Data Rich	1	1	Maternal alcohol consumption during pregnancy (proportion)	96
Congenital anomalies	Male	0-6 days	65-69 years	Data Rich	1	1	Birth prevalence of congenital chromosomal anomalies	375
Congenital anomalies	Male	0-6 days	65-69 years	Data Rich	1	1	Live Births 35+ (proportion)	439
Congenital anomalies	Male	0-6 days	65-69 years	Data Rich	-1	2	Legality of Abortion	205
Congenital anomalies	Male	0-6 days	65-69 years	Data Rich	-1	2	Healthcare access and quality index	245
Congenital anomalies	Male	0-6 days	65-69 years	Data Rich	-1	2	Antenatal Care (1 visit) Coverage (proportion)	--
Congenital anomalies	Male	0-6 days	65-69 years	Data Rich	-1	2	Antenatal Care (4 visits) Coverage (proportion)	--
Congenital anomalies	Male	0-6 days	65-69 years	Data Rich	-1	2	Composite fortification standard and folic acid inclusion	--
Congenital anomalies	Male	0-6 days	65-69 years	Data Rich	-1	2	In-Facility Delivery (proportion)	--
Congenital anomalies	Male	0-6 days	65-69 years	Data Rich	1	2	Age-standardized SEV for Smoking	222
Congenital anomalies	Male	0-6 days	65-69 years	Data Rich	-1	3	Maternal Education (years per capita)	13
Congenital anomalies	Male	0-6 days	65-69 years	Data Rich	-1	3	Socio-demographic Index	63
Congenital anomalies	Male	0-6 days	65-69 years	Data Rich	1	3	Age-standardized SEV for Low vegetables	2
Congenital anomalies	Male	0-6 days	65-69 years	Data Rich	1	3	Age-standardized SEV for Low fruit	42
Congenital anomalies	Male	0-6 days	65-69 years	Data Rich	1	3	Age-standardized SEV for Ambient particulate matter	209
Congenital anomalies	Male	0-6 days	65-69 years	Data Rich	1	3	Liters of alcohol consumed per capita	322
Congenital anomalies	Male	0-6 days	65-69 years	Data Rich	1	3	Age-standardized SEV for Household air pollution	--
Congenital anomalies	Male	0-6 days	65-69 years	Global	-1	1	Folic acid unadjusted (ug)	173
Congenital anomalies	Male	0-6 days	65-69 years	Global	1	1	Birth prevalence of CHD	45
Congenital anomalies	Male	0-6 days	65-69 years	Global	1	1	Birth prevalence of congenital chromosomal anomalies	218
Congenital anomalies	Male	0-6 days	65-69 years	Global	1	1	Maternal alcohol consumption during pregnancy (proportion)	425
Congenital anomalies	Male	0-6 days	65-69 years	Global	1	1	Live Births 35+ (proportion)	782
Congenital anomalies	Male	0-6 days	65-69 years	Global	-1	2	Composite fortification standard and folic acid inclusion	9
Congenital anomalies	Male	0-6 days	65-69 years	Global	-1	2	In-Facility Delivery (proportion)	16
Congenital anomalies	Male	0-6 days	65-69 years	Global	-1	2	Healthcare access and quality index	86
Congenital anomalies	Male	0-6 days	65-69 years	Global	-1	2	Antenatal Care (1 visit) Coverage (proportion)	--
Congenital anomalies	Male	0-6 days	65-69 years	Global	-1	2	Antenatal Care (4 visits) Coverage (proportion)	--
Congenital anomalies	Male	0-6 days	65-69 years	Global	-1	2	Legality of Abortion	--
Congenital anomalies	Male	0-6 days	65-69 years	Global	1	2	Age-standardized SEV for Smoking	102
Congenital anomalies	Male	0-6 days	65-69 years	Global	-1	3	Socio-demographic Index	31
Congenital anomalies	Male	0-6 days	65-69 years	Global	-1	3	Maternal Education (years per capita)	47
Congenital anomalies	Male	0-6 days	65-69 years	Global	1	3	Age-standardized SEV for Household air pollution	12
Congenital anomalies	Male	0-6 days	65-69 years	Global	1	3	Liters of alcohol consumed per capita	46
Congenital anomalies	Male	0-6 days	65-69 years	Global	1	3	Age-standardized SEV for Low fruit	56
Congenital anomalies	Male	0-6 days	65-69 years	Global	1	3	Outdoor Air Pollution (PM2.5)	414
Congenital anomalies	Male	0-6 days	65-69 years	Global	1	3	Age-standardized SEV for Low vegetables	--
Neural tube defects	Female	0-6 days	65-69 years	Data Rich	-1	1	In-Facility Delivery (proportion)	91
Neural tube defects	Female	0-6 days	65-69 years	Data Rich	-1	1	Socio-demographic Index	341

CODEm covariates used, level of covariate, and expected direction of covariate by cause, sex, and age

Cause	Sex	Age Start	Age End	Model Version Type	Direction	Level	Covariate Name	Number of Draws
Neural tube defects	Female	0-6 days	65-69 years	Data Rich	-1	1	Folic acid unadjusted (ug)	659
Neural tube defects	Female	0-6 days	65-69 years	Data Rich	-1	1	Composite fortification standard and folic acid inclusion	--
Neural tube defects	Female	0-6 days	65-69 years	Data Rich	-1	2	Antenatal Care (4 visits) Coverage (proportion)	28
Neural tube defects	Female	0-6 days	65-69 years	Data Rich	-1	2	Healthcare access and quality index	155
Neural tube defects	Female	0-6 days	65-69 years	Data Rich	-1	2	Legality of Abortion	215
Neural tube defects	Female	0-6 days	65-69 years	Data Rich	-1	2	Antenatal Care (1 visit) Coverage (proportion)	--
Neural tube defects	Female	0-6 days	65-69 years	Data Rich	1	2	Age-standardized SEV for Smoking	3
Neural tube defects	Female	0-6 days	65-69 years	Data Rich	-1	3	Maternal Education (years per capita)	102
Neural tube defects	Female	0-6 days	65-69 years	Data Rich	1	3	Age-standardized SEV for Low vegetables	21
Neural tube defects	Female	0-6 days	65-69 years	Data Rich	1	3	Age-standardized SEV for Low fruit	92
Neural tube defects	Female	0-6 days	65-69 years	Data Rich	1	3	Maternal alcohol consumption during pregnancy (proportion)	149
Neural tube defects	Female	0-6 days	65-69 years	Data Rich	1	3	Age-standardized SEV for High fasting plasma glucose	320
Neural tube defects	Female	0-6 days	65-69 years	Data Rich	1	3	Liters of alcohol consumed per capita	456
Neural tube defects	Female	0-6 days	65-69 years	Data Rich	1	3	Age-standardized SEV for Household air pollution	--
Neural tube defects	Female	0-6 days	65-69 years	Global	-1	1	Composite fortification standard and folic acid inclusion	5
Neural tube defects	Female	0-6 days	65-69 years	Global	-1	1	In-Facility Delivery (proportion)	104
Neural tube defects	Female	0-6 days	65-69 years	Global	-1	1	Socio-demographic Index	278
Neural tube defects	Female	0-6 days	65-69 years	Global	-1	1	Folic acid unadjusted (ug)	410
Neural tube defects	Female	0-6 days	65-69 years	Global	-1	2	Antenatal Care (4 visits) Coverage (proportion)	1
Neural tube defects	Female	0-6 days	65-69 years	Global	-1	2	Healthcare access and quality index	269
Neural tube defects	Female	0-6 days	65-69 years	Global	-1	2	Legality of Abortion	723
Neural tube defects	Female	0-6 days	65-69 years	Global	-1	2	Antenatal Care (1 visit) Coverage (proportion)	--
Neural tube defects	Female	0-6 days	65-69 years	Global	1	2	Age-standardized SEV for Smoking	138
Neural tube defects	Female	0-6 days	65-69 years	Global	-1	3	Maternal Education (years per capita)	51
Neural tube defects	Female	0-6 days	65-69 years	Global	1	3	Age-standardized SEV for Household air pollution	0
Neural tube defects	Female	0-6 days	65-69 years	Global	1	3	Age-standardized SEV for Low vegetables	0
Neural tube defects	Female	0-6 days	65-69 years	Global	1	3	Age-standardized SEV for Low fruit	90
Neural tube defects	Female	0-6 days	65-69 years	Global	1	3	Age-standardized SEV for High fasting plasma glucose	297
Neural tube defects	Female	0-6 days	65-69 years	Global	1	3	Liters of alcohol consumed per capita	400
Neural tube defects	Female	0-6 days	65-69 years	Global	1	3	Maternal alcohol consumption during pregnancy (proportion)	--
Neural tube defects	Male	0-6 days	65-69 years	Data Rich	-1	1	In-Facility Delivery (proportion)	28
Neural tube defects	Male	0-6 days	65-69 years	Data Rich	-1	1	Socio-demographic Index	190
Neural tube defects	Male	0-6 days	65-69 years	Data Rich	-1	1	Folic acid unadjusted (ug)	808
Neural tube defects	Male	0-6 days	65-69 years	Data Rich	-1	1	Composite fortification standard and folic acid inclusion	--
Neural tube defects	Male	0-6 days	65-69 years	Data Rich	-1	2	Antenatal Care (4 visits) Coverage (proportion)	0
Neural tube defects	Male	0-6 days	65-69 years	Data Rich	-1	2	Healthcare access and quality index	199
Neural tube defects	Male	0-6 days	65-69 years	Data Rich	-1	2	Legality of Abortion	368
Neural tube defects	Male	0-6 days	65-69 years	Data Rich	-1	2	Antenatal Care (1 visit) Coverage (proportion)	--
Neural tube defects	Male	0-6 days	65-69 years	Data Rich	1	2	Age-standardized SEV for Smoking	19
Neural tube defects	Male	0-6 days	65-69 years	Data Rich	-1	3	Maternal Education (years per capita)	10
Neural tube defects	Male	0-6 days	65-69 years	Data Rich	1	3	Age-standardized SEV for Low fruit	1
Neural tube defects	Male	0-6 days	65-69 years	Data Rich	1	3	Age-standardized SEV for Low vegetables	15
Neural tube defects	Male	0-6 days	65-69 years	Data Rich	1	3	Maternal alcohol consumption during pregnancy (proportion)	159
Neural tube defects	Male	0-6 days	65-69 years	Data Rich	1	3	Age-standardized SEV for High fasting plasma glucose	238
Neural tube defects	Male	0-6 days	65-69 years	Data Rich	1	3	Liters of alcohol consumed per capita	443
Neural tube defects	Male	0-6 days	65-69 years	Data Rich	1	3	Age-standardized SEV for Household air pollution	--
Neural tube defects	Male	0-6 days	65-69 years	Global	-1	1	Composite fortification standard and folic acid inclusion	0
Neural tube defects	Male	0-6 days	65-69 years	Global	-1	1	In-Facility Delivery (proportion)	127
Neural tube defects	Male	0-6 days	65-69 years	Global	-1	1	Socio-demographic Index	291
Neural tube defects	Male	0-6 days	65-69 years	Global	-1	1	Folic acid unadjusted (ug)	916
Neural tube defects	Male	0-6 days	65-69 years	Global	-1	2	Antenatal Care (1 visit) Coverage (proportion)	1
Neural tube defects	Male	0-6 days	65-69 years	Global	-1	2	Antenatal Care (4 visits) Coverage (proportion)	12
Neural tube defects	Male	0-6 days	65-69 years	Global	-1	2	Healthcare access and quality index	312
Neural tube defects	Male	0-6 days	65-69 years	Global	-1	2	Legality of Abortion	362
Neural tube defects	Male	0-6 days	65-69 years	Global	1	2	Age-standardized SEV for Smoking	397
Neural tube defects	Male	0-6 days	65-69 years	Global	-1	3	Maternal Education (years per capita)	25
Neural tube defects	Male	0-6 days	65-69 years	Global	1	3	Age-standardized SEV for Household air pollution	0
Neural tube defects	Male	0-6 days	65-69 years	Global	1	3	Age-standardized SEV for Low vegetables	21
Neural tube defects	Male	0-6 days	65-69 years	Global	1	3	Age-standardized SEV for Low fruit	29
Neural tube defects	Male	0-6 days	65-69 years	Global	1	3	Maternal alcohol consumption during pregnancy (proportion)	121
Neural tube defects	Male	0-6 days	65-69 years	Global	1	3	Liters of alcohol consumed per capita	142
Neural tube defects	Male	0-6 days	65-69 years	Global	1	3	Age-standardized SEV for High fasting plasma glucose	268
Congenital heart anomalies	Female	0-6 days	65-69 years	Data Rich	1	1	Birth prevalence of CHD	1000
Congenital heart anomalies	Female	0-6 days	65-69 years	Data Rich	1	1	Maternal alcohol consumption during pregnancy (proportion)	--
Congenital heart anomalies	Female	0-6 days	65-69 years	Data Rich	-1	2	Antenatal Care (1 visit) Coverage (proportion)	8
Congenital heart anomalies	Female	0-6 days	65-69 years	Data Rich	-1	2	In-Facility Delivery (proportion)	50
Congenital heart anomalies	Female	0-6 days	65-69 years	Data Rich	-1	2	Socio-demographic Index	136
Congenital heart anomalies	Female	0-6 days	65-69 years	Data Rich	-1	2	Legality of Abortion	144
Congenital heart anomalies	Female	0-6 days	65-69 years	Data Rich	-1	2	Healthcare access and quality index	--
Congenital heart anomalies	Female	0-6 days	65-69 years	Data Rich	1	2	Age-standardized SEV for Smoking	350
Congenital heart anomalies	Female	0-6 days	65-69 years	Data Rich	1	2	Age-standardized SEV for High fasting plasma glucose	--
Congenital heart anomalies	Female	0-6 days	65-69 years	Data Rich	-1	3	Skilled Birth Attendance (proportion)	31
Congenital heart anomalies	Female	0-6 days	65-69 years	Data Rich	-1	3	Antenatal Care (4 visits) Coverage (proportion)	37
Congenital heart anomalies	Female	0-6 days	65-69 years	Data Rich	-1	3	Maternal Education (years per capita)	114
Congenital heart anomalies	Female	0-6 days	65-69 years	Data Rich	1	3	Liters of alcohol consumed per capita	441
Congenital heart anomalies	Female	0-6 days	65-69 years	Data Rich	1	3	Live Births 35+ (proportion)	--
Congenital heart anomalies	Female	0-6 days	65-69 years	Global	1	1	Birth prevalence of CHD	--
Congenital heart anomalies	Female	0-6 days	65-69 years	Global	1	1	Maternal alcohol consumption during pregnancy (proportion)	--
Congenital heart anomalies	Female	0-6 days	65-69 years	Global	-1	2	Legality of Abortion	619
Congenital heart anomalies	Female	0-6 days	65-69 years	Global	-1	2	Socio-demographic Index	619
Congenital heart anomalies	Female	0-6 days	65-69 years	Global	-1	2	Antenatal Care (1 visit) Coverage (proportion)	--
Congenital heart anomalies	Female	0-6 days	65-69 years	Global	-1	2	Healthcare access and quality index	--
Congenital heart anomalies	Female	0-6 days	65-69 years	Global	-1	2	In-Facility Delivery (proportion)	--
Congenital heart anomalies	Female	0-6 days	65-69 years	Global	1	2	Age-standardized SEV for High fasting plasma glucose	--
Congenital heart anomalies	Female	0-6 days	65-69 years	Global	1	2	Age-standardized SEV for Smoking	--
Congenital heart anomalies	Female	0-6 days	65-69 years	Global	-1	3	Skilled Birth Attendance (proportion)	142
Congenital heart anomalies	Female	0-6 days	65-69 years	Global	-1	3	Maternal Education (years per capita)	239
Congenital heart anomalies	Female	0-6 days	65-69 years	Global	-1	3	Antenatal Care (4 visits) Coverage (proportion)	--
Congenital heart anomalies	Female	0-6 days	65-69 years	Global	1	3	Liters of alcohol consumed per capita	342
Congenital heart anomalies	Female	0-6 days	65-69 years	Global	1	3	Live Births 35+ (proportion)	--
Congenital heart anomalies	Male	0-6 days	65-69 years	Data Rich	1	1	Birth prevalence of CHD	--
Congenital heart anomalies	Male	0-6 days	65-69 years	Data Rich	1	1	Maternal alcohol consumption during pregnancy (proportion)	--
Congenital heart anomalies	Male	0-6 days	65-69 years	Data Rich	-1	2	Legality of Abortion	304
Congenital heart anomalies	Male	0-6 days	65-69 years	Data Rich	-1	2	Healthcare access and quality index	317
Congenital heart anomalies	Male	0-6 days	65-69 years	Data Rich	-1	2	Antenatal Care (1 visit) Coverage (proportion)	--
Congenital heart anomalies	Male	0-6 days	65-69 years	Data Rich	-1	2	In-Facility Delivery (proportion)	--
Congenital heart anomalies	Male	0-6 days	65-69 years	Data Rich	-1	2	Socio-demographic Index	--
Congenital heart anomalies	Male	0-6 days	65-69 years	Data Rich	1	2	Age-standardized SEV for High fasting plasma glucose	--
Congenital heart anomalies	Male	0-6 days	65-69 years	Data Rich	1	2	Age-standardized SEV for Smoking	--
Congenital heart anomalies	Male	0-6 days	65-69 years	Data Rich	-1	3	Maternal Education (years per capita)	443
Congenital heart anomalies	Male	0-6 days	65-69 years	Data Rich	-1	3	Antenatal Care (4 visits) Coverage (proportion)	--
Congenital heart anomalies	Male	0-6 days	65-69 years	Data Rich	-1	3	Skilled Birth Attendance (proportion)	--
Congenital heart anomalies	Male	0-6 days	65-69 years	Data Rich	1	3	Liters of alcohol consumed per capita	346
Congenital heart anomalies	Male	0-6 days	65-69 years	Data Rich	1	3	Live Births 35+ (proportion)	--
Congenital heart anomalies	Male	0-6 days	65-69 years	Global	1	1	Birth prevalence of CHD	--
Congenital heart anomalies	Male	0-6 days	65-69 years	Global	1	1	Maternal alcohol consumption during pregnancy (proportion)	--
Congenital heart anomalies	Male	0-6 days	65-69 years	Global	-1	2	Antenatal Care (1 visit) Coverage (proportion)	22

CODEm covariates used, level of covariate, and expected direction of covariate by cause, sex, and age

Cause	Sex	Age Start	Age End	Model Version Type	Direction	Level	Covariate Name	Number of Draws
Congenital heart anomalies	Male	0-6 days	65-69 years	Global	-1	2	In-Facility Delivery (proportion)	53
Congenital heart anomalies	Male	0-6 days	65-69 years	Global	-1	2	Healthcare access and quality index	127
Congenital heart anomalies	Male	0-6 days	65-69 years	Global	-1	2	Legality of Abortion	--
Congenital heart anomalies	Male	0-6 days	65-69 years	Global	-1	2	Socio-demographic Index	--
Congenital heart anomalies	Male	0-6 days	65-69 years	Global	1	2	Age-standardized SEV for Smoking	639
Congenital heart anomalies	Male	0-6 days	65-69 years	Global	1	2	Age-standardized SEV for High fasting plasma glucose	--
Congenital heart anomalies	Male	0-6 days	65-69 years	Global	-1	3	Skilled Birth Attendance (proportion)	17
Congenital heart anomalies	Male	0-6 days	65-69 years	Global	-1	3	Antenatal Care (4 visits) Coverage (proportion)	--
Congenital heart anomalies	Male	0-6 days	65-69 years	Global	-1	3	Maternal Education (years per capita)	--
Congenital heart anomalies	Male	0-6 days	65-69 years	Global	1	3	Live Births 35+ (proportion)	219
Congenital heart anomalies	Male	0-6 days	65-69 years	Global	1	3	Liters of alcohol consumed per capita	398
Orofacial clefts	Female	0-6 days	2-4 years	Data Rich	-1	1	Composite fortification standard and folic acid inclusion	113
Orofacial clefts	Female	0-6 days	2-4 years	Data Rich	-1	1	Socio-demographic Index	552
Orofacial clefts	Female	0-6 days	2-4 years	Data Rich	-1	1	Folic acid unadjusted (ug)	671
Orofacial clefts	Female	0-6 days	2-4 years	Data Rich	-1	2	Legality of Abortion	32
Orofacial clefts	Female	0-6 days	2-4 years	Data Rich	-1	2	Skilled Birth Attendance (proportion)	88
Orofacial clefts	Female	0-6 days	2-4 years	Data Rich	-1	2	Healthcare access and quality index	490
Orofacial clefts	Female	0-6 days	2-4 years	Data Rich	1	2	Age-standardized SEV for Smoking	546
Orofacial clefts	Female	0-6 days	2-4 years	Data Rich	1	2	Age-standardized SEV for High fasting plasma glucose	--
Orofacial clefts	Female	0-6 days	2-4 years	Data Rich	1	2	Maternal alcohol consumption during pregnancy (proportion)	--
Orofacial clefts	Female	0-6 days	2-4 years	Data Rich	-1	3	Antenatal Care (1 visit) Coverage (proportion)	11
Orofacial clefts	Female	0-6 days	2-4 years	Data Rich	-1	3	Antenatal Care (4 visits) Coverage (proportion)	26
Orofacial clefts	Female	0-6 days	2-4 years	Data Rich	-1	3	Maternal Education (years per capita)	314
Orofacial clefts	Female	0-6 days	2-4 years	Data Rich	1	3	Indoor Air Pollution (All Cooking Fuels)	45
Orofacial clefts	Female	0-6 days	2-4 years	Data Rich	1	3	Age-standardized SEV for Low fruit	241
Orofacial clefts	Female	0-6 days	2-4 years	Data Rich	1	3	Liters of alcohol consumed per capita	483
Orofacial clefts	Female	0-6 days	2-4 years	Data Rich	1	3	Age-standardized SEV for Low vegetables	--
Orofacial clefts	Female	0-6 days	2-4 years	Global	-1	1	Composite fortification standard and folic acid inclusion	413
Orofacial clefts	Female	0-6 days	2-4 years	Global	-1	1	Socio-demographic Index	863
Orofacial clefts	Female	0-6 days	2-4 years	Global	-1	2	Legality of Abortion	4
Orofacial clefts	Female	0-6 days	2-4 years	Global	-1	2	Skilled Birth Attendance (proportion)	4
Orofacial clefts	Female	0-6 days	2-4 years	Global	-1	2	Healthcare access and quality index	595
Orofacial clefts	Female	0-6 days	2-4 years	Global	1	2	Age-standardized SEV for Smoking	513
Orofacial clefts	Female	0-6 days	2-4 years	Global	1	2	Age-standardized SEV for High fasting plasma glucose	--
Orofacial clefts	Female	0-6 days	2-4 years	Global	1	2	Maternal alcohol consumption during pregnancy (proportion)	--
Orofacial clefts	Female	0-6 days	2-4 years	Global	-1	3	Antenatal Care (1 visit) Coverage (proportion)	0
Orofacial clefts	Female	0-6 days	2-4 years	Global	-1	3	Antenatal Care (4 visits) Coverage (proportion)	1
Orofacial clefts	Female	0-6 days	2-4 years	Global	-1	3	Maternal Education (years per capita)	35
Orofacial clefts	Female	0-6 days	2-4 years	Global	1	3	Age-standardized SEV for Household air pollution	0
Orofacial clefts	Female	0-6 days	2-4 years	Global	1	3	Age-standardized SEV for Low fruit	202
Orofacial clefts	Female	0-6 days	2-4 years	Global	1	3	Liters of alcohol consumed per capita	656
Orofacial clefts	Female	0-6 days	2-4 years	Global	1	3	Age-standardized SEV for Low vegetables	--
Orofacial clefts	Male	0-6 days	2-4 years	Data Rich	-1	1	Composite fortification standard and folic acid inclusion	405
Orofacial clefts	Male	0-6 days	2-4 years	Data Rich	-1	1	Folic acid unadjusted (ug)	612
Orofacial clefts	Male	0-6 days	2-4 years	Data Rich	-1	1	Socio-demographic Index	789
Orofacial clefts	Male	0-6 days	2-4 years	Data Rich	-1	2	Skilled Birth Attendance (proportion)	27
Orofacial clefts	Male	0-6 days	2-4 years	Data Rich	-1	2	Legality of Abortion	194
Orofacial clefts	Male	0-6 days	2-4 years	Data Rich	-1	2	Healthcare access and quality index	366
Orofacial clefts	Male	0-6 days	2-4 years	Data Rich	1	2	Age-standardized SEV for Smoking	422
Orofacial clefts	Male	0-6 days	2-4 years	Data Rich	1	2	Age-standardized SEV for High fasting plasma glucose	--
Orofacial clefts	Male	0-6 days	2-4 years	Data Rich	1	2	Maternal alcohol consumption during pregnancy (proportion)	--
Orofacial clefts	Male	0-6 days	2-4 years	Data Rich	-1	3	Antenatal Care (4 visits) Coverage (proportion)	24
Orofacial clefts	Male	0-6 days	2-4 years	Data Rich	-1	3	Antenatal Care (1 visit) Coverage (proportion)	25
Orofacial clefts	Male	0-6 days	2-4 years	Data Rich	-1	3	Maternal Education (years per capita)	71
Orofacial clefts	Male	0-6 days	2-4 years	Data Rich	1	3	Indoor Air Pollution (All Cooking Fuels)	62
Orofacial clefts	Male	0-6 days	2-4 years	Data Rich	1	3	Age-standardized SEV for Low fruit	289
Orofacial clefts	Male	0-6 days	2-4 years	Data Rich	1	3	Liters of alcohol consumed per capita	420
Orofacial clefts	Male	0-6 days	2-4 years	Data Rich	1	3	Age-standardized SEV for Low vegetables	--
Orofacial clefts	Male	0-6 days	2-4 years	Global	-1	1	Composite fortification standard and folic acid inclusion	586
Orofacial clefts	Male	0-6 days	2-4 years	Global	-1	1	Socio-demographic Index	717
Orofacial clefts	Male	0-6 days	2-4 years	Global	-1	2	Skilled Birth Attendance (proportion)	54
Orofacial clefts	Male	0-6 days	2-4 years	Global	-1	2	Healthcare access and quality index	524
Orofacial clefts	Male	0-6 days	2-4 years	Global	-1	2	Legality of Abortion	672
Orofacial clefts	Male	0-6 days	2-4 years	Global	1	2	Age-standardized SEV for Smoking	567
Orofacial clefts	Male	0-6 days	2-4 years	Global	1	2	Age-standardized SEV for High fasting plasma glucose	--
Orofacial clefts	Male	0-6 days	2-4 years	Global	1	2	Maternal alcohol consumption during pregnancy (proportion)	--
Orofacial clefts	Male	0-6 days	2-4 years	Global	-1	3	Antenatal Care (1 visit) Coverage (proportion)	26
Orofacial clefts	Male	0-6 days	2-4 years	Global	-1	3	Antenatal Care (4 visits) Coverage (proportion)	26
Orofacial clefts	Male	0-6 days	2-4 years	Global	-1	3	Maternal Education (years per capita)	301
Orofacial clefts	Male	0-6 days	2-4 years	Global	1	3	Age-standardized SEV for Household air pollution	35
Orofacial clefts	Male	0-6 days	2-4 years	Global	1	3	Liters of alcohol consumed per capita	392
Orofacial clefts	Male	0-6 days	2-4 years	Global	1	3	Age-standardized SEV for Low fruit	410
Orofacial clefts	Male	0-6 days	2-4 years	Global	1	3	Age-standardized SEV for Low vegetables	--
Down's syndrome	Female	0-6 days	65-69 years	Data Rich	-1	1	Legality of Abortion	294
Down's syndrome	Female	0-6 days	65-69 years	Data Rich	1	1	Live Births 40+ (proportion)	224
Down's syndrome	Female	0-6 days	65-69 years	Data Rich	1	1	Birth prevalence of congenital chromosomal anomalies	634
Down's syndrome	Female	0-6 days	65-69 years	Data Rich	1	1	Live Births 35+ (proportion)	693
Down's syndrome	Female	0-6 days	65-69 years	Data Rich	-1	2	Healthcare access and quality index	--
Down's syndrome	Female	0-6 days	65-69 years	Data Rich	-1	2	In-Facility Delivery (proportion)	--
Down's syndrome	Female	0-6 days	65-69 years	Data Rich	-1	2	Socio-demographic Index	--
Down's syndrome	Female	0-6 days	65-69 years	Data Rich	-1	3	Antenatal Care (1 visit) Coverage (proportion)	--
Down's syndrome	Female	0-6 days	65-69 years	Data Rich	-1	3	Antenatal Care (4 visits) Coverage (proportion)	--
Down's syndrome	Female	0-6 days	65-69 years	Data Rich	-1	3	Maternal Education (years per capita)	--
Down's syndrome	Female	0-6 days	65-69 years	Data Rich	1	3	Liters of alcohol consumed per capita	257
Down's syndrome	Female	0-6 days	65-69 years	Data Rich	1	3	Age-standardized SEV for Household air pollution	--
Down's syndrome	Female	0-6 days	65-69 years	Data Rich	1	3	Age-standardized SEV for Low vegetables	--
Down's syndrome	Female	0-6 days	65-69 years	Data Rich	1	3	Age-standardized SEV for Smoking	--
Down's syndrome	Female	0-6 days	65-69 years	Data Rich	1	3	Maternal alcohol consumption during pregnancy (proportion)	--
Down's syndrome	Female	0-6 days	65-69 years	Global	-1	1	Legality of Abortion	305
Down's syndrome	Female	0-6 days	65-69 years	Global	1	1	Live Births 40+ (proportion)	161
Down's syndrome	Female	0-6 days	65-69 years	Global	1	1	Birth prevalence of congenital chromosomal anomalies	793
Down's syndrome	Female	0-6 days	65-69 years	Global	1	1	Live Births 35+ (proportion)	829
Down's syndrome	Female	0-6 days	65-69 years	Global	-1	2	Healthcare access and quality index	--
Down's syndrome	Female	0-6 days	65-69 years	Global	-1	2	In-Facility Delivery (proportion)	--
Down's syndrome	Female	0-6 days	65-69 years	Global	-1	2	Socio-demographic Index	--
Down's syndrome	Female	0-6 days	65-69 years	Global	-1	3	Antenatal Care (1 visit) Coverage (proportion)	--
Down's syndrome	Female	0-6 days	65-69 years	Global	-1	3	Antenatal Care (4 visits) Coverage (proportion)	--
Down's syndrome	Female	0-6 days	65-69 years	Global	-1	3	Maternal Education (years per capita)	--
Down's syndrome	Female	0-6 days	65-69 years	Global	1	3	Liters of alcohol consumed per capita	11
Down's syndrome	Female	0-6 days	65-69 years	Global	1	3	Age-standardized SEV for Low vegetables	270
Down's syndrome	Female	0-6 days	65-69 years	Global	1	3	Age-standardized SEV for Household air pollution	--
Down's syndrome	Female	0-6 days	65-69 years	Global	1	3	Age-standardized SEV for Smoking	--
Down's syndrome	Female	0-6 days	65-69 years	Global	1	3	Maternal alcohol consumption during pregnancy (proportion)	--
Down's syndrome	Male	0-6 days	65-69 years	Data Rich	-1	1	Legality of Abortion	319
Down's syndrome	Male	0-6 days	65-69 years	Data Rich	1	1	Live Births 40+ (proportion)	201
Down's syndrome	Male	0-6 days	65-69 years	Data Rich	1	1	Birth prevalence of congenital chromosomal anomalies	583
Down's syndrome	Male	0-6 days	65-69 years	Data Rich	1	1	Live Births 35+ (proportion)	698
Down's syndrome	Male	0-6 days	65-69 years	Data Rich	-1	2	Healthcare access and quality index	--
Down's syndrome	Male	0-6 days	65-69 years	Data Rich	-1	2	In-Facility Delivery (proportion)	--

CODEm covariates used, level of covariate, and expected direction of covariate by cause, sex, and age

Cause	Sex	Age Start	Age End	Model Version Type	Direction	Level	Covariate Name	Number of Draws
Down's syndrome	Male	0-6 days	65-69 years	Data Rich	-1	2	Socio-demographic Index	--
Down's syndrome	Male	0-6 days	65-69 years	Data Rich	-1	3	Antenatal Care (1 visit) Coverage (proportion)	--
Down's syndrome	Male	0-6 days	65-69 years	Data Rich	-1	3	Antenatal Care (4 visits) Coverage (proportion)	--
Down's syndrome	Male	0-6 days	65-69 years	Data Rich	-1	3	Maternal Education (years per capita)	--
Down's syndrome	Male	0-6 days	65-69 years	Data Rich	1	3	Liters of alcohol consumed per capita	341
Down's syndrome	Male	0-6 days	65-69 years	Data Rich	1	3	Age-standardized SEV for Household air pollution	--
Down's syndrome	Male	0-6 days	65-69 years	Data Rich	1	3	Age-standardized SEV for Low vegetables	--
Down's syndrome	Male	0-6 days	65-69 years	Data Rich	1	3	Age-standardized SEV for Smoking	--
Down's syndrome	Male	0-6 days	65-69 years	Data Rich	1	3	Maternal alcohol consumption during pregnancy (proportion)	--
Down's syndrome	Male	0-6 days	65-69 years	Global	-1	1	Legality of Abortion	530
Down's syndrome	Male	0-6 days	65-69 years	Global	1	1	Live Births 40+ (proportion)	376
Down's syndrome	Male	0-6 days	65-69 years	Global	1	1	Live Births 35+ (proportion)	552
Down's syndrome	Male	0-6 days	65-69 years	Global	1	1	Birth prevalence of congenital chromosomal anomalies	783
Down's syndrome	Male	0-6 days	65-69 years	Global	-1	2	Healthcare access and quality index	--
Down's syndrome	Male	0-6 days	65-69 years	Global	-1	2	In-Facility Delivery (proportion)	--
Down's syndrome	Male	0-6 days	65-69 years	Global	-1	2	Socio-demographic Index	--
Down's syndrome	Male	0-6 days	65-69 years	Global	-1	3	Antenatal Care (1 visit) Coverage (proportion)	--
Down's syndrome	Male	0-6 days	65-69 years	Global	-1	3	Antenatal Care (4 visits) Coverage (proportion)	--
Down's syndrome	Male	0-6 days	65-69 years	Global	-1	3	Maternal Education (years per capita)	--
Down's syndrome	Male	0-6 days	65-69 years	Global	1	3	Age-standardized SEV for Low vegetables	36
Down's syndrome	Male	0-6 days	65-69 years	Global	1	3	Liters of alcohol consumed per capita	382
Down's syndrome	Male	0-6 days	65-69 years	Global	1	3	Age-standardized SEV for Household air pollution	--
Down's syndrome	Male	0-6 days	65-69 years	Global	1	3	Age-standardized SEV for Smoking	--
Down's syndrome	Male	0-6 days	65-69 years	Global	1	3	Maternal alcohol consumption during pregnancy (proportion)	--
Other chromosomal abnormalities	Female	0-6 days	65-69 years	Data Rich	-1	1	Legality of Abortion	649
Other chromosomal abnormalities	Female	0-6 days	65-69 years	Data Rich	1	1	Live Births 40+ (proportion)	12
Other chromosomal abnormalities	Female	0-6 days	65-69 years	Data Rich	1	1	Live Births 35+ (proportion)	988
Other chromosomal abnormalities	Female	0-6 days	65-69 years	Data Rich	-1	2	Antenatal Care (1 visit) Coverage (proportion)	--
Other chromosomal abnormalities	Female	0-6 days	65-69 years	Data Rich	-1	2	Antenatal Care (4 visits) Coverage (proportion)	--
Other chromosomal abnormalities	Female	0-6 days	65-69 years	Data Rich	-1	2	Healthcare access and quality index	--
Other chromosomal abnormalities	Female	0-6 days	65-69 years	Data Rich	-1	2	In-Facility Delivery (proportion)	--
Other chromosomal abnormalities	Female	0-6 days	65-69 years	Data Rich	-1	2	LDI (I$ per capita)	--
Other chromosomal abnormalities	Female	0-6 days	65-69 years	Data Rich	1	2	Maternal alcohol consumption during pregnancy (proportion)	--
Other chromosomal abnormalities	Female	0-6 days	65-69 years	Data Rich	-1	3	Maternal Education (years per capita)	--
Other chromosomal abnormalities	Female	0-6 days	65-69 years	Data Rich	-1	3	Skilled Birth Attendance (proportion)	--
Other chromosomal abnormalities	Female	0-6 days	65-69 years	Data Rich	-1	3	Socio-demographic Index	--
Other chromosomal abnormalities	Female	0-6 days	65-69 years	Data Rich	1	3	Liters of alcohol consumed per capita	127
Other chromosomal abnormalities	Female	0-6 days	65-69 years	Data Rich	1	3	Age-standardized SEV for Household air pollution	--
Other chromosomal abnormalities	Female	0-6 days	65-69 years	Data Rich	1	3	Age-standardized SEV for Smoking	--
Other chromosomal abnormalities	Female	0-6 days	65-69 years	Global	-1	1	Legality of Abortion	578
Other chromosomal abnormalities	Female	0-6 days	65-69 years	Global	1	1	Live Births 40+ (proportion)	214
Other chromosomal abnormalities	Female	0-6 days	65-69 years	Global	1	1	Live Births 35+ (proportion)	786
Other chromosomal abnormalities	Female	0-6 days	65-69 years	Global	-1	2	Antenatal Care (1 visit) Coverage (proportion)	--
Other chromosomal abnormalities	Female	0-6 days	65-69 years	Global	-1	2	Antenatal Care (4 visits) Coverage (proportion)	--
Other chromosomal abnormalities	Female	0-6 days	65-69 years	Global	-1	2	Healthcare access and quality index	--
Other chromosomal abnormalities	Female	0-6 days	65-69 years	Global	-1	2	In-Facility Delivery (proportion)	--
Other chromosomal abnormalities	Female	0-6 days	65-69 years	Global	-1	2	LDI (I$ per capita)	--
Other chromosomal abnormalities	Female	0-6 days	65-69 years	Global	1	2	Maternal alcohol consumption during pregnancy (proportion)	--
Other chromosomal abnormalities	Female	0-6 days	65-69 years	Global	-1	3	Maternal Education (years per capita)	--
Other chromosomal abnormalities	Female	0-6 days	65-69 years	Global	-1	3	Skilled Birth Attendance (proportion)	--
Other chromosomal abnormalities	Female	0-6 days	65-69 years	Global	-1	3	Socio-demographic Index	--
Other chromosomal abnormalities	Female	0-6 days	65-69 years	Global	1	3	Liters of alcohol consumed per capita	225
Other chromosomal abnormalities	Female	0-6 days	65-69 years	Global	1	3	Age-standardized SEV for Household air pollution	--
Other chromosomal abnormalities	Female	0-6 days	65-69 years	Global	1	3	Age-standardized SEV for Smoking	--
Other chromosomal abnormalities	Male	0-6 days	65-69 years	Data Rich	-1	1	Legality of Abortion	389
Other chromosomal abnormalities	Male	0-6 days	65-69 years	Data Rich	1	1	Live Births 40+ (proportion)	42
Other chromosomal abnormalities	Male	0-6 days	65-69 years	Data Rich	1	1	Live Births 35+ (proportion)	958
Other chromosomal abnormalities	Male	0-6 days	65-69 years	Data Rich	-1	2	Antenatal Care (1 visit) Coverage (proportion)	--
Other chromosomal abnormalities	Male	0-6 days	65-69 years	Data Rich	-1	2	Antenatal Care (4 visits) Coverage (proportion)	--
Other chromosomal abnormalities	Male	0-6 days	65-69 years	Data Rich	-1	2	Healthcare access and quality index	--
Other chromosomal abnormalities	Male	0-6 days	65-69 years	Data Rich	-1	2	In-Facility Delivery (proportion)	--
Other chromosomal abnormalities	Male	0-6 days	65-69 years	Data Rich	-1	2	LDI (I$ per capita)	--
Other chromosomal abnormalities	Male	0-6 days	65-69 years	Data Rich	1	2	Maternal alcohol consumption during pregnancy (proportion)	--
Other chromosomal abnormalities	Male	0-6 days	65-69 years	Data Rich	-1	3	Maternal Education (years per capita)	--
Other chromosomal abnormalities	Male	0-6 days	65-69 years	Data Rich	-1	3	Skilled Birth Attendance (proportion)	--
Other chromosomal abnormalities	Male	0-6 days	65-69 years	Data Rich	-1	3	Socio-demographic Index	--
Other chromosomal abnormalities	Male	0-6 days	65-69 years	Data Rich	1	3	Liters of alcohol consumed per capita	327
Other chromosomal abnormalities	Male	0-6 days	65-69 years	Data Rich	1	3	Age-standardized SEV for Household air pollution	--
Other chromosomal abnormalities	Male	0-6 days	65-69 years	Data Rich	1	3	Age-standardized SEV for Smoking	--
Other chromosomal abnormalities	Male	0-6 days	65-69 years	Global	-1	1	Legality of Abortion	274
Other chromosomal abnormalities	Male	0-6 days	65-69 years	Global	1	1	Live Births 40+ (proportion)	403
Other chromosomal abnormalities	Male	0-6 days	65-69 years	Global	1	1	Live Births 35+ (proportion)	597
Other chromosomal abnormalities	Male	0-6 days	65-69 years	Global	-1	2	Antenatal Care (1 visit) Coverage (proportion)	--
Other chromosomal abnormalities	Male	0-6 days	65-69 years	Global	-1	2	Antenatal Care (4 visits) Coverage (proportion)	--
Other chromosomal abnormalities	Male	0-6 days	65-69 years	Global	-1	2	Healthcare access and quality index	--
Other chromosomal abnormalities	Male	0-6 days	65-69 years	Global	-1	2	In-Facility Delivery (proportion)	--
Other chromosomal abnormalities	Male	0-6 days	65-69 years	Global	-1	2	LDI (I$ per capita)	--
Other chromosomal abnormalities	Male	0-6 days	65-69 years	Global	1	2	Maternal alcohol consumption during pregnancy (proportion)	--
Other chromosomal abnormalities	Male	0-6 days	65-69 years	Global	-1	3	Maternal Education (years per capita)	--
Other chromosomal abnormalities	Male	0-6 days	65-69 years	Global	-1	3	Skilled Birth Attendance (proportion)	--
Other chromosomal abnormalities	Male	0-6 days	65-69 years	Global	-1	3	Socio-demographic Index	--
Other chromosomal abnormalities	Male	0-6 days	65-69 years	Global	1	3	Liters of alcohol consumed per capita	58
Other chromosomal abnormalities	Male	0-6 days	65-69 years	Global	1	3	Age-standardized SEV for Household air pollution	--
Other chromosomal abnormalities	Male	0-6 days	65-69 years	Global	1	3	Age-standardized SEV for Smoking	--
Congenital musculoskeletal and limb anomalies	Female	0-6 days	65-69 years	Data Rich	-1	1	Legality of Abortion	718
Congenital musculoskeletal and limb anomalies	Female	0-6 days	65-69 years	Data Rich	1	1	Maternal alcohol consumption during pregnancy (proportion)	--
Congenital musculoskeletal and limb anomalies	Female	0-6 days	65-69 years	Data Rich	-1	2	Healthcare access and quality index	--
Congenital musculoskeletal and limb anomalies	Female	0-6 days	65-69 years	Data Rich	-1	2	In-Facility Delivery (proportion)	--
Congenital musculoskeletal and limb anomalies	Female	0-6 days	65-69 years	Data Rich	-1	2	Socio-demographic Index	--
Congenital musculoskeletal and limb anomalies	Female	0-6 days	65-69 years	Data Rich	1	2	Age-standardized SEV for High fasting plasma glucose	--
Congenital musculoskeletal and limb anomalies	Female	0-6 days	65-69 years	Data Rich	1	2	Age-standardized SEV for Household air pollution	--
Congenital musculoskeletal and limb anomalies	Female	0-6 days	65-69 years	Data Rich	1	2	Age-standardized SEV for Smoking	--
Congenital musculoskeletal and limb anomalies	Female	0-6 days	65-69 years	Data Rich	-1	3	Antenatal Care (1 visit) Coverage (proportion)	--
Congenital musculoskeletal and limb anomalies	Female	0-6 days	65-69 years	Data Rich	-1	3	Antenatal Care (4 visits) Coverage (proportion)	--
Congenital musculoskeletal and limb anomalies	Female	0-6 days	65-69 years	Data Rich	-1	3	LDI (I$ per capita)	--
Congenital musculoskeletal and limb anomalies	Female	0-6 days	65-69 years	Data Rich	-1	3	Maternal Education (years per capita)	--
Congenital musculoskeletal and limb anomalies	Female	0-6 days	65-69 years	Data Rich	1	3	Liters of alcohol consumed per capita	282
Congenital musculoskeletal and limb anomalies	Female	0-6 days	65-69 years	Data Rich	1	3	Age-standardized SEV for Low fruit	337
Congenital musculoskeletal and limb anomalies	Female	0-6 days	65-69 years	Data Rich	1	3	Age-standardized SEV for Low vegetables	--
Congenital musculoskeletal and limb anomalies	Female	0-6 days	65-69 years	Global	-1	1	Legality of Abortion	549
Congenital musculoskeletal and limb anomalies	Female	0-6 days	65-69 years	Global	1	1	Maternal alcohol consumption during pregnancy (proportion)	--
Congenital musculoskeletal and limb anomalies	Female	0-6 days	65-69 years	Global	-1	2	Healthcare access and quality index	109
Congenital musculoskeletal and limb anomalies	Female	0-6 days	65-69 years	Global	-1	2	Socio-demographic Index	151
Congenital musculoskeletal and limb anomalies	Female	0-6 days	65-69 years	Global	-1	2	In-Facility Delivery (proportion)	214
Congenital musculoskeletal and limb anomalies	Female	0-6 days	65-69 years	Global	1	2	Age-standardized SEV for Smoking	63
Congenital musculoskeletal and limb anomalies	Female	0-6 days	65-69 years	Global	1	2	Age-standardized SEV for High fasting plasma glucose	312
Congenital musculoskeletal and limb anomalies	Female	0-6 days	65-69 years	Global	1	2	Age-standardized SEV for Household air pollution	--
Congenital musculoskeletal and limb anomalies	Female	0-6 days	65-69 years	Global	-1	3	Antenatal Care (4 visits) Coverage (proportion)	25
Congenital musculoskeletal and limb anomalies	Female	0-6 days	65-69 years	Global	-1	3	Antenatal Care (1 visit) Coverage (proportion)	28

CODEm covariates used, level of covariate, and expected direction of covariate by cause, sex, and age

Cause	Sex	Age Start	Age End	Model Version Type	Direction	Level	Covariate Name	Number of Draws
Congenital musculoskeletal and limb anomalies	Female	0-6 days	65-69 years	Global	-1	3	Maternal Education (years per capita)	170
Congenital musculoskeletal and limb anomalies	Female	0-6 days	65-69 years	Global	-1	3	LDI (I$ per capita)	--
Congenital musculoskeletal and limb anomalies	Female	0-6 days	65-69 years	Global	1	3	Age-standardized SEV for Low vegetables	19
Congenital musculoskeletal and limb anomalies	Female	0-6 days	65-69 years	Global	1	3	Age-standardized SEV for Low fruit	137
Congenital musculoskeletal and limb anomalies	Female	0-6 days	65-69 years	Global	1	3	Liters of alcohol consumed per capita	255
Congenital musculoskeletal and limb anomalies	Male	0-6 days	65-69 years	Data Rich	-1	1	Legality of Abortion	810
Congenital musculoskeletal and limb anomalies	Male	0-6 days	65-69 years	Data Rich	1	1	Maternal alcohol consumption during pregnancy (proportion)	--
Congenital musculoskeletal and limb anomalies	Male	0-6 days	65-69 years	Data Rich	-1	2	Socio-demographic Index	223
Congenital musculoskeletal and limb anomalies	Male	0-6 days	65-69 years	Data Rich	-1	2	Healthcare access and quality index	324
Congenital musculoskeletal and limb anomalies	Male	0-6 days	65-69 years	Data Rich	-1	2	In-Facility Delivery (proportion)	--
Congenital musculoskeletal and limb anomalies	Male	0-6 days	65-69 years	Data Rich	1	2	Age-standardized SEV for High fasting plasma glucose	190
Congenital musculoskeletal and limb anomalies	Male	0-6 days	65-69 years	Data Rich	1	2	Age-standardized SEV for Smoking	547
Congenital musculoskeletal and limb anomalies	Male	0-6 days	65-69 years	Data Rich	1	2	Age-standardized SEV for Household air pollution	--
Congenital musculoskeletal and limb anomalies	Male	0-6 days	65-69 years	Data Rich	-1	3	Maternal Education (years per capita)	86
Congenital musculoskeletal and limb anomalies	Male	0-6 days	65-69 years	Data Rich	-1	3	Antenatal Care (1 visit) Coverage (proportion)	--
Congenital musculoskeletal and limb anomalies	Male	0-6 days	65-69 years	Data Rich	-1	3	Antenatal Care (4 visits) Coverage (proportion)	--
Congenital musculoskeletal and limb anomalies	Male	0-6 days	65-69 years	Data Rich	-1	3	LDI (I$ per capita)	--
Congenital musculoskeletal and limb anomalies	Male	0-6 days	65-69 years	Data Rich	1	3	Age-standardized SEV for Low vegetables	166
Congenital musculoskeletal and limb anomalies	Male	0-6 days	65-69 years	Data Rich	1	3	Liters of alcohol consumed per capita	171
Congenital musculoskeletal and limb anomalies	Male	0-6 days	65-69 years	Data Rich	1	3	Age-standardized SEV for Low fruit	--
Congenital musculoskeletal and limb anomalies	Male	0-6 days	65-69 years	Global	-1	1	Legality of Abortion	647
Congenital musculoskeletal and limb anomalies	Male	0-6 days	65-69 years	Global	1	1	Maternal alcohol consumption during pregnancy (proportion)	--
Congenital musculoskeletal and limb anomalies	Male	0-6 days	65-69 years	Global	-1	2	In-Facility Delivery (proportion)	252
Congenital musculoskeletal and limb anomalies	Male	0-6 days	65-69 years	Global	-1	2	Socio-demographic Index	252
Congenital musculoskeletal and limb anomalies	Male	0-6 days	65-69 years	Global	-1	2	Healthcare access and quality index	--
Congenital musculoskeletal and limb anomalies	Male	0-6 days	65-69 years	Global	1	2	Age-standardized SEV for High fasting plasma glucose	195
Congenital musculoskeletal and limb anomalies	Male	0-6 days	65-69 years	Global	1	2	Age-standardized SEV for Smoking	252
Congenital musculoskeletal and limb anomalies	Male	0-6 days	65-69 years	Global	1	2	Age-standardized SEV for Household air pollution	--
Congenital musculoskeletal and limb anomalies	Male	0-6 days	65-69 years	Global	-1	3	Antenatal Care (1 visit) Coverage (proportion)	--
Congenital musculoskeletal and limb anomalies	Male	0-6 days	65-69 years	Global	-1	3	Antenatal Care (4 visits) Coverage (proportion)	--
Congenital musculoskeletal and limb anomalies	Male	0-6 days	65-69 years	Global	-1	3	LDI (I$ per capita)	--
Congenital musculoskeletal and limb anomalies	Male	0-6 days	65-69 years	Global	-1	3	Maternal Education (years per capita)	--
Congenital musculoskeletal and limb anomalies	Male	0-6 days	65-69 years	Global	1	3	Age-standardized SEV for Low fruit	104
Congenital musculoskeletal and limb anomalies	Male	0-6 days	65-69 years	Global	1	3	Liters of alcohol consumed per capita	158
Congenital musculoskeletal and limb anomalies	Male	0-6 days	65-69 years	Global	1	3	Age-standardized SEV for Low vegetables	--
Urogenital congenital anomalies	Female	0-6 days	65-69 years	Data Rich	1	1	Smoking Prevalence (Reproductive Age Standardized)	326
Urogenital congenital anomalies	Female	0-6 days	65-69 years	Data Rich	1	1	Age-standardized SEV for Smoking	392
Urogenital congenital anomalies	Female	0-6 days	65-69 years	Data Rich	1	1	Maternal alcohol consumption during pregnancy (proportion)	--
Urogenital congenital anomalies	Female	0-6 days	65-69 years	Data Rich	-1	2	Healthcare access and quality index	--
Urogenital congenital anomalies	Female	0-6 days	65-69 years	Data Rich	-1	2	In-Facility Delivery (proportion)	--
Urogenital congenital anomalies	Female	0-6 days	65-69 years	Data Rich	-1	2	Socio-demographic Index	--
Urogenital congenital anomalies	Female	0-6 days	65-69 years	Data Rich	1	2	Age-standardized SEV for Ambient particulate matter	--
Urogenital congenital anomalies	Female	0-6 days	65-69 years	Data Rich	1	2	Age-standardized SEV for Household air pollution	--
Urogenital congenital anomalies	Female	0-6 days	65-69 years	Data Rich	-1	3	Antenatal Care (1 visit) Coverage (proportion)	--
Urogenital congenital anomalies	Female	0-6 days	65-69 years	Data Rich	-1	3	Antenatal Care (4 visits) Coverage (proportion)	--
Urogenital congenital anomalies	Female	0-6 days	65-69 years	Data Rich	-1	3	LDI (I$ per capita)	--
Urogenital congenital anomalies	Female	0-6 days	65-69 years	Data Rich	-1	3	Maternal Education (years per capita)	--
Urogenital congenital anomalies	Female	0-6 days	65-69 years	Data Rich	1	3	Liters of alcohol consumed per capita	282
Urogenital congenital anomalies	Female	0-6 days	65-69 years	Global	1	1	Age-standardized SEV for Smoking	--
Urogenital congenital anomalies	Female	0-6 days	65-69 years	Global	1	1	Maternal alcohol consumption during pregnancy (proportion)	--
Urogenital congenital anomalies	Female	0-6 days	65-69 years	Global	-1	2	Socio-demographic Index	148
Urogenital congenital anomalies	Female	0-6 days	65-69 years	Global	-1	2	Healthcare access and quality index	187
Urogenital congenital anomalies	Female	0-6 days	65-69 years	Global	-1	2	In-Facility Delivery (proportion)	--
Urogenital congenital anomalies	Female	0-6 days	65-69 years	Global	1	2	Age-standardized SEV for Household air pollution	137
Urogenital congenital anomalies	Female	0-6 days	65-69 years	Global	1	2	Age-standardized SEV for Ambient particulate matter	857
Urogenital congenital anomalies	Female	0-6 days	65-69 years	Global	1	2	Diabetes Age-Standardized Prevalence (proportion)	--
Urogenital congenital anomalies	Female	0-6 days	65-69 years	Global	-1	3	Antenatal Care (1 visit) Coverage (proportion)	--
Urogenital congenital anomalies	Female	0-6 days	65-69 years	Global	-1	3	Antenatal Care (4 visits) Coverage (proportion)	--
Urogenital congenital anomalies	Female	0-6 days	65-69 years	Global	-1	3	LDI (I$ per capita)	--
Urogenital congenital anomalies	Female	0-6 days	65-69 years	Global	-1	3	Maternal Education (years per capita)	--
Urogenital congenital anomalies	Female	0-6 days	65-69 years	Global	1	3	Liters of alcohol consumed per capita	314
Urogenital congenital anomalies	Male	0-6 days	65-69 years	Data Rich	1	1	Age-standardized SEV for Smoking	--
Urogenital congenital anomalies	Male	0-6 days	65-69 years	Data Rich	1	1	Maternal alcohol consumption during pregnancy (proportion)	--
Urogenital congenital anomalies	Male	0-6 days	65-69 years	Data Rich	-1	2	Healthcare access and quality index	225
Urogenital congenital anomalies	Male	0-6 days	65-69 years	Data Rich	-1	2	In-Facility Delivery (proportion)	--
Urogenital congenital anomalies	Male	0-6 days	65-69 years	Data Rich	-1	2	Socio-demographic Index	--
Urogenital congenital anomalies	Male	0-6 days	65-69 years	Data Rich	1	2	Age-standardized SEV for Ambient particulate matter	547
Urogenital congenital anomalies	Male	0-6 days	65-69 years	Data Rich	1	2	Diabetes Age-Standardized Prevalence (proportion)	772
Urogenital congenital anomalies	Male	0-6 days	65-69 years	Data Rich	1	2	Age-standardized SEV for Household air pollution	--
Urogenital congenital anomalies	Male	0-6 days	65-69 years	Data Rich	-1	3	Antenatal Care (1 visit) Coverage (proportion)	--
Urogenital congenital anomalies	Male	0-6 days	65-69 years	Data Rich	-1	3	Antenatal Care (4 visits) Coverage (proportion)	--
Urogenital congenital anomalies	Male	0-6 days	65-69 years	Data Rich	-1	3	LDI (I$ per capita)	--
Urogenital congenital anomalies	Male	0-6 days	65-69 years	Data Rich	-1	3	Maternal Education (years per capita)	--
Urogenital congenital anomalies	Male	0-6 days	65-69 years	Data Rich	1	3	Liters of alcohol consumed per capita	403
Urogenital congenital anomalies	Male	0-6 days	65-69 years	Global	1	1	Age-standardized SEV for Smoking	412
Urogenital congenital anomalies	Male	0-6 days	65-69 years	Global	1	1	Maternal alcohol consumption during pregnancy (proportion)	--
Urogenital congenital anomalies	Male	0-6 days	65-69 years	Global	-1	2	Healthcare access and quality index	--
Urogenital congenital anomalies	Male	0-6 days	65-69 years	Global	-1	2	In-Facility Delivery (proportion)	--
Urogenital congenital anomalies	Male	0-6 days	65-69 years	Global	-1	2	Socio-demographic Index	--
Urogenital congenital anomalies	Male	0-6 days	65-69 years	Global	1	2	Age-standardized SEV for Ambient particulate matter	428
Urogenital congenital anomalies	Male	0-6 days	65-69 years	Global	1	2	Diabetes Age-Standardized Prevalence (proportion)	615
Urogenital congenital anomalies	Male	0-6 days	65-69 years	Global	1	2	Age-standardized SEV for Household air pollution	--
Urogenital congenital anomalies	Male	0-6 days	65-69 years	Global	-1	3	Antenatal Care (1 visit) Coverage (proportion)	--
Urogenital congenital anomalies	Male	0-6 days	65-69 years	Global	-1	3	Antenatal Care (4 visits) Coverage (proportion)	--
Urogenital congenital anomalies	Male	0-6 days	65-69 years	Global	-1	3	LDI (I$ per capita)	--
Urogenital congenital anomalies	Male	0-6 days	65-69 years	Global	-1	3	Maternal Education (years per capita)	--
Urogenital congenital anomalies	Male	0-6 days	65-69 years	Global	1	3	Liters of alcohol consumed per capita	352
Digestive congenital anomalies	Female	0-6 days	65-69 years	Data Rich	1	1	Age-standardized SEV for Smoking	1000
Digestive congenital anomalies	Female	0-6 days	65-69 years	Data Rich	1	1	Maternal alcohol consumption during pregnancy (proportion)	--
Digestive congenital anomalies	Female	0-6 days	65-69 years	Data Rich	-1	2	Healthcare access and quality index	--
Digestive congenital anomalies	Female	0-6 days	65-69 years	Data Rich	-1	2	In-Facility Delivery (proportion)	--
Digestive congenital anomalies	Female	0-6 days	65-69 years	Data Rich	-1	2	Socio-demographic Index	--
Digestive congenital anomalies	Female	0-6 days	65-69 years	Data Rich	1	2	Age-standardized SEV for High body-mass index	249
Digestive congenital anomalies	Female	0-6 days	65-69 years	Data Rich	1	2	Age-standardized SEV for High fasting plasma glucose	490
Digestive congenital anomalies	Female	0-6 days	65-69 years	Data Rich	1	2	Age-standardized SEV for Household air pollution	--
Digestive congenital anomalies	Female	0-6 days	65-69 years	Data Rich	-1	3	Maternal Education (years per capita)	326
Digestive congenital anomalies	Female	0-6 days	65-69 years	Data Rich	-1	3	Antenatal Care (1 visit) Coverage (proportion)	--
Digestive congenital anomalies	Female	0-6 days	65-69 years	Data Rich	-1	3	Antenatal Care (4 visits) Coverage (proportion)	--
Digestive congenital anomalies	Female	0-6 days	65-69 years	Data Rich	-1	3	LDI (I$ per capita)	--
Digestive congenital anomalies	Female	0-6 days	65-69 years	Data Rich	-1	3	Maternal care and immunization	--
Digestive congenital anomalies	Female	0-6 days	65-69 years	Data Rich	1	3	Age-standardized SEV for Low fruit	84
Digestive congenital anomalies	Female	0-6 days	65-69 years	Data Rich	1	3	Liters of alcohol consumed per capita	103
Digestive congenital anomalies	Female	0-6 days	65-69 years	Data Rich	1	3	Age-standardized SEV for Low vegetables	--
Digestive congenital anomalies	Female	0-6 days	65-69 years	Global	1	1	Age-standardized SEV for Smoking	--
Digestive congenital anomalies	Female	0-6 days	65-69 years	Global	1	1	Maternal alcohol consumption during pregnancy (proportion)	--
Digestive congenital anomalies	Female	0-6 days	65-69 years	Global	-1	2	Healthcare access and quality index	412

CODEm covariates used, level of covariate, and expected direction of covariate by cause, sex, and age								
Cause	Sex	Age Start	Age End	Model Version Type	Direction	Level	Covariate Name	Number of Draws
Digestive congenital anomalies	Female	0-6 days	65-69 years	Global	-1	2	In-Facility Delivery (proportion)	--
Digestive congenital anomalies	Female	0-6 days	65-69 years	Global	-1	2	Socio-demographic Index	--
Digestive congenital anomalies	Female	0-6 days	65-69 years	Global	1	2	Age-standardized SEV for High body-mass index	428
Digestive congenital anomalies	Female	0-6 days	65-69 years	Global	1	2	Age-standardized SEV for High fasting plasma glucose	--
Digestive congenital anomalies	Female	0-6 days	65-69 years	Global	1	2	Age-standardized SEV for Household air pollution	--
Digestive congenital anomalies	Female	0-6 days	65-69 years	Global	-1	3	Antenatal Care (1 visit) Coverage (proportion)	--
Digestive congenital anomalies	Female	0-6 days	65-69 years	Global	-1	3	Antenatal Care (4 visits) Coverage (proportion)	--
Digestive congenital anomalies	Female	0-6 days	65-69 years	Global	-1	3	LDI (I$ per capita)	--
Digestive congenital anomalies	Female	0-6 days	65-69 years	Global	-1	3	Maternal Education (years per capita)	--
Digestive congenital anomalies	Female	0-6 days	65-69 years	Global	-1	3	Maternal care and immunization	--
Digestive congenital anomalies	Female	0-6 days	65-69 years	Global	1	3	Liters of alcohol consumed per capita	577
Digestive congenital anomalies	Female	0-6 days	65-69 years	Global	1	3	Age-standardized SEV for Low fruit	--
Digestive congenital anomalies	Female	0-6 days	65-69 years	Global	1	3	Age-standardized SEV for Low vegetables	--
Digestive congenital anomalies	Male	0-6 days	65-69 years	Data Rich	1	1	Age-standardized SEV for Smoking	--
Digestive congenital anomalies	Male	0-6 days	65-69 years	Data Rich	1	1	Maternal alcohol consumption during pregnancy (proportion)	--
Digestive congenital anomalies	Male	0-6 days	65-69 years	Data Rich	-1	2	Healthcare access and quality index	--
Digestive congenital anomalies	Male	0-6 days	65-69 years	Data Rich	-1	2	In-Facility Delivery (proportion)	--
Digestive congenital anomalies	Male	0-6 days	65-69 years	Data Rich	-1	2	Socio-demographic Index	--
Digestive congenital anomalies	Male	0-6 days	65-69 years	Data Rich	1	2	Age-standardized SEV for High body-mass index	326
Digestive congenital anomalies	Male	0-6 days	65-69 years	Data Rich	1	2	Age-standardized SEV for High fasting plasma glucose	--
Digestive congenital anomalies	Male	0-6 days	65-69 years	Data Rich	1	2	Age-standardized SEV for Household air pollution	--
Digestive congenital anomalies	Male	0-6 days	65-69 years	Data Rich	-1	3	Antenatal Care (1 visit) Coverage (proportion)	--
Digestive congenital anomalies	Male	0-6 days	65-69 years	Data Rich	-1	3	Antenatal Care (4 visits) Coverage (proportion)	--
Digestive congenital anomalies	Male	0-6 days	65-69 years	Data Rich	-1	3	LDI (I$ per capita)	--
Digestive congenital anomalies	Male	0-6 days	65-69 years	Data Rich	-1	3	Maternal Education (years per capita)	--
Digestive congenital anomalies	Male	0-6 days	65-69 years	Data Rich	-1	3	Maternal care and immunization	--
Digestive congenital anomalies	Male	0-6 days	65-69 years	Data Rich	1	3	Liters of alcohol consumed per capita	267
Digestive congenital anomalies	Male	0-6 days	65-69 years	Data Rich	1	3	Age-standardized SEV for Low fruit	--
Digestive congenital anomalies	Male	0-6 days	65-69 years	Data Rich	1	3	Age-standardized SEV for Low vegetables	--
Digestive congenital anomalies	Male	0-6 days	65-69 years	Global	1	1	Age-standardized SEV for Smoking	--
Digestive congenital anomalies	Male	0-6 days	65-69 years	Global	1	1	Maternal alcohol consumption during pregnancy (proportion)	--
Digestive congenital anomalies	Male	0-6 days	65-69 years	Global	-1	2	Healthcare access and quality index	--
Digestive congenital anomalies	Male	0-6 days	65-69 years	Global	-1	2	In-Facility Delivery (proportion)	--
Digestive congenital anomalies	Male	0-6 days	65-69 years	Global	-1	2	Socio-demographic Index	--
Digestive congenital anomalies	Male	0-6 days	65-69 years	Global	1	2	Age-standardized SEV for High body-mass index	--
Digestive congenital anomalies	Male	0-6 days	65-69 years	Global	1	2	Age-standardized SEV for High fasting plasma glucose	--
Digestive congenital anomalies	Male	0-6 days	65-69 years	Global	1	2	Age-standardized SEV for Household air pollution	--
Digestive congenital anomalies	Male	0-6 days	65-69 years	Global	-1	3	Antenatal Care (1 visit) Coverage (proportion)	--
Digestive congenital anomalies	Male	0-6 days	65-69 years	Global	-1	3	Antenatal Care (4 visits) Coverage (proportion)	--
Digestive congenital anomalies	Male	0-6 days	65-69 years	Global	-1	3	LDI (I$ per capita)	--
Digestive congenital anomalies	Male	0-6 days	65-69 years	Global	-1	3	Maternal Education (years per capita)	--
Digestive congenital anomalies	Male	0-6 days	65-69 years	Global	-1	3	Maternal care and immunization	--
Digestive congenital anomalies	Male	0-6 days	65-69 years	Global	1	3	Liters of alcohol consumed per capita	398
Digestive congenital anomalies	Male	0-6 days	65-69 years	Global	1	3	Age-standardized SEV for Low fruit	602
Digestive congenital anomalies	Male	0-6 days	65-69 years	Global	1	3	Age-standardized SEV for Low vegetables	--
Other congenital anomalies	Female	0-6 days	65-69 years	Data Rich	1	1	Live Births 35+ (proportion)	593
Other congenital anomalies	Female	0-6 days	65-69 years	Data Rich	1	1	Maternal alcohol consumption during pregnancy (proportion)	--
Other congenital anomalies	Female	0-6 days	65-69 years	Data Rich	-1	2	Healthcare access and quality index	--
Other congenital anomalies	Female	0-6 days	65-69 years	Data Rich	-1	2	In-Facility Delivery (proportion)	--
Other congenital anomalies	Female	0-6 days	65-69 years	Data Rich	-1	2	Legality of Abortion	--
Other congenital anomalies	Female	0-6 days	65-69 years	Data Rich	1	2	Age-standardized SEV for Household air pollution	--
Other congenital anomalies	Female	0-6 days	65-69 years	Data Rich	1	2	Age-standardized SEV for Smoking	--
Other congenital anomalies	Female	0-6 days	65-69 years	Data Rich	-1	3	Antenatal Care (1 visit) Coverage (proportion)	--
Other congenital anomalies	Female	0-6 days	65-69 years	Data Rich	-1	3	Antenatal Care (4 visits) Coverage (proportion)	--
Other congenital anomalies	Female	0-6 days	65-69 years	Data Rich	-1	3	LDI (I$ per capita)	--
Other congenital anomalies	Female	0-6 days	65-69 years	Data Rich	-1	3	Maternal Education (years per capita)	--
Other congenital anomalies	Female	0-6 days	65-69 years	Data Rich	-1	3	Socio-demographic Index	--
Other congenital anomalies	Female	0-6 days	65-69 years	Data Rich	1	3	Age-standardized SEV for High fasting plasma glucose	326
Other congenital anomalies	Female	0-6 days	65-69 years	Data Rich	1	3	Liters of alcohol consumed per capita	733
Other congenital anomalies	Female	0-6 days	65-69 years	Global	1	1	Live Births 35+ (proportion)	--
Other congenital anomalies	Female	0-6 days	65-69 years	Global	1	1	Maternal alcohol consumption during pregnancy (proportion)	--
Other congenital anomalies	Female	0-6 days	65-69 years	Global	-1	2	Healthcare access and quality index	270
Other congenital anomalies	Female	0-6 days	65-69 years	Global	-1	2	Legality of Abortion	270
Other congenital anomalies	Female	0-6 days	65-69 years	Global	-1	2	In-Facility Delivery (proportion)	--
Other congenital anomalies	Female	0-6 days	65-69 years	Global	1	2	Age-standardized SEV for Smoking	290
Other congenital anomalies	Female	0-6 days	65-69 years	Global	1	2	Age-standardized SEV for Household air pollution	--
Other congenital anomalies	Female	0-6 days	65-69 years	Global	-1	3	Antenatal Care (1 visit) Coverage (proportion)	--
Other congenital anomalies	Female	0-6 days	65-69 years	Global	-1	3	Antenatal Care (4 visits) Coverage (proportion)	--
Other congenital anomalies	Female	0-6 days	65-69 years	Global	-1	3	LDI (I$ per capita)	--
Other congenital anomalies	Female	0-6 days	65-69 years	Global	-1	3	Maternal Education (years per capita)	--
Other congenital anomalies	Female	0-6 days	65-69 years	Global	-1	3	Socio-demographic Index	--
Other congenital anomalies	Female	0-6 days	65-69 years	Global	1	3	Liters of alcohol consumed per capita	211
Other congenital anomalies	Female	0-6 days	65-69 years	Global	1	3	Age-standardized SEV for High fasting plasma glucose	308
Other congenital anomalies	Male	0-6 days	65-69 years	Data Rich	1	1	Live Births 35+ (proportion)	--
Other congenital anomalies	Male	0-6 days	65-69 years	Data Rich	1	1	Maternal alcohol consumption during pregnancy (proportion)	--
Other congenital anomalies	Male	0-6 days	65-69 years	Data Rich	-1	2	Legality of Abortion	718
Other congenital anomalies	Male	0-6 days	65-69 years	Data Rich	-1	2	Healthcare access and quality index	--
Other congenital anomalies	Male	0-6 days	65-69 years	Data Rich	-1	2	In-Facility Delivery (proportion)	--
Other congenital anomalies	Male	0-6 days	65-69 years	Data Rich	1	2	Age-standardized SEV for Household air pollution	--
Other congenital anomalies	Male	0-6 days	65-69 years	Data Rich	1	2	Age-standardized SEV for Smoking	--
Other congenital anomalies	Male	0-6 days	65-69 years	Data Rich	-1	3	Antenatal Care (1 visit) Coverage (proportion)	--
Other congenital anomalies	Male	0-6 days	65-69 years	Data Rich	-1	3	Antenatal Care (4 visits) Coverage (proportion)	--
Other congenital anomalies	Male	0-6 days	65-69 years	Data Rich	-1	3	LDI (I$ per capita)	--
Other congenital anomalies	Male	0-6 days	65-69 years	Data Rich	-1	3	Maternal Education (years per capita)	--
Other congenital anomalies	Male	0-6 days	65-69 years	Data Rich	-1	3	Socio-demographic Index	--
Other congenital anomalies	Male	0-6 days	65-69 years	Data Rich	1	3	Liters of alcohol consumed per capita	282
Other congenital anomalies	Male	0-6 days	65-69 years	Data Rich	1	3	Age-standardized SEV for High fasting plasma glucose	373
Other congenital anomalies	Male	0-6 days	65-69 years	Global	1	1	Live Births 35+ (proportion)	--
Other congenital anomalies	Male	0-6 days	65-69 years	Global	1	1	Maternal alcohol consumption during pregnancy (proportion)	--
Other congenital anomalies	Male	0-6 days	65-69 years	Global	-1	2	Healthcare access and quality index	476
Other congenital anomalies	Male	0-6 days	65-69 years	Global	-1	2	Legality of Abortion	476
Other congenital anomalies	Male	0-6 days	65-69 years	Global	-1	2	In-Facility Delivery (proportion)	--
Other congenital anomalies	Male	0-6 days	65-69 years	Global	1	2	Age-standardized SEV for Household air pollution	--
Other congenital anomalies	Male	0-6 days	65-69 years	Global	1	2	Age-standardized SEV for Smoking	--
Other congenital anomalies	Male	0-6 days	65-69 years	Global	-1	3	Antenatal Care (1 visit) Coverage (proportion)	--
Other congenital anomalies	Male	0-6 days	65-69 years	Global	-1	3	Antenatal Care (4 visits) Coverage (proportion)	--
Other congenital anomalies	Male	0-6 days	65-69 years	Global	-1	3	LDI (I$ per capita)	--
Other congenital anomalies	Male	0-6 days	65-69 years	Global	-1	3	Maternal Education (years per capita)	--
Other congenital anomalies	Male	0-6 days	65-69 years	Global	-1	3	Socio-demographic Index	--
Other congenital anomalies	Male	0-6 days	65-69 years	Global	1	3	Age-standardized SEV for High fasting plasma glucose	417
Other congenital anomalies	Male	0-6 days	65-69 years	Global	1	3	Liters of alcohol consumed per capita	524
Urinary diseases and male infertility	Female	0-6 days	95+ years	Data Rich	-1	2	Healthcare access and quality index	246
Urinary diseases and male infertility	Female	0-6 days	95+ years	Data Rich	1	2	90th percentile climatic temperature in the given country-year.	347
Urinary diseases and male infertility	Female	0-6 days	95+ years	Data Rich	1	2	Sanitation (proportion with access)	622
Urinary diseases and male infertility	Female	0-6 days	95+ years	Data Rich	1	2	Mean BMI	884
Urinary diseases and male infertility	Female	0-6 days	95+ years	Data Rich	-1	3	Education (years per capita)	14
Urinary diseases and male infertility	Female	0-6 days	95+ years	Data Rich	-1	3	Socio-demographic Index	104

CODEm covariates used, level of covariate, and expected direction of covariate by cause, sex, and age

Cause	Sex	Age Start	Age End	Model Version Type	Direction	Level	Covariate Name	Number of Draws
Urinary diseases and male infertility	Female	0-6 days	95+ years	Data Rich	-1	3	LDI (I$ per capita)	--
Urinary diseases and male infertility	Female	0-6 days	95+ years	Global	-1	2	Healthcare access and quality index	337
Urinary diseases and male infertility	Female	0-6 days	95+ years	Global	1	2	Sanitation (proportion with access)	508
Urinary diseases and male infertility	Female	0-6 days	95+ years	Global	1	2	Mean BMI	883
Urinary diseases and male infertility	Female	0-6 days	95+ years	Global	1	2	90th percentile climatic temperature in the given country-year.	--
Urinary diseases and male infertility	Female	0-6 days	95+ years	Global	-1	3	Education (years per capita)	178
Urinary diseases and male infertility	Female	0-6 days	95+ years	Global	-1	3	Socio-demographic Index	190
Urinary diseases and male infertility	Female	0-6 days	95+ years	Global	-1	3	LDI (I$ per capita)	--
Urinary diseases and male infertility	Male	0-6 days	95+ years	Data Rich	-1	2	Healthcare access and quality index	65
Urinary diseases and male infertility	Male	0-6 days	95+ years	Data Rich	1	2	90th percentile climatic temperature in the given country-year.	261
Urinary diseases and male infertility	Male	0-6 days	95+ years	Data Rich	1	2	Sanitation (proportion with access)	717
Urinary diseases and male infertility	Male	0-6 days	95+ years	Data Rich	1	2	Mean BMI	896
Urinary diseases and male infertility	Male	0-6 days	95+ years	Data Rich	-1	3	Education (years per capita)	11
Urinary diseases and male infertility	Male	0-6 days	95+ years	Data Rich	-1	3	Socio-demographic Index	265
Urinary diseases and male infertility	Male	0-6 days	95+ years	Data Rich	-1	3	LDI (I$ per capita)	--
Urinary diseases and male infertility	Male	0-6 days	95+ years	Global	-1	2	Healthcare access and quality index	542
Urinary diseases and male infertility	Male	0-6 days	95+ years	Global	1	2	90th percentile climatic temperature in the given country-year.	268
Urinary diseases and male infertility	Male	0-6 days	95+ years	Global	1	2	Sanitation (proportion with access)	558
Urinary diseases and male infertility	Male	0-6 days	95+ years	Global	1	2	Mean BMI	637
Urinary diseases and male infertility	Male	0-6 days	95+ years	Global	-1	3	Socio-demographic Index	151
Urinary diseases and male infertility	Male	0-6 days	95+ years	Global	-1	3	Education (years per capita)	192
Urinary diseases and male infertility	Male	0-6 days	95+ years	Global	-1	3	LDI (I$ per capita)	--
Urinary tract infections and interstitial nephritis	Female	0-6 days	95+ years	Data Rich	1	1	Sanitation (proportion with access)	--
Urinary tract infections and interstitial nephritis	Female	0-6 days	95+ years	Data Rich	-1	2	Healthcare access and quality index	1000
Urinary tract infections and interstitial nephritis	Female	0-6 days	95+ years	Data Rich	-1	2	Education (years per capita)	--
Urinary tract infections and interstitial nephritis	Female	0-6 days	95+ years	Data Rich	-1	2	LDI (I$ per capita)	--
Urinary tract infections and interstitial nephritis	Female	0-6 days	95+ years	Data Rich	-1	3	Socio-demographic Index	--
Urinary tract infections and interstitial nephritis	Female	0-6 days	95+ years	Global	1	1	Sanitation (proportion with access)	--
Urinary tract infections and interstitial nephritis	Female	0-6 days	95+ years	Global	-1	2	Education (years per capita)	--
Urinary tract infections and interstitial nephritis	Female	0-6 days	95+ years	Global	-1	2	Healthcare access and quality index	--
Urinary tract infections and interstitial nephritis	Female	0-6 days	95+ years	Global	-1	2	LDI (I$ per capita)	--
Urinary tract infections and interstitial nephritis	Female	0-6 days	95+ years	Global	-1	3	Socio-demographic Index	726
Urinary tract infections and interstitial nephritis	Male	0-6 days	95+ years	Data Rich	1	1	Sanitation (proportion with access)	1000
Urinary tract infections and interstitial nephritis	Male	0-6 days	95+ years	Data Rich	-1	2	Education (years per capita)	--
Urinary tract infections and interstitial nephritis	Male	0-6 days	95+ years	Data Rich	-1	2	Healthcare access and quality index	--
Urinary tract infections and interstitial nephritis	Male	0-6 days	95+ years	Data Rich	-1	2	LDI (I$ per capita)	--
Urinary tract infections and interstitial nephritis	Male	0-6 days	95+ years	Data Rich	-1	3	Socio-demographic Index	273
Urinary tract infections and interstitial nephritis	Male	0-6 days	95+ years	Global	1	1	Sanitation (proportion with access)	588
Urinary tract infections and interstitial nephritis	Male	0-6 days	95+ years	Global	-1	2	Healthcare access and quality index	293
Urinary tract infections and interstitial nephritis	Male	0-6 days	95+ years	Global	-1	2	Education (years per capita)	395
Urinary tract infections and interstitial nephritis	Male	0-6 days	95+ years	Global	-1	2	LDI (I$ per capita)	--
Urinary tract infections and interstitial nephritis	Male	0-6 days	95+ years	Global	-1	3	Socio-demographic Index	107
Urolithiasis	Female	12-23 months	95+ years	Data Rich	1	1	90th percentile climatic temperature in the given country-year.	678
Urolithiasis	Female	12-23 months	95+ years	Data Rich	1	1	red meats unadjusted(g)	749
Urolithiasis	Female	12-23 months	95+ years	Data Rich	-1	2	Healthcare access and quality index	69
Urolithiasis	Female	12-23 months	95+ years	Data Rich	-1	2	vegetables unadjusted(g)	545
Urolithiasis	Female	12-23 months	95+ years	Data Rich	-1	2	fruits unadjusted(g)	--
Urolithiasis	Female	12-23 months	95+ years	Data Rich	-1	3	Education (years per capita)	--
Urolithiasis	Female	12-23 months	95+ years	Data Rich	-1	3	LDI (I$ per capita)	--
Urolithiasis	Female	12-23 months	95+ years	Data Rich	-1	3	Socio-demographic Index	--
Urolithiasis	Female	12-23 months	95+ years	Global	1	1	90th percentile climatic temperature in the given country-year.	133
Urolithiasis	Female	12-23 months	95+ years	Global	1	1	red meats unadjusted(g)	--
Urolithiasis	Female	12-23 months	95+ years	Global	-1	2	fruits unadjusted(g)	170
Urolithiasis	Female	12-23 months	95+ years	Global	-1	2	Healthcare access and quality index	184
Urolithiasis	Female	12-23 months	95+ years	Global	-1	2	vegetables unadjusted(g)	244
Urolithiasis	Female	12-23 months	95+ years	Global	-1	3	Socio-demographic Index	269
Urolithiasis	Female	12-23 months	95+ years	Global	-1	3	Education (years per capita)	--
Urolithiasis	Female	12-23 months	95+ years	Global	-1	3	LDI (I$ per capita)	--
Urolithiasis	Male	12-23 months	95+ years	Data Rich	1	1	90th percentile climatic temperature in the given country-year.	595
Urolithiasis	Male	12-23 months	95+ years	Data Rich	1	1	red meats unadjusted(g)	632
Urolithiasis	Male	12-23 months	95+ years	Data Rich	-1	2	Healthcare access and quality index	112
Urolithiasis	Male	12-23 months	95+ years	Data Rich	-1	2	vegetables unadjusted(g)	308
Urolithiasis	Male	12-23 months	95+ years	Data Rich	-1	2	fruits unadjusted(g)	--
Urolithiasis	Male	12-23 months	95+ years	Data Rich	-1	3	Education (years per capita)	65
Urolithiasis	Male	12-23 months	95+ years	Data Rich	-1	3	Socio-demographic Index	151
Urolithiasis	Male	12-23 months	95+ years	Data Rich	-1	3	LDI (I$ per capita)	--
Urolithiasis	Male	12-23 months	95+ years	Global	1	1	90th percentile climatic temperature in the given country-year.	1000
Urolithiasis	Male	12-23 months	95+ years	Global	1	1	Age- and sex-specific SEV for High red meat	--
Urolithiasis	Male	12-23 months	95+ years	Global	-1	2	Healthcare access and quality index	182
Urolithiasis	Male	12-23 months	95+ years	Global	1	2	Age- and sex-specific SEV for Low vegetables	100
Urolithiasis	Male	12-23 months	95+ years	Global	1	2	Age- and sex-specific SEV for Low fruit	299
Urolithiasis	Male	12-23 months	95+ years	Global	-1	3	Education (years per capita)	144
Urolithiasis	Male	12-23 months	95+ years	Global	-1	3	LDI (I$ per capita)	--
Urolithiasis	Male	12-23 months	95+ years	Global	-1	3	Socio-demographic Index	--
Other urinary diseases	Female	0-6 days	95+ years	Data Rich	1	1	Mean BMI	--
Other urinary diseases	Female	0-6 days	95+ years	Data Rich	-1	2	Healthcare access and quality index	243
Other urinary diseases	Female	0-6 days	95+ years	Data Rich	-1	2	LDI (I$ per capita)	571
Other urinary diseases	Female	0-6 days	95+ years	Data Rich	-1	2	Education (years per capita)	--
Other urinary diseases	Female	0-6 days	95+ years	Data Rich	-1	3	Socio-demographic Index	328
Other urinary diseases	Female	0-6 days	95+ years	Global	1	1	Mean BMI	--
Other urinary diseases	Female	0-6 days	95+ years	Global	-1	2	Healthcare access and quality index	726
Other urinary diseases	Female	0-6 days	95+ years	Global	-1	2	Education (years per capita)	--
Other urinary diseases	Female	0-6 days	95+ years	Global	-1	2	LDI (I$ per capita)	--
Other urinary diseases	Female	0-6 days	95+ years	Global	-1	3	Socio-demographic Index	274
Other urinary diseases	Male	0-6 days	95+ years	Data Rich	1	1	Mean BMI	--
Other urinary diseases	Male	0-6 days	95+ years	Data Rich	-1	2	Healthcare access and quality index	325
Other urinary diseases	Male	0-6 days	95+ years	Data Rich	-1	2	LDI (I$ per capita)	427
Other urinary diseases	Male	0-6 days	95+ years	Data Rich	-1	2	Education (years per capita)	--
Other urinary diseases	Male	0-6 days	95+ years	Data Rich	-1	3	Socio-demographic Index	248
Other urinary diseases	Male	0-6 days	95+ years	Global	1	1	Mean BMI	--
Other urinary diseases	Male	0-6 days	95+ years	Global	-1	2	Healthcare access and quality index	267
Other urinary diseases	Male	0-6 days	95+ years	Global	-1	2	Education (years per capita)	452
Other urinary diseases	Male	0-6 days	95+ years	Global	-1	2	LDI (I$ per capita)	511
Other urinary diseases	Male	0-6 days	95+ years	Global	-1	3	Socio-demographic Index	222
Gynecological diseases	Female	10-14 years	95+ years	Data Rich	-1	1	Age- and sex-specific SEV for Smoking	506
Gynecological diseases	Female	10-14 years	95+ years	Data Rich	-1	2	Maternal care and immunization	162
Gynecological diseases	Female	10-14 years	95+ years	Data Rich	-1	2	Healthcare access and quality index	444
Gynecological diseases	Female	10-14 years	95+ years	Data Rich	-1	2	Skilled Birth Attendance (proportion)	448
Gynecological diseases	Female	10-14 years	95+ years	Data Rich	1	2	Total Fertility Rate	328
Gynecological diseases	Female	10-14 years	95+ years	Data Rich	1	2	Live Births 35+ (proportion)	--
Gynecological diseases	Female	10-14 years	95+ years	Data Rich	-1	3	Education (years per capita)	123
Gynecological diseases	Female	10-14 years	95+ years	Data Rich	-1	3	Socio-demographic Index	212
Gynecological diseases	Female	10-14 years	95+ years	Data Rich	-1	3	LDI (I$ per capita)	--
Gynecological diseases	Female	10-14 years	95+ years	Global	-1	1	Age- and sex-specific SEV for Smoking	1000
Gynecological diseases	Female	10-14 years	95+ years	Global	-1	2	Maternal care and immunization	123
Gynecological diseases	Female	10-14 years	95+ years	Global	-1	2	Healthcare access and quality index	218
Gynecological diseases	Female	10-14 years	95+ years	Global	-1	2	Skilled Birth Attendance (proportion)	--
Gynecological diseases	Female	10-14 years	95+ years	Global	1	2	Total Fertility Rate	171
Gynecological diseases	Female	10-14 years	95+ years	Global	1	2	Live Births 35+ (proportion)	512
Gynecological diseases	Female	10-14 years	95+ years	Global	-1	3	Education (years per capita)	242
Gynecological diseases	Female	10-14 years	95+ years	Global	-1	3	Socio-demographic Index	242
Gynecological diseases	Female	10-14 years	95+ years	Global	-1	3	LDI (I$ per capita)	--
Uterine fibroids	Female	10-14 years	95+ years	Data Rich	-1	1	Age- and sex-specific SEV for Smoking	535
Uterine fibroids	Female	10-14 years	95+ years	Data Rich	-1	2	Skilled Birth Attendance (proportion)	85
Uterine fibroids	Female	10-14 years	95+ years	Data Rich	-1	2	Maternal care and immunization	147
Uterine fibroids	Female	10-14 years	95+ years	Data Rich	-1	2	Healthcare access and quality index	446
Uterine fibroids	Female	10-14 years	95+ years	Data Rich	1	2	Total Fertility Rate	28
Uterine fibroids	Female	10-14 years	95+ years	Data Rich	1	2	Live Births 35+ (proportion)	--
Uterine fibroids	Female	10-14 years	95+ years	Data Rich	-1	3	Education (years per capita)	194
Uterine fibroids	Female	10-14 years	95+ years	Data Rich	-1	3	Socio-demographic Index	194

CODEm covariates used, level of covariate, and expected direction of covariate by cause, sex, and age

Cause	Sex	Age Start	Age End	Model Version Type	Direction	Level	Covariate Name	Number of Draws
Uterine fibroids	Female	10-14 years	95+ years	Data Rich	-1	3	LDI (I$ per capita)	--
Uterine fibroids	Female	10-14 years	95+ years	Global	-1	1	Age- and sex-specific SEV for Smoking	439
Uterine fibroids	Female	10-14 years	95+ years	Global	-1	2	Skilled Birth Attendance (proportion)	92
Uterine fibroids	Female	10-14 years	95+ years	Global	-1	2	Healthcare access and quality index	160
Uterine fibroids	Female	10-14 years	95+ years	Global	-1	2	Maternal care and immunization	--
Uterine fibroids	Female	10-14 years	95+ years	Global	1	2	Total Fertility Rate	325
Uterine fibroids	Female	10-14 years	95+ years	Global	1	2	Live Births 35+ (proportion)	--
Uterine fibroids	Female	10-14 years	95+ years	Global	-1	3	Socio-demographic Index	111
Uterine fibroids	Female	10-14 years	95+ years	Global	-1	3	Education (years per capita)	347
Uterine fibroids	Female	10-14 years	95+ years	Global	-1	3	LDI (I$ per capita)	--
Endometriosis	Female	10-14 years	50-54 years	Data Rich	-1	1	Age- and sex-specific SEV for Smoking	586
Endometriosis	Female	10-14 years	50-54 years	Data Rich	-1	2	Healthcare access and quality index	414
Endometriosis	Female	10-14 years	50-54 years	Data Rich	-1	2	Maternal care and immunization	--
Endometriosis	Female	10-14 years	50-54 years	Data Rich	-1	2	Skilled Birth Attendance (proportion)	--
Endometriosis	Female	10-14 years	50-54 years	Data Rich	1	2	Live Births 35+ (proportion)	224
Endometriosis	Female	10-14 years	50-54 years	Data Rich	1	2	Total Fertility Rate	--
Endometriosis	Female	10-14 years	50-54 years	Data Rich	-1	3	Education (years per capita)	--
Endometriosis	Female	10-14 years	50-54 years	Data Rich	-1	3	LDI (I$ per capita)	--
Endometriosis	Female	10-14 years	50-54 years	Data Rich	-1	3	Socio-demographic Index	--
Endometriosis	Female	10-14 years	50-54 years	Global	-1	1	Age- and sex-specific SEV for Smoking	593
Endometriosis	Female	10-14 years	50-54 years	Global	-1	2	Healthcare access and quality index	407
Endometriosis	Female	10-14 years	50-54 years	Global	-1	2	Maternal care and immunization	--
Endometriosis	Female	10-14 years	50-54 years	Global	-1	2	Skilled Birth Attendance (proportion)	--
Endometriosis	Female	10-14 years	50-54 years	Global	1	2	Live Births 35+ (proportion)	267
Endometriosis	Female	10-14 years	50-54 years	Global	1	2	Total Fertility Rate	--
Endometriosis	Female	10-14 years	50-54 years	Global	-1	3	Education (years per capita)	--
Endometriosis	Female	10-14 years	50-54 years	Global	-1	3	LDI (I$ per capita)	--
Endometriosis	Female	10-14 years	50-54 years	Global	-1	3	Socio-demographic Index	--
Genital prolapse	Female	10-14 years	95+ years	Data Rich	-1	1	Age- and sex-specific SEV for Smoking	--
Genital prolapse	Female	10-14 years	95+ years	Data Rich	-1	2	Skilled Birth Attendance (proportion)	545
Genital prolapse	Female	10-14 years	95+ years	Data Rich	-1	2	Healthcare access and quality index	--
Genital prolapse	Female	10-14 years	95+ years	Data Rich	-1	2	Maternal care and immunization	--
Genital prolapse	Female	10-14 years	95+ years	Data Rich	1	2	Total Fertility Rate	40
Genital prolapse	Female	10-14 years	95+ years	Data Rich	1	2	Live Births 35+ (proportion)	--
Genital prolapse	Female	10-14 years	95+ years	Data Rich	-1	3	Socio-demographic Index	207
Genital prolapse	Female	10-14 years	95+ years	Data Rich	-1	3	Education (years per capita)	390
Genital prolapse	Female	10-14 years	95+ years	Data Rich	-1	3	LDI (I$ per capita)	--
Genital prolapse	Female	10-14 years	95+ years	Global	-1	1	Age- and sex-specific SEV for Smoking	1000
Genital prolapse	Female	10-14 years	95+ years	Global	-1	2	Maternal care and immunization	29
Genital prolapse	Female	10-14 years	95+ years	Global	-1	2	Healthcare access and quality index	236
Genital prolapse	Female	10-14 years	95+ years	Global	-1	2	Skilled Birth Attendance (proportion)	472
Genital prolapse	Female	10-14 years	95+ years	Global	1	2	Total Fertility Rate	437
Genital prolapse	Female	10-14 years	95+ years	Global	1	2	Live Births 35+ (proportion)	--
Genital prolapse	Female	10-14 years	95+ years	Global	-1	3	Socio-demographic Index	143
Genital prolapse	Female	10-14 years	95+ years	Global	-1	3	Education (years per capita)	444
Genital prolapse	Female	10-14 years	95+ years	Global	-1	3	LDI (I$ per capita)	--
Other gynecological diseases	Female	10-14 years	95+ years	Data Rich	-1	1	Age- and sex-specific SEV for Smoking	1000
Other gynecological diseases	Female	10-14 years	95+ years	Data Rich	-1	2	Healthcare access and quality index	118
Other gynecological diseases	Female	10-14 years	95+ years	Data Rich	-1	2	Maternal care and immunization	272
Other gynecological diseases	Female	10-14 years	95+ years	Data Rich	-1	2	Skilled Birth Attendance (proportion)	322
Other gynecological diseases	Female	10-14 years	95+ years	Data Rich	1	2	Live Births 35+ (proportion)	476
Other gynecological diseases	Female	10-14 years	95+ years	Data Rich	1	2	Total Fertility Rate	594
Other gynecological diseases	Female	10-14 years	95+ years	Data Rich	-1	3	Education (years per capita)	69
Other gynecological diseases	Female	10-14 years	95+ years	Data Rich	-1	3	Socio-demographic Index	90
Other gynecological diseases	Female	10-14 years	95+ years	Data Rich	-1	3	LDI (I$ per capita)	--
Other gynecological diseases	Female	10-14 years	95+ years	Global	-1	1	Age- and sex-specific SEV for Smoking	1000
Other gynecological diseases	Female	10-14 years	95+ years	Global	-1	2	Maternal care and immunization	173
Other gynecological diseases	Female	10-14 years	95+ years	Global	-1	2	Skilled Birth Attendance (proportion)	459
Other gynecological diseases	Female	10-14 years	95+ years	Global	-1	2	Healthcare access and quality index	--
Other gynecological diseases	Female	10-14 years	95+ years	Global	1	2	Live Births 35+ (proportion)	--
Other gynecological diseases	Female	10-14 years	95+ years	Global	1	2	Total Fertility Rate	--
Other gynecological diseases	Female	10-14 years	95+ years	Global	-1	3	Education (years per capita)	96
Other gynecological diseases	Female	10-14 years	95+ years	Global	-1	3	Socio-demographic Index	285
Other gynecological diseases	Female	10-14 years	95+ years	Global	-1	3	LDI (I$ per capita)	--
Hemoglobinopathies and hemolytic anaemias	Female	0-6 days	95+ years	Data Rich	1	1	Hemoglobinopathies Prevalence x Excess Mortality	328
Hemoglobinopathies and hemolytic anaemias	Female	0-6 days	95+ years	Data Rich	1	1	Hemoglobinopathies Prevalence x Excess Mortality (excluding G6PD deficiency)	354
Hemoglobinopathies and hemolytic anaemias	Female	0-6 days	95+ years	Data Rich	1	1	Malaria Lysenko PFPR 1 (Holoendemic)	422
Hemoglobinopathies and hemolytic anaemias	Female	0-6 days	95+ years	Data Rich	1	1	Hemoglobin C (sickle type C) trait	--
Hemoglobinopathies and hemolytic anaemias	Female	0-6 days	95+ years	Data Rich	1	1	Hemoglobin S (sickle type S) trait	--
Hemoglobinopathies and hemolytic anaemias	Female	0-6 days	95+ years	Data Rich	-1	2	Maternal care and immunization	0
Hemoglobinopathies and hemolytic anaemias	Female	0-6 days	95+ years	Data Rich	-1	2	Healthcare access and quality index	16
Hemoglobinopathies and hemolytic anaemias	Female	0-6 days	95+ years	Data Rich	-1	3	Education (years per capita)	0
Hemoglobinopathies and hemolytic anaemias	Female	0-6 days	95+ years	Data Rich	-1	3	Socio-demographic Index	2
Hemoglobinopathies and hemolytic anaemias	Female	0-6 days	95+ years	Data Rich	-1	3	Latitude Over 45 (proportion)	491
Hemoglobinopathies and hemolytic anaemias	Female	0-6 days	95+ years	Data Rich	-1	3	LDI (I$ per capita)	--
Hemoglobinopathies and hemolytic anaemias	Female	0-6 days	95+ years	Data Rich	-1	3	Latitude 30 to 45 (proportion)	--
Hemoglobinopathies and hemolytic anaemias	Female	0-6 days	95+ years	Data Rich	1	3	Latitude Under 15 (proportion)	143
Hemoglobinopathies and hemolytic anaemias	Female	0-6 days	95+ years	Data Rich	1	3	Latitude 15 to 30 (proportion)	--
Hemoglobinopathies and hemolytic anaemias	Female	0-6 days	95+ years	Global	1	1	Malaria Lysenko PFPR 1 (Holoendemic)	175
Hemoglobinopathies and hemolytic anaemias	Female	0-6 days	95+ years	Global	1	1	Hemoglobin C (sickle type C) trait	--
Hemoglobinopathies and hemolytic anaemias	Female	0-6 days	95+ years	Global	1	1	Hemoglobin S (sickle type S) trait	--
Hemoglobinopathies and hemolytic anaemias	Female	0-6 days	95+ years	Global	-1	2	Maternal care and immunization	28
Hemoglobinopathies and hemolytic anaemias	Female	0-6 days	95+ years	Global	-1	2	Healthcare access and quality index	329
Hemoglobinopathies and hemolytic anaemias	Female	0-6 days	95+ years	Global	-1	3	Socio-demographic Index	54
Hemoglobinopathies and hemolytic anaemias	Female	0-6 days	95+ years	Global	-1	3	Education (years per capita)	68
Hemoglobinopathies and hemolytic anaemias	Female	0-6 days	95+ years	Global	-1	3	Latitude Over 45 (proportion)	421
Hemoglobinopathies and hemolytic anaemias	Female	0-6 days	95+ years	Global	-1	3	LDI (I$ per capita)	--
Hemoglobinopathies and hemolytic anaemias	Female	0-6 days	95+ years	Global	-1	3	Latitude 30 to 45 (proportion)	--
Hemoglobinopathies and hemolytic anaemias	Female	0-6 days	95+ years	Global	1	3	Latitude Under 15 (proportion)	65
Hemoglobinopathies and hemolytic anaemias	Female	0-6 days	95+ years	Global	1	3	Latitude 15 to 30 (proportion)	--
Hemoglobinopathies and hemolytic anaemias	Male	0-6 days	95+ years	Data Rich	1	1	Hemoglobinopathies Prevalence x Excess Mortality	90
Hemoglobinopathies and hemolytic anaemias	Male	0-6 days	95+ years	Data Rich	1	1	Hemoglobinopathies Prevalence x Excess Mortality (excluding G6PD deficiency)	238
Hemoglobinopathies and hemolytic anaemias	Male	0-6 days	95+ years	Data Rich	1	1	Hemoglobin C (sickle type C) trait	--
Hemoglobinopathies and hemolytic anaemias	Male	0-6 days	95+ years	Data Rich	1	1	Hemoglobin S (sickle type S) trait	--
Hemoglobinopathies and hemolytic anaemias	Male	0-6 days	95+ years	Data Rich	1	1	Malaria Lysenko PFPR 1 (Holoendemic)	--
Hemoglobinopathies and hemolytic anaemias	Male	0-6 days	95+ years	Data Rich	-1	2	Maternal care and immunization	0
Hemoglobinopathies and hemolytic anaemias	Male	0-6 days	95+ years	Data Rich	-1	2	Healthcare access and quality index	415
Hemoglobinopathies and hemolytic anaemias	Male	0-6 days	95+ years	Data Rich	-1	3	Socio-demographic Index	42
Hemoglobinopathies and hemolytic anaemias	Male	0-6 days	95+ years	Data Rich	-1	3	Education (years per capita)	104
Hemoglobinopathies and hemolytic anaemias	Male	0-6 days	95+ years	Data Rich	-1	3	Latitude Over 45 (proportion)	394
Hemoglobinopathies and hemolytic anaemias	Male	0-6 days	95+ years	Data Rich	-1	3	LDI (I$ per capita)	--
Hemoglobinopathies and hemolytic anaemias	Male	0-6 days	95+ years	Data Rich	-1	3	Latitude 30 to 45 (proportion)	--
Hemoglobinopathies and hemolytic anaemias	Male	0-6 days	95+ years	Data Rich	1	3	Latitude Under 15 (proportion)	317
Hemoglobinopathies and hemolytic anaemias	Male	0-6 days	95+ years	Data Rich	1	3	Latitude 15 to 30 (proportion)	--
Hemoglobinopathies and hemolytic anaemias	Male	0-6 days	95+ years	Global	1	1	Hemoglobin C (sickle type C) trait	--
Hemoglobinopathies and hemolytic anaemias	Male	0-6 days	95+ years	Global	1	1	Hemoglobin S (sickle type S) trait	--
Hemoglobinopathies and hemolytic anaemias	Male	0-6 days	95+ years	Global	1	1	Malaria Lysenko PFPR 1 (Holoendemic)	--
Hemoglobinopathies and hemolytic anaemias	Male	0-6 days	95+ years	Global	-1	2	Maternal care and immunization	9
Hemoglobinopathies and hemolytic anaemias	Male	0-6 days	95+ years	Global	-1	2	Healthcare access and quality index	252
Hemoglobinopathies and hemolytic anaemias	Male	0-6 days	95+ years	Global	-1	3	Education (years per capita)	7
Hemoglobinopathies and hemolytic anaemias	Male	0-6 days	95+ years	Global	-1	3	Socio-demographic Index	71
Hemoglobinopathies and hemolytic anaemias	Male	0-6 days	95+ years	Global	-1	3	Latitude Over 45 (proportion)	236
Hemoglobinopathies and hemolytic anaemias	Male	0-6 days	95+ years	Global	-1	3	LDI (I$ per capita)	--
Hemoglobinopathies and hemolytic anaemias	Male	0-6 days	95+ years	Global	-1	3	Latitude 30 to 45 (proportion)	--
Hemoglobinopathies and hemolytic anaemias	Male	0-6 days	95+ years	Global	1	3	Latitude Under 15 (proportion)	357
Hemoglobinopathies and hemolytic anaemias	Male	0-6 days	95+ years	Global	1	3	Latitude 15 to 30 (proportion)	--
Endocrine, metabolic, blood, and immune disorders	Female	0-6 days	95+ years	Data Rich	1	1	Mean BMI	703
Endocrine, metabolic, blood, and immune disorders	Female	0-6 days	95+ years	Data Rich	-1	2	Healthcare access and quality index	396
Endocrine, metabolic, blood, and immune disorders	Female	0-6 days	95+ years	Data Rich	1	2	Liters of alcohol consumed per capita	297
Endocrine, metabolic, blood, and immune disorders	Female	0-6 days	95+ years	Data Rich	1	2	Low-Density Lipoprotein (mmol/L)	--
Endocrine, metabolic, blood, and immune disorders	Female	0-6 days	95+ years	Data Rich	-1	3	Education (years per capita)	131
Endocrine, metabolic, blood, and immune disorders	Female	0-6 days	95+ years	Data Rich	-1	3	Socio-demographic Index	225

CODEm covariates used, level of covariate, and expected direction of covariate by cause, sex, and age

Cause	Sex	Age Start	Age End	Model Version Type	Direction	Level	Covariate Name	Number of Draws
Endocrine, metabolic, blood, and immune disorders	Female	0-6 days	95+ years	Data Rich	-1	3	LDI (I$ per capita)	--
Endocrine, metabolic, blood, and immune disorders	Female	0-6 days	95+ years	Global	1	1	Mean BMI	--
Endocrine, metabolic, blood, and immune disorders	Female	0-6 days	95+ years	Global	-1	2	Healthcare access and quality index	381
Endocrine, metabolic, blood, and immune disorders	Female	0-6 days	95+ years	Global	1	2	Liters of alcohol consumed per capita	255
Endocrine, metabolic, blood, and immune disorders	Female	0-6 days	95+ years	Global	1	2	Low-Density Lipoprotein (mmol/L)	--
Endocrine, metabolic, blood, and immune disorders	Female	0-6 days	95+ years	Global	-1	3	Education (years per capita)	100
Endocrine, metabolic, blood, and immune disorders	Female	0-6 days	95+ years	Global	-1	3	Socio-demographic Index	138
Endocrine, metabolic, blood, and immune disorders	Female	0-6 days	95+ years	Global	-1	3	LDI (I$ per capita)	--
Endocrine, metabolic, blood, and immune disorders	Male	0-6 days	95+ years	Data Rich	1	1	Mean BMI	--
Endocrine, metabolic, blood, and immune disorders	Male	0-6 days	95+ years	Data Rich	-1	2	Healthcare access and quality index	100
Endocrine, metabolic, blood, and immune disorders	Male	0-6 days	95+ years	Data Rich	1	2	Liters of alcohol consumed per capita	--
Endocrine, metabolic, blood, and immune disorders	Male	0-6 days	95+ years	Data Rich	1	2	Low-Density Lipoprotein (mmol/L)	--
Endocrine, metabolic, blood, and immune disorders	Male	0-6 days	95+ years	Data Rich	-1	3	Socio-demographic Index	76
Endocrine, metabolic, blood, and immune disorders	Male	0-6 days	95+ years	Data Rich	-1	3	Education (years per capita)	171
Endocrine, metabolic, blood, and immune disorders	Male	0-6 days	95+ years	Data Rich	-1	3	LDI (I$ per capita)	--
Endocrine, metabolic, blood, and immune disorders	Male	0-6 days	95+ years	Global	1	1	Mean BMI	--
Endocrine, metabolic, blood, and immune disorders	Male	0-6 days	95+ years	Global	-1	2	Healthcare access and quality index	310
Endocrine, metabolic, blood, and immune disorders	Male	0-6 days	95+ years	Global	1	2	Liters of alcohol consumed per capita	--
Endocrine, metabolic, blood, and immune disorders	Male	0-6 days	95+ years	Global	1	2	Low-Density Lipoprotein (mmol/L)	--
Endocrine, metabolic, blood, and immune disorders	Male	0-6 days	95+ years	Global	-1	3	Education (years per capita)	65
Endocrine, metabolic, blood, and immune disorders	Male	0-6 days	95+ years	Global	-1	3	Socio-demographic Index	277
Endocrine, metabolic, blood, and immune disorders	Male	0-6 days	95+ years	Global	-1	3	LDI (I$ per capita)	--
Thyroid diseases	Female	0-6 days	95+ years	Data Rich	-1	1	Healthcare access and quality index	--
Thyroid diseases	Female	0-6 days	95+ years	Data Rich	1	1	Absolute value of average latitude	--
Thyroid diseases	Female	0-6 days	95+ years	Data Rich	1	2	Diabetes Age-Standardized Prevalence (proportion)	1000
Thyroid diseases	Female	0-6 days	95+ years	Data Rich	-1	3	Education (years per capita)	--
Thyroid diseases	Female	0-6 days	95+ years	Data Rich	-1	3	LDI (I$ per capita)	--
Thyroid diseases	Female	0-6 days	95+ years	Data Rich	-1	3	Socio-demographic Index	--
Thyroid diseases	Female	0-6 days	95+ years	Data Rich	1	3	Mean BMI	632
Thyroid diseases	Female	0-6 days	95+ years	Data Rich	1	3	Cumulative Cigarettes (10 Years)	--
Thyroid diseases	Female	0-6 days	95+ years	Data Rich	1	3	Cumulative Cigarettes (5 Years)	--
Thyroid diseases	Female	0-6 days	95+ years	Data Rich	1	3	Smoking Prevalence	--
Thyroid diseases	Female	0-6 days	95+ years	Global	-1	1	Healthcare access and quality index	718
Thyroid diseases	Female	0-6 days	95+ years	Global	1	1	Absolute value of average latitude	--
Thyroid diseases	Female	0-6 days	95+ years	Global	1	2	Diabetes Age-Standardized Prevalence (proportion)	384
Thyroid diseases	Female	0-6 days	95+ years	Global	-1	3	Education (years per capita)	--
Thyroid diseases	Female	0-6 days	95+ years	Global	-1	3	LDI (I$ per capita)	--
Thyroid diseases	Female	0-6 days	95+ years	Global	-1	3	Socio-demographic Index	--
Thyroid diseases	Female	0-6 days	95+ years	Global	1	3	Mean BMI	706
Thyroid diseases	Female	0-6 days	95+ years	Global	1	3	Cumulative Cigarettes (10 Years)	--
Thyroid diseases	Female	0-6 days	95+ years	Global	1	3	Cumulative Cigarettes (5 Years)	--
Thyroid diseases	Female	0-6 days	95+ years	Global	1	3	Smoking Prevalence	--
Thyroid diseases	Male	0-6 days	95+ years	Data Rich	-1	1	Healthcare access and quality index	--
Thyroid diseases	Male	0-6 days	95+ years	Data Rich	1	1	Absolute value of average latitude	--
Thyroid diseases	Male	0-6 days	95+ years	Data Rich	1	2	Diabetes Age-Standardized Prevalence (proportion)	410
Thyroid diseases	Male	0-6 days	95+ years	Data Rich	-1	3	Education (years per capita)	--
Thyroid diseases	Male	0-6 days	95+ years	Data Rich	-1	3	LDI (I$ per capita)	--
Thyroid diseases	Male	0-6 days	95+ years	Data Rich	-1	3	Socio-demographic Index	--
Thyroid diseases	Male	0-6 days	95+ years	Data Rich	1	3	Mean BMI	874
Thyroid diseases	Male	0-6 days	95+ years	Data Rich	1	3	Cumulative Cigarettes (10 Years)	--
Thyroid diseases	Male	0-6 days	95+ years	Data Rich	1	3	Cumulative Cigarettes (5 Years)	--
Thyroid diseases	Male	0-6 days	95+ years	Data Rich	1	3	Smoking Prevalence	--
Thyroid diseases	Male	0-6 days	95+ years	Global	-1	1	Healthcare access and quality index	674
Thyroid diseases	Male	0-6 days	95+ years	Global	1	1	Absolute value of average latitude	--
Thyroid diseases	Male	0-6 days	95+ years	Global	1	2	Diabetes Age-Standardized Prevalence (proportion)	477
Thyroid diseases	Male	0-6 days	95+ years	Global	-1	3	Education (years per capita)	--
Thyroid diseases	Male	0-6 days	95+ years	Global	-1	3	LDI (I$ per capita)	--
Thyroid diseases	Male	0-6 days	95+ years	Global	-1	3	Socio-demographic Index	--
Thyroid diseases	Male	0-6 days	95+ years	Global	1	3	Mean BMI	593
Thyroid diseases	Male	0-6 days	95+ years	Global	1	3	Cumulative Cigarettes (10 Years)	--
Thyroid diseases	Male	0-6 days	95+ years	Global	1	3	Cumulative Cigarettes (5 Years)	--
Thyroid diseases	Male	0-6 days	95+ years	Global	1	3	Smoking Prevalence	--
Other endocrine, metabolic, blood, and immune disorders	Female	0-6 days	95+ years	Data Rich	1	1	Mean BMI	--
Other endocrine, metabolic, blood, and immune disorders	Female	0-6 days	95+ years	Data Rich	-1	2	Healthcare access and quality index	403
Other endocrine, metabolic, blood, and immune disorders	Female	0-6 days	95+ years	Data Rich	1	2	Liters of alcohol consumed per capita	--
Other endocrine, metabolic, blood, and immune disorders	Female	0-6 days	95+ years	Data Rich	1	2	Low-Density Lipoprotein (mmol/L)	--
Other endocrine, metabolic, blood, and immune disorders	Female	0-6 days	95+ years	Data Rich	-1	3	Education (years per capita)	26
Other endocrine, metabolic, blood, and immune disorders	Female	0-6 days	95+ years	Data Rich	-1	3	Socio-demographic Index	206
Other endocrine, metabolic, blood, and immune disorders	Female	0-6 days	95+ years	Data Rich	-1	3	LDI (I$ per capita)	--
Other endocrine, metabolic, blood, and immune disorders	Female	0-6 days	95+ years	Global	1	1	Mean BMI	--
Other endocrine, metabolic, blood, and immune disorders	Female	0-6 days	95+ years	Global	-1	2	Healthcare access and quality index	328
Other endocrine, metabolic, blood, and immune disorders	Female	0-6 days	95+ years	Global	1	2	Liters of alcohol consumed per capita	398
Other endocrine, metabolic, blood, and immune disorders	Female	0-6 days	95+ years	Global	1	2	Low-Density Lipoprotein (mmol/L)	--
Other endocrine, metabolic, blood, and immune disorders	Female	0-6 days	95+ years	Global	-1	3	Education (years per capita)	274
Other endocrine, metabolic, blood, and immune disorders	Female	0-6 days	95+ years	Global	-1	3	LDI (I$ per capita)	--
Other endocrine, metabolic, blood, and immune disorders	Female	0-6 days	95+ years	Global	-1	3	Socio-demographic Index	--
Other endocrine, metabolic, blood, and immune disorders	Male	0-6 days	95+ years	Data Rich	1	1	Mean BMI	--
Other endocrine, metabolic, blood, and immune disorders	Male	0-6 days	95+ years	Data Rich	-1	2	Healthcare access and quality index	227
Other endocrine, metabolic, blood, and immune disorders	Male	0-6 days	95+ years	Data Rich	1	2	Liters of alcohol consumed per capita	--
Other endocrine, metabolic, blood, and immune disorders	Male	0-6 days	95+ years	Data Rich	1	2	Low-Density Lipoprotein (mmol/L)	--
Other endocrine, metabolic, blood, and immune disorders	Male	0-6 days	95+ years	Data Rich	-1	3	Education (years per capita)	77
Other endocrine, metabolic, blood, and immune disorders	Male	0-6 days	95+ years	Data Rich	-1	3	Socio-demographic Index	173
Other endocrine, metabolic, blood, and immune disorders	Male	0-6 days	95+ years	Data Rich	-1	3	LDI (I$ per capita)	--
Other endocrine, metabolic, blood, and immune disorders	Male	0-6 days	95+ years	Global	1	1	Mean BMI	--
Other endocrine, metabolic, blood, and immune disorders	Male	0-6 days	95+ years	Global	-1	2	Healthcare access and quality index	398
Other endocrine, metabolic, blood, and immune disorders	Male	0-6 days	95+ years	Global	1	2	Liters of alcohol consumed per capita	--
Other endocrine, metabolic, blood, and immune disorders	Male	0-6 days	95+ years	Global	1	2	Low-Density Lipoprotein (mmol/L)	--
Other endocrine, metabolic, blood, and immune disorders	Male	0-6 days	95+ years	Global	-1	3	Socio-demographic Index	328
Other endocrine, metabolic, blood, and immune disorders	Male	0-6 days	95+ years	Global	-1	3	Education (years per capita)	602
Other endocrine, metabolic, blood, and immune disorders	Male	0-6 days	95+ years	Global	-1	3	LDI (I$ per capita)	--
Sudden infant death syndrome	Female	7-27 days	6-11 months	Data Rich	-1	1	In-Facility Delivery (proportion)	391
Sudden infant death syndrome	Female	7-27 days	6-11 months	Data Rich	-1	2	Skilled Birth Attendance (proportion)	53
Sudden infant death syndrome	Female	7-27 days	6-11 months	Data Rich	-1	2	Healthcare access and quality index	--
Sudden infant death syndrome	Female	7-27 days	6-11 months	Data Rich	-1	2	Maternal care and immunization	--
Sudden infant death syndrome	Female	7-27 days	6-11 months	Data Rich	-1	3	Education (years per capita)	--
Sudden infant death syndrome	Female	7-27 days	6-11 months	Data Rich	1	3	Socio-demographic Index	609
Sudden infant death syndrome	Female	7-27 days	6-11 months	Data Rich	1	3	LDI (I$ per capita)	--
Sudden infant death syndrome	Female	7-27 days	6-11 months	Data Rich	1	3	Total Fertility Rate	--
Sudden infant death syndrome	Female	7-27 days	6-11 months	Global	-1	1	In-Facility Delivery (proportion)	674
Sudden infant death syndrome	Female	7-27 days	6-11 months	Global	-1	2	Skilled Birth Attendance (proportion)	462
Sudden infant death syndrome	Female	7-27 days	6-11 months	Global	-1	2	Healthcare access and quality index	--
Sudden infant death syndrome	Female	7-27 days	6-11 months	Global	-1	2	Maternal care and immunization	--
Sudden infant death syndrome	Female	7-27 days	6-11 months	Global	-1	3	Education (years per capita)	--
Sudden infant death syndrome	Female	7-27 days	6-11 months	Global	1	3	Socio-demographic Index	326
Sudden infant death syndrome	Female	7-27 days	6-11 months	Global	1	3	LDI (I$ per capita)	--
Sudden infant death syndrome	Female	7-27 days	6-11 months	Global	1	3	Total Fertility Rate	--
Sudden infant death syndrome	Male	7-27 days	6-11 months	Data Rich	-1	1	In-Facility Delivery (proportion)	219
Sudden infant death syndrome	Male	7-27 days	6-11 months	Data Rich	-1	2	Skilled Birth Attendance (proportion)	173
Sudden infant death syndrome	Male	7-27 days	6-11 months	Data Rich	-1	2	Healthcare access and quality index	--
Sudden infant death syndrome	Male	7-27 days	6-11 months	Data Rich	-1	2	Maternal care and immunization	--

CODEm covariates used, level of covariate, and expected direction of covariate by cause, sex, and age								
Cause	Sex	Age Start	Age End	Model Version Type	Direction	Level	Covariate Name	Number of Draws
Sudden infant death syndrome	Male	7-27 days	6-11 months	Data Rich	-1	3	Education (years per capita)	--
Sudden infant death syndrome	Male	7-27 days	6-11 months	Data Rich	1	3	Socio-demographic Index	781
Sudden infant death syndrome	Male	7-27 days	6-11 months	Data Rich	1	3	LDI (I$ per capita)	--
Sudden infant death syndrome	Male	7-27 days	6-11 months	Data Rich	1	3	Total Fertility Rate	--
Sudden infant death syndrome	Male	7-27 days	6-11 months	Global	-1	1	In-Facility Delivery (proportion)	674
Sudden infant death syndrome	Male	7-27 days	6-11 months	Global	-1	2	Skilled Birth Attendance (proportion)	210
Sudden infant death syndrome	Male	7-27 days	6-11 months	Global	-1	2	Healthcare access and quality index	--
Sudden infant death syndrome	Male	7-27 days	6-11 months	Global	-1	2	Maternal care and immunization	--
Sudden infant death syndrome	Male	7-27 days	6-11 months	Global	-1	3	Education (years per capita)	--
Sudden infant death syndrome	Male	7-27 days	6-11 months	Global	1	3	Socio-demographic Index	326
Sudden infant death syndrome	Male	7-27 days	6-11 months	Global	1	3	LDI (I$ per capita)	--
Sudden infant death syndrome	Male	7-27 days	6-11 months	Global	1	3	Total Fertility Rate	--
Transport injuries	Female	0-6 days	95+ years	Data Rich	1	1	BAC law youth drivers (quartile)	284
Transport injuries	Female	0-6 days	95+ years	Data Rich	1	1	Vehicles - 2 wheels fraction (proportion)	340
Transport injuries	Female	0-6 days	95+ years	Data Rich	1	1	BAC law professional drivers (quartile)	716
Transport injuries	Female	0-6 days	95+ years	Data Rich	1	1	BAC law general population (quartile)	--
Transport injuries	Female	0-6 days	95+ years	Data Rich	1	1	Liters of alcohol consumed per capita	--
Transport injuries	Female	0-6 days	95+ years	Data Rich	1	1	Speed limit law rural (quartile)	--
Transport injuries	Female	0-6 days	95+ years	Data Rich	1	1	Speed limit law urban (quartile)	--
Transport injuries	Female	0-6 days	95+ years	Data Rich	1	1	Vehicles - 2+4 wheels (per capita)	--
Transport injuries	Female	0-6 days	95+ years	Data Rich	-1	2	Education (years per capita)	11
Transport injuries	Female	0-6 days	95+ years	Data Rich	-1	2	Healthcare access and quality index	34
Transport injuries	Female	0-6 days	95+ years	Data Rich	-1	2	Socio-demographic Index	40
Transport injuries	Female	0-6 days	95+ years	Data Rich	-1	2	LDI (I$ per capita)	--
Transport injuries	Female	0-6 days	95+ years	Data Rich	1	2	Population 15 to 30 (proportion)	24
Transport injuries	Female	0-6 days	95+ years	Data Rich	1	2	Population Density (300-500 ppl/sqkm, proportion)	99
Transport injuries	Female	0-6 days	95+ years	Data Rich	1	2	Population Density (500-1000 ppl/sqkm, proportion)	121
Transport injuries	Female	0-6 days	95+ years	Data Rich	1	2	Population-weighted mean temperature	--
Transport injuries	Female	0-6 days	95+ years	Data Rich	1	3	Rainfall Quintile 5 (proportion)	--
Transport injuries	Female	0-6 days	95+ years	Global	1	1	Vehicles - 2+4 wheels (per capita)	235
Transport injuries	Female	0-6 days	95+ years	Global	1	1	BAC law professional drivers (quartile)	313
Transport injuries	Female	0-6 days	95+ years	Global	1	1	BAC law youth drivers (quartile)	319
Transport injuries	Female	0-6 days	95+ years	Global	1	1	Vehicles - 2 wheels fraction (proportion)	410
Transport injuries	Female	0-6 days	95+ years	Global	1	1	Liters of alcohol consumed per capita	452
Transport injuries	Female	0-6 days	95+ years	Global	1	1	BAC law general population (quartile)	--
Transport injuries	Female	0-6 days	95+ years	Global	1	1	Speed limit law rural (quartile)	--
Transport injuries	Female	0-6 days	95+ years	Global	1	1	Speed limit law urban (quartile)	--
Transport injuries	Female	0-6 days	95+ years	Global	-1	2	Socio-demographic Index	41
Transport injuries	Female	0-6 days	95+ years	Global	-1	2	Healthcare access and quality index	42
Transport injuries	Female	0-6 days	95+ years	Global	-1	2	Education (years per capita)	66
Transport injuries	Female	0-6 days	95+ years	Global	-1	2	LDI (I$ per capita)	--
Transport injuries	Female	0-6 days	95+ years	Global	1	2	Population Density (500-1000 ppl/sqkm, proportion)	147
Transport injuries	Female	0-6 days	95+ years	Global	1	2	Population Density (300-500 ppl/sqkm, proportion)	207
Transport injuries	Female	0-6 days	95+ years	Global	1	2	Population-weighted mean temperature	220
Transport injuries	Female	0-6 days	95+ years	Global	1	2	Population 15 to 30 (proportion)	450
Transport injuries	Female	0-6 days	95+ years	Global	1	3	Rainfall Quintile 5 (proportion)	--
Transport injuries	Male	0-6 days	95+ years	Data Rich	1	1	BAC law youth drivers (quartile)	550
Transport injuries	Male	0-6 days	95+ years	Data Rich	1	1	BAC law professional drivers (quartile)	762
Transport injuries	Male	0-6 days	95+ years	Data Rich	1	1	Vehicles - 2 wheels fraction (proportion)	882
Transport injuries	Male	0-6 days	95+ years	Data Rich	1	1	BAC law general population (quartile)	--
Transport injuries	Male	0-6 days	95+ years	Data Rich	1	1	Liters of alcohol consumed per capita	--
Transport injuries	Male	0-6 days	95+ years	Data Rich	1	1	Speed limit law rural (quartile)	--
Transport injuries	Male	0-6 days	95+ years	Data Rich	1	1	Speed limit law urban (quartile)	--
Transport injuries	Male	0-6 days	95+ years	Data Rich	1	1	Vehicles - 2+4 wheels (per capita)	--
Transport injuries	Male	0-6 days	95+ years	Data Rich	-1	2	Education (years per capita)	18
Transport injuries	Male	0-6 days	95+ years	Data Rich	-1	2	Healthcare access and quality index	42
Transport injuries	Male	0-6 days	95+ years	Data Rich	-1	2	Socio-demographic Index	56
Transport injuries	Male	0-6 days	95+ years	Data Rich	-1	2	LDI (I$ per capita)	--
Transport injuries	Male	0-6 days	95+ years	Data Rich	1	2	Population-weighted mean temperature	0
Transport injuries	Male	0-6 days	95+ years	Data Rich	1	2	Population Density (300-500 ppl/sqkm, proportion)	42
Transport injuries	Male	0-6 days	95+ years	Data Rich	1	2	Population Density (500-1000 ppl/sqkm, proportion)	75
Transport injuries	Male	0-6 days	95+ years	Data Rich	1	2	Population 15 to 30 (proportion)	116
Transport injuries	Male	0-6 days	95+ years	Data Rich	1	3	Rainfall Quintile 5 (proportion)	3
Transport injuries	Male	0-6 days	95+ years	Global	1	1	Vehicles - 2+4 wheels (per capita)	191
Transport injuries	Male	0-6 days	95+ years	Global	1	1	BAC law youth drivers (quartile)	439
Transport injuries	Male	0-6 days	95+ years	Global	1	1	BAC law professional drivers (quartile)	561
Transport injuries	Male	0-6 days	95+ years	Global	1	1	Vehicles - 2 wheels fraction (proportion)	714
Transport injuries	Male	0-6 days	95+ years	Global	1	1	BAC law general population (quartile)	--
Transport injuries	Male	0-6 days	95+ years	Global	1	1	Liters of alcohol consumed per capita	--
Transport injuries	Male	0-6 days	95+ years	Global	1	1	Speed limit law rural (quartile)	--
Transport injuries	Male	0-6 days	95+ years	Global	1	1	Speed limit law urban (quartile)	--
Transport injuries	Male	0-6 days	95+ years	Global	-1	2	Healthcare access and quality index	2
Transport injuries	Male	0-6 days	95+ years	Global	-1	2	Education (years per capita)	3
Transport injuries	Male	0-6 days	95+ years	Global	-1	2	Socio-demographic Index	18
Transport injuries	Male	0-6 days	95+ years	Global	-1	2	LDI (I$ per capita)	--
Transport injuries	Male	0-6 days	95+ years	Global	1	2	Population Density (500-1000 ppl/sqkm, proportion)	227
Transport injuries	Male	0-6 days	95+ years	Global	1	2	Population Density (300-500 ppl/sqkm, proportion)	530
Transport injuries	Male	0-6 days	95+ years	Global	1	2	Population 15 to 30 (proportion)	619
Transport injuries	Male	0-6 days	95+ years	Global	1	2	Population-weighted mean temperature	--
Transport injuries	Male	0-6 days	95+ years	Global	1	3	Rainfall Quintile 5 (proportion)	--
Road injuries	Female	0-6 days	95+ years	Data Rich	1	1	BAC law professional drivers (quartile)	250
Road injuries	Female	0-6 days	95+ years	Data Rich	1	1	BAC law youth drivers (quartile)	378
Road injuries	Female	0-6 days	95+ years	Data Rich	1	1	Vehicles - 2 wheels fraction (proportion)	649
Road injuries	Female	0-6 days	95+ years	Data Rich	1	1	BAC law general population (quartile)	--
Road injuries	Female	0-6 days	95+ years	Data Rich	1	1	Liters of alcohol consumed per capita	--
Road injuries	Female	0-6 days	95+ years	Data Rich	1	1	Log-transformed SEV scalar: Road Inj	--
Road injuries	Female	0-6 days	95+ years	Data Rich	1	1	Speed limit law rural (quartile)	--
Road injuries	Female	0-6 days	95+ years	Data Rich	1	1	Speed limit law urban (quartile)	--
Road injuries	Female	0-6 days	95+ years	Data Rich	1	1	Vehicles - 2 wheels (per capita)	--
Road injuries	Female	0-6 days	95+ years	Data Rich	1	1	Vehicles - 2+4 wheels (per capita)	--
Road injuries	Female	0-6 days	95+ years	Data Rich	1	1	Vehicles - 4 wheels (per capita)	--
Road injuries	Female	0-6 days	95+ years	Data Rich	-1	2	Education (years per capita)	12
Road injuries	Female	0-6 days	95+ years	Data Rich	-1	2	Socio-demographic Index	41
Road injuries	Female	0-6 days	95+ years	Data Rich	-1	2	Healthcare access and quality index	--
Road injuries	Female	0-6 days	95+ years	Data Rich	-1	2	LDI (I$ per capita)	--
Road injuries	Female	0-6 days	95+ years	Data Rich	1	2	Population 15 to 30 (proportion)	20
Road injuries	Female	0-6 days	95+ years	Data Rich	1	2	Population Density (500-1000 ppl/sqkm, proportion)	65
Road injuries	Female	0-6 days	95+ years	Data Rich	1	2	Population Density (300-500 ppl/sqkm, proportion)	73
Road injuries	Female	0-6 days	95+ years	Data Rich	1	2	Population-weighted mean temperature	--
Road injuries	Female	0-6 days	95+ years	Data Rich	1	3	Rainfall Quintile 5 (proportion)	1
Road injuries	Female	0-6 days	95+ years	Global	1	1	Vehicles - 4 wheels (per capita)	118
Road injuries	Female	0-6 days	95+ years	Global	1	1	Vehicles - 2+4 wheels (per capita)	131
Road injuries	Female	0-6 days	95+ years	Global	1	1	Vehicles - 2 wheels fraction (proportion)	290
Road injuries	Female	0-6 days	95+ years	Global	1	1	BAC law professional drivers (quartile)	428
Road injuries	Female	0-6 days	95+ years	Global	1	1	BAC law youth drivers (quartile)	430
Road injuries	Female	0-6 days	95+ years	Global	1	1	Vehicles - 2 wheels (per capita)	488
Road injuries	Female	0-6 days	95+ years	Global	1	1	BAC law general population (quartile)	--
Road injuries	Female	0-6 days	95+ years	Global	1	1	Liters of alcohol consumed per capita	--
Road injuries	Female	0-6 days	95+ years	Global	1	1	Log-transformed SEV scalar: Road Inj	--
Road injuries	Female	0-6 days	95+ years	Global	1	1	Speed limit law rural (quartile)	--
Road injuries	Female	0-6 days	95+ years	Global	1	1	Speed limit law urban (quartile)	--
Road injuries	Female	0-6 days	95+ years	Global	-1	2	Healthcare access and quality index	0
Road injuries	Female	0-6 days	95+ years	Global	1	2	Population Density (500-1000 ppl/sqkm, proportion)	96
Road injuries	Female	0-6 days	95+ years	Global	1	2	Population Density (300-500 ppl/sqkm, proportion)	211
Road injuries	Female	0-6 days	95+ years	Global	1	2	Population 15 to 30 (proportion)	322
Road injuries	Female	0-6 days	95+ years	Global	1	2	Population-weighted mean temperature	414
Road injuries	Female	0-6 days	95+ years	Global	-1	3	Education (years per capita)	211
Road injuries	Female	0-6 days	95+ years	Global	-1	3	Socio-demographic Index	275
Road injuries	Female	0-6 days	95+ years	Global	-1	3	LDI (I$ per capita)	--

CODEm covariates used, level of covariate, and expected direction of covariate by cause, sex, and age								
**Cause**	**Sex**	**Age Start**	**Age End**	**Model Version Type**	**Direction**	**Level**	**Covariate Name**	**Number of Draws**
Road injuries	Female	0-6 days	95+ years	Global	1	3	Rainfall Quintile 5 (proportion)	6
Road injuries	Male	0-6 days	95+ years	Data Rich	1	1	BAC law professional drivers (quartile)	259
Road injuries	Male	0-6 days	95+ years	Data Rich	1	1	BAC law youth drivers (quartile)	511
Road injuries	Male	0-6 days	95+ years	Data Rich	1	1	Vehicles - 2 wheels fraction (proportion)	844
Road injuries	Male	0-6 days	95+ years	Data Rich	1	1	BAC law general population (quartile)	--
Road injuries	Male	0-6 days	95+ years	Data Rich	1	1	Liters of alcohol consumed per capita	--
Road injuries	Male	0-6 days	95+ years	Data Rich	1	1	Log-transformed SEV scalar: Road Inj	--
Road injuries	Male	0-6 days	95+ years	Data Rich	1	1	Speed limit law rural (quartile)	--
Road injuries	Male	0-6 days	95+ years	Data Rich	1	1	Speed limit law urban (quartile)	--
Road injuries	Male	0-6 days	95+ years	Data Rich	1	1	Vehicles - 2 wheels (per capita)	--
Road injuries	Male	0-6 days	95+ years	Data Rich	1	1	Vehicles - 2+4 wheels (per capita)	--
Road injuries	Male	0-6 days	95+ years	Data Rich	1	1	Vehicles - 4 wheels (per capita)	--
Road injuries	Male	0-6 days	95+ years	Data Rich	-1	2	Healthcare access and quality index	0
Road injuries	Male	0-6 days	95+ years	Data Rich	-1	2	Education (years per capita)	1
Road injuries	Male	0-6 days	95+ years	Data Rich	-1	2	Socio-demographic Index	5
Road injuries	Male	0-6 days	95+ years	Data Rich	-1	2	LDI (I$ per capita)	--
Road injuries	Male	0-6 days	95+ years	Data Rich	1	2	Population 15 to 30 (proportion)	12
Road injuries	Male	0-6 days	95+ years	Data Rich	1	2	Population Density (300-500 ppl/sqkm, proportion)	21
Road injuries	Male	0-6 days	95+ years	Data Rich	1	2	Population Density (500-1000 ppl/sqkm, proportion)	28
Road injuries	Male	0-6 days	95+ years	Data Rich	1	2	Population-weighted mean temperature	456
Road injuries	Male	0-6 days	95+ years	Data Rich	1	3	Rainfall Quintile 5 (proportion)	0
Road injuries	Male	0-6 days	95+ years	Global	1	1	Vehicles - 2 wheels fraction (proportion)	96
Road injuries	Male	0-6 days	95+ years	Global	1	1	Vehicles - 2+4 wheels (per capita)	106
Road injuries	Male	0-6 days	95+ years	Global	1	1	Vehicles - 2 wheels (per capita)	798
Road injuries	Male	0-6 days	95+ years	Global	1	1	Liters of alcohol consumed per capita	--
Road injuries	Male	0-6 days	95+ years	Global	1	1	Log-transformed SEV scalar: Road Inj	--
Road injuries	Male	0-6 days	95+ years	Global	1	1	Vehicles - 4 wheels (per capita)	--
Road injuries	Male	0-6 days	95+ years	Global	-1	2	Healthcare access and quality index	99
Road injuries	Male	0-6 days	95+ years	Global	1	2	Population Density (300-500 ppl/sqkm, proportion)	24
Road injuries	Male	0-6 days	95+ years	Global	1	2	Population 15 to 30 (proportion)	165
Road injuries	Male	0-6 days	95+ years	Global	1	2	BAC law youth drivers (quartile)	299
Road injuries	Male	0-6 days	95+ years	Global	1	2	BAC law professional drivers (quartile)	474
Road injuries	Male	0-6 days	95+ years	Global	1	2	Population Density (500-1000 ppl/sqkm, proportion)	828
Road injuries	Male	0-6 days	95+ years	Global	1	2	BAC law general population (quartile)	--
Road injuries	Male	0-6 days	95+ years	Global	1	2	Population-weighted mean temperature	--
Road injuries	Male	0-6 days	95+ years	Global	1	2	Speed limit law rural (quartile)	--
Road injuries	Male	0-6 days	95+ years	Global	1	2	Speed limit law urban (quartile)	--
Road injuries	Male	0-6 days	95+ years	Global	-1	3	Education (years per capita)	316
Road injuries	Male	0-6 days	95+ years	Global	-1	3	Socio-demographic Index	399
Road injuries	Male	0-6 days	95+ years	Global	-1	3	LDI (I$ per capita)	--
Road injuries	Male	0-6 days	95+ years	Global	1	3	Rainfall Quintile 5 (proportion)	--
Pedestrian road injuries	Female	0-6 days	95+ years	Data Rich	1	1	BAC law professional drivers (quartile)	0
Pedestrian road injuries	Female	0-6 days	95+ years	Data Rich	1	1	Liters of alcohol consumed per capita	7
Pedestrian road injuries	Female	0-6 days	95+ years	Data Rich	1	1	BAC law youth drivers (quartile)	993
Pedestrian road injuries	Female	0-6 days	95+ years	Data Rich	1	1	BAC law general population (quartile)	--
Pedestrian road injuries	Female	0-6 days	95+ years	Data Rich	1	1	Log-transformed SEV scalar: Pedest	--
Pedestrian road injuries	Female	0-6 days	95+ years	Data Rich	1	1	Speed limit law rural (quartile)	--
Pedestrian road injuries	Female	0-6 days	95+ years	Data Rich	1	1	Speed limit law urban (quartile)	--
Pedestrian road injuries	Female	0-6 days	95+ years	Data Rich	1	1	Vehicles - 2 wheels fraction (proportion)	--
Pedestrian road injuries	Female	0-6 days	95+ years	Data Rich	1	1	Vehicles - 2+4 wheels (per capita)	--
Pedestrian road injuries	Female	0-6 days	95+ years	Data Rich	-1	2	Socio-demographic Index	0
Pedestrian road injuries	Female	0-6 days	95+ years	Data Rich	-1	2	Healthcare access and quality index	190
Pedestrian road injuries	Female	0-6 days	95+ years	Data Rich	-1	2	Education (years per capita)	1000
Pedestrian road injuries	Female	0-6 days	95+ years	Data Rich	-1	2	LDI (I$ per capita)	--
Pedestrian road injuries	Female	0-6 days	95+ years	Data Rich	1	2	Population Density (300-500 ppl/sqkm, proportion)	0
Pedestrian road injuries	Female	0-6 days	95+ years	Data Rich	1	2	Population Density (500-1000 ppl/sqkm, proportion)	0
Pedestrian road injuries	Female	0-6 days	95+ years	Data Rich	1	2	Population-weighted mean temperature	803
Pedestrian road injuries	Female	0-6 days	95+ years	Data Rich	1	2	Population 15 to 30 (proportion)	1000
Pedestrian road injuries	Female	0-6 days	95+ years	Data Rich	1	3	Rainfall Quintile 5 (proportion)	32
Pedestrian road injuries	Female	0-6 days	95+ years	Global	1	1	Vehicles - 2 wheels fraction (proportion)	0
Pedestrian road injuries	Female	0-6 days	95+ years	Global	1	1	BAC law youth drivers (quartile)	297
Pedestrian road injuries	Female	0-6 days	95+ years	Global	1	1	BAC law professional drivers (quartile)	313
Pedestrian road injuries	Female	0-6 days	95+ years	Global	1	1	Liters of alcohol consumed per capita	709
Pedestrian road injuries	Female	0-6 days	95+ years	Global	1	1	BAC law general population (quartile)	--
Pedestrian road injuries	Female	0-6 days	95+ years	Global	1	1	Log-transformed SEV scalar: Pedest	--
Pedestrian road injuries	Female	0-6 days	95+ years	Global	1	1	Speed limit law rural (quartile)	--
Pedestrian road injuries	Female	0-6 days	95+ years	Global	1	1	Speed limit law urban (quartile)	--
Pedestrian road injuries	Female	0-6 days	95+ years	Global	1	1	Vehicles - 2+4 wheels (per capita)	--
Pedestrian road injuries	Female	0-6 days	95+ years	Global	-1	2	Socio-demographic Index	279
Pedestrian road injuries	Female	0-6 days	95+ years	Global	-1	2	Healthcare access and quality index	684
Pedestrian road injuries	Female	0-6 days	95+ years	Global	-1	2	Education (years per capita)	744
Pedestrian road injuries	Female	0-6 days	95+ years	Global	-1	2	LDI (I$ per capita)	--
Pedestrian road injuries	Female	0-6 days	95+ years	Global	1	2	Population Density (500-1000 ppl/sqkm, proportion)	299
Pedestrian road injuries	Female	0-6 days	95+ years	Global	1	2	Population Density (300-500 ppl/sqkm, proportion)	388
Pedestrian road injuries	Female	0-6 days	95+ years	Global	1	2	Population 15 to 30 (proportion)	730
Pedestrian road injuries	Female	0-6 days	95+ years	Global	1	2	Population-weighted mean temperature	819
Pedestrian road injuries	Female	0-6 days	95+ years	Global	1	3	Rainfall Quintile 5 (proportion)	409
Pedestrian road injuries	Male	0-6 days	95+ years	Data Rich	1	1	BAC law professional drivers (quartile)	0
Pedestrian road injuries	Male	0-6 days	95+ years	Data Rich	1	1	Liters of alcohol consumed per capita	0
Pedestrian road injuries	Male	0-6 days	95+ years	Data Rich	1	1	BAC law youth drivers (quartile)	251
Pedestrian road injuries	Male	0-6 days	95+ years	Data Rich	1	1	Population-weighted mean temperature	999
Pedestrian road injuries	Male	0-6 days	95+ years	Data Rich	1	1	BAC law general population (quartile)	--
Pedestrian road injuries	Male	0-6 days	95+ years	Data Rich	1	1	Log-transformed SEV scalar: Pedest	--
Pedestrian road injuries	Male	0-6 days	95+ years	Data Rich	1	1	Speed limit law rural (quartile)	--
Pedestrian road injuries	Male	0-6 days	95+ years	Data Rich	1	1	Speed limit law urban (quartile)	--
Pedestrian road injuries	Male	0-6 days	95+ years	Data Rich	1	1	Vehicles - 2 wheels fraction (proportion)	--
Pedestrian road injuries	Male	0-6 days	95+ years	Data Rich	1	1	Vehicles - 2+4 wheels (per capita)	--
Pedestrian road injuries	Male	0-6 days	95+ years	Data Rich	-1	2	Education (years per capita)	472
Pedestrian road injuries	Male	0-6 days	95+ years	Data Rich	-1	2	Healthcare access and quality index	621
Pedestrian road injuries	Male	0-6 days	95+ years	Data Rich	-1	2	Socio-demographic Index	884
Pedestrian road injuries	Male	0-6 days	95+ years	Data Rich	-1	2	LDI (I$ per capita)	--
Pedestrian road injuries	Male	0-6 days	95+ years	Data Rich	1	2	Population Density (500-1000 ppl/sqkm, proportion)	1
Pedestrian road injuries	Male	0-6 days	95+ years	Data Rich	1	2	Population Density (300-500 ppl/sqkm, proportion)	22
Pedestrian road injuries	Male	0-6 days	95+ years	Data Rich	1	2	Population 15 to 30 (proportion)	1000
Pedestrian road injuries	Male	0-6 days	95+ years	Data Rich	1	3	Rainfall Quintile 5 (proportion)	41
Pedestrian road injuries	Male	0-6 days	95+ years	Global	1	1	Vehicles - 2 wheels fraction (proportion)	14
Pedestrian road injuries	Male	0-6 days	95+ years	Global	1	1	BAC law youth drivers (quartile)	288
Pedestrian road injuries	Male	0-6 days	95+ years	Global	1	1	BAC law professional drivers (quartile)	986
Pedestrian road injuries	Male	0-6 days	95+ years	Global	1	1	BAC law general population (quartile)	--
Pedestrian road injuries	Male	0-6 days	95+ years	Global	1	1	Liters of alcohol consumed per capita	--
Pedestrian road injuries	Male	0-6 days	95+ years	Global	1	1	Log-transformed SEV scalar: Pedest	--
Pedestrian road injuries	Male	0-6 days	95+ years	Global	1	1	Speed limit law rural (quartile)	--
Pedestrian road injuries	Male	0-6 days	95+ years	Global	1	1	Speed limit law urban (quartile)	--
Pedestrian road injuries	Male	0-6 days	95+ years	Global	1	1	Vehicles - 2+4 wheels (per capita)	--
Pedestrian road injuries	Male	0-6 days	95+ years	Global	-1	2	Socio-demographic Index	288
Pedestrian road injuries	Male	0-6 days	95+ years	Global	-1	2	Healthcare access and quality index	437
Pedestrian road injuries	Male	0-6 days	95+ years	Global	-1	2	Education (years per capita)	526
Pedestrian road injuries	Male	0-6 days	95+ years	Global	-1	2	LDI (I$ per capita)	--
Pedestrian road injuries	Male	0-6 days	95+ years	Global	1	2	Population Density (300-500 ppl/sqkm, proportion)	335
Pedestrian road injuries	Male	0-6 days	95+ years	Global	1	2	Population Density (500-1000 ppl/sqkm, proportion)	390
Pedestrian road injuries	Male	0-6 days	95+ years	Global	1	2	Population 15 to 30 (proportion)	747
Pedestrian road injuries	Male	0-6 days	95+ years	Global	1	2	Population-weighted mean temperature	--
Pedestrian road injuries	Male	0-6 days	95+ years	Global	1	3	Rainfall Quintile 5 (proportion)	--
Cyclist road injuries	Female	12-23 months	90-94 years	Data Rich	1	1	BAC law general population (quartile)	0
Cyclist road injuries	Female	12-23 months	90-94 years	Data Rich	1	1	Vehicles - 2 wheels fraction (proportion)	0
Cyclist road injuries	Female	12-23 months	90-94 years	Data Rich	1	1	BAC law professional drivers (quartile)	99
Cyclist road injuries	Female	12-23 months	90-94 years	Data Rich	1	1	Vehicles - 2+4 wheels (per capita)	497
Cyclist road injuries	Female	12-23 months	90-94 years	Data Rich	1	1	BAC law youth drivers (quartile)	593
Cyclist road injuries	Female	12-23 months	90-94 years	Data Rich	1	1	Liters of alcohol consumed per capita	1000

CODEm covariates used, level of covariate, and expected direction of covariate by cause, sex, and age

Cause	Sex	Age Start	Age End	Model Version Type	Direction	Level	Covariate Name	Number of Draws
Cyclist road injuries	Female	12-23 months	90-94 years	Data Rich	1	1	Log-transformed SEV scalar: Cyclist	--
Cyclist road injuries	Female	12-23 months	90-94 years	Data Rich	1	1	Speed limit law rural (quartile)	--
Cyclist road injuries	Female	12-23 months	90-94 years	Data Rich	1	1	Speed limit law urban (quartile)	--
Cyclist road injuries	Female	12-23 months	90-94 years	Data Rich	-1	2	Education (years per capita)	0
Cyclist road injuries	Female	12-23 months	90-94 years	Data Rich	-1	2	Healthcare access and quality index	0
Cyclist road injuries	Female	12-23 months	90-94 years	Data Rich	-1	2	Socio-demographic Index	0
Cyclist road injuries	Female	12-23 months	90-94 years	Data Rich	-1	2	LDI (I$ per capita)	--
Cyclist road injuries	Female	12-23 months	90-94 years	Data Rich	1	2	Population 15 to 30 (proportion)	262
Cyclist road injuries	Female	12-23 months	90-94 years	Data Rich	1	2	Population Density (300-500 ppl/sqkm, proportion)	418
Cyclist road injuries	Female	12-23 months	90-94 years	Data Rich	1	2	Population-weighted mean temperature	447
Cyclist road injuries	Female	12-23 months	90-94 years	Data Rich	1	2	Population Density (500-1000 ppl/sqkm, proportion)	561
Cyclist road injuries	Female	12-23 months	90-94 years	Data Rich	1	3	Rainfall Quintile 5 (proportion)	--
Cyclist road injuries	Female	12-23 months	90-94 years	Global	1	1	BAC law youth drivers (quartile)	178
Cyclist road injuries	Female	12-23 months	90-94 years	Global	1	1	BAC law general population (quartile)	258
Cyclist road injuries	Female	12-23 months	90-94 years	Global	1	1	Vehicles - 2 wheels fraction (proportion)	272
Cyclist road injuries	Female	12-23 months	90-94 years	Global	1	1	BAC law professional drivers (quartile)	371
Cyclist road injuries	Female	12-23 months	90-94 years	Global	1	1	Vehicles - 2+4 wheels (per capita)	452
Cyclist road injuries	Female	12-23 months	90-94 years	Global	1	1	Liters of alcohol consumed per capita	1000
Cyclist road injuries	Female	12-23 months	90-94 years	Global	1	1	Log-transformed SEV scalar: Cyclist	--
Cyclist road injuries	Female	12-23 months	90-94 years	Global	1	1	Speed limit law rural (quartile)	--
Cyclist road injuries	Female	12-23 months	90-94 years	Global	1	1	Speed limit law urban (quartile)	--
Cyclist road injuries	Female	12-23 months	90-94 years	Global	-1	2	Education (years per capita)	0
Cyclist road injuries	Female	12-23 months	90-94 years	Global	-1	2	Socio-demographic Index	0
Cyclist road injuries	Female	12-23 months	90-94 years	Global	-1	2	Healthcare access and quality index	--
Cyclist road injuries	Female	12-23 months	90-94 years	Global	-1	2	LDI (I$ per capita)	--
Cyclist road injuries	Female	12-23 months	90-94 years	Global	1	2	Population 15 to 30 (proportion)	47
Cyclist road injuries	Female	12-23 months	90-94 years	Global	1	2	Population Density (300-500 ppl/sqkm, proportion)	380
Cyclist road injuries	Female	12-23 months	90-94 years	Global	1	2	Population Density (500-1000 ppl/sqkm, proportion)	619
Cyclist road injuries	Female	12-23 months	90-94 years	Global	1	2	Population-weighted mean temperature	684
Cyclist road injuries	Female	12-23 months	90-94 years	Global	1	3	Rainfall Quintile 5 (proportion)	--
Cyclist road injuries	Male	12-23 months	90-94 years	Data Rich	1	1	BAC law professional drivers (quartile)	0
Cyclist road injuries	Male	12-23 months	90-94 years	Data Rich	1	1	BAC law youth drivers (quartile)	0
Cyclist road injuries	Male	12-23 months	90-94 years	Data Rich	1	1	Vehicles - 2 wheels fraction (proportion)	0
Cyclist road injuries	Male	12-23 months	90-94 years	Data Rich	1	1	BAC law general population (quartile)	165
Cyclist road injuries	Male	12-23 months	90-94 years	Data Rich	1	1	Vehicles - 2+4 wheels (per capita)	999
Cyclist road injuries	Male	12-23 months	90-94 years	Data Rich	1	1	Liters of alcohol consumed per capita	1000
Cyclist road injuries	Male	12-23 months	90-94 years	Data Rich	1	1	Log-transformed SEV scalar: Cyclist	--
Cyclist road injuries	Male	12-23 months	90-94 years	Data Rich	1	1	Speed limit law rural (quartile)	--
Cyclist road injuries	Male	12-23 months	90-94 years	Data Rich	1	1	Speed limit law urban (quartile)	--
Cyclist road injuries	Male	12-23 months	90-94 years	Data Rich	-1	2	Education (years per capita)	0
Cyclist road injuries	Male	12-23 months	90-94 years	Data Rich	-1	2	Healthcare access and quality index	0
Cyclist road injuries	Male	12-23 months	90-94 years	Data Rich	-1	2	Socio-demographic Index	0
Cyclist road injuries	Male	12-23 months	90-94 years	Data Rich	-1	2	LDI (I$ per capita)	--
Cyclist road injuries	Male	12-23 months	90-94 years	Data Rich	1	2	Population 15 to 30 (proportion)	0
Cyclist road injuries	Male	12-23 months	90-94 years	Data Rich	1	2	Population Density (300-500 ppl/sqkm, proportion)	160
Cyclist road injuries	Male	12-23 months	90-94 years	Data Rich	1	2	Population Density (500-1000 ppl/sqkm, proportion)	803
Cyclist road injuries	Male	12-23 months	90-94 years	Data Rich	1	2	Population-weighted mean temperature	804
Cyclist road injuries	Male	12-23 months	90-94 years	Data Rich	1	3	Rainfall Quintile 5 (proportion)	0
Cyclist road injuries	Male	12-23 months	90-94 years	Global	1	1	BAC law professional drivers (quartile)	7
Cyclist road injuries	Male	12-23 months	90-94 years	Global	1	1	BAC law youth drivers (quartile)	206
Cyclist road injuries	Male	12-23 months	90-94 years	Global	1	1	BAC law general population (quartile)	299
Cyclist road injuries	Male	12-23 months	90-94 years	Global	1	1	Vehicles - 2 wheels fraction (proportion)	492
Cyclist road injuries	Male	12-23 months	90-94 years	Global	1	1	Vehicles - 2+4 wheels (per capita)	796
Cyclist road injuries	Male	12-23 months	90-94 years	Global	1	1	Liters of alcohol consumed per capita	995
Cyclist road injuries	Male	12-23 months	90-94 years	Global	1	1	Log-transformed SEV scalar: Cyclist	--
Cyclist road injuries	Male	12-23 months	90-94 years	Global	1	1	Speed limit law rural (quartile)	--
Cyclist road injuries	Male	12-23 months	90-94 years	Global	1	1	Speed limit law urban (quartile)	--
Cyclist road injuries	Male	12-23 months	90-94 years	Global	-1	2	Healthcare access and quality index	0
Cyclist road injuries	Male	12-23 months	90-94 years	Global	-1	2	Socio-demographic Index	0
Cyclist road injuries	Male	12-23 months	90-94 years	Global	-1	2	Education (years per capita)	--
Cyclist road injuries	Male	12-23 months	90-94 years	Global	-1	2	LDI (I$ per capita)	--
Cyclist road injuries	Male	12-23 months	90-94 years	Global	1	2	Population 15 to 30 (proportion)	0
Cyclist road injuries	Male	12-23 months	90-94 years	Global	1	2	Population Density (500-1000 ppl/sqkm, proportion)	201
Cyclist road injuries	Male	12-23 months	90-94 years	Global	1	2	Population-weighted mean temperature	367
Cyclist road injuries	Male	12-23 months	90-94 years	Global	1	2	Population Density (300-500 ppl/sqkm, proportion)	459
Cyclist road injuries	Male	12-23 months	90-94 years	Global	1	3	Rainfall Quintile 5 (proportion)	--
Motorcyclist road injuries	Female	0-6 days	95+ years	Data Rich	1	1	BAC law professional drivers (quartile)	1
Motorcyclist road injuries	Female	0-6 days	95+ years	Data Rich	1	1	BAC law youth drivers (quartile)	37
Motorcyclist road injuries	Female	0-6 days	95+ years	Data Rich	1	1	Vehicles - 2 wheels fraction (proportion)	38
Motorcyclist road injuries	Female	0-6 days	95+ years	Data Rich	1	1	BAC law general population (quartile)	--
Motorcyclist road injuries	Female	0-6 days	95+ years	Data Rich	1	1	Liters of alcohol consumed per capita	--
Motorcyclist road injuries	Female	0-6 days	95+ years	Data Rich	1	1	Log-transformed SEV scalar: Mot Cyc	--
Motorcyclist road injuries	Female	0-6 days	95+ years	Data Rich	1	1	Speed limit law rural (quartile)	--
Motorcyclist road injuries	Female	0-6 days	95+ years	Data Rich	1	1	Speed limit law urban (quartile)	--
Motorcyclist road injuries	Female	0-6 days	95+ years	Data Rich	-1	2	Education (years per capita)	0
Motorcyclist road injuries	Female	0-6 days	95+ years	Data Rich	-1	2	Socio-demographic Index	7
Motorcyclist road injuries	Female	0-6 days	95+ years	Data Rich	-1	2	Healthcare access and quality index	38
Motorcyclist road injuries	Female	0-6 days	95+ years	Data Rich	-1	2	LDI (I$ per capita)	--
Motorcyclist road injuries	Female	0-6 days	95+ years	Data Rich	1	2	Population 15 to 30 (proportion)	38
Motorcyclist road injuries	Female	0-6 days	95+ years	Data Rich	1	2	Population Density (300-500 ppl/sqkm, proportion)	160
Motorcyclist road injuries	Female	0-6 days	95+ years	Data Rich	1	2	Population Density (500-1000 ppl/sqkm, proportion)	840
Motorcyclist road injuries	Female	0-6 days	95+ years	Data Rich	1	2	Population-weighted mean temperature	--
Motorcyclist road injuries	Female	0-6 days	95+ years	Data Rich	1	3	Rainfall Quintile 5 (proportion)	--
Motorcyclist road injuries	Female	0-6 days	95+ years	Global	1	1	BAC law professional drivers (quartile)	25
Motorcyclist road injuries	Female	0-6 days	95+ years	Global	1	1	Vehicles - 2 wheels fraction (proportion)	513
Motorcyclist road injuries	Female	0-6 days	95+ years	Global	1	1	BAC law youth drivers (quartile)	686
Motorcyclist road injuries	Female	0-6 days	95+ years	Global	1	1	BAC law general population (quartile)	--
Motorcyclist road injuries	Female	0-6 days	95+ years	Global	1	1	Liters of alcohol consumed per capita	--
Motorcyclist road injuries	Female	0-6 days	95+ years	Global	1	1	Log-transformed SEV scalar: Mot Cyc	--
Motorcyclist road injuries	Female	0-6 days	95+ years	Global	1	1	Speed limit law rural (quartile)	--
Motorcyclist road injuries	Female	0-6 days	95+ years	Global	1	1	Speed limit law urban (quartile)	--
Motorcyclist road injuries	Female	0-6 days	95+ years	Global	-1	2	Education (years per capita)	85
Motorcyclist road injuries	Female	0-6 days	95+ years	Global	-1	2	Socio-demographic Index	131
Motorcyclist road injuries	Female	0-6 days	95+ years	Global	-1	2	Healthcare access and quality index	168
Motorcyclist road injuries	Female	0-6 days	95+ years	Global	-1	2	LDI (I$ per capita)	--
Motorcyclist road injuries	Female	0-6 days	95+ years	Global	1	2	Population Density (300-500 ppl/sqkm, proportion)	187
Motorcyclist road injuries	Female	0-6 days	95+ years	Global	1	2	Population 15 to 30 (proportion)	340
Motorcyclist road injuries	Female	0-6 days	95+ years	Global	1	2	Population Density (500-1000 ppl/sqkm, proportion)	382
Motorcyclist road injuries	Female	0-6 days	95+ years	Global	1	2	Population-weighted mean temperature	707
Motorcyclist road injuries	Female	0-6 days	95+ years	Global	1	3	Rainfall Quintile 5 (proportion)	--
Motorcyclist road injuries	Male	0-6 days	95+ years	Data Rich	1	1	BAC law youth drivers (quartile)	1
Motorcyclist road injuries	Male	0-6 days	95+ years	Data Rich	1	1	Vehicles - 2 wheels fraction (proportion)	199
Motorcyclist road injuries	Male	0-6 days	95+ years	Data Rich	1	1	BAC law professional drivers (quartile)	--
Motorcyclist road injuries	Male	0-6 days	95+ years	Data Rich	1	1	BAC law general population (quartile)	--
Motorcyclist road injuries	Male	0-6 days	95+ years	Data Rich	1	1	Liters of alcohol consumed per capita	--
Motorcyclist road injuries	Male	0-6 days	95+ years	Data Rich	1	1	Log-transformed SEV scalar: Mot Cyc	--
Motorcyclist road injuries	Male	0-6 days	95+ years	Data Rich	1	1	Speed limit law rural (quartile)	--
Motorcyclist road injuries	Male	0-6 days	95+ years	Data Rich	1	1	Speed limit law urban (quartile)	--
Motorcyclist road injuries	Male	0-6 days	95+ years	Data Rich	-1	2	Education (years per capita)	0
Motorcyclist road injuries	Male	0-6 days	95+ years	Data Rich	-1	2	Socio-demographic Index	6
Motorcyclist road injuries	Male	0-6 days	95+ years	Data Rich	-1	2	Healthcare access and quality index	192
Motorcyclist road injuries	Male	0-6 days	95+ years	Data Rich	-1	2	LDI (I$ per capita)	--
Motorcyclist road injuries	Male	0-6 days	95+ years	Data Rich	1	2	Population 15 to 30 (proportion)	1
Motorcyclist road injuries	Male	0-6 days	95+ years	Data Rich	1	2	Population Density (300-500 ppl/sqkm, proportion)	1
Motorcyclist road injuries	Male	0-6 days	95+ years	Data Rich	1	2	Population-weighted mean temperature	38
Motorcyclist road injuries	Male	0-6 days	95+ years	Data Rich	1	2	Population Density (500-1000 ppl/sqkm, proportion)	961
Motorcyclist road injuries	Male	0-6 days	95+ years	Data Rich	1	3	Rainfall Quintile 5 (proportion)	--
Motorcyclist road injuries	Male	0-6 days	95+ years	Global	1	1	Vehicles - 2 wheels fraction (proportion)	476

CODEm covariates used, level of covariate, and expected direction of covariate by cause, sex, and age

Cause	Sex	Age Start	Age End	Model Version Type	Direction	Level	Covariate Name	Number of Draws
Motorcyclist road injuries	Male	0-6 days	95+ years	Global	1	1	BAC law general population (quartile)	828
Motorcyclist road injuries	Male	0-6 days	95+ years	Global	1	1	BAC law professional drivers (quartile)	--
Motorcyclist road injuries	Male	0-6 days	95+ years	Global	1	1	BAC law youth drivers (quartile)	--
Motorcyclist road injuries	Male	0-6 days	95+ years	Global	1	1	Liters of alcohol consumed per capita	--
Motorcyclist road injuries	Male	0-6 days	95+ years	Global	1	1	Log-transformed SEV scalar: Mot Cyc	--
Motorcyclist road injuries	Male	0-6 days	95+ years	Global	1	1	Speed limit law rural (quartile)	--
Motorcyclist road injuries	Male	0-6 days	95+ years	Global	1	1	Speed limit law urban (quartile)	--
Motorcyclist road injuries	Male	0-6 days	95+ years	Global	-1	2	Education (years per capita)	59
Motorcyclist road injuries	Male	0-6 days	95+ years	Global	-1	2	Socio-demographic Index	66
Motorcyclist road injuries	Male	0-6 days	95+ years	Global	-1	2	Healthcare access and quality index	125
Motorcyclist road injuries	Male	0-6 days	95+ years	Global	-1	2	LDI (I$ per capita)	--
Motorcyclist road injuries	Male	0-6 days	95+ years	Global	1	2	Population 15 to 30 (proportion)	125
Motorcyclist road injuries	Male	0-6 days	95+ years	Global	1	2	Population-weighted mean temperature	219
Motorcyclist road injuries	Male	0-6 days	95+ years	Global	1	2	Population Density (500-1000 ppl/sqkm, proportion)	425
Motorcyclist road injuries	Male	0-6 days	95+ years	Global	1	2	Population Density (300-500 ppl/sqkm, proportion)	429
Motorcyclist road injuries	Male	0-6 days	95+ years	Global	1	3	Rainfall Quintile 5 (proportion)	--
Motor vehicle road injuries	Female	0-6 days	95+ years	Data Rich	1	1	BAC law youth drivers (quartile)	333
Motor vehicle road injuries	Female	0-6 days	95+ years	Data Rich	1	1	BAC law professional drivers (quartile)	445
Motor vehicle road injuries	Female	0-6 days	95+ years	Data Rich	1	1	Vehicles - 4 wheels (per capita)	555
Motor vehicle road injuries	Female	0-6 days	95+ years	Data Rich	1	1	BAC law general population (quartile)	--
Motor vehicle road injuries	Female	0-6 days	95+ years	Data Rich	1	1	Liters of alcohol consumed per capita	--
Motor vehicle road injuries	Female	0-6 days	95+ years	Data Rich	1	1	Log-transformed SEV scalar: Mot Veh	--
Motor vehicle road injuries	Female	0-6 days	95+ years	Data Rich	1	1	Speed limit law rural (quartile)	--
Motor vehicle road injuries	Female	0-6 days	95+ years	Data Rich	1	1	Speed limit law urban (quartile)	--
Motor vehicle road injuries	Female	0-6 days	95+ years	Data Rich	-1	2	Healthcare access and quality index	148
Motor vehicle road injuries	Female	0-6 days	95+ years	Data Rich	-1	2	Socio-demographic Index	333
Motor vehicle road injuries	Female	0-6 days	95+ years	Data Rich	-1	2	Education (years per capita)	370
Motor vehicle road injuries	Female	0-6 days	95+ years	Data Rich	-1	2	LDI (I$ per capita)	--
Motor vehicle road injuries	Female	0-6 days	95+ years	Data Rich	1	2	Population-weighted mean temperature	259
Motor vehicle road injuries	Female	0-6 days	95+ years	Data Rich	1	2	Population Density (300-500 ppl/sqkm, proportion)	333
Motor vehicle road injuries	Female	0-6 days	95+ years	Data Rich	1	2	Population Density (500-1000 ppl/sqkm, proportion)	333
Motor vehicle road injuries	Female	0-6 days	95+ years	Data Rich	1	2	Population 15 to 30 (proportion)	444
Motor vehicle road injuries	Female	0-6 days	95+ years	Data Rich	1	3	Rainfall Quintile 5 (proportion)	--
Motor vehicle road injuries	Female	0-6 days	95+ years	Global	1	1	BAC law professional drivers (quartile)	300
Motor vehicle road injuries	Female	0-6 days	95+ years	Global	1	1	Vehicles - 4 wheels (per capita)	700
Motor vehicle road injuries	Female	0-6 days	95+ years	Global	1	1	BAC law general population (quartile)	--
Motor vehicle road injuries	Female	0-6 days	95+ years	Global	1	1	BAC law youth drivers (quartile)	--
Motor vehicle road injuries	Female	0-6 days	95+ years	Global	1	1	Liters of alcohol consumed per capita	--
Motor vehicle road injuries	Female	0-6 days	95+ years	Global	1	1	Log-transformed SEV scalar: Mot Veh	--
Motor vehicle road injuries	Female	0-6 days	95+ years	Global	1	1	Speed limit law rural (quartile)	--
Motor vehicle road injuries	Female	0-6 days	95+ years	Global	1	1	Speed limit law urban (quartile)	--
Motor vehicle road injuries	Female	0-6 days	95+ years	Global	-1	2	Socio-demographic Index	222
Motor vehicle road injuries	Female	0-6 days	95+ years	Global	-1	2	Healthcare access and quality index	225
Motor vehicle road injuries	Female	0-6 days	95+ years	Global	-1	2	Education (years per capita)	423
Motor vehicle road injuries	Female	0-6 days	95+ years	Global	-1	2	LDI (I$ per capita)	--
Motor vehicle road injuries	Female	0-6 days	95+ years	Global	1	2	Population Density (300-500 ppl/sqkm, proportion)	108
Motor vehicle road injuries	Female	0-6 days	95+ years	Global	1	2	Population 15 to 30 (proportion)	419
Motor vehicle road injuries	Female	0-6 days	95+ years	Global	1	2	Population Density (500-1000 ppl/sqkm, proportion)	676
Motor vehicle road injuries	Female	0-6 days	95+ years	Global	1	2	Population-weighted mean temperature	--
Motor vehicle road injuries	Female	0-6 days	95+ years	Global	1	3	Rainfall Quintile 5 (proportion)	--
Motor vehicle road injuries	Male	0-6 days	95+ years	Data Rich	1	1	BAC law youth drivers (quartile)	6
Motor vehicle road injuries	Male	0-6 days	95+ years	Data Rich	1	1	BAC law professional drivers (quartile)	32
Motor vehicle road injuries	Male	0-6 days	95+ years	Data Rich	1	1	Vehicles - 4 wheels (per capita)	962
Motor vehicle road injuries	Male	0-6 days	95+ years	Data Rich	1	1	BAC law general population (quartile)	--
Motor vehicle road injuries	Male	0-6 days	95+ years	Data Rich	1	1	Liters of alcohol consumed per capita	--
Motor vehicle road injuries	Male	0-6 days	95+ years	Data Rich	1	1	Log-transformed SEV scalar: Mot Veh	--
Motor vehicle road injuries	Male	0-6 days	95+ years	Data Rich	1	1	Speed limit law rural (quartile)	--
Motor vehicle road injuries	Male	0-6 days	95+ years	Data Rich	1	1	Speed limit law urban (quartile)	--
Motor vehicle road injuries	Male	0-6 days	95+ years	Data Rich	-1	2	Healthcare access and quality index	0
Motor vehicle road injuries	Male	0-6 days	95+ years	Data Rich	-1	2	Socio-demographic Index	1
Motor vehicle road injuries	Male	0-6 days	95+ years	Data Rich	-1	2	Education (years per capita)	160
Motor vehicle road injuries	Male	0-6 days	95+ years	Data Rich	-1	2	LDI (I$ per capita)	--
Motor vehicle road injuries	Male	0-6 days	95+ years	Data Rich	1	2	Population Density (500-1000 ppl/sqkm, proportion)	0
Motor vehicle road injuries	Male	0-6 days	95+ years	Data Rich	1	2	Population-weighted mean temperature	160
Motor vehicle road injuries	Male	0-6 days	95+ years	Data Rich	1	2	Population 15 to 30 (proportion)	161
Motor vehicle road injuries	Male	0-6 days	95+ years	Data Rich	1	2	Population Density (300-500 ppl/sqkm, proportion)	161
Motor vehicle road injuries	Male	0-6 days	95+ years	Data Rich	1	3	Rainfall Quintile 5 (proportion)	--
Motor vehicle road injuries	Male	0-6 days	95+ years	Global	1	1	Vehicles - 4 wheels (per capita)	761
Motor vehicle road injuries	Male	0-6 days	95+ years	Global	1	1	BAC law youth drivers (quartile)	1000
Motor vehicle road injuries	Male	0-6 days	95+ years	Global	1	1	BAC law professional drivers (quartile)	--
Motor vehicle road injuries	Male	0-6 days	95+ years	Global	1	1	BAC law general population (quartile)	--
Motor vehicle road injuries	Male	0-6 days	95+ years	Global	1	1	Liters of alcohol consumed per capita	--
Motor vehicle road injuries	Male	0-6 days	95+ years	Global	1	1	Log-transformed SEV scalar: Mot Veh	--
Motor vehicle road injuries	Male	0-6 days	95+ years	Global	1	1	Speed limit law rural (quartile)	--
Motor vehicle road injuries	Male	0-6 days	95+ years	Global	1	1	Speed limit law urban (quartile)	--
Motor vehicle road injuries	Male	0-6 days	95+ years	Global	-1	2	Socio-demographic Index	219
Motor vehicle road injuries	Male	0-6 days	95+ years	Global	-1	2	Healthcare access and quality index	520
Motor vehicle road injuries	Male	0-6 days	95+ years	Global	-1	2	Education (years per capita)	543
Motor vehicle road injuries	Male	0-6 days	95+ years	Global	-1	2	LDI (I$ per capita)	--
Motor vehicle road injuries	Male	0-6 days	95+ years	Global	1	2	Population Density (300-500 ppl/sqkm, proportion)	431
Motor vehicle road injuries	Male	0-6 days	95+ years	Global	1	2	Population 15 to 30 (proportion)	608
Motor vehicle road injuries	Male	0-6 days	95+ years	Global	1	2	Population Density (500-1000 ppl/sqkm, proportion)	874
Motor vehicle road injuries	Male	0-6 days	95+ years	Global	1	2	Population-weighted mean temperature	--
Motor vehicle road injuries	Male	0-6 days	95+ years	Global	1	3	Rainfall Quintile 5 (proportion)	--
Other road injuries	Female	0-6 days	95+ years	Data Rich	1	1	Vehicles - 2+4 wheels (per capita)	994
Other road injuries	Female	0-6 days	95+ years	Data Rich	1	1	Vehicles - 2 wheels fraction (proportion)	1000
Other road injuries	Female	0-6 days	95+ years	Data Rich	1	1	BAC law professional drivers (quartile)	--
Other road injuries	Female	0-6 days	95+ years	Data Rich	1	1	BAC law general population (quartile)	--
Other road injuries	Female	0-6 days	95+ years	Data Rich	1	1	BAC law youth drivers (quartile)	--
Other road injuries	Female	0-6 days	95+ years	Data Rich	1	1	Liters of alcohol consumed per capita	--
Other road injuries	Female	0-6 days	95+ years	Data Rich	1	1	Log-transformed SEV scalar: Oth Road	--
Other road injuries	Female	0-6 days	95+ years	Data Rich	1	1	Speed limit law rural (quartile)	--
Other road injuries	Female	0-6 days	95+ years	Data Rich	1	1	Speed limit law urban (quartile)	--
Other road injuries	Female	0-6 days	95+ years	Data Rich	-1	2	Healthcare access and quality index	0
Other road injuries	Female	0-6 days	95+ years	Data Rich	-1	2	Education (years per capita)	1
Other road injuries	Female	0-6 days	95+ years	Data Rich	-1	2	LDI (I$ per capita)	--
Other road injuries	Female	0-6 days	95+ years	Data Rich	1	2	Population 15 to 30 (proportion)	0
Other road injuries	Female	0-6 days	95+ years	Data Rich	1	2	Population-weighted mean temperature	--
Other road injuries	Female	0-6 days	95+ years	Data Rich	-1	3	Socio-demographic Index	967
Other road injuries	Female	0-6 days	95+ years	Data Rich	1	3	Rainfall Quintile 5 (proportion)	--
Other road injuries	Female	0-6 days	95+ years	Global	1	1	BAC law youth drivers (quartile)	36
Other road injuries	Female	0-6 days	95+ years	Global	1	1	BAC law professional drivers (quartile)	82
Other road injuries	Female	0-6 days	95+ years	Global	1	1	Vehicles - 2+4 wheels (per capita)	331
Other road injuries	Female	0-6 days	95+ years	Global	1	1	Vehicles - 2 wheels fraction (proportion)	1000
Other road injuries	Female	0-6 days	95+ years	Global	1	1	BAC law general population (quartile)	--
Other road injuries	Female	0-6 days	95+ years	Global	1	1	Liters of alcohol consumed per capita	--
Other road injuries	Female	0-6 days	95+ years	Global	1	1	Log-transformed SEV scalar: Oth Road	--
Other road injuries	Female	0-6 days	95+ years	Global	1	1	Speed limit law rural (quartile)	--
Other road injuries	Female	0-6 days	95+ years	Global	1	1	Speed limit law urban (quartile)	--
Other road injuries	Female	0-6 days	95+ years	Global	-1	2	Education (years per capita)	178
Other road injuries	Female	0-6 days	95+ years	Global	-1	2	Healthcare access and quality index	186
Other road injuries	Female	0-6 days	95+ years	Global	-1	2	LDI (I$ per capita)	--
Other road injuries	Female	0-6 days	95+ years	Global	1	2	Population 15 to 30 (proportion)	394
Other road injuries	Female	0-6 days	95+ years	Global	1	2	Population-weighted mean temperature	--
Other road injuries	Female	0-6 days	95+ years	Global	-1	3	Socio-demographic Index	173
Other road injuries	Female	0-6 days	95+ years	Global	1	3	Rainfall Quintile 5 (proportion)	--
Other road injuries	Male	0-6 days	95+ years	Data Rich	1	1	Vehicles - 2 wheels fraction (proportion)	807
Other road injuries	Male	0-6 days	95+ years	Data Rich	1	1	Vehicles - 2+4 wheels (per capita)	1000
Other road injuries	Male	0-6 days	95+ years	Data Rich	1	1	BAC law professional drivers (quartile)	--
Other road injuries	Male	0-6 days	95+ years	Data Rich	1	1	BAC law general population (quartile)	--

CODEm covariates used, level of covariate, and expected direction of covariate by cause, sex, and age

Cause	Sex	Age Start	Age End	Model Version Type	Direction	Level	Covariate Name	Number of Draws
Other road injuries	Male	0-6 days	95+ years	Data Rich	1	1	BAC law youth drivers (quartile)	--
Other road injuries	Male	0-6 days	95+ years	Data Rich	1	1	Liters of alcohol consumed per capita	--
Other road injuries	Male	0-6 days	95+ years	Data Rich	1	1	Log-transformed SEV scalar: Oth Road	--
Other road injuries	Male	0-6 days	95+ years	Data Rich	1	1	Speed limit law rural (quartile)	--
Other road injuries	Male	0-6 days	95+ years	Data Rich	1	1	Speed limit law urban (quartile)	--
Other road injuries	Male	0-6 days	95+ years	Data Rich	-1	2	Education (years per capita)	0
Other road injuries	Male	0-6 days	95+ years	Data Rich	-1	2	Healthcare access and quality index	6
Other road injuries	Male	0-6 days	95+ years	Data Rich	-1	2	LDI (I$ per capita)	--
Other road injuries	Male	0-6 days	95+ years	Data Rich	1	2	Population 15 to 30 (proportion)	39
Other road injuries	Male	0-6 days	95+ years	Data Rich	1	2	Population-weighted mean temperature	--
Other road injuries	Male	0-6 days	95+ years	Data Rich	-1	3	Socio-demographic Index	993
Other road injuries	Male	0-6 days	95+ years	Data Rich	1	3	Rainfall Quintile 5 (proportion)	--
Other road injuries	Male	0-6 days	95+ years	Global	1	1	BAC law professional drivers (quartile)	0
Other road injuries	Male	0-6 days	95+ years	Global	1	1	BAC law general population (quartile)	189
Other road injuries	Male	0-6 days	95+ years	Global	1	1	Vehicles - 2 wheels fraction (proportion)	438
Other road injuries	Male	0-6 days	95+ years	Global	1	1	BAC law youth drivers (quartile)	498
Other road injuries	Male	0-6 days	95+ years	Global	1	1	Vehicles - 2+4 wheels (per capita)	903
Other road injuries	Male	0-6 days	95+ years	Global	1	1	Liters of alcohol consumed per capita	--
Other road injuries	Male	0-6 days	95+ years	Global	1	1	Log-transformed SEV scalar: Oth Road	--
Other road injuries	Male	0-6 days	95+ years	Global	1	1	Speed limit law rural (quartile)	--
Other road injuries	Male	0-6 days	95+ years	Global	1	1	Speed limit law urban (quartile)	--
Other road injuries	Male	0-6 days	95+ years	Global	-1	2	Socio-demographic Index	332
Other road injuries	Male	0-6 days	95+ years	Global	-1	2	Healthcare access and quality index	444
Other road injuries	Male	0-6 days	95+ years	Global	-1	2	Education (years per capita)	630
Other road injuries	Male	0-6 days	95+ years	Global	-1	2	LDI (I$ per capita)	--
Other road injuries	Male	0-6 days	95+ years	Global	1	2	Population 15 to 30 (proportion)	582
Other road injuries	Male	0-6 days	95+ years	Global	1	2	Population-weighted mean temperature	--
Other road injuries	Male	0-6 days	95+ years	Global	1	3	Rainfall Quintile 5 (proportion)	--
Other transport injuries	Female	0-6 days	95+ years	Data Rich	1	1	Vehicles - 2+4 wheels (per capita)	0
Other transport injuries	Female	0-6 days	95+ years	Data Rich	1	1	BAC law professional drivers (quartile)	1
Other transport injuries	Female	0-6 days	95+ years	Data Rich	1	1	Log-transformed SEV scalar: Oth Trans	167
Other transport injuries	Female	0-6 days	95+ years	Data Rich	1	1	BAC law youth drivers (quartile)	192
Other transport injuries	Female	0-6 days	95+ years	Data Rich	1	1	Vehicles - 2 wheels fraction (proportion)	1000
Other transport injuries	Female	0-6 days	95+ years	Data Rich	1	1	BAC law general population (quartile)	--
Other transport injuries	Female	0-6 days	95+ years	Data Rich	1	1	Liters of alcohol consumed per capita	--
Other transport injuries	Female	0-6 days	95+ years	Data Rich	1	1	Speed limit law rural (quartile)	--
Other transport injuries	Female	0-6 days	95+ years	Data Rich	1	1	Speed limit law urban (quartile)	--
Other transport injuries	Female	0-6 days	95+ years	Data Rich	-1	2	Education (years per capita)	0
Other transport injuries	Female	0-6 days	95+ years	Data Rich	-1	2	Healthcare access and quality index	0
Other transport injuries	Female	0-6 days	95+ years	Data Rich	-1	2	Socio-demographic Index	0
Other transport injuries	Female	0-6 days	95+ years	Data Rich	-1	2	LDI (I$ per capita)	--
Other transport injuries	Female	0-6 days	95+ years	Data Rich	1	2	Population 15 to 30 (proportion)	0
Other transport injuries	Female	0-6 days	95+ years	Data Rich	1	2	Population Density (500-1000 ppl/sqkm, proportion)	0
Other transport injuries	Female	0-6 days	95+ years	Data Rich	1	2	Population Density (300-500 ppl/sqkm, proportion)	801
Other transport injuries	Female	0-6 days	95+ years	Data Rich	1	2	Population-weighted mean temperature	--
Other transport injuries	Female	0-6 days	95+ years	Data Rich	1	3	Rainfall Quintile 5 (proportion)	--
Other transport injuries	Female	0-6 days	95+ years	Global	1	1	BAC law general population (quartile)	263
Other transport injuries	Female	0-6 days	95+ years	Global	1	1	Vehicles - 2 wheels fraction (proportion)	284
Other transport injuries	Female	0-6 days	95+ years	Global	1	1	BAC law youth drivers (quartile)	329
Other transport injuries	Female	0-6 days	95+ years	Global	1	1	Vehicles - 2+4 wheels (per capita)	370
Other transport injuries	Female	0-6 days	95+ years	Global	1	1	BAC law professional drivers (quartile)	384
Other transport injuries	Female	0-6 days	95+ years	Global	1	1	Liters of alcohol consumed per capita	--
Other transport injuries	Female	0-6 days	95+ years	Global	1	1	Log-transformed SEV scalar: Oth Trans	--
Other transport injuries	Female	0-6 days	95+ years	Global	1	1	Speed limit law rural (quartile)	--
Other transport injuries	Female	0-6 days	95+ years	Global	1	1	Speed limit law urban (quartile)	--
Other transport injuries	Female	0-6 days	95+ years	Global	-1	2	Education (years per capita)	32
Other transport injuries	Female	0-6 days	95+ years	Global	-1	2	Socio-demographic Index	128
Other transport injuries	Female	0-6 days	95+ years	Global	-1	2	Healthcare access and quality index	279
Other transport injuries	Female	0-6 days	95+ years	Global	-1	2	LDI (I$ per capita)	--
Other transport injuries	Female	0-6 days	95+ years	Global	1	2	Population 15 to 30 (proportion)	15
Other transport injuries	Female	0-6 days	95+ years	Global	1	2	Population Density (500-1000 ppl/sqkm, proportion)	130
Other transport injuries	Female	0-6 days	95+ years	Global	1	2	Population Density (300-500 ppl/sqkm, proportion)	254
Other transport injuries	Female	0-6 days	95+ years	Global	1	2	Population-weighted mean temperature	--
Other transport injuries	Female	0-6 days	95+ years	Global	1	3	Rainfall Quintile 5 (proportion)	--
Other transport injuries	Male	0-6 days	95+ years	Data Rich	1	1	BAC law professional drivers (quartile)	0
Other transport injuries	Male	0-6 days	95+ years	Data Rich	1	1	BAC law youth drivers (quartile)	1
Other transport injuries	Male	0-6 days	95+ years	Data Rich	1	1	Vehicles - 2+4 wheels (per capita)	38
Other transport injuries	Male	0-6 days	95+ years	Data Rich	1	1	Vehicles - 2 wheels fraction (proportion)	999
Other transport injuries	Male	0-6 days	95+ years	Data Rich	1	1	BAC law general population (quartile)	--
Other transport injuries	Male	0-6 days	95+ years	Data Rich	1	1	Liters of alcohol consumed per capita	--
Other transport injuries	Male	0-6 days	95+ years	Data Rich	1	1	Log-transformed SEV scalar: Oth Trans	--
Other transport injuries	Male	0-6 days	95+ years	Data Rich	1	1	Speed limit law rural (quartile)	--
Other transport injuries	Male	0-6 days	95+ years	Data Rich	1	1	Speed limit law urban (quartile)	--
Other transport injuries	Male	0-6 days	95+ years	Data Rich	-1	2	Education (years per capita)	0
Other transport injuries	Male	0-6 days	95+ years	Data Rich	-1	2	Healthcare access and quality index	0
Other transport injuries	Male	0-6 days	95+ years	Data Rich	-1	2	Socio-demographic Index	1
Other transport injuries	Male	0-6 days	95+ years	Data Rich	-1	2	LDI (I$ per capita)	--
Other transport injuries	Male	0-6 days	95+ years	Data Rich	1	2	Population 15 to 30 (proportion)	7
Other transport injuries	Male	0-6 days	95+ years	Data Rich	1	2	Population Density (300-500 ppl/sqkm, proportion)	7
Other transport injuries	Male	0-6 days	95+ years	Data Rich	1	2	Population Density (500-1000 ppl/sqkm, proportion)	7
Other transport injuries	Male	0-6 days	95+ years	Data Rich	1	2	Population-weighted mean temperature	--
Other transport injuries	Male	0-6 days	95+ years	Data Rich	1	3	Rainfall Quintile 5 (proportion)	--
Other transport injuries	Male	0-6 days	95+ years	Global	1	1	BAC law professional drivers (quartile)	65
Other transport injuries	Male	0-6 days	95+ years	Global	1	1	BAC law youth drivers (quartile)	213
Other transport injuries	Male	0-6 days	95+ years	Global	1	1	BAC law general population (quartile)	484
Other transport injuries	Male	0-6 days	95+ years	Global	1	1	Vehicles - 2 wheels fraction (proportion)	647
Other transport injuries	Male	0-6 days	95+ years	Global	1	1	Vehicles - 2+4 wheels (per capita)	938
Other transport injuries	Male	0-6 days	95+ years	Global	1	1	Liters of alcohol consumed per capita	--
Other transport injuries	Male	0-6 days	95+ years	Global	1	1	Log-transformed SEV scalar: Oth Trans	--
Other transport injuries	Male	0-6 days	95+ years	Global	1	1	Speed limit law rural (quartile)	--
Other transport injuries	Male	0-6 days	95+ years	Global	1	1	Speed limit law urban (quartile)	--
Other transport injuries	Male	0-6 days	95+ years	Global	-1	2	Healthcare access and quality index	90
Other transport injuries	Male	0-6 days	95+ years	Global	-1	2	Education (years per capita)	136
Other transport injuries	Male	0-6 days	95+ years	Global	-1	2	Socio-demographic Index	176
Other transport injuries	Male	0-6 days	95+ years	Global	-1	2	LDI (I$ per capita)	--
Other transport injuries	Male	0-6 days	95+ years	Global	1	2	Population 15 to 30 (proportion)	95
Other transport injuries	Male	0-6 days	95+ years	Global	1	2	Population Density (300-500 ppl/sqkm, proportion)	111
Other transport injuries	Male	0-6 days	95+ years	Global	1	2	Population Density (500-1000 ppl/sqkm, proportion)	354
Other transport injuries	Male	0-6 days	95+ years	Global	1	2	Population-weighted mean temperature	--
Other transport injuries	Male	0-6 days	95+ years	Global	1	3	Rainfall Quintile 5 (proportion)	--
Falls	Female	0-6 days	95+ years	Data Rich	-1	1	Education (years per capita)	5
Falls	Female	0-6 days	95+ years	Data Rich	1	1	Liters of alcohol consumed per capita	20
Falls	Female	0-6 days	95+ years	Data Rich	1	1	Log-transformed SEV scalar: Falls	--
Falls	Female	0-6 days	95+ years	Data Rich	-1	2	Population-weighted mean temperature	922
Falls	Female	0-6 days	95+ years	Data Rich	-1	2	Healthcare access and quality index	--
Falls	Female	0-6 days	95+ years	Data Rich	-1	3	Socio-demographic Index	16
Falls	Female	0-6 days	95+ years	Data Rich	-1	3	LDI (I$ per capita)	--
Falls	Female	0-6 days	95+ years	Data Rich	1	3	Elevation Over 1500m (proportion)	791
Falls	Female	0-6 days	95+ years	Global	-1	1	Education (years per capita)	487
Falls	Female	0-6 days	95+ years	Global	1	1	Liters of alcohol consumed per capita	513
Falls	Female	0-6 days	95+ years	Global	1	1	Log-transformed SEV scalar: Falls	--
Falls	Female	0-6 days	95+ years	Global	-1	2	Healthcare access and quality index	--
Falls	Female	0-6 days	95+ years	Global	-1	2	Population-weighted mean temperature	--
Falls	Female	0-6 days	95+ years	Global	-1	3	Socio-demographic Index	193
Falls	Female	0-6 days	95+ years	Global	-1	3	LDI (I$ per capita)	--
Falls	Female	0-6 days	95+ years	Global	1	3	Elevation Over 1500m (proportion)	565
Falls	Male	0-6 days	95+ years	Data Rich	-1	1	Education (years per capita)	2
Falls	Male	0-6 days	95+ years	Data Rich	1	1	Liters of alcohol consumed per capita	998
Falls	Male	0-6 days	95+ years	Data Rich	1	1	Log-transformed SEV scalar: Falls	--
Falls	Male	0-6 days	95+ years	Data Rich	-1	2	Healthcare access and quality index	53
Falls	Male	0-6 days	95+ years	Data Rich	-1	2	Population-weighted mean temperature	391

CODEm covariates used, level of covariate, and expected direction of covariate by cause, sex, and age

Cause	Sex	Age Start	Age End	Model Version Type	Direction	Level	Covariate Name	Number of Draws
Falls	Male	0-6 days	95+ years	Data Rich	-1	3	Socio-demographic Index	6
Falls	Male	0-6 days	95+ years	Data Rich	-1	3	LDI (I$ per capita)	--
Falls	Male	0-6 days	95+ years	Data Rich	1	3	Elevation Over 1500m (proportion)	284
Falls	Male	0-6 days	95+ years	Global	-1	1	Education (years per capita)	674
Falls	Male	0-6 days	95+ years	Global	1	1	Liters of alcohol consumed per capita	--
Falls	Male	0-6 days	95+ years	Global	1	1	Log-transformed SEV scalar: Falls	--
Falls	Male	0-6 days	95+ years	Global	-1	2	Healthcare access and quality index	--
Falls	Male	0-6 days	95+ years	Global	-1	2	Population-weighted mean temperature	--
Falls	Male	0-6 days	95+ years	Global	-1	3	Socio-demographic Index	237
Falls	Male	0-6 days	95+ years	Global	-1	3	LDI (I$ per capita)	--
Falls	Male	0-6 days	95+ years	Global	1	3	Elevation Over 1500m (proportion)	511
Drowning	Female	0-6 days	95+ years	Data Rich	-1	1	Landlocked Nation (binary)	840
Drowning	Female	0-6 days	95+ years	Data Rich	-1	1	Rainfall Quintile 1 (proportion)	--
Drowning	Female	0-6 days	95+ years	Data Rich	1	1	Population-weighted mean temperature	0
Drowning	Female	0-6 days	95+ years	Data Rich	1	1	Rainfall Quintile 5 (proportion)	0
Drowning	Female	0-6 days	95+ years	Data Rich	1	1	Log-transformed SEV scalar: Drown	38
Drowning	Female	0-6 days	95+ years	Data Rich	1	1	Coastal Population within 10km (proportion)	191
Drowning	Female	0-6 days	95+ years	Data Rich	1	2	Elevation Under 100m (proportion)	6
Drowning	Female	0-6 days	95+ years	Data Rich	-1	3	Education (years per capita)	159
Drowning	Female	0-6 days	95+ years	Data Rich	-1	3	Socio-demographic Index	841
Drowning	Female	0-6 days	95+ years	Data Rich	-1	3	LDI (I$ per capita)	--
Drowning	Female	0-6 days	95+ years	Global	-1	1	Landlocked Nation (binary)	83
Drowning	Female	0-6 days	95+ years	Global	-1	1	Rainfall Quintile 1 (proportion)	--
Drowning	Female	0-6 days	95+ years	Global	1	1	Population-weighted mean temperature	215
Drowning	Female	0-6 days	95+ years	Global	1	1	Rainfall Quintile 5 (proportion)	261
Drowning	Female	0-6 days	95+ years	Global	1	1	Log-transformed SEV scalar: Drown	410
Drowning	Female	0-6 days	95+ years	Global	1	1	Coastal Population within 10km (proportion)	704
Drowning	Female	0-6 days	95+ years	Global	1	2	Elevation Under 100m (proportion)	36
Drowning	Female	0-6 days	95+ years	Global	-1	3	Socio-demographic Index	176
Drowning	Female	0-6 days	95+ years	Global	-1	3	Education (years per capita)	261
Drowning	Female	0-6 days	95+ years	Global	-1	3	LDI (I$ per capita)	--
Drowning	Male	0-6 days	95+ years	Data Rich	-1	1	Rainfall Quintile 1 (proportion)	0
Drowning	Male	0-6 days	95+ years	Data Rich	-1	1	Landlocked Nation (binary)	7
Drowning	Male	0-6 days	95+ years	Data Rich	1	1	Rainfall Quintile 5 (proportion)	0
Drowning	Male	0-6 days	95+ years	Data Rich	1	1	Log-transformed SEV scalar: Drown	31
Drowning	Male	0-6 days	95+ years	Data Rich	1	1	Coastal Population within 10km (proportion)	197
Drowning	Male	0-6 days	95+ years	Data Rich	1	1	Population-weighted mean temperature	999
Drowning	Male	0-6 days	95+ years	Data Rich	1	2	Elevation Under 100m (proportion)	6
Drowning	Male	0-6 days	95+ years	Data Rich	-1	3	Education (years per capita)	0
Drowning	Male	0-6 days	95+ years	Data Rich	-1	3	Socio-demographic Index	1000
Drowning	Male	0-6 days	95+ years	Data Rich	-1	3	LDI (I$ per capita)	--
Drowning	Male	0-6 days	95+ years	Global	-1	1	Landlocked Nation (binary)	27
Drowning	Male	0-6 days	95+ years	Global	-1	1	Rainfall Quintile 1 (proportion)	--
Drowning	Male	0-6 days	95+ years	Global	1	1	Rainfall Quintile 5 (proportion)	138
Drowning	Male	0-6 days	95+ years	Global	1	1	Coastal Population within 10km (proportion)	209
Drowning	Male	0-6 days	95+ years	Global	1	1	Log-transformed SEV scalar: Drown	265
Drowning	Male	0-6 days	95+ years	Global	1	1	Population-weighted mean temperature	704
Drowning	Male	0-6 days	95+ years	Global	1	2	Elevation Under 100m (proportion)	605
Drowning	Male	0-6 days	95+ years	Global	-1	3	Education (years per capita)	231
Drowning	Male	0-6 days	95+ years	Global	-1	3	Socio-demographic Index	315
Drowning	Male	0-6 days	95+ years	Global	-1	3	LDI (I$ per capita)	--
Fire, heat, and hot substances	Female	0-6 days	95+ years	Data Rich	1	1	Log-transformed SEV scalar: Fire	--
Fire, heat, and hot substances	Female	0-6 days	95+ years	Data Rich	1	1	Population-weighted mean temperature	--
Fire, heat, and hot substances	Female	0-6 days	95+ years	Data Rich	-1	2	Healthcare access and quality index	21
Fire, heat, and hot substances	Female	0-6 days	95+ years	Data Rich	1	2	Indoor Air Pollution (All Cooking Fuels)	737
Fire, heat, and hot substances	Female	0-6 days	95+ years	Data Rich	1	2	Population Density (over 1000 ppl/sqkm, proportion)	--
Fire, heat, and hot substances	Female	0-6 days	95+ years	Data Rich	1	2	Tobacco (cigarettes per capita)	--
Fire, heat, and hot substances	Female	0-6 days	95+ years	Data Rich	-1	3	Socio-demographic Index	2
Fire, heat, and hot substances	Female	0-6 days	95+ years	Data Rich	-1	3	Education (years per capita)	111
Fire, heat, and hot substances	Female	0-6 days	95+ years	Data Rich	-1	3	LDI (I$ per capita)	--
Fire, heat, and hot substances	Female	0-6 days	95+ years	Global	1	1	Population-weighted mean temperature	1000
Fire, heat, and hot substances	Female	0-6 days	95+ years	Global	1	1	Log-transformed SEV scalar: Fire	--
Fire, heat, and hot substances	Female	0-6 days	95+ years	Global	-1	2	Healthcare access and quality index	258
Fire, heat, and hot substances	Female	0-6 days	95+ years	Global	1	2	Indoor Air Pollution (All Cooking Fuels)	50
Fire, heat, and hot substances	Female	0-6 days	95+ years	Global	1	2	Tobacco (cigarettes per capita)	517
Fire, heat, and hot substances	Female	0-6 days	95+ years	Global	1	2	Population Density (over 1000 ppl/sqkm, proportion)	--
Fire, heat, and hot substances	Female	0-6 days	95+ years	Global	-1	3	Socio-demographic Index	336
Fire, heat, and hot substances	Female	0-6 days	95+ years	Global	-1	3	Education (years per capita)	356
Fire, heat, and hot substances	Female	0-6 days	95+ years	Global	-1	3	LDI (I$ per capita)	--
Fire, heat, and hot substances	Male	0-6 days	95+ years	Data Rich	1	1	Log-transformed SEV scalar: Fire	--
Fire, heat, and hot substances	Male	0-6 days	95+ years	Data Rich	1	1	Population-weighted mean temperature	--
Fire, heat, and hot substances	Male	0-6 days	95+ years	Data Rich	-1	2	Healthcare access and quality index	59
Fire, heat, and hot substances	Male	0-6 days	95+ years	Data Rich	1	2	Indoor Air Pollution (All Cooking Fuels)	59
Fire, heat, and hot substances	Male	0-6 days	95+ years	Data Rich	1	2	Tobacco (cigarettes per capita)	59
Fire, heat, and hot substances	Male	0-6 days	95+ years	Data Rich	1	2	Population Density (over 1000 ppl/sqkm, proportion)	--
Fire, heat, and hot substances	Male	0-6 days	95+ years	Data Rich	-1	3	Education (years per capita)	0
Fire, heat, and hot substances	Male	0-6 days	95+ years	Data Rich	-1	3	Socio-demographic Index	800
Fire, heat, and hot substances	Male	0-6 days	95+ years	Data Rich	-1	3	LDI (I$ per capita)	--
Fire, heat, and hot substances	Male	0-6 days	95+ years	Global	1	1	Population-weighted mean temperature	703
Fire, heat, and hot substances	Male	0-6 days	95+ years	Global	1	1	Log-transformed SEV scalar: Fire	--
Fire, heat, and hot substances	Male	0-6 days	95+ years	Global	-1	2	Healthcare access and quality index	600
Fire, heat, and hot substances	Male	0-6 days	95+ years	Global	1	2	Indoor Air Pollution (All Cooking Fuels)	50
Fire, heat, and hot substances	Male	0-6 days	95+ years	Global	1	2	Tobacco (cigarettes per capita)	174
Fire, heat, and hot substances	Male	0-6 days	95+ years	Global	1	2	Population Density (over 1000 ppl/sqkm, proportion)	--
Fire, heat, and hot substances	Male	0-6 days	95+ years	Global	-1	3	Socio-demographic Index	48
Fire, heat, and hot substances	Male	0-6 days	95+ years	Global	-1	3	Education (years per capita)	346
Fire, heat, and hot substances	Male	0-6 days	95+ years	Global	-1	3	LDI (I$ per capita)	--
Poisonings	Female	0-6 days	95+ years	Data Rich	1	1	Liters of alcohol consumed per capita	--
Poisonings	Female	0-6 days	95+ years	Data Rich	1	1	Log-transformed SEV scalar: Poison	--
Poisonings	Female	0-6 days	95+ years	Data Rich	1	1	Population-weighted mean temperature	--
Poisonings	Female	0-6 days	95+ years	Data Rich	-1	2	Healthcare access and quality index	0
Poisonings	Female	0-6 days	95+ years	Data Rich	-1	2	Population Density (over 1000 ppl/sqkm, proportion)	46
Poisonings	Female	0-6 days	95+ years	Data Rich	1	2	Population Density (under 150 ppl/sqkm, proportion)	111
Poisonings	Female	0-6 days	95+ years	Data Rich	-1	3	Education (years per capita)	6
Poisonings	Female	0-6 days	95+ years	Data Rich	-1	3	Socio-demographic Index	261
Poisonings	Female	0-6 days	95+ years	Data Rich	-1	3	LDI (I$ per capita)	--
Poisonings	Female	0-6 days	95+ years	Global	1	1	Liters of alcohol consumed per capita	--
Poisonings	Female	0-6 days	95+ years	Global	1	1	Log-transformed SEV scalar: Poison	--
Poisonings	Female	0-6 days	95+ years	Global	1	1	Population-weighted mean temperature	--
Poisonings	Female	0-6 days	95+ years	Global	-1	2	Population Density (over 1000 ppl/sqkm, proportion)	175
Poisonings	Female	0-6 days	95+ years	Global	-1	2	Healthcare access and quality index	336
Poisonings	Female	0-6 days	95+ years	Global	1	2	Population Density (under 150 ppl/sqkm, proportion)	--
Poisonings	Female	0-6 days	95+ years	Global	-1	3	Education (years per capita)	281
Poisonings	Female	0-6 days	95+ years	Global	-1	3	Socio-demographic Index	411
Poisonings	Female	0-6 days	95+ years	Global	-1	3	LDI (I$ per capita)	--
Poisonings	Male	0-6 days	95+ years	Data Rich	1	1	Log-transformed SEV scalar: Poison	1000
Poisonings	Male	0-6 days	95+ years	Data Rich	1	1	Liters of alcohol consumed per capita	--
Poisonings	Male	0-6 days	95+ years	Data Rich	1	1	Population-weighted mean temperature	--
Poisonings	Male	0-6 days	95+ years	Data Rich	-1	2	Population Density (over 1000 ppl/sqkm, proportion)	47
Poisonings	Male	0-6 days	95+ years	Data Rich	-1	2	Healthcare access and quality index	625
Poisonings	Male	0-6 days	95+ years	Data Rich	1	2	Population Density (under 150 ppl/sqkm, proportion)	578
Poisonings	Male	0-6 days	95+ years	Data Rich	-1	3	Socio-demographic Index	202
Poisonings	Male	0-6 days	95+ years	Data Rich	-1	3	Education (years per capita)	253
Poisonings	Male	0-6 days	95+ years	Data Rich	-1	3	LDI (I$ per capita)	--
Poisonings	Male	0-6 days	95+ years	Global	1	1	Liters of alcohol consumed per capita	--
Poisonings	Male	0-6 days	95+ years	Global	1	1	Log-transformed SEV scalar: Poison	--
Poisonings	Male	0-6 days	95+ years	Global	1	1	Population-weighted mean temperature	--
Poisonings	Male	0-6 days	95+ years	Global	-1	2	Population Density (over 1000 ppl/sqkm, proportion)	113
Poisonings	Male	0-6 days	95+ years	Global	-1	2	Healthcare access and quality index	306

CODEm covariates used, level of covariate, and expected direction of covariate by cause, sex, and age

Cause	Sex	Age Start	Age End	Model Version Type	Direction	Level	Covariate Name	Number of Draws
Poisonings	Male	0-6 days	95+ years	Global	1	2	Population Density (under 150 ppl/sqkm, proportion)	288
Poisonings	Male	0-6 days	95+ years	Global	-1	3	Socio-demographic Index	266
Poisonings	Male	0-6 days	95+ years	Global	-1	3	Education (years per capita)	543
Poisonings	Male	0-6 days	95+ years	Global	-1	3	LDI (I$ per capita)	--
Poisoning by carbon monoxide	Female	0-6 days	95+ years	Data Rich	1	1	Log-transformed SEV scalar: Inj Pois CO	993
Poisoning by carbon monoxide	Female	0-6 days	95+ years	Data Rich	1	1	Liters of alcohol consumed per capita	--
Poisoning by carbon monoxide	Female	0-6 days	95+ years	Data Rich	-1	2	Population-weighted mean temperature	839
Poisoning by carbon monoxide	Female	0-6 days	95+ years	Data Rich	-1	3	Socio-demographic Index	1
Poisoning by carbon monoxide	Female	0-6 days	95+ years	Data Rich	-1	3	Education (years per capita)	6
Poisoning by carbon monoxide	Female	0-6 days	95+ years	Data Rich	-1	3	Healthcare access and quality index	7
Poisoning by carbon monoxide	Female	0-6 days	95+ years	Data Rich	-1	3	LDI (I$ per capita)	--
Poisoning by carbon monoxide	Female	0-6 days	95+ years	Global	1	1	Liters of alcohol consumed per capita	428
Poisoning by carbon monoxide	Female	0-6 days	95+ years	Global	1	1	Log-transformed SEV scalar: Inj Pois CO	--
Poisoning by carbon monoxide	Female	0-6 days	95+ years	Global	-1	2	Population-weighted mean temperature	636
Poisoning by carbon monoxide	Female	0-6 days	95+ years	Global	-1	3	Education (years per capita)	160
Poisoning by carbon monoxide	Female	0-6 days	95+ years	Global	-1	3	Socio-demographic Index	229
Poisoning by carbon monoxide	Female	0-6 days	95+ years	Global	-1	3	Healthcare access and quality index	--
Poisoning by carbon monoxide	Female	0-6 days	95+ years	Global	-1	3	LDI (I$ per capita)	--
Poisoning by carbon monoxide	Male	0-6 days	95+ years	Data Rich	1	1	Log-transformed SEV scalar: Inj Pois CO	991
Poisoning by carbon monoxide	Male	0-6 days	95+ years	Data Rich	1	1	Liters of alcohol consumed per capita	--
Poisoning by carbon monoxide	Male	0-6 days	95+ years	Data Rich	-1	2	Population-weighted mean temperature	961
Poisoning by carbon monoxide	Male	0-6 days	95+ years	Data Rich	-1	3	Socio-demographic Index	0
Poisoning by carbon monoxide	Male	0-6 days	95+ years	Data Rich	-1	3	Education (years per capita)	9
Poisoning by carbon monoxide	Male	0-6 days	95+ years	Data Rich	-1	3	Healthcare access and quality index	9
Poisoning by carbon monoxide	Male	0-6 days	95+ years	Data Rich	-1	3	LDI (I$ per capita)	--
Poisoning by carbon monoxide	Male	0-6 days	95+ years	Global	1	1	Liters of alcohol consumed per capita	255
Poisoning by carbon monoxide	Male	0-6 days	95+ years	Global	1	1	Log-transformed SEV scalar: Inj Pois CO	--
Poisoning by carbon monoxide	Male	0-6 days	95+ years	Global	-1	2	Population-weighted mean temperature	459
Poisoning by carbon monoxide	Male	0-6 days	95+ years	Global	-1	3	Healthcare access and quality index	166
Poisoning by carbon monoxide	Male	0-6 days	95+ years	Global	-1	3	Socio-demographic Index	328
Poisoning by carbon monoxide	Male	0-6 days	95+ years	Global	-1	3	Education (years per capita)	--
Poisoning by carbon monoxide	Male	0-6 days	95+ years	Global	-1	3	LDI (I$ per capita)	--
Poisoning by other means	Female	0-6 days	95+ years	Data Rich	1	1	Liters of alcohol consumed per capita	1000
Poisoning by other means	Female	0-6 days	95+ years	Data Rich	1	1	Log-transformed SEV scalar: Inj Pois Oth	--
Poisoning by other means	Female	0-6 days	95+ years	Data Rich	1	1	Population-weighted mean temperature	--
Poisoning by other means	Female	0-6 days	95+ years	Data Rich	-1	3	Socio-demographic Index	161
Poisoning by other means	Female	0-6 days	95+ years	Data Rich	-1	3	Education (years per capita)	806
Poisoning by other means	Female	0-6 days	95+ years	Data Rich	-1	3	Healthcare access and quality index	--
Poisoning by other means	Female	0-6 days	95+ years	Data Rich	-1	3	LDI (I$ per capita)	--
Poisoning by other means	Female	0-6 days	95+ years	Global	1	1	Population-weighted mean temperature	300
Poisoning by other means	Female	0-6 days	95+ years	Global	1	1	Liters of alcohol consumed per capita	528
Poisoning by other means	Female	0-6 days	95+ years	Global	1	1	Log-transformed SEV scalar: Inj Pois Oth	--
Poisoning by other means	Female	0-6 days	95+ years	Global	-1	3	Healthcare access and quality index	66
Poisoning by other means	Female	0-6 days	95+ years	Global	-1	3	Socio-demographic Index	392
Poisoning by other means	Female	0-6 days	95+ years	Global	-1	3	Education (years per capita)	442
Poisoning by other means	Female	0-6 days	95+ years	Global	-1	3	LDI (I$ per capita)	--
Poisoning by other means	Male	0-6 days	95+ years	Data Rich	1	1	Liters of alcohol consumed per capita	1000
Poisoning by other means	Male	0-6 days	95+ years	Data Rich	1	1	Log-transformed SEV scalar: Inj Pois Oth	--
Poisoning by other means	Male	0-6 days	95+ years	Data Rich	1	1	Population-weighted mean temperature	--
Poisoning by other means	Male	0-6 days	95+ years	Data Rich	-1	3	Socio-demographic Index	331
Poisoning by other means	Male	0-6 days	95+ years	Data Rich	-1	3	Education (years per capita)	931
Poisoning by other means	Male	0-6 days	95+ years	Data Rich	-1	3	Healthcare access and quality index	931
Poisoning by other means	Male	0-6 days	95+ years	Data Rich	-1	3	LDI (I$ per capita)	--
Poisoning by other means	Male	0-6 days	95+ years	Global	1	1	Liters of alcohol consumed per capita	--
Poisoning by other means	Male	0-6 days	95+ years	Global	1	1	Log-transformed SEV scalar: Inj Pois Oth	--
Poisoning by other means	Male	0-6 days	95+ years	Global	1	1	Population-weighted mean temperature	--
Poisoning by other means	Male	0-6 days	95+ years	Global	-1	3	Healthcare access and quality index	271
Poisoning by other means	Male	0-6 days	95+ years	Global	-1	3	Socio-demographic Index	421
Poisoning by other means	Male	0-6 days	95+ years	Global	-1	3	Education (years per capita)	672
Poisoning by other means	Male	0-6 days	95+ years	Global	-1	3	LDI (I$ per capita)	--
Exposure to mechanical forces	Female	0-6 days	95+ years	Data Rich	1	1	Population-weighted mean temperature	--
Exposure to mechanical forces	Female	0-6 days	95+ years	Data Rich	-1	2	Healthcare access and quality index	0
Exposure to mechanical forces	Female	0-6 days	95+ years	Data Rich	-1	2	Population Density (over 1000 ppl/sqkm, proportion)	6
Exposure to mechanical forces	Female	0-6 days	95+ years	Data Rich	1	2	Population Density (under 150 ppl/sqkm, proportion)	33
Exposure to mechanical forces	Female	0-6 days	95+ years	Data Rich	-1	3	Education (years per capita)	161
Exposure to mechanical forces	Female	0-6 days	95+ years	Data Rich	-1	3	Socio-demographic Index	801
Exposure to mechanical forces	Female	0-6 days	95+ years	Data Rich	-1	3	LDI (I$ per capita)	--
Exposure to mechanical forces	Female	0-6 days	95+ years	Global	1	1	Population-weighted mean temperature	--
Exposure to mechanical forces	Female	0-6 days	95+ years	Global	-1	2	Healthcare access and quality index	159
Exposure to mechanical forces	Female	0-6 days	95+ years	Global	-1	2	Population Density (over 1000 ppl/sqkm, proportion)	202
Exposure to mechanical forces	Female	0-6 days	95+ years	Global	1	2	Population Density (under 150 ppl/sqkm, proportion)	545
Exposure to mechanical forces	Female	0-6 days	95+ years	Global	-1	3	Education (years per capita)	157
Exposure to mechanical forces	Female	0-6 days	95+ years	Global	-1	3	Socio-demographic Index	359
Exposure to mechanical forces	Female	0-6 days	95+ years	Global	-1	3	LDI (I$ per capita)	--
Exposure to mechanical forces	Male	0-6 days	95+ years	Data Rich	1	1	Population-weighted mean temperature	--
Exposure to mechanical forces	Male	0-6 days	95+ years	Data Rich	-1	2	Population Density (over 1000 ppl/sqkm, proportion)	161
Exposure to mechanical forces	Male	0-6 days	95+ years	Data Rich	-1	2	Healthcare access and quality index	--
Exposure to mechanical forces	Male	0-6 days	95+ years	Data Rich	1	2	Population Density (under 150 ppl/sqkm, proportion)	38
Exposure to mechanical forces	Male	0-6 days	95+ years	Data Rich	-1	3	Education (years per capita)	7
Exposure to mechanical forces	Male	0-6 days	95+ years	Data Rich	-1	3	Socio-demographic Index	961
Exposure to mechanical forces	Male	0-6 days	95+ years	Data Rich	-1	3	LDI (I$ per capita)	--
Exposure to mechanical forces	Male	0-6 days	95+ years	Global	1	1	Population-weighted mean temperature	--
Exposure to mechanical forces	Male	0-6 days	95+ years	Global	-1	2	Healthcare access and quality index	393
Exposure to mechanical forces	Male	0-6 days	95+ years	Global	-1	2	Population Density (under 150 ppl/sqkm, proportion)	--
Exposure to mechanical forces	Male	0-6 days	95+ years	Global	1	2	Population Density (over 1000 ppl/sqkm, proportion)	--
Exposure to mechanical forces	Male	0-6 days	95+ years	Global	-1	3	Education (years per capita)	352
Exposure to mechanical forces	Male	0-6 days	95+ years	Global	-1	3	Socio-demographic Index	399
Exposure to mechanical forces	Male	0-6 days	95+ years	Global	-1	3	LDI (I$ per capita)	--
Unintentional firearm injuries	Female	0-6 days	95+ years	Data Rich	1	1	Population-weighted mean temperature	1000
Unintentional firearm injuries	Female	0-6 days	95+ years	Data Rich	1	1	Log-transformed SEV scalar: Mech Gun	--
Unintentional firearm injuries	Female	0-6 days	95+ years	Data Rich	-1	2	Healthcare access and quality index	261
Unintentional firearm injuries	Female	0-6 days	95+ years	Data Rich	-1	3	Socio-demographic Index	38
Unintentional firearm injuries	Female	0-6 days	95+ years	Data Rich	-1	3	Education (years per capita)	102
Unintentional firearm injuries	Female	0-6 days	95+ years	Data Rich	-1	3	Population Density (over 1000 ppl/sqkm, proportion)	240
Unintentional firearm injuries	Female	0-6 days	95+ years	Data Rich	-1	3	LDI (I$ per capita)	--
Unintentional firearm injuries	Female	0-6 days	95+ years	Data Rich	1	3	Population Density (under 150 ppl/sqkm, proportion)	--
Unintentional firearm injuries	Female	0-6 days	95+ years	Global	1	1	Log-transformed SEV scalar: Mech Gun	535
Unintentional firearm injuries	Female	0-6 days	95+ years	Global	1	1	Population-weighted mean temperature	764
Unintentional firearm injuries	Female	0-6 days	95+ years	Global	-1	2	Healthcare access and quality index	622
Unintentional firearm injuries	Female	0-6 days	95+ years	Global	-1	3	Socio-demographic Index	194
Unintentional firearm injuries	Female	0-6 days	95+ years	Global	-1	3	Population Density (over 1000 ppl/sqkm, proportion)	285
Unintentional firearm injuries	Female	0-6 days	95+ years	Global	-1	3	Education (years per capita)	343
Unintentional firearm injuries	Female	0-6 days	95+ years	Global	-1	3	LDI (I$ per capita)	--
Unintentional firearm injuries	Female	0-6 days	95+ years	Global	1	3	Population Density (under 150 ppl/sqkm, proportion)	78
Unintentional firearm injuries	Male	0-6 days	95+ years	Data Rich	1	1	Population-weighted mean temperature	1000
Unintentional firearm injuries	Male	0-6 days	95+ years	Data Rich	1	1	Log-transformed SEV scalar: Mech Gun	--
Unintentional firearm injuries	Male	0-6 days	95+ years	Data Rich	-1	2	Healthcare access and quality index	999
Unintentional firearm injuries	Male	0-6 days	95+ years	Data Rich	-1	3	Population Density (over 1000 ppl/sqkm, proportion)	7
Unintentional firearm injuries	Male	0-6 days	95+ years	Data Rich	-1	3	Education (years per capita)	--
Unintentional firearm injuries	Male	0-6 days	95+ years	Data Rich	-1	3	LDI (I$ per capita)	--
Unintentional firearm injuries	Male	0-6 days	95+ years	Data Rich	-1	3	Socio-demographic Index	--
Unintentional firearm injuries	Male	0-6 days	95+ years	Data Rich	1	3	Population Density (under 150 ppl/sqkm, proportion)	160
Unintentional firearm injuries	Male	0-6 days	95+ years	Global	1	1	Log-transformed SEV scalar: Mech Gun	62
Unintentional firearm injuries	Male	0-6 days	95+ years	Global	1	1	Population-weighted mean temperature	541
Unintentional firearm injuries	Male	0-6 days	95+ years	Global	-1	2	Healthcare access and quality index	511
Unintentional firearm injuries	Male	0-6 days	95+ years	Global	-1	3	Education (years per capita)	102
Unintentional firearm injuries	Male	0-6 days	95+ years	Global	-1	3	Socio-demographic Index	271

CODEm covariates used, level of covariate, and expected direction of covariate by cause, sex, and age									
**Cause**	**Sex**	**Age Start**	**Age End**	**Model Version Type**	**Direction**	**Level**	**Covariate Name**	**Number of Draws**
Unintentional firearm injuries	Male	0-6 days	95+ years	Global	-1	3	Population Density (over 1000 ppl/sqkm, proportion)	475
Unintentional firearm injuries	Male	0-6 days	95+ years	Global	-1	3	LDI (I$ per capita)	--
Unintentional firearm injuries	Male	0-6 days	95+ years	Global	1	3	Population Density (under 150 ppl/sqkm, proportion)	403
Other exposure to mechanical forces	Female	0-6 days	95+ years	Data Rich	1	1	Log-transformed SEV scalar: Oth Mech	7
Other exposure to mechanical forces	Female	0-6 days	95+ years	Data Rich	1	1	Population-weighted mean temperature	--
Other exposure to mechanical forces	Female	0-6 days	95+ years	Data Rich	-1	2	Population Density (over 1000 ppl/sqkm, proportion)	32
Other exposure to mechanical forces	Female	0-6 days	95+ years	Data Rich	-1	2	Healthcare access and quality index	--
Other exposure to mechanical forces	Female	0-6 days	95+ years	Data Rich	1	2	Population Density (under 150 ppl/sqkm, proportion)	38
Other exposure to mechanical forces	Female	0-6 days	95+ years	Data Rich	-1	3	Socio-demographic Index	160
Other exposure to mechanical forces	Female	0-6 days	95+ years	Data Rich	-1	3	Education (years per capita)	--
Other exposure to mechanical forces	Female	0-6 days	95+ years	Data Rich	-1	3	LDI (I$ per capita)	--
Other exposure to mechanical forces	Female	0-6 days	95+ years	Global	1	1	Log-transformed SEV scalar: Oth Mech	--
Other exposure to mechanical forces	Female	0-6 days	95+ years	Global	1	1	Population-weighted mean temperature	--
Other exposure to mechanical forces	Female	0-6 days	95+ years	Global	-1	2	Population Density (over 1000 ppl/sqkm, proportion)	345
Other exposure to mechanical forces	Female	0-6 days	95+ years	Global	-1	2	Healthcare access and quality index	--
Other exposure to mechanical forces	Female	0-6 days	95+ years	Global	1	2	Population Density (under 150 ppl/sqkm, proportion)	425
Other exposure to mechanical forces	Female	0-6 days	95+ years	Global	-1	3	Socio-demographic Index	332
Other exposure to mechanical forces	Female	0-6 days	95+ years	Global	-1	3	Education (years per capita)	545
Other exposure to mechanical forces	Female	0-6 days	95+ years	Global	-1	3	LDI (I$ per capita)	--
Other exposure to mechanical forces	Male	0-6 days	95+ years	Data Rich	1	1	Log-transformed SEV scalar: Oth Mech	--
Other exposure to mechanical forces	Male	0-6 days	95+ years	Data Rich	1	1	Population-weighted mean temperature	--
Other exposure to mechanical forces	Male	0-6 days	95+ years	Data Rich	-1	2	Population Density (over 1000 ppl/sqkm, proportion)	0
Other exposure to mechanical forces	Male	0-6 days	95+ years	Data Rich	-1	2	Healthcare access and quality index	6
Other exposure to mechanical forces	Male	0-6 days	95+ years	Data Rich	1	2	Population Density (under 150 ppl/sqkm, proportion)	33
Other exposure to mechanical forces	Male	0-6 days	95+ years	Data Rich	-1	3	Socio-demographic Index	32
Other exposure to mechanical forces	Male	0-6 days	95+ years	Data Rich	-1	3	Education (years per capita)	160
Other exposure to mechanical forces	Male	0-6 days	95+ years	Data Rich	-1	3	LDI (I$ per capita)	--
Other exposure to mechanical forces	Male	0-6 days	95+ years	Global	1	1	Log-transformed SEV scalar: Oth Mech	--
Other exposure to mechanical forces	Male	0-6 days	95+ years	Global	1	1	Population-weighted mean temperature	--
Other exposure to mechanical forces	Male	0-6 days	95+ years	Global	-1	2	Healthcare access and quality index	116
Other exposure to mechanical forces	Male	0-6 days	95+ years	Global	-1	2	Population Density (over 1000 ppl/sqkm, proportion)	328
Other exposure to mechanical forces	Male	0-6 days	95+ years	Global	1	2	Population Density (under 150 ppl/sqkm, proportion)	273
Other exposure to mechanical forces	Male	0-6 days	95+ years	Global	-1	3	Education (years per capita)	199
Other exposure to mechanical forces	Male	0-6 days	95+ years	Global	-1	3	Socio-demographic Index	332
Other exposure to mechanical forces	Male	0-6 days	95+ years	Global	-1	3	LDI (I$ per capita)	--
Adverse effects of medical treatment	Female	0-6 days	95+ years	Data Rich	-1	1	Education (years per capita)	790
Adverse effects of medical treatment	Female	0-6 days	95+ years	Data Rich	1	1	Population-weighted mean temperature	210
Adverse effects of medical treatment	Female	0-6 days	95+ years	Data Rich	-1	2	Healthcare access and quality index	--
Adverse effects of medical treatment	Female	0-6 days	95+ years	Data Rich	-1	3	Socio-demographic Index	475
Adverse effects of medical treatment	Female	0-6 days	95+ years	Data Rich	1	3	LDI (I$ per capita)	--
Adverse effects of medical treatment	Female	0-6 days	95+ years	Global	-1	1	Education (years per capita)	592
Adverse effects of medical treatment	Female	0-6 days	95+ years	Global	1	1	Population-weighted mean temperature	541
Adverse effects of medical treatment	Female	0-6 days	95+ years	Global	-1	2	Healthcare access and quality index	199
Adverse effects of medical treatment	Female	0-6 days	95+ years	Global	-1	3	Socio-demographic Index	322
Adverse effects of medical treatment	Female	0-6 days	95+ years	Global	1	3	LDI (I$ per capita)	--
Adverse effects of medical treatment	Male	0-6 days	95+ years	Data Rich	-1	1	Education (years per capita)	1000
Adverse effects of medical treatment	Male	0-6 days	95+ years	Data Rich	1	1	Population-weighted mean temperature	187
Adverse effects of medical treatment	Male	0-6 days	95+ years	Data Rich	-1	2	Healthcare access and quality index	2
Adverse effects of medical treatment	Male	0-6 days	95+ years	Data Rich	-1	3	Socio-demographic Index	13
Adverse effects of medical treatment	Male	0-6 days	95+ years	Data Rich	1	3	LDI (I$ per capita)	--
Adverse effects of medical treatment	Male	0-6 days	95+ years	Global	-1	1	Education (years per capita)	572
Adverse effects of medical treatment	Male	0-6 days	95+ years	Global	1	1	Population-weighted mean temperature	780
Adverse effects of medical treatment	Male	0-6 days	95+ years	Global	1	2	Healthcare access and quality index	--
Adverse effects of medical treatment	Male	0-6 days	95+ years	Global	-1	3	Socio-demographic Index	417
Adverse effects of medical treatment	Male	0-6 days	95+ years	Global	1	3	LDI (I$ per capita)	--
Animal contact	Female	0-6 days	95+ years	Data Rich	1	1	Population-weighted mean temperature	1000
Animal contact	Female	0-6 days	95+ years	Data Rich	1	1	Liters of alcohol consumed per capita	--
Animal contact	Female	0-6 days	95+ years	Data Rich	1	1	Log-transformed SEV scalar: Animal	--
Animal contact	Female	0-6 days	95+ years	Data Rich	-1	2	Healthcare access and quality index	--
Animal contact	Female	0-6 days	95+ years	Data Rich	1	2	Population 15 to 30 (proportion)	50
Animal contact	Female	0-6 days	95+ years	Data Rich	-1	3	Population Density (over 1000 ppl/sqkm, proportion)	3
Animal contact	Female	0-6 days	95+ years	Data Rich	-1	3	Education (years per capita)	93
Animal contact	Female	0-6 days	95+ years	Data Rich	-1	3	Elevation Over 1500m (proportion)	--
Animal contact	Female	0-6 days	95+ years	Data Rich	-1	3	LDI (I$ per capita)	--
Animal contact	Female	0-6 days	95+ years	Data Rich	-1	3	Socio-demographic Index	--
Animal contact	Female	0-6 days	95+ years	Data Rich	1	3	Population Density (under 150 ppl/sqkm, proportion)	552
Animal contact	Female	0-6 days	95+ years	Data Rich	1	3	Elevation Under 100m (proportion)	--
Animal contact	Female	0-6 days	95+ years	Global	1	1	Population-weighted mean temperature	1000
Animal contact	Female	0-6 days	95+ years	Global	1	1	Liters of alcohol consumed per capita	--
Animal contact	Female	0-6 days	95+ years	Global	1	1	Log-transformed SEV scalar: Animal	--
Animal contact	Female	0-6 days	95+ years	Global	-1	2	Healthcare access and quality index	331
Animal contact	Female	0-6 days	95+ years	Global	1	2	Population 15 to 30 (proportion)	66
Animal contact	Female	0-6 days	95+ years	Global	-1	3	Education (years per capita)	230
Animal contact	Female	0-6 days	95+ years	Global	-1	3	Socio-demographic Index	368
Animal contact	Female	0-6 days	95+ years	Global	-1	3	Population Density (over 1000 ppl/sqkm, proportion)	618
Animal contact	Female	0-6 days	95+ years	Global	-1	3	Elevation Over 1500m (proportion)	--
Animal contact	Female	0-6 days	95+ years	Global	-1	3	LDI (I$ per capita)	--
Animal contact	Female	0-6 days	95+ years	Global	1	3	Population Density (under 150 ppl/sqkm, proportion)	229
Animal contact	Female	0-6 days	95+ years	Global	1	3	Elevation Under 100m (proportion)	--
Animal contact	Male	0-6 days	95+ years	Data Rich	1	1	Population-weighted mean temperature	180
Animal contact	Male	0-6 days	95+ years	Data Rich	1	1	Liters of alcohol consumed per capita	--
Animal contact	Male	0-6 days	95+ years	Data Rich	1	1	Log-transformed SEV scalar: Animal	--
Animal contact	Male	0-6 days	95+ years	Data Rich	-1	2	Healthcare access and quality index	42
Animal contact	Male	0-6 days	95+ years	Data Rich	1	2	Population 15 to 30 (proportion)	27
Animal contact	Male	0-6 days	95+ years	Data Rich	-1	3	Socio-demographic Index	150
Animal contact	Male	0-6 days	95+ years	Data Rich	-1	3	Education (years per capita)	255
Animal contact	Male	0-6 days	95+ years	Data Rich	-1	3	Population Density (over 1000 ppl/sqkm, proportion)	459
Animal contact	Male	0-6 days	95+ years	Data Rich	-1	3	Elevation Over 1500m (proportion)	--
Animal contact	Male	0-6 days	95+ years	Data Rich	-1	3	LDI (I$ per capita)	--
Animal contact	Male	0-6 days	95+ years	Data Rich	1	3	Population Density (under 150 ppl/sqkm, proportion)	392
Animal contact	Male	0-6 days	95+ years	Data Rich	1	3	Elevation Under 100m (proportion)	--
Animal contact	Male	0-6 days	95+ years	Global	1	1	Population-weighted mean temperature	275
Animal contact	Male	0-6 days	95+ years	Global	1	1	Log-transformed SEV scalar: Animal	760
Animal contact	Male	0-6 days	95+ years	Global	1	1	Liters of alcohol consumed per capita	--
Animal contact	Male	0-6 days	95+ years	Global	-1	2	Healthcare access and quality index	293
Animal contact	Male	0-6 days	95+ years	Global	1	2	Population 15 to 30 (proportion)	185
Animal contact	Male	0-6 days	95+ years	Global	-1	3	Education (years per capita)	92
Animal contact	Male	0-6 days	95+ years	Global	-1	3	Socio-demographic Index	94
Animal contact	Male	0-6 days	95+ years	Global	-1	3	Population Density (over 1000 ppl/sqkm, proportion)	540
Animal contact	Male	0-6 days	95+ years	Global	-1	3	Elevation Over 1500m (proportion)	--
Animal contact	Male	0-6 days	95+ years	Global	-1	3	LDI (I$ per capita)	--
Animal contact	Male	0-6 days	95+ years	Global	1	3	Population Density (under 150 ppl/sqkm, proportion)	379
Animal contact	Male	0-6 days	95+ years	Global	1	3	Elevation Under 100m (proportion)	--
Venomous animal contact	Female	0-6 days	95+ years	Data Rich	1	1	Proportion of population vulnerable to venomous snakebites	33
Venomous animal contact	Female	0-6 days	95+ years	Data Rich	1	1	Mean number of venomous snake species	967
Venomous animal contact	Female	0-6 days	95+ years	Data Rich	1	1	Log-transformed SEV scalar: Venom	--
Venomous animal contact	Female	0-6 days	95+ years	Data Rich	-1	2	Absolute value of average latitude	0
Venomous animal contact	Female	0-6 days	95+ years	Data Rich	-1	2	Healthcare access and quality index	0
Venomous animal contact	Female	0-6 days	95+ years	Data Rich	-1	2	Urbanicity	967
Venomous animal contact	Female	0-6 days	95+ years	Data Rich	1	2	Population-weighted mean temperature	0
Venomous animal contact	Female	0-6 days	95+ years	Data Rich	1	2	Proportion of population involved in agricultural activities	0
Venomous animal contact	Female	0-6 days	95+ years	Data Rich	1	2	Rainfall Population-Weighted (mm/yr)	0
Venomous animal contact	Female	0-6 days	95+ years	Data Rich	1	2	Liters of alcohol consumed per capita	--
Venomous animal contact	Female	0-6 days	95+ years	Data Rich	1	2	Sahel Region of Africa (binary)	--
Venomous animal contact	Female	0-6 days	95+ years	Data Rich	-1	3	Elevation Under 100m (proportion)	0

CODEm covariates used, level of covariate, and expected direction of covariate by cause, sex, and age

Cause	Sex	Age Start	Age End	Model Version Type	Direction	Level	Covariate Name	Number of Draws
Venomous animal contact	Female	0-6 days	95+ years	Data Rich	-1	3	Socio-demographic Index	0
Venomous animal contact	Female	0-6 days	95+ years	Data Rich	-1	3	Elevation Over 1500m (proportion)	38
Venomous animal contact	Female	0-6 days	95+ years	Data Rich	-1	3	Population Density (over 1000 ppl/sqkm, proportion)	834
Venomous animal contact	Female	0-6 days	95+ years	Data Rich	-1	3	Education (years per capita)	967
Venomous animal contact	Female	0-6 days	95+ years	Data Rich	-1	3	LDI (I$ per capita)	--
Venomous animal contact	Female	0-6 days	95+ years	Data Rich	1	3	Population Density (under 150 ppl/sqkm, proportion)	160
Venomous animal contact	Female	0-6 days	95+ years	Global	1	1	Log-transformed SEV scalar: Venom	327
Venomous animal contact	Female	0-6 days	95+ years	Global	1	1	Mean number of venomous snake species	542
Venomous animal contact	Female	0-6 days	95+ years	Global	1	1	Proportion of population vulnerable to venomous snakebites	799
Venomous animal contact	Female	0-6 days	95+ years	Global	-1	2	Absolute value of average latitude	186
Venomous animal contact	Female	0-6 days	95+ years	Global	-1	2	Healthcare access and quality index	538
Venomous animal contact	Female	0-6 days	95+ years	Global	-1	2	Urbanicity	658
Venomous animal contact	Female	0-6 days	95+ years	Global	1	2	Rainfall Population-Weighted (mm/yr)	347
Venomous animal contact	Female	0-6 days	95+ years	Global	1	2	Proportion of population involved in agricultural activities	370
Venomous animal contact	Female	0-6 days	95+ years	Global	1	2	Population-weighted mean temperature	579
Venomous animal contact	Female	0-6 days	95+ years	Global	1	2	Liters of alcohol consumed per capita	--
Venomous animal contact	Female	0-6 days	95+ years	Global	1	2	Sahel Region of Africa (binary)	--
Venomous animal contact	Female	0-6 days	95+ years	Global	-1	3	Socio-demographic Index	158
Venomous animal contact	Female	0-6 days	95+ years	Global	-1	3	Population Density (over 1000 ppl/sqkm, proportion)	161
Venomous animal contact	Female	0-6 days	95+ years	Global	-1	3	Elevation Over 1500m (proportion)	199
Venomous animal contact	Female	0-6 days	95+ years	Global	-1	3	Education (years per capita)	266
Venomous animal contact	Female	0-6 days	95+ years	Global	-1	3	Elevation Under 100m (proportion)	325
Venomous animal contact	Female	0-6 days	95+ years	Global	-1	3	LDI (I$ per capita)	--
Venomous animal contact	Female	0-6 days	95+ years	Global	1	3	Population Density (under 150 ppl/sqkm, proportion)	236
Venomous animal contact	Male	0-6 days	95+ years	Data Rich	1	1	Proportion of population vulnerable to venomous snakebites	923
Venomous animal contact	Male	0-6 days	95+ years	Data Rich	1	1	Log-transformed SEV scalar: Venom	--
Venomous animal contact	Male	0-6 days	95+ years	Data Rich	1	1	Mean number of venomous snake species	--
Venomous animal contact	Male	0-6 days	95+ years	Data Rich	-1	2	Healthcare access and quality index	5
Venomous animal contact	Male	0-6 days	95+ years	Data Rich	-1	2	Urbanicity	29
Venomous animal contact	Male	0-6 days	95+ years	Data Rich	-1	2	Absolute value of average latitude	218
Venomous animal contact	Male	0-6 days	95+ years	Data Rich	1	2	Proportion of population involved in agricultural activities	32
Venomous animal contact	Male	0-6 days	95+ years	Data Rich	1	2	Liters of alcohol consumed per capita	--
Venomous animal contact	Male	0-6 days	95+ years	Data Rich	1	2	Population-weighted mean temperature	--
Venomous animal contact	Male	0-6 days	95+ years	Data Rich	1	2	Rainfall Population-Weighted (mm/yr)	--
Venomous animal contact	Male	0-6 days	95+ years	Data Rich	1	2	Sahel Region of Africa (binary)	--
Venomous animal contact	Male	0-6 days	95+ years	Data Rich	-1	3	Elevation Over 1500m (proportion)	4
Venomous animal contact	Male	0-6 days	95+ years	Data Rich	-1	3	Socio-demographic Index	7
Venomous animal contact	Male	0-6 days	95+ years	Data Rich	-1	3	Education (years per capita)	273
Venomous animal contact	Male	0-6 days	95+ years	Data Rich	-1	3	Elevation Under 100m (proportion)	499
Venomous animal contact	Male	0-6 days	95+ years	Data Rich	-1	3	Population Density (over 1000 ppl/sqkm, proportion)	718
Venomous animal contact	Male	0-6 days	95+ years	Data Rich	-1	3	LDI (I$ per capita)	--
Venomous animal contact	Male	0-6 days	95+ years	Data Rich	1	3	Population Density (under 150 ppl/sqkm, proportion)	195
Venomous animal contact	Male	0-6 days	95+ years	Global	1	1	Log-transformed SEV scalar: Venom	234
Venomous animal contact	Male	0-6 days	95+ years	Global	1	1	Mean number of venomous snake species	607
Venomous animal contact	Male	0-6 days	95+ years	Global	1	1	Proportion of population vulnerable to venomous snakebites	952
Venomous animal contact	Male	0-6 days	95+ years	Global	-1	2	Absolute value of average latitude	45
Venomous animal contact	Male	0-6 days	95+ years	Global	-1	2	Urbanicity	334
Venomous animal contact	Male	0-6 days	95+ years	Global	-1	2	Healthcare access and quality index	367
Venomous animal contact	Male	0-6 days	95+ years	Global	1	2	Population-weighted mean temperature	329
Venomous animal contact	Male	0-6 days	95+ years	Global	1	2	Rainfall Population-Weighted (mm/yr)	370
Venomous animal contact	Male	0-6 days	95+ years	Global	1	2	Proportion of population involved in agricultural activities	377
Venomous animal contact	Male	0-6 days	95+ years	Global	1	2	Liters of alcohol consumed per capita	--
Venomous animal contact	Male	0-6 days	95+ years	Global	-1	3	Socio-demographic Index	145
Venomous animal contact	Male	0-6 days	95+ years	Global	-1	3	Elevation Over 1500m (proportion)	230
Venomous animal contact	Male	0-6 days	95+ years	Global	-1	3	Education (years per capita)	231
Venomous animal contact	Male	0-6 days	95+ years	Global	-1	3	Elevation Under 100m (proportion)	232
Venomous animal contact	Male	0-6 days	95+ years	Global	-1	3	Population Density (over 1000 ppl/sqkm, proportion)	354
Venomous animal contact	Male	0-6 days	95+ years	Global	-1	3	LDI (I$ per capita)	--
Venomous animal contact	Male	0-6 days	95+ years	Global	1	3	Population Density (under 150 ppl/sqkm, proportion)	21
Non-venomous animal contact	Female	0-6 days	95+ years	Data Rich	1	1	Liters of alcohol consumed per capita	--
Non-venomous animal contact	Female	0-6 days	95+ years	Data Rich	1	1	Log-transformed SEV scalar: Non Ven	--
Non-venomous animal contact	Female	0-6 days	95+ years	Data Rich	1	1	Population-weighted mean temperature	--
Non-venomous animal contact	Female	0-6 days	95+ years	Data Rich	-1	2	Healthcare access and quality index	38
Non-venomous animal contact	Female	0-6 days	95+ years	Data Rich	-1	3	Socio-demographic Index	160
Non-venomous animal contact	Female	0-6 days	95+ years	Data Rich	-1	3	Population Density (over 1000 ppl/sqkm, proportion)	193
Non-venomous animal contact	Female	0-6 days	95+ years	Data Rich	-1	3	Education (years per capita)	--
Non-venomous animal contact	Female	0-6 days	95+ years	Data Rich	-1	3	Elevation Over 1500m (proportion)	--
Non-venomous animal contact	Female	0-6 days	95+ years	Data Rich	-1	3	LDI (I$ per capita)	--
Non-venomous animal contact	Female	0-6 days	95+ years	Data Rich	1	3	Population Density (under 150 ppl/sqkm, proportion)	961
Non-venomous animal contact	Female	0-6 days	95+ years	Data Rich	1	3	Elevation Under 100m (proportion)	--
Non-venomous animal contact	Female	0-6 days	95+ years	Global	1	1	Log-transformed SEV scalar: Non Ven	816
Non-venomous animal contact	Female	0-6 days	95+ years	Global	1	1	Liters of alcohol consumed per capita	--
Non-venomous animal contact	Female	0-6 days	95+ years	Global	1	1	Population-weighted mean temperature	--
Non-venomous animal contact	Female	0-6 days	95+ years	Global	-1	2	Healthcare access and quality index	317
Non-venomous animal contact	Female	0-6 days	95+ years	Global	-1	3	Education (years per capita)	240
Non-venomous animal contact	Female	0-6 days	95+ years	Global	-1	3	Socio-demographic Index	317
Non-venomous animal contact	Female	0-6 days	95+ years	Global	-1	3	Population Density (over 1000 ppl/sqkm, proportion)	705
Non-venomous animal contact	Female	0-6 days	95+ years	Global	-1	3	Elevation Over 1500m (proportion)	--
Non-venomous animal contact	Female	0-6 days	95+ years	Global	-1	3	LDI (I$ per capita)	--
Non-venomous animal contact	Female	0-6 days	95+ years	Global	1	3	Population Density (under 150 ppl/sqkm, proportion)	782
Non-venomous animal contact	Female	0-6 days	95+ years	Global	1	3	Elevation Under 100m (proportion)	--
Non-venomous animal contact	Male	0-6 days	95+ years	Data Rich	1	1	Liters of alcohol consumed per capita	--
Non-venomous animal contact	Male	0-6 days	95+ years	Data Rich	1	1	Log-transformed SEV scalar: Non Ven	--
Non-venomous animal contact	Male	0-6 days	95+ years	Data Rich	1	1	Population-weighted mean temperature	--
Non-venomous animal contact	Male	0-6 days	95+ years	Data Rich	-1	2	Healthcare access and quality index	135
Non-venomous animal contact	Male	0-6 days	95+ years	Data Rich	-1	3	Socio-demographic Index	226
Non-venomous animal contact	Male	0-6 days	95+ years	Data Rich	-1	3	Population Density (over 1000 ppl/sqkm, proportion)	393
Non-venomous animal contact	Male	0-6 days	95+ years	Data Rich	-1	3	Education (years per capita)	--
Non-venomous animal contact	Male	0-6 days	95+ years	Data Rich	-1	3	Elevation Over 1500m (proportion)	--
Non-venomous animal contact	Male	0-6 days	95+ years	Data Rich	-1	3	LDI (I$ per capita)	--
Non-venomous animal contact	Male	0-6 days	95+ years	Data Rich	1	3	Population Density (under 150 ppl/sqkm, proportion)	514
Non-venomous animal contact	Male	0-6 days	95+ years	Data Rich	1	3	Elevation Under 100m (proportion)	--
Non-venomous animal contact	Male	0-6 days	95+ years	Global	-1	1	Population Density (over 1000 ppl/sqkm, proportion)	638
Non-venomous animal contact	Male	0-6 days	95+ years	Global	-1	1	Elevation Over 1500m (proportion)	--
Non-venomous animal contact	Male	0-6 days	95+ years	Global	1	1	Population-weighted mean temperature	378
Non-venomous animal contact	Male	0-6 days	95+ years	Global	1	1	Log-transformed SEV scalar: Non Ven	618
Non-venomous animal contact	Male	0-6 days	95+ years	Global	1	1	Population Density (under 150 ppl/sqkm, proportion)	956
Non-venomous animal contact	Male	0-6 days	95+ years	Global	1	1	Elevation Under 100m (proportion)	--
Non-venomous animal contact	Male	0-6 days	95+ years	Global	1	1	Liters of alcohol consumed per capita	--
Non-venomous animal contact	Male	0-6 days	95+ years	Global	-1	3	Socio-demographic Index	64
Non-venomous animal contact	Male	0-6 days	95+ years	Global	-1	3	Healthcare access and quality index	114
Non-venomous animal contact	Male	0-6 days	95+ years	Global	-1	3	Education (years per capita)	115
Non-venomous animal contact	Male	0-6 days	95+ years	Global	-1	3	LDI (I$ per capita)	--
Foreign body	Female	0-6 days	95+ years	Data Rich	1	1	LDI (I$ per capita)	140
Foreign body	Female	0-6 days	95+ years	Data Rich	1	1	Education (years per capita)	359
Foreign body	Female	0-6 days	95+ years	Data Rich	1	1	Liters of alcohol consumed per capita	618
Foreign body	Female	0-6 days	95+ years	Data Rich	1	1	Population Over 65 (proportion)	977
Foreign body	Female	0-6 days	95+ years	Data Rich	1	1	Indoor Air Pollution (All Cooking Fuels)	--
Foreign body	Female	0-6 days	95+ years	Data Rich	1	1	Population-weighted mean temperature	--
Foreign body	Female	0-6 days	95+ years	Data Rich	-1	2	Healthcare access and quality index	0
Foreign body	Female	0-6 days	95+ years	Data Rich	-1	3	Socio-demographic Index	0
Foreign body	Female	0-6 days	95+ years	Global	1	1	Indoor Air Pollution (All Cooking Fuels)	55
Foreign body	Female	0-6 days	95+ years	Global	1	1	Liters of alcohol consumed per capita	166
Foreign body	Female	0-6 days	95+ years	Global	1	1	Population-weighted mean temperature	407

CODEm covariates used, level of covariate, and expected direction of covariate by cause, sex, and age

Cause	Sex	Age Start	Age End	Model Version Type	Direction	Level	Covariate Name	Number of Draws
Foreign body	Female	0-6 days	95+ years	Global	1	1	Population Over 65 (proportion)	483
Foreign body	Female	0-6 days	95+ years	Global	1	1	LDI (I$ per capita)	504
Foreign body	Female	0-6 days	95+ years	Global	1	1	Education (years per capita)	595
Foreign body	Female	0-6 days	95+ years	Global	-1	2	Healthcare access and quality index	38
Foreign body	Female	0-6 days	95+ years	Global	-1	3	Socio-demographic Index	24
Foreign body	Male	0-6 days	95+ years	Data Rich	1	1	Indoor Air Pollution (All Cooking Fuels)	3
Foreign body	Male	0-6 days	95+ years	Data Rich	1	1	LDI (I$ per capita)	92
Foreign body	Male	0-6 days	95+ years	Data Rich	1	1	Liters of alcohol consumed per capita	616
Foreign body	Male	0-6 days	95+ years	Data Rich	1	1	Education (years per capita)	693
Foreign body	Male	0-6 days	95+ years	Data Rich	1	1	Population Over 65 (proportion)	824
Foreign body	Male	0-6 days	95+ years	Data Rich	1	1	Population-weighted mean temperature	--
Foreign body	Male	0-6 days	95+ years	Data Rich	-1	2	Healthcare access and quality index	8
Foreign body	Male	0-6 days	95+ years	Data Rich	-1	3	Socio-demographic Index	--
Foreign body	Male	0-6 days	95+ years	Global	1	1	Indoor Air Pollution (All Cooking Fuels)	177
Foreign body	Male	0-6 days	95+ years	Global	1	1	LDI (I$ per capita)	544
Foreign body	Male	0-6 days	95+ years	Global	1	1	Education (years per capita)	664
Foreign body	Male	0-6 days	95+ years	Global	1	1	Liters of alcohol consumed per capita	682
Foreign body	Male	0-6 days	95+ years	Global	1	1	Population Over 65 (proportion)	884
Foreign body	Male	0-6 days	95+ years	Global	1	1	Population-weighted mean temperature	--
Foreign body	Male	0-6 days	95+ years	Global	-1	2	Healthcare access and quality index	36
Foreign body	Male	0-6 days	95+ years	Global	-1	3	Socio-demographic Index	--
Pulmonary aspiration and foreign body in airway	Female	0-6 days	95+ years	Data Rich	-1	1	Education (years per capita)	--
Pulmonary aspiration and foreign body in airway	Female	0-6 days	95+ years	Data Rich	1	1	Liters of alcohol consumed per capita	1000
Pulmonary aspiration and foreign body in airway	Female	0-6 days	95+ years	Data Rich	1	1	Log-transformed SEV scalar: F Body Asp	--
Pulmonary aspiration and foreign body in airway	Female	0-6 days	95+ years	Data Rich	1	1	Population-weighted mean temperature	--
Pulmonary aspiration and foreign body in airway	Female	0-6 days	95+ years	Data Rich	-1	2	Healthcare access and quality index	102
Pulmonary aspiration and foreign body in airway	Female	0-6 days	95+ years	Data Rich	1	2	Mean BMI	102
Pulmonary aspiration and foreign body in airway	Female	0-6 days	95+ years	Data Rich	-1	3	LDI (I$ per capita)	--
Pulmonary aspiration and foreign body in airway	Female	0-6 days	95+ years	Data Rich	-1	3	Socio-demographic Index	--
Pulmonary aspiration and foreign body in airway	Female	0-6 days	95+ years	Global	1	1	Liters of alcohol consumed per capita	393
Pulmonary aspiration and foreign body in airway	Female	0-6 days	95+ years	Global	1	1	Population-weighted mean temperature	867
Pulmonary aspiration and foreign body in airway	Female	0-6 days	95+ years	Global	1	1	Log-transformed SEV scalar: F Body Asp	--
Pulmonary aspiration and foreign body in airway	Female	0-6 days	95+ years	Global	-1	2	Healthcare access and quality index	79
Pulmonary aspiration and foreign body in airway	Female	0-6 days	95+ years	Global	1	2	Alcohol binge drinker proportion, age-standardized	207
Pulmonary aspiration and foreign body in airway	Female	0-6 days	95+ years	Global	1	2	Mean BMI	372
Pulmonary aspiration and foreign body in airway	Female	0-6 days	95+ years	Global	-1	3	Socio-demographic Index	39
Pulmonary aspiration and foreign body in airway	Female	0-6 days	95+ years	Global	-1	3	Education (years per capita)	48
Pulmonary aspiration and foreign body in airway	Female	0-6 days	95+ years	Global	-1	3	LDI (I$ per capita)	--
Pulmonary aspiration and foreign body in airway	Male	0-6 days	95+ years	Data Rich	-1	1	Education (years per capita)	63
Pulmonary aspiration and foreign body in airway	Male	0-6 days	95+ years	Data Rich	1	1	Liters of alcohol consumed per capita	1000
Pulmonary aspiration and foreign body in airway	Male	0-6 days	95+ years	Data Rich	1	1	Log-transformed SEV scalar: F Body Asp	--
Pulmonary aspiration and foreign body in airway	Male	0-6 days	95+ years	Data Rich	1	1	Population-weighted mean temperature	--
Pulmonary aspiration and foreign body in airway	Male	0-6 days	95+ years	Data Rich	-1	2	Healthcare access and quality index	15
Pulmonary aspiration and foreign body in airway	Male	0-6 days	95+ years	Data Rich	1	2	Mean BMI	527
Pulmonary aspiration and foreign body in airway	Male	0-6 days	95+ years	Data Rich	-1	3	Socio-demographic Index	31
Pulmonary aspiration and foreign body in airway	Male	0-6 days	95+ years	Data Rich	-1	3	LDI (I$ per capita)	--
Pulmonary aspiration and foreign body in airway	Male	0-6 days	95+ years	Global	-1	1	Education (years per capita)	537
Pulmonary aspiration and foreign body in airway	Male	0-6 days	95+ years	Global	1	1	Liters of alcohol consumed per capita	780
Pulmonary aspiration and foreign body in airway	Male	0-6 days	95+ years	Global	1	1	Log-transformed SEV scalar: F Body Asp	--
Pulmonary aspiration and foreign body in airway	Male	0-6 days	95+ years	Global	1	1	Population-weighted mean temperature	--
Pulmonary aspiration and foreign body in airway	Male	0-6 days	95+ years	Global	-1	2	Healthcare access and quality index	173
Pulmonary aspiration and foreign body in airway	Male	0-6 days	95+ years	Global	1	2	Mean BMI	528
Pulmonary aspiration and foreign body in airway	Male	0-6 days	95+ years	Global	-1	3	LDI (I$ per capita)	--
Pulmonary aspiration and foreign body in airway	Male	0-6 days	95+ years	Global	-1	3	Socio-demographic Index	--
Foreign body in other body part	Female	0-6 days	95+ years	Data Rich	1	1	Liters of alcohol consumed per capita	--
Foreign body in other body part	Female	0-6 days	95+ years	Data Rich	1	1	Log-transformed SEV scalar: Oth F Body	--
Foreign body in other body part	Female	0-6 days	95+ years	Data Rich	1	1	Population-weighted mean temperature	--
Foreign body in other body part	Female	0-6 days	95+ years	Data Rich	-1	2	Healthcare access and quality index	--
Foreign body in other body part	Female	0-6 days	95+ years	Data Rich	-1	3	Socio-demographic Index	1000
Foreign body in other body part	Female	0-6 days	95+ years	Data Rich	-1	3	Education (years per capita)	--
Foreign body in other body part	Female	0-6 days	95+ years	Data Rich	-1	3	LDI (I$ per capita)	--
Foreign body in other body part	Female	0-6 days	95+ years	Global	1	1	Log-transformed SEV scalar: Oth F Body	392
Foreign body in other body part	Female	0-6 days	95+ years	Global	1	1	Population-weighted mean temperature	837
Foreign body in other body part	Female	0-6 days	95+ years	Global	1	1	Liters of alcohol consumed per capita	--
Foreign body in other body part	Female	0-6 days	95+ years	Global	-1	2	Healthcare access and quality index	196
Foreign body in other body part	Female	0-6 days	95+ years	Global	-1	3	Socio-demographic Index	176
Foreign body in other body part	Female	0-6 days	95+ years	Global	-1	3	Education (years per capita)	201
Foreign body in other body part	Female	0-6 days	95+ years	Global	-1	3	LDI (I$ per capita)	--
Foreign body in other body part	Male	0-6 days	95+ years	Data Rich	1	1	Liters of alcohol consumed per capita	801
Foreign body in other body part	Male	0-6 days	95+ years	Data Rich	1	1	Population-weighted mean temperature	968
Foreign body in other body part	Male	0-6 days	95+ years	Data Rich	1	1	Log-transformed SEV scalar: Oth F Body	993
Foreign body in other body part	Male	0-6 days	95+ years	Data Rich	-1	2	Healthcare access and quality index	--
Foreign body in other body part	Male	0-6 days	95+ years	Data Rich	-1	3	Socio-demographic Index	6
Foreign body in other body part	Male	0-6 days	95+ years	Data Rich	-1	3	Education (years per capita)	--
Foreign body in other body part	Male	0-6 days	95+ years	Data Rich	-1	3	LDI (I$ per capita)	--
Foreign body in other body part	Male	0-6 days	95+ years	Global	1	1	Log-transformed SEV scalar: Oth F Body	739
Foreign body in other body part	Male	0-6 days	95+ years	Global	1	1	Population-weighted mean temperature	1000
Foreign body in other body part	Male	0-6 days	95+ years	Global	1	1	Liters of alcohol consumed per capita	--
Foreign body in other body part	Male	0-6 days	95+ years	Global	-1	2	Healthcare access and quality index	150
Foreign body in other body part	Male	0-6 days	95+ years	Global	-1	3	Education (years per capita)	103
Foreign body in other body part	Male	0-6 days	95+ years	Global	-1	3	Socio-demographic Index	124
Foreign body in other body part	Male	0-6 days	95+ years	Global	-1	3	LDI (I$ per capita)	--
Electrocution	Female	0-6 days	95+ years	Data Rich	1	1	Log-transformed SEV scalar: Oth Unint	--
Electrocution	Female	0-6 days	95+ years	Data Rich	1	1	Vehicles - 2 wheels (per capita)	--
Electrocution	Female	0-6 days	95+ years	Data Rich	1	1	Vehicles - 4 wheels (per capita)	--
Electrocution	Female	0-6 days	95+ years	Data Rich	-1	2	Healthcare access and quality index	538
Electrocution	Female	0-6 days	95+ years	Data Rich	-1	3	Education (years per capita)	92
Electrocution	Female	0-6 days	95+ years	Data Rich	-1	3	Socio-demographic Index	133
Electrocution	Female	0-6 days	95+ years	Data Rich	-1	3	Population Density (over 1000 ppl/sqkm, proportion)	404
Electrocution	Female	0-6 days	95+ years	Data Rich	-1	3	LDI (I$ per capita)	--
Electrocution	Female	0-6 days	95+ years	Data Rich	1	3	Population Density (under 150 ppl/sqkm, proportion)	346
Electrocution	Female	0-6 days	95+ years	Global	1	1	Log-transformed SEV scalar: Oth Unint	--
Electrocution	Female	0-6 days	95+ years	Global	1	1	Vehicles - 2 wheels (per capita)	--
Electrocution	Female	0-6 days	95+ years	Global	1	1	Vehicles - 4 wheels (per capita)	--
Electrocution	Female	0-6 days	95+ years	Global	-1	2	Healthcare access and quality index	455
Electrocution	Female	0-6 days	95+ years	Global	-1	3	Education (years per capita)	187
Electrocution	Female	0-6 days	95+ years	Global	-1	3	Population Density (over 1000 ppl/sqkm, proportion)	205
Electrocution	Female	0-6 days	95+ years	Global	-1	3	Socio-demographic Index	304
Electrocution	Female	0-6 days	95+ years	Global	-1	3	LDI (I$ per capita)	--
Electrocution	Female	0-6 days	95+ years	Global	1	3	Population Density (under 150 ppl/sqkm, proportion)	86
Electrocution	Male	0-6 days	95+ years	Data Rich	1	1	Log-transformed SEV scalar: Oth Unint	--
Electrocution	Male	0-6 days	95+ years	Data Rich	1	1	Vehicles - 2 wheels (per capita)	--
Electrocution	Male	0-6 days	95+ years	Data Rich	1	1	Vehicles - 4 wheels (per capita)	--
Electrocution	Male	0-6 days	95+ years	Data Rich	-1	2	Healthcare access and quality index	598
Electrocution	Male	0-6 days	95+ years	Data Rich	-1	3	Socio-demographic Index	112
Electrocution	Male	0-6 days	95+ years	Data Rich	-1	3	Population Density (over 1000 ppl/sqkm, proportion)	283
Electrocution	Male	0-6 days	95+ years	Data Rich	-1	3	Education (years per capita)	332
Electrocution	Male	0-6 days	95+ years	Data Rich	-1	3	LDI (I$ per capita)	--
Electrocution	Male	0-6 days	95+ years	Data Rich	1	3	Population Density (under 150 ppl/sqkm, proportion)	336
Electrocution	Male	0-6 days	95+ years	Global	1	1	Log-transformed SEV scalar: Oth Unint	--
Electrocution	Male	0-6 days	95+ years	Global	1	1	Vehicles - 2 wheels (per capita)	--
Electrocution	Male	0-6 days	95+ years	Global	1	1	Vehicles - 4 wheels (per capita)	--
Electrocution	Male	0-6 days	95+ years	Global	-1	2	Healthcare access and quality index	--
Electrocution	Male	0-6 days	95+ years	Global	-1	3	Education (years per capita)	280
Electrocution	Male	0-6 days	95+ years	Global	-1	3	Population Density (over 1000 ppl/sqkm, proportion)	421
Electrocution	Male	0-6 days	95+ years	Global	-1	3	Socio-demographic Index	454
Electrocution	Male	0-6 days	95+ years	Global	-1	3	LDI (I$ per capita)	--
Electrocution	Male	0-6 days	95+ years	Global	1	3	Population Density (under 150 ppl/sqkm, proportion)	100
Environmental heat and cold exposure	Female	0-6 days	95+ years	Data Rich	-1	2	Healthcare access and quality index	193
Environmental heat and cold exposure	Female	0-6 days	95+ years	Data Rich	-1	3	Education (years per capita)	--

CODEm covariates used, level of covariate, and expected direction of covariate by cause, sex, and age								
**Cause**	**Sex**	**Age Start**	**Age End**	**Model Version Type**	**Direction**	**Level**	**Covariate Name**	**Number of Draws**
Environmental heat and cold exposure	Female	0-6 days	95+ years	Data Rich	-1	3	LDI (I$ per capita)	--
Environmental heat and cold exposure	Female	0-6 days	95+ years	Data Rich	-1	3	Population Density (150-300 ppl/sqkm, proportion)	--
Environmental heat and cold exposure	Female	0-6 days	95+ years	Data Rich	-1	3	Sanitation (proportion with access)	--
Environmental heat and cold exposure	Female	0-6 days	95+ years	Data Rich	-1	3	Socio-demographic Index	--
Environmental heat and cold exposure	Female	0-6 days	95+ years	Data Rich	1	3	Elevation Over 1500m (proportion)	839
Environmental heat and cold exposure	Female	0-6 days	95+ years	Data Rich	1	3	90th percentile climatic temperature in the given country-year.	--
Environmental heat and cold exposure	Female	0-6 days	95+ years	Data Rich	1	3	Elevation 500 to 1500m (proportion)	--
Environmental heat and cold exposure	Female	0-6 days	95+ years	Data Rich	1	3	Population-weighted mean temperature	--
Environmental heat and cold exposure	Female	0-6 days	95+ years	Data Rich	1	3	Rainfall (Quintiles 4-5)	--
Environmental heat and cold exposure	Female	0-6 days	95+ years	Global	-1	2	Healthcare access and quality index	1000
Environmental heat and cold exposure	Female	0-6 days	95+ years	Global	-1	3	Education (years per capita)	100
Environmental heat and cold exposure	Female	0-6 days	95+ years	Global	-1	3	LDI (I$ per capita)	120
Environmental heat and cold exposure	Female	0-6 days	95+ years	Global	-1	3	Population Density (150-300 ppl/sqkm, proportion)	--
Environmental heat and cold exposure	Female	0-6 days	95+ years	Global	-1	3	Sanitation (proportion with access)	--
Environmental heat and cold exposure	Female	0-6 days	95+ years	Global	-1	3	Socio-demographic Index	--
Environmental heat and cold exposure	Female	0-6 days	95+ years	Global	1	3	90th percentile climatic temperature in the given country-year.	173
Environmental heat and cold exposure	Female	0-6 days	95+ years	Global	1	3	Elevation 500 to 1500m (proportion)	208
Environmental heat and cold exposure	Female	0-6 days	95+ years	Global	1	3	Population-weighted mean temperature	328
Environmental heat and cold exposure	Female	0-6 days	95+ years	Global	1	3	Elevation Over 1500m (proportion)	--
Environmental heat and cold exposure	Female	0-6 days	95+ years	Global	1	3	Rainfall (Quintiles 4-5)	--
Environmental heat and cold exposure	Male	0-6 days	95+ years	Data Rich	-1	2	Healthcare access and quality index	941
Environmental heat and cold exposure	Male	0-6 days	95+ years	Data Rich	-1	3	Sanitation (proportion with access)	59
Environmental heat and cold exposure	Male	0-6 days	95+ years	Data Rich	-1	3	Socio-demographic Index	59
Environmental heat and cold exposure	Male	0-6 days	95+ years	Data Rich	-1	3	Education (years per capita)	--
Environmental heat and cold exposure	Male	0-6 days	95+ years	Data Rich	-1	3	LDI (I$ per capita)	--
Environmental heat and cold exposure	Male	0-6 days	95+ years	Data Rich	-1	3	Population Density (150-300 ppl/sqkm, proportion)	--
Environmental heat and cold exposure	Male	0-6 days	95+ years	Data Rich	1	3	90th percentile climatic temperature in the given country-year.	59
Environmental heat and cold exposure	Male	0-6 days	95+ years	Data Rich	1	3	Elevation 500 to 1500m (proportion)	59
Environmental heat and cold exposure	Male	0-6 days	95+ years	Data Rich	1	3	Elevation Over 1500m (proportion)	792
Environmental heat and cold exposure	Male	0-6 days	95+ years	Data Rich	1	3	Population-weighted mean temperature	--
Environmental heat and cold exposure	Male	0-6 days	95+ years	Data Rich	1	3	Rainfall (Quintiles 4-5)	--
Environmental heat and cold exposure	Male	0-6 days	95+ years	Global	-1	2	Healthcare access and quality index	593
Environmental heat and cold exposure	Male	0-6 days	95+ years	Global	-1	3	LDI (I$ per capita)	185
Environmental heat and cold exposure	Male	0-6 days	95+ years	Global	-1	3	Education (years per capita)	--
Environmental heat and cold exposure	Male	0-6 days	95+ years	Global	-1	3	Population Density (150-300 ppl/sqkm, proportion)	--
Environmental heat and cold exposure	Male	0-6 days	95+ years	Global	-1	3	Sanitation (proportion with access)	--
Environmental heat and cold exposure	Male	0-6 days	95+ years	Global	-1	3	Socio-demographic Index	--
Environmental heat and cold exposure	Male	0-6 days	95+ years	Global	1	3	90th percentile climatic temperature in the given country-year.	309
Environmental heat and cold exposure	Male	0-6 days	95+ years	Global	1	3	Elevation 500 to 1500m (proportion)	531
Environmental heat and cold exposure	Male	0-6 days	95+ years	Global	1	3	Elevation Over 1500m (proportion)	--
Environmental heat and cold exposure	Male	0-6 days	95+ years	Global	1	3	Population-weighted mean temperature	--
Environmental heat and cold exposure	Male	0-6 days	95+ years	Global	1	3	Rainfall (Quintiles 4-5)	--
Other unintentional injuries	Female	0-6 days	95+ years	Data Rich	1	1	Liters of alcohol consumed per capita	1000
Other unintentional injuries	Female	0-6 days	95+ years	Data Rich	1	1	Log-transformed SEV scalar: Oth Unint	--
Other unintentional injuries	Female	0-6 days	95+ years	Data Rich	1	1	Population-weighted mean temperature	--
Other unintentional injuries	Female	0-6 days	95+ years	Data Rich	1	1	Vehicles - 2 wheels (per capita)	--
Other unintentional injuries	Female	0-6 days	95+ years	Data Rich	1	1	Vehicles - 4 wheels (per capita)	--
Other unintentional injuries	Female	0-6 days	95+ years	Data Rich	-1	2	Healthcare access and quality index	1000
Other unintentional injuries	Female	0-6 days	95+ years	Data Rich	-1	3	Socio-demographic Index	7
Other unintentional injuries	Female	0-6 days	95+ years	Data Rich	-1	3	Population Density (over 1000 ppl/sqkm, proportion)	33
Other unintentional injuries	Female	0-6 days	95+ years	Data Rich	-1	3	Education (years per capita)	192
Other unintentional injuries	Female	0-6 days	95+ years	Data Rich	-1	3	LDI (I$ per capita)	--
Other unintentional injuries	Female	0-6 days	95+ years	Data Rich	1	3	Population Density (under 150 ppl/sqkm, proportion)	166
Other unintentional injuries	Female	0-6 days	95+ years	Global	1	1	Liters of alcohol consumed per capita	--
Other unintentional injuries	Female	0-6 days	95+ years	Global	1	1	Log-transformed SEV scalar: Oth Unint	--
Other unintentional injuries	Female	0-6 days	95+ years	Global	1	1	Population-weighted mean temperature	--
Other unintentional injuries	Female	0-6 days	95+ years	Global	1	1	Vehicles - 2 wheels (per capita)	--
Other unintentional injuries	Female	0-6 days	95+ years	Global	1	1	Vehicles - 4 wheels (per capita)	--
Other unintentional injuries	Female	0-6 days	95+ years	Global	-1	2	Healthcare access and quality index	564
Other unintentional injuries	Female	0-6 days	95+ years	Global	-1	3	Socio-demographic Index	488
Other unintentional injuries	Female	0-6 days	95+ years	Global	-1	3	Education (years per capita)	548
Other unintentional injuries	Female	0-6 days	95+ years	Global	-1	3	Population Density (over 1000 ppl/sqkm, proportion)	586
Other unintentional injuries	Female	0-6 days	95+ years	Global	-1	3	LDI (I$ per capita)	--
Other unintentional injuries	Female	0-6 days	95+ years	Global	1	3	Population Density (under 150 ppl/sqkm, proportion)	540
Other unintentional injuries	Male	0-6 days	95+ years	Data Rich	1	1	Liters of alcohol consumed per capita	999
Other unintentional injuries	Male	0-6 days	95+ years	Data Rich	1	1	Log-transformed SEV scalar: Oth Unint	--
Other unintentional injuries	Male	0-6 days	95+ years	Data Rich	1	1	Population-weighted mean temperature	--
Other unintentional injuries	Male	0-6 days	95+ years	Data Rich	1	1	Vehicles - 2 wheels (per capita)	--
Other unintentional injuries	Male	0-6 days	95+ years	Data Rich	1	1	Vehicles - 4 wheels (per capita)	--
Other unintentional injuries	Male	0-6 days	95+ years	Data Rich	-1	2	Healthcare access and quality index	820
Other unintentional injuries	Male	0-6 days	95+ years	Data Rich	-1	3	Education (years per capita)	38
Other unintentional injuries	Male	0-6 days	95+ years	Data Rich	-1	3	Socio-demographic Index	172
Other unintentional injuries	Male	0-6 days	95+ years	Data Rich	-1	3	Population Density (over 1000 ppl/sqkm, proportion)	180
Other unintentional injuries	Male	0-6 days	95+ years	Data Rich	-1	3	LDI (I$ per capita)	--
Other unintentional injuries	Male	0-6 days	95+ years	Data Rich	1	3	Population Density (under 150 ppl/sqkm, proportion)	38
Other unintentional injuries	Male	0-6 days	95+ years	Global	1	1	Liters of alcohol consumed per capita	--
Other unintentional injuries	Male	0-6 days	95+ years	Global	1	1	Log-transformed SEV scalar: Oth Unint	--
Other unintentional injuries	Male	0-6 days	95+ years	Global	1	1	Population-weighted mean temperature	--
Other unintentional injuries	Male	0-6 days	95+ years	Global	1	1	Vehicles - 2 wheels (per capita)	--
Other unintentional injuries	Male	0-6 days	95+ years	Global	1	1	Vehicles - 4 wheels (per capita)	--
Other unintentional injuries	Male	0-6 days	95+ years	Global	-1	2	Healthcare access and quality index	333
Other unintentional injuries	Male	0-6 days	95+ years	Global	-1	3	Socio-demographic Index	455
Other unintentional injuries	Male	0-6 days	95+ years	Global	-1	3	Population Density (over 1000 ppl/sqkm, proportion)	505
Other unintentional injuries	Male	0-6 days	95+ years	Global	-1	3	Education (years per capita)	520
Other unintentional injuries	Male	0-6 days	95+ years	Global	-1	3	LDI (I$ per capita)	--
Other unintentional injuries	Male	0-6 days	95+ years	Global	1	3	Population Density (under 150 ppl/sqkm, proportion)	447
Self-harm	Female	10-14 years	95+ years	Data Rich	1	1	12-month non-partner sexual violence	187
Self-harm	Female	10-14 years	95+ years	Data Rich	1	1	Liters of alcohol consumed per capita	791
Self-harm	Female	10-14 years	95+ years	Data Rich	1	1	Major depressive disorder	813
Self-harm	Female	10-14 years	95+ years	Data Rich	1	1	Log-transformed SEV scalar: Self Harm	--
Self-harm	Female	10-14 years	95+ years	Data Rich	1	1	Population-weighted mean temperature	--
Self-harm	Female	10-14 years	95+ years	Data Rich	-1	2	Healthcare access and quality index	1
Self-harm	Female	10-14 years	95+ years	Data Rich	-1	2	Population Density (over 1000 ppl/sqkm, proportion)	8
Self-harm	Female	10-14 years	95+ years	Data Rich	-1	2	Population Density (300-500 ppl/sqkm, proportion)	568
Self-harm	Female	10-14 years	95+ years	Data Rich	-1	2	Population Density (500-1000 ppl/sqkm, proportion)	--
Self-harm	Female	10-14 years	95+ years	Data Rich	1	2	Population Density (150-300 ppl/sqkm, proportion)	0
Self-harm	Female	10-14 years	95+ years	Data Rich	1	2	Population Density (under 150 ppl/sqkm, proportion)	0
Self-harm	Female	10-14 years	95+ years	Data Rich	-1	3	Socio-demographic Index	3
Self-harm	Female	10-14 years	95+ years	Data Rich	-1	3	Education (years per capita)	53
Self-harm	Female	10-14 years	95+ years	Data Rich	-1	3	LDI (I$ per capita)	--
Self-harm	Female	10-14 years	95+ years	Global	1	1	Population-weighted mean temperature	321
Self-harm	Female	10-14 years	95+ years	Global	1	1	Major depressive disorder	679
Self-harm	Female	10-14 years	95+ years	Global	1	1	12-month non-partner sexual violence	--
Self-harm	Female	10-14 years	95+ years	Global	1	1	Liters of alcohol consumed per capita	--
Self-harm	Female	10-14 years	95+ years	Global	1	1	Log-transformed SEV scalar: Self Harm	--
Self-harm	Female	10-14 years	95+ years	Global	-1	2	Population Density (over 1000 ppl/sqkm, proportion)	64
Self-harm	Female	10-14 years	95+ years	Global	-1	2	Healthcare access and quality index	--
Self-harm	Female	10-14 years	95+ years	Global	-1	2	Population Density (300-500 ppl/sqkm, proportion)	--
Self-harm	Female	10-14 years	95+ years	Global	-1	2	Population Density (500-1000 ppl/sqkm, proportion)	--
Self-harm	Female	10-14 years	95+ years	Global	1	2	Population Density (under 150 ppl/sqkm, proportion)	20
Self-harm	Female	10-14 years	95+ years	Global	1	2	Population Density (150-300 ppl/sqkm, proportion)	386
Self-harm	Female	10-14 years	95+ years	Global	-1	3	Socio-demographic Index	28
Self-harm	Female	10-14 years	95+ years	Global	-1	3	Education (years per capita)	145

CODEm covariates used, level of covariate, and expected direction of covariate by cause, sex, and age

Cause	Sex	Age Start	Age End	Model Version Type	Direction	Level	Covariate Name	Number of Draws
Self-harm	Female	10-14 years	95+ years	Global	-1	3	LDI (I$ per capita)	--
Self-harm	Male	10-14 years	95+ years	Data Rich	1	1	12-month non-partner sexual violence	581
Self-harm	Male	10-14 years	95+ years	Data Rich	1	1	Major depressive disorder	668
Self-harm	Male	10-14 years	95+ years	Data Rich	1	1	Liters of alcohol consumed per capita	--
Self-harm	Male	10-14 years	95+ years	Data Rich	1	1	Muslim Religion (proportion of population)	--
Self-harm	Male	10-14 years	95+ years	Data Rich	1	1	Population-weighted mean temperature	--
Self-harm	Male	10-14 years	95+ years	Data Rich	-1	2	Healthcare access and quality index	83
Self-harm	Male	10-14 years	95+ years	Data Rich	-1	2	Population Density (over 1000 ppl/sqkm, proportion)	83
Self-harm	Male	10-14 years	95+ years	Data Rich	-1	2	Population Density (300-500 ppl/sqkm, proportion)	--
Self-harm	Male	10-14 years	95+ years	Data Rich	-1	2	Population Density (500-1000 ppl/sqkm, proportion)	--
Self-harm	Male	10-14 years	95+ years	Data Rich	1	2	Population Density (150-300 ppl/sqkm, proportion)	249
Self-harm	Male	10-14 years	95+ years	Data Rich	1	2	Population Density (under 150 ppl/sqkm, proportion)	249
Self-harm	Male	10-14 years	95+ years	Data Rich	-1	3	Education (years per capita)	83
Self-harm	Male	10-14 years	95+ years	Data Rich	-1	3	Socio-demographic Index	83
Self-harm	Male	10-14 years	95+ years	Data Rich	-1	3	LDI (I$ per capita)	--
Self-harm	Male	10-14 years	95+ years	Global	1	1	Major depressive disorder	109
Self-harm	Male	10-14 years	95+ years	Global	1	1	Population-weighted mean temperature	602
Self-harm	Male	10-14 years	95+ years	Global	1	1	12-month non-partner sexual violence	803
Self-harm	Male	10-14 years	95+ years	Global	1	1	Liters of alcohol consumed per capita	--
Self-harm	Male	10-14 years	95+ years	Global	-1	2	Population Density (over 1000 ppl/sqkm, proportion)	341
Self-harm	Male	10-14 years	95+ years	Global	-1	2	Muslim Religion (proportion of population)	654
Self-harm	Male	10-14 years	95+ years	Global	-1	2	Healthcare access and quality index	--
Self-harm	Male	10-14 years	95+ years	Global	-1	2	Population Density (300-500 ppl/sqkm, proportion)	--
Self-harm	Male	10-14 years	95+ years	Global	-1	2	Population Density (500-1000 ppl/sqkm, proportion)	--
Self-harm	Male	10-14 years	95+ years	Global	1	2	Population Density (150-300 ppl/sqkm, proportion)	468
Self-harm	Male	10-14 years	95+ years	Global	1	2	Population Density (under 150 ppl/sqkm, proportion)	--
Self-harm	Male	10-14 years	95+ years	Global	-1	3	Education (years per capita)	33
Self-harm	Male	10-14 years	95+ years	Global	-1	3	Socio-demographic Index	100
Self-harm	Male	10-14 years	95+ years	Global	-1	3	LDI (I$ per capita)	--
Self-harm by firearm	Female	10-14 years	95+ years	Data Rich	1	1	Major depressive disorder	1000
Self-harm by firearm	Female	10-14 years	95+ years	Data Rich	1	1	12-month non-partner sexual violence	--
Self-harm by firearm	Female	10-14 years	95+ years	Data Rich	1	1	Liters of alcohol consumed per capita	--
Self-harm by firearm	Female	10-14 years	95+ years	Data Rich	1	1	Log-transformed SEV scalar: Self Harm	--
Self-harm by firearm	Female	10-14 years	95+ years	Data Rich	1	1	Population-weighted mean temperature	--
Self-harm by firearm	Female	10-14 years	95+ years	Data Rich	-1	2	Population Density (over 1000 ppl/sqkm, proportion)	1
Self-harm by firearm	Female	10-14 years	95+ years	Data Rich	-1	2	Healthcare access and quality index	--
Self-harm by firearm	Female	10-14 years	95+ years	Data Rich	-1	2	Population Density (300-500 ppl/sqkm, proportion)	--
Self-harm by firearm	Female	10-14 years	95+ years	Data Rich	-1	2	Population Density (500-1000 ppl/sqkm, proportion)	--
Self-harm by firearm	Female	10-14 years	95+ years	Data Rich	1	2	Population Density (under 150 ppl/sqkm, proportion)	0
Self-harm by firearm	Female	10-14 years	95+ years	Data Rich	1	2	Population Density (150-300 ppl/sqkm, proportion)	198
Self-harm by firearm	Female	10-14 years	95+ years	Data Rich	-1	3	Education (years per capita)	32
Self-harm by firearm	Female	10-14 years	95+ years	Data Rich	-1	3	Socio-demographic Index	161
Self-harm by firearm	Female	10-14 years	95+ years	Data Rich	-1	3	LDI (I$ per capita)	--
Self-harm by firearm	Female	10-14 years	95+ years	Global	1	1	Major depressive disorder	627
Self-harm by firearm	Female	10-14 years	95+ years	Global	1	1	12-month non-partner sexual violence	--
Self-harm by firearm	Female	10-14 years	95+ years	Global	1	1	Liters of alcohol consumed per capita	--
Self-harm by firearm	Female	10-14 years	95+ years	Global	1	1	Log-transformed SEV scalar: Self Harm	--
Self-harm by firearm	Female	10-14 years	95+ years	Global	1	1	Population-weighted mean temperature	--
Self-harm by firearm	Female	10-14 years	95+ years	Global	-1	2	Population Density (over 1000 ppl/sqkm, proportion)	643
Self-harm by firearm	Female	10-14 years	95+ years	Global	-1	2	Healthcare access and quality index	--
Self-harm by firearm	Female	10-14 years	95+ years	Global	-1	2	Population Density (300-500 ppl/sqkm, proportion)	--
Self-harm by firearm	Female	10-14 years	95+ years	Global	-1	2	Population Density (500-1000 ppl/sqkm, proportion)	--
Self-harm by firearm	Female	10-14 years	95+ years	Global	1	2	Population Density (under 150 ppl/sqkm, proportion)	385
Self-harm by firearm	Female	10-14 years	95+ years	Global	1	2	Population Density (150-300 ppl/sqkm, proportion)	--
Self-harm by firearm	Female	10-14 years	95+ years	Global	-1	3	Socio-demographic Index	143
Self-harm by firearm	Female	10-14 years	95+ years	Global	-1	3	Education (years per capita)	401
Self-harm by firearm	Female	10-14 years	95+ years	Global	-1	3	LDI (I$ per capita)	--
Self-harm by firearm	Male	10-14 years	95+ years	Data Rich	1	1	Major depressive disorder	1000
Self-harm by firearm	Male	10-14 years	95+ years	Data Rich	1	1	12-month non-partner sexual violence	--
Self-harm by firearm	Male	10-14 years	95+ years	Data Rich	1	1	Liters of alcohol consumed per capita	--
Self-harm by firearm	Male	10-14 years	95+ years	Data Rich	1	1	Log-transformed SEV scalar: Self Harm	--
Self-harm by firearm	Male	10-14 years	95+ years	Data Rich	1	1	Population-weighted mean temperature	--
Self-harm by firearm	Male	10-14 years	95+ years	Data Rich	-1	2	Healthcare access and quality index	0
Self-harm by firearm	Male	10-14 years	95+ years	Data Rich	-1	2	Population Density (over 1000 ppl/sqkm, proportion)	0
Self-harm by firearm	Male	10-14 years	95+ years	Data Rich	-1	2	Population Density (300-500 ppl/sqkm, proportion)	--
Self-harm by firearm	Male	10-14 years	95+ years	Data Rich	-1	2	Population Density (500-1000 ppl/sqkm, proportion)	--
Self-harm by firearm	Male	10-14 years	95+ years	Data Rich	1	2	Population Density (under 150 ppl/sqkm, proportion)	0
Self-harm by firearm	Male	10-14 years	95+ years	Data Rich	1	2	Population Density (150-300 ppl/sqkm, proportion)	193
Self-harm by firearm	Male	10-14 years	95+ years	Data Rich	-1	3	Socio-demographic Index	38
Self-harm by firearm	Male	10-14 years	95+ years	Data Rich	-1	3	Education (years per capita)	--
Self-harm by firearm	Male	10-14 years	95+ years	Data Rich	-1	3	LDI (I$ per capita)	--
Self-harm by firearm	Male	10-14 years	95+ years	Global	1	1	12-month non-partner sexual violence	--
Self-harm by firearm	Male	10-14 years	95+ years	Global	1	1	Liters of alcohol consumed per capita	--
Self-harm by firearm	Male	10-14 years	95+ years	Global	1	1	Log-transformed SEV scalar: Self Harm	--
Self-harm by firearm	Male	10-14 years	95+ years	Global	1	1	Major depressive disorder	--
Self-harm by firearm	Male	10-14 years	95+ years	Global	1	1	Population-weighted mean temperature	--
Self-harm by firearm	Male	10-14 years	95+ years	Global	-1	2	Population Density (over 1000 ppl/sqkm, proportion)	475
Self-harm by firearm	Male	10-14 years	95+ years	Global	-1	2	Healthcare access and quality index	706
Self-harm by firearm	Male	10-14 years	95+ years	Global	-1	2	Population Density (300-500 ppl/sqkm, proportion)	--
Self-harm by firearm	Male	10-14 years	95+ years	Global	-1	2	Population Density (500-1000 ppl/sqkm, proportion)	--
Self-harm by firearm	Male	10-14 years	95+ years	Global	1	2	Population Density (150-300 ppl/sqkm, proportion)	424
Self-harm by firearm	Male	10-14 years	95+ years	Global	1	2	Population Density (under 150 ppl/sqkm, proportion)	513
Self-harm by firearm	Male	10-14 years	95+ years	Global	-1	3	Socio-demographic Index	161
Self-harm by firearm	Male	10-14 years	95+ years	Global	-1	3	Education (years per capita)	--
Self-harm by firearm	Male	10-14 years	95+ years	Global	-1	3	LDI (I$ per capita)	--
Self-harm by other specified means	Female	10-14 years	95+ years	Data Rich	1	1	12-month non-partner sexual violence	78
Self-harm by other specified means	Female	10-14 years	95+ years	Data Rich	1	1	Liters of alcohol consumed per capita	408
Self-harm by other specified means	Female	10-14 years	95+ years	Data Rich	1	1	Log-transformed SEV scalar: Self Harm	600
Self-harm by other specified means	Female	10-14 years	95+ years	Data Rich	1	1	Major depressive disorder	891
Self-harm by other specified means	Female	10-14 years	95+ years	Data Rich	1	1	Population-weighted mean temperature	--
Self-harm by other specified means	Female	10-14 years	95+ years	Data Rich	-1	2	Healthcare access and quality index	0
Self-harm by other specified means	Female	10-14 years	95+ years	Data Rich	-1	2	Population Density (300-500 ppl/sqkm, proportion)	113
Self-harm by other specified means	Female	10-14 years	95+ years	Data Rich	-1	2	Population Density (500-1000 ppl/sqkm, proportion)	195
Self-harm by other specified means	Female	10-14 years	95+ years	Data Rich	-1	2	Population Density (over 1000 ppl/sqkm, proportion)	--
Self-harm by other specified means	Female	10-14 years	95+ years	Data Rich	1	2	Population Density (150-300 ppl/sqkm, proportion)	--
Self-harm by other specified means	Female	10-14 years	95+ years	Data Rich	1	2	Population Density (under 150 ppl/sqkm, proportion)	--
Self-harm by other specified means	Female	10-14 years	95+ years	Data Rich	-1	3	Education (years per capita)	0
Self-harm by other specified means	Female	10-14 years	95+ years	Data Rich	-1	3	Socio-demographic Index	0
Self-harm by other specified means	Female	10-14 years	95+ years	Data Rich	-1	3	LDI (I$ per capita)	--
Self-harm by other specified means	Female	10-14 years	95+ years	Global	1	1	Liters of alcohol consumed per capita	364
Self-harm by other specified means	Female	10-14 years	95+ years	Global	1	1	Log-transformed SEV scalar: Self Harm	373
Self-harm by other specified means	Female	10-14 years	95+ years	Global	1	1	Major depressive disorder	734
Self-harm by other specified means	Female	10-14 years	95+ years	Global	1	1	12-month non-partner sexual violence	--
Self-harm by other specified means	Female	10-14 years	95+ years	Global	1	1	Population-weighted mean temperature	--
Self-harm by other specified means	Female	10-14 years	95+ years	Global	-1	2	Healthcare access and quality index	15
Self-harm by other specified means	Female	10-14 years	95+ years	Global	-1	2	Population Density (over 1000 ppl/sqkm, proportion)	34
Self-harm by other specified means	Female	10-14 years	95+ years	Global	-1	2	Population Density (300-500 ppl/sqkm, proportion)	--

CODEm covariates used, level of covariate, and expected direction of covariate by cause, sex, and age								
**Cause**	**Sex**	**Age Start**	**Age End**	**Model Version Type**	**Direction**	**Level**	**Covariate Name**	**Number of Draws**
Self-harm by other specified means	Female	10-14 years	95+ years	Global	-1	2	Population Density (500-1000 ppl/sqkm, proportion)	--
Self-harm by other specified means	Female	10-14 years	95+ years	Global	1	2	Population Density (150-300 ppl/sqkm, proportion)	123
Self-harm by other specified means	Female	10-14 years	95+ years	Global	1	2	Population Density (under 150 ppl/sqkm, proportion)	--
Self-harm by other specified means	Female	10-14 years	95+ years	Global	-1	3	Socio-demographic Index	121
Self-harm by other specified means	Female	10-14 years	95+ years	Global	-1	3	Education (years per capita)	199
Self-harm by other specified means	Female	10-14 years	95+ years	Global	-1	3	LDI (I$ per capita)	--
Self-harm by other specified means	Male	10-14 years	95+ years	Data Rich	1	1	Liters of alcohol consumed per capita	0
Self-harm by other specified means	Male	10-14 years	95+ years	Data Rich	1	1	Major depressive disorder	621
Self-harm by other specified means	Male	10-14 years	95+ years	Data Rich	1	1	Log-transformed SEV scalar: Self Harm	941
Self-harm by other specified means	Male	10-14 years	95+ years	Data Rich	1	1	12-month non-partner sexual violence	1000
Self-harm by other specified means	Male	10-14 years	95+ years	Data Rich	1	1	Population-weighted mean temperature	--
Self-harm by other specified means	Male	10-14 years	95+ years	Data Rich	-1	2	Healthcare access and quality index	0
Self-harm by other specified means	Male	10-14 years	95+ years	Data Rich	-1	2	Population Density (300-500 ppl/sqkm, proportion)	0
Self-harm by other specified means	Male	10-14 years	95+ years	Data Rich	-1	2	Population Density (500-1000 ppl/sqkm, proportion)	104
Self-harm by other specified means	Male	10-14 years	95+ years	Data Rich	-1	2	Population Density (over 1000 ppl/sqkm, proportion)	--
Self-harm by other specified means	Male	10-14 years	95+ years	Data Rich	1	2	Population Density (150-300 ppl/sqkm, proportion)	--
Self-harm by other specified means	Male	10-14 years	95+ years	Data Rich	1	2	Population Density (under 150 ppl/sqkm, proportion)	--
Self-harm by other specified means	Male	10-14 years	95+ years	Data Rich	-1	3	Education (years per capita)	0
Self-harm by other specified means	Male	10-14 years	95+ years	Data Rich	-1	3	Socio-demographic Index	0
Self-harm by other specified means	Male	10-14 years	95+ years	Data Rich	-1	3	LDI (I$ per capita)	--
Self-harm by other specified means	Male	10-14 years	95+ years	Global	1	1	Liters of alcohol consumed per capita	466
Self-harm by other specified means	Male	10-14 years	95+ years	Global	1	1	Log-transformed SEV scalar: Self Harm	490
Self-harm by other specified means	Male	10-14 years	95+ years	Global	1	1	12-month non-partner sexual violence	636
Self-harm by other specified means	Male	10-14 years	95+ years	Global	1	1	Major depressive disorder	731
Self-harm by other specified means	Male	10-14 years	95+ years	Global	1	1	Population-weighted mean temperature	--
Self-harm by other specified means	Male	10-14 years	95+ years	Global	-1	2	Population Density (over 1000 ppl/sqkm, proportion)	12
Self-harm by other specified means	Male	10-14 years	95+ years	Global	-1	2	Healthcare access and quality index	55
Self-harm by other specified means	Male	10-14 years	95+ years	Global	-1	2	Population Density (300-500 ppl/sqkm, proportion)	205
Self-harm by other specified means	Male	10-14 years	95+ years	Global	-1	2	Population Density (500-1000 ppl/sqkm, proportion)	206
Self-harm by other specified means	Male	10-14 years	95+ years	Global	1	2	Population Density (150-300 ppl/sqkm, proportion)	55
Self-harm by other specified means	Male	10-14 years	95+ years	Global	1	2	Population Density (under 150 ppl/sqkm, proportion)	--
Self-harm by other specified means	Male	10-14 years	95+ years	Global	-1	3	Socio-demographic Index	29
Self-harm by other specified means	Male	10-14 years	95+ years	Global	-1	3	Education (years per capita)	42
Self-harm by other specified means	Male	10-14 years	95+ years	Global	-1	3	LDI (I$ per capita)	--
Interpersonal violence	Female	0-6 days	95+ years	Data Rich	1	1	Log-transformed SEV scalar: Violence	7
Interpersonal violence	Female	0-6 days	95+ years	Data Rich	1	1	Education Relative Inequality (Gini)	25
Interpersonal violence	Female	0-6 days	95+ years	Data Rich	1	1	Liters of alcohol consumed per capita	125
Interpersonal violence	Female	0-6 days	95+ years	Data Rich	1	1	Population-weighted mean temperature	202
Interpersonal violence	Female	0-6 days	95+ years	Data Rich	1	1	Population 15 to 30 males (proportion)	975
Interpersonal violence	Female	0-6 days	95+ years	Data Rich	-1	2	Healthcare access and quality index	31
Interpersonal violence	Female	0-6 days	95+ years	Data Rich	1	2	Population Density (over 1000 ppl/sqkm, proportion)	250
Interpersonal violence	Female	0-6 days	95+ years	Data Rich	-1	3	Education (years per capita)	0
Interpersonal violence	Female	0-6 days	95+ years	Data Rich	-1	3	Socio-demographic Index	1
Interpersonal violence	Female	0-6 days	95+ years	Data Rich	-1	3	LDI (I$ per capita)	--
Interpersonal violence	Female	0-6 days	95+ years	Global	1	1	Log-transformed SEV scalar: Violence	9
Interpersonal violence	Female	0-6 days	95+ years	Global	1	1	Education Relative Inequality (Gini)	35
Interpersonal violence	Female	0-6 days	95+ years	Global	1	1	Population-weighted mean temperature	666
Interpersonal violence	Female	0-6 days	95+ years	Global	1	1	Population 15 to 30 males (proportion)	685
Interpersonal violence	Female	0-6 days	95+ years	Global	1	1	Liters of alcohol consumed per capita	--
Interpersonal violence	Female	0-6 days	95+ years	Global	-1	2	Healthcare access and quality index	176
Interpersonal violence	Female	0-6 days	95+ years	Global	1	2	Population Density (over 1000 ppl/sqkm, proportion)	330
Interpersonal violence	Female	0-6 days	95+ years	Global	-1	3	Socio-demographic Index	84
Interpersonal violence	Female	0-6 days	95+ years	Global	-1	3	Education (years per capita)	166
Interpersonal violence	Female	0-6 days	95+ years	Global	-1	3	LDI (I$ per capita)	--
Interpersonal violence	Male	0-6 days	95+ years	Data Rich	1	1	Education Relative Inequality (Gini)	0
Interpersonal violence	Male	0-6 days	95+ years	Data Rich	1	1	Population 15 to 30 males (proportion)	7
Interpersonal violence	Male	0-6 days	95+ years	Data Rich	1	1	Liters of alcohol consumed per capita	32
Interpersonal violence	Male	0-6 days	95+ years	Data Rich	1	1	Population-weighted mean temperature	1000
Interpersonal violence	Male	0-6 days	95+ years	Data Rich	1	1	Log-transformed SEV scalar: Violence	--
Interpersonal violence	Male	0-6 days	95+ years	Data Rich	-1	2	Healthcare access and quality index	0
Interpersonal violence	Male	0-6 days	95+ years	Data Rich	1	2	Population Density (over 1000 ppl/sqkm, proportion)	807
Interpersonal violence	Male	0-6 days	95+ years	Data Rich	-1	3	Education (years per capita)	0
Interpersonal violence	Male	0-6 days	95+ years	Data Rich	-1	3	Socio-demographic Index	0
Interpersonal violence	Male	0-6 days	95+ years	Data Rich	-1	3	LDI (I$ per capita)	--
Interpersonal violence	Male	0-6 days	95+ years	Global	1	1	Education Relative Inequality (Gini)	189
Interpersonal violence	Male	0-6 days	95+ years	Global	1	1	Population 15 to 30 males (proportion)	437
Interpersonal violence	Male	0-6 days	95+ years	Global	1	1	Population-weighted mean temperature	589
Interpersonal violence	Male	0-6 days	95+ years	Global	1	1	Liters of alcohol consumed per capita	--
Interpersonal violence	Male	0-6 days	95+ years	Global	1	1	Log-transformed SEV scalar: Violence	--
Interpersonal violence	Male	0-6 days	95+ years	Global	-1	2	Healthcare access and quality index	100
Interpersonal violence	Male	0-6 days	95+ years	Global	1	2	Population Density (over 1000 ppl/sqkm, proportion)	328
Interpersonal violence	Male	0-6 days	95+ years	Global	-1	3	Education (years per capita)	26
Interpersonal violence	Male	0-6 days	95+ years	Global	-1	3	Socio-demographic Index	137
Interpersonal violence	Male	0-6 days	95+ years	Global	-1	3	LDI (I$ per capita)	--
Assault by firearm	Female	0-6 days	95+ years	Data Rich	1	1	Education Relative Inequality (Gini)	39
Assault by firearm	Female	0-6 days	95+ years	Data Rich	1	1	Population 15 to 30 males (proportion)	621
Assault by firearm	Female	0-6 days	95+ years	Data Rich	1	1	Population-weighted mean temperature	891
Assault by firearm	Female	0-6 days	95+ years	Data Rich	1	1	Liters of alcohol consumed per capita	--
Assault by firearm	Female	0-6 days	95+ years	Data Rich	1	1	Log-transformed SEV scalar: Viol Gun	--
Assault by firearm	Female	0-6 days	95+ years	Data Rich	-1	2	Healthcare access and quality index	250
Assault by firearm	Female	0-6 days	95+ years	Data Rich	1	2	Population Density (over 1000 ppl/sqkm, proportion)	--
Assault by firearm	Female	0-6 days	95+ years	Data Rich	-1	3	Socio-demographic Index	65
Assault by firearm	Female	0-6 days	95+ years	Data Rich	-1	3	Education (years per capita)	--
Assault by firearm	Female	0-6 days	95+ years	Data Rich	-1	3	LDI (I$ per capita)	--
Assault by firearm	Female	0-6 days	95+ years	Global	1	1	Education Relative Inequality (Gini)	192
Assault by firearm	Female	0-6 days	95+ years	Global	1	1	Population 15 to 30 males (proportion)	323
Assault by firearm	Female	0-6 days	95+ years	Global	1	1	Population-weighted mean temperature	936
Assault by firearm	Female	0-6 days	95+ years	Global	1	1	Liters of alcohol consumed per capita	--
Assault by firearm	Female	0-6 days	95+ years	Global	1	1	Log-transformed SEV scalar: Viol Gun	--
Assault by firearm	Female	0-6 days	95+ years	Global	-1	2	Healthcare access and quality index	--
Assault by firearm	Female	0-6 days	95+ years	Global	1	2	Population Density (over 1000 ppl/sqkm, proportion)	331
Assault by firearm	Female	0-6 days	95+ years	Global	-1	3	Education (years per capita)	92
Assault by firearm	Female	0-6 days	95+ years	Global	-1	3	LDI (I$ per capita)	--
Assault by firearm	Female	0-6 days	95+ years	Global	-1	3	Socio-demographic Index	--
Assault by firearm	Male	0-6 days	95+ years	Data Rich	1	1	Liters of alcohol consumed per capita	0
Assault by firearm	Male	0-6 days	95+ years	Data Rich	1	1	Log-transformed SEV scalar: Viol Gun	82
Assault by firearm	Male	0-6 days	95+ years	Data Rich	1	1	Education Relative Inequality (Gini)	106
Assault by firearm	Male	0-6 days	95+ years	Data Rich	1	1	Population-weighted mean temperature	894
Assault by firearm	Male	0-6 days	95+ years	Data Rich	1	1	Population 15 to 30 males (proportion)	1000
Assault by firearm	Male	0-6 days	95+ years	Data Rich	-1	2	Healthcare access and quality index	2
Assault by firearm	Male	0-6 days	95+ years	Data Rich	1	2	Population Density (over 1000 ppl/sqkm, proportion)	744
Assault by firearm	Male	0-6 days	95+ years	Data Rich	-1	3	Education (years per capita)	0
Assault by firearm	Male	0-6 days	95+ years	Data Rich	-1	3	Socio-demographic Index	0
Assault by firearm	Male	0-6 days	95+ years	Data Rich	-1	3	LDI (I$ per capita)	--
Assault by firearm	Male	0-6 days	95+ years	Global	1	1	Education Relative Inequality (Gini)	5
Assault by firearm	Male	0-6 days	95+ years	Global	1	1	Liters of alcohol consumed per capita	92
Assault by firearm	Male	0-6 days	95+ years	Global	1	1	Population 15 to 30 males (proportion)	362
Assault by firearm	Male	0-6 days	95+ years	Global	1	1	Log-transformed SEV scalar: Viol Gun	368
Assault by firearm	Male	0-6 days	95+ years	Global	1	1	Population-weighted mean temperature	490
Assault by firearm	Male	0-6 days	95+ years	Global	-1	2	Healthcare access and quality index	613
Assault by firearm	Male	0-6 days	95+ years	Global	1	2	Population Density (over 1000 ppl/sqkm, proportion)	790
Assault by firearm	Male	0-6 days	95+ years	Global	-1	3	Socio-demographic Index	25
Assault by firearm	Male	0-6 days	95+ years	Global	-1	3	Education (years per capita)	140
Assault by firearm	Male	0-6 days	95+ years	Global	-1	3	LDI (I$ per capita)	--
Assault by sharp object	Female	0-6 days	95+ years	Data Rich	1	1	Liters of alcohol consumed per capita	0

CODEm covariates used, level of covariate, and expected direction of covariate by cause, sex, and age

Cause	Sex	Age Start	Age End	Model Version Type	Direction	Level	Covariate Name	Number of Draws
Assault by sharp object	Female	0-6 days	95+ years	Data Rich	1	1	Log-transformed SEV scalar: Viol Knife	0
Assault by sharp object	Female	0-6 days	95+ years	Data Rich	1	1	Education Relative Inequality (Gini)	157
Assault by sharp object	Female	0-6 days	95+ years	Data Rich	1	1	Population 15 to 30 males (proportion)	1000
Assault by sharp object	Female	0-6 days	95+ years	Data Rich	-1	2	Healthcare access and quality index	--
Assault by sharp object	Female	0-6 days	95+ years	Data Rich	1	2	Population-weighted mean temperature	0
Assault by sharp object	Female	0-6 days	95+ years	Data Rich	1	2	Population Density (over 1000 ppl/sqkm, proportion)	705
Assault by sharp object	Female	0-6 days	95+ years	Data Rich	-1	3	Socio-demographic Index	0
Assault by sharp object	Female	0-6 days	95+ years	Data Rich	-1	3	Education (years per capita)	38
Assault by sharp object	Female	0-6 days	95+ years	Data Rich	-1	3	LDI (I$ per capita)	--
Assault by sharp object	Female	0-6 days	95+ years	Global	1	1	Log-transformed SEV scalar: Viol Knife	0
Assault by sharp object	Female	0-6 days	95+ years	Global	1	1	Education Relative Inequality (Gini)	177
Assault by sharp object	Female	0-6 days	95+ years	Global	1	1	Population 15 to 30 males (proportion)	326
Assault by sharp object	Female	0-6 days	95+ years	Global	1	1	Liters of alcohol consumed per capita	690
Assault by sharp object	Female	0-6 days	95+ years	Global	1	1	Population-weighted mean temperature	897
Assault by sharp object	Female	0-6 days	95+ years	Global	-1	2	Healthcare access and quality index	94
Assault by sharp object	Female	0-6 days	95+ years	Global	1	2	Population Density (over 1000 ppl/sqkm, proportion)	279
Assault by sharp object	Female	0-6 days	95+ years	Global	-1	3	Socio-demographic Index	74
Assault by sharp object	Female	0-6 days	95+ years	Global	-1	3	Education (years per capita)	122
Assault by sharp object	Female	0-6 days	95+ years	Global	-1	3	LDI (I$ per capita)	--
Assault by sharp object	Male	0-6 days	95+ years	Data Rich	1	1	Liters of alcohol consumed per capita	82
Assault by sharp object	Male	0-6 days	95+ years	Data Rich	1	1	Log-transformed SEV scalar: Viol Knife	221
Assault by sharp object	Male	0-6 days	95+ years	Data Rich	1	1	Population-weighted mean temperature	226
Assault by sharp object	Male	0-6 days	95+ years	Data Rich	1	1	Education Relative Inequality (Gini)	745
Assault by sharp object	Male	0-6 days	95+ years	Data Rich	1	1	Population 15 to 30 males (proportion)	811
Assault by sharp object	Male	0-6 days	95+ years	Data Rich	-1	2	Healthcare access and quality index	105
Assault by sharp object	Male	0-6 days	95+ years	Data Rich	1	2	Population Density (over 1000 ppl/sqkm, proportion)	141
Assault by sharp object	Male	0-6 days	95+ years	Data Rich	-1	3	Socio-demographic Index	9
Assault by sharp object	Male	0-6 days	95+ years	Data Rich	-1	3	Education (years per capita)	75
Assault by sharp object	Male	0-6 days	95+ years	Data Rich	-1	3	LDI (I$ per capita)	--
Assault by sharp object	Male	0-6 days	95+ years	Global	1	1	Liters of alcohol consumed per capita	0
Assault by sharp object	Male	0-6 days	95+ years	Global	1	1	Log-transformed SEV scalar: Viol Knife	513
Assault by sharp object	Male	0-6 days	95+ years	Global	1	1	Population-weighted mean temperature	815
Assault by sharp object	Male	0-6 days	95+ years	Global	1	1	Education Relative Inequality (Gini)	903
Assault by sharp object	Male	0-6 days	95+ years	Global	1	1	Population 15 to 30 males (proportion)	998
Assault by sharp object	Male	0-6 days	95+ years	Global	-1	2	Healthcare access and quality index	553
Assault by sharp object	Male	0-6 days	95+ years	Global	1	2	Population Density (over 1000 ppl/sqkm, proportion)	374
Assault by sharp object	Male	0-6 days	95+ years	Global	-1	3	Education (years per capita)	21
Assault by sharp object	Male	0-6 days	95+ years	Global	-1	3	Socio-demographic Index	174
Assault by sharp object	Male	0-6 days	95+ years	Global	-1	3	LDI (I$ per capita)	--
Assault by other means	Female	0-6 days	95+ years	Data Rich	1	1	Education Relative Inequality (Gini)	0
Assault by other means	Female	0-6 days	95+ years	Data Rich	1	1	Log-transformed SEV scalar: Oth Viol	0
Assault by other means	Female	0-6 days	95+ years	Data Rich	1	1	Liters of alcohol consumed per capita	15
Assault by other means	Female	0-6 days	95+ years	Data Rich	1	1	Population-weighted mean temperature	80
Assault by other means	Female	0-6 days	95+ years	Data Rich	1	1	Population 15 to 30 males (proportion)	921
Assault by other means	Female	0-6 days	95+ years	Data Rich	-1	2	Healthcare access and quality index	258
Assault by other means	Female	0-6 days	95+ years	Data Rich	1	2	Population Density (over 1000 ppl/sqkm, proportion)	655
Assault by other means	Female	0-6 days	95+ years	Data Rich	-1	3	Education (years per capita)	175
Assault by other means	Female	0-6 days	95+ years	Data Rich	-1	3	Socio-demographic Index	356
Assault by other means	Female	0-6 days	95+ years	Data Rich	-1	3	LDI (I$ per capita)	--
Assault by other means	Female	0-6 days	95+ years	Global	1	1	Log-transformed SEV scalar: Oth Viol	0
Assault by other means	Female	0-6 days	95+ years	Global	1	1	Education Relative Inequality (Gini)	434
Assault by other means	Female	0-6 days	95+ years	Global	1	1	Population-weighted mean temperature	836
Assault by other means	Female	0-6 days	95+ years	Global	1	1	Population 15 to 30 males (proportion)	945
Assault by other means	Female	0-6 days	95+ years	Global	1	1	Liters of alcohol consumed per capita	--
Assault by other means	Female	0-6 days	95+ years	Global	-1	2	Healthcare access and quality index	80
Assault by other means	Female	0-6 days	95+ years	Global	1	2	Population Density (over 1000 ppl/sqkm, proportion)	342
Assault by other means	Female	0-6 days	95+ years	Global	-1	3	Education (years per capita)	98
Assault by other means	Female	0-6 days	95+ years	Global	-1	3	Socio-demographic Index	286
Assault by other means	Female	0-6 days	95+ years	Global	-1	3	LDI (I$ per capita)	--
Assault by other means	Male	0-6 days	95+ years	Data Rich	1	1	Population 15 to 30 males (proportion)	0
Assault by other means	Male	0-6 days	95+ years	Data Rich	1	1	Education Relative Inequality (Gini)	161
Assault by other means	Male	0-6 days	95+ years	Data Rich	1	1	Population-weighted mean temperature	999
Assault by other means	Male	0-6 days	95+ years	Data Rich	1	1	Liters of alcohol consumed per capita	--
Assault by other means	Male	0-6 days	95+ years	Data Rich	1	1	Log-transformed SEV scalar: Oth Viol	--
Assault by other means	Male	0-6 days	95+ years	Data Rich	-1	2	Healthcare access and quality index	0
Assault by other means	Male	0-6 days	95+ years	Data Rich	1	2	Population Density (over 1000 ppl/sqkm, proportion)	0
Assault by other means	Male	0-6 days	95+ years	Data Rich	-1	3	Socio-demographic Index	0
Assault by other means	Male	0-6 days	95+ years	Data Rich	-1	3	Education (years per capita)	6
Assault by other means	Male	0-6 days	95+ years	Data Rich	-1	3	LDI (I$ per capita)	--
Assault by other means	Male	0-6 days	95+ years	Global	1	1	Education Relative Inequality (Gini)	220
Assault by other means	Male	0-6 days	95+ years	Global	1	1	Population-weighted mean temperature	587
Assault by other means	Male	0-6 days	95+ years	Global	1	1	Population 15 to 30 males (proportion)	708
Assault by other means	Male	0-6 days	95+ years	Global	1	1	Liters of alcohol consumed per capita	--
Assault by other means	Male	0-6 days	95+ years	Global	1	1	Log-transformed SEV scalar: Oth Viol	--
Assault by other means	Male	0-6 days	95+ years	Global	-1	2	Healthcare access and quality index	85
Assault by other means	Male	0-6 days	95+ years	Global	1	2	Population Density (over 1000 ppl/sqkm, proportion)	292
Assault by other means	Male	0-6 days	95+ years	Global	-1	3	Education (years per capita)	115
Assault by other means	Male	0-6 days	95+ years	Global	-1	3	Socio-demographic Index	146
Assault by other means	Male	0-6 days	95+ years	Global	-1	3	LDI (I$ per capita)	--
Executions and police conflict	Female	1-5 months	95+ years	Data Rich	1	1	Population-weighted mean temperature	1000
Executions and police conflict	Female	1-5 months	95+ years	Data Rich	1	1	Liters of alcohol consumed per capita	--
Executions and police conflict	Female	1-5 months	95+ years	Data Rich	1	1	Population 15 to 30 males (proportion)	--
Executions and police conflict	Female	1-5 months	95+ years	Data Rich	-1	2	Healthcare access and quality index	46
Executions and police conflict	Female	1-5 months	95+ years	Data Rich	-1	2	Socio-demographic Index	--
Executions and police conflict	Female	1-5 months	95+ years	Data Rich	1	2	Population Density (over 1000 ppl/sqkm, proportion)	1
Executions and police conflict	Female	1-5 months	95+ years	Data Rich	-1	3	LDI (I$ per capita)	--
Executions and police conflict	Female	1-5 months	95+ years	Data Rich	1	3	Education (years per capita)	180
Executions and police conflict	Female	1-5 months	95+ years	Global	1	1	Population-weighted mean temperature	445
Executions and police conflict	Female	1-5 months	95+ years	Global	1	1	Liters of alcohol consumed per capita	--
Executions and police conflict	Female	1-5 months	95+ years	Global	1	1	Population 15 to 30 males (proportion)	--
Executions and police conflict	Female	1-5 months	95+ years	Global	-1	2	Socio-demographic Index	86
Executions and police conflict	Female	1-5 months	95+ years	Global	-1	2	Healthcare access and quality index	227
Executions and police conflict	Female	1-5 months	95+ years	Global	1	2	Population Density (over 1000 ppl/sqkm, proportion)	375
Executions and police conflict	Female	1-5 months	95+ years	Global	-1	3	LDI (I$ per capita)	60
Executions and police conflict	Female	1-5 months	95+ years	Global	1	3	Education (years per capita)	437
Executions and police conflict	Male	1-5 months	95+ years	Data Rich	1	1	Population-weighted mean temperature	1000
Executions and police conflict	Male	1-5 months	95+ years	Data Rich	1	1	Education Relative Inequality (Gini)	--
Executions and police conflict	Male	1-5 months	95+ years	Data Rich	1	1	Liters of alcohol consumed per capita	--
Executions and police conflict	Male	1-5 months	95+ years	Data Rich	1	1	Population 15 to 30 males (proportion)	--
Executions and police conflict	Male	1-5 months	95+ years	Data Rich	-1	2	Healthcare access and quality index	134
Executions and police conflict	Male	1-5 months	95+ years	Data Rich	-1	2	Socio-demographic Index	--
Executions and police conflict	Male	1-5 months	95+ years	Data Rich	1	2	Population Density (over 1000 ppl/sqkm, proportion)	752
Executions and police conflict	Male	1-5 months	95+ years	Data Rich	-1	3	LDI (I$ per capita)	--
Executions and police conflict	Male	1-5 months	95+ years	Data Rich	1	3	Education (years per capita)	59
Executions and police conflict	Male	1-5 months	95+ years	Global	1	1	Liters of alcohol consumed per capita	138
Executions and police conflict	Male	1-5 months	95+ years	Global	1	1	Population-weighted mean temperature	732
Executions and police conflict	Male	1-5 months	95+ years	Global	1	1	Population 15 to 30 males (proportion)	--
Executions and police conflict	Male	1-5 months	95+ years	Global	-1	2	Healthcare access and quality index	--
Executions and police conflict	Male	1-5 months	95+ years	Global	-1	2	Socio-demographic Index	--
Executions and police conflict	Male	1-5 months	95+ years	Global	1	2	Population Density (over 1000 ppl/sqkm, proportion)	510
Executions and police conflict	Male	1-5 months	95+ years	Global	-1	3	LDI (I$ per capita)	66
Executions and police conflict	Male	1-5 months	95+ years	Global	1	3	Education (years per capita)	472

Appendix Table S13: CODEm predictive validity results by cause, model type, sex, and age

Cause	Sex	Age Start	Age End	RMSE In-Sample	RMSE Out-of-Sample	Trend In-Sample	Trend Out-of-Sample	Coverage In-Sample	Coverage Out-of-Sample
Sexually transmitted infections excluding HIV [Global]	Female	10-14 years	95+ years	0.581793	0.772112	0.467983	0.447775	0.99058	0.981726
Sexually transmitted infections excluding HIV [Global]	Male	10-14 years	95+ years	0.990104	1.58213	0.678406	0.640179	0.971911	0.899134
Sexually transmitted infections excluding HIV [Data Rich]	Female	10-14 years	95+ years	0.252189	0.393846	0.21036	0.286279	0.99818	0.997526
Sexually transmitted infections excluding HIV [Data Rich]	Male	10-14 years	95+ years	0.679702	0.920385	0.573083	0.500618	0.975839	0.966603
Lower respiratory infections [Global]	Female	0-6 days	2-4 years	0.201955	0.312116	0.150155	0.147852	0.999827	0.995392
Lower respiratory infections [Global]	Female	5-9 years	95+ years	0.231987	0.352347	0.141167	0.146675	0.999682	0.990411
Lower respiratory infections [Global]	Male	0-6 days	2-4 years	0.205351	0.308331	0.149788	0.141355	0.999808	0.995068
Lower respiratory infections [Global]	Male	5-9 years	95+ years	0.22664	0.369574	0.142515	0.151718	0.999735	0.988401
Lower respiratory infections [Data Rich]	Female	0-6 days	2-4 years	0.0976013	0.223303	0.0731488	0.12517	0.999934	0.999359
Lower respiratory infections [Data Rich]	Female	5-9 years	95+ years	0.108987	0.246613	0.0892778	0.162482	0.999969	0.999568
Lower respiratory infections [Data Rich]	Male	0-6 days	2-4 years	0.0972832	0.223076	0.071776	0.127022	0.999898	0.999178
Lower respiratory infections [Data Rich]	Male	5-9 years	95+ years	0.105236	0.241421	0.08758	0.162267	0.99998	0.999607
Upper respiratory infections [Global]	Female	0-6 days	95+ years	0.632479	1.05705	0.372446	0.361979	0.990544	0.969325
Upper respiratory infections [Global]	Male	0-6 days	95+ years	0.640749	1.03381	0.384547	0.367377	0.988912	0.971833
Upper respiratory infections [Data Rich]	Female	0-6 days	95+ years	0.339779	0.62241	0.280886	0.277026	0.99713	0.994079
Upper respiratory infections [Data Rich]	Male	0-6 days	95+ years	0.343716	0.54825	0.288077	0.271153	0.995826	0.992448
Otitis media [Global]	Female	0-6 days	95+ years	0.989067	1.98914	0.78354	0.76997	0.9675	0.903024
Otitis media [Global]	Male	0-6 days	95+ years	1.00285	1.9889	0.79538	0.759221	0.968362	0.884782
Otitis media [Data Rich]	Female	0-6 days	95+ years	0.833246	1.82454	0.703894	0.693884	0.966767	0.927686
Otitis media [Data Rich]	Male	0-6 days	95+ years	0.796611	1.76629	0.693491	0.670862	0.967374	0.934413
Diarrhoeal diseases [Global]	Female	0-6 days	2-4 years	0.355335	0.888941	0.257129	0.250336	0.999866	0.980093
Diarrhoeal diseases [Global]	Female	5-9 years	95+ years	0.613329	0.890544	0.185972	0.198046	0.999812	0.984629
Diarrhoeal diseases [Global]	Male	0-6 days	2-4 years	0.725816	0.831011	0.253668	0.243057	0.99811	0.987062
Diarrhoeal diseases [Global]	Male	5-9 years	95+ years	0.600262	0.854947	0.190352	0.201777	0.999609	0.985049
Diarrhoeal diseases [Data Rich]	Female	0-6 days	2-4 years	0.158594	0.413882	0.103057	0.199915	0.999981	0.999456
Diarrhoeal diseases [Data Rich]	Female	5-9 years	95+ years	0.135132	0.403882	0.104327	0.205275	0.999751	0.999456
Diarrhoeal diseases [Data Rich]	Male	0-6 days	2-4 years	0.149826	0.38299	0.0995731	0.186262	0.999991	0.999243
Diarrhoeal diseases [Data Rich]	Male	5-9 years	95+ years	0.137563	0.38308	0.105108	0.212532	0.999767	0.999197
Rabies [Global]	Female	1-5 months	95+ years	0.869887	1.66374	0.667415	0.701537	0.982026	0.92017
Rabies [Global]	Male	1-5 months	95+ years	0.819336	1.61841	0.623106	0.644999	0.986857	0.917409
Rabies [Data Rich]	Female	1-5 months	95+ years	0.504302	1.23724	0.418815	0.670808	0.99496	0.96875
Rabies [Data Rich]	Male	1-5 months	95+ years	0.487547	1.25962	0.396773	0.623104	0.994654	0.966109
Other neglected tropical diseases [Global]	Female	0-6 days	95+ years	0.506141	1.11781	0.332547	0.35036	0.994237	0.971642
Other neglected tropical diseases [Global]	Male	0-6 days	95+ years	0.532768	1.06953	0.372473	0.374925	0.993057	0.96906
Other neglected tropical diseases [Data Rich]	Female	0-6 days	95+ years	0.336029	0.83544	0.253344	0.301946	0.997175	0.993737
Other neglected tropical diseases [Data Rich]	Male	0-6 days	95+ years	0.374907	0.790882	0.301384	0.330766	0.996149	0.991953
Meningitis [Global]	Female	0-6 days	2-4 years	0.322408	0.441303	0.263057	0.217388	0.998732	0.99588
Meningitis [Global]	Female	5-9 years	95+ years	0.268106	0.407559	0.1548	0.152534	0.99908	0.994633
Meningitis [Global]	Male	0-6 days	2-4 years	0.303452	0.404611	0.207282	0.185994	0.999399	0.99683
Meningitis [Global]	Male	5-9 years	95+ years	0.271358	0.422144	0.157827	0.160772	0.999104	0.994635
Meningitis [Data Rich]	Female	0-6 days	2-4 years	0.0975549	0.222892	0.0788691	0.126019	1	0.999987
Meningitis [Data Rich]	Female	5-9 years	95+ years	0.1079	0.213619	0.0878048	0.132311	0.999997	0.999979
Meningitis [Data Rich]	Male	0-6 days	2-4 years	0.101521	0.239791	0.0809412	0.12949	1	0.999957
Meningitis [Data Rich]	Male	5-9 years	95+ years	0.105166	0.222703	0.0874155	0.135459	1	0.999989
Encephalitis [Global]	Female	0-6 days	95+ years	0.470079	0.829564	0.325106	0.366561	0.993416	0.980829
Encephalitis [Global]	Male	0-6 days	95+ years	0.483084	0.777007	0.311959	0.328569	0.995021	0.982013
Encephalitis [Data Rich]	Female	0-6 days	95+ years	0.297312	0.574585	0.239186	0.371364	0.997561	0.99422
Encephalitis [Data Rich]	Male	0-6 days	95+ years	0.27397	0.552917	0.221194	0.318531	0.998026	0.995179
Tetanus [Global]	Female	0-6 days	6-11 months	1.03053	1.55798	0.927175	0.796847	0.978147	0.944787
Tetanus [Global]	Female	12-23 months	95+ years	1.06403	1.73041	0.767132	0.77817	0.986872	0.939133
Tetanus [Global]	Male	0-6 days	6-11 months	1.0386	1.60192	0.997774	0.982552	0.98008	0.942651
Tetanus [Global]	Male	12-23 months	95+ years	0.966534	1.56645	0.775975	0.805873	0.985013	0.922728
Tetanus [Data Rich]	Female	0-6 days	6-11 months	0.741627	1.01188	0.632168	0.677317	0.984316	0.963913
Tetanus [Data Rich]	Female	12-23 months	95+ years	0.834025	1.22714	0.716411	0.78407	0.981245	0.95773
Tetanus [Data Rich]	Male	0-6 days	6-11 months	0.712395	1.01653	0.622453	0.724875	0.98721	0.971709
Tetanus [Data Rich]	Male	12-23 months	95+ years	0.776492	1.1165	0.680569	0.813536	0.98655	0.970145
Acute hepatitis [Global]	Female	1-5 months	95+ years	0.513917	0.976475	0.378972	0.415863	0.997565	0.968317
Acute hepatitis [Global]	Male	1-5 months	95+ years	0.482348	0.894612	0.35674	0.398075	0.997743	0.97388
Acute hepatitis [Data Rich]	Female	1-5 months	95+ years	0.436994	0.769622	0.366514	0.485498	0.998349	0.994185
Acute hepatitis [Data Rich]	Male	1-5 months	95+ years	0.410544	0.741317	0.344073	0.542681	0.997926	0.994519
Acute hepatitis A [Global]	Female	1-5 months	95+ years	0.727581	1.27369	0.568696	0.596399	0.995476	0.930184
Acute hepatitis A [Global]	Male	1-5 months	95+ years	0.713911	1.28778	0.540364	0.569378	0.995628	0.928346
Acute hepatitis A [Data Rich]	Female	1-5 months	95+ years	0.641981	0.998701	0.548921	0.628383	0.996641	0.985739
Acute hepatitis A [Data Rich]	Male	1-5 months	95+ years	0.607803	1.04975	0.51545	0.626909	0.99701	0.986085
Acute hepatitis B [Global]	Female	1-5 months	95+ years	0.602256	1.41134	0.450936	0.513054	0.996467	0.900257
Acute hepatitis B [Global]	Male	1-5 months	95+ years	0.645504	1.43492	0.499849	0.583082	0.990106	0.901053
Acute hepatitis B [Data Rich]	Female	1-5 months	95+ years	0.513735	1.56158	0.424964	0.590848	0.997387	0.977536
Acute hepatitis B [Data Rich]	Male	1-5 months	95+ years	0.559509	1.61182	0.476512	0.720154	0.990761	0.980572
Acute hepatitis C [Global]	Female	1-5 months	95+ years	0.51865	1.01105	0.330646	0.358196	0.999311	0.951785
Acute hepatitis C [Global]	Male	1-5 months	95+ years	0.562224	1.00988	0.315548	0.320137	0.999619	0.969546
Acute hepatitis C [Data Rich]	Female	1-5 months	95+ years	0.238758	0.887517	0.211585	0.392594	0.99979	0.995969
Acute hepatitis C [Data Rich]	Male	1-5 months	95+ years	0.228453	0.835563	0.193156	0.351691	0.999874	0.997719
Acute hepatitis E [Global]	Female	1-5 months	95+ years	0.765866	1.13395	0.57767	0.561354	0.998304	0.966945
Acute hepatitis E [Global]	Male	1-5 months	95+ years	0.721379	1.18638	0.543935	0.53201	0.999485	0.958666
Acute hepatitis E [Data Rich]	Female	1-5 months	95+ years	0.642759	0.916043	0.544365	0.462696	0.998248	0.977586
Acute hepatitis E [Data Rich]	Male	1-5 months	95+ years	0.595119	0.883942	0.529499	0.446534	0.999363	0.991919
Other unspecified infectious diseases [Global]	Female	0-6 days	95+ years	0.604204	0.766262	0.531464	0.500109	0.992693	0.982815
Other unspecified infectious diseases [Global]	Male	0-6 days	95+ years	0.58046	0.746129	0.522767	0.46565	0.993721	0.981245
Other unspecified infectious diseases [Data Rich]	Female	0-6 days	95+ years	0.128789	0.368344	0.090375	0.175903	0.999993	0.999854
Other unspecified infectious diseases [Data Rich]	Male	0-6 days	95+ years	0.125408	0.338449	0.0901981	0.188054	0.999985	0.999809
Neonatal disorders [Global]	Female	0-6 days	2-4 years	0.171099	0.235976	0.104184	0.104978	0.999869	0.998962
Neonatal disorders [Global]	Male	0-6 days	2-4 years	0.173019	0.246354	0.10509	0.100689	0.999939	0.999085
Neonatal disorders [Data Rich]	Female	0-6 days	2-4 years	0.0824093	0.167801	0.0573891	0.0951784	0.999971	0.999849
Neonatal disorders [Data Rich]	Male	0-6 days	2-4 years	0.0839022	0.167771	0.0588337	0.0986354	0.999962	0.999889
Neonatal preterm birth [Global]	Female	0-6 days	2-4 years	0.187765	0.247449	0.119718	0.116266	0.999703	0.998601
Neonatal preterm birth [Global]	Male	0-6 days	2-4 years	0.189525	0.257696	0.120771	0.115852	0.999657	0.998412
Neonatal preterm birth [Data Rich]	Female	0-6 days	2-4 years	0.0889009	0.207343	0.0644058	0.100283	0.99999	0.999649
Neonatal preterm birth [Data Rich]	Male	0-6 days	2-4 years	0.0889205	0.209367	0.0639016	0.100655	0.999969	0.999817
Neonatal encephalopathy due to birth asphyxia and trauma [Global]	Female	0-6 days	2-4 years	0.204951	0.34075	0.126137	0.125454	0.999966	0.998949
Neonatal encephalopathy due to birth asphyxia and trauma [Global]	Male	0-6 days	2-4 years	0.209624	0.327927	0.129877	0.124298	0.999859	0.998998
Neonatal encephalopathy due to birth asphyxia and trauma [Data Rich]	Female	0-6 days	2-4 years	0.116563	0.279324	0.073385	0.111402	0.999979	0.999505
Neonatal encephalopathy due to birth asphyxia and trauma [Data Rich]	Male	0-6 days	2-4 years	0.149405	0.321563	0.0777752	0.115384	0.99999	0.999546
Neonatal sepsis and other neonatal infections [Global]	Female	0-6 days	2-4 years	0.386142	0.604738	0.280545	0.301671	0.99621	0.992456
Neonatal sepsis and other neonatal infections [Global]	Male	0-6 days	2-4 years	0.328086	0.552141	0.234707	0.24901	0.998609	0.99556
Neonatal sepsis and other neonatal infections [Data Rich]	Female	0-6 days	2-4 years	0.188491	0.515472	0.15193	0.244201	0.999049	0.995561
Neonatal sepsis and other neonatal infections [Data Rich]	Male	0-6 days	2-4 years	0.181313	0.48135	0.139065	0.197354	0.999323	0.996613
Hemolytic disease and other neonatal jaundice [Global]	Female	0-6 days	2-4 years	0.895938	1.34731	0.698029	0.689739	0.981675	0.902489
Hemolytic disease and other neonatal jaundice [Global]	Male	0-6 days	2-4 years	0.768136	1.1197	0.570881	0.547527	0.977828	0.938482
Hemolytic disease and other neonatal jaundice [Data Rich]	Female	0-6 days	2-4 years	0.849337	1.07867	0.688823	0.606031	0.979981	0.956934
Hemolytic disease and other neonatal jaundice [Data Rich]	Male	0-6 days	2-4 years	0.64725	0.844399	0.553658	0.529078	0.982906	0.969576
Other neonatal disorders [Global]	Female	0-6 days	2-4 years	0.292205	0.484289	0.181011	0.172029	0.99951	0.995836
Other neonatal disorders [Global]	Male	0-6 days	2-4 years	0.28256	0.497284	0.171096	0.175353	0.999551	0.994966
Other neonatal disorders [Data Rich]	Female	0-6 days	2-4 years	0.133177	0.36818	0.097004	0.161962	0.999938	0.99901
Other neonatal disorders [Data Rich]	Male	0-6 days	2-4 years	0.133698	0.377341	0.0967647	0.173264	0.999878	0.999366
Nutritional deficiencies [Global]	Female	1-5 months	95+ years	0.254289	0.565265	0.168977	0.163667	0.99918	0.986704
Nutritional deficiencies [Global]	Male	1-5 months	95+ years	0.259912	0.576657	0.174517	0.174454	0.999008	0.987735
Nutritional deficiencies [Data Rich]	Female	1-5 months	95+ years	0.116175	0.260688	0.0810534	0.152097	0.999991	0.9998
Nutritional deficiencies [Data Rich]	Male	1-5 months	95+ years	0.126614	0.268939	0.0909788	0.170649	0.999974	0.999719
Protein-energy malnutrition [Global]	Female	1-5 months	2-4 years	0.286961	0.722616	0.171669	0.189833	0.99969	0.980251
Protein-energy malnutrition [Global]	Female	5-9 years	95+ years	0.32682	0.733188	0.185333	0.193541	0.997886	0.97543
Protein-energy malnutrition [Global]	Male	1-5 months	2-4 years	0.287365	0.700955	0.17714	0.191233	0.999542	0.979869
Protein-energy malnutrition [Global]	Male	5-9 years	95+ years	0.314571	0.755181	0.192585	0.201825	0.998643	0.975782
Protein-energy malnutrition [Data Rich]	Female	1-5 months	2-4 years	0.129518	0.447528	0.0957517	0.20183	1	0.999311
Protein-energy malnutrition [Data Rich]	Female	5-9 years	95+ years	0.136419	0.419636	0.0996262	0.20465	0.999973	0.99951
Protein-energy malnutrition [Data Rich]	Male	1-5 months	2-4 years	0.138162	0.515905	0.100139	0.202024	1	0.999722
Protein-energy malnutrition [Data Rich]	Male	5-9 years	95+ years	0.147648	0.480358	0.110829	0.213968	0.999968	0.999568
Other nutritional deficiencies [Global]	Female	1-5 months	95+ years	0.366312	0.674668	0.232406	0.222145	0.99762	0.986517
Other nutritional deficiencies [Global]	Male	1-5 months	95+ years	0.346048	0.735509	0.232292	0.227	0.997631	0.982157
Other nutritional deficiencies [Data Rich]	Female	1-5 months	95+ years	0.245747	0.428824	0.163636	0.172145	0.999286	0.998062
Other nutritional deficiencies [Data Rich]	Male	1-5 months	95+ years	0.25695	0.464291	0.180917	0.214203	0.99899	0.997704
Lip and oral cavity cancer [Global]	Female	15-19 years	95+ years	0.256125	0.355837	0.200878	0.200461	0.999391	0.997759
Lip and oral cavity cancer [Global]	Male	15-19 years	95+ years	0.245151	0.368538	0.197485	0.194456	0.999823	0.994966
Lip and oral cavity cancer [Data Rich]	Female	15-19 years	95+ years	0.164134	0.237499	0.134436	0.171212	0.999869	0.999632
Lip and oral cavity cancer [Data Rich]	Male	15-19 years	95+ years	0.175588	0.249384	0.144059	0.180003	0.999805	0.999452
Nasopharynx cancer [Global]	Female	5-9 years	95+ years	0.348461	0.532573	0.300122	0.302497	0.997353	0.990966
Nasopharynx cancer [Global]	Male	5-9 years	95+ years	0.298252	0.489388	0.251377	0.245859	0.999466	0.991316
Nasopharynx cancer [Data Rich]	Female	5-9 years	95+ years	0.322111	0.40984	0.295335	0.382419	0.997583	0.996185
Nasopharynx cancer [Data Rich]	Male	5-9 years	95+ years	0.256092	0.317816	0.229993	0.256	0.999779	0.999516
Other pharynx cancer [Global]	Female	20-24 years	95+ years	0.314638	0.470765	0.247236	0.265905	0.998521	0.995517
Other pharynx cancer [Global]	Male	20-24 years	95+ years	0.268027	0.478376	0.208852	0.211364	0.999296	0.99139
Other pharynx cancer [Data Rich]	Female	20-24 years	95+ years	0.232436	0.333183	0.206232	0.274087	0.998925	0.996798
Other pharynx cancer [Data Rich]	Male	20-24 years	95+ years	0.18187	0.272597	0.155351	0.193709	0.999751	0.999373
Oesophageal cancer [Global]	Female	20-24 years	95+ years	0.248071	0.423095	0.193286	0.191115	0.999013	0.985491
Oesophageal cancer [Global]	Male	20-24 years	95+ years	0.226034	0.374916	0.181463	0.18105	0.997933	0.983043
Oesophageal cancer [Data Rich]	Female	20-24 years	95+ years	0.17415	0.24563	0.150126	0.192417	0.99929	0.99866

CODEm predictive validity results by cause, model type, sex, and age									
**Cause**	**Sex**	**Age Start**	**Age End**	**RMSE In-Sample**	**RMSE Out-of-Sample**	**Trend In-Sample**	**Trend Out-of-Sample**	**Coverage In-Sample**	**Coverage Out-of-Sample**
Oesophageal cancer [Data Rich]	Male	20-24 years	95+ years	0.157962	0.23139	0.135373	0.178381	0.999509	0.998605
Stomach cancer [Global]	Female	15-19 years	95+ years	0.209356	0.30649	0.169006	0.166391	0.998408	0.984432
Stomach cancer [Global]	Male	15-19 years	95+ years	0.221288	0.322174	0.17218	0.171668	0.998361	0.979615
Stomach cancer [Data Rich]	Female	15-19 years	95+ years	0.13748	0.194761	0.119289	0.153123	0.99866	0.996972
Stomach cancer [Data Rich]	Male	15-19 years	95+ years	0.139961	0.197638	0.12165	0.15224	0.998608	0.996773
Colon and rectum cancer [Global]	Female	15-19 years	95+ years	0.21234	0.285836	0.176941	0.17531	0.998926	0.993019
Colon and rectum cancer [Global]	Male	15-19 years	95+ years	0.225594	0.295015	0.185829	0.180955	0.99892	0.992111
Colon and rectum cancer [Data Rich]	Female	15-19 years	95+ years	0.136925	0.195853	0.116362	0.156951	0.99887	0.99728
Colon and rectum cancer [Data Rich]	Male	15-19 years	95+ years	0.143537	0.205279	0.122299	0.157625	0.99863	0.997126
Gallbladder and biliary tract cancer [Global]	Female	20-24 years	95+ years	0.284089	0.43622	0.209653	0.21353	0.997473	0.990636
Gallbladder and biliary tract cancer [Global]	Male	20-24 years	95+ years	0.413657	0.535975	0.309999	0.312726	0.996372	0.993139
Gallbladder and biliary tract cancer [Data Rich]	Female	20-24 years	95+ years	0.14901	0.224855	0.12508	0.163398	0.99917	0.998276
Gallbladder and biliary tract cancer [Data Rich]	Male	20-24 years	95+ years	0.176315	0.258078	0.154415	0.18893	0.998972	0.998161
Pancreatic cancer [Global]	Female	15-19 years	95+ years	0.229375	0.304218	0.189857	0.19529	0.998838	0.994475
Pancreatic cancer [Global]	Male	15-19 years	95+ years	0.235515	0.303661	0.193528	0.194612	0.998883	0.995817
Pancreatic cancer [Data Rich]	Female	15-19 years	95+ years	0.164638	0.239947	0.147064	0.194925	0.998535	0.996839
Pancreatic cancer [Data Rich]	Male	15-19 years	95+ years	0.153468	0.225409	0.133425	0.167162	0.998557	0.996917
Larynx cancer [Global]	Female	20-24 years	95+ years	0.327174	0.497487	0.257627	0.250843	0.999049	0.992909
Larynx cancer [Global]	Male	20-24 years	95+ years	0.23137	0.347141	0.184251	0.183537	0.999632	0.989815
Larynx cancer [Data Rich]	Female	20-24 years	95+ years	0.220803	0.301109	0.195363	0.240641	0.999507	0.999076
Larynx cancer [Data Rich]	Male	20-24 years	95+ years	0.160909	0.224909	0.13578	0.169436	0.999723	0.999244
Tracheal, bronchus, and lung cancer [Global]	Female	15-19 years	95+ years	0.237207	0.358027	0.188821	0.190969	0.998862	0.98531
Tracheal, bronchus, and lung cancer [Global]	Male	15-19 years	95+ years	0.238231	0.328252	0.183886	0.181453	0.998122	0.979653
Tracheal, bronchus, and lung cancer [Data Rich]	Female	15-19 years	95+ years	0.154234	0.229663	0.132576	0.186681	0.998776	0.996952
Tracheal, bronchus, and lung cancer [Data Rich]	Male	15-19 years	95+ years	0.147142	0.211796	0.12546	0.163387	0.997989	0.994813
Malignant skin melanoma [Global]	Female	15-19 years	95+ years	0.337939	0.47225	0.256357	0.260271	0.998603	0.994364
Malignant skin melanoma [Global]	Male	15-19 years	95+ years	0.35136	0.480523	0.255388	0.252384	0.999055	0.994576
Malignant skin melanoma [Data Rich]	Female	15-19 years	95+ years	0.196021	0.277747	0.160934	0.207039	0.998913	0.998111
Malignant skin melanoma [Data Rich]	Male	15-19 years	95+ years	0.223496	0.329916	0.184776	0.231426	0.999394	0.998639
Non-melanoma skin cancer (squamous-cell carcinoma) [Global]	Female	20-24 years	95+ years	0.420313	0.602826	0.229413	0.220153	0.992422	0.982008
Non-melanoma skin cancer (squamous-cell carcinoma) [Global]	Male	20-24 years	95+ years	0.46471	0.638663	0.232113	0.222428	0.991695	0.982228
Non-melanoma skin cancer (squamous-cell carcinoma) [Data Rich]	Female	20-24 years	95+ years	0.106962	0.240861	0.0874003	0.143534	0.999997	0.999854
Non-melanoma skin cancer (squamous-cell carcinoma) [Data Rich]	Male	20-24 years	95+ years	0.113479	0.252163	0.0891332	0.155853	0.999986	0.999805
Soft tissue and other extraosseous sarcomas [Global]	Female	0-6 days	95+ years	0.382597	0.481327	0.296399	0.285787	0.998903	0.997229
Soft tissue and other extraosseous sarcomas [Global]	Male	0-6 days	95+ years	0.403041	0.499339	0.283579	0.276157	0.998782	0.996507
Soft tissue and other extraosseous sarcomas [Data Rich]	Female	0-6 days	95+ years	0.301434	0.397389	0.271871	0.265833	0.999725	0.999338
Soft tissue and other extraosseous sarcomas [Data Rich]	Male	0-6 days	95+ years	0.289639	0.392729	0.260193	0.256262	0.999591	0.999129
Malignant neoplasm of bone and articular cartilage [Global]	Female	12-23 months	95+ years	0.391657	0.512153	0.281173	0.281723	0.998077	0.994202
Malignant neoplasm of bone and articular cartilage [Global]	Male	12-23 months	95+ years	0.417041	0.548914	0.27975	0.284674	0.998098	0.994069
Malignant neoplasm of bone and articular cartilage [Data Rich]	Female	12-23 months	95+ years	0.288589	0.368742	0.218172	0.265399	0.998992	0.998725
Malignant neoplasm of bone and articular cartilage [Data Rich]	Male	12-23 months	95+ years	0.274317	0.365431	0.217947	0.27053	0.999153	0.998853
Breast cancer [Global]	Female	15-19 years	95+ years	0.240811	0.306985	0.196825	0.190222	0.998341	0.990631
Breast cancer [Global]	Male	15-19 years	95+ years	0.42863	0.586202	0.337211	0.333331	0.997216	0.990026
Breast cancer [Data Rich]	Female	15-19 years	95+ years	0.162273	0.232013	0.136275	0.181459	0.998425	0.99608
Breast cancer [Data Rich]	Male	15-19 years	95+ years	0.325716	0.452038	0.265552	0.313302	0.997704	0.995685
Cervical cancer [Global]	Female	15-19 years	95+ years	0.217256	0.327248	0.178692	0.182284	0.999631	0.993819
Cervical cancer [Data Rich]	Female	15-19 years	95+ years	0.167896	0.241093	0.145345	0.193498	0.999459	0.998452
Uterine cancer [Global]	Female	20-24 years	95+ years	0.290588	0.36062	0.240044	0.220232	0.999311	0.996159
Uterine cancer [Data Rich]	Female	20-24 years	95+ years	0.160858	0.221894	0.138989	0.165508	0.999817	0.999427
Ovarian cancer [Global]	Female	15-19 years	95+ years	0.237037	0.328415	0.187162	0.191584	0.999401	0.995675
Ovarian cancer [Data Rich]	Female	15-19 years	95+ years	0.167568	0.248925	0.142382	0.185209	0.998765	0.996804
Prostate cancer [Global]	Male	20-24 years	95+ years	0.365799	0.435396	0.30256	0.299856	0.997802	0.988953
Prostate cancer [Data Rich]	Male	20-24 years	95+ years	0.225513	0.305004	0.196611	0.233855	0.996073	0.992752
Testicular cancer [Global]	Male	15-19 years	95+ years	0.411861	0.6036	0.336137	0.348501	0.996125	0.993634
Testicular cancer [Data Rich]	Male	15-19 years	95+ years	0.242046	0.340754	0.219921	0.246193	0.999415	0.998967
Kidney cancer [Global]	Female	0-6 days	95+ years	0.31447	0.420194	0.261454	0.24842	0.999099	0.995313
Kidney cancer [Global]	Male	0-6 days	95+ years	0.296272	0.4109	0.25045	0.26804	0.999227	0.995434
Kidney cancer [Data Rich]	Female	0-6 days	95+ years	0.211131	0.352969	0.175208	0.242258	0.999562	0.998904
Kidney cancer [Data Rich]	Male	0-6 days	95+ years	0.227457	0.331014	0.20299	0.262968	0.999485	0.998583
Bladder cancer [Global]	Female	15-19 years	95+ years	0.240868	0.343994	0.194053	0.193686	0.99941	0.996333
Bladder cancer [Global]	Male	15-19 years	95+ years	0.249031	0.324854	0.205511	0.207364	0.99929	0.993807
Bladder cancer [Data Rich]	Female	15-19 years	95+ years	0.156611	0.229835	0.131104	0.165074	0.999603	0.999075
Bladder cancer [Data Rich]	Male	15-19 years	95+ years	0.187111	0.24995	0.160739	0.19367	0.999044	0.997667
Brain and nervous system cancer [Global]	Female	0-6 days	95+ years	0.271671	0.396856	0.206577	0.208052	0.999304	0.996247
Brain and nervous system cancer [Global]	Male	0-6 days	95+ years	0.273497	0.365601	0.202649	0.198195	0.999355	0.997282
Brain and nervous system cancer [Data Rich]	Female	0-6 days	95+ years	0.190659	0.274238	0.163524	0.196311	0.999239	0.998413
Brain and nervous system cancer [Data Rich]	Male	0-6 days	95+ years	0.193646	0.277333	0.1677	0.190969	0.999255	0.998337
Retinoblastoma [Global]	Female	0-6 days	5-9 years	1.26506	1.67599	0.998545	0.964099	0.978205	0.93782
Retinoblastoma [Global]	Male	0-6 days	5-9 years	1.19445	1.56343	0.946241	0.932603	0.977217	0.940863
Retinoblastoma [Data Rich]	Female	0-6 days	5-9 years	1.05859	1.38762	0.929115	0.993483	0.975673	0.953076
Retinoblastoma [Data Rich]	Male	0-6 days	5-9 years	0.99093	1.25384	0.877249	0.947354	0.977339	0.950845
Other eye cancers [Global]	Female	10-14 years	95+ years	0.680855	0.786395	0.545134	0.492728	0.988647	0.981581
Other eye cancers [Global]	Male	10-14 years	95+ years	0.718687	0.777541	0.571015	0.514446	0.986174	0.97994
Other eye cancers [Data Rich]	Female	10-14 years	95+ years	0.391224	0.442837	0.326542	0.270254	0.99751	0.995588
Other eye cancers [Data Rich]	Male	10-14 years	95+ years	0.337763	0.427677	0.294666	0.292028	0.998507	0.996805
Neuroblastoma and other peripheral nervous cell tumours [Global]	Female	0-6 days	95+ years	0.532862	0.662074	0.401684	0.364634	0.994799	0.989846
Neuroblastoma and other peripheral nervous cell tumours [Global]	Male	0-6 days	95+ years	0.54644	0.787158	0.443655	0.437182	0.993744	0.984324
Neuroblastoma and other peripheral nervous cell tumours [Data Rich]	Female	0-6 days	95+ years	0.37097	0.488746	0.328893	0.280394	0.998946	0.9978
Neuroblastoma and other peripheral nervous cell tumours [Data Rich]	Male	0-6 days	95+ years	0.424442	0.572662	0.383319	0.330689	0.998277	0.996269
Thyroid cancer [Global]	Female	5-9 years	95+ years	0.460661	0.569419	0.364875	0.386416	0.99612	0.993101
Thyroid cancer [Global]	Male	5-9 years	95+ years	0.424624	0.554965	0.325346	0.338382	0.996574	0.99494
Thyroid cancer [Data Rich]	Female	5-9 years	95+ years	0.317551	0.414232	0.282198	0.303369	0.998337	0.996338
Thyroid cancer [Data Rich]	Male	5-9 years	95+ years	0.249684	0.358592	0.214556	0.263764	0.999601	0.999297
Mesothelioma [Global]	Female	20-24 years	95+ years	0.256339	0.443389	0.178768	0.186988	0.998745	0.9948
Mesothelioma [Global]	Male	20-24 years	95+ years	0.218945	0.400858	0.156001	0.18494	0.999427	0.997552
Mesothelioma [Data Rich]	Female	20-24 years	95+ years	0.115512	0.301206	0.0827518	0.217638	0.999982	0.99963
Mesothelioma [Data Rich]	Male	20-24 years	95+ years	0.0958742	0.234412	0.0729859	0.164817	0.99997	0.999865
Hodgkin lymphoma [Global]	Female	2-4 years	95+ years	0.428654	0.565177	0.319959	0.327645	0.996661	0.991316
Hodgkin lymphoma [Global]	Male	2-4 years	95+ years	0.46432	0.597488	0.337932	0.339831	0.99573	0.99002
Hodgkin lymphoma [Data Rich]	Female	2-4 years	95+ years	0.279389	0.390281	0.253744	0.341598	0.997107	0.99598
Hodgkin lymphoma [Data Rich]	Male	2-4 years	95+ years	0.312978	0.420786	0.287889	0.359054	0.994418	0.99241
Non-Hodgkin's lymphoma [Global]	Female	12-23 months	95+ years	0.258559	0.366856	0.212164	0.207807	0.999624	0.995788
Non-Hodgkin's lymphoma [Global]	Male	12-23 months	95+ years	0.246064	0.351103	0.190526	0.188789	0.999555	0.995412
Non-Hodgkin's lymphoma [Data Rich]	Female	12-23 months	95+ years	0.185001	0.275314	0.162952	0.199887	0.999321	0.998608
Non-Hodgkin's lymphoma [Data Rich]	Male	12-23 months	95+ years	0.175139	0.261065	0.150654	0.185233	0.999285	0.998593
Other non-Hodgkin lymphoma [Global]	Female	12-23 months	95+ years	0.341658	0.423567	0.279439	0.263393	0.999211	0.996931
Other non-Hodgkin lymphoma [Global]	Male	12-23 months	95+ years	0.358551	0.419613	0.291911	0.264257	0.996655	0.993481
Other non-Hodgkin lymphoma [Data Rich]	Female	12-23 months	95+ years	0.206908	0.348726	0.180833	0.202055	0.999749	0.99954
Other non-Hodgkin lymphoma [Data Rich]	Male	12-23 months	95+ years	0.201223	0.330453	0.176395	0.189718	0.999638	0.999335
Multiple myeloma [Global]	Female	20-24 years	95+ years	0.373231	0.489289	0.277401	0.27929	0.995635	0.991411
Multiple myeloma [Global]	Male	20-24 years	95+ years	0.346417	0.458536	0.259054	0.265139	0.996404	0.990968
Multiple myeloma [Data Rich]	Female	20-24 years	95+ years	0.192932	0.269674	0.164366	0.194556	0.999591	0.999308
Multiple myeloma [Data Rich]	Male	20-24 years	95+ years	0.185717	0.264326	0.153258	0.184912	0.999641	0.999299
Leukaemia [Global]	Female	0-6 days	95+ years	0.281379	0.339702	0.255165	0.249788	0.999532	0.9973
Leukaemia [Global]	Male	0-6 days	95+ years	0.269474	0.324466	0.219487	0.214477	0.999644	0.997873
Leukaemia [Data Rich]	Female	0-6 days	95+ years	0.179808	0.24673	0.159588	0.193247	0.999458	0.998828
Leukaemia [Data Rich]	Male	0-6 days	95+ years	0.176704	0.245469	0.153932	0.190994	0.999653	0.999112
Acute lymphoid leukaemia [Global]	Female	0-6 days	95+ years	0.54212	0.702714	0.356456	0.318997	0.996696	0.993122
Acute lymphoid leukaemia [Global]	Male	0-6 days	95+ years	0.540451	0.716983	0.381341	0.324596	0.998091	0.992439
Acute lymphoid leukaemia [Data Rich]	Female	0-6 days	95+ years	0.299944	0.553202	0.23533	0.276631	0.999089	0.993447
Acute lymphoid leukaemia [Data Rich]	Male	0-6 days	95+ years	0.273216	0.430368	0.251435	0.262255	0.999387	0.99778
Chronic lymphoid leukaemia [Global]	Female	20-24 years	95+ years	0.578008	1.13695	0.467795	0.466149	0.996371	0.973589
Chronic lymphoid leukaemia [Global]	Male	20-24 years	95+ years	0.494588	0.734093	0.397579	0.332934	0.997768	0.99125
Chronic lymphoid leukaemia [Data Rich]	Female	20-24 years	95+ years	0.357853	0.467338	0.332577	0.279815	0.998182	0.995288
Chronic lymphoid leukaemia [Data Rich]	Male	20-24 years	95+ years	0.329046	0.387517	0.308809	0.253543	0.998758	0.997585
Acute myeloid leukaemia [Global]	Female	0-6 days	95+ years	0.452892	0.559827	0.322621	0.298238	0.995917	0.992446
Acute myeloid leukaemia [Global]	Male	0-6 days	95+ years	0.470322	0.634969	0.325422	0.29714	0.99654	0.991806
Acute myeloid leukaemia [Data Rich]	Female	0-6 days	95+ years	0.21518	0.382517	0.188705	0.196488	0.99943	0.998874
Acute myeloid leukaemia [Data Rich]	Male	0-6 days	95+ years	0.242082	0.380178	0.216392	0.22976	0.998831	0.997714
Chronic myeloid leukaemia [Global]	Female	0-6 days	95+ years	0.665631	0.955437	0.523533	0.468923	0.993721	0.98191
Chronic myeloid leukaemia [Global]	Male	0-6 days	95+ years	0.615174	0.893365	0.487145	0.476944	0.995704	0.988857
Chronic myeloid leukaemia [Data Rich]	Female	0-6 days	95+ years	0.470684	0.867777	0.441147	0.474122	0.995376	0.986993
Chronic myeloid leukaemia [Data Rich]	Male	0-6 days	95+ years	0.460927	0.750818	0.440176	0.456168	0.994814	0.988758
Other leukaemia [Global]	Female	0-6 days	95+ years	0.743694	1.17624	0.51597	0.517044	0.995178	0.972861
Other leukaemia [Global]	Male	0-6 days	95+ years	0.634721	1.0713	0.405431	0.423443	0.996256	0.974445
Other leukaemia [Data Rich]	Female	0-6 days	95+ years	0.52196	1.10827	0.444919	0.464963	0.997328	0.980133
Other leukaemia [Data Rich]	Male	0-6 days	95+ years	0.430103	0.947296	0.360009	0.34305	0.9973	0.985317
Other malignant cancers [Global]	Female	0-6 days	95+ years	0.360656	0.415402	0.296127	0.292735	0.999031	0.995564
Other malignant cancers [Global]	Male	0-6 days	95+ years	0.346961	0.429359	0.259927	0.261217	0.998469	0.995271
Other malignant cancers [Data Rich]	Female	0-6 days	95+ years	0.231226	0.314288	0.204879	0.239302	0.999562	0.998898
Other malignant cancers [Data Rich]	Male	0-6 days	95+ years	0.220723	0.310986	0.189653	0.239376	0.999641	0.999143
Myelodysplastic, myeloproliferative, and other hematopoietic neoplasms [Global]	Female	0-6 days	95+ years	0.397896	0.806098	0.250484	0.300054	0.996301	0.976354
Myelodysplastic, myeloproliferative, and other hematopoietic neoplasms [Global]	Male	0-6 days	95+ years	0.543784	0.916086	0.226708	0.261133	0.997864	0.981602

CODEm predictive validity results by cause, model type, sex, and age

Cause	Sex	Age Start	Age End	RMSE In-Sample	RMSE Out-of-Sample	Trend In-Sample	Trend Out-of-Sample	Coverage In-Sample	Coverage Out-of-Sample
Myelodysplastic, myeloproliferative, and other hematopoietic neoplasms [Data Rich]	Female	0-6 days	95+ years	0.226508	0.776854	0.129668	0.257845	0.999324	0.998326
Myelodysplastic, myeloproliferative, and other hematopoietic neoplasms [Data Rich]	Male	0-6 days	95+ years	0.173662	0.768863	0.121072	0.279318	0.999698	0.999071
Cardiovascular diseases [Global]	Female	0-6 days	95+ years	0.142892	0.218964	0.100826	0.102116	0.999732	0.99262
Cardiovascular diseases [Global]	Male	0-6 days	95+ years	0.144472	0.226928	0.0999496	0.0989903	0.999698	0.988117
Cardiovascular diseases [Data Rich]	Female	0-6 days	95+ years	0.0829391	0.154437	0.066502	0.102258	0.999978	0.999651
Cardiovascular diseases [Data Rich]	Male	0-6 days	95+ years	0.081988	0.153259	0.0646696	0.100762	0.999987	0.999405
Rheumatic heart disease [Global]	Female	12-23 months	95+ years	0.1996	0.38606	0.12448	0.125181	0.999886	0.99064
Rheumatic heart disease [Global]	Male	12-23 months	95+ years	0.212687	0.413725	0.135057	0.137304	0.999929	0.99237
Rheumatic heart disease [Data Rich]	Female	12-23 months	95+ years	0.10954	0.274468	0.0787165	0.119659	0.999995	0.999678
Rheumatic heart disease [Data Rich]	Male	12-23 months	95+ years	0.115665	0.275785	0.0833168	0.12441	1	0.999766
Ischaemic heart disease [Global]	Female	15-19 years	95+ years	0.143579	0.254042	0.100622	0.10115	0.99956	0.977678
Ischaemic heart disease [Global]	Male	15-19 years	95+ years	0.1385	0.256977	0.0959974	0.0970444	0.999573	0.976782
Ischaemic heart disease [Data Rich]	Female	15-19 years	95+ years	0.0809436	0.148387	0.0671845	0.100738	0.999965	0.999141
Ischaemic heart disease [Data Rich]	Male	15-19 years	95+ years	0.0785197	0.146581	0.0639568	0.0988609	0.999984	0.998606
Stroke [Global]	Female	0-6 days	95+ years	0.161202	0.26477	0.108477	0.109216	0.999793	0.991561
Stroke [Global]	Male	0-6 days	95+ years	0.169532	0.274115	0.111638	0.111336	0.999713	0.992153
Stroke [Data Rich]	Female	0-6 days	95+ years	0.0975736	0.185592	0.0728896	0.10927	0.99998	0.999696
Stroke [Data Rich]	Male	0-6 days	95+ years	0.0985635	0.190106	0.0731154	0.109461	0.999978	0.999625
Ischaemic stroke [Global]	Female	0-6 days	95+ years	0.16329	0.289806	0.102071	0.10386	0.999886	0.998486
Ischaemic stroke [Global]	Male	0-6 days	95+ years	0.167059	0.28451	0.104327	0.105492	0.999883	0.998368
Ischaemic stroke [Data Rich]	Female	0-6 days	95+ years	0.099448	0.254867	0.0689693	0.0982263	1	0.999933
Ischaemic stroke [Data Rich]	Male	0-6 days	95+ years	0.0950877	0.215608	0.0696697	0.0981692	0.999995	0.99989
Intracerebral hemorrhage [Global]	Female	0-6 days	95+ years	0.184303	0.310849	0.108305	0.110352	0.999709	0.994178
Intracerebral hemorrhage [Global]	Male	0-6 days	95+ years	0.167167	0.28839	0.106691	0.10782	0.99962	0.993445
Intracerebral hemorrhage [Data Rich]	Female	0-6 days	95+ years	0.108866	0.465393	0.0729309	0.107652	0.999971	0.999539
Intracerebral hemorrhage [Data Rich]	Male	0-6 days	95+ years	0.0951443	0.419758	0.07067	0.10384	0.999986	0.999823
Subarachnoid hemorrhage [Global]	Female	0-6 days	95+ years	0.212886	0.340995	0.120557	0.118838	0.99929	0.993815
Subarachnoid hemorrhage [Global]	Male	0-6 days	95+ years	0.289065	0.400997	0.127498	0.125633	0.999503	0.994879
Subarachnoid hemorrhage [Data Rich]	Female	0-6 days	95+ years	0.114538	0.274712	0.0709359	0.110165	0.999968	0.999564
Subarachnoid hemorrhage [Data Rich]	Male	0-6 days	95+ years	0.121183	0.328578	0.0731448	0.120493	0.999993	0.999767
Hypertensive heart disease [Global]	Female	15-19 years	95+ years	0.226211	0.47191	0.131671	0.134168	0.999347	0.974893
Hypertensive heart disease [Global]	Male	15-19 years	95+ years	0.233886	0.469943	0.142086	0.145051	0.998965	0.973908
Hypertensive heart disease [Data Rich]	Female	15-19 years	95+ years	0.093107	0.24417	0.071388	0.115559	0.999923	0.999311
Hypertensive heart disease [Data Rich]	Male	15-19 years	95+ years	0.103144	0.271719	0.0787306	0.131035	0.999943	0.999176
Non-rheumatic valvular heart disease [Global]	Female	15-19 years	95+ years	0.267477	0.475834	0.14561	0.145365	0.999891	0.98956
Non-rheumatic valvular heart disease [Global]	Male	15-19 years	95+ years	0.200314	0.453712	0.130919	0.130335	0.999869	0.984991
Non-rheumatic valvular heart disease [Data Rich]	Female	15-19 years	95+ years	0.0925707	0.268337	0.0751472	0.135276	0.999984	0.999877
Non-rheumatic valvular heart disease [Data Rich]	Male	15-19 years	95+ years	0.0924648	0.264762	0.0748463	0.129788	0.999984	0.999797
Non-rheumatic calcific aortic valve disease [Global]	Female	15-19 years	95+ years	0.243111	0.618436	0.128396	0.132378	0.999918	0.981712
Non-rheumatic calcific aortic valve disease [Global]	Male	15-19 years	95+ years	0.210305	0.571282	0.127249	0.127861	0.999961	0.983593
Non-rheumatic calcific aortic valve disease [Data Rich]	Female	15-19 years	95+ years	0.0983489	0.296316	0.0747953	0.133464	0.999983	0.999891
Non-rheumatic calcific aortic valve disease [Data Rich]	Male	15-19 years	95+ years	0.0905364	0.286603	0.0730469	0.127477	0.999993	0.999895
Non-rheumatic degenerative mitral valve disease [Global]	Female	15-19 years	95+ years	0.38181	0.675823	0.169084	0.156785	0.999089	0.984299
Non-rheumatic degenerative mitral valve disease [Global]	Male	15-19 years	95+ years	0.31582	0.602357	0.169127	0.151742	0.999439	0.985904
Non-rheumatic degenerative mitral valve disease [Data Rich]	Female	15-19 years	95+ years	0.0963277	0.303016	0.074243	0.120896	1	0.999908
Non-rheumatic degenerative mitral valve disease [Data Rich]	Male	15-19 years	95+ years	0.102201	0.279246	0.0776384	0.124449	1	0.999943
Other non-rheumatic valve diseases [Global]	Female	15-19 years	95+ years	0.994711	1.37322	0.618105	0.61853	0.977492	0.91823
Other non-rheumatic valve diseases [Global]	Male	15-19 years	95+ years	1.08369	1.47514	0.685873	0.67283	0.979926	0.907494
Other non-rheumatic valve diseases [Data Rich]	Female	15-19 years	95+ years	0.660096	0.898816	0.563128	0.592311	0.978106	0.965897
Other non-rheumatic valve diseases [Data Rich]	Male	15-19 years	95+ years	0.688924	1.0055	0.625703	0.623982	0.98113	0.965726
Cardiomyopathy and myocarditis [Global]	Female	0-6 days	95+ years	0.270655	0.614704	0.171724	0.181108	0.99943	0.981387
Cardiomyopathy and myocarditis [Global]	Male	0-6 days	95+ years	0.249554	0.512983	0.154195	0.157908	0.999472	0.980386
Cardiomyopathy and myocarditis [Data Rich]	Female	0-6 days	95+ years	0.117852	0.360638	0.0945569	0.164918	0.99993	0.999454
Cardiomyopathy and myocarditis [Data Rich]	Male	0-6 days	95+ years	0.117426	0.326577	0.0889963	0.15419	0.999893	0.999297
Myocarditis [Global]	Female	0-6 days	95+ years	0.461172	0.920062	0.231096	0.232138	0.999137	0.977357
Myocarditis [Global]	Male	0-6 days	95+ years	0.478569	0.830311	0.237531	0.232561	0.998243	0.980191
Myocarditis [Data Rich]	Female	0-6 days	95+ years	0.21283	0.619531	0.167369	0.232195	0.999952	0.996715
Myocarditis [Data Rich]	Male	0-6 days	95+ years	0.219198	0.652003	0.175168	0.274144	0.999716	0.997884
Alcoholic cardiomyopathy [Global]	Female	15-19 years	95+ years	0.417551	0.823058	0.289243	0.299605	0.997812	0.983706
Alcoholic cardiomyopathy [Global]	Male	15-19 years	95+ years	0.383132	0.698889	0.231249	0.241358	0.999246	0.993265
Alcoholic cardiomyopathy [Data Rich]	Female	15-19 years	95+ years	0.237361	0.555139	0.190141	0.193134	0.999634	0.998076
Alcoholic cardiomyopathy [Data Rich]	Male	15-19 years	95+ years	0.190463	0.443819	0.148105	0.146197	0.999412	0.998493
Other cardiomyopathy [Global]	Female	0-6 days	95+ years	0.245875	0.63924	0.158553	0.167293	0.999936	0.984946
Other cardiomyopathy [Global]	Male	0-6 days	95+ years	0.231857	0.545127	0.153257	0.158791	0.999852	0.985818
Other cardiomyopathy [Data Rich]	Female	0-6 days	95+ years	0.122055	0.385822	0.0961929	0.17098	0.999993	0.999918
Other cardiomyopathy [Data Rich]	Male	0-6 days	95+ years	0.117016	0.340446	0.0914513	0.152793	0.999982	0.999844
Pulmonary Arterial Hypertension [Global]	Female	0-6 days	95+ years	0.335602	0.642696	0.197972	0.204087	0.999878	0.990906
Pulmonary Arterial Hypertension [Global]	Male	0-6 days	95+ years	0.340174	0.692926	0.217623	0.217332	0.999468	0.982964
Pulmonary Arterial Hypertension [Data Rich]	Female	0-6 days	95+ years	0.185585	0.490386	0.147923	0.190564	0.999996	0.996157
Pulmonary Arterial Hypertension [Data Rich]	Male	0-6 days	95+ years	0.207305	0.565705	0.16781	0.214867	0.99973	0.991332
Atrial fibrillation and flutter [Global]	Female	30-34 years	95+ years	0.123357	0.200395	0.0555143	0.0559709	0.999904	0.997719
Atrial fibrillation and flutter [Global]	Male	30-34 years	95+ years	0.110644	0.198258	0.0577197	0.0570308	0.999889	0.997889
Atrial fibrillation and flutter [Data Rich]	Female	30-34 years	95+ years	0.0667011	0.154171	0.0358559	0.0546686	0.999966	0.99978
Atrial fibrillation and flutter [Data Rich]	Male	30-34 years	95+ years	0.0504349	0.144197	0.0363068	0.0521697	0.999962	0.999846
Aortic aneurysm [Global]	Female	15-19 years	95+ years	0.308444	0.497269	0.184435	0.186177	0.997249	0.989017
Aortic aneurysm [Global]	Male	15-19 years	95+ years	0.2428	0.441674	0.153287	0.158444	0.998027	0.986863
Aortic aneurysm [Data Rich]	Female	15-19 years	95+ years	0.102303	0.260096	0.0826643	0.136585	0.999922	0.999668
Aortic aneurysm [Data Rich]	Male	15-19 years	95+ years	0.0944321	0.237037	0.0772852	0.12357	0.999929	0.999604
Lower extremity peripheral arterial disease [Global]	Female	40-44 years	95+ years	0.423771	0.77322	0.270675	0.240333	0.992224	0.976069
Lower extremity peripheral arterial disease [Global]	Male	40-44 years	95+ years	0.39236	0.681035	0.248703	0.221537	0.992009	0.973876
Lower extremity peripheral arterial disease [Data Rich]	Female	40-44 years	95+ years	0.10631	0.457764	0.0826424	0.182394	0.999989	0.997909
Lower extremity peripheral arterial disease [Data Rich]	Male	40-44 years	95+ years	0.0991722	0.423015	0.0824088	0.179746	0.999989	0.997846
Endocarditis [Global]	Female	0-6 days	95+ years	0.399137	0.694731	0.154272	0.167082	0.999907	0.994062
Endocarditis [Global]	Male	0-6 days	95+ years	0.434463	0.692213	0.167667	0.173835	0.999502	0.991894
Endocarditis [Data Rich]	Female	0-6 days	95+ years	0.136577	0.597438	0.0820887	0.160995	0.999995	0.998593
Endocarditis [Data Rich]	Male	0-6 days	95+ years	0.124896	0.547937	0.0763941	0.154106	0.999998	0.999348
Other cardiovascular and circulatory diseases [Global]	Female	0-6 days	95+ years	0.240747	0.382977	0.144405	0.146033	0.999041	0.992874
Other cardiovascular and circulatory diseases [Global]	Male	0-6 days	95+ years	0.228107	0.380998	0.148285	0.149948	0.998635	0.99334
Other cardiovascular and circulatory diseases [Data Rich]	Female	0-6 days	95+ years	0.105135	0.247851	0.0789161	0.13017	0.999968	0.999796
Other cardiovascular and circulatory diseases [Data Rich]	Male	0-6 days	95+ years	0.0986865	0.214792	0.0794136	0.127775	0.999975	0.999849
Chronic respiratory diseases [Global]	Female	0-6 days	95+ years	0.281558	1.15691	0.139165	0.138142	0.999414	0.961815
Chronic respiratory diseases [Global]	Male	0-6 days	95+ years	0.264681	1.12793	0.145128	0.142754	0.999153	0.957108
Chronic respiratory diseases [Data Rich]	Female	0-6 days	95+ years	0.162604	2.20986	0.0872561	0.236282	0.99998	0.99429
Chronic respiratory diseases [Data Rich]	Male	0-6 days	95+ years	0.187845	2.13906	0.0824904	0.221577	0.999978	0.993386
Chronic obstructive pulmonary disease [Global]	Female	12-23 months	95+ years	0.170445	0.421674	0.113651	0.118633	0.999701	0.974778
Chronic obstructive pulmonary disease [Global]	Male	12-23 months	95+ years	0.158792	0.309616	0.109406	0.112778	0.999719	0.979811
Chronic obstructive pulmonary disease [Data Rich]	Female	12-23 months	95+ years	0.0982075	0.267337	0.0777095	0.129811	0.999925	0.999604
Chronic obstructive pulmonary disease [Data Rich]	Male	12-23 months	95+ years	0.0936086	0.205728	0.0740888	0.113838	0.999942	0.999502
Pneumoconiosis [Global]	Female	15-19 years	95+ years	0.677197	1.38384	0.373924	0.399994	0.994359	0.928287
Pneumoconiosis [Global]	Male	15-19 years	95+ years	0.458275	1.09896	0.280711	0.254534	0.993877	0.93944
Pneumoconiosis [Data Rich]	Female	15-19 years	95+ years	0.410425	0.799509	0.321757	0.36854	0.994956	0.990126
Pneumoconiosis [Data Rich]	Male	15-19 years	95+ years	0.266292	0.475631	0.178621	0.18023	0.99881	0.997968
Silicosis [Global]	Female	15-19 years	95+ years	0.795366	1.39321	0.563755	0.577824	0.993226	0.944381
Silicosis [Global]	Male	15-19 years	95+ years	0.624255	1.36863	0.361428	0.348698	0.993059	0.902856
Silicosis [Data Rich]	Female	15-19 years	95+ years	0.62138	1.18547	0.51353	0.604157	0.993002	0.963022
Silicosis [Data Rich]	Male	15-19 years	95+ years	0.385265	0.862226	0.287167	0.258249	0.995588	0.991499
Asbestosis [Global]	Female	15-19 years	95+ years	1.00393	1.59086	0.512638	0.532673	0.992849	0.914978
Asbestosis [Global]	Male	15-19 years	95+ years	0.786751	1.21411	0.361024	0.345888	0.993504	0.956504
Asbestosis [Data Rich]	Female	15-19 years	95+ years	0.555442	1.25206	0.455267	0.52406	0.992777	0.977321
Asbestosis [Data Rich]	Male	15-19 years	95+ years	0.379407	0.979232	0.260451	0.297188	0.997514	0.994054
Coal workers pneumoconiosis [Global]	Female	15-19 years	95+ years	0.858359	1.47355	0.641604	0.661969	0.992921	0.952365
Coal workers pneumoconiosis [Global]	Male	15-19 years	95+ years	0.617712	1.40889	0.425425	0.412974	0.989379	0.911156
Coal workers pneumoconiosis [Data Rich]	Female	15-19 years	95+ years	0.671658	1.29083	0.57679	0.656722	0.991825	0.940708
Coal workers pneumoconiosis [Data Rich]	Male	15-19 years	95+ years	0.459605	0.857569	0.365087	0.311787	0.992554	0.986185
Other pneumoconiosis [Global]	Female	15-19 years	95+ years	0.767805	1.73918	0.570997	0.582655	0.985707	0.855334
Other pneumoconiosis [Global]	Male	15-19 years	95+ years	0.788055	1.57267	0.565155	0.552421	0.983509	0.847703
Other pneumoconiosis [Data Rich]	Female	15-19 years	95+ years	0.627472	1.15347	0.522501	0.528003	0.9849	0.971846
Other pneumoconiosis [Data Rich]	Male	15-19 years	95+ years	0.606688	0.99048	0.508943	0.42466	0.986948	0.976509
Asthma [Global]	Female	12-23 months	95+ years	0.190377	0.416242	0.131148	0.132175	0.999947	0.989773
Asthma [Global]	Male	12-23 months	95+ years	0.215771	0.477786	0.148833	0.147108	0.999797	0.981641
Asthma [Data Rich]	Female	12-23 months	95+ years	0.11014	0.301678	0.0877116	0.130305	0.999992	0.999813
Asthma [Data Rich]	Male	12-23 months	95+ years	0.125995	0.335921	0.102667	0.138258	0.999981	0.999727
Interstitial lung disease and pulmonary sarcoidosis [Global]	Female	12-23 months	95+ years	0.559254	0.797551	0.25702	0.242643	0.993328	0.982762
Interstitial lung disease and pulmonary sarcoidosis [Global]	Male	12-23 months	95+ years	0.576794	0.845045	0.240095	0.244889	0.993897	0.981094
Interstitial lung disease and pulmonary sarcoidosis [Data Rich]	Female	12-23 months	95+ years	0.180133	0.570766	0.113439	0.177482	0.999039	0.997341
Interstitial lung disease and pulmonary sarcoidosis [Data Rich]	Male	12-23 months	95+ years	0.192157	0.596343	0.11923	0.18743	0.999405	0.995428
Other chronic respiratory diseases [Global]	Female	0-6 days	95+ years	0.494788	0.79986	0.296979	0.32987	0.998163	0.982607
Other chronic respiratory diseases [Global]	Male	0-6 days	95+ years	0.483126	0.746569	0.264811	0.282703	0.999718	0.9897
Other chronic respiratory diseases [Data Rich]	Female	0-6 days	95+ years	0.309409	0.837132	0.233025	0.289179	0.999852	0.997868
Other chronic respiratory diseases [Data Rich]	Male	0-6 days	95+ years	0.299088	0.80294	0.227078	0.278443	0.999772	0.999064
Digestive diseases [Global]	Female	0-6 days	95+ years	0.183793	0.33604	0.138797	0.142925	0.999718	0.98755
Digestive diseases [Global]	Male	0-6 days	95+ years	0.185381	0.33432	0.135476	0.139134	0.999699	0.986197

CODEm predictive validity results by cause, model type, sex, and age

Cause	Sex	Age Start	Age End	RMSE In-Sample	RMSE Out-of-Sample	Trend In-Sample	Trend Out-of-Sample	Coverage In-Sample	Coverage Out-of-Sample
Digestive diseases [Data Rich]	Female	0-6 days	95+ years	0.0986135	0.200207	0.0730545	0.119196	0.999996	0.999417
Digestive diseases [Data Rich]	Male	0-6 days	95+ years	0.0963002	0.19528	0.0727615	0.119503	0.999998	0.999482
Cirrhosis and other chronic liver diseases [Global]	Female	12-23 months	95+ years	0.183713	0.387491	0.120845	0.125221	0.99978	0.988338
Cirrhosis and other chronic liver diseases [Global]	Male	12-23 months	95+ years	0.18844	0.416978	0.117836	0.125177	0.999546	0.98084
Cirrhosis and other chronic liver diseases [Data Rich]	Female	12-23 months	95+ years	0.0976833	0.212814	0.0772222	0.128021	0.999995	0.999589
Cirrhosis and other chronic liver diseases [Data Rich]	Male	12-23 months	95+ years	0.0980887	0.232574	0.0774607	0.134069	0.999992	0.999398
Upper digestive system diseases [Global]	Female	6-11 months	95+ years	0.227983	0.406601	0.1302	0.127589	0.998811	0.991342
Upper digestive system diseases [Global]	Male	6-11 months	95+ years	0.223144	0.392796	0.126777	0.127827	0.998731	0.991898
Upper digestive system diseases [Data Rich]	Female	6-11 months	95+ years	0.115211	0.351002	0.0787737	0.121593	0.99997	0.999622
Upper digestive system diseases [Data Rich]	Male	6-11 months	95+ years	0.118045	0.406722	0.0802686	0.12205	0.999983	0.999486
Peptic ulcer disease [Global]	Female	6-11 months	95+ years	0.196922	0.35675	0.122965	0.1249	0.999645	0.992299
Peptic ulcer disease [Global]	Male	6-11 months	95+ years	0.188652	0.319503	0.122707	0.124502	0.999678	0.993068
Peptic ulcer disease [Data Rich]	Female	6-11 months	95+ years	0.111137	0.270638	0.0787831	0.114302	0.99998	0.999463
Peptic ulcer disease [Data Rich]	Male	6-11 months	95+ years	0.112102	0.238878	0.0801932	0.112015	0.999994	0.99947
Gastritis and duodenitis [Global]	Female	6-11 months	95+ years	0.267159	0.63834	0.174525	0.168944	0.999272	0.983769
Gastritis and duodenitis [Global]	Male	6-11 months	95+ years	0.315495	0.649006	0.189193	0.18034	0.998788	0.987134
Gastritis and duodenitis [Data Rich]	Female	6-11 months	95+ years	0.176457	0.450067	0.135158	0.158415	0.99987	0.999653
Gastritis and duodenitis [Data Rich]	Male	6-11 months	95+ years	0.185142	0.466984	0.140238	0.183188	0.999791	0.999364
Appendicitis [Global]	Female	12-23 months	95+ years	0.265133	0.464673	0.169708	0.170234	0.999679	0.996641
Appendicitis [Global]	Male	12-23 months	95+ years	0.231255	0.410208	0.155586	0.154729	0.99971	0.99656
Appendicitis [Data Rich]	Female	12-23 months	95+ years	0.170946	0.303026	0.134751	0.178654	0.999891	0.999765
Appendicitis [Data Rich]	Male	12-23 months	95+ years	0.168154	0.27353	0.132243	0.159459	0.999972	0.999813
Paralytic ileus and intestinal obstruction [Global]	Female	0-6 days	95+ years	0.261047	0.409151	0.143593	0.139832	0.999695	0.997045
Paralytic ileus and intestinal obstruction [Global]	Male	0-6 days	95+ years	0.27012	0.399063	0.144142	0.149681	0.999728	0.998322
Paralytic ileus and intestinal obstruction [Data Rich]	Female	0-6 days	95+ years	0.113272	0.269363	0.0753164	0.125745	1	0.999871
Paralytic ileus and intestinal obstruction [Data Rich]	Male	0-6 days	95+ years	0.111701	0.268227	0.0783659	0.133002	1	0.999909
Inguinal, femoral, and abdominal hernia [Global]	Female	0-6 days	95+ years	0.33662	0.498543	0.230601	0.212686	0.996508	0.991066
Inguinal, femoral, and abdominal hernia [Global]	Male	0-6 days	95+ years	0.344552	0.507692	0.200018	0.19236	0.99769	0.992897
Inguinal, femoral, and abdominal hernia [Data Rich]	Female	0-6 days	95+ years	0.117031	0.276495	0.0759853	0.1196	0.999986	0.999662
Inguinal, femoral, and abdominal hernia [Data Rich]	Male	0-6 days	95+ years	0.152188	0.356498	0.0822622	0.127074	0.99998	0.997832
Inflammatory bowel disease [Global]	Female	2-4 years	95+ years	0.417342	0.588912	0.244867	0.23826	0.997251	0.992643
Inflammatory bowel disease [Global]	Male	2-4 years	95+ years	0.369119	0.545572	0.232382	0.232034	0.997934	0.993812
Inflammatory bowel disease [Data Rich]	Female	2-4 years	95+ years	0.119268	0.40402	0.0968456	0.162669	0.999972	0.998553
Inflammatory bowel disease [Data Rich]	Male	2-4 years	95+ years	0.123817	0.369693	0.0974516	0.16281	0.999973	0.999279
Vascular intestinal disorders [Global]	Female	2-4 years	95+ years	0.24778	0.452353	0.159493	0.150524	0.999011	0.992313
Vascular intestinal disorders [Global]	Male	2-4 years	95+ years	0.284311	0.518229	0.180568	0.160435	0.998566	0.989456
Vascular intestinal disorders [Data Rich]	Female	2-4 years	95+ years	0.106021	0.295166	0.0713645	0.114674	0.999997	0.999726
Vascular intestinal disorders [Data Rich]	Male	2-4 years	95+ years	0.127352	0.297008	0.0728581	0.111854	0.999988	0.998372
Gallbladder and biliary diseases [Global]	Female	2-4 years	95+ years	0.333228	0.49281	0.142465	0.144303	0.999312	0.99153
Gallbladder and biliary diseases [Global]	Male	2-4 years	95+ years	0.326339	0.471323	0.147575	0.150056	0.999197	0.99284
Gallbladder and biliary diseases [Data Rich]	Female	2-4 years	95+ years	0.13559	0.331605	0.0762074	0.13669	0.999989	0.998591
Gallbladder and biliary diseases [Data Rich]	Male	2-4 years	95+ years	0.12808	0.268636	0.0780008	0.134507	0.999981	0.998974
Pancreatitis [Global]	Female	2-4 years	95+ years	0.269532	0.401562	0.154777	0.144458	0.998499	0.994885
Pancreatitis [Global]	Male	2-4 years	95+ years	0.291099	0.435269	0.14283	0.147496	0.998737	0.993705
Pancreatitis [Data Rich]	Female	2-4 years	95+ years	0.110305	0.255474	0.0769018	0.127203	0.999992	0.999386
Pancreatitis [Data Rich]	Male	2-4 years	95+ years	0.104285	0.249242	0.0779305	0.130637	0.999997	0.999718
Other digestive diseases [Global]	Female	12-23 months	95+ years	0.267377	0.431581	0.151733	0.153997	0.999469	0.994674
Other digestive diseases [Global]	Male	12-23 months	95+ years	0.277022	0.42331	0.164771	0.164701	0.997535	0.994948
Other digestive diseases [Data Rich]	Female	12-23 months	95+ years	0.0953045	0.215289	0.0712349	0.121877	0.999976	0.999798
Other digestive diseases [Data Rich]	Male	12-23 months	95+ years	0.0950565	0.211582	0.0712201	0.12411	0.99999	0.999884
Parkinson's disease [Global]	Female	20-24 years	95+ years	0.157618	0.25188	0.0563705	0.059928	0.999901	0.998702
Parkinson's disease [Global]	Male	20-24 years	95+ years	0.157342	0.26855	0.0543088	0.0569697	0.999876	0.997838
Parkinson's disease [Data Rich]	Female	20-24 years	95+ years	0.0602835	0.293611	0.0363323	0.0692658	0.999962	0.999777
Parkinson's disease [Data Rich]	Male	20-24 years	95+ years	0.0910628	0.240186	0.0356768	0.0705164	0.999983	0.999846
Idiopathic epilepsy [Global]	Female	0-6 days	95+ years	0.304257	0.524541	0.151015	0.151685	0.999564	0.99284
Idiopathic epilepsy [Global]	Male	0-6 days	95+ years	0.299813	0.571841	0.147272	0.154595	0.999639	0.992291
Idiopathic epilepsy [Data Rich]	Female	0-6 days	95+ years	0.121275	0.267469	0.0895491	0.151773	0.999993	0.999918
Idiopathic epilepsy [Data Rich]	Male	0-6 days	95+ years	0.116119	0.255126	0.0854211	0.146224	0.999998	0.999895
Multiple sclerosis [Global]	Female	5-9 years	95+ years	0.266683	0.751115	0.186931	0.195913	0.998675	0.990897
Multiple sclerosis [Global]	Male	5-9 years	95+ years	0.344753	0.869945	0.325429	0.324247	0.996953	0.978493
Multiple sclerosis [Data Rich]	Female	5-9 years	95+ years	0.237246	0.500612	0.152166	0.269609	0.998936	0.997146
Multiple sclerosis [Data Rich]	Male	5-9 years	95+ years	0.219883	0.394661	0.153259	0.1947	0.999396	0.998226
Motor neuron disease [Global]	Female	0-6 days	95+ years	0.49089	0.976618	0.293525	0.302516	0.990182	0.959242
Motor neuron disease [Global]	Male	0-6 days	95+ years	0.567857	0.946834	0.337443	0.318502	0.991332	0.968171
Motor neuron disease [Data Rich]	Female	0-6 days	95+ years	0.139959	0.621725	0.108326	0.215835	0.999741	0.99887
Motor neuron disease [Data Rich]	Male	0-6 days	95+ years	0.133962	0.58036	0.104637	0.214496	0.999837	0.999123
Other neurological disorders [Global]	Female	0-6 days	95+ years	0.378662	0.584038	0.236089	0.253497	0.993651	0.985653
Other neurological disorders [Global]	Male	0-6 days	95+ years	0.378424	0.552817	0.235515	0.244389	0.992371	0.986734
Other neurological disorders [Data Rich]	Female	0-6 days	95+ years	0.111556	0.298072	0.0851111	0.142626	0.999984	0.999841
Other neurological disorders [Data Rich]	Male	0-6 days	95+ years	0.11225	0.297042	0.0837732	0.142723	0.999989	0.999723
Anorexia nervosa [Global]	Female	5-9 years	45-49 years	0.975381	2.24866	0.810508	0.826848	0.96801	0.808397
Anorexia nervosa [Global]	Male	5-9 years	45-49 years	1.47577	2.65064	1.1102	1.16935	0.937422	0.767958
Anorexia nervosa [Data Rich]	Female	5-9 years	45-49 years	0.787266	1.93611	0.675197	0.861294	0.975385	0.938889
Anorexia nervosa [Data Rich]	Male	5-9 years	45-49 years	1.23711	2.13347	1.0201	1.16177	0.942809	0.904272
Alcohol use disorders [Global]	Female	15-19 years	95+ years	0.256627	0.672928	0.168591	0.158669	0.998541	0.980478
Alcohol use disorders [Global]	Male	15-19 years	95+ years	0.2563	0.608547	0.154616	0.162797	0.999018	0.973409
Alcohol use disorders [Data Rich]	Female	15-19 years	95+ years	0.11007	0.258001	0.0866506	0.161485	0.9999	0.999328
Alcohol use disorders [Data Rich]	Male	15-19 years	95+ years	0.101175	0.268221	0.0814895	0.157609	0.999928	0.99816
Drug use disorders [Global]	Female	15-19 years	95+ years	0.307994	0.685792	0.202222	0.192678	0.99847	0.986281
Drug use disorders [Global]	Male	15-19 years	95+ years	0.311927	0.72089	0.21066	0.222838	0.999126	0.98994
Drug use disorders [Data Rich]	Female	15-19 years	95+ years	0.184306	0.301249	0.139086	0.208631	0.999877	0.999824
Drug use disorders [Data Rich]	Male	15-19 years	95+ years	0.19617	0.33757	0.142383	0.218221	0.999875	0.999791
Opioid use disorders [Global]	Female	15-19 years	95+ years	0.32258	0.731773	0.229785	0.227447	0.998064	0.983937
Opioid use disorders [Global]	Male	15-19 years	95+ years	0.2918	0.732235	0.189279	0.188065	0.99913	0.986662
Opioid use disorders [Data Rich]	Female	15-19 years	95+ years	0.224516	0.471602	0.183253	0.263648	0.999381	0.998453
Opioid use disorders [Data Rich]	Male	15-19 years	95+ years	0.200382	0.607824	0.136138	0.208988	0.999867	0.99966
Cocaine use disorders [Global]	Female	15-19 years	95+ years	0.342365	0.751537	0.248551	0.245203	0.997748	0.980501
Cocaine use disorders [Global]	Male	15-19 years	95+ years	0.309933	0.746804	0.214728	0.205079	0.998832	0.987865
Cocaine use disorders [Data Rich]	Female	15-19 years	95+ years	0.218441	0.472718	0.168909	0.20193	0.999316	0.99899
Cocaine use disorders [Data Rich]	Male	15-19 years	95+ years	0.185424	0.41515	0.137439	0.180407	0.999791	0.999436
Amphetamine use disorders [Global]	Female	15-19 years	95+ years	0.345165	0.760782	0.235271	0.223848	0.997853	0.986481
Amphetamine use disorders [Global]	Male	15-19 years	95+ years	0.37776	0.785637	0.252553	0.257664	0.998132	0.985837
Amphetamine use disorders [Data Rich]	Female	15-19 years	95+ years	0.206273	0.486721	0.163369	0.174353	0.999347	0.998025
Amphetamine use disorders [Data Rich]	Male	15-19 years	95+ years	0.249479	0.55852	0.185562	0.241341	0.999483	0.998021
Other drug use disorders [Global]	Female	15-19 years	95+ years	0.278584	0.600607	0.183836	0.166984	0.998343	0.991042
Other drug use disorders [Global]	Male	15-19 years	95+ years	0.271599	0.574388	0.179579	0.161874	0.999254	0.992676
Other drug use disorders [Data Rich]	Female	15-19 years	95+ years	0.167992	0.392132	0.130001	0.192934	0.999833	0.999526
Other drug use disorders [Data Rich]	Male	15-19 years	95+ years	0.162695	0.345606	0.119861	0.177553	0.999938	0.999766
Diabetes mellitus [Global]	Female	0-6 days	10-14 years	0.37295	0.526072	0.13538	0.136736	0.999645	0.998215
Diabetes mellitus [Global]	Female	15-19 years	95+ years	0.211912	0.384817	0.13002	0.13291	0.9964	0.976058
Diabetes mellitus [Global]	Male	0-6 days	10-14 years	0.280586	0.391201	0.117476	0.115829	0.999654	0.999116
Diabetes mellitus [Global]	Male	15-19 years	95+ years	0.207378	0.361296	0.131367	0.13221	0.997903	0.980349
Diabetes mellitus [Data Rich]	Female	0-6 days	10-14 years	0.124685	0.328042	0.0720178	0.128846	1	0.999886
Diabetes mellitus [Data Rich]	Female	15-19 years	95+ years	0.0939124	0.207715	0.0762533	0.127031	0.999966	0.99933
Diabetes mellitus [Data Rich]	Male	0-6 days	10-14 years	0.120652	0.276308	0.0677484	0.119795	1	0.999642
Diabetes mellitus [Data Rich]	Male	15-19 years	95+ years	0.0950108	0.210545	0.0777947	0.135508	0.999978	0.999538
Diabetes mellitus type 1 [Global]	Female	0-6 days	95+ years	0.25684	0.41145	0.112919	0.109636	0.999913	0.996012
Diabetes mellitus type 1 [Global]	Male	0-6 days	95+ years	0.241546	0.38584	0.104069	0.101658	0.999962	0.997546
Diabetes mellitus type 1 [Data Rich]	Female	0-6 days	95+ years	0.111878	0.3284	0.0795159	0.0906577	0.999982	0.99996
Diabetes mellitus type 1 [Data Rich]	Male	0-6 days	95+ years	0.102282	0.263323	0.0728284	0.0856082	0.999995	0.999977
Diabetes mellitus type 2 [Global]	Female	15-19 years	95+ years	0.218476	0.401829	0.124401	0.124636	0.999498	0.991019
Diabetes mellitus type 2 [Global]	Male	15-19 years	95+ years	0.208773	0.38185	0.124034	0.125431	0.999407	0.991724
Diabetes mellitus type 2 [Data Rich]	Female	15-19 years	95+ years	0.0883426	0.286262	0.0709429	0.115767	1	0.999979
Diabetes mellitus type 2 [Data Rich]	Male	15-19 years	95+ years	0.0880464	0.261985	0.0707518	0.117352	1	0.999984
Chronic kidney disease [Global]	Female	0-6 days	95+ years	0.196207	0.392103	0.113451	0.117563	0.9995	0.990302
Chronic kidney disease [Global]	Male	0-6 days	95+ years	0.203372	0.387673	0.117298	0.122369	0.999738	0.990545
Chronic kidney disease [Data Rich]	Female	0-6 days	95+ years	0.0967364	0.264776	0.0713447	0.131904	0.999976	0.999735
Chronic kidney disease [Data Rich]	Male	0-6 days	95+ years	0.09966	0.272898	0.0735468	0.134833	0.999974	0.999732
Acute glomerulonephritis [Global]	Female	0-6 days	95+ years	0.652767	1.14477	0.441298	0.437246	0.986737	0.954328
Acute glomerulonephritis [Global]	Male	0-6 days	95+ years	0.729197	1.26551	0.472373	0.465499	0.98524	0.95215
Acute glomerulonephritis [Data Rich]	Female	0-6 days	95+ years	0.478862	0.82889	0.397208	0.354194	0.991436	0.984697
Acute glomerulonephritis [Data Rich]	Male	0-6 days	95+ years	0.514076	0.816821	0.419619	0.394987	0.989283	0.982261
Skin and subcutaneous diseases [Global]	Female	0-6 days	95+ years	0.270614	0.54955	0.135553	0.138801	0.999175	0.990918
Skin and subcutaneous diseases [Global]	Male	0-6 days	95+ years	0.249815	0.527122	0.139161	0.154652	0.999367	0.995067
Skin and subcutaneous diseases [Data Rich]	Female	0-6 days	95+ years	0.144722	0.380938	0.0813266	0.155504	0.999977	0.999336
Skin and subcutaneous diseases [Data Rich]	Male	0-6 days	95+ years	0.123181	0.372607	0.0815006	0.157609	0.999982	0.999852
Bacterial skin diseases [Global]	Female	0-6 days	95+ years	0.194493	0.760132	0.150054	0.171383	0.999699	0.998224
Bacterial skin diseases [Global]	Male	0-6 days	95+ years	0.199798	0.718313	0.155417	0.18576	0.999864	0.998564
Bacterial skin diseases [Data Rich]	Female	0-6 days	95+ years	0.186604	0.50104	0.0990131	0.190982	0.999977	0.998861
Bacterial skin diseases [Data Rich]	Male	0-6 days	95+ years	0.169606	0.509492	0.104201	0.212424	0.999971	0.99978
Cellulitis [Global]	Female	0-6 days	95+ years	0.392712	0.910948	0.194778	0.209831	0.998921	0.976897
Cellulitis [Global]	Male	0-6 days	95+ years	0.357229	0.778526	0.207492	0.220173	0.998939	0.977573
Cellulitis [Data Rich]	Female	0-6 days	95+ years	0.227104	0.780343	0.168597	0.218567	0.999457	0.998114
Cellulitis [Data Rich]	Male	0-6 days	95+ years	0.243568	0.865407	0.188937	0.251958	0.999	0.99759
Pyoderma [Global]	Female	0-6 days	95+ years	0.3144	0.790329	0.18856	0.217088	0.999666	0.981853
Pyoderma [Global]	Male	0-6 days	95+ years	0.301244	0.751511	0.189749	0.213019	0.999666	0.985334
Pyoderma [Data Rich]	Female	0-6 days	95+ years	0.21918	0.725556	0.158322	0.256071	0.99983	0.993678
Pyoderma [Data Rich]	Male	0-6 days	95+ years	0.214921	0.689412	0.157466	0.259669	0.999797	0.997082
Decubitus ulcer [Global]	Female	12-23 months	95+ years	0.536878	1.01615	0.19707	0.213965	0.997217	0.972617
Decubitus ulcer [Global]	Male	12-23 months	95+ years	0.543059	0.976353	0.25954	0.276602	0.995913	0.962391

CODEm predictive validity results by cause, model type, sex, and age									
Cause	Sex	Age Start	Age End	RMSE In-Sample	RMSE Out-of-Sample	Trend In-Sample	Trend Out-of-Sample	Coverage In-Sample	Coverage Out-of-Sample
Decubitus ulcer [Data Rich]	Female	12-23 months	95+ years	0.218086	0.552037	0.12634	0.142779	0.999701	0.999041
Decubitus ulcer [Data Rich]	Male	12-23 months	95+ years	0.244085	0.520134	0.152546	0.179454	0.999403	0.998068
Other skin and subcutaneous diseases [Global]	Female	0-6 days	95+ years	0.397073	0.809885	0.247525	0.253234	0.99605	0.976785
Other skin and subcutaneous diseases [Global]	Male	0-6 days	95+ years	0.455273	0.908128	0.330151	0.344104	0.993507	0.971799
Other skin and subcutaneous diseases [Data Rich]	Female	0-6 days	95+ years	0.303328	0.546795	0.218793	0.268604	0.998253	0.995557
Other skin and subcutaneous diseases [Data Rich]	Male	0-6 days	95+ years	0.377316	0.636918	0.303842	0.351157	0.995091	0.993215
Musculoskeletal disorders [Global]	Female	1-5 months	95+ years	0.266727	0.466476	0.138383	0.142876	0.999043	0.988754
Musculoskeletal disorders [Global]	Male	1-5 months	95+ years	0.302519	0.461595	0.179218	0.16957	0.998566	0.990683
Musculoskeletal disorders [Data Rich]	Female	1-5 months	95+ years	0.116442	0.297363	0.0802287	0.145468	0.999896	0.998821
Musculoskeletal disorders [Data Rich]	Male	1-5 months	95+ years	0.103295	0.252297	0.0839314	0.14668	0.999974	0.999716
Rheumatoid arthritis [Global]	Female	5-9 years	95+ years	0.277802	0.629161	0.176468	0.168706	0.998147	0.986854
Rheumatoid arthritis [Global]	Male	5-9 years	95+ years	0.292465	0.685716	0.188511	0.184633	0.998186	0.987577
Rheumatoid arthritis [Data Rich]	Female	5-9 years	95+ years	0.127343	0.349182	0.0798311	0.14384	0.99989	0.998296
Rheumatoid arthritis [Data Rich]	Male	5-9 years	95+ years	0.131301	0.344645	0.0903425	0.153642	0.999935	0.999011
Other musculoskeletal disorders [Global]	Female	1-5 months	95+ years	0.236993	0.445343	0.143362	0.152685	0.999783	0.995659
Other musculoskeletal disorders [Global]	Male	1-5 months	95+ years	0.287004	0.44402	0.173846	0.168969	0.998756	0.995549
Other musculoskeletal disorders [Data Rich]	Female	1-5 months	95+ years	0.116945	0.39564	0.0839529	0.161374	0.999921	0.999363
Other musculoskeletal disorders [Data Rich]	Male	1-5 months	95+ years	0.110039	0.298176	0.0872516	0.155021	0.999927	0.999778
Congenital anomalies [Global]	Female	0-6 days	65-69 years	0.189172	0.264702	0.12382	0.118818	0.99978	0.998559
Congenital anomalies [Global]	Male	0-6 days	65-69 years	0.185299	0.270271	0.121414	0.11885	0.999861	0.998474
Congenital anomalies [Data Rich]	Female	0-6 days	65-69 years	0.0893763	0.179809	0.0742003	0.114815	0.999997	0.999828
Congenital anomalies [Data Rich]	Male	0-6 days	65-69 years	0.0960474	0.197297	0.077458	0.122308	0.999994	0.9998
Neural tube defects [Global]	Female	0-6 days	65-69 years	0.31239	0.561341	0.200886	0.210886	0.998267	0.993932
Neural tube defects [Global]	Male	0-6 days	65-69 years	0.325073	0.551168	0.226288	0.228726	0.997657	0.994991
Neural tube defects [Data Rich]	Female	0-6 days	65-69 years	0.229987	0.464836	0.171003	0.209206	0.998948	0.99775
Neural tube defects [Data Rich]	Male	0-6 days	65-69 years	0.253518	0.423844	0.198723	0.181514	0.998141	0.996365
Congenital heart anomalies [Global]	Female	0-6 days	65-69 years	0.243982	0.394889	0.144791	0.144434	0.999701	0.997691
Congenital heart anomalies [Global]	Male	0-6 days	65-69 years	0.199544	0.284903	0.125109	0.121359	0.999759	0.998902
Congenital heart anomalies [Data Rich]	Female	0-6 days	65-69 years	0.106942	0.22235	0.083168	0.122246	1	0.999954
Congenital heart anomalies [Data Rich]	Male	0-6 days	65-69 years	0.147279	0.301335	0.0946692	0.140997	1	0.999727
Orofacial clefts [Global]	Female	0-6 days	2-4 years	1.4287	2.07904	1.22394	1.21241	0.937757	0.832977
Orofacial clefts [Global]	Male	0-6 days	2-4 years	1.38775	1.96119	1.09049	1.06694	0.963273	0.862802
Orofacial clefts [Data Rich]	Female	0-6 days	2-4 years	1.39184	1.83739	1.22151	1.13561	0.926921	0.876626
Orofacial clefts [Data Rich]	Male	0-6 days	2-4 years	1.26119	1.78992	1.06735	1.02605	0.943842	0.901461
Down's syndrome [Global]	Female	0-6 days	65-69 years	0.303147	0.792967	0.191436	0.211934	0.999919	0.977316
Down's syndrome [Global]	Male	0-6 days	65-69 years	0.31975	0.827842	0.195802	0.207891	0.999609	0.977469
Down's syndrome [Data Rich]	Female	0-6 days	65-69 years	0.218988	0.69755	0.161785	0.226728	0.999954	0.999883
Down's syndrome [Data Rich]	Male	0-6 days	65-69 years	0.224272	0.752658	0.16951	0.24497	0.999653	0.999629
Other chromosomal abnormalities [Global]	Female	0-6 days	65-69 years	0.289819	0.606943	0.170363	0.176146	0.999783	0.992826
Other chromosomal abnormalities [Global]	Male	0-6 days	65-69 years	0.279746	0.60354	0.183215	0.184759	0.999538	0.993579
Other chromosomal abnormalities [Data Rich]	Female	0-6 days	65-69 years	0.190382	0.434248	0.136981	0.176148	0.999847	0.999206
Other chromosomal abnormalities [Data Rich]	Male	0-6 days	65-69 years	0.193863	0.458905	0.146951	0.184284	0.999704	0.999572
Congenital musculoskeletal and limb anomalies [Global]	Female	0-6 days	65-69 years	0.395625	0.593882	0.316732	0.291077	0.996837	0.992835
Congenital musculoskeletal and limb anomalies [Global]	Male	0-6 days	65-69 years	0.388537	0.548363	0.303069	0.293097	0.997033	0.995989
Congenital musculoskeletal and limb anomalies [Data Rich]	Female	0-6 days	65-69 years	0.384989	0.469452	0.297465	0.290378	0.996938	0.99655
Congenital musculoskeletal and limb anomalies [Data Rich]	Male	0-6 days	65-69 years	0.32119	0.456423	0.272466	0.27441	0.99719	0.995957
Urogenital congenital anomalies [Global]	Female	0-6 days	65-69 years	0.764489	1.02339	0.643468	0.643913	0.986049	0.972643
Urogenital congenital anomalies [Global]	Male	0-6 days	65-69 years	0.681686	0.931402	0.564647	0.574919	0.989268	0.981775
Urogenital congenital anomalies [Data Rich]	Female	0-6 days	65-69 years	0.643184	0.782937	0.449904	0.534307	0.993933	0.991358
Urogenital congenital anomalies [Data Rich]	Male	0-6 days	65-69 years	0.452055	0.549823	0.348352	0.371079	0.995045	0.99331
Digestive congenital anomalies [Global]	Female	0-6 days	65-69 years	0.292246	0.428481	0.157855	0.15235	0.99935	0.998386
Digestive congenital anomalies [Global]	Male	0-6 days	65-69 years	0.325871	0.435162	0.175473	0.169127	0.999127	0.998284
Digestive congenital anomalies [Data Rich]	Female	0-6 days	65-69 years	0.21231	0.362446	0.141099	0.166366	0.999866	0.999423
Digestive congenital anomalies [Data Rich]	Male	0-6 days	65-69 years	0.256659	0.308961	0.151124	0.147117	0.999384	0.999099
Other congenital anomalies [Global]	Female	0-6 days	65-69 years	0.210946	0.35285	0.140789	0.145462	0.999806	0.998898
Other congenital anomalies [Global]	Male	0-6 days	65-69 years	0.242518	0.352649	0.136053	0.131262	0.999801	0.998437
Other congenital anomalies [Data Rich]	Female	0-6 days	65-69 years	0.163637	0.255608	0.0955966	0.128223	1	0.999977
Other congenital anomalies [Data Rich]	Male	0-6 days	65-69 years	0.172811	0.285497	0.0970132	0.134837	1	0.999981
Urinary diseases and male infertility [Global]	Female	0-6 days	95+ years	0.271114	0.521127	0.134332	0.140044	0.999619	0.990271
Urinary diseases and male infertility [Global]	Male	0-6 days	95+ years	0.258457	0.515542	0.139071	0.144125	0.999646	0.992903
Urinary diseases and male infertility [Data Rich]	Female	0-6 days	95+ years	0.111721	0.262471	0.0803922	0.143712	0.999996	0.999757
Urinary diseases and male infertility [Data Rich]	Male	0-6 days	95+ years	0.106537	0.244809	0.0807014	0.137726	0.999989	0.999659
Urinary tract infections and interstitial nephritis [Global]	Female	0-6 days	95+ years	0.38181	0.609742	0.162364	0.171671	0.999724	0.988478
Urinary tract infections and interstitial nephritis [Global]	Male	0-6 days	95+ years	0.321062	0.554234	0.156575	0.162967	0.99983	0.992796
Urinary tract infections and interstitial nephritis [Data Rich]	Female	0-6 days	95+ years	0.133069	0.400333	0.091372	0.170794	0.999993	0.999697
Urinary tract infections and interstitial nephritis [Data Rich]	Male	0-6 days	95+ years	0.124338	0.373452	0.0863684	0.160201	0.999993	0.999713
Urolithiasis [Global]	Female	12-23 months	95+ years	0.390403	0.64292	0.216601	0.218775	0.997433	0.990748
Urolithiasis [Global]	Male	12-23 months	95+ years	0.367686	0.655879	0.224783	0.215511	0.997597	0.991918
Urolithiasis [Data Rich]	Female	12-23 months	95+ years	0.178793	0.340588	0.140486	0.15717	0.999647	0.999373
Urolithiasis [Data Rich]	Male	12-23 months	95+ years	0.183164	0.373757	0.140566	0.164772	0.999737	0.999375
Other urinary diseases [Global]	Female	0-6 days	95+ years	0.629713	0.89895	0.362064	0.356495	0.991994	0.97721
Other urinary diseases [Global]	Male	0-6 days	95+ years	0.457467	0.679958	0.249286	0.252972	0.996306	0.987628
Other urinary diseases [Data Rich]	Female	0-6 days	95+ years	0.335293	0.61211	0.258364	0.222664	0.997368	0.995456
Other urinary diseases [Data Rich]	Male	0-6 days	95+ years	0.217589	0.48103	0.163797	0.179627	0.9995	0.999046
Gynecological diseases [Global]	Female	10-14 years	95+ years	0.362445	0.613914	0.233661	0.22659	0.997422	0.988527
Gynecological diseases [Data Rich]	Female	10-14 years	95+ years	0.214848	0.350358	0.175536	0.201747	0.998616	0.998063
Uterine fibroids [Global]	Female	10-14 years	95+ years	0.565968	1.05491	0.365195	0.363697	0.994236	0.946417
Uterine fibroids [Data Rich]	Female	10-14 years	95+ years	0.403764	0.796649	0.334611	0.334371	0.994724	0.986705
Endometriosis [Global]	Female	10-14 years	50-54 years	1.11765	1.69162	0.796731	0.817245	0.991178	0.941676
Endometriosis [Data Rich]	Female	10-14 years	50-54 years	0.962654	1.25369	0.741667	0.742619	0.990819	0.97631
Genital prolapse [Global]	Female	10-14 years	95+ years	0.705331	1.28983	0.597602	0.610232	0.97884	0.938051
Genital prolapse [Data Rich]	Female	10-14 years	95+ years	0.568599	1.04476	0.493591	0.478114	0.982389	0.971975
Other gynecological diseases [Global]	Female	10-14 years	95+ years	0.387244	0.670356	0.291332	0.279201	0.995864	0.986283
Other gynecological diseases [Data Rich]	Female	10-14 years	95+ years	0.310594	0.552654	0.259737	0.259054	0.997919	0.990328
Hemoglobinopathies and hemolytic anaemias [Global]	Female	0-6 days	95+ years	0.254066	0.409301	0.148032	0.133891	0.999503	0.997839
Hemoglobinopathies and hemolytic anaemias [Global]	Male	0-6 days	95+ years	0.240008	0.423973	0.170449	0.159183	0.999065	0.996899
Hemoglobinopathies and hemolytic anaemias [Data Rich]	Female	0-6 days	95+ years	0.127549	0.269018	0.0796946	0.126548	0.999998	0.999314
Hemoglobinopathies and hemolytic anaemias [Data Rich]	Male	0-6 days	95+ years	0.112875	0.238146	0.0860288	0.125481	0.999998	0.999925
Endocrine, metabolic, blood, and immune disorders [Global]	Female	0-6 days	95+ years	0.282135	0.434468	0.151612	0.152879	0.999134	0.994476
Endocrine, metabolic, blood, and immune disorders [Global]	Male	0-6 days	95+ years	0.247469	0.42259	0.150391	0.159086	0.999395	0.995217
Endocrine, metabolic, blood, and immune disorders [Data Rich]	Female	0-6 days	95+ years	0.106572	0.245711	0.0828625	0.147446	0.999993	0.999801
Endocrine, metabolic, blood, and immune disorders [Data Rich]	Male	0-6 days	95+ years	0.104154	0.237265	0.0837468	0.151123	0.999991	0.999865
Sudden infant death syndrome [Global]	Female	7-27 days	6-11 months	0.564867	0.856379	0.363083	0.37982	0.997154	0.984334
Sudden infant death syndrome [Global]	Male	7-27 days	6-11 months	0.549268	0.896919	0.350761	0.373874	0.997051	0.983723
Sudden infant death syndrome [Data Rich]	Female	7-27 days	6-11 months	0.38285	0.846253	0.31808	0.462897	0.996314	0.989125
Sudden infant death syndrome [Data Rich]	Male	7-27 days	6-11 months	0.360215	0.82039	0.298669	0.468942	0.996737	0.990989
Transport injuries [Global]	Female	0-6 days	95+ years	0.206686	0.351569	0.156575	0.149811	0.999653	0.995814
Transport injuries [Global]	Male	0-6 days	95+ years	0.198921	0.316254	0.1546	0.156277	0.999488	0.991469
Transport injuries [Data Rich]	Female	0-6 days	95+ years	0.0985164	0.190805	0.078926	0.126653	0.999974	0.999761
Transport injuries [Data Rich]	Male	0-6 days	95+ years	0.0987227	0.188492	0.0773134	0.121804	0.999957	0.999472
Road injuries [Global]	Female	0-6 days	95+ years	0.231389	0.400775	0.146501	0.151521	0.998232	0.992402
Road injuries [Global]	Male	0-6 days	95+ years	0.218891	0.364307	0.138883	0.141515	0.99851	0.99078
Road injuries [Data Rich]	Female	0-6 days	95+ years	0.0991374	0.197952	0.0793593	0.124722	0.999987	0.999767
Road injuries [Data Rich]	Male	0-6 days	95+ years	0.100048	0.196302	0.0779856	0.122077	0.999954	0.999789
Pedestrian road injuries [Global]	Female	0-6 days	95+ years	0.252449	0.434011	0.154016	0.161492	0.998286	0.995261
Pedestrian road injuries [Global]	Male	0-6 days	95+ years	0.273497	0.463444	0.151668	0.153866	0.998892	0.995119
Pedestrian road injuries [Data Rich]	Female	0-6 days	95+ years	0.102007	0.28926	0.0812846	0.115037	1	0.999958
Pedestrian road injuries [Data Rich]	Male	0-6 days	95+ years	0.101917	0.287078	0.0801615	0.115488	1	0.99995
Cyclist road injuries [Global]	Female	12-23 months	90-94 years	0.508827	0.805658	0.303251	0.286185	0.989207	0.981937
Cyclist road injuries [Global]	Male	12-23 months	90-94 years	0.360988	0.579428	0.20732	0.194828	0.99302	0.985628
Cyclist road injuries [Data Rich]	Female	12-23 months	90-94 years	0.189701	0.445926	0.153006	0.164516	0.999806	0.999543
Cyclist road injuries [Data Rich]	Male	12-23 months	90-94 years	0.146366	0.405528	0.112337	0.138853	0.999965	0.999908
Motorcyclist road injuries [Global]	Female	0-6 days	95+ years	0.322695	0.697857	0.207091	0.213382	0.997821	0.990471
Motorcyclist road injuries [Global]	Male	0-6 days	95+ years	0.296487	0.515833	0.152656	0.160386	0.998248	0.99152
Motorcyclist road injuries [Data Rich]	Female	0-6 days	95+ years	0.149161	0.69256	0.113034	0.205332	0.999943	0.99895
Motorcyclist road injuries [Data Rich]	Male	0-6 days	95+ years	0.108414	0.402619	0.0765461	0.129456	0.999988	0.999864
Motor vehicle road injuries [Global]	Female	0-6 days	95+ years	0.234553	0.432198	0.147466	0.149226	0.998219	0.993597
Motor vehicle road injuries [Global]	Male	0-6 days	95+ years	0.235852	0.405794	0.141872	0.141462	0.998512	0.990717
Motor vehicle road injuries [Data Rich]	Female	0-6 days	95+ years	0.0991658	0.299928	0.0786099	0.122619	0.999981	0.999715
Motor vehicle road injuries [Data Rich]	Male	0-6 days	95+ years	0.10227	0.279669	0.078197	0.118133	0.999972	0.999781
Other road injuries [Global]	Female	0-6 days	95+ years	0.610365	0.97511	0.343155	0.360084	0.991318	0.976167
Other road injuries [Global]	Male	0-6 days	95+ years	0.593452	0.964198	0.331762	0.358168	0.98979	0.963923
Other road injuries [Data Rich]	Female	0-6 days	95+ years	0.300375	0.896554	0.221881	0.363057	0.999612	0.991254
Other road injuries [Data Rich]	Male	0-6 days	95+ years	0.299966	0.959081	0.222646	0.371656	0.999638	0.985912
Other transport injuries [Global]	Female	0-6 days	95+ years	0.323751	0.584202	0.227169	0.239594	0.997657	0.987969
Other transport injuries [Global]	Male	0-6 days	95+ years	0.264476	0.508582	0.1666	0.166619	0.99819	0.991221
Other transport injuries [Data Rich]	Female	0-6 days	95+ years	0.146798	0.377742	0.125161	0.244612	0.999964	0.99965
Other transport injuries [Data Rich]	Male	0-6 days	95+ years	0.121248	0.3404	0.0876823	0.148865	0.999941	0.99976
Falls [Global]	Female	0-6 days	95+ years	0.251245	0.396103	0.153676	0.154808	0.999677	0.994107
Falls [Global]	Male	0-6 days	95+ years	0.240922	0.357605	0.136661	0.133485	0.999744	0.994721
Falls [Data Rich]	Female	0-6 days	95+ years	0.097602	0.197968	0.0734055	0.121509	0.999998	0.999943
Falls [Data Rich]	Male	0-6 days	95+ years	0.0931816	0.184652	0.0728627	0.121571	1	0.999945
Drowning [Global]	Female	0-6 days	95+ years	0.238259	0.418015	0.180948	0.168785	0.999212	0.99516
Drowning [Global]	Male	0-6 days	95+ years	0.211869	0.360236	0.172727	0.164128	0.999605	0.993808

CODEm predictive validity results by cause, model type, sex, and age									
Cause	Sex	Age Start	Age End	RMSE In-Sample	RMSE Out-of-Sample	Trend In-Sample	Trend Out-of-Sample	Coverage In-Sample	Coverage Out-of-Sample
Drowning [Data Rich]	Female	0-6 days	95+ years	0.113362	0.232679	0.0868692	0.14171	0.999989	0.999672
Drowning [Data Rich]	Male	0-6 days	95+ years	0.10634	0.203183	0.0828581	0.131	0.999985	0.999503
Fire, heat, and hot substances [Global]	Female	0-6 days	95+ years	0.251725	0.402223	0.175409	0.160981	0.998524	0.995409
Fire, heat, and hot substances [Global]	Male	0-6 days	95+ years	0.279596	0.414553	0.189205	0.160435	0.999098	0.996441
Fire, heat, and hot substances [Data Rich]	Female	0-6 days	95+ years	0.100028	0.194774	0.0812037	0.128372	0.999991	0.999868
Fire, heat, and hot substances [Data Rich]	Male	0-6 days	95+ years	0.105254	0.197574	0.0827381	0.131332	0.999987	0.9998
Poisonings [Global]	Female	0-6 days	95+ years	0.395944	0.608608	0.251698	0.238014	0.998863	0.993743
Poisonings [Global]	Male	0-6 days	95+ years	0.338329	0.578612	0.249975	0.242675	0.999066	0.992864
Poisonings [Data Rich]	Female	0-6 days	95+ years	0.126811	0.29145	0.102608	0.179991	0.999989	0.999676
Poisonings [Data Rich]	Male	0-6 days	95+ years	0.117559	0.255183	0.094621	0.152956	0.999976	0.999814
Poisoning by carbon monoxide [Global]	Female	0-6 days	95+ years	0.413346	0.719882	0.265882	0.263329	0.998709	0.992113
Poisoning by carbon monoxide [Global]	Male	0-6 days	95+ years	0.377433	0.63944	0.223457	0.226754	0.998347	0.992009
Poisoning by carbon monoxide [Data Rich]	Female	0-6 days	95+ years	0.257792	0.385033	0.21149	0.271939	0.998487	0.996339
Poisoning by carbon monoxide [Data Rich]	Male	0-6 days	95+ years	0.213626	0.301313	0.174932	0.206034	0.999342	0.99834
Poisoning by other means [Global]	Female	0-6 days	95+ years	0.38681	0.740535	0.221662	0.224026	0.99747	0.980154
Poisoning by other means [Global]	Male	0-6 days	95+ years	0.422296	0.800191	0.204143	0.213388	0.998974	0.979476
Poisoning by other means [Data Rich]	Female	0-6 days	95+ years	0.200794	0.58623	0.160576	0.207737	0.999629	0.999187
Poisoning by other means [Data Rich]	Male	0-6 days	95+ years	0.215724	0.674915	0.165077	0.173753	0.999822	0.999512
Exposure to mechanical forces [Global]	Female	0-6 days	95+ years	0.372249	0.524753	0.364638	0.323877	0.999071	0.995263
Exposure to mechanical forces [Global]	Male	0-6 days	95+ years	0.361604	0.511006	0.331843	0.296206	0.999323	0.992828
Exposure to mechanical forces [Data Rich]	Female	0-6 days	95+ years	0.131717	0.285381	0.0913498	0.160589	0.999956	0.999616
Exposure to mechanical forces [Data Rich]	Male	0-6 days	95+ years	0.117494	0.247024	0.0837333	0.133027	0.999889	0.999592
Unintentional firearm injuries [Global]	Female	0-6 days	95+ years	0.528522	0.984585	0.351512	0.36392	0.997436	0.973149
Unintentional firearm injuries [Global]	Male	0-6 days	95+ years	0.462638	0.841717	0.286832	0.289746	0.996447	0.980758
Unintentional firearm injuries [Data Rich]	Female	0-6 days	95+ years	0.317495	0.705872	0.260872	0.360213	0.999123	0.997848
Unintentional firearm injuries [Data Rich]	Male	0-6 days	95+ years	0.274065	0.582571	0.22442	0.277323	0.997824	0.996476
Other exposure to mechanical forces [Global]	Female	0-6 days	95+ years	0.404124	0.579458	0.388514	0.342068	0.999183	0.996336
Other exposure to mechanical forces [Global]	Male	0-6 days	95+ years	0.363011	0.537685	0.349766	0.333419	0.999265	0.994877
Other exposure to mechanical forces [Data Rich]	Female	0-6 days	95+ years	0.140558	0.368493	0.0975766	0.17749	0.999945	0.999536
Other exposure to mechanical forces [Data Rich]	Male	0-6 days	95+ years	0.123398	0.323734	0.0851836	0.136914	0.999948	0.999841
Adverse effects of medical treatment [Global]	Female	0-6 days	95+ years	0.23256	0.40773	0.138933	0.144043	0.999918	0.995619
Adverse effects of medical treatment [Global]	Male	0-6 days	95+ years	0.284608	0.492857	0.149742	0.155454	0.999882	0.994658
Adverse effects of medical treatment [Data Rich]	Female	0-6 days	95+ years	0.118941	0.282655	0.0829911	0.150526	1	0.99991
Adverse effects of medical treatment [Data Rich]	Male	0-6 days	95+ years	0.115364	0.297602	0.0859243	0.158242	0.999996	0.99988
Animal contact [Global]	Female	0-6 days	95+ years	0.372105	0.757253	0.259088	0.279065	0.997653	0.983307
Animal contact [Global]	Male	0-6 days	95+ years	0.311041	0.685074	0.204663	0.212275	0.998515	0.99009
Animal contact [Data Rich]	Female	0-6 days	95+ years	0.290878	0.50817	0.235039	0.340944	0.998695	0.997097
Animal contact [Data Rich]	Male	0-6 days	95+ years	0.216175	0.440638	0.17343	0.22804	0.999715	0.99918
Venomous animal contact [Global]	Female	0-6 days	95+ years	0.577893	0.932934	0.435773	0.468974	0.995339	0.962696
Venomous animal contact [Global]	Male	0-6 days	95+ years	0.469249	0.836421	0.378996	0.408837	0.99409	0.969729
Venomous animal contact [Data Rich]	Female	0-6 days	95+ years	0.501911	0.898669	0.416256	0.554202	0.996709	0.992519
Venomous animal contact [Data Rich]	Male	0-6 days	95+ years	0.412138	0.829012	0.36465	0.502814	0.995443	0.991844
Non-venomous animal contact [Global]	Female	0-6 days	95+ years	0.472955	0.905757	0.3288	0.348468	0.995485	0.97324
Non-venomous animal contact [Global]	Male	0-6 days	95+ years	0.380875	0.810429	0.265361	0.267849	0.995941	0.978538
Non-venomous animal contact [Data Rich]	Female	0-6 days	95+ years	0.350691	0.707791	0.2943	0.421245	0.997965	0.994028
Non-venomous animal contact [Data Rich]	Male	0-6 days	95+ years	0.268233	0.653325	0.21885	0.280077	0.999096	0.99819
Foreign body [Global]	Female	0-6 days	95+ years	0.220904	0.422581	0.12976	0.132358	0.999367	0.99425
Foreign body [Global]	Male	0-6 days	95+ years	0.230317	0.358353	0.134131	0.128334	0.999444	0.993378
Foreign body [Data Rich]	Female	0-6 days	95+ years	0.0943893	0.220453	0.0722108	0.128766	0.999993	0.999825
Foreign body [Data Rich]	Male	0-6 days	95+ years	0.095133	0.207412	0.0721348	0.123193	0.999967	0.999657
Pulmonary aspiration and foreign body in airway [Global]	Female	0-6 days	95+ years	0.210489	0.404755	0.129912	0.128936	0.999731	0.995679
Pulmonary aspiration and foreign body in airway [Global]	Male	0-6 days	95+ years	0.238455	0.387641	0.126158	0.126693	0.999953	0.993906
Pulmonary aspiration and foreign body in airway [Data Rich]	Female	0-6 days	95+ years	0.102753	0.338332	0.0745187	0.128089	0.999993	0.99983
Pulmonary aspiration and foreign body in airway [Data Rich]	Male	0-6 days	95+ years	0.0951713	0.302969	0.072688	0.119032	0.999983	0.999762
Foreign body in other body part [Global]	Female	0-6 days	95+ years	0.513232	0.895174	0.348	0.367603	0.995477	0.976899
Foreign body in other body part [Global]	Male	0-6 days	95+ years	0.52634	0.867045	0.332447	0.341323	0.995143	0.983281
Foreign body in other body part [Data Rich]	Female	0-6 days	95+ years	0.346674	0.634162	0.285369	0.360743	0.997927	0.995368
Foreign body in other body part [Data Rich]	Male	0-6 days	95+ years	0.338071	0.616571	0.276546	0.349411	0.997947	0.996055
Environmental heat and cold exposure [Global]	Female	0-6 days	95+ years	0.459565	0.830256	0.261649	0.270804	0.997411	0.987817
Environmental heat and cold exposure [Global]	Male	0-6 days	95+ years	0.453512	0.706214	0.237786	0.24415	0.997811	0.991365
Environmental heat and cold exposure [Data Rich]	Female	0-6 days	95+ years	0.147922	0.418758	0.11698	0.244182	0.999872	0.999048
Environmental heat and cold exposure [Data Rich]	Male	0-6 days	95+ years	0.142262	0.359629	0.102504	0.203784	0.999923	0.999404
Other unintentional injuries [Global]	Female	0-6 days	95+ years	0.415989	0.660213	0.212758	0.227035	0.998243	0.985837
Other unintentional injuries [Global]	Male	0-6 days	95+ years	0.366753	0.616113	0.196689	0.218621	0.99829	0.986495
Other unintentional injuries [Data Rich]	Female	0-6 days	95+ years	0.206843	0.393304	0.158779	0.232946	0.999826	0.999399
Other unintentional injuries [Data Rich]	Male	0-6 days	95+ years	0.200382	0.408906	0.140704	0.261455	0.999876	0.99936
Self-harm [Global]	Female	10-14 years	95+ years	0.193919	0.357102	0.132518	0.131065	0.999674	0.987295
Self-harm [Global]	Male	10-14 years	95+ years	0.182852	0.331292	0.125459	0.129534	0.999762	0.981373
Self-harm [Data Rich]	Female	10-14 years	95+ years	0.100758	0.210588	0.0815021	0.140265	0.999997	0.999503
Self-harm [Data Rich]	Male	10-14 years	95+ years	0.101531	0.215338	0.0800531	0.147486	0.999988	0.999012
Self-harm by firearm [Global]	Female	10-14 years	95+ years	0.446663	0.970559	0.30175	0.281394	0.997629	0.974641
Self-harm by firearm [Global]	Male	10-14 years	95+ years	0.303139	0.585996	0.174613	0.160404	0.999424	0.97996
Self-harm by firearm [Data Rich]	Female	10-14 years	95+ years	0.192997	0.588935	0.157909	0.246574	0.999703	0.998038
Self-harm by firearm [Data Rich]	Male	10-14 years	95+ years	0.107825	0.313195	0.0862407	0.135288	0.999984	0.999541
Self-harm by other specified means [Global]	Female	10-14 years	95+ years	0.174975	0.377762	0.125089	0.132131	0.999952	0.989104
Self-harm by other specified means [Global]	Male	10-14 years	95+ years	0.188983	0.360892	0.122548	0.127999	0.999944	0.985846
Self-harm by other specified means [Data Rich]	Female	10-14 years	95+ years	0.103929	0.329018	0.0834723	0.14254	0.999987	0.999822
Self-harm by other specified means [Data Rich]	Male	10-14 years	95+ years	0.107987	0.321648	0.0827059	0.145602	0.999987	0.999664
Interpersonal violence [Global]	Female	0-6 days	95+ years	0.284721	0.468764	0.225065	0.220847	0.998755	0.992634
Interpersonal violence [Global]	Male	0-6 days	95+ years	0.281714	0.47801	0.215525	0.212589	0.998312	0.983314
Interpersonal violence [Data Rich]	Female	0-6 days	95+ years	0.170556	0.292361	0.17055	0.227063	0.998636	0.995578
Interpersonal violence [Data Rich]	Male	0-6 days	95+ years	0.164102	0.298074	0.158657	0.218109	0.998918	0.996259
Assault by firearm [Global]	Female	0-6 days	95+ years	0.457037	0.952874	0.320207	0.325336	0.994648	0.968072
Assault by firearm [Global]	Male	0-6 days	95+ years	0.33142	0.734514	0.232398	0.234826	0.998631	0.976987
Assault by firearm [Data Rich]	Female	0-6 days	95+ years	0.223595	0.688713	0.180446	0.283028	0.999451	0.995305
Assault by firearm [Data Rich]	Male	0-6 days	95+ years	0.162327	0.537567	0.126734	0.217862	0.999877	0.99814
Assault by sharp object [Global]	Female	0-6 days	95+ years	0.30218	0.603757	0.206385	0.217306	0.998359	0.989361
Assault by sharp object [Global]	Male	0-6 days	95+ years	0.257818	0.508555	0.166741	0.176703	0.999213	0.989505
Assault by sharp object [Data Rich]	Female	0-6 days	95+ years	0.146931	0.580975	0.11919	0.229075	0.999829	0.999001
Assault by sharp object [Data Rich]	Male	0-6 days	95+ years	0.132827	0.368476	0.0958938	0.170967	0.999926	0.999562
Assault by other means [Global]	Female	0-6 days	95+ years	0.237399	0.389646	0.150001	0.157476	0.999255	0.997178
Assault by other means [Global]	Male	0-6 days	95+ years	0.217138	0.389727	0.135181	0.134117	0.999575	0.995676
Assault by other means [Data Rich]	Female	0-6 days	95+ years	0.117354	0.316536	0.089819	0.160659	0.999998	0.999714
Assault by other means [Data Rich]	Male	0-6 days	95+ years	0.108335	0.336473	0.0769759	0.136725	0.999964	0.999764
Executions and police conflict [Global]	Female	1-5 months	95+ years	0.734795	1.23078	0.577684	0.573843	0.994207	0.964949
Executions and police conflict [Global]	Male	1-5 months	95+ years	0.876239	1.49126	0.685366	0.693594	0.992741	0.913153
Executions and police conflict [Data Rich]	Female	1-5 months	95+ years	0.591761	0.969238	0.55526	0.532272	0.992793	0.974932
Executions and police conflict [Data Rich]	Male	1-5 months	95+ years	0.803338	1.20293	0.717632	0.667244	0.991032	0.955769

Appendix Table S14: Comparison of GBD 2019 and GBD 2021 covariates and level of covariates used in cause of death modeling									
Cause	Model Version Type	Sex	Covariate	Level 1 GBD 2019	Level 2 GBD 2019	Level 3 GBD 2019	Level 1 GBD 2021	Level 2 GBD 2021	Level 3 GBD 2021
Sexually transmitted infections excluding HIV	Data Rich	Female	LDI (I$ per capita)			X			X
Sexually transmitted infections excluding HIV	Global	Female	LDI (I$ per capita)			X			X
Sexually transmitted infections excluding HIV	Data Rich	Female	Antenatal Care (1 visit) Coverage (proportion)			X			X
Sexually transmitted infections excluding HIV	Global	Female	Antenatal Care (1 visit) Coverage (proportion)			X			X
Sexually transmitted infections excluding HIV	Data Rich	Female	Antenatal Care (4 visits) Coverage (proportion)			X			X
Sexually transmitted infections excluding HIV	Global	Female	Antenatal Care (4 visits) Coverage (proportion)			X			X
Sexually transmitted infections excluding HIV	Data Rich	Female	Education (years per capita)		X			X	
Sexually transmitted infections excluding HIV	Global	Female	Education (years per capita)		X			X	
Sexually transmitted infections excluding HIV	Data Rich	Female	Healthcare access and quality index		X			X	
Sexually transmitted infections excluding HIV	Global	Female	Healthcare access and quality index		X			X	
Sexually transmitted infections excluding HIV	Data Rich	Female	Total Fertility Rate		X			X	
Sexually transmitted infections excluding HIV	Global	Female	Total Fertility Rate		X			X	
Sexually transmitted infections excluding HIV	Data Rich	Female	Maternal care and immunization		X			X	
Sexually transmitted infections excluding HIV	Global	Female	Maternal care and immunization		X			X	
Sexually transmitted infections excluding HIV	Data Rich	Female	Legality of Abortion		X			X	
Sexually transmitted infections excluding HIV	Global	Female	Legality of Abortion		X			X	
Sexually transmitted infections excluding HIV	Data Rich	Female	Age-Specific Fertility Rate		X			X	
Sexually transmitted infections excluding HIV	Global	Female	Age-Specific Fertility Rate		X			X	
Sexually transmitted infections excluding HIV	Data Rich	Female	Syphilis prevalence (proportion)	X			X		
Sexually transmitted infections excluding HIV	Global	Female	Syphilis prevalence (proportion)	X			X		
Sexually transmitted infections excluding HIV	Data Rich	Male	LDI (I$ per capita)			X			X
Sexually transmitted infections excluding HIV	Global	Male	LDI (I$ per capita)			X			X
Sexually transmitted infections excluding HIV	Data Rich	Male	Antenatal Care (1 visit) Coverage (proportion)			X			X
Sexually transmitted infections excluding HIV	Global	Male	Antenatal Care (1 visit) Coverage (proportion)			X			X
Sexually transmitted infections excluding HIV	Data Rich	Male	Antenatal Care (4 visits) Coverage (proportion)			X			X
Sexually transmitted infections excluding HIV	Global	Male	Antenatal Care (4 visits) Coverage (proportion)			X			X
Sexually transmitted infections excluding HIV	Data Rich	Male	Education (years per capita)		X			X	
Sexually transmitted infections excluding HIV	Global	Male	Education (years per capita)		X			X	
Sexually transmitted infections excluding HIV	Data Rich	Male	Healthcare access and quality index		X			X	
Sexually transmitted infections excluding HIV	Global	Male	Healthcare access and quality index		X			X	
Sexually transmitted infections excluding HIV	Data Rich	Male	Total Fertility Rate		X			X	
Sexually transmitted infections excluding HIV	Global	Male	Total Fertility Rate		X			X	
Sexually transmitted infections excluding HIV	Data Rich	Male	Maternal care and immunization		X			X	
Sexually transmitted infections excluding HIV	Global	Male	Maternal care and immunization		X			X	
Sexually transmitted infections excluding HIV	Data Rich	Male	Legality of Abortion		X			X	
Sexually transmitted infections excluding HIV	Global	Male	Legality of Abortion		X			X	
Sexually transmitted infections excluding HIV	Data Rich	Male	Age-Specific Fertility Rate		X			X	
Sexually transmitted infections excluding HIV	Global	Male	Age-Specific Fertility Rate		X			X	
Sexually transmitted infections excluding HIV	Data Rich	Male	Syphilis prevalence (proportion)	X			X		
Sexually transmitted infections excluding HIV	Global	Male	Syphilis prevalence (proportion)	X			X		
Lower respiratory infections	Global	Female	Education (years per capita)			X			X
Lower respiratory infections	Data Rich	Female	Education (years per capita)			X			X
Lower respiratory infections	Global	Female	LDI (I$ per capita)			X			X
Lower respiratory infections	Data Rich	Female	LDI (I$ per capita)			X			X
Lower respiratory infections	Global	Female	Socio-demographic Index			X			X
Lower respiratory infections	Data Rich	Female	Socio-demographic Index			X			X
Lower respiratory infections	Data Rich	Female	Socio-demographic Index			X			X
Lower respiratory infections	Global	Female	Socio-demographic Index			X			X
Lower respiratory infections	Data Rich	Female	Population Density (over 1000 ppl/sqkm, proportion)			X			X
Lower respiratory infections	Global	Female	Population Density (over 1000 ppl/sqkm, proportion)			X			X
Lower respiratory infections	Data Rich	Female	Population Density (under 150 ppl/sqkm, proportion)			X			X
Lower respiratory infections	Data Rich	Female	Maternal Education (years per capita)			X			X
Lower respiratory infections	Global	Female	Maternal Education (years per capita)			X			X
Lower respiratory infections	Global	Female	Age- and sex-specific SEV for Unsafe sanitation			X			X
Lower respiratory infections	Data Rich	Female	Age- and sex-specific SEV for Unsafe sanitation			X			X
Lower respiratory infections	Data Rich	Female	Age- and sex-specific SEV for Unsafe sanitation			X			X
Lower respiratory infections	Global	Female	Age- and sex-specific SEV for Unsafe sanitation			X			X
Lower respiratory infections	Global	Female	Healthcare access and quality index		X			X	
Lower respiratory infections	Data Rich	Female	Healthcare access and quality index		X			X	
Lower respiratory infections	Data Rich	Female	Healthcare access and quality index		X			X	
Lower respiratory infections	Global	Female	Healthcare access and quality index		X			X	
Lower respiratory infections	Data Rich	Female	Outdoor Air Pollution (PM2.5)		X			X	
Lower respiratory infections	Global	Female	Outdoor Air Pollution (PM2.5)		X			X	
Lower respiratory infections	Global	Female	Age-standardized proportion adult underweight		X			X	
Lower respiratory infections	Data Rich	Female	Age-standardized proportion adult underweight		X			X	
Lower respiratory infections	Global	Female	No access to handwashing facility		X			X	
Lower respiratory infections	Data Rich	Female	No access to handwashing facility		X			X	
Lower respiratory infections	Data Rich	Female	Secondhand smoke		X			X	
Lower respiratory infections	Global	Female	Secondhand smoke		X			X	
Lower respiratory infections	Data Rich	Female	Vitamin A Deficiency Prevalence (age-standardized)		X			X	
Lower respiratory infections	Data Rich	Female	Zinc deficiency		X			X	
Lower respiratory infections	Global	Female	Zinc deficiency		X			X	
Lower respiratory infections	Data Rich	Female	Age-standardized SEV for Handwashing		X			X	
Lower respiratory infections	Global	Female	Age-standardized SEV for Handwashing		X			X	
Lower respiratory infections	Data Rich	Female	Age- and sex-specific SEV for Ambient particulate matter		X			X	
Lower respiratory infections	Global	Female	Age- and sex-specific SEV for Ambient particulate matter		X			X	
Lower respiratory infections	Data Rich	Female	Age- and sex-specific SEV for Household air pollution		X			X	
Lower respiratory infections	Global	Female	Age- and sex-specific SEV for Household air pollution		X			X	
Lower respiratory infections	Global	Female	Smoking Prevalence	X			X		
Lower respiratory infections	Data Rich	Female	Smoking Prevalence	X			X		
Lower respiratory infections	Global	Female	Indoor Air Pollution (All Cooking Fuels)	X			X		
Lower respiratory infections	Data Rich	Female	Indoor Air Pollution (All Cooking Fuels)	X			X		
Lower respiratory infections	Data Rich	Female	Indoor Air Pollution (All Cooking Fuels)	X			X		
Lower respiratory infections	Global	Female	Indoor Air Pollution (All Cooking Fuels)	X			X		
Lower respiratory infections	Global	Female	Outdoor Air Pollution (PM2.5)	X			X		
Lower respiratory infections	Data Rich	Female	Outdoor Air Pollution (PM2.5)	X			X		
Lower respiratory infections	Data Rich	Female	Age- and sex-specific SEV for Child underweight	X			X		
Lower respiratory infections	Global	Female	Age- and sex-specific SEV for Child underweight	X			X		
Lower respiratory infections	Global	Female	Log-transformed SEV scalar: LRI	X			X		
Lower respiratory infections	Data Rich	Female	Log-transformed SEV scalar: LRI	X			X		
Lower respiratory infections	Data Rich	Female	Log-transformed SEV scalar: LRI	X			X		
Lower respiratory infections	Global	Female	Log-transformed SEV scalar: LRI	X			X		
Lower respiratory infections	Global	Female	Secondhand smoke	X			X		
Lower respiratory infections	Data Rich	Female	Secondhand smoke	X			X		
Lower respiratory infections	Data Rich	Female	Antibiotics for LRI	X			X		
Lower respiratory infections	Global	Female	Antibiotics for LRI	X			X		
Lower respiratory infections	Data Rich	Female	Age- and sex-specific SEV for Child stunting	X			X		
Lower respiratory infections	Global	Female	Age- and sex-specific SEV for Child stunting	X			X		
Lower respiratory infections	Data Rich	Female	Age- and sex-specific SEV for Child wasting	X			X		
Lower respiratory infections	Global	Female	Age- and sex-specific SEV for Child wasting	X			X		
Lower respiratory infections	Global	Female	DTP3 lagged five year coverage (proportion)					X	
Lower respiratory infections	Data Rich	Female	DTP3 lagged five year coverage (proportion)					X	
Lower respiratory infections	Data Rich	Female	DTP3 lagged five year coverage (proportion)					X	
Lower respiratory infections	Global	Female	DTP3 lagged five year coverage (proportion)					X	
Lower respiratory infections	Global	Female	PCV3 lagged five year coverage (proportion)					X	
Lower respiratory infections	Data Rich	Female	PCV3 lagged five year coverage (proportion)					X	
Lower respiratory infections	Data Rich	Female	PCV3 lagged five year coverage (proportion)				X		
Lower respiratory infections	Global	Female	PCV3 lagged five year coverage (proportion)				X		
Lower respiratory infections	Data Rich	Female	Hib3 lagged five year coverage (proportion)				X		
Lower respiratory infections	Global	Female	Hib3 lagged five year coverage (proportion)				X		
Lower respiratory infections	Global	Male	Education (years per capita)			X			X
Lower respiratory infections	Data Rich	Male	Education (years per capita)			X			X
Lower respiratory infections	Global	Male	LDI (I$ per capita)			X			X
Lower respiratory infections	Data Rich	Male	LDI (I$ per capita)			X			X
Lower respiratory infections	Global	Male	Socio-demographic Index			X			X
Lower respiratory infections	Data Rich	Male	Socio-demographic Index			X			X
Lower respiratory infections	Global	Male	Socio-demographic Index			X			X
Lower respiratory infections	Data Rich	Male	Socio-demographic Index			X			X
Lower respiratory infections	Global	Male	Population Density (over 1000 ppl/sqkm, proportion)			X			X
Lower respiratory infections	Data Rich	Male	Population Density (over 1000 ppl/sqkm, proportion)			X			X
Lower respiratory infections	Global	Male	Maternal Education (years per capita)			X			X
Lower respiratory infections	Data Rich	Male	Maternal Education (years per capita)			X			X
Lower respiratory infections	Global	Male	Age- and sex-specific SEV for Unsafe sanitation			X			X
Lower respiratory infections	Data Rich	Male	Age- and sex-specific SEV for Unsafe sanitation			X			X
Lower respiratory infections	Global	Male	Age- and sex-specific SEV for Unsafe sanitation			X			X

Comparison of GBD 2019 and GBD 2021 covariates and level of covariates used in cause of death modeling									
Cause	Model Version Type	Sex	Covariate	Level 1 GBD 2019	Level 2 GBD 2019	Level 3 GBD 2019	Level 1 GBD 2021	Level 2 GBD 2021	Level 3 GBD 2021
Lower respiratory infections	Data Rich	Male	Age- and sex-specific SEV for Unsafe sanitation			X			X
Lower respiratory infections	Global	Male	Healthcare access and quality index		X			X	
Lower respiratory infections	Data Rich	Male	Healthcare access and quality index		X			X	
Lower respiratory infections	Global	Male	Healthcare access and quality index		X			X	
Lower respiratory infections	Data Rich	Male	Healthcare access and quality index		X			X	
Lower respiratory infections	Global	Male	Outdoor Air Pollution (PM2.5)		X			X	
Lower respiratory infections	Data Rich	Male	Outdoor Air Pollution (PM2.5)		X			X	
Lower respiratory infections	Global	Male	Age-standardized proportion adult underweight		X			X	
Lower respiratory infections	Data Rich	Male	Age-standardized proportion adult underweight		X			X	
Lower respiratory infections	Global	Male	No access to handwashing facility		X			X	
Lower respiratory infections	Data Rich	Male	No access to handwashing facility		X			X	
Lower respiratory infections	Global	Male	Secondhand smoke		X			X	
Lower respiratory infections	Data Rich	Male	Secondhand smoke		X			X	
Lower respiratory infections	Global	Male	Zinc deficiency		X			X	
Lower respiratory infections	Data Rich	Male	Zinc deficiency		X			X	
Lower respiratory infections	Global	Male	Age-standardized SEV for Handwashing		X			X	
Lower respiratory infections	Data Rich	Male	Age-standardized SEV for Handwashing		X			X	
Lower respiratory infections	Global	Male	Age- and sex-specific SEV for Ambient particulate matter		X			X	
Lower respiratory infections	Data Rich	Male	Age- and sex-specific SEV for Ambient particulate matter		X			X	
Lower respiratory infections	Global	Male	Age- and sex-specific SEV for Household air pollution		X			X	
Lower respiratory infections	Data Rich	Male	Age- and sex-specific SEV for Household air pollution		X			X	
Lower respiratory infections	Global	Male	Smoking Prevalence	X			X		
Lower respiratory infections	Data Rich	Male	Smoking Prevalence	X			X		
Lower respiratory infections	Global	Male	Indoor Air Pollution (All Cooking Fuels)	X			X		
Lower respiratory infections	Data Rich	Male	Indoor Air Pollution (All Cooking Fuels)	X			X		
Lower respiratory infections	Global	Male	Indoor Air Pollution (All Cooking Fuels)	X			X		
Lower respiratory infections	Data Rich	Male	Indoor Air Pollution (All Cooking Fuels)	X			X		
Lower respiratory infections	Global	Male	Outdoor Air Pollution (PM2.5)	X			X		
Lower respiratory infections	Data Rich	Male	Outdoor Air Pollution (PM2.5)	X			X		
Lower respiratory infections	Global	Male	Age- and sex-specific SEV for Child underweight	X			X		
Lower respiratory infections	Data Rich	Male	Age- and sex-specific SEV for Child underweight	X			X		
Lower respiratory infections	Global	Male	Log-transformed SEV scalar: LRI	X			X		
Lower respiratory infections	Data Rich	Male	Log-transformed SEV scalar: LRI	X			X		
Lower respiratory infections	Global	Male	Log-transformed SEV scalar: LRI	X			X		
Lower respiratory infections	Data Rich	Male	Log-transformed SEV scalar: LRI	X			X		
Lower respiratory infections	Global	Male	Secondhand smoke	X			X		
Lower respiratory infections	Data Rich	Male	Secondhand smoke	X			X		
Lower respiratory infections	Global	Male	Antibiotics for LRI	X			X		
Lower respiratory infections	Data Rich	Male	Antibiotics for LRI	X			X		
Lower respiratory infections	Global	Male	Age- and sex-specific SEV for Child stunting	X			X		
Lower respiratory infections	Data Rich	Male	Age- and sex-specific SEV for Child stunting	X			X		
Lower respiratory infections	Global	Male	Age- and sex-specific SEV for Child wasting	X			X		
Lower respiratory infections	Data Rich	Male	Age- and sex-specific SEV for Child wasting	X			X		
Lower respiratory infections	Global	Male	DTP3 lagged five year coverage (proportion)					X	
Lower respiratory infections	Data Rich	Male	DTP3 lagged five year coverage (proportion)					X	
Lower respiratory infections	Global	Male	DTP3 lagged five year coverage (proportion)					X	
Lower respiratory infections	Data Rich	Male	DTP3 lagged five year coverage (proportion)					X	
Lower respiratory infections	Global	Male	PCV3 lagged five year coverage (proportion)					X	
Lower respiratory infections	Data Rich	Male	PCV3 lagged five year coverage (proportion)					X	
Lower respiratory infections	Global	Male	PCV3 lagged five year coverage (proportion)				X		
Lower respiratory infections	Data Rich	Male	PCV3 lagged five year coverage (proportion)				X		
Lower respiratory infections	Global	Male	Hib3 lagged five year coverage (proportion)				X		
Lower respiratory infections	Data Rich	Male	Hib3 lagged five year coverage (proportion)				X		
Upper respiratory infections	Data Rich	Female	Education (years per capita)			X			X
Upper respiratory infections	Global	Female	Education (years per capita)			X			X
Upper respiratory infections	Data Rich	Female	LDI (I$ per capita)			X			X
Upper respiratory infections	Global	Female	LDI (I$ per capita)			X			X
Upper respiratory infections	Data Rich	Female	Socio-demographic Index			X			X
Upper respiratory infections	Global	Female	Socio-demographic Index			X			X
Upper respiratory infections	Data Rich	Female	Healthcare access and quality index		X			X	
Upper respiratory infections	Global	Female	Healthcare access and quality index		X			X	
Upper respiratory infections	Data Rich	Female	Indoor Air Pollution (All Cooking Fuels)		X			X	
Upper respiratory infections	Global	Female	Indoor Air Pollution (All Cooking Fuels)		X			X	
Upper respiratory infections	Data Rich	Female	Outdoor Air Pollution (PM2.5)		X			X	
Upper respiratory infections	Global	Female	Outdoor Air Pollution (PM2.5)		X			X	
Upper respiratory infections	Data Rich	Female	Smoking Prevalence	X			X		
Upper respiratory infections	Global	Female	Smoking Prevalence	X			X		
Upper respiratory infections	Data Rich	Male	Education (years per capita)			X			X
Upper respiratory infections	Global	Male	Education (years per capita)			X			X
Upper respiratory infections	Data Rich	Male	LDI (I$ per capita)			X			X
Upper respiratory infections	Global	Male	LDI (I$ per capita)			X			X
Upper respiratory infections	Data Rich	Male	Socio-demographic Index			X			X
Upper respiratory infections	Global	Male	Socio-demographic Index			X			X
Upper respiratory infections	Data Rich	Male	Healthcare access and quality index		X			X	
Upper respiratory infections	Global	Male	Healthcare access and quality index		X			X	
Upper respiratory infections	Data Rich	Male	Indoor Air Pollution (All Cooking Fuels)		X			X	
Upper respiratory infections	Global	Male	Indoor Air Pollution (All Cooking Fuels)		X			X	
Upper respiratory infections	Data Rich	Male	Outdoor Air Pollution (PM2.5)		X			X	
Upper respiratory infections	Global	Male	Outdoor Air Pollution (PM2.5)		X			X	
Upper respiratory infections	Data Rich	Male	Smoking Prevalence	X			X		
Upper respiratory infections	Global	Male	Smoking Prevalence	X			X		
Otitis media	Global	Female	Education (years per capita)			X			X
Otitis media	Data Rich	Female	Education (years per capita)			X			X
Otitis media	Global	Female	LDI (I$ per capita)			X			X
Otitis media	Data Rich	Female	LDI (I$ per capita)			X			X
Otitis media	Global	Female	Socio-demographic Index			X			X
Otitis media	Data Rich	Female	Socio-demographic Index			X			X
Otitis media	Global	Female	Healthcare access and quality index		X			X	
Otitis media	Data Rich	Female	Healthcare access and quality index		X			X	
Otitis media	Global	Female	Indoor Air Pollution (All Cooking Fuels)		X			X	
Otitis media	Data Rich	Female	Indoor Air Pollution (All Cooking Fuels)		X			X	
Otitis media	Global	Female	Outdoor Air Pollution (PM2.5)		X			X	
Otitis media	Data Rich	Female	Outdoor Air Pollution (PM2.5)		X			X	
Otitis media	Global	Female	Smoking Prevalence	X			X		
Otitis media	Data Rich	Female	Smoking Prevalence	X			X		
Otitis media	Global	Female	Log-transformed SEV scalar: Otitis	X			X		
Otitis media	Data Rich	Female	Log-transformed SEV scalar: Otitis	X			X		
Otitis media	Global	Male	Education (years per capita)			X			X
Otitis media	Data Rich	Male	Education (years per capita)			X			X
Otitis media	Global	Male	LDI (I$ per capita)			X			X
Otitis media	Data Rich	Male	LDI (I$ per capita)			X			X
Otitis media	Global	Male	Socio-demographic Index			X			X
Otitis media	Data Rich	Male	Socio-demographic Index			X			X
Otitis media	Global	Male	Healthcare access and quality index		X			X	
Otitis media	Data Rich	Male	Healthcare access and quality index		X			X	
Otitis media	Global	Male	Indoor Air Pollution (All Cooking Fuels)		X			X	
Otitis media	Data Rich	Male	Indoor Air Pollution (All Cooking Fuels)		X			X	
Otitis media	Global	Male	Outdoor Air Pollution (PM2.5)		X			X	
Otitis media	Data Rich	Male	Outdoor Air Pollution (PM2.5)		X			X	
Otitis media	Global	Male	Smoking Prevalence	X			X		
Otitis media	Data Rich	Male	Smoking Prevalence	X			X		
Otitis media	Global	Male	Log-transformed SEV scalar: Otitis	X			X		
Otitis media	Data Rich	Male	Log-transformed SEV scalar: Otitis	X			X		
Diarrhoeal diseases	Global	Female	Education (years per capita)			X			X
Diarrhoeal diseases	Data Rich	Female	Education (years per capita)			X			X
Diarrhoeal diseases	Global	Female	LDI (I$ per capita)			X			X
Diarrhoeal diseases	Data Rich	Female	LDI (I$ per capita)			X			X
Diarrhoeal diseases	Global	Female	LDI (I$ per capita)			X			X
Diarrhoeal diseases	Data Rich	Female	LDI (I$ per capita)			X			X
Diarrhoeal diseases	Global	Female	Maternal Education (years per capita)			X			X
Diarrhoeal diseases	Data Rich	Female	Maternal Education (years per capita)			X			X
Diarrhoeal diseases	Global	Female	Age-standardized proportion adult underweight			X			X
Diarrhoeal diseases	Data Rich	Female	Age-standardized proportion adult underweight			X			X
Diarrhoeal diseases	Global	Female	No access to handwashing facility			X			X
Diarrhoeal diseases	Data Rich	Female	No access to handwashing facility			X			X
Diarrhoeal diseases	Global	Female	Socio-demographic Index			X			X
Diarrhoeal diseases	Data Rich	Female	Socio-demographic Index			X			X
Diarrhoeal diseases	Global	Female	Socio-demographic Index			X			X
Diarrhoeal diseases	Data Rich	Female	Socio-demographic Index			X			X
Diarrhoeal diseases	Global	Female	Healthcare access and quality index			X		X	
Diarrhoeal diseases	Data Rich	Female	Healthcare access and quality index		X			X	
Diarrhoeal diseases	Global	Female	Healthcare access and quality index		X			X	
Diarrhoeal diseases	Data Rich	Female	Healthcare access and quality index		X			X	

Comparison of GBD 2019 and GBD 2021 covariates and level of covariates used in cause of death modeling

Cause	Model Version Type	Sex	Covariate	Level 1 GBD 2019	Level 2 GBD 2019	Level 3 GBD 2019	Level 1 GBD 2021	Level 2 GBD 2021	Level 3 GBD 2021
Diarrhoeal diseases	Global	Female	Zinc deficiency		X			X	
Diarrhoeal diseases	Data Rich	Female	Zinc deficiency		X			X	
Diarrhoeal diseases	Global	Female	Rotavirus coverage (proportion)		X			X	
Diarrhoeal diseases	Data Rich	Female	Rotavirus coverage (proportion)		X			X	
Diarrhoeal diseases	Global	Female	ORS (oral rehydration)		X			X	
Diarrhoeal diseases	Data Rich	Female	ORS (oral rehydration)		X			X	
Diarrhoeal diseases	Global	Female	Zinc treatment for diarrhea		X			X	
Diarrhoeal diseases	Data Rich	Female	Zinc treatment for diarrhea		X			X	
Diarrhoeal diseases	Global	Female	Sanitation (proportion with access)	X			X		
Diarrhoeal diseases	Data Rich	Female	Sanitation (proportion with access)	X			X		
Diarrhoeal diseases	Global	Female	Sanitation (proportion with access)	X			X		
Diarrhoeal diseases	Data Rich	Female	Sanitation (proportion with access)	X			X		
Diarrhoeal diseases	Global	Female	Improved Water Source (proportion with access)	X			X		
Diarrhoeal diseases	Data Rich	Female	Improved Water Source (proportion with access)	X			X		
Diarrhoeal diseases	Global	Female	Improved Water Source (proportion with access)	X			X		
Diarrhoeal diseases	Data Rich	Female	Improved Water Source (proportion with access)	X			X		
Diarrhoeal diseases	Global	Female	Rotavirus coverage (proportion)	X			X		
Diarrhoeal diseases	Data Rich	Female	Rotavirus coverage (proportion)	X			X		
Diarrhoeal diseases	Global	Female	ORS (oral rehydration)	X			X		
Diarrhoeal diseases	Data Rich	Female	ORS (oral rehydration)	X			X		
Diarrhoeal diseases	Global	Female	No access to handwashing facility						X
Diarrhoeal diseases	Data Rich	Female	No access to handwashing facility						X
Diarrhoeal diseases	Global	Male	Education (years per capita)			X			X
Diarrhoeal diseases	Data Rich	Male	Education (years per capita)			X			X
Diarrhoeal diseases	Global	Male	LDI (I$ per capita)			X			X
Diarrhoeal diseases	Data Rich	Male	LDI (I$ per capita)			X			X
Diarrhoeal diseases	Global	Male	LDI (I$ per capita)			X			X
Diarrhoeal diseases	Data Rich	Male	LDI (I$ per capita)			X			X
Diarrhoeal diseases	Global	Male	Maternal Education (years per capita)			X			X
Diarrhoeal diseases	Data Rich	Male	Maternal Education (years per capita)			X			X
Diarrhoeal diseases	Global	Male	Age-standardized proportion adult underweight			X			X
Diarrhoeal diseases	Data Rich	Male	Age-standardized proportion adult underweight			X			X
Diarrhoeal diseases	Global	Male	No access to handwashing facility			X			X
Diarrhoeal diseases	Data Rich	Male	No access to handwashing facility			X			X
Diarrhoeal diseases	Global	Male	Socio-demographic Index			X			X
Diarrhoeal diseases	Data Rich	Male	Socio-demographic Index			X			X
Diarrhoeal diseases	Global	Male	Socio-demographic Index			X			X
Diarrhoeal diseases	Data Rich	Male	Socio-demographic Index			X			X
Diarrhoeal diseases	Global	Male	Healthcare access and quality index			X		X	
Diarrhoeal diseases	Data Rich	Male	Healthcare access and quality index		X			X	
Diarrhoeal diseases	Global	Male	Healthcare access and quality index		X			X	
Diarrhoeal diseases	Data Rich	Male	Healthcare access and quality index		X			X	
Diarrhoeal diseases	Global	Male	Zinc deficiency		X			X	
Diarrhoeal diseases	Data Rich	Male	Zinc deficiency		X			X	
Diarrhoeal diseases	Global	Male	Rotavirus coverage (proportion)		X			X	
Diarrhoeal diseases	Data Rich	Male	Rotavirus coverage (proportion)		X			X	
Diarrhoeal diseases	Global	Male	ORS (oral rehydration)		X			X	
Diarrhoeal diseases	Data Rich	Male	ORS (oral rehydration)		X			X	
Diarrhoeal diseases	Global	Male	Zinc treatment for diarrhea		X			X	
Diarrhoeal diseases	Data Rich	Male	Zinc treatment for diarrhea		X			X	
Diarrhoeal diseases	Global	Male	Sanitation (proportion with access)	X			X		
Diarrhoeal diseases	Data Rich	Male	Sanitation (proportion with access)	X			X		
Diarrhoeal diseases	Global	Male	Sanitation (proportion with access)	X			X		
Diarrhoeal diseases	Data Rich	Male	Sanitation (proportion with access)	X			X		
Diarrhoeal diseases	Global	Male	Improved Water Source (proportion with access)	X			X		
Diarrhoeal diseases	Data Rich	Male	Improved Water Source (proportion with access)	X			X		
Diarrhoeal diseases	Global	Male	Improved Water Source (proportion with access)	X			X		
Diarrhoeal diseases	Data Rich	Male	Improved Water Source (proportion with access)	X			X		
Diarrhoeal diseases	Global	Male	Rotavirus coverage (proportion)	X			X		
Diarrhoeal diseases	Data Rich	Male	Rotavirus coverage (proportion)	X			X		
Diarrhoeal diseases	Global	Male	ORS (oral rehydration)	X			X		
Diarrhoeal diseases	Data Rich	Male	ORS (oral rehydration)	X			X		
Diarrhoeal diseases	Global	Male	No access to handwashing facility						X
Diarrhoeal diseases	Data Rich	Male	No access to handwashing facility						X
Rabies	Global	Female	Population Density (500-1000 ppl/sqkm, proportion)						X
Rabies	Data Rich	Female	Population Density (500-1000 ppl/sqkm, proportion)						X
Rabies	Global	Female	Population Density (under 150 ppl/sqkm, proportion)						X
Rabies	Data Rich	Female	Population Density (under 150 ppl/sqkm, proportion)						X
Rabies	Global	Female	Skilled Birth Attendance (proportion)					X	
Rabies	Data Rich	Female	Skilled Birth Attendance (proportion)					X	
Rabies	Global	Female	Healthcare access and quality index					X	
Rabies	Data Rich	Female	Healthcare access and quality index					X	
Rabies	Data Rich	Female	Maternal care and immunization					X	
Rabies	Global	Female	In-Facility Delivery (proportion)				X		
Rabies	Data Rich	Female	In-Facility Delivery (proportion)				X		
Rabies	Global	Female	Socio-demographic Index				X		
Rabies	Data Rich	Female	Socio-demographic Index				X		
Rabies	Global	Female	Antenatal Care (4 visits) Coverage (proportion)				X		
Rabies	Data Rich	Female	Antenatal Care (4 visits) Coverage (proportion)				X		
Rabies	Global	Male	Population Density (500-1000 ppl/sqkm, proportion)						X
Rabies	Data Rich	Male	Population Density (500-1000 ppl/sqkm, proportion)						X
Rabies	Global	Male	Population Density (under 150 ppl/sqkm, proportion)						X
Rabies	Data Rich	Male	Population Density (under 150 ppl/sqkm, proportion)						X
Rabies	Global	Male	Skilled Birth Attendance (proportion)					X	
Rabies	Data Rich	Male	Skilled Birth Attendance (proportion)					X	
Rabies	Global	Male	Healthcare access and quality index					X	
Rabies	Data Rich	Male	Healthcare access and quality index					X	
Rabies	Data Rich	Male	Maternal care and immunization					X	
Rabies	Global	Male	In-Facility Delivery (proportion)				X		
Rabies	Data Rich	Male	In-Facility Delivery (proportion)				X		
Rabies	Global	Male	Socio-demographic Index				X		
Rabies	Data Rich	Male	Socio-demographic Index				X		
Rabies	Global	Male	Antenatal Care (4 visits) Coverage (proportion)				X		
Rabies	Data Rich	Male	Antenatal Care (4 visits) Coverage (proportion)				X		
Other neglected tropical diseases	Data Rich	Female	Education (years per capita)						X
Other neglected tropical diseases	Global	Female	Education (years per capita)						X
Other neglected tropical diseases	Data Rich	Female	LDI (I$ per capita)						X
Other neglected tropical diseases	Global	Female	LDI (I$ per capita)						X
Other neglected tropical diseases	Data Rich	Female	Socio-demographic Index						X
Other neglected tropical diseases	Global	Female	Socio-demographic Index						X
Other neglected tropical diseases	Data Rich	Female	Sanitation (proportion with access)					X	
Other neglected tropical diseases	Global	Female	Sanitation (proportion with access)					X	
Other neglected tropical diseases	Data Rich	Female	Rainfall Quintile 5 (proportion)					X	
Other neglected tropical diseases	Global	Female	Rainfall Quintile 5 (proportion)					X	
Other neglected tropical diseases	Data Rich	Female	Healthcare access and quality index				X		
Other neglected tropical diseases	Global	Female	Healthcare access and quality index				X		
Other neglected tropical diseases	Data Rich	Female	Latitude Under 15 (proportion)				X		
Other neglected tropical diseases	Global	Female	Latitude Under 15 (proportion)				X		
Other neglected tropical diseases	Data Rich	Male	Education (years per capita)						X
Other neglected tropical diseases	Global	Male	Education (years per capita)						X
Other neglected tropical diseases	Data Rich	Male	LDI (I$ per capita)						X
Other neglected tropical diseases	Global	Male	LDI (I$ per capita)						X
Other neglected tropical diseases	Data Rich	Male	Socio-demographic Index						X
Other neglected tropical diseases	Global	Male	Socio-demographic Index						X
Other neglected tropical diseases	Data Rich	Male	Sanitation (proportion with access)					X	
Other neglected tropical diseases	Global	Male	Sanitation (proportion with access)					X	
Other neglected tropical diseases	Data Rich	Male	Rainfall Quintile 5 (proportion)					X	
Other neglected tropical diseases	Global	Male	Rainfall Quintile 5 (proportion)					X	
Other neglected tropical diseases	Data Rich	Male	Healthcare access and quality index				X		
Other neglected tropical diseases	Global	Male	Healthcare access and quality index				X		
Other neglected tropical diseases	Data Rich	Male	Latitude Under 15 (proportion)				X		
Other neglected tropical diseases	Global	Male	Latitude Under 15 (proportion)				X		
Meningitis	Global	Female	LDI (I$ per capita)			X			X
Meningitis	Data Rich	Female	LDI (I$ per capita)			X			X
Meningitis	Global	Female	LDI (I$ per capita)			X			X
Meningitis	Data Rich	Female	LDI (I$ per capita)			X			X
Meningitis	Global	Female	Socio-demographic Index			X			X
Meningitis	Data Rich	Female	Socio-demographic Index			X			X
Meningitis	Global	Female	Socio-demographic Index			X			X
Meningitis	Data Rich	Female	Socio-demographic Index			X			X

Comparison of GBD 2019 and GBD 2021 covariates and level of covariates used in cause of death modeling									
Cause	Model Version Type	Sex	Covariate	Level 1 GBD 2019	Level 2 GBD 2019	Level 3 GBD 2019	Level 1 GBD 2021	Level 2 GBD 2021	Level 3 GBD 2021
Meningitis	Global	Female	Sanitation (proportion with access)			X			X
Meningitis	Data Rich	Female	Sanitation (proportion with access)			X			X
Meningitis	Global	Female	Sanitation (proportion with access)			X			X
Meningitis	Data Rich	Female	Sanitation (proportion with access)			X			X
Meningitis	Global	Female	Maternal Education (years per capita)			X			X
Meningitis	Data Rich	Female	Maternal Education (years per capita)			X			X
Meningitis	Global	Female	Maternal Education (years per capita)			X			X
Meningitis	Data Rich	Female	Maternal Education (years per capita)			X			X
Meningitis	Global	Female	Healthcare access and quality index		X			X	
Meningitis	Data Rich	Female	Healthcare access and quality index		X			X	
Meningitis	Global	Female	Healthcare access and quality index		X			X	
Meningitis	Data Rich	Female	Healthcare access and quality index		X			X	
Meningitis	Global	Female	Improved Water Source (proportion with access)		X			X	
Meningitis	Data Rich	Female	Improved Water Source (proportion with access)		X			X	
Meningitis	Global	Female	Improved Water Source (proportion with access)		X			X	
Meningitis	Data Rich	Female	Improved Water Source (proportion with access)		X			X	
Meningitis	Global	Female	Maternal care and immunization		X			X	
Meningitis	Data Rich	Female	Maternal care and immunization		X			X	
Meningitis	Global	Female	Maternal care and immunization		X			X	
Meningitis	Data Rich	Female	Maternal care and immunization		X			X	
Meningitis	Global	Female	Age- and sex-specific SEV for Child underweight		X			X	
Meningitis	Data Rich	Female	Age- and sex-specific SEV for Child underweight		X			X	
Meningitis	Global	Female	Age-standardized SEV for Child underweight		X			X	
Meningitis	Data Rich	Female	Age-standardized SEV for Child underweight		X			X	
Meningitis	Global	Female	meningitis belt (proportion)	X			X		
Meningitis	Data Rich	Female	meningitis belt (proportion)	X			X		
Meningitis	Global	Female	meningitis belt (proportion)	X			X		
Meningitis	Data Rich	Female	meningitis belt (proportion)	X			X		
Meningitis	Global	Female	Proportion of total population covered by menafrivac initiative (meningitis meningococcal type A vaccine)	X			X		
Meningitis	Data Rich	Female	Proportion of total population covered by menafrivac initiative (meningitis meningococcal type A vaccine)	X			X		
Meningitis	Global	Female	Proportion of total population covered by menafrivac initiative (meningitis meningococcal type A vaccine)	X			X		
Meningitis	Data Rich	Female	Proportion of total population covered by menafrivac initiative (meningitis meningococcal type A vaccine)	X			X		
Meningitis	Global	Female	PCV3 lagged five year coverage (proportion)				X		
Meningitis	Data Rich	Female	PCV3 lagged five year coverage (proportion)				X		
Meningitis	Global	Female	PCV3 lagged five year coverage (proportion)				X		
Meningitis	Data Rich	Female	PCV3 lagged five year coverage (proportion)				X		
Meningitis	Global	Female	Hib3 transformed: population-level coverage, including indirect effects				X		
Meningitis	Data Rich	Female	Hib3 transformed: population-level coverage, including indirect effects				X		
Meningitis	Global	Female	Hib3 transformed: population-level coverage, including indirect effects				X		
Meningitis	Data Rich	Female	Hib3 transformed: population-level coverage, including indirect effects				X		
Meningitis	Global	Male	LDI (I$ per capita)			X			X
Meningitis	Data Rich	Male	LDI (I$ per capita)			X			X
Meningitis	Global	Male	LDI (I$ per capita)			X			X
Meningitis	Data Rich	Male	LDI (I$ per capita)			X			X
Meningitis	Global	Male	Socio-demographic Index			X			X
Meningitis	Data Rich	Male	Socio-demographic Index			X			X
Meningitis	Global	Male	Socio-demographic Index			X			X
Meningitis	Data Rich	Male	Socio-demographic Index			X			X
Meningitis	Global	Male	Sanitation (proportion with access)			X			X
Meningitis	Data Rich	Male	Sanitation (proportion with access)			X			X
Meningitis	Global	Male	Sanitation (proportion with access)			X			X
Meningitis	Data Rich	Male	Sanitation (proportion with access)			X			X
Meningitis	Global	Male	Maternal Education (years per capita)			X			X
Meningitis	Data Rich	Male	Maternal Education (years per capita)			X			X
Meningitis	Global	Male	Maternal Education (years per capita)			X			X
Meningitis	Data Rich	Male	Maternal Education (years per capita)			X			X
Meningitis	Global	Male	Healthcare access and quality index		X			X	
Meningitis	Data Rich	Male	Healthcare access and quality index		X			X	
Meningitis	Global	Male	Healthcare access and quality index		X			X	
Meningitis	Data Rich	Male	Healthcare access and quality index		X			X	
Meningitis	Global	Male	Improved Water Source (proportion with access)		X			X	
Meningitis	Data Rich	Male	Improved Water Source (proportion with access)		X			X	
Meningitis	Global	Male	Improved Water Source (proportion with access)		X			X	
Meningitis	Data Rich	Male	Improved Water Source (proportion with access)		X			X	
Meningitis	Global	Male	Maternal care and immunization		X			X	
Meningitis	Data Rich	Male	Maternal care and immunization		X			X	
Meningitis	Global	Male	Maternal care and immunization		X			X	
Meningitis	Data Rich	Male	Maternal care and immunization		X			X	
Meningitis	Global	Male	Age- and sex-specific SEV for Child underweight		X			X	
Meningitis	Data Rich	Male	Age- and sex-specific SEV for Child underweight		X			X	
Meningitis	Global	Male	Age-standardized SEV for Child underweight		X			X	
Meningitis	Data Rich	Male	Age-standardized SEV for Child underweight		X			X	
Meningitis	Global	Male	meningitis belt (proportion)	X			X		
Meningitis	Data Rich	Male	meningitis belt (proportion)	X			X		
Meningitis	Global	Male	meningitis belt (proportion)	X			X		
Meningitis	Data Rich	Male	meningitis belt (proportion)	X			X		
Meningitis	Global	Male	Proportion of total population covered by menafrivac initiative (meningitis meningococcal type A vaccine)	X			X		
Meningitis	Data Rich	Male	Proportion of total population covered by menafrivac initiative (meningitis meningococcal type A vaccine)	X			X		
Meningitis	Global	Male	Proportion of total population covered by menafrivac initiative (meningitis meningococcal type A vaccine)	X			X		
Meningitis	Data Rich	Male	Proportion of total population covered by menafrivac initiative (meningitis meningococcal type A vaccine)	X			X		
Meningitis	Global	Male	PCV3 lagged five year coverage (proportion)				X		
Meningitis	Data Rich	Male	PCV3 lagged five year coverage (proportion)				X		
Meningitis	Global	Male	PCV3 lagged five year coverage (proportion)				X		
Meningitis	Data Rich	Male	PCV3 lagged five year coverage (proportion)				X		
Meningitis	Global	Male	Hib3 transformed: population-level coverage, including indirect effects				X		
Meningitis	Data Rich	Male	Hib3 transformed: population-level coverage, including indirect effects				X		
Meningitis	Global	Male	Hib3 transformed: population-level coverage, including indirect effects				X		
Meningitis	Data Rich	Male	Hib3 transformed: population-level coverage, including indirect effects				X		
Encephalitis	Global	Female	Socio-demographic Index			X			X
Encephalitis	Data Rich	Female	Socio-demographic Index			X			X
Encephalitis	Global	Female	Sanitation (proportion with access)			X			X
Encephalitis	Data Rich	Female	Sanitation (proportion with access)			X			X
Encephalitis	Global	Female	Improved Water Source (proportion with access)			X			X
Encephalitis	Data Rich	Female	Improved Water Source (proportion with access)			X			X
Encephalitis	Global	Female	DTP3 Coverage (proportion)			X			X
Encephalitis	Data Rich	Female	DTP3 Coverage (proportion)			X			X
Encephalitis	Global	Female	Maternal Education (years per capita)			X			X
Encephalitis	Data Rich	Female	Maternal Education (years per capita)			X			X
Encephalitis	Global	Female	In-Facility Delivery (proportion)			X			X
Encephalitis	Data Rich	Female	In-Facility Delivery (proportion)			X			X
Encephalitis	Global	Female	LDI (I$ per capita)		X			X	
Encephalitis	Data Rich	Female	LDI (I$ per capita)		X			X	
Encephalitis	Global	Female	Healthcare access and quality index		X			X	
Encephalitis	Data Rich	Female	Healthcare access and quality index		X			X	
Encephalitis	Global	Female	Maternal care and immunization		X			X	
Encephalitis	Data Rich	Female	Maternal care and immunization		X			X	
Encephalitis	Global	Female	Age- and sex-specific SEV for Child underweight	X			X		
Encephalitis	Data Rich	Female	Age- and sex-specific SEV for Child underweight	X			X		
Encephalitis	Global	Female	Japanese encelphalitis endemic area (binary)	X			X		
Encephalitis	Data Rich	Female	Japanese encelphalitis endemic area (binary)	X			X		
Encephalitis	Data Rich	Male	Socio-demographic Index			X			X
Encephalitis	Global	Male	Socio-demographic Index			X			X
Encephalitis	Data Rich	Male	Sanitation (proportion with access)			X			X
Encephalitis	Global	Male	Sanitation (proportion with access)			X			X

Comparison of GBD 2019 and GBD 2021 covariates and level of covariates used in cause of death modeling

Cause	Model Version Type	Sex	Covariate	Level 1 GBD 2019	Level 2 GBD 2019	Level 3 GBD 2019	Level 1 GBD 2021	Level 2 GBD 2021	Level 3 GBD 2021
Encephalitis	Data Rich	Male	Improved Water Source (proportion with access)			X			X
Encephalitis	Global	Male	Improved Water Source (proportion with access)			X			X
Encephalitis	Data Rich	Male	DTP3 Coverage (proportion)			X			X
Encephalitis	Global	Male	DTP3 Coverage (proportion)			X			X
Encephalitis	Data Rich	Male	Maternal Education (years per capita)			X			X
Encephalitis	Global	Male	Maternal Education (years per capita)			X			X
Encephalitis	Data Rich	Male	In-Facility Delivery (proportion)			X			X
Encephalitis	Global	Male	In-Facility Delivery (proportion)			X			X
Encephalitis	Data Rich	Male	LDI (I$ per capita)		X			X	
Encephalitis	Global	Male	LDI (I$ per capita)		X			X	
Encephalitis	Data Rich	Male	Healthcare access and quality index		X			X	
Encephalitis	Global	Male	Healthcare access and quality index		X			X	
Encephalitis	Data Rich	Male	Maternal care and immunization		X			X	
Encephalitis	Global	Male	Maternal care and immunization		X			X	
Encephalitis	Data Rich	Male	Age- and sex-specific SEV for Child underweight	X			X		
Encephalitis	Global	Male	Age- and sex-specific SEV for Child underweight	X			X		
Encephalitis	Data Rich	Male	Japanese encephalitis endemic area (binary)	X			X		
Encephalitis	Global	Male	Japanese encephalitis endemic area (binary)	X			X		
Tetanus	Data Rich	Female	Education (years per capita)			X			X
Tetanus	Global	Female	Education (years per capita)			X			X
Tetanus	Data Rich	Female	Education (years per capita)			X			X
Tetanus	Global	Female	Education (years per capita)			X			X
Tetanus	Data Rich	Female	LDI (I$ per capita)			X			X
Tetanus	Global	Female	LDI (I$ per capita)			X			X
Tetanus	Data Rich	Female	LDI (I$ per capita)			X			X
Tetanus	Global	Female	LDI (I$ per capita)			X			X
Tetanus	Data Rich	Female	Socio-demographic Index			X			X
Tetanus	Global	Female	Socio-demographic Index			X			X
Tetanus	Data Rich	Female	Socio-demographic Index			X			X
Tetanus	Global	Female	Socio-demographic Index			X			X
Tetanus	Data Rich	Female	Sanitation (proportion with access)			X			X
Tetanus	Global	Female	Sanitation (proportion with access)			X			X
Tetanus	Data Rich	Female	In-Facility Delivery (proportion)		X			X	
Tetanus	Global	Female	In-Facility Delivery (proportion)		X			X	
Tetanus	Data Rich	Female	Skilled Birth Attendance (proportion)		X			X	
Tetanus	Global	Female	Skilled Birth Attendance (proportion)		X			X	
Tetanus	Data Rich	Female	Healthcare access and quality index		X			X	
Tetanus	Global	Female	Healthcare access and quality index		X			X	
Tetanus	Data Rich	Female	Healthcare access and quality index		X			X	
Tetanus	Global	Female	Healthcare access and quality index		X			X	
Tetanus	Data Rich	Female	tetanus toxoid maternal protection at birth	X			X		
Tetanus	Global	Female	tetanus toxoid maternal protection at birth	X			X		
Tetanus	Data Rich	Female	DTP3 lagged five year coverage (proportion)				X		
Tetanus	Global	Female	DTP3 lagged five year coverage (proportion)				X		
Tetanus	Data Rich	Female	DTP3 lagged five year coverage (proportion)				X		
Tetanus	Global	Female	DTP3 lagged five year coverage (proportion)				X		
Tetanus	Data Rich	Male	Education (years per capita)			X			X
Tetanus	Global	Male	Education (years per capita)			X			X
Tetanus	Data Rich	Male	Education (years per capita)			X			X
Tetanus	Global	Male	Education (years per capita)			X			X
Tetanus	Data Rich	Male	LDI (I$ per capita)			X			X
Tetanus	Global	Male	LDI (I$ per capita)			X			X
Tetanus	Data Rich	Male	LDI (I$ per capita)			X			X
Tetanus	Global	Male	LDI (I$ per capita)			X			X
Tetanus	Data Rich	Male	Socio-demographic Index			X			X
Tetanus	Global	Male	Socio-demographic Index			X			X
Tetanus	Data Rich	Male	Socio-demographic Index			X			X
Tetanus	Global	Male	Socio-demographic Index			X			X
Tetanus	Data Rich	Male	Sanitation (proportion with access)			X			X
Tetanus	Global	Male	Sanitation (proportion with access)			X			X
Tetanus	Data Rich	Male	In-Facility Delivery (proportion)		X			X	
Tetanus	Global	Male	In-Facility Delivery (proportion)		X			X	
Tetanus	Data Rich	Male	Skilled Birth Attendance (proportion)		X			X	
Tetanus	Global	Male	Skilled Birth Attendance (proportion)		X			X	
Tetanus	Data Rich	Male	Healthcare access and quality index		X			X	
Tetanus	Global	Male	Healthcare access and quality index		X			X	
Tetanus	Data Rich	Male	Healthcare access and quality index		X			X	
Tetanus	Global	Male	Healthcare access and quality index		X			X	
Tetanus	Data Rich	Male	tetanus toxoid maternal protection at birth	X			X		
Tetanus	Global	Male	tetanus toxoid maternal protection at birth	X			X		
Tetanus	Data Rich	Male	DTP3 lagged five year coverage (proportion)				X		
Tetanus	Global	Male	DTP3 lagged five year coverage (proportion)				X		
Tetanus	Data Rich	Male	DTP3 lagged five year coverage (proportion)				X		
Tetanus	Global	Male	DTP3 lagged five year coverage (proportion)				X		
Acute hepatitis	Data Rich	Female	Education (years per capita)			X			X
Acute hepatitis	Global	Female	Education (years per capita)			X			X
Acute hepatitis	Data Rich	Female	LDI (I$ per capita)			X			X
Acute hepatitis	Global	Female	LDI (I$ per capita)			X			X
Acute hepatitis	Data Rich	Female	Socio-demographic Index		X			X	
Acute hepatitis	Data Rich	Female	Healthcare access and quality index		X			X	
Acute hepatitis	Global	Female	Healthcare access and quality index		X			X	
Acute hepatitis	Data Rich	Female	Intravenous drug use (proportion by age)		X			X	
Acute hepatitis	Global	Female	Intravenous drug use (proportion by age)		X			X	
Acute hepatitis	Data Rich	Female	Hepatitis B vaccine coverage (proportion), aged through time		X			X	
Acute hepatitis	Global	Female	Hepatitis B vaccine coverage (proportion), aged through time		X			X	
Acute hepatitis	Data Rich	Female	Age- and sex-specific SEV for Unsafe sanitation		X			X	
Acute hepatitis	Global	Female	Age- and sex-specific SEV for Unsafe sanitation		X			X	
Acute hepatitis	Data Rich	Female	Age- and sex-specific SEV for Unsafe water		X			X	
Acute hepatitis	Global	Female	Age- and sex-specific SEV for Unsafe water		X			X	
Acute hepatitis	Global	Female	Socio-demographic Index		X			X	
Acute hepatitis	Data Rich	Female	Log-transformed SEV scalar: Hep	X			X		
Acute hepatitis	Global	Female	Log-transformed SEV scalar: Hep	X			X		
Acute hepatitis	Data Rich	Female	Hepatitis A Seroprevalence (anti-HAV) age standardized	X			X		
Acute hepatitis	Global	Female	Hepatitis A Seroprevalence (anti-HAV) age standardized	X			X		
Acute hepatitis	Data Rich	Female	Hepatitis C Seroprevalence (anti-HCV) age standardized	X			X		
Acute hepatitis	Global	Female	Hepatitis C Seroprevalence (anti-HCV) age standardized	X			X		
Acute hepatitis	Data Rich	Female	Hepatitis E Seroprevalence (anti-HEV) age standardized	X			X		
Acute hepatitis	Global	Female	Hepatitis E Seroprevalence (anti-HEV) age standardized	X			X		
Acute hepatitis	Data Rich	Female	Vaccine adjusted HbSAg serporevalence age standardized				X		
Acute hepatitis	Global	Female	Vaccine adjusted HbSAg serporevalence age standardized				X		
Acute hepatitis	Data Rich	Male	Education (years per capita)			X			X
Acute hepatitis	Global	Male	Education (years per capita)			X			X
Acute hepatitis	Data Rich	Male	LDI (I$ per capita)			X			X
Acute hepatitis	Global	Male	LDI (I$ per capita)			X			X
Acute hepatitis	Data Rich	Male	Socio-demographic Index		X			X	
Acute hepatitis	Data Rich	Male	Healthcare access and quality index		X			X	
Acute hepatitis	Global	Male	Healthcare access and quality index		X			X	
Acute hepatitis	Data Rich	Male	Intravenous drug use (proportion by age)		X			X	
Acute hepatitis	Global	Male	Intravenous drug use (proportion by age)		X			X	
Acute hepatitis	Data Rich	Male	Hepatitis B vaccine coverage (proportion), aged through time		X			X	
Acute hepatitis	Global	Male	Hepatitis B vaccine coverage (proportion), aged through time		X			X	
Acute hepatitis	Data Rich	Male	Age- and sex-specific SEV for Unsafe sanitation		X			X	
Acute hepatitis	Global	Male	Age- and sex-specific SEV for Unsafe sanitation		X			X	
Acute hepatitis	Data Rich	Male	Age- and sex-specific SEV for Unsafe water		X			X	
Acute hepatitis	Global	Male	Age- and sex-specific SEV for Unsafe water		X			X	
Acute hepatitis	Global	Male	Socio-demographic Index		X			X	
Acute hepatitis	Data Rich	Male	Log-transformed SEV scalar: Hep	X			X		
Acute hepatitis	Global	Male	Log-transformed SEV scalar: Hep	X			X		
Acute hepatitis	Data Rich	Male	Hepatitis A Seroprevalence (anti-HAV) age standardized	X			X		
Acute hepatitis	Global	Male	Hepatitis A Seroprevalence (anti-HAV) age standardized	X			X		
Acute hepatitis	Data Rich	Male	Hepatitis C Seroprevalence (anti-HCV) age standardized	X			X		
Acute hepatitis	Global	Male	Hepatitis C Seroprevalence (anti-HCV) age standardized	X			X		
Acute hepatitis	Data Rich	Male	Hepatitis E Seroprevalence (anti-HEV) age standardized	X			X		
Acute hepatitis	Global	Male	Hepatitis E Seroprevalence (anti-HEV) age standardized	X			X		
Acute hepatitis	Data Rich	Male	Vaccine adjusted HbSAg serporevalence age standardized				X		
Acute hepatitis	Global	Male	Vaccine adjusted HbSAg serporevalence age standardized				X		
Acute hepatitis A	Data Rich	Female	Education (years per capita)			X			X

Comparison of GBD 2019 and GBD 2021 covariates and level of covariates used in cause of death modeling									
Cause	Model Version Type	Sex	Covariate	Level 1 GBD 2019	Level 2 GBD 2019	Level 3 GBD 2019	Level 1 GBD 2021	Level 2 GBD 2021	Level 3 GBD 2021
Acute hepatitis A	Global	Female	Education (years per capita)			X			X
Acute hepatitis A	Data Rich	Female	LDI (I$ per capita)			X			X
Acute hepatitis A	Global	Female	LDI (I$ per capita)			X			X
Acute hepatitis A	Data Rich	Female	Socio-demographic Index		X			X	
Acute hepatitis A	Global	Female	Socio-demographic Index		X			X	
Acute hepatitis A	Data Rich	Female	Healthcare access and quality index		X			X	
Acute hepatitis A	Global	Female	Healthcare access and quality index		X			X	
Acute hepatitis A	Data Rich	Female	Age- and sex-specific SEV for Unsafe sanitation		X			X	
Acute hepatitis A	Global	Female	Age- and sex-specific SEV for Unsafe sanitation		X			X	
Acute hepatitis A	Data Rich	Female	Age- and sex-specific SEV for Unsafe water		X			X	
Acute hepatitis A	Global	Female	Age- and sex-specific SEV for Unsafe water		X			X	
Acute hepatitis A	Data Rich	Female	Log-transformed SEV scalar: Hep	X			X		
Acute hepatitis A	Global	Female	Log-transformed SEV scalar: Hep	X			X		
Acute hepatitis A	Data Rich	Female	Hepatitis A Seroprevalence (anti-HAV) age standardized	X			X		
Acute hepatitis A	Global	Female	Hepatitis A Seroprevalence (anti-HAV) age standardized	X			X		
Acute hepatitis A	Data Rich	Male	Education (years per capita)			X			X
Acute hepatitis A	Global	Male	Education (years per capita)			X			X
Acute hepatitis A	Data Rich	Male	LDI (I$ per capita)			X			X
Acute hepatitis A	Global	Male	LDI (I$ per capita)			X			X
Acute hepatitis A	Data Rich	Male	Socio-demographic Index		X			X	
Acute hepatitis A	Global	Male	Socio-demographic Index		X			X	
Acute hepatitis A	Data Rich	Male	Healthcare access and quality index		X			X	
Acute hepatitis A	Global	Male	Healthcare access and quality index		X			X	
Acute hepatitis A	Data Rich	Male	Age- and sex-specific SEV for Unsafe sanitation		X			X	
Acute hepatitis A	Global	Male	Age- and sex-specific SEV for Unsafe sanitation		X			X	
Acute hepatitis A	Data Rich	Male	Age- and sex-specific SEV for Unsafe water		X			X	
Acute hepatitis A	Global	Male	Age- and sex-specific SEV for Unsafe water		X			X	
Acute hepatitis A	Data Rich	Male	Log-transformed SEV scalar: Hep	X			X		
Acute hepatitis A	Global	Male	Log-transformed SEV scalar: Hep	X			X		
Acute hepatitis A	Data Rich	Male	Hepatitis A Seroprevalence (anti-HAV) age standardized	X			X		
Acute hepatitis A	Global	Male	Hepatitis A Seroprevalence (anti-HAV) age standardized	X			X		
Acute hepatitis B	Data Rich	Female	Education (years per capita)			X			X
Acute hepatitis B	Global	Female	Education (years per capita)			X			X
Acute hepatitis B	Data Rich	Female	LDI (I$ per capita)			X			X
Acute hepatitis B	Global	Female	LDI (I$ per capita)			X			X
Acute hepatitis B	Data Rich	Female	Socio-demographic Index		X			X	
Acute hepatitis B	Global	Female	Socio-demographic Index		X			X	
Acute hepatitis B	Data Rich	Female	Healthcare access and quality index		X			X	
Acute hepatitis B	Global	Female	Healthcare access and quality index		X			X	
Acute hepatitis B	Data Rich	Female	Intravenous drug use (proportion by age)		X		X		
Acute hepatitis B	Global	Female	Intravenous drug use (proportion by age)		X		X		
Acute hepatitis B	Data Rich	Female	Hepatitis B vaccine coverage (proportion), aged through time		X		X		
Acute hepatitis B	Global	Female	Hepatitis B vaccine coverage (proportion), aged through time		X		X		
Acute hepatitis B	Data Rich	Female	Log-transformed SEV scalar: Hep	X				X	
Acute hepatitis B	Global	Female	Log-transformed SEV scalar: Hep	X				X	
Acute hepatitis B	Data Rich	Female	Vaccine adjusted HbSAg serporevalence age standardized				X		
Acute hepatitis B	Global	Female	Vaccine adjusted HbSAg serporevalence age standardized				X		
Acute hepatitis B	Data Rich	Male	Education (years per capita)			X			X
Acute hepatitis B	Global	Male	Education (years per capita)			X			X
Acute hepatitis B	Data Rich	Male	LDI (I$ per capita)			X			X
Acute hepatitis B	Global	Male	LDI (I$ per capita)			X			X
Acute hepatitis B	Data Rich	Male	Socio-demographic Index		X			X	
Acute hepatitis B	Global	Male	Socio-demographic Index		X			X	
Acute hepatitis B	Data Rich	Male	Healthcare access and quality index		X			X	
Acute hepatitis B	Global	Male	Healthcare access and quality index		X			X	
Acute hepatitis B	Data Rich	Male	Intravenous drug use (proportion by age)		X		X		
Acute hepatitis B	Global	Male	Intravenous drug use (proportion by age)		X		X		
Acute hepatitis B	Data Rich	Male	Hepatitis B vaccine coverage (proportion), aged through time		X		X		
Acute hepatitis B	Global	Male	Hepatitis B vaccine coverage (proportion), aged through time		X		X		
Acute hepatitis B	Data Rich	Male	Log-transformed SEV scalar: Hep	X				X	
Acute hepatitis B	Global	Male	Log-transformed SEV scalar: Hep	X				X	
Acute hepatitis B	Data Rich	Male	Vaccine adjusted HbSAg serporevalence age standardized				X		
Acute hepatitis B	Global	Male	Vaccine adjusted HbSAg serporevalence age standardized				X		
Acute hepatitis C	Data Rich	Female	Education (years per capita)			X			X
Acute hepatitis C	Global	Female	Education (years per capita)			X			X
Acute hepatitis C	Data Rich	Female	LDI (I$ per capita)			X			X
Acute hepatitis C	Global	Female	LDI (I$ per capita)			X			X
Acute hepatitis C	Data Rich	Female	Socio-demographic Index		X			X	
Acute hepatitis C	Global	Female	Socio-demographic Index		X			X	
Acute hepatitis C	Data Rich	Female	Healthcare access and quality index		X			X	
Acute hepatitis C	Global	Female	Healthcare access and quality index		X			X	
Acute hepatitis C	Data Rich	Female	Intravenous drug use (proportion by age)		X			X	
Acute hepatitis C	Global	Female	Intravenous drug use (proportion by age)		X			X	
Acute hepatitis C	Data Rich	Female	Log-transformed SEV scalar: Hep	X			X		
Acute hepatitis C	Global	Female	Log-transformed SEV scalar: Hep	X			X		
Acute hepatitis C	Data Rich	Female	Hepatitis C Seroprevalence (anti-HCV) age standardized	X			X		
Acute hepatitis C	Global	Female	Hepatitis C Seroprevalence (anti-HCV) age standardized	X			X		
Acute hepatitis C	Data Rich	Male	Education (years per capita)			X			X
Acute hepatitis C	Global	Male	Education (years per capita)			X			X
Acute hepatitis C	Data Rich	Male	LDI (I$ per capita)			X			X
Acute hepatitis C	Global	Male	LDI (I$ per capita)			X			X
Acute hepatitis C	Data Rich	Male	Socio-demographic Index		X			X	
Acute hepatitis C	Global	Male	Socio-demographic Index		X			X	
Acute hepatitis C	Data Rich	Male	Healthcare access and quality index		X			X	
Acute hepatitis C	Global	Male	Healthcare access and quality index		X			X	
Acute hepatitis C	Data Rich	Male	Intravenous drug use (proportion by age)		X		X		
Acute hepatitis C	Global	Male	Intravenous drug use (proportion by age)		X		X		
Acute hepatitis C	Data Rich	Male	Log-transformed SEV scalar: Hep	X				X	
Acute hepatitis C	Global	Male	Log-transformed SEV scalar: Hep	X				X	
Acute hepatitis C	Data Rich	Male	Hepatitis C Seroprevalence (anti-HCV) age standardized	X			X		
Acute hepatitis C	Global	Male	Hepatitis C Seroprevalence (anti-HCV) age standardized	X			X		
Acute hepatitis E	Data Rich	Female	Education (years per capita)			X			X
Acute hepatitis E	Global	Female	Education (years per capita)			X			X
Acute hepatitis E	Data Rich	Female	LDI (I$ per capita)			X			X
Acute hepatitis E	Global	Female	LDI (I$ per capita)			X			X
Acute hepatitis E	Data Rich	Female	Socio-demographic Index		X			X	
Acute hepatitis E	Global	Female	Socio-demographic Index		X			X	
Acute hepatitis E	Data Rich	Female	Healthcare access and quality index		X			X	
Acute hepatitis E	Global	Female	Healthcare access and quality index		X			X	
Acute hepatitis E	Data Rich	Female	Age- and sex-specific SEV for Unsafe sanitation		X			X	
Acute hepatitis E	Global	Female	Age- and sex-specific SEV for Unsafe sanitation		X			X	
Acute hepatitis E	Data Rich	Female	Age- and sex-specific SEV for Unsafe water		X			X	
Acute hepatitis E	Global	Female	Age- and sex-specific SEV for Unsafe water		X			X	
Acute hepatitis E	Data Rich	Female	Log-transformed SEV scalar: Hep	X			X		
Acute hepatitis E	Global	Female	Log-transformed SEV scalar: Hep	X			X		
Acute hepatitis E	Data Rich	Female	Hepatitis E Seroprevalence (anti-HEV) age standardized	X			X		
Acute hepatitis E	Global	Female	Hepatitis E Seroprevalence (anti-HEV) age standardized	X			X		
Acute hepatitis E	Global	Female	Proportion of the population living in the classic monsoon region					X	
Acute hepatitis E	Data Rich	Female	Proportion of the population living in the classic monsoon region				X		
Acute hepatitis E	Data Rich	Male	Education (years per capita)			X			X
Acute hepatitis E	Global	Male	Education (years per capita)			X			X
Acute hepatitis E	Data Rich	Male	LDI (I$ per capita)			X			X
Acute hepatitis E	Global	Male	LDI (I$ per capita)			X			X
Acute hepatitis E	Data Rich	Male	Socio-demographic Index		X			X	
Acute hepatitis E	Global	Male	Socio-demographic Index		X			X	
Acute hepatitis E	Data Rich	Male	Healthcare access and quality index		X			X	
Acute hepatitis E	Global	Male	Healthcare access and quality index		X			X	
Acute hepatitis E	Data Rich	Male	Age- and sex-specific SEV for Unsafe sanitation		X			X	
Acute hepatitis E	Global	Male	Age- and sex-specific SEV for Unsafe sanitation		X			X	
Acute hepatitis E	Data Rich	Male	Age- and sex-specific SEV for Unsafe water		X			X	
Acute hepatitis E	Global	Male	Age- and sex-specific SEV for Unsafe water		X			X	
Acute hepatitis E	Data Rich	Male	Log-transformed SEV scalar: Hep	X			X		
Acute hepatitis E	Global	Male	Log-transformed SEV scalar: Hep	X			X		
Acute hepatitis E	Data Rich	Male	Hepatitis E Seroprevalence (anti-HEV) age standardized	X			X		
Acute hepatitis E	Global	Male	Hepatitis E Seroprevalence (anti-HEV) age standardized	X			X		
Acute hepatitis E	Data Rich	Male	Proportion of the population living in the classic monsoon region					X	

Comparison of GBD 2019 and GBD 2021 covariates and level of covariates used in cause of death modeling									
Cause	Model Version Type	Sex	Covariate	Level 1 GBD 2019	Level 2 GBD 2019	Level 3 GBD 2019	Level 1 GBD 2021	Level 2 GBD 2021	Level 3 GBD 2021
Acute hepatitis E	Global	Male	Proportion of the population living in the classic monsoon region					X	
Other unspecified infectious diseases	Data Rich	Female	Socio-demographic Index			X			X
Other unspecified infectious diseases	Global	Female	Socio-demographic Index			X			X
Other unspecified infectious diseases	Data Rich	Female	Antenatal Care (1 visit) Coverage (proportion)			X			X
Other unspecified infectious diseases	Global	Female	Antenatal Care (1 visit) Coverage (proportion)			X			X
Other unspecified infectious diseases	Data Rich	Female	Healthcare access and quality index		X			X	
Other unspecified infectious diseases	Global	Female	Healthcare access and quality index		X			X	
Other unspecified infectious diseases	Data Rich	Female	Sanitation (proportion with access)		X			X	
Other unspecified infectious diseases	Global	Female	Sanitation (proportion with access)		X			X	
Other unspecified infectious diseases	Data Rich	Female	Improved Water Source (proportion with access)		X			X	
Other unspecified infectious diseases	Global	Female	Improved Water Source (proportion with access)		X			X	
Other unspecified infectious diseases	Data Rich	Female	DTP3 Coverage (proportion)	X			X		
Other unspecified infectious diseases	Global	Female	DTP3 Coverage (proportion)	X			X		
Other unspecified infectious diseases	Data Rich	Male	Socio-demographic Index			X			X
Other unspecified infectious diseases	Global	Male	Socio-demographic Index			X			X
Other unspecified infectious diseases	Data Rich	Male	Antenatal Care (1 visit) Coverage (proportion)			X			X
Other unspecified infectious diseases	Global	Male	Antenatal Care (1 visit) Coverage (proportion)			X			X
Other unspecified infectious diseases	Data Rich	Male	Healthcare access and quality index		X			X	
Other unspecified infectious diseases	Global	Male	Healthcare access and quality index		X			X	
Other unspecified infectious diseases	Data Rich	Male	Sanitation (proportion with access)		X			X	
Other unspecified infectious diseases	Global	Male	Sanitation (proportion with access)		X			X	
Other unspecified infectious diseases	Data Rich	Male	Improved Water Source (proportion with access)		X			X	
Other unspecified infectious diseases	Global	Male	Improved Water Source (proportion with access)		X			X	
Other unspecified infectious diseases	Data Rich	Male	DTP3 Coverage (proportion)	X			X		
Other unspecified infectious diseases	Global	Male	DTP3 Coverage (proportion)	X			X		
Neonatal disorders	Global	Female	In-Facility Delivery (proportion)			X			X
Neonatal disorders	Data Rich	Female	In-Facility Delivery (proportion)			X			X
Neonatal disorders	Global	Female	LDI (I$ per capita)			X			X
Neonatal disorders	Data Rich	Female	LDI (I$ per capita)			X			X
Neonatal disorders	Global	Female	Skilled Birth Attendance (proportion)			X			X
Neonatal disorders	Data Rich	Female	Skilled Birth Attendance (proportion)			X			X
Neonatal disorders	Global	Female	Total Fertility Rate			X			X
Neonatal disorders	Data Rich	Female	Total Fertility Rate			X			X
Neonatal disorders	Global	Female	Antenatal Care (4 visits) Coverage (proportion)			X			X
Neonatal disorders	Data Rich	Female	Antenatal Care (4 visits) Coverage (proportion)			X			X
Neonatal disorders	Global	Female	Antenatal Care (1 visit) Coverage (proportion)			X			X
Neonatal disorders	Data Rich	Female	Antenatal Care (1 visit) Coverage (proportion)			X			X
Neonatal disorders	Global	Female	Socio-demographic Index		X			X	
Neonatal disorders	Data Rich	Female	Socio-demographic Index		X			X	
Neonatal disorders	Global	Female	Healthcare access and quality index		X			X	
Neonatal disorders	Data Rich	Female	Healthcare access and quality index		X			X	
Neonatal disorders	Global	Female	Live Births 35+ (proportion)		X			X	
Neonatal disorders	Data Rich	Female	Live Births 35+ (proportion)		X			X	
Neonatal disorders	Global	Female	Proportion of the population with at least 6 years of education, maternal		X			X	
Neonatal disorders	Data Rich	Female	Proportion of the population with at least 6 years of education, maternal		X			X	
Neonatal disorders	Global	Female	Proportion of the population with at least 12 years of education, maternal		X			X	
Neonatal disorders	Data Rich	Female	Proportion of the population with at least 12 years of education, maternal		X			X	
Neonatal disorders	Global	Female	Maternal care and immunization	X			X		
Neonatal disorders	Data Rich	Female	Maternal care and immunization	X			X		
Neonatal disorders	Global	Female	Age-standardized SEV for Ambient particulate matter	X			X		
Neonatal disorders	Data Rich	Female	Age-standardized SEV for Ambient particulate matter	X			X		
Neonatal disorders	Global	Female	Age-standardized SEV for Household air pollution	X			X		
Neonatal disorders	Data Rich	Female	Age-standardized SEV for Household air pollution	X			X		
Neonatal disorders	Global	Female	Age-standardized SEV for Short gestation	X			X		
Neonatal disorders	Data Rich	Female	Age-standardized SEV for Short gestation	X			X		
Neonatal disorders	Global	Female	Age-standardized SEV for Low birth weight	X			X		
Neonatal disorders	Data Rich	Female	Age-standardized SEV for Low birth weight	X			X		
Neonatal disorders	Global	Female	Age-standardized SEV for Smoking	X			X		
Neonatal disorders	Data Rich	Female	Age-standardized SEV for Smoking	X			X		
Neonatal disorders	Global	Male	In-Facility Delivery (proportion)			X			X
Neonatal disorders	Data Rich	Male	In-Facility Delivery (proportion)			X			X
Neonatal disorders	Global	Male	LDI (I$ per capita)			X			X
Neonatal disorders	Data Rich	Male	LDI (I$ per capita)			X			X
Neonatal disorders	Global	Male	Skilled Birth Attendance (proportion)			X			X
Neonatal disorders	Data Rich	Male	Skilled Birth Attendance (proportion)			X			X
Neonatal disorders	Global	Male	Total Fertility Rate			X			X
Neonatal disorders	Data Rich	Male	Total Fertility Rate			X			X
Neonatal disorders	Global	Male	Antenatal Care (4 visits) Coverage (proportion)			X			X
Neonatal disorders	Data Rich	Male	Antenatal Care (4 visits) Coverage (proportion)			X			X
Neonatal disorders	Global	Male	Antenatal Care (1 visit) Coverage (proportion)			X			X
Neonatal disorders	Data Rich	Male	Antenatal Care (1 visit) Coverage (proportion)			X			X
Neonatal disorders	Global	Male	Socio-demographic Index		X			X	
Neonatal disorders	Data Rich	Male	Socio-demographic Index		X			X	
Neonatal disorders	Global	Male	Healthcare access and quality index		X			X	
Neonatal disorders	Data Rich	Male	Healthcare access and quality index		X			X	
Neonatal disorders	Global	Male	Live Births 35+ (proportion)		X			X	
Neonatal disorders	Data Rich	Male	Live Births 35+ (proportion)		X			X	
Neonatal disorders	Global	Male	Proportion of the population with at least 6 years of education, maternal		X			X	
Neonatal disorders	Data Rich	Male	Proportion of the population with at least 6 years of education, maternal		X			X	
Neonatal disorders	Global	Male	Proportion of the population with at least 12 years of education, maternal		X			X	
Neonatal disorders	Data Rich	Male	Proportion of the population with at least 12 years of education, maternal		X			X	
Neonatal disorders	Global	Male	Maternal care and immunization	X			X		
Neonatal disorders	Data Rich	Male	Maternal care and immunization	X			X		
Neonatal disorders	Global	Male	Age-standardized SEV for Ambient particulate matter	X			X		
Neonatal disorders	Data Rich	Male	Age-standardized SEV for Ambient particulate matter	X			X		
Neonatal disorders	Global	Male	Age-standardized SEV for Household air pollution	X			X		
Neonatal disorders	Data Rich	Male	Age-standardized SEV for Household air pollution	X			X		
Neonatal disorders	Global	Male	Age-standardized SEV for Short gestation	X			X		
Neonatal disorders	Data Rich	Male	Age-standardized SEV for Short gestation	X			X		
Neonatal disorders	Global	Male	Age-standardized SEV for Low birth weight	X			X		
Neonatal disorders	Data Rich	Male	Age-standardized SEV for Low birth weight	X			X		
Neonatal disorders	Global	Male	Age-standardized SEV for Smoking	X			X		
Neonatal disorders	Data Rich	Male	Age-standardized SEV for Smoking	X			X		
Neonatal preterm birth	Global	Female	In-Facility Delivery (proportion)			X			X
Neonatal preterm birth	Data Rich	Female	In-Facility Delivery (proportion)			X			X
Neonatal preterm birth	Global	Female	LDI (I$ per capita)			X			X
Neonatal preterm birth	Data Rich	Female	LDI (I$ per capita)			X			X
Neonatal preterm birth	Global	Female	Skilled Birth Attendance (proportion)			X			X
Neonatal preterm birth	Data Rich	Female	Skilled Birth Attendance (proportion)			X			X
Neonatal preterm birth	Global	Female	Total Fertility Rate			X			X
Neonatal preterm birth	Data Rich	Female	Total Fertility Rate			X			X
Neonatal preterm birth	Global	Female	Antenatal Care (4 visits) Coverage (proportion)			X			X
Neonatal preterm birth	Data Rich	Female	Antenatal Care (4 visits) Coverage (proportion)			X			X
Neonatal preterm birth	Global	Female	Antenatal Care (1 visit) Coverage (proportion)			X			X
Neonatal preterm birth	Data Rich	Female	Antenatal Care (1 visit) Coverage (proportion)			X			X
Neonatal preterm birth	Global	Female	Socio-demographic Index		X			X	
Neonatal preterm birth	Data Rich	Female	Socio-demographic Index		X			X	
Neonatal preterm birth	Global	Female	Healthcare access and quality index		X			X	
Neonatal preterm birth	Data Rich	Female	Healthcare access and quality index		X			X	
Neonatal preterm birth	Global	Female	Live Births 35+ (proportion)		X			X	
Neonatal preterm birth	Data Rich	Female	Live Births 35+ (proportion)		X			X	
Neonatal preterm birth	Global	Female	Proportion of the population with at least 6 years of education, maternal		X			X	
Neonatal preterm birth	Data Rich	Female	Proportion of the population with at least 6 years of education, maternal		X			X	
Neonatal preterm birth	Global	Female	Proportion of the population with at least 12 years of education, maternal		X			X	
Neonatal preterm birth	Data Rich	Female	Proportion of the population with at least 12 years of education, maternal		X			X	
Neonatal preterm birth	Global	Female	Maternal care and immunization	X			X		
Neonatal preterm birth	Data Rich	Female	Maternal care and immunization	X			X		
Neonatal preterm birth	Global	Female	Age-standardized SEV for Ambient particulate matter	X			X		
Neonatal preterm birth	Data Rich	Female	Age-standardized SEV for Ambient particulate matter	X			X		
Neonatal preterm birth	Global	Female	Age-standardized SEV for Household air pollution	X			X		

Comparison of GBD 2019 and GBD 2021 covariates and level of covariates used in cause of death modeling									
Cause	Model Version Type	Sex	Covariate	Level 1 GBD 2019	Level 2 GBD 2019	Level 3 GBD 2019	Level 1 GBD 2021	Level 2 GBD 2021	Level 3 GBD 2021
Neonatal preterm birth	Data Rich	Female	Age-standardized SEV for Household air pollution	X			X		
Neonatal preterm birth	Global	Female	Age-standardized SEV for Short gestation	X			X		
Neonatal preterm birth	Data Rich	Female	Age-standardized SEV for Short gestation	X			X		
Neonatal preterm birth	Global	Female	Age-standardized SEV for Low birth weight	X			X		
Neonatal preterm birth	Data Rich	Female	Age-standardized SEV for Low birth weight	X			X		
Neonatal preterm birth	Global	Female	Age-standardized SEV for Smoking	X			X		
Neonatal preterm birth	Data Rich	Female	Age-standardized SEV for Smoking	X			X		
Neonatal preterm birth	Global	Male	In-Facility Delivery (proportion)			X			X
Neonatal preterm birth	Data Rich	Male	In-Facility Delivery (proportion)			X			X
Neonatal preterm birth	Global	Male	LDI (I$ per capita)			X			X
Neonatal preterm birth	Data Rich	Male	LDI (I$ per capita)			X			X
Neonatal preterm birth	Global	Male	Skilled Birth Attendance (proportion)			X			X
Neonatal preterm birth	Data Rich	Male	Skilled Birth Attendance (proportion)			X			X
Neonatal preterm birth	Global	Male	Total Fertility Rate			X			X
Neonatal preterm birth	Data Rich	Male	Total Fertility Rate			X			X
Neonatal preterm birth	Global	Male	Antenatal Care (4 visits) Coverage (proportion)			X			X
Neonatal preterm birth	Data Rich	Male	Antenatal Care (4 visits) Coverage (proportion)			X			X
Neonatal preterm birth	Global	Male	Antenatal Care (1 visit) Coverage (proportion)			X			X
Neonatal preterm birth	Data Rich	Male	Antenatal Care (1 visit) Coverage (proportion)			X			X
Neonatal preterm birth	Global	Male	Socio-demographic Index		X			X	
Neonatal preterm birth	Data Rich	Male	Socio-demographic Index		X			X	
Neonatal preterm birth	Global	Male	Healthcare access and quality index		X			X	
Neonatal preterm birth	Data Rich	Male	Healthcare access and quality index		X			X	
Neonatal preterm birth	Global	Male	Live Births 35+ (proportion)		X			X	
Neonatal preterm birth	Data Rich	Male	Live Births 35+ (proportion)		X			X	
Neonatal preterm birth	Global	Male	Proportion of the population with at least 6 years of education, maternal		X			X	
Neonatal preterm birth	Data Rich	Male	Proportion of the population with at least 6 years of education, maternal		X			X	
Neonatal preterm birth	Global	Male	Proportion of the population with at least 12 years of education, maternal		X			X	
Neonatal preterm birth	Data Rich	Male	Proportion of the population with at least 12 years of education, maternal		X			X	
Neonatal preterm birth	Global	Male	Maternal care and immunization	X			X		
Neonatal preterm birth	Data Rich	Male	Maternal care and immunization	X			X		
Neonatal preterm birth	Global	Male	Age-standardized SEV for Ambient particulate matter	X			X		
Neonatal preterm birth	Data Rich	Male	Age-standardized SEV for Ambient particulate matter	X			X		
Neonatal preterm birth	Global	Male	Age-standardized SEV for Household air pollution	X			X		
Neonatal preterm birth	Data Rich	Male	Age-standardized SEV for Household air pollution	X			X		
Neonatal preterm birth	Global	Male	Age-standardized SEV for Short gestation	X			X		
Neonatal preterm birth	Data Rich	Male	Age-standardized SEV for Short gestation	X			X		
Neonatal preterm birth	Global	Male	Age-standardized SEV for Low birth weight	X			X		
Neonatal preterm birth	Data Rich	Male	Age-standardized SEV for Low birth weight	X			X		
Neonatal preterm birth	Global	Male	Age-standardized SEV for Smoking	X			X		
Neonatal preterm birth	Data Rich	Male	Age-standardized SEV for Smoking	X			X		
Neonatal encephalopathy due to birth asphyxia and trauma	Global	Female	In-Facility Delivery (proportion)			X			X
Neonatal encephalopathy due to birth asphyxia and trauma	Data Rich	Female	In-Facility Delivery (proportion)			X			X
Neonatal encephalopathy due to birth asphyxia and trauma	Global	Female	LDI (I$ per capita)			X			X
Neonatal encephalopathy due to birth asphyxia and trauma	Data Rich	Female	LDI (I$ per capita)			X			X
Neonatal encephalopathy due to birth asphyxia and trauma	Global	Female	Skilled Birth Attendance (proportion)			X			X
Neonatal encephalopathy due to birth asphyxia and trauma	Data Rich	Female	Skilled Birth Attendance (proportion)			X			X
Neonatal encephalopathy due to birth asphyxia and trauma	Global	Female	Total Fertility Rate			X			X
Neonatal encephalopathy due to birth asphyxia and trauma	Data Rich	Female	Total Fertility Rate			X			X
Neonatal encephalopathy due to birth asphyxia and trauma	Global	Female	Antenatal Care (4 visits) Coverage (proportion)			X			X
Neonatal encephalopathy due to birth asphyxia and trauma	Data Rich	Female	Antenatal Care (4 visits) Coverage (proportion)			X			X
Neonatal encephalopathy due to birth asphyxia and trauma	Data Rich	Female	Antenatal Care (1 visit) Coverage (proportion)			X			X
Neonatal encephalopathy due to birth asphyxia and trauma	Global	Female	Socio-demographic Index		X			X	
Neonatal encephalopathy due to birth asphyxia and trauma	Data Rich	Female	Socio-demographic Index		X			X	
Neonatal encephalopathy due to birth asphyxia and trauma	Global	Female	Healthcare access and quality index		X			X	
Neonatal encephalopathy due to birth asphyxia and trauma	Data Rich	Female	Healthcare access and quality index		X			X	
Neonatal encephalopathy due to birth asphyxia and trauma	Global	Female	Live Births 35+ (proportion)		X			X	
Neonatal encephalopathy due to birth asphyxia and trauma	Data Rich	Female	Live Births 35+ (proportion)		X			X	
Neonatal encephalopathy due to birth asphyxia and trauma	Global	Female	Proportion of the population with at least 6 years of education, maternal		X			X	
Neonatal encephalopathy due to birth asphyxia and trauma	Data Rich	Female	Proportion of the population with at least 6 years of education, maternal		X			X	
Neonatal encephalopathy due to birth asphyxia and trauma	Global	Female	Proportion of the population with at least 12 years of education, maternal		X			X	
Neonatal encephalopathy due to birth asphyxia and trauma	Data Rich	Female	Proportion of the population with at least 12 years of education, maternal		X			X	
Neonatal encephalopathy due to birth asphyxia and trauma	Global	Female	Maternal care and immunization	X			X		
Neonatal encephalopathy due to birth asphyxia and trauma	Data Rich	Female	Maternal care and immunization	X			X		
Neonatal encephalopathy due to birth asphyxia and trauma	Global	Female	Antenatal Care (1 visit) Coverage (proportion)	X			X		
Neonatal encephalopathy due to birth asphyxia and trauma	Global	Female	Age-standardized SEV for Ambient particulate matter	X			X		
Neonatal encephalopathy due to birth asphyxia and trauma	Data Rich	Female	Age-standardized SEV for Ambient particulate matter	X			X		
Neonatal encephalopathy due to birth asphyxia and trauma	Global	Female	Age-standardized SEV for Household air pollution	X			X		
Neonatal encephalopathy due to birth asphyxia and trauma	Data Rich	Female	Age-standardized SEV for Household air pollution	X			X		
Neonatal encephalopathy due to birth asphyxia and trauma	Global	Female	Age-standardized SEV for Short gestation	X			X		
Neonatal encephalopathy due to birth asphyxia and trauma	Data Rich	Female	Age-standardized SEV for Short gestation	X			X		
Neonatal encephalopathy due to birth asphyxia and trauma	Global	Female	Age-standardized SEV for Low birth weight	X			X		
Neonatal encephalopathy due to birth asphyxia and trauma	Data Rich	Female	Age-standardized SEV for Low birth weight	X			X		
Neonatal encephalopathy due to birth asphyxia and trauma	Global	Female	Age-standardized SEV for Smoking	X			X		
Neonatal encephalopathy due to birth asphyxia and trauma	Data Rich	Female	Age-standardized SEV for Smoking	X			X		
Neonatal encephalopathy due to birth asphyxia and trauma	Global	Male	In-Facility Delivery (proportion)			X			X
Neonatal encephalopathy due to birth asphyxia and trauma	Data Rich	Male	In-Facility Delivery (proportion)			X			X
Neonatal encephalopathy due to birth asphyxia and trauma	Global	Male	LDI (I$ per capita)			X			X
Neonatal encephalopathy due to birth asphyxia and trauma	Data Rich	Male	LDI (I$ per capita)			X			X
Neonatal encephalopathy due to birth asphyxia and trauma	Global	Male	Skilled Birth Attendance (proportion)			X			X
Neonatal encephalopathy due to birth asphyxia and trauma	Data Rich	Male	Skilled Birth Attendance (proportion)			X			X
Neonatal encephalopathy due to birth asphyxia and trauma	Global	Male	Total Fertility Rate			X			X
Neonatal encephalopathy due to birth asphyxia and trauma	Data Rich	Male	Total Fertility Rate			X			X
Neonatal encephalopathy due to birth asphyxia and trauma	Global	Male	Antenatal Care (4 visits) Coverage (proportion)			X			X
Neonatal encephalopathy due to birth asphyxia and trauma	Data Rich	Male	Antenatal Care (4 visits) Coverage (proportion)			X			X
Neonatal encephalopathy due to birth asphyxia and trauma	Data Rich	Male	Antenatal Care (1 visit) Coverage (proportion)			X			X
Neonatal encephalopathy due to birth asphyxia and trauma	Global	Male	Socio-demographic Index		X			X	
Neonatal encephalopathy due to birth asphyxia and trauma	Data Rich	Male	Socio-demographic Index		X			X	
Neonatal encephalopathy due to birth asphyxia and trauma	Global	Male	Healthcare access and quality index		X			X	
Neonatal encephalopathy due to birth asphyxia and trauma	Data Rich	Male	Healthcare access and quality index		X			X	
Neonatal encephalopathy due to birth asphyxia and trauma	Global	Male	Live Births 35+ (proportion)		X			X	
Neonatal encephalopathy due to birth asphyxia and trauma	Data Rich	Male	Live Births 35+ (proportion)		X			X	
Neonatal encephalopathy due to birth asphyxia and trauma	Global	Male	Proportion of the population with at least 6 years of education, maternal		X			X	
Neonatal encephalopathy due to birth asphyxia and trauma	Data Rich	Male	Proportion of the population with at least 6 years of education, maternal		X			X	
Neonatal encephalopathy due to birth asphyxia and trauma	Global	Male	Proportion of the population with at least 12 years of education, maternal		X			X	
Neonatal encephalopathy due to birth asphyxia and trauma	Data Rich	Male	Proportion of the population with at least 12 years of education, maternal		X			X	
Neonatal encephalopathy due to birth asphyxia and trauma	Global	Male	Maternal care and immunization	X			X		
Neonatal encephalopathy due to birth asphyxia and trauma	Data Rich	Male	Maternal care and immunization	X			X		
Neonatal encephalopathy due to birth asphyxia and trauma	Global	Male	Antenatal Care (1 visit) Coverage (proportion)	X			X		
Neonatal encephalopathy due to birth asphyxia and trauma	Global	Male	Age-standardized SEV for Ambient particulate matter	X			X		
Neonatal encephalopathy due to birth asphyxia and trauma	Data Rich	Male	Age-standardized SEV for Ambient particulate matter	X			X		
Neonatal encephalopathy due to birth asphyxia and trauma	Global	Male	Age-standardized SEV for Household air pollution	X			X		
Neonatal encephalopathy due to birth asphyxia and trauma	Data Rich	Male	Age-standardized SEV for Household air pollution	X			X		
Neonatal encephalopathy due to birth asphyxia and trauma	Global	Male	Age-standardized SEV for Short gestation	X			X		
Neonatal encephalopathy due to birth asphyxia and trauma	Data Rich	Male	Age-standardized SEV for Short gestation	X			X		

Comparison of GBD 2019 and GBD 2021 covariates and level of covariates used in cause of death modeling

Cause	Model Version Type	Sex	Covariate	Level 1 GBD 2019	Level 2 GBD 2019	Level 3 GBD 2019	Level 1 GBD 2021	Level 2 GBD 2021	Level 3 GBD 2021
Neonatal encephalopathy due to birth asphyxia and trauma	Global	Male	Age-standardized SEV for Low birth weight	X			X		
Neonatal encephalopathy due to birth asphyxia and trauma	Data Rich	Male	Age-standardized SEV for Low birth weight	X			X		
Neonatal encephalopathy due to birth asphyxia and trauma	Global	Male	Age-standardized SEV for Smoking	X			X		
Neonatal encephalopathy due to birth asphyxia and trauma	Data Rich	Male	Age-standardized SEV for Smoking	X			X		
Neonatal sepsis and other neonatal infections	Data Rich	Female	LDI (I$ per capita)			X			X
Neonatal sepsis and other neonatal infections	Global	Female	LDI (I$ per capita)			X			X
Neonatal sepsis and other neonatal infections	Data Rich	Female	Total Fertility Rate			X			X
Neonatal sepsis and other neonatal infections	Global	Female	Total Fertility Rate			X			X
Neonatal sepsis and other neonatal infections	Data Rich	Female	In-Facility Delivery (proportion)			X			X
Neonatal sepsis and other neonatal infections	Global	Female	In-Facility Delivery (proportion)			X			X
Neonatal sepsis and other neonatal infections	Data Rich	Female	Antenatal Care (1 visit) Coverage (proportion)			X			X
Neonatal sepsis and other neonatal infections	Global	Female	Antenatal Care (1 visit) Coverage (proportion)			X			X
Neonatal sepsis and other neonatal infections	Data Rich	Female	Antenatal Care (4 visits) Coverage (proportion)			X			X
Neonatal sepsis and other neonatal infections	Global	Female	Antenatal Care (4 visits) Coverage (proportion)			X			X
Neonatal sepsis and other neonatal infections	Data Rich	Female	Skilled Birth Attendance (proportion)			X			X
Neonatal sepsis and other neonatal infections	Global	Female	Skilled Birth Attendance (proportion)			X			X
Neonatal sepsis and other neonatal infections	Data Rich	Female	Healthcare access and quality index		X			X	
Neonatal sepsis and other neonatal infections	Global	Female	Healthcare access and quality index		X			X	
Neonatal sepsis and other neonatal infections	Data Rich	Female	Live Births 35+ (proportion)		X			X	
Neonatal sepsis and other neonatal infections	Global	Female	Live Births 35+ (proportion)		X			X	
Neonatal sepsis and other neonatal infections	Data Rich	Female	Proportion of the population with at least 6 years of education, maternal		X			X	
Neonatal sepsis and other neonatal infections	Global	Female	Proportion of the population with at least 6 years of education, maternal		X			X	
Neonatal sepsis and other neonatal infections	Data Rich	Female	Proportion of the population with at least 12 years of education, maternal		X			X	
Neonatal sepsis and other neonatal infections	Global	Female	Proportion of the population with at least 12 years of education, maternal		X			X	
Neonatal sepsis and other neonatal infections	Data Rich	Female	Socio-demographic Index		X			X	
Neonatal sepsis and other neonatal infections	Global	Female	Socio-demographic Index		X			X	
Neonatal sepsis and other neonatal infections	Data Rich	Female	Maternal care and immunization	X			X		
Neonatal sepsis and other neonatal infections	Global	Female	Maternal care and immunization	X			X		
Neonatal sepsis and other neonatal infections	Data Rich	Female	Age-standardized SEV for Ambient particulate matter	X			X		
Neonatal sepsis and other neonatal infections	Global	Female	Age-standardized SEV for Ambient particulate matter	X			X		
Neonatal sepsis and other neonatal infections	Data Rich	Female	Age-standardized SEV for Household air pollution	X			X		
Neonatal sepsis and other neonatal infections	Global	Female	Age-standardized SEV for Household air pollution	X			X		
Neonatal sepsis and other neonatal infections	Data Rich	Female	Age-standardized SEV for Smoking	X			X		
Neonatal sepsis and other neonatal infections	Global	Female	Age-standardized SEV for Smoking	X			X		
Neonatal sepsis and other neonatal infections	Data Rich	Female	Age- and sex-specific SEV for Short gestation	X			X		
Neonatal sepsis and other neonatal infections	Global	Female	Age- and sex-specific SEV for Short gestation	X			X		
Neonatal sepsis and other neonatal infections	Data Rich	Female	Age- and sex-specific SEV for Low birth weight	X			X		
Neonatal sepsis and other neonatal infections	Global	Female	Age- and sex-specific SEV for Low birth weight	X			X		
Neonatal sepsis and other neonatal infections	Data Rich	Male	LDI (I$ per capita)			X			X
Neonatal sepsis and other neonatal infections	Global	Male	LDI (I$ per capita)			X			X
Neonatal sepsis and other neonatal infections	Data Rich	Male	Total Fertility Rate			X			X
Neonatal sepsis and other neonatal infections	Global	Male	Total Fertility Rate			X			X
Neonatal sepsis and other neonatal infections	Data Rich	Male	In-Facility Delivery (proportion)			X			X
Neonatal sepsis and other neonatal infections	Global	Male	In-Facility Delivery (proportion)			X			X
Neonatal sepsis and other neonatal infections	Data Rich	Male	Antenatal Care (1 visit) Coverage (proportion)			X			X
Neonatal sepsis and other neonatal infections	Global	Male	Antenatal Care (1 visit) Coverage (proportion)			X			X
Neonatal sepsis and other neonatal infections	Data Rich	Male	Antenatal Care (4 visits) Coverage (proportion)			X			X
Neonatal sepsis and other neonatal infections	Global	Male	Antenatal Care (4 visits) Coverage (proportion)			X			X
Neonatal sepsis and other neonatal infections	Data Rich	Male	Skilled Birth Attendance (proportion)			X			X
Neonatal sepsis and other neonatal infections	Global	Male	Skilled Birth Attendance (proportion)			X			X
Neonatal sepsis and other neonatal infections	Data Rich	Male	Healthcare access and quality index		X			X	
Neonatal sepsis and other neonatal infections	Global	Male	Healthcare access and quality index		X			X	
Neonatal sepsis and other neonatal infections	Data Rich	Male	Live Births 35+ (proportion)		X			X	
Neonatal sepsis and other neonatal infections	Global	Male	Live Births 35+ (proportion)		X			X	
Neonatal sepsis and other neonatal infections	Data Rich	Male	Proportion of the population with at least 6 years of education, maternal		X			X	
Neonatal sepsis and other neonatal infections	Global	Male	Proportion of the population with at least 6 years of education, maternal		X			X	
Neonatal sepsis and other neonatal infections	Data Rich	Male	Proportion of the population with at least 12 years of education, maternal		X			X	
Neonatal sepsis and other neonatal infections	Global	Male	Proportion of the population with at least 12 years of education, maternal		X			X	
Neonatal sepsis and other neonatal infections	Data Rich	Male	Socio-demographic Index		X			X	
Neonatal sepsis and other neonatal infections	Global	Male	Socio-demographic Index		X			X	
Neonatal sepsis and other neonatal infections	Data Rich	Male	Maternal care and immunization	X			X		
Neonatal sepsis and other neonatal infections	Global	Male	Maternal care and immunization	X			X		
Neonatal sepsis and other neonatal infections	Data Rich	Male	Age-standardized SEV for Ambient particulate matter	X			X		
Neonatal sepsis and other neonatal infections	Global	Male	Age-standardized SEV for Ambient particulate matter	X			X		
Neonatal sepsis and other neonatal infections	Data Rich	Male	Age-standardized SEV for Household air pollution	X			X		
Neonatal sepsis and other neonatal infections	Global	Male	Age-standardized SEV for Household air pollution	X			X		
Neonatal sepsis and other neonatal infections	Data Rich	Male	Age-standardized SEV for Short gestation	X			X		
Neonatal sepsis and other neonatal infections	Global	Male	Age-standardized SEV for Short gestation	X			X		
Neonatal sepsis and other neonatal infections	Data Rich	Male	Age-standardized SEV for Low birth weight	X			X		
Neonatal sepsis and other neonatal infections	Global	Male	Age-standardized SEV for Low birth weight	X			X		
Neonatal sepsis and other neonatal infections	Data Rich	Male	Age-standardized SEV for Smoking	X			X		
Neonatal sepsis and other neonatal infections	Global	Male	Age-standardized SEV for Smoking	X			X		
Hemolytic disease and other neonatal jaundice	Global	Female	In-Facility Delivery (proportion)			X			X
Hemolytic disease and other neonatal jaundice	Data Rich	Female	In-Facility Delivery (proportion)			X			X
Hemolytic disease and other neonatal jaundice	Global	Female	LDI (I$ per capita)			X			X
Hemolytic disease and other neonatal jaundice	Data Rich	Female	LDI (I$ per capita)			X			X
Hemolytic disease and other neonatal jaundice	Global	Female	Skilled Birth Attendance (proportion)			X			X
Hemolytic disease and other neonatal jaundice	Data Rich	Female	Skilled Birth Attendance (proportion)			X			X
Hemolytic disease and other neonatal jaundice	Global	Female	Total Fertility Rate			X			X
Hemolytic disease and other neonatal jaundice	Data Rich	Female	Total Fertility Rate			X			X
Hemolytic disease and other neonatal jaundice	Global	Female	Antenatal Care (4 visits) Coverage (proportion)			X			X
Hemolytic disease and other neonatal jaundice	Data Rich	Female	Antenatal Care (4 visits) Coverage (proportion)			X			X
Hemolytic disease and other neonatal jaundice	Global	Female	Antenatal Care (1 visit) Coverage (proportion)			X			X
Hemolytic disease and other neonatal jaundice	Data Rich	Female	Antenatal Care (1 visit) Coverage (proportion)			X			X
Hemolytic disease and other neonatal jaundice	Global	Female	Socio-demographic Index		X			X	
Hemolytic disease and other neonatal jaundice	Data Rich	Female	Socio-demographic Index		X			X	
Hemolytic disease and other neonatal jaundice	Global	Female	Healthcare access and quality index		X			X	
Hemolytic disease and other neonatal jaundice	Data Rich	Female	Healthcare access and quality index		X			X	
Hemolytic disease and other neonatal jaundice	Global	Female	Live Births 35+ (proportion)		X			X	
Hemolytic disease and other neonatal jaundice	Data Rich	Female	Live Births 35+ (proportion)		X			X	
Hemolytic disease and other neonatal jaundice	Global	Female	Proportion of the population with at least 6 years of education, maternal		X			X	
Hemolytic disease and other neonatal jaundice	Data Rich	Female	Proportion of the population with at least 6 years of education, maternal		X			X	
Hemolytic disease and other neonatal jaundice	Global	Female	Proportion of the population with at least 12 years of education, maternal		X			X	
Hemolytic disease and other neonatal jaundice	Data Rich	Female	Proportion of the population with at least 12 years of education, maternal		X			X	
Hemolytic disease and other neonatal jaundice	Global	Female	Maternal care and immunization	X			X		
Hemolytic disease and other neonatal jaundice	Data Rich	Female	Maternal care and immunization	X			X		
Hemolytic disease and other neonatal jaundice	Global	Female	Age-standardized SEV for Ambient particulate matter	X			X		
Hemolytic disease and other neonatal jaundice	Data Rich	Female	Age-standardized SEV for Ambient particulate matter	X			X		
Hemolytic disease and other neonatal jaundice	Global	Female	Age-standardized SEV for Household air pollution	X			X		
Hemolytic disease and other neonatal jaundice	Data Rich	Female	Age-standardized SEV for Household air pollution	X			X		
Hemolytic disease and other neonatal jaundice	Global	Female	Age-standardized SEV for Short gestation	X			X		
Hemolytic disease and other neonatal jaundice	Data Rich	Female	Age-standardized SEV for Short gestation	X			X		
Hemolytic disease and other neonatal jaundice	Global	Female	Age-standardized SEV for Low birth weight	X			X		
Hemolytic disease and other neonatal jaundice	Data Rich	Female	Age-standardized SEV for Low birth weight	X			X		
Hemolytic disease and other neonatal jaundice	Global	Female	Age-standardized SEV for Smoking	X			X		
Hemolytic disease and other neonatal jaundice	Data Rich	Female	Age-standardized SEV for Smoking	X			X		
Hemolytic disease and other neonatal jaundice	Global	Male	In-Facility Delivery (proportion)			X			X
Hemolytic disease and other neonatal jaundice	Data Rich	Male	In-Facility Delivery (proportion)			X			X
Hemolytic disease and other neonatal jaundice	Global	Male	LDI (I$ per capita)			X			X
Hemolytic disease and other neonatal jaundice	Data Rich	Male	LDI (I$ per capita)			X			X
Hemolytic disease and other neonatal jaundice	Global	Male	Skilled Birth Attendance (proportion)			X			X
Hemolytic disease and other neonatal jaundice	Data Rich	Male	Skilled Birth Attendance (proportion)			X			X
Hemolytic disease and other neonatal jaundice	Global	Male	Total Fertility Rate			X			X
Hemolytic disease and other neonatal jaundice	Data Rich	Male	Total Fertility Rate			X			X
Hemolytic disease and other neonatal jaundice	Global	Male	Antenatal Care (4 visits) Coverage (proportion)			X			X
Hemolytic disease and other neonatal jaundice	Data Rich	Male	Antenatal Care (4 visits) Coverage (proportion)			X			X
Hemolytic disease and other neonatal jaundice	Global	Male	Antenatal Care (1 visit) Coverage (proportion)			X			X
Hemolytic disease and other neonatal jaundice	Data Rich	Male	Antenatal Care (1 visit) Coverage (proportion)			X			X

**Comparison of GBD 2019 and GBD 2021 covariates and level of covariates used in cause of death modeling**									
**Cause**	**Model Version Type**	**Sex**	**Covariate**	**Level 1 GBD 2019**	**Level 2 GBD 2019**	**Level 3 GBD 2019**	**Level 1 GBD 2021**	**Level 2 GBD 2021**	**Level 3 GBD 2021**
Hemolytic disease and other neonatal jaundice	Global	Male	Socio-demographic Index		X			X	
Hemolytic disease and other neonatal jaundice	Data Rich	Male	Socio-demographic Index		X			X	
Hemolytic disease and other neonatal jaundice	Global	Male	Healthcare access and quality index		X			X	
Hemolytic disease and other neonatal jaundice	Data Rich	Male	Healthcare access and quality index		X			X	
Hemolytic disease and other neonatal jaundice	Global	Male	Live Births 35+ (proportion)		X			X	
Hemolytic disease and other neonatal jaundice	Data Rich	Male	Live Births 35+ (proportion)		X			X	
Hemolytic disease and other neonatal jaundice	Global	Male	Proportion of the population with at least 6 years of education, maternal		X			X	
Hemolytic disease and other neonatal jaundice	Data Rich	Male	Proportion of the population with at least 6 years of education, maternal		X			X	
Hemolytic disease and other neonatal jaundice	Global	Male	Proportion of the population with at least 12 years of education, maternal		X			X	
Hemolytic disease and other neonatal jaundice	Data Rich	Male	Proportion of the population with at least 12 years of education, maternal		X			X	
Hemolytic disease and other neonatal jaundice	Global	Male	Maternal care and immunization	X			X		
Hemolytic disease and other neonatal jaundice	Data Rich	Male	Maternal care and immunization	X			X		
Hemolytic disease and other neonatal jaundice	Global	Male	Age-standardized SEV for Ambient particulate matter	X			X		
Hemolytic disease and other neonatal jaundice	Data Rich	Male	Age-standardized SEV for Ambient particulate matter	X			X		
Hemolytic disease and other neonatal jaundice	Global	Male	Age-standardized SEV for Household air pollution	X			X		
Hemolytic disease and other neonatal jaundice	Data Rich	Male	Age-standardized SEV for Household air pollution	X			X		
Hemolytic disease and other neonatal jaundice	Global	Male	Age-standardized SEV for Short gestation	X			X		
Hemolytic disease and other neonatal jaundice	Data Rich	Male	Age-standardized SEV for Short gestation	X			X		
Hemolytic disease and other neonatal jaundice	Global	Male	Age-standardized SEV for Low birth weight	X			X		
Hemolytic disease and other neonatal jaundice	Data Rich	Male	Age-standardized SEV for Low birth weight	X			X		
Hemolytic disease and other neonatal jaundice	Global	Male	Age-standardized SEV for Smoking	X			X		
Hemolytic disease and other neonatal jaundice	Data Rich	Male	Age-standardized SEV for Smoking	X			X		
Other neonatal disorders	Data Rich	Female	LDI (I$ per capita)			X			X
Other neonatal disorders	Global	Female	LDI (I$ per capita)			X			X
Other neonatal disorders	Data Rich	Female	Total Fertility Rate			X			X
Other neonatal disorders	Global	Female	Total Fertility Rate			X			X
Other neonatal disorders	Data Rich	Female	In-Facility Delivery (proportion)			X			X
Other neonatal disorders	Global	Female	In-Facility Delivery (proportion)			X			X
Other neonatal disorders	Data Rich	Female	Antenatal Care (1 visit) Coverage (proportion)			X			X
Other neonatal disorders	Global	Female	Antenatal Care (1 visit) Coverage (proportion)			X			X
Other neonatal disorders	Data Rich	Female	Antenatal Care (4 visits) Coverage (proportion)			X			X
Other neonatal disorders	Global	Female	Antenatal Care (4 visits) Coverage (proportion)			X			X
Other neonatal disorders	Data Rich	Female	Skilled Birth Attendance (proportion)			X			X
Other neonatal disorders	Global	Female	Skilled Birth Attendance (proportion)			X			X
Other neonatal disorders	Data Rich	Female	Healthcare access and quality index		X			X	
Other neonatal disorders	Global	Female	Healthcare access and quality index		X			X	
Other neonatal disorders	Data Rich	Female	Live Births 35+ (proportion)		X			X	
Other neonatal disorders	Global	Female	Live Births 35+ (proportion)		X			X	
Other neonatal disorders	Data Rich	Female	Proportion of the population with at least 6 years of education, maternal		X			X	
Other neonatal disorders	Global	Female	Proportion of the population with at least 6 years of education, maternal		X			X	
Other neonatal disorders	Data Rich	Female	Proportion of the population with at least 12 years of education, maternal		X			X	
Other neonatal disorders	Global	Female	Proportion of the population with at least 12 years of education, maternal		X			X	
Other neonatal disorders	Data Rich	Female	Socio-demographic Index		X			X	
Other neonatal disorders	Global	Female	Socio-demographic Index		X			X	
Other neonatal disorders	Data Rich	Female	Maternal care and immunization	X			X		
Other neonatal disorders	Global	Female	Maternal care and immunization	X			X		
Other neonatal disorders	Data Rich	Female	Age-standardized SEV for Ambient particulate matter	X			X		
Other neonatal disorders	Global	Female	Age-standardized SEV for Ambient particulate matter	X			X		
Other neonatal disorders	Data Rich	Female	Age-standardized SEV for Household air pollution	X			X		
Other neonatal disorders	Global	Female	Age-standardized SEV for Household air pollution	X			X		
Other neonatal disorders	Data Rich	Female	Age-standardized SEV for Short gestation	X			X		
Other neonatal disorders	Global	Female	Age-standardized SEV for Short gestation	X			X		
Other neonatal disorders	Data Rich	Female	Age-standardized SEV for Low birth weight	X			X		
Other neonatal disorders	Global	Female	Age-standardized SEV for Low birth weight	X			X		
Other neonatal disorders	Data Rich	Female	Age-standardized SEV for Smoking	X			X		
Other neonatal disorders	Global	Female	Age-standardized SEV for Smoking	X			X		
Other neonatal disorders	Data Rich	Male	LDI (I$ per capita)			X			X
Other neonatal disorders	Global	Male	LDI (I$ per capita)			X			X
Other neonatal disorders	Data Rich	Male	Total Fertility Rate			X			X
Other neonatal disorders	Global	Male	Total Fertility Rate			X			X
Other neonatal disorders	Data Rich	Male	In-Facility Delivery (proportion)			X			X
Other neonatal disorders	Global	Male	In-Facility Delivery (proportion)			X			X
Other neonatal disorders	Data Rich	Male	Antenatal Care (1 visit) Coverage (proportion)			X			X
Other neonatal disorders	Global	Male	Antenatal Care (1 visit) Coverage (proportion)			X			X
Other neonatal disorders	Data Rich	Male	Antenatal Care (4 visits) Coverage (proportion)			X			X
Other neonatal disorders	Global	Male	Antenatal Care (4 visits) Coverage (proportion)			X			X
Other neonatal disorders	Data Rich	Male	Skilled Birth Attendance (proportion)			X			X
Other neonatal disorders	Global	Male	Skilled Birth Attendance (proportion)			X			X
Other neonatal disorders	Data Rich	Male	Healthcare access and quality index		X			X	
Other neonatal disorders	Global	Male	Healthcare access and quality index		X			X	
Other neonatal disorders	Data Rich	Male	Live Births 35+ (proportion)		X			X	
Other neonatal disorders	Global	Male	Live Births 35+ (proportion)		X			X	
Other neonatal disorders	Data Rich	Male	Proportion of the population with at least 6 years of education, maternal		X			X	
Other neonatal disorders	Global	Male	Proportion of the population with at least 6 years of education, maternal		X			X	
Other neonatal disorders	Data Rich	Male	Proportion of the population with at least 12 years of education, maternal		X			X	
Other neonatal disorders	Global	Male	Proportion of the population with at least 12 years of education, maternal		X			X	
Other neonatal disorders	Data Rich	Male	Socio-demographic Index		X			X	
Other neonatal disorders	Global	Male	Socio-demographic Index		X			X	
Other neonatal disorders	Data Rich	Male	Maternal care and immunization	X			X		
Other neonatal disorders	Global	Male	Maternal care and immunization	X			X		
Other neonatal disorders	Data Rich	Male	Age-standardized SEV for Ambient particulate matter	X			X		
Other neonatal disorders	Global	Male	Age-standardized SEV for Ambient particulate matter	X			X		
Other neonatal disorders	Data Rich	Male	Age-standardized SEV for Household air pollution	X			X		
Other neonatal disorders	Global	Male	Age-standardized SEV for Household air pollution	X			X		
Other neonatal disorders	Data Rich	Male	Age-standardized SEV for Short gestation	X			X		
Other neonatal disorders	Global	Male	Age-standardized SEV for Short gestation	X			X		
Other neonatal disorders	Data Rich	Male	Age-standardized SEV for Low birth weight	X			X		
Other neonatal disorders	Global	Male	Age-standardized SEV for Low birth weight	X			X		
Other neonatal disorders	Data Rich	Male	Age-standardized SEV for Smoking	X			X		
Other neonatal disorders	Global	Male	Age-standardized SEV for Smoking	X			X		
Nutritional deficiencies	Global	Female	Education (years per capita)			X			X
Nutritional deficiencies	Data Rich	Female	Education (years per capita)			X			X
Nutritional deficiencies	Global	Female	LDI (I$ per capita)			X			X
Nutritional deficiencies	Data Rich	Female	LDI (I$ per capita)			X			X
Nutritional deficiencies	Data Rich	Female	Maternal Education (years per capita)			X			X
Nutritional deficiencies	Data Rich	Female	Socio-demographic Index			X			X
Nutritional deficiencies	Global	Female	Socio-demographic Index			X		X	
Nutritional deficiencies	Global	Female	Age- and sex-specific SEV for Alcohol use		X				X
Nutritional deficiencies	Data Rich	Female	Age- and sex-specific SEV for Alcohol use		X				X
Nutritional deficiencies	Global	Female	Healthcare access and quality index		X			X	
Nutritional deficiencies	Data Rich	Female	Healthcare access and quality index		X			X	
Nutritional deficiencies	Global	Female	Maternal care and immunization		X			X	
Nutritional deficiencies	Data Rich	Female	Maternal care and immunization		X			X	
Nutritional deficiencies	Global	Female	Age- and sex-specific SEV for Unsafe sanitation		X			X	
Nutritional deficiencies	Global	Female	Rainfall Quintile 1 (proportion)		X			X	
Nutritional deficiencies	Data Rich	Female	Rainfall Quintile 1 (proportion)		X			X	
Nutritional deficiencies	Global	Female	Age- and sex-specific SEV for Unsafe water		X			X	
Nutritional deficiencies	Global	Female	Rainfall Quintile 2 (proportion)		X			X	
Nutritional deficiencies	Data Rich	Female	Rainfall Quintile 2 (proportion)		X			X	
Nutritional deficiencies	Global	Female	Log-transformed SEV scalar: Diarrhea		X		X		
Nutritional deficiencies	Data Rich	Female	Log-transformed SEV scalar: Diarrhea		X		X		
Nutritional deficiencies	Global	Female	Age-standardized SEV for Child underweight	X			X		
Nutritional deficiencies	Data Rich	Female	Age-standardized SEV for Child underweight	X			X		
Nutritional deficiencies	Global	Female	energy unadjusted(kcal)	X			X		
Nutritional deficiencies	Data Rich	Female	energy unadjusted(kcal)	X			X		
Nutritional deficiencies	Global	Female	Age-Standardize Prevalence of Severe Anemia	X			X		
Nutritional deficiencies	Data Rich	Female	Age-Standardize Prevalence of Severe Anemia	X			X		
Nutritional deficiencies	Global	Female	Age-standardized SEV for Child wasting	X			X		
Nutritional deficiencies	Data Rich	Female	Age-standardized SEV for Child wasting	X			X		
Nutritional deficiencies	Global	Female	Proportion of households using iodized salt (adjusted)	X			X		
Nutritional deficiencies	Data Rich	Female	Proportion of households using iodized salt (adjusted)	X			X		
Nutritional deficiencies	Global	Female	Maternal Education (years per capita)						X

Comparison of GBD 2019 and GBD 2021 covariates and level of covariates used in cause of death modeling

Cause	Model Version Type	Sex	Covariate	Level 1 GBD 2019	Level 2 GBD 2019	Level 3 GBD 2019	Level 1 GBD 2021	Level 2 GBD 2021	Level 3 GBD 2021
Nutritional deficiencies	Data Rich	Female	Sanitation (proportion with access)					X	
Nutritional deficiencies	Data Rich	Female	Improved Water Source (proportion with access)					X	
Nutritional deficiencies	Global	Female	Mortality Rate Due to Death Shocks in Last 10 Years (per 1 person)					X	
Nutritional deficiencies	Data Rich	Female	Mortality Rate Due to Death Shocks in Last 10 Years (per 1 person)					X	
Nutritional deficiencies	Global	Male	Education (years per capita)			X			X
Nutritional deficiencies	Data Rich	Male	Education (years per capita)			X			X
Nutritional deficiencies	Global	Male	LDI (I$ per capita)			X			X
Nutritional deficiencies	Data Rich	Male	LDI (I$ per capita)			X			X
Nutritional deficiencies	Global	Male	Maternal Education (years per capita)			X			X
Nutritional deficiencies	Data Rich	Male	Maternal Education (years per capita)			X			X
Nutritional deficiencies	Data Rich	Male	Socio-demographic Index			X			X
Nutritional deficiencies	Global	Male	Socio-demographic Index			X		X	
Nutritional deficiencies	Global	Male	Age- and sex-specific SEV for Alcohol use		X				X
Nutritional deficiencies	Data Rich	Male	Age- and sex-specific SEV for Alcohol use		X				X
Nutritional deficiencies	Global	Male	Healthcare access and quality index		X			X	
Nutritional deficiencies	Data Rich	Male	Healthcare access and quality index		X			X	
Nutritional deficiencies	Data Rich	Male	Sanitation (proportion with access)		X			X	
Nutritional deficiencies	Data Rich	Male	Improved Water Source (proportion with access)		X			X	
Nutritional deficiencies	Global	Male	Maternal care and immunization		X			X	
Nutritional deficiencies	Data Rich	Male	Maternal care and immunization		X			X	
Nutritional deficiencies	Global	Male	Age- and sex-specific SEV for Unsafe sanitation		X			X	
Nutritional deficiencies	Global	Male	Rainfall Quintile 1 (proportion)		X			X	
Nutritional deficiencies	Data Rich	Male	Rainfall Quintile 1 (proportion)		X			X	
Nutritional deficiencies	Global	Male	Age- and sex-specific SEV for Unsafe water		X			X	
Nutritional deficiencies	Global	Male	Rainfall Quintile 2 (proportion)		X			X	
Nutritional deficiencies	Data Rich	Male	Rainfall Quintile 2 (proportion)		X			X	
Nutritional deficiencies	Global	Male	Log-transformed SEV scalar: Diarrhea		X		X		
Nutritional deficiencies	Global	Male	Age-standardized SEV for Child underweight	X			X		
Nutritional deficiencies	Data Rich	Male	Age-standardized SEV for Child underweight	X			X		
Nutritional deficiencies	Global	Male	energy unadjusted(kcal)	X			X		
Nutritional deficiencies	Data Rich	Male	energy unadjusted(kcal)	X			X		
Nutritional deficiencies	Global	Male	Age-Standardize Prevalence of Severe Anemia	X			X		
Nutritional deficiencies	Data Rich	Male	Age-Standardize Prevalence of Severe Anemia	X			X		
Nutritional deficiencies	Global	Male	Age-standardized SEV for Child wasting	X			X		
Nutritional deficiencies	Data Rich	Male	Age-standardized SEV for Child wasting	X			X		
Nutritional deficiencies	Global	Male	Proportion of households using iodized salt (adjusted)	X			X		
Nutritional deficiencies	Data Rich	Male	Proportion of households using iodized salt (adjusted)	X			X		
Nutritional deficiencies	Global	Male	Mortality Rate Due to Death Shocks in Last 10 Years (per 1 person)					X	
Nutritional deficiencies	Data Rich	Male	Mortality Rate Due to Death Shocks in Last 10 Years (per 1 person)					X	
Nutritional deficiencies	Data Rich	Male	Log-transformed SEV scalar: Diarrhea				X		
Protein-energy malnutrition	Data Rich	Female	Education (years per capita)			X			X
Protein-energy malnutrition	Global	Female	Education (years per capita)			X			X
Protein-energy malnutrition	Global	Female	Education (years per capita)			X			X
Protein-energy malnutrition	Data Rich	Female	Education (years per capita)			X			X
Protein-energy malnutrition	Data Rich	Female	LDI (I$ per capita)			X			X
Protein-energy malnutrition	Global	Female	LDI (I$ per capita)			X			X
Protein-energy malnutrition	Global	Female	LDI (I$ per capita)			X			X
Protein-energy malnutrition	Data Rich	Female	LDI (I$ per capita)			X			X
Protein-energy malnutrition	Data Rich	Female	Socio-demographic Index			X			X
Protein-energy malnutrition	Data Rich	Female	Socio-demographic Index			X		X	
Protein-energy malnutrition	Global	Female	Socio-demographic Index			X		X	
Protein-energy malnutrition	Global	Female	Socio-demographic Index			X		X	
Protein-energy malnutrition	Data Rich	Female	Age- and sex-specific SEV for Alcohol use		X				X
Protein-energy malnutrition	Global	Female	Age- and sex-specific SEV for Alcohol use		X				X
Protein-energy malnutrition	Global	Female	Age- and sex-specific SEV for Alcohol use		X				X
Protein-energy malnutrition	Data Rich	Female	Age- and sex-specific SEV for Alcohol use		X				X
Protein-energy malnutrition	Data Rich	Female	Healthcare access and quality index		X			X	
Protein-energy malnutrition	Global	Female	Healthcare access and quality index		X			X	
Protein-energy malnutrition	Global	Female	Healthcare access and quality index		X			X	
Protein-energy malnutrition	Data Rich	Female	Healthcare access and quality index		X			X	
Protein-energy malnutrition	Data Rich	Female	Maternal care and immunization		X			X	
Protein-energy malnutrition	Global	Female	Maternal care and immunization		X			X	
Protein-energy malnutrition	Global	Female	Maternal care and immunization		X			X	
Protein-energy malnutrition	Data Rich	Female	Maternal care and immunization		X			X	
Protein-energy malnutrition	Data Rich	Female	Age- and sex-specific SEV for Unsafe sanitation		X			X	
Protein-energy malnutrition	Global	Female	Age- and sex-specific SEV for Unsafe sanitation		X			X	
Protein-energy malnutrition	Global	Female	Age- and sex-specific SEV for Unsafe sanitation		X			X	
Protein-energy malnutrition	Data Rich	Female	Age- and sex-specific SEV for Unsafe sanitation		X			X	
Protein-energy malnutrition	Data Rich	Female	Rainfall Quintile 1 (proportion)		X			X	
Protein-energy malnutrition	Global	Female	Rainfall Quintile 1 (proportion)		X			X	
Protein-energy malnutrition	Global	Female	Rainfall Quintile 1 (proportion)		X			X	
Protein-energy malnutrition	Data Rich	Female	Rainfall Quintile 1 (proportion)		X			X	
Protein-energy malnutrition	Data Rich	Female	Age- and sex-specific SEV for Unsafe water		X			X	
Protein-energy malnutrition	Global	Female	Age- and sex-specific SEV for Unsafe water		X			X	
Protein-energy malnutrition	Global	Female	Age- and sex-specific SEV for Unsafe water		X			X	
Protein-energy malnutrition	Data Rich	Female	Age- and sex-specific SEV for Unsafe water		X			X	
Protein-energy malnutrition	Data Rich	Female	Rainfall Quintile 2 (proportion)		X			X	
Protein-energy malnutrition	Global	Female	Rainfall Quintile 2 (proportion)		X			X	
Protein-energy malnutrition	Global	Female	Rainfall Quintile 2 (proportion)		X			X	
Protein-energy malnutrition	Data Rich	Female	Rainfall Quintile 2 (proportion)		X			X	
Protein-energy malnutrition	Data Rich	Female	Log-transformed SEV scalar: Diarrhea		X		X		
Protein-energy malnutrition	Global	Female	Log-transformed SEV scalar: Diarrhea		X		X		
Protein-energy malnutrition	Global	Female	Log-transformed SEV scalar: Diarrhea		X		X		
Protein-energy malnutrition	Data Rich	Female	Log-transformed SEV scalar: Diarrhea		X		X		
Protein-energy malnutrition	Data Rich	Female	Age- and sex-specific SEV for Child wasting	X			X		
Protein-energy malnutrition	Global	Female	Age- and sex-specific SEV for Child wasting	X			X		
Protein-energy malnutrition	Data Rich	Female	energy unadjusted(kcal)	X			X		
Protein-energy malnutrition	Global	Female	energy unadjusted(kcal)	X			X		
Protein-energy malnutrition	Global	Female	energy unadjusted(kcal)	X			X		
Protein-energy malnutrition	Data Rich	Female	energy unadjusted(kcal)	X			X		
Protein-energy malnutrition	Data Rich	Female	Age-Standardize Prevalence of Severe Anemia	X			X		
Protein-energy malnutrition	Global	Female	Age-Standardize Prevalence of Severe Anemia	X			X		
Protein-energy malnutrition	Global	Female	Age-Standardize Prevalence of Severe Anemia	X			X		
Protein-energy malnutrition	Data Rich	Female	Age-Standardize Prevalence of Severe Anemia	X			X		
Protein-energy malnutrition	Data Rich	Female	Malnutrition Shock mortality rate	X			X		
Protein-energy malnutrition	Data Rich	Female	Age-standardized SEV for Child wasting	X			X		
Protein-energy malnutrition	Global	Female	Maternal Education (years per capita)						X
Protein-energy malnutrition	Data Rich	Female	Maternal Education (years per capita)						X
Protein-energy malnutrition	Data Rich	Female	Mortality Rate Due to Death Shocks in Last 10 Years (per 1 person)					X	
Protein-energy malnutrition	Global	Female	Mortality Rate Due to Death Shocks in Last 10 Years (per 1 person)					X	
Protein-energy malnutrition	Global	Female	Mortality Rate Due to Death Shocks in Last 10 Years (per 1 person)					X	
Protein-energy malnutrition	Data Rich	Female	Mortality Rate Due to Death Shocks in Last 10 Years (per 1 person)					X	
Protein-energy malnutrition	Data Rich	Male	Education (years per capita)			X			X
Protein-energy malnutrition	Global	Male	Education (years per capita)			X			X
Protein-energy malnutrition	Global	Male	Education (years per capita)			X			X
Protein-energy malnutrition	Data Rich	Male	Education (years per capita)			X			X
Protein-energy malnutrition	Data Rich	Male	LDI (I$ per capita)			X			X
Protein-energy malnutrition	Global	Male	LDI (I$ per capita)			X			X
Protein-energy malnutrition	Global	Male	LDI (I$ per capita)			X			X
Protein-energy malnutrition	Data Rich	Male	LDI (I$ per capita)			X			X
Protein-energy malnutrition	Data Rich	Male	Socio-demographic Index			X			X
Protein-energy malnutrition	Global	Male	Maternal Education (years per capita)			X			X
Protein-energy malnutrition	Data Rich	Male	Socio-demographic Index			X		X	
Protein-energy malnutrition	Global	Male	Socio-demographic Index			X		X	
Protein-energy malnutrition	Global	Male	Socio-demographic Index			X		X	
Protein-energy malnutrition	Data Rich	Male	Age- and sex-specific SEV for Alcohol use		X				X
Protein-energy malnutrition	Global	Male	Age- and sex-specific SEV for Alcohol use		X				X
Protein-energy malnutrition	Global	Male	Age- and sex-specific SEV for Alcohol use		X				X
Protein-energy malnutrition	Data Rich	Male	Age- and sex-specific SEV for Alcohol use		X				X
Protein-energy malnutrition	Data Rich	Male	Healthcare access and quality index		X			X	
Protein-energy malnutrition	Global	Male	Healthcare access and quality index		X			X	
Protein-energy malnutrition	Global	Male	Healthcare access and quality index		X			X	
Protein-energy malnutrition	Data Rich	Male	Healthcare access and quality index		X			X	
Protein-energy malnutrition	Data Rich	Male	Maternal care and immunization		X			X	
Protein-energy malnutrition	Global	Male	Maternal care and immunization		X			X	
Protein-energy malnutrition	Global	Male	Maternal care and immunization		X			X	
Protein-energy malnutrition	Data Rich	Male	Maternal care and immunization		X			X	
Protein-energy malnutrition	Data Rich	Male	Age- and sex-specific SEV for Unsafe sanitation		X			X	

Comparison of GBD 2019 and GBD 2021 covariates and level of covariates used in cause of death modeling

Cause	Model Version Type	Sex	Covariate	Level 1 GBD 2019	Level 2 GBD 2019	Level 3 GBD 2019	Level 1 GBD 2021	Level 2 GBD 2021	Level 3 GBD 2021
Protein-energy malnutrition	Global	Male	Age- and sex-specific SEV for Unsafe sanitation		X			X	
Protein-energy malnutrition	Global	Male	Age- and sex-specific SEV for Unsafe sanitation		X			X	
Protein-energy malnutrition	Data Rich	Male	Age- and sex-specific SEV for Unsafe sanitation		X			X	
Protein-energy malnutrition	Data Rich	Male	Rainfall Quintile 1 (proportion)		X			X	
Protein-energy malnutrition	Global	Male	Rainfall Quintile 1 (proportion)		X			X	
Protein-energy malnutrition	Global	Male	Rainfall Quintile 1 (proportion)		X			X	
Protein-energy malnutrition	Data Rich	Male	Rainfall Quintile 1 (proportion)		X			X	
Protein-energy malnutrition	Data Rich	Male	Age- and sex-specific SEV for Unsafe water		X			X	
Protein-energy malnutrition	Global	Male	Age- and sex-specific SEV for Unsafe water		X			X	
Protein-energy malnutrition	Global	Male	Age- and sex-specific SEV for Unsafe water		X			X	
Protein-energy malnutrition	Data Rich	Male	Age- and sex-specific SEV for Unsafe water		X			X	
Protein-energy malnutrition	Data Rich	Male	Rainfall Quintile 2 (proportion)		X			X	
Protein-energy malnutrition	Global	Male	Rainfall Quintile 2 (proportion)		X			X	
Protein-energy malnutrition	Global	Male	Rainfall Quintile 2 (proportion)		X			X	
Protein-energy malnutrition	Data Rich	Male	Rainfall Quintile 2 (proportion)		X			X	
Protein-energy malnutrition	Data Rich	Male	Log-transformed SEV scalar: Diarrhea		X		X		
Protein-energy malnutrition	Global	Male	Log-transformed SEV scalar: Diarrhea		X		X		
Protein-energy malnutrition	Global	Male	Log-transformed SEV scalar: Diarrhea		X		X		
Protein-energy malnutrition	Data Rich	Male	Log-transformed SEV scalar: Diarrhea		X		X		
Protein-energy malnutrition	Data Rich	Male	Age- and sex-specific SEV for Child wasting	X			X		
Protein-energy malnutrition	Global	Male	Age- and sex-specific SEV for Child wasting	X			X		
Protein-energy malnutrition	Data Rich	Male	energy unadjusted(kcal)	X			X		
Protein-energy malnutrition	Global	Male	energy unadjusted(kcal)	X			X		
Protein-energy malnutrition	Global	Male	energy unadjusted(kcal)	X			X		
Protein-energy malnutrition	Data Rich	Male	energy unadjusted(kcal)	X			X		
Protein-energy malnutrition	Data Rich	Male	Age-Standardize Prevalence of Severe Anemia	X			X		
Protein-energy malnutrition	Global	Male	Age-Standardize Prevalence of Severe Anemia	X			X		
Protein-energy malnutrition	Global	Male	Age-Standardize Prevalence of Severe Anemia	X			X		
Protein-energy malnutrition	Data Rich	Male	Age-Standardize Prevalence of Severe Anemia	X			X		
Protein-energy malnutrition	Data Rich	Male	Malnutrition Shock mortality rate	X			X		
Protein-energy malnutrition	Data Rich	Male	Maternal Education (years per capita)						X
Protein-energy malnutrition	Data Rich	Male	Mortality Rate Due to Death Shocks in Last 10 Years (per 1 person)					X	
Protein-energy malnutrition	Global	Male	Mortality Rate Due to Death Shocks in Last 10 Years (per 1 person)					X	
Protein-energy malnutrition	Global	Male	Mortality Rate Due to Death Shocks in Last 10 Years (per 1 person)					X	
Protein-energy malnutrition	Data Rich	Male	Mortality Rate Due to Death Shocks in Last 10 Years (per 1 person)					X	
Protein-energy malnutrition	Data Rich	Male	Age-standardized SEV for Child wasting				X		
Other nutritional deficiencies	Global	Female	Education (years per capita)			X			X
Other nutritional deficiencies	Data Rich	Female	Education (years per capita)			X			X
Other nutritional deficiencies	Global	Female	LDI (I$ per capita)			X			X
Other nutritional deficiencies	Data Rich	Female	LDI (I$ per capita)			X			X
Other nutritional deficiencies	Global	Female	Socio-demographic Index			X		X	
Other nutritional deficiencies	Data Rich	Female	Socio-demographic Index			X		X	
Other nutritional deficiencies	Global	Female	Age- and sex-specific SEV for Alcohol use		X				X
Other nutritional deficiencies	Data Rich	Female	Age- and sex-specific SEV for Alcohol use		X				X
Other nutritional deficiencies	Global	Female	Healthcare access and quality index		X			X	
Other nutritional deficiencies	Data Rich	Female	Healthcare access and quality index		X			X	
Other nutritional deficiencies	Global	Female	Maternal care and immunization		X			X	
Other nutritional deficiencies	Data Rich	Female	Maternal care and immunization		X			X	
Other nutritional deficiencies	Global	Female	Age- and sex-specific SEV for Unsafe sanitation		X			X	
Other nutritional deficiencies	Data Rich	Female	Age- and sex-specific SEV for Unsafe sanitation		X			X	
Other nutritional deficiencies	Global	Female	Rainfall Quintile 1 (proportion)		X			X	
Other nutritional deficiencies	Data Rich	Female	Rainfall Quintile 1 (proportion)		X			X	
Other nutritional deficiencies	Global	Female	Age- and sex-specific SEV for Unsafe water		X			X	
Other nutritional deficiencies	Data Rich	Female	Age- and sex-specific SEV for Unsafe water		X			X	
Other nutritional deficiencies	Data Rich	Female	energy unadjusted(kcal)		X			X	
Other nutritional deficiencies	Global	Female	Rainfall Quintile 2 (proportion)		X			X	
Other nutritional deficiencies	Data Rich	Female	Rainfall Quintile 2 (proportion)		X			X	
Other nutritional deficiencies	Global	Female	energy unadjusted(kcal)		X		X		
Other nutritional deficiencies	Global	Female	Log-transformed SEV scalar: Diarrhea		X		X		
Other nutritional deficiencies	Data Rich	Female	Log-transformed SEV scalar: Diarrhea		X		X		
Other nutritional deficiencies	Global	Female	Age-standardized SEV for Child underweight	X			X		
Other nutritional deficiencies	Data Rich	Female	Age-standardized SEV for Child underweight	X			X		
Other nutritional deficiencies	Global	Female	Age-Standardize Prevalence of Severe Anemia	X			X		
Other nutritional deficiencies	Data Rich	Female	Age-Standardize Prevalence of Severe Anemia	X			X		
Other nutritional deficiencies	Global	Female	Maternal Education (years per capita)						X
Other nutritional deficiencies	Data Rich	Female	Maternal Education (years per capita)						X
Other nutritional deficiencies	Global	Female	Mortality Rate Due to Death Shocks in Last 10 Years (per 1 person)					X	
Other nutritional deficiencies	Data Rich	Female	Mortality Rate Due to Death Shocks in Last 10 Years (per 1 person)					X	
Other nutritional deficiencies	Global	Male	Education (years per capita)			X			X
Other nutritional deficiencies	Data Rich	Male	Education (years per capita)			X			X
Other nutritional deficiencies	Global	Male	LDI (I$ per capita)			X			X
Other nutritional deficiencies	Data Rich	Male	LDI (I$ per capita)			X			X
Other nutritional deficiencies	Global	Male	Socio-demographic Index			X		X	
Other nutritional deficiencies	Data Rich	Male	Socio-demographic Index			X		X	
Other nutritional deficiencies	Global	Male	Age- and sex-specific SEV for Alcohol use		X				X
Other nutritional deficiencies	Data Rich	Male	Age- and sex-specific SEV for Alcohol use		X				X
Other nutritional deficiencies	Global	Male	Healthcare access and quality index		X			X	
Other nutritional deficiencies	Data Rich	Male	Healthcare access and quality index		X			X	
Other nutritional deficiencies	Global	Male	Maternal care and immunization		X			X	
Other nutritional deficiencies	Data Rich	Male	Maternal care and immunization		X			X	
Other nutritional deficiencies	Global	Male	Age- and sex-specific SEV for Unsafe sanitation		X			X	
Other nutritional deficiencies	Data Rich	Male	Age- and sex-specific SEV for Unsafe sanitation		X			X	
Other nutritional deficiencies	Global	Male	Rainfall Quintile 1 (proportion)		X			X	
Other nutritional deficiencies	Data Rich	Male	Rainfall Quintile 1 (proportion)		X			X	
Other nutritional deficiencies	Global	Male	Age- and sex-specific SEV for Unsafe water		X			X	
Other nutritional deficiencies	Data Rich	Male	Age- and sex-specific SEV for Unsafe water		X			X	
Other nutritional deficiencies	Data Rich	Male	energy unadjusted(kcal)		X			X	
Other nutritional deficiencies	Global	Male	Rainfall Quintile 2 (proportion)		X			X	
Other nutritional deficiencies	Data Rich	Male	Rainfall Quintile 2 (proportion)		X			X	
Other nutritional deficiencies	Global	Male	energy unadjusted(kcal)		X		X		
Other nutritional deficiencies	Global	Male	Log-transformed SEV scalar: Diarrhea		X		X		
Other nutritional deficiencies	Data Rich	Male	Log-transformed SEV scalar: Diarrhea		X		X		
Other nutritional deficiencies	Global	Male	Age-standardized SEV for Child underweight	X			X		
Other nutritional deficiencies	Data Rich	Male	Age-standardized SEV for Child underweight	X			X		
Other nutritional deficiencies	Global	Male	Age-Standardize Prevalence of Severe Anemia	X			X		
Other nutritional deficiencies	Data Rich	Male	Age-Standardize Prevalence of Severe Anemia	X			X		
Other nutritional deficiencies	Global	Male	Maternal Education (years per capita)						X
Other nutritional deficiencies	Data Rich	Male	Maternal Education (years per capita)						X
Other nutritional deficiencies	Global	Male	Mortality Rate Due to Death Shocks in Last 10 Years (per 1 person)					X	
Other nutritional deficiencies	Data Rich	Male	Mortality Rate Due to Death Shocks in Last 10 Years (per 1 person)					X	
Lip and oral cavity cancer	Global	Female	Education (years per capita)			X			X
Lip and oral cavity cancer	Data Rich	Female	Education (years per capita)			X			X
Lip and oral cavity cancer	Global	Female	LDI (I$ per capita)			X			X
Lip and oral cavity cancer	Data Rich	Female	LDI (I$ per capita)			X			X
Lip and oral cavity cancer	Global	Female	Socio-demographic Index			X			X
Lip and oral cavity cancer	Data Rich	Female	Socio-demographic Index			X			X
Lip and oral cavity cancer	Global	Female	Healthcare access and quality index		X			X	
Lip and oral cavity cancer	Data Rich	Female	Healthcare access and quality index		X			X	
Lip and oral cavity cancer	Global	Female	Age- and sex-specific SEV for Low fruit		X			X	
Lip and oral cavity cancer	Data Rich	Female	Age- and sex-specific SEV for Low fruit		X			X	
Lip and oral cavity cancer	Global	Female	Age- and sex-specific SEV for Low vegetables		X			X	
Lip and oral cavity cancer	Data Rich	Female	Age- and sex-specific SEV for Low vegetables		X			X	
Lip and oral cavity cancer	Global	Female	Age- and sex-specific SEV for High red meat		X			X	
Lip and oral cavity cancer	Data Rich	Female	Age- and sex-specific SEV for High red meat		X			X	
Lip and oral cavity cancer	Global	Female	Liters of alcohol consumed per capita	X			X		
Lip and oral cavity cancer	Data Rich	Female	Liters of alcohol consumed per capita	X			X		
Lip and oral cavity cancer	Global	Female	Cumulative Cigarettes (10 Years)	X			X		
Lip and oral cavity cancer	Data Rich	Female	Cumulative Cigarettes (10 Years)	X			X		
Lip and oral cavity cancer	Global	Female	Tobacco (cigarettes per capita)	X			X		
Lip and oral cavity cancer	Data Rich	Female	Tobacco (cigarettes per capita)	X			X		
Lip and oral cavity cancer	Global	Female	Cumulative Cigarettes (20 Years)	X			X		
Lip and oral cavity cancer	Data Rich	Female	Cumulative Cigarettes (20 Years)	X			X		
Lip and oral cavity cancer	Global	Female	Log-transformed SEV scalar: Lip Oral C	X			X		
Lip and oral cavity cancer	Data Rich	Female	Log-transformed SEV scalar: Lip Oral C	X			X		
Lip and oral cavity cancer	Global	Male	Education (years per capita)			X			X
Lip and oral cavity cancer	Data Rich	Male	Education (years per capita)			X			X

Comparison of GBD 2019 and GBD 2021 covariates and level of covariates used in cause of death modeling									
**Cause**	**Model Version Type**	**Sex**	**Covariate**	**Level 1 GBD 2019**	**Level 2 GBD 2019**	**Level 3 GBD 2019**	**Level 1 GBD 2021**	**Level 2 GBD 2021**	**Level 3 GBD 2021**
Lip and oral cavity cancer	Global	Male	LDI (I$ per capita)			X			X
Lip and oral cavity cancer	Data Rich	Male	LDI (I$ per capita)			X			X
Lip and oral cavity cancer	Global	Male	Socio-demographic Index			X			X
Lip and oral cavity cancer	Data Rich	Male	Socio-demographic Index			X			X
Lip and oral cavity cancer	Global	Male	Healthcare access and quality index		X			X	
Lip and oral cavity cancer	Data Rich	Male	Healthcare access and quality index		X			X	
Lip and oral cavity cancer	Global	Male	Age- and sex-specific SEV for Low fruit		X			X	
Lip and oral cavity cancer	Data Rich	Male	Age- and sex-specific SEV for Low fruit		X			X	
Lip and oral cavity cancer	Global	Male	Age- and sex-specific SEV for Low vegetables		X			X	
Lip and oral cavity cancer	Data Rich	Male	Age- and sex-specific SEV for Low vegetables		X			X	
Lip and oral cavity cancer	Global	Male	Age- and sex-specific SEV for High red meat		X			X	
Lip and oral cavity cancer	Data Rich	Male	Age- and sex-specific SEV for High red meat		X			X	
Lip and oral cavity cancer	Global	Male	Liters of alcohol consumed per capita	X			X		
Lip and oral cavity cancer	Data Rich	Male	Liters of alcohol consumed per capita	X			X		
Lip and oral cavity cancer	Global	Male	Cumulative Cigarettes (10 Years)	X			X		
Lip and oral cavity cancer	Data Rich	Male	Cumulative Cigarettes (10 Years)	X			X		
Lip and oral cavity cancer	Global	Male	Tobacco (cigarettes per capita)	X			X		
Lip and oral cavity cancer	Data Rich	Male	Tobacco (cigarettes per capita)	X			X		
Lip and oral cavity cancer	Global	Male	Cumulative Cigarettes (20 Years)	X			X		
Lip and oral cavity cancer	Data Rich	Male	Cumulative Cigarettes (20 Years)	X			X		
Lip and oral cavity cancer	Global	Male	Log-transformed SEV scalar: Lip Oral C	X			X		
Lip and oral cavity cancer	Data Rich	Male	Log-transformed SEV scalar: Lip Oral C	X			X		
Nasopharynx cancer	Global	Female	Education (years per capita)			X			X
Nasopharynx cancer	Data Rich	Female	Education (years per capita)			X			X
Nasopharynx cancer	Global	Female	LDI (I$ per capita)			X			X
Nasopharynx cancer	Data Rich	Female	LDI (I$ per capita)			X			X
Nasopharynx cancer	Global	Female	Age- and sex-specific SEV for Low fruit			X			X
Nasopharynx cancer	Data Rich	Female	Age- and sex-specific SEV for Low fruit			X			X
Nasopharynx cancer	Global	Female	Socio-demographic Index			X			X
Nasopharynx cancer	Data Rich	Female	Socio-demographic Index			X			X
Nasopharynx cancer	Global	Female	Healthcare access and quality index		X			X	
Nasopharynx cancer	Data Rich	Female	Healthcare access and quality index		X			X	
Nasopharynx cancer	Global	Female	Population Density (over 1000 ppl/sqkm, proportion)		X			X	
Nasopharynx cancer	Data Rich	Female	Population Density (over 1000 ppl/sqkm, proportion)		X			X	
Nasopharynx cancer	Global	Female	Age- and sex-specific SEV for Low vegetables		X			X	
Nasopharynx cancer	Data Rich	Female	Age- and sex-specific SEV for Low vegetables		X			X	
Nasopharynx cancer	Global	Female	Liters of alcohol consumed per capita	X			X		
Nasopharynx cancer	Data Rich	Female	Liters of alcohol consumed per capita	X			X		
Nasopharynx cancer	Global	Female	Cumulative Cigarettes (10 Years)	X			X		
Nasopharynx cancer	Data Rich	Female	Cumulative Cigarettes (10 Years)	X			X		
Nasopharynx cancer	Global	Female	Tobacco (cigarettes per capita)	X			X		
Nasopharynx cancer	Data Rich	Female	Tobacco (cigarettes per capita)	X			X		
Nasopharynx cancer	Global	Female	Cumulative Cigarettes (20 Years)	X			X		
Nasopharynx cancer	Data Rich	Female	Cumulative Cigarettes (20 Years)	X			X		
Nasopharynx cancer	Global	Female	Log-transformed SEV scalar: Nasoph C	X			X		
Nasopharynx cancer	Data Rich	Female	Log-transformed SEV scalar: Nasoph C	X			X		
Nasopharynx cancer	Global	Male	Education (years per capita)			X			X
Nasopharynx cancer	Data Rich	Male	Education (years per capita)			X			X
Nasopharynx cancer	Global	Male	LDI (I$ per capita)			X			X
Nasopharynx cancer	Data Rich	Male	LDI (I$ per capita)			X			X
Nasopharynx cancer	Global	Male	Age- and sex-specific SEV for Low fruit			X			X
Nasopharynx cancer	Data Rich	Male	Age- and sex-specific SEV for Low fruit			X			X
Nasopharynx cancer	Global	Male	Socio-demographic Index			X			X
Nasopharynx cancer	Data Rich	Male	Socio-demographic Index			X			X
Nasopharynx cancer	Global	Male	Healthcare access and quality index		X			X	
Nasopharynx cancer	Data Rich	Male	Healthcare access and quality index		X			X	
Nasopharynx cancer	Global	Male	Population Density (over 1000 ppl/sqkm, proportion)		X			X	
Nasopharynx cancer	Data Rich	Male	Population Density (over 1000 ppl/sqkm, proportion)		X			X	
Nasopharynx cancer	Global	Male	Age- and sex-specific SEV for Low vegetables		X			X	
Nasopharynx cancer	Data Rich	Male	Age- and sex-specific SEV for Low vegetables		X			X	
Nasopharynx cancer	Global	Male	Liters of alcohol consumed per capita	X			X		
Nasopharynx cancer	Data Rich	Male	Liters of alcohol consumed per capita	X			X		
Nasopharynx cancer	Global	Male	Cumulative Cigarettes (10 Years)	X			X		
Nasopharynx cancer	Data Rich	Male	Cumulative Cigarettes (10 Years)	X			X		
Nasopharynx cancer	Global	Male	Tobacco (cigarettes per capita)	X			X		
Nasopharynx cancer	Data Rich	Male	Tobacco (cigarettes per capita)	X			X		
Nasopharynx cancer	Global	Male	Cumulative Cigarettes (20 Years)	X			X		
Nasopharynx cancer	Data Rich	Male	Cumulative Cigarettes (20 Years)	X			X		
Nasopharynx cancer	Global	Male	Log-transformed SEV scalar: Nasoph C	X			X		
Nasopharynx cancer	Data Rich	Male	Log-transformed SEV scalar: Nasoph C	X			X		
Other pharynx cancer	Global	Female	Education (years per capita)			X			X
Other pharynx cancer	Data Rich	Female	Education (years per capita)			X			X
Other pharynx cancer	Global	Female	LDI (I$ per capita)			X			X
Other pharynx cancer	Data Rich	Female	LDI (I$ per capita)			X			X
Other pharynx cancer	Global	Female	Socio-demographic Index			X			X
Other pharynx cancer	Data Rich	Female	Socio-demographic Index			X			X
Other pharynx cancer	Global	Female	Healthcare access and quality index		X			X	
Other pharynx cancer	Data Rich	Female	Healthcare access and quality index		X			X	
Other pharynx cancer	Global	Female	Cumulative Cigarettes (5 Years)		X			X	
Other pharynx cancer	Data Rich	Female	Cumulative Cigarettes (5 Years)		X			X	
Other pharynx cancer	Global	Female	Population Density (over 1000 ppl/sqkm, proportion)		X			X	
Other pharynx cancer	Data Rich	Female	Population Density (over 1000 ppl/sqkm, proportion)		X			X	
Other pharynx cancer	Global	Female	Population Density (under 150 ppl/sqkm, proportion)		X			X	
Other pharynx cancer	Data Rich	Female	Population Density (under 150 ppl/sqkm, proportion)		X			X	
Other pharynx cancer	Global	Female	Age- and sex-specific SEV for Low fruit		X			X	
Other pharynx cancer	Data Rich	Female	Age- and sex-specific SEV for Low fruit		X			X	
Other pharynx cancer	Global	Female	Age- and sex-specific SEV for Low vegetables		X			X	
Other pharynx cancer	Data Rich	Female	Age- and sex-specific SEV for Low vegetables		X			X	
Other pharynx cancer	Global	Female	Smoking Prevalence	X			X		
Other pharynx cancer	Data Rich	Female	Smoking Prevalence	X			X		
Other pharynx cancer	Global	Female	Liters of alcohol consumed per capita	X			X		
Other pharynx cancer	Data Rich	Female	Liters of alcohol consumed per capita	X			X		
Other pharynx cancer	Global	Female	Log-transformed SEV scalar: Oth Phar C	X			X		
Other pharynx cancer	Data Rich	Female	Log-transformed SEV scalar: Oth Phar C	X			X		
Other pharynx cancer	Global	Male	Education (years per capita)			X			X
Other pharynx cancer	Data Rich	Male	Education (years per capita)			X			X
Other pharynx cancer	Global	Male	LDI (I$ per capita)			X			X
Other pharynx cancer	Data Rich	Male	LDI (I$ per capita)			X			X
Other pharynx cancer	Global	Male	Socio-demographic Index			X			X
Other pharynx cancer	Data Rich	Male	Socio-demographic Index			X			X
Other pharynx cancer	Global	Male	Healthcare access and quality index		X			X	
Other pharynx cancer	Data Rich	Male	Healthcare access and quality index		X			X	
Other pharynx cancer	Global	Male	Cumulative Cigarettes (5 Years)		X			X	
Other pharynx cancer	Data Rich	Male	Cumulative Cigarettes (5 Years)		X			X	
Other pharynx cancer	Global	Male	Population Density (over 1000 ppl/sqkm, proportion)		X			X	
Other pharynx cancer	Data Rich	Male	Population Density (over 1000 ppl/sqkm, proportion)		X			X	
Other pharynx cancer	Global	Male	Population Density (under 150 ppl/sqkm, proportion)		X			X	
Other pharynx cancer	Data Rich	Male	Population Density (under 150 ppl/sqkm, proportion)		X			X	
Other pharynx cancer	Global	Male	Age- and sex-specific SEV for Low fruit		X			X	
Other pharynx cancer	Data Rich	Male	Age- and sex-specific SEV for Low fruit		X			X	
Other pharynx cancer	Global	Male	Age- and sex-specific SEV for Low vegetables		X			X	
Other pharynx cancer	Data Rich	Male	Age- and sex-specific SEV for Low vegetables		X			X	
Other pharynx cancer	Global	Male	Smoking Prevalence	X			X		
Other pharynx cancer	Data Rich	Male	Smoking Prevalence	X			X		
Other pharynx cancer	Global	Male	Liters of alcohol consumed per capita	X			X		
Other pharynx cancer	Data Rich	Male	Liters of alcohol consumed per capita	X			X		
Other pharynx cancer	Global	Male	Log-transformed SEV scalar: Oth Phar C	X			X		
Other pharynx cancer	Data Rich	Male	Log-transformed SEV scalar: Oth Phar C	X			X		
Oesophageal cancer	Global	Female	Education (years per capita)			X			X
Oesophageal cancer	Data Rich	Female	Education (years per capita)			X			X
Oesophageal cancer	Global	Female	LDI (I$ per capita)			X			X
Oesophageal cancer	Data Rich	Female	LDI (I$ per capita)			X			X
Oesophageal cancer	Global	Female	Sanitation (proportion with access)			X			X
Oesophageal cancer	Data Rich	Female	Sanitation (proportion with access)			X			X
Oesophageal cancer	Global	Female	Improved Water Source (proportion with access)			X			X
Oesophageal cancer	Data Rich	Female	Improved Water Source (proportion with access)			X			X
Oesophageal cancer	Global	Female	Socio-demographic Index			X			X
Oesophageal cancer	Data Rich	Female	Socio-demographic Index			X			X

Comparison of GBD 2019 and GBD 2021 covariates and level of covariates used in cause of death modeling

Cause	Model Version Type	Sex	Covariate	Level 1 GBD 2019	Level 2 GBD 2019	Level 3 GBD 2019	Level 1 GBD 2021	Level 2 GBD 2021	Level 3 GBD 2021
Oesophageal cancer	Global	Female	Healthcare access and quality index		X			X	
Oesophageal cancer	Data Rich	Female	Healthcare access and quality index		X			X	
Oesophageal cancer	Global	Female	Tobacco (cigarettes per capita)		X			X	
Oesophageal cancer	Data Rich	Female	Tobacco (cigarettes per capita)		X			X	
Oesophageal cancer	Global	Female	Age- and sex-specific SEV for Low fruit		X			X	
Oesophageal cancer	Data Rich	Female	Age- and sex-specific SEV for Low fruit		X			X	
Oesophageal cancer	Global	Female	Age- and sex-specific SEV for Low vegetables		X			X	
Oesophageal cancer	Data Rich	Female	Age- and sex-specific SEV for Low vegetables		X			X	
Oesophageal cancer	Global	Female	Indoor Air Pollution (All Cooking Fuels)		X			X	
Oesophageal cancer	Data Rich	Female	Indoor Air Pollution (All Cooking Fuels)		X			X	
Oesophageal cancer	Global	Female	Mean BMI	X			X		
Oesophageal cancer	Data Rich	Female	Mean BMI	X			X		
Oesophageal cancer	Global	Female	Smoking Prevalence	X			X		
Oesophageal cancer	Data Rich	Female	Smoking Prevalence	X			X		
Oesophageal cancer	Global	Female	Liters of alcohol consumed per capita	X			X		
Oesophageal cancer	Data Rich	Female	Liters of alcohol consumed per capita	X			X		
Oesophageal cancer	Global	Female	Log-transformed age-standardized SEV scalar: Esophag C	X			X		
Oesophageal cancer	Data Rich	Female	Log-transformed age-standardized SEV scalar: Esophag C	X			X		
Oesophageal cancer	Global	Male	Education (years per capita)			X			X
Oesophageal cancer	Data Rich	Male	Education (years per capita)			X			X
Oesophageal cancer	Global	Male	LDI (I$ per capita)			X			X
Oesophageal cancer	Data Rich	Male	LDI (I$ per capita)			X			X
Oesophageal cancer	Global	Male	Sanitation (proportion with access)			X			X
Oesophageal cancer	Data Rich	Male	Sanitation (proportion with access)			X			X
Oesophageal cancer	Global	Male	Improved Water Source (proportion with access)			X			X
Oesophageal cancer	Data Rich	Male	Improved Water Source (proportion with access)			X			X
Oesophageal cancer	Global	Male	Socio-demographic Index			X			X
Oesophageal cancer	Data Rich	Male	Socio-demographic Index			X			X
Oesophageal cancer	Global	Male	Healthcare access and quality index		X			X	
Oesophageal cancer	Data Rich	Male	Healthcare access and quality index		X			X	
Oesophageal cancer	Global	Male	Tobacco (cigarettes per capita)		X			X	
Oesophageal cancer	Data Rich	Male	Tobacco (cigarettes per capita)		X			X	
Oesophageal cancer	Global	Male	Age- and sex-specific SEV for Low fruit		X			X	
Oesophageal cancer	Data Rich	Male	Age- and sex-specific SEV for Low fruit		X			X	
Oesophageal cancer	Global	Male	Age- and sex-specific SEV for Low vegetables		X			X	
Oesophageal cancer	Data Rich	Male	Age- and sex-specific SEV for Low vegetables		X			X	
Oesophageal cancer	Global	Male	Indoor Air Pollution (All Cooking Fuels)		X			X	
Oesophageal cancer	Data Rich	Male	Indoor Air Pollution (All Cooking Fuels)		X			X	
Oesophageal cancer	Global	Male	Mean BMI	X			X		
Oesophageal cancer	Data Rich	Male	Mean BMI	X			X		
Oesophageal cancer	Global	Male	Smoking Prevalence	X			X		
Oesophageal cancer	Data Rich	Male	Smoking Prevalence	X			X		
Oesophageal cancer	Global	Male	Liters of alcohol consumed per capita	X			X		
Oesophageal cancer	Data Rich	Male	Liters of alcohol consumed per capita	X			X		
Oesophageal cancer	Global	Male	Log-transformed age-standardized SEV scalar: Esophag C	X			X		
Oesophageal cancer	Data Rich	Male	Log-transformed age-standardized SEV scalar: Esophag C	X			X		
Stomach cancer	Global	Female	Education (years per capita)			X			X
Stomach cancer	Data Rich	Female	Education (years per capita)			X			X
Stomach cancer	Global	Female	LDI (I$ per capita)			X			X
Stomach cancer	Data Rich	Female	LDI (I$ per capita)			X			X
Stomach cancer	Global	Female	Age- and sex-specific SEV for Low fruit			X			X
Stomach cancer	Data Rich	Female	Age- and sex-specific SEV for Low fruit			X			X
Stomach cancer	Global	Female	Age- and sex-specific SEV for Low vegetables			X			X
Stomach cancer	Data Rich	Female	Age- and sex-specific SEV for Low vegetables			X			X
Stomach cancer	Global	Female	Socio-demographic Index			X			X
Stomach cancer	Data Rich	Female	Socio-demographic Index			X			X
Stomach cancer	Global	Female	Healthcare access and quality index		X			X	
Stomach cancer	Data Rich	Female	Healthcare access and quality index		X			X	
Stomach cancer	Global	Female	Mean BMI		X			X	
Stomach cancer	Data Rich	Female	Mean BMI		X			X	
Stomach cancer	Global	Female	Cumulative Cigarettes (20 Years)		X			X	
Stomach cancer	Data Rich	Female	Cumulative Cigarettes (20 Years)		X			X	
Stomach cancer	Global	Female	Sanitation (proportion with access)		X			X	
Stomach cancer	Data Rich	Female	Sanitation (proportion with access)		X			X	
Stomach cancer	Global	Female	Improved Water Source (proportion with access)		X			X	
Stomach cancer	Data Rich	Female	Improved Water Source (proportion with access)		X			X	
Stomach cancer	Global	Female	Age- and sex-specific SEV for Unsafe sanitation		X			X	
Stomach cancer	Data Rich	Female	Age- and sex-specific SEV for Unsafe sanitation		X			X	
Stomach cancer	Global	Female	Age- and sex-specific SEV for Unsafe water		X			X	
Stomach cancer	Data Rich	Female	Age- and sex-specific SEV for Unsafe water		X			X	
Stomach cancer	Global	Female	Tobacco (cigarettes per capita)	X			X		
Stomach cancer	Data Rich	Female	Tobacco (cigarettes per capita)	X			X		
Stomach cancer	Global	Female	Log-transformed SEV scalar: Stomach C	X			X		
Stomach cancer	Data Rich	Female	Log-transformed SEV scalar: Stomach C	X			X		
Stomach cancer	Global	Female	Diet high in sodium	X			X		
Stomach cancer	Data Rich	Female	Diet high in sodium	X			X		
Stomach cancer	Global	Male	Education (years per capita)			X			X
Stomach cancer	Data Rich	Male	Education (years per capita)			X			X
Stomach cancer	Global	Male	LDI (I$ per capita)			X			X
Stomach cancer	Data Rich	Male	LDI (I$ per capita)			X			X
Stomach cancer	Global	Male	Age- and sex-specific SEV for Low fruit			X			X
Stomach cancer	Data Rich	Male	Age- and sex-specific SEV for Low fruit			X			X
Stomach cancer	Global	Male	Age- and sex-specific SEV for Low vegetables			X			X
Stomach cancer	Data Rich	Male	Age- and sex-specific SEV for Low vegetables			X			X
Stomach cancer	Global	Male	Socio-demographic Index			X			X
Stomach cancer	Data Rich	Male	Socio-demographic Index			X			X
Stomach cancer	Global	Male	Healthcare access and quality index		X			X	
Stomach cancer	Data Rich	Male	Healthcare access and quality index		X			X	
Stomach cancer	Global	Male	Mean BMI		X			X	
Stomach cancer	Data Rich	Male	Mean BMI		X			X	
Stomach cancer	Global	Male	Cumulative Cigarettes (20 Years)		X			X	
Stomach cancer	Data Rich	Male	Cumulative Cigarettes (20 Years)		X			X	
Stomach cancer	Global	Male	Sanitation (proportion with access)		X			X	
Stomach cancer	Data Rich	Male	Sanitation (proportion with access)		X			X	
Stomach cancer	Global	Male	Improved Water Source (proportion with access)		X			X	
Stomach cancer	Data Rich	Male	Improved Water Source (proportion with access)		X			X	
Stomach cancer	Global	Male	Age- and sex-specific SEV for Unsafe sanitation		X			X	
Stomach cancer	Data Rich	Male	Age- and sex-specific SEV for Unsafe sanitation		X			X	
Stomach cancer	Global	Male	Age- and sex-specific SEV for Unsafe water		X			X	
Stomach cancer	Data Rich	Male	Age- and sex-specific SEV for Unsafe water		X			X	
Stomach cancer	Global	Male	Tobacco (cigarettes per capita)	X			X		
Stomach cancer	Data Rich	Male	Tobacco (cigarettes per capita)	X			X		
Stomach cancer	Global	Male	Log-transformed SEV scalar: Stomach C	X			X		
Stomach cancer	Data Rich	Male	Log-transformed SEV scalar: Stomach C	X			X		
Stomach cancer	Global	Male	Diet high in sodium	X			X		
Stomach cancer	Data Rich	Male	Diet high in sodium	X			X		
Colon and rectum cancer	Global	Female	Education (years per capita)			X			X
Colon and rectum cancer	Data Rich	Female	Education (years per capita)			X			X
Colon and rectum cancer	Global	Female	LDI (I$ per capita)			X			X
Colon and rectum cancer	Data Rich	Female	LDI (I$ per capita)			X			X
Colon and rectum cancer	Global	Female	Healthcare access and quality index			X			X
Colon and rectum cancer	Data Rich	Female	Healthcare access and quality index			X			X
Colon and rectum cancer	Global	Female	Age- and sex-specific SEV for Low fruit			X			X
Colon and rectum cancer	Data Rich	Female	Age- and sex-specific SEV for Low fruit			X			X
Colon and rectum cancer	Global	Female	Age- and sex-specific SEV for Low nuts and seeds			X			X
Colon and rectum cancer	Data Rich	Female	Age- and sex-specific SEV for Low nuts and seeds			X			X
Colon and rectum cancer	Global	Female	Socio-demographic Index			X			X
Colon and rectum cancer	Data Rich	Female	Socio-demographic Index			X			X
Colon and rectum cancer	Global	Female	Age- and sex-specific SEV for Low milk			X			X
Colon and rectum cancer	Data Rich	Female	Age- and sex-specific SEV for Low milk			X			X
Colon and rectum cancer	Global	Female	Liters of alcohol consumed per capita		X			X	
Colon and rectum cancer	Data Rich	Female	Liters of alcohol consumed per capita		X			X	
Colon and rectum cancer	Global	Female	Cumulative Cigarettes (5 Years)		X			X	
Colon and rectum cancer	Data Rich	Female	Cumulative Cigarettes (5 Years)		X			X	
Colon and rectum cancer	Global	Female	Diabetes Fasting Plasma Glucose (mmol/L), age-standardized 25+		X			X	
Colon and rectum cancer	Data Rich	Female	Diabetes Fasting Plasma Glucose (mmol/L), age-standardized 25+		X			X	
Colon and rectum cancer	Global	Female	Age- and sex-specific SEV for Low vegetables		X			X	

Comparison of GBD 2019 and GBD 2021 covariates and level of covariates used in cause of death modeling

Cause	Model Version Type	Sex	Covariate	Level 1 GBD 2019	Level 2 GBD 2019	Level 3 GBD 2019	Level 1 GBD 2021	Level 2 GBD 2021	Level 3 GBD 2021
Colon and rectum cancer	Data Rich	Female	Age- and sex-specific SEV for Low vegetables		X			X	
Colon and rectum cancer	Global	Female	pufa adjusted(percent)		X			X	
Colon and rectum cancer	Data Rich	Female	pufa adjusted(percent)		X			X	
Colon and rectum cancer	Global	Female	Age- and sex-specific SEV for Low fiber		X			X	
Colon and rectum cancer	Data Rich	Female	Age- and sex-specific SEV for Low fiber		X			X	
Colon and rectum cancer	Global	Female	Age- and sex-specific SEV for Low calcium		X			X	
Colon and rectum cancer	Data Rich	Female	Age- and sex-specific SEV for Low calcium		X			X	
Colon and rectum cancer	Global	Female	Mean BMI	X			X		
Colon and rectum cancer	Data Rich	Female	Mean BMI	X			X		
Colon and rectum cancer	Global	Female	Tobacco (cigarettes per capita)	X			X		
Colon and rectum cancer	Data Rich	Female	Tobacco (cigarettes per capita)	X			X		
Colon and rectum cancer	Global	Female	Age- and sex-specific SEV for High red meat	X			X		
Colon and rectum cancer	Data Rich	Female	Age- and sex-specific SEV for High red meat	X			X		
Colon and rectum cancer	Global	Female	Log-transformed SEV scalar: Colorect C	X			X		
Colon and rectum cancer	Data Rich	Female	Log-transformed SEV scalar: Colorect C	X			X		
Colon and rectum cancer	Global	Female	Total Physical Activity (MET-min/week), Age-specific	X			X		
Colon and rectum cancer	Data Rich	Female	Total Physical Activity (MET-min/week), Age-specific	X			X		
Colon and rectum cancer	Global	Male	Education (years per capita)			X			X
Colon and rectum cancer	Data Rich	Male	Education (years per capita)			X			X
Colon and rectum cancer	Global	Male	LDI (I$ per capita)			X			X
Colon and rectum cancer	Data Rich	Male	LDI (I$ per capita)			X			X
Colon and rectum cancer	Global	Male	Healthcare access and quality index			X			X
Colon and rectum cancer	Data Rich	Male	Healthcare access and quality index			X			X
Colon and rectum cancer	Global	Male	Age- and sex-specific SEV for Low fruit			X			X
Colon and rectum cancer	Data Rich	Male	Age- and sex-specific SEV for Low fruit			X			X
Colon and rectum cancer	Global	Male	Age- and sex-specific SEV for Low nuts and seeds			X			X
Colon and rectum cancer	Data Rich	Male	Age- and sex-specific SEV for Low nuts and seeds			X			X
Colon and rectum cancer	Global	Male	Socio-demographic Index			X			X
Colon and rectum cancer	Data Rich	Male	Socio-demographic Index			X			X
Colon and rectum cancer	Global	Male	Age- and sex-specific SEV for Low milk			X			X
Colon and rectum cancer	Data Rich	Male	Age- and sex-specific SEV for Low milk			X			X
Colon and rectum cancer	Global	Male	Liters of alcohol consumed per capita		X			X	
Colon and rectum cancer	Data Rich	Male	Liters of alcohol consumed per capita		X			X	
Colon and rectum cancer	Data Rich	Male	Cumulative Cigarettes (5 Years)		X			X	
Colon and rectum cancer	Global	Male	Cumulative Cigarettes (20 Years)		X			X	
Colon and rectum cancer	Global	Male	Diabetes Fasting Plasma Glucose (mmol/L), age-standardized 25+		X			X	
Colon and rectum cancer	Data Rich	Male	Diabetes Fasting Plasma Glucose (mmol/L), age-standardized 25+		X			X	
Colon and rectum cancer	Global	Male	Age- and sex-specific SEV for Low vegetables		X			X	
Colon and rectum cancer	Data Rich	Male	Age- and sex-specific SEV for Low vegetables		X			X	
Colon and rectum cancer	Global	Male	pufa adjusted(percent)		X			X	
Colon and rectum cancer	Data Rich	Male	pufa adjusted(percent)		X			X	
Colon and rectum cancer	Global	Male	Age- and sex-specific SEV for Low fiber		X			X	
Colon and rectum cancer	Data Rich	Male	Age- and sex-specific SEV for Low fiber		X			X	
Colon and rectum cancer	Global	Male	Age- and sex-specific SEV for Low calcium		X			X	
Colon and rectum cancer	Data Rich	Male	Age- and sex-specific SEV for Low calcium		X			X	
Colon and rectum cancer	Global	Male	Mean BMI	X			X		
Colon and rectum cancer	Data Rich	Male	Mean BMI	X			X		
Colon and rectum cancer	Global	Male	Tobacco (cigarettes per capita)	X			X		
Colon and rectum cancer	Data Rich	Male	Tobacco (cigarettes per capita)	X			X		
Colon and rectum cancer	Global	Male	Age- and sex-specific SEV for High red meat	X			X		
Colon and rectum cancer	Data Rich	Male	Age- and sex-specific SEV for High red meat	X			X		
Colon and rectum cancer	Global	Male	Log-transformed SEV scalar: Colorect C	X			X		
Colon and rectum cancer	Data Rich	Male	Log-transformed SEV scalar: Colorect C	X			X		
Colon and rectum cancer	Global	Male	Total Physical Activity (MET-min/week), Age-specific	X			X		
Colon and rectum cancer	Data Rich	Male	Total Physical Activity (MET-min/week), Age-specific	X			X		
Gallbladder and biliary tract cancer	Data Rich	Female	Education (years per capita)			X			X
Gallbladder and biliary tract cancer	Global	Female	Education (years per capita)			X			X
Gallbladder and biliary tract cancer	Data Rich	Female	LDI (I$ per capita)			X			X
Gallbladder and biliary tract cancer	Global	Female	LDI (I$ per capita)			X			X
Gallbladder and biliary tract cancer	Data Rich	Female	Socio-demographic Index			X			X
Gallbladder and biliary tract cancer	Global	Female	Socio-demographic Index			X			X
Gallbladder and biliary tract cancer	Data Rich	Female	Healthcare access and quality index		X			X	
Gallbladder and biliary tract cancer	Global	Female	Healthcare access and quality index		X			X	
Gallbladder and biliary tract cancer	Data Rich	Female	Smoking Prevalence		X			X	
Gallbladder and biliary tract cancer	Global	Female	Smoking Prevalence		X			X	
Gallbladder and biliary tract cancer	Data Rich	Female	Liters of alcohol consumed per capita		X			X	
Gallbladder and biliary tract cancer	Global	Female	Liters of alcohol consumed per capita		X			X	
Gallbladder and biliary tract cancer	Data Rich	Female	Cumulative Cigarettes (5 Years)		X			X	
Gallbladder and biliary tract cancer	Global	Female	Cumulative Cigarettes (5 Years)		X			X	
Gallbladder and biliary tract cancer	Data Rich	Female	Cumulative Cigarettes (10 Years)		X			X	
Gallbladder and biliary tract cancer	Global	Female	Cumulative Cigarettes (10 Years)		X			X	
Gallbladder and biliary tract cancer	Data Rich	Female	Tobacco (cigarettes per capita)		X			X	
Gallbladder and biliary tract cancer	Global	Female	Tobacco (cigarettes per capita)		X			X	
Gallbladder and biliary tract cancer	Data Rich	Female	Diabetes Age-Standardized Prevalence (proportion)		X			X	
Gallbladder and biliary tract cancer	Global	Female	Diabetes Age-Standardized Prevalence (proportion)		X			X	
Gallbladder and biliary tract cancer	Data Rich	Female	Age- and sex-specific SEV for Low fruit		X			X	
Gallbladder and biliary tract cancer	Global	Female	Age- and sex-specific SEV for Low fruit		X			X	
Gallbladder and biliary tract cancer	Data Rich	Female	Age- and sex-specific SEV for Low vegetables		X			X	
Gallbladder and biliary tract cancer	Global	Female	Age- and sex-specific SEV for Low vegetables		X			X	
Gallbladder and biliary tract cancer	Data Rich	Female	Mean BMI	X			X		
Gallbladder and biliary tract cancer	Global	Female	Mean BMI	X			X		
Gallbladder and biliary tract cancer	Data Rich	Female	Log-transformed SEV scalar: Gallblad C	X			X		
Gallbladder and biliary tract cancer	Global	Female	Log-transformed SEV scalar: Gallblad C	X			X		
Gallbladder and biliary tract cancer	Data Rich	Male	Education (years per capita)			X			X
Gallbladder and biliary tract cancer	Global	Male	Education (years per capita)			X			X
Gallbladder and biliary tract cancer	Data Rich	Male	LDI (I$ per capita)			X			X
Gallbladder and biliary tract cancer	Global	Male	LDI (I$ per capita)			X			X
Gallbladder and biliary tract cancer	Data Rich	Male	Socio-demographic Index			X			X
Gallbladder and biliary tract cancer	Global	Male	Socio-demographic Index			X			X
Gallbladder and biliary tract cancer	Data Rich	Male	Healthcare access and quality index		X			X	
Gallbladder and biliary tract cancer	Global	Male	Healthcare access and quality index		X			X	
Gallbladder and biliary tract cancer	Data Rich	Male	Smoking Prevalence		X			X	
Gallbladder and biliary tract cancer	Global	Male	Smoking Prevalence		X			X	
Gallbladder and biliary tract cancer	Data Rich	Male	Liters of alcohol consumed per capita		X			X	
Gallbladder and biliary tract cancer	Global	Male	Liters of alcohol consumed per capita		X			X	
Gallbladder and biliary tract cancer	Data Rich	Male	Cumulative Cigarettes (5 Years)		X			X	
Gallbladder and biliary tract cancer	Global	Male	Cumulative Cigarettes (5 Years)		X			X	
Gallbladder and biliary tract cancer	Data Rich	Male	Cumulative Cigarettes (10 Years)		X			X	
Gallbladder and biliary tract cancer	Global	Male	Cumulative Cigarettes (10 Years)		X			X	
Gallbladder and biliary tract cancer	Data Rich	Male	Tobacco (cigarettes per capita)		X			X	
Gallbladder and biliary tract cancer	Global	Male	Tobacco (cigarettes per capita)		X			X	
Gallbladder and biliary tract cancer	Data Rich	Male	Diabetes Age-Standardized Prevalence (proportion)		X			X	
Gallbladder and biliary tract cancer	Global	Male	Diabetes Age-Standardized Prevalence (proportion)		X			X	
Gallbladder and biliary tract cancer	Data Rich	Male	Age- and sex-specific SEV for Low fruit		X			X	
Gallbladder and biliary tract cancer	Global	Male	Age- and sex-specific SEV for Low fruit		X			X	
Gallbladder and biliary tract cancer	Data Rich	Male	Age- and sex-specific SEV for Low vegetables		X			X	
Gallbladder and biliary tract cancer	Global	Male	Age- and sex-specific SEV for Low vegetables		X			X	
Gallbladder and biliary tract cancer	Data Rich	Male	Mean BMI	X			X		
Gallbladder and biliary tract cancer	Global	Male	Mean BMI	X			X		
Gallbladder and biliary tract cancer	Data Rich	Male	Log-transformed SEV scalar: Gallblad C	X			X		
Gallbladder and biliary tract cancer	Global	Male	Log-transformed SEV scalar: Gallblad C	X			X		
Pancreatic cancer	Data Rich	Female	Education (years per capita)			X			X
Pancreatic cancer	Global	Female	Education (years per capita)			X			X
Pancreatic cancer	Data Rich	Female	LDI (I$ per capita)			X			X
Pancreatic cancer	Global	Female	LDI (I$ per capita)			X			X
Pancreatic cancer	Data Rich	Female	Socio-demographic Index			X			X
Pancreatic cancer	Global	Female	Socio-demographic Index			X			X
Pancreatic cancer	Data Rich	Female	Age- and sex-specific SEV for Low fruit			X			X
Pancreatic cancer	Global	Female	Age- and sex-specific SEV for Low fruit			X			X
Pancreatic cancer	Data Rich	Female	Age- and sex-specific SEV for Low vegetables			X			X
Pancreatic cancer	Global	Female	Age- and sex-specific SEV for Low vegetables			X			X
Pancreatic cancer	Data Rich	Female	Healthcare access and quality index		X			X	
Pancreatic cancer	Global	Female	Healthcare access and quality index		X			X	
Pancreatic cancer	Data Rich	Female	Liters of alcohol consumed per capita		X			X	
Pancreatic cancer	Global	Female	Liters of alcohol consumed per capita		X			X	
Pancreatic cancer	Data Rich	Female	Diabetes Fasting Plasma Glucose (mmol/L), age-standardized 25+		X			X	
Pancreatic cancer	Global	Female	Diabetes Fasting Plasma Glucose (mmol/L), age-standardized 25+		X			X	

Comparison of GBD 2019 and GBD 2021 covariates and level of covariates used in cause of death modeling

Cause	Model Version Type	Sex	Covariate	Level 1 GBD 2019	Level 2 GBD 2019	Level 3 GBD 2019	Level 1 GBD 2021	Level 2 GBD 2021	Level 3 GBD 2021
Pancreatic cancer	Data Rich	Female	Diabetes Age-Standardized Prevalence (proportion)		X			X	
Pancreatic cancer	Global	Female	Diabetes Age-Standardized Prevalence (proportion)		X			X	
Pancreatic cancer	Data Rich	Female	Age- and sex-specific SEV for High red meat		X			X	
Pancreatic cancer	Global	Female	Age- and sex-specific SEV for High red meat		X			X	
Pancreatic cancer	Data Rich	Female	energy unadjusted(kcal)		X			X	
Pancreatic cancer	Global	Female	energy unadjusted(kcal)		X			X	
Pancreatic cancer	Data Rich	Female	Mean BMI	X			X		
Pancreatic cancer	Global	Female	Mean BMI	X			X		
Pancreatic cancer	Data Rich	Female	Cumulative Cigarettes (10 Years)	X			X		
Pancreatic cancer	Global	Female	Cumulative Cigarettes (10 Years)	X			X		
Pancreatic cancer	Data Rich	Female	Tobacco (cigarettes per capita)	X			X		
Pancreatic cancer	Global	Female	Tobacco (cigarettes per capita)	X			X		
Pancreatic cancer	Data Rich	Female	Cumulative Cigarettes (20 Years)	X			X		
Pancreatic cancer	Global	Female	Cumulative Cigarettes (20 Years)	X			X		
Pancreatic cancer	Data Rich	Female	Log-transformed SEV scalar: Pancreas C	X			X		
Pancreatic cancer	Global	Female	Log-transformed SEV scalar: Pancreas C	X			X		
Pancreatic cancer	Data Rich	Male	Education (years per capita)			X			X
Pancreatic cancer	Global	Male	Education (years per capita)			X			X
Pancreatic cancer	Data Rich	Male	LDI (I$ per capita)			X			X
Pancreatic cancer	Global	Male	LDI (I$ per capita)			X			X
Pancreatic cancer	Data Rich	Male	Socio-demographic Index			X			X
Pancreatic cancer	Global	Male	Socio-demographic Index			X			X
Pancreatic cancer	Data Rich	Male	Age- and sex-specific SEV for Low fruit			X			X
Pancreatic cancer	Global	Male	Age- and sex-specific SEV for Low fruit			X			X
Pancreatic cancer	Data Rich	Male	Age- and sex-specific SEV for Low vegetables			X			X
Pancreatic cancer	Global	Male	Age- and sex-specific SEV for Low vegetables			X			X
Pancreatic cancer	Data Rich	Male	Healthcare access and quality index		X			X	
Pancreatic cancer	Global	Male	Healthcare access and quality index		X			X	
Pancreatic cancer	Data Rich	Male	Liters of alcohol consumed per capita		X			X	
Pancreatic cancer	Global	Male	Liters of alcohol consumed per capita		X			X	
Pancreatic cancer	Data Rich	Male	Diabetes Fasting Plasma Glucose (mmol/L), age-standardized 25+		X			X	
Pancreatic cancer	Global	Male	Diabetes Fasting Plasma Glucose (mmol/L), age-standardized 25+		X			X	
Pancreatic cancer	Data Rich	Male	Diabetes Age-Standardized Prevalence (proportion)		X			X	
Pancreatic cancer	Global	Male	Diabetes Age-Standardized Prevalence (proportion)		X			X	
Pancreatic cancer	Data Rich	Male	Age- and sex-specific SEV for High red meat		X			X	
Pancreatic cancer	Global	Male	Age- and sex-specific SEV for High red meat		X			X	
Pancreatic cancer	Data Rich	Male	energy unadjusted(kcal)		X			X	
Pancreatic cancer	Global	Male	energy unadjusted(kcal)		X			X	
Pancreatic cancer	Data Rich	Male	Mean BMI	X			X		
Pancreatic cancer	Global	Male	Mean BMI	X			X		
Pancreatic cancer	Data Rich	Male	Cumulative Cigarettes (10 Years)	X			X		
Pancreatic cancer	Global	Male	Cumulative Cigarettes (10 Years)	X			X		
Pancreatic cancer	Data Rich	Male	Tobacco (cigarettes per capita)	X			X		
Pancreatic cancer	Global	Male	Tobacco (cigarettes per capita)	X			X		
Pancreatic cancer	Data Rich	Male	Cumulative Cigarettes (20 Years)	X			X		
Pancreatic cancer	Global	Male	Cumulative Cigarettes (20 Years)	X			X		
Pancreatic cancer	Data Rich	Male	Log-transformed SEV scalar: Pancreas C	X			X		
Pancreatic cancer	Global	Male	Log-transformed SEV scalar: Pancreas C	X			X		
Larynx cancer	Global	Female	LDI (I$ per capita)			X			X
Larynx cancer	Data Rich	Female	LDI (I$ per capita)			X			X
Larynx cancer	Global	Female	Age- and sex-specific SEV for Low fruit			X			X
Larynx cancer	Global	Female	Socio-demographic Index			X			X
Larynx cancer	Data Rich	Female	Socio-demographic Index			X			X
Larynx cancer	Data Rich	Female	Age- and sex-specific SEV for Low fruit			X		X	
Larynx cancer	Data Rich	Female	Age- and sex-specific SEV for Low vegetables		X				X
Larynx cancer	Global	Female	Healthcare access and quality index		X			X	
Larynx cancer	Data Rich	Female	Healthcare access and quality index		X			X	
Larynx cancer	Global	Female	Smoking Prevalence		X			X	
Larynx cancer	Data Rich	Female	Smoking Prevalence		X			X	
Larynx cancer	Global	Female	Cumulative Cigarettes (10 Years)		X			X	
Larynx cancer	Data Rich	Female	Cumulative Cigarettes (10 Years)		X			X	
Larynx cancer	Global	Female	Cumulative Cigarettes (20 Years)		X			X	
Larynx cancer	Data Rich	Female	Cumulative Cigarettes (20 Years)		X			X	
Larynx cancer	Global	Female	Population Density (over 1000 ppl/sqkm, proportion)		X			X	
Larynx cancer	Data Rich	Female	Population Density (over 1000 ppl/sqkm, proportion)		X			X	
Larynx cancer	Global	Female	Asbestos consumption (metric tons per year per capita)		X			X	
Larynx cancer	Data Rich	Female	Asbestos consumption (metric tons per year per capita)		X			X	
Larynx cancer	Global	Female	Age- and sex-specific SEV for Low vegetables		X			X	
Larynx cancer	Global	Female	Liters of alcohol consumed per capita	X			X		
Larynx cancer	Data Rich	Female	Liters of alcohol consumed per capita	X			X		
Larynx cancer	Global	Female	Log-transformed SEV scalar: Larynx C	X			X		
Larynx cancer	Data Rich	Female	Log-transformed SEV scalar: Larynx C	X			X		
Larynx cancer	Global	Male	LDI (I$ per capita)			X			X
Larynx cancer	Data Rich	Male	LDI (I$ per capita)			X			X
Larynx cancer	Global	Male	Age- and sex-specific SEV for Low fruit			X			X
Larynx cancer	Data Rich	Male	Age- and sex-specific SEV for Low vegetables			X			X
Larynx cancer	Global	Male	Socio-demographic Index			X			X
Larynx cancer	Data Rich	Male	Socio-demographic Index			X			X
Larynx cancer	Global	Male	Healthcare access and quality index		X			X	
Larynx cancer	Data Rich	Male	Healthcare access and quality index		X			X	
Larynx cancer	Global	Male	Smoking Prevalence		X			X	
Larynx cancer	Data Rich	Male	Smoking Prevalence		X			X	
Larynx cancer	Global	Male	Cumulative Cigarettes (10 Years)		X			X	
Larynx cancer	Data Rich	Male	Cumulative Cigarettes (10 Years)		X			X	
Larynx cancer	Global	Male	Cumulative Cigarettes (20 Years)		X			X	
Larynx cancer	Data Rich	Male	Cumulative Cigarettes (20 Years)		X			X	
Larynx cancer	Global	Male	Population Density (over 1000 ppl/sqkm, proportion)		X			X	
Larynx cancer	Data Rich	Male	Population Density (over 1000 ppl/sqkm, proportion)		X			X	
Larynx cancer	Global	Male	Asbestos consumption (metric tons per year per capita)		X			X	
Larynx cancer	Data Rich	Male	Asbestos consumption (metric tons per year per capita)		X			X	
Larynx cancer	Data Rich	Male	Age- and sex-specific SEV for Low fruit		X			X	
Larynx cancer	Global	Male	Age- and sex-specific SEV for Low vegetables		X			X	
Larynx cancer	Global	Male	Liters of alcohol consumed per capita	X			X		
Larynx cancer	Data Rich	Male	Liters of alcohol consumed per capita	X			X		
Larynx cancer	Global	Male	Log-transformed SEV scalar: Larynx C	X			X		
Larynx cancer	Data Rich	Male	Log-transformed SEV scalar: Larynx C	X			X		
Tracheal, bronchus, and lung cancer	Global	Female	Education (years per capita)			X			X
Tracheal, bronchus, and lung cancer	Data Rich	Female	Education (years per capita)			X			X
Tracheal, bronchus, and lung cancer	Global	Female	LDI (I$ per capita)			X			X
Tracheal, bronchus, and lung cancer	Data Rich	Female	LDI (I$ per capita)			X			X
Tracheal, bronchus, and lung cancer	Global	Female	Socio-demographic Index			X			X
Tracheal, bronchus, and lung cancer	Data Rich	Female	Socio-demographic Index			X			X
Tracheal, bronchus, and lung cancer	Global	Female	Healthcare access and quality index		X			X	
Tracheal, bronchus, and lung cancer	Data Rich	Female	Healthcare access and quality index		X			X	
Tracheal, bronchus, and lung cancer	Global	Female	Cumulative Cigarettes (10 Years)		X			X	
Tracheal, bronchus, and lung cancer	Data Rich	Female	Cumulative Cigarettes (10 Years)		X			X	
Tracheal, bronchus, and lung cancer	Global	Female	Cumulative Cigarettes (20 Years)		X			X	
Tracheal, bronchus, and lung cancer	Data Rich	Female	Cumulative Cigarettes (20 Years)		X			X	
Tracheal, bronchus, and lung cancer	Global	Female	Diabetes Fasting Plasma Glucose (mmol/L), age-standardized 25+		X			X	
Tracheal, bronchus, and lung cancer	Data Rich	Female	Diabetes Fasting Plasma Glucose (mmol/L), age-standardized 25+		X			X	
Tracheal, bronchus, and lung cancer	Global	Female	Secondhand smoke		X			X	
Tracheal, bronchus, and lung cancer	Data Rich	Female	Secondhand smoke		X			X	
Tracheal, bronchus, and lung cancer	Global	Female	Indoor Air Pollution (All Cooking Fuels)		X			X	
Tracheal, bronchus, and lung cancer	Data Rich	Female	Indoor Air Pollution (All Cooking Fuels)		X			X	
Tracheal, bronchus, and lung cancer	Global	Female	Outdoor Air Pollution (PM2.5)		X			X	
Tracheal, bronchus, and lung cancer	Data Rich	Female	Outdoor Air Pollution (PM2.5)		X			X	
Tracheal, bronchus, and lung cancer	Global	Female	Residential radon		X			X	
Tracheal, bronchus, and lung cancer	Data Rich	Female	Residential radon		X			X	
Tracheal, bronchus, and lung cancer	Global	Female	Smoking Prevalence	X			X		
Tracheal, bronchus, and lung cancer	Data Rich	Female	Smoking Prevalence	X			X		
Tracheal, bronchus, and lung cancer	Global	Female	Asbestos consumption (metric tons per year per capita)	X			X		
Tracheal, bronchus, and lung cancer	Data Rich	Female	Asbestos consumption (metric tons per year per capita)	X			X		
Tracheal, bronchus, and lung cancer	Global	Female	Log-transformed SEV scalar: Lung C	X			X		
Tracheal, bronchus, and lung cancer	Data Rich	Female	Log-transformed SEV scalar: Lung C	X			X		
Tracheal, bronchus, and lung cancer	Global	Female	Log-transformed age-standardized SEV scalar: Lung C	X			X		
Tracheal, bronchus, and lung cancer	Data Rich	Female	Log-transformed age-standardized SEV scalar: Lung C	X			X		

Comparison of GBD 2019 and GBD 2021 covariates and level of covariates used in cause of death modeling									
Cause	Model Version Type	Sex	Covariate	Level 1 GBD 2019	Level 2 GBD 2019	Level 3 GBD 2019	Level 1 GBD 2021	Level 2 GBD 2021	Level 3 GBD 2021
Tracheal, bronchus, and lung cancer	Global	Male	Education (years per capita)			X			X
Tracheal, bronchus, and lung cancer	Data Rich	Male	Education (years per capita)			X			X
Tracheal, bronchus, and lung cancer	Global	Male	LDI (I$ per capita)			X			X
Tracheal, bronchus, and lung cancer	Data Rich	Male	LDI (I$ per capita)			X			X
Tracheal, bronchus, and lung cancer	Global	Male	Socio-demographic Index			X			X
Tracheal, bronchus, and lung cancer	Data Rich	Male	Socio-demographic Index			X			X
Tracheal, bronchus, and lung cancer	Global	Male	Healthcare access and quality index		X			X	
Tracheal, bronchus, and lung cancer	Data Rich	Male	Healthcare access and quality index		X			X	
Tracheal, bronchus, and lung cancer	Global	Male	Cumulative Cigarettes (10 Years)		X			X	
Tracheal, bronchus, and lung cancer	Data Rich	Male	Cumulative Cigarettes (10 Years)		X			X	
Tracheal, bronchus, and lung cancer	Global	Male	Cumulative Cigarettes (20 Years)		X			X	
Tracheal, bronchus, and lung cancer	Data Rich	Male	Cumulative Cigarettes (20 Years)		X			X	
Tracheal, bronchus, and lung cancer	Global	Male	Diabetes Fasting Plasma Glucose (mmol/L), age-standardized 25+		X			X	
Tracheal, bronchus, and lung cancer	Data Rich	Male	Diabetes Fasting Plasma Glucose (mmol/L), age-standardized 25+		X			X	
Tracheal, bronchus, and lung cancer	Global	Male	Secondhand smoke		X			X	
Tracheal, bronchus, and lung cancer	Data Rich	Male	Secondhand smoke		X			X	
Tracheal, bronchus, and lung cancer	Global	Male	Indoor Air Pollution (All Cooking Fuels)		X			X	
Tracheal, bronchus, and lung cancer	Data Rich	Male	Indoor Air Pollution (All Cooking Fuels)		X			X	
Tracheal, bronchus, and lung cancer	Global	Male	Outdoor Air Pollution (PM2.5)		X			X	
Tracheal, bronchus, and lung cancer	Data Rich	Male	Outdoor Air Pollution (PM2.5)		X			X	
Tracheal, bronchus, and lung cancer	Global	Male	Residential radon		X			X	
Tracheal, bronchus, and lung cancer	Data Rich	Male	Residential radon		X			X	
Tracheal, bronchus, and lung cancer	Global	Male	Smoking Prevalence	X			X		
Tracheal, bronchus, and lung cancer	Data Rich	Male	Smoking Prevalence	X			X		
Tracheal, bronchus, and lung cancer	Global	Male	Asbestos consumption (metric tons per year per capita)	X			X		
Tracheal, bronchus, and lung cancer	Data Rich	Male	Asbestos consumption (metric tons per year per capita)	X			X		
Tracheal, bronchus, and lung cancer	Global	Male	Log-transformed SEV scalar: Lung C	X			X		
Tracheal, bronchus, and lung cancer	Data Rich	Male	Log-transformed SEV scalar: Lung C	X			X		
Tracheal, bronchus, and lung cancer	Global	Male	Log-transformed age-standardized SEV scalar: Lung C	X			X		
Tracheal, bronchus, and lung cancer	Data Rich	Male	Log-transformed age-standardized SEV scalar: Lung C	X			X		
Malignant skin melanoma	Global	Female	Education (years per capita)			X			X
Malignant skin melanoma	Data Rich	Female	Education (years per capita)			X			X
Malignant skin melanoma	Global	Female	LDI (I$ per capita)			X			X
Malignant skin melanoma	Data Rich	Female	LDI (I$ per capita)			X			X
Malignant skin melanoma	Global	Female	Socio-demographic Index			X			X
Malignant skin melanoma	Data Rich	Female	Socio-demographic Index			X			X
Malignant skin melanoma	Global	Female	Healthcare access and quality index		X			X	
Malignant skin melanoma	Data Rich	Female	Healthcare access and quality index		X			X	
Malignant skin melanoma	Global	Female	Latitude Under 15 (proportion)		X			X	
Malignant skin melanoma	Data Rich	Female	Latitude Under 15 (proportion)		X			X	
Malignant skin melanoma	Global	Female	Latitude 15 to 30 (proportion)		X			X	
Malignant skin melanoma	Data Rich	Female	Latitude 15 to 30 (proportion)		X			X	
Malignant skin melanoma	Global	Female	Latitude 30 to 45 (proportion)		X			X	
Malignant skin melanoma	Data Rich	Female	Latitude 30 to 45 (proportion)		X			X	
Malignant skin melanoma	Global	Female	Latitude Over 45 (proportion)		X			X	
Malignant skin melanoma	Data Rich	Female	Latitude Over 45 (proportion)		X			X	
Malignant skin melanoma	Global	Female	Liters of alcohol consumed per capita	X			X		
Malignant skin melanoma	Data Rich	Female	Liters of alcohol consumed per capita	X			X		
Malignant skin melanoma	Global	Male	Education (years per capita)			X			X
Malignant skin melanoma	Data Rich	Male	Education (years per capita)			X			X
Malignant skin melanoma	Global	Male	LDI (I$ per capita)			X			X
Malignant skin melanoma	Data Rich	Male	LDI (I$ per capita)			X			X
Malignant skin melanoma	Global	Male	Socio-demographic Index			X			X
Malignant skin melanoma	Data Rich	Male	Socio-demographic Index			X			X
Malignant skin melanoma	Global	Male	Healthcare access and quality index		X			X	
Malignant skin melanoma	Data Rich	Male	Healthcare access and quality index		X			X	
Malignant skin melanoma	Global	Male	Latitude Under 15 (proportion)		X			X	
Malignant skin melanoma	Data Rich	Male	Latitude Under 15 (proportion)		X			X	
Malignant skin melanoma	Global	Male	Latitude 15 to 30 (proportion)		X			X	
Malignant skin melanoma	Data Rich	Male	Latitude 15 to 30 (proportion)		X			X	
Malignant skin melanoma	Global	Male	Latitude 30 to 45 (proportion)		X			X	
Malignant skin melanoma	Data Rich	Male	Latitude 30 to 45 (proportion)		X			X	
Malignant skin melanoma	Global	Male	Latitude Over 45 (proportion)		X			X	
Malignant skin melanoma	Data Rich	Male	Latitude Over 45 (proportion)		X			X	
Malignant skin melanoma	Global	Male	Liters of alcohol consumed per capita	X			X		
Malignant skin melanoma	Data Rich	Male	Liters of alcohol consumed per capita	X			X		
Non-melanoma skin cancer (squamous-cell carcinoma)	Data Rich	Female	Education (years per capita)			X			X
Non-melanoma skin cancer (squamous-cell carcinoma)	Global	Female	Education (years per capita)			X			X
Non-melanoma skin cancer (squamous-cell carcinoma)	Data Rich	Female	LDI (I$ per capita)			X			X
Non-melanoma skin cancer (squamous-cell carcinoma)	Global	Female	LDI (I$ per capita)			X			X
Non-melanoma skin cancer (squamous-cell carcinoma)	Data Rich	Female	Socio-demographic Index			X			X
Non-melanoma skin cancer (squamous-cell carcinoma)	Global	Female	Socio-demographic Index			X			X
Non-melanoma skin cancer (squamous-cell carcinoma)	Data Rich	Female	Healthcare access and quality index		X			X	
Non-melanoma skin cancer (squamous-cell carcinoma)	Global	Female	Healthcare access and quality index		X			X	
Non-melanoma skin cancer (squamous-cell carcinoma)	Data Rich	Female	Average latitude		X			X	
Non-melanoma skin cancer (squamous-cell carcinoma)	Global	Female	Average latitude		X			X	
Non-melanoma skin cancer (squamous-cell carcinoma)	Data Rich	Female	Smoking Prevalence	X			X		
Non-melanoma skin cancer (squamous-cell carcinoma)	Global	Female	Smoking Prevalence	X			X		
Non-melanoma skin cancer (squamous-cell carcinoma)	Data Rich	Female	Cumulative Cigarettes (5 Years)	X			X		
Non-melanoma skin cancer (squamous-cell carcinoma)	Global	Female	Cumulative Cigarettes (5 Years)	X			X		
Non-melanoma skin cancer (squamous-cell carcinoma)	Data Rich	Female	Cumulative Cigarettes (10 Years)	X			X		
Non-melanoma skin cancer (squamous-cell carcinoma)	Global	Female	Cumulative Cigarettes (10 Years)	X			X		
Non-melanoma skin cancer (squamous-cell carcinoma)	Data Rich	Female	Cumulative Cigarettes (15 Years)	X			X		
Non-melanoma skin cancer (squamous-cell carcinoma)	Global	Female	Cumulative Cigarettes (15 Years)	X			X		
Non-melanoma skin cancer (squamous-cell carcinoma)	Data Rich	Male	Education (years per capita)			X			X
Non-melanoma skin cancer (squamous-cell carcinoma)	Global	Male	Education (years per capita)			X			X
Non-melanoma skin cancer (squamous-cell carcinoma)	Data Rich	Male	LDI (I$ per capita)			X			X
Non-melanoma skin cancer (squamous-cell carcinoma)	Global	Male	LDI (I$ per capita)			X			X
Non-melanoma skin cancer (squamous-cell carcinoma)	Data Rich	Male	Socio-demographic Index			X			X
Non-melanoma skin cancer (squamous-cell carcinoma)	Global	Male	Socio-demographic Index			X			X
Non-melanoma skin cancer (squamous-cell carcinoma)	Data Rich	Male	Healthcare access and quality index		X			X	
Non-melanoma skin cancer (squamous-cell carcinoma)	Global	Male	Healthcare access and quality index		X			X	
Non-melanoma skin cancer (squamous-cell carcinoma)	Data Rich	Male	Average latitude		X			X	
Non-melanoma skin cancer (squamous-cell carcinoma)	Global	Male	Average latitude		X			X	
Non-melanoma skin cancer (squamous-cell carcinoma)	Data Rich	Male	Smoking Prevalence	X			X		
Non-melanoma skin cancer (squamous-cell carcinoma)	Global	Male	Smoking Prevalence	X			X		
Non-melanoma skin cancer (squamous-cell carcinoma)	Data Rich	Male	Cumulative Cigarettes (5 Years)	X			X		
Non-melanoma skin cancer (squamous-cell carcinoma)	Global	Male	Cumulative Cigarettes (5 Years)	X			X		
Non-melanoma skin cancer (squamous-cell carcinoma)	Data Rich	Male	Cumulative Cigarettes (10 Years)	X			X		
Non-melanoma skin cancer (squamous-cell carcinoma)	Global	Male	Cumulative Cigarettes (10 Years)	X			X		
Non-melanoma skin cancer (squamous-cell carcinoma)	Data Rich	Male	Cumulative Cigarettes (15 Years)	X			X		
Non-melanoma skin cancer (squamous-cell carcinoma)	Global	Male	Cumulative Cigarettes (15 Years)	X			X		
Soft tissue and other extraosseous sarcomas	Global	Female	Education (years per capita)						X
Soft tissue and other extraosseous sarcomas	Data Rich	Female	Education (years per capita)						X
Soft tissue and other extraosseous sarcomas	Global	Female	Liters of alcohol consumed per capita						X
Soft tissue and other extraosseous sarcomas	Data Rich	Female	Liters of alcohol consumed per capita						X
Soft tissue and other extraosseous sarcomas	Global	Female	Maternal care and immunization						X
Soft tissue and other extraosseous sarcomas	Data Rich	Female	Maternal care and immunization						X
Soft tissue and other extraosseous sarcomas	Global	Female	Log-transformed SEV scalar: HIV						X
Soft tissue and other extraosseous sarcomas	Data Rich	Female	Log-transformed SEV scalar: HIV						X
Soft tissue and other extraosseous sarcomas	Global	Female	Log-transformed age-standardized SEV scalar: HIV						X
Soft tissue and other extraosseous sarcomas	Data Rich	Female	Log-transformed age-standardized SEV scalar: HIV						X
Soft tissue and other extraosseous sarcomas	Global	Female	LDI (I$ per capita)					X	
Soft tissue and other extraosseous sarcomas	Data Rich	Female	LDI (I$ per capita)					X	
Soft tissue and other extraosseous sarcomas	Global	Female	Healthcare access and quality index					X	
Soft tissue and other extraosseous sarcomas	Data Rich	Female	Healthcare access and quality index					X	
Soft tissue and other extraosseous sarcomas	Global	Female	Socio-demographic Index					X	
Soft tissue and other extraosseous sarcomas	Data Rich	Female	Socio-demographic Index					X	
Soft tissue and other extraosseous sarcomas	Global	Female	Universal health coverage					X	
Soft tissue and other extraosseous sarcomas	Data Rich	Female	Universal health coverage					X	

Comparison of GBD 2019 and GBD 2021 covariates and level of covariates used in cause of death modeling

Cause	Model Version Type	Sex	Covariate	Level 1 GBD 2019	Level 2 GBD 2019	Level 3 GBD 2019	Level 1 GBD 2021	Level 2 GBD 2021	Level 3 GBD 2021
Soft tissue and other extraosseous sarcomas	Global	Male	Education (years per capita)						X
Soft tissue and other extraosseous sarcomas	Data Rich	Male	Education (years per capita)						X
Soft tissue and other extraosseous sarcomas	Global	Male	Liters of alcohol consumed per capita						X
Soft tissue and other extraosseous sarcomas	Data Rich	Male	Liters of alcohol consumed per capita						X
Soft tissue and other extraosseous sarcomas	Global	Male	Maternal care and immunization						X
Soft tissue and other extraosseous sarcomas	Data Rich	Male	Maternal care and immunization						X
Soft tissue and other extraosseous sarcomas	Global	Male	Log-transformed SEV scalar: HIV						X
Soft tissue and other extraosseous sarcomas	Data Rich	Male	Log-transformed SEV scalar: HIV						X
Soft tissue and other extraosseous sarcomas	Global	Male	Log-transformed age-standardized SEV scalar: HIV						X
Soft tissue and other extraosseous sarcomas	Data Rich	Male	Log-transformed age-standardized SEV scalar: HIV						X
Soft tissue and other extraosseous sarcomas	Global	Male	LDI (I$ per capita)					X	
Soft tissue and other extraosseous sarcomas	Data Rich	Male	LDI (I$ per capita)					X	
Soft tissue and other extraosseous sarcomas	Global	Male	Healthcare access and quality index					X	
Soft tissue and other extraosseous sarcomas	Data Rich	Male	Healthcare access and quality index					X	
Soft tissue and other extraosseous sarcomas	Global	Male	Socio-demographic Index					X	
Soft tissue and other extraosseous sarcomas	Data Rich	Male	Socio-demographic Index					X	
Soft tissue and other extraosseous sarcomas	Global	Male	Universal health coverage					X	
Soft tissue and other extraosseous sarcomas	Data Rich	Male	Universal health coverage					X	
Malignant neoplasm of bone and articular cartilage	Data Rich	Female	Education (years per capita)						X
Malignant neoplasm of bone and articular cartilage	Global	Female	Education (years per capita)						X
Malignant neoplasm of bone and articular cartilage	Data Rich	Female	Smoking Prevalence						X
Malignant neoplasm of bone and articular cartilage	Global	Female	Smoking Prevalence						X
Malignant neoplasm of bone and articular cartilage	Data Rich	Female	Maternal care and immunization						X
Malignant neoplasm of bone and articular cartilage	Global	Female	Maternal care and immunization						X
Malignant neoplasm of bone and articular cartilage	Data Rich	Female	Health worker density						X
Malignant neoplasm of bone and articular cartilage	Global	Female	Health worker density						X
Malignant neoplasm of bone and articular cartilage	Data Rich	Female	Log-transformed SEV scalar: Osteoarth						X
Malignant neoplasm of bone and articular cartilage	Global	Female	Log-transformed SEV scalar: Osteoarth						X
Malignant neoplasm of bone and articular cartilage	Data Rich	Female	Log-transformed age-standardized SEV scalar: Osteoarth						X
Malignant neoplasm of bone and articular cartilage	Global	Female	Log-transformed age-standardized SEV scalar: Osteoarth						X
Malignant neoplasm of bone and articular cartilage	Data Rich	Female	Age- and sex-specific SEV for Low bone mineral density						X
Malignant neoplasm of bone and articular cartilage	Global	Female	Age- and sex-specific SEV for Low bone mineral density						X
Malignant neoplasm of bone and articular cartilage	Data Rich	Female	LDI (I$ per capita)					X	
Malignant neoplasm of bone and articular cartilage	Global	Female	LDI (I$ per capita)					X	
Malignant neoplasm of bone and articular cartilage	Data Rich	Female	Healthcare access and quality index					X	
Malignant neoplasm of bone and articular cartilage	Global	Female	Healthcare access and quality index					X	
Malignant neoplasm of bone and articular cartilage	Data Rich	Female	Socio-demographic Index					X	
Malignant neoplasm of bone and articular cartilage	Global	Female	Socio-demographic Index					X	
Malignant neoplasm of bone and articular cartilage	Data Rich	Female	Universal health coverage					X	
Malignant neoplasm of bone and articular cartilage	Global	Female	Universal health coverage					X	
Malignant neoplasm of bone and articular cartilage	Data Rich	Male	Education (years per capita)						X
Malignant neoplasm of bone and articular cartilage	Global	Male	Education (years per capita)						X
Malignant neoplasm of bone and articular cartilage	Data Rich	Male	Smoking Prevalence						X
Malignant neoplasm of bone and articular cartilage	Global	Male	Smoking Prevalence						X
Malignant neoplasm of bone and articular cartilage	Data Rich	Male	Maternal care and immunization						X
Malignant neoplasm of bone and articular cartilage	Global	Male	Maternal care and immunization						X
Malignant neoplasm of bone and articular cartilage	Data Rich	Male	Health worker density						X
Malignant neoplasm of bone and articular cartilage	Global	Male	Health worker density						X
Malignant neoplasm of bone and articular cartilage	Data Rich	Male	Log-transformed SEV scalar: Osteoarth						X
Malignant neoplasm of bone and articular cartilage	Global	Male	Log-transformed SEV scalar: Osteoarth						X
Malignant neoplasm of bone and articular cartilage	Data Rich	Male	Log-transformed age-standardized SEV scalar: Osteoarth						X
Malignant neoplasm of bone and articular cartilage	Global	Male	Log-transformed age-standardized SEV scalar: Osteoarth						X
Malignant neoplasm of bone and articular cartilage	Data Rich	Male	Age- and sex-specific SEV for Low bone mineral density						X
Malignant neoplasm of bone and articular cartilage	Global	Male	Age- and sex-specific SEV for Low bone mineral density						X
Malignant neoplasm of bone and articular cartilage	Data Rich	Male	LDI (I$ per capita)					X	
Malignant neoplasm of bone and articular cartilage	Global	Male	LDI (I$ per capita)					X	
Malignant neoplasm of bone and articular cartilage	Data Rich	Male	Healthcare access and quality index					X	
Malignant neoplasm of bone and articular cartilage	Global	Male	Healthcare access and quality index					X	
Malignant neoplasm of bone and articular cartilage	Data Rich	Male	Socio-demographic Index					X	
Malignant neoplasm of bone and articular cartilage	Global	Male	Socio-demographic Index					X	
Malignant neoplasm of bone and articular cartilage	Data Rich	Male	Universal health coverage					X	
Malignant neoplasm of bone and articular cartilage	Global	Male	Universal health coverage					X	
Breast cancer	Global	Female	LDI (I$ per capita)			X			X
Breast cancer	Data Rich	Female	LDI (I$ per capita)			X			X
Breast cancer	Global	Female	Socio-demographic Index			X			X
Breast cancer	Data Rich	Female	Socio-demographic Index			X			X
Breast cancer	Global	Female	Healthcare access and quality index		X			X	
Breast cancer	Data Rich	Female	Healthcare access and quality index		X			X	
Breast cancer	Global	Female	Smoking Prevalence		X			X	
Breast cancer	Data Rich	Female	Smoking Prevalence		X			X	
Breast cancer	Global	Female	Cumulative Cigarettes (10 Years)		X			X	
Breast cancer	Data Rich	Female	Cumulative Cigarettes (10 Years)		X			X	
Breast cancer	Global	Female	Cumulative Cigarettes (20 Years)		X			X	
Breast cancer	Data Rich	Female	Cumulative Cigarettes (20 Years)		X			X	
Breast cancer	Global	Female	Diabetes Fasting Plasma Glucose (mmol/L), age-standardized 25+		X			X	
Breast cancer	Data Rich	Female	Diabetes Fasting Plasma Glucose (mmol/L), age-standardized 25+		X			X	
Breast cancer	Global	Female	Age- and sex-specific SEV for Low fruit		X			X	
Breast cancer	Data Rich	Female	Age- and sex-specific SEV for Low fruit		X			X	
Breast cancer	Global	Female	Age- and sex-specific SEV for Low vegetables		X			X	
Breast cancer	Data Rich	Female	Age- and sex-specific SEV for Low vegetables		X			X	
Breast cancer	Global	Female	Age-Specific Fertility Rate		X			X	
Breast cancer	Data Rich	Female	Age-Specific Fertility Rate		X			X	
Breast cancer	Global	Female	Total Fertility Rate		X			X	
Breast cancer	Data Rich	Female	Total Fertility Rate		X			X	
Breast cancer	Global	Female	Mean BMI	X			X		
Breast cancer	Data Rich	Female	Mean BMI	X			X		
Breast cancer	Global	Female	Liters of alcohol consumed per capita	X			X		
Breast cancer	Data Rich	Female	Liters of alcohol consumed per capita	X			X		
Breast cancer	Global	Female	Log-transformed SEV scalar: Breast C	X			X		
Breast cancer	Data Rich	Female	Log-transformed SEV scalar: Breast C	X			X		
Breast cancer	Global	Male	LDI (I$ per capita)			X			X
Breast cancer	Data Rich	Male	LDI (I$ per capita)			X			X
Breast cancer	Global	Male	Socio-demographic Index			X			X
Breast cancer	Data Rich	Male	Socio-demographic Index			X			X
Breast cancer	Global	Male	Healthcare access and quality index		X			X	
Breast cancer	Data Rich	Male	Healthcare access and quality index		X			X	
Breast cancer	Global	Male	Smoking Prevalence		X			X	
Breast cancer	Data Rich	Male	Smoking Prevalence		X			X	
Breast cancer	Global	Male	Cumulative Cigarettes (10 Years)		X			X	
Breast cancer	Data Rich	Male	Cumulative Cigarettes (10 Years)		X			X	
Breast cancer	Global	Male	Cumulative Cigarettes (20 Years)		X			X	
Breast cancer	Data Rich	Male	Cumulative Cigarettes (20 Years)		X			X	
Breast cancer	Global	Male	Diabetes Fasting Plasma Glucose (mmol/L), age-standardized 25+		X			X	
Breast cancer	Data Rich	Male	Diabetes Fasting Plasma Glucose (mmol/L), age-standardized 25+		X			X	
Breast cancer	Global	Male	Age- and sex-specific SEV for Low fruit		X			X	
Breast cancer	Data Rich	Male	Age- and sex-specific SEV for Low fruit		X			X	
Breast cancer	Global	Male	Age- and sex-specific SEV for Low vegetables		X			X	
Breast cancer	Data Rich	Male	Age- and sex-specific SEV for Low vegetables		X			X	
Breast cancer	Global	Male	Mean BMI	X			X		
Breast cancer	Data Rich	Male	Mean BMI	X			X		
Breast cancer	Global	Male	Liters of alcohol consumed per capita	X			X		
Breast cancer	Data Rich	Male	Liters of alcohol consumed per capita	X			X		
Breast cancer	Global	Male	Log-transformed SEV scalar: Breast C	X			X		
Breast cancer	Data Rich	Male	Log-transformed SEV scalar: Breast C	X			X		
Cervical cancer	Global	Female	Education (years per capita)			X			X
Cervical cancer	Data Rich	Female	Education (years per capita)			X			X
Cervical cancer	Global	Female	LDI (I$ per capita)			X			X
Cervical cancer	Data Rich	Female	LDI (I$ per capita)			X			X
Cervical cancer	Global	Female	Socio-demographic Index			X			X
Cervical cancer	Data Rich	Female	Socio-demographic Index			X			X
Cervical cancer	Global	Female	Healthcare access and quality index		X			X	
Cervical cancer	Data Rich	Female	Healthcare access and quality index		X			X	
Cervical cancer	Global	Female	Smoking Prevalence		X			X	
Cervical cancer	Data Rich	Female	Smoking Prevalence		X			X	
Cervical cancer	Global	Female	Age- and sex-specific SEV for Low fruit		X			X	
Cervical cancer	Data Rich	Female	Age- and sex-specific SEV for Low fruit		X			X	
Cervical cancer	Global	Female	Age- and sex-specific SEV for Low vegetables		X			X	
Cervical cancer	Data Rich	Female	Age- and sex-specific SEV for Low vegetables		X			X	
Cervical cancer	Global	Female	Age-Specific Fertility Rate		X			X	
Cervical cancer	Data Rich	Female	Age-Specific Fertility Rate		X			X	
Cervical cancer	Global	Female	Total Fertility Rate		X			X	

Comparison of GBD 2019 and GBD 2021 covariates and level of covariates used in cause of death modeling									
**Cause**	**Model Version Type**	**Sex**	**Covariate**	**Level 1 GBD 2019**	**Level 2 GBD 2019**	**Level 3 GBD 2019**	**Level 1 GBD 2021**	**Level 2 GBD 2021**	**Level 3 GBD 2021**
Cervical cancer	Data Rich	Female	Total Fertility Rate		X			X	
Cervical cancer	Global	Female	Cumulative Cigarettes (5 Years)	X			X		
Cervical cancer	Data Rich	Female	Cumulative Cigarettes (5 Years)	X			X		
Cervical cancer	Global	Female	HIV age-standardized prevalence	X			X		
Cervical cancer	Data Rich	Female	HIV age-standardized prevalence	X			X		
Cervical cancer	Global	Female	Log-transformed SEV scalar: HIV				X		
Cervical cancer	Data Rich	Female	Log-transformed SEV scalar: HIV				X		
Cervical cancer	Global	Female	Log-transformed age-standardized SEV scalar: HIV				X		
Cervical cancer	Data Rich	Female	Log-transformed age-standardized SEV scalar: HIV				X		
Uterine cancer	Global	Female	Education (years per capita)			X			X
Uterine cancer	Data Rich	Female	Education (years per capita)			X			X
Uterine cancer	Global	Female	LDI (I$ per capita)			X			X
Uterine cancer	Data Rich	Female	LDI (I$ per capita)			X			X
Uterine cancer	Global	Female	Socio-demographic Index			X			X
Uterine cancer	Data Rich	Female	Socio-demographic Index			X			X
Uterine cancer	Global	Female	Healthcare access and quality index		X			X	
Uterine cancer	Data Rich	Female	Healthcare access and quality index		X			X	
Uterine cancer	Global	Female	Smoking Prevalence		X			X	
Uterine cancer	Data Rich	Female	Smoking Prevalence		X			X	
Uterine cancer	Global	Female	Cumulative Cigarettes (5 Years)		X			X	
Uterine cancer	Data Rich	Female	Cumulative Cigarettes (5 Years)		X			X	
Uterine cancer	Global	Female	Cumulative Cigarettes (10 Years)		X			X	
Uterine cancer	Data Rich	Female	Cumulative Cigarettes (10 Years)		X			X	
Uterine cancer	Global	Female	Tobacco (cigarettes per capita)		X			X	
Uterine cancer	Data Rich	Female	Tobacco (cigarettes per capita)		X			X	
Uterine cancer	Global	Female	Diabetes Age-Standardized Prevalence (proportion)		X			X	
Uterine cancer	Data Rich	Female	Diabetes Age-Standardized Prevalence (proportion)		X			X	
Uterine cancer	Global	Female	Age- and sex-specific SEV for Low fruit		X			X	
Uterine cancer	Data Rich	Female	Age- and sex-specific SEV for Low fruit		X			X	
Uterine cancer	Global	Female	Age- and sex-specific SEV for Low vegetables		X			X	
Uterine cancer	Data Rich	Female	Age- and sex-specific SEV for Low vegetables		X			X	
Uterine cancer	Global	Female	Total Fertility Rate		X			X	
Uterine cancer	Data Rich	Female	Total Fertility Rate		X			X	
Uterine cancer	Global	Female	Mean BMI	X			X		
Uterine cancer	Data Rich	Female	Mean BMI	X			X		
Uterine cancer	Global	Female	Log-transformed SEV scalar: Uterus C	X			X		
Uterine cancer	Data Rich	Female	Log-transformed SEV scalar: Uterus C	X			X		
Ovarian cancer	Data Rich	Female	Education (years per capita)			X			X
Ovarian cancer	Global	Female	Education (years per capita)			X			X
Ovarian cancer	Data Rich	Female	LDI (I$ per capita)			X			X
Ovarian cancer	Global	Female	LDI (I$ per capita)			X			X
Ovarian cancer	Data Rich	Female	Socio-demographic Index			X			X
Ovarian cancer	Global	Female	Socio-demographic Index			X			X
Ovarian cancer	Data Rich	Female	Age- and sex-specific SEV for Low fruit			X			X
Ovarian cancer	Global	Female	Age- and sex-specific SEV for Low fruit			X			X
Ovarian cancer	Data Rich	Female	Age- and sex-specific SEV for Low vegetables			X			X
Ovarian cancer	Global	Female	Age- and sex-specific SEV for Low vegetables			X			X
Ovarian cancer	Data Rich	Female	Healthcare access and quality index		X			X	
Ovarian cancer	Global	Female	Healthcare access and quality index		X			X	
Ovarian cancer	Data Rich	Female	Mean BMI		X			X	
Ovarian cancer	Global	Female	Mean BMI		X			X	
Ovarian cancer	Data Rich	Female	Smoking Prevalence		X			X	
Ovarian cancer	Global	Female	Smoking Prevalence		X			X	
Ovarian cancer	Data Rich	Female	Cumulative Cigarettes (10 Years)		X			X	
Ovarian cancer	Global	Female	Cumulative Cigarettes (10 Years)		X			X	
Ovarian cancer	Data Rich	Female	Cumulative Cigarettes (20 Years)		X			X	
Ovarian cancer	Global	Female	Cumulative Cigarettes (20 Years)		X			X	
Ovarian cancer	Data Rich	Female	Diabetes Age-Standardized Prevalence (proportion)		X			X	
Ovarian cancer	Global	Female	Diabetes Age-Standardized Prevalence (proportion)		X			X	
Ovarian cancer	Data Rich	Female	Asbestos consumption (metric tons per year per capita)		X			X	
Ovarian cancer	Global	Female	Asbestos consumption (metric tons per year per capita)		X			X	
Ovarian cancer	Data Rich	Female	Total Fertility Rate		X			X	
Ovarian cancer	Global	Female	Total Fertility Rate		X			X	
Ovarian cancer	Data Rich	Female	energy unadjusted(kcal)		X			X	
Ovarian cancer	Global	Female	energy unadjusted(kcal)		X			X	
Ovarian cancer	Data Rich	Female	Contraception (Modern) Prevalence (proportion)		X			X	
Ovarian cancer	Global	Female	Contraception (Modern) Prevalence (proportion)		X			X	
Ovarian cancer	Data Rich	Female	Liters of alcohol consumed per capita	X			X		
Ovarian cancer	Global	Female	Liters of alcohol consumed per capita	X			X		
Ovarian cancer	Data Rich	Female	Log-transformed SEV scalar: Ovary C	X			X		
Ovarian cancer	Global	Female	Log-transformed SEV scalar: Ovary C	X			X		
Prostate cancer	Global	Male	Education (years per capita)			X			X
Prostate cancer	Data Rich	Male	Education (years per capita)			X			X
Prostate cancer	Global	Male	LDI (I$ per capita)			X			X
Prostate cancer	Data Rich	Male	LDI (I$ per capita)			X			X
Prostate cancer	Global	Male	Socio-demographic Index			X			X
Prostate cancer	Data Rich	Male	Socio-demographic Index			X			X
Prostate cancer	Global	Male	Healthcare access and quality index		X			X	
Prostate cancer	Data Rich	Male	Healthcare access and quality index		X			X	
Prostate cancer	Global	Male	Smoking Prevalence		X			X	
Prostate cancer	Data Rich	Male	Smoking Prevalence		X			X	
Prostate cancer	Global	Male	Log-transformed SEV scalar: Prostate C	X			X		
Prostate cancer	Data Rich	Male	Log-transformed SEV scalar: Prostate C	X			X		
Testicular cancer	Global	Male	Education (years per capita)			X			X
Testicular cancer	Data Rich	Male	Education (years per capita)			X			X
Testicular cancer	Global	Male	LDI (I$ per capita)			X			X
Testicular cancer	Data Rich	Male	LDI (I$ per capita)			X			X
Testicular cancer	Global	Male	Socio-demographic Index			X			X
Testicular cancer	Data Rich	Male	Socio-demographic Index			X			X
Testicular cancer	Global	Male	Healthcare access and quality index		X			X	
Testicular cancer	Data Rich	Male	Healthcare access and quality index		X			X	
Testicular cancer	Global	Male	Smoking Prevalence		X			X	
Testicular cancer	Data Rich	Male	Smoking Prevalence		X			X	
Testicular cancer	Global	Male	Cumulative Cigarettes (5 Years)		X			X	
Testicular cancer	Data Rich	Male	Cumulative Cigarettes (5 Years)		X			X	
Testicular cancer	Global	Male	Cumulative Cigarettes (10 Years)		X			X	
Testicular cancer	Data Rich	Male	Cumulative Cigarettes (10 Years)		X			X	
Testicular cancer	Global	Male	Cumulative Cigarettes (15 Years)		X			X	
Testicular cancer	Data Rich	Male	Cumulative Cigarettes (15 Years)		X			X	
Testicular cancer	Global	Male	Tobacco (cigarettes per capita)		X			X	
Testicular cancer	Data Rich	Male	Tobacco (cigarettes per capita)		X			X	
Testicular cancer	Global	Male	Cumulative Cigarettes (20 Years)		X			X	
Testicular cancer	Data Rich	Male	Cumulative Cigarettes (20 Years)		X			X	
Testicular cancer	Global	Male	Age- and sex-specific SEV for Low fruit		X			X	
Testicular cancer	Data Rich	Male	Age- and sex-specific SEV for Low fruit		X			X	
Testicular cancer	Global	Male	Age- and sex-specific SEV for Low vegetables		X			X	
Testicular cancer	Data Rich	Male	Age- and sex-specific SEV for Low vegetables		X			X	
Kidney cancer	Global	Female	Education (years per capita)			X			X
Kidney cancer	Data Rich	Female	Education (years per capita)			X			X
Kidney cancer	Global	Female	LDI (I$ per capita)			X			X
Kidney cancer	Data Rich	Female	LDI (I$ per capita)			X			X
Kidney cancer	Global	Female	Socio-demographic Index			X			X
Kidney cancer	Data Rich	Female	Socio-demographic Index			X			X
Kidney cancer	Global	Female	Healthcare access and quality index		X			X	
Kidney cancer	Data Rich	Female	Healthcare access and quality index		X			X	
Kidney cancer	Global	Female	Systolic Blood Pressure (mmHg)		X			X	
Kidney cancer	Data Rich	Female	Systolic Blood Pressure (mmHg)		X			X	
Kidney cancer	Global	Female	Liters of alcohol consumed per capita		X			X	
Kidney cancer	Data Rich	Female	Liters of alcohol consumed per capita		X			X	
Kidney cancer	Global	Female	Diabetes Age-Standardized Prevalence (proportion)		X			X	
Kidney cancer	Data Rich	Female	Diabetes Age-Standardized Prevalence (proportion)		X			X	
Kidney cancer	Global	Female	Mean BMI	X			X		
Kidney cancer	Data Rich	Female	Mean BMI	X			X		
Kidney cancer	Global	Female	Cumulative Cigarettes (10 Years)	X			X		
Kidney cancer	Data Rich	Female	Cumulative Cigarettes (10 Years)	X			X		
Kidney cancer	Global	Female	Tobacco (cigarettes per capita)	X			X		
Kidney cancer	Data Rich	Female	Tobacco (cigarettes per capita)	X			X		
Kidney cancer	Global	Female	Log-transformed SEV scalar: Kidney C	X			X		
Kidney cancer	Data Rich	Female	Log-transformed SEV scalar: Kidney C	X			X		
Kidney cancer	Global	Male	Education (years per capita)			X			X
Kidney cancer	Data Rich	Male	Education (years per capita)			X			X
Kidney cancer	Global	Male	LDI (I$ per capita)			X			X

Comparison of GBD 2019 and GBD 2021 covariates and level of covariates used in cause of death modeling									
**Cause**	**Model Version Type**	**Sex**	**Covariate**	**Level 1 GBD 2019**	**Level 2 GBD 2019**	**Level 3 GBD 2019**	**Level 1 GBD 2021**	**Level 2 GBD 2021**	**Level 3 GBD 2021**
Kidney cancer	Data Rich	Male	LDI (I$ per capita)			X			X
Kidney cancer	Global	Male	Socio-demographic Index			X			X
Kidney cancer	Data Rich	Male	Socio-demographic Index			X			X
Kidney cancer	Global	Male	Healthcare access and quality index		X			X	
Kidney cancer	Data Rich	Male	Healthcare access and quality index		X			X	
Kidney cancer	Global	Male	Systolic Blood Pressure (mmHg)		X			X	
Kidney cancer	Data Rich	Male	Systolic Blood Pressure (mmHg)		X			X	
Kidney cancer	Global	Male	Liters of alcohol consumed per capita		X			X	
Kidney cancer	Data Rich	Male	Liters of alcohol consumed per capita		X			X	
Kidney cancer	Global	Male	Diabetes Age-Standardized Prevalence (proportion)		X			X	
Kidney cancer	Data Rich	Male	Diabetes Age-Standardized Prevalence (proportion)		X			X	
Kidney cancer	Global	Male	Mean BMI	X			X		
Kidney cancer	Data Rich	Male	Mean BMI	X			X		
Kidney cancer	Global	Male	Cumulative Cigarettes (10 Years)	X			X		
Kidney cancer	Data Rich	Male	Cumulative Cigarettes (10 Years)	X			X		
Kidney cancer	Global	Male	Tobacco (cigarettes per capita)	X			X		
Kidney cancer	Data Rich	Male	Tobacco (cigarettes per capita)	X			X		
Kidney cancer	Global	Male	Log-transformed SEV scalar: Kidney C	X			X		
Kidney cancer	Data Rich	Male	Log-transformed SEV scalar: Kidney C	X			X		
Bladder cancer	Global	Female	LDI (I$ per capita)			X			X
Bladder cancer	Data Rich	Female	LDI (I$ per capita)			X			X
Bladder cancer	Global	Female	Age- and sex-specific SEV for Low fruit			X			X
Bladder cancer	Data Rich	Female	Age- and sex-specific SEV for Low fruit			X			X
Bladder cancer	Global	Female	Socio-demographic Index			X			X
Bladder cancer	Data Rich	Female	Socio-demographic Index			X			X
Bladder cancer	Global	Female	Healthcare access and quality index		X			X	
Bladder cancer	Data Rich	Female	Healthcare access and quality index		X			X	
Bladder cancer	Global	Female	Liters of alcohol consumed per capita		X			X	
Bladder cancer	Data Rich	Female	Liters of alcohol consumed per capita		X			X	
Bladder cancer	Global	Female	Cumulative Cigarettes (10 Years)		X			X	
Bladder cancer	Data Rich	Female	Cumulative Cigarettes (10 Years)		X			X	
Bladder cancer	Global	Female	Diabetes Fasting Plasma Glucose (mmol/L), age-standardized 25+		X			X	
Bladder cancer	Data Rich	Female	Diabetes Fasting Plasma Glucose (mmol/L), age-standardized 25+		X			X	
Bladder cancer	Global	Female	Age- and sex-specific SEV for Low vegetables		X			X	
Bladder cancer	Data Rich	Female	Age- and sex-specific SEV for Low vegetables		X			X	
Bladder cancer	Global	Female	Smoking Prevalence	X			X		
Bladder cancer	Data Rich	Female	Smoking Prevalence	X			X		
Bladder cancer	Global	Female	Log-transformed SEV scalar: Bladder C	X			X		
Bladder cancer	Data Rich	Female	Log-transformed SEV scalar: Bladder C	X			X		
Bladder cancer	Global	Female	Schistosomiasis Prevalence Results				X		
Bladder cancer	Data Rich	Female	Schistosomiasis Prevalence Results				X		
Bladder cancer	Global	Male	LDI (I$ per capita)			X			X
Bladder cancer	Data Rich	Male	LDI (I$ per capita)			X			X
Bladder cancer	Global	Male	Age- and sex-specific SEV for Low fruit			X			X
Bladder cancer	Data Rich	Male	Age- and sex-specific SEV for Low fruit			X			X
Bladder cancer	Global	Male	Socio-demographic Index			X			X
Bladder cancer	Data Rich	Male	Socio-demographic Index			X			X
Bladder cancer	Global	Male	Healthcare access and quality index		X			X	
Bladder cancer	Data Rich	Male	Healthcare access and quality index		X			X	
Bladder cancer	Global	Male	Liters of alcohol consumed per capita		X			X	
Bladder cancer	Data Rich	Male	Liters of alcohol consumed per capita		X			X	
Bladder cancer	Global	Male	Cumulative Cigarettes (10 Years)		X			X	
Bladder cancer	Data Rich	Male	Cumulative Cigarettes (10 Years)		X			X	
Bladder cancer	Global	Male	Diabetes Fasting Plasma Glucose (mmol/L), age-standardized 25+		X			X	
Bladder cancer	Data Rich	Male	Diabetes Fasting Plasma Glucose (mmol/L), age-standardized 25+		X			X	
Bladder cancer	Global	Male	Age- and sex-specific SEV for Low vegetables		X			X	
Bladder cancer	Data Rich	Male	Age- and sex-specific SEV for Low vegetables		X			X	
Bladder cancer	Global	Male	Smoking Prevalence	X			X		
Bladder cancer	Data Rich	Male	Smoking Prevalence	X			X		
Bladder cancer	Global	Male	Log-transformed SEV scalar: Bladder C	X			X		
Bladder cancer	Data Rich	Male	Log-transformed SEV scalar: Bladder C	X			X		
Bladder cancer	Global	Male	Schistosomiasis Prevalence Results				X		
Bladder cancer	Data Rich	Male	Schistosomiasis Prevalence Results				X		
Brain and nervous system cancer	Data Rich	Female	Education (years per capita)			X			X
Brain and nervous system cancer	Global	Female	Education (years per capita)			X			X
Brain and nervous system cancer	Data Rich	Female	LDI (I$ per capita)			X			X
Brain and nervous system cancer	Global	Female	LDI (I$ per capita)			X			X
Brain and nervous system cancer	Data Rich	Female	Socio-demographic Index			X			X
Brain and nervous system cancer	Global	Female	Socio-demographic Index			X			X
Brain and nervous system cancer	Data Rich	Female	Healthcare access and quality index		X			X	
Brain and nervous system cancer	Global	Female	Healthcare access and quality index		X			X	
Brain and nervous system cancer	Data Rich	Female	Low-Density Lipoprotein (mmol/L)		X			X	
Brain and nervous system cancer	Global	Female	Low-Density Lipoprotein (mmol/L)		X			X	
Brain and nervous system cancer	Data Rich	Female	Systolic Blood Pressure (mmHg)		X			X	
Brain and nervous system cancer	Global	Female	Systolic Blood Pressure (mmHg)		X			X	
Brain and nervous system cancer	Data Rich	Female	Age- and sex-specific SEV for Low fruit		X			X	
Brain and nervous system cancer	Global	Female	Age- and sex-specific SEV for Low fruit		X			X	
Brain and nervous system cancer	Data Rich	Female	Age- and sex-specific SEV for Low vegetables		X			X	
Brain and nervous system cancer	Global	Female	Age- and sex-specific SEV for Low vegetables		X			X	
Brain and nervous system cancer	Data Rich	Female	Age- and sex-specific SEV for High red meat		X			X	
Brain and nervous system cancer	Global	Female	Age- and sex-specific SEV for High red meat		X			X	
Brain and nervous system cancer	Data Rich	Female	Smoking Prevalence	X			X		
Brain and nervous system cancer	Global	Female	Smoking Prevalence	X			X		
Brain and nervous system cancer	Data Rich	Female	Liters of alcohol consumed per capita	X			X		
Brain and nervous system cancer	Global	Female	Liters of alcohol consumed per capita	X			X		
Brain and nervous system cancer	Data Rich	Female	Cumulative Cigarettes (10 Years)	X			X		
Brain and nervous system cancer	Global	Female	Cumulative Cigarettes (10 Years)	X			X		
Brain and nervous system cancer	Data Rich	Male	Education (years per capita)			X			X
Brain and nervous system cancer	Global	Male	Education (years per capita)			X			X
Brain and nervous system cancer	Data Rich	Male	LDI (I$ per capita)			X			X
Brain and nervous system cancer	Global	Male	LDI (I$ per capita)			X			X
Brain and nervous system cancer	Data Rich	Male	Socio-demographic Index			X			X
Brain and nervous system cancer	Global	Male	Socio-demographic Index			X			X
Brain and nervous system cancer	Data Rich	Male	Healthcare access and quality index		X			X	
Brain and nervous system cancer	Global	Male	Healthcare access and quality index		X			X	
Brain and nervous system cancer	Data Rich	Male	Low-Density Lipoprotein (mmol/L)		X			X	
Brain and nervous system cancer	Global	Male	Low-Density Lipoprotein (mmol/L)		X			X	
Brain and nervous system cancer	Data Rich	Male	Systolic Blood Pressure (mmHg)		X			X	
Brain and nervous system cancer	Global	Male	Systolic Blood Pressure (mmHg)		X			X	
Brain and nervous system cancer	Data Rich	Male	Age- and sex-specific SEV for Low fruit		X			X	
Brain and nervous system cancer	Global	Male	Age- and sex-specific SEV for Low fruit		X			X	
Brain and nervous system cancer	Data Rich	Male	Age- and sex-specific SEV for Low vegetables		X			X	
Brain and nervous system cancer	Global	Male	Age- and sex-specific SEV for Low vegetables		X			X	
Brain and nervous system cancer	Data Rich	Male	Age- and sex-specific SEV for High red meat		X			X	
Brain and nervous system cancer	Global	Male	Age- and sex-specific SEV for High red meat		X			X	
Brain and nervous system cancer	Data Rich	Male	Smoking Prevalence	X			X		
Brain and nervous system cancer	Global	Male	Smoking Prevalence	X			X		
Brain and nervous system cancer	Data Rich	Male	Liters of alcohol consumed per capita	X			X		
Brain and nervous system cancer	Global	Male	Liters of alcohol consumed per capita	X			X		
Brain and nervous system cancer	Data Rich	Male	Cumulative Cigarettes (10 Years)	X			X		
Brain and nervous system cancer	Global	Male	Cumulative Cigarettes (10 Years)	X			X		
Retinoblastoma	Global	Female	Education (years per capita)						X
Retinoblastoma	Data Rich	Female	Education (years per capita)						X
Retinoblastoma	Global	Female	LDI (I$ per capita)						X
Retinoblastoma	Data Rich	Female	LDI (I$ per capita)						X
Retinoblastoma	Global	Female	Maternal care and immunization						X
Retinoblastoma	Data Rich	Female	Maternal care and immunization						X
Retinoblastoma	Global	Female	Socio-demographic Index						X
Retinoblastoma	Data Rich	Female	Socio-demographic Index						X
Retinoblastoma	Global	Female	Healthcare access and quality index					X	
Retinoblastoma	Data Rich	Female	Healthcare access and quality index					X	
Retinoblastoma	Global	Female	Universal health coverage					X	
Retinoblastoma	Data Rich	Female	Universal health coverage					X	
Retinoblastoma	Global	Male	Education (years per capita)						X
Retinoblastoma	Data Rich	Male	Education (years per capita)						X
Retinoblastoma	Global	Male	LDI (I$ per capita)						X
Retinoblastoma	Data Rich	Male	LDI (I$ per capita)						X
Retinoblastoma	Global	Male	Maternal care and immunization						X
Retinoblastoma	Data Rich	Male	Maternal care and immunization						X
Retinoblastoma	Global	Male	Socio-demographic Index						X
Retinoblastoma	Data Rich	Male	Socio-demographic Index						X
Retinoblastoma	Global	Male	Healthcare access and quality index					X	

Comparison of GBD 2019 and GBD 2021 covariates and level of covariates used in cause of death modeling

Cause	Model Version Type	Sex	Covariate	Level 1 GBD 2019	Level 2 GBD 2019	Level 3 GBD 2019	Level 1 GBD 2021	Level 2 GBD 2021	Level 3 GBD 2021
Retinoblastoma	Data Rich	Male	Healthcare access and quality index					X	
Retinoblastoma	Global	Male	Universal health coverage					X	
Retinoblastoma	Data Rich	Male	Universal health coverage					X	
Other eye cancers	Global	Female	Education (years per capita)						X
Other eye cancers	Data Rich	Female	Education (years per capita)						X
Other eye cancers	Global	Female	LDI (I$ per capita)						X
Other eye cancers	Data Rich	Female	LDI (I$ per capita)						X
Other eye cancers	Global	Female	Socio-demographic Index						X
Other eye cancers	Data Rich	Female	Socio-demographic Index						X
Other eye cancers	Global	Female	Healthcare access and quality index					X	
Other eye cancers	Data Rich	Female	Healthcare access and quality index					X	
Other eye cancers	Global	Female	Universal health coverage					X	
Other eye cancers	Data Rich	Female	Universal health coverage					X	
Other eye cancers	Global	Female	Age-standardized melanoma					X	
Other eye cancers	Data Rich	Female	Age-standardized melanoma					X	
Other eye cancers	Global	Male	Education (years per capita)						X
Other eye cancers	Data Rich	Male	Education (years per capita)						X
Other eye cancers	Global	Male	LDI (I$ per capita)						X
Other eye cancers	Data Rich	Male	LDI (I$ per capita)						X
Other eye cancers	Global	Male	Socio-demographic Index						X
Other eye cancers	Data Rich	Male	Socio-demographic Index						X
Other eye cancers	Global	Male	Healthcare access and quality index					X	
Other eye cancers	Data Rich	Male	Healthcare access and quality index					X	
Other eye cancers	Global	Male	Universal health coverage					X	
Other eye cancers	Data Rich	Male	Universal health coverage					X	
Other eye cancers	Global	Male	Age-standardized melanoma					X	
Other eye cancers	Data Rich	Male	Age-standardized melanoma					X	
Neuroblastoma and other peripheral nervous cell tumours	Global	Female	Education (years per capita)						X
Neuroblastoma and other peripheral nervous cell tumours	Data Rich	Female	Education (years per capita)						X
Neuroblastoma and other peripheral nervous cell tumours	Global	Female	LDI (I$ per capita)						X
Neuroblastoma and other peripheral nervous cell tumours	Data Rich	Female	LDI (I$ per capita)						X
Neuroblastoma and other peripheral nervous cell tumours	Global	Female	Healthcare access and quality index						X
Neuroblastoma and other peripheral nervous cell tumours	Data Rich	Female	Healthcare access and quality index						X
Neuroblastoma and other peripheral nervous cell tumours	Global	Female	Smoking Prevalence						X
Neuroblastoma and other peripheral nervous cell tumours	Data Rich	Female	Smoking Prevalence						X
Neuroblastoma and other peripheral nervous cell tumours	Global	Female	Maternal care and immunization						X
Neuroblastoma and other peripheral nervous cell tumours	Data Rich	Female	Maternal care and immunization						X
Neuroblastoma and other peripheral nervous cell tumours	Global	Female	Socio-demographic Index						X
Neuroblastoma and other peripheral nervous cell tumours	Data Rich	Female	Socio-demographic Index						X
Neuroblastoma and other peripheral nervous cell tumours	Global	Female	Universal health coverage						X
Neuroblastoma and other peripheral nervous cell tumours	Data Rich	Female	Universal health coverage						X
Neuroblastoma and other peripheral nervous cell tumours	Global	Female	Health worker density						X
Neuroblastoma and other peripheral nervous cell tumours	Data Rich	Female	Health worker density						X
Neuroblastoma and other peripheral nervous cell tumours	Global	Male	Education (years per capita)						X
Neuroblastoma and other peripheral nervous cell tumours	Data Rich	Male	Education (years per capita)						X
Neuroblastoma and other peripheral nervous cell tumours	Global	Male	LDI (I$ per capita)						X
Neuroblastoma and other peripheral nervous cell tumours	Data Rich	Male	LDI (I$ per capita)						X
Neuroblastoma and other peripheral nervous cell tumours	Global	Male	Healthcare access and quality index						X
Neuroblastoma and other peripheral nervous cell tumours	Data Rich	Male	Healthcare access and quality index						X
Neuroblastoma and other peripheral nervous cell tumours	Global	Male	Smoking Prevalence						X
Neuroblastoma and other peripheral nervous cell tumours	Data Rich	Male	Smoking Prevalence						X
Neuroblastoma and other peripheral nervous cell tumours	Global	Male	Maternal care and immunization						X
Neuroblastoma and other peripheral nervous cell tumours	Data Rich	Male	Maternal care and immunization						X
Neuroblastoma and other peripheral nervous cell tumours	Global	Male	Socio-demographic Index						X
Neuroblastoma and other peripheral nervous cell tumours	Data Rich	Male	Socio-demographic Index						X
Neuroblastoma and other peripheral nervous cell tumours	Global	Male	Universal health coverage						X
Neuroblastoma and other peripheral nervous cell tumours	Data Rich	Male	Universal health coverage						X
Neuroblastoma and other peripheral nervous cell tumours	Global	Male	Health worker density						X
Neuroblastoma and other peripheral nervous cell tumours	Data Rich	Male	Health worker density						X
Thyroid cancer	Global	Female	Education (years per capita)			X			X
Thyroid cancer	Data Rich	Female	Education (years per capita)			X			X
Thyroid cancer	Global	Female	LDI (I$ per capita)			X			X
Thyroid cancer	Data Rich	Female	LDI (I$ per capita)			X			X
Thyroid cancer	Global	Female	Sanitation (proportion with access)			X			X
Thyroid cancer	Data Rich	Female	Sanitation (proportion with access)			X			X
Thyroid cancer	Global	Female	Improved Water Source (proportion with access)			X			X
Thyroid cancer	Data Rich	Female	Improved Water Source (proportion with access)			X			X
Thyroid cancer	Global	Female	Age- and sex-specific SEV for Low fruit			X			X
Thyroid cancer	Data Rich	Female	Age- and sex-specific SEV for Low fruit			X			X
Thyroid cancer	Global	Female	Socio-demographic Index			X			X
Thyroid cancer	Data Rich	Female	Socio-demographic Index			X			X
Thyroid cancer	Global	Female	Healthcare access and quality index		X			X	
Thyroid cancer	Data Rich	Female	Healthcare access and quality index		X			X	
Thyroid cancer	Global	Female	Mean BMI		X			X	
Thyroid cancer	Data Rich	Female	Mean BMI		X			X	
Thyroid cancer	Global	Female	Tobacco (cigarettes per capita)		X			X	
Thyroid cancer	Data Rich	Female	Tobacco (cigarettes per capita)		X			X	
Thyroid cancer	Global	Female	Age- and sex-specific SEV for Low vegetables		X			X	
Thyroid cancer	Data Rich	Female	Age- and sex-specific SEV for Low vegetables		X			X	
Thyroid cancer	Global	Female	Age- and sex-specific SEV for High red meat		X			X	
Thyroid cancer	Data Rich	Female	Age- and sex-specific SEV for High red meat		X			X	
Thyroid cancer	Global	Female	Liters of alcohol consumed per capita	X			X		
Thyroid cancer	Data Rich	Female	Liters of alcohol consumed per capita	X			X		
Thyroid cancer	Global	Female	Log-transformed SEV scalar: Thyroid C	X			X		
Thyroid cancer	Data Rich	Female	Log-transformed SEV scalar: Thyroid C	X			X		
Thyroid cancer	Global	Male	Education (years per capita)			X			X
Thyroid cancer	Data Rich	Male	Education (years per capita)			X			X
Thyroid cancer	Global	Male	LDI (I$ per capita)			X			X
Thyroid cancer	Data Rich	Male	LDI (I$ per capita)			X			X
Thyroid cancer	Global	Male	Sanitation (proportion with access)			X			X
Thyroid cancer	Data Rich	Male	Sanitation (proportion with access)			X			X
Thyroid cancer	Global	Male	Improved Water Source (proportion with access)			X			X
Thyroid cancer	Data Rich	Male	Improved Water Source (proportion with access)			X			X
Thyroid cancer	Global	Male	Age- and sex-specific SEV for Low fruit			X			X
Thyroid cancer	Data Rich	Male	Age- and sex-specific SEV for Low fruit			X			X
Thyroid cancer	Global	Male	Socio-demographic Index			X			X
Thyroid cancer	Data Rich	Male	Socio-demographic Index			X			X
Thyroid cancer	Global	Male	Healthcare access and quality index		X			X	
Thyroid cancer	Data Rich	Male	Healthcare access and quality index		X			X	
Thyroid cancer	Global	Male	Mean BMI		X			X	
Thyroid cancer	Data Rich	Male	Mean BMI		X			X	
Thyroid cancer	Global	Male	Tobacco (cigarettes per capita)		X			X	
Thyroid cancer	Data Rich	Male	Tobacco (cigarettes per capita)		X			X	
Thyroid cancer	Global	Male	Age- and sex-specific SEV for Low vegetables		X			X	
Thyroid cancer	Data Rich	Male	Age- and sex-specific SEV for Low vegetables		X			X	
Thyroid cancer	Global	Male	Age- and sex-specific SEV for High red meat		X			X	
Thyroid cancer	Data Rich	Male	Age- and sex-specific SEV for High red meat		X			X	
Thyroid cancer	Global	Male	Liters of alcohol consumed per capita	X			X		
Thyroid cancer	Data Rich	Male	Liters of alcohol consumed per capita	X			X		
Thyroid cancer	Global	Male	Log-transformed SEV scalar: Thyroid C	X			X		
Thyroid cancer	Data Rich	Male	Log-transformed SEV scalar: Thyroid C	X			X		
Mesothelioma	Global	Female	Education (years per capita)			X			X
Mesothelioma	Data Rich	Female	Education (years per capita)			X			X
Mesothelioma	Global	Female	LDI (I$ per capita)			X			X
Mesothelioma	Data Rich	Female	LDI (I$ per capita)			X			X
Mesothelioma	Global	Female	Socio-demographic Index			X			X
Mesothelioma	Data Rich	Female	Socio-demographic Index			X			X
Mesothelioma	Global	Female	Healthcare access and quality index		X			X	
Mesothelioma	Data Rich	Female	Healthcare access and quality index		X			X	
Mesothelioma	Global	Female	Cumulative Cigarettes (5 Years)		X			X	

Comparison of GBD 2019 and GBD 2021 covariates and level of covariates used in cause of death modeling

Cause	Model Version Type	Sex	Covariate	Level 1 GBD 2019	Level 2 GBD 2019	Level 3 GBD 2019	Level 1 GBD 2021	Level 2 GBD 2021	Level 3 GBD 2021
Mesothelioma	Data Rich	Female	Cumulative Cigarettes (5 Years)		X			X	
Mesothelioma	Global	Female	Indoor Air Pollution (All Cooking Fuels)		X			X	
Mesothelioma	Data Rich	Female	Indoor Air Pollution (All Cooking Fuels)		X			X	
Mesothelioma	Global	Female	Population Density (over 1000 ppl/sqkm, proportion)		X			X	
Mesothelioma	Data Rich	Female	Population Density (over 1000 ppl/sqkm, proportion)		X			X	
Mesothelioma	Global	Female	Gold production (binary)		X			X	
Mesothelioma	Data Rich	Female	Gold production (binary)		X			X	
Mesothelioma	Global	Female	Smoking Prevalence	X			X		
Mesothelioma	Data Rich	Female	Smoking Prevalence	X			X		
Mesothelioma	Global	Female	Asbestos consumption (metric tons per year per capita)	X			X		
Mesothelioma	Data Rich	Female	Asbestos consumption (metric tons per year per capita)	X			X		
Mesothelioma	Global	Female	Age- and sex-specific SEV for Occupational asbestos				X		
Mesothelioma	Data Rich	Female	Age- and sex-specific SEV for Occupational asbestos				X		
Mesothelioma	Global	Female	Age-standardized SEV for Occupational asbestos				X		
Mesothelioma	Data Rich	Female	Age-standardized SEV for Occupational asbestos				X		
Mesothelioma	Global	Male	Education (years per capita)			X			X
Mesothelioma	Data Rich	Male	Education (years per capita)			X			X
Mesothelioma	Global	Male	LDI (I$ per capita)			X			X
Mesothelioma	Data Rich	Male	LDI (I$ per capita)			X			X
Mesothelioma	Global	Male	Socio-demographic Index			X			X
Mesothelioma	Data Rich	Male	Socio-demographic Index			X			X
Mesothelioma	Global	Male	Healthcare access and quality index		X			X	
Mesothelioma	Data Rich	Male	Healthcare access and quality index		X			X	
Mesothelioma	Global	Male	Cumulative Cigarettes (5 Years)		X			X	
Mesothelioma	Data Rich	Male	Cumulative Cigarettes (5 Years)		X			X	
Mesothelioma	Global	Male	Indoor Air Pollution (All Cooking Fuels)		X			X	
Mesothelioma	Data Rich	Male	Indoor Air Pollution (All Cooking Fuels)		X			X	
Mesothelioma	Global	Male	Population Density (over 1000 ppl/sqkm, proportion)		X			X	
Mesothelioma	Data Rich	Male	Population Density (over 1000 ppl/sqkm, proportion)		X			X	
Mesothelioma	Global	Male	Gold production (binary)		X			X	
Mesothelioma	Data Rich	Male	Gold production (binary)		X			X	
Mesothelioma	Global	Male	Smoking Prevalence	X			X		
Mesothelioma	Data Rich	Male	Smoking Prevalence	X			X		
Mesothelioma	Global	Male	Asbestos consumption (metric tons per year per capita)	X			X		
Mesothelioma	Data Rich	Male	Asbestos consumption (metric tons per year per capita)	X			X		
Mesothelioma	Global	Male	Age- and sex-specific SEV for Occupational asbestos				X		
Mesothelioma	Data Rich	Male	Age- and sex-specific SEV for Occupational asbestos				X		
Mesothelioma	Global	Male	Age-standardized SEV for Occupational asbestos				X		
Mesothelioma	Data Rich	Male	Age-standardized SEV for Occupational asbestos				X		
Hodgkin lymphoma	Data Rich	Female	Education (years per capita)			X			X
Hodgkin lymphoma	Global	Female	Education (years per capita)			X			X
Hodgkin lymphoma	Data Rich	Female	LDI (I$ per capita)			X			X
Hodgkin lymphoma	Global	Female	LDI (I$ per capita)			X			X
Hodgkin lymphoma	Data Rich	Female	Socio-demographic Index			X			X
Hodgkin lymphoma	Global	Female	Socio-demographic Index			X			X
Hodgkin lymphoma	Data Rich	Female	Healthcare access and quality index		X			X	
Hodgkin lymphoma	Global	Female	Healthcare access and quality index		X			X	
Hodgkin lymphoma	Data Rich	Male	Education (years per capita)			X			X
Hodgkin lymphoma	Global	Male	Education (years per capita)			X			X
Hodgkin lymphoma	Data Rich	Male	LDI (I$ per capita)			X			X
Hodgkin lymphoma	Global	Male	LDI (I$ per capita)			X			X
Hodgkin lymphoma	Data Rich	Male	Socio-demographic Index			X			X
Hodgkin lymphoma	Global	Male	Socio-demographic Index			X			X
Hodgkin lymphoma	Data Rich	Male	Healthcare access and quality index		X			X	
Hodgkin lymphoma	Global	Male	Healthcare access and quality index		X			X	
Non-Hodgkin's lymphoma	Global	Female	LDI (I$ per capita)			X			X
Non-Hodgkin's lymphoma	Data Rich	Female	LDI (I$ per capita)			X			X
Non-Hodgkin's lymphoma	Global	Female	Socio-demographic Index			X			X
Non-Hodgkin's lymphoma	Data Rich	Female	Socio-demographic Index			X			X
Non-Hodgkin's lymphoma	Global	Female	Total Fertility Rate			X			X
Non-Hodgkin's lymphoma	Data Rich	Female	Total Fertility Rate			X			X
Non-Hodgkin's lymphoma	Global	Female	Healthcare access and quality index		X			X	
Non-Hodgkin's lymphoma	Data Rich	Female	Healthcare access and quality index		X			X	
Non-Hodgkin's lymphoma	Global	Female	Mean BMI		X			X	
Non-Hodgkin's lymphoma	Data Rich	Female	Mean BMI		X			X	
Non-Hodgkin's lymphoma	Global	Female	Liters of alcohol consumed per capita		X			X	
Non-Hodgkin's lymphoma	Data Rich	Female	Liters of alcohol consumed per capita		X			X	
Non-Hodgkin's lymphoma	Global	Female	Cumulative Cigarettes (5 Years)		X			X	
Non-Hodgkin's lymphoma	Data Rich	Female	Cumulative Cigarettes (5 Years)		X			X	
Non-Hodgkin's lymphoma	Global	Female	Cumulative Cigarettes (10 Years)		X			X	
Non-Hodgkin's lymphoma	Data Rich	Female	Cumulative Cigarettes (10 Years)		X			X	
Non-Hodgkin's lymphoma	Global	Female	Cumulative Cigarettes (15 Years)		X			X	
Non-Hodgkin's lymphoma	Data Rich	Female	Cumulative Cigarettes (15 Years)		X			X	
Non-Hodgkin's lymphoma	Global	Female	Cumulative Cigarettes (20 Years)		X			X	
Non-Hodgkin's lymphoma	Data Rich	Female	Cumulative Cigarettes (20 Years)		X			X	
Non-Hodgkin's lymphoma	Global	Female	Education (years per capita)						X
Non-Hodgkin's lymphoma	Data Rich	Female	Education (years per capita)						X
Non-Hodgkin's lymphoma	Global	Female	Log-transformed SEV scalar: HIV						X
Non-Hodgkin's lymphoma	Data Rich	Female	Log-transformed SEV scalar: HIV						X
Non-Hodgkin's lymphoma	Global	Female	Log-transformed age-standardized SEV scalar: HIV						X
Non-Hodgkin's lymphoma	Data Rich	Female	Log-transformed age-standardized SEV scalar: HIV						X
Non-Hodgkin's lymphoma	Global	Female	Universal health coverage					X	
Non-Hodgkin's lymphoma	Data Rich	Female	Universal health coverage					X	
Non-Hodgkin's lymphoma	Global	Male	LDI (I$ per capita)			X			X
Non-Hodgkin's lymphoma	Data Rich	Male	LDI (I$ per capita)			X			X
Non-Hodgkin's lymphoma	Global	Male	Socio-demographic Index			X			X
Non-Hodgkin's lymphoma	Data Rich	Male	Socio-demographic Index			X			X
Non-Hodgkin's lymphoma	Global	Male	Healthcare access and quality index		X			X	
Non-Hodgkin's lymphoma	Data Rich	Male	Healthcare access and quality index		X			X	
Non-Hodgkin's lymphoma	Global	Male	Mean BMI		X			X	
Non-Hodgkin's lymphoma	Data Rich	Male	Mean BMI		X			X	
Non-Hodgkin's lymphoma	Global	Male	Liters of alcohol consumed per capita		X			X	
Non-Hodgkin's lymphoma	Data Rich	Male	Liters of alcohol consumed per capita		X			X	
Non-Hodgkin's lymphoma	Global	Male	Cumulative Cigarettes (5 Years)		X			X	
Non-Hodgkin's lymphoma	Data Rich	Male	Cumulative Cigarettes (5 Years)		X			X	
Non-Hodgkin's lymphoma	Global	Male	Cumulative Cigarettes (10 Years)		X			X	
Non-Hodgkin's lymphoma	Data Rich	Male	Cumulative Cigarettes (10 Years)		X			X	
Non-Hodgkin's lymphoma	Global	Male	Cumulative Cigarettes (15 Years)		X			X	
Non-Hodgkin's lymphoma	Data Rich	Male	Cumulative Cigarettes (15 Years)		X			X	
Non-Hodgkin's lymphoma	Global	Male	Cumulative Cigarettes (20 Years)		X			X	
Non-Hodgkin's lymphoma	Data Rich	Male	Cumulative Cigarettes (20 Years)		X			X	
Non-Hodgkin's lymphoma	Global	Male	Education (years per capita)						X
Non-Hodgkin's lymphoma	Data Rich	Male	Education (years per capita)						X
Non-Hodgkin's lymphoma	Global	Male	Log-transformed SEV scalar: HIV						X
Non-Hodgkin's lymphoma	Data Rich	Male	Log-transformed SEV scalar: HIV						X
Non-Hodgkin's lymphoma	Global	Male	Log-transformed age-standardized SEV scalar: HIV						X
Non-Hodgkin's lymphoma	Data Rich	Male	Log-transformed age-standardized SEV scalar: HIV						X
Non-Hodgkin's lymphoma	Global	Male	Universal health coverage					X	
Non-Hodgkin's lymphoma	Data Rich	Male	Universal health coverage					X	
Other non-Hodgkin lymphoma	Data Rich	Female	Education (years per capita)						X
Other non-Hodgkin lymphoma	Global	Female	Education (years per capita)						X
Other non-Hodgkin lymphoma	Data Rich	Female	LDI (I$ per capita)						X
Other non-Hodgkin lymphoma	Global	Female	LDI (I$ per capita)						X
Other non-Hodgkin lymphoma	Global	Female	Total Fertility Rate						X
Other non-Hodgkin lymphoma	Data Rich	Female	Socio-demographic Index						X
Other non-Hodgkin lymphoma	Global	Female	Socio-demographic Index						X
Other non-Hodgkin lymphoma	Data Rich	Female	Log-transformed SEV scalar: HIV						X
Other non-Hodgkin lymphoma	Global	Female	Log-transformed SEV scalar: HIV						X
Other non-Hodgkin lymphoma	Data Rich	Female	Log-transformed age-standardized SEV scalar: HIV						X
Other non-Hodgkin lymphoma	Global	Female	Log-transformed age-standardized SEV scalar: HIV						X
Other non-Hodgkin lymphoma	Data Rich	Female	Total Fertility Rate						X
Other non-Hodgkin lymphoma	Data Rich	Female	Healthcare access and quality index					X	
Other non-Hodgkin lymphoma	Global	Female	Healthcare access and quality index					X	
Other non-Hodgkin lymphoma	Data Rich	Female	Mean BMI					X	
Other non-Hodgkin lymphoma	Global	Female	Mean BMI					X	
Other non-Hodgkin lymphoma	Data Rich	Female	Liters of alcohol consumed per capita					X	
Other non-Hodgkin lymphoma	Global	Female	Liters of alcohol consumed per capita					X	
Other non-Hodgkin lymphoma	Data Rich	Female	Cumulative Cigarettes (5 Years)					X	
Other non-Hodgkin lymphoma	Global	Female	Cumulative Cigarettes (5 Years)					X	
Other non-Hodgkin lymphoma	Data Rich	Female	Cumulative Cigarettes (10 Years)					X	

Comparison of GBD 2019 and GBD 2021 covariates and level of covariates used in cause of death modeling

Cause	Model Version Type	Sex	Covariate	Level 1 GBD 2019	Level 2 GBD 2019	Level 3 GBD 2019	Level 1 GBD 2021	Level 2 GBD 2021	Level 3 GBD 2021
Other non-Hodgkin lymphoma	Global	Female	Cumulative Cigarettes (10 Years)					X	
Other non-Hodgkin lymphoma	Data Rich	Female	Cumulative Cigarettes (15 Years)					X	
Other non-Hodgkin lymphoma	Global	Female	Cumulative Cigarettes (15 Years)					X	
Other non-Hodgkin lymphoma	Data Rich	Female	Cumulative Cigarettes (20 Years)					X	
Other non-Hodgkin lymphoma	Global	Female	Cumulative Cigarettes (20 Years)					X	
Other non-Hodgkin lymphoma	Data Rich	Female	Universal health coverage					X	
Other non-Hodgkin lymphoma	Global	Female	Universal health coverage					X	
Other non-Hodgkin lymphoma	Data Rich	Male	Education (years per capita)						X
Other non-Hodgkin lymphoma	Global	Male	Education (years per capita)						X
Other non-Hodgkin lymphoma	Data Rich	Male	LDI (I$ per capita)						X
Other non-Hodgkin lymphoma	Global	Male	LDI (I$ per capita)						X
Other non-Hodgkin lymphoma	Data Rich	Male	Socio-demographic Index						X
Other non-Hodgkin lymphoma	Global	Male	Socio-demographic Index						X
Other non-Hodgkin lymphoma	Data Rich	Male	Log-transformed SEV scalar: HIV						X
Other non-Hodgkin lymphoma	Global	Male	Log-transformed SEV scalar: HIV						X
Other non-Hodgkin lymphoma	Data Rich	Male	Log-transformed age-standardized SEV scalar: HIV						X
Other non-Hodgkin lymphoma	Global	Male	Log-transformed age-standardized SEV scalar: HIV						X
Other non-Hodgkin lymphoma	Data Rich	Male	Healthcare access and quality index					X	
Other non-Hodgkin lymphoma	Global	Male	Healthcare access and quality index					X	
Other non-Hodgkin lymphoma	Data Rich	Male	Mean BMI					X	
Other non-Hodgkin lymphoma	Global	Male	Mean BMI					X	
Other non-Hodgkin lymphoma	Data Rich	Male	Liters of alcohol consumed per capita					X	
Other non-Hodgkin lymphoma	Global	Male	Liters of alcohol consumed per capita					X	
Other non-Hodgkin lymphoma	Data Rich	Male	Cumulative Cigarettes (5 Years)					X	
Other non-Hodgkin lymphoma	Global	Male	Cumulative Cigarettes (5 Years)					X	
Other non-Hodgkin lymphoma	Data Rich	Male	Cumulative Cigarettes (10 Years)					X	
Other non-Hodgkin lymphoma	Global	Male	Cumulative Cigarettes (10 Years)					X	
Other non-Hodgkin lymphoma	Data Rich	Male	Cumulative Cigarettes (15 Years)					X	
Other non-Hodgkin lymphoma	Global	Male	Cumulative Cigarettes (15 Years)					X	
Other non-Hodgkin lymphoma	Data Rich	Male	Cumulative Cigarettes (20 Years)					X	
Other non-Hodgkin lymphoma	Global	Male	Cumulative Cigarettes (20 Years)					X	
Other non-Hodgkin lymphoma	Data Rich	Male	Universal health coverage					X	
Other non-Hodgkin lymphoma	Global	Male	Universal health coverage					X	
Multiple myeloma	Global	Female	Education (years per capita)			X			X
Multiple myeloma	Data Rich	Female	Education (years per capita)			X			X
Multiple myeloma	Global	Female	LDI (I$ per capita)			X			X
Multiple myeloma	Data Rich	Female	LDI (I$ per capita)			X			X
Multiple myeloma	Global	Female	Socio-demographic Index			X			X
Multiple myeloma	Data Rich	Female	Socio-demographic Index			X			X
Multiple myeloma	Global	Female	Healthcare access and quality index		X			X	
Multiple myeloma	Data Rich	Female	Healthcare access and quality index		X			X	
Multiple myeloma	Global	Female	Mean BMI		X			X	
Multiple myeloma	Data Rich	Female	Mean BMI		X			X	
Multiple myeloma	Global	Female	Sanitation (proportion with access)		X			X	
Multiple myeloma	Data Rich	Female	Sanitation (proportion with access)		X			X	
Multiple myeloma	Global	Female	Improved Water Source (proportion with access)		X			X	
Multiple myeloma	Data Rich	Female	Improved Water Source (proportion with access)		X			X	
Multiple myeloma	Global	Female	Age- and sex-specific SEV for Low fruit		X			X	
Multiple myeloma	Data Rich	Female	Age- and sex-specific SEV for Low fruit		X			X	
Multiple myeloma	Global	Female	Age- and sex-specific SEV for Low vegetables		X			X	
Multiple myeloma	Data Rich	Female	Age- and sex-specific SEV for Low vegetables		X			X	
Multiple myeloma	Global	Female	Age- and sex-specific SEV for High red meat		X			X	
Multiple myeloma	Data Rich	Female	Age- and sex-specific SEV for High red meat		X			X	
Multiple myeloma	Global	Female	Smoking Prevalence	X			X		
Multiple myeloma	Data Rich	Female	Smoking Prevalence	X			X		
Multiple myeloma	Global	Female	Liters of alcohol consumed per capita	X			X		
Multiple myeloma	Data Rich	Female	Liters of alcohol consumed per capita	X			X		
Multiple myeloma	Global	Female	Tobacco (cigarettes per capita)	X			X		
Multiple myeloma	Data Rich	Female	Tobacco (cigarettes per capita)	X			X		
Multiple myeloma	Global	Male	Education (years per capita)			X			X
Multiple myeloma	Data Rich	Male	Education (years per capita)			X			X
Multiple myeloma	Global	Male	LDI (I$ per capita)			X			X
Multiple myeloma	Data Rich	Male	LDI (I$ per capita)			X			X
Multiple myeloma	Global	Male	Socio-demographic Index			X			X
Multiple myeloma	Data Rich	Male	Socio-demographic Index			X			X
Multiple myeloma	Global	Male	Healthcare access and quality index		X			X	
Multiple myeloma	Data Rich	Male	Healthcare access and quality index		X			X	
Multiple myeloma	Global	Male	Mean BMI		X			X	
Multiple myeloma	Data Rich	Male	Mean BMI		X			X	
Multiple myeloma	Global	Male	Sanitation (proportion with access)		X			X	
Multiple myeloma	Data Rich	Male	Sanitation (proportion with access)		X			X	
Multiple myeloma	Global	Male	Improved Water Source (proportion with access)		X			X	
Multiple myeloma	Data Rich	Male	Improved Water Source (proportion with access)		X			X	
Multiple myeloma	Global	Male	Age- and sex-specific SEV for Low fruit		X			X	
Multiple myeloma	Data Rich	Male	Age- and sex-specific SEV for Low fruit		X			X	
Multiple myeloma	Global	Male	Age- and sex-specific SEV for Low vegetables		X			X	
Multiple myeloma	Data Rich	Male	Age- and sex-specific SEV for Low vegetables		X			X	
Multiple myeloma	Global	Male	Age- and sex-specific SEV for High red meat		X			X	
Multiple myeloma	Data Rich	Male	Age- and sex-specific SEV for High red meat		X			X	
Multiple myeloma	Global	Male	Smoking Prevalence	X			X		
Multiple myeloma	Data Rich	Male	Smoking Prevalence	X			X		
Multiple myeloma	Global	Male	Liters of alcohol consumed per capita	X			X		
Multiple myeloma	Data Rich	Male	Liters of alcohol consumed per capita	X			X		
Multiple myeloma	Global	Male	Tobacco (cigarettes per capita)	X			X		
Multiple myeloma	Data Rich	Male	Tobacco (cigarettes per capita)	X			X		
Leukaemia	Data Rich	Female	Education (years per capita)			X			X
Leukaemia	Global	Female	Education (years per capita)			X			X
Leukaemia	Data Rich	Female	LDI (I$ per capita)			X			X
Leukaemia	Global	Female	LDI (I$ per capita)			X			X
Leukaemia	Data Rich	Female	Socio-demographic Index			X			X
Leukaemia	Global	Female	Socio-demographic Index			X			X
Leukaemia	Data Rich	Female	Healthcare access and quality index		X			X	
Leukaemia	Global	Female	Healthcare access and quality index		X			X	
Leukaemia	Data Rich	Female	Mean BMI		X			X	
Leukaemia	Global	Female	Mean BMI		X			X	
Leukaemia	Data Rich	Female	Liters of alcohol consumed per capita		X			X	
Leukaemia	Global	Female	Liters of alcohol consumed per capita		X			X	
Leukaemia	Data Rich	Female	Cumulative Cigarettes (10 Years)		X			X	
Leukaemia	Global	Female	Cumulative Cigarettes (10 Years)		X			X	
Leukaemia	Data Rich	Female	Tobacco (cigarettes per capita)		X			X	
Leukaemia	Global	Female	Tobacco (cigarettes per capita)		X			X	
Leukaemia	Data Rich	Female	Cumulative Cigarettes (20 Years)		X			X	
Leukaemia	Global	Female	Cumulative Cigarettes (20 Years)		X			X	
Leukaemia	Data Rich	Female	Log-transformed SEV scalar: Leukemia	X			X		
Leukaemia	Global	Female	Log-transformed SEV scalar: Leukemia	X			X		
Leukaemia	Data Rich	Female	Log-transformed age-standardized SEV scalar: Leukemia	X			X		
Leukaemia	Global	Female	Log-transformed age-standardized SEV scalar: Leukemia	X			X		
Leukaemia	Data Rich	Male	Education (years per capita)			X			X
Leukaemia	Global	Male	Education (years per capita)			X			X
Leukaemia	Data Rich	Male	LDI (I$ per capita)			X			X
Leukaemia	Global	Male	LDI (I$ per capita)			X			X
Leukaemia	Data Rich	Male	Socio-demographic Index			X			X
Leukaemia	Global	Male	Socio-demographic Index			X			X
Leukaemia	Data Rich	Male	Healthcare access and quality index		X			X	
Leukaemia	Global	Male	Healthcare access and quality index		X			X	
Leukaemia	Data Rich	Male	Mean BMI		X			X	
Leukaemia	Global	Male	Mean BMI		X			X	
Leukaemia	Data Rich	Male	Liters of alcohol consumed per capita		X			X	
Leukaemia	Global	Male	Liters of alcohol consumed per capita		X			X	
Leukaemia	Data Rich	Male	Cumulative Cigarettes (10 Years)		X			X	
Leukaemia	Global	Male	Cumulative Cigarettes (10 Years)		X			X	
Leukaemia	Data Rich	Male	Tobacco (cigarettes per capita)		X			X	
Leukaemia	Global	Male	Tobacco (cigarettes per capita)		X			X	
Leukaemia	Data Rich	Male	Cumulative Cigarettes (20 Years)		X			X	
Leukaemia	Global	Male	Cumulative Cigarettes (20 Years)		X			X	
Leukaemia	Data Rich	Male	Log-transformed SEV scalar: Leukemia	X			X		
Leukaemia	Global	Male	Log-transformed SEV scalar: Leukemia	X			X		
Leukaemia	Data Rich	Male	Log-transformed age-standardized SEV scalar: Leukemia	X			X		
Leukaemia	Global	Male	Log-transformed age-standardized SEV scalar: Leukemia	X			X		
Acute lymphoid leukaemia	Global	Female	Education (years per capita)			X			X
Acute lymphoid leukaemia	Data Rich	Female	Education (years per capita)			X			X
Acute lymphoid leukaemia	Global	Female	LDI (I$ per capita)			X			X

Comparison of GBD 2019 and GBD 2021 covariates and level of covariates used in cause of death modeling									
**Cause**	**Model Version Type**	**Sex**	**Covariate**	**Level 1 GBD 2019**	**Level 2 GBD 2019**	**Level 3 GBD 2019**	**Level 1 GBD 2021**	**Level 2 GBD 2021**	**Level 3 GBD 2021**
Acute lymphoid leukaemia	Data Rich	Female	LDI (I$ per capita)			X			X
Acute lymphoid leukaemia	Global	Female	Socio-demographic Index			X			X
Acute lymphoid leukaemia	Data Rich	Female	Socio-demographic Index			X			X
Acute lymphoid leukaemia	Global	Female	Healthcare access and quality index		X			X	
Acute lymphoid leukaemia	Data Rich	Female	Healthcare access and quality index		X			X	
Acute lymphoid leukaemia	Global	Female	Mean BMI		X			X	
Acute lymphoid leukaemia	Data Rich	Female	Mean BMI		X			X	
Acute lymphoid leukaemia	Global	Female	Log-transformed SEV scalar: Leukemia	X			X		
Acute lymphoid leukaemia	Data Rich	Female	Log-transformed SEV scalar: Leukemia	X			X		
Acute lymphoid leukaemia	Global	Female	Log-transformed age-standardized SEV scalar: Leukemia	X			X		
Acute lymphoid leukaemia	Data Rich	Female	Log-transformed age-standardized SEV scalar: Leukemia	X			X		
Acute lymphoid leukaemia	Global	Male	Education (years per capita)			X			X
Acute lymphoid leukaemia	Data Rich	Male	Education (years per capita)			X			X
Acute lymphoid leukaemia	Global	Male	LDI (I$ per capita)			X			X
Acute lymphoid leukaemia	Data Rich	Male	LDI (I$ per capita)			X			X
Acute lymphoid leukaemia	Global	Male	Socio-demographic Index			X			X
Acute lymphoid leukaemia	Data Rich	Male	Socio-demographic Index			X			X
Acute lymphoid leukaemia	Global	Male	Healthcare access and quality index		X			X	
Acute lymphoid leukaemia	Data Rich	Male	Healthcare access and quality index		X			X	
Acute lymphoid leukaemia	Global	Male	Mean BMI		X			X	
Acute lymphoid leukaemia	Data Rich	Male	Mean BMI		X			X	
Acute lymphoid leukaemia	Global	Male	Log-transformed SEV scalar: Leukemia	X			X		
Acute lymphoid leukaemia	Data Rich	Male	Log-transformed SEV scalar: Leukemia	X			X		
Acute lymphoid leukaemia	Global	Male	Log-transformed age-standardized SEV scalar: Leukemia	X			X		
Acute lymphoid leukaemia	Data Rich	Male	Log-transformed age-standardized SEV scalar: Leukemia	X			X		
Chronic lymphoid leukaemia	Global	Female	Education (years per capita)			X			X
Chronic lymphoid leukaemia	Data Rich	Female	Education (years per capita)			X			X
Chronic lymphoid leukaemia	Global	Female	LDI (I$ per capita)			X			X
Chronic lymphoid leukaemia	Data Rich	Female	LDI (I$ per capita)			X			X
Chronic lymphoid leukaemia	Global	Female	Socio-demographic Index			X			X
Chronic lymphoid leukaemia	Data Rich	Female	Socio-demographic Index			X			X
Chronic lymphoid leukaemia	Data Rich	Female	Healthcare access and quality index		X			X	
Chronic lymphoid leukaemia	Global	Female	Mean BMI		X			X	
Chronic lymphoid leukaemia	Data Rich	Female	Mean BMI		X			X	
Chronic lymphoid leukaemia	Global	Female	Smoking Prevalence		X			X	
Chronic lymphoid leukaemia	Data Rich	Female	Smoking Prevalence		X			X	
Chronic lymphoid leukaemia	Global	Female	Liters of alcohol consumed per capita		X			X	
Chronic lymphoid leukaemia	Data Rich	Female	Liters of alcohol consumed per capita		X			X	
Chronic lymphoid leukaemia	Global	Female	Cumulative Cigarettes (5 Years)		X			X	
Chronic lymphoid leukaemia	Data Rich	Female	Cumulative Cigarettes (5 Years)		X			X	
Chronic lymphoid leukaemia	Global	Female	Cumulative Cigarettes (10 Years)		X			X	
Chronic lymphoid leukaemia	Data Rich	Female	Cumulative Cigarettes (10 Years)		X			X	
Chronic lymphoid leukaemia	Global	Female	Cumulative Cigarettes (15 Years)		X			X	
Chronic lymphoid leukaemia	Data Rich	Female	Cumulative Cigarettes (15 Years)		X			X	
Chronic lymphoid leukaemia	Global	Female	Tobacco (cigarettes per capita)		X			X	
Chronic lymphoid leukaemia	Data Rich	Female	Tobacco (cigarettes per capita)		X			X	
Chronic lymphoid leukaemia	Global	Female	Cumulative Cigarettes (20 Years)		X			X	
Chronic lymphoid leukaemia	Data Rich	Female	Cumulative Cigarettes (20 Years)		X			X	
Chronic lymphoid leukaemia	Global	Female	Healthcare access and quality index	X				X	
Chronic lymphoid leukaemia	Global	Female	Log-transformed SEV scalar: Leukemia	X			X		
Chronic lymphoid leukaemia	Data Rich	Female	Log-transformed SEV scalar: Leukemia	X			X		
Chronic lymphoid leukaemia	Global	Female	Log-transformed age-standardized SEV scalar: Leukemia	X			X		
Chronic lymphoid leukaemia	Data Rich	Female	Log-transformed age-standardized SEV scalar: Leukemia	X			X		
Chronic lymphoid leukaemia	Global	Male	Education (years per capita)			X			X
Chronic lymphoid leukaemia	Data Rich	Male	Education (years per capita)			X			X
Chronic lymphoid leukaemia	Global	Male	LDI (I$ per capita)			X			X
Chronic lymphoid leukaemia	Data Rich	Male	LDI (I$ per capita)			X			X
Chronic lymphoid leukaemia	Global	Male	Socio-demographic Index			X			X
Chronic lymphoid leukaemia	Data Rich	Male	Socio-demographic Index			X			X
Chronic lymphoid leukaemia	Global	Male	Healthcare access and quality index		X			X	
Chronic lymphoid leukaemia	Data Rich	Male	Healthcare access and quality index		X			X	
Chronic lymphoid leukaemia	Global	Male	Mean BMI		X			X	
Chronic lymphoid leukaemia	Data Rich	Male	Mean BMI		X			X	
Chronic lymphoid leukaemia	Global	Male	Smoking Prevalence		X			X	
Chronic lymphoid leukaemia	Data Rich	Male	Smoking Prevalence		X			X	
Chronic lymphoid leukaemia	Global	Male	Liters of alcohol consumed per capita		X			X	
Chronic lymphoid leukaemia	Data Rich	Male	Liters of alcohol consumed per capita		X			X	
Chronic lymphoid leukaemia	Global	Male	Cumulative Cigarettes (5 Years)		X			X	
Chronic lymphoid leukaemia	Data Rich	Male	Cumulative Cigarettes (5 Years)		X			X	
Chronic lymphoid leukaemia	Global	Male	Cumulative Cigarettes (10 Years)		X			X	
Chronic lymphoid leukaemia	Data Rich	Male	Cumulative Cigarettes (10 Years)		X			X	
Chronic lymphoid leukaemia	Global	Male	Cumulative Cigarettes (15 Years)		X			X	
Chronic lymphoid leukaemia	Data Rich	Male	Cumulative Cigarettes (15 Years)		X			X	
Chronic lymphoid leukaemia	Global	Male	Tobacco (cigarettes per capita)		X			X	
Chronic lymphoid leukaemia	Data Rich	Male	Tobacco (cigarettes per capita)		X			X	
Chronic lymphoid leukaemia	Global	Male	Cumulative Cigarettes (20 Years)		X			X	
Chronic lymphoid leukaemia	Data Rich	Male	Cumulative Cigarettes (20 Years)		X			X	
Chronic lymphoid leukaemia	Global	Male	Log-transformed SEV scalar: Leukemia	X			X		
Chronic lymphoid leukaemia	Data Rich	Male	Log-transformed SEV scalar: Leukemia	X			X		
Chronic lymphoid leukaemia	Global	Male	Log-transformed age-standardized SEV scalar: Leukemia	X			X		
Chronic lymphoid leukaemia	Data Rich	Male	Log-transformed age-standardized SEV scalar: Leukemia	X			X		
Acute myeloid leukaemia	Global	Female	Education (years per capita)			X			X
Acute myeloid leukaemia	Data Rich	Female	Education (years per capita)			X			X
Acute myeloid leukaemia	Global	Female	LDI (I$ per capita)			X			X
Acute myeloid leukaemia	Data Rich	Female	LDI (I$ per capita)			X			X
Acute myeloid leukaemia	Global	Female	Socio-demographic Index			X			X
Acute myeloid leukaemia	Data Rich	Female	Socio-demographic Index			X			X
Acute myeloid leukaemia	Global	Female	Healthcare access and quality index		X			X	
Acute myeloid leukaemia	Data Rich	Female	Healthcare access and quality index		X			X	
Acute myeloid leukaemia	Global	Female	Mean BMI		X			X	
Acute myeloid leukaemia	Data Rich	Female	Mean BMI		X			X	
Acute myeloid leukaemia	Global	Female	Smoking Prevalence		X			X	
Acute myeloid leukaemia	Data Rich	Female	Smoking Prevalence		X			X	
Acute myeloid leukaemia	Global	Female	Liters of alcohol consumed per capita		X			X	
Acute myeloid leukaemia	Data Rich	Female	Liters of alcohol consumed per capita		X			X	
Acute myeloid leukaemia	Global	Female	Cumulative Cigarettes (10 Years)		X			X	
Acute myeloid leukaemia	Data Rich	Female	Cumulative Cigarettes (10 Years)		X			X	
Acute myeloid leukaemia	Global	Female	Cumulative Cigarettes (20 Years)		X			X	
Acute myeloid leukaemia	Data Rich	Female	Cumulative Cigarettes (20 Years)		X			X	
Acute myeloid leukaemia	Global	Female	Log-transformed SEV scalar: Leukemia	X			X		
Acute myeloid leukaemia	Data Rich	Female	Log-transformed SEV scalar: Leukemia	X			X		
Acute myeloid leukaemia	Global	Female	Log-transformed age-standardized SEV scalar: Leukemia	X			X		
Acute myeloid leukaemia	Data Rich	Female	Log-transformed age-standardized SEV scalar: Leukemia	X			X		
Acute myeloid leukaemia	Global	Male	Education (years per capita)			X			X
Acute myeloid leukaemia	Data Rich	Male	Education (years per capita)			X			X
Acute myeloid leukaemia	Global	Male	LDI (I$ per capita)			X			X
Acute myeloid leukaemia	Data Rich	Male	LDI (I$ per capita)			X			X
Acute myeloid leukaemia	Global	Male	Socio-demographic Index			X			X
Acute myeloid leukaemia	Data Rich	Male	Socio-demographic Index			X			X
Acute myeloid leukaemia	Global	Male	Healthcare access and quality index		X			X	
Acute myeloid leukaemia	Data Rich	Male	Healthcare access and quality index		X			X	
Acute myeloid leukaemia	Global	Male	Mean BMI		X			X	
Acute myeloid leukaemia	Data Rich	Male	Mean BMI		X			X	
Acute myeloid leukaemia	Global	Male	Smoking Prevalence		X			X	
Acute myeloid leukaemia	Data Rich	Male	Smoking Prevalence		X			X	
Acute myeloid leukaemia	Global	Male	Liters of alcohol consumed per capita		X			X	
Acute myeloid leukaemia	Data Rich	Male	Liters of alcohol consumed per capita		X			X	
Acute myeloid leukaemia	Global	Male	Cumulative Cigarettes (10 Years)		X			X	
Acute myeloid leukaemia	Data Rich	Male	Cumulative Cigarettes (10 Years)		X			X	
Acute myeloid leukaemia	Global	Male	Cumulative Cigarettes (20 Years)		X			X	
Acute myeloid leukaemia	Data Rich	Male	Cumulative Cigarettes (20 Years)		X			X	
Acute myeloid leukaemia	Global	Male	Log-transformed SEV scalar: Leukemia	X			X		
Acute myeloid leukaemia	Data Rich	Male	Log-transformed SEV scalar: Leukemia	X			X		
Acute myeloid leukaemia	Global	Male	Log-transformed age-standardized SEV scalar: Leukemia	X			X		
Acute myeloid leukaemia	Data Rich	Male	Log-transformed age-standardized SEV scalar: Leukemia	X			X		
Chronic myeloid leukaemia	Data Rich	Female	Education (years per capita)			X			X
Chronic myeloid leukaemia	Global	Female	Education (years per capita)			X			X
Chronic myeloid leukaemia	Data Rich	Female	LDI (I$ per capita)			X			X
Chronic myeloid leukaemia	Global	Female	LDI (I$ per capita)			X			X
Chronic myeloid leukaemia	Data Rich	Female	Socio-demographic Index			X			X
Chronic myeloid leukaemia	Global	Female	Socio-demographic Index			X			X
Chronic myeloid leukaemia	Data Rich	Female	Healthcare access and quality index		X			X	
Chronic myeloid leukaemia	Global	Female	Healthcare access and quality index		X			X	
Chronic myeloid leukaemia	Data Rich	Female	Mean BMI		X			X	
Chronic myeloid leukaemia	Global	Female	Mean BMI		X			X	

Comparison of GBD 2019 and GBD 2021 covariates and level of covariates used in cause of death modeling									
Cause	Model Version Type	Sex	Covariate	Level 1 GBD 2019	Level 2 GBD 2019	Level 3 GBD 2019	Level 1 GBD 2021	Level 2 GBD 2021	Level 3 GBD 2021
Chronic myeloid leukaemia	Data Rich	Female	Smoking Prevalence		X			X	
Chronic myeloid leukaemia	Global	Female	Smoking Prevalence		X			X	
Chronic myeloid leukaemia	Data Rich	Female	Liters of alcohol consumed per capita		X			X	
Chronic myeloid leukaemia	Global	Female	Liters of alcohol consumed per capita		X			X	
Chronic myeloid leukaemia	Data Rich	Female	Cumulative Cigarettes (5 Years)		X			X	
Chronic myeloid leukaemia	Global	Female	Cumulative Cigarettes (5 Years)		X			X	
Chronic myeloid leukaemia	Data Rich	Female	Cumulative Cigarettes (10 Years)		X			X	
Chronic myeloid leukaemia	Global	Female	Cumulative Cigarettes (10 Years)		X			X	
Chronic myeloid leukaemia	Data Rich	Female	Cumulative Cigarettes (15 Years)		X			X	
Chronic myeloid leukaemia	Global	Female	Cumulative Cigarettes (15 Years)		X			X	
Chronic myeloid leukaemia	Data Rich	Female	Tobacco (cigarettes per capita)		X			X	
Chronic myeloid leukaemia	Global	Female	Tobacco (cigarettes per capita)		X			X	
Chronic myeloid leukaemia	Data Rich	Female	Cumulative Cigarettes (20 Years)		X			X	
Chronic myeloid leukaemia	Global	Female	Cumulative Cigarettes (20 Years)		X			X	
Chronic myeloid leukaemia	Data Rich	Female	Log-transformed SEV scalar: Leukemia	X			X		
Chronic myeloid leukaemia	Global	Female	Log-transformed SEV scalar: Leukemia	X			X		
Chronic myeloid leukaemia	Data Rich	Female	Log-transformed age-standardized SEV scalar: Leukemia	X			X		
Chronic myeloid leukaemia	Global	Female	Log-transformed age-standardized SEV scalar: Leukemia	X			X		
Chronic myeloid leukaemia	Data Rich	Male	Education (years per capita)			X			X
Chronic myeloid leukaemia	Global	Male	Education (years per capita)			X			X
Chronic myeloid leukaemia	Data Rich	Male	LDI (I$ per capita)			X			X
Chronic myeloid leukaemia	Global	Male	LDI (I$ per capita)			X			X
Chronic myeloid leukaemia	Data Rich	Male	Socio-demographic Index			X			X
Chronic myeloid leukaemia	Global	Male	Socio-demographic Index			X			X
Chronic myeloid leukaemia	Data Rich	Male	Healthcare access and quality index		X			X	
Chronic myeloid leukaemia	Global	Male	Healthcare access and quality index		X			X	
Chronic myeloid leukaemia	Data Rich	Male	Mean BMI		X			X	
Chronic myeloid leukaemia	Global	Male	Mean BMI		X			X	
Chronic myeloid leukaemia	Data Rich	Male	Smoking Prevalence		X			X	
Chronic myeloid leukaemia	Global	Male	Smoking Prevalence		X			X	
Chronic myeloid leukaemia	Data Rich	Male	Liters of alcohol consumed per capita		X			X	
Chronic myeloid leukaemia	Global	Male	Liters of alcohol consumed per capita		X			X	
Chronic myeloid leukaemia	Data Rich	Male	Cumulative Cigarettes (5 Years)		X			X	
Chronic myeloid leukaemia	Global	Male	Cumulative Cigarettes (5 Years)		X			X	
Chronic myeloid leukaemia	Data Rich	Male	Cumulative Cigarettes (10 Years)		X			X	
Chronic myeloid leukaemia	Global	Male	Cumulative Cigarettes (10 Years)		X			X	
Chronic myeloid leukaemia	Data Rich	Male	Cumulative Cigarettes (15 Years)		X			X	
Chronic myeloid leukaemia	Global	Male	Cumulative Cigarettes (15 Years)		X			X	
Chronic myeloid leukaemia	Data Rich	Male	Tobacco (cigarettes per capita)		X			X	
Chronic myeloid leukaemia	Global	Male	Tobacco (cigarettes per capita)		X			X	
Chronic myeloid leukaemia	Data Rich	Male	Cumulative Cigarettes (20 Years)		X			X	
Chronic myeloid leukaemia	Global	Male	Cumulative Cigarettes (20 Years)		X			X	
Chronic myeloid leukaemia	Data Rich	Male	Log-transformed SEV scalar: Leukemia	X			X		
Chronic myeloid leukaemia	Global	Male	Log-transformed SEV scalar: Leukemia	X			X		
Chronic myeloid leukaemia	Data Rich	Male	Log-transformed age-standardized SEV scalar: Leukemia	X			X		
Chronic myeloid leukaemia	Global	Male	Log-transformed age-standardized SEV scalar: Leukemia	X			X		
Other leukaemia	Global	Female	Education (years per capita)			X			X
Other leukaemia	Data Rich	Female	Education (years per capita)			X			X
Other leukaemia	Global	Female	LDI (I$ per capita)			X			X
Other leukaemia	Data Rich	Female	LDI (I$ per capita)			X			X
Other leukaemia	Global	Female	Socio-demographic Index			X			X
Other leukaemia	Data Rich	Female	Socio-demographic Index			X			X
Other leukaemia	Global	Female	Healthcare access and quality index		X			X	
Other leukaemia	Data Rich	Female	Healthcare access and quality index		X			X	
Other leukaemia	Global	Female	Mean BMI		X			X	
Other leukaemia	Data Rich	Female	Mean BMI		X			X	
Other leukaemia	Global	Female	Liters of alcohol consumed per capita		X			X	
Other leukaemia	Global	Female	Cumulative Cigarettes (10 Years)		X			X	
Other leukaemia	Data Rich	Female	Cumulative Cigarettes (10 Years)		X			X	
Other leukaemia	Global	Female	Tobacco (cigarettes per capita)		X			X	
Other leukaemia	Data Rich	Female	Tobacco (cigarettes per capita)		X			X	
Other leukaemia	Global	Female	Cumulative Cigarettes (20 Years)		X			X	
Other leukaemia	Data Rich	Female	Cumulative Cigarettes (20 Years)		X			X	
Other leukaemia	Data Rich	Female	Liters of alcohol consumed per capita	X			X		
Other leukaemia	Global	Female	Log-transformed SEV scalar: Leukemia	X			X		
Other leukaemia	Data Rich	Female	Log-transformed SEV scalar: Leukemia	X			X		
Other leukaemia	Global	Female	Log-transformed age-standardized SEV scalar: Leukemia	X			X		
Other leukaemia	Data Rich	Female	Log-transformed age-standardized SEV scalar: Leukemia	X			X		
Other leukaemia	Global	Male	Education (years per capita)			X			X
Other leukaemia	Data Rich	Male	Education (years per capita)			X			X
Other leukaemia	Global	Male	LDI (I$ per capita)			X			X
Other leukaemia	Data Rich	Male	LDI (I$ per capita)			X			X
Other leukaemia	Global	Male	Socio-demographic Index			X			X
Other leukaemia	Data Rich	Male	Socio-demographic Index			X			X
Other leukaemia	Global	Male	Healthcare access and quality index		X			X	
Other leukaemia	Data Rich	Male	Healthcare access and quality index		X			X	
Other leukaemia	Global	Male	Mean BMI		X			X	
Other leukaemia	Data Rich	Male	Mean BMI		X			X	
Other leukaemia	Global	Male	Liters of alcohol consumed per capita		X			X	
Other leukaemia	Data Rich	Male	Liters of alcohol consumed per capita		X			X	
Other leukaemia	Global	Male	Cumulative Cigarettes (10 Years)		X			X	
Other leukaemia	Data Rich	Male	Cumulative Cigarettes (10 Years)		X			X	
Other leukaemia	Global	Male	Tobacco (cigarettes per capita)		X			X	
Other leukaemia	Data Rich	Male	Tobacco (cigarettes per capita)		X			X	
Other leukaemia	Global	Male	Cumulative Cigarettes (20 Years)		X			X	
Other leukaemia	Data Rich	Male	Cumulative Cigarettes (20 Years)		X			X	
Other leukaemia	Global	Male	Log-transformed SEV scalar: Leukemia	X			X		
Other leukaemia	Data Rich	Male	Log-transformed SEV scalar: Leukemia	X			X		
Other leukaemia	Global	Male	Log-transformed age-standardized SEV scalar: Leukemia	X			X		
Other leukaemia	Data Rich	Male	Log-transformed age-standardized SEV scalar: Leukemia	X			X		
Other malignant cancers	Data Rich	Female	Education (years per capita)			X			X
Other malignant cancers	Global	Female	Education (years per capita)			X			X
Other malignant cancers	Data Rich	Female	LDI (I$ per capita)			X			X
Other malignant cancers	Global	Female	LDI (I$ per capita)			X			X
Other malignant cancers	Data Rich	Female	Socio-demographic Index			X			X
Other malignant cancers	Global	Female	Socio-demographic Index			X			X
Other malignant cancers	Data Rich	Female	Healthcare access and quality index		X			X	
Other malignant cancers	Global	Female	Healthcare access and quality index		X			X	
Other malignant cancers	Data Rich	Female	Age- and sex-specific SEV for Low fruit		X			X	
Other malignant cancers	Global	Female	Age- and sex-specific SEV for Low fruit		X			X	
Other malignant cancers	Data Rich	Female	Age- and sex-specific SEV for Low vegetables		X			X	
Other malignant cancers	Global	Female	Age- and sex-specific SEV for Low vegetables		X			X	
Other malignant cancers	Data Rich	Female	Age- and sex-specific SEV for Low nuts and seeds		X			X	
Other malignant cancers	Global	Female	Age- and sex-specific SEV for Low nuts and seeds		X			X	
Other malignant cancers	Data Rich	Female	pufa adjusted(percent)		X			X	
Other malignant cancers	Global	Female	pufa adjusted(percent)		X			X	
Other malignant cancers	Data Rich	Female	Smoking Prevalence	X			X		
Other malignant cancers	Global	Female	Smoking Prevalence	X			X		
Other malignant cancers	Data Rich	Female	Tobacco (cigarettes per capita)	X			X		
Other malignant cancers	Global	Female	Tobacco (cigarettes per capita)	X			X		
Other malignant cancers	Data Rich	Male	Education (years per capita)			X			X
Other malignant cancers	Global	Male	Education (years per capita)			X			X
Other malignant cancers	Data Rich	Male	LDI (I$ per capita)			X			X
Other malignant cancers	Global	Male	LDI (I$ per capita)			X			X
Other malignant cancers	Data Rich	Male	Socio-demographic Index			X			X
Other malignant cancers	Global	Male	Socio-demographic Index			X			X
Other malignant cancers	Data Rich	Male	Healthcare access and quality index		X			X	
Other malignant cancers	Global	Male	Healthcare access and quality index		X			X	
Other malignant cancers	Data Rich	Male	Age- and sex-specific SEV for Low fruit		X			X	
Other malignant cancers	Global	Male	Age- and sex-specific SEV for Low fruit		X			X	
Other malignant cancers	Data Rich	Male	Age- and sex-specific SEV for Low vegetables		X			X	
Other malignant cancers	Global	Male	Age- and sex-specific SEV for Low vegetables		X			X	
Other malignant cancers	Data Rich	Male	Age- and sex-specific SEV for Low nuts and seeds		X			X	
Other malignant cancers	Global	Male	Age- and sex-specific SEV for Low nuts and seeds		X			X	
Other malignant cancers	Data Rich	Male	pufa adjusted(percent)		X			X	
Other malignant cancers	Global	Male	pufa adjusted(percent)		X			X	
Other malignant cancers	Data Rich	Male	Smoking Prevalence	X			X		
Other malignant cancers	Global	Male	Smoking Prevalence	X			X		
Other malignant cancers	Data Rich	Male	Tobacco (cigarettes per capita)	X			X		
Other malignant cancers	Global	Male	Tobacco (cigarettes per capita)	X			X		
Myelodysplastic, myeloproliferative, and other hematopoietic neoplasms	Data Rich	Female	Education (years per capita)			X			X
Myelodysplastic, myeloproliferative, and other hematopoietic neoplasms	Global	Female	Education (years per capita)			X			X

Comparison of GBD 2019 and GBD 2021 covariates and level of covariates used in cause of death modeling

Cause	Model Version Type	Sex	Covariate	Level 1 GBD 2019	Level 2 GBD 2019	Level 3 GBD 2019	Level 1 GBD 2021	Level 2 GBD 2021	Level 3 GBD 2021
Myelodysplastic, myeloproliferative, and other hematopoietic neoplasms	Data Rich	Female	LDI (I$ per capita)			X			X
Myelodysplastic, myeloproliferative, and other hematopoietic neoplasms	Global	Female	LDI (I$ per capita)			X			X
Myelodysplastic, myeloproliferative, and other hematopoietic neoplasms	Data Rich	Female	Socio-demographic Index			X			X
Myelodysplastic, myeloproliferative, and other hematopoietic neoplasms	Global	Female	Socio-demographic Index			X			X
Myelodysplastic, myeloproliferative, and other hematopoietic neoplasms	Data Rich	Female	Healthcare access and quality index		X			X	
Myelodysplastic, myeloproliferative, and other hematopoietic neoplasms	Global	Female	Healthcare access and quality index		X			X	
Myelodysplastic, myeloproliferative, and other hematopoietic neoplasms	Data Rich	Female	Smoking Prevalence		X			X	
Myelodysplastic, myeloproliferative, and other hematopoietic neoplasms	Global	Female	Smoking Prevalence		X			X	
Myelodysplastic, myeloproliferative, and other hematopoietic neoplasms	Data Rich	Female	Liters of alcohol consumed per capita		X			X	
Myelodysplastic, myeloproliferative, and other hematopoietic neoplasms	Global	Female	Liters of alcohol consumed per capita		X			X	
Myelodysplastic, myeloproliferative, and other hematopoietic neoplasms	Data Rich	Female	Cumulative Cigarettes (5 Years)		X			X	
Myelodysplastic, myeloproliferative, and other hematopoietic neoplasms	Global	Female	Cumulative Cigarettes (5 Years)		X			X	
Myelodysplastic, myeloproliferative, and other hematopoietic neoplasms	Data Rich	Female	Cumulative Cigarettes (10 Years)		X			X	
Myelodysplastic, myeloproliferative, and other hematopoietic neoplasms	Global	Female	Cumulative Cigarettes (10 Years)		X			X	
Myelodysplastic, myeloproliferative, and other hematopoietic neoplasms	Data Rich	Female	Cumulative Cigarettes (15 Years)		X			X	
Myelodysplastic, myeloproliferative, and other hematopoietic neoplasms	Global	Female	Cumulative Cigarettes (15 Years)		X			X	
Myelodysplastic, myeloproliferative, and other hematopoietic neoplasms	Data Rich	Female	Tobacco (cigarettes per capita)		X			X	
Myelodysplastic, myeloproliferative, and other hematopoietic neoplasms	Global	Female	Tobacco (cigarettes per capita)		X			X	
Myelodysplastic, myeloproliferative, and other hematopoietic neoplasms	Data Rich	Female	Cumulative Cigarettes (20 Years)		X			X	
Myelodysplastic, myeloproliferative, and other hematopoietic neoplasms	Global	Female	Cumulative Cigarettes (20 Years)		X			X	
Myelodysplastic, myeloproliferative, and other hematopoietic neoplasms	Data Rich	Female	Log-transformed SEV scalar: Leukemia	X			X		
Myelodysplastic, myeloproliferative, and other hematopoietic neoplasms	Global	Female	Log-transformed SEV scalar: Leukemia	X			X		
Myelodysplastic, myeloproliferative, and other hematopoietic neoplasms	Data Rich	Female	Log-transformed age-standardized SEV scalar: Leukemia	X			X		
Myelodysplastic, myeloproliferative, and other hematopoietic neoplasms	Global	Female	Log-transformed age-standardized SEV scalar: Leukemia	X			X		
Myelodysplastic, myeloproliferative, and other hematopoietic neoplasms	Data Rich	Male	Education (years per capita)			X			X
Myelodysplastic, myeloproliferative, and other hematopoietic neoplasms	Global	Male	Education (years per capita)			X			X
Myelodysplastic, myeloproliferative, and other hematopoietic neoplasms	Data Rich	Male	LDI (I$ per capita)			X			X
Myelodysplastic, myeloproliferative, and other hematopoietic neoplasms	Global	Male	LDI (I$ per capita)			X			X
Myelodysplastic, myeloproliferative, and other hematopoietic neoplasms	Data Rich	Male	Socio-demographic Index			X			X
Myelodysplastic, myeloproliferative, and other hematopoietic neoplasms	Global	Male	Socio-demographic Index			X			X
Myelodysplastic, myeloproliferative, and other hematopoietic neoplasms	Data Rich	Male	Healthcare access and quality index		X			X	
Myelodysplastic, myeloproliferative, and other hematopoietic neoplasms	Global	Male	Healthcare access and quality index		X			X	
Myelodysplastic, myeloproliferative, and other hematopoietic neoplasms	Data Rich	Male	Smoking Prevalence		X			X	
Myelodysplastic, myeloproliferative, and other hematopoietic neoplasms	Global	Male	Smoking Prevalence		X			X	
Myelodysplastic, myeloproliferative, and other hematopoietic neoplasms	Data Rich	Male	Liters of alcohol consumed per capita		X			X	
Myelodysplastic, myeloproliferative, and other hematopoietic neoplasms	Global	Male	Liters of alcohol consumed per capita		X			X	
Myelodysplastic, myeloproliferative, and other hematopoietic neoplasms	Data Rich	Male	Cumulative Cigarettes (5 Years)		X			X	
Myelodysplastic, myeloproliferative, and other hematopoietic neoplasms	Global	Male	Cumulative Cigarettes (5 Years)		X			X	
Myelodysplastic, myeloproliferative, and other hematopoietic neoplasms	Data Rich	Male	Cumulative Cigarettes (10 Years)		X			X	
Myelodysplastic, myeloproliferative, and other hematopoietic neoplasms	Global	Male	Cumulative Cigarettes (10 Years)		X			X	
Myelodysplastic, myeloproliferative, and other hematopoietic neoplasms	Data Rich	Male	Cumulative Cigarettes (15 Years)		X			X	
Myelodysplastic, myeloproliferative, and other hematopoietic neoplasms	Global	Male	Cumulative Cigarettes (15 Years)		X			X	
Myelodysplastic, myeloproliferative, and other hematopoietic neoplasms	Data Rich	Male	Tobacco (cigarettes per capita)		X			X	
Myelodysplastic, myeloproliferative, and other hematopoietic neoplasms	Global	Male	Tobacco (cigarettes per capita)		X			X	
Myelodysplastic, myeloproliferative, and other hematopoietic neoplasms	Data Rich	Male	Cumulative Cigarettes (20 Years)		X			X	
Myelodysplastic, myeloproliferative, and other hematopoietic neoplasms	Global	Male	Cumulative Cigarettes (20 Years)		X			X	
Myelodysplastic, myeloproliferative, and other hematopoietic neoplasms	Data Rich	Male	Log-transformed SEV scalar: Leukemia	X			X		
Myelodysplastic, myeloproliferative, and other hematopoietic neoplasms	Global	Male	Log-transformed SEV scalar: Leukemia	X			X		
Myelodysplastic, myeloproliferative, and other hematopoietic neoplasms	Data Rich	Male	Log-transformed age-standardized SEV scalar: Leukemia	X			X		
Myelodysplastic, myeloproliferative, and other hematopoietic neoplasms	Global	Male	Log-transformed age-standardized SEV scalar: Leukemia	X			X		
Cardiovascular diseases	Global	Female	LDI (I$ per capita)			X			X
Cardiovascular diseases	Data Rich	Female	LDI (I$ per capita)			X			X
Cardiovascular diseases	Global	Female	Liters of alcohol consumed per capita			X			X
Cardiovascular diseases	Data Rich	Female	Liters of alcohol consumed per capita			X			X
Cardiovascular diseases	Global	Female	Age- and sex-specific SEV for Low fruit			X			X
Cardiovascular diseases	Data Rich	Female	Age- and sex-specific SEV for Low fruit			X			X
Cardiovascular diseases	Global	Female	Age- and sex-specific SEV for Low vegetables			X			X
Cardiovascular diseases	Data Rich	Female	Age- and sex-specific SEV for Low vegetables			X			X
Cardiovascular diseases	Global	Female	Age- and sex-specific SEV for Low PUFA			X			X
Cardiovascular diseases	Data Rich	Female	Age- and sex-specific SEV for Low PUFA			X			X
Cardiovascular diseases	Global	Female	Age- and sex-specific SEV for Low nuts and seeds			X			X
Cardiovascular diseases	Data Rich	Female	Age- and sex-specific SEV for Low nuts and seeds			X			X
Cardiovascular diseases	Global	Female	Age- and sex-specific SEV for Low omega-3			X			X
Cardiovascular diseases	Data Rich	Female	Age- and sex-specific SEV for Low omega-3			X			X
Cardiovascular diseases	Global	Female	Pulses legumes unadjusted(g)			X			X
Cardiovascular diseases	Data Rich	Female	Pulses legumes unadjusted(g)			X			X
Cardiovascular diseases	Global	Female	Diet high in trans fatty acids			X			X
Cardiovascular diseases	Data Rich	Female	Diet high in trans fatty acids			X			X
Cardiovascular diseases	Global	Female	Healthcare access and quality index		X			X	
Cardiovascular diseases	Data Rich	Female	Healthcare access and quality index		X			X	
Cardiovascular diseases	Global	Female	Mean BMI		X			X	
Cardiovascular diseases	Data Rich	Female	Mean BMI		X			X	
Cardiovascular diseases	Global	Female	Indoor Air Pollution (All Cooking Fuels)		X			X	
Cardiovascular diseases	Data Rich	Female	Indoor Air Pollution (All Cooking Fuels)		X			X	
Cardiovascular diseases	Global	Female	Outdoor Air Pollution (PM2.5)		X			X	
Cardiovascular diseases	Data Rich	Female	Outdoor Air Pollution (PM2.5)		X			X	
Cardiovascular diseases	Global	Female	Elevation Over 1500m (proportion)		X			X	
Cardiovascular diseases	Data Rich	Female	Elevation Over 1500m (proportion)		X			X	
Cardiovascular diseases	Global	Female	Diabetes Fasting Plasma Glucose (mmol/L), age-standardized 25+		X			X	
Cardiovascular diseases	Data Rich	Female	Diabetes Fasting Plasma Glucose (mmol/L), age-standardized 25+		X			X	
Cardiovascular diseases	Global	Female	Low-Density Lipoprotein (mmol/L)	X			X		
Cardiovascular diseases	Data Rich	Female	Low-Density Lipoprotein (mmol/L)	X			X		
Cardiovascular diseases	Global	Female	Systolic Blood Pressure (mmHg)	X			X		
Cardiovascular diseases	Data Rich	Female	Systolic Blood Pressure (mmHg)	X			X		
Cardiovascular diseases	Global	Female	Smoking Prevalence	X			X		
Cardiovascular diseases	Data Rich	Female	Smoking Prevalence	X			X		
Cardiovascular diseases	Global	Male	LDI (I$ per capita)			X			X
Cardiovascular diseases	Data Rich	Male	LDI (I$ per capita)			X			X
Cardiovascular diseases	Global	Male	Liters of alcohol consumed per capita			X			X
Cardiovascular diseases	Data Rich	Male	Liters of alcohol consumed per capita			X			X
Cardiovascular diseases	Global	Male	Age- and sex-specific SEV for Low fruit			X			X
Cardiovascular diseases	Data Rich	Male	Age- and sex-specific SEV for Low fruit			X			X
Cardiovascular diseases	Global	Male	Age- and sex-specific SEV for Low vegetables			X			X
Cardiovascular diseases	Data Rich	Male	Age- and sex-specific SEV for Low vegetables			X			X
Cardiovascular diseases	Global	Male	Age- and sex-specific SEV for Low PUFA			X			X
Cardiovascular diseases	Data Rich	Male	Age- and sex-specific SEV for Low PUFA			X			X
Cardiovascular diseases	Global	Male	Age- and sex-specific SEV for Low nuts and seeds			X			X
Cardiovascular diseases	Data Rich	Male	Age- and sex-specific SEV for Low nuts and seeds			X			X
Cardiovascular diseases	Global	Male	Age- and sex-specific SEV for Low omega-3			X			X
Cardiovascular diseases	Data Rich	Male	Age- and sex-specific SEV for Low omega-3			X			X
Cardiovascular diseases	Global	Male	Pulses legumes unadjusted(g)			X			X
Cardiovascular diseases	Data Rich	Male	Pulses legumes unadjusted(g)			X			X
Cardiovascular diseases	Global	Male	Diet high in trans fatty acids			X			X
Cardiovascular diseases	Data Rich	Male	Diet high in trans fatty acids			X			X
Cardiovascular diseases	Global	Male	Healthcare access and quality index		X			X	
Cardiovascular diseases	Data Rich	Male	Healthcare access and quality index		X			X	
Cardiovascular diseases	Global	Male	Mean BMI		X			X	
Cardiovascular diseases	Data Rich	Male	Mean BMI		X			X	
Cardiovascular diseases	Global	Male	Indoor Air Pollution (All Cooking Fuels)		X			X	
Cardiovascular diseases	Data Rich	Male	Indoor Air Pollution (All Cooking Fuels)		X			X	
Cardiovascular diseases	Global	Male	Outdoor Air Pollution (PM2.5)		X			X	

Comparison of GBD 2019 and GBD 2021 covariates and level of covariates used in cause of death modeling

Cause	Model Version Type	Sex	Covariate	Level 1 GBD 2019	Level 2 GBD 2019	Level 3 GBD 2019	Level 1 GBD 2021	Level 2 GBD 2021	Level 3 GBD 2021
Cardiovascular diseases	Data Rich	Male	Outdoor Air Pollution (PM2.5)		X			X	
Cardiovascular diseases	Global	Male	Elevation Over 1500m (proportion)		X			X	
Cardiovascular diseases	Data Rich	Male	Elevation Over 1500m (proportion)		X			X	
Cardiovascular diseases	Global	Male	Diabetes Fasting Plasma Glucose (mmol/L), age-standardized 25+		X			X	
Cardiovascular diseases	Data Rich	Male	Diabetes Fasting Plasma Glucose (mmol/L), age-standardized 25+		X			X	
Cardiovascular diseases	Global	Male	Low-Density Lipoprotein (mmol/L)	X			X		
Cardiovascular diseases	Data Rich	Male	Low-Density Lipoprotein (mmol/L)	X			X		
Cardiovascular diseases	Global	Male	Systolic Blood Pressure (mmHg)	X			X		
Cardiovascular diseases	Data Rich	Male	Systolic Blood Pressure (mmHg)	X			X		
Cardiovascular diseases	Global	Male	Smoking Prevalence	X			X		
Cardiovascular diseases	Data Rich	Male	Smoking Prevalence	X			X		
Rheumatic heart disease	Data Rich	Female	Education (years per capita)			X			X
Rheumatic heart disease	Global	Female	Education (years per capita)			X			X
Rheumatic heart disease	Data Rich	Female	LDI (I$ per capita)			X			X
Rheumatic heart disease	Global	Female	LDI (I$ per capita)			X			X
Rheumatic heart disease	Data Rich	Female	Healthcare access and quality index		X			X	
Rheumatic heart disease	Global	Female	Healthcare access and quality index		X			X	
Rheumatic heart disease	Data Rich	Female	Sanitation (proportion with access)	X			X		
Rheumatic heart disease	Global	Female	Sanitation (proportion with access)	X			X		
Rheumatic heart disease	Data Rich	Female	Improved Water Source (proportion with access)	X			X		
Rheumatic heart disease	Global	Female	Improved Water Source (proportion with access)	X			X		
Rheumatic heart disease	Data Rich	Female	Age- and sex-specific SEV for Child underweight	X			X		
Rheumatic heart disease	Global	Female	Age- and sex-specific SEV for Child underweight	X			X		
Rheumatic heart disease	Data Rich	Female	Log-transformed SEV scalar: RHD	X			X		
Rheumatic heart disease	Global	Female	Log-transformed SEV scalar: RHD	X			X		
Rheumatic heart disease	Data Rich	Male	Education (years per capita)			X			X
Rheumatic heart disease	Global	Male	Education (years per capita)			X			X
Rheumatic heart disease	Data Rich	Male	LDI (I$ per capita)			X			X
Rheumatic heart disease	Global	Male	LDI (I$ per capita)			X			X
Rheumatic heart disease	Global	Male	Socio-demographic Index			X			X
Rheumatic heart disease	Data Rich	Male	Healthcare access and quality index		X			X	
Rheumatic heart disease	Global	Male	Healthcare access and quality index		X			X	
Rheumatic heart disease	Data Rich	Male	Sanitation (proportion with access)	X			X		
Rheumatic heart disease	Global	Male	Sanitation (proportion with access)	X			X		
Rheumatic heart disease	Data Rich	Male	Improved Water Source (proportion with access)	X			X		
Rheumatic heart disease	Global	Male	Improved Water Source (proportion with access)	X			X		
Rheumatic heart disease	Data Rich	Male	Age- and sex-specific SEV for Child underweight	X			X		
Rheumatic heart disease	Global	Male	Age- and sex-specific SEV for Child underweight	X			X		
Rheumatic heart disease	Data Rich	Male	Log-transformed SEV scalar: RHD	X			X		
Rheumatic heart disease	Global	Male	Log-transformed SEV scalar: RHD	X			X		
Ischaemic heart disease	Data Rich	Female	LDI (I$ per capita)			X			X
Ischaemic heart disease	Global	Female	LDI (I$ per capita)			X			X
Ischaemic heart disease	Data Rich	Female	Liters of alcohol consumed per capita			X			X
Ischaemic heart disease	Global	Female	Liters of alcohol consumed per capita			X			X
Ischaemic heart disease	Data Rich	Female	Age- and sex-specific SEV for Low fruit			X			X
Ischaemic heart disease	Global	Female	Age- and sex-specific SEV for Low fruit			X			X
Ischaemic heart disease	Data Rich	Female	Age- and sex-specific SEV for Low vegetables			X			X
Ischaemic heart disease	Global	Female	Age- and sex-specific SEV for Low vegetables			X			X
Ischaemic heart disease	Data Rich	Female	Age- and sex-specific SEV for Low PUFA			X			X
Ischaemic heart disease	Global	Female	Age- and sex-specific SEV for Low PUFA			X			X
Ischaemic heart disease	Data Rich	Female	Age- and sex-specific SEV for Low nuts and seeds			X			X
Ischaemic heart disease	Global	Female	Age- and sex-specific SEV for Low nuts and seeds			X			X
Ischaemic heart disease	Data Rich	Female	Age- and sex-specific SEV for Low omega-3			X			X
Ischaemic heart disease	Global	Female	Age- and sex-specific SEV for Low omega-3			X			X
Ischaemic heart disease	Data Rich	Female	Pulses legumes unadjusted(g)			X			X
Ischaemic heart disease	Global	Female	Pulses legumes unadjusted(g)			X			X
Ischaemic heart disease	Data Rich	Female	Diet high in trans fatty acids			X			X
Ischaemic heart disease	Global	Female	Diet high in trans fatty acids			X			X
Ischaemic heart disease	Data Rich	Female	Healthcare access and quality index		X			X	
Ischaemic heart disease	Global	Female	Healthcare access and quality index		X			X	
Ischaemic heart disease	Data Rich	Female	Mean BMI		X			X	
Ischaemic heart disease	Global	Female	Mean BMI		X			X	
Ischaemic heart disease	Data Rich	Female	Indoor Air Pollution (All Cooking Fuels)		X			X	
Ischaemic heart disease	Global	Female	Indoor Air Pollution (All Cooking Fuels)		X			X	
Ischaemic heart disease	Data Rich	Female	Outdoor Air Pollution (PM2.5)		X			X	
Ischaemic heart disease	Global	Female	Outdoor Air Pollution (PM2.5)		X			X	
Ischaemic heart disease	Data Rich	Female	Elevation Over 1500m (proportion)		X			X	
Ischaemic heart disease	Global	Female	Elevation Over 1500m (proportion)		X			X	
Ischaemic heart disease	Data Rich	Female	Diabetes Fasting Plasma Glucose (mmol/L), age-standardized 25+		X			X	
Ischaemic heart disease	Global	Female	Diabetes Fasting Plasma Glucose (mmol/L), age-standardized 25+		X			X	
Ischaemic heart disease	Data Rich	Female	Low-Density Lipoprotein (mmol/L)	X			X		
Ischaemic heart disease	Global	Female	Low-Density Lipoprotein (mmol/L)	X			X		
Ischaemic heart disease	Data Rich	Female	Systolic Blood Pressure (mmHg)	X			X		
Ischaemic heart disease	Global	Female	Systolic Blood Pressure (mmHg)	X			X		
Ischaemic heart disease	Data Rich	Female	Smoking Prevalence	X			X		
Ischaemic heart disease	Global	Female	Smoking Prevalence	X			X		
Ischaemic heart disease	Data Rich	Female	Log-transformed SEV scalar: IHD	X			X		
Ischaemic heart disease	Global	Female	Log-transformed SEV scalar: IHD	X			X		
Ischaemic heart disease	Data Rich	Male	LDI (I$ per capita)			X			X
Ischaemic heart disease	Global	Male	LDI (I$ per capita)			X			X
Ischaemic heart disease	Data Rich	Male	Liters of alcohol consumed per capita			X			X
Ischaemic heart disease	Global	Male	Liters of alcohol consumed per capita			X			X
Ischaemic heart disease	Data Rich	Male	Age- and sex-specific SEV for Low fruit			X			X
Ischaemic heart disease	Global	Male	Age- and sex-specific SEV for Low fruit			X			X
Ischaemic heart disease	Data Rich	Male	Age- and sex-specific SEV for Low vegetables			X			X
Ischaemic heart disease	Global	Male	Age- and sex-specific SEV for Low vegetables			X			X
Ischaemic heart disease	Data Rich	Male	Age- and sex-specific SEV for Low PUFA			X			X
Ischaemic heart disease	Global	Male	Age- and sex-specific SEV for Low PUFA			X			X
Ischaemic heart disease	Data Rich	Male	Age- and sex-specific SEV for Low nuts and seeds			X			X
Ischaemic heart disease	Global	Male	Age- and sex-specific SEV for Low nuts and seeds			X			X
Ischaemic heart disease	Data Rich	Male	Age- and sex-specific SEV for Low omega-3			X			X
Ischaemic heart disease	Global	Male	Age- and sex-specific SEV for Low omega-3			X			X
Ischaemic heart disease	Data Rich	Male	Pulses legumes unadjusted(g)			X			X
Ischaemic heart disease	Global	Male	Pulses legumes unadjusted(g)			X			X
Ischaemic heart disease	Data Rich	Male	Diet high in trans fatty acids			X			X
Ischaemic heart disease	Global	Male	Diet high in trans fatty acids			X			X
Ischaemic heart disease	Data Rich	Male	Healthcare access and quality index		X			X	
Ischaemic heart disease	Global	Male	Healthcare access and quality index		X			X	
Ischaemic heart disease	Data Rich	Male	Mean BMI		X			X	
Ischaemic heart disease	Global	Male	Mean BMI		X			X	
Ischaemic heart disease	Data Rich	Male	Indoor Air Pollution (All Cooking Fuels)		X			X	
Ischaemic heart disease	Global	Male	Indoor Air Pollution (All Cooking Fuels)		X			X	
Ischaemic heart disease	Data Rich	Male	Outdoor Air Pollution (PM2.5)		X			X	
Ischaemic heart disease	Global	Male	Outdoor Air Pollution (PM2.5)		X			X	
Ischaemic heart disease	Data Rich	Male	Elevation Over 1500m (proportion)		X			X	
Ischaemic heart disease	Global	Male	Elevation Over 1500m (proportion)		X			X	
Ischaemic heart disease	Data Rich	Male	Diabetes Fasting Plasma Glucose (mmol/L), age-standardized 25+		X			X	
Ischaemic heart disease	Global	Male	Diabetes Fasting Plasma Glucose (mmol/L), age-standardized 25+		X			X	
Ischaemic heart disease	Data Rich	Male	Low-Density Lipoprotein (mmol/L)	X			X		
Ischaemic heart disease	Global	Male	Low-Density Lipoprotein (mmol/L)	X			X		
Ischaemic heart disease	Data Rich	Male	Systolic Blood Pressure (mmHg)	X			X		
Ischaemic heart disease	Global	Male	Systolic Blood Pressure (mmHg)	X			X		
Ischaemic heart disease	Data Rich	Male	Smoking Prevalence	X			X		
Ischaemic heart disease	Global	Male	Smoking Prevalence	X			X		
Ischaemic heart disease	Data Rich	Male	Log-transformed SEV scalar: IHD	X			X		
Ischaemic heart disease	Global	Male	Log-transformed SEV scalar: IHD	X			X		
Stroke	Data Rich	Female	LDI (I$ per capita)			X			X
Stroke	Global	Female	LDI (I$ per capita)			X			X
Stroke	Data Rich	Female	Liters of alcohol consumed per capita			X			X
Stroke	Global	Female	Liters of alcohol consumed per capita			X			X
Stroke	Data Rich	Female	Age- and sex-specific SEV for Low fruit			X			X
Stroke	Global	Female	Age- and sex-specific SEV for Low fruit			X			X
Stroke	Data Rich	Female	Age- and sex-specific SEV for Low vegetables			X			X
Stroke	Global	Female	Age- and sex-specific SEV for Low vegetables			X			X
Stroke	Data Rich	Female	Age- and sex-specific SEV for Low PUFA			X			X
Stroke	Global	Female	Age- and sex-specific SEV for Low PUFA			X			X
Stroke	Data Rich	Female	Age- and sex-specific SEV for Low nuts and seeds			X			X

Comparison of GBD 2019 and GBD 2021 covariates and level of covariates used in cause of death modeling									
Cause	Model Version Type	Sex	Covariate	Level 1 GBD 2019	Level 2 GBD 2019	Level 3 GBD 2019	Level 1 GBD 2021	Level 2 GBD 2021	Level 3 GBD 2021
Stroke	Global	Female	Age- and sex-specific SEV for Low nuts and seeds			X			X
Stroke	Data Rich	Female	Age- and sex-specific SEV for Low omega-3			X			X
Stroke	Global	Female	Age- and sex-specific SEV for Low omega-3			X			X
Stroke	Data Rich	Female	Pulses legumes unadjusted(g)			X			X
Stroke	Global	Female	Pulses legumes unadjusted(g)			X			X
Stroke	Data Rich	Female	Diet high in trans fatty acids			X			X
Stroke	Global	Female	Diet high in trans fatty acids			X			X
Stroke	Data Rich	Female	Healthcare access and quality index		X			X	
Stroke	Global	Female	Healthcare access and quality index		X			X	
Stroke	Data Rich	Female	Mean BMI		X			X	
Stroke	Global	Female	Mean BMI		X			X	
Stroke	Data Rich	Female	Indoor Air Pollution (All Cooking Fuels)		X			X	
Stroke	Global	Female	Indoor Air Pollution (All Cooking Fuels)		X			X	
Stroke	Data Rich	Female	Outdoor Air Pollution (PM2.5)		X			X	
Stroke	Global	Female	Outdoor Air Pollution (PM2.5)		X			X	
Stroke	Data Rich	Female	Elevation Over 1500m (proportion)		X			X	
Stroke	Global	Female	Elevation Over 1500m (proportion)		X			X	
Stroke	Data Rich	Female	Diabetes Fasting Plasma Glucose (mmol/L), age-standardized 25+		X			X	
Stroke	Global	Female	Diabetes Fasting Plasma Glucose (mmol/L), age-standardized 25+		X			X	
Stroke	Data Rich	Female	Low-Density Lipoprotein (mmol/L)	X			X		
Stroke	Global	Female	Low-Density Lipoprotein (mmol/L)	X			X		
Stroke	Data Rich	Female	Systolic Blood Pressure (mmHg)	X			X		
Stroke	Global	Female	Systolic Blood Pressure (mmHg)	X			X		
Stroke	Data Rich	Female	Smoking Prevalence	X			X		
Stroke	Global	Female	Smoking Prevalence	X			X		
Stroke	Data Rich	Female	Log-transformed SEV scalar: Stroke	X			X		
Stroke	Global	Female	Log-transformed SEV scalar: Stroke	X			X		
Stroke	Data Rich	Male	LDI (I$ per capita)			X			X
Stroke	Global	Male	LDI (I$ per capita)			X			X
Stroke	Data Rich	Male	Liters of alcohol consumed per capita			X			X
Stroke	Global	Male	Liters of alcohol consumed per capita			X			X
Stroke	Data Rich	Male	Age- and sex-specific SEV for Low fruit			X			X
Stroke	Global	Male	Age- and sex-specific SEV for Low fruit			X			X
Stroke	Data Rich	Male	Age- and sex-specific SEV for Low vegetables			X			X
Stroke	Global	Male	Age- and sex-specific SEV for Low vegetables			X			X
Stroke	Data Rich	Male	Age- and sex-specific SEV for Low PUFA			X			X
Stroke	Global	Male	Age- and sex-specific SEV for Low PUFA			X			X
Stroke	Data Rich	Male	Age- and sex-specific SEV for Low nuts and seeds			X			X
Stroke	Global	Male	Age- and sex-specific SEV for Low nuts and seeds			X			X
Stroke	Data Rich	Male	Age- and sex-specific SEV for Low omega-3			X			X
Stroke	Global	Male	Age- and sex-specific SEV for Low omega-3			X			X
Stroke	Data Rich	Male	Pulses legumes unadjusted(g)			X			X
Stroke	Global	Male	Pulses legumes unadjusted(g)			X			X
Stroke	Data Rich	Male	Diet high in trans fatty acids			X			X
Stroke	Global	Male	Diet high in trans fatty acids			X			X
Stroke	Data Rich	Male	Healthcare access and quality index		X			X	
Stroke	Global	Male	Healthcare access and quality index		X			X	
Stroke	Data Rich	Male	Mean BMI		X			X	
Stroke	Global	Male	Mean BMI		X			X	
Stroke	Data Rich	Male	Indoor Air Pollution (All Cooking Fuels)		X			X	
Stroke	Global	Male	Indoor Air Pollution (All Cooking Fuels)		X			X	
Stroke	Data Rich	Male	Outdoor Air Pollution (PM2.5)		X			X	
Stroke	Global	Male	Outdoor Air Pollution (PM2.5)		X			X	
Stroke	Data Rich	Male	Elevation Over 1500m (proportion)		X			X	
Stroke	Global	Male	Elevation Over 1500m (proportion)		X			X	
Stroke	Data Rich	Male	Diabetes Fasting Plasma Glucose (mmol/L), age-standardized 25+		X			X	
Stroke	Global	Male	Diabetes Fasting Plasma Glucose (mmol/L), age-standardized 25+		X			X	
Stroke	Data Rich	Male	Low-Density Lipoprotein (mmol/L)	X			X		
Stroke	Global	Male	Low-Density Lipoprotein (mmol/L)	X			X		
Stroke	Data Rich	Male	Systolic Blood Pressure (mmHg)	X			X		
Stroke	Global	Male	Systolic Blood Pressure (mmHg)	X			X		
Stroke	Data Rich	Male	Smoking Prevalence	X			X		
Stroke	Global	Male	Smoking Prevalence	X			X		
Stroke	Data Rich	Male	Log-transformed SEV scalar: Stroke	X			X		
Stroke	Global	Male	Log-transformed SEV scalar: Stroke	X			X		
Ischaemic stroke	Data Rich	Female	LDI (I$ per capita)			X			X
Ischaemic stroke	Global	Female	LDI (I$ per capita)			X			X
Ischaemic stroke	Data Rich	Female	Liters of alcohol consumed per capita			X			X
Ischaemic stroke	Global	Female	Liters of alcohol consumed per capita			X			X
Ischaemic stroke	Data Rich	Female	Age- and sex-specific SEV for Low fruit			X			X
Ischaemic stroke	Global	Female	Age- and sex-specific SEV for Low fruit			X			X
Ischaemic stroke	Data Rich	Female	Age- and sex-specific SEV for Low vegetables			X			X
Ischaemic stroke	Global	Female	Age- and sex-specific SEV for Low vegetables			X			X
Ischaemic stroke	Data Rich	Female	Age- and sex-specific SEV for Low PUFA			X			X
Ischaemic stroke	Global	Female	Age- and sex-specific SEV for Low PUFA			X			X
Ischaemic stroke	Data Rich	Female	Age- and sex-specific SEV for Low nuts and seeds			X			X
Ischaemic stroke	Global	Female	Age- and sex-specific SEV for Low nuts and seeds			X			X
Ischaemic stroke	Data Rich	Female	Age- and sex-specific SEV for Low omega-3			X			X
Ischaemic stroke	Global	Female	Age- and sex-specific SEV for Low omega-3			X			X
Ischaemic stroke	Data Rich	Female	Pulses legumes unadjusted(g)			X			X
Ischaemic stroke	Global	Female	Pulses legumes unadjusted(g)			X			X
Ischaemic stroke	Data Rich	Female	Diet high in trans fatty acids			X			X
Ischaemic stroke	Global	Female	Diet high in trans fatty acids			X			X
Ischaemic stroke	Data Rich	Female	Healthcare access and quality index		X			X	
Ischaemic stroke	Global	Female	Healthcare access and quality index		X			X	
Ischaemic stroke	Data Rich	Female	Mean BMI		X			X	
Ischaemic stroke	Global	Female	Mean BMI		X			X	
Ischaemic stroke	Data Rich	Female	Indoor Air Pollution (All Cooking Fuels)		X			X	
Ischaemic stroke	Global	Female	Indoor Air Pollution (All Cooking Fuels)		X			X	
Ischaemic stroke	Data Rich	Female	Outdoor Air Pollution (PM2.5)		X			X	
Ischaemic stroke	Global	Female	Outdoor Air Pollution (PM2.5)		X			X	
Ischaemic stroke	Data Rich	Female	Elevation Over 1500m (proportion)		X			X	
Ischaemic stroke	Global	Female	Elevation Over 1500m (proportion)		X			X	
Ischaemic stroke	Data Rich	Female	Diabetes Fasting Plasma Glucose (mmol/L), age-standardized 25+		X			X	
Ischaemic stroke	Global	Female	Diabetes Fasting Plasma Glucose (mmol/L), age-standardized 25+		X			X	
Ischaemic stroke	Data Rich	Female	Low-Density Lipoprotein (mmol/L)	X			X		
Ischaemic stroke	Global	Female	Low-Density Lipoprotein (mmol/L)	X			X		
Ischaemic stroke	Data Rich	Female	Systolic Blood Pressure (mmHg)	X			X		
Ischaemic stroke	Global	Female	Systolic Blood Pressure (mmHg)	X			X		
Ischaemic stroke	Data Rich	Female	Smoking Prevalence	X			X		
Ischaemic stroke	Global	Female	Smoking Prevalence	X			X		
Ischaemic stroke	Data Rich	Female	Log-transformed SEV scalar: Isch Stroke	X			X		
Ischaemic stroke	Global	Female	Log-transformed SEV scalar: Isch Stroke	X			X		
Ischaemic stroke	Data Rich	Male	LDI (I$ per capita)			X			X
Ischaemic stroke	Global	Male	LDI (I$ per capita)			X			X
Ischaemic stroke	Data Rich	Male	Liters of alcohol consumed per capita			X			X
Ischaemic stroke	Global	Male	Liters of alcohol consumed per capita			X			X
Ischaemic stroke	Data Rich	Male	Age- and sex-specific SEV for Low fruit			X			X
Ischaemic stroke	Global	Male	Age- and sex-specific SEV for Low fruit			X			X
Ischaemic stroke	Data Rich	Male	Age- and sex-specific SEV for Low vegetables			X			X
Ischaemic stroke	Global	Male	Age- and sex-specific SEV for Low vegetables			X			X
Ischaemic stroke	Data Rich	Male	Age- and sex-specific SEV for Low PUFA			X			X
Ischaemic stroke	Global	Male	Age- and sex-specific SEV for Low PUFA			X			X
Ischaemic stroke	Data Rich	Male	Age- and sex-specific SEV for Low nuts and seeds			X			X
Ischaemic stroke	Global	Male	Age- and sex-specific SEV for Low nuts and seeds			X			X
Ischaemic stroke	Data Rich	Male	Age- and sex-specific SEV for Low omega-3			X			X
Ischaemic stroke	Global	Male	Age- and sex-specific SEV for Low omega-3			X			X
Ischaemic stroke	Data Rich	Male	Pulses legumes unadjusted(g)			X			X
Ischaemic stroke	Global	Male	Pulses legumes unadjusted(g)			X			X
Ischaemic stroke	Data Rich	Male	Diet high in trans fatty acids			X			X
Ischaemic stroke	Global	Male	Diet high in trans fatty acids			X			X
Ischaemic stroke	Data Rich	Male	Healthcare access and quality index		X			X	
Ischaemic stroke	Global	Male	Healthcare access and quality index		X			X	
Ischaemic stroke	Data Rich	Male	Mean BMI		X			X	
Ischaemic stroke	Global	Male	Mean BMI		X			X	
Ischaemic stroke	Data Rich	Male	Indoor Air Pollution (All Cooking Fuels)		X			X	
Ischaemic stroke	Global	Male	Indoor Air Pollution (All Cooking Fuels)		X			X	
Ischaemic stroke	Data Rich	Male	Outdoor Air Pollution (PM2.5)		X			X	

Comparison of GBD 2019 and GBD 2021 covariates and level of covariates used in cause of death modeling

Cause	Model Version Type	Sex	Covariate	Level 1 GBD 2019	Level 2 GBD 2019	Level 3 GBD 2019	Level 1 GBD 2021	Level 2 GBD 2021	Level 3 GBD 2021
Ischaemic stroke	Global	Male	Outdoor Air Pollution (PM2.5)		X			X	
Ischaemic stroke	Data Rich	Male	Elevation Over 1500m (proportion)		X			X	
Ischaemic stroke	Global	Male	Elevation Over 1500m (proportion)		X			X	
Ischaemic stroke	Data Rich	Male	Diabetes Fasting Plasma Glucose (mmol/L), age-standardized 25+		X			X	
Ischaemic stroke	Global	Male	Diabetes Fasting Plasma Glucose (mmol/L), age-standardized 25+		X			X	
Ischaemic stroke	Data Rich	Male	Low-Density Lipoprotein (mmol/L)	X			X		
Ischaemic stroke	Global	Male	Low-Density Lipoprotein (mmol/L)	X			X		
Ischaemic stroke	Data Rich	Male	Systolic Blood Pressure (mmHg)	X			X		
Ischaemic stroke	Global	Male	Systolic Blood Pressure (mmHg)	X			X		
Ischaemic stroke	Data Rich	Male	Smoking Prevalence	X			X		
Ischaemic stroke	Global	Male	Smoking Prevalence	X			X		
Ischaemic stroke	Data Rich	Male	Log-transformed SEV scalar: Isch Stroke	X			X		
Ischaemic stroke	Global	Male	Log-transformed SEV scalar: Isch Stroke	X			X		
Intracerebral hemorrhage	Data Rich	Female	LDI (I$ per capita)			X			X
Intracerebral hemorrhage	Global	Female	LDI (I$ per capita)			X			X
Intracerebral hemorrhage	Data Rich	Female	Low-Density Lipoprotein (mmol/L)			X			X
Intracerebral hemorrhage	Global	Female	Low-Density Lipoprotein (mmol/L)			X			X
Intracerebral hemorrhage	Data Rich	Female	Liters of alcohol consumed per capita			X			X
Intracerebral hemorrhage	Global	Female	Liters of alcohol consumed per capita			X			X
Intracerebral hemorrhage	Data Rich	Female	Age- and sex-specific SEV for Low fruit			X			X
Intracerebral hemorrhage	Global	Female	Age- and sex-specific SEV for Low fruit			X			X
Intracerebral hemorrhage	Global	Female	Age- and sex-specific SEV for Low vegetables			X			X
Intracerebral hemorrhage	Data Rich	Female	Age- and sex-specific SEV for Low PUFA			X			X
Intracerebral hemorrhage	Global	Female	Age- and sex-specific SEV for Low PUFA			X			X
Intracerebral hemorrhage	Data Rich	Female	Age- and sex-specific SEV for Low nuts and seeds			X			X
Intracerebral hemorrhage	Global	Female	Age- and sex-specific SEV for Low nuts and seeds			X			X
Intracerebral hemorrhage	Data Rich	Female	Age- and sex-specific SEV for Low omega-3			X			X
Intracerebral hemorrhage	Global	Female	Age- and sex-specific SEV for Low omega-3			X			X
Intracerebral hemorrhage	Data Rich	Female	Pulses legumes unadjusted(g)			X			X
Intracerebral hemorrhage	Global	Female	Pulses legumes unadjusted(g)			X			X
Intracerebral hemorrhage	Data Rich	Female	Diet high in trans fatty acids			X			X
Intracerebral hemorrhage	Global	Female	Diet high in trans fatty acids			X			X
Intracerebral hemorrhage	Data Rich	Female	Healthcare access and quality index		X			X	
Intracerebral hemorrhage	Global	Female	Healthcare access and quality index		X			X	
Intracerebral hemorrhage	Data Rich	Female	Mean BMI		X			X	
Intracerebral hemorrhage	Global	Female	Mean BMI		X			X	
Intracerebral hemorrhage	Data Rich	Female	Indoor Air Pollution (All Cooking Fuels)		X			X	
Intracerebral hemorrhage	Global	Female	Indoor Air Pollution (All Cooking Fuels)		X			X	
Intracerebral hemorrhage	Data Rich	Female	Outdoor Air Pollution (PM2.5)		X			X	
Intracerebral hemorrhage	Global	Female	Outdoor Air Pollution (PM2.5)		X			X	
Intracerebral hemorrhage	Data Rich	Female	Elevation Over 1500m (proportion)		X			X	
Intracerebral hemorrhage	Global	Female	Elevation Over 1500m (proportion)		X			X	
Intracerebral hemorrhage	Data Rich	Female	Diabetes Fasting Plasma Glucose (mmol/L), age-standardized 25+		X			X	
Intracerebral hemorrhage	Global	Female	Diabetes Fasting Plasma Glucose (mmol/L), age-standardized 25+		X			X	
Intracerebral hemorrhage	Data Rich	Female	Systolic Blood Pressure (mmHg)	X			X		
Intracerebral hemorrhage	Global	Female	Systolic Blood Pressure (mmHg)	X			X		
Intracerebral hemorrhage	Data Rich	Female	Smoking Prevalence	X			X		
Intracerebral hemorrhage	Global	Female	Smoking Prevalence	X			X		
Intracerebral hemorrhage	Data Rich	Female	Log-transformed SEV scalar: Intrahem Stroke	X			X		
Intracerebral hemorrhage	Global	Female	Log-transformed SEV scalar: Intrahem Stroke	X			X		
Intracerebral hemorrhage	Data Rich	Male	LDI (I$ per capita)			X			X
Intracerebral hemorrhage	Global	Male	LDI (I$ per capita)			X			X
Intracerebral hemorrhage	Data Rich	Male	Low-Density Lipoprotein (mmol/L)			X			X
Intracerebral hemorrhage	Global	Male	Low-Density Lipoprotein (mmol/L)			X			X
Intracerebral hemorrhage	Data Rich	Male	Liters of alcohol consumed per capita			X			X
Intracerebral hemorrhage	Global	Male	Liters of alcohol consumed per capita			X			X
Intracerebral hemorrhage	Data Rich	Male	Age- and sex-specific SEV for Low fruit			X			X
Intracerebral hemorrhage	Global	Male	Age- and sex-specific SEV for Low fruit			X			X
Intracerebral hemorrhage	Data Rich	Male	Age- and sex-specific SEV for Low vegetables			X			X
Intracerebral hemorrhage	Global	Male	Age- and sex-specific SEV for Low vegetables			X			X
Intracerebral hemorrhage	Data Rich	Male	Age- and sex-specific SEV for Low PUFA			X			X
Intracerebral hemorrhage	Global	Male	Age- and sex-specific SEV for Low PUFA			X			X
Intracerebral hemorrhage	Data Rich	Male	Age- and sex-specific SEV for Low nuts and seeds			X			X
Intracerebral hemorrhage	Global	Male	Age- and sex-specific SEV for Low nuts and seeds			X			X
Intracerebral hemorrhage	Data Rich	Male	Age- and sex-specific SEV for Low omega-3			X			X
Intracerebral hemorrhage	Global	Male	Age- and sex-specific SEV for Low omega-3			X			X
Intracerebral hemorrhage	Data Rich	Male	Pulses legumes unadjusted(g)			X			X
Intracerebral hemorrhage	Global	Male	Pulses legumes unadjusted(g)			X			X
Intracerebral hemorrhage	Data Rich	Male	Diet high in trans fatty acids			X			X
Intracerebral hemorrhage	Global	Male	Diet high in trans fatty acids			X			X
Intracerebral hemorrhage	Data Rich	Male	Healthcare access and quality index		X			X	
Intracerebral hemorrhage	Global	Male	Healthcare access and quality index		X			X	
Intracerebral hemorrhage	Data Rich	Male	Mean BMI		X			X	
Intracerebral hemorrhage	Global	Male	Mean BMI		X			X	
Intracerebral hemorrhage	Data Rich	Male	Indoor Air Pollution (All Cooking Fuels)		X			X	
Intracerebral hemorrhage	Global	Male	Indoor Air Pollution (All Cooking Fuels)		X			X	
Intracerebral hemorrhage	Data Rich	Male	Outdoor Air Pollution (PM2.5)		X			X	
Intracerebral hemorrhage	Global	Male	Outdoor Air Pollution (PM2.5)		X			X	
Intracerebral hemorrhage	Data Rich	Male	Elevation Over 1500m (proportion)		X			X	
Intracerebral hemorrhage	Global	Male	Elevation Over 1500m (proportion)		X			X	
Intracerebral hemorrhage	Data Rich	Male	Diabetes Fasting Plasma Glucose (mmol/L), age-standardized 25+		X			X	
Intracerebral hemorrhage	Global	Male	Diabetes Fasting Plasma Glucose (mmol/L), age-standardized 25+		X			X	
Intracerebral hemorrhage	Data Rich	Male	Systolic Blood Pressure (mmHg)	X			X		
Intracerebral hemorrhage	Global	Male	Systolic Blood Pressure (mmHg)	X			X		
Intracerebral hemorrhage	Data Rich	Male	Smoking Prevalence	X			X		
Intracerebral hemorrhage	Global	Male	Smoking Prevalence	X			X		
Intracerebral hemorrhage	Data Rich	Male	Log-transformed SEV scalar: Intrahem Stroke	X			X		
Intracerebral hemorrhage	Global	Male	Log-transformed SEV scalar: Intrahem Stroke	X			X		
Subarachnoid hemorrhage	Data Rich	Female	LDI (I$ per capita)			X			X
Subarachnoid hemorrhage	Global	Female	LDI (I$ per capita)			X			X
Subarachnoid hemorrhage	Data Rich	Female	Liters of alcohol consumed per capita			X			X
Subarachnoid hemorrhage	Global	Female	Liters of alcohol consumed per capita			X			X
Subarachnoid hemorrhage	Data Rich	Female	Healthcare access and quality index		X			X	
Subarachnoid hemorrhage	Global	Female	Healthcare access and quality index		X			X	
Subarachnoid hemorrhage	Data Rich	Female	Systolic Blood Pressure (mmHg)	X			X		
Subarachnoid hemorrhage	Global	Female	Systolic Blood Pressure (mmHg)	X			X		
Subarachnoid hemorrhage	Data Rich	Female	Smoking Prevalence	X			X		
Subarachnoid hemorrhage	Global	Female	Smoking Prevalence	X			X		
Subarachnoid hemorrhage	Data Rich	Female	Log-transformed SEV scalar: Sub Hem	X			X		
Subarachnoid hemorrhage	Global	Female	Log-transformed SEV scalar: Sub Hem	X			X		
Subarachnoid hemorrhage	Data Rich	Male	LDI (I$ per capita)			X			X
Subarachnoid hemorrhage	Global	Male	LDI (I$ per capita)			X			X
Subarachnoid hemorrhage	Data Rich	Male	Liters of alcohol consumed per capita			X			X
Subarachnoid hemorrhage	Global	Male	Liters of alcohol consumed per capita			X			X
Subarachnoid hemorrhage	Data Rich	Male	Healthcare access and quality index		X			X	
Subarachnoid hemorrhage	Global	Male	Healthcare access and quality index		X			X	
Subarachnoid hemorrhage	Data Rich	Male	Systolic Blood Pressure (mmHg)	X			X		
Subarachnoid hemorrhage	Global	Male	Systolic Blood Pressure (mmHg)	X			X		
Subarachnoid hemorrhage	Data Rich	Male	Smoking Prevalence	X			X		
Subarachnoid hemorrhage	Global	Male	Smoking Prevalence	X			X		
Subarachnoid hemorrhage	Data Rich	Male	Log-transformed SEV scalar: Sub Hem	X			X		
Subarachnoid hemorrhage	Global	Male	Log-transformed SEV scalar: Sub Hem	X			X		
Hypertensive heart disease	Global	Female	LDI (I$ per capita)			X			X
Hypertensive heart disease	Data Rich	Female	LDI (I$ per capita)			X			X
Hypertensive heart disease	Global	Female	Socio-demographic Index			X			X
Hypertensive heart disease	Data Rich	Female	Socio-demographic Index			X			X
Hypertensive heart disease	Global	Female	Liters of alcohol consumed per capita			X			X
Hypertensive heart disease	Data Rich	Female	Liters of alcohol consumed per capita			X			X
Hypertensive heart disease	Global	Female	Age- and sex-specific SEV for Low fruit			X			X
Hypertensive heart disease	Data Rich	Female	Age- and sex-specific SEV for Low fruit			X			X
Hypertensive heart disease	Global	Female	Age- and sex-specific SEV for Low vegetables			X			X
Hypertensive heart disease	Data Rich	Female	Age- and sex-specific SEV for Low vegetables			X			X
Hypertensive heart disease	Global	Female	Age- and sex-specific SEV for Low PUFA			X			X
Hypertensive heart disease	Data Rich	Female	Age- and sex-specific SEV for Low PUFA			X			X
Hypertensive heart disease	Global	Female	Age- and sex-specific SEV for Low nuts and seeds			X			X
Hypertensive heart disease	Data Rich	Female	Age- and sex-specific SEV for Low nuts and seeds			X			X
Hypertensive heart disease	Global	Female	Age- and sex-specific SEV for Low omega-3			X			X
Hypertensive heart disease	Data Rich	Female	Age- and sex-specific SEV for Low omega-3			X			X

Comparison of GBD 2019 and GBD 2021 covariates and level of covariates used in cause of death modeling

Cause	Model Version Type	Sex	Covariate	Level 1 GBD 2019	Level 2 GBD 2019	Level 3 GBD 2019	Level 1 GBD 2021	Level 2 GBD 2021	Level 3 GBD 2021
Hypertensive heart disease	Global	Female	Pulses legumes unadjusted(g)			X			X
Hypertensive heart disease	Data Rich	Female	Pulses legumes unadjusted(g)			X			X
Hypertensive heart disease	Global	Female	Diet high in trans fatty acids			X			X
Hypertensive heart disease	Data Rich	Female	Diet high in trans fatty acids			X			X
Hypertensive heart disease	Global	Female	Healthcare access and quality index		X			X	
Hypertensive heart disease	Data Rich	Female	Healthcare access and quality index		X			X	
Hypertensive heart disease	Global	Female	Mean BMI		X			X	
Hypertensive heart disease	Data Rich	Female	Mean BMI		X			X	
Hypertensive heart disease	Global	Female	Low-Density Lipoprotein (mmol/L)		X			X	
Hypertensive heart disease	Data Rich	Female	Low-Density Lipoprotein (mmol/L)		X			X	
Hypertensive heart disease	Global	Female	Smoking Prevalence		X			X	
Hypertensive heart disease	Data Rich	Female	Smoking Prevalence		X			X	
Hypertensive heart disease	Global	Female	Systolic Blood Pressure (mmHg)	X			X		
Hypertensive heart disease	Data Rich	Female	Systolic Blood Pressure (mmHg)	X			X		
Hypertensive heart disease	Global	Male	LDI (I$ per capita)			X			X
Hypertensive heart disease	Data Rich	Male	LDI (I$ per capita)			X			X
Hypertensive heart disease	Global	Male	Socio-demographic Index			X			X
Hypertensive heart disease	Data Rich	Male	Socio-demographic Index			X			X
Hypertensive heart disease	Global	Male	Liters of alcohol consumed per capita			X			X
Hypertensive heart disease	Data Rich	Male	Liters of alcohol consumed per capita			X			X
Hypertensive heart disease	Global	Male	Age- and sex-specific SEV for Low fruit			X			X
Hypertensive heart disease	Data Rich	Male	Age- and sex-specific SEV for Low fruit			X			X
Hypertensive heart disease	Global	Male	Age- and sex-specific SEV for Low vegetables			X			X
Hypertensive heart disease	Data Rich	Male	Age- and sex-specific SEV for Low vegetables			X			X
Hypertensive heart disease	Global	Male	Age- and sex-specific SEV for Low PUFA			X			X
Hypertensive heart disease	Data Rich	Male	Age- and sex-specific SEV for Low PUFA			X			X
Hypertensive heart disease	Global	Male	Age- and sex-specific SEV for Low nuts and seeds			X			X
Hypertensive heart disease	Data Rich	Male	Age- and sex-specific SEV for Low nuts and seeds			X			X
Hypertensive heart disease	Global	Male	Age- and sex-specific SEV for Low omega-3			X			X
Hypertensive heart disease	Data Rich	Male	Age- and sex-specific SEV for Low omega-3			X			X
Hypertensive heart disease	Global	Male	Pulses legumes unadjusted(g)			X			X
Hypertensive heart disease	Data Rich	Male	Pulses legumes unadjusted(g)			X			X
Hypertensive heart disease	Global	Male	Diet high in trans fatty acids			X			X
Hypertensive heart disease	Data Rich	Male	Diet high in trans fatty acids			X			X
Hypertensive heart disease	Global	Male	Healthcare access and quality index		X			X	
Hypertensive heart disease	Data Rich	Male	Healthcare access and quality index		X			X	
Hypertensive heart disease	Global	Male	Mean BMI		X			X	
Hypertensive heart disease	Data Rich	Male	Mean BMI		X			X	
Hypertensive heart disease	Global	Male	Low-Density Lipoprotein (mmol/L)		X			X	
Hypertensive heart disease	Data Rich	Male	Low-Density Lipoprotein (mmol/L)		X			X	
Hypertensive heart disease	Global	Male	Smoking Prevalence		X			X	
Hypertensive heart disease	Data Rich	Male	Smoking Prevalence		X			X	
Hypertensive heart disease	Global	Male	Systolic Blood Pressure (mmHg)	X			X		
Hypertensive heart disease	Data Rich	Male	Systolic Blood Pressure (mmHg)	X			X		
Non-rheumatic valvular heart disease	Data Rich	Female	LDI (I$ per capita)			X			X
Non-rheumatic valvular heart disease	Global	Female	LDI (I$ per capita)			X			X
Non-rheumatic valvular heart disease	Data Rich	Female	Socio-demographic Index			X			X
Non-rheumatic valvular heart disease	Global	Female	Socio-demographic Index			X			X
Non-rheumatic valvular heart disease	Data Rich	Female	Liters of alcohol consumed per capita			X			X
Non-rheumatic valvular heart disease	Global	Female	Liters of alcohol consumed per capita			X			X
Non-rheumatic valvular heart disease	Data Rich	Female	Healthcare access and quality index		X			X	
Non-rheumatic valvular heart disease	Global	Female	Healthcare access and quality index		X			X	
Non-rheumatic valvular heart disease	Data Rich	Female	Mean BMI		X			X	
Non-rheumatic valvular heart disease	Global	Female	Mean BMI		X			X	
Non-rheumatic valvular heart disease	Data Rich	Female	Low-Density Lipoprotein (mmol/L)		X			X	
Non-rheumatic valvular heart disease	Global	Female	Low-Density Lipoprotein (mmol/L)		X			X	
Non-rheumatic valvular heart disease	Data Rich	Female	Systolic Blood Pressure (mmHg)	X			X		
Non-rheumatic valvular heart disease	Global	Female	Systolic Blood Pressure (mmHg)	X			X		
Non-rheumatic valvular heart disease	Data Rich	Female	Smoking Prevalence	X			X		
Non-rheumatic valvular heart disease	Global	Female	Smoking Prevalence	X			X		
Non-rheumatic valvular heart disease	Data Rich	Female	Log-transformed SEV scalar: Valvular	X			X		
Non-rheumatic valvular heart disease	Global	Female	Log-transformed SEV scalar: Valvular	X			X		
Non-rheumatic valvular heart disease	Data Rich	Male	LDI (I$ per capita)			X			X
Non-rheumatic valvular heart disease	Global	Male	LDI (I$ per capita)			X			X
Non-rheumatic valvular heart disease	Data Rich	Male	Socio-demographic Index			X			X
Non-rheumatic valvular heart disease	Global	Male	Socio-demographic Index			X			X
Non-rheumatic valvular heart disease	Data Rich	Male	Liters of alcohol consumed per capita			X			X
Non-rheumatic valvular heart disease	Global	Male	Liters of alcohol consumed per capita			X			X
Non-rheumatic valvular heart disease	Data Rich	Male	Healthcare access and quality index		X			X	
Non-rheumatic valvular heart disease	Global	Male	Healthcare access and quality index		X			X	
Non-rheumatic valvular heart disease	Data Rich	Male	Mean BMI		X			X	
Non-rheumatic valvular heart disease	Global	Male	Mean BMI		X			X	
Non-rheumatic valvular heart disease	Data Rich	Male	Low-Density Lipoprotein (mmol/L)		X			X	
Non-rheumatic valvular heart disease	Global	Male	Low-Density Lipoprotein (mmol/L)		X			X	
Non-rheumatic valvular heart disease	Data Rich	Male	Systolic Blood Pressure (mmHg)	X			X		
Non-rheumatic valvular heart disease	Global	Male	Systolic Blood Pressure (mmHg)	X			X		
Non-rheumatic valvular heart disease	Data Rich	Male	Smoking Prevalence	X			X		
Non-rheumatic valvular heart disease	Global	Male	Smoking Prevalence	X			X		
Non-rheumatic valvular heart disease	Data Rich	Male	Log-transformed SEV scalar: Valvular	X			X		
Non-rheumatic valvular heart disease	Global	Male	Log-transformed SEV scalar: Valvular	X			X		
Non-rheumatic calcific aortic valve disease	Data Rich	Female	LDI (I$ per capita)			X			X
Non-rheumatic calcific aortic valve disease	Global	Female	LDI (I$ per capita)			X			X
Non-rheumatic calcific aortic valve disease	Data Rich	Female	Socio-demographic Index			X			X
Non-rheumatic calcific aortic valve disease	Global	Female	Socio-demographic Index			X			X
Non-rheumatic calcific aortic valve disease	Data Rich	Female	Liters of alcohol consumed per capita			X			X
Non-rheumatic calcific aortic valve disease	Global	Female	Liters of alcohol consumed per capita			X			X
Non-rheumatic calcific aortic valve disease	Data Rich	Female	Healthcare access and quality index		X			X	
Non-rheumatic calcific aortic valve disease	Global	Female	Healthcare access and quality index		X			X	
Non-rheumatic calcific aortic valve disease	Data Rich	Female	Low-Density Lipoprotein (mmol/L)		X			X	
Non-rheumatic calcific aortic valve disease	Global	Female	Low-Density Lipoprotein (mmol/L)		X			X	
Non-rheumatic calcific aortic valve disease	Data Rich	Female	Diabetes Fasting Plasma Glucose (mmol/L), age-standardized 25+		X			X	
Non-rheumatic calcific aortic valve disease	Global	Female	Diabetes Fasting Plasma Glucose (mmol/L), age-standardized 25+		X			X	
Non-rheumatic calcific aortic valve disease	Data Rich	Female	Mean BMI	X			X		
Non-rheumatic calcific aortic valve disease	Global	Female	Mean BMI	X			X		
Non-rheumatic calcific aortic valve disease	Data Rich	Female	Systolic Blood Pressure (mmHg)	X			X		
Non-rheumatic calcific aortic valve disease	Global	Female	Systolic Blood Pressure (mmHg)	X			X		
Non-rheumatic calcific aortic valve disease	Data Rich	Female	Smoking Prevalence	X			X		
Non-rheumatic calcific aortic valve disease	Global	Female	Smoking Prevalence	X			X		
Non-rheumatic calcific aortic valve disease	Data Rich	Female	Log-transformed SEV scalar: CAVD	X			X		
Non-rheumatic calcific aortic valve disease	Global	Female	Log-transformed SEV scalar: CAVD	X			X		
Non-rheumatic calcific aortic valve disease	Data Rich	Male	LDI (I$ per capita)			X			X
Non-rheumatic calcific aortic valve disease	Global	Male	LDI (I$ per capita)			X			X
Non-rheumatic calcific aortic valve disease	Data Rich	Male	Socio-demographic Index			X			X
Non-rheumatic calcific aortic valve disease	Global	Male	Socio-demographic Index			X			X
Non-rheumatic calcific aortic valve disease	Data Rich	Male	Liters of alcohol consumed per capita			X			X
Non-rheumatic calcific aortic valve disease	Global	Male	Liters of alcohol consumed per capita			X			X
Non-rheumatic calcific aortic valve disease	Data Rich	Male	Healthcare access and quality index		X			X	
Non-rheumatic calcific aortic valve disease	Global	Male	Healthcare access and quality index		X			X	
Non-rheumatic calcific aortic valve disease	Data Rich	Male	Low-Density Lipoprotein (mmol/L)		X			X	
Non-rheumatic calcific aortic valve disease	Global	Male	Low-Density Lipoprotein (mmol/L)		X			X	
Non-rheumatic calcific aortic valve disease	Data Rich	Male	Diabetes Fasting Plasma Glucose (mmol/L), age-standardized 25+		X			X	
Non-rheumatic calcific aortic valve disease	Global	Male	Diabetes Fasting Plasma Glucose (mmol/L), age-standardized 25+		X			X	
Non-rheumatic calcific aortic valve disease	Data Rich	Male	Mean BMI	X			X		
Non-rheumatic calcific aortic valve disease	Global	Male	Mean BMI	X			X		
Non-rheumatic calcific aortic valve disease	Data Rich	Male	Systolic Blood Pressure (mmHg)	X			X		
Non-rheumatic calcific aortic valve disease	Global	Male	Systolic Blood Pressure (mmHg)	X			X		
Non-rheumatic calcific aortic valve disease	Data Rich	Male	Smoking Prevalence	X			X		
Non-rheumatic calcific aortic valve disease	Global	Male	Smoking Prevalence	X			X		
Non-rheumatic calcific aortic valve disease	Data Rich	Male	Log-transformed SEV scalar: CAVD	X			X		
Non-rheumatic calcific aortic valve disease	Global	Male	Log-transformed SEV scalar: CAVD	X			X		
Non-rheumatic degenerative mitral valve disease	Data Rich	Female	LDI (I$ per capita)	X			X		
Non-rheumatic degenerative mitral valve disease	Global	Female	LDI (I$ per capita)	X			X		
Non-rheumatic degenerative mitral valve disease	Data Rich	Female	Socio-demographic Index	X			X		
Non-rheumatic degenerative mitral valve disease	Global	Female	Socio-demographic Index	X			X		
Non-rheumatic degenerative mitral valve disease	Data Rich	Female	Healthcare access and quality index	X			X		
Non-rheumatic degenerative mitral valve disease	Global	Female	Healthcare access and quality index	X			X		
Non-rheumatic degenerative mitral valve disease	Data Rich	Male	LDI (I$ per capita)	X			X		
Non-rheumatic degenerative mitral valve disease	Global	Male	LDI (I$ per capita)	X			X		
Non-rheumatic degenerative mitral valve disease	Data Rich	Male	Socio-demographic Index	X			X		
Non-rheumatic degenerative mitral valve disease	Global	Male	Socio-demographic Index	X			X		
Non-rheumatic degenerative mitral valve disease	Data Rich	Male	Healthcare access and quality index	X			X		
Non-rheumatic degenerative mitral valve disease	Global	Male	Healthcare access and quality index	X			X		
Other non-rheumatic valve diseases	Data Rich	Female	Socio-demographic Index		X			X	
Other non-rheumatic valve diseases	Global	Female	Socio-demographic Index		X			X	
Other non-rheumatic valve diseases	Data Rich	Female	Healthcare access and quality index	X			X		

Comparison of GBD 2019 and GBD 2021 covariates and level of covariates used in cause of death modeling

Cause	Model Version Type	Sex	Covariate	Level 1 GBD 2019	Level 2 GBD 2019	Level 3 GBD 2019	Level 1 GBD 2021	Level 2 GBD 2021	Level 3 GBD 2021
Other non-rheumatic valve diseases	Global	Female	Healthcare access and quality index	X			X		
Other non-rheumatic valve diseases	Data Rich	Female	Log-transformed SEV scalar: Valvular	X			X		
Other non-rheumatic valve diseases	Global	Female	Log-transformed SEV scalar: Valvular	X			X		
Other non-rheumatic valve diseases	Data Rich	Male	Socio-demographic Index		X			X	
Other non-rheumatic valve diseases	Global	Male	Socio-demographic Index		X			X	
Other non-rheumatic valve diseases	Data Rich	Male	Healthcare access and quality index	X			X		
Other non-rheumatic valve diseases	Global	Male	Healthcare access and quality index	X			X		
Other non-rheumatic valve diseases	Data Rich	Male	Log-transformed SEV scalar: Valvular	X			X		
Other non-rheumatic valve diseases	Global	Male	Log-transformed SEV scalar: Valvular	X			X		
Cardiomyopathy and myocarditis	Global	Female	LDI (I$ per capita)			X			X
Cardiomyopathy and myocarditis	Data Rich	Female	LDI (I$ per capita)			X			X
Cardiomyopathy and myocarditis	Global	Female	Socio-demographic Index			X			X
Cardiomyopathy and myocarditis	Data Rich	Female	Socio-demographic Index			X			X
Cardiomyopathy and myocarditis	Global	Female	Healthcare access and quality index		X			X	
Cardiomyopathy and myocarditis	Data Rich	Female	Healthcare access and quality index		X			X	
Cardiomyopathy and myocarditis	Global	Female	Mean BMI		X			X	
Cardiomyopathy and myocarditis	Data Rich	Female	Mean BMI		X			X	
Cardiomyopathy and myocarditis	Global	Female	Systolic Blood Pressure (mmHg)		X			X	
Cardiomyopathy and myocarditis	Data Rich	Female	Systolic Blood Pressure (mmHg)		X			X	
Cardiomyopathy and myocarditis	Global	Female	Smoking Prevalence	X			X		
Cardiomyopathy and myocarditis	Data Rich	Female	Smoking Prevalence	X			X		
Cardiomyopathy and myocarditis	Global	Female	Log-transformed SEV scalar: CMP	X			X		
Cardiomyopathy and myocarditis	Data Rich	Female	Log-transformed SEV scalar: CMP	X			X		
Cardiomyopathy and myocarditis	Global	Male	LDI (I$ per capita)			X			X
Cardiomyopathy and myocarditis	Data Rich	Male	LDI (I$ per capita)			X			X
Cardiomyopathy and myocarditis	Global	Male	Socio-demographic Index			X			X
Cardiomyopathy and myocarditis	Data Rich	Male	Socio-demographic Index			X			X
Cardiomyopathy and myocarditis	Global	Male	Healthcare access and quality index		X			X	
Cardiomyopathy and myocarditis	Data Rich	Male	Healthcare access and quality index		X			X	
Cardiomyopathy and myocarditis	Global	Male	Mean BMI		X			X	
Cardiomyopathy and myocarditis	Data Rich	Male	Mean BMI		X			X	
Cardiomyopathy and myocarditis	Global	Male	Systolic Blood Pressure (mmHg)		X			X	
Cardiomyopathy and myocarditis	Data Rich	Male	Systolic Blood Pressure (mmHg)		X			X	
Cardiomyopathy and myocarditis	Global	Male	Smoking Prevalence	X			X		
Cardiomyopathy and myocarditis	Data Rich	Male	Smoking Prevalence	X			X		
Cardiomyopathy and myocarditis	Global	Male	Log-transformed SEV scalar: CMP	X			X		
Cardiomyopathy and myocarditis	Data Rich	Male	Log-transformed SEV scalar: CMP	X			X		
Myocarditis	Data Rich	Female	LDI (I$ per capita)			X			X
Myocarditis	Global	Female	LDI (I$ per capita)			X			X
Myocarditis	Data Rich	Female	Socio-demographic Index			X			X
Myocarditis	Global	Female	Socio-demographic Index			X			X
Myocarditis	Data Rich	Female	Healthcare access and quality index		X			X	
Myocarditis	Global	Female	Healthcare access and quality index		X			X	
Myocarditis	Data Rich	Male	LDI (I$ per capita)			X			X
Myocarditis	Global	Male	LDI (I$ per capita)			X			X
Myocarditis	Data Rich	Male	Socio-demographic Index			X			X
Myocarditis	Global	Male	Socio-demographic Index			X			X
Myocarditis	Data Rich	Male	Healthcare access and quality index		X			X	
Myocarditis	Global	Male	Healthcare access and quality index		X			X	
Alcoholic cardiomyopathy	Global	Female	LDI (I$ per capita)			X			X
Alcoholic cardiomyopathy	Data Rich	Female	LDI (I$ per capita)			X			X
Alcoholic cardiomyopathy	Global	Female	Healthcare access and quality index		X			X	
Alcoholic cardiomyopathy	Data Rich	Female	Healthcare access and quality index		X			X	
Alcoholic cardiomyopathy	Global	Female	Smoking Prevalence	X			X		
Alcoholic cardiomyopathy	Data Rich	Female	Smoking Prevalence	X			X		
Alcoholic cardiomyopathy	Global	Female	Log-transformed SEV scalar: CMP	X			X		
Alcoholic cardiomyopathy	Data Rich	Female	Log-transformed SEV scalar: CMP	X			X		
Alcoholic cardiomyopathy	Global	Female	Age- and sex-specific SEV for Alcohol use				X		
Alcoholic cardiomyopathy	Data Rich	Female	Age- and sex-specific SEV for Alcohol use				X		
Alcoholic cardiomyopathy	Global	Male	LDI (I$ per capita)			X			X
Alcoholic cardiomyopathy	Data Rich	Male	LDI (I$ per capita)			X			X
Alcoholic cardiomyopathy	Global	Male	Healthcare access and quality index		X			X	
Alcoholic cardiomyopathy	Data Rich	Male	Healthcare access and quality index		X			X	
Alcoholic cardiomyopathy	Global	Male	Smoking Prevalence	X			X		
Alcoholic cardiomyopathy	Data Rich	Male	Smoking Prevalence	X			X		
Alcoholic cardiomyopathy	Global	Male	Log-transformed SEV scalar: CMP	X			X		
Alcoholic cardiomyopathy	Data Rich	Male	Log-transformed SEV scalar: CMP	X			X		
Alcoholic cardiomyopathy	Global	Male	Age- and sex-specific SEV for Alcohol use				X		
Alcoholic cardiomyopathy	Data Rich	Male	Age- and sex-specific SEV for Alcohol use				X		
Other cardiomyopathy	Data Rich	Female	LDI (I$ per capita)			X			X
Other cardiomyopathy	Global	Female	LDI (I$ per capita)			X			X
Other cardiomyopathy	Data Rich	Female	Socio-demographic Index			X			X
Other cardiomyopathy	Global	Female	Socio-demographic Index			X			X
Other cardiomyopathy	Data Rich	Female	Healthcare access and quality index		X			X	
Other cardiomyopathy	Global	Female	Healthcare access and quality index		X			X	
Other cardiomyopathy	Data Rich	Female	Mean BMI		X			X	
Other cardiomyopathy	Global	Female	Mean BMI		X			X	
Other cardiomyopathy	Data Rich	Female	Systolic Blood Pressure (mmHg)	X			X		
Other cardiomyopathy	Global	Female	Systolic Blood Pressure (mmHg)	X			X		
Other cardiomyopathy	Data Rich	Female	Smoking Prevalence	X			X		
Other cardiomyopathy	Global	Female	Smoking Prevalence	X			X		
Other cardiomyopathy	Data Rich	Female	Log-transformed SEV scalar: CMP	X			X		
Other cardiomyopathy	Global	Female	Log-transformed SEV scalar: CMP	X			X		
Other cardiomyopathy	Data Rich	Male	LDI (I$ per capita)			X			X
Other cardiomyopathy	Global	Male	LDI (I$ per capita)			X			X
Other cardiomyopathy	Data Rich	Male	Socio-demographic Index			X			X
Other cardiomyopathy	Global	Male	Socio-demographic Index			X			X
Other cardiomyopathy	Data Rich	Male	Healthcare access and quality index		X			X	
Other cardiomyopathy	Global	Male	Healthcare access and quality index		X			X	
Other cardiomyopathy	Data Rich	Male	Mean BMI		X			X	
Other cardiomyopathy	Global	Male	Mean BMI		X			X	
Other cardiomyopathy	Data Rich	Male	Systolic Blood Pressure (mmHg)	X			X		
Other cardiomyopathy	Global	Male	Systolic Blood Pressure (mmHg)	X			X		
Other cardiomyopathy	Data Rich	Male	Smoking Prevalence	X			X		
Other cardiomyopathy	Global	Male	Smoking Prevalence	X			X		
Other cardiomyopathy	Data Rich	Male	Log-transformed SEV scalar: CMP	X			X		
Other cardiomyopathy	Global	Male	Log-transformed SEV scalar: CMP	X			X		
Pulmonary Arterial Hypertension	Data Rich	Female	Socio-demographic Index				X		
Pulmonary Arterial Hypertension	Global	Female	Socio-demographic Index				X		
Pulmonary Arterial Hypertension	Data Rich	Female	Healthcare access and quality index				X		
Pulmonary Arterial Hypertension	Global	Female	Healthcare access and quality index				X		
Pulmonary Arterial Hypertension	Data Rich	Female	Log-transformed SEV scalar: HIV				X		
Pulmonary Arterial Hypertension	Global	Female	Log-transformed SEV scalar: HIV				X		
Pulmonary Arterial Hypertension	Data Rich	Female	Schistosomiasis Prevalence Results				X		
Pulmonary Arterial Hypertension	Global	Female	Schistosomiasis Prevalence Results				X		
Pulmonary Arterial Hypertension	Data Rich	Male	Socio-demographic Index				X		
Pulmonary Arterial Hypertension	Global	Male	Socio-demographic Index				X		
Pulmonary Arterial Hypertension	Data Rich	Male	Healthcare access and quality index				X		
Pulmonary Arterial Hypertension	Global	Male	Healthcare access and quality index				X		
Pulmonary Arterial Hypertension	Data Rich	Male	Log-transformed SEV scalar: HIV				X		
Pulmonary Arterial Hypertension	Global	Male	Log-transformed SEV scalar: HIV				X		
Pulmonary Arterial Hypertension	Data Rich	Male	Schistosomiasis Prevalence Results				X		
Pulmonary Arterial Hypertension	Global	Male	Schistosomiasis Prevalence Results				X		
Atrial fibrillation and flutter	Data Rich	Female	LDI (I$ per capita)			X			X
Atrial fibrillation and flutter	Global	Female	LDI (I$ per capita)			X			X
Atrial fibrillation and flutter	Data Rich	Female	Socio-demographic Index			X			X
Atrial fibrillation and flutter	Global	Female	Socio-demographic Index			X			X
Atrial fibrillation and flutter	Data Rich	Female	Liters of alcohol consumed per capita			X			X
Atrial fibrillation and flutter	Global	Female	Liters of alcohol consumed per capita			X			X
Atrial fibrillation and flutter	Data Rich	Female	Age- and sex-specific SEV for Low fruit			X			X
Atrial fibrillation and flutter	Global	Female	Age- and sex-specific SEV for Low fruit			X			X
Atrial fibrillation and flutter	Data Rich	Female	Age- and sex-specific SEV for Low vegetables			X			X
Atrial fibrillation and flutter	Global	Female	Age- and sex-specific SEV for Low vegetables			X			X
Atrial fibrillation and flutter	Data Rich	Female	Age- and sex-specific SEV for Low PUFA			X			X
Atrial fibrillation and flutter	Global	Female	Age- and sex-specific SEV for Low PUFA			X			X
Atrial fibrillation and flutter	Data Rich	Female	Age- and sex-specific SEV for Low nuts and seeds			X			X
Atrial fibrillation and flutter	Global	Female	Age- and sex-specific SEV for Low nuts and seeds			X			X
Atrial fibrillation and flutter	Data Rich	Female	Age- and sex-specific SEV for Low omega-3			X			X
Atrial fibrillation and flutter	Global	Female	Age- and sex-specific SEV for Low omega-3			X			X
Atrial fibrillation and flutter	Data Rich	Female	Pulses legumes unadjusted(g)			X			X
Atrial fibrillation and flutter	Global	Female	Pulses legumes unadjusted(g)			X			X
Atrial fibrillation and flutter	Data Rich	Female	Diet high in trans fatty acids			X			X
Atrial fibrillation and flutter	Global	Female	Diet high in trans fatty acids			X			X
Atrial fibrillation and flutter	Data Rich	Female	Healthcare access and quality index		X			X	
Atrial fibrillation and flutter	Global	Female	Healthcare access and quality index		X			X	
Atrial fibrillation and flutter	Data Rich	Female	Mean BMI		X			X	
Atrial fibrillation and flutter	Global	Female	Mean BMI		X			X	

Comparison of GBD 2019 and GBD 2021 covariates and level of covariates used in cause of death modeling									
Cause	Model Version Type	Sex	Covariate	Level 1 GBD 2019	Level 2 GBD 2019	Level 3 GBD 2019	Level 1 GBD 2021	Level 2 GBD 2021	Level 3 GBD 2021
Atrial fibrillation and flutter	Data Rich	Female	Low-Density Lipoprotein (mmol/L)		X			X	
Atrial fibrillation and flutter	Global	Female	Low-Density Lipoprotein (mmol/L)		X			X	
Atrial fibrillation and flutter	Data Rich	Female	Diabetes Fasting Plasma Glucose (mmol/L), age-standardized 25+		X			X	
Atrial fibrillation and flutter	Global	Female	Diabetes Fasting Plasma Glucose (mmol/L), age-standardized 25+		X			X	
Atrial fibrillation and flutter	Data Rich	Female	Systolic Blood Pressure (mmHg)	X			X		
Atrial fibrillation and flutter	Global	Female	Systolic Blood Pressure (mmHg)	X			X		
Atrial fibrillation and flutter	Data Rich	Female	Smoking Prevalence	X			X		
Atrial fibrillation and flutter	Global	Female	Smoking Prevalence	X			X		
Atrial fibrillation and flutter	Data Rich	Female	Log-transformed SEV scalar: A Fib	X			X		
Atrial fibrillation and flutter	Global	Female	Log-transformed SEV scalar: A Fib	X			X		
Atrial fibrillation and flutter	Data Rich	Male	LDI (I$ per capita)			X			X
Atrial fibrillation and flutter	Global	Male	LDI (I$ per capita)			X			X
Atrial fibrillation and flutter	Data Rich	Male	Socio-demographic Index			X			X
Atrial fibrillation and flutter	Global	Male	Socio-demographic Index			X			X
Atrial fibrillation and flutter	Data Rich	Male	Liters of alcohol consumed per capita			X			X
Atrial fibrillation and flutter	Global	Male	Liters of alcohol consumed per capita			X			X
Atrial fibrillation and flutter	Data Rich	Male	Age- and sex-specific SEV for Low fruit			X			X
Atrial fibrillation and flutter	Global	Male	Age- and sex-specific SEV for Low fruit			X			X
Atrial fibrillation and flutter	Data Rich	Male	Age- and sex-specific SEV for Low vegetables			X			X
Atrial fibrillation and flutter	Global	Male	Age- and sex-specific SEV for Low vegetables			X			X
Atrial fibrillation and flutter	Data Rich	Male	Age- and sex-specific SEV for Low PUFA			X			X
Atrial fibrillation and flutter	Global	Male	Age- and sex-specific SEV for Low PUFA			X			X
Atrial fibrillation and flutter	Data Rich	Male	Age- and sex-specific SEV for Low nuts and seeds			X			X
Atrial fibrillation and flutter	Global	Male	Age- and sex-specific SEV for Low nuts and seeds			X			X
Atrial fibrillation and flutter	Data Rich	Male	Age- and sex-specific SEV for Low omega-3			X			X
Atrial fibrillation and flutter	Global	Male	Age- and sex-specific SEV for Low omega-3			X			X
Atrial fibrillation and flutter	Data Rich	Male	Pulses legumes unadjusted(g)			X			X
Atrial fibrillation and flutter	Global	Male	Pulses legumes unadjusted(g)			X			X
Atrial fibrillation and flutter	Data Rich	Male	Diet high in trans fatty acids			X			X
Atrial fibrillation and flutter	Global	Male	Diet high in trans fatty acids			X			X
Atrial fibrillation and flutter	Data Rich	Male	Healthcare access and quality index		X			X	
Atrial fibrillation and flutter	Global	Male	Healthcare access and quality index		X			X	
Atrial fibrillation and flutter	Data Rich	Male	Mean BMI		X			X	
Atrial fibrillation and flutter	Global	Male	Mean BMI		X			X	
Atrial fibrillation and flutter	Data Rich	Male	Low-Density Lipoprotein (mmol/L)		X			X	
Atrial fibrillation and flutter	Global	Male	Low-Density Lipoprotein (mmol/L)		X			X	
Atrial fibrillation and flutter	Data Rich	Male	Diabetes Fasting Plasma Glucose (mmol/L), age-standardized 25+		X			X	
Atrial fibrillation and flutter	Global	Male	Diabetes Fasting Plasma Glucose (mmol/L), age-standardized 25+		X			X	
Atrial fibrillation and flutter	Data Rich	Male	Systolic Blood Pressure (mmHg)	X			X		
Atrial fibrillation and flutter	Global	Male	Systolic Blood Pressure (mmHg)	X			X		
Atrial fibrillation and flutter	Data Rich	Male	Smoking Prevalence	X			X		
Atrial fibrillation and flutter	Global	Male	Smoking Prevalence	X			X		
Atrial fibrillation and flutter	Data Rich	Male	Log-transformed SEV scalar: A Fib	X			X		
Atrial fibrillation and flutter	Global	Male	Log-transformed SEV scalar: A Fib	X			X		
Aortic aneurysm	Data Rich	Female	LDI (I$ per capita)			X			X
Aortic aneurysm	Global	Female	LDI (I$ per capita)			X			X
Aortic aneurysm	Data Rich	Female	Socio-demographic Index			X			X
Aortic aneurysm	Global	Female	Socio-demographic Index			X			X
Aortic aneurysm	Data Rich	Female	Liters of alcohol consumed per capita			X			X
Aortic aneurysm	Global	Female	Liters of alcohol consumed per capita			X			X
Aortic aneurysm	Data Rich	Female	Age- and sex-specific SEV for Low fruit			X			X
Aortic aneurysm	Global	Female	Age- and sex-specific SEV for Low fruit			X			X
Aortic aneurysm	Data Rich	Female	Age- and sex-specific SEV for Low vegetables			X			X
Aortic aneurysm	Global	Female	Age- and sex-specific SEV for Low vegetables			X			X
Aortic aneurysm	Data Rich	Female	Age- and sex-specific SEV for Low PUFA			X			X
Aortic aneurysm	Global	Female	Age- and sex-specific SEV for Low PUFA			X			X
Aortic aneurysm	Data Rich	Female	Age- and sex-specific SEV for Low nuts and seeds			X			X
Aortic aneurysm	Global	Female	Age- and sex-specific SEV for Low nuts and seeds			X			X
Aortic aneurysm	Data Rich	Female	Age- and sex-specific SEV for Low omega-3			X			X
Aortic aneurysm	Global	Female	Age- and sex-specific SEV for Low omega-3			X			X
Aortic aneurysm	Data Rich	Female	Pulses legumes unadjusted(g)			X			X
Aortic aneurysm	Global	Female	Pulses legumes unadjusted(g)			X			X
Aortic aneurysm	Data Rich	Female	Healthcare access and quality index		X			X	
Aortic aneurysm	Global	Female	Healthcare access and quality index		X			X	
Aortic aneurysm	Data Rich	Female	Mean BMI		X			X	
Aortic aneurysm	Global	Female	Mean BMI		X			X	
Aortic aneurysm	Data Rich	Female	Low-Density Lipoprotein (mmol/L)	X			X		
Aortic aneurysm	Global	Female	Low-Density Lipoprotein (mmol/L)	X			X		
Aortic aneurysm	Data Rich	Female	Systolic Blood Pressure (mmHg)	X			X		
Aortic aneurysm	Global	Female	Systolic Blood Pressure (mmHg)	X			X		
Aortic aneurysm	Data Rich	Female	Cumulative Cigarettes (10 Years)	X			X		
Aortic aneurysm	Global	Female	Cumulative Cigarettes (10 Years)	X			X		
Aortic aneurysm	Data Rich	Female	Log-transformed SEV scalar: Aort An	X			X		
Aortic aneurysm	Global	Female	Log-transformed SEV scalar: Aort An	X			X		
Aortic aneurysm	Data Rich	Male	LDI (I$ per capita)			X			X
Aortic aneurysm	Global	Male	LDI (I$ per capita)			X			X
Aortic aneurysm	Data Rich	Male	Socio-demographic Index			X			X
Aortic aneurysm	Global	Male	Socio-demographic Index			X			X
Aortic aneurysm	Data Rich	Male	Liters of alcohol consumed per capita			X			X
Aortic aneurysm	Global	Male	Liters of alcohol consumed per capita			X			X
Aortic aneurysm	Data Rich	Male	Age- and sex-specific SEV for Low fruit			X			X
Aortic aneurysm	Global	Male	Age- and sex-specific SEV for Low fruit			X			X
Aortic aneurysm	Data Rich	Male	Age- and sex-specific SEV for Low vegetables			X			X
Aortic aneurysm	Global	Male	Age- and sex-specific SEV for Low vegetables			X			X
Aortic aneurysm	Data Rich	Male	Age- and sex-specific SEV for Low PUFA			X			X
Aortic aneurysm	Global	Male	Age- and sex-specific SEV for Low PUFA			X			X
Aortic aneurysm	Data Rich	Male	Age- and sex-specific SEV for Low nuts and seeds			X			X
Aortic aneurysm	Global	Male	Age- and sex-specific SEV for Low nuts and seeds			X			X
Aortic aneurysm	Data Rich	Male	Age- and sex-specific SEV for Low omega-3			X			X
Aortic aneurysm	Global	Male	Age- and sex-specific SEV for Low omega-3			X			X
Aortic aneurysm	Data Rich	Male	Pulses legumes unadjusted(g)			X			X
Aortic aneurysm	Global	Male	Pulses legumes unadjusted(g)			X			X
Aortic aneurysm	Data Rich	Male	Healthcare access and quality index		X			X	
Aortic aneurysm	Global	Male	Healthcare access and quality index		X			X	
Aortic aneurysm	Data Rich	Male	Mean BMI		X			X	
Aortic aneurysm	Global	Male	Mean BMI		X			X	
Aortic aneurysm	Data Rich	Male	Low-Density Lipoprotein (mmol/L)	X			X		
Aortic aneurysm	Global	Male	Low-Density Lipoprotein (mmol/L)	X			X		
Aortic aneurysm	Data Rich	Male	Systolic Blood Pressure (mmHg)	X			X		
Aortic aneurysm	Global	Male	Systolic Blood Pressure (mmHg)	X			X		
Aortic aneurysm	Data Rich	Male	Cumulative Cigarettes (10 Years)	X			X		
Aortic aneurysm	Global	Male	Cumulative Cigarettes (10 Years)	X			X		
Aortic aneurysm	Data Rich	Male	Log-transformed SEV scalar: Aort An	X			X		
Aortic aneurysm	Global	Male	Log-transformed SEV scalar: Aort An	X			X		
Lower extremity peripheral arterial disease	Global	Female	LDI (I$ per capita)			X			X
Lower extremity peripheral arterial disease	Data Rich	Female	LDI (I$ per capita)			X			X
Lower extremity peripheral arterial disease	Global	Female	Socio-demographic Index			X			X
Lower extremity peripheral arterial disease	Data Rich	Female	Socio-demographic Index			X			X
Lower extremity peripheral arterial disease	Global	Female	Liters of alcohol consumed per capita			X			X
Lower extremity peripheral arterial disease	Data Rich	Female	Liters of alcohol consumed per capita			X			X
Lower extremity peripheral arterial disease	Global	Female	Age- and sex-specific SEV for Low fruit			X			X
Lower extremity peripheral arterial disease	Data Rich	Female	Age- and sex-specific SEV for Low fruit			X			X
Lower extremity peripheral arterial disease	Global	Female	Age- and sex-specific SEV for Low vegetables			X			X
Lower extremity peripheral arterial disease	Data Rich	Female	Age- and sex-specific SEV for Low vegetables			X			X
Lower extremity peripheral arterial disease	Global	Female	Age- and sex-specific SEV for Low PUFA			X			X
Lower extremity peripheral arterial disease	Data Rich	Female	Age- and sex-specific SEV for Low PUFA			X			X
Lower extremity peripheral arterial disease	Global	Female	Age- and sex-specific SEV for Low nuts and seeds			X			X
Lower extremity peripheral arterial disease	Data Rich	Female	Age- and sex-specific SEV for Low nuts and seeds			X			X
Lower extremity peripheral arterial disease	Global	Female	Age- and sex-specific SEV for Low omega-3			X			X
Lower extremity peripheral arterial disease	Data Rich	Female	Age- and sex-specific SEV for Low omega-3			X			X
Lower extremity peripheral arterial disease	Global	Female	Pulses legumes unadjusted(g)			X			X
Lower extremity peripheral arterial disease	Data Rich	Female	Pulses legumes unadjusted(g)			X			X
Lower extremity peripheral arterial disease	Global	Female	Healthcare access and quality index		X			X	
Lower extremity peripheral arterial disease	Data Rich	Female	Healthcare access and quality index		X			X	
Lower extremity peripheral arterial disease	Global	Female	Mean BMI		X			X	
Lower extremity peripheral arterial disease	Data Rich	Female	Mean BMI		X			X	
Lower extremity peripheral arterial disease	Global	Female	Diabetes Fasting Plasma Glucose (mmol/L), age-standardized 25+		X			X	

Comparison of GBD 2019 and GBD 2021 covariates and level of covariates used in cause of death modeling

Cause	Model Version Type	Sex	Covariate	Level 1 GBD 2019	Level 2 GBD 2019	Level 3 GBD 2019	Level 1 GBD 2021	Level 2 GBD 2021	Level 3 GBD 2021
Lower extremity peripheral arterial disease	Data Rich	Female	Diabetes Fasting Plasma Glucose (mmol/L), age-standardized 25+		X			X	
Lower extremity peripheral arterial disease	Global	Female	Low-Density Lipoprotein (mmol/L)	X			X		
Lower extremity peripheral arterial disease	Data Rich	Female	Low-Density Lipoprotein (mmol/L)	X			X		
Lower extremity peripheral arterial disease	Global	Female	Systolic Blood Pressure (mmHg)	X			X		
Lower extremity peripheral arterial disease	Data Rich	Female	Systolic Blood Pressure (mmHg)	X			X		
Lower extremity peripheral arterial disease	Global	Female	Smoking Prevalence	X			X		
Lower extremity peripheral arterial disease	Data Rich	Female	Smoking Prevalence	X			X		
Lower extremity peripheral arterial disease	Global	Female	Log-transformed SEV scalar: PAD	X			X		
Lower extremity peripheral arterial disease	Data Rich	Female	Log-transformed SEV scalar: PAD	X			X		
Lower extremity peripheral arterial disease	Global	Male	LDI (I$ per capita)			X			X
Lower extremity peripheral arterial disease	Data Rich	Male	LDI (I$ per capita)			X			X
Lower extremity peripheral arterial disease	Global	Male	Socio-demographic Index			X			X
Lower extremity peripheral arterial disease	Data Rich	Male	Socio-demographic Index			X			X
Lower extremity peripheral arterial disease	Global	Male	Liters of alcohol consumed per capita			X			X
Lower extremity peripheral arterial disease	Data Rich	Male	Liters of alcohol consumed per capita			X			X
Lower extremity peripheral arterial disease	Global	Male	Age- and sex-specific SEV for Low fruit			X			X
Lower extremity peripheral arterial disease	Data Rich	Male	Age- and sex-specific SEV for Low fruit			X			X
Lower extremity peripheral arterial disease	Global	Male	Age- and sex-specific SEV for Low vegetables			X			X
Lower extremity peripheral arterial disease	Data Rich	Male	Age- and sex-specific SEV for Low vegetables			X			X
Lower extremity peripheral arterial disease	Global	Male	Age- and sex-specific SEV for Low PUFA			X			X
Lower extremity peripheral arterial disease	Data Rich	Male	Age- and sex-specific SEV for Low PUFA			X			X
Lower extremity peripheral arterial disease	Global	Male	Age- and sex-specific SEV for Low nuts and seeds			X			X
Lower extremity peripheral arterial disease	Data Rich	Male	Age- and sex-specific SEV for Low nuts and seeds			X			X
Lower extremity peripheral arterial disease	Global	Male	Age- and sex-specific SEV for Low omega-3			X			X
Lower extremity peripheral arterial disease	Data Rich	Male	Age- and sex-specific SEV for Low omega-3			X			X
Lower extremity peripheral arterial disease	Global	Male	Pulses legumes unadjusted(g)			X			X
Lower extremity peripheral arterial disease	Data Rich	Male	Pulses legumes unadjusted(g)			X			X
Lower extremity peripheral arterial disease	Global	Male	Healthcare access and quality index		X			X	
Lower extremity peripheral arterial disease	Data Rich	Male	Healthcare access and quality index		X			X	
Lower extremity peripheral arterial disease	Global	Male	Mean BMI		X			X	
Lower extremity peripheral arterial disease	Data Rich	Male	Mean BMI		X			X	
Lower extremity peripheral arterial disease	Global	Male	Diabetes Fasting Plasma Glucose (mmol/L), age-standardized 25+		X			X	
Lower extremity peripheral arterial disease	Data Rich	Male	Diabetes Fasting Plasma Glucose (mmol/L), age-standardized 25+		X			X	
Lower extremity peripheral arterial disease	Global	Male	Low-Density Lipoprotein (mmol/L)	X			X		
Lower extremity peripheral arterial disease	Data Rich	Male	Low-Density Lipoprotein (mmol/L)	X			X		
Lower extremity peripheral arterial disease	Global	Male	Systolic Blood Pressure (mmHg)	X			X		
Lower extremity peripheral arterial disease	Data Rich	Male	Systolic Blood Pressure (mmHg)	X			X		
Lower extremity peripheral arterial disease	Global	Male	Smoking Prevalence	X			X		
Lower extremity peripheral arterial disease	Data Rich	Male	Smoking Prevalence	X			X		
Lower extremity peripheral arterial disease	Global	Male	Log-transformed SEV scalar: PAD	X			X		
Lower extremity peripheral arterial disease	Data Rich	Male	Log-transformed SEV scalar: PAD	X			X		
Endocarditis	Data Rich	Female	LDI (I$ per capita)			X			X
Endocarditis	Global	Female	LDI (I$ per capita)			X			X
Endocarditis	Data Rich	Female	Socio-demographic Index			X			X
Endocarditis	Global	Female	Socio-demographic Index			X			X
Endocarditis	Data Rich	Female	Healthcare access and quality index		X			X	
Endocarditis	Global	Female	Healthcare access and quality index		X			X	
Endocarditis	Data Rich	Female	Sanitation (proportion with access)	X			X		
Endocarditis	Global	Female	Sanitation (proportion with access)	X			X		
Endocarditis	Data Rich	Female	Improved Water Source (proportion with access)	X			X		
Endocarditis	Global	Female	Improved Water Source (proportion with access)	X			X		
Endocarditis	Data Rich	Female	Log-transformed SEV scalar: Endocar	X			X		
Endocarditis	Global	Female	Log-transformed SEV scalar: Endocar	X			X		
Endocarditis	Data Rich	Male	LDI (I$ per capita)			X			X
Endocarditis	Global	Male	LDI (I$ per capita)			X			X
Endocarditis	Data Rich	Male	Socio-demographic Index			X			X
Endocarditis	Global	Male	Socio-demographic Index			X			X
Endocarditis	Data Rich	Male	Healthcare access and quality index		X			X	
Endocarditis	Global	Male	Healthcare access and quality index		X			X	
Endocarditis	Data Rich	Male	Sanitation (proportion with access)	X			X		
Endocarditis	Global	Male	Sanitation (proportion with access)	X			X		
Endocarditis	Data Rich	Male	Improved Water Source (proportion with access)	X			X		
Endocarditis	Global	Male	Improved Water Source (proportion with access)	X			X		
Endocarditis	Data Rich	Male	Log-transformed SEV scalar: Endocar	X			X		
Endocarditis	Global	Male	Log-transformed SEV scalar: Endocar	X			X		
Other cardiovascular and circulatory diseases	Global	Female	LDI (I$ per capita)			X			X
Other cardiovascular and circulatory diseases	Data Rich	Female	LDI (I$ per capita)			X			X
Other cardiovascular and circulatory diseases	Global	Female	Socio-demographic Index			X			X
Other cardiovascular and circulatory diseases	Data Rich	Female	Socio-demographic Index			X			X
Other cardiovascular and circulatory diseases	Global	Female	Liters of alcohol consumed per capita			X			X
Other cardiovascular and circulatory diseases	Data Rich	Female	Liters of alcohol consumed per capita			X			X
Other cardiovascular and circulatory diseases	Global	Female	Healthcare access and quality index		X			X	
Other cardiovascular and circulatory diseases	Data Rich	Female	Healthcare access and quality index		X			X	
Other cardiovascular and circulatory diseases	Global	Female	Mean BMI		X			X	
Other cardiovascular and circulatory diseases	Data Rich	Female	Mean BMI		X			X	
Other cardiovascular and circulatory diseases	Global	Female	Indoor Air Pollution (All Cooking Fuels)		X			X	
Other cardiovascular and circulatory diseases	Data Rich	Female	Indoor Air Pollution (All Cooking Fuels)		X			X	
Other cardiovascular and circulatory diseases	Global	Female	Outdoor Air Pollution (PM2.5)		X			X	
Other cardiovascular and circulatory diseases	Data Rich	Female	Outdoor Air Pollution (PM2.5)		X			X	
Other cardiovascular and circulatory diseases	Global	Female	Elevation Over 1500m (proportion)		X			X	
Other cardiovascular and circulatory diseases	Data Rich	Female	Elevation Over 1500m (proportion)		X			X	
Other cardiovascular and circulatory diseases	Global	Female	Diabetes Fasting Plasma Glucose (mmol/L), age-standardized 25+		X			X	
Other cardiovascular and circulatory diseases	Data Rich	Female	Diabetes Fasting Plasma Glucose (mmol/L), age-standardized 25+		X			X	
Other cardiovascular and circulatory diseases	Global	Female	Low-Density Lipoprotein (mmol/L)	X			X		
Other cardiovascular and circulatory diseases	Data Rich	Female	Low-Density Lipoprotein (mmol/L)	X			X		
Other cardiovascular and circulatory diseases	Global	Female	Systolic Blood Pressure (mmHg)	X			X		
Other cardiovascular and circulatory diseases	Data Rich	Female	Systolic Blood Pressure (mmHg)	X			X		
Other cardiovascular and circulatory diseases	Global	Female	Smoking Prevalence	X			X		
Other cardiovascular and circulatory diseases	Data Rich	Female	Smoking Prevalence	X			X		
Other cardiovascular and circulatory diseases	Global	Female	Log-transformed SEV scalar: Oth Cardio	X			X		
Other cardiovascular and circulatory diseases	Data Rich	Female	Log-transformed SEV scalar: Oth Cardio	X			X		
Other cardiovascular and circulatory diseases	Global	Female	Age- and sex-specific SEV for Low fruit						X
Other cardiovascular and circulatory diseases	Data Rich	Female	Age- and sex-specific SEV for Low fruit						X
Other cardiovascular and circulatory diseases	Global	Female	Age- and sex-specific SEV for Low vegetables						X
Other cardiovascular and circulatory diseases	Data Rich	Female	Age- and sex-specific SEV for Low vegetables						X
Other cardiovascular and circulatory diseases	Global	Female	Age- and sex-specific SEV for Low PUFA						X
Other cardiovascular and circulatory diseases	Data Rich	Female	Age- and sex-specific SEV for Low PUFA						X
Other cardiovascular and circulatory diseases	Global	Female	Age- and sex-specific SEV for Low legumes						X
Other cardiovascular and circulatory diseases	Data Rich	Female	Age- and sex-specific SEV for Low legumes						X
Other cardiovascular and circulatory diseases	Global	Female	Age- and sex-specific SEV for Low nuts and seeds						X
Other cardiovascular and circulatory diseases	Global	Female	Age- and sex-specific SEV for Low omega-3						X
Other cardiovascular and circulatory diseases	Data Rich	Female	Age- and sex-specific SEV for Low omega-3						X
Other cardiovascular and circulatory diseases	Global	Male	LDI (I$ per capita)			X			X
Other cardiovascular and circulatory diseases	Data Rich	Male	LDI (I$ per capita)			X			X
Other cardiovascular and circulatory diseases	Global	Male	Socio-demographic Index			X			X
Other cardiovascular and circulatory diseases	Data Rich	Male	Socio-demographic Index			X			X
Other cardiovascular and circulatory diseases	Global	Male	Liters of alcohol consumed per capita			X			X
Other cardiovascular and circulatory diseases	Data Rich	Male	Liters of alcohol consumed per capita			X			X
Other cardiovascular and circulatory diseases	Global	Male	Age- and sex-specific SEV for Low nuts and seeds			X			X
Other cardiovascular and circulatory diseases	Global	Male	Healthcare access and quality index		X			X	
Other cardiovascular and circulatory diseases	Data Rich	Male	Healthcare access and quality index		X			X	
Other cardiovascular and circulatory diseases	Global	Male	Mean BMI		X			X	
Other cardiovascular and circulatory diseases	Data Rich	Male	Mean BMI		X			X	
Other cardiovascular and circulatory diseases	Global	Male	Indoor Air Pollution (All Cooking Fuels)		X			X	
Other cardiovascular and circulatory diseases	Data Rich	Male	Indoor Air Pollution (All Cooking Fuels)		X			X	
Other cardiovascular and circulatory diseases	Global	Male	Outdoor Air Pollution (PM2.5)		X			X	
Other cardiovascular and circulatory diseases	Data Rich	Male	Outdoor Air Pollution (PM2.5)		X			X	
Other cardiovascular and circulatory diseases	Global	Male	Elevation Over 1500m (proportion)		X			X	
Other cardiovascular and circulatory diseases	Data Rich	Male	Elevation Over 1500m (proportion)		X			X	
Other cardiovascular and circulatory diseases	Global	Male	Diabetes Fasting Plasma Glucose (mmol/L), age-standardized 25+		X			X	
Other cardiovascular and circulatory diseases	Data Rich	Male	Diabetes Fasting Plasma Glucose (mmol/L), age-standardized 25+		X			X	
Other cardiovascular and circulatory diseases	Global	Male	Low-Density Lipoprotein (mmol/L)	X			X		
Other cardiovascular and circulatory diseases	Data Rich	Male	Low-Density Lipoprotein (mmol/L)	X			X		
Other cardiovascular and circulatory diseases	Global	Male	Systolic Blood Pressure (mmHg)	X			X		
Other cardiovascular and circulatory diseases	Data Rich	Male	Systolic Blood Pressure (mmHg)	X			X		
Other cardiovascular and circulatory diseases	Global	Male	Smoking Prevalence	X			X		
Other cardiovascular and circulatory diseases	Data Rich	Male	Smoking Prevalence	X			X		
Other cardiovascular and circulatory diseases	Global	Male	Log-transformed SEV scalar: Oth Cardio	X			X		
Other cardiovascular and circulatory diseases	Data Rich	Male	Log-transformed SEV scalar: Oth Cardio	X			X		
Other cardiovascular and circulatory diseases	Global	Male	Age- and sex-specific SEV for Low fruit						X

Comparison of GBD 2019 and GBD 2021 covariates and level of covariates used in cause of death modeling									
Cause	Model Version Type	Sex	Covariate	Level 1 GBD 2019	Level 2 GBD 2019	Level 3 GBD 2019	Level 1 GBD 2021	Level 2 GBD 2021	Level 3 GBD 2021
Other cardiovascular and circulatory diseases	Data Rich	Male	Age- and sex-specific SEV for Low fruit						X
Other cardiovascular and circulatory diseases	Global	Male	Age- and sex-specific SEV for Low vegetables						X
Other cardiovascular and circulatory diseases	Data Rich	Male	Age- and sex-specific SEV for Low vegetables						X
Other cardiovascular and circulatory diseases	Global	Male	Age- and sex-specific SEV for Low PUFA						X
Other cardiovascular and circulatory diseases	Data Rich	Male	Age- and sex-specific SEV for Low PUFA						X
Other cardiovascular and circulatory diseases	Global	Male	Age- and sex-specific SEV for Low legumes						X
Other cardiovascular and circulatory diseases	Data Rich	Male	Age- and sex-specific SEV for Low legumes						X
Other cardiovascular and circulatory diseases	Global	Male	Age- and sex-specific SEV for Low omega-3						X
Other cardiovascular and circulatory diseases	Data Rich	Male	Age- and sex-specific SEV for Low omega-3						X
Chronic respiratory diseases	Global	Female	Education (years per capita)			X			X
Chronic respiratory diseases	Data Rich	Female	Education (years per capita)			X			X
Chronic respiratory diseases	Global	Female	LDI (I$ per capita)			X			X
Chronic respiratory diseases	Data Rich	Female	LDI (I$ per capita)			X			X
Chronic respiratory diseases	Global	Female	Socio-demographic Index			X			X
Chronic respiratory diseases	Global	Female	Population Density (over 1000 ppl/sqkm, proportion)			X			X
Chronic respiratory diseases	Data Rich	Female	Population Density (over 1000 ppl/sqkm, proportion)			X			X
Chronic respiratory diseases	Global	Female	Elevation 500 to 1500m (proportion)			X			X
Chronic respiratory diseases	Data Rich	Female	Elevation 500 to 1500m (proportion)			X			X
Chronic respiratory diseases	Data Rich	Female	Socio-demographic Index			X			X
Chronic respiratory diseases	Global	Female	Smoking Prevalence		X			X	
Chronic respiratory diseases	Data Rich	Female	Smoking Prevalence		X			X	
Chronic respiratory diseases	Global	Female	Indoor Air Pollution (All Cooking Fuels)		X			X	
Chronic respiratory diseases	Data Rich	Female	Indoor Air Pollution (All Cooking Fuels)		X			X	
Chronic respiratory diseases	Global	Female	Outdoor Air Pollution (PM2.5)		X			X	
Chronic respiratory diseases	Data Rich	Female	Outdoor Air Pollution (PM2.5)		X			X	
Chronic respiratory diseases	Global	Female	Elevation Over 1500m (proportion)		X			X	
Chronic respiratory diseases	Data Rich	Female	Elevation Over 1500m (proportion)		X			X	
Chronic respiratory diseases	Data Rich	Female	Healthcare access and quality index	X				X	
Chronic respiratory diseases	Global	Female	Healthcare access and quality index	X			X		
Chronic respiratory diseases	Global	Female	Cumulative Cigarettes (5 Years)	X			X		
Chronic respiratory diseases	Data Rich	Female	Cumulative Cigarettes (5 Years)	X			X		
Chronic respiratory diseases	Global	Female	Cumulative Cigarettes (10 Years)	X			X		
Chronic respiratory diseases	Data Rich	Female	Cumulative Cigarettes (10 Years)	X			X		
Chronic respiratory diseases	Global	Male	Education (years per capita)			X			X
Chronic respiratory diseases	Data Rich	Male	Education (years per capita)			X			X
Chronic respiratory diseases	Global	Male	LDI (I$ per capita)			X			X
Chronic respiratory diseases	Data Rich	Male	LDI (I$ per capita)			X			X
Chronic respiratory diseases	Global	Male	Socio-demographic Index			X			X
Chronic respiratory diseases	Global	Male	Population Density (over 1000 ppl/sqkm, proportion)			X			X
Chronic respiratory diseases	Data Rich	Male	Population Density (over 1000 ppl/sqkm, proportion)			X			X
Chronic respiratory diseases	Global	Male	Elevation 500 to 1500m (proportion)			X			X
Chronic respiratory diseases	Data Rich	Male	Elevation 500 to 1500m (proportion)			X			X
Chronic respiratory diseases	Data Rich	Male	Socio-demographic Index			X			X
Chronic respiratory diseases	Global	Male	Healthcare access and quality index		X			X	
Chronic respiratory diseases	Data Rich	Male	Healthcare access and quality index		X			X	
Chronic respiratory diseases	Data Rich	Male	Smoking Prevalence		X			X	
Chronic respiratory diseases	Data Rich	Male	Indoor Air Pollution (All Cooking Fuels)		X			X	
Chronic respiratory diseases	Global	Male	Outdoor Air Pollution (PM2.5)		X			X	
Chronic respiratory diseases	Data Rich	Male	Outdoor Air Pollution (PM2.5)		X			X	
Chronic respiratory diseases	Global	Male	Elevation Over 1500m (proportion)		X			X	
Chronic respiratory diseases	Data Rich	Male	Elevation Over 1500m (proportion)		X			X	
Chronic respiratory diseases	Global	Male	Smoking Prevalence	X			X		
Chronic respiratory diseases	Global	Male	Cumulative Cigarettes (5 Years)	X			X		
Chronic respiratory diseases	Data Rich	Male	Cumulative Cigarettes (5 Years)	X			X		
Chronic respiratory diseases	Global	Male	Indoor Air Pollution (All Cooking Fuels)	X			X		
Chronic respiratory diseases	Global	Male	Cumulative Cigarettes (10 Years)	X			X		
Chronic respiratory diseases	Data Rich	Male	Cumulative Cigarettes (10 Years)	X			X		
Chronic obstructive pulmonary disease	Data Rich	Female	Education (years per capita)			X			X
Chronic obstructive pulmonary disease	Global	Female	Education (years per capita)			X			X
Chronic obstructive pulmonary disease	Data Rich	Female	LDI (I$ per capita)			X			X
Chronic obstructive pulmonary disease	Global	Female	LDI (I$ per capita)			X			X
Chronic obstructive pulmonary disease	Data Rich	Female	Socio-demographic Index			X			X
Chronic obstructive pulmonary disease	Global	Female	Socio-demographic Index			X			X
Chronic obstructive pulmonary disease	Data Rich	Female	Smoking Prevalence		X			X	
Chronic obstructive pulmonary disease	Global	Female	Smoking Prevalence		X			X	
Chronic obstructive pulmonary disease	Data Rich	Female	Indoor Air Pollution (All Cooking Fuels)		X			X	
Chronic obstructive pulmonary disease	Global	Female	Indoor Air Pollution (All Cooking Fuels)		X			X	
Chronic obstructive pulmonary disease	Data Rich	Female	Healthcare access and quality index	X			X		
Chronic obstructive pulmonary disease	Global	Female	Healthcare access and quality index	X			X		
Chronic obstructive pulmonary disease	Data Rich	Female	Cumulative Cigarettes (5 Years)	X			X		
Chronic obstructive pulmonary disease	Global	Female	Cumulative Cigarettes (5 Years)	X			X		
Chronic obstructive pulmonary disease	Data Rich	Female	Cumulative Cigarettes (10 Years)	X			X		
Chronic obstructive pulmonary disease	Global	Female	Cumulative Cigarettes (10 Years)	X			X		
Chronic obstructive pulmonary disease	Data Rich	Female	Outdoor Air Pollution (PM2.5)	X			X		
Chronic obstructive pulmonary disease	Global	Female	Outdoor Air Pollution (PM2.5)	X			X		
Chronic obstructive pulmonary disease	Data Rich	Female	Elevation Over 1500m (proportion)	X			X		
Chronic obstructive pulmonary disease	Global	Female	Elevation Over 1500m (proportion)	X			X		
Chronic obstructive pulmonary disease	Data Rich	Female	Log-transformed SEV scalar: COPD	X			X		
Chronic obstructive pulmonary disease	Global	Female	Log-transformed SEV scalar: COPD	X			X		
Chronic obstructive pulmonary disease	Data Rich	Male	Education (years per capita)			X			X
Chronic obstructive pulmonary disease	Global	Male	Education (years per capita)			X			X
Chronic obstructive pulmonary disease	Data Rich	Male	LDI (I$ per capita)			X			X
Chronic obstructive pulmonary disease	Global	Male	LDI (I$ per capita)			X			X
Chronic obstructive pulmonary disease	Data Rich	Male	Socio-demographic Index			X			X
Chronic obstructive pulmonary disease	Global	Male	Socio-demographic Index			X			X
Chronic obstructive pulmonary disease	Data Rich	Male	Healthcare access and quality index		X			X	
Chronic obstructive pulmonary disease	Global	Male	Healthcare access and quality index		X			X	
Chronic obstructive pulmonary disease	Data Rich	Male	Smoking Prevalence		X			X	
Chronic obstructive pulmonary disease	Global	Male	Smoking Prevalence		X			X	
Chronic obstructive pulmonary disease	Data Rich	Male	Indoor Air Pollution (All Cooking Fuels)		X			X	
Chronic obstructive pulmonary disease	Global	Male	Indoor Air Pollution (All Cooking Fuels)		X			X	
Chronic obstructive pulmonary disease	Data Rich	Male	Cumulative Cigarettes (5 Years)	X			X		
Chronic obstructive pulmonary disease	Global	Male	Cumulative Cigarettes (5 Years)	X			X		
Chronic obstructive pulmonary disease	Data Rich	Male	Cumulative Cigarettes (10 Years)	X			X		
Chronic obstructive pulmonary disease	Global	Male	Cumulative Cigarettes (10 Years)	X			X		
Chronic obstructive pulmonary disease	Data Rich	Male	Cumulative Cigarettes (20 Years)	X			X		
Chronic obstructive pulmonary disease	Global	Male	Cumulative Cigarettes (20 Years)	X			X		
Chronic obstructive pulmonary disease	Data Rich	Male	Outdoor Air Pollution (PM2.5)	X			X		
Chronic obstructive pulmonary disease	Global	Male	Outdoor Air Pollution (PM2.5)	X			X		
Chronic obstructive pulmonary disease	Data Rich	Male	Elevation Over 1500m (proportion)	X			X		
Chronic obstructive pulmonary disease	Global	Male	Elevation Over 1500m (proportion)	X			X		
Chronic obstructive pulmonary disease	Data Rich	Male	Log-transformed SEV scalar: COPD	X			X		
Chronic obstructive pulmonary disease	Global	Male	Log-transformed SEV scalar: COPD	X			X		
Pneumoconiosis	Global	Female	Education (years per capita)			X			X
Pneumoconiosis	Data Rich	Female	Education (years per capita)			X			X
Pneumoconiosis	Global	Female	LDI (I$ per capita)			X			X
Pneumoconiosis	Data Rich	Female	LDI (I$ per capita)			X			X
Pneumoconiosis	Global	Female	Socio-demographic Index			X			X
Pneumoconiosis	Data Rich	Female	Socio-demographic Index			X			X
Pneumoconiosis	Global	Female	Healthcare access and quality index		X			X	
Pneumoconiosis	Data Rich	Female	Healthcare access and quality index		X			X	
Pneumoconiosis	Global	Female	Smoking Prevalence		X			X	
Pneumoconiosis	Data Rich	Female	Smoking Prevalence		X			X	
Pneumoconiosis	Global	Female	Cumulative Cigarettes (5 Years)		X			X	
Pneumoconiosis	Data Rich	Female	Cumulative Cigarettes (5 Years)		X			X	
Pneumoconiosis	Global	Female	Indoor Air Pollution (All Cooking Fuels)		X			X	
Pneumoconiosis	Data Rich	Female	Indoor Air Pollution (All Cooking Fuels)		X			X	
Pneumoconiosis	Global	Female	Log-transformed coal production (per capita)	X			X		
Pneumoconiosis	Data Rich	Female	Log-transformed coal production (per capita)	X			X		
Pneumoconiosis	Global	Female	Asbestos consumption (metric tons per year per capita)	X			X		
Pneumoconiosis	Data Rich	Female	Asbestos consumption (metric tons per year per capita)	X			X		
Pneumoconiosis	Global	Female	Gold production (kg) per capita, smoothed with 20-year lag	X			X		
Pneumoconiosis	Data Rich	Female	Gold production (kg) per capita, smoothed with 20-year lag	X			X		
Pneumoconiosis	Data Rich	Female	Age- and sex-specific SEV for Occupational asbestos	X			X		
Pneumoconiosis	Data Rich	Female	Age- and sex-specific SEV for Occupational beryllium	X			X		
Pneumoconiosis	Data Rich	Female	Age- and sex-specific SEV for Occupational silica	X			X		
Pneumoconiosis	Global	Male	Education (years per capita)			X			X
Pneumoconiosis	Data Rich	Male	Education (years per capita)			X			X
Pneumoconiosis	Global	Male	LDI (I$ per capita)			X			X
Pneumoconiosis	Data Rich	Male	LDI (I$ per capita)			X			X
Pneumoconiosis	Global	Male	Socio-demographic Index			X			X
Pneumoconiosis	Data Rich	Male	Socio-demographic Index			X			X

Comparison of GBD 2019 and GBD 2021 covariates and level of covariates used in cause of death modeling

Cause	Model Version Type	Sex	Covariate	Level 1 GBD 2019	Level 2 GBD 2019	Level 3 GBD 2019	Level 1 GBD 2021	Level 2 GBD 2021	Level 3 GBD 2021
Pneumoconiosis	Global	Male	Healthcare access and quality index		X			X	
Pneumoconiosis	Data Rich	Male	Healthcare access and quality index		X			X	
Pneumoconiosis	Global	Male	Smoking Prevalence		X			X	
Pneumoconiosis	Data Rich	Male	Smoking Prevalence		X			X	
Pneumoconiosis	Global	Male	Cumulative Cigarettes (5 Years)		X			X	
Pneumoconiosis	Data Rich	Male	Cumulative Cigarettes (5 Years)		X			X	
Pneumoconiosis	Global	Male	Indoor Air Pollution (All Cooking Fuels)		X			X	
Pneumoconiosis	Data Rich	Male	Indoor Air Pollution (All Cooking Fuels)		X			X	
Pneumoconiosis	Global	Male	Log-transformed coal production (per capita)	X			X		
Pneumoconiosis	Data Rich	Male	Log-transformed coal production (per capita)	X			X		
Pneumoconiosis	Global	Male	Asbestos consumption (metric tons per year per capita)	X			X		
Pneumoconiosis	Data Rich	Male	Asbestos consumption (metric tons per year per capita)	X			X		
Pneumoconiosis	Global	Male	Gold production (kg) per capita, smoothed with 20-year lag	X			X		
Pneumoconiosis	Data Rich	Male	Gold production (kg) per capita, smoothed with 20-year lag	X			X		
Pneumoconiosis	Data Rich	Male	Age- and sex-specific SEV for Occupational asbestos	X			X		
Pneumoconiosis	Data Rich	Male	Age- and sex-specific SEV for Occupational beryllium	X			X		
Pneumoconiosis	Data Rich	Male	Age- and sex-specific SEV for Occupational silica	X			X		
Silicosis	Global	Female	Education (years per capita)			X			X
Silicosis	Data Rich	Female	Education (years per capita)			X			X
Silicosis	Global	Female	LDI (I$ per capita)			X			X
Silicosis	Data Rich	Female	LDI (I$ per capita)			X			X
Silicosis	Global	Female	Socio-demographic Index			X			X
Silicosis	Data Rich	Female	Socio-demographic Index			X			X
Silicosis	Global	Female	Healthcare access and quality index		X			X	
Silicosis	Data Rich	Female	Healthcare access and quality index		X			X	
Silicosis	Global	Female	Smoking Prevalence		X			X	
Silicosis	Data Rich	Female	Smoking Prevalence		X			X	
Silicosis	Global	Female	Cumulative Cigarettes (5 Years)		X			X	
Silicosis	Data Rich	Female	Cumulative Cigarettes (5 Years)		X			X	
Silicosis	Global	Female	Indoor Air Pollution (All Cooking Fuels)		X			X	
Silicosis	Data Rich	Female	Indoor Air Pollution (All Cooking Fuels)		X			X	
Silicosis	Global	Female	Gold production (kg) per capita, smoothed with 20-year lag	X			X		
Silicosis	Data Rich	Female	Gold production (kg) per capita, smoothed with 20-year lag	X			X		
Silicosis	Data Rich	Female	Age- and sex-specific SEV for Occupational silica	X			X		
Silicosis	Global	Male	Education (years per capita)			X			X
Silicosis	Data Rich	Male	Education (years per capita)			X			X
Silicosis	Global	Male	LDI (I$ per capita)			X			X
Silicosis	Data Rich	Male	LDI (I$ per capita)			X			X
Silicosis	Global	Male	Socio-demographic Index			X			X
Silicosis	Data Rich	Male	Socio-demographic Index			X			X
Silicosis	Global	Male	Healthcare access and quality index		X			X	
Silicosis	Data Rich	Male	Healthcare access and quality index		X			X	
Silicosis	Global	Male	Smoking Prevalence		X			X	
Silicosis	Data Rich	Male	Smoking Prevalence		X			X	
Silicosis	Global	Male	Cumulative Cigarettes (5 Years)		X			X	
Silicosis	Data Rich	Male	Cumulative Cigarettes (5 Years)		X			X	
Silicosis	Global	Male	Indoor Air Pollution (All Cooking Fuels)		X			X	
Silicosis	Data Rich	Male	Indoor Air Pollution (All Cooking Fuels)		X			X	
Silicosis	Global	Male	Gold production (kg) per capita, smoothed with 20-year lag	X			X		
Silicosis	Data Rich	Male	Gold production (kg) per capita, smoothed with 20-year lag	X			X		
Silicosis	Data Rich	Male	Age- and sex-specific SEV for Occupational silica	X			X		
Asbestosis	Data Rich	Female	Education (years per capita)			X			X
Asbestosis	Global	Female	Education (years per capita)			X			X
Asbestosis	Data Rich	Female	LDI (I$ per capita)			X			X
Asbestosis	Global	Female	LDI (I$ per capita)			X			X
Asbestosis	Data Rich	Female	Socio-demographic Index			X			X
Asbestosis	Global	Female	Socio-demographic Index			X			X
Asbestosis	Data Rich	Female	Healthcare access and quality index		X			X	
Asbestosis	Global	Female	Healthcare access and quality index		X			X	
Asbestosis	Data Rich	Female	Smoking Prevalence		X			X	
Asbestosis	Global	Female	Smoking Prevalence		X			X	
Asbestosis	Data Rich	Female	Cumulative Cigarettes (5 Years)		X			X	
Asbestosis	Global	Female	Cumulative Cigarettes (5 Years)		X			X	
Asbestosis	Data Rich	Female	Indoor Air Pollution (All Cooking Fuels)		X			X	
Asbestosis	Global	Female	Indoor Air Pollution (All Cooking Fuels)		X			X	
Asbestosis	Data Rich	Female	Cumulative Cigarettes (10 Years)		X			X	
Asbestosis	Global	Female	Cumulative Cigarettes (10 Years)		X			X	
Asbestosis	Data Rich	Female	Elevation Over 1500m (proportion)		X			X	
Asbestosis	Global	Female	Elevation Over 1500m (proportion)		X			X	
Asbestosis	Data Rich	Female	Elevation 500 to 1500m (proportion)		X			X	
Asbestosis	Global	Female	Elevation 500 to 1500m (proportion)		X			X	
Asbestosis	Data Rich	Female	Asbestos consumption (metric tons per year per capita)	X			X		
Asbestosis	Global	Female	Asbestos consumption (metric tons per year per capita)	X			X		
Asbestosis	Global	Male	Education (years per capita)			X			X
Asbestosis	Data Rich	Male	Education (years per capita)			X			X
Asbestosis	Global	Male	LDI (I$ per capita)			X			X
Asbestosis	Data Rich	Male	LDI (I$ per capita)			X			X
Asbestosis	Global	Male	Socio-demographic Index			X			X
Asbestosis	Data Rich	Male	Socio-demographic Index			X			X
Asbestosis	Global	Male	Healthcare access and quality index		X			X	
Asbestosis	Data Rich	Male	Healthcare access and quality index		X			X	
Asbestosis	Global	Male	Smoking Prevalence		X			X	
Asbestosis	Global	Male	Cumulative Cigarettes (5 Years)		X			X	
Asbestosis	Data Rich	Male	Cumulative Cigarettes (5 Years)		X			X	
Asbestosis	Global	Male	Indoor Air Pollution (All Cooking Fuels)		X			X	
Asbestosis	Data Rich	Male	Indoor Air Pollution (All Cooking Fuels)		X			X	
Asbestosis	Global	Male	Elevation Over 1500m (proportion)		X			X	
Asbestosis	Data Rich	Male	Elevation Over 1500m (proportion)		X			X	
Asbestosis	Global	Male	Elevation 500 to 1500m (proportion)		X			X	
Asbestosis	Data Rich	Male	Elevation 500 to 1500m (proportion)		X			X	
Asbestosis	Data Rich	Male	Smoking Prevalence	X			X		
Asbestosis	Global	Male	Asbestos consumption (metric tons per year per capita)	X			X		
Asbestosis	Data Rich	Male	Asbestos consumption (metric tons per year per capita)	X			X		
Asbestosis	Data Rich	Male	Age- and sex-specific SEV for Occupational asbestos	X			X		
Coal workers pneumoconiosis	Data Rich	Female	Education (years per capita)			X			X
Coal workers pneumoconiosis	Global	Female	Education (years per capita)			X			X
Coal workers pneumoconiosis	Data Rich	Female	LDI (I$ per capita)			X			X
Coal workers pneumoconiosis	Global	Female	LDI (I$ per capita)			X			X
Coal workers pneumoconiosis	Data Rich	Female	Socio-demographic Index			X			X
Coal workers pneumoconiosis	Global	Female	Socio-demographic Index			X			X
Coal workers pneumoconiosis	Data Rich	Female	Healthcare access and quality index		X			X	
Coal workers pneumoconiosis	Global	Female	Healthcare access and quality index		X			X	
Coal workers pneumoconiosis	Data Rich	Female	Smoking Prevalence		X			X	
Coal workers pneumoconiosis	Global	Female	Smoking Prevalence		X			X	
Coal workers pneumoconiosis	Data Rich	Female	Cumulative Cigarettes (5 Years)		X			X	
Coal workers pneumoconiosis	Global	Female	Cumulative Cigarettes (5 Years)		X			X	
Coal workers pneumoconiosis	Data Rich	Female	Indoor Air Pollution (All Cooking Fuels)		X			X	
Coal workers pneumoconiosis	Global	Female	Indoor Air Pollution (All Cooking Fuels)		X			X	
Coal workers pneumoconiosis	Data Rich	Female	Log-transformed coal production (per capita)	X			X		
Coal workers pneumoconiosis	Global	Female	Log-transformed coal production (per capita)	X			X		
Coal workers pneumoconiosis	Data Rich	Male	Education (years per capita)			X			X
Coal workers pneumoconiosis	Global	Male	Education (years per capita)			X			X
Coal workers pneumoconiosis	Data Rich	Male	LDI (I$ per capita)			X			X
Coal workers pneumoconiosis	Global	Male	LDI (I$ per capita)			X			X
Coal workers pneumoconiosis	Data Rich	Male	Socio-demographic Index			X			X
Coal workers pneumoconiosis	Global	Male	Socio-demographic Index			X			X
Coal workers pneumoconiosis	Data Rich	Male	Healthcare access and quality index		X			X	
Coal workers pneumoconiosis	Global	Male	Healthcare access and quality index		X			X	
Coal workers pneumoconiosis	Data Rich	Male	Smoking Prevalence		X			X	
Coal workers pneumoconiosis	Global	Male	Smoking Prevalence		X			X	
Coal workers pneumoconiosis	Data Rich	Male	Cumulative Cigarettes (5 Years)		X			X	
Coal workers pneumoconiosis	Global	Male	Cumulative Cigarettes (5 Years)		X			X	
Coal workers pneumoconiosis	Data Rich	Male	Indoor Air Pollution (All Cooking Fuels)		X			X	
Coal workers pneumoconiosis	Global	Male	Indoor Air Pollution (All Cooking Fuels)		X			X	
Coal workers pneumoconiosis	Data Rich	Male	Log-transformed coal production (per capita)	X			X		
Coal workers pneumoconiosis	Global	Male	Log-transformed coal production (per capita)	X			X		
Other pneumoconiosis	Global	Female	Education (years per capita)			X			X
Other pneumoconiosis	Data Rich	Female	Education (years per capita)			X			X
Other pneumoconiosis	Global	Female	LDI (I$ per capita)			X			X

Comparison of GBD 2019 and GBD 2021 covariates and level of covariates used in cause of death modeling									
**Cause**	**Model Version Type**	**Sex**	**Covariate**	**Level 1 GBD 2019**	**Level 2 GBD 2019**	**Level 3 GBD 2019**	**Level 1 GBD 2021**	**Level 2 GBD 2021**	**Level 3 GBD 2021**
Other pneumoconiosis	Data Rich	Female	LDI (I$ per capita)			X			X
Other pneumoconiosis	Global	Female	Socio-demographic Index			X			X
Other pneumoconiosis	Data Rich	Female	Socio-demographic Index			X			X
Other pneumoconiosis	Global	Female	Healthcare access and quality index		X			X	
Other pneumoconiosis	Data Rich	Female	Healthcare access and quality index		X			X	
Other pneumoconiosis	Global	Female	Smoking Prevalence		X			X	
Other pneumoconiosis	Data Rich	Female	Smoking Prevalence		X			X	
Other pneumoconiosis	Global	Female	Cumulative Cigarettes (5 Years)		X			X	
Other pneumoconiosis	Data Rich	Female	Cumulative Cigarettes (5 Years)		X			X	
Other pneumoconiosis	Global	Female	Indoor Air Pollution (All Cooking Fuels)		X			X	
Other pneumoconiosis	Data Rich	Female	Indoor Air Pollution (All Cooking Fuels)		X			X	
Other pneumoconiosis	Data Rich	Female	Age- and sex-specific SEV for Occupational beryllium	X			X		
Other pneumoconiosis	Global	Female	Tuberculosis prevalence (age-standardized)	X			X		
Other pneumoconiosis	Data Rich	Female	Tuberculosis prevalence (age-standardized)	X			X		
Other pneumoconiosis	Global	Female	Occupation Professionals	X			X		
Other pneumoconiosis	Data Rich	Female	Occupation Professionals	X			X		
Other pneumoconiosis	Global	Male	Education (years per capita)			X			X
Other pneumoconiosis	Data Rich	Male	Education (years per capita)			X			X
Other pneumoconiosis	Global	Male	LDI (I$ per capita)			X			X
Other pneumoconiosis	Data Rich	Male	LDI (I$ per capita)			X			X
Other pneumoconiosis	Global	Male	Socio-demographic Index			X			X
Other pneumoconiosis	Data Rich	Male	Socio-demographic Index			X			X
Other pneumoconiosis	Global	Male	Healthcare access and quality index		X			X	
Other pneumoconiosis	Data Rich	Male	Healthcare access and quality index		X			X	
Other pneumoconiosis	Global	Male	Smoking Prevalence		X			X	
Other pneumoconiosis	Data Rich	Male	Smoking Prevalence		X			X	
Other pneumoconiosis	Global	Male	Cumulative Cigarettes (5 Years)		X			X	
Other pneumoconiosis	Data Rich	Male	Cumulative Cigarettes (5 Years)		X			X	
Other pneumoconiosis	Global	Male	Indoor Air Pollution (All Cooking Fuels)		X			X	
Other pneumoconiosis	Data Rich	Male	Indoor Air Pollution (All Cooking Fuels)		X			X	
Other pneumoconiosis	Data Rich	Male	Age- and sex-specific SEV for Occupational beryllium	X			X		
Other pneumoconiosis	Global	Male	Tuberculosis prevalence (age-standardized)	X			X		
Other pneumoconiosis	Data Rich	Male	Tuberculosis prevalence (age-standardized)	X			X		
Other pneumoconiosis	Global	Male	Occupation Professionals	X			X		
Other pneumoconiosis	Data Rich	Male	Occupation Professionals	X			X		
Asthma	Data Rich	Female	Education (years per capita)			X			X
Asthma	Global	Female	Education (years per capita)			X			X
Asthma	Data Rich	Female	LDI (I$ per capita)			X			X
Asthma	Global	Female	LDI (I$ per capita)			X			X
Asthma	Data Rich	Female	Socio-demographic Index			X			X
Asthma	Global	Female	Socio-demographic Index			X			X
Asthma	Data Rich	Female	Smoking Prevalence		X			X	
Asthma	Global	Female	Smoking Prevalence		X			X	
Asthma	Data Rich	Female	Indoor Air Pollution (All Cooking Fuels)		X			X	
Asthma	Global	Female	Indoor Air Pollution (All Cooking Fuels)		X			X	
Asthma	Data Rich	Female	Outdoor Air Pollution (PM2.5)		X			X	
Asthma	Global	Female	Outdoor Air Pollution (PM2.5)		X			X	
Asthma	Data Rich	Female	Healthcare access and quality index	X			X		
Asthma	Global	Female	Healthcare access and quality index	X			X		
Asthma	Data Rich	Female	Cumulative Cigarettes (5 Years)	X			X		
Asthma	Global	Female	Cumulative Cigarettes (5 Years)	X			X		
Asthma	Data Rich	Female	Cumulative Cigarettes (10 Years)	X			X		
Asthma	Global	Female	Cumulative Cigarettes (10 Years)	X			X		
Asthma	Data Rich	Female	Log-transformed SEV scalar: Asthma	X			X		
Asthma	Global	Female	Log-transformed SEV scalar: Asthma	X			X		
Asthma	Data Rich	Male	Education (years per capita)			X			X
Asthma	Global	Male	Education (years per capita)			X			X
Asthma	Data Rich	Male	LDI (I$ per capita)			X			X
Asthma	Global	Male	LDI (I$ per capita)			X			X
Asthma	Data Rich	Male	Socio-demographic Index			X			X
Asthma	Global	Male	Socio-demographic Index			X			X
Asthma	Data Rich	Male	Smoking Prevalence		X			X	
Asthma	Global	Male	Smoking Prevalence		X			X	
Asthma	Data Rich	Male	Indoor Air Pollution (All Cooking Fuels)		X			X	
Asthma	Global	Male	Indoor Air Pollution (All Cooking Fuels)		X			X	
Asthma	Data Rich	Male	Outdoor Air Pollution (PM2.5)		X			X	
Asthma	Global	Male	Outdoor Air Pollution (PM2.5)		X			X	
Asthma	Data Rich	Male	Healthcare access and quality index	X			X		
Asthma	Global	Male	Healthcare access and quality index	X			X		
Asthma	Data Rich	Male	Cumulative Cigarettes (5 Years)	X			X		
Asthma	Global	Male	Cumulative Cigarettes (5 Years)	X			X		
Asthma	Data Rich	Male	Cumulative Cigarettes (10 Years)	X			X		
Asthma	Global	Male	Cumulative Cigarettes (10 Years)	X			X		
Asthma	Data Rich	Male	Log-transformed SEV scalar: Asthma	X			X		
Asthma	Global	Male	Log-transformed SEV scalar: Asthma	X			X		
Interstitial lung disease and pulmonary sarcoidosis	Global	Female	Education (years per capita)			X			X
Interstitial lung disease and pulmonary sarcoidosis	Data Rich	Female	Education (years per capita)			X			X
Interstitial lung disease and pulmonary sarcoidosis	Global	Female	LDI (I$ per capita)			X			X
Interstitial lung disease and pulmonary sarcoidosis	Data Rich	Female	LDI (I$ per capita)			X			X
Interstitial lung disease and pulmonary sarcoidosis	Global	Female	Socio-demographic Index			X			X
Interstitial lung disease and pulmonary sarcoidosis	Data Rich	Female	Socio-demographic Index			X			X
Interstitial lung disease and pulmonary sarcoidosis	Global	Female	Healthcare access and quality index		X			X	
Interstitial lung disease and pulmonary sarcoidosis	Data Rich	Female	Healthcare access and quality index		X			X	
Interstitial lung disease and pulmonary sarcoidosis	Data Rich	Female	Indoor Air Pollution (All Cooking Fuels)		X			X	
Interstitial lung disease and pulmonary sarcoidosis	Data Rich	Female	Outdoor Air Pollution (PM2.5)		X			X	
Interstitial lung disease and pulmonary sarcoidosis	Global	Female	Elevation Over 1500m (proportion)		X			X	
Interstitial lung disease and pulmonary sarcoidosis	Data Rich	Female	Elevation Over 1500m (proportion)		X			X	
Interstitial lung disease and pulmonary sarcoidosis	Global	Female	Elevation 500 to 1500m (proportion)		X			X	
Interstitial lung disease and pulmonary sarcoidosis	Data Rich	Female	Elevation 500 to 1500m (proportion)		X			X	
Interstitial lung disease and pulmonary sarcoidosis	Global	Female	Smoking Prevalence	X			X		
Interstitial lung disease and pulmonary sarcoidosis	Data Rich	Female	Smoking Prevalence	X			X		
Interstitial lung disease and pulmonary sarcoidosis	Global	Female	Cumulative Cigarettes (5 Years)	X			X		
Interstitial lung disease and pulmonary sarcoidosis	Data Rich	Female	Cumulative Cigarettes (5 Years)	X			X		
Interstitial lung disease and pulmonary sarcoidosis	Global	Female	Indoor Air Pollution (All Cooking Fuels)	X			X		
Interstitial lung disease and pulmonary sarcoidosis	Global	Female	Cumulative Cigarettes (10 Years)	X			X		
Interstitial lung disease and pulmonary sarcoidosis	Data Rich	Female	Cumulative Cigarettes (10 Years)	X			X		
Interstitial lung disease and pulmonary sarcoidosis	Global	Female	Outdoor Air Pollution (PM2.5)	X			X		
Interstitial lung disease and pulmonary sarcoidosis	Global	Female	Occupation Professionals	X			X		
Interstitial lung disease and pulmonary sarcoidosis	Data Rich	Female	Occupation Professionals	X			X		
Interstitial lung disease and pulmonary sarcoidosis	Global	Male	Education (years per capita)			X			X
Interstitial lung disease and pulmonary sarcoidosis	Data Rich	Male	Education (years per capita)			X			X
Interstitial lung disease and pulmonary sarcoidosis	Global	Male	LDI (I$ per capita)			X			X
Interstitial lung disease and pulmonary sarcoidosis	Data Rich	Male	LDI (I$ per capita)			X			X
Interstitial lung disease and pulmonary sarcoidosis	Global	Male	Socio-demographic Index			X			X
Interstitial lung disease and pulmonary sarcoidosis	Data Rich	Male	Socio-demographic Index			X			X
Interstitial lung disease and pulmonary sarcoidosis	Global	Male	Healthcare access and quality index		X			X	
Interstitial lung disease and pulmonary sarcoidosis	Data Rich	Male	Healthcare access and quality index		X			X	
Interstitial lung disease and pulmonary sarcoidosis	Global	Male	Indoor Air Pollution (All Cooking Fuels)		X			X	
Interstitial lung disease and pulmonary sarcoidosis	Data Rich	Male	Indoor Air Pollution (All Cooking Fuels)		X			X	
Interstitial lung disease and pulmonary sarcoidosis	Global	Male	Outdoor Air Pollution (PM2.5)		X			X	
Interstitial lung disease and pulmonary sarcoidosis	Data Rich	Male	Outdoor Air Pollution (PM2.5)		X			X	
Interstitial lung disease and pulmonary sarcoidosis	Global	Male	Elevation Over 1500m (proportion)		X			X	
Interstitial lung disease and pulmonary sarcoidosis	Data Rich	Male	Elevation Over 1500m (proportion)		X			X	
Interstitial lung disease and pulmonary sarcoidosis	Global	Male	Elevation 500 to 1500m (proportion)		X			X	
Interstitial lung disease and pulmonary sarcoidosis	Data Rich	Male	Elevation 500 to 1500m (proportion)		X			X	
Interstitial lung disease and pulmonary sarcoidosis	Global	Male	Smoking Prevalence	X			X		
Interstitial lung disease and pulmonary sarcoidosis	Data Rich	Male	Smoking Prevalence	X			X		
Interstitial lung disease and pulmonary sarcoidosis	Global	Male	Cumulative Cigarettes (5 Years)	X			X		
Interstitial lung disease and pulmonary sarcoidosis	Data Rich	Male	Cumulative Cigarettes (5 Years)	X			X		
Interstitial lung disease and pulmonary sarcoidosis	Global	Male	Cumulative Cigarettes (10 Years)	X			X		
Interstitial lung disease and pulmonary sarcoidosis	Data Rich	Male	Cumulative Cigarettes (10 Years)	X			X		
Interstitial lung disease and pulmonary sarcoidosis	Global	Male	Occupation Professionals	X			X		
Interstitial lung disease and pulmonary sarcoidosis	Data Rich	Male	Occupation Professionals	X			X		
Other chronic respiratory diseases	Data Rich	Female	Education (years per capita)			X			X
Other chronic respiratory diseases	Global	Female	Education (years per capita)			X			X
Other chronic respiratory diseases	Data Rich	Female	LDI (I$ per capita)			X			X
Other chronic respiratory diseases	Global	Female	LDI (I$ per capita)			X			X
Other chronic respiratory diseases	Data Rich	Female	Socio-demographic Index			X			X
Other chronic respiratory diseases	Global	Female	Socio-demographic Index			X			X
Other chronic respiratory diseases	Data Rich	Female	Healthcare access and quality index		X			X	
Other chronic respiratory diseases	Global	Female	Healthcare access and quality index		X			X	
Other chronic respiratory diseases	Data Rich	Female	Elevation Over 1500m (proportion)		X			X	
Other chronic respiratory diseases	Global	Female	Elevation Over 1500m (proportion)		X			X	
Other chronic respiratory diseases	Data Rich	Female	Population Density (over 1000 ppl/sqkm, proportion)		X			X	
Other chronic respiratory diseases	Global	Female	Population Density (over 1000 ppl/sqkm, proportion)		X			X	
Other chronic respiratory diseases	Data Rich	Female	Elevation 500 to 1500m (proportion)		X			X	
Other chronic respiratory diseases	Global	Female	Elevation 500 to 1500m (proportion)		X			X	
Other chronic respiratory diseases	Data Rich	Female	Smoking Prevalence	X			X		

Comparison of GBD 2019 and GBD 2021 covariates and level of covariates used in cause of death modeling

Cause	Model Version Type	Sex	Covariate	Level 1 GBD 2019	Level 2 GBD 2019	Level 3 GBD 2019	Level 1 GBD 2021	Level 2 GBD 2021	Level 3 GBD 2021
Other chronic respiratory diseases	Global	Female	Smoking Prevalence	X			X		
Other chronic respiratory diseases	Data Rich	Female	Cumulative Cigarettes (5 Years)	X			X		
Other chronic respiratory diseases	Global	Female	Cumulative Cigarettes (5 Years)	X			X		
Other chronic respiratory diseases	Data Rich	Female	Indoor Air Pollution (All Cooking Fuels)	X			X		
Other chronic respiratory diseases	Global	Female	Indoor Air Pollution (All Cooking Fuels)	X			X		
Other chronic respiratory diseases	Data Rich	Female	Outdoor Air Pollution (PM2.5)	X			X		
Other chronic respiratory diseases	Global	Female	Outdoor Air Pollution (PM2.5)	X			X		
Other chronic respiratory diseases	Data Rich	Male	Education (years per capita)			X			X
Other chronic respiratory diseases	Global	Male	Education (years per capita)			X			X
Other chronic respiratory diseases	Data Rich	Male	LDI (I$ per capita)			X			X
Other chronic respiratory diseases	Global	Male	LDI (I$ per capita)			X			X
Other chronic respiratory diseases	Data Rich	Male	Socio-demographic Index			X			X
Other chronic respiratory diseases	Global	Male	Socio-demographic Index			X			X
Other chronic respiratory diseases	Data Rich	Male	Healthcare access and quality index		X			X	
Other chronic respiratory diseases	Global	Male	Healthcare access and quality index		X			X	
Other chronic respiratory diseases	Data Rich	Male	Elevation Over 1500m (proportion)		X			X	
Other chronic respiratory diseases	Global	Male	Elevation Over 1500m (proportion)		X			X	
Other chronic respiratory diseases	Data Rich	Male	Population Density (over 1000 ppl/sqkm, proportion)		X			X	
Other chronic respiratory diseases	Global	Male	Population Density (over 1000 ppl/sqkm, proportion)		X			X	
Other chronic respiratory diseases	Data Rich	Male	Elevation 500 to 1500m (proportion)		X			X	
Other chronic respiratory diseases	Global	Male	Elevation 500 to 1500m (proportion)		X			X	
Other chronic respiratory diseases	Data Rich	Male	Smoking Prevalence	X			X		
Other chronic respiratory diseases	Global	Male	Smoking Prevalence	X			X		
Other chronic respiratory diseases	Data Rich	Male	Cumulative Cigarettes (5 Years)	X			X		
Other chronic respiratory diseases	Global	Male	Cumulative Cigarettes (5 Years)	X			X		
Other chronic respiratory diseases	Data Rich	Male	Indoor Air Pollution (All Cooking Fuels)	X			X		
Other chronic respiratory diseases	Global	Male	Indoor Air Pollution (All Cooking Fuels)	X			X		
Other chronic respiratory diseases	Data Rich	Male	Outdoor Air Pollution (PM2.5)	X			X		
Other chronic respiratory diseases	Global	Male	Outdoor Air Pollution (PM2.5)	X			X		
Digestive diseases	Global	Female	Education (years per capita)			X			X
Digestive diseases	Data Rich	Female	Education (years per capita)			X			X
Digestive diseases	Global	Female	LDI (I$ per capita)			X			X
Digestive diseases	Data Rich	Female	LDI (I$ per capita)			X			X
Digestive diseases	Global	Female	Socio-demographic Index			X			X
Digestive diseases	Data Rich	Female	Socio-demographic Index			X			X
Digestive diseases	Global	Female	Healthcare access and quality index		X			X	
Digestive diseases	Data Rich	Female	Healthcare access and quality index		X			X	
Digestive diseases	Global	Female	Mean BMI		X			X	
Digestive diseases	Global	Female	Age- and sex-specific SEV for Low fruit		X			X	
Digestive diseases	Data Rich	Female	Age- and sex-specific SEV for Low fruit		X			X	
Digestive diseases	Global	Female	Age- and sex-specific SEV for Low vegetables		X			X	
Digestive diseases	Data Rich	Female	Age- and sex-specific SEV for Low vegetables		X			X	
Digestive diseases	Global	Female	Age- and sex-specific SEV for High red meat		X			X	
Digestive diseases	Data Rich	Female	Age- and sex-specific SEV for High red meat		X			X	
Digestive diseases	Data Rich	Female	Mean BMI	X			X		
Digestive diseases	Global	Female	Smoking Prevalence	X			X		
Digestive diseases	Data Rich	Female	Smoking Prevalence	X			X		
Digestive diseases	Global	Female	Liters of alcohol consumed per capita	X			X		
Digestive diseases	Data Rich	Female	Liters of alcohol consumed per capita	X			X		
Digestive diseases	Global	Female	Cumulative Cigarettes (5 Years)	X			X		
Digestive diseases	Data Rich	Female	Cumulative Cigarettes (5 Years)	X			X		
Digestive diseases	Global	Female	Cumulative Cigarettes (10 Years)	X			X		
Digestive diseases	Data Rich	Female	Cumulative Cigarettes (10 Years)	X			X		
Digestive diseases	Global	Female	Sanitation (proportion with access)	X			X		
Digestive diseases	Data Rich	Female	Sanitation (proportion with access)	X			X		
Digestive diseases	Global	Male	Education (years per capita)			X			X
Digestive diseases	Data Rich	Male	Education (years per capita)			X			X
Digestive diseases	Global	Male	LDI (I$ per capita)			X			X
Digestive diseases	Data Rich	Male	LDI (I$ per capita)			X			X
Digestive diseases	Global	Male	Socio-demographic Index			X			X
Digestive diseases	Data Rich	Male	Socio-demographic Index			X			X
Digestive diseases	Global	Male	Healthcare access and quality index		X			X	
Digestive diseases	Data Rich	Male	Healthcare access and quality index		X			X	
Digestive diseases	Global	Male	Mean BMI		X			X	
Digestive diseases	Data Rich	Male	Mean BMI		X			X	
Digestive diseases	Global	Male	Age- and sex-specific SEV for Low fruit		X			X	
Digestive diseases	Data Rich	Male	Age- and sex-specific SEV for Low fruit		X			X	
Digestive diseases	Global	Male	Age- and sex-specific SEV for Low vegetables		X			X	
Digestive diseases	Data Rich	Male	Age- and sex-specific SEV for Low vegetables		X			X	
Digestive diseases	Global	Male	Age- and sex-specific SEV for High red meat		X			X	
Digestive diseases	Data Rich	Male	Age- and sex-specific SEV for High red meat		X			X	
Digestive diseases	Global	Male	Smoking Prevalence	X			X		
Digestive diseases	Data Rich	Male	Smoking Prevalence	X			X		
Digestive diseases	Global	Male	Liters of alcohol consumed per capita	X			X		
Digestive diseases	Data Rich	Male	Liters of alcohol consumed per capita	X			X		
Digestive diseases	Global	Male	Cumulative Cigarettes (5 Years)	X			X		
Digestive diseases	Data Rich	Male	Cumulative Cigarettes (5 Years)	X			X		
Digestive diseases	Global	Male	Cumulative Cigarettes (10 Years)	X			X		
Digestive diseases	Data Rich	Male	Cumulative Cigarettes (10 Years)	X			X		
Digestive diseases	Global	Male	Sanitation (proportion with access)	X			X		
Digestive diseases	Data Rich	Male	Sanitation (proportion with access)	X			X		
Cirrhosis and other chronic liver diseases	Data Rich	Female	Education (years per capita)			X			X
Cirrhosis and other chronic liver diseases	Global	Female	Education (years per capita)			X			X
Cirrhosis and other chronic liver diseases	Data Rich	Female	LDI (I$ per capita)			X			X
Cirrhosis and other chronic liver diseases	Global	Female	LDI (I$ per capita)			X			X
Cirrhosis and other chronic liver diseases	Data Rich	Female	Socio-demographic Index			X			X
Cirrhosis and other chronic liver diseases	Global	Female	Socio-demographic Index			X			X
Cirrhosis and other chronic liver diseases	Data Rich	Female	Healthcare access and quality index		X			X	
Cirrhosis and other chronic liver diseases	Global	Female	Healthcare access and quality index		X			X	
Cirrhosis and other chronic liver diseases	Data Rich	Female	Mean BMI		X			X	
Cirrhosis and other chronic liver diseases	Global	Female	Mean BMI		X			X	
Cirrhosis and other chronic liver diseases	Data Rich	Female	Diabetes Age-Standardized Prevalence (proportion)		X			X	
Cirrhosis and other chronic liver diseases	Global	Female	Diabetes Age-Standardized Prevalence (proportion)		X			X	
Cirrhosis and other chronic liver diseases	Data Rich	Female	Intravenous drug use (proportion by age)		X			X	
Cirrhosis and other chronic liver diseases	Global	Female	Intravenous drug use (proportion by age)		X			X	
Cirrhosis and other chronic liver diseases	Data Rich	Female	Liters of alcohol consumed per capita	X			X		
Cirrhosis and other chronic liver diseases	Global	Female	Liters of alcohol consumed per capita	X			X		
Cirrhosis and other chronic liver diseases	Data Rich	Female	Hepatitis B vaccine coverage (proportion), aged through time	X			X		
Cirrhosis and other chronic liver diseases	Global	Female	Hepatitis B vaccine coverage (proportion), aged through time	X			X		
Cirrhosis and other chronic liver diseases	Data Rich	Female	Chronic Hepatitis C age standardized				X		
Cirrhosis and other chronic liver diseases	Global	Female	Chronic Hepatitis C age standardized				X		
Cirrhosis and other chronic liver diseases	Data Rich	Female	Vaccine adjusted HbSAg serporevalence age standardized				X		
Cirrhosis and other chronic liver diseases	Global	Female	Vaccine adjusted HbSAg serporevalence age standardized				X		
Cirrhosis and other chronic liver diseases	Data Rich	Male	Education (years per capita)			X			X
Cirrhosis and other chronic liver diseases	Global	Male	Education (years per capita)			X			X
Cirrhosis and other chronic liver diseases	Data Rich	Male	LDI (I$ per capita)			X			X
Cirrhosis and other chronic liver diseases	Global	Male	LDI (I$ per capita)			X			X
Cirrhosis and other chronic liver diseases	Data Rich	Male	Socio-demographic Index			X			X
Cirrhosis and other chronic liver diseases	Global	Male	Socio-demographic Index			X			X
Cirrhosis and other chronic liver diseases	Data Rich	Male	Healthcare access and quality index		X			X	
Cirrhosis and other chronic liver diseases	Global	Male	Healthcare access and quality index		X			X	
Cirrhosis and other chronic liver diseases	Data Rich	Male	Mean BMI		X			X	
Cirrhosis and other chronic liver diseases	Global	Male	Mean BMI		X			X	
Cirrhosis and other chronic liver diseases	Data Rich	Male	Diabetes Age-Standardized Prevalence (proportion)		X			X	
Cirrhosis and other chronic liver diseases	Global	Male	Diabetes Age-Standardized Prevalence (proportion)		X			X	
Cirrhosis and other chronic liver diseases	Data Rich	Male	Intravenous drug use (proportion by age)		X			X	
Cirrhosis and other chronic liver diseases	Global	Male	Intravenous drug use (proportion by age)		X			X	
Cirrhosis and other chronic liver diseases	Data Rich	Male	Liters of alcohol consumed per capita	X			X		
Cirrhosis and other chronic liver diseases	Global	Male	Liters of alcohol consumed per capita	X			X		
Cirrhosis and other chronic liver diseases	Data Rich	Male	Hepatitis B vaccine coverage (proportion), aged through time	X			X		
Cirrhosis and other chronic liver diseases	Global	Male	Hepatitis B vaccine coverage (proportion), aged through time	X			X		
Cirrhosis and other chronic liver diseases	Data Rich	Male	Chronic Hepatitis C age standardized				X		
Cirrhosis and other chronic liver diseases	Global	Male	Chronic Hepatitis C age standardized				X		
Cirrhosis and other chronic liver diseases	Data Rich	Male	Vaccine adjusted HbSAg serporevalence age standardized				X		
Cirrhosis and other chronic liver diseases	Global	Male	Vaccine adjusted HbSAg serporevalence age standardized				X		
Upper digestive system diseases	Global	Female	Education (years per capita)			X			X
Upper digestive system diseases	Data Rich	Female	Education (years per capita)			X			X
Upper digestive system diseases	Global	Female	LDI (I$ per capita)			X			X
Upper digestive system diseases	Data Rich	Female	LDI (I$ per capita)			X			X
Upper digestive system diseases	Global	Female	Socio-demographic Index			X			X

Comparison of GBD 2019 and GBD 2021 covariates and level of covariates used in cause of death modeling

Cause	Model Version Type	Sex	Covariate	Level 1 GBD 2019	Level 2 GBD 2019	Level 3 GBD 2019	Level 1 GBD 2021	Level 2 GBD 2021	Level 3 GBD 2021
Upper digestive system diseases	Data Rich	Female	Socio-demographic Index			X			X
Upper digestive system diseases	Global	Female	Healthcare access and quality index		X			X	
Upper digestive system diseases	Data Rich	Female	Healthcare access and quality index		X			X	
Upper digestive system diseases	Global	Female	Liters of alcohol consumed per capita		X			X	
Upper digestive system diseases	Data Rich	Female	Liters of alcohol consumed per capita		X			X	
Upper digestive system diseases	Global	Female	vegetables unadjusted(g)		X			X	
Upper digestive system diseases	Data Rich	Female	vegetables unadjusted(g)		X			X	
Upper digestive system diseases	Global	Female	Smoking Prevalence	X			X		
Upper digestive system diseases	Data Rich	Female	Smoking Prevalence	X			X		
Upper digestive system diseases	Global	Female	Cumulative Cigarettes (5 Years)	X			X		
Upper digestive system diseases	Data Rich	Female	Cumulative Cigarettes (5 Years)	X			X		
Upper digestive system diseases	Global	Female	Cumulative Cigarettes (10 Years)	X			X		
Upper digestive system diseases	Data Rich	Female	Cumulative Cigarettes (10 Years)	X			X		
Upper digestive system diseases	Global	Female	Sanitation (proportion with access)	X			X		
Upper digestive system diseases	Data Rich	Female	Sanitation (proportion with access)	X			X		
Upper digestive system diseases	Global	Female	Age- and sex-specific SEV for Unsafe water	X			X		
Upper digestive system diseases	Data Rich	Female	Age- and sex-specific SEV for Unsafe water	X			X		
Upper digestive system diseases	Global	Male	Education (years per capita)			X			X
Upper digestive system diseases	Data Rich	Male	Education (years per capita)			X			X
Upper digestive system diseases	Global	Male	LDI (I$ per capita)			X			X
Upper digestive system diseases	Data Rich	Male	LDI (I$ per capita)			X			X
Upper digestive system diseases	Global	Male	Socio-demographic Index			X			X
Upper digestive system diseases	Data Rich	Male	Socio-demographic Index			X			X
Upper digestive system diseases	Global	Male	Healthcare access and quality index		X			X	
Upper digestive system diseases	Data Rich	Male	Healthcare access and quality index		X			X	
Upper digestive system diseases	Global	Male	Liters of alcohol consumed per capita		X			X	
Upper digestive system diseases	Data Rich	Male	Liters of alcohol consumed per capita		X			X	
Upper digestive system diseases	Global	Male	vegetables unadjusted(g)		X			X	
Upper digestive system diseases	Data Rich	Male	vegetables unadjusted(g)		X			X	
Upper digestive system diseases	Global	Male	Smoking Prevalence	X			X		
Upper digestive system diseases	Data Rich	Male	Smoking Prevalence	X			X		
Upper digestive system diseases	Global	Male	Cumulative Cigarettes (5 Years)	X			X		
Upper digestive system diseases	Data Rich	Male	Cumulative Cigarettes (5 Years)	X			X		
Upper digestive system diseases	Global	Male	Cumulative Cigarettes (10 Years)	X			X		
Upper digestive system diseases	Data Rich	Male	Cumulative Cigarettes (10 Years)	X			X		
Upper digestive system diseases	Global	Male	Sanitation (proportion with access)	X			X		
Upper digestive system diseases	Data Rich	Male	Sanitation (proportion with access)	X			X		
Upper digestive system diseases	Global	Male	Age- and sex-specific SEV for Unsafe water	X			X		
Upper digestive system diseases	Data Rich	Male	Age- and sex-specific SEV for Unsafe water	X			X		
Peptic ulcer disease	Global	Female	Education (years per capita)			X			X
Peptic ulcer disease	Data Rich	Female	Education (years per capita)			X			X
Peptic ulcer disease	Global	Female	LDI (I$ per capita)			X			X
Peptic ulcer disease	Data Rich	Female	LDI (I$ per capita)			X			X
Peptic ulcer disease	Global	Female	Socio-demographic Index			X			X
Peptic ulcer disease	Data Rich	Female	Socio-demographic Index			X			X
Peptic ulcer disease	Global	Female	Healthcare access and quality index		X			X	
Peptic ulcer disease	Data Rich	Female	Healthcare access and quality index		X			X	
Peptic ulcer disease	Global	Female	Liters of alcohol consumed per capita		X			X	
Peptic ulcer disease	Data Rich	Female	Liters of alcohol consumed per capita		X			X	
Peptic ulcer disease	Global	Female	vegetables unadjusted(g)		X			X	
Peptic ulcer disease	Data Rich	Female	vegetables unadjusted(g)		X			X	
Peptic ulcer disease	Global	Female	Smoking Prevalence	X			X		
Peptic ulcer disease	Data Rich	Female	Smoking Prevalence	X			X		
Peptic ulcer disease	Global	Female	Cumulative Cigarettes (5 Years)	X			X		
Peptic ulcer disease	Data Rich	Female	Cumulative Cigarettes (5 Years)	X			X		
Peptic ulcer disease	Global	Female	Cumulative Cigarettes (10 Years)	X			X		
Peptic ulcer disease	Data Rich	Female	Cumulative Cigarettes (10 Years)	X			X		
Peptic ulcer disease	Global	Female	Sanitation (proportion with access)	X			X		
Peptic ulcer disease	Data Rich	Female	Sanitation (proportion with access)	X			X		
Peptic ulcer disease	Global	Female	Age- and sex-specific SEV for Unsafe water	X			X		
Peptic ulcer disease	Data Rich	Female	Age- and sex-specific SEV for Unsafe water	X			X		
Peptic ulcer disease	Global	Male	Education (years per capita)			X			X
Peptic ulcer disease	Data Rich	Male	Education (years per capita)			X			X
Peptic ulcer disease	Global	Male	LDI (I$ per capita)			X			X
Peptic ulcer disease	Data Rich	Male	LDI (I$ per capita)			X			X
Peptic ulcer disease	Global	Male	Socio-demographic Index			X			X
Peptic ulcer disease	Data Rich	Male	Socio-demographic Index			X			X
Peptic ulcer disease	Global	Male	Healthcare access and quality index		X			X	
Peptic ulcer disease	Data Rich	Male	Healthcare access and quality index		X			X	
Peptic ulcer disease	Global	Male	Liters of alcohol consumed per capita		X			X	
Peptic ulcer disease	Data Rich	Male	Liters of alcohol consumed per capita		X			X	
Peptic ulcer disease	Global	Male	vegetables unadjusted(g)		X			X	
Peptic ulcer disease	Data Rich	Male	vegetables unadjusted(g)		X			X	
Peptic ulcer disease	Global	Male	Smoking Prevalence	X			X		
Peptic ulcer disease	Data Rich	Male	Smoking Prevalence	X			X		
Peptic ulcer disease	Global	Male	Cumulative Cigarettes (5 Years)	X			X		
Peptic ulcer disease	Data Rich	Male	Cumulative Cigarettes (5 Years)	X			X		
Peptic ulcer disease	Global	Male	Cumulative Cigarettes (10 Years)	X			X		
Peptic ulcer disease	Data Rich	Male	Cumulative Cigarettes (10 Years)	X			X		
Peptic ulcer disease	Global	Male	Sanitation (proportion with access)	X			X		
Peptic ulcer disease	Data Rich	Male	Sanitation (proportion with access)	X			X		
Peptic ulcer disease	Global	Male	Age- and sex-specific SEV for Unsafe water	X			X		
Peptic ulcer disease	Data Rich	Male	Age- and sex-specific SEV for Unsafe water	X			X		
Gastritis and duodenitis	Global	Female	Education (years per capita)			X			X
Gastritis and duodenitis	Data Rich	Female	Education (years per capita)			X			X
Gastritis and duodenitis	Global	Female	LDI (I$ per capita)			X			X
Gastritis and duodenitis	Data Rich	Female	LDI (I$ per capita)			X			X
Gastritis and duodenitis	Global	Female	Socio-demographic Index			X			X
Gastritis and duodenitis	Data Rich	Female	Socio-demographic Index			X			X
Gastritis and duodenitis	Global	Female	Healthcare access and quality index		X			X	
Gastritis and duodenitis	Data Rich	Female	Healthcare access and quality index		X			X	
Gastritis and duodenitis	Global	Female	Smoking Prevalence		X			X	
Gastritis and duodenitis	Data Rich	Female	Smoking Prevalence		X			X	
Gastritis and duodenitis	Global	Female	Liters of alcohol consumed per capita		X			X	
Gastritis and duodenitis	Data Rich	Female	Liters of alcohol consumed per capita		X			X	
Gastritis and duodenitis	Global	Female	Cumulative Cigarettes (5 Years)		X			X	
Gastritis and duodenitis	Data Rich	Female	Cumulative Cigarettes (5 Years)		X			X	
Gastritis and duodenitis	Global	Female	Cumulative Cigarettes (10 Years)		X			X	
Gastritis and duodenitis	Data Rich	Female	Cumulative Cigarettes (10 Years)		X			X	
Gastritis and duodenitis	Global	Female	vegetables unadjusted(g)		X			X	
Gastritis and duodenitis	Data Rich	Female	vegetables unadjusted(g)		X			X	
Gastritis and duodenitis	Global	Female	Sanitation (proportion with access)	X			X		
Gastritis and duodenitis	Data Rich	Female	Sanitation (proportion with access)	X			X		
Gastritis and duodenitis	Global	Female	Age- and sex-specific SEV for Unsafe water	X			X		
Gastritis and duodenitis	Data Rich	Female	Age- and sex-specific SEV for Unsafe water	X			X		
Gastritis and duodenitis	Global	Male	Education (years per capita)			X			X
Gastritis and duodenitis	Data Rich	Male	Education (years per capita)			X			X
Gastritis and duodenitis	Global	Male	LDI (I$ per capita)			X			X
Gastritis and duodenitis	Data Rich	Male	LDI (I$ per capita)			X			X
Gastritis and duodenitis	Global	Male	Socio-demographic Index			X			X
Gastritis and duodenitis	Data Rich	Male	Socio-demographic Index			X			X
Gastritis and duodenitis	Global	Male	Healthcare access and quality index		X			X	
Gastritis and duodenitis	Data Rich	Male	Healthcare access and quality index		X			X	
Gastritis and duodenitis	Global	Male	Smoking Prevalence		X			X	
Gastritis and duodenitis	Data Rich	Male	Smoking Prevalence		X			X	
Gastritis and duodenitis	Global	Male	Liters of alcohol consumed per capita		X			X	
Gastritis and duodenitis	Data Rich	Male	Liters of alcohol consumed per capita		X			X	
Gastritis and duodenitis	Global	Male	Cumulative Cigarettes (5 Years)		X			X	
Gastritis and duodenitis	Data Rich	Male	Cumulative Cigarettes (5 Years)		X			X	
Gastritis and duodenitis	Global	Male	Cumulative Cigarettes (10 Years)		X			X	
Gastritis and duodenitis	Data Rich	Male	Cumulative Cigarettes (10 Years)		X			X	
Gastritis and duodenitis	Global	Male	vegetables unadjusted(g)		X			X	
Gastritis and duodenitis	Data Rich	Male	vegetables unadjusted(g)		X			X	
Gastritis and duodenitis	Global	Male	Sanitation (proportion with access)	X			X		
Gastritis and duodenitis	Data Rich	Male	Sanitation (proportion with access)	X			X		
Gastritis and duodenitis	Global	Male	Age- and sex-specific SEV for Unsafe water	X			X		
Gastritis and duodenitis	Data Rich	Male	Age- and sex-specific SEV for Unsafe water	X			X		
Appendicitis	Data Rich	Female	Education (years per capita)			X			X
Appendicitis	Global	Female	Education (years per capita)			X			X
Appendicitis	Data Rich	Female	LDI (I$ per capita)			X			X
Appendicitis	Global	Female	LDI (I$ per capita)			X			X
Appendicitis	Data Rich	Female	Socio-demographic Index			X			X
Appendicitis	Global	Female	Socio-demographic Index			X			X
Appendicitis	Data Rich	Female	Healthcare access and quality index		X			X	
Appendicitis	Global	Female	Healthcare access and quality index		X			X	
Appendicitis	Data Rich	Female	Age- and sex-specific SEV for Low fruit		X			X	
Appendicitis	Global	Female	Age- and sex-specific SEV for Low fruit		X			X	
Appendicitis	Data Rich	Female	Age- and sex-specific SEV for Low vegetables		X			X	
Appendicitis	Global	Female	Age- and sex-specific SEV for Low vegetables		X			X	

Comparison of GBD 2019 and GBD 2021 covariates and level of covariates used in cause of death modeling

Cause	Model Version Type	Sex	Covariate	Level 1 GBD 2019	Level 2 GBD 2019	Level 3 GBD 2019	Level 1 GBD 2021	Level 2 GBD 2021	Level 3 GBD 2021
Appendicitis	Data Rich	Male	Education (years per capita)			X			X
Appendicitis	Global	Male	Education (years per capita)			X			X
Appendicitis	Data Rich	Male	LDI (I$ per capita)			X			X
Appendicitis	Global	Male	LDI (I$ per capita)			X			X
Appendicitis	Data Rich	Male	Socio-demographic Index			X			X
Appendicitis	Global	Male	Socio-demographic Index			X			X
Appendicitis	Data Rich	Male	Healthcare access and quality index		X			X	
Appendicitis	Global	Male	Healthcare access and quality index		X			X	
Appendicitis	Data Rich	Male	Age- and sex-specific SEV for Low fruit		X			X	
Appendicitis	Global	Male	Age- and sex-specific SEV for Low fruit		X			X	
Appendicitis	Data Rich	Male	Age- and sex-specific SEV for Low vegetables		X			X	
Appendicitis	Global	Male	Age- and sex-specific SEV for Low vegetables		X			X	
Paralytic ileus and intestinal obstruction	Data Rich	Female	Education (years per capita)			X			X
Paralytic ileus and intestinal obstruction	Global	Female	Education (years per capita)			X			X
Paralytic ileus and intestinal obstruction	Data Rich	Female	LDI (I$ per capita)			X			X
Paralytic ileus and intestinal obstruction	Global	Female	LDI (I$ per capita)			X			X
Paralytic ileus and intestinal obstruction	Data Rich	Female	Socio-demographic Index			X			X
Paralytic ileus and intestinal obstruction	Global	Female	Socio-demographic Index			X			X
Paralytic ileus and intestinal obstruction	Data Rich	Female	Healthcare access and quality index		X			X	
Paralytic ileus and intestinal obstruction	Global	Female	Healthcare access and quality index		X			X	
Paralytic ileus and intestinal obstruction	Data Rich	Female	fruits unadjusted(g)		X			X	
Paralytic ileus and intestinal obstruction	Global	Female	fruits unadjusted(g)		X			X	
Paralytic ileus and intestinal obstruction	Data Rich	Female	vegetables unadjusted(g)		X			X	
Paralytic ileus and intestinal obstruction	Global	Female	vegetables unadjusted(g)		X			X	
Paralytic ileus and intestinal obstruction	Data Rich	Male	Education (years per capita)			X			X
Paralytic ileus and intestinal obstruction	Global	Male	Education (years per capita)			X			X
Paralytic ileus and intestinal obstruction	Data Rich	Male	LDI (I$ per capita)			X			X
Paralytic ileus and intestinal obstruction	Global	Male	LDI (I$ per capita)			X			X
Paralytic ileus and intestinal obstruction	Data Rich	Male	Socio-demographic Index			X			X
Paralytic ileus and intestinal obstruction	Global	Male	Socio-demographic Index			X			X
Paralytic ileus and intestinal obstruction	Data Rich	Male	Healthcare access and quality index		X			X	
Paralytic ileus and intestinal obstruction	Global	Male	Healthcare access and quality index		X			X	
Paralytic ileus and intestinal obstruction	Data Rich	Male	fruits unadjusted(g)		X			X	
Paralytic ileus and intestinal obstruction	Global	Male	fruits unadjusted(g)		X			X	
Paralytic ileus and intestinal obstruction	Data Rich	Male	vegetables unadjusted(g)		X			X	
Paralytic ileus and intestinal obstruction	Global	Male	vegetables unadjusted(g)		X			X	
Inguinal, femoral, and abdominal hernia	Global	Female	Education (years per capita)			X			X
Inguinal, femoral, and abdominal hernia	Data Rich	Female	Education (years per capita)			X			X
Inguinal, femoral, and abdominal hernia	Global	Female	LDI (I$ per capita)			X			X
Inguinal, femoral, and abdominal hernia	Data Rich	Female	LDI (I$ per capita)			X			X
Inguinal, femoral, and abdominal hernia	Global	Female	Socio-demographic Index			X			X
Inguinal, femoral, and abdominal hernia	Data Rich	Female	Socio-demographic Index			X			X
Inguinal, femoral, and abdominal hernia	Global	Female	Healthcare access and quality index		X			X	
Inguinal, femoral, and abdominal hernia	Data Rich	Female	Healthcare access and quality index		X			X	
Inguinal, femoral, and abdominal hernia	Global	Female	Mean BMI	X			X		
Inguinal, femoral, and abdominal hernia	Data Rich	Female	Mean BMI	X			X		
Inguinal, femoral, and abdominal hernia	Global	Female	Smoking Prevalence	X			X		
Inguinal, femoral, and abdominal hernia	Data Rich	Female	Smoking Prevalence	X			X		
Inguinal, femoral, and abdominal hernia	Global	Female	Cumulative Cigarettes (5 Years)	X			X		
Inguinal, femoral, and abdominal hernia	Data Rich	Female	Cumulative Cigarettes (5 Years)	X			X		
Inguinal, femoral, and abdominal hernia	Global	Female	Cumulative Cigarettes (10 Years)	X			X		
Inguinal, femoral, and abdominal hernia	Data Rich	Female	Cumulative Cigarettes (10 Years)	X			X		
Inguinal, femoral, and abdominal hernia	Global	Male	Education (years per capita)			X			X
Inguinal, femoral, and abdominal hernia	Data Rich	Male	Education (years per capita)			X			X
Inguinal, femoral, and abdominal hernia	Global	Male	LDI (I$ per capita)			X			X
Inguinal, femoral, and abdominal hernia	Data Rich	Male	LDI (I$ per capita)			X			X
Inguinal, femoral, and abdominal hernia	Global	Male	Socio-demographic Index			X			X
Inguinal, femoral, and abdominal hernia	Data Rich	Male	Socio-demographic Index			X			X
Inguinal, femoral, and abdominal hernia	Global	Male	Healthcare access and quality index		X			X	
Inguinal, femoral, and abdominal hernia	Data Rich	Male	Healthcare access and quality index		X			X	
Inguinal, femoral, and abdominal hernia	Global	Male	Mean BMI	X			X		
Inguinal, femoral, and abdominal hernia	Data Rich	Male	Mean BMI	X			X		
Inguinal, femoral, and abdominal hernia	Global	Male	Smoking Prevalence	X			X		
Inguinal, femoral, and abdominal hernia	Data Rich	Male	Smoking Prevalence	X			X		
Inguinal, femoral, and abdominal hernia	Global	Male	Cumulative Cigarettes (5 Years)	X			X		
Inguinal, femoral, and abdominal hernia	Data Rich	Male	Cumulative Cigarettes (5 Years)	X			X		
Inguinal, femoral, and abdominal hernia	Global	Male	Cumulative Cigarettes (10 Years)	X			X		
Inguinal, femoral, and abdominal hernia	Data Rich	Male	Cumulative Cigarettes (10 Years)	X			X		
Inflammatory bowel disease	Global	Female	Education (years per capita)			X			X
Inflammatory bowel disease	Data Rich	Female	Education (years per capita)			X			X
Inflammatory bowel disease	Global	Female	LDI (I$ per capita)			X			X
Inflammatory bowel disease	Data Rich	Female	LDI (I$ per capita)			X			X
Inflammatory bowel disease	Global	Female	Socio-demographic Index			X			X
Inflammatory bowel disease	Data Rich	Female	Socio-demographic Index			X			X
Inflammatory bowel disease	Global	Female	Healthcare access and quality index		X			X	
Inflammatory bowel disease	Data Rich	Female	Healthcare access and quality index		X			X	
Inflammatory bowel disease	Global	Female	Latitude 15 to 30 (proportion)		X			X	
Inflammatory bowel disease	Data Rich	Female	Latitude 15 to 30 (proportion)		X			X	
Inflammatory bowel disease	Global	Female	Latitude 30 to 45 (proportion)		X			X	
Inflammatory bowel disease	Data Rich	Female	Latitude 30 to 45 (proportion)		X			X	
Inflammatory bowel disease	Global	Female	Latitude Over 45 (proportion)		X			X	
Inflammatory bowel disease	Data Rich	Female	Latitude Over 45 (proportion)		X			X	
Inflammatory bowel disease	Global	Female	Age- and sex-specific SEV for Low fruit	X			X		
Inflammatory bowel disease	Data Rich	Female	Age- and sex-specific SEV for Low fruit	X			X		
Inflammatory bowel disease	Global	Female	Age- and sex-specific SEV for Low vegetables	X			X		
Inflammatory bowel disease	Data Rich	Female	Age- and sex-specific SEV for Low vegetables	X			X		
Inflammatory bowel disease	Global	Female	Age- and sex-specific SEV for High red meat	X			X		
Inflammatory bowel disease	Data Rich	Female	Age- and sex-specific SEV for High red meat	X			X		
Inflammatory bowel disease	Global	Female	Age- and sex-specific SEV for Low PUFA	X			X		
Inflammatory bowel disease	Data Rich	Female	Age- and sex-specific SEV for Low PUFA	X			X		
Inflammatory bowel disease	Global	Male	Education (years per capita)			X			X
Inflammatory bowel disease	Data Rich	Male	Education (years per capita)			X			X
Inflammatory bowel disease	Global	Male	LDI (I$ per capita)			X			X
Inflammatory bowel disease	Data Rich	Male	LDI (I$ per capita)			X			X
Inflammatory bowel disease	Global	Male	Socio-demographic Index			X			X
Inflammatory bowel disease	Data Rich	Male	Socio-demographic Index			X			X
Inflammatory bowel disease	Global	Male	Healthcare access and quality index		X			X	
Inflammatory bowel disease	Data Rich	Male	Healthcare access and quality index		X			X	
Inflammatory bowel disease	Global	Male	Latitude 15 to 30 (proportion)		X			X	
Inflammatory bowel disease	Data Rich	Male	Latitude 15 to 30 (proportion)		X			X	
Inflammatory bowel disease	Global	Male	Latitude 30 to 45 (proportion)		X			X	
Inflammatory bowel disease	Data Rich	Male	Latitude 30 to 45 (proportion)		X			X	
Inflammatory bowel disease	Global	Male	Latitude Over 45 (proportion)		X			X	
Inflammatory bowel disease	Data Rich	Male	Latitude Over 45 (proportion)		X			X	
Inflammatory bowel disease	Global	Male	Age- and sex-specific SEV for Low fruit	X			X		
Inflammatory bowel disease	Data Rich	Male	Age- and sex-specific SEV for Low fruit	X			X		
Inflammatory bowel disease	Global	Male	Age- and sex-specific SEV for Low vegetables	X			X		
Inflammatory bowel disease	Data Rich	Male	Age- and sex-specific SEV for Low vegetables	X			X		
Inflammatory bowel disease	Global	Male	Age- and sex-specific SEV for High red meat	X			X		
Inflammatory bowel disease	Data Rich	Male	Age- and sex-specific SEV for High red meat	X			X		
Inflammatory bowel disease	Global	Male	Age- and sex-specific SEV for Low PUFA	X			X		
Inflammatory bowel disease	Data Rich	Male	Age- and sex-specific SEV for Low PUFA	X			X		
Vascular intestinal disorders	Data Rich	Female	Education (years per capita)			X			X
Vascular intestinal disorders	Global	Female	Education (years per capita)			X			X
Vascular intestinal disorders	Data Rich	Female	LDI (I$ per capita)			X			X
Vascular intestinal disorders	Global	Female	LDI (I$ per capita)			X			X
Vascular intestinal disorders	Data Rich	Female	Liters of alcohol consumed per capita			X			X
Vascular intestinal disorders	Global	Female	Liters of alcohol consumed per capita			X			X
Vascular intestinal disorders	Data Rich	Female	Total Fertility Rate			X			X
Vascular intestinal disorders	Global	Female	Total Fertility Rate			X			X
Vascular intestinal disorders	Data Rich	Female	Age- and sex-specific SEV for Low fruit			X			X
Vascular intestinal disorders	Global	Female	Age- and sex-specific SEV for Low fruit			X			X
Vascular intestinal disorders	Data Rich	Female	Age- and sex-specific SEV for Low vegetables			X			X
Vascular intestinal disorders	Global	Female	Age- and sex-specific SEV for Low vegetables			X			X
Vascular intestinal disorders	Data Rich	Female	Age- and sex-specific SEV for Low PUFA			X			X
Vascular intestinal disorders	Global	Female	Age- and sex-specific SEV for Low PUFA			X			X
Vascular intestinal disorders	Data Rich	Female	Age- and sex-specific SEV for Low nuts and seeds			X			X
Vascular intestinal disorders	Global	Female	Age- and sex-specific SEV for Low nuts and seeds			X			X
Vascular intestinal disorders	Data Rich	Female	Age- and sex-specific SEV for Low omega-3			X			X
Vascular intestinal disorders	Global	Female	Age- and sex-specific SEV for Low omega-3			X			X
Vascular intestinal disorders	Data Rich	Female	Pulses legumes unadjusted(g)			X			X
Vascular intestinal disorders	Global	Female	Pulses legumes unadjusted(g)			X			X
Vascular intestinal disorders	Data Rich	Female	Socio-demographic Index			X			X

Comparison of GBD 2019 and GBD 2021 covariates and level of covariates used in cause of death modeling

Cause	Model Version Type	Sex	Covariate	Level 1 GBD 2019	Level 2 GBD 2019	Level 3 GBD 2019	Level 1 GBD 2021	Level 2 GBD 2021	Level 3 GBD 2021
Vascular intestinal disorders	Data Rich	Female	Healthcare access and quality index		X			X	
Vascular intestinal disorders	Global	Female	Healthcare access and quality index		X			X	
Vascular intestinal disorders	Data Rich	Female	Mean BMI		X			X	
Vascular intestinal disorders	Global	Female	Mean BMI		X			X	
Vascular intestinal disorders	Data Rich	Female	Smoking Prevalence		X			X	
Vascular intestinal disorders	Global	Female	Smoking Prevalence		X			X	
Vascular intestinal disorders	Data Rich	Female	Low-Density Lipoprotein (mmol/L)	X			X		
Vascular intestinal disorders	Global	Female	Low-Density Lipoprotein (mmol/L)	X			X		
Vascular intestinal disorders	Data Rich	Female	Systolic Blood Pressure (mmHg)	X			X		
Vascular intestinal disorders	Global	Female	Systolic Blood Pressure (mmHg)	X			X		
Vascular intestinal disorders	Data Rich	Female	Diabetes Fasting Plasma Glucose (mmol/L), age-standardized 25+	X			X		
Vascular intestinal disorders	Global	Female	Diabetes Fasting Plasma Glucose (mmol/L), age-standardized 25+	X			X		
Vascular intestinal disorders	Data Rich	Male	Education (years per capita)			X			X
Vascular intestinal disorders	Global	Male	Education (years per capita)			X			X
Vascular intestinal disorders	Data Rich	Male	LDI (I$ per capita)			X			X
Vascular intestinal disorders	Global	Male	LDI (I$ per capita)			X			X
Vascular intestinal disorders	Data Rich	Male	Liters of alcohol consumed per capita			X			X
Vascular intestinal disorders	Global	Male	Liters of alcohol consumed per capita			X			X
Vascular intestinal disorders	Data Rich	Male	Total Fertility Rate			X			X
Vascular intestinal disorders	Global	Male	Total Fertility Rate			X			X
Vascular intestinal disorders	Data Rich	Male	Age- and sex-specific SEV for Low fruit			X			X
Vascular intestinal disorders	Global	Male	Age- and sex-specific SEV for Low fruit			X			X
Vascular intestinal disorders	Data Rich	Male	Age- and sex-specific SEV for Low vegetables			X			X
Vascular intestinal disorders	Global	Male	Age- and sex-specific SEV for Low vegetables			X			X
Vascular intestinal disorders	Data Rich	Male	Age- and sex-specific SEV for Low PUFA			X			X
Vascular intestinal disorders	Global	Male	Age- and sex-specific SEV for Low PUFA			X			X
Vascular intestinal disorders	Data Rich	Male	Age- and sex-specific SEV for Low nuts and seeds			X			X
Vascular intestinal disorders	Global	Male	Age- and sex-specific SEV for Low nuts and seeds			X			X
Vascular intestinal disorders	Data Rich	Male	Age- and sex-specific SEV for Low omega-3			X			X
Vascular intestinal disorders	Global	Male	Age- and sex-specific SEV for Low omega-3			X			X
Vascular intestinal disorders	Data Rich	Male	Pulses legumes unadjusted(g)			X			X
Vascular intestinal disorders	Global	Male	Pulses legumes unadjusted(g)			X			X
Vascular intestinal disorders	Data Rich	Male	Socio-demographic Index			X			X
Vascular intestinal disorders	Data Rich	Male	Healthcare access and quality index		X			X	
Vascular intestinal disorders	Global	Male	Healthcare access and quality index		X			X	
Vascular intestinal disorders	Data Rich	Male	Mean BMI		X			X	
Vascular intestinal disorders	Global	Male	Mean BMI		X			X	
Vascular intestinal disorders	Data Rich	Male	Smoking Prevalence		X			X	
Vascular intestinal disorders	Global	Male	Smoking Prevalence		X			X	
Vascular intestinal disorders	Data Rich	Male	Low-Density Lipoprotein (mmol/L)	X			X		
Vascular intestinal disorders	Global	Male	Low-Density Lipoprotein (mmol/L)	X			X		
Vascular intestinal disorders	Data Rich	Male	Systolic Blood Pressure (mmHg)	X			X		
Vascular intestinal disorders	Global	Male	Systolic Blood Pressure (mmHg)	X			X		
Vascular intestinal disorders	Data Rich	Male	Diabetes Fasting Plasma Glucose (mmol/L), age-standardized 25+	X			X		
Vascular intestinal disorders	Global	Male	Diabetes Fasting Plasma Glucose (mmol/L), age-standardized 25+	X			X		
Gallbladder and biliary diseases	Global	Female	Education (years per capita)			X			X
Gallbladder and biliary diseases	Data Rich	Female	Education (years per capita)			X			X
Gallbladder and biliary diseases	Global	Female	LDI (I$ per capita)			X			X
Gallbladder and biliary diseases	Data Rich	Female	LDI (I$ per capita)			X			X
Gallbladder and biliary diseases	Global	Female	Socio-demographic Index			X			X
Gallbladder and biliary diseases	Data Rich	Female	Socio-demographic Index			X			X
Gallbladder and biliary diseases	Global	Female	Healthcare access and quality index		X			X	
Gallbladder and biliary diseases	Data Rich	Female	Healthcare access and quality index		X			X	
Gallbladder and biliary diseases	Global	Female	Liters of alcohol consumed per capita		X			X	
Gallbladder and biliary diseases	Data Rich	Female	Liters of alcohol consumed per capita		X			X	
Gallbladder and biliary diseases	Global	Female	Population Over 65 (proportion)		X			X	
Gallbladder and biliary diseases	Data Rich	Female	Population Over 65 (proportion)		X			X	
Gallbladder and biliary diseases	Global	Female	Age- and sex-specific SEV for High red meat		X			X	
Gallbladder and biliary diseases	Data Rich	Female	Age- and sex-specific SEV for High red meat		X			X	
Gallbladder and biliary diseases	Global	Female	Mean BMI	X			X		
Gallbladder and biliary diseases	Data Rich	Female	Mean BMI	X			X		
Gallbladder and biliary diseases	Global	Female	Age- and sex-specific SEV for Low PUFA	X			X		
Gallbladder and biliary diseases	Data Rich	Female	Age- and sex-specific SEV for Low PUFA	X			X		
Gallbladder and biliary diseases	Global	Male	Education (years per capita)			X			X
Gallbladder and biliary diseases	Data Rich	Male	Education (years per capita)			X			X
Gallbladder and biliary diseases	Global	Male	LDI (I$ per capita)			X			X
Gallbladder and biliary diseases	Data Rich	Male	LDI (I$ per capita)			X			X
Gallbladder and biliary diseases	Global	Male	Socio-demographic Index			X			X
Gallbladder and biliary diseases	Data Rich	Male	Socio-demographic Index			X			X
Gallbladder and biliary diseases	Global	Male	Healthcare access and quality index		X			X	
Gallbladder and biliary diseases	Data Rich	Male	Healthcare access and quality index		X			X	
Gallbladder and biliary diseases	Global	Male	Liters of alcohol consumed per capita		X			X	
Gallbladder and biliary diseases	Data Rich	Male	Liters of alcohol consumed per capita		X			X	
Gallbladder and biliary diseases	Global	Male	Population Over 65 (proportion)		X			X	
Gallbladder and biliary diseases	Data Rich	Male	Population Over 65 (proportion)		X			X	
Gallbladder and biliary diseases	Global	Male	Age- and sex-specific SEV for High red meat		X			X	
Gallbladder and biliary diseases	Data Rich	Male	Age- and sex-specific SEV for High red meat		X			X	
Gallbladder and biliary diseases	Global	Male	Mean BMI	X			X		
Gallbladder and biliary diseases	Data Rich	Male	Mean BMI	X			X		
Gallbladder and biliary diseases	Global	Male	Age- and sex-specific SEV for Low PUFA	X			X		
Gallbladder and biliary diseases	Data Rich	Male	Age- and sex-specific SEV for Low PUFA	X			X		
Pancreatitis	Data Rich	Female	Education (years per capita)			X			X
Pancreatitis	Global	Female	Education (years per capita)			X			X
Pancreatitis	Data Rich	Female	LDI (I$ per capita)			X			X
Pancreatitis	Global	Female	LDI (I$ per capita)			X			X
Pancreatitis	Data Rich	Female	Socio-demographic Index			X			X
Pancreatitis	Global	Female	Socio-demographic Index			X			X
Pancreatitis	Data Rich	Female	Healthcare access and quality index		X			X	
Pancreatitis	Global	Female	Healthcare access and quality index		X			X	
Pancreatitis	Data Rich	Female	Mean BMI		X			X	
Pancreatitis	Global	Female	Mean BMI		X			X	
Pancreatitis	Data Rich	Female	Liters of alcohol consumed per capita	X			X		
Pancreatitis	Global	Female	Liters of alcohol consumed per capita	X			X		
Pancreatitis	Data Rich	Female	Log-transformed SEV scalar: Pancreatit	X			X		
Pancreatitis	Global	Female	Log-transformed SEV scalar: Pancreatit	X			X		
Pancreatitis	Data Rich	Male	Education (years per capita)			X			X
Pancreatitis	Global	Male	Education (years per capita)			X			X
Pancreatitis	Data Rich	Male	LDI (I$ per capita)			X			X
Pancreatitis	Global	Male	LDI (I$ per capita)			X			X
Pancreatitis	Data Rich	Male	Socio-demographic Index			X			X
Pancreatitis	Global	Male	Socio-demographic Index			X			X
Pancreatitis	Data Rich	Male	Healthcare access and quality index		X			X	
Pancreatitis	Global	Male	Healthcare access and quality index		X			X	
Pancreatitis	Data Rich	Male	Mean BMI		X			X	
Pancreatitis	Global	Male	Mean BMI		X			X	
Pancreatitis	Data Rich	Male	Liters of alcohol consumed per capita	X			X		
Pancreatitis	Global	Male	Liters of alcohol consumed per capita	X			X		
Pancreatitis	Data Rich	Male	Log-transformed SEV scalar: Pancreatit	X			X		
Pancreatitis	Global	Male	Log-transformed SEV scalar: Pancreatit	X			X		
Other digestive diseases	Data Rich	Female	Education (years per capita)			X			X
Other digestive diseases	Global	Female	Education (years per capita)			X			X
Other digestive diseases	Data Rich	Female	LDI (I$ per capita)			X			X
Other digestive diseases	Global	Female	LDI (I$ per capita)			X			X
Other digestive diseases	Data Rich	Female	Socio-demographic Index			X			X
Other digestive diseases	Global	Female	Socio-demographic Index			X			X
Other digestive diseases	Data Rich	Female	Healthcare access and quality index		X			X	
Other digestive diseases	Global	Female	Healthcare access and quality index		X			X	
Other digestive diseases	Data Rich	Female	Mean BMI		X			X	
Other digestive diseases	Global	Female	Mean BMI		X			X	
Other digestive diseases	Data Rich	Female	Sanitation (proportion with access)		X			X	
Other digestive diseases	Global	Female	Sanitation (proportion with access)		X			X	
Other digestive diseases	Data Rich	Female	Diabetes Age-Standardized Prevalence (proportion)		X			X	
Other digestive diseases	Global	Female	Diabetes Age-Standardized Prevalence (proportion)		X			X	
Other digestive diseases	Data Rich	Female	Improved Water Source (proportion with access)		X			X	
Other digestive diseases	Global	Female	Improved Water Source (proportion with access)		X			X	
Other digestive diseases	Data Rich	Female	Age- and sex-specific SEV for Low fruit		X			X	
Other digestive diseases	Global	Female	Age- and sex-specific SEV for Low fruit		X			X	
Other digestive diseases	Data Rich	Female	Age- and sex-specific SEV for Low vegetables		X			X	
Other digestive diseases	Global	Female	Age- and sex-specific SEV for Low vegetables		X			X	
Other digestive diseases	Data Rich	Female	Age- and sex-specific SEV for High red meat		X			X	

Comparison of GBD 2019 and GBD 2021 covariates and level of covariates used in cause of death modeling									
Cause	Model Version Type	Sex	Covariate	Level 1 GBD 2019	Level 2 GBD 2019	Level 3 GBD 2019	Level 1 GBD 2021	Level 2 GBD 2021	Level 3 GBD 2021
Other digestive diseases	Global	Female	Age- and sex-specific SEV for High red meat		X			X	
Other digestive diseases	Data Rich	Female	Age- and sex-specific SEV for Low PUFA		X			X	
Other digestive diseases	Global	Female	Age- and sex-specific SEV for Low PUFA		X			X	
Other digestive diseases	Data Rich	Female	Smoking Prevalence	X			X		
Other digestive diseases	Global	Female	Smoking Prevalence	X			X		
Other digestive diseases	Data Rich	Female	Liters of alcohol consumed per capita	X			X		
Other digestive diseases	Global	Female	Liters of alcohol consumed per capita	X			X		
Other digestive diseases	Data Rich	Female	Cumulative Cigarettes (5 Years)	X			X		
Other digestive diseases	Global	Female	Cumulative Cigarettes (5 Years)	X			X		
Other digestive diseases	Data Rich	Female	Cumulative Cigarettes (10 Years)	X			X		
Other digestive diseases	Global	Female	Cumulative Cigarettes (10 Years)	X			X		
Other digestive diseases	Data Rich	Male	Education (years per capita)			X			X
Other digestive diseases	Global	Male	Education (years per capita)			X			X
Other digestive diseases	Data Rich	Male	LDI (I$ per capita)			X			X
Other digestive diseases	Global	Male	LDI (I$ per capita)			X			X
Other digestive diseases	Data Rich	Male	Socio-demographic Index			X			X
Other digestive diseases	Global	Male	Socio-demographic Index			X			X
Other digestive diseases	Data Rich	Male	Healthcare access and quality index		X			X	
Other digestive diseases	Global	Male	Healthcare access and quality index		X			X	
Other digestive diseases	Data Rich	Male	Mean BMI		X			X	
Other digestive diseases	Global	Male	Mean BMI		X			X	
Other digestive diseases	Data Rich	Male	Sanitation (proportion with access)		X			X	
Other digestive diseases	Global	Male	Sanitation (proportion with access)		X			X	
Other digestive diseases	Data Rich	Male	Diabetes Age-Standardized Prevalence (proportion)		X			X	
Other digestive diseases	Global	Male	Diabetes Age-Standardized Prevalence (proportion)		X			X	
Other digestive diseases	Data Rich	Male	Improved Water Source (proportion with access)		X			X	
Other digestive diseases	Global	Male	Improved Water Source (proportion with access)		X			X	
Other digestive diseases	Data Rich	Male	Age- and sex-specific SEV for Low fruit		X			X	
Other digestive diseases	Global	Male	Age- and sex-specific SEV for Low fruit		X			X	
Other digestive diseases	Data Rich	Male	Age- and sex-specific SEV for Low vegetables		X			X	
Other digestive diseases	Global	Male	Age- and sex-specific SEV for Low vegetables		X			X	
Other digestive diseases	Data Rich	Male	Age- and sex-specific SEV for High red meat		X			X	
Other digestive diseases	Global	Male	Age- and sex-specific SEV for High red meat		X			X	
Other digestive diseases	Data Rich	Male	Age- and sex-specific SEV for Low PUFA		X			X	
Other digestive diseases	Global	Male	Age- and sex-specific SEV for Low PUFA		X			X	
Other digestive diseases	Data Rich	Male	Smoking Prevalence	X			X		
Other digestive diseases	Global	Male	Smoking Prevalence	X			X		
Other digestive diseases	Data Rich	Male	Liters of alcohol consumed per capita	X			X		
Other digestive diseases	Data Rich	Male	Cumulative Cigarettes (5 Years)	X			X		
Other digestive diseases	Global	Male	Cumulative Cigarettes (5 Years)	X			X		
Other digestive diseases	Data Rich	Male	Cumulative Cigarettes (10 Years)	X			X		
Other digestive diseases	Global	Male	Cumulative Cigarettes (10 Years)	X			X		
Parkinson's disease	Global	Female	Education (years per capita)			X			X
Parkinson's disease	Data Rich	Female	Education (years per capita)			X			X
Parkinson's disease	Global	Female	LDI (I$ per capita)			X			X
Parkinson's disease	Data Rich	Female	LDI (I$ per capita)			X			X
Parkinson's disease	Global	Female	Socio-demographic Index			X			X
Parkinson's disease	Data Rich	Female	Socio-demographic Index			X			X
Parkinson's disease	Global	Female	Healthcare access and quality index		X			X	
Parkinson's disease	Data Rich	Female	Healthcare access and quality index		X			X	
Parkinson's disease	Global	Female	Low-Density Lipoprotein (mmol/L)		X			X	
Parkinson's disease	Data Rich	Female	Low-Density Lipoprotein (mmol/L)		X			X	
Parkinson's disease	Global	Female	Sanitation (proportion with access)		X			X	
Parkinson's disease	Data Rich	Female	Sanitation (proportion with access)		X			X	
Parkinson's disease	Global	Female	Absolute value of average latitude		X			X	
Parkinson's disease	Data Rich	Female	Absolute value of average latitude		X			X	
Parkinson's disease	Global	Female	Improved Water Source (proportion with access)		X			X	
Parkinson's disease	Data Rich	Female	Improved Water Source (proportion with access)		X			X	
Parkinson's disease	Global	Female	Cumulative Cigarettes (10 Years)	X			X		
Parkinson's disease	Data Rich	Female	Cumulative Cigarettes (10 Years)	X			X		
Parkinson's disease	Global	Female	Age- and sex-specific SEV for Low fruit	X			X		
Parkinson's disease	Data Rich	Female	Age- and sex-specific SEV for Low fruit	X			X		
Parkinson's disease	Global	Male	Education (years per capita)			X			X
Parkinson's disease	Data Rich	Male	Education (years per capita)			X			X
Parkinson's disease	Global	Male	LDI (I$ per capita)			X			X
Parkinson's disease	Data Rich	Male	LDI (I$ per capita)			X			X
Parkinson's disease	Global	Male	Socio-demographic Index			X			X
Parkinson's disease	Data Rich	Male	Socio-demographic Index			X			X
Parkinson's disease	Global	Male	Healthcare access and quality index		X			X	
Parkinson's disease	Data Rich	Male	Healthcare access and quality index		X			X	
Parkinson's disease	Global	Male	Low-Density Lipoprotein (mmol/L)		X			X	
Parkinson's disease	Data Rich	Male	Low-Density Lipoprotein (mmol/L)		X			X	
Parkinson's disease	Global	Male	Sanitation (proportion with access)		X			X	
Parkinson's disease	Data Rich	Male	Sanitation (proportion with access)		X			X	
Parkinson's disease	Global	Male	Absolute value of average latitude		X			X	
Parkinson's disease	Data Rich	Male	Absolute value of average latitude		X			X	
Parkinson's disease	Global	Male	Improved Water Source (proportion with access)		X			X	
Parkinson's disease	Data Rich	Male	Improved Water Source (proportion with access)		X			X	
Parkinson's disease	Global	Male	Cumulative Cigarettes (10 Years)	X			X		
Parkinson's disease	Data Rich	Male	Cumulative Cigarettes (10 Years)	X			X		
Parkinson's disease	Global	Male	Age- and sex-specific SEV for Low fruit	X			X		
Parkinson's disease	Data Rich	Male	Age- and sex-specific SEV for Low fruit	X			X		
Idiopathic epilepsy	Data Rich	Female	Education (years per capita)			X			X
Idiopathic epilepsy	Global	Female	Education (years per capita)			X			X
Idiopathic epilepsy	Data Rich	Female	LDI (I$ per capita)			X			X
Idiopathic epilepsy	Global	Female	LDI (I$ per capita)			X			X
Idiopathic epilepsy	Data Rich	Female	Socio-demographic Index			X			X
Idiopathic epilepsy	Global	Female	Socio-demographic Index			X			X
Idiopathic epilepsy	Data Rich	Female	Cumulative Cigarettes (5 Years)			X			X
Idiopathic epilepsy	Global	Female	Cumulative Cigarettes (5 Years)			X			X
Idiopathic epilepsy	Data Rich	Female	Cumulative Cigarettes (10 Years)			X			X
Idiopathic epilepsy	Global	Female	Cumulative Cigarettes (10 Years)			X			X
Idiopathic epilepsy	Data Rich	Female	Healthcare access and quality index		X			X	
Idiopathic epilepsy	Global	Female	Healthcare access and quality index		X			X	
Idiopathic epilepsy	Data Rich	Female	Mean BMI		X			X	
Idiopathic epilepsy	Global	Female	Mean BMI		X			X	
Idiopathic epilepsy	Data Rich	Female	Low-Density Lipoprotein (mmol/L)		X			X	
Idiopathic epilepsy	Global	Female	Low-Density Lipoprotein (mmol/L)		X			X	
Idiopathic epilepsy	Data Rich	Female	Systolic Blood Pressure (mmHg)	X			X		
Idiopathic epilepsy	Global	Female	Systolic Blood Pressure (mmHg)	X			X		
Idiopathic epilepsy	Data Rich	Female	Pigs (per capita)	X			X		
Idiopathic epilepsy	Global	Female	Pigs (per capita)	X			X		
Idiopathic epilepsy	Data Rich	Female	Log-transformed SEV scalar: Idiopathic epilepsy	X			X		
Idiopathic epilepsy	Global	Female	Log-transformed SEV scalar: Idiopathic epilepsy	X			X		
Idiopathic epilepsy	Data Rich	Male	Education (years per capita)			X			X
Idiopathic epilepsy	Global	Male	Education (years per capita)			X			X
Idiopathic epilepsy	Data Rich	Male	LDI (I$ per capita)			X			X
Idiopathic epilepsy	Global	Male	LDI (I$ per capita)			X			X
Idiopathic epilepsy	Data Rich	Male	Socio-demographic Index			X			X
Idiopathic epilepsy	Global	Male	Socio-demographic Index			X			X
Idiopathic epilepsy	Data Rich	Male	Cumulative Cigarettes (5 Years)			X			X
Idiopathic epilepsy	Global	Male	Cumulative Cigarettes (5 Years)			X			X
Idiopathic epilepsy	Data Rich	Male	Cumulative Cigarettes (10 Years)			X			X
Idiopathic epilepsy	Global	Male	Cumulative Cigarettes (10 Years)			X			X
Idiopathic epilepsy	Data Rich	Male	Healthcare access and quality index		X			X	
Idiopathic epilepsy	Global	Male	Healthcare access and quality index		X			X	
Idiopathic epilepsy	Data Rich	Male	Mean BMI		X			X	
Idiopathic epilepsy	Global	Male	Mean BMI		X			X	
Idiopathic epilepsy	Data Rich	Male	Low-Density Lipoprotein (mmol/L)		X			X	
Idiopathic epilepsy	Global	Male	Low-Density Lipoprotein (mmol/L)		X			X	
Idiopathic epilepsy	Data Rich	Male	Systolic Blood Pressure (mmHg)	X			X		
Idiopathic epilepsy	Global	Male	Systolic Blood Pressure (mmHg)	X			X		
Idiopathic epilepsy	Data Rich	Male	Pigs (per capita)	X			X		
Idiopathic epilepsy	Global	Male	Pigs (per capita)	X			X		
Idiopathic epilepsy	Data Rich	Male	Log-transformed SEV scalar: Idiopathic epilepsy	X			X		
Idiopathic epilepsy	Global	Male	Log-transformed SEV scalar: Idiopathic epilepsy	X			X		
Multiple sclerosis	Data Rich	Female	Education (years per capita)			X			X
Multiple sclerosis	Global	Female	Education (years per capita)			X			X
Multiple sclerosis	Data Rich	Female	LDI (I$ per capita)			X			X
Multiple sclerosis	Global	Female	LDI (I$ per capita)			X			X
Multiple sclerosis	Data Rich	Female	Socio-demographic Index			X			X
Multiple sclerosis	Global	Female	Socio-demographic Index			X			X

Comparison of GBD 2019 and GBD 2021 covariates and level of covariates used in cause of death modeling									
Cause	Model Version Type	Sex	Covariate	Level 1 GBD 2019	Level 2 GBD 2019	Level 3 GBD 2019	Level 1 GBD 2021	Level 2 GBD 2021	Level 3 GBD 2021
Multiple sclerosis	Data Rich	Female	Smoking Prevalence			X			X
Multiple sclerosis	Global	Female	Smoking Prevalence			X			X
Multiple sclerosis	Data Rich	Female	Cumulative Cigarettes (5 Years)			X			X
Multiple sclerosis	Global	Female	Cumulative Cigarettes (5 Years)			X			X
Multiple sclerosis	Data Rich	Female	Cumulative Cigarettes (10 Years)			X			X
Multiple sclerosis	Global	Female	Cumulative Cigarettes (10 Years)			X			X
Multiple sclerosis	Data Rich	Female	Healthcare access and quality index		X			X	
Multiple sclerosis	Global	Female	Healthcare access and quality index		X			X	
Multiple sclerosis	Data Rich	Female	Low-Density Lipoprotein (mmol/L)		X			X	
Multiple sclerosis	Global	Female	Low-Density Lipoprotein (mmol/L)		X			X	
Multiple sclerosis	Data Rich	Female	Absolute value of average latitude	X			X		
Multiple sclerosis	Global	Female	Absolute value of average latitude	X			X		
Multiple sclerosis	Data Rich	Male	Education (years per capita)			X			X
Multiple sclerosis	Global	Male	Education (years per capita)			X			X
Multiple sclerosis	Data Rich	Male	LDI (I$ per capita)			X			X
Multiple sclerosis	Global	Male	LDI (I$ per capita)			X			X
Multiple sclerosis	Data Rich	Male	Socio-demographic Index			X			X
Multiple sclerosis	Global	Male	Socio-demographic Index			X			X
Multiple sclerosis	Data Rich	Male	Smoking Prevalence			X			X
Multiple sclerosis	Global	Male	Smoking Prevalence			X			X
Multiple sclerosis	Data Rich	Male	Cumulative Cigarettes (5 Years)			X			X
Multiple sclerosis	Global	Male	Cumulative Cigarettes (5 Years)			X			X
Multiple sclerosis	Data Rich	Male	Cumulative Cigarettes (10 Years)			X			X
Multiple sclerosis	Global	Male	Cumulative Cigarettes (10 Years)			X			X
Multiple sclerosis	Data Rich	Male	Healthcare access and quality index		X			X	
Multiple sclerosis	Global	Male	Healthcare access and quality index		X			X	
Multiple sclerosis	Data Rich	Male	Low-Density Lipoprotein (mmol/L)		X			X	
Multiple sclerosis	Global	Male	Low-Density Lipoprotein (mmol/L)		X			X	
Multiple sclerosis	Data Rich	Male	Absolute value of average latitude	X			X		
Multiple sclerosis	Global	Male	Absolute value of average latitude	X			X		
Motor neuron disease	Global	Female	Education (years per capita)			X			X
Motor neuron disease	Data Rich	Female	Education (years per capita)			X			X
Motor neuron disease	Global	Female	LDI (I$ per capita)			X			X
Motor neuron disease	Data Rich	Female	LDI (I$ per capita)			X			X
Motor neuron disease	Global	Female	Healthcare access and quality index		X			X	
Motor neuron disease	Data Rich	Female	Healthcare access and quality index		X			X	
Motor neuron disease	Global	Female	Population-weighted mean temperature		X			X	
Motor neuron disease	Data Rich	Female	Population-weighted mean temperature		X			X	
Motor neuron disease	Global	Female	Sanitation (proportion with access)		X			X	
Motor neuron disease	Data Rich	Female	Sanitation (proportion with access)		X			X	
Motor neuron disease	Global	Female	Improved Water Source (proportion with access)		X			X	
Motor neuron disease	Data Rich	Female	Improved Water Source (proportion with access)		X			X	
Motor neuron disease	Global	Female	Socio-demographic Index	X			X		
Motor neuron disease	Data Rich	Female	Socio-demographic Index	X			X		
Motor neuron disease	Global	Female	Mean BMI	X			X		
Motor neuron disease	Data Rich	Female	Mean BMI	X			X		
Motor neuron disease	Global	Female	Low-Density Lipoprotein (mmol/L)	X			X		
Motor neuron disease	Data Rich	Female	Low-Density Lipoprotein (mmol/L)	X			X		
Motor neuron disease	Global	Female	Diabetes Fasting Plasma Glucose (mmol/L), age-standardized 25+	X			X		
Motor neuron disease	Data Rich	Female	Diabetes Fasting Plasma Glucose (mmol/L), age-standardized 25+	X			X		
Motor neuron disease	Global	Female	Absolute value of average latitude	X			X		
Motor neuron disease	Data Rich	Female	Absolute value of average latitude	X			X		
Motor neuron disease	Global	Female	Age- and sex-specific SEV for Low fruit	X			X		
Motor neuron disease	Data Rich	Female	Age- and sex-specific SEV for Low fruit	X			X		
Motor neuron disease	Global	Male	Education (years per capita)			X			X
Motor neuron disease	Data Rich	Male	Education (years per capita)			X			X
Motor neuron disease	Global	Male	LDI (I$ per capita)			X			X
Motor neuron disease	Data Rich	Male	LDI (I$ per capita)			X			X
Motor neuron disease	Global	Male	Healthcare access and quality index		X			X	
Motor neuron disease	Data Rich	Male	Healthcare access and quality index		X			X	
Motor neuron disease	Global	Male	Population-weighted mean temperature		X			X	
Motor neuron disease	Data Rich	Male	Population-weighted mean temperature		X			X	
Motor neuron disease	Global	Male	Sanitation (proportion with access)		X			X	
Motor neuron disease	Data Rich	Male	Sanitation (proportion with access)		X			X	
Motor neuron disease	Global	Male	Improved Water Source (proportion with access)		X			X	
Motor neuron disease	Data Rich	Male	Improved Water Source (proportion with access)		X			X	
Motor neuron disease	Global	Male	Socio-demographic Index	X			X		
Motor neuron disease	Data Rich	Male	Socio-demographic Index	X			X		
Motor neuron disease	Global	Male	Mean BMI	X			X		
Motor neuron disease	Data Rich	Male	Mean BMI	X			X		
Motor neuron disease	Global	Male	Low-Density Lipoprotein (mmol/L)	X			X		
Motor neuron disease	Data Rich	Male	Low-Density Lipoprotein (mmol/L)	X			X		
Motor neuron disease	Global	Male	Diabetes Fasting Plasma Glucose (mmol/L), age-standardized 25+	X			X		
Motor neuron disease	Data Rich	Male	Diabetes Fasting Plasma Glucose (mmol/L), age-standardized 25+	X			X		
Motor neuron disease	Global	Male	Absolute value of average latitude	X			X		
Motor neuron disease	Data Rich	Male	Absolute value of average latitude	X			X		
Motor neuron disease	Global	Male	Age- and sex-specific SEV for Low fruit	X			X		
Motor neuron disease	Data Rich	Male	Age- and sex-specific SEV for Low fruit	X			X		
Other neurological disorders	Global	Female	Education (years per capita)			X			X
Other neurological disorders	Data Rich	Female	Education (years per capita)			X			X
Other neurological disorders	Global	Female	LDI (I$ per capita)			X			X
Other neurological disorders	Data Rich	Female	LDI (I$ per capita)			X			X
Other neurological disorders	Global	Female	Smoking Prevalence			X			X
Other neurological disorders	Data Rich	Female	Smoking Prevalence			X			X
Other neurological disorders	Global	Female	Cumulative Cigarettes (5 Years)			X			X
Other neurological disorders	Data Rich	Female	Cumulative Cigarettes (5 Years)			X			X
Other neurological disorders	Global	Female	Cumulative Cigarettes (10 Years)			X			X
Other neurological disorders	Data Rich	Female	Cumulative Cigarettes (10 Years)			X			X
Other neurological disorders	Global	Female	Socio-demographic Index			X			X
Other neurological disorders	Data Rich	Female	Socio-demographic Index			X			X
Other neurological disorders	Global	Female	Healthcare access and quality index		X			X	
Other neurological disorders	Data Rich	Female	Healthcare access and quality index		X			X	
Other neurological disorders	Global	Female	Population Density (over 1000 ppl/sqkm, proportion)		X			X	
Other neurological disorders	Data Rich	Female	Population Density (over 1000 ppl/sqkm, proportion)		X			X	
Other neurological disorders	Global	Female	Age- and sex-specific SEV for Low fruit		X			X	
Other neurological disorders	Data Rich	Female	Age- and sex-specific SEV for Low fruit		X			X	
Other neurological disorders	Global	Female	Mean BMI	X			X		
Other neurological disorders	Data Rich	Female	Mean BMI	X			X		
Other neurological disorders	Global	Female	Low-Density Lipoprotein (mmol/L)	X			X		
Other neurological disorders	Data Rich	Female	Low-Density Lipoprotein (mmol/L)	X			X		
Other neurological disorders	Global	Female	Systolic Blood Pressure (mmHg)	X			X		
Other neurological disorders	Data Rich	Female	Systolic Blood Pressure (mmHg)	X			X		
Other neurological disorders	Data Rich	Female	Age- and sex-specific SEV for Child underweight	X			X		
Other neurological disorders	Global	Female	Age- and sex-specific SEV for High red meat	X			X		
Other neurological disorders	Data Rich	Female	Age- and sex-specific SEV for High red meat	X			X		
Other neurological disorders	Global	Female	Pigs (per capita)	X			X		
Other neurological disorders	Data Rich	Female	Pigs (per capita)	X			X		
Other neurological disorders	Global	Male	Education (years per capita)			X			X
Other neurological disorders	Data Rich	Male	Education (years per capita)			X			X
Other neurological disorders	Global	Male	LDI (I$ per capita)			X			X
Other neurological disorders	Data Rich	Male	LDI (I$ per capita)			X			X
Other neurological disorders	Global	Male	Smoking Prevalence			X			X
Other neurological disorders	Data Rich	Male	Smoking Prevalence			X			X
Other neurological disorders	Global	Male	Cumulative Cigarettes (5 Years)			X			X
Other neurological disorders	Data Rich	Male	Cumulative Cigarettes (5 Years)			X			X
Other neurological disorders	Global	Male	Cumulative Cigarettes (10 Years)			X			X
Other neurological disorders	Data Rich	Male	Cumulative Cigarettes (10 Years)			X			X
Other neurological disorders	Global	Male	Socio-demographic Index			X			X
Other neurological disorders	Data Rich	Male	Socio-demographic Index			X			X
Other neurological disorders	Global	Male	Healthcare access and quality index		X			X	
Other neurological disorders	Data Rich	Male	Healthcare access and quality index		X			X	
Other neurological disorders	Global	Male	Liters of alcohol consumed per capita		X			X	
Other neurological disorders	Global	Male	Population Density (over 1000 ppl/sqkm, proportion)		X			X	
Other neurological disorders	Data Rich	Male	Population Density (over 1000 ppl/sqkm, proportion)		X			X	
Other neurological disorders	Global	Male	Age- and sex-specific SEV for Low fruit		X			X	
Other neurological disorders	Data Rich	Male	Age- and sex-specific SEV for Low fruit		X			X	
Other neurological disorders	Global	Male	Mean BMI	X			X		
Other neurological disorders	Data Rich	Male	Mean BMI	X			X		
Other neurological disorders	Global	Male	Low-Density Lipoprotein (mmol/L)	X			X		
Other neurological disorders	Data Rich	Male	Low-Density Lipoprotein (mmol/L)	X			X		
Other neurological disorders	Global	Male	Systolic Blood Pressure (mmHg)	X			X		
Other neurological disorders	Data Rich	Male	Systolic Blood Pressure (mmHg)	X			X		
Other neurological disorders	Global	Male	Age- and sex-specific SEV for Child underweight	X			X		

Comparison of GBD 2019 and GBD 2021 covariates and level of covariates used in cause of death modeling									
**Cause**	**Model Version Type**	**Sex**	**Covariate**	**Level 1 GBD 2019**	**Level 2 GBD 2019**	**Level 3 GBD 2019**	**Level 1 GBD 2021**	**Level 2 GBD 2021**	**Level 3 GBD 2021**
Other neurological disorders	Data Rich	Male	Age- and sex-specific SEV for Child underweight	X			X		
Other neurological disorders	Global	Male	Age- and sex-specific SEV for High red meat	X			X		
Other neurological disorders	Data Rich	Male	Age- and sex-specific SEV for High red meat	X			X		
Other neurological disorders	Global	Male	Pigs (per capita)	X			X		
Other neurological disorders	Data Rich	Male	Pigs (per capita)	X			X		
Anorexia nervosa	Global	Female	Socio-demographic Index			X			X
Anorexia nervosa	Data Rich	Female	Socio-demographic Index			X			X
Anorexia nervosa	Global	Female	Healthcare access and quality index		X			X	
Anorexia nervosa	Data Rich	Female	Healthcare access and quality index		X			X	
Anorexia nervosa	Global	Female	Education (years per capita)	X			X		
Anorexia nervosa	Data Rich	Female	Education (years per capita)	X			X		
Anorexia nervosa	Global	Female	LDI (I$ per capita)	X			X		
Anorexia nervosa	Data Rich	Female	LDI (I$ per capita)	X			X		
Anorexia nervosa	Global	Female	Sanitation (proportion with access)	X			X		
Anorexia nervosa	Data Rich	Female	Sanitation (proportion with access)	X			X		
Anorexia nervosa	Global	Female	Maternal Education (years per capita)	X			X		
Anorexia nervosa	Data Rich	Female	Maternal Education (years per capita)	X			X		
Anorexia nervosa	Global	Female	Age- and sex-specific SEV for Child underweight	X			X		
Anorexia nervosa	Data Rich	Female	Age- and sex-specific SEV for Child underweight	X			X		
Anorexia nervosa	Global	Male	Socio-demographic Index			X			X
Anorexia nervosa	Data Rich	Male	Socio-demographic Index			X			X
Anorexia nervosa	Global	Male	Healthcare access and quality index		X			X	
Anorexia nervosa	Data Rich	Male	Healthcare access and quality index		X			X	
Anorexia nervosa	Global	Male	Education (years per capita)	X			X		
Anorexia nervosa	Data Rich	Male	Education (years per capita)	X			X		
Anorexia nervosa	Global	Male	LDI (I$ per capita)	X			X		
Anorexia nervosa	Data Rich	Male	LDI (I$ per capita)	X			X		
Anorexia nervosa	Global	Male	Sanitation (proportion with access)	X			X		
Anorexia nervosa	Data Rich	Male	Sanitation (proportion with access)	X			X		
Anorexia nervosa	Global	Male	Maternal Education (years per capita)	X			X		
Anorexia nervosa	Data Rich	Male	Maternal Education (years per capita)	X			X		
Anorexia nervosa	Global	Male	Age- and sex-specific SEV for Child underweight	X			X		
Anorexia nervosa	Data Rich	Male	Age- and sex-specific SEV for Child underweight	X			X		
Alcohol use disorders	Global	Female	Education (years per capita)			X			X
Alcohol use disorders	Data Rich	Female	Education (years per capita)			X			X
Alcohol use disorders	Global	Female	LDI (I$ per capita)			X			X
Alcohol use disorders	Data Rich	Female	LDI (I$ per capita)			X			X
Alcohol use disorders	Global	Female	Socio-demographic Index			X			X
Alcohol use disorders	Data Rich	Female	Socio-demographic Index			X			X
Alcohol use disorders	Global	Female	Healthcare access and quality index		X			X	
Alcohol use disorders	Data Rich	Female	Healthcare access and quality index		X			X	
Alcohol use disorders	Global	Female	Smoking Prevalence		X			X	
Alcohol use disorders	Data Rich	Female	Smoking Prevalence		X			X	
Alcohol use disorders	Global	Female	Cumulative Cigarettes (10 Years)		X			X	
Alcohol use disorders	Data Rich	Female	Cumulative Cigarettes (10 Years)		X			X	
Alcohol use disorders	Global	Female	Liters of alcohol consumed per capita	X			X		
Alcohol use disorders	Data Rich	Female	Liters of alcohol consumed per capita	X			X		
Alcohol use disorders	Global	Female	Alcohol binge drinker proportion, age-standardized	X			X		
Alcohol use disorders	Data Rich	Female	Alcohol binge drinker proportion, age-standardized	X			X		
Alcohol use disorders	Global	Female	Alcohol drinker proportion, age-standardized	X			X		
Alcohol use disorders	Data Rich	Female	Alcohol drinker proportion, age-standardized	X			X		
Alcohol use disorders	Global	Female	Alcohol consumption, age standardized, in grams per day	X			X		
Alcohol use disorders	Data Rich	Female	Alcohol consumption, age standardized, in grams per day	X			X		
Alcohol use disorders	Data Rich	Male	Education (years per capita)			X			X
Alcohol use disorders	Global	Male	Education (years per capita)			X			X
Alcohol use disorders	Data Rich	Male	LDI (I$ per capita)			X			X
Alcohol use disorders	Global	Male	LDI (I$ per capita)			X			X
Alcohol use disorders	Data Rich	Male	Socio-demographic Index			X			X
Alcohol use disorders	Global	Male	Socio-demographic Index			X			X
Alcohol use disorders	Data Rich	Male	Healthcare access and quality index		X			X	
Alcohol use disorders	Global	Male	Healthcare access and quality index		X			X	
Alcohol use disorders	Data Rich	Male	Smoking Prevalence		X			X	
Alcohol use disorders	Global	Male	Smoking Prevalence		X			X	
Alcohol use disorders	Data Rich	Male	Cumulative Cigarettes (10 Years)		X			X	
Alcohol use disorders	Global	Male	Cumulative Cigarettes (10 Years)		X			X	
Alcohol use disorders	Data Rich	Male	Liters of alcohol consumed per capita	X			X		
Alcohol use disorders	Global	Male	Liters of alcohol consumed per capita	X			X		
Alcohol use disorders	Data Rich	Male	Alcohol binge drinker proportion, age-standardized	X			X		
Alcohol use disorders	Global	Male	Alcohol binge drinker proportion, age-standardized	X			X		
Alcohol use disorders	Data Rich	Male	Alcohol drinker proportion, age-standardized	X			X		
Alcohol use disorders	Global	Male	Alcohol drinker proportion, age-standardized	X			X		
Alcohol use disorders	Data Rich	Male	Alcohol consumption, age standardized, in grams per day	X			X		
Alcohol use disorders	Global	Male	Alcohol consumption, age standardized, in grams per day	X			X		
Drug use disorders	Global	Female	Education (years per capita)			X			X
Drug use disorders	Data Rich	Female	Education (years per capita)			X			X
Drug use disorders	Global	Female	LDI (I$ per capita)			X			X
Drug use disorders	Data Rich	Female	LDI (I$ per capita)			X			X
Drug use disorders	Global	Female	Socio-demographic Index			X			X
Drug use disorders	Data Rich	Female	Socio-demographic Index			X			X
Drug use disorders	Global	Female	Healthcare access and quality index		X			X	
Drug use disorders	Data Rich	Female	Healthcare access and quality index		X			X	
Drug use disorders	Global	Female	Smoking Prevalence		X			X	
Drug use disorders	Data Rich	Female	Smoking Prevalence		X			X	
Drug use disorders	Global	Female	Cumulative Cigarettes (5 Years)		X			X	
Drug use disorders	Data Rich	Female	Cumulative Cigarettes (5 Years)		X			X	
Drug use disorders	Global	Female	Cumulative Cigarettes (10 Years)		X			X	
Drug use disorders	Data Rich	Female	Cumulative Cigarettes (10 Years)		X			X	
Drug use disorders	Global	Female	Intravenous drug use (proportion by age)	X			X		
Drug use disorders	Data Rich	Female	Intravenous drug use (proportion by age)	X			X		
Drug use disorders	Global	Female	Intravenous drug use (age-standardized proportion)	X			X		
Drug use disorders	Data Rich	Female	Intravenous drug use (age-standardized proportion)	X			X		
Drug use disorders	Global	Female	Opioids per million population per day (10 year lag)	X			X		
Drug use disorders	Data Rich	Female	Opioids per million population per day (10 year lag)	X			X		
Drug use disorders	Global	Male	Education (years per capita)			X			X
Drug use disorders	Data Rich	Male	Education (years per capita)			X			X
Drug use disorders	Global	Male	LDI (I$ per capita)			X			X
Drug use disorders	Data Rich	Male	LDI (I$ per capita)			X			X
Drug use disorders	Global	Male	Socio-demographic Index			X			X
Drug use disorders	Data Rich	Male	Socio-demographic Index			X			X
Drug use disorders	Global	Male	Healthcare access and quality index		X			X	
Drug use disorders	Data Rich	Male	Healthcare access and quality index		X			X	
Drug use disorders	Global	Male	Smoking Prevalence		X			X	
Drug use disorders	Data Rich	Male	Smoking Prevalence		X			X	
Drug use disorders	Global	Male	Cumulative Cigarettes (5 Years)		X			X	
Drug use disorders	Data Rich	Male	Cumulative Cigarettes (5 Years)		X			X	
Drug use disorders	Global	Male	Cumulative Cigarettes (10 Years)		X			X	
Drug use disorders	Data Rich	Male	Cumulative Cigarettes (10 Years)		X			X	
Drug use disorders	Global	Male	Intravenous drug use (proportion by age)	X			X		
Drug use disorders	Data Rich	Male	Intravenous drug use (proportion by age)	X			X		
Drug use disorders	Global	Male	Intravenous drug use (age-standardized proportion)	X			X		
Drug use disorders	Data Rich	Male	Intravenous drug use (age-standardized proportion)	X			X		
Drug use disorders	Global	Male	Opioids per million population per day (10 year lag)	X			X		
Drug use disorders	Data Rich	Male	Opioids per million population per day (10 year lag)	X			X		
Opioid use disorders	Data Rich	Female	Education (years per capita)			X			X
Opioid use disorders	Global	Female	Education (years per capita)			X			X
Opioid use disorders	Data Rich	Female	LDI (I$ per capita)			X			X
Opioid use disorders	Global	Female	LDI (I$ per capita)			X			X
Opioid use disorders	Data Rich	Female	Socio-demographic Index			X			X
Opioid use disorders	Global	Female	Socio-demographic Index			X			X
Opioid use disorders	Data Rich	Female	Healthcare access and quality index		X			X	
Opioid use disorders	Global	Female	Healthcare access and quality index		X			X	
Opioid use disorders	Data Rich	Female	Smoking Prevalence		X			X	
Opioid use disorders	Global	Female	Smoking Prevalence		X			X	
Opioid use disorders	Data Rich	Female	Cumulative Cigarettes (5 Years)		X			X	
Opioid use disorders	Global	Female	Cumulative Cigarettes (5 Years)		X			X	
Opioid use disorders	Data Rich	Female	Cumulative Cigarettes (10 Years)		X			X	
Opioid use disorders	Global	Female	Cumulative Cigarettes (10 Years)		X			X	

Comparison of GBD 2019 and GBD 2021 covariates and level of covariates used in cause of death modeling									
**Cause**	**Model Version Type**	**Sex**	**Covariate**	**Level 1 GBD 2019**	**Level 2 GBD 2019**	**Level 3 GBD 2019**	**Level 1 GBD 2021**	**Level 2 GBD 2021**	**Level 3 GBD 2021**
Opioid use disorders	Data Rich	Female	Opioids per million population per day (10 year lag)	X			X		
Opioid use disorders	Global	Female	Opioids per million population per day (10 year lag)	X			X		
Opioid use disorders	Data Rich	Female	Intravenous drug use (proportion by age)				X		
Opioid use disorders	Global	Female	Intravenous drug use (proportion by age)				X		
Opioid use disorders	Data Rich	Female	Intravenous drug use (age-standardized proportion)				X		
Opioid use disorders	Global	Female	Intravenous drug use (age-standardized proportion)				X		
Opioid use disorders	Data Rich	Male	Smoking Prevalence		X			X	
Opioid use disorders	Global	Male	Smoking Prevalence		X			X	
Opioid use disorders	Data Rich	Male	Cumulative Cigarettes (5 Years)		X			X	
Opioid use disorders	Global	Male	Cumulative Cigarettes (5 Years)		X			X	
Opioid use disorders	Data Rich	Male	Cumulative Cigarettes (10 Years)		X			X	
Opioid use disorders	Global	Male	Cumulative Cigarettes (10 Years)		X			X	
Opioid use disorders	Data Rich	Male	Intravenous drug use (proportion by age)	X			X		
Opioid use disorders	Global	Male	Intravenous drug use (proportion by age)	X			X		
Opioid use disorders	Data Rich	Male	Intravenous drug use (age-standardized proportion)	X			X		
Opioid use disorders	Global	Male	Intravenous drug use (age-standardized proportion)	X			X		
Opioid use disorders	Data Rich	Male	Opioids per million population per day (10 year lag)	X			X		
Opioid use disorders	Global	Male	Opioids per million population per day (10 year lag)	X			X		
Cocaine use disorders	Global	Female	Education (years per capita)			X			X
Cocaine use disorders	Data Rich	Female	Education (years per capita)			X			X
Cocaine use disorders	Global	Female	LDI (I$ per capita)			X			X
Cocaine use disorders	Data Rich	Female	LDI (I$ per capita)			X			X
Cocaine use disorders	Global	Female	Socio-demographic Index			X			X
Cocaine use disorders	Data Rich	Female	Socio-demographic Index			X			X
Cocaine use disorders	Global	Female	Healthcare access and quality index		X			X	
Cocaine use disorders	Data Rich	Female	Healthcare access and quality index		X			X	
Cocaine use disorders	Global	Female	Smoking Prevalence	X			X		
Cocaine use disorders	Data Rich	Female	Smoking Prevalence	X			X		
Cocaine use disorders	Global	Female	Liters of alcohol consumed per capita	X			X		
Cocaine use disorders	Data Rich	Female	Liters of alcohol consumed per capita	X			X		
Cocaine use disorders	Global	Female	Cumulative Cigarettes (5 Years)	X			X		
Cocaine use disorders	Data Rich	Female	Cumulative Cigarettes (5 Years)	X			X		
Cocaine use disorders	Global	Female	Cumulative Cigarettes (10 Years)	X			X		
Cocaine use disorders	Data Rich	Female	Cumulative Cigarettes (10 Years)	X			X		
Cocaine use disorders	Global	Female	Tobacco (cigarettes per capita)	X			X		
Cocaine use disorders	Data Rich	Female	Tobacco (cigarettes per capita)	X			X		
Cocaine use disorders	Global	Female	Intravenous drug use (proportion by age)	X			X		
Cocaine use disorders	Data Rich	Female	Intravenous drug use (proportion by age)	X			X		
Cocaine use disorders	Global	Female	Intravenous drug use (age-standardized proportion)	X			X		
Cocaine use disorders	Data Rich	Female	Intravenous drug use (age-standardized proportion)	X			X		
Cocaine use disorders	Global	Female	Alcohol drinker proportion, age-standardized	X			X		
Cocaine use disorders	Data Rich	Female	Alcohol drinker proportion, age-standardized	X			X		
Cocaine use disorders	Global	Male	Education (years per capita)			X			X
Cocaine use disorders	Data Rich	Male	Education (years per capita)			X			X
Cocaine use disorders	Global	Male	LDI (I$ per capita)			X			X
Cocaine use disorders	Data Rich	Male	LDI (I$ per capita)			X			X
Cocaine use disorders	Global	Male	Socio-demographic Index			X			X
Cocaine use disorders	Data Rich	Male	Socio-demographic Index			X			X
Cocaine use disorders	Global	Male	Healthcare access and quality index		X			X	
Cocaine use disorders	Data Rich	Male	Healthcare access and quality index		X			X	
Cocaine use disorders	Global	Male	Smoking Prevalence	X			X		
Cocaine use disorders	Data Rich	Male	Smoking Prevalence	X			X		
Cocaine use disorders	Global	Male	Liters of alcohol consumed per capita	X			X		
Cocaine use disorders	Data Rich	Male	Liters of alcohol consumed per capita	X			X		
Cocaine use disorders	Global	Male	Cumulative Cigarettes (5 Years)	X			X		
Cocaine use disorders	Data Rich	Male	Cumulative Cigarettes (5 Years)	X			X		
Cocaine use disorders	Global	Male	Cumulative Cigarettes (10 Years)	X			X		
Cocaine use disorders	Data Rich	Male	Cumulative Cigarettes (10 Years)	X			X		
Cocaine use disorders	Global	Male	Tobacco (cigarettes per capita)	X			X		
Cocaine use disorders	Data Rich	Male	Tobacco (cigarettes per capita)	X			X		
Cocaine use disorders	Global	Male	Intravenous drug use (proportion by age)	X			X		
Cocaine use disorders	Data Rich	Male	Intravenous drug use (proportion by age)	X			X		
Cocaine use disorders	Global	Male	Intravenous drug use (age-standardized proportion)	X			X		
Cocaine use disorders	Data Rich	Male	Intravenous drug use (age-standardized proportion)	X			X		
Cocaine use disorders	Global	Male	Alcohol drinker proportion, age-standardized	X			X		
Cocaine use disorders	Data Rich	Male	Alcohol drinker proportion, age-standardized	X			X		
Amphetamine use disorders	Global	Female	Education (years per capita)			X			X
Amphetamine use disorders	Data Rich	Female	Education (years per capita)			X			X
Amphetamine use disorders	Global	Female	LDI (I$ per capita)			X			X
Amphetamine use disorders	Data Rich	Female	LDI (I$ per capita)			X			X
Amphetamine use disorders	Global	Female	Socio-demographic Index			X			X
Amphetamine use disorders	Data Rich	Female	Socio-demographic Index			X			X
Amphetamine use disorders	Global	Female	Healthcare access and quality index		X			X	
Amphetamine use disorders	Data Rich	Female	Healthcare access and quality index		X			X	
Amphetamine use disorders	Global	Female	Smoking Prevalence	X			X		
Amphetamine use disorders	Data Rich	Female	Smoking Prevalence	X			X		
Amphetamine use disorders	Global	Female	Liters of alcohol consumed per capita	X			X		
Amphetamine use disorders	Data Rich	Female	Liters of alcohol consumed per capita	X			X		
Amphetamine use disorders	Global	Female	Cumulative Cigarettes (5 Years)	X			X		
Amphetamine use disorders	Data Rich	Female	Cumulative Cigarettes (5 Years)	X			X		
Amphetamine use disorders	Global	Female	Cumulative Cigarettes (10 Years)	X			X		
Amphetamine use disorders	Data Rich	Female	Cumulative Cigarettes (10 Years)	X			X		
Amphetamine use disorders	Global	Female	Tobacco (cigarettes per capita)	X			X		
Amphetamine use disorders	Data Rich	Female	Tobacco (cigarettes per capita)	X			X		
Amphetamine use disorders	Global	Female	Intravenous drug use (proportion by age)	X			X		
Amphetamine use disorders	Data Rich	Female	Intravenous drug use (proportion by age)	X			X		
Amphetamine use disorders	Global	Female	Intravenous drug use (age-standardized proportion)	X			X		
Amphetamine use disorders	Data Rich	Female	Intravenous drug use (age-standardized proportion)	X			X		
Amphetamine use disorders	Global	Female	Alcohol drinker proportion, age-standardized	X			X		
Amphetamine use disorders	Data Rich	Female	Alcohol drinker proportion, age-standardized	X			X		
Amphetamine use disorders	Data Rich	Male	Education (years per capita)			X			X
Amphetamine use disorders	Global	Male	Education (years per capita)			X			X
Amphetamine use disorders	Data Rich	Male	LDI (I$ per capita)			X			X
Amphetamine use disorders	Global	Male	LDI (I$ per capita)			X			X
Amphetamine use disorders	Data Rich	Male	Socio-demographic Index			X			X
Amphetamine use disorders	Global	Male	Socio-demographic Index			X			X
Amphetamine use disorders	Data Rich	Male	Healthcare access and quality index		X			X	
Amphetamine use disorders	Global	Male	Healthcare access and quality index		X			X	
Amphetamine use disorders	Data Rich	Male	Smoking Prevalence	X			X		
Amphetamine use disorders	Global	Male	Smoking Prevalence	X			X		
Amphetamine use disorders	Data Rich	Male	Liters of alcohol consumed per capita	X			X		
Amphetamine use disorders	Global	Male	Liters of alcohol consumed per capita	X			X		
Amphetamine use disorders	Data Rich	Male	Cumulative Cigarettes (5 Years)	X			X		
Amphetamine use disorders	Global	Male	Cumulative Cigarettes (5 Years)	X			X		
Amphetamine use disorders	Data Rich	Male	Cumulative Cigarettes (10 Years)	X			X		
Amphetamine use disorders	Global	Male	Cumulative Cigarettes (10 Years)	X			X		
Amphetamine use disorders	Data Rich	Male	Tobacco (cigarettes per capita)	X			X		
Amphetamine use disorders	Global	Male	Tobacco (cigarettes per capita)	X			X		
Amphetamine use disorders	Data Rich	Male	Intravenous drug use (proportion by age)	X			X		
Amphetamine use disorders	Global	Male	Intravenous drug use (proportion by age)	X			X		
Amphetamine use disorders	Data Rich	Male	Intravenous drug use (age-standardized proportion)	X			X		
Amphetamine use disorders	Global	Male	Intravenous drug use (age-standardized proportion)	X			X		
Amphetamine use disorders	Data Rich	Male	Alcohol drinker proportion, age-standardized	X			X		
Amphetamine use disorders	Global	Male	Alcohol drinker proportion, age-standardized	X			X		
Other drug use disorders	Global	Female	Education (years per capita)			X			X
Other drug use disorders	Data Rich	Female	Education (years per capita)			X			X
Other drug use disorders	Global	Female	LDI (I$ per capita)			X			X
Other drug use disorders	Data Rich	Female	LDI (I$ per capita)			X			X
Other drug use disorders	Global	Female	Socio-demographic Index			X			X
Other drug use disorders	Data Rich	Female	Socio-demographic Index			X			X
Other drug use disorders	Global	Female	Healthcare access and quality index		X			X	
Other drug use disorders	Data Rich	Female	Healthcare access and quality index		X			X	
Other drug use disorders	Global	Female	Smoking Prevalence	X			X		
Other drug use disorders	Data Rich	Female	Smoking Prevalence	X			X		
Other drug use disorders	Global	Female	Liters of alcohol consumed per capita	X			X		
Other drug use disorders	Data Rich	Female	Liters of alcohol consumed per capita	X			X		
Other drug use disorders	Global	Female	Cumulative Cigarettes (5 Years)	X			X		
Other drug use disorders	Data Rich	Female	Cumulative Cigarettes (5 Years)	X			X		
Other drug use disorders	Global	Female	Cumulative Cigarettes (10 Years)	X			X		

Comparison of GBD 2019 and GBD 2021 covariates and level of covariates used in cause of death modeling

Cause	Model Version Type	Sex	Covariate	Level 1 GBD 2019	Level 2 GBD 2019	Level 3 GBD 2019	Level 1 GBD 2021	Level 2 GBD 2021	Level 3 GBD 2021
Other drug use disorders	Data Rich	Female	Cumulative Cigarettes (10 Years)	X			X		
Other drug use disorders	Global	Female	Tobacco (cigarettes per capita)	X			X		
Other drug use disorders	Data Rich	Female	Tobacco (cigarettes per capita)	X			X		
Other drug use disorders	Global	Female	Intravenous drug use (proportion by age)	X			X		
Other drug use disorders	Data Rich	Female	Intravenous drug use (proportion by age)	X			X		
Other drug use disorders	Global	Female	Intravenous drug use (age-standardized proportion)	X			X		
Other drug use disorders	Data Rich	Female	Intravenous drug use (age-standardized proportion)	X			X		
Other drug use disorders	Global	Female	Alcohol drinker proportion, age-standardized	X			X		
Other drug use disorders	Data Rich	Female	Alcohol drinker proportion, age-standardized	X			X		
Other drug use disorders	Global	Male	Education (years per capita)			X			X
Other drug use disorders	Data Rich	Male	Education (years per capita)			X			X
Other drug use disorders	Global	Male	LDI (I$ per capita)			X			X
Other drug use disorders	Data Rich	Male	LDI (I$ per capita)			X			X
Other drug use disorders	Global	Male	Socio-demographic Index			X			X
Other drug use disorders	Data Rich	Male	Socio-demographic Index			X			X
Other drug use disorders	Global	Male	Healthcare access and quality index		X			X	
Other drug use disorders	Data Rich	Male	Healthcare access and quality index		X			X	
Other drug use disorders	Global	Male	Smoking Prevalence	X			X		
Other drug use disorders	Data Rich	Male	Smoking Prevalence	X			X		
Other drug use disorders	Global	Male	Liters of alcohol consumed per capita	X			X		
Other drug use disorders	Data Rich	Male	Liters of alcohol consumed per capita	X			X		
Other drug use disorders	Global	Male	Cumulative Cigarettes (5 Years)	X			X		
Other drug use disorders	Data Rich	Male	Cumulative Cigarettes (5 Years)	X			X		
Other drug use disorders	Global	Male	Cumulative Cigarettes (10 Years)	X			X		
Other drug use disorders	Data Rich	Male	Cumulative Cigarettes (10 Years)	X			X		
Other drug use disorders	Global	Male	Tobacco (cigarettes per capita)	X			X		
Other drug use disorders	Data Rich	Male	Tobacco (cigarettes per capita)	X			X		
Other drug use disorders	Global	Male	Intravenous drug use (proportion by age)	X			X		
Other drug use disorders	Data Rich	Male	Intravenous drug use (proportion by age)	X			X		
Other drug use disorders	Global	Male	Intravenous drug use (age-standardized proportion)	X			X		
Other drug use disorders	Data Rich	Male	Intravenous drug use (age-standardized proportion)	X			X		
Other drug use disorders	Global	Male	Alcohol drinker proportion, age-standardized	X			X		
Other drug use disorders	Data Rich	Male	Alcohol drinker proportion, age-standardized	X			X		
Diabetes mellitus	Data Rich	Female	Education (years per capita)			X			X
Diabetes mellitus	Global	Female	Education (years per capita)			X			X
Diabetes mellitus	Global	Female	Education (years per capita)			X			X
Diabetes mellitus	Data Rich	Female	Education (years per capita)			X			X
Diabetes mellitus	Global	Female	LDI (I$ per capita)			X			X
Diabetes mellitus	Data Rich	Female	LDI (I$ per capita)			X			X
Diabetes mellitus	Data Rich	Female	Socio-demographic Index			X			X
Diabetes mellitus	Global	Female	Socio-demographic Index			X			X
Diabetes mellitus	Global	Female	Healthcare access and quality index			X			X
Diabetes mellitus	Data Rich	Female	Healthcare access and quality index			X			X
Diabetes mellitus	Global	Female	Low-Density Lipoprotein (mmol/L)		X			X	
Diabetes mellitus	Data Rich	Female	Low-Density Lipoprotein (mmol/L)		X			X	
Diabetes mellitus	Global	Female	Systolic Blood Pressure (mmHg)		X			X	
Diabetes mellitus	Data Rich	Female	Systolic Blood Pressure (mmHg)		X			X	
Diabetes mellitus	Data Rich	Female	Absolute value of average latitude		X			X	
Diabetes mellitus	Global	Female	Absolute value of average latitude		X			X	
Diabetes mellitus	Data Rich	Female	Live Births 35+ (proportion)		X			X	
Diabetes mellitus	Global	Female	Live Births 35+ (proportion)		X			X	
Diabetes mellitus	Data Rich	Female	Live Births 40+ (proportion)		X			X	
Diabetes mellitus	Global	Female	Live Births 40+ (proportion)		X			X	
Diabetes mellitus	Global	Female	Age- and sex-specific SEV for Low fruit		X			X	
Diabetes mellitus	Data Rich	Female	Age- and sex-specific SEV for Low fruit		X			X	
Diabetes mellitus	Global	Female	Age- and sex-specific SEV for Low vegetables		X			X	
Diabetes mellitus	Data Rich	Female	Age- and sex-specific SEV for Low vegetables		X			X	
Diabetes mellitus	Global	Female	sugar unadjusted(g)		X			X	
Diabetes mellitus	Data Rich	Female	sugar unadjusted(g)		X			X	
Diabetes mellitus	Global	Female	Age- and sex-specific SEV for Alcohol use		X			X	
Diabetes mellitus	Data Rich	Female	Age- and sex-specific SEV for Alcohol use		X			X	
Diabetes mellitus	Data Rich	Female	Healthcare access and quality index	X			X		
Diabetes mellitus	Global	Female	Healthcare access and quality index	X			X		
Diabetes mellitus	Global	Female	Mean BMI	X			X		
Diabetes mellitus	Data Rich	Female	Mean BMI	X			X		
Diabetes mellitus	Global	Female	Diabetes Fasting Plasma Glucose (mmol/L), age-standardized 25+	X			X		
Diabetes mellitus	Data Rich	Female	Diabetes Fasting Plasma Glucose (mmol/L), age-standardized 25+	X			X		
Diabetes mellitus	Global	Female	Diabetes Age-Standardized Prevalence (proportion)	X			X		
Diabetes mellitus	Data Rich	Female	Diabetes Age-Standardized Prevalence (proportion)	X			X		
Diabetes mellitus	Global	Female	Prevalence of obesity	X			X		
Diabetes mellitus	Data Rich	Female	Prevalence of obesity	X			X		
Diabetes mellitus	Data Rich	Male	Education (years per capita)			X			X
Diabetes mellitus	Global	Male	Education (years per capita)			X			X
Diabetes mellitus	Global	Male	Education (years per capita)			X			X
Diabetes mellitus	Data Rich	Male	Education (years per capita)			X			X
Diabetes mellitus	Global	Male	LDI (I$ per capita)			X			X
Diabetes mellitus	Data Rich	Male	LDI (I$ per capita)			X			X
Diabetes mellitus	Data Rich	Male	Socio-demographic Index			X			X
Diabetes mellitus	Global	Male	Socio-demographic Index			X			X
Diabetes mellitus	Global	Male	Healthcare access and quality index			X			X
Diabetes mellitus	Data Rich	Male	Healthcare access and quality index			X			X
Diabetes mellitus	Global	Male	Low-Density Lipoprotein (mmol/L)		X			X	
Diabetes mellitus	Data Rich	Male	Low-Density Lipoprotein (mmol/L)		X			X	
Diabetes mellitus	Global	Male	Systolic Blood Pressure (mmHg)		X			X	
Diabetes mellitus	Data Rich	Male	Systolic Blood Pressure (mmHg)		X			X	
Diabetes mellitus	Data Rich	Male	Absolute value of average latitude		X			X	
Diabetes mellitus	Global	Male	Absolute value of average latitude		X			X	
Diabetes mellitus	Data Rich	Male	Live Births 35+ (proportion)		X			X	
Diabetes mellitus	Global	Male	Live Births 35+ (proportion)		X			X	
Diabetes mellitus	Data Rich	Male	Live Births 40+ (proportion)		X			X	
Diabetes mellitus	Global	Male	Live Births 40+ (proportion)		X			X	
Diabetes mellitus	Global	Male	Age- and sex-specific SEV for Low fruit		X			X	
Diabetes mellitus	Data Rich	Male	Age- and sex-specific SEV for Low fruit		X			X	
Diabetes mellitus	Global	Male	Age- and sex-specific SEV for Low vegetables		X			X	
Diabetes mellitus	Data Rich	Male	Age- and sex-specific SEV for Low vegetables		X			X	
Diabetes mellitus	Global	Male	sugar unadjusted(g)		X			X	
Diabetes mellitus	Data Rich	Male	sugar unadjusted(g)		X			X	
Diabetes mellitus	Global	Male	Age- and sex-specific SEV for Alcohol use		X			X	
Diabetes mellitus	Data Rich	Male	Age- and sex-specific SEV for Alcohol use		X			X	
Diabetes mellitus	Data Rich	Male	Healthcare access and quality index	X			X		
Diabetes mellitus	Global	Male	Healthcare access and quality index	X			X		
Diabetes mellitus	Global	Male	Mean BMI	X			X		
Diabetes mellitus	Data Rich	Male	Mean BMI	X			X		
Diabetes mellitus	Global	Male	Diabetes Fasting Plasma Glucose (mmol/L), age-standardized 25+	X			X		
Diabetes mellitus	Data Rich	Male	Diabetes Fasting Plasma Glucose (mmol/L), age-standardized 25+	X			X		
Diabetes mellitus	Global	Male	Diabetes Age-Standardized Prevalence (proportion)	X			X		
Diabetes mellitus	Data Rich	Male	Diabetes Age-Standardized Prevalence (proportion)	X			X		
Diabetes mellitus	Global	Male	Prevalence of obesity	X			X		
Diabetes mellitus	Data Rich	Male	Prevalence of obesity	X			X		
Diabetes mellitus type 1	Data Rich	Female	Education (years per capita)			X			X
Diabetes mellitus type 1	Global	Female	Education (years per capita)			X			X
Diabetes mellitus type 1	Data Rich	Female	Socio-demographic Index			X			X
Diabetes mellitus type 1	Global	Female	Socio-demographic Index			X			X
Diabetes mellitus type 1	Data Rich	Female	Absolute value of average latitude		X			X	
Diabetes mellitus type 1	Global	Female	Absolute value of average latitude		X			X	
Diabetes mellitus type 1	Data Rich	Female	Live Births 35+ (proportion)		X			X	
Diabetes mellitus type 1	Global	Female	Live Births 35+ (proportion)		X			X	
Diabetes mellitus type 1	Data Rich	Female	Live Births 40+ (proportion)		X			X	
Diabetes mellitus type 1	Global	Female	Live Births 40+ (proportion)		X			X	
Diabetes mellitus type 1	Data Rich	Female	Healthcare access and quality index	X			X		
Diabetes mellitus type 1	Global	Female	Healthcare access and quality index	X			X		
Diabetes mellitus type 1	Data Rich	Male	Education (years per capita)			X			X
Diabetes mellitus type 1	Global	Male	Education (years per capita)			X			X
Diabetes mellitus type 1	Data Rich	Male	Socio-demographic Index			X			X
Diabetes mellitus type 1	Global	Male	Socio-demographic Index			X			X
Diabetes mellitus type 1	Data Rich	Male	Absolute value of average latitude		X			X	
Diabetes mellitus type 1	Global	Male	Absolute value of average latitude		X			X	
Diabetes mellitus type 1	Data Rich	Male	Live Births 35+ (proportion)		X			X	
Diabetes mellitus type 1	Global	Male	Live Births 35+ (proportion)		X			X	
Diabetes mellitus type 1	Data Rich	Male	Live Births 40+ (proportion)		X			X	
Diabetes mellitus type 1	Global	Male	Live Births 40+ (proportion)		X			X	

Comparison of GBD 2019 and GBD 2021 covariates and level of covariates used in cause of death modeling

Cause	Model Version Type	Sex	Covariate	Level 1 GBD 2019	Level 2 GBD 2019	Level 3 GBD 2019	Level 1 GBD 2021	Level 2 GBD 2021	Level 3 GBD 2021
Diabetes mellitus type 1	Data Rich	Male	Healthcare access and quality index	X			X		
Diabetes mellitus type 1	Global	Male	Healthcare access and quality index	X			X		
Diabetes mellitus type 2	Global	Female	Education (years per capita)			X			X
Diabetes mellitus type 2	Data Rich	Female	Education (years per capita)			X			X
Diabetes mellitus type 2	Global	Female	LDI (I$ per capita)			X			X
Diabetes mellitus type 2	Data Rich	Female	LDI (I$ per capita)			X			X
Diabetes mellitus type 2	Global	Female	Healthcare access and quality index			X			X
Diabetes mellitus type 2	Data Rich	Female	Healthcare access and quality index			X			X
Diabetes mellitus type 2	Global	Female	Low-Density Lipoprotein (mmol/L)		X			X	
Diabetes mellitus type 2	Data Rich	Female	Low-Density Lipoprotein (mmol/L)		X			X	
Diabetes mellitus type 2	Global	Female	Systolic Blood Pressure (mmHg)		X			X	
Diabetes mellitus type 2	Data Rich	Female	Systolic Blood Pressure (mmHg)		X			X	
Diabetes mellitus type 2	Global	Female	Age- and sex-specific SEV for Low fruit		X			X	
Diabetes mellitus type 2	Data Rich	Female	Age- and sex-specific SEV for Low fruit		X			X	
Diabetes mellitus type 2	Global	Female	Age- and sex-specific SEV for Low vegetables		X			X	
Diabetes mellitus type 2	Data Rich	Female	Age- and sex-specific SEV for Low vegetables		X			X	
Diabetes mellitus type 2	Global	Female	sugar unadjusted(g)		X			X	
Diabetes mellitus type 2	Data Rich	Female	sugar unadjusted(g)		X			X	
Diabetes mellitus type 2	Global	Female	Age- and sex-specific SEV for Alcohol use		X			X	
Diabetes mellitus type 2	Data Rich	Female	Age- and sex-specific SEV for Alcohol use		X			X	
Diabetes mellitus type 2	Global	Female	Mean BMI	X			X		
Diabetes mellitus type 2	Data Rich	Female	Mean BMI	X			X		
Diabetes mellitus type 2	Global	Female	Diabetes Fasting Plasma Glucose (mmol/L), age-standardized 25+	X			X		
Diabetes mellitus type 2	Data Rich	Female	Diabetes Fasting Plasma Glucose (mmol/L), age-standardized 25+	X			X		
Diabetes mellitus type 2	Global	Female	Diabetes Age-Standardized Prevalence (proportion)	X			X		
Diabetes mellitus type 2	Data Rich	Female	Diabetes Age-Standardized Prevalence (proportion)	X			X		
Diabetes mellitus type 2	Global	Female	Prevalence of obesity	X			X		
Diabetes mellitus type 2	Data Rich	Female	Prevalence of obesity	X			X		
Diabetes mellitus type 2	Global	Male	Education (years per capita)			X			X
Diabetes mellitus type 2	Data Rich	Male	Education (years per capita)			X			X
Diabetes mellitus type 2	Global	Male	LDI (I$ per capita)			X			X
Diabetes mellitus type 2	Data Rich	Male	LDI (I$ per capita)			X			X
Diabetes mellitus type 2	Global	Male	Healthcare access and quality index			X			X
Diabetes mellitus type 2	Data Rich	Male	Healthcare access and quality index			X			X
Diabetes mellitus type 2	Global	Male	Low-Density Lipoprotein (mmol/L)		X			X	
Diabetes mellitus type 2	Data Rich	Male	Low-Density Lipoprotein (mmol/L)		X			X	
Diabetes mellitus type 2	Global	Male	Systolic Blood Pressure (mmHg)		X			X	
Diabetes mellitus type 2	Data Rich	Male	Systolic Blood Pressure (mmHg)		X			X	
Diabetes mellitus type 2	Global	Male	Age- and sex-specific SEV for Low fruit		X			X	
Diabetes mellitus type 2	Data Rich	Male	Age- and sex-specific SEV for Low fruit		X			X	
Diabetes mellitus type 2	Global	Male	Age- and sex-specific SEV for Low vegetables		X			X	
Diabetes mellitus type 2	Data Rich	Male	Age- and sex-specific SEV for Low vegetables		X			X	
Diabetes mellitus type 2	Global	Male	sugar unadjusted(g)		X			X	
Diabetes mellitus type 2	Data Rich	Male	sugar unadjusted(g)		X			X	
Diabetes mellitus type 2	Global	Male	Age- and sex-specific SEV for Alcohol use		X			X	
Diabetes mellitus type 2	Data Rich	Male	Age- and sex-specific SEV for Alcohol use		X			X	
Diabetes mellitus type 2	Global	Male	Mean BMI	X			X		
Diabetes mellitus type 2	Data Rich	Male	Mean BMI	X			X		
Diabetes mellitus type 2	Global	Male	Diabetes Fasting Plasma Glucose (mmol/L), age-standardized 25+	X			X		
Diabetes mellitus type 2	Data Rich	Male	Diabetes Fasting Plasma Glucose (mmol/L), age-standardized 25+	X			X		
Diabetes mellitus type 2	Global	Male	Diabetes Age-Standardized Prevalence (proportion)	X			X		
Diabetes mellitus type 2	Data Rich	Male	Diabetes Age-Standardized Prevalence (proportion)	X			X		
Diabetes mellitus type 2	Global	Male	Prevalence of obesity	X			X		
Diabetes mellitus type 2	Data Rich	Male	Prevalence of obesity	X			X		
Chronic kidney disease	Data Rich	Female	Education (years per capita)			X			X
Chronic kidney disease	Global	Female	Education (years per capita)			X			X
Chronic kidney disease	Data Rich	Female	LDI (I$ per capita)			X			X
Chronic kidney disease	Global	Female	LDI (I$ per capita)			X			X
Chronic kidney disease	Data Rich	Female	Socio-demographic Index			X			X
Chronic kidney disease	Global	Female	Socio-demographic Index			X			X
Chronic kidney disease	Data Rich	Female	Low-Density Lipoprotein (mmol/L)		X			X	
Chronic kidney disease	Global	Female	Low-Density Lipoprotein (mmol/L)		X			X	
Chronic kidney disease	Data Rich	Female	red meats unadjusted(g)		X			X	
Chronic kidney disease	Global	Female	red meats unadjusted(g)		X			X	
Chronic kidney disease	Data Rich	Female	energy unadjusted(kcal)		X			X	
Chronic kidney disease	Global	Female	energy unadjusted(kcal)		X			X	
Chronic kidney disease	Data Rich	Female	Healthcare access and quality index	X			X		
Chronic kidney disease	Global	Female	Healthcare access and quality index	X			X		
Chronic kidney disease	Data Rich	Female	Mean BMI	X			X		
Chronic kidney disease	Global	Female	Mean BMI	X			X		
Chronic kidney disease	Data Rich	Female	Systolic Blood Pressure (mmHg)	X			X		
Chronic kidney disease	Global	Female	Systolic Blood Pressure (mmHg)	X			X		
Chronic kidney disease	Data Rich	Female	Diabetes Fasting Plasma Glucose (mmol/L), age-standardized 25+	X			X		
Chronic kidney disease	Global	Female	Diabetes Fasting Plasma Glucose (mmol/L), age-standardized 25+	X			X		
Chronic kidney disease	Data Rich	Female	Diabetes Age-Standardized Prevalence (proportion)	X			X		
Chronic kidney disease	Global	Female	Diabetes Age-Standardized Prevalence (proportion)	X			X		
Chronic kidney disease	Global	Male	Education (years per capita)			X			X
Chronic kidney disease	Data Rich	Male	Education (years per capita)			X			X
Chronic kidney disease	Global	Male	LDI (I$ per capita)			X			X
Chronic kidney disease	Data Rich	Male	LDI (I$ per capita)			X			X
Chronic kidney disease	Global	Male	Socio-demographic Index			X			X
Chronic kidney disease	Data Rich	Male	Socio-demographic Index			X			X
Chronic kidney disease	Global	Male	Low-Density Lipoprotein (mmol/L)		X			X	
Chronic kidney disease	Data Rich	Male	Low-Density Lipoprotein (mmol/L)		X			X	
Chronic kidney disease	Global	Male	red meats unadjusted(g)		X			X	
Chronic kidney disease	Data Rich	Male	red meats unadjusted(g)		X			X	
Chronic kidney disease	Global	Male	energy unadjusted(kcal)		X			X	
Chronic kidney disease	Data Rich	Male	energy unadjusted(kcal)		X			X	
Chronic kidney disease	Global	Male	Healthcare access and quality index	X			X		
Chronic kidney disease	Data Rich	Male	Healthcare access and quality index	X			X		
Chronic kidney disease	Global	Male	Mean BMI	X			X		
Chronic kidney disease	Data Rich	Male	Mean BMI	X			X		
Chronic kidney disease	Global	Male	Systolic Blood Pressure (mmHg)	X			X		
Chronic kidney disease	Data Rich	Male	Systolic Blood Pressure (mmHg)	X			X		
Chronic kidney disease	Global	Male	Diabetes Fasting Plasma Glucose (mmol/L), age-standardized 25+	X			X		
Chronic kidney disease	Data Rich	Male	Diabetes Fasting Plasma Glucose (mmol/L), age-standardized 25+	X			X		
Chronic kidney disease	Global	Male	Diabetes Age-Standardized Prevalence (proportion)	X			X		
Chronic kidney disease	Data Rich	Male	Diabetes Age-Standardized Prevalence (proportion)	X			X		
Acute glomerulonephritis	Global	Female	Education (years per capita)			X			X
Acute glomerulonephritis	Data Rich	Female	Education (years per capita)			X			X
Acute glomerulonephritis	Global	Female	LDI (I$ per capita)			X			X
Acute glomerulonephritis	Data Rich	Female	LDI (I$ per capita)			X			X
Acute glomerulonephritis	Global	Female	Socio-demographic Index			X			X
Acute glomerulonephritis	Data Rich	Female	Socio-demographic Index			X			X
Acute glomerulonephritis	Global	Female	Healthcare access and quality index		X			X	
Acute glomerulonephritis	Data Rich	Female	Healthcare access and quality index		X			X	
Acute glomerulonephritis	Global	Female	Systolic Blood Pressure (mmHg)		X			X	
Acute glomerulonephritis	Data Rich	Female	Systolic Blood Pressure (mmHg)		X			X	
Acute glomerulonephritis	Global	Female	Sanitation (proportion with access)		X			X	
Acute glomerulonephritis	Data Rich	Female	Sanitation (proportion with access)		X			X	
Acute glomerulonephritis	Global	Female	Improved Water Source (proportion with access)		X			X	
Acute glomerulonephritis	Data Rich	Female	Improved Water Source (proportion with access)		X			X	
Acute glomerulonephritis	Global	Male	Education (years per capita)			X			X
Acute glomerulonephritis	Data Rich	Male	Education (years per capita)			X			X
Acute glomerulonephritis	Global	Male	LDI (I$ per capita)			X			X
Acute glomerulonephritis	Data Rich	Male	LDI (I$ per capita)			X			X
Acute glomerulonephritis	Global	Male	Socio-demographic Index			X			X
Acute glomerulonephritis	Data Rich	Male	Socio-demographic Index			X			X
Acute glomerulonephritis	Global	Male	Healthcare access and quality index		X			X	
Acute glomerulonephritis	Data Rich	Male	Healthcare access and quality index		X			X	
Acute glomerulonephritis	Global	Male	Systolic Blood Pressure (mmHg)		X			X	
Acute glomerulonephritis	Data Rich	Male	Systolic Blood Pressure (mmHg)		X			X	
Acute glomerulonephritis	Global	Male	Sanitation (proportion with access)		X			X	
Acute glomerulonephritis	Data Rich	Male	Sanitation (proportion with access)		X			X	
Acute glomerulonephritis	Global	Male	Improved Water Source (proportion with access)		X			X	
Acute glomerulonephritis	Data Rich	Male	Improved Water Source (proportion with access)		X			X	
Skin and subcutaneous diseases	Global	Female	Education (years per capita)			X			X
Skin and subcutaneous diseases	Data Rich	Female	Education (years per capita)			X			X
Skin and subcutaneous diseases	Global	Female	LDI (I$ per capita)			X			X

Comparison of GBD 2019 and GBD 2021 covariates and level of covariates used in cause of death modeling									
Cause	Model Version Type	Sex	Covariate	Level 1 GBD 2019	Level 2 GBD 2019	Level 3 GBD 2019	Level 1 GBD 2021	Level 2 GBD 2021	Level 3 GBD 2021
Skin and subcutaneous diseases	Data Rich	Female	LDI (I$ per capita)			X			X
Skin and subcutaneous diseases	Global	Female	Socio-demographic Index			X			X
Skin and subcutaneous diseases	Data Rich	Female	Socio-demographic Index			X			X
Skin and subcutaneous diseases	Global	Female	Smoking Prevalence		X			X	
Skin and subcutaneous diseases	Data Rich	Female	Smoking Prevalence		X			X	
Skin and subcutaneous diseases	Global	Female	Liters of alcohol consumed per capita		X			X	
Skin and subcutaneous diseases	Data Rich	Female	Liters of alcohol consumed per capita		X			X	
Skin and subcutaneous diseases	Global	Female	Cumulative Cigarettes (5 Years)		X			X	
Skin and subcutaneous diseases	Data Rich	Female	Cumulative Cigarettes (5 Years)		X			X	
Skin and subcutaneous diseases	Global	Female	Cumulative Cigarettes (10 Years)		X			X	
Skin and subcutaneous diseases	Data Rich	Female	Cumulative Cigarettes (10 Years)		X			X	
Skin and subcutaneous diseases	Global	Female	Healthcare access and quality index	X			X		
Skin and subcutaneous diseases	Data Rich	Female	Healthcare access and quality index	X			X		
Skin and subcutaneous diseases	Global	Female	Prevalence of overweight and obesity	X			X		
Skin and subcutaneous diseases	Data Rich	Female	Prevalence of overweight and obesity	X			X		
Skin and subcutaneous diseases	Global	Female	Diabetes Fasting Plasma Glucose (mmol/L), by age	X			X		
Skin and subcutaneous diseases	Data Rich	Female	Diabetes Fasting Plasma Glucose (mmol/L), by age	X			X		
Skin and subcutaneous diseases	Global	Female	Improved Water Source (proportion with access)	X			X		
Skin and subcutaneous diseases	Data Rich	Female	Improved Water Source (proportion with access)	X			X		
Skin and subcutaneous diseases	Global	Female	Age- and sex-specific SEV for Unsafe sanitation	X			X		
Skin and subcutaneous diseases	Data Rich	Female	Age- and sex-specific SEV for Unsafe sanitation	X			X		
Skin and subcutaneous diseases	Global	Male	Education (years per capita)			X			X
Skin and subcutaneous diseases	Data Rich	Male	Education (years per capita)			X			X
Skin and subcutaneous diseases	Global	Male	LDI (I$ per capita)			X			X
Skin and subcutaneous diseases	Data Rich	Male	LDI (I$ per capita)			X			X
Skin and subcutaneous diseases	Global	Male	Socio-demographic Index			X			X
Skin and subcutaneous diseases	Data Rich	Male	Socio-demographic Index			X			X
Skin and subcutaneous diseases	Global	Male	Smoking Prevalence		X			X	
Skin and subcutaneous diseases	Data Rich	Male	Smoking Prevalence		X			X	
Skin and subcutaneous diseases	Global	Male	Liters of alcohol consumed per capita		X			X	
Skin and subcutaneous diseases	Data Rich	Male	Liters of alcohol consumed per capita		X			X	
Skin and subcutaneous diseases	Global	Male	Cumulative Cigarettes (5 Years)		X			X	
Skin and subcutaneous diseases	Data Rich	Male	Cumulative Cigarettes (5 Years)		X			X	
Skin and subcutaneous diseases	Global	Male	Cumulative Cigarettes (10 Years)		X			X	
Skin and subcutaneous diseases	Data Rich	Male	Cumulative Cigarettes (10 Years)		X			X	
Skin and subcutaneous diseases	Global	Male	Healthcare access and quality index	X			X		
Skin and subcutaneous diseases	Data Rich	Male	Healthcare access and quality index	X			X		
Skin and subcutaneous diseases	Global	Male	Prevalence of overweight and obesity	X			X		
Skin and subcutaneous diseases	Data Rich	Male	Prevalence of overweight and obesity	X			X		
Skin and subcutaneous diseases	Global	Male	Diabetes Fasting Plasma Glucose (mmol/L), by age	X			X		
Skin and subcutaneous diseases	Data Rich	Male	Diabetes Fasting Plasma Glucose (mmol/L), by age	X			X		
Skin and subcutaneous diseases	Global	Male	Improved Water Source (proportion with access)	X			X		
Skin and subcutaneous diseases	Data Rich	Male	Improved Water Source (proportion with access)	X			X		
Skin and subcutaneous diseases	Global	Male	Age- and sex-specific SEV for Unsafe sanitation	X			X		
Skin and subcutaneous diseases	Data Rich	Male	Age- and sex-specific SEV for Unsafe sanitation	X			X		
Bacterial skin diseases	Global	Female	Education (years per capita)			X			X
Bacterial skin diseases	Data Rich	Female	Education (years per capita)			X			X
Bacterial skin diseases	Global	Female	LDI (I$ per capita)			X			X
Bacterial skin diseases	Data Rich	Female	LDI (I$ per capita)			X			X
Bacterial skin diseases	Global	Female	Socio-demographic Index			X			X
Bacterial skin diseases	Data Rich	Female	Socio-demographic Index			X			X
Bacterial skin diseases	Global	Female	Smoking Prevalence		X			X	
Bacterial skin diseases	Data Rich	Female	Smoking Prevalence		X			X	
Bacterial skin diseases	Global	Female	Liters of alcohol consumed per capita		X			X	
Bacterial skin diseases	Data Rich	Female	Liters of alcohol consumed per capita		X			X	
Bacterial skin diseases	Global	Female	Cumulative Cigarettes (5 Years)		X			X	
Bacterial skin diseases	Data Rich	Female	Cumulative Cigarettes (5 Years)		X			X	
Bacterial skin diseases	Global	Female	Cumulative Cigarettes (10 Years)		X			X	
Bacterial skin diseases	Data Rich	Female	Cumulative Cigarettes (10 Years)		X			X	
Bacterial skin diseases	Global	Female	Healthcare access and quality index	X			X		
Bacterial skin diseases	Data Rich	Female	Healthcare access and quality index	X			X		
Bacterial skin diseases	Global	Female	Prevalence of overweight and obesity	X			X		
Bacterial skin diseases	Data Rich	Female	Prevalence of overweight and obesity	X			X		
Bacterial skin diseases	Global	Female	Diabetes Fasting Plasma Glucose (mmol/L), by age	X			X		
Bacterial skin diseases	Data Rich	Female	Diabetes Fasting Plasma Glucose (mmol/L), by age	X			X		
Bacterial skin diseases	Global	Female	Improved Water Source (proportion with access)	X			X		
Bacterial skin diseases	Data Rich	Female	Improved Water Source (proportion with access)	X			X		
Bacterial skin diseases	Global	Female	Age- and sex-specific SEV for Unsafe sanitation	X			X		
Bacterial skin diseases	Data Rich	Female	Age- and sex-specific SEV for Unsafe sanitation	X			X		
Bacterial skin diseases	Global	Male	Education (years per capita)			X			X
Bacterial skin diseases	Data Rich	Male	Education (years per capita)			X			X
Bacterial skin diseases	Global	Male	LDI (I$ per capita)			X			X
Bacterial skin diseases	Data Rich	Male	LDI (I$ per capita)			X			X
Bacterial skin diseases	Global	Male	Socio-demographic Index			X			X
Bacterial skin diseases	Data Rich	Male	Socio-demographic Index			X			X
Bacterial skin diseases	Global	Male	Smoking Prevalence		X			X	
Bacterial skin diseases	Data Rich	Male	Smoking Prevalence		X			X	
Bacterial skin diseases	Global	Male	Liters of alcohol consumed per capita		X			X	
Bacterial skin diseases	Data Rich	Male	Liters of alcohol consumed per capita		X			X	
Bacterial skin diseases	Global	Male	Cumulative Cigarettes (5 Years)		X			X	
Bacterial skin diseases	Data Rich	Male	Cumulative Cigarettes (5 Years)		X			X	
Bacterial skin diseases	Global	Male	Cumulative Cigarettes (10 Years)		X			X	
Bacterial skin diseases	Data Rich	Male	Cumulative Cigarettes (10 Years)		X			X	
Bacterial skin diseases	Global	Male	Healthcare access and quality index	X			X		
Bacterial skin diseases	Data Rich	Male	Healthcare access and quality index	X			X		
Bacterial skin diseases	Global	Male	Prevalence of overweight and obesity	X			X		
Bacterial skin diseases	Data Rich	Male	Prevalence of overweight and obesity	X			X		
Bacterial skin diseases	Global	Male	Diabetes Fasting Plasma Glucose (mmol/L), by age	X			X		
Bacterial skin diseases	Data Rich	Male	Diabetes Fasting Plasma Glucose (mmol/L), by age	X			X		
Bacterial skin diseases	Global	Male	Improved Water Source (proportion with access)	X			X		
Bacterial skin diseases	Data Rich	Male	Improved Water Source (proportion with access)	X			X		
Bacterial skin diseases	Global	Male	Age- and sex-specific SEV for Unsafe sanitation	X			X		
Bacterial skin diseases	Data Rich	Male	Age- and sex-specific SEV for Unsafe sanitation	X			X		
Cellulitis	Data Rich	Female	Education (years per capita)			X			X
Cellulitis	Global	Female	Education (years per capita)			X			X
Cellulitis	Data Rich	Female	LDI (I$ per capita)		X			X	
Cellulitis	Global	Female	LDI (I$ per capita)		X			X	
Cellulitis	Data Rich	Female	Healthcare access and quality index	X			X		
Cellulitis	Global	Female	Healthcare access and quality index	X			X		
Cellulitis	Data Rich	Female	Prevalence of overweight and obesity	X			X		
Cellulitis	Global	Female	Prevalence of overweight and obesity	X			X		
Cellulitis	Data Rich	Female	Diabetes Fasting Plasma Glucose (mmol/L), by age	X			X		
Cellulitis	Global	Female	Diabetes Fasting Plasma Glucose (mmol/L), by age	X			X		
Cellulitis	Data Rich	Male	Education (years per capita)			X			X
Cellulitis	Global	Male	Education (years per capita)			X			X
Cellulitis	Data Rich	Male	LDI (I$ per capita)		X			X	
Cellulitis	Global	Male	LDI (I$ per capita)		X			X	
Cellulitis	Data Rich	Male	Healthcare access and quality index	X			X		
Cellulitis	Global	Male	Healthcare access and quality index	X			X		
Cellulitis	Data Rich	Male	Prevalence of overweight and obesity	X			X		
Cellulitis	Global	Male	Prevalence of overweight and obesity	X			X		
Cellulitis	Data Rich	Male	Diabetes Fasting Plasma Glucose (mmol/L), by age	X			X		
Cellulitis	Global	Male	Diabetes Fasting Plasma Glucose (mmol/L), by age	X			X		
Pyoderma	Global	Female	Education (years per capita)			X			X
Pyoderma	Data Rich	Female	Education (years per capita)			X			X
Pyoderma	Global	Female	LDI (I$ per capita)			X			X
Pyoderma	Data Rich	Female	LDI (I$ per capita)			X			X
Pyoderma	Global	Female	Socio-demographic Index			X			X
Pyoderma	Data Rich	Female	Socio-demographic Index			X			X
Pyoderma	Global	Female	Smoking Prevalence		X			X	
Pyoderma	Data Rich	Female	Smoking Prevalence		X			X	
Pyoderma	Global	Female	Liters of alcohol consumed per capita		X			X	
Pyoderma	Data Rich	Female	Liters of alcohol consumed per capita		X			X	
Pyoderma	Global	Female	Cumulative Cigarettes (5 Years)		X			X	
Pyoderma	Data Rich	Female	Cumulative Cigarettes (5 Years)		X			X	
Pyoderma	Global	Female	Cumulative Cigarettes (10 Years)		X			X	
Pyoderma	Data Rich	Female	Cumulative Cigarettes (10 Years)		X			X	

Comparison of GBD 2019 and GBD 2021 covariates and level of covariates used in cause of death modeling									
Cause	Model Version Type	Sex	Covariate	Level 1 GBD 2019	Level 2 GBD 2019	Level 3 GBD 2019	Level 1 GBD 2021	Level 2 GBD 2021	Level 3 GBD 2021
Pyoderma	Global	Female	Healthcare access and quality index	X			X		
Pyoderma	Data Rich	Female	Healthcare access and quality index	X			X		
Pyoderma	Global	Female	Prevalence of overweight and obesity	X			X		
Pyoderma	Data Rich	Female	Prevalence of overweight and obesity	X			X		
Pyoderma	Global	Female	Diabetes Fasting Plasma Glucose (mmol/L), by age	X			X		
Pyoderma	Data Rich	Female	Diabetes Fasting Plasma Glucose (mmol/L), by age	X			X		
Pyoderma	Global	Female	Improved Water Source (proportion with access)	X			X		
Pyoderma	Data Rich	Female	Improved Water Source (proportion with access)	X			X		
Pyoderma	Global	Female	Age- and sex-specific SEV for Unsafe sanitation	X			X		
Pyoderma	Data Rich	Female	Age- and sex-specific SEV for Unsafe sanitation	X			X		
Pyoderma	Global	Male	Education (years per capita)			X			X
Pyoderma	Data Rich	Male	Education (years per capita)			X			X
Pyoderma	Global	Male	LDI (I$ per capita)			X			X
Pyoderma	Data Rich	Male	LDI (I$ per capita)			X			X
Pyoderma	Global	Male	Socio-demographic Index			X			X
Pyoderma	Data Rich	Male	Socio-demographic Index			X			X
Pyoderma	Global	Male	Smoking Prevalence		X			X	
Pyoderma	Data Rich	Male	Smoking Prevalence		X			X	
Pyoderma	Global	Male	Liters of alcohol consumed per capita		X			X	
Pyoderma	Data Rich	Male	Liters of alcohol consumed per capita		X			X	
Pyoderma	Global	Male	Cumulative Cigarettes (5 Years)		X			X	
Pyoderma	Data Rich	Male	Cumulative Cigarettes (5 Years)		X			X	
Pyoderma	Global	Male	Cumulative Cigarettes (10 Years)		X			X	
Pyoderma	Data Rich	Male	Cumulative Cigarettes (10 Years)		X			X	
Pyoderma	Global	Male	Healthcare access and quality index	X			X		
Pyoderma	Data Rich	Male	Healthcare access and quality index	X			X		
Pyoderma	Global	Male	Prevalence of overweight and obesity	X			X		
Pyoderma	Data Rich	Male	Prevalence of overweight and obesity	X			X		
Pyoderma	Global	Male	Diabetes Fasting Plasma Glucose (mmol/L), by age	X			X		
Pyoderma	Data Rich	Male	Diabetes Fasting Plasma Glucose (mmol/L), by age	X			X		
Pyoderma	Global	Male	Improved Water Source (proportion with access)	X			X		
Pyoderma	Data Rich	Male	Improved Water Source (proportion with access)	X			X		
Pyoderma	Global	Male	Age- and sex-specific SEV for Unsafe sanitation	X			X		
Pyoderma	Data Rich	Male	Age- and sex-specific SEV for Unsafe sanitation	X			X		
Decubitus ulcer	Global	Female	Education (years per capita)			X			X
Decubitus ulcer	Data Rich	Female	Education (years per capita)			X			X
Decubitus ulcer	Global	Female	LDI (I$ per capita)			X			X
Decubitus ulcer	Data Rich	Female	LDI (I$ per capita)			X			X
Decubitus ulcer	Global	Female	Socio-demographic Index			X			X
Decubitus ulcer	Data Rich	Female	Socio-demographic Index			X			X
Decubitus ulcer	Global	Female	Age- and sex-specific SEV for Unsafe sanitation			X			X
Decubitus ulcer	Data Rich	Female	Age- and sex-specific SEV for Unsafe sanitation			X			X
Decubitus ulcer	Global	Female	Healthcare access and quality index		X			X	
Decubitus ulcer	Data Rich	Female	Healthcare access and quality index		X			X	
Decubitus ulcer	Global	Female	Smoking Prevalence		X			X	
Decubitus ulcer	Data Rich	Female	Smoking Prevalence		X			X	
Decubitus ulcer	Data Rich	Female	Liters of alcohol consumed per capita		X			X	
Decubitus ulcer	Global	Female	Cumulative Cigarettes (5 Years)		X			X	
Decubitus ulcer	Data Rich	Female	Cumulative Cigarettes (5 Years)		X			X	
Decubitus ulcer	Global	Female	Cumulative Cigarettes (10 Years)		X			X	
Decubitus ulcer	Data Rich	Female	Cumulative Cigarettes (10 Years)		X			X	
Decubitus ulcer	Global	Female	Liters of alcohol consumed per capita	X			X		
Decubitus ulcer	Global	Female	Diabetes Fasting Plasma Glucose (mmol/L), age-standardized 25+	X			X		
Decubitus ulcer	Data Rich	Female	Diabetes Fasting Plasma Glucose (mmol/L), age-standardized 25+	X			X		
Decubitus ulcer	Global	Female	Improved Water Source (proportion with access)	X			X		
Decubitus ulcer	Data Rich	Female	Improved Water Source (proportion with access)	X			X		
Decubitus ulcer	Global	Female	Prevalence of obesity	X			X		
Decubitus ulcer	Data Rich	Female	Prevalence of obesity	X			X		
Decubitus ulcer	Global	Male	Education (years per capita)			X			X
Decubitus ulcer	Data Rich	Male	Education (years per capita)			X			X
Decubitus ulcer	Global	Male	LDI (I$ per capita)			X			X
Decubitus ulcer	Data Rich	Male	LDI (I$ per capita)			X			X
Decubitus ulcer	Global	Male	Socio-demographic Index			X			X
Decubitus ulcer	Data Rich	Male	Socio-demographic Index			X			X
Decubitus ulcer	Global	Male	Age- and sex-specific SEV for Unsafe sanitation			X			X
Decubitus ulcer	Data Rich	Male	Age- and sex-specific SEV for Unsafe sanitation			X			X
Decubitus ulcer	Global	Male	Healthcare access and quality index		X			X	
Decubitus ulcer	Data Rich	Male	Healthcare access and quality index		X			X	
Decubitus ulcer	Global	Male	Smoking Prevalence		X			X	
Decubitus ulcer	Data Rich	Male	Smoking Prevalence		X			X	
Decubitus ulcer	Data Rich	Male	Liters of alcohol consumed per capita		X			X	
Decubitus ulcer	Global	Male	Cumulative Cigarettes (5 Years)		X			X	
Decubitus ulcer	Data Rich	Male	Cumulative Cigarettes (5 Years)		X			X	
Decubitus ulcer	Global	Male	Cumulative Cigarettes (10 Years)		X			X	
Decubitus ulcer	Data Rich	Male	Cumulative Cigarettes (10 Years)		X			X	
Decubitus ulcer	Global	Male	Liters of alcohol consumed per capita	X			X		
Decubitus ulcer	Global	Male	Diabetes Fasting Plasma Glucose (mmol/L), age-standardized 25+	X			X		
Decubitus ulcer	Data Rich	Male	Diabetes Fasting Plasma Glucose (mmol/L), age-standardized 25+	X			X		
Decubitus ulcer	Global	Male	Improved Water Source (proportion with access)	X			X		
Decubitus ulcer	Data Rich	Male	Improved Water Source (proportion with access)	X			X		
Decubitus ulcer	Global	Male	Prevalence of obesity	X			X		
Decubitus ulcer	Data Rich	Male	Prevalence of obesity	X			X		
Other skin and subcutaneous diseases	Global	Female	Education (years per capita)			X			X
Other skin and subcutaneous diseases	Data Rich	Female	Education (years per capita)			X			X
Other skin and subcutaneous diseases	Global	Female	LDI (I$ per capita)			X			X
Other skin and subcutaneous diseases	Data Rich	Female	LDI (I$ per capita)			X			X
Other skin and subcutaneous diseases	Global	Female	Socio-demographic Index			X			X
Other skin and subcutaneous diseases	Data Rich	Female	Socio-demographic Index			X			X
Other skin and subcutaneous diseases	Global	Female	Smoking Prevalence		X			X	
Other skin and subcutaneous diseases	Data Rich	Female	Smoking Prevalence		X			X	
Other skin and subcutaneous diseases	Global	Female	Liters of alcohol consumed per capita		X			X	
Other skin and subcutaneous diseases	Data Rich	Female	Liters of alcohol consumed per capita		X			X	
Other skin and subcutaneous diseases	Global	Female	Cumulative Cigarettes (5 Years)		X			X	
Other skin and subcutaneous diseases	Data Rich	Female	Cumulative Cigarettes (5 Years)		X			X	
Other skin and subcutaneous diseases	Global	Female	Cumulative Cigarettes (10 Years)		X			X	
Other skin and subcutaneous diseases	Data Rich	Female	Cumulative Cigarettes (10 Years)		X			X	
Other skin and subcutaneous diseases	Global	Female	Healthcare access and quality index	X			X		
Other skin and subcutaneous diseases	Data Rich	Female	Healthcare access and quality index	X			X		
Other skin and subcutaneous diseases	Global	Female	Prevalence of overweight and obesity	X			X		
Other skin and subcutaneous diseases	Data Rich	Female	Prevalence of overweight and obesity	X			X		
Other skin and subcutaneous diseases	Global	Female	Diabetes Fasting Plasma Glucose (mmol/L), by age	X			X		
Other skin and subcutaneous diseases	Data Rich	Female	Diabetes Fasting Plasma Glucose (mmol/L), by age	X			X		
Other skin and subcutaneous diseases	Global	Female	Improved Water Source (proportion with access)	X			X		
Other skin and subcutaneous diseases	Data Rich	Female	Improved Water Source (proportion with access)	X			X		
Other skin and subcutaneous diseases	Global	Female	Age- and sex-specific SEV for Unsafe sanitation	X			X		
Other skin and subcutaneous diseases	Data Rich	Female	Age- and sex-specific SEV for Unsafe sanitation	X			X		
Other skin and subcutaneous diseases	Global	Female	Age-standardized SEV for Child underweight	X			X		
Other skin and subcutaneous diseases	Data Rich	Female	Age-standardized SEV for Child underweight	X			X		
Other skin and subcutaneous diseases	Global	Male	Education (years per capita)			X			X
Other skin and subcutaneous diseases	Data Rich	Male	Education (years per capita)			X			X
Other skin and subcutaneous diseases	Global	Male	LDI (I$ per capita)			X			X
Other skin and subcutaneous diseases	Data Rich	Male	LDI (I$ per capita)			X			X
Other skin and subcutaneous diseases	Global	Male	Socio-demographic Index			X			X
Other skin and subcutaneous diseases	Data Rich	Male	Socio-demographic Index			X			X
Other skin and subcutaneous diseases	Global	Male	Smoking Prevalence		X			X	
Other skin and subcutaneous diseases	Data Rich	Male	Smoking Prevalence		X			X	
Other skin and subcutaneous diseases	Global	Male	Liters of alcohol consumed per capita		X			X	
Other skin and subcutaneous diseases	Data Rich	Male	Liters of alcohol consumed per capita		X			X	
Other skin and subcutaneous diseases	Global	Male	Cumulative Cigarettes (5 Years)		X			X	
Other skin and subcutaneous diseases	Data Rich	Male	Cumulative Cigarettes (5 Years)		X			X	
Other skin and subcutaneous diseases	Global	Male	Cumulative Cigarettes (10 Years)		X			X	
Other skin and subcutaneous diseases	Data Rich	Male	Cumulative Cigarettes (10 Years)		X			X	
Other skin and subcutaneous diseases	Global	Male	Healthcare access and quality index	X			X		
Other skin and subcutaneous diseases	Data Rich	Male	Healthcare access and quality index	X			X		
Other skin and subcutaneous diseases	Global	Male	Prevalence of overweight and obesity	X			X		
Other skin and subcutaneous diseases	Data Rich	Male	Prevalence of overweight and obesity	X			X		

Comparison of GBD 2019 and GBD 2021 covariates and level of covariates used in cause of death modeling

Cause	Model Version Type	Sex	Covariate	Level 1 GBD 2019	Level 2 GBD 2019	Level 3 GBD 2019	Level 1 GBD 2021	Level 2 GBD 2021	Level 3 GBD 2021
Other skin and subcutaneous diseases	Global	Male	Diabetes Fasting Plasma Glucose (mmol/L), by age	X			X		
Other skin and subcutaneous diseases	Data Rich	Male	Diabetes Fasting Plasma Glucose (mmol/L), by age	X			X		
Other skin and subcutaneous diseases	Global	Male	Improved Water Source (proportion with access)	X			X		
Other skin and subcutaneous diseases	Data Rich	Male	Improved Water Source (proportion with access)	X			X		
Other skin and subcutaneous diseases	Global	Male	Age- and sex-specific SEV for Unsafe sanitation	X			X		
Other skin and subcutaneous diseases	Data Rich	Male	Age- and sex-specific SEV for Unsafe sanitation	X			X		
Other skin and subcutaneous diseases	Global	Male	Age-standardized SEV for Child underweight	X			X		
Other skin and subcutaneous diseases	Data Rich	Male	Age-standardized SEV for Child underweight	X			X		
Musculoskeletal disorders	Data Rich	Female	Socio-demographic Index			X			X
Musculoskeletal disorders	Global	Female	Socio-demographic Index			X			X
Musculoskeletal disorders	Data Rich	Female	Education (years per capita)		X			X	
Musculoskeletal disorders	Global	Female	Education (years per capita)		X			X	
Musculoskeletal disorders	Data Rich	Female	LDI (I$ per capita)		X			X	
Musculoskeletal disorders	Global	Female	LDI (I$ per capita)		X			X	
Musculoskeletal disorders	Data Rich	Female	Healthcare access and quality index		X			X	
Musculoskeletal disorders	Global	Female	Healthcare access and quality index		X			X	
Musculoskeletal disorders	Data Rich	Female	Low-Density Lipoprotein (mmol/L)		X			X	
Musculoskeletal disorders	Global	Female	Low-Density Lipoprotein (mmol/L)		X			X	
Musculoskeletal disorders	Data Rich	Female	Smoking Prevalence		X			X	
Musculoskeletal disorders	Global	Female	Smoking Prevalence		X			X	
Musculoskeletal disorders	Data Rich	Female	Cumulative Cigarettes (5 Years)		X			X	
Musculoskeletal disorders	Global	Female	Cumulative Cigarettes (5 Years)		X			X	
Musculoskeletal disorders	Data Rich	Female	Cumulative Cigarettes (10 Years)		X			X	
Musculoskeletal disorders	Global	Female	Cumulative Cigarettes (10 Years)		X			X	
Musculoskeletal disorders	Data Rich	Female	Low bone mineral density		X			X	
Musculoskeletal disorders	Global	Female	Low bone mineral density		X			X	
Musculoskeletal disorders	Data Rich	Female	Age-standardized bone mineral density among population age 60+ years		X			X	
Musculoskeletal disorders	Global	Female	Age-standardized bone mineral density among population age 60+ years		X			X	
Musculoskeletal disorders	Data Rich	Female	Mean BMI	X			X		
Musculoskeletal disorders	Global	Female	Mean BMI	X			X		
Musculoskeletal disorders	Data Rich	Female	Liters of alcohol consumed per capita	X			X		
Musculoskeletal disorders	Global	Female	Liters of alcohol consumed per capita	X			X		
Musculoskeletal disorders	Global	Female	vegetables unadjusted(g)	X			X		
Musculoskeletal disorders	Data Rich	Male	Socio-demographic Index			X			X
Musculoskeletal disorders	Global	Male	Socio-demographic Index			X			X
Musculoskeletal disorders	Data Rich	Male	Education (years per capita)		X			X	
Musculoskeletal disorders	Global	Male	Education (years per capita)		X			X	
Musculoskeletal disorders	Data Rich	Male	LDI (I$ per capita)		X			X	
Musculoskeletal disorders	Global	Male	LDI (I$ per capita)		X			X	
Musculoskeletal disorders	Data Rich	Male	Healthcare access and quality index		X			X	
Musculoskeletal disorders	Global	Male	Healthcare access and quality index		X			X	
Musculoskeletal disorders	Data Rich	Male	Low-Density Lipoprotein (mmol/L)		X			X	
Musculoskeletal disorders	Global	Male	Low-Density Lipoprotein (mmol/L)		X			X	
Musculoskeletal disorders	Data Rich	Male	Smoking Prevalence		X			X	
Musculoskeletal disorders	Global	Male	Smoking Prevalence		X			X	
Musculoskeletal disorders	Data Rich	Male	Cumulative Cigarettes (5 Years)		X			X	
Musculoskeletal disorders	Global	Male	Cumulative Cigarettes (5 Years)		X			X	
Musculoskeletal disorders	Data Rich	Male	Cumulative Cigarettes (10 Years)		X			X	
Musculoskeletal disorders	Global	Male	Cumulative Cigarettes (10 Years)		X			X	
Musculoskeletal disorders	Data Rich	Male	Low bone mineral density		X			X	
Musculoskeletal disorders	Data Rich	Male	Age-standardized bone mineral density among population age 60+ years		X			X	
Musculoskeletal disorders	Data Rich	Male	Mean BMI	X			X		
Musculoskeletal disorders	Global	Male	Mean BMI	X			X		
Musculoskeletal disorders	Data Rich	Male	Liters of alcohol consumed per capita	X			X		
Musculoskeletal disorders	Global	Male	Liters of alcohol consumed per capita	X			X		
Musculoskeletal disorders	Data Rich	Male	vegetables unadjusted(g)	X			X		
Musculoskeletal disorders	Global	Male	vegetables unadjusted(g)	X			X		
Rheumatoid arthritis	Data Rich	Female	Education (years per capita)			X			X
Rheumatoid arthritis	Global	Female	Education (years per capita)			X			X
Rheumatoid arthritis	Data Rich	Female	LDI (I$ per capita)			X			X
Rheumatoid arthritis	Global	Female	LDI (I$ per capita)			X			X
Rheumatoid arthritis	Data Rich	Female	Socio-demographic Index			X			X
Rheumatoid arthritis	Global	Female	Socio-demographic Index			X			X
Rheumatoid arthritis	Data Rich	Female	Mean BMI		X			X	
Rheumatoid arthritis	Global	Female	Mean BMI		X			X	
Rheumatoid arthritis	Data Rich	Female	Low-Density Lipoprotein (mmol/L)		X			X	
Rheumatoid arthritis	Global	Female	Low-Density Lipoprotein (mmol/L)		X			X	
Rheumatoid arthritis	Data Rich	Female	Healthcare access and quality index	X			X		
Rheumatoid arthritis	Global	Female	Healthcare access and quality index	X			X		
Rheumatoid arthritis	Data Rich	Female	Smoking Prevalence	X			X		
Rheumatoid arthritis	Global	Female	Smoking Prevalence	X			X		
Rheumatoid arthritis	Data Rich	Female	Liters of alcohol consumed per capita	X			X		
Rheumatoid arthritis	Global	Female	Liters of alcohol consumed per capita	X			X		
Rheumatoid arthritis	Data Rich	Female	Cumulative Cigarettes (5 Years)	X			X		
Rheumatoid arthritis	Global	Female	Cumulative Cigarettes (5 Years)	X			X		
Rheumatoid arthritis	Data Rich	Female	Cumulative Cigarettes (10 Years)	X			X		
Rheumatoid arthritis	Global	Female	Cumulative Cigarettes (10 Years)	X			X		
Rheumatoid arthritis	Data Rich	Female	milk unadjusted(g)	X			X		
Rheumatoid arthritis	Global	Female	milk unadjusted(g)	X			X		
Rheumatoid arthritis	Data Rich	Male	Education (years per capita)			X			X
Rheumatoid arthritis	Global	Male	Education (years per capita)			X			X
Rheumatoid arthritis	Data Rich	Male	LDI (I$ per capita)			X			X
Rheumatoid arthritis	Global	Male	LDI (I$ per capita)			X			X
Rheumatoid arthritis	Data Rich	Male	Socio-demographic Index			X			X
Rheumatoid arthritis	Global	Male	Socio-demographic Index			X			X
Rheumatoid arthritis	Data Rich	Male	Mean BMI		X			X	
Rheumatoid arthritis	Global	Male	Mean BMI		X			X	
Rheumatoid arthritis	Data Rich	Male	Low-Density Lipoprotein (mmol/L)		X			X	
Rheumatoid arthritis	Global	Male	Low-Density Lipoprotein (mmol/L)		X			X	
Rheumatoid arthritis	Data Rich	Male	Healthcare access and quality index	X			X		
Rheumatoid arthritis	Global	Male	Healthcare access and quality index	X			X		
Rheumatoid arthritis	Data Rich	Male	Smoking Prevalence	X			X		
Rheumatoid arthritis	Global	Male	Smoking Prevalence	X			X		
Rheumatoid arthritis	Data Rich	Male	Liters of alcohol consumed per capita	X			X		
Rheumatoid arthritis	Global	Male	Liters of alcohol consumed per capita	X			X		
Rheumatoid arthritis	Data Rich	Male	Cumulative Cigarettes (5 Years)	X			X		
Rheumatoid arthritis	Global	Male	Cumulative Cigarettes (5 Years)	X			X		
Rheumatoid arthritis	Data Rich	Male	Cumulative Cigarettes (10 Years)	X			X		
Rheumatoid arthritis	Global	Male	Cumulative Cigarettes (10 Years)	X			X		
Rheumatoid arthritis	Data Rich	Male	milk unadjusted(g)	X			X		
Rheumatoid arthritis	Global	Male	milk unadjusted(g)	X			X		
Other musculoskeletal disorders	Data Rich	Female	Socio-demographic Index			X			X
Other musculoskeletal disorders	Global	Female	Socio-demographic Index			X			X
Other musculoskeletal disorders	Data Rich	Female	Education (years per capita)		X			X	
Other musculoskeletal disorders	Global	Female	Education (years per capita)		X			X	
Other musculoskeletal disorders	Data Rich	Female	LDI (I$ per capita)		X			X	
Other musculoskeletal disorders	Global	Female	LDI (I$ per capita)		X			X	
Other musculoskeletal disorders	Data Rich	Female	Healthcare access and quality index		X			X	
Other musculoskeletal disorders	Global	Female	Healthcare access and quality index		X			X	
Other musculoskeletal disorders	Data Rich	Female	Low-Density Lipoprotein (mmol/L)		X			X	
Other musculoskeletal disorders	Global	Female	Low-Density Lipoprotein (mmol/L)		X			X	
Other musculoskeletal disorders	Data Rich	Female	Smoking Prevalence		X			X	
Other musculoskeletal disorders	Global	Female	Smoking Prevalence		X			X	
Other musculoskeletal disorders	Data Rich	Female	Cumulative Cigarettes (5 Years)		X			X	
Other musculoskeletal disorders	Global	Female	Cumulative Cigarettes (5 Years)		X			X	
Other musculoskeletal disorders	Data Rich	Female	Cumulative Cigarettes (10 Years)		X			X	
Other musculoskeletal disorders	Global	Female	Cumulative Cigarettes (10 Years)		X			X	
Other musculoskeletal disorders	Data Rich	Female	Mean BMI	X			X		
Other musculoskeletal disorders	Global	Female	Mean BMI	X			X		
Other musculoskeletal disorders	Data Rich	Female	Liters of alcohol consumed per capita	X			X		
Other musculoskeletal disorders	Global	Female	Liters of alcohol consumed per capita	X			X		
Other musculoskeletal disorders	Data Rich	Female	vegetables unadjusted(g)	X			X		
Other musculoskeletal disorders	Global	Female	vegetables unadjusted(g)	X			X		
Other musculoskeletal disorders	Data Rich	Male	Socio-demographic Index			X			X
Other musculoskeletal disorders	Global	Male	Socio-demographic Index			X			X
Other musculoskeletal disorders	Data Rich	Male	Education (years per capita)		X			X	
Other musculoskeletal disorders	Global	Male	Education (years per capita)		X			X	
Other musculoskeletal disorders	Data Rich	Male	LDI (I$ per capita)		X			X	
Other musculoskeletal disorders	Global	Male	LDI (I$ per capita)		X			X	
Other musculoskeletal disorders	Data Rich	Male	Healthcare access and quality index		X			X	
Other musculoskeletal disorders	Global	Male	Healthcare access and quality index		X			X	
Other musculoskeletal disorders	Data Rich	Male	Low-Density Lipoprotein (mmol/L)		X			X	
Other musculoskeletal disorders	Global	Male	Low-Density Lipoprotein (mmol/L)		X			X	
Other musculoskeletal disorders	Data Rich	Male	Smoking Prevalence		X			X	
Other musculoskeletal disorders	Global	Male	Smoking Prevalence		X			X	

Comparison of GBD 2019 and GBD 2021 covariates and level of covariates used in cause of death modeling									
Cause	Model Version Type	Sex	Covariate	Level 1 GBD 2019	Level 2 GBD 2019	Level 3 GBD 2019	Level 1 GBD 2021	Level 2 GBD 2021	Level 3 GBD 2021
Other musculoskeletal disorders	Data Rich	Male	Cumulative Cigarettes (5 Years)		X			X	
Other musculoskeletal disorders	Global	Male	Cumulative Cigarettes (5 Years)		X			X	
Other musculoskeletal disorders	Data Rich	Male	Cumulative Cigarettes (10 Years)		X			X	
Other musculoskeletal disorders	Global	Male	Cumulative Cigarettes (10 Years)		X			X	
Other musculoskeletal disorders	Data Rich	Male	Mean BMI	X			X		
Other musculoskeletal disorders	Global	Male	Mean BMI	X			X		
Other musculoskeletal disorders	Data Rich	Male	Liters of alcohol consumed per capita	X			X		
Other musculoskeletal disorders	Global	Male	Liters of alcohol consumed per capita	X			X		
Other musculoskeletal disorders	Data Rich	Male	vegetables unadjusted(g)	X			X		
Other musculoskeletal disorders	Global	Male	vegetables unadjusted(g)	X			X		
Congenital anomalies	Data Rich	Female	Socio-demographic Index			X			X
Congenital anomalies	Global	Female	Socio-demographic Index			X			X
Congenital anomalies	Data Rich	Female	Liters of alcohol consumed per capita			X			X
Congenital anomalies	Global	Female	Liters of alcohol consumed per capita			X			X
Congenital anomalies	Data Rich	Female	Maternal Education (years per capita)			X			X
Congenital anomalies	Global	Female	Maternal Education (years per capita)			X			X
Congenital anomalies	Data Rich	Female	Age-standardized SEV for Ambient particulate matter			X			X
Congenital anomalies	Global	Female	Age-standardized SEV for Ambient particulate matter			X			X
Congenital anomalies	Data Rich	Female	Age-standardized SEV for Household air pollution			X			X
Congenital anomalies	Global	Female	Age-standardized SEV for Household air pollution			X			X
Congenital anomalies	Data Rich	Female	Age-standardized SEV for Low fruit			X			X
Congenital anomalies	Global	Female	Age-standardized SEV for Low fruit			X			X
Congenital anomalies	Data Rich	Female	Age-standardized SEV for Low vegetables			X			X
Congenital anomalies	Global	Female	Age-standardized SEV for Low vegetables			X			X
Congenital anomalies	Data Rich	Female	Healthcare access and quality index		X			X	
Congenital anomalies	Global	Female	Healthcare access and quality index		X			X	
Congenital anomalies	Data Rich	Female	Antenatal Care (4 visits) Coverage (proportion)		X			X	
Congenital anomalies	Global	Female	Antenatal Care (4 visits) Coverage (proportion)		X			X	
Congenital anomalies	Data Rich	Female	Antenatal Care (1 visit) Coverage (proportion)		X			X	
Congenital anomalies	Global	Female	Antenatal Care (1 visit) Coverage (proportion)		X			X	
Congenital anomalies	Data Rich	Female	Age-standardized SEV for Smoking		X			X	
Congenital anomalies	Global	Female	Age-standardized SEV for Smoking		X			X	
Congenital anomalies	Data Rich	Female	Legality of Abortion		X			X	
Congenital anomalies	Global	Female	Legality of Abortion		X			X	
Congenital anomalies	Data Rich	Female	In-Facility Delivery (proportion)	X				X	
Congenital anomalies	Global	Female	In-Facility Delivery (proportion)	X				X	
Congenital anomalies	Data Rich	Female	Composite fortification standard and folic acid inclusion	X				X	
Congenital anomalies	Global	Female	Composite fortification standard and folic acid inclusion	X				X	
Congenital anomalies	Data Rich	Female	Live Births 35+ (proportion)	X			X		
Congenital anomalies	Global	Female	Live Births 35+ (proportion)	X			X		
Congenital anomalies	Data Rich	Female	Maternal alcohol consumption during pregnancy (proportion)	X			X		
Congenital anomalies	Global	Female	Maternal alcohol consumption during pregnancy (proportion)	X			X		
Congenital anomalies	Data Rich	Female	Folic acid unadjusted (ug)	X			X		
Congenital anomalies	Global	Female	Folic acid unadjusted (ug)	X			X		
Congenital anomalies	Data Rich	Female	Birth prevalence of congenital chromosomal anomalies	X			X		
Congenital anomalies	Global	Female	Birth prevalence of congenital chromosomal anomalies	X			X		
Congenital anomalies	Data Rich	Female	Birth prevalence of CHD	X			X		
Congenital anomalies	Global	Female	Birth prevalence of CHD	X			X		
Congenital anomalies	Global	Male	Socio-demographic Index			X			X
Congenital anomalies	Data Rich	Male	Socio-demographic Index			X			X
Congenital anomalies	Global	Male	Liters of alcohol consumed per capita			X			X
Congenital anomalies	Data Rich	Male	Liters of alcohol consumed per capita			X			X
Congenital anomalies	Global	Male	Outdoor Air Pollution (PM2.5)			X			X
Congenital anomalies	Global	Male	Maternal Education (years per capita)			X			X
Congenital anomalies	Data Rich	Male	Maternal Education (years per capita)			X			X
Congenital anomalies	Data Rich	Male	Age-standardized SEV for Ambient particulate matter			X			X
Congenital anomalies	Global	Male	Age-standardized SEV for Household air pollution			X			X
Congenital anomalies	Data Rich	Male	Age-standardized SEV for Household air pollution			X			X
Congenital anomalies	Global	Male	Age-standardized SEV for Low fruit			X			X
Congenital anomalies	Data Rich	Male	Age-standardized SEV for Low fruit			X			X
Congenital anomalies	Global	Male	Age-standardized SEV for Low vegetables			X			X
Congenital anomalies	Data Rich	Male	Age-standardized SEV for Low vegetables			X			X
Congenital anomalies	Global	Male	Healthcare access and quality index		X			X	
Congenital anomalies	Data Rich	Male	Healthcare access and quality index		X			X	
Congenital anomalies	Global	Male	Antenatal Care (4 visits) Coverage (proportion)		X			X	
Congenital anomalies	Data Rich	Male	Antenatal Care (4 visits) Coverage (proportion)		X			X	
Congenital anomalies	Global	Male	Antenatal Care (1 visit) Coverage (proportion)		X			X	
Congenital anomalies	Data Rich	Male	Antenatal Care (1 visit) Coverage (proportion)		X			X	
Congenital anomalies	Global	Male	Age-standardized SEV for Smoking		X			X	
Congenital anomalies	Data Rich	Male	Age-standardized SEV for Smoking		X			X	
Congenital anomalies	Global	Male	Legality of Abortion		X			X	
Congenital anomalies	Data Rich	Male	Legality of Abortion		X			X	
Congenital anomalies	Global	Male	In-Facility Delivery (proportion)	X				X	
Congenital anomalies	Data Rich	Male	In-Facility Delivery (proportion)	X				X	
Congenital anomalies	Global	Male	Composite fortification standard and folic acid inclusion	X				X	
Congenital anomalies	Data Rich	Male	Composite fortification standard and folic acid inclusion	X				X	
Congenital anomalies	Global	Male	Live Births 35+ (proportion)	X			X		
Congenital anomalies	Data Rich	Male	Live Births 35+ (proportion)	X			X		
Congenital anomalies	Global	Male	Maternal alcohol consumption during pregnancy (proportion)	X			X		
Congenital anomalies	Data Rich	Male	Maternal alcohol consumption during pregnancy (proportion)	X			X		
Congenital anomalies	Global	Male	Folic acid unadjusted (ug)	X			X		
Congenital anomalies	Data Rich	Male	Folic acid unadjusted (ug)	X			X		
Congenital anomalies	Global	Male	Birth prevalence of congenital chromosomal anomalies	X			X		
Congenital anomalies	Data Rich	Male	Birth prevalence of congenital chromosomal anomalies	X			X		
Congenital anomalies	Global	Male	Birth prevalence of CHD	X			X		
Congenital anomalies	Data Rich	Male	Birth prevalence of CHD	X			X		
Neural tube defects	Global	Female	Liters of alcohol consumed per capita			X			X
Neural tube defects	Data Rich	Female	Liters of alcohol consumed per capita			X			X
Neural tube defects	Global	Female	Maternal Education (years per capita)			X			X
Neural tube defects	Data Rich	Female	Maternal Education (years per capita)			X			X
Neural tube defects	Global	Female	Age-standardized SEV for Household air pollution			X			X
Neural tube defects	Data Rich	Female	Age-standardized SEV for Household air pollution			X			X
Neural tube defects	Global	Female	Maternal alcohol consumption during pregnancy (proportion)			X			X
Neural tube defects	Data Rich	Female	Maternal alcohol consumption during pregnancy (proportion)			X			X
Neural tube defects	Global	Female	Age-standardized SEV for Low fruit			X			X
Neural tube defects	Data Rich	Female	Age-standardized SEV for Low fruit			X			X
Neural tube defects	Global	Female	Age-standardized SEV for Low vegetables			X			X
Neural tube defects	Data Rich	Female	Age-standardized SEV for Low vegetables			X			X
Neural tube defects	Global	Female	Age-standardized SEV for High fasting plasma glucose			X			X
Neural tube defects	Data Rich	Female	Age-standardized SEV for High fasting plasma glucose			X			X
Neural tube defects	Global	Female	Healthcare access and quality index		X			X	
Neural tube defects	Data Rich	Female	Healthcare access and quality index		X			X	
Neural tube defects	Global	Female	Antenatal Care (4 visits) Coverage (proportion)		X			X	
Neural tube defects	Data Rich	Female	Antenatal Care (4 visits) Coverage (proportion)		X			X	
Neural tube defects	Global	Female	Antenatal Care (1 visit) Coverage (proportion)		X			X	
Neural tube defects	Data Rich	Female	Antenatal Care (1 visit) Coverage (proportion)		X			X	
Neural tube defects	Global	Female	Age-standardized SEV for Smoking		X			X	
Neural tube defects	Data Rich	Female	Age-standardized SEV for Smoking		X			X	
Neural tube defects	Global	Female	Legality of Abortion		X			X	
Neural tube defects	Data Rich	Female	Legality of Abortion		X			X	
Neural tube defects	Global	Female	In-Facility Delivery (proportion)	X			X		
Neural tube defects	Data Rich	Female	In-Facility Delivery (proportion)	X			X		
Neural tube defects	Global	Female	Socio-demographic Index	X			X		
Neural tube defects	Data Rich	Female	Socio-demographic Index	X			X		
Neural tube defects	Global	Female	Folic acid unadjusted (ug)	X			X		
Neural tube defects	Data Rich	Female	Folic acid unadjusted (ug)	X			X		
Neural tube defects	Global	Female	Composite fortification standard and folic acid inclusion	X			X		
Neural tube defects	Data Rich	Female	Composite fortification standard and folic acid inclusion	X			X		
Neural tube defects	Data Rich	Male	Liters of alcohol consumed per capita			X			X
Neural tube defects	Global	Male	Liters of alcohol consumed per capita			X			X
Neural tube defects	Data Rich	Male	Maternal Education (years per capita)			X			X
Neural tube defects	Global	Male	Maternal Education (years per capita)			X			X

Comparison of GBD 2019 and GBD 2021 covariates and level of covariates used in cause of death modeling									
Cause	Model Version Type	Sex	Covariate	Level 1 GBD 2019	Level 2 GBD 2019	Level 3 GBD 2019	Level 1 GBD 2021	Level 2 GBD 2021	Level 3 GBD 2021
Neural tube defects	Data Rich	Male	Age-standardized SEV for Household air pollution			X			X
Neural tube defects	Global	Male	Age-standardized SEV for Household air pollution			X			X
Neural tube defects	Data Rich	Male	Maternal alcohol consumption during pregnancy (proportion)			X			X
Neural tube defects	Global	Male	Maternal alcohol consumption during pregnancy (proportion)			X			X
Neural tube defects	Data Rich	Male	Age-standardized SEV for Low fruit			X			X
Neural tube defects	Global	Male	Age-standardized SEV for Low fruit			X			X
Neural tube defects	Data Rich	Male	Age-standardized SEV for Low vegetables			X			X
Neural tube defects	Global	Male	Age-standardized SEV for Low vegetables			X			X
Neural tube defects	Data Rich	Male	Age-standardized SEV for High fasting plasma glucose			X			X
Neural tube defects	Global	Male	Age-standardized SEV for High fasting plasma glucose			X			X
Neural tube defects	Data Rich	Male	Healthcare access and quality index		X			X	
Neural tube defects	Global	Male	Healthcare access and quality index		X			X	
Neural tube defects	Data Rich	Male	Antenatal Care (4 visits) Coverage (proportion)		X			X	
Neural tube defects	Global	Male	Antenatal Care (4 visits) Coverage (proportion)		X			X	
Neural tube defects	Data Rich	Male	Antenatal Care (1 visit) Coverage (proportion)		X			X	
Neural tube defects	Global	Male	Antenatal Care (1 visit) Coverage (proportion)		X			X	
Neural tube defects	Global	Male	Age-standardized SEV for Smoking		X			X	
Neural tube defects	Data Rich	Male	Legality of Abortion		X			X	
Neural tube defects	Global	Male	Legality of Abortion		X			X	
Neural tube defects	Data Rich	Male	Age-standardized SEV for Smoking	X				X	
Neural tube defects	Data Rich	Male	In-Facility Delivery (proportion)	X			X		
Neural tube defects	Global	Male	In-Facility Delivery (proportion)	X			X		
Neural tube defects	Data Rich	Male	Socio-demographic Index	X			X		
Neural tube defects	Global	Male	Socio-demographic Index	X			X		
Neural tube defects	Data Rich	Male	Folic acid unadjusted (ug)	X			X		
Neural tube defects	Global	Male	Folic acid unadjusted (ug)	X			X		
Neural tube defects	Data Rich	Male	Composite fortification standard and folic acid inclusion	X			X		
Neural tube defects	Global	Male	Composite fortification standard and folic acid inclusion	X			X		
Congenital heart anomalies	Data Rich	Female	Skilled Birth Attendance (proportion)			X			X
Congenital heart anomalies	Global	Female	Skilled Birth Attendance (proportion)			X			X
Congenital heart anomalies	Data Rich	Female	Liters of alcohol consumed per capita			X			X
Congenital heart anomalies	Global	Female	Liters of alcohol consumed per capita			X			X
Congenital heart anomalies	Data Rich	Female	Live Births 35+ (proportion)			X			X
Congenital heart anomalies	Global	Female	Live Births 35+ (proportion)			X			X
Congenital heart anomalies	Data Rich	Female	Antenatal Care (4 visits) Coverage (proportion)			X			X
Congenital heart anomalies	Global	Female	Antenatal Care (4 visits) Coverage (proportion)			X			X
Congenital heart anomalies	Data Rich	Female	Maternal Education (years per capita)			X			X
Congenital heart anomalies	Global	Female	Maternal Education (years per capita)			X			X
Congenital heart anomalies	Data Rich	Female	In-Facility Delivery (proportion)		X			X	
Congenital heart anomalies	Global	Female	In-Facility Delivery (proportion)		X			X	
Congenital heart anomalies	Data Rich	Female	Socio-demographic Index		X			X	
Congenital heart anomalies	Global	Female	Socio-demographic Index		X			X	
Congenital heart anomalies	Data Rich	Female	Healthcare access and quality index		X			X	
Congenital heart anomalies	Global	Female	Healthcare access and quality index		X			X	
Congenital heart anomalies	Data Rich	Female	Antenatal Care (1 visit) Coverage (proportion)		X			X	
Congenital heart anomalies	Global	Female	Antenatal Care (1 visit) Coverage (proportion)		X			X	
Congenital heart anomalies	Data Rich	Female	Age-standardized SEV for Smoking		X			X	
Congenital heart anomalies	Global	Female	Age-standardized SEV for Smoking		X			X	
Congenital heart anomalies	Data Rich	Female	Legality of Abortion		X			X	
Congenital heart anomalies	Global	Female	Legality of Abortion		X			X	
Congenital heart anomalies	Data Rich	Female	Age-standardized SEV for High fasting plasma glucose		X			X	
Congenital heart anomalies	Global	Female	Age-standardized SEV for High fasting plasma glucose		X			X	
Congenital heart anomalies	Data Rich	Female	Maternal alcohol consumption during pregnancy (proportion)	X			X		
Congenital heart anomalies	Global	Female	Maternal alcohol consumption during pregnancy (proportion)	X			X		
Congenital heart anomalies	Data Rich	Female	Birth prevalence of CHD	X			X		
Congenital heart anomalies	Global	Female	Birth prevalence of CHD	X			X		
Congenital heart anomalies	Data Rich	Male	Skilled Birth Attendance (proportion)			X			X
Congenital heart anomalies	Global	Male	Skilled Birth Attendance (proportion)			X			X
Congenital heart anomalies	Data Rich	Male	Liters of alcohol consumed per capita			X			X
Congenital heart anomalies	Global	Male	Liters of alcohol consumed per capita			X			X
Congenital heart anomalies	Data Rich	Male	Live Births 35+ (proportion)			X			X
Congenital heart anomalies	Global	Male	Live Births 35+ (proportion)			X			X
Congenital heart anomalies	Data Rich	Male	Antenatal Care (4 visits) Coverage (proportion)			X			X
Congenital heart anomalies	Global	Male	Antenatal Care (4 visits) Coverage (proportion)			X			X
Congenital heart anomalies	Data Rich	Male	Maternal Education (years per capita)			X			X
Congenital heart anomalies	Global	Male	Maternal Education (years per capita)			X			X
Congenital heart anomalies	Data Rich	Male	In-Facility Delivery (proportion)		X			X	
Congenital heart anomalies	Global	Male	In-Facility Delivery (proportion)		X			X	
Congenital heart anomalies	Data Rich	Male	Socio-demographic Index		X			X	
Congenital heart anomalies	Global	Male	Socio-demographic Index		X			X	
Congenital heart anomalies	Data Rich	Male	Healthcare access and quality index		X			X	
Congenital heart anomalies	Global	Male	Healthcare access and quality index		X			X	
Congenital heart anomalies	Data Rich	Male	Antenatal Care (1 visit) Coverage (proportion)		X			X	
Congenital heart anomalies	Global	Male	Antenatal Care (1 visit) Coverage (proportion)		X			X	
Congenital heart anomalies	Data Rich	Male	Age-standardized SEV for Smoking		X			X	
Congenital heart anomalies	Global	Male	Age-standardized SEV for Smoking		X			X	
Congenital heart anomalies	Data Rich	Male	Legality of Abortion		X			X	
Congenital heart anomalies	Global	Male	Legality of Abortion		X			X	
Congenital heart anomalies	Data Rich	Male	Age-standardized SEV for High fasting plasma glucose		X			X	
Congenital heart anomalies	Global	Male	Age-standardized SEV for High fasting plasma glucose		X			X	
Congenital heart anomalies	Data Rich	Male	Maternal alcohol consumption during pregnancy (proportion)	X			X		
Congenital heart anomalies	Global	Male	Maternal alcohol consumption during pregnancy (proportion)	X			X		
Congenital heart anomalies	Data Rich	Male	Birth prevalence of CHD	X			X		
Congenital heart anomalies	Global	Male	Birth prevalence of CHD	X			X		
Orofacial clefts	Data Rich	Female	Liters of alcohol consumed per capita			X			X
Orofacial clefts	Global	Female	Liters of alcohol consumed per capita			X			X
Orofacial clefts	Data Rich	Female	Antenatal Care (4 visits) Coverage (proportion)			X			X
Orofacial clefts	Global	Female	Antenatal Care (4 visits) Coverage (proportion)			X			X
Orofacial clefts	Data Rich	Female	Antenatal Care (1 visit) Coverage (proportion)			X			X
Orofacial clefts	Global	Female	Antenatal Care (1 visit) Coverage (proportion)			X			X
Orofacial clefts	Data Rich	Female	Maternal Education (years per capita)			X			X
Orofacial clefts	Global	Female	Maternal Education (years per capita)			X			X
Orofacial clefts	Global	Female	Age-standardized SEV for Household air pollution			X			X
Orofacial clefts	Data Rich	Female	Age-standardized SEV for Low fruit			X			X
Orofacial clefts	Global	Female	Age-standardized SEV for Low fruit			X			X
Orofacial clefts	Data Rich	Female	Age-standardized SEV for Low vegetables			X			X
Orofacial clefts	Global	Female	Age-standardized SEV for Low vegetables			X			X
Orofacial clefts	Data Rich	Female	Skilled Birth Attendance (proportion)		X			X	
Orofacial clefts	Global	Female	Skilled Birth Attendance (proportion)		X			X	
Orofacial clefts	Data Rich	Female	Healthcare access and quality index		X			X	
Orofacial clefts	Global	Female	Healthcare access and quality index		X			X	
Orofacial clefts	Data Rich	Female	Age-standardized SEV for Smoking		X			X	
Orofacial clefts	Global	Female	Age-standardized SEV for Smoking		X			X	
Orofacial clefts	Data Rich	Female	Legality of Abortion		X			X	
Orofacial clefts	Global	Female	Legality of Abortion		X			X	
Orofacial clefts	Data Rich	Female	Maternal alcohol consumption during pregnancy (proportion)		X			X	
Orofacial clefts	Global	Female	Maternal alcohol consumption during pregnancy (proportion)		X			X	
Orofacial clefts	Data Rich	Female	Age-standardized SEV for High fasting plasma glucose		X			X	
Orofacial clefts	Global	Female	Age-standardized SEV for High fasting plasma glucose		X			X	
Orofacial clefts	Data Rich	Female	Indoor Air Pollution (All Cooking Fuels)	X					X
Orofacial clefts	Data Rich	Female	Socio-demographic Index	X			X		
Orofacial clefts	Global	Female	Socio-demographic Index	X			X		
Orofacial clefts	Data Rich	Female	Composite fortification standard and folic acid inclusion	X			X		
Orofacial clefts	Global	Female	Composite fortification standard and folic acid inclusion	X			X		
Orofacial clefts	Data Rich	Female	Folic acid unadjusted (ug)				X		
Orofacial clefts	Global	Male	Liters of alcohol consumed per capita			X			X
Orofacial clefts	Data Rich	Male	Liters of alcohol consumed per capita			X			X
Orofacial clefts	Data Rich	Male	Indoor Air Pollution (All Cooking Fuels)			X			X
Orofacial clefts	Global	Male	Antenatal Care (4 visits) Coverage (proportion)			X			X
Orofacial clefts	Data Rich	Male	Antenatal Care (4 visits) Coverage (proportion)			X			X
Orofacial clefts	Global	Male	Antenatal Care (1 visit) Coverage (proportion)			X			X

Comparison of GBD 2019 and GBD 2021 covariates and level of covariates used in cause of death modeling

Cause	Model Version Type	Sex	Covariate	Level 1 GBD 2019	Level 2 GBD 2019	Level 3 GBD 2019	Level 1 GBD 2021	Level 2 GBD 2021	Level 3 GBD 2021
Orofacial clefts	Data Rich	Male	Antenatal Care (1 visit) Coverage (proportion)			X			X
Orofacial clefts	Global	Male	Maternal Education (years per capita)			X			X
Orofacial clefts	Data Rich	Male	Maternal Education (years per capita)			X			X
Orofacial clefts	Global	Male	Age-standardized SEV for Household air pollution			X			X
Orofacial clefts	Global	Male	Age-standardized SEV for Low fruit			X			X
Orofacial clefts	Data Rich	Male	Age-standardized SEV for Low fruit			X			X
Orofacial clefts	Global	Male	Age-standardized SEV for Low vegetables			X			X
Orofacial clefts	Data Rich	Male	Age-standardized SEV for Low vegetables			X			X
Orofacial clefts	Global	Male	Skilled Birth Attendance (proportion)		X			X	
Orofacial clefts	Data Rich	Male	Skilled Birth Attendance (proportion)		X			X	
Orofacial clefts	Global	Male	Healthcare access and quality index		X			X	
Orofacial clefts	Data Rich	Male	Healthcare access and quality index		X			X	
Orofacial clefts	Global	Male	Age-standardized SEV for Smoking		X			X	
Orofacial clefts	Data Rich	Male	Age-standardized SEV for Smoking		X			X	
Orofacial clefts	Global	Male	Legality of Abortion		X			X	
Orofacial clefts	Data Rich	Male	Legality of Abortion		X			X	
Orofacial clefts	Global	Male	Maternal alcohol consumption during pregnancy (proportion)		X			X	
Orofacial clefts	Data Rich	Male	Maternal alcohol consumption during pregnancy (proportion)		X			X	
Orofacial clefts	Global	Male	Age-standardized SEV for High fasting plasma glucose		X			X	
Orofacial clefts	Data Rich	Male	Age-standardized SEV for High fasting plasma glucose		X			X	
Orofacial clefts	Global	Male	Socio-demographic Index	X			X		
Orofacial clefts	Data Rich	Male	Socio-demographic Index	X			X		
Orofacial clefts	Data Rich	Male	Folic acid unadjusted (ug)	X			X		
Orofacial clefts	Global	Male	Composite fortification standard and folic acid inclusion	X			X		
Orofacial clefts	Data Rich	Male	Composite fortification standard and folic acid inclusion	X			X		
Down's syndrome	Data Rich	Female	Liters of alcohol consumed per capita			X			X
Down's syndrome	Global	Female	Liters of alcohol consumed per capita			X			X
Down's syndrome	Data Rich	Female	Antenatal Care (4 visits) Coverage (proportion)			X			X
Down's syndrome	Global	Female	Antenatal Care (4 visits) Coverage (proportion)			X			X
Down's syndrome	Data Rich	Female	Antenatal Care (1 visit) Coverage (proportion)			X			X
Down's syndrome	Global	Female	Antenatal Care (1 visit) Coverage (proportion)			X			X
Down's syndrome	Data Rich	Female	Maternal Education (years per capita)			X			X
Down's syndrome	Global	Female	Maternal Education (years per capita)			X			X
Down's syndrome	Data Rich	Female	Age-standardized SEV for Household air pollution			X			X
Down's syndrome	Global	Female	Age-standardized SEV for Household air pollution			X			X
Down's syndrome	Data Rich	Female	Age-standardized SEV for Smoking			X			X
Down's syndrome	Global	Female	Age-standardized SEV for Smoking			X			X
Down's syndrome	Data Rich	Female	Maternal alcohol consumption during pregnancy (proportion)			X			X
Down's syndrome	Global	Female	Maternal alcohol consumption during pregnancy (proportion)			X			X
Down's syndrome	Data Rich	Female	Age-standardized SEV for Low vegetables			X			X
Down's syndrome	Global	Female	Age-standardized SEV for Low vegetables			X			X
Down's syndrome	Data Rich	Female	In-Facility Delivery (proportion)		X			X	
Down's syndrome	Global	Female	In-Facility Delivery (proportion)		X			X	
Down's syndrome	Data Rich	Female	Socio-demographic Index		X			X	
Down's syndrome	Global	Female	Socio-demographic Index		X			X	
Down's syndrome	Data Rich	Female	Healthcare access and quality index		X			X	
Down's syndrome	Global	Female	Healthcare access and quality index		X			X	
Down's syndrome	Data Rich	Female	Live Births 35+ (proportion)	X			X		
Down's syndrome	Global	Female	Live Births 35+ (proportion)	X			X		
Down's syndrome	Data Rich	Female	Live Births 40+ (proportion)	X			X		
Down's syndrome	Global	Female	Live Births 40+ (proportion)	X			X		
Down's syndrome	Data Rich	Female	Legality of Abortion	X			X		
Down's syndrome	Global	Female	Legality of Abortion	X			X		
Down's syndrome	Data Rich	Female	Birth prevalence of congenital chromosomal anomalies	X			X		
Down's syndrome	Global	Female	Birth prevalence of congenital chromosomal anomalies	X			X		
Down's syndrome	Data Rich	Male	Liters of alcohol consumed per capita			X			X
Down's syndrome	Global	Male	Liters of alcohol consumed per capita			X			X
Down's syndrome	Data Rich	Male	Antenatal Care (4 visits) Coverage (proportion)			X			X
Down's syndrome	Global	Male	Antenatal Care (4 visits) Coverage (proportion)			X			X
Down's syndrome	Data Rich	Male	Antenatal Care (1 visit) Coverage (proportion)			X			X
Down's syndrome	Global	Male	Antenatal Care (1 visit) Coverage (proportion)			X			X
Down's syndrome	Data Rich	Male	Maternal Education (years per capita)			X			X
Down's syndrome	Global	Male	Maternal Education (years per capita)			X			X
Down's syndrome	Data Rich	Male	Age-standardized SEV for Household air pollution			X			X
Down's syndrome	Global	Male	Age-standardized SEV for Household air pollution			X			X
Down's syndrome	Data Rich	Male	Age-standardized SEV for Smoking			X			X
Down's syndrome	Global	Male	Age-standardized SEV for Smoking			X			X
Down's syndrome	Data Rich	Male	Maternal alcohol consumption during pregnancy (proportion)			X			X
Down's syndrome	Global	Male	Maternal alcohol consumption during pregnancy (proportion)			X			X
Down's syndrome	Data Rich	Male	Age-standardized SEV for Low vegetables			X			X
Down's syndrome	Global	Male	Age-standardized SEV for Low vegetables			X			X
Down's syndrome	Data Rich	Male	In-Facility Delivery (proportion)		X			X	
Down's syndrome	Global	Male	In-Facility Delivery (proportion)		X			X	
Down's syndrome	Data Rich	Male	Socio-demographic Index		X			X	
Down's syndrome	Global	Male	Socio-demographic Index		X			X	
Down's syndrome	Data Rich	Male	Healthcare access and quality index		X			X	
Down's syndrome	Global	Male	Healthcare access and quality index		X			X	
Down's syndrome	Data Rich	Male	Live Births 35+ (proportion)	X			X		
Down's syndrome	Global	Male	Live Births 35+ (proportion)	X			X		
Down's syndrome	Data Rich	Male	Live Births 40+ (proportion)	X			X		
Down's syndrome	Global	Male	Live Births 40+ (proportion)	X			X		
Down's syndrome	Data Rich	Male	Legality of Abortion	X			X		
Down's syndrome	Global	Male	Legality of Abortion	X			X		
Down's syndrome	Data Rich	Male	Birth prevalence of congenital chromosomal anomalies	X			X		
Down's syndrome	Global	Male	Birth prevalence of congenital chromosomal anomalies	X			X		
Other chromosomal abnormalities	Global	Female	Skilled Birth Attendance (proportion)			X			X
Other chromosomal abnormalities	Data Rich	Female	Skilled Birth Attendance (proportion)			X			X
Other chromosomal abnormalities	Global	Female	Socio-demographic Index			X			X
Other chromosomal abnormalities	Data Rich	Female	Socio-demographic Index			X			X
Other chromosomal abnormalities	Global	Female	Liters of alcohol consumed per capita			X			X
Other chromosomal abnormalities	Data Rich	Female	Liters of alcohol consumed per capita			X			X
Other chromosomal abnormalities	Global	Female	Maternal Education (years per capita)			X			X
Other chromosomal abnormalities	Data Rich	Female	Maternal Education (years per capita)			X			X
Other chromosomal abnormalities	Global	Female	Age-standardized SEV for Household air pollution			X			X
Other chromosomal abnormalities	Data Rich	Female	Age-standardized SEV for Household air pollution			X			X
Other chromosomal abnormalities	Global	Female	Age-standardized SEV for Smoking			X			X
Other chromosomal abnormalities	Data Rich	Female	Age-standardized SEV for Smoking			X			X
Other chromosomal abnormalities	Global	Female	In-Facility Delivery (proportion)		X			X	
Other chromosomal abnormalities	Data Rich	Female	In-Facility Delivery (proportion)		X			X	
Other chromosomal abnormalities	Global	Female	LDI (I$ per capita)		X			X	
Other chromosomal abnormalities	Data Rich	Female	LDI (I$ per capita)		X			X	
Other chromosomal abnormalities	Global	Female	Healthcare access and quality index		X			X	
Other chromosomal abnormalities	Data Rich	Female	Healthcare access and quality index		X			X	
Other chromosomal abnormalities	Global	Female	Antenatal Care (4 visits) Coverage (proportion)		X			X	
Other chromosomal abnormalities	Data Rich	Female	Antenatal Care (4 visits) Coverage (proportion)		X			X	
Other chromosomal abnormalities	Global	Female	Antenatal Care (1 visit) Coverage (proportion)		X			X	
Other chromosomal abnormalities	Data Rich	Female	Antenatal Care (1 visit) Coverage (proportion)		X			X	
Other chromosomal abnormalities	Global	Female	Maternal alcohol consumption during pregnancy (proportion)		X			X	
Other chromosomal abnormalities	Data Rich	Female	Maternal alcohol consumption during pregnancy (proportion)		X			X	
Other chromosomal abnormalities	Global	Female	Live Births 35+ (proportion)	X			X		
Other chromosomal abnormalities	Data Rich	Female	Live Births 35+ (proportion)	X			X		
Other chromosomal abnormalities	Global	Female	Live Births 40+ (proportion)	X			X		
Other chromosomal abnormalities	Data Rich	Female	Live Births 40+ (proportion)	X			X		
Other chromosomal abnormalities	Global	Female	Legality of Abortion	X			X		
Other chromosomal abnormalities	Data Rich	Female	Legality of Abortion	X			X		
Other chromosomal abnormalities	Global	Male	Skilled Birth Attendance (proportion)			X			X
Other chromosomal abnormalities	Data Rich	Male	Skilled Birth Attendance (proportion)			X			X
Other chromosomal abnormalities	Global	Male	Socio-demographic Index			X			X
Other chromosomal abnormalities	Data Rich	Male	Socio-demographic Index			X			X
Other chromosomal abnormalities	Global	Male	Liters of alcohol consumed per capita			X			X
Other chromosomal abnormalities	Data Rich	Male	Liters of alcohol consumed per capita			X			X
Other chromosomal abnormalities	Global	Male	Maternal Education (years per capita)			X			X
Other chromosomal abnormalities	Data Rich	Male	Maternal Education (years per capita)			X			X

Comparison of GBD 2019 and GBD 2021 covariates and level of covariates used in cause of death modeling

Cause	Model Version Type	Sex	Covariate	Level 1 GBD 2019	Level 2 GBD 2019	Level 3 GBD 2019	Level 1 GBD 2021	Level 2 GBD 2021	Level 3 GBD 2021
Other chromosomal abnormalities	Global	Male	Age-standardized SEV for Household air pollution			X			X
Other chromosomal abnormalities	Data Rich	Male	Age-standardized SEV for Household air pollution			X			X
Other chromosomal abnormalities	Global	Male	Age-standardized SEV for Smoking			X			X
Other chromosomal abnormalities	Data Rich	Male	Age-standardized SEV for Smoking			X			X
Other chromosomal abnormalities	Global	Male	In-Facility Delivery (proportion)		X			X	
Other chromosomal abnormalities	Data Rich	Male	In-Facility Delivery (proportion)		X			X	
Other chromosomal abnormalities	Global	Male	LDI (I$ per capita)		X			X	
Other chromosomal abnormalities	Data Rich	Male	LDI (I$ per capita)		X			X	
Other chromosomal abnormalities	Global	Male	Healthcare access and quality index		X			X	
Other chromosomal abnormalities	Data Rich	Male	Healthcare access and quality index		X			X	
Other chromosomal abnormalities	Global	Male	Antenatal Care (4 visits) Coverage (proportion)		X			X	
Other chromosomal abnormalities	Data Rich	Male	Antenatal Care (4 visits) Coverage (proportion)		X			X	
Other chromosomal abnormalities	Global	Male	Antenatal Care (1 visit) Coverage (proportion)		X			X	
Other chromosomal abnormalities	Data Rich	Male	Antenatal Care (1 visit) Coverage (proportion)		X			X	
Other chromosomal abnormalities	Global	Male	Maternal alcohol consumption during pregnancy (proportion)		X			X	
Other chromosomal abnormalities	Data Rich	Male	Maternal alcohol consumption during pregnancy (proportion)		X			X	
Other chromosomal abnormalities	Global	Male	Live Births 35+ (proportion)	X			X		
Other chromosomal abnormalities	Data Rich	Male	Live Births 35+ (proportion)	X			X		
Other chromosomal abnormalities	Global	Male	Live Births 40+ (proportion)	X			X		
Other chromosomal abnormalities	Data Rich	Male	Live Births 40+ (proportion)	X			X		
Other chromosomal abnormalities	Global	Male	Legality of Abortion	X			X		
Other chromosomal abnormalities	Data Rich	Male	Legality of Abortion	X			X		
Congenital musculoskeletal and limb anomalies	Global	Female	LDI (I$ per capita)			X			X
Congenital musculoskeletal and limb anomalies	Data Rich	Female	LDI (I$ per capita)			X			X
Congenital musculoskeletal and limb anomalies	Global	Female	Liters of alcohol consumed per capita			X			X
Congenital musculoskeletal and limb anomalies	Data Rich	Female	Liters of alcohol consumed per capita			X			X
Congenital musculoskeletal and limb anomalies	Global	Female	Antenatal Care (4 visits) Coverage (proportion)			X			X
Congenital musculoskeletal and limb anomalies	Data Rich	Female	Antenatal Care (4 visits) Coverage (proportion)			X			X
Congenital musculoskeletal and limb anomalies	Global	Female	Antenatal Care (1 visit) Coverage (proportion)			X			X
Congenital musculoskeletal and limb anomalies	Data Rich	Female	Antenatal Care (1 visit) Coverage (proportion)			X			X
Congenital musculoskeletal and limb anomalies	Global	Female	Maternal Education (years per capita)			X			X
Congenital musculoskeletal and limb anomalies	Data Rich	Female	Maternal Education (years per capita)			X			X
Congenital musculoskeletal and limb anomalies	Global	Female	Age-standardized SEV for Low fruit			X			X
Congenital musculoskeletal and limb anomalies	Data Rich	Female	Age-standardized SEV for Low fruit			X			X
Congenital musculoskeletal and limb anomalies	Global	Female	Age-standardized SEV for Low vegetables			X			X
Congenital musculoskeletal and limb anomalies	Data Rich	Female	Age-standardized SEV for Low vegetables			X			X
Congenital musculoskeletal and limb anomalies	Global	Female	In-Facility Delivery (proportion)		X			X	
Congenital musculoskeletal and limb anomalies	Data Rich	Female	In-Facility Delivery (proportion)		X			X	
Congenital musculoskeletal and limb anomalies	Global	Female	Socio-demographic Index		X			X	
Congenital musculoskeletal and limb anomalies	Data Rich	Female	Socio-demographic Index		X			X	
Congenital musculoskeletal and limb anomalies	Global	Female	Healthcare access and quality index		X			X	
Congenital musculoskeletal and limb anomalies	Data Rich	Female	Healthcare access and quality index		X			X	
Congenital musculoskeletal and limb anomalies	Global	Female	Age-standardized SEV for Household air pollution		X			X	
Congenital musculoskeletal and limb anomalies	Data Rich	Female	Age-standardized SEV for Household air pollution		X			X	
Congenital musculoskeletal and limb anomalies	Global	Female	Age-standardized SEV for Smoking		X			X	
Congenital musculoskeletal and limb anomalies	Data Rich	Female	Age-standardized SEV for Smoking		X			X	
Congenital musculoskeletal and limb anomalies	Global	Female	Age-standardized SEV for High fasting plasma glucose		X			X	
Congenital musculoskeletal and limb anomalies	Data Rich	Female	Age-standardized SEV for High fasting plasma glucose		X			X	
Congenital musculoskeletal and limb anomalies	Global	Female	Legality of Abortion	X			X		
Congenital musculoskeletal and limb anomalies	Data Rich	Female	Legality of Abortion	X			X		
Congenital musculoskeletal and limb anomalies	Global	Female	Maternal alcohol consumption during pregnancy (proportion)	X			X		
Congenital musculoskeletal and limb anomalies	Data Rich	Female	Maternal alcohol consumption during pregnancy (proportion)	X			X		
Congenital musculoskeletal and limb anomalies	Global	Male	LDI (I$ per capita)			X			X
Congenital musculoskeletal and limb anomalies	Data Rich	Male	LDI (I$ per capita)			X			X
Congenital musculoskeletal and limb anomalies	Global	Male	Liters of alcohol consumed per capita			X			X
Congenital musculoskeletal and limb anomalies	Data Rich	Male	Liters of alcohol consumed per capita			X			X
Congenital musculoskeletal and limb anomalies	Global	Male	Antenatal Care (4 visits) Coverage (proportion)			X			X
Congenital musculoskeletal and limb anomalies	Data Rich	Male	Antenatal Care (4 visits) Coverage (proportion)			X			X
Congenital musculoskeletal and limb anomalies	Global	Male	Antenatal Care (1 visit) Coverage (proportion)			X			X
Congenital musculoskeletal and limb anomalies	Data Rich	Male	Antenatal Care (1 visit) Coverage (proportion)			X			X
Congenital musculoskeletal and limb anomalies	Global	Male	Maternal Education (years per capita)			X			X
Congenital musculoskeletal and limb anomalies	Data Rich	Male	Maternal Education (years per capita)			X			X
Congenital musculoskeletal and limb anomalies	Global	Male	Age-standardized SEV for Low fruit			X			X
Congenital musculoskeletal and limb anomalies	Data Rich	Male	Age-standardized SEV for Low fruit			X			X
Congenital musculoskeletal and limb anomalies	Global	Male	Age-standardized SEV for Low vegetables			X			X
Congenital musculoskeletal and limb anomalies	Data Rich	Male	Age-standardized SEV for Low vegetables			X			X
Congenital musculoskeletal and limb anomalies	Global	Male	In-Facility Delivery (proportion)		X			X	
Congenital musculoskeletal and limb anomalies	Data Rich	Male	In-Facility Delivery (proportion)		X			X	
Congenital musculoskeletal and limb anomalies	Global	Male	Socio-demographic Index		X			X	
Congenital musculoskeletal and limb anomalies	Data Rich	Male	Socio-demographic Index		X			X	
Congenital musculoskeletal and limb anomalies	Global	Male	Healthcare access and quality index		X			X	
Congenital musculoskeletal and limb anomalies	Data Rich	Male	Healthcare access and quality index		X			X	
Congenital musculoskeletal and limb anomalies	Global	Male	Age-standardized SEV for Household air pollution		X			X	
Congenital musculoskeletal and limb anomalies	Data Rich	Male	Age-standardized SEV for Household air pollution		X			X	
Congenital musculoskeletal and limb anomalies	Global	Male	Age-standardized SEV for Smoking		X			X	
Congenital musculoskeletal and limb anomalies	Data Rich	Male	Age-standardized SEV for Smoking		X			X	
Congenital musculoskeletal and limb anomalies	Global	Male	Age-standardized SEV for High fasting plasma glucose		X			X	
Congenital musculoskeletal and limb anomalies	Data Rich	Male	Age-standardized SEV for High fasting plasma glucose		X			X	
Congenital musculoskeletal and limb anomalies	Global	Male	Legality of Abortion	X			X		
Congenital musculoskeletal and limb anomalies	Data Rich	Male	Legality of Abortion	X			X		
Congenital musculoskeletal and limb anomalies	Global	Male	Maternal alcohol consumption during pregnancy (proportion)	X			X		
Congenital musculoskeletal and limb anomalies	Data Rich	Male	Maternal alcohol consumption during pregnancy (proportion)	X			X		
Urogenital congenital anomalies	Data Rich	Female	LDI (I$ per capita)			X			X
Urogenital congenital anomalies	Global	Female	LDI (I$ per capita)			X			X
Urogenital congenital anomalies	Data Rich	Female	Liters of alcohol consumed per capita			X			X
Urogenital congenital anomalies	Global	Female	Liters of alcohol consumed per capita			X			X
Urogenital congenital anomalies	Data Rich	Female	Antenatal Care (4 visits) Coverage (proportion)			X			X
Urogenital congenital anomalies	Global	Female	Antenatal Care (4 visits) Coverage (proportion)			X			X
Urogenital congenital anomalies	Data Rich	Female	Antenatal Care (1 visit) Coverage (proportion)			X			X
Urogenital congenital anomalies	Global	Female	Antenatal Care (1 visit) Coverage (proportion)			X			X
Urogenital congenital anomalies	Data Rich	Female	Maternal Education (years per capita)			X			X
Urogenital congenital anomalies	Global	Female	Maternal Education (years per capita)			X			X
Urogenital congenital anomalies	Data Rich	Female	In-Facility Delivery (proportion)		X			X	
Urogenital congenital anomalies	Global	Female	In-Facility Delivery (proportion)		X			X	
Urogenital congenital anomalies	Data Rich	Female	Socio-demographic Index		X			X	
Urogenital congenital anomalies	Global	Female	Socio-demographic Index		X			X	
Urogenital congenital anomalies	Data Rich	Female	Healthcare access and quality index		X			X	
Urogenital congenital anomalies	Global	Female	Healthcare access and quality index		X			X	
Urogenital congenital anomalies	Global	Female	Diabetes Age-Standardized Prevalence (proportion)		X			X	
Urogenital congenital anomalies	Data Rich	Female	Age-standardized SEV for Ambient particulate matter		X			X	
Urogenital congenital anomalies	Global	Female	Age-standardized SEV for Ambient particulate matter		X			X	
Urogenital congenital anomalies	Data Rich	Female	Age-standardized SEV for Household air pollution		X			X	
Urogenital congenital anomalies	Global	Female	Age-standardized SEV for Household air pollution		X			X	
Urogenital congenital anomalies	Data Rich	Female	Age-standardized SEV for Smoking	X			X		
Urogenital congenital anomalies	Global	Female	Age-standardized SEV for Smoking	X			X		
Urogenital congenital anomalies	Data Rich	Female	Maternal alcohol consumption during pregnancy (proportion)	X			X		
Urogenital congenital anomalies	Global	Female	Maternal alcohol consumption during pregnancy (proportion)	X			X		
Urogenital congenital anomalies	Data Rich	Female	Smoking Prevalence (Reproductive Age Standardized)	X			X		
Urogenital congenital anomalies	Data Rich	Male	LDI (I$ per capita)			X			X
Urogenital congenital anomalies	Global	Male	LDI (I$ per capita)			X			X
Urogenital congenital anomalies	Data Rich	Male	Liters of alcohol consumed per capita			X			X
Urogenital congenital anomalies	Global	Male	Liters of alcohol consumed per capita			X			X
Urogenital congenital anomalies	Data Rich	Male	Antenatal Care (4 visits) Coverage (proportion)			X			X
Urogenital congenital anomalies	Global	Male	Antenatal Care (4 visits) Coverage (proportion)			X			X
Urogenital congenital anomalies	Data Rich	Male	Antenatal Care (1 visit) Coverage (proportion)			X			X
Urogenital congenital anomalies	Global	Male	Antenatal Care (1 visit) Coverage (proportion)			X			X
Urogenital congenital anomalies	Data Rich	Male	Maternal Education (years per capita)			X			X
Urogenital congenital anomalies	Global	Male	Maternal Education (years per capita)			X			X
Urogenital congenital anomalies	Data Rich	Male	In-Facility Delivery (proportion)		X			X	
Urogenital congenital anomalies	Global	Male	In-Facility Delivery (proportion)		X			X	

Comparison of GBD 2019 and GBD 2021 covariates and level of covariates used in cause of death modeling									
**Cause**	**Model Version Type**	**Sex**	**Covariate**	**Level 1 GBD 2019**	**Level 2 GBD 2019**	**Level 3 GBD 2019**	**Level 1 GBD 2021**	**Level 2 GBD 2021**	**Level 3 GBD 2021**
Urogenital congenital anomalies	Data Rich	Male	Socio-demographic Index		X			X	
Urogenital congenital anomalies	Global	Male	Socio-demographic Index		X			X	
Urogenital congenital anomalies	Data Rich	Male	Healthcare access and quality index		X			X	
Urogenital congenital anomalies	Global	Male	Healthcare access and quality index		X			X	
Urogenital congenital anomalies	Data Rich	Male	Diabetes Age-Standardized Prevalence (proportion)		X			X	
Urogenital congenital anomalies	Global	Male	Diabetes Age-Standardized Prevalence (proportion)		X			X	
Urogenital congenital anomalies	Data Rich	Male	Age-standardized SEV for Ambient particulate matter		X			X	
Urogenital congenital anomalies	Global	Male	Age-standardized SEV for Ambient particulate matter		X			X	
Urogenital congenital anomalies	Data Rich	Male	Age-standardized SEV for Household air pollution		X			X	
Urogenital congenital anomalies	Global	Male	Age-standardized SEV for Household air pollution		X			X	
Urogenital congenital anomalies	Data Rich	Male	Age-standardized SEV for Smoking	X			X		
Urogenital congenital anomalies	Global	Male	Age-standardized SEV for Smoking	X			X		
Urogenital congenital anomalies	Data Rich	Male	Maternal alcohol consumption during pregnancy (proportion)	X			X		
Urogenital congenital anomalies	Global	Male	Maternal alcohol consumption during pregnancy (proportion)	X			X		
Digestive congenital anomalies	Global	Female	LDI (I$ per capita)			X			X
Digestive congenital anomalies	Data Rich	Female	LDI (I$ per capita)			X			X
Digestive congenital anomalies	Global	Female	Liters of alcohol consumed per capita			X			X
Digestive congenital anomalies	Data Rich	Female	Liters of alcohol consumed per capita			X			X
Digestive congenital anomalies	Global	Female	Maternal care and immunization			X			X
Digestive congenital anomalies	Data Rich	Female	Maternal care and immunization			X			X
Digestive congenital anomalies	Global	Female	Antenatal Care (4 visits) Coverage (proportion)			X			X
Digestive congenital anomalies	Data Rich	Female	Antenatal Care (4 visits) Coverage (proportion)			X			X
Digestive congenital anomalies	Global	Female	Antenatal Care (1 visit) Coverage (proportion)			X			X
Digestive congenital anomalies	Data Rich	Female	Antenatal Care (1 visit) Coverage (proportion)			X			X
Digestive congenital anomalies	Global	Female	Maternal Education (years per capita)			X			X
Digestive congenital anomalies	Data Rich	Female	Maternal Education (years per capita)			X			X
Digestive congenital anomalies	Global	Female	Age-standardized SEV for Low fruit			X			X
Digestive congenital anomalies	Data Rich	Female	Age-standardized SEV for Low fruit			X			X
Digestive congenital anomalies	Global	Female	Age-standardized SEV for Low vegetables			X			X
Digestive congenital anomalies	Data Rich	Female	Age-standardized SEV for Low vegetables			X			X
Digestive congenital anomalies	Global	Female	In-Facility Delivery (proportion)		X			X	
Digestive congenital anomalies	Data Rich	Female	In-Facility Delivery (proportion)		X			X	
Digestive congenital anomalies	Global	Female	Socio-demographic Index		X			X	
Digestive congenital anomalies	Data Rich	Female	Socio-demographic Index		X			X	
Digestive congenital anomalies	Global	Female	Healthcare access and quality index		X			X	
Digestive congenital anomalies	Data Rich	Female	Healthcare access and quality index		X			X	
Digestive congenital anomalies	Global	Female	Age-standardized SEV for Household air pollution		X			X	
Digestive congenital anomalies	Data Rich	Female	Age-standardized SEV for Household air pollution		X			X	
Digestive congenital anomalies	Global	Female	Age-standardized SEV for High fasting plasma glucose		X			X	
Digestive congenital anomalies	Data Rich	Female	Age-standardized SEV for High fasting plasma glucose		X			X	
Digestive congenital anomalies	Global	Female	Age-standardized SEV for High body-mass index		X			X	
Digestive congenital anomalies	Data Rich	Female	Age-standardized SEV for High body-mass index		X			X	
Digestive congenital anomalies	Global	Female	Age-standardized SEV for Smoking	X			X		
Digestive congenital anomalies	Data Rich	Female	Age-standardized SEV for Smoking	X			X		
Digestive congenital anomalies	Global	Female	Maternal alcohol consumption during pregnancy (proportion)	X			X		
Digestive congenital anomalies	Data Rich	Female	Maternal alcohol consumption during pregnancy (proportion)	X			X		
Digestive congenital anomalies	Global	Male	LDI (I$ per capita)			X			X
Digestive congenital anomalies	Data Rich	Male	LDI (I$ per capita)			X			X
Digestive congenital anomalies	Global	Male	Liters of alcohol consumed per capita			X			X
Digestive congenital anomalies	Data Rich	Male	Liters of alcohol consumed per capita			X			X
Digestive congenital anomalies	Global	Male	Maternal care and immunization			X			X
Digestive congenital anomalies	Data Rich	Male	Maternal care and immunization			X			X
Digestive congenital anomalies	Global	Male	Antenatal Care (4 visits) Coverage (proportion)			X			X
Digestive congenital anomalies	Data Rich	Male	Antenatal Care (4 visits) Coverage (proportion)			X			X
Digestive congenital anomalies	Global	Male	Antenatal Care (1 visit) Coverage (proportion)			X			X
Digestive congenital anomalies	Data Rich	Male	Antenatal Care (1 visit) Coverage (proportion)			X			X
Digestive congenital anomalies	Global	Male	Maternal Education (years per capita)			X			X
Digestive congenital anomalies	Data Rich	Male	Maternal Education (years per capita)			X			X
Digestive congenital anomalies	Global	Male	Age-standardized SEV for Low fruit			X			X
Digestive congenital anomalies	Data Rich	Male	Age-standardized SEV for Low fruit			X			X
Digestive congenital anomalies	Global	Male	Age-standardized SEV for Low vegetables			X			X
Digestive congenital anomalies	Data Rich	Male	Age-standardized SEV for Low vegetables			X			X
Digestive congenital anomalies	Global	Male	In-Facility Delivery (proportion)		X			X	
Digestive congenital anomalies	Data Rich	Male	In-Facility Delivery (proportion)		X			X	
Digestive congenital anomalies	Global	Male	Socio-demographic Index		X			X	
Digestive congenital anomalies	Data Rich	Male	Socio-demographic Index		X			X	
Digestive congenital anomalies	Global	Male	Healthcare access and quality index		X			X	
Digestive congenital anomalies	Data Rich	Male	Healthcare access and quality index		X			X	
Digestive congenital anomalies	Global	Male	Age-standardized SEV for Household air pollution		X			X	
Digestive congenital anomalies	Data Rich	Male	Age-standardized SEV for Household air pollution		X			X	
Digestive congenital anomalies	Global	Male	Age-standardized SEV for High fasting plasma glucose		X			X	
Digestive congenital anomalies	Global	Male	Age-standardized SEV for High body-mass index		X			X	
Digestive congenital anomalies	Global	Male	Age-standardized SEV for Smoking	X			X		
Digestive congenital anomalies	Data Rich	Male	Age-standardized SEV for Smoking	X			X		
Digestive congenital anomalies	Global	Male	Maternal alcohol consumption during pregnancy (proportion)	X			X		
Digestive congenital anomalies	Data Rich	Male	Maternal alcohol consumption during pregnancy (proportion)	X			X		
Digestive congenital anomalies	Data Rich	Male	Age-standardized SEV for High fasting plasma glucose					X	
Digestive congenital anomalies	Data Rich	Male	Age-standardized SEV for High body-mass index					X	
Other congenital anomalies	Global	Female	LDI (I$ per capita)			X			X
Other congenital anomalies	Data Rich	Female	LDI (I$ per capita)			X			X
Other congenital anomalies	Global	Female	Socio-demographic Index			X			X
Other congenital anomalies	Data Rich	Female	Socio-demographic Index			X			X
Other congenital anomalies	Global	Female	Liters of alcohol consumed per capita			X			X
Other congenital anomalies	Data Rich	Female	Liters of alcohol consumed per capita			X			X
Other congenital anomalies	Global	Female	Antenatal Care (4 visits) Coverage (proportion)			X			X
Other congenital anomalies	Data Rich	Female	Antenatal Care (4 visits) Coverage (proportion)			X			X
Other congenital anomalies	Global	Female	Antenatal Care (1 visit) Coverage (proportion)			X			X
Other congenital anomalies	Data Rich	Female	Antenatal Care (1 visit) Coverage (proportion)			X			X
Other congenital anomalies	Global	Female	Maternal Education (years per capita)			X			X
Other congenital anomalies	Data Rich	Female	Maternal Education (years per capita)			X			X
Other congenital anomalies	Global	Female	Age-standardized SEV for High fasting plasma glucose			X			X
Other congenital anomalies	Data Rich	Female	Age-standardized SEV for High fasting plasma glucose			X			X
Other congenital anomalies	Global	Female	In-Facility Delivery (proportion)		X			X	
Other congenital anomalies	Data Rich	Female	In-Facility Delivery (proportion)		X			X	
Other congenital anomalies	Global	Female	Healthcare access and quality index		X			X	
Other congenital anomalies	Data Rich	Female	Healthcare access and quality index		X			X	
Other congenital anomalies	Global	Female	Age-standardized SEV for Household air pollution		X			X	
Other congenital anomalies	Data Rich	Female	Age-standardized SEV for Household air pollution		X			X	
Other congenital anomalies	Global	Female	Age-standardized SEV for Smoking		X			X	
Other congenital anomalies	Data Rich	Female	Age-standardized SEV for Smoking		X			X	
Other congenital anomalies	Global	Female	Legality of Abortion		X			X	
Other congenital anomalies	Data Rich	Female	Legality of Abortion		X			X	
Other congenital anomalies	Global	Female	Live Births 35+ (proportion)	X			X		
Other congenital anomalies	Data Rich	Female	Live Births 35+ (proportion)	X			X		
Other congenital anomalies	Global	Female	Maternal alcohol consumption during pregnancy (proportion)	X			X		
Other congenital anomalies	Data Rich	Female	Maternal alcohol consumption during pregnancy (proportion)	X			X		
Other congenital anomalies	Global	Male	LDI (I$ per capita)			X			X
Other congenital anomalies	Data Rich	Male	LDI (I$ per capita)			X			X
Other congenital anomalies	Global	Male	Socio-demographic Index			X			X
Other congenital anomalies	Data Rich	Male	Socio-demographic Index			X			X
Other congenital anomalies	Global	Male	Liters of alcohol consumed per capita			X			X
Other congenital anomalies	Data Rich	Male	Liters of alcohol consumed per capita			X			X
Other congenital anomalies	Global	Male	Antenatal Care (4 visits) Coverage (proportion)			X			X
Other congenital anomalies	Data Rich	Male	Antenatal Care (4 visits) Coverage (proportion)			X			X
Other congenital anomalies	Global	Male	Antenatal Care (1 visit) Coverage (proportion)			X			X
Other congenital anomalies	Data Rich	Male	Antenatal Care (1 visit) Coverage (proportion)			X			X
Other congenital anomalies	Global	Male	Maternal Education (years per capita)			X			X
Other congenital anomalies	Data Rich	Male	Maternal Education (years per capita)			X			X
Other congenital anomalies	Global	Male	Age-standardized SEV for High fasting plasma glucose			X			X

Comparison of GBD 2019 and GBD 2021 covariates and level of covariates used in cause of death modeling

Cause	Model Version Type	Sex	Covariate	Level 1 GBD 2019	Level 2 GBD 2019	Level 3 GBD 2019	Level 1 GBD 2021	Level 2 GBD 2021	Level 3 GBD 2021
Other congenital anomalies	Data Rich	Male	Age-standardized SEV for High fasting plasma glucose			X			X
Other congenital anomalies	Global	Male	In-Facility Delivery (proportion)		X			X	
Other congenital anomalies	Data Rich	Male	In-Facility Delivery (proportion)		X			X	
Other congenital anomalies	Global	Male	Healthcare access and quality index		X			X	
Other congenital anomalies	Data Rich	Male	Healthcare access and quality index		X			X	
Other congenital anomalies	Global	Male	Age-standardized SEV for Household air pollution		X			X	
Other congenital anomalies	Data Rich	Male	Age-standardized SEV for Household air pollution		X			X	
Other congenital anomalies	Data Rich	Male	Age-standardized SEV for Smoking		X			X	
Other congenital anomalies	Global	Male	Legality of Abortion		X			X	
Other congenital anomalies	Data Rich	Male	Legality of Abortion		X			X	
Other congenital anomalies	Global	Male	Live Births 35+ (proportion)	X			X		
Other congenital anomalies	Data Rich	Male	Live Births 35+ (proportion)	X			X		
Other congenital anomalies	Global	Male	Maternal alcohol consumption during pregnancy (proportion)	X			X		
Other congenital anomalies	Data Rich	Male	Maternal alcohol consumption during pregnancy (proportion)	X			X		
Other congenital anomalies	Global	Male	Age-standardized SEV for Smoking					X	
Urinary diseases and male infertility	Global	Female	Education (years per capita)			X			X
Urinary diseases and male infertility	Data Rich	Female	Education (years per capita)			X			X
Urinary diseases and male infertility	Global	Female	LDI (I$ per capita)			X			X
Urinary diseases and male infertility	Data Rich	Female	LDI (I$ per capita)			X			X
Urinary diseases and male infertility	Global	Female	Socio-demographic Index			X			X
Urinary diseases and male infertility	Data Rich	Female	Socio-demographic Index			X			X
Urinary diseases and male infertility	Global	Female	Healthcare access and quality index		X			X	
Urinary diseases and male infertility	Data Rich	Female	Healthcare access and quality index		X			X	
Urinary diseases and male infertility	Global	Female	Mean BMI		X			X	
Urinary diseases and male infertility	Data Rich	Female	Mean BMI		X			X	
Urinary diseases and male infertility	Global	Female	Sanitation (proportion with access)		X			X	
Urinary diseases and male infertility	Data Rich	Female	Sanitation (proportion with access)		X			X	
Urinary diseases and male infertility	Global	Female	90th percentile climatic temperature in the given country-year.		X			X	
Urinary diseases and male infertility	Data Rich	Female	90th percentile climatic temperature in the given country-year.		X			X	
Urinary diseases and male infertility	Global	Male	Education (years per capita)			X			X
Urinary diseases and male infertility	Data Rich	Male	Education (years per capita)			X			X
Urinary diseases and male infertility	Global	Male	LDI (I$ per capita)			X			X
Urinary diseases and male infertility	Data Rich	Male	LDI (I$ per capita)			X			X
Urinary diseases and male infertility	Global	Male	Socio-demographic Index			X			X
Urinary diseases and male infertility	Data Rich	Male	Socio-demographic Index			X			X
Urinary diseases and male infertility	Global	Male	Healthcare access and quality index		X			X	
Urinary diseases and male infertility	Data Rich	Male	Healthcare access and quality index		X			X	
Urinary diseases and male infertility	Global	Male	Mean BMI		X			X	
Urinary diseases and male infertility	Data Rich	Male	Mean BMI		X			X	
Urinary diseases and male infertility	Global	Male	Sanitation (proportion with access)		X			X	
Urinary diseases and male infertility	Data Rich	Male	Sanitation (proportion with access)		X			X	
Urinary diseases and male infertility	Global	Male	90th percentile climatic temperature in the given country-year.		X			X	
Urinary diseases and male infertility	Data Rich	Male	90th percentile climatic temperature in the given country-year.		X			X	
Urinary tract infections and interstitial nephritis	Data Rich	Female	Socio-demographic Index			X			X
Urinary tract infections and interstitial nephritis	Global	Female	Socio-demographic Index			X			X
Urinary tract infections and interstitial nephritis	Data Rich	Female	Education (years per capita)		X			X	
Urinary tract infections and interstitial nephritis	Global	Female	Education (years per capita)		X			X	
Urinary tract infections and interstitial nephritis	Data Rich	Female	LDI (I$ per capita)		X			X	
Urinary tract infections and interstitial nephritis	Global	Female	LDI (I$ per capita)		X			X	
Urinary tract infections and interstitial nephritis	Data Rich	Female	Healthcare access and quality index		X			X	
Urinary tract infections and interstitial nephritis	Global	Female	Healthcare access and quality index		X			X	
Urinary tract infections and interstitial nephritis	Data Rich	Female	Sanitation (proportion with access)	X			X		
Urinary tract infections and interstitial nephritis	Global	Female	Sanitation (proportion with access)	X			X		
Urinary tract infections and interstitial nephritis	Data Rich	Male	Socio-demographic Index			X			X
Urinary tract infections and interstitial nephritis	Global	Male	Socio-demographic Index			X			X
Urinary tract infections and interstitial nephritis	Data Rich	Male	Education (years per capita)		X			X	
Urinary tract infections and interstitial nephritis	Global	Male	Education (years per capita)		X			X	
Urinary tract infections and interstitial nephritis	Data Rich	Male	LDI (I$ per capita)		X			X	
Urinary tract infections and interstitial nephritis	Global	Male	LDI (I$ per capita)		X			X	
Urinary tract infections and interstitial nephritis	Data Rich	Male	Healthcare access and quality index		X			X	
Urinary tract infections and interstitial nephritis	Global	Male	Healthcare access and quality index		X			X	
Urinary tract infections and interstitial nephritis	Data Rich	Male	Sanitation (proportion with access)	X			X		
Urinary tract infections and interstitial nephritis	Global	Male	Sanitation (proportion with access)	X			X		
Urolithiasis	Data Rich	Female	Education (years per capita)			X			X
Urolithiasis	Global	Female	Education (years per capita)			X			X
Urolithiasis	Data Rich	Female	LDI (I$ per capita)			X			X
Urolithiasis	Global	Female	LDI (I$ per capita)			X			X
Urolithiasis	Data Rich	Female	Socio-demographic Index			X			X
Urolithiasis	Global	Female	Socio-demographic Index			X			X
Urolithiasis	Data Rich	Female	Healthcare access and quality index		X			X	
Urolithiasis	Global	Female	Healthcare access and quality index		X			X	
Urolithiasis	Data Rich	Female	fruits unadjusted(g)		X			X	
Urolithiasis	Global	Female	fruits unadjusted(g)		X			X	
Urolithiasis	Data Rich	Female	vegetables unadjusted(g)		X			X	
Urolithiasis	Global	Female	vegetables unadjusted(g)		X			X	
Urolithiasis	Data Rich	Female	90th percentile climatic temperature in the given country-year.	X			X		
Urolithiasis	Global	Female	90th percentile climatic temperature in the given country-year.	X			X		
Urolithiasis	Data Rich	Female	red meats unadjusted(g)	X			X		
Urolithiasis	Global	Female	red meats unadjusted(g)	X			X		
Urolithiasis	Data Rich	Male	Education (years per capita)			X			X
Urolithiasis	Global	Male	Education (years per capita)			X			X
Urolithiasis	Data Rich	Male	LDI (I$ per capita)			X			X
Urolithiasis	Global	Male	LDI (I$ per capita)			X			X
Urolithiasis	Data Rich	Male	Socio-demographic Index			X			X
Urolithiasis	Global	Male	Socio-demographic Index			X			X
Urolithiasis	Data Rich	Male	Healthcare access and quality index		X			X	
Urolithiasis	Global	Male	Healthcare access and quality index		X			X	
Urolithiasis	Data Rich	Male	fruits unadjusted(g)		X			X	
Urolithiasis	Data Rich	Male	vegetables unadjusted(g)		X			X	
Urolithiasis	Global	Male	Age- and sex-specific SEV for Low fruit		X			X	
Urolithiasis	Global	Male	Age- and sex-specific SEV for Low vegetables		X			X	
Urolithiasis	Data Rich	Male	90th percentile climatic temperature in the given country-year.	X			X		
Urolithiasis	Global	Male	90th percentile climatic temperature in the given country-year.	X			X		
Urolithiasis	Global	Male	Age- and sex-specific SEV for High red meat	X			X		
Urolithiasis	Data Rich	Male	red meats unadjusted(g)	X			X		
Other urinary diseases	Data Rich	Female	Socio-demographic Index			X			X
Other urinary diseases	Global	Female	Socio-demographic Index			X			X
Other urinary diseases	Data Rich	Female	Education (years per capita)		X			X	
Other urinary diseases	Global	Female	Education (years per capita)		X			X	
Other urinary diseases	Data Rich	Female	LDI (I$ per capita)		X			X	
Other urinary diseases	Global	Female	LDI (I$ per capita)		X			X	
Other urinary diseases	Data Rich	Female	Healthcare access and quality index		X			X	
Other urinary diseases	Global	Female	Healthcare access and quality index		X			X	
Other urinary diseases	Data Rich	Female	Mean BMI	X			X		
Other urinary diseases	Global	Female	Mean BMI	X			X		
Other urinary diseases	Data Rich	Male	Socio-demographic Index			X			X
Other urinary diseases	Global	Male	Socio-demographic Index			X			X
Other urinary diseases	Data Rich	Male	Education (years per capita)		X			X	
Other urinary diseases	Global	Male	Education (years per capita)		X			X	
Other urinary diseases	Data Rich	Male	LDI (I$ per capita)		X			X	
Other urinary diseases	Global	Male	LDI (I$ per capita)		X			X	
Other urinary diseases	Data Rich	Male	Healthcare access and quality index		X			X	
Other urinary diseases	Global	Male	Healthcare access and quality index		X			X	
Other urinary diseases	Data Rich	Male	Mean BMI	X			X		
Other urinary diseases	Global	Male	Mean BMI	X			X		
Gynecological diseases	Data Rich	Female	Education (years per capita)			X			X
Gynecological diseases	Global	Female	Education (years per capita)			X			X
Gynecological diseases	Data Rich	Female	LDI (I$ per capita)			X			X
Gynecological diseases	Global	Female	LDI (I$ per capita)			X			X
Gynecological diseases	Data Rich	Female	Socio-demographic Index			X			X
Gynecological diseases	Global	Female	Socio-demographic Index			X			X
Gynecological diseases	Data Rich	Female	Skilled Birth Attendance (proportion)		X			X	
Gynecological diseases	Global	Female	Skilled Birth Attendance (proportion)		X			X	
Gynecological diseases	Data Rich	Female	Healthcare access and quality index		X			X	
Gynecological diseases	Global	Female	Healthcare access and quality index		X			X	
Gynecological diseases	Data Rich	Female	Total Fertility Rate		X			X	
Gynecological diseases	Global	Female	Total Fertility Rate		X			X	
Gynecological diseases	Data Rich	Female	Maternal care and immunization		X			X	
Gynecological diseases	Global	Female	Maternal care and immunization		X			X	
Gynecological diseases	Data Rich	Female	Live Births 35+ (proportion)		X			X	
Gynecological diseases	Global	Female	Live Births 35+ (proportion)		X			X	
Gynecological diseases	Data Rich	Female	Age- and sex-specific SEV for Smoking	X			X		
Gynecological diseases	Global	Female	Age- and sex-specific SEV for Smoking	X			X		

Comparison of GBD 2019 and GBD 2021 covariates and level of covariates used in cause of death modeling

Cause	Model Version Type	Sex	Covariate	Level 1 GBD 2019	Level 2 GBD 2019	Level 3 GBD 2019	Level 1 GBD 2021	Level 2 GBD 2021	Level 3 GBD 2021
Uterine fibroids	Data Rich	Female	Education (years per capita)			X			X
Uterine fibroids	Global	Female	Education (years per capita)			X			X
Uterine fibroids	Data Rich	Female	LDI (I$ per capita)			X			X
Uterine fibroids	Global	Female	LDI (I$ per capita)			X			X
Uterine fibroids	Data Rich	Female	Socio-demographic Index			X			X
Uterine fibroids	Global	Female	Socio-demographic Index			X			X
Uterine fibroids	Data Rich	Female	Skilled Birth Attendance (proportion)		X			X	
Uterine fibroids	Global	Female	Skilled Birth Attendance (proportion)		X			X	
Uterine fibroids	Data Rich	Female	Healthcare access and quality index		X			X	
Uterine fibroids	Global	Female	Healthcare access and quality index		X			X	
Uterine fibroids	Data Rich	Female	Total Fertility Rate		X			X	
Uterine fibroids	Global	Female	Total Fertility Rate		X			X	
Uterine fibroids	Data Rich	Female	Maternal care and immunization		X			X	
Uterine fibroids	Global	Female	Maternal care and immunization		X			X	
Uterine fibroids	Data Rich	Female	Live Births 35+ (proportion)		X			X	
Uterine fibroids	Global	Female	Live Births 35+ (proportion)		X			X	
Uterine fibroids	Data Rich	Female	Age- and sex-specific SEV for Smoking	X			X		
Uterine fibroids	Global	Female	Age- and sex-specific SEV for Smoking	X			X		
Endometriosis	Global	Female	Education (years per capita)			X			X
Endometriosis	Data Rich	Female	Education (years per capita)			X			X
Endometriosis	Global	Female	LDI (I$ per capita)			X			X
Endometriosis	Data Rich	Female	LDI (I$ per capita)			X			X
Endometriosis	Global	Female	Socio-demographic Index			X			X
Endometriosis	Data Rich	Female	Socio-demographic Index			X			X
Endometriosis	Global	Female	Skilled Birth Attendance (proportion)		X			X	
Endometriosis	Data Rich	Female	Skilled Birth Attendance (proportion)		X			X	
Endometriosis	Global	Female	Healthcare access and quality index		X			X	
Endometriosis	Data Rich	Female	Healthcare access and quality index		X			X	
Endometriosis	Global	Female	Total Fertility Rate		X			X	
Endometriosis	Data Rich	Female	Total Fertility Rate		X			X	
Endometriosis	Global	Female	Maternal care and immunization		X			X	
Endometriosis	Data Rich	Female	Maternal care and immunization		X			X	
Endometriosis	Global	Female	Live Births 35+ (proportion)		X			X	
Endometriosis	Data Rich	Female	Live Births 35+ (proportion)		X			X	
Endometriosis	Global	Female	Age- and sex-specific SEV for Smoking	X			X		
Endometriosis	Data Rich	Female	Age- and sex-specific SEV for Smoking	X			X		
Genital prolapse	Data Rich	Female	Education (years per capita)			X			X
Genital prolapse	Global	Female	Education (years per capita)			X			X
Genital prolapse	Data Rich	Female	LDI (I$ per capita)			X			X
Genital prolapse	Global	Female	LDI (I$ per capita)			X			X
Genital prolapse	Data Rich	Female	Socio-demographic Index			X			X
Genital prolapse	Global	Female	Socio-demographic Index			X			X
Genital prolapse	Data Rich	Female	Skilled Birth Attendance (proportion)		X			X	
Genital prolapse	Global	Female	Skilled Birth Attendance (proportion)		X			X	
Genital prolapse	Data Rich	Female	Healthcare access and quality index		X			X	
Genital prolapse	Global	Female	Healthcare access and quality index		X			X	
Genital prolapse	Data Rich	Female	Total Fertility Rate		X			X	
Genital prolapse	Global	Female	Total Fertility Rate		X			X	
Genital prolapse	Data Rich	Female	Maternal care and immunization		X			X	
Genital prolapse	Global	Female	Maternal care and immunization		X			X	
Genital prolapse	Data Rich	Female	Live Births 35+ (proportion)		X			X	
Genital prolapse	Global	Female	Live Births 35+ (proportion)		X			X	
Genital prolapse	Data Rich	Female	Age- and sex-specific SEV for Smoking	X			X		
Genital prolapse	Global	Female	Age- and sex-specific SEV for Smoking	X			X		
Other gynecological diseases	Data Rich	Female	Education (years per capita)			X			X
Other gynecological diseases	Global	Female	Education (years per capita)			X			X
Other gynecological diseases	Data Rich	Female	LDI (I$ per capita)			X			X
Other gynecological diseases	Global	Female	LDI (I$ per capita)			X			X
Other gynecological diseases	Data Rich	Female	Socio-demographic Index			X			X
Other gynecological diseases	Global	Female	Socio-demographic Index			X			X
Other gynecological diseases	Data Rich	Female	Skilled Birth Attendance (proportion)		X			X	
Other gynecological diseases	Global	Female	Skilled Birth Attendance (proportion)		X			X	
Other gynecological diseases	Data Rich	Female	Healthcare access and quality index		X			X	
Other gynecological diseases	Global	Female	Healthcare access and quality index		X			X	
Other gynecological diseases	Data Rich	Female	Total Fertility Rate		X			X	
Other gynecological diseases	Global	Female	Total Fertility Rate		X			X	
Other gynecological diseases	Data Rich	Female	Maternal care and immunization		X			X	
Other gynecological diseases	Global	Female	Maternal care and immunization		X			X	
Other gynecological diseases	Data Rich	Female	Live Births 35+ (proportion)		X			X	
Other gynecological diseases	Global	Female	Live Births 35+ (proportion)		X			X	
Other gynecological diseases	Data Rich	Female	Age- and sex-specific SEV for Smoking	X			X		
Other gynecological diseases	Global	Female	Age- and sex-specific SEV for Smoking	X			X		
Hemoglobinopathies and hemolytic anaemias	Data Rich	Female	Education (years per capita)			X			X
Hemoglobinopathies and hemolytic anaemias	Global	Female	Education (years per capita)			X			X
Hemoglobinopathies and hemolytic anaemias	Data Rich	Female	LDI (I$ per capita)			X			X
Hemoglobinopathies and hemolytic anaemias	Global	Female	LDI (I$ per capita)			X			X
Hemoglobinopathies and hemolytic anaemias	Data Rich	Female	Socio-demographic Index			X			X
Hemoglobinopathies and hemolytic anaemias	Global	Female	Socio-demographic Index			X			X
Hemoglobinopathies and hemolytic anaemias	Data Rich	Female	Latitude Under 15 (proportion)			X			X
Hemoglobinopathies and hemolytic anaemias	Global	Female	Latitude Under 15 (proportion)			X			X
Hemoglobinopathies and hemolytic anaemias	Data Rich	Female	Latitude 15 to 30 (proportion)			X			X
Hemoglobinopathies and hemolytic anaemias	Global	Female	Latitude 15 to 30 (proportion)			X			X
Hemoglobinopathies and hemolytic anaemias	Data Rich	Female	Latitude 30 to 45 (proportion)			X			X
Hemoglobinopathies and hemolytic anaemias	Global	Female	Latitude 30 to 45 (proportion)			X			X
Hemoglobinopathies and hemolytic anaemias	Data Rich	Female	Latitude Over 45 (proportion)			X			X
Hemoglobinopathies and hemolytic anaemias	Global	Female	Latitude Over 45 (proportion)			X			X
Hemoglobinopathies and hemolytic anaemias	Data Rich	Female	Healthcare access and quality index		X			X	
Hemoglobinopathies and hemolytic anaemias	Global	Female	Healthcare access and quality index		X			X	
Hemoglobinopathies and hemolytic anaemias	Data Rich	Female	Maternal care and immunization		X			X	
Hemoglobinopathies and hemolytic anaemias	Global	Female	Maternal care and immunization		X			X	
Hemoglobinopathies and hemolytic anaemias	Data Rich	Female	Malaria Lysenko PFPR 1 (Holoendemic)	X			X		
Hemoglobinopathies and hemolytic anaemias	Global	Female	Malaria Lysenko PFPR 1 (Holoendemic)	X			X		
Hemoglobinopathies and hemolytic anaemias	Data Rich	Female	Hemoglobinopathies Prevalence x Excess Mortality	X			X		
Hemoglobinopathies and hemolytic anaemias	Data Rich	Female	Hemoglobinopathies Prevalence x Excess Mortality (excluding G6PD deficiency)	X			X		
Hemoglobinopathies and hemolytic anaemias	Data Rich	Female	Hemoglobin C (sickle type C) trait	X			X		
Hemoglobinopathies and hemolytic anaemias	Global	Female	Hemoglobin C (sickle type C) trait	X			X		
Hemoglobinopathies and hemolytic anaemias	Data Rich	Female	Hemoglobin S (sickle type S) trait	X			X		
Hemoglobinopathies and hemolytic anaemias	Global	Female	Hemoglobin S (sickle type S) trait	X			X		
Hemoglobinopathies and hemolytic anaemias	Data Rich	Male	Education (years per capita)			X			X
Hemoglobinopathies and hemolytic anaemias	Global	Male	Education (years per capita)			X			X
Hemoglobinopathies and hemolytic anaemias	Data Rich	Male	LDI (I$ per capita)			X			X
Hemoglobinopathies and hemolytic anaemias	Global	Male	LDI (I$ per capita)			X			X
Hemoglobinopathies and hemolytic anaemias	Data Rich	Male	Socio-demographic Index			X			X
Hemoglobinopathies and hemolytic anaemias	Global	Male	Socio-demographic Index			X			X
Hemoglobinopathies and hemolytic anaemias	Data Rich	Male	Latitude Under 15 (proportion)			X			X
Hemoglobinopathies and hemolytic anaemias	Global	Male	Latitude Under 15 (proportion)			X			X
Hemoglobinopathies and hemolytic anaemias	Data Rich	Male	Latitude 15 to 30 (proportion)			X			X
Hemoglobinopathies and hemolytic anaemias	Global	Male	Latitude 15 to 30 (proportion)			X			X
Hemoglobinopathies and hemolytic anaemias	Data Rich	Male	Latitude 30 to 45 (proportion)			X			X
Hemoglobinopathies and hemolytic anaemias	Global	Male	Latitude 30 to 45 (proportion)			X			X
Hemoglobinopathies and hemolytic anaemias	Data Rich	Male	Latitude Over 45 (proportion)			X			X
Hemoglobinopathies and hemolytic anaemias	Global	Male	Latitude Over 45 (proportion)			X			X
Hemoglobinopathies and hemolytic anaemias	Data Rich	Male	Healthcare access and quality index		X			X	
Hemoglobinopathies and hemolytic anaemias	Global	Male	Healthcare access and quality index		X			X	
Hemoglobinopathies and hemolytic anaemias	Data Rich	Male	Maternal care and immunization		X			X	
Hemoglobinopathies and hemolytic anaemias	Global	Male	Maternal care and immunization		X			X	
Hemoglobinopathies and hemolytic anaemias	Data Rich	Male	Malaria Lysenko PFPR 1 (Holoendemic)	X			X		
Hemoglobinopathies and hemolytic anaemias	Global	Male	Malaria Lysenko PFPR 1 (Holoendemic)	X			X		
Hemoglobinopathies and hemolytic anaemias	Data Rich	Male	Hemoglobinopathies Prevalence x Excess Mortality	X			X		
Hemoglobinopathies and hemolytic anaemias	Data Rich	Male	Hemoglobinopathies Prevalence x Excess Mortality (excluding G6PD deficiency)	X			X		
Hemoglobinopathies and hemolytic anaemias	Data Rich	Male	Hemoglobin C (sickle type C) trait	X			X		
Hemoglobinopathies and hemolytic anaemias	Global	Male	Hemoglobin C (sickle type C) trait	X			X		
Hemoglobinopathies and hemolytic anaemias	Data Rich	Male	Hemoglobin S (sickle type S) trait	X			X		
Hemoglobinopathies and hemolytic anaemias	Global	Male	Hemoglobin S (sickle type S) trait	X			X		
Endocrine, metabolic, blood, and immune disorders	Data Rich	Female	Education (years per capita)			X			X
Endocrine, metabolic, blood, and immune disorders	Global	Female	Education (years per capita)			X			X
Endocrine, metabolic, blood, and immune disorders	Data Rich	Female	LDI (I$ per capita)			X			X
Endocrine, metabolic, blood, and immune disorders	Global	Female	LDI (I$ per capita)			X			X
Endocrine, metabolic, blood, and immune disorders	Data Rich	Female	Socio-demographic Index			X			X
Endocrine, metabolic, blood, and immune disorders	Global	Female	Socio-demographic Index			X			X
Endocrine, metabolic, blood, and immune disorders	Data Rich	Female	Healthcare access and quality index		X			X	
Endocrine, metabolic, blood, and immune disorders	Global	Female	Healthcare access and quality index		X			X	
Endocrine, metabolic, blood, and immune disorders	Data Rich	Female	Low-Density Lipoprotein (mmol/L)		X			X	
Endocrine, metabolic, blood, and immune disorders	Global	Female	Low-Density Lipoprotein (mmol/L)		X			X	
Endocrine, metabolic, blood, and immune disorders	Data Rich	Female	Liters of alcohol consumed per capita		X			X	
Endocrine, metabolic, blood, and immune disorders	Global	Female	Liters of alcohol consumed per capita		X			X	
Endocrine, metabolic, blood, and immune disorders	Data Rich	Female	Mean BMI	X			X		
Endocrine, metabolic, blood, and immune disorders	Global	Female	Mean BMI	X			X		

Comparison of GBD 2019 and GBD 2021 covariates and level of covariates used in cause of death modeling									
**Cause**	**Model Version Type**	**Sex**	**Covariate**	**Level 1 GBD 2019**	**Level 2 GBD 2019**	**Level 3 GBD 2019**	**Level 1 GBD 2021**	**Level 2 GBD 2021**	**Level 3 GBD 2021**
Endocrine, metabolic, blood, and immune disorders	Data Rich	Male	Education (years per capita)			X			X
Endocrine, metabolic, blood, and immune disorders	Global	Male	Education (years per capita)			X			X
Endocrine, metabolic, blood, and immune disorders	Data Rich	Male	LDI (I$ per capita)			X			X
Endocrine, metabolic, blood, and immune disorders	Global	Male	LDI (I$ per capita)			X			X
Endocrine, metabolic, blood, and immune disorders	Data Rich	Male	Socio-demographic Index			X			X
Endocrine, metabolic, blood, and immune disorders	Global	Male	Socio-demographic Index			X			X
Endocrine, metabolic, blood, and immune disorders	Data Rich	Male	Healthcare access and quality index		X			X	
Endocrine, metabolic, blood, and immune disorders	Global	Male	Healthcare access and quality index		X			X	
Endocrine, metabolic, blood, and immune disorders	Data Rich	Male	Low-Density Lipoprotein (mmol/L)		X			X	
Endocrine, metabolic, blood, and immune disorders	Global	Male	Low-Density Lipoprotein (mmol/L)		X			X	
Endocrine, metabolic, blood, and immune disorders	Data Rich	Male	Liters of alcohol consumed per capita		X			X	
Endocrine, metabolic, blood, and immune disorders	Global	Male	Liters of alcohol consumed per capita		X			X	
Endocrine, metabolic, blood, and immune disorders	Data Rich	Male	Mean BMI	X			X		
Endocrine, metabolic, blood, and immune disorders	Global	Male	Mean BMI	X			X		
Sudden infant death syndrome	Data Rich	Female	Education (years per capita)			X			X
Sudden infant death syndrome	Global	Female	Education (years per capita)			X			X
Sudden infant death syndrome	Data Rich	Female	LDI (I$ per capita)			X			X
Sudden infant death syndrome	Global	Female	LDI (I$ per capita)			X			X
Sudden infant death syndrome	Data Rich	Female	Socio-demographic Index			X			X
Sudden infant death syndrome	Global	Female	Socio-demographic Index			X			X
Sudden infant death syndrome	Data Rich	Female	Total Fertility Rate			X			X
Sudden infant death syndrome	Global	Female	Total Fertility Rate			X			X
Sudden infant death syndrome	Data Rich	Female	Skilled Birth Attendance (proportion)		X			X	
Sudden infant death syndrome	Global	Female	Skilled Birth Attendance (proportion)		X			X	
Sudden infant death syndrome	Data Rich	Female	Healthcare access and quality index		X			X	
Sudden infant death syndrome	Global	Female	Healthcare access and quality index		X			X	
Sudden infant death syndrome	Data Rich	Female	Maternal care and immunization		X			X	
Sudden infant death syndrome	Global	Female	Maternal care and immunization		X			X	
Sudden infant death syndrome	Data Rich	Female	In-Facility Delivery (proportion)	X			X		
Sudden infant death syndrome	Global	Female	In-Facility Delivery (proportion)	X			X		
Sudden infant death syndrome	Data Rich	Male	Education (years per capita)			X			X
Sudden infant death syndrome	Global	Male	Education (years per capita)			X			X
Sudden infant death syndrome	Data Rich	Male	LDI (I$ per capita)			X			X
Sudden infant death syndrome	Global	Male	LDI (I$ per capita)			X			X
Sudden infant death syndrome	Data Rich	Male	Socio-demographic Index			X			X
Sudden infant death syndrome	Global	Male	Socio-demographic Index			X			X
Sudden infant death syndrome	Data Rich	Male	Total Fertility Rate			X			X
Sudden infant death syndrome	Global	Male	Total Fertility Rate			X			X
Sudden infant death syndrome	Data Rich	Male	Skilled Birth Attendance (proportion)		X			X	
Sudden infant death syndrome	Global	Male	Skilled Birth Attendance (proportion)		X			X	
Sudden infant death syndrome	Data Rich	Male	Healthcare access and quality index		X			X	
Sudden infant death syndrome	Global	Male	Healthcare access and quality index		X			X	
Sudden infant death syndrome	Data Rich	Male	Maternal care and immunization		X			X	
Sudden infant death syndrome	Global	Male	Maternal care and immunization		X			X	
Sudden infant death syndrome	Data Rich	Male	In-Facility Delivery (proportion)	X			X		
Sudden infant death syndrome	Global	Male	In-Facility Delivery (proportion)	X			X		
Transport injuries	Global	Female	Rainfall Quintile 5 (proportion)			X			X
Transport injuries	Data Rich	Female	Rainfall Quintile 5 (proportion)			X			X
Transport injuries	Global	Female	Education (years per capita)		X			X	
Transport injuries	Data Rich	Female	Education (years per capita)		X			X	
Transport injuries	Global	Female	LDI (I$ per capita)		X			X	
Transport injuries	Data Rich	Female	LDI (I$ per capita)		X			X	
Transport injuries	Global	Female	Socio-demographic Index		X			X	
Transport injuries	Data Rich	Female	Socio-demographic Index		X			X	
Transport injuries	Global	Female	Healthcare access and quality index		X			X	
Transport injuries	Data Rich	Female	Healthcare access and quality index		X			X	
Transport injuries	Global	Female	Population-weighted mean temperature		X			X	
Transport injuries	Data Rich	Female	Population-weighted mean temperature		X			X	
Transport injuries	Global	Female	Population Density (300-500 ppl/sqkm, proportion)		X			X	
Transport injuries	Data Rich	Female	Population Density (300-500 ppl/sqkm, proportion)		X			X	
Transport injuries	Global	Female	Population Density (500-1000 ppl/sqkm, proportion)		X			X	
Transport injuries	Data Rich	Female	Population Density (500-1000 ppl/sqkm, proportion)		X			X	
Transport injuries	Global	Female	Population 15 to 30 (proportion)		X			X	
Transport injuries	Data Rich	Female	Population 15 to 30 (proportion)		X			X	
Transport injuries	Global	Female	Liters of alcohol consumed per capita	X			X		
Transport injuries	Data Rich	Female	Liters of alcohol consumed per capita	X			X		
Transport injuries	Global	Female	Vehicles - 2 wheels fraction (proportion)	X			X		
Transport injuries	Data Rich	Female	Vehicles - 2 wheels fraction (proportion)	X			X		
Transport injuries	Global	Female	BAC law general population (quartile)	X			X		
Transport injuries	Data Rich	Female	BAC law general population (quartile)	X			X		
Transport injuries	Global	Female	BAC law youth drivers (quartile)	X			X		
Transport injuries	Data Rich	Female	BAC law youth drivers (quartile)	X			X		
Transport injuries	Global	Female	BAC law professional drivers (quartile)	X			X		
Transport injuries	Data Rich	Female	BAC law professional drivers (quartile)	X			X		
Transport injuries	Global	Female	Speed limit law urban (quartile)	X			X		
Transport injuries	Data Rich	Female	Speed limit law urban (quartile)	X			X		
Transport injuries	Global	Female	Speed limit law rural (quartile)	X			X		
Transport injuries	Data Rich	Female	Speed limit law rural (quartile)	X			X		
Transport injuries	Global	Female	Vehicles - 2+4 wheels (per capita)	X			X		
Transport injuries	Data Rich	Female	Vehicles - 2+4 wheels (per capita)	X			X		
Transport injuries	Global	Male	Rainfall Quintile 5 (proportion)			X			X
Transport injuries	Data Rich	Male	Rainfall Quintile 5 (proportion)			X			X
Transport injuries	Global	Male	Education (years per capita)		X			X	
Transport injuries	Data Rich	Male	Education (years per capita)		X			X	
Transport injuries	Global	Male	LDI (I$ per capita)		X			X	
Transport injuries	Data Rich	Male	LDI (I$ per capita)		X			X	
Transport injuries	Global	Male	Socio-demographic Index		X			X	
Transport injuries	Data Rich	Male	Socio-demographic Index		X			X	
Transport injuries	Global	Male	Healthcare access and quality index		X			X	
Transport injuries	Data Rich	Male	Healthcare access and quality index		X			X	
Transport injuries	Global	Male	Population-weighted mean temperature		X			X	
Transport injuries	Data Rich	Male	Population-weighted mean temperature		X			X	
Transport injuries	Global	Male	Population Density (300-500 ppl/sqkm, proportion)		X			X	
Transport injuries	Data Rich	Male	Population Density (300-500 ppl/sqkm, proportion)		X			X	
Transport injuries	Global	Male	Population Density (500-1000 ppl/sqkm, proportion)		X			X	
Transport injuries	Data Rich	Male	Population Density (500-1000 ppl/sqkm, proportion)		X			X	
Transport injuries	Global	Male	Population 15 to 30 (proportion)		X			X	
Transport injuries	Data Rich	Male	Population 15 to 30 (proportion)		X			X	
Transport injuries	Global	Male	Liters of alcohol consumed per capita	X			X		
Transport injuries	Data Rich	Male	Liters of alcohol consumed per capita	X			X		
Transport injuries	Global	Male	Vehicles - 2 wheels fraction (proportion)	X			X		
Transport injuries	Data Rich	Male	Vehicles - 2 wheels fraction (proportion)	X			X		
Transport injuries	Global	Male	BAC law general population (quartile)	X			X		
Transport injuries	Data Rich	Male	BAC law general population (quartile)	X			X		
Transport injuries	Global	Male	BAC law youth drivers (quartile)	X			X		
Transport injuries	Data Rich	Male	BAC law youth drivers (quartile)	X			X		
Transport injuries	Global	Male	BAC law professional drivers (quartile)	X			X		
Transport injuries	Data Rich	Male	BAC law professional drivers (quartile)	X			X		
Transport injuries	Global	Male	Speed limit law urban (quartile)	X			X		
Transport injuries	Data Rich	Male	Speed limit law urban (quartile)	X			X		
Transport injuries	Global	Male	Speed limit law rural (quartile)	X			X		
Transport injuries	Data Rich	Male	Speed limit law rural (quartile)	X			X		
Transport injuries	Global	Male	Vehicles - 2+4 wheels (per capita)	X			X		
Transport injuries	Data Rich	Male	Vehicles - 2+4 wheels (per capita)	X			X		
Road injuries	Global	Female	Education (years per capita)			X			X
Road injuries	Global	Female	LDI (I$ per capita)			X			X
Road injuries	Global	Female	Socio-demographic Index			X			X
Road injuries	Data Rich	Female	Rainfall Quintile 5 (proportion)			X			X
Road injuries	Global	Female	Rainfall Quintile 5 (proportion)			X			X
Road injuries	Data Rich	Female	Education (years per capita)		X			X	
Road injuries	Data Rich	Female	LDI (I$ per capita)		X			X	
Road injuries	Data Rich	Female	Socio-demographic Index		X			X	
Road injuries	Data Rich	Female	Healthcare access and quality index		X			X	
Road injuries	Global	Female	Healthcare access and quality index		X			X	
Road injuries	Data Rich	Female	Population-weighted mean temperature		X			X	
Road injuries	Global	Female	Population-weighted mean temperature		X			X	
Road injuries	Data Rich	Female	Population Density (300-500 ppl/sqkm, proportion)		X			X	
Road injuries	Global	Female	Population Density (300-500 ppl/sqkm, proportion)		X			X	
Road injuries	Data Rich	Female	Population Density (500-1000 ppl/sqkm, proportion)		X			X	
Road injuries	Global	Female	Population Density (500-1000 ppl/sqkm, proportion)		X			X	
Road injuries	Data Rich	Female	Population 15 to 30 (proportion)		X			X	
Road injuries	Global	Female	Population 15 to 30 (proportion)		X			X	
Road injuries	Data Rich	Female	Liters of alcohol consumed per capita	X			X		
Road injuries	Global	Female	Liters of alcohol consumed per capita	X			X		
Road injuries	Data Rich	Female	Vehicles - 2 wheels fraction (proportion)	X			X		

Comparison of GBD 2019 and GBD 2021 covariates and level of covariates used in cause of death modeling

Cause	Model Version Type	Sex	Covariate	Level 1 GBD 2019	Level 2 GBD 2019	Level 3 GBD 2019	Level 1 GBD 2021	Level 2 GBD 2021	Level 3 GBD 2021
Road injuries	Global	Female	Vehicles - 2 wheels fraction (proportion)	X			X		
Road injuries	Data Rich	Female	BAC law general population (quartile)	X			X		
Road injuries	Global	Female	BAC law general population (quartile)	X			X		
Road injuries	Data Rich	Female	BAC law youth drivers (quartile)	X			X		
Road injuries	Global	Female	BAC law youth drivers (quartile)	X			X		
Road injuries	Data Rich	Female	BAC law professional drivers (quartile)	X			X		
Road injuries	Global	Female	BAC law professional drivers (quartile)	X			X		
Road injuries	Data Rich	Female	Speed limit law urban (quartile)	X			X		
Road injuries	Global	Female	Speed limit law urban (quartile)	X			X		
Road injuries	Data Rich	Female	Speed limit law rural (quartile)	X			X		
Road injuries	Global	Female	Speed limit law rural (quartile)	X			X		
Road injuries	Data Rich	Female	Vehicles - 2+4 wheels (per capita)	X			X		
Road injuries	Global	Female	Vehicles - 2+4 wheels (per capita)	X			X		
Road injuries	Data Rich	Female	Vehicles - 2 wheels (per capita)	X			X		
Road injuries	Global	Female	Vehicles - 2 wheels (per capita)	X			X		
Road injuries	Data Rich	Female	Vehicles - 4 wheels (per capita)	X			X		
Road injuries	Global	Female	Vehicles - 4 wheels (per capita)	X			X		
Road injuries	Data Rich	Female	Log-transformed SEV scalar: Road Inj	X			X		
Road injuries	Global	Female	Log-transformed SEV scalar: Road Inj	X			X		
Road injuries	Global	Male	Education (years per capita)			X			X
Road injuries	Global	Male	LDI (I$ per capita)			X			X
Road injuries	Global	Male	Socio-demographic Index			X			X
Road injuries	Data Rich	Male	Rainfall Quintile 5 (proportion)			X			X
Road injuries	Data Rich	Male	Education (years per capita)		X			X	
Road injuries	Data Rich	Male	LDI (I$ per capita)		X			X	
Road injuries	Data Rich	Male	Socio-demographic Index		X			X	
Road injuries	Global	Male	Healthcare access and quality index		X			X	
Road injuries	Data Rich	Male	Healthcare access and quality index		X			X	
Road injuries	Global	Male	Population-weighted mean temperature		X			X	
Road injuries	Data Rich	Male	Population-weighted mean temperature		X			X	
Road injuries	Global	Male	Population Density (300-500 ppl/sqkm, proportion)		X			X	
Road injuries	Data Rich	Male	Population Density (300-500 ppl/sqkm, proportion)		X			X	
Road injuries	Global	Male	Population Density (500-1000 ppl/sqkm, proportion)		X			X	
Road injuries	Data Rich	Male	Population Density (500-1000 ppl/sqkm, proportion)		X			X	
Road injuries	Global	Male	Population 15 to 30 (proportion)		X			X	
Road injuries	Data Rich	Male	Population 15 to 30 (proportion)		X			X	
Road injuries	Global	Male	BAC law general population (quartile)		X			X	
Road injuries	Global	Male	BAC law youth drivers (quartile)		X			X	
Road injuries	Global	Male	BAC law professional drivers (quartile)		X			X	
Road injuries	Global	Male	Speed limit law urban (quartile)		X			X	
Road injuries	Global	Male	Speed limit law rural (quartile)		X			X	
Road injuries	Data Rich	Male	BAC law general population (quartile)		X		X		
Road injuries	Data Rich	Male	BAC law youth drivers (quartile)		X		X		
Road injuries	Data Rich	Male	BAC law professional drivers (quartile)		X		X		
Road injuries	Data Rich	Male	Speed limit law urban (quartile)		X		X		
Road injuries	Data Rich	Male	Speed limit law rural (quartile)		X		X		
Road injuries	Global	Male	Liters of alcohol consumed per capita	X			X		
Road injuries	Data Rich	Male	Liters of alcohol consumed per capita	X			X		
Road injuries	Global	Male	Vehicles - 2 wheels fraction (proportion)	X			X		
Road injuries	Data Rich	Male	Vehicles - 2 wheels fraction (proportion)	X			X		
Road injuries	Global	Male	Vehicles - 2+4 wheels (per capita)	X			X		
Road injuries	Data Rich	Male	Vehicles - 2+4 wheels (per capita)	X			X		
Road injuries	Global	Male	Vehicles - 2 wheels (per capita)	X			X		
Road injuries	Data Rich	Male	Vehicles - 2 wheels (per capita)	X			X		
Road injuries	Global	Male	Vehicles - 4 wheels (per capita)	X			X		
Road injuries	Data Rich	Male	Vehicles - 4 wheels (per capita)	X			X		
Road injuries	Global	Male	Log-transformed SEV scalar: Road Inj	X			X		
Road injuries	Data Rich	Male	Log-transformed SEV scalar: Road Inj	X			X		
Road injuries	Global	Male	Rainfall Quintile 5 (proportion)						X
Pedestrian road injuries	Data Rich	Female	Rainfall Quintile 5 (proportion)			X			X
Pedestrian road injuries	Global	Female	Rainfall Quintile 5 (proportion)			X			X
Pedestrian road injuries	Data Rich	Female	Education (years per capita)		X			X	
Pedestrian road injuries	Global	Female	Education (years per capita)		X			X	
Pedestrian road injuries	Data Rich	Female	LDI (I$ per capita)		X			X	
Pedestrian road injuries	Global	Female	LDI (I$ per capita)		X			X	
Pedestrian road injuries	Data Rich	Female	Socio-demographic Index		X			X	
Pedestrian road injuries	Global	Female	Socio-demographic Index		X			X	
Pedestrian road injuries	Data Rich	Female	Healthcare access and quality index		X			X	
Pedestrian road injuries	Global	Female	Healthcare access and quality index		X			X	
Pedestrian road injuries	Data Rich	Female	Population-weighted mean temperature		X			X	
Pedestrian road injuries	Global	Female	Population-weighted mean temperature		X			X	
Pedestrian road injuries	Data Rich	Female	Population Density (300-500 ppl/sqkm, proportion)		X			X	
Pedestrian road injuries	Global	Female	Population Density (300-500 ppl/sqkm, proportion)		X			X	
Pedestrian road injuries	Data Rich	Female	Population Density (500-1000 ppl/sqkm, proportion)		X			X	
Pedestrian road injuries	Global	Female	Population Density (500-1000 ppl/sqkm, proportion)		X			X	
Pedestrian road injuries	Data Rich	Female	Population 15 to 30 (proportion)		X			X	
Pedestrian road injuries	Global	Female	Population 15 to 30 (proportion)		X			X	
Pedestrian road injuries	Data Rich	Female	Liters of alcohol consumed per capita	X			X		
Pedestrian road injuries	Global	Female	Liters of alcohol consumed per capita	X			X		
Pedestrian road injuries	Data Rich	Female	Vehicles - 2 wheels fraction (proportion)	X			X		
Pedestrian road injuries	Global	Female	Vehicles - 2 wheels fraction (proportion)	X			X		
Pedestrian road injuries	Data Rich	Female	BAC law general population (quartile)	X			X		
Pedestrian road injuries	Global	Female	BAC law general population (quartile)	X			X		
Pedestrian road injuries	Data Rich	Female	BAC law youth drivers (quartile)	X			X		
Pedestrian road injuries	Global	Female	BAC law youth drivers (quartile)	X			X		
Pedestrian road injuries	Data Rich	Female	BAC law professional drivers (quartile)	X			X		
Pedestrian road injuries	Global	Female	BAC law professional drivers (quartile)	X			X		
Pedestrian road injuries	Data Rich	Female	Speed limit law urban (quartile)	X			X		
Pedestrian road injuries	Global	Female	Speed limit law urban (quartile)	X			X		
Pedestrian road injuries	Data Rich	Female	Speed limit law rural (quartile)	X			X		
Pedestrian road injuries	Global	Female	Speed limit law rural (quartile)	X			X		
Pedestrian road injuries	Data Rich	Female	Vehicles - 2+4 wheels (per capita)	X			X		
Pedestrian road injuries	Global	Female	Vehicles - 2+4 wheels (per capita)	X			X		
Pedestrian road injuries	Data Rich	Female	Log-transformed SEV scalar: Pedest	X			X		
Pedestrian road injuries	Global	Female	Log-transformed SEV scalar: Pedest	X			X		
Pedestrian road injuries	Global	Male	Rainfall Quintile 5 (proportion)			X			X
Pedestrian road injuries	Data Rich	Male	Rainfall Quintile 5 (proportion)			X			X
Pedestrian road injuries	Global	Male	Education (years per capita)		X			X	
Pedestrian road injuries	Data Rich	Male	Education (years per capita)		X			X	
Pedestrian road injuries	Global	Male	LDI (I$ per capita)		X			X	
Pedestrian road injuries	Data Rich	Male	LDI (I$ per capita)		X			X	
Pedestrian road injuries	Global	Male	Socio-demographic Index		X			X	
Pedestrian road injuries	Data Rich	Male	Socio-demographic Index		X			X	
Pedestrian road injuries	Global	Male	Healthcare access and quality index		X			X	
Pedestrian road injuries	Data Rich	Male	Healthcare access and quality index		X			X	
Pedestrian road injuries	Global	Male	Population-weighted mean temperature		X			X	
Pedestrian road injuries	Global	Male	Population Density (300-500 ppl/sqkm, proportion)		X			X	
Pedestrian road injuries	Data Rich	Male	Population Density (300-500 ppl/sqkm, proportion)		X			X	
Pedestrian road injuries	Global	Male	Population Density (500-1000 ppl/sqkm, proportion)		X			X	
Pedestrian road injuries	Data Rich	Male	Population Density (500-1000 ppl/sqkm, proportion)		X			X	
Pedestrian road injuries	Global	Male	Population 15 to 30 (proportion)		X			X	
Pedestrian road injuries	Data Rich	Male	Population 15 to 30 (proportion)		X			X	
Pedestrian road injuries	Global	Male	Liters of alcohol consumed per capita	X			X		
Pedestrian road injuries	Data Rich	Male	Liters of alcohol consumed per capita	X			X		
Pedestrian road injuries	Data Rich	Male	Population-weighted mean temperature	X			X		
Pedestrian road injuries	Global	Male	Vehicles - 2 wheels fraction (proportion)	X			X		
Pedestrian road injuries	Data Rich	Male	Vehicles - 2 wheels fraction (proportion)	X			X		
Pedestrian road injuries	Global	Male	BAC law general population (quartile)	X			X		
Pedestrian road injuries	Data Rich	Male	BAC law general population (quartile)	X			X		
Pedestrian road injuries	Global	Male	BAC law youth drivers (quartile)	X			X		
Pedestrian road injuries	Data Rich	Male	BAC law youth drivers (quartile)	X			X		
Pedestrian road injuries	Global	Male	BAC law professional drivers (quartile)	X			X		
Pedestrian road injuries	Data Rich	Male	BAC law professional drivers (quartile)	X			X		
Pedestrian road injuries	Global	Male	Speed limit law urban (quartile)	X			X		
Pedestrian road injuries	Data Rich	Male	Speed limit law urban (quartile)	X			X		
Pedestrian road injuries	Global	Male	Speed limit law rural (quartile)	X			X		
Pedestrian road injuries	Data Rich	Male	Speed limit law rural (quartile)	X			X		
Pedestrian road injuries	Global	Male	Vehicles - 2+4 wheels (per capita)	X			X		
Pedestrian road injuries	Data Rich	Male	Vehicles - 2+4 wheels (per capita)	X			X		
Pedestrian road injuries	Global	Male	Log-transformed SEV scalar: Pedest	X			X		
Pedestrian road injuries	Data Rich	Male	Log-transformed SEV scalar: Pedest	X			X		
Cyclist road injuries	Data Rich	Female	Rainfall Quintile 5 (proportion)			X			X
Cyclist road injuries	Global	Female	Rainfall Quintile 5 (proportion)			X			X
Cyclist road injuries	Data Rich	Female	Education (years per capita)		X			X	
Cyclist road injuries	Global	Female	Education (years per capita)		X			X	

Comparison of GBD 2019 and GBD 2021 covariates and level of covariates used in cause of death modeling

Cause	Model Version Type	Sex	Covariate	Level 1 GBD 2019	Level 2 GBD 2019	Level 3 GBD 2019	Level 1 GBD 2021	Level 2 GBD 2021	Level 3 GBD 2021
Cyclist road injuries	Data Rich	Female	LDI (I$ per capita)		X			X	
Cyclist road injuries	Global	Female	LDI (I$ per capita)		X			X	
Cyclist road injuries	Data Rich	Female	Socio-demographic Index		X			X	
Cyclist road injuries	Global	Female	Socio-demographic Index		X			X	
Cyclist road injuries	Data Rich	Female	Healthcare access and quality index		X			X	
Cyclist road injuries	Global	Female	Healthcare access and quality index		X			X	
Cyclist road injuries	Data Rich	Female	Population-weighted mean temperature		X			X	
Cyclist road injuries	Global	Female	Population-weighted mean temperature		X			X	
Cyclist road injuries	Data Rich	Female	Population Density (300-500 ppl/sqkm, proportion)		X			X	
Cyclist road injuries	Global	Female	Population Density (300-500 ppl/sqkm, proportion)		X			X	
Cyclist road injuries	Data Rich	Female	Population Density (500-1000 ppl/sqkm, proportion)		X			X	
Cyclist road injuries	Global	Female	Population Density (500-1000 ppl/sqkm, proportion)		X			X	
Cyclist road injuries	Data Rich	Female	Population 15 to 30 (proportion)		X			X	
Cyclist road injuries	Global	Female	Population 15 to 30 (proportion)		X			X	
Cyclist road injuries	Data Rich	Female	Liters of alcohol consumed per capita	X			X		
Cyclist road injuries	Global	Female	Liters of alcohol consumed per capita	X			X		
Cyclist road injuries	Data Rich	Female	Vehicles - 2 wheels fraction (proportion)	X			X		
Cyclist road injuries	Global	Female	Vehicles - 2 wheels fraction (proportion)	X			X		
Cyclist road injuries	Data Rich	Female	BAC law general population (quartile)	X			X		
Cyclist road injuries	Global	Female	BAC law general population (quartile)	X			X		
Cyclist road injuries	Data Rich	Female	BAC law youth drivers (quartile)	X			X		
Cyclist road injuries	Global	Female	BAC law youth drivers (quartile)	X			X		
Cyclist road injuries	Data Rich	Female	BAC law professional drivers (quartile)	X			X		
Cyclist road injuries	Global	Female	BAC law professional drivers (quartile)	X			X		
Cyclist road injuries	Data Rich	Female	Speed limit law urban (quartile)	X			X		
Cyclist road injuries	Global	Female	Speed limit law urban (quartile)	X			X		
Cyclist road injuries	Data Rich	Female	Speed limit law rural (quartile)	X			X		
Cyclist road injuries	Global	Female	Speed limit law rural (quartile)	X			X		
Cyclist road injuries	Data Rich	Female	Vehicles - 2+4 wheels (per capita)	X			X		
Cyclist road injuries	Global	Female	Vehicles - 2+4 wheels (per capita)	X			X		
Cyclist road injuries	Data Rich	Female	Log-transformed SEV scalar: Cyclist	X			X		
Cyclist road injuries	Global	Female	Log-transformed SEV scalar: Cyclist	X			X		
Cyclist road injuries	Data Rich	Male	Rainfall Quintile 5 (proportion)			X			X
Cyclist road injuries	Global	Male	Rainfall Quintile 5 (proportion)			X			X
Cyclist road injuries	Data Rich	Male	Education (years per capita)		X			X	
Cyclist road injuries	Global	Male	Education (years per capita)		X			X	
Cyclist road injuries	Data Rich	Male	LDI (I$ per capita)		X			X	
Cyclist road injuries	Global	Male	LDI (I$ per capita)		X			X	
Cyclist road injuries	Data Rich	Male	Socio-demographic Index		X			X	
Cyclist road injuries	Global	Male	Socio-demographic Index		X			X	
Cyclist road injuries	Data Rich	Male	Healthcare access and quality index		X			X	
Cyclist road injuries	Global	Male	Healthcare access and quality index		X			X	
Cyclist road injuries	Data Rich	Male	Population-weighted mean temperature		X			X	
Cyclist road injuries	Global	Male	Population-weighted mean temperature		X			X	
Cyclist road injuries	Data Rich	Male	Population Density (300-500 ppl/sqkm, proportion)		X			X	
Cyclist road injuries	Global	Male	Population Density (300-500 ppl/sqkm, proportion)		X			X	
Cyclist road injuries	Data Rich	Male	Population Density (500-1000 ppl/sqkm, proportion)		X			X	
Cyclist road injuries	Global	Male	Population Density (500-1000 ppl/sqkm, proportion)		X			X	
Cyclist road injuries	Data Rich	Male	Population 15 to 30 (proportion)		X			X	
Cyclist road injuries	Global	Male	Population 15 to 30 (proportion)		X			X	
Cyclist road injuries	Data Rich	Male	Liters of alcohol consumed per capita	X			X		
Cyclist road injuries	Global	Male	Liters of alcohol consumed per capita	X			X		
Cyclist road injuries	Data Rich	Male	Vehicles - 2 wheels fraction (proportion)	X			X		
Cyclist road injuries	Global	Male	Vehicles - 2 wheels fraction (proportion)	X			X		
Cyclist road injuries	Data Rich	Male	BAC law general population (quartile)	X			X		
Cyclist road injuries	Global	Male	BAC law general population (quartile)	X			X		
Cyclist road injuries	Data Rich	Male	BAC law youth drivers (quartile)	X			X		
Cyclist road injuries	Global	Male	BAC law youth drivers (quartile)	X			X		
Cyclist road injuries	Data Rich	Male	BAC law professional drivers (quartile)	X			X		
Cyclist road injuries	Global	Male	BAC law professional drivers (quartile)	X			X		
Cyclist road injuries	Data Rich	Male	Speed limit law urban (quartile)	X			X		
Cyclist road injuries	Global	Male	Speed limit law urban (quartile)	X			X		
Cyclist road injuries	Data Rich	Male	Speed limit law rural (quartile)	X			X		
Cyclist road injuries	Global	Male	Speed limit law rural (quartile)	X			X		
Cyclist road injuries	Data Rich	Male	Vehicles - 2+4 wheels (per capita)	X			X		
Cyclist road injuries	Global	Male	Vehicles - 2+4 wheels (per capita)	X			X		
Cyclist road injuries	Data Rich	Male	Log-transformed SEV scalar: Cyclist	X			X		
Cyclist road injuries	Global	Male	Log-transformed SEV scalar: Cyclist	X			X		
Motorcyclist road injuries	Data Rich	Female	Rainfall Quintile 5 (proportion)			X			X
Motorcyclist road injuries	Global	Female	Rainfall Quintile 5 (proportion)			X			X
Motorcyclist road injuries	Data Rich	Female	Education (years per capita)		X			X	
Motorcyclist road injuries	Global	Female	Education (years per capita)		X			X	
Motorcyclist road injuries	Data Rich	Female	LDI (I$ per capita)		X			X	
Motorcyclist road injuries	Global	Female	LDI (I$ per capita)		X			X	
Motorcyclist road injuries	Data Rich	Female	Socio-demographic Index		X			X	
Motorcyclist road injuries	Global	Female	Socio-demographic Index		X			X	
Motorcyclist road injuries	Data Rich	Female	Healthcare access and quality index		X			X	
Motorcyclist road injuries	Global	Female	Healthcare access and quality index		X			X	
Motorcyclist road injuries	Data Rich	Female	Population-weighted mean temperature		X			X	
Motorcyclist road injuries	Global	Female	Population-weighted mean temperature		X			X	
Motorcyclist road injuries	Data Rich	Female	Population Density (300-500 ppl/sqkm, proportion)		X			X	
Motorcyclist road injuries	Global	Female	Population Density (300-500 ppl/sqkm, proportion)		X			X	
Motorcyclist road injuries	Data Rich	Female	Population Density (500-1000 ppl/sqkm, proportion)		X			X	
Motorcyclist road injuries	Global	Female	Population Density (500-1000 ppl/sqkm, proportion)		X			X	
Motorcyclist road injuries	Data Rich	Female	Population 15 to 30 (proportion)		X			X	
Motorcyclist road injuries	Global	Female	Population 15 to 30 (proportion)		X			X	
Motorcyclist road injuries	Data Rich	Female	Liters of alcohol consumed per capita	X			X		
Motorcyclist road injuries	Global	Female	Liters of alcohol consumed per capita	X			X		
Motorcyclist road injuries	Data Rich	Female	Vehicles - 2 wheels fraction (proportion)	X			X		
Motorcyclist road injuries	Global	Female	Vehicles - 2 wheels fraction (proportion)	X			X		
Motorcyclist road injuries	Data Rich	Female	Log-transformed SEV scalar: Mot Cyc	X			X		
Motorcyclist road injuries	Global	Female	Log-transformed SEV scalar: Mot Cyc	X			X		
Motorcyclist road injuries	Data Rich	Female	BAC law general population (quartile)	X			X		
Motorcyclist road injuries	Global	Female	BAC law general population (quartile)	X			X		
Motorcyclist road injuries	Data Rich	Female	BAC law youth drivers (quartile)	X			X		
Motorcyclist road injuries	Global	Female	BAC law youth drivers (quartile)	X			X		
Motorcyclist road injuries	Data Rich	Female	BAC law professional drivers (quartile)	X			X		
Motorcyclist road injuries	Global	Female	BAC law professional drivers (quartile)	X			X		
Motorcyclist road injuries	Data Rich	Female	Speed limit law urban (quartile)	X			X		
Motorcyclist road injuries	Global	Female	Speed limit law urban (quartile)	X			X		
Motorcyclist road injuries	Data Rich	Female	Speed limit law rural (quartile)	X			X		
Motorcyclist road injuries	Global	Female	Speed limit law rural (quartile)	X			X		
Motorcyclist road injuries	Data Rich	Male	Rainfall Quintile 5 (proportion)			X			X
Motorcyclist road injuries	Global	Male	Rainfall Quintile 5 (proportion)			X			X
Motorcyclist road injuries	Data Rich	Male	Education (years per capita)		X			X	
Motorcyclist road injuries	Global	Male	Education (years per capita)		X			X	
Motorcyclist road injuries	Data Rich	Male	LDI (I$ per capita)		X			X	
Motorcyclist road injuries	Global	Male	LDI (I$ per capita)		X			X	
Motorcyclist road injuries	Data Rich	Male	Socio-demographic Index		X			X	
Motorcyclist road injuries	Global	Male	Socio-demographic Index		X			X	
Motorcyclist road injuries	Data Rich	Male	Healthcare access and quality index		X			X	
Motorcyclist road injuries	Global	Male	Healthcare access and quality index		X			X	
Motorcyclist road injuries	Data Rich	Male	Population-weighted mean temperature		X			X	
Motorcyclist road injuries	Global	Male	Population-weighted mean temperature		X			X	
Motorcyclist road injuries	Data Rich	Male	Population Density (300-500 ppl/sqkm, proportion)		X			X	
Motorcyclist road injuries	Global	Male	Population Density (300-500 ppl/sqkm, proportion)		X			X	
Motorcyclist road injuries	Data Rich	Male	Population Density (500-1000 ppl/sqkm, proportion)		X			X	
Motorcyclist road injuries	Global	Male	Population Density (500-1000 ppl/sqkm, proportion)		X			X	
Motorcyclist road injuries	Data Rich	Male	Population 15 to 30 (proportion)		X			X	
Motorcyclist road injuries	Global	Male	Population 15 to 30 (proportion)		X			X	
Motorcyclist road injuries	Data Rich	Male	Liters of alcohol consumed per capita	X			X		
Motorcyclist road injuries	Global	Male	Liters of alcohol consumed per capita	X			X		
Motorcyclist road injuries	Data Rich	Male	Vehicles - 2 wheels fraction (proportion)	X			X		
Motorcyclist road injuries	Global	Male	Vehicles - 2 wheels fraction (proportion)	X			X		
Motorcyclist road injuries	Data Rich	Male	Log-transformed SEV scalar: Mot Cyc	X			X		
Motorcyclist road injuries	Global	Male	Log-transformed SEV scalar: Mot Cyc	X			X		
Motorcyclist road injuries	Data Rich	Male	BAC law general population (quartile)	X			X		
Motorcyclist road injuries	Global	Male	BAC law general population (quartile)	X			X		
Motorcyclist road injuries	Data Rich	Male	BAC law youth drivers (quartile)	X			X		
Motorcyclist road injuries	Global	Male	BAC law youth drivers (quartile)	X			X		
Motorcyclist road injuries	Data Rich	Male	BAC law professional drivers (quartile)	X			X		
Motorcyclist road injuries	Global	Male	BAC law professional drivers (quartile)	X			X		
Motorcyclist road injuries	Data Rich	Male	Speed limit law urban (quartile)	X			X		

Comparison of GBD 2019 and GBD 2021 covariates and level of covariates used in cause of death modeling

Cause	Model Version Type	Sex	Covariate	Level 1 GBD 2019	Level 2 GBD 2019	Level 3 GBD 2019	Level 1 GBD 2021	Level 2 GBD 2021	Level 3 GBD 2021
Motorcyclist road injuries	Global	Male	Speed limit law urban (quartile)	X			X		
Motorcyclist road injuries	Data Rich	Male	Speed limit law rural (quartile)	X			X		
Motorcyclist road injuries	Global	Male	Speed limit law rural (quartile)	X			X		
Motor vehicle road injuries	Data Rich	Female	Rainfall Quintile 5 (proportion)			X			X
Motor vehicle road injuries	Global	Female	Rainfall Quintile 5 (proportion)			X			X
Motor vehicle road injuries	Data Rich	Female	Education (years per capita)		X			X	
Motor vehicle road injuries	Global	Female	Education (years per capita)		X			X	
Motor vehicle road injuries	Data Rich	Female	LDI (I$ per capita)		X			X	
Motor vehicle road injuries	Global	Female	LDI (I$ per capita)		X			X	
Motor vehicle road injuries	Data Rich	Female	Socio-demographic Index		X			X	
Motor vehicle road injuries	Global	Female	Socio-demographic Index		X			X	
Motor vehicle road injuries	Data Rich	Female	Healthcare access and quality index		X			X	
Motor vehicle road injuries	Global	Female	Healthcare access and quality index		X			X	
Motor vehicle road injuries	Data Rich	Female	Population-weighted mean temperature		X			X	
Motor vehicle road injuries	Global	Female	Population-weighted mean temperature		X			X	
Motor vehicle road injuries	Data Rich	Female	Population Density (300-500 ppl/sqkm, proportion)		X			X	
Motor vehicle road injuries	Global	Female	Population Density (300-500 ppl/sqkm, proportion)		X			X	
Motor vehicle road injuries	Data Rich	Female	Population Density (500-1000 ppl/sqkm, proportion)		X			X	
Motor vehicle road injuries	Global	Female	Population Density (500-1000 ppl/sqkm, proportion)		X			X	
Motor vehicle road injuries	Data Rich	Female	Population 15 to 30 (proportion)		X			X	
Motor vehicle road injuries	Global	Female	Population 15 to 30 (proportion)		X			X	
Motor vehicle road injuries	Data Rich	Female	Liters of alcohol consumed per capita	X			X		
Motor vehicle road injuries	Global	Female	Liters of alcohol consumed per capita	X			X		
Motor vehicle road injuries	Data Rich	Female	BAC law general population (quartile)	X			X		
Motor vehicle road injuries	Global	Female	BAC law general population (quartile)	X			X		
Motor vehicle road injuries	Data Rich	Female	BAC law youth drivers (quartile)	X			X		
Motor vehicle road injuries	Global	Female	BAC law youth drivers (quartile)	X			X		
Motor vehicle road injuries	Data Rich	Female	BAC law professional drivers (quartile)	X			X		
Motor vehicle road injuries	Global	Female	BAC law professional drivers (quartile)	X			X		
Motor vehicle road injuries	Data Rich	Female	Speed limit law urban (quartile)	X			X		
Motor vehicle road injuries	Global	Female	Speed limit law urban (quartile)	X			X		
Motor vehicle road injuries	Data Rich	Female	Speed limit law rural (quartile)	X			X		
Motor vehicle road injuries	Global	Female	Speed limit law rural (quartile)	X			X		
Motor vehicle road injuries	Data Rich	Female	Vehicles - 4 wheels (per capita)	X			X		
Motor vehicle road injuries	Global	Female	Vehicles - 4 wheels (per capita)	X			X		
Motor vehicle road injuries	Data Rich	Female	Log-transformed SEV scalar: Mot Veh	X			X		
Motor vehicle road injuries	Global	Female	Log-transformed SEV scalar: Mot Veh	X			X		
Motor vehicle road injuries	Data Rich	Male	Rainfall Quintile 5 (proportion)			X			X
Motor vehicle road injuries	Global	Male	Rainfall Quintile 5 (proportion)			X			X
Motor vehicle road injuries	Data Rich	Male	Education (years per capita)		X			X	
Motor vehicle road injuries	Global	Male	Education (years per capita)		X			X	
Motor vehicle road injuries	Data Rich	Male	LDI (I$ per capita)		X			X	
Motor vehicle road injuries	Global	Male	LDI (I$ per capita)		X			X	
Motor vehicle road injuries	Data Rich	Male	Socio-demographic Index		X			X	
Motor vehicle road injuries	Global	Male	Socio-demographic Index		X			X	
Motor vehicle road injuries	Data Rich	Male	Healthcare access and quality index		X			X	
Motor vehicle road injuries	Global	Male	Healthcare access and quality index		X			X	
Motor vehicle road injuries	Data Rich	Male	Population-weighted mean temperature		X			X	
Motor vehicle road injuries	Global	Male	Population-weighted mean temperature		X			X	
Motor vehicle road injuries	Data Rich	Male	Population Density (300-500 ppl/sqkm, proportion)		X			X	
Motor vehicle road injuries	Global	Male	Population Density (300-500 ppl/sqkm, proportion)		X			X	
Motor vehicle road injuries	Data Rich	Male	Population Density (500-1000 ppl/sqkm, proportion)		X			X	
Motor vehicle road injuries	Global	Male	Population Density (500-1000 ppl/sqkm, proportion)		X			X	
Motor vehicle road injuries	Data Rich	Male	Population 15 to 30 (proportion)		X			X	
Motor vehicle road injuries	Global	Male	Population 15 to 30 (proportion)		X			X	
Motor vehicle road injuries	Data Rich	Male	Liters of alcohol consumed per capita	X			X		
Motor vehicle road injuries	Global	Male	Liters of alcohol consumed per capita	X			X		
Motor vehicle road injuries	Data Rich	Male	BAC law general population (quartile)	X			X		
Motor vehicle road injuries	Global	Male	BAC law general population (quartile)	X			X		
Motor vehicle road injuries	Data Rich	Male	BAC law youth drivers (quartile)	X			X		
Motor vehicle road injuries	Global	Male	BAC law youth drivers (quartile)	X			X		
Motor vehicle road injuries	Data Rich	Male	BAC law professional drivers (quartile)	X			X		
Motor vehicle road injuries	Global	Male	BAC law professional drivers (quartile)	X			X		
Motor vehicle road injuries	Data Rich	Male	Speed limit law urban (quartile)	X			X		
Motor vehicle road injuries	Global	Male	Speed limit law urban (quartile)	X			X		
Motor vehicle road injuries	Data Rich	Male	Speed limit law rural (quartile)	X			X		
Motor vehicle road injuries	Global	Male	Speed limit law rural (quartile)	X			X		
Motor vehicle road injuries	Data Rich	Male	Vehicles - 4 wheels (per capita)	X			X		
Motor vehicle road injuries	Global	Male	Vehicles - 4 wheels (per capita)	X			X		
Motor vehicle road injuries	Data Rich	Male	Log-transformed SEV scalar: Mot Veh	X			X		
Motor vehicle road injuries	Global	Male	Log-transformed SEV scalar: Mot Veh	X			X		
Other road injuries	Data Rich	Female	Socio-demographic Index			X			X
Other road injuries	Global	Female	Socio-demographic Index			X			X
Other road injuries	Data Rich	Female	Rainfall Quintile 5 (proportion)			X			X
Other road injuries	Global	Female	Rainfall Quintile 5 (proportion)			X			X
Other road injuries	Data Rich	Female	Education (years per capita)		X			X	
Other road injuries	Global	Female	Education (years per capita)		X			X	
Other road injuries	Data Rich	Female	LDI (I$ per capita)		X			X	
Other road injuries	Global	Female	LDI (I$ per capita)		X			X	
Other road injuries	Data Rich	Female	Healthcare access and quality index		X			X	
Other road injuries	Global	Female	Healthcare access and quality index		X			X	
Other road injuries	Data Rich	Female	Population-weighted mean temperature		X			X	
Other road injuries	Global	Female	Population-weighted mean temperature		X			X	
Other road injuries	Data Rich	Female	Population 15 to 30 (proportion)		X			X	
Other road injuries	Global	Female	Population 15 to 30 (proportion)		X			X	
Other road injuries	Data Rich	Female	Liters of alcohol consumed per capita	X			X		
Other road injuries	Global	Female	Liters of alcohol consumed per capita	X			X		
Other road injuries	Data Rich	Female	Vehicles - 2 wheels fraction (proportion)	X			X		
Other road injuries	Global	Female	Vehicles - 2 wheels fraction (proportion)	X			X		
Other road injuries	Data Rich	Female	BAC law general population (quartile)	X			X		
Other road injuries	Global	Female	BAC law general population (quartile)	X			X		
Other road injuries	Data Rich	Female	BAC law youth drivers (quartile)	X			X		
Other road injuries	Global	Female	BAC law youth drivers (quartile)	X			X		
Other road injuries	Data Rich	Female	BAC law professional drivers (quartile)	X			X		
Other road injuries	Global	Female	BAC law professional drivers (quartile)	X			X		
Other road injuries	Data Rich	Female	Speed limit law urban (quartile)	X			X		
Other road injuries	Global	Female	Speed limit law urban (quartile)	X			X		
Other road injuries	Data Rich	Female	Speed limit law rural (quartile)	X			X		
Other road injuries	Global	Female	Speed limit law rural (quartile)	X			X		
Other road injuries	Data Rich	Female	Vehicles - 2+4 wheels (per capita)	X			X		
Other road injuries	Global	Female	Vehicles - 2+4 wheels (per capita)	X			X		
Other road injuries	Data Rich	Female	Log-transformed SEV scalar: Oth Road	X			X		
Other road injuries	Global	Female	Log-transformed SEV scalar: Oth Road	X			X		
Other road injuries	Data Rich	Male	Socio-demographic Index			X			X
Other road injuries	Data Rich	Male	Rainfall Quintile 5 (proportion)			X			X
Other road injuries	Global	Male	Rainfall Quintile 5 (proportion)			X			X
Other road injuries	Data Rich	Male	Education (years per capita)		X			X	
Other road injuries	Global	Male	Education (years per capita)		X			X	
Other road injuries	Data Rich	Male	LDI (I$ per capita)		X			X	
Other road injuries	Global	Male	LDI (I$ per capita)		X			X	
Other road injuries	Global	Male	Socio-demographic Index		X			X	
Other road injuries	Data Rich	Male	Healthcare access and quality index		X			X	
Other road injuries	Global	Male	Healthcare access and quality index		X			X	
Other road injuries	Data Rich	Male	Population-weighted mean temperature		X			X	
Other road injuries	Global	Male	Population-weighted mean temperature		X			X	
Other road injuries	Data Rich	Male	Population 15 to 30 (proportion)		X			X	
Other road injuries	Global	Male	Population 15 to 30 (proportion)		X			X	
Other road injuries	Data Rich	Male	Liters of alcohol consumed per capita	X			X		
Other road injuries	Global	Male	Liters of alcohol consumed per capita	X			X		
Other road injuries	Data Rich	Male	Vehicles - 2 wheels fraction (proportion)	X			X		
Other road injuries	Global	Male	Vehicles - 2 wheels fraction (proportion)	X			X		
Other road injuries	Data Rich	Male	BAC law general population (quartile)	X			X		
Other road injuries	Global	Male	BAC law general population (quartile)	X			X		
Other road injuries	Data Rich	Male	BAC law youth drivers (quartile)	X			X		
Other road injuries	Global	Male	BAC law youth drivers (quartile)	X			X		
Other road injuries	Data Rich	Male	BAC law professional drivers (quartile)	X			X		
Other road injuries	Global	Male	BAC law professional drivers (quartile)	X			X		
Other road injuries	Data Rich	Male	Speed limit law urban (quartile)	X			X		
Other road injuries	Global	Male	Speed limit law urban (quartile)	X			X		
Other road injuries	Data Rich	Male	Speed limit law rural (quartile)	X			X		
Other road injuries	Global	Male	Speed limit law rural (quartile)	X			X		
Other road injuries	Data Rich	Male	Vehicles - 2+4 wheels (per capita)	X			X		
Other road injuries	Global	Male	Vehicles - 2+4 wheels (per capita)	X			X		
Other road injuries	Data Rich	Male	Log-transformed SEV scalar: Oth Road	X			X		
Other road injuries	Global	Male	Log-transformed SEV scalar: Oth Road	X			X		
Other transport injuries	Global	Female	Rainfall Quintile 5 (proportion)			X			X

Comparison of GBD 2019 and GBD 2021 covariates and level of covariates used in cause of death modeling

Cause	Model Version Type	Sex	Covariate	Level 1 GBD 2019	Level 2 GBD 2019	Level 3 GBD 2019	Level 1 GBD 2021	Level 2 GBD 2021	Level 3 GBD 2021
Other transport injuries	Data Rich	Female	Rainfall Quintile 5 (proportion)			X			X
Other transport injuries	Global	Female	Education (years per capita)		X			X	
Other transport injuries	Data Rich	Female	Education (years per capita)		X			X	
Other transport injuries	Global	Female	LDI (I$ per capita)		X			X	
Other transport injuries	Data Rich	Female	LDI (I$ per capita)		X			X	
Other transport injuries	Global	Female	Socio-demographic Index		X			X	
Other transport injuries	Data Rich	Female	Socio-demographic Index		X			X	
Other transport injuries	Global	Female	Healthcare access and quality index		X			X	
Other transport injuries	Data Rich	Female	Healthcare access and quality index		X			X	
Other transport injuries	Global	Female	Population-weighted mean temperature		X			X	
Other transport injuries	Data Rich	Female	Population-weighted mean temperature		X			X	
Other transport injuries	Global	Female	Population Density (300-500 ppl/sqkm, proportion)		X			X	
Other transport injuries	Data Rich	Female	Population Density (300-500 ppl/sqkm, proportion)		X			X	
Other transport injuries	Global	Female	Population Density (500-1000 ppl/sqkm, proportion)		X			X	
Other transport injuries	Data Rich	Female	Population Density (500-1000 ppl/sqkm, proportion)		X			X	
Other transport injuries	Global	Female	Population 15 to 30 (proportion)		X			X	
Other transport injuries	Data Rich	Female	Population 15 to 30 (proportion)		X			X	
Other transport injuries	Global	Female	Liters of alcohol consumed per capita	X			X		
Other transport injuries	Data Rich	Female	Liters of alcohol consumed per capita	X			X		
Other transport injuries	Global	Female	Vehicles - 2 wheels fraction (proportion)	X			X		
Other transport injuries	Data Rich	Female	Vehicles - 2 wheels fraction (proportion)	X			X		
Other transport injuries	Global	Female	BAC law general population (quartile)	X			X		
Other transport injuries	Data Rich	Female	BAC law general population (quartile)	X			X		
Other transport injuries	Global	Female	BAC law youth drivers (quartile)	X			X		
Other transport injuries	Data Rich	Female	BAC law youth drivers (quartile)	X			X		
Other transport injuries	Global	Female	BAC law professional drivers (quartile)	X			X		
Other transport injuries	Data Rich	Female	BAC law professional drivers (quartile)	X			X		
Other transport injuries	Global	Female	Speed limit law urban (quartile)	X			X		
Other transport injuries	Data Rich	Female	Speed limit law urban (quartile)	X			X		
Other transport injuries	Global	Female	Speed limit law rural (quartile)	X			X		
Other transport injuries	Data Rich	Female	Speed limit law rural (quartile)	X			X		
Other transport injuries	Global	Female	Vehicles - 2+4 wheels (per capita)	X			X		
Other transport injuries	Data Rich	Female	Vehicles - 2+4 wheels (per capita)	X			X		
Other transport injuries	Global	Female	Log-transformed SEV scalar: Oth Trans	X			X		
Other transport injuries	Data Rich	Female	Log-transformed SEV scalar: Oth Trans	X			X		
Other transport injuries	Global	Male	Rainfall Quintile 5 (proportion)			X			X
Other transport injuries	Data Rich	Male	Rainfall Quintile 5 (proportion)			X			X
Other transport injuries	Global	Male	Education (years per capita)		X			X	
Other transport injuries	Data Rich	Male	Education (years per capita)		X			X	
Other transport injuries	Global	Male	LDI (I$ per capita)		X			X	
Other transport injuries	Data Rich	Male	LDI (I$ per capita)		X			X	
Other transport injuries	Global	Male	Socio-demographic Index		X			X	
Other transport injuries	Data Rich	Male	Socio-demographic Index		X			X	
Other transport injuries	Global	Male	Healthcare access and quality index		X			X	
Other transport injuries	Data Rich	Male	Healthcare access and quality index		X			X	
Other transport injuries	Global	Male	Population-weighted mean temperature		X			X	
Other transport injuries	Data Rich	Male	Population-weighted mean temperature		X			X	
Other transport injuries	Global	Male	Population Density (300-500 ppl/sqkm, proportion)		X			X	
Other transport injuries	Data Rich	Male	Population Density (300-500 ppl/sqkm, proportion)		X			X	
Other transport injuries	Global	Male	Population Density (500-1000 ppl/sqkm, proportion)		X			X	
Other transport injuries	Data Rich	Male	Population Density (500-1000 ppl/sqkm, proportion)		X			X	
Other transport injuries	Global	Male	Population 15 to 30 (proportion)		X			X	
Other transport injuries	Data Rich	Male	Population 15 to 30 (proportion)		X			X	
Other transport injuries	Global	Male	Liters of alcohol consumed per capita	X			X		
Other transport injuries	Data Rich	Male	Liters of alcohol consumed per capita	X			X		
Other transport injuries	Global	Male	Vehicles - 2 wheels fraction (proportion)	X			X		
Other transport injuries	Data Rich	Male	Vehicles - 2 wheels fraction (proportion)	X			X		
Other transport injuries	Global	Male	BAC law general population (quartile)	X			X		
Other transport injuries	Data Rich	Male	BAC law general population (quartile)	X			X		
Other transport injuries	Global	Male	BAC law youth drivers (quartile)	X			X		
Other transport injuries	Data Rich	Male	BAC law youth drivers (quartile)	X			X		
Other transport injuries	Global	Male	BAC law professional drivers (quartile)	X			X		
Other transport injuries	Data Rich	Male	BAC law professional drivers (quartile)	X			X		
Other transport injuries	Global	Male	Speed limit law urban (quartile)	X			X		
Other transport injuries	Data Rich	Male	Speed limit law urban (quartile)	X			X		
Other transport injuries	Global	Male	Speed limit law rural (quartile)	X			X		
Other transport injuries	Data Rich	Male	Speed limit law rural (quartile)	X			X		
Other transport injuries	Global	Male	Vehicles - 2+4 wheels (per capita)	X			X		
Other transport injuries	Data Rich	Male	Vehicles - 2+4 wheels (per capita)	X			X		
Other transport injuries	Global	Male	Log-transformed SEV scalar: Oth Trans	X			X		
Other transport injuries	Data Rich	Male	Log-transformed SEV scalar: Oth Trans	X			X		
Falls	Data Rich	Female	LDI (I$ per capita)			X			X
Falls	Global	Female	LDI (I$ per capita)			X			X
Falls	Data Rich	Female	Socio-demographic Index			X			X
Falls	Global	Female	Socio-demographic Index			X			X
Falls	Data Rich	Female	Elevation Over 1500m (proportion)			X			X
Falls	Global	Female	Elevation Over 1500m (proportion)			X			X
Falls	Data Rich	Female	Healthcare access and quality index		X			X	
Falls	Global	Female	Healthcare access and quality index		X			X	
Falls	Data Rich	Female	Population-weighted mean temperature		X			X	
Falls	Global	Female	Population-weighted mean temperature		X			X	
Falls	Data Rich	Female	Education (years per capita)	X			X		
Falls	Global	Female	Education (years per capita)	X			X		
Falls	Data Rich	Female	Liters of alcohol consumed per capita	X			X		
Falls	Global	Female	Liters of alcohol consumed per capita	X			X		
Falls	Data Rich	Female	Log-transformed SEV scalar: Falls	X			X		
Falls	Global	Female	Log-transformed SEV scalar: Falls	X			X		
Falls	Data Rich	Male	LDI (I$ per capita)			X			X
Falls	Global	Male	LDI (I$ per capita)			X			X
Falls	Data Rich	Male	Socio-demographic Index			X			X
Falls	Global	Male	Socio-demographic Index			X			X
Falls	Data Rich	Male	Elevation Over 1500m (proportion)			X			X
Falls	Global	Male	Elevation Over 1500m (proportion)			X			X
Falls	Data Rich	Male	Healthcare access and quality index		X			X	
Falls	Global	Male	Healthcare access and quality index		X			X	
Falls	Data Rich	Male	Population-weighted mean temperature		X			X	
Falls	Global	Male	Population-weighted mean temperature		X			X	
Falls	Data Rich	Male	Education (years per capita)	X			X		
Falls	Global	Male	Education (years per capita)	X			X		
Falls	Data Rich	Male	Liters of alcohol consumed per capita	X			X		
Falls	Global	Male	Liters of alcohol consumed per capita	X			X		
Falls	Data Rich	Male	Log-transformed SEV scalar: Falls	X			X		
Falls	Global	Male	Log-transformed SEV scalar: Falls	X			X		
Drowning	Data Rich	Female	Education (years per capita)			X			X
Drowning	Global	Female	Education (years per capita)			X			X
Drowning	Data Rich	Female	LDI (I$ per capita)			X			X
Drowning	Global	Female	LDI (I$ per capita)			X			X
Drowning	Data Rich	Female	Socio-demographic Index			X			X
Drowning	Global	Female	Socio-demographic Index			X			X
Drowning	Data Rich	Female	Elevation Under 100m (proportion)		X			X	
Drowning	Global	Female	Elevation Under 100m (proportion)		X			X	
Drowning	Data Rich	Female	Population-weighted mean temperature	X			X		
Drowning	Global	Female	Population-weighted mean temperature	X			X		
Drowning	Data Rich	Female	Rainfall Quintile 5 (proportion)	X			X		
Drowning	Global	Female	Rainfall Quintile 5 (proportion)	X			X		
Drowning	Data Rich	Female	Coastal Population within 10km (proportion)	X			X		
Drowning	Global	Female	Coastal Population within 10km (proportion)	X			X		
Drowning	Data Rich	Female	Landlocked Nation (binary)	X			X		
Drowning	Global	Female	Landlocked Nation (binary)	X			X		
Drowning	Data Rich	Female	Rainfall Quintile 1 (proportion)	X			X		
Drowning	Global	Female	Rainfall Quintile 1 (proportion)	X			X		
Drowning	Data Rich	Female	Log-transformed SEV scalar: Drown	X			X		
Drowning	Global	Female	Log-transformed SEV scalar: Drown	X			X		
Drowning	Data Rich	Male	Education (years per capita)			X			X
Drowning	Global	Male	Education (years per capita)			X			X
Drowning	Data Rich	Male	LDI (I$ per capita)			X			X
Drowning	Global	Male	LDI (I$ per capita)			X			X
Drowning	Data Rich	Male	Socio-demographic Index			X			X
Drowning	Global	Male	Socio-demographic Index			X			X
Drowning	Data Rich	Male	Elevation Under 100m (proportion)		X			X	
Drowning	Global	Male	Elevation Under 100m (proportion)		X			X	
Drowning	Data Rich	Male	Population-weighted mean temperature	X			X		
Drowning	Global	Male	Population-weighted mean temperature	X			X		
Drowning	Data Rich	Male	Rainfall Quintile 5 (proportion)	X			X		
Drowning	Global	Male	Rainfall Quintile 5 (proportion)	X			X		
Drowning	Data Rich	Male	Coastal Population within 10km (proportion)	X			X		

Comparison of GBD 2019 and GBD 2021 covariates and level of covariates used in cause of death modeling

Cause	Model Version Type	Sex	Covariate	Level 1 GBD 2019	Level 2 GBD 2019	Level 3 GBD 2019	Level 1 GBD 2021	Level 2 GBD 2021	Level 3 GBD 2021
Drowning	Global	Male	Coastal Population within 10km (proportion)	X			X		
Drowning	Data Rich	Male	Landlocked Nation (binary)	X			X		
Drowning	Global	Male	Landlocked Nation (binary)	X			X		
Drowning	Data Rich	Male	Rainfall Quintile 1 (proportion)	X			X		
Drowning	Global	Male	Rainfall Quintile 1 (proportion)	X			X		
Drowning	Data Rich	Male	Log-transformed SEV scalar: Drown	X			X		
Drowning	Global	Male	Log-transformed SEV scalar: Drown	X			X		
Fire, heat, and hot substances	Data Rich	Female	Education (years per capita)			X			X
Fire, heat, and hot substances	Global	Female	Education (years per capita)			X			X
Fire, heat, and hot substances	Data Rich	Female	LDI (I$ per capita)			X			X
Fire, heat, and hot substances	Global	Female	LDI (I$ per capita)			X			X
Fire, heat, and hot substances	Data Rich	Female	Socio-demographic Index			X			X
Fire, heat, and hot substances	Global	Female	Socio-demographic Index			X			X
Fire, heat, and hot substances	Data Rich	Female	Healthcare access and quality index		X			X	
Fire, heat, and hot substances	Global	Female	Healthcare access and quality index		X			X	
Fire, heat, and hot substances	Data Rich	Female	Indoor Air Pollution (All Cooking Fuels)		X			X	
Fire, heat, and hot substances	Global	Female	Indoor Air Pollution (All Cooking Fuels)		X			X	
Fire, heat, and hot substances	Data Rich	Female	Tobacco (cigarettes per capita)		X			X	
Fire, heat, and hot substances	Global	Female	Tobacco (cigarettes per capita)		X			X	
Fire, heat, and hot substances	Data Rich	Female	Population Density (over 1000 ppl/sqkm, proportion)		X			X	
Fire, heat, and hot substances	Global	Female	Population Density (over 1000 ppl/sqkm, proportion)		X			X	
Fire, heat, and hot substances	Data Rich	Female	Population-weighted mean temperature	X			X		
Fire, heat, and hot substances	Global	Female	Population-weighted mean temperature	X			X		
Fire, heat, and hot substances	Data Rich	Female	Log-transformed SEV scalar: Fire	X			X		
Fire, heat, and hot substances	Global	Female	Log-transformed SEV scalar: Fire	X			X		
Fire, heat, and hot substances	Data Rich	Male	Education (years per capita)			X			X
Fire, heat, and hot substances	Global	Male	Education (years per capita)			X			X
Fire, heat, and hot substances	Data Rich	Male	LDI (I$ per capita)			X			X
Fire, heat, and hot substances	Global	Male	LDI (I$ per capita)			X			X
Fire, heat, and hot substances	Data Rich	Male	Socio-demographic Index			X			X
Fire, heat, and hot substances	Global	Male	Socio-demographic Index			X			X
Fire, heat, and hot substances	Data Rich	Male	Healthcare access and quality index		X			X	
Fire, heat, and hot substances	Global	Male	Healthcare access and quality index		X			X	
Fire, heat, and hot substances	Data Rich	Male	Indoor Air Pollution (All Cooking Fuels)		X			X	
Fire, heat, and hot substances	Global	Male	Indoor Air Pollution (All Cooking Fuels)		X			X	
Fire, heat, and hot substances	Data Rich	Male	Tobacco (cigarettes per capita)		X			X	
Fire, heat, and hot substances	Global	Male	Tobacco (cigarettes per capita)		X			X	
Fire, heat, and hot substances	Data Rich	Male	Population Density (over 1000 ppl/sqkm, proportion)		X			X	
Fire, heat, and hot substances	Global	Male	Population Density (over 1000 ppl/sqkm, proportion)		X			X	
Fire, heat, and hot substances	Data Rich	Male	Population-weighted mean temperature	X			X		
Fire, heat, and hot substances	Global	Male	Population-weighted mean temperature	X			X		
Fire, heat, and hot substances	Data Rich	Male	Log-transformed SEV scalar: Fire	X			X		
Fire, heat, and hot substances	Global	Male	Log-transformed SEV scalar: Fire	X			X		
Poisonings	Data Rich	Female	Education (years per capita)			X			X
Poisonings	Global	Female	Education (years per capita)			X			X
Poisonings	Data Rich	Female	LDI (I$ per capita)			X			X
Poisonings	Global	Female	LDI (I$ per capita)			X			X
Poisonings	Data Rich	Female	Socio-demographic Index			X			X
Poisonings	Global	Female	Socio-demographic Index			X			X
Poisonings	Data Rich	Female	Healthcare access and quality index		X			X	
Poisonings	Global	Female	Healthcare access and quality index		X			X	
Poisonings	Data Rich	Female	Population Density (over 1000 ppl/sqkm, proportion)		X			X	
Poisonings	Global	Female	Population Density (over 1000 ppl/sqkm, proportion)		X			X	
Poisonings	Data Rich	Female	Population Density (under 150 ppl/sqkm, proportion)		X			X	
Poisonings	Global	Female	Population Density (under 150 ppl/sqkm, proportion)		X			X	
Poisonings	Data Rich	Female	Liters of alcohol consumed per capita	X			X		
Poisonings	Global	Female	Liters of alcohol consumed per capita	X			X		
Poisonings	Data Rich	Female	Population-weighted mean temperature	X			X		
Poisonings	Global	Female	Population-weighted mean temperature	X			X		
Poisonings	Data Rich	Female	Log-transformed SEV scalar: Poison	X			X		
Poisonings	Global	Female	Log-transformed SEV scalar: Poison	X			X		
Poisonings	Global	Male	Education (years per capita)			X			X
Poisonings	Data Rich	Male	Education (years per capita)			X			X
Poisonings	Global	Male	LDI (I$ per capita)			X			X
Poisonings	Data Rich	Male	LDI (I$ per capita)			X			X
Poisonings	Global	Male	Socio-demographic Index			X			X
Poisonings	Data Rich	Male	Socio-demographic Index			X			X
Poisonings	Global	Male	Healthcare access and quality index		X			X	
Poisonings	Data Rich	Male	Healthcare access and quality index		X			X	
Poisonings	Global	Male	Population Density (over 1000 ppl/sqkm, proportion)		X			X	
Poisonings	Data Rich	Male	Population Density (over 1000 ppl/sqkm, proportion)		X			X	
Poisonings	Global	Male	Population Density (under 150 ppl/sqkm, proportion)		X			X	
Poisonings	Data Rich	Male	Population Density (under 150 ppl/sqkm, proportion)		X			X	
Poisonings	Global	Male	Liters of alcohol consumed per capita	X			X		
Poisonings	Data Rich	Male	Liters of alcohol consumed per capita	X			X		
Poisonings	Global	Male	Population-weighted mean temperature	X			X		
Poisonings	Data Rich	Male	Population-weighted mean temperature	X			X		
Poisonings	Global	Male	Log-transformed SEV scalar: Poison	X			X		
Poisonings	Data Rich	Male	Log-transformed SEV scalar: Poison	X			X		
Poisoning by carbon monoxide	Global	Female	Education (years per capita)			X			X
Poisoning by carbon monoxide	Data Rich	Female	Education (years per capita)			X			X
Poisoning by carbon monoxide	Global	Female	LDI (I$ per capita)			X			X
Poisoning by carbon monoxide	Data Rich	Female	LDI (I$ per capita)			X			X
Poisoning by carbon monoxide	Global	Female	Socio-demographic Index			X			X
Poisoning by carbon monoxide	Data Rich	Female	Socio-demographic Index			X			X
Poisoning by carbon monoxide	Global	Female	Healthcare access and quality index			X			X
Poisoning by carbon monoxide	Data Rich	Female	Healthcare access and quality index			X			X
Poisoning by carbon monoxide	Global	Female	Population-weighted mean temperature		X			X	
Poisoning by carbon monoxide	Data Rich	Female	Population-weighted mean temperature		X			X	
Poisoning by carbon monoxide	Global	Female	Liters of alcohol consumed per capita	X			X		
Poisoning by carbon monoxide	Data Rich	Female	Liters of alcohol consumed per capita	X			X		
Poisoning by carbon monoxide	Global	Female	Log-transformed SEV scalar: Inj Pois CO	X			X		
Poisoning by carbon monoxide	Data Rich	Female	Log-transformed SEV scalar: Inj Pois CO	X			X		
Poisoning by carbon monoxide	Global	Male	Education (years per capita)			X			X
Poisoning by carbon monoxide	Data Rich	Male	Education (years per capita)			X			X
Poisoning by carbon monoxide	Global	Male	LDI (I$ per capita)			X			X
Poisoning by carbon monoxide	Data Rich	Male	LDI (I$ per capita)			X			X
Poisoning by carbon monoxide	Global	Male	Socio-demographic Index			X			X
Poisoning by carbon monoxide	Data Rich	Male	Socio-demographic Index			X			X
Poisoning by carbon monoxide	Global	Male	Healthcare access and quality index			X			X
Poisoning by carbon monoxide	Data Rich	Male	Healthcare access and quality index			X			X
Poisoning by carbon monoxide	Global	Male	Population-weighted mean temperature		X			X	
Poisoning by carbon monoxide	Data Rich	Male	Population-weighted mean temperature		X			X	
Poisoning by carbon monoxide	Global	Male	Liters of alcohol consumed per capita	X			X		
Poisoning by carbon monoxide	Data Rich	Male	Liters of alcohol consumed per capita	X			X		
Poisoning by carbon monoxide	Global	Male	Log-transformed SEV scalar: Inj Pois CO	X			X		
Poisoning by carbon monoxide	Data Rich	Male	Log-transformed SEV scalar: Inj Pois CO	X			X		
Poisoning by other means	Global	Female	Education (years per capita)			X			X
Poisoning by other means	Data Rich	Female	Education (years per capita)			X			X
Poisoning by other means	Global	Female	LDI (I$ per capita)			X			X
Poisoning by other means	Data Rich	Female	LDI (I$ per capita)			X			X
Poisoning by other means	Global	Female	Socio-demographic Index			X			X
Poisoning by other means	Data Rich	Female	Socio-demographic Index			X			X
Poisoning by other means	Global	Female	Healthcare access and quality index			X			X
Poisoning by other means	Data Rich	Female	Healthcare access and quality index			X			X
Poisoning by other means	Global	Female	Liters of alcohol consumed per capita	X			X		
Poisoning by other means	Data Rich	Female	Liters of alcohol consumed per capita	X			X		
Poisoning by other means	Global	Female	Population-weighted mean temperature	X			X		
Poisoning by other means	Data Rich	Female	Population-weighted mean temperature	X			X		
Poisoning by other means	Global	Female	Log-transformed SEV scalar: Inj Pois Oth	X			X		
Poisoning by other means	Data Rich	Female	Log-transformed SEV scalar: Inj Pois Oth	X			X		
Poisoning by other means	Data Rich	Male	Education (years per capita)			X			X
Poisoning by other means	Global	Male	Education (years per capita)			X			X
Poisoning by other means	Data Rich	Male	LDI (I$ per capita)			X			X
Poisoning by other means	Global	Male	LDI (I$ per capita)			X			X
Poisoning by other means	Data Rich	Male	Socio-demographic Index			X			X
Poisoning by other means	Global	Male	Socio-demographic Index			X			X
Poisoning by other means	Data Rich	Male	Healthcare access and quality index			X			X
Poisoning by other means	Global	Male	Healthcare access and quality index			X			X
Poisoning by other means	Data Rich	Male	Liters of alcohol consumed per capita	X			X		
Poisoning by other means	Global	Male	Liters of alcohol consumed per capita	X			X		
Poisoning by other means	Data Rich	Male	Population-weighted mean temperature	X			X		
Poisoning by other means	Global	Male	Population-weighted mean temperature	X			X		
Poisoning by other means	Data Rich	Male	Log-transformed SEV scalar: Inj Pois Oth	X			X		
Poisoning by other means	Global	Male	Log-transformed SEV scalar: Inj Pois Oth	X			X		

Comparison of GBD 2019 and GBD 2021 covariates and level of covariates used in cause of death modeling									
**Cause**	**Model Version Type**	**Sex**	**Covariate**	**Level 1 GBD 2019**	**Level 2 GBD 2019**	**Level 3 GBD 2019**	**Level 1 GBD 2021**	**Level 2 GBD 2021**	**Level 3 GBD 2021**
Exposure to mechanical forces	Data Rich	Female	Education (years per capita)			X			X
Exposure to mechanical forces	Global	Female	Education (years per capita)			X			X
Exposure to mechanical forces	Data Rich	Female	LDI (I$ per capita)			X			X
Exposure to mechanical forces	Global	Female	LDI (I$ per capita)			X			X
Exposure to mechanical forces	Data Rich	Female	Socio-demographic Index			X			X
Exposure to mechanical forces	Global	Female	Socio-demographic Index			X			X
Exposure to mechanical forces	Data Rich	Female	Healthcare access and quality index		X			X	
Exposure to mechanical forces	Global	Female	Healthcare access and quality index		X			X	
Exposure to mechanical forces	Data Rich	Female	Population Density (over 1000 ppl/sqkm, proportion)		X			X	
Exposure to mechanical forces	Global	Female	Population Density (over 1000 ppl/sqkm, proportion)		X			X	
Exposure to mechanical forces	Data Rich	Female	Population Density (under 150 ppl/sqkm, proportion)		X			X	
Exposure to mechanical forces	Global	Female	Population Density (under 150 ppl/sqkm, proportion)		X			X	
Exposure to mechanical forces	Data Rich	Female	Population-weighted mean temperature	X			X		
Exposure to mechanical forces	Global	Female	Population-weighted mean temperature	X			X		
Exposure to mechanical forces	Data Rich	Male	Education (years per capita)			X			X
Exposure to mechanical forces	Global	Male	Education (years per capita)			X			X
Exposure to mechanical forces	Data Rich	Male	LDI (I$ per capita)			X			X
Exposure to mechanical forces	Global	Male	LDI (I$ per capita)			X			X
Exposure to mechanical forces	Data Rich	Male	Socio-demographic Index			X			X
Exposure to mechanical forces	Global	Male	Socio-demographic Index			X			X
Exposure to mechanical forces	Data Rich	Male	Healthcare access and quality index		X			X	
Exposure to mechanical forces	Global	Male	Healthcare access and quality index		X			X	
Exposure to mechanical forces	Data Rich	Male	Population Density (over 1000 ppl/sqkm, proportion)		X			X	
Exposure to mechanical forces	Global	Male	Population Density (over 1000 ppl/sqkm, proportion)		X			X	
Exposure to mechanical forces	Data Rich	Male	Population Density (under 150 ppl/sqkm, proportion)		X			X	
Exposure to mechanical forces	Global	Male	Population Density (under 150 ppl/sqkm, proportion)		X			X	
Exposure to mechanical forces	Data Rich	Male	Population-weighted mean temperature	X			X		
Exposure to mechanical forces	Global	Male	Population-weighted mean temperature	X			X		
Unintentional firearm injuries	Data Rich	Female	Education (years per capita)			X			X
Unintentional firearm injuries	Global	Female	Education (years per capita)			X			X
Unintentional firearm injuries	Data Rich	Female	LDI (I$ per capita)			X			X
Unintentional firearm injuries	Global	Female	LDI (I$ per capita)			X			X
Unintentional firearm injuries	Data Rich	Female	Socio-demographic Index			X			X
Unintentional firearm injuries	Global	Female	Socio-demographic Index			X			X
Unintentional firearm injuries	Data Rich	Female	Population Density (over 1000 ppl/sqkm, proportion)			X			X
Unintentional firearm injuries	Global	Female	Population Density (over 1000 ppl/sqkm, proportion)			X			X
Unintentional firearm injuries	Data Rich	Female	Population Density (under 150 ppl/sqkm, proportion)			X			X
Unintentional firearm injuries	Global	Female	Population Density (under 150 ppl/sqkm, proportion)			X			X
Unintentional firearm injuries	Data Rich	Female	Healthcare access and quality index		X			X	
Unintentional firearm injuries	Global	Female	Healthcare access and quality index		X			X	
Unintentional firearm injuries	Data Rich	Female	Population-weighted mean temperature	X			X		
Unintentional firearm injuries	Global	Female	Population-weighted mean temperature	X			X		
Unintentional firearm injuries	Data Rich	Female	Log-transformed SEV scalar: Mech Gun	X			X		
Unintentional firearm injuries	Global	Female	Log-transformed SEV scalar: Mech Gun	X			X		
Unintentional firearm injuries	Data Rich	Male	Education (years per capita)			X			X
Unintentional firearm injuries	Global	Male	Education (years per capita)			X			X
Unintentional firearm injuries	Data Rich	Male	LDI (I$ per capita)			X			X
Unintentional firearm injuries	Global	Male	LDI (I$ per capita)			X			X
Unintentional firearm injuries	Data Rich	Male	Socio-demographic Index			X			X
Unintentional firearm injuries	Global	Male	Socio-demographic Index			X			X
Unintentional firearm injuries	Data Rich	Male	Population Density (over 1000 ppl/sqkm, proportion)			X			X
Unintentional firearm injuries	Global	Male	Population Density (over 1000 ppl/sqkm, proportion)			X			X
Unintentional firearm injuries	Data Rich	Male	Population Density (under 150 ppl/sqkm, proportion)			X			X
Unintentional firearm injuries	Global	Male	Population Density (under 150 ppl/sqkm, proportion)			X			X
Unintentional firearm injuries	Data Rich	Male	Healthcare access and quality index		X			X	
Unintentional firearm injuries	Global	Male	Healthcare access and quality index		X			X	
Unintentional firearm injuries	Data Rich	Male	Population-weighted mean temperature	X			X		
Unintentional firearm injuries	Global	Male	Population-weighted mean temperature	X			X		
Unintentional firearm injuries	Data Rich	Male	Log-transformed SEV scalar: Mech Gun	X			X		
Unintentional firearm injuries	Global	Male	Log-transformed SEV scalar: Mech Gun	X			X		
Other exposure to mechanical forces	Data Rich	Female	Education (years per capita)			X			X
Other exposure to mechanical forces	Global	Female	Education (years per capita)			X			X
Other exposure to mechanical forces	Data Rich	Female	LDI (I$ per capita)			X			X
Other exposure to mechanical forces	Global	Female	LDI (I$ per capita)			X			X
Other exposure to mechanical forces	Data Rich	Female	Socio-demographic Index			X			X
Other exposure to mechanical forces	Global	Female	Socio-demographic Index			X			X
Other exposure to mechanical forces	Data Rich	Female	Healthcare access and quality index		X			X	
Other exposure to mechanical forces	Global	Female	Healthcare access and quality index		X			X	
Other exposure to mechanical forces	Data Rich	Female	Population Density (over 1000 ppl/sqkm, proportion)		X			X	
Other exposure to mechanical forces	Global	Female	Population Density (over 1000 ppl/sqkm, proportion)		X			X	
Other exposure to mechanical forces	Data Rich	Female	Population Density (under 150 ppl/sqkm, proportion)		X			X	
Other exposure to mechanical forces	Global	Female	Population Density (under 150 ppl/sqkm, proportion)		X			X	
Other exposure to mechanical forces	Data Rich	Female	Population-weighted mean temperature	X			X		
Other exposure to mechanical forces	Global	Female	Population-weighted mean temperature	X			X		
Other exposure to mechanical forces	Data Rich	Female	Log-transformed SEV scalar: Oth Mech	X			X		
Other exposure to mechanical forces	Global	Female	Log-transformed SEV scalar: Oth Mech	X			X		
Other exposure to mechanical forces	Data Rich	Male	Education (years per capita)			X			X
Other exposure to mechanical forces	Global	Male	Education (years per capita)			X			X
Other exposure to mechanical forces	Data Rich	Male	LDI (I$ per capita)			X			X
Other exposure to mechanical forces	Global	Male	LDI (I$ per capita)			X			X
Other exposure to mechanical forces	Data Rich	Male	Socio-demographic Index			X			X
Other exposure to mechanical forces	Global	Male	Socio-demographic Index			X			X
Other exposure to mechanical forces	Data Rich	Male	Healthcare access and quality index		X			X	
Other exposure to mechanical forces	Global	Male	Healthcare access and quality index		X			X	
Other exposure to mechanical forces	Data Rich	Male	Population Density (over 1000 ppl/sqkm, proportion)		X			X	
Other exposure to mechanical forces	Global	Male	Population Density (over 1000 ppl/sqkm, proportion)		X			X	
Other exposure to mechanical forces	Data Rich	Male	Population Density (under 150 ppl/sqkm, proportion)		X			X	
Other exposure to mechanical forces	Global	Male	Population Density (under 150 ppl/sqkm, proportion)		X			X	
Other exposure to mechanical forces	Data Rich	Male	Population-weighted mean temperature	X			X		
Other exposure to mechanical forces	Global	Male	Population-weighted mean temperature	X			X		
Other exposure to mechanical forces	Data Rich	Male	Log-transformed SEV scalar: Oth Mech	X			X		
Other exposure to mechanical forces	Global	Male	Log-transformed SEV scalar: Oth Mech	X			X		
Adverse effects of medical treatment	Data Rich	Female	LDI (I$ per capita)			X			X
Adverse effects of medical treatment	Global	Female	LDI (I$ per capita)			X			X
Adverse effects of medical treatment	Data Rich	Female	Socio-demographic Index			X			X
Adverse effects of medical treatment	Global	Female	Socio-demographic Index			X			X
Adverse effects of medical treatment	Data Rich	Female	Healthcare access and quality index		X			X	
Adverse effects of medical treatment	Global	Female	Healthcare access and quality index		X			X	
Adverse effects of medical treatment	Data Rich	Female	Education (years per capita)	X			X		
Adverse effects of medical treatment	Global	Female	Education (years per capita)	X			X		
Adverse effects of medical treatment	Data Rich	Female	Population-weighted mean temperature	X			X		
Adverse effects of medical treatment	Global	Female	Population-weighted mean temperature	X			X		
Adverse effects of medical treatment	Data Rich	Male	LDI (I$ per capita)			X			X
Adverse effects of medical treatment	Global	Male	LDI (I$ per capita)			X			X
Adverse effects of medical treatment	Data Rich	Male	Socio-demographic Index			X			X
Adverse effects of medical treatment	Global	Male	Socio-demographic Index			X			X
Adverse effects of medical treatment	Data Rich	Male	Healthcare access and quality index		X			X	
Adverse effects of medical treatment	Global	Male	Healthcare access and quality index		X			X	
Adverse effects of medical treatment	Data Rich	Male	Education (years per capita)	X			X		
Adverse effects of medical treatment	Global	Male	Education (years per capita)	X			X		
Adverse effects of medical treatment	Data Rich	Male	Population-weighted mean temperature	X			X		
Adverse effects of medical treatment	Global	Male	Population-weighted mean temperature	X			X		
Animal contact	Data Rich	Female	Education (years per capita)			X			X
Animal contact	Global	Female	Education (years per capita)			X			X
Animal contact	Data Rich	Female	LDI (I$ per capita)			X			X
Animal contact	Global	Female	LDI (I$ per capita)			X			X
Animal contact	Data Rich	Female	Socio-demographic Index			X			X
Animal contact	Global	Female	Socio-demographic Index			X			X
Animal contact	Data Rich	Female	Elevation Over 1500m (proportion)			X			X
Animal contact	Global	Female	Elevation Over 1500m (proportion)			X			X
Animal contact	Data Rich	Female	Population Density (over 1000 ppl/sqkm, proportion)			X			X
Animal contact	Global	Female	Population Density (over 1000 ppl/sqkm, proportion)			X			X
Animal contact	Data Rich	Female	Population Density (under 150 ppl/sqkm, proportion)			X			X
Animal contact	Global	Female	Population Density (under 150 ppl/sqkm, proportion)			X			X
Animal contact	Data Rich	Female	Elevation Under 100m (proportion)			X			X
Animal contact	Global	Female	Elevation Under 100m (proportion)			X			X
Animal contact	Data Rich	Female	Healthcare access and quality index		X			X	

Comparison of GBD 2019 and GBD 2021 covariates and level of covariates used in cause of death modeling

Cause	Model Version Type	Sex	Covariate	Level 1 GBD 2019	Level 2 GBD 2019	Level 3 GBD 2019	Level 1 GBD 2021	Level 2 GBD 2021	Level 3 GBD 2021
Animal contact	Global	Female	Healthcare access and quality index		X			X	
Animal contact	Data Rich	Female	Population 15 to 30 (proportion)		X			X	
Animal contact	Global	Female	Population 15 to 30 (proportion)		X			X	
Animal contact	Data Rich	Female	Liters of alcohol consumed per capita	X			X		
Animal contact	Global	Female	Liters of alcohol consumed per capita	X			X		
Animal contact	Data Rich	Female	Population-weighted mean temperature	X			X		
Animal contact	Global	Female	Population-weighted mean temperature	X			X		
Animal contact	Data Rich	Female	Log-transformed SEV scalar: Animal	X			X		
Animal contact	Global	Female	Log-transformed SEV scalar: Animal	X			X		
Animal contact	Data Rich	Male	Education (years per capita)			X			X
Animal contact	Global	Male	Education (years per capita)			X			X
Animal contact	Data Rich	Male	LDI (I$ per capita)			X			X
Animal contact	Global	Male	LDI (I$ per capita)			X			X
Animal contact	Data Rich	Male	Socio-demographic Index			X			X
Animal contact	Global	Male	Socio-demographic Index			X			X
Animal contact	Data Rich	Male	Elevation Over 1500m (proportion)			X			X
Animal contact	Global	Male	Elevation Over 1500m (proportion)			X			X
Animal contact	Data Rich	Male	Population Density (over 1000 ppl/sqkm, proportion)			X			X
Animal contact	Global	Male	Population Density (over 1000 ppl/sqkm, proportion)			X			X
Animal contact	Data Rich	Male	Population Density (under 150 ppl/sqkm, proportion)			X			X
Animal contact	Global	Male	Population Density (under 150 ppl/sqkm, proportion)			X			X
Animal contact	Data Rich	Male	Elevation Under 100m (proportion)			X			X
Animal contact	Global	Male	Elevation Under 100m (proportion)			X			X
Animal contact	Data Rich	Male	Healthcare access and quality index		X			X	
Animal contact	Global	Male	Healthcare access and quality index		X			X	
Animal contact	Data Rich	Male	Population 15 to 30 (proportion)		X			X	
Animal contact	Global	Male	Population 15 to 30 (proportion)		X			X	
Animal contact	Data Rich	Male	Liters of alcohol consumed per capita	X			X		
Animal contact	Global	Male	Liters of alcohol consumed per capita	X			X		
Animal contact	Data Rich	Male	Population-weighted mean temperature	X			X		
Animal contact	Global	Male	Population-weighted mean temperature	X			X		
Animal contact	Data Rich	Male	Log-transformed SEV scalar: Animal	X			X		
Animal contact	Global	Male	Log-transformed SEV scalar: Animal	X			X		
Venomous animal contact	Data Rich	Female	Education (years per capita)			X			X
Venomous animal contact	Global	Female	Education (years per capita)			X			X
Venomous animal contact	Data Rich	Female	LDI (I$ per capita)			X			X
Venomous animal contact	Global	Female	LDI (I$ per capita)			X			X
Venomous animal contact	Data Rich	Female	Socio-demographic Index			X			X
Venomous animal contact	Global	Female	Socio-demographic Index			X			X
Venomous animal contact	Data Rich	Female	Elevation Over 1500m (proportion)			X			X
Venomous animal contact	Global	Female	Elevation Over 1500m (proportion)			X			X
Venomous animal contact	Data Rich	Female	Population Density (over 1000 ppl/sqkm, proportion)			X			X
Venomous animal contact	Global	Female	Population Density (over 1000 ppl/sqkm, proportion)			X			X
Venomous animal contact	Data Rich	Female	Population Density (under 150 ppl/sqkm, proportion)			X			X
Venomous animal contact	Global	Female	Population Density (under 150 ppl/sqkm, proportion)			X			X
Venomous animal contact	Data Rich	Female	Elevation Under 100m (proportion)			X			X
Venomous animal contact	Global	Female	Elevation Under 100m (proportion)			X			X
Venomous animal contact	Data Rich	Female	Healthcare access and quality index		X			X	
Venomous animal contact	Global	Female	Healthcare access and quality index		X			X	
Venomous animal contact	Data Rich	Female	Liters of alcohol consumed per capita	X				X	
Venomous animal contact	Global	Female	Liters of alcohol consumed per capita	X				X	
Venomous animal contact	Data Rich	Female	Population-weighted mean temperature	X				X	
Venomous animal contact	Global	Female	Population-weighted mean temperature	X				X	
Venomous animal contact	Data Rich	Female	Absolute value of average latitude	X				X	
Venomous animal contact	Global	Female	Absolute value of average latitude	X				X	
Venomous animal contact	Data Rich	Female	Rainfall Population-Weighted (mm/yr)	X				X	
Venomous animal contact	Global	Female	Rainfall Population-Weighted (mm/yr)	X				X	
Venomous animal contact	Data Rich	Female	Sahel Region of Africa (binary)	X				X	
Venomous animal contact	Global	Female	Sahel Region of Africa (binary)	X				X	
Venomous animal contact	Data Rich	Female	Urbanicity	X				X	
Venomous animal contact	Global	Female	Urbanicity	X				X	
Venomous animal contact	Data Rich	Female	Proportion of population involved in agricultural activities	X				X	
Venomous animal contact	Global	Female	Proportion of population involved in agricultural activities	X				X	
Venomous animal contact	Data Rich	Female	Log-transformed SEV scalar: Venom	X			X		
Venomous animal contact	Global	Female	Log-transformed SEV scalar: Venom	X			X		
Venomous animal contact	Data Rich	Female	Proportion of population vulnerable to venomous snakebites	X			X		
Venomous animal contact	Global	Female	Proportion of population vulnerable to venomous snakebites	X			X		
Venomous animal contact	Data Rich	Female	Mean number of venomous snake species	X			X		
Venomous animal contact	Global	Female	Mean number of venomous snake species	X			X		
Venomous animal contact	Data Rich	Male	Education (years per capita)			X			X
Venomous animal contact	Global	Male	Education (years per capita)			X			X
Venomous animal contact	Data Rich	Male	LDI (I$ per capita)			X			X
Venomous animal contact	Global	Male	LDI (I$ per capita)			X			X
Venomous animal contact	Data Rich	Male	Socio-demographic Index			X			X
Venomous animal contact	Global	Male	Socio-demographic Index			X			X
Venomous animal contact	Data Rich	Male	Elevation Over 1500m (proportion)			X			X
Venomous animal contact	Global	Male	Elevation Over 1500m (proportion)			X			X
Venomous animal contact	Data Rich	Male	Population Density (over 1000 ppl/sqkm, proportion)			X			X
Venomous animal contact	Global	Male	Population Density (over 1000 ppl/sqkm, proportion)			X			X
Venomous animal contact	Data Rich	Male	Population Density (under 150 ppl/sqkm, proportion)			X			X
Venomous animal contact	Global	Male	Population Density (under 150 ppl/sqkm, proportion)			X			X
Venomous animal contact	Data Rich	Male	Elevation Under 100m (proportion)			X			X
Venomous animal contact	Global	Male	Elevation Under 100m (proportion)			X			X
Venomous animal contact	Data Rich	Male	Healthcare access and quality index		X			X	
Venomous animal contact	Global	Male	Healthcare access and quality index		X			X	
Venomous animal contact	Data Rich	Male	Liters of alcohol consumed per capita	X				X	
Venomous animal contact	Global	Male	Liters of alcohol consumed per capita	X				X	
Venomous animal contact	Data Rich	Male	Population-weighted mean temperature	X				X	
Venomous animal contact	Global	Male	Population-weighted mean temperature	X				X	
Venomous animal contact	Data Rich	Male	Absolute value of average latitude	X				X	
Venomous animal contact	Global	Male	Absolute value of average latitude	X				X	
Venomous animal contact	Data Rich	Male	Rainfall Population-Weighted (mm/yr)	X				X	
Venomous animal contact	Global	Male	Rainfall Population-Weighted (mm/yr)	X				X	
Venomous animal contact	Data Rich	Male	Sahel Region of Africa (binary)	X				X	
Venomous animal contact	Data Rich	Male	Urbanicity	X				X	
Venomous animal contact	Global	Male	Urbanicity	X				X	
Venomous animal contact	Data Rich	Male	Proportion of population involved in agricultural activities	X				X	
Venomous animal contact	Global	Male	Proportion of population involved in agricultural activities	X				X	
Venomous animal contact	Data Rich	Male	Log-transformed SEV scalar: Venom	X			X		
Venomous animal contact	Global	Male	Log-transformed SEV scalar: Venom	X			X		
Venomous animal contact	Data Rich	Male	Proportion of population vulnerable to venomous snakebites	X			X		
Venomous animal contact	Global	Male	Proportion of population vulnerable to venomous snakebites	X			X		
Venomous animal contact	Data Rich	Male	Mean number of venomous snake species	X			X		
Venomous animal contact	Global	Male	Mean number of venomous snake species	X			X		
Non-venomous animal contact	Data Rich	Female	Education (years per capita)			X			X
Non-venomous animal contact	Global	Female	Education (years per capita)			X			X
Non-venomous animal contact	Data Rich	Female	LDI (I$ per capita)			X			X
Non-venomous animal contact	Global	Female	LDI (I$ per capita)			X			X
Non-venomous animal contact	Data Rich	Female	Socio-demographic Index			X			X
Non-venomous animal contact	Global	Female	Socio-demographic Index			X			X
Non-venomous animal contact	Data Rich	Female	Elevation Over 1500m (proportion)			X			X
Non-venomous animal contact	Global	Female	Elevation Over 1500m (proportion)			X			X
Non-venomous animal contact	Data Rich	Female	Population Density (over 1000 ppl/sqkm, proportion)			X			X
Non-venomous animal contact	Global	Female	Population Density (over 1000 ppl/sqkm, proportion)			X			X
Non-venomous animal contact	Data Rich	Female	Population Density (under 150 ppl/sqkm, proportion)			X			X
Non-venomous animal contact	Global	Female	Population Density (under 150 ppl/sqkm, proportion)			X			X
Non-venomous animal contact	Data Rich	Female	Elevation Under 100m (proportion)			X			X
Non-venomous animal contact	Global	Female	Elevation Under 100m (proportion)			X			X
Non-venomous animal contact	Data Rich	Female	Healthcare access and quality index		X			X	
Non-venomous animal contact	Global	Female	Healthcare access and quality index		X			X	
Non-venomous animal contact	Data Rich	Female	Liters of alcohol consumed per capita	X			X		
Non-venomous animal contact	Global	Female	Liters of alcohol consumed per capita	X			X		
Non-venomous animal contact	Data Rich	Female	Population-weighted mean temperature	X			X		
Non-venomous animal contact	Global	Female	Population-weighted mean temperature	X			X		
Non-venomous animal contact	Data Rich	Female	Log-transformed SEV scalar: Non Ven	X			X		
Non-venomous animal contact	Global	Female	Log-transformed SEV scalar: Non Ven	X			X		
Non-venomous animal contact	Data Rich	Male	Education (years per capita)			X			X
Non-venomous animal contact	Global	Male	Education (years per capita)			X			X
Non-venomous animal contact	Data Rich	Male	LDI (I$ per capita)			X			X

Comparison of GBD 2019 and GBD 2021 covariates and level of covariates used in cause of death modeling									
Cause	Model Version Type	Sex	Covariate	Level 1 GBD 2019	Level 2 GBD 2019	Level 3 GBD 2019	Level 1 GBD 2021	Level 2 GBD 2021	Level 3 GBD 2021
Non-venomous animal contact	Global	Male	LDI (I$ per capita)			X			X
Non-venomous animal contact	Data Rich	Male	Socio-demographic Index			X			X
Non-venomous animal contact	Global	Male	Socio-demographic Index			X			X
Non-venomous animal contact	Global	Male	Healthcare access and quality index			X			X
Non-venomous animal contact	Data Rich	Male	Elevation Over 1500m (proportion)			X			X
Non-venomous animal contact	Data Rich	Male	Population Density (over 1000 ppl/sqkm, proportion)			X			X
Non-venomous animal contact	Data Rich	Male	Population Density (under 150 ppl/sqkm, proportion)			X			X
Non-venomous animal contact	Data Rich	Male	Elevation Under 100m (proportion)			X			X
Non-venomous animal contact	Data Rich	Male	Healthcare access and quality index		X			X	
Non-venomous animal contact	Data Rich	Male	Liters of alcohol consumed per capita	X			X		
Non-venomous animal contact	Global	Male	Liters of alcohol consumed per capita	X			X		
Non-venomous animal contact	Global	Male	Elevation Over 1500m (proportion)	X			X		
Non-venomous animal contact	Data Rich	Male	Population-weighted mean temperature	X			X		
Non-venomous animal contact	Global	Male	Population-weighted mean temperature	X			X		
Non-venomous animal contact	Global	Male	Population Density (over 1000 ppl/sqkm, proportion)	X			X		
Non-venomous animal contact	Global	Male	Population Density (under 150 ppl/sqkm, proportion)	X			X		
Non-venomous animal contact	Global	Male	Elevation Under 100m (proportion)	X			X		
Non-venomous animal contact	Data Rich	Male	Log-transformed SEV scalar: Non Ven	X			X		
Non-venomous animal contact	Global	Male	Log-transformed SEV scalar: Non Ven	X			X		
Foreign body	Data Rich	Female	Socio-demographic Index			X			X
Foreign body	Global	Female	Socio-demographic Index			X			X
Foreign body	Data Rich	Female	Healthcare access and quality index		X			X	
Foreign body	Global	Female	Healthcare access and quality index		X			X	
Foreign body	Data Rich	Female	Education (years per capita)	X			X		
Foreign body	Global	Female	Education (years per capita)	X			X		
Foreign body	Data Rich	Female	LDI (I$ per capita)	X			X		
Foreign body	Global	Female	LDI (I$ per capita)	X			X		
Foreign body	Data Rich	Female	Liters of alcohol consumed per capita	X			X		
Foreign body	Global	Female	Liters of alcohol consumed per capita	X			X		
Foreign body	Data Rich	Female	Indoor Air Pollution (All Cooking Fuels)	X			X		
Foreign body	Global	Female	Indoor Air Pollution (All Cooking Fuels)	X			X		
Foreign body	Data Rich	Female	Population-weighted mean temperature	X			X		
Foreign body	Global	Female	Population-weighted mean temperature	X			X		
Foreign body	Data Rich	Female	Population Over 65 (proportion)	X			X		
Foreign body	Global	Female	Population Over 65 (proportion)	X			X		
Foreign body	Data Rich	Male	Socio-demographic Index			X			X
Foreign body	Global	Male	Socio-demographic Index			X			X
Foreign body	Data Rich	Male	Healthcare access and quality index		X			X	
Foreign body	Global	Male	Healthcare access and quality index		X			X	
Foreign body	Data Rich	Male	Education (years per capita)	X			X		
Foreign body	Global	Male	Education (years per capita)	X			X		
Foreign body	Data Rich	Male	LDI (I$ per capita)	X			X		
Foreign body	Global	Male	LDI (I$ per capita)	X			X		
Foreign body	Data Rich	Male	Liters of alcohol consumed per capita	X			X		
Foreign body	Global	Male	Liters of alcohol consumed per capita	X			X		
Foreign body	Data Rich	Male	Indoor Air Pollution (All Cooking Fuels)	X			X		
Foreign body	Global	Male	Indoor Air Pollution (All Cooking Fuels)	X			X		
Foreign body	Data Rich	Male	Population-weighted mean temperature	X			X		
Foreign body	Global	Male	Population-weighted mean temperature	X			X		
Foreign body	Data Rich	Male	Population Over 65 (proportion)	X			X		
Foreign body	Global	Male	Population Over 65 (proportion)	X			X		
Pulmonary aspiration and foreign body in airway	Global	Female	Education (years per capita)			X			X
Pulmonary aspiration and foreign body in airway	Data Rich	Female	LDI (I$ per capita)			X			X
Pulmonary aspiration and foreign body in airway	Global	Female	LDI (I$ per capita)			X			X
Pulmonary aspiration and foreign body in airway	Data Rich	Female	Socio-demographic Index			X			X
Pulmonary aspiration and foreign body in airway	Global	Female	Socio-demographic Index			X			X
Pulmonary aspiration and foreign body in airway	Data Rich	Female	Healthcare access and quality index		X			X	
Pulmonary aspiration and foreign body in airway	Global	Female	Healthcare access and quality index		X			X	
Pulmonary aspiration and foreign body in airway	Data Rich	Female	Mean BMI		X			X	
Pulmonary aspiration and foreign body in airway	Global	Female	Mean BMI		X			X	
Pulmonary aspiration and foreign body in airway	Global	Female	Alcohol binge drinker proportion, age-standardized		X			X	
Pulmonary aspiration and foreign body in airway	Data Rich	Female	Education (years per capita)	X			X		
Pulmonary aspiration and foreign body in airway	Data Rich	Female	Liters of alcohol consumed per capita	X			X		
Pulmonary aspiration and foreign body in airway	Global	Female	Liters of alcohol consumed per capita	X			X		
Pulmonary aspiration and foreign body in airway	Data Rich	Female	Population-weighted mean temperature	X			X		
Pulmonary aspiration and foreign body in airway	Global	Female	Population-weighted mean temperature	X			X		
Pulmonary aspiration and foreign body in airway	Data Rich	Female	Log-transformed SEV scalar: F Body Asp	X			X		
Pulmonary aspiration and foreign body in airway	Global	Female	Log-transformed SEV scalar: F Body Asp	X			X		
Pulmonary aspiration and foreign body in airway	Global	Male	LDI (I$ per capita)			X			X
Pulmonary aspiration and foreign body in airway	Data Rich	Male	LDI (I$ per capita)			X			X
Pulmonary aspiration and foreign body in airway	Global	Male	Socio-demographic Index			X			X
Pulmonary aspiration and foreign body in airway	Data Rich	Male	Socio-demographic Index			X			X
Pulmonary aspiration and foreign body in airway	Global	Male	Healthcare access and quality index		X			X	
Pulmonary aspiration and foreign body in airway	Data Rich	Male	Healthcare access and quality index		X			X	
Pulmonary aspiration and foreign body in airway	Global	Male	Mean BMI		X			X	
Pulmonary aspiration and foreign body in airway	Data Rich	Male	Mean BMI		X			X	
Pulmonary aspiration and foreign body in airway	Global	Male	Education (years per capita)	X			X		
Pulmonary aspiration and foreign body in airway	Data Rich	Male	Education (years per capita)	X			X		
Pulmonary aspiration and foreign body in airway	Global	Male	Liters of alcohol consumed per capita	X			X		
Pulmonary aspiration and foreign body in airway	Data Rich	Male	Liters of alcohol consumed per capita	X			X		
Pulmonary aspiration and foreign body in airway	Global	Male	Population-weighted mean temperature	X			X		
Pulmonary aspiration and foreign body in airway	Data Rich	Male	Population-weighted mean temperature	X			X		
Pulmonary aspiration and foreign body in airway	Global	Male	Log-transformed SEV scalar: F Body Asp	X			X		
Pulmonary aspiration and foreign body in airway	Data Rich	Male	Log-transformed SEV scalar: F Body Asp	X			X		
Foreign body in other body part	Global	Female	Education (years per capita)			X			X
Foreign body in other body part	Data Rich	Female	Education (years per capita)			X			X
Foreign body in other body part	Global	Female	LDI (I$ per capita)			X			X
Foreign body in other body part	Data Rich	Female	LDI (I$ per capita)			X			X
Foreign body in other body part	Global	Female	Socio-demographic Index			X			X
Foreign body in other body part	Data Rich	Female	Socio-demographic Index			X			X
Foreign body in other body part	Global	Female	Healthcare access and quality index		X			X	
Foreign body in other body part	Data Rich	Female	Healthcare access and quality index		X			X	
Foreign body in other body part	Global	Female	Liters of alcohol consumed per capita	X			X		
Foreign body in other body part	Data Rich	Female	Liters of alcohol consumed per capita	X			X		
Foreign body in other body part	Global	Female	Population-weighted mean temperature	X			X		
Foreign body in other body part	Data Rich	Female	Population-weighted mean temperature	X			X		
Foreign body in other body part	Global	Female	Log-transformed SEV scalar: Oth F Body	X			X		
Foreign body in other body part	Data Rich	Female	Log-transformed SEV scalar: Oth F Body	X			X		
Foreign body in other body part	Data Rich	Male	Education (years per capita)			X			X
Foreign body in other body part	Global	Male	Education (years per capita)			X			X
Foreign body in other body part	Data Rich	Male	LDI (I$ per capita)			X			X
Foreign body in other body part	Global	Male	LDI (I$ per capita)			X			X
Foreign body in other body part	Data Rich	Male	Socio-demographic Index			X			X
Foreign body in other body part	Global	Male	Socio-demographic Index			X			X
Foreign body in other body part	Data Rich	Male	Healthcare access and quality index		X			X	
Foreign body in other body part	Global	Male	Healthcare access and quality index		X			X	
Foreign body in other body part	Data Rich	Male	Liters of alcohol consumed per capita	X			X		
Foreign body in other body part	Global	Male	Liters of alcohol consumed per capita	X			X		
Foreign body in other body part	Data Rich	Male	Population-weighted mean temperature	X			X		
Foreign body in other body part	Global	Male	Population-weighted mean temperature	X			X		
Foreign body in other body part	Data Rich	Male	Log-transformed SEV scalar: Oth F Body	X			X		
Foreign body in other body part	Global	Male	Log-transformed SEV scalar: Oth F Body	X			X		
Environmental heat and cold exposure	Data Rich	Female	Education (years per capita)			X			X
Environmental heat and cold exposure	Global	Female	Education (years per capita)			X			X
Environmental heat and cold exposure	Data Rich	Female	LDI (I$ per capita)			X			X
Environmental heat and cold exposure	Global	Female	LDI (I$ per capita)			X			X
Environmental heat and cold exposure	Data Rich	Female	Socio-demographic Index			X			X
Environmental heat and cold exposure	Global	Female	Socio-demographic Index			X			X
Environmental heat and cold exposure	Data Rich	Female	Elevation Over 1500m (proportion)			X			X
Environmental heat and cold exposure	Global	Female	Elevation Over 1500m (proportion)			X			X
Environmental heat and cold exposure	Data Rich	Female	Population-weighted mean temperature			X			X
Environmental heat and cold exposure	Global	Female	Population-weighted mean temperature			X			X
Environmental heat and cold exposure	Data Rich	Female	Population Density (150-300 ppl/sqkm, proportion)			X			X
Environmental heat and cold exposure	Global	Female	Population Density (150-300 ppl/sqkm, proportion)			X			X
Environmental heat and cold exposure	Data Rich	Female	Elevation 500 to 1500m (proportion)			X			X
Environmental heat and cold exposure	Global	Female	Elevation 500 to 1500m (proportion)			X			X
Environmental heat and cold exposure	Data Rich	Female	Rainfall (Quintiles 4-5)			X			X
Environmental heat and cold exposure	Global	Female	Rainfall (Quintiles 4-5)			X			X
Environmental heat and cold exposure	Data Rich	Female	Sanitation (proportion with access)			X			X
Environmental heat and cold exposure	Global	Female	Sanitation (proportion with access)			X			X
Environmental heat and cold exposure	Data Rich	Female	90th percentile climatic temperature in the given country-year.			X			X
Environmental heat and cold exposure	Global	Female	90th percentile climatic temperature in the given country-year.			X			X
Environmental heat and cold exposure	Data Rich	Female	Healthcare access and quality index		X			X	
Environmental heat and cold exposure	Global	Female	Healthcare access and quality index		X			X	
Environmental heat and cold exposure	Data Rich	Male	Education (years per capita)			X			X
Environmental heat and cold exposure	Global	Male	Education (years per capita)			X			X

Comparison of GBD 2019 and GBD 2021 covariates and level of covariates used in cause of death modeling									
Cause	Model Version Type	Sex	Covariate	Level 1 GBD 2019	Level 2 GBD 2019	Level 3 GBD 2019	Level 1 GBD 2021	Level 2 GBD 2021	Level 3 GBD 2021
Environmental heat and cold exposure	Data Rich	Male	LDI (I$ per capita)			X			X
Environmental heat and cold exposure	Global	Male	LDI (I$ per capita)			X			X
Environmental heat and cold exposure	Data Rich	Male	Socio-demographic Index			X			X
Environmental heat and cold exposure	Global	Male	Socio-demographic Index			X			X
Environmental heat and cold exposure	Data Rich	Male	Elevation Over 1500m (proportion)			X			X
Environmental heat and cold exposure	Global	Male	Elevation Over 1500m (proportion)			X			X
Environmental heat and cold exposure	Data Rich	Male	Population-weighted mean temperature			X			X
Environmental heat and cold exposure	Global	Male	Population-weighted mean temperature			X			X
Environmental heat and cold exposure	Data Rich	Male	Population Density (150-300 ppl/sqkm, proportion)			X			X
Environmental heat and cold exposure	Global	Male	Population Density (150-300 ppl/sqkm, proportion)			X			X
Environmental heat and cold exposure	Data Rich	Male	Elevation 500 to 1500m (proportion)			X			X
Environmental heat and cold exposure	Global	Male	Elevation 500 to 1500m (proportion)			X			X
Environmental heat and cold exposure	Data Rich	Male	Rainfall (Quintiles 4-5)			X			X
Environmental heat and cold exposure	Global	Male	Rainfall (Quintiles 4-5)			X			X
Environmental heat and cold exposure	Data Rich	Male	Sanitation (proportion with access)			X			X
Environmental heat and cold exposure	Global	Male	Sanitation (proportion with access)			X			X
Environmental heat and cold exposure	Data Rich	Male	90th percentile climatic temperature in the given country-year.			X			X
Environmental heat and cold exposure	Global	Male	90th percentile climatic temperature in the given country-year.			X			X
Environmental heat and cold exposure	Data Rich	Male	Healthcare access and quality index		X			X	
Environmental heat and cold exposure	Global	Male	Healthcare access and quality index		X			X	
Other unintentional injuries	Data Rich	Female	Education (years per capita)			X			X
Other unintentional injuries	Global	Female	Education (years per capita)			X			X
Other unintentional injuries	Data Rich	Female	LDI (I$ per capita)			X			X
Other unintentional injuries	Global	Female	LDI (I$ per capita)			X			X
Other unintentional injuries	Data Rich	Female	Socio-demographic Index			X			X
Other unintentional injuries	Global	Female	Socio-demographic Index			X			X
Other unintentional injuries	Data Rich	Female	Population Density (over 1000 ppl/sqkm, proportion)			X			X
Other unintentional injuries	Global	Female	Population Density (over 1000 ppl/sqkm, proportion)			X			X
Other unintentional injuries	Data Rich	Female	Population Density (under 150 ppl/sqkm, proportion)			X			X
Other unintentional injuries	Global	Female	Population Density (under 150 ppl/sqkm, proportion)			X			X
Other unintentional injuries	Data Rich	Female	Healthcare access and quality index		X			X	
Other unintentional injuries	Global	Female	Healthcare access and quality index		X			X	
Other unintentional injuries	Data Rich	Female	Liters of alcohol consumed per capita	X			X		
Other unintentional injuries	Global	Female	Liters of alcohol consumed per capita	X			X		
Other unintentional injuries	Data Rich	Female	Population-weighted mean temperature	X			X		
Other unintentional injuries	Global	Female	Population-weighted mean temperature	X			X		
Other unintentional injuries	Data Rich	Female	Vehicles - 2 wheels (per capita)	X			X		
Other unintentional injuries	Global	Female	Vehicles - 2 wheels (per capita)	X			X		
Other unintentional injuries	Data Rich	Female	Vehicles - 4 wheels (per capita)	X			X		
Other unintentional injuries	Global	Female	Vehicles - 4 wheels (per capita)	X			X		
Other unintentional injuries	Data Rich	Female	Log-transformed SEV scalar: Oth Unint	X			X		
Other unintentional injuries	Global	Female	Log-transformed SEV scalar: Oth Unint	X			X		
Other unintentional injuries	Data Rich	Male	Education (years per capita)			X			X
Other unintentional injuries	Global	Male	Education (years per capita)			X			X
Other unintentional injuries	Data Rich	Male	LDI (I$ per capita)			X			X
Other unintentional injuries	Global	Male	LDI (I$ per capita)			X			X
Other unintentional injuries	Data Rich	Male	Socio-demographic Index			X			X
Other unintentional injuries	Global	Male	Socio-demographic Index			X			X
Other unintentional injuries	Data Rich	Male	Population Density (over 1000 ppl/sqkm, proportion)			X			X
Other unintentional injuries	Global	Male	Population Density (over 1000 ppl/sqkm, proportion)			X			X
Other unintentional injuries	Data Rich	Male	Population Density (under 150 ppl/sqkm, proportion)			X			X
Other unintentional injuries	Global	Male	Population Density (under 150 ppl/sqkm, proportion)			X			X
Other unintentional injuries	Data Rich	Male	Healthcare access and quality index		X			X	
Other unintentional injuries	Global	Male	Healthcare access and quality index		X			X	
Other unintentional injuries	Data Rich	Male	Liters of alcohol consumed per capita	X			X		
Other unintentional injuries	Data Rich	Male	Population-weighted mean temperature	X			X		
Other unintentional injuries	Data Rich	Male	Vehicles - 2 wheels (per capita)	X			X		
Other unintentional injuries	Global	Male	Vehicles - 2 wheels (per capita)	X			X		
Other unintentional injuries	Data Rich	Male	Vehicles - 4 wheels (per capita)	X			X		
Other unintentional injuries	Global	Male	Vehicles - 4 wheels (per capita)	X			X		
Other unintentional injuries	Data Rich	Male	Log-transformed SEV scalar: Oth Unint	X			X		
Other unintentional injuries	Global	Male	Log-transformed SEV scalar: Oth Unint	X			X		
Other unintentional injuries	Global	Male	Liters of alcohol consumed per capita				X		
Other unintentional injuries	Global	Male	Population-weighted mean temperature				X		
Self-harm	Data Rich	Female	Education (years per capita)			X			X
Self-harm	Global	Female	Education (years per capita)			X			X
Self-harm	Data Rich	Female	LDI (I$ per capita)			X			X
Self-harm	Global	Female	LDI (I$ per capita)			X			X
Self-harm	Data Rich	Female	Socio-demographic Index			X			X
Self-harm	Global	Female	Socio-demographic Index			X			X
Self-harm	Data Rich	Female	Healthcare access and quality index		X			X	
Self-harm	Global	Female	Healthcare access and quality index		X			X	
Self-harm	Data Rich	Female	Population Density (150-300 ppl/sqkm, proportion)		X			X	
Self-harm	Global	Female	Population Density (150-300 ppl/sqkm, proportion)		X			X	
Self-harm	Data Rich	Female	Population Density (300-500 ppl/sqkm, proportion)		X			X	
Self-harm	Global	Female	Population Density (300-500 ppl/sqkm, proportion)		X			X	
Self-harm	Data Rich	Female	Population Density (500-1000 ppl/sqkm, proportion)		X			X	
Self-harm	Global	Female	Population Density (500-1000 ppl/sqkm, proportion)		X			X	
Self-harm	Data Rich	Female	Population Density (over 1000 ppl/sqkm, proportion)		X			X	
Self-harm	Global	Female	Population Density (over 1000 ppl/sqkm, proportion)		X			X	
Self-harm	Data Rich	Female	Population Density (under 150 ppl/sqkm, proportion)		X			X	
Self-harm	Global	Female	Population Density (under 150 ppl/sqkm, proportion)		X			X	
Self-harm	Data Rich	Female	Liters of alcohol consumed per capita	X			X		
Self-harm	Global	Female	Liters of alcohol consumed per capita	X			X		
Self-harm	Data Rich	Female	Population-weighted mean temperature	X			X		
Self-harm	Global	Female	Population-weighted mean temperature	X			X		
Self-harm	Data Rich	Female	Log-transformed SEV scalar: Self Harm	X			X		
Self-harm	Global	Female	Log-transformed SEV scalar: Self Harm	X			X		
Self-harm	Data Rich	Female	Major depressive disorder	X			X		
Self-harm	Global	Female	Major depressive disorder	X			X		
Self-harm	Data Rich	Female	12-month non-partner sexual violence	X			X		
Self-harm	Global	Female	12-month non-partner sexual violence	X			X		
Self-harm	Data Rich	Male	Education (years per capita)			X			X
Self-harm	Global	Male	Education (years per capita)			X			X
Self-harm	Data Rich	Male	LDI (I$ per capita)			X			X
Self-harm	Global	Male	LDI (I$ per capita)			X			X
Self-harm	Data Rich	Male	Socio-demographic Index			X			X
Self-harm	Global	Male	Socio-demographic Index			X			X
Self-harm	Data Rich	Male	Healthcare access and quality index		X			X	
Self-harm	Global	Male	Healthcare access and quality index		X			X	
Self-harm	Data Rich	Male	Population Density (150-300 ppl/sqkm, proportion)		X			X	
Self-harm	Global	Male	Population Density (150-300 ppl/sqkm, proportion)		X			X	
Self-harm	Data Rich	Male	Population Density (300-500 ppl/sqkm, proportion)		X			X	
Self-harm	Global	Male	Population Density (300-500 ppl/sqkm, proportion)		X			X	
Self-harm	Data Rich	Male	Population Density (500-1000 ppl/sqkm, proportion)		X			X	
Self-harm	Global	Male	Population Density (500-1000 ppl/sqkm, proportion)		X			X	
Self-harm	Data Rich	Male	Population Density (over 1000 ppl/sqkm, proportion)		X			X	
Self-harm	Global	Male	Population Density (over 1000 ppl/sqkm, proportion)		X			X	
Self-harm	Data Rich	Male	Population Density (under 150 ppl/sqkm, proportion)		X			X	
Self-harm	Global	Male	Population Density (under 150 ppl/sqkm, proportion)		X			X	
Self-harm	Global	Male	Muslim Religion (proportion of population)		X			X	
Self-harm	Data Rich	Male	Liters of alcohol consumed per capita	X			X		
Self-harm	Global	Male	Liters of alcohol consumed per capita	X			X		
Self-harm	Data Rich	Male	Population-weighted mean temperature	X			X		
Self-harm	Global	Male	Population-weighted mean temperature	X			X		
Self-harm	Data Rich	Male	Major depressive disorder	X			X		
Self-harm	Global	Male	Major depressive disorder	X			X		
Self-harm	Data Rich	Male	12-month non-partner sexual violence	X			X		
Self-harm	Global	Male	12-month non-partner sexual violence	X			X		
Self-harm	Data Rich	Male	Muslim Religion (proportion of population)	X			X		
Self-harm by firearm	Data Rich	Female	Education (years per capita)			X			X
Self-harm by firearm	Global	Female	Education (years per capita)			X			X
Self-harm by firearm	Data Rich	Female	LDI (I$ per capita)			X			X
Self-harm by firearm	Global	Female	LDI (I$ per capita)			X			X
Self-harm by firearm	Data Rich	Female	Socio-demographic Index			X			X
Self-harm by firearm	Global	Female	Socio-demographic Index			X			X

Comparison of GBD 2019 and GBD 2021 covariates and level of covariates used in cause of death modeling

Cause	Model Version Type	Sex	Covariate	Level 1 GBD 2019	Level 2 GBD 2019	Level 3 GBD 2019	Level 1 GBD 2021	Level 2 GBD 2021	Level 3 GBD 2021
Self-harm by firearm	Data Rich	Female	Healthcare access and quality index		X			X	
Self-harm by firearm	Global	Female	Healthcare access and quality index		X			X	
Self-harm by firearm	Data Rich	Female	Population Density (150-300 ppl/sqkm, proportion)		X			X	
Self-harm by firearm	Global	Female	Population Density (150-300 ppl/sqkm, proportion)		X			X	
Self-harm by firearm	Data Rich	Female	Population Density (300-500 ppl/sqkm, proportion)		X			X	
Self-harm by firearm	Global	Female	Population Density (300-500 ppl/sqkm, proportion)		X			X	
Self-harm by firearm	Data Rich	Female	Population Density (500-1000 ppl/sqkm, proportion)		X			X	
Self-harm by firearm	Global	Female	Population Density (500-1000 ppl/sqkm, proportion)		X			X	
Self-harm by firearm	Data Rich	Female	Population Density (over 1000 ppl/sqkm, proportion)		X			X	
Self-harm by firearm	Global	Female	Population Density (over 1000 ppl/sqkm, proportion)		X			X	
Self-harm by firearm	Data Rich	Female	Population Density (under 150 ppl/sqkm, proportion)		X			X	
Self-harm by firearm	Global	Female	Population Density (under 150 ppl/sqkm, proportion)		X			X	
Self-harm by firearm	Data Rich	Female	Liters of alcohol consumed per capita	X			X		
Self-harm by firearm	Global	Female	Liters of alcohol consumed per capita	X			X		
Self-harm by firearm	Data Rich	Female	Population-weighted mean temperature	X			X		
Self-harm by firearm	Global	Female	Population-weighted mean temperature	X			X		
Self-harm by firearm	Data Rich	Female	Log-transformed SEV scalar: Self Harm	X			X		
Self-harm by firearm	Global	Female	Log-transformed SEV scalar: Self Harm	X			X		
Self-harm by firearm	Data Rich	Female	Major depressive disorder	X			X		
Self-harm by firearm	Global	Female	Major depressive disorder	X			X		
Self-harm by firearm	Data Rich	Female	12-month non-partner sexual violence	X			X		
Self-harm by firearm	Global	Female	12-month non-partner sexual violence	X			X		
Self-harm by firearm	Data Rich	Male	Education (years per capita)			X			X
Self-harm by firearm	Global	Male	Education (years per capita)			X			X
Self-harm by firearm	Data Rich	Male	LDI (I$ per capita)			X			X
Self-harm by firearm	Global	Male	LDI (I$ per capita)			X			X
Self-harm by firearm	Data Rich	Male	Socio-demographic Index			X			X
Self-harm by firearm	Global	Male	Socio-demographic Index			X			X
Self-harm by firearm	Data Rich	Male	Healthcare access and quality index		X			X	
Self-harm by firearm	Global	Male	Healthcare access and quality index		X			X	
Self-harm by firearm	Data Rich	Male	Population Density (150-300 ppl/sqkm, proportion)		X			X	
Self-harm by firearm	Global	Male	Population Density (150-300 ppl/sqkm, proportion)		X			X	
Self-harm by firearm	Data Rich	Male	Population Density (300-500 ppl/sqkm, proportion)		X			X	
Self-harm by firearm	Global	Male	Population Density (300-500 ppl/sqkm, proportion)		X			X	
Self-harm by firearm	Data Rich	Male	Population Density (500-1000 ppl/sqkm, proportion)		X			X	
Self-harm by firearm	Global	Male	Population Density (500-1000 ppl/sqkm, proportion)		X			X	
Self-harm by firearm	Data Rich	Male	Population Density (over 1000 ppl/sqkm, proportion)		X			X	
Self-harm by firearm	Global	Male	Population Density (over 1000 ppl/sqkm, proportion)		X			X	
Self-harm by firearm	Data Rich	Male	Population Density (under 150 ppl/sqkm, proportion)		X			X	
Self-harm by firearm	Global	Male	Population Density (under 150 ppl/sqkm, proportion)		X			X	
Self-harm by firearm	Data Rich	Male	Liters of alcohol consumed per capita	X			X		
Self-harm by firearm	Global	Male	Liters of alcohol consumed per capita	X			X		
Self-harm by firearm	Data Rich	Male	Population-weighted mean temperature	X			X		
Self-harm by firearm	Global	Male	Population-weighted mean temperature	X			X		
Self-harm by firearm	Data Rich	Male	Log-transformed SEV scalar: Self Harm	X			X		
Self-harm by firearm	Global	Male	Log-transformed SEV scalar: Self Harm	X			X		
Self-harm by firearm	Data Rich	Male	Major depressive disorder	X			X		
Self-harm by firearm	Global	Male	Major depressive disorder	X			X		
Self-harm by firearm	Data Rich	Male	12-month non-partner sexual violence	X			X		
Self-harm by firearm	Global	Male	12-month non-partner sexual violence	X			X		
Self-harm by other specified means	Data Rich	Female	Education (years per capita)			X			X
Self-harm by other specified means	Global	Female	Education (years per capita)			X			X
Self-harm by other specified means	Data Rich	Female	LDI (I$ per capita)			X			X
Self-harm by other specified means	Global	Female	LDI (I$ per capita)			X			X
Self-harm by other specified means	Data Rich	Female	Socio-demographic Index			X			X
Self-harm by other specified means	Global	Female	Socio-demographic Index			X			X
Self-harm by other specified means	Data Rich	Female	Healthcare access and quality index		X			X	
Self-harm by other specified means	Global	Female	Healthcare access and quality index		X			X	
Self-harm by other specified means	Data Rich	Female	Population Density (150-300 ppl/sqkm, proportion)		X			X	
Self-harm by other specified means	Global	Female	Population Density (150-300 ppl/sqkm, proportion)		X			X	
Self-harm by other specified means	Data Rich	Female	Population Density (300-500 ppl/sqkm, proportion)		X			X	
Self-harm by other specified means	Global	Female	Population Density (300-500 ppl/sqkm, proportion)		X			X	
Self-harm by other specified means	Data Rich	Female	Population Density (500-1000 ppl/sqkm, proportion)		X			X	
Self-harm by other specified means	Global	Female	Population Density (500-1000 ppl/sqkm, proportion)		X			X	
Self-harm by other specified means	Data Rich	Female	Population Density (over 1000 ppl/sqkm, proportion)		X			X	
Self-harm by other specified means	Global	Female	Population Density (over 1000 ppl/sqkm, proportion)		X			X	
Self-harm by other specified means	Data Rich	Female	Population Density (under 150 ppl/sqkm, proportion)		X			X	
Self-harm by other specified means	Global	Female	Population Density (under 150 ppl/sqkm, proportion)		X			X	
Self-harm by other specified means	Data Rich	Female	Liters of alcohol consumed per capita	X			X		
Self-harm by other specified means	Global	Female	Liters of alcohol consumed per capita	X			X		
Self-harm by other specified means	Data Rich	Female	Population-weighted mean temperature	X			X		
Self-harm by other specified means	Global	Female	Population-weighted mean temperature	X			X		
Self-harm by other specified means	Data Rich	Female	Log-transformed SEV scalar: Self Harm	X			X		
Self-harm by other specified means	Global	Female	Log-transformed SEV scalar: Self Harm	X			X		
Self-harm by other specified means	Data Rich	Female	Major depressive disorder	X			X		
Self-harm by other specified means	Global	Female	Major depressive disorder	X			X		
Self-harm by other specified means	Data Rich	Female	12-month non-partner sexual violence	X			X		
Self-harm by other specified means	Global	Female	12-month non-partner sexual violence	X			X		
Self-harm by other specified means	Data Rich	Male	Education (years per capita)			X			X
Self-harm by other specified means	Global	Male	Education (years per capita)			X			X
Self-harm by other specified means	Data Rich	Male	LDI (I$ per capita)			X			X
Self-harm by other specified means	Global	Male	LDI (I$ per capita)			X			X
Self-harm by other specified means	Data Rich	Male	Socio-demographic Index			X			X
Self-harm by other specified means	Global	Male	Socio-demographic Index			X			X
Self-harm by other specified means	Data Rich	Male	Healthcare access and quality index		X			X	
Self-harm by other specified means	Global	Male	Healthcare access and quality index		X			X	
Self-harm by other specified means	Data Rich	Male	Population Density (150-300 ppl/sqkm, proportion)		X			X	
Self-harm by other specified means	Global	Male	Population Density (150-300 ppl/sqkm, proportion)		X			X	
Self-harm by other specified means	Data Rich	Male	Population Density (300-500 ppl/sqkm, proportion)		X			X	
Self-harm by other specified means	Global	Male	Population Density (300-500 ppl/sqkm, proportion)		X			X	
Self-harm by other specified means	Data Rich	Male	Population Density (500-1000 ppl/sqkm, proportion)		X			X	
Self-harm by other specified means	Global	Male	Population Density (500-1000 ppl/sqkm, proportion)		X			X	
Self-harm by other specified means	Data Rich	Male	Population Density (over 1000 ppl/sqkm, proportion)		X			X	
Self-harm by other specified means	Global	Male	Population Density (over 1000 ppl/sqkm, proportion)		X			X	
Self-harm by other specified means	Data Rich	Male	Population Density (under 150 ppl/sqkm, proportion)		X			X	
Self-harm by other specified means	Global	Male	Population Density (under 150 ppl/sqkm, proportion)		X			X	
Self-harm by other specified means	Data Rich	Male	Liters of alcohol consumed per capita	X			X		
Self-harm by other specified means	Global	Male	Liters of alcohol consumed per capita	X			X		
Self-harm by other specified means	Data Rich	Male	Population-weighted mean temperature	X			X		
Self-harm by other specified means	Global	Male	Population-weighted mean temperature	X			X		
Self-harm by other specified means	Data Rich	Male	Log-transformed SEV scalar: Self Harm	X			X		
Self-harm by other specified means	Global	Male	Log-transformed SEV scalar: Self Harm	X			X		
Self-harm by other specified means	Data Rich	Male	Major depressive disorder	X			X		
Self-harm by other specified means	Global	Male	Major depressive disorder	X			X		
Self-harm by other specified means	Data Rich	Male	12-month non-partner sexual violence	X			X		
Self-harm by other specified means	Global	Male	12-month non-partner sexual violence	X			X		
Interpersonal violence	Global	Female	Education (years per capita)			X			X
Interpersonal violence	Data Rich	Female	Education (years per capita)			X			X
Interpersonal violence	Global	Female	LDI (I$ per capita)			X			X
Interpersonal violence	Data Rich	Female	LDI (I$ per capita)			X			X
Interpersonal violence	Global	Female	Socio-demographic Index			X			X
Interpersonal violence	Data Rich	Female	Socio-demographic Index			X			X
Interpersonal violence	Global	Female	Healthcare access and quality index		X			X	
Interpersonal violence	Data Rich	Female	Healthcare access and quality index		X			X	
Interpersonal violence	Global	Female	Population Density (over 1000 ppl/sqkm, proportion)		X			X	
Interpersonal violence	Data Rich	Female	Population Density (over 1000 ppl/sqkm, proportion)		X			X	
Interpersonal violence	Global	Female	Liters of alcohol consumed per capita	X			X		
Interpersonal violence	Data Rich	Female	Liters of alcohol consumed per capita	X			X		
Interpersonal violence	Global	Female	Population-weighted mean temperature	X			X		
Interpersonal violence	Data Rich	Female	Population-weighted mean temperature	X			X		
Interpersonal violence	Global	Female	Education Relative Inequality (Gini)	X			X		

Comparison of GBD 2019 and GBD 2021 covariates and level of covariates used in cause of death modeling									
Cause	Model Version Type	Sex	Covariate	Level 1 GBD 2019	Level 2 GBD 2019	Level 3 GBD 2019	Level 1 GBD 2021	Level 2 GBD 2021	Level 3 GBD 2021
Interpersonal violence	Data Rich	Female	Education Relative Inequality (Gini)	X			X		
Interpersonal violence	Global	Female	Population 15 to 30 males (proportion)	X			X		
Interpersonal violence	Data Rich	Female	Population 15 to 30 males (proportion)	X			X		
Interpersonal violence	Global	Female	Log-transformed SEV scalar: Violence	X			X		
Interpersonal violence	Data Rich	Female	Log-transformed SEV scalar: Violence	X			X		
Interpersonal violence	Global	Male	Education (years per capita)			X			X
Interpersonal violence	Data Rich	Male	Education (years per capita)			X			X
Interpersonal violence	Global	Male	LDI (I$ per capita)			X			X
Interpersonal violence	Data Rich	Male	LDI (I$ per capita)			X			X
Interpersonal violence	Global	Male	Socio-demographic Index			X			X
Interpersonal violence	Data Rich	Male	Socio-demographic Index			X			X
Interpersonal violence	Global	Male	Healthcare access and quality index		X			X	
Interpersonal violence	Data Rich	Male	Healthcare access and quality index		X			X	
Interpersonal violence	Global	Male	Population Density (over 1000 ppl/sqkm, proportion)		X			X	
Interpersonal violence	Data Rich	Male	Population Density (over 1000 ppl/sqkm, proportion)		X			X	
Interpersonal violence	Global	Male	Liters of alcohol consumed per capita	X			X		
Interpersonal violence	Data Rich	Male	Liters of alcohol consumed per capita	X			X		
Interpersonal violence	Global	Male	Population-weighted mean temperature	X			X		
Interpersonal violence	Data Rich	Male	Population-weighted mean temperature	X			X		
Interpersonal violence	Global	Male	Education Relative Inequality (Gini)	X			X		
Interpersonal violence	Data Rich	Male	Education Relative Inequality (Gini)	X			X		
Interpersonal violence	Global	Male	Population 15 to 30 males (proportion)	X			X		
Interpersonal violence	Data Rich	Male	Population 15 to 30 males (proportion)	X			X		
Interpersonal violence	Global	Male	Log-transformed SEV scalar: Violence	X			X		
Interpersonal violence	Data Rich	Male	Log-transformed SEV scalar: Violence	X			X		
Assault by firearm	Data Rich	Female	Education (years per capita)			X			X
Assault by firearm	Global	Female	Education (years per capita)			X			X
Assault by firearm	Data Rich	Female	LDI (I$ per capita)			X			X
Assault by firearm	Global	Female	LDI (I$ per capita)			X			X
Assault by firearm	Data Rich	Female	Socio-demographic Index			X			X
Assault by firearm	Global	Female	Socio-demographic Index			X			X
Assault by firearm	Data Rich	Female	Healthcare access and quality index		X			X	
Assault by firearm	Global	Female	Healthcare access and quality index		X			X	
Assault by firearm	Data Rich	Female	Population Density (over 1000 ppl/sqkm, proportion)		X			X	
Assault by firearm	Global	Female	Population Density (over 1000 ppl/sqkm, proportion)		X			X	
Assault by firearm	Data Rich	Female	Liters of alcohol consumed per capita	X			X		
Assault by firearm	Global	Female	Liters of alcohol consumed per capita	X			X		
Assault by firearm	Data Rich	Female	Population-weighted mean temperature	X			X		
Assault by firearm	Global	Female	Population-weighted mean temperature	X			X		
Assault by firearm	Data Rich	Female	Log-transformed SEV scalar: Viol Gun	X			X		
Assault by firearm	Global	Female	Log-transformed SEV scalar: Viol Gun	X			X		
Assault by firearm	Data Rich	Female	Education Relative Inequality (Gini)	X			X		
Assault by firearm	Global	Female	Education Relative Inequality (Gini)	X			X		
Assault by firearm	Data Rich	Female	Population 15 to 30 males (proportion)	X			X		
Assault by firearm	Global	Female	Population 15 to 30 males (proportion)	X			X		
Assault by firearm	Data Rich	Male	Education (years per capita)			X			X
Assault by firearm	Global	Male	Education (years per capita)			X			X
Assault by firearm	Data Rich	Male	LDI (I$ per capita)			X			X
Assault by firearm	Global	Male	LDI (I$ per capita)			X			X
Assault by firearm	Data Rich	Male	Socio-demographic Index			X			X
Assault by firearm	Global	Male	Socio-demographic Index			X			X
Assault by firearm	Data Rich	Male	Healthcare access and quality index		X			X	
Assault by firearm	Global	Male	Healthcare access and quality index		X			X	
Assault by firearm	Data Rich	Male	Population Density (over 1000 ppl/sqkm, proportion)		X			X	
Assault by firearm	Global	Male	Population Density (over 1000 ppl/sqkm, proportion)		X			X	
Assault by firearm	Data Rich	Male	Liters of alcohol consumed per capita	X			X		
Assault by firearm	Global	Male	Liters of alcohol consumed per capita	X			X		
Assault by firearm	Data Rich	Male	Population-weighted mean temperature	X			X		
Assault by firearm	Global	Male	Population-weighted mean temperature	X			X		
Assault by firearm	Data Rich	Male	Log-transformed SEV scalar: Viol Gun	X			X		
Assault by firearm	Global	Male	Log-transformed SEV scalar: Viol Gun	X			X		
Assault by firearm	Data Rich	Male	Education Relative Inequality (Gini)	X			X		
Assault by firearm	Global	Male	Education Relative Inequality (Gini)	X			X		
Assault by firearm	Data Rich	Male	Population 15 to 30 males (proportion)	X			X		
Assault by firearm	Global	Male	Population 15 to 30 males (proportion)	X			X		
Assault by sharp object	Global	Female	Education (years per capita)			X			X
Assault by sharp object	Data Rich	Female	Education (years per capita)			X			X
Assault by sharp object	Global	Female	LDI (I$ per capita)			X			X
Assault by sharp object	Data Rich	Female	LDI (I$ per capita)			X			X
Assault by sharp object	Global	Female	Socio-demographic Index			X			X
Assault by sharp object	Data Rich	Female	Socio-demographic Index			X			X
Assault by sharp object	Global	Female	Healthcare access and quality index		X			X	
Assault by sharp object	Data Rich	Female	Healthcare access and quality index		X			X	
Assault by sharp object	Data Rich	Female	Population-weighted mean temperature		X			X	
Assault by sharp object	Global	Female	Population Density (over 1000 ppl/sqkm, proportion)		X			X	
Assault by sharp object	Data Rich	Female	Population Density (over 1000 ppl/sqkm, proportion)		X			X	
Assault by sharp object	Global	Female	Liters of alcohol consumed per capita	X			X		
Assault by sharp object	Data Rich	Female	Liters of alcohol consumed per capita	X			X		
Assault by sharp object	Global	Female	Population-weighted mean temperature	X			X		
Assault by sharp object	Global	Female	Education Relative Inequality (Gini)	X			X		
Assault by sharp object	Data Rich	Female	Education Relative Inequality (Gini)	X			X		
Assault by sharp object	Global	Female	Population 15 to 30 males (proportion)	X			X		
Assault by sharp object	Data Rich	Female	Population 15 to 30 males (proportion)	X			X		
Assault by sharp object	Global	Female	Log-transformed SEV scalar: Viol Knife	X			X		
Assault by sharp object	Data Rich	Female	Log-transformed SEV scalar: Viol Knife	X			X		
Assault by sharp object	Global	Male	Education (years per capita)			X			X
Assault by sharp object	Data Rich	Male	Education (years per capita)			X			X
Assault by sharp object	Global	Male	LDI (I$ per capita)			X			X
Assault by sharp object	Data Rich	Male	LDI (I$ per capita)			X			X
Assault by sharp object	Global	Male	Socio-demographic Index			X			X
Assault by sharp object	Data Rich	Male	Socio-demographic Index			X			X
Assault by sharp object	Global	Male	Healthcare access and quality index		X			X	
Assault by sharp object	Data Rich	Male	Healthcare access and quality index		X			X	
Assault by sharp object	Global	Male	Population Density (over 1000 ppl/sqkm, proportion)		X			X	
Assault by sharp object	Data Rich	Male	Population Density (over 1000 ppl/sqkm, proportion)		X			X	
Assault by sharp object	Global	Male	Liters of alcohol consumed per capita	X			X		
Assault by sharp object	Data Rich	Male	Liters of alcohol consumed per capita	X			X		
Assault by sharp object	Global	Male	Population-weighted mean temperature	X			X		
Assault by sharp object	Data Rich	Male	Population-weighted mean temperature	X			X		
Assault by sharp object	Global	Male	Education Relative Inequality (Gini)	X			X		
Assault by sharp object	Data Rich	Male	Education Relative Inequality (Gini)	X			X		
Assault by sharp object	Global	Male	Population 15 to 30 males (proportion)	X			X		
Assault by sharp object	Data Rich	Male	Population 15 to 30 males (proportion)	X			X		
Assault by sharp object	Global	Male	Log-transformed SEV scalar: Viol Knife	X			X		
Assault by sharp object	Data Rich	Male	Log-transformed SEV scalar: Viol Knife	X			X		
Assault by other means	Global	Female	Education (years per capita)			X			X
Assault by other means	Data Rich	Female	Education (years per capita)			X			X
Assault by other means	Global	Female	LDI (I$ per capita)			X			X
Assault by other means	Data Rich	Female	LDI (I$ per capita)			X			X
Assault by other means	Global	Female	Socio-demographic Index			X			X
Assault by other means	Data Rich	Female	Socio-demographic Index			X			X
Assault by other means	Global	Female	Healthcare access and quality index		X			X	
Assault by other means	Data Rich	Female	Healthcare access and quality index		X			X	
Assault by other means	Global	Female	Population Density (over 1000 ppl/sqkm, proportion)		X			X	
Assault by other means	Data Rich	Female	Population Density (over 1000 ppl/sqkm, proportion)		X			X	
Assault by other means	Global	Female	Liters of alcohol consumed per capita	X			X		
Assault by other means	Data Rich	Female	Liters of alcohol consumed per capita	X			X		
Assault by other means	Global	Female	Population-weighted mean temperature	X			X		
Assault by other means	Data Rich	Female	Population-weighted mean temperature	X			X		
Assault by other means	Global	Female	Education Relative Inequality (Gini)	X			X		
Assault by other means	Data Rich	Female	Education Relative Inequality (Gini)	X			X		
Assault by other means	Global	Female	Population 15 to 30 males (proportion)	X			X		
Assault by other means	Data Rich	Female	Population 15 to 30 males (proportion)	X			X		
Assault by other means	Global	Female	Log-transformed SEV scalar: Oth Viol	X			X		
Assault by other means	Data Rich	Female	Log-transformed SEV scalar: Oth Viol	X			X		
Assault by other means	Global	Male	Education (years per capita)			X			X
Assault by other means	Data Rich	Male	Education (years per capita)			X			X
Assault by other means	Global	Male	LDI (I$ per capita)			X			X
Assault by other means	Data Rich	Male	LDI (I$ per capita)			X			X
Assault by other means	Global	Male	Socio-demographic Index			X			X
Assault by other means	Data Rich	Male	Socio-demographic Index			X			X
Assault by other means	Global	Male	Healthcare access and quality index		X			X	
Assault by other means	Data Rich	Male	Healthcare access and quality index		X			X	
Assault by other means	Global	Male	Population Density (over 1000 ppl/sqkm, proportion)		X			X	

Comparison of GBD 2019 and GBD 2021 covariates and level of covariates used in cause of death modeling									
Cause	Model Version Type	Sex	Covariate	Level 1 GBD 2019	Level 2 GBD 2019	Level 3 GBD 2019	Level 1 GBD 2021	Level 2 GBD 2021	Level 3 GBD 2021
Assault by other means	Data Rich	Male	Population Density (over 1000 ppl/sqkm, proportion)		X			X	
Assault by other means	Global	Male	Liters of alcohol consumed per capita	X			X		
Assault by other means	Data Rich	Male	Liters of alcohol consumed per capita	X			X		
Assault by other means	Global	Male	Population-weighted mean temperature	X			X		
Assault by other means	Data Rich	Male	Population-weighted mean temperature	X			X		
Assault by other means	Global	Male	Education Relative Inequality (Gini)	X			X		
Assault by other means	Data Rich	Male	Education Relative Inequality (Gini)	X			X		
Assault by other means	Global	Male	Population 15 to 30 males (proportion)	X			X		
Assault by other means	Data Rich	Male	Population 15 to 30 males (proportion)	X			X		
Assault by other means	Global	Male	Log-transformed SEV scalar: Oth Viol	X			X		
Assault by other means	Data Rich	Male	Log-transformed SEV scalar: Oth Viol	X			X		
Executions and police conflict	Global	Female	Education (years per capita)			X			X
Executions and police conflict	Data Rich	Female	Education (years per capita)			X			X
Executions and police conflict	Global	Female	LDI (I$ per capita)			X			X
Executions and police conflict	Data Rich	Female	LDI (I$ per capita)			X			X
Executions and police conflict	Global	Female	Socio-demographic Index		X			X	
Executions and police conflict	Data Rich	Female	Socio-demographic Index		X			X	
Executions and police conflict	Global	Female	Healthcare access and quality index		X			X	
Executions and police conflict	Data Rich	Female	Healthcare access and quality index		X			X	
Executions and police conflict	Global	Female	Population Density (over 1000 ppl/sqkm, proportion)		X			X	
Executions and police conflict	Data Rich	Female	Population Density (over 1000 ppl/sqkm, proportion)		X			X	
Executions and police conflict	Global	Female	Liters of alcohol consumed per capita	X			X		
Executions and police conflict	Data Rich	Female	Liters of alcohol consumed per capita	X			X		
Executions and police conflict	Global	Female	Population-weighted mean temperature	X			X		
Executions and police conflict	Data Rich	Female	Population-weighted mean temperature	X			X		
Executions and police conflict	Global	Female	Population 15 to 30 males (proportion)	X			X		
Executions and police conflict	Data Rich	Female	Population 15 to 30 males (proportion)	X			X		
Executions and police conflict	Global	Male	Education (years per capita)			X			X
Executions and police conflict	Data Rich	Male	Education (years per capita)			X			X
Executions and police conflict	Global	Male	LDI (I$ per capita)			X			X
Executions and police conflict	Data Rich	Male	LDI (I$ per capita)			X			X
Executions and police conflict	Global	Male	Socio-demographic Index		X			X	
Executions and police conflict	Data Rich	Male	Socio-demographic Index		X			X	
Executions and police conflict	Global	Male	Healthcare access and quality index		X			X	
Executions and police conflict	Data Rich	Male	Healthcare access and quality index		X			X	
Executions and police conflict	Global	Male	Population Density (over 1000 ppl/sqkm, proportion)		X			X	
Executions and police conflict	Data Rich	Male	Population Density (over 1000 ppl/sqkm, proportion)		X			X	
Executions and police conflict	Global	Male	Liters of alcohol consumed per capita	X			X		
Executions and police conflict	Data Rich	Male	Liters of alcohol consumed per capita	X			X		
Executions and police conflict	Global	Male	Population-weighted mean temperature	X			X		
Executions and police conflict	Data Rich	Male	Population-weighted mean temperature	X			X		
Executions and police conflict	Data Rich	Male	Education Relative Inequality (Gini)	X			X		
Executions and police conflict	Global	Male	Population 15 to 30 males (proportion)	X			X		
Executions and police conflict	Data Rich	Male	Population 15 to 30 males (proportion)	X			X		

Appendix Table S15: Theoretical minimum risk life table, which is a life table composed of the minimum mortality rate observed across GBD national locations with population at least 5 million, from 2010 to 2021. m_x: mortality rate in the age group; a_x: average years lived by those who died in the age group; q_x: probability of death in the age group, conditional on surviving to the start of the age group; l_x: number of persons surviving to the start of the age group; d_x: number of deaths in the age group; L_x: number of person-years lived in the age group; T_x: number of person-years lived in and after the age group; e_x: average number of years of life remaining for those who survive to the start of the age group.

Age group	Location	Year	Sex	m_x	a_x	q_x	l_x	d_x	L_x	T_x	e_x
0-7d	Singapore	2019	Female	0.03049148	0.00958811	5.85E-04	1	5.85E-04	0.01917247	89.9580397	89.9580397
7-28d	Finland	2013	Female	0.00454259	0.02876587	2.61E-04	0.9994154	2.61E-04	0.0574931	89.9388673	89.991476
1-6m	Singapore	2019	Male	8.58E-04	0.21231587	3.64E-04	0.99915424	3.64E-04	0.42422109	89.8813742	89.9574569
6-12m	Finland	2015	Female	1.19E-04	0.24931261	5.92E-05	0.99879029	5.91E-05	0.4980122	89.4571531	89.5655011
1y	Denmark	2019	Female	2.11E-04	0.5	2.11E-04	0.99873117	2.11E-04	0.99862569	88.9591409	89.072158
2y	Netherlands	2019	Female	5.11E-05	0.5	5.11E-05	0.99852022	5.11E-05	0.99849469	87.9605152	88.0908707
3	Netherlands	2019	Female	4.25E-05	0.5	4.25E-05	0.99846916	4.24E-05	0.99844794	86.9620205	87.0953494
4	Netherlands	2019	Female	3.93E-05	0.5	3.93E-05	0.99842673	3.92E-05	0.99840712	85.9635725	86.0990298
5	Finland	2019	Female	4.04E-05	0.5	4.04E-05	0.99838751	4.03E-05	0.99836735	84.9651654	85.1023919
6	Finland	2019	Female	3.69E-05	0.5	3.69E-05	0.99834719	3.69E-05	0.99832876	83.9667981	84.1058089
7	Finland	2019	Female	3.15E-05	0.5	3.15E-05	0.99831034	3.15E-05	0.9982946	82.9684693	83.1088953
8	Finland	2019	Female	3.01E-05	0.5	3.01E-05	0.99827886	3.00E-05	0.99826386	81.9701747	82.1115002
9	Finland	2019	Female	2.77E-05	0.5	2.77E-05	0.99824886	2.76E-05	0.99823505	80.9719109	81.1139531
10	Denmark	2017	Female	3.91E-05	0.5	3.91E-05	0.99822124	3.91E-05	0.9982017	79.9736758	80.1161834
11	Denmark	2017	Female	3.90E-05	0.5	3.90E-05	0.99818216	3.90E-05	0.99816268	78.9754741	79.1193
12	Denmark	2017	Female	4.24E-05	0.5	4.24E-05	0.9981432	4.24E-05	0.99812201	77.9773114	78.1223692
13	Denmark	2017	Female	4.80E-05	0.5	4.80E-05	0.99810083	4.80E-05	0.99807686	76.9791894	77.125664
14	Denmark	2017	Female	6.16E-05	0.5	6.16E-05	0.99805288	6.15E-05	0.99802212	75.9811126	76.1293455
15	Denmark	2013	Female	9.20E-05	0.5	9.20E-05	0.99799137	9.18E-05	0.99794547	74.9830904	75.1340069
16	Singapore	2010	Female	1.00E-04	0.5	1.00E-04	0.99789957	9.99E-05	0.99784962	73.985145	74.1408729
17	Italy	2019	Female	1.12E-04	0.5	1.12E-04	0.99779967	1.12E-04	0.99774369	72.9872953	73.1482455
18	Italy	2019	Female	1.18E-04	0.5	1.18E-04	0.99768772	1.18E-04	0.99762872	71.9895517	72.1563976

19	Italy	2019	Female	1.23E-04	0.5	1.23E-04	0.99756972	1.23E-04	0.99750822	70.9919229	71.1648732
20	Italy	2019	Female	1.28E-04	0.5	1.28E-04	0.99744672	1.27E-04	0.99738301	69.9944147	70.1735877
21	Singapore	2014	Female	1.27E-04	0.5	1.27E-04	0.99731931	1.27E-04	0.99725601	68.9970317	69.1824886
22	Singapore	2014	Female	1.25E-04	0.5	1.25E-04	0.99719272	1.25E-04	0.9971303	67.9997757	68.1912075
23	Singapore	2014	Female	1.29E-04	0.5	1.29E-04	0.99706788	1.29E-04	0.99700355	67.0026454	67.199683
24	Singapore	2014	Female	1.38E-04	0.5	1.38E-04	0.99693923	1.38E-04	0.99687022	66.0056418	66.2082904
25	Singapore	2018	Female	1.37E-04	0.5	1.37E-04	0.9968012	1.36E-04	0.99673312	65.0087716	65.2173888
26	Singapore	2018	Female	1.34E-04	0.5	1.34E-04	0.99666503	1.34E-04	0.99659823	64.0120385	64.2262312
27	Singapore	2018	Female	1.38E-04	0.5	1.38E-04	0.99653143	1.38E-04	0.99646265	63.0154403	63.2347744
28	Singapore	2018	Female	1.49E-04	0.5	1.49E-04	0.99639386	1.48E-04	0.99631981	62.0189776	62.2434362
29	Singapore	2018	Female	1.66E-04	0.5	1.66E-04	0.99624576	1.65E-04	0.99616316	61.0226578	61.252615
30	Singapore	2018	Female	1.88E-04	0.5	1.88E-04	0.99608057	1.87E-04	0.99598704	60.0264947	60.2626902
31	Singapore	2013	Female	1.94E-04	0.5	1.94E-04	0.99589352	1.93E-04	0.99579704	59.0305076	59.2739148
32	Singapore	2013	Female	1.97E-04	0.5	1.97E-04	0.99570057	1.97E-04	0.99560227	58.0347106	58.2853042
33	Singapore	2014	Female	2.01E-04	0.5	2.01E-04	0.99550397	2.00E-04	0.99540392	57.0391083	57.296716
34	Singapore	2014	Female	1.98E-04	0.5	1.98E-04	0.99530388	1.97E-04	0.9952055	56.0437044	56.3081344
35	Singapore	2015	Female	1.93E-04	0.5	1.93E-04	0.99510713	1.92E-04	0.9950111	55.0484989	55.3191685
36	Singapore	2015	Female	2.03E-04	0.5	2.03E-04	0.99491507	2.02E-04	0.99481395	54.0534878	54.3297507
37	Singapore	2016	Female	2.36E-04	0.5	2.36E-04	0.99471282	2.34E-04	0.99459566	53.0586738	53.3406958
38	Singapore	2019	Female	2.61E-04	0.5	2.61E-04	0.99447851	2.60E-04	0.99434855	52.0640782	52.3531456
39	Singapore	2019	Female	2.68E-04	0.5	2.68E-04	0.99421858	2.66E-04	0.99408541	51.0697296	51.3667019
40	Singapore	2019	Female	2.81E-04	0.5	2.81E-04	0.99395224	2.79E-04	0.99381277	50.0756442	50.3803323
41	Singapore	2019	Female	3.17E-04	0.5	3.17E-04	0.9936733	3.15E-04	0.99351578	49.0818314	49.3943345
42	Singapore	2019	Female	3.82E-04	0.5	3.82E-04	0.99335826	3.79E-04	0.99316876	48.0883157	48.4098414
43	Singapore	2019	Female	4.74E-04	0.5	4.74E-04	0.99297927	4.71E-04	0.99274388	47.0951469	47.4281271
44	Singapore	2019	Female	5.95E-04	0.5	5.95E-04	0.99250849	5.90E-04	0.99221329	46.102403	46.4503864
45	Switzerland	2019	Female	7.29E-04	0.5	7.29E-04	0.99191809	7.23E-04	0.99155663	45.1101897	45.4777365
46	Switzerland	2019	Female	8.00E-04	0.5	8.00E-04	0.99119517	7.93E-04	0.99079886	44.1186331	44.510541

47	Switzerland	2019	Female	8.88E-04	0.5	8.87E-04	0.99040256	8.79E-04	0.98996312	43.1278342	43.5457622
48	Switzerland	2019	Female	9.93E-04	0.5	9.92E-04	0.98952369	9.82E-04	0.98903285	42.1378711	42.5839941
49	Singapore	2019	Female	0.00109431	0.5	0.00109372	0.98854201	0.00108118	0.98800141	41.1488383	41.6257863
50	Singapore	2019	Female	0.00113646	0.5	0.00113581	0.98746082	0.00112157	0.98690004	40.1608368	40.6708154
51	Singapore	2019	Female	0.00121541	0.5	0.00121468	0.98633925	0.00119808	0.98574021	39.1739368	39.7164939
52	Singapore	2019	Female	0.00134355	0.5	0.00134265	0.98514117	0.0013227	0.98447982	38.1881966	38.7641872
53	Republic of Korea	2019	Female	0.00150149	0.5	0.00150037	0.98381847	0.00147609	0.98308042	37.2037168	37.8156317
54	Republic of Korea	2019	Female	0.00161723	0.5	0.00161592	0.98234238	0.00158739	0.98154868	36.2206364	36.871703
55	Republic of Korea	2017	Female	0.00172893	0.5	0.00172744	0.98075499	0.00169419	0.97990789	35.2390877	35.9305719
56	Republic of Korea	2017	Female	0.00183116	0.5	0.00182948	0.9790608	0.00179117	0.97816521	34.2591798	34.9918819
57	Republic of Korea	2017	Female	0.00194805	0.5	0.00194616	0.97726962	0.00190192	0.97631866	33.2810146	34.0550998
58	Republic of Korea	2017	Female	0.00207977	0.5	0.00207761	0.9753677	0.00202643	0.97435449	32.3046959	33.1205306
59	Republic of Korea	2019	Female	0.00218223	0.5	0.00217985	0.97334127	0.00212174	0.9722804	31.3303414	32.1884443
60	Republic of Korea	2019	Female	0.00228216	0.5	0.00227956	0.97121953	0.00221395	0.97011256	30.358061	31.2576714
61	Republic of Korea	2019	Female	0.00243398	0.5	0.00243102	0.96900558	0.00235567	0.96782775	29.3879485	30.3279454
62	Republic of Korea	2019	Female	0.00265032	0.5	0.00264681	0.96664991	0.00255854	0.96537064	28.4201207	29.4006345
63	Republic of Korea	2019	Female	0.00293198	0.5	0.00292769	0.96409137	0.00282256	0.96268009	27.4547501	28.4773321
64	Republic of Korea	2019	Female	0.00327992	0.5	0.00327455	0.96126881	0.00314772	0.95969495	26.49207	27.5594815
65	United Arab Emirates	2017	Female	0.00204806	0.5	0.0020455	0.95812109	0.00195983	0.95714117	25.532375	26.6483802
66	Republic of Korea	2019	Female	0.00411926	0.5	0.00411079	0.95616126	0.00393058	0.95419597	24.5752339	25.7019762
67	Republic of Korea	2019	Female	0.00456791	0.5	0.00455749	0.95223068	0.00433978	0.95006079	23.6210379	24.8060039
68	Republic of Korea	2019	Female	0.00503446	0.5	0.00502181	0.94789089	0.00476013	0.94551083	22.6709771	23.9172855
69	Republic of Korea	2019	Female	0.00551986	0.5	0.00550465	0.94313077	0.0051916	0.94053496	21.7254663	23.0354761
70	Republic of Korea	2019	Female	0.00607383	0.5	0.00605542	0.93793916	0.00567962	0.93509935	20.7849313	22.1602127

71	Republic of Korea	2019	Female	0.00689273	0.5	0.00686903	0.93225954	0.00640372	0.92905768	19.849832	21.2921735
72	Republic of Korea	2019	Female	0.00803484	0.5	0.00800265	0.92585582	0.0074093	0.92215117	18.9207743	20.4359834
73	Japan	2019	Female	0.00898096	0.5	0.00894075	0.91844653	0.0082116	0.91434072	17.9986231	19.5968111
74	Japan	2019	Female	0.0099716	0.5	0.00992205	0.91023492	0.0090314	0.90571923	17.0842824	18.7690913
75	Japan	2019	Female	0.01114908	0.5	0.01108716	0.90120353	0.00999179	0.89620763	16.1785631	17.9521747
76	Japan	2019	Female	0.01263005	0.5	0.01255062	0.89121174	0.01118526	0.88561911	15.2823555	17.1478391
77	Japan	2019	Female	0.01445654	0.5	0.01435254	0.88002648	0.01263062	0.87371117	14.3967364	16.3594354
78	Japan	2019	Female	0.01665131	0.5	0.01651344	0.86739586	0.01432369	0.86023402	13.5230252	15.5903732
79	Japan	2019	Female	0.01926879	0.5	0.01908434	0.85307217	0.01628032	0.84493201	12.6627912	14.8437514
80	Japan	2019	Female	0.02232351	0.5	0.02207618	0.83679186	0.01847317	0.82755527	11.8178592	14.1228181
81	Japan	2019	Female	0.02583649	0.5	0.02550558	0.81831869	0.02087169	0.80788284	10.9903039	13.430347
82	Japan	2019	Female	0.02986324	0.5	0.02942174	0.797447	0.02346228	0.78571586	10.1824211	12.7687747
83	Japan	2019	Female	0.03450507	0.5	0.03391655	0.77398472	0.02625089	0.76085927	9.39670523	12.1406857
84	Japan	2019	Female	0.0399001	0.5	0.03911456	0.74773383	0.02924728	0.73311018	8.63584595	11.5493584
85	Japan	2019	Female	0.04616719	0.5	0.04511768	0.71848654	0.03241645	0.70227832	7.90273577	10.9991424
86	Japan	2019	Female	0.0533005	0.5	0.05190491	0.6860701	0.03561041	0.66826489	7.20045745	10.4952212
87	Singapore	2019	Female	0.06021443	0.5	0.05843716	0.65045969	0.03801102	0.63145418	6.53219256	10.0424249
88	Singapore	2019	Female	0.06752354	0.5	0.06529403	0.61244867	0.03998924	0.59245405	5.90073838	9.63466601
89	Singapore	2019	Female	0.07684742	0.5	0.07396859	0.57245943	0.04234402	0.55128742	5.30828433	9.27276948
90	Singapore	2019	Female	0.08828077	0.5	0.08449592	0.53011541	0.04479259	0.50771912	4.75699691	8.97351178
91	Singapore	2019	Female	0.10004469	0.5	0.09520269	0.48532283	0.04620404	0.46222081	4.24927779	8.75556961
92	Turkmenistan	2012	Female	0.10993406	0.5	0.10410649	0.43911879	0.04571512	0.41626123	3.78705698	8.62421991
93	Turkmenistan	2012	Female	0.11259205	0.5	0.10648459	0.39340367	0.04189143	0.37245796	3.37079575	8.56828751
94	Turkmenistan	2012	Female	0.11347253	0.5	0.10727089	0.35151224	0.03770703	0.33265873	2.9983378	8.52982465
95	Algeria	2019	Female	0.10267119	0.5	0.09757581	0.31380521	0.0306198	0.29849531	2.66567907	8.49469343
96	Turkmenistan	2012	Female	0.11035506	0.5	0.10448325	0.28318542	0.02958813	0.26839135	2.36718375	8.35913019
97	Turkmenistan	2012	Female	0.10856429	0.5	0.10287803	0.25359728	0.02608959	0.24055249	2.09879241	8.27608396

98	Colombia	2014	Male	0.10444678	0.5	0.09917648	0.22750769	0.02256341	0.21622599	1.85823992	8.16781129
99	Malaysia	2015	Female	0.09709322	0.5	0.09252826	0.20494428	0.01896314	0.19546271	1.64201393	8.01200167
100	Turkmenistan	2012	Female	0.10397765	0.5	0.09875396	0.18598114	0.01836637	0.17679796	1.44655122	7.77794559
101	Turkmenistan	2012	Female	0.10641068	0.5	0.10094399	0.16761477	0.0169197	0.15915492	1.26975326	7.57542585
102	Turkmenistan	2012	Female	0.1090353	0.5	0.10330051	0.15069507	0.01556688	0.14291163	1.11059834	7.36983878
103	Turkmenistan	2012	Female	0.11183401	0.5	0.10580653	0.13512819	0.01429744	0.12797947	0.96768671	7.16124979
104	Turkmenistan	2012	Female	0.11481489	0.5	0.10846796	0.12083074	0.01310626	0.11427761	0.83970725	6.94945024
105	Colombia	2014	Male	0.0958616	0.5	0.09140827	0.10772448	0.00984691	0.10280103	0.72542963	6.73412054
106	Colombia	2014	Male	0.09640388	0.5	0.09190078	0.09787757	0.00899502	0.09338006	0.62262861	6.36130011
107	Colombia	2014	Male	0.09704439	0.5	0.09248216	0.08888255	0.00822005	0.08477252	0.52924855	5.95447101
108	Colombia	2014	Male	0.09778765	0.5	0.09315634	0.0806625	0.00751422	0.07690539	0.44447603	5.51031821
109	Colombia	2014	Male	0.09862266	0.5	0.09391316	0.07314827	0.00686959	0.06971348	0.36757064	5.02500772
110+	Colombia	2014	Male	0.22251836	4.49578615	1	0.06627869	0.06627869	0.29785716	0.29785716	4.49401111

Section 11: CoD cause-specific modelling descriptions

GBD 2021 cause of death appendix write-ups in order:

1. Acute glomerulonephritis
2. African trypanosomiasis
3. Alcohol use disorders
4. Alcoholic cardiomyopathy
5. Alzheimer's disease and other dementias
6. Amphetamine use disorders
7. Anorexia nervosa
8. Aortic aneurysm
9. Appendicitis
10. Ascariasis
11. Asthma
12. Bacterial skin diseases
13. Cancer
14. Cardiomyopathy and myocarditis
15. Cardiovascular diseases
16. Cellulitis
17. Chagas disease
18. Chronic kidney disease
19. Chronic obstructive pulmonary disease
20. Chronic respiratory diseases
21. Cirrhosis and other chronic liver diseases
22. Cocaine use disorders
23. Congenital birth defects
24. COVID-19
25. COVID Impact on Select Infectious Syndromes
26. Cystic echinococcosis
27. Cysticercosis
28. Decubitus ulcer
29. Dementia
30. Dengue
31. Diabetes mellitus
32. Diarrhoeal diseases
33. Digestive diseases
34. Diphtheria
35. Drug use disorders
36. Drug-resistant tuberculosis, and HIV/AIDS–drug-susceptible tuberculosis
37. Eating disorders
38. Ebola virus disease
39. Encephalitis
40. Endocarditis
41. Endocrine, metabolic, blood, and immune disorders
42. Gallbladder and biliary diseases
43. Gastritis and duodenitis

44. Gynaecological diseases
45. Haemoglobinopathies and haemolytic anaemias
46. Hepatitis
47. HIV/AIDS
48. HIV/AIDs–multidrug-resistant tuberculosis without extensive drug resistance, HIV/AIDS–extensively
49. Hypertensive heart disease
50. Idiopathic epilepsy
51. Inflammatory bowel disease
52. Inguinal, femoral, and abdominal hernia
53. Injuries
54. Interstitial lung disease and pulmonary sarcoidosis
55. Intracerebral haemorrhage
56. Invasive non-typhoidal Salmonella (iNTS)
57. Ischaemic heart disease
58. Ischaemic stroke
59. Lower respiratory infections
60. Malaria
61. Maternal disorders
62. Measles
63. Meningitis
64. Motor neuron disease
65. Multiple sclerosis
66. Musculoskeletal disorders
67. Myocarditis
68. Neonatal disorders
69. Neoplasms
70. Non-rheumatic valvular heart disease
71. Nutritional deficiencies
72. Opioid use disorders
73. Other cardiomyopathy
74. Other cardiovascular and circulatory diseases
75. Other chronic respiratory diseases
76. Other digestive diseases
77. Other drug use disorders
78. Other intestinal infectious diseases
79. Other musculoskeletal disorders
80. Other neglected tropical diseases
81. Other neurological disorders
82. Other skin and subcutaneous diseases
83. Other unspecified infectious diseases
84. Other urinary diseases
85. Otitis media
86. Pancreatitis

87. Paralytic ileus and intestinal obstruction
88. Paratyphoid fever
89. Parkinson disease
90. Peptic ulcer disease
91. Peripheral artery disease
92. Pertussis
93. Pneumoconiosis: silicosis, asbestosis, coal worker's pneumoconiosis, and other pneumoconiosis
94. Pulmonary arterial hypertension
95. Pyoderma
96. Rabies
97. Rheumatic heart disease
98. Rheumatoid arthritis
99. Schistosomiasis
100. Sexually transmitted diseases excluding HIV
101. Shocks or Fatal discontinuities
102. Skin and subcutaneous diseases
103. Stroke
104. Subarachnoid haemorrhage
105. Sudden infant death syndrome
106. Tetanus
107. Tuberculosis
108. Typhoid fever
109. Upper digestive system diseases
110. Upper respiratory infections
111. Urinary diseases and male infertility
112. Urinary tract infection and interstitial nephritis
113. Urolithiasis
114. Varicella and herpes zoster
115. Vascular intestinal disorders
116. Visceral leishmaniasis
117. Yellow fever
118. Zika virus disease

Acute glomerulonephritis

Flowchart

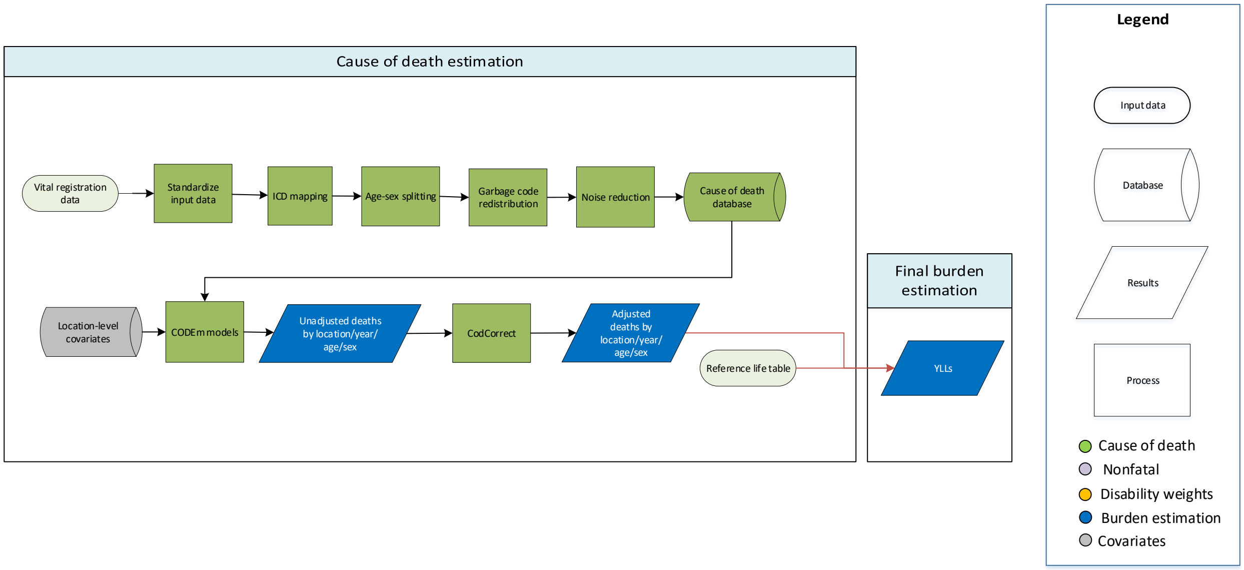

Input data and methodological summary for acute glomerulonephritis

Input data

Data used to estimate mortality of acute glomerulonephritis consisted of vital registration data from the cause of death (COD) database (see appendix section on ICD mapping for details). Outliers were identified by systematic examination of datapoints for all location-years. Data were excluded if they violated well-established age or time trends, and data in instances where garbage code redistribution and noise reduction, in combination with small sample sizes, resulted in unreasonable cause fractions.

Modelling strategy

The estimation strategy used for fatal acute glomerulonephritis is largely similar to methods used in GBD 2019. A standard CODEm model with location-level covariates was used to model deaths due to acute glomerulonephritis (see appendix section on CODEm method for details). Separate models were conducted for male and female mortality, and age-restrictions for death estimations included 0 days for lower bound (in GBD 2019, the lower bound was set at 28 days) and 95+ years for upper bound. We hybridised separate global and data-rich models to acquire unadjusted results, which we adjusted using CoDCorrect and compared to the reference life table to calculate final YLLs due to acute glomerulonephritis.

In GBD 2021, we updated the linear floor value in our CODEm models to allow the model to be influenced by lower data, which resulted from changes to COD data processing. These data processing changes include changing the method of estimating the non-zero floor and population size cutoffs for when to noise-reduce at the country versus region level, and are described in greater detail in the appendix section GBD 2021 Causes of Death database. Apart from these, no other substantive changes were made in GBD 2021 from the modelling strategy used in GBD 2019.

The following table has the full list of covariates used for fatal acute glomerulonephritis.

Table 1. Covariates used in acute glomerulonephritis mortality modelling

Level	Covariate	Direction
2	Mean systolic blood pressure (mmHg)	+
	Sanitation (proportion with access)	-
	Improved water sources (proportion with access)	-
	Healthcare Access and Quality Index	-
3	Socio-demographic Index	-
	Education (years per capita)	-
	Log LDI ($I per capita)	-

Human African trypanosomiasis (HAT)

Flowchart

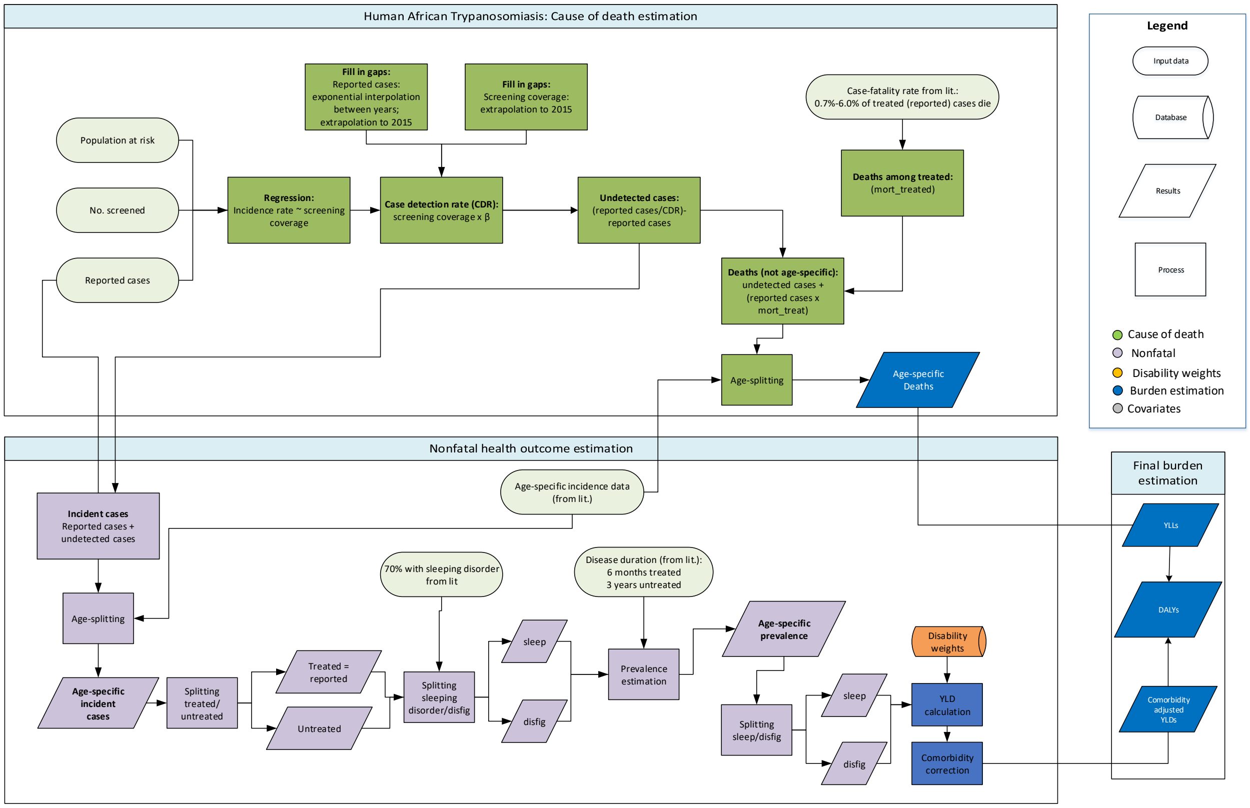

Input data and methodological summary

Case definition

Human African trypanosomiasis (HAT), also known as sleeping sickness, is a vector-borne disease which is transmitted by the bite of the tsetse fly. It is caused by the parasite *Trypanosoma brucei* with two subspecies, namely *T.b. rhodesience* (makes up less than 5% of total HAT cases) and *T.b. gambiense*. Cases are diagnosed through laboratory methods which rest on finding the parasite in body fluid or tissue by microscopy. In highly endemic or epidemic areas where the likelihood of false positives in serological tests is deemed lower, a seropositive individual is considered affected even in the absence of parasitological confirmation. The ICD-10 codes for HAT are B56.0, B56.1, and B56.9.

Input data

Model inputs

Data sources for GBD 2021:

Annual case totals 1980–2018: National-level annual case totals from 1990–2018 were obtained from WHO's publicly available dataset, available here:
http://apps.who.int/gho/data/node.main.A1635?lang=en

Subnational data:

Kenya: Deaths due to HAT were attributed to Busia county. Identification of subnational locations for Kenyan case data were obtained via studies published in the peer-reviewed literature[1] and review of maps published via the WHO HAT Atlas:
http://www.who.int/entity/trypanosomiasis_african/country/Kenya_whole_0014.jpg?ua=1

Nigeria: Review of historical data on the distribution of HAT indicated that cases have been reported from Delta State. All Nigeria estimates were then applied to that location.

Age/sex data: Data on the age and sex distribution of HAT were last updated for GBD 2017. Cases were extracted from the peer-reviewed literature via a systematic review of sources identified in PubMed using the following search string:

((African trypanosomiasis[Title/Abstract] AND (incidence[Title/Abstract] OR burden[Title/Abstract] OR prevalence[Title/Abstract] OR community[Title/Abstract])) AND ("1990"[Date – Publication] : "2017"[Date – Publication]))

This yielded 219 studies, of which only three met the inclusion criteria and were extracted. The inclusion criteria were:

Studies representative of the national population
Population-based studies
Studies with primary data on incidence
Studies of human African trypanosomiasis (excluded studies on animal African trypanosomiasis)

1 Population at risk estimates 1980–2015: population at risk estimates from GBD 2010 ArcGIS analysis using geocoded case notifications for 2000 to 2009[2] and population Count Grid estimates from Gridded Population of the World 3.

2 Screening coverage: Data on active versus passive screening coverage were obtained from a Weekly Epidemiological Report[3] identifying the population screened from 1997 to 2004 at the national level.

3 Geographical restrictions: Data file of all GBD locations, defining location as either endemic or non-endemic for HAT. Estimates are not produced for non-endemic countries, nor are they generated for countries with a history of HAT transmission but no data reported by WHO from 1990–2018.

Modelling strategy

Geographical restrictions

For countries historically considered endemic for HAT, but which have no reported case data or estimate of the population at risk, estimates are not produced. These countries include Botswana, Ethiopia, Guinea-Bissau, and Rwanda.

Among countries where population at risk data are available, if no cases were reported to WHO, we assume the incidence of HAT is zero for those years and generate model estimates accordingly.

Modelling steps

The cause of death model for HAT is implemented as follows:

The incidence of reported HAT cases among the population at risk was calculated as the total number of reported cases divided by the population at risk estimates generated by the GBD working group for the period 1980–2015. Population at risk estimates for 2016–2021 were generated by assuming an annual 2% rate of population growth.

1 To estimate the number of cases that were likely undetected by country and year, a multilevel mixed-effects linear regression of log-transformed incidence rate (ratio of reported HAT cases to population at risk) on log-transformed screening coverage[3] (ratio of number screened for HAT to population at risk), with country random effects, was performed. Gaps were then filled using interpolation between years and extrapolation from 2019 to 2021 for reported cases. This model generates a beta-coefficient which is used to estimate the case detection rate (see step 4).

For country-years in which no screening coverage data were reported:

Among countries with data reported, 1997–2004, the proportion of the at-risk population screened from 1997 was used retrospectively for the period 1980–1996 and the screening coverage from 2004 was carried forward from 2005 to 2021.

For countries with no screening data reported, the mean screening coverage for the region was used to impute a value over time.

2 To construct an estimate of total deaths, we first assume that all detected cases receive treatment, and that mortality among the treated occurs for a small proportion of cases. Deaths among detected cases are estimated by generating 1000 draws of mortality among treated cases, assuming that between 0.7% and 6.0% of all reported (and therefore assumed to have received treatment) cases die.[4-6]

3 We then assume that all undetected cases experience mortality. This is estimated via generation of 1000 draws of the case detection rate (CDR), given the expected screening coverage from the regression (in step 2). Undetected deaths were then estimated as the difference between the ratio of reported cases to CDR and reported cases (reported cases/CDR – reported cases).

4 Estimates of death were obtained by adding the deaths among treated cases to the total number of undetected cases. Without information on sex-specific incidence or deaths, death rates between both sexes were equal.

5 Finally, an age-pattern was applied to the mortality estimates using the incidence studies from Sudan,[7] DRC,[8] and Uganda.[9] The age-pattern in GBD 2021 employed a cubic spline to account for the higher risk of infection among working-age adults.

Changes from GBD 2019 to GBD 2021

We have made no substantive changes in the modelling strategy from GBD 2019.

References

1. Rutto JJ, Osano O, Thuranira EG, Kurgat RK, Odenyo VA. Socio-economic and cultural determinants of human african trypanosomiasis at the Kenya - Uganda transboundary. *PLoS Negl Trop Dis* 2013; **7**(4): e2186.

2. Simarro PP, Cecchi G, Paone M, et al. The Atlas of human African trypanosomiasis: a contribution to global mapping of neglected tropical diseases. *Int J Health Geogr* 2010; **9**: 57.

3. World Health O. Human African trypanosomiasis (sleeping sickness): epidemiological update. *Weekly epidemiological record* 2006; **February 24**(8): 69-80.

4. Kato CD, Nanteza A, Mugasa C, Edyelu A, Matovu E, Alibu VP. Clinical profiles, disease outcome and co-morbidities among T. b. rhodesiense sleeping sickness patients in Uganda. *PLoS One* 2015; **10**(2): e0118370.

5. Balasegaram M, Harris S, Checchi F, Hamel C, Karunakara U. Treatment outcomes and risk factors for relapse in patients with early-stage human African trypanosomiasis (HAT) in the Republic of the Congo. *Bull World Health Organ* 2006; **84**(10): 777-82.

6. Odiit M, Kansiime F, Enyaru JC. Duration of symptoms and case fatality of sleeping sickness caused by Trypanosoma brucei rhodesiense in Tororo, Uganda. *East Afr Med J* 1997; **74**(12): 792-5.

7. Moore A, Richer M, Enrile M, Losio E, Roberts J, Levy D. Resurgence of sleeping sickness in Tambura County, Sudan. *Am J Trop Med Hyg* 1999; **61**(2): 315-8.

8. Lutumba P, Makieya E, Shaw A, Meheus F, Boelaert M. Human African trypanosomiasis in a rural community, Democratic Republic of Congo. *Emerg Infect Dis* 2007; **13**(2): 248-54.

9. Fevre EM, Odiit M, Coleman PG, Woolhouse ME, Welburn SC. Estimating the burden of rhodesiense sleeping sickness during an outbreak in Serere, eastern Uganda. *BMC Public Health* 2008; **8**: 96.

Alcohol use disorders

Flowchart

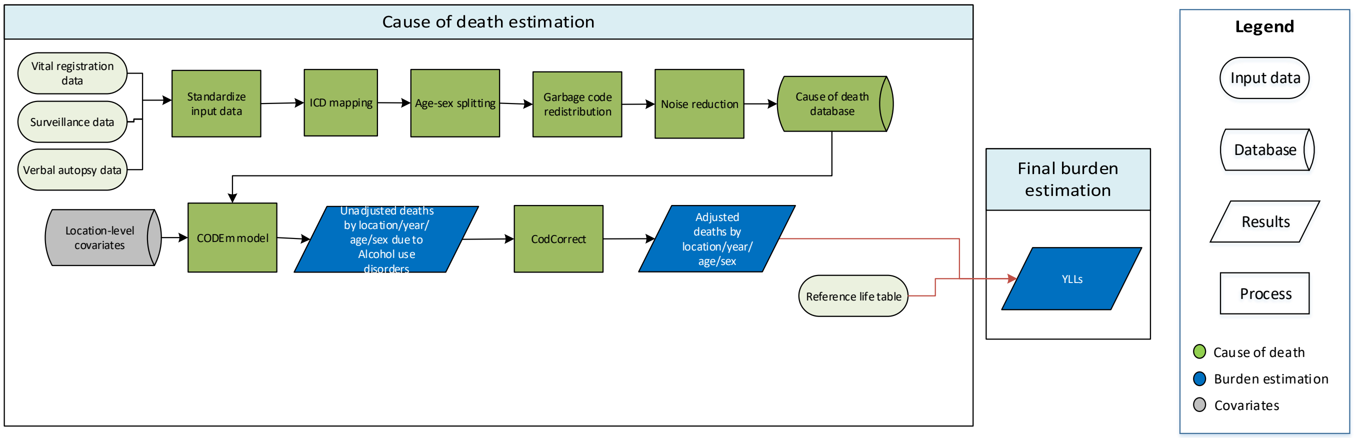

Input data and methodological summary for alcohol use disorders

Input data

All data were from vital registration, China surveillance, and verbal autopsy sources. Some data were outliered from countries with sparse yet heterogeneous data if they created implausible fluctuations in deaths and regional patterns, verified with in-country collaborator and subject experts. As an example, Medical Certification of Cause of Death data from India were excluded for alcohol use disorders due to the extremely low estimates. All data came from the following ICD-10 codes: E24.4, F10, G31.2, G62.1, G72.1, P04.3, Q86.0, R78.0, X45, X65, Y15.

Modelling strategy

The standard Cause of Death Ensemble modelling (CODEm)[1] approach (detailed in reference appendix section 3.1) was used to estimate deaths due to alcohol use disorders. Model covariate inclusion was based on empirical evidence and expert feedback, which resulted in a set of model covariates that reflected alcohol consumption, smoking, education, health system access, domestic income, and Socio-demographic Index (SDI).

Key changes from GBD 2021:

- There were no major modelling changes this round.

Table 1: Covariates used in alcohol use disorders mortality model

Level	Covariate	Direction
1	Alcohol consumption (litres per capita)	+
	Alcohol binge drinking	+
	Alcohol consumption, age-standardised, in grams per day	+

	Alcohol drinker proportion, age-standardised	+
2	Cumulative cigarettes (10 years)	+
	Smoking prevalence	+
	Healthcare Access and Quality Index	-
3	Log LDI (I$ per capita)	-
	Education (years per capita)	-
	Socio-demographic Index	-

Covariate Influences:

The following plots show the influence of each covariate on the four CODEm models (male global, male data-rich, female global, and female data-rich). A positive standardised beta (to the right) means that the covariate was associated with increased death. A negative standardised beta (to the left) means the covariate was associated with decreased death.

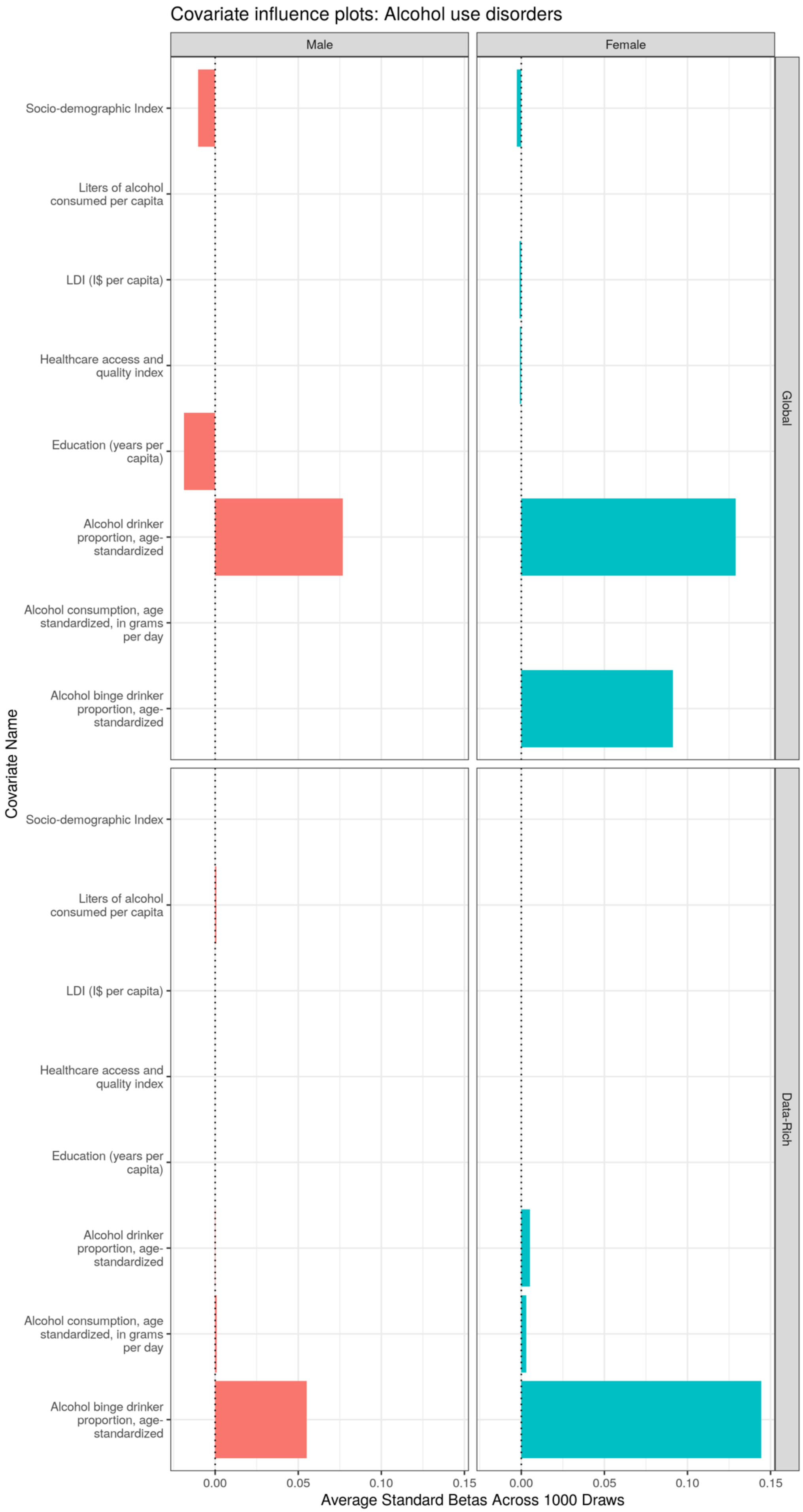

Covariate influence plots: Alcohol use disorders
Male
Female
Global
Data-Rich
Covariate Name
Socio-demographic Index
Liters of alcohol consumed per capita
LDI (I$ per capita)
Healthcare access and quality index
Education (years per capita)
Alcohol drinker proportion, age-standardized
Alcohol consumption, age standardized, in grams per day
Alcohol binge drinker proportion, age-standardized
0.00
0.05
0.10
0.15
Average Standard Betas Across 1000 Draws

[1]Vos T, Lim SS, Abbafati C, *et al.* Global burden of 369 diseases and injuries in 204 countries and territories, 1990–2019: a systematic analysis for the Global Burden of Disease Study 2019. *The Lancet* 2020; 396**:** 1204–22. doi: https://doi.org/10.1016/S0140-6736(20)30925-9

Alcoholic cardiomyopathy

Flowchart

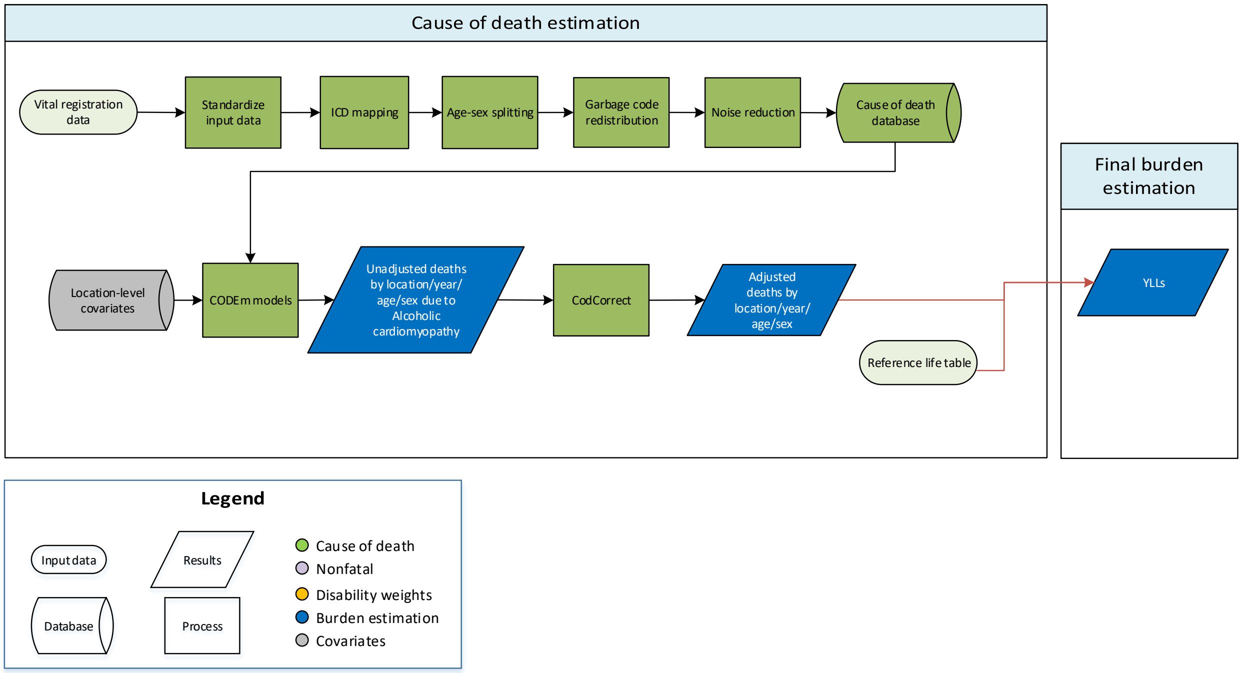

Input data and methodological summary for alcoholic cardiomyopathy

Input data

Vital registration data were used to model deaths due to alcoholic cardiomyopathy. We outliered ICD9 datapoints in Cyprus that were implausibly high and discontinuous with the rest of the time series. We also dropped ICD9BTL datapoints in locations in central and eastern Europe where we were unable to disaggregate them appropriately. We also outliered all datapoints in Bosnia and Herzegovina, Slovenia, Greece, and Egypt due to implausibly high cause fractions likely caused by regional patterns in the use of ICD codes for cardiomyopathies. Additionally, we outliered tabulated ICD10 datapoints in locations where unreliable estimates caused an abrupt inconsistency with detailed ICD10 data.

Modelling strategy

We used a standard CODEm approach to model deaths from alcoholic cardiomyopathy. The covariates selected for inclusion in the CODEm modelling process can be found in the table below. For GBD 2021, we replaced the alcohol (litres per capita) covariate with the age- and sex-specific summary exposure value for alcohol consumption. In addition, we changed the ICD mapping of ICD9 code 425.4 ("other primary cardiomyopathies") from being partially redistributed to alcoholic cardiomyopathy into solely other cardiomyopathy. This had the result of decreasing the number of deaths due to alcoholic cardiomyopathy. In addition, for GBD 2021, the method used to reduce the noise in the data, implemented after redistribution to handle both the stochastic variation across time and space and the occurrence of small number of deaths in each location/year/age/sex, was updated. This new empirical Bayesian noise reduction algorithm uses grouped data by region and data type as prior to better reflect regional patterns. A detailed description on the updates to noise reduction can be found in the cause of death methods section of the appendix.

Table 1. Covariates used in alcoholic cardiomyopathy mortality modelling

Level	Covariate	Direction
1	Summary exposure value, CMP	1
	Smoking prevalence	1
	Summary exposure value, Alcohol consumption	1
2	Healthcare Access and Quality Index	-1
3	Log-transformed lag distributed income per capita (I$)	-1

Amphetamine use disorder

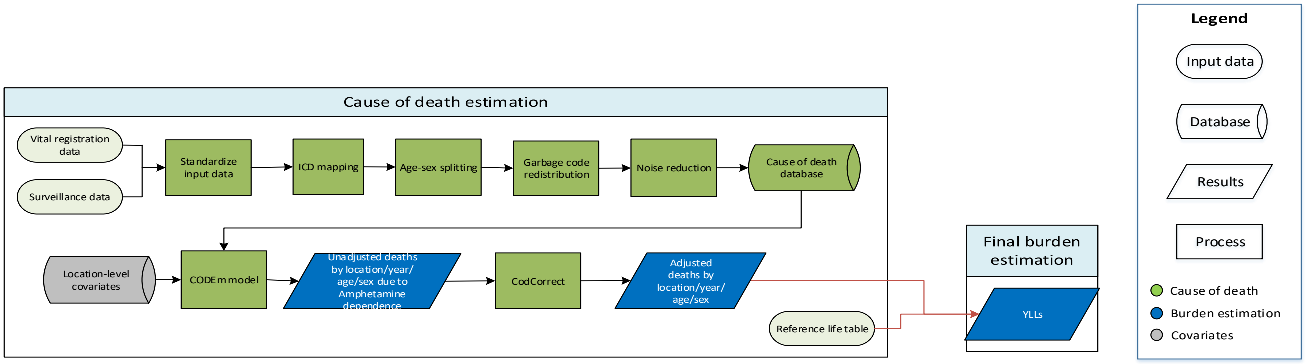

Input data and methodological summary for amphetamine use disorders

Input data

All data were from vital registration and surveillance sources. Data from countries with sparse yet heterogeneous data were excluded as the data exaggerated fluctuations in deaths and gave implausible regional patterns, according to in-country and subject matter experts. Excluded data were typically from lower-income countries. A full description of changes to coding and redistribution are described in the write-up focusing on drug use disorders.

Modelling strategy

The standard Cause of Death Ensemble modelling (CODEm)[1] approach (detailed in reference appendix section 3.1) was used to estimate deaths due to cocaine use disorders. Model covariate inclusion was based on empirical evidence and expert feedback, which resulted in a set of model covariates that reflected alcohol consumption, smoking, education, health system access, domestic income, and Socio-demographic Index (SDI) (Table 1).

Key changes from GBD 2021:

- The intravenous drug use covariate incorporated additional data and increased time smoothing, which increased estimates in the United States and Western Europe and made the yearly change more consistent over time.

Table 1: Covariates used in amphetamine use CODEm model

Level	Covariate	Direction
1	Alcohol (litres per capita)	+
	Current drinking prevalence	+
	Intravenous drug use, age-standardised	+
	Intravenous drug use, age-specific	+
	Cumulative cigarettes (10 years)	+
	Cumulative cigarettes (5 years)	+
	Cigarettes per capita	+
	Smoking prevalence	+
2	Healthcare Access and Quality Index	-
3	log LDI (I$ per capita)	+
	education (years per capita)	+
	Socio-demographic Index	+

Amphetamine use disorder is a “child’ disease that is fit into an overall “parent” drug use disorders model. The unadjusted death estimates from amphetamine use disorders are summed alongside other “child” causes (opioid, cocaine, other drugs) and fit to the distribution of deaths in an overall drug use disorders “parent” model as part of the CoDCorrect adjustment process.[1] This results in deaths recorded using non-specific coding systems, such as verbal autopsy, being included in the parent model and redistributed to the child models proportionately.

Covariate influences:

The following plots show the influence of each covariate on the four CODEm models (male global, male data-rich, female global, and female data-rich). A positive standardised beta (to the right) means that the covariate was associated with increased death. A negative standardised beta (to the left) means the covariate was associated with decreased death.

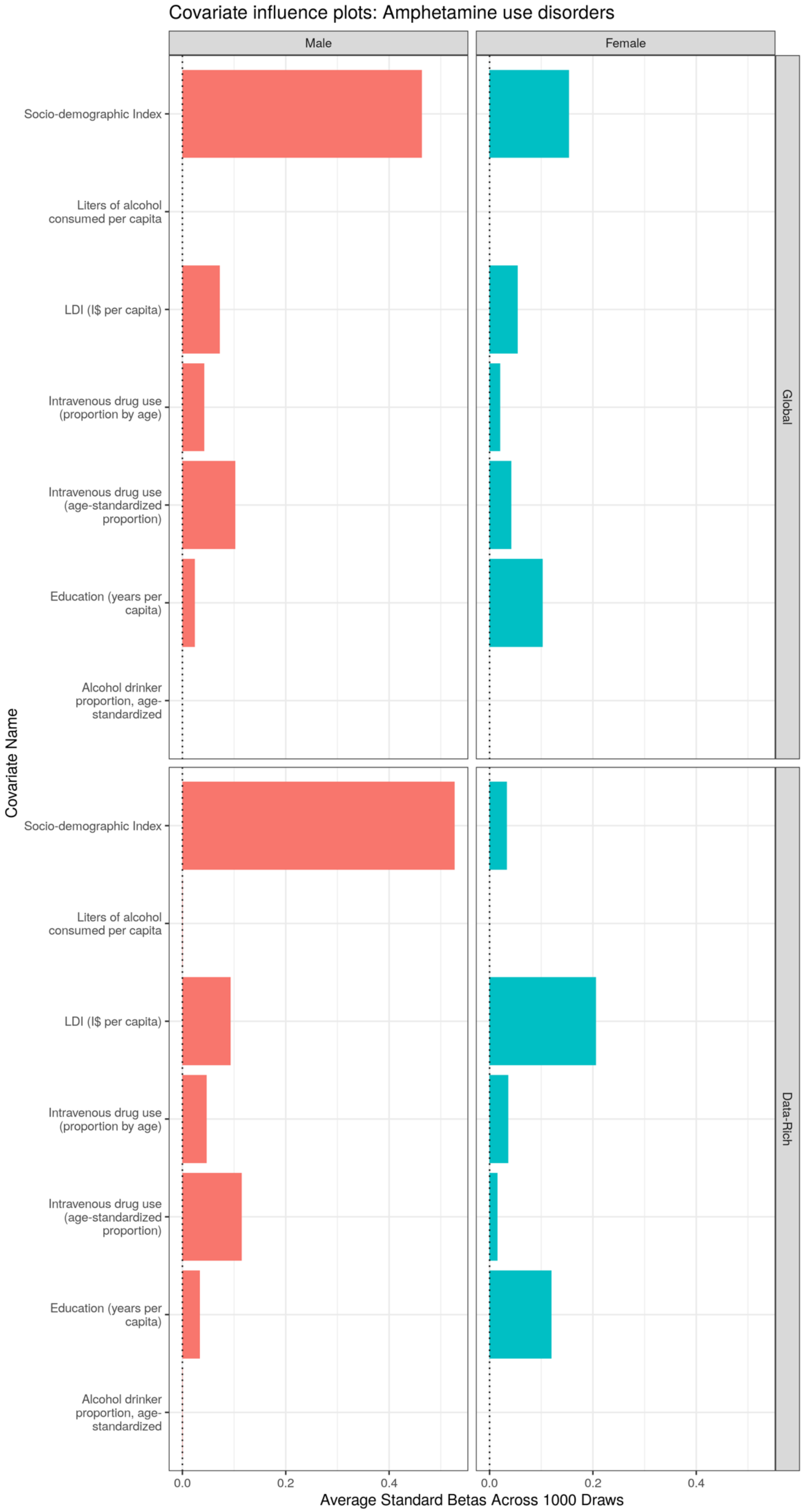
Covariate influence plots: Amphetamine use disorders
Male
Female
Global
Data-Rich
Socio-demographic Index
Liters of alcohol consumed per capita
LDI (I$ per capita)
Intravenous drug use (proportion by age)
Intravenous drug use (age-standardized proportion)
Education (years per capita)
Alcohol drinker proportion, age-standardized
Covariate Name
0.0
0.2
0.4
Average Standard Betas Across 1000 Draws

[1] Vos T, Lim SS, Abbafati C, *et al.* Global burden of 369 diseases and injuries in 204 countries and territories, 1990–2019: a systematic analysis for the Global Burden of Disease Study 2019. *The Lancet* 2020; **396**: 1204–22. doi: https://doi.org/10.1016/S0140-6736(20)30925-9

Anorexia nervosa

Flowchart

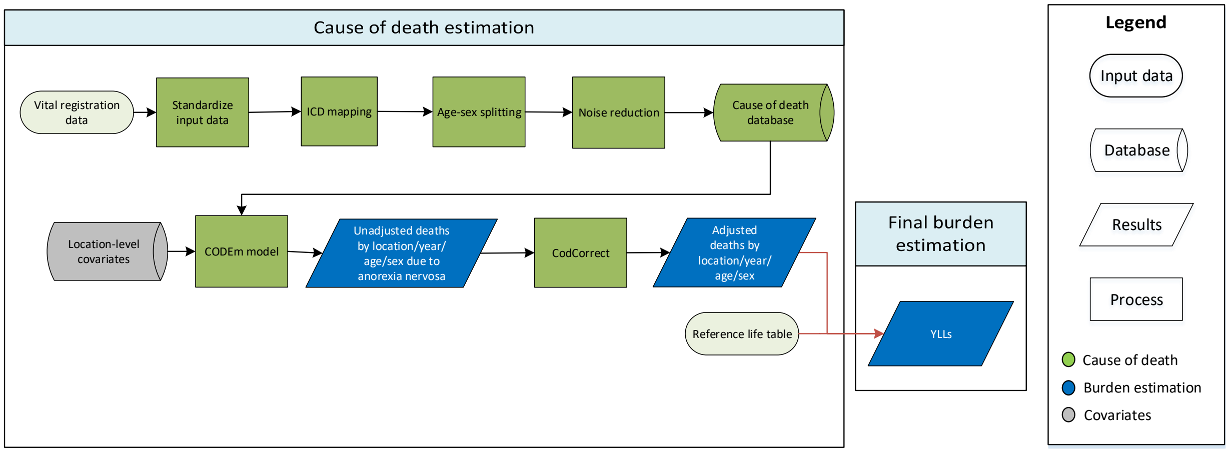

Input data and methodological summary for anorexia nervosa

Data used to estimate anorexia nervosa mortality included centrally prepped vital registration data from the cause of death (COD) database.

Modelling strategy

We have made no substantive changes in the modelling strategy from GBD 2019.

Anorexia nervosa was modelled using the standard CODEm approach and came under the eating disorders parent model. Age was restricted to deaths occurring between 5 and 49 years based on expert advice and patterns of prevalence seen in the non-fatal model. Several covariates were applied to this model and are listed in the table below, along with the direction in which they were applied.

Level	Covariate	Direction
1	Education (years per capita)	+
	Log LDI (I$ per capita)	+
	Age- and sex-specific SEV for child underweight	-
	Sanitation (proportion with access)	+
	Maternal education (years per capita)	+
2	Healthcare Access and Quality Index	-
3	Socio-demographic Index	+

In GBD 2013, anorexia nervosa deaths were extrapolated from the eating disorders model, which was modelled through a negative binomial approach. This approach was changed in GBD 2015, with anorexia nervosa being modelled as a standard CODEm model, as no additional benefit was seen from using the custom modelling approach. GBD 2021 continued to utilise the same approach.

A decision was made not to redistribute any deaths from garbage codes to anorexia nervosa given that deaths due to dehydration in low- and middle-income countries are likely to bias the age, sex, and geographical distribution of deaths observed. For example, testing of this process showed that while only a relatively small proportion of dehydration garbage code deaths were redistributed to anorexia nervosa, this added a comparatively large number of deaths to the model, particularly in regions with higher rates of infectious diseases. The redistributed deaths were also applied equally between males and females despite the prevalence of anorexia nervosa known to be up to ten times higher in females.

Aortic aneurysm

Flowchart

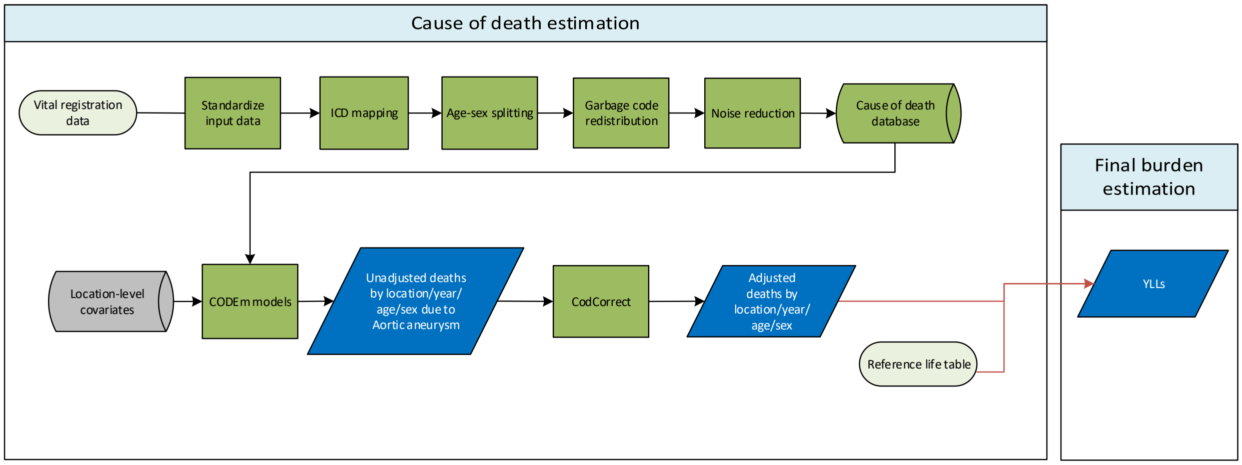

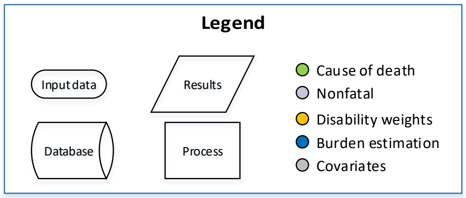

Case definition

Aortic aneurysms represent full-thickness dilation of the aorta, usually due to atherosclerosis, elevated blood pressure, or inflammation of the blood vessel. We include both abdominal and thoracic aortic aneurysms in our estimates of cause-specific mortality.

Input data and methodological summary for aortic aneurysm

Input data

Vital registration data were used to model cause-specific mortality for aortic aneurysm. We outliered data in Oman as they were improbably high in comparison with the rest of the region. We also outliered ICD8 data that were discontinuous with the rest of the time series and created implausible time trends. In addition, we outliered a subset of vital registration datapoints in Latin America due to implausibly high values at the oldest age groups that resulted in inconsistencies in time trends. Data from Estonia for population aged 20–24 years and Ukraine was also outliered due to implausibly low values.

Modelling strategy

We used a standard CODEm approach to model deaths from aortic aneurysm. The covariates selected for inclusion in the CODEm modelling process can be found in the table below. For GBD 2021, substantive changes to the redistribution and noise reduction methods were made. Specifically, a new approach to redistribute deaths coded to hypertension was implemented using data sources which included information on the chain of events leading to death. This update resulted in an increase in the number of deaths that were re-assigned to cardiovascular diseases. Similarly, the method used to reduce the noise in the data, implemented after redistribution to handle both the stochastic variation

across time and space and the occurrence of small number of deaths in each location/year/age/sex, was updated. This new empirical Bayesian noise reduction algorithm uses grouped data by region and data type as prior to better reflect regional patterns. A detailed description on the updates to noise reduction can be found in the cause of death methods section of the appendix.

Table 1. Covariates used in aortic aneurysm mortality modelling

Level	Covariate	Direction
1	Summary exposure variable, aortic aneurysm	1
	Cholesterol (total, mean per capita)	1
	Cumulative cigarettes (10 yrs)	1
	Systolic blood pressure (mm Hg)	1
2	Mean BMI	1
	Healthcare access and quality index	-1
3	Lag distributed income per capita (I$)	-1
	Socio-demographic Index	1
	Summary exposure value omega-3	1
	Summary exposure value fruits	1
	Summary exposure value vegetables	1
	Summary exposure value nuts and seeds	1
	Pulses/legumes (kcal/capita, un-adjusted)	-1
	Summary exposure value PUFA	1
	Alcohol (litres per capita)	1

Appendicitis

Flowchart

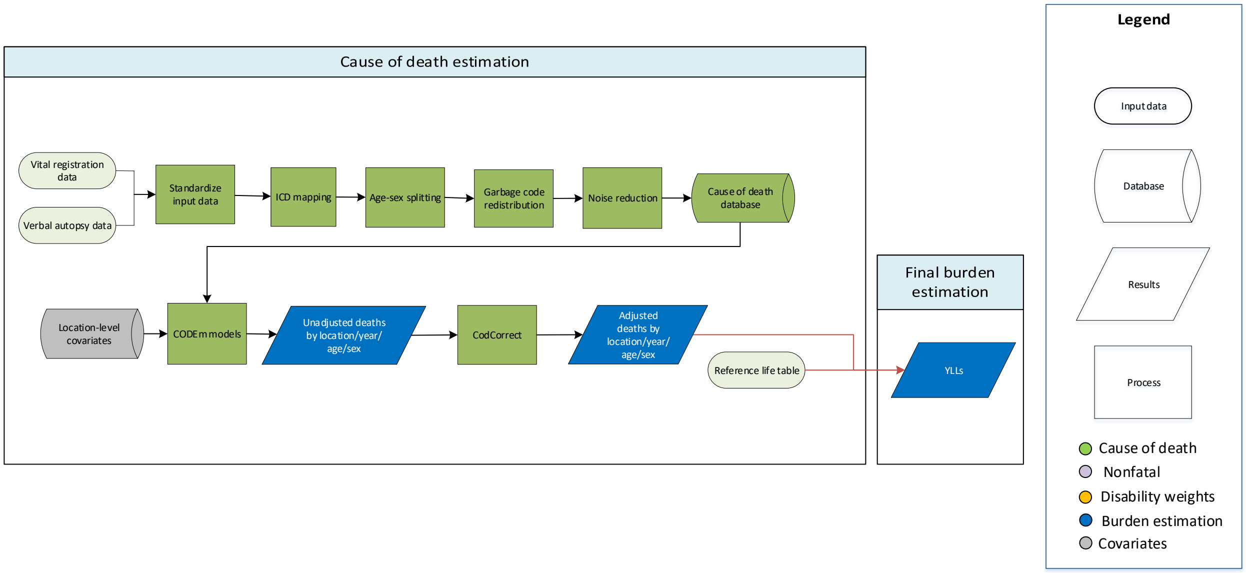

Input data and methodological summary for appendicitis

Input data

Data used to estimate mortality of appendicitis consisted of vital registration and verbal autopsy data from the cause of death (COD) database (see appendix section on ICD mapping for details). Outliers were identified by systematic examination of datapoints for all location-years. Data were excluded if they violated well-established age or time trends, and data in instances where garbage code redistribution and noise reduction, in combination with small sample sizes, resulted in unreasonable cause fractions. Methods for assigning outlier status were consistent across both vital registration and verbal autopsy data.

Modelling strategy

The estimation strategy used for fatal appendicitis is largely similar to methods used in GBD 2019. A standard CODEm model with location-level covariates was used to model deaths due to appendicitis with age restrictions for death estimations of 12 months for lower bound and 95+ years for upper bound (see appendix section on CODEm method for details). Separate models were conducted for male and female mortality. We hybridised separate global and data-rich models to acquire unadjusted results, which we adjusted using CoDCorrect and compared to the reference life table to calculate final YLLs due to appendicitis.

In GBD 2021, we updated the linear floor value in our CODEm models to allow the model to be influenced by lower data, which resulted from changes to COD data processing. These data processing changes include changing the method of estimating the non-zero floor and population size cutoffs for when to noise-reduce at the country versus region level, and are described in greater detail in the appendix section "GBD 2021 Causes of Death database". Apart from these, no other substantive changes were made in GBD 2021 from the modelling strategy used in GBD 2019.

The following table has the full list of covariates used for appendicitis.

Table 1. Covariates used in appendicitis mortality modelling

Level	Covariate	Direction
2	Age-sex-specific scaled exposure variable for low fruit consumption	+
	Age-sex-specific scaled exposure variable for low vegetable consumption	+
	Healthcare Access and Quality Index	-
3	Socio-demographic Index	-
	Education (years per capita)	-
	Log LDI ($I per capita)	-

Adjustment in CoDCorrect included fitting unadjusted death estimates for all other specific and non-specific digestive diseases to overall digestive disease deaths, which was then adjusted with all other causes to sum to all-cause counts of death.

Ascariasis

Flowchart

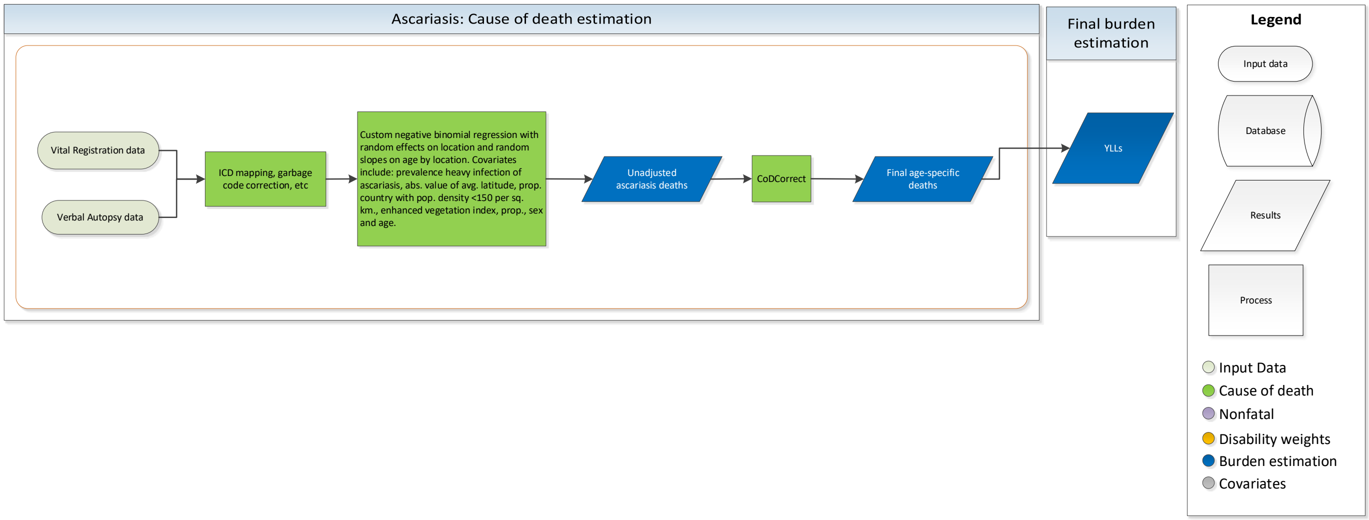

Input data and methodological summary for ascariasis

Input data

To estimate mortality due to ascariasis, country-year-age-sex-specific verbal autopsy and vital registration data were used. Covariates used included prevalence of heavy infection of ascariasis, the absolute value of average latitude, the proportion of the country with population density under 150 people per square kilometer, enhanced vegetation index, sex, and age.

Geographical restrictions

We conducted a literature review (last updated for GBD 2017) to determine the geographical extent of the disease and classify locations based on whether the disease is absent or present in each year. Locations that were geographically restricted in any given year did not have estimates made for them. Of note, we did not attempt a complete systematic review, since a single high-quality source could offer sufficient evidence of presence. Evidence of absence or presence was not available for every location for each year. Assumptions made for missing years took into consideration the epidemiological characteristics of the disease.

If evidence indicated disease presence for two non-consecutive years, we assumed presence for all years between the two. If evidence indicated disease absence for two non-consecutive years, we assumed absence for all years between the two. If evidence indicated a change in status (ie, from absent to present, or present to absent) between two non-consecutive years, then we conducted targeted searches to ascertain the relevant year of introduction or elimination for that location. In the cases where presence or absence information was missing for the start or end years of our study interval without evidence of any introduction or elimination events within the interval, we applied the status of the first and last presence/absence observations, respectively, to all years between the interval bound and the observation year. Table 1 shows the search strings and associated yield for each of the databases queried.

Table 1. Geographical restriction search strings

Database	Search string	Yield
PubMed	(Ascariasis[Title/Abstract] OR Ascaris[Title/Abstract] OR "A. lumbricoides"[Title/Abstract] OR Ascaris[MeSH] OR Trichuris[Title/Abstract] OR Trichuriasis[Title/Abstract] OR "Whip Worm"[Title/Abstract] OR "T. trichura"[Title/Abstract] OR Trichuris[MeSH] OR Hookworm[Title/Abstract] OR "A. duodenale"[Title/Abstract] OR "Ancylostoma duodenale"[Title/Abstract] OR ancylostomiasis[Title/Abstract] OR "N. americanus"[Title/Abstract] OR "Necator americanus"[Title/Abstract] OR necatoriasis[Title/Abstract] OR Ancylostoma [MeSH] OR Necator[MeSH]) AND (prevalence[Title/Abstract] OR incidence[Title/Abstract] OR epidemiology[Title/Abstract] OR surveillance[Title/Abstract]) NOT(Animals[MeSH] NOT Humans[MeSH])	2376
Web of Science	(Ascariasis OR Ascaris OR A. lumbricoides OR Trichuris OR Trichuriasis OR Whip Worm OR T. trichura OR Hookworm OR A. duodenale OR Ancylostoma duodenale OR anclyostomiasis OR N. americanus OR Necator americanus OR necatoriasis) AND TOPIC:(prevalence OR incidence OR epidemiology OR surveillance) NOTTOPIC: ((Animals NOT Humans)) Timespan: 1980-2016. Indexes: SCI-EXPANDED, SSCI, A&HCI, ESCI.	2266
SCOPUS	TITLE-ABS_KEY (ascariasis OR ascaris OR a. lumbricoides OR trichuris OR trichuriasis OR whip worm OR t. trichura OR hookworm OR a. duodenale OR ancylostoma duodenale OR anclyostomiasis OR n. americanus OR necator americanus OR necatoriasis) AND PUBYEAR>1979	29

These papers were used to classify location-years for all locations and years present in the literature. Additionally, systematic literature reviews, meta-analyses, national health statistics publications, and collaborator input were used to classify location-years not present in the literature review wherever possible.

Modelling strategy

A negative binomial model was used to estimate deaths from ascariasis with random intercepts for locations and random slopes for age groups by location. A multivariate normal distribution using the mean and variance-covariance matrix from the model was used to generate 1000 draws of deaths due to ascariasis. The final model was selected based on how well the estimated number fit the input data and how plausible the predicted distribution of disease was over time and with age.

Asthma

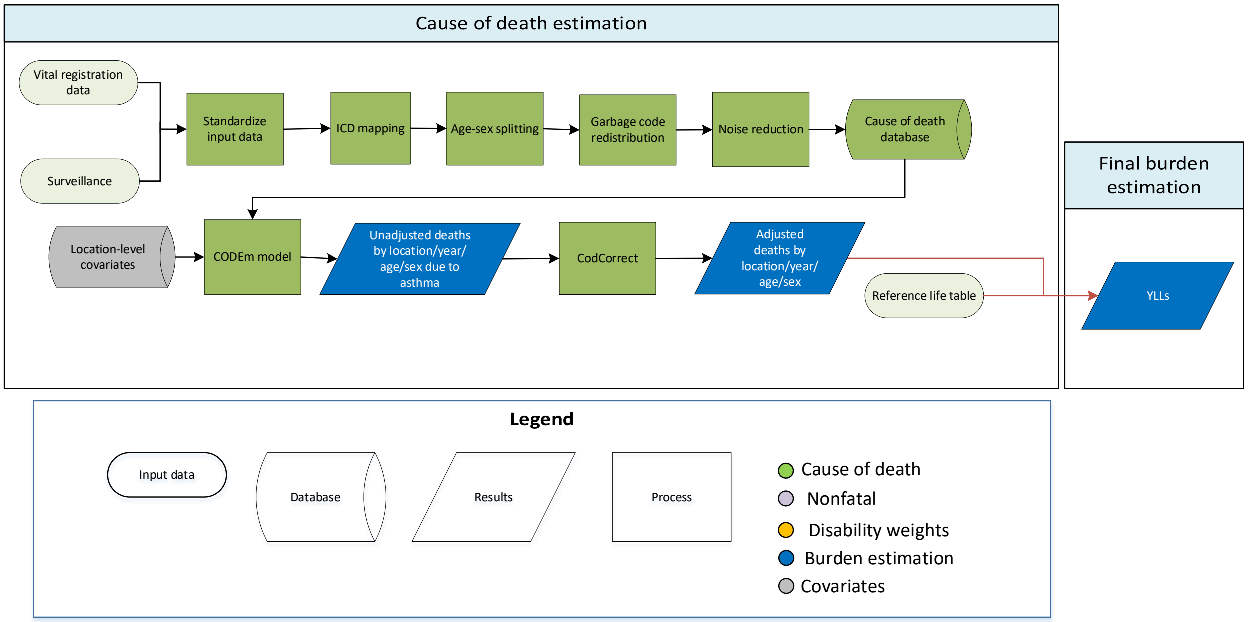

Input data

Data used to estimate asthma mortality included vital registration and surveillance data from the cause of death (COD) database. Verbal autopsy data were not included and were instead mapped to an overall chronic respiratory model. Our outlier criteria excluded datapoints that (1) were implausibly high or low relative to global or regional patterns, (2) substantially conflicted with established age or temporal patterns, or (3) significantly conflicted with other data sources conducted from the same locations or locations with similar characteristics (ie, Socio-demographic Index).

Modelling strategy

There were no substantive changes to the modelling approach this round. The standard Cause of Death Ensemble modelling (CODEm) [1] approach (detailed in reference appendix section 3.1) was used to estimate deaths due to asthma. Separate models were conducted for male and female mortality, and the age range for both models was 1 to 95+ years.

The following table lists the covariates included in the model. This requires that the covariate selected for the model must have the directional relationship with chronic respiratory deaths. For GBD 2021, no significant updates were made to covariate selections. Covariate directions were selected based on the strength of the evidence.

**Level**	**Covariate**	**Direction**
1	Log-transformed SEV scalar: asthma	+
	Cumulative cigarettes (10 years)	+
	Cumulative cigarettes (5 years)	+
	Healthcare Access and Quality Index	-
2	Smoking prevalence	+
	Indoor air pollution (all cooking fuels)	+
	Outdoor air pollution ($PM_{2.5}$)	+
3	Lagged 10-year LDI (I$ per capita)	-
	Education (years per capita)	-
	Socio-demographic Index	-

In CoDCorrect, the unadjusted death estimates for asthma are combined with those for chronic obstructive pulmonary disease, interstitial lung disease and pulmonary sarcoidosis, pneumoconiosis, and other chronic respiratory diseases and fit to the distribution of deaths in an overall chronic respiratory disease envelope model. This results in deaths recorded using non-specific coding systems, such as verbal autopsy, being included in the parent model and redistributed to the child models proportionately.

Covariate influences:

The following plots show the influence of each covariate on the four CODEm models (male global, male data-rich, female global, and female data-rich). A positive standardised beta (to the right) means that the covariate was associated with increased death. A negative standardised beta (to the left) means the covariate was associated with decreased death.

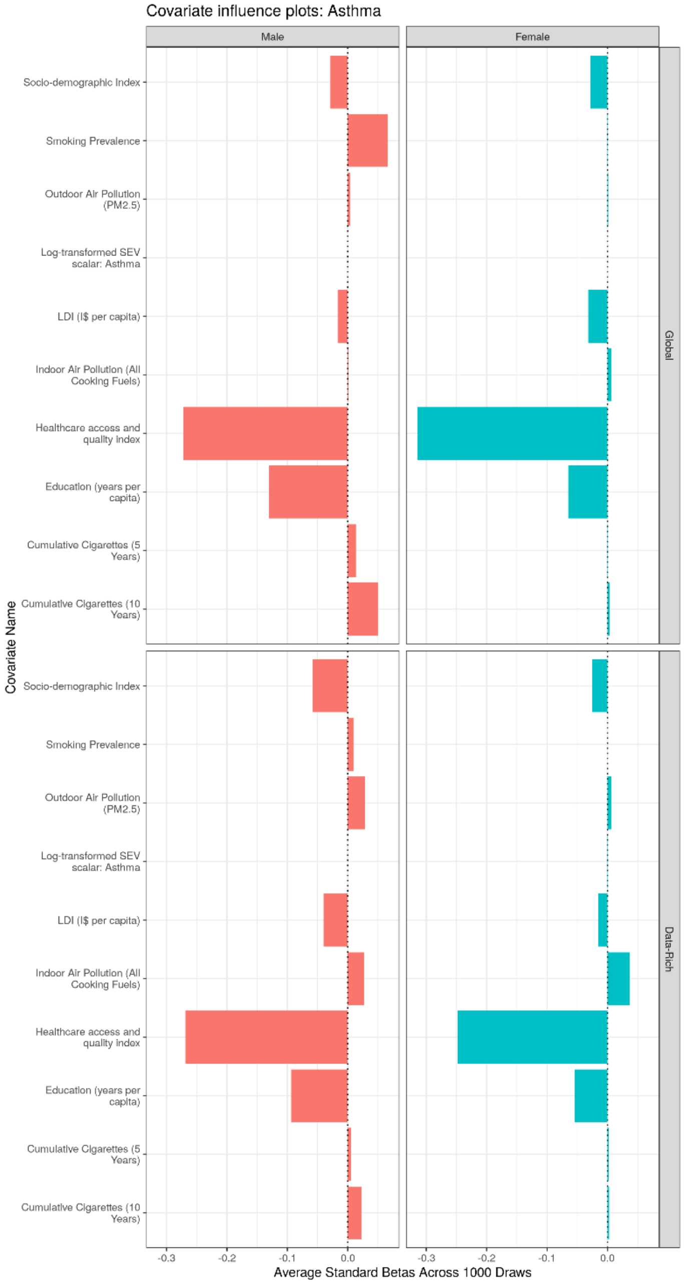

[1]Vos T, Lim SS, Abbafati C, *et al.* Global burden of 369 diseases and injuries in 204 countries and territories, 1990–2019: a systematic analysis for the Global Burden of Disease Study 2019. *The Lancet* 2020; 396**:** 1204–22. doi: https://doi.org/10.1016/S0140-6736(20)30925-9

Bacterial skin diseases

Flowchart

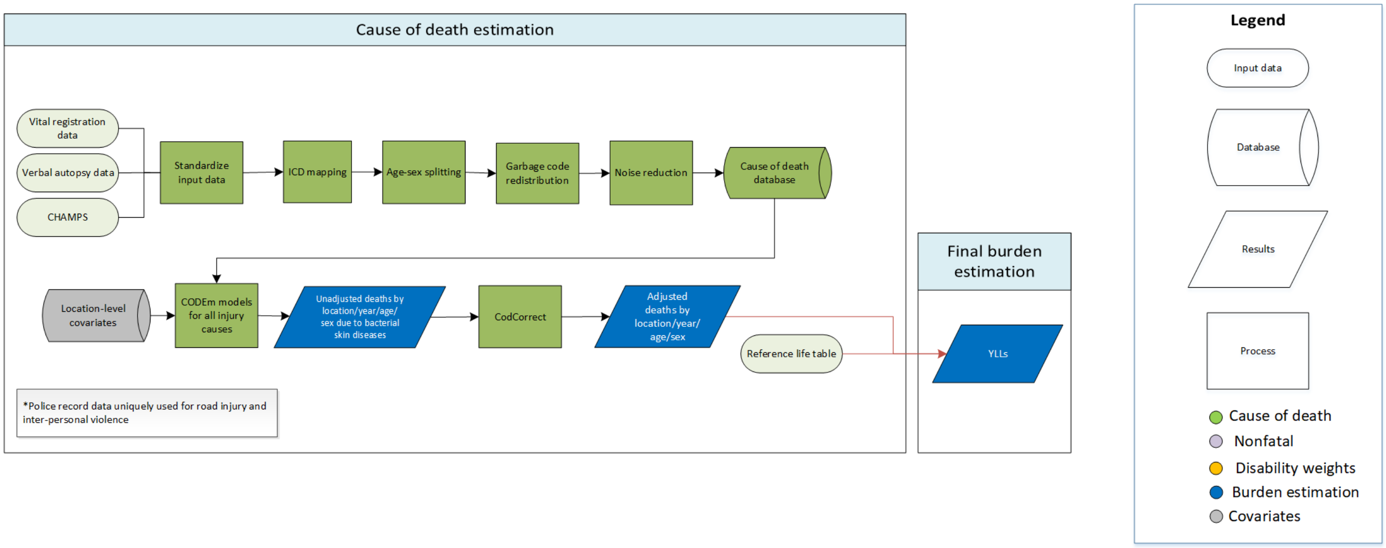

Input data and methodological summary for bacterial skin diseases

Input data

Data used to estimate bacterial diseases consisted of vital registration, verbal autopsy, and Chinese Disease Surveillance Points (DSP) data from the cause of death (COD) database. Outlier criteria excluded datapoints that were implausibly high or low relative to global or regional patterns and data from countries with small populations.

Modelling strategy

This is a parent model of pyoderma and cellulitis. The standard CODEm modelling approach was used to estimate deaths due to bacterial skin diseases. CODEm parameters were a combination of those from pyoderma and cellulitis.

There were no significant changes in the modelling process between GBD 2019 and GBD 2021.

Table 1. Covariates used in bacterial skin mortality modelling

Level	Covariate	Direction
1	Summary exposure value (SEV) scalar for unsafe sanitation	+
	Prevalence of overweight and obesity	+
	Healthcare Access and Quality Index	-
	Diabetes fasting plasma glucose (mmol/L), by age	+
	Improved water source (proportion with access)	-
	Alcohol (litres per capita)	+

2	Cumulative cigarettes (5 years)	+
	Cumulative cigarettes (10 years)	+
	Smoking prevalence	+
3	Education (years per capita)	-
	Lag distributed income (per capita)	-
	Socio-demographic Index	-

Cancers

Input data and methodological summary for all cancers except for non-melanoma skin cancer

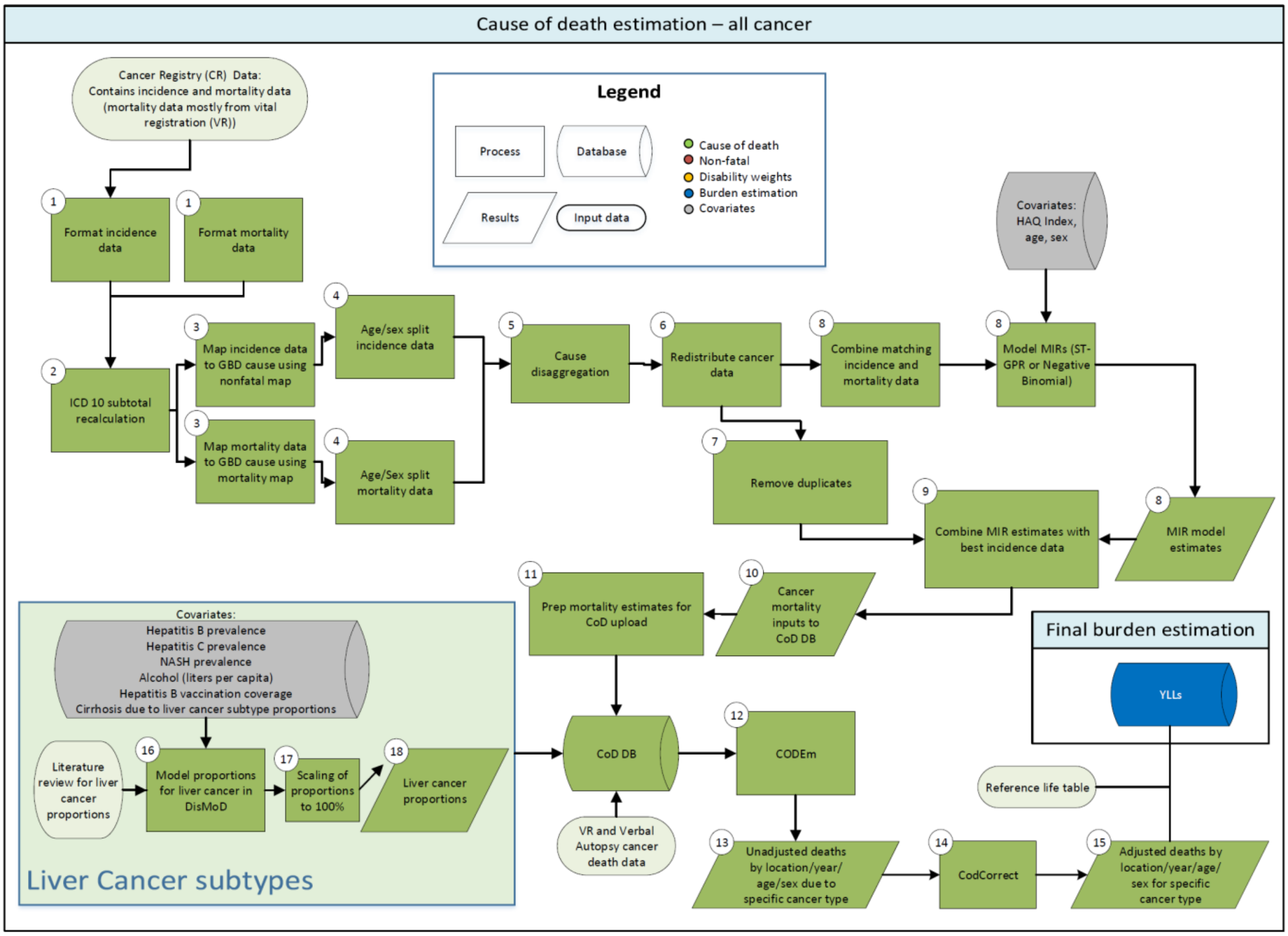

Abbreviations: COD, causes of death; CODEm, cause of death ensemble model; DB, database; DisMod-MR, disease model - Bayesian meta-regression, HAQ Index, healthcare access and quality index; ICD, International classification of diseases; MIR, mortality-to-incidence ratio; NASH, nonalcoholic steatohepatitis; ST-GPR, spaciotemporal Gaussian process regression; VR, vital registration; YLL, years of life lost

Data

The GBD cause of death (CoD) database contains cancer mortality data originating from multiple sources, including vital registration (VR), verbal autopsy (VA), and cancer registry (CR) data. The cancer registry mortality estimates that are uploaded into the CoD database stem from cancer registry incidence data that have been transformed to mortality estimates through the use of mortality-to-incidence ratios (MIRs).

Data-seeking processes

Cancer mortality data in the cause of death database other than cancer registry data

Sources for cancer mortality data other than cancer registry data are described in the CoD section of the Capstone Appendix.

Cancer registry data

We used data from GBD 2019 and added cancer registry data from Algeria, Argentina, Bolivia, Brunei, Chile, China, Cyprus, France, Georgia, Germany, Honduras, Hungary, India, Iran, Italy, Japan, Mexico, Mozambique, Namibia, Nepal, Niger, Nigeria, Norway, Pakistan, Peru, Poland, Russia, Rwanda, Singapore, South Africa, Spain, Switzerland, Thailand, the UK, and the USA.

Inclusion and exclusion criteria

Only population-based cancer registries were included, with inclusion criteria that they included all cancers (ie, were not specialty registries for a subset of cancer types), reported data for all age groups (with the exception of paediatric cancer registries), and reported data for both sexes. Pathology-based and hospital-based cancer registries were excluded. Redundant cancer registry data were excluded from either the final incidence data input or the MIR model input if a more detailed source (eg, providing more detailed age or diagnostic groups) was available for the same population. Preference was given to registries with national coverage over those with subnational coverage, except those from countries where the GBD study provides subnational estimates. Data were excluded if the coverage population was unknown, except for in high Socio-demographic Index (SDI) quintile locations with full geographical coverage where the GBD estimated population could be substituted.

Bias of categories of input data

Cancer registry data can be biased in multiple ways. A high proportion of ill-defined cancer cases in the cancer registry data requires redistribution of these cases to other cancers, which introduces a potential for bias. Changes between coding systems can lead to artificial differences in disease estimates; however, we adjust for this bias by mapping the different coding systems to GBD cancer causes. Underreporting of cancers that require advanced diagnostic techniques (eg, leukaemia, brain, pancreatic, and liver cancer) can be an issue in cancer registries from low-income countries. On the other hand, misclassification of metastatic sites as primary cancer can lead to overestimation of cancer sites that are common sites for metastases (eg, brain cancer). Since many cancer registries are located in urban areas, the representativeness of the registry for the general non-urban population can also be problematic. The accuracy of mortality data reported by a subset of cancer registries usually depends on the quality of the vital registration system. If the vital registration system is incomplete or of poor quality, the mortality-to-incidence ratio can be biased to lower ratios.

Data for liver cancer aetiology splits

Additional data are utilised to proportionally split the age ≥10 years liver cancer total (which excludes hepatoblastoma) into the five aetiology groups included in the GBD: 1) liver cancer due to hepatitis B, 2) liver cancer due to hepatitis C, 3) liver cancer due to alcohol, 4) liver cancer due to non-alcoholic steatohepatitis (NASH), and 5) liver cancer due to other causes. To find data to inform these proportions, a systematic literature search was performed in PubMed on 10/24/2016 using the following search string:

> “("liver neoplasms"[All Fields] OR "HCC"[All Fields] OR "liver cancer"[All Fields] OR "Carcinoma, Hepatocellular"[Mesh]) AND (("hepatitis B"[All Fields] OR "Hepatitis B"[Mesh] OR "Hepatitis B virus"[Mesh] OR "Hepatitis B Antibodies"[Mesh] OR "Hepatitis B Antigens"[Mesh]) OR ("hepatitis C"[All Fields] OR "Hepatitis C"[Mesh] OR "hepatitis C antibodies"[MESH] OR "Hepatitis C Antigens"[Mesh] OR "Hepacivirus"[Mesh]) OR ("alcohol"[All Fields] OR "Alcohol Drinking"[Mesh] OR "Alcohol-Related Disorders"[Mesh] OR "Alcoholism"[Mesh] OR "Alcohol-Induced Disorders"[Mesh])) NOT (animals[MeSH] NOT humans[MeSH])”.

Studies were included if they provided proportion data that were representative for the location covered. Several studies not identified through this search but that were included in the meta-analysis by de Martel and colleagues were included.[1] We also included the study by Hong and colleagues after the authors provided us with additional data on the overlap in aetiologies.[2]

For each study, the proportions of liver cancer due to the five specific aetiologies were calculated. Cases were considered to be due to NASH when the manuscript explicitly listed the aetiology to be NASH or non-alcoholic fatty liver disease (NAFLD). Cases where the aetiology was listed as "cryptogenic", "idiopathic", or "unknown" were assessed as either implicitly NASH (included within the "NASH" category when alcohol, hepatitis B, and hepatitis C were reported), or of ambiguous aetiology (included within the "other causes" category when any of alcohol, hepatitis B, or hepatitis C could not be ruled out). These implicit NASH proportions were adjusted to the explicit NASH proportions using the GBD meta-regression, Bayesian, regularised, trimmed (MR-BRT) method, which is detailed elsewhere in the GBD summary papers. In manuscripts where the aetiology for a case was not known but major categories could not be ruled out (for example, if the study tested for hepatitis B and C but did not assess alcohol use), only the explicitly defined proportions were included (in this example, including proportions for hepatitis B and C, and excluding any remainder). Any remaining named aetiologies were included under a combined "other" group (for example, haemochromatosis, autoimmune hepatitis, Wilson's disease, etc.). If multiple aetiologies were reported for any cases, these were assigned proportionally to the individual aetiologies reported in the study. Sex-combined sources were split into sex-specific proportions using the GBD MR-BRT method detailed elsewhere the GBD summary papers.

Methods

Steps of analysis and data transformation processes

Cancer registry data go through multiple processing steps before entering the CoD database.

1. Formatting incidence and mortality data. First, the original data are transformed into standardised files, which included standardisation of format, categorisation, and registry names (#1 in flowchart).

2. Subtotal recalculation. Some cancer registries report individual codes as well as aggregated totals. An example of this would be where the registry data report C18, C19, and C20 individually, and also the aggregated group of C18–C20 (colon and rectum cancer). The data processing step, "subtotal recalculation" (#2 in flowchart), verifies these totals and subtracts the values of any individual codes from the aggregates.

3. Mapping data to GBD causes. In the third step (#3 in the flowchart), cancer registry incidence data and cancer registry mortality data are mapped to GBD causes. A different map is used for incidence and for mortality data because of the assumption that there are no deaths for certain cancers. One example is basal cell carcinoma of the skin. In the cancer registry incidence data, basal cell carcinoma is mapped to non-melanoma skin cancer (basal cell carcinoma). However, if basal cell skin cancer is recorded in the cancer registry mortality data, the deaths are instead mapped to non-melanoma skin cancer (squamous cell carcinoma) under the assumption that they were actually misclassified squamous cell skin cancers. Another example is benign or in situ neoplasms. Because cancer registries do not collect non-malignant neoplasms in a standardised way, any benign or in situ neoplasms reported in a cancer registry incidence dataset are dropped from that dataset. The same neoplasms reported in a cancer registry mortality dataset are instead mapped to the respective invasive cancer. For example, cases of "ductal

carcinoma in situ" in a cancer registry incidence dataset are dropped from the dataset, while deaths from "ductal carcinoma in situ" in a cancer registry mortality dataset are mapped to breast cancer. The exception is codes for benign tumours of the brain.

4. Age/sex splitting. In the fourth data processing step (#4 in the flowchart) cancer registry data are standardised to the GBD age groups. For each cancer, the minimum age group estimated was determined as the youngest age group where SEER reported at least 50 cases over the period 1990 to 2015.[3] Global age-specific incidence rates are generated using select comprehensive cancer registry datasets, such as from SEER,[4] NORDCAN,[5] CI5,[6–16] and IICC.[17] The use of high-quality population-based cancer registry data to generate these rates is a change for GBD 2021; in GBD 2019, incidence age rates were based on hospital inpatient data. Age-specific mortality rates are generated using age weights from CoD VR data, as described elsewhere in the GBD summary papers. For incidence or mortality datasets that require age-splitting, global age-specific proportions are then generated by applying the age-specific rates to the overall registry population to produce the expected number of cases (or deaths for a mortality dataset) for that registry by age. The expected number of cases (or deaths) for each sex, age, and cancer were normalised to 1, creating final, age-specific proportions. These proportions were then applied to the total number of cases (or deaths) by sex and cancer to get the GBD age group-specific number of cases (or deaths) related to that dataset.

In the rare case that the cancer registry only contains data for both sexes combined, the age-specific cases or deaths are split and reassigned to separate sexes using the same weights that are used for the age-splitting process. Starting from the expected number of deaths, global proportions are generated by sex for each age. For example, if for ages 15–19 years old there are 6 expected deaths for males and 4 expected deaths for females from cause of death data, then 60% of the combined-sex deaths for ages 15–19 years would be assigned to males and the remaining 40% would be assigned to females.

5. Cause disaggregation. In the fifth step (#5 in the flowchart) data for cause entries that are aggregates of GBD causes were redistributed across those GBD causes. Examples of these aggregated causes include some cancer registries reporting ICD-10 codes C00–C14 together as "lip, oral cavity, and pharyngeal cancer". These groups are broken down into sub-causes that can be individually mapped to single GBD causes. In this example, the more specific ICD-10 codes within C00–C14 are "lip and oral cavity cancer" (C00–C08), "nasopharyngeal cancer" (C11), "cancer of other parts of the pharynx" (C09–C10, C12–C13), and "malignant neoplasm of other and ill-defined sites in the lip, oral cavity, and pharynx" (C14). To redistribute the data, weights were created using the same "rate-applied-to-population" method employed in age-sex splitting (see step four above). For the undefined code (C14 in the example) an "average all cancer" weight was used, calculated on the high-quality cancer registry data from SEER,[3] NORDCAN,[5] and CI5[6–16] by dividing the sum of the cases across these registries by the combined population across these registries. Then, proportions were generated by sub-cause for each aggregate cause as in the sex-splitting example above (see step 4). The total number of cases from the aggregated group (C00–C14) was recalculated for each subgroup and the undefined code (C14). C14 was then redistributed as a "garbage code" in step six. For some exceptions, C44 (non-melanoma skin cancer), C46 (Kaposi's sarcoma), and C74 (malignant neoplasm of adrenal gland), fixed proportions were used to redistribute into GBD causes. Non-melanoma skin cancer processing is described below under the section specific to these cancers. C46 entries were primarily redistributed to HIV according to age

(100% for age <15 years, 95% for age 15–49 years, and 90% for age ≥50 years), with the remainder redistributed to the GBD cause, “Soft tissue and other extraosseous sarcomas”. C74 entries were redistributed to “neuroblastoma and other peripheral nervous cell tumours” and “other malignant neoplasms”, with percentages varying by age (younger age groups with a greater proportion redistributed to “neuroblastoma and other peripheral nervous cell tumours”).

6. Redistribution. In the sixth step (#6 in the flowchart), unspecified ICD codes (“garbage codes”) such as “ill-defined cancer site” (for example, C76 or C80) are redistributed across relevant causes estimated within the GBD hierarchy. Redistribution of cancer registry incidence and mortality data mirrored the redistribution process and maps used in the cause of death database, as detailed elsewhere in the GBD summary papers.

7. Removal of duplicates. In the seventh step (#7 in the flowchart) duplicate or redundant data sources were removed from the processed cancer registry dataset. Duplicate sources were present if, for example, a cancer registry was part of the CI5[6–16] database but we also had data from that registry directly. Redundancies occurred and were removed as described in “Inclusion and exclusion criteria”, where more detailed data were available, or when national registry data could replace regionally representative data. From here, two parallel selection processes were run: one to generate input data for the mortality-to-incidence ratio (MIR) models, and one to generate incidence for final mortality estimation. When creating the final incidence input, higher priority was given to registry data from the most standardised source, whereas for the MIR model input, only sources that reported both incidence and mortality were used.

8. Combine matching incidence and mortality data and model MIRs. In the eighth step (#8 in the flowchart) the processed incidence and mortality data from cancer registries were matched by cancer, age, sex, year, and location to generate MIRs. These MIRs were used as input for further modelling, using one of two approaches, depending on the cancer.

As in previous GBD cycles, MIRs for most cancers were estimated with a three-step modelling approach using the general GBD spatiotemporal Gaussian process regression (ST-GPR) approach. These used logit-transformed MIR as the outcome, with covariates for sex, categorical age group, and Healthcare Access and Quality (HAQ) Index as a covariate in the linear mixed-effects model.[18]

$$\text{logit}\left(MIR_{c,a,s,t}\right) = \alpha + \beta_1(HAQIndex)_{c,t} + \sum_{a}^{A} \beta_2 I_a + \beta_3 I_s + \epsilon_{c,a,s,t}$$

MIR: mortality-to-incidence ratio
c: country (or subnational for subnationally modelled locations), a: age group, t: time (years); s: sex
HAQIndex: Healthcare Access and Quality Index
I: indicator variable
$\epsilon_{c,a,s,t}$: error term

Results from the final linear model were used as input for spatiotemporal smoothing and a Gaussian process regression. The ST-GPR model has three main hyper-parameters that control for smoothing across time, age, and geography.[19] These hyper-parameter values were unchanged for GBD 2021. The time adjustment parameter lambda (λ) aims to borrow strength from neighbouring time points (ie, the

value in this year is highly correlated with the value in the previous year but less so further back in time) and was set to 0.05. The age adjustment parameter omega (ω) borrows strength from data in neighbouring age groups and was set to 0.5. The space adjustment parameter zeta (ξ) aims to borrow strength across the hierarchy of geographical locations and was set to 0.01. For the remaining parameters in the Gaussian process regression, we set amplitude to 1 (influences fluctuation from the mean function) and set the scale value to 10 (influences the time distance over which points are correlated). Additional details on ST-GPR are described elsewhere in the GBD summary papers. These models were used to obtain MIR estimates for all combinations of GBD age, sex, year, cause, and location. Datapoints were outliered manually if they clearly influenced the model in an unrealistic way. For example, a datapoint was marked as an outlier if it created a single-year, single age group spike in model predictions that was inconsistent with the trend suggested by surrounding datapoints.

For eight of the nine cancer causes that are newly estimated in GBD 2021, we modelled MIRs using a negative binomial regression approach. The exception was "other non-Hodgkin lymphoma", which was modelled using the ST-GPR methods described above due to greater data availability for this cause. The negative binomial approach was used for most of the newly estimated cancer causes because it allows modelling of count data with overdispersion (meaning the mean and variance are allowed to differ in the underlying distribution), which was determined to be needed due to the relatively rare deaths for these cancer causes. MIRs were estimated for each age-sex-year-location using a negative binomial regression run in R (version 3.5.0) using glm.nb from the MASS package. We used categorical age and HAQ Index as covariates and offset by the logarithm of cases.

For all causes that existed in GBD 2019, data-cleaning steps for MIR estimation were the same as for GBD 2019. For each cancer, MIRs from locations in HAQ Index quintiles 1–4 were dropped if they were below the median of MIRs from locations in HAQ Index quintile 5. We also dropped MIRs from locations in HAQ Index quintiles 1–4 if the MIRs were above an outlier threshold calculated as the third quartile + 1.5 * IQR (inter-quartile range). We dropped all MIR data that were based on fewer than 15 incident cases to avoid excessive variation in the ratio due to small numbers. An exception to this threshold was made for mesothelioma and acute myeloid leukaemia, where instead we dropped MIRs that were based on fewer than ten cases because of lower data availability for these two cancers. For the lower end of the age spectrum where cancers are generally rarer, we also aggregated incidence and mortality to the youngest five-year age bin where SEER[3] reported at least 50 cases from 1990 to 2015, to avoid unstable MIR predictions in young age groups because of too few cases or deaths. The MIR estimates in this SEER-based minimum age bin were then copied down to all younger GBD age groups estimated for that cancer.

For the nine new cancer causes first estimated in GBD 2021, additional data processing steps were used to help stabilise the input data and MIR estimates. First, data were aggregated across sexes and across bins of ten calendar years. Data were then only excluded if there were 0 cases. As cancer registry mortality data were limited for new cancer causes Burkitt lymphoma and retinoblastoma, we supplemented with mortality data from vital registration systems. For these two causes, cancer registry incidence was matched with vital registration mortality by age-sex-year-location. These cancer registry–vital registration matched inputs were processed the same as the standard matched inputs.

Since MIRs can be above 1, especially in older age groups and for cancers with low cure rates, we used the 95th percentile (by age group) of the cleaned dataset (detailed above) to cap the MIR input data.

These "upper cap" values were used to allow MIRs over 1 in some age groups but to constrain the MIRs to a maximum level. The addition of new data for GBD 2021 led to slightly different upper caps compared to GBD 2019 (see upper cap values for GBD 2021 below). New for GBD 2021, the upper caps for paediatric age groups (under 20 years) were increased to 1 (regardless of the 95th percentile) to allow for more model flexibility in the distribution of MIRs across locations.

**Age group (years):**	**0–4**	**5–9**	**10–14**	**15–19**	**20–24**	**25–29**	**30–34**	**35–39**	**40–44**	**45–49**
**Upper cap:**	1.00	1.00	1.00	1.00	1.04	0.949	0.936	0.888	0.928	0.950

**Age group (years):**	**50–54**	**55–59**	**60–64**	**65–69**	**70–74**	**75–79**	**80–84**	**85–89**	**90–94**	**95+**
**Upper cap:**	0.962	0.992	1.02	1.06	1.11	1.18	1.27	1.36	1.48	1.61

Any MIR values over this upper cap were Winsorised to the cap value. To run the logit model in ST-GPR, the input data were first divided by the upper caps to get proportional data ranging from 0 to 1. Model predictions from ST-GPR were then rescaled back to MIRs by multiplying the scaled predictions by the upper caps. To constrain the MIRs at the lower end, we used the fifth percentile of the cancer and age-specific cleaned MIR input data to Winsorise all model predictions below this lower cap.

9. Generate mortality estimates from incidence and MIRs. Final estimated MIRs were matched with the cleaned cancer registry incidence dataset finalised in the ninth step (#9 in the flowchart) to generate mortality estimates (#10 in the flowchart):

$$MIR_{estimates} * \ incidence_{registry} = mortality_{CR\ inputs}$$

These mortality estimates were then smoothed by a Bayesian noise-reduction algorithm to deal with zero counts; this is also applied to the CoD data inputs (VR and VA data), as detailed elsewhere in the GBD summary papers. These data were uploaded into the CoD database as CR data (#11 in the flowchart). Cancer-specific mortality modelling then followed the general CODEm process using the totality of VA, VR, and CR data inputs.

Liver cancer aetiology split models

The proportion data from the liver cancer systematic literature review (see above) were used as input for five separate DisMod-MR 2.1 models to determine the proportion of liver cancers due to the five subgroups for all locations, sexes, years, and age groups (step #16 in the flowchart). For liver cancer due to alcohol, a prior value of 0 percent was set for ages 0 to 5 years. For liver cancer due to hepatitis B and hepatitis C, a prior value of 0 percent was set between ages 0 and 0.01 years. Covariates differed by model and direction. The liver cancer due to alcohol model included positive covariates for the litres of alcohol consumed per capita, the age-standardised proportion of alcohol drinkers, and the proportion of cirrhosis due to alcohol. The liver cancer due to hepatitis B model included positive covariates for age-standardised vaccine-adjusted HBsAg seroprevalence and the proportion of cirrhosis due to hepatitis B, and a negative covariate for 10-year lagged hepatitis B 3-dose vaccine coverage. The liver cancer due to

hepatitis C model included positive covariates for age-standardised chronic hepatitis C and the proportion of cirrhosis due to hepatitis C. The liver cancer due to NASH model included positive covariates for mean body-mass index, the age-standardised prevalence of obesity, the prevalence of NASH and non-alcoholic fatty liver disease, and the proportion of cirrhosis due to NASH. The liver cancer due to other causes model included a positive covariate for the proportion of cirrhosis due to other causes.

To ensure consistency between cirrhosis and liver cancer estimates and to take advantage of the data for the related causes (eg, "liver cancer due to hepatitis C" and the related cause "cirrhosis due to hepatitis C"), we generated covariates from initial liver cancer proportion model estimates that were then used in the cirrhosis aetiology proportion models. Estimates from the cirrhosis aetiology proportion models were then used to make covariates for use in the final liver cancer aetiology models. More information about cirrhosis modelling can be found in the "Cirrhosis" section of this Appendix.

Since the five aetiology proportion models were run independently of each other, the final proportion estimates were scaled to sum to 100% within each age, sex, year, and location, by dividing each proportion by the sum of the five (step # 17). For the liver cancer subtype mortality estimates, we multiplied the parent cause "liver cancer" deaths (excluding deaths in ages less than 10, which are assigned to hepatoblastoma, see below) by the corresponding scaled proportions (step # 18). Single cause estimates were later adjusted to fit into the separately modelled all-cause mortality in the CoDCorrect process. CoDCorrect also combines these five subtypes with the hepatoblastoma estimates (see below) to create a new total liver cancer estimate across all ages.

Hepatoblastoma estimation

For GBD 2021, all mortality estimates under age 10 from the liver cancer parent model were assigned to hepatoblastoma. No aetiology was assigned to these under-10 hepatoblastoma estimates. While hepatoblastoma is not the only type of liver cancer that children under 10 can die from, the GBD currently only estimates Level 4 mortality from liver cancer under 10 years in the cause hepatoblastoma. Independent modelling using observed hepatoblastoma data and consideration of other liver cancer types under 10 years of age are anticipated in the future, depending on data availability.

Results

Interpretation of results

Cancer mortality estimates for GBD 2021 can differ from the GBD 2019 results for multiple reasons. New and updated cancer mortality data were added from vital registration system data, verbal autopsy studies, and cancer registry incidence data, and MIRs were informed by a substantial amount of new paediatric cancer registry data. The addition of new cancers for GBD 2021 led to mapping changes, with many cancer cases and deaths that were previously mapped to the cause "other malignant neoplasms" now mapped to one of the new cancer causes. These new causes required new models to be developed, and in some cases new approaches to their estimation because of the rarer events and sparser data (eg, Step 8 above).

The other group producing global and country-level cancer mortality estimates is the International Agency for Research on Cancer (IARC) with their GLOBOCAN[20] estimates. Substantially different methods between the GBD study and GLOBOCAN can lead to differences in results. For GLOBOCAN, estimates are produced separately at the national level, using several different regression or imputation

models differentially by country depending on the data available.[21] For the GBD, cancer estimation occurs globally across all locations following a consistent, well-documented ensemble modelling approach that includes relevant covariate data, which allows for cross-validation of models as well as determination of uncertainty. Another major difference is the ability in the GBD study to adjust single cause estimates to the all-cause mortality envelope, which is determined independently. This allows correction for the underdiagnosis of cancer in countries with inadequate diagnostic resources. Redistribution of a fraction of undefined causes of death to certain cancers is another methodological advantage of the GBD study as compared to GLOBOCAN, and estimates for cancer mortality can therefore differ substantially in countries with a large proportion of undefined causes of death in their vital registration data or a large proportion of undefined cancer cases in their cancer registry data. There are also differences in the inclusion and categorisation of cancer types reported; for instance, basal cell carcinoma cases are included in GBD total cancer incidence estimates but excluded in GLOBOCAN incidence estimates; and a handful of cancers are individually reported in GBD which are not reported separately in GLOBOCAN (eg, malignant neoplasm of bone and articular cartilage) or are reported in GLOBOCAN (eg, penis cancer), which in the GBD are included within the "other malignant neoplasms" cause.[20]

Limitations

There are certain limitations to consider when interpreting the GBD mortality cancer estimates. First, even though every effort is made to include the most recently available data for each country, data-seeking resources are not limitless and new data cannot always be accessed as soon as they are made available. It is therefore possible that the GBD study does not include all available data sources for cancer incidence or cancer mortality. Second, different redistribution methods can potentially change the cancer estimates substantially if the data sources used for the estimated location contain a large number of undefined causes; however, neglecting to account for these undefined deaths would likely introduce an even greater bias in the disease estimates. Third, using mortality-to-incidence ratios to transform cancer registry incidence data to mortality estimates requires accurate MIRs. For GBD 2021, we have made further refinements to the estimation of MIRs, but the method remains sensitive to under-diagnosis of cancer cases or under-ascertainment of cancer deaths. However, given that the majority of data used for cancer mortality estimation come from vital registration data and not cancer registry data, this is not a major limitation.

Non-melanoma skin cancer (squamous cell carcinoma)

In the GBD framework, non-melanoma skin cancer (NMSC) estimates include both squamous cell carcinoma (both incidence and mortality) and basal cell carcinoma (incidence only). This section describes the methods for squamous cell carcinoma estimation, while a description of methods for basal cell carcinoma estimates can be found elsewhere in the GBD summary papers.

Data

Data-seeking processes

Since squamous cell carcinomas are very infrequently recorded by cancer registries, only vital registration system data were used as input for the squamous cell carcinoma mortality modelling.

Inclusion and exclusion criteria

Inclusion and exclusion criteria followed the same methods as for the vital registration data sources, as described elsewhere in the GBD summary papers.

Bias of categories of input data

The potential biases of the input data are the same as for other cancers (see above).

Methods

Overall methodological process

Vital registration system data were used as input to model deaths due to squamous cell skin cancer in CODEm.

Steps of analysis and data transformation processes

Since mortality estimates for non-melanoma skin cancer are produced for squamous cell carcinoma under the assumption that basal cell carcinoma causes almost no deaths, all mortality reported as ICD-10 code "C44" or ICD-9 code "173" were mapped to the GBD cause "squamous cell carcinoma".

Model selection

The modelling strategy for non-melanoma skin cancer (squamous cell carcinoma) followed the general CODEm process.

Model performance and sensitivity

The modelling performance and sensitivity for non-melanoma skin cancer (squamous cell carcinoma) mirrored that of the general CODEm process.

Uncertainty intervals

Uncertainty was determined using standard CODEm methodology, as described in the GBD summary papers.

Results

Interpretation of results

Although the data availability for non-melanoma skin cancer is a challenge, it is a common incident cancer and thus has been included in the GBD framework since GBD 2016. Non-melanoma skin cancer (NMSC) incidence and mortality estimates are not widely available from other sources. GLOBOCAN,[20,21] for example, reported cases and deaths due to non-melanoma skin cancer for the first time in their 2018 release; these GLOBOCAN NMSC new case estimates exclude basal cell carcinoma, while GLOBOCAN NMSC death estimates include all types of NMSC.

Limitations

Cancer registry data for non-melanoma skin cancer incidence have to be interpreted with caution due to a substantial amount of under-reporting, or rules that only the first non-melanoma skin cancer has to be registered. Many cancer registries therefore do not report non-melanoma skin cancers at all. Information regarding whether or not cancer registries capture non-melanoma skin cancer is not consistently available. Therefore, no cancer registry data were used to estimate deaths due to squamous cell carcinoma of the skin. For vital registration data, we make the assumption that there are no deaths due to basal cell non-melanoma skin cancer, and therefore all deaths attributed to basal cell carcinoma were included instead as squamous cell carcinoma.

Covariates by cancer cause

Acute lymphoid leukaemia

Level	Covariate	Direction
1	*Log-transformed age-standardised SEV scalar: Leukaemia*	+
	Log-transformed SEV scalar: Leukaemia	+
2	*Mean BMI*	+
	Healthcare Access and Quality Index	–
3	*Education (years per capita)*	–
	LDI (I$ per capita)	–
	Socio-demographic Index	+

Acute myeloid leukaemia

Level	Covariate	Direction
1	*Log-transformed age-standardised SEV scalar: Leukaemia*	+
	Log-transformed SEV scalar: Leukaemia	+
2	*Litres of alcohol consumed per capita*	+
	Mean BMI	+
	Cumulative cigarettes (10 years)	+
	Cumulative cigarettes (20 years)	+
	Tobacco (cigarettes per capita)	+
	Healthcare Access and Quality Index	–
3	*Education (years per capita)*	–
	LDI (I$ per capita)	+
	Socio-demographic Index	+

Bladder cancer

Level	Covariate	Direction
1	*Schistosomiasis prevalence (proportion)*	+
	Smoking prevalence	+
	Log-transformed SEV scalar: Bladder cancer	+
2	*Litres of alcohol consumed per capita*	+
	Diabetes fasting plasma glucose (mmol/L), age-standardised 25+	+
	Age- and sex-specific SEV for low vegetables	+
	Healthcare Access and Quality Index	–
	Cumulative cigarettes (10 years)	+
3	*Age- and sex-specific SEV for low fruits*	+
	LDI (I$ per capita)	+
	Socio-demographic Index	+

Brain and central nervous system cancer

Level	Covariate	Direction
1	*Litres of alcohol consumed per capita*	+
	Cumulative cigarettes (10 years)	+
	Smoking prevalence	+
2	*Cholesterol (total, mean per capita)*	+
	Systolic blood pressure (mmHg)	+
	Age- and sex-specific SEV for high red meat	+
	Age- and sex-specific SEV for low vegetables	+
	Age- and sex-specific SEV for low fruit	+
	Healthcare Access and Quality Index	–
3	*Education (years per capita)*	–
	LDI (I$ per capita)	–
	Socio-demographic Index	+

Breast cancer

Level	Covariate	Direction
1	*Litres of alcohol consumed per capita*	+
	Mean BMI	+
	Log-transformed SEV scalar: Breast cancer	+
2	*Age-specific fertility rate*	–
	Total fertility rate	–
	Age- and sex-specific SEV for low fruit	+
	Age- and sex-specific SEV for low vegetables	+
	Cumulative cigarettes (10 years)	+
	Cumulative cigarettes (20 years)	+
	Smoking prevalence	+
	Diabetes fasting plasma glucose (mmol/L), age-standardised 25+	+
	Healthcare Access and Quality Index	–
3	*LDI (I$ per capita)*	–
	Socio-demographic Index	+

Burkitt lymphoma

Level	Covariate	Direction
2	*Log-transformed SEV scalar: HIV*	+
	Log-transformed age-standardised SEV scalar: HIV	+
	Malaria incidence map	+
	Malaria incidence adjusted for antimalarial coverage and drug effectiveness	+
	Universal health coverage	–
	Healthcare Access and Quality Index	–
3	*Maternal care and immunisation*	–
	Education (years per capita)	–
	LDI (I$ per capita)	–
	Socio-demographic Index	–

Cervical cancer

Level	Covariate	Direction
1	*Cumulative cigarettes (5 years)*	+
	Log-transformed age-standardised SEV scalar: HIV	+
	HIV age-standardised prevalence	+
	Log-transformed SEV scalar: HIV	+
2	*Age-specific fertility rate*	+
	Total fertility rate	+
	Smoking prevalence	+
	Age- and sex-specific SEV for low fruit	+
	Age- and sex-specific SEV for low vegetables	+
	Healthcare Access and Quality Index	–
3	*Education (years per capita)*	–
	LDI (I$ per capita)	–
	Socio-demographic Index	–

Chronic lymphoid leukaemia

Level	Covariate	Direction
1	*Log-transformed age-standardised SEV scalar: Leukaemia*	+
	Log-transformed SEV scalar: Leukaemia	+
2	*Litres of alcohol consumed per capita*	+
	Mean BMI	+
	Cumulative cigarettes (5 years)	+
	Cumulative cigarettes (10 years)	+
	Cumulative cigarettes (15 years)	+
	Cumulative cigarettes (20 years)	+
	Tobacco (cigarettes per capita)	+
	Smoking prevalence	+
	Healthcare Access and Quality Index	–
3	*Education (years per capita)*	–
	LDI (I$ per capita)	–
	Socio-demographic Index	+

Chronic myeloid leukaemia

Level	Covariate	Direction
1	*Log-transformed age-standardised SEV scalar: Leukaemia*	+
	Log-transformed SEV scalar: Leukaemia	+
2	*Litres of alcohol consumed per capita*	+
	Mean BMI	+
	Cumulative cigarettes (5 years)	+
	Cumulative cigarettes (10 years)	+
	Cumulative cigarettes (15 years)	+
	Cumulative cigarettes (20 years)	+
	Tobacco (cigarettes per capita)	+
	Smoking prevalence	+
	Healthcare Access and Quality Index	–
3	*Education (years per capita)*	–
	LDI (I$ per capita)	+
	Socio-demographic Index	–

Colon and rectum cancer

Level	*Covariate*	*Direction*
1	*Mean BMI*	+
	Tobacco (cigarettes per capita)	+
	Total physical activity (MET-min/week), age-specific	–
	Log-transformed SEV scalar: Colorectal cancer	+
	Age- and sex-specific SEV for high red meat	+
2	*Litres of alcohol consumed per capita*	+
	PUFA adjusted (percent)	–
	Age- and sex-specific SEV for low vegetables	+
	Age- and sex-specific SEV for low fibre	+
	Age- and sex-specific SEV for low calcium	+
	Cumulative cigarettes (5 years)	+
	Diabetes fasting plasma glucose (mmol/L), age-standardised 25+	+
3	*Education (years per capita)*	–
	Age- and sex-specific SEV for low milk	+
	Age- and sex-specific SEV for low fruit	+
	Age- and sex-specific SEV for low nuts and seeds	+
	Healthcare Access and Quality Index	–
	LDI (I$ per capita)	+
	Socio-demographic Index	+

Eye cancer

Level	*Covariate*	*Direction*
2	*Universal health coverage*	–
	Age-standardised melanoma	+
	Healthcare Access and Quality	–
3	*Index LDI (I$ per capita)*	–
	Socio-demographic Index	–
	Education (years per capita)	–

Gallbladder and biliary tract cancer

Level	*Covariate*	*Direction*
1	*Log-transformed SEV scalar: Gallbladder cancer*	+
	Mean BMI	+
2	*Litres of alcohol consumed per capita*	+
	Cumulative cigarettes (5 years)	+
	Cumulative cigarettes (10 years)	+
	Smoking prevalence	+
	Tobacco (cigarettes per capita)	+
	Age- and sex-specific SEV for low fruit	+
	Age- and sex-specific SEV for low vegetables	+
	Diabetes age-standardised prevalence (proportion)	+
	Healthcare Access and Quality Index	–
3	*Education (years per capita)*	–
	LDI (I$ per capita)	+
	Socio-demographic Index	-

Hepatoblastoma (liver cancer parent)

Level	Covariate	Direction
1	*Litres of alcohol consumed per capita*	+
	HIV age-standardised prevalence	+
	Vaccine adjusted HBsAg seroprevalence age-standardised	+
	Hepatitis C seroprevalence (anti-HCV) age-standardised	+
	Log-transformed SEV scalar: Liver cancer	+
2	*Hepatitis B 3-dose coverage (proportion)*	–
	Hepatitis B vaccine coverage (proportion), aged through time	–
	Intravenous drug use (age-standardised proportion)	+
	Cumulative cigarettes (20 years)	+
	Mean BMI	+
	Tobacco (cigarettes per capita)	+
	Healthcare Access and Quality Index	–
	Diabetes fasting plasma glucose (mmol/L), age-standardised 25+	+
3	*Education (years per capita)*	–
	Age- and sex-specific SEV for high red meat	+
	LDI (I$ per capita)	–
	Socio-demographic Index	–

Hodgkin lymphoma

Level	Covariate	Direction
2	*Healthcare Access and Quality Index*	–
3	*Education (years per capita)*	–
	LDI (I$ per capita)	–
	Socio-demographic Index	–

Kidney cancer

Level	Covariate	Direction
1	*Tobacco (cigarettes per capita)*	+
	Cumulative cigarettes (10 years)	+
	Mean BMI	+
	Log-transformed SEV scalar: Kidney cancer	+
2	*Litres of alcohol consumed per capita*	+
	Diabetes age-standardised prevalence (proportion)	+
	Systolic blood pressure (mmHg)	+
	Healthcare Access and Quality Index	–
3	*Education (years per capita)*	–
	LDI (I$ per capita)	+
	Socio-demographic Index	+

Larynx cancer

Level	Covariate	Direction
1	*Litres of alcohol consumed per capita*	+
	Log-transformed SEV scalar: Larynx Cancer	+
2	*Smoking prevalence*	+
	Asbestos consumption (metric tons per year per capita)	+
	Age- and sex-specific SEV for low vegetables	+
	Cumulative cigarettes (10 years)	+
	Cumulative cigarettes (20 years)	+
	Population density (over 1000 ppl/sqkm, proportion)	+
	Healthcare Access and Quality Index	–
3	*Age- and sex-specific SEV for low fruit*	+
	LDI (I$ per capita)	+
	Socio-demographic Index	+

Leukaemia

Level	Covariate	Direction
1	*Log-transformed age-standardised SEV scalar: Leukaemia*	+
	Log-transformed SEV scalar: Leukaemia	+
2	*Litres of alcohol consumed per capita*	+
	Mean BMI	+
	Cumulative cigarettes (10 years)	+
	Cumulative cigarettes (20 years)	+
	Tobacco (cigarettes per capita)	+
	Healthcare Access and Quality Index	–
3	*Education (years per capita)*	–
	LDI (I$ per capita)	+
	Socio-demographic Index	–

Lip and oral cavity cancer

Level	Covariate	Direction
1	*Litres of alcohol consumed per capita*	+
	Cumulative cigarettes (10 years)	+
	Cumulative cigarettes (20 years)	+
	Tobacco (cigarettes per capita)	+
	Log-transformed SEV scalar: Mouth cancer	+
2	*Age- and sex-specific SEV for high red meat*	+
	Age- and sex-specific SEV for low vegetables	+
	Age- and sex-specific SEV for low fruit	+
	Healthcare Access and Quality Index	–
3	*Education (years per capita)*	–
	LDI (I$ per capita)	+
	Socio-demographic Index	+

Liver cancer (Level 3 parent model)

Level	Covariate	Direction
1	Litres of alcohol consumed per capita	+
	HIV age-standardised prevalence	+
	Vaccine adjusted HBsAg seroprevalence age-standardised	+
	Hepatitis C seroprevalence (anti-HCV) age-standardised	+
	Log-transformed SEV scalar: Liver cancer	+
2	Hepatitis B 3-dose coverage (proportion)	–
	Hepatitis B vaccine coverage (proportion), aged through time	–
	Intravenous drug use (age-standardised proportion)	+
	Cumulative cigarettes (20 years)	+
	Mean BMI	+
	Tobacco (cigarettes per capita)	+
	Healthcare Access and Quality Index	–
	Diabetes fasting plasma glucose (mmol/L), age-standardised 25+	+
3	Education (years per capita)	–
	Age- and sex-specific SEV for high red meat	+
	LDI (I$ per capita)	–
	Socio-demographic Index	–

Malignant neoplasm of bone and articular cartilage

Level	Covariate	Direction
2	Universal health coverage	–
	Socio-demographic Index	–
	LDI (I$ per capita)	–
	Healthcare Access and Quality Index	–
3	Smoking prevalence	+
	Health worker density	–
	Education (years per capita)	–
	Age- and sex-specific SEV for low bone mineral density	+
	Maternal care and immunisation	–
	Log-transformed SEV scalar: Osteoarthritis	+
	Log-transformed age-standardised SEV scalar: Osteoarthritis	+

Malignant skin melanoma

Level	Covariate	Direction
1	Litres of alcohol consumed per capita	+
2	Latitude under 15 (proportion)	–
	Latitude 15 to 30 (proportion)	–
	Latitude 30 to 45 (proportion)	–
	Latitude over 45 (proportion)	–
	Healthcare Access and Quality Index	–
3	Education (years per capita)	–
	LDI (I$ per capita)	–
	Socio-demographic Index	+

Mesothelioma

Level	Covariate	Direction
1	Asbestos consumption (metric tons per year per capita)	+
	Age- and sex-specific SEV for Occupational asbestos	+
	Age-standardised SEV for occupational asbestos	+
	Smoking prevalence	+
2	Gold production (binary)	+
	Indoor air pollution (all cooking fuels)	+
	Population density (over 1000 ppl/sqkm, proportion)	+
	Healthcare Access and Quality Index	–
	Cumulative cigarettes (5 years)	+
3	Education (years per capita)	–
	LDI (I$ per capita)	–
	Socio-demographic Index	+

Multiple myeloma

Level	Covariate	Direction
1	Litres of alcohol consumed per capita	+
	Smoking prevalence	+
	Tobacco (cigarettes per capita)	+
2	Age- and sex-specific SEV for low vegetables	+
	Age- and sex-specific SEV for low fruits	+
	Age- and sex-specific SEV for high red meat	+
	Mean BMI	+
	Sanitation (proportion with access)	–
	Improved water source (proportion with access)	–
	Healthcare Access and Quality Index	–
3	Education (years per capita)	–
	LDI (I$ per capita)	+
	Socio-demographic Index	+

Myelodysplastic, myeloproliferative, and other hematopoietic neoplasms

Level	Covariate	Direction
1	Log-transformed age-standardised SEV scalar: Leukaemia	+
	Log-transformed SEV scalar: Leukaemia	+
2	Litres of alcohol consumed per capita	+
	Cumulative cigarettes (5 years)	+
	Cumulative cigarettes (10 years)	+
	Cumulative cigarettes (15 years)	+
	Cumulative cigarettes (20 years)	+
	Smoking prevalence	+
	Tobacco (cigarettes per capita)	+
	Healthcare Access and Quality Index	–
3	Education (years per capita)	–
	LDI (I$ per capita)	+
	Socio-demographic Index	+

Nasopharynx cancer

Level	Covariate	Direction
1	*Litres of alcohol consumed per capita*	+
	Cumulative cigarettes (10 years)	+
	Cumulative cigarettes (20 years)	+
	Tobacco (cigarettes per capita)	+
	Log-transformed SEV scalar: Nasopharynx cancer	+
2	*Age- and sex-specific SEV for low vegetables*	+
	Population density (over 1000 ppl/sqkm, proportion)	+
	Healthcare Access and Quality Index	–
3	*Education (years per capita)*	–
	Age- and sex-specific SEV for low fruit	+
	LDI (I$ per capita)	–
	Socio-demographic Index	+

Neuroblastoma and other peripheral nervous cell tumours

Level	Covariate	Direction
3	*Smoking prevalence*	+
	Health worker density	–
	Healthcare Access and Quality Index	–
	Education (years per capita)	–
	LDI (I$ per capita)	+
	Socio-demographic Index	+
	Maternal care and immunisation	–
	Universal health coverage	–

Non-Hodgkin lymphoma

Level	Covariate	Direction
2	*Cumulative cigarettes (5 years)*	+
	Cumulative cigarettes (10 years)	+
	Cumulative cigarettes (15 years)	+
	Cumulative cigarettes (20 years)	+
	Litres of alcohol consumed per capita	+
	Mean BMI	+
	Universal health coverage	–
	Healthcare Access and Quality Index	–
3	*Log-transformed SEV scalar: HIV*	+
	Log-transformed age-standardised SEV scalar: HIV	+
	Total fertility rate	–
	Education (years per capita)	–
	LDI (I$ per capita)	–
	Socio-demographic Index	–

Non-melanoma skin cancer & squamous cell carcinoma

Level	Covariate	Direction
1	*Cumulative cigarettes (5 years)*	+
	Cumulative cigarettes (10 years)	+
	Cumulative cigarettes (15 years)	+
	Smoking prevalence	+
2	*Average latitude*	–
	Healthcare Access and Quality Index	–
3	*Education (years per capita)*	–
	LDI (I$ per capita)	–
	Socio-demographic Index	+

Oesophageal cancer

Level	Covariate	Direction
1	*Litres of alcohol consumed per capita*	+
	Log-transformed age-standardised SEV scalar: Oesophageal cancer	+
	Mean BMI	+
	Smoking prevalence	+
2	*Indoor air pollution (all cooking fuels)*	+
	Tobacco (cigarettes per capita)	+
	Age- and sex-specific SEV for low vegetables	+
	Age- and sex-specific SEV for low fruit	+
	Healthcare Access and Quality Index	–
3	*Education (years per capita)*	–
	Sanitation (proportion with access)	–
	Improved water source (proportion with access)	–
	LDI (I$ per capita)	+
	Socio-demographic Index	+

Other benign and in situ neoplasms

Level	Covariate	Direction
2	*Tobacco (cigarettes per capita)*	+
	Cumulative cigarettes (10 years)	+
	Smoking prevalence	+
	Healthcare Access and Quality Index	–
3	*LDI (I$ per capita)*	–
	Socio-demographic Index	–
	Litres of alcohol consumed per capita	+
	Education (years per capita)	–

Other eye cancers

Level	Covariate	Direction
2	*Universal health coverage*	–
	Age-standardised melanoma	+
	Healthcare Access and Quality	–
3	*Index LDI (I$ per capita)*	–
	Socio-demographic Index	–
	Education (years per capita)	–

Other leukaemia

Level	Covariate	Direction
1	*Log-transformed age-standardised SEV scalar: Leukaemia*	+
	Litres of alcohol consumed per capita	+
	Log-transformed SEV scalar: Leukaemia	+
2	*Mean BMI*	+
	Cumulative cigarettes (10 years)	+
	Cumulative cigarettes (20 years)	+
	Tobacco (cigarettes per capita)	+
	Healthcare Access and Quality Index	–
3	*Education (years per capita)*	–
	LDI (I$ per capita)	+
	Socio-demographic Index	–

Other malignant neoplasms

Level	Covariate	Direction
1	*Smoking prevalence*	+
	Tobacco (cigarettes per capita)	+
2	*Age- and sex-specific SEV for low vegetables*	+
	Age- and sex-specific SEV for low fruits	+
	Age- and sex-specific SEV for low nuts and seeds	+
	PUFA adjusted (percent)	–
	Healthcare Access and Quality Index	–
3	*Education (years per capita)*	–
	LDI (I$ per capita)	+
	Socio-demographic Index	+

Other neoplasms

Level	Covariate	Direction
3	*Healthcare Access and Quality Index*	–
	Education (years per capita)	–
	LDI (I$ per capita)	+
	Socio-demographic Index	–
	Tobacco (cigarettes per capita)	+
	Cumulative cigarettes (10 years)	+
	Cholesterol (total, mean per capita)	+
	Smoking prevalence	+
	Maternal care and immunisation	–
	Log-transformed SEV scalar: Leukaemia	+
	Log-transformed age-standardised SEV scalar: Leukaemia	+
	Universal health coverage	–
	Litres of alcohol consumed per capita	+

Other non-Hodgkin lymphoma

Level	Covariate	Direction
2	*Cumulative cigarettes (5 years)*	+
	Cumulative cigarettes (10 years)	+
	Cumulative cigarettes (15 years)	+
	Cumulative cigarettes (20 years)	+
	Litres of alcohol consumed per capita	+
	Mean BMI	+
	Universal health coverage	–
	Healthcare Access and Quality Index	–
3	*Log-transformed SEV scalar: HIV*	+
	Log-transformed age-standardised SEV scalar: HIV	+
	Education (years per capita)	–
	LDI (I$ per capita)	–
	Socio-demographic Index	–

Other pharynx cancer

Level	Covariate	Direction
1	*Litres of alcohol consumed per capita*	+
	Smoking prevalence	+
	Log-transformed SEV scalar: Other pharynx cancer	+
2	*Cumulative cigarettes (5 years)*	+
	Age- and sex-specific SEV for low fruit	+
	Age- and sex-specific SEV for low vegetables	+
	Population density (over 1000 ppl/sqkm, proportion)	+
	Population density (under 150 ppl/sqkm, proportion)	+
	Healthcare Access and Quality Index	–
3	*Education (years per capita)*	–
	LDI (I$ per capita)	+
	Socio-demographic Index	+

Ovarian cancer

Level	Covariate	Direction
1	Litres of alcohol consumed per capita	+
	Log-transformed SEV scalar: Ovary cancer	+
2	Asbestos consumption (metric tons per year per capita)	+
	Smoking prevalence	+
	Total fertility rate	–
	Energy unadjusted (kcal)	+
	Mean BMI	+
	Contraception (modern) prevalence (proportion)	–
	Diabetes age-standardised prevalence (proportion)	+
	Cumulative cigarettes (10 years)	+
	Cumulative cigarettes (20 years)	+
	Healthcare Access and Quality Index	–
3	Education (years per capita)	–
	Age- and sex-specific SEV for low fruits	+
	Age- and sex-specific SEV for low vegetables	+
	LDI (I$ per capita)	–
	Socio-demographic Index	+

Pancreatic cancer

Level	Covariate	Direction
1	Cumulative cigarettes (10 years)	+
	Cumulative cigarettes (20 years)	+
	Tobacco (cigarettes per capita)	+
	Log-transformed SEV scalar: Pancreas cancer	+
	Mean BMI	+
2	Age- and sex-specific SEV for high red meat	+
	Litres of alcohol consumed per capita	+
	Energy unadjusted (kcal)	+
	Diabetes fasting plasma glucose (mmol/L), age-standardised 25+	+
	Diabetes age-standardised prevalence (proportion)	+
	Healthcare Access and Quality Index	–
3	Education (years per capita)	–
	Age- and sex-specific SEV for low fruit	+
	LDI (I$ per capita)	+
	Socio-demographic Index	+
	Age- and sex-specific SEV for low vegetables	+

Prostate cancer

Level	Covariate	Direction
1	Log-transformed SEV scalar: Prostate cancer	+
2	Smoking prevalence	+
	Healthcare Access and Quality Index	–
3	Education (years per capita)	–
	LDI (I$ per capita)	–
	Socio-demographic Index	+

Retinoblastoma

Level	Covariate	Direction
2	*Universal health coverage*	–
	Healthcare Access and Quality	–
3	*LDI (I$ per capita)*	–
	Maternal care and immunisation	–
	Socio-demographic Index	–
	Education (years per capita)	–

Soft tissue and other extraosseous sarcomas

Level	Covariate	Direction
2	*Healthcare Access and Quality Index*	–
	LDI (I$ per capita)	–
	Socio-demographic Index	–
	Universal health coverage	–
3	*Education (years per capita)*	–
	Maternal care and immunisation	–
	Log-transformed SEV scalar: HIV	+
	Log-transformed age-standardised SEV scalar: HIV	+
	Litres of alcohol consumed per capita	+

Stomach cancer

Level	Covariate	Direction
1	*Diet high in sodium*	+
	Tobacco (cigarettes per capita)	+
	Log-transformed SEV scalar: Stomach cancer	+
2	*Cumulative cigarettes (20 years)*	+
	Age- and sex-specific SEV for unsafe water	+
	Age- and sex-specific SEV for unsafe sanitation	+
	Mean BMI	+
	Sanitation (proportion with access)	–
	Improved water source (proportion with access)	–
	Healthcare Access and Quality Index	–
3	*Education (years per capita)*	–
	Age- and sex-specific SEV for low fruit	+
	Age- and sex-specific SEV for low vegetables	+
	LDI (I$ per capita)	+
	Socio-demographic Index	–

Testicular cancer

Level	Covariate	Direction
2	*Cumulative cigarettes (5 years)*	+
	Cumulative cigarettes (10 years)	+
	Cumulative cigarettes (15 years)	+
	Cumulative cigarettes (20 years)	+
	Tobacco (cigarettes per capita)	+
	Smoking prevalence	+
	Age- and sex-specific SEV for low fruits	+
	Age- and sex-specific SEV for low vegetables	+
	Healthcare Access and Quality Index	–
3	*Education (years per capita)*	–
	LDI (I$ per capita)	+
	Socio-demographic Index	+

Thyroid cancer

Level	Covariate	Direction
1	*Litres of alcohol consumed per capita*	+
	Log-transformed SEV scalar: Thyroid cancer	+
2	*Age- and sex-specific SEV for low vegetables*	+
	Age- and sex-specific SEV for high red meat	+
	Tobacco (cigarettes per capita)	+
	Mean BMI	+
	Healthcare Access and Quality Index	–
3	*Education (years per capita)*	–
	Sanitation (proportion with access)	–
	Improved water source (proportion with access)	–
	Age- and sex-specific SEV for low fruits	+
	LDI (I$ per capita)	+
	Socio-demographic Index	+

Tracheal, bronchus and lung cancer

Level	Covariate	Direction
1	*Asbestos consumption (metric tons per year per capita)*	+
	Smoking prevalence	+
	Log-transformed SEV scalar: Lung cancer	+
	Log-transformed age-standardised SEV scalar: Lung cancer	+
2	*Indoor air pollution (all cooking fuels)*	+
	Second-hand smoke	+
	Cumulative cigarettes (10 years)	+
	Cumulative cigarettes (20 years)	+
	Outdoor air pollution ($PM_{2.5}$)	+
	Residential radon	+
	Diabetes fasting plasma glucose (mmol/L), age-standardised 25+	+
	Healthcare Access and Quality Index	–
3	*Education (years per capita)*	–
	LDI (I$ per capita)	+
	Socio-demographic Index	+

Uterine cancer

Level	Covariate	Direction
1	Log-transformed SEV scalar: Uterus cancer	+
	Mean BMI	+
2	Cumulative cigarettes (5 years)	+
	Cumulative cigarettes (10 years)	+
	Smoking prevalence	+
	Tobacco (cigarettes per capita)	+
	Diabetes age-standardised prevalence (proportion)	+
	Total fertility rate	–
	Age- and sex-specific SEV for low fruit	+
	Age- and sex-specific SEV for low vegetables	+
	Healthcare Access and Quality Index	–
3	Education (years per capita)	–
	LDI (I$ per capita)	+
	Socio-demographic Index	+

References

1 de Martel C, Maucort-Boulch D, Plummer M, Franceschi S. World-wide relative contribution of hepatitis B and C viruses in hepatocellular carcinoma. *Hepatology* 2015; **62**: 1190–200.

2 Hong TP, Gow P, Fink M, *et al.* Novel population-based study finding higher than reported hepatocellular carcinoma incidence suggests an updated approach is needed. *Hepatology* 2016; **63**: 1205–12.

3 Surveillance, Epidemiology, and End Results (SEER) Program (www.seer.cancer.gov) SEER*Stat Database: Incidence - SEER 18 Regs Research Data + Hurricane Katrina Impacted Louisiana Cases, Nov 2017 Sub (1973-2015 varying) - Linked To County Attributes - Total U.S., 1969-2016 Counties, National Cancer Institute, DCCPS, Surveillance Research Program, released April 2018, based on the November 2017 submission.

4 Surveillance, Epidemiology, and End Results (SEER) Program (www.Seer.Cancer.Gov) SEER*Stat Database: Incidence - SEER 18.

5 Engholm G, Ferlay J, Christensen N, *et al.* NORDCAN--a Nordic tool for cancer information, planning, quality control and research. *Acta Oncol* 2010; **49**: 725–36.

6 R D, P P, JAH W. Cancer Incidence in Five Continents Volume I. https://publications.iarc.fr/Non-Series-Publications/Other-Non-Series-Publications/Cancer-Incidence-In-Five-Continents-Volume-I-1966 (accessed Feb 24, 2021).

7 Doll R, Muir C, Waterhouse J. Cancer Incidence in Five Continents: Volume II–1970. Springer Science & Business Media, 2012.

8 Waterhouse J, Muir C, Correa P, Powell J. Cancer Incidence in Five Continents III. Lyon: IARC; 1976.

9 Waterhouse J, Muir C, Shanmugaratnam K, Powell J. Cancer Incidence in Five Continents IV. Lyon: IARC; 1982.

10 Muir C, Mack T, Powell J, Whelan S. Cancer Incidence in Five Continents V. Lyon: IARC; 1987.

11 Parkin D, Muir C, Whelan S, Gao Y, Ferlay J, Powell J. Cancer Incidence in Five Continents VI. Lyon: IARC; 1992.

12 Parkin D, Whelan S, Ferlay J, Raymond L, Young J. Cancer Incidence in Five Continents VII. Lyon: IARC; 1997.

13 Parkin D, Whelan S, Ferlay J, Teppo L, Thomas D. Cancer Incidence in Five Continents VIII. Lyon: IARC; 2002.

14 Curado M, Edwards B, Shin H, et al. Cancer Incidence in Five Continents IX. Lyon: IARC; 2007. http://www.iarc.fr/en/publ.

15 Forman D, Bray F, Brewster D, et al. Cancer Incidence in Five Continents X. http://ci5.iarc.fr. Published 2013.

16 Bray F, Colombet M, Mery L, Piñeros M, Znaor A, Zanetti R and Ferlay J, editors (2017). Cancer Incidence in Five Contine.

17 Steliarova-Foucher E, Colombet M, Ries LAG, *et al.* International incidence of childhood cancer, 2001–10: a population-based registry study. *The Lancet Oncology* 2017; **18**: 719–31.

18 Barber RM, Fullman N, Sorensen RJD, *et al.* Healthcare Access and Quality Index based on mortality from causes amenable to personal health care in 195 countries and territories, 1990–2015: a novel analysis from the Global Burden of Disease Study 2015. *The Lancet* 2017; **390**: 231–66.

19 Murray CJL, Aravkin AY, Zheng P, *et al.* Global burden of 87 risk factors in 204 countries and territories, 1990–2019: a systematic analysis for the Global Burden of Disease Study 2019. *The Lancet* 2020; **396**: 1223–49.

20 Sung H, Ferlay J, Siegel RL, *et al.* Global Cancer Statistics 2020: GLOBOCAN Estimates of Incidence and Mortality Worldwide for 36 Cancers in 185 Countries. *CA: A Cancer Journal for Clinicians* 2021; **71**: 209–49.

21 Ferlay J, Colombet M, Soerjomataram I, *et al.* Estimating the global cancer incidence and mortality in 2018: GLOBOCAN sources and methods. *International Journal of Cancer* 2019; **144**: 1941–53.

Cardiomyopathy and myocarditis

Flowchart

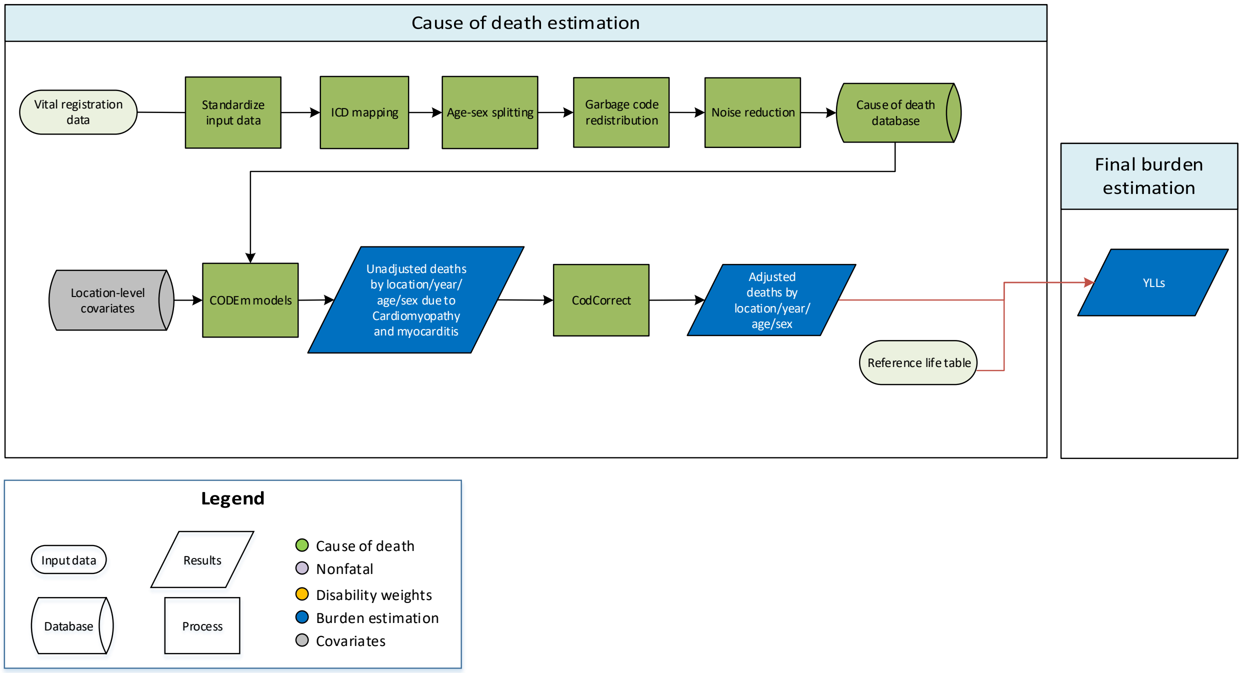

Input data and methodological summary for cardiomyopathy and myocarditis

Input data

Vital registration data were used to model deaths due to cardiomyopathy and myocarditis. We outliered datapoints in central Asia, central Europe, and eastern Europe due to implausibly high values which we attributed to variation in local coding practices. Other countries outliered due to implausible values include Egypt, Sri Lanka, and Cook Islands. We also outliered ICD8 and ICD9BTL datapoints in countries where they were discontinuous with other data in the time series or were implausibly high or low. In addition, we outliered a lone datapoint in India which was implausibly low compared to other data in the region.

Modelling strategy

We used a standard CODEm approach to model deaths from cardiomyopathy and myocarditis. The covariates selected for inclusion in the CODEm modelling process can be found in the table below. For GBD 2021, the method used to reduce the noise in the data, implemented after redistribution to handle both, the stochastic variation across time and space and the occurrence of small number of deaths in each location/year/age/sex, was updated. This new empirical Bayesian noise reduction algorithm uses grouped data by region and data type as prior to better reflect regional patterns. A detailed description on the updates to noise reduction can be found in the cause of death methods section of the appendix.

Table 1. Covariates used in cardiomyopathy and myocarditis mortality modelling

Level	Covariate	Direction
1	Summary exposure variable, CMP	1
	Smoking prevalence	1
2	Body mass index (kg/m^2)	1
	Healthcare Access and Quality Index	-1
	Systolic blood pressure (mm Hg)	1
3	Log transformed lag distributed income per capita (I$)	-1
	Socio-demographic Index	-1

Cardiovascular diseases

Flowchart

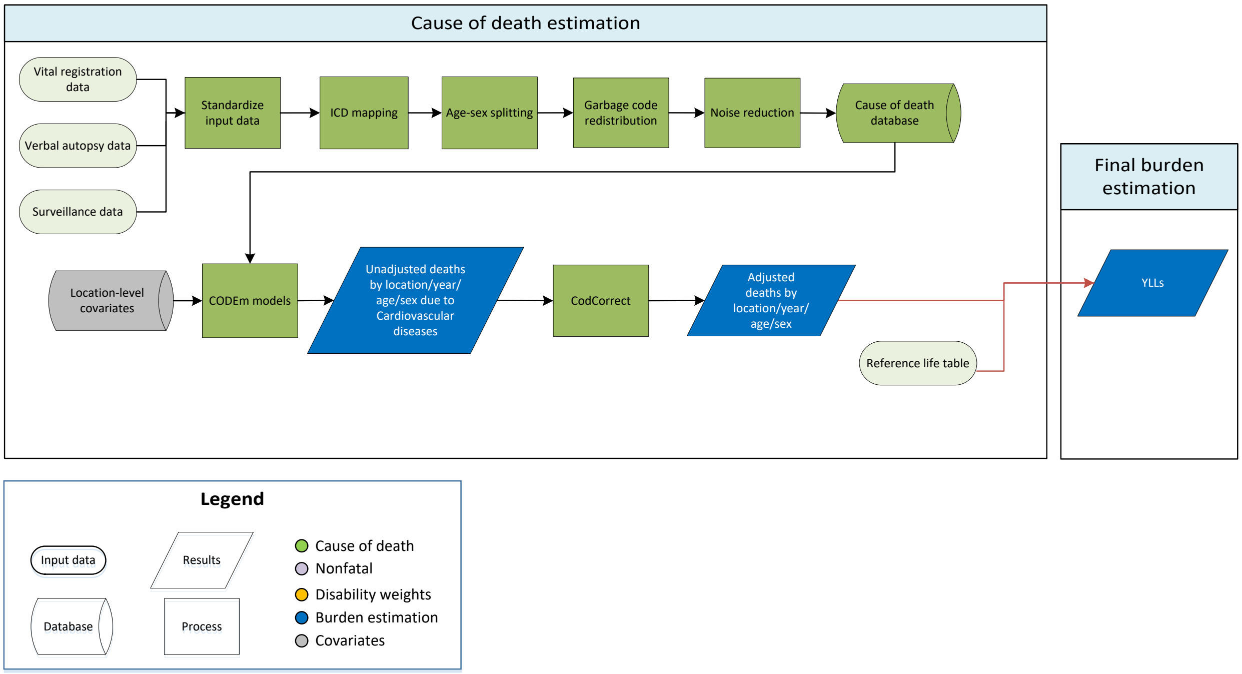

Input data and methodological summary for cardiovascular diseases

Input data

Vital registration and verbal autopsy data were used to model the parent cardiovascular envelope. For GBD 2021, all verbal autopsy data sources included in the cardiovascular envelope were systematically reviewed. In order to maximise the reliability of the included data, verbal autopsy studies that did not meet the World Health Organization standards were excluded.[1] In addition, non-representative subnational verbal autopsies from a number of Indian states and verbal autopsy data in Nepal and Papua New Guinea that were implausible in terms of time and age trends, were outliered.

Data sources that were implausibly low in all age groups as well as ICD8 and ICD9BTL datapoints that were inconsistent with the rest of the data and created implausible time trends were also outliered. In addition, implausibly high ICD10 data from the 150 England Upper Tier Local Authorities 2014–2018 were outliered.

Modelling strategy

We used a standard CODEm approach to model deaths from cardiovascular diseases. For GBD 2021, a new approach to redistribute deaths coded to hypertension was implemented using data sources which included information on the chain of events leading to death. This update resulted in an increase in the

number of deaths that were re-assigned to cardiovascular diseases. Similarly, the method used to reduce the noise in the data, implemented after redistribution to handle both the stochastic variation across time and space and the occurrence of small number of deaths in each location/year/age/sex, was updated. This new empirical Bayesian noise reduction algorithm uses grouped data by region and data type as prior to better reflect regional patterns. A detailed description on the updates to noise reduction can be found in the cause of death methods section of the appendix.

The covariates included in the ensemble modelling process are listed in the table below. The summary exposure value scalar for CVD was dropped as these scalars were not produced for Level 2 causes after GBD 2019.

Apart from these changes to the covariates and the updates to the redistribution of deaths coded to hypertension, there are no substantive changes from the approach used in GBD 2019.

Table: Covariates used in cardiovascular diseases mortality modelling

Level	Covariate	Direction
1	Cholesterol (total, mean per capita)	1
	Smoking prevalence	1
	Systolic blood pressure (mm Hg)	1
2	Mean BMI	1
	Elevation over 1500m (proportion)	-1
	Fasting plasma glucose (mmol/L)	1
	Outdoor pollution ($PM_{2.5}$)	1
	Indoor air pollution (all fuel types)	1
	Healthcare access and quality index	-1
3	Lag distributed income per capita (I$)	-1
	Summary exposure value, omega-3 fatty acids	1
	Summary exposure value, fruits	1
	Summary exposure value, vegetables	1
	Summary exposure value, nuts and seeds	1
	Pulses/legumes (kcal/capita, unadjusted)	-1
	Summary exposure value, PUFA adjusted (percent)	1
	Alcohol (litres per capita)	1
	Trans fatty acid	1

References

1 WHO | Methodological trends in studies based on verbal autopsies before and after published guidelines. https://www.who.int/bulletin/volumes/87/9/07-049288/en/ (accessed April 22, 2021).

Cellulitis

Flowchart

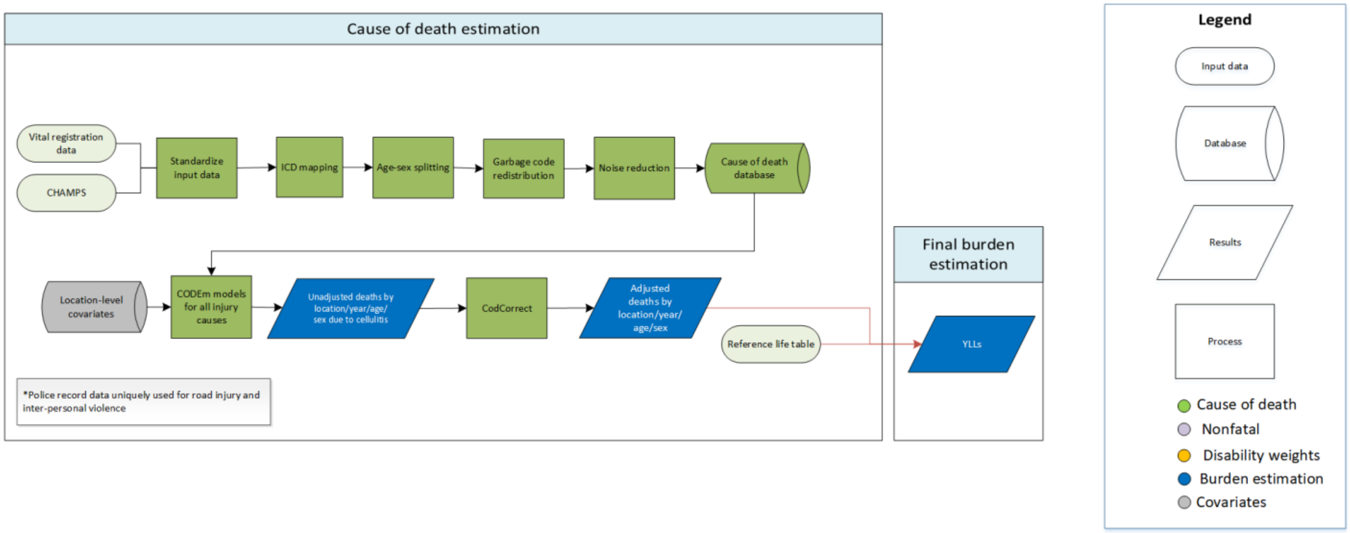

Input data and methodological summary for cellulitis

Input data

Data used to estimate cellulitis mortality consisted of vital registration and Chinese disease surveillance point (DSP) data from the cause of death (COD) database. Outlier criteria excluded datapoints that were implausibly high or low relative to global or regional patterns and data from countries with small populations.

Modelling strategy

We modelled deaths due to cellulitis with a standard CODEm model using the cause of death database and location-level covariates as inputs. The model followed standard parameters. We hybridised separate global and data-rich models to acquire unadjusted results, which we finalised and adjusted using CoDCorrect to reach final years of life lost (YLLs) due to cellulitis.

There were no significant changes in the modelling process between GBD 2019 and GBD 2021.

Table 1. Covariates used in cellulitis mortality modelling

Level	Covariate	Direction
1	Healthcare Access and Quality Index	-
	Diabetes fasting plasma glucose (mmol/L), by age	+
	Prevalence of overweight and obesity	+
2	Lag distributed income (per capita)	-
3	Education (years per capita)	-

Chagas disease

Flowchart

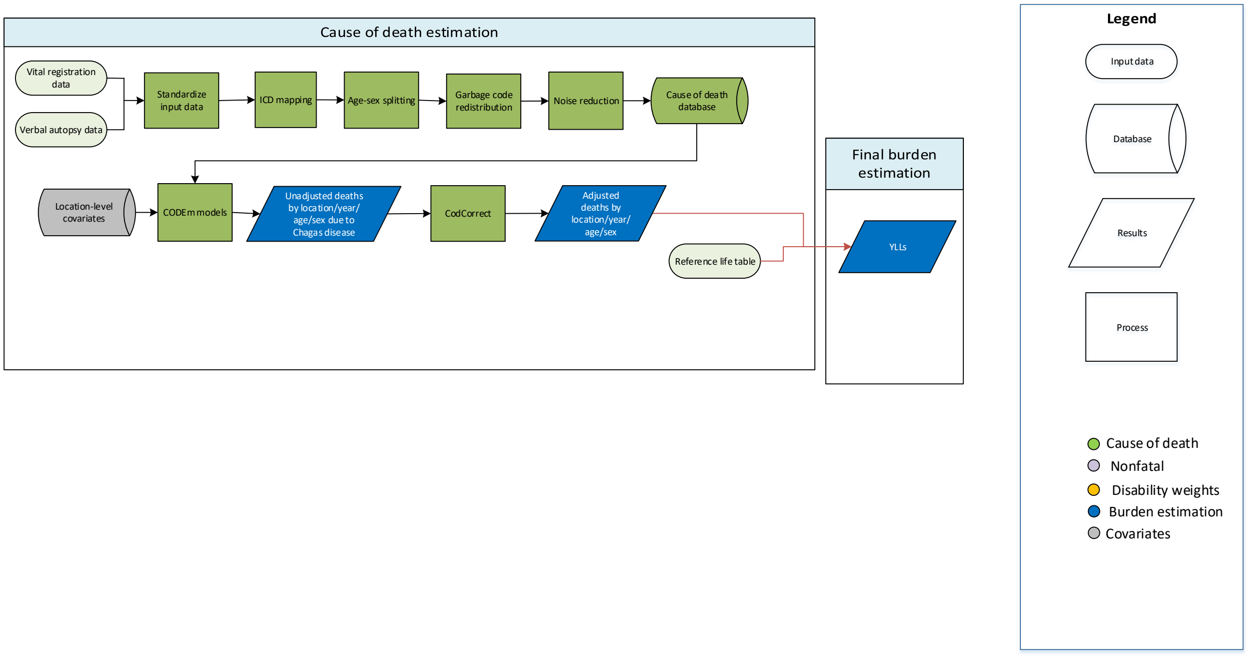

Input data

We modelled Chagas mortality using all available data in the cause of death (CoD) database. Datapoints were outliered if they reported an improbable number of Chagas deaths (eg, zero Chagas deaths in a hyper-endemic country) or if their inclusion in the model yielded distorted trends.

Modelling strategy

We modelled Chagas mortality using a CODEm model of all Chagas-endemic countries of Latin America using all data in the CoD database. Estimates of Chagas mortality in endemic countries were drawn from the CODEm model. Estimates of mortality in countries without known endemic transmission were added as imported cases if reported through vital registration systems.

The CODEm models included three covariates:

Level	Covariate	Direction
2	Healthcare Access and Quality Index	-
	Socio-demographic Index	-

Changes from GBD 2019

As noted elsewhere in this appendix, there were changes in the way CoD data were processed in CODEm, specifically in applying a linear floor rate and calculation of sample variance. Due to these changes, the CODEm model for Chagas switched to using only spatiotemporal Gaussian process regression (ST-GPR) models to build the predictions, whereas in GBD 2019 the ensemble model was a

combination of both ST-GPR and linear mixed effects models. Additionally, the Chagas prevalence covariate was removed from covariate selection. These changes allowed the ensemble model to better reflect observed temporal trends in the underlying data.

Chronic kidney disease

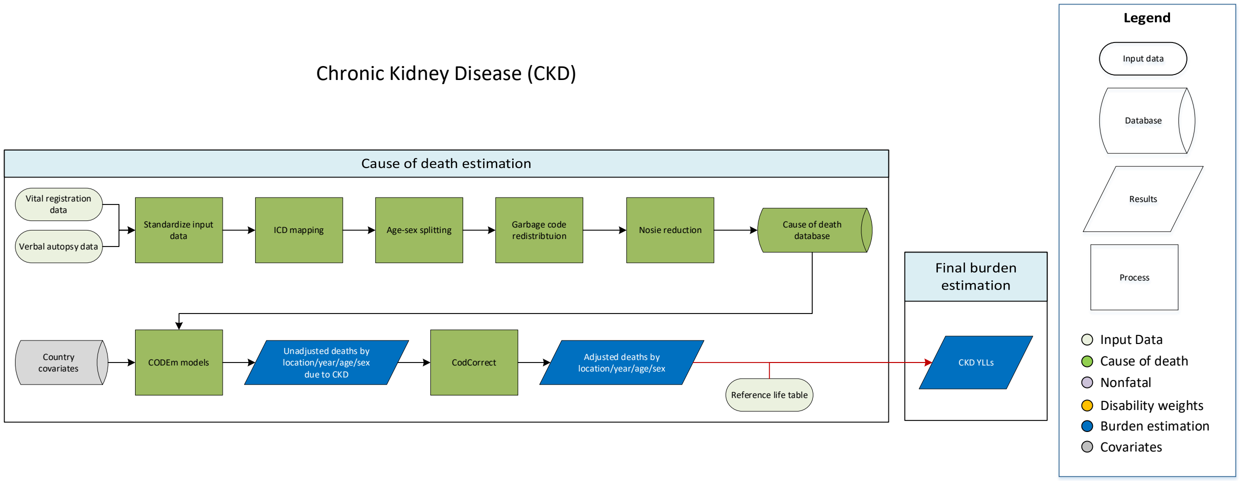

Input data

Vital registration and verbal autopsy data were used to model mortality due to chronic kidney disease. Data were standardised and mapped according to the GBD causes of death ICD mapping method. These data were then age-sex split, and appropriate redistribution of garbage code data was performed. Datapoints that violated well-established age or time trends or that resulted in extremely high or low cause fractions were marked as outliers and excluded.

Modelling strategy

We have made no substantive changes in the modelling strategy from GBD 2019 for fatal chronic kidney disease. A standard Cause of Death Ensemble model (CODEm)[1] with location-level covariates was used to model deaths due to chronic kidney disease.

The full list of covariates used in the GBD 2021 model is displayed below.

Level	Covariate	Direction
1	Diabetes fasting plasma glucose (mmol/L)	+
	Diabetes age-standardised prevalence (proportion)	+
	Mean systolic blood pressure (mmHg)	+
	Mean BMI	+
	Healthcare Access and Quality Index	–
2	Mean cholesterol	+
	Total calories available per capita per day	+
	Red meat unadjusted (kcal per capita)	+
3	Socio-demographic Index	–
	Education (years per capita)	–
	LDI (I$ per capita)	–

Covariate influences:

The following plots show the influence of each covariate on the four CODEm models (male global, male data-rich, female global, and female data-rich). A positive standardised beta (to the right) means that the covariate was associated with increased death. A negative standardised beta (to the left) means the covariate was associated with decreased death.

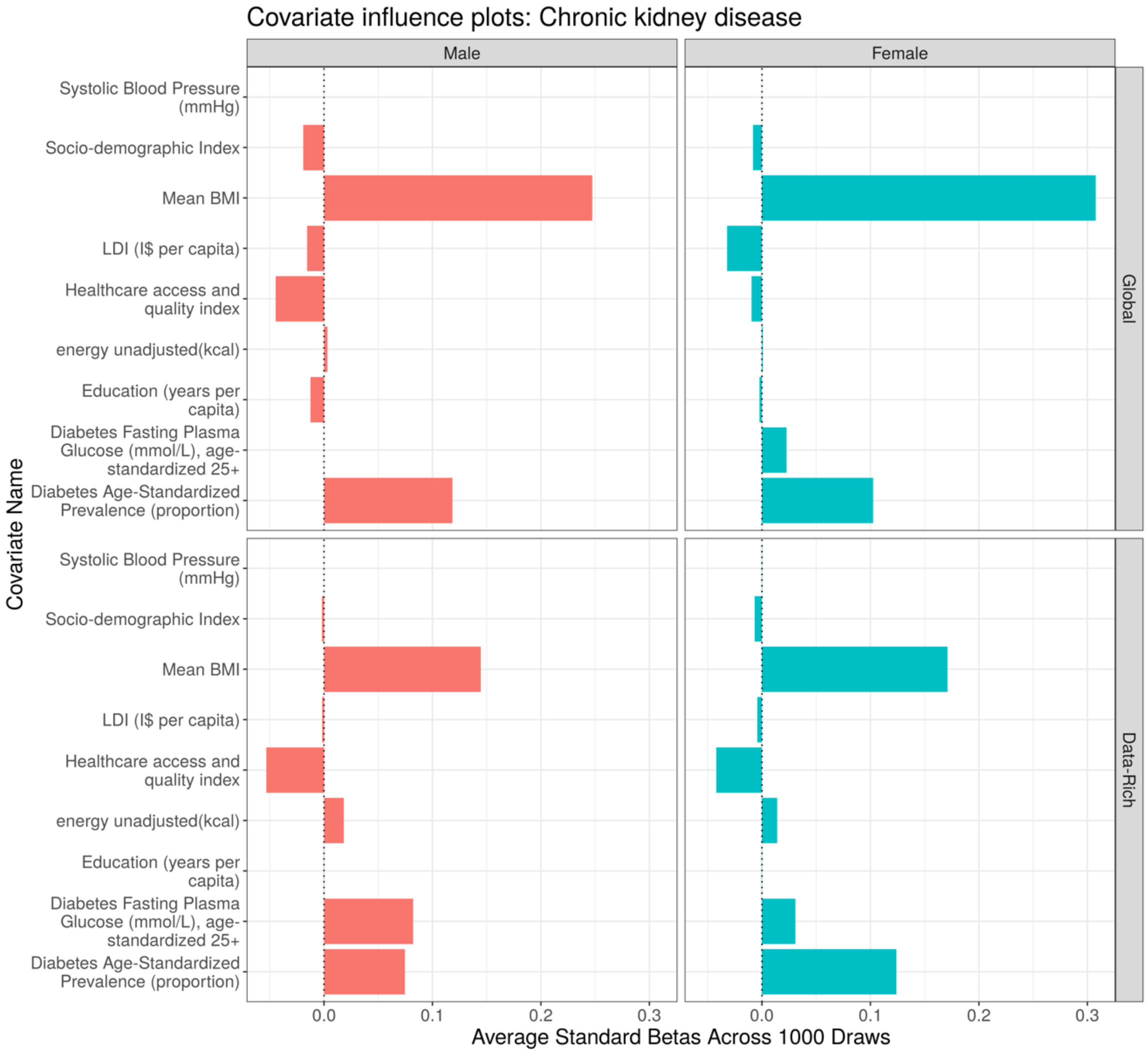

Chronic kidney disease subtypes

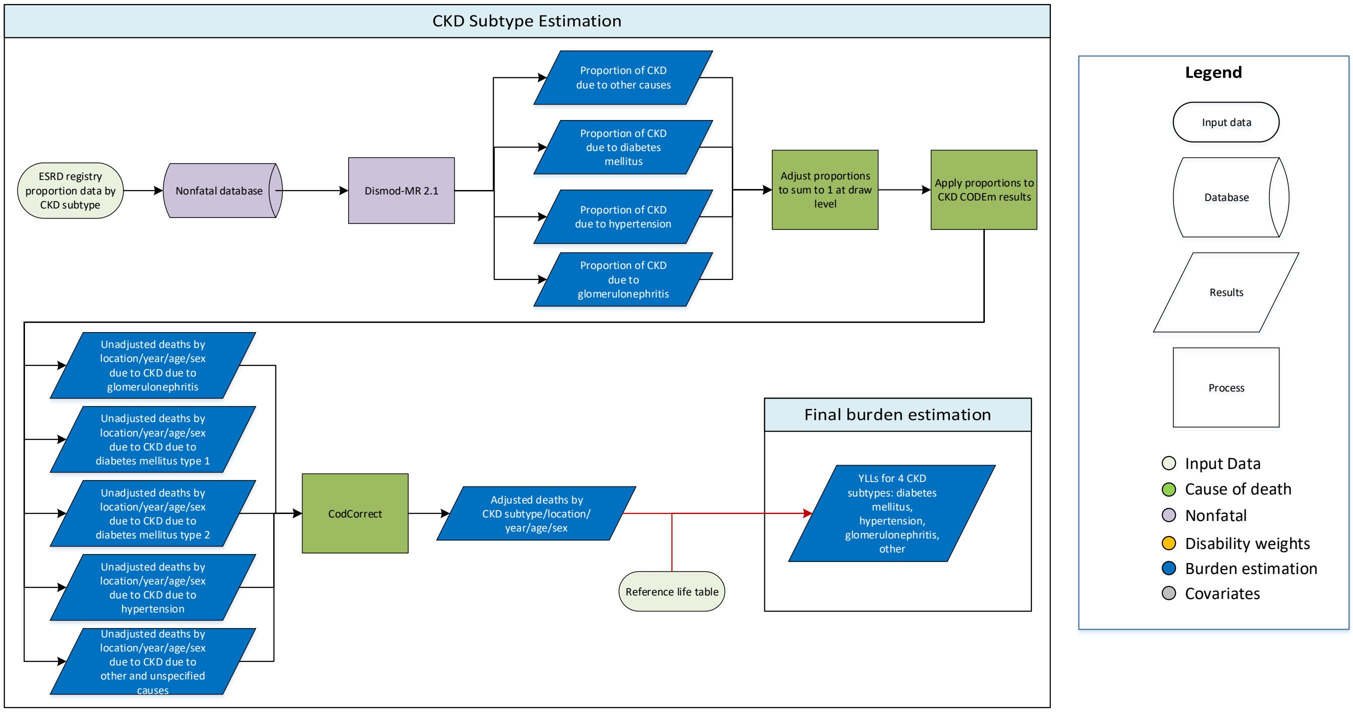

Input data

We estimated deaths due to five subtypes of chronic kidney disease: diabetes mellitus (DM) type 1, diabetes mellitus (DM) type 2, hypertension, glomerulonephritis, and other causes. Deaths due to congenital kidney anomalies (cystic kidney disease and reflux hydronephrosis) were included in the latter category. Data from end-stage renal disease registries were used to estimate the proportion of CKD mortality attributable to each CKD subtype. Age-specific data on the proportion of ESRD by subtype was available from the United States, Australia, New Zealand, Nigeria, and Russia. Vital registration (VR) data were excluded from subtype-specific estimates, as aetiology coding in VR sources was considered to be of highly variable quality between countries.

Modelling strategy

We utilised data primarily from end-stage kidney registries that included CKD aetiologies to model CKD-death aetiology proportions. Data for CKD due to overall DM were more widely available than data by type of DM. To make use of all available data, we modelled the proportion of CKD due to overall DM, DM type 1, and DM type 2 through a disease model—Bayesian meta-regression[1] (DisMod-MR 2.1) with diabetes prevalence and mean systolic blood pressure as country-level covariates to obtain estimates of each by location, year, age, and sex. The proportions of CKD due to DM type 1 and DM type 2 were then scaled to sum to the proportion of overall DM by location, year, age, and sex.

Then the results from all five aetiology-specific models were adjusted proportionally so that estimates across the aetiologies sum to 100%. These adjusted proportions were applied to the overall CKD CODEm model to obtain estimates of CKD mortality due to each aetiology.

Table 1. Country-level covariates for end-stage registry proportion CKD due to hypertension

Model name	Covariate	Value	Exponentiated
CKD registry proportion YLDs due to hypertension	Mean systolic blood pressure	0.067 (0.0037–0.17)	1.07 (1.00–1.19)

Citations

1. Vos T, Lim SS, Abbafati C, *et al.* Global burden of 369 diseases and injuries in 204 countries and territories, 1990–2019: a systematic analysis for the Global Burden of Disease Study 2019. *The Lancet* 2020; **396**: 1204–22. doi: https://doi.org/10.1016/S0140-6736(20)30925-9

Chronic obstructive pulmonary disease

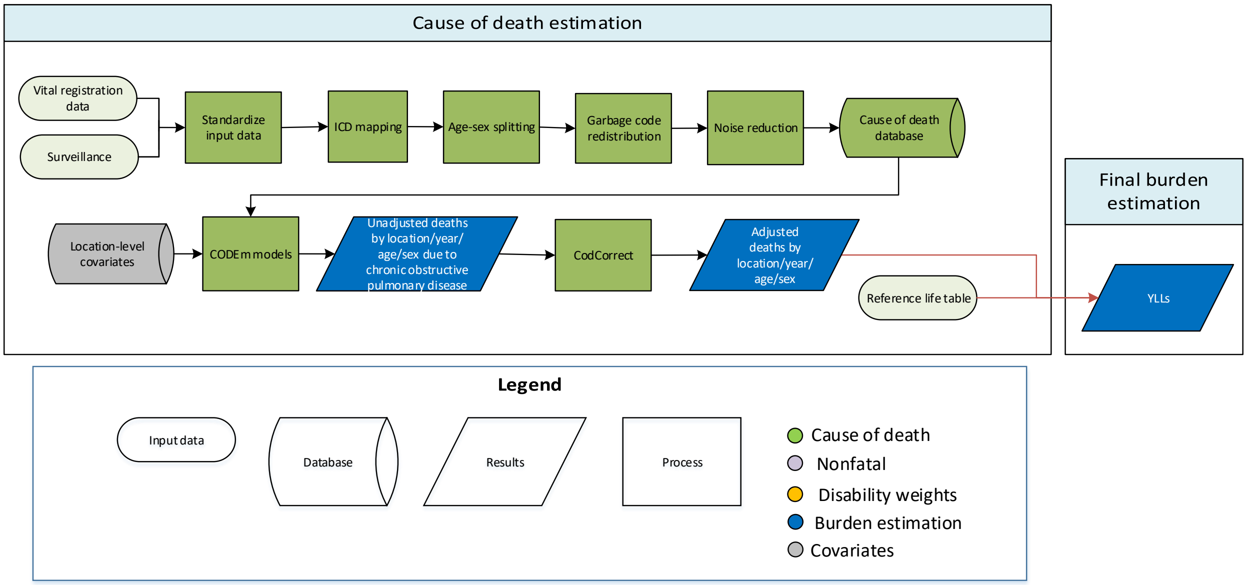

Input data

Data used to estimate chronic obstructive pulmonary disease (COPD) mortality included vital registration and surveillance data from the cause of death (COD) database. Verbal autopsy data were not included and were instead mapped to an overall chronic respiratory disease model. Our outlier criteria excluded datapoints that (1) were implausibly high or low, (2) substantially conflicted with established age or temporal patterns, or (3) substantially conflicted with other data sources conducted from the same locations or locations with similar characteristics (ie, Socio-demographic Index).

Modelling strategy

There were no substantive changes to the modelling approach this round. The standard Cause of Death Ensemble modelling (CODEm) [1] approach (detailed in reference appendix section 3.1) was used to estimate deaths due to COPD. Separate models were conducted for male and female mortality, and the age range for both models was 15 to 95+ years.

The following table lists the covariates included in the model. This requires that the covariate selected for the model must have the directional relationship with COPD. For GBD 2021, no significant updates were made to covariate selections. Covariate directions were selected based on the strength of the evidence.

**Level**	**Covariate**	**Direction**
1	Log-transformed SEV scalar: COPD	+
	Cumulative cigarettes (10 years)	+
	Cumulative cigarettes (5 years)	+
	Cumulative cigarettes (20 years)	+
	Elevation over 1500 m (proportion)	+
	Outdoor air pollution ($PM_{2.5}$)	+
2	Smoking prevalence	+
	Indoor air pollution (all cooking fuels)	+
	Healthcare Access and Quality Index	-
3	Socio-demographic Index	-
	Lagged 10-year income per capita (I$ per capita)	-
	Education (years per capita)	-

The unadjusted death estimates from COPD are summed alongside other chronic respiratory disease causes (asthma, interstitial lung disease and pulmonary sarcoidosis, and pneumoconiosis) and fit to the distribution of deaths in an overall chronic respiratory disease envelope model as part of the CoDCorrect adjustment process. This results in deaths recorded using non-specific coding systems, such as verbal autopsy, being included in the parent model and redistributed to the child models proportionately.

Covariate influences

The following plots show the influence of each covariate on the four CODEm models (male global, male data-rich, female global, and female data-rich). A positive standardised beta (to the right) means that the covariate was associated with increased death. A negative standardised beta (to the left) means the covariate was associated with decreased death.

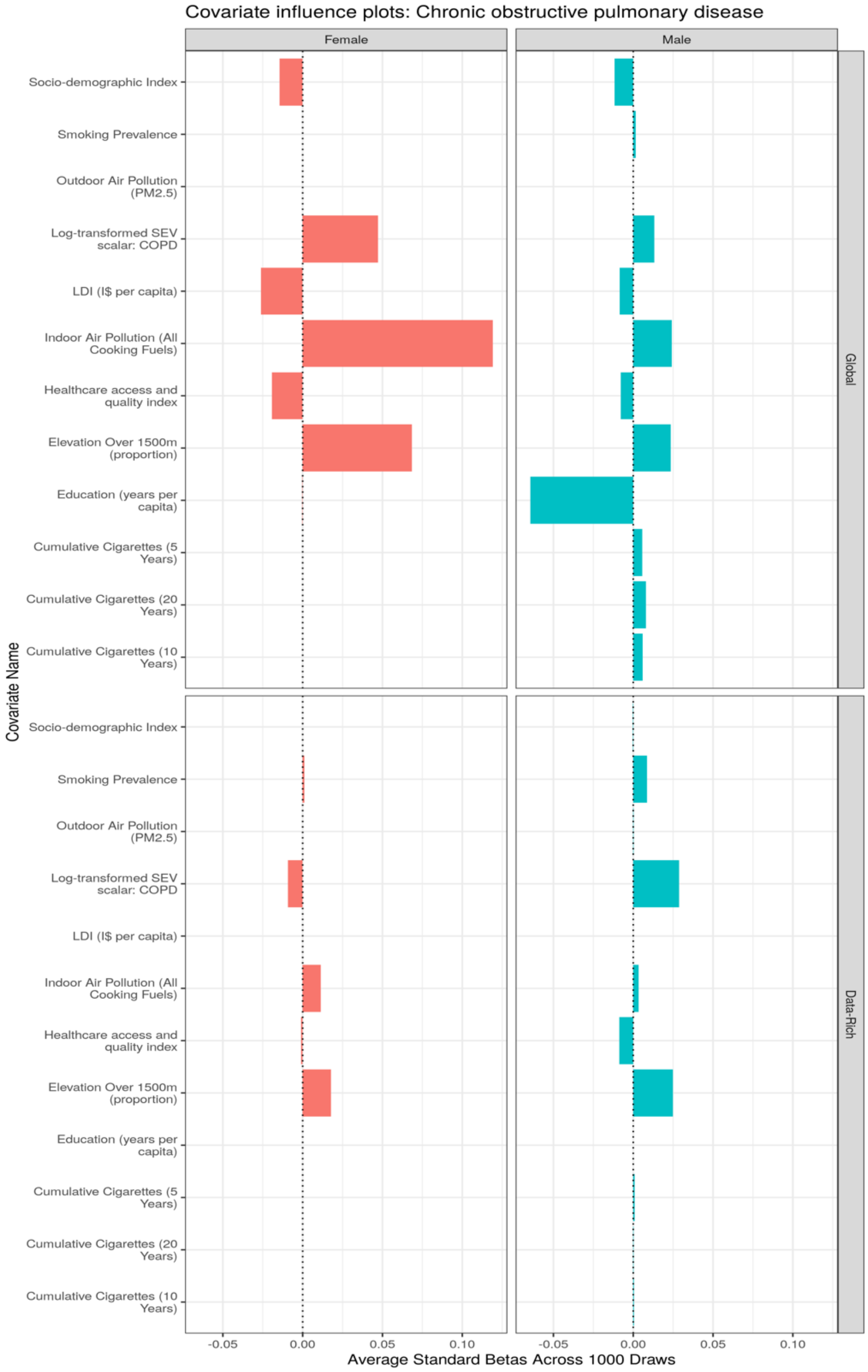

[1]Vos T, Lim SS, Abbafati C, *et al.* Global burden of 369 diseases and injuries in 204 countries and territories, 1990–2019: a systematic analysis for the Global Burden of Disease Study 2019. *The Lancet* 2020; **396**: 1204–22. doi: https://doi.org/10.1016/S0140-6736(20)30925-9

Chronic respiratory diseases

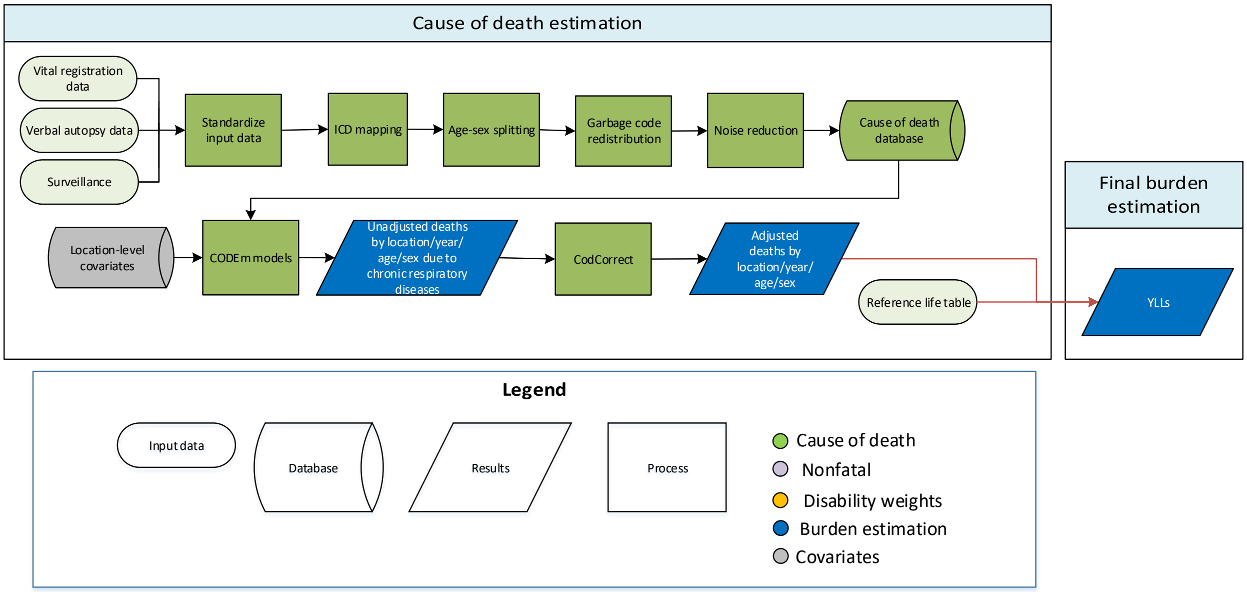

Input data

Sources used to estimate chronic respiratory disease mortality included vital registration, verbal autopsy, and surveillance data from China. Our outlier criteria excluded datapoints that (1) were implausibly high or low, (2) substantially conflicted with established age or temporal patterns, or (3) significantly conflicted with other data sources conducted from the same locations or locations with similar characteristics (ie, Socio-demographic Index).

Modelling strategy

There were no substantive changes to the modelling approach this round. The standard Cause of Death Ensemble modelling (CODEm) [1] approach (detailed in reference appendix section 3.1) was used to estimate deaths due to chronic respiratory diseases. Separate models were conducted for male and female mortality, and the age range for both models was 1 to 95+ years.

The following table lists the covariates included in the model. This requires that the covariate selected for the model must have the directional relationship with chronic respiratory deaths. For GBD 2021, no significant updates were made covariate selections. Covariate directions were selected based on the strength of the evidence.

**Level**	**Covariate**	**Direction**
1	Indoor air pollution (all cooking fuels)	+
	Cumulative cigarettes (10 years)	+
	Cumulative cigarettes (5 years)	+

	Smoking prevalence	+
2	Healthcare Access and Quality Index	-
	Outdoor air pollution ($PM_{2.5}$)	+
	Population above 1500 m elevation (proportion)	+
3	LDI (I$ per capita)	-
	Education (years per capita)	-
	Socio-demographic Index	-
	Population between 500 and 1500 m elevation (proportion)	+
	Population density over 1000 people/kilometer^2 (proportion)	+

Chronic respiratory diseases served as an envelope to the following causes:

- chronic obstructive pulmonary disease
- pneumoconiosis (silicosis, asbestosis, coal worker's pneumoconiosis, other pneumoconiosis)
- asthma
- interstitial lung disease and pulmonary sarcoidosis
- other chronic respiratory diseases

The unadjusted death estimates for all these individual chronic respiratory disease causes are summed and fit to the distribution of deaths estimated for the envelope during the CoDCorrect adjustment process. This results in deaths recorded using non-specific coding systems, such as verbal autopsy, being included in the parent model and redistributed to the child models proportionately. This approach assumes that deaths reported in non-specific data sources have the same underlying distribution of specific causes as deaths reported in more specific data sources.

Covariate influences

The following plots show the influence of each covariate on the four CODEm models (male global, male data-rich, female global, and female data-rich). A positive standardised beta (to the right) means that the covariate was associated with increased death. A negative standardised beta (to the left) means the covariate was associated with decreased death.

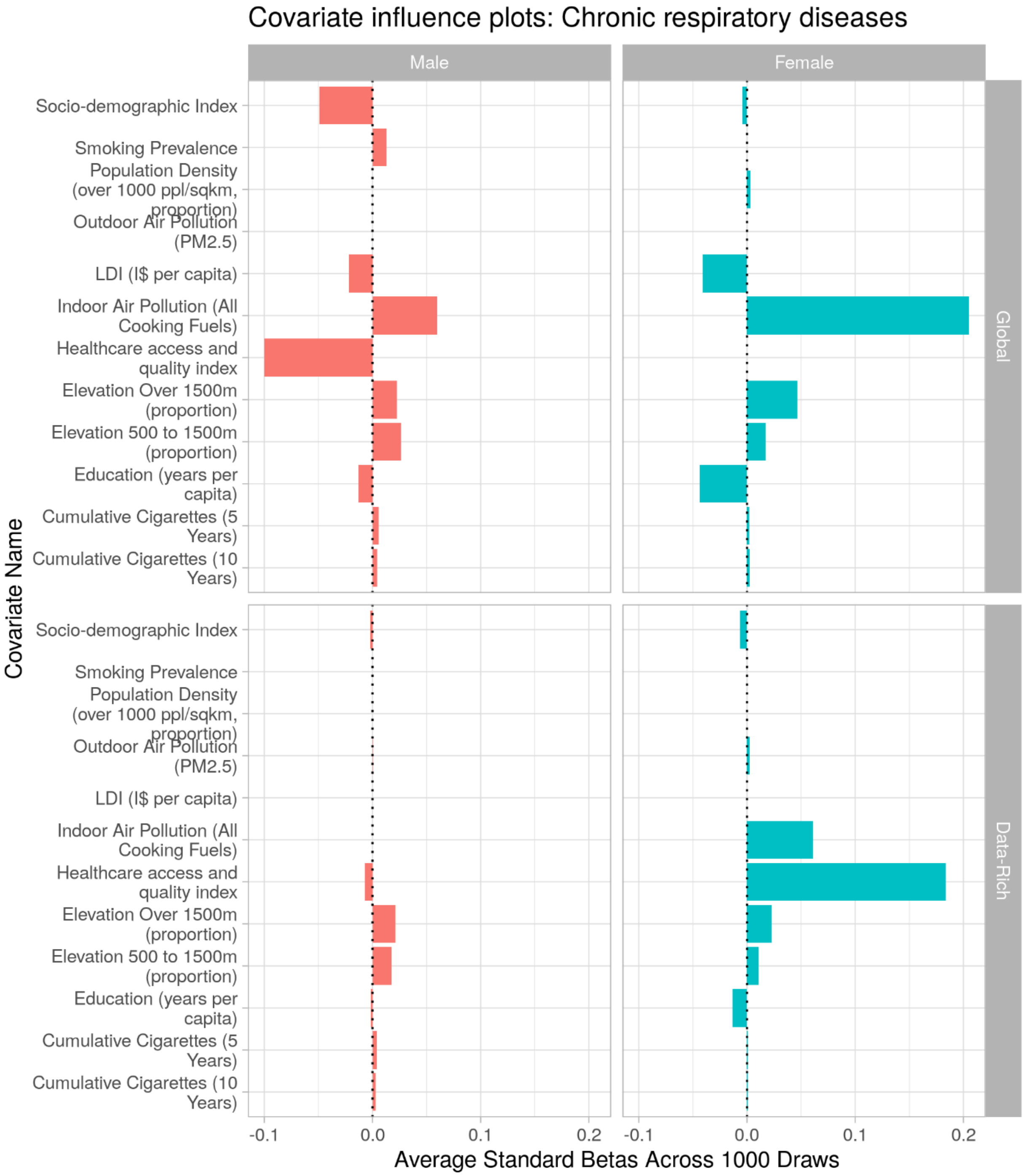

[1]Vos T, Lim SS, Abbafati C, *et al.* Global burden of 369 diseases and injuries in 204 countries and territories, 1990–2019: a systematic analysis for the Global Burden of Disease Study 2019. *The Lancet* 2020; **396**: 1204–22. doi: https://doi.org/10.1016/S0140-6736(20)30925-9

Cirrhosis

Flowchart

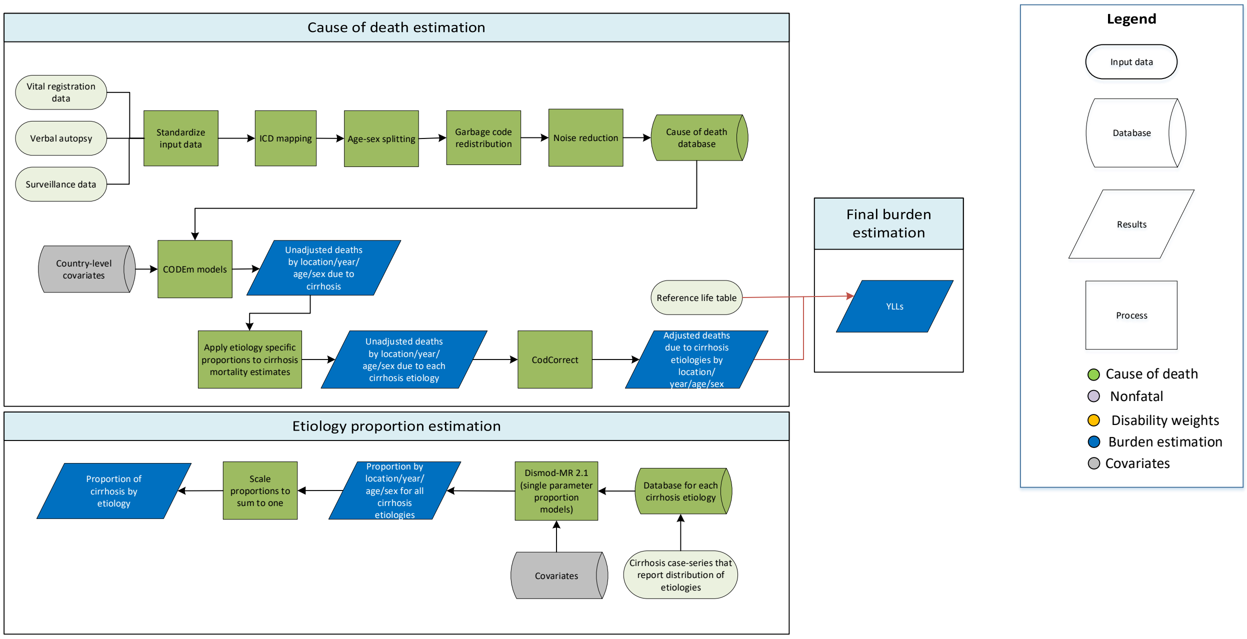

Input data and methodological summary for cirrhosis

Input data

We modelled cirrhosis mortality using vital registration and verbal autopsy data in the cause of death database. See the appendix section on causes of death data preparation for detailed description of this database. A complete list of ICD codes can be found in the appendix section on International Classification of Diseases (ICD) codes mapped to the Global Burden of Disease cause list for causes of death.

Table 1: List of International Classification of Diseases (ICD) codes mapped to cirrhosis

ICD system	Codes
10	B18,B18.0,B18.1,B18.2,B18.8,B18.9,I85,I85.0,I85.00,I85.01,I85.1,I85.10,I85.11,I85.9,I98.2,K70,K70.0,K70.1,K70.10,K70.11,K70.2,K70.3,K70.30,K70.31,K71.7,K73,K73.0,K73.1,K73.2,K73.8,K73.9,K74,K74.0,K74.1,K74.2,K74.3,K74.4,K74.5,K74.6,K74.60,K74.69,K74.7,K74.8,K74.9,K75,K75.2,K75.4,K75.8,K75.81,K75.89,K75.9,K76,K76.0,K76.1,K76.2,K76.4,K76.5,K76.6,K76.7,K76.8,K76.81,K76.89,K76.9,K77.8
9	070.22,070.23,070.54,456.0,456.1,456.2,456.20,456.21,571,571.0,571.1,571.2,571.3,571.4,571.40,571.5,571.6,571.8,571.9,572.2,572.3,572.4,572.5,572.6,572.8,572.9,573,573.0,573.4,573.5,573.8,573.9

Outliers were identified by systematic examination of datapoints for all location-years. Data were excluded if they violated well-established time or age trends, and data in instances where garbage code

redistribution and noise reduction, in combination with small sample sizes, resulted in unreasonable cause fractions.

Additionally, we use data from cirrhosis case-series that report the proportion of cirrhosis cases attributed to alcohol, hepatitis B, hepatitis C, NASH, and other causes. In GBD 2021, 11 new case-series studies were added from a literature review using the search string below. Given time limitations, we expedited the search by looking for results that reported the terms “cirrhosis” and “cases” from the search hits. Studies that did not have these terms in the title/abstract were deferred to GBD 2021 for screening. See the non-fatal methods appendix on cirrhosis estimation for additional details on the case-series data.

(((((((hepatitis b[Title/Abstract] OR "hepatitis b antibod*"[Title/Abstract] OR "hepatitis b antigens"[Title/Abstract] OR hbsag[Title/Abstract])) OR (hepatitis c[Title/Abstract] OR "hepatitis c antibod*"[Title/Abstract] OR "hepatitis c antigens"[Title/Abstract] OR "anti-hcv"[Title/Abstract] OR HCV-RNA[Title/Abstract]))))) AND (alcohol* OR "alcoholic disorders" OR cirrhosis))) AND (NAFLD OR "non-alcoholic fatty liver disease" OR NAFL)

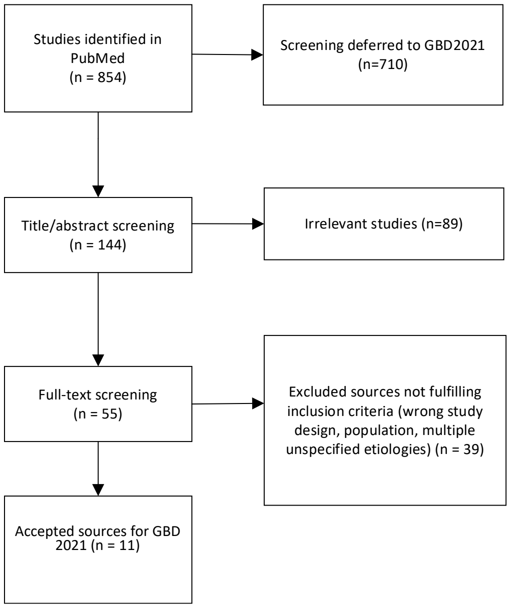

Modelling strategy

We modelled total cirrhosis mortality using a standard CODEm approach, restricting to ages 1 to 95+. Further details can be found in the appendix section on cause of death modelling methods. Predictive covariates entered for selection in this CODEm model are shown in Table 2. In GBD 2021, schistosomiasis was removed from possible covariate selection.

In GBD 2021, we updated the linear floor value in our CODEm models to allow the model to be influenced by lower data, which resulted from changes to COD data processing. These data processing changes include changing the method of estimating the non-zero floor and population size cutoffs for when to noise-reduce at the country versus region level, and are described in greater detail in the appendix section on the GBD 2021 Causes of Death database.

Table 2: Covariates used in CODEm model for cirrhosis and other chronic liver diseases (parent)

Level	Covariate	Direction
1	Litres of alcohol consumed per capita	+
	Vaccine-adjusted HbSAg seroprevalence, age-standardised	+
	Chronic hepatitis C, age-standardised	+
	Hepatitis B vaccine coverage proportion, aged through time	-
2	Mean BMI	+
	Healthcare Access and Quality Index	-
	Diabetes prevalence, age-standardised	+
	Intravenous drug use (proportion by age)	+
3	Education (years per capita)	-
	Lag distributed income (LDI) (ln transformation)	-
	Socio-demographic Index	-

Proportions of cirrhosis due to alcohol, cirrhosis due to hepatitis B, cirrhosis due to hepatitis C, cirrhosis due to other causes, and cirrhosis due to NASH/NAFLD were modelled using DisMod-MR 2.1. Proportions from the five aetiology models were then rescaled to sum to one (at the draw level) and used to split the total cirrhosis mortality estimates from CODEm. The summary of DisMod model covariates is listed in Tables 3-6.

Table 3: Covariates used in the proportion of cirrhosis due to hepatitis B DisMod-MR meta-regression model

Covariate	Exponentiated beta (95% uncertainty interval)
Vaccine-adjusted HBsAg seroprevalence, age-standardised	1.64 (1.06–2.57)
Proportion of liver cancer due to hepatitis B (age-standardised)	1.28 (1.02–1.72)
Hepatitis B vaccine coverage (proportion), aged through time	0.55 (0.38–0.88)
Proportion of cirrhosis due to alcohol	0.46 (0.37–0.65)
Proportion of cirrhosis due to hepatitis C	0.65 (0.45–0.93)
Proportion of cirrhosis due to other causes	0.68 (0.47–0.94)
Proportion of cirrhosis due to NASH	0.59 (0.39–0.94)

Table 4: Covariates used in the proportion of cirrhosis due to hepatitis C DisMod-MR meta-regression model

Covariate	Exponentiated beta (95% uncertainty interval)
Chronic hepatitis C, age-standardsed	1.79 (1.10–2.63)
Proportion of liver cancer due to hepatitis C (Age Standardized)	1.86 (1.19–2.63)
Proportion of cirrhosis due to alcohol	0.41 (0.37–0.50)
Proportion of cirrhosis due to hepatitis B	0.53 (0.38–0.80)
Proportion of cirrhosis due to other causes	0.94 (0.82–1.00)
Proportion of cirrhosis due to NASH	0.63 (0.41–0.94)

Table 5: Covariates used in the proportion of cirrhosis due to alcohol DisMod-MR meta-regression model

Covariate	Exponentiated beta (95% uncertainty interval)
Litres of alcohol consumed per capita	1.01 (1.00–1.03)
Alcohol drinker proportion, age-standardised	1.58 (1.14–2.19)
Proportion of liver cancer due to alcohol (age-standardised)	1.39 (1.02–2.17)

Table 6: Covariates used in the proportion of cirrhosis due to other causes DisMod-MR meta-regression model

Covariate	Exponentiated beta (95% uncertainty interval)
Proportion of liver cancer due to other causes (age-standardised)	1.91 (1.22–2.64)

Table 7: Covariates used in the proportion of cirrhosis due to NASH DisMod-MR meta-regression model

Covariate	Exponentiated beta (95% uncertainty interval)
Mean BMI	1.27 (1.06–1.54)
Prevalence of obesity	2.19 (1.06–5.45)
NAFLD/NASH prevalence	3.22 (1.27–6.78)
Proportion of liver cancer due to NASH (age-standardised)	2.30 (1.07–5.75)

We have made no substantive changes to the modelling strategy of adjusting cryptogenic cases to NASH cases since GBD 2019. Epidemiological studies and hepatologists have indicated that cryptogenic cases of cirrhosis may be un-identified cases of cirrhosis due to NASH. In GBD 2017, when a cirrhosis case-series identified all of our aetiologies of interest as well as cryptogenic cirrhosis, cryptogenic cases were extracted as "other causes", but when a case-series did not explicitly identify NASH, cases reported as "cryptogenic" were extracted as NASH. In GBD 2019 we analysed case-series studies that reported both NASH and cryptogenic cases, modelling the proportion due to NASH (out of NASH plus cryptogenic) in MR-BRT (meta-regression—Bayesian, regularized, trimmed). We then identified the case-series in our database that reported cryptogenic, but not NASH, as an aetiology of cirrhosis, and extracted a proportion due to NASH and a proportion due to other causes based on the proportion modelled in MR-BRT. We added additional data sources in the meta-regression, but overall the strategy remained similar to that of GBD 2019.

Table 8: Proportion of cryptogenic cases in studies that did not specify NASH believed to be NASH, as modeled in MR-BRT

Data input	Beta coefficient, logit (95% CI)	Gamma
Proportion of cryptogenic cases out of cryptogenic cases plus NASH cases reported in the same study	0.465 (0.231–0.698)	0.111

Cocaine use disorder

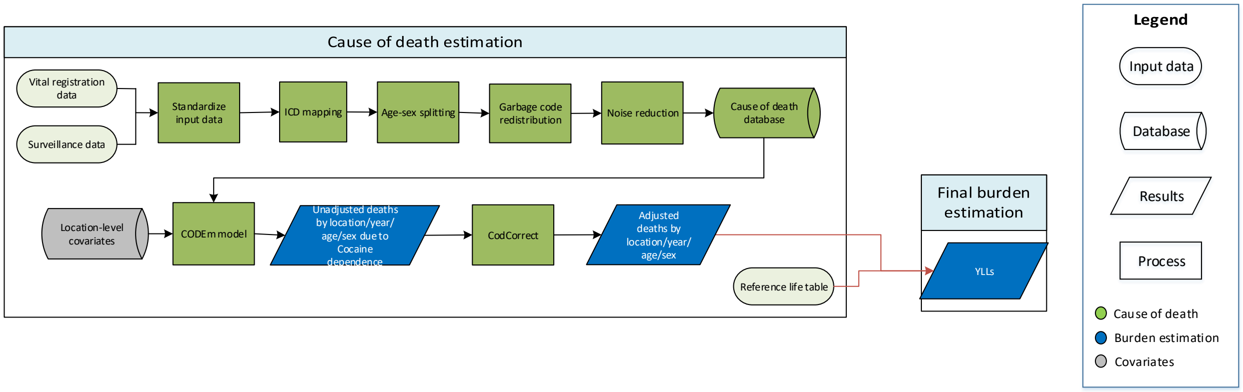

Input data and methodological summary for cocaine use disorders

Input data
All data were from vital registration and surveillance sources. Data from countries with sparse yet heterogeneous data were excluded as the data exaggerated fluctuations in deaths and gave implausible regional patterns, according to in-country and subject matter experts. Excluded data were typically from low- and middle-income countries. A full description of changes to coding and redistribution are described in the write-up on drug use disorders.

Modelling strategy
The standard Cause of Death Ensemble modelling (CODEm)[1] approach (detailed in reference appendix section 3.1) was used to estimate deaths due to cocaine use disorders. Model covariate inclusion was based on empirical evidence and expert feedback, which resulted in a set of model covariates that reflected alcohol consumption, smoking, education, health system access, income per capita, and Socio-demographic Index (SDI) (Table 1).

Key changes from GBD 2021:

- The intravenous drug use covariate incorporated additional data and increased time smoothing, which increased estimates in the United States and Western Europe and made the yearly change more consistent over time.

Table 1: Covariates used in cocaine use CODEm model

Level	Covariate	Direction
1	Alcohol (litres per capita)	+
	Current drinking prevalence	+
	Intravenous drug use, age-standardised	+
	Intravenous drug use, age-specific	+
	Cumulative cigarettes (10 years)	+
	Cumulative cigarettes (5 years)	+
	Cigarettes per capita	+
	Smoking prevalence	+
2	Healthcare Access and Quality Index	-
3	Log LDI (I$ per capita)	+
	Education (years per capita)	+
	Socio-demographic Index	+

Cocaine use disorder is a “child” disease that is fit into an overall “parent” drug use disorders model. The unadjusted death estimates from cocaine use disorders are summed alongside other “child” causes (opioid, amphetamine, other drugs) and fit to the distribution of deaths in an overall drug use disorders “parent” model as part of the CoDCorrect adjustment process.[1] This results in deaths recorded using non-specific coding systems, such as verbal autopsy, being included in the parent model and redistributed to the child models proportionately.

Covariate influences:

The following plots show the influence of each covariate on the four CODEm models (male global, male data-rich, female global, and female data-rich). A positive standardised beta (to the right) means that the covariate was associated with increased death. A negative standardised beta (to the left) means the covariate was associated with decreased death.

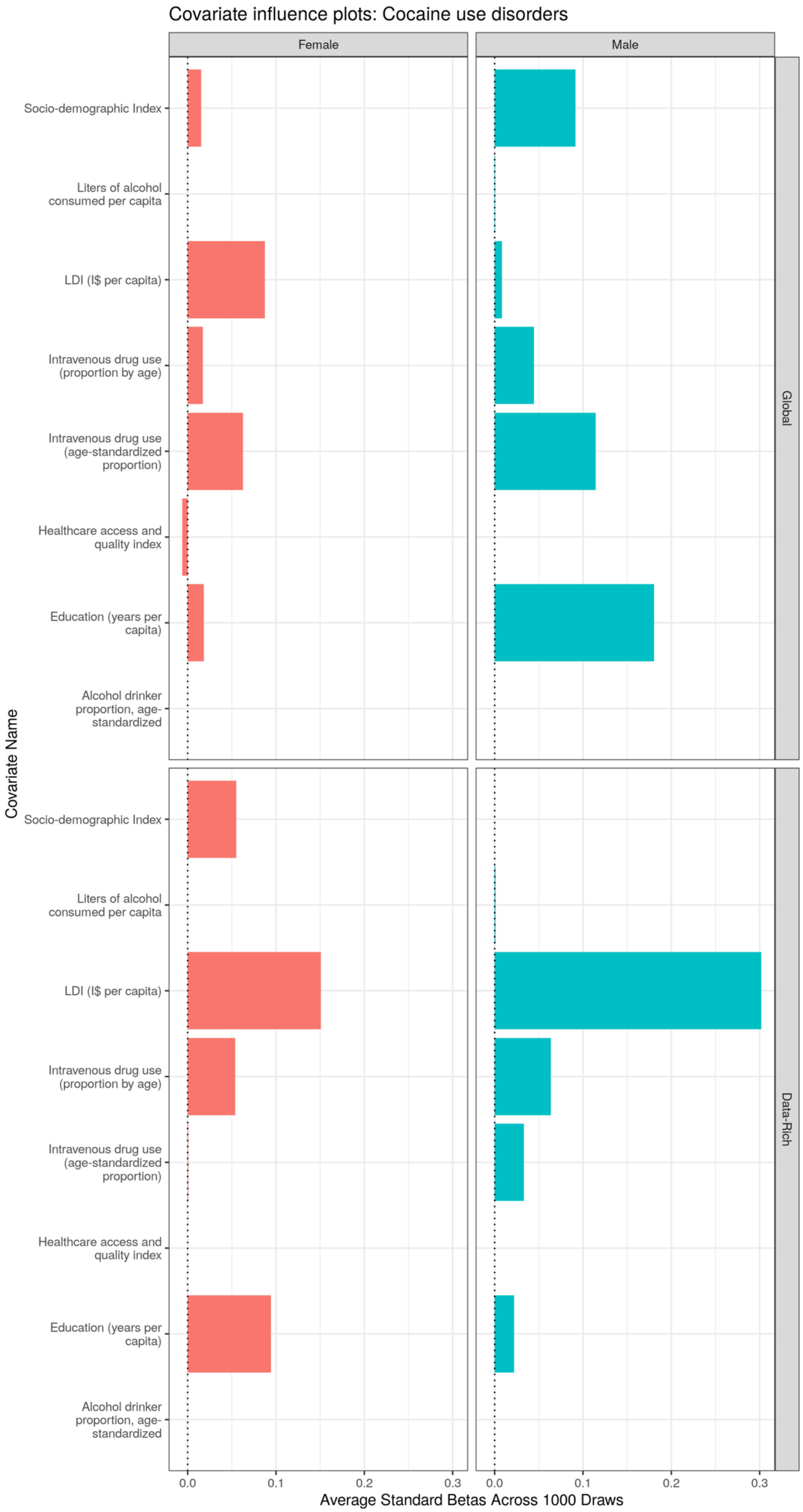

Covariate influence plots: Cocaine use disorders
Female
Male
Global
Data-Rich
Covariate Name
Socio-demographic Index
Liters of alcohol consumed per capita
LDI (I$ per capita)
Intravenous drug use (proportion by age)
Intravenous drug use (age-standardized proportion)
Healthcare access and quality index
Education (years per capita)
Alcohol drinker proportion, age-standardized
0.0
0.1
0.2
0.3
Average Standard Betas Across 1000 Draws

[1] Vos T, Lim SS, Abbafati C, *et al.* Global burden of 369 diseases and injuries in 204 countries and territories, 1990–2019: a systematic analysis for the Global Burden of Disease Study 2019. *The Lancet* 2020; **396**: 1204–22. doi: https://doi.org/10.1016/S0140-6736(20)30925-9

Congenital birth defects: neural tube defects, congenital heart anomalies, orofacial clefts, Down syndrome, Turner syndrome, Klinefelter syndrome, other chromosomal disorders, congenital musculoskeletal anomalies, urogenital congenital anomalies, digestive congenital anomalies, and other congenital birth defects.

Flowchart

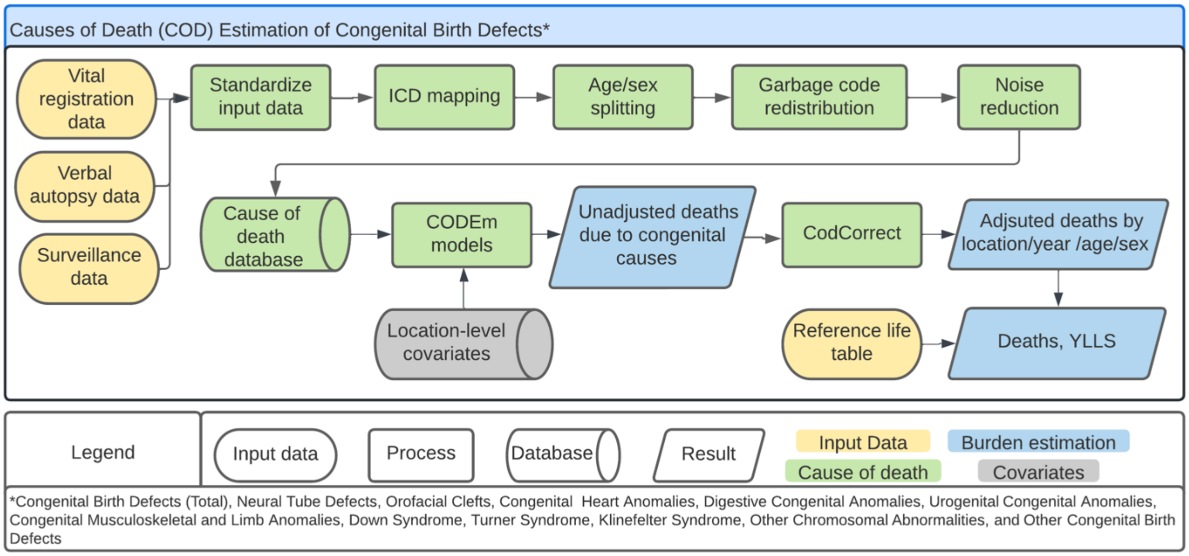

Input data

For GBD 2021, input data for estimating mortality due to congenital anomalies were centrally extracted, processed, and stored in the cause of death (CoD) database. Vital registration (VR) was the dominant data type, followed by verbal autopsy (VA) and surveillance. Those CoD data sources that specified the subcause of birth defect were included in estimation of both the parent congenital anomalies model as well as in subtype-specific models.

For GBD 2021, data exclusions were limited. The majority of VA data were outliered in those over 5 years old as the age patterns were unreliable and led to poor model performance in the under-5 age groups. We also excluded some data sources from the parent model where only a subset of subcauses were specified (e.g., congenital heart disease, neural tube defects, and other congenital anomalies) and the sum of the subcauses clearly represented systematic under-reporting of one of the subcauses. Systematic underreporting was suspected when sex- and age-specific rates were more than an order of magnitude lower than neighbouring or comparable locations. Data sources for those locations were still included by default for subcause-specific models because under-reporting of the total was not assumed to necessarily be associated with under-reporting of all of the component conditions.

Modelling strategy

All types of congenital anomalies were estimated using cause of death ensemble modelling (CODEm) for GBD 2021, as was done for previous iterations of the GBD study. Specific causes included neural tube defects, congenital heart anomalies, orofacial clefts, Down syndrome, other chromosomal anomalies, congenital musculoskeletal anomalies, urogenital congenital anomalies, digestive congenital anomalies, and other congenital birth defects. We assumed no mortality from either Klinefelter syndrome or Turner syndrome, for which we model non-fatal outcomes only. For GBD 2021, we modelled congenital anomalies as a cause of death for ages 0–69 years only, assuming that all mortality from congenital conditions occurs before age 70 years of age.

For GBD 2016, we added three new causes to the congenital anomalies: congenital musculoskeletal and limb anomalies; urogenital congenital anomalies; and digestive congenital anomalies. We have made no additions to the causes of congenital anomalies since GBD 2016.

Table 1: Covariates tested for CODEm model of overall congenital birth defects

Covariate	Transformation	Level	Direction
Maternal alcohol consumption during pregnancy (proportion)	None	1	+
In-facility delivery (proportion)	None	1	-
Live births 35+ (proportion)	None	1	+
Folic acid unadjusted (ug)	None	1	-
Folic acid fortification index	None	1	-
Birth prevalence of congenital heart disease	None	1	+
Birth prevalence of chromosomal anomalies	None	1	+
Legality of abortion	None	2	-
Antenatal care (1 visit) coverage (proportion)	None	2	-
Age-standardised summary exposure value (SEV) of smoking	None	2	+
Antenatal care (4 visits) coverage (proportion)	None	2	-
Healthcare Access and Quality Index	None	2	-
Maternal education (years per capita)	None	3	-
Alcohol (litres per capita)	None	3	+
Age-standardised SEV of low fruits	None	3	+
Outdoor air pollution (PM2.5)	None	3	+
Age-standardised SEV of household air pollution	None	3	+
Socio-demographic Index	None	3	-
Age-standardised SEV of low vegetables	None	3	+

Table 2: Covariates tested for CODEm model of neural tube defects

Covariate	Transformation	Level	Direction
In-facility delivery (proportion)	None	1	-
Folic acid unadjusted (ug)	None	1	-
Folic acid fortification index	None	1	-
Healthcare Access and Quality Index	None	2	-
Antenatal care (1 visit) coverage (proportion)	None	2	-
Antenatal care (4 visits) coverage (proportion)	None	2	-
Age-standardised SEV of smoking	None	2	+
Age-standardised SEV of low fruits	None	3	+
Age-standardised SEV of low vegetables	None	3	+
Maternal education (years per capita)	None	3	-

Socio-demographic Index	None	3	-
Legality of abortion	None	2	-
Maternal alcohol consumption during pregnancy (proportion)	None	3	+
Age-standardised SEV of household air pollution	None	3	+
Age-standardised SEV of fasting plasma glucose	None	3	+
Litres of alcohol consumed per capita	None	3	+

Table 3: Covariates selected for CODEm model of congenital heart anomalies

Covariate	Transformation	Level	Direction
Maternal alcohol consumption during pregnancy (proportion)	None	1	+
Birth prevalence of congenital heart disease	None	1	+
Socio-demographic Index	Log	2	-
Age-standardised SEV of smoking	None	2	+
Age-standardised SEV of diabetes	None	2	+
Healthcare Access and Quality Index	None	2	-
Legality of abortion	None	2	-
Antenatal care (1 visit) coverage (proportion)	None	2	-
In-facility delivery (proportion)	None	2	-
Maternal education (years per capita)	None	3	-
Alcohol (litres per capita)	None	3	+
Antenatal care (4 visits) coverage (proportion)	None	3	-
Skilled birth attendance (proportion)	None	3	-
Live births 35+ (proportion)	None	3	+

Table 4: Covariates selected for CODEm model of cleft lip and cleft palate

Covariate	Transformation	Level	Direction
Socio-demographic Index	None	1	-
Folic acid fortification index	None	1	-
Age-standardised SEV of diabetes	None	2	+
Maternal alcohol consumption during pregnancy (proportion)	None	2	+
Healthcare Access and Quality Index	None	2	-
Legality of abortion	None	2	-
Skilled birth attendance (proportion)	None	2	-
Age-standardised SEV of smoking	None	2	+
Age-standardised SEV of household air pollution	None	3	+
Age-standardised SEV of low vegetables	None	3	+
Alcohol (litres per capita)	None	3	+
Antenatal care (4 visits) coverage (proportion)	None	3	-
Maternal education (years per capita)	None	3	-
Age-standardised SEV of low fruits	None	3	+
Antenatal care (1 visit) coverage (proportion)	None	3	-

Table 5: Covariates selected for CODEm model of Down syndrome

Covariate	Transformation	Level	Direction
Livebirths 35+ (proportion)	None	1	+
Legality of abortion	None	1	-
Livebirths 40+ (proportion)	None	1	+
Birth prevalence of chromosomal anomalies	None	1	+
Socio-demographic Index	None	2	-
In-facility delivery (proportion)	None	2	-

Healthcare Access and Quality Index	None	2	-
Maternal alcohol consumption during pregnancy (proportion)	None	3	+
Antenatal care (1 visit) coverage (proportion)	None	3	-
Maternal education (years per capita)	None	3	-
Age-standardised SEV of household air pollution	None	3	+
Antenatal care (4 visits) coverage (proportion)	None	3	-
Age-standardised SEV of low vegetables	None	3	-
Age-standardised SEV of smoking	None	3	+
Litres of alcohol consumed per capita	None	3	+

Table 6: Covariates selected for CODEm model of other chromosomal abnormalities

Covariate	Transformation	Level	Direction
Livebirths 35+ (proportion)	None	1	+
Livebirths 40+ (proportion)	None	1	+
Legality of abortion	None	1	-
Lag distributed income (LDI) (I$ per capita)	Log	2	-
Healthcare Access and Quality Index	None	2	-
Antenatal care (4 visits) coverage (proportion)	None	2	-
Antenatal care (1 visit) coverage (proportion)	None	2	-
In-facility delivery (proportion)	None	2	-
Maternal alcohol consumption during pregnancy (proportion)	None	2	+
Socio-demographic Index	None	3	-
Alcohol (litres per capita)	None	3	+
Age-standardised SEV of smoking	None	3	+
Age-standardised SEV of household air pollution	None	3	+
Maternal education (years per capita)	None	3	-
Skilled birth attendance (proportion)	None	3	-

Table 7: Covariates selected for CODEm model of congenital musculoskeletal and limb anomalies

Covariate	Transformation	Level	Direction
Maternal alcohol consumption during pregnancy (proportion)	None	1	+
Legality of abortion	None	1	-
In-facility delivery (proportion)	None	2	-
Ag-standardised SEV of diabetes	None	2	+
Socio-demographic Index	None	2	-
Healthcare Access and Quality Index	None	2	-
Age-standardised SEV of household air pollution	None	2	+
Age-standardised SEV of smoking	None	2	+
Antenatal care (4 visits) coverage (proportion)	None	3	-
Alcohol (litres per capita)	None	3	+
Age-standardised SEV of low fruits	None	3	+
Age-standardised SEV of low vegetables	None	3	+
Maternal education (years per capita)	None	3	-
Antenatal care (1 visit) coverage (proportion)	None	3	-
LDI per capita	Log	3	-

Table 8: Covariates selected for CODEm model of urogenital congenital anomalies

Covariate	Transformation	Level	Direction
Age-standardised SEV of smoking	None	1	+

Maternal alcohol consumption during pregnancy (proportion)	None	1	+
Healthcare Access and Quality Index	None	2	-
Diabetes age-standardised prevalence (proportion)	None	2	+
Socio-demographic Index	None	2	-
Age-standardised SEV of outdoor air pollution	None	2	+
In-facility delivery (proportion)	None	2	-
Age-standardised SEV of household air pollution	None	2	+
Antenatal care (1 visit) coverage (proportion)	None	3	-
Alcohol (litres per capita)	None	3	+
Maternal education (years per capita)	None	3	-
LDI (I$ per capita)	Log	3	-
Antenatal care (4 visits) coverage (proportion)	None	3	-

Table 9: Covariates selected for CODEm model of digestive congenital anomalies

Covariate	Transformation	Level	Direction
Maternal alcohol consumption during pregnancy (proportion)	None	1	+
Age-standardised SEV of smoking	None	1	+
Age-standardised SEV of household air pollution	None	2	+
Diabetes age-standardised prevalence (proportion)	None	2	+
Age-standardised SEV of diabetes	None	2	+
Socio-demographic Index	None	2	-
Age-standardised SEV of obesity	None	2	+
In-facility delivery (proportion)	None	2	-
Healthcare Access and Quality Index	None	2	-
Alcohol (litres per capita)	None	3	+
Maternal education (years per capita)	None	3	-
Age-standardised SEV of low vegetables	None	3	+
Antenatal care (1 visit) coverage (proportion)	None	3	-
Antenatal care (4 visits) coverage (proportion)	None	3	-
Age-standardised SEV of low fruits	None	3	+
LDI (I$ per capita)	Log	3	-
MCI	None	3	-

Table 10: Covariates selected for CODEm model of other congenital birth defects

Covariate	Transformation	Level	Direction
Maternal alcohol consumption during pregnancy (proportion)	None	1	+
Livebirths 35+ (proportion)	None	1	+
Maternal education (years per capita)	None	2	-
Legality of abortion	None	2	-
In-facility delivery (proportion)	None	2	-
Age-standardised SEV of household air pollution	None	2	+
Healthcare Access and Quality Index	None	2	-
Antenatal care (1 visit) coverage (proportion)	None	3	-
Age-standardised SEV of diabetes	None	3	+
LDI (I$ per capita)	Log	3	-
Socio-demographic Index	None	3	-
Antenatal care (4 visits) coverage (proportion)	None	3	-
Alcohol (litres per capita)	None	3	+

COVID-19

Flowchart

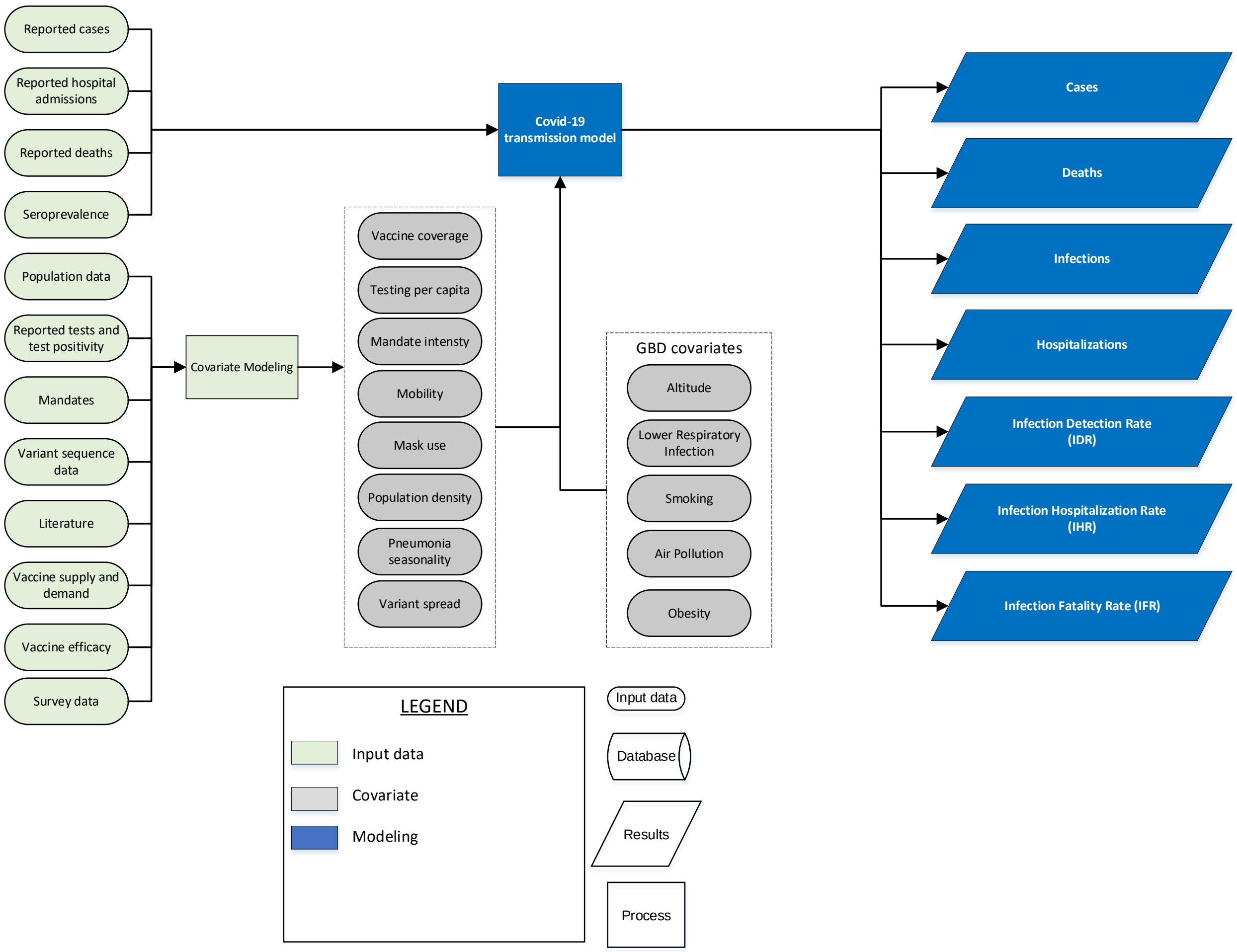

Input data and methodological summary for COVID-19

1. Input data

COVID-19 was estimated outside of the Causes of Death database. All data used to model COVID-19 are available on the GHDx (https://internal-ghdx.healthdata.org/record/ihme-data/covid-19-estimates-december-16-2022). At least 4596 individual data sources were used to model COVID-19 outcomes, including national reports of COVID cases and deaths, surveys including serosurveys, and administrative records of vaccination, among other types of data, and are described in more detail in appendix 1.

Due to the novel nature of COVID-19, we did not conduct literature reviews for all inputs to the model; in many cases, literature was not available. For past SARS-CoV-2 infection protection against re-infection (Stein, et al. 2023) and vaccine protection against re-infection, we conducted the literature reviews described below.

For immunity derived from past infections, we conducted a living systematic review and included data published from inception up to September 30, 2022, for studies that reported results on protection from past COVID-19 infection. We searched peer-reviewed publications, reports, preprints, medRxiv, and news articles. We routinely searched PubMed, Web of Science, medRxiv, SSRN, and the bibliographies of the included papers using the following keywords: “COVID-19”, “SARS-CoV-2”, “natural immunity”, “previous infection”, “past infection”, “protection”, and “reinfection”. The search was not limited to any language.

The protocol of this study is registered at PROSPERO international database (number CRD42022303850). This study complies with the Guidelines for Accurate and Transparent Health Estimates Reporting. See GATHER checklist (table 1) and the PRISMA recommendations (table 2). All code used in the analyses is available at GitHub (https://github.com/ihmeuw/past_covid_infection_meta_analysis. Date accessed: February 10, 2023)

Table 1: GATHER checklist

Item #	Checklist item	Reported location
Objectives and funding		
1	Define the indicator(s), populations (including age, sex, and geographic entities), and time period(s) for which estimates were made.	*Methods (Study selection and data extraction); Appendix Table S3 page* 9
2	List the funding sources for the work.	*Summary*
Data Inputs		
For all data inputs from multiple sources that are synthesized as part of the study:		
3	Describe how the data were identified and how the data were accessed.	*Methods (Overview)*
4	Specify the inclusion and exclusion criteria. Identify all ad-hoc exclusions.	*Methods (Inclusion and exclusion criteria)*
5	Provide information on all included data sources and their main characteristics. For each data source used, report reference information or contact name/institution, population represented, data collection method, year(s) of data collection, sex and age range, diagnostic criteria or measurement method, and sample size, as relevant.	*Appendix Table S3 page 9*
6	Identify and describe any categories of input data that have potentially important biases (e.g., based on characteristics listed in item 5).	*Methods (Risk of bias assessment); (appendix, tables S4, S5, and S6 page 125-129*
For data inputs that contribute to the analysis but were not synthesized as part of the study:		
7	Describe and give sources for any other data inputs.	*NA*
For all data inputs:		
8	Provide all data inputs in a file format from which data can be efficiently extracted (e.g., a spreadsheet rather than a PDF), including all relevant meta-data listed in item 5. For any data inputs that cannot be shared because of ethical or legal reasons, such as third-party ownership, provide a contact name or the name of the institution that retains the right to the data.	*Appendix, table S3 page* 9
Data analysis		
9	Provide a conceptual overview of the data analysis method. A diagram may be helpful.	*Methods (Data analysis)*
10	Provide a detailed description of all steps of the analysis, including mathematical formulae. This description should cover, as relevant, data cleaning, data pre-processing, data adjustments and weighting of data sources, and mathematical or statistical model(s).	*Methods (Data analysis)*
11	Describe how candidate models were evaluated and how the final model(s) were selected.	*NA*

12	Provide the results of an evaluation of model performance, if done, as well as the results of any relevant sensitivity analysis.	*Appendix, figure S3 and S4 page 104*
13	Describe methods for calculating uncertainty of the estimates. State which sources of uncertainty were, and were not, accounted for in the uncertainty analysis.	*Methods (Data analysis)*
14	State how analytic or statistical source code used to generate estimates can be accessed.	*GitHub URL(s) will be made available*
Results and Discussion		
15	Provide published estimates in a file format from which data can be efficiently extracted.	*Appendix, table S3 page 9*
16	Report a quantitative measure of the uncertainty of the estimates (e.g. uncertainty intervals).	*Results*
17	Interpret results in light of existing evidence. If updating a previous set of estimates, describe the reasons for changes in estimates.	*Discussion*
18	Discuss limitations of the estimates. Include a discussion of any modelling assumptions or data limitations that affect interpretation of the estimates.	*Discussion*

Table 2: PRISMA 2020 checklist

Section and topic	Item #	Checklist item	Location where item is reported
TITLE			
Title	1	Identify the report as a systematic review.	Paper title
ABSTRACT			
Abstract	2	See the PRISMA 2020 for Abstracts checklist.	N/A
INTRODUCTION			
Rationale	3	Describe the rationale for the review in the context of existing knowledge.	Main text introduction, paragraph 3
Objectives	4	Provide an explicit statement of the objective(s) or question(s) the review addresses.	Final sentence of main text introduction
METHODS			
Eligibility criteria	5	Specify the inclusion and exclusion criteria for the review and how studies were grouped for the syntheses.	Main text methods "inclusion and exclusion criteria" section
Information sources	6	Specify all databases, registers, websites, organisations, reference lists and other sources searched or consulted to identify studies. Specify the date when each source was last searched or consulted.	Main text methods "overview," paragraph 1
Search strategy	7	Present the full search strategies for all databases, registers and websites, including any filters and limits used.	Main text methods "overview," paragraph 1
Selection process	8	Specify the methods used to decide whether a study met the inclusion criteria of the review, including how many reviewers screened each record and each report retrieved, whether they worked independently, and if applicable, details of automation tools used in the process.	Main text methods "study selection and data extraction" section, paragraph 1
Data collection process	9	Specify the methods used to collect data from reports, including how many reviewers collected data from each report, whether they worked independently, any processes for obtaining or confirming data from study investigators, and if applicable, details of automation tools used in the process.	Main text methods "study selection and data extraction" section
Data items	10a	List and define all outcomes for which data were sought. Specify whether all results that were compatible with each outcome domain in each study were sought (e.g. for all measures, time points, analyses), and if not, the methods used to decide which results to collect.	Main text methods "outcomes assessed" section
	10b	List and define all other variables for which data were sought (e.g. participant and intervention characteristics, funding sources). Describe any assumptions made about any missing or unclear information.	Main text methods "study selection and data extraction" section

Study risk of bias assessment	11	Specify the methods used to assess risk of bias in the included studies, including details of the tool(s) used, how many reviewers assessed each study and whether they worked independently, and if applicable, details of automation tools used in the process.	Main text methods “risk of bias assessment” section
Effect measures	12	Specify for each outcome the effect measure(s) (e.g. risk ratio, mean difference) used in the synthesis or presentation of results.	Main text methods “data analysis” section
Synthesis methods	13a	Describe the processes used to decide which studies were eligible for each synthesis (e.g. tabulating the study intervention characteristics and comparing against the planned groups for each synthesis (item #5)).	N/A
	13b	Describe any methods required to prepare the data for presentation or synthesis, such as handling of missing summary statistics, or data conversions.	N/A
	13c	Describe any methods used to tabulate or visually display results of individual studies and syntheses.	Main text methods “data analysis” section
	13d	Describe any methods used to synthesize results and provide a rationale for the choice(s). If meta-analysis was performed, describe the model(s), method(s) to identify the presence and extent of statistical heterogeneity, and software package(s) used.	Main text methods “data analysis” section
	13e	Describe any methods used to explore possible causes of heterogeneity among study results (e.g. subgroup analysis, meta-regression).	Main text methods “data analysis” section
	13f	Describe any sensitivity analyses conducted to assess robustness of the synthesized results.	Main text methods “data analysis” section
Reporting bias assessment	14	Describe any methods used to assess risk of bias due to missing results in a synthesis (arising from reporting biases).	Main text methods “Risk of bias assessment” section
Certainty assessment	15	Describe any methods used to assess certainty (or confidence) in the body of evidence for an outcome.	Main text methods “data analysis” section
RESULTS			
Study selection	16a	Describe the results of the search and selection process, from the number of records identified in the search to the number of studies included in the review, ideally using a flow diagram.	Main text results, paragraphs 1 and 2
	16b	Cite studies that might appear to meet the inclusion criteria, but which were excluded, and explain why they were excluded.	N/A
Study characteristics	17	Cite each included study and present its characteristics.	Appendix Table S3
Risk of bias in studies	18	Present assessments of risk of bias for each included study.	Appendix tables S4, S5, and S6
Results of individual studies	19	For all outcomes, present, for each study: (a) summary statistics for each group (where appropriate) and (b) an effect estimate and its precision (e.g. confidence/credible interval), ideally using structured tables or plots.	Appendix table S3
Results of syntheses	20a	For each synthesis, briefly summarise the characteristics and risk of bias among contributing studies.	Main text results, paragraph 9
	20b	Present results of all statistical syntheses conducted. If meta-analysis was done, present for each the summary estimate and its precision (e.g. confidence/credible interval) and measures of statistical heterogeneity. If comparing groups, describe the direction of the effect.	Main text results, paragraphs 3–8
	20c	Present results of all investigations of possible causes of heterogeneity among study results.	NA
	20d	Present results of all sensitivity analyses conducted to assess the robustness of the synthesized results.	Main text results, paragraph 10
Reporting biases	21	Present assessments of risk of bias due to missing results (arising from reporting biases) for each synthesis assessed.	NA
Certainty of evidence	22	Present assessments of certainty (or confidence) in the body of evidence for each outcome assessed.	95% uncertainty intervals (UIs) are presented for all mean estimates in the main text results section and in all figures, as relevant
DISCUSSION			
Discussion	23a	Provide a general interpretation of the results in the context of other evidence.	Main text discussion, particularly paragraph 2 and 3

	23b	Discuss any limitations of the evidence included in the review.	Main text discussion, paragraph 7-9
	23c	Discuss any limitations of the review processes used.	Main text discussion, paragraph 7-9
	23d	Discuss implications of the results for practice, policy, and future research.	Main text discussion, paragraph 4-6; next steps; conclusion
OTHER INFORMATION			
Registration and protocol	24a	Provide registration information for the review, including register name and registration number, or state that the review was not registered.	Main text methods “overview,” second paragraph
	24b	Indicate where the review protocol can be accessed, or state that a protocol was not prepared.	Main text methods “overview,” second paragraph
	24c	Describe and explain any amendments to information provided at registration or in the protocol.	N/A
Support	25	Describe sources of financial or non-financial support for the review, and the role of the funders or sponsors in the review.	Main text methods “role of the funding source” section, acknowledgments section
Competing interests	26	Declare any competing interests of review authors.	Main text declaration of interests section
Availability of data, code and other materials	27	Report which of the following are publicly available and where they can be found: template data collection forms; data extracted from included studies; data used for all analyses; analytic code; any other materials used in the review.	Main text data sharing section, methods “overview,” Appendix table S3

Any study with results for the protective effect of COVID-19 natural immunity in individuals who were non-vaccinated in comparison with those who were non-vaccinated and COVID-19-naive were included in our analysis. We also included studies that included individuals who were vaccinated but controlled for vaccination status. We included retrospective and prospective cohort studies, and test-negative case-control studies. Any study that included results only for the protective effectiveness of natural immunity in combination with vaccination (ie, hybrid immunity) was excluded from the analysis.

For vaccine-derived immunity, we conducted a living systematic review, using literature published from June 2021 through December 2022, routinely searching peer-reviewed publications, reports, preprints, medRxiv, and news articles using the search strategy “vaccine AND efficacy OR effectiveness” (for medRxiv alert), in any language.

The protocol of this review is registered at Prospero international database (https://www.crd.york.ac.uk/prospero/display_record.php?RecordID=303850). This study complies with the Guidelines for Accurate and Transparent Health Estimates Reporting (Table 1) and the Preferred Reporting Items for Systematic Reviews and Meta-Analyses (PRISMA) recommendations (Table 2).

All studies that had data on vaccine effectiveness and vaccine effectiveness considering time since vaccination by variants and vaccine types as listed in the intervention(s), exposure(s) section for full vaccination and booster dose were included in the analysis. The included studies were test-negative case-control studies; retrospective and prospective cohort studies; and phase 3, observer-blinded, randomised, placebo-controlled trials. Exclusion criteria include (i) studies that had vaccine effectiveness or vaccine effectiveness considering time since vaccination not segregated by variant and vaccine type, (ii) studies that were done for vulnerable populations only, such as pregnant or immunocompromised

participants, (iii) studies that included variants and vaccine types other than the ones listed in the intervention(s), exposure(s) section, (iv) studies that included only first-dose results, (v) studies that included people with previous infections, and (vi) studies where participants received their full vaccination from two different vaccine types.

2. Modelling strategy

Overview of SEI transmission model

We estimated COVID-19 outcomes using a collection of interconnected sub-models. The core model in the process was a susceptible-exposed-infectious (SEI) transmission model that accounts for vaccination, boosters, multiple infections, antiviral treatments, and the differential waning of vaccine- and infection-derived immunity against infection and severe disease. The transmission model is structured to accept three parameterisations, each of which is used at a different phase of the modelling process. The first parameterisation takes as inputs a paired epidemiological measure (infections, hospitalisations, or deaths) and estimation of the ratio (infection–detection, infection–hospitalisation, or infection–fatality, respectively) of that measure to infections among the COVID-naive and unvaccinated population. This parameterisation is used to produce measure-specific estimates of transmission intensity and infections. The second parameterisation accepts a single estimate of transmission intensity and all available information about infections, hospitalisations, and deaths. The transmission intensity estimate is derived from an average of the measure-specific estimates produced with the first model parameterisation. This second parameterisation is used to produce final historical estimates of infections, hospitalisations, and deaths, and of the infection–detection ratio (IDR), infection–hospitalisation ratio (IHR), and infection–fatality ratio (IFR), for each modelled location. The third model parameterisation takes transmission intensity and the epidemiological ratios as inputs and produces cases, hospitalisations, and deaths as outputs.

Time periods, locations, age groups, and outcome measures

Our historical model begins in December 2019, when the first COVID infections emerged in the Wuhan province of China. COVID-19 infections, hospital admissions, and deaths are estimated for all ages and sexes combined. Estimates are presented at the global and WHO regional levels, for 176 countries and territories and for 206 subnational locations in 14 of those countries and territories.

Estimating key model drivers

Before implementing our epidemiological SEI model, we began by estimating several key model drivers. First, we modelled the invasion date and rate of invasion of the most prevalent SARS-CoV-2 variants using data primarily sourced from the GISAID Initiative and performed a secondary analysis to match invasion dates with reported cases, deaths, and hospital admissions. Second, using data from Our World in Data, Linksbridge, the Duke Global Health Innovation Center (https://launchandscalefaster.org/COVID-19), the COVID-19 Trends and Impact Surveys for the USA (https://delphi.cmu.edu/covidcast/survey-results/) and globally (https://covidmap.umd.edu/), and other sources detailed in the source tables included in the methods appendix, we modelled the supply, delivery, and demand of available vaccines against SARS-CoV-2 to estimate and forecast the number of full vaccine courses and booster doses delivered in each location by brand. Third, we estimated brand-specific waning vaccine efficacy against each variant and used these estimates to transform vaccine

delivery into transmission risk-reduction curves. Fourth, we estimated the waning of infection-derived immunity and protection from severe disease. Fifth, we estimated the ratio of location-specific weekly pneumonia deaths to the annual average as a proxy for seasonal trends in COVID-19 transmission based on weekly vital registration data. Sixth, using reports from government health authorities and data from Our World in Data, we estimated the per capita testing rate and forecasted its growth up to a location-specific threshold. Seventh, we compiled a comprehensive database of the application of 21 detailed NPIs (non-pharmaceutical interventions, eg, closing primary school or non-essential retail) representing six NPI categories (eg, education and business closures) from January 2020 to October 2022 that are standardised across all modelled countries and subnational units. Eighth, using survey data from the USA and Global COVID-19 Trends and Impact Surveys, the PREMISE Behavior Survey, and the YouGov COVID-19 Behaviour Tracker Survey, we estimated the percentage of the population regularly wearing masks (averaged across different mask types and settings based on survey participants' own interpretations of what "always" wearing a mask means), and projected mask use by location. Full data and modelling details for the model drivers are available in the methods section. In addition to the model drivers we estimated, we leveraged demographic data and several time-invariant covariates from the Global Burden of Diseases, Injuries, and Risk Factors Study (GBD) 2019 to better inform location-level heterogeneity in many of our sub-models.

Estimating historical COVID-19 outcomes by location

Model drivers in hand, our next task was to estimate historical transmission intensity in each location using all available reported data. We parameterised our model of variant-specific IDR, IHR, and IFR relative to the IDR, IHR, and IFR among the infection- and vaccine-naive population experiencing an infection with ancestral-type (D614G) COVID-19. We then paired a database of bias-corrected cases, hospital admissions, and excess deaths with SARS-CoV-2 seroprevalence surveys adjusted for waning antibody sensitivity, vaccination, and escape variant reinfection. These paired data were subset to the first period of the pandemic, when no vaccines or variants were present, and used to produce an initial empirical estimate of the infection- and vaccine-naive IDR, IHR, and IFR using a statistical model of the ratios that incorporated several of the model drivers to fill data gaps. The input measure and resulting ratio from each ratio model were then run through the first parameterisation of our transmission model using all historical data, allowing an initial estimate of the fraction of COVID-19 infections, hospital admissions, and deaths due to ancestral COVID-19 among the infection- and vaccine-naive population. These naive measures were then paired with the full seroprevalence dataset and run through the ratio estimation process a second time to produce a robust empirical estimate of the epidemiological ratios. The updated ratios and original measures were run again through the transmission model to produce the final measure-specific estimates of transmission intensity. The next task was to use measure-specific models to produce a single, coherent estimate of historical COVID-19 outcomes in all locations. To do so, the measure-specific estimates of transmission intensity were averaged into a single transmission intensity per location and then input with all available infection, hospitalisation, and death estimates into the second parameterisation of the transmission system. This produced our final historical estimates of infections, hospital admissions, and deaths due to all variants among all vaccine- and SARS-CoV-2-exposure population subgroups. This step also implicitly produced our final estimates of the IDR, IHR, and IFR for each location so that each was consistent with the combined estimate of transmission intensity.

References

Stein C, et al. Past SARS-CoV-2 infection protection against re-infection: a systematic review and meta-analysis. *The Lancet*. 2023; 401(10379):833-842. https://doi.org/10.1016/S0140-6736(22)02465-5.

Stevens GA, Alkema L, Black RE, et al. Guidelines for accurate and transparent health estimates reporting: the GATHER statement. *The Lancet*. 2016; 388: e19-e23.

Estimating COVID-19 impact on select infectious syndromes

COVID-19 has strained health-care systems around the world and limited capacity to deliver routine immunisations, priming populations for outbreaks of infectious disease. Conversely, physical distancing measures, masking, and school closures have the potential to interrupt usual transmission patterns of other infectious diseases, as they do for COVID-19. Considering these competing ways in which COVID-19 could influence other diseases, in combination with many countries reporting greatly reduced incidence of influenza and measles, we sought to capture the impact of COVID-19 in our estimates of other infectious diseases for 2020 and 2021.

Data

We reviewed national-level case notification data from ministry of health websites, media reports, and published literature for measles, pertussis, diphtheria, tetanus, varicella, diarrhoeal disease, influenza, respiratory syncytial virus, and infections due to *S. pneumoniae*, *H. influenzae*, and *N. meningitidis* to look for evidence of disruption. For measles and influenza, we relied on case notifications reported directly by countries to WHO regional offices; these causes had the most complete geographical and temporal coverage. Because of this completeness in reporting, we utilised them as indicator causes for further modelling, as described below.

Modelling

We began by evaluating each cause for evidence of disruption. For each cause, to determine whether a disruption occurred in 2020, we conducted a random effect meta-analysis with restricted maximum likelihood estimation using the metafor package in R. Each point was the ratio of cases observed in 2020 to the cases observed over the average of 2017–2019. Given the relative completeness of measles and influenza data, we developed a primary model for these causes and then, for causes other than measles and influenza, evaluated whether the reduction modelled for measles or influenza could be applied directly to the other cause. To do this, we examined the change in case notifications between 2020 and previous years for a cause relative to the change in case notifications between 2020 and previous years for measles and influenza. When determining whether to adjust each cause, we considered the size and statistical significance of the observed effect, the consistency and quality of the available data, and epidemiological plausibility. At the time of estimation, these factors supported adjustment of only pertussis and RSV, using estimates of disruption derived from the influenza disruption model results (see below). As we receive more data, we plan to re-examine additional causes and aetiologies to apply disruption if warranted.

We developed a multi-step modelling process to estimate the effect of non-pharmaceutical interventions (NPIs) associated with the COVID-19 pandemic on the incidence of influenza and measles in 2020 and 2021. First, we interpolated the number of reported cases of influenza and measles in 2020 and 2021, by month. We leveraged the RegMod framework, a Poisson model that estimates the underlying rate of infection in each month as a function of a seasonal pattern and an underlying temporal trend. The temporal trend was reflected as a piecewise linear spline with knots at the start of each year. We placed the last knot of the underlying time trend in January 2021 for measles and influenza. We used monthly data through March 2022 (the last month of available data at the time of modeling) to fit the model, starting in January 2010 for influenza and January 2011 for measles. The

RegMod model results are 1000 sets of estimates of the number of reported cases in each month and inputs to the next phase of modelling. We excluded RegMod results from any country missing at least six months of data in any year within 2017–2021 to reduce the risk of outbreaks occurring and subsiding during the periods of missing data.

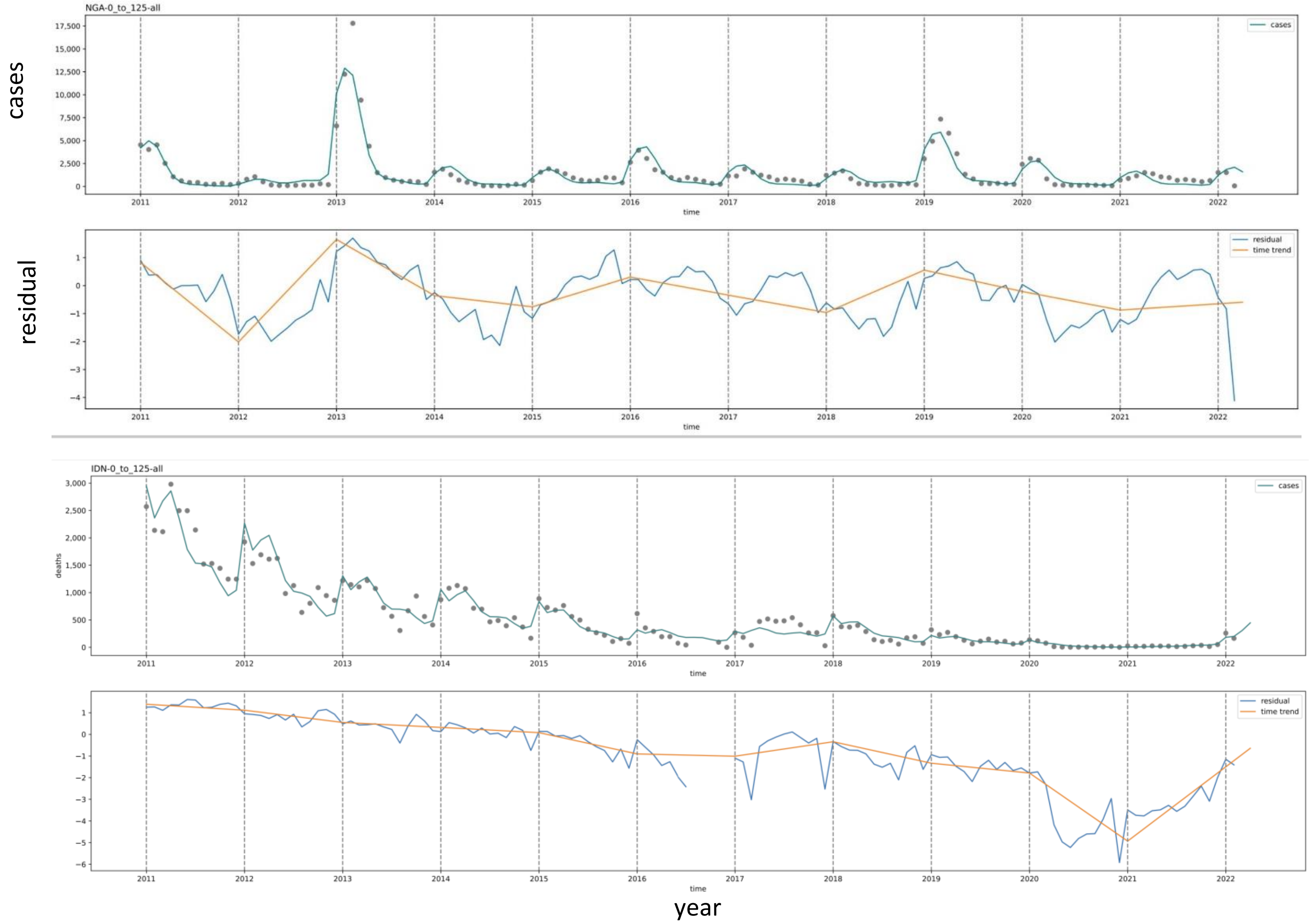

In the second step of the modelling process, we calculated the underreporting ratio (URR) in the pre-pandemic reference period, for each location, for both influenza and measles, by dividing the interpolated number of reported cases from RegMod by the GBD estimated number of incident cases of influenza or measles. For influenza, we used a reference period of 2017–2019 when calculating the URR; for measles, we used 2015–2019. We used a longer period for measles because of greater year-to-year variation in the long-term time trends in cases.

cases

residual

year

Third, we estimated the pandemic-free counterfactual number of reported cases, meaning, the number of reported cases we would have expected during 2020 and 2021 in the hypothetical pandemic-free scenario. We did this by multiplying the URR by the number of cases of LRI due to influenza or measles, for 2020 and 2021, that GBD models would have estimated in a pandemic-free scenario.

Lastly, we calculated a disruption scalar for each location for 2020 and 2021. This scalar was computed by dividing the interpolated number of reported cases from RegMod (result of first step) by the counterfactual disruption-free number of reported cases (result of third step). This value was calculated by year in all cases except for measles in 2020, where it was calculated by month to account for the timing of the onset of the pandemic in relation to typical seasonal variation in measles epidemiology.

Adjusting 2020 estimates for these expected seasonal variations in measles epidemiology required an additional step to move between annual and monthly time scales. RegMod estimates were produced at the monthly time scale, requiring the conversion of annual estimates of counterfactual reported cases to monthly estimates to allow for the monthly calculation necessary. To account for seasonality, we calculated a seasonality weight for each month for measles. For each month from January 2017 to December 2019, we divided the RegMod measles case estimates from that location-month by the average monthly cases across months in that year. This gave a set of seasonality weights for each location-month, for each year. We then averaged each month's seasonality weight across the three years to yield a three-year average seasonality weight for each location-month. We used these seasonality weights to estimate the counterfactual COVID-free number of reported cases for each month during the pandemic for each location, dividing the location's annual counterfactual COVID-free number of reported cases measles cases by 12 and multiplying by the seasonality weight. For locations without a full time-series of data, we used the average seasonality weight from locations with similar latitude. Monthly disruption scalars were calculated by dividing the new monthly estimates of counterfactual reported cases by RegMod's estimate for the same month. Disruption scalars were set to 1 for January, February, and March 2020 to remove the influence of outbreaks observed in early 2020 in the absence of COVID-19 on the overall disruption ratio. We then converted our monthly disruption estimates for measles in 2020 to annual disruption estimates by calculating a seasonality-weighted average of the monthly disruption estimates for each location.

This approach yielded annual disruption scalars for 2020 and 2021 for influenza and measles, by location. These ratios can be interpreted as estimates of the relative change in influenza and measles incidence that occurred during the COVID pandemic in 2020 and 2021 for each location, relative to expected trends from the GBD models in a counterfactual COVID-free scenario. For countries with no data, the median disruption scalar in the region was used. All operations were performed at the 1000 draw level.

Measles adjustment

For locations in the Latin America and the Caribbean, high-income, and central Europe, eastern Europe, and central Asia super-regions and any locations outside these super-regions with WHO-verified measles elimination, as well as select locations with known strong measles surveillance systems (China and Jordan), we used measles case notifications directly for our burden estimates, assuming complete reporting. This practice is consistent with our measles incidence estimation framework in years without COVID-19. For all other locations, we generated counterfactual COVID-free measles incidence and

prevalence estimates using our standard measles estimation approach (described elsewhere in this appendix), substituting counterfactual estimates of vaccine coverage in the absence of COVID-19 as the vaccine coverage covariate for the years 2020 and 2021. These counterfactual COVID-free measles estimates were then multiplied by the location- and year-specific measles disruption scalar described above to derive COVID-inclusive incidence and prevalence estimates for each year and location. At the time of this analysis, there were insufficient data to estimate whether and to what degree COVID-19 may have affected measles case-fatality rates. We therefore did not incorporate an additional COVID effect on measles case-fatality rate. Maintaining our usual natural history model framework for measles, fatal estimates were scaled using the same disruption ratios applied to incidence and prevalence. Additional data and analyses will be required in the future to better assess the potential impact of the COVID-19 pandemic on case-fatality rates, including for measles.

LRI adjustment

We conducted a meta-analysis to compare location-specific disruptions for RSV to measles and influenza and found that the disruption in RSV cases in 2020 was analogous to that observed for influenza. For each location/age/sex for which LRI is estimated, influenza and RSV cases were scaled using the annualised ratios as calculated for influenza. Other aetiology-attributed cases of LRI were not scaled at this time.

Next, we calculated how the disruption scalars for influenza and RSV would apply to the overall LRI estimates. Because the etiological fraction of LRI due to RSV and influenza varies by age and sex, this calculation was performed by sex at the most granular age group level, for each country and year. It was also performed separately for deaths and cases since the etiological fraction of LRI due to RSV and influenza is different for deaths and cases. For a given country-year, the influenza disruption scalar was multiplied by the number of LRI influenza and RSV case/death counts, as pulled from GBD counterfactual estimates, to get adjusted flu and RSV counts. Then, we calculated the number of LRI cases/deaths to "remove" from the counterfactual number of LRI cases/deaths in the adjusted scenario as: the sum of counterfactual flu count and RSV count, minus the sum of COVID-adjusted flu count and RSV count. Finally, we calculate the LRI scalar for each country-age-sex-year as the LRI cases/deaths count from GBD counterfactual estimates, minus the number of LRI cases/deaths to "remove", all divided by the counterfactual LRI cases/deaths count.

To adjust incidence and prevalence estimates for a given cause, we simply multiplied these estimates by the annual disruption ratio for that cause, calculated as described above. To adjust mortality estimates for a given cause, scalars are applied to an intermediate set of mortality results (counterfactual LRI death count) to create a count of LRI deaths to subtract using the formula below:

LRI deaths to subtract = (Counterfactual LRI death count * (LRI scalar – 1))

These values are subtracted from counterfactual LRI deaths to get adjusted LRI deaths. This operation is performed at the 1000 draw level for each location, age, sex, and year. This process is applied to final estimates the same way as other causes known in the GBD framework as fatal discontinuities.

Pertussis adjustment

We conducted a meta-analysis to compare location-specific disruptions for pertussis to measles and influenza and found that the disruption in pertussis cases in 2020 was analogous to that observed for influenza. All locations' incidence and prevalence estimates for 2020 and 2021 were scaled using the annualised ratios as calculated for influenza.

Limitations

A key limitation of this framework is that it relies exclusively on case notification data from national and multinational surveillance networks. It cannot separate the effects of true decreases in disease incidence from the effects of decreased reporting. Currently, we cannot adjust for the assumption that case notifications reflect true decreases in disease incidence because we do not have any data without changes in reporting, or data on reporting patterns themselves; however, we hope to address this in the future. In addition, we have only adjusted estimates for influenza, measles, RSV, and pertussis in this release due to a scarcity of data. New research also suggests substantial decreases in other LRI and meningitis-causing pathogens, specifically *N. meningitidis*, *S. pneumoniae*, and *H. influenzae*; we plan to incorporate this source, and continue our data seeking, to improve our adjustments for additional diseases in later releases. For future years, additional data and revisions to this modelling framework will be needed to allow for more flexibility in capturing disease resurgence.

For fatal estimates, once created, scalars are applied to an intermediate set of CoDCorrect results (prior to adding shocks) to create a set of positive or negative shocks using the formula below.

$$\text{Shock} = (\text{cc\_draw} * (\text{scalar\_draw} - 1))$$

For non-fatal estimates, once created, scalars are applied to select disease estimates before going through additional central GBD processes such as EPIC and Burdenator. Additional information on those processes can be found in this appendix.

Cystic echinococcosis

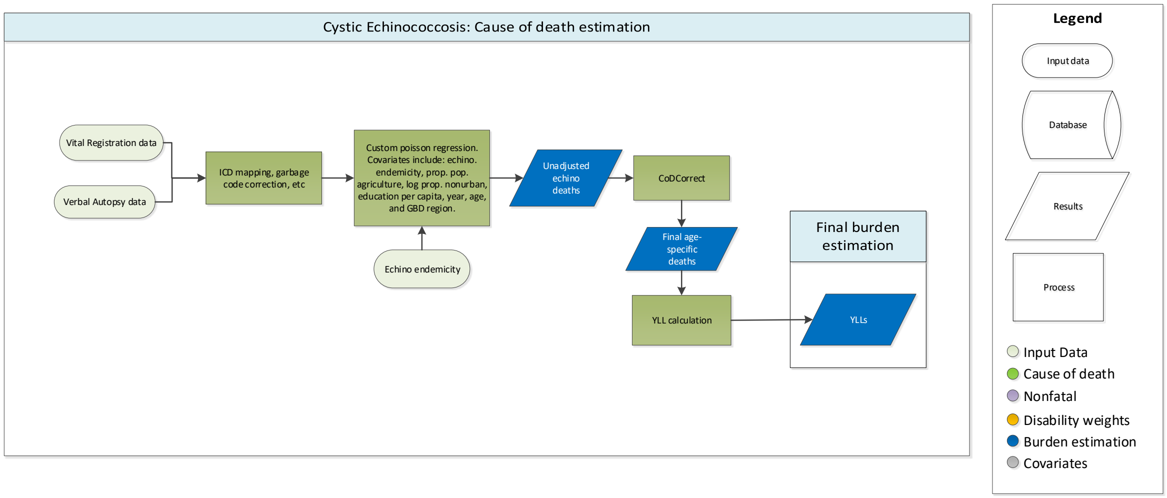

Input data

Geographical restrictions

We conducted a literature review to determine the geographical extent of the disease and classify locations based on whether the disease is absent or present in each year. Locations that were geographically restricted in any given year did not have estimates made for them but could have imported cases attributed to them at a later stage. Of note, we did not attempt a complete systematic review, since a single high-quality source could offer sufficient evidence of presence. Evidence of absence or presence was not available for every location for each year, and so assumptions were made for missing years by taking into consideration the epidemiological characteristics of the disease. If evidence indicated disease presence at a given point in time, we assumed presence for all years. If evidence indicated disease absence, we assumed absence for all years. If evidence indicated a change in status (ie, from absent to present, or present to absent) between two non-consecutive years, then we conducted targeted searches to ascertain the relevant year of introduction or elimination for that location. In the cases where presence or absence information was missing from the start or end years of our study interval (1990–2021) without evidence of any introduction or elimination events within the interval, we applied the status of the first and last presence/absence observations, respectively, to all years between the interval bound and the observation year. For cystic echinococcosis (CE), we reviewed all references pertaining to CE in "Global Distribution of Alveolar and Cystic Echinococcosis" by Deplazes and colleagues[1] and supplemented with targeted searches to classify location-years in PubMed and the GHDx.

Data sources

Mortality due to cystic echinococcosis was modelled using vital registration data and covariates. The Mortality and Cause of Death team provided country-year-age-sex-specific vital registration. Of note, the ICD codes mapped to cystic echinococcosis are:

Table 1: ICD-9 codes mapped to CE

ICD code	ICD name
**122**	Echinococcosis
**122.0**	*Echinococcus granulosus* infection of liver
**122.1**	*Echinococcus granulosus* infection of lung
**122.2**	*Echinococcus granulosus* infection of thyroid
**122.3**	*Echinococcus granulosus* infection, other
**122.4**	*Echinococcus granulosus* infection, unspecified
**122.8**	Echinococcosis unspecified, of liver
**122.9**	Echinococcosis other and unspecified

Table 2: ICD-10 codes mapped to CE

ICD code	ICD name
**B67.0**	*Echinococcus granulosus* infection of liver
**B67.1**	*Echinococcus granulosus* infection of lung
**B67.2**	*Echinococcus granulosus* infection of bone
**B67.3**	*Echinococcus granulosus* infection, other and multiple sites
**B67.31**	*Echinococcus granulosus* infection, thyroid gland
**B67.32**	*Echinococcus granulosus* infection, multiple sites
**B67.39**	*Echinococcus granulosus* infection, other sites
**B67.4**	*Echinococcus granulosus* infection, unspecified
**B67.8**	Echinococcosis, unspecified, of liver
**B67.9**	Echinococcosis, other and unspecified
**B67.90**	Echinococcosis, unspecified
**B67.99**	Other echinococcosis

Due to the scarcity of hospital data, especially in endemic areas, we incorporated covariates to drive global distribution of deaths in the model.

We created a categorical cystic echinococcosis endemicity covariate based on expert opinion and an endemicity map published by WHO [2]. We assigned GBD locations to one of four categories: probable absence, rare and/or sporadic transmission, suspected and/or confirmed transmission, and high endemic areas.

We based further selection of covariates on a meta-analysis of potential risk factors associated with cystic echinococcosis [3]. According to the meta-analysis, statistically significant potential risk factors include living in rural endemic areas, slaughtering, feeding dogs with viscera, and low income. Hence, we also included two other covariates: the proportion of the population participating in agricultural activities and the log of proportion non-urban.

Modelling strategy

We implemented a Poisson regression model to estimate deaths due to cystic echinococcosis. The Poisson regression was selected due to its suitability for modelling count data that are not over-dispersed. Covariates for the model, including echinococcosis endemicity, log of lag distributed income per capita, proportion of the population participating in agricultural activities, and education (years per capita), were incorporated into the model to influence the global trend due to paucity of data. Random effects were used on location with random slopes on age by location. A multivariate normal distribution using the mean and variance-covariance matrix from the model was used to generate 1000 draws of deaths due to cystic echinococcosis. The final model was selected based on how well the estimated numbers fit the input data and how plausible the predicted distribution of disease was over time and with age.

Changes from GBD 2019 to GBD 2021

We have made no substantive changes in the modelling strategy from GBD 2019.

References

1. Deplazes P, Rinaldi L, Rojas CA, Torgerson PR, Harandi MF, Romig T, Antolova D, Schurer JM, Lahmar S, Cringoli GJ, Magambo J. Global distribution of alveolar and cystic echinococcosis. Advances in parasitology. 2017 Jan 1;95:315-493.
2. World Health Organization (2010). Global Echinococcosis Granulosus and Cystic Echinococcosis (hydatisosis) Worldwide 2009.
3. Possenti A, Manzano-Román R, Sánchez-Ovejero C, et al. Potential Risk Factors Associated with Human Cystic Echinococcosis: Systematic Review and Meta-analysis. Flisser A, ed. *PLoS Neglected Tropical Diseases*. 2016;10(11):e0005114. doi:10.1371/journal.pntd.0005114.

Cysticercosis

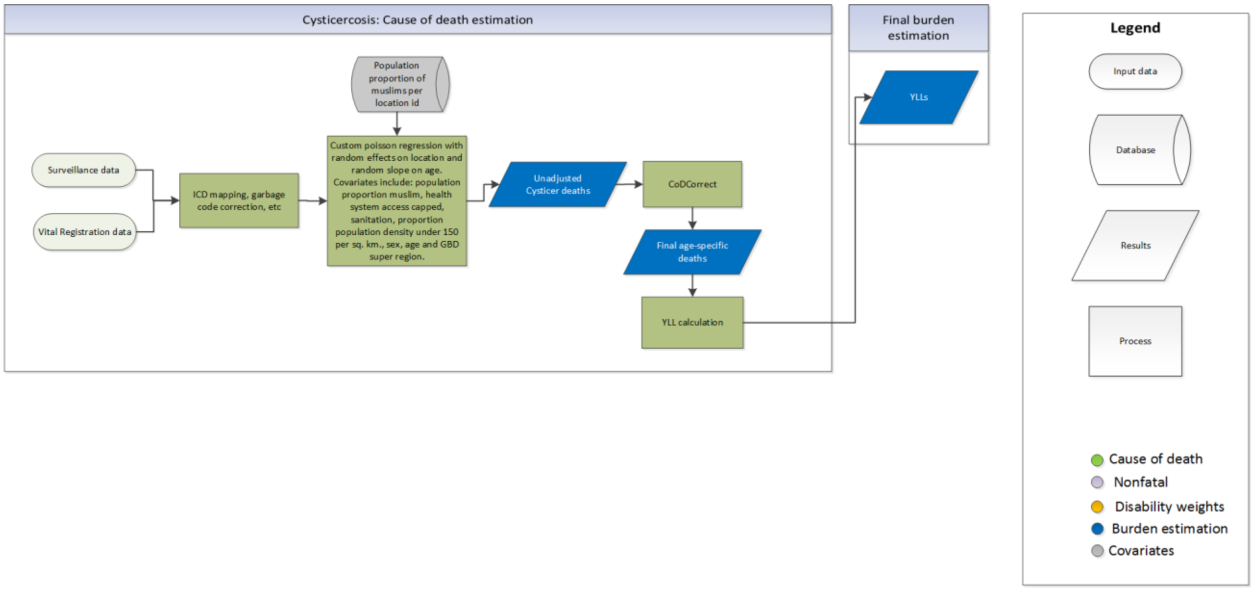

Input data

The model for mortality due to cysticercosis relied on vital registration and surveillance data from endemic countries. In addition, we used data from the Pew Research Center on percentage of population that is Muslim by country. The primary covariates adjusted for in the model were proportion of the population that is Muslim; a composite indicator of maternal care and immunisation coverage (combining estimates of antenatal care, in-facility delivery skilled birth attendance, and vaccine [DTP3 and MCV1] coverage); proportion of the population with access to sanitation; proportion of the country with population density under 150 people per square kilometer; sex; age; and GBD super-region.

Geographical restrictions

We conducted a literature review to determine the geographical extent of the disease and classify locations based on whether the disease is absent or present in each year. Locations that were geographically restricted in any given year did not have estimates made for them but could have imported cases attributed to them at a later stage. Of note, we did not attempt a complete systematic review, since a single high-quality source could offer sufficient evidence of presence. Evidence of absence or presence was not available for every location for each year, and so assumptions were made for missing years by taking into consideration the epidemiological characteristics of the disease. If evidence indicated disease presence for two non-consecutive years, we assumed presence for all years between the two. If evidence indicated disease absence for two non-consecutive years, we assumed absence for all years between the two. If evidence indicated a change in status (ie, from absent to present, or present to absent) between two non-consecutive years, then we conducted targeted searches to ascertain the relevant year of introduction or elimination for that location. In the cases where presence or absence information was missing for the start or end years of our study interval (1990–2021) without evidence of any introduction or elimination events within the interval, we applied

the status of the first and last presence/absence observations, respectively, to all years between the interval bound and the observation year. For cysticercosis, we performed targeted searches to classify location-years in PubMed and Google Scholar. Our map was populated by 21 peer-reviewed articles and meta-analyses and WHO reports.

Modelling strategy

Globally, deaths due to cysticercosis are relatively low. Therefore, a Poisson model was used to model cysticercosis deaths due to its suitability for count data. This model choice was validated by tests for overdispersion. Random effects were used on location with random slopes on age by location. A multivariate normal distribution using the mean and variance-covariance matrix from the model was used to generate 1000 draws of deaths due to cysticercosis.

Since the Pew Research Center has data on proportion of Muslims only by country, we applied the national proportions to subnational locations. We understand that this does not account for sometimes large, expected differences in proportions of Muslims within a country, but were limited by data availability.

Changes from GBD 2019 to GBD 2021

We have made no substantive changes in the modelling strategy from GBD 2019.

Decubitus ulcer

Flowchart

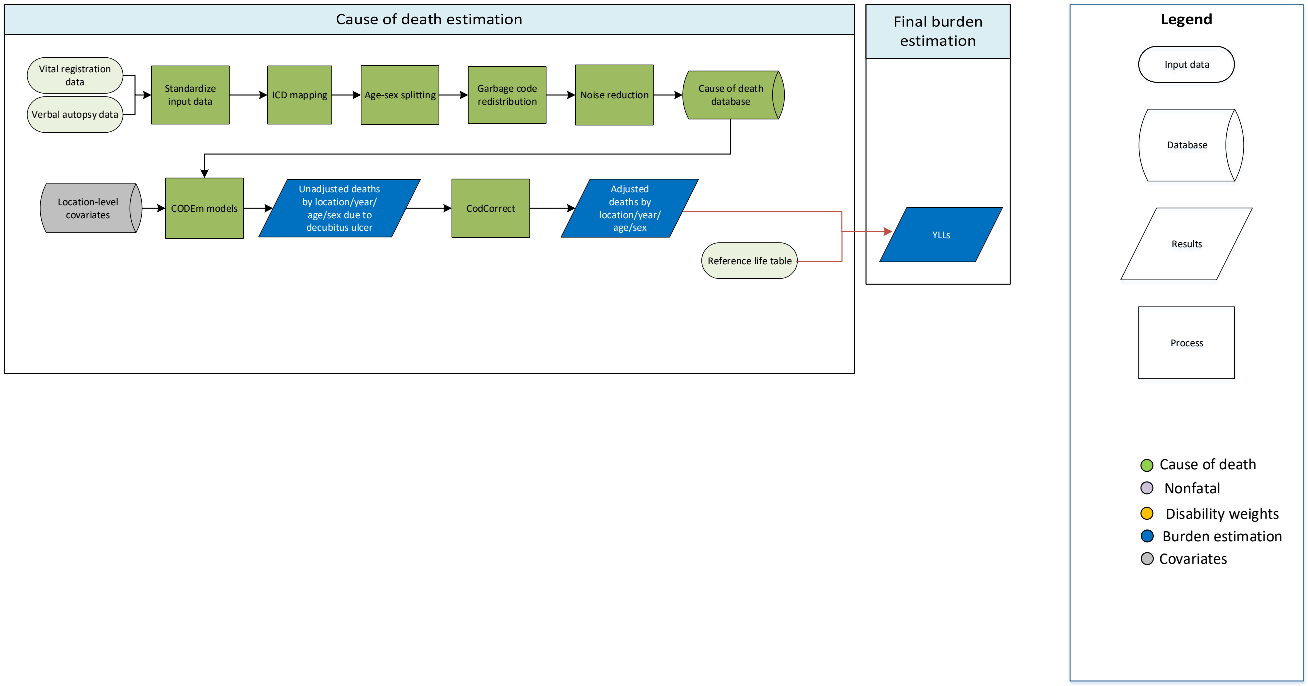

Input data and methodological summary for decubitus ulcer

Input data

Data used to estimate decubitus ulcer mortality consisted of vital registration sources and verbal autopsy sources from the cause of death (COD) database. Outlier criteria excluded datapoints that were implausibly high or low relative to global or regional patterns and data from countries with small populations.

Modelling strategy

We modelled deaths due to decubitus ulcer with a standard CODEm model using the cause of death database and location-level covariates as inputs. The model followed standard parameters. We hybridised separate global and data-rich models to acquire unadjusted results, which we finalised and adjusted using CoDCorrect to reach final years of life lost (YLLs) due to decubitus ulcer. Decubitus ulcer death estimates were also corrected for misclassification of Alzheimer's and Parkinson's disease deaths.

There were no significant changes in the modelling process between GBD 2019 and GBD 2021.

Table 1. Covariates used in decubitus ulcer mortality modelling

Level	Covariate	Direction
	Alcohol (liters per capita)	+

1	Prevalence of overweight and obesity	+
	Diabetes fasting plasma glucose (mmol/L), by age	+
	Improved water source (proportion with access)	-
2	Healthcare Access and Quality Index	–
	Cumulative cigarettes (5 years)	+
	Cumulative cigarettes (10 years)	+
	Smoking prevalence	+
3	Education (years per capita)	-
	Summary exposure variable (SEV) scalar for unsafe sanitation	+
	Socio-demographic Index	-
	Lag distributed income (per capita)	-

Alzheimer's disease and other dementias

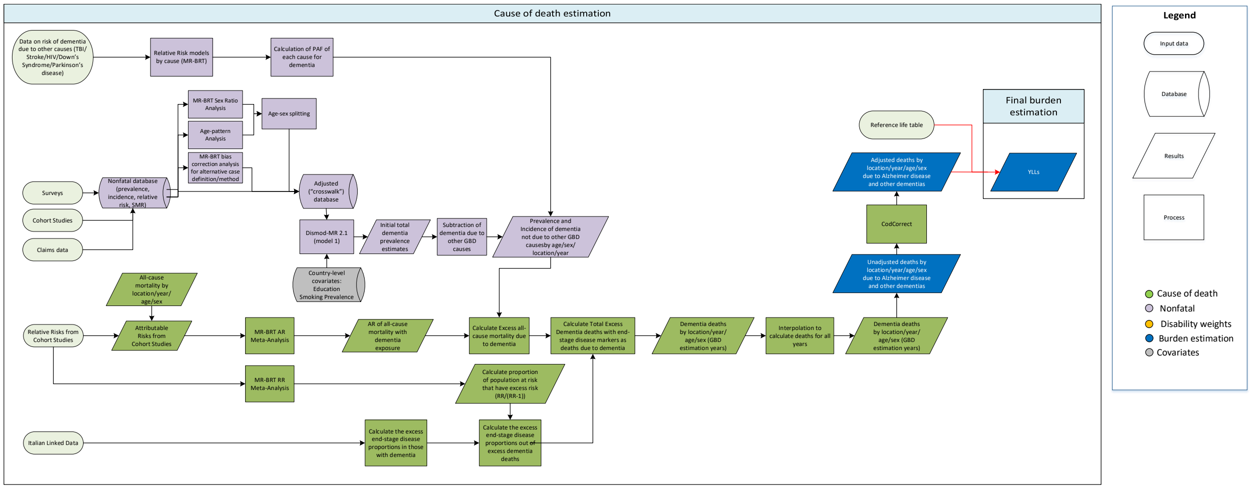

Input data

In GBD 2019 onward, fatal modelling was redesigned to remove reliance on vital registration data (described in more detail in the "Modelling strategy" section). Instead, two new source types were extracted:

(1) Literature on the relative risk of all-cause mortality given the exposure of dementia. Relative risk sources were identified through a systematic review using search terms[2] in PubMed. This yielded 4470 total hits, of which 34 studies were marked for extraction. Overall, the data were heterogeneous and varied in the exposure category measured (all dementia, Alzheimer's disease, cognitive impairment) and in the different factors controlled for in analyses.

(2) Linked vital registration and hospitalisation data. We used mortality records linked to inpatient records, covering all deaths from 2003 to 2017 in the Emilia-Romagna region of Italy.

Table 1: Results of systematic review on all-cause excess mortality with dementia

N		60
Region name (%)	East Asia	4 (6.7)
	Eastern sub-Saharan Africa	2 (3.3)
	High-income Asia Pacific	4 (6.7)
	High-income North America	22 (36.7)
	North Africa and Middle East	1 (1.7)
	Tropical Latin America	1 (1.7)
	Western Europe	26 (43.3)
Exposure (%)	Alzheimer's disease	11 (18.3)
	Cognitive impairment	10 (16.7)

	Other dementia	35 (58.3)
	Vascular dementia	4 (6.7)
Conducted in clinical setting (%)	Clinical setting	10 (16.7)
	Population representative	50 (83.3)
Controlled for education (%)	Controlled	32 (53.3)
	No control	28 (46.7)
Controlled for basic CVD info (%)	Controlled	33 (55.0)
	No control	27 (45.0)
Extensive CVD control (%)	Controlled	15 (25.0)
	No control	45 (75.0)
Controlled for smoking and alcohol (%)	Controlled	11 (18.3)
	No control	49 (81.7)
Controlled for factors in causal pathway (%)	Controlled	13 (21.7)
	No control	47 (78.3)

Modelling strategy

Overview

Dementia mortality rates have increased more than five-fold since 1980 in high-quality vital registration systems such as in the USA and Scandinavia. We have not seen an equivalent increase in prevalence and incidence data sources. If at all, there has been a modest decline in incidence and prevalence of dementia in studies in the UK and the USA.[1,2] Also, the greater than 20-fold variation in mortality rates of dementia between countries is much greater than the four-fold difference in prevalence and incidence between countries. As it is unlikely that case fatality from dementia has dramatically increased over the time period and that it would differ by a very large margin between countries, the hypothesis is that certifying and coding practices have changed over time and at a different pace between countries. To avoid spurious large trends over time in the fatal component of the burden of dementia, we decided for GBD 2013 to make dementia mortality rates consistent with the most recent rates relative to prevalence of countries that are most likely to certify or code dementia as an underlying cause of death. This approach was applied again for GBD 2017 with some modifications. For GBD 2019 onward, the fatal modelling process was redesigned to avoid the need for using estimates only from the highest dementia mortality locations. This was accomplished with an attributable risk model based on a systematic review of cohort studies and relative risk data, and end-stage disease proportions from linked hospital and death records. The modelling process is described below.

Modelling steps

Relative risk data

[1] Akushevich I, Kravchenko J, Ukraintseva S, Arbeev K, Yashin AI. Time trends of incidence of age-associated diseases in the US elderly population: Medicare-based analysis. *Age and ageing*. 2013 Jul 1;42(4):494-500.

[2] Matthews FE, Arthur A, Barnes LE, Bond J, Jagger C, Robinson L, Brayne C, Medical Research Council Cognitive Function and Ageing Collaboration. A two-decade comparison of prevalence of dementia in individuals aged 65 years and older from three geographical areas of England: results of the Cognitive Function and Ageing Study I and II. *The Lancet*. 2013 Nov 1;382(9902):1405-12.

First, using relative risk data extracted from studies identified by systematic review, we calculated attributable risk and the GBD estimate of all-cause mortality rate for a given study location and time, using the following formula:

$$Attributable\ Risk = (Relative\ Risk - 1) * All\text{-}Cause\ Mortality$$

We then conducted a meta-analysis on the attributable risk data, using covariates for age, sex, exposure category (all dementia, Alzheimer's disease, cognitive impairment), whether the study was conducted in a clinical sample, and categories indicating different types of variables that were controlled for in the component studies (educational attainment, cardiovascular disease comorbidities, smoking and alcohol consumption, and daily activities or residence in a nursing home). Relative risks were estimated using a second Bayesian bias-reduction meta-regression model and the same studies identified through systematic review. Regression results for relative risk and attributable risk analyses are displayed below.

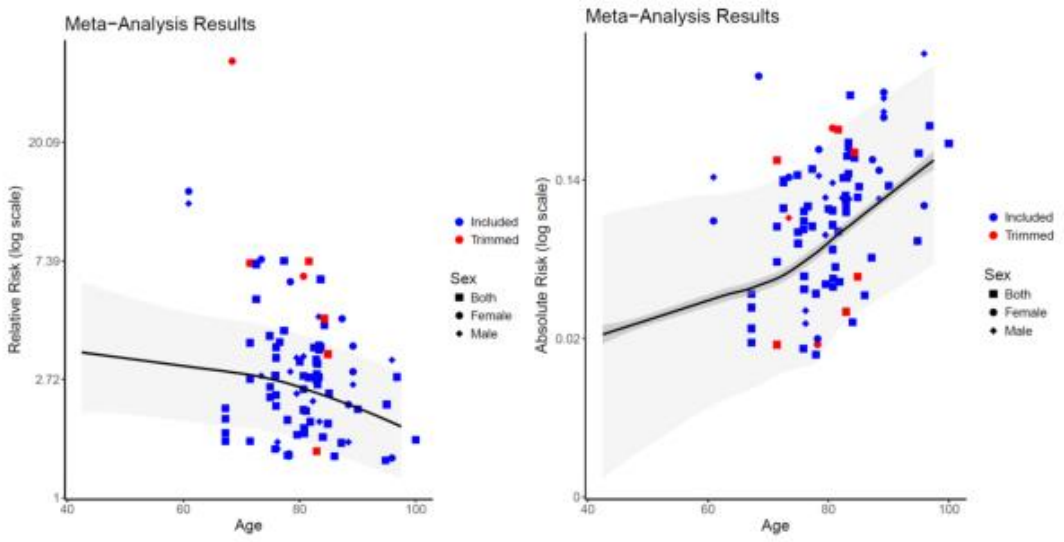

Meta-regression results were used to calculate the total number of excess deaths due to dementia as the product of our prevalence estimates (post-adjustment for dementia caused by other GBD diseases) and our estimates of attributable risk. See the non-fatal write-up on dementia for details on prevalence calculations.

Linked data

The excess deaths calculated through the multiplication of attributable risk and prevalence represent the total number of excess deaths due to having dementia, which likely includes deaths due to other conditions, such as cardiovascular diseases, that are more common in those with dementia as compared to the general population due to common underlying risk factors such as blood pressure, smoking, and lower educational attainment. In order to subset this total number of excess dementia deaths to calculate the number of deaths that were caused by dementia, we completed an analysis of linked clinical and mortality data. We used mortality records linked to inpatient records, covering all deaths from 2003 to 2017 in the Emilia-Romagna region of Italy. Using these data, we looked for markers of severe, end-stage disease in the clinical records up to one year before death.

To select these markers, for each ICD code that appeared in the data we calculated the difference in the proportion of individuals who died with dementia and had a record of each code in the year before death and the proportion of individuals who died without dementia and had a record of the same code in the year before death. We reviewed the 150 codes with the highest difference and selected codes that indicated end-stage disease, excluding codes for conditions such as cardiovascular disease. Codes for decubitus ulcer, malnutrition, sepsis, pneumonia, urinary tract infections, falling from bed, senility, dehydration, sodium imbalance, muscular wasting, bronchitis, dysphagia, hip fracture, and bedridden status were used as indicators of severe disease.

In order to determine the proportion of excess deaths that were caused by dementia, we calculated the proportion of dementia deaths that had clinical markers of end-stage disease in the year before death, above and beyond the occurrence of end-stage disease markers in those who died without dementia. The subtraction of the proportions with end-stage disease markers in those without dementia from the proportions in those with dementia represents the proportion of individuals who are assumed to have died with severe, end-stage dementia out of total deaths in those with dementia.

Figure 2: Percent of individuals who died with and without dementia with end stage conditions in the last year before death

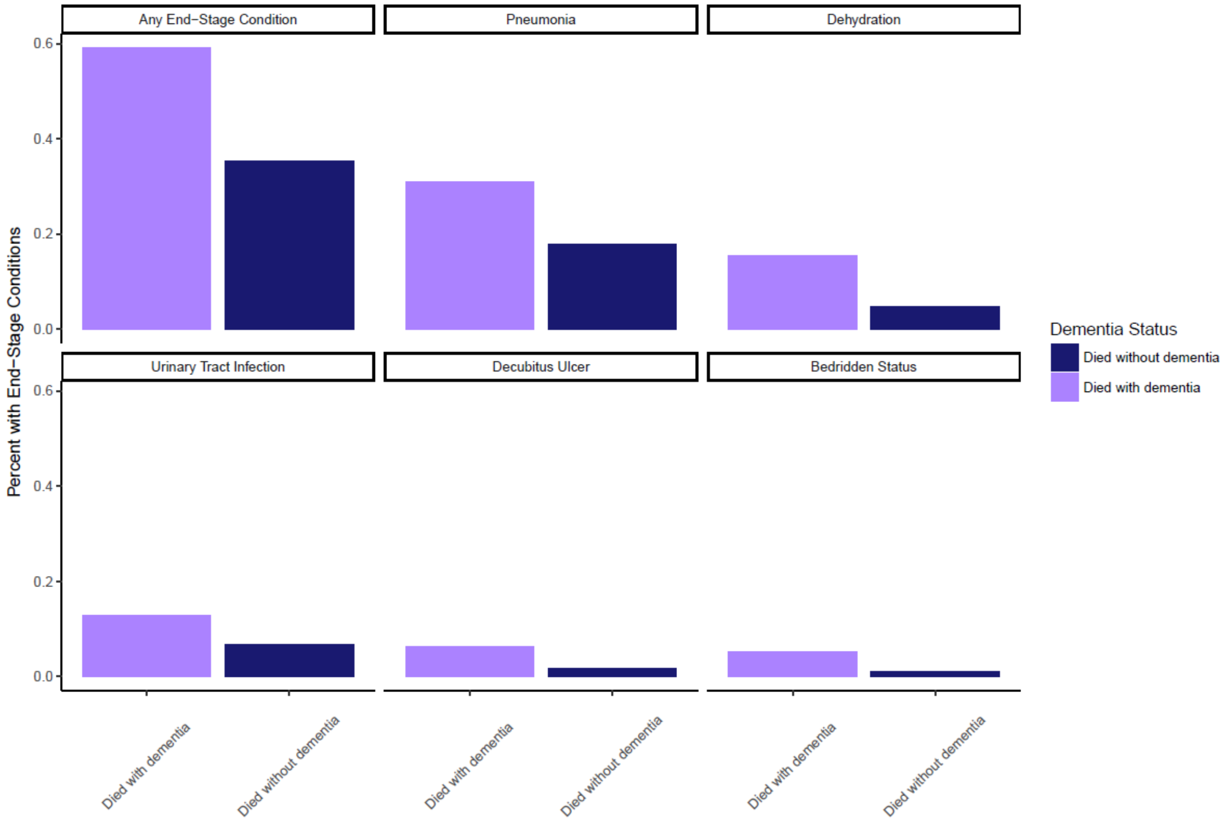

Calculation of deaths due to dementia

In order to apply these estimates to the total excess deaths we then adjusted these proportions to calculate the proportion of individuals who died with severe, end-stage dementia out of excess dementia deaths using the formula:

$$\frac{Died\ with\ Severe\ Disease}{Excess\ Dementia\ Deaths} = \frac{Died\ with\ Severe\ Disease}{Total\ Dementia\ Deaths} * \frac{Relative\ Risk}{Relative\ Risk - 1}$$

We then calculated the number of deaths due to dementia as the product of total excess dementia deaths and the proportion of those who died with severe disease out of excess dementia deaths. These final estimates of deaths due to dementia were then used to adjust data on causes of death from all other causes in vital registration systems.

Interpolation for all years

Finally, we used log-linear interpolation to interpolate these results (limited to 1990, 1995, 2000, 2005, 2010, 2015, 2017, 2019, 2020, 2021, and 2022) to create estimates for the entire time series from 1980 to 2022. Socio-demographic Index was used as a covariate to extrapolate back to the year 1980.

Dengue

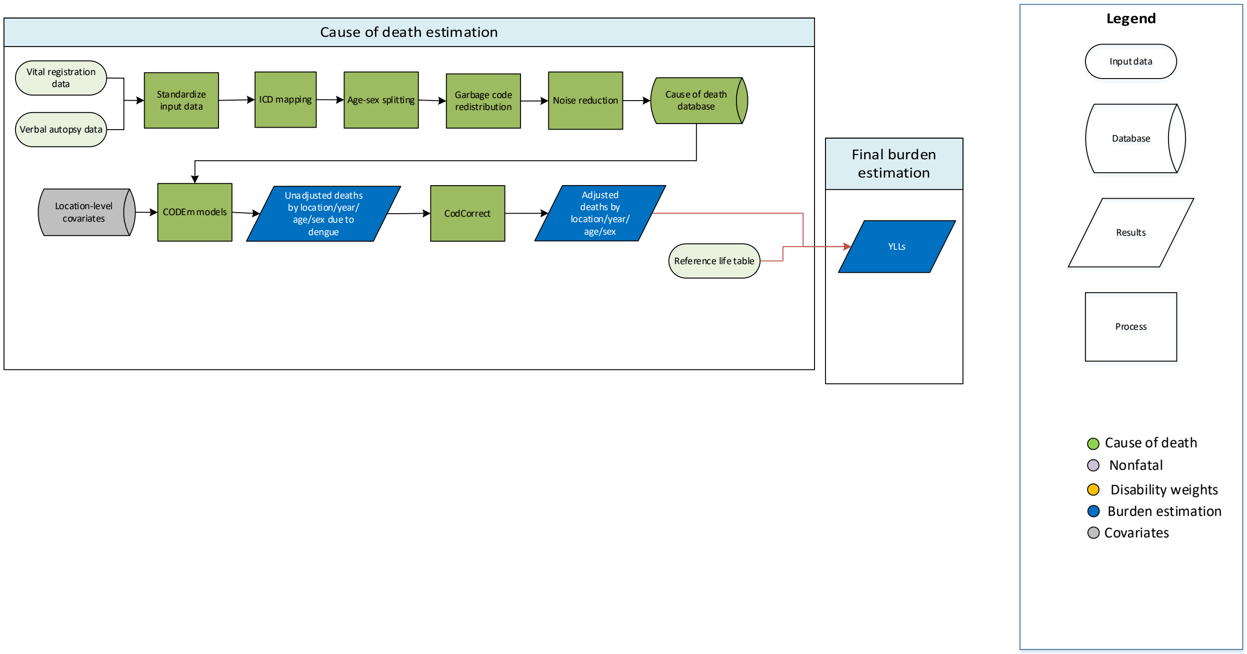

Input data

We modelled dengue mortality using all available data in the cause of death database. Datapoints were outliered if they reported an improbably low number of dengue deaths (eg, zero dengue deaths in a hyper-endemic country) or an improbably high number of dengue deaths.

Modelling strategy

We modelled dengue mortality using three-model hybrid approach: 1) a CODEm model of all dengue-endemic locations using all data in the CoD database for these locations; 2) a shocks model to account for outbreaks; and 3) estimates of mortality from imported cases in non-endemic, data-rich countries. Where dengue deaths were reported in non-endemic data-rich countries, we produced non-zero estimates by drawing from a beta distribution based on number of reported deaths and the underlying sample size.

We use country-level covariates to inform our model. The *Level* is the associated strength of relationship between the covariate and LRI mortality, ranked from 1 (proximally related) to 3 (distally related). The direction is the forced direction of the association between the covariate and dengue mortality (Table 1).

Table 1. CODEm model covariates and directions

Level	Covariate	Direction
1	Population density (over 1000 ppl/km^2, proportion)	+
	Population weighted probability of dengue transmission	+
2	Health system access	-
	Latitude under 15 (proportion)	+
	Elevation under 100 m (proportion)	+
	Rainfall quintile 4 (proportion)	+
	Rainfall quintile 5 (proportion)	+
	Dengue outbreaks (binary)	+
3	Education (years per capita)	-
	LDI (1$ per capita)	-

Geographical restrictions

We last updated the geographical restrictions for GBD 2019. The geographical restrictions determine whether a location is considered non-endemic (and, therefore, will have estimates based on the imported case model) in a given year. We derived our geographical restrictions for 2010 from Brady and colleagues(1). We have also refreshed our literature review to determine locations and years in which dengue was introduced or eliminated, to allow for time-varying geographical restrictions.

Changes from GBD 2019 to GBD 2021

As noted in Foreman and colleagues (2), there were changes in the way CoD data were processed in CODEm, specifically in applying a linear floor rate and calculation of sample variance. Due to these changes, the CODEm model for dengue switched to using only spatiotemporal Gaussian process regression (ST-GPR) models to build the predictions, whereas in GBD 2019 the ensemble model was a combination of both ST-GPR and linear mixed effects models. Additionally, in GBD 2019, to estimate dengue mortality in endemic locations, a CODEm model was fit to data from all locations, regardless of dengue endemicity, and predictions from this model were used for endemic locations. In GBD 2021, we restricted the CODEm model to fit only to data from dengue-endemic locations. These changes allowed the ensemble model to better reflect observed temporal trends in the underlying data.

References

1. Brady OJ, Gething PW, Bhatt S, Messina JP, Brownstein JS, Hoen AG, et al. Refining the Global Spatial Limits of Dengue Virus Transmission by Evidence-Based Consensus. PLoS Negl Trop Dis. 2012 Aug 7;6(8):e1760.

2. Foreman KJ, Lozano R, Lopez AD, Murray CJL. Modeling causes of death: an integrated approach using CODEm. *Popul Health Metr* 2012; **10:** 1.

Diabetes mellitus

Diabetes mellitus mortality was estimated for overall diabetes mellitus, diabetes mellitus type 1, and diabetes mellitus type 2 in GBD 2021.

Overall diabetes mellitus

Flowchart

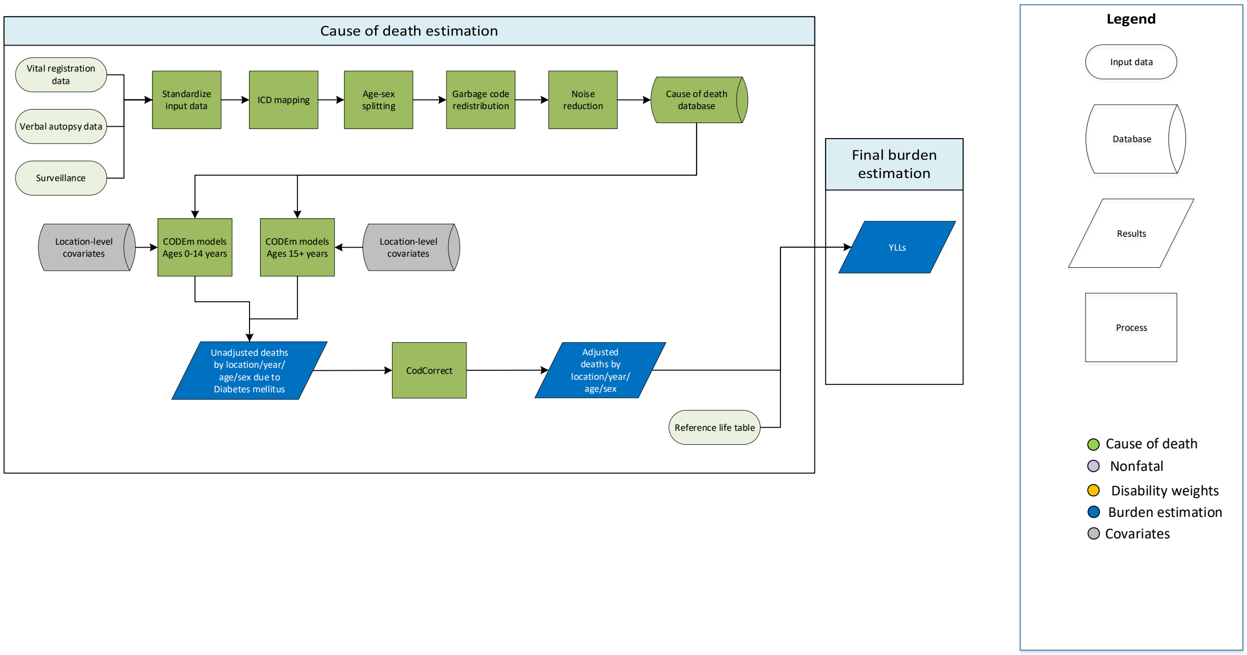

Input data and methodological summary for diabetes mellitus

Input data

Overall diabetes mellitus mortality was estimated using deaths directly attributed to diabetes mellitus. We used verbal autopsy and vital registration data as inputs into the model.

Verbal autopsy data: We outliered datapoints from sources where there were zero deaths estimated in an age group as this was not realistic for deaths due to diabetes and we determined that these data sources were unreliable.

Vital registration data: We outliered all data from the India Medical Certification of Cause of Death report since the source of the data were unreliable according to expert opinion. We also outliered ICD-9-BTL datapoints that were inconsistent with the rest of the data series and created unlikely time trends.

Modelling strategy

The Cause of Death Ensemble model (CODEm)[1] was used for deaths due to diabetes mellitus estimation. Additional information on CODEm methods can be found in appendix 1, section 3 of the reference article.

In the overall diabetes mellitus model, we used two models to estimate overall diabetes deaths with different age restrictions. This is because deaths in younger age groups are almost exclusively due to type 1 diabetes, while deaths in older ages are primarily due to type 2 diabetes. This allowed us to select predictive covariates that are specific to the pathophysiology of diabetes type 1 and type 2. We set the younger age model from 0 to 14 years and the older age model from 15 to 95+ years. We determined the age threshold based on evidence of the onset of diabetes type 2 occurring at younger ages.

Covariate selection

The following table lists the covariates included in the model. This requires that the covariate selected for the model must have the directional relationship with diabetes mellitus deaths. For GBD 2021, no significant updates were made for diabetes mellitus covariate selection. Covariate directions were selected based on the strength of the evidence.

Table 1. Covariates used in diabetes mellitus mortality modelling

Model	Level	Covariate	Direction
0-14 years	1	Healthcare Access and Quality Index	-
	2	Latitude	+
	2	Percentage of births occurring in women >35 years old	+
	2	Percentage of births occurring in women >40 years old	+
	3	Socio-demographic Index	-
	3	Education years per capita	-
15+ years	1	Age-standardised mean fasting plasma glucose (mmol/L)	+
	1	Age-standardised prevalence of diabetes	+
	1	Mean BMI	+
	1	Prevalence of obesity	+
	2	Mean cholesterol	+
	2	Mean systolic blood pressure	+
	2	Age- and sex-specific summary exposure variable for low fruit	-
	2	Unadjusted grams of sugar	+
	2	Age- and sex-specific summary exposure variable for low vegetables	-
	2	Age- and sex-specific summary exposure variable for alcohol use	+
	3	Healthcare Access and Quality Index	-
	3	Education years per capita	-
	3	Lag-distributed income per capita	+

Covariate influences

The following plots show the influence of each covariate on the four CODEm models (male global, male data-rich, female global, and female data-rich). A positive standardised beta (to the right) means that the covariate was associated with increased death. A negative standardised beta (to the left) means the covariate was associated with decreased death.

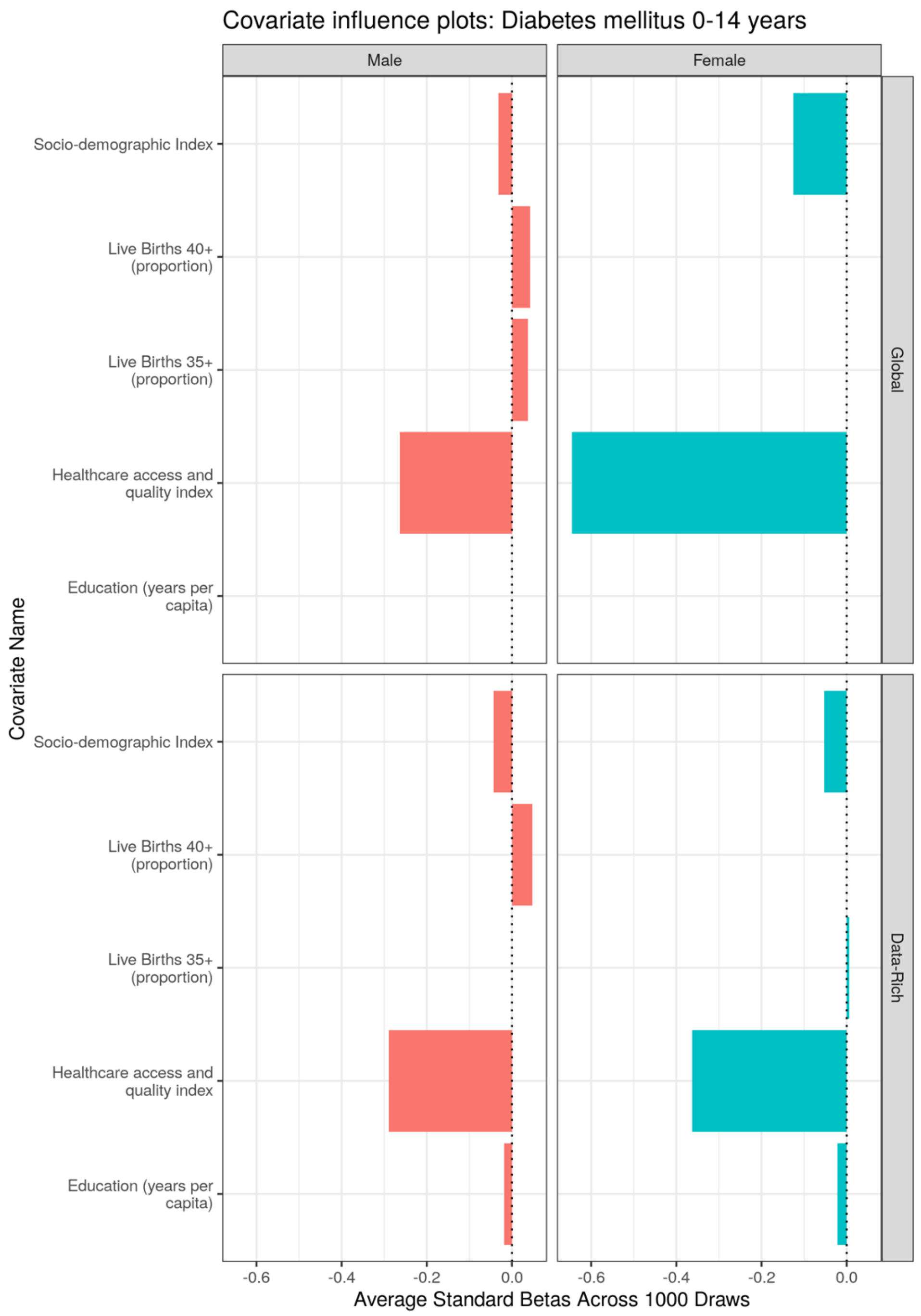

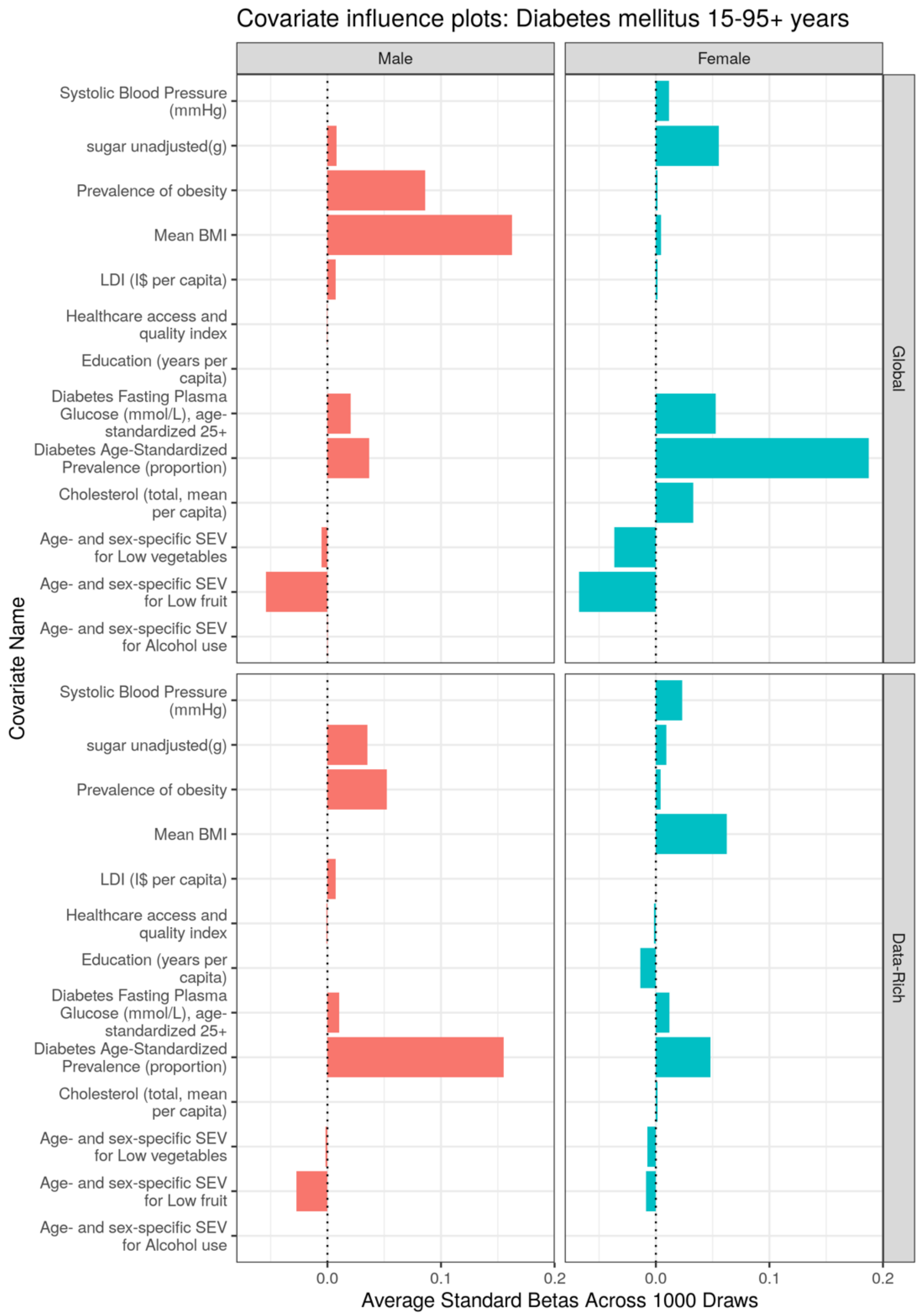
Covariate influence plots: Diabetes mellitus 15-95+ years
Male
Female
Global
Data-Rich
Covariate Name
Systolic Blood Pressure (mmHg)
sugar unadjusted(g)
Prevalence of obesity
Mean BMI
LDI (I$ per capita)
Healthcare access and quality index
Education (years per capita)
Diabetes Fasting Plasma Glucose (mmol/L), age-standardized 25+
Diabetes Age-Standardized Prevalence (proportion)
Cholesterol (total, mean per capita)
Age- and sex-specific SEV for Low vegetables
Age- and sex-specific SEV for Low fruit
Age- and sex-specific SEV for Alcohol use
0.0
0.1
0.2
Average Standard Betas Across 1000 Draws

Diabetes mellitus type 1 and type 2

Flowchart

Diabetes mellitus type 1

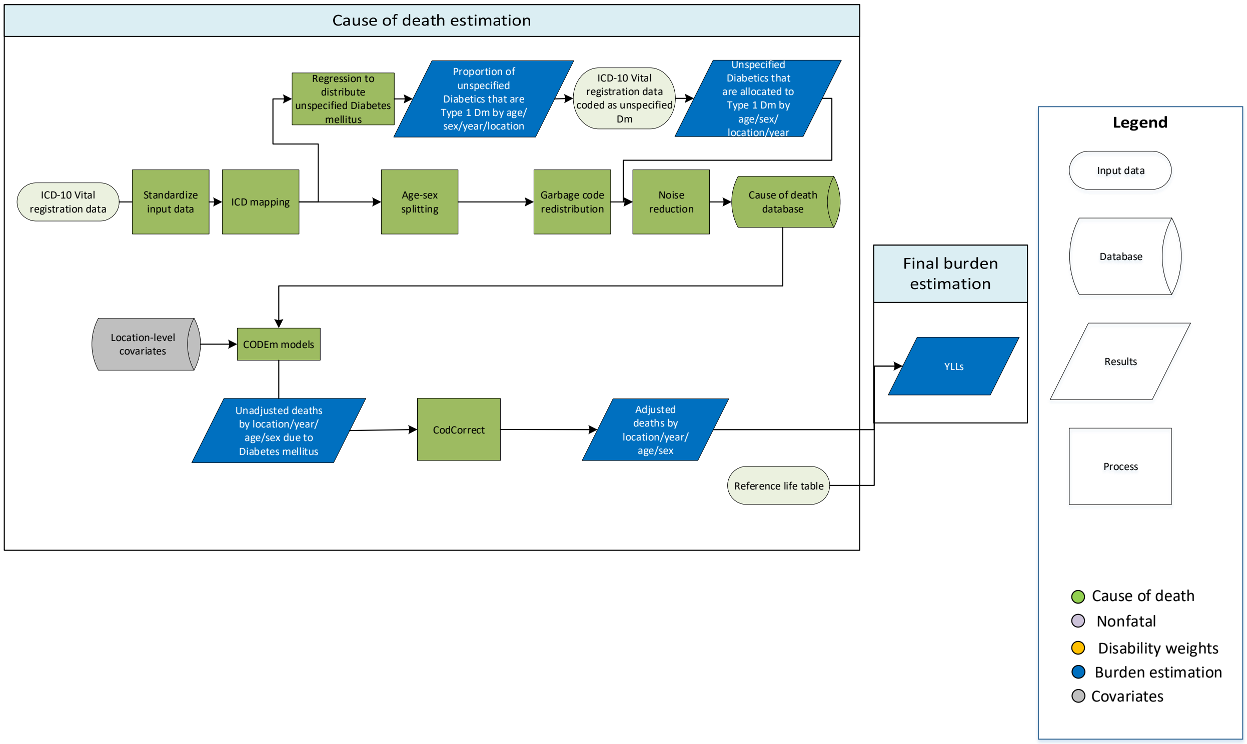

Diabetes mellitus type 2

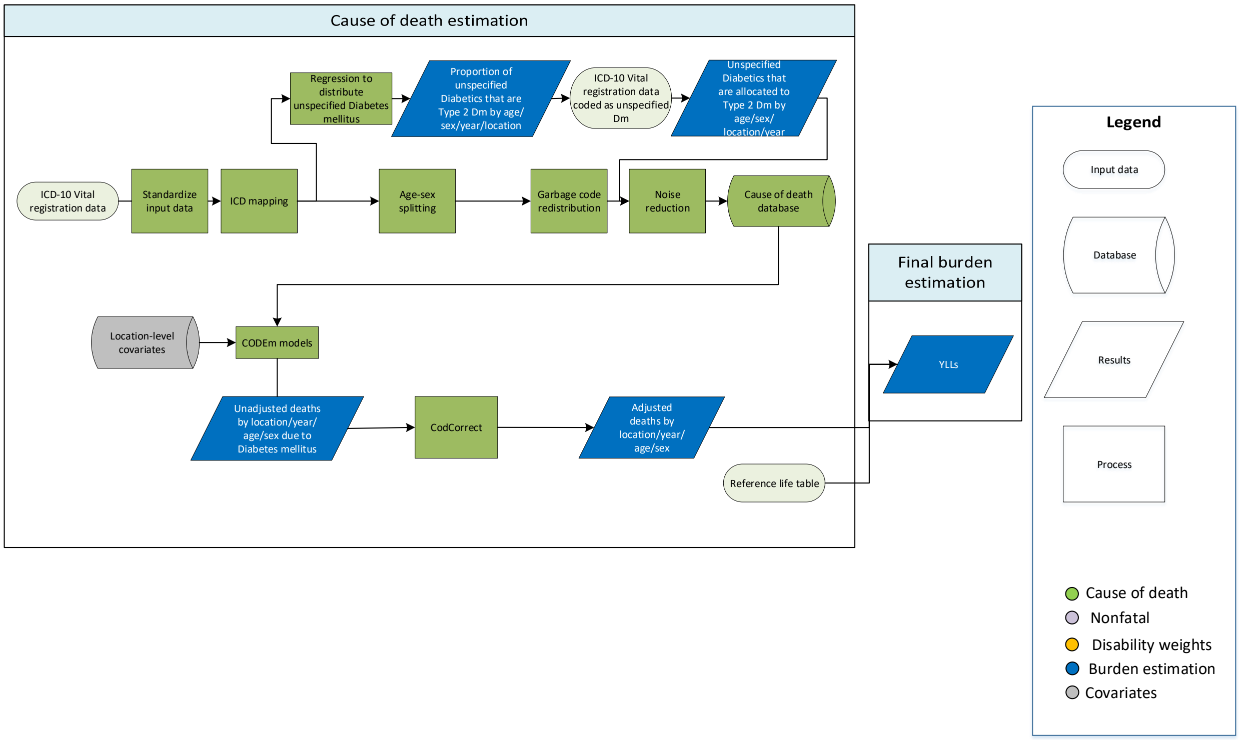

Input data and methodological summary for type 1 and type 2 diabetes mellitus

Input data

Type-specific diabetes mellitus mortality was estimated using deaths from vital registration sources in ICD-10 codes only. Diabetes type-specific information was not available in ICD-9 codes or deaths determined by verbal autopsy.

Modelling strategy

CODEm was used for deaths due to diabetes mellitus type-specific estimation.

Deaths in younger age groups are almost exclusively due to type 1 diabetes, while deaths in older ages are primarily due to type 2 diabetes. To account for this age pattern, we set the age range of the diabetes type 1 model to 0–95+ years and the age range of the diabetes type 2 model to 15–95+ years. We used the same covariates in the diabetes type 1 model and diabetes type 2 model as the 0–14 year and 15–95+ year in the overall diabetes models, respectively.

There were two unique data manipulation steps that occurred to prepare the data as part of the modelling process.

1. We assumed that all deaths <15 years were due to type 1 regardless of the ICD-10 code assigned to the death. We imposed 100% attribution of diabetes mellitus deaths in <15 years to type 1 diabetes mellitus.
2. ICD-10 diabetes data were reported as type 1, type 2, or unspecified. We assumed that all deaths in persons >50 years were unspecified regardless of the ICD-10 code assigned to the death because we found an unreasonably high proportion of deaths due to diabetes were assigned to type 1 diabetes. We developed a regression to estimate the fraction of unspecified diabetes mellitus that was type 1 and type 2. We only used data from 703 country-years to inform the regression. This is because these country-years had more than 50% of the deaths typed to type 1 or type 2 AND at least 70% of type-specific deaths in people >25 years were coded to type 2. Since there was a separate regression to estimate the proportion of type 1 diabetes mellitus and type 2 diabetes mellitus, we scaled the predicted proportions to 1. These scaled proportions were then applied to number of deaths coded to unspecified diabetes in each location, year, sex where ICD-10 data were reported.

<u>Regression equations</u>:

Type 1:

$$logit\left(\frac{number\ type\ 1\ DM}{number\ total\ DM}\right) \sim logit\left(\frac{number\ unspecified\ DM}{number\ total\ DM}\right) + \beta_1 age\ group + \beta_2 age\text{-}st\ prev\ obesity * age\ group + age\text{-}st\ prev\ obesity$$

Type 2:

$$logit\left(\frac{number\ type\ 2\ DM}{number\ total\ DM}\right) \sim logit\left(\frac{number\ unspecified\ DM}{number\ total\ DM}\right) + \beta_1 age\ group + \beta_2 age\text{-}st\ prev\ obesity * age\ group + age\text{-}st\ prev\ obesity$$

Covariate selection

The following are the covariates included in the model. We selected the same covariates for the type 1 diabetes model as the 0–14-year diabetes parent model and the type 2 diabetes model as the 15–95+ year diabetes parent model. For GBD 2021, no significant updates were made for the type-specific diabetes covariate selection. Covariate directions were selected based on the strength of the evidence.

Table 2. Covariates used in diabetes mellitus type 1 and type 2 mortality modelling

Model	Level	Covariate	Direction
Type 1	1	Healthcare Access and Quality Index	-
	2	Latitude	+
	2	Percentage of births occurring in women >35 years old	+
	2	Percentage of births occurring in women >40 years old	+
	3	Socio-demographic Index	-
	3	Education years per capita	-
Type 2	1	Age-standardised mean fasting plasma glucose (mmol/L)	+
	1	Age-standardised prevalence of diabetes	+
	1	Mean BMI	+
	1	Prevalence of obesity	+
	2	Mean cholesterol	+
	2	Mean systolic blood pressure	+
	2	Age- and sex-specific summary exposure variable for low fruit	-
	2	Unadjusted grams of sugar	+
	2	Age- and sex-specific summary exposure variable for low vegetables	-
	2	Age- and sex-specific summary exposure variable for alcohol use	+
	3	Healthcare Access and Quality Index	-
	3	Education years per capita	-
	3	Lag-distributed income per capita	+

Covariate influences

The following plots show the influence of each covariate on the four CODEm models (male global, male data-rich, female global, and female data-rich). A positive standardised beta (to the right) means that the covariate was associated with increased death. A negative standardised beta (to the left) means the covariate was associated with decreased death.

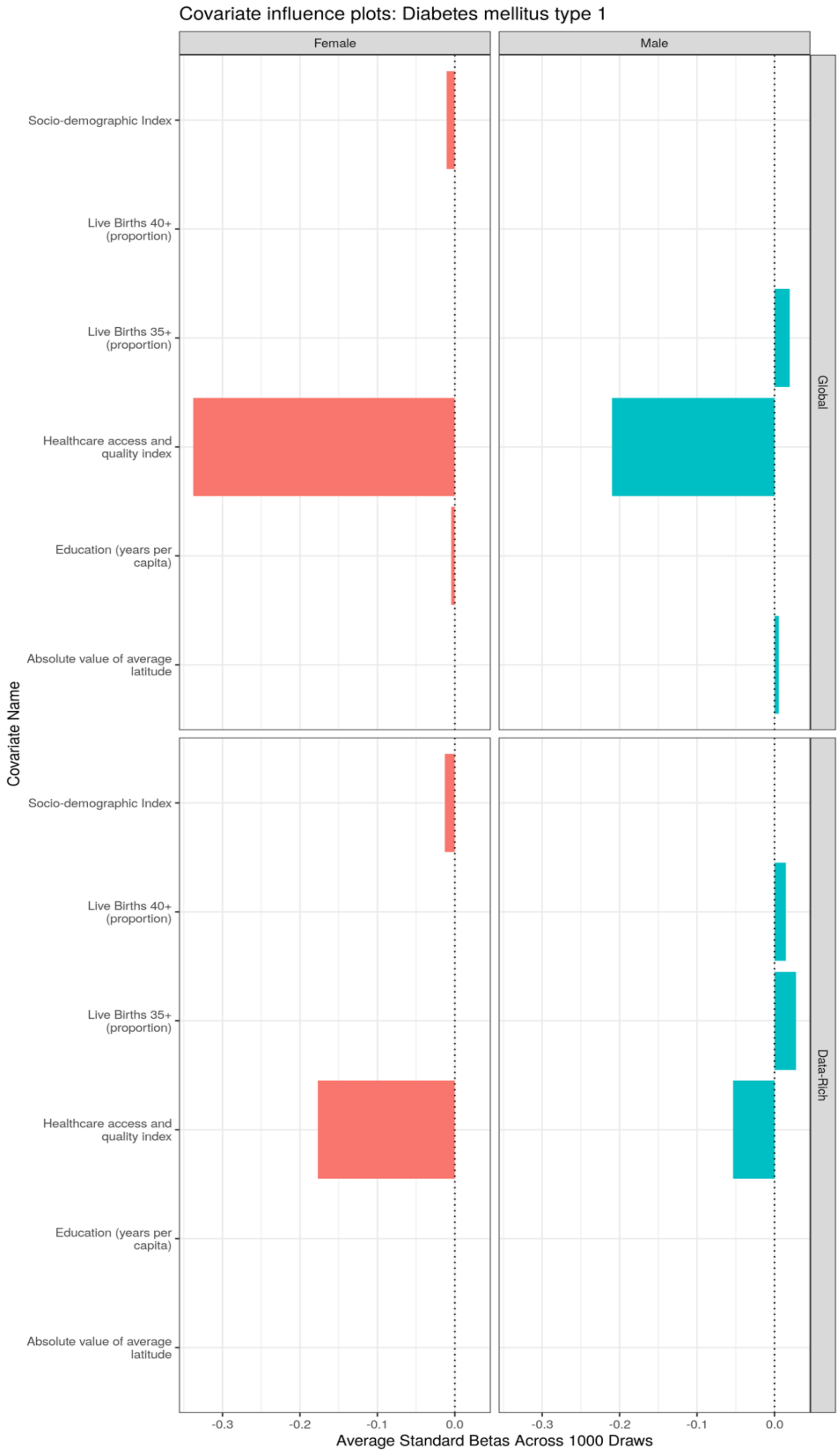

Covariate influence plots: Diabetes mellitus type 1
Female
Male
Global
Data-Rich
Socio-demographic Index
Live Births 40+ (proportion)
Live Births 35+ (proportion)
Healthcare access and quality index
Education (years per capita)
Absolute value of average latitude
Covariate Name
-0.3
-0.2
-0.1
0.0
Average Standard Betas Across 1000 Draws

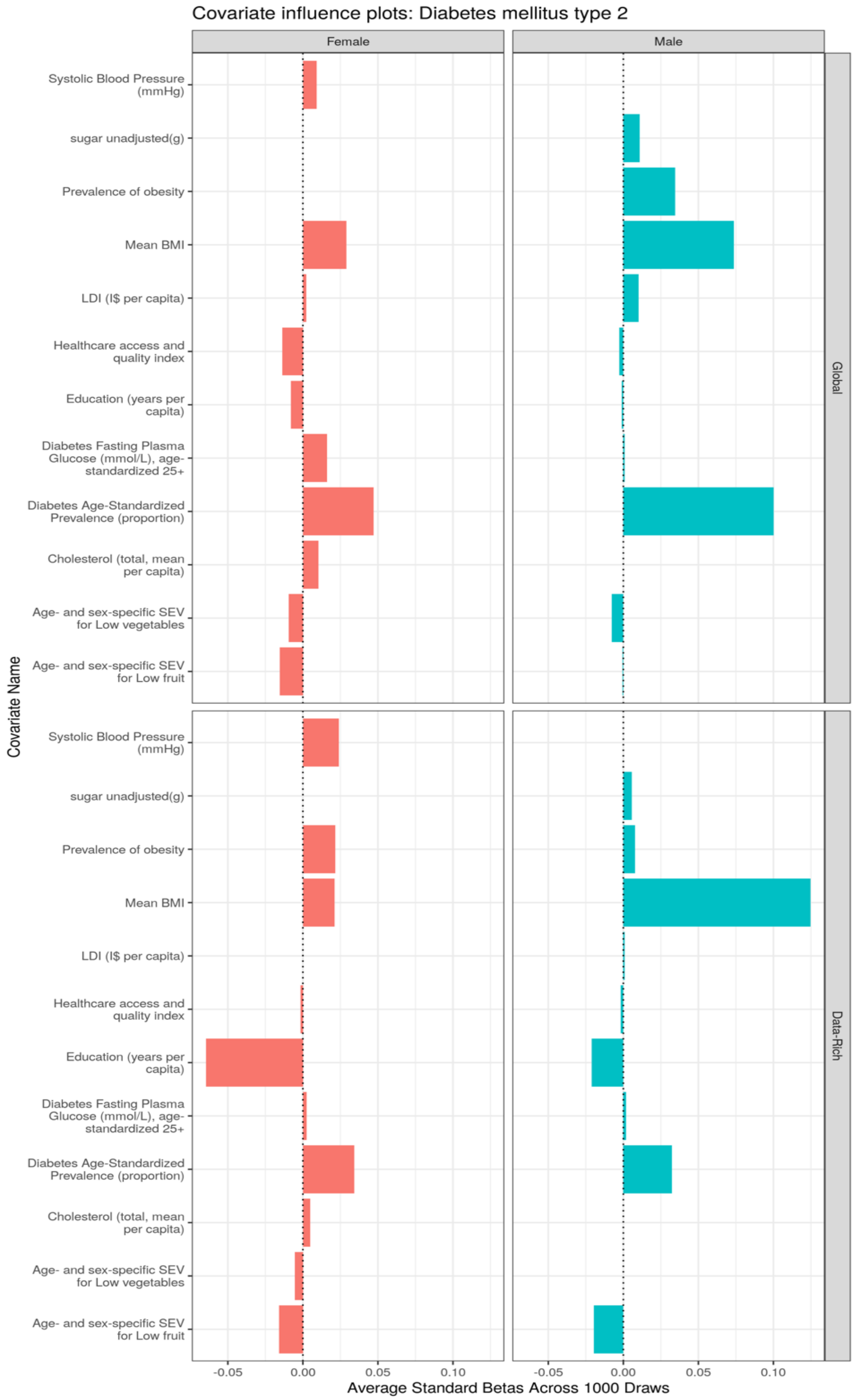
Covariate influence plots: Diabetes mellitus type 2
Female
Male
Global
Data-Rich
Covariate Name
Systolic Blood Pressure (mmHg)
sugar unadjusted(g)
Prevalence of obesity
Mean BMI
LDI (I$ per capita)
Healthcare access and quality index
Education (years per capita)
Diabetes Fasting Plasma Glucose (mmol/L), age-standardized 25+
Diabetes Age-Standardized Prevalence (proportion)
Cholesterol (total, mean per capita)
Age- and sex-specific SEV for Low vegetables
Age- and sex-specific SEV for Low fruit
-0.05
0.00
0.05
0.10
Average Standard Betas Across 1000 Draws

References

[1]GBD 2019 Diseases and Injuries Collaborators. Global burden of 369 diseases and injuries in 204 countries and territories, 1990–2019: a systematic analysis for the Global Burden of Disease Study 2019. Lancet 2020; 396: 1204–22. doi: https://doi.org/10.1016/S0140-6736(20)30925-9

Diarrhoeal diseases

Flowchart

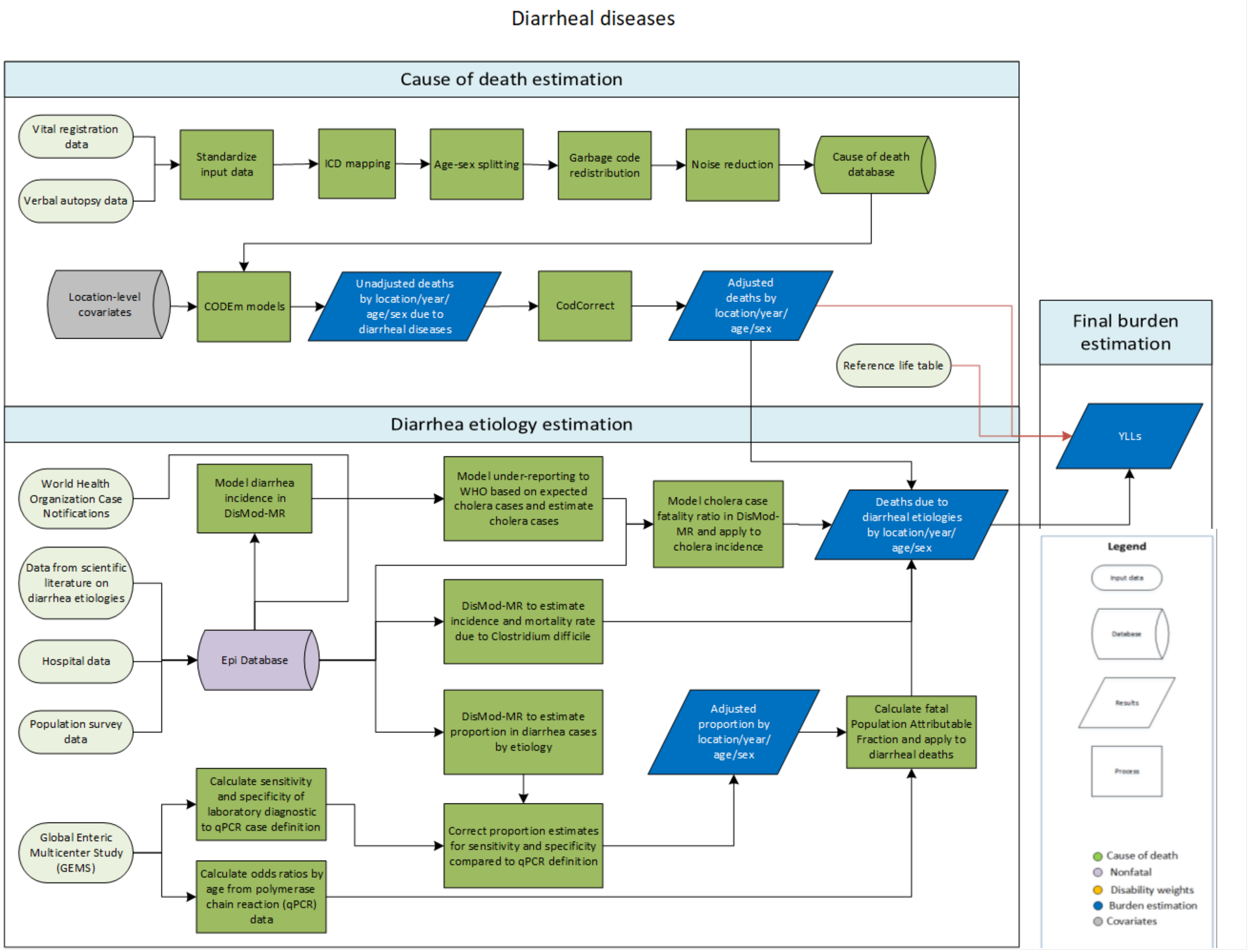

Diarrhoeal diseases are a cause of death in GBD. We also estimated the attributable deaths from 13 diarrhoeal aetiologies using an independent modelling strategy. These pathways are shown in the flowchart above and will be described in this report.

Input data

Cause of death. We used all available data from vital registration systems, surveillance systems, and verbal autopsy. Datapoints that violated well-established age or time trends were determined to be outliers. We also excluded early neonatal mortality data in the Philippines (1994–1998), India Civil Registration System data, and medically certified cause of death (MCCD) data in all states (1986–2013).

Aetiologies. The second type of data describe diarrhoea aetiologies. There are 13 aetiologies in GBD 2019 for diarrhoea: adenovirus, *Aeromonas, Campylobacter, Vibrio cholerae, Clostridium difficile, Cryptosporidium*, *Entamoeba histolytica,* typical enteropathogenic *E. coli* (typical EPEC), heat-stable toxin-producing enterotoxigenic *E. coli* (ST-ETEC), norovirus, rotavirus, non-

typhoidal Salmonella, and Shigella. We extracted data on all aetiologies except *C. difficile* from scientific literature that reported the proportion of diarrhoea cases that tested positive for each pathogen. We completed a systematic literature review covering the period May 2018 to February 2019 for diarrhoea prevalence, incidence, and all diarrhoea aetiologies. Inclusion criteria included diarrhoea as the case definition, studies with a sample size of at least 100, and studies with at least one year of follow-up. We excluded studies that reported on diarrhoeal outbreaks exclusively and those that used acute gastroenteritis with or without diarrhoea.

We searched articles using a PubMed search term that combined nonspecific and aetiology-specific diarrhoea in February 2019 using the following search string:

(diarrhoea[title/abstract] OR diarrhea[title/abstract]) AND (2018/07/30:2019/2/7[PDat]) AND Humans[MeSH Terms] AND (incidence[title/abstract] OR prevalence[title/abstract] OR epidemiology[title/abstract] OR salmonella[title/abstract] OR aeromona[title/abstract] OR shigell*[title/abstract] OR enteropathogenic[title/abstract] OR enterotoxigenic[title/abstract] OR campylobacter[title/abstract] OR amoebiasis[title/abstract] OR entamoeb*[title/abstract] OR cryptosporid*[title/abstract] OR rotavirus[title/abstract] OR norovirus[title/abstract] OR adenovirus[title/abstract] OR etiology[title/abstract]) NOT (appendicitis[title/abstract] OR esophag*[title/abstract] OR surger*[title/abstract] OR gastritis[title/abstract] OR liver[title/abstract] OR case report[title] OR case-report[title] OR therapy[title] OR treatment[title] Crohn[title/abstract] OR "inflammatory bowel"[title/abstract] OR irritable[title/abstract] OR travel*[title] OR Outbreak[title] OR Review[ptyp] OR vomiting[title/abstract).*

We identified 82 studies, of which three met our inclusion criteria. We extracted data for location, sex, year, and age.

We used the Global Enteric Multicenter Study (GEMS), a seven-site, case-control study of moderate-to-severe diarrhoea in children under 5 years,[1] and the MAL-ED study,[2] a multi-site birth cohort, to calculate odds ratios for the diarrhoeal pathogens. We analysed raw data for a systematic reanalysis, representative of the distribution of cases and controls by age and site that were tested for the presence of pathogen using quantitative polymerase chain reaction (qPCR).[3]

Data that did not use qPCR for detection were adjusted for sensitivity and specificity prior to modelling in order to standardise data regardless of detection method. Adjusting these data prior to modelling allowed us to adjust only data that did not use qPCR, as well as better control for values at extreme bounds, and capture uncertainty in modelling.

Modelling strategy

Cause of death. Diarrhoeal disease mortality was estimated in the Cause of Death Ensemble modelling platform (CODEm). We estimated diarrhoea mortality separately for males and

females and for children under 5 years and older than 5 years. We used country-level covariates to inform our CODEm models (**Table 1**).

In past GBD cycles, estimates of rotavirus vaccine coverage among infants in the modelled year were used as the primary covariate for this linear regression. In GBD 2021, we now use a lagged mean of rotavirus vaccine coverage calculated over a rolling, five-year interval in order to capture population-level vaccine-derived immunity among under-5-year-olds, including coverage both in the current year and in recent years.

Table 1. Covariates used in diarrhoea mortality modelling. Table 1A shows the covariates used in the 0–4 years model, and Table 2B shows the covariates used in the 5–95+ years model. The *Level* represents the strength of the association between the covariate and diarrhoea mortality from 1 (proximally related) to 3 (distally related). The *Direction* indicates the positive or negative association between the covariate and diarrhoea mortality. **Table 1A. Covariates used in the 0–4 years model**

Level	Covariate	Direction
1	Oral rehydration solution treatment	-
	Safe sanitation access	-
	Improved water source access	-
	Rotavirus vaccine coverage, lagged	-
	Unsafe sanitation SEV	+
	Unsafe water SEV	+
	Child underweight SEV	+
	Child stunting SEV	+
	Child wasting SEV	+
2	Vitamin A deficiency	+
	Zinc deficiency	+
	Zinc treatment for diarrhoea	-
	Healthcare Access and Quality Index	-
	No handwashing facility access	-
3	Lag distributed income (LDI) per capita	-
	Maternal education years	-
	Nonexclusive breastfeeding SEV	-
	Socio-demographic Index (SDI)	-
	Population density greater than 1000/km^2	+

Table 1B. Covariates used in the 5–95+ years model

Level	Covariate	Direction
1	Diarrhoea summary exposure value (SEV)	+
	Unsafe sanitation SEV	+
	Unsafe water SEV	+
	Safe sanitation access	-

	Improved water source access	-
2	Healthcare Access and Quality Index	-
	Rotavirus vaccine coverage, lagged	-
	No handwashing facility access	+
	Oral rehydration access	-
3	Education years per capita	-
	LDI per capita	-
	Adult underweight	+
	SDI	-
	Population density greater than 1000/km^2	+

Aetiologies. We estimated diarrhoeal disease aetiologies independently from overall diarrhoea mortality using a counterfactual strategy for enteric adenovirus, *Aeromonas*, *Entamoeba histolytica* (amoebiasis), *Campylobacter*, *Cryptosporidium*, typical EPEC, enterotoxigenic *Escherichia coli* (ETEC), norovirus, non-typhoidal Salmonella infections, rotavirus, and Shigella. *Vibrio cholerae* and *C. difficile* were modelled separately.

Diarrhoeal aetiologies are attributed to diarrhoeal deaths using a counterfactual approach. We calculated a population attributable fraction (PAF) from the proportion of severe diarrhoea cases that are positive for each aetiology. The PAF represents the relative reduction in diarrhoea mortality if there was no exposure to a given aetiology. As diarrhoea can be caused by multiple pathogens and the pathogens may co-infect, PAFs can overlap and are not scaled to sum to 100%. We calculated the PAF from the proportion of severe diarrhoea cases that are positive for each aetiology. We assumed that hospitalised diarrhoea cases are a proxy of severe and fatal cases. We used the following formula to estimate PAF:[4]

$$PAF = Proportion * (1 - \frac{1}{OR})$$

Where *Proportion* is the proportion of diarrhoea cases positive for an aetiology and *OR* is the odds ratio of diarrhoea given the presence of the pathogen.

We dichotomised the continuous qPCR test result using the value of the cycle threshold (Ct) that most accurately discriminated between cases and controls. The Ct values range from 0 to 35 cycles, representing the relative concentration of the target gene in the stool sample. A low value indicates a higher concentration of the pathogen while a value of 35 indicates the absence of the target in the sample. We used the lower Ct value when we had multiple Ct values for the cutpoint. The case definition for each pathogen is a Ct value that is below the established cutoff point.

We used a mixed effects conditional logistic regression model to calculate the odds ratio for under 1 year and 1–4 years old for each of our pathogens. The stool samples from cases and controls in GEMS were used exclusively to calculate these odds ratios as we assumed that the

association between pathogens and moderate-to-severe diarrhoea is a proxy for fatal outcomes. The odds ratio for 1–4 years was applied to all GBD age groups over 5 years. There were three pathogen-age odds ratios that were not statistically significant: *Aeromonas* and amoebiasis in under 1 year and *Campylobacter* in 1–4 years. The mean value of the odds ratio was above 1 in all three cases, so we transformed the odds ratios for these three exceptions only in log space such that exponentiated values could not be below 1. The transformation was:

Odds ratio = exp(log(OR) – 1)) + 1

We modelled the proportion data using the Bayesian meta-regression tool DisMod-MR to estimate the proportion of positive diarrhoea cases for each separate aetiology by location/year/age/sex and to adjust for the covariates. We used the estimated sensitivity and specificity of the original laboratory diagnostic test results from the pooled GEMS and MAL-ED qPCR stool samples compared to the qPCR test result to adjust our proportion before we modelled the proportions:[5]

$$Proportion_{True} = \frac{(Proportion_{Observed} + Specificity - 1)}{(Sensitivity + Specificity - 1)}$$

We used this correction to account for the fact that the proportions we used are based on a new test that is not consistent with the laboratory-based case definition (qPCR versus GEMS conventional laboratory testing for pathogens).[6] Because differences in the type of PCR used in the original (non-reference qPCR diagnostic) between GEMS and MAL-ED in detecting norovirus, we combined the sensitivity and specificity results for norovirus such that 50% of the draws were coming from GEMS test results exclusively and 50% of the draws were coming from MAL-ED test results exclusively. Additionally, because the original laboratory diagnostic technique used for *Campylobacter* in MAL-ED was one not commonly used, we only used GEMS to determine the sensitivity and specificity of bacterial culture compared to qPCR in detecting *Campylobacter.*[7]

Our literature review extracted the proportion of any EPEC without differentiating between typical (tEPEC) and atypical (aEPEC). In order to be consistent with the odds ratios that we obtained, we adjusted our proportion estimates of any EPEC to typical EPEC only. This adjustment was informed by a subset of our literature review that reported both atypical and typical EPEC. We estimated a ratio by super-region of tEPEC to any EPEC and adjusted our proportion estimates accordingly. We found that the majority of EPEC diarrhoea cases were positive for atypical EPEC, consistent with other published work.[8] We applied the same approach to differentiate between heat-stable toxin (ST) and heat-labile toxin producing (LT) ETEC. For the first time, GBD 2019 split these serotypes so that estimates in GBD 2019 represent the diarrhoeal disease burden attributable to ST-ETEC. This was based on work showing that ST-ETEC was much more pathogenic than LT-ETEC. As our proportion data were extracted for any ETEC, we determined a proportion of all ETEC that produced ST from the

GEMS and MAL-ED studies and applied that ratio to our input data so that they represented ST-ETEC only. We re-estimated the sensitivity and specificity values as well as the odds ratios for our new definition of ST-ETEC.

For *Vibrio cholerae* (cholera), we used the literature review to estimate the expected number of cholera cases for each country-year using the incidence of diarrhoea (estimated using DisMod-MR) and the proportion of diarrhoea cases that are positive for cholera. We assigned cholera PAF using odds ratios from the qPCR results to estimate a number of cholera-attributable cases. We compared this expected number of cholera cases to the number reported to the World Health Organization at the country-year level.[9] We modelled the under-reporting fraction to correct the cholera case notification data for all countries using health system access and the diarrhoea SEV scalar to predict total cholera cases. We used the age-specific proportion of positive cholera samples in DisMod-MR and our incidence estimates to predict the number of cholera cases for each age/sex/year/location. Finally, we modelled the case fatality ratio of cholera using DisMod-MR and estimated the number of cholera deaths.

For *C. difficile*, we modelled incidence and mortality in DisMod-MR for each age, sex, year, location. DisMod-MR is a Bayesian meta-regression tool that uses spatiotemporal information as priors to estimate prevalence, incidence, remission, and mortality for *C. difficile* infection. DisMod-MR uses a compartmental model to relate prevalence, incidence, remission, and mortality. We set remission in our model to 1 month.

For rotavirus, we made a change to the process of estimating attributable fraction to explicitly account for rotavirus vaccine efficacy in GBD 2019. The impact of the rotavirus vaccine is dependent on modelled vaccine coverage for a location-year and on the rotavirus vaccine efficacy (VE). There are numerous studies that demonstrate a difference in VE by location.[10] We determined that SDI was the best predictor of rotavirus VE, and we used a meta-regression with this covariate to predict the rotavirus VE by location where the VE was higher in areas with larger SDI values and followed a logit-linear distribution.

For GBD 2019, we explicitly incorporated the results from our analysis of VE to produce more robust estimates of the proportion of diarrhoea that has rotavirus over time and space. We assumed that the impact of the vaccine can be represented as one minus the product of the estimated vaccine coverage and VE.

$$Vaccine\ impact = 1 - vaccine\ coverage * vaccine\ efficacy$$

Both of these values vary in time and space but not by age. To avoid discontinuities in our model, we adjusted the input proportion data to remove the impact of the rotavirus vaccine by dividing the observed proportion by the vaccine impact.

$$Rotavirus\ proportion_{Adjusted} = \frac{Rotavirus\ proportion}{1 - Cov_{RotaV} * VE_{Modeled}}$$

The result is the modelled proportion of diarrhoea positive for rotavirus in the absence of the vaccine. This modelled value is then multiplied by the impact of the rotavirus vaccine to determine the estimated proportion of diarrhoea positive for rotavirus in the presence of the vaccine. Our modified attributable fraction is then:

$$DisModPAF = Modelled\ Proportion\ (from\ DisMod)\ * \left(1 - \frac{1}{OR}\right)$$

The last step is to account for the expected impact of the rotavirus vaccine. We do this using the equation below:

$$PAF_{Rota} = DisModPAF * \frac{(1 - Cov_{RotaV} * VE_{Modeled})}{(1 - DisModPAF * Cov_{RotaV} * VE_{Modeled})}$$

Where the final attributable fraction for rotavirus is the product of the PAF estimated in DisMod-MR and the expected reduction in that PAF given modelled vaccine coverage and modelled VE by location-year, and this value is only applied to children 28 days to 5 years old. The product of the rotavirus attributable fraction and the number of deaths or cases of diarrhoea is the number of deaths and cases caused by rotavirus.

References

1 Kotloff KL, Nataro JP, Blackwelder WC, *et al.* Burden and aetiology of diarrhoeal disease in infants and young children in developing countries (the Global Enteric Multicenter Study, GEMS): a prospective, case-control study. *Lancet Lond Engl* 2013; **382**: 209–22.

2 Platts-Mills J, Liu J, Rogawski E. Aetiology, burden and clinical characteristics of diarrhoea in children in low-resource settings using quantitative molecular diagnostics: results from the MAL-ED cohort study. *Lancet Glob Health* 2018; : Accepted.

3 Liu J, Gratz J, Amour C, *et al.* A laboratory-developed TaqMan Array Card for simultaneous detection of 19 enteropathogens. *J Clin Microbiol* 2013; **51**: 472–80.

4 Miettinen OS. Proportion of disease caused or prevented by a given exposure, trait or intervention. *Am J Epidemiol* 1974; **99**: 325–32.

5 Reiczigel J, Földi J, Ozsvári L. Exact confidence limits for prevalence of a disease with an imperfect diagnostic test. *Epidemiol Infect* 2010; **138**: 1674–8.

6 Platts-Mills JA, Operario DJ, Houpt ER. Molecular diagnosis of diarrhea: current status and future potential. *Curr Infect Dis Rep* 2012; **14**: 41–6.

7 Platts-Mills JA, Liu J, Gratz J, *et al.* Detection of Campylobacter in stool and determination of significance by culture, enzyme immunoassay, and PCR in developing countries. *J Clin Microbiol* 2014; **52**: 1074–80.

8 Ochoa TJ, Barletta F, Contreras C, Mercado E. New insights into the epidemiology of enteropathogenic Escherichia coli infection. *Trans R Soc Trop Med Hyg* 2008; **102**: 852–6.

9 World Health Organization. Global Health Observatory data repository: Cholera. 2016. http://apps.who.int/gho/data/node.main.174?lang=en (accessed Aug 25, 2016).

10 Lamberti LM, Ashraf S, Walker CLF, Black RE. A Systematic Review of the Effect of Rotavirus Vaccination on Diarrhea Outcomes Among Children Younger Than 5 Years. *Pediatr Infect Dis J* 2016; **35**: 992–8.

Digestive diseases

Flowchart

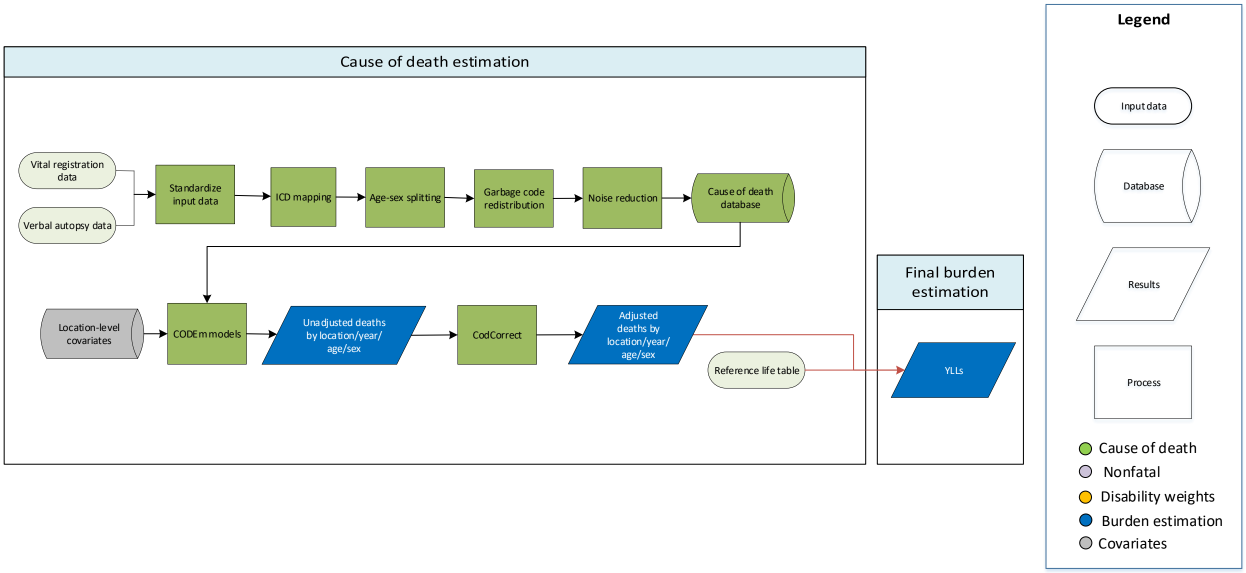

Input data and methodological summary for digestive diseases

Input data

Data used to estimate mortality of digestive diseases consisted of vital registration data and verbal autopsy data from the cause of death (COD) database (see appendix section on ICD mapping for details). The data in digestive diseases consisted of aggregated data from all other specific digestive diseases (ie, cirrhosis and other chronic liver diseases; upper digestive system diseases; appendicitis; vascular intestinal disorders; paralytic ileus and intestinal obstruction; inguinal, femoral and abdominal hernias; inflammatory bowel disease; gallbladder and biliary diseases; pancreatitis) as well as unique datapoints from deaths reported with a set of non-specific digestive disease codes.

Outliers were identified by systematic examination of datapoints for all location-years. Data were excluded if they violated well-established age or time trends, and data in instances where garbage code redistribution and noise reduction, in combination with small sample sizes, resulted in unreasonable cause fractions. Methods for assigning outlier status were consistent across both vital registration and verbal autopsy data.

Modelling strategy

The estimation strategy used for fatal digestive diseases is largely similar to methods used in GBD 2019. A standard CODEm model with location-level covariates was used to model deaths due to digestive diseases (see appendix section on CODEm method for details). Separate models were conducted for male and female mortality, and age restrictions for death estimations included 0 days for lower bound and 95+ years for upper bound. We hybridised separate global and data-rich models to acquire unadjusted results, which we adjusted using CoDCorrect and compared to the reference life table to calculate final YLLs due to digestive diseases.

In GBD 2021, we updated the linear floor value in our CODEm models to allow the model to be influenced by lower data, which resulted from changes to COD data processing. These data processing changes include changing the method of estimating the non-zero floor and population size cutoffs for when to noise-reduce at the country versus region level, and are described in greater detail in the appendix section "GBD 2021 Causes of Death database". Apart from these, no other substantive changes were made in GBD 2021 from the modelling strategy used in GBD 2019.

The following table has the full list of covariates used for fatal digestive diseases.

Table 1. Covariates used in digestive diseases mortality modelling

Level	Covariate	Direction
1	Sanitation (proportion with access)	-
	Cumulative cigarettes (10 years)	+
	Cumulative cigarettes (5 years)	+
	Smoking prevalence	+
	Alcohol (litres per capita)	+
2	Mean BMI	+
	Age-sex-specific scaled exposure variable for low fruit consumption	+
	Age-sex-specific scaled exposure variable for low vegetable consumption	+
	Age-sex-specific scaled exposure variable for high red meat consumption	+
	Healthcare Access and Quality Index	-
3	Socio-demographic Index	-
	Education (year per capita)	-
	Log LDI ($I per capita)	-

Adjustment in CoDCorrect included fitting unadjusted death estimates for all other specific and non-specific digestive diseases to overall digestive disease deaths, which was then adjusted with all other causes to sum to all-cause counts of death.

Diphtheria

Flowchart

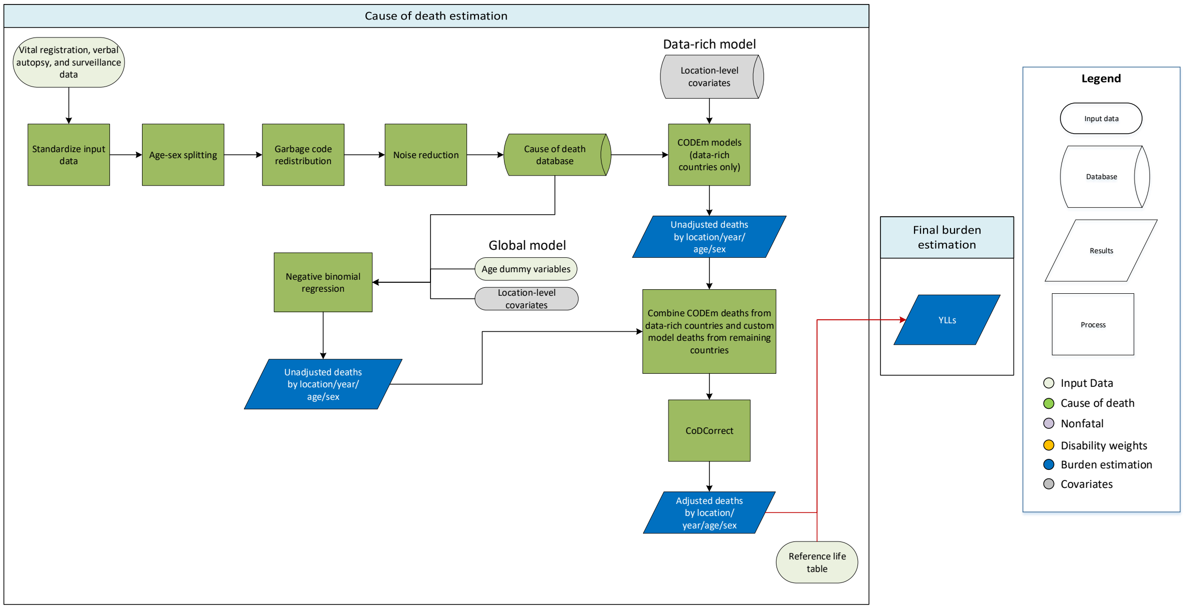

Input data

Diphtheria cause of death (CoD) data for GBD 2021 included vital registration, verbal autopsy, and surveillance sources from all locations as available. We excluded CoD data if they were highly incongruent with other available data from the same location or locations with similar sociodemographic characteristics.

Modelling strategy

We used two distinct methods to estimate diphtheria mortality for different countries based on the quality of vital registration data available. We used a Cause of Death Ensemble modelling strategy (CODEm) for countries with well-defined vital registration (ie, “data-rich” countries), and for remaining countries a custom count negative binomial regression model. Each approach is further described below. For all countries, we produced estimates for age groups between post-neonatal and 59 years of age.

1. Data-rich countries

New this cycle, changes were made to the CODEm modeling process and CoD data preparation central framework, as described in another section of this appendix. In order to help the model continue to track diphtheria’s strong temporal trend after these central changes, we changed our CODEm modelling approach. In GBD 2019, we used an ensemble of models in CODEm that estimated counts of deaths, mortality rates, and cause fractions, using both linear mixed effects and spatiotemporal Gaussian

process regression (ST-GPR) models. For GBD 2021, we used only ST-GPR models of mortality rates and cause fractions in CODEm, as the ensemble of space-time models had lower out-of-sample root mean squared error (RMSE) and better out-of-sample coverage of the data than the full ensemble that included linear mixed effects and counts-based models. For data-rich locations, we used the covariates outlined in Table 1 to inform CODEm predictions. Average DTP3 coverage over the previous five years was used as a covariate instead of the estimates of routine DTP3 coverage among infants in the modelled year that was used in previous GBD cycles. This change was motivated by fluctuations in the annual coverage estimates driven by vaccine stockouts, disruptions due to conflict, or other single-year events. The lagged covariate allows the influence of these events to be distributed across time in our final diphtheria model, better reflecting the expected relationship between coverage and mortality.

Table 1. Covariates. Summary of covariates used in the data-rich diphtheria cause of death model

Level	Covariate	Direction
1	Average diphtheria-tetanus-pertussis third-dose vaccination coverage (DTP3) over the past five years	-
	Healthcare Access and Quality (HAQ) Index	-
	Age- and sex-specific SEV for child wasting	+
3	Lag-distributed income (LDI)	-
	Socio-demographic Index (SDI)	-
	Mean years of education per capita	-

2. Custom count model

Our custom counts mortality model for all non-data-rich locations also used COD data as available by location. We excluded data with extremely high cause fractions (ie, greater than the 99.9th percentile of all diphtheria cause fractions). Using a negative binomial regression with a log link, cause fractions representing the number of deaths due to diphtheria as a proportion of the all-cause mortality envelope were regressed using five-year rolling diphtheria-pertussis-tetanus third-dose (DTP3) vaccine coverage as a covariate, with dummy variables for each GBD age group as predictors. For GBD 2021, we added Healthcare Access and Quality Index as an additional covariate to capture the between-country variation in mortality not accounted for by DTP3 coverage:

$$Y_{ij} = \beta_0 + \beta_1\, DTP3_{ij} + \beta_2\, HAQ_{ij} + \beta_a\, age_a + e_{ij},$$

where Y_{ij} is the log-transformed cause fraction (counts of deaths with an offset of the total number of deaths); β_0 is the fixed-effect intercept; β_1 is the fixed-effects slope on vaccine coverage; β_2 is the fixed-effects slope on healthcare access and quality; β_a is the fixed-effects slope on age_a, the dummy variable for each GBD age group in the estimation; e_{ij} is the residual; i is the year; and j is the location.

Uncertainty was estimated by predicting 1000 draws based on the variance-covariance matrix, and a random sample of the dispersion parameter from a gamma distribution. Results were summarised as the mean of all draws and an associated 95% uncertainty interval (the 2.5th and 97.5th percentile of all draws).

Drug use disorders

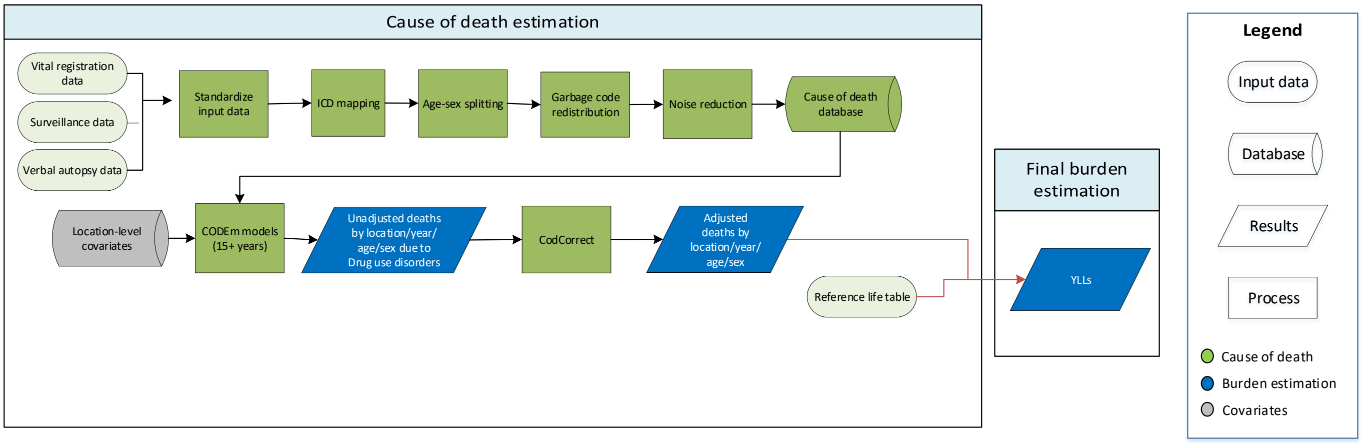

Input data and methodological summary for drug use disorders

Input data

All data were from vital registration, verbal autopsy, and surveillance sources. Data from countries with sparse yet heterogeneous data were excluded as the data exaggerated fluctuations in deaths and gave implausible regional patterns determined by in-country experts and subject experts. Excluded data were primarily from low-income countries.

An ongoing challenges with the input data available for the drug use disorders model are ICD codes that are considered garbage codes.[1] Garbage codes most relevant to drug use disorders include ICD codes for accidental poisonings (X40-44 and X49), exposure to unspecified factors (X59), and external causes of undetermined intent (Y34). As in past rounds, we have used multiple cause of death (MCOD) records to inform redistribution packages[1]. Drug-specific redistribution follows an algorithm based on the fatality of different substances when considering a combination of drugs (Table 1).

Table 1. Algorithm for the selection and assignment of a substance or drug use cause of death for deaths coded to an underlying cause of unintentional poisoning using multiple cause of death data

selection algorithm						
Other cause / Other cause	Opioids	Cannabis	Cocaine	Amphetamines	alcohol	Psychoactive and psychedelic drug
Opioids	Opioids	Opioids	Opioids	Opioids	Opioids	Opioids
Cannabis	Opioids	Cannabis	Cocaine	Amphetamines	alcohol	Psychoactive and psychedelic drug
Cocaine	Opioids	Cocaine	Cocaine	Amphetamines	Cocaine	Cocaine
Amphetamines	Opioids	Amphetamines	Amphetamines	Amphetamines	Amphetamines	Amphetamines
alcohol	Opioids	alcohol	Cocaine	Amphetamines	alcohol	Psychoactive and psychedelic drug
Psychoactive and psychedelic drug	Opioids	Psychoactive and psychedelic drug	Cocaine	Amphetamines	Psychoactive and psychedelic drug	Psychoactive and psychedelic drug

Modelling strategy

The standard Cause of Death Ensemble modelling (CODEm)[2] approach (detailed in reference appendix section 3.1) was used to estimate deaths due to drug use disorders. Level 1 covariates include intravenous drug use prevalence and opioid consumption per million inhabitants per day. The latter covariate is derived from data from the International Narcotics Control Board (INCB), which measures *"defined daily doses for statistical purposes" (S-DDD,* and is considered an approximate measure to rank consumption in different countries.

Due to the extremely small number of drug deaths being recorded, drug models are restricted to ages 15 and older. To capture drug deaths among ages under 15, deaths recorded in vital registration for ages younger than 15 were directly added during post-processing steps, rather than being modelled.

Key changes from GBD 2021:

- The intravenous drug use covariate incorporated additional data and increased time smoothing, which increased estimates in the United States and Western Europe and made the yearly change more consistent over time.

Table 2. Covariates used in drug use disorders CODEm model

Level	Covariate	Direction
1	Intravenous drug use, age-standardised	+
	Intravenous drug use, age-specific	+
	Opioid standard doses per million per day (10-year lag)	+

2	Cumulative cigarettes (10 years)	+
	Cumulative cigarettes (5 years)	+
	Opium cultivation bin	+
	Smoking prevalence	+
	Healthcare Access and Quality Index	-
3	Log LDI (I$ per capita)	+
	Education (years per capita)	+
	Socio-demographic Index	+

Drug use disorders served as a "parent" to the following causes:

- Amphetamine use disorders
- Opioid use disorders
- Cocaine use disorders
- Other drug use disorders

The unadjusted death estimates for all these "child" causes are summed and fit to the distribution of deaths estimated for the "parent" during the CoDCorrect adjustment process.[2] This results in deaths recorded using non-specific coding systems, such as verbal autopsy, that are included in the parent model can be redistributed to the child models proportionately. This approach assumes that deaths reported in non-specific data sources have the same underlying distribution of specific causes as deaths reported in more specific data sources.

Covariate influences:

The following plots show the influence of each covariate on the four CODEm models (male global, male data-rich, female global, and female data-rich). A positive standardised beta (to the right) means that the covariate was associated with increased death. A negative standardised beta (to the left) means the covariate was associated with decreased death.

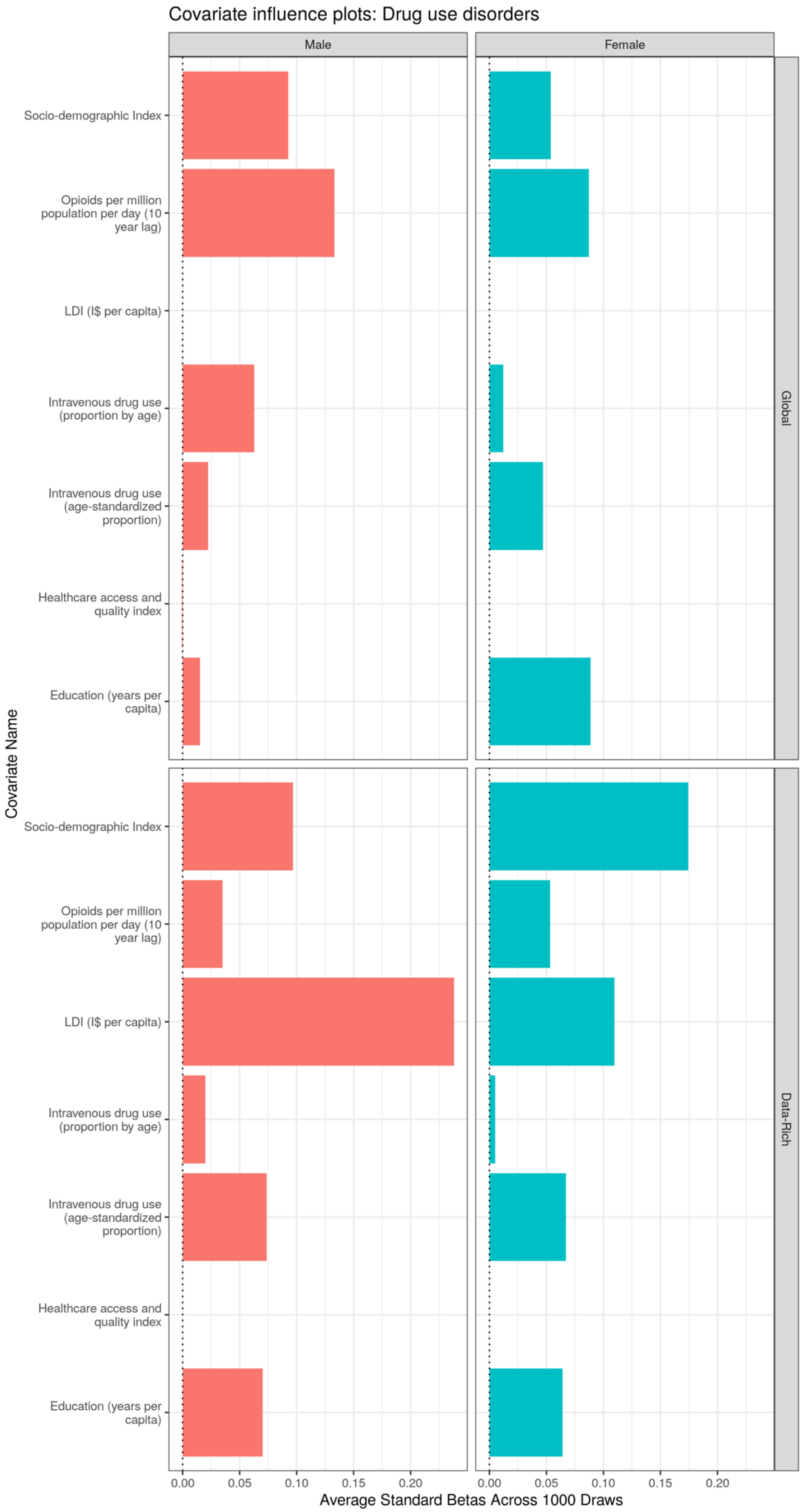
Covariate influence plots: Drug use disorders
Male
Female
Global
Data-Rich
Covariate Name
Socio-demographic Index
Opioids per million population per day (10 year lag)
LDI (I$ per capita)
Intravenous drug use (proportion by age)
Intravenous drug use (age-standardized proportion)
Healthcare access and quality index
Education (years per capita)
0.00
0.05
0.10
0.15
0.20
Average Standard Betas Across 1000 Draws

[1] Johnson, S.C., Cunningham, M., Dippenaar, I.N. *et al.* Public health utility of cause of death data: applying empirical algorithms to improve data quality. *BMC Med Inform Decis Mak* **21,** 175 (2021). https://doi.org/10.1186/s12911-021-01501-1

[2] Vos T, Lim SS, Abbafati C, *et al.* Global burden of 369 diseases and injuries in 204 countries and territories, 1990–2019: a systematic analysis for the Global Burden of Disease Study 2019. *The Lancet* 2020; **396**: 1204–22. doi: https://doi.org/10.1016/S0140-6736(20)30925-9

Multidrug-resistant tuberculosis, extensively drug-resistant tuberculosis, and drug-susceptible tuberculosis

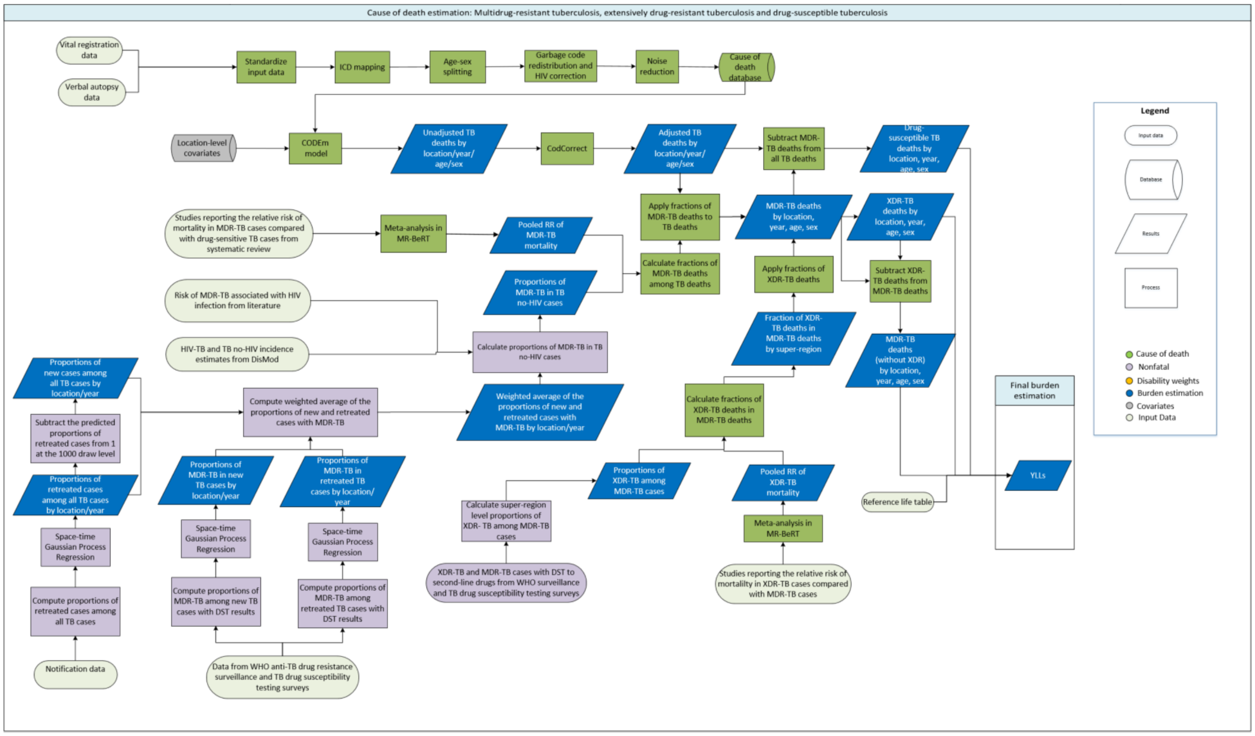

Input data

Input data include: (i) the number of drug-resistant cases by type (multidrug-resistant tuberculosis [MDR-TB], extensively drug-resistant tuberculosis [XDR-TB], all TB cases with a drug-susceptible testing [DST] result for isoniazid and rifampicin, and MDR-TB cases with DST for second-line drugs) from routine surveillance and surveys reported to the World Health Organization, (ii) data from studies (identified through our systematic review) reporting on the relative risk of death in MDR-TB cases compared with non-MDR TB (drug-susceptible TB) cases, and the relative risk of death in XDR-TB cases compared with MDR-TB cases, and (iii) the risk of MDR-TB associated with HIV infection from the literature.[1]

PRISMA diagram of MDR-TB mortality relative risk in GBD 2021

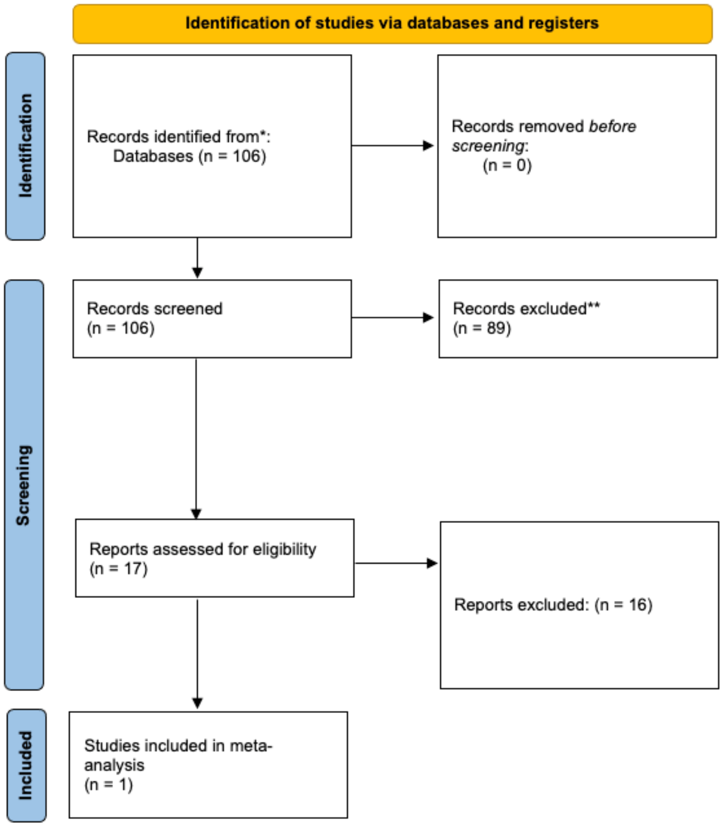

From: Page MJ, McKenzie JE, Bossuyt PM, Boutron I, Hoffmann TC, Mulrow CD, et al. The PRISMA 2020 statement: an updated guideline for reporting systematic reviews. BMJ 2021;372:n71. doi: 10.1136/bmj.n71

For more information, visit: http://www.prisma-statement.org/

Modelling strategy

We conducted a systematic review and meta-analysis of studies reporting the relative risk of death in MDR-TB cases compared with drug-susceptible TB cases. We ran spatiotemporal Gaussian process regressions to predict the proportions of new TB cases with MDR-TB, proportions of retreated TB cases with MDR-TB, and proportions of retreated cases among all TB cases for all locations and years. We also calculated the proportions of new TB cases among all TB cases. We then computed the weighted average of the proportions of new and retreated cases with MDR-TB at the 1000-draw level. We then used the weighted average proportions of MDR-TB, along with the HIV-TB and TB no-HIV incidence estimates (from our modelling of non-fatal TB), and the relative risk of MDR-TB associated with HIV infection from the literature[1] to compute the proportions of MDR-TB cases among HIV-negative TB cases $(P_{MDRnoHIVc,y,a,s})$ by location, year, age, and sex using the following formula:

$$P_{MDRnoHIVc,y,a,s} = \frac{MDR_{c,y}}{\left(1 + \left(RR_{HIV}\frac{HIVTB_{c,y,a,s}}{TBnoHIV_{c,y,a,s}}\right)\right) TBnoHIV_{c,y,a,s}}$$

where $MDR_{c,y}$ is the number of all MDR-TB cases among HIV-positive and HIV-negative individuals by location and year, RR_{HIV} is the relative risk of MDR-TB associated with HIV infection, $HIVTB_{c,y,a,s}$ is the number of HIV-TB incident cases by location, year, age, and sex, and $TBnoHIV_{c,y,a,s}$ is the number of TB no-HIV incident cases by location, year, age, and sex.

We then computed the fraction of MDR-TB deaths among all HIV-negative TB deaths ($D_{MDRnoHIVc,y,a,s}$) using the following formula:

$$D_{MDRnoHIVc,y,a,s} = \frac{P_{MDRnoHIVc,y,a,s}RR_{MDR}}{P_{MDRnoHIVc,y,a,s}RR_{MDR} + 1 - P_{MDRnoHIVc,y,a,s}}$$

where RR_{MDR} is the relative risk of death in MDR-TB cases compared with drug-susceptible TB cases. In GBD 2021, the pooled relative risk was derived from a meta-analysis in the meta-regression with Bayesian priors, regularisation, and trimming (MR-BRT) model. After derivation of the pooled relative risk, we then applied the predicted HIV-MDR-TB death fractions to all HIV-TB death estimates to generate HIV-MDR-TB deaths by location, year, age, and sex. Next, we subtracted MDR-TB deaths from all TB deaths to generate drug-susceptible TB deaths by location, year, age, and sex.

To separate out XDR-TB from MDR-TB, we aggregated the XDR-TB cases and MDR-TB cases (with DST for second-line drugs) up to the super-region level and calculated the super-region-level proportions of XDR-TB among MDR-TB cases. Next, we computed the super-region-specific fractions of XDR-TB deaths among all MDR-TB deaths (D_{XDRsr}) using the following formula:

$$D_{XDRsr} = \frac{P_{XDRsr}RR_{XDR}}{P_{XDRsr}RR_{XDR} + 1 - P_{XDRsr}}$$

where P_{XDRsr} is the proportion of XDR-TB among MDR-TB cases for each super-region, and RR_{XDR} is the pooled relative risk of mortality in XDR-TB cases compared with MDR-TB cases. Similar to the pooled relative risk for MDR-TB, the derivation of the pooled relative risk of mortality in XDR-TB was computed with a meta-analysis in the MR-BRT model for GBD 2021. These fractions were then applied to MDR-TB deaths in corresponding countries within the super-regions to produce XDR-TB deaths by location, age, and sex for the most recent year of estimation. We linearly extrapolated XDR-TB mortality rates back, assuming the mortality rates were zero in 1992, one year before 1993 when XDR-TB was first recorded in USA surveillance data.[2] Finally, we subtracted XDR-TB deaths from MDR-TB deaths to generate MDR-TB (without extensive drug resistance) deaths by location, year, age, and sex.

References

1. Mesfin YM, Hailemariam D, Biadglign S, Kibret KT. Association between HIV/AIDS and multi-drug resistance tuberculosis: a systematic review and meta-analysis. PLoS One. 2014;9(1):e82235.
2. Centers for Disease Control and Prevention (CDC). Extensively Drug-Resistant Tuberculosis --- United States, 1993—2006. MMWR. 2007; 56(11);250-253

Eating disorders

Flowchart

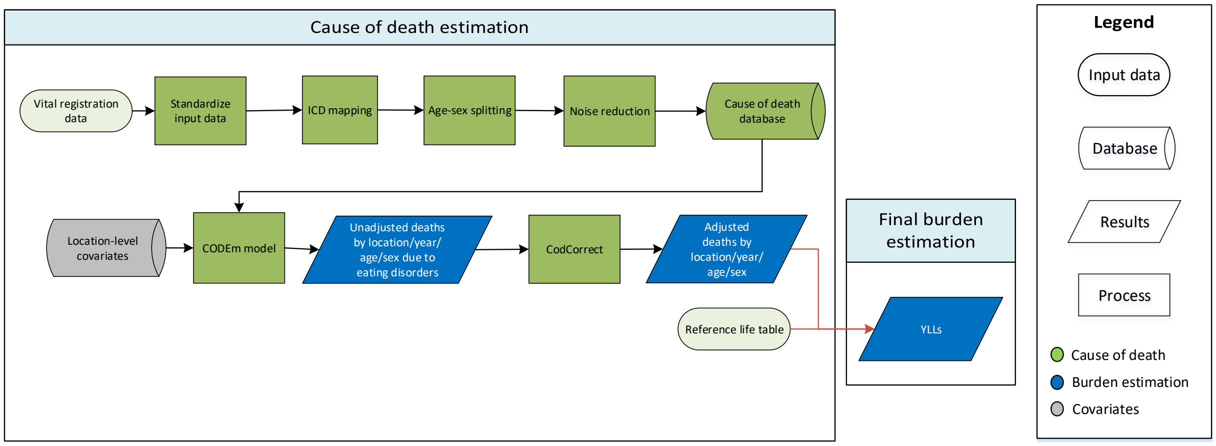

Input data and methodological summary for eating disorders

Data used to estimate eating disorders mortality included vital registration data from the cause of death (COD) database.

Modelling strategy

In GBD 2019, eating disorders were modelled using a standard CODEm modelling approach and encompassing the two child models of anorexia nervosa and bulimia nervosa. In GBD 2021, a decision was made to remove bulimia nervosa as a cause of death due to the very limited data available to inform this model. Mortality data for bulimia nervosa are limited, making any cause of death analysis difficult within the GBD framework and the final estimates difficult to interpret. There was also no clear evidence from our systematic review of the epidemiological literature to suggest that bulimia nervosa is associated with a statistically significant risk of death. This decision was based on feedback and discussion with collaborators in regards to the GBD 2019 results.

In the anorexia nervosa model, age was restricted to deaths occurring between 5 and 49 years based on expert advice and patterns of prevalence seen in the non-fatal model. Several covariates were applied to this model and are listed in the table below, along with the direction in which they were applied.

Table 1. Covariates used in eating disorders mortality modelling

Level	Covariate	Direction
1	Education (years per capita)	+
	Log LDI (I$ per capita)	+
	Age- and sex-specific SEV for child underweight	-

	Sanitation (proportion with access)	+
	Maternal education (years per capita)	+
2	Healthcare Access and Quality Index	-
3	Socio-demographic Index	+

In GBD 2013, eating disorders were modelled as a negative binomial model using a custom approach. This approach was changed in GBD 2015, with eating disorders being modelled as a standard CODEm model, as no additional benefit was seen from using the custom modelling approach. GBD 2021 continued to utilise the same approach.

A decision was made not to redistribute any deaths from garbage codes to eating disorders given that deaths due to dehydration in low- and middle-income countries are likely to bias the age, sex, and geographical distribution of deaths observed. For example, testing of this process showed that while only a relatively small proportion of dehydration garbage code deaths were redistributed to eating disorders, this added a comparatively large number of deaths to the model, particularly in regions with higher rates of infectious diseases. The redistributed deaths were also applied equally between males and females despite the prevalence of eating disorders known to be significantly higher among females.

Ebola virus disease

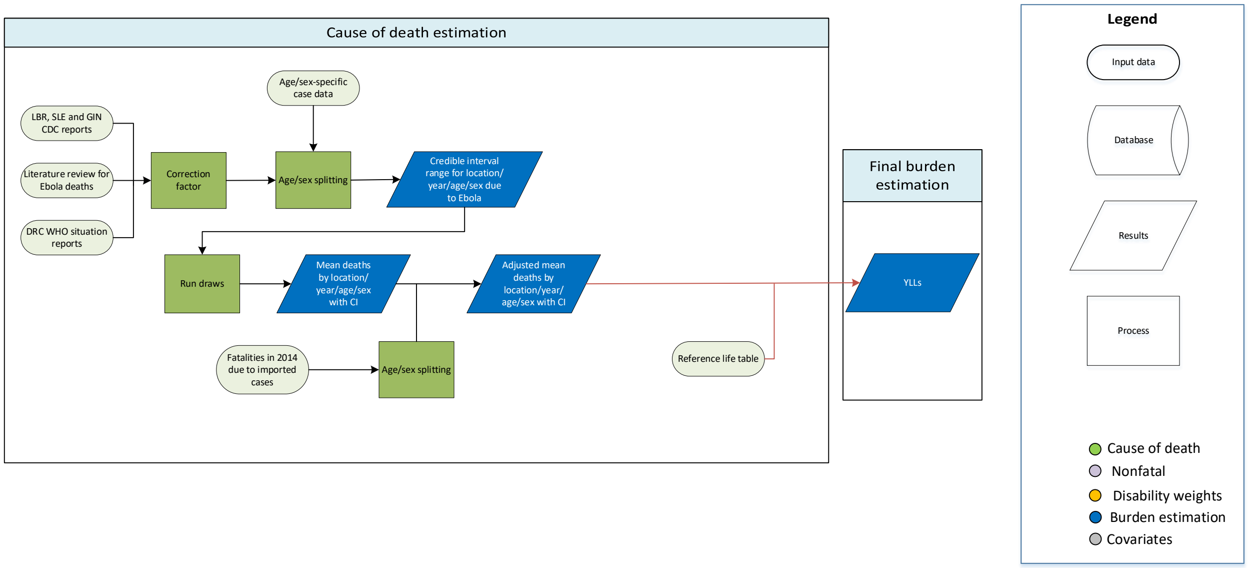

Input data

The input data for deaths due to Ebola virus disease (EVD) came in three forms: (i) total case count tallies provided by the Center for Disease Control (CDC), (ii) total case count tallies for the Democratic Republic of the Congo (DRC) outbreak from 2018 to 2020 provided by the World Health Organization (WHO) and (iii) literature searches for reported deaths due to EVD not captured by the aforementioned outbreaks. This is further explained below:

i. *CDC estimates for the West African outbreak, 2014-2016*

The final tallies as reported by the CDC, were used for each of the three worst affected countries (Liberia, Guinea, and Sierra Leone). Death data from Guinea ranged from February 18, 2014, until September 27, 2015, with data from Liberia ranging from March 20, 2014, to May 4, 2015, and data from Sierra Leone ranging from May 21 until September 28, 2015. In order to capture the small number of fatalities outside of these core three countries, WHO situation reports supplemented the CDC estimates. Fatalities were reported in the US (specifically Texas), Mali and Nigeria[1]. All deaths occurred in 2014. Additional age- and sex-specific information could only be obtained for the death that occurred in the US.

CDC estimates for the Guinea & DRC outbreaks, 2021

The final tallies as reported by the CDC, were used for each country. Death data from Guinea ranged from February 14, 2021 until June 19, 2021, and data from DRC for two outbreaks, ranging from February 7, 2021 until May 3, 2021 and October 8, 2021 until December 16, 2021[8]. All deaths occurred in 2021.

ii. *WHO estimates for DRC, 2018-2020*

Final case counts in the DRC as reported by WHO External Situation reports were used. This included the outbreak in the Equateur Province[2] in 2018, and the outbreak from 2018-2020 in Ituri, North Kivu and South Kivu Provinces[3,4,5]. The latter outbreak included a case reported

for Uganda[6]. This DRC data was further supplemented by CDC case counts of the Equateur Province outbreak[7] from June 1, 2020 to November 18, 2020.

iii. *Literature searches for reported deaths due to Ebola outside of the above outbreaks*
Using a previous review of historical outbreaks[9,10], original articles describing the progression of historical outbreaks were consulted, which was supplemented by additional searches. This resulted in datasets describing each outbreak with variable degrees of detail – some fully describing the age- and sex-specific breakdown of all deaths [eg, Rosello and colleagues[11]] and others simply providing the final total. Only confirmed or probable deaths were included; suspected EVD deaths were omitted. Outbreaks that spanned multiple years, in the absence of sufficient data providing an accurate breakdown, were apportioned between the years by evenly assigning a uniform number of deaths to each month of the outbreak's duration.

A full tabulation of death metadata availability listed in Table 1.

Table 1. Death Metadata				
Outbreak	Number of deaths	Sex metadata	Age metadata	Year metadata
Côte d'Ivoire 1994	No deaths	N/A	N/A	N/A
Gabon 1994/1995	Georges 1999	Imputed	Imputed	Georges 1999
Democratic Republic of the Congo 1995	Rosello 2015	Rosello 2015	Rosello 2015 [94.5% coverage]	Rosello 2015
Gabon 1996	Milleliri 2004	Imputed	Imputed	Milleliri 2004
Gabon 1996/1997	Milleliri 2004	Imputed	Imputed	Imputed
Uganda 2000/2001	Okware 2002	Imputed	Imputed	Imputed
Congo 2002/2003	Kuhn 2008	Imputed	Imputed	Imputed
Congo 2003	Boumandouki 2005	Imputed	Imputed	Boumandouki 2005
South Sudan 2004	WHO 2004	WHO 2004	WHO 2004 [42.86% coverage]	WHO 2004
Congo 2005	Nkoghe 2011	Nkoghe 2011	Nkoghe 2011	Nkoghe 2011
Democratic Republic of the Congo 2007	Rosello 2015	Rosello 2015	Rosello 2015	Rosello 2015
Uganda 2007	Wamala 2010	Wamala 2010	Imputed	Wamala 2010
Democratic Republic of the Congo 2008	Rosello 2015	Rosello 2015	Rosello 2015	Rosello 2015
Uganda 2011	Shoemaker 2012	Shoemaker 2012	Shoemaker 2012	Shoemaker 2012
Democratic Republic of the Congo 2012	Rosello 2015	Rosello 2015	Rosello 2015	Rosello 2015
Uganda 2012	Albarino 2013	Imputed	Imputed	Albarino 2013
Uganda 2012/2013	Albarino 2013	Imputed	Imputed	Imputed

West Africa 2013/2015	WHO/CDC	Imputed	Imputed	WHO/CDC
Democratic Republic of the Congo 2014	Rosello 2015	Rosello 2015	Rosello 2015	Rosello 2015
Democratic Republic of the Congo, Equateur province 2018	WHO 2018	Imputed	Imputed	WHO 2018
Democratic Republic of the Congo, North Kivu 2018/2019/2020	WHO 2018, WHO 2019, WHO 2020	Imputed	Imputed	WHO 2018, WHO 2019, WHO 2020
Democratic Republic of the Congo, Equateur province 2020	CDC 2020	Imputed	Imputed	CDC 2020
Guinea, N'Zérékoré prefecture 2021	CDC 2021	Imputed	Imputed	CDC 2021
Democratic Republic of the Congo, North Kivu province 2021	CDC 2021	Imputed	Imputed	CDC 2021

Modelling strategy

Data on deaths resulting from imported cases from 2014 were used as specific count data as it was assumed to be an accurate representation of the cases and outbreaks in these countries, all of which were on high alert for importation of cases[12,13].

The other input data were processed prior to inclusion in GBD to account for any potential underreporting of deaths. A meta-analysis of existing underreporting studies from the literature was performed, using a random effects model with a DerSimonian-Laird estimator. A variety of sources were included, capturing several different estimation processes, all identified by literature review. The figure below shows the different effect sizes of the various studies[14-19], as well as the resulting GBD 2016 correction factor, with the GBD 2015 correction factor for reference. The correction factor ranged from 1.4580 to 2.5475, with a mean of 2.0027. For GBD 2021 the GBD 2016 factor was used.

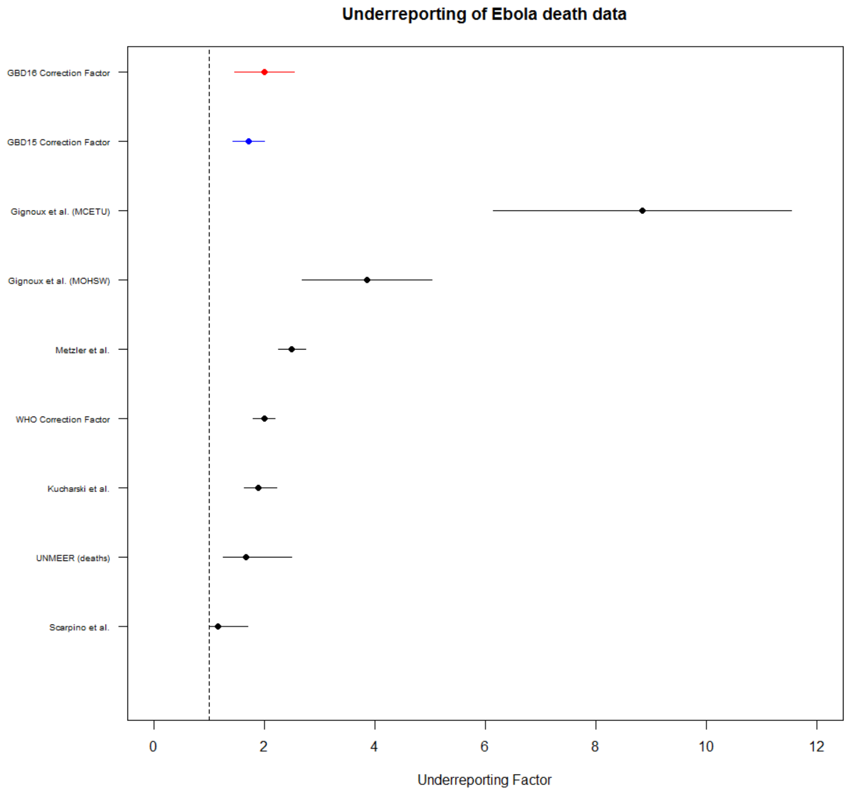

In order to capture this potential variation, all input data were multiplied by the lower and upper limit of this estimated correction factor; these numbers then provided the lower and upper bounds from which draw values were taken. For outbreaks where no data were supplied for age and/or sex, the pattern observed in the age- and sex-specific case data was used to apportion these total values.

One thousand draws were taken from a normal distribution fitted between these lower and upper bound values, which generated mean estimates stratified by age, sex, location, and year along with credible intervals for these numbers. These estimates were then adjusted by including the count data for imported cases from 2014.

Data on Ebola outbreaks prior to 2014 are sparse, and as a result many values derived from the West African outbreak were assumed to be valid for historical outbreaks as well. This may mask significant differences that exist between these outbreaks, some of which were caused by different species of *Ebolavirus*. To minimize this problem, we chose to implement a data-driven approach – for those outbreaks where sufficiently detailed historical data could be obtained, these were used in preference to any assumed age/sex breakdown.

References

1 World Health Organization. Ebola Situation Reports. 2016. Interview (accessed March 14, 2016).

2 World Health Organization (WHO). WHO Ebola Situation Report 2018 - Number 17. 2018.

3 World Health Organization (WHO). WHO Ebola Situation Report 2019 - Number 69. 2019.

4 World Health Organization (WHO). WHO Ebola Situation Report 2019 - Number 73. 2019.

5 World Health Organization (WHO). WHO Ebola Situation Report 2019 - Number 98. 2020.

6 World Health Organization (WHO). WHO Ebola Situation Report 2019 - Number 45. 2019.

7 Centers for Disease Control and Prevention (CDC). Ebola (Ebola Virus Disease). 2020 Democratic Republic of the Congo, Equateur Province. 2020. https://www-cdc-gov.offcampus.lib.washington.edu/vhf/ebola/outbreaks/drc/2020-june.html (accessed January 11, 2021).

8 Centers for Disease Control and Prevention (CDC). Ebola (Ebola Virus Disease). 2021 Democratic Republic of the Congo, North Kivu Province & Guinea, N'Zérékoré prefecture. 2021. https://www.cdc.gov/vhf/ebola/history/chronology.html (accessed July 27, 2022).

9 Pigott DM, Golding N, Mylne A, *et al.* Mapping the zoonotic niche of Ebola virus disease in Africa. *Elife* 2014; **3**: e04395.

10 Mylne A, Brady OJ, Huang Z, *et al.* A comprehensive database of the geographic spread of past human Ebola outbreaks. *Sci Data* 2014; **1**: 140042.

11 Rosello A, Mossoko M, Flasche S, *et al.* Ebola virus disease in the Democratic Republic of the Congo, 1976-2014. *Elife* 2015; **4**. DOI:10.7554/eLife.09015.

12 Fasina FO, Shittu A, Lazarus D, *et al.* Transmission dynamics and control of Ebola virus disease outbreak in Nigeria, July to September 2014. *Euro Surveill* 2014; **19**: 20920.

13 Althaus CL, Low N, Musa EO, Shuaib F, Gsteiger S. Ebola virus disease outbreak in Nigeria: Transmission dynamics and rapid control. *Epidemics* 2015; **11**: 80–4.

14 Gignoux E, Idowu R, Bawo L, *et al.* Use of Capture-Recapture to Estimate Underreporting of Ebola Virus Disease, Montserrado County, Liberia. *Emerg Infect Dis* 2015; **21**: 2265–7.

15 Meltzer MI, Atkins CY, Santibanez S, *et al.* Estimating the future number of cases in the Ebola epidemic--Liberia and Sierra Leone, 2014-2015. *MMWR Suppl* 2014; **63**: 1–14.

16 Scarpino S V, Iamarino A, Wells C, *et al.* Epidemiological and viral genomic sequence analysis of the 2014 ebola outbreak reveals clustered transmission. *Clin Infect Dis* 2015; **60**: 1079–82.

17 Kucharski AJ, Camacho A, Flasche S, Glover RE, Edmunds WJ, Funk S. Measuring the impact of Ebola control measures in Sierra Leone. *Proc Natl Acad Sci U S A* 2015; **112**: 14366–71.

18 UNMEER. Sierra Leone: Ebola emergency Weekly Situation Report No. 7. 2014 https://www.humanitarianresponse.info/system/files/documents/files/UNMEER_NERC_SitRep_07Dec.pdf.

19 Enserink M. How many Ebola cases are there really? | Science | AAAS. 2014. http://www.sciencemag.org/news/2014/10/how-many-ebola-cases-are-there-really (accessed Jan 28, 2017).

Encephalitis

Flowchart

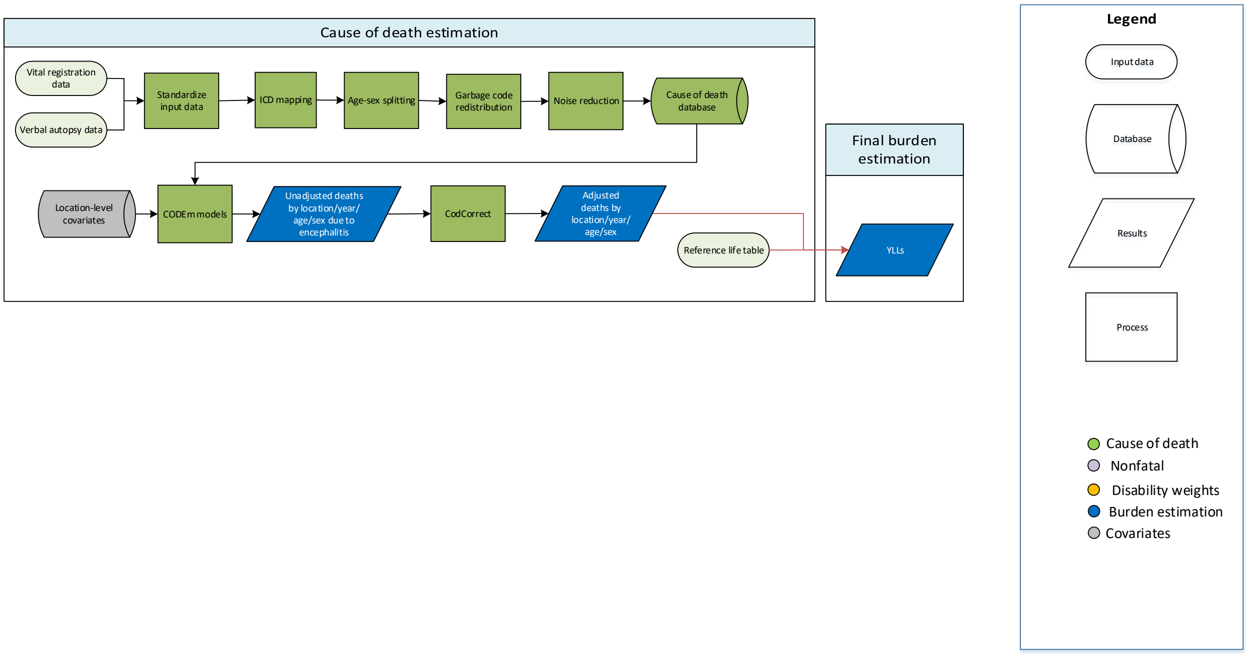

Input data and methodological summary for encephalitis

Input data

For GBD 2021, vital registration and verbal autopsy data were used to model this cause. We outliered data in instances where garbage code redistribution and noise reduction, in combination with small sample sizes, resulted in unreasonable cause fractions when compared to regional, super-regional, and global rates, and data that violated well-established time or age trends. Outliering methods were consistent across both vital registration and verbal autopsy data.

Modelling strategy

We modelled deaths due to encephalitis with a standard CODEm model using the cause of death database and location-level covariates as inputs. We hybridised separate global and data-rich models to acquire unadjusted results, which were adjusted using CodCorrect to reach final years of life lost due to encephalitis.

There were no substantive changes from GBD 2019 in terms of modelling strategy. A full list of covariate inputs in the published model can be found in Table 1. Covariates were weighted and selected based on the ensemble model process.

Table 1. Covariates used in encephalitis mortality modelling

Level	Covariate	Direction
1	Japanese encephalitis endemic area binary	+
	Age- and sex-specific summary exposure value (SEV) for child underweight	+
2	Log-transformed lag distributed income	-
	Healthcare Access and Quality Index	-
	Maternal care and immunisation	-
3	Squared proportion of in-facility deliveries	-
	Socio-demographic Index	-
	Logit-transformed sanitation (proportion with access)	-
	Logit-transformed water (proportion with access)	-
	DTP3 coverage	-
	Maternal education (years per capita)	-

Endocarditis

Flowchart

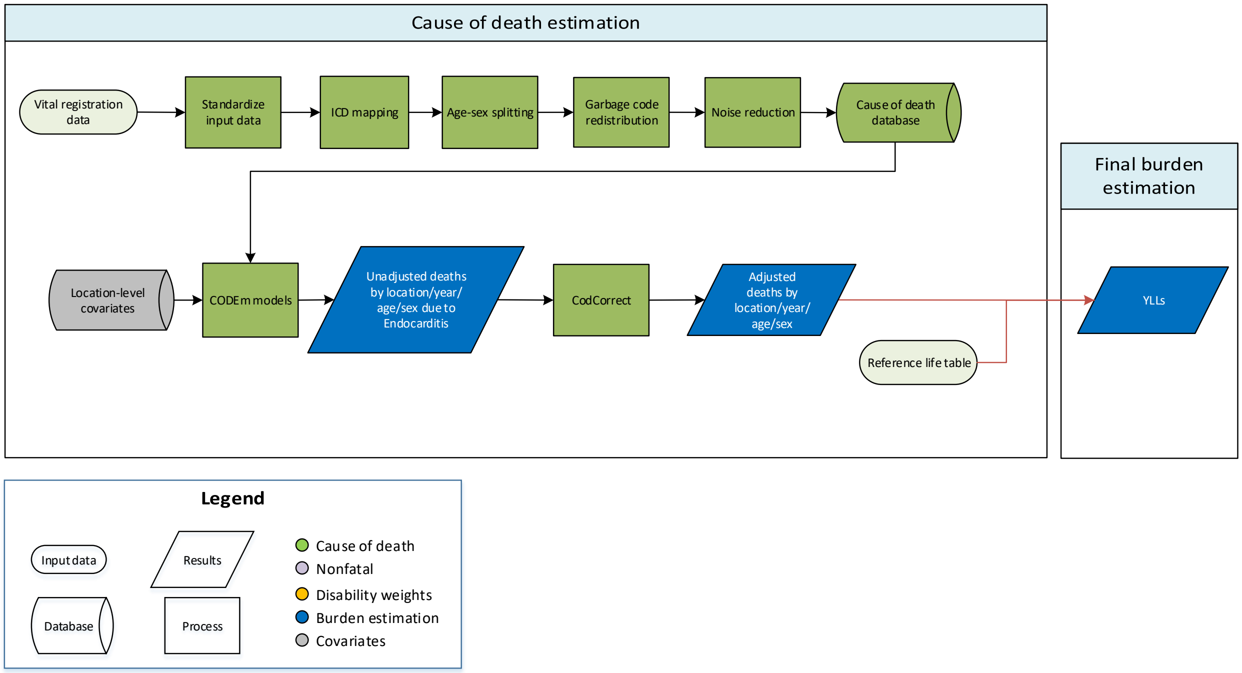

Input data and methodological summary for endocarditis

Input data

Vital registration data were used to model endocarditis. We outliered data in Mozambique as these were non-representative for sub-Saharan Africa and were causing regional estimates to be implausibly low. In addition, data from Cabo Verde, Ghana, Kiribati, Fiji, American Samoa, Guam, Palau were also outliered due to poor data quality leading to implausible results. We also outliered ICD8 data that were discontinuous from the rest of the data series and created an implausible time trend.

Modelling strategy

We used a standard CODEm approach to model deaths from endocarditis. For GBD 2021, the method used to reduce the noise in the data, implemented after redistribution to handle both the stochastic variation across time and space and the occurrence of small number of deaths in each location/year/age/sex, was updated. This new empirical Bayesian noise reduction algorithm uses grouped data by region and data type as prior to better reflect regional patterns. A detailed description on the updates to noise reduction can be found in the cause of death methods section of the appendix.

Covariates selected for inclusion in the CODEm ensemble modelling process are listed in the table below.

Table 1. Covariates used in endocarditis mortality modelling

Level	Covariate	Direction
1	Sanitation (proportion with access)	1

	Improved water (proportion)	1
	Summary exposure value, endocarditis	1
2	Healthcare Access and Quality Index	-1
3	Socio-demographic Index	-1
	Log-transformed lag distributed income per capital (I$)	-1

Endocrine, metabolic, blood, and immune disorders

Flowchart

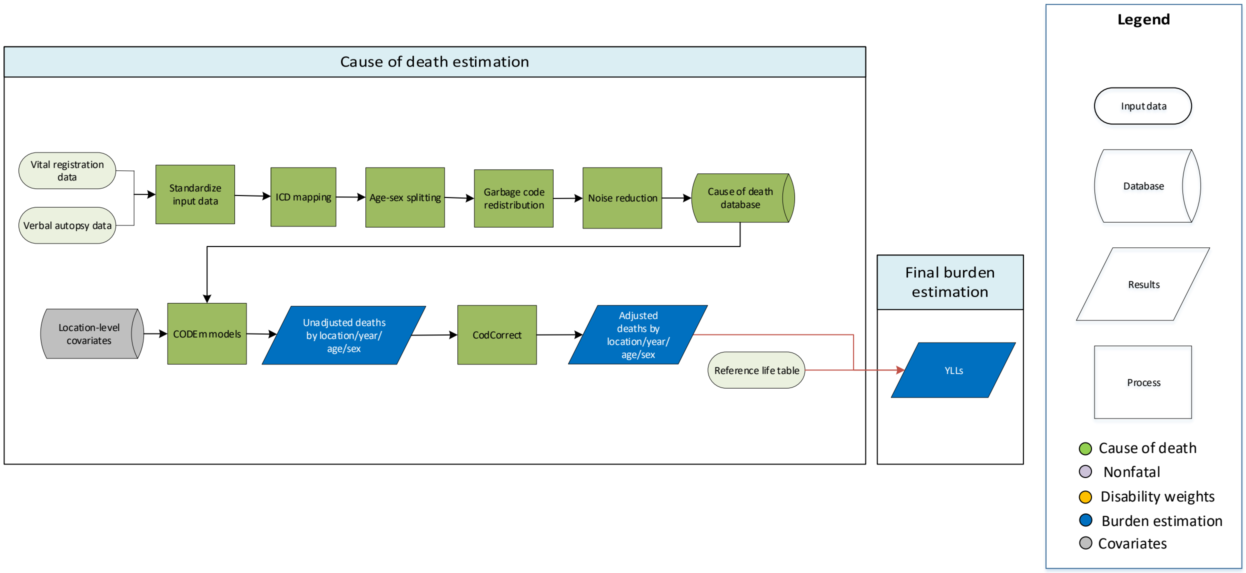

Input data and methodological summary for endocrine, metabolic, blood, and immune disorders

Input data

Vital registration and verbal autopsy data from the cause of death (COD) database were used to model mortality due to endocrine, metabolic, blood, and immune disorders (see appendix section on ICD mapping for details). Relative to GBD 2019, in GBD 2021 we re-mapped codes for a small number of secondary endocrine, immune, or metabolic disorders to their underlying causes (ICD codes C7, D3 codes and E67). We also re-mapped the following codes to garbage codes (ICD codes D69, D70, D72, D75, E26, E83).

Outliers were identified by systematic examination of datapoints for all location-years. Data were excluded if they violated well-established age or time trends, and data in instances where garbage code redistribution and noise reduction, in combination with small sample sizes, resulted in unreasonable cause fractions. Methods for assigning outlier status were consistent across both vital registration and verbal autopsy data.

Modelling strategy

The estimation strategy used for fatal endocrine, blood, metabolic, and immune disorders (EMBID) is largely similar to methods used in GBD 2019. A standard CODEm model with location-level covariates was used to model deaths due to EMBID (see appendix section on CODEm method for details). Separate models were conducted for male and female mortality with the age-restrictions of 0 days for lower bound and 95+ years for upper bound. We hybridised separate global and data-rich models to acquire unadjusted results, which we adjusted using CoDCorrect and compared to the reference life table to calculate final YLLs due to EMBID.

In GBD 2021, we updated the linear floor value in our CODEm models to allow the model to be influenced by lower data, which resulted from changes to COD data processing. These data processing changes include changing the method of estimating the non-zero floor and population size cutoffs for when to noise-reduce at the country versus region level, and are described in greater detail in the appendix section GBD 2021 Causes of Death database. Apart from these, no other substantive changes were made in GBD 2021 from the modelling strategy used in GBD 2019.

The following table has the full list of covariates used for fatal EMBID.

Table 1. Covariates used in endocrine, blood, metabolic, and immune disorders mortality modelling

Level	Covariate	Direction
1	Mean BMI	+
2	Mean cholesterol	+
	Alcohol (litres per capita)	+
	Healthcare Access and Quality Index	-
3	Socio-demographic Index	-
	Education (year per capita)	-
	Log LDI ($I per capita)	-

In GBD 2021, we introduced two Level 4 child causes of EMBID in the GBD disease hierarchy: thyroid disorders and other EMBID that excludes thyroid disorders. The fatal models of these two child causes were modelled in the same manner as EMBID. The following tables have the full list of covariates used for thyroid disorders and other EMBID.

Table 2. Covariates used in thyroid disorders mortality modelling

Level	Covariate	Direction
1	Absolute value of average latitude	+
	Healthcare Access and Quality Index	-
2	Diabetes age-standardised prevalence (proportion)	+
3	Cumulative cigarettes (10 years)	+
	Cumulative cigarettes (5 years)	+
	Smoking prevalence	+
	Mean BMI	+
	Socio-demographic Index	-
	Education (year per capita)	-
	Log LDI ($I per capita)	-

Table 3. Covariates used in other endocrine, blood, metabolic, and immune disorders mortality modelling

Level	Covariate	Direction
1	Mean BMI	+
2	Mean cholesterol	+
	Alcohol (litres per capita)	+

	Healthcare Access and Quality Index	-
3	Socio-demographic Index	-
	Education (year per capita)	-
	Log LDI ($I per capita)	-

Adjustment in CoDCorrect included fitting unadjusted death estimates for Level 4 child causes of EMBID (ie, thyroid disorders and other EMBID) to overall EMBID deaths, which were then adjusted with all other causes to sum to all-cause counts of death.

Gallbladder and biliary diseases

Flowchart

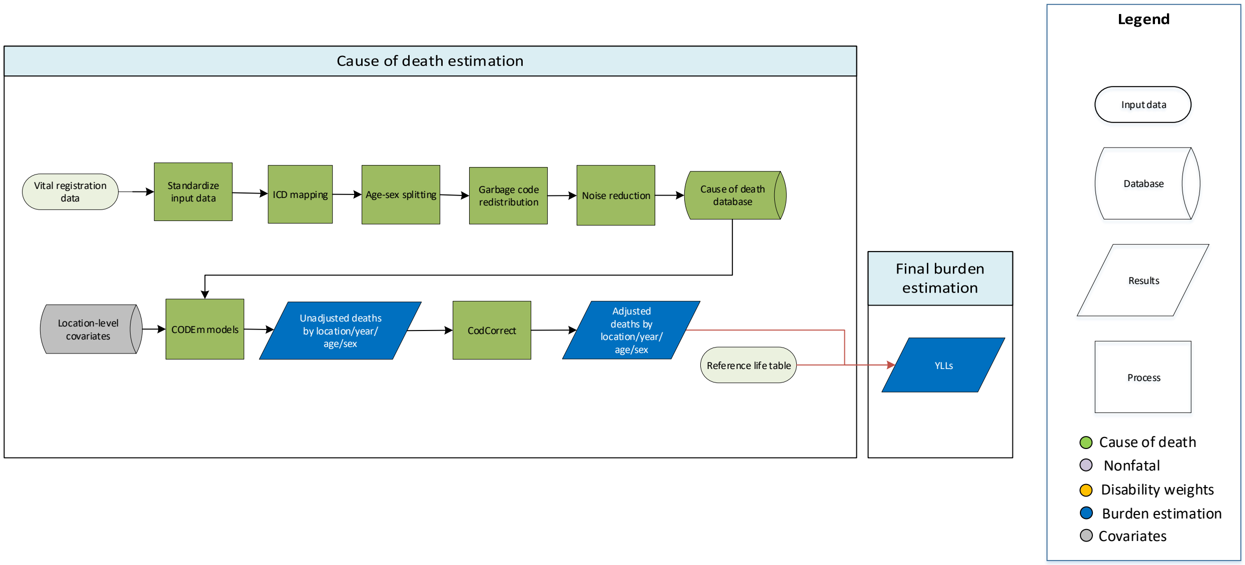

Input data and methodological summary for gallbladder and biliary diseases

Input data

Data used to estimate mortality of gallbladder and biliary diseases consisted of vital registration data from the cause of death (COD) database (see appendix section on ICD mapping for details). Outliers were identified by systematic examination of datapoints for all location-years. Data were excluded if they violated well-established age or time trends, and data in instances where garbage code redistribution and noise reduction, in combination with small sample sizes, resulted in unreasonable cause fractions.

Modelling strategy

The estimation strategy used for fatal gallbladder and biliary diseases is largely similar to methods used in GBD 2019. A standard CODEm model with location-level covariates was used to model deaths due to gallbladder and biliary diseases (see appendix section on CODEm method for details). Separate models were conducted for male and female mortality, and age restrictions for death estimations included 2 years for lower bound (in GBD 2019, the lower bound was set at 1 year) and 95+ years for upper bound. We hybridised separate global and data-rich models to acquire unadjusted results, which we adjusted using CoDCorrect and compared to the reference life table to calculate final YLLs due to gallbladder and biliary diseases.

In GBD 2021, we updated the linear floor value in our CODEm models to allow the model to be influenced by lower data, which resulted from changes to COD data processing. These data processing changes include changing the method of estimating the non-zero floor and population size cutoffs for when to noise-reduce at the country versus region level, and are described in greater detail in the appendix section "GBD 2021 Causes of Death database". Apart from these, no other substantive changes were made in GBD 2021 from the modeling strategy used in GBD 2019.

The following table has the full list of covariates used for fatal estimation of gallbladder and biliary diseases.

Table 1. Covariates used in gallbladder and biliary diseases mortality modelling

Level	Covariate	Direction
1	Age-sex-specific scaled exposure variable for low polyunsaturated fatty acids	+
	BMI (mean)	+
2	Alcohol (litres per capita)	+
	Healthcare Access and Quality Index	-
	Age-sex-specific scaled exposure variable for high red meat consumption	+
	Population over 65 (proportion)	+
3	Socio-demographic Index	-
	Education (years per capita)	-
	Log LDI ($I per capita)	-

Adjustment in CoDCorrect included fitting unadjusted death estimates for all other specific and non-specific digestive diseases to overall digestive disease deaths, which was then adjusted with all other causes to sum to all-cause counts of death.

Gastritis and duodenitis

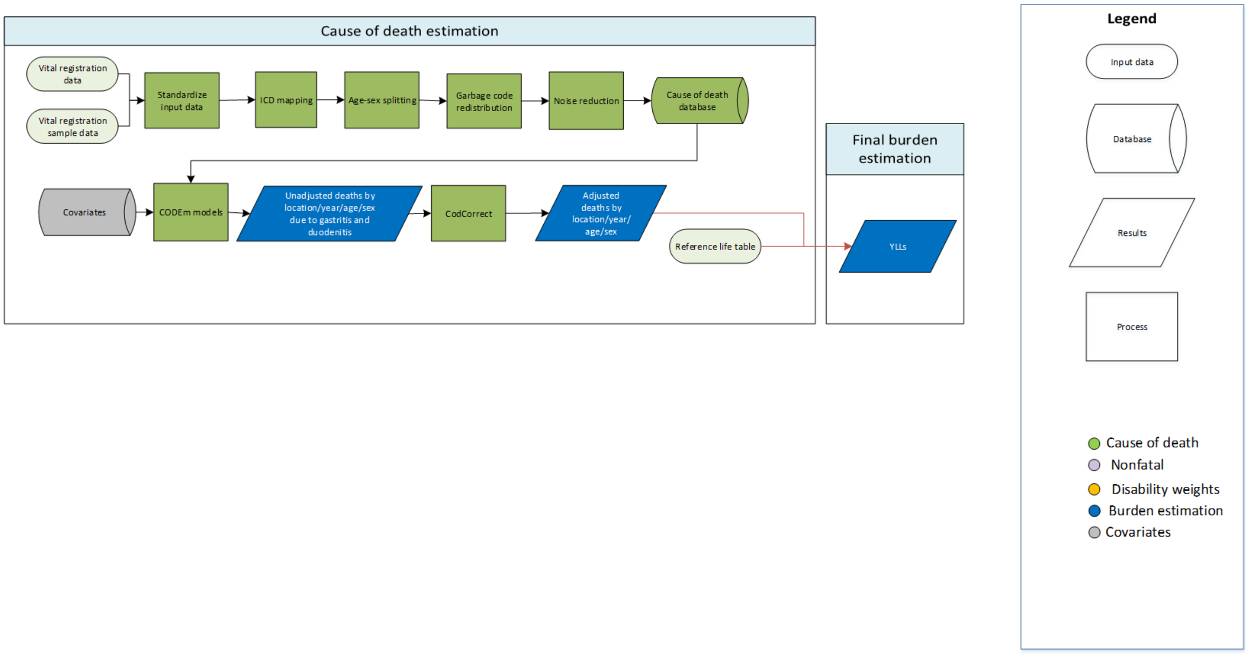

Input data

Data used to estimate unadjusted mortality of gastritis and duodenitis consisted of vital registration data and vital registration sample data from those sources in the cause of death (COD) database that use ICD9 or ICD10 codes and report un-tabulated (individual) deaths. The diagnostic codes that map to gastritis and duodenitis can be found in the "List of International Classification of Diseases (ICD) codes mapped to the Global Burden of Disease cause list for causes of death" found elsewhere in this appendix.

Details of COD data processing, including changes in processing introduced in GBD 2021, are described in detail in the "GBD 2021 Causes of Death database" section of this appendix. These data processing changes include changing the method of estimating the non-zero floor and population size cutoffs for when to noise-reduce at the country versus region level.

Outliers were identified by systematic examination of datapoints for all location-years. Data were excluded if they violated well-established time or age trends, and data in instances where garbage code redistribution and noise reduction, in combination with small sample sizes, resulted in unreasonable cause fractions or unreasonable time, age, or spatial trends; data from Tibet, Yunnan, Iran, Qatar, and Kiribati were excluded for these reasons. Data in Ghana, Bahrain, Palestine, and Grenada that were excluded for these reasons in GBD 2019 were included in GBD 2021 because advances in noise reduction improved the plausibility of these data. In situations where unreasonable temporal and spatial trends were observed at transitions between data sources, higher-quality data sources were retained and lower-quality sources were excluded; this affected subnational locations in India, where vital registration data biased toward in-hospital deaths (MCCD) were available for urban locations only. Data for young-adult age groups in South African subnational locations, which were excluded in previous rounds due to concerns about misclassified HIV deaths, were corrected for misclassification and used in GBD 2021. Two datapoints from Mali were excluded because they were only available for a single age group.

Modelling strategy

We modelled deaths due to gastritis and duodenitis with a standard CODEm model. (The CODEm modelling tool is described in the "Cause of death modelling methods: CODEm" section of this appendix.) The gastritis and duodenitis CODEm model employed the same parameter settings in GBD 2021 as in GBD 2019, with the exception that we updated the linear floor value to allow the model to be influenced by lower data, which resulted from changes to COD data processing.

Covariates entered into CODEm, their level of priority for testing, and their permitted directions were the same in GBD 2021 as in GBD 2019. A complete list is provided in the table below.

Covariate	Level	Direction
Sanitation, proportion with access	1	-1
Scaled exposure variable for unsafe water source	1	1
Smoking prevalence	2	1
Cumulative cigarettes (10 years)	2	1
Cumulative cigarettes (5 years)	2	1
Litres of alcohol consumed per capita	2	1
Vegetables (grams, unadjusted)	2	-1
Healthcare Access and Quality Index	2	-1
Lag distributed income (per capita)	3	-1
Education (years per capita)	3	-1
Socio-demographic Index	3	-1

In CoDCorrect estimates for peptic ulcer disease and gastritis and duodenitis were first adjusted to sum to all upper digestive disease deaths and then to sum to all-cause mortality with all other causes.

Gynaecological diseases

Flowchart

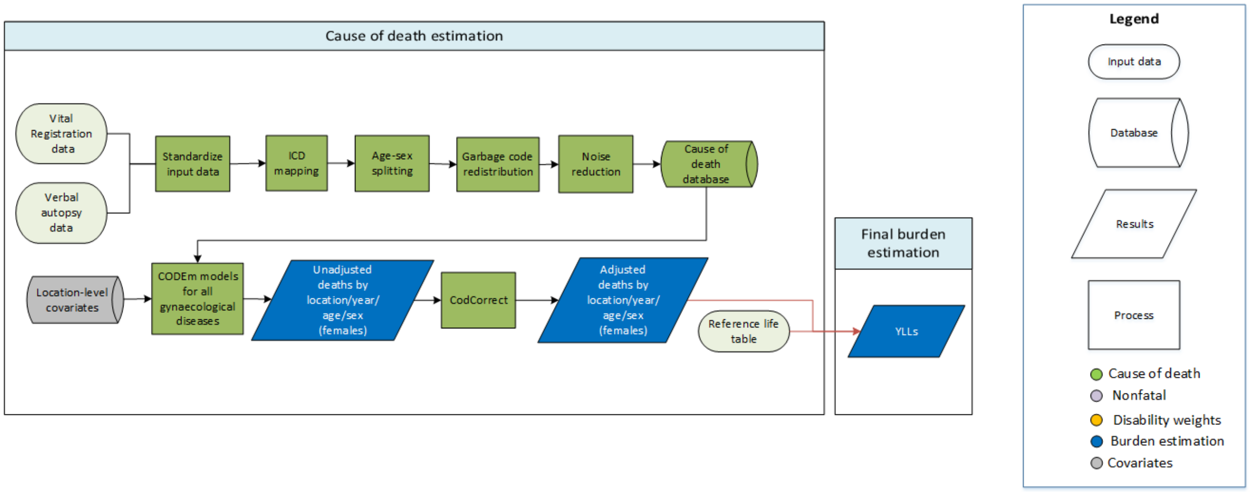

Input data

For GBD 2021, vital registration and verbal autopsy data were used to estimate deaths for gynaecological diseases in aggregate, and for the following sub-causes: uterine fibroids, endometriosis, genital prolapse, and other gynaecological disorders. Other gynaecological disorders includes inflammatory disease of cervix uteri, diseases of Bartholin's gland, other inflammation of vagina and vulva, vulvovaginal ulceration and inflammation, and non-inflammatory disorders of ovary, fallopian tube, and broad ligament. As in previous GBD cycles, we reassigned deaths due to leiomyomas and other benign uterine tumors to uterine fibroids, and we assumed no deaths from premenstrual syndrome, primary infertility, and polycystic ovarian syndrome, which we model as non-fatal outcomes only. The International Classification of Diseases (ICD) codes that map to each cause can be found in the list of figures and tables for this appendix.

Outliers were identified by systematic examination of datapoints for all location-years. Data were excluded if they violated well-established time or age trends, and data in instances where garbage code redistribution and noise reduction, in combination with small sample sizes, resulted in unreasonable cause fractions. Methods for assigning outlier status were consistent across vital registration and verbal autopsy data types.

Modelling strategy

For gynaecological diseases in aggregate and for each sub-cause, we completed data-rich (DR) and global models for females aged 10 years and older in the Cause of Death Ensemble modelling (CODEm) tool. More information about this tool can be found in the "Cause of death modelling methods: CODEm" section of this appendix. Models for each of the gynaecological diseases included the nine covariates in the table below for possible selection in their final models.

In GBD 2021, we updated the linear floor value in our CODEm models to allow the model to be influenced by lower data, which resulted from changes to COD data processing. These data processing changes include changing the method of estimating the non-zero floor and population size cutoffs for when to noise-reduce at the country versus region level, and are described in greater detail in the appendix section “GBD 2021 Causes of Death database”.

Table 1. Covariates used in gynaecological diseases mortality modelling

Level	Covariate	Direction
1	Age- and sex-specific SEV for smoking	-
2	Percentage of births in women over 35 years	+
	Skilled birth attendance proportion	-
	Total fertility rate	+
	Healthcare Access and Quality Index	-
	Maternal care & immunisation	-
3	Education, years per capita	-
	Lag-distributed income per capita	-
	Socio-demographic Index	-

Haemoglobinopathies and haemolytic anaemias

This write-up covers the following sub-causes: sickle cell disorders, thalassaemias, glucose-6-phosphate dehydrogenase (G6PD) deficiency, and other haemoglobinopathies and haemolytic anaemias

Flowchart

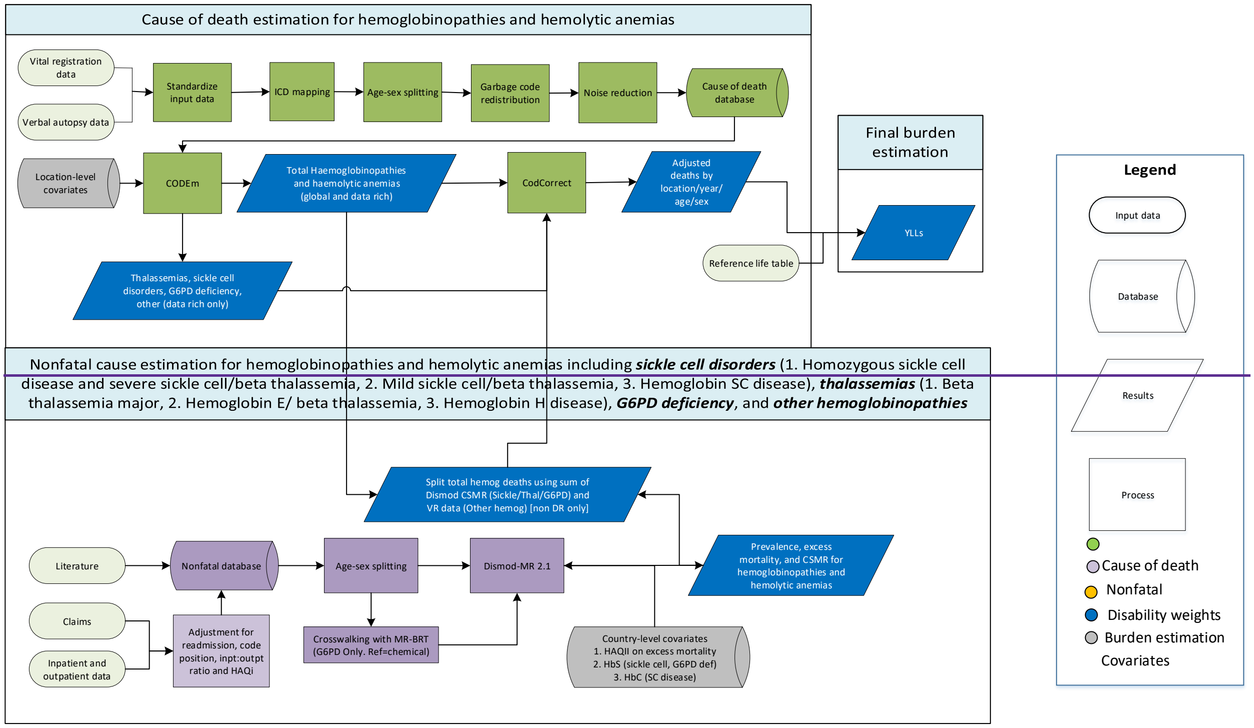

Cause of death estimation for hemoglobinopathies and hemolytic anemias
Vital registration data
Verbal autopsy data
Standardize input data
ICD mapping
Age-sex splitting
Garbage code redistribution
Noise reduction
Cause of death database
Location-level covariates
CODEm
Total Haemoglobinopathies and haemolytic anemias (global and data rich)
CodCorrect
Adjusted deaths by location/year/ age/sex
Thalassemias, sickle cell disorders, G6PD deficiency, other (data rich only)
Reference life table
Final burden estimation
YLLs
Nonfatal cause estimation for hemoglobinopathies and hemolytic anemias including ***sickle cell disorders*** (1. Homozygous sickle cell disease and severe sickle cell/beta thalassemia, 2. Mild sickle cell/beta thalassemia, 3. Hemoglobin SC disease), ***thalassemias*** (1. Beta thalassemia major, 2. Hemoglobin E/ beta thalassemia, 3. Hemoglobin H disease), ***G6PD deficiency***, and ***other hemoglobinopathies***
Split total hemog deaths using sum of Dismod CSMR (Sickle/Thal/G6PD) and VR data (Other hemog) [non DR only]
Literature
Nonfatal database
Age-sex splitting
Dismod-MR 2.1
Prevalence, excess mortality, and CSMR for hemoglobinopathies and hemolytic anemias
Claims
Inpatient and outpatient data
Adjustment for readmission, code position, inpt:outpt ratio and HAQi
Crosswalking with MR-BRT (G6PD Only. Ref=chemical)
Country-level covariates 1. HAQII on excess mortality 2. HbS (sickle cell, G6PD def) 3. HbC (SC disease)
Legend
Input data
Database
Results
Process
Cause of death
Nonfatal
Disability weights
Burden estimation
Covariates

Haemoglobinopathies and haemolytic anaemias

This write-up covers the following sub-causes: sickle cell disorders, thalassaemias, glucose-6-phosphate dehydrogenase (G6PD) deficiency, and other haemoglobinopathies and haemolytic anaemias

Flowchart

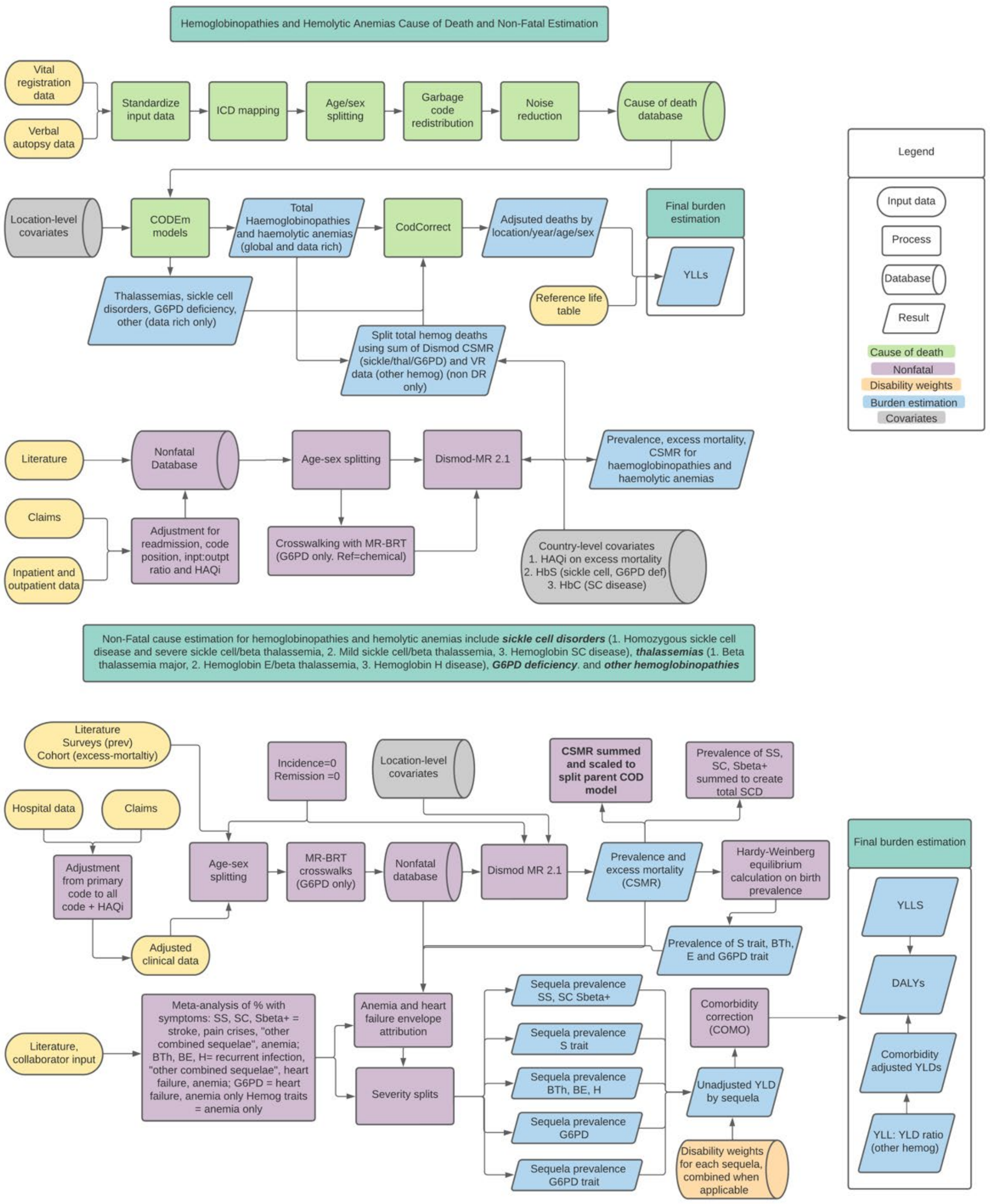

Input data and methodological summary

For GBD 2021, our approach was as follows: Cause of death Ensemble modelling (CODEm) models were developed for all of haemoglobinopathies and haemolytic anaemias combined across all age groups and years. CODEm models were run separately for males and females; one model was run for all locations (global) and a separate one for all “data-rich” locations and described elsewhere. For sub-causes of haemoglobinopathies and haemolytic anaemias, our approach was the same as GBD 2019. First, we

used vital registration (VR) cause of death data from the CoD database coded as “Other haemoglobinopathies” (input 1) to estimate a year-pooled, data-rich average mortality rate. Next, we interpolated cause-specific mortality estimates from the seven non-fatal DisMod-MR 2.1 haemoglobinopathy models (beta-thalassaemia major, haemoglobin E/beta-thalassaemia, haemoglobin H disease, homozygous sickle cell and severe sickle cell/beta-thalassaemia, haemoglobin SC disease, mild sickle cell/beta-thalassaemia, and G6PD deficiency) to obtain a set of complete estimation years (1980–2022) for each (input 2). We then calculated the proportion of total haemoglobinopathies cause-specific mortality due to sickle cell disease by summing the three sickle cell disorder models of transformed input 2, and dividing by the sum of transformed inputs of 1 and 2. Finally, we applied this proportion to CSMR estimates from the CODEm total haemoglobinopathies model (input 3) to create a set of scaled results attributable to sickle cell disorders in data sparse locations.

Input data

Input data to CODEm models were centrally processed along with all other specific causes of death and stored in the cause of death (COD) database. Data processing steps are described elsewhere. It should be noted that updates to garbage code redistribution algorithms in GBD 2021 had substantial impact on the CODEm input data in some location-year-age-sex combinations. Outliers were identified as those data where age patterns or temporal patterns were inconsistent with neighbouring age groups or locations, or where sparse data were predicting implausible overall temporal or age patterns for a given location. Covariates used in each of the CODEm models, along with their level and direction, are shown in the table below. Most notably, prevalence of haemoglobin S trait and haemoglobin C trait, as estimated by the Malaria Atlas Project, were added as covariates to the total CODEm model and the sub-cause models for sickle cell disorders. Other haemoglobinopathies and haemolytic anaemias has several covariates unique to it, reflecting the risk factors for aplastic anaemias that constitute a large proportion of this cause category.

Table 1. Covariates used in <u>haemoglobinopathies and haemolytic anaemias</u> CODEm models (data-rich and global models)

Level	Covariate	Direction	Cause
1	Sickle S trait from Malaria Atlas Project	+	Total (squared), sickle (linear)
	Sickle C trait from Malaria Atlas Project	+	Total (squared), sickle (linear)
	Lysenko 1 (holoendemic) proportion	+	Total, thal
	Haemoglobinopathies prevalence * excess mortality	+	All
	Sickle cell and thalassaemias prevalence * excess mortality	+	All
	SEV – Leukaemia	+	Other
	SEV – WaSH (water)	+	Other
	SEV – WaSH (sanitation)	+	Other
2	Maternal care and immunisation (MCI)	-	Total, sickle
	Healthcare Access and Quality Index	-	All
	SEV – drugs/alcohol (age-standardised)	+	Other
	SEV – high BMI (age-specific)	+	Other
3	Lag-distributed income (LN-transformed)	-	All
	Population proportion (0-15 latitude)	+	Total, sickle, thal, G6PD
	Population proportion (15-30 latitude)	+	Total, sickle, thal, G6PD
	Population proportion (30-45 latitude)	-	Total, thal, G6PD
	Population proportion (45+ latitude)	-	Total, sickle, thal, G6PD

	Education (years per capita)	-	Total, other
	Education (proportion w 6+ years schooling)	-	Sickle, thal, G6PD, other
	Education (proportion w 12+ years schooling)	-	Sickle, thal, G6PD, other
	Socio-demographic Index	-	All

***Level refers to a categorical assessment of the strength of mechanistic relationship between the covariate and mortality (1 = more likely; 3 = less likely); direction refers to the direction of the relationship (1 = positive correlation; -1 = negative correlation).**

Modelling strategy

As mentioned above, DisMod-MR 2.1 was used to estimate sickle cell disorders, thalassaemias, and G6PD deficiency age- and sex-specific prevalence and mortality for each location and year in the GBD. More details on this modelling process, including input data processing, are described in the corresponding non-fatal appendix section. Briefly, each datum for sickle cell disease models was used for one of three mutually exclusive conditions: 1) homozygous sickle cell disease and severe sickle cell/beta-thalassaemia, 2) mild sickle cell/beta-thalassaemia, or 3) haemoglobin SC disease. We similarly extracted data for thalassaemias using three mutually exclusive disease states: 1) beta-thalassaemia major, 2) haemoglobin E/beta-thalassaemia, and 3) haemoglobin H disease. G6PD deficiency was estimated as a single model. Cause-specific mortality rates for other haemoglobinopathies and haemolytic anaemias, lacking more specific data, were assumed to be geographically uniform, but did vary by age and sex; the levels and trends were informed by analysis of VR data from the COD database.

Case definitions for each of the types of thalassemias and sickle cell were based on genotype. G6PD deficiency is an X-linked recessive genetic disease, and our reference definition was based on quantitative decline in G6PD activity reagent (ie, chemical) testing. Three sources of data were used for DisMod-MR 2.1 models: literature (generally from community prevalence surveys, birth screening, and cohort studies), claims data, and ICD-9 & ICD-10 hospital discharge data that were adjusted for ICD code position, readmission, inpatient-to-outpatient ratio, and location-specific Healthcare Access and Quality Index. We added data from select geographies identified by GBD collaborators for GBD 2019. Of note, there were no hospital data available for haemoglobin E/beta-thalassaemia, haemoglobin H disease, or G6PD deficiency. Our last comprehensive literature review was completed in GBD 2016, where we identified data on prevalence, excess mortality rate, or with-condition mortality rate. Age-specific survival probabilities from cohort studies were converted to corresponding with-condition mortality rates.

The primary limitation of our estimation is data availability, especially in the locations thought to have the highest burden. We elected a hybrid approach of CODEm and DisMod-MR 2.1 to improve the quality of estimates in data-poor locations, but in most of these location data are still relatively sparse for non-fatal models, which leads to relatively large uncertainty. Further adding to the uncertainty is the fact that the mechanism of death in many with haemoglobinopathies is due to infectious agents such as malaria, lower respiratory infections, and diarrhoea, or due to cardiovascular diseases such as ischaemic heart disease or stroke, and are associated with increased risk of death during pregnancy. In locations with poor diagnostic capabilities and high infectious burden, it is thus very plausible that mortality due to haemoglobinopathies may be even higher. Secondly, our specification of seven distinct entities for DisMod-MR 2.1 models does not align perfectly with the cause categories in the central COD prep, which limits the extent to which CSMR data from the COD database can inform non-fatal models.

Acute hepatitis

Flowchart

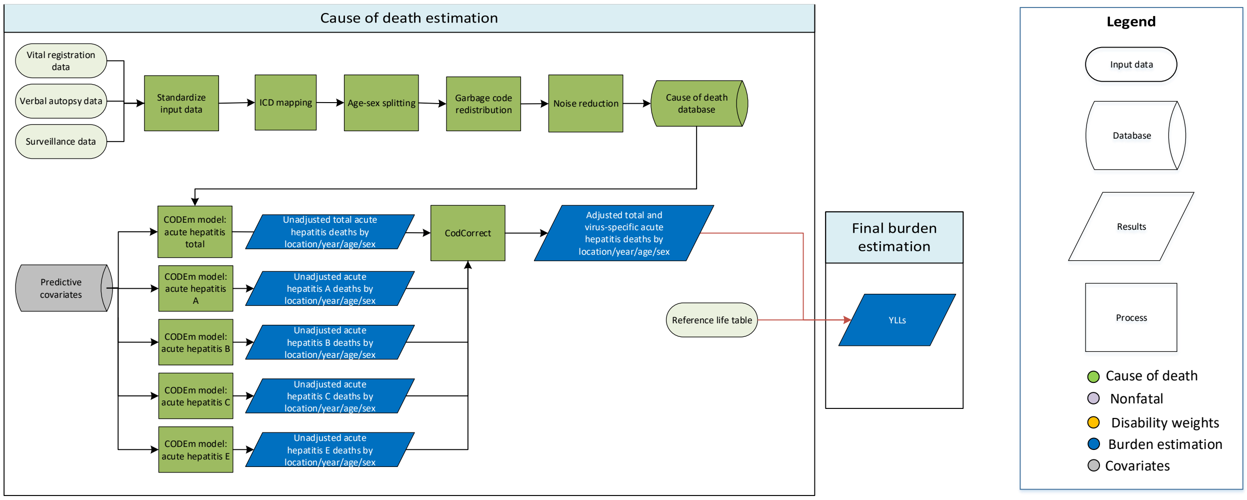

Input data and methodological summary for acute hepatitis

"Acute hepatitis" in GBD methodology refers to acute viral hepatitis caused by the hepatitis A, B, C, or E viruses.

Input data

We modelled acute hepatitis mortality using vital registration, surveillance, and verbal autopsy data from the cause of death database. The table below shows the ICD codes mapped directly to acute hepatitides. All viral hepatitis deaths were included in the database for total acute hepatitis; those that specified virus type (A, B, or E) were mapped directly to separate databases by viral type. Additionally, acute delta infections of hepatitis B carrier deaths were mapped to acute hepatitis B.

Table 1: List of International Classification of Disease (ICD) codes for acute hepatitis

Cause	ICD system	Codes
Acute hepatitis A	10	B15,B15.0,B15.9
Acute hepatitis A	9	070.0,070.1
Acute hepatitis B	10	B16,B16.0,B16.1,B16.2,B17.0,B19.1,B19.10,B19.11,P35.3
Acute hepatitis B	9	070.2,070.20,070.21,070.42,070.52
Acute hepatitis E	10	B17.2
Acute hepatitis E	9	070.43,070.53

No substantive changes in hepatitis redistribution were made since GBD 2019. We continued to redistribute unspecified hepatitis deaths based on the analysis in GBD 2019, in which we found that when unspecified hepatitis ICD codes are assigned as underlying cause of death, ICD codes reflecting the presence of chronic liver disease were frequently found in the cause of death chain. Given this, hepatitis unspecified deaths were redistributed mostly to cirrhosis and other chronic liver diseases, and a small

proportion were redistributed to acute hepatitis B and C. This contrasts GBD 2017 and earlier rounds, when all hepatitis B unspecified deaths were redistributed to acute hepatitis deaths. Unspecified acute viral hepatitis deaths were included in the database for total acute hepatitis and distributed proportionately to the databases for acute hepatitis due to hepatitis A, B, C, and E.

Outliers were identified by systematic examination of datapoints for all location-years. Data were excluded if they violated well-established time or age trends, and data in instances where garbage code redistribution and noise reduction, in combination with small sample sizes, resulted in unreasonable cause fractions. Methods for assigning outlier status were consistent across data types.

Modelling strategy

The models used to estimate acute hepatitis mortality employed the GBD's standard approach of running two models – 1) a global CODEm model of all locations, using all data in the CoD database; and 2) a CODEm model restricted to data-rich countries – and hybridising the results. (See appendix section on CODEm method for details.)

In GBD 2021, we updated the linear floor value in our CODEm models to allow the model to be influenced by lower data, which resulted from changes to COD data processing. These data processing changes include changing the method of estimating the non-zero floor and population-size cutoffs for when to noise-reduce at the country versus region level, and are described in greater detail in the appendix section on GBD 2021 Causes of Death database.

We modelled acute hepatitis deaths encompassing all hepatitis virus types (A, B, C, and E) in a parent CODEm model and also modelled acute hepatitis A, B, C, and E in separate CODEm models. The virus-specific acute hepatitis deaths were then rescaled to fit within the envelope defined by the parent acute hepatitis CODEm model through the CoDCorrect process.

The following tables report the covariates included for possible selection in each model.

Table 2: Covariates used in parent acute hepatitis mortality modelling

Level	Covariate	Direction
1	SEV scalar age-standardised hepatitis	+
	Vaccine-adjusted HBsAg seroprevalence, age-standardised	+
	Seroprevalence (anti-HCV), age-standardised	+
	Seroprevalence (anti-HAV), age-standardised	+
	Seroprevalence (anti-HEV), age-standardised	+
2	Healthcare Access and Quality Index	-
	SEV unsafe sanitation	+
	SEV unsafe water	+
	Socio-demographic Index	-
	Hep B vaccine coverage proportion, aged through time	-
	Injection drug use proportion by age	+
3	Education (years per capita)	-
	Lag distributed income (LDI) (ln transformation)	-

Table 3: Covariates used in acute hepatitis A mortality modelling

Level	Covariate	Direction
1	SEV scalar (hepatitis)	+
	Seroprevalence (anti-HAV), age-standardised	+
2	Healthcare Access and Quality Index	-
	SEV unsafe sanitation	+
	SEV unsafe water	+
	Socio-demographic Index	-
3	Education (years per capita)	-
	Lag distributed income (LDI) (ln transformation)	-

Table 4: ***Covariates used in acute hepatitis B mortality modelling***

Level	Covariate	Direction
1	Vaccine adjusted HBsAg seroprevalence, age-standardised	+
	Injection drug use proportion by age	+
	Hep B vaccine coverage proportion, aged through time	-
2	Healthcare Access and Quality Index	-
	Socio-demographic Index	-
	SEV scalar (hepatitis)	+
3	Education (years per capita)	-
	Lag distributed income (LDI) (ln transformation)	-

Table 5: Covariates used in acute hepatitis C mortality modelling

Level	Covariate	Direction
1	Seroprevalence (anti-HCV), age-standardised	+
	Injection drug use proportion by age	+
2	Healthcare Access and Quality Index	-
	Socio-demographic Index	-
	SEV scalar (hepatitis)	+
3	Education (years per capita)	-
	Lag distributed income (LDI) (ln transformation)	-

Table 6: Covariates used in acute hepatitis E mortality modelling

Level	Covariate	Direction
1	SEV scalar (hepatitis)	+
	Seroprevalence (anti-HEV), age-standardised	+
2	Healthcare Access and Quality Index	-
	SEV unsafe sanitation	+

	SEV unsafe water	+
	Socio-demographic Index	-
	Proportion of the population living in the classic monsoon region	+
3	Education (years per capita)	-
	Lag distributed income (LDI) (ln transformation)	-

HIV/AIDS

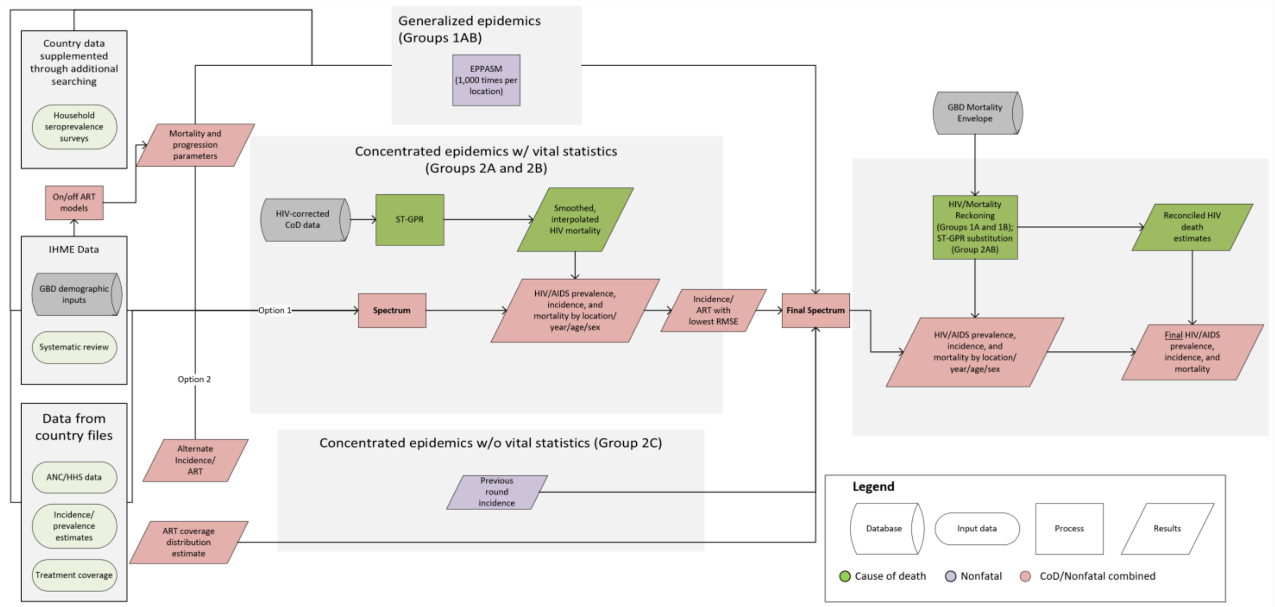

Case definition

Infection with the human immunodeficiency virus (HIV) causes influenza-like symptoms during the acute period following infection and can lead to acquired immunodeficiency syndrome (AIDS) if untreated. HIV attacks the immune system of its host, leaving infected individuals more susceptible to opportunistic infections like tuberculosis. Although there are two different subtypes of HIV, HIV-1 and HIV-2, no distinction is made in our estimation process or presentation of results. For HIV, ICD-10 codes are B20-B24, C46-C469, D84.9; ICD-9 codes are 042-044, 112-118 (after 1980), 130 (after 1980), 136.3-136.8 (after 1980), 176.0-176.9 (after 1980), 279 (after 1980); and ICD-9 BTL codes are B184-B185.

Input data

Household seroprevalence surveys
Geographically representative HIV seroprevalence survey results were used as inputs to the model for countries with generalised HIV epidemics where available.

GBD demographic inputs
Location-specific population, fertility, migration, and HIV-free survival rates from GBD 2021 were used as inputs in modelling all locations.

Data from countries
The files compiled by UNAIDS for their HIV/AIDS estimation process were our main source of data for producing estimates of HIV burden. The files are often built by within-country experts with the support of UNAIDS, which publishes estimates annually on behalf of countries and only shares their files when permission is granted. The files contain the HIV-specific information which is needed to run the Spectrum,[1] and Estimation and Projection Package-Age Sex[2] (EPP-ASM) models.

Spectrum and EPP-ASM require the following input data: AIDS mortality among people living with HIV with and without ART, CD4 progression among people living with HIV not on ART, ART coverage among adults and children, cotrimoxazole coverage among children, coverage of breastfeeding among women living with HIV, prevention of mother-to-child transmission coverage, and CD4 thresholds for treatment eligibility. EPP-ASM additionally uses HIV prevalence data from surveillance sites and representative surveys. Antenatal care (ANC), incidence, prevalence, and treatment coverage data from UNAIDS were used in modelling for all locations. We extracted all of these data from the proprietary format used by UNAIDS.

Changes for GBD 2021

We supplemented the antenatal care and treatment coverage data available through processing done by the Local Burden of Disease team[3] and retrieving data on adult antiretroviral (ART) treatment coverage rates from country reports, respectively. The addition of ANC sites affected 33 countries, while ART data were added in 45 countries. During the Local Burden of Disease alignment process, the antenatal care clinic prevalence estimates were corrected in a number of facets. There were 17 estimates with placeholder sample sizes that were corrected, duplicate observations in Togo were removed, 123 additional observations were added, 1491 non-ANC observations were removed, and 232 points were outliered.

We did not have country UNAIDS files for 40 locations, many of them countries with small populations and/or low HIV prevalence. As in previous rounds, we generated regional averages of all needed inputs in these locations. This enabled us to run Spectrum for every GBD location.

Vital registration data

We used all available sources of vital registration and sample registration data from the GBD Causes of Death database after garbage code redistribution and HIV/AIDS mis-coding correction[4] in Group 2 countries and India. There are two different cause of death data sources for HIV/AIDS in China: the Disease Surveillance Points (DSP) system and the Notifiable Infectious Disease Reporting (NIDR) system. Both systems are administered by the Chinese Center for Disease Control and Prevention, but the reported number of deaths due to HIV is significantly lower in DSP. Therefore, we have used the provincial-level ratio of deaths due to HIV/AIDS from NIDR to those from DSP, choosing the larger ratio between years 2013 and 2014, and scaled the reported deaths in the DSP system, which is in turn used in the spatiotemporal Gaussian process regression (ST-GPR). ST-GPR deaths were used as final deaths in group 2A and group 2B.

On-ART literature data

Data were identified by using search string: "hiv"[MeSH Terms] OR "hiv"[All Fields]) AND ("mortality"[Subheading] OR "mortality"[All Fields] OR "mortality"[MeSH Terms]) AND antiretroviral[All Fields] AND ("therapy"[Subheading] OR "therapy"[All Fields] OR "therapeutics"[MeSH Terms] OR "therapeutics"[All Fields]) in PubMed.

To be included, studies must include only HIV-positive people over the age of 15 who receive antiretroviral therapy (ART) but who were ART-naïve prior to the study. In addition, studies must report either a duration-specific (time since initiation of ART) mortality proportion or a hazard ratio across age or sex, and must not include children.

For duration-specific survival data, studies must report uncertainty on mortality estimates or provide stratum-specific sample sizes and must include duration-specific data to allow for calculation of 0–6, 7–

12, or 13–24-month conditional mortality. In addition, studies must either report separate mortality and loss-to-follow-up (LTFU) curves, be corrected for LTFU using vital registration data or double sampling, or be conducted in a high-income setting. Finally, studies must report the percentage of participants who are male and the median age of participants.

Hazard ratio data for ages or sexes can only be used if the hazard ratios are controlled for other variables of interest (age, sex, and CD4 category). In GBD 2021, we included 61 studies, 13 of which were new this cycle. Of these studies, we added ten to inform the estimation age-sex hazard ratios, and three studies informed LFTU curves.

PRISMA flow diagram for GBD 2021 on-ART systematic review

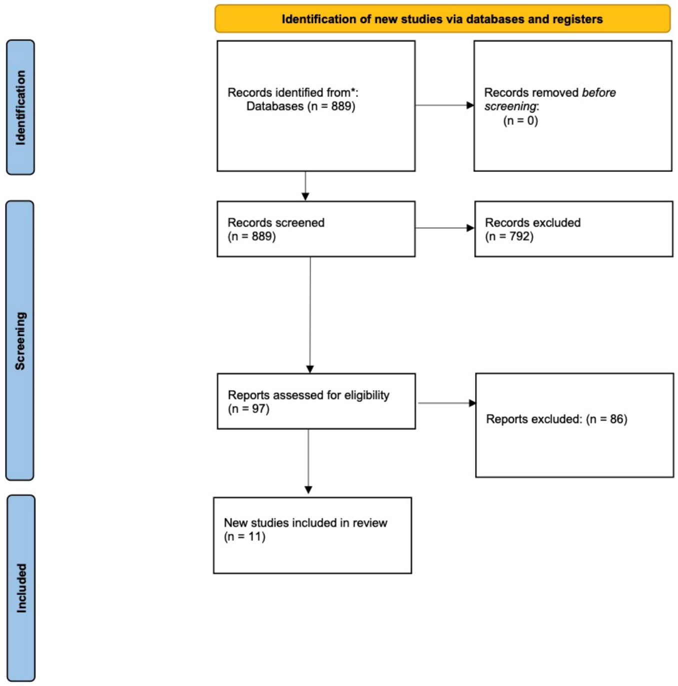

*Note: This systematic review was an update to the GBD 2019 review and doubled as a historical review of sources to capture previously missed studies. As a result, the number of sources being reviewed and excluded are ongoing.

From: Page MJ, McKenzie JE, Bossuyt PM, Boutron I, Hoffmann TC, Mulrow CD, et al. The PRISMA 2020 statement: an updated guideline for reporting systematic reviews. BMJ 2021;372:n71. doi: 10.1136/bmj.n71

For more information, visit: http://www.prisma-statement.org/

Off-ART literature data

In GBD 2013, we systematically reviewed the literature on mortality without ART to characterise uncertainty in the progression and death rates. We searched terms related to pre-ART or ART-naïve survival since seroconversion.[5] After screening, we identified 13 cohort studies that included the cohorts used by UNAIDS, from which we extracted survival at each one-year point after infection. Screening for additional, recently published studies in GBD 2015, GBD 2016, and GBD 2017 identified no new cohort studies for inclusion in this analysis. We did not search for new studies in GBD 2019 or GBD 2021.

Severity splits and disability weights

The basis of the GBD disability weight survey assessments are lay descriptions of sequelae highlighting major functional consequences and symptoms. The lay descriptions and disability weights for HIV/AIDS severity levels are shown below.

Severity level	Lay description	DW (95% CI)
Symptomatic HIV	Has weight loss, fatigue, and frequent infections.	0.274 (0.184–0.377)
AIDS with antiretroviral treatment	Has occasional fevers and infections. The person takes daily medication that sometimes causes diarrhoea.	0.078 (0.052–0.111)
AIDS without antiretroviral treatment	Has severe weight loss, weakness, fatigue, cough and fever, and frequent infections, skin rashes, and diarrhoea.	0.582 (0.406–0.743)

Modelling strategy

Countries were divided into groups: Groups 1A, 1B, and 2A, 2B, and 2C.

Group 1 includes countries with HIV prevalence data from antenatal clinics or nationally- or subnationally-representative population-based seroprevalence surveys. Group 1A included countries with a peak of at least 0.5% prevalence, and Group 1B includes countries with a peak of at least 0.25% prevalence and vital registration completeness less than 65%.

The remaining countries made up Group 2,which are further subdivided in Group 2A, 2B and 2C based on availability of vital registration data. Group 2A consisted of countries with high-quality vital registration data; Group 2B consisted of countries with any available vital registration data, which was generally lower-quality; and Group 2C countries were those without any vital registration data. Quality was measured based on a star rating system as described elsewhere.[6]

Both groups of countries relied on the same approach to modelling on- and off-ART mortality, as described below.

On-ART

Changes for GBD 2021
In GBD 2021, we replaced the use of DisMod-MR[7] in favour of the meta-regression—Bayesian, regularised, trimmed (MR-BRT) model.[7] This model is a mixed effects meta-regression that accounts for between-study heterogeneity and bias. We ran a total of 90 models to arrive at our final on-ART mortality results: one for each age group (15–25, 25–35, 35–45, 45–55, or 55–100), sex (male or female), duration since ART initiation (0–6, 7–12 or 13–24 months) and super-region (sub-Saharan Africa, high-income, or other) strata.

We corrected reported probabilities of death for loss to follow-up using an approach developed by Verguet and colleagues.[8] Verguet and colleagues used tracing and follow-up studies to empirically estimate the relationship between death in LTFU and the rate of LTFU.

To create estimates of sex-specific hazard ratios, we use the *metan* function in Stata to create estimates of relative risks separately by region, using female age groups as the reference group. The age and sex hazard ratios were applied to the study-level mortality rates, accounting for the distribution of ages and sexes in the mortality data. We then subtracted HIV-free mortality from the model life table process to calculate study-level age-sex HIV-specific mortality.

We then used MR-BRT to synthesise the age-sex-split study-level data into estimates of conditional probability of death over initial CD4 count. We replaced our on-ART mortality rates with those estimated off treatment if they were higher.

Off-ART

Following UNAIDS assumptions, no-ART mortality is modelled as shown in the figure below.

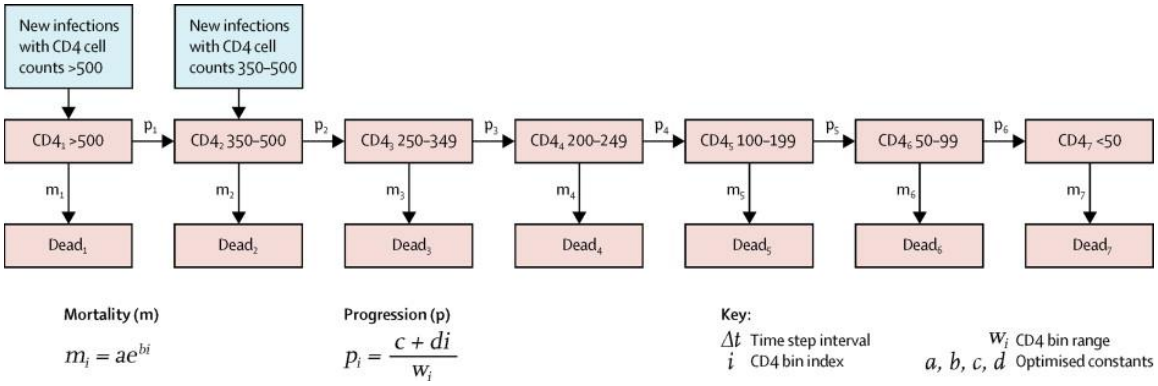

The death and progression rates between CD4 categories vary by age according to four age groups: 15–24 years, 25–34 years, 35–44 years, and 45 years or older. We modelled the logit of the conditional probability of death between years in these studies using the following formula:

$$logit\ (m_{ijk}) = \beta_0 + \sum_{i=1}^{4} \beta_{1i}\, a_i + \sum_{j=1}^{12} \beta_{2j} t_j + u_k + \varepsilon_{ijk}$$

In the formula, *m* is conditional probability of death from year t_j to t_{j+1}, a_i is an indicator variable for age group at seroconversion (15–24 years, 25–34 years, 35–44 years, and 45 years or older), t_j is an indicator variable of year since seroconversion, and u_K is a study-level random effect.

By sampling the variance-covariance matrix of the regression coefficients and the study-level random effect, we generated 1000 survival curves for each age group that capture the systematic variation in survival across the available studies. For each of the 1000 survival curves, we used a framework modelled after the UNAIDS optimisation framework in which we find a set of progression and death rates that minimises the sum of the squared errors for the fit to the survival curve.[9,10]

We estimate mortality for each region in its own DisMod model based on data from the IeDEA cohort collaboration,[11] and include a covariate for year as mortality among the LFTU has been found to decline

in recent years.[12] Finally, in cases where on-ART rates were higher, we replaced our estimated on-ART mortality rates by rates off ART to account for progression to lower CD4 categories. This ensured individuals would not experience higher mortality when they entered treatment in Spectrum or EPP-ASM.

GBD 2021 burden estimation overview

We used two different components to derive year-, age- and sex-specific estimates of HIV incidence, prevalence, and mortality depending on locations' availability of data and extent of HIV burden, as described below:

1. EPP-ASM was used to estimate incidence, prevalence, and mortality that are consistent with serosurveillance data from antenatal care clinics and/or prevalence surveys.

2. Spectrum is a compartmental HIV progression model used to generate age-sex-specific incidence, prevalence, and death rates from input incidence and prevalence curves and assumptions about intervention scale-up and local variation in epidemiology. This model was used in conjunction with EPP-ASM for India and for all Group 2 countries.

EPP-ASM model

For GBD 2021, we continued to use our modified version of EPP-ASM both to improve the fit to data and to generate paediatric estimates. We built a paediatric module in EPP-ASM that mirrored early updates to the paediatric module in Spectrum.[13] This child module included CD4 progression and CD4-specific mortality rates taken from a model fit to survival data from IeDEA and child initiation of ART based on ART distribution data from IeDEA. Perinatal and breastfeeding transmission was calculated as a function of prevalence among pregnant women and PMTCT programme data. We were thus able to utilise EPP-ASM to produce HIV incidence, prevalence, and mortality estimates for all ages. Additionally, we improved fit to prevalence data through allowing flexibility in the age distribution of incidence over time. We parameterised the ratio of incidence among ages 15–24:25+ as a constant before year 2000 and a linear regression thereafter. This allowed for the shifts in the age distribution of incidence observed over the course of the HIV epidemic to be reflected in our results. Finally, we utilised GBD demographic inputs and substituted in our own assumptions about HIV progression rates and on/off-ART mortality.

To incorporate uncertainty in our demographic and progression parameters, we run EPP-ASM with separate draws of CD4 progression, on- and off-ART mortality rates, fertility, and HIV-free mortality. This process produced 1000 posterior distributions for each of the locations that make up Group 1. For every location in the group, we sampled one draw from each of the sets of EPP-ASM results to create a final distribution. By sampling one draw from each set, we ensured that the distribution of mortality parameters dictating the relationship between incidence and prevalence aligned with those used in the GBD demographics estimates.

We also continued to use the approach implemented in GBD 2019 to address selection bias resulting from temporal and geographical variation in ANC reporting. The ANC data which EPP-ASM uses cannot be assumed as representative of HIV prevalence in the full population. This is especially the case when there are minimal or no nationally representative prevalence surveys to anchor estimates, as in the early epidemic.[14]

EPP-ASM has embedded approaches to adjust for the bias associated with using prevalence among ANC-site-attending pregnant women to estimate prevalence among the both-sexes population. For the bias between pregnant women and the national both-sexes population, it makes assumptions around the difference in total fertility rate among HIV-positive and HIV-negative women, and the difference in prevalence between men and women. For the bias associated with the data coming from ANC sites, the specification of the likelihood of observed ANC data includes random intercepts for each clinic. The random intercepts allow each site's baseline prevalence to vary randomly around the overall mean prevalence. In other words, factors that could drive differences between sites' HIV prevalence levels are "adjusted" for.

However, the embedded approach does not explicitly account for the fact that the location of the clinic in space may also drive its HIV prevalence level. For example, we might expect rural sites to be more correlated than urban sites. Thus, to further adjust for this bias, we used an offset term that represents the difference in the prevalence among the national, both-sexes population and the prevalence among the female, pregnant population associated with an ANC site location. The offset term was derived for each location as the difference between the adjusted prevalence in a given site-year and the adjusted national prevalence in that year. These estimates are adjusted for covariates that are thought to influence prevalence, for example, access to health-care facilities, malaria incidence, and male circumcision.

Thus, our final strategy for estimating the likelihood of the observed ANC data was:

$$\boldsymbol{W_{st} = \varphi^{-1}(\rho_t) + \vartheta_{st} + u_s + e_{st}}$$

$$\boldsymbol{e_{st} \sim N(0, \sigma_{st}^2)}$$

$$\boldsymbol{u_s \sim N(0, \sigma_s^2)}$$

Where:

W_{st} = the probit transformed prevalence at site *s* and time *t*
ρ_t= the national prevalence adjusted to represent prevalence among pregnant women from the model simulation
ϑ_{st} = the offset term representing the difference between the adjusted prevalence in a given site-year and the adjusted national prevalence in that year
φ^{-1} = probit transformation
e_{st} = site-specific error term
u_s = site-specific intercept

Spectrum

For GBD 2013, we created an exact replica of Spectrum in Python. This enabled us to run thousands of iterations of the model at once on our computing cluster and allowed for more flexible input data structures. Additionally, we scaled all input values by a uniformly sampled factor between 0.9 and 1.1 to generate estimates with realistic ranges of uncertainty. For example, if treatment retention rates across CD4 categories were 0.906, 0.759, 0.787, 0.795, 0.785, 0.756, 0.813, and 0.700, we multiplied each number by an array of equivalent size that contained factors ranging from 0.9 to 1.1. At each draw, the array would contain different, randomly selected factors in the same range. Further, we previously improved our sex-specific modelling strategy in Spectrum by sex-splitting incidence based on a model fit to the sex ratio of prevalence observed in countries with representative surveys and updated the

Spectrum paediatric module to reflect changes made by UNAIDS.[13] Our child module was revised to include CD4 progression and CD4-specific mortality rates taken from a model fit to survival data from IeDEA. Finally, we updated child initiation of ART to include data on ART distribution from IeDEA. These changes were retained in GBD 2021.

ART coverage distribution

Spectrum determines the number of people initiating ART treatment across each CD4 category based on eligibility criteria, and the number of expected deaths and untreated people. In other words, groups with a large proportion of people living with HIV and high numbers of expected deaths initiated the most individuals into treatment.

We improved the basis for this distribution using survey microdata and country-level wealth information. Three relevant surveys were available: Uganda AIS 2011 and Kenya AIS 2007 and 2012. These surveys conducted CD4 count measurements and include a question regarding the amount of time that an individual receiving ART had been enrolled in treatment. Survey data provide cross-sectional CD4 count information; however, the Spectrum modelling framework tracks individuals by categorical CD4 count at the initiation of treatment. In order to crosswalk the cross-sectional survey data into estimates of CD4 count at treatment initiation, we built a model using relevant cohort data which tracked changes in CD4 count after initiation of treatment to translate an individual's current CD4 count and duration on treatment into CD4 count at initiation of treatment. The functional form for changes in CD4 count as a function of duration on treatment was a natural spline on duration with knots at 3, 12, 24, and 36 months, and an interaction between initial CD4 count and duration.

After crosswalking, we predicted the probability of being on treatment as a function of individual income (measured through an asset-based index), stratified by CD4 count, age, and sex. The results of this prediction were translated into country-specific age-sex-year-CD4 count probabilities of coverage using a conversion factor between individual income and lag-distributed GDP per capita. We used stochastic frontier analysis to constrain the maximum possible coverage for a given degree of income and CD4 count.

Predicted probabilities of coverage were input to Spectrum to inform the distribution, and not the overall level, of ART treatment by CD4 count. Within Spectrum, the probabilities of coverage are converted to counts of expected individuals on treatment in each CD4 count group. These are scaled to the distribution across CD4 count groups to match the input data on the number of people on ART coming from UNAIDS country files. In cases where the predicted number of individuals initiating treatment exceeds the total number of untreated individuals in a CD4 count group, we reallocate treatment evenly to other CD4 count groups.

Countries with seroprevalence surveys and antenatal clinic data (Groups 1ABC)

53 countries – as well as subnational locations in India, Kenya, Ethiopia, Nigeria, and South Africa – were included in Group 1 with available antenatal care clinic (ANC) data and/or least one geographically representative HIV seroprevalence survey. For all these locations we used EPP-ASM, which was updated to incorporate the new ANC bias adjustment.

In EPP-ASM, the transmission rate, r(t), is a simple transmission model applied at each time step (1/10 of a year) to the population. 'r' represents the number of new cases expected to emanate from a single case. Over 3000 iterations, a new r(t) is drawn, the full epidemic is determined and compared to the observed prevalence data to determine its likelihood. Beyond the end of the data, a prior distribution on r(t) helps to determine how we should expect the epidemic to behave. This assumption was different in

EPP-ASM versus EPP. In EPP-ASM in most countries, we extended a random walk into the future based on the 'r-hybrid' r(t). The r-hybrid assumes a logistic decay until the year 2003, a linear interpolation until year 2008, and a random walk form after this.

Changes for GBD 2021

For India, Comoros, São Tomé and Príncipe, and Mauritania, we used EPP-ASM to model HIV burden for GBD 2021. For India, we used EPP-ASM in combination with Spectrum, to be able to capitalise on SRS data. We also used an 'equilibrium prior,' for r(t) rather than 'r-hybrid', which provided a better fit to the comparatively lower magnitude of the epidemic. The equilibrium prior extends into the future with a rate of change following a normal distribution with a mean equal to the value of r expected when the proportion of the population infected is saturated, ie, the epidemic has stabilised.

The HIV/mortality reckoning process is intended as a method of reconciling separate estimates of HIV mortality (and its resulting effect on estimates of HIV-free and all-cause mortality) in Group 1 countries by averaging estimates of HIV mortality from the model life table process and EPP-ASM. Additional details on the reckoning can be found elsewhere.[15]

Since EPP-ASM produces HIV incidence, prevalence, and deaths that are consistent with one another over time, the reckoning process results in death numbers that are no longer consistent with the incidence and prevalence produced in Spectrum. To recreate this consistency, we recalculated incidence for all Group 1 locations using reckoned deaths and prevalence produced by EPP-ASM. The updated incidence is calculated by aggregating counts of new infections, HIV deaths from EPP-ASM, and HIV deaths after reckoning at the year-sex level. The difference between reckoned HIV deaths and HIV deaths from EPP-ASM is added to EPP-ASM incidence, and we calculate the ratio between updated incidence and EPP-ASM incidence. Age-specific counts of new infections are then scaled by their corresponding sex-year ratios.

Countries with vital registration data (Groups 2A and 2B)

Vital registration is one of the highest-quality sources of data on HIV burden in many countries, so generating estimates that are consistent with these data with necessary adjustment to account for any potential underreporting is critical. We identified 121 countries – as well as 760 subnational locations from China, Japan, Indonesia, India, Mexico, Sweden, the Philippines, Poland, Italy, the UK, Ukraine, Russia, New Zealand, Iran, Norway, and the USA – with usable points of vital registration data, verbal autopsy (VA) data, or sample registration system (SRS) data. In India, Vietnam, and Indonesia, we used SRS and VA data, respectively, as input mortality for CIBA. For India, we extracted the resulting age-sex distribution of incidence but scaled the level to match the adult incidence rate estimated from EPP for each state.

We imputed missing years of data to generate a complete time series for HIV from the estimated start year of the epidemic using ST-GPR. We analysed mortality trends using ST-GPR starting in 1981, the year that HIV was first identified in the USA.[16] For ST-GPR, we adjusted the lambda (time weight) and GPR scale according to the completeness of vital registration data, with 4- and 5-star quality VR using parameters designed to follow the data more closely. We produced separate splines by country/age group, up to the peak year of death rate. We then ran a linear regression with fixed effects on region, age, and sex. Following this, we ran space-time residual smoothing, in which time, age, and space weights are used to inform smoothing of the residuals between datapoints and the linear regression estimate. From this process, we generated space-time estimates with the applied weights, along with the median absolute deviation (MAD) of the space-time estimates from the data. The MAD was

calculated at various levels of the geographical hierarchy (eg, subnational and national), and was added into the data variance term. The data variance and space-time estimates were then analysed using Gaussian process regression to return a final estimate of mortality along with uncertainty.

Although Spectrum produces HIV mortality estimates that are within the realm of possibility in most countries using the incidence curves provided in the UNAIDS country files, it is a deterministic model that has not yet been integrated into an optimisable framework. Therefore, in order to "fit" it to vital registration data, we need to adjust input incidence. In contrast to GBD 2019 and previous cycles, in addition to adjusting input incidence, we determined the most plausible best treatment input based on fit to vital registration as well.

Changes for GBD 2021

For GBD 2021, we then created a grid of incidence and treatment options and reran Spectrum for each using each of these options, rather than using the CIBA-adjusted incidence for our final Spectrum run in all locations. The incidence options included the CIBA-adjusted incidence and the non-CIBA-adjusted incidence from the initial Spectrum run, both using the most recent data and the last cycle, in addition to incidence data available from public-use UNAIDS files. The adult ART options included the data available from public-use UNAIDS files. Where these data were provided in terms of the number of people on treatment, we created additional treatment options by dividing the number on treatment by prevalence, as estimated by the current and previous GBD cycles. We ran Spectrum on every combination of incidence and treatment options and determined the root mean squared error of the resulting mortality relative to the vital registration data.

Finally, to produce location-, year-, age-, and sex-specific estimates of HIV incidence, prevalence, and mortality, we ran a final Spectrum run using the incidence and treatment option that resulted in the best fit to VR data, or the lowest RMSE.

Countries without survey data and vital registration data (Group 2C)
32 countries had neither geographically representative seroprevalence surveys nor reliable vital registration systems. To produce estimates of HIV burden in these countries, we used Spectrum to produce estimates of burden. As above, the estimates of incidence, prevalence, and mortality were incorporated into the rest of the machinery via the reckoning process.

Changes for GBD 2021
Group 2C countries no longer sampled bias adjustment ratios from other Group 2 countries within the same super-region.

References

1 Stover J, Glaubius R, Mofenson L, *et al.* Updates to the Spectrum/AIM model for estimating key HIV indicators at national and subnational levels. *AIDS* 2019; **33 Suppl 3**: S227–34.

2 Eaton JW, Brown T, Puckett R, *et al.* The Estimation and Projection Package Age-Sex Model and the r-hybrid model: new tools for estimating HIV incidence trends in sub-Saharan Africa. *AIDS* 2019; **33**: S235–44.

3 Dwyer-Lindgren L, Cork MA, Sligar A, *et al.* Mapping HIV prevalence in sub-Saharan Africa between 2000 and 2017. *Nature* 2019; **570**: 189–93.

4 Kyu HH, Jahagirdar D, Cunningham M, *et al.* Accounting for misclassified and unknown cause of death data in vital registration systems for estimating trends in HIV mortality. *Journal of the International AIDS Society* 2021; **24**: e25791.

5 Murray CJL, Ortblad KF, Guinovart C, *et al.* Global, regional, and national incidence and mortality for HIV, tuberculosis, and malaria during 1990-2013: a systematic analysis for the Global Burden of Disease Study 2013. *Lancet* 2014; **384**: 1005–70.

6 Vos T, Lim SS, Abbafati C, *et al.* Global burden of 369 diseases and injuries in 204 countries and territories, 1990–2019: a systematic analysis for the Global Burden of Disease Study 2019. *The Lancet* 2020; **396**: 1204–22.

7 Zheng P, Barber R, Sorensen RJD, Murray CJL, Aravkin AY. Trimmed Constrained Mixed Effects Models: Formulations and Algorithms. *Journal of Computational and Graphical Statistics* 2021; **30**: 544–56.

8 Verguet S, Lim SS, Murray CJL, Gakidou E, Salomon JA. Incorporating Loss to Follow-up in Estimates of Survival Among HIV-Infected Individuals in Sub-Saharan Africa Enrolled in Antiretroviral Therapy Programs. *The Journal of Infectious Diseases* 2013; **207**: 72–9.

9 Ghys PD, Żaba B, Prins M. Survival and mortality of people infected with HIV in low and middle income countries: results from the extended ALPHA network. *AIDS (London, England)* 2007; **21**: S1.

10 Hallett TB, Zaba B, Todd J, *et al.* Estimating Incidence from Prevalence in Generalised HIV Epidemics: Methods and Validation. *PLOS Medicine* 2008; **5**: e80.

11 International epidemiologic Databases to Evaluate AIDS (IeDEA). https://www.nichd.nih.gov/. https://www.nichd.nih.gov/research/supported/iedea (accessed Feb 22, 2022).

12 Zürcher K, Mooser A, Anderegg N, *et al.* Outcomes of HIV-positive patients lost to follow-up in African treatment programmes. *Trop Med Int Health* 2017; **22**: 375–87.

13 MAHY M, PENAZZATO M, CIARANELLO A, *et al.* Improving estimates of children living with HIV from the Spectrum AIDS Impact Model. *AIDS* 2017; **31**: S13–22.

14 Ng M, Gakidou E, Murray CJ, Lim SS. A comparison of missing data procedures for addressing selection bias in HIV sentinel surveillance data. *Population Health Metrics* 2013; **11**: 12.

15 GBD 2019 Demographics Collaborators. Global age-sex-specific fertility, mortality, healthy life expectancy (HALE), and population estimates in 204 countries and territories, 1950–2019: a comprehensive demographic analysis for the Global Burden of Disease Study 2019. *The Lancet* 2020; **396**: 1160–203.

16 Pneumocystis Pneumonia --- Los Angeles. https://www.cdc.gov/mmwr/preview/mmwrhtml/june_5.htm (accessed Feb 22, 2022).

HIV/AIDS—multidrug-resistant tuberculosis without extensive drug resistance, HIV/AIDS—extensively drug-resistant tuberculosis, and HIV/AIDS—drug-susceptible tuberculosis

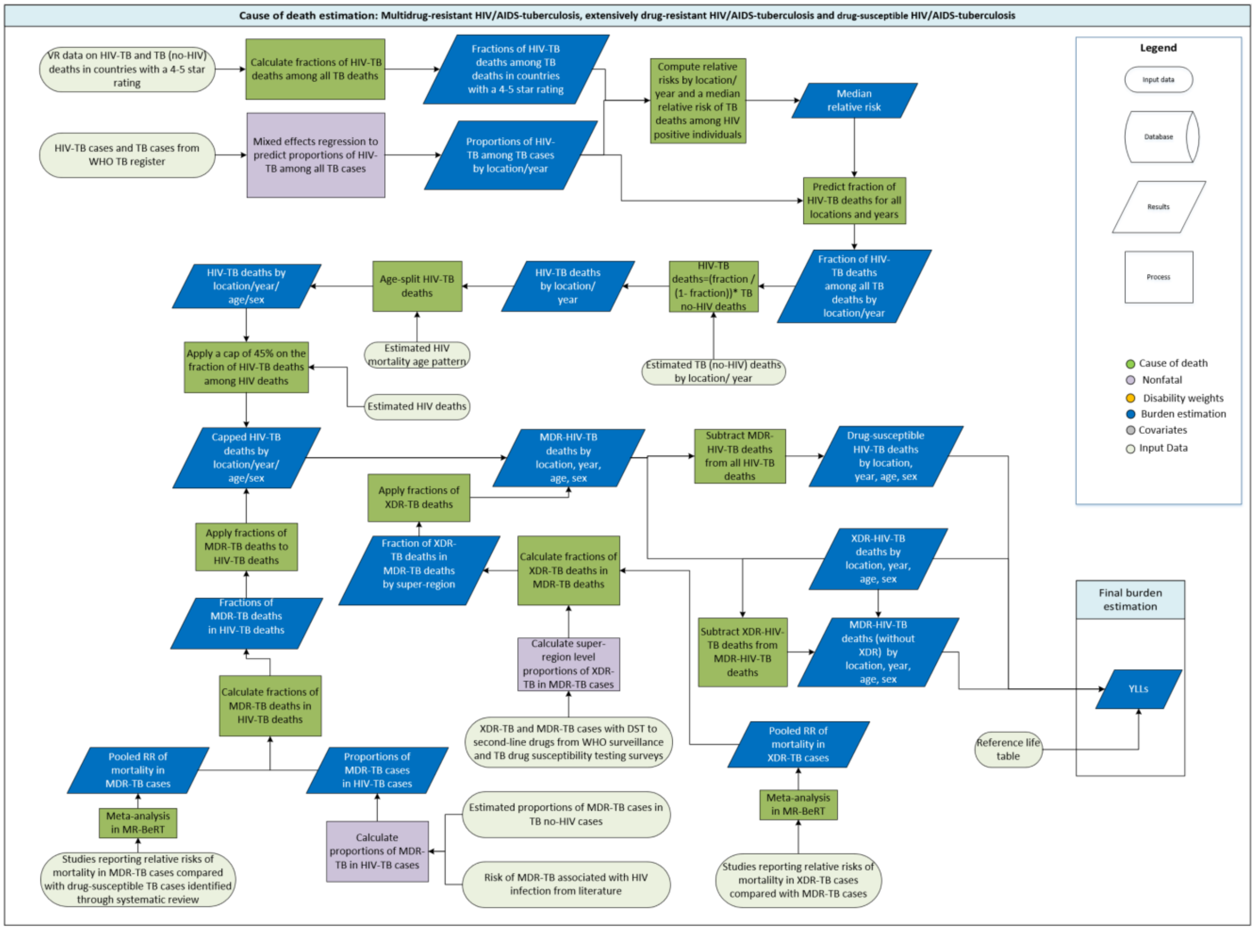

Input data

Input data for HIV/AIDS-tuberculosis (HIV-TB) mortality estimation include (i) 1277 site-years of vital registration data from countries with a four- or five-star rating where cause of death data for directly coded HIV-TB and tuberculosis (TB) were available, and (ii) the number of TB cases (new and re-treatment) recorded as HIV-positive and the number of TB cases (new and re-treatment) with an HIV test result recorded in the TB register from the World Health Organization (WHO). We excluded data from countries with ten HIV-TB deaths or less. We also excluded data that were largely conflicting with the majority of data for other years from the same country.

Input data for estimation of multidrug-resistant and extensively drug-resistant HIV-TB include: (i) the number of drug-resistant cases by type (multidrug-resistant tuberculosis [MDR-TB], extensively drug-resistant tuberculosis [XDR-TB], all TB cases with a drug sensitivity testing [DST] result for isoniazid and rifampicin, and MDR-TB cases with DST for second-line drugs) from routine surveillance and surveys reported to WHO. Additional input data include relative risks of mortality in MDR-TB cases compared

with drug-susceptible TB cases, and relative risks of mortality in XDR-TB cases compared with MDR-TB cases reported by studies identified through our systematic review, and the risk of MDR-TB associated with HIV infection from the literature.[1]

Prisma diagram of MDR-TB mortality relative risk in GBD 2021

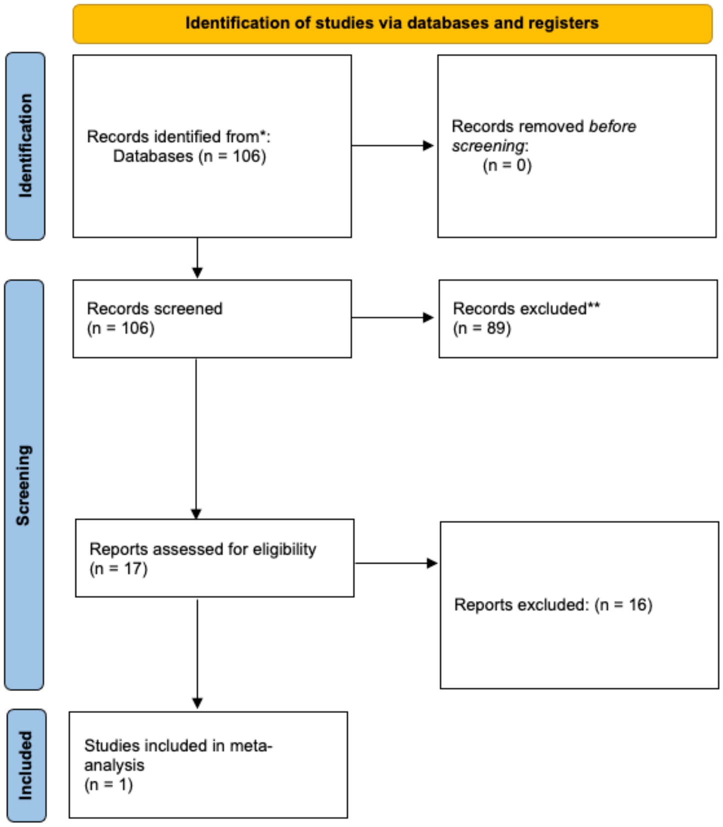

From: Page MJ, McKenzie JE, Bossuyt PM, Boutron I, Hoffmann TC, Mulrow CD, et al. The PRISMA 2020 statement: an updated guideline for reporting systematic reviews. BMJ 2021;372:n71. doi: 10.1136/bmj.n71

For more information, visit: http://www.prisma-statement.org/

Modelling strategy

To determine TB deaths in HIV-positive individuals, we first computed the fraction of HIV-TB deaths among all TB deaths using vital registration data from countries with a four- or five-star rating. We also calculated the proportion of TB cases that are HIV-positive (ie, number of TB cases recorded as HIV-positive/number of TB cases with an HIV test result recorded in the WHO TB register). We used these proportions as input data for a mixed effects regression to predict the proportions of HIV-TB cases among all TB cases for all locations and years using an adult HIV death rate covariate. We estimated the fraction of HIV-TB deaths among all TB deaths in each location and year $(D_{c,y})$, defined by

$$D_{c,y} = \frac{P_{c,y}RR}{P_{c,y}RR + 1 - P_{c,y}}$$

where $P_{c,y}$ is the proportion of HIV-TB cases among all TB cases and *RR* is the relative risk of TB deaths in HIV positive individuals, defined by:

$$RR = \frac{D_{c,y}P_{c,y} - D_{c,y}}{D_{c,y}P_{c,y} - P_{c,y}}$$

We took the median relative risk (RR) from each calculation. We then applied the median RR and the predicted proportions of HIV-TB cases among all TB cases to get the fractions of HIV-TB deaths among all TB deaths for all locations and years. Location-year-specific HIV-TB deaths were then calculated using the following equation:

$$Deaths_{HIV-TB} = \frac{D_{c,y}}{1 - D_{c,y}} Deaths_{TB}$$

where $Deaths_{TB}$ is location-year-specific deaths from the CODEm TB no-HIV model. Finally, we applied the age-sex pattern of the HIV mortality estimates to these HIV-TB deaths to generate location-year-age-sex-specific HIV-TB deaths. As the HIV-TB deaths were estimated based on the fraction of HIV-TB deaths among all TB deaths, the total number of HIV-TB deaths could exceed the total number of HIV deaths in some locations. To avoid this, we applied a cap of 45% on the fraction of HIV-TB deaths among HIV deaths, based on a review by Cox and colleagues, 2010,[2] and a systematic review and meta-analysis by Ford and colleagues, 2016.[3]

To split HIV-TB into HIV-MDR-TB and HIV-drug-susceptible-TB, we first calculated the proportion of HIV-MDR-TB among all HIV-TB cases ($P_{MDR-HIVc,y,a,s}$) for each location, year, age, and sex using the following formula:

$$P_{MDR-HIVc,y,a,s} = P_{MDRnoHIVc,y,a,s} RR_{HIV}$$

where $P_{MDRnoHIVc,y,a,s}$ is the estimated proportion of MDR-TB among HIV-negative TB cases for each location, year, age, and sex (see MDR-TB modelling strategy for the detail) and RR_{HIV} is the relative risk of MDR-TB associated with HIV infection.

We then computed the fraction of HIV-MDR-TB deaths among all HIV-TB deaths ($D_{MDR-HIVc,y,a,s}$) using the following formula:

$$D_{MDR-HIVc,y,a,s} = \frac{P_{MDR-HIVc,y,a,s} RR_{MDR}}{P_{MDR-HIVc,y,a,s} RR_{MDR} + 1 - P_{MDR-HIVc,y,a,s}}$$

where RR_{MDR} is the pooled relative risk of mortality in MDR-TB cases compared with drug-susceptible TB cases. In GBD 2021, the pooled relative risk was derived from a meta-analysis in the meta-regression with Bayesian priors, regularisation, and trimming (MR-BRT) model. After derivation of the pooled relative risk, we then applied the predicted HIV-MDR-TB death fractions to all HIV-TB death estimates to

generate HIV-MDR-TB deaths by location, year, age, and sex. Next, we subtracted HIV-MDR-TB deaths from all HIV-TB deaths at the 1000 draw level to generate drug-susceptible HIV-TB deaths by location, year, age, and sex.

To separate out HIV-XDR-TB from HIV-MDR-TB, we aggregated the XDR-TB cases and MDR-TB cases (with DST for second-line drugs) up to the super-region level and calculated the super-region-level proportions of XDR-TB among MDR-TB cases. Next, we computed the super-region-specific fraction of XDR-TB deaths among all MDR-TB deaths (D_{XDRsr}) using the following formula:

$$D_{XDRsr} = \frac{P_{XDRsr} RR_{XDR}}{P_{XDRsr} RR_{XDR} + 1 - P_{XDRsr}}$$

where P_{XDRsr} is the proportion of XDR-TB among MDR-TB cases for each super-region, and RR_{XDR} is the pooled relative risk of mortality in XDR-TB cases compared with MDR-TB cases. Similar to the pooled relative risk for MDR-TB, the derivation of the pooled relative risk of mortality in XDR-TB was computed with a meta-analysis in the MR-BRT model for GBD 2021. The fractions were then applied to MDR-TB deaths in corresponding countries within the super-regions to produce XDR-TB deaths by location, age, and sex for the most recent year of estimation. We linearly extrapolated XDR-TB mortality rates back, assuming the mortality rates were zero in 1992, one year before 1993 when XDR-TB was first recorded in USA surveillance data.[4] Finally, we subtracted HIV-XDR-TB deaths from HIV-MDR-TB deaths to generate HIV-MDR-TB (without extensive drug resistance) deaths by location, year, age, and sex.

References

1. Mesfin YM, Hailemariam D, Biadglign S, Kibret KT. Association between HIV/AIDS and multi-drug resistance tuberculosis: a systematic review and meta-analysis. PLoS One. 2014;9(1):e82235.
2. Cox JA, Lukande RL, Lucas S, Nelson AM, Van Marck E, Colebunders R. Autopsy causes of death in HIV-positive individuals in sub-Saharan Africa and correlation with clinical diagnoses. *AIDS Rev* 2010; **12**: 183–94.
3. Ford N, Matteelli A, Shubber Z, *et al.* TB as a cause of hospitalization and in-hospital mortality among people living with HIV worldwide: a systematic review and meta-analysis. *J Int AIDS Soc* 2016; **19**: 20714.
4. Centers for Disease Control and Prevention (CDC). Extensively Drug-Resistant Tuberculosis --- United States, 1993—2006. MMWR. 2007; 56(11);250-253

Hypertensive heart disease

Flowchart

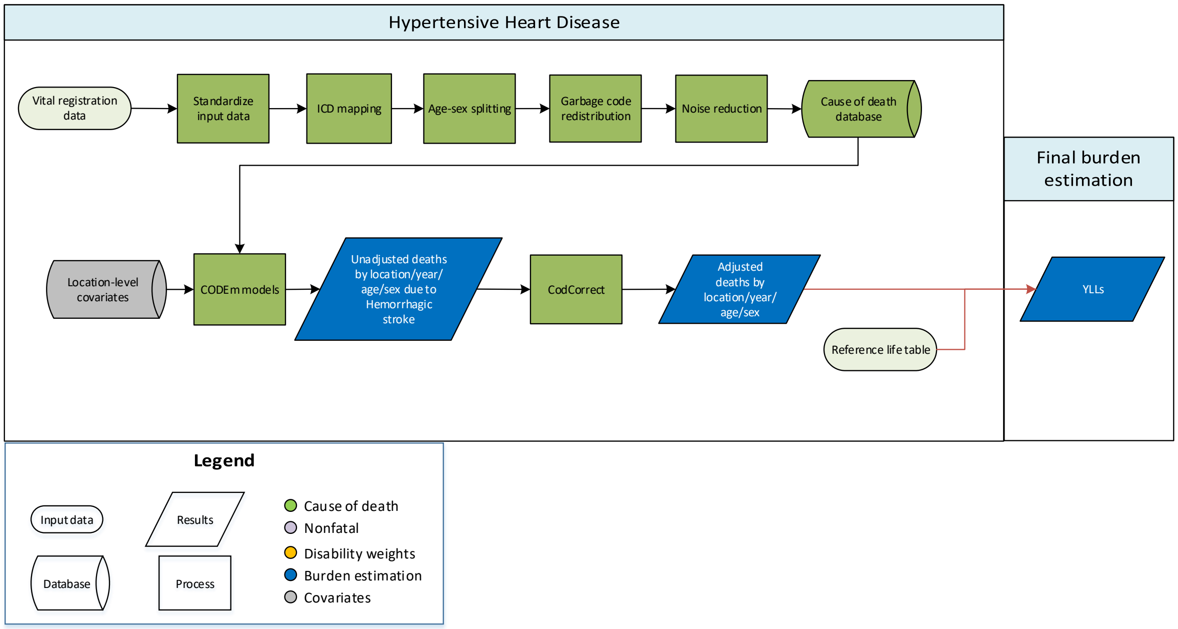

Case definition

Hypertensive heart disease is structural heart disease defined by its underlying cause (systemic hypertension), resulting in left ventricular hypertrophy, diastolic dysfunction, and clinical heart failure (following Framingham, ESC, or similar definitions of HF) with either preservation or reduction in systolic function. HHD may be an underlying cause of death or result in disability related to heart failure. All non-fatal disease burden for hypertensive heart disease is captured as part of the heart failure modelling process.

Input data and methodological summary for hypertensive heart disease

Input data

Vital registration data were used to model cause-specific mortality for hypertensive heart disease. We outliered ICD9BTL datapoints, which were inconsistent with the rest of the data and created implausible time trends. In addition, we outliered vital registration data from Grenada in 2017 and Saudi Arabia, Ukraine, and Armenia in 2018 for being implausibly low across all age groups. Additionally, ICD8 datapoints from Norway and Sweden which caused implausible time trends were outliered.

Modelling strategy

We used a standard CODEm approach to model deaths from hypertensive heart disease. For GBD 2021, a new approach to redistribute deaths coded to hypertension was implemented using data sources which included information on the chain of events leading to death. This update resulted in an increase in the number of deaths that were re-assigned to hypertensive heart disease. Similarly, the method used

to reduce the noise in the data, implemented after redistribution to handle both the stochastic variation across time and space and the occurrence of small number of deaths in each location/year/age/sex, was updated. This new empirical Bayesian noise reduction algorithm uses grouped data by region and data type as prior to better reflect regional patterns. A detailed description on the updates to redistribution and noise reduction can be found in the cause of death methods section of the appendix.

The covariates included in the ensemble modelling process are listed in the table below.

Apart from the changes to the redistribution of deaths coded to hypertension, there are no substantive changes from the approach used in GBD 2019.

Table 1. Covariates used in hypertensive heart disease mortality modelling

Level	Covariate	Direction
1	Systolic blood pressure (mm Hg)	1
2	Cholesterol (total, mean per capita)	1
	Smoking prevalence	1
	Mean BMI	1
	Healthcare access and quality index	-1
3	Lag distributed income per capita (I$)	-1
	Socio-demographic Index	1
	Alcohol (litres per capita)	1
	Summary exposure value, omega-3	1
	Summary exposure value, fruits	1
	Summary exposure value, nuts and seeds	1
	Summary exposure value, PUFA	1
	Summary exposure value, vegetables	1
	Pulses/legumes (kcal/capita, unadjusted)	-1
	Trans fatty acid (percent)	1

Idiopathic epilepsy

Flowchart

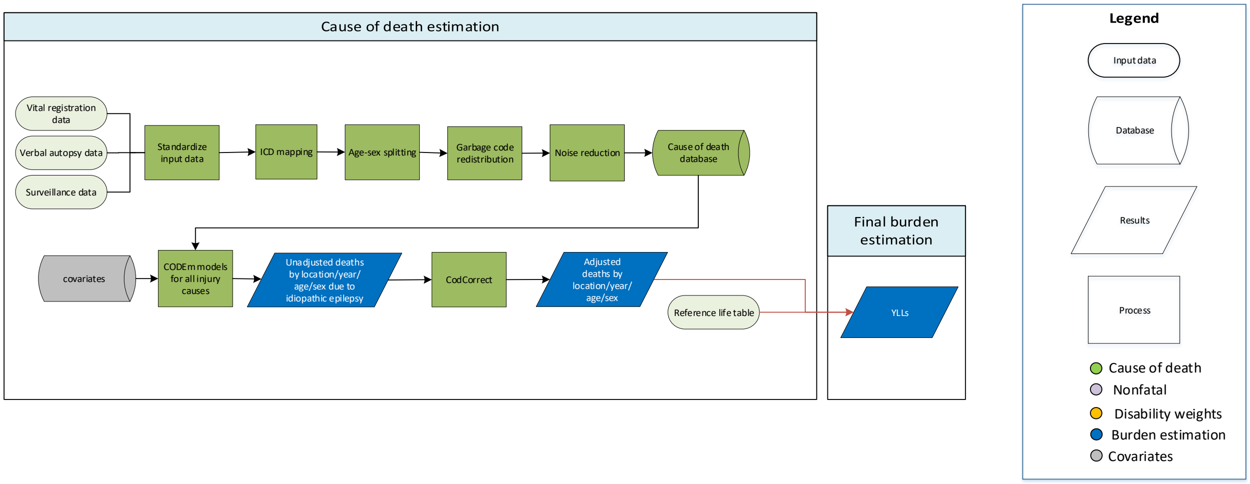

Input data and methodological summary for idiopathic epilepsy

Input data

Data used to estimate epilepsy mortality included vital registration (VR), verbal autopsy, and China mortality surveillance data from the cause of death (COD) database. Our outlier criteria were to exclude datapoints that were (1) implausibly high or low relative to global or regional patterns, (2) substantially conflicted with established age or temporal patterns, or (3) substantially conflicted with other data sources based in the same locations or locations with similar characteristics (ie, Socio-demographic Index).

Modelling strategy

The standard Cause of Death Ensemble modelling (CODEm)[1] approach (detailed in reference appendix section 3.1) was used to estimate deaths due to idiopathic epilepsy. Separate models were conducted for male and female mortality, and the age range for both models was 28 days to 95+ years. Unadjusted death estimates were adjusted using CoDCorrect to produce final estimates of years of life lost (YLLs).[1] See appendix section 3.1 of the reference for further information.

The following table lists the covariates included in the model. This requires that the covariate selected for the model must have the directional relationship with idiopathic epilepsy deaths. For GBD 2021, no significant updates were made for idiopathic epilepsy covariate selection. Covariate directions were selected based on the strength of the evidence.

Table 1. Covariates used in idiopathic epilepsy mortality modelling

Level	Covariate	Direction
1	Pigs (per capita)	+
	SEV scalar: epilepsy	+
	Mean systolic blood pressure (mmHg)	+
2	Healthcare Access and Quality Index	-
	Mean body-mass index	+
	Mean serum total cholesterol (mmol/L)	+

3	Cumulative cigarettes (10 years)	+
	Cumulative cigarettes (5 years)	+
	Education (years per capita)	-
	Log LDI (per capita)	-
	Socio-demographic Index	-

The following plot shows the influence of each covariate on the four CODEm models (male global, male data-rich, female global, and female data-rich). A positive standardised beta (to the right) means that the covariate was associated with increased death. A negative standardised beta (to the left) means the covariate was associated with decreased death.

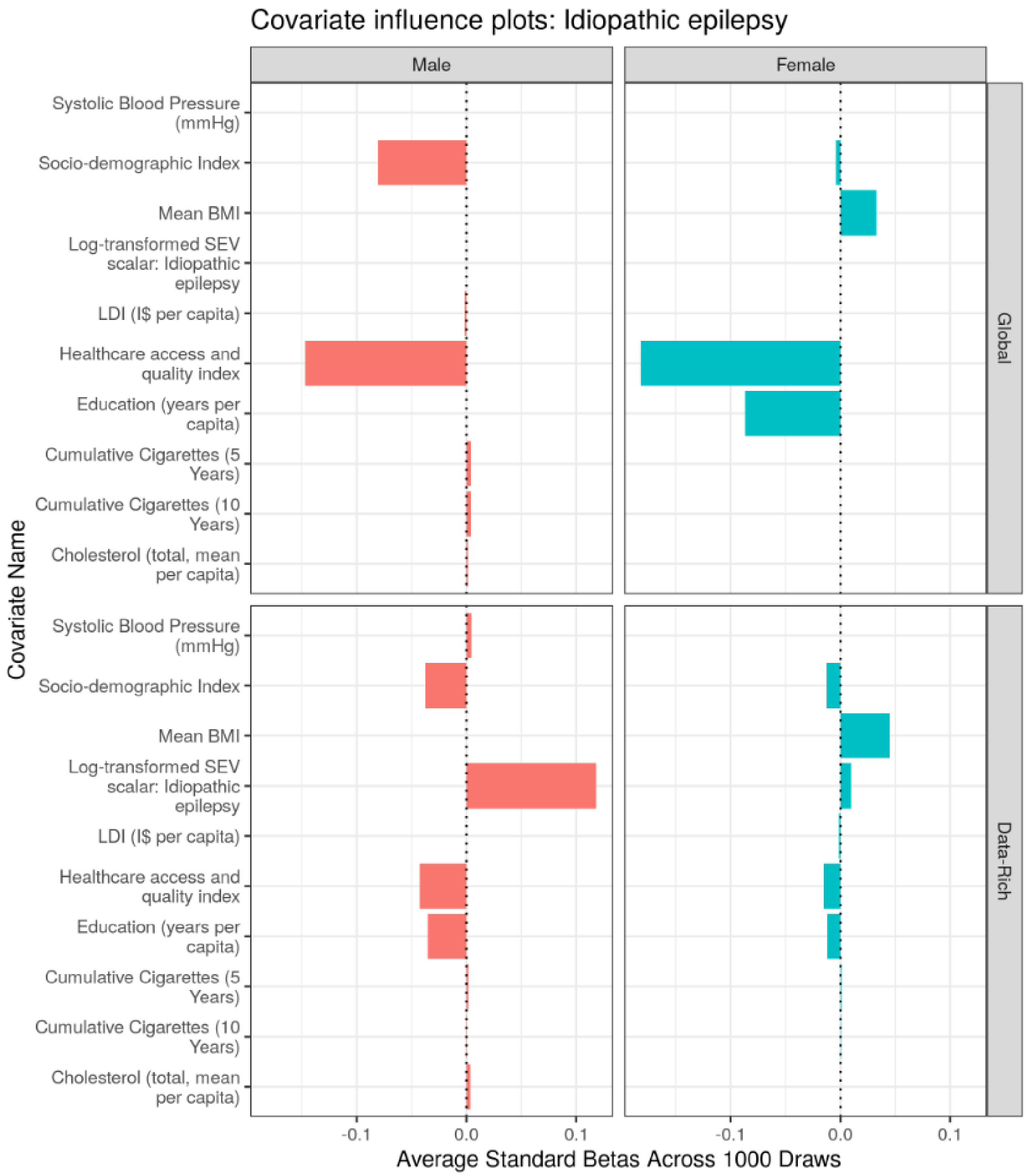

[1]Vos T, Lim SS, Abbafati C, *et al.* Global burden of 369 diseases and injuries in 204 countries and territories, 1990–2019: a systematic analysis for the Global Burden of Disease Study 2019. *The Lancet* 2020; **396**: 1204–22. doi: https://doi.org/10.1016/S0140-6736(20)30925-9

Inflammatory bowel disease

Flowchart

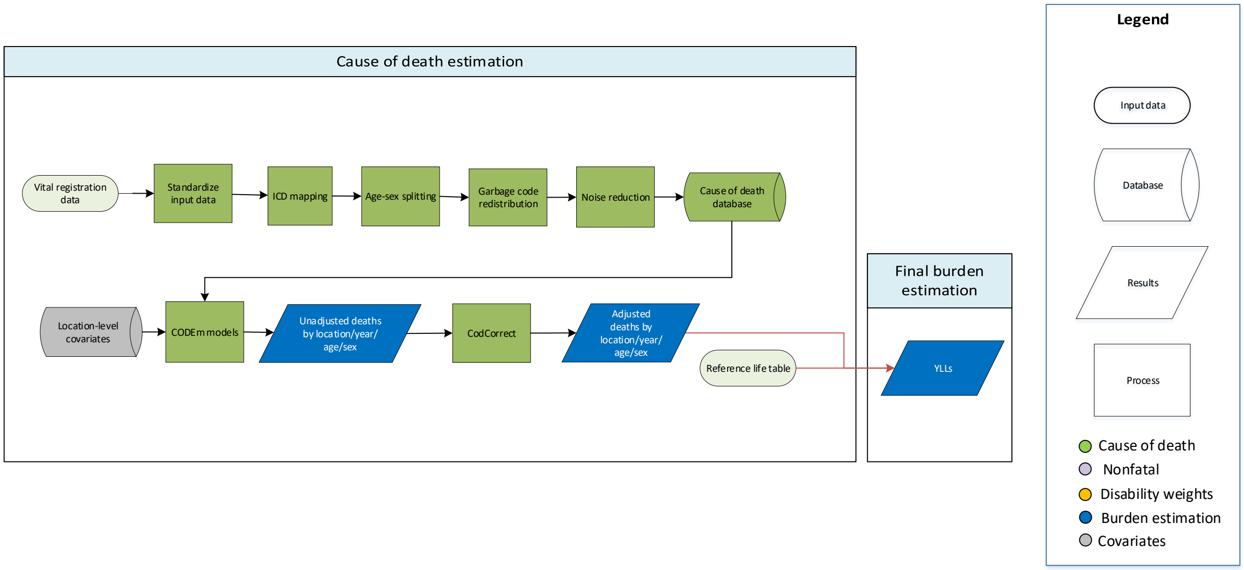

Input data and methodological summary for inflammatory bowel diseases

Input data

Data used to estimate mortality of inflammatory bowel disease consisted of vital registration data from the cause of death (COD) database (see appendix section on ICD mapping for details). Outliers were identified by systematic examination of datapoints for all location-years. Data were excluded if they violated well-established age or time trends, and data in instances where garbage code redistribution and noise reduction, in combination with small sample sizes, resulted in unreasonable cause fractions.

Modelling strategy

The estimation strategy used for fatal inflammatory bowel disease is largely similar to methods used in GBD 2019. A standard CODEm model with location-level covariates was used to model deaths due to inflammatory bowel disease (see appendix section on CODEm method for details). Separate models were conducted for male and female mortality with age restrictions for death estimations of 2 years for lower bound (in GBD 2019, the lower bound was set at 1 year) and 95+ for upper bound. Separate models were conducted for male and female mortality. We hybridised separate global and data-rich models to acquire unadjusted results, which we adjusted using CoDCorrect and compared to the reference life table to calculate final YLLs due to inflammatory bowel disease.

In GBD 2021, we updated the linear floor value in our CODEm models to allow the model to be influenced by lower data, which resulted from changes to COD data processing. These data processing changes include changing the method of estimating the non-zero floor and population size cutoffs for when to noise-reduce at the country versus region level, and are described in greater detail in the appendix section “GBD 2021 Causes of Death database”. Apart from these, no other substantive changes were made in GBD 2021 from the modelling strategy used in GBD 2019.

The following table has the full list of covariates used for inflammatory bowel disease.

Table 1. Covariates used in inflammatory bowel disease mortality modelling

Level	Covariate	Direction
1	Age-sex-specific scaled exposure variable for low polyunsaturated fatty acids consumption	+
	Age-sex-specific scaled exposure variable for low fruit consumption	+
	Age-sex-specific scaled exposure variable for low vegetable consumption	+
	Age-sex-specific scaled exposure variable for high red meat consumption	+
2	Healthcare Access and Quality Index	-
	Latitude 15 to 30 (proportion)	-
	Latitude 30 to 45 (proportion)	+
	Latitude 45 plus (proportion)	+
3	Socio-demographic Index	+
	Education (years per capita)	-
	Log LDI ($I per capita)	+

Adjustment in CoDCorrect included fitting unadjusted death estimates for Level 4 child causes of inflammatory bowel disease (ie, Crohn's disease and ulcerative colitis) to overall inflammatory bowel disease deaths, which were then adjusted with all other causes to sum to all-cause counts of death.

Inguinal, femoral, and abdominal hernia

Flowchart

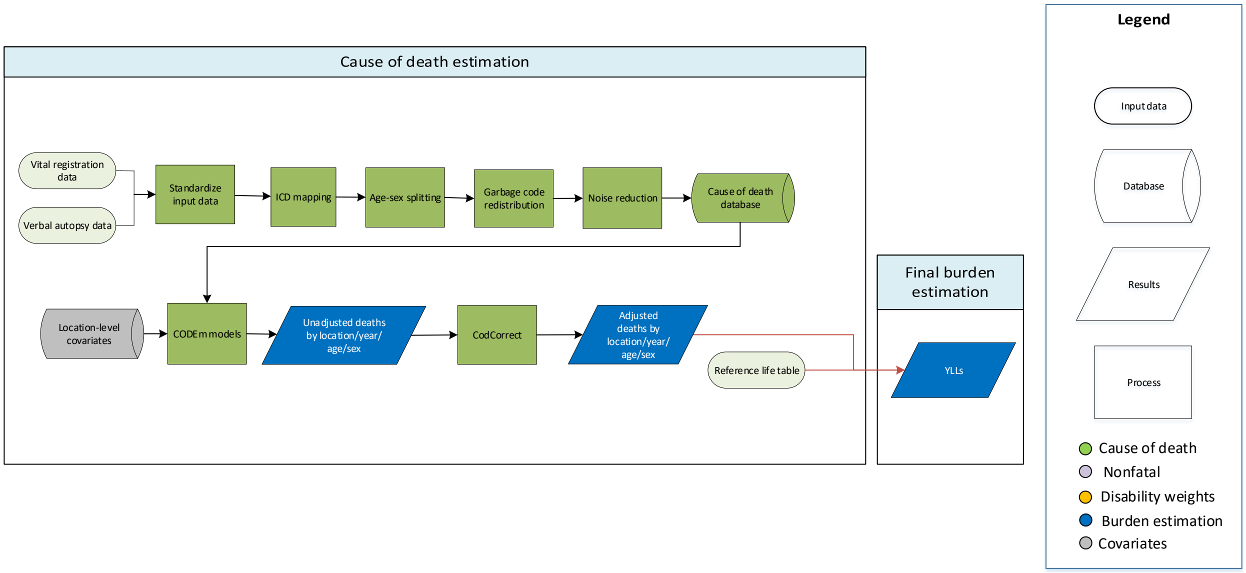

Input data and methodological summary for inguinal, femoral, and abdominal hernia

Input data

Data used to estimate mortality of inguinal, femoral, and abdominal hernia consisted of vital registration and verbal autopsy data from the cause of death (COD) database (see appendix section on ICD mapping for details). Outliers were identified by systematic examination of datapoints for all location-years. Data were excluded if they violated well-established age or time trends, and data in instances where garbage code redistribution and noise reduction, in combination with small sample sizes, resulted in unreasonable cause fractions. Methods for assigning outlier status were consistent across both vital registration and verbal autopsy data.

Modelling strategy

The estimation strategy used for fatal inguinal, femoral, and abdominal hernia is largely similar to methods used in GBD 2019. A standard CODEm model with location-level covariates was used to model deaths due to inguinal, femoral, and abdominal hernia (see appendix section on CODEm method for details). Separate models were conducted for male and female mortality. We hybridised separate global and data-rich models to acquire unadjusted results, which we adjusted using CoDCorrect and compared to the reference life table to calculate final YLLs due to inguinal, femoral, and abdominal hernia.

In GBD 2021, we updated the linear floor value in our CODEm models to allow the model to be influenced by lower data, which resulted from changes to COD data processing. These data processing changes include changing the method of estimating the non-zero floor and population size cutoffs for when to noise-reduce at the country versus region level, and are described in greater detail in the appendix section "GBD 221 Causes of Death database". Apart from these, no other substantive changes were made in GBD 2021 from the modelling strategy used in GBD 2019.

The following table has the full list of covariates used for fatal inguinal, femoral, and abdominal hernia.

Table 1. Covariates used in inguinal, femoral, and abdominal hernia mortality modelling

Level	Covariate	Direction
1	BMI (mean)	-
	Cumulative cigarettes (10 years)	+
	Cumulative cigarettes (5 years)	+
	Smoking prevalence	+
2	Healthcare Access and Quality Index	-
3	Socio-demographic Index	+
	Education (years per capita)	-
	Log LDI ($I per capita)	-

Adjustment in CoDCorrect included fitting unadjusted death estimates for all other specific and non-specific digestive diseases to overall digestive disease deaths, which was then adjusted with all other causes to sum to all-cause counts of death.

Injuries

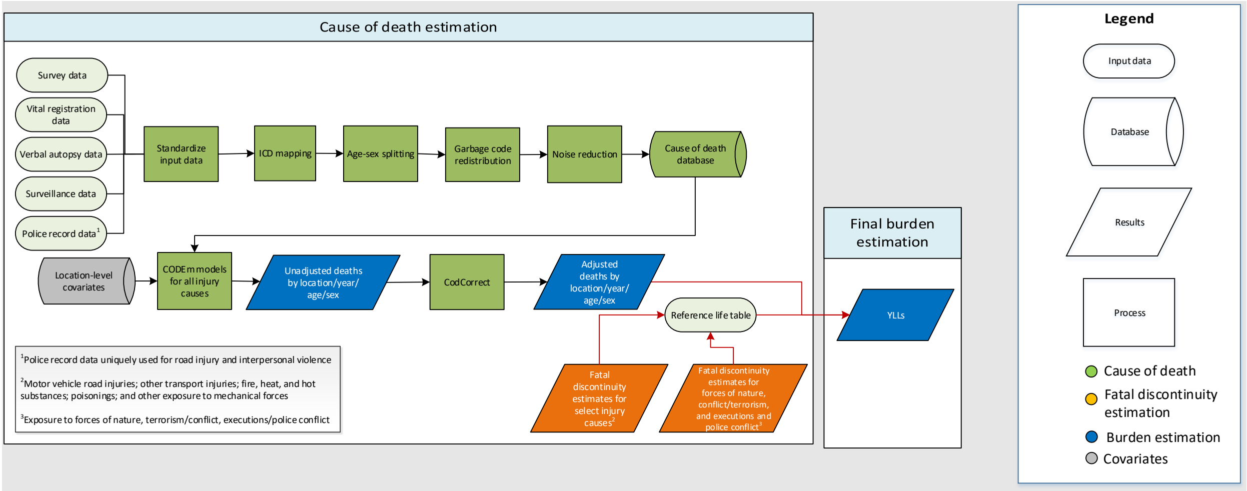

Overview

Injuries are a Level 1 condition within the GBD hierarchy. We made estimates for 36 injuries which are grouped into three categories: transport injuries, unintentional injuries, and self-harm and interpersonal violence. The table below lists each of the injuries for which we make estimates.

Table 1: Name of 36 injuries where GBD makes fatal estimates

**Transport injuries**	**Unintentional injuries**	**Self-harm and interpersonal violence**
Transport injuries	Falls	Self-harm
Road injuries	Drowning	Self-harm by firearm
Pedestrian road injuries	Fire, heat, and hot substances	Self-harm by other specified means
Cyclist road injuries	Poisonings	Interpersonal violence
Motorcyclist road injuries	Poisoning by carbon monoxide	Physical violence by firearm
Motor vehicle road injuries	Poisoning by other means	Physical violence by sharp object
Other road injuries	Exposure to mechanical forces	Physical violence by other means
Other transport injuries	Unintentional firearm injuries	Conflict and terrorism
	Other exposure to mechanical forces	Executions and police conflict
	Adverse effects of medical treatment	
	Animal contact	
	Venomous animal contact	
	Non-venomous animal contact	
	Foreign body	
	Pulmonary aspiration and foreign body in airway	
	Foreign body in other body part	
	Environmental exposure to heat and cold	
	Exposure to forces of nature	
	Other unintentional injuries	

Input data

We estimated injury mortality using data from vital registration, verbal autopsy, mortality surveillance, censuses, surveys, and police record data. Police and crime reports were data sources uniquely used for the estimation of deaths from transport and road traffic injuries, and interpersonal violence. The police data were collected from published studies, national agencies, and institutional surveys such as the United Nations Crime Trends Survey and the WHO Global Status Report on Road Safety Survey. Police records were not used for countries with vital registration data unless the recorded number of road injury and interpersonal violence deaths from police records exceeded deaths in the vital registration data.

Infrequently, datapoints were marked as outliers. Outlier criteria excluded datapoints that (1) were implausibly high or low relative to global or regional patterns based on subject-matter or in-country experts, (2) substantially conflicted with established age or temporal patterns, or (3) significantly conflicted with higher-quality data sources conducted from the same locations or locations with similar characteristics (ie, Socio-demographic Index).

Modelling strategy

Overview

In GBD 2021, the standard CODEm modelling approach was applied to estimate deaths due to all causes of injury, excluding fatal discontinuities (see "Fatal discontinuities" section below). Refer to the table at the end of this section for a complete list of the cause-of-injury categories, modelling strategies, and covariate changes from GBD 2021.

GBD injury codes and categories

The International Classification of Diseases (ICD) was used to classify injuries. In GBD, injury incidence and death are defined as ICD-9 codes E000–E999 and ICD-10 chapters V to Y. There is one exception: deaths and cases of alcohol poisoning and drug overdoses are classified under drug and alcohol use disorders and modelled outside of the injury framework. Injury causes were organised into 28 mutually exclusive and collectively exhaustive external cause-of-injury categories.

Preparation of data

The preparation of cause-of-death data includes age splitting, age-sex splitting, smoothing, and outlier detection. These steps are described in detail by Naghavi et al and Lozano et al.[1,2,3] We also found that some deaths are assigned an ICD code that links to a condition that does not lead to death (eg, senility), are overly vague and could be attributed to more than one cause of death (ie,"Exposure to unspecified factor" [X59 in ICD-10 and E887 in ICD-9]), and all undetermined intent codes (Y10-Y34 in ICD-10 and E980-E988 in ICD-9), or is an intermediate cause of death (ie, septicemia or peritonitis) or as an ill-defined and unknown cause of mortality (R99). We consider these codes as "garbage codes" and redistribute them based on methods described in GBD 1990.[4] Approximately 1% of total deaths in countries with vital registration data are assigned to these garbage code categories.

In countries with non-detail ICD code data, cause-of-injury categories were proportionally split into sublevel cause-of-injury categories. The sublevel cause-of-injury causes were created in the CoDCorrect process. For GBD iterations of 2015, 2016, 2017, 2019, and 2021, the proportions were based on post-mortem investigation of injury deaths as described in the paper by Matzopoulos et al. 2015.[5]

We added police data for road injuries and interpersonal violence in countries with sparse or absent cause of death data even though we know from countries with near-complete vital registration data that police records tend to underestimate the true level of deaths. However, we applied police data estimates in instances where reported deaths were higher than vital registration numbers. In these select instances, we were advised by in-country experts that the police data were more complete than vital registration data.

Due to nationwide protests by the Black Lives Matter movement in the USA in 2020 and increased national attention on the epidemic of police violence against Black Americans, input data on police conflict and executions for the USA were reviewed for completeness. We determined that the USA National Vital Statistics System (NVSS) systematically under-reports deaths due to police violence by about 50% every year. In order to quantify this bias, we ran a network meta-regression on NVSS data with direct comparisons by state and year to Mapping Police Violence (MPV),[7] an alternate open-source database that catalogs deaths due to police violence, and indirect comparisons to an additional source, Fatal Encounters (FE).[8] We believe that they more accurately capture deaths due to police violence because of they use open-source methodologies to identify police violence deaths, rather than relying on death certificates.[9,10,11] The regression included a fixed effect on state to capture different under-reporting rates across states but assumed that under-reporting rates are constant across age, sex, and year. Additionally, since MPV does not attempt to capture police killed by civilians and neither MPV nor FE attempts to capture executions, death counts from the FBI's Law Enforcement Officers Killed and Assaulted database and the Death Penalty Information Center (DPIC) were added to these data sources in order to conform them to the GBD definition of executions and police conflict.[12,13] During data processing, we added police conflict deaths in USA NVSS by pulling deaths from a selection of redistribution packages and GBD causes that are likely to contain the miscoded deaths. We took away deaths from interpersonal violence and related garbage packages first, then from GBD causes falls and exposure to mechanical forces and garbage packages exposure to unspecified factor (X59) and ill-defined. These groups of causes were selected based on literature review that found homicide as the main source of miscoded police conflict deaths.[9,14,15] Record linkage between NVSS and open-source databases has shown that interpersonal violence is the most common underlying cause of death listed on death certificates for mis-assigned police violence deaths.[6]

Injuries estimated as fatal discontinuities

Fatal discontinuity events, defined as spikes in a time series of deaths that are unexpected and introduce a "shock" of deaths that is not predictable through modelling, were estimated for ten injury causes that are also modelled in CODEm. These causes included "Other transport injuries", "Fire, heat, and hot substances", "Poisoning by other means", "Other exposure to mechanical forces", "Non-venomous animal contact", "Environmental heat and cold exposure", "Physical violence by firearm", "Physical violence by sharp object", "Physical violence by other means", and "Executions and police conflict". Final fatal discontinuity estimations for these causes were merged with CODEm results post-CoDCorrect to produce final cause of death results.

"Exposure to forces of nature," and "Conflict and terrorism" are injuries that were modelled outside of the CODEm process only using fatal discontinuity estimation. Details on the fatal discontinuity estimation process can be found in its own section of this appendix.

Covariates

The following covariates were tested for each injury cause of death model.

TRANSPORT INJURIES

Table 2: Covariate changes from GBD 2019 to GBD 2021

ID	Cause	Modelling strategy	Covariate changes from GBD 2019
1	Transport injuries	CODEm	
1.1	Road injuries	CODEm	
1.1.1	Pedestrian road injuries	CODEm	
1.1.2	Cyclist road injuries	CODEm	
1.1.3	Motorcyclist road injuries	CODEm	Added to all models: Vehicles – 2 wheels fraction (proportion)
1.1.4	Motor vehicle road injuries	CODEm	
1.1.5	Other road injuries	CODEm	
1.2	Other transport injuries	CODEm and fatal discontinuity estimation	Added to female global model: Vehicles – 2 wheels fraction (proportion)

Table 2.1: Transport injuries covariate levels and directions

Covariate	Transport Injuries		Road injuries		Pedestrian Road injuries		Cyclist road injuries		Motorcycle road injuries		Motor vehicle road injuries		Other road injuries		Other transport injuries	
	Level	Direction	Level	Direction	Level	Direction	Level	Direction	Level	Direction	Level	Direction	Level	Direction	Level	Direction
BAC law professional drivers (quartile)	1	1	1[a]	1	1	1	1	1	1	1	1	1	1	1	1	1
BAC law general population (quartile)	1	1	1[a]	1	1	1	1	1	1	1	1	1	1	1	1	1
BAC law youth drivers (quartile)	1	1	1[a]	1	1	1	1	1	1	1	1	1	1	1	1	1
Litres of alcohol consumed per capita	1	1	1	1	1	1	1	1	1	1	1	1	1	1	1	1
Speed limit law rural (quartile)	1	1	1[a]	1	1	1	1	1	1	1	1	1	1	1	1	1
Speed limit law urban (quartile)	1	1	1[a]	1	1	1	1	1	1	1	1	1	1	1	1	1
Vehicles - 2 wheels (per capita)	NA	NA	1	1	NA	NA	NA	NA	NA	NA	NA	NA	NA	NA	NA	NA

Vehicles - 2 wheels fraction (proportion)	1	1	1	1	1	1	1	1	1	1	NA	NA	1	1	1	1
Vehicles - 2+4 wheels (per capita)	1	1	1	1	1	1	1	1	NA	NA	NA	NA	1	1	1	1
Vehicles - 4 wheels (per capita)	NA	NA	1	1	NA	NA	NA	NA	NA	NA	NA	NA	NA	NA	NA	NA
Education (years per capita)	2	-1	2[b]	-1	2	-1	2	-1	2	-1	2	-1	2	-1	2	-1
Healthcare Access and Quality Index	2	-1	2	-1	2	-1	2	-1	2	-1	2	-1	2	-1	2	-1
LDI (I$ per capita)	2	-1	2[b]	-1	2	-1	2	-1	2	-1	2	-1	2	-1	2	-1
Population 15 to 30 (proportion)	2	1	2	1	2	1	2	1	2	1	2	1	2	1	2	1
Population density (300-500 ppl/sqkm, proportion)	2	1	2	1	2	1	2	1	2	1	2	1	NA	NA	2	1
Population density (500-1000 ppl/sqkm, proportion)	2	1	2	1	2	1	2	1	2	1	2	1	NA	NA	2	1
Population-weighted mean temperature	2	1	2	1	2[c]	1	2	1	2	1	2	1	2	1	2	1
Socio-demographic Index	2	-1	2[b]	-1	2	-1	2	-1	2	-1	2	-1	3[e]	-1	2	-1
Rainfall quintile 5 (proportion)	3	1	3[d]	1	3	1	3	1	3	1	3	1	3	1	3	1
Log-transformed SEV scalar: Road Inj	NA	NA	1	1	NA	NA	NA	NA	NA	NA	1	1	NA	NA	1	1
Log-transformed SEV scalar: Pedest	NA	NA	NA	NA	1	1	NA	NA	NA	NA	NA	NA	NA	NA	NA	NA
Log-transformed SEV scalar: Cyclist	NA	NA	NA	NA	NA	NA	1	1	NA	NA	NA	NA	NA	NA	NA	NA
Log-transformed SEV scalar: Mot Cyc	NA	NA	NA	NA	NA	NA	NA	NA	1	1	NA	NA	NA	NA	NA	NA
Log-transformed SEV scalar: Mot Veh	NA	NA	NA	NA	NA	NA	NA	NA	NA	NA	1	1	NA	NA	NA	NA
Log-transformed SEV scalar: Oth Road	NA	NA	NA	NA	NA	NA	NA	NA	NA	NA	NA	NA	1	1	NA	NA
Log-transformed SEV scalar: Oth Trans	NA	NA	NA	NA	NA	NA	NA	NA	NA	NA	NA	NA	NA	NA	1	1

a: Used at Level 1 in female models, Level 2 in males
b: Used at Level 3 in global models, Level 2 in data-rich models
c: Used at Level 1 in male data-rich model. Level 2 in other three models.
d: Not used in male global model
e: Used at Level 2 in male global model, Level 3 for the other three models

Covariate level is grouped by strong biological link (1), strong evidence of a relationship (2), or weak relationship (3). The direction indicates whether the covariate and cause of death change in the same direction (1) or opposite (-1).

Figure 2a: Transport injuries covariate influence plot

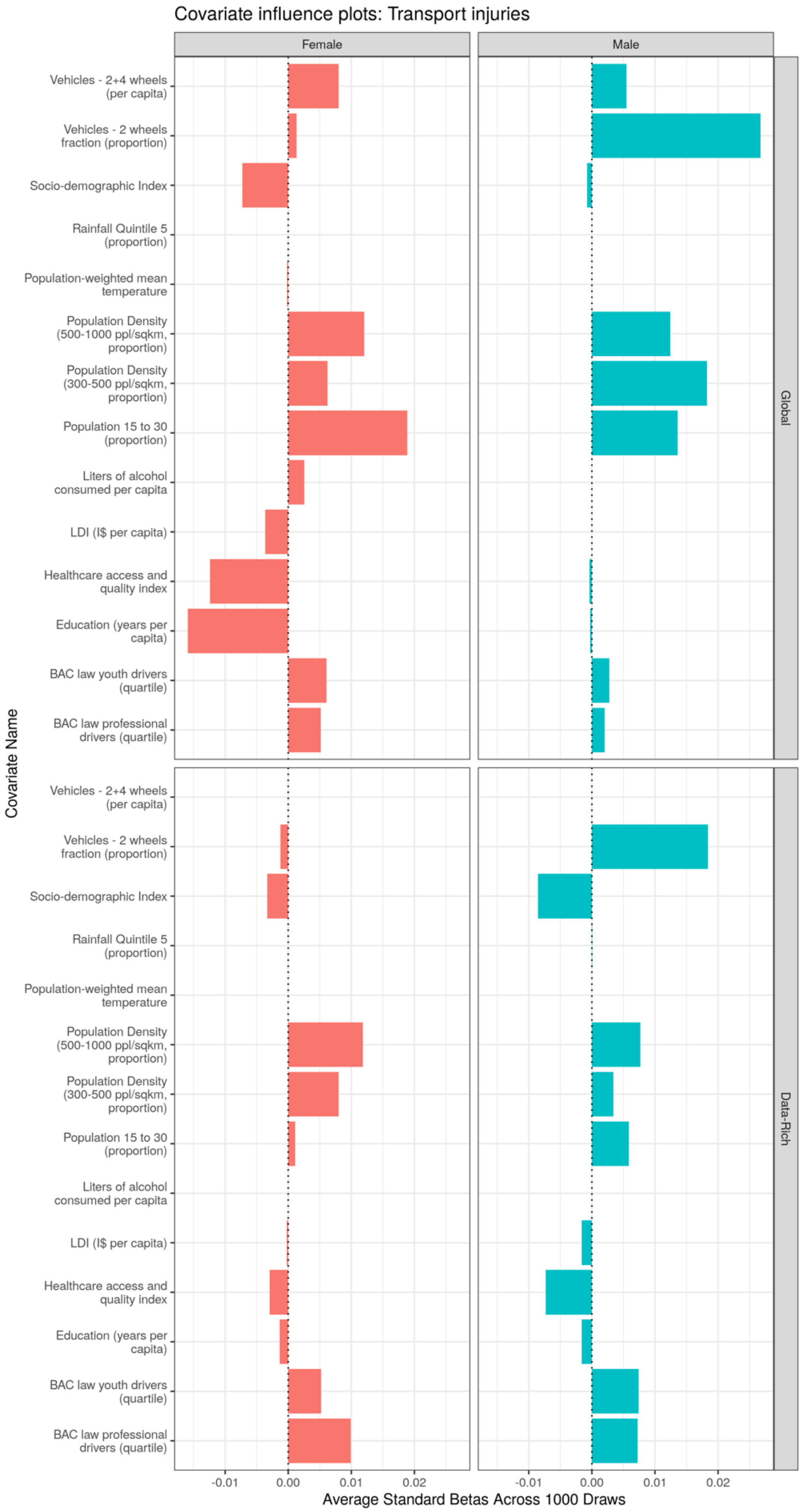

Figure 2b: Road injuries covariate influence plot

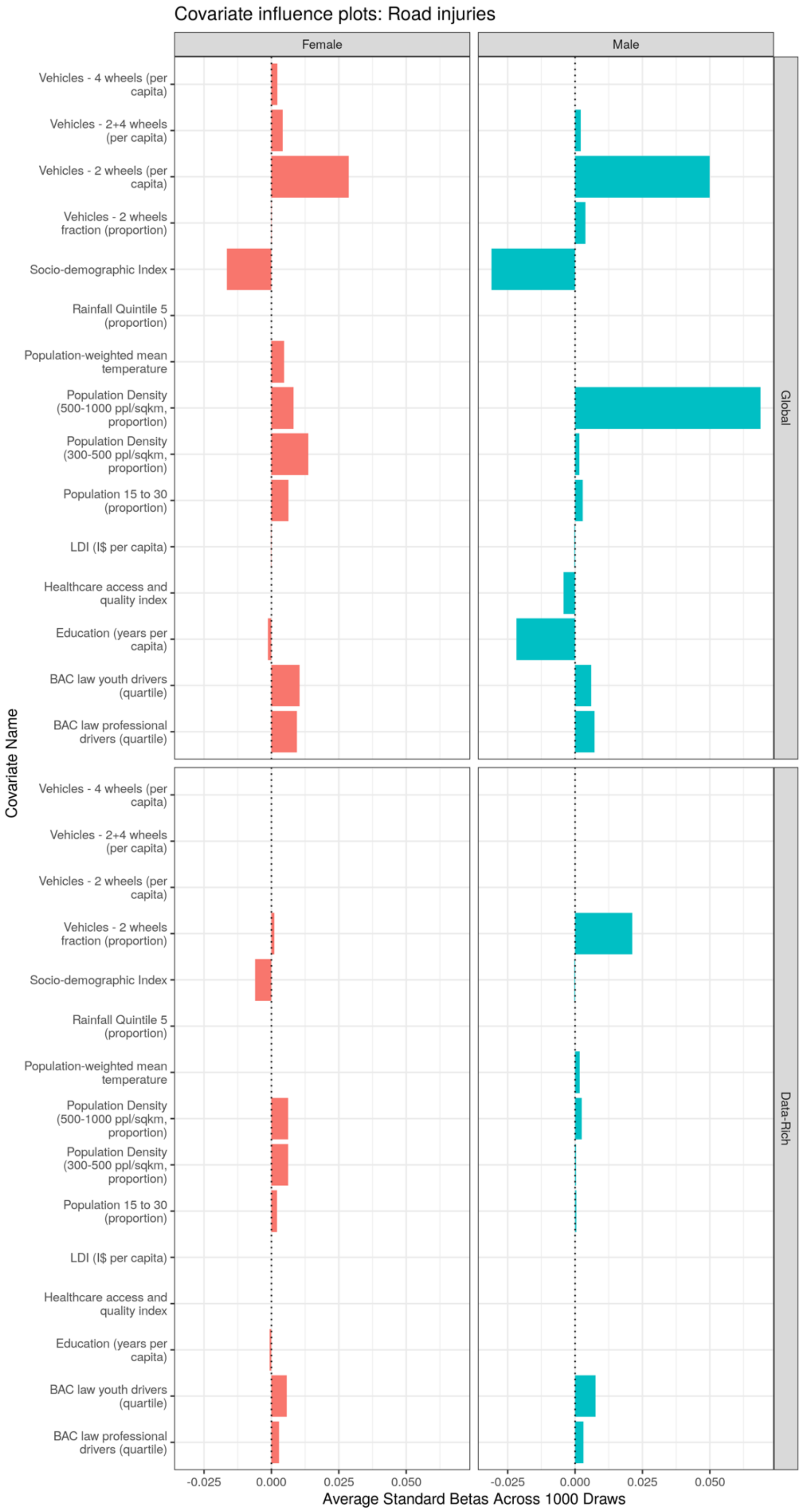

UNINTENTIONAL INJURIES

Table 3: Covariate changes from GBD 2019 to GBD 2021

ID	Cause	Modelling strategy	Covariate changes from GBD 2019
2	Unintentional injuries	Not modelled at parent cause level	
2.1	Falls	CODEm	
2.2	Drowning	CODEm	
2.3	Fire, heat, and hot substances	CODEm and fatal discontinuity estimation	
2.4	Poisonings	CODEm	
2.4.1	Poisoning by carbon monoxide	CODEm	Added: Litres of alcohol consumed per capita
2.4.2	Poisoning by other means	CODEm and fatal discontinuity estimation	Added: Litres of alcohol consumed per capita
2.5	Exposure to mechanical forces	CODEm	
2.5.1	Unintentional firearm injuries	CODEm	
2.5.2	Other exposure to mechanical forces	CODEm and fatal discontinuity estimation	
2.6	Adverse effects of medical treatment	CODEm	Dropped: Litres of alcohol consumed per capita
2.7	Animal contact	CODEm	
2.7.1	Venomous animal contact	CODEm	Changed from Level 1 to Level 2: Litres of alcohol consumed per capita; Absolute value of average latitude; Population-weighted mean temperature; Rainfall population-weighted (mm/yr); Proportion of population involved in agricultural activities; Urbanicity; Sahel Region of Africa (binary) for female models and male data-rich model<br><br>Dropped: Litres of alcohol consumed per capita for all models; Sahel Region of Africa (binary) for male global model

2.7.2	Non-venomous animal contact	CODEm and fatal discontinuity estimation	Changed to Level 3 for female models and male data-rich models: Elevation Over 1500m (proportion); Elevation Under 100m (proportion; Population density (over 1000 ppl/sqkm, proportion); Population density (under 150 ppl/sqkm, proportion)<br><br>Changed to Level 1 for male global models: Population density (over 1000 ppl/sqkm, proportion); Population density (under 150 ppl/sqkm, proportion)
2.8	Foreign body	CODEm	
2.8.1	Pulmonary aspiration and foreign body in airway	CODEm	
2.8.2	Foreign body in other body part	CODEm	
2.9	Environmental exposure to heat and cold	CODEm and fatal discontinuity estimation	
2.10	Exposure to forces of nature	Fatal discontinuity estimation	
2.11	Other unintentional injuries	CODEm and fatal discontinuity estimation	

Table 3.1: Unintentional injuries covariate levels and directions

Covariate	Falls		Drowning		Fire, heat, and hot substances	
	Level	Directions	Level	Directions	Level	Directions
Education (years per capita)	1	-1	3	-1	3	-1
Litres of alcohol consumed per capita	1	1	NA	NA	NA	NA
Log-transformed SEV scalar: Falls	1	1	NA	NA	NA	NA
Healthcare Access and Quality Index	2	-1	NA	NA	2	-1
Population-weighted mean temperature	2	-1	1	1	1	1

Elevation over 1500m (proportion)	3	1	NA	NA	NA	NA
LDI (I$ per capita)	3	-1	3	-1	3	-1
Socio-demographic Index	3	-1	3	-1	3	-1
Coastal population within 10km (proportion)	NA	NA	1	1	NA	NA
Landlocked nation (binary)	NA	NA	1	-1	NA	NA
Log-transformed SEV scalar: Drown	NA	NA	1	1	NA	NA
Rainfall quintile 1 (proportion)	NA	NA	1	-1	NA	NA
Rainfall quintile 5 (proportion)	NA	NA	1	1	NA	NA
Elevation under 100m (proportion)	NA	NA	2	1	NA	NA
Log-transformed SEV scalar: Fire	NA	NA	NA	NA	1	1
Indoor air pollution (all cooking fuels)	NA	NA	NA	NA	2	1
Population density (over 1000 ppl/sqkm, proportion)	NA	NA	NA	NA	2	1
Tobacco (cigarettes per capita)	NA	NA	NA	NA	2	1

Covariate level is grouped by strong biological link (1), strong evidence of a relationship (2), or weak relationship (3). The direction indicates whether the covariate and cause of death change in the same direction (1) or opposite (-1).

Table 3.2: Poisonings covariate levels and directions

Covariate	Poisonings		Poisonings by carbon monoxide		Poisoning by other means	
	Level	Directions	Level	Directions	Level	Directions
Education (years per capita)	3	-1	3	-1	3	-1
Litres of alcohol consumed per capita	NA	NA	1	1	1	1
Healthcare Access and Quality Index	2	-1	3	-1	3	-1
Population-weighted mean temperature	1	1	2	-1	1	1
Elevation over 1500m (proportion)	NA	NA	NA	NA	NA	NA
LDI (I$ per capita)	3	-1	3	-1	3	-1
Socio-demographic Index	3	-1	3	-1	3	-1

Population density (over 1000 ppl/sqkm, proportion)	2	-1	NA	NA	NA	NA
Log-transformed SEV scalar: Poison	1	1	NA	NA	NA	NA
Opium cultivation (binary)	1	1	NA	NA	NA	NA
Population density (under 150 ppl/sqkm, proportion)	2	1	NA	NA	NA	NA
Log-transformed SEV scalar: Inj pois CO	NA	NA	1	1	NA	NA
Log-transformed SEV scalar: Inj pois oth	NA	NA	NA	NA	1	1

Covariate level is grouped by strong biological link (1), strong evidence of a relationship (2), or weak relationship (3). The direction indicates whether the covariate and cause of death change in the same direction (1) or opposite (-1).

Table 3.3: Mechanical forces covariate levels and directions

Covariate	Exposure to mechanical forces		Firearm injuries		Other exposure to mechanical forces	
	Level	Directions	Level	Directions	Level	Directions
Education (years per capita)	3	-1	3	-1	3	-1
Healthcare Access and Quality Index	2	-1	2	-1	2	-1
Population-weighted mean temperature	1	1	1	1	1	1
LDI (I$ per capita)	3	-1	3	-1	3	-1
Socio-demographic Index	3	-1	3	-1	3	-1
Population density (over 1000 ppl/sqkm, proportion)	2	-1	3	-1	2	-1
Population density (under 150 ppl/sqkm, proportion)	2	1	2	-1	2	1
Log-transformed SEV scalar: Mech gun	NA	NA	1	1	NA	NA
Log-transformed SEV scalar: Oth mech	NA.	NA	NA	NA	1	1

Covariate level is grouped by strong biological link (1), strong evidence of a relationship (2), or weak relationship (3). The direction indicates whether the covariate and cause of death change in the same direction (1) or opposite (-1).

Table 3.4: Animal contact covariate levels and directions

Covariate	Animal contact		Venomous animal contact		Non-venomous animal contact	
	Level	Directions	Level	Directions	Level	Directions

Education (years per capita)	3	-1	3	-1	3	-1
Litres of alcohol consumed per capita	1	1	NA	NA	1	1
Healthcare Access and Quality Index	2	-1	2	1	2[l]	-1
Population-weighted mean temperature	1	1	2	1	1	1
Elevation over 1500m (proportion)	3	-1	3	-1	3[g]	-1
LDI (I$ per capita)	3	-1	3	-1	3	-1
Socio-demographic Index	3	-1	3	-1	3	-1
Elevation under 100m (proportion)	3	1	3	-1	3[g]	1
Population density (over 1000 ppl/sqkm, proportion)	3	-1	3	-1	3[g]	-1
Population density (under 150 ppl/sqkm, proportion)	3	1	3	1	3[g]	1
Log-transformed SEV scalar: Animal	1	1	NA	NA	NA	NA
Population 15 to 30 (proportion)	2	1	NA	NA	NA	NA
Log-transformed SEV scalar: Venom	NA	NA	1	1	NA	NA
Mean number of venomous snake species	NA	NA	1	1	NA	NA
Proportion of population vulnerable to snake species	NA	NA	1	1	NA	NA
Absolute value of average latitude	NA	NA	2	-1	NA	NA
Rainfall population-weighted (mm/yr)	NA	NA	2	1	NA	NA
Proportion of population involved in agricultural activities	NA	NA	2	1	NA	NA
Sahel region of Africa (binary)	NA	NA	2[d]	1	NA	NA
Urbanicity	NA	NA	2	-1	NA	NA
Log-transformed SEV scalar: Non ven	NA	NA	NA	NA	1	1

d: Not used in male global model
g: Used at Level 1 in male global model, Level 3 for the other three models
l: Used at Level 3 in male global model

Covariate level is grouped by strong biological link (1), strong evidence of a relationship (2), or weak relationship (3). The direction indicates whether the covariate and cause of death change in the same direction (1) or opposite (-1).

Table 3.5: Foreign body covariate levels and directions

Covariate	Foreign body		Pulmonary aspiration and foreign body in airway		Foreign body in other body part	
	Level	Directions	Level	Directions	Level	Directions
Education (years per capita)	1	1	1[j]	-1	3	-1
Litres of alcohol consumed per capita	1	1	1	1	1	1
Healthcare Access and Quality Index	2	-1	2	-1	2	-1
Population-weighted mean temperature	1	1	1	1	1	1
LDI (I$ per capita)	1	1	3	-1	3	-1
Socio-demographic Index	3	-1	3	-1	3	-1
Indoor air pollution (all cooking fuels)	1	1	NA	NA	NA	NA
Population over 65 (proportion)	1	1	NA	NA	NA	NA
Log-transformed SEV scalar: Foreign body asp	NA	NA	1	1	NA	NA
Alcohol binge drinker proportion, age-standardised	NA	NA	2[k]	1	NA	NA
Mean BMI	NA	NA	2	1	NA	NA
Log-transformed SEV scalar: Other foreign body	NA	NA	NA	NA	1	1

j: Used at Level 3 in the female global model
k: Only used in the female global model
Covariate level is grouped by strong biological link (1), strong evidence of a relationship (2), or weak relationship (3). The direction indicates whether the covariate and cause of death change in the same direction (1) or opposite (-1).

Table 3.6: Other unintentional injuries covariates level and directions

Covariate	Adverse effects of medical treatment		Other unintentional injuries		Environmental exposure to heat and cold	
	Level	Directions	Level	Directions	Level	Directions
Education (years per capita)	1	-1	3	-1	3	-1
Healthcare Access and Quality Index	2	-1	1[d]	1	2	-1
Population-weighted mean temperature	1	1	2	-1	3	1

LDI (I$ per capita)	3	1	1[d]	1	3	-1
Socio-demographic Index	3	-1	3	-1	3	-1
Population density (over 1000 ppl/sqkm, proportion)	NA	NA	3	-1	NA	NA
Population density (under 150 ppl/sqkm, proportion)	NA	NA	3	-1	NA	NA
Log-transformed SEV scalar: Oth unint	NA	NA	3	1	NA	NA
Vehicles - 2 wheels (per capita)	NA	NA	1	1	NA	NA
Vehicles - 4 wheels (per capita)	NA	NA	1	1	NA	NA
Elevation over 1500m (proportion)	NA	NA	1	1	3	1
90th percentile climatic temperature in the given country-year	NA	NA	NA	NA	3	1
Elevation 500 to 1500m (proportion)	NA	NA	NA	NA	3	1
Population density (150-300 ppl/sqkm, proportion)	NA	NA	NA	NA	3	-1
Rainfall (quintiles 4-5)	NA	NA	NA	NA	3	1
Sanitation (proportion with access)	NA	NA	NA	NA	3	-1

d: Not used in male global model
Covariate level is grouped by strong biological link (1), strong evidence of a relationship (2), or weak relationship (3). The direction indicates whether the covariate and cause of death change in the same direction (1) or opposite (-1).

Figure 3a: Drowning covariate influence plot

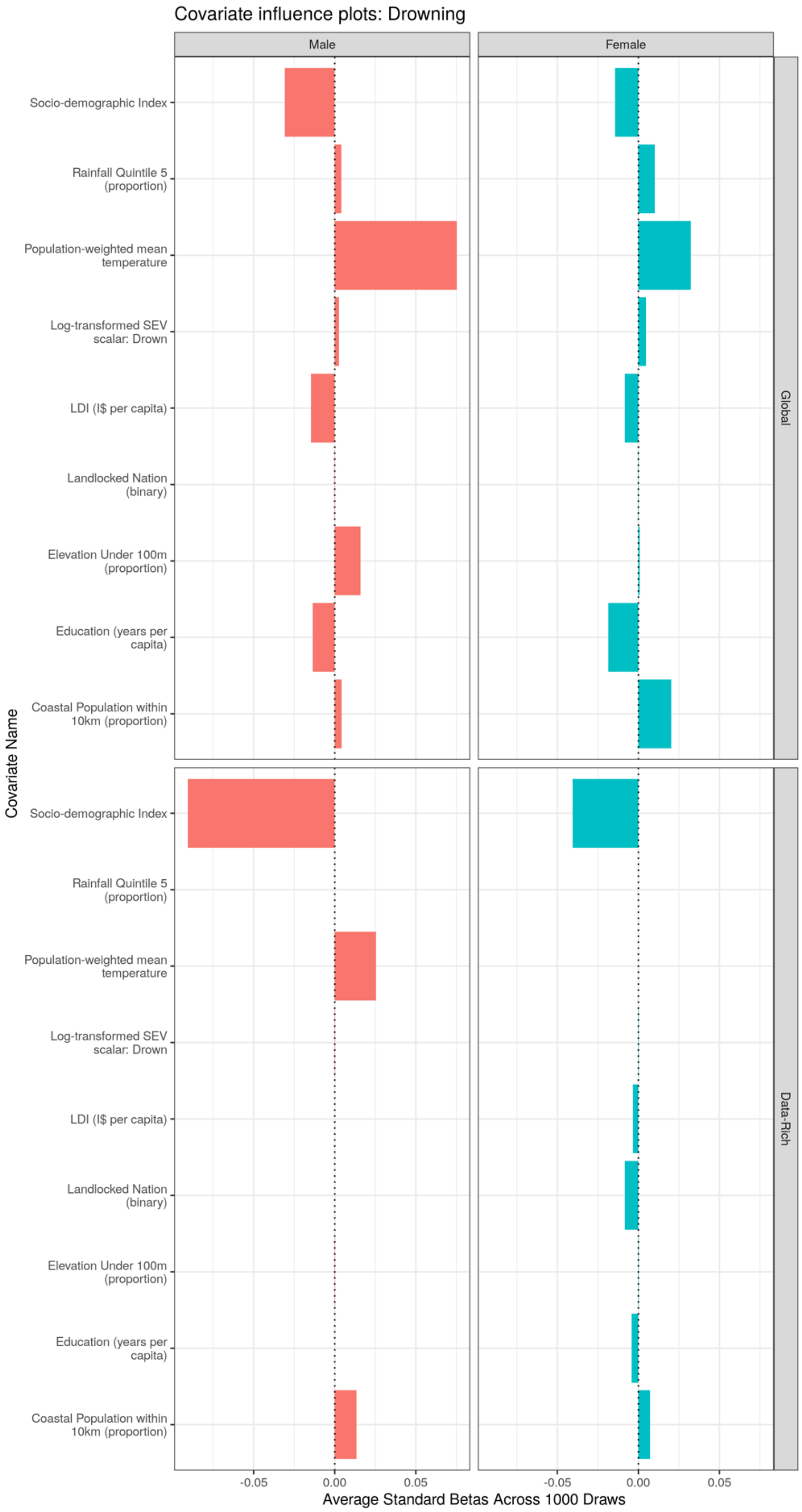

Figure 3b: Falls covariate influence plot

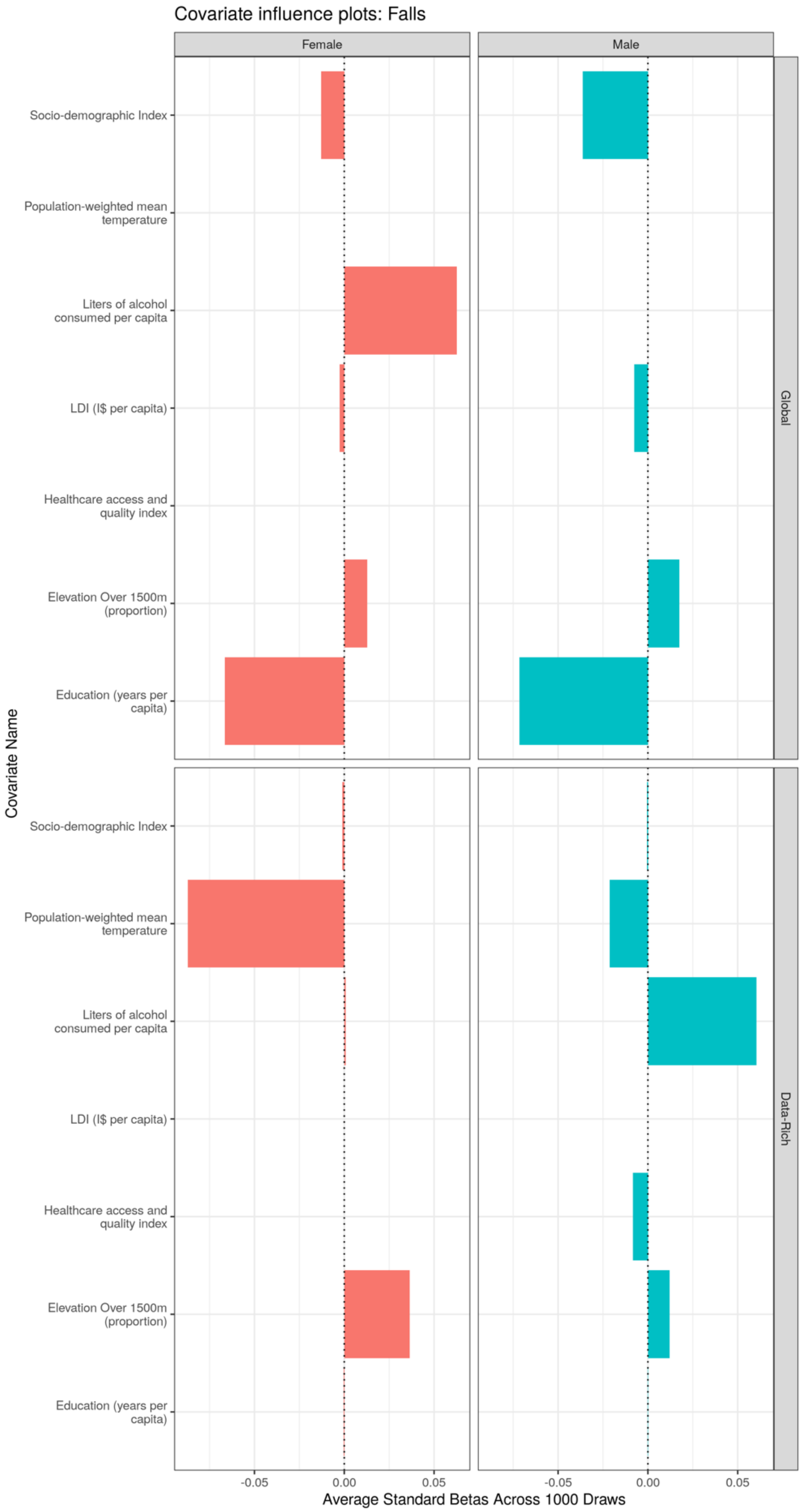

Figure 3c: Fire, heat, and hot substances covariate influence plot

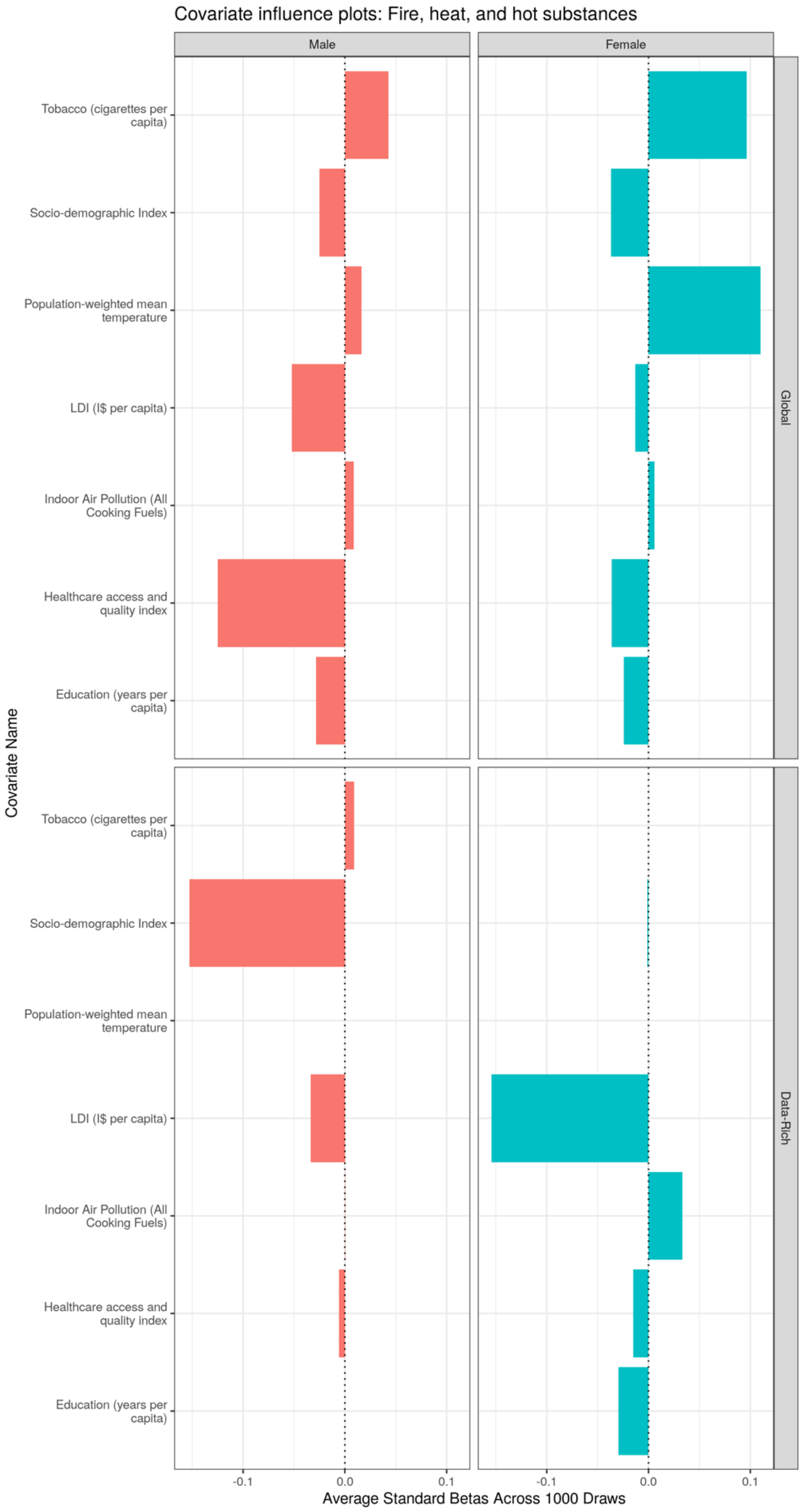

Figure 3d: Poisonings covariate influence plot

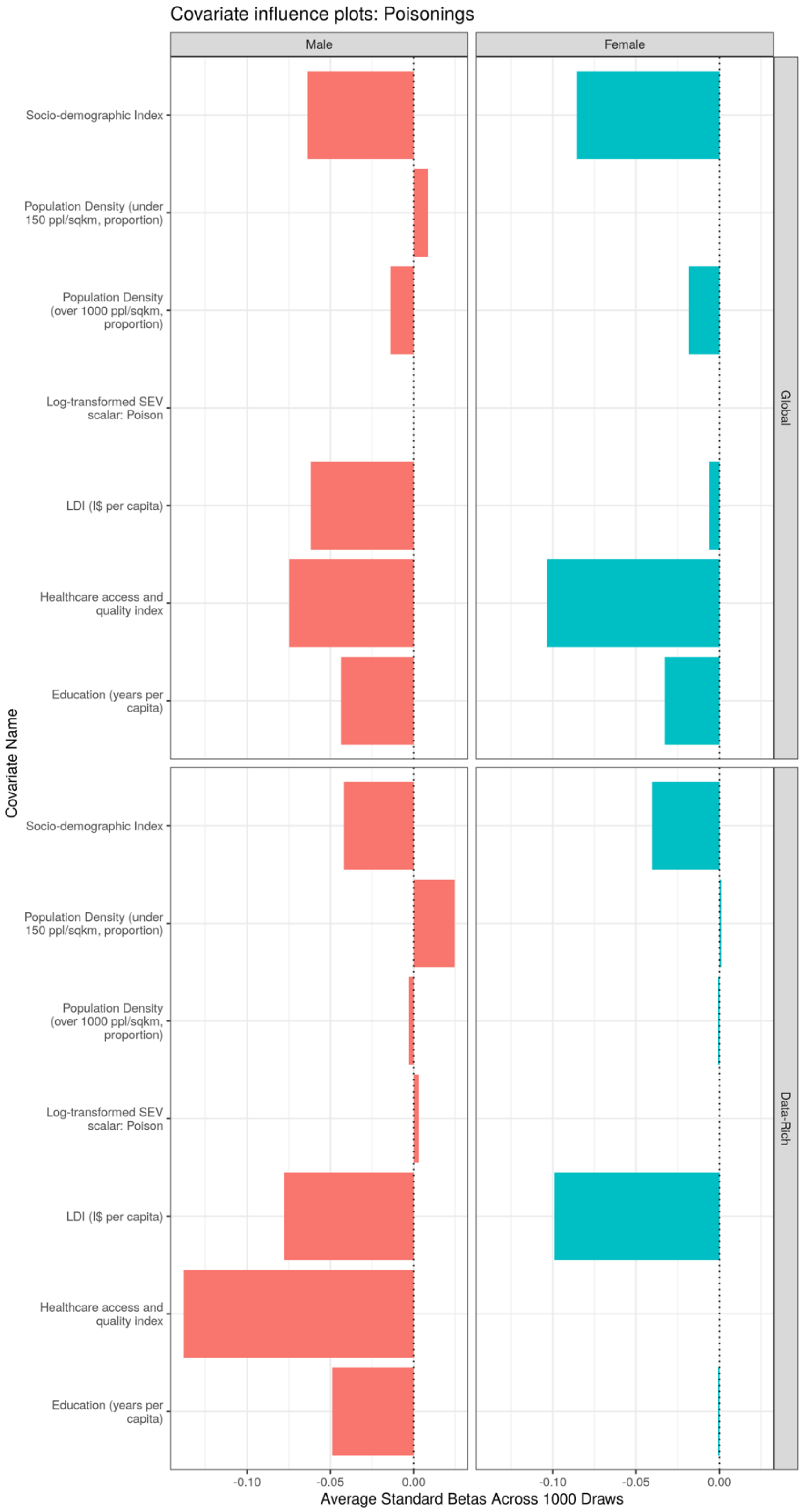

Figure 3e: Exposure to mechanical forces covariate influence plot

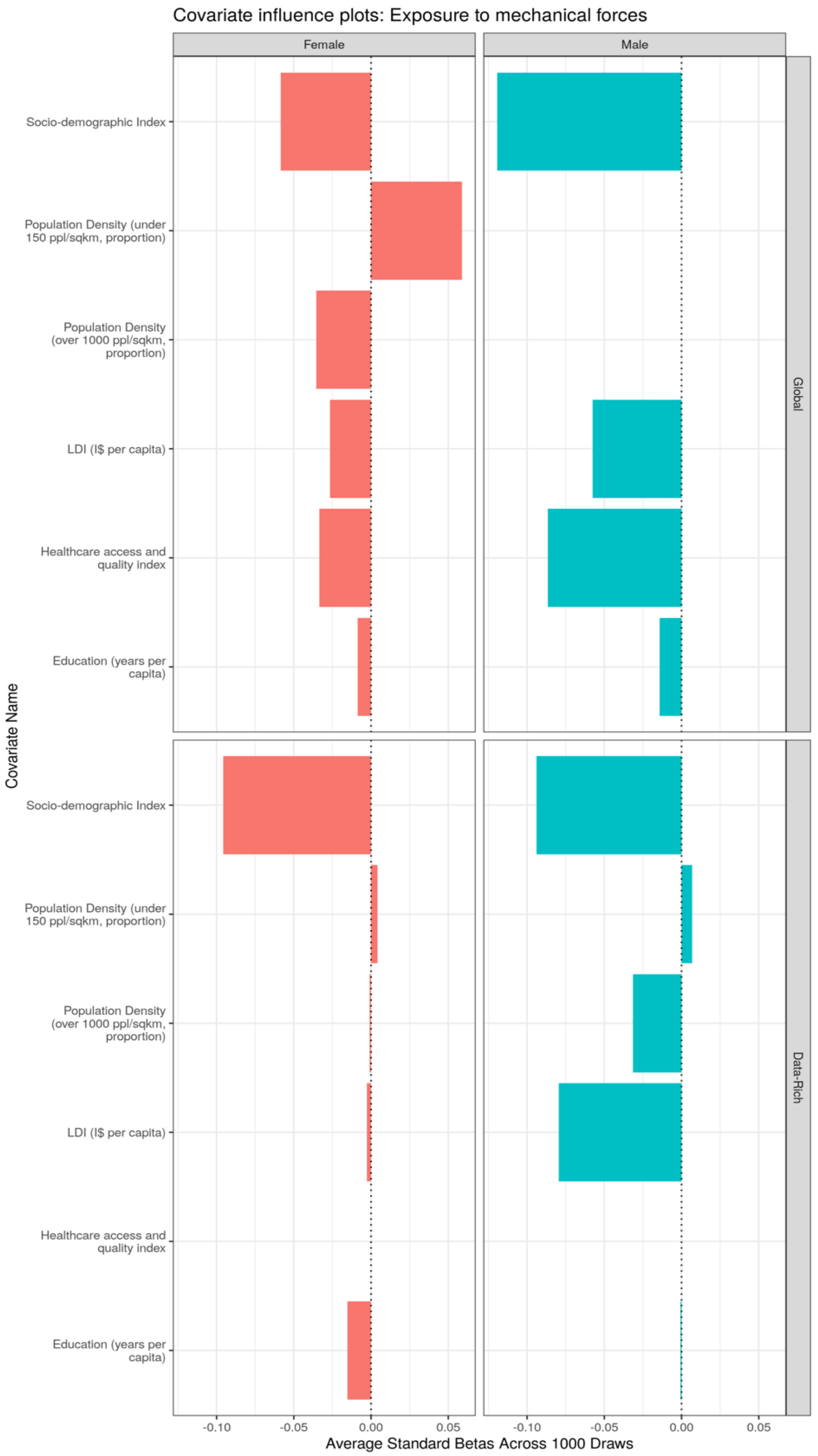

Figure 3f: Animal contact covariate influence plot

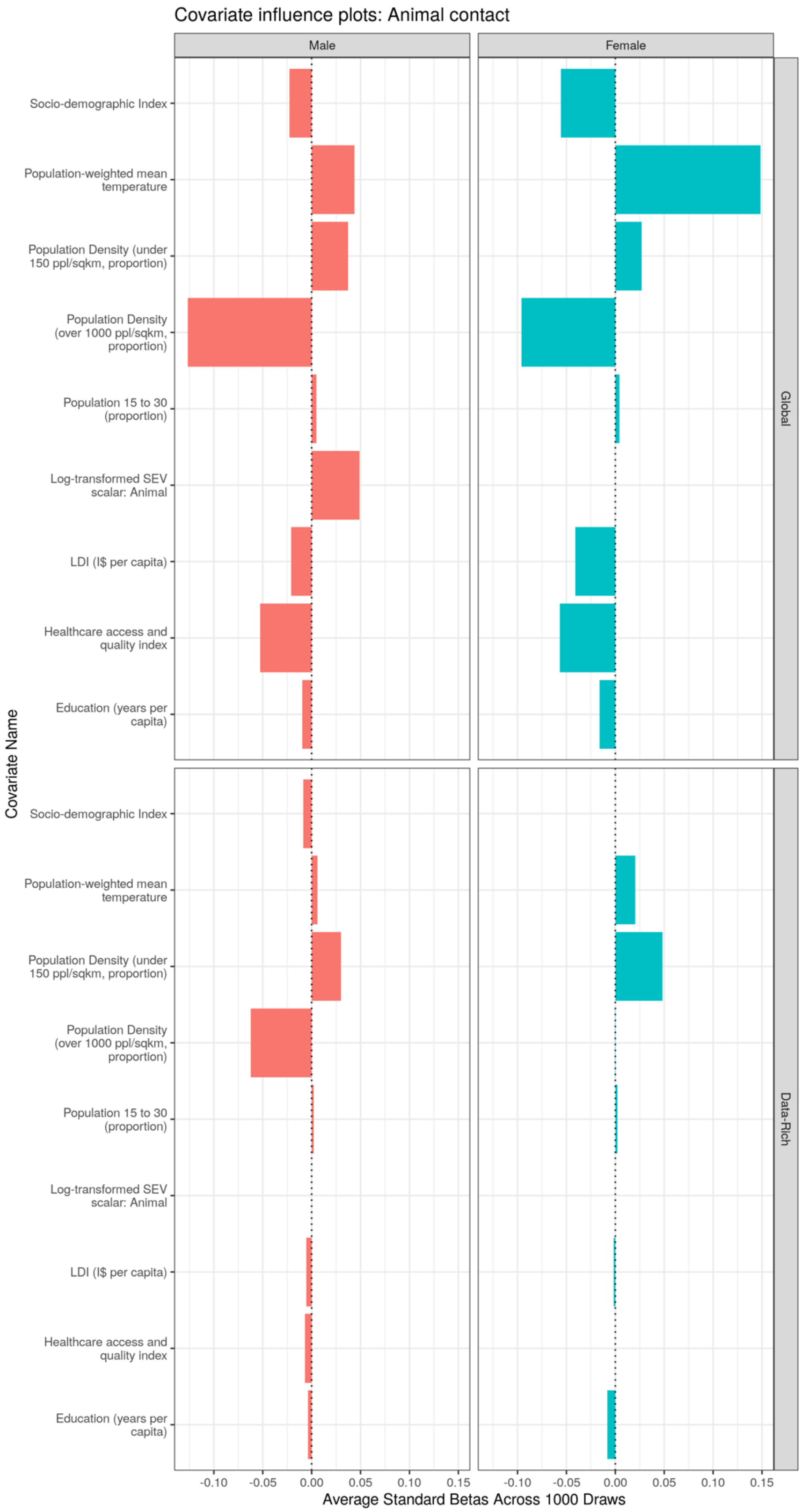

Figure 3g: Foreign body covariate influence plot

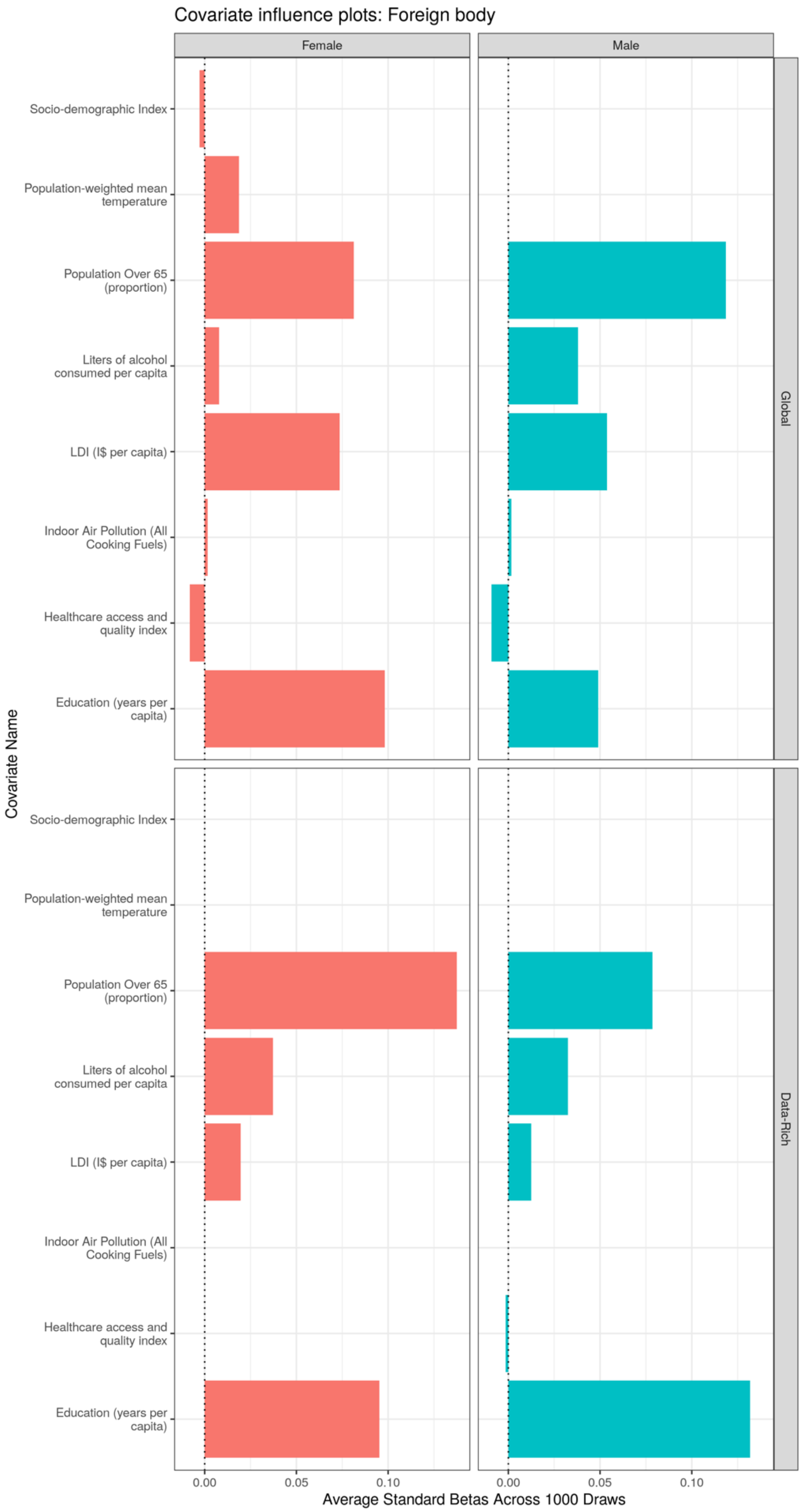

Figure 3h: Other unintentional injuries covariate influence plot

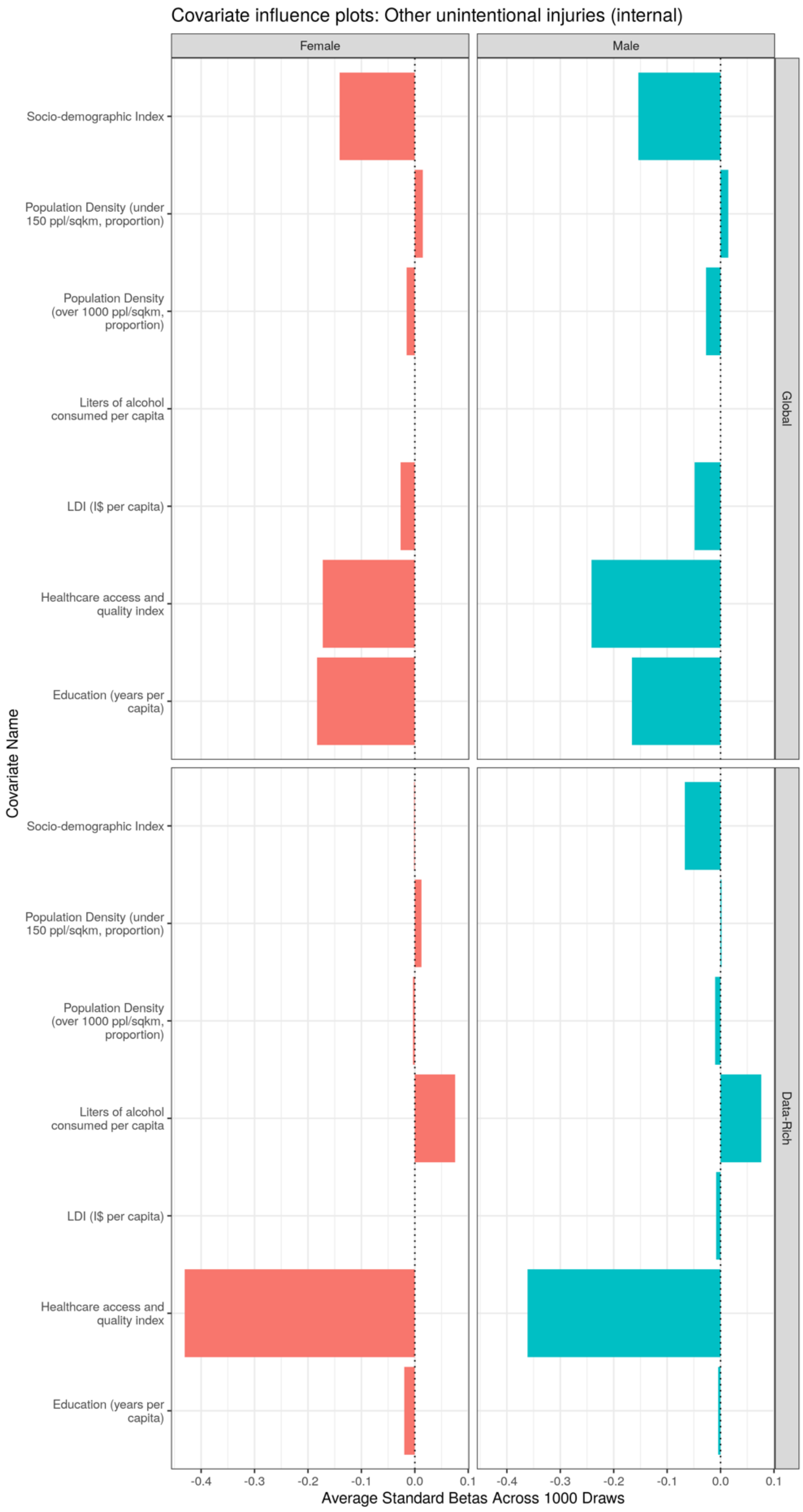

Figure 3i: Adverse effects of medical treatment covariate influence plot

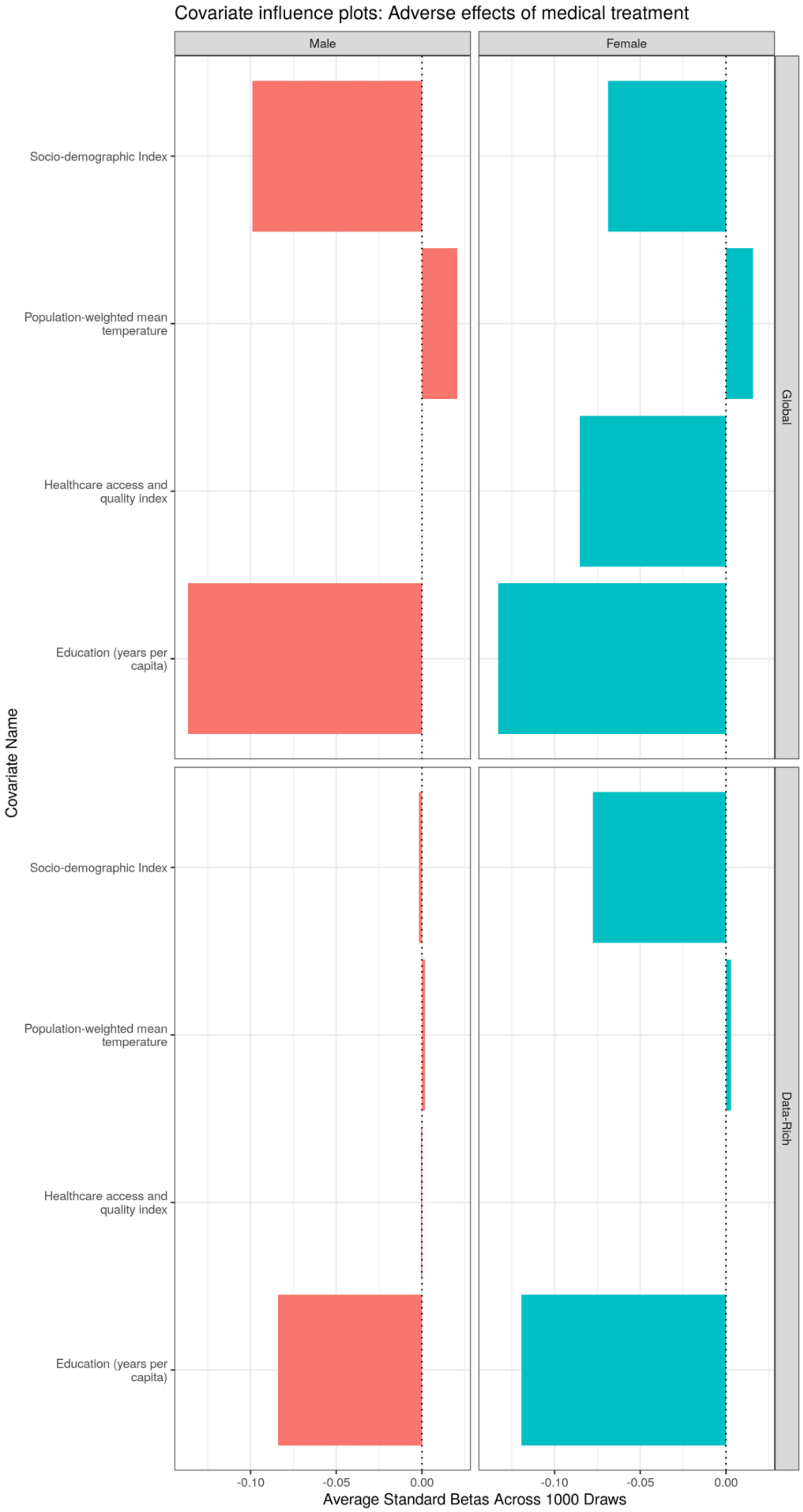

SELF-HARM AND INTERPERSONAL VIOLENCE

Table 4: Covariate changes from GBD 2019 to GBD 2021

ID	Cause	Modelling strategy	Covariate changes from GBD 2019
3	Self-harm and interpersonal violence	Not modelled at parent cause level	
3.1	Self-harm	CODEm	
3.1.1	Self-harm by firearm	CODEm	
3.1.2	Self-harm by other specified means	CODEm	
3.2	Interpersonal violence	CODEm	Dropped: Opium cultivation (binary)
3.2.1	Physical violence by firearm	CODEm and fatal discontinuity estimation	Dropped: Opium cultivation (binary)
3.2.2	Physical violence by sharp object	CODEm and fatal discontinuity estimation	Dropped: Opium cultivation (binary)<br><br>Changed to Level 1 in female global model: Population-weighted mean temperature<br><br>Changed to Level 2 in female data-rich model: Population-weighted mean temperature
3.2.3	Physical violence by other means	CODEm and fatal discontinuity estimation	Dropped: Opium cultivation (binary)
3.3	Conflict and terrorism	Fatal discontinuity estimation	
3.4	Executions and police conflict	CODEm and fatal discontinuity estimation	

Table 4.1. Self-harm covariate levels and directions

Covariate	Self-harm		Self-harm by firearm		Other specified means	
	Level	Direction	Level	Direction	Level	Direction
12-month non-partner sexual violence	1	1	1	1	1	1
Litres of alcohol consumed per capita	1	1	1	1	1	1
Log-transformed SEV scalar: Self-harm	1[h]	1	1	1	1	1
Major depressive disorder	1	1	1	1	1	1
Muslim religion (proportion of population)	1[i]	1	NA	NA	NA	NA
Population-weighted mean temperature	1	1	1	1	1	1
Healthcare Access and Quality Index	2	-1	2	-1	2	-1
Population density (150-300 ppl/sqkm, proportion)	2	1	2	1	2	1
Population density (300-500 ppl/sqkm, proportion)	2	-1	2	-1	2	-1
Population density (500-1000 ppl/sqkm, proportion)	2	-1	2	-1	2	-1
Population density (over 1000 ppl/sqkm, proportion)	2	-1	2	-1	2	-1
Population density (under 150 ppl/sqkm, proportion)	2	1	2	1	2	1
Education (years per capita)	3	-1	3	-1	3	-1
LDI (I$ per capita)	3	-1	3	-1	3	-1
Socio-demographic Index	3	-1	3	-1	3	-1

h: Only used in female models
i: Used at Level 2 in male global model, used at Level 1 in male data-rich model. Not used in female model.

Covariate level is grouped by strong biological link (1), strong evidence of a relationship (2), or weak relationship (3). The direction indicates whether the covariate and cause of death change in the same direction (1) or opposite (-1).

Table 4.2: Interpersonal violence covariate level and directions

Covariate	Interpersonal violence		Execution and police conflict	
	Level	Direction	Level	Direction
Litres of alcohol consumed per capita	NA	NA	1	1
Log-transformed SEV scalar: Self harm	1	1	NA	NA
Healthcare Access and Quality Index	1	1	2	-1
Population density (150-300 ppl/sqkm, proportion)	2	-1	1	1
Population density (over 1000 ppl/sqkm, proportion)	NA	NA	2	1
Population density (under 150 ppl/sqkm, proportion)	2	1	NA	NA
Education (years per capita)	NA	NA	3	1
LDI (I$ per capita)	3	-1	3	-1
Socio-demographic Index	3	-1	3	-1
Education relative inequality (Gini)	3	-1	1s	1
Log-transformed SEV scalar: Violence	1	1	NA	NA
Population 15 to 30 males (proportion)	1	1	1	1
Log-transformed SEV scalar: Viol gun	1	1	NA	NA

Covariate level is grouped by strong biological link (1), strong evidence of a relationship (2), or weak relationship (3). The direction indicates whether the covariate and cause of death change in the same direction (1) or opposite (-1).

Table 4.3: Physical violence covariate level and directions

Covariate	Physical violence by firearm		Physical violence by sharp object		Physical violence by other means	
	Level	Direction	Level	Direction	Level	Direction
Litres of alcohol consumed per capita	1	1	1	1	1	1
Population-weighted mean temperature	1	1	1[f]	1	1	1
Healthcare Access and Quality Index	2	-1	2	-1	2	-1
Population density (over 1000 ppl/sqkm, proportion)	2	1	2	1	2	1

Education (years per capita)	3	-1	3	-1	3	-1
LDI (I$ per capita)	3	-1	3	-1	3	-1
Socio-demographic Index	3	-1	3	-1	3	-1
Education relative inequality (Gini)	1	1	1	1	1	1
Population 15 to 30 males (proportion)	1	1	1	1	1	1
Log-transformed SEV scalar: Viol gun	1	1	NA	NA	NA	NA
Log-transformed SEV scalar: Viol knife	NA	NA	1	1	NA	NA
Log-transformed SEV scalar: Oth viol	NA	NA	NA	NA	1	1

f: Not used in female global model

Covariate level is grouped by strong biological link (1), strong evidence of a relationship (2), or weak relationship (3). The direction indicates whether the covariate and cause of death change in the same direction (1) or opposite (-1).

Figure 4a: Self-harm covariate influence plot

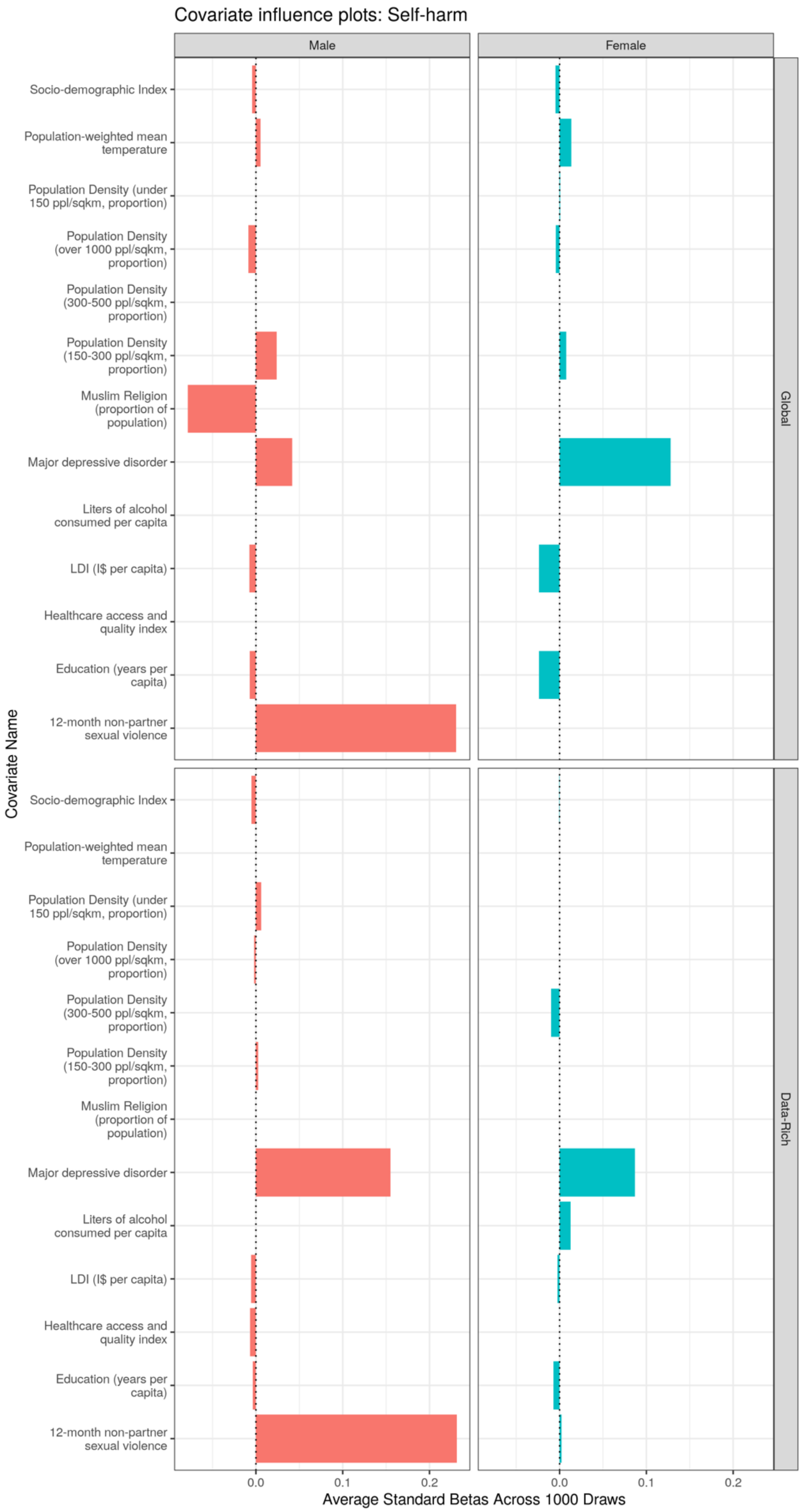

Figure 4b: Interpersonal violence covariate influence plot

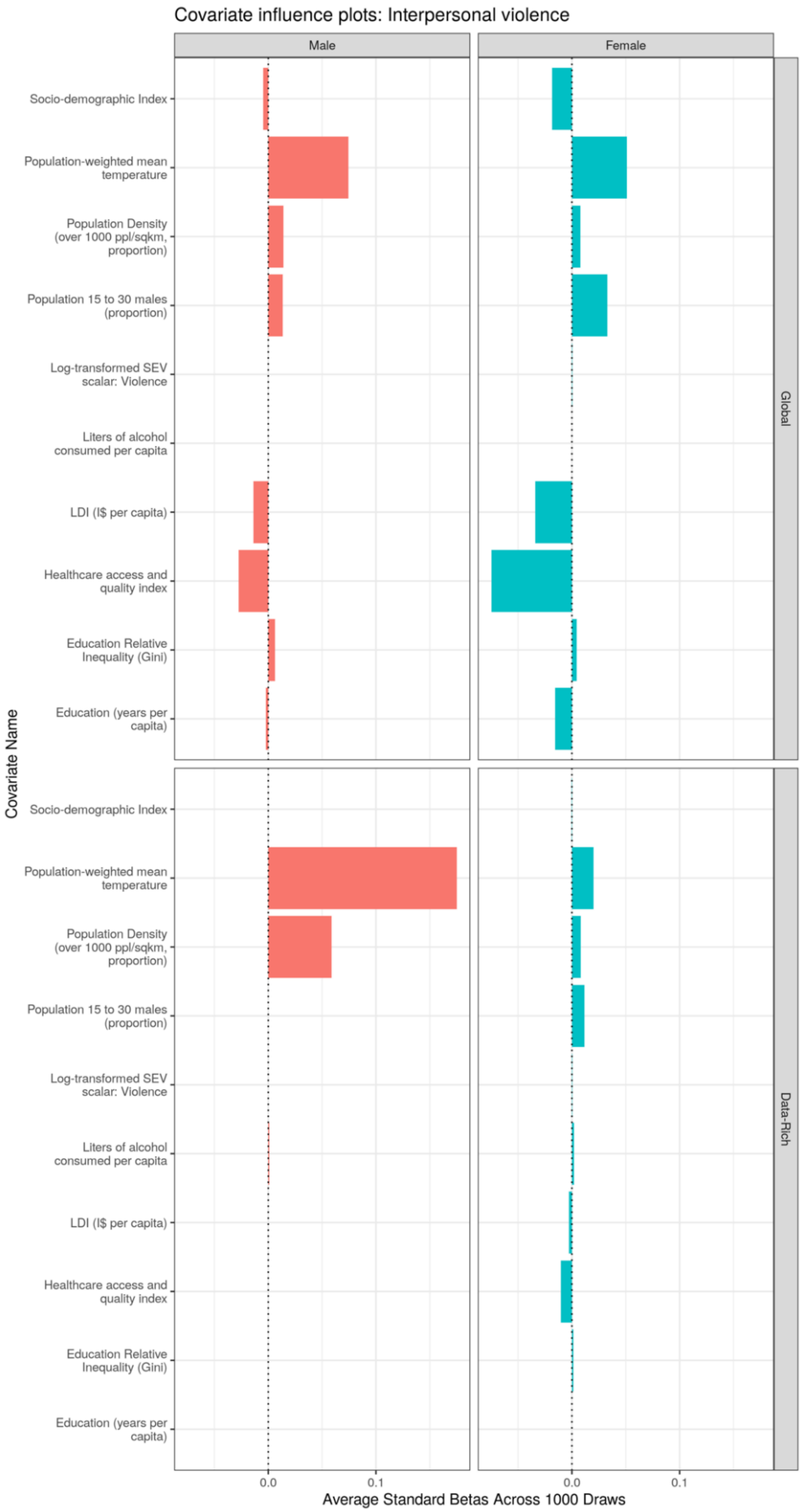

Figure 4c: Environmental heat and cold exposure covariate influence plot

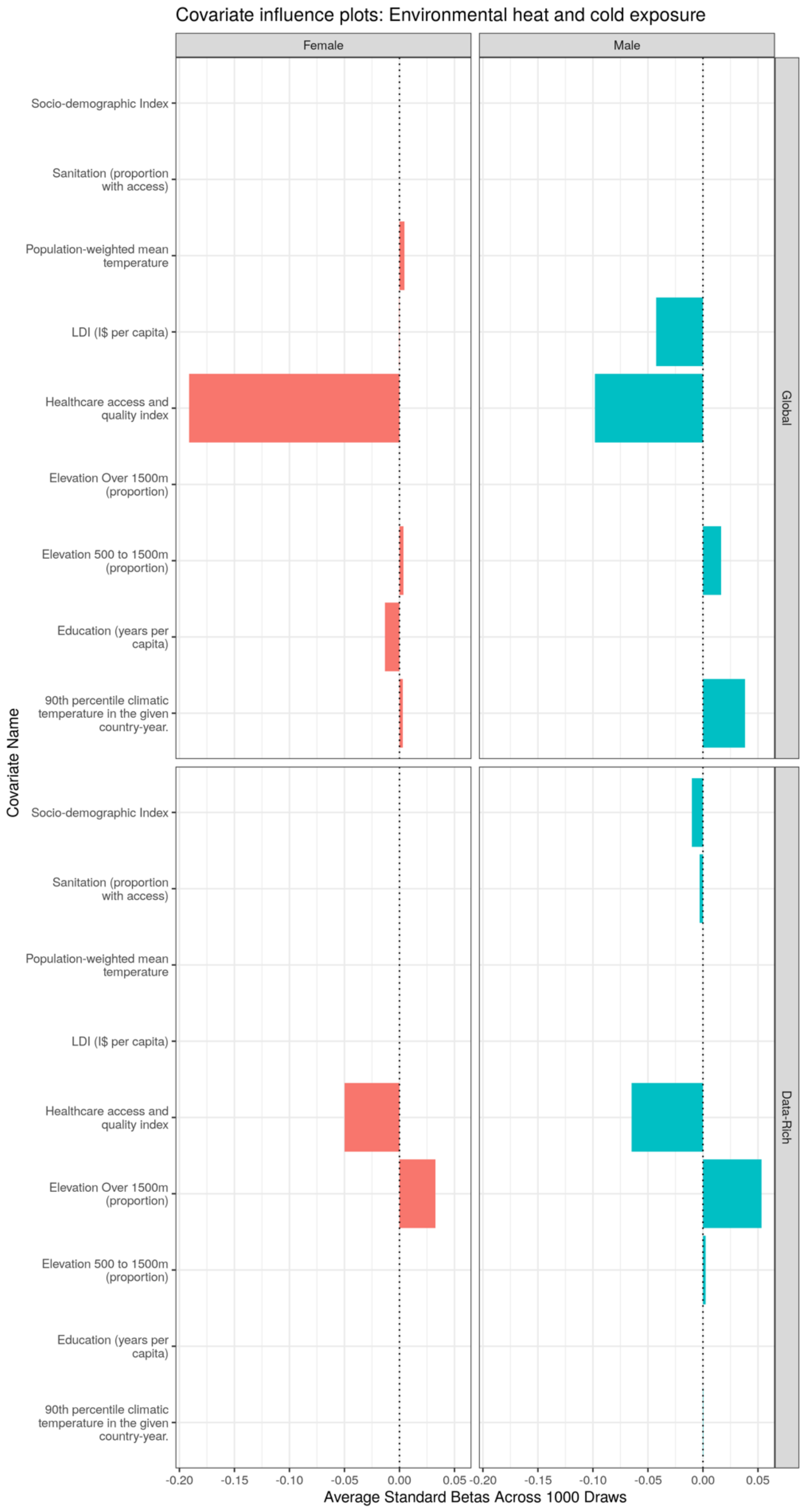

Figure 4d: Police conflict and executions covariate influence plot

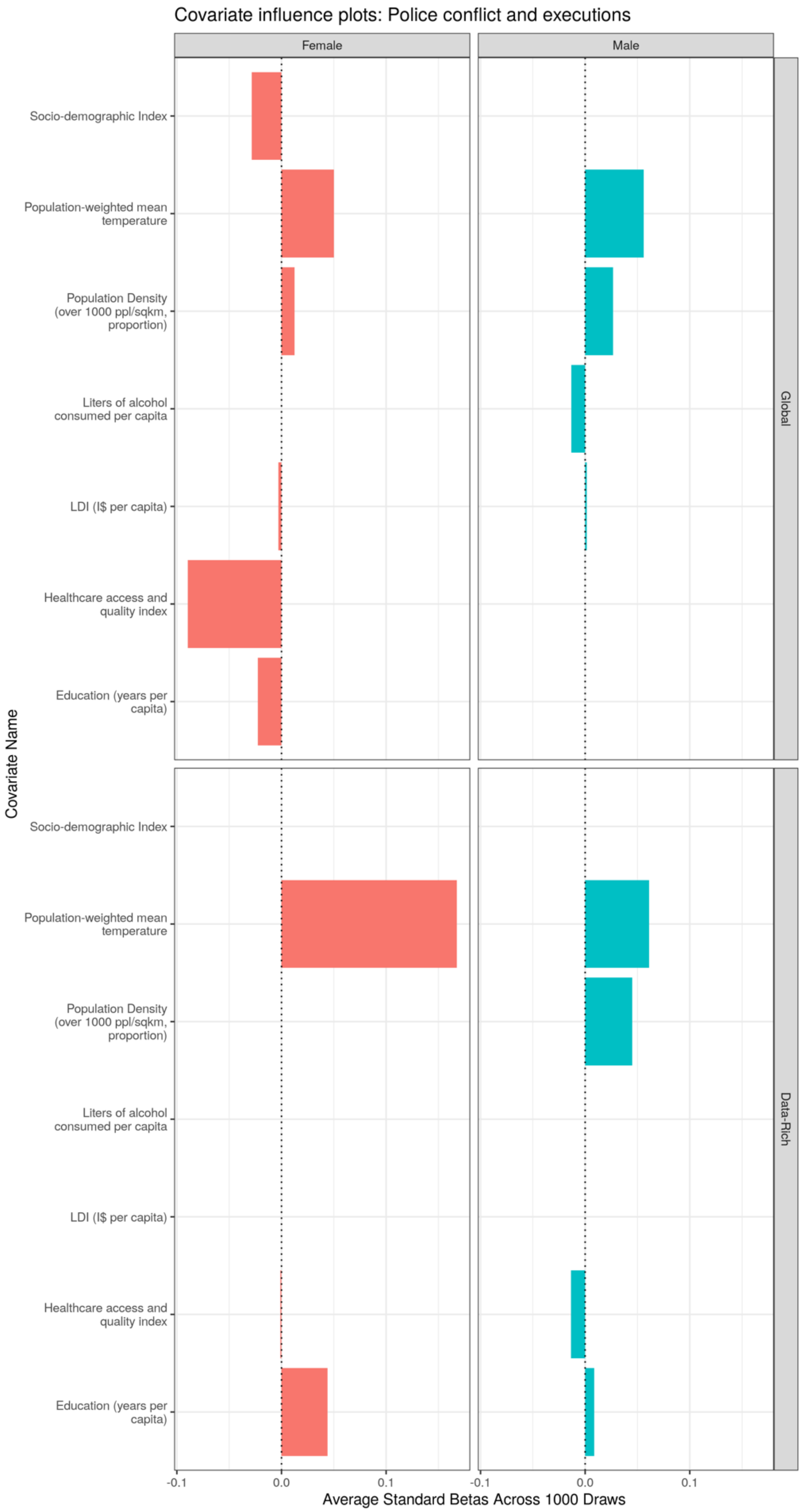

Covariate levels and direction superscript

a: Used at Level 1 in female models, Level 2 in males.
b: Used at Level 3 in global models, Level 2 in data-rich models.
c: Used at Level 1 in male data-rich model. Level 2 in other three models.
d: Not used in male global model.
e: Used at Level 2 in male global model, Level 3 for the other three models.
f: Not used in female global model.
g: Used at Level 1 in male global model, Level 3 for the other three models.
h: Only used in female models.
i: Used at Level 2 in male global model, used at Level 1 in male data-rich model. Not used in female model.
j: Used at Level 3 in the female global model.
k: Only used in the female global model.
l: Used at Level 3 in male global model.
m: Not used in global models.

References

1 Lozano R, Naghavi M, Foreman K, *et al.* Global and regional mortality from 235 causes of death for 20 age groups in 1990 and 2010: a systematic analysis for the Global Burden of Disease Study 2010. *The Lancet* 2012; **380**: 2095–128.

2 Global, regional, and national age–sex specific all-cause and cause-specific mortality for 240 causes of death, 1990–2013: a systematic analysis for the Global Burden of Disease Study 2013. *The Lancet* 2015; **385**: 117–71.

3 Global, regional, and national life expectancy, all-cause mortality, and cause-specific mortality for 249 causes of death, 1980-2015: a systematic analysis for the Global Burden of Disease Study 2015. The Lancet 2016; **388**: 1459-1544.

4 Murray CJL, Lopez AD, Harvard School of Public Health, World Health Organization, World Bank. The global burden of disease: a comprehensive assessment of mortality and disability from diseases, injuries, and risk factors in 1990 and projected to 2020. Cambridge, MA: Published by the Harvard School of Public Health on behalf of the World Health Organization and the World Bank : Distributed by Harvard University Press, 1996.

5 Matzopoulos R, Prinsloo M, Wyk VP, Gwebushe N, Mathews S, et al. Injury-related mortality in South Africa: a retrospective descriptive study of postmortem investigations. *Bull World Health Organ* 2015; **93**: 303–13.

6 Feldman JM, Gruskin S, Coull BA, Krieger N (2017) Quantifying underreporting of law-enforcement-related deaths in United States vital statistics and news-media-based data sources: A capture–recapture analysis. PLOS Medicine 14(10): e1002399.

7. Samuel S, DeRay M. Mapping Police Violence (United States). 2021.

8. Burghart B. Fatal Encounters Database (United States). 2021.

9. Banks D, Ruddle P, Kennedy E, Planty M/ Arrest-Related Deaths Program Redesign Study. *Bureau of Justice Statistics* 2016. No. NCJ 250112.

10. Feldman JM, Gruskin S, Coull BA, Krieger N. Killed by Police: Validity of Media-Based Data and Misclassification of Death Certificates in Massachusetts, 2004–2016. *American Journal of Public Health* 2017. 107, no. 10: pp. 1624-1626.

11. Ozkan T, Worrall JL, Zettler H. Validating media-driven and crowdsourced police shooting data: a research note. *Journal of Crime and Justice* 2018. 41:3, 334-345.

12. United States Department of Justice, Federal Bureau of Investigation. Uniform Crime Reporting Program Data: Law Enforcement Officers Killed and Assaulted (LEOKA). 2019.

13. Death Penalty Information Center (DPIC). United States Death Penalty Execution Database. Washington, DC, United States of America: Death Penalty Information Center (DPIC). 2020.

14. Loftin C, Wiersema B, McDowall D, Dobrin A. Underreporting of Justifiable Homicides Committed by Police Officers in the United States, 1976–1998. *American Journal of Public Health* 2003. 93, no. 7: pp. 1117-1121.

15. Loftin C, McDowall D, Xie M. Underreporting of Homicides by Police Officers in the United States, 1976–2013. *Homicide Studies* 2017. 21(2):159-174.

Interstitial lung disease and pulmonary sarcoidosis

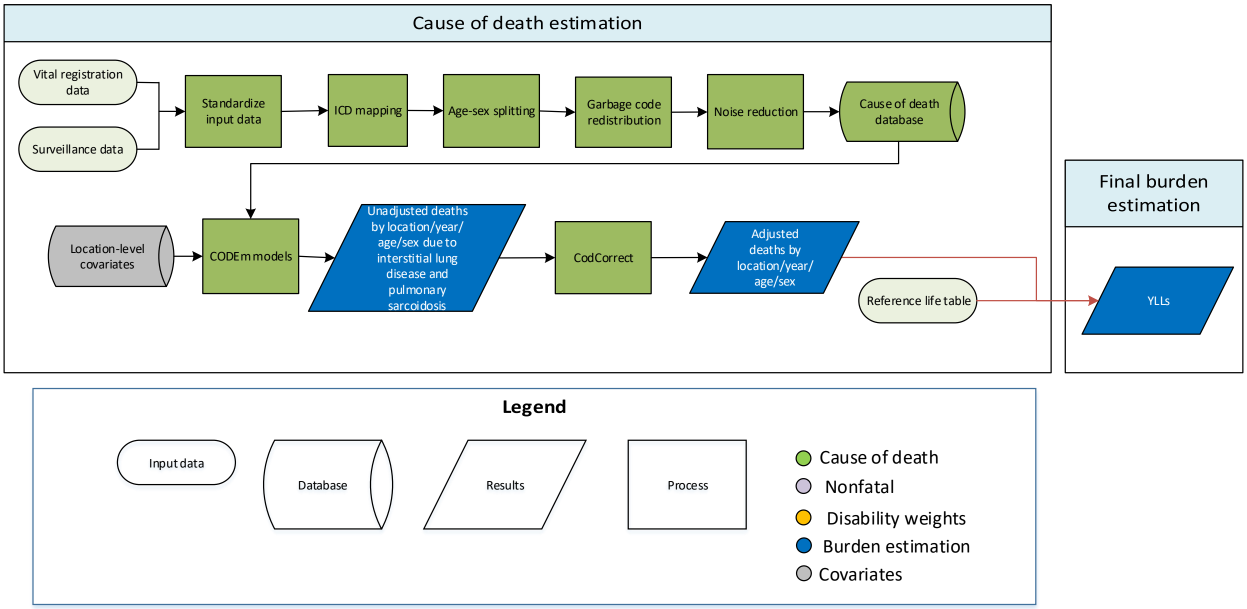

Input data

Data used to estimate interstitial lung disease and pulmonary sarcoidosis (ILD) mortality included vital registration and surveillance data from the cause of death (COD) database. Our outlier criteria excluded datapoints that (1) were implausibly high or low, (2) substantially conflicted with established age or temporal patterns, or (3) substantially conflicted with other data sources conducted from the same locations or locations with similar characteristics (ie, Socio-demographic Index).

Modelling strategy

There were no substantive changes to the modelling approach this round. The standard Cause of Death Ensemble modelling (CODEm) [1] approach (detailed in reference appendix section 3.1) was used to estimate deaths due to interstitial lung disease and pulmonary sarcoidosis. Separate models were conducted for male and female mortality, and the age range for both models was 1 to 95+ years. For GBD 2021, we removed the summary exposure value: interstitial lung disease and occupational professionals covariates due to a lack of predictive quality.

The following table lists the covariates included in the model. This requires that the covariate selected for the model must have the directional relationship with ILD. For GBD 2021, no significant updates were made to covariate selections. Covariate directions were selected based on the strength of the evidence.

Level	Covariate	Direction
1	Smoking prevalence	+
	Cumulative cigarettes (5 years)	+

2	Elevation over 1500 m (proportion)	+
	Elevation between 500 and 1500 m (proportion)	+
	Indoor air pollution (all cooking fuels)	+
	Outdoor air pollution ($PM_{2.5}$)	+
	Healthcare Access and Quality Index	-
3	Log LDI (I$ per capita)	-
	Education (years per capita)	-
	Socio-demographic Index	-

The unadjusted death estimates from interstitial lung disease and pulmonary sarcoidosis are summed alongside other chronic respiratory disease causes (chronic obstructive pulmonary disease, asthma, and pneumoconiosis) and fit to the distribution of deaths in an overall chronic respiratory disease envelope model as part of the CoDCorrect adjustment process. This results in deaths recorded using non-specific coding systems, such as verbal autopsy, being included in the parent model and redistributed to the child models proportionately.

Covariate influences:

The following plots show the influence of each covariate on the four CODEm models (male global, male data-rich, female global, and female data-rich). A positive standardised beta (to the right) means that the covariate was associated with increased death. A negative standardised beta (to the left) means the covariate was associated with decreased death.

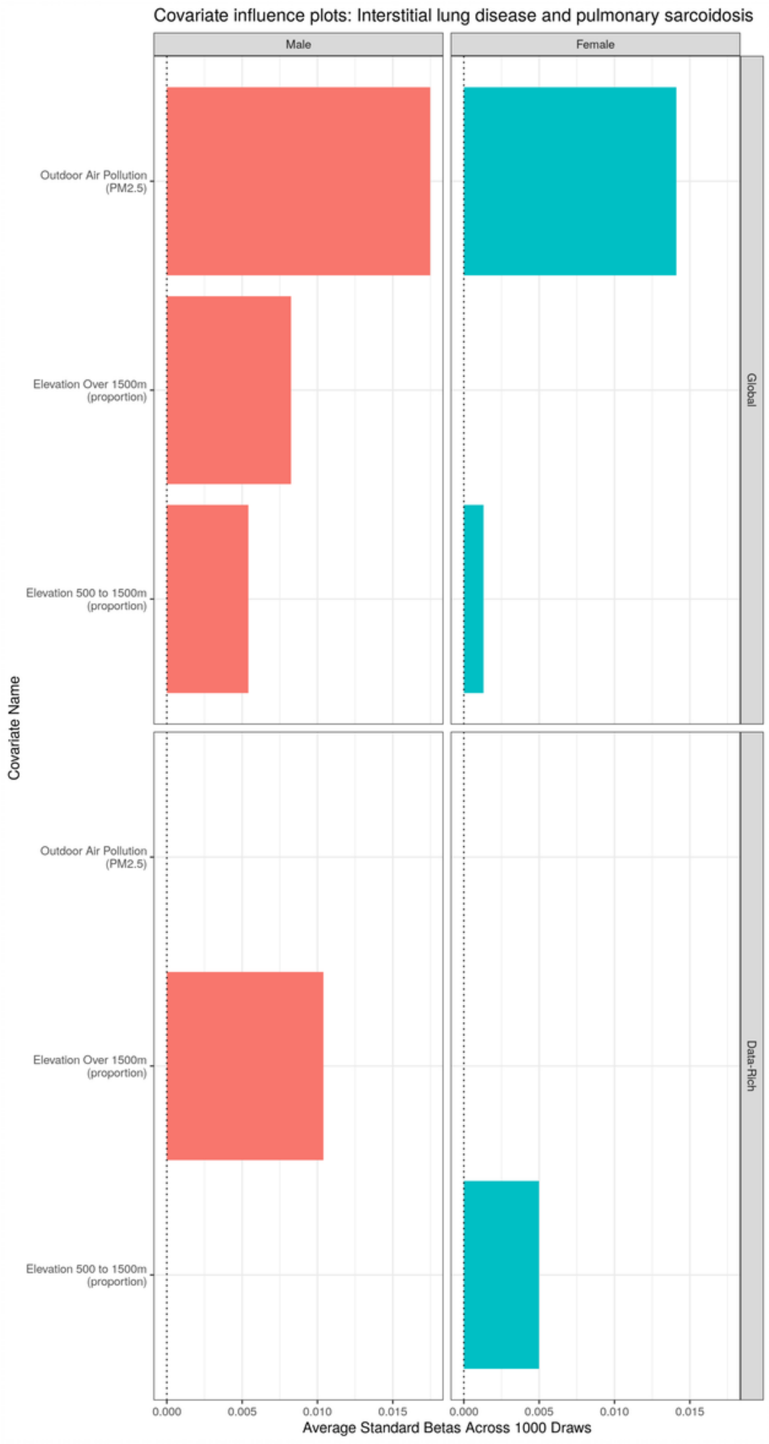

[1]Vos T, Lim SS, Abbafati C, *et al.* Global burden of 369 diseases and injuries in 204 countries and territories, 1990–2019: a systematic analysis for the Global Burden of Disease Study 2019. *The Lancet* 2020**;** 396: 1204–22. doi: https://doi.org/10.1016/S0140-6736(20)30925-9

Intracerebral haemorrhage

Flowchart

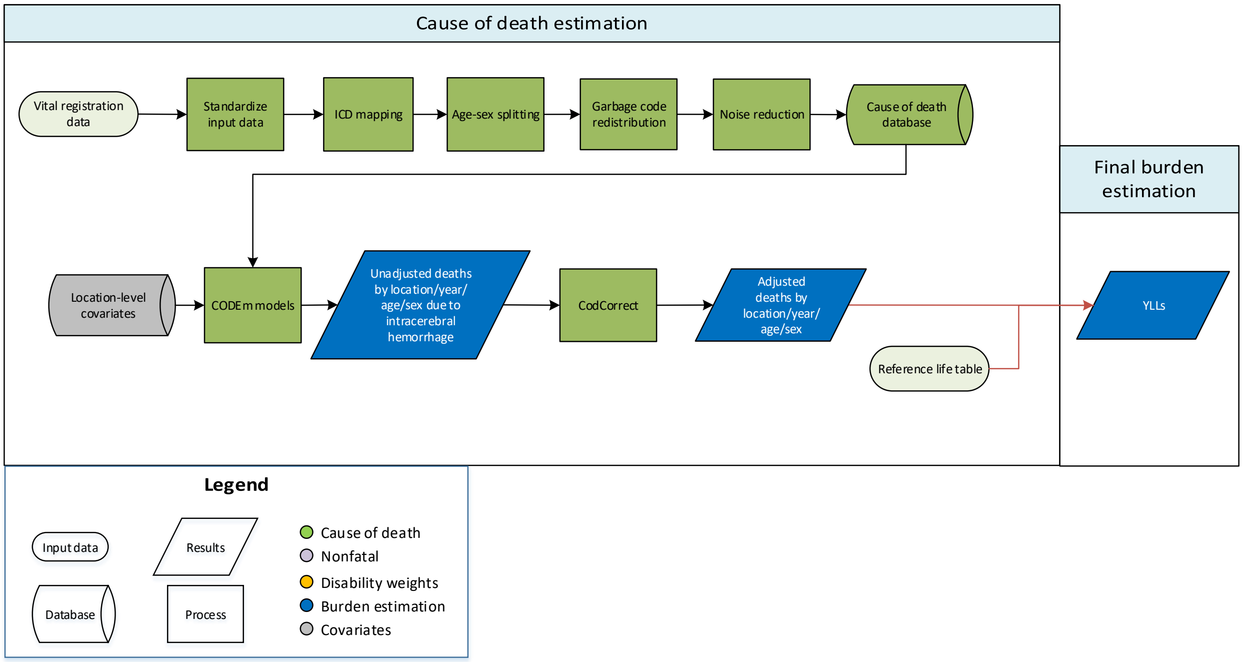

Input data and methodological summary for intracerebral haemorrhage

Input data

Vital registration data were used to model intracerebral haemorrhage. We outliered ICD8 datapoints which were inconsistent with the rest of the data and created implausible time trends. In addition, we outliered vital registration datapoints in certain Latin American countries due to implausibly high values at the oldest age groups resulting in inconsistencies in time trends. We also outliered implausibly high ICD10 vital registration data in Ghana, Cabo Verde, and Palestine.

Modelling strategy

We used a standard CODEm approach to model deaths from intracerebral haemorrhage. For GBD 2021, we updated the redistribution of deaths attributed to hypertension to an analysis of multiple cause of death datasets containing information on the entire chain of causes listed on the death certificate from seven countries with high quality data. In GBD 2019, the redistribution of hypertension deaths was done by regressing redistribution codes against non-redistribution codes; details on this approach can be found elsewhere in the appendix. Using the multiple cause of death approach to redistribution allows us to observe the true underlying cause of death where the reported cause of death is labelled as hypertension. For details on the multiple cause of death redistribution methods, refer to the appendix section on redistribution. This had the effect of increasing the number of hypertension deaths attributed to stroke in general; an increase in post-redistributed deaths was observed for intracerebral haemorrhage specifically. For example, in GBD 2019 we redistributed 185 deaths originally coded to hypertension in the United States for all ages and sexes in the year 2016 to intracerebral haemorrhage. In GBD 2021, the number of deaths redistributed from hypertension to intracerebral haemorrhage in the same demographic increased to 2,896 deaths. Another example to consider is Brazil for all ages and sexes in 2016, where the total number of originally coded hypertension deaths that were redistributed

to intracerebral haemorrhage in GBD 2019 was 199 deaths compared to the GBD 2021 total number of 4,709 redistributed hypertension deaths. For GBD 2021 we updated our approach to noise reduction of the cause of death data so that stochastic time series of data would resemble patterns seen at regional levels more closely. Additional details on the updates to noise reduction can be found in the cause of death methods section of the appendix.

Table: Selected covariates for CODEm models, intracerebral haemorrhage

Level	Covariate	Direction
1	Summary exposure variable, intracerebral haemorrhage	1
	Smoking prevalence	1
	Systolic blood pressure (mm Hg)	1
2	Mean BMI	1
	Elevation over 1,500m (proportion)	-1
	Fasting plasma glucose	1
	Outdoor pollution ($PM_{2.5}$)	1
	Indoor air pollution	1
	Healthcare Access and Quality Index	-1
3	Log-transformed lag distributed income per capita (I$)	-1
	Summary exposure value, omega-3	1
	Summary exposure value, fruits	1
	Summary exposure value, vegetables	1
	Summary exposure value, nuts and seeds	1
	Pulses/legumes (kcal/capita, unadjusted)	-1
	Summary exposure value, PUFA adjusted (percent)	1
	Alcohol (litres per capita)	1
	LDL Cholesterol (mean per capita)	-1
	Trans fatty acid	1

Invasive non-typhoidal salmonella (iNTS)

Flowchart

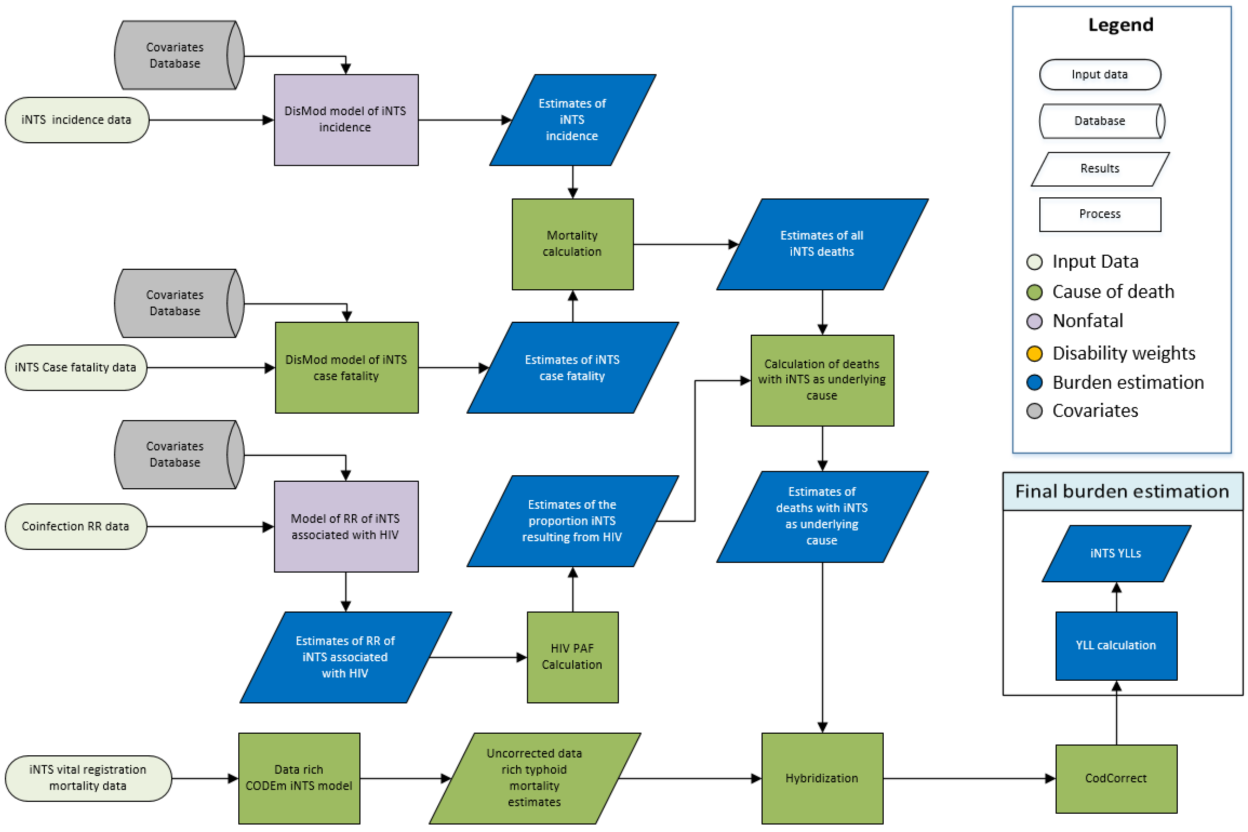

Input data and methodological summary for iNTS

Input data

Our CODEm model used all available data in the cause of death database from data-rich countries. No data were outliered for this cause. Incidence estimates for the natural history model are modelled using an incidence dataset based principally on prospective cohort studies and facility-based surveillance. Similarly, data on case fatality and co-infection come from prospective cohort studies and facility-based surveillance.

Modelling strategy

We model iNTS deaths using a hybrid modelling strategy with two components: 1) for data-rich locations, we estimate iNTS mortality using a CODEm model of CoD data; and 2) in all other locations (ie, not data-rich) we use a natural history model in which we derive deaths as the product of cases and case fatality.

The CODEm model included three covariates:

Level	Covariate	Direction
1	SEV unsafe water	+
	Malaria incidence adjusted for antimalarial coverage and drug effectiveness	+
	HIV mortality rate	+

For the natural history model, we estimate iNTS deaths as the product of cases and case fatality. Incidence was modelled with DisMod-MR 2.1, using the HIV mortality rate, malaria incidence adjusted for antimalarial coverage and drug effectiveness, and the summary exposure value (SEV), unsafe water, as covariates. We estimated the relative risk of iNTS comparing people with HIV to those without using a negative binomial model with log-age and log of the summary exposure value (SEV) for water as predictors. We used the resulting relative risk estimates and HIV prevalence estimates to calculate the proportion of iNTS that was attributable to HIV in each location, year, age, and sex. Using these proportions, we divided iNTS cases into those that were attributable to HIV and those that were not. We modelled case fatality by age and Socio-demographic Index (SDI) separately for those with and without HIV using a generalised additive model, parameterising age with P-splines, and estimated mortality as the product of incidence and case fatality. Where iNTS occurs among those with HIV, we assume that iNTS is an opportunistic infection and that HIV is therefore the underlying cause of death. We therefore estimate deaths with iNTS as the underlying cause as total iNTS deaths times the proportion of cases not attributable to HIV.

We have made no substantive changes to our modelling strategy between GBD 2019 and 2021.

Figure 1: PRISMA 2009 flow diagram

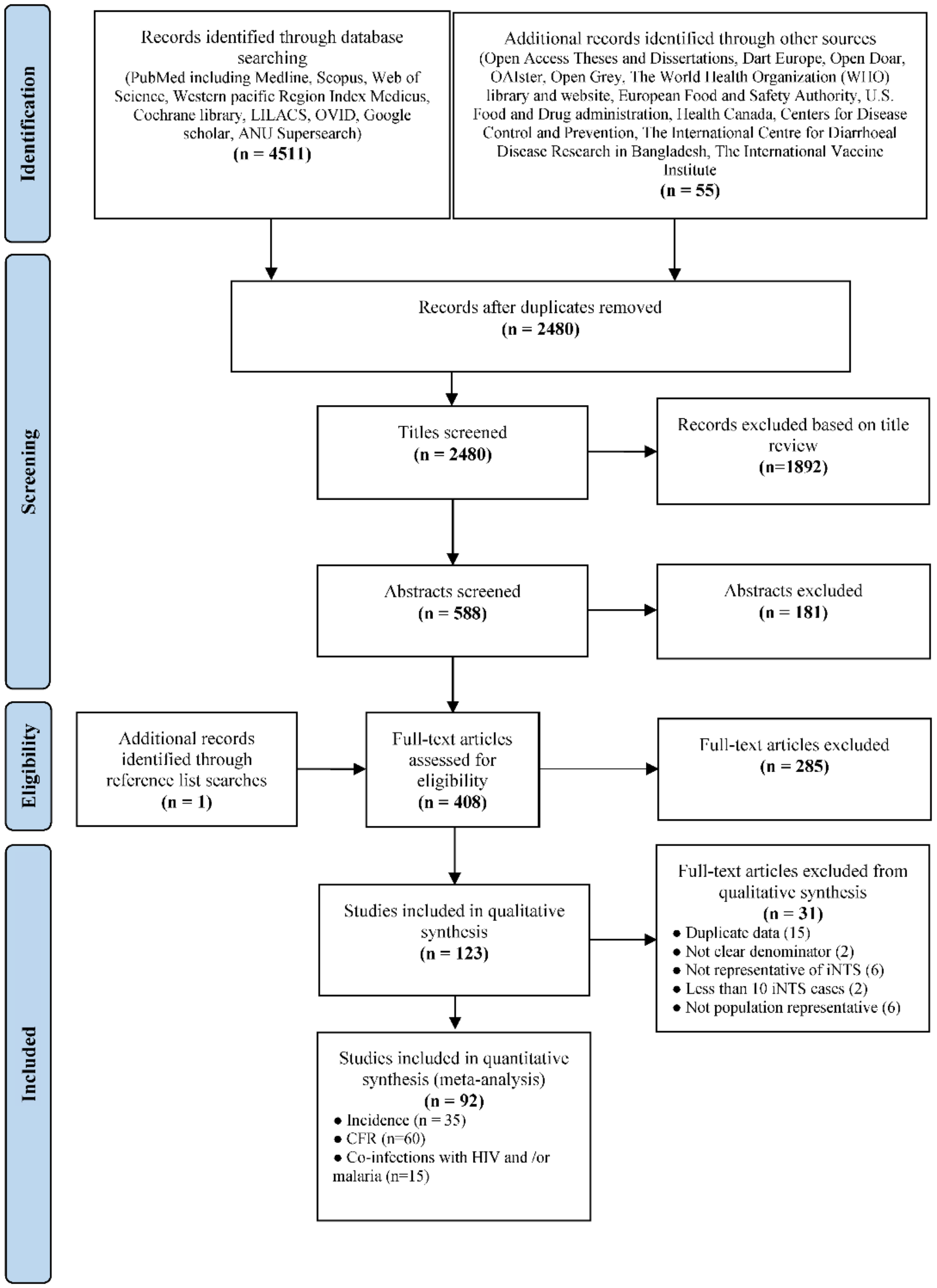

Ischaemic heart disease

Flowchart

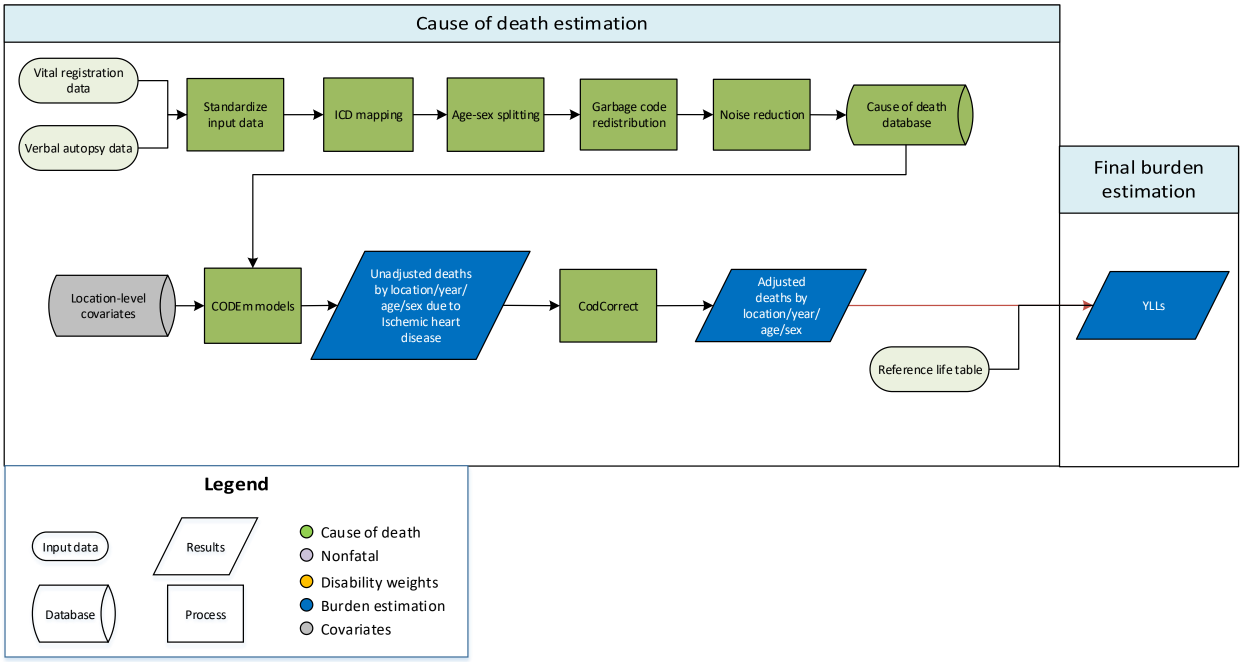

Input data and methodological summary for ischaemic heart disease

Input data

Vital registration and verbal autopsy data were used to model ischaemic heart disease.

For GBD 2021, all verbal autopsy data sources included in the ischaemic heart disease model were systematically reviewed. In order to maximise the reliability of data included in the model, verbal autopsy studies that did not meet the World Health Organization standards were excluded.[1] Verbal autopsy studies which included only populations under 30 years were also excluded. In addition, we outliered non-representative subnational verbal autopsies from a number of Indian states and verbal autopsy data in Nepal and Papua New Guinea that were implausible in terms of time and age trends. We also outliered verbal autopsy data in countries and subnational locations where high-quality vital registration data were available.

After evaluating the available vital registration data, we outliered ICD8 and ICD9BTL datapoints from Germany and Mauritius which were inconsistent with the rest of the data and created implausible time trends. We also outliered vital registration data in a number of Indian states identified by experts as poor-quality.

Modelling strategy

We used a standard CODEm approach to model deaths from ischaemic heart disease. For GBD 2021, a new approach to redistribute deaths coded to hypertension was implemented using data sources which included information on the chain of events leading to death. This update resulted in a decrease in the number of deaths that were re-assigned to ischaemic heart disease for locations that use the hypertension ICD code frequently. For GBD 2021 we updated our approach to noise reduction of the cause of death data so that stochastic time series of data would resemble patterns seen at regional levels more closely. Additional details on the updates to noise reduction can be found in the cause of death methods section of the appendix.

The covariates included in the ensemble modelling process are listed in the table below. No changes to the covariates were made for GBD 2021.

Apart from the changes to the redistribution of deaths coded to hypertension and noise reduction, there are no substantive changes from the approach used in GBD 2019.

Table 1: Covariates used in cardiovascular diseases mortality modelling

Level	Covariate	Direction
1	Summary exposure value, IHD	1
	Cholesterol (total, mean per capita)	1
	Smoking prevalence	1
	Systolic blood pressure (mm Hg)	1
2	Mean BMI	1
	Elevation over 1500m (proportion)	-1
	Fasting plasma glucose	1
	Outdoor pollution ($PM_{2.5}$)	1
	Indoor air pollution	1
	Healthcare access and quality index	-1
3	Lag distributed income per capita (I$)	-1
	Summary exposure value, omega-3	1
	Summary exposure value, fruits	1
	Summary exposure value, vegetables	1
	Summary exposure value, nuts and seeds	1
	Pulses/legumes (kcal/capita, unadjusted)	-1
	Summary exposure value, PUFA (percent, adjusted)	1
	Alcohol (litres per capita)	1
	Trans fatty acid	1

References

1 WHO | Methodological trends in studies based on verbal autopsies before and after published guidelines. https://www.who.int/bulletin/volumes/87/9/07-049288/en/ (accessed April 22, 2021).

Ischaemic stroke

Flowchart

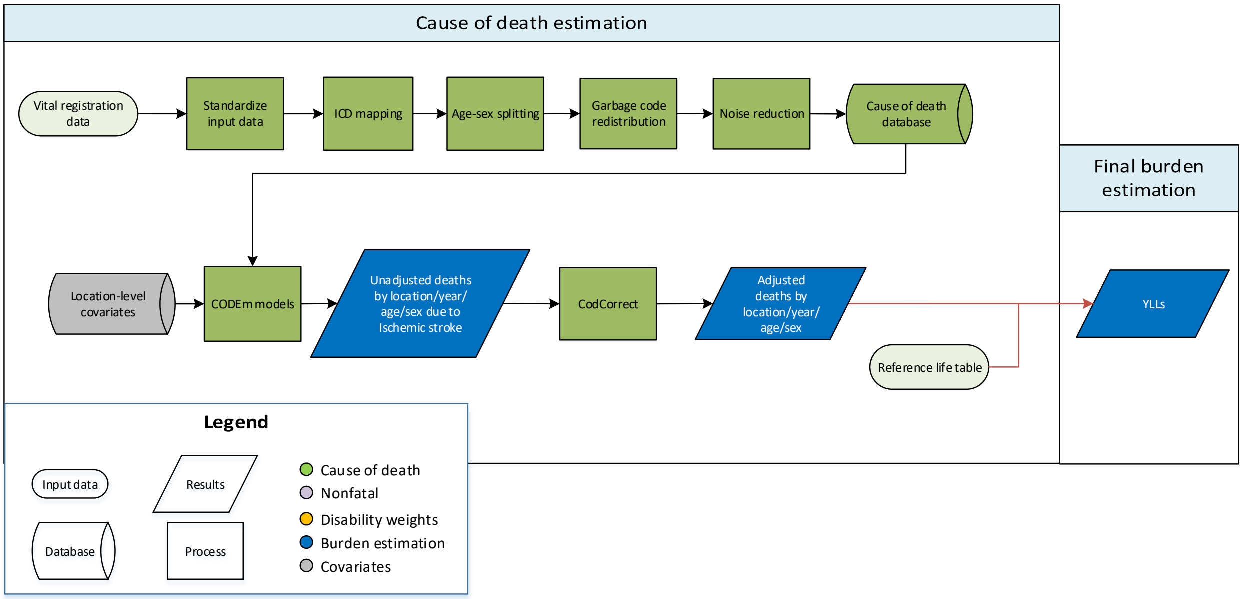

Input data and methodological summary for ischaemic stroke

Input data

Vital registration data were used to model deaths from ischaemic stroke. We outliered ICD8 datapoints which were inconsistent with the rest of the data and created implausible time trends. In addition, we outliered ICD 10 datapoints from sources which were implausibly low in all age groups and datapoints that were causing the regional estimates to be improbably high.

Modelling strategy

We used a standard CODEm approach to model deaths from ischaemic stroke. For GBD 2021, we updated the redistribution of deaths attributed to hypertension to an analysis of multiple cause of death datasets containing information on the entire chain of causes listed on the death certificate from seven countries with high quality data. In GBD 2019, the redistribution of hypertension deaths was done by regressing redistribution codes against non-redistribution codes; details on this approach can be found elsewhere in the appendix. Using the multiple cause of death approach to redistribution allows us to observe the true underlying cause of death where the reported cause of death is labelled as hypertension. For details on the multiple cause of death redistribution methods, refer to the appendix section on redistribution. This had the effect of increasing the number of hypertension deaths attributed to stroke in general, a minor increase in post-redistributed deaths was observed for ischaemic stroke specifically. For example, in GBD 2019 we redistributed 2534 deaths originally coded to hypertension in the United States for all ages and sexes in the year 2016 to ischaemic stroke. In GBD 2021, the number of deaths redistributed from hypertension to ischaemic stroke in the same demographic increased to 2600 deaths. For GBD 2021 we updated our approach to noise reduction of the cause of death data so that stochastic time series of data would resemble patterns seen at regional levels more closely. Additional details on the updates to noise reduction can be found in the cause of death methods section of the appendix.

Table 1. Covariates used in ischaemic stroke mortality modelling

Level	Covariate	Direction
1	Summary exposure variable, ischaemic stroke	1
	LDL Cholesterol (mean per capita)	1
	Smoking prevalence	1
	Systolic blood pressure (mm Hg)	1
2	Mean BMI	1
	Elevation over 1,500m (proportion)	-1
	Fasting plasma glucose	1
	Outdoor pollution ($PM_{2.5}$)	1
	Indoor air pollution	1
	Healthcare Access and Quality Index	-1
3	Log-transformed lag distributed income per capita (I$)	-1
	Summary exposure value, omega-3	1
	Summary exposure value, fruits	1
	Summary exposure value, vegetables	1
	Summary exposure value, nuts and seeds	1
	Pulses/legumes (kcal/capita, unadjusted)	-1
	Summary exposure value, PUFA adjusted (percent)	1
	Alcohol (litres per capita)	1
	Trans fatty acid	1

Lower respiratory infections (LRI)

Flowchart

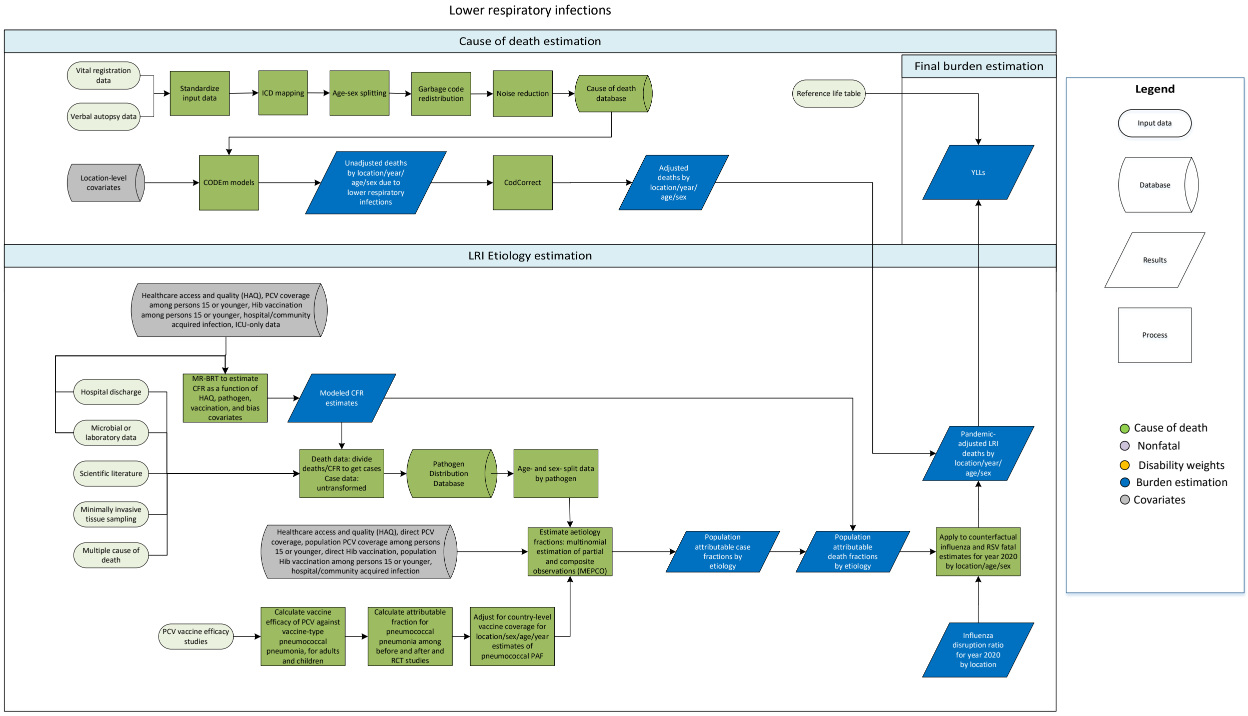

Input data and methodological summary for lower respiratory infections

Input data

Overall LRI

Lower respiratory infection (LRI) mortality was estimated in CODEm. We estimated LRI mortality separately for males and females and for children under 5 years and 5 years and older. We used all available data from vital registration systems, surveillance systems, and verbal autopsy. We checked for and excluded outliers from our data by country or region. We also excluded ICD-9-coded mortality data in Sri Lanka (1982, 1987–1992), ICD-9-coded neonatal mortality data in Guatemala (1980, 1981, 1984, 2000–2004), and medically coded cause of death data and Civil Registration System data in many Indian states (1986–2013).

Aetiologies

Input data for aetiology estimation consisted of multiple cause of death, vital registration, hospital discharge, and microbial data, as well as the aforementioned systematic literature review and a separate, targeted review pulling data from citations found in meta-analyses. For data sources that provided ICD codes (multiple cause of death, vital registration, hospital discharge, and some microbial data), these codes were used to identify patients with lower respiratory tract infections and the culprit pathogen, when detailed. For the microbial data that did not provide ICD codes, we identified pathogens associated with LRI based on the type of sample that was collected from the patient. Samples we

deemed were related to LRI included sputum, aspirates from the lower respiratory tract, and pleural fluid. We excluded samples from the eyes, ears, nose, or throat.

Table 1: ICD codes used to identify LRI with known aetiology

**Type of LRI**	**ICD-10 code(s)**	**ICD-9 code(s)**
LRI due to *Bordetella pertussis*	A37-A37.9	033-033.9, 484.3
LRI due to *Legionella spp.*	A48.1-A48.2	--
LRI due to *Actinomyces*	--	039.1
LRI due to *Chlamydia spp.*	A70, J16.0, P23.1	073-073.9, 483.1, 484.2
LRI due to *Streptococcus pneumoniae*	J13-J13.9, J15.4, J20.2	481-481.9, 482.3
LRI due to *Haemophilus influenzae*	J14-J14.0, J20.1	482.2
LRI due to *Klebsiella pneumoniae*	J15.0	482.0
LRI due to *Pseudomonas spp.*	--	482.1
LRI due to *Pseudomonas aeruginosa*	J15.1, P23.5	--
LRI due to *Staphylococcus aureus*	J15.2, P23.2	482.4
LRI due to Group B *Streptococcus*	J15.3, P23.3	--
LRI due to *Escherichia coli*	J15.5, P23.4	--
LRI due to *Mycoplasma pneumoniae*	J15.7, J20.0	483.0
LRI due to *Francisella tularensis*	--	484.4
LRI due to *Bacillus anthracis*	--	484.5
LRI due to virus	--	079.6-079.7, 480-480.9, 484.0-484.1, 487-489
LRI due to coronaviruses	B34.2, B97.2, J12.8	--
LRI due to respiratory syncytial virus	B97.4, J12.1, J20.5, J21.0	--
LRI due to influenza viruses	J09-J11.8	--
LRI due to parainfluenza viruses	J12.2, J20.4	--
LRI due to adenoviruses	J12.0	--
LRI due to rhinoviruses	J20.6	--

LRI due to other virus	J12, J12.3, J12.9, J17.0, J17.2-J17.8, J20.3, J20.7-J20.8, J21.1	--

Data on pathogens cultured from human infections were solicited from a wide array of international stakeholders (representing every inhabited continent). These included research hospitals, surveillance networks, and infection databases maintained by private laboratories and medical technology companies. For a full list of non-literature sources used for our estimates, please refer to the following article appendix (section 2).[i]

Due to the documented challenge in the microbiological identification of some LRI culprit pathogens,[ii,iii] we supplemented these data with estimates of the PAF of pneumonia due to *Streptococcus pneumoniae* (pneumococcus), which was calculated based on vaccine efficacy data reported in 18 high-quality vaccine probe studies.

We conducted a systematic literature review of PCV efficacy studies until January 2020. For PCV studies, we extracted, if available, the distribution of pneumococcal pneumonia serotypes and the serotypes included in the PCV used in the study. Four new PCV studies were identified for GBD 2021, which were all extracted only from PCV efficacy studies. PCV trial data are also frequently limited to younger populations. To understand the contribution of pneumococcal pneumonia in older populations, we also included PCV efficacy studies that used before-after approaches.

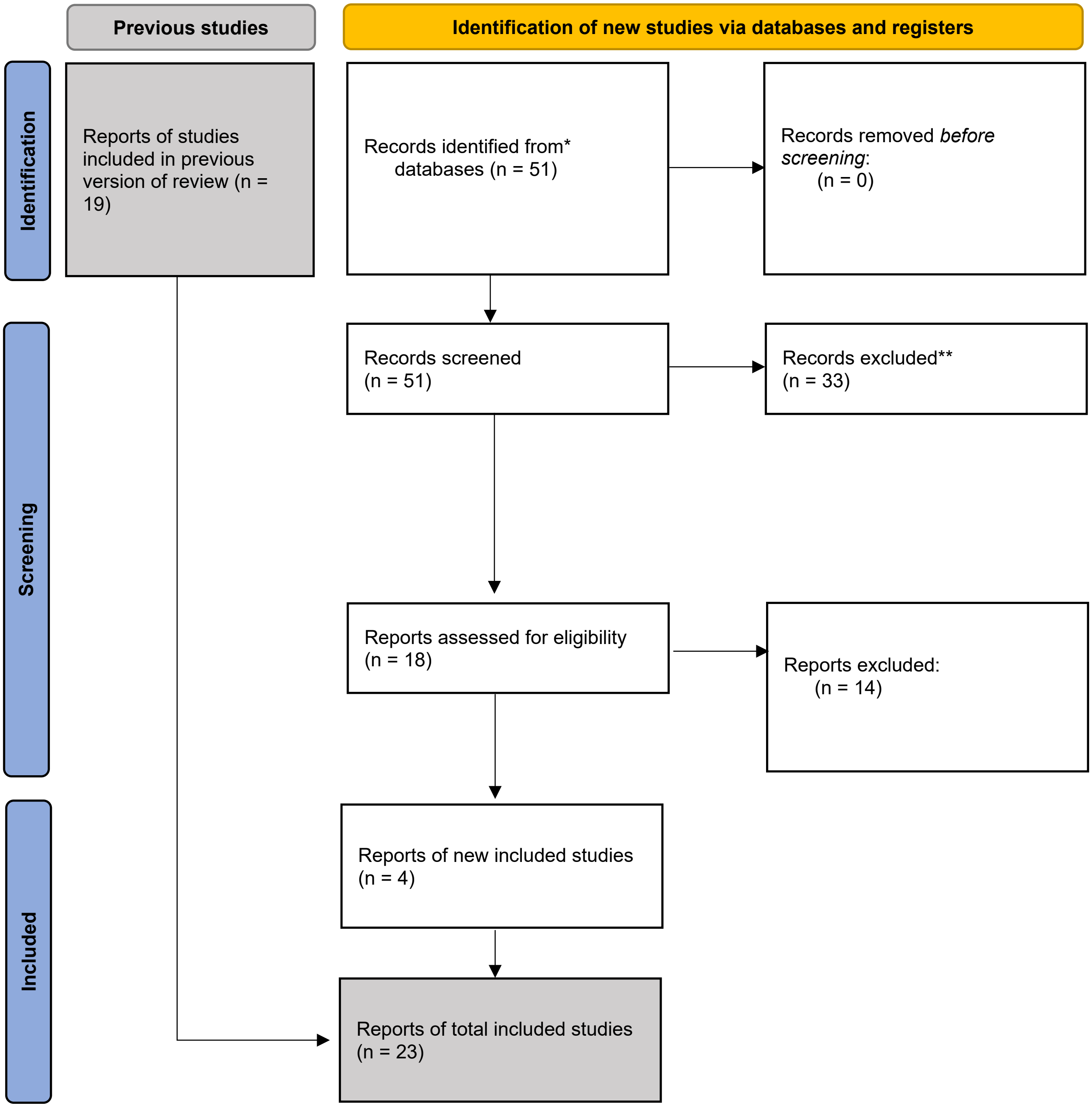

Figure 1. PCV vaccine efficacy systematic review flowchart

Modelling strategy

Overall LRI

LRI fatal modelling occurs using CODEm. Because of starkly different patterns, LRI CODEm models include under 5 years and 5–95+ years. Like all models of mortality in GBD, LRI mortality models are single-cause, requiring in effect that the sum of all mortality models must be equal to the all-cause mortality envelope. We correct LRI mortality estimates, and other causes of mortality, by rescaling them according to the uncertainty around the cause-specific mortality rate. This process is called CoDCorrect and is essential to ensure internal consistency among causes of death.

In past GBD cycles, estimates of PCV3, Hib3, and DTP3 vaccine coverage among infants in the modelled year were used as the primary covariate for this linear regression. In GBD 2021, we now use a lagged mean of PCV3, Hib3, and DTP3 vaccine coverage calculated over a rolling, five-year interval to capture population-level vaccine-derived immunity among under-5-year-olds, including coverage both in the current year and in recent years.

Table 1. Covariates used in LRI mortality modelling. Table 1A is for children under 5 and Table 1B shows the covariates used for ages 5–95+. The *Level* is the associated strength of relationship between the covariate and LRI mortality, ranked from 1 (proximally related) to 3 (distally related). *Direction* is the direction of the association between the covariate and LRI mortality.

Table 2A. Covariates used in under 5 years model

Level	Covariate	Direction
1	Childhood stunting summary exposure value (SEV)	+
	Childhood underweight SEV	+
	Childhood wasting SEV	+
	Indoor air pollution	+
	LRI SEV	+
	Antibiotics for LRI	-
	Hib3 vaccine coverage proportion, lagged	-
	PCV3 vaccine coverage proportion, lagged	-
2	Secondhand smoking prevalence	+
	Zinc deficiency	+
	DTP3 vaccine coverage proportion, lagged	-
	Healthcare Access and Quality Index	-
	Ambient particulate matter SEV	+
	Household air pollution SEV	+
	Outdoor air pollution ($PM_{2.5}$)	+
	Handwashing SEV	+
3	Sanitation SEV	+
	Population density >1000/km^2	+
	Maternal education	-
	Socio-demographic Index	-

Table 2B. Covariates used in 5–95+ years model

Level	Covariate	Direction
**1**	Indoor air pollution	+
	LRI SEV	+
	Outdoor air pollution ($PM_{2.5}$)	+
	Secondhand smoking prevalence	+
	Smoking prevalence	+
**2**	DTP3 vaccine coverage proportion, lagged	-
	Adult underweight	+
	Healthcare Access and Quality Index	-
	PCV3 vaccine coverage proportion, lagged	-
	Handwashing access	+
**3**	Education years per capita	-
	Lag distributed income per capita	-
	Socio-demographic Index	-
	Sanitation SEV	+

We adjusted overall LRI mortality estimates for 2020 and 2021 to account for the reductions in influenza and RSV mortality associated with the COVID-19 pandemic, as described elsewhere in this appendix.

Aetiology estimation

To generate aetiology fraction estimates for fatal lower respiratory infections, we took our aetiology fractions estimated for non-fatal LRI and multiplied them by a set of pathogen-specific case fatality rates (CFRs). CFRs were estimated using ICD-coded hospital data and microbial data with patient discharge status using a cases-offset Poisson regression model.[iv] We predicted CFRs as a function of pathogen, crude age (neonatal, post neonatal-5 years, 5–50 years, 50–70 years, and 70 years and older), an interaction term between pathogen and the proportions of the population age 15 or younger that had received PCV and *Haemophilus influenzae* type B vaccinations[v], Healthcare Access and Quality Index (HAQ Index), and bias covariates for data source (for the largest data sources). Separate models were run for CFRs associated with hospital-acquired and community-acquired LRI, and for the aetiology results reported here, only community-acquired CFRs were used. We additionally controlled for data provided from ICU-only sources (which would be biased towards higher CFRs) and data with “unknown” setting of infection origin (which was included in the community-acquired models to supplement input data).

The CFR model was run using the RegMod python package. The RegMod package implements and extends the generalized linear modeling (GLM) framework. It allows:

- User-specified likelihoods, capturing standard exponential family examples such as continuous, Poisson, and binomial, as well as quasi-likelihoods, and other user-defined extensions.
- User-specified models for predicting parameters, based on link functions, covariates, and splines.
- Priors, constraints, and trimming.

We utilized a Poisson family model, encoding the number of deaths as our Y variable. The Poisson probability distribution takes the form:

$$P(y_i|\lambda_i) = \frac{1}{y_i!}\exp(-\lambda_i)\,\lambda_i^{y_i} = \frac{1}{y_i!}\exp\left(-\lambda_i + y_i\log(\lambda_i)\right)$$

which suggest a parameterization

$$\log(\lambda_i) = c_i + \langle x_i, \beta\rangle.$$

Here, the link function is the exponential map, and $\langle x_i, \beta\rangle$ is a linear predictor that can use direct covariates or splines. The quantity c_i is an offset, which we use for observation-specific normalisation of the number of cases, thereby allowing our model to estimate rates.

The negative log likelihood estimation problem for β becomes:

$$\min_{\beta}\sum_{i}\exp\left(c_i + \langle x_i, \beta\rangle\right) - y_i(c_i + \langle x_i, \beta\rangle)$$

Where we can place constraints and priors on the β coefficients. The following priors were used:

- Prior on β for pathogen:vaccination interaction: We assumed vaccination would have no impact on CFRs of unrelated pathogens, and for all combinations of the pathogen:vaccination interaction that were not *Streptococcus pneumoniae*:PCV vaccination or *Haemophilus influenzae*:Hib vaccination we coerced the βs to 0 using model priors. For the *Streptococcus pneumoniae*:PCV vaccination and *Haemophilus influenzae*:Hib vaccination interaction terms, we employed a negativity prior to enforce case-fatality rates for these pathogens to decrease as vaccination was introduced.

- Prior on β for large data source dummy-variables: data source was included to account for source heterogeneity, however many input data sources covered only a single country, leading to low variability in HAQ Index within each data source. Such collinearity adversely influenced the accuracy of the estimated effect of HAQ Index, which was instrumental in extrapolating trends from the input data to global results. To emphasise the contribution of HAQ Index over data-source in the modelled estimates, we implemented a Gaussian prior (mean 0, standard error 0.1) on the βs for data source variables.

Non-fatal pathogen proportions $p_{i,j}$ for a given demographic group i and pathogen j were then converted to deaths using the CFRs estimates for demographic group i as follows:

$$p_{i,j}^{deaths} = \frac{p_{i,j} \times CFR_i}{\sum_{\hat{j}} p_{i,\hat{j}} \times CFR_i}$$

We adjusted influenza and RSV mortality estimates for 2020 and 2021 to account for the reductions in influenza and RSV cases associated with the COVID-19 pandemic, as described elsewhere in this appendix.

References

[i] Murray CJ, Ikuta KS, Sharara F, Swetschinski L, Aguilar GR, Gray A, Han C, Bisignano C, Rao P, Wool E, Johnson SC. Global burden of bacterial antimicrobial resistance in 2019: a systematic analysis. *The Lancet*. 2022 Feb 12;399(10325):629-55.

[ii] Ewig S, Schlochtermeier M, Goïke N, Niederman MS. Applying sputum as a diagnostic tool in pneumonia: limited yield, minimal impact on treatment decisions. *Chest*. 2002 May 1;121(5):1486-92.

[iii] Ogawa H, Kitsios GD, Iwata M, Terasawa T. Sputum Gram stain for bacterial pathogen diagnosis in community-acquired pneumonia: a systematic review and Bayesian meta-analysis of diagnostic accuracy and yield. *Clinical Infectious Diseases*. 2020 Jul 27;71(3):499-513.

[iv] Zheng P, Barber R, Sorensen RJ, Murray CJ, Aravkin AY. Trimmed constrained mixed effects models: formulations and algorithms. *Journal of Computational and Graphical Statistics*. 2021 Sep 16;30(3):544-56.

[v] Galles NC, Liu PY, Updike RL, Fullman N, Nguyen J, Rolfe S, Sbarra AN, Schipp MF, Marks A, Abady GG, Abbas KM. Measuring routine childhood vaccination coverage in 204 countries and territories, 1980–2019: a systematic analysis for the Global Burden of Disease Study 2020, Release 1. *The Lancet*. 2021 Aug 7;398(10299):503-21.

Estimating COVID-19 impact on select infectious syndromes

COVID-19 has strained health-care systems around the world and limited capacity to deliver routine immunisations, priming populations for outbreaks of infectious disease. Conversely, physical distancing measures, masking, and school closures have the potential to interrupt usual transmission patterns of other infectious diseases, as they do for COVID-19. Considering these competing ways in which COVID-19 could influence other diseases, in combination with many countries reporting greatly reduced incidence of influenza and measles, we sought to capture the impact of COVID-19 in our estimates of other infectious diseases for 2020 and 2021.

Data

We reviewed national-level case notification data from ministry of health websites, media reports, and published literature for measles, pertussis, diphtheria, tetanus, varicella, diarrhoeal disease, influenza, respiratory syncytial virus, and infections due to *S. pneumoniae*, *H. influenzae*, and *N. meningitidis* to look for evidence of disruption. For measles and influenza, we relied on case notifications reported directly by countries to WHO regional offices; these causes had the most complete geographical and temporal coverage. Because of this completeness in reporting, we utilised them as indicator causes for further modelling, as described below.

Modelling

We began by evaluating each cause for evidence of disruption. For each cause, to determine whether a disruption occurred in 2020, we conducted a random effect meta-analysis with restricted maximum likelihood estimation using the metafor package in R. Each point was the ratio of cases observed in 2020 to the cases observed over the average of 2017–2019. Given the relative completeness of measles and influenza data, we developed a primary model for these causes and then, for causes other than measles and influenza, evaluated whether the reduction modelled for measles or influenza could be applied directly to the other cause. To do this, we examined the change in case notifications between 2020 and previous years for a cause relative to the change in case notifications between 2020 and previous years for measles and influenza. When determining whether to adjust each cause, we considered the size and statistical significance of the observed effect, the consistency and quality of the available data, and epidemiological plausibility. At the time of estimation, these factors supported adjustment of only pertussis and RSV, using estimates of disruption derived from the influenza disruption model results (see below). As we receive more data, we plan to re-examine additional causes and aetiologies to apply disruption if warranted.

We developed a multi-step modelling process to estimate the effect of non-pharmaceutical interventions (NPIs) associated with the COVID-19 pandemic on the incidence of influenza and measles in 2020 and 2021. First, we interpolated the number of reported cases of influenza and measles in 2020 and 2021, by month. We leveraged the RegMod framework, a Poisson model that estimates the underlying rate of infection in each month as a function of a seasonal pattern and an underlying temporal trend. The temporal trend was reflected as a piecewise linear spline with knots at the start of each year. We placed the last knot of the underlying time trend in January 2021 for measles and influenza. We used monthly data through March 2022 (the last month of available data at the time of modeling) to fit the model, starting in January 2010 for influenza and January 2011 for measles. The

RegMod model results are 1000 sets of estimates of the number of reported cases in each month and inputs to the next phase of modelling. We excluded RegMod results from any country missing at least six months of data in any year within 2017–2021 to reduce the risk of outbreaks occurring and subsiding during the periods of missing data.

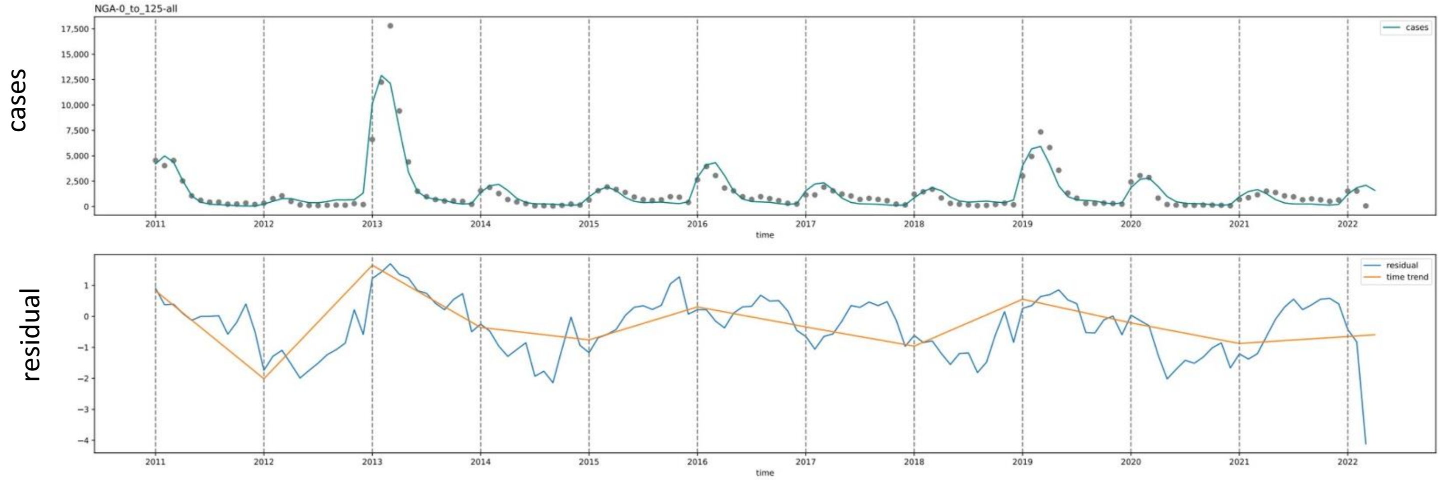

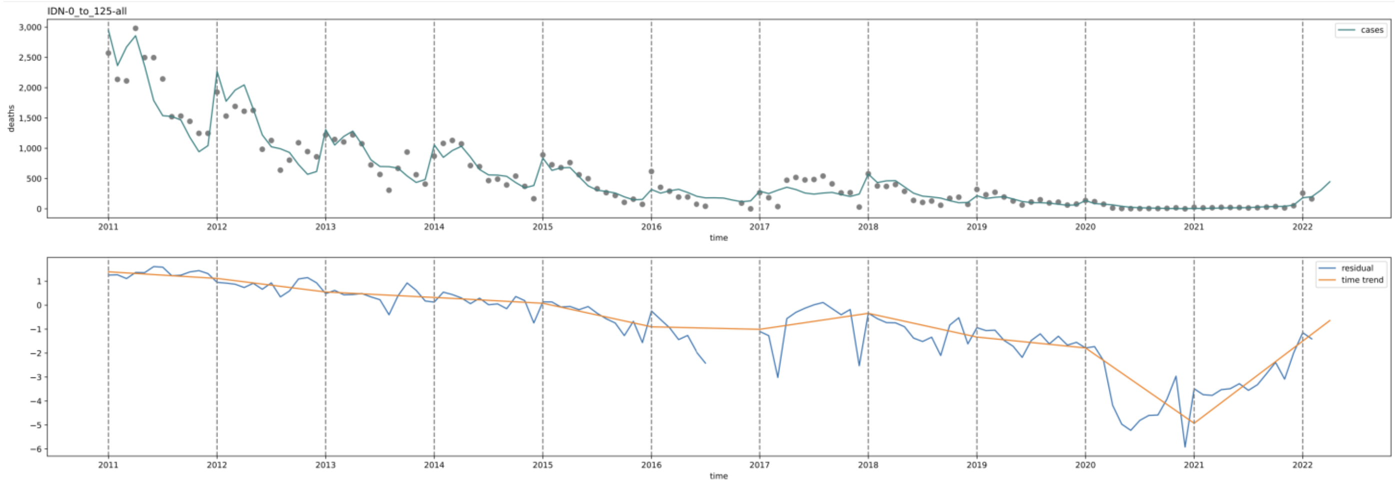

year

In the second step of the modelling process, we calculated the underreporting ratio (URR) in the pre-pandemic reference period, for each location, for both influenza and measles, by dividing the interpolated number of reported cases from RegMod by the GBD estimated number of incident cases of influenza or measles. For influenza, we used a reference period of 2017–2019 when calculating the URR; for measles, we used 2015–2019. We used a longer period for measles because of greater year-to-year variation in the long-term time trends in cases.

cases

residual

year

Third, we estimated the pandemic-free counterfactual number of reported cases, meaning, the number of reported cases we would have expected during 2020 and 2021 in the hypothetical pandemic-free scenario. We did this by multiplying the URR by the number of cases of LRI due to influenza or measles, for 2020 and 2021, that GBD models would have estimated in a pandemic-free scenario.

Lastly, we calculated a disruption scalar for each location for 2020 and 2021. This scalar was computed by dividing the interpolated number of reported cases from RegMod (result of first step) by the counterfactual disruption-free number of reported cases (result of third step). This value was calculated by year in all cases except for measles in 2020, where it was calculated by month to account for the timing of the onset of the pandemic in relation to typical seasonal variation in measles epidemiology.

Adjusting 2020 estimates for these expected seasonal variations in measles epidemiology required an additional step to move between annual and monthly time scales. RegMod estimates were produced at the monthly time scale, requiring the conversion of annual estimates of counterfactual reported cases to monthly estimates to allow for the monthly calculation necessary. To account for seasonality, we calculated a seasonality weight for each month for measles. For each month from January 2017 to December 2019, we divided the RegMod measles case estimates from that location-month by the average monthly cases across months in that year. This gave a set of seasonality weights for each location-month, for each year. We then averaged each month's seasonality weight across the three years to yield a three-year average seasonality weight for each location-month. We used these seasonality weights to estimate the counterfactual COVID-free number of reported cases for each month during the pandemic for each location, dividing the location's annual counterfactual COVID-free number of reported cases measles cases by 12 and multiplying by the seasonality weight. For locations without a full time-series of data, we used the average seasonality weight from locations with similar latitude. Monthly disruption scalars were calculated by dividing the new monthly estimates of counterfactual reported cases by RegMod's estimate for the same month. Disruption scalars were set to 1 for January, February, and March 2020 to remove the influence of outbreaks observed in early 2020 in the absence of COVID-19 on the overall disruption ratio. We then converted our monthly disruption estimates for measles in 2020 to annual disruption estimates by calculating a seasonality-weighted average of the monthly disruption estimates for each location.

This approach yielded annual disruption scalars for 2020 and 2021 for influenza and measles, by location. These ratios can be interpreted as estimates of the relative change in influenza and measles incidence that occurred during the COVID pandemic in 2020 and 2021 for each location, relative to expected trends from the GBD models in a counterfactual COVID-free scenario. For countries with no data, the median disruption scalar in the region was used. All operations were performed at the 1000 draw level.

Measles adjustment

For locations in the Latin America and the Caribbean, high-income, and central Europe, eastern Europe, and central Asia super-regions and any locations outside these super-regions with WHO-verified measles elimination, as well as select locations with known strong measles surveillance systems (China and Jordan), we used measles case notifications directly for our burden estimates, assuming complete reporting. This practice is consistent with our measles incidence estimation framework in years without COVID-19. For all other locations, we generated counterfactual COVID-free measles incidence and

prevalence estimates using our standard measles estimation approach (described elsewhere in this appendix), substituting counterfactual estimates of vaccine coverage in the absence of COVID-19 as the vaccine coverage covariate for the years 2020 and 2021. These counterfactual COVID-free measles estimates were then multiplied by the location- and year-specific measles disruption scalar described above to derive COVID-inclusive incidence and prevalence estimates for each year and location. At the time of this analysis, there were insufficient data to estimate whether and to what degree COVID-19 may have affected measles case-fatality rates. We therefore did not incorporate an additional COVID effect on measles case-fatality rate. Maintaining our usual natural history model framework for measles, fatal estimates were scaled using the same disruption ratios applied to incidence and prevalence. Additional data and analyses will be required in the future to better assess the potential impact of the COVID-19 pandemic on case-fatality rates, including for measles.

LRI adjustment

We conducted a meta-analysis to compare location-specific disruptions for RSV to measles and influenza and found that the disruption in RSV cases in 2020 was analogous to that observed for influenza. For each location/age/sex for which LRI is estimated, influenza and RSV cases were scaled using the annualised ratios as calculated for influenza. Other aetiology-attributed cases of LRI were not scaled at this time.

Next, we calculated how the disruption scalars for influenza and RSV would apply to the overall LRI estimates. Because the etiological fraction of LRI due to RSV and influenza varies by age and sex, this calculation was performed by sex at the most granular age group level, for each country and year. It was also performed separately for deaths and cases since the etiological fraction of LRI due to RSV and influenza is different for deaths and cases. For a given country-year, the influenza disruption scalar was multiplied by the number of LRI influenza and RSV case/death counts, as pulled from GBD counterfactual estimates, to get adjusted flu and RSV counts. Then, we calculated the number of LRI cases/deaths to "remove" from the counterfactual number of LRI cases/deaths in the adjusted scenario as: the sum of counterfactual flu count and RSV count, minus the sum of COVID-adjusted flu count and RSV count. Finally, we calculate the LRI scalar for each country-age-sex-year as the LRI cases/deaths count from GBD counterfactual estimates, minus the number of LRI cases/deaths to "remove", all divided by the counterfactual LRI cases/deaths count.

To adjust incidence and prevalence estimates for a given cause, we simply multiplied these estimates by the annual disruption ratio for that cause, calculated as described above. To adjust mortality estimates for a given cause, scalars are applied to an intermediate set of mortality results (counterfactual LRI death count) to create a count of LRI deaths to subtract using the formula below:

LRI deaths to subtract = (Counterfactual LRI death count * (LRI scalar – 1))

These values are subtracted from counterfactual LRI deaths to get adjusted LRI deaths. This operation is performed at the 1000 draw level for each location, age, sex, and year. This process is applied to final estimates the same way as other causes known in the GBD framework as fatal discontinuities.

Pertussis adjustment

We conducted a meta-analysis to compare location-specific disruptions for pertussis to measles and influenza and found that the disruption in pertussis cases in 2020 was analogous to that observed for influenza. All locations' incidence and prevalence estimates for 2020 and 2021 were scaled using the annualised ratios as calculated for influenza.

Limitations

A key limitation of this framework is that it relies exclusively on case notification data from national and multinational surveillance networks. It cannot separate the effects of true decreases in disease incidence from the effects of decreased reporting. Currently, we cannot adjust for the assumption that case notifications reflect true decreases in disease incidence because we do not have any data without changes in reporting, or data on reporting patterns themselves; however, we hope to address this in the future. In addition, we have only adjusted estimates for influenza, measles, RSV, and pertussis in this release due to a scarcity of data. New research also suggests substantial decreases in other LRI and meningitis-causing pathogens, specifically *N. meningitidis*, *S. pneumoniae*, and *H. influenzae*; we plan to incorporate this source, and continue our data seeking, to improve our adjustments for additional diseases in later releases. For future years, additional data and revisions to this modelling framework will be needed to allow for more flexibility in capturing disease resurgence.

For fatal estimates, once created, scalars are applied to an intermediate set of CoDCorrect results (prior to adding shocks) to create a set of positive or negative shocks using the formula below.

$$\text{Shock} = (\text{cc\_draw} * (\text{scalar\_draw} - 1))$$

For non-fatal estimates, once created, scalars are applied to select disease estimates before going through additional central GBD processes such as EPIC and Burdenator. Additional information on those processes can be found in this appendix.

Maternal disorders

Flowchart

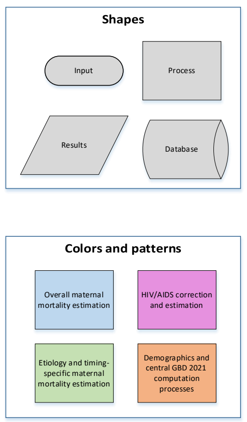

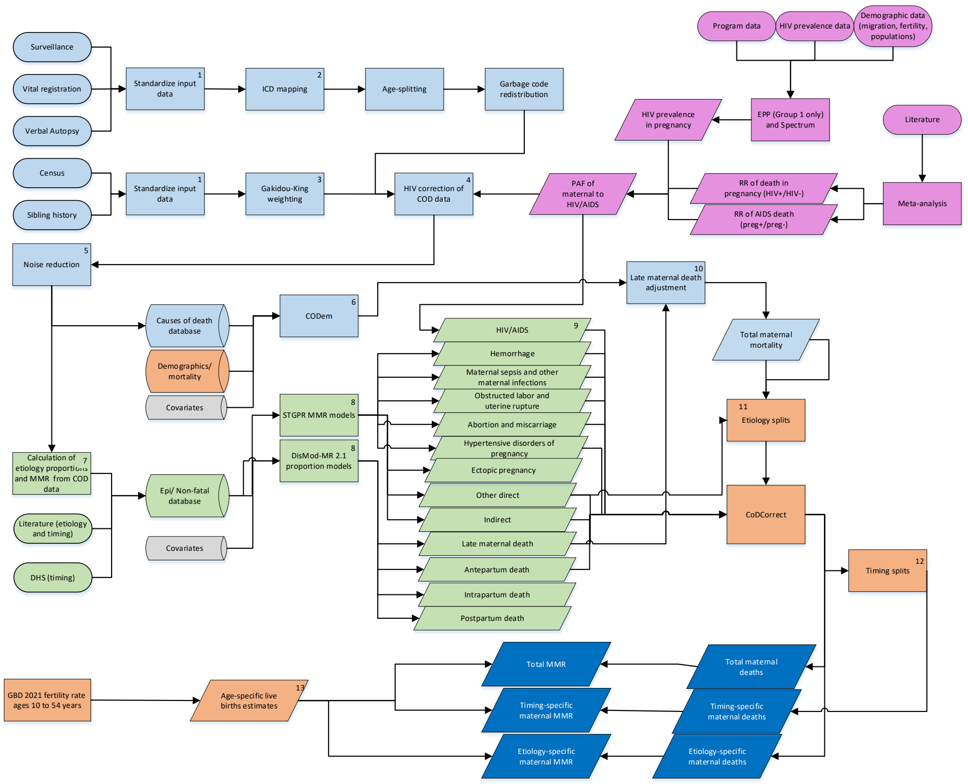

Ovals represent data inputs, square boxes represent analytical steps, cylinders represent databases, and parallelograms represent intermediate and final results. The flowchart is color-coded by major estimation component: data preparation and overall maternal mortality in blue; cause- and timing-specific estimation in green; analysis and data specific to the role of HIV/AIDS in maternal mortality in pink; steps related to demographic and computational processes that ensure internal consistency in orange, and final estimates in dark blue. GBD = Global Burden of Disease, MMR = maternal mortality ratio, DHS = Demographic and Health Survey, COD = causes of death, RR = relative risk, HIV = Human immunodeficiency virus, AIDS = Acquired Immunodeficiency Syndrome, EPP = Estimation and Projection Package, WPP = World Population Prospects, Epi = epidemiology.

Definitions

In GBD, deaths due to maternal disorders (aka maternal deaths) comprise all deaths due to obstetric complications (direct maternal deaths) or concurrent disorders exacerbated by pregnancy (indirect maternal deaths) up to one year after the end of the pregnancy, irrespective of the method by which pregnancy ended and excluding accidental and incidental deaths. Within this envelope, we estimate the following sub-causes of maternal death: maternal haemorrhage, maternal sepsis and other maternal infections, maternal hypertensive disorders, obstructed labor and uterine rupture, abortion and miscarriage, ectopic pregnancy, other direct maternal disorders, maternal deaths aggravated by HIV/AIDS, (other) indirect maternal deaths, and late maternal deaths.

Input data

Models of maternal mortality were informed by centrally prepped data stored in the cause of death (COD) database, including data sources that comprehensively assign cause of death to an entire sample of deaths and are used for other cause-specific mortality estimates in GBD (vital registration, verbal autopsy), as well as sources that specifically measure maternal deaths (censuses, sibling histories from large household surveys, maternal mortality surveillance systems or confidential enquiries, and other targeted surveys). These data came from the COD database, other data sources and reports from the Global Health Data Exchange, and published studies identified through the search below.

Our systematic literature review for maternal disorders is completed annually and encompasses all aspects of maternal disorder burden estimation, including overall maternal mortality, cause-specific maternal mortality, incidence of pregnancy complications by type, relative risk of mortality in pregnancy in HIV-positive versus HIV-negative women, and relative risk of mortality in HIV-positive women who are pregnant versus non-pregnant. We updated this search on January 29, 2020, using the following search string:

(((("Postpartum Hemorrhage" OR "Uterine Hemorrhage") OR (maternal[Title/Abstract] OR pregnan*[Title/Abstract] OR mothers) AND (haemorrhag*[Title/Abstract] OR hemorrhag*[Title/Abstract]) NOT "case report"[All fields]) OR (("induced abortion" OR "Therapeutic abortion" OR "legal Abortion" OR "medical abortion" OR "miscarriage" OR "Abortion, Induced"[Mesh] OR "Abortion, Therapeutic"[Mesh] OR "Abortion, Legal"[Mesh] OR "ectopic Pregnancy") NOT ("case report"[Title/Abstract] OR "birth defect"[Title/Abstract] OR congenital[Title/Abstract])) OR ("obstructed labour" OR "obstructed labor" OR "labour dystocia" OR "labor dystocia" OR dystocia OR "cephalopelvic disproportion" OR "cephalo-pelvic disproportion") OR (("obstetric fistula" OR "vesicovaginal fistula") OR "rectovaginal fistula") OR (("Puerperal Infection"[Mesh] OR "Puerperal Infection" OR ((maternal[Title/Abstract] OR pregnan*[Title/Abstract]) AND (Sepsis OR infection[Title/Abstract]))) NOT "case report") OR ((pre-eclampsia[Title/Abstract] OR preeclampsia[Title/Abstract] OR eclampsia[Title/Abstract] OR Pre-Eclampsia[Mesh] OR Eclampsia[Mesh] OR "Hypertension, Pregnancy-Induced"[Mesh] OR "pregnancy induced hypertension"[Title/Abstract] OR "gestational hypertension"[Title/Abstract] OR "Hypertensive disorders of pregnancy"[Title/Abstract]) NOT ("case report" OR "kidney donor"[Title/Abstract] OR "kidney donors"[Title/Abstract] OR polymorphism*[Title/Abstract] OR endotheli*[Title/Abstract]))) OR ((("maternal mortality"[Title/Abstract] OR "maternal death"[Title/Abstract] OR "maternal deaths"[Title/Abstract] OR "MM"[Title/Abstract] OR "confidential enquiry"[Title/Abstract] OR "confidential inquiry"[Title/Abstract] OR ((obstetric[Title/Abstract] OR pregnan*[Title/Abstract]) AND (etiology[Title/Abstract] OR cause[Title/Abstract] OR pattern[Title/Abstract]) AND (death[Title/Abstract] OR mortality[Title/Abstract]))) NOT (fetal[Title/Abstract] OR newborn*[Title/Abstract] OR neonatal[Title/Abstract] OR "case report" [Title/Abstract] OR "case study" [Title/Abstract] OR pathogenesis[Title/Abstract] OR thromboprophylaxis[Title/Abstract])) OR ((("maternal mortality"[Title/Abstract] OR "maternal death"[Title/Abstract] OR "maternal deaths"[Title/Abstract] OR "MMR"[Title/Abstract]) AND ("Afghanistan"[Title/Abstract] OR "Albania"[Title/Abstract] OR "Algeria"[Title/Abstract] OR "Andorra"[Title/Abstract] OR "Angola"[Title/Abstract] OR "Antigua and Barbuda"[Title/Abstract] OR "Argentina"[Title/Abstract] OR "Armenia"[Title/Abstract] OR "Azerbaijan"[Title/Abstract] OR "Bahrain"[Title/Abstract] OR "Bangladesh"[Title/Abstract] OR "Barbados"[Title/Abstract] OR "Belarus"[Title/Abstract] OR "Belize"[Title/Abstract] OR "Benin"[Title/Abstract] OR "Bhutan"[Title/Abstract] OR "Bolivia"[Title/Abstract] OR "Bosnia and Herzegovina"[Title/Abstract] OR "Botswana"[Title/Abstract] OR "Brazil"[Title/Abstract] OR "Brunei"[Title/Abstract] OR "Bulgaria"[Title/Abstract] OR "Burkina Faso"[Title/Abstract] OR "Burundi"[Title/Abstract] OR "Cambodia"[Title/Abstract] OR "Cameroon"[Title/Abstract] OR "Cape Verde"[Title/Abstract] OR "Central African Republic"[Title/Abstract] OR "Chad"[Title/Abstract] OR "China"[Title/Abstract] OR "Colombia"[Title/Abstract] OR "Comoros"[Title/Abstract] OR "Congo"[Title/Abstract] OR "Costa Rica"[Title/Abstract] OR "Croatia"[Title/Abstract] OR "Cuba"[Title/Abstract] OR "Cyprus"[Title/Abstract] OR "Côte d'Ivoire"[Title/Abstract] OR "Democratic Republic of the Congo"[Title/Abstract] OR "Djibouti"[Title/Abstract] OR "Dominica"[Title/Abstract] OR "Dominican Republic"[Title/Abstract] OR "Ecuador"[Title/Abstract] OR "Egypt"[Title/Abstract] OR "El Salvador"[Title/Abstract] OR "Equatorial Guinea"[Title/Abstract] OR "Eritrea"[Title/Abstract] OR "Ethiopia"[Title/Abstract] OR "Federated States of Micronesia"[Title/Abstract] OR "Fiji"[Title/Abstract] OR "Gabon"[Title/Abstract] OR "Georgia"[Title/Abstract] OR "Ghana"[Title/Abstract] OR "Grenada"[Title/Abstract] OR "Guatemala"[Title/Abstract] OR "Guinea"[Title/Abstract] OR "Guinea-Bissau"[Title/Abstract] OR "Guyana"[Title/Abstract] OR "Haiti"[Title/Abstract] OR "Honduras"[Title/Abstract] OR "India"[Title/Abstract] OR "Indonesia"[Title/Abstract] OR "Iran"[Title/Abstract] OR "Iraq"[Title/Abstract] OR "Jamaica"[Title/Abstract] OR "Jordan"[Title/Abstract] OR "Kazakhstan"[Title/Abstract] OR "Kenya"[Title/Abstract] OR "Kiribati"[Title/Abstract] OR "Kuwait"[Title/Abstract] OR "Kyrgyzstan"[Title/Abstract] OR "Laos"[Title/Abstract] OR "Latvia"[Title/Abstract] OR "Lebanon"[Title/Abstract] OR "Lesotho"[Title/Abstract] OR "Liberia"[Title/Abstract] OR "Libya"[Title/Abstract] OR "Lithuania"[Title/Abstract] OR "Macedonia"[Title/Abstract] OR "Madagascar"[Title/Abstract] OR "Malawi"[Title/Abstract] OR "Malaysia"[Title/Abstract] OR "Maldives"[Title/Abstract] OR "Mali"[Title/Abstract] OR "Malta"[Title/Abstract] OR "Marshall Islands"[Title/Abstract] OR "Mauritania"[Title/Abstract] OR "Mauritius"[Title/Abstract] OR "Moldova"[Title/Abstract] OR "Mongolia"[Title/Abstract] OR "Montenegro"[Title/Abstract] OR "Morocco"[Title/Abstract] OR "Mozambique"[Title/Abstract] OR "Myanmar"[Title/Abstract] OR "Namibia"[Title/Abstract] OR "Nepal"[Title/Abstract] OR "Nicaragua"[Title/Abstract] OR "Niger"[Title/Abstract] OR "Nigeria"[Title/Abstract] OR "North Korea"[Title/Abstract] OR "Oman"[Title/Abstract] OR "Pakistan"[Title/Abstract] OR "Palestine"[Title/Abstract] OR "Panama"[Title/Abstract] OR "Papua New Guinea"[Title/Abstract] OR "Paraguay"[Title/Abstract] OR "Peru"[Title/Abstract] OR "Philippines"[Title/Abstract] OR "Qatar"[Title/Abstract] OR "Romania"[Title/Abstract] OR "Russia"[Title/Abstract] OR "Rwanda"[Title/Abstract] OR "Saint Lucia"[Title/Abstract] OR "Saint Vincent and the Grenadines"[Title/Abstract] OR "Samoa"[Title/Abstract] OR "Saudi Arabia"[Title/Abstract] OR "Senegal"[Title/Abstract] OR "Serbia"[Title/Abstract] OR "Seychelles"[Title/Abstract] OR "Sierra Leone"[Title/Abstract] OR "Singapore"[Title/Abstract] OR "Solomon Islands"[Title/Abstract] OR "Somalia"[Title/Abstract] OR "South Africa"[Title/Abstract] OR "South Sudan"[Title/Abstract] OR "Sri Lanka"[Title/Abstract] OR "Sudan"[Title/Abstract] OR "Suriname"[Title/Abstract] OR "Swaziland"[Title/Abstract] OR "Syria"[Title/Abstract] OR "São Tomé and Principe"[Title/Abstract] OR "Taiwan"[Title/Abstract] OR "Tajikistan"[Title/Abstract] OR "Tanzania"[Title/Abstract] OR "Thailand"[Title/Abstract] OR "The Bahamas"[Title/Abstract] OR "The Gambia"[Title/Abstract] OR "Timor-Leste"[Title/Abstract] OR "Togo"[Title/Abstract] OR "Tonga"[Title/Abstract] OR "Trinidad and Tobago"[Title/Abstract] OR "Tunisia"[Title/Abstract] OR "Turkmenistan"[Title/Abstract] OR "Uganda"[Title/Abstract] OR "Ukraine"[Title/Abstract] OR "United Arab Emirates"[Title/Abstract] OR "Uruguay"[Title/Abstract] OR "Uzbekistan"[Title/Abstract] OR "Vanuatu"[Title/Abstract] OR "Venezuela"[Title/Abstract] OR "Vietnam"[Title/Abstract] OR "Yemen"[Title/Abstract] OR "Zambia"[Title/Abstract] OR "Zimbabwe"[Title/Abstract])) NOT ("demographic and health survey"[Title/Abstract] OR "demographic and health surveys "[Title/Abstract] OR DHS[Title/Abstract] OR "reproductive health survey"[Title/Abstract] OR "reproductive health surveys"[Title/Abstract] OR RHS[Title/Abstract])) OR ((HIV[Title/Abstract] OR "Acquired Immunodeficiency Syndrome"[Title/Abstract] OR AIDS[Title/Abstract]) AND (pregnan*[Title/Abstract] OR "postpartum"[Title/Abstract] OR "post partum"[Title/Abstract]) AND ("mortality"[Title/Abstract] OR "death"[Title/Abstract]) NOT "case report")) AND (2019/05/10[PDat] : 3000[PDat]) NOT (animals[MeSH] NOT humans[MeSH]))

For GBD 2021, a total of 5563 new published reports were identified and reviewed for their title and abstract. Of the 235 sources selected for full text review, 66 met criteria for inclusion for one or more indicator of maternal disorder burden. This round, there were no new sources identified for maternal deaths aggravated by HIV.

Figure 1: PRISMA 2020 Flow Diagram

From: Page MJ, McKenzie JE, Bossuyt PM, Boutron I, Hoffmann TC, Mulrow CD, et al. The PRISMA 2020 statement: an updated guideline for reporting systematic reviews. BMJ 2021;372:n71. doi: 10.1136/bmj.n71

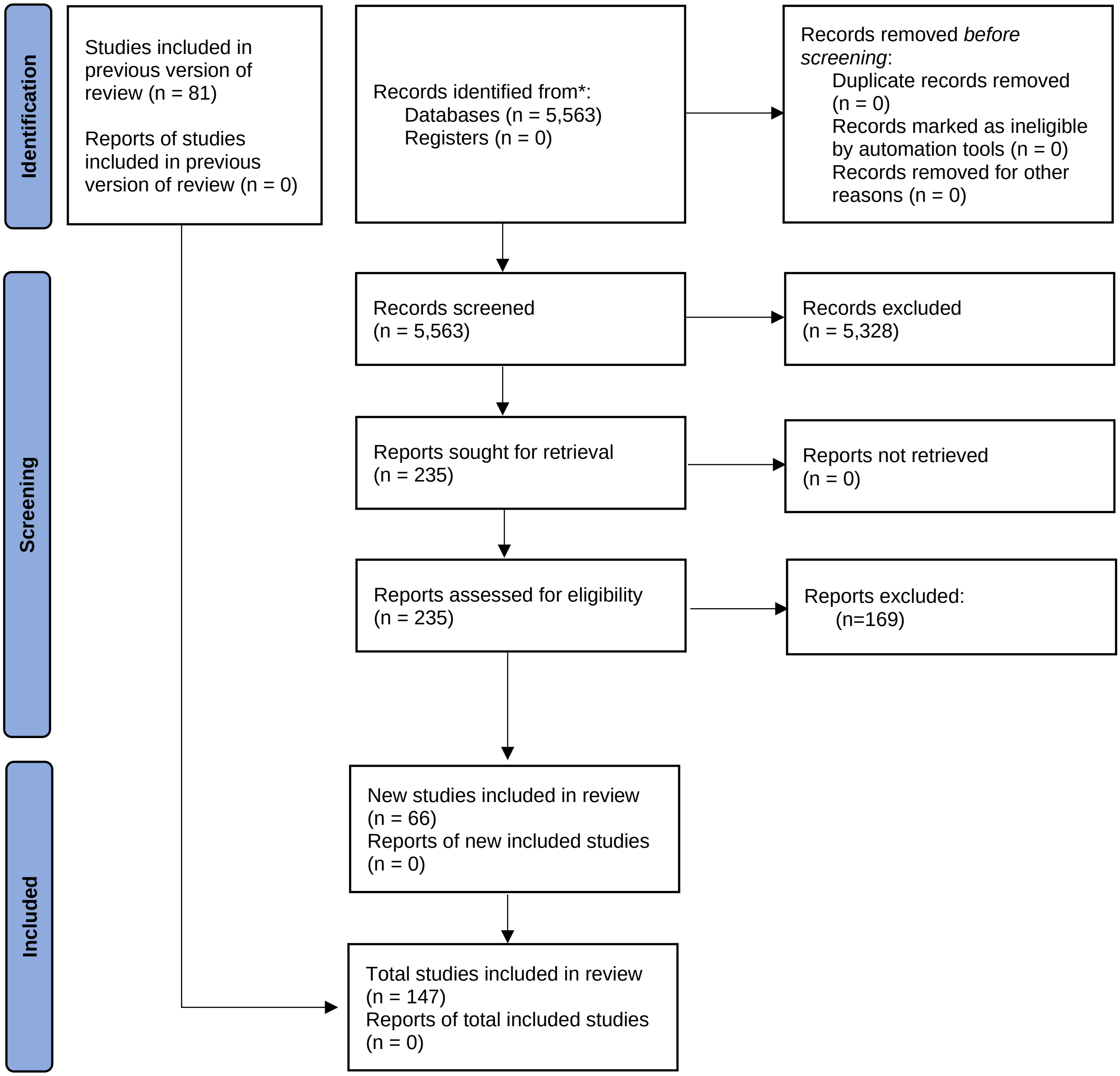

Overall maternal mortality measurements were extracted as cause fraction (number of maternal deaths divided by all deaths of women of reproductive age) to permit modelling using the Cause of Death Ensemble modelling (CODEm) tool, as described below. All sub-cause-specific maternal mortality data were extracted as maternal mortality ratio (MMR; cause-specific deaths per livebirth) for input into the spatiotemporal Gaussian process regression (ST-GPR) modelling tool. All sources that reported overall

maternal mortality as MMR, or sub-cause-specific maternal mortality as cause fraction, or either outcome as a population rate, were converted to the preferred metric using all-cause mortality, population, and age-specific fertility results estimated in GBD 2021.

All data were corrected for incidental HIV deaths by combining estimated HIV prevalence in pregnancy with relative risk (RR) of mortality during pregnancy for HIV-positive women to calculate a population attributable fraction (PAFs) that was then divided between incidental and maternal deaths based on RR of death in HIV-positive women during pregnancy. Incidental HIV deaths were removed from sibling history and census data, while maternal HIV deaths were added to vital registration, verbal autopsy, and surveillance data. This process is described in more detail in the appendix section on HIV/AIDS estimation.

One exception was late maternal death, where only raw, unprocessed COD data were included from the COD database, and only for the subset of locations where the proportion of late maternal deaths coded in VR exceeded the lowest published rate from a comprehensive study.[1] Our assumption is that any location that has never reported a late maternal death in its VR does not capture any late maternal deaths. These data were supplemented with late maternal death data, all of which were extracted and prepped as proportion of the total. For the subset of locations where they were reliably coded in raw VR. All cause-specific MMR and proportion (late only) data were uploaded to the non-fatal database.

All data from all geographies were reviewed in CODEm models. Outliers were identified as those data where age patterns or temporal patterns were inconsistent with neighbouring age groups or locations or where sparse data were predicting implausible overall temporal or age patterns for a given location.

Modelling strategy

Overall maternal mortality

Overall maternal mortality was estimated with CODEm. Covariates included in this model and their level and directionality are show in the table below:

Table 1: Covariates used in CODEm models of overall maternal mortality

Level	Covariate	Direction
Level 1	Age-specific fertility rate	+
	Total fertility rate (log-transformed)	+
	Maternal education (years per capita)	–
	In-facility delivery (proportion)	–
	Skilled birth attendance (proportion)	–
	Neonatal mortality ratio (log-transformed)	+
	Age-specific HIV mortality in females 10-54 (log-transformed)	+
Level 2	Antenatal care 1-visit coverage (proportion)	-
	Antenatal care 4-visits coverage (proportion)	-
	Age-standardised wasting (weight-for-height) summary exposure value (SEV)	+
	Age-standardised stunting (height-for-age) SEV	+
	Healthcare Access and Quality Index	-
	Age- and sex-specific SEV for high body-mass index (BMI)	+
	Age- and sex-specific SEV for high blood pressure (SBP)	+

	Underweight women of reproductive age	+
Level 3	Socio-demographic Index<br>Mortality shock (cumulative rate in last 10 years)<br>LDI (log-transformed)<br>Hospital beds (per 1,000 population)	-<br>+<br>-<br>–

Cause-specific maternal mortality

We used spatiotemporal Gaussian process regression (ST-GPR) to estimate MMRs for each of the eight maternal sub-causes. This modelling strategy requires data to be in standard GBD age groups. To achieve this, we used the global age pattern of the COD data for each cause and applied it to all data that were not in the standard GBD age groups. ST-GPR also requires variance for each datapoint. In order to compute variance, we ran a Lowess regression on the data by year and used the variance of the residuals resulting from the difference between the data and the predicted values.

The first step in the past has been a mixed-effects ordinary least squares regression of the quantity of interest and a specified set of location-level covariates. Since GBD 2019, we have revised this first step to instead be informed by an ensemble of regressions where weighting of each component model was based on out-of-sample coverage prediction performance. This approach allowed us to test a larger number of covariates and also specify the directionality of relationships between location-level covariates and the outcome of interest. Country covariates were specific for each sub-cause model, as shown in the table below:

Table 2: Covariates used in generation of ensemble stage 1 predictions of cause-specific maternal mortality ST-GPR models

**Maternal sub-cause**	**Country-level covariates**	**Direction**
Maternal haemorrhage	In-facility delivery (proportion)<br>Skilled birth attendance (proportion)<br>Age- and sex-specific SEV for unsafe sanitation<br>Neonatal mortality ratio (log-transformed)<br>Maternal education<br>Healthcare Access and Quality Index	-<br>-<br>+<br>+<br>-<br>-
Maternal hypertensive disorders	Age- and sex-specific SEV for fasting plasma glucose (FPG)<br>Age- and sex-specific SEV for high body-mass index (BMI)<br>Age- and sex-specific SEV for high blood pressure (SBP)<br>Neonatal mortality ratio (log-transformed)<br>Hospital beds (per 1000 population)<br>Antenatal care 1-visit coverage (proportion)<br>Antenatal care 4-visits coverage (proportion)<br>Healthcare Access and Quality Index	+<br>+<br>+<br>+<br>-<br>-<br>-<br>-
Obstructed labour and uterine rupture	In-facility delivery (proportion)<br>Skilled birth attendance (proportion)<br>Underweight women of reproductive age<br>Neonatal mortality ratio (log-transformed)<br>Hospital beds (per 1000 population)<br>Age-standardised wasting (weight-for-height) SEV<br>Age-standardised stunting (height-for-age) SEV	-<br>-<br>+<br>+<br>-<br>+<br>+
Abortion and miscarriage	Abortion legality<br>Antenatal care 1-visit coverage (proportion)	-<br>-

	Antenatal care 4-visits coverage (proportion)<br>Hospital beds (per 1,000 population)<br>Maternal education<br>Healthcare Access and Quality Index	-<br>-<br>-<br>-
Ectopic pregnancy	Abortion legality<br>Pelvic inflammatory disease age-standardised prevalence<br>Antenatal care 1-visit coverage (proportion)<br>Antenatal care 4-visits coverage (proportion)<br>Hospital beds (per 1,000 population)<br>Maternal education<br>Healthcare Access and Quality Index	-<br>+<br>-<br>-<br>-<br>-<br>-
Maternal sepsis and other maternal infections	In-facility delivery (proportion)<br>Skilled birth attendance (proportion)<br>Age- and sex-specific SEV for unsafe sanitation<br>Age- and sex-specific SEV for fasting plasma glucose (FPG)<br>Antenatal care 1-visit coverage (proportion)<br>Antenatal care 4-visits coverage (proportion)<br>LDI (log-transformed)<br>Healthcare Access and Quality Index	-<br>-<br>+<br>+<br>-<br>-<br>-<br>-
Other maternal deaths	In-facility delivery (proportion)<br>Skilled birth attendance (proportion)<br>Antenatal care 1-visit coverage (proportion)<br>Antenatal care 4-visits coverage (proportion)<br>LDI (log-transformed)<br>Age- and sex-specific SEV for high body-mass index (BMI)<br>Maternal education<br>Healthcare Access and Quality Index	-<br>-<br>-<br>-<br>-<br>+<br>-<br>-
Indirect maternal deaths	In-facility delivery (proportion)<br>Skilled birth attendance (proportion)<br>Antenatal care 1-visit coverage (proportion)<br>Antenatal care 4-visits coverage (proportion)<br>LDI (log-transformed)<br>Age- and sex-specific SEV for high body-mass index (BMI)<br>Maternal education<br>Healthcare Access and Quality Index	-<br>-<br>-<br>-<br>-<br>+<br>-<br>-

Late maternal death and model processing

Aetiology-specific estimates were derived by scaling the results from the ST-GPR sub-cause-specific models scaled in relation to each other to equal one and then multiplying them by the total maternal deaths, corrected for late maternal deaths, for that age group, location, and year. A single parameter proportion model was run in DisMod-MR 2.1 for late maternal deaths using the data described above. The proportions coming for the VR data sources were taken before any of the central data processing. We used the Healthcare Access and Quality Index as a country-level covariate for the model.

Measles

Model flowchart

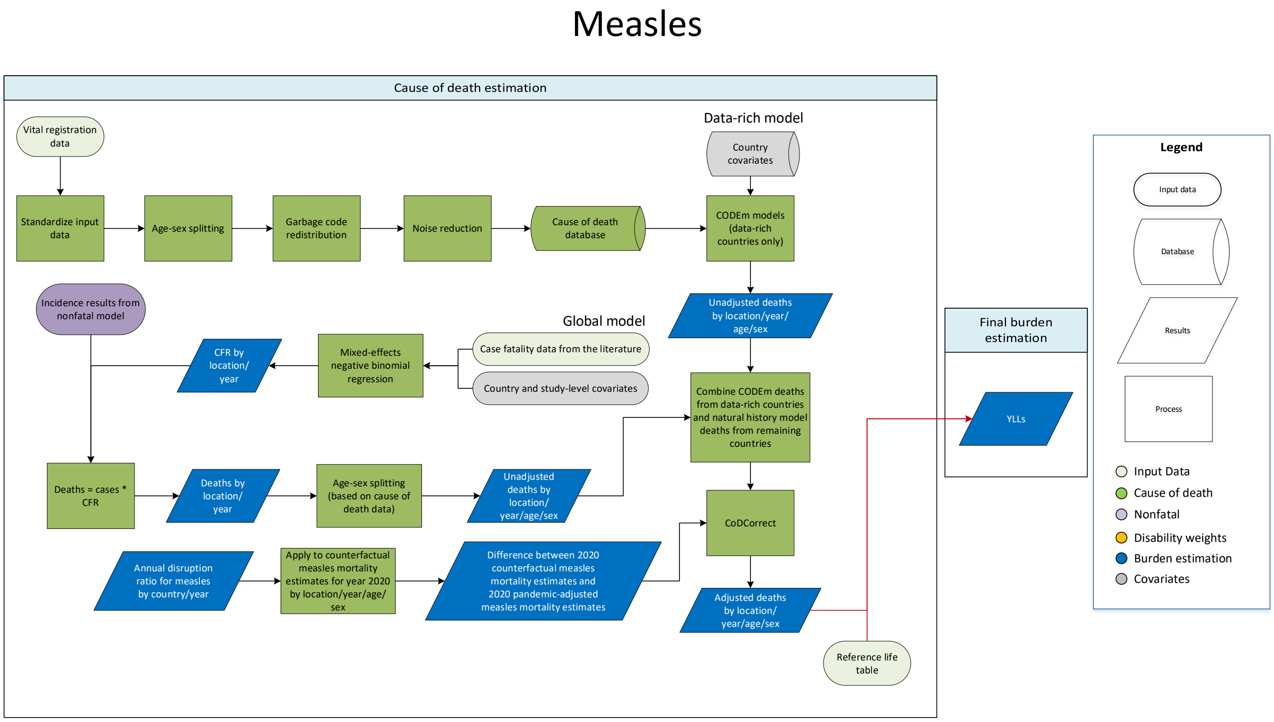

Modelling strategy overview

The GBD 2021 measles mortality estimates were generated in one of two ways depending on the quality of available vital registration data for the country. For countries with well-defined vital registration (ie, "data-rich" countries), we used a Cause of Death Ensemble model (CODEm). For the remaining countries, we leveraged a natural history model approach, drawing from preceding non-fatal case estimates. For all countries, we produced estimates for all age groups between 6 months and 64 years.

Data-rich countries

For data-rich countries modeled in CODEm, we used the covariates listed in Table 1 to inform predictions. New this cycle, a moving average of coverage of the first dose of measles-containing vaccine (MCV1) over the last five years was used as a covariate in the CODEm models. In previous GBD cycles, estimates of routine MCV1 coverage in only the year being modelled were used as a covariate. These moving-average estimates better capture changes in coverage in the population over time, allowing the influence of a single-year decline in coverage (eg, due to a vaccine stockout or conflict) to be spread across time rather than only informing the model's predictions in the year of the disruption.

Table 1. Covariates. Summary of covariates used in the data-rich measles cause of death model

Level	Covariate	Direction
1	Average measles-containing vaccination dose one (MCV1) coverage over the last five years	-
2	Healthcare Access and Quality (HAQ) Index	-
3	Socio-demographic Index (SDI)	-
	Mean years of education per capita	-

Natural history model

A natural history model is used to estimate measles mortality in non-data-rich locations where mortality data are sparse. GBD estimates of non-fatal measles cases are combined with estimates of measles case-fatality rate (CFR) generated by an intermediate, custom CFR model to produce this output. As described in the non-fatal measles modelling methods text, case notifications informing the measles non-fatal model come from the World Health Organization (WHO) Joint Reporting Form (JRF) and additional case notification sources identified by collaborators. The measles CFR data are compiled through systematic reviews of the literature, and this search was last updated in GBD 2019. This search was conducted in PubMed using the following search string: *((((measles[MeSH Terms] OR measles) AND (mortality[MeSH Terms] OR mortality OR "case fatality rate" OR "case fatality ratio" OR "case fatality"))) AND ("2016"[Date - Publication] : "2019"[Date - Publication]))*.

With the available measles CFR input data, we make location- and year-specific death estimates using a negative binomial model with Socio-demographic Index (SDI) as a country-level covariate, additionally accounting for three indicators (hospital-based or not; outbreak or not; and rural or urban/mixed) as study-level covariates, with country random effects:

$$Y_{ij} = \beta_0 + \beta_1 SDI_{ij} + \beta_2 hospital_{ij} + \beta_3 outbreak_{ij} + \beta_4 rural_{ij} + u_j + e_{ij}$$

where Y_{ij} is the number of deaths (using measles cases as the offset term); β_0 is the fixed-effect intercept; β_1, β_2, β_3, and β_4 are the fixed-effects slopes on the Socio-demographic Index (SDI) and hospital, outbreak, and rurality study-level covariates; u_j is country-level random effects; e_{ij} is the residual; i is the year; and j is the location. New in GBD 2021, we weight the input study data by the sample size when fitting the CFR model. Surveillance or other population-level data are assigned a weight of the median sample size of the non-population-level data. This weighting scheme was selected over sample size weighting for all studies and over an equal weighting for all studies because it resulted in the lowest RMSE. Further updates to the CFR model come from updated CFR model input data. We began to include studies, previously excluded, that report a CFR over 0.1 and studies from locations in the high-income super-region that report a CFR over 0.025. When predicting CFR from this model for each location and year, covariates are set to reflect non-hospital-based, non-outbreak, and urban (non-rural) settings. For countries without data, we use the mean of all the country-level random effects in the region as the random effect for CFR prediction. Uncertainty was estimated by taking 1000 iterations of the predictions based on the variance-covariance matrix and uncertainty in country random effects. Uncertainty was estimated by taking 1000 iterations of the predictions based on the variance-covariance matrix and uncertainty in country random effects.

Measles log-transformed incidence – modelled independently – is generated from a mixed effects linear regression model predicting measles cases as a function of vaccination coverage (rolling means of MCV1 and MCV2 over the preceding five years, and five-year lagged SIA coverage) given WHO case notification data. Combining these estimates of incidence for every estimated location-year with location- and year-specific estimates of measles CFR, measles deaths were calculated as:

$$deaths = incidence * CFR$$

This calculation was replicated at the draw level 1000 times, producing draw-level estimates of total measles deaths for each location and year, which were then split by age and sex using an age-sex distribution based on global-level age- and sex-specific patterns found in the cause of death data. All draw-level estimates were then summarised as the mean of the draws along with a 95% uncertainty interval (the 2.5th and 97.5th percentile of all draws). We adjusted measles death estimates for 2020 and 2021 to account for the reductions in measles cases associated with the COVID-19 pandemic, as described elsewhere in this appendix.

Meningitis

Flowchart

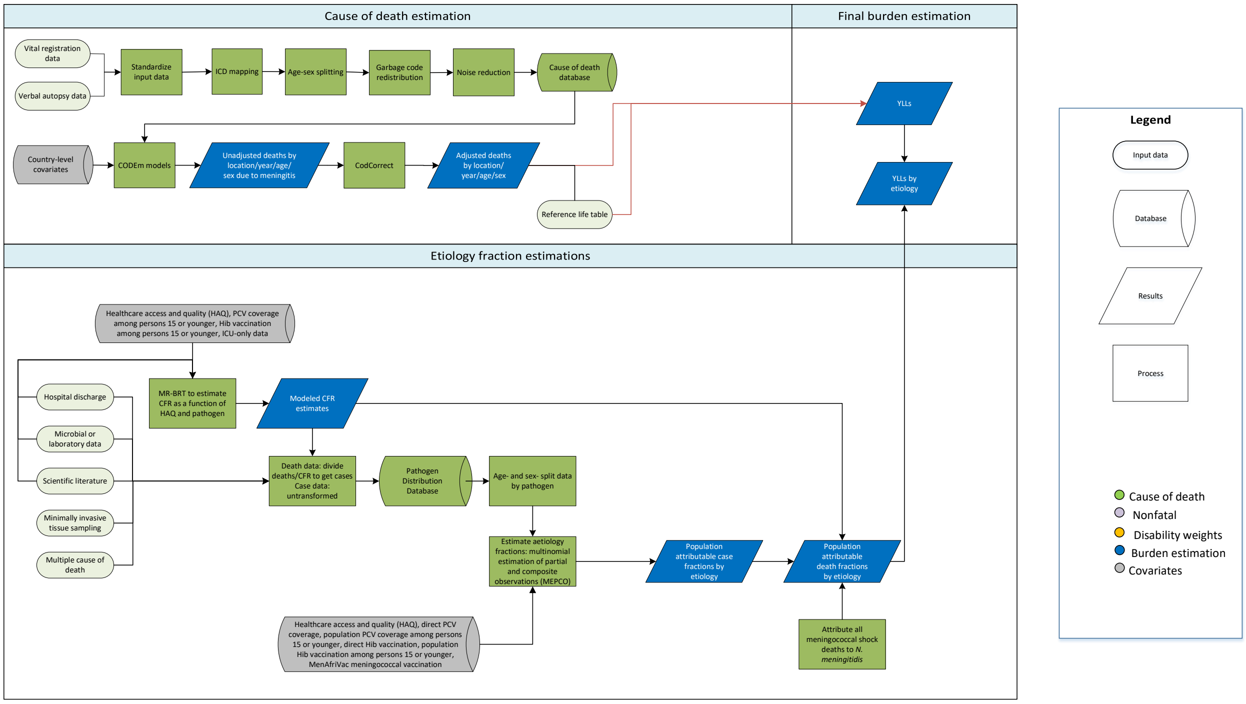

Input data and methodological summary for meningitis

Input data

Overall meningitis

Input data for the overall meningitis model came from the cause of death database, which includes vital registration (VR) and verbal autopsy (VA) data. We outliered data in instances where garbage code redistribution and noise reduction, in combination with small sample sizes, resulted in unreasonable cause fractions when compared to regional, super-regional, and global rates, and data that violated well-established time or age trends. Outliering methods were consistent across both VR and VA data.

Aetiology proportions

The PubMed search string below was used to look for the incidence of meningitis cases, the aetiology proportion for meningitis aetiologies, and the case fatality rates of meningitis aetiologies (described in more detail in next section).

("meningitis"[MeSH Terms] OR "meningitis"[Title/Abstract]) AND ("case fatality rate"[Title/Abstract] OR “case fatality ratio”[Title/Abstract] OR “mortality”[MeSH] OR "mortality"[Title/Abstract] OR "fatal*"[Title/Abstract] OR “inciden*”[Title/Abstract] OR “cases”[Title/Abstract]) AND ("Meningitis, Haemophilus"[MeSH Terms] OR "Haemophilus"[Title/Abstract] OR “Hib meningitis”[Title/Abstract] OR

"Meningitis, Pneumococcal"[MeSH Terms] OR "Pneumo*"[Title/Abstract] OR "Meningitis, Meningococcal"[MeSH Terms] OR "Meningococcal"[Title/Abstract] OR “Neisseria meningitidis”[MeSH Terms] OR “Neisseria meningitidis”[Title/Abstract] OR "Meningitis, Viral"[MeSH Terms] OR "Viral Meningitis"[Title/Abstract] OR "Streptococcus agalactiae"[MeSH Terms] OR "Streptococcus agalactiae"[Title/Abstract] OR “Group B Strep*”[Title/Abstract] OR “GBS”[Title/Abstract] NOT ("animals"[MeSH Terms] NOT "humans"[MeSH Terms]) AND (1990[DP] : 3000[DP]))

We ran this search string on 3/11/2020 for GBD 2021.

The inclusion criteria stipulated that (1) the publication year must be between 1980 and the present year; (2) “caseness” was based on presence of bacterial pathogen in blood (with additional clinical presentation of meningitis) or cerebrospinal fluid, as diagnosed by culture, antigen test, polymerase chain reaction test, or latex agglutination test, or gram staining; (3) sufficient information must be provided on study method and sample characteristics to assess the quality of the study; and (4) study samples must be representative of the general population. No limitation was set on the language of publication. We identified 265 studies after title-abstract screening, of which 133 met our inclusion criteria and were extracted. We excluded studies that were unrepresentative of the general population, studies that used animals as subjects, and studies (for incidence) with study population under 100.

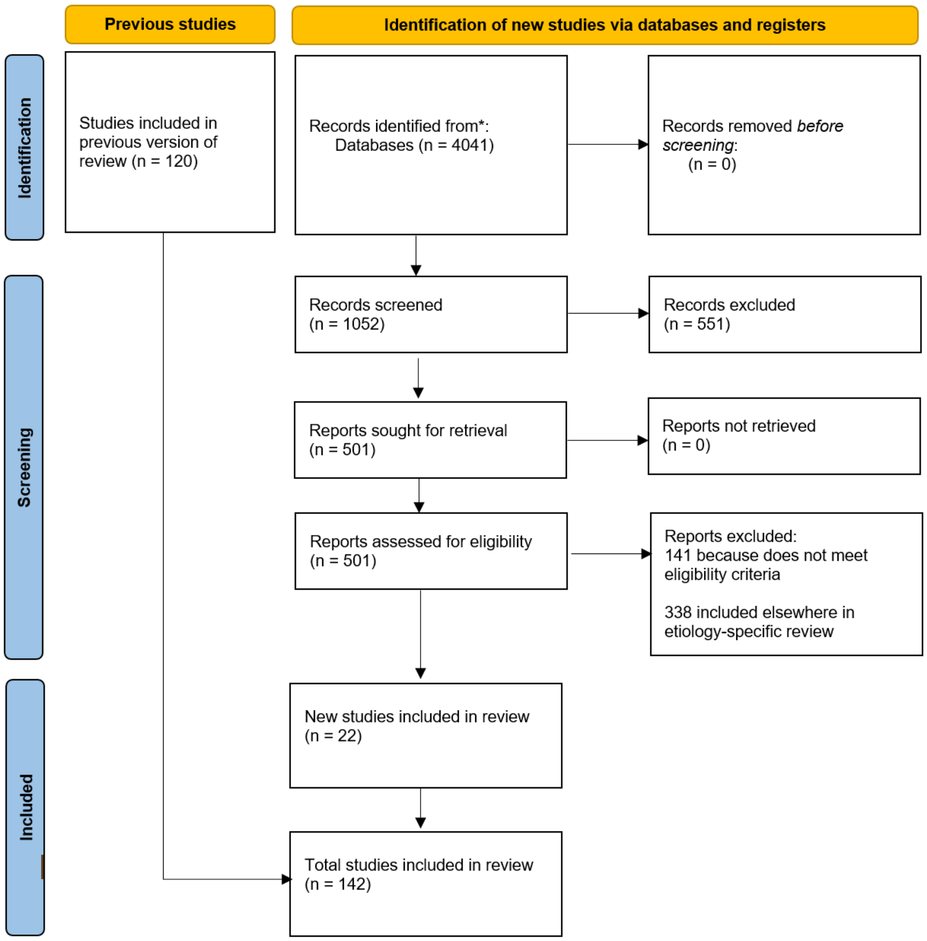

Figure 1: PRISMA diagram for meningitis 2021 systematic review for incidence

Input data for aetiology estimation consisted of multiple cause of death, vital registration, hospital discharge, and microbial data, as well as the aforementioned systematic literature review and a separate, targeted review pulling data from citations found in meta-analyses. For data sources that provided ICD codes (multiple cause of death, vital registration, hospital discharge, and some microbial data), these codes were used to identify patients with meningitis and the culprit pathogen, when detailed. For the microbial data that did not provide ICD codes, we identified pathogens associated with meningitis using cerebrospinal fluid samples. The table below documents the ICD codes used to identify meningitis cases with known etiology.

**Type of meningitis**	**ICD 10 code(s)**	**ICD9 code(s)**
Meningitis due to *Listeria*	A32.1	--
Meningitis due to *Neisseria meningitidis*	A39-A39.0	036-036.1, 320.5-320.8
Meningitis due to *Haemophilus*	G00.0	320.0
Meningitis due to *Streptococcus pneumoniae*	G00.1	320.1
Meningitis due to Group B *Streptococcus*	G00.2	320.2
Meningitis due to *Staphylococcus aureus*	G00.3	320.3
Meningitis due to virus	A87-A87.9, G03.0	047-049.9

Data on pathogens cultured from human infections were solicited from a wide array of international stakeholders (representing every inhabited continent). These included research hospitals, surveillance networks, and infection databases maintained by private laboratories and medical technology companies. For a full list of non-literature sources used for our estimates, please refer to the referenced article appendix (section 2).[1]

Modelling strategy

We modelled deaths due to all meningitis with two CODEm models, separately for each sex and two age categories – under 5 and 5 years and above. The mortality trends differ substantially between children and adults, and a significant number of data sources only have data for children under 5. The two models used the same covariates (with the exception of the covariate for underweight, which is age-specific in the under-5 model and age-standardised in the 5+ model) and otherwise standard CODEm parameters. The final sex-specific models for deaths due to all meningitis were a hybridised model of separate global and data-rich models for males and females.

In past GBD cycles, estimates of PCV3 vaccine coverage among infants in the modelled year were used as the primary covariate for this linear regression. In GBD 2021, we now use a lagged mean of PCV3 vaccine coverage calculated over a rolling, five-year interval in order to capture population-level vaccine-derived immunity among under-5-year-olds, including coverage both in the current year and in recent years. For Hib3 vaccine coverage, we use a version of the lagged Hib3 covariate with an

additional transformation applied to account for indirect effects from herd immunity. The Hib vaccine is one of the most effective vaccines in use, with 95% to 100% efficacy.[2] This results in a highly pronounced herd immunity effect, meaning that the reduction in invasive Hib disease seen in the population is greater than what would be expected with direct coverage alone. This effect was quantified by Majumder and colleagues, who performed a meta-analysis to determine an indirect effect multiplier for vaccine coverage.[3] We used the equations from the Wolfson method for expected protection from invasive Hib provided in this meta-analysis to transform the Hib3 covariate.

Table 1. Covariates used in meningitis mortality modelling (0–4 years, 5–95+ years)

Covariate name	Level	Direction
Meningitis belt (proportion of population in belt)	1	+
MenAfriVac coverage	1	-
Hib3 vaccine coverage proportion, indirect	1	-
PCV3 vaccine coverage proportion, lagged	1	-
Age- and sex-specific summary exposure value (SEV) for child underweight	2	+
Logit-transformed water (proportion with access)	2	-
Maternal care and immunisation	2	-
Healthcare Access and Quality (HAQ) Index	2	-
Log-transformed lag-distributed income	3	-
Sanitation (proportion with access)	3	-
Maternal education (years per capita)	3	-
Socio-demographic Index	3	-

Aetiology estimation

To generate aetiology fraction estimates for fatal meningitis, we took our aetiology fractions estimated for non-fatal meningitis and multiplied them by a set of pathogen-specific case fatality rates (CFRs). CFRs were estimated using ICD-coded hospital data, microbial data with patient discharge status, and a systematic literature review examining mortality associated with various pathogens. We employed the MR-BRT framework (meta-regression, Bayesian, regularised, trimmed) to predict CFRs as a function of pathogen, crude age (neonatal, post neonatal–5 years, 5–50 years, 50–70 years, and 70 years and older), HAQ Index, an interaction term between pathogen and the proportions of the population age 15 or younger that had received PCV and *Haemophilus influenzae* type B vaccinations,[4] and a data source random effect. We additionally controlled for data provided from ICU-only sources (which would be biased toward higher CFRs). The general specification of the model is as follows:

$$logit(y_i) = X_i\beta + u_i 1 + \epsilon_i, \qquad \epsilon_i \sim N(0, \Sigma_i), \qquad u_i \sim N(0, \gamma)$$

where

- y_i contains CFRs for data source i
- Design matrix X_i contains as columns the model covariates
- β are fixed effect multipliers
- ϵ_i are observation error terms with known variances
- u_i are data source-specific random intercepts with unknown covariance γ

The modelling environment allows specification of priors on γ, for which we used the following:

- Prior on β for pathogen:vaccination interaction: We assumed vaccination would have no impact on CFRs of unrelated pathogens, and for all combinations of the pathogen:vaccination interaction that were not *Streptococcus pneumoniae*:PCV vaccination or *Haemophilus influenzae*:Hib vaccination, we coerced the βs to 0 using model priors. For the *Streptococcus pneumoniae*:PCV vaccination and *Haemophilus influenzae*:Hib vaccination interaction terms, we employed a negativity prior to enforce case-fatality rates for these pathogens to decrease as vaccination was introduced.
- Prior on γ, data source random effect: Many input data sources cover only a single country, leading to low variability in HAQ Index within each data source. To emphasise the contribution of HAQ Index over data source in the modelled estimates, we implemented a strong Gaussian prior (mean 0, standard error 0.001) on γ.

There was significant variability with respect to the quality of input data we were able to collect for CFR estimation for different pathogens. For those pathogens with "rich" data, defined by our method as having at least ten high-quality datapoints below a moderate HAQ Index (0.7), we modelled a unique effect of HAQ Index, achieved by interacting the HAQ Index fixed-effect with the pathogen-specific fixed-effect. This process allowed the relative deadliness of pathogens to vary depending on a location's HAQ Index. For those pathogens with fewer than ten high-quality datapoints below 0.7 HAQ Index, or those whose results in the interaction models indicated an unrealistically large influence of HAQ Index (eg, 70% CFR in low HAQ Index countries, 1% CFR in high HAQ Index countries), we modelled a pathogen-specific intercept with an HAQ Index fixed-effect shared across the pathogens. As a consequence of the single fixed-effect on HAQ Index, a pathogen that was predicted to be the deadliest in low HAQ Index countries would also be predicted to be the deadliest in high HAQ Index countries. The table below details which pathogens were modelled with each of the above methods.

Modelled with distinct intercept and effect of HAQ Index	Modelled with distinct intercept, pooled effect of HAQ Index
Haemophilus influenzae, Streptococcus pneumoniae, and viral meningitis	*Escherichia coli,* group B *Streptococcus, Klebsiella pneumoniae, Listeria monocytogenes, Neisseria meningitidis,* and *Staphylococcus aureus*

To estimate the CFRs for those infections associated with residual "other pathogens", we pooled all bacterial data together and estimated a single CFR curve from age, HAQ Index, and the data source heterogeneity covariates. Non-fatal pathogen proportions $p_{i,j}$ for a given demographic group i and pathogen j were then converted to deaths using the CFRs estimates for demographic group i as follows:

$$p_{i,j}^{deaths} = \frac{p_{i,j} \times CFR_i}{\sum_{\hat{j}} p_{i,\hat{j}} \times CFR_i}$$

A more thorough account of these methods, including model validation, has been described previously elsewhere.[5]

References

---

[1] Murray CJ, Ikuta KS, Sharara F, Swetschinski L, Aguilar GR, Gray A, Han C, Bisignano C, Rao P, Wool E, Johnson SC. Global burden of bacterial antimicrobial resistance in 2019: a systematic analysis. *The Lancet*. 2022 Feb 12;399(10325):629-55.

[2] Hamborsky J, Kroger A, editors. Epidemiology and prevention of vaccine-preventable diseases, E-Book: The Pink Book. Public Health Foundation; 2015 Oct 19. Chapter 8: *Haemophilus influenzae*.

[3] Majumder A. *Quantifying the Indirect Effects of* Haemophilus Influenzae *type b Vaccination in Children Under 5 Years-Old* (Doctoral dissertation, Johns Hopkins University).

[4] Galles NC, Liu PY, Updike RL, Fullman N, Nguyen J, Rolfe S, Sbarra AN, Schipp MF, Marks A, Abady GG, Abbas KM. Measuring routine childhood vaccination coverage in 204 countries and territories, 1980–2019: a systematic analysis for the Global Burden of Disease Study 2020, Release 1. *The Lancet*. 2021 Aug 7;398(10299):503-21.

[5] Murray CJL, Ikuta K, Sharara F, et al. Global burden of bacterial antimicrobial resistance in 2019: a systematic analysis. *The Lancet* 2022; 399: 629–55.

Motor neuron disease

Flowchart

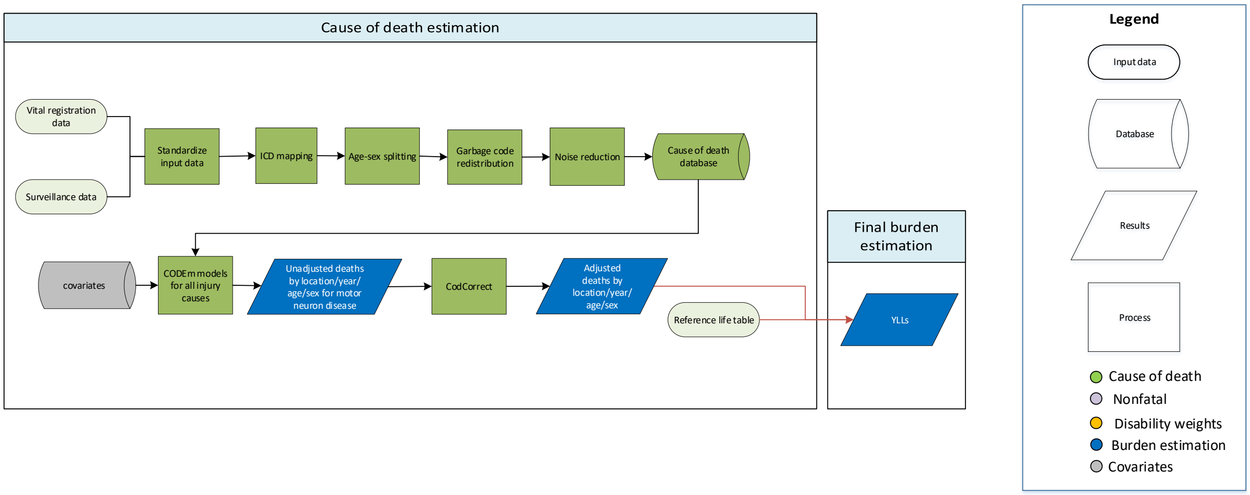

Input data and methodological summary for motor neuron disease

Input data

Data used to estimate motor neuron disease included vital registration and surveillance data from the cause of death (CoD) database. Our outlier criteria were to exclude datapoints that (1) were implausibly high or low, (2) substantially conflicted with established age or temporal patterns, or (3) substantially conflicted with other data sources from the same locations or locations with similar characteristics (ie, Socio-demographic Index). In GBD 2021, this affected Kazakhstan, where ICD-9-BTL-tabulated vital registration data were available for 1991–2003 and ICD-10-coded vital registration were available for 2013 onward. The raw ICD-9-BTL data for 1991 were 14-fold higher than raw ICD-9-BTL (1992–2003) and ICD-10 (2013 onward), causing an implausible time pattern via noise reduction data processing methods for ICD-9-BTL data. For that reason, the ICD-9-BTL data were excluded and the ICD-10 data retained.

Modelling strategy

The standard Cause of Death Ensemble modelling (CODEm)[1] approach (described in reference appendix section 3.1) was used to estimate deaths due to motor neuron disease. Separate models were conducted for male and female mortality, and the age range for both models was 0 days to 95+ years. Unadjusted death estimates were adjusted using CoDCorrect to produce final estimates of years of life lost (YLLs).[1] See appendix section 3.1 of the reference article for further information.

The following table lists the covariates included in the model. This requires that the covariate selected for the model must have the directional relationship with motor neuron disease deaths. For GBD 2021, no significant updates were made for motor neuron disease covariate selection. Covariate directions were selected based on the strength of the evidence.

Table 1. Covariates used in motor neuron disease mortality modelling

Level	Covariate	Direction
	Mean total body-mass index (kg/m^2)	-
	Mean serum total cholesterol (mmol/L)	-
	Absolute value of average latitude	+
	Mean diabetes fasting plasma glucose (mmol/L)	+
	Fruit consumption (grams per day, adjusted)	-
	Socio-demographic Index	+
	Healthcare Access and Quality Index	-
2	Population-weighted mean temperature	-
	Sanitation (proportion with access)	+
	Improved water source (proportion with access)	-
3	Education (years per capita)	+
	Log-transformed LDI (per capita)	+

The following plots show the influence of each covariate on the four CODEm models (male global, male data-rich, female global, and female data-rich). A positive standardised beta (to the right) means that the covariate was associated with increased death. A negative standardised beta (to the left) means the covariate was associated with decreased death.

[1]Vos T, Lim SS, Abbafati C, *et al.* Global burden of 369 diseases and injuries in 204 countries and territories, 1990–2019: a systematic analysis for the Global Burden of Disease Study 2019. *The Lancet* 2020; **396**: 1204–22. doi: https://doi.org/10.1016/S0140-6736(20)30925-9

Covariate influence plots: Motor neuron disease

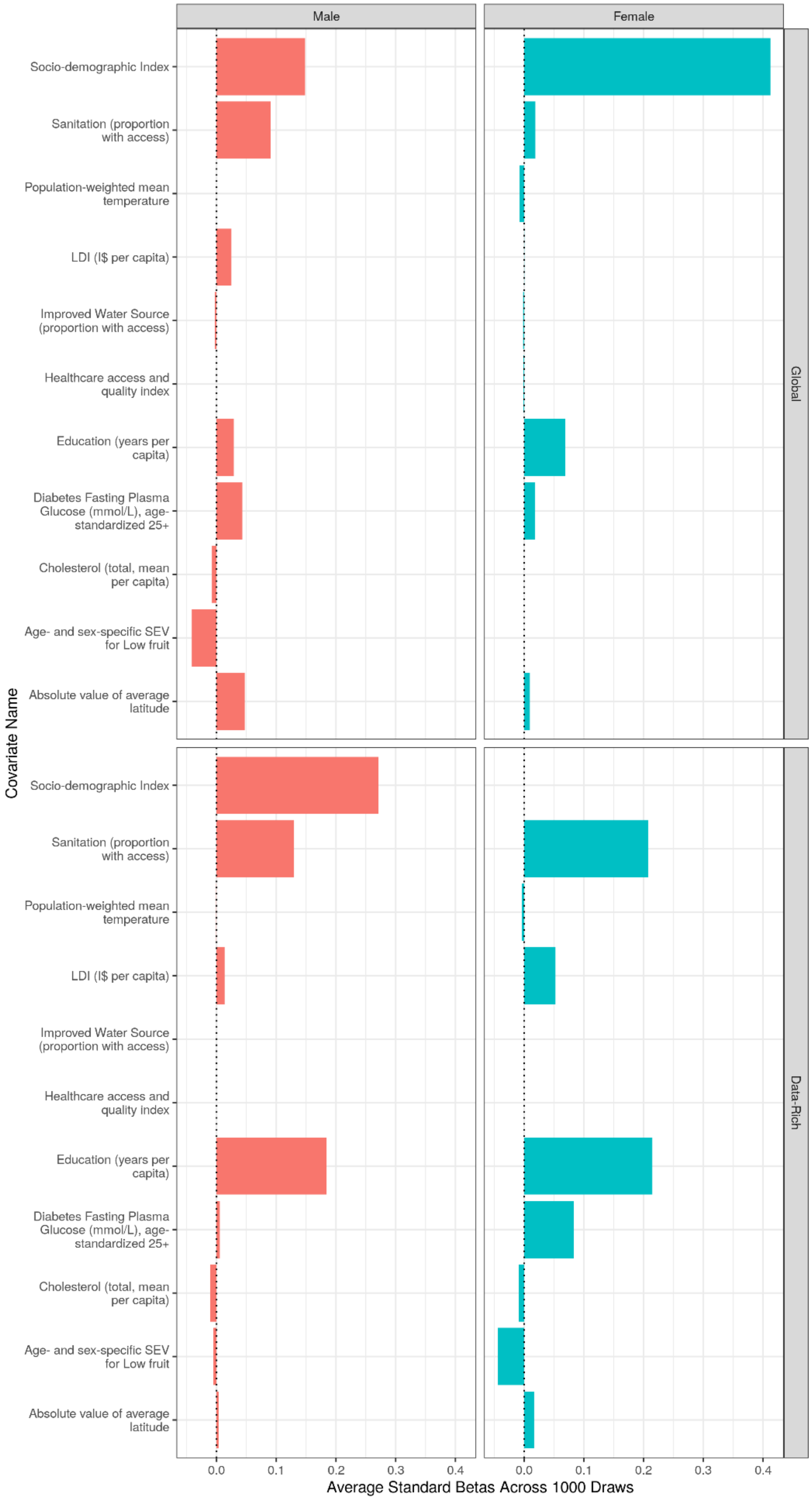

Multiple sclerosis

Flowchart

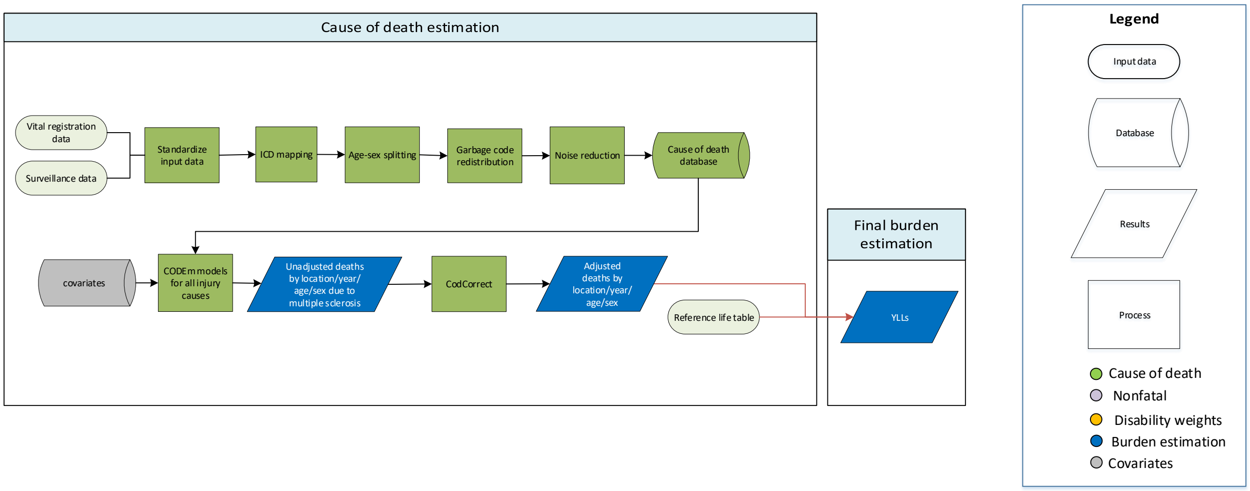

Input data and methodological summary for multiple sclerosis

Input data

Data used to estimate multiple sclerosis included vital registration and surveillance data from the cause of death (COD) database. Our outlier criteria were to exclude datapoints that (1) were implausibly high or low, (2) substantially conflicted with established age or temporal patterns, or (3) substantially conflicted with other data sources conducted from the same locations or locations with similar characteristics (ie, Socio-demographic Index). In particular, where data processing could not resolve discrepancies between different coding systems for the same location over time, one system was selected as more reliable and the other was excluded. In GBD 2021, this affected Kazakhstan, where ICD-9-BTL-tabulated vital registration data were available for 1991–2003 and ICD-10-coded vital registration were available for 2013 onward. The raw ICD-10 data for 2013 onward were five-fold higher than the raw ICD-9-BTL (1992–2003), causing an implausible time pattern for multiple sclerosis. As such, the ICD-10-coded data were excluded.

Modelling strategy

The standard Cause of Death Ensemble modelling (CODEm)[1] approach (detailed in reference appendix section 3.1) was used to estimate deaths due to multiple sclerosis. Separate models were conducted for male and female mortality, and the age range for both models was 28 days to 95+ years. Unadjusted death estimates were adjusted using CoDCorrect to produce final estimates of years of life lost (YLLs).[1] See appendix section 3.1 of the reference article for further information.

The following table lists the covariates included in the model. This requires that the covariate selected for the model must have the directional relationship with multiple sclerosis deaths. For GBD 2021, no significant updates were made for multiple sclerosis covariate selection. Covariate directions were selected based on the strength of the evidence.

Table 1. Covariates used in multiple sclerosis mortality modelling

Level	Covariate	Direction
1	Absolute value of average latitude	+
2	Mean serum total cholesterol (mmol/L)	+
	Healthcare Access and Quality Index	-
3	Cumulative cigarettes (10 years)	+
	Cumulative cigarettes (5 years)	+
	Education (years per capita)	-
	Log-transformed LDI (per capita)	-
	Smoking prevalence	+
	Socio-demographic Index	+

The following plot shows the influence of each covariate on the four CODEm models (male global, male data-rich, female global, and female data-rich). A positive standardised beta (to the right) means that the covariate was associated with increased death. A negative standardised beta (to the left) means the covariate was associated with decreased death.

[1]Vos T, Lim SS, Abbafati C, *et al.* Global burden of 369 diseases and injuries in 204 countries and territories, 1990–2019: a systematic analysis for the Global Burden of Disease Study 2019. *The Lancet* 2020; **396**: 1204–22. doi: https://doi.org/10.1016/S0140-6736(20)30925-9

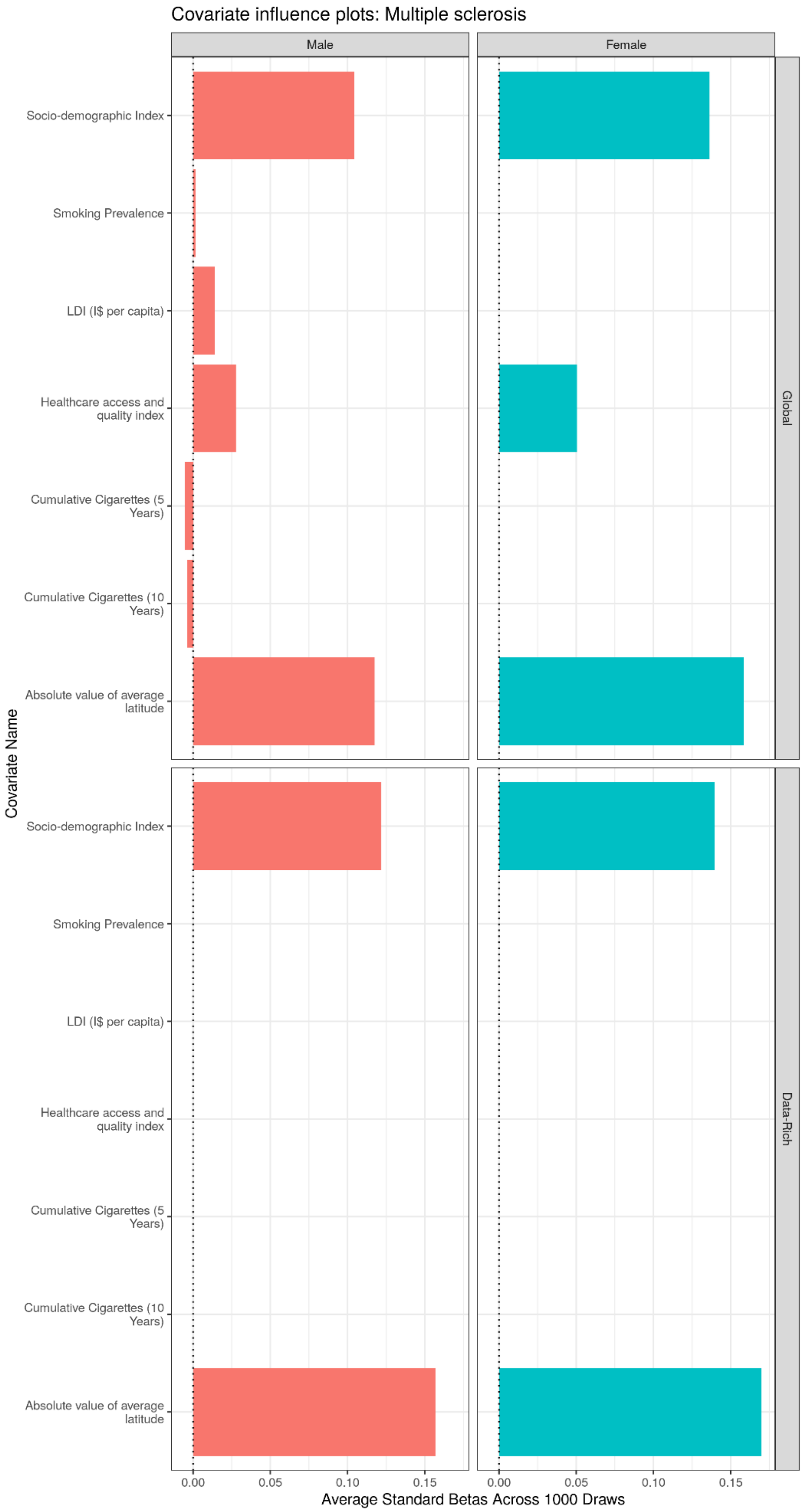
Covariate influence plots: Multiple sclerosis
Male
Female
Global
Data-Rich
Covariate Name
Socio-demographic Index
Smoking Prevalence
LDI (I$ per capita)
Healthcare access and quality index
Cumulative Cigarettes (5 Years)
Cumulative Cigarettes (10 Years)
Absolute value of average latitude
Socio-demographic Index
Smoking Prevalence
LDI (I$ per capita)
Healthcare access and quality index
Cumulative Cigarettes (5 Years)
Cumulative Cigarettes (10 Years)
Absolute value of average latitude
0.00
0.05
0.10
0.15
0.00
0.05
0.10
0.15
Average Standard Betas Across 1000 Draws

Musculoskeletal disorders

Flowchart

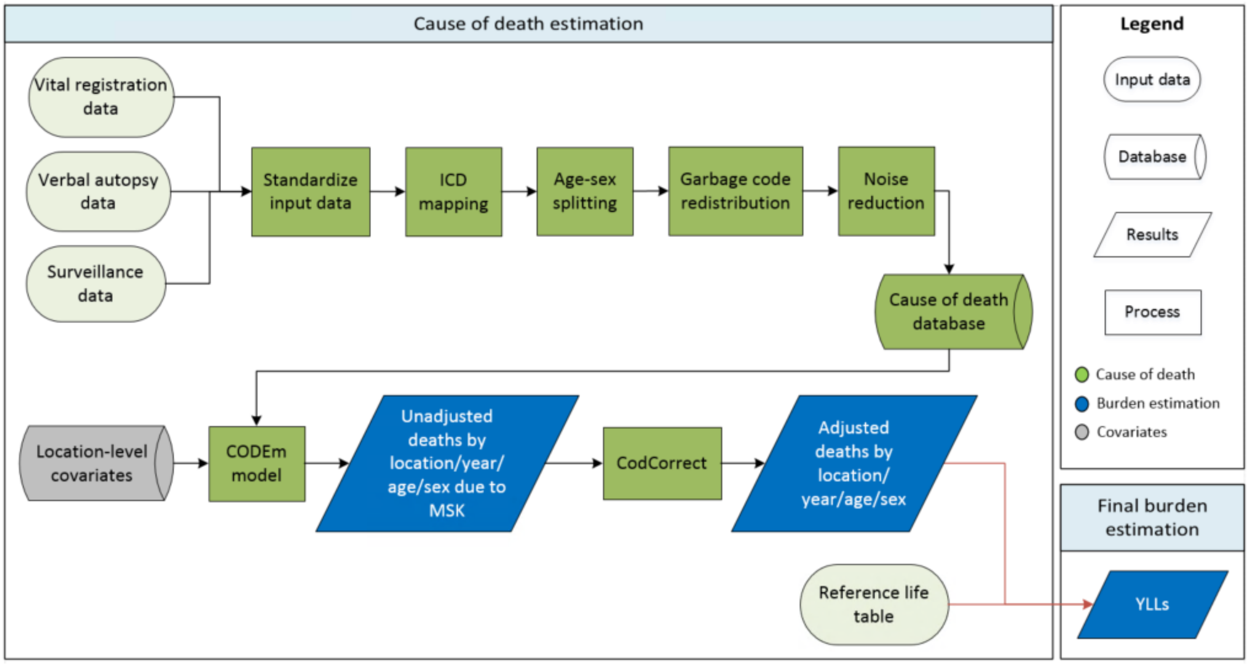

Input data and methodological summary for musculoskeletal disorders

Input data

Data used to estimate mortality from musculoskeletal disorders (MSK) included vital registration (VR) and China disease surveillance data from the cause of death (COD) database. Our outlier criteria excluded (1) datapoints that were implausibly high or low relative to global or regional patterns according to subject matter experts, (2) substantially conflicted with established age or temporal patterns, or (3) significantly conflicted with other data sources based from the same locations or locations with similar characteristics (ie, Socio-demographic Index), and (4) from verbal autopsy sources due to the inability of verbal autopsy to accurately capture most musculoskeletal conditions.

In GBD 2021, we un-outliered Philippines subnational VR data, as these data were no longer implausibly high globally according to subject matter experts. This resulted in an increase in Philippines MSK prevalence. We additionally outliered all Greenland data for males, as estimates were implausibly high due to a small number of non-zero estimates. Incoming VA data from Zambia, Bangladesh, Nairobi, Indonesia, and Ghana were outliered in accordance with the GBD 2019 approach. Data from Tajikistan and Kyrgyzstan were outliered due to implausibly low values globally according to subject matter experts. ICD-8A data for Finland were outliered due to coding differences with ICD9, which had introduced discontinuity in the time trend estimate, and being implausibly low according to subject matter experts. ICD9-BTL data were outliered for Chile, Uruguay, and Argentina due to the fact that they

introduced discontinuity in the time trend estimates and were implausibly low according to subject matter experts.

Modelling strategy

The standard CODEm (Cause of Death Ensemble model) modelling approach was applied to estimate deaths due to musculoskeletal disorders.[1] We applied mostly the same covariates used in GBD 2019, with a few changes.[2] The CODEm model for musculoskeletal disorders is limited by a lack of strong predictive covariates. Many are selected as a proxy for Socio-demographic Index (SDI), as many musculoskeletal disorders are autoimmune conditions which tend to have increasing prevalence with SDI. The following table lists the covariates included in the model. This requires that the covariate selected for the model must have the directional relationship with musculoskeletal disorder deaths. Covariate directions were selected based on the strength of the evidence.

Table 1. Covariates used in musculoskeletal disorder mortality modelling

Level	Covariate	Direction
1	Mean BMI	+
	Vegetables (g), unadjusted	+
	Alcohol consumption (litres per capita)	+
2	Cumulative cigarettes (10 years)	+
	Cumulative cigarettes (5 years)	+
	Education (years per capita)	+
	Log-transformed LDI: lag-distributed income ($ per capita)	+
	Mean cholesterol[2]	+
	Smoking prevalence	+
	Healthcare Access and Quality Index	-
	Age-standardised low bone mineral density	+
	Low bone mineral density	+
	Age-standardised bone mineral density among population age 60+ years	+
3	SDI: Socio-demographic Index	+

Covariate influences:

The following plot shows the influence of each covariate on the four CODEm models (male global, male data-rich, female global, and female data-rich). A positive standardised beta (to the right) means that

[1] *Vos T, Lim SS, Abbafati C, et al. Global Burden of 369 Diseases and Injuries in 204 Countries and Territories, 1990–2019: A Systematic Analysis for the Global Burden of Disease Study 2019. The Lancet 2020; 396: 1204–22. Doi: Https://Doi.Org/10.1016/S0140-6736(20)30925-9. Details Found in Appendix 1, Section 3.*, n.d.

[2] *Vos T, Lim SS, Abbafati C, et al. Global Burden of 369 Diseases and Injuries in 204 Countries and Territories, 1990–2019: A Systematic Analysis for the Global Burden of Disease Study 2019. The Lancet 2020; 396: 1204–22. Doi: Https://Doi.Org/10.1016/S0140-6736(20)30925-9. Details Found in Appendix 1, Section 3.*

the covariate was associated with increased death. A negative standardised beta (to the left) means the covariate was associated with decreased death.

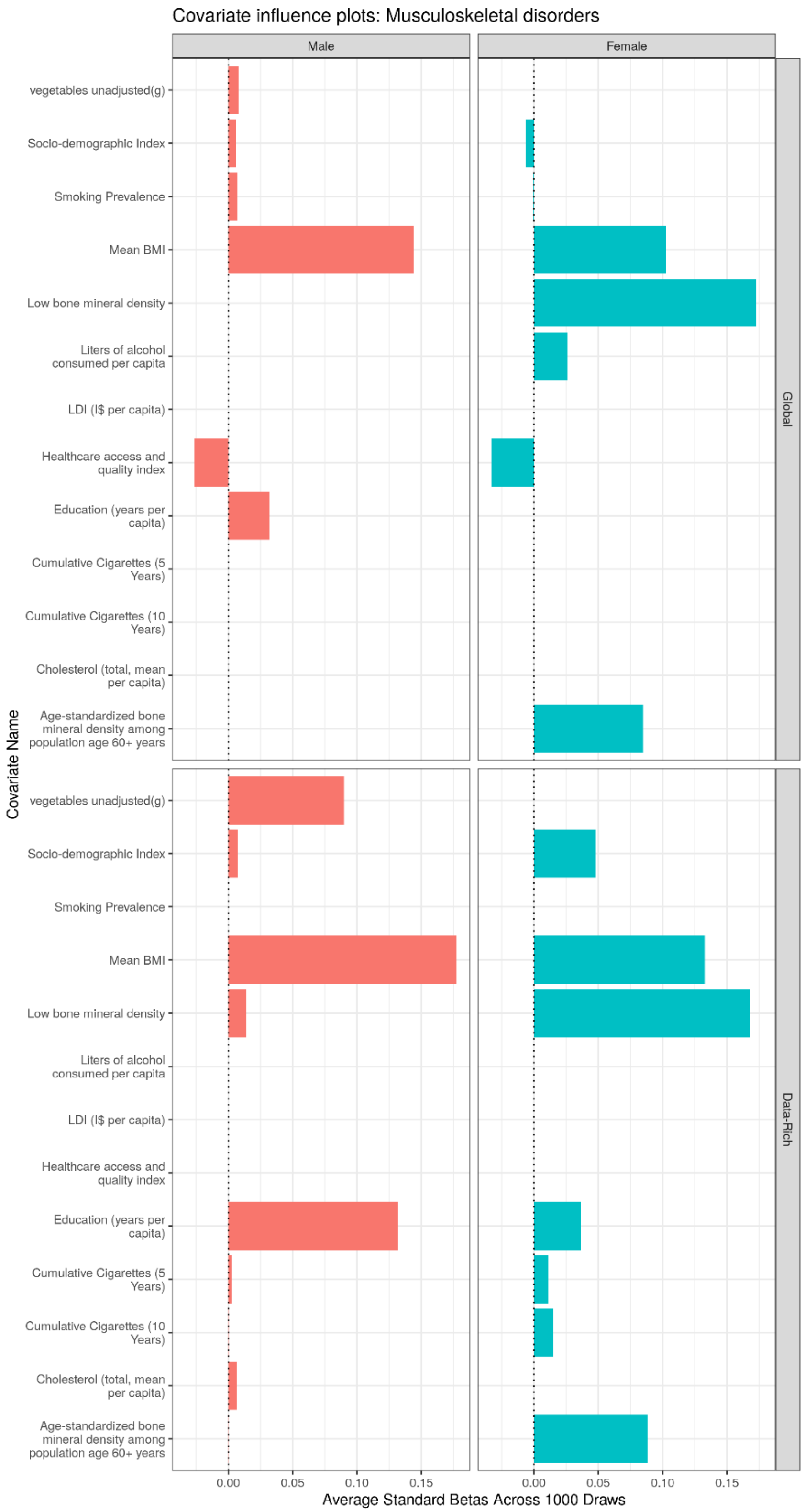

Myocarditis

Flowchart

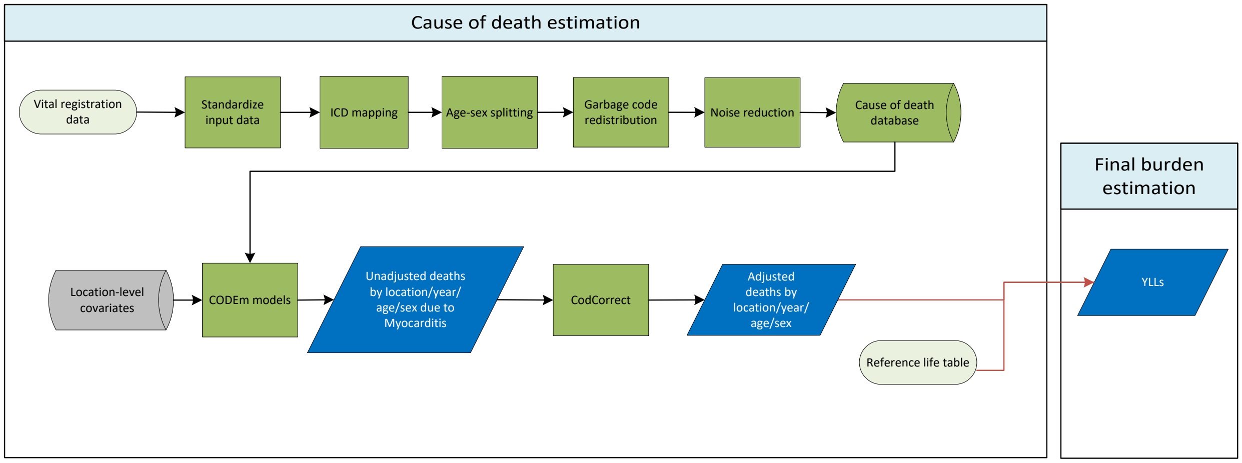

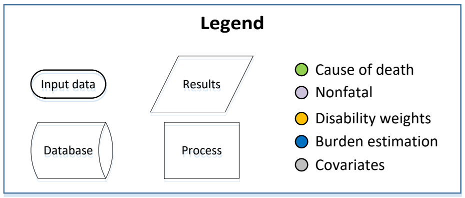

Input data and methodological summary for myocarditis

Input data

Vital registration data were used to model deaths due to myocarditis. We outliered ICD 10 data in Egypt and Bosnia and Herzegovina due to an implausibly high cause fraction compared to other locations in the region. In addition, data from Guyana, Cook Islands, Philippines, and western sub-Saharan Africa (Cabo Verde and Ghana) were outliered due to implausibly high values and time trends.

Modelling strategy

We used a standard CODEm approach to model deaths from myocarditis. The covariates selected for evaluation in the CODEm ensemble modelling process can be found in the table below. For GBD 2021, we removed the summary exposure variable covariate for cardiomyopathy and myocarditis (CMP) and systolic blood pressure (mm Hg) covariate from the CODEm model following a review of the association between mortality and these covariates which found the associations were not significant in predicting mortality. In addition, we changed the direction of the health access and quality index and lag distributed income per capital variables from -1 to 1 following a review of the association between VR data and these variables in our dataset. This change of direction was determined by observing statistically significant positive direction of the health access and quality and lag distributed income covariates in a linear mixed effects model controlled for age and location at the $p = .05$ level. We hypothesise that this positive association between myocarditis death and health access and quality and lag distributed income is a result of the ability of health systems to detect myocarditis and arrive at a diagnosis. In addition to previously mentioned methodological changes, the method used to reduce the noise in the data, implemented after redistribution to handle both the stochastic variation across time and space and the occurrence of small number of deaths in each location/year/age/sex, was also updated. This new empirical Bayesian noise reduction algorithm uses grouped data by region and data

type as prior to better reflect regional patterns. A detailed description on the updates to noise reduction can be found in the cause of death methods section of the appendix.

Table 1. Covariates used in myocarditis mortality modelling

Level	Covariate	Direction
2	Healthcare Access and Quality Index	1
	Log-transformed lag distributed income per capital (I$)	1
3	Socio-demographic index	1

Neonatal disorders

Flowchart

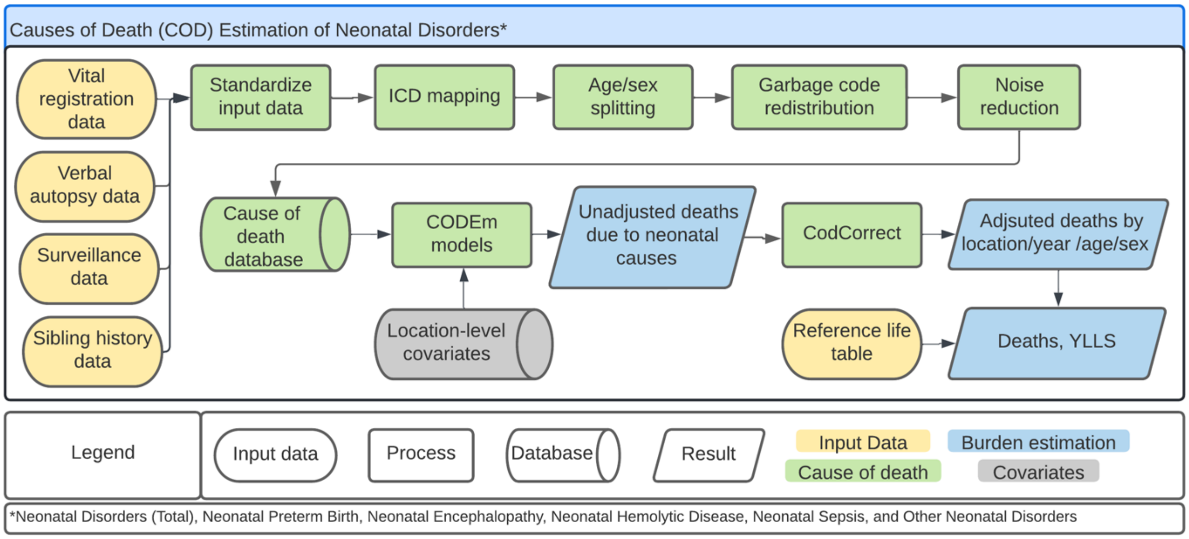

Input data and methodological summary for neonatal disorders

Mortality for five causes is modelled within "neonatal disorders": neonatal preterm birth complications, neonatal encephalopathy due to birth asphyxia and trauma, neonatal sepsis and other neonatal infections, haemolytic disease and other neonatal jaundice, and other neonatal disorders. An overall neonatal disorders "parent" envelope is also estimated, to which all neonatal causes are squeezed.

Of note, in GBD 2021, the ICD-10 codes for "small for gestational age" (P05.1) were assigned to the "neonatal preterm birth" category; past GBD rounds assigned them to "other neonatal disorders".

Input data

Vital registration and surveillance were the majority of data sources used for GBD 2021 to estimate number of deaths from each condition. Only deaths among males and females under age 5 were modelled, in four separate age groups: early neonatal period, late neonatal period, post-neonatal period, and 1-4 years. Datapoints were selected as outliers if they were implausibly high, low, or significantly conflicted with established age or temporal patterns.

As of GBD 2019, neonatal disorders began using data from the Child Health and Mortality Prevention Surveillance (CHAMPS) Program in Bangladesh, Kenya, Mozambique, South Africa, and Mali, which provides minimally invasive tissue sampling (MITS) data on under-5 causes of death. In GBD 2021, more MITS data were added to the models.

Though verbal autopsy data were available, validation studies suggest that verbal autopsy methods tend to be less accurate for cause of death ascertainment in the neonatal age groups.[1-4] Thus, for GBD 2021 the majority of verbal autopsy data were excluded. Verbal autopsy data were used, however, in the Indian states, in which they were the only data source.

Modelling strategy

The standard CODEm modelling approach was used to model each of the neonatal conditions. All neonatal causes used the following pool of covariates in covariate selection:

Table 1. Covariates used in neonatal disorders mortality modelling

Level	Covariate	Direction
1	Maternal care and immunisation	-
	Age-standardised SEV for ambient particulate matter	+
	Age-standardised SEV for household air pollution	+
	Age-standardised SEV for short gestation	+
	Age-standardised SEV for low birthweight	+
	Age-standardised SEV for smoking	+
2	Proportion of the population with at least 12 years of education, maternal	-
	Proportion of the population with at least 6 years of education, maternal	-
	Livebirths 35+ (proportion)	+
	Socio-demographic Index	-
	Healthcare Access and Quality Index	-
3	Antenatal care (1 visit) coverage (proportion)	-
	Antenatal care (4 visits) coverage (proportion)	-
	In-facility delivery (proportion)	-
	LDI (I$ per capita)	-
	Skilled birth attendance (proportion)	-
	Total fertility rate	+

References

1. Anker M, Black RE, Coldham C, *et al.* A Standard Verbal Autopsy Method for Investigating Causes of Death in Infants and Children. Geneva, Switzerland: World Health Organization Department of Communicable Disease Surveillance and Response; The Johns Hopkins School of Hygiene and Public Health; The London School of Hygiene and Tropical Medicine, 1999.

2. Kalter HD, Gray RH, Black RE, Gultiano SA. Validation of postmortem interviews to ascertain selected causes of death in children. *Int J Epidemiol* 1990; **19**: 380–6.

3. Quigley MA, Armstrong Schellenberg JR, Snow RW. Algorithms for verbal autopsies: a validation study in Kenyan children. *Bull World Health Organ* 1996; **74**: 147–54.

4. Snow RW, Armstrong JR, Forster D, *et al.* Childhood deaths in Africa: uses and limitations of verbal autopsies. *The Lancet* 1992; **340**: 351–5.

Non-rheumatic valvular heart disease

Non-rheumatic calcific aortic valve disease
Non-rheumatic degenerative mitral valve disease
Other non-rheumatic valvular heart diseases

Flowchart

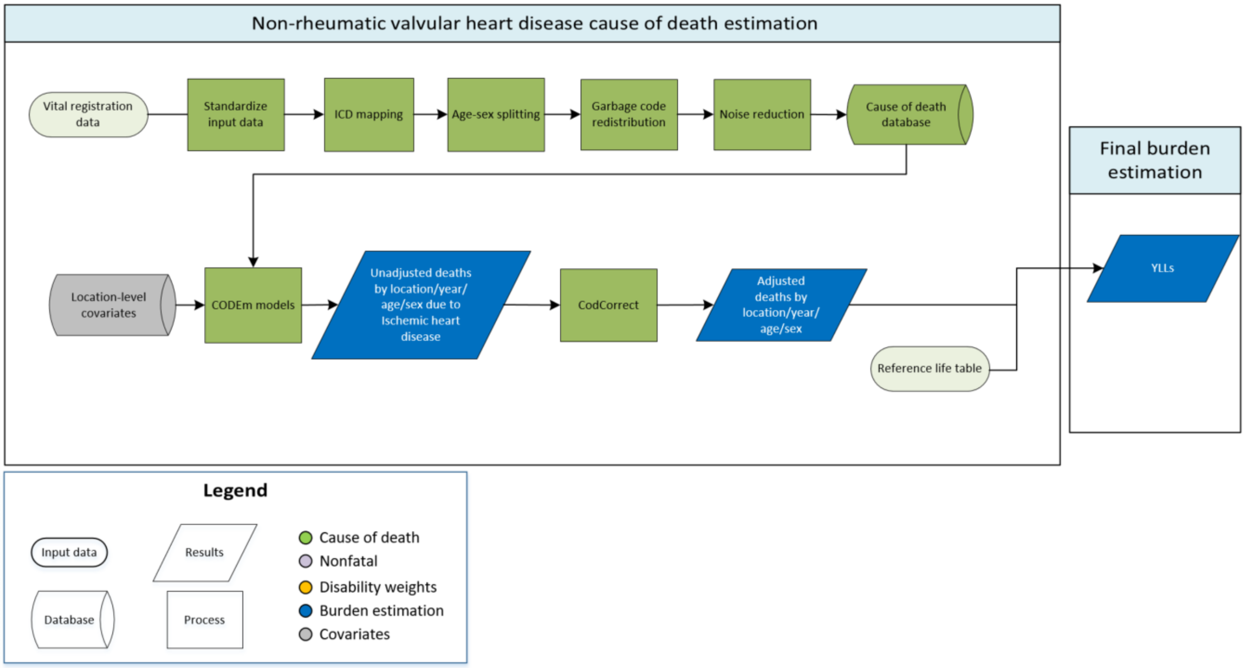

Input data and methodological summary for non-rheumatic valvular heart disease

Input data

Vital registration data were used to model non-rheumatic valvular heart disease, non-rheumatic calcific valve disease, non-rheumatic degenerative mitral valve disease, and other non-rheumatic valvular diseases. We outliered ICD8, ICD9BTL, and tabulated ICD10 datapoints which were inconsistent with the rest of the data and created implausible time trends. Datapoints from sources which were implausibly low in all age groups and datapoints that were causing the regional estimates to be improbably high were outliered. In addition, ICD9 data from Poland, Czechia, Greenland, and ICD10 data from France and Greenland with implausible values and discontinuous with the rest of the time series were outliered.

Modelling strategy

We used a standard CODEm approach to model deaths from non-rheumatic valvular heart disease, non-rheumatic calcific valve disease, non-rheumatic degenerative mitral valve disease, and other non-rheumatic valvular diseases. The covariates used in the GBD 2021 models, along with their importance levels, and imposed directions are reported by cause in the tables below. For GBD 2021, we changed the p value threshold used to select the covariates in the model for other non-rheumatic valvular diseases from 0.05 to 0.08. In addition, the methods used to redistribute hypertension deaths were updated for

GBD 2021. We switch from regressing redistribution codes against non-redistribution codes to the multiple cause of death redistribution approach. For details on the multiple cause of death redistribution methods, refer to the appendix section on redistribution. The method to estimate the minimum cause fraction or "the floor" across all location/years for a given cause/age/sex was also updated for GBD 2021. For causes like non-rheumatic valvular heart disease with a minimum raw cause fraction of 0, floors were generated for each cause/age/sex such that all floors for a given cause add up to one death globally per year. The distribution of the floors within a cause was determined by the cause of death age/sex weights. In addition, we updated our approach to noise reduction of the cause of death data so that very stochastic time series of data would resemble patterns seen at regional levels more closely. Details on this approach can be found elsewhere in the appendix.

Updating redistribution to using multiple cause of death analysis resulted in minimum changes in the number of hypertension deaths reassigned to non-rheumatic valve disease. However, updating the methods to estimate the floor led to a reduction in the number of deaths assigned to non-rheumatic valvular heart disease in comparison to GBD 2019.

Table 1. Covariates used in non-rheumatic valvular heart disease mortality modelling

Level	Covariate	Direction
1	Smoking prevalence	1
	Summary exposure value, non-rheumatic valve disease	1
	Systolic blood pressure (mm Hg)	1
2	Cholesterol (total, mean per capita)	1
	Mean BMI (kg/m^2)	1
	Healthcare Access and Quality Index	-1
3	Lag distributed income per capita (I$)	-1
	Socio-demographic Index	1
	Alcohol (litres per capita)	1

Table 2. Covariates used in non-rheumatic calcific aortic valve disease mortality modelling

Level	Covariate	Direction
1	Smoking prevalence	1
	Summary exposure value, non-rheumatic calcific aortic valve disease	1
	Systolic blood pressure (mm Hg)	1
2	Cholesterol (total, mean per capita)	1
	Mean BMI (kg/m^2)	1
	Healthcare Access and Quality Index	-1
	Fasting plasma glucose (mmol/L)	1
3	Lag distributed income per capita (I$)	-1
	Socio-demographic Index	1
	Alcohol (litres per capita)	1

Table 3. Covariates used in non-rheumatic degenerative mitral valve disease mortality modelling

Level	Covariate	Direction
1	Healthcare Access and Quality Index	-1
	Lag distributed income per capita (I$)	1
	Socio-demographic Index	1

Table 4. Covariates used in other non-rheumatic valvular heart diseases mortality modelling

Level	Covariate	Direction
1	Summary exposure value, non-rheumatic valve disease	1
	Healthcare Access and Quality Index	1
2	Socio-demographic Index	1

Nutritional deficiencies: *Parent nutritional deficiencies, protein-energy malnutrition, and other nutritional deficiencies*

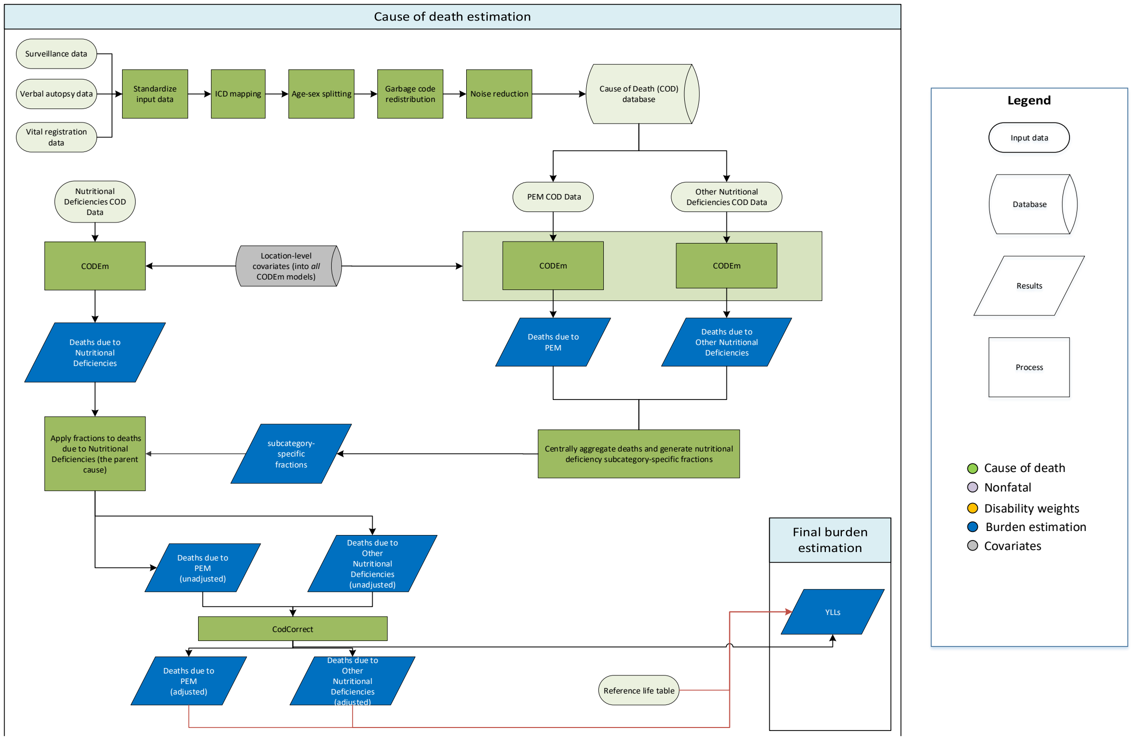

Input data and methodological summary for nutritional deficiencies

Input data

Vital registration (VR), verbal autopsy (VA), and surveillance data were used to model deaths due to nutritional deficiencies. We outliered data that were largely conflicting with the majority of data from other studies conducted either in the same countries or different countries (with similar socio-demographic characteristics) in the same region. ICD codes, which can be interpreted as case definitions, for each of the nutritional deficiencies are listed in Table 1 below.

Table 1. ICD-10 codes included in the nutritional deficiency models

GBD cause	ICD-10 code
Protein-energy malnutrition	E40-E46.9 (Kwashiorkor, marasmus, specified and unspecified protein-calorie malnutrition)
Other nutritional deficiencies	D51-D52.0 (vitamin B12 deficiency anaemia and folate deficiency anaemia)
Other nutritional deficiencies	D52.8-D53.9 (other nutritional anaemias)
Other nutritional deficiencies	D64.3 (other sideroblastic anaemias)

Other nutritional deficiencies	E51-E61.9 (thiamine, niacin, other B group vitamins, ascorbic acid, vitamin D, other vitamin, dietary calcium, dietary selenium, dietary zinc, and other nutrient element deficiencies)
Other nutritional deficiencies	E63-E64.0 (other nutritional deficiencies and sequelae of protein-calorie malnutrition)
Other nutritional deficiencies	E64.2-E64.9 (sequelae of vitamin C deficiency, rickets, other nutritional deficiencies, and unspecified nutritional deficiencies)
Other nutritional deficiencies	M12.1-M12.19 (Kashin-Beck disease)
Garbage code	D50, D50.0 and D50.9 (unspecified anaemia)

Modelling strategy

Data and data processing methods were updated centrally by the cause of death team for GBD 2021. Apart from these updates, we did not make any modelling strategy changes this cycle.

We estimated mortality for the nutritional deficiencies in two steps. CODEm was first used to generate mortality estimates for total nutritional deficiencies. The sub-categories of nutritional deficiencies, protein-energy malnutrition, and other nutritional deficiencies were modelled individually. Protein-energy malnutrition was modelled separately for age groups under 5 and over 5 so that the data trends and patterns in children under 5 were accurately captured. Estimates from the two nutritional sub-categories were then scaled at the 1000-draw level in CoDCorrect to match that for total nutritional deficiencies.

The CODEm covariates (including level and direction) used for each of the models are listed in the table below.

Table 2. Covariates used in mortality modelling

Nutritional deficiencies (overall)		
**Level**	**Covariate**	**Direction**
1	Age-standardised prevalence of severe anaemia	+
	Age-standardised SEV for child underweight	+
	Age-standardised SEV for child wasting	+
	Proportion of households using iodised salt	–
	Total kcal per person per day availability	–
2	Population living in the first world quintile (least) of annual rainfall	+
	Population living in the second world quintile (second least) of annual rainfall	+
	Unsafe sanitation SEV	+
	Unsafe water SEV	+
	Log-transformed diarrhoeal diseases SEV	+
	Mortality rate due to war shocks	+
	Healthcare Access and Quality Index	–
	Age- and sex-specific SEV for alcohol and drug use	+
	Maternal care and immunisation	–
3	Education (years per capita)	–

	Lag-distributed income per capita	–
	Socio-demographic Index	–
	Maternal education (years per capita)	–
**Protein-energy malnutrition**		
**Level**	**Covariate**	**Direction**
1	Age-standardised prevalence of severe anaemia	+
	Total kcal per person per day availability	–
	Age-standardised SEV for child wasting	+
2	Population living in the first world quintile (least) of annual rainfall	+
	Population living in the second world quintile (second least) of annual rainfall	+
	Unsafe sanitation SEV	+
	Unsafe water SEV	+
	Log-transformed diarrhoeal diseases SEV	+
	Mortality rate due to war shocks	+
	Healthcare Access and Quality Index	–
	Age- and sex-specific SEV for alcohol and alcohol use	+
	Maternal care and immunisation	–
3	Antenatal care (4 visits) coverage proportion	–
	Education (years per capita)	–
	Lag-distributed income per capita	–
	Socio-demographic Index	–
**Other nutritional deficiencies**		
**Level**	**Covariate**	**Direction**
1	Age-standardised prevalence of severe anaemia	+
	Total kcal per person per day availability	–
	Age-standardised SEV for child underweight	+
2	Population living in the first world quintile (least) of annual rainfall	+
	Population living in the second world quintile (second least) of annual rainfall	+
	Unsafe sanitation SEV	+
	Unsafe water SEV	+
	Log-transformed diarrhoeal diseases SEV	+
	Mortality rate due to war shocks	+
	Healthcare Access and Quality Index	–
	Age- and sex-specific SEV for alcohol use	+
	Maternal care and immunisation	–
3	Education (years per capita)	–
	Lag-distributed income per capita	–
	Socio-demographic Index	–

Opioid use disorders

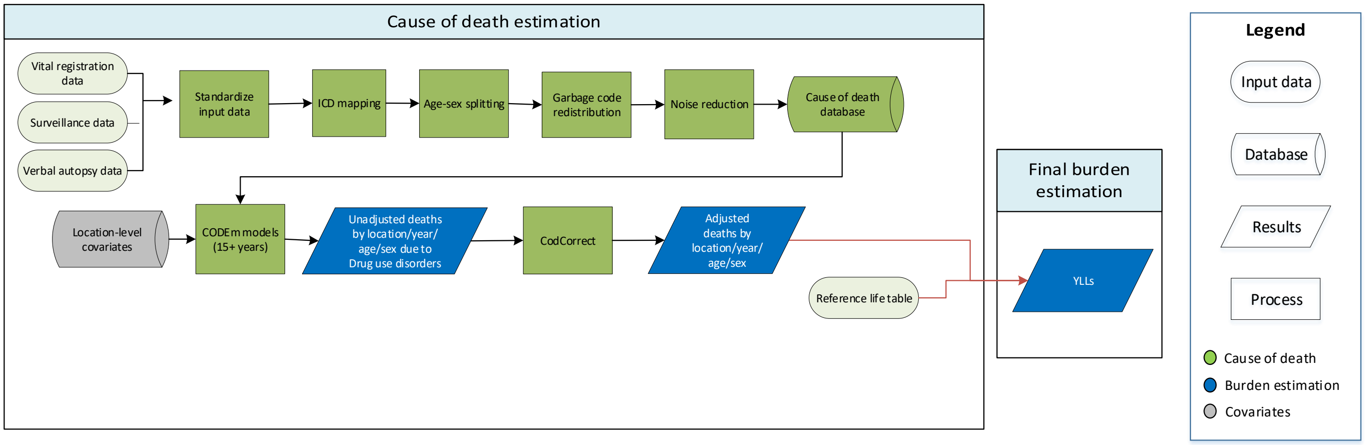

Input data and methodological summary for opioid use disorders

Input data

All input data were from vital registration and surveillance sources. Data from countries with sparse yet heterogeneous data were excluded as the data exaggerated fluctuations in deaths and gave implausible regional patterns, according to in-country and subject matter experts. Excluded data were typically from low- and middle-income countries. The locations for which there was the most data included North America, Australia, Western Europe, and parts of Latin America.

Modelling strategy

The standard Cause of Death Ensemble modelling (CODEm)[1] approach (detailed in reference appendix section 3.1) was used to estimate deaths due to opioid use disorders. Several covariates are particularly important for the opioid use disorder models to be able to capture the rapid increases in opioid use disorder deaths recently observed in the United States. These include intravenous drug use prevalence from the model used to estimate exposure for the drug use as a risk analyses, and opioid consumption per million inhabitants per day. The latter covariate was derived from data from the International Narcotics Control Board (INCB) which measures *"defined daily doses for statistical purposes" (S-DDD),* which translates all different opioids of different types and dosages into comparable units to quantify consumption in different countries.

Key changes from GBD 2021:

- The intravenous drug use covariate incorporated additional data and increased time smoothing, which increased estimates in the United States and Western Europe and made the yearly change more consistent over time.
- We removed Socio-demographic Index, education, and LDI covariates that were causing estimates to diverge from the data in the USA and Canada.

Table 1: Covariates used in opioid use CODEm model

Level	Covariate	Direction
1	Intravenous drug use, age-standardised	+
	Intravenous drug use, age-specific	+
	Opioid standard doses per million per day (10-year lag)	+
2	Cumulative cigarettes (10 years)	+
	Cumulative cigarettes (5 years)	+
	Opium cultivation bin	+
	Smoking prevalence	+
	Healthcare Access and Quality Index	-

Opioid use disorder is a “child’ disease that is fit into an overall “parent” drug use disorders model. The unadjusted death estimates from opioid use disorders are summed alongside other “child” causes (opioid, cocaine, amphetamines, and other drugs) and fit to the distribution of deaths in an overall drug use disorders “parent” model as part of the CoDCorrect adjustment process. This results in deaths recorded using non-specific coding systems, such as verbal autopsy, being included in the parent model and redistributed to the child models proportionately.

Covariate influences:

The following plots show the influence of each covariate on the four CODEm models (male global, male data-rich, female global, and female data-rich). A positive standardised beta (to the right) means that the covariate was associated with increased death. A negative standardised beta (to the left) means the covariate was associated with decreased death.

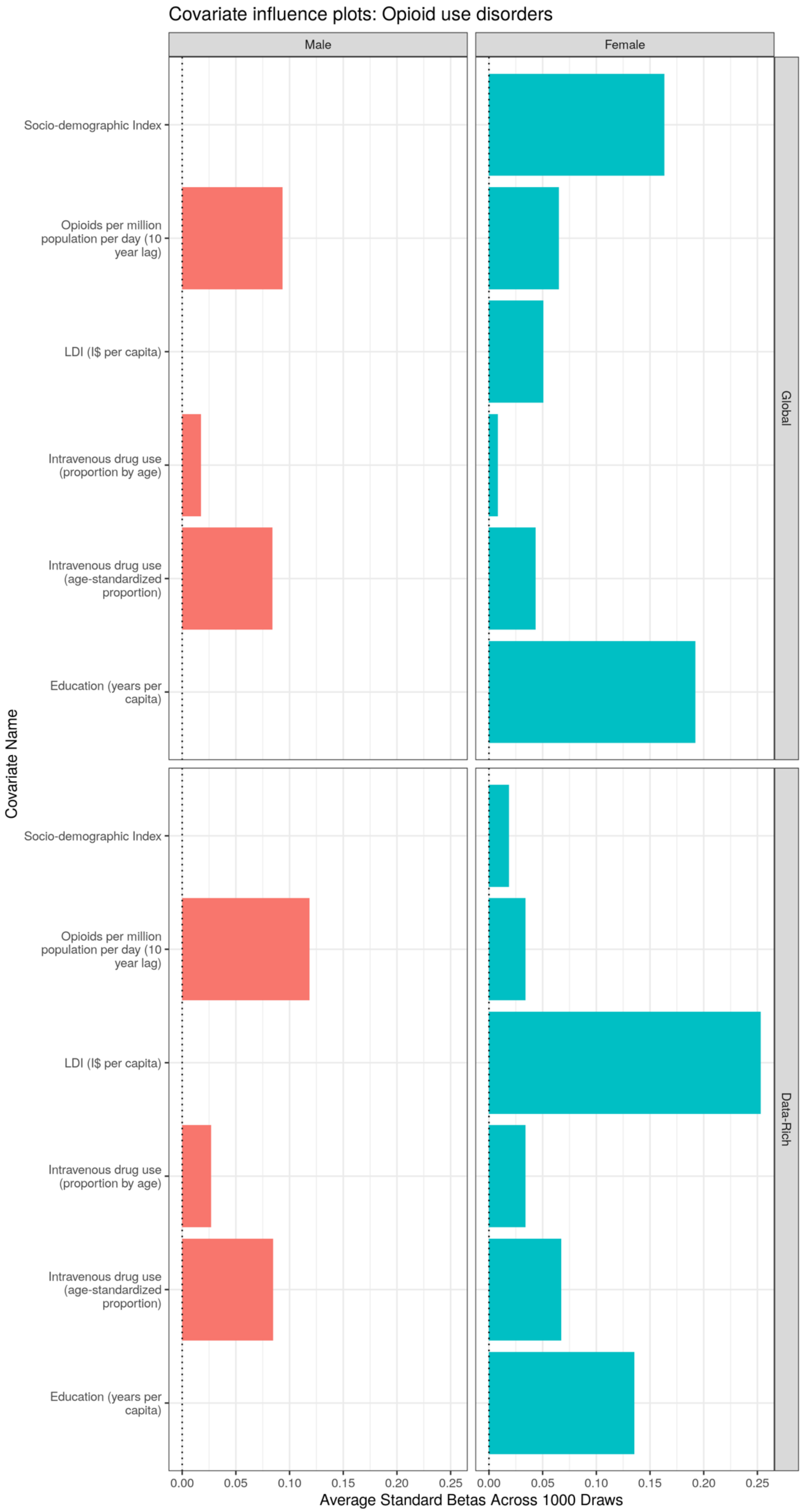

Covariate influence plots: Opioid use disorders
Male
Female
Global
Data-Rich
Covariate Name
Socio-demographic Index
Opioids per million population per day (10 year lag)
LDI (I$ per capita)
Intravenous drug use (proportion by age)
Intravenous drug use (age-standardized proportion)
Education (years per capita)
0.00
0.05
0.10
0.15
0.20
0.25
Average Standard Betas Across 1000 Draws

[1] Vos T, Lim SS, Abbafati C, *et al.* Global burden of 369 diseases and injuries in 204 countries and territories, 1990–2019: a systematic analysis for the Global Burden of Disease Study 2019. *The Lancet* 2020; 396**:** 1204–22. doi: https://doi.org/10.1016/S0140-6736(20)30925-9

Other cardiomyopathy

Flowchart

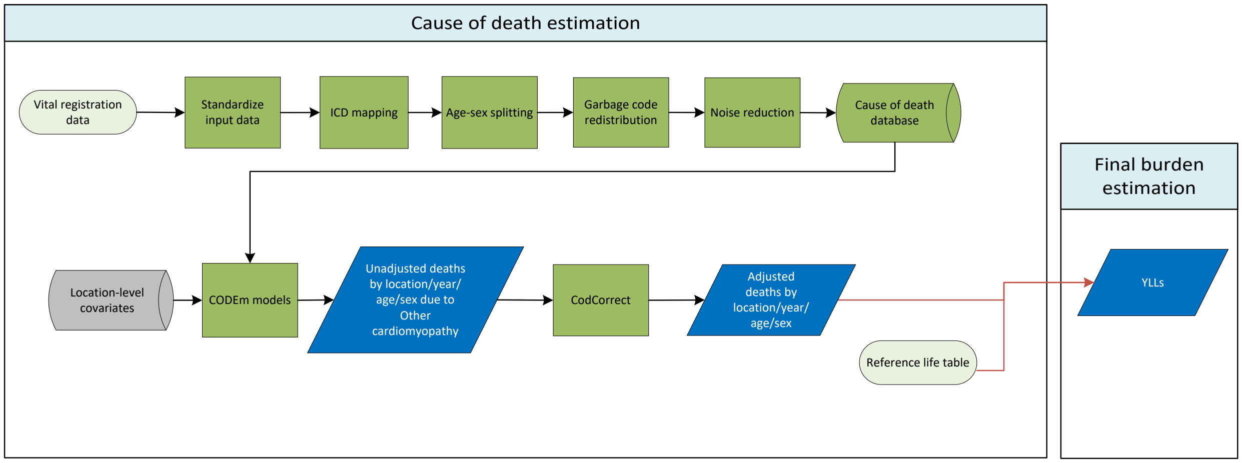

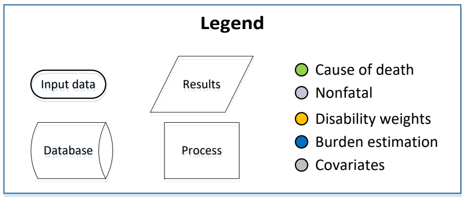

Input data and methodological summary for other cardiomyopathy

Input data

Vital registration data were used to model deaths due to other cardiomyopathy. We outliered datapoints in central Asia and central and eastern Europe and Egypt due to implausibly high values, which we attributed to variation in local coding practices after review with experts. In addition, we outliered a datapoint in India that was implausibly low compared to other locations in south Asia.

Modelling strategy

We used a standard CODEm approach to model deaths from other cardiomyopathy. The covariates selected for inclusion in the CODEm modelling process can be found in the table below. For GBD 2021, we changed the ICD mapping of ICD9 code 425.4 ("other primary cardiomyopathies") from being partially redistributed to alcoholic cardiomyopathy into solely other cardiomyopathy. This resulted in decreasing the number of deaths due to alcoholic cardiomyopathy and increasing the number of deaths attributed to other cardiomyopathy as the underlying cause of death. In addition, the method used to reduce the noise in the data, implemented after redistribution to handle both the stochastic variation across time and space and the occurrence of small number of deaths in each location/year/age/sex, was updated. This new empirical Bayesian noise reduction algorithm uses grouped data by region and data type as a prior to better reflect regional patterns. A detailed description on the updates to noise reduction can be found in the cause of death methods section of the appendix.

Table 1. Covariates used in other cardiomyopathy mortality modelling

Level	Covariate	Direction
1	Summary exposure variable, CMP	1
	Systolic blood pressure (mm Hg)	1
	Smoking prevalence	1
2	Body mass index (kg/m^2)	1
	Healthcare Access and Quality Index	-1
3	Log-transformed lag distributed income per capita (I$)	-1
	Socio-demographic Index	1

Other cardiovascular and circulatory diseases

Flowchart

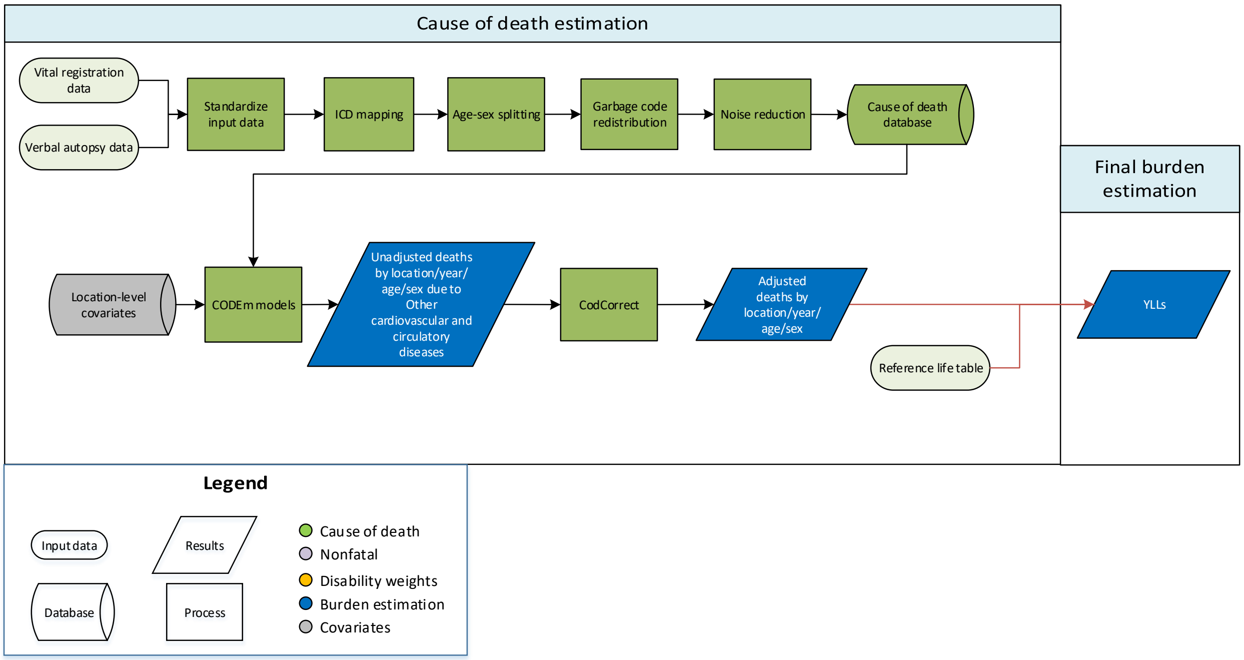

Input data and methodological summary

Input data

Vital registration and verbal autopsy data were used to model other cardiovascular and circulatory diseases. In 2020, ICD codes for pulmonary arterial hypertension (ICD-10 code I27.0 and ICD-9 code 416) were removed from this cause, as pulmonary arterial hypertension is modelled separately. We outliered ICD8 and ICD9 BTL datapoints that were inconsistent with the rest of the data and created implausible time trends. We also outliered ICD8 datapoints which were not nationally representative. In addition, we outliered ICD 10 datapoints from Oman in all age groups that were causing the regional estimates to be improbably high.

Modelling strategy

We used a standard CODEm approach to model deaths from other circulatory and cardiovascular diseases. Covariates selected for inclusion in the ensemble model are listed in the table below. Multiple cause of death data were used to redistribute deaths originally coded to heart failure and other intermediate or implausible causes of death. For details on the multiple cause of death redistribution methods, refer to the appendix section on redistribution. In GBD 2021, adjusted dietary covariates for consumption of fruits, omega-3 fatty acids, vegetables, nuts and seeds, and polyunsaturated fatty acids were replaced with the summary exposure value scalars for diet low in each of these factors. The direction for each dietary covariate was changed from -1 to 1 to as our *a priori* assumption is that low levels of intake of these dietary factors are associated with increasing mortality risk from other

cardiovascular disease. For GBD 2021 we updated our approach to noise reduction of the cause of death data so that stochastic time series of data would resemble patterns seen at regional levels more closely. Additional details on the updates to noise reduction can be found in the cause of death methods section of the appendix. No other significant changes were made from GBD 2019.

Table 1. Covariates used in other cardiovascular diseases mortality modelling

Level	Covariate	Direction
1	Summary exposure value (SEV) other CVD	1
	LDL Cholesterol (mean per capita)	1
	Smoking prevalence	1
	Systolic blood pressure (mm Hg)	1
2	Mean Body mass index (kg/m^2)	1
	Elevation over 1,500m (proportion)	-1
	Fasting plasma glucose	1
	Outdoor pollution ($PM_{2.5}$)	1
	Indoor air pollution	1
	Healthcare Access and Quality Index	-1
3	Log-transformed lag distributed income per capita (I$)	-1
	Summary exposure value, omega-3	1
	Summary exposure value, fruits	1
	Summary exposure value, vegetables	1
	Summary exposure value, nuts and seeds	-1
	Summary exposure value, low legumes	1
	Summary exposure value, PUFA adjusted (percent)	1
	Alcohol (litres per capita)	1

Other chronic respiratory diseases

In addition to the chronic respiratory diseases described above, there are other types of chronic respiratory diseases with a range of severities and associated sequelae. Because these chronic respiratory diseases are diverse in their underlying causes and risk factors as well as in their associated health outcomes, modelling them together in a DisMod-MR model would not produce reliable estimates of prevalence. Instead, we calculated the YLDs caused by other chronic respiratory diseases directly using a YLD/YLL ratio as a "place holder".

We calculated the ratio of YLDs to YLLs across the specified chronic respiratory diseases for which non-fatal outcomes were modelled, using YLL estimates from the GBD 2021 cause of death (CoD) analysis. We then multiplied this YLD/YLL ratio by the YLL estimates for other chronic respiratory diseases.

Other digestive diseases

Flowchart

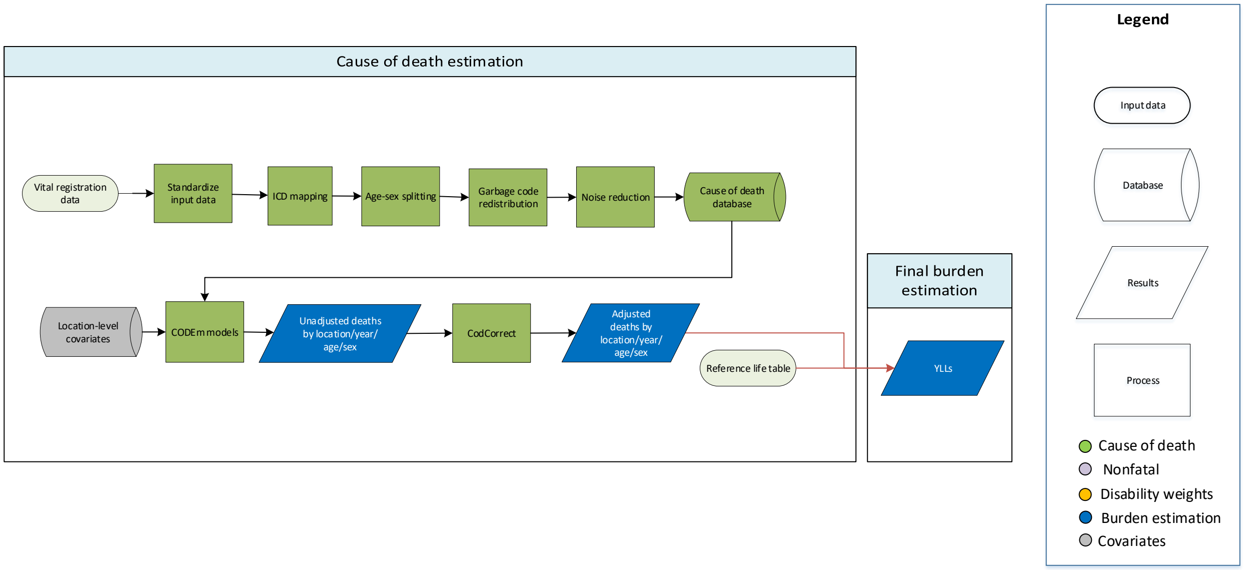

Input data and methodological summary for other digestive diseases

Input data

Data used to estimate mortality of other digestive diseases consisted of vital registration data from the cause of death (COD) database (see appendix section on ICD mapping for details). The data in other digestive diseases consist of unique datapoints from deaths reported with a set of non-specific digestive disease codes (see appendix section on ICD mapping for details). Outliers were identified by systematic examination of datapoints for all location-years. Data were excluded if they violated well-established age or time trends, and data in instances where garbage code redistribution and noise reduction, in combination with small sample sizes, resulted in unreasonable cause fractions.

Modelling strategy

The estimation strategy used for fatal other digestive diseases is largely similar to methods used in GBD 2019. A standard CODEm model with location-level covariates was used to model deaths due to other digestive diseases (see appendix section on CODEm method for details). Separate models were conducted for male and female mortality, and age restrictions for death estimations included 12 months for lower bound and 95+ years for upper bound. We hybridised separate global and data-rich models to acquire unadjusted results, which we adjusted using CoDCorrect and compared to the reference life table to calculate final YLLs due to other digestive diseases.

In GBD 2021, we updated the linear floor value in our CODEm models to allow the model to be influenced by lower data, which resulted from changes to COD data processing. These data processing changes include changing the method of estimating the non-zero floor and population size cutoffs for when to noise-reduce at the country versus region level, and are described in greater detail in the appendix section “GBD 2021 Causes of Death database”. Apart from these, no other substantive changes were made in GBD 2021 from the modelling strategy used in GBD 2019.

The following table has the full list of covariates used for other digestive diseases.

Table 1. Covariates used in other digestive diseases mortality modelling

Level	Covariate	Direction
1	Cumulative cigarettes (10 years)	+
	Cumulative cigarettes (5 years)	+
	Smoking prevalence	+
	Alcohol (litres per capita)	+
2	Diabetes age-standardised prevalence (proportion)	+
	BMI (mean)	+
	Sanitation (proportion with access)	-
	Improved water source (proportion with access)	-
	Age-sex-specific scaled exposure variable for low polyunsaturated fatty acids consumption	+
	Age-sex-specific scaled exposure variable for low fruit consumption	+
	Age-sex-specific scaled exposure variable for low vegetable consumption	+
	Age-sex-specific scaled exposure variable for high red meat consumption	+
	Healthcare Access and Quality Index	-
3	Socio-demographic Index	-
	Education (years per capita)	-
	Log LDI ($I per capita)	-

Adjustment in CoDCorrect included fitting unadjusted death estimates for all other specific and non-specific digestive diseases to overall digestive disease deaths, which was then adjusted with all other causes to sum to all-cause counts of death.

Other drug use disorders

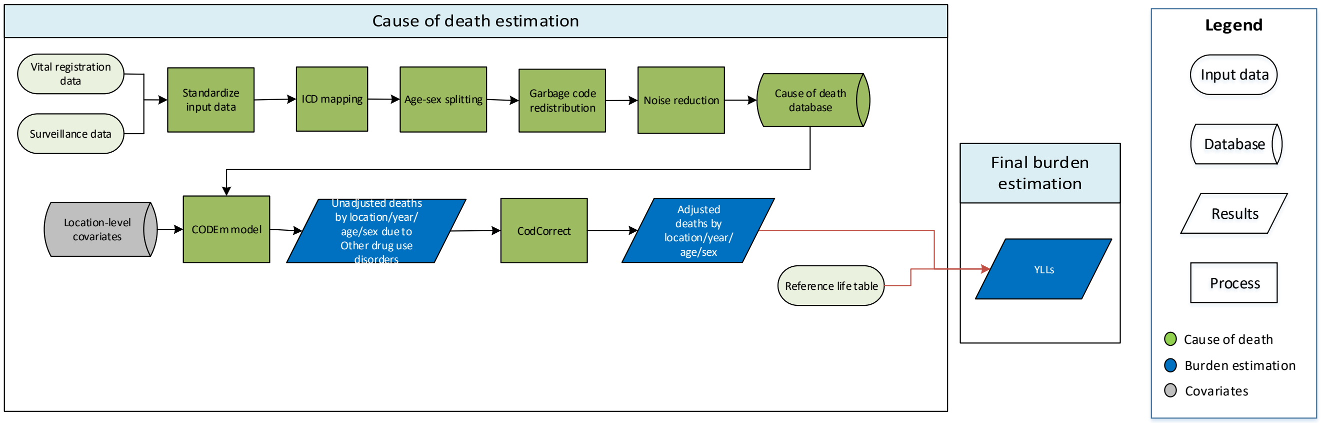

Input data and methodological summary for other drug use disorders

Input data

All data were from vital registration and surveillance sources. Data from countries with sparse yet heterogeneous data were excluded as the data exaggerated fluctuations in deaths and gave implausible regional patterns. Excluded data were typically from lower-income countries. A full description of changes to coding and redistribution are described in the appendix section focusing on aggregate drug use disorders

Modelling strategy

The standard Cause of Death Ensemble modelling (CODEm)[1] approach (detailed in reference appendix section 3.1) was used to estimate deaths due to cocaine use disorders. Model covariate inclusion was based on empirical evidence and expert feedback, which resulted in a set of model covariates that reflected alcohol consumption, smoking, education, health system access, domestic income, and Socio-demographic Index (SDI) (Table 1).

Key changes from GBD 2021:

- The intravenous drug use covariate incorporated additional data and increased time smoothing, which increased estimates in the United States and Western Europe and made the yearly change more consistent over time.

Table 1: Covariates used in other drug use CODEm model

Level	Covariate	Direction
1	Alcohol (litres per capita)	+
	Current drinking prevalence	+
	Intravenous drug use, age-standardised	+
	Intravenous drug use, age-specific	+
	Cumulative cigarettes (10 years)	+
	Cumulative cigarettes (5 years)	+
	Cigarettes per capita	+
	Smoking prevalence	+
2	Healthcare Access and Quality Index	-
3	log LDI (I$ per capita)	+
	education (years per capita)	+
	Socio-demographic Index	+

Other drug use disorder is a “child’ disease that is fit into an overall “parent” drug use disorders model. The unadjusted death estimates from other drug use disorders are summed alongside other “child” causes (opioid, amphetamine, cocaine) and fit to the distribution of deaths in an overall drug use disorders “parent” model as part of the CoDCorrect adjustment process.[1] This results in deaths recorded using non-specific coding systems, such as verbal autopsy, being included in the parent model and redistributed to the child models proportionately.

Covariate influences:

The following plots show the influence of each covariate on the four CODEm models (male global, male data-rich, female global, and female data-rich). A positive standardised beta (to the right) means that the covariate was associated with increased death. A negative standardised beta (to the left) means the covariate was associated with decreased death.

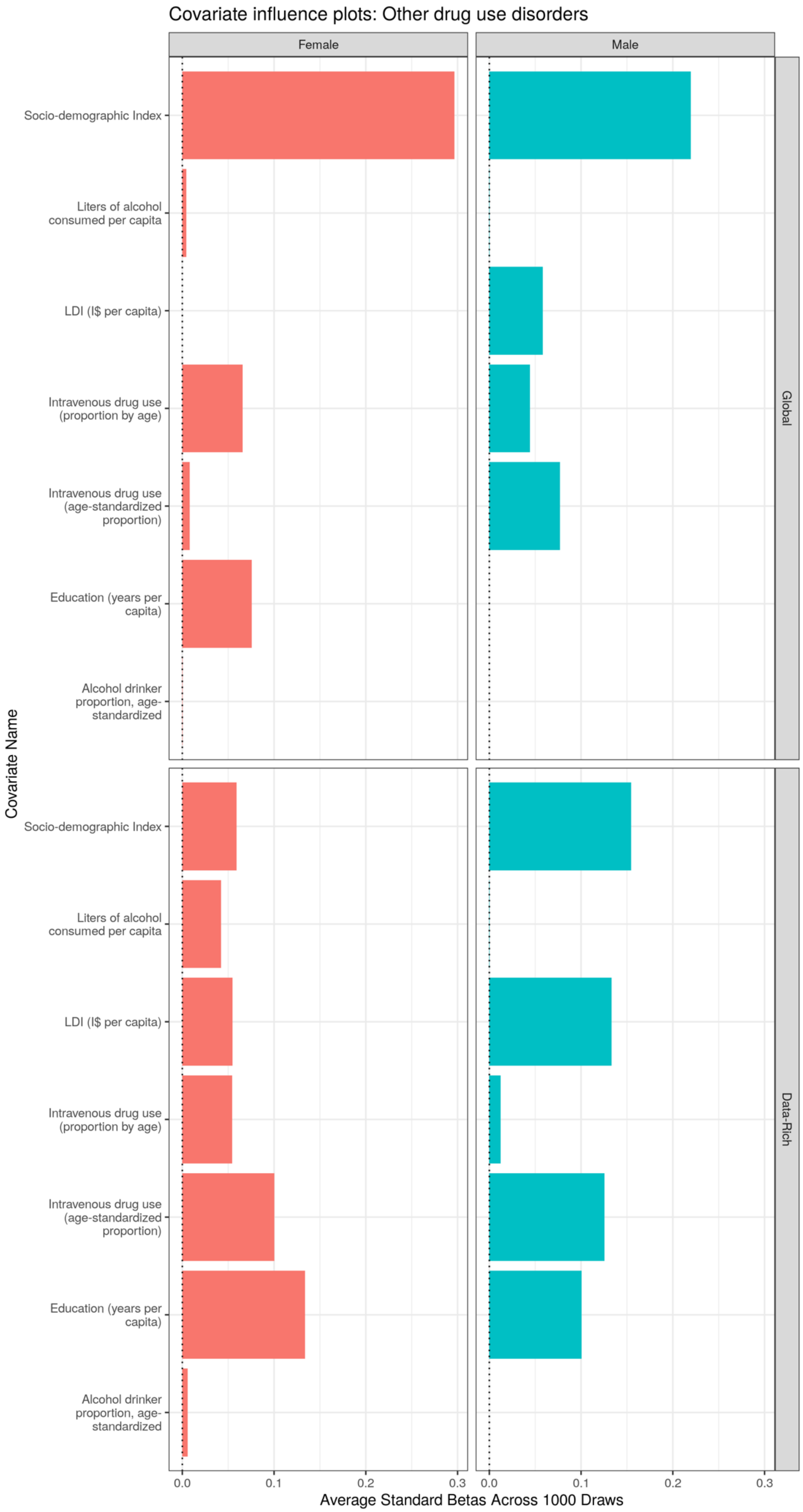

Covariate influence plots: Other drug use disorders
Female
Male
Global
Data-Rich
Covariate Name
Socio-demographic Index
Liters of alcohol consumed per capita
LDI (I$ per capita)
Intravenous drug use (proportion by age)
Intravenous drug use (age-standardized proportion)
Education (years per capita)
Alcohol drinker proportion, age-standardized
0.0
0.1
0.2
0.3
Average Standard Betas Across 1000 Draws

[1] Vos T, Lim SS, Abbafati C, *et al.* Global burden of 369 diseases and injuries in 204 countries and territories, 1990–2019: a systematic analysis for the Global Burden of Disease Study 2019. *The Lancet* 2020; **396**: 1204–22. doi: https://doi.org/10.1016/S0140-6736(20)30925-9

Other intestinal infectious diseases

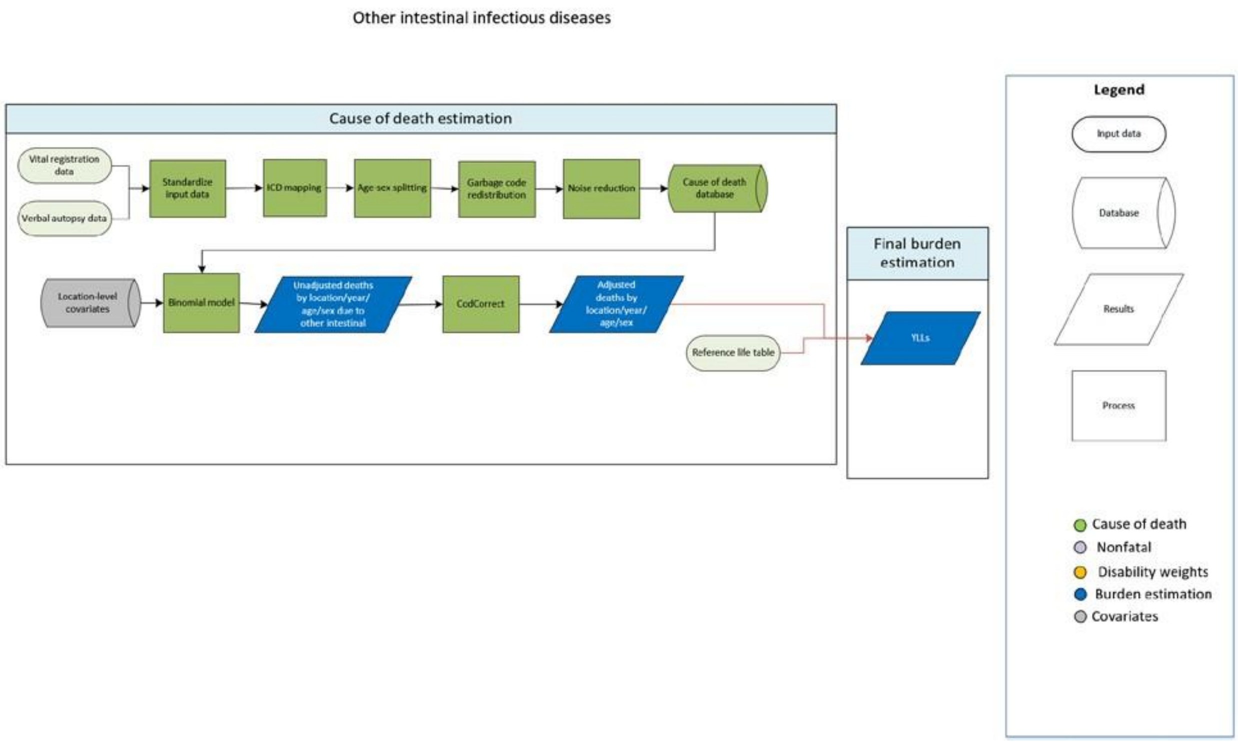

Input data

We modelled other intestinal infectious disease mortality using all available data in the cause of death (CoD) database. Datapoints were outliered if they reported an improbable number of deaths or if their inclusion in the model yielded distorted trends. In some cases, multiple data sources for the same location differed dramatically in both their quality and reported other intestinal infectious disease mortality (eg, a verbal autopsy and vital registration source). In these cases, the lower-quality data source was outliered.

Modelling strategy

We modelled other intestinal infectious disease mortality using a custom binomial model of all data in the CoD database. The custom model was used because of very small death counts. We used the number of cause-specific deaths as the outcome, with the all-cause mortality envelope as the exposure term. We included the square root of Socio-demographic Index, age group, and sex as covariates, and included a random effect on region.

We have made no substantive changes to the modelling strategy in GBD 2021.

Other musculoskeletal disorders

Flowchart

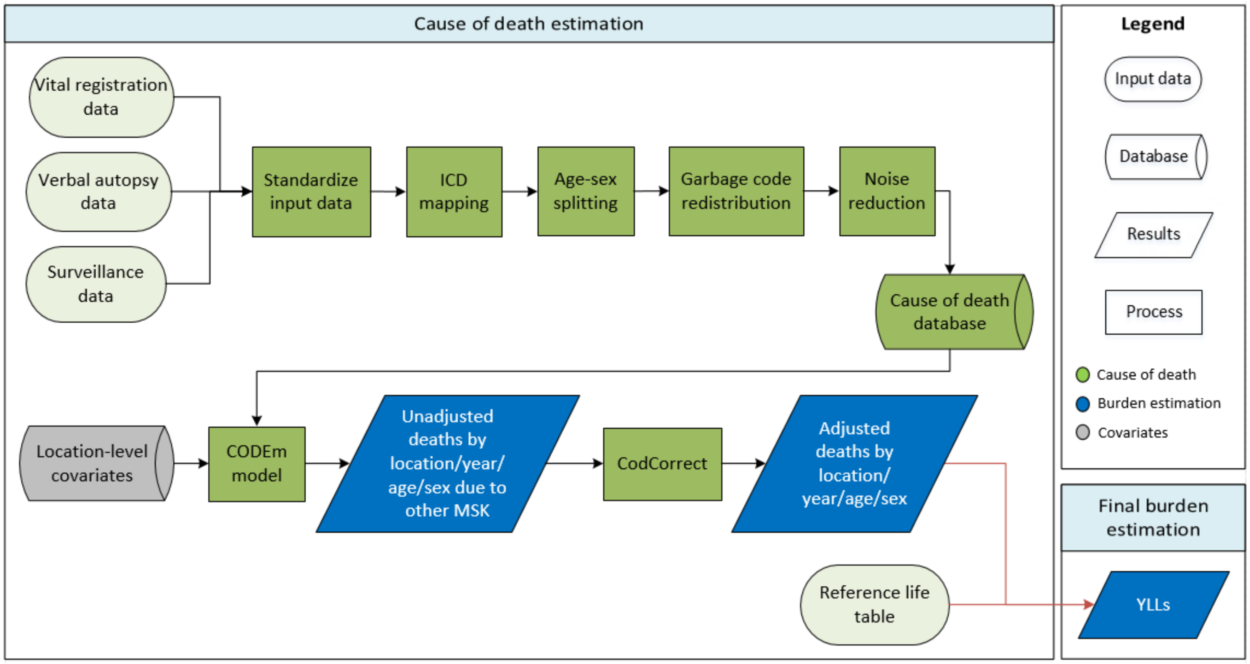

Input data and methodological summary for other musculoskeletal disorders

Input data

Data used to estimate mortality of other musculoskeletal disorders (MSK) included vital registration and China disease surveillance data from the cause of death database. Our outlier criteria excluded datapoints that were (1) implausibly high or low relative to global or regional patterns according to subject matter experts, (2) substantially conflicted with established age or temporal patterns, (3) significantly conflicted with other data sources from the same locations or locations with similar characteristics (ie, Socio-demographic Index), or (4) from verbal autopsy sources due to the inability of verbal autopsy to accurately capture most musculoskeletal conditions.

Globally, 60% of all ICD-10-coded deaths for other MSK were coded to autoimmune disorders (like systemic lupus erythematosus and systemic sclerosis), 21% to osteoporosis, 7% to pyogenic arthritis, and 4% to spinal deformities.

Modelling strategy

The standard CODEm (Cause of Death Ensemble model) modelling approach was applied to estimate deaths due to other musculoskeletal disorders, and general methods are described elsewhere.[1] We applied the same covariates used in GBD 2019. The CODEm model for other musculoskeletal disorders is

[1] *Vos T, Lim SS, Abbafati C, et al. Global Burden of 369 Diseases and Injuries in 204 Countries and Territories, 1990–2019: A Systematic Analysis for the Global Burden of Disease Study 2019. The Lancet 2020; 396: 1204–22. Doi: Https://Doi.Org/10.1016/S0140-6736(20)30925-9. Details Found in Appendix 1, Section 3*.

limited by a lack of strong predictive covariates. Many are selected as a proxy for Socio-demographic Index (SDI), as many other musculoskeletal disorders are autoimmune conditions whose prevalence is expected to increase with SDI. The following table lists the covariates included in the model. This requires that the covariate selected for the model must have the directional relationship with other musculoskeletal disorder deaths. Covariate directions were selected based on the strength of the evidence.

Table 1. Covariates used in other MSK mortality modelling

Level	Covariate	Direction
1	Mean BMI	+
	Vegetables (g), unadjusted	-
	Alcohol consumption (litres per capita)	+
2	Cumulative cigarettes (10 years)	+
	Cumulative cigarettes (5 years)	+
	Education (years per capita)	+
	Log-transformed LDI: lag-distributed income ($ per capita)	+
	Mean cholesterol[2]	+
	Smoking prevalence	+
	Healthcare Access and Quality Index	-
3	SDI: Socio-demographic Index	+

[2] This covariate is quantified based on LDL cholesterol.

Covariate influences:

The following plot shows the influence of each covariate on the four CODEm models (male global, male data-rich, female global, and female data-rich). A positive standardised beta (to the right) means that the covariate was associated with increased death. A negative standardised beta (to the left) means the covariate was associated with decreased death.

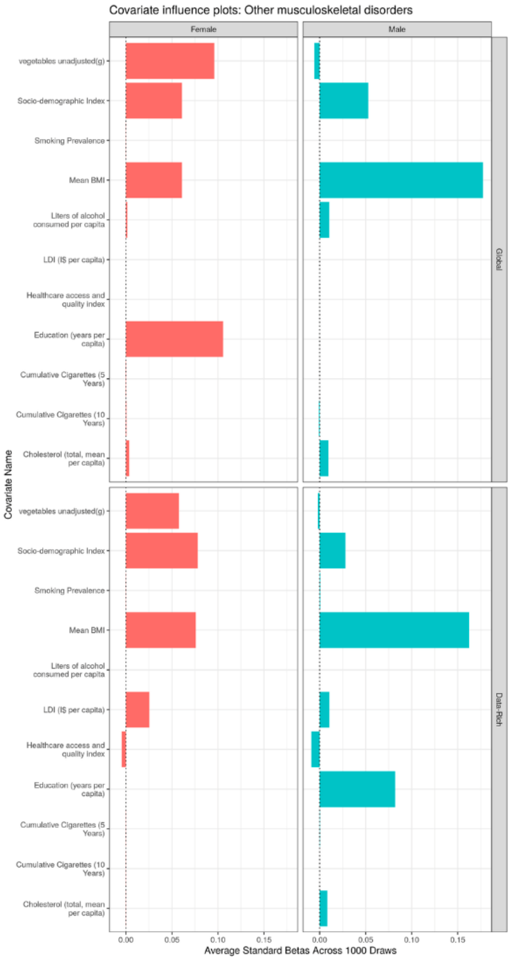

Other neurological disorders

Flowchart

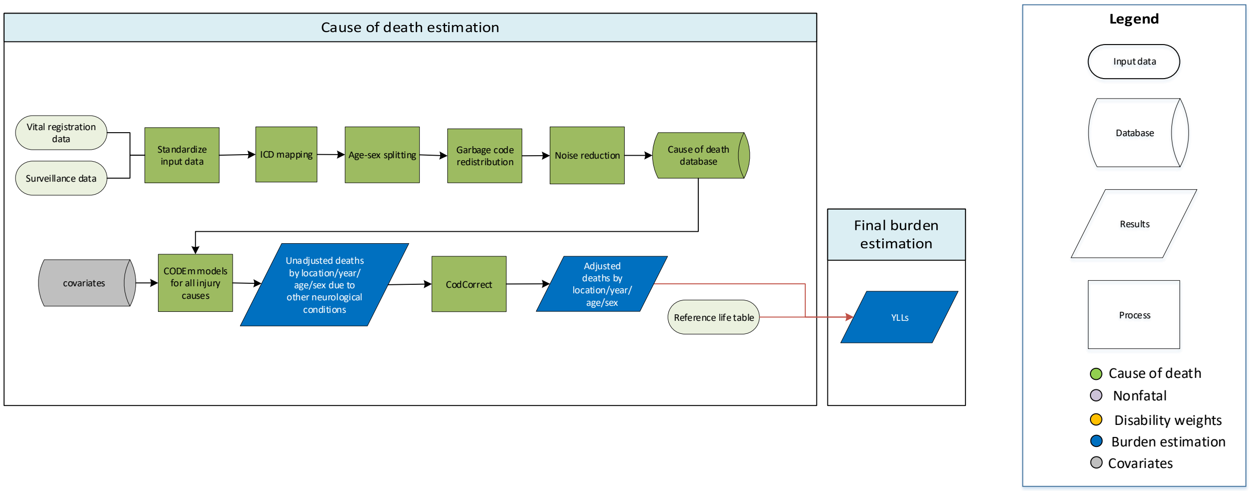

Input data and methodological summary for other neurological disorders

Input data

Data used to estimate other neurological disorders included vital registration and surveillance data from the cause of death (CoD) database. Our outlier criteria were to exclude datapoints that (1) were implausibly high or low, (2) substantially conflicted with established age or temporal patterns, or (3) significantly conflicted with other data sources conducted from the same locations or locations with similar characteristics (ie, Socio-demographic Index). In particular,

- Data excluded as outliers in GBD 2019 continued to be excluded in GBD 2021.
- ICD-10 data were available for Kazakhstan for 2013 onward but were marked as outliers as the raw data were ten-fold greater than the previously modelled mean.
- Similarly, Brunei data from 2011 to 2018 were marked as outliers because they were more than three-fold higher than the median for countries in the high-income Asia Pacific countries.

Modelling strategy

The standard Cause of Death Ensemble model (CODEm)[1] modelling approach (as described in the reference appendix section 3.1) was used to estimate deaths due to other neurological conditions. Separate models were conducted for male and female mortality, and the age range for both models was 28 days to 95+ years. The full list of covariates used in GBD 2021 is displayed below. Unadjusted death estimates were adjusted using CoDCorrect to produce final estimates of years of life lost (YLLs).[1] See appendix section 3.1 of the reference article.

The following table lists the covariates included in the model. This requires that the covariate selected for the model must have the directional relationship with other neurological disorders deaths. For GBD

2020, no significant updates were made for other neurological disorders covariate selection. Covariate directions were selected based on the strength of the evidence.

Table 1. Covariates used in other neurological disorders mortality modelling

Level	Covariate	Direction
1	Mean total body-mass index	+
	Mean serum total cholesterol (mmol/L)	+
	Mean systolic blood pressure (mm/Hg)	+
	Pigs per capita	+
	Age-standardised SEV for underweight children	+
	Red meat consumption, adjusted	+
2	Population density over 1000 per square kilometer pct	+
	Healthcare Access and Quality Index	-
	Fruit consumption (grams per day, adjusted)	-
3	Cumulative cigarettes (10 years)	+
	Cumulative cigarettes (5 years)	+
	Education (years per capita)	-
	Log-transformed LDI (per capita)	-
	Smoking prevalence	+
	Socio-demographic Index	+

The following plots show the influence of each covariate on the four CODEm models (male global, male data-rich, female global, and female data-rich). A positive standardised beta (to the right) means that the covariate was associated with increased death. A negative standardised beta (to the left) means the covariate was associated with decreased death.

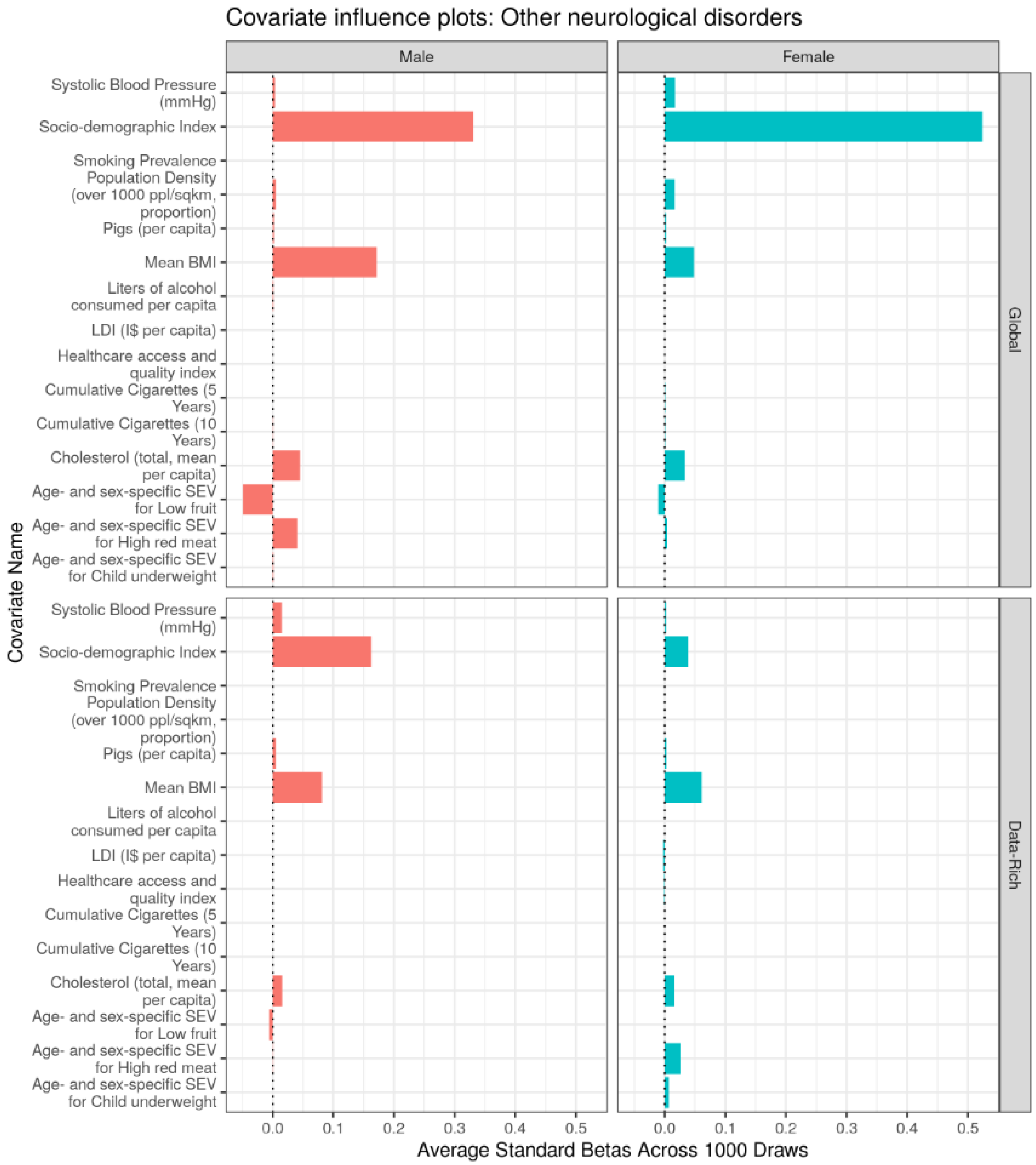

1 Vos T, Lim SS, Abbafati C, *et al.* Global burden of 369 diseases and injuries in 204 countries and territories, 1990–2019: a systematic analysis for the Global Burden of Disease Study 2019. *The Lancet* 2020; **396**: 1204–22. doi: https://doi.org/10.1016/S0140-6736(20)30925-9

Other neglected tropical diseases (NTDs)

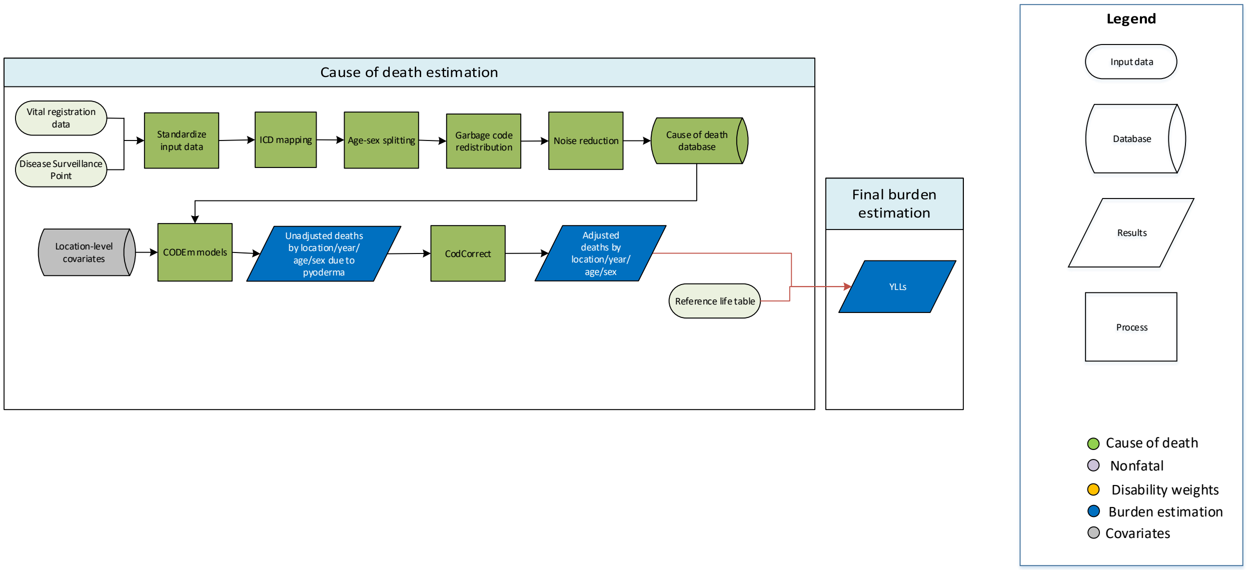

There are many diverse types of neglected tropical diseases, which are encompassed by the following ICD 10 codes:

A68 Relapsing fevers

A68.0 Louse-borne relapsing fever

A68.1 Tick-borne relapsing fever

A68.9 Relapsing fever, unspecified

A69.2 Lyme disease

A69.20 Lyme disease, unspecified

A69.21 Meningitis due to Lyme disease

A69.22 Other neurologic disorders in Lyme disease

A69.23 Arthritis due to Lyme disease

A69.29 Other conditions associated with Lyme disease

A69.5 There is not this code in ICD-10 site, but we have this in mortality data

A69.8 Other specified spirochetal infections

A69.9 Spirochetal infection, unspecified

A75 Typhus fever

A75.0 Epidemic louse-borne typhus fever due to *Rickettsia prowazekii*

A75.1 Recrudescent typhus [Brill's disease]

A75.2 Typhus fever due to *Rickettsia typhi*

A75.3 Typhus fever due to *Rickettsia tsutsugamushi*

A75.9 Typhus fever, unspecified

A77 Spotted fever [tick-borne rickettsioses]

A77.0 Spotted fever due to *Rickettsia rickettsii*

A77.1 Spotted fever due to *Rickettsia conorii*

A77.2 Spotted fever due to *Rickettsia siberica*

A77.3 Spotted fever due to *Rickettsia australis*

A77.4 Ehrlichiosis

A77.40 Ehrlichiosis, unspecified

A77.41 Ehrlichiosis chafeensis [*E. chafeensis*]

A77.49 Other ehrlichiosis

A77.8 Other spotted fevers

A77.9 Spotted fever, unspecified

A78 Q fever

A79 Other rickettsioses

A79.0 Trench fever

A79.1 Rickettsial pox due to *Rickettsia akari*

A79.8 Other specified rickettsioses

A79.81 Rickettsiosis due to *Ehrlichia sennetsu*

A79.89 Other specified rickettsioses

A79.9 Rickettsiosis, unspecified

A92 Other mosquito-borne viral fevers

A92.0 Chikungunya virus disease

A92.1 O'nyong-nyong fever

A92.2 Venezuelan equine fever

A92.3 West Nile virus infection

A92.30 West Nile virus infection, unspecified

A92.31 West Nile virus infection with encephalitis

A92.32 West Nile virus infection with other neurologic manifestation

A92.39 West Nile virus infection with other complications

A92.4 Rift Valley fever

A92.8 Other specified mosquito-borne viral fevers

A92.9 Mosquito-borne viral fever, unspecified

A93 Other arthropod-borne viral fevers, not elsewhere classified

A93.0 Oropouche virus disease

A93.1 Sandfly fever

A93.2 Colorado tick fever

A93.8 Other specified arthropod-borne viral fevers

A94 Unspecified arthropod-borne viral fever

A94.0 Unspecified arthropod-borne viral fever

A96 Arenaviral hemorrhagic fever

A96.0 Junin hemorrhagic fever

A96.1 Machupo hemorrhagic fever

A96.2 Lassa fever

A96.8 Other arenaviral hemorrhagic fevers

A96.9 Arenaviral hemorrhagic fever, unspecified

A98 Other viral hemorrhagic fevers, not elsewhere classified

A98.0 Crimean-Congo hemorrhagic fever

A98.1 Omsk hemorrhagic fever

A98.2 Kyasanur Forest disease

A98.3 Marburg virus disease

A98.5 Hemorrhagic fever with renal syndrome

A98.8 Other specified viral hemorrhagic fevers

B33.0 Epidemic myalgia

B33.1 Ross River disease

B60 Other protozoal diseases, not elsewhere classified

B60.0 Babesiosis

B60.1 Acanthamebiasis

B60.10 Acanthamebiasis, unspecified

B60.11 Meningoencephalitis due to *Acanthamoeba* (*culbertsoni*)

B60.12 Conjunctivitis due to *Acanthamoeba*

B60.13 Keratoconjunctivitis due to *Acanthamoeba*

B60.19 Other acanthamebic disease

B60.2 Naegleriasis

B60.8 Other specified protozoal diseases

B67.5 *Echinococcus multilocularis* infection of liver

B67.6 *Echinococcus multilocularis* infection, other and multiple sites

B67.61 *Echinococcus multilocularis* infection, multiple sites

B67.69 *Echinococcus multilocularis* infection, other sites

B67.7 *Echinococcus multilocularis* infection, unspecified

B70 Diphyllobothriasis and sparganosis

B70.0 Diphyllobothriasis

B70.1 Sparganosis

B71 Other cestode infections

B71.0 Hymenolepiasis

B71.1 Dipylidiasis

B71.8 Other specified cestode infections

B71.9 Cestode infection, unspecified

B74.3 Loiasis

B74.4 Mansonelliasis

B74.8 Other filariases

B74.9 Filariasis, unspecified

B75 Trichinellosis

B83 Other helminthiases

B83.0 Visceral larva migrans

B83.1 Gnathostomiasis

B83.2 Angiostrongyliasis due to *Parastrongylus cantonensis*

B83.3 Syngamiasis

B83.4 Internal hirudiniasis

B83.8 Other specified helminthiases

P37.1 Congenital toxoplasmosis

Input data

We modelled other neglected tropical disease mortality using all available data in the cause of death database. Datapoints were outliered if they reported an improbable number of deaths or if their inclusion in the model yielded distorted trends.

Modelling strategy

We modelled other neglected tropical disease mortality using a two-model hybrid approach: 1) a global CODEm model of all locations, using all data in the CoD database; and 2) a CODEm model restricted to data-rich countries.

Level	Covariate	Direction
1	Healthcare Access and Quality Index	–
	Proportion of the population living between 0 and 15 degrees latitude	+
2	Proportion of the population living in the 5th quintile of rainfall	+
	Sanitation	–
3	Education (years per capita)	–
	Lag-distributed income (per capita)	–
	Socio-demographic Index	–

Changes from GBD 2019 to GBD 2021

We have made no substantive changes in the modelling strategy for other neglected tropical diseases from GBD 2019.

Other skin and subcutaneous diseases

Flowchart

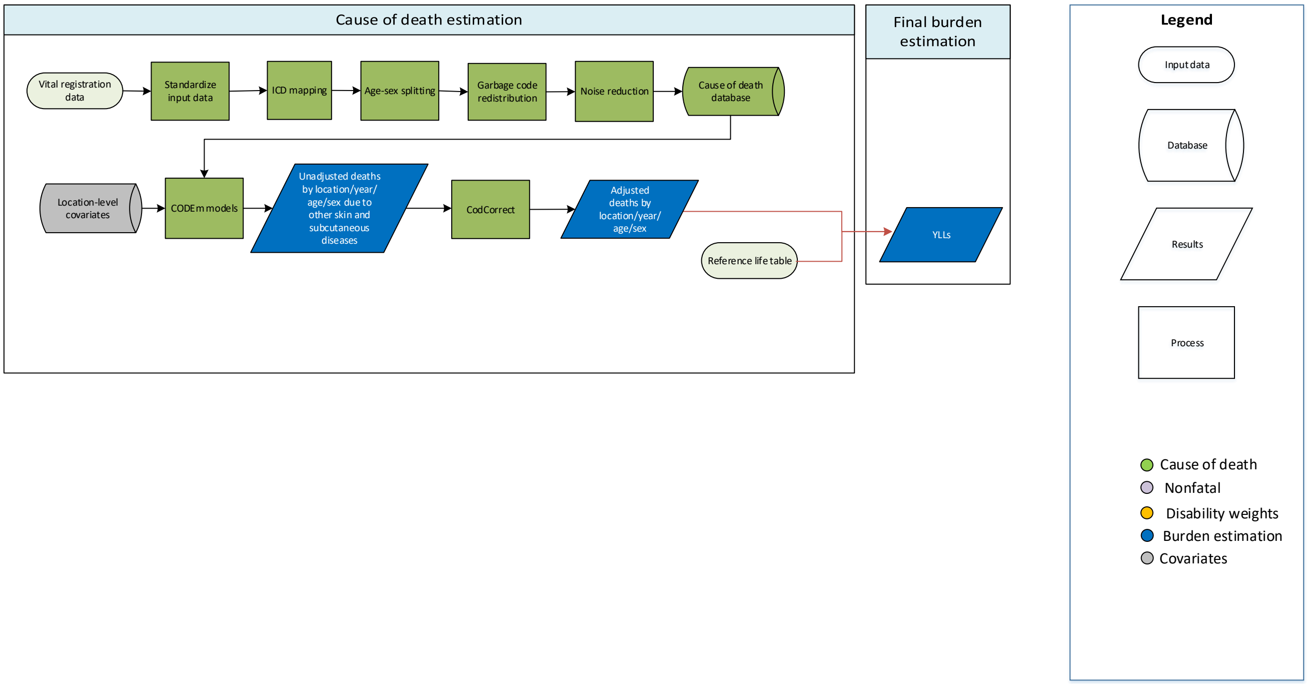

Input data and methodological summary for other skin and subcutaneous diseases

Input data

Data used to estimate mortality due to other skin and subcutaneous diseases consisted of vital registration data from the cause of death (COD) database. We outliered data in instances where garbage code redistribution and noise reduction, in combination with small sample sizes, resulted in unreasonable cause fractions. We also outlliered data that violated well-established time or age trends.

Modelling strategy

We modelled deaths due to other skin and subcutaneous diseases with a standard CODEm model using the COD database and location-level covariates as inputs. The model followed standard parameters. We hybridised separate global and data-rich models to acquire unadjusted results, which we finalised and adjusted using CoDCorrect to reach final years of life lost due to other skin diseases.

There were no significant changes in the modelling process between GBD 2019 and GBD 2021.

Table 1. Covariates used in other skin and subcutaneous diseases mortality modelling

Level	Covariate	Direction
1	Age-standardised summary exposure value (SEV) for child underweight	+

	Improved water source (proportion with access)	-
	SEV scalar for unsafe sanitation	+
	Diabetes fasting plasma glucose (mmol/L), by age	+
	Healthcare Access and Quality Index	-
	Prevalence of overweight and obesity	+
2	Smoking prevalence	+
	Alcohol (litres per capita)	+
	Cumulative cigarettes (5 years)	+
	Cumulative cigarettes (10 years)	+
3	Education (years per capita)	-
	Lag distributed income (per capita)	-
	Socio-demographic Index	-

Other unspecified infectious diseases

Flowchart

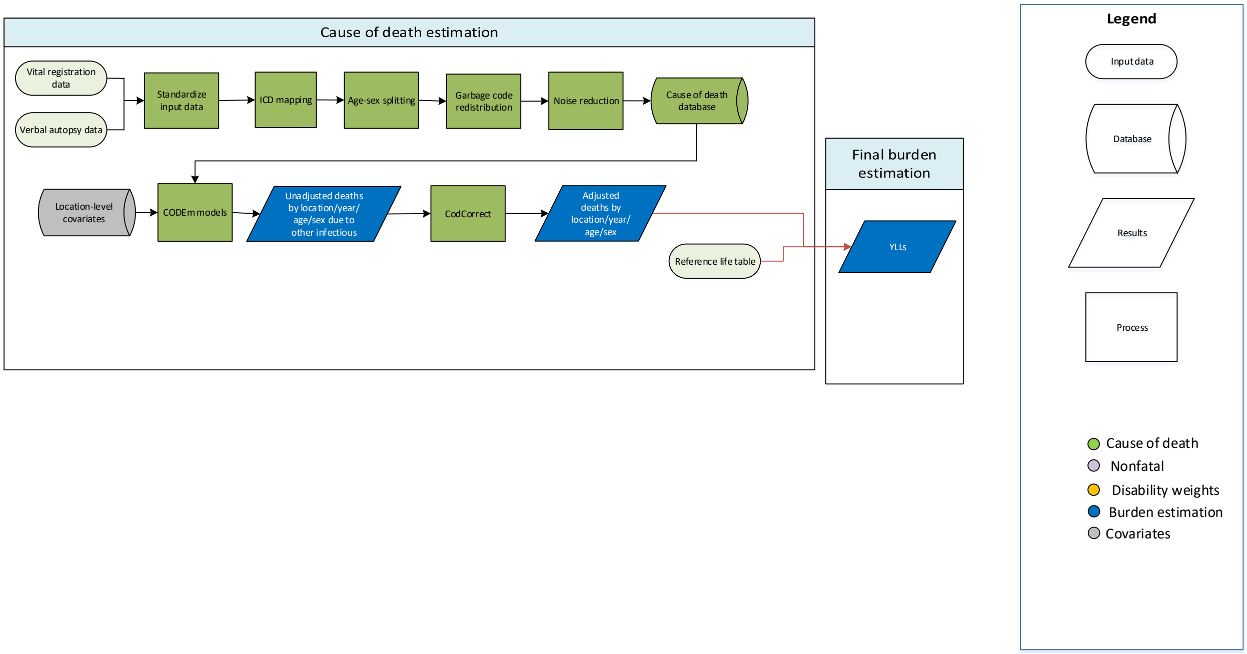

Input data and methodological summary for other unspecified infectious diseases

Input data

We modelled other infectious disease mortality using all available data in the cause of death database. Datapoints were outliered if they reported an improbable number of deaths or if their inclusion in the model yielded distorted trends.

Modelling strategy

We modelled other unspecified infectious disease mortality using a two-model hybrid approach: 1) a global CODEm model of all locations, using all data in the CoD database; and 2) a CODEm model restricted to data-rich countries. We have made no substantive changes to the modelling strategy since GBD 2017.

Table 1. Covariates used in other unspecified infectious diseases mortality modelling

Covariate name	Level	Direction
ANC proportion	3	-
DPT3 coverage	1	-
Sanitation proportion	2	-
Clear water proportion	2	-
Socio-demographic Index	3	-
Healthcare Access and Quality Index	2	-

Other urinary diseases

Flowchart

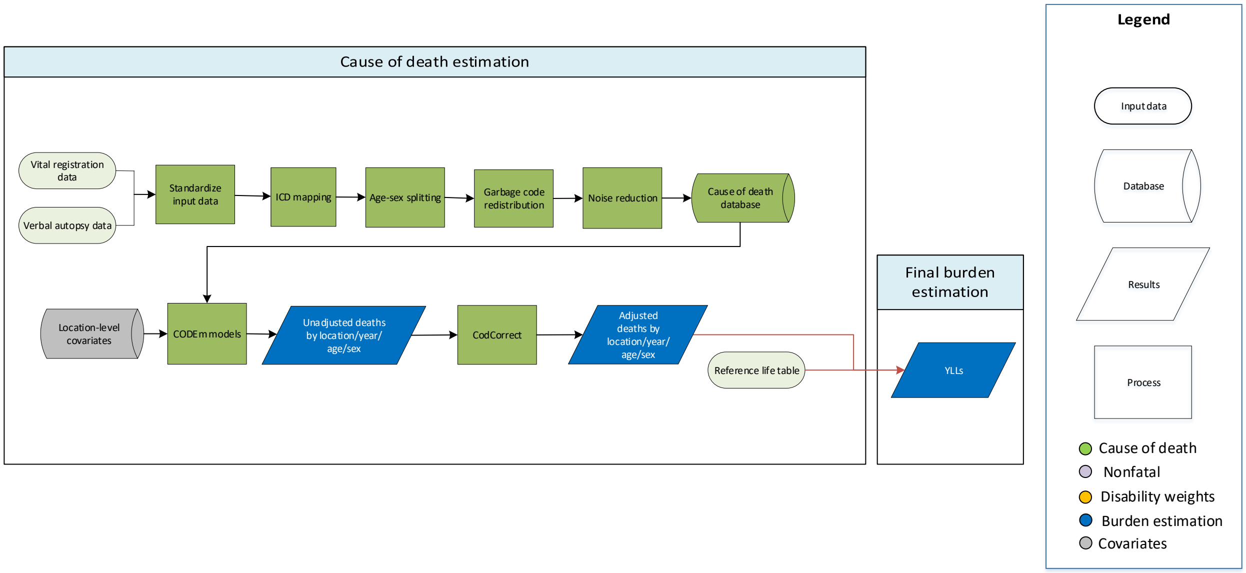

Input data and methodological summary for other urinary diseases

Input data

Data used to estimate mortality of other urinary diseases consisted of vital registration and verbal autopsy data from the cause of death (COD) database. The data in other urinary diseases consist of unique datapoints from deaths reported with a set of non-specific urinary disease codes (see appendix section on ICD mapping for details). Outliers were identified by systematic examination of datapoints for all location-years. Data were excluded if they violated well-established age or time trends, and data in instances where garbage code redistribution and noise reduction, in combination with small sample sizes, resulted in unreasonable cause fractions. Methods for assigning outlier status were consistent across both vital registration and verbal autopsy data.

Modelling strategy

The estimation strategy used for other urinary diseases is largely similar to methods used in GBD 2019. A standard CODEm model with location-level covariates was used to model deaths due to other urinary diseases (see appendix section on CODEm method for details). Separate models were conducted for male and female mortality, and age-restrictions for death estimations included 0 days for lower bound, 95+ years for upper bound. We hybridised separate global and data-rich models to acquire unadjusted results, which we adjusted using CoDCorrect and compared to the reference life table to calculate final YLLs due to other urinary diseases.

In GBD 2021, we updated the linear floor value in our CODEm models to allow the model to be influenced by lower data, which resulted from changes to COD data processing. These data processing changes include changing the method of estimating the non-zero floor and population size cutoffs for when to noise-reduce at the country versus region level, and are described in greater detail in the

appendix section GBD 2021 Causes of Death database. Apart from these, no other substantive changes were made in GBD 2021 from the modelling strategy used in GBD 2019.

The following table has the full list of covariates used for other urinary diseases.

Table 1. Covariates used in other urinary diseases mortality modelling

Level	Covariate	Direction
1	Mean BMI	+
2	Education (years per capita)	-
	Log LDI ($I per capita)	-
	Healthcare Access and Quality Index	-
3	Socio-demographic Index	-

Adjustment in CoDCorrect included fitting unadjusted death estimates for all other specific and non-specific urinary diseases to overall urinary disease deaths, which were then adjusted with all other causes to sum to all-cause counts of death.

Otitis media

Flowchart

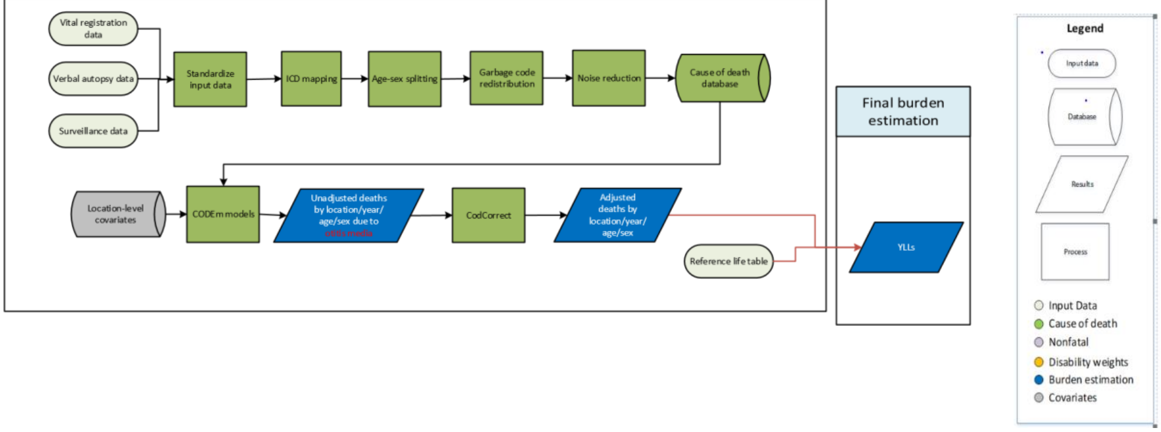

Input data and methodological summary for otitis media

Input data

Vital registration, verbal autopsy, and surveillance data were used. Outliers were identified by systematic examination of datapoints. Datapoints that violated well-established age or time trends were inconsistent with other country- or region-specific points, or that resulted in extremely high or low mortality rates were determined to be outliers.

Modelling strategy

A general CODEm modelling strategy was used. There were no substantive changes from GBD 2019 in terms of modelling strategy. The covariates used are displayed in Table 1.

Table 1. Covariates used in otitis media mortality modelling

Level	Covariate	Direction
1	Otitis summary exposure value (SEV)	+
	Smoking prevalence	+
2	Indoor pollution	+
	Healthcare Access and Quality Index	-
	Outdoor pollution ($PM_{2.5}$)	+
3	Socio-demographic Index (SDI)	-
	Log-transformed lag distributed income	-
	Education (years per capita)	-

Pancreatitis

Flowchart

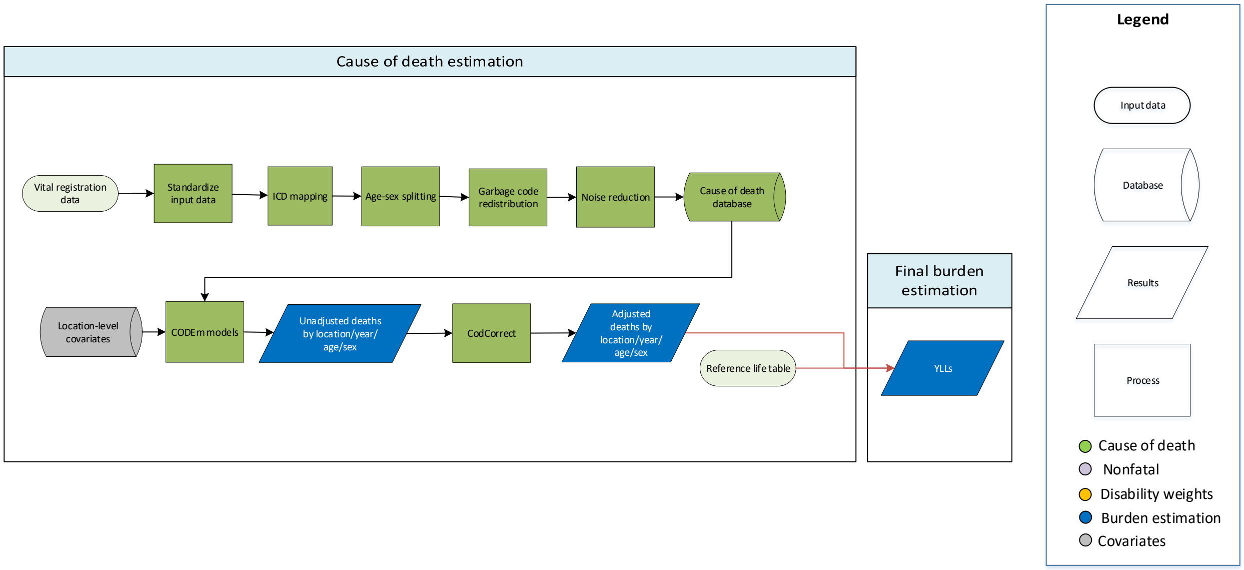

Input data and methodological summary for pancreatitis

Input data

Data used to estimate mortality of pancreatitis consisted of vital registration data from the cause of death (COD) database (see appendix section on ICD mapping for details). Outliers were identified by systematic examination of datapoints for all location-years. Data were excluded if they violated well-established age or time trends, and data in instances where garbage code redistribution and noise reduction, in combination with small sample sizes, resulted in unreasonable cause fractions.

Modelling strategy

The estimation strategy used for fatal pancreatitis is largely similar to methods used in GBD 2019. A standard CODEm model with location-level covariates was used to model deaths due to pancreatitis (see appendix section on CODEm method for details). Separate models were conducted for male and female mortality, and age restrictions for death estimations included 2 years for lower bound (in GBD 2019, the lower bound was set at 1 year) and 95+ for upper bound. We hybridised separate global and data-rich models to acquire unadjusted results, which we adjusted using CoDCorrect and compared to the reference life table to calculate final YLLs due to pancreatitis.

In GBD 2021, we updated the linear floor value in our CODEm models to allow the model to be influenced by lower data, which resulted from changes to COD data processing. These data processing changes include changing the method of estimating the non-zero floor and population size cutoffs for when to noise-reduce at the country versus region level, and are described in greater detail in the appendix section "GBD 2021 Causes of Death database". Apart from these, no other substantive changes were made in GBD 2021 from the modelling strategy used in GBD 2019.

The following table has the full list of covariates used for pancreatitis.

Table 1. Covariates used in pancreatitis mortality modelling

Level	Covariate	Direction
1	Log-transformed scaled exposure variable for pancreatitis	+
	Alcohol (litres per capita)	+
2	Healthcare Access and Quality Index	-
	BMI (mean)	+
3	Socio-demographic Index	-
	Education (years per capita)	-
	Log LDI ($I per capita)	-

Adjustment in CoDCorrect included fitting unadjusted death estimates for all other specific and non-specific digestive diseases to overall digestive disease deaths, which was then adjusted with all other causes to sum to all-cause counts of death.

Paralytic ileus and intestinal obstruction

Flowchart

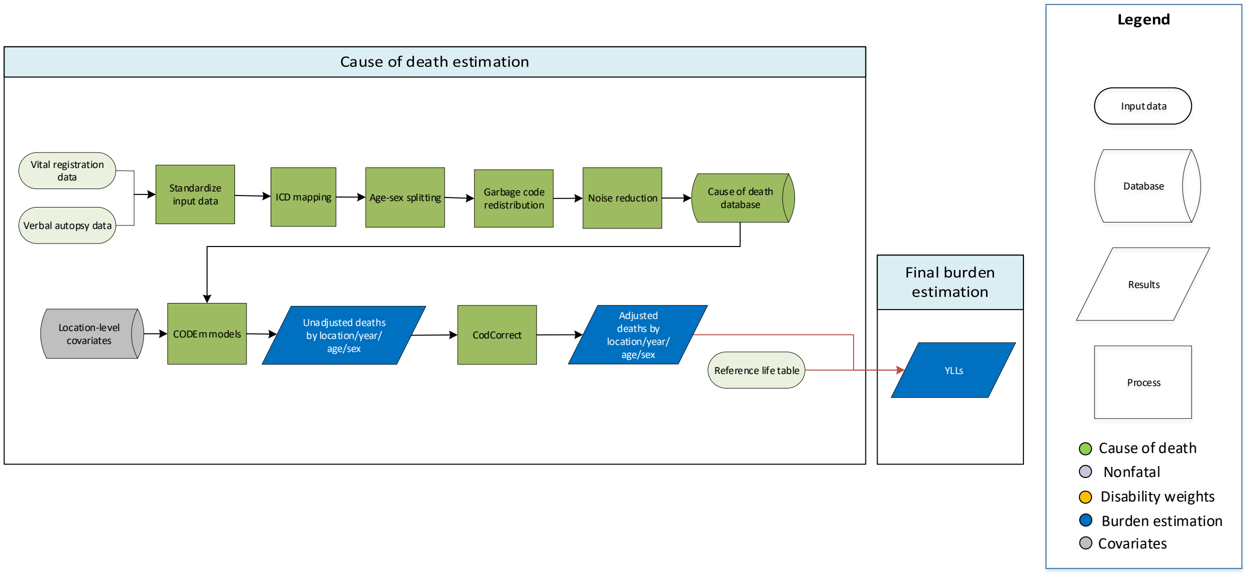

Input data

Data used to estimate mortality of paralytic ileus and intestinal obstruction consisted of vital registration and verbal autopsy data from the cause of death (COD) database (see appendix section on ICD mapping for details). Outliers were identified by systematic examination of datapoints for all location-years. Data were excluded if they violated well-established age or time trends, and data in instances where garbage code redistribution and noise reduction, in combination with small sample sizes, resulted in unreasonable cause fractions. Methods for assigning outlier status were consistent across both vital registration and verbal autopsy data.

Modelling strategy

The estimation strategy used for fatal paralytic ileus and intestinal obstruction is largely similar to methods used in GBD 2019. A standard CODEm model with location-level covariates was used to model deaths due to paralytic ileus and intestinal obstruction diseases (see appendix section on CODEm method for details). Separate models were conducted for male and female mortality, with age restrictions for death estimations of 0 years for lower bound (instead of 1 year as in GBD 2019) and 95+ years for upper bound (see appendix section on CODEm method for details). Separate models were conducted for male and female mortality. We hybridised separate global and data-rich models to acquire unadjusted results, which we adjusted using CoDCorrect and compared to the reference life table to calculate final YLLs due to paralytic ileus and intestinal obstruction.

In GBD 2021, we updated the linear floor value in our CODEm models to allow the model to be influenced by lower data, which resulted from changes to COD data processing. These data processing changes include changing the method of estimating the non-zero floor and population size cutoffs for when to noise-reduce at the country versus region level, and are described in greater detail in the appendix section “GBD 2021 Causes of Death database”. Apart from these, no other substantive changes were made in GBD 2021 from the modelling strategy used in GBD 2019.

The following table has the full list of covariates used for paralytic ileus and intestinal obstruction.

Table 1. Covariates used in paralytic ileus and intestinal obstruction mortality modelling

Level	Covariate	Direction
2	Fruit consumption (unadjusted, kcal per capita)	-
	Vegetable consumption (unadjusted, kcal per capita)	-
	Healthcare Access and Quality Index	-
3	Socio-demographic Index	-
	Education (years per capita)	-
	Log LDI ($I per capita)	-

Adjustment in CoDCorrect included fitting unadjusted death estimates for all other specific and non-specific digestive diseases to overall digestive disease deaths, which was then adjusted with all other causes to sum to all-cause counts of death.

Paratyphoid fever

Flowchart

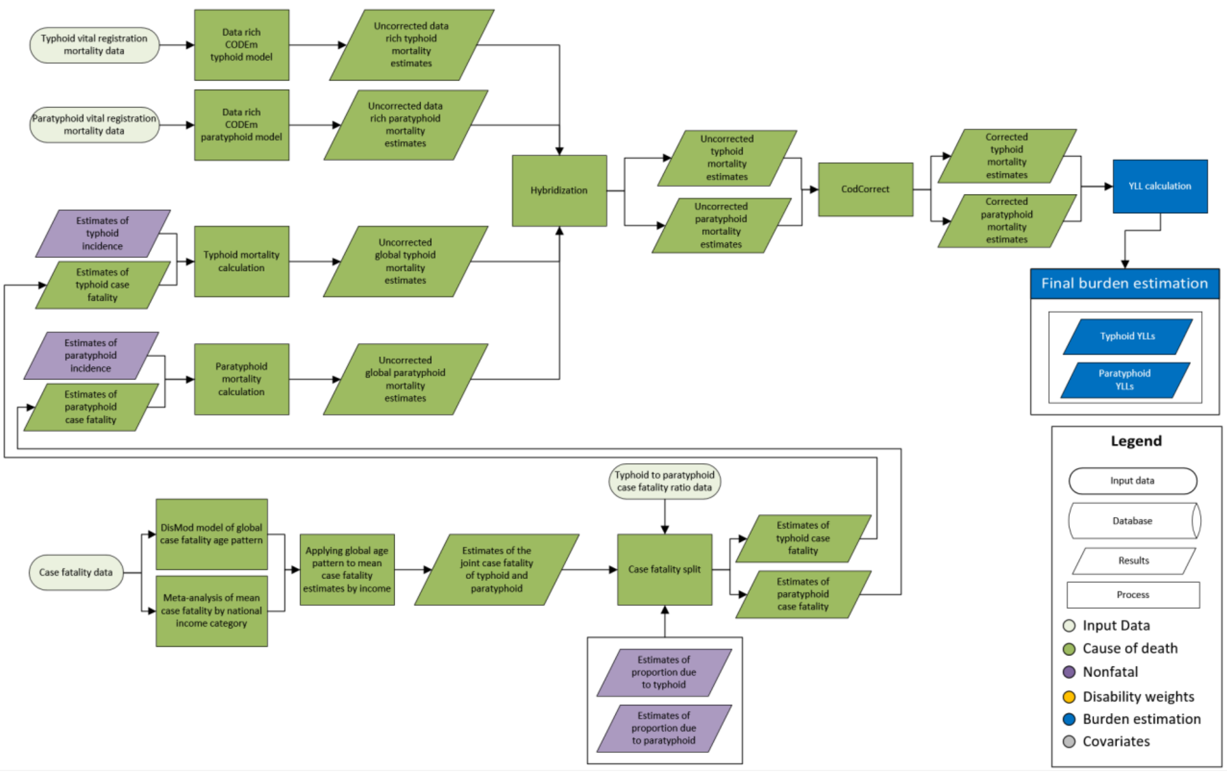

Input data and methodological summary for paratyphoid fever

Input data

Our CODEm model used all available data in the cause of death database from data-rich countries. No data were outliered for this cause. For the natural history model, our incidence dataset included a combination of data from prospective cohort studies and national surveillance systems. Similarly, data on proportions due to typhoid and paratyphoid included a combination of prospective cohort studies and national surveillance systems. Case fatality data were from national surveillance systems and hospital databases.

Modelling strategy

We model paratyphoid deaths using a hybrid modelling strategy with two components: 1) for data-rich locations, we estimate paratyphoid mortality using a CODEm model of CoD data; and 2) in all other locations (ie, not data-rich) we use a natural history model in which we derive deaths as the product of cases and case fatality.

The CODEm model included six covariates:

Level	Covariate	Direction
1	Sanitation (proportion with access)	-
	Improved water source (proportion of the population with access)	-
	Proportion of the population living in the Indian Ocean monsoon belt	+
	SEV unsafe water	+
	SEV unsafe sanitation	+
2	Healthcare Access and Quality Index	-

For the natural history model, we first model total incidence of typhoid and paratyphoid combined. Second, we model the proportion of this total due to typhoid and the proportion due to paratyphoid. Third, we estimate case fatality by age and national income category for typhoid and paratyphoid combined. Fourth, we use data on the relative fatality of typhoid and paratyphoid to split the joint case fatality estimates into typhoid- and paratyphoid-specific case fatality estimates. Finally, we estimate cause-specific mortality rates as the product of incidence and case fatality.

Total incidence was modelled using DisMod-MR 2.1, using the proportion of the population with access to clean water, and the proportion of the population living in the Indian Ocean monsoon belt as covariates. We performed a crosswalk using a study-level covariate indicating sources that were based on passive versus active surveillance, with active surveillance as the reference. This adjusts for incomplete case capture by passive surveillance. Incidence data were inflated to account for poor diagnostic sensitivity, based on a meta-analysis of the sensitivity of blood culture, the most common diagnostic used for typhoid and paratyphoid. Similarly, we used two DisMod models to estimate aetiologic proportions: one for the proportion of total incidence due to typhoid, and one for the proportion due to paratyphoid.

Case fatality data were too limited to allow for a complete DisMod model, or to allow for varying estimates by time and space. We had sufficient data, however, to estimate case fatality by age and by three categories of national income. We used DisMod to extract a global age pattern in case fatality, and meta-regression to estimate the mean case fatality by income category. Finally, we estimated the relative risk of death from typhoid relative to paratyphoid based on data from Chinese surveillance and used that relative risk to estimate case fatality separately for typhoid and paratyphoid, by age and income.

Finally, we estimated paratyphoid mortality as the product of total incidence, the proportion of the total due to paratyphoid, and case fatality for paratyphoid. We propagated uncertainty through every step of the modelling process by pulling 1000 draws from the distribution of each model component (eg, incidence, proportion due to paratyphoid, overall case fatality, case fatality age pattern, relative fatalness of typhoid versus paratyphoid), and performing all calculations at the draw level.

We have made no substantive changes to our modelling strategy between GBD 2019 and 2021.

Parkinson's disease

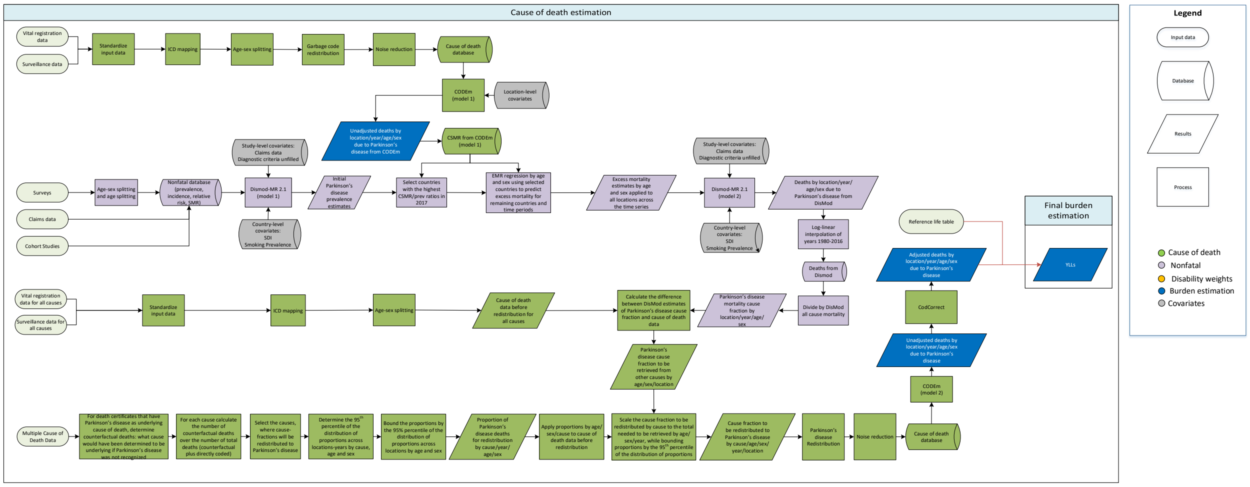

Input data

In GBD 2021, data used to estimate deaths due to Parkinson's disease included mortality data from vital registration (VR) systems and non-fatal and mortality data from surveys and claims sources as described in the search string below.

An updated systematic review was conducted from September 2015 to August 2017, and search terms were set to capture studies for Parkinson's disease: (Parkinson disease[Title/Abstract] OR Parkinson's disease[Title/Abstract]) AND (epidemiology[Title/Abstract] OR prevalence[Title/Abstract] OR incidence[Title/Abstract]) AND ("2015/09/31"[PDAT] : "2017/08/23"[PDAT]). Inclusion criteria comprised studies that reported prevalence, incidence, remission rate, excess mortality rate, relative risk of mortality, standardised mortality ratio, or with-condition mortality rate. Studies with no clearly defined sample or that drew from specific clinic/patient organisations were excluded. We also added USA claims data for 2011 and 2012–2015. No further prevalence or incidence data were added in GBD 2021 to inform fatal modelling. While we collect incidence data, we do not use these data to inform our estimates of Parkinson's disease because it is difficult to determine onset of Parkinson's disease (versus parkinsonism) and therefore we preferentially use prevalence data.

Modelling strategy

Overview

Parkinson's disease mortality rates have more than doubled since 1980 in high-quality VR systems such as in the USA, Canada, Australia, France, Germany, the United Kingdom, and Finland, while other European countries like the Netherlands, Sweden, and Norway have not seen such increases over time. We have not seen an equivalent increase in prevalence and incidence data sources. Additionally, the greater than 15-fold variation in mortality rates of Parkinson's disease between countries is much greater the three-fold difference in prevalence and incidence between high-income countries. As it is unlikely that case fatality from Parkinson's disease has dramatically increased over the time period and

that it would differ by a very large margin between countries, the hypothesis is that certifying and coding practices have changed over time and at a different pace between countries. For GBD 2016 onward, we decided to employ a modelling strategy that avoids spurious large trends over time in the fatal component of the burden of Parkinson's disease by making Parkinson's mortality rates consistent with the rates observed in estimation years, relative to prevalence in countries that are most likely to certify or code Parkinson's disease as an underlying cause of death.

Modelling steps

Fatal modelling for Parkinson's disease is described in the following steps. The initial steps were not re-run in GBD 2021 and so the Multiple Cause of Death (MCOD) Parkinson's disease inputs were identical to those used in the GBD 2019 capstone publication.[1]

First, we ran a Cause of Death Ensemble model[1] (CODEm; additional information on methods can be found in appendix 1, section 3 of the citation) for Parkinson's disease and extracted the mortality rates by age, sex, and geography. The covariates used in this intermediary model are displayed in the table that follows this paragraph; some have a direction of zero because this model was run early in the previous GBD cycle. The final Parkinson's model for GBD 2021 has a negative or positive direction specified for all covariates (see Table 1).

Table 1

Level	Covariate	Direction
1	Cumulative cigarette consumption (10 years)	-
2	Absolute latitude	+
	Cholesterol (total, mean per capita)	+
	Sanitation (proportion with access)	0
	Improved water source (proportion with access)	0
	Fruit consumption adjusted (g)	-
	Healthcare Access and Quality Index	-
3	Education (years per capita)	-
	Socio-demographic Index	+
	Lag distributed income	0

Second, we ran a DisMod-MR 2.1 model with all data on incidence, prevalence, and mortality risk (relative risk, standardised mortality rate, or with-condition mortality rates) and a setting of zero remission and extracted prevalence by age, sex, and geography. Studies where the case definition of two of the four cardinal symptoms of Parkinson's disease (tremors/trembling, bradykinesia, stiffness of limbs and torso, posture instability) was not filled were adjusted to studies using the reference case definition. No random effects were used in the model in order to prevent spurious inflation of regional differences due to differences in measurement and measurement error.

Third, we selected the seven countries (France, England, the USA, the Netherlands, Finland, Scotland, and Wales) with the highest cause-specific mortality rate (from step 1) to prevalence (from step 2) ratio in 2017, which also had an age-standardised prevalence rate greater than 0.0005, and a population greater than 1 million.

Fourth, we used a linear effects regression with dummies on age group and sex to predict excess mortality (EMR) (ie, the ratio of cause-specific mortality rate and prevalence) by age and sex, the results of which are found in the Tables 2 and 3.

Table 2: Fixed effect coefficients of EMR regression. Outcome: ln(EMR)

Independent variables	Coef	Std. error	P value	95% confidence interval	
Male	0.288	0.036	0.000	0.218	0.358
Age 40–59	-3.25	0.076	0.000	-3.399	-3.101
Age 60–64	-2.557	0.076	0.000	-2.706	-2.407
Age 65–69	-2.021	0.076	0.000	-2.17	-1.871
Age 70–74	-1.42	0.076	0.000	-1.57	-1.271
Age 75–80	-0.898	0.076	0.000	-1.047	-0.749
Age 80–84	-0.502	0.076	0.000	-0.651	-0.352
Age 85–89	-0.248	0.076	0.001	-0.397	-0.099
Age 90–94	-0.047	0.076	0.537	-0.196	0.102
Constant	-2.357	0.057	0.000	-2.469	-2.246

Table 3: Predicted EMR values by age and sex (95% CI)

	Male	Female
Age 40–59	0.005 (0.004–0.005)	0.004 (0.003–0.004)
Age 60–64	0.01 (0.009–0.011)	0.007 (0.007–0.008)
Age 65–69	0.017 (0.015–0.019)	0.013 (0.011–0.014)
Age 70–74	0.031 (0.027–0.034)	0.023 (0.02–0.025)
Age 75–80	0.051 (0.046–0.057)	0.039 (0.035–0.043)
Age 80–84	0.076 (0.068–0.085)	0.058 (0.052–0.064)
Age 85–89	0.099 (0.089–0.111)	0.074 (0.066–0.083)
Age 90–94	0.12 (0.108–0.135)	0.09 (0.081–0.1)
Age 95+	0.126 (0.113–0.142)	0.095 (0.085–0.106)

Fifth, these estimates were added to a second DisMod-MR 2.1 model. For the countries included in the regression, we allowed them to retain their original EMR values when the age-standardised EMR for a country was higher than the age-standardised EMR prediction generated from the regression. These countries retained their age- and sex-specific ratios and entered those also as pertaining to the full estimation period. Smoking prevalence was used as a country-level covariate. We excluded data for standardised mortality ratio, with-condition mortality rate, and relative risk as we wanted to estimate cause-specific mortality rates that were consistent with the level of excess mortality from the seven chosen countries.

Sixth, we took the predictions of cause-specific mortality by age, sex, geography, and year that DisMod-MR 2.1 calculated as being consistent with the data on incidence, prevalence, and the priors on excess mortality from step five. Because DisMod-MR 2.1 produces estimates in five-year intervals only, we expanded the time series by log-linear interpolation; values for 1980–1990 were generated using a regression on the entire time series with Socio-demographic Index included as a predictor. We divided this cause-specific mortality by the all-cause mortality used in DisMod-MR 2.1 to calculate the

Parkinson's disease cause-fraction based on prevalence data and the excess mortality derived from countries most likely to code to Parkinson's disease as a cause of death.

Seventh, we calculated the difference between this cause-fraction derived from DisMod and the cause-fraction derived from the cause of death data prep process before redistribution in order to get the amount of cause-fraction that needed to be retrieved from other causes through the Parkinson's disease redistribution process.

Eighth, in order to calculate where these Parkinson's disease deaths should be retrieved from, we analysed MCOD data. We only used data from the USA, and asserted that the data from 2010 to 2015, during which the increases in coding to Parkinson's disease as a cause of death levelled off, are the reference data.

Ninth, for deaths where Parkinson's disease is the underlying cause of death in the years 2010–2015, we calculated what the underlying cause of death would have been in the counterfactual scenario in which Parkinson's disease had not been recognised. In order to calculate this counterfactual, we examined the causes listed in part one of the chain of the death certificate. For each death certificate chain, we looked across the entire dataset from 1980 to 2015 and determined what the distribution of underlying causes of death was in individuals with that particular death certificate chain. Then, we assigned the counterfactual deaths proportionally to the causes that are listed as underlying in these death certificates. If, over the time period, there were fewer than 1000 death certificates that had exactly the same death certificate chain, then we included all death certificate chains that had those same causes, but which could additionally include other causes in the chain as well. To assign counterfactual deaths for these chains, we further subsetted the data to death certificate chains where any of the causes in the original death certificate chain were listed as underlying, determined the distribution of underlying causes of death among just this subset, and then assigned counterfactual deaths proportionally in the same manner.

Tenth, once we determined the counterfactual causes of death stemming from all Parkinson's disease deaths from 2010 to 2015, we calculated the proportion of deaths by cause that should be Parkinson's disease deaths according to the reference data by taking the counterfactual deaths for each cause and dividing by the sum of the counterfactual deaths for that cause plus the directly coded deaths for that cause.

Eleventh, we applied the proportions to cause of death data in cause-fraction space and scaled the cause fractions to the total mortality cause-fraction to be retrieved based on the DisMod-MR 2.1 model. We set caps on the percentage of deaths that were moved by age, sex, and cause. The caps were determined by finding the 95th percentile of the percentages of deaths moved in each age-sex-cause category across all high-quality data source locations. The causes of death data were then processed using general redistribution strategies and noise reduction described.

Finally, the data derived from this process were used in a final CODEm model, using the same covariates as the original CODEm model. These covariates were adjusted for this model in GBD 2019 onward so that every covariate had a specified directionality (see Table 4 below), and with some adjustments for level. These results were then adjusted through CoDCorrect and become the final cause of death estimates for Parkinson's disease.

Table 4

Level	Covariate	Direction
1	Cumulative cigarette consumption (10 years)	-
	Fruit consumption, adjusted (g)	-
2	Absolute latitude	+
	Cholesterol (total, mean per capita)	+
	Sanitation (proportion with access)	+
	Improved water source (proportion with access)	+
	Healthcare Access and Quality Index	-
3	Education (years per capita)	-
	Socio-demographic Index	+
	Lag distributed income	+

The plots in Figure 1 show the influence of each covariate on the four CODEm models (male global, male data-rich, female global, and female data-rich). A positive standardised beta (to the right) means that the covariate was associated with increased death. A negative standardised beta (to the left) means the covariate was associated with decreased death.

Figure 1

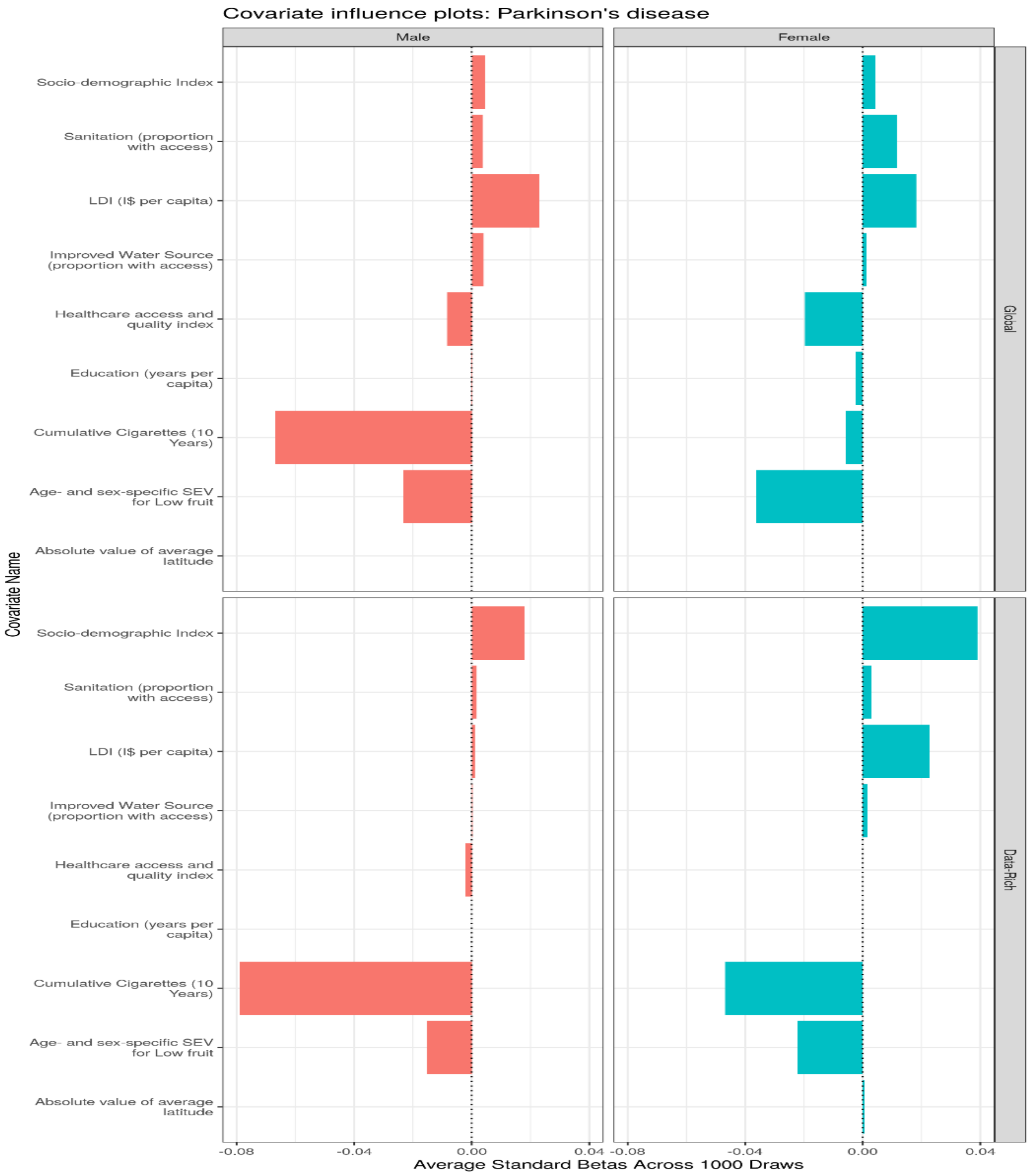

Reference

[1]Vos T, Lim SS, Abbafati C, et al. Global burden of 369 diseases and injuries in 204 countries and territories, 1990–2019: a systematic analysis for the Global Burden of Disease Study 2019. *The Lancet* 2020; 396: 1204–22. doi: https://doi.org/10.1016/S0140-6736(20)30925-9

Peptic ulcer disease

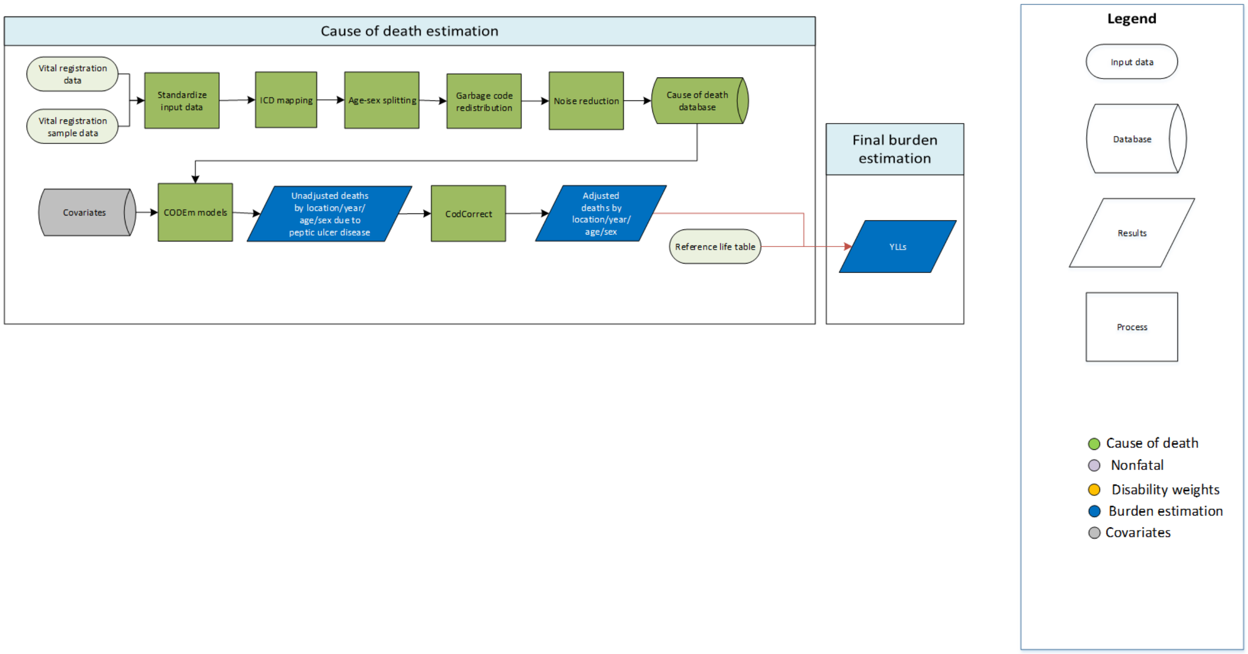

Input data

Data used to estimate unadjusted mortality of peptic ulcer disease consisted of vital registration data and vital registration sample data from those sources in the cause of death (COD) database that use ICD9 or ICD10 codes and report un-tabulated (individual) deaths. The diagnostic codes that map to peptic ulcer disease can be found in the "List of International Classification of Diseases (ICD) codes mapped to the Global Burden of Disease cause list for causes of death" found elsewhere in this appendix.

Details of COD data processing, including changes in processing introduced in GBD 2021, are described in detail in the "GBD 2021 Causes of Death database" section of this appendix. These data processing changes include changing the method of estimating the non-zero floor and population size cutoffs for when to noise-reduce at the country versus region level.

Outliers were identified by systematic examination of datapoints for all location-years. Data were excluded if they violated well-established time or age trends, and data in instances where garbage code redistribution and noise reduction, in combination with small sample sizes, resulted in unreasonable cause fractions or unreasonable time, age, or spatial trends; data from Tibet, Kiribati, Iran, and Mozambique were excluded for these reasons. Data in Fiji, Palestine, and Stockholm that were excluded for these reasons in GBD 2019 were included in GBD 2021 because advances in noise reduction improved the plausibility of these data. In situations where unreasonable temporal and spatial trends were observed at transitions between data sources, higher-quality data sources were retained and lower-quality sources were excluded; this affected subnational locations in India, where vital registration data biased toward in-hospital deaths (MCCD) were available for urban locations only. Two datapoints from Mali were excluded because they were only available for a single age group.

Modelling strategy

We modelled deaths due to peptic ulcer disease with a standard CODEm model. (The CODEm modelling tool is described in the "Cause of death modelling methods: CODEm" section of this appendix.) The peptic ulcer disease CODEm model employed the same parameter settings in GBD 2021 as in GBD 2019, with the exception that we updated the linear floor value to allow the model to be influenced by lower data, which resulted from changes to COD data processing.

Covariates entered into CODEm, their level of priority for testing, and their permitted directions were the same in GBD 2021 as in GBD 2019. A complete list is provided in the table below.

**Covariate**	**Level**	**Direction**
Sanitation, proportion with access	1	-1
Scaled exposure variable for unsafe water source	1	1
Smoking prevalence	1	1
Cumulative cigarettes (10 years)	1	1
Cumulative cigarettes (5 years)	1	1
Litres of alcohol consumed per capita	2	1
Vegetables (grams, unadjusted)	2	-1
Healthcare Access and Quality Index	2	-1
Lag distributed income (per capita)	3	-1
Education (years per capita)	3	-1
Socio-demographic Index	3	-1

In CoDCorrect estimates for peptic ulcer disease and gastritis and duodenitis were first adjusted to sum to all upper digestive disease deaths, and then to sum to all-cause mortality with all other causes.

Peripheral artery disease

Flowchart

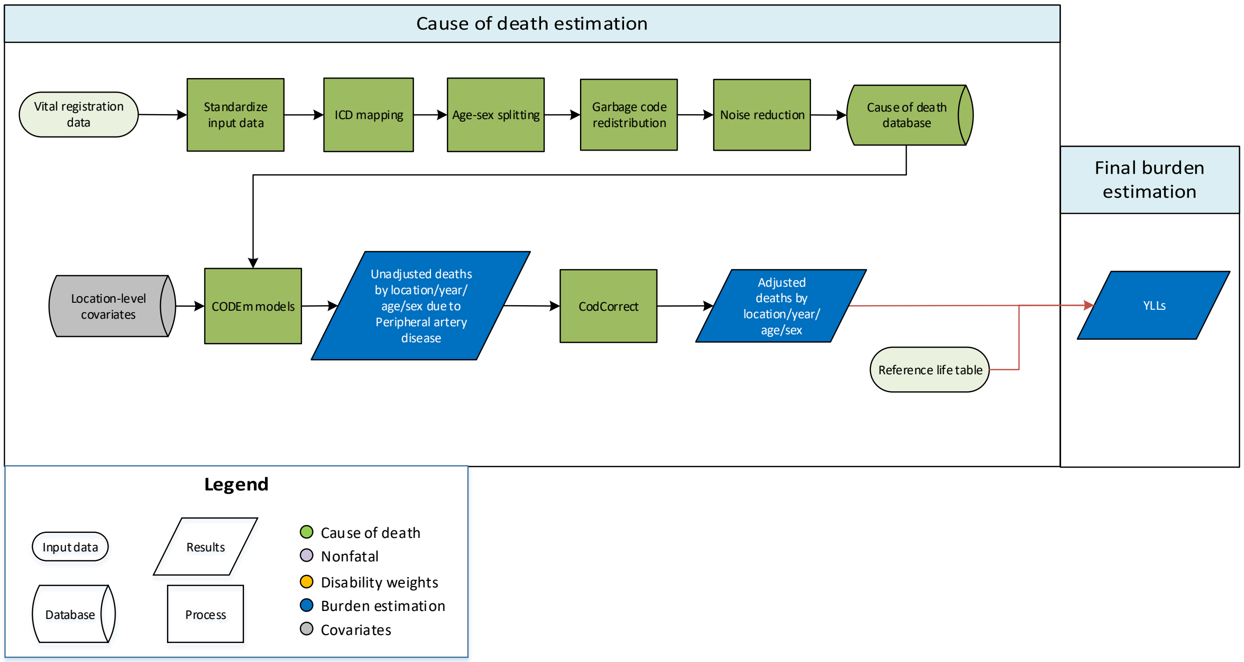

Input data and methodological summary for peripheral artery disease

Vital registration data were used to model peripheral artery disease. We outliered all datapoints with less than 1 death in Egypt per expert review. Data from Australia, Greenland, American Samoa, Guam, Kiribati, Palau, Greece, England Upper Tier Local Authorities, Finland, Estonia, Latvia, Lithuania, Republic of Moldova, Russian Federation, and other eastern European countries, were outliered due to implausibly low values and narrowed uncertainty intervals. In addition, ICD8 datapoints from Norway and Sweden with implausible values and discontinuous with the rest of the time series were outliered.

Modelling strategy

We used a standard CODEm approach to model deaths from peripheral artery disease. For GBD 2021, a new approach to redistribute deaths coded to hypertension was implemented using data sources which included information on the chain of events leading to death. This update resulted in an increase in the number of deaths that were re-assigned to cardiovascular diseases. Similarly, the method used to reduce the noise in the data, implemented after redistribution to handle both the stochastic variation across time and space and the occurrence of small number of deaths in each location/year/age/sex, was updated. This new empirical Bayesian noise reduction algorithm uses grouped data by region and data type as prior to better reflect regional patterns. A detailed description on the updates to noise reduction can be found in the cause of death methods section of the appendix. The covariates included in the ensemble modelling process are listed in the table below.

Table 1. Covariates used in peripheral artery disease mortality modelling

Level	Covariate	Direction
1	Summary exposure variable, PAD	1
	Systolic blood pressure (mm Hg)	1
	Cholesterol (total, mean per capita)	1
	Smoking prevalence	1
2	Mean body mass index (kg/m^2)	-1
	Healthcare access and quality index	1
	Diabetes fasting plasma glucose (mmol/L)	-1
3	Lag distributed income per capita (I$)	-1
	Socio-demographic Index	1
	Summary exposure value, omega-3	1
	Summary exposure value, fruits	1
	Summary exposure value, vegetables	1
	Summary exposure value, nuts and seeds	1
	Pulses/legumes (kcal/capita, unadjusted)	-1
	Summary exposure value, polyunsaturated fatty acids	1
	Alcohol (litres per capita)	1

Pertussis (whooping cough)

Flowchart

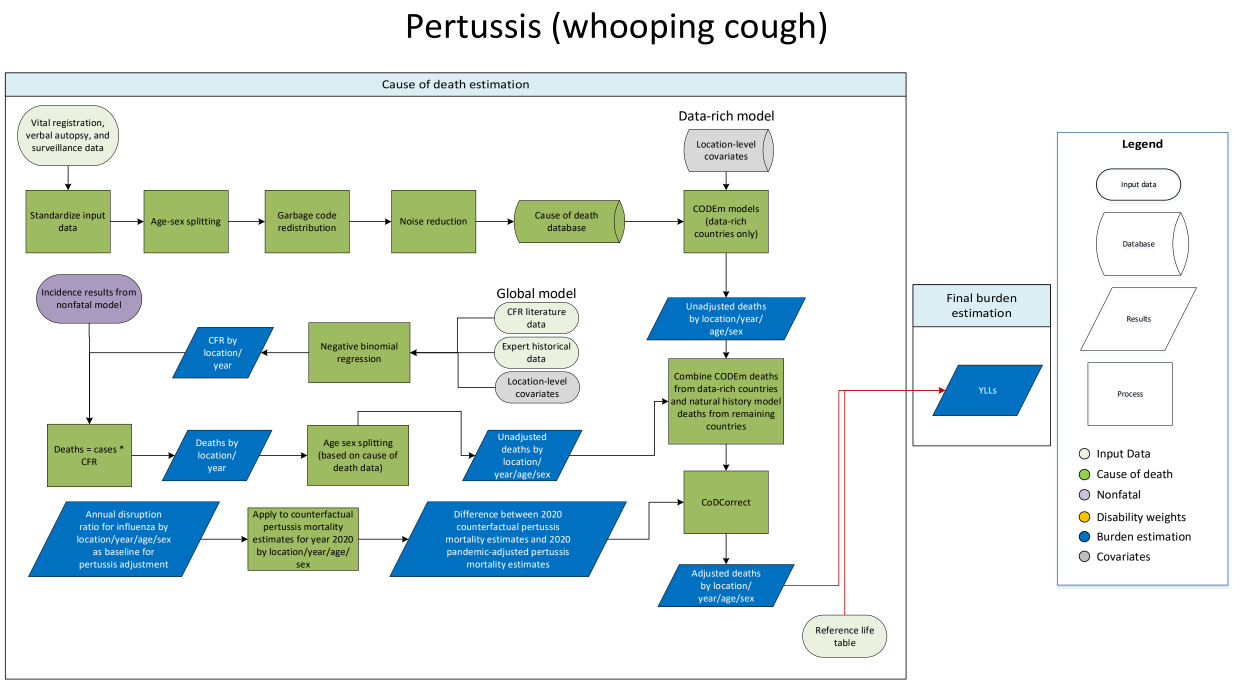

Modelling strategy overview

We used two strategies to develop GBD 2021 pertussis mortality estimates, depending on the quality of available vital registration data for the country. For countries with well-defined vital registration (ie, “data-rich” countries), we used a Cause of Death Ensemble model (CODEm). For the remaining countries, we leveraged a natural history model approach, drawing from preceding non-fatal case estimates. For all countries, we produced estimates for all age groups between post-neonatal and 59 years.

1. Data-rich countries

For data-rich countries modelled in CODEm, we used the covariates listed in Table 1 to inform predictions. New this cycle, changes were made to the CODEm modelling process and CoD data preparation central framework, as described in another section of this appendix. In order to help the model continue to track pertussis’s strong temporal trend after these central changes, we increased the linear floor value, amplitude, and psi parameters. To further improve the fit, we also changed the types of sub-models considered for inclusion in the final ensemble model in CODEm. In GBD 2019, we used a standard CODEm modelling framework, which includes both linear mixed effects models and spatiotemporal Gaussian process regression (ST-GPR) models in the final ensemble model. In GBD 2021, we only included ST-GPR models in the final CODEm ensemble. The ST-GPR-only ensemble with increased linear floor, amplitude, and psi had lower out-of-sample RMSE and better out-of-sample data coverage than the ensemble model with GBD 2019 settings. Additionally, in GBD 2019 and earlier GBD estimates, we used estimates of routine DTP3 coverage in infants in the year being modelled as a

covariate. In GBD 2021, we instead used estimates of average DTP3 coverage over the last five years. This change was motivated by fluctuations in the annual coverage estimates driven by vaccine stockouts, disruptions due to conflict, or other single-year events. The five-year average covariate allows the influence of these events to be distributed across time in our final pertussis model, better reflecting the expected relationship between coverage and pertussis epidemiology over time.

Table 1. Covariates. Summary of covariates used in the data-rich pertussis cause of death model

Level	Covariate	Direction
1	Average diphtheria-tetanus-pertussis third-dose (DTP3) vaccination coverage over the last five years	-
	Age- and sex-specific SEV for child underweight	+
	Healthcare Access and Quality (HAQ) Index	-
3	Lag-distributed income (LDI)	-
	Socio-demographic Index (SDI)	-
	Mean years of education per capita	-

2. *Natural history model*

The pertussis natural history model uses GBD estimates of non-fatal pertussis cases and an intermediate, custom model of pertussis case fatality rate (CFR) to produce estimates in non-data-rich locations where pertussis mortality data are sparse. As described in the non-fatal pertussis modelling text, case notifications informing the pertussis non-fatal model come from the World Health Organization (WHO) Joint Reporting Form (JRF) and historical documentation of pertussis cases and vaccination from the UK. The pertussis CFR data are compiled through systematic reviews of the literature. This systematic review was not updated for GBD 2021.

With the available pertussis CFR input data, we make location- and year-specific estimates using a negative binomial model with the Healthcare Access and Quality (HAQ) Index as a covariate:

$$Y_{ij} = \beta_0 + \beta_1 HAQ_{ij} + u_j + e_{ij}\,,$$

Pertussis log-transformed incidence – modelled independently – is generated from a mixed effects linear regression model predicting pertussis cases as a function of vaccination coverage. Combining these estimates of incidence for every estimated location and year with location-/year-specific estimates of pertussis CFR, pertussis deaths were calculated as:

$$deaths = incidence * CFR\ .$$

This calculation was replicated at the draw level 1000 times in order to produce estimates of total deaths by location and year and associated uncertainty. These draw-level estimates were age- and sex-split using an age-sex distribution based on global-level age- and sex-specific patterns found in the cause of death data that were updated for GBD 2021, then summarised as the mean of the draws and a 95% uncertainty interval (the 2.5^{th} and 97.5^{th} percentile of all draws). We adjusted pertussis death estimates

for 2020 and 2021 to account for the reductions in pertussis cases associated with the COVID-19 pandemic, as described elsewhere in this appendix.

Pneumoconiosis diseases: silicosis, asbestosis, coal worker's pneumoconiosis, and other pneumoconiosis

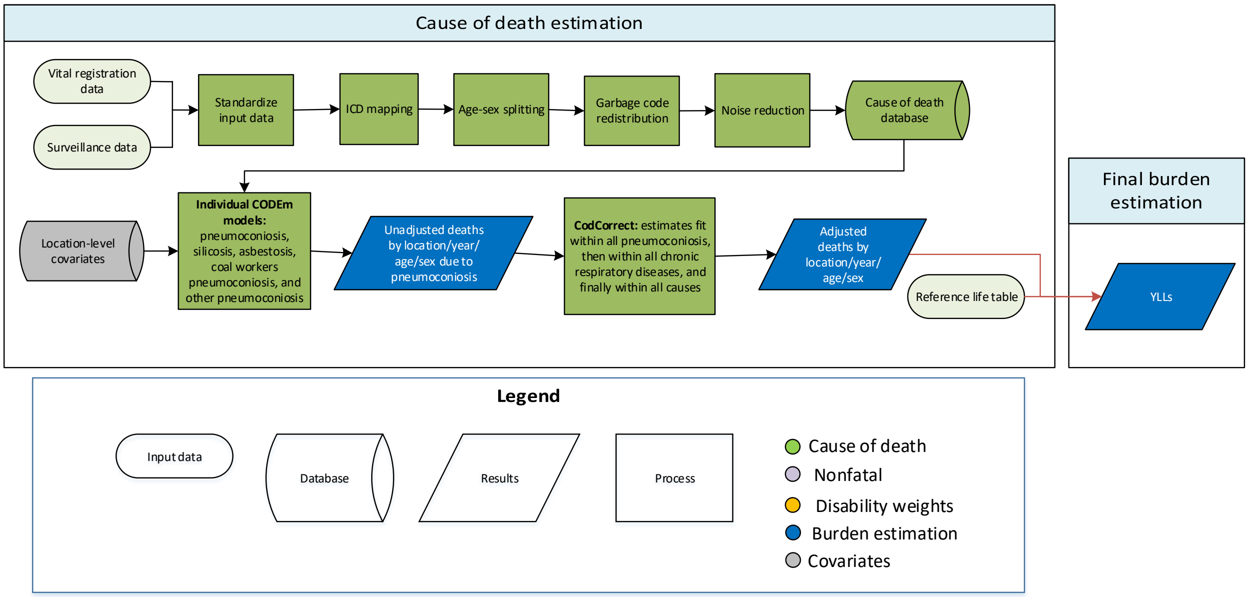

Input data

Data used to estimate pneumoconiosis mortality included vital registration and China mortality surveillance data from the cause of death (COD) database. Our outlier criteria excluded datapoints that (1) were implausibly high or low based on previous data or expert collaborator knowledge, (2) substantially conflicted with established age or temporal patterns, or (3) substantially conflicted with the majority of data from the same locations or locations with similar characteristics (ie, Socio-demographic Index).

Modelling strategy

The standard Cause of Death Ensemble modelling (CODEm) [1] approach (detailed in reference appendix section 3.1) was used to estimate deaths due pneumoconiosis. Separate models were conducted for male and female mortality, and the age range for both models was 15 to 95+ years. We run separate models for total pneumoconiosis, silicosis, asbestosis, coal worker's pneumoconiosis, and other pneumoconiosis.

The total pneumoconiosis model serves as an envelope model for silicosis, asbestosis, coal worker's pneumoconiosis, and other pneumoconiosis models. The mortality estimates from pneumoconiosis disease models were then fit into the chronic respiratory envelope, which is the parent cause for pneumoconiosis disease. Finally, the chronic respiratory disease envelope is fit into the all-cause mortality envelope.

In GBD 2021, we corrected an error in distribution of unspecified pneumoconiosis codes in Taiwan (province of China). This had the effect of reducing overall other pneumoconiosis deaths in Taiwan while slightly increasing asbestosis and silicosis deaths. We also removed age- and sex-specific SEV covariates for occupational asbestos, beryllium, and silica exposure due to a lack of predictive power.

The following table lists the covariates included in the model. This requires that the covariate selected for the model must have the directional relationship with pneumoconiosis. Covariate directions were selected based on the strength of the evidence.

Level	Covariate	Direction
1	Asbestos consumption per capita*	+
	Coal production per capita*	+
	Gold production per capita*	+
2	Smoking prevalence	+
	Indoor air pollution (all cooking fuels)	+
	Cumulative cigarettes (5 years)	+
	Healthcare Access and Quality Index	-
3	LDI (I$ per capita)	-
	Education (years per capita)	-
	Socio-demographic Index	-

* Asbestos, coal, and gold covariates are each used only in a subset of the pneumoconiosis models, as follows: all three are included in the parent all pneumoconiosis model, asbestos consumption is included in the asbestosis model, coal production is included in the coal worker's pneumoconiosis model, and gold production is included in the silicosis model.

Covariate influences:

The following plots show the influence of each covariate on the four CODEm models (male global, male data-rich, female global, and female data-rich). A positive standardised beta (to the right) means that the covariate was associated with increased death. A negative standardised beta (to the left) means the covariate was associated with decreased death.

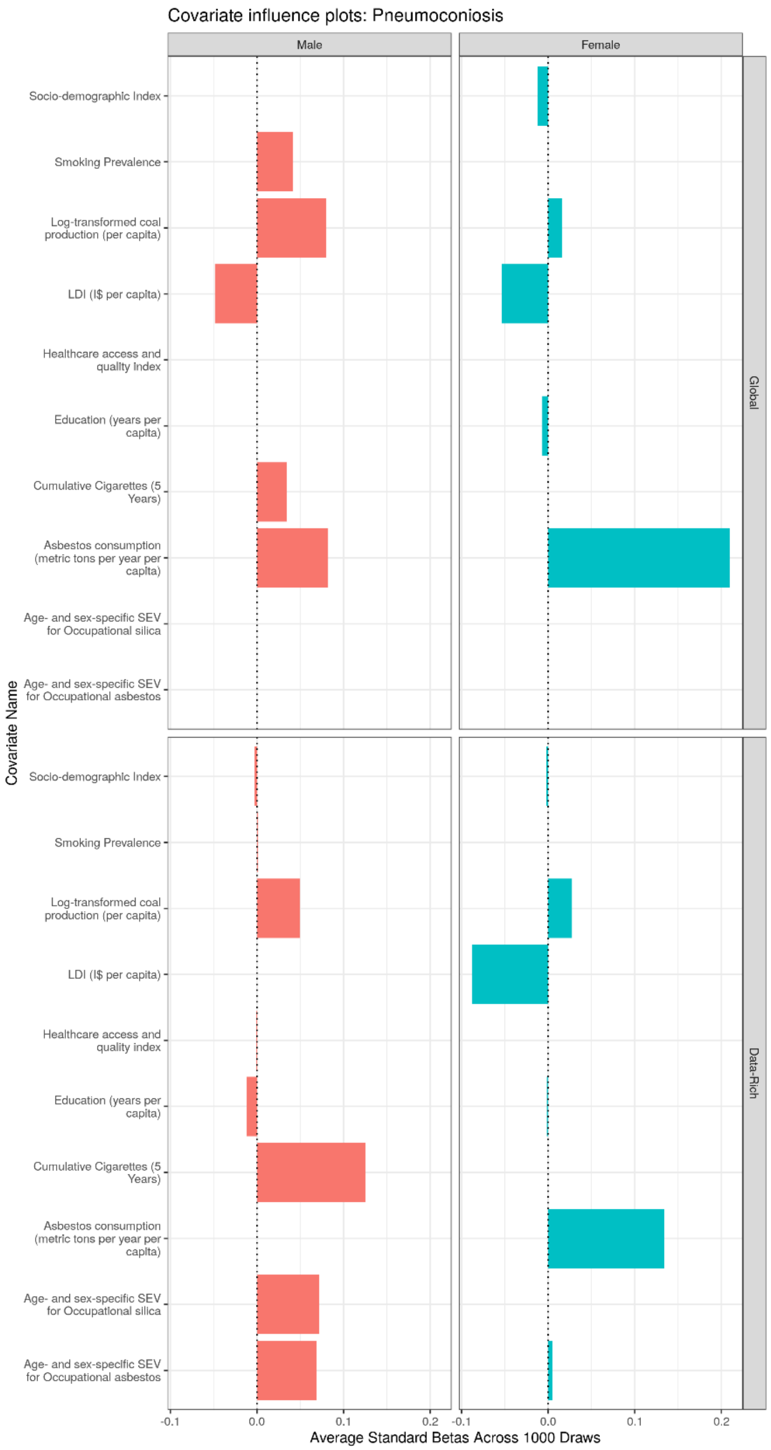

Covariate influence plots: Pneumoconiosis
Male
Female
Global
Data-Rich
Covariate Name
Socio-demographic Index
Smoking Prevalence
Log-transformed coal production (per capita)
LDI (I$ per capita)
Healthcare access and quality index
Education (years per capita)
Cumulative Cigarettes (5 Years)
Asbestos consumption (metric tons per year per capita)
Age- and sex-specific SEV for Occupational silica
Age- and sex-specific SEV for Occupational asbestos
-0.1
0.0
0.1
0.2
Average Standard Betas Across 1000 Draws

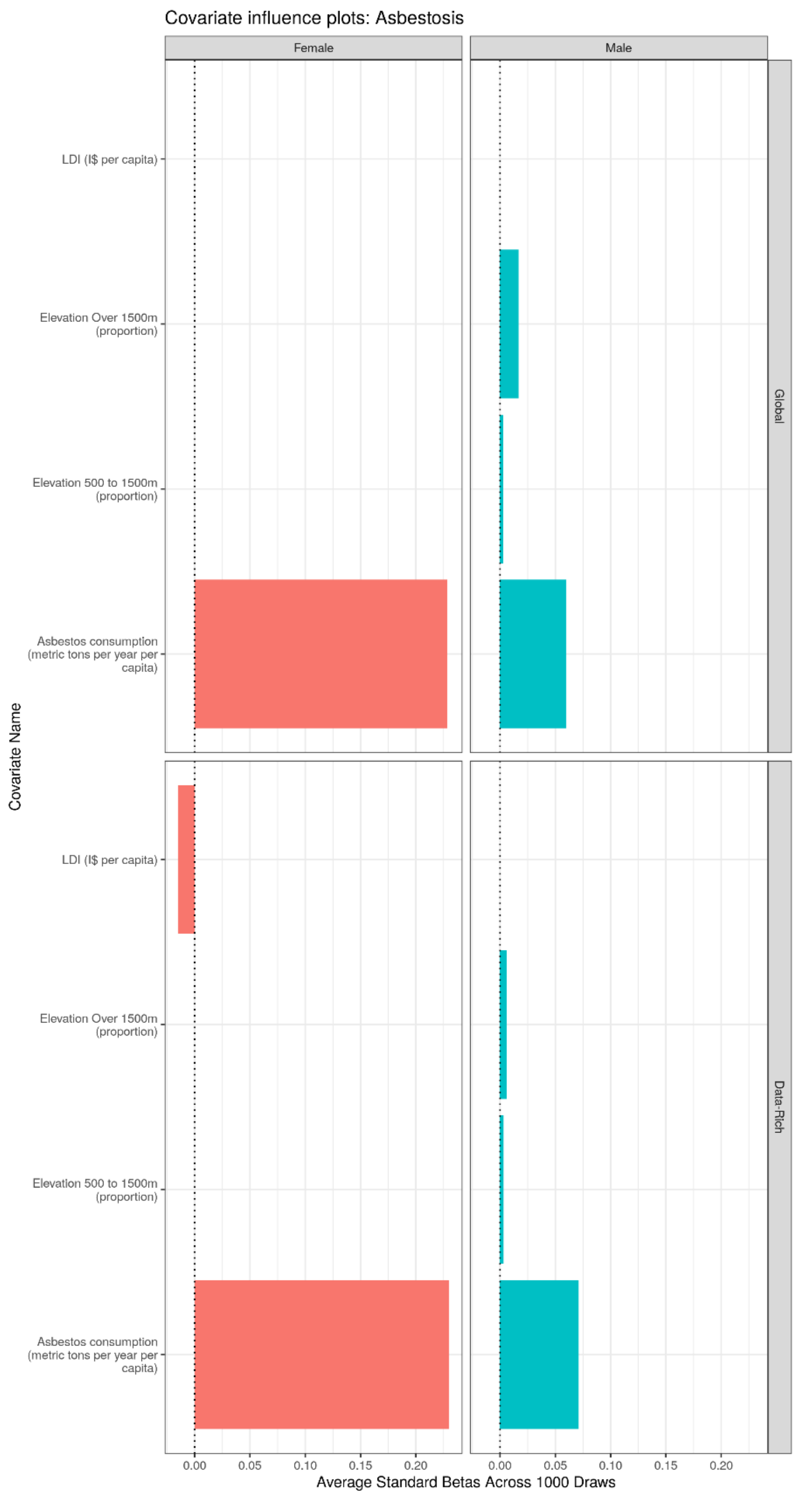

Covariate influence plots: Asbestosis
Female
Male
Global
Data-Rich
Covariate Name
LDI (I$ per capita)
Elevation Over 1500m (proportion)
Elevation 500 to 1500m (proportion)
Asbestos consumption (metric tons per year per capita)
0.00
0.05
0.10
0.15
0.20
Average Standard Betas Across 1000 Draws

Covariate influence plots: Coal workers pneumoconiosis

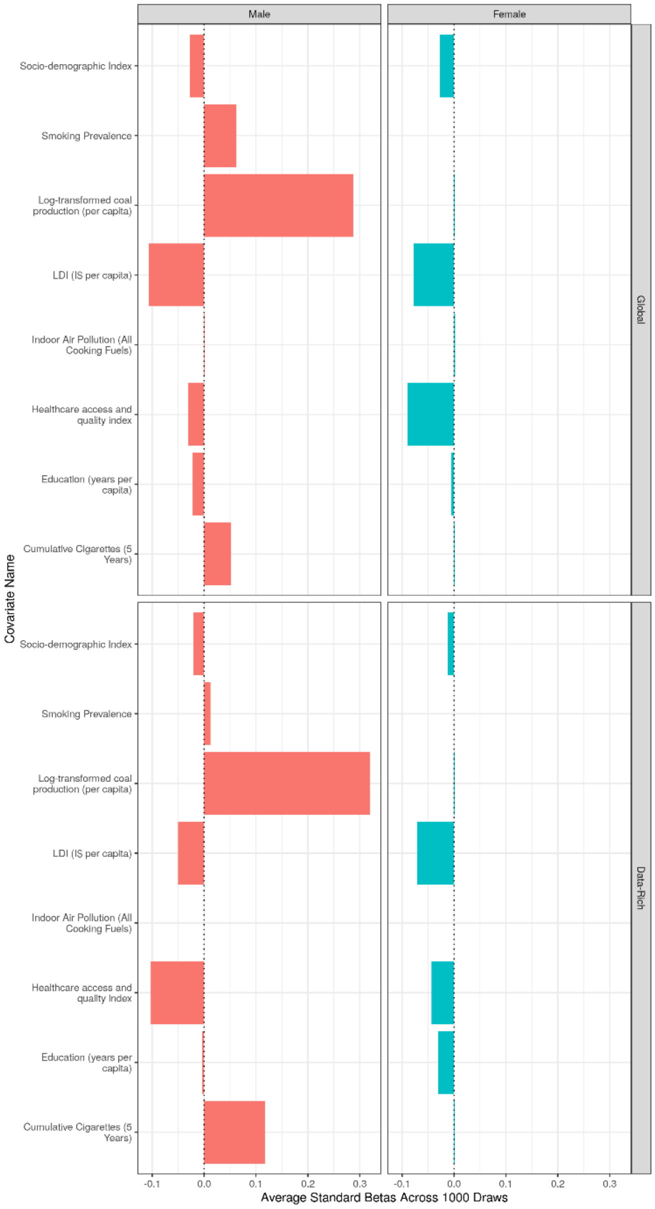

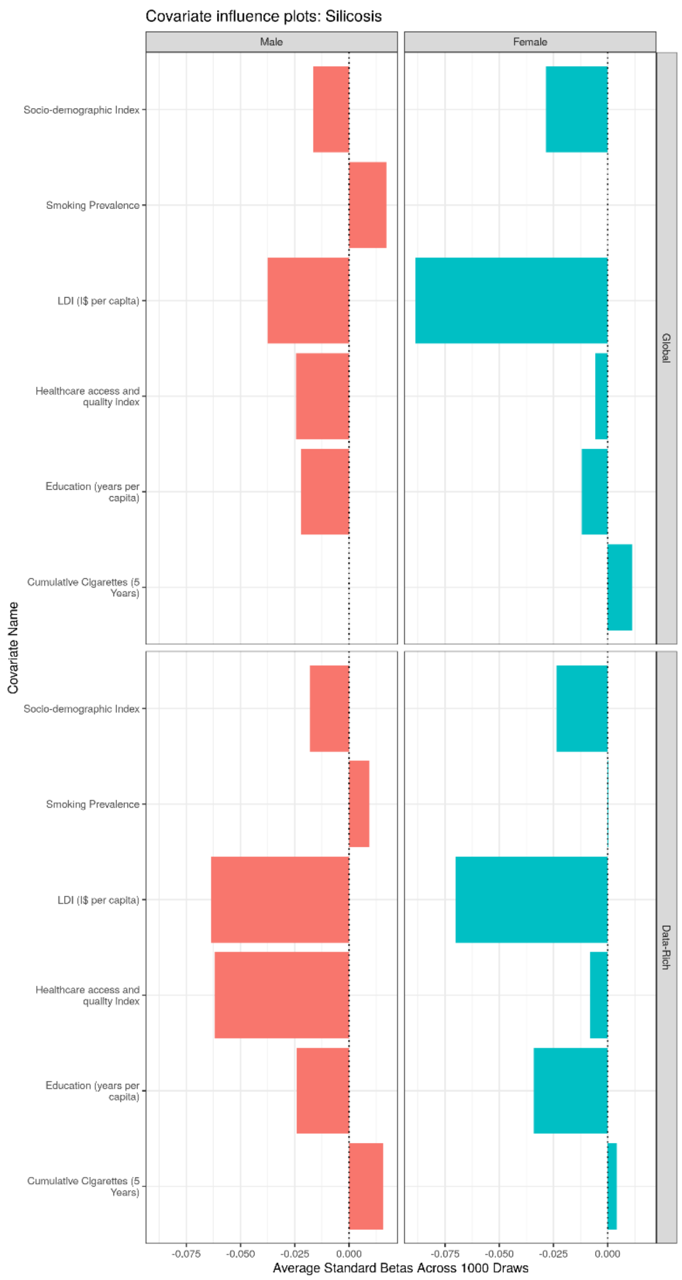
Covariate influence plots: Silicosis
Male
Female
Global
Data-Rich
Covariate Name
Socio-demographic Index
Smoking Prevalence
LDI (I$ per capita)
Healthcare access and quality Index
Education (years per capita)
Cumulative Cigarettes (5 Years)
-0.075
-0.050
-0.025
0.000
Average Standard Betas Across 1000 Draws

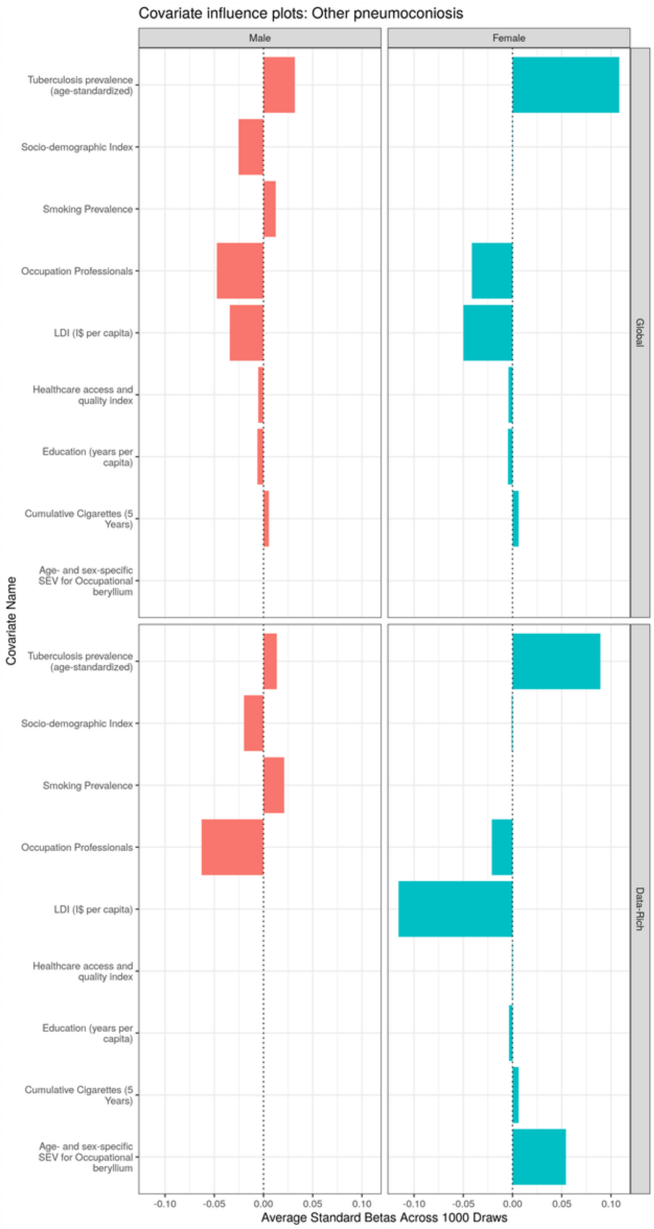

[1]Vos T, Lim SS, Abbafati C, *et al.* Global burden of 369 diseases and injuries in 204 countries and territories, 1990–2019: a systematic analysis for the Global Burden of Disease Study 2019. *The Lancet* 2020; **396**: 1204–22. doi: https://doi.org/10.1016/S0140-6736(20)30925-9

Pulmonary arterial hypertension

Flowchart

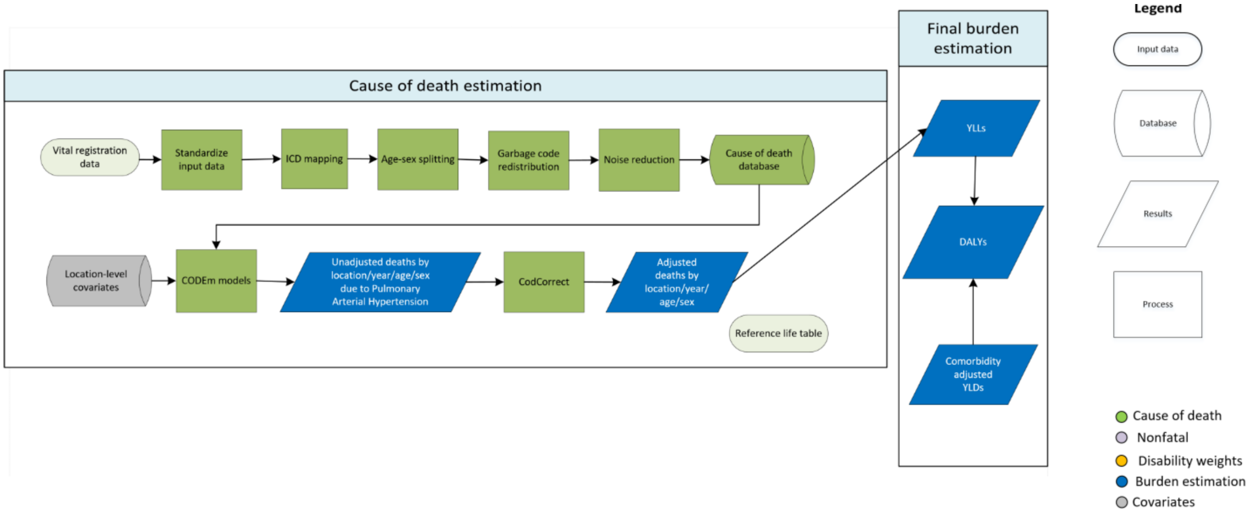

Input data and methodological summary for pulmonary arterial hypertension

Input data

Vital registration data were used to model pulmonary arterial hypertension. ICD codes 416 and I27.0 were used. Clinical guidelines, available ICD codes, and ICD coding practices have changed over time for PAH; after review of the literature and raw data, these codes were chosen as they capture PAH and not pulmonary hypertension groups 2-5. Specifically, we excluded the code I27.2, which may include other pulmonary diseases in addition to PAH. We outliered ICD8, ICD9 BTL, and ICD10 tabulated datapoints, which we believe do not have sufficient detail to distinguish PAH from other pulmonary diseases. In countries where the introduction of I27.0 caused a drastic change in PAH mortality estimates, either in level or temporal trend, we excluded data before the introduction of I27.0. Figure 1 illustrates this change in the US; data before 2003 were excluded, and I27.0 alone represents PAH.

Figure 1: Rate of PAH, as estimated by three ICD codes, in the United States, Belgium, and France

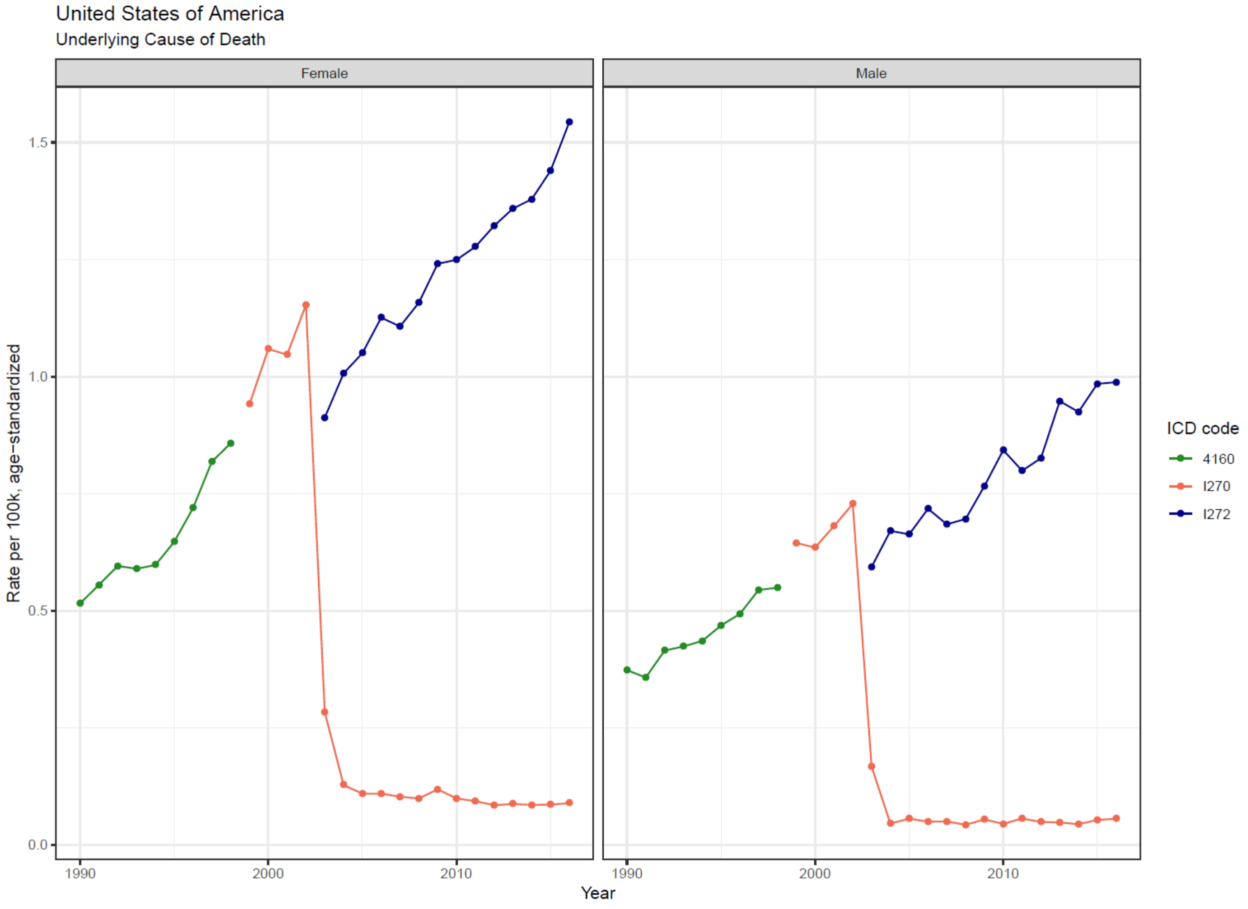

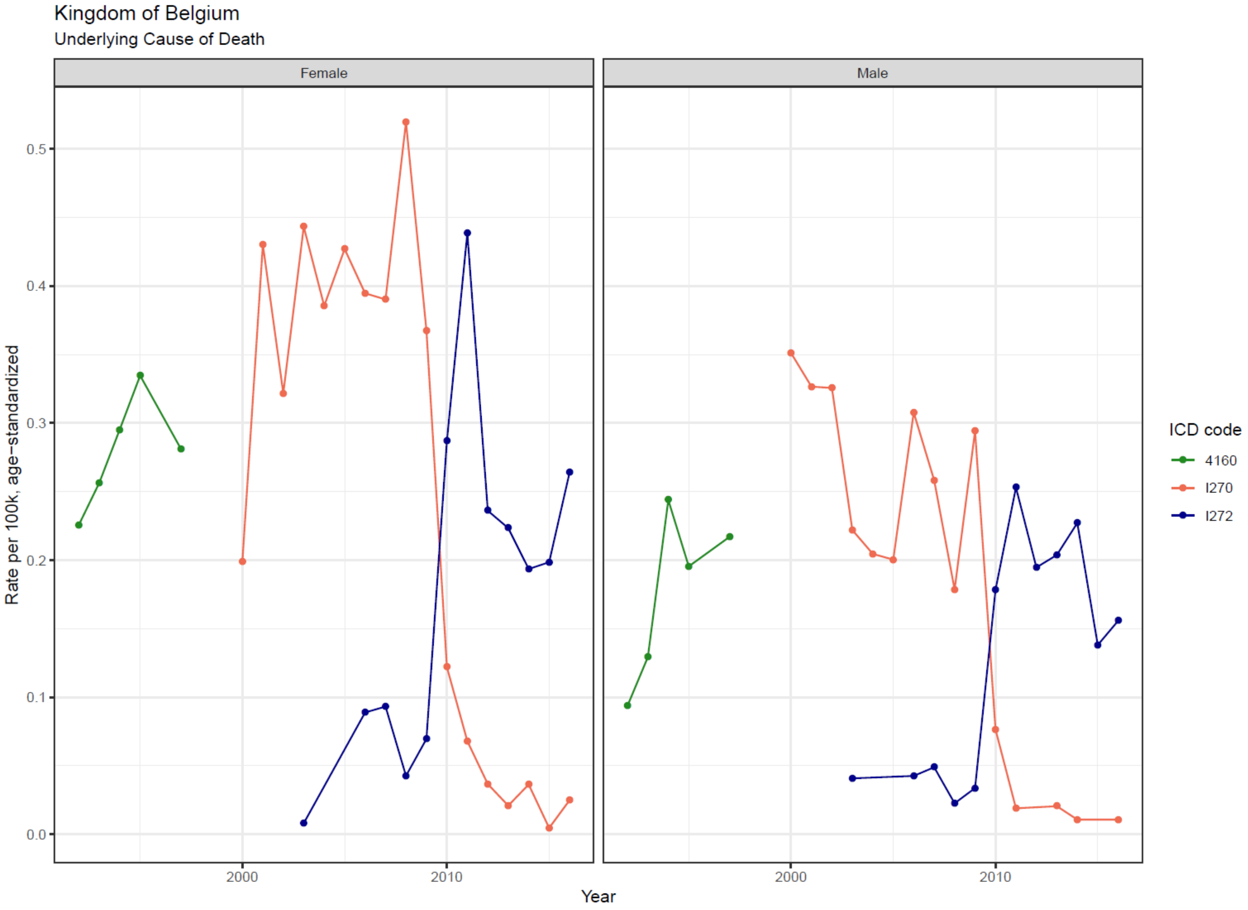
Kingdom of Belgium
Underlying Cause of Death
Female
Male
Rate per 100k, age−standardized
0.0
0.1
0.2
0.3
0.4
0.5
2000
2010
Year
ICD code
4160
I270
I272

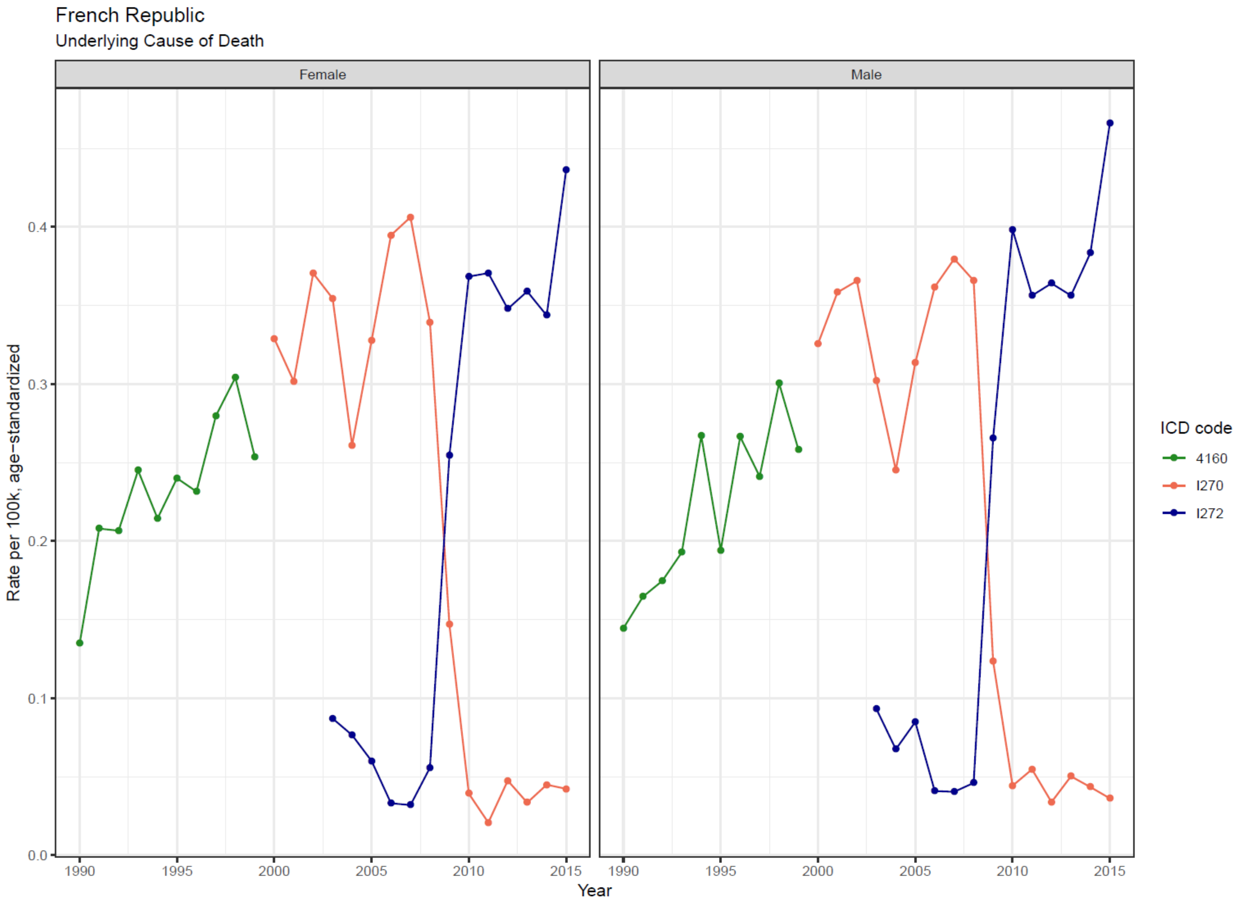

Modelling strategy

We used a standard CODEm approach to model deaths from pulmonary arterial hypertension. The covariates used along with their transformations, importance levels, and imposed directions are reported by cause in the tables below. Schistosomiasis and HIV were chosen as covariates because these diseases are underlying causes of PAH, and they are major drivers of PAH mortality in some locations. SDI and HAQ Index were assigned a negative direction to reflect how treatment, screening, and medication can lower mortality of PAH in locations with high SDI or HAQ Index values. For GBD 2021, we switched from an ensemble of exponential counts models to an ensemble of spacetime models that use exponential smoothing. This improved model fit and reduced uncertainty of estimates for PAH. In addition, we updated our approach to noise reduction of the cause of death data so that stochastic time series of data would resemble patterns seen at regional levels more closely. Additional details on the updates to noise reduction can be found in the cause of death methods section of the appendix.

Table 1. Covariates used in pulmonary arterial hypertension mortality modelling

Level	Covariate	Direction
1	Prevalence of Schistosomiasis	1
	Summary Exposure Value (SEV), HIV	1
	Socio-demographic Index	-1

	Healthcare access and quality index	-1

Pyoderma

Flowchart

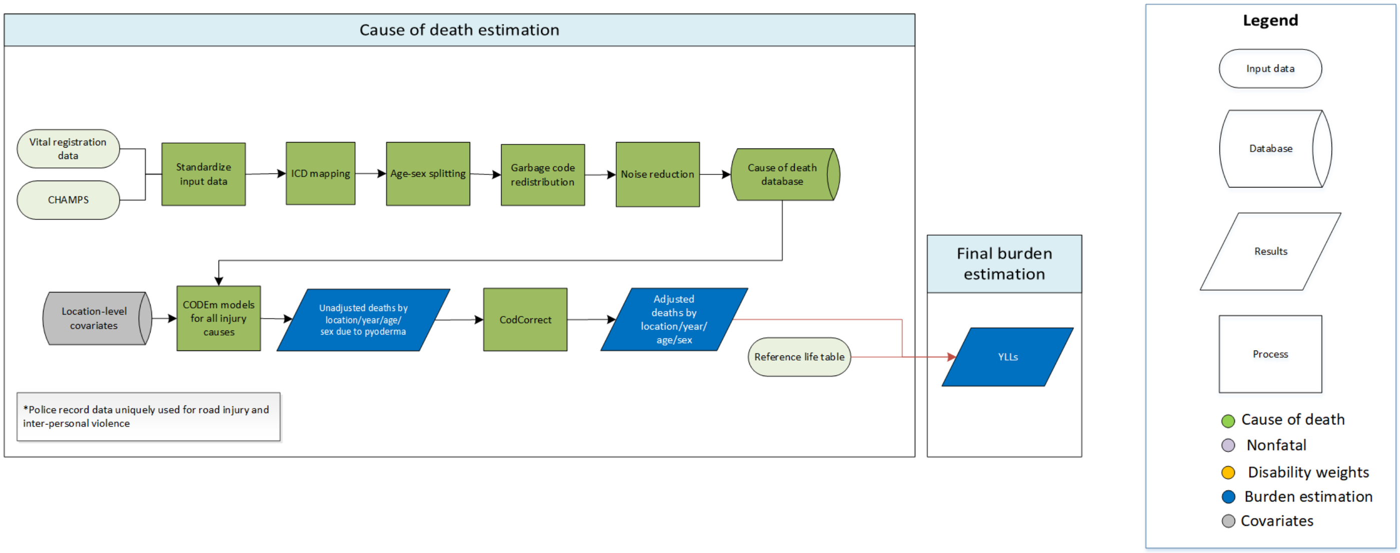

Input data and methodological summary for pyoderma

Input data

Data used to estimate pyoderma mortality included centrally prepped vital registration and verbal autopsy data from the cause of death (COD) database. Outlier criteria excluded datapoints that were implausibly high or low relative to global or regional patterns and data from countries with small populations.

Modelling strategy

We modelled deaths due to pyoderma with a standard CODEm model using the COD database and location-level covariates as inputs. The model followed standard parameters. We hybridised separate global and data-rich models to acquire unadjusted results, which we finalised and adjusted using CoDCorrect to reach final years of life lost due to pyoderma.

There were no significant changes in the modelling process between GBD 2019 and GBD 2021.

Table 1. Covariates used in pyoderma mortality modelling

Level	Covariate	Direction
1	Improved water source (proportion with access)	-
	Prevalence of overweight and obesity	+
	Healthcare Access and Quality Index	-
	Diabetes fasting plasma glucose (mmol/L), by age	+
	Unsafe sanitation (summary exposure value)	+
2	Alcohol (litres per capita)	+
	Cumulative cigarettes (10 years)	+
	Cumulative cigarettes (5 years)	+

	Smoking prevalence	+
3	Lag distributed income (per capita)	-
	Education (years per capita)	-
	Socio-demographic Index	-

Rabies

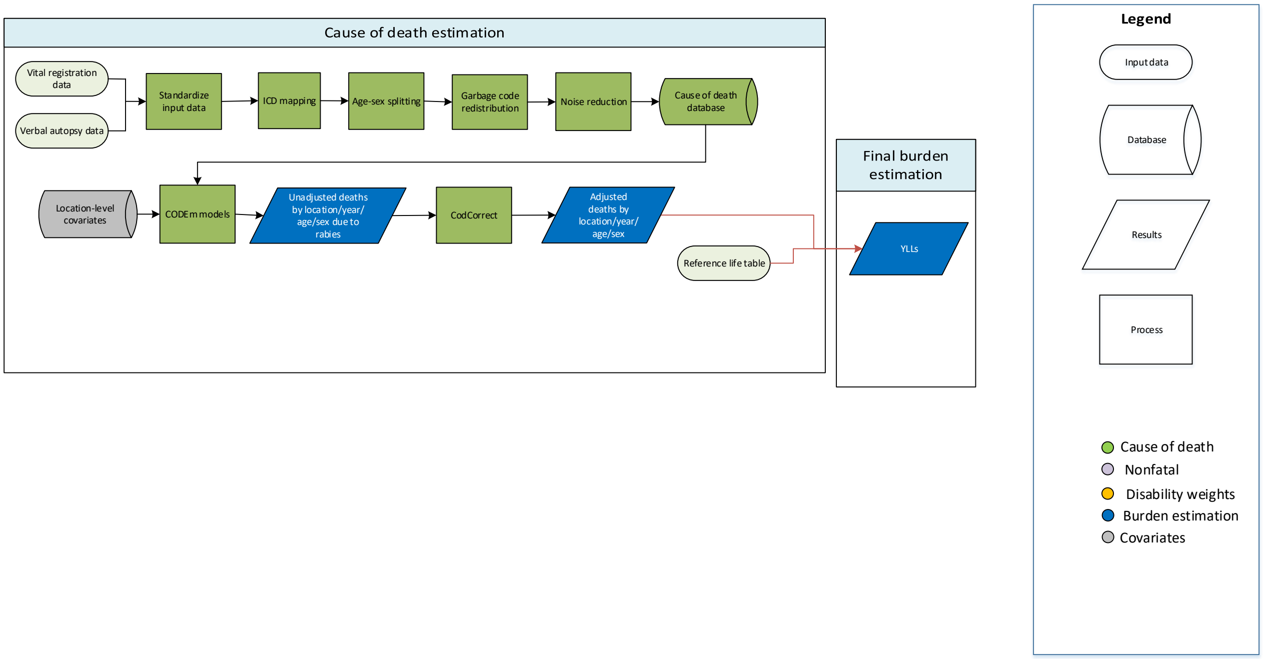

Input data

We modelled rabies mortality using all available data in the cause of death database. Datapoints were outliered if they reported an improbable number of rabies deaths (eg, zero rabies deaths in a hyper-endemic country) or if their inclusion in the model yielded distorted trends. In some cases, multiple data sources for the same location differed dramatically in both their quality and reported rabies mortality (eg, a verbal autopsy and vital registration source). In these cases the lower-quality data source was outliered.

Modelling strategy

We modelled rabies mortality using a two-model hybrid approach: 1) a global (GLB) CODEm model of all locations, using all data in the CoD database; and 2) a CODEm model restricted to data-rich (DR) countries. The CODEm models included eight covariates:

Level	Covariate	Direction	GLB or DR
1	Antenatal care coverage (4 visits)	-	GLB, DR
	Socio-demographic Index	-	GLB, DR
	In-facility delivery coverage	-	GLB, DR
2	Healthcare Access and Quality Index	-	GLB, DR
	Skilled birth attendance coverage	-	GLB, DR
	Maternal care and immunisation	-	DR
3	Population density, 500–1000 per km^2	+	GLB, DR
	Population density, <150 per km^2	+	GLB, DR

Changes since GBD 2019

There were no significant changes in modelling strategy from GBD 2019 to GBD 2021. There were some changes to the input data for the rabies fatal model in GBD 2021. Mainly, previously outliered Survey of Causes of Death (SCD) data in India were restored for all years and ages, and both sexes.

In addition, there were changes in the way CoD data were processed in CODEm (described in detail in another section of this appendix), specifically in applying a linear floor rate and calculation of sample variance. Due to these changes, the CODEm model for rabies implemented various changes to CODEm settings allowing the model to better reflect observed temporal trends in the underlying data. See below for a comprehensive list.

Setting	GBD 2019	GBD 2021
Types of models included in ensemble selection	Combination of ST-GPR and linear mixed effects models of rates and cause fractions	Combination of ST-GPR and linear mixed effects models of rates, cause fractions, and counts
Temporal correlation (ie, number of years over which have correlation for a cause)	10	5
SDI, covariate selection level	3 (ie, weak or unknown relationship to outcome)	1 (ie, strong biological link to outcome)

Rheumatic heart disease

Flowchart

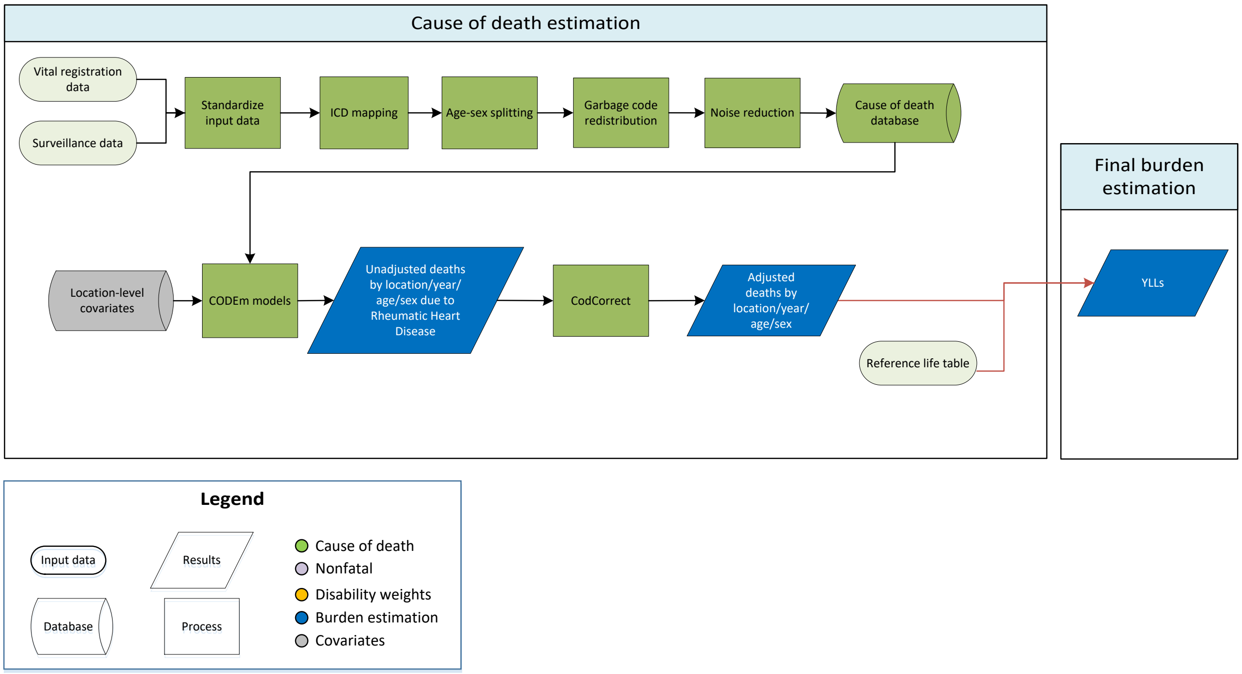

Input data and methodological summary for rheumatic heart disease

Input data

Vital registration and surveillance data were used to model rheumatic heart disease (RHD). We outliered ICD8 and ICD9 BTL datapoints which were inconsistent with the rest of the data and created implausible time trends. We also outliered datapoints which were too high after the redistribution process in a number of age groups. We also outliered ICD8 and ICD9 data from Belgium, Finland, Bulgaria, Mauritius, and Seychelles that were discontinuous with the rest of the time series and created implausible time trends.

In previous GBD cycles, we had included verbal autopsy data in a subset of locations where RHD is endemic. After further review in GBD 2021, we systematically outliered all verbal autopsy data across all locations from the model. This change was made because we judged, after further review and consultation with experts, that verbal autopsy results would be unreliable due to the difficulty distinguishing RHD-attributable deaths from other causes of cardiac death. For this purpose, we removed 31 data sources from 124 locations.

The locations with VA data that were removed include: Aceh, Addis Ababa, Andhra Pradesh, Rural, Andhra Pradesh, Urban, Angola, Arunachal Pradesh, Rural, Arunachal Pradesh, Urban, Assam, Rural, Assam, Urban, Bali, Balochistan, Bangka-Belitung Islands, Bangladesh, Banten, Bengkulu, Bihar, Rural, Bihar, Urban, Brazil, Burkina Faso, Central Java, Central Kalimantan, Central Sulawesi, Chhattisgarh,

Rural, Chhattisgarh, Urban, Delhi, Rural, Delhi, Urban, East Java, East Kalimantan, East Nusa Tenggara, Ethiopia, Ghana, Goa, Rural, Goa, Urban, Gorontalo, Gujarat, Rural, Gujarat, Urban, Haryana, Rural, Haryana, Urban, Himachal Pradesh, Rural, Himachal Pradesh, Urban, India, Indonesia, Jakarta, Jambi, Jammu and Kashmir, Rural, Jammu and Kashmir, Urban, Jharkhand, Rural, Jharkhand, Urban, Jordan, Karnataka, Rural, Karnataka, Urban, Kenya, Kerala, Rural, Kerala, Urban, Khyber Pakhtunkhwa, Kisumu, KwaZulu-Natal, Lampung, Madhya Pradesh, Rural, Madhya Pradesh, Urban, Maharashtra, Rural, Maharashtra, Urban, Malawi, Manipur, Rural, Manipur, Urban, Meghalaya, Rural, Meghalaya, Urban, Mizoram, Rural, Mizoram, Urban, Mozambique, Mpumalanga, Myanmar, Nagaland, Rural, Nagaland, Urban, Nepal, North Sulawesi, North Sumatra, Odisha, Rural, Odisha, Urban, Oromia, Pakistan, Papua, Punjab, Punjab, Rural, Punjab, Urban, Rajasthan, Rural, Rajasthan, Urban, Riau, Riau Islands, Rio Grande do Sul, Sikkim, Rural, Sikkim, Urban, Sindh, South Africa, South Kalimantan, South Sulawesi, South Sumatra, Southeast Sulawesi, Southern Nations, Nationalities, and Peoples, Tamil Nadu, Rural, Tamil Nadu, Urban, Telangana, Rural, Telangana, Urban, Thailand, Tripura, Rural, Tripura, Urban, Turkey, Union Territories other than Delhi, Rural, Union Territories other than Delhi, Urban, United Republic of Tanzania, Uttar Pradesh, Rural, Uttar Pradesh, Urban, Uttarakhand, Rural, Uttarakhand, Urban, Viet Nam, West Bengal, Rural, West Bengal, Urban, West Java, West Kalimantan, West Nusa Tenggara, West Sulawesi, West Sumatra, and Yogyakarta.

Modelling strategy

We used a standard CODEm approach to model deaths from RHD. In addition to the exclusion of verbal autopsy data described above, for GBD 2021, a new approach to redistribute deaths coded to hypertension was implemented using data sources which included information on the chain of events leading to death. This update resulted in an increase in the number of deaths that were re-assigned to cardiovascular diseases. Similarly, the method used to reduce the noise in the data, implemented after redistribution to handle both the stochastic variation across time and space and the occurrence of small number of deaths in each location/year/age/sex, was updated. This new empirical Bayesian noise reduction algorithm uses grouped data by region and data type as prior to better reflect regional patterns and include previously excluded data from data sparse locations such as Philippines subnational locations and Palau. A detailed description on the updates to noise reduction can be found in the cause of death methods section of the appendix.

Table 1: Covariates used in rheumatic heart disease mortality modelling

Level	Covariate	Direction
1	Rheumatic heat disease SEV scalar	1
	Improved water (proportion with access)	-1
	Age- and sex-specific SEV scalar for child underweight	1
	Sanitation (proportion with access)	-1
2	Healthcare access and quality index	-1
3	Lag distributed income per capita (I$)	-1
	Socio-demographic Index	-1
	Education (years per capita)	-1

Rheumatoid arthritis

Flowchart

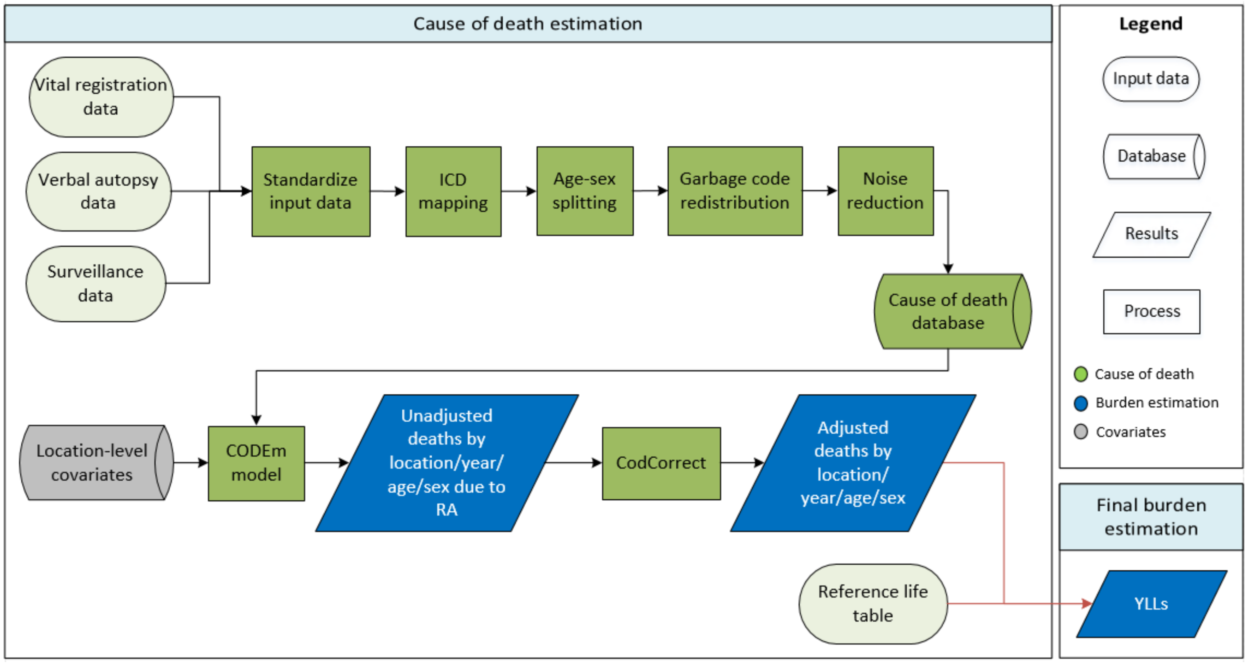

Input data and methodological summary for rheumatoid arthritis

Input data

Data used to estimate rheumatoid arthritis mortality included vital registration and China disease surveillance data from the cause of death database. Our outlier criteria were to exclude datapoints that were (1) implausibly high or low relative to global or regional patterns according to subject matter experts, (2) substantially conflicted with established age or temporal patterns, or (3) significantly conflicted with other data sources based from the same locations or locations with similar characteristics (ie, Socio-demographic Index), and (4) from verbal autopsy sources due to the inability of verbal autopsy to accurately capture most musculoskeletal conditions. Data for males in Oman, Qatar, and Saudi Arabia were outliered because they were implausibly low, according to subject matter experts.

Modelling strategy

The standard CODEm (Cause of Death Ensemble model)[1] modelling approach was applied to estimate deaths due to rheumatoid arthritis. We mostly applied the same covariates used in GBD 2019, with a few changes such as including the “milk, unadjusted” covariate in lieu of the deprecated “milk, adjusted”

[1] *Vos T, Lim SS, Abbafati C, et al. Global Burden of 369 Diseases and Injuries in 204 Countries and Territories, 1990–2019: A Systematic Analysis for the Global Burden of Disease Study 2019. The Lancet 2020; 396: 1204–22. Doi: Https://Doi.Org/10.1016/S0140-6736(20)30925-9. Details Found in Appendix 1, Section 3.*, n.d.

covariate. General methods are described elsewhere.[2] The CODEm model for rheumatoid arthritis is limited by a lack of strong predictive covariates. Many are selected as a proxy for Socio-demographic Index (SDI), as auto-immune conditions are expected to increase with SDI. The following table lists the covariates included in the model. This requires that the covariate selected for the model must have the directional relationship with rheumatoid arthritis deaths. Covariate directions were selected based on the strength of the evidence.

Table 1. Covariates used in rheumatoid arthritis mortality modelling

Level	Covariate	Direction
1	Cumulative cigarettes (10 years)	+
	Cumulative cigarettes (5 years)	+
	Smoking prevalence	+
	Milk (g), unadjusted	
	Healthcare Access and Quality Index	-
	Alcohol consumption (litres per capita)	+
2	Mean BMI	+
	Mean cholesterol[3]	+
3	Education (years per capita)	+
	Log-transformed LDI: lag-distributed income ($ per capita)	+
	SDI: Socio-demographic Index	-

Covariate Influences:

The following plot shows the influence of each covariate on the four CODEm models (male global, male data-rich, female global, and female data-rich). A positive standardised beta (to the right) means that the covariate was associated with increased death. A negative standardised beta (to the left) means the covariate was associated with decreased death.

[2] *Vos T, Lim SS, Abbafati C, et al. Global Burden of 369 Diseases and Injuries in 204 Countries and Territories, 1990–2019: A Systematic Analysis for the Global Burden of Disease Study 2019. The Lancet 2020; 396: 1204–22. Doi: Https://Doi.Org/10.1016/S0140-6736(20)30925-9. Details Found in Appendix 1, Section 3.*

[3] This covariate refers to LDL cholesterol.

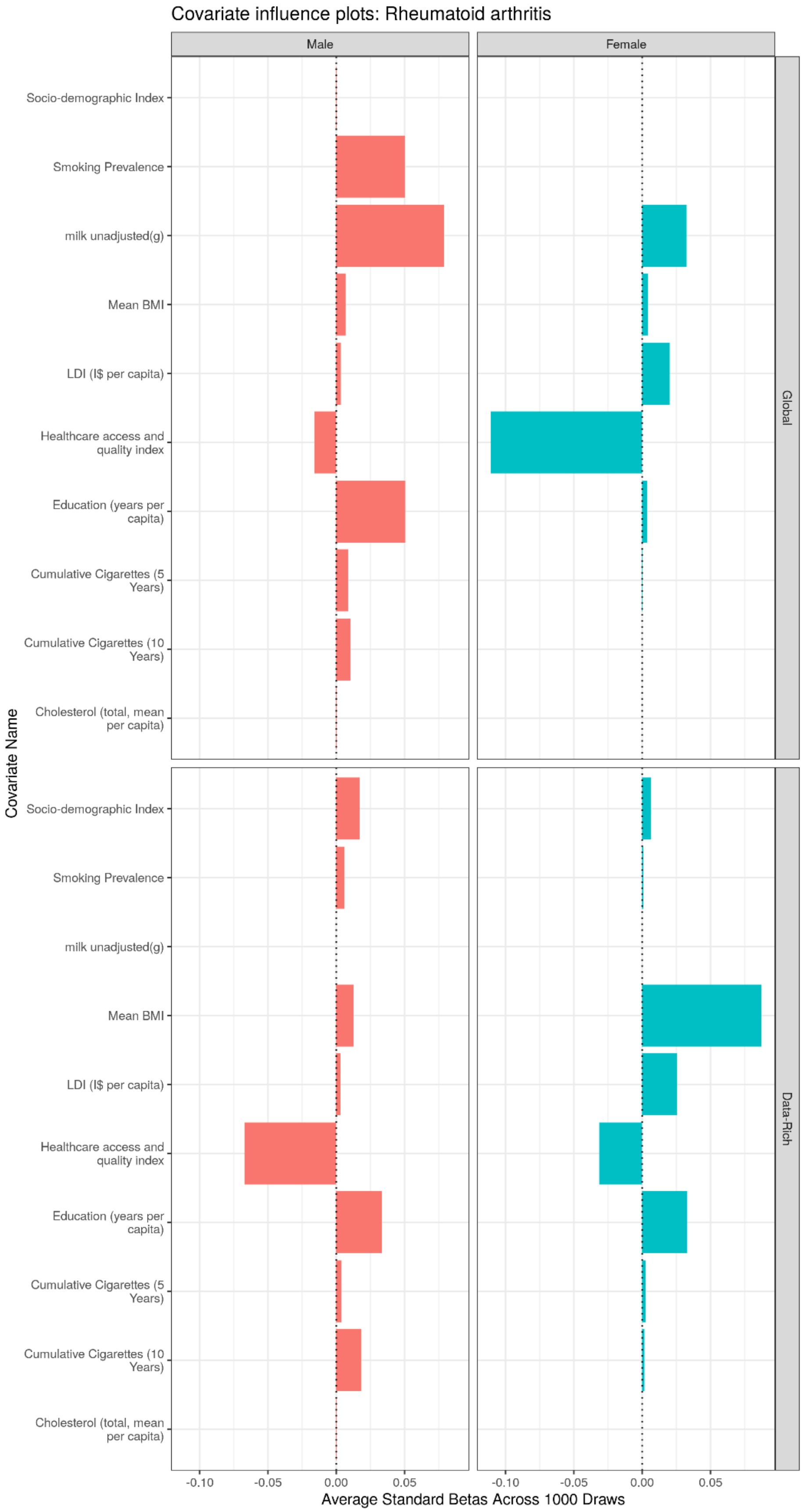
Covariate influence plots: Rheumatoid arthritis
Male
Female
Global
Data-Rich
Covariate Name
Socio-demographic Index
Smoking Prevalence
milk unadjusted(g)
Mean BMI
LDI (I$ per capita)
Healthcare access and quality index
Education (years per capita)
Cumulative Cigarettes (5 Years)
Cumulative Cigarettes (10 Years)
Cholesterol (total, mean per capita)
-0.10
-0.05
0.00
0.05
Average Standard Betas Across 1000 Draws

Schistosomiasis

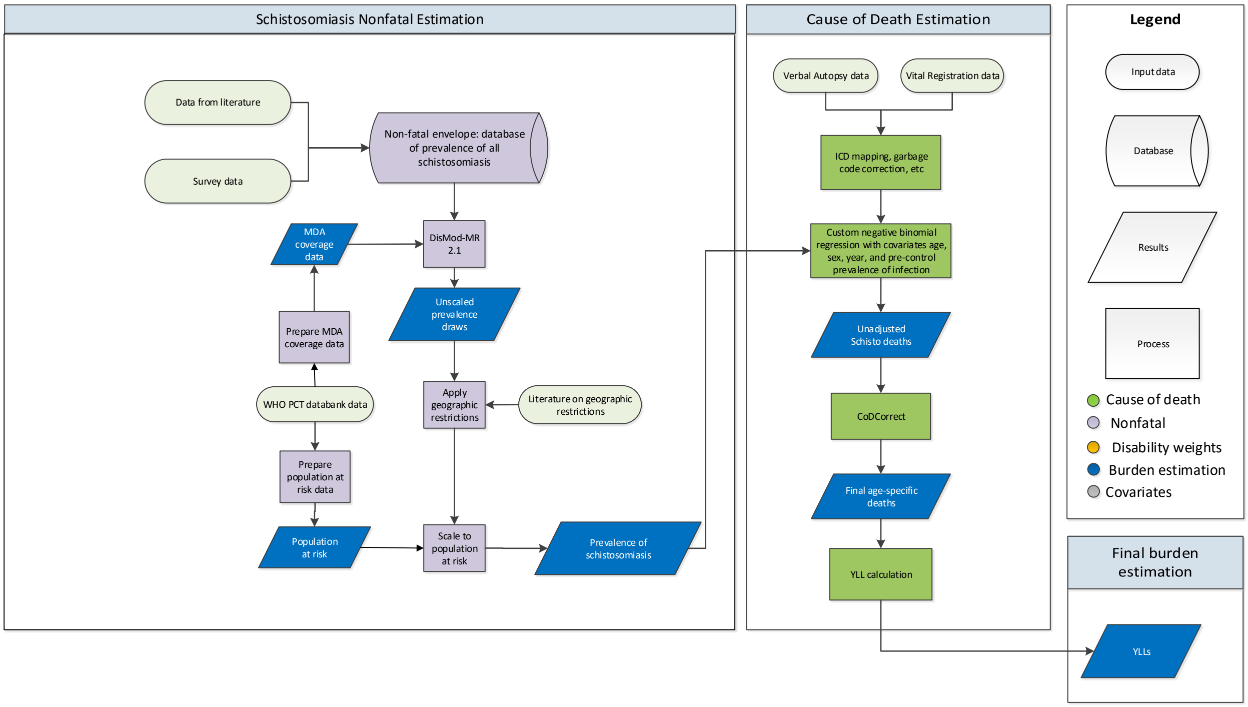

Input data

To estimate mortality due to schistosomiasis, data on deaths and prevalence of infection were used. The prevalence data were prepared for GBD 2021, and further information on prevalence data is available in the non-fatal write-up for this cause. Country-year-age-sex-specific verbal autopsy and vital registration data were used in the mortality model.

Geographical restrictions

We conducted a literature review (last updated for GBD 2017) to determine the geographical extent of the disease and classify locations based on whether the disease is absent or present in each year. Locations that were geographically restricted in any given year did not have estimates made for them but could have imported cases attributed to them at a later stage. Evidence of absence or presence was not available for every location for each year, and so assumptions were made for missing years by taking into consideration the epidemiological characteristics of the disease. If evidence indicated disease presence for two non-consecutive years, we assumed presence for all years between the two. If evidence indicated disease absence for two non-consecutive years, we assumed absence for all years between the two. If evidence indicated a change in status (ie, from absent to present, or present to absent) between two non-consecutive years, then we conducted targeted searches to ascertain the relevant year of introduction or elimination for that location. In the cases where presence or absence information was missing for the start or end years of our study interval (1990–2021) without evidence of any introduction or elimination events within the interval, we applied the status of the first and last presence/absence observations, respectively, to all years between the interval bound and the

observation year. For schistosomiasis, we used a combination of Chitsulo and colleagues' *The global status of schistosomiasis and its control* (1) and WHO's *Preventative chemotherapy in human helminthiasis* report (2) as a baseline. Where country-level endemicity statuses conflicted between the two sources, we searched PubMed and Google Scholar for country- and subnational-specific endemicity status. Our search yielded 22 sources that were used to develop our annual geographical restriction map for schistosomiasis.

Modelling strategy

To estimate deaths due to schistosomiasis, a negative binomial regression model of country-year-age-sex-specific deaths on natural log-transformed age-standardised schistosomiasis infection prevalence with a 15-year lag was used. The negative binomial regression was selected due to its suitability for modelling count data. In addition, there are relatively low numbers of deaths attributable to schistosomiasis. Indicator variables for endemic Brazil subnationals and South Africa subnationals were used to allow the model to follow data in those areas. A multivariate normal distribution using the mean and variance-covariance matrix from the model was used to generate 1000 draws of deaths due to schistosomiasis.

Models were evaluated by assessing the AIC and plotting the predicted deaths against time, age, and sex. In addition, the Cause of Death visualisation tool was used to evaluate time trends across locations, age, and sex. A map of the global distribution of schistosomiasis across age groups was also used to assess the changes in death rates over time. The final model was selected based on how well the estimated numbers fit the input data and how plausible the predicted distribution of disease was over time and with age.

Changes from GBD 2019 to GBD 2021

We have made no substantive changes in the modelling strategy from GBD 2019.

References

(1) Chitsulo, L., Engels, D., Montresor, A., & Savioli, L. (2000). The global status of schistosomiasis and its control. Acta Tropica, 77(1), 41-51. doi:10.1016/s0001-706x(00)00122-4
(2) World Health Organization (2006). Preventive chemotherapy in human helminthiasis: coordinated use of anthelminthic drugs in control interventions : a manual for health professionals and programme managers.

Sexually transmitted infections excluding HIV

Total, chlamydia, gonorrhoea, syphilis, and other

Mortality due to sexually transmitted infections excluding HIV in adults (adult STIs) and mortality due to congenital syphilis are estimated separately and aggregated to calculate total fatal burden for sexually transmitted infections excluding HIV.

Flowchart – adult STIs

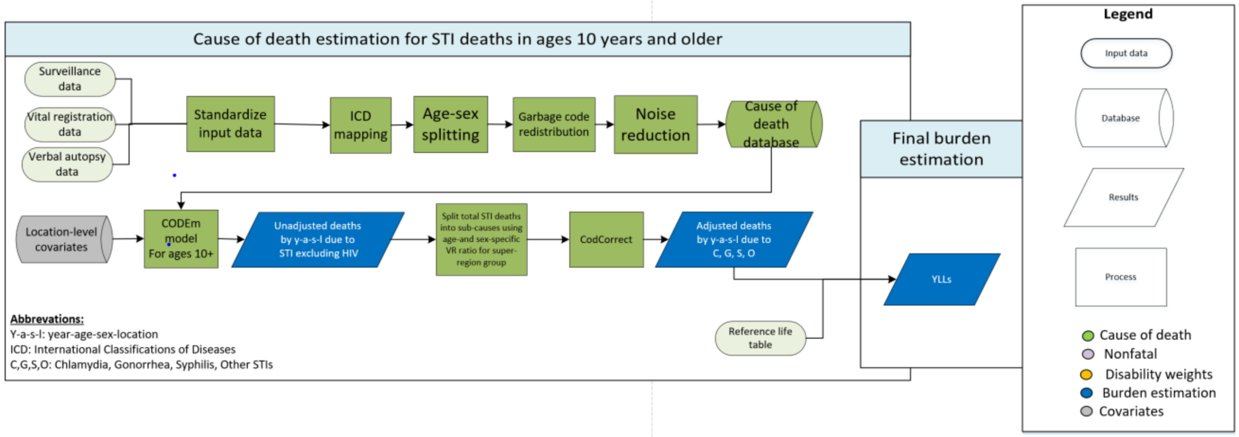

Input data – adult STIs

Total adult deaths due to STI excluding HIV were modelled in aggregate for males and females 10 years and older using centrally processed vital registration, verbal autopsy, and surveillance data from the cause of death (COD) database. These data included deaths from all geographies and coding systems for syphilis, chlamydial infection, gonococcal infection, and other STIs excluding HIV. Please see the list of International Classification of Diseases (ICD) codes mapped to the Global Burden of Disease cause list for causes of death elsewhere in the appendix. Outliers were identified by systematic examination of all datapoints for all location-years. Data were excluded if they violated well-established time or age trends, and data in instances where garbage code redistribution and noise reduction, in combination with small sample sizes, resulted in unreasonable cause fractions. Methods for assigning outlier status were consistent across vital registration, verbal autopsy, and surveillance data types.

To produce estimates of deaths specifically due to syphilis, chlamydial infection, gonococcal infection, and other STIs, estimates from the total model were divided according to proportions that were estimated from all available cause-specific vital registration data.

Modelling strategy – adult STIs

We completed data-rich (DR) and global CODEm models for ages 10 years and older for males and females separately. Ten covariates were entered for possible selection in each CODEm model, including 1) prevalence of positive syphilis serology; 2) coverage of one antenatal care (ANC) visit; 3) coverage of four or more ANC visits; 4) age-specific fertility rate; 5) total fertility rate; 6) maternal care & immunisation (a covariate based on a principal components analysis of ANC, in-facility delivery, skilled birth attendance, and vaccine coverage); 7) Healthcare Access and Quality (HAQ) Index, 8) lag-

distributed income per capita (LDI); 9) years of education per capita; and 10) abortion legality (a categorical rating of abortion laws that range from 1 [always illegal] to 7 [always legal on demand]).

Table 1: Covariates used in STI mortality modelling

Level	Covariate	Direction
1	Syphilis prevalence	+
2	Abortion legality	-
	Age-specific fertility rate	+
	Education (years per capita)	-
	Total fertility rate	+
	Maternal care & immunisation	-
	Healthcare Access and Quality Index	-
3	Antenatal care coverage, 1+ visits	-
	Antenatal care coverage, 4+ visits	-
	Lag-distributed income	-

Data-rich and global models for each sex were then hybridised. Outputs of these models were then split into the sub-causes using vital registration (VR) data from the COD database. Trichomoniasis and HSV-2 were assumed not to cause mortality. Chlamydia was assumed to not to cause death in males. Therefore, for males, the STI CODEm model was split into deaths due to syphilis, gonorrhoea or other STI. For females, the STI CODEm model was split into deaths due to syphilis, gonorrhoea, chlamydia, or other STI.

In GBD 2017, cause-specific VR data were summed by age group and sex, then scaled to the total STI death model in order to calculate proportions for each specific infection. These proportions were then applied to all locations. Beginning in GBD 2019 and continuing in GBD 2021, to account for geographical variation in proportions, cause-specific VR data were summed by age group, sex, and super-region, then scaled to the total. Unfortunately, the COD database had very sparse data on STI causes of death in sub-Saharan Africa and north Africa and the Middle East, which resulted in implausible proportions estimated for these super-regions. As a result, the decision was made to calculate cause-specific proportions by age, sex, and two super-region groups.

Table 2: Super-region groups for STI sub-cause proportions

Super-region group	Super-regions included
ALMA	• *Southeast Asia, east Asia & Oceania*<br>• *Latin America & Caribbean*<br>• *North Africa & Middle East*<br>• *South Asia*<br>• *Sub-Saharan Africa*
CECAH	• *Central Europe, eastern Europe, and central Asia*<br>• *High income*

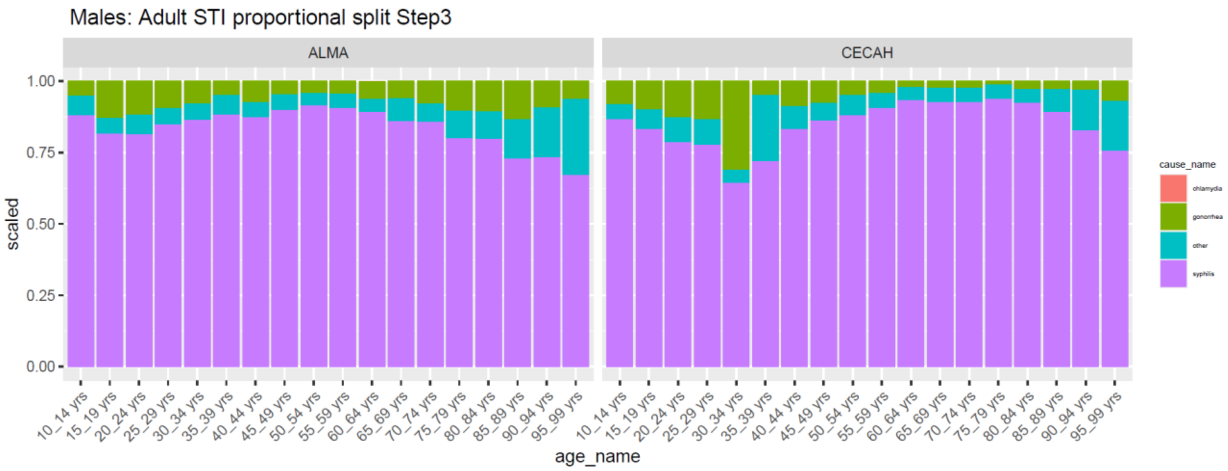

Males: Adult STI proportional split Step3
ALMA
CECAH
scaled
1.00
0.75
0.50
0.25
0.00
10_14 yrs
15_19 yrs
20_24 yrs
25_29 yrs
30_34 yrs
35_39 yrs
40_44 yrs
45_49 yrs
50_54 yrs
55_59 yrs
60_64 yrs
65_69 yrs
70_74 yrs
75_79 yrs
80_84 yrs
85_89 yrs
90_94 yrs
95_99 yrs
age_name
cause_name

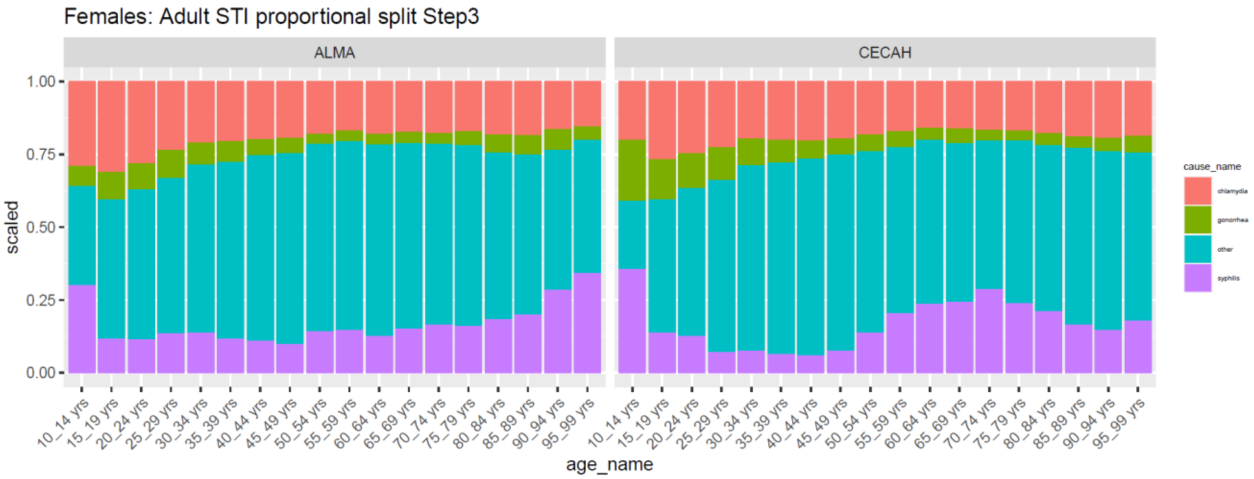

Females: Adult STI proportional split Step3
ALMA
CECAH
scaled
1.00
0.75
0.50
0.25
0.00
10_14 yrs
15_19 yrs
20_24 yrs
25_29 yrs
30_34 yrs
35_39 yrs
40_44 yrs
45_49 yrs
50_54 yrs
55_59 yrs
60_64 yrs
65_69 yrs
70_74 yrs
75_79 yrs
80_84 yrs
85_89 yrs
90_94 yrs
95_99 yrs
age_name
cause_name

Flowchart – congenital syphilis

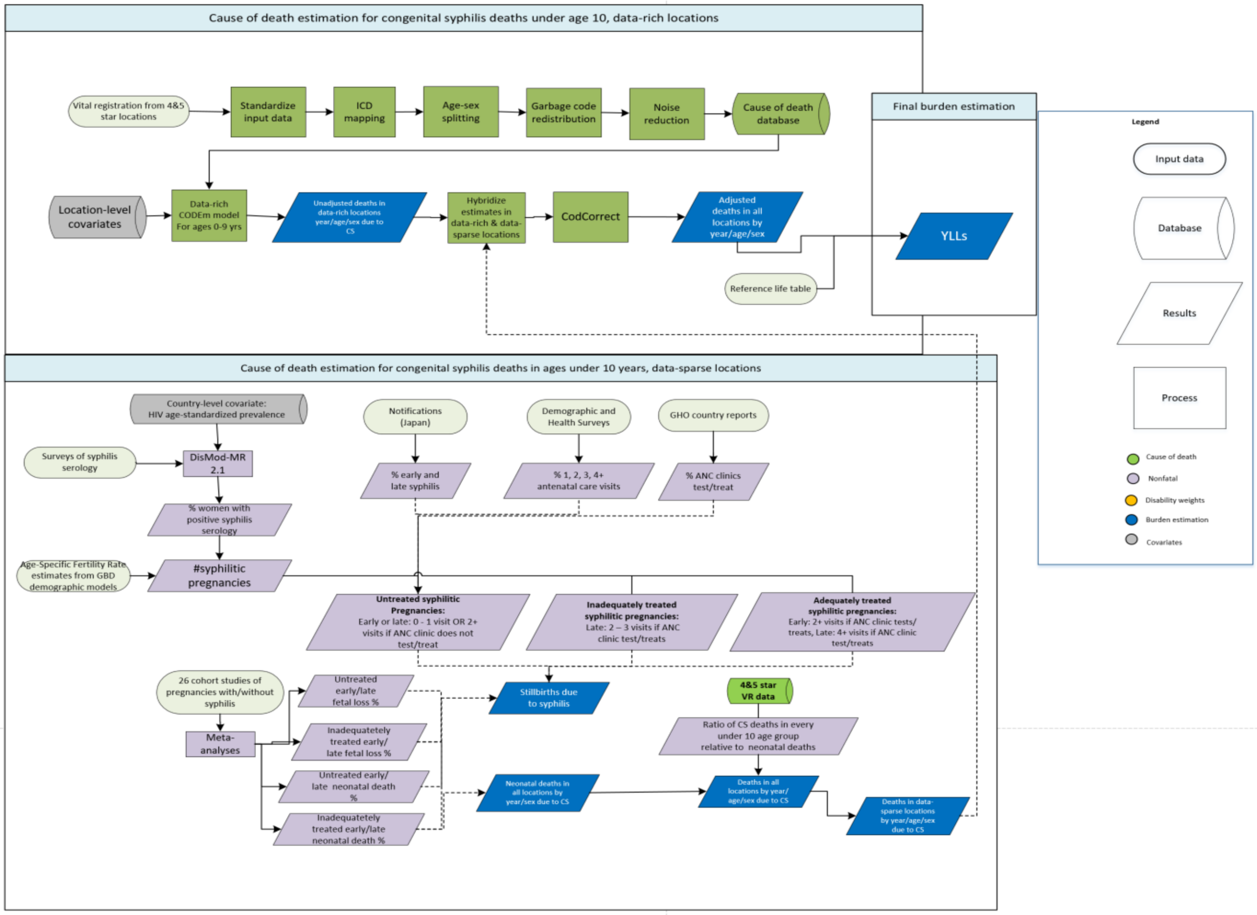

Congenital syphilis arises from the transmission of syphilis from mother to child, in the womb or during childbirth. We model deaths due to congenital syphilis for males and females aged 0 to 9 years. Of all STIs excluding HIV, only syphilis is regarded as causing deaths in children under 10 years. In GBD 2017, congenital syphilis deaths were estimated in all locations with a natural history model. However, after GBD 2017, we found that our natural history model exceeded the number of deaths recorded by countries with high-quality vital registration (VR) and a record of investment into the eradication of congenital syphilis. To produce more plausible estimates based on data considered to be highly complete and reliable, we decided that congenital syphilis deaths in data-rich countries would be estimated in a CODEm model. We continue to use the natural history model to produce estimates for countries with no or lesser quality VR (data-sparse). Outputs for data-sparse countries produced in the natural history model are combined with outputs for data-rich countries produced in the CODEm model, then passed on to the CoDCorrect process as a hybrid model and included in final GBD estimates of mortality due to congenital syphilis. Please see the “cause of death modelling methods: central computation” section of the appendix for more information. In the sections below, the input data and the modelling strategy for each method are described.

Input data – congenital syphilis

CODEm

Deaths due to congenital syphilis (CS) in data-rich countries were modelled using centrally processed vital registration data from the cause of death (COD) database. Please see the list of International Classification of Diseases (ICD) codes mapped to the Global Burden of Disease cause list for causes of death elsewhere in the appendix. The process of marking outliers for this model was similar to that described for adult STIs above.

Natural history

Five different inputs were used to model the natural history of congenital syphilis. Inputs were drawn from both data-rich and data-sparse locations, and the model produced outputs for all location-years. Outputs for data-sparse location-years were passed on to the hybrid model that went into CoDCorrect and subsequently included in final GBD estimates of mortality due to congenital syphilis. Our first inputs were estimates of positive syphilis serology in women of reproductive age pulled from our non-fatal DisMod model of syphilis seroprevalence. A more detailed description of these estimates can be found in the non-fatal methods appendix for sexually transmitted infections (STIs) under the syphilis subheading. Our second inputs were age-specific fertility rates (ASFRs) estimated in the GBD 2021 demographic analyses. Please see the GBD 2021 demographics capstone paper for methodological information about the ASFRs. Third, we used GBD estimates of the number of antenatal care (ANC) visits per pregnant woman. Fourth, we used published data from the Global Health Observatory on the proportion of women that access ANC services that are tested for syphilis[1] and the proportion of positive ANC attendees that receive treatment for syphilis.[2] Fifth, we used cohort studies on the risk of fetal loss and neonatal death in syphilitic women. In GBD 2017, 11 studies were collected through recommendations from our GBD collaborator network. In GBD 2019, we conducted a systematic review of congenital syphilis. The search string below was run on April 4, 2019, through PubMed. It returned 1675 articles. After title/abstract review, 442 articles remained for full-text screening. Of these, 165 were deemed eligible for data extraction. These sources included information on the following: syphilis during pregnancy, stillbirth, spontaneous abortion, preterm birth, low birthweight, neonatal death, transmission of CS, and sequela of CS. 15 of the articles on stillbirth, spontaneous abortion, and neonatal death were combined with the 11 studies from GBD 2017 and included in a meta-analysis of excess neonatal death and fetal loss. Of the remaining 150 articles, sources on the vertical transmission of CS or the sequela of CS are utilised in the non-fatal estimation of congenital syphilis. Sources on syphilis during pregnancy are utilised in the non-fatal estimation of adult syphilis seroprevalence. Please see the sexually transmitted infections (STIs) section of the non-fatal appendix for further information on methodology. Articles on preterm birth and low birthweight aren't currently used in CS estimation but are catalogued in IHME's database, the Global Health Data Exchange (GHDx).

(syphilis[tiab] OR "treponema pallidum"[tiab]) AND ((pregnan[tiab] OR fetal[tiab] OR foetal[tiab] or fetus*[tiab] OR foetus*[tiab] OR neonat*[tiab] OR infan*[tiab] OR newborn*[tiab] OR congenital[tiab]) OR ((vertical*[tiab] OR maternal[tiab] OR mother[tiab] OR fetomaternal[tiab]) AND transmi*[tiab])) AND (outcomes[tiab] OR sequela*[tiab] OR manifestation*[tiab] OR morbidity*[tiab] OR diagnos*[tiab] OR hutchinson*[tiab])*

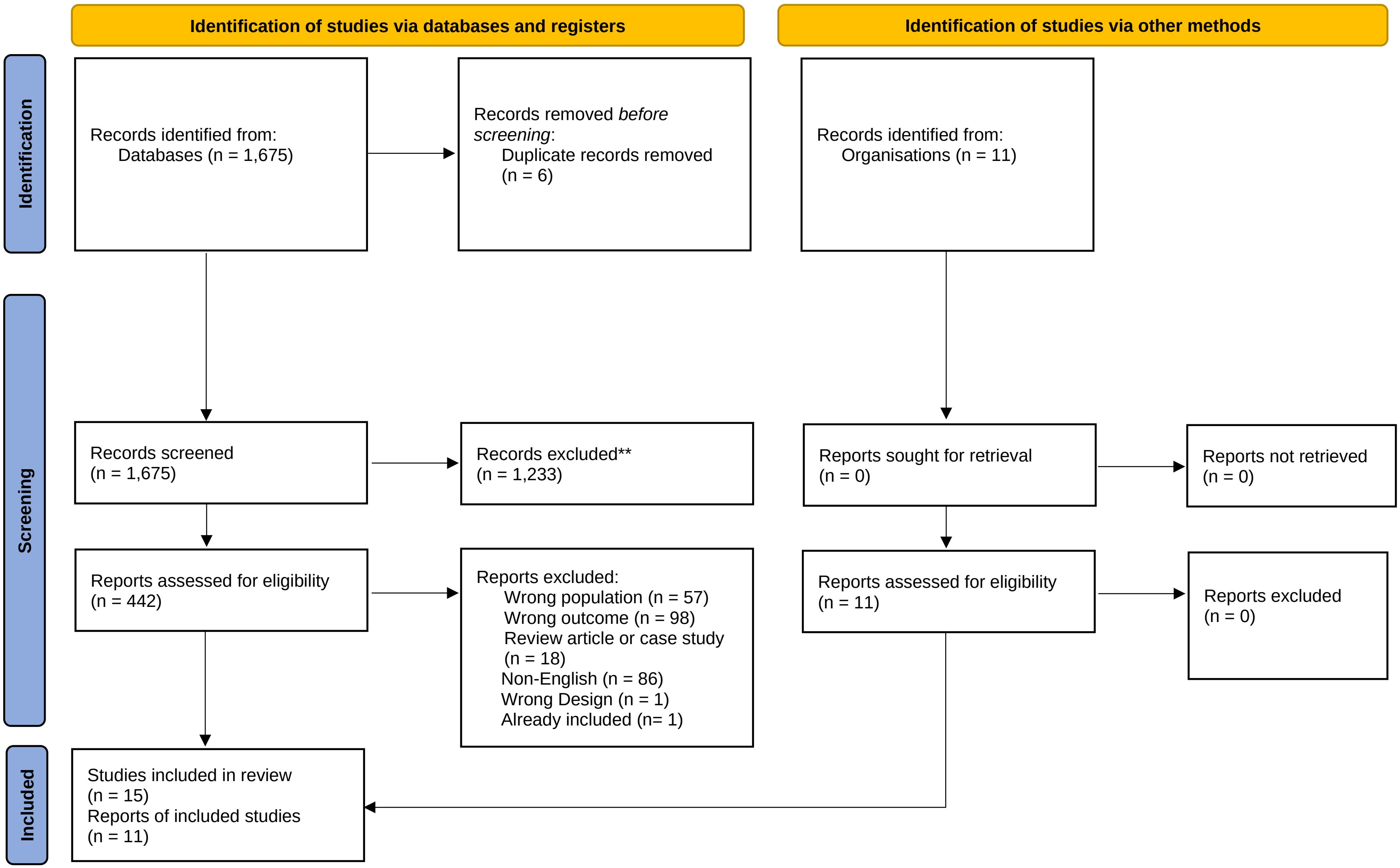
Identification of studies via databases and registers
Identification of studies via other methods
Identification
Screening
Included
Records identified from:
Databases (n = 1,675)
Records removed *before screening*:
Duplicate records removed (n = 6)
Records identified from:
Organisations (n = 11)
Records screened
(n = 1,675)
Records excluded**
(n = 1,233)
Reports sought for retrieval
(n = 0)
Reports not retrieved
(n = 0)
Reports assessed for eligibility
(n = 442)
Reports excluded:
Wrong population (n = 57)
Wrong outcome (n = 98)
Review article or case study (n = 18)
Non-English (n = 86)
Wrong Design (n = 1)
Already included (n= 1)
Reports assessed for eligibility
(n = 11)
Reports excluded
(n = 0)
Studies included in review
(n = 15)
Reports of included studies
(n = 11)

Modelling strategy – congenital syphilis

CODEm

We completed a data-rich CODEm model for ages 0–9 years for males and females separately. Please see the cause of death modelling methods: CODEm section of the appendix for more information. Ten covariates were entered for possible selection in each CODEm model, including 1) female age-standardised prevalence of positive syphilis serology; 2) coverage of one antenatal care (ANC) visit; 3) coverage of four or more ANC visits; 4) maternal care & immunisation (a covariate based on a principal components analysis of ANC, in-facility delivery, skilled birth attendance, and vaccine coverage); 5) abortion legality, an index that includes a categorical rating of abortion laws that ranges from 1 (always illegal) to 7 (always legal on demand); 6) age-specific fertility rate (ASFR); 7) total fertility rate (TFR), 8) years of education per capita; 9) Healthcare Access and Quality (HAQ) index; and 10) lag-distributed income (LDI).

Table 3: Covariates used in congenital syphilis data-rich CODEm model

Level	Covariate	Direction
1	Syphilis prevalence	+
	Antenatal care coverage, 1+ visits	-
	Antenatal care coverage, 4+ visits	-
	Maternal care & immunisation	-
2	Abortion legality	-
	Age-specific fertility rate	+
	Total fertility rate	+
3	Years of education	-
	Healthcare Access and Quality Index	-
	Lag-distributed income	-

Natural history

Our natural history model for congenital syphilis mortality begins with the estimation of pregnancies that are at risk. To calculate this, we multiply the prevalence of positive syphilis serology in women of childbearing age by age-specific fertility rates.

Next, we incorporate five separate measures that allow us to estimate the number of fetal and neonatal deaths in children of infected mothers. These are: 1) the proportion of antenatal (ANC) clinics that both test and treat for syphilis, 2) the number of times that a mother visits an ANC clinic during pregnancy, 3) the stage of disease in infected mothers, 4) excess risk of stillbirth and neonatal death in syphilitic pregnancies by treatment status and stage, and 5) ratios of syphilis death for every age group up to 10 years of age, relative to neonatal deaths.

1) ANC testing and treatment data are obtained from 132 countries via the Global Health Observatory. The first of these measures is the proportion of ANC attendees who are tested for syphilis at their first visit. The second is the proportion of infected women who receive treatment if they test positive for syphilis. These data are entered into a ST-GPR model to estimate these measures for all year-age-location combinations with Socio-demographic Index (SDI) as a covariate.

2) The distribution of the number of skilled antenatal care visits during pregnancy is produced by internal GBD analyses of maternal care. There are three categories: 1 visit, 2-3 visits, and 4+ visits.[3]
3) Detailed notification data from Japan on the stage of syphilis infection in pregnant women diagnosed during antenatal screening.

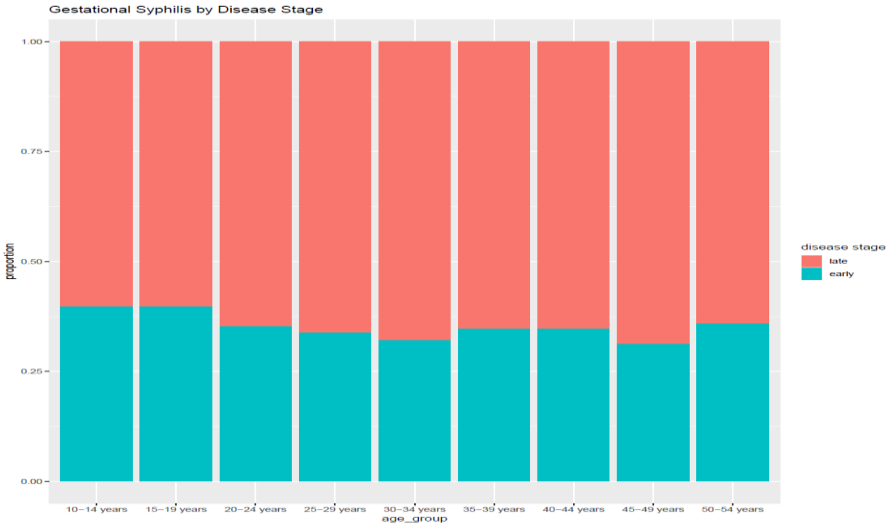

4) The excess risk of stillbirth and neonatal death in syphilitic versus non-syphilitic pregnancies as estimated in a meta-analysis described below.
5) 4- and 5-star vital registration data on deaths from congenital syphilis for males and females in every age group up to 10 years (early neonatal, late neonatal, 1-5 months, 6-11 months, 12-23 months, 2-4 years, 5-9 years). Using these data, we calculate a ratio of deaths for every age group relative to neonatal deaths.

Measures 1-4 are used to estimate total fetal loss and neonatal death from congenital syphilis. The fifth measure allows us to disaggregate neonatal deaths into the early and late neonatal groups, and to estimate the number of deaths in infants in the post-neonatal stage up until age 9 years.

Delving into the methods behind measure 4, the excess risk of fetal loss and neonatal death for syphilitic mothers relative to non-syphilitic mothers was estimated using a meta-analysis of 26 cohort studies. Risks were calculated specified by treatment status of the mother. The year of study, and hence, baseline rates of fetal loss and neonatal death, varied across the cohort studies included in the meta-analysis, but these differences were accounted for by calculating excess risk as the rate in syphilitic pregnancies minus the rate observed in non-syphilitic pregnancies from the same study. For the subset of studies that only reported rates of fetal loss and neonatal death in syphilitic pregnancies, excess risk rates were calculated by subtracting off the year-age-location-specific all-cause stillbirth and all-cause neonatal mortality rates estimated by the GBD demographics team. Please see the GBD 2021 demographics capstone paper for methodological information on these rates. Forest plots of the excess risk data from cohort studies are below. Values of mortality from women of unknown treatment status were excluded from the analysis.

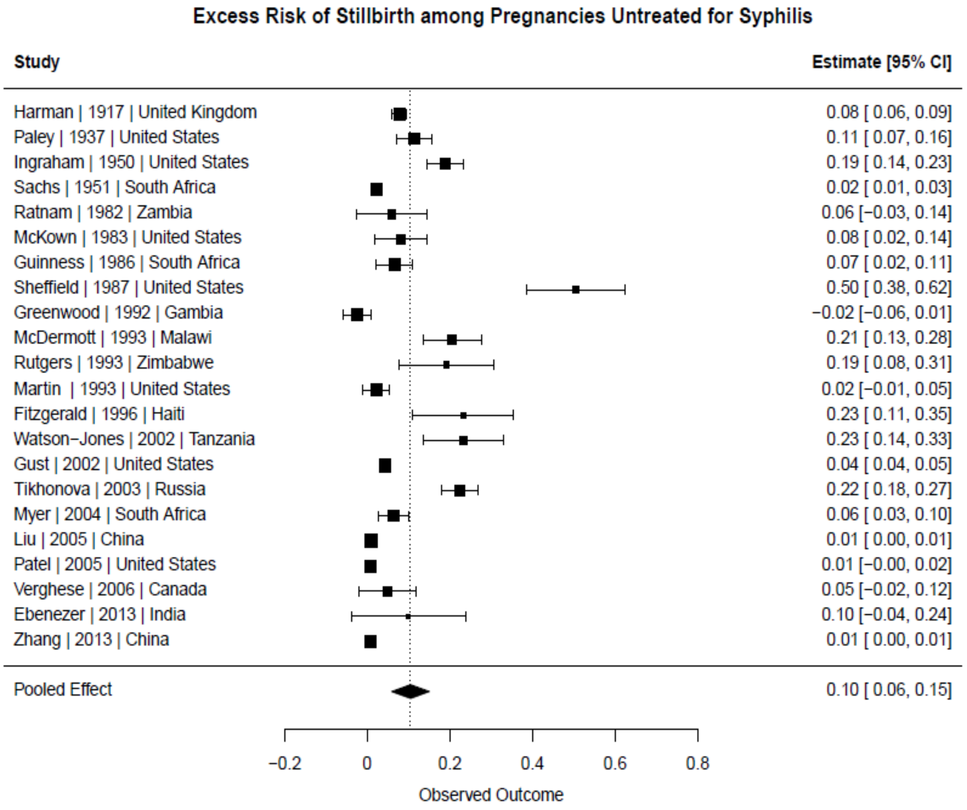
Excess Risk of Stillbirth among Pregnancies Untreated for Syphilis
Study
Estimate [95% CI]
Harman | 1917 | United Kingdom
0.08 [0.06, 0.09]
Paley | 1937 | United States
0.11 [0.07, 0.16]
Ingraham | 1950 | United States
0.19 [0.14, 0.23]
Sachs | 1951 | South Africa
0.02 [0.01, 0.03]
Ratnam | 1982 | Zambia
0.06 [−0.03, 0.14]
McKown | 1983 | United States
0.08 [0.02, 0.14]
Guinness | 1986 | South Africa
0.07 [0.02, 0.11]
Sheffield | 1987 | United States
0.50 [0.38, 0.62]
Greenwood | 1992 | Gambia
−0.02 [−0.06, 0.01]
McDermott | 1993 | Malawi
0.21 [0.13, 0.28]
Rutgers | 1993 | Zimbabwe
0.19 [0.08, 0.31]
Martin | 1993 | United States
0.02 [−0.01, 0.05]
Fitzgerald | 1996 | Haiti
0.23 [0.11, 0.35]
Watson−Jones | 2002 | Tanzania
0.23 [0.14, 0.33]
Gust | 2002 | United States
0.04 [0.04, 0.05]
Tikhonova | 2003 | Russia
0.22 [0.18, 0.27]
Myer | 2004 | South Africa
0.06 [0.03, 0.10]
Liu | 2005 | China
0.01 [0.00, 0.01]
Patel | 2005 | United States
0.01 [−0.00, 0.02]
Verghese | 2006 | Canada
0.05 [−0.02, 0.12]
Ebenezer | 2013 | India
0.10 [−0.04, 0.24]
Zhang | 2013 | China
0.01 [0.00, 0.01]
Pooled Effect
0.10 [0.06, 0.15]
−0.2
0
0.2
0.4
0.6
0.8
Observed Outcome

Excess Risk of Stillbirth among Pregnancies Inadequately Treated for Syphilis

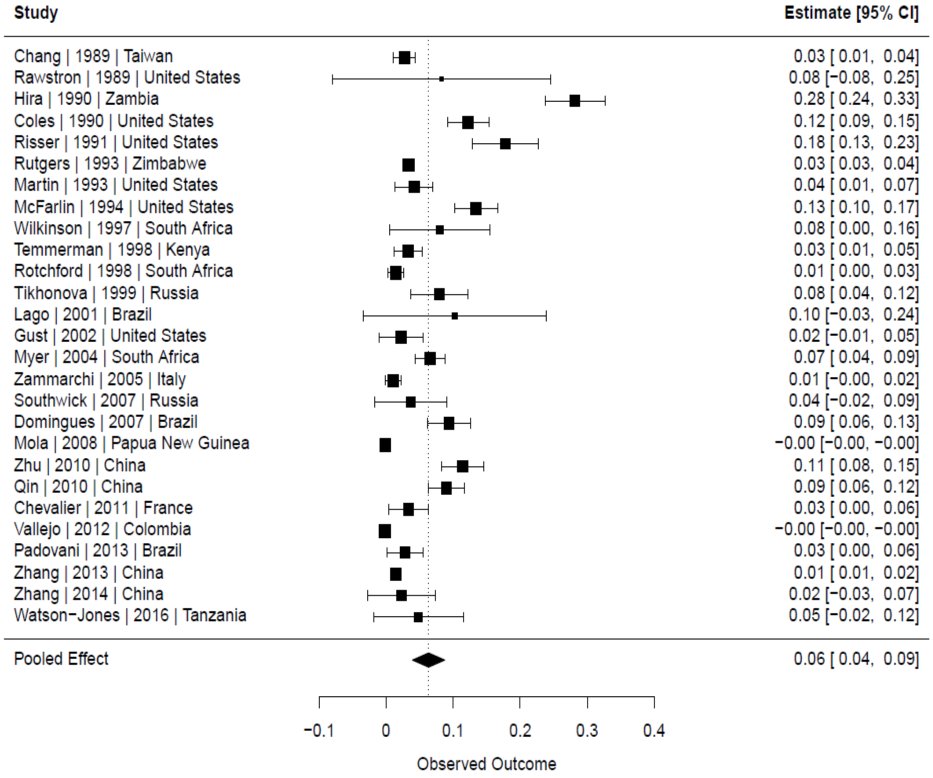

Excess Risk of Neonatal Death among Pregnancies Untreated for Syphilis

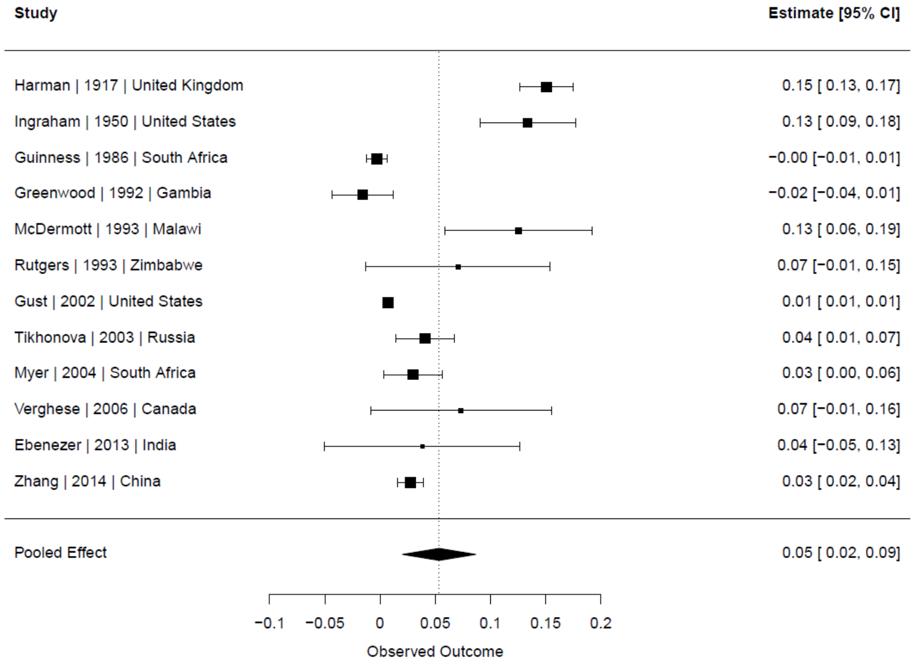

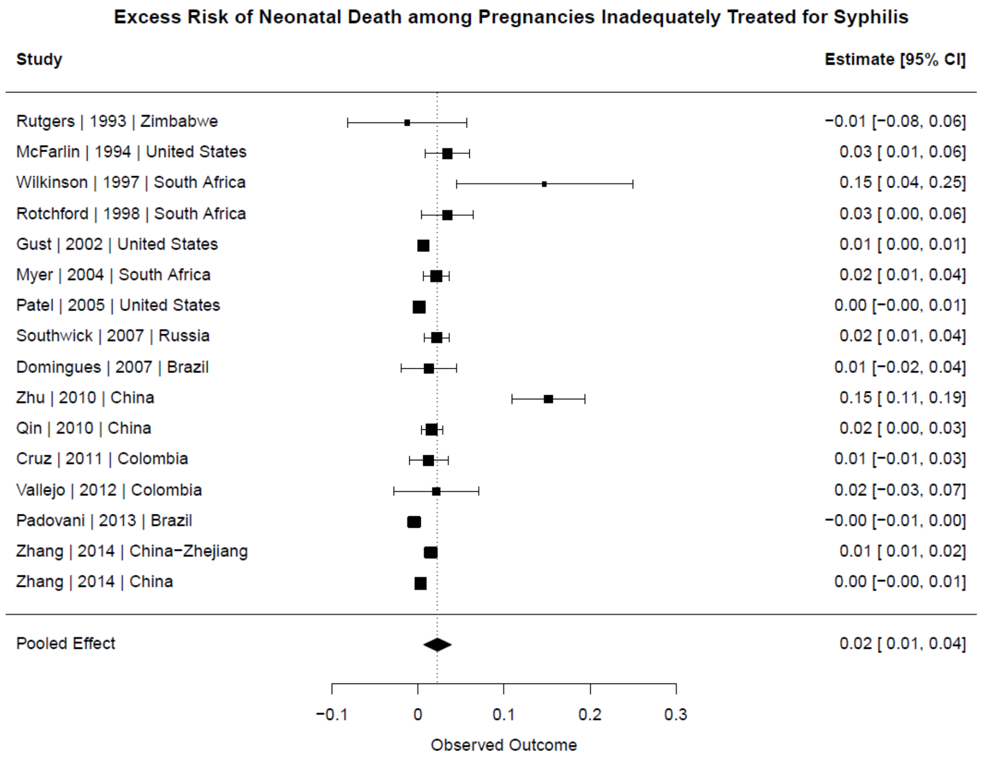

No excess mortality or fetal loss was assumed for adequately treated cases of maternal syphilis. A comparison of the neonatal mortality rates between adequately treated women and uninfected women showed a smaller proportion of babies from adequately treated women died than babies from uninfected women.

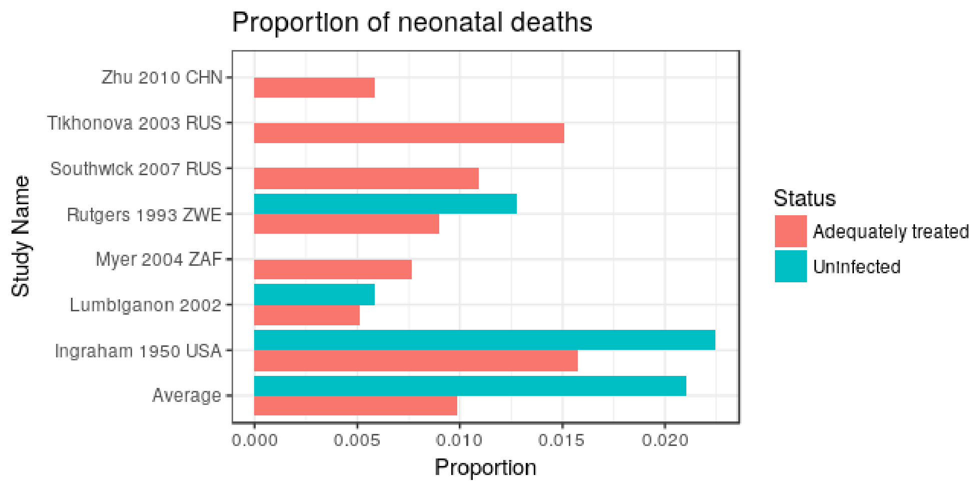

To combine these measures and obtain the final death counts:

We multiplied the number of syphilitic pregnancies by the proportions of mothers attending antenatal clinics at least one, two, or four times during pregnancy, the probability of attending a clinic that tests

and treats, and the proportions of early and late syphilis in pregnant women. This gave us the number of pregnancies of untreated status, inadequately treated status, or adequately treated status. These three groups are estimated because treatment status impacts the risk of fetal loss and neonatal death. The recommendation throughout literature is that individuals with early syphilis infection require one dose of penicillin to be adequately treated, while those with late syphilis infection are recommended three doses of penicillin for adequate treatment. We assume that women need to attend an ANC clinic at least two times – once to undergo syphilis testing, and a second time to receive test results and begin treatment. Thus, for those with early infection, 0-1 ANC visits indicates untreated status, and two or more visits indicate adequately treated status. For those with late infection, 0-1 visits indicates untreated status, 2-3 visits indicate inadequately treated status, and 4+ visits indicate adequately treatment status.

After the number of pregnancies in each treatment group are calculated, we multiply the untreated and the inadequately treated categories by the excess risk of fetal loss specific to each treatment category. This produces the number of excess stillbirths in pregnancies of each treatment category. We subtract the number of excess stillbirths in each treatment category from the pregnancies of each respective treatment category to get the number of livebirths born from pregnancies of each treatment category. We then multiply the livebirths born from untreated and inadequately treated pregnancies by the excess risk of neonatal death specific to each treatment category. This produces the number of neonatal deaths for each treatment category. The numbers of neonatal deaths are then summed for all treatment categories to get the total number of neonatal deaths due to congenital syphilis. Finally, we distribute neonatal deaths across the early and late neonatal age groups, and estimate the number of deaths for the 1-5 months, 6-11 months, 12-23 months, 2-4 years, and 5-9 years age groups. In GBD 2017, ratios for each age group relative to neonatal deaths were calculated using vital registration (VR) data from all location-years. However, this produced implausible differences between males and females in the estimated ratios. To solve this, starting in GBD 2019 and continuing in GBD 2021, only 4- and 5-star VR data were used to calculate ratios of deaths for every age group relative to neonatal deaths. A further explanation of the star rating system can be found in the "GBD 2021 Causes of Death database: Causes of death data star rating calculation" section of the appendix. We multiply the ratios calculated from high-quality VR data by our estimated number of neonatal deaths.

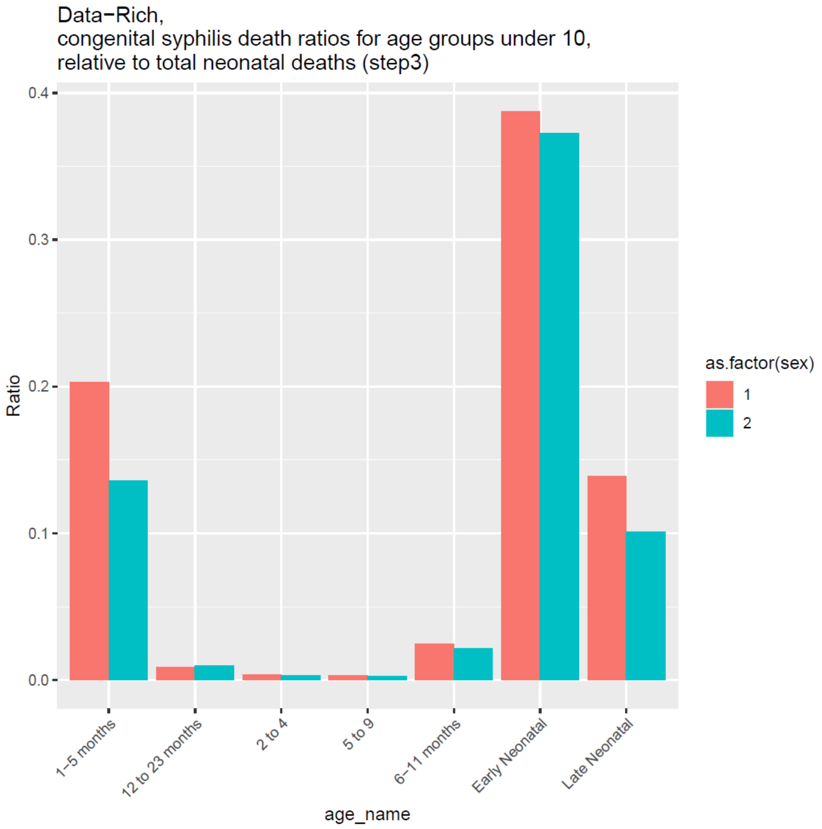

Subsequently, the sex- and age-specific congenital syphilis deaths estimated in the natural history model for data-sparse location-years were hybridised with the deaths estimated in the CODEm model for data-rich locations, and the hybrid model results were uploaded to the causes of death database and entered into the CoDCorrect process.

References

1. Global Health Observatory. "Women Accessing Antenatal Care (ANC) Services Who Were Tested for Syphilis (%)." World Health Organization, www.who.int/data/gho/data/indicators/indicator-details/GHO/women-accessing-antenatal-care-(anc)-services-who-were-tested-for-syphilis-(-). Accessed July 2017.
2. Global Health Observatory. "Antenatal Care attendees positive for syphilis who received treatment (%)." World Health Organization, https://www.who.int/data/gho/data/indicators/indicator-details/GHO/antenatal-care-attendees-positive-for-syphilis-who-received-treatment-(-). Accessed July 2017.
3. Gardner, William M. *Global, Regional, and National Estimates of Complete Coverage of Maternal Obstetric Care Interventions, 1990-2019*. Diss. 2020.

Fatal discontinuities

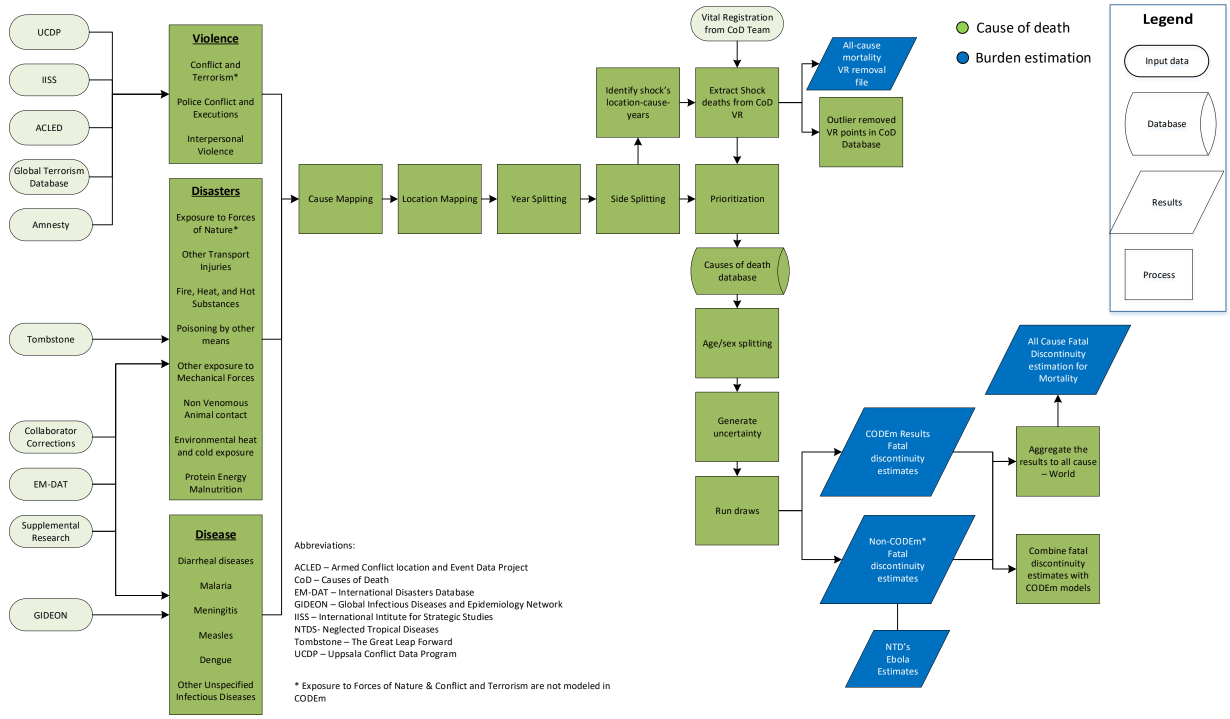

Fatal discontinuities are defined as events that are stochastic in nature and cannot be modelled because they do not have a predictable time trend. Some causes have both fatal discontinuities, as well as a continuous background mortality that has a smooth time trend and can be modelled; these include police violence and executions; interpersonal violence; other transport injuries; fire, heat, and hot substances; poisoning by other means; other exposure to mechanical forces; non-venomous animal contact; environmental heat and cold exposure; protein-energy malnutrition; diarrhoeal disease; malaria; meningitis; encephalitis; diphtheria; measles; dengue; and other unspecified infectious disease. Causes without a continuous background mortality that are exclusively estimated using the fatal discontinuity method are conflict and terrorism, and exposure to forces of nature. Any other causes are not captured in fatal discontinuities.

Input data

Overall

Input data for fatal discontinuities are compiled from a range of sources, including country vital registration (VR) data; international databases that capture several cause-specific fatal discontinuities; and supplemental data in the presence of known issues with data quality or representativeness, or time lags in reporting. A Twitter scrape was used in place of a systematic literature review as a way to identify supplemental input data for missing fatal discontinuities. Below more detail is provided on the different input data sources by sub-causes of fatal discontinuities.

Exposure to forces of nature

In GBD 2021, exposure to forces of nature is defined as *"A force which is beyond human control".*[12] The Centre for Research on the Epidemiology of Disasters' International Disaster Database (EM-DAT)[7] served as the primary non-VR source of fatal discontinuities due to exposure to forces of nature (ie, natural disasters, lightning, earthquake, volcanic eruption, avalanche, storms, and floods). Data from EM-DAT were last accessed December 2, 2020. Supplemental online research was conducted for events where EM-DAT and VR were not up to date.

Partial discontinuity (CODEm)

For causes modelled in CODEm that have fatal discontinuities hiding in the time trend, a process was established to avoid duplication of fatal discontinuity deaths in CODEm and the fatal discontinuity estimates. First, location-cause-years were identified through outside non-VR sources. If these location-cause-years also had VR death estimates that were greater than the average of the immediate surrounding years, the difference between the identified year and the average of the surrounding years was included in the relevant cause for the fatal discontinuities database. The extracted deaths for all fatal discontinuity causes from VR are then subtracted from the all-cause VR data used in the all-cause mortality estimation process.

Police conflict and executions

In GBD 2021, police conflict and executions is defined as "*The lawful use or threatened use of force or violence against individual or group of people or property in an attempt to achieve political or socioeconomic objectives for a state".*[15] Data for police conflict and executions mainly came from Amnesty International, but other sources such as UCDP, ACLED, and VR that reported deaths due to legal intervention were also cause-mapped to police conflict and executions.

Homicide

In GBD 2021, homicide is defined as "*The use of violence against an individual or group of people without the motivation of political, religious, or ideological objectives."*[15] Data for homicide come from VR, IISS, GED, ACLED, and other supplements. Events are mapped to homicide where the notes found in the raw data indicate gang violence. Deaths from IISS, GED, and ACLED were then split among three homicide sub-types; physical violence by firearms, physical violence by sharp object, and physical violence by other means, based on the rates calculated from VR by country if available, and by region if country VR was unavailable.

Protein-energy malnutrition (PEM)

Protein-energy malnutrition is defined as *"a lack of dietary protein and/or energy"*[16] and covers famines as well as severe droughts. The primary source for PEM, other than VR, is EM-DAT. Supplemental online

research was conducted for events where EM-DAT and VR were not up to date. The Tombstone report was used to estimate deaths attributed to the famine during the Great Leap Forward in China in the 1960s.[8]

Other injury causes

Other injury causes include other transport injuries (eg, plane, train, and boat accidents); poisonings; fire, heat, and hot substances; and other exposure to mechanical forces (eg, building collapse). The primary data source other than VR for these events is EM-DAT. Supplemental online research was conducted for events where EM-DAT and VR were not up to date.

Meningococcal meningitis and other diseases

In GBD 2021, fatal discontinuities due to a subset of infectious diseases were estimated, including meningococcal meningitis (or meningococcal infection), and diarrhoeal disease caused by cholera, dengue, and malaria. These infectious diseases were first included on the fatal discontinuity cause list for GBD 2016 because (1) their current modelling strategies with the Cause of Death Ensemble model (CODEm) do not optimally capture the potentially highly variable – or epidemic – mortality levels and trends characteristic of these two causes; and (2) they can contribute to significant total fatalities in a given location-year. Other infectious diseases for which the latter is true – high death rates in the presence of an outbreak or epidemic – are currently modelled with alternative cause of death methods (eg, natural history models for measles and yellow fever), which allow for greater variation year-over-year if or when outbreaks occur.

The Global Infectious Diseases and Epidemiology Network (GIDEON) and EM-DAT served as the primary data sources for collating cholera and meningococcal meningitis or meningococcal infection death reports.[9,10] For any year that cholera or meningococcal meningitis deaths were recorded in a country or territory covered by the GBD, reported deaths were directly extracted from 1950 to 2020. If GIDEON or EMDAT had reporting gaps in cholera or meningococcal meningitis deaths, and the World Health Organization (WHO) reports had coverage for those years, the WHO reports were used. For the Yemen cholera outbreak in 2016 and 2017, estimates from local collaborators were used in the absence of other data sources.

Location mapping

Every event in the fatal discontinuities database was mapped to a GBD location using a four-step process that includes the following steps in succession: manual mapping, string matching, GPS overlay, and geocoding. If an event was manually mapped, the location was assigned without the use of any other map types. In manual mapping, events are manually assigned to locations by matching the location provided in the raw data to a GBD location. During string matching, an event's location strings are directly compared to the GBD ASCII location names. During GPS overlay, events that have GPS coordinates provided are overlaid onto a map of GBD locations. If the event is placed over a GBD most-detailed location, the event is assigned to that location. During geocoding, the event's location string is

entered into Open Street Maps, which returns GPS coordinates. These coordinates are processed using GPS overlay to return GBD locations. This hierarchy provides results where the results of manual mappings are considered the most reliable, followed successively by string matching, GPS coordinates, and then geocoding.

Side splitting

Many fatal discontinuities, such as war, have deaths that are reported across multiple locations. In these instances, deaths are split across the population from both locations, unless estimates by side are provided by the source itself or if weights are provided by another source. If the resulting locations are at the most detailed level according to GBD, no further splitting is needed. If a location is not most detailed, the deaths are distributed among the child locations by population.

Prioritisation

Choosing between multiple sources for same event (prioritisation)

Where multiple sources reported shock deaths for the same location-year-cause, a cause-specific prioritisation scheme was followed that reflected the available detail in the cause-specific datasets. For example, the Georeferenced Event Dataset from UCDP was prioritised above all other non-VR sources because it included detail on how deaths were distributed between multiple actors and locations in each conflict event. In most cases, VR from 4- or 5-star locations was used where available. In some cases, VR from 4- or 5-star locations was not chosen if there were well-known data quality issues or discrepancies in the cause of death data reporting related to a particular event (eg, supplemental death data for Louisiana were used for Hurricane Katrina because of established data reporting issues).

Age-sex splitting

All compiled data were run through the causes of death age-sex splitting process, except for where we had strong supplemental information on the age distribution of specific, large events, such as United States mortality in the Vietnam War and Iranian mortality from the Iran-Iraq conflict in the early 1980s.

Assigning uncertainty and generating draws

Uncertainty analysis

Uncertainty intervals for deaths due to conflict and terrorism were generated using UCDP high and low death estimates, except in the case of Iraq for 2003–2016. During this time period, deaths due to conflict and terrorism in Iraq were estimated using a combination of supplemental sources. The source found with the lowest number of deaths, Iraq Body Count (IBC),[2] was used as the lower bound of the uncertainty interval from 2003 to 2016. Estimates from the Iraq Mortality Study (IMS) by Hagopian et al[3]

from 2003 to 2006, the deadliest years of the war, were used to scale deaths to generate the upper uncertainty interval limits using the following formula:

$$deaths_{GBD\ 2017,\ high} = deaths_{IBC} \cdot \overline{\left[\frac{deaths_{IMS}}{deaths_{IBC}}\right]}_{2003-2006}$$

GBD 2021 used the average ratio between IMS and IBC reported deaths between 2003 and 2006, multiplied by the number of deaths reported by the IBC. This high estimate was carried forward through 2017 under the assumption that the Iraq Body Count similarly undercounts the number of deaths due to the ongoing civil war in Iraq. The final, best estimate for conflict and terrorism deaths in Iraq from 2003 to 2016 is the midpoint of the high and low estimates given above.

In cases where low and high estimates were not included in the available data, the regional average uncertainty interval was applied to the available death estimate across all fatal discontinuity causes.

A log-normal distribution was assumed, using mean death rates and standard error based on high and low estimates. In the case that standard error was less than 10e-8, the draws were set equal to the mean rate. 1000 draws were sampled from this log-normal distribution. These 1000 draws were then converted back to count space and used for final calculations of means and uncertainty intervals.

Changes from GBD 2019

In GBD 2021, methods were implemented to allow more for more complicated data manipulation. Now information from a variety of sources can influence how an event's deaths are split among several locations. When the main source does not provide death counts by side, this leads to more accurate side splitting instead of solely relying on population.

Work was also done to handle the deaths of migrants who die en route and split migrants into their respective countries of origin using patterns found from the Missing Migrants Project.[11] Previously, reports of these deaths had little to no information on where migrants originated and only provided where the migrants died.

References

1 UCDP/PRIO Armed Conflict Dataset Codebook. Uppsala Conflict Data Program (UCDP); Centre for the Study of Civil Wars, International Peace Research Institute, Oslo (PRIO), 2013.

2 Iraq Body Count. https://www.iraqbodycount.org/database/ (accessed May 8, 2017).

3 Hagopian A, Flaxman AD, Takaro TK, *et al.* Mortality in Iraq Associated with the 2003–2011 War and Occupation: Findings from a National Cluster Sample Survey by the University Collaborative Iraq Mortality Study. *PLOS Medicine* 2013; **10**: e1001533.

4 Jarvis C. The Rise and Fall of Albania's Pyramid Schemes. *F&D* 2000; **37**. http://www.imf.org/external/pubs/ft/fandd/2000/03/jarvis.htm.

5 Obermeyer Z, Murray CJL, Gakidou E. Fifty years of violent war deaths from Vietnam to Bosnia: analysis of data from the world health survey programme. *BMJ* 2008; **336**: 1482–6.

6 Milton L. Rwanda, 1994: International incompetence produces genocide. 1994 https://ezproxy.uwc.edu/login?url=http://search.proquest.com/docview/234405747?accountid=42411.

7 Centre for Research on the Epidemiology of Disasters (CRED). EM-DAT: The OFDA/CRED International Disaster Database. Brussels, Belgium: Catholic University of Leuven

8 Jisheng Y, Friedman E, Guo J, Mosher S. Tombstone: The Great Chinese Famine, 1958-1962. New York: Farrar, Straus and Giroux (Macmillan), 2012.

9 Inc GI, Berger DS. Cholera: Global Status: 2017 edition. GIDEON Informatics Inc, 2017.

10 Inc GI, Berger DS. Bacterial Meningitis: Global Status: 2017 edition. GIDEON Informatics Inc, 2017.

11 Missing Migrants Project. 2021 [cited 2021May19]. Available from: https://missingmigrants.iom.int/

12 Act of God [Internet]. Wikipedia. Wikimedia Foundation; 2021 [cited 2021May19]. Available from: https://en.wikipedia.org/wiki/Act_of_God

13 Nato. NATO's military concept for defence against terrorism [Internet]. NATO. [cited 2021May19]. Available from: https://www.nato.int/cps/en/natohq/topics_69482.htm

14 projects Cto W. War [Internet]. Wikiquote. Wikimedia Foundation, Inc.; 2021 [cited 2021May19]. Available from: https://en.wikiquote.org/wiki/War

15 Alex Schmid, *Terrorism - The Definitional Problem*, 36 Case W. Res. J. Int'l L. 375 (2004) Available at: https://scholarlycommons.law.case.edu/jil/vol36/iss2/8 –

17 Protein-Energy Malnutrition Definition. Merck Manual Professional Edition; 2007 [cited 2021May19]. Available from: http://healthinsight.org/Internal/assets/Nursing%20Home/PRU%20-%20Protein%20Energy%20and%20Malnutrition.pdf

Skin and subcutaneous diseases

Flowchart

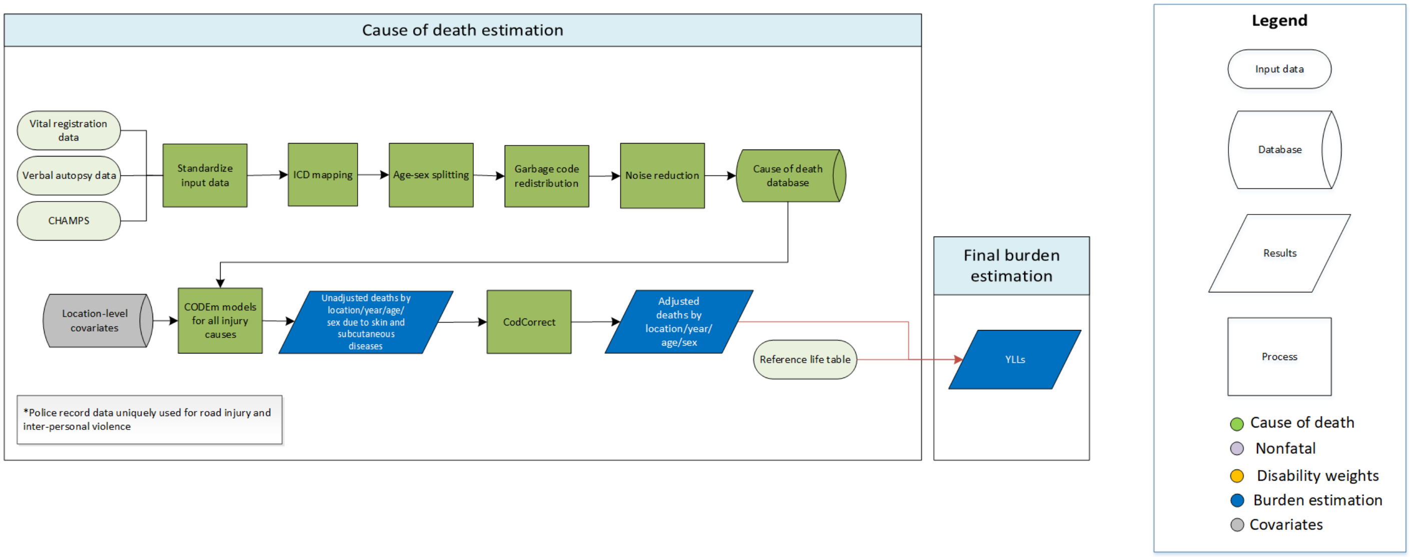

Input data and methodological summary for skin and subcutaneous diseases

Input data

Data used to estimate mortality of skin and subcutaneous diseases consisted of vital registration data and verbal autopsy data from the cause of death (COD) database. We marked data as outliers in instances where garbage code redistribution and noise reduction—in combination with small sample sizes—resulted in unreasonable cause fractions, as well as data that violated well-established time or age trends. The data in skin and subcutaneous diseases consist of aggregated data from all other specific skin diseases (cellulitis, pyoderma, decubitus ulcer) as well as unique datapoints from unspecified codes of skin and subcutaneous disease.

Modelling strategy

We modelled deaths due to skin and subcutaneous diseases with a standard CODEm model using the cause of death database and location-level covariates as inputs. The model followed standard parameters, with the exception that the start age of the model was 28 days instead of 0. We hybridised separate global and data-rich models to acquire unadjusted results, which we finalised and adjusted using CoDCorrect to reach final years of life lost (YLLs) due to skin and subcutaneous diseases.

There were no significant changes in the modelling process between GBD 2019 and GBD 2021.

Table 1. Covariates used in skin and subcutaneous diseases mortality modelling

Level	Covariate	Direction
1	Summary exposure value (SEV) scalar for unsafe sanitation	+
	Prevalence of overweight and obesity	+
	Healthcare Access and Quality Index	-
	Diabetes fasting plasma glucose (mmol/L), by age	+
	Improved water source (proportion with access)	-
2	Alcohol consumed (litres per capita)	+
	Cumulative cigarettes (5 years)	+
	Cumulative cigarettes (10 years)	+
	Smoking prevalence	+
3	Education (years per capita)	-
	Lag distributed income (per capita)	-
	Socio-demographic Index	-

Stroke

Flowchart

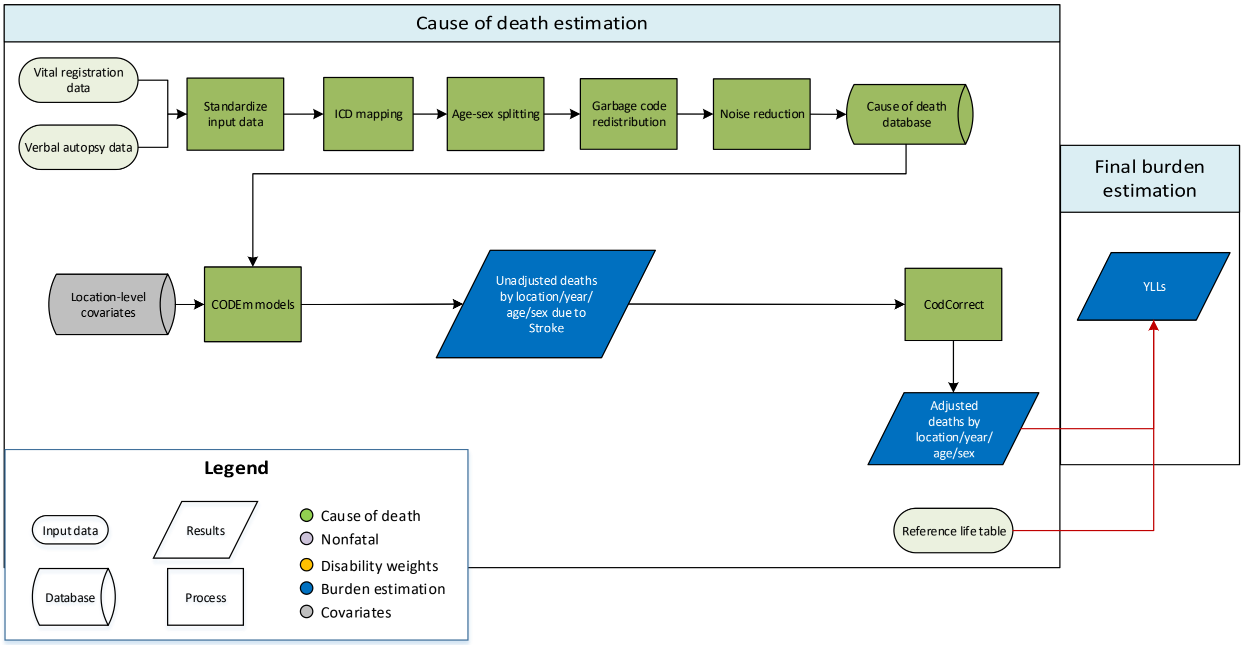

Input Data and Methodological Summary for stroke

Input data

Verbal autopsy and vital registration data were used to model cerebrovascular disease (stroke). For GBD 2021, we reviewed all available verbal autopsy reports of death for stroke. We outliered any studies where studied populations were not representative of the broader location. We did not use verbal autopsy data for ages under 15 years old. We also outliered ICD8, ICD9BTL, and tabulated ICD10 datapoints which were inconsistent with the rest of the data and created implausible time trends. In addition, we outliered ICD 10 datapoints from sources which were implausibly low in all age groups and datapoints that were causing the regional estimates to be improbably high.

Modelling strategy

We used a standard CODEm approach to model deaths from stroke. The covariates included in the ensemble modelling process are listed in the table below. For GBD 2021, we updated the redistribution of deaths attributed to hypertension to an analysis of multiple cause of death datasets containing information on the entire chain of causes listed on the death certificate from seven countries with high quality data. In GBD 2019, the redistribution of hypertension deaths was done by regressing redistribution codes against non-redistribution codes; details on this approach can be found elsewhere in the appendix. Using the multiple cause of death approach to redistribution allows us to observe the true underlying cause of death where the reported cause of death is labelled as hypertension. For details on the multiple cause of death redistribution methods, refer to the appendix section on redistribution. Updating redistribution to using multiple cause of death analysis resulted in increasing the number of hypertension deaths reassigned to stroke as the underlying cause of death. For example,

in GBD 2019 we redistributed 5,622 deaths originally coded to hypertension in the United States for all ages and sexes in the year 2016 to stroke. In GBD 2021, the number of deaths redistributed from hypertension to stroke in the same demographic increased to 6,134 deaths. Another example to consider is Brazil for all ages and sexes in 2016, where the total number of originally coded hypertension deaths that were redistributed to stroke in GBD 2019 was 5,639 deaths compared to the GBD 2021 total number of 10,561 redistributed hypertension deaths. This was the largest and most substantial update to redistribution methods regarding stroke and the stroke subtypes. For GBD 2021 we updated our approach to noise reduction of the cause of death data so that stochastic time series of data would resemble patterns seen at regional levels more closely. Additional details on the updates to noise reduction can be found in the cause of death methods section of the appendix.

Table 1. Covariates used in stroke mortality modelling

Level	Covariate	Direction
1	Summary exposure variable, stroke	1
	LDL Cholesterol (mean per capita)	1
	Smoking prevalence	1
	Systolic blood pressure (mm Hg)	1
2	Mean Body mass index (kg/m^2)	1
	Elevation over 1,500m (proportion)	-1
	Fasting plasma glucose	1
	Outdoor pollution ($PM_{2.5}$)	1
	Indoor air pollution	1
	Healthcare Access and Quality Index	-1
3	Log-transformed lag distributed income per capita (I$)	-1
	Summary exposure value, omega-3	1
	Summary exposure value, fruits	1
	Summary exposure value, vegetables	1
	Summary exposure value, nuts and seeds	1
	Pulses/legumes (kcal/capita, unadjusted)	-1
	Summary exposure value, PUFA adjusted (percent)	1
	Alcohol (litres per capita)	1
	Trans fatty acid	1

Subarachnoid haemorrhage

Flowchart

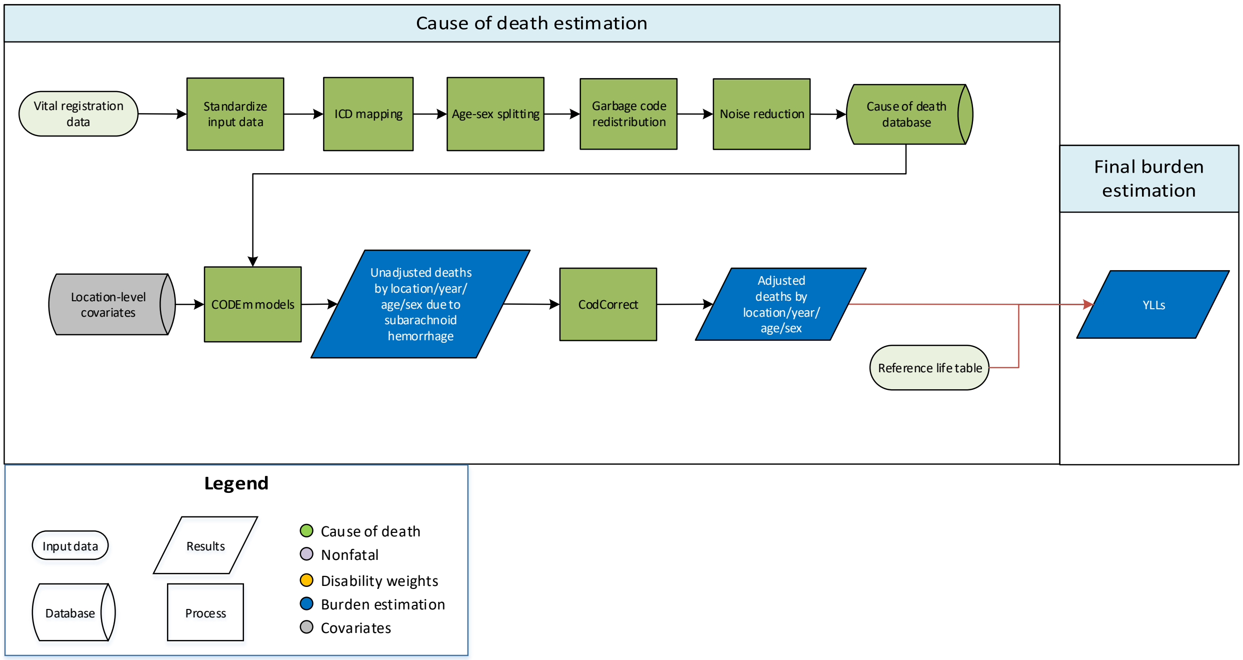

Input Data and Methodological Summary for subarachnoid haemorrhage

Input data

Vital registration data were used to model subarachnoid haemorrhage. We outliered ICD8 datapoints which were inconsistent with the rest of the data and created implausible time trends. In addition, we outliered vital registration data in Tibet, Ghana, and Cabo Verde that were implausibly high for all years and age groups. We also outliered vital registration data in Tajikistan and Palestine that were implausibly low.

Modelling strategy

We used a standard CODEm approach to model deaths from subarachnoid haemorrhage. The covariates chosen for inclusion in the ensemble modelling process are listed in the table below. For GBD 2021, we updated the redistribution of deaths attributed to hypertension to an analysis of multiple cause of death datasets containing information on the entire chain of causes listed on the death certificate from seven countries with high quality data. In GBD 2019, the redistribution of hypertension deaths was done by regressing redistribution codes against non-redistribution codes; details on this approach can be found elsewhere in the appendix. Using the multiple cause of death approach to redistribution allows us to observe the true underlying cause of death where the reported cause of death is labelled as hypertension. For details on the multiple cause of death redistribution methods, refer to the appendix section on redistribution. This had the effect of increasing the number of hypertension deaths attributed to stroke in general; there were both increases and decreases in post-redistributed deaths observed for subarachnoid haemorrhage specifically dependent on location. This better reflects our ability to capture location variation with this new approach. For example, in GBD 2019 we redistributed 2904 deaths originally coded to hypertension in the United States for all ages and sexes in the year 2016 to

subarachnoid haemorrhage. In GBD 2021, the number of deaths redistributed from hypertension to stroke in the same demographic decreased to 547 deaths. Another example to consider is Brazil for all ages and sexes in 2016, where the total number of originally coded hypertension deaths that were redistributed to subarachnoid haemorrhage in GBD 2019 was 149 deaths compared to the GBD 2021 total number of 850 redistributed hypertension deaths. For GBD 2021 we updated our approach to noise reduction of the cause of death data so that stochastic time series of data would resemble patterns seen at regional levels more closely. Additional details on the updates to noise reduction can be found in the cause of death methods section of the appendix.

Table 1. Covariates used in subarachnoid haemorrhage mortality modelling

Level	Covariate	Direction
1	Summary exposure variable, subarachnoid haemorrhage	1
	Smoking prevalence	1
	Systolic blood pressure (mm Hg)	1
2	Healthcare Access and Quality Index	-1
3	Log-transformed lag distributed income per capita (I$)	-1
	Alcohol (litres per capita)	1

Sudden infant death syndrome (SIDS)

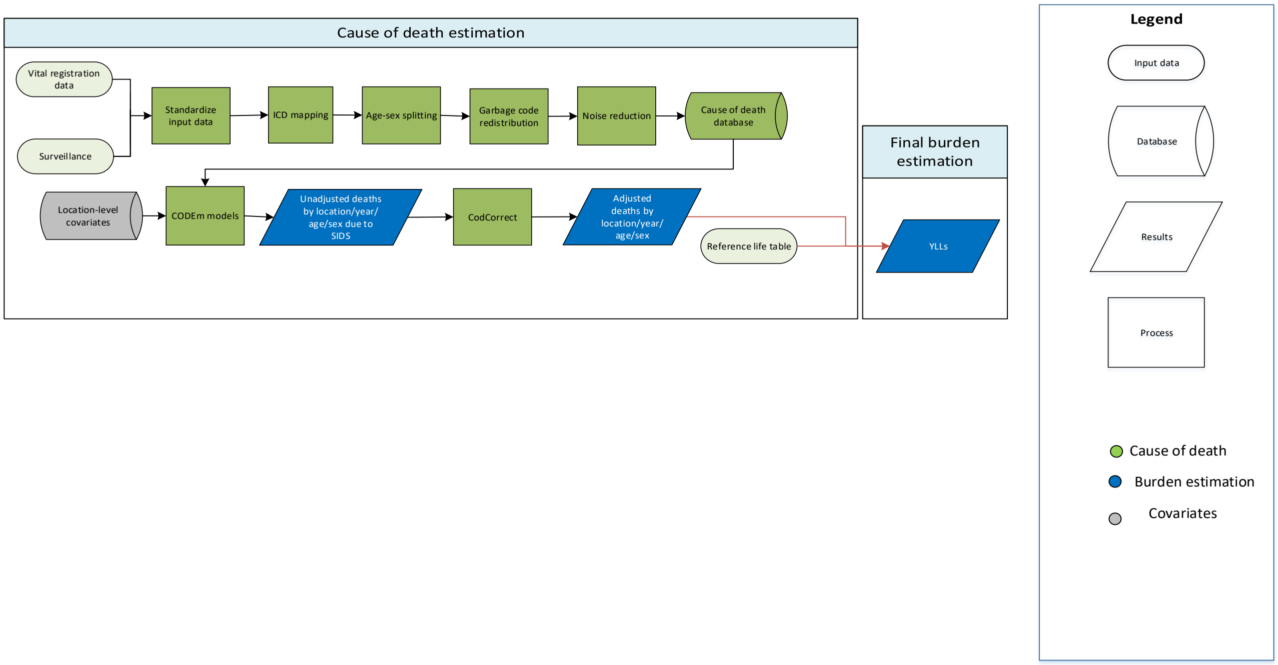

Input data

Vital registration data were used to estimate deaths due to sudden infant death syndrome (SIDS). Datapoints were selected as outliers if they met the following criteria: (1) implausibly high values relative to country time trends or global or regional patterns, based on the assumption that there are not "outbreaks" of SIDS, or (2) substantial conflict with established age or temporal patterns. In addition, for GBD 2017, all deaths assigned to SIDS outside of countries with 4- and 5-star data were reassigned to neonatal disorders. SIDS can only be ascertained as a cause of death by autopsy, which is unlikely to have been used outside of 4- and 5-star countries. All deaths coded to SIDS in verbal autopsy data were mapped to neonatal disorders.

Modelling strategy

The standard CODEm modelling approach was applied to estimate deaths due to SIDS. We ran CODEm models for ages 7–27 days, 1–5 months, and 6–11 months because we believe that deaths assigned to SIDS in other age groups are mis-assigned, and these are therefore treated as garbage codes. Surveillance data and verbal autopsy data were not used as inputs to this model because these sources do not use data collection methods that can accurately diagnose deaths due to SIDS.

Notable differences between the GBD 2013 and GBD 2015 strategy included updates across the board to smoking-related covariates, total fertility rate, and Socio-demographic Index covariates. The addition of American Samoa to the Oceania region was also of note, as well as the shift to including more ICD

detail codes in the input data for some countries that previously reported only aggregated codes. There were no significant changes in strategy from GBD 2015 to GBD 2021.

Covariates in GBD 2021 are shown in the following table.

			Data-rich		Global	
Level	Covariate	Direction	Acceptance in males	Acceptance in females	Acceptance in males	Acceptance in females
1	In-facility delivery (proportion)	-	Y	Y	Y	Y
2	Maternal care and immunisation	-	N	N	N	N
	Skilled birth attendance (proportion)	-	Y	Y	Y	Y
	Healthcare Access and Quality Index	-	N	N	N	N
3	Lag distributed income (I$ per capita)	+	N	N	N	N
	Education (years per capita)	-	N	N	N	N
	Total fertility rate	+	Y	Y	Y	Y
	Socio-demographic Index	+	Y	Y	Y	Y

Tetanus

Flowchart

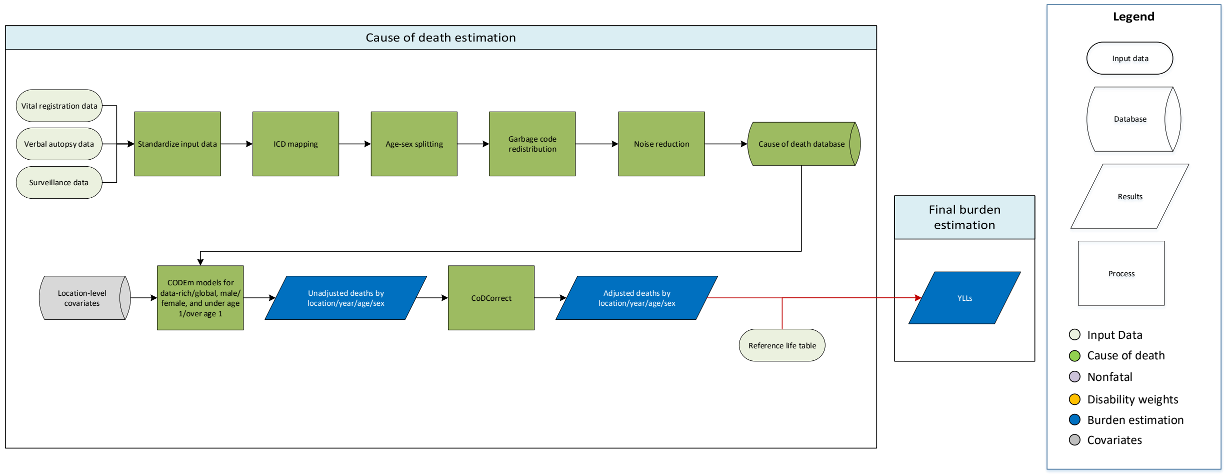

Input data

Tetanus cause of death (COD) data for GBD 2021 included vital registration, verbal autopsy, and surveillance sources from all locations as available. We excluded prepared COD data if they were highly incongruent with other available data from the same location or locations of similar sociodemographic characteristics.

Modelling strategy

We used a Cause of Death Ensemble modelling approach (CODEm) to compute age-, sex-, location-, and year-specific estimates. Given the relative rarity of tetanus mortality, we modelled directly in count-space. These models in count space had lower out-of-sample root mean squared error (RMSE) than rate-space models and thus were frequently the top models selected in the ensemble.

Separate, sex-specific models were run for neonatal tetanus (under-1-year age groups) and all other tetanus (1 year to 95+ age groups). We also stratified models by vital registration data quality, running both "data-rich" and global models for each age- and sex-specific group. Following model completion, the data-rich and global model outputs were combined to produce a single set of estimates for all locations by sex and age (under-1 and over-1 age groups).

Table 1a lists the covariates used in the data-rich and global under-1 models, and table 1b the covariates in the over-1 model. We replaced the annual estimates of routine-DTP3 coverage in infants that we used as a covariate in past GBD cycles with an average of coverage over the last five years. This change was motivated by fluctuations in the annual coverage estimates driven by vaccine stockouts, disruptions due to conflict, or other single-year events. The five-year average covariate allows the influence of these events to be distributed across time in our final tetanus model, better reflecting the expected relationship between coverage and tetanus epidemiology over time. For the tetanus toxoid coverage

covariate used in the under-1 tetanus model as a proxy for protection by maternal antibodies at birth, we continue to use a single-year estimate.

Table 1a. Covariates. Summary of covariates used in the under-1 tetanus cause of death model

Level	Covariate	Direction
1	Average diphtheria-tetanus-pertussis third-dose (DTP3) vaccination coverage over the last five years	-
	Tetanus toxoid coverage	-
2	In-facility deliveries (proportion)	-
	Skilled birth attendance (proportion)	-
	Healthcare Access and Quality (HAQ) Index	-
3	Lag-distributed income (LDI)	-
	Socio-demographic Index (SDI)	-
	Mean years of education per capita	-

Table 1b. Covariates. Summary of covariates used in the over-1 tetanus cause of death model

Level	Covariate	Direction
1	Average diphtheria-tetanus-pertussis third-dose (DTP3) vaccination coverage over the last five years	-
2	Healthcare Access and Quality (HAQ) Index	-
3	Sanitation access (proportion)	-
	Lag-distributed income (LDI)	-
	Socio-demographic Index (SDI)	-
	Mean years of education per capita	-

Tuberculosis

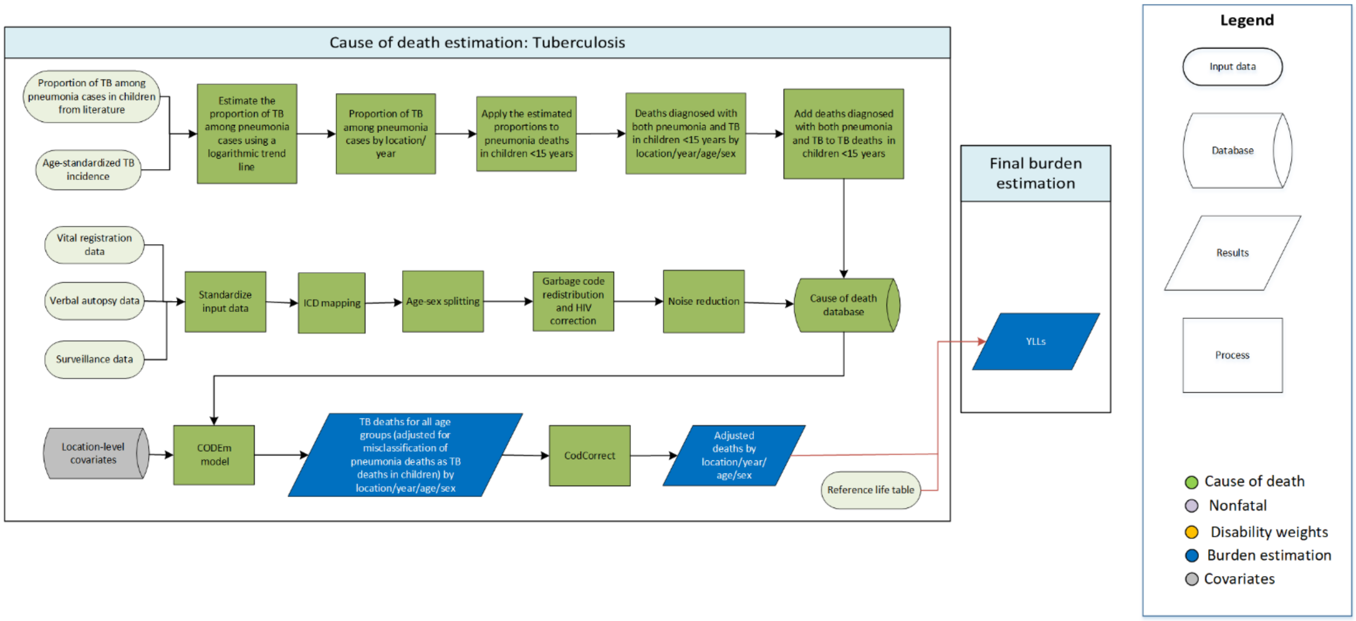

Input data

Input data for modelling tuberculosis (TB) mortality among HIV-negative individuals include vital registration, verbal autopsy, and surveillance data. Vital registration data were adjusted for garbage coding (including ill-defined codes and the use of intermediate causes) following GBD algorithms and misclassified HIV deaths (ie, HIV deaths being assigned to other underlying causes of death such as tuberculosis or diarrhoea because of stigma or misdiagnosis).

Verbal autopsy data in countries with age-standardised HIV prevalence greater than 5% were removed because of a high probability of misclassification, as verbal autopsy studies have poor validity in distinguishing HIV deaths from HIV-TB deaths.

Modelling strategy

A general CODEm modelling strategy was used. In GBD 2021, we made a small change with regard to the alcohol litres per capita covariate where we exchanged it for an all-age and both-sex equivalent that aligns better with the covariate framework for CODEm. We continued to use the TB strain prevalence-weighted transmission risk and cigarettes per capita covariate that were introduced in GBD 2017. Other location-level covariates included in the CODEm model were the same as in previous GBD cycles: adult underweight proportion, alcohol (litres per capita), diabetes (fasting plasma glucose mmol/L), education (years per capita), Healthcare Access and Quality Index, lag-distributed income, indoor air pollution, outdoor air pollution, population density, prevalence of active tuberculosis, prevalence of latent tuberculosis infection, smoking prevalence, Socio-demographic Index, and a summary exposure variable reflecting the average exposure to all of the risk factors.

Covariate table

	Covariate	Direction
Level 1	TB prevalence<br>Latent TB infection prevalence<br>SEV scalar<br>Litres of alcohol consumed per capita<br>Smoking prevalence<br>Cigarettes per capita<br>Fasting plasma glucose<br>TB strain prevalence-weighted transmission risk	+<br>+<br>+<br>+<br>+<br>+<br>+<br>+
Level 2	HAQ Index<br>Adult underweight proportion<br>Indoor air pollution<br>Outdoor air pollution<br>Population density	-<br>+<br>+<br>+<br>+
Level 3	Log LDI<br>Education (years per capita)<br>Socio-demographic Index (SDI)	-<br>-<br>-

Correcting for a potential misclassification of tuberculosis deaths as pneumonia deaths in children

Since GBD 2017, we have addressed the potential for misclassification of TB deaths as pneumonia deaths among children in locations with high TB burden. First, we estimated the proportion of tuberculosis among pneumonia cases as a function of age-standardised TB incidence using data from eight clinical studies[2,3,4,5,6,7,8,9] reporting the proportion of pneumonia cases that had tuberculosis (or the data to calculate them) and the age-standardised TB incidence estimates. We used a logarithmic trend line to fit these data. In GBD 2021, we applied the estimated proportions to pneumonia deaths reported in data among children younger than 15 years to compute the number of deaths diagnosed with both pneumonia and TB, which were then added to child TB data. Following this correction in our input data, the CODEm model was run to provide location-year-age-sex specific estimates. This is a departure from GBD 2017, where the estimated proportions were applied after CODEm. Finally, the CODEm estimates were adjusted using CoDCorrect, which ensures that the number of deaths from each cause add up to all-cause mortality deaths for a given year.

References

1. Graham SM, Sismanidis C, Menzies HJ, Marais BJ, Detjen AK, Black RE. Importance of tuberculosis control to address child survival. *Lancet* 2014; **383**(9928): 1605-7.
2. Adegbola RA, Falade AG, Sam BE, et al. The etiology of pneumonia in malnourished and well-nourished Gambian children. Pediatr Infect Dis J 1994; 13: 975–82.

3. Chisti MJ, Graham SM, Duke T, et al. A prospective study of the prevalence of tuberculosis and bacteraemia in Bangladeshi children with severe malnutrition and pneumonia including an evaluation of Xpert MTB/RIF assay. PloS One 2014; 9: e93776.
4. Madhi SA, Petersen K, Madhi A, Khoosal M, Klugman KP. Increased disease burden and antibiotic resistance of bacteria causing severe community-acquired lower respiratory tract infections in human immunodeficiency virus type 1-infected children. Clin Infect Dis 2000; 31: 170–76.
5. McNally LM, Jeena PM, Gajee K, et al. Effect of age, polymicrobial disease, and maternal HIV status on treatment response and cause of severe pneumonia in South African children: a prospective descriptive study. Lancet 2007; 369: 1440–51.
6. Moore DP, Klugman KP, Madhi SA. Role of Streptococcus pneumoniae in hospitalisation for acute community-acquired pneumonia associated with culture-confirmed Mycobacterium tuberculosis in children: a pneumococcal conjugate vaccine probe study. Pediatr Infect Dis J 2010; 29: 1099–104.
7. Nantongo JM, Wobudeya E, Mupere E, et al. High incidence of pulmonary tuberculosis in children admitted with severe pneumonia in Uganda. BMC Pediatr 2013; 13: 16.
8. Zar HJ, Hanslo D, Tannenbaum E, et al. Aetiology and outcome of pneumonia in human immunodeficiency virus-infected children hospitalized in South Africa. Acta Paediatr 2001; 90: 119–25.
9. Moore DP, Higdon MM, Hammitt LL, Prosperi C, DeLuca AN, Da Silva P, Baillie VL, Adrian PV, Mudau A, Deloria Knoll M, Feikin DR. The incremental value of repeated induced sputum and gastric aspirate samples for the diagnosis of pulmonary tuberculosis in young children with acute community-acquired pneumonia. Clinical Infectious Diseases. 2017 May 27;64(suppl_3):S309-16.

TB strain prevalence-weighted transmission risk covariate

In GBD 2017, we incorporated a TB covariate that incorporated data on the global distribution of TB strains and the relative risk of transmission associated with those strains. We continued the use of this covariate in GBD 2019. For this covariate, we defined TB strains according to the seven phylogenetic lineages of the *Mycobacterium tuberculosis* complex (MTBC) identified by S. Gagneaux and colleagues.[1] We determined the global distribution of these strains using a systematic review of human TB molecular epidemiology studies from 1990 to 2017 in PubMed and Scopus, as described in greater detail elsewhere.[2] All studies that used population-based sampling methods or collected isolates from all culture-positive TB cases in a given location and time period were included. All genotypes that could be converted to phylogenetic lineages were extracted, including genotypes determined by spoligotyping, MIRU-VNTR typing, and PCR or whole-genome sequencing. Studies of sub-populations, such as prison populations or drug-resistant cases only, were excluded. In total, 206 studies representing 85 countries and over 200,000 bacterial isolates were included. In GBD 2019, the systematic review was updated, which yielded an additional 18 studies published between 2017 and 2019, but we did not update it in GBD 2021 due to lack of bandwidth. A map of these strains highlighted the widespread global distribution of Euro-American Lineage 4 strains and East Asian Lineage 2 strains, and the geographical restriction of Lineage 5 and 6 strains to West Africa. Thirty of these studies also reported transmission chains associated with bacterial genotypes, as defined by genetic clustering.[3]

We used spatiotemporal Gaussian process regression (ST-GPR) to model the distribution of each strain in each GBD location across all ages and sexes, as described in greater detail elsewhere.[4] The covariates tested in each model included HIV age-standardised prevalence, population density, and a custom-made human movement covariate. The human movement covariate took into account (1) immigration and emigration patterns[5] and (2) airplane passenger flow[6] to and from each country. In the ST-GPR models we assumed strong correlation and smoothing over both space and time. We then used a random-effects meta-analysis to determine the relative risk (RR) of transmission associated with each strain, as defined by genetic clustering. We used the most widespread strains, Euro-American Lineage 4 strains, as the reference group. We found that East Asian Lineage 2 strains were associated with increased risk of transmission overall (relative risk [95% CI] = 1.24 [1.07, 1.45]), while West African Lineage 5 and 6 strains were associated with reduced transmission (relative risk [95% CI] = 0.61 [0.43, 0.86]). We used the following formula to calculate a TB strain prevalence-weighted risk of transmission based on these estimates:

$$\sum_{i=1}^{n} Pr_i RR_i$$

i=TB strain; Pr=proportion; RR=relative risk

References

1. Comas I, Coscolla M, Luo T, *et al.* Out-of-Africa migration and Neolithic coexpansion of *Mycobacterium tuberculosis* with modern humans. *Nat Genet* 2013; **45**: 1176–82.
2. Wiens KE, Woyczynski LP, Ledesma JR, *et al*. Global variation in bacterial strains that cause tuberculosis disease: a systematic review and meta-analysis. *BMC Medicine 2018*; 16:196.
3. Dheda K, Gumbo T, Maartens G, *et al.* The epidemiology, pathogenesis, transmission, diagnosis, and management of multidrug-resistant, extensively drug-resistant, and incurable tuberculosis. *Lancet Respir Med* 2017; **5**: 291–360.
4. Manuscript in preparation.
5. United Nations Population Division. United Nations Trends in International Migrant Stock: The 2015 Revision. New York City, United States: United Nations Population Division, 2015.
6. Huang Z, Wu X, Garcia AJ, *et al*. An open-access modeled passenger flow matrix for the global air network in 2010. *PLoS ONE* **8(5)**: e64317.

Typhoid fever

Flowchart

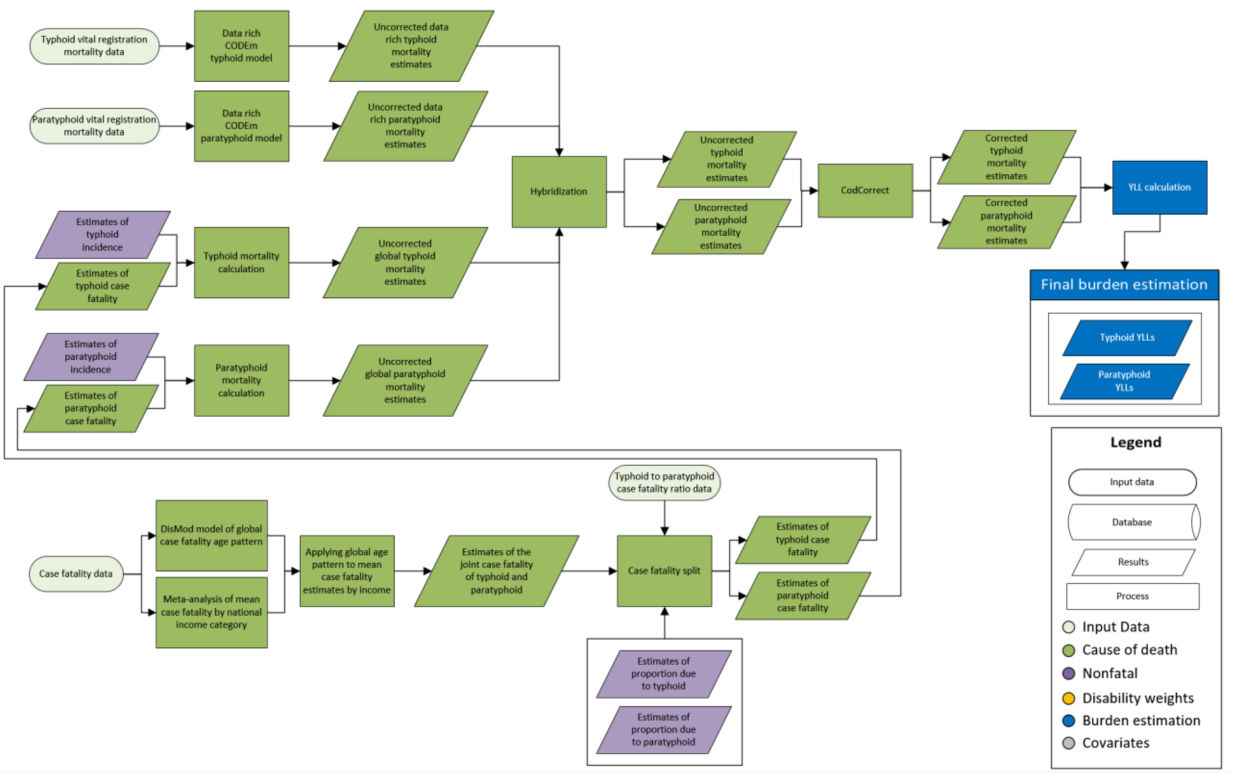

Input data and methodological summary for typhoid fever

Input data

Our CODEm model used all available data in the cause of death database from data-rich countries. No data were outliered for this cause. For the natural history model, our incidence dataset included a combination of data from prospective cohort studies and national surveillance systems. Similarly, data on proportions due to typhoid and paratyphoid included a combination of prospective cohort studies and national surveillance systems. Case fatality data were from national surveillance systems and hospital databases.

Modelling strategy

We model typhoid deaths using a hybrid modelling strategy with two components: 1) for data-rich locations we estimate typhoid mortality using a CODEm model of CoD data; and 2) in all other locations (ie, not data-rich) we use a natural history model in which we derive deaths as the product of cases and case fatality.

The CODEm model included six covariates:

Level	Covariate	Direction
1	Sanitation (proportion with access)	-
	Improved water source (proportion of the population with access)	-
	Proportion of the population living in the Indian Ocean monsoon belt	+
	SEV unsafe water	+
	SEV unsafe sanitation	+
2	Healthcare Access and Quality Index	-

For the natural history model, we first model total incidence of typhoid and paratyphoid combined. Second, we model the proportion of this total due to typhoid and the proportion due to paratyphoid. Third, we estimate case fatality by age and national income category for typhoid and paratyphoid combined. Fourth, we use data on the relative fatality of typhoid and paratyphoid to split the joint case fatality estimates into typhoid- and paratyphoid-specific case fatality estimates. Finally, we estimate cause-specific mortality rates as the product of incidence and case fatality.

Total incidence was modelled using DisMod-MR 2.1 using the proportion of the population with access to clean water, and the proportion of the population living in the Indian Ocean monsoon belt as covariates. We performed a crosswalk using a study-level covariate indicating sources that were based on passive versus active surveillance, with active surveillance as the reference. This adjusts for incomplete case capture by passive surveillance. Incidence data were inflated to account for poor diagnostic sensitivity, based on a meta-analysis of the sensitivity of blood culture, the most common diagnostic used for typhoid. Similarly, we used two DisMod models to estimate aetiological proportions: one for the proportion of total incidence due to typhoid, and one for the proportion due to paratyphoid.

Case fatality data were too limited to allow for a complete DisMod model, or to allow for varying estimates by time and space. We had sufficient data, however, to estimate case fatality by age and by three categories of national income. We used DisMod to extract a global age-pattern in case fatality, and meta-regression to estimate the mean case fatality by income category. Finally, we estimated the relative risk of death from typhoid relative to paratyphoid based on data from Chinese surveillance and used that relative risk to estimate case fatality separately for typhoid and paratyphoid, by age and income.

Finally, we estimated typhoid mortality as the product of total incidence, the proportion of the total due to typhoid, and case fatality for typhoid. We propagated uncertainty through every step of the modelling process by pulling 1,000 draws from the distribution of each model component (eg, incidence, proportion due to typhoid, overall case fatality, case fatality age pattern, relative fatalness of typhoid versus paratyphoid), and performing all calculations at the draw level.

We have made no substantive changes to our modelling strategy between GBD 2019 and 2021.

Upper digestive diseases

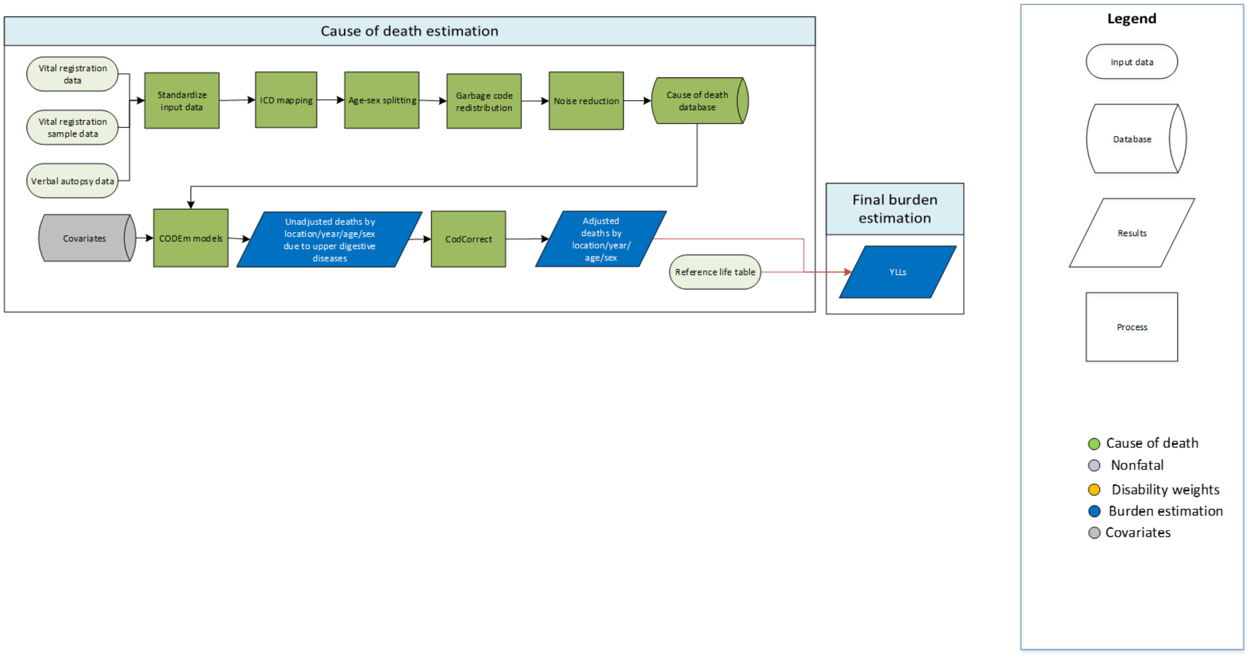

Input data

Data used to estimate mortality due to upper digestive diseases consisted of vital registration data, vital registration sample data, and verbal autopsy data from the cause of death (COD) database. The diagnostic codes that map to upper digestive disease can be found in the "List of International Classification of Diseases (ICD) codes mapped to the Global Burden of Disease cause list for causes of death" found elsewhere in this appendix. Upper digestive disease data aggregate deaths due to peptic ulcer disease and gastritis and duodenitis, which are also modelled separately. For sources of data that were considered too low-quality to definitively assign peptic ulcer or gastritis deaths to one of these two causes, data were included only in the upper digestive disease dataset.

Details of COD data processing, including changes in processing introduced in GBD 2021, are described in detail in the "GBD 2021 Causes of Death database" section of this appendix. These data processing changes include changing the method of estimating the non-zero floor and population size cutoffs for when to noise-reduce at the country versus region level.

Outliers were identified by systematic examination of datapoints for all location-years. Data were excluded if they violated well-established time or age trends, and data in instances where garbage code redistribution and noise reduction, in combination with small sample sizes, resulted in unreasonable cause fractions or unreasonable time, age, or spatial trends; data from Tibet, Kiribati, Cook Islands, and urban Tripura were excluded for these reasons. In situations where unreasonable temporal and spatial trends were observed at transitions between data sources, higher-quality data sources were retained and lower-quality sources were excluded; this affected subnational locations in India, where vital registration data biased toward in-hospital deaths were available for urban locations only (MCCD), whereas high-quality verbal autopsy data with representative sampling were available for both urban and rural locations. A small number of datapoints for Riau Islands were also excluded because they were only available for females in four age-groups.

Modelling strategy

We modelled deaths due to upper digestive diseases with a standard CODEm model. (The CODEm modelling tool is described in the "Cause of death modelling methods: CODEm" section of this appendix.) The upper digestive diseases CODEm model employed the same parameter settings in GBD 2021 as in GBD 2019, with the exception that we updated the linear floor value to allow the model to be influenced by lower data, which resulted from changes to COD data processing.

Covariates entered into CODEm, their level of priority for testing, and their permitted directions were the same in GBD 2021 as in GBD 2019. A complete list is provided in the table below.

Covariate	Level	Direction
Sanitation, proportion with access	1	-1
Scaled exposure variable for unsafe water source	1	1
Smoking prevalence	1	1
Cumulative cigarettes (10 years)	1	1
Cumulative cigarettes (5 years)	1	1
Litres of alcohol consumed per capita	2	1
Vegetables (grams, unadjusted)	2	-1
Healthcare Access and Quality Index	2	-1
Lag distributed income (per capita)	3	-1
Education (years per capita)	3	-1
Socio-demographic Index	3	-1

Adjustment in CoDCorrect included fitting estimates for peptic ulcer disease and gastritis and duodenitis to all upper digestive disease deaths first before the adjustment with all other causes to sum to all-cause counts of death.

Upper respiratory infections

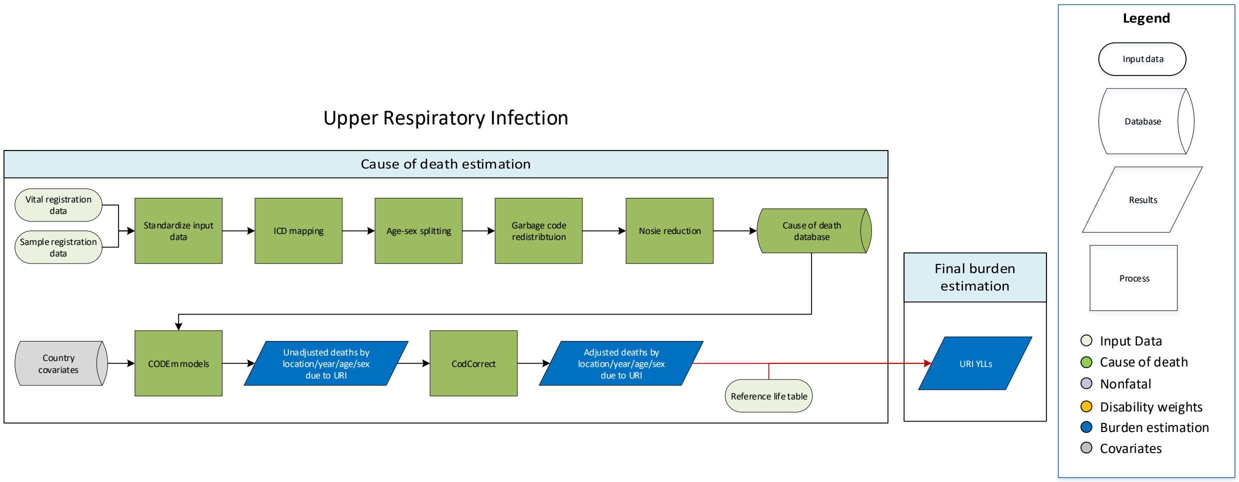

Input data and methodological summary for upper respiratory infections

Input data

Vital registration and surveillance data from the cause of death (CoD) database were used. Outliers were identified by systematic examination of datapoints. Datapoints that violated well-established age or time trends, were inconsistent with other country- or region-specific points, or that resulted in extremely high or low mortality rates were determined to be outliers.

Modelling strategy

A generic CODEm approach was used to estimate mortality due to upper respiratory infections (URI) in GBD 2021. In GBD 2016, mortality from URI was modelled using a negative binomial regression. It was determined that a negative binomial regression was an appropriate approach for estimating URI due to a small number of deaths due to URI in the CoD database. However, due to changes in how we redistribute cause of death codes, more deaths were attributed to URI in the CoD database, and thus it was determined that a generic CODEm approach was feasible for estimating URI mortality in GBD 2017. The covariates used are displayed below. We have made no substantive changes to the modelling strategy in 2021.

Level	Covariate	Direction
1	Smoking prevalence	+
2	Indoor pollution	+
	Outdoor pollution ($PM_{2.5}$)	+
	Healthcare Access and Quality Index	-
3	Socio-demographic Index	-
	Lag distributed income	-
	Education (years per capita)	-

Urinary diseases and male infertility

Flowchart

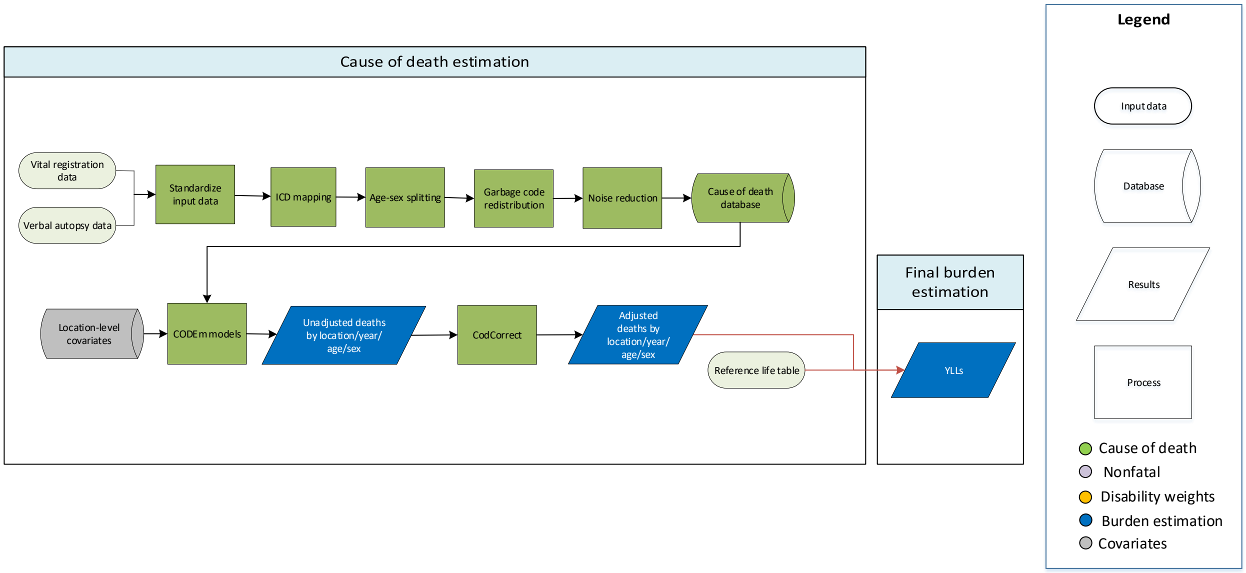

Input data and methodological summary for urinary diseases and male infertility

Input data

Data used to estimate mortality of urinary diseases and male infertility consisted of vital registration data and verbal autopsy data from the cause of death (COD) database (see appendix section on ICD mapping for details). The data in urinary diseases consist of aggregated data from all other specific urinary diseases (ie, urolithiasis, urinary tract infections), as well as unique datapoints from deaths reported with a set of non-specific urinary disease codes (ie, renal osteodystrophy, bladder-neck obstruction). Outliers were identified by systematic examination of datapoints for all location-years. Data were excluded if they violated well-established age or time trends, and data in instances where garbage code redistribution and noise reduction, in combination with small sample sizes, resulted in unreasonable cause fractions. Methods for assigning outlier status were consistent across both vital registration and verbal autopsy data.

Modelling strategy

The estimation strategy used for fatal urinary diseases and male infertility is largely similar to methods used in GBD 2019. A standard CODEm model with location-level covariates was used to model deaths due to urinary diseases and male infertility (see appendix section on CODEm method for details). Separate models were conducted for male and female mortality, and age-restrictions for death estimations included 0 days for lower bound and 95+ years for upper bound. We hybridised separate global and data-rich models to acquire unadjusted results, which we adjusted using CoDCorrect and compared to the reference life table to calculate final YLLs due to urinary diseases and male infertility.

In GBD 2021, we updated the linear floor value in our CODEm models to allow the model to be influenced by lower data, which resulted from changes to COD data processing. These data processing changes include changing the method of estimating the non-zero floor and population size cutoffs for

when to noise-reduce at the country versus region level, and are described in greater detail in the appendix section GBD 2021 Causes of Death database. Apart from these, no other substantive changes were made in GBD 2021 from the modelling strategy used in GBD 2019.

The following table has the full list of covariates used for fatal urinary diseases and male infertility.

Table 1. Covariates used in urinary diseases and male infertility mortality modelling

Level	Covariate	Direction
2	Temperature (90th percentile)	+
	Sanitation (proportion with access)	+
	Mean BMI	+
	Healthcare Access and Quality Index	-
3	Socio-demographic Index	-
	Education (years per capita)	-
	Log LDI ($I per capita)	-

Adjustment in CoDCorrect included fitting unadjusted death estimates for all other specific and non-specific urinary diseases to overall urinary disease deaths, which were then adjusted with all other causes to sum to all-cause counts of death.

Urinary tract infection

Flowchart

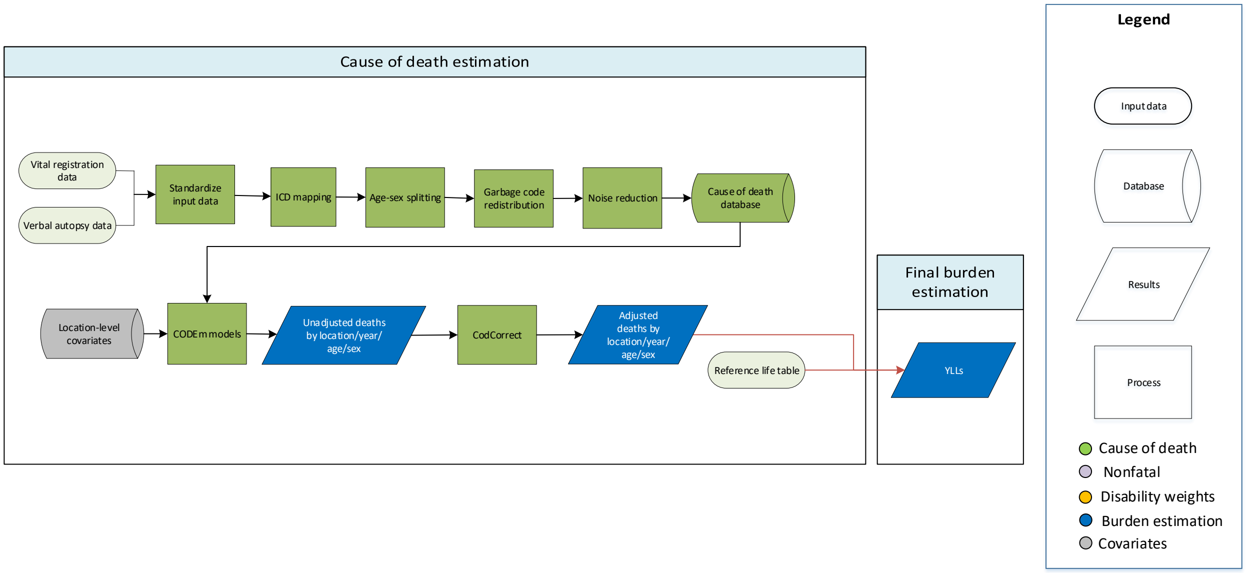

Input data and methodological summary for urinary tract infection

Input data

Data used to estimate mortality of urinary tract infection consisted of vital registration data and verbal autopsy data from the cause of death (COD) database (see appendix section on ICD mapping for details). Outliers were identified by systematic examination of datapoints for all location-years. Data were excluded if they violated well-established age or time trends, and data in instances where garbage code redistribution and noise reduction, in combination with small sample sizes, resulted in unreasonable cause fractions. Methods for assigning outlier status were consistent across both vital registration and verbal autopsy data.

Modelling strategy

The estimation strategy used for fatal urinary tract infection is largely similar to methods used in GBD 2019. A standard CODEm model with location-level covariates was used to model deaths due to urinary tract infection (see appendix section on CODEm method for details). Separate models were conducted for male and female mortality, and age-restrictions for death estimations included 0 days for lower bound and 95+ years for upper bound. We hybridised separate global and data-rich models to acquire unadjusted results, which we adjusted using CoDCorrect and compared to the reference life table to calculate final YLLs due to urinary tract infection.

In GBD 2021, we updated the linear floor value in our CODEm models to allow the model to be influenced by lower data, which resulted from changes to COD data processing. These data processing changes include changing the method of estimating the non-zero floor and population size cutoffs for when to noise-reduce at the country versus region level, and are described in greater detail in the

appendix section GBD 2021 Causes of Death database. Apart from these, no other substantive changes were made in GBD 2021 from the modelling strategy used in GBD 2019.

The following table has the full list of covariates used for fatal urinary tract infection.

Table 1. Covariates used in urinary tract infection mortality modelling

Level	Covariate	Direction
1	Sanitation (proportion with access)	+
2	Education (years per capita)	-
	Log LDI ($I per capita)	-
	Healthcare Access and Quality Index	-
3	Socio-demographic Index	-

Adjustment in CoDCorrect included fitting unadjusted death estimates for all other specific and non-specific urinary diseases to overall urinary disease deaths, which were then adjusted with all other causes to sum to all-cause counts of death.

Urolithiasis

Flowchart

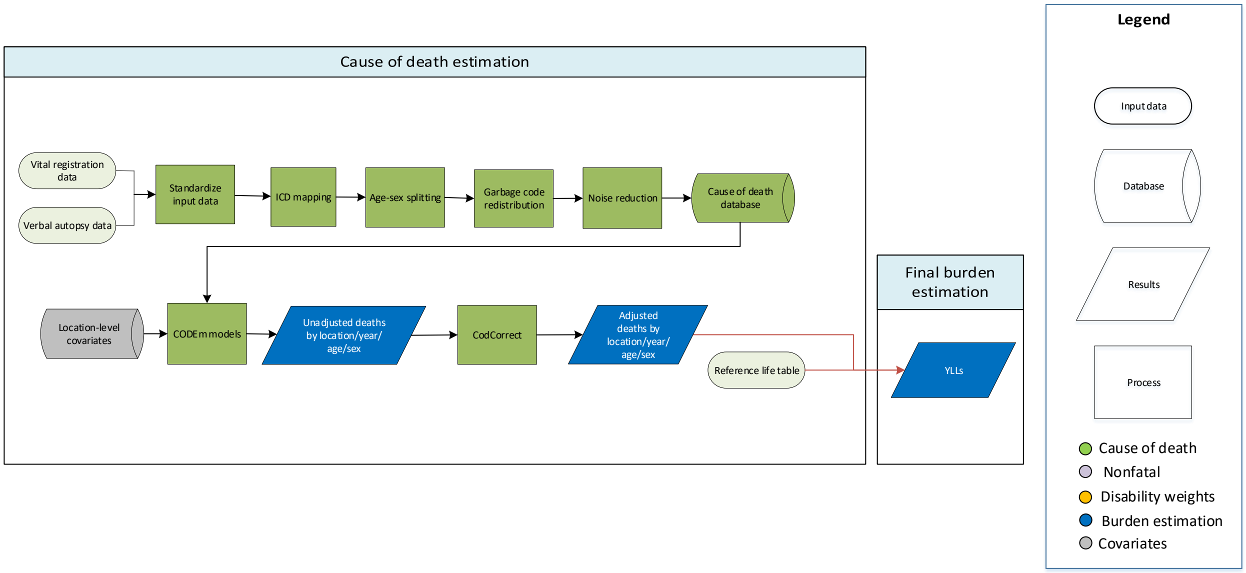

Input data and methodological summary for urolithiasis

Input data

Data used to estimate mortality of urolithiasis consisted of vital registration data and verbal autopsy data from the cause of death (COD) database (see appendix section on ICD mapping for details). Outliers were identified by systematic examination of datapoints for all location-years. Data were excluded if they violated well-established age or time trends, and data in instances where garbage code redistribution and noise reduction, in combination with small sample sizes, resulted in unreasonable cause fractions. Methods for assigning outlier status were consistent across both vital registration and verbal autopsy data.

Modelling strategy

The estimation strategy used for fatal urolithiasis is largely similar to methods used in GBD 2019. A standard CODEm model with location-level covariates was used to model deaths due to urolithiasis (see appendix section on CODEm method for details). Separate models were conducted for male and female mortality, and age-restrictions for death estimations included 12 months for lower bound and 95+ years for upper bound. We hybridised separate global and data-rich models to acquire unadjusted results, which we adjusted using CoDCorrect and compared to the reference life table to calculate final YLLs due to due to urolithiasis.

In GBD 2021, we updated the linear floor value in our CODEm models to allow the model to be influenced by lower data, which resulted from changes to COD data processing. These data processing changes include changing the method of estimating the non-zero floor and population size cutoffs for when to noise-reduce at the country versus region level, and are described in greater detail in the appendix section GBD 2021 Causes of Death database. Apart from these, no other substantive changes were made in GBD 2021 from the modelling strategy used in GBD 2019.

The following table has the full list of covariates used for fatal urolithiasis.

Table 1. Covariates used in urolithiasis mortality modelling

Level	Covariate	Direction
1	Temperature (90th percentile)	+
	Red meat consumption (unadjusted, kcal per capita)	+
2	Fruit consumption (unadjusted, kcal per capita)	-
	Vegetable consumption (unadjusted, kcal per capita)	-
	Healthcare Access and Quality Index	-
3	Socio-demographic Index	-
	Education (years per capita)	-
	Log LDI ($I per capita)	-

Adjustment in CoDCorrect included fitting unadjusted death estimates for all other specific and non-specific urinary diseases to overall urinary disease deaths, which were then adjusted with all other causes to sum to all-cause counts of death.

Varicella

Flowchart

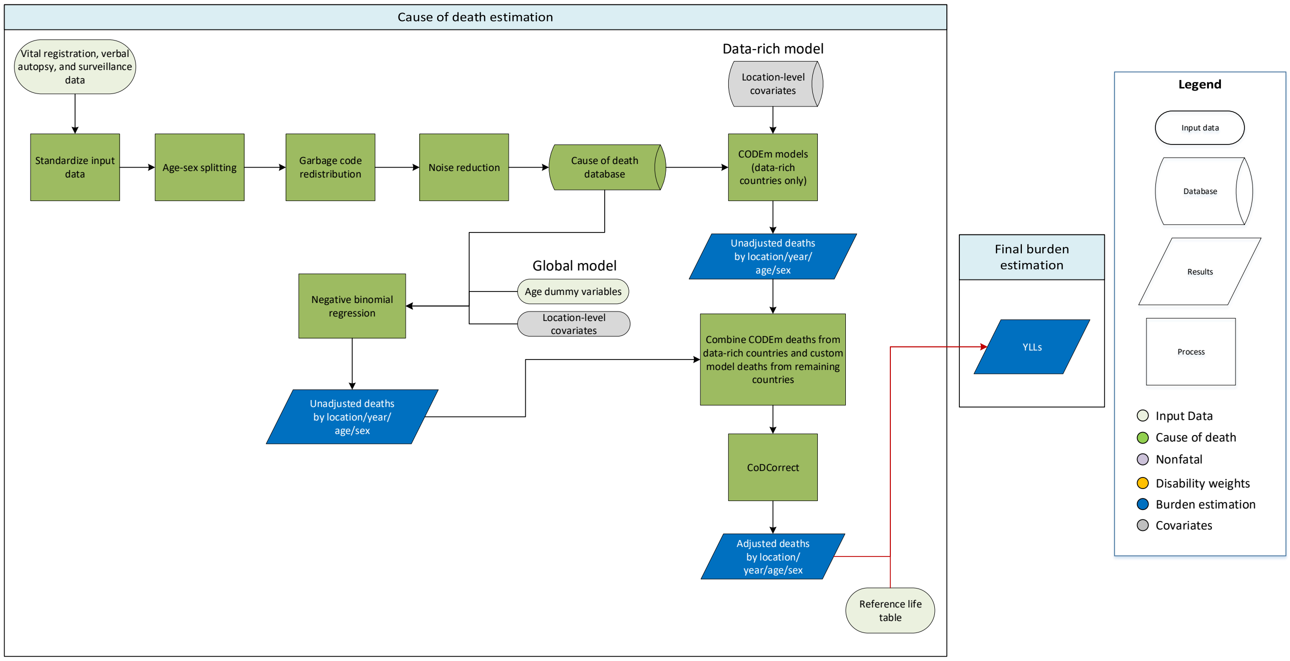

Input data

Varicella cause of death (COD) data for GBD 2021 included vital registration, verbal autopsy, and surveillance sources from all locations as available. We excluded COD data if they were highly incongruent with other available data from the same location or locations of similar sociodemographic characteristics.

Modelling strategy overview

The modeling strategy for varicella did not significantly change from GBD 2019. We used two distinct methods to estimate varicella mortality based on the quality of vital registration data available for each country. We used a counts-based Cause of Death Ensemble modelling strategy (CODEm) for countries with well-defined vital registration (ie, “data-rich” countries), and for remaining countries a custom count negative binomial regression model. Each approach is further described below.

1. Data-rich countries

For data-rich countries, the covariates listed in Table 1 were used to inform CODEm predictions.

Table 1. Covariates. Summary of covariates used in the data-rich varicella cause of death model

Level	Covariate	Direction
1	Healthcare Access and Quality (HAQ) Index	-
	Age- and sex-specific SEV for child underweight	+
	Age- and sex-specific SEV for child wasting	+

3	Lag-distributed income (LDI)	-
	Mean years of education per capita	-
	Sanitation access (proportion)	-
	Population density over 1000 people per square kilometer (proportion)	+
	Socio-demographic Index (SDI)	-

2. *Custom count model*

Our custom counts mortality model for all non-data-rich locations fit a negative binomial regression to CoD data, as available by location, to model varicella mortality. We modelled counts of deaths due to varicella using the Healthcare Access and Quality (HAQ) Index and age dummy variables with the offset set to the location-, year-, age-, and sex-specific populations:

$$Y_{ij} = \beta_0 + \beta_1\ HAQ_{ij} + age_{a\ ij} + e_{ij},$$

where Y_{ij} is the log-transformed number of varicella deaths offset by population size; β_0 is the fixed-effect intercept; β_1 is the fixed-effects slope on location- and year-specific HAQ_{ij}; $age_{a\ ij}$ is a dummy variable for each GBD age group in the estimation; e_{ij} is the residual; *i* is the year; and *j* is the location. We exclude studies that report a varicella-specific mortality fraction (the fraction of deaths due to varicella) that is higher than the 99th percentile of all reported varicella-specific mortality fractions. Uncertainty was estimated by taking 1000 samples of the predictions based on the variance-covariance matrix and a random sample of the dispersion parameter from a gamma distribution.

Vascular intestinal disorders

Flowchart

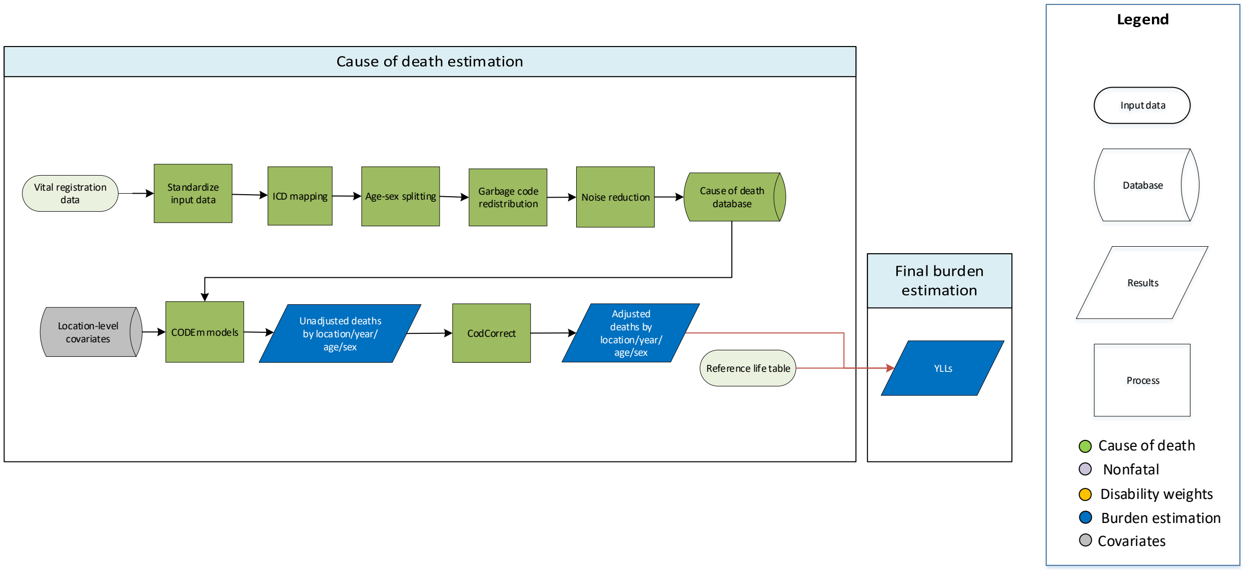

Input data and methodological summary for vascular intestinal disorders

Input data

Data used to estimate mortality of vascular intestinal disorders consisted of vital registration data from the cause of death (COD) database (see appendix section on ICD mapping for details). Outliers were identified by systematic examination of datapoints for all location-years. Data were excluded if they violated well-established age or time trends, and data in instances where garbage code redistribution and noise reduction, in combination with small sample sizes, resulted in unreasonable cause fractions.

Modelling strategy

The estimation strategy used for fatal vascular intestinal disorders is largely similar to methods used in GBD 2019. A standard CODEm model with location-level covariates was used to model deaths due to vascular intestinal disorders (see appendix section on CODEm method for details). Separate models were conducted for male and female mortality, and age restrictions for death estimations included 2 years for lower bound (in GBD 2019, the lower bound was set at 1 year) and 95+ for upper bound. We hybridised separate global and data-rich models to acquire unadjusted results, which we adjusted using CoDCorrect and compared to the reference life table to calculate final YLLs due to vascular intestinal disorders.

In GBD 2021, we updated the linear floor value in our CODEm models to allow the model to be influenced by lower data, which resulted from changes to COD data processing. These data processing changes include changing the method of estimating the non-zero floor and population size cutoffs for when to noise-reduce at the country versus region level, and are described in greater detail in the appendix section “GBD 2021 Causes of Death database”. Apart from these, no other substantive changes were made in GBD 2021 from the modelling strategy used in GBD 2019.

The following table has the full list of covariates used for vascular intestinal disorders.

Table 1. Covariates used in vascular intestinal disorders mortality modelling

Level	Covariate	Direction
1	Fasting plasma glucose	+
	Cholesterol (total, mean per capita)	+
	Systolic blood pressures (mmHg)	+
2	BMI (mean)	+
	Smoking prevalence	+
	Healthcare Access and Quality Index	-
3	Socio-demographic Index	-
	Education (years per capita)	-
	Log LDI ($I per capita)	-
	Pulses/legumes (kcal/capita, adjusted)	-
	Age-sex-specific scaled exposure variable for low fruit consumption	+
	Age-sex-specific scaled exposure variable for low vegetable consumption	+
	Age-sex-specific scaled exposure variable for high red meat consumption	+
	Age-sex-specific scaled exposure variable for low fish consumption	+
	Age-sex-specific scaled exposure variable for low nut consumption	+
	Consumption of high trans-unsaturated fatty acids	+
	Alcohol (litres per capita)	+

Adjustment in CoDCorrect included fitting unadjusted death estimates for all other specific and non-specific digestive diseases to overall digestive disease deaths, which was then adjusted with all other causes to sum to all-cause counts of death.

Visceral leishmaniasis

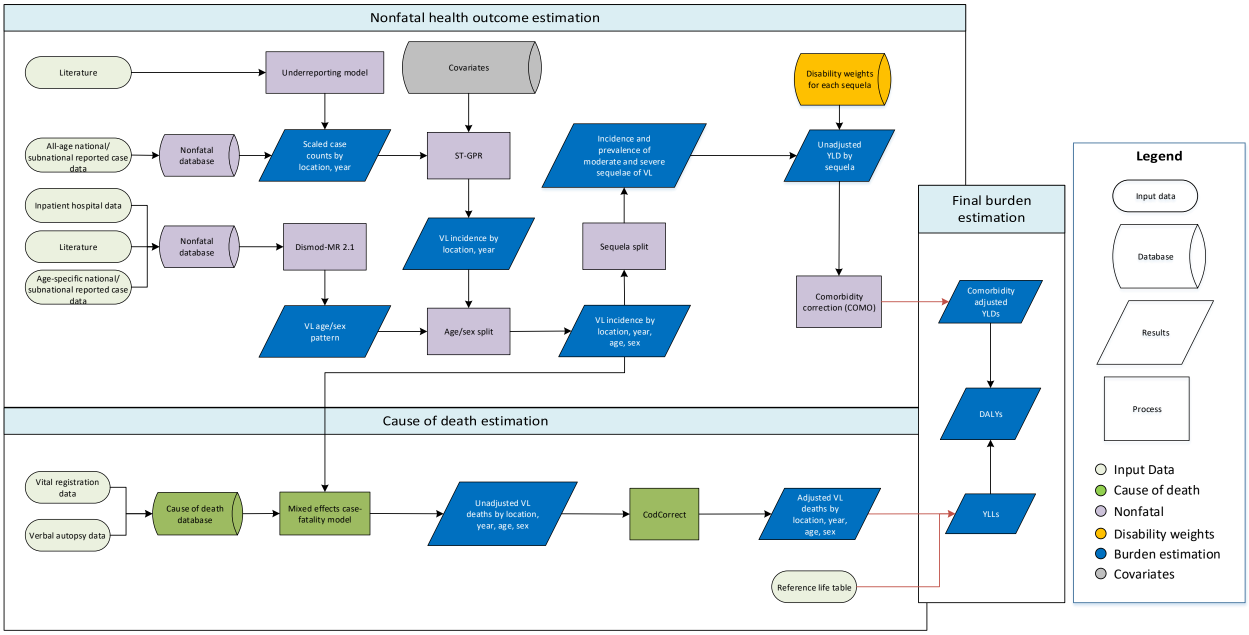

Case definition

Visceral leishmaniasis (VL) is the most serious manifestation of disease caused by the *Leishmania* parasite, transmitted through the bite of phlebotomine sandflies. Those infected typically present with fever, weight loss, anaemia, leukopenia, thrombocytopenia, and enlargement of the spleen and liver. If left untreated, it can be fatal. Transmission varies by geographical region, with a variety of reservoir hosts implicated, and different vector species associated, maintaining both zoonotic and anthroponotic transmission cycles. The ICD-9 code related to visceral leishmaniasis is 085.0, and the ICD-10 code is B55.0.

Description of general methodology

The fatal estimation process for visceral leishmaniasis builds from incident case notification data, described in more detail in another section of this appendix. Briefly, incident case data that are representative of the GBD geographical location are adjusted for under-reporting. The upscaled all-age, both-sex case counts are modelled using spatiotemporal Gaussian process regression (ST-GPR) in order to impute for missing location-year combinations as well as to account for further biases and inaccuracies in reporting. Datasets that disaggregate VL cases by age and sex are modelled using DisMod-MR to produce a global age-sex split, which is applied to the all-age, both-sex envelope estimates resulting from ST-GPR. The mean incidence estimates are compared with estimated death counts to generate a case-fatality rate model that is subsequently used to estimate deaths for each age, sex, location, and year.

Input data – mortality

Deaths were extracted from a variety of sources, ranging from vital registration (VR) records to verbal autopsy (VA) assessments. Deaths assigned to visceral leishmaniasis were processed following central cause of death processing, outlined elsewhere.

Method – YLL estimation

Deaths were modelled using a mixed effect model parameterising case-fatality rate, with data derived from taking attributed-death data and dividing it by the mean predicted incident cases.

$$Logit(Case\ Fatality\ Rate) \sim Age + Sex + (Age|Super\ Region/Region)$$

Only data from countries defined as present or protocol present were used, as these represent locations that are generalisable to all endemic regions for VL. The deaths in non-endemic countries, while not used in the case-fatality rate model, are subsequently added back into the death envelope as-is. For African and European countries as well as South Sudan from 1990–1994, we assumed custom case-fatality rates. These assumptions were more consistent with external literature of visceral leishmaniasis case-fatality rates. For African case-fatality rates, 1000 draws were taken from a uniform distribution between 0.10 and 0.30 (Alvar and colleagues 2012, Martins-Melo and colleagues 2014). For European case-fatality rates, including endemic Italian subnationals, we drew 1000 draws from a uniform distribution between 0.06 and 0.10 (Martins-Melo and colleagues 2014), and we assumed a 0.69 case fatality rate for South Sudan between the years 1990 and 1994, based on data reported during the VL epidemic from the late 1980s to 1994 (Seamen and colleagues 1996).

Case-fatality rate estimates had high uncertainty in some geographies. In general, female mean case-fatality rates were higher than male case-fatality rates. Typically an all-age estimate of 10% case-fatality rate is discussed when looking at visceral leishmaniasis (Alvar and colleagues 2012). Final fatal estimates of VL were calculated as the product of incident case estimates and case fatality.

Limitations

Known limitations for the VL model will be the focus in future GBD rounds and engagement with collaborators. Given the focus on location-representative estimates, the existing model is based upon national case counts. This excludes a large resource of published literature and grey literature focused on site-specific surveillance or surveys. In the future, there is a need to identify an independent resource to aid in quantifying the population at risk.

Age-sex patterns are highly reflective of the countries from which data are obtained. Importantly, there is a large skew in information coming from Brazil. This information has potential biases due to the nature of the data inputs (notification and hospital data) and the corresponding age-sex variation in health-seeking behaviours which may not be generalisable to other settings.

References

1. Alvar J, Vélez ID, Bern C, *et al.* Leishmaniasis Worldwide and Global Estimates of Its Incidence. *PLoS One* 2012; **7**. DOI:10.1371/journal.pone.0035671.
2. Martins-Melo FR, Lima M da S, Ramos AN, Alencar CH, Heukelbach J. Mortality and case fatality due to visceral leishmaniasis in Brazil: a nationwide analysis of epidemiology, trends and spatial patterns. *PLoS One* 2014; **9**: e93770.
3. Seaman J, Mercer AJ, Sondorp E. The epidemic of visceral leishmaniasis in western Upper Nile, southern Sudan: course and impact from 1984 to 1994. *Int J Epidemiol* 1996; **25**: 862–71.

Yellow fever

Flowchart

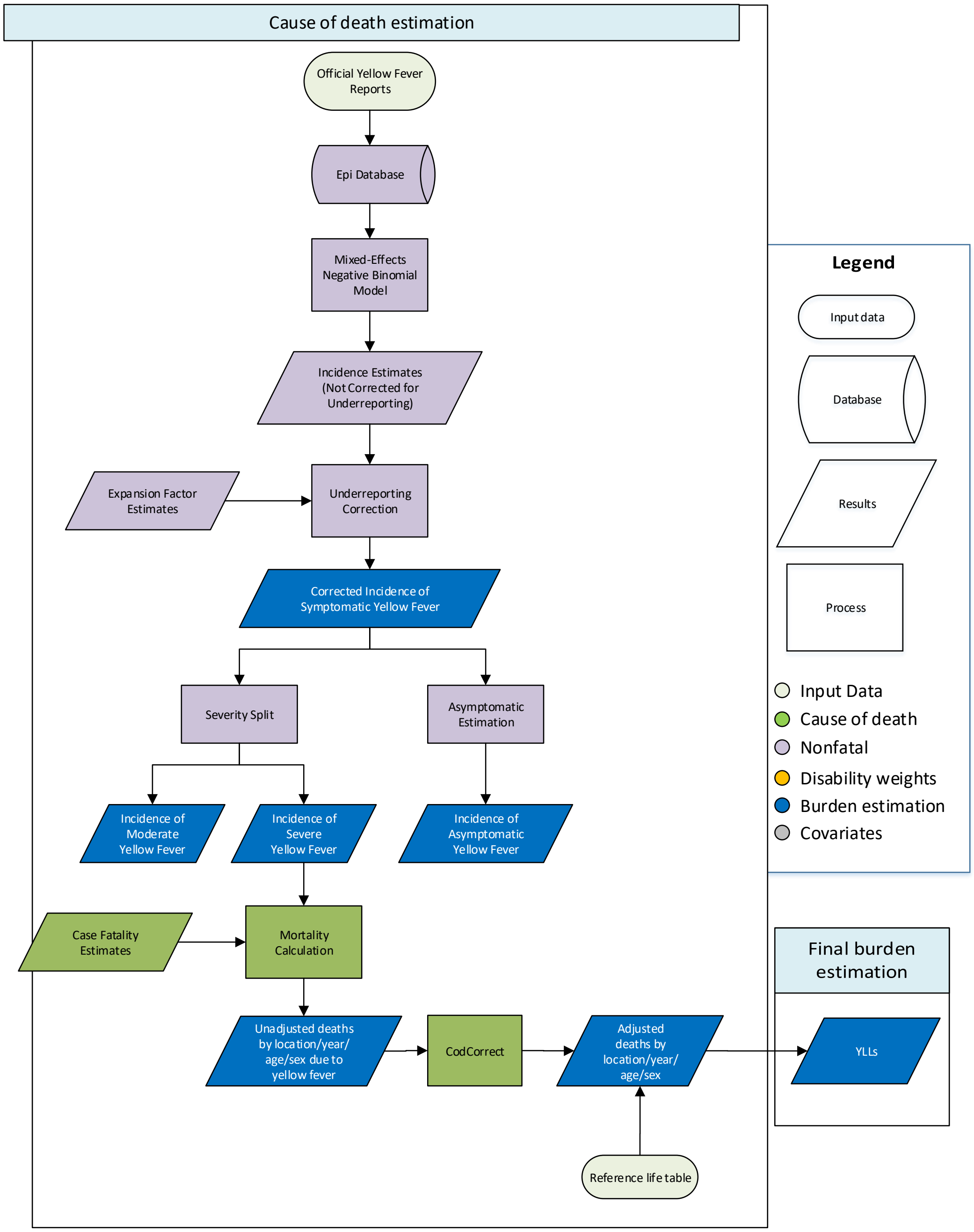

Input data and methodological summary for yellow fever

Input data

Case data come from official case reports filed with the World Health Organization. Data on case fatality come from published studies of yellow fever fatality. Data on deaths in non-endemic countries are restricted to only vital registration data.

Modelling strategy

We model yellow fever deaths using a hybrid approach. For countries in which yellow fever is endemic, we use a natural history approach in which we estimate deaths as the product of severe incident cases and case fatality. For non-endemic countries, we allow for deaths among imported cases where we have vital registration data indicating yellow fever deaths. That is, we assume no yellow fever deaths in non-endemic countries; however, where yellow fever deaths are reported in vital registration data, we accept those as true imported yellow fever deaths.

We model reported cases using a mixed-effects negative binomial model, described in detail elsewhere in this appendix. Based on published estimates, we assume that 27% of symptomatic cases will be severe.[1] We performed a meta-analysis of case fatality using data from published studies of yellow fever fatality (see Table 1 below). Studies tend to report deaths among those with severe infection (eg, hospitalised cases), rather than among all cases. We assume that no deaths occur with asymptomatic infection or among those with only moderate symptoms. With that, we estimate deaths as the product of severe cases and case fatality.

Table 1. Metadata for case-fatality assumptions. Each record lists a citation, GBD location of relevance, year, and output values used in modelling.

Citation	GBD location	Time period	Case fatality rate (# deaths/# reported cases)
Reiter *et al.* First recorded outbreak of yellow fever in Kenya, 1992–1993[2]	Kenya	1992–1993	5/26
Soghaier *et al*. Yellow Fever outbreak in Darfur, Sudan in October 2012[3]	Sudan	2012	5/7
Thonnon *et al.* Re-emergence of yellow fever in Senegal in 1995[4]	Senegal	1995	15/79
Tuboi *et al.* Clinical and epidemiological characteristics of yellow fever in Brazil: analysis of reported cases 1998–2002[5]	Brazil	1998–2002	11/251
Vasconcelos *et al.* Epidemic of jungle yellow fever in Brazil, 2000: implications of climatic alterations in disease spread[6]	Brazil	2000	39/77
Vasconcelos *et al.* An epidemic of sylvatic yellow fever in the southeast region of Maranhao State, Brazil,	Brazil	1993–1994	13/74

1993–1994: epidemiologic and entomologic findings[7]			
Wamala *et al.* Epidemiological and laboratory characterization of a yellow fever outbreak in northern Uganda[8]	Uganda	2010–2011	7/13

We accept deaths reported in vital registration data as true imported deaths. As in GBD 2021, we adjust total death estimates to account for the high case burden observed in the 2017–2018 outbreak in Brazil. We used reported deaths from Brazilian vital registration data from 2017 to derive an age and sex distribution of these deaths, and simulated uncertainty for case totals from a Poisson distribution to inflate modelled death estimates to account for this outbreak.

Changes since GBD 2019

We have made no substantive modelling changes in GBD 2021.

References

1. Johansson MA, Vasconcelos PFC, Staples JE. The whole iceberg: estimating the incidence of yellow fever virus infection from the number of severe cases. *Trans R Soc Trop Med Hyg* 2014; 108: 482–7.
2. Reiter P, Cordellier R, Ouma JO, *et al.* First recorded outbreak of yellow fever in Kenya, 1992-1993. II. Entomologic investigations. *Am J Trop Med Hyg* 1998; **59**: 650–6.
3. Soghaier MA, Hagar A, Abbas MA, Elmangory MM, Eltahir KM, Sall AA. Yellow Fever outbreak in Darfur, Sudan in October 2012; the initial outbreak investigation report. *J Infect Public Health* 2013; **6**: 370–6.
4. Thonnon J, Fontenille D, Tall A, *et al.* Re-emergence of yellow fever in Senegal in 1995. *Am J Trop Med Hyg* 1998; **59**: 108–14.
5. Tuboi SH, Costa ZGA, da Costa Vasconcelos PF, Hatch D. Clinical and epidemiological characteristics of yellow fever in Brazil: analysis of reported cases 1998-2002. *Trans R Soc Trop Med Hyg* 2007; **101**: 169–75.
6. Vasconcelos PF, Costa ZG, Travassos Da Rosa ES, *et al.* Epidemic of jungle yellow fever in Brazil, 2000: implications of climatic alterations in disease spread. *J Med Virol* 2001; **65**: 598–604.
7. Vasconcelos PF, Rodrigues SG, Degallier N, *et al.* An epidemic of sylvatic yellow fever in the southeast region of Maranhao State, Brazil, 1993-1994: epidemiologic and entomologic findings. *Am J Trop Med Hyg* 1997; **57**: 132–7.
8. Wamala JF, Malimbo M, Okot CL, *et al.* Epidemiological and laboratory characterization of a yellow fever outbreak in northern Uganda, October 2010-January 2011. *Int J Infect Dis* 2012; **16**: e536-542.

Zika virus disease

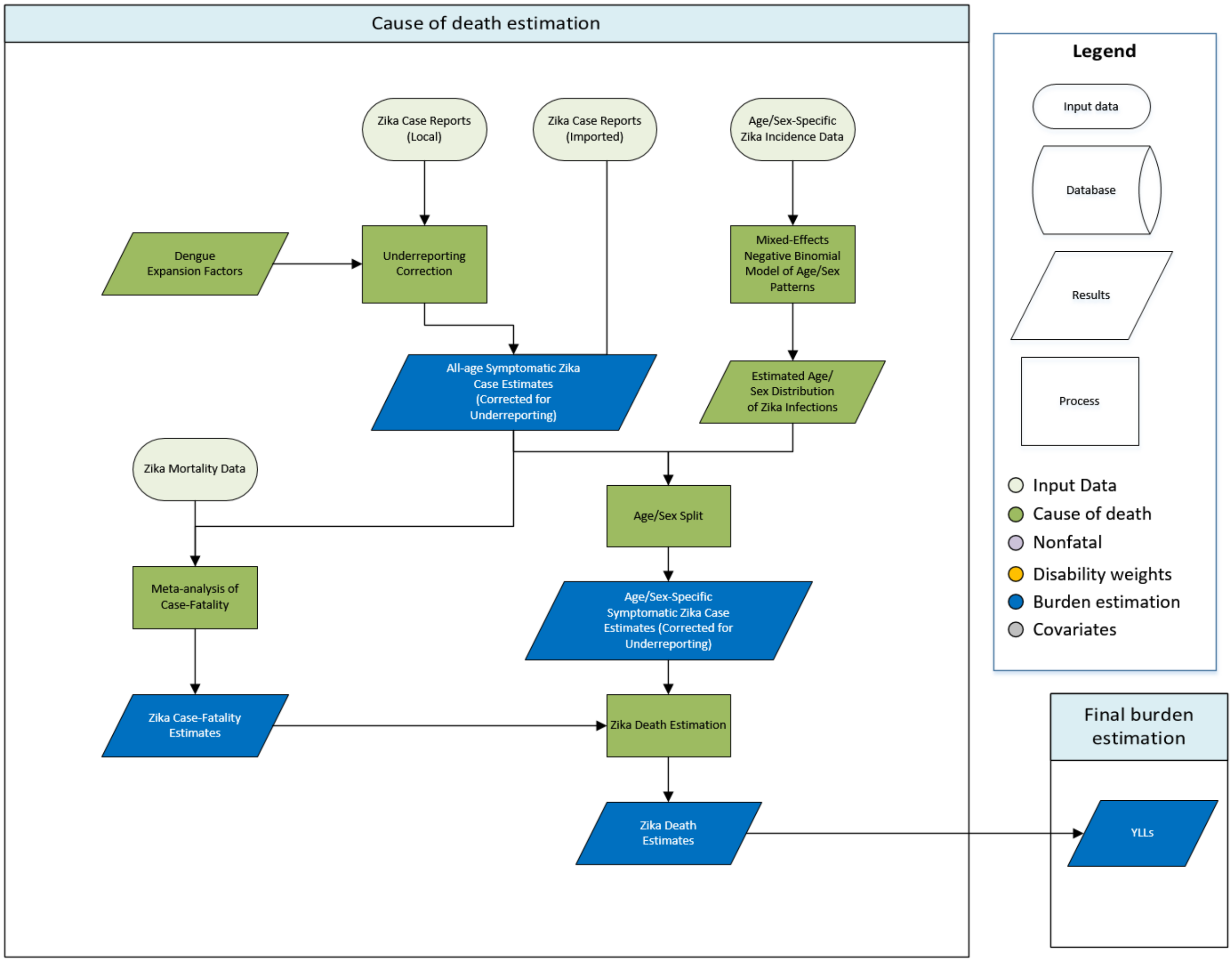

Input data

The death data come from official reports, primarily from PAHO, in which deaths attributed to Zika virus infection were reported for the period 2015–2018. Overall, a total of 22 deaths were reported in Brazil, Suriname, and Puerto Rico during this period. Of these cases, the majority were among adult males. The incidence data come from our GBD 2021 Zika symptomatic incidence case estimates.

Modelling strategy

We modelled Zika deaths using a global case-fatality rate (CFR) model. The numerator was the all-ages–both-sex total deaths reported in the period from all endemic locations. The denominator was the total all-ages–both-sex number of symptomatic incident Zika cases from 2015 to 2017 (years in which we had reported deaths) from all endemic locations. We used a binomial distribution to generate 1000 draws of a global, all-age, both-sex CFR. Then, we multiplied the CFR draws by draws of age-sex-specific symptomatic Zika incidence case counts, by location and year, to generate draws of counts of deaths, by

age, sex, location, and year. For locations/years where the reported number of deaths was higher than the estimated value from this approach, we used a location-year-specific case-fatality rate rather than the global case-fatality rate.

Changes from GBD 2019 to GBD 2021

For GBD 2021, we updated the modelling approach to estimate CFR as described above, then calculated the number of deaths by age, sex, location, and year by multiplying the number of incident symptomatic Zika cases by this CFR while propagating uncertainty.
We did not apply any adjustments for the COVID-19 pandemic to Zika due to a lack of available data quantifying the impacts of the pandemic on NTD epidemiology.
