# Supplementary material for: Global burden of 288 causes of death and life expectancy decomposition in 204 countries and territories and 811 subnational locations, 1990–2021: a systematic analysis for the Global Burden of Disease Study 2021
Source: Lancet. 2024 May 18;403(10440):2100–32. doi: 10.1016/S0140-6736(24)00367-2 (PMC11126520; doi:10.1016/S0140-6736(24)00367-2)
Supplement: Supplementary appendix 3 [file mmc3.pdf]

THE LANCET

Supplementary appendix 3

This appendix formed part of the original submission and has been peer reviewed. We post it as supplied by the authors.

Supplement to: GBD 2021 Causes of Death Collaborators. Global burden of 288 causes of death and life expectancy decomposition in 204 countries and territories and 811 subnational locations, 1990–2021: a systematic analysis for the Global Burden of Disease Study 2021. *Lancet* 2024; **403:** 2100–32.

Appendix 3: Authorship appendix to “Global burden of 288 causes of death and life expectancy decomposition in 204 countries and territories and 811 subnational locations, 1990– 2021: a systematic analysis for the Global Burden of Disease Study 2021”

This appendix provides further authorship detail for “Global burden of 288 causes of death and life expectancy decomposition in 204 countries and territories and 811 subnational locations, 1990– 2021: a systematic analysis for the Global Burden of Disease Study 2021.”

Table of Contents

GBD 2021 CoD Collaborators **2**

Affiliations **9**

Authors' Contributions **57**

GBD 2021 Causes of Death Collaborators

Mohsen Naghavi*, Kanyin Liane Ong*, Amirali Aali, Hazim S Ababneh, Yohannes Habtegiorgis Abate, Cristiana Abbafati, Rouzbeh Abbasgholizadeh, Mohammadreza Abbasian, Mohsen Abbasi-Kangevari, Hedayat Abbastabar, Samar Abd ElHafeez, Michael Abdelmasseh, Sherief Abd-Elsalam, Ahmed Abdelwahab, Mohammad Abdollahi, Mohammad-Amin Abdollahifar, Meriem Abdoun, Deldar Morad Abdulah, Auwal Abdullahi, Mesfin Abebe, Samrawit Shawel Abebe, Aidin Abedi, Kedir Hussein Abegaz, E S Abhilash, Hassan Abidi, Olumide Abiodun, Richard Gyan Aboagye, Hassan Abolhassani, Meysam Abolmaali, Mohamed Abouzid, Girma Beressa Aboye, Lucas Guimarães Abreu, Woldu Aberhe Abrha, Dariush Abtahi, Samir Abu Rumeileh, Hasan Abualruz, Bilyaminu Abubakar, Eman Abu-Gharbieh, Niveen ME Abu-Rmeileh, Salahdein Aburuz, Ahmed Abu-Zaid, Manfred Mario Kokou Accrombessi, Tadele Girum Adal, Abdu A Adamu, Isaac Yeboah Addo, Giovanni Addolorato, Akindele Olupelumi Adebiyi, Victor Adekanmbi, Abiola Victor Adepoju, Charles Oluwaseun Adetunji, Juliana Bunmi Adetunji, Temitayo Esther Adeyeoluwa, Daniel Adedayo Adeyinka, Olorunsola Israel Adeyomoye, Biruk Adie Admass, Qorinah Estiningtyas Sakilah Adnani, Saryia Adra, Aanuoluwapo Adeyimika Afolabi, Muhammad Sohail Afzal, Saira Afzal, Suneth Buddhika Agampodi, Pradyumna Agasthi, Manik Aggarwal, Shahin Aghamiri, Feleke Doyore Agide, Antonella Agodi, Anurag Agrawal, Williams Agyemang-Duah, Bright Opoku Ahinkorah, Aqeel Ahmad, Danish Ahmad, Firdos Ahmad, Muayyad M Ahmad, Sajjad Ahmad, Shahzaib Ahmad, Tauseef Ahmad, Keivan Ahmadi, Amir Mahmoud Ahmadzade, Ali Ahmed, Ayman Ahmed, Haroon Ahmed, Luai A Ahmed, Mehrunnisha Sharif Ahmed, Meqdad Saleh Ahmed, Muktar Beshir Ahmed, Syed Anees Ahmed, Marjan Ajami, Budi Aji, Essona Matatom Akara, Hossein Akbarialiabad, Karolina Akinosoglou, Tomi Akinyemiju, Mohammed Ahmed Akkaif, Samuel Akyirem, Hanadi Al Hamad, Syed Mahfuz Al Hasan, Fares Alahdab, Samer O Alalalmeh, Tariq A Alalwan, Ziyad Al-Aly, Khurshid Alam, Manjurul Alam, Noore Alam, Rasmieh Mustafa Al-amer, Fahad Mashhour Alanezi, Turki M Alanzi, Sayer Al-Azzam, Almaza Albakri, Mohammed Albashtawy, Mohammad T AlBataineh, Jacqueline Elizabeth Alcalde-Rabanal, Khalifah A Aldawsari, Wafa A Aldhaleei, Robert W Aldridge, Haileselasie Berhane Alema, Mulubirhan Assefa Alemayohu, Sharifullah Alemi, Yihun Mulugeta Alemu, Adel Ali Saeed Al-Gheethi, Khalid F Alhabib, Fadwa Alhalaiqa Naji Alhalaiqa, Mohammed Khaled Al-Hanawi, Abid Ali, Amjad Ali, Liaqat Ali, Mohammed Usman Ali, Rafat Ali, Shahid Ali, Syed Shujait Shujait Ali, Gianfranco Alicandro, Sheikh Mohammad Alif, Reyhaneh Alikhani, Yousef Alimohamadi, Ahmednur Adem Aliyi, Mohammad A M Aljasir, Syed Mohamed Aljunid, François Alla, Peter Allebeck, Sabah Al-Marwani, Sadeq Ali Ali Al-Maweri, Joseph Uy Almazan, Hesham M Al-Mekhlafi, Louay Almidani, Omar Almidani, Mahmoud A Alomari, Basem Al-Omari, Jordi Alonso, Jaber S Alqahtani, Shehabaldin Alqalyoobi, Ahmed Yaseen Alqutaibi, Salman Khalifah Al-Sabah, Zaid Altaany, Awais Altaf, Jaffar A Al-Tawfiq, Khalid A Altirkawi, Deborah Oyine Aluh, Nelson Alvis-Guzman, Hassan Alwafi, Yaser Mohammed Al-Worafi, Hany Aly, Safwat Aly, Karem H Alzoubi, Reza Amani, Azmeraw T Amare, Prince M Amegbor, Edward Kwabena Ameyaw, Tarek Tawfik Amin, Alireza Amindarolzarbi, Sohrab Amiri, Mohammad Hosein Amirzade-Iranaq, Hubert Amu, Dickson A Amugsi, Ganiyu Adeniyi Amusa, Robert Ancuceanu, Deanna Anderlini, David B Anderson, Pedro Prata Andrade, Catalina Liliana Andrei, Tudorel Andrei, Colin Angus, Abhishek Anil, Sneha Anil, Amir Anoushiravani, Hossein Ansari, Ansariadi Ansariadi, Alireza Ansari-Moghaddam, Catherine M Antony, Ernoiz Antriyandarti, Davood Anvari, Saeid Anvari, Saleha Anwar, Sumadi Lukman Anwar, Razique Anwer, Anayochukwu Edward Anyasodor, Muhammad Aqeel, Juan Pablo Arab, Jalal Arabloo, Mosab Arafat, Aleksandr Y Aravkin, Demelash Areda, Abdulfatai Aremu, Olatunde Aremu, Hany Ariffin, Mesay Arkew, Benedetta Armocida, Michael Benjamin Arndt, Johan Ärnlöv, Mahwish Arooj, Anton A Artamonov, Judie Arulappan,

Raphael Taiwo Aruleba, Ashokan Arumugam, Malke Asaad, Mohsen Asadi-Lari, Akeza Awealom Asgedom, Mona Asghariahmadabad, Mohammad Asghari-Jafarabadi, Muhammad Ashraf, Armin Aslani, Thomas Astell-Burt, Mohammad Athar, Seyyed Shamsadin Athari, Bantalem Tilaye Tilaye Atinafu, Habtamu Wondmagegn Atlaw, Prince Atorkey, Maha Moh'd Wahbi Atout, Alok Atreya, Avinash Aujayeb, Marcel Ausloos, Abolfazl Avan, Atalel Fentahun Awedew, Amlaku Mulat Aweke, Beatriz Paulina Ayala Quintanilla, Haleh Ayatollahi, Jose L Ayuso-Mateos, Seyed Mohammad Ayyoubzadeh, Sina Azadnajafabad, Rui M S Azevedo, Ahmed Y Azzam, Darshan B B, Abraham Samuel Babu, Muhammad Badar, Ashish D Badiye, Soroush Baghdadi, Nasser Bagheri, Sara Bagherieh, Sulaiman Bah, Saeed Bahadorikhalili, Najmeh Bahmanziari, Ruhai Bai, Atif Amin Baig, Jennifer L Baker, Abdulaziz T Bako, Ravleen Kaur Bakshi, Senthilkumar Balakrishnan, Madhan Balasubramanian, Ovidiu Constantin Baltatu, Kiran Bam, Maciej Banach, Soham Bandyopadhyay, Palash Chandra Banik, Hansi Bansal, Kannu Bansal, Franca Barbic, Martina Barchitta, Mainak Bardhan, Erfan Bardideh, Suzanne Lyn Barker-Collo, Till Winfried Bärnighausen, Francesco Barone-Adesi, Hiba Jawdat Barqawi, Lope H Barrero, Amadou Barrow, Sandra Barteit, Lingkan Barua, Zarrin Basharat, Azadeh Bashiri, Afisu Basiru, Pritish Baskaran, Buddha Basnyat, Quique Bassat, João Diogo Basso, Ann V L Basting, Sanjay Basu, Kavita Batra, Bernhard T Baune, Mohsen Bayati, Nebiyou Simegnew Bayileyegn, Thomas Beaney, Neeraj Bedi, Massimiliano Beghi, Emad Behboudi, Priyamadhaba Behera, Amir Hossein Behnoush, Masoud Behzadifar, Maryam Beiranvand, Diana Fernanda Bejarano Ramirez, Yannick Béjot, Sefealem Assefa Belay, Chalie Mulu Belete, Michelle L Bell, Muhammad Bashir Bello, Olorunjuwon Omolaja Bello, Luis Belo, Apostolos Beloukas, Rose Grace Bender, Isabela M Bensenor, Azizullah Beran, Zombor Berezvai, Alemshet Yirga Berhie, Betyna N Berice, Robert S Bernstein, Gregory J Bertolacci, Paulo J G Bettencourt, Kebede A Beyene, Devidas S Bhagat, Akshaya Srikanth Bhagavathula, Neeraj Bhala, Ashish Bhalla, Dinesh Bhandari, Kayleigh Bhangdia, Nikha Bhardwaj, Pankaj Bhardwaj, Prarthna V Bhardwaj, Ashish Bhargava, Sonu Bhaskar, Vivek Bhat, Gurjit Kaur Bhatti, Jasvinder Singh Bhatti, Manpreet S Bhatti, Rajbir Bhatti, Zulfiqar A Bhutta, Boris Bikbov, Jessica Devin Bishai, Catherine Bisignano, Francesca Bisulli, Atanu Biswas, Bijit Biswas, Saeid Bitaraf, Bikes Destaw Bitew, Veera R Bitra, Tone Bjørge, Micheal Kofi Boachie, Mary Sefa Boampong, Anca Vasilica Bobirca, Virginia Bodolica, Aadam Olalekan Bodunrin, Eyob Ketema Bogale, Kassawmar Angaw Bogale, Somayeh Bohlouli, Obasanjo Afolabi Bolarinwa, Archith Boloor, Milad Bonakdar Hashemi, Aime Bonny, Kaustubh Bora, Berrak Bora Basara, Hamed Borhany, Arturo Borzutzky, Souad Bouaoud, Antoine Boustany, Christopher Boxe, Edward J Boyko, Oliver J Brady, Dejana Braithwaite, Luisa C Brant, Michael Brauer, Alexandra Brazinova, Javier Brazo-Sayavera, Nicholas J K Breitborde, Susanne Breitner, Hermann Brenner, Andrey Nikolaevich Briko, Nikolay Ivanovich Briko, Gabrielle Britton, Julie Brown, Traolach Brugha, Norma B Bulamu, Lemma N Bulto, Danilo Buonsenso, Richard A Burns, Reinhard Busse, Yasser Bustanji, Nadeem Shafique Butt, Zahid A Butt, Florentino Luciano Caetano dos Santos, Daniela Calina, Luis Alberto Cámera, Luciana Aparecida Campos, Ismael R Campos-Nonato, Chao Cao, Yin Cao, Angelo Capodici, Rosario Cárdenas, Sinclair Carr, Giulia Carreras, Juan J Carrero, Andrea Carugno, Cristina G Carvalheiro, Felix Carvalho, Márcia Carvalho, Joao Mauricio Castaldelli-Maia, Carlos A Castañeda-Orjuela, Giulio Castelpietra, Ferrán Catalá-López, Alberico L Catapano, Maria Sofia Cattaruzza, Christopher R Cederroth, Luca Cegolon, Francieli Cembranel, Muthia Cenderadewi, Kelly M Cercy, Ester Cerin, Muge Cevik, Joshua Chadwick, Yaacoub Chahine, Chiranjib Chakraborty, Promit Ananyo Chakraborty, Jeffrey Shi Kai Chan, Raymond N C Chan, Rama Mohan Chandika, Eeshwar K Chandrasekar, Chin-Kuo Chang, Jung-Chen Chang, Gashaw Sisay Chanie, Periklis Charalampous, Vijay Kumar Chattu, Pankaj Chaturvedi, Victoria Chatzimavridou-Grigoriadou, Akhilanand Chaurasia, Angela W Chen, An-Tian Chen, Catherine S Chen, Haowei Chen, Meng Xuan Chen,

Simiao Chen, Ching-Yu Cheng, Esther T W Cheng, Nicolas Cherbuin, Wondimye Ashenafi Cheru, Ju-Huei Chien, Odgerel Chimed-Ochir, Ritesh Chimoriya, Patrick R Ching, Jesus Lorenzo Chirinos-Caceres, Abdulaal Chitheer, William C S Cho, Bryan Chong, Hitesh Chopra, Sonali Gajanan Choudhari, Rajiv Chowdhury, Devasahayam J Christopher, Isaac Sunday Chukwu, Eric Chung, Erin Chung, Eunice Chung, Sheng-Chia Chung, Muhammad Chutiyami, Zinhle Cindi, Iolanda Cioffi, Mareli M Claassens, Rafael M Claro, Kaleb Coberly, Rebecca M Cogen, Alyssa Columbus, Haley Comfort, Joao Conde, Samuele Cortese, Paolo Angelo Cortesi, Vera Marisa Costa, Simona Costanzo, Ewerton Cousin, Rosa A S Couto, Richard G Cowden, Kenneth Michael Cramer, Michael H Criqui, Natália Cruz-Martins, Silvia Magali Cuadra-Hernández, Garland T Culbreth, Patricia Cullen, Matthew Cunningham, Maria paula Curado, Sriharsha Dadana, Omid Dadras, Siyu Dai, Xiaochen Dai, Zhaoli Dai, Lachlan L Dalli, Giovanni Damiani, Jiregna Darega Gela, Jai K Das, Saswati Das, Subasish Das, Ana Maria Dascalu, Nihar Ranjan Dash, Mohsen Dashti, Anna Dastiridou, Gail Davey, Claudio Alberto Dávila-Cervantes, Nicole Davis Weaver, Kairat Davletov, Diego De Leo, Katie de Luca, Aklilu Tamire Debele, Shayom Debopadhaya, Louisa Degenhardt, Azizallah Dehghan, Lee Deitesfeld, Cristian Del Bo', Ivan Delgado-Enciso, Berecha Hundessa Demessa, Andreas K Demetriades, Ke Deng, Xinlei Deng, Edgar Denova-Gutiérrez, Niloofar Deravi, Nebiyu Dereje, Nikolaos Dervenis, Emina Dervišević, Don C Des Jarlais, Hardik Dineshbhai Desai, Rupak Desai, Vinoth Gnana Chellaiyan Devanbu, Syed Masudur Rahman Dewan, Arkadeep Dhali, Kuldeep Dhama, Meghnath Dhimal, Sameer Dhingra, Vishal R Dhulipala, Diana Dias da Silva, Daniel Diaz, Michael J Diaz, Adriana Dima, Delaney D Ding, Huanghe Ding, Ricardo Jorge Dinis-Oliveira, M Ashworth Dirac, Shirin Djalalinia, Thao Huynh Phuong Do, Camila Bruneli do Prado, Saeid Doaei, Masoud Dodangeh, Milad Dodangeh, Sushil Dohare, Klara Georgieva Dokova, Christiane Dolecek, Regina-Mae Villanueva Dominguez, Wanyue Dong, Deepa Dongarwar, Mario D'Oria, Fariba Dorostkar, E Ray Dorsey, Wendel Mombaque dos Santos, Rajkumar Doshi, Leila Doshmangir, Robert Kokou Dowou, Tim Robert Driscoll, Haneil Larson Dsouza, Viola Dsouza, Mi Du, John Dube, Bruce B Duncan, Andre Rodrigues Duraes, Senbagam Duraisamy, Oyewole Christopher Durojaiye, Laura Dwyer-Lindgren, Paulina Agnieszka Dzianach, Arkadiusz Marian Dziedzic, Abdel Rahman E'mar, Ejemai Eboreime, Alireza Ebrahimi, Chidiebere Peter Echieh, Hisham Atan Edinur, David Edvardsson, Kristina Edvardsson, Defi Efendi, Ferry Efendi, Diyan Ermawan Effendi, Terje Andreas Eikemo, Ebrahim Eini, Michael Ekholuenetale, Temitope Cyrus Ekundayo, Iman El Sayed, Iffat Elbarazi, Teshome Bekele Elema, Noha Mousaad Elemam, Frank J Elgar, Islam Y Elgendy, Ghada Metwally Tawfik ElGohary, Hala Rashad Elhabashy, Muhammed Elhadi, Waseem El-Huneidi, Legesse Tesfaye Elilo, Omar Abdelsadek Abdou Elmeligy, Mohamed A Elmonem, Mohammed Elshaer, Ibrahim Elsohaby, Theophilus I Emeto, Luchuo Engelbert Bain, Ryenchindorj Erkhembayar, Christopher Imokhuede Esezobor, Babak Eshrati, Sharareh Eskandarieh, Juan Espinosa-Montero, Habtamu Esubalew, Farshid Etaee, Natalia Fabin, Adewale Oluwaseun Fadaka, Adeniyi Francis Fagbamigbe, Ayesha Fahim, Saman Fahimi, Aliasghar Fakhri-Demeshghieh, Luca Falzone, Mohammad Fareed, Carla Sofia e Sá Farinha, MoezAlIslam Ezzat Mahmoud Faris, Pawan Sirwan Faris, Andre Faro, Abidemi Omolara Fasanmi, Ali Fatehizadeh, Hamed Fattahi, Nelsensius Klau Fauk, Pooria Fazeli, Valery L Feigin, Alireza Feizkhah, Ginenus Fekadu, Xiaoru Feng, Seyed-Mohammad Fereshtehnejad, Abdullah Hamid Feroze, Daniela Ferrante, Alize J Ferrari, Nuno Ferreira, Getahun Fetensa, Bikila Regassa Feyisa, Irina Filip, Florian Fischer, Joanne Flavel, David Flood, Bobirca Teodor Florin, Nataliya A Foigt, Morenike Oluwatoyin Folayan, Artem Alekseevich Fomenkov, Behzad Foroutan, Masoud Foroutan, Ingeborg Forthun, Daniela Fortuna, Matteo Foschi, Kayode Raphael Fowobaje, Kate Louise Francis, Richard Charles Franklin, Alberto Freitas, Joseph Friedman, Sara D Friedman, Takeshi Fukumoto, John E Fuller, Blima Fux, Peter Andras Gaal,

Muktar A Gadanya, Abhay Motiramji Gaidhane, Santosh Gaihre, Emmanuela Gakidou, Yaseen Galali, Natalie C Galles, Silvano Gallus, Mandukhai Ganbat, Aravind P Gandhi, Balasankar Ganesan, Mohammad Arfat Ganiyani, MA Garcia-Gordillo, William M Gardner, Jalaj Garg, Naval Garg, Rupesh K Gautam, Semiu Olatunde Gbadamosi, Tilaye Gebru Gebi, Miglas W Gebregergis, Mesfin Gebrehiwot, Teferi Gebru Gebremeskel, Simona Roxana Georgescu, Tamirat Getachew, Peter W Gething, Molla Getie, Keyghobad Ghadiri, Sulmaz Ghahramani, Khalid Yaser Ghailan, Mohammad-Reza Ghasemi, Ghazal Ghasempour Dabaghi, Afsaneh Ghasemzadeh, Ahmad Ghashghaee, Fariba Ghassemi, Ramy Mohamed Ghazy, Ajnish Ghimire, Sama Ghoba, Maryam Gholamalizadeh, Asadollah Gholamian, Ali Gholamrezanezhad, Nasim Gholizadeh, Mahsa Ghorbani, Pooyan Ghorbani Vajargah, Aloke Gopal Ghoshal, Paramjit Singh Gill, Tiffany K Gill, Richard F Gillum, Themba G Ginindza, Alem Girmay, James C Glasbey, Elena V Gnedovskaya, Laszlo Göbölös, Myron Anthony Godinho, Amit Goel, Ali Golchin, Mohamad Goldust, Mahaveer Golechha, Pouya Goleij, Nelson G M Gomes, Philimon N Gona, Sameer Vali Gopalani, Giuseppe Gorini, Houman Goudarzi, Alessandra C Goulart, Bárbara Niegia Garcia Goulart, Anmol Goyal, Ayman Grada, Simon Matthew Graham, Michal Grivna, Giuseppe Grosso, Shi-Yang Guan, Giovanni Guarducci, Mohammed Ibrahim Mohialdeen Gubari, Mesay Dechasa Gudeta, Avirup Guha, Stefano Guicciardi, Rafael Alves Guimarães, Snigdha Gulati, Damitha Asanga Gunawardane, Sasidhar Gunturu, Cui Guo, Anish Kumar Gupta, Bhawna Gupta, Manoj Kumar Gupta, Mohak Gupta, Rajat Das Gupta, Rajeev Gupta, Sapna Gupta, Veer Bala Gupta, Vijai Kumar Gupta, Vivek Kumar Gupta, Lami Gurmessa, Reyna Alma Gutiérrez, Farrokh Habibzadeh, Parham Habibzadeh, Rasool Haddadi, Mostafa Hadei, Najah R Hadi, Nils Haep, Nima Hafezi-Nejad, Demewoz Haile, Alemayehu Hailu, Arvin Haj-Mirzaian, Esam S Halboub, Brian J Hall, Sebastian Haller, Rabih Halwani, Randah R Hamadeh, Sajid Hameed, Samer Hamidi, Erin B Hamilton, Chieh Han, Qiuxia Han, Asif Hanif, Nasrin Hanifi, Graeme J Hankey, Fahad Hanna, Md Abdul Hannan, Md Nuruzzaman Haque, Harapan Harapan, Arief Hargono, Josep Maria Haro, Ahmed I Hasaballah, Ikramul Hasan, M Tasdik Hasan, Hamidreza Hasani, Mohammad Hasanian, Abdiwahab Hashi, Md Saquib Hasnain, Ikrama Hassan, Soheil Hassanipour, Hadi Hassankhani, Johannes Haubold, Rasmus J Havmoeller, Simon I Hay, Jiawei He, Jeffrey J Hebert, Omar E Hegazi, Golnaz Heidari, Mohammad Heidari, Mahsa Heidari-Foroozan, Bartosz Helfer, Delia Hendrie, Brenda Yuliana Herrera-Serna, Claudiu Herteliu, Hamed Hesami, Kamal Hezam, Catherine L Hill, Yuta Hiraike, Ramesh Holla, Nobuyuki Horita, Md Mahbub Hossain, Sahadat Hossain, Mohammad-Salar Hosseini, Hassan Hosseinzadeh, Mehdi Hosseinzadeh, Ahmad Hosseinzadeh Adli, Mihaela Hostiuc, Sorin Hostiuc, Mohamed Hsairi, Vivian Chia-rong Hsieh, Rebecca L Hsu, Chengxi Hu, Junjie Huang, Michael Hultström, Ayesha Humayun, Tsegaye Gebreyes Hundie, Javid Hussain, M Azhar Hussain, Nawfal R Hussein, Foziya Mohammed Hussien, Hong-Han Huynh, Bing-Fang Hwang, Segun Emmanuel Ibitoye, Khalid S Ibrahim, Pulwasha Maria Iftikhar, Desta Ijo, Adalia I Ikiroma, Kevin S Ikuta, Paul Chukwudi Ikwegbue, Olayinka Stephen Ilesanmi, Irena M Ilic, Milena D Ilic, Mohammad Tarique Imam, Mustapha Immurana, Sumant Inamdar, Endang Indriasih, Muhammad Iqhrammullah, Arnaud Iradukunda, Kenneth Chukwuemeka Iregbu, Md Rabiul Islam, Sheikh Mohammed Shariful Islam, Farhad Islami, Faisal Ismail, Nahlah Elkudssiah Ismail, Hiroyasu Iso, Gaetano Isola, Masao Iwagami, Chidozie C D Iwu, Ihoghosa Osamuyi Iyamu, Mahalaxmi Iyer, Linda Merin J, Jalil Jaafari, Louis Jacob, Kathryn H Jacobsen, Farhad Jadidi-Niaragh, Morteza Jafarinia, Abdollah Jafarzadeh, Khushleen Jaggi, Kasra Jahankhani, Nader Jahanmehr, Haitham Jahrami, Nityanand Jain, Ammar Abdulrahman Jairoun, Abhishek Jaiswal, Elham Jamshidi, Mark M Janko, Abubakar Ibrahim Jatau, Sabzali Javadov, Tahereh Javaheri, Sathish Kumar Jayapal, Shubha Jayaram, Rime Jebai, Sun Ha Jee, Jayakumar Jeganathan, Anil K Jha, Ravi Prakash Jha, Heng Jiang, Yingzhao Jin, Olatunji Johnson,

Mohammad Jokar, Jost B Jonas, Tamas Joo, Abel Joseph, Nitin Joseph, Charity Ehimwenma Joshua, Grace Joshy, Jacek Jerzy Jozwiak, Mikk Jürisson, Vaishali K, Billingsley Kaambwa, Ali Kabir, Zubair Kabir, Vidya Kadashetti, Dler Hussein Kadir, Rizwan Kalani, Laleh R Kalankesh, Leila R Kalankesh, Feroze Kaliyadan, Sanjay Kalra, Vineet Kumar Kamal, Sivesh Kathir Kamarajah, Rajesh Kamath, Zahra Kamiab, Naser Kamyari, Thanigaivelan Kanagasabai, Tanuj Kanchan, Himal Kandel, Arun R Kanmanthareddy, Edmund Wedam Kanmiki, Kehinde Kazeem Kanmodi, Suthanthira Kannan S, Sushil Kumar Kansal, Rami S Kantar, Neeti Kapoor, Mehrdad Karajizadeh, Shama D Karanth, Reema A Karasneh, Ibraheem M Karaye, André Karch, Asima Karim, Salah Eddin Karimi, Arman Karimi Behnagh, Faizan Zaffar Kashoo, Qalandar Hussein Abdulkarim Kasnazani, Hengameh Kasraei, Nicholas J Kassebaum, Molly B Kassel, Joonas H Kauppila, Navjot Kaur, Norito Kawakami, Gbenga A Kayode, Foad Kazemi, Sina Kazemian, Tahseen Haider Kazmi, Getu Mosisa Kebebew, Adera Debella Kebede, Fassikaw Kebede, Tibebeselassie S Keflie, Peter Njenga Keiyoro, Cathleen Keller, Jaimon Terence Kelly, John H Kempen, Jessica A Kerr, Emmanuelle Kesse-Guyot, Himanshu Khajuria, Amirmohammad Khalaji, Nauman Khalid, Anees Ahmed Khalil, Alireza Khalilian, Faham Khamesipour, Ajmal Khan, Asaduzzaman Khan, Gulfaraz Khan, Ikramullah Khan, Imteyaz A Khan, M Nuruzzaman Khan, Maseer Khan, Mohammad Jobair Khan, Moien AB Khan, Zeeshan Ali Khan, Mahammed Ziauddin Khan suheb, Shaghayegh Khanmohammadi, Khaled Khatab, Fatemeh Khatami, Haitham Khatatbeh, Moawiah Mohammad Khatatbeh, Armin Khavandegar, Hamid Reza Khayat Kashani, Feriha Fatima Khidri, Elaheh Khodadoust, Mohammad Khorgamphar, Moein Khormali, Zahra Khorrami, Ahmad Khosravi, Mohammad Ali Khosravi, Zemene Demelash Kifle, Grace Kim, Jihee Kim, Kwanghyun Kim, Min Seo Kim, Yun Jin Kim, Ruth W Kimokoti, Kasey E Kinzel, Adnan Kisa, Sezer Kisa, Desmond Klu, Ann Kristin Skrindo Knudsen, Jonathan M Kocarnik, Sonali Kochhar, Timea Kocsis, David S Q Koh, Ali-Asghar Kolahi, Kairi Kolves, Farzad Kompani, Gerbrand Koren, Soewarta Kosen, Karel Kostev, Parvaiz A Koul, Sindhura Lakshmi Koulmane Laxminarayana, Kewal Krishan, Hare Krishna, Varun Krishna, Vijay Krishnamoorthy, Yuvaraj Krishnamoorthy, Kris J Krohn, Barthelemy Kuate Defo, Burcu Kucuk Bicer, Md Abdul Kuddus, Mohammed Kuddus, Ilari Kuitunen, Mukhtar Kulimbet, Vishnutheertha Kulkarni, Akshay Kumar, Ashish Kumar, Harish Kumar, Manasi Kumar, Rakesh Kumar, Madhulata Kumari, Fantahun Tarekegn Kumie, Satyajit Kundu, Om P Kurmi, Asep Kusnali, Dian Kusuma, Alexander Kwarteng, Ilias Kyriopoulos, Hmwe Hmwe Kyu, Carlo La Vecchia, Ben Lacey, Muhammad Awwal Ladan, Lucie Laflamme, Abraham K Lagat, Anton C J Lager, Abdelilah Lahmar, Daphne Teck Ching Lai, Dharmesh Kumar Lal, Ratilal Lalloo, Tea Lallukka, Hilton Lam, Judit Lám, Kelsey R Landrum, Francesco Lanfranchi, Justin J Lang, Berthold Langguth, Van Charles Lansingh, Ariane Laplante-Lévesque, Bagher Larijani, Anders O Larsson, Savita Lasrado, Zohra S Lassi, Kamaluddin Latief, Kaveh Latifinaibin, Paolo Lauriola, Nhi Huu Hanh Le, Thao Thi Thu Le, Trang Diep Thanh Le, Caterina Ledda, Jorge R Ledesma, Munjae Lee, Paul H Lee, Seung Won Lee, Shaun Wen Huey Lee, Wei-Chen Lee, Yo Han Lee, Kate E LeGrand, James Leigh, Elvynna Leong, Temesgen L Lerango, Ming-Chieh Li, Wei Li, Xiaopan Li, Yichong Li, Zhihui Li, Virendra S Ligade, Andrew Tiyamike Makhiringa Likaka, Lee-Ling Lim, Stephen S Lim, Megan Lindstrom, Christine Linehan, Chaojie Liu, Gang Liu, Jue Liu, Runben Liu, Shiwei Liu, Xiaofeng Liu, Xuefeng Liu, Erand Llanaj, Michael J Loftus, Rubén López-Bueno, Platon D Lopukhov, Arianna Maever Loreche, Stefan Lorkowski, Paulo A Lotufo, Rafael Lozano, Jailos Lubinda, Giancarlo Lucchetti, Alessandra Lugo, Raimundas Lunevicius, Zheng Feei Ma, Kelsey Lynn Maass, Nikolaos Machairas, Monika Machoy, Farzan Madadizadeh, Christian Madsen, Áurea M Madureira-Carvalho, Azzam A Maghazachi, Sandeep B Maharaj, Soleiman Mahjoub, Mansour Adam Mahmoud, Alireza Mahmoudi, Elham Mahmoudi, Razzagh Mahmoudi, Azeem Majeed, Irsa Fatima Makhdoom, Elaheh Malakan Rad, Venkatesh

Maled, Reza Malekzadeh, Armaan K Malhotra, Kashish Malhotra, Ahmad Azam Malik, Iram Malik, Deborah Carvalho Malta, Abdullah A Mamun, Pejman Mansouri, Mohammad Ali Mansournia, Lorenzo Giovanni Mantovani, Sajid Maqsood, Bishnu P Marasini, Hamid Reza Marateb, Joemer C Maravilla, Agustina M Marconi, Parham Mardi, Mirko Marino, Abdoljalal Marjani, Gabriel Martinez, Bernardo Alfonso Martinez-Guerra, Ramon Martinez-Piedra, Daniela Martini, Santi Martini, Francisco Rogerlândio Martins-Melo, Miquel Martorell, Wolfgang Marx, Sharmeen Maryam, Roy Rillera Marzo, Anthony Masaka, Awoke Masrie, Stephanie Mathieson, Alexander G Mathioudakis, Manu Raj Mathur, Jishanth Mattumpuram, Richard Matzopoulos, Richard James Maude, Andrea Maugeri, Pallab K Maulik, Mahsa Mayeli, Maryam Mazaheri, Mohsen Mazidi, John J McGrath, Martin McKee, Anna Laura W McKowen, Susan A McLaughlin, Steven M McPhail, Enkeleint A Mechili, John Robert Carabeo Medina, Rishi P Mediratta, Jitendra Kumar Meena, Rahul Mehra, Kamran Mehrabani-Zeinabad, Entezar Mehrabi Nasab, Tesfahun Mekene Meto, Gebrekiros Gebremichael Meles, Max Alberto Mendez Mendez-Lopez, Walter Mendoza, Ritesh G Menezes, Belayneh Mengist, Alexios-Fotios A Mentis, Sultan Ayoub Meo, Haftu Asmerom Meresa, Atte Meretoja, Tuomo J Meretoja, Abera M Mersha, Bezawit Afework Mesfin, Tomislav Mestrovic, Kukulege Chamila Dinushi Mettananda, Sachith Mettananda, Peter Meylakhs, Adquate Mhlanga, Laurette Mhlanga, Tianyue Mi, Tomasz Miazgowski, Georgia Micha, Irmina Maria Michalek, Ted R Miller, Edward J Mills, Le Huu Nhat Minh, GK Mini, Pouya Mir Mohammad Sadeghi, Andreea Mirica, Antonio Mirijello, Erkin M Mirrakhimov, Mizan Kiros Mirutse, Maryam Mirzaei, Awoke Misganaw, Ashim Mishra, Sanjeev Misra, Philip B Mitchell, Prasanna Mithra, Chaitanya Mittal, Mohammadreza Mobayen, Madeline E Moberg, Ashraf Mohamadkhani, Jama Mohamed, Mouhand F H Mohamed, Nouh Saad Mohamed, Sakineh Mohammad-Alizadeh-Charandabi, Soheil Mohammadi, Abdollah Mohammadian-Hafshejani, Noushin Mohammadifard, Hassen Mohammed, Hussen Mohammed, Mustapha Mohammed, Salahuddin Mohammed, Shafiu Mohammed, Viswanathan Mohan, Hoda Mojiri-Forushani, Amin Mokari, Ali H Mokdad, Sabrina Molinaro, Mariam Molokhia, Sara Momtazmanesh, Lorenzo Monasta, Stefania Mondello, Mohammad Ali Moni, AmirAli Moodi Ghalibaf, Maryam Moradi, Yousef Moradi, Maziar Moradi-Lakeh, Maliheh Moradzadeh, Paula Moraga, Lidia Morawska, Rafael Silveira Moreira, Negar Morovatdar, Shane Douglas Morrison, Jakub Morze, Jonathan F Mosser, Rohith Motappa, Vincent Mougin, Simin Mouodi, Parsa Mousavi, Seyed Ehsan Mousavi, Amin Mousavi Khaneghah, Emmanuel A Mpolya, Matías Mrejen, Sumaira Mubarik, Lorenzo Muccioli, Ulrich Otto Mueller, Faraz Mughal, Sumoni Mukherjee, Francesk Mulita, Kavita Munjal, Efrén Murillo-Zamora, Fungai Musaigwa, Khaled M Musallam, Ahmad Mustafa, Ghulam Mustafa, Saravanan Muthupandian, Raman Muthusamy, Muhammad Muzaffar, Woojae Myung, Ahamarshan Jayaraman Nagarajan, Gabriele Nagel, Pirouz Naghavi, Aliya Naheed, Ganesh R Naik, Gurudatta Naik, Firzan Nainu, Sanjeev Nair, Hastyar Hama Rashid Najmuldeen, Noureddin Nakhostin Ansari, Vinay Nangia, Atta Abbas Naqvi, Sreenivas Narasimha Swamy, Aparna Ichalangod Narayana, Shumaila Nargus, Bruno Ramos Nascimento, Gustavo G Nascimento, Samar Nasehi, Abdulqadir J Nashwan, Zuhair S Natto, Javaid Nauman, Muhammad Naveed, Biswa Prakash Nayak, Vinod C Nayak, Athare Nazri-Panjaki, Rawlance Ndejjo, Sabina Onyinye Nduaguba, Hadush Negash, Ionut Negoi, Ruxandra Irina Negoi, Serban Mircea Negru, Seyed Aria Nejadghaderi, Chakib Nejjari, Evangelia Nena, Samata Nepal, Marie Ng, Haruna Asura Nggada, Georges Nguefack-Tsague, Josephine W Ngunjiri, Anh Hoang Nguyen, Dang H Nguyen, Hau Thi Hien Nguyen, Phat Tuan Nguyen, Van Thanh Nguyen, Robina Khan Niazi, Katie R Nielsen, Yeshambel T Nigatu, Taxiarchis Konstantinos Nikolouzakis, Ali Nikoobar, Fatemeh Nikoomanesh, Amin Reza Nikpoor, Dina Nur Anggraini Ningrum, Chukwudi A Nnaji, Lawrence Achilles Nnyanzi, Efaq Ali Noman, Shuhei Nomura, Mamoona Noreen, Nafise Noroozi, Bo

Norrving, Jean Jacques Noubiap, Amanda Novotney, Chisom Adaobi Nri-Ezedi, George Ntaios, Mpiko Ntsekhe, Virginia Nuñez-Samudio, Dieta Nurrika, Jerry John Nutor, Bogdan Oancea, Kehinde O Obamiro, Mary Aigbiremo Oboh, Ismail A Odetokun, Nkechi Martina Odogwu, Martin James O'Donnell, Michael Safo Oduro, Akinyemi O D Ofakunrin, Abiola Ogunkoya, Ayodipupo Sikiru Oguntade, In-Hwan Oh, Hassan Okati-Aliabad, Sylvester Reuben Okeke, Akinkunmi Paul Okekunle, Osaretin Christabel Okonji, Andrew T Olagunju, Muideen Tunbosun Olaiya, Matthew Idowu Olatubi, Gláucia Maria Moraes Oliveira, Isaac Iyinoluwa Olufadewa, Bolajoko Olubukunola Olusanya, Jacob Olusegun Olusanya, Yinka Doris Oluwafemi, Hany A Omar, Ahmed Omar Bali, Goran Latif Omer, Maureene Auma Ondayo, Sokking Ong, Obinna E Onwujekwe, Kenneth Ikenna Onyedibe, Michal Ordak, Orish Ebere Orisakwe, Verner N Orish, Doris V Ortega-Altamirano, Alberto Ortiz, Wael M S Osman, Samuel M Ostroff, Uchechukwu Levi Osuagwu, Adrian Otoiu, Nikita Otstavnov, Stanislav S Otstavnov, Amel Ouyahia, Guoqing Ouyang, Mayowa O Owolabi, Yaz Ozten, Mahesh Padukudru P A, Alicia Padron-Monedero, Jagadish Rao Padubidri, Pramod Kumar Pal, Tamás Palicz, Claudia Palladino, Raffaele Palladino, Raul Felipe Palma-Alvarez, Feng Pan, Hai-Feng Pan, Adrian Pana, Paramjot Panda, Songhomitra Panda-Jonas, Seithikurippu R Pandi-Perumal, Helena Ullyartha Pangaribuan, Georgios D Panos, Leonidas D Panos, Ioannis Pantazopoulos, Anca Mihaela Pantea Stoian, Paraskevi Papadopoulou, Romil R Parikh, Seoyeon Park, Ashwaghosha Parthasarathi, Ava Pashaei, Maja Pasovic, Roberto Passera, Deepak Kumar Pasupula, Hemal M Patel, Jay Patel, Sangram Kishor Patel, Shankargouda Patil, Dimitrios Patoulias, Venkata Suresh Patthipati, Uttam Paudel, Hamidreza Pazoki Toroudi, Spencer A Pease, Amy E Peden, Paolo Pedersini, Umberto Pensato, Veincent Christian Filipino Pepito, Emmanuel K Peprah, Prince Peprah, João Perdigão, Marcos Pereira, Mario F P Peres, Arokiasamy Perianayagam, Norberto Perico, Richard G Pestell, Konrad Pesudovs, Fanny Emily Petermann-Rocha, William A Petri, Hoang Tran Pham, Anil K Philip, Michael R Phillips, Daniela Pierannunzio, Manon Pigeolet, David M Pigott, Thomas Pilgrim, Zahra Zahid Piracha, Michael A Piradov, Saeed Pirouzpanah, Nishad Plakkal, Evgenii Plotnikov, Vivek Podder, Dimitri Poddighe, Suzanne Polinder, Kevan R Polkinghorne, Ramesh Poluru, Ville T Ponkilainen, Fabio Porru, Maarten J Postma, Govinda Raj Poudel, Akram Pourshams, Naeimeh Pourtaheri, Sergio I Prada, Pranil Man Singh Pradhan, Thejeswar N Prakasham, Manya Prasad, Akila Prashant, Elton Junio Sady Prates, Daniel Prieto Alhambra, TINA PRISCILLA, Natalie Pritchett, Bharathi M Purohit, Jagadeesh Puvvula, Nameer Hashim Qasim, Ibrahim Qattea, Asma Saleem Qazi, Gangzhen Qian, Suli Qiu, Maryam Faiz Qureshi, Mehrdad Rabiee Rad, Amir Radfar, Raghu Anekal Radhakrishnan, Venkatraman Radhakrishnan, Hadi Raeisi Shahraki, Quinn Rafferty, Alberto Raggi, Pankaja Raghav Raghav, Nasiru Raheem, Fakher Rahim, Md Jillur Rahim, Vafa Rahimi-Movaghar, Md Mosfequr Rahman, Mohammad Hifz Ur Rahman, Mosiur Rahman, Muhammad Aziz Rahman, Amir Masoud Rahmani, Shayan Rahmani, Vahid Rahmanian, Sathish Rajaa, Prashant Rajput, Ivo Rakovac, Shakthi Kumaran Ramasamy, Sheena Ramazanu, Kritika Rana, Chhabi Lal Ranabhat, Nemanja Rancic, Amey Rane, Chythra R Rao, Indu Ramachandra Rao, Mithun Rao, Sowmya J Rao, Drona Prakash Rasali, Davide Rasella, Sina Rashedi, Vahid Rashedi, Mohammad-Mahdi Rashidi, Ashkan Rasouli-Saravani, Azad Rasul, Giridhara Rathnaiah Babu, Santosh Kumar Rauniyar, Ramin Ravangard, Nakul Ravikumar, David Laith Rawaf, Salman Rawaf, Lal Rawal, Reza Rawassizadeh, Bharat Rawlley, Rabail Zehra Raza, Christian Razo, Elrashdy Moustafa Mohamed Redwan, Faizan Ur Rehman, Lennart Reifels, Robert C Reiner Jr, Giuseppe Remuzzi, Luis Felipe Reyes, Maryam Rezaei, Nazila Rezaei, Negar Rezaei, Mohsen Rezaeian, Taeho Gregory Rhee, Mavra A Riaz, Antonio Luiz P Ribeiro, Jennifer Rickard, Hannah R Riva, Hannah Elizabeth Robinson-Oden, Célia Fortuna Rodrigues, Mónica Rodrigues, Leonardo Roever, Emma Lynn Best Rogowski, Peter Rohloff, Debby Syahru Romadlon,

Esperanza Romero-Rodríguez, Michele Romoli, Luca Ronfani, Gholamreza Roshandel, Gregory A Roth, Himanshu Sekhar Rout, Nitai Roy, Priyanka Roy, Enrico Rubagotti, Guilherme de Andrade Ruela, Susan Fred Rumisha, Tilleye Runghien, Godfrey M Rwegerera, Andrzej Rynkiewicz, Chandan S N, Aly M A Saad, Zahra Saadatian, Korosh Saber, Maha Mohamed Saber-Ayad, Morteza SaberiKamarposhti, Siamak Sabour, Simona Sacco, Perminder S Sachdev, Rajesh Sachdeva, Basema Saddik, Adam Saddler, Bashdar Abuzed Sadee, Ehsan Sadeghi, Erfan Sadeghi, Farideh Sadeghian, Mohammad Reza Saeb, Umar Saeed, Fahimeh Safaeinejad, Sher Zaman Safi, Rajesh Sagar, Amene Saghazadeh, Dominic Sagoe, Fatemeh Saheb Sharif-Askari, Narjes Saheb Sharif-Askari, Amirhossein Sahebkar, Soumya Swaroop Sahoo, Umakanta Sahoo, Monalisha Sahu, Zahra Saif, Mirza Rizwan Sajid, Joseph W Sakshaug, Nasir Salam, Payman Salamati, Afeez Abolarinwa Salami, Luciane B Salaroli, Mohamed A Saleh, Sana Salehi, Marwa Rashad Salem, Mohammed Z Y Salem, Sohrab Salimi, Hossein Samadi Kafil, Sara Samadzadeh, Saad Samargandy, Yoseph Leonardo Samodra, Abdallah M Samy, Juan Sanabria, Francesca Sanna, Damian Francesco Santomauro, Itamar S Santos, Milena M Santric-Milicevic, Bruno Piassi Sao Jose, Made Ary Sarasmita, Sivan Yegnanarayana Iyer Saraswathy, Aswini Saravanan, Babak Saravi, Yaser Sarikhani, Tanmay Sarkar, Rodrigo Sarmiento-Suárez, Gargi Sachin Sarode, Sachin C Sarode, Arash Sarveazad, Brijesh Sathian, Thirunavukkarasu Sathish, Maheswar Satpathy, Abu Sayeed, Md Abu Sayeed, Mete Saylan, Mehdi Sayyah, Nikolaos Scarmeas, Benedikt Michael Schaarschmidt, Markus P Schlaich, Winfried Schlee, Maria Inês Schmidt, Ione Jayce Ceola Schneider, Art Schuermans, Austin E Schumacher, Aletta Elisabeth Schutte, Michaël Schwarzinger, David C Schwebel, Falk Schwendicke, Mario Šekerija, Siddharthan Selvaraj, Sabyasachi Senapati, Subramanian Senthilkumaran, Sadaf G Sepanlou, Dragos Serban, Yashendra Sethi, Feng Sha, Maryam Shabany, Amir Shafaat, Mahan Shafie, Nilay S Shah, Pritik A Shah, Syed Mahboob Shah, Saeed Shahabi, Ataollah Shahbandi, Izza Shahid, Samiah Shahid, Wajeehah Shahid, Hamid R Shahsavari, Moyad Jamal Shahwan, Ahmed Shaikh, Masood Ali Shaikh, Alireza Shakeri, Ali S Shalash, Sunder Sham, Muhammad Aaqib Shamim, Mehran Shams-Beyranvand, Hina Shamshad, Mohammad Anas Shamsi, Mohd Shanawaz, Abhishek Shankar, Sadaf Sharfaei, Amin Sharifan, Javad Sharifi-Rad, Rajesh Sharma, Saurab Sharma, Ujjawal Sharma, Vishal Sharma, Rajesh P Shastry, Amin Shavandi, Maryam Shayan, Amr Mohamed Elsayed Shehabeldine, Aziz Sheikh, Rahim Ali Sheikhi, Jiabin Shen, Adithi Shetty, B Suresh Kumar Shetty, Pavanchand H Shetty, Peilin Shi, Kenji Shibuya, Desalegn Shiferaw, Mika Shigematsu, Min-Jeong Shin, Youn Ho Shin, Rahman Shiri, Reza Shirkoohi, Nebiyu Aniley Shitaye, Aminu Shittu, Ivy Shiue, K M Shivakumar, Velizar Shivarov, Farhad Shokraneh, Azad Shokri, Sina Shool, Seyed Afshin Shorofi, Sunil Shrestha, Kerem Shuval, Emmanuel Edwar Siddig, João Pedro Silva, Luís Manuel Lopes Rodrigues Silva, Soraia Silva, Colin R Simpson, Anjali Singal, Abhinav Singh, Balbir Bagicha Singh, Garima Singh, Jasbir Singh, Narinder Pal Singh, Paramdeep Singh, Surjit Singh, Dhirendra Narain Sinha, Robert Sinto, Md Shahjahan Siraj, Sarah Brooke Sirota, Freddy Sitas, Shravan Sivakumar, Valentin Yurievich Skryabin, Anna Aleksandrovna Skryabina, David A Sleet, Bogdan Socea, Anton Sokhan, Ranjan Solanki, Shipra Solanki, Hamidreza Soleimani, Sameh S M Soliman, Suhang Song, Yimeng Song, Reed J D Sorensen, Joan B Soriano, Ireneous N Soyiri, Michael Spartalis, Sandra Spearman, Chandrashekhar T Sreeramareddy, Vijay Kumar Srivastava, Jeffrey D Stanaway, Muhammad Haroon Stanikzai, Benjamin A Stark, Joseph R Starnes, Antonina V Starodubova, Caroline Stein, Dan J Stein, Fridolin Steinbeis, Caitlyn Steiner, Jaimie D Steinmetz, Paschalis Steiropoulos, Aleksandar Stevanović, Leo Stockfelt, Mark A Stokes, Stefan Stortecky, Vetriselvan Subramaniyan, Muhammad Suleman, Rizwan Suliankatchi Abdulkader, Abida Sultana, Haitong Zhe Sun, Jing Sun, Johan Sundström, David Sunkersing, Katharina S Sunnerhagen, Chandan Kumar Swain, Lukasz Szarpak, Mindy D Szeto, Miklós Szócska, Payam Tabaee Damavandi, Rafael

Tabarés-Seisdedos, Seyyed Mohammad Tabatabaei, Ozra Tabatabaei Malazy, Seyed-Amir Tabatabaeizadeh, Shima Tabatabai, Mohammad Tabish, Jyothi Tadkamadla, Santosh Kumar Tadakamadla, Yasaman Taheri Abkenar, Moslem Taheri Soodejani, Jabeen Taiba, Ken Takahashi, Iman M Talaat, Ashis Talukder, Mircea Tampa, Jacques Lukenze Tamuzi, Ker-Kan Tan, Sarmila Tandukar, Haosu Tang, Hong K Tang, Ingan Ukur Tarigan, Mengistie Kassahun Tariku, Md Tariqujjaman, Elvis Enowbeyang Tarkang, Razieh Tavakoli Oliaee, Seyed Mohammad Tavangar, Nuno Taveira, Yibekal Manaye Tefera, Mohamad-Hani Temsah, Reem Mohamad Hani Temsah, Masayuki Teramoto, Riki Tesler, Enoch Teye-Kwadjo, Rishu Thakur, Pugazhenthan Thangaraju, Kavumpurathu Raman Thankappan, Samar Tharwat, Rasiah Thayakaran, Nihal Thomas, Nikhil Kenny Thomas, Azalea M Thomson, Amanda G Thrift, Chern Choong Chern Thum, Lau Caspar Thygesen, Jing Tian, Ales Tichopad, Jansje Henny Vera Ticoalu, Tala Tillawi, Tenaw Yimer Tiruye, Mariya Vladimirovna Titova, Marcello Tonelli, Roman Topor-Madry, Adetunji T Toriola, Anna E Torre, Mathilde Touvier, Marcos Roberto Tovani-Palone, Jasmine T Tran, Nghia Minh Tran, Domenico Trico, Samuel Joseph Tromans, Thien Tan Tri Tai Truyen, Aristidis Tsatsakis, Guesh Mebrahtom Tsegay, Evangelia Eirini Tsermpini, Munkhtuya Tumurkhuu, Kang Tung, Stefanos Tyrovolas, Sayed Mohammad Nazim Uddin, Aniefiok John Udoakang, Arit Udoh, Atta Ullah, Irfan Ullah, Saeed Ullah, Sana Ullah, Srikanth Umakanthan, Chukwuma David Umeokonkwo, Brigid Unim, Bhaskaran Unnikrishnan, Carolyn Anne Unsworth, Era Upadhyay, Daniele Urso, Jibrin Sammani Usman, Seyed Mohammad Vahabi, Asokan Govindaraj Vaithinathan, Rohollah Valizadeh, Sarah M Van de Velde, Jef Van den Eynde, Orsolya Varga, Priya Vart, Shoban Babu Varthya, Tommi Juhani Vasankari, Milena Vasic, Siavash Vaziri, Balachandar Vellingiri, Narayanaswamy Venketasubramanian, Nicholas Alexander Verghese, Madhur Verma, Massimiliano Veroux, Georgios-Ioannis Verras, Dominique Vervoort, Jorge Hugo Villafañe, Gabriela Ines Villanueva, Manish Vinayak, Francesco S Violante, Maria Viskadourou, Sergey Konstantinovitch Vladimirov, Vasily Vlassov, Bay Vo, Stein Emil Vollset, Avina Vongpradith, Theo Vos, Isidora S Vujcic, Rade Vukovic, Hatem A Wafa, Yasir Waheed, Richard G Wamai, Cong Wang, Ning Wang, Shu Wang, Song Wang, Yanzhong Wang, Yuan-Pang Wang, Muhammad Waqas, Paul Ward, Emebet Gashaw Wassie, Stefanie Watson, Stephanie Louise Watson Watson, Kosala Gayan Weerakoon, Melissa Y Wei, Robert G Weintraub, Daniel J Weiss, Ronny Westerman, Joanna L Whisnant, Taweewat Wiangkham, Dakshitha Praneeth Wickramasinghe, Nuwan Darshana Wickramasinghe, Angga Wilandika, Caroline Wilkerson, Peter Willeit, Shadrach Wilson, Marcin W Wojewodzic, Axel Walter Wolf, Charles D A Wolfe, Yohannes Addisu Wondimagegene, Yen Jun Wong, Utoomporn Wongsin, Ai-Min Wu, Chenkai Wu, Felicia Wu, Xinsheng Wu, Zenghong Wu, Juan Xia, Hong Xiao, Yang Xie, Suowen Xu, Wang-Dong Xu, Xiaoyue Xu, Yvonne Yiru Xu, Ali Yadollahpour, Kazumasa Yamagishi, Danting Yang, Lin Yang, Yuichiro Yano, Yao Yao, Habib Yaribeygi, Pengpeng Ye, Sisay Shewasinad Yehualashet, Metin Yesiltepe, Subah Abderehim Yesuf, Saber Yezli, Siyan Yi, Amanuel Yigezu, Arzu Yiğit, Vahit Yiğit, Paul Yip, Malede Berihun Yismaw, Yazachew Yismaw, Dong Keon Yon, Naohiro Yonemoto, Seok-Jun Yoon, Yuyi You, Mustafa Z Younis, Zabihollah Yousefi, Chuanhua Yu, Yong Yu, Faith H Yuh, Siddhesh Zadey, Vesna Zadnik, Nima Zafari, Fathiah Zakham, Nazar Zaki, Sojib Bin Zaman, Nelson Zamora, Ramin Zand, Moein Zangiabadian, Heather J Zar, Iman Zare, Armin Zarrintan, Mohammed G M Zeariya, Zahra Zeinali, Haijun Zhang, Jianrong Zhang, Jingya Zhang, Liqun Zhang, Yunquan Zhang, Zhi-Jiang Zhang, Hanqing Zhao, Chenwen Zhong, Juexiao Zhou, Bin Zhu, Lei Zhu, Makan Ziafati, Magdalena Zielińska, Osama A Zitoun, Mohammad Zoladl, Zhiyong Zou, Liesl J Zuhlke, Alimuddin Zumla, Elric Zweck, Samer H Zyoud, Eve E Wool†, and Christopher J L Murray†.

*Joint first authors; †Joint senior authors

Affiliations

Institute for Health Metrics and Evaluation (Prof M Naghavi PhD, K L Ong PhD, C M Antony MA, A Y Aravkin PhD, M B Arndt PhD, A V L Basting MPH, R G Bender MSc, B N Berice MPH, G J Bertolacci BS, K Bhangdia MS, J D Bishai BA, C Bisignano MPH, Prof M Brauer DSc, K M Cercy BS, C S Chen BA, E Chung MD, E Chung MSc, K Coberly BS, R M Cogen BA, H Comfort MPH, E Cousin PhD, G T Culbreth PhD, M Cunningham MSc, X Dai PhD, N Davis Weaver MPH, Prof L Degenhardt PhD, L Deitesfeld MA, M A Dirac MD, R V Dominguez BS, L Dwyer-Lindgren PhD, Prof V L Feigin PhD, A J Ferrari PhD, J E Fuller MLIS, Prof E Gakidou PhD, N C Galles MPH, W M Gardner MPH, S Ghoba MS, D Haile MPH, E B Hamilton MPH, C Han BA, Prof S I Hay FMedSci, J He MSc, R L Hsu, K S Ikuta MD, N J Kassebaum MD, M B Kassel BA, C Keller MPH, K E Kinzel MSPH, J M Kocarnik PhD, K J Krohn MPH, H H Kyu PhD, J R Ledesma MPH, K E LeGrand MPH, Prof S S Lim PhD, M Lindstrom PhD, Prof R Lozano MD, K L Maass PhD, A L W McKowen MA, S A McLaughlin PhD, T Mestrovic PhD, M E Moberg MS, A H Mokdad PhD, J F Mosser MD, V Mougin BA, A Novotney MPH, S M Ostroff PhD, Y Ozten MS, M Pasovic MEd, S A Pease BS, D M Pigott PhD, N Pritchett DrPH, Q Rafferty BA, C Razo PhD, R C Reiner Jr PhD, H E Robinson-Oden MLIS, G A Roth MD, T Runghien MSc, D F Santomauro PhD, A E Schumacher PhD, S B Sirota MA, R J D Sorensen PhD, S Spearman MS, J D Stanaway PhD, B A Stark MA, C Stein PhD, C Steiner MPH, A M Thomson BA, A E Torre BS, N A Verghese BA, Prof S E Vollset DrPH, A Vongpradith BA, Prof T Vos PhD, S Watson MS, J L Whisnant MPH, C Wilkerson MPH, S Wilson BS, Y Xu MPH, F H Yuh MPA, E E Wool MPH, Prof C J L Murray DPhil), Department of Health Metrics Sciences, School of Medicine (Prof M Naghavi PhD, A Y Aravkin PhD, E Cousin PhD, X Dai PhD, M A Dirac MD, L Dwyer-Lindgren PhD, Prof E Gakidou PhD, Prof S I Hay FMedSci, N J Kassebaum MD, H H Kyu PhD, Prof S S Lim PhD, Prof R Lozano MD, A Misganaw PhD, A H Mokdad PhD, E A Mpolya PhD, D M Pigott PhD, R C Reiner Jr PhD, G A Roth MD, J D Stanaway PhD, C Stein PhD, Prof S E Vollset DrPH, Prof T Vos PhD, Prof C J L Murray DPhil), Department of Applied Mathematics (A Y Aravkin PhD), Department of Global Health (M B Arndt PhD, S Kochhar MD, K R Nielsen MD, R J D Sorensen PhD, Z Zeinali MD), School of Medicine (Prof E J Boyko MD), Department of Internal Medicine (Y Chahine MD), Department of Cardiology (Y Chahine MD), Department of Health Systems and Population Health (A W Chen MSc), Department of Pediatrics (E Chung MD, K R Nielsen MD), Department of Family Medicine (M A Dirac MD), Department of Neurology (R Kalani MD), Department of Anesthesiology & Pain Medicine (N J Kassebaum MD, V Krishnamoorthy MD), Division of Plastic and Reconstructive Surgery (S D Morrison MD), Henry M Jackson School of International Studies (S M Ostroff PhD), Division of Cardiology (G A Roth MD), University of Washington, Seattle, WA, USA; Faculty of Medicine (A Aali MD), Department of Neuroscience (A Ahmadzade MD), Dental Research Center (E Bardideh DDS), Orthodontics Department (M Ghorbani DDS), Clinical Research Development Unit (N Morovatdar MD), Applied Biomedical Research Center (A Sahebkar PhD), Biotechnology Research Center (A Sahebkar PhD), Department of Medical Informatics (S Tabatabaei PhD), Clinial Research Development Unit (S Tabatabaei PhD), Department of Medical Genetics (N Zafari MD), Mashhad University of Medical Sciences, Mashhad, Iran; Radiation Oncology (H S Ababneh MD), Department of Orthopaedic Surgery (A Ebrahimi MD), Division of Cardiology (I Y Elgendy MD, D H Nguyen BS), Department of Radiology (A Haj-Mirzaian MD, X Liu PhD), Cardiovascular Research Center (A Schuermans BSc), Massachusetts General Hospital, Boston, MA, USA; Department of Clinical Governance and Quality Improvement (Y H Abate MSc), Aleta Wondo Hospital, Aleta Wondo, Ethiopia; Department of Juridical and Economic Studies (C Abbafati PhD), Department of Public Health and Infectious Diseases (M S Cattaruzza PhD), La Sapienza University, Rome, Italy; Doheny Eye Institute (R

Abbasgholizadeh MD), Doheny Image Reading and Research Lab (DIRRL) (L Almidani MSc), Center for Social Medicine (J Friedman PhD), Department of Environmental Health Sciences (M Khorgamphar BA), Department of Health Policy Management (M Khorgamphar BA), General Internal Medicine and Health Services Research (M Y Wei MD), University of California Los Angeles, Los Angeles, CA, USA; Department of Orthopedic Surgery (M Abbasian MD), Department of Pediatrics (S Aly MD), T.H. Chan School of Public Health (Prof T W Bärnighausen MD, P M S Pradhan MD, E Zweck MD), Center for Primary Care (S Basu PhD), Harvard Business School (F Caetano dos Santos PhD), Department of Epidemiology (S Carr MS), Department of Health Policy and Management (H Ding MPH), Division of Cardiology (I Y Elgendy MD), Department of Neurological Surgery at Brigham and Women's Hospital (A H Feroze MD), Department of Ophthalmology (Prof J H Kempen MD), Department of Global Health and Population (Z Li PhD, P Rohloff MD), Department of Health Policy and Oral Epidemiology (Z S Natto DrPH), Department of Global Health and Social Medicine (M Pigeolet MD), Beth Israel Deaconess Medical Center (S Sharfaei MD), Division of General Internal Medicine (Prof A Sheikh MD), Harvard University, Boston, MA, USA; Department of Orthopaedic Surgery (M Abbasian MD), Department of Anesthesiology (D Abtahi MD, S Salimi MD, A Shakeri MD), Department of Biotechnology (S Aghamiri PhD), National Nutrition and Food Technology Research Institute (M Ajami PhD), Urology Department (M Bonakdar Hashemi MD), Internal Medicine Department of SBMU (H Borhany MD), School of Medicine (N Deravi MD, M Heidari-Foroozan BSc, S Nejadghaderi MD, S Rahmani MD, M Zangiabadian MD), Department of Community Nutrition (S Doaei PhD, A Mokari PhD), Medical Genetics (M Ghasemi PhD), Cancer Research Center (M Gholamalizadeh PhD), Obesity Research Center (A Haj-Mirzaian MD), Urology and Nephrology Research Center (H Hesami MD), Ophtalmic Reserch Centre (H Hesami MD), Department of Immunology (K Jahankhani MSc, A Rasouli-Saravani PhD), Department of Health Policy and Management (N Jahanmehr PhD), Safety Promotion and Injury Prevention Research Center (N Jahanmehr PhD), Department of Neurosurgery (H Khayat Kashani MD), Ophthalmology and Vision Science (Z Khorrami PhD), Social Determinants of Health Research Center (A Kolahi MD, A Nikoobar DipSc, M Rashidi MD), Department of Epidemiology (S Sabour PhD), Traditional Medicine and Materia Medica Research Center (F Safaeinejad PhD), Ophthalmic Research Center (ORC) (M Shayan MD), Emergency Department (S Shool MD), Department of Medical Education (S Tabatabai PhD), Shahid Beheshti University of Medical Sciences, Tehran, Iran (S Nasehi MSc); Non-communicable Diseases Research Center (M Abbasi-Kangevari MD, S Azadnajafabad MD, S Momtazmanesh MD, P Mousavi MD, S Rahmani MD, M Rashidi MD, N Rezaei MD, N Rezaei PhD), Advanced Diagnostic and Interventional Radiology Research Center (H Abbastabar PhD), The Institute of Pharmaceutical Sciences (TIPS) (Prof M Abdollahi PhD), School of Pharmacy (Prof M Abdollahi PhD), Research Center for Immunodeficiencies (H Abolhassani PhD, A Saghazadeh MD), Tehran Heart Center (R Alikhani MD, E Mehrabi Nasab MD), Universal Scientific Education and Research Network (USERN) (M Amirzade-Iranaq DDS), Digestive Diseases Research Institute (A Anoushiravani MD, S Fahimi MD, Prof R Malekzadeh MD, A Mohamadkhani PhD, Prof A Pourshams MD, S G Sepanlou MD), Department of Health Information Management (S Ayyoubzadeh PhD), Translational Ophthalmology Research Center (N Bahmanziari PhD), School of Medicine (A Behnoush BS, N Hafezi-Nejad MD, A Khalaji BS, S Khanmohammadi MD, M Mayeli MD, S Mohammadi MD, S Momtazmanesh MD), Iranian Research Center for HIV/AIDS (O Dadras DrPH), Multiple Sclerosis Research Center (S Eskandarieh PhD), Department of Ophthalmology (Prof F Ghassemi MD, A Mahmoudi MD), Department of Health in Emergencies and Disasters (M Hadei PhD), Department of Virology (A Hosseinzadeh Adli PhD), Cardiac Primary Prevention Research Center (S Kazemian

MD), Department of Cardiac Electrophysiology (S Kazemian MD), Center for Research and Training in Skin Diseases and Leprosy (F Khamesipour PhD), Urology Research Center (Prof F Khatami PhD), Sina Trauma and Surgery Research Center (A Khavandegar MD, M Khormali MD, Prof V Rahimi-Movaghar MD, F Sadeghian PhD, Prof P Salamati MD, S Shool MD), Children's Medical Center (F Kompani MD), Endocrinology and Metabolism Research Institute (Prof B Larijani FACE, N Rezaei PhD, O Tabatabaei Malazy PhD), Department of Cardiology (E Mahmoudi MD, P Mansouri MD, S Rashedi MD), Department of Pediatric Cardiology (Prof E Malakan Rad MD), Department of Epidemiology and Biostatistics (M Mansournia PhD), Department of Physiotherapy (Prof N Nakhostin Ansari PhD), Research Center for War-affected People (Prof N Nakhostin Ansari PhD), Department of Pharmacology (N Noroozi DVM), Sina Trauma Research Center (M Shabany PhD), Department of Neurology (M Shafie MD), Department of Medicine (A Shahbandi MD), Department of Pharmaceutical Care (A Sharifan PharmD), Research Center for Rational Use of Drugs (A Sharifan PharmD), Cancer Research Center (R Shirkoohi PhD), Cancer Biology Research Center (R Shirkoohi PhD), Department of Pathology (Prof S Tavangar MD), Faculty of Medicine (S Vahabi MD), Tehran University of Medical Sciences, Tehran, Iran; Department of Epidemiology (S Abd ElHafeez DrPH), Biomedical Informatics and Medical Statistics Department (I El Sayed PhD), Pediatric Dentistry and Dental Public Health Department (Prof O A A Elmeligy PhD), Department of Tropical Health and Parasitology (R M Ghazy PhD), Department of Pathology (Prof I M Talaat PhD), Alexandria University, Alexandria, Egypt; Department of Surgery (M Abdelmasseh MD, Prof J Sanabria MD), Marshall University, Huntington, WV, USA; Department of Tropical Medicine and Infectious Diseases (S Abd-Elsalam PhD), Tanta University, Tanta, Egypt; Department of Internal Medicine (A Abdelwahab MD), Section of General Internal Medicine (Prof A Agrawal PhD), Baylor College of Medicine, Houston, TX, USA; Department of Small Animal Clinical Sciences (M Abdollahifar PhD), Department of Community Health and Epidemiology (D A Adeyinka PhD), University of Saskatchewan, Saskatoon, SK, Canada; Department of Medicine (Prof M Abdoun B.Med.Sc.), University of Setif Algeria, Sétif, Algeria; Community and Maternity Nursing Unit (D M Abdulah MPH), Department of Pathology and Microbiology (M S Ahmed MSc), University of Duhok, Duhok, Iraq; Department of Physiotherapy (A Abdullahi PhD), Department of Community Medicine (Prof M A Gadanya FMCPH), Department of Nursing Science (M Ladan PhD), Bayero University Kano, Kano, Nigeria; Department of Rehabilitation Sciences (A Abdullahi PhD, M U Ali MSc, M Khan MPH, J S Usman PhD), School of Nursing (S Tyrovolas PhD), Hong Kong Polytechnic University, Hong Kong, China; Department of Midwifery (M Abebe MSc), Department of Public Health (T L Lerango MPH, Y A Wondimagegene PhD), Dilla University, Dilla, Ethiopia; Department of Public Health (S S Abebe MPH), Department of Medical Laboratory Sciences (M Arkew MSc), School of Public Health (W A Cheru PhD, A Masrie MPH), Department of Health Policy and Management (A T Debele MSc), Health Sciences Department of Oncology Nursing (T G Gebi MSc), School of Nursing and Midwifery (T Getachew MSc, A D Kebede MSc), Department of Clinical Pharmacy (M D Gudeta MSc), School of Medical Laboratory Sciences (H A Meresa MSc), Haramaya University, Harar, Ethiopia; Department of Neurosurgery (A Abedi MD), Keck School of Medicine (A Abedi MD), Department of Radiology (A Gholamrezanezhad MD), Mark and Mary Stevens Neuroimaging and Informatics Institute (S Salehi MD), University of Southern California, Los Angeles, CA, USA; Department of Biostatistics (K H Abegaz PhD), Near East University, Nicosia / TRNC, Turkiye; Department of Biostatistics and Health Informatics (K H Abegaz PhD), Madda Walabu University, Bale Robe, Ethiopia; Department of Botany (E S Abhilash PhD), Sree Narayana Guru College Chelannur, Kozhikode, India; Laboratory Technology Sciences Department (H Abidi PhD), Department of Nursing (M Zoladl PhD), Yasuj University of Medical Sciences, Yasuj, Iran;

Department of Community Medicine (O Abiodun MPH), Babcock University, Ilishan-Remo, Nigeria; Department of Family and Community Health (R G Aboagye MPH), Department of Population and Behavioural Sciences (H Amu PhD, E E Tarkang PhD), Department of Health Policy Planning and Management (M K Boachie PhD), Department of Medical Epidemiology and Biostatistics (R K Dowou MPhil), Institute of Health Research (M Immurana PhD, D Klu PhD), Department of Microbiology and Immunology (V N Orish PhD), University of Health and Allied Sciences, Ho, Ghana; Department of Medical Biochemistry and Biophysics (H Abolhassani PhD), Department of Global Public Health (Prof P Allebeck MD, Prof L Laflamme PhD), Department of Neurobiology, Care Sciences and Society (Prof J Ärnlöv PhD, S Fereshtehnejad PhD), Department of Medical Epidemiology and Biostatistics (Prof J J Carrero PhD), Department of Physiology and Pharmacology (C R Cederroth PhD), Department of Molecular Medicine and Surgery (Prof J H Kauppila MD), Aging Research Centre (A C J Lager PhD), Karolinska Institute, Stockholm, Sweden; Department of Neurosurgery (M Abolmaali MD), Pars Advanced and Minimally Invasive Medical Manners Research Center (Y Alimohamadi PhD), Health Management and Economics Research Center (J Arabloo PhD, H Ayatollahi PhD), Department of Epidemiology (M Asadi-Lari PhD), Department of Health Information Management (H Ayatollahi PhD), School of Medicine (M Dodangeh MD), Department of Medical Laboratory Sciences (F Dorostkar PhD), Preventive Medicine and Public Health Research Center (B Eshrati PhD, M Moradi-Lakeh MD), Department of Ophthalmology (H Hasani MD, M Ziafati MD), Minimally Invasive Surgery Research Center (A Kabir MD), Endocrine Research Center (A Karimi Behnagh MD), Department of Echocardiography (A Karimi Behnagh MD), Eye Research Center (H Kasraei MD), Educational Development Center (E Khodadoust MD), Department of Anesthesiology (K Latifinaibin MD), Gastrointestinal and Liver Diseases Research Center (M Moradi-Lakeh MD), Department of Physiology (H Pazoki Toroudi PhD), Physiology Research Center (H Pazoki Toroudi PhD), Colorectal Research Center (A Sarveazad PhD), Iran University of Medical Sciences, Tehran, Iran (M Moradi MD); Khatam Al-anbia Hospital (M Abolmaali MD), Shefa Neuroscience Research Center, Tehran, Iran; Department of Physical Pharmacy and Pharmacokinetics (M Abouzid PharmD), Poznan University of Medical Sciences, Poznan, Poland; Department of Public Health (G B Aboye MSc), Madda Walabu University, Addis Ababa, Ethiopia; Department of Nutrition and Dietetics (G B Aboye MSc), USAID-JSI Digital Health Activity (B H Demessa MPH), Jimma University, Addis Ababa, Ethiopia; Department of Pediatric Dentistry (Prof L G Abreu PhD), Department of Internal Medicine (Prof L C Brant PhD, Prof A P Ribeiro MD), Department of Nutrition (Prof R M Claro PhD), Department of Maternal and Child Nursing and Public Health (Prof D C Malta PhD, E J S Prates BS), Department of Clinical Medicine (Prof B R Nascimento PhD), Clinical Hospital (Prof B R Nascimento PhD), Centre of Telehealth (Prof A P Ribeiro MD), Department of Infectious Diseases and Tropical Medicine (B P Sao Jose PhD), Federal University of Minas Gerais, Belo Horizonte, Brazil; Department of Adult Health Nursing (W A Abrha MSc), Department of Nursing (A Girmay MSc, G M Tsegay MSc), Aksum University, Aksum, Ethiopia; Department of Neurology (S Abu Rumeileh MD), Martin Luther University Halle-Wittenberg, Halle (Saale), Germany; Department of Nursing (H Abualruz PhD), Al Zaytoonah University of Jordan, Amman, Jordan; Department of Pharmacology and Toxicology (B Abubakar PhD), Department of Veterinary Microbiology (M B Bello PhD), Department of Veterinary Public Health and Preventive Medicine (A Shittu MSc), Usmanu Danfodiyo University, Sokoto, Sokoto, Nigeria; Nigerian Institute of Medical Research (B Abubakar PhD), Nigerian Institute of Medical Research, Lagos, Nigeria; Clinical Sciences Department (E Abu-Gharbieh PhD, S Adra MD, H J Barqawi MPhil, N R Dash MD, Prof R Halwani PhD, Prof A A Maghazachi PhD, M M Saber-Ayad MD, N Saheb Sharif-Askari PhD, Prof I M Talaat PhD), College of Medicine (F Ahmad PhD,

Prof R Halwani PhD, Prof B Saddik PhD, M A Saleh PhD), Department of Pharmacy Practice and Pharmacotherapeutics (Prof K H Alzoubi PhD, Prof H A Omar PhD), Department of Physiotherapy (A Arumugam PhD), Department of Basic Biomedical Sciences (Y Bustanji PhD), Sharjah Institute for Medical Research (N M Elemam PhD), Department of Basic Medical Sciences (W El-Huneidi PhD, A Karim PhD), Department of Clinical Nutrition and Dietetics (M E M Faris PhD), Department of Finance and Economics (Prof M Hussain PhD), Sharjah Institute of Medical Sciences (F Saheb Sharif-Askari PhD), Department of Medicinal Chemistry (S S M Soliman PhD), University of Sharjah, Sharjah, United Arab Emirates (K A Altirkawi MD); Institute of Community and Public Health (Prof N M Abu-Rmeileh PhD), Birzeit University, Ramallah, Palestine; Department of Therapeutics (Prof S Aburuz PhD), Institute of Public Health (L A Ahmed PhD, I Elbarazi DrPH, Prof S M Shah PhD), College of Medicine and Health Sciences (Prof M Grivna PhD, J Nauman PhD), Department of Medical Microbiology & Immunology (Prof G Khan PhD), Family Medicine Department (M A Khan MSc), Department of Food, Nutrition and Health (Prof S Maqsood PhD), Department of Computer Science and Software Engineering (Prof N Zaki PhD), United Arab Emirates University, Al Ain, United Arab Emirates; College of Pharmacy (Prof S Aburuz PhD), Department of Clinical Nursing (Prof M M Ahmad PhD), University of Jordan, Amman, Jordan; Department of Surgery (A Abu-Zaid MD), College of Pharmacy (R M H Temsah PharmD), Alfaisal University, Riyadh, Saudi Arabia; College of Graduate Health Sciences (A Abu-Zaid MD), Department of Neurology (R Zand MD), University of Tennessee, Memphis, TN, USA; Department of Disease Control (M M K Accrombessi PhD), Department of Infectious Disease Epidemiology (O J Brady PhD), Department of Non-Communicable Disease Epidemiology (M Iwagami PhD), Department of Health Services Research and Policy (Prof M McKee DSc), London School of Hygiene & Tropical Medicine, London, UK; Department of Clinical Research (M M K Accrombessi PhD), Clinical Research Institute of Benin (IRCB), Abomey-Calavi, Benin; Department of Public Health (T G Adal MPH), Wolkite University, Wolkite, Ethiopia; Department of Global Health (A A Adamu PhD), South African Centre for Epidemiological Modelling and Analysis (SACEMA) (L Mhlanga PhD), Department of Epidemiology (J L Tamuzi MSc), Department of Industrial Psychology (E Teye-Kwadjo PhD), Stellenbosch University, Cape Town, South Africa; Cochrane South Africa (A A Adamu PhD), Burden of Disease Research Unit (R Matzopoulos PhD), Risk and Resilience in Mental Disorders Unit (Prof D J Stein MD), South African Medical Research Council, Cape Town, South Africa (C A Nnaji MPH); Centre for Social Research in Health (I Y Addo PhD, S R Okeke PhD), St George and Sutherland Clinical School (H Akbarialiabad MD), National Drug and Alcohol Research Centre (Prof L Degenhardt PhD), School of Medicine (P K Maulik PhD), School of Psychiatry (Prof P B Mitchell MD), School of Public Health and Community Medicine (A E Peden PhD, Prof A E Schutte PhD), Centre for Primary Health Care and Equity (CPHCE) (P Peprah MSc, F Sitas PhD), School of Optometry and Vision Science (Prof K Pesudovs PhD), Faculty of Medicine and Health (S Sharma PhD), The George Institute for Global Health (P Ye MPH), University of New South Wales, Sydney, NSW, Australia; Quality and Systems Performance Unit (I Y Addo PhD), Cancer Institute NSW, Sydney, NSW, Australia; Internal Medicine and Alcohol Related Disease Unit (Prof G Addolorato MD), Department of Woman and Child Health and Public Health (D Buonsenso MD), Fondazione Policlinico Universitario A Gemelli IRCCS (Agostino Gemelli University Polyclinic IRCCS), Rome, Italy; Department of Medical and Surgical Sciences (Prof G Addolorato MD), Università Cattolica di Roma (Catholic University of Rome), Rome, Italy; Department of Community Medicine (A O Adebiyi MD, A A Afolabi MPH, O S Ilesanmi PhD), Department of Veterinary Medicine (T E Adeyeoluwa PhD), Department of Epidemiology and Medical Statistics (M Ekholuenetale MSc, A F Fagbamigbe PhD, K R Fowobaje MSc), Faculty of Public Health (M

Ekholuenetale MSc, I I Olufadewa MHS), Department of Health Promotion and Education (S E Ibitoye MPH, A Ogunkoya MPH), College of Medicine (A P Okekunle PhD), Department of Medicine (Prof M O Owolabi DrM), University of Ibadan, Ibadan, Nigeria; Department of Community Medicine (A O Adebiyi MD, O S Ilesanmi PhD), Department of Medicine (A S Oguntade MSc, Prof M O Owolabi DrM), Department of Oral and Maxillofacial Surgery (A A Salami BDS), University College Hospital, Ibadan, Ibadan, Nigeria; Department of Obstetrics and Gynecology (V Adekanmbi PhD), University of Texas Medical Branch, Galveston, TX, USA; Department of HIV and Infectious Diseases (A V Adepoju MD), Jhpiego, Abuja, Nigeria; Department of Adolescent Research and Care (A V Adepoju MD), Adolescent Friendly Research Initiative and Care, Ado Ekiti, Nigeria; Department of Microbiology (Prof C O Adetunji PhD), Edo State University Uzairue, Iyamho, Nigeria; Department of Biochemistry (J B Adetunji PhD), Osun State University, Osogbo, Nigeria; Department of Biosciences and Biotechnology (T E Adeyeoluwa PhD, A J Udoakang PhD), Department of Physiology (O I Adeyomoye PhD), Department of Microbiology (O O Bello PhD, Y D Oluwafemi PhD), Department of Biological Sciences (T C Ekundayo PhD), University of Medical Sciences, Ondo, Ondo, Nigeria; Department of Public Health (D A Adeyinka PhD), Federal Ministry of Health, Abuja, Nigeria; Anesthesia (B A Admass MSc), Institute of Public Health (B D Bitew PhD), Department of Environmental and Occupational Health and Safety (B D Bitew PhD), Department of Clinical Pharmacy (G S Chanie MSc), Department of Pharmacology (Z D Kifle MSc), University of Gondar, Gondar, Ethiopia; Faculty of Medicine (Q E S Adnani PhD), Center of Excellence in Higher Education for Pharmaceutical Care Innovation (Prof M J Postma PhD), Universitas Padjadjaran (Padjadjaran University), Bandung, Indonesia; Department of Life Sciences (M S Afzal PhD), University of Management and Technology, Lahore, Pakistan; Department of Community Medicine (Prof S Afzal PhD), King Edward Memorial Hospital, Lahore, Pakistan; Department of Public Health (Prof S Afzal PhD), Public Health Institute, Lahore, Pakistan; Department of Community Medicine (Prof S B Agampodi MD, N D Wickramasinghe MD), Department of Parasitology (Prof K G Weerakoon PhD), Rajarata University of Sri Lanka, Anuradhapura, Sri Lanka; New Initiatives (Prof S B Agampodi MD), International Vaccine Institute, Seoul, South Korea; Department of Cardiovascular Medicine (P Agasthi MD), Mayo Clinic, Scottsdale, AZ, USA; Department of Internal Medicine (M Aggarwal MD, A Boustany MD, M Gupta MD), Department of Pediatrics (Prof H Aly MD, A E'mar MD), Lerner Research Institute (X Liu PhD), Cleveland Clinic, Cleveland, OH, USA; Department of Health Education and Health Promotion (F D Agide PhD), Department of Public Health (N Dereje PhD, L T Elilo MPH), Wachemo University, Hossana, Ethiopia; Department of Medical and Surgical Sciences and Advanced Technologies "GF Ingrassia" (Prof A Agodi PhD, M Barchitta PhD, A Maugeri PhD, Prof M Veroux PhD), Department of Biomedical and Biotechnological Sciences (L Falzone PhD, G Grosso PhD), Department of General Surgery and Medical-Surgical Specialties (Prof G Isola PhD), Department of Clinical and Experimental Medicine (C Ledda PhD), University of Catania, Catania, Italy; Trivedi School of Biosciences (Prof A Agrawal PhD), Ashoka University, Sonipat, Haryana 131029, India; Department of Geography and Planning (W Agyemang-Duah MSc), Department of Biomedical and Molecular Sciences (A Nikpoor PhD), Queen's University, Kingston, ON, Canada; School of Public Health (B O Ahinkorah MPhil), School of Nursing and Midwifery (M Chutiyami PhD), University of Technology Sydney, Sydney, NSW, Australia; Department of Medical Biochemistry (A Ahmad PhD), Department of Pediatrics (Prof G Mustafa MD), Department of Pharmacology (M Tabish MPharm), Shaqra University, Shaqra, Saudi Arabia; School of Medicine and Psychology (D Ahmad PhD), National Centre for Epidemiology and Population Health (Y Alemu MPH, G Joshy PhD), Research School of Population Health (N Bagheri PhD, R A Burns PhD, Prof N

Cherbuin PhD), Australian National University, Canberra, ACT, Australia; Public Health Foundation of India, Gandhinagar, India (D Ahmad PhD); Department of Health and Biological Sciences (S Ahmad PhD), Abasyn University, Peshawar, Pakistan; Department of Natural Sciences (S Ahmad PhD), Gilbert and Rose-Marie Chagoury School of Medicine (L Roever PhD), Lebanese American University, Beirut, Lebanon; Department of Medical Oncology (S Ahmad MD), Department of Medicine (M Ganiyani MD), Miami Cancer Institute, Miami, FL, USA; Department of Community Medicine and Preventive Health (S Ahmad MD), King Edward Medical University Lahore, Lahore, Pakistan; Department of Epidemiology and Health Statistics (T Ahmad MS), Southeast University, Nanjing, China; School of Public Health (K Ahmadi PhD, S Basu PhD), Department of Primary Care and Public Health (T Beaney MSc, Prof A Majeed MD, R Palladino MD, Prof S Rawaf MD), WHO Collaborating Centre for Public Health Education and Training (D L Rawaf MRCS), Imperial College London, London, UK; Faculty of Pharmaceutical Sciences (K Ahmadi PhD), UCSI University, Kuala Lumpur, Malaysia; Department of Pharmacy Practice (A Ahmed PhD), Riphah Institute of Pharmaceutical Sciences, Islamabad, Pakistan; Division of Infectious Diseases and Global Public Health (IDGPH) (A Ahmed PhD), University of California, San Diego, CA, USA; Institute of Endemic Diseases (A Ahmed MSc), Unit of Basic Medical Sciences (E E Siddig MD), University of Khartoum, Khartoum, Sudan; Swiss Tropical and Public Health Institute (A Ahmed MSc), University of Basel, Basel, Switzerland; Department of Biosciences (H Ahmed PhD), COMSATS Institute of Information Technology, Islamabad, Pakistan; Department of Nursing (M S Ahmed MSc), Majmaah University, Al Majmaah, Saudi Arabia; Department of Epidemiology (M B Ahmed MPH, D Shiferaw MPH), Department of Surgery (N S Bayileyegn MD), Jimma University, Jimma, Ethiopia; Australian Center for Precision Health (M B Ahmed MPH), Department of Allied Health and Human Performance (T Y Tiruye PhD), University of South Australia, Adelaide, SA, Australia; Brody School of Medicine (S Ahmed PhD), Department of Internal Medicine (S Alqalyoobi MD), Department of Computer Science (A O Bodunrin MSc), Department of Physiology (M Tumurkhuu PhD), Diabetes & Obesity Institute and Physiology (K Tung PhD), East Carolina University, Greenville, NC, USA (R T Aruleba PhD); Department of Food and Nutrition Policy and Planning Research (M Ajami PhD), National Institute of Nutrition, Tehran, Iran; Faculty of Medicine and Public Health (B Aji DrPH), Jenderal Soedirman University, Purwokerto, Indonesia; Moyen Mono Health District (E Akara MD), Ministry of Health, Tohoun, Togo; Department of Internal Medicine (K Akinosoglou PhD), University of Patras, Patras, Greece; Department of Internal Medicine and Infectious Diseases (K Akinosoglou PhD), University General Hospital of Patras, Patras, Greece; Department of Population Health Sciences (T Akinyemiju PhD), Duke Global Health Institute (T Akinyemiju PhD, M M Janko PhD, C Wu PhD), Department of Anesthesiology (V Krishnamoorthy MD), Center for the Study of Aging and Human Development (Y Yao MD), Duke University, Durham, NC, USA; Department of Cardiology (M A Akkaif PhD), Fudan University, Shanghai, China; Yale School of Nursing (S Akyirem MRes), School of the Environment (Prof M L Bell PhD, Y Song PhD), School of Medicine (R G Bender MSc), Department of Internal Medicine (F Etaee MD), Department of Dermatology (M Goldust MD), Department of Psychiatry (W Li PhD, T G Rhee PhD), Department of Radiology and Biomedical Imaging (X Liu PhD), Yale University, New Haven, CT, USA; Department of Geriatric and Long Term Care (H Al Hamad MD, B Sathian PhD), Rumailah Hospital (H Al Hamad MD), Department of Nursing Education & Research (A J Nashwan MSc), Hamad Medical Corporation, Doha, Qatar; Division of Public Health Sciences (S Al Hasan PhD), Washington University School of Medicine, St Louis, MO, USA; McWilliams School of Biomedical Informatics (F Alahdab MSc), UTHealth, Houston, TX, USA; Department of Biomedical Informatics, Biostatistics, and Epidemiology (F Alahdab MSc), University of Missouri, Columbia,

MO, USA; Department of Clinical Sciences (S O Alalalmeh BPharm., O E Hegazi BPharm.), Center for Medical and Bio-Allied Health Sciences Research (Prof M J Shahwan PhD, M A Shamsi PhD, S H Zyoud PhD), Ajman University, Ajman, United Arab Emirates; Department of Biology (T A Alalwan PhD), University of Bahrain, Sakhir, Bahrain; John T. Milliken Department of Internal Medicine (Z Al-Aly MD), Department of Surgery (Y Cao DSc, Prof A T Toriola MD, C Wang MPH), Brown School (C Wang MPH), Washington University in St. Louis, St. Louis, MO, USA; Clinical Epidemiology Center (Z Al-Aly MD), US Department of Veterans Affairs (VA), St Louis, MO, USA; Murdoch Business School (K Alam PhD), Murdoch University, Perth, WA, Australia; Department of Bioengineering (M Alam PhD), Department of Nutrition and Food Studies (S Tyrovolas PhD), George Mason University, Fairfax, VA, USA; Prevention Division (N Alam MPH), Department of Medicine (V Kulkarni MS), Digital Health and Informatics Directorate (Prof S M McPhail PhD), Queensland Health, Brisbane, QLD, Australia; Centre for Environment and Population Health (N Alam MPH), Griffith University, Nathan, QLD, Australia; School of Nursing (R M Al-amer PhD), Department of Basic Medical Sciences (R A Karasneh PhD, M M Khatatbeh PhD), Yarmouk University, Irbid, Jordan; School of Nursing and Midwifery (R M Al-amer PhD), Translational Health Research Institute (R Chimoriya PhD), Department of Engineering (G R Naik PhD), Western Sydney University, Sydney, NSW, Australia; Department of Health Information Management and Technology (T M Alanzi PhD), Department of Public Health (Prof S Bah PhD), Division of Forensic Medicine (Prof R G Menezes MD), Imam Abdulrahman Bin Faisal University, Dammam, Saudi Arabia (F M Alanezi PhD); Department of Clinical Pharmacy (Prof S Al-Azzam PharmD, Prof K H Alzoubi PhD), Department of Rehabilitation Sciences and Physical Therapy (Prof M A Alomari PhD), Jordan University of Science and Technology, Irbid, Jordan; Department of Medicine (A Albakri MD), Royal Jordanian Medical Services, Amman, Jordan; Department of Community and Mental Health (Prof M Albashtawy PhD), Al al-Bayt University, Mafraq, Jordan; Faculty of Medicine (Prof M T AlBataineh PhD), Yarmouk University, Irbid, Jordan; Center for Health Systems Research (J E Alcalde-Rabanal PhD, S M Cuadra-Hernández PhD, D V Ortega-Altamirano DrPH), Center for Nutrition and Health Research (I R Campos-Nonato PhD, E Denova-Gutiérrez DSc), National Institute of Public Health, Cuernavaca, Mexico; Department of Pediatrics (K A Aldawsari MD), Nicklaus Children's Hospital, Miami, FL, USA; Heart Center (K A Aldawsari MD), Biostatics, Epidemiology, and Science Computing Department (S Yezli PhD), King Faisal Specialist Hospital & Research Center, Riyadh, Saudi Arabia; Division of Gastroenterology and Hepatology (W A Aldhaleei MD), Mayo Clinic, Jacksonville, FL, USA; Institute of Health Informatics (R W Aldridge PhD), Department of Health Informatics (S Chung PhD), Department of Behavioural Science and Health (S Hossain MS), Institute of Epidemiology and Health Care (J Kim MSc), Division of Psychology and Language Sciences (M Kumar PhD), Institute of Cardiovascular Science (A S Oguntade MSc), Department of Population Health Sciences (D Sunkersing PhD), Department of Infection (Prof A Zumla PhD), University College London, London, UK; Department of Public Health (H B Alema MPH), Aksum University, Axum, Ethiopia; Department of Epidemiology (M Alemayohu MPH), Department of Environmental Health (A A Asgedom PhD), School of Public Health (G G Meles MPH), Mekelle University, Mekelle, Ethiopia; Unit of Epidemiology & Medical Statistics (M Alemayohu MPH), University of Verona, Verona, Italy; Global Health Entrepreneurship (S Alemi PhD), Tokyo Medical and Dental University, Tokyo, Japan; Department of Epidemiology and Biostatistics (Y Alemu MPH, K A Bogale MPH), College of Medicine and Health Science (A Amare PhD), Department of Midwifery (A M Aweke MSc), College of Medicine and Health Sciences (S A Belay MSc), School of Health Science (A Y Berhie MSc), Department of Health Promotion and Behavioural Science (E K Bogale MPH), Anaesthesiology (F T Kumie MSc), Department of Surgery

(N A Shitaye MD), Department of Pharmacy (M Yismaw MSc), Department of Pharmacology (Y Yismaw MSc), Bahir Dar University, Bahir Dar, Ethiopia; Global Centre for Environmental Remediation (A A S Al-Gheethi PhD), School of Medicine and Public Health (P Atorkey MPhil), University of Newcastle, Newcastle, NSW, Australia; Cooperative Research Centre for Contamination Assessment and Remediation of the Environment, Newcastle, NSW, Australia (A A S Al-Gheethi PhD); Department of Cardiac Sciences (Prof K F Alhabib MD), Section of Adult Hematology (Prof G M T ElGohary MD), Department of Physiology (Prof S A Meo PhD), Pediatric Intensive Care Unit (M Temsah MD), King Saud University, Riyadh, Saudi Arabia; College of Nursing (Prof F A N Alhalaiqa PhD), College of Dental Medicine (S A A Al-Maweri PhD), Department of Physical Education (Prof M A Alomari PhD), QU Health (M Mohammed PhD), Department of Population Medicine (Prof G Rathnaiah Babu PhD), Qatar University, Doha, Qatar; Psychological Sciences Association, Amman, Jordan (Prof F A N Alhalaiqa PhD); Department of Health Services and Hospital Administration (M K Al-Hanawi PhD), Health Economics Research Group (M K Al-Hanawi PhD), Department of Family and Community Medicine (N S Butt PhD), Department of Pediatric Dentistry (Prof O A A Elmeligy PhD), Rabigh Faculty of Medicine (A A Malik PhD), Department of Dental Public Health (Z S Natto DrPH), Department of Community Medicine (S Samargandy PhD), King Abdulaziz University, Jeddah, Saudi Arabia; Department of Zoology (A Ali PhD), Department of Botany (Prof I Khan PhD), Abdul Wali Khan University Mardan, Mardan, Pakistan; Department of Biotechnology and Genetic Engineering (A Ali PhD, M Waqas PhD), Hazara University Mansehra, Mansehra, Pakistan; Department of Biological Sciences (L Ali PhD, A S Qazi PhD, R Z Raza PhD), National University of Medical Sciences (NUMS), Rawalpindi, Pakistan; Department of Medical Rehabilitation (Physiotherapy) (M U Ali MSc), Department of Human Pathology (Prof H A Nggada MD), University of Maiduguri, Maiduguri, Nigeria; Department of Biosciences (R Ali MPhil, N Salam PhD), Centre for Interdisciplinary Research in Basic Sciences (CIRBSc) (S Anwar PhD, M A Shamsi PhD), Jamia Millia Islamia, New Delhi, India; Centre for Biotechnology and Microbiology (S Ali PhD), Center for Biotechnology and Microbiology (S S Ali PhD, M Suleman PhD), University of Swat, Charbagh, Pakistan; Department of Pathophysiology and Transplantation (G Alicandro PhD), Università degli Studi di Milano (University of Milan), Milan, Italy; Cystic Fibrosis Center (G Alicandro PhD), Fondazione IRCCS Ospedale Maggiore Policlinico IRCCS "Ca' Granda Maggiore Policlinico" Hospital Foundation, Milan, Italy; School of Public Health and Preventive Medicine (S M Alif PhD), School of Public Health and Preventative Medicine (Prof M Asghari-Jafarabadi PhD), Department of Human Centered Computing (M Hasan MSc), Department of Infectious Diseases (M J Loftus MBBS), Department of Medicine (Prof K R Polkinghorne PhD, Prof A G Thrift PhD), Monash University, Melbourne, VIC, Australia; Department of Public Health (A A Aliyi MPH), Madda Walabu University, Goba, Ethiopia; Medical Laboratories (M A M Aljasir PhD), Qassim University, Buraydah, Saudi Arabia; Department of Molecular and Clinical Pharmacology (M A M Aljasir PhD), Institute of Infection and Global Health (Prof A Beloukas PhD), Liverpool Orthopaedic and Trauma Service (S M Graham PhD), Department of Surgery (Prof R Lunevicius DSc), Institute of Population Health Sciences (M R Mathur PhD), University of Liverpool, Liverpool, UK; Department of Health Policy and Management (Prof S M Aljunid PhD), Department of Surgery (S K Al-Sabah MD), Kuwait University, Kuwait, Kuwait; International Centre for Casemix and Clinical Coding (Prof S M Aljunid PhD), National University of Malaysia, Bandar Tun Razak, Malaysia; Bordeaux School of Public Health (Prof F Alla PhD), University of Bordeaux, Bordeaux, France; Department of Dentistry (S Al-Marwani MSc), Independent Consultant, Sana'a, Yemen; Public Health and Community Medicine (S Al-Marwani MSc), Independent Consultant, Irbid, Jordan; Department of Medicine (J U Almazan PhD, Prof D Poddighe PhD), Nazarbayev University, Astana, Kazakhstan; Department of

Parasitology (Prof H M Al-Mekhlafi PhD), Department of Paediatrics (Prof H Ariffin MD), University of Malaya Medical Centre (Prof H Ariffin MD), Department of Medicine (L Lim MRCP), University of Malaya, Kuala Lumpur, Malaysia; Department of Parasitology (Prof H M Al-Mekhlafi PhD), Sana'a University, Sana'a, Yemen; Wilmer Eye Institute (L Almidani MSc), Department of Radiology and Radiological Science (A Amindarolzarbi MD, N Hafezi-Nejad MD), Department of Biostatistics (A Columbus MS), Department of Epidemiology (T G Hundie MD), Department of Neurosurgery (F Kazemi MD), Department of Health Policy and Management (D Vervoort MD), Division of Cardiology (M Viskadourou MD), Department of International Health (H Zhang MS), Johns Hopkins University, Baltimore, MD, USA (E Jamshidi PharmD); Department of Urology (O Almidani MSc), Department of Cardiac Surgery (L Göbölös PhD), Cleveland Clinic Abu Dhabi, Abu Dhabi, United Arab Emirates; Nuffield Department of Surgical Sciences (O Almidani MSc, S Bandyopadhyay BA), Nuffield Department of Medicine (B Basnyat MD, Prof R J Maude PhD), Oxford Centre for Global Health Research (C Dolecek PhD), Nuffield Department of Population Health (B Lacey PhD), University of Oxford, Oxford, UK; Department of Epidemiology and Population Health (B Al-Omari PhD), Khalifa University of Science, Technology & Research, Abu Dhabi, United Arab Emirates; Research Program of Epidemiology and Public Health (J Alonso MD), Pompeu Fabra University, Barcelona, Spain; Department of Experimental and Health Sciences (J Alonso MD), Biomedical Research Networking Center in Epidemiology and Public Health (CiberESP), Madrid, Spain; Department of Respiratory Care (J S Alqahtani PhD), Prince Sultan Military College of Health Sciences, Dammam, Saudi Arabia; Independent Consultant, Greenville, NC, USA (S Alqalyoobi MD); Department of Prosthodontics and Implant Dentistry (A Alqutaibi PhD), Taibah University, Medinah, Saudi Arabia; Department of Prosthodontics (A Alqutaibi PhD), Ibb University, Ibb, Yemen; Jaber Al Ahmad Al Sabah Hospital (S K Al-Sabah MD), Ministry of Health, Kuwait, Kuwait; Departent of Basic sciences (Z Altaany PhD), Yarmouk Univeristy, Irbid, Jordan; Institute of Molecular Biology and Biotechnology (A Altaf PhD, Prof M Ashraf PhD, S Shahid PhD), University College of Medicine & Dentistry (Prof M Arooj PhD), Department of Oral Biology (A Fahim PhD), University Institute of Public Health (S Hameed MPH, A Hanif PhD, A A Malik PhD, S Nargus PhD), University Institute of Diet and Nutritional Sciences (A Khalil PhD), Department of Technology (M Muzaffar MBA), Research Centre for Health Sciences (RCHS) (M Muzaffar MBA, S Shahid PhD), Department of Physics (W Shahid PhD), The University of Lahore, Lahore, Pakistan (M A Riaz Mcom); Department of Specialty Internal Medicine (Prof J A Al-Tawfiq MD), Johns Hopkins Aramco Healthcare, Dhahran, Saudi Arabia; Department of Medicine (Prof J A Al-Tawfiq MD), Indiana University School of Medicine, Indianapolis, IN, USA; Lisbon Institute of Global Mental Health (D O Aluh MSc), Nova University of Lisbon, Lisboa, Nigeria; Clinical Pharmacy and Pharmacy Management (D O Aluh MSc), University of Nigeria Nsukka, Nsukka, Nigeria; Research Group in Hospital Management and Health Policies (Prof N Alvis-Guzman PhD), Universidad de la Costa (University of the Coast), Barranquilla, Colombia; Research Group in Health Economics (Prof N Alvis-Guzman PhD), University of Cartagena, Cartagena, Colombia; Department of Clinical Pharmacology and Toxicology (H Alwafi PhD), Department of Medical Genetics (M Athar PhD), Science and Technology Unit (M Athar PhD), Institute of Center and Research Studies (F Rehman PhD), Umm Al-Qura University, Makkah, Saudi Arabia; Department of Medical Sciences (Prof Y M Al-Worafi PhD), Azal University for Human Development, Sana'a, Yemen; Department of Clinical Sciences (Prof Y M Al-Worafi PhD), University of Science and Technology of Fujairah, Fujairah, United Arab Emirates; Department of Pediatric Cardiology (S Aly MD), Boston Children's Hospital, Boston, MA, USA; Interdisciplinary Graduate Program in Human Toxicology (R Amani DVM), University of Iowa, Iowa City, IA, USA; Health Policy Research Center (R Amani DVM, S

Ghahramani MD, H Kasraei MD, Y Sarikhani PhD, S Shahabi PhD), Health Information Management (A Bashiri PhD), Health Human Resources Research Center (M Bayati PhD), Trauma Research Center (P Fazeli MSc, M Karajizadeh PhD), Department of Medical Immunology (P Fazeli MSc), Shiraz Neuroscience Research Center (M Jafarinia PhD, R Tavakoli Oliaee PhD), Non-communicable Disease Research Center (Prof R Malekzadeh MD, S G Sepanlou MD), Department of Epidemiology and Biostatistics (H Raeisi Shahraki PhD), Department of Health Services Management (R Ravangard PhD), Department of Biostatistics (E Sadeghi PhD), Shiraz University of Medical Sciences, Shiraz, Iran; School of Medicine (A Amare PhD), School of Public Health (M Du MSc, V Podder HSC), Adelaide Medical School (T K Gill PhD, Prof C L Hill MD), Robinson Research Institute (Z S Lassi PhD, H Mohammed PhD), Centre for Heart Rhythm Disorders (J Noubiap MD), University of Adelaide, Adelaide, SA, Australia; School of Global Public Health (P M Amegbor PhD, S D Friedman BA, E K Peprah PhD), Department of Child and Adolescent Psychiatry (Prof S Cortese PhD), The Center for Drug Use and HIV Research (CDUHR) (P Meylakhs PhD), New York University, New York, NY, USA; School of Graduate Studies (E K Ameyaw MPhil), Lingnan University, Hong Kong, China; Public Health and Community Medicine Department (Prof T T Amin MD), Department of Neurophysiology (Prof H R Elhabashy MD), Cairo University, Cairo, Egypt; Medicine, Quran and Hadith Research Center (S Amiri PhD), Baqiyatallah University of Medical Sciences, Tehran, Iran; Department of Maternal and Child Wellbeing (D A Amugsi PhD), African Population and Health Research Center, Nairobi, Kenya; Department of Medicine (G A Amusa MD), Department of Pediatrics (A O D Ofakunrin MSc), University of Jos, Jos, Nigeria; Department of Internal Medicine (G A Amusa MD), Department of Pediatrics (A O D Ofakunrin MSc), Jos University Teaching Hospital, Jos, Nigeria; Faculty of Pharmacy (Prof R Ancuceanu PhD), Department of Cardiology (C Andrei PhD), Department of Internal Medicine and Rheumatology (A V Bobirca PhD), Ophthalmology Department (A Dascalu PhD), Department of General Surgery (B T Florin PhD, I Negoi PhD, D Serban PhD, B Socea PhD), Department of Dermatology and Venereology (Prof S R Georgescu PhD), Department of Internal Medicine (M Hostiuc PhD), Department of Legal Medicine and Bioethics (S Hostiuc PhD), Department of Anatomy and Embryology (R I Negoi PhD), Department of Diabetes, Nutrition and Metabolic Diseases (A Pantea Stoian PhD), Department of Dermatology (M Tampa PhD), Carol Davila University of Medicine and Pharmacy, Bucharest, Romania; Centre for Sensorimotor Performance (D Anderlini MD), Department of Urology (Prof E Chung MD), School of Public Health (A J Ferrari PhD, D F Santomauro PhD), Institute for Social Science Research (E Kanmiki MPH, A A Mamun PhD, J C Maravilla PhD), School of Health and Rehabilitation Sciences (A Khan PhD, M Moni PhD), School of Dentistry (R Lalloo PhD), Queensland Brain Institute (Prof J J McGrath MD), The University of Queensland, Brisbane, QLD, Australia (J T Kelly PhD); Neurology Department (D Anderlini MD), Royal Brisbane and Women's Hospital, Brisbane, QLD, Australia; Faculty of Medicine (D B Anderson PhD), School of Architecture, Design, and Planning (Prof T Astell-Burt PhD), Menzies Centre for Health Policy and Economics (M Balasubramanian PhD), School of Pharmacy and Charles Perkins Centre (Z Dai PhD), School of Public Health (Prof T R Driscoll PhD, H K Tang PhD), Sydney Medical School (S Islam PhD), Save Sight Institute (H Kandel PhD, Prof S L W Watson PhD, Y You PhD), Department of Public Health (M Khan PhD), Asbestos Diseases Research Institute (J Leigh MD), Kolling Institute (S Mathieson PhD), Sydney Musculoskeletal Health (S Mathieson PhD), School of Veterinary Science (B B Singh PhD), Menzies Centre for Health Policy (F Sitas PhD), University of Sydney, Sydney, NSW, Australia; Department of Health Care Management (P P Andrade MD, Prof R Busse PhD, S Mohammed PhD), Technical University of Berlin, Berlin, Germany; European University, Lisbon, Portugal (P P Andrade MD); Department of Statistics and

Econometrics (Prof T Andrei PhD, Prof M Ausloos PhD, Prof C Herteliu PhD, A Mirica PhD, A Otoiu PhD), Faculty of Management (A Dima PhD), Bucharest University of Economic Studies, Bucharest, Romania; School of Health and Related Research (C Angus MSc), Department of Infection and Tropical Medicine (O C Durojaiye MPH), Department of Psychology (A Yadollahpour PhD), University of Sheffield, Sheffield, UK; Department of Pharmacology (A Anil MD, M Shamim MBBS, S Singh MD, S B Varthya MD), Department of Community Medicine and Family Medicine (P Baskaran MD, P Bhardwaj MD, M K Gupta MD, Prof P R Raghav MD), Department of Anatomy (Prof N Bhardwaj MD, H Krishna MD), School of Public Health (P Bhardwaj MD), Department of Forensic Medicine and Toxicology (T Kanchan MD), Department of Surgical Oncology (Prof S Misra MCh), Department of Pharmacology and Research (A Saravanan MD), Department of Community Medicine (G Singh MD), All India Institute of Medical Sciences, Jodhpur, India; All India Institute of Medical Sciences, Bhubaneswar, India (A Anil MD); Department of Obstetrics and Gynecology (S Anil MBBS), Ernakulam Medical Centre, Palarivattom, Kochi, India; Department of Epidemiology and Biostatistics (Prof H Ansari PhD, Prof A Ansari-Moghaddam PhD), Department of Health Promotion (A Nazri-Panjaki MSc), Health Promotion Research Center (H Okati-Aliabad PhD), Zahedan University of Medical Sciences, Zahedan, Iran; School of Public Health (A Ansariadi PhD), Faculty of Pharmacy (F Nainu PhD), Hasanuddin University, Makassar, Indonesia; Agribusiness Study Program (E Antriyandarti DrAgrSc), Sebelas Maret University, Surakarta, Indonesia; Department of Parasitology (D Anvari PhD), Department of Dermatology (N Gholizadeh MD), Department of Medical-Surgical Nursing (S Shorofi PhD), Department of Environmental Health (Prof Z Yousefi PhD), Mazandaran University of Medical Sciences, Sari, Iran; Department of Parasitology (D Anvari PhD), Department of Pharmacology (Prof B Foroutan PhD), Iranshahr University of Medical Sciences, Iranshahr, Iran; Regenerative Medicine, Organ Procurement, and Transplantation Multi-diciplinary Center (S Anvari MD), School of Health (S Doaei PhD), Department of Social Medicine and Epidemiology (A Feizkhah MD), Department of Medical-Surgical Nursing (P Ghorbani Vajargah MSc), Gastrointestinal and Liver Diseases Research Center (S Hassanipour PhD), Caspian Digestive Disease Research Center (S Hassanipour PhD), Department of Environmental Health Engineering (J Jaafari PhD), Guilan University of Medical Sciences, Rasht, Iran; School of Chemical and Life Sciences (SCLS) (S Anwar PhD), Jamia Hamdard, New Delhi, India; Department of Surgery (S Anwar PhD), Gadjah Mada University, Yogyakarta, Indonesia; Department of Pathology (R Anwer PhD), Imam Mohammad Ibn Saud Islamic University, Riyadh, Saudi Arabia; School of Dentistry and Medical Sciences (A E Anyasodor PhD), Charles Sturt University, Orange, NSW, Australia; Department of Psychology (M Aqeel PhD, M Aqeel PhD), Foundation University Islamabad, Rawalpandi, Pakistan; Department of Medicine (J Arab MD), Department of Epidemiology and Biostatistics (F Barbic PhD), Western University, London, ON, Canada; Gastroenterology Department (J Arab MD), Pediatric Infectious Diseases and Immunology (A Borzutzky MD), Pontifical Catholic University of Chile, Santiago, Chile; College of Pharmacy (M Arafat PhD), Al Ain University, Abu Dhabi, United Arab Emirates; College of Art and Science (D Areda PhD), Ottawa University, Surprise, AZ, USA; School of Life Sciences (D Areda PhD), Arizona State University, Tempe, AZ, USA; Department of Veterinary Pharmacology and Toxicology (A Aremu PhD), Department of Veterinary Physiology and Biochemistry (A Basiru PhD), Department of Veterinary Public Health and Preventive Medicine (I A Odetokun PhD), University of Ilorin, Ilorin, Nigeria; Department of Public Health (O Aremu PhD), Birmingham City University, Birmingham, UK; Department of Cardiovascular, Endocrine-metabolic Diseases and Aging (B Armocida MSc, B Unim PhD), National Institute of Health, Rome, Italy; Division of Tropical and Humanitarian Medicine (B Armocida MSc), University of Geneva, Geneva, Switzerland; School of

Health and Social Studies (Prof J Ärnlöv PhD), Dalarna University, Falun, Sweden; Department of Biophysics (A A Artamonov PhD), K.A. Timiryazev Institute of Plant Physiology (M V Titova PhD), Russian Academy of Sciences, Moscow, Russia; Department of Maternal and Child Health (J Arulappan DSc), Sultan Qaboos University, Muscat, Oman; Department of Community Medicine and Rehabilitation (A Arumugam PhD), Department of Nursing (Prof D Edvardsson PhD), Umeå University, Umea, Sweden; Department of Plastic Surgery (M Asaad MD), Health Science Center (D Dongarwar MS), University of Texas, Houston, TX, USA; International Relations Department (M Asadi-Lari PhD), Development of Research and Technology Center (S Djalalinia PhD), Centre for Primary Health Care Network Management (H Fattahi PhD), Ministry of Health and Medical Education, Tehran, Iran; Neurological Surgery Department (M Asghariahmadabad MD), School of Nursing (J Nutor PhD), Department of Epidemiology and Biostatistics (M Teramoto MD), University of California San Francisco, San Francisco, CA, USA; Cabrini Research (Prof M Asghari-Jafarabadi PhD), Cabrini Health, Malvern, VIC, Australia; Department of Aging Research Institute (A Aslani MD), Department of Radiology (M Dashti MD, A Ghasemzadeh MD, A Zarrintan MD), Department of Health Policy and Management (L Doshmangir PhD), School of Nursing and Midwifery (H Hassankhani PhD), Research Center for Evidence-Based Medicine (M Hosseini MD), Department of Virology (A Hosseinzadeh Adli PhD), Department of Immunology (F Jadidi-Niaragh PhD), School of Management and Medical Informatics (L R Kalankesh PhD), Social Determinants of Health Research Center (S Karimi PhD, Prof S Mohammad-Alizadeh-Charandabi PhD), Midwifery Department (Prof S Mohammad-Alizadeh-Charandabi PhD), Department of Community Medicine (S Mousavi MD), Molecular Medicine Research Center (S Pirouzpanah PhD), Drug Applied Research Center (H Samadi Kafil PhD), Tabriz University of Medical Sciences, Tabriz, Iran; Department of Immunology (S Athari PhD), Department of Critical Care and Emergency Nursing (N Hanifi PhD), Zanjan University of Medical Sciences, Zanjan, Iran; School of Nursing and Midwifery (B T Atinafu MSc), Department of Pediatrics and Child Health Nursing (S S Yehualashet MSc), Debre Berhan University, Debre Berhan, Ethiopia; Department of Biomedical sciences (H W Atlaw MSc), Department of Public Health (H Esubalew MPH), Department of Nursing (A M Mersha MSc), Department of Clinical Midwifery (B A Mesfin B.Med.Sc.), Arba Minch University, Arba Minch, Ethiopia; Hunter New England Population Health, Wallsend, NSW, Australia (P Atorkey MPhil); Faculty of Nursing (M M W Atout PhD), Philadelphia University, Amman, Jordan; Department of Forensic Medicine (A Atreya MD), Lumbini Medical College, Palpa, Nepal; Northumbria HealthCare NHS Foundation Trust, Newcastle upon Tyne, UK (A Aujayeb MBBS); School of Business (Prof M Ausloos PhD), Department of Health Sciences (Prof T Brugha MD, P H Lee PhD, S J Tromans PhD), University of Leicester, Leicester, UK; Robarts Research Institute (A Avan MD), The University of Western Ontario, London, ON, Canada; Department of Surgery (A F Awedew MD), Addis Ababa University, Debre Tabor, Ethiopia; The Judith Lumley Centre (B Ayala Quintanilla PhD), School of Nursing and Midwifery (Prof D Edvardsson PhD, F Efendi PhD, M Rahman PhD), Department of Public Health (H Jiang PhD, Prof C Liu PhD), La Trobe University, Melbourne, VIC, Australia; San Martin de Porres University, Lima, Peru (B Ayala Quintanilla PhD); Department of Psychiatry (Prof J L Ayuso-Mateos PhD), Department of Medicine (Prof A Ortiz MD), Hospital Universitario de La Princesa (Princess University Hospital) (Prof J B Soriano MD), Universidad Autónoma de Madrid (Autonomous University of Madrid), Madrid, Spain; Biomedical Research Networking Center for Mental Health Network (CIBERSAM) (Prof J L Ayuso-Mateos PhD), National School of Public Health (F Catalá-López PhD, A Padron-Monedero PhD), Institute of Health Carlos III, Madrid, Spain; Department of Sciences (Prof R M S Azevedo PhD), Therapeutic and Diagnostic Technologies Department (Prof N Cruz-Martins PhD), Toxicology Research Unit (TOXRUN) (Prof D Dias da Silva

PhD, Á M Madureira-Carvalho PhD), Cooperativa de Ensino Superior Politécnico e Universitário (CESPU) (University Polytechnic Higher Education Cooperative), Gandra, Portugal; Department of Neurovascular Research (A Y Azzam MBBCh), Nested Knowledge, Inc., Saint Paul, MN, USA; Faculty of Medicine (A Y Azzam MBBCh), October 6 University, 6th of October City, Egypt; Kasturba Medical College, Mangalore (D B B MD, R Holla MD, M Rao MD), Department of Physiotherapy (A S Babu PhD, Prof V K PhD), Department of Health Policy (V Dsouza MSc), Prasanna School of Public health (R Kamath MHA), Department of Pharmacy Management (V S Ligade PhD), Department of Forensic Medicine (A Mishra MD), Manipal College of Dental Sciences (Prof A I Narayana PhD, Prof R A Radhakrishnan PhD), Department of Forensic Medicine and Toxicology (Prof V C Nayak MD), Manipal TATA Medical College (M Rahman PhD), Department of Community Medicine (C R Rao MD), Department of Nephrology (I Rao DM), Manipal Academy of Higher Education, Manipal, India; Department of Medicine (A S Babu PhD), Melbourne School of Population and Global Health (H Jiang PhD, L Reifels PhD), School of Health Sciences (A Meretoja MD), Department of General Practice (J Zhang MD), University of Melbourne, Melbourne, VIC, Australia; Gomal Center of Biochemistry and Biotechnology (M Badar PhD), Gomal University, Dera Ismail Khan, Pakistan; Department of Forensic Science (A D Badiye PhD, H Bansal MSc, N Kapoor PhD), Government Institute of Forensic Science, Nagpur, India; Division of Orthopaedics (S Baghdadi MD), Children's Hospital of Philadelphia, Philadelphia, PA, USA; Health Research Institute (N Bagheri PhD), University of Canberra, Canberra, ACT, Australia; School of Medicine (S Bagherieh BSc, G Ghasempour Dabaghi MD, M Rabiee Rad MD), Department of Environmental Health Engineering (A Fatehizadeh PhD), Cardiac Rehabilitation Research Center (K Mehrabani-Zeinabad PhD), Department of General Surgery (P Mir Mohammad Sadeghi MD), Isfahan Cardiovascular Research Institute (N Mohammadifard PhD), Department of Medical Physics (K Saber PhD), Musculoskeletal Research Center (A Shafaat MS), Isfahan University of Medical Sciences, Isfahan, Iran; NanoElectronics and Photonics Systems (MEPHOS) (S Bahadorikhalili PhD), Universitat Rovira i Virgili, Tarragona, Spain; School of Public Affairs (R Bai MD), Nanjing University of Science and Technology, Nanjing, China; International Medical School (A A Baig PhD), Management and Science University, Alam, Malaysia; Center for Clinical Research and Prevention (J L Baker PhD), Bispebjerg University Hospital, Frederiksberg, Denmark; Department of Neurosurgery (A T Bako PhD), Houston Methodist Hospital, Houston, TX, USA; Maternal and Child Health Unit (R K Bakshi MD), Department of Biostatistics (V K Kamal PhD), Indian Council of Medical Research, New Delhi, India (D K Lal MD); Division of Biological Sciences (S Balakrishnan PhD), Tamil Nadu State Council for Science and Technology, Chennai, India; Health Care Management Department (M Balasubramanian PhD), Flinders Health and Medical Research Institute (N B Bulamu PhD), College of Nursing and Health Sciences (L N Bulto PhD), College of Medicine and Public Health (T G Gebremeskel PhD, B Kaambwa PhD, G R Naik PhD), Health Economics Unit (B Kaambwa PhD), Department of Nursing and Health Sciences (S Shorofi PhD), Flinders University, Adelaide, SA, Australia; Center of Innovation, Technology and Education (CITE) (Prof O C Baltatu PhD), Institute of Biomedical Engineering (Prof L A Campos PhD), Anhembi Morumbi University, Sao Jose dos Campos, Brazil; Department of Medicine (K Bam MPH, M T Olaiya PhD), School of Nursing and Midwifery (D Bhandari PhD), Stroke and Ageing Research, Victorian Heart Institute (L L Dalli PhD), Department of Neuroscience (Prof C A Unsworth PhD), Monash University, Clayton, VIC, Australia; Department of Hypertension (Prof M Banach PhD), Medical University of Lodz, Lodz, Poland; Polish Mothers' Memorial Hospital Research Institute, Lodz, Poland (Prof M Banach PhD); Department of Neurosurgery (S Bandyopadhyay BA), School of Psychology (Prof S Cortese PhD), Faculty of Medicine (R Thayakaran PhD), University of

Southampton, Southampton, UK; Department of Non-communicable Diseases (P C Banik MPhil, L Barua MPH), Bangladesh University of Health Sciences, Dhaka, Bangladesh; Department of Medicine (K Bansal MD), Department of Neurology (S Sivakumar MD), University of Massachusetts Medical School, Worcester, MA, USA; Department of Medicine (K Bansal MD), Department of Cardiovascular Medicine (A K Jha MD), Saint Vincent Hospital, Worcester, MA, USA; Department of Biomedical Sciences (F Barbic PhD), Humanitas University, Milan, Italy; Miami Cancer Institute (M Bardhan MD), Baptist Health South Florida, Miami, FL, USA; School of Psychology (Prof S L Barker-Collo PhD), School of Pharmacy (K A Beyene PhD), University of Auckland, Auckland, New Zealand; Heidelberg Institute of Global Health (HIGH) (Prof T W Bärnighausen MD, S Barteit PhD, S Chen DSc), Heidelberg University, Heidelberg, Germany; Department of Translational Medicine (F Barone-Adesi PhD), University of Eastern Piedmont, Novara, Italy; Department of Industrial Engineering (Prof L H Barrero DSc), Pontifical Javeriana University, Bogota, Colombia; Department of Epidemiology (A Barrow MPH, D Braithwaite PhD, D D Ding BS, D Yang MPH), College of Medicine (M J Diaz BS), UF Health Cancer Center (S D Karanth PhD), Department of Computer and Information Science and Engineering (P Naghavi MSc), University of Florida, Gainesville, FL, USA; Department of Public & Environmental Health (A Barrow MPH), University of The Gambia, Brikama, The Gambia; Alpha Genomics, Islamabad, Pakistan (Z Basharat PhD); Department of Tuberculosis (B Basnyat MD), Birat Nepal Medical Trust, Kathmandu, Nepal; Barcelona Institute for Global Health (Prof Q Bassat MD), Universitat de Barcelona (University of Barcelona), Barcelona, Spain; Catalan Institution for Research and Advanced Studies (ICREA), Barcelona, Spain (Prof Q Bassat MD); Faculty of Pharmacy (J D Basso PharmD, S Silva MSc), Coimbra Chemistry Centre (J D Basso PharmD), Department of Geography and Demography (M Rodrigues PhD), Coimbra Institute for Biomedical Imaging and Translational Research (S Silva MSc), University of Coimbra, Coimbra, Portugal; Department of Medical Education (K Batra PhD), University of Nevada Las Vegas, Las Vegas, NV, USA; Department of Psychiatry (Prof B T Baune PhD), Institute for Epidemiology and Social Medicine (A Karch MD), University of Münster, Münster, Germany; Department of Psychiatry (Prof B T Baune PhD), Melbourne Medical School, Melbourne, VIC, Australia; School of Public Health (Prof N Bedi MD), Dr. D. Y. Patil University, Mumbai, India; Clinical Nutrition (R M Chandika PhD), Department of Epidemiology (S Dohare MD, K Y Ghailan PhD, M Khan MD), Department of Maxillofacial Surgery and Diagnostic Sciences (E S Halboub PhD), Department of Health Education and Promotion (M Shanawaz MD), Jazan University, Jazan, Saudi Arabia (Prof N Bedi MD); Department of Mental Health (M Beghi MD), AUSL Romagna, Ravenna, Italy; Department of Basic Sciences (E Behboudi PhD), Khoy University of Medical Sciences, Khoy, Iran; Department of Community Medicine and Family Medicine (P Behera MD), All India Institute of Medical Sciences, BHUBANESWAR, India; Department of Epidemiology (M Heidari-Foroozan BSc, S Khanmohammadi MD, S Nejadghaderi MD, S Rashedi MD, H Soleimani MD), Endocrinology and Metabolism Research Institute (A Khalaji BS), Non-Communicable Diseases Research Center (NCDRC), Tehran, Iran (A Behnoush BS); Social Determinants of Health Research Center (M Behzadifar PhD), Lorestan University of Medical Sciences, Khorramabad, Iran; Division of Pulmonary, Critical Care, and Sleep (M Beiranvand PhD), University of Florida, Jacksonville, FL, USA; Department of Medicine (D F Bejarano Ramirez BN), El Bosque University, Bogota, Colombia; Transplant Service (D F Bejarano Ramirez BN), University Hospital Foundation Santa Fe de Bogotá, Bogota, Colombia; Department of Neurology (Prof Y Béjot PhD), University Hospital of Dijon, Dijon, France; Dijon Stroke Registry (Prof Y Béjot PhD), University of Burgundy, Dijon, France; National Data Management Center (NDMC) for Health, Burden of Disease Unit (C M Belete MSc, W A Cheru PhD), National Data Management Center for Health (A Misganaw PhD), Ethiopian Public Health

Institute, Addis Ababa, Ethiopia; Land Administration and Use Bureau (C M Belete MSc), Academia Sinica, Bahir Dar, Ethiopia; Infectious Disease Research Department (M B Bello PhD), King Abdullah International Medical Research Center, Riyadh, Saudi Arabia; Department of Biological Sciences (L Belo PhD), Research Unit on Applied Molecular Biosciences (UCIBIO) (L Belo PhD, Prof F Carvalho PhD, V M Costa PhD, Prof D Dias da Silva PhD, J P Silva PhD), Associated Laboratory for Green Chemistry (LAQV) (M Carvalho PhD, N G M Gomes PhD), Department of Chemical Sciences (R A S Couto MD), Institute for Research and Innovation in Health (Prof N Cruz-Martins PhD), Public Health and Forensic Sciences, and Medical Education Department (Prof R J Dinis-Oliveira PhD), Department of Community Medicine, Information and Health Decision Sciences (A Freitas PhD), Department of Chemistry (N G M Gomes PhD), Department of Chemical Engineering (Prof C F Rodrigues PhD), University of Porto, Porto, Portugal; Department of Biomedical Sciences (Prof A Beloukas PhD), University of West Attica, Athens, Greece; Department of Internal Medicine (I M Bensenor PhD, I S Santos PhD), Department of Psychiatry (Prof J Castaldelli-Maia PhD, Prof M F P Peres MD, Y Wang PhD), Center for Clinical and Epidemiological Research (I S Santos PhD), University of São Paulo, São Paulo, Brazil; School of Medicine (A Beran MD, J T Tran BS), Indiana University, Indianapolis, IN, USA; Institute of Marketing (Z Berezvai PhD), Corvinus University of Budapest, Budapest, Hungary; Competition Economics and Market Research Section (Z Berezvai PhD), Hungarian Competition Authority, Budapest, Hungary; Hubert Department of Global Health (R S Bernstein MD), School of Medicine (A O Fasanmi PhD, E L B Rogowski MPH), Department of Family and Preventive Medicine (T Sathish PhD), Rollins School of Public Health (Prof D A Sleet PhD), Emory University, Atlanta, GA, USA; Butte County Department of Public Health, Chico, CA, USA (R S Bernstein MD); Faculty of Medicine (P J G Bettencourt PhD), Catholic University of Portugal, Rio de Mouro, Portugal; Department of Pharmaceutical and Administrative Sciences (K A Beyene PhD), University of Health Sciences and Pharmacy in St. Louis, St Louis, MO, USA; Department of Forensic Chemistry (D S Bhagat PhD), Government Institute of Forensic Science, Aurangabad, Aurangabad, India; Department of Public Health (A S Bhagavathula PhD), North Dakota State University, Fargo, ND, USA; Institutes of Applied Health Research and Translational Medicine (N Bhala PhD), Queen Elizabeth Hospital Birmingham, Birmingham, UK; Institute of Applied Health Research (N Bhala PhD), NIHR Global Health Research Unit on Global Surgery (J C Glasbey MSc), University of Birmingham, Birmingham, UK; Department of Internal Medicine (Prof A Bhalla MD), Post Graduate Institute of Medical Education and Research, Chandigarh, India; Public Health Research Laboratory (D Bhandari PhD), Department of Biotechnology (B P Marasini PhD), Faculty of Humanities and Social Sciences (U Paudel PhD), Department of Community Medicine (P M S Pradhan MD), Tribhuvan University, Kathmandu, Nepal; Department of Hematology Oncology (P V Bhardwaj MD), University of Massachusetts Medical School, Springfield, MA, USA; Department of Internal Medicine (A Bhargava MD), Wayne State University, Detroit, MI, USA; Global Health Neurology Lab (S Bhaskar PhD), NSW Brain Clot Bank, Sydney, NSW, Australia; Department of Neurology and Neurophysiology (S Bhaskar PhD), South West Sydney Local Heath District and Liverpool Hospital, Sydney, NSW, Australia; Department of Internal Medicine (V Bhat MBBS), St. John's National Academy of Health Sciences, Bangalore, India; Medical Lab Technology (G K Bhatti PhD), University Centre for Research and Development (S Kalra DM), Chandigarh University, Mohali, India; Department of Human Genetics and Molecular Medicine (Prof J S Bhatti PhD, S Senapati PhD, U Sharma PhD), Department of Zoology (B Vellingiri PhD), Central University of Punjab, Bathinda, India; Department of Botanical and Environmental Sciences (Prof M S Bhatti PhD), Department of Pharmaceutical Sciences (R Bhatti PhD), Guru Nanak Dev University, Amritsar, India; Centre for Global Child Health (Prof Z A Bhutta PhD), Temerty Faculty of Medicine

(V Chattu MD), Division of Neurology (S Fereshtehnejad PhD), Department of Neurosurgery (A K Malhotra MD), University of Toronto, Toronto, ON, Canada; Centre of Excellence in Women & Child Health (Prof Z A Bhutta PhD), Division of Women and Child Health (J K Das MD), Department of Pediatrics (Z S Lassi PhD), Department of Family Medicine (Prof S M Shah PhD), Aga Khan University, Karachi, Pakistan; Scientific-Tools.Org, Bergamo, Italy (B Bikbov MD); Department of Biomedical and NeuroMotor Sciences (Prof F Bisulli PhD), Department of Biomedical and Neuromotor Sciences (A Capodici MD, S Guicciardi MD, L Muccioli MD), Department of Medical and Surgical Sciences (Prof F S Violante MD), University of Bologna, Bologna, Italy; UOC Clinica Neurologica (Prof F Bisulli PhD), IRCCS Istituto delle Scienze Neurologiche di Bologna (Institute of Neurological Sciences of Bologna), Bologna, Italy; Department of Neurology (Prof A Biswas DM), Department of GI Surgery (A Dhali MBBS), Institute of Post-Graduate Medical Education and Research and Seth Sukhlal Karnani Memorial Hospital, Kolkata, India; Community & Family Medicine (B Biswas MD), All India Institute of Medical Sciences, Deoghar, India; Department of Biostatistics and Epidemiology (Prof S Bitaraf PhD), Department of Orthodontics (E Eini DDS), Education Development Center (M Sayyah MD), Ahvaz Jundishapur University of Medical Sciences, Ahvaz, Iran; Faculty of Health Sciences (V R Bitra PhD), Department of Internal Medicine (G M Rwegerera MD), University of Botswana, Gaborone, Botswana; Department of Global Public Health and Primary Care (Prof T Bjørge PhD, A Hailu PhD), Department of Psychosocial Science (D Sagoe PhD), University of Bergen, Bergen, Norway; Department of Research (M W Wojewodzic PhD), Cancer Registry of Norway, Oslo, Norway (Prof T Bjørge PhD); SAMRC Centre for Health Economics and Decision Science (PRICELESS SA) (M K Boachie PhD), University of the Witwatersrand, Johannesburg, South Africa; Department of Sociology and Social Work (M S Boampong PhD), Department of Biochemistry and Biotechnology (A Kwarteng PhD), Kwame Nkrumah University of Science and Technology, Kumasi, Ghana; School of Business Administration (Prof V Bodolica PhD), American University of Sharjah, Sharjah, United Arab Emirates; Department of Veterinary Medicine (S Bohlouli PhD), Islamic Azad University, Kermanshah, Iran; Discipline of Public Health Medicine (O A Bolarinwa MSc, T G Ginindza PhD), School of Nursing and Public Health (E E Tarkang PhD), University of KwaZulu-Natal, Durban, South Africa; Department of Internal Medicine (A Boloor MD), Department of Forensic Medicine and Toxicology (H L Dsouza MD, V Krishna MD, Prof B K Shetty MD, P H Shetty MD), Department of General Medicine (J Jeganathan MD), Department of Community Medicine (N Joseph MD, P Mithra MD, R Motappa MD), Department of Obstetrics and Gynaecology (A Shetty MS), Kasturba Medical College, Mangalore (Prof B Unnikrishnan MD), Manipal Academy of Higher Education, Mangalore, India; Faculty of Medicine and Pharmaceutical Sciences (A Bonny MD), University of Douala, Douala, Cameroon; Department of Cardiology (A Bonny MD), Centre Hospitalier Montfermeil (Montfermeil Hospital Center), Montfermeil, France; Regional Medical Research Centre, North East Region (K Bora MD), Indian Council of Medical Research, Dibrugarh, India; General Directorate of Health Information Systems (B Bora Basara PhD), Ministry of Health, Ankara, Turkiye; Department of Medicine (Prof S Bouaoud MD), Faculty of Medicine (Prof A Ouyahia PhD), University Ferhat Abbas of Setif, Setif, Algeria; Department of Epidemiology and Preventive Medicine (Prof S Bouaoud MD), University Hospital Saadna Abdenour, Setif, Algeria; Department of Earth, Environment, and Equity (C Boxe PhD), Division of General Internal Medicine (R F Gillum MD), Department of Community and Family Medicine (R F Gillum MD), Howard University, Washington, DC, USA; General Medicine Service (Prof E J Boyko MD), Department of Veterans Affairs, Seattle, WA, USA; Cancer Population Sciences Program (D Braithwaite PhD), University of Florida Health Cancer Center, Gainesville, FL, USA; School of Population and Public Health (Prof M Brauer DSc, P A Chakraborty

MPH, I O Iyamu MD, D P Rasali PhD), School of Nursing (A Pashaei MSc), University of British Columbia, Vancouver, BC, Canada; Institute of Epidemiology (A Brazinova MD), Comenius University, Bratislava, Slovakia; Department of Sports and Computer Science (J Brazo-Sayavera PhD), Universidad Pablo de Olavid (Pablo de Olavide University), Seville, Spain; Department of Psychiatry and Behavioral Health (Prof N J K Breitborde PhD), Department of Psychology (Prof N J K Breitborde PhD), Division of Cardiovascular Medicine (A Guha MD), Ohio State University, Columbus, OH, USA; Institute for Medical Information Processing, Biometry, and Epidemiology (S Breitner DSc), Ludwig Maximilian University of Munich, Munich, Germany; Institute of Epidemiology (S Breitner DSc), Helmholtz Zentrum München German Research Center for Environmental Health, Neuherberg, Germany; Division of Clinical Epidemiology and Aging Research (Prof H Brenner MD), German Cancer Research Center, Heidelberg, Germany; Biomedical Department (A N Briko PhD), Bauman Moscow State Technical University, Moscow, Russia; Department of Epidemiology and Evidence-Based Medicine (Prof N I Briko DSc, P D Lopukhov PhD), I.M. Sechenov First Moscow State Medical University, Moscow, Russia; Department of Neuroscience (G Britton PhD), University of Panama, Ancon, Panama; Infectious Diseases Department (G Britton PhD), Gorgas Memorial Institute for Health Studies, Panama City, Panama; Department of Injury (J Brown PhD), Global Women's Health Program (P Cullen PhD), The George Institute for Global Health, Newtown, NSW, Australia; Faculty of Medicine (J Brown PhD), School of Population Health (P Cullen PhD, Z Dai PhD, X Xu PhD), School of Psychiatry (Prof P S Sachdev MD), University of New South Wales, Kensington, NSW, Australia; Global Health Research Institute (D Buonsenso MD), Università Cattolica del Sacro Cuore (Catholic University of Sacred Heart), Rome, Italy; Department of Biopharmaceutics and Clinical Pharmacy (Y Bustanji PhD), The University of Jordan, Amman, Jordan; School of Public Health Sciences (Z A Butt PhD, O A Zitoun MD), University of Waterloo, Waterloo, ON, Canada; Al Shifa School of Public Health (Z A Butt PhD), Al Shifa Trust Eye Hospital, Rawalpindi, Pakistan; Department of Clinical Pharmacy (Prof D Calina PhD), University of Medicine and Pharmacy of Craiova, Craiova, Romania; Department of Internal Medicine (Prof L A Cámera MD), Hospital Italiano de Buenos Aires (Italian Hospital of Buenos Aires), Buenos Aires, Argentina; Board of Directors (Prof L A Cámera MD), Argentine Society of Medicine, Buenos Aires, Argentina; College of Health Sciences (Prof L A Campos PhD), Abu Dhabi University, Abu Dhabi, United Arab Emirates; Dana-Farber Cancer Institute, Boston, MA, USA (C Cao MPH); Management and Healthcare (EMbeDS) (A Capodici MD), Sant'Anna School of Advanced Studies, Pisa, Italy; Department of Health Care (Prof R Cárdenas DSc), Metropolitan Autonomous University, Mexico City, Mexico; Oncological Network, Prevention and Research Institute (G Gorini MD), Institute for Cancer Research, Prevention and Clinical Network, Florence, Italy (G Carreras PhD); Dermatology Unit (A Carugno MD), Azienda Socio Sanitaria Territoriale Papa Giovanni XXIII (Territorial Healthcare Company Pope John XXIII), Bergamo, Italy; Department of Pediatrics (C G Carvalheiro PhD), University of São Paulo, Ribeirao Preto, Brazil; Department of Pediatrics (C G Carvalheiro PhD), Clinical Hospital of Ribeirão Preto, Ribeirão Preto, Brazil; Instituto de Investigação, Inovação e Desenvolvimento (Institute of Research Innovation and Development) (M Carvalho PhD), University Fernando Pessoa, Porto, Portugal; Colombian National Health Observatory (C A Castañeda-Orjuela MD), National Institute of Health, Bogota, Colombia; Epidemiology and Public Health Evaluation Group (C A Castañeda-Orjuela MD), National University of Colombia, Bogota, Colombia; Department of Medicine (G Castelpietra PhD), University of Udine, Udine, Italy; Department of Mental Health (G Castelpietra PhD), Healthcare Agency "Friuli Occidentale", Pordenone, Italy; Clinical Epidemiology Program (F Catalá-López PhD), Ottawa Hospital Research Institute, Ottawa, ON, Canada; Department of Pharmacological and Biomolecular

Sciences (Prof A L Catapano PhD), IRCCS Istituto Ortopedico Galeazzi (Galeazzi Orthopedic Institute IRCCS) (G Damiani MD), Department of Clinical Sciences and Community Health (Prof C La Vecchia MD), Department of Food, Environmental and Nutritional Sciences (Prof D Martini PhD), University of Milan, Milan, Italy; MultiMedica Sesto San Giovanni IRCCS, Sesto S. Giovanni, Italy (Prof A L Catapano PhD); Department of Otolaryngology, Head and Neck Surgery (C R Cederroth PhD), University of Tu¨bingen, Tu¨bingen, Germany; Department of Medical, Surgical & Health Sciences (L Cegolon PhD), Department of Medical, Surgical, and Health Sciences (Prof M D'Oria MD), University of Trieste, Trieste, Italy; Public Health Unit (L Cegolon PhD), University Health Agency Giuliano-Isontina (ASUGI), Trieste, Italy; Department of Nutrition (Prof F Cembranel DSc), Federal University of Santa Catarina, Florianópolis, Brazil; College of Public Health, Medical, and Veterinary Sciences (M Cenderadewi MPHTM, Prof R C Franklin PhD, A E Peden PhD), Department of Public Health and Tropical Medicine (T I Emeto PhD), James Cook University, Townsville, QLD, Australia (K O Obamiro PhD); Department of Public Health (M Cenderadewi MPHTM), University of Mataram, Mataram, Indonesia; Mary MacKillop Institute for Health Research (Prof E Cerin PhD), Faculty of Health Sciences (G R Poudel PhD), Australian Catholic University, Melbourne, VIC, Australia; School of Public Health (Prof E Cerin PhD), Department of Periodontology and Implant Dentistry (K Deng PhD), Department of Urban Planning and Design (C Guo PhD), Centre for Suicide Research and Prevention (Prof P Yip PhD), Department of Social Work and Social Administration (Prof P Yip PhD), University of Hong Kong, Hong Kong, China; Infection and Global Health Research (M Cevik MD), University of St Andrews, St Andrews, UK; Regional Infectious Diseases Unit (M Cevik MD), Department of Clinical Effectiveness (A I Ikiroma PhD), NHS National Services Scotland, Edinburgh, UK; ICMR School of Public Health (J Chadwick MD), Division of Epidemiology and Biostatistics (V K Kamal PhD), National Institute of Epidemiology, Chennai, India; Department of Biotechnology (Prof C Chakraborty PhD), Adamas University, Kolkata, India; Skeletal Aging & Orthopedic Surgery (Prof C Chakraborty PhD), Hallym University, Chuncheon, South Korea; Heart Failure and Structural Heart Disease Unit (J Chan MBChB), Cardiovascular Analytics Group, Hong Kong, China; Department of Medicine and Therapeutic (R N C Chan MBChB), Prince of Wales Hospital, Hong Kong, China; Department of Anesthesiology and Perioperative Medicine (E K Chandrasekar MD), School of Medicine (Prof S Xu PhD), University of Rochester, Rochester, NY, USA (E Dorsey MD); Institute of Epidemiology and Preventive Medicine (C Chang PhD), National Taiwan University, Taipei City, Taiwan; Department of Psychological Medicine (C Chang PhD), Department of Twin Research and Genetic Epidemiology (M Mazidi PhD), Faculty of Life Sciences and Medicine (M Molokhia PhD), Institute of Psychiatry, Psychology & Neuroscience (D Urso MD), School of Population Health and Environmental Sciences (H A Wafa MPH, Y Wang PhD, Prof C D A Wolfe MD), King's College London, London, UK; College of Medicine (J Chang PhD), National Taiwan University, Taipei, Taiwan; Department of Nursing (J Chang PhD), National Taiwan University Hospital, Taipei, Taiwan; Department of Public Health (P Charalampous PhD, S Polinder PhD, F Porru MD), Department of Medical Informatics (Prof D Prieto Alhambra PhD), Erasmus University Medical Center, Rotterdam, Netherlands; Department of Community Medicine (V Chattu MD), Datta Meghe Institute of Medical Sciences, Sawangi, India; Center for Cancer Epidemiology (Prof P Chaturvedi MD), Homi Bhabha National Institute (HBNI), Mumbai, India; Department of Endocrinology (V Chatzimavridou-Grigoriadou MD), Department of Mathematics (O Johnson PhD), Division of Immunology, Immunity to Infection and Respiratory Medicine (A G Mathioudakis PhD), Division of Psychology and Mental Health (F Mughal FRCGP), University of Manchester, Manchester, UK; Department of Endocrinology (V Chatzimavridou-Grigoriadou MD), Christie Hospital NHS Foundation Trust, Manchester, UK; Oral Medicine and

Radiology (A Chaurasia MD), King George's Medical University, Lucknow, India; Fuwai Hospital (A Chen PhD), Chinese Academy of Medical Sciences & Peking Union Medical College, Beijing, China; Department of Computer Science (A Chen PhD), University of Texas Austin, Austin, TX, USA; Clinical Research Center (H Chen MB), Southern Medical University, Guangzhou, China; Department of Internal Medicine (D Flood MD), University of Michigan, Ann Arbor, MI, USA (M Chen BDS); Ocular Epidemiology Research Group (Prof C Cheng MD), Singapore Eye Research Institute, Singapore, Singapore; Ophthalmology & Visual Sciences Academic Clinical Program (Prof C Cheng MD), National Dental Research Institute Singapore (G G Nascimento PhD), Duke-NUS Medical School, Singapore, Singapore; Department of Paediatrics (E T W Cheng MBChB, S Dai PhD), School of Pharmacy (G Fekadu MSc), Jockey Club School of Public Health and Primary Care (J Huang MD, C Zhong MD), Department of Medicine & Therapeutics (Y Jin MD), Department of Medicine and Therapeutics (L Lim MRCP), The Chinese University of Hong Kong, Hong Kong, China; Department of Laboratory Medicine (J Chien PhD), Taichung Tzu-Chi Hospital Buddhist Tzu-Chi Medical Foundation, Tanzih, Taiwan; Department of Medical Laboratory Science and Biotechnology (J Chien PhD), Central Taiwan University of Science and Technology, Taichung, Taiwan; Department of Public Health and Health Policy (O Chimed-Ochir PhD), Hiroshima University, Hiroshima, Japan; Westmead Clinical School (R Chimoriya PhD), University of Sydney, Sydney, Australia; Division of Infectious Diseases (P R Ching MD), School of Medicine (H Ding MPH), Virginia Commonwealth University, Richmond, VA, USA; Department of Public Health, Administration, and Social Sciences (J L Chirinos-Caceres DrPH), Cayetano Heredia University, Lima, Peru; Iraq Field Epidemiology Training Program (I-FETP) (A Chitheer MD), Ministry of Health, Baghdad, Iraq; Department of Clinical Oncology (W C S Cho PhD), Queen Elizabeth Hospital, Hong Kong, China; Department of Medicine (B Chong MBBS), Saw Swee Hock School of Public Health (Prof D S Q Koh PhD, Prof D S Q Koh PhD, S Yi PhD), School of Medicine (M Ng PhD), Leadership Institute for Global Health Transformation (LIGHT) (S Ramazanu PhD), Department of Surgery (K Tan PhD), Yong Loo Lin School of Medicine (Prof N Venketasubramanian MBBS), National University of Singapore, Singapore, Singapore; Department of Biosciences (H Chopra PhD), Center for Global Health Research (M Fareed PhD, S Muthupandian PhD), Department of Public Health Dentistry (Prof G Mini PhD), Saveetha Dental College and Hospitals (M R Tovani-Palone PhD), Saveetha Institute of Medical and Technical Sciences (SIMATS), Chennai, India; Department of Community Medicine (Prof S G Choudhari MD, Prof A M Gaidhane MD), Datta Meghe Institute of Medical Sciences, Wardha, India; Department of Epidemiology (S O Gbadamosi MD, R Jebai MPH), Florida International University, Miami, FL, USA (Prof R Chowdhury PhD); Department of Epidemiology (Prof R Chowdhury PhD), Institute of Social and Preventive Medicine (M Ganbat MPH), Department of Neurology (L D Panos MD), Department of Emergency Medicine (I Pantazopoulos PhD), Department of Cardiology (T Pilgrim MD, S Stortecky MD), University of Bern, Bern, Switzerland; Department of Pulmonary Medicine (Prof D J Christopher MD), Department of Endocrinology, Diabetes and Metabolism (Prof N Thomas PhD), Christian Medical College and Hospital (CMC), Vellore, India; Department of Paediatric Surgery (I S Chukwu B.Med.Sc.), Federal Medical Centre, Umuahia, Nigeria; Department of AndroUrology (Prof E Chung MD), AndroUrology Centre, Brisbane, QLD, Australia; Health Data Research UK, London, UK (S Chung PhD); Department of Genetics (Z Cindi PhD), School of Veterinary Medicine (F Musaigwa PhD), Department of Biostatistics Epidemiology and Informatics (J Puvvula PhD), University of Pennsylvania, Philadelphia, PA, USA; Department of Food, Environmental and Nutritional Sciences (I Cioffi PhD, C Del Bo' PhD, M Marino PhD), University of Milan, Milano, Italy; Department of Biochemistry and Microbiology (M M Claassens PhD), University of Namibia,

Windhoek, Namibia; Department of Paediatrics and Child Health (M M Claassens PhD), Stellenbosch University, Tygerberg, South Africa; Nova Medical School (J Conde PhD), Nova University of Lisbon, Lisbon, Portugal; School of Medicine and Surgery (P A Cortesi PhD, Prof L G Mantovani DSc), University of Milan Bicocca, Monza, Italy; Department of Epidemiology and Prevention (S Costanzo PhD), IRCCS Neuromed, Pozzilli, Italy; Department of Psychology (R G Cowden PhD), University of the Free State, Park West, South Africa; Department of Psychology (K M Cramer PhD), Office of Institutional Analysis (J Dube MA), University of Windsor, Windsor, ON, Canada; Department of Family Medicine and Public Health (Prof M H Criqui MD), University of California San Diego, La Jolla, CA, USA; Department of Epidemiology (Prof M p Curado PhD), Accamargo Cancer Center, São Paulo, Brazil; Department of Internal Medicine (S Dadana MD), Cheyenne Regional Medical Center, Cheyenne, WY, USA; School of Clinical Medicine (S Dai PhD), Hangzhou Normal University, Hangzhou, China; Department of Dermatology (G Damiani MD), Lerner College of Medicine (L Göbölös PhD), Harrington Heart and Vascular Institute (A Guha MD), Department of Quantitative Health Science (X Liu PhD), Department of Neonatology (I Qattea MD), Department of Nutrition and Preventive Medicine (Prof J Sanabria MD), Case Western Reserve University, Cleveland, OH, USA; Department of Public Health (J Darega Gela MPH), Ambo University, Ambo, Ethiopia; Department of Biochemistry (S Das MD), Ministry of Health and Welfare, New Delhi, India; Ingram School of Engineering (S Das PhD), Texas State University, San Marcos, TX, USA; Ophthalmology Department (A Dascalu PhD), Emergency University Hospital Bucharest, Bucuresti, Romania; 2nd University Ophthalmology Department (A Dastiridou MD), Department of Ophthalmology (N Dervenis MD), Second Department of Cardiology (D Patoulias PhD), Aristotle University of Thessaloniki, Thessaloniki, Greece; Ophthalmology Department (A Dastiridou MD), University of Thessaly, Greece; Department of Global Health and Infection (Prof G Davey MD), Brighton and Sussex Medical School, Brighton, UK; School of Public Health (Prof G Davey MD, N Dereje PhD), Center for Food Science and Nutrition (T B Elema MA), Medical Laboratory Science (M Getie MSc), Emergency Department (D Ijo MSc), Department of Health Management Information Systems (D Ijo MSc), Addis Ababa University, Addis Ababa, Ethiopia; Department of Population and Development (C A Dávila-Cervantes PhD), Latin American Faculty of Social Sciences Mexico, Mexico City, Mexico; Health Research Institute (K Davletov PhD), Asfendiyarov Kazakh National Medical University, Almaty, Kazakhstan; Australian Institute for Suicide Research and Prevention (Prof D De Leo DSc, Prof K Kolves PhD), Griffith University, Mount Gravatt, QLD, Australia; Discipline of Chiropractic (K de Luca PhD), CQ University, Brisbane, QLD, Australia; Medical College (S Debopadhaya BS), Albany Medical College, Albany, NY, USA; Department of Epidemiology and Community Medicine (A Dehghan PhD), Fasa University of Medical Sciences, Fasa, Iran; School of Medicine (I Delgado-Enciso DSc), University of Colima, Colima, Mexico; Department of Research (I Delgado-Enciso DSc), Colima State Health Services, Colima, Mexico; Department of Neurosurgery (A K Demetriades MD), Global Health Governance Programme (J Patel BSc), Centre for Medical Informatics (Prof A Sheikh MD), Usher Institute (Prof C R Simpson PhD), College of Medicine and Veterinary Medicine (G Verras MD), University of Edinburgh, Edinburgh, UK; Department of Neurosurgery (A K Demetriades MD), National Health Service Scotland, Edinburgh, UK; Epidemiology Branch (X Deng PhD), National Institute of Health, Durham, NC, USA; St Paul's Eye Unit (N Dervenis MD), Royal Liverpool University Hospital, Liverpool, UK; Department of Forensic Medicine (E Dervišević PhD), University of Sarajevo, Sarajevo, Bosnia and Herzegovina; Department of Forensic Medicine (E Dervišević PhD), Universiti Kebangsaan Malaysia Medical Centre, Sarajevo, Bosnia and Herzegovina; Department of Psychiatry (Prof D C Des Jarlais PhD, S Gunturu MD), The Zena and Michael A. Wiener Cardiovascular

Institute (V R Dhulipala MD), Institute of Critical Care Medicine (A Shaikh MD), Department of Cardiology (M Vinayak MD), Icahn School of Medicine at Mount Sinai, New York, NY, USA (A Shaikh MD); Graduate Medical Education (H D Desai MD), Gujarat Adani Institute of Medical Sciences, Bhuj, India; Division of Cardiology (R Desai MBBS), Atlanta Veterans Affairs Medical Center, Decatur, GA, USA; Department of Community Medicine (V G C Devanbu MD), Chettinad Academy of Research and Education, Chennai, India; Department of Pharmacy (S Dewan PhD, M R Islam PhD), University of Asia Pacific, Dhaka, Bangladesh; Pharmacology Department (S Dewan PhD), Center for Life Sciences Research Bangladesh, Dhaka, Bangladesh; Division of Pathology (K Dhama PhD), ICAR-Indian Veterinary Research Institute, Bareilly, India; Research Department (M Dhimal PhD, B P Marasini PhD, U Paudel PhD), Research Section (A Ghimire BSc), Nepal Health Research Council, Kathmandu, Nepal; Department of Pharmacy Practice (S Dhingra PhD), National Institute of Pharmaceutical Education and Research, Hajipur, India; Faculty of Science (Prof D Diaz PhD), School of Medicine (Prof R Lozano MD), National Autonomous University of Mexico, Mexico City, Mexico; Toxicology Research Unit (TOXRUN) (Prof R J Dinis-Oliveira PhD), Advanced Polytechnic and University Cooperative (CESPU), Gandra, Portugal; Department of Medicine (T H Do MD), Can Tho University of Medicine and Pharmacy, Can Tho, Viet Nam; Center for Health Sciences (C B do Prado MSc), Federal University of Espírito Santo, Vitória, Brazil; Department of Biostatistics (M Dodangeh Mcom), Independent Consultant, Tehran, Iran; Department of Social Medicine and Health Care Organisation (K G Dokova PhD), Medical University "Prof. Dr. Paraskev Stoyanov", Varna, Bulgaria; Mahidol Oxford Tropical Medicine Research Unit (C Dolecek PhD), Mahidol University, Bangkok, Thailand; School of Elderly Care Services and Management (W Dong MD), Nanjing University of Chinese Medicine, Nanjing, China; Cardio-Thoraco-Vascular Department (Prof M D'Oria MD), Azienda Sanitaria Universitaria Giuliano Isontina, Trieste, Italy; Departamento de Responsabilidade Social (Department of Social Responsibility) (W M dos Santos PhD), Hospital Alemão Oswaldo Cruz (Oswaldo Cruz German Hospital), São Paulo, Brazil; Brazilian Centre for Evidence-based Healthcare (W M dos Santos PhD), Joanna Briggs Institute, São Paulo, Brazil; Department of Cardiology (R Doshi MD), St. Joseph's University Medical Center, Paterson, NJ, USA; Department of Forensic Medicine and Toxicology (H L Dsouza MD), Kasturba Medical College Mangalore, Mangalore, India; Department of Periodontology (M Du MSc), Shandong University, Jinan, China; Postgraduate Program in Epidemiology (Prof B B Duncan MD, Prof B N G Goulart DSc, Prof M I Schmidt MD), Federal University of Rio Grande do Sul, Porto Alegre, Brazil; School of Medicine (Prof A R Duraes PhD), Institute of Collective Health (Prof M Pereira PhD, Prof D Rasella PhD), Federal University of Bahia, Salvador, Brazil; Department of Internal Medicine (Prof A R Duraes PhD), Escola Bahiana de Medicina e Saúde Pública (Bahiana School of Medicine and Public Health), Salvador, Brazil; Department of Biotechnology (S Duraisamy PhD), SRM Institute of Science and Technology (SRMIST), Kattankulathur, India; Child Health Analytics Research Program (P A Dzianach PhD, Prof P W Gething PhD, F Sanna PhD, D J Weiss PhD), Geospatial Health and Development Team (J Lubinda PhD, A Saddler PhD), The Malaria Atlas Project (S F Rumisha PhD), Telethon Kids Institute, Perth, WA, Australia; Department of Conservative Dentistry with Endodontics (A M Dziedzic DSc), Medical University of Silesia, Katowice, Poland; Department of Psychiatry (E Eboreime PhD, E Tsermpini PhD), Dalhousie University, Halifax, NS, Canada; Department of Psychiatry (E Eboreime PhD), University of Alberta, Edmonton, AB, Canada; Division of Cardiothoracic Vascular Surgery (C P Echieh FWACS), University of Calabar, Calabar, Nigeria; Division of Cardiothoracic Surgery (C P Echieh FWACS), University of Arizona, Tucson, AZ, USA; School of Health Sciences (H A Edinur PhD), Universiti Sains Malaysia (University of Science Malaysia), Kubang Kerian, Malaysia; College of Science, Health and Engineering (K Edvardsson

PhD), La Trobe University, Bundoora, VIC, Australia; Department Pediatric Nursing (D Efendi MSN), Faculty of Public Health (D Kusuma DSc), Centre for Family Welfare (K Latief Master of Epidemiology), University of Indonesia, Depok, Indonesia; Neonatal Intensive Care Unit (D Efendi MSN), University of Indonesia Hospital, Depok, Indonesia; Department of Community Health Nursing (F Efendi PhD), Department of Epidemiology (A Hargono DMD), Faculty of Public Health (S Martini PhD), Universitas Airlangga (Airlangga University), Surabaya, Indonesia; Research Center for Public Health and Nutrition (D E Effendi MA), National Research and Innovation Agency Republic of Indonesia (BRIN), Jakarta, Indonesia (A Kusnali LLB, H U Pangaribuan MSc); Centre for Global Health Inequalities Research (CHAIN) (Prof T Eikemo PhD), Department of Circulation and Medical Imaging (J Nauman PhD), Norwegian University of Science and Technology, Trondheim, Norway; Department of Food Science and Nutrition (T B Elema MA), Arsi University, Asella, Ethiopia; School of Population and Global Health (Prof F J Elgar PhD), McGill University, Montreal, QC, Canada; Department of Internal Medicine and Hematology Unit (Prof G M T ElGohary MD), Department of Entomology (A M Samy PhD), Medical Ain Shams Research Institute (MASRI) (A M Samy PhD), Neurology Department (Prof A S Shalash PhD), Ain Shams University, Cairo, Egypt; Faculty of Medicine (M Elhadi MD), University of Tripoli, Tripoli, Libya; Egypt Center for Research and Regenerative Medicine (ECRRM), Cairo, Egypt (M A Elmonem PhD); Department of Clinical Pathology (M Elshaer MD), Faculty of Pharmacy (M A Saleh PhD), Rheumatology and Immunology Unit (S Tharwat MD), Mansoura University, Mansoura, Egypt; Department of Infectious Diseases and Public Health (I Elsohaby PhD), City University of Hong Kong, Hong Kong, China; Department of Animal Medicine (I Elsohaby PhD), Cardiovascular Department (Prof A M A Saad MD), Zagazig University, Zagazig, Egypt; Lincoln International Institute for Rural Health (L Engelbert Bain PhD), University of Lincoln, Lincoln, UK; Department of International Cyber Education (R Erkhembayar MD), Mongolian National University of Medical Sciences, Ulaanbaatar, Mongolia; Department of Paediatrics (C I Esezobor MB), Department of Psychiatry (A T Olagunju MD), University of Lagos, Lagos, Nigeria; Department of Paediatrics (C I Esezobor MB), Lagos University Teaching Hospital, Lagos, Nigeria; Department of Obesity, Diabetes and Cardiovascular Risk (Prof J Espinosa-Montero PhD), National Institute of Public Health Mexico, Cuernavaca, Mexico; Independent Consultant, Bologna, Italy (N Fabin MD); Department of Anesthesia (A O Fadaka PhD), Cincinnati Children's Hospital Medical Center, Cincinnati, OH, USA; Department of Biotechnology (A O Fadaka PhD), School of Pharmacy (O C Okonji MSc), University of the Western Cape, Cape Town, South Africa; Research Centre for Healthcare and Community (A F Fagbamigbe PhD), Faculty of Health and Life Sciences (O P Kurmi PhD), Coventry University, Coventry, UK; Department of Food Hygiene and Quality Control (A Fakhri-Demeshghieh DVM), University of Tehran, Tehran, Iran; Epidemiology and Biostatistics Unit (L Falzone PhD), IRCCS Pascale, Naples, Italy; Dissemination Division (C S e Farinha MSc), National Institute of Statistics, Lisbon, Portugal; Activity Planning and Control Unit (C S e Farinha MSc), Directorate-General of Health (DGS), Lisbon, Portugal; Department of Biology (P S Faris PhD), Department of Food Technology (Y Galali ResM, B A Sadee PhD), Department of Statistics (D H Kadir PhD), Salahaddin University-Erbil, Erbil, Iraq; Department of Biology (P S Faris PhD), Department of Nutrition and Dietetics (Y Galali ResM, B A Sadee PhD), Cihan University-Erbil, Erbil, Iraq; Department of Psychology (Prof A Faro PhD), Federal University of Sergipe, São Cristóvão, Brazil; Satcher Health Leadership Institute (A O Fasanmi PhD), Morehouse School of Medicine, Atlanta, GA, USA; Centre for Health Policy Research (Prof P Ward PhD), Torrens University Australia, Adelaide, SA, Australia (N K Fauk MSc); Institute of Resource Governance and Social Change, Kupang, Indonesia (N K Fauk MSc); National Institute for Stroke and Applied Neurosciences (Prof V L Feigin PhD), Auckland University of Technology,

Auckland, New Zealand; Third Department of Neurology (E V Gnedovskaya PhD), Research Center of Neurology, Moscow, Russia (Prof V L Feigin PhD, Prof M A Piradov DSc); Department of Pharmacy (G Fekadu MSc), Department of Nursing (G Fetensa MSc, L Gurmessa MSc, G M Kebebew MSc), Institute of Health Sciences (B R Feyisa MPH), Wollega University, Nekemte, Ethiopia; School of Medicine (X Feng PhD), Department of Psychology (C Hu PhD), Tsinghua Vanke School of Public Health (Z Li PhD), Tsinghua University, Beijing, China; Department of Translational Medicine (D Ferrante PhD), University of Piemonte Orientale, Italy, Novara, Italy; Department of Social Sciences (Prof N Ferreira PhD), University of Nicosia, Nicosia, Cyprus; Department of Psychiatry (I Filip MD), Kaiser Permanente, Fontana, CA, USA; School of Health Sciences (I Filip MD), A.T. Still University, Mesa, AZ, USA; Institute of Public Health (F Fischer PhD), Departement of Surgery (N Haep MD), Department of Neurology (S Samadzadeh MD), Department of Infectious Diseases and Respiratory Medicine (F Steinbeis MD), Charité Universitätsmedizin Berlin (Charité Medical University Berlin), Berlin, Germany; School of Social Sciences (J Flavel PhD), Stretton Health Equity, Adelaide, SA, Australia; Center for Research in Indigenous Health (D Flood MD), Maya Health Alliance, Tecpán, Guatemala; Institute of Gerontology (N A Foigt PhD), National Academy of Medical Sciences of Ukraine, Kyiv, Ukraine; Department of Child Dental Health (Prof M O Folayan FWACS), Obafemi Awolowo University, Ile-Ife, Nigeria; Clinical Science Department (Prof M O Folayan FWACS), Nigerian Institute of Medical Research, Yaba, Nigeria; Department of Cell Biology and Biotechnology (A A Fomenkov PhD), K.A. Timiryazev Institute of Plant Physiology, Moscow, Russia; Department of Medical Parasitology (M Foroutan PhD), Faculty of Medicine (M Foroutan PhD), Department of Public Health (N Kamyari PhD), Department of Pharmacology (H Mojiri-Forushani PhD), Abadan University of Medical Sciences, Abadan, Iran; Department of Disease Burden (I Forthun PhD, A S Knudsen PhD, C Madsen PhD), GBD Collaborating Unit (Prof S E Vollset DrPH), Norwegian Institute of Public Health, Bergen, Norway; Healthcare Innovation Department (D Fortuna MSc), Regional Agency for Health and Social Care of Emilia-Romagna, Bologna, Italy; Department of Biotechnological and Applied Clinical Sciences (DISCAB) (M Foschi MD), Department of Neurology (Prof S Sacco MD), University of L'Aquila, L'Aquila, Italy; Department of Neuroscience (M Foschi MD), Hospital Santa Maria delle Croci, Ravenna, Italy; Child Survival Unit (K R Fowobaje MSc), Centre for African Newborn Health and Nutrition, Ibadan, Nigeria; Centre for Adolescent Health (K L Francis MBiostat, J A Kerr PhD), Department of Critical Care and Neurosciences (Prof R G Weintraub MB), Murdoch Childrens Research Institute, Parkville, VIC, Australia; Center for Health Technology and Services Research (CINTESIS), Porto, Portugal (A Freitas PhD); Department of Dermatology (T Fukumoto PhD), Kobe University, Kobe, Japan; Department of Pathology (Prof B Fux PhD), Department of Integrated Health Education (Prof L B Salaroli PhD), Federal University of Espirito Santo, Vitória, Brazil; Health Services Management Training Centre (P A Gaal PhD, T Joo PhD, J Lám PhD, T Palicz MD), Karoly Racz Doctoral School of Clinical Medicine (T Kocsis MSc), Faculty of Health and Public Administration (M Szócska PhD), Semmelweis University, Budapest, Hungary; Department of Applied Social Sciences (P A Gaal PhD), Sapientia Hungarian University of Transylvania, Târgu-Mureş, Romania; Department of Community Medicine (Prof M A Gadanya FMCPH), Aminu Kano Teaching Hospital, Kano, Nigeria; Institute of Applied Health Sciences (S Gaihre PhD), University of Aberdeen, Aberdeen, UK; Department of Environmental Health Sciences (S Gallus DSc, A Lugo PhD), Mario Negri Institute for Pharmacological Research, Milan, Italy; Department of Research Publication (M Ganbat MPH), Zaigal Research Institute, Ulaanbaatar, Mongolia, Mongolia; Department of Community Medicine and Family Medicine (A P Gandhi MD), All India Institute of Medical Sciences, Nagpur, India; Institute of Health and Wellbeing (B Ganesan PhD), Federation

University, Churchill, VIC, Australia; Department of General Medicine (M Ganiyani MD), Grant Medical College & Sir J.J. Group of Hospitals, Mumbai, India; Faculty of Business and Management (M Garcia-Gordillo PhD), Universidad Autonóma de Chile (Autonomous University of Chile), Talca, Chile; Division of Cardiovascular Medicine (J Garg MD), Medical College of Wisconsin, Milwaukee, WI, USA; University School of Management and Entrepreneurship (N Garg PhD, R Sharma PhD), Delhi Technological University, Delhi, India; Department of Pharmacology (Prof R K Gautam PhD), Indore Institute of Pharmacy, Indore, India; Department of Midwifery (M W Gebregergis MSc), Department of Medical Laboratory Sciences (H Negash MSc), Adigrat University, Adigrat, Ethiopia; Department of Environmental Health (M Gebrehiwot DSc), Department of Public Health (F M Hussien MPH, F M Hussien MPH), Wollo University, Dessie, Ethiopia; Reproductive and Family Health (T G Gebremeskel PhD), Axum College of Health Science, Axum, Ethiopia; Department of Dermatology (Prof S R Georgescu PhD), "Victor Babes" Clinical Hospital of Infectious and Tropical Diseases, Bucharest, Romania; School of Population Health (Prof P W Gething PhD, D J Weiss PhD), School of Public Health (D Hendrie PhD, T R Miller PhD), Curtin University, Perth, WA, Australia; Infectious Disease Research Center (Prof K Ghadiri MD), Pediatric Department (Prof K Ghadiri MD), Universal Scientific Education and Research Network (USERN) (P Goleij MSc), Department of Rehabilitation and Sports Medicine (M Mirzaei MSc), Research Center for Environmental Determinants of Health (Prof E Sadeghi PhD), Department of Infectious Disease (Prof S Vaziri MD), Kermanshah University of Medical Sciences, Kermanshah, Iran; Center of Health Management (K Y Ghailan PhD), Aden University, Aden, Yemen; School of Public Health (A Ghashghaee BSc), Department of Food Hygiene and Safety (Prof R Mahmoudi PhD), Qazvin University of Medical Sciences, Qazvin, Iran; Young Researchers and Elite Club (A Gholamian MSc), Islamic Azad University, Rasht, Iran; Department of Biology (A Gholamian MSc), Islamic Azad University, Tehran, Iran; Respiratory Medicine Department (Prof A G Ghoshal MD), National Allergy Asthma Bronchitis Institute, Kolkata, India; Department of Respiratory Medicine (Prof A G Ghoshal MD), Fortis Hospital, Kolkata, India; Warwick Medical School (Prof P S Gill DM), University of Warwick, Coventry, UK (J W Sakshaug PhD); Westmead Applied Research Centre (M A Godinho MBBS), University of Sydney, Westmead, NSW, Australia; Department of Hepatology (Prof A Goel DM), Sanjay Gandhi Postgraduate Institute of Medical Sciences, Lucknow, India; Department of Applied Cell Sciences (A Golchin PhD), Cellular and Molecular Medicine Institute (A Golchin PhD), Urmia University of Medical Sciences, Urmia, Iran (R Valizadeh PhD); Department of Health Systems and Policy Research (M Golechha PhD), Indian Institute of Public Health, Gandhinagar, India; Department of Genetics (P Goleij MSc), Sana Institute of Higher Education, Sari, Iran; Department of Exercise and Health Sciences (P N Gona PhD), University of Massachusetts Boston, Boston, MA, USA; Hudson College of Public Health (S V Gopalani MPH), University of Oklahoma Health Sciences Center, Oklahoma City, OK, USA; Department of Health and Social Affairs (S V Gopalani MPH), Government of the Federated States of Micronesia, Palikir, Federated States of Micronesia; Department of Respiratory Medicine (H Goudarzi PhD), Center for Environmental and Health Sciences (H Goudarzi PhD), Hokkaido University, Sapporo, Japan; Department of Epidemiology (Prof A C Goulart PhD), Universidade de São Paulo (University of São Paulo), São Paulo, Brazil; Blood and Marrow Transplantation and Cellular Therapy Program (A Goyal MD), Division of Pediatric Hospital Medicine (R P Mediratta MD), Stanford University, Palo Alto, CA, USA; Department of Dermatology (A Grada MD), Health Informatic Lab (T Javaheri PhD), Department of Computer Science (R Rawassizadeh PhD), Boston University, Boston, MA, USA; Nuffield Department of Orthopaedics, Rheumatology, and Musculoskeletal Sciences (S M Graham PhD, Prof D Prieto Alhambra PhD), Nuffield Department of Medicine (T Runghien MSc), Nuffield

Department of Primary Care Health Sciences (T Tillawi MD), Oxford University, Oxford, UK; Department of Public Health and Preventive Medicine (Prof M Grivna PhD), Charles University, Prague, Czech Republic; Department of Epidemiology and Biostatistics (S Guan MD), Anhui Medicla University, Hefei, China; Post Graduate School of Public Health (G Guarducci MD), University of Siena, Siena, Italy; Department of Family and Community Medicine (M I M Gubari PhD), University Of Sulaimani, Sulaimani, Iraq; Health Directorate (S Guicciardi MD), Local Health Authority of Bologna, Bologna, Italy; Faculty of Nursing (Prof R A Guimarães PhD), Federal University of Goiás, Goiânia, Brazil; Department of General Surgery (S Gulati MD), Dignity Health, Phoenix, AZ, USA; Department of Community Medicine (D A Gunawardane MD), University of Peradeniya, Kandy, Sri Lanka; Department of Psychiatry (S Gunturu MD), Bronxcare Health System, Bronx, NY, USA; Department of Internal Medicine (A K Gupta PharmD), Faculty of Medicine and Health Sciences (Prof N P Singh MD), Shree Guru Gobind Singh Tricentenary University, Gurugram, India; Non-communicable Division (NCD) (A K Gupta PharmD), Indian Council of Medical Research, Delhi, India; Department of Public Health (B Gupta PhD), Department of Health and Education (F Hanna PhD), Torrens University Australia, Melbourne, VIC, Australia; Department of Epidemiology and Biostatistics (R Gupta MPH), Department of Health Promotion, Education, and Behavior (T Mi PhD), University of South Carolina, Columbia, SC, USA; Centre for Noncommunicable Diseases and Nutrition (R Gupta MPH), BRAC University, Dhaka, Bangladesh; Department of Preventive Cardiology (Prof R Gupta MD), Eternal Heart Care Centre & Research Institute, Jaipur, India; Department of Medicine (Prof R Gupta MD), Mahatma Gandhi University Medical Sciences, Jaipur, India; Department of Toxicology (S Gupta MSc), Shriram Institute for Industrial Research, Delhi, India; School of Medicine (V Gupta PhD), Institute for Mental and Physical Health and Clinical Translation (IMPACT) (W Marx PhD), Deakin University, Geelong, VIC, Australia; School of Biotechnology (V Gupta PhD), Dublin City University, Glasnevin, Ireland; Faculty of Medicine Health and Human Sciences (Prof V K Gupta PhD), Macquarie Medical School (Y You PhD), Macquarie University, Sydney, NSW, Australia; Department of Epidemiology and Psychosocial Research (R A Gutiérrez PhD), Ramón de la Fuente Muñiz National Institute of Psychiatry, Mexico City, Mexico; Global Virus Network, Middle East Region, Shiraz, Iran (F Habibzadeh MD); School of Medicine (P Habibzadeh MD), University of Maryland, Baltimore, MD, USA; Department of Pharmacology and Toxicology (R Haddadi PhD), Hamadan University of Medical Sciences, Hamadan, Iran; Department of Clinical Pharmacology and Medicine (Prof N R Hadi PhD), University of Kufa, Najaf, Iraq; Clinician Scientist Program (N Haep MD), Berlin Institute of Health, Berlin, Germany; NYU Shanghai, Shanghai, China (B J Hall PhD); Department of Infectious Disease Epidemiology (S Haller MD), Robert Koch Institute, Berlin, Germany; Department of Public Health (S Haller MD), Charité Institute of Public Health, Berlin, Germany; Department of Family and Community Medicine (Prof R R Hamadeh PhD), College of Medicine and Medical Sciences (H Jahrami PhD), Arabian Gulf University, Manama, Bahrain; School of Health and Environmental Studies (Prof S Hamidi DrPH), Hamdan Bin Mohammed Smart University, Dubai, United Arab Emirates; Department of Nephrology (Q Han PhD), Beijing Chao-yang Hospital, Capital Medical University, Beijing, China; Centre for Neuromuscular and Neurological Disorders (Prof G J Hankey MD), The University of Western Australia, Perth, WA, Australia; Perron Institute for Neurological and Translational Science, Perth, WA, Australia (Prof G J Hankey MD); Department of Biochemistry and Molecular Biology (Prof M Hannan PhD), Bangladesh Agricultural University, Mymensingh, Bangladesh; Department of Anatomy (Prof M Hannan PhD), Dongguk University, Gyeongju, South Korea; Department of Population Science and Human Resource Development (Prof M Haque PhD, Prof M Rahman PhD, M Rahman DrPH), Department of Mathematics (M Kuddus PhD), University

of Rajshahi, Rajshahi, Bangladesh; Medical Research Unit (H Harapan PhD), Universitas Syiah Kuala (Syiah Kuala University), Banda Aceh, Indonesia; Research Unit (J M Haro MD), University of Barcelona, Barcelona, Spain; Biomedical Research Networking Center for Mental Health Network (CiberSAM), Barcelona, Spain (J M Haro MD); Department of Zoology and Entomology (A I Hasaballah PhD, M G M Zeariya PhD), Botany and Microbiology Department (A M E Shehabeldine PhD), Al-Azhar University, Cairo, Egypt; Department of Pharmaceutical Technology (I Hasan MPharm), University of Dhaka, Dhaka, Bangladesh; Department of Radiology (M Hasanian MD), Arak University of Medical Sciences, Arak, Iran; Department of Public Health (A Hashi PhD), Jigjiga University, Jigjiga, Ethiopia; Department of Pharmacy (Prof M S Hasnain PhD), Palamau Institute of Pharmacy, Daltonganj, India; Public Health Department (I Hassan MPH), Dalhatu Araf Specialist Hospital, Lafia, Nigeria; Department of Public Health (I Hassan MPH), Federal University of Lafia, Lafia, Nigeria; Independent Consultant, Tabriz, Iran (H Hassankhani PhD); Department of Diagnostic and Interventional Radiology and Neuroradiology (J Haubold MD, Prof B M Schaarschmidt MD), Institute of Artificial Intelligence in Medicine (J Haubold MD), University Hospital Essen, Essen, Germany; Skaane University Hospital (R J Havmoeller PhD), Skaane County Council, Malmö, Sweden; Faculty of Kinesiology (Prof J J Hebert PhD), University of New Brunswick, Fredericton, NB, Canada; School of Allied Health (Prof J J Hebert PhD), Murdoch University, Murdoch, WA, Australia; Independent Consultant, Santa Clara, CA, USA (G Heidari MD); Community-Oriented Nursing Midwifery Research Center (M Heidari PhD), Department of Epidemiology and Biostatistics (A Mohammadian-Hafshejani PhD), Department of Health in Disasters and Emergencies (R Sheikhi B.Hlth.Sci), Shahrekord University of Medical Sciences, Shahrekord, Iran; Institute of Psychology (B Helfer PhD), University of Wroclaw, Wroclaw, Poland; Meta Research Centre (B Helfer PhD), University of Wrocław, Wroclaw, Poland; Departamento de Salud Oral (Department of Oral Health) (B Y Herrera-Serna PhD), Universidad Autónoma de Manizales (Autonomous University of Manizales), Manizales, Colombia; School of Business (Prof C Herteliu PhD), London South Bank University, London, UK; Department of Microbiology (K Hezam PhD), Department of Applied Microbiology (E A Noman PhD), Taiz University, Taiz, Yemen; School of Medicine (K Hezam PhD), Nankai University, Tianjin, China; Rheumatology Department (Prof C L Hill MD), The Queen Elizabeth Hospital, Woodville, SA, Australia; Division for Health Service Promotion (Y Hiraike PhD), Department of Mental Health (Prof N Kawakami PhD), Department of Global Health Policy (S Nomura PhD, S K Rauniyar PhD), University of Tokyo, Tokyo, Japan; Department of Pulmonology (N Horita PhD), Yokohama City University, Yokohama, Japan; National Human Genome Research Institute (NHGRI) (N Horita PhD), National Institutes of Health, Bethesda, MD, USA; Social and Environmental Health Research (M Hossain MPH), Nature Study Society of Bangladesh, Khulna, Bangladesh; Department of Health Promotion and Community Health Sciences (M Hossain MPH), Texas A&M University, College Station, TX, USA; Department of Public Health and Informatics (S Hossain MS), Jahangirnagar University, Dhaka, Bangladesh; School of Health and Society (H Hosseinzadeh PhD), University of Wollongong, Wollongong, NSW, Australia; Institute of Research and Development (Prof M Hosseinzadeh PhD), Faculty of Medicine (H T H Nguyen MD), Institue for Research and Training in Medicine, Biology and Pharmacy (H T H Nguyen MD), Duy Tan University, Da Nang, Viet Nam; Department of Computer Science (Prof M Hosseinzadeh PhD), Diplomacy and Public Relations Department (A Omar Bali PhD), University of Human Development, Sulaymaniyah, Iraq; Department of Clinical Legal Medicine (S Hostiuc PhD), National Institute of Legal Medicine Mina Minovici, Bucharest, Romania; Faculty of Medicine of Tunis (Prof M Hsairi MPH), University Tunis El Manar, Tunis, Tunisia; Department of Health Services Administration (V Hsieh PhD), Department of Occupational Safety and Health (Prof B

Hwang PhD), China Medical University, Taichung, Taiwan; Department of Surgical Sciences (M Hultström PhD), Department of Medical Cell Biology (M Hultström PhD), Department of Medical Sciences (Prof A O Larsson PhD, Prof J Sundström PhD), Uppsala University, Uppsala, Sweden; Department of Public Health and Community Medicine (Prof A Humayun PhD), Shaikh Zayed Postgraduate Medical Institute, Lahore, Pakistan; Department of Biological Sciences and Chemistry (Prof J Hussain PhD), Natural and Medical Sciences Research Center (A Khan PhD, A Ullah MS, S Ullah MSc, M Waqas PhD), School of Pharmacy (A K Philip PhD), University of Nizwa, Nizwa, Oman; Department of Social Sciences and Business (Prof M Hussain PhD), Roskilde University, Roskilde, Denmark; Department of Biomolecular Sciences (N R Hussein PhD), Department of Biology (K S Ibrahim PhD), University of Zakho, Zakho, Iraq; International Master Program for Translational Science (H Huynh BS), Department of Global Health and Health Security (K Latief Master of Epidemiology), International Ph.D. Program in Medicine (L Minh MD), Research Center for Artificial Intelligence in Medicine (L Minh MD), Graduate Institute of Biomedical Informatics (D N A Ningrum MPH), School of Public Health (Y L Samodra MPH, Y L Samodra MPH), Department of Clinical Pharmacy (M A Sarasmita PharmD), Global Health and Health Security Department (U Wongsin PhD), Taipei Medical University, Taipei, Taiwan; Department of Occupational Therapy (Prof B Hwang PhD), Asia University, Taiwan, Taichung, Taiwan; Health Policy and Management Department (P M Iftikhar MD), City University of New York, New York, NY, USA; Division of Infectious Diseases (K S Ikuta MD), Veterans Affairs Greater Los Angeles, Los Angeles, CA, USA; Department of Pathology (P C Ikwegbue MSc), School of Public Health and Family Medicine (R Matzopoulos PhD, C A Nnaji MPH), Division of Cardiology (Prof M Ntsekhe PhD), Department of Paediatrics and Child Health (Prof H J Zar PhD, Prof L J Zuhlke PhD), Department of Medicine (Prof L J Zuhlke PhD), University of Cape Town, Cape Town, South Africa; Faculty of Medicine (I M Ilic PhD, Prof M M Santric-Milicevic PhD, A Stevanović MD, I S Vujcic PhD), School of Public Health and Health Management (Prof M M Santric-Milicevic PhD), School of Medicine (R Vukovic PhD), University of Belgrade, Belgrade, Serbia; Department of Epidemiology (Prof M D Ilic PhD), University of Kragujevac, Kragujevac, Serbia; College Of Pharmacy (M Imam PhD), Prince Sattam Bin Abdulaziz University, Al Kharj, Saudi Arabia; Division of Gastroenterology and Hepatology (S Inamdar MD), University of Arkansas for Medical Sciences, Little Rock, AR, USA; National Research and Innovation Agency, Jakarta, Indonesia (E Indriasih PhD, I U Tarigan PhD); Faculty of Public Health (M Iqhrammullah PhD), Universitas Muhammadiyah Aceh, Banda Aceh, Indonesia; Department of Medicine (A Iradukunda MD), University of Burundi, Bujumbura, Burundi; Research Department (A Iradukunda MD), ARNECH Research and Consulting Office, Bujumbura, Burundi; Department of Medical Microbiology (K C Iregbu MD), University of Abuja, Abuja, Nigeria; Department of Medical Microbiology (K C Iregbu MD), National Hospital, Abuja, Nigeria; Institute for Physical Activity and Nutrition (S Islam PhD), Department of Psychology (M A Stokes PhD), Deakin University, Burwood, VIC, Australia; Department of Surveillance and Health Services Research (F Islami PhD), American Cancer Society, Atlanta, GA, USA; Clinical Laboratory (F Ismail PhD), Tobruk University, Tobruk, Libya; Blood Transmitted Diseases (F Ismail PhD), National Center for Disease Control, Tobruk, Libya; Department of Clinical Pharmacy & Pharmacy Practice (Prof N Ismail PhD), Asian Institute of Medicine, Science and Technology, Kedah, Malaysia; Malaysian Academy of Pharmacy, Puchong, Malaysia (Prof N Ismail PhD); Public Health Department of Social Medicine (Prof H Iso MD), Graduate School of Medicine (Prof K Yamagishi MD), Osaka University, Suita, Japan; Department of Health Services Research (M Iwagami PhD), Research and Development Center for Health Services (Prof K Yamagishi MD), University of Tsukuba, Tsukuba, Japan; School of Health Systems and Public Health (C C D Iwu MPH),

University of Pretoria, Pretoria, South Africa; Knowledge Translation Program (I O Iyamu MD), Centre for Health Evaluation and Outcome Sciences, Vancouver, BC, Canada; Department of Biotechnology (M Iyer PhD), Karpagam Academy of Higher Education (Deemed to be University), Coimbatore, India; Department of Orthodontics & Dentofacial Orthopedics (L J BDS), Department of Oral Pathology and Microbiology (Prof G S Sarode PhD, Prof S C Sarode PhD), Dr. D. Y. Patil University, Pune, India; Research and Development Unit (L Jacob MD), Biomedical Research Networking Center for Mental Health Network (CiberSAM), Sant Boi de Llobregat, Spain; Faculty of Medicine (L Jacob MD), University of Versailles Saint-Quentin-en-Yvelines, Montigny-le-Bretonneux, France; Department of Health Studies (K H Jacobsen PhD), University of Richmond, Richmond, VA, USA; Department of Immunology (Prof A Jafarzadeh PhD), Research Center for Hydatid Disease in Iran (F Khamesipour PhD), Kerman University of Medical Sciences, Kerman, Iran; Department of Immunology (Prof A Jafarzadeh PhD), Family Medicine Department (Z Kamiab MD), Clinical Research Development Unit (Z Kamiab MD), Department of Epidemiology and Biostatistics (Prof M Rezaeian PhD), Rafsanjan University of Medical Sciences, Rafsanjan, Iran; Department of Nephrology (K Jaggi MD), San Mateo Medical Center, San Mateo, CA, USA; Department of Nephrology (K Jaggi MD), Mills Peninsula Medical Center, Burlingame, CA, USA; Department of Psychiatry (Z Saif MBA), Ministry of Health, Manama, Bahrain (H Jahrami PhD); Statistics Unit (N Jain MD), Riga Stradins University, Riga, Latvia; Department of Health and Safety (A A Jairoun PhD), Dubai Municipality, Dubai, United Arab Emirates; Centre for Community Medicine (A Jaiswal MD), Department of Preventive Oncology (J K Meena MD), Centre for Dental Education and Research (B M Purohit MDS), Department of Psychiatry (Prof R Sagar MD), Department of Radiation Oncology (A Shankar MD), All India Institute of Medical Sciences, New Delhi, India; School of Pharmacy and Pharmacology (A Jatau PhD), Menzies Institute for Medical Research (F Pan PhD, J Tian PhD), University of Tasmania, Hobart, TAS, Australia; Department of Physiology (Prof S Javadov PhD), University of Puerto Rico Medical Sciences Campus, San Juan, Puerto Rico; Centre of Studies and Research (S Jayapal PhD), Ministry of Health, Muscat, Oman; Department of Biochemistry (Prof S Jayaram MD), Government Medical College, Mysuru, India; Department of Epidemiology and Health Promotion (Prof S Jee PhD), Department of Preventive Medicine (K Kim MD), Yonsei University, Seoul, South Korea; Department of Community Medicine (R P Jha MSc), Dr. Baba Saheb Ambedkar Medical College & Hospital, Delhi, India; Department of Community Medicine (R P Jha MSc), Banaras Hindu University, Varanasi, India; Zoonoses Research Center (M Jokar DVM), Islamic Azad University, Karaj, Iran; Department of Clinical Sciences (M Jokar DVM), Department of Public Health (Y Sarikhani PhD), Jahrom University of Medical Sciences, Jahrom, Iran; Institute of Molecular and Clinical Ophthalmology Basel, Basel, Switzerland (Prof J B Jonas MD); Department of Ophthalmology (Prof J B Jonas MD), Heidelberg University, Mannheim, Germany; Hungarian Health Management Association (T Palicz MD), Hungarian Health Management Association, Budapest, Hungary (T Joo PhD); Department of Gastroenterology and Hepatology (A Joseph MD), Department of Biomedical Data Science (S Park MD), Department of Radiology (S Ramasamy MD), Stanford University, Stanford, CA, USA; Department of Economics (C E Joshua BSc), National Open University, Benin City, Nigeria; Department of Family Medicine and Public Health (J J Jozwiak PhD), University of Opole, Opole, Poland; Institute of Family Medicine and Public Health (M Jürisson PhD), University of Tartu, Tartu, Estonia; School of Public Health (Z Kabir PhD), University College Cork, Cork, Ireland; Department of Oral and Maxillofacial Pathology (V Kadashetti MDS), Department of Public Health Dentistry (Prof K M Shivakumar PhD), Krishna Vishwa Vidyapeeth (Deemed to be University), Karad, India; Social Determinants of Health Research Center (L R Kalankesh PhD), Faculty of Medicine (Z Saadatian PhD), Infectious Diseases Research

Center (Z Saadatian PhD), Gonabad University of Medical Sciences, Gonabad, Iran; Department of Dermatology (F Kaliyadan MD), King Faisal University, Hofuf, Saudi Arabia; Department of Endocrinology (S Kalra DM), Bharti Hospital Karnal, Karnal, India; Northern Oesophagogastric Cancer Unit (S K Kamarajah BSc), Newcastle University, Newcastle, UK; Care and Public Health Research Institute (CAPHRI) (R Kamath MHA), Maastricht University, Maastricht, Netherlands; School of Graduate Studies (T Kanagasabai PhD), Meharry Medical College, Nashville, TN, USA; Sydney Eye Hospital (H Kandel PhD), South Eastern Sydney Local Health District, Sydney, NSW, Australia; Division of Cardiology (A R Kanmanthareddy MD), Creighton University, Omaha, NE, USA; College of Public Health (A R Kanmanthareddy MD), Department of Environmental, Agricultural and Occupational Health (J Taiba MPH), University of Nebraska Medical Center, Omaha, NE, USA; Regional Institute for Population Studies (E Kanmiki MPH), University of Ghana, Accra, Ghana; Faculty of Dentistry (K K Kanmodi MPH), University of Puthisastra, Phnom Penh, Cambodia; Office of the Executive Director (K K Kanmodi MPH), Campaign for Health and Neck Cancer Education (CHANCE) Programme (A A Salami BDS), Cephas Health Research Initiative Inc, Ibadan, Nigeria; Department of Community Medicine (S Kannan S MD), ESIC Medical College and Hospital Chennai, Chennai, India; Dr. S S Bhatnagar University Institute of Chemical Engg. & Technology (Prof S K Kansal PhD), Department of Anthropology (Prof K Krishan PhD), Institute of Forensic Science & Criminology (V Sharma PhD), Panjab University, Chandigarh, India; The Hansjörg Wyss Department of Plastic and Reconstructive Surgery (R S Kantar MD), Nab'a Al-Hayat Foundation for Medical Sciences and Health Care, New York, NY, USA; Cleft Lip and Palate Surgery Division (R S Kantar MD), Global Smile Foundation, Norwood, MA, USA; School of Health Professions and Human Services (I M Karaye MD), Hofstra University, Hempstead, NY, USA; Department of Anesthesiology (I M Karaye MD), Montefiore Medical Center, Bronx, NY, USA; Department of Physical Therapy and Health Rehabilitation (F Z Kashoo MSc), Majmaah University, Majmaah, Saudi Arabia; Gastrointestinal Center (Q H A Kasnazani MD), Anwar Shyxa Medical City, Sulaimanyah, Iraq; Department of Digestive Surgery (Q H A Kasnazani MD), Kurdistan Higher Council For Medical Specialities, Sulaimanyah, Iraq; Surgery Research Unit (Prof J H Kauppila MD), Center for Environmental and Respiratory Health Research (I Shiue PhD), Martti Ahtisaari Institute (I Shiue PhD), University of Oulu, Oulu, Finland; Department of ENT (N Kaur MS), Dr. B. R. Ambedkar State Institute of Medical Sciences (AIMS), Mohali, India; International Research Center of Excellence (G A Kayode PhD), Institute of Human Virology Nigeria, Abuja, Nigeria; Julius Centre for Health Sciences and Primary Care (G A Kayode PhD), Copernicus Institute of Sustainable Development (G Koren PhD), Utrecht University, Utrecht, Netherlands; Community Medicine Department (T H Kazmi FCPS), Shalamar Medical & Dental College, Lahore, Pakistan; Public Health (F Kebede MPH), Woldia University, Woldia, Ethiopia; Institute of Biological Chemistry and Nutrition (T S Keflie PhD), University Hohenheim, Stuttgart, Germany; Open, Distance and eLearning Campus (Prof P N Keiyoro PhD), Department of Psychiatry (M Kumar PhD), School of Public Health (R G Wamai PhD), University of Nairobi, Nairobi, Kenya; Eye Unit (Prof J H Kempen MD), MyungSung Medical College, Addis Ababa, Ethiopia; Department of Psychological Medicine (J A Kerr PhD), University of Otago, Christchurch, New Zealand; Department of Human Nutrition (E Kesse-Guyot PhD), National Research Institute for Agriculture, Food and Environment, Jouy-en-Josas, France; University Sorbonne Paris Nord (E Kesse-Guyot PhD), Department of Health, Medicine and Human Biology (M Touvier PhD), Sorbonne Paris Nord University, Bobigny, France; Amity Institute of Forensic Sciences (H Khajuria PhD, B P Nayak PhD), Amity Institute of Pharmacy (K Munjal PhD), Amity University, Noida, India; College of Health Sciences (N Khalid PhD), Abu Dhabi University, Adu Dhabi, United Arab Emirates; Department of Biostatistics (Prof A Khalilian

PhD), Mazandaran University of Medical Sciences, Mazandaran, Iran; Department of Pediatrics (I A Khan MD), Center for Pharmacoepidemiology and Treatment Science (A Parthasarathi MD), Rutgers University, New Brunswick, NJ, USA; Population Science Department (M Khan PhD), Jatiya Kabi Kazi Nazrul Islam University, Mymensingh, Bangladesh; Primary Care Department (M A Khan MSc), NHS North West London, London, UK; Department of Medicine (Z A Khan MD), Independent Consultant, Hyderabad, India; Department of Critical Care Medicine (M Z Khan suheb MD), St. Luke's Aurora Medical Center, Milwaukee, WI, USA; College of Health, Wellbeing and Life Sciences (Prof K Khatab PhD), Sheffield Hallam University, Sheffield, UK; College of Arts and Sciences (Prof K Khatab PhD), Ohio University, Zanesville, OH, USA; Faculty of Nursing (H Khatatbeh PhD), Jerash University, Jerash, Jordan; Department of Biochemistry (F Khidri PhD), Liaquat University Of Medical and Health Sciences, Jamshoro, Pakistan; Department of Epidemiology (A Khosravi PhD), Center for Health Related Social and Behavioral Sciences Research (F Sadeghian PhD), Shahroud University of Medical Sciences, Shahroud, Iran; Molecular Medicine Department (M Khosravi PhD), Pasteur Institute of Iran, Tehran, Iran; Department of Pediatrics (G Kim MD), Case Western Reserve University School of Medicine, Cleveland, OH, USA; Division of Pediatric Hospital Medicine (G Kim MD), University Hospitals Rainbow Babies and Children's Hospital, Cleveland, OH, USA; Cardiovascular Disease Initiative (M Kim MD), Broad Institute of MIT and Harvard, Cambridge, MA, USA; School of Traditional Chinese Medicine (Y Kim PhD), Xiamen University Malaysia, Sepang, Malaysia; Millennium Prevention, Westwood, MA, USA (R W Kimokoti MD); School of Health Sciences (Prof A Kisa PhD), Kristiania University College, Oslo, Norway; Department of International Health and Sustainable Development (Prof A Kisa PhD), Tulane University, New Orleans, LA, USA; Department of Nursing and Health Promotion (S Kisa PhD), Faculty of Health Sciences (Prof A W Wolf PhD), Oslo Metropolitan University, Oslo, Norway; Global Healthcare Consulting, New Delhi, India (S Kochhar MD); Independent Consultant, Jakarta, Indonesia (S Kosen MD); Department of Epidemiology (Prof K Kostev PhD), IQVIA, Frankfurt, Germany; Department of Gynecology (Prof K Kostev PhD), Philipps-Universität Marburg, Marburg, Germany; Department of Internal and Pulmonary Medicine (Prof P A Koul MD), Sheri Kashmir Institute of Medical Sciences, Srinagar, India; Kasturba Medical College, Mangalore (S Koulmane Laxminarayana MD), Manipal Academy of Higher Education, Udupi, India; Evidence Synthesis Unit (Y Krishnamoorthy MD), Partnership for Research, Opportunity, Planning, Upskilling, and Leadership (PROPUL) Evidence, Chennai, India; Department of Demography (Prof B Kuate Defo PhD), Department of Social and Preventive Medicine (Prof B Kuate Defo PhD), University of Montreal, Montreal, QC, Canada; Faculty of Medicine (B Kucuk Bicer PhD), Gazi University, Ankara, Turkiye; Department of Biochemistry (Prof M Kuddus PhD), College of Public Health & Health Informatics (R Kumar PhD), Department of Public Health (M G M Zeariya PhD), University of Hail, Hail, Saudi Arabia; Department of Pediatrics (I Kuitunen PhD), Kuopio University Hospital, Kuopio, Finland; Institute of Clinical Medicine (I Kuitunen PhD), University of Eastern Finland, Kuopio, Finland; Department of Health Research (M Kulimbet MSc), Atchabarov Scientific Research Institute of Fundamental and Applied Medicine (M Kulimbet MSc), Kazakh National Medical University, Almaty, Kazakhstan; Cardiothoracic Surgery (A Kumar MD), UN Mehta Institute of Cardiology and Research Center, Ahmedabad, India; Cardiothoracic Surgery (A Kumar MD), Medanta Hospital, Gurugram, India; Department of Internal Medicine (A Kumar MD), Cabrini Institute, Akron, OH, USA; Department of Food Technology (Prof H Kumar PhD), Shri Vishwakarma Skill University, Palwal, India; Department of Biotechnology (Prof H Kumar PhD), Amity Institute of Biotechnology (M Kumari PhD, E Upadhyay PhD), Amity University Rajasthan, Jaipur, India; Global Health Institute (S Kundu MPH), North South University, Dhaka, Bangladesh;

Department of Nutrition and Food Science (S Kundu MPH), Department of Biochemistry and Food Analysis (N Roy PhD), Department of Post-Harvest Technology and Marketing (A Sayeed MSc), Patuakhali Science and Technology University, Patuakhali, Bangladesh; Department of Medicine (O P Kurmi PhD), Department of Health Research Methods, Evidence and Impact (E J Mills PhD), Department of Psychiatry and Behavioural Neurosciences (A T Olagunju MD), McMaster University, Hamilton, ON, Canada; Department of Health Services Research and Management (D Kusuma DSc), City University of London, London, UK; Department of Health Policy (I Kyriopoulos PhD), London School of Economics and Political Science, London, UK; National Institute for Health Research (NIHR) Oxford Biomedical Research Centre, Oxford, UK (B Lacey PhD); Institute for Social and Health Sciences (Prof L Laflamme PhD), University of South Africa, Pretoria, South Africa; International Maize and Wheat Improvement Center (CIMMYT), El Batan, Mexico (A K Lagat BS); Department of Basic Sciences (A K Lagat BS), Jomo Kenyatta University of Agriculture and Technology, Nairobi, Kenya; Stockholm County Council Surveillance and Analysis Centre for Epidemiology and Community Medicine, Stockholm, Sweden (A C J Lager PhD); Department of Family Medicine (A Lahmar MD), University of Medicine, Oujda, Morocco; School of Digital Science (D T C Lai PhD), Institute of Applied Data Analytics (D T C Lai PhD), Faculty of Science (E Leong PhD), Universiti Brunei Darussalam (University of Brunei Darussalam), Bandar Seri Begawan, Brunei; Department of Public Health (Prof T Lallukka PhD), Department of Virology (F Zakham PhD), University of Helsinki, Helsinki, Finland (T J Meretoja MD); Institute of Health Policy and Development Studies (Prof H Lam PhD), National Institutes of Health, Manila, Philippines; NEVES Society for Patient Safety, Budapest, Hungary (J Lám PhD); Department of Epidemiology (K R Landrum MSc), University of North Carolina at Chapel Hill, Chapel Hill, NC, USA; Duke Global Health Institute (K R Landrum MSc), Duke University, Durham, USA; Department of Health Sciences (DISSAL) (F Lanfranchi MD), University of Genoa, Genoa, Italy; Centre for Surveillance and Applied Research (J J Lang PhD), Public Health Agency of Canada, Ottawa, ON, Canada; Department of Psychiatry and Psychotherapy (B Langguth PhD, W Schlee PhD), University of Regensburg, Regensburg, Germany; Chief Medical Office (Prof V C Lansingh PhD), HelpMeSee, New York, NY, USA; Mexican Institute of Ophthalmology, Queretaro, Mexico (Prof V C Lansingh PhD); Department of Behavioural Sciences and Learning (A Laplante-Lévesque PhD), Linköping University, Linköping, Sweden; Department of Clinical Chemistry and Pharmacology (Prof A O Larsson PhD), Uppsala University Hospital, Uppsala, Sweden; Department of Otorhinolaryngology (S Lasrado MS), Father Muller Medical College, Mangalore, India; International Society Doctors for the Environment, Arezzo, Italy (P Lauriola MD); Faculty of Medicine (N Le MD), Department of General Medicine (V T Nguyen MD), University of Medicine and Pharmacy at Ho Chi Minh City, Ho Chi Minh City, Viet Nam (T T Le MD, T D T Le MD); Department of Cardiovascular Research (N Le MD), Methodist Hospital, Merrillville, IN, USA; Independent Consultant, Ho Chi Minh City, Viet Nam (T D T Le MD); Department of Medical Science (M Lee PhD), Ajou University School of Medicine, Suwon, South Korea; Department of Precision Medicine (Prof S W Lee MD), Sungkyunkwan University, Suwon-si, South Korea; School of Pharmacy (S W H Lee PhD), Monash University, Bandar Sunway, Malaysia; School of Pharmacy (S W H Lee PhD), Taylor's University Lakeside Campus, Subang Jaya, Malaysia; The Department of Family Medicine (W Lee PhD), University of Texas, Galveston, TX, USA; Department of Preventive Medicine (Prof Y Lee PhD, Prof S Yoon PhD), Korea University, Seoul, South Korea (Prof M Shin PhD); Department of Health Promotion and Health Education (M Li PhD), National Taiwan Normal University, Taipei, Taiwan; Department of Health Management Center (X Li PhD), Fudan University, Shanghai, China; National Clinical Research Center for Cardiovascular Diseases (Y Li PhD), Chinese Academy of Medical

Sciences, Shenzhen, China; Directorate of Quality Management and Digital Health (A T M Likaka MPH), Ministry of Health, Lilongwe, Malawi; UCD Centre for Disability Studies (C Linehan PhD), University College Dublin, Dublin, Ireland; School of Life Sciences (G Liu PhD), University of Technology Sydney, Ultimo, NSW, Australia; Centre for Inflammation (G Liu PhD), Centenary Institute, Camperdown, NSW, Australia; Department of Epidemiology and Biostatistics (Prof J Liu PhD), China Center for Health Development Studies (Y Yao MD), School of Public Health (H Zhang MS), Institute of Child and Adolescent Health (Z Zou MD), Peking University, Beijing, China; Center for Evidence-Based Medicine and Clinical Research (R Liu MD), School of Public Health and Management (Y Yu MS), Hubei University of Medicine, Shiyan, China; National Center for Chronic and Noncommunicable Disease Control and Prevention (N Wang PhD, P Ye MPH), Chinese Center for Disease Control and Prevention, Beijing, China (Prof S Liu PhD); Department of Molecular Epidemiology (E Llanaj PhD), German Institute of Human Nutrition Potsdam-Rehbrücke, Potsdam, Germany; German Center for Diabetes Research (DZD), München-Neuherberg, Germany (E Llanaj PhD); Department of Infectious Diseases (M J Loftus MBBS), Alfred Health, Melbourne, VIC, Australia; Department of Physical Medicine and Nursing (R López-Bueno PhD), University of Zaragoza, Zaragoza, Spain; Department of Musculoskeletal disorders (R López-Bueno PhD), National Research Centre for the Working Environment, Copenhagen, Denmark; National Institutes of Health (A Loreche BS), Department of Epidemiology and Biostatistics (J C Medina MD), University of the Philippines Manila, Manila, Philippines; School of Medicine and Public Health (A Loreche BS), Center for Research and Innovation (V F Pepito MSc), Ateneo De Manila University, Pasig City, Philippines; Institute of Nutritional Sciences (Prof S Lorkowski PhD), Friedrich Schiller University Jena, Jena, Germany; Competence Cluster for Nutrition and Cardiovascular Health (nutriCARD), Jena, Germany (Prof S Lorkowski PhD); Department of Medicine (Prof P A Lotufo DrPH), University of Sao Paulo, Sao Paulo, Brazil; School of Medicine (Prof G Lucchetti PhD), Federal University of Juiz de Fora, Juiz de Fora, Brazil; Department of General Surgery (Prof R Lunevicius DSc), Liverpool University Hospitals NHS Foundation Trust, Liverpool, UK; Centre for Public Health and Wellbeing (Z Ma PhD), University of the West of England, Bristol, UK; 2nd Department of Propaedeutic Surgery (N Machairas PhD), Department of Biophysics (Prof P Papadopoulou PhD), 3rd Department of Cardiology (M Spartalis PhD), University of Athens, Athens, Greece; Periodontal Department (Prof M Machoy PhD), Department of Propedeutics of Internal Diseases & Arterial Hypertension (Prof T Miazgowski MD), Pomeranian Medical University, Szczecin, Poland; Department of Biostatistics and Epidemiology (F Madadizadeh PhD), Yazd University of Medical Sciences, Yazd, Iran; Laboratório de Farmacognosia (LAQV) (Associated Laboratory for Green Chemistry (Á M Madureira-Carvalho PhD), Universidade do Porto (University of Porto), Porto, Portugal; School of Pharmacy (S B Maharaj DBA), University of the West Indies, St. Augustine, Trinidad and Tobago; Planetary Health Alliance, Boston, MA, USA (S B Maharaj DBA); Cellular and Molecular Biology Research Center (Prof S Mahjoub PhD), Department of Clinical Biochemistry (Prof S Mahjoub PhD), Social Determinants of Health Research Center (S Mouodi PhD), Babol University of Medical Sciences, Babol, Iran; Department of Clinical and Hospital Pharmacy (M A Mahmoud PhD), Taibah University, Al-Madinah Al-Munawarrah, Saudi Arabia; Department of Psychology (I F Makhdoom PhD), University of Sargodha, Sargodha, Pakistan; Department of Forensic Medicine (Prof V Maled MD), Shri Dharmasthala Manjunatheshwara University, Dharwad, India; Department of Forensic Medicine (Prof V Maled MD), Department of Infectious Diseases and Microbiology (P A Shah MBBS), Rajiv Gandhi University of Health Sciences, Bangalore, India; Department of Internal Medicine (K Malhotra MBBS), Dayanand Medical College and Hospital, Ludhiana, India; Department of Electrical Engineering (I Malik PhD), Prince Sattam

bin Abdulaziz University, Al Kharj, Saudi Arabia; Laboratory of Public Health (Prof L G Mantovani DSc), Instituto Auxologico Italiano IRCCS (Italian Auxological Institute), Milan, Italy; Biomedical Engineering Research Center (CREB) (H Marateb PhD), Universitat Politècnica de Catalunya (Barcelona Tech - UPC), Barcelona, Spain; Department of Artificial Intelligence (H Marateb PhD), Smart University of Medical Sciences, Tehran, Iran; University Health Services (A M Marconi MD), University of Wisconsin- Madison, Madison, WI, USA; Centro de Estudio e Investigación para la prevención y el tratamiento de las adicciones (Center for the Study and Investigation of Addiction Prevention and Treatment) (A M Marconi MD), Universidad de Buenos Aires (University of Buenos Aires), Buenos Aires, Argentina; Non-communicable Diseases Research Center (P Mardi MD), School of Medicine (M Shams-Beyranvand MSc), Alborz University of Medical Sciences, Karaj, Iran; Department of Biochemistry (A Marjani PhD), Joint, Bone, Connective tissue, Rheumatology Research Center (JBCRC) (M Moradzadeh PhD), Golestan Research Center of Gastroenterology and Hepatology (G Roshandel PhD), Golestan University of Medical Sciences, Gorgan, Iran; Department of Economics (Prof G Martinez PhD), Autonomous Technology Institute of Mexico, Mexico City, Mexico; Department of Infectious Diseases (B A Martinez-Guerra MSc), Instituto Nacional de Nutrición Salvador Zubirán (Salvador Zubiran National Institute of Medical Sciences and Nutrition), Mexico City, Mexico; Noncommunicable Diseases and Mental Health Department (R Martinez-Piedra BSc), Pan American Health Organization, Washington, DC, USA; Indonesian Public Health Association, Surabaya, Indonesia (S Martini PhD); Campus Fortaleza (F R Martins-Melo PhD), Federal Institute of Education, Science and Technology of Ceará, Fortaleza, Brazil; Department of Nutrition and Dietetics (M Martorell PhD), Centre for Healthy Living (M Martorell PhD), University of Concepción, Concepción, Chile; Department of Pharmacy (S Maryam PharmD), Bahauddin Zakariya University, Multan, Pakistan; Faculty of Humanities and Health Sciences (Prof R R Marzo MD), Curtin University, Malaysia, Sarawak, Malaysia; Jeffrey Cheah School of Medicine and Health Sciences (Prof R R Marzo MD), School of Pharmacy (Y Wong PhD), Monash University, Subang Jaya, Malaysia; Faculty of Health and Education (A Masaka MSc), Botho University-Botswana, Gaborone, Botswana; North West Lung Centre (A G Mathioudakis PhD), Manchester University NHS Foundation Trust, Manchester, UK; Health Policy Research (M R Mathur PhD), Public Health Foundation of India, Gurugram, India; Department of Medicine (J Mattumpuram MD), University of Louisville, Louisville, KY, USA; Department of Epidemiology (Prof R J Maude PhD), Mahidol Oxford Tropical Medicine Research Unit, Bangkok, Thailand; Research Division (P K Maulik PhD), The George Institute for Global Health, New Delhi, India; Department of Social Medicine and Family (M Mazaheri PhD), Dezful University of Medical Sciences, Dezful, Iran; National Centre for Register-based Research (Prof J J McGrath MD), Aarhus University, Aarhus, Denmark; Australian Centre for Health Services Innovation (Prof S M McPhail PhD), Queensland University of Technology, Kelvin Grove, QLD, Australia; Department of Healthcare (E A Mechili PhD), University of Vlora, Vlora City, Albania; Clinic of Social and Family Medicine (E A Mechili PhD), Laboratory of Toxicology (T K Nikolouzakis PhD), Department of Medicine (Prof A Tsatsakis DSc), University of Crete, Heraklion, Greece; Department of Global Health (J C Medina MD), University of the Ryukyus, Nishihara, Japan; Department of Food Science and Technology (R Mehra PhD), Maharishi Markandeshwar (Deemed to be University), Ambala, India; Department of Public Health (T Mekene Meto MPH), Arba Minch University, Arbaminch, Ethiopia; Department of Medical Oncology and Hematology (M A M Mendez-Lopez PhD), Kantonsspital St. Gallen, St. Gallen, Switzerland; Peru Country Office (W Mendoza MD), United Nations Population Fund (UNFPA), Lima, Peru; Department of Public Health (B Mengist MPH), Department of Epidemiology and Biostatistics (M K Tariku MPH), Public Health Department (T Y Tiruye PhD), Department of Human Nutrition and

Food Sciences (E G Wassie MSc), Debre Markos University, Debre Markos, Ethiopia; International Dx Department (A A Mentis MD), BGI Genomics, Copenhagen, Denmark; Neurology Unit (A Meretoja MD), Breast Surgery Unit (T J Meretoja MD), Helsinki University Hospital, Helsinki, Finland; University Centre Varazdin (T Mestrovic PhD), University North, Varazdin, Croatia; Department of Pharmacology (Prof K D Mettananda PhD), Department of Paediatrics (Prof S Mettananda DPhil), University of Kelaniya, Ragama, Sri Lanka; Clinical Medicine Department (Prof K D Mettananda PhD), North Colombo Teadhing Hospital, Ragama, Sri Lanka; University Paediatrics Unit (Prof S Mettananda DPhil), Colombo North Teaching Hospital, Ragama, Sri Lanka; International Centre for Health Economics, Management, and Policy (P Meylakhs PhD), National Research University Higher School of Economics, St. Petersburg, Russia; Stritch School of Medicine (A Mhlanga PhD), Loyola University Chicago, Chicago, IL, USA; Department of Preventive Medicine (L Mhlanga PhD), Department of Medicine (N S Shah MD), Northwestern University, Chicago, IL, USA; Anaesthesiology Department (G Micha PhD), Helena Venizelou General and Maternity Hospital, Athens, Greece; Department of Epidemiology (I Michalek PhD), National Cancer Registry (I Michalek PhD), Maria Sklodowska-Curie National Research Institute of Oncology, Warsaw, Poland; Pacific Institute for Research & Evaluation, Calverton, MD, USA (T R Miller PhD); Global Institute of Public Health (Prof G Mini PhD), Ananthapuri Hospitals and Research Institute, Trivandrum, India; Department of Medical Sciences (A Mirijello MD), IRCCS Casa Sollievo della Sofferenza General Hospital (IRCCS Home for the Relief of Suffering General Hospital), San Giovanni Rotondo, Italy; Internal Medicine Programme (Prof E M Mirrakhimov PhD), Kyrgyz State Medical Academy, Bishkek, Kyrgyzstan; Department of Atherosclerosis and Coronary Heart Disease (Prof E M Mirrakhimov PhD), National Center of Cardiology and Internal Disease, Bishkek, Kyrgyzstan; Office of the Minster (M K Mirutse MPH), Federal Ministry of Health, Addis Ababa, Ethiopia; Department of Forensic Medicine and Toxicology (C Mittal MD), Dr. B. C. Roy Multi-Specialty Medical Research Centre, Kharagpur, India; Burn and Regenerative Medicine Research Center (Prof M Mobayen MD), Guilan University of Medical Sciences, rasht, Iran; College of Applied and Natural Science (J Mohamed MSc), University of Hargeisa, Hargeisa, Somalia; Department of Internal Medicine (M F H Mohamed MSc), Brown University, Providence, RI, USA; Molecular Biology Unit (N S Mohamed MSc), Bio-Statistical and Molecular Biology Department (N S Mohamed MSc), Sirius Training and Research Centre, Khartoum, Sudan; Department of Public Health (H Mohammed MPH, Y M Tefera MPH), Dire Dawa University, Dire Dawa, Ethiopia; Department of Pharmaceutical Sciences (S Mohammed PhD), Notre Dame of Maryland University, Baltimore, MD, USA; Department of Pharmacy (S Mohammed PhD), Mizan-Tepi University, Mizan, Ethiopia; Health Systems and Policy Research Unit (S Mohammed PhD), Ahmadu Bello University, Zaria, Nigeria; Department of Diabetology (V Mohan DSc), Madras Diabetes Research Foundation, Chennai, India; Department of Diabeteology (V Mohan DSc), Dr. Mohan's Diabetes Specialities Centre, Chennai, India; Institute of Clinical Physiology (S Molinaro PhD), National Research Council, Pisa, Italy; Clinical Epidemiology and Public Health Research Unit (L Monasta DSc, L Ronfani PhD), Burlo Garofolo Institute for Maternal and Child Health, Trieste, Italy; Department of Biomedical and Dental Sciences and Morphofunctional Imaging (Prof S Mondello MD), Messina University, Messina, Italy; Faculty of Medicine (A Moodi Ghalibaf MD), Infectious Diseases Research Center (F Nikoomanesh PhD), Medical Toxicology & Drug Abuse Research Center (M Rezaei MD), Birjand University of Medical Sciences, Birjand, Iran; Department of Epidemiology and Biostatistics (Y Moradi PhD), Social Determinants of Health Research Center (A Shokri PhD), Kurdistan University of Medical Sciences, Sanandaj, Iran; Computer, Electrical, and Mathematical Sciences and Engineering Division (P Moraga PhD), King Abdullah University of Science and Technology,

Thuwal, Saudi Arabia; International Laboratory for Air Quality and Health (Prof L Morawska PhD), School of Public Health and Social Work (N Wang PhD), Queensland University of Technology, Brisbane, QLD, Australia; Department of Public Health (Prof R S Moreira PhD), Oswaldo Cruz Foundation, Recife, Brazil; Department of Public Health (Prof R S Moreira PhD), Federal University of Pernambuco, Recife, Brazil; Department of Biology and Biological Engineering (J Morze PhD), Chalmers University of Technology, Gothenburg, Sweden; College of Medical Sciences (J Morze PhD), SGMK Copernicus University, Warsaw, Poland; Epidemiology Department (S Mousavi MD), Aging Research Institute, Tabriz, Iran; Department of Fruit and Vegetable Product Technology (Prof A Mousavi Khaneghah PhD), Prof. Wacław Dąbrowski Institute of Agricultural and Food Biotechnology State Research Institute, Warsaw, Poland; Department of Health and Biomedical Sciences (E A Mpolya PhD), Nelson Mandela African Institution of Science and Technology, Arusha, Tanzania; Research Department (M Mrejen PhD), Instituto de Estudos para Políticas de Saúde (IEPS), São Paulo, Brazil; Unit of Pharmacotherapy, Epidemiology and Economy (S Mubarik MS), University Medical Center Groningen (Prof M J Postma PhD), Department of Internal Medicine (P Vart PhD), University of Groningen, Groningen, Netherlands; Competence Center of Mortality-Follow-Up of the German National Cohort (R Westerman DSc), Federal Institute for Population Research, Wiesbaden, Germany (Prof U O Mueller MD); Center for Population and Health, Wiesbaden, Germany (Prof U O Mueller MD); School of Medicine (F Mughal FRCGP), Keele University, Keele, UK; Knowledge Management Department (S Mukherjee PhD), Prahlad Omkarwati Foundation (POF), Mumbai, India; Independent Consultant, New Delhi, India (S Mukherjee PhD); Department of Surgery (F Mulita PhD, G Verras MD), General University Hospital of Patras, Patras, Greece; Faculty of Medicine (F Mulita PhD), Department of Internal Medicine (G Ntaios PhD), Department of Emergency Medicine (I Pantazopoulos PhD), University of Thessaly, Larissa, Greece; Clinical Epidemiology Research Unit (E Murillo-Zamora PhD), Mexican Institute of Social Security, Villa de Alvarez, Mexico; Postgraduate in Medical Sciences (E Murillo-Zamora PhD), Universidad de Colima, Colima, Mexico; Research & Innovation Department (Prof K M Musallam MD), Burjeel Medical City, Abu Dhabi, United Arab Emirates; Department of Internal Medicine (A Mustafa MD), Staten Island University Hospital Northwell Health, Staten Island, NY, USA; Department of Pediatrics & Pediatric Pulmonology (Prof G Mustafa MD), Institute of Mother & Child Care, Multan, Pakistan; Prince Fahad bin Sultan Chair for Biomedical Research (S Muthupandian PhD), University of Tabuk, Tabuk, Saudi Arabia; Centre for Infectious Diseases (Prof R Muthusamy PhD), Saveetha Medical College and Hospitals (S R Pandi-Perumal MSc), Centre of Molecular Medicine and Diagnostics (COMManD) (Prof S Patil PhD), Saveetha University, Chennai, India; Department of Neuropsychiatry (W Myung PhD), Department of Food and Nutrition (A P Okekunle PhD), Seoul National University, Seoul, South Korea; Research and Analytics Department (A J Nagarajan MTech), Initiative for Financing Health and Human Development, Chennai, India; Department of Research and Analytics (A J Nagarajan MTech), Bioinsilico Technologies, Chennai, India; Institute of Epidemiology and Medical Biometry (Prof G Nagel PhD), Ulm University, Ulm, Germany; Initiative for Non Communicable Diseases (A Naheed PhD), Maternal and Child Health Division (A Sayeed MSc, M Siraj MSc), Nutrition and Clinical Services Division (M Tariqujjaman MSc), International Centre for Diarrhoeal Disease Research, Bangladesh, Dhaka, Bangladesh; Comprehensive Cancer Center (G Naik MPH), Department of Health Policy & Organization (M Rahim MA), Department of Health Services Administration (M Rahim MA), Department of Psychology (D C Schwebel PhD), University of Alabama at Birmingham, Birmingham, AL, USA; Department of Pulmonary Medicine (S Nair MD), Government Medical College Trivandrum, Trivandrum, India; Health Action by People, Trivandrum, India (S Nair MD); Department of Medical

Laboratory Analysis (H H Najmuldeen PhD), Cihan University Sulaimaniya Research Center (CUSRC) (N H Qasim PhD), Cihan University Sulaymaniya, Sulaymaniyah, Iraq; Suraj Eye Institute, Nagpur, India (V Nangia MD); School of Pharmacy (A Naqvi PhD), University of Reading, Reading, UK; Mysore Medical College and Research Institute (Prof S Narasimha Swamy MD), Government Medical College, Mysore, India; Department of Biotechnology (M Naveed PhD), University of Central Punjab, Lahore, Pakistan; Department of Disease Control and Environmental Health (R Ndejjo MSc), Makerere University, Kampala, Uganda; School of Pharmacy (S O Nduaguba PhD), West Virginia University, Morgantown, WV, USA; Department of General Surgery (I Negoi PhD), Fourth Department of General Surgery (D Serban PhD), Emergency University Hospital Bucharest, Bucharest, Romania; Department of Cardiology (R I Negoi PhD), Cardio-Aid, Bucharest, Romania; Department of Oncology (S Negru MD), Victor Babes University of Medicine and Pharmacy, Timisoara, Romania; Faculty of Medicine (Prof C Nejjari PhD), Euromed University of Fes, Fez, Morocco; Faculty of Medicine (Prof C Nejjari PhD), University Sidi Mohammed Ben Abdellah, Fez, Morocco; Department of Medicine (E Nena MD, P Steiropoulos MD), Democritus University of Thrace, Alexandroupolis, Greece; Department of Community Medicine (S Nepal MD), Kathmandu University, Palpa, Nepal; Department of Histopathology (Prof H A Nggada MD), University of Maiduguri Teaching Hospital, Maiduguri, Nigeria; Department of Public Health (G Nguefack-Tsague PhD), University of Yaoundé I, Yaoundé, Cameroon; Department of Biological Sciences (J W Ngunjiri DrPH), University of Embu, Embu, Kenya; Department of General Medicine (A H Nguyen MD), Thai Binh University of Medicine and Pharmacy, Thai Binh City, Viet Nam; Department of Medical Engineering (D H Nguyen BS), University of South Florida, Tampa, FL, USA; Department of Surgery (P T Nguyen MD), Danang Family Hospital, Danang, Viet Nam; International Islamic University Islamabad, Islamabad, Pakistan (R K Niazi PhD); Institute for Mental Health and Policy (Y T Nigatu PhD), Centre for Addiction and Mental Health, Toronto, ON, Canada; Department of General Surgery (T K Nikolouzakis PhD), University Hospital of Heraklion, Heraklion, Crete, Greece; Public Health Department (D N A Ningrum MPH), Universitas Negeri Semarang (State University of Semarang), Kota Semarang, Indonesia; Center for Public Health (L A Nnyanzi PhD), Teesside University, Middlesbrough, UK; Faculty of Applied Sciences and Technology (E A Noman PhD), Universiti Tun Hussein Onn Malaysia, Johor, Malaysia; Department of Health Policy and Management (S Nomura PhD), Keio University, Tokyo, Japan; Department of Microbiology and Molecular Genetics (M Noreen PhD), The Women University Multan, Multan, Pakistan; Department of Clinical Sciences (Prof B Norrving PhD), Lund University, Lund, Sweden; Department of Paediatrics (C A Nri-Ezedi MD), Nnamdi Azikiwe University, Awka, Nigeria; The Cardiac Clinic (Prof M Ntsekhe PhD), Groote Schuur Hospital, Cape Town, South Africa; Unit of Microbiology and Public Health (V Nuñez-Samudio PhD), Institute of Medical Sciences, Las Tablas, Panama; Department of Public Health (V Nuñez-Samudio PhD), Ministry of Health, Herrera, Panama; Department of Public Health (D Nurrika PhD), Banten School of Health Science, South Tangerang, Indonesia; Ministry of Research, Technology and Higher Education (D Nurrika PhD), Higher Education Service Institutions (LL-DIKTI) Region IV, Bandung, Indonesia; Department of Applied Economics and Quantitative Analysis (Prof B Oancea PhD), University of Bucharest, Bucharest, Romania; Disease Control and Elimination (M A Oboh PhD), Medical Research Council Unit, The Gambia, Banjul, The Gambia; Division of General Internal Medicine (N M Odogwu PhD), Mayo Clinic, Rochester, MN, USA; School of Public Health (N M Odogwu PhD), Department of Epidemiology and Community Health (R R Parikh MD), Department of Surgery (J Rickard MD), University of Minnesota, Minneapolis, MN, USA; Department of Medicine (M J O'Donnell PhD), National University of Ireland - Galway, Galway, Ireland; PSSM Data Sciences, Pfizer Research &

Development (M Oduro PhD), Pfizer Inc., Groton, CT, USA; Department of Preventive Medicine (I Oh PhD), Department of Pediatrics (Prof D Yon MD), Kyung Hee University, Seoul, South Korea; Independent Consultant, Sydney, NSW, Australia (S R Okeke PhD); Department of Nursing Science (M I Olatubi PhD), Bowen University, Iwo, Nigeria; Cardiology Department (G M M Oliveira PhD), Federal University of Rio de Janeiro, Rio de Janeiro, Brazil; Slum and Rural Health Initiative Research Academy (I I Olufadewa MHS), Slum and Rural Health Initiative, Ibadan, Nigeria; Centre for Healthy Start Initiative, Lagos, Nigeria (B O Olusanya PhD, J O Olusanya MBA); Department of Pharmacology and Toxicology (Prof H A Omar PhD), Beni-Suef University, Beni-Suef, Egypt; Surgery Department (G L Omer MD), Sulaimani University, Sulaimani, Iraq; ENT Department (G L Omer MD), Tor Vergata University of Rome, Rome, Italy; School of Public Health (M A Ondayo PhD), Moi University, Eldoret, Kenya; Department of Environmental Health and Biology (M A Ondayo PhD), University of Eldoret, Eldoret, Kenya; Non-communicable Disease Prevention Unit (S Ong FAMS), Ministry of Health, Bandar Seri Begawan, Brunei; Early Detection & Cancer Prevention Services (S Ong FAMS), Pantai Jerudong Specialist Centre, Bandar Seri Begawan, Brunei; Department of Pharmacology and Therapeutics (Prof O E Onwujekwe PhD), University of Nigeria Nsukka, Enugu, Nigeria; Department of Biomedical Sciences (K I Onyedibe PhD), Mercer University School of Medicine, Macon, GA, USA; Department of Pharmacotherapy and Pharmaceutical Care (M Ordak PhD), Department of Biochemistry and Pharmacogenomics (M Zielińska MPharm), Medical University of Warsaw, Warsaw, Poland; University of Port Harcourt, Port Harcourt, Nigeria (Prof O E Orisakwe PhD); Sickle Cell Unit (V N Orish PhD), Ho Teaching Hospital, Ho Municipality, Ghana; Department of Nephrology and Hypertension (Prof A Ortiz MD), The Institute for Health Research Foundation Jiménez Díaz University Hospital, Madrid, Spain; , Department of Biology (W M S Osman PhD), Khalifa University, Abu Dhabi, United Arab Emirates; School of Medicine (U L Osuagwu PhD), Translation Health Research Institute (K Rana PhD), Western Sydney University, Campbelltown, NSW, Australia; Department of Optometry and Vision Science (U L Osuagwu PhD), University of KwaZulu-Natal, KwaZulu-Natal, South Africa; Laboratory of Public Health Indicators Analysis and Health Digitalization (N Otstavnov BA, S S Otstavnov PhD), Department of Information Technologies and Management (S K Vladimirov PhD), Moscow Institute of Physics and Technology, Dolgoprudny, Russia; Department of Project Management (S S Otstavnov PhD), Department of Health Care Administration and Economics (Prof V Vlassov MD), National Research University Higher School of Economics, Moscow, Russia; Division of Infectious Diseases (Prof A Ouyahia PhD), University Hospital of Setif, Setif, Algeria; Department of General Surgery (G Ouyang MD), Central South University, ChangSha, China; Department of Respiratory Medicine (Prof M P P A DNB), Department of Oral and Maxillofacial Surgery (C S N PhD), Jagadguru Sri Shivarathreeswara University, Mysore, India; Department of Forensic Medicine and Toxicology (J Padubidri MD), Kasturba Medical College, Mangalore, Mangalore, India; Department of Neurology (Prof P K Pal DM), National Institute of Mental Health and Neurosciences, Bengalore, India; Research Institute for Medicines (C Palladino PhD, Prof J Perdigão PhD, Prof N Taveira PhD), Universidade de Lisboa (University of Lisbon), Lisbon, Portugal; Department of Public Health (R Palladino MD), University of Naples Federico II, Naples, Italy; Department of Mental Health (R F Palma-Alvarez PhD), Hospital Universitari Vall d'Hebron (Vall d'Hebron University Hospital), Barcelona, Spain; Department of Psychiatry, Mental Health and Addictions (R F Palma-Alvarez PhD), Vall d'Hebron Institut de Recerca (Vall d'Hebron Research Institute), Barcelona, Spain; Department of Epidemiology and Biostatistics (Prof H Pan PhD), Anhui Medical University, Hefei, China; Department of Public Health (A Pana PhD), Babes Bolyai University, Cluj Napoca, Romania; Department of Health Metrics (A Pana PhD), Center for Health Outcomes & Evaluation, Bucharest,

Romania; School of Public Health (P Panda MPH), Asian Institute of Public Health University, Bhubaneswar, India; Privatpraxis, Heidelberg, Germany (S Panda-Jonas MD); Division of Research and Development (S R Pandi-Perumal MSc), Lovely Professional University, Phagwara, India; Department of Ophthalmology (G D Panos PhD), Nottingham University Hospitals Queen's Medical Centre Campus, Nottingham, UK; Division of Ophthalmology & Visual Sciences (G D Panos PhD), People in Psychiatry and Applied Psychology (F Shokraneh PhD), University of Nottingham, Nottingham, UK; Department of Science and Mathematics (Prof P Papadopoulou PhD), Deree-The American College of Greece, Athens, Greece; Research Center (A Parthasarathi MD), Allergy Asthma and Chest Center, Mysore, India; Department of Medical Sciences (R Passera PhD), University of Torino, Torino, Italy; Department of Imaging (R Passera PhD), AOU Città della Salute e della Scienza di Torino, Torino, Italy; Cardiology Department (D Pasupula MD), MercyOne North Iowa Medical Center, Mason City, IA, USA; Department of Physiotherapy (H M Patel PhD), Charotar University of Science and Technology, Anand, India; School of Dentistry (J Patel BSc), University of Leeds, Leeds, UK; Department of Poverty, Gender and Youth (S K Patel PhD), Population Council, New Delhi, India; College of Dental Medicine (Prof S Patil PhD), Roseman University of Health Sciences, South Jordan, UT, USA; Second Department of Internal Medicine (D Patoulias PhD), European Interbalkan Medical Center, Thessaloniki, Greece; Department of Internal Medicine (V Patthipati MD), Advent Health, Palm Coast, FL, USA; Department of Hospital Medicine (V Patthipati MD), Sound Physicians, Palm Coast, FL, USA; Clinical Research Department (P Pedersini MSc, J H Villafañe PhD), IRCCS Fondazione Don Carlo Gnocchi, Milan, Italy; Department of Neurology (U Pensato MD), IRCCS Humanitas Research Hospital, Milan, Italy; International Institute for Educational Planning (IIEP) (Prof M F P Peres MD), Albert Einstein Hospital, São Paulo, Brazil; Department of Development Studies (Prof A Perianayagam PhD), Department of Fertility Studies (U Sahoo PhD), International Institute for Population Sciences, Mumbai, India; Mario Negri Institute for Pharmacological Research, Bergamo, Italy (N Perico MD, Prof G Remuzzi MD); Pennsylvania Cancer and Regenerative Medicine Center (R G Pestell MD), Baruch S Blumberg Institute, Doylestown, PA, USA; Department of Medicine (R G Pestell MD), Xavier University School of Medicine, Woodbury, NY, USA; Facultad de Medicina (F E Petermann-Rocha PhD), Universidad Diego Portales (Diego Portales University), Santiago, Chile; School of Cardiovascular and Metabolic Health (F E Petermann-Rocha PhD), University of Glasgow, Glasgow, UK; School of Medicine (W A Petri MD), University of Virginia, Charlottesville, VA, USA; School of Medicine (H Pham MD), Department of Epidemiology (H K Tang PhD), Pham Ngoc Thach University of Medicine, Ho Chi Minh City, Viet Nam; Shanghai Mental Health Center (Prof M R Phillips MD), Shanghai Jiao Tong University, Shanghai, China; Department of Psychiatry (Prof M R Phillips MD), Department of Neurology (Prof N Scarmeas PhD), Columbia University, New York, NY, USA; National Centre for Disease Prevention and Health Promotion (D Pierannunzio PhD), National Institute of Health, Roma, Italy; Department of Pediatric Orthopedic Surgery (M Pigeolet MD), Hôpital Necker - Enfants Malades, Paris, France; International Center of Medical Sciences Research, Islamabad, Pakistan (Z Z Piracha PhD); Department of Neonatology (N Plakkal MD), Jawaharlal Institute of Postgraduate Medical Education and Research, Puducherry, India; Research School of Chemistry and Applied Biomedical Sciences (E Plotnikov PhD), Tomsk Polytechnic University, Tomsk, Russia; Mental Health Research Institute (E Plotnikov PhD), Tomsk National Research Medical Center of the Russian Academy of Sciences, Tomsk, Russia; Medical College (V Podder HSC), Tairunnessa Memorial Medical College and Hospital, Gazipur, Bangladesh; Clinical Academic Department of Pediatrics (Prof D Poddighe PhD), University Medical Center (UMC), Astana, Kazakhstan; Department of Data Management and Analysis (R Poluru PhD), The INCLEN Trust International,

New Delhi, India; Department of Ortopedics and Traumatology (V T Ponkilainen PhD), University of Tampere, Tampere, Finland; Non-communicable Diseases Research Center (N Pourtaheri PhD), Bam University of Medical Sciences, Bam, Iran; Centro de Investigaciones Clinicas (Clinical Research Center) (S I Prada PhD), Fundación Valle del Lili (Valle del Lili Foundation), Cali, Colombia; Universidad ICESI, (S I Prada PhD); Division of Medical Oncology and Hematology (Prof T N Prakasham DM), Department of Dermatology venereology and leprosy-DVL (Prof T PRISCILLA MD), Apollo Institute of Medical Sciences and Research, Hyderabad, India; Department of Clinical Research and Epidemiology (M Prasad MD), Institute of Liver and Biliary Sciences, New Delhi, New Delhi, India; Department of Biochemistry (Prof A Prashant PhD), Jagadguru Sri Shivarathreeswara University, Mysuru, India; Department of Cardiology (G Qian MS), Third Military Medical University, Chongqing, China; The Department of Gynecology (S Qiu MD), Sun Yat-sen University, Guangzhou, China; Medical Sciences Education Department (M Qureshi MD), Western University of Health Sciences, Pomona, CA, USA; Cardiovascular Research Center (M Rabiee Rad MD), Isfahan Cariovascular Research Institute, Isfahan, Iran; College of Medicine (A Radfar MD), University of Central Florida, Orlando, FL, USA; Department of Medical Oncology (Prof V Radhakrishnan MD), Cancer Institute (W.I.A), Chennai, India; UO Neurologia, Salute Pubblica e Disabilità (The Neurology, Public Health and Disability Unit) (A Raggi PhD), Fondazione IRCCS Istituto Neurologico Carlo Besta (IRCCS Foundation Carlo Besta Neurological Institute), Milan, Italy; Pathology Department (N Raheem FMCPath), Mobiddo Adama University Teaching Hospital -Yola, Yola, Nigeria; Department of Health Sciences (Prof F Rahim PhD), Cihan University-Sulaymaniyah, Sulaymaniyah, Iraq; Cihan University Sulaimaniya Research Center (CUSRC), Sulaymaniyah, Iraq (Prof F Rahim PhD); Institute of Health and Wellbeing (M Rahman PhD), Federation University Australia, Berwick, VIC, Australia; Future Technology Research Center (A Rahmani PhD), National Yunlin University of Science and Technology, Yunlin, Taiwan; Department of Public Health (V Rahmanian PhD), Torbat Jam Faculty of Medical Sciences, Torbat Jam, Iran; Department of Community Medicine (S Rajaa MD), Employees' State Insurance Model Hospital, Chennai, India; Centre for Chronic Disease Control, New Delhi, India (P Rajput PhD); European Office for the Prevention and Control of Noncommunicable Diseases (I Rakovac PhD), World Health Organization (WHO), Moscow, Russia; Research Department (C L Ranabhat PhD), Science, Technology and Natural Resources Department (S Tandukar PhD), Policy Research Institute, Kathmandu, Nepal; Health and Public Policy Department (C L Ranabhat PhD), Global Center for Research and Development, Kathmandu, Nepal; Centre for Clinical Pharmacology (N Rancic PhD), University of Defence in Belgrade, Belgrade, Serbia; Centre for Clinical Pharmacology (N Rancic PhD), Medical College of Georgia at Augusta University, Belgrade, Serbia; Health Economics and Outcomes Research Department (A Rane MS), Agios Pharmaceuticals, Cambridge, MA, USA; Department of Pharmaceutical Economics and Policy (A Rane MS), Massachusetts College of Pharmacy and Health Sciences, Boston, MA, USA; Department of Oral Pathology (S Rao MDS), Sharavathi Dental College and Hospital, Shimogga, India; Data Analytic Services (D P Rasali PhD), British Columbia Centre for Disease Control, Vancouver, BC, Canada; University of Social Welfare and Rehabilitation Sciences, Tehran, Iran (V Rashedi PhD); Department of Geography (A Rasul PhD), Soran University, Soran, Iraq; Section of Pulmonary and Critical Care Medicine (N Ravikumar MD), University of Chicago, Chicago, IL, USA; Inovus Medical, St Helens, UK (D L Rawaf MRCS); Academic Public Health England (Prof S Rawaf MD), Public Health England, London, UK; School of Health, Medical and Applied Sciences (L Rawal PhD), CQ University, Sydney, NSW, Australia; Internal Medicine Department (B Rawlley MD), State University of New York, Syracuse, NY, USA; Department of Biological Sciences (Prof E M M Redwan PhD), King Abdulaziz University, Jeddah, Egypt;

Department of Protein Research (Prof E M M Redwan PhD), Research and Academic Institution, Alexandria, Egypt; Grenoble Computer Science Laboratory (LIG) (F Rehman PhD), University of Grenoble Alpes, Grenoble, France; Unisabana Center for Translational Science (L F Reyes PhD), Universidad de La Sabana (Savannah University), Chia, Colombia; Critical Care Department (L F Reyes PhD), Clinica Universidad De La Sabana (Savannah University Clinic), Chia, Colombia; Department of Public Health Sciences (T G Rhee PhD), University of Connecticut, Farmington, CT, USA; Department of Surgery (J Rickard MD), University Teaching Hospital of Kigali, Kigali, Rwanda; Department of Medical Education (H R Riva MPAS), Texas Tech University, El Paso, TX, USA; Department of Dermatology (H R Riva MPAS), University of Colorado Denver, Aurora, CO, USA; 1H-TOXRUN - One Health Toxicology Research Unit (Prof C F Rodrigues PhD), Instituto Universitário de Ciências da Saúde (CESPU), Paredes, Portugal; Department of Clinical Research (L Roever PhD), Federal University of Uberlândia, Uberlândia, Brazil; Institute for Health Metrics and Evaluation (E L B Rogowski MPH), University of Washington, Seattle, USA; Center for Indigenous Health Research (P Rohloff MD), Wuqu' Kawoq Maya Health Alliance, Tecpan, Guatemala; Faculty of Nursing (D S Romadlon PhD), Chulalongkorn University, Bangkok, Thailand; Clinical and Epidemiological Research in Primary Care (GICEAP) (E Romero-Rodríguez PhD), Maimonides Biomedical Research Institute of Cordoba (IMIBIC), Cordoba, Spain; Maurizio Bufalini Hospital, Cesena, Italy (M Romoli MD); Department of Analytical and Applied Economics (Prof H S Rout PhD, C K Swain MPhil), UGC Centre of Advanced Study in Psychology (M Satpathy PhD), Utkal University, Bhubaneswar, India; Department of Labour (P Roy PhD), Directorate of Factories, Government of West Bengal, Kolkata, India; Centro de Investigación Palmira (Palmira Research Center) (E Rubagotti PhD), Corporación Colombiana de Investigación Agropecuaria AGROSAVIA (Colombian Agricultural Research Corporation), Bogota, Colombia; Advanced Campus Governador Valadares (Prof G d Ruela MSc), Juiz de For a Federal University, Governador Valadares, Brazil; Nursing Department (Prof G d Ruela MSc), Universidade Presidente Antônio Carlos (President Antônio Carlos University), Governador Valadares, Brazil; Department of Health Statistics (S F Rumisha PhD), National Institute for Medical Research, Dar es Salaam, Tanzania; Department of Cardiology and Internal Medicine (Prof A Rynkiewicz PhD), University of Warmia and Mazury, Olsztyn, Poland; Department of Medical Pharmacology (M M Saber-Ayad MD), Public Health and Community Medicine Department (M R Salem MD), Cairo University, Giza, Egypt; Faculty of Computing and Informatics (M SaberiKamarposhti PhD), Multimedia University, Cyberjaya, Malaysia; Neuropsychiatric Institute (Prof P S Sachdev MD), Prince of Wales Hospital, Randwick, NSW, Australia; Department of Cardiology (R Sachdeva MD), Department of Veterans Affairs, Decatur, GA, USA; Department of Cardiology (R Sachdeva MD), Medical College of Georgia at Augusta University, Augusta, GA, USA; Department of Pharmaceutical Chemistry (Prof M R Saeb PhD), Medical University of Gdańsk, Gdańsk, Poland; Multidisciplinary Laboratory Foundation University School of Health Sciences (FUSH) (Prof U Saeed PhD), Foundation University, Islamabad, Pakistan; International Center of Medical Sciences Research (ICMSR), Islamabad, Pakistan (Prof U Saeed PhD); Faculty of Medicine, Bioscience and Nursing (S Z Safi PhD), MAHSA University, Selangor, Malaysia; Interdisciplinary Research Centre in Biomedical Materials (IRCBM) (S Z Safi PhD), COMSATS Institute of Information Technology, Lahore, Pakistan; Department of Community Medicine and Family Medicine (S S Sahoo MD, M Verma MD), Department of Anatomy (A Singal PhD), Department of Radiodiagnosis (P Singh MD), All India Institute of Medical Sciences, Bathinda, India; Department of Preventive & Social Medicine (M Sahu MD), All India Institute of Hygiene & Public Health, Kolkata, India; Department of Statistics (M R Sajid PhD), University of Gujrat, Pakistan, Gujrat, Pakistan; Institute for Employment Research, Nuremberg,

Germany (J W Sakshaug PhD); Technology Management Department (Prof M Z Y Salem PhD), University College of Applied Sciences, Gaza, Palestine; School of Economics and Management (Prof M Z Y Salem PhD), University of Kassel, Kassel, Germany; Department of Neurology (S Samadzadeh MD), University of Southern Denmark, Odense, Denmark; Policy and Epidemiology Group (D F Santomauro PhD), Queensland Centre for Mental Health Research, Wacol, QLD, Australia; Pharmacy Study Program (M A Sarasmita PharmD), Udayana University, Badung, Indonesia; Independent Consultant, Thiruvananthapuram, India (S Y Saraswathy PhD); Indira Gandhi Medical College and Research Institute, Puducherry, India (A Saravanan MD); Department of Orthpaedics and Trauma Surgery (B Saravi PhD), University of Freiburg, Freiburg, Germany; Department of Orthopaedics (B Saravi PhD), Loretto Hospital Freiburg, Freiburg, Germany; Department of Food Processing Technology (T Sarkar PhD), West Bengal State Council of Technical Education, Malda, India; Department of Health and Society (Prof R Sarmiento-Suárez MPH), University of Applied and Environmental Sciences, Bogota, Colombia; National School of Public Health (Prof R Sarmiento-Suárez MPH), Carlos III Health Institute, Madrid, Spain; Faculty of Health & Social Sciences (B Sathian PhD), Bournemouth University, Bournemouth, UK; Udyam-Global Association for Sustainable Development, Bhubaneswar, India (M Satpathy PhD); National Centre for Epidemiology and Population Health (M Sayeed MS, A Talukder MSc), Australian National University, Acton, ACT, Australia; Market Access Division (M Saylan MD), Bayer, Istanbul, Turkiye; Department of Neurology (Prof N Scarmeas PhD), National and Kapodistrian University of Athens, Athens, Greece; Dobney Hypertension Centre (Prof M P Schlaich MD), University of Western Australia, Perth, WA, Australia; Hypertension and Kidney Disease Laboratory (Prof M P Schlaich MD), Baker Heart and Diabetes Institute, Melbourne, VIC, Australia; Department of Health Sciences (I J C Schneider PhD), Federal University of Santa Catarina, Araranguá, Brazil; Department of Cardiovascular Sciences (A Schuermans BSc, J Van den Eynde BSc), Katholieke Universiteit Leuven, Leuven, Belgium; Cardiovascular Program (X Xu PhD), The George Institute for Global Health, Sydney, NSW, Australia (Prof A E Schutte PhD, Prof J Sundström PhD); Department of Methodology and Innovation in Prevention (M Schwarzinger MD), University Hospital of Bordeaux, France, Bordeaux, France; University of Bordeaux (M Schwarzinger MD), The National Institute of Health and Medical Research (Inserm), Bordeaux, France; Clinic for Conservative Dentistry and Periodontology (Prof F Schwendicke PhD), University Hospital of the Ludwig-Maximilians-University Munich, Munich, Germany; Department of Medical Statistics (M Šekerija PhD), University of Zagreb, Zagreb, Croatia; Department of Epidemiology and Prevention of Chronic Noncommunicable Diseases (M Šekerija PhD), Croatian Institute of Public Health, Zagreb, Croatia; Faculty of Dentistry (S Selvaraj PhD), AIMST University, Bedong, Malaysia; Emergency Department (S Senthilkumaran MD), Manian Medical Centre, Erode, India; Department of Medicine and Surgery (Y Sethi MBBBS), Government Doon Medical College, Dehradun, India; Center for Biomedical Information Technology (F Sha PhD), Shenzhen Institutes of Advanced Technology, Shenzhen, China; Department of Community Health (M Shabany PhD), Aja University of medical sciences, Tehran, Iran; HepatoPancreatoBiliary Surgery and Liver Transplant Department (P A Shah MBBS), Healthcare Global Limited Cancer Care Hospital, Bangalore, India; Division of Preventive Cardiology (I Shahid MBBS), Houston Methodist Academic Institute, Houston, TX, USA; Department of Chemistry (H Shahsavari PhD), Institute for Advanced Studies in Basic Sciences (IASBS), Zanjan, Iran; Independent Consultant, Karachi, Pakistan (M A Shaikh MD); Department of Pathology and Laboratory Medicine (S Sham MD), Northwell Health, New York, NY, USA; Research Institute of Pharmaceutical Sciences (H Shamshad PhD), International Center for Chemical and Biological Sciences (S Ullah MSc), University of Karachi, Karachi, Pakistan; Department of Clinical Review and

Safety (S Sharfaei MD), Baim Institute for Clinical Research, Boston, MA, USA; Facultad de Medicine (Faculty of Medicine) (J Sharifi-Rad PhD), Universidad del Azuay (University of Azuay), Cuenca, Ecuador; Department of Physiotherapy (S Sharma PhD), Kathmandu University, Dhulikhel, Nepal; Department of Microbiology (R P Shastry PhD), Yenepoya University, Mangalore, India; Department of Engineering (A Shavandi PhD), Free University of Brussels, Brussels, Belgium; Department of Ophthalmology (M Shayan MD), Harvard Medical School, Boston, MA, USA; Psychology Department (J Shen PhD), University of Massachusetts Lowell, Boston, MA, USA; Friedman School of Nutrition Science and Policy (P Shi PhD), Tufts University, Boston, MA, USA; Tokyo Foundation for Policy Research, Tokyo, Japan (Prof K Shibuya MD); Department of Public Health (D Shiferaw MPH), Dambi Dollo University, Dembi Dollo, Ethiopia; National Institute of Infectious Diseases, Tokyo, Japan (M Shigematsu PhD); Department of Pediatrics (Prof Y Shin PhD), CHA University, Seoul, South Korea; Department of Pediatrics (Prof Y Shin PhD), CHA Gangnam Medical Center, Seoul, South Korea; Finnish Institute of Occupational Health, Helsinki, Finland (R Shiri PhD); Department of Clinical Immunology and Hematology (V Shivarov PhD), Sofiamed University Hospital, Sofia, Bulgaria; Department of Genetics (V Shivarov PhD), Sofia University "St. Kliment Ohridiski", Sofia, Bulgaria; Department of Public Health and Primary Care (F Shokraneh PhD, Prof P Willeit PhD), Yusuf Hamied Department of Chemistry (H Z Sun PhD), University of Cambridge, Cambridge, UK; School of Pharmacy (S Shrestha PharmD), Monash University, Selangor Darul Ehsan, Malaysia; The Cooper Institute, Dallas, TX, USA (K Shuval PhD); Department of Medical Microbiology and Infectious Diseases (E E Siddig MD), Erasmus University, Rotterdam, Netherlands; Center of Potential and Innovation of Natural Resources (Prof L M R Silva PhD), Polytechnic Institute of Guarda, Guarda, Portugal; Health Sciences Research Centre (Prof L M R Silva PhD), University of Beira Interior, Covilhã, Portugal; School of Health (Prof C R Simpson PhD), Victoria University of Wellington, Wellington, New Zealand; Department of Dentistry (A Singh MD), All India Institute of Medical Sciences, Bhopal, India; School of Public Health & Zoonoses (B B Singh PhD), Guru Angad Dev Veterinary & Animal Sciences University, Ludhiana, India; Department of Community Medicine (G Singh MD), Lady Hardinge Medical College, New Delhi, India; Department of Paediatrics (J Singh MD), All India Institute of Medical Sciences, Bilaspur, India; Department of Epidemiology (D N Sinha PhD), School of Preventive Oncology, Patna, India; Department of Epidemiology (D N Sinha PhD), Healis Sekhsaria Institute for Public Health, Mumbai, India; Department of Internal Medicine (R Sinto MD), University of Indonesia, Jakarta Pusat, Indonesia; Department of Internal Medicine (R Sinto MD), Dr. Cipto Mangunkusumo National Hospital, Jakarta Pusat, Indonesia; Clinical Branch (V Y Skryabin MD), Moscow Research and Practical Centre on Addictions, Moscow, Russia; Addiction Psychiatry Department (V Y Skryabin MD), Russian Medical Academy of Continuous Professional Education, Moscow, Russia; Department of Infectious Diseases and Epidemiology (A A Skryabina MD), Department of Internal Disease (A V Starodubova DSc), Pirogov Russian National Research Medical University, Moscow, Russia; Division of Injury Prevention (Prof D A Sleet PhD), The Bizzell Group, Atlanta, GA, USA; Department of Surgery (B Socea PhD), "Sf. Pantelimon" Emergency Clinical Hospital Bucharest, Bucharest, Romania; Department of Infectious Diseases (A Sokhan PhD), Kharkiv National Medical University, Kharkiv, Ukraine; Department of Systemic Pathology (R Solanki MD), Touro College of Osteopathic Medicine, Middletown, NY, USA; Department of Pathology (R Solanki MD), American University of the Caribbean School of Medicine, Cupecoy, Saint Martin; Department of Biochemistry (S Solanki MD), Other, Barbados; Department of Health Policy and Management (S Song PhD), University of Georgia College of Public Health, Athens, GA, USA; Centro de Investigación Biomédica en Red Enfermedades Respiratorias (CIBERES) (Center for Biomedical Research in

Respiratory Diseases Network), Madrid, Spain (Prof J B Soriano MD); Hull York Medical School (I N Soyiri PhD), University of Hull, Hull City, UK; Division of Community Medicine (C T Sreeramareddy MD), International Medical University, Kuala Lumpur, Malaysia; Amity Institute of Biotechnology (V K Srivastava PhD), Amity University Rajasthan, Jaipur, Jaipur, India; Department of Biological Sciences (V K Srivastava PhD), Indian Institute of Science Education and Research, Bhopal, Bhopal, India; Public Health Department (M Stanikzai MPH), Kandahar University, Kandahar, Afghanistan; Department of Pediatric Cardiology (J R Starnes MD), Vanderbilt University Medical Center, Nashville, TN, USA; Department of Research and Learning (J R Starnes MD), Lwala Community Alliance, Rongo, Kenya; Nutrition and Dietetics Department (A V Starodubova DSc), Federal Research Institute of Nutrition, Biotechnology and Food Safety, Moscow, Russia; Institute for Health Metrics and Evaluation (J D Steinmetz PhD), University of Washington, Seattle, WA, USA; Occupational and Environmental Medicine Department (L Stockfelt PhD), Institute of Neuroscience and Physiology (Prof K S Sunnerhagen PhD), Institute of Health and Care Sciences (Prof A W Wolf PhD), University of Gothenburg, Gothenburg, Sweden; School of Medicine (V Subramaniyan PhD), Monash University, Sunway, Malaysia; School of Life Sciences (M Suleman PhD), Xiamen University, China, Xiamen, China; National Institute of Epidemiology (R Suliankatchi Abdulkader MD), Indian Council of Medical Research, Chennai, India; Mental Health Research (A Sultana MD), Independent Consultant, Khulna, Bangladesh; Division of Global Mental Health (A Sultana MD), EviSyn Health, Khulna, Bangladesh; Rural Health Research Institute (Prof J Sun PhD), Charles Sturt University, Bathurst, NSW, Australia; Institute of Integrated Intelligence and Systems (Prof J Sun PhD), Griffith University, QLD, Australia; Department of Neurocare (Prof K S Sunnerhagen PhD), Sabzevar University of Medical Sciences, Gothenburg, Sweden; Department of Clinical Outcomes (Prof L Szarpak PhD), Maria Sklodowska-Curie Medical Academy, Warsaw, Poland; Department of Clinical Research and Development (Prof L Szarpak PhD), LUXMED Group, Warsaw, Poland; Department of Dermatology (M D Szeto BS), University of Colorado, Aurora, CO, USA; Department of Neurology (P Tabaee Damavandi MD), Neurocenter of Southern Switzerland (NSI), Lugano, Switzerland; Department of Medicine (Prof R Tabarés-Seisdedos PhD), University of Valencia, Valencia, Spain; Carlos III Health Institute (Prof R Tabarés-Seisdedos PhD), Biomedical Research Networking Center for Mental Health Network (CiberSAM), Madrid, Spain; Department of Basic Medical Sciences (S Tabatabaeizadeh PhD), Department of Internal Medicine (S Tabatabaeizadeh PhD), Islamic Azad University, Mashhad, Iran; Dentistry and Oral Health, Rural Clinical Sciences (J Tadkamadla PhD), La Trobe University, Bendigo, VIC, Australia; School of Dentistry and Oral Health (S K Tadakamadla PhD), Griffith University, Gold Coast, QLD, Australia; Living Systems Institute (Y Taheri Abkenar PharmD), Department of Health and Community Sciences (A Udoh PhD), University of Exeter, Exeter, UK; Department of Biostatistics and Epidemiology (M Taheri Soodejani PhD), Shahid Sadoughi University of Medical Sciences, Yazd, Iran; University of Western Australia, Perth, NSW, Australia (Prof K Takahashi PhD); University of Occupational and Environmental Health, Kitakyushu, Japan (Prof K Takahashi PhD); Statistics Discipline (A Talukder MSc), Khulna University, Khulna, Bangladesh; Department of Dermato-Venereology (M Tampa PhD), Dr. Victor Babes Clinical Hospital of Infectious Diseases and Tropical Diseases, Bucharest, Romania; Department of Medicine (J L Tamuzi MSc), Northlands Medical Group, Omuthiya, Namibia; State Key Laboratory of Numerical Modeling for Atmospheric Sciences and Geophysical Fluid Dynamics (LASG) (H Tang PhD), Chinese Academy of Sciences, Beijing, China; University Institute "Egas Moniz", Monte da Caparica, Portugal (Prof N Taveira PhD); Health Management Department (R Tesler PhD), Ariel University, Ariel, Israel; Department of Psychology (E Teye-Kwadjo PhD), University of Ghana, Legon, Ghana; Wellbeing Preventable and Chronic

Diseases Division (R Thakur PhD), Menzies School of Health Research, Alice Springs, NT, Australia; Charles Darwin University, Alice Springs, NT, Australia (R Thakur PhD); Department of Pharmacology (P Thangaraju MD), All India Institute of Medical Sciences, Raipur, India; Public Health Department (Prof K R Thankappan MD), Amrita Institute of Medical Sciences, Kochi, India; Institute of Applied Health Research (R Thayakaran PhD), University of Birmingham, Birmingham, UK, UK; Department of Gastroenterology (N K Thomas MD), PSG Institute of Medical Sciences and Research, Coimbatore, India; Department of Psychiatry (C C Thum MB), Hospital Sultan Abdul Aziz Shah Universiti Putra Malaysia, Serdang, Malaysia; National Institute of Public Health (Prof L C Thygesen PhD), University of Southern Denmark, Copenhagen, Denmark; Faculty of Biomedical Engineering (A Tichopad PhD), Czech Technical University, Prague, Czech Republic; Faculty of Public Health (J H V Ticoalu MPH), Universitas Sam Ratulangi, Manado, Indonesia; Laboratory of Public Health Indicators Analysis and Health Digitalization (M V Titova PhD), Moscow Institute of Physics and Technology, Moscow, Russia; Department of Medicine (Prof M Tonelli MD), Department of Oncology (L Yang PhD), University of Calgary, Calgary, AB, Canada; Institute of Public Health (R Topor-Madry PhD), Jagiellonian University Medical College, Kraków, Poland; Agency for Health Technology Assessment and Tariff System, Warsaw, Poland (R Topor-Madry PhD); Nutritional Epidemiology Research Team (EREN) (M Touvier PhD), National Institute for Health and Medical Research (INSERM), Paris, France; SRM College of Pharmacy (M R Tovani-Palone PhD), SRM Institute of Science and Technology (SRMIST), Chennai, India; Department of Health (N M Tran MD), Children's Hospital 1, Ho Chi Minh City, Viet Nam; Department of Surgical, Medical, Molecular Pathology and Critical Care Medicine (D Trico MD), University of Pisa, Pisa, Italy; Adult Learning Disability Service (S J Tromans PhD), Leicestershire Partnership National Health Service Trust, Leicester, UK; School of Medicine (T T Truyen MD), Nam Can Tho University, Can Tho, Viet Nam; Environmental Sciences Program (S Uddin PhD), Asian University for Women, Chittagong, Bangladesh; Department of Geography (S Uddin PhD), University of Victoria, Victoria, BC, Canada; Department of Rehabilitation and Health Sciences (I Ullah PhD), Iqra University, Islamabad, Pakistan; Department of Zoology (S Ullah PhD), Division of Science and Technology (S Ullah PhD), University of Education, Lahore, Lahore, Pakistan; Department of Paraclinical Sciences (S Umakanthan MD), The University of the West Indies, St. Augustine, Trinidad and Tobago; Department of Community Medicine (C D Umeokonkwo MPH), Alex Ekwueme Federal University Teaching Hospital Abakaliki, Abakaliki, Nigeria; Institute of Health and Wellbeing (Prof C A Unsworth PhD), Federation University Australia, Churchill, VIC, Australia; Center for Neurodegenerative Diseases and the Aging Brain (D Urso MD), University of Bari, Tricase, Italy; Department of Physiotherapy (J S Usman PhD), Bayero University, Kano, Kano, Nigeria; College of Health and Sport Sciences (A G Vaithinathan MSc), University of Bahrain, Salmanya, Bahrain; Department of Social Sciences (Prof S M Van de Velde PhD), Universiteit Antwerpen, Antwerpen, Belgium; Department of Public Health and Epidemiology (O Varga PhD), University of Debrecen, Debrecen, Hungary; UKK Institute, Tampere, Finland (Prof T J Vasankari MD); Faculty of Medicine and Health Technology (Prof T J Vasankari MD), Tampere University, Tampere, Finland; Institute of Public Health of Serbia, Belgrade, Serbia (M Vasic PhD); Department of Human Genetics & Molecular Biology (B Vellingiri PhD), Bharathiar University, Coimbatore, India; Raffles Neuroscience Centre (Prof N Venketasubramanian MBBS), Raffles Hospital, Singapore, Singapore; Department Pediatric Hematology and Oncology (G I Villanueva MD), Hospital de Clinicas Jose de San Martin (Jose de San Martín Clinical Hospital), Cuidad Autonoma de Buenos Aires, Argentina; Occupational Health Unit (Prof F S Violante MD), Sant'Orsola Malpighi Hospital, Bologna, Italy; Department of Molecular Epidemiology (S K Vladimirov PhD), Research Institute for Systems

Biology and Medicine, Moscow, Russia; Faculty of Information Technology (B Vo PhD), HUTECH University, Ho Chi Minh City, Viet Nam; Department of Pediatric Endocrinology (R Vukovic PhD), Mother and Child Healthcare Institute of Serbia "Dr Vukan Cupic", Belgrade, Serbia; Office of Research, Innovation, and Commercialization (ORIC) (Prof Y Waheed PhD), Shaheed Zulfiqar Ali Bhutto Medical University (SZABMU), Islamabad, Pakistan; Gilbert and Rose-Marie Chagoury School of Medicine (Prof Y Waheed PhD), Lebanese American University, Byblos, Lebanon; Department of Cultures, Societies and Global Studies (R G Wamai PhD), Northeastern University, Boston, MA, USA; Department of Neurosurgery (S Wang MD), School of Public Health (J Xia PhD), Capital Medical University, Beijing, China; Department of Gastroenterology (S Wang PhD), Shanghai Jiao Tong University Affiliated Sixth People's Hospital, Shanghai, China; Department of Medicine (M Y Wei MD), Greater Los Angeles VA Healthcare System, Los Angeles, CA, USA; Cardiology Department (Prof R G Weintraub MB), Royal Children's Hospital, Melbourne, VIC, Australia; Department of Physical Therapy (T Wiangkham PhD), Naresuan University, Phitsanulok, Thailand; Department of Surgery (D P Wickramasinghe MD), University of Colombo, Colombo, Sri Lanka; Department of Nursing (A Wilandika MKep), Universitas Aisyiyah Bandung, Bandung, Indonesia; Department of Medical Statistics, Informatics and Health Economics (Prof P Willeit PhD), Medical University Innsbruck, Innsbruck, Austria; Department of Chemical Toxicology (M W Wojewodzic PhD), Norwegian Institute of Public Health, Oslo, Norway; Department of Nutrition (D H Woldegebreal MPH), University of California Davis, Davis, CA, USA; NIHR Biomedical Research Centre (Prof C D A Wolfe MD), Guy's and St. Thomas' Hospital and Kings College London, London, UK; The Second Affiliated Hospital (Prof A Wu MD), Wenzhou Medical University, Wenzhou, China; Global Health Research Center (C Wu PhD), Duke Kunshan University, Kunshan, China; Department of Food Science and Human Nutrition (Prof F Wu PhD), Michigan State University, East Lansing, MI, USA; School of Public Health (Shenzhen) (X Wu MPH), Sun Yat-sen University, Shenzhen, China; Division of Gastroenterology (Prof Z Wu PhD), Huazhong University of Science and Technology, Wuhan, China; School of Public Health (H Xiao PhD), Zhejiang University, Zhejiang, China; Department of Public Health Science (H Xiao PhD), Fred Hutchinson Cancer Research Center, Seattle, WA, USA; Department of Behavior and Operation Management (Y Xie MD), Beijing Advanced Innovation Center for Big Data-based Precision Medicine, Beijing, China; Department of Endocrinology (Prof S Xu PhD), University of Science and Technology of China, Hefei, China; School of Public Health (Prof W Xu MD), Southwest Medical University, luzhou, China; Department of Cancer Epidemiology and Prevention Research (L Yang PhD), Alberta Health Services, Calgary, AB, Canada; Faculty of Medicine (Y Yano MD), Department of Public Health (N Yonemoto PhD), Juntendo University, Tokyo, Japan; Research Center of Physiology (H Yaribeygi PhD), Semnan University of Medical Sciences, Semnan, Iran; Department of Pharmacology, Physiology & Neuroscience (M Yesiltepe PhD), Rutgers University, Newark, Turkiye; Clinical Investigation Unit (M Yesiltepe PhD), Ankara City Hospital, Ankara, Turkiye; Department of Family Medicine (S A Yesuf MSc), St. Peter's Specialized Hospital, Addis Ababa, Ethiopia; Independent Consultant, Addis Ababa, Ethiopia (S A Yesuf MSc); KHANA Center for Population Health Research, Phnom Penh, Cambodia (S Yi PhD); Trinity College Institute for Neuroscience (A Yigezu MPH), School of Medicine (A Yigezu MPH), Trinity College Dublin, Dublin, Ireland; Department of Health Management (A Yiğit PhD, V Yiğit PhD), Süleyman Demirel Üniversitesi (Süleyman Demirel University), Isparta, Turkiye; Pharmacy Department (Y Yismaw MSc), Alkan Health Science, Business and Technology College, Bahir Dar, Ethiopia; Department of Neuropsychopharmacology (N Yonemoto PhD), National Center of Neurology and Psychiatry, Kodaira, Japan; Department of Health Policy and Management (Prof M Z Younis PhD), Jackson State University, Jackson, MS,

USA; School of Business & Economics (Prof M Z Younis PhD), Universiti Putra Malaysia (University of Putra Malaysia), Kuala Lumpur, Malaysia; Department of Epidemiology and Biostatistics (Prof C Yu PhD), School of Medicine (Z Zhang PhD), Wuhan University, Wuhan, China; Association for Socially Applicable Research (ASAR), Pune, India (S Zadey MS); Department of Emergency Medicine (S Zadey MS), Global Emergency Medicine Innovation and Implementation (GEMINI) Research Center, Durham, NC, USA; Epidemiology and Cancer Registry Sector (Prof V Zadnik PhD), Institute of Oncology Ljubljana, Ljubljana, Slovenia; Faculty of Medicine and Health Sciences (F Zakham PhD), Hodeidah University, Hodeidah, Yemen; Department of Health Sciences (S Zaman MSc), James Madison University, Harrisonburg, VA, USA; Hospital San Juan de Dios, Tarija, Bolivia (N Zamora MD); Department of Neuroscience (R Zand MD), Geisinger Health System, Danville, PA, USA; Unit on Child & Adolescent Health (Prof H J Zar PhD), Medical Research Council South Africa, Cape Town, South Africa; Research and Development Department (I Zare BSc), Sina Medical Biochemistry Technologies, Shiraz, Iran; Victorian Comprehensive Cancer Centre, Melbourne, VIC, Australia (J Zhang MD); School of Public Policy and Administration (J Zhang BA), Xi 'an Jiaotong University, Xi'an, China; Medical Oncology Department of Gastrointestinal Cancer (L Zhang MS), Cancer Hospital of Dalian University of Technology, Shenyang, China; School of Biomedical Engineering, Faculty of Medicine (L Zhang MS), Dalian University of Technology, Dalian, China; School of Public Health (Y Zhang PhD), Hubei Province Key Laboratory of Occupational Hazard Identification and Control (Y Zhang PhD), Wuhan University of Science and Technology, Wuhan, China; College of Traditional Chinese Medicine (H Zhao MD), Hebei University, Baoding, China; Computational Bioscience Research Center (J Zhou PhD), King Abdullah University of Science and Technology, Jeddah, Saudi Arabia; School of Public Health and Emergency Management (B Zhu PhD), Southern University of Science and Technology, Shenzhen, China; School of Life Sciences (L Zhu PhD), Yunnan University, Kunming, China; College of Medicine (O A Zitoun MD), Sulaiman Alrajhi University, Al Bukairiyah, Saudi Arabia; NIHR-Biomedical Research Centre (NIHR-BRC) (Prof A Zumla PhD), University College London Hospitals, London, UK; Department of Cardiology, Pulmonology, and Vascular Medicine (E Zweck MD), Heinrich-Heine-University, Duesseldorf, Germany; School of Physics (S H Zyoud PhD), Universiti Sains Malaysia (University of Science Malaysia), Penang, Malaysia

Authors' Contributions

Managing the overall research enterprise

Nicole Davis Weaver, Simon I Hay, Christopher Murray, Mohsen Naghavi, Kanyin Liane Ong, and Eve Wool.

Writing the first draft of the manuscript

Matthew Cunningham, Nicole Davis Weaver, Mohsen Naghavi, and Eve E Wool.

Primary responsibility for applying analytical methods to produce estimates

Gregory J Bertolacci, Matthew Cunningham, Chieh Han, Rebecca Hsu, and Vincent Mougin.

Primary responsibility for seeking, cataloguing, extracting, or cleaning data; designing or coding figures and tables

Gregory J Bertolacci, Matthew Cunningham, Sama Ghoba, Rebecca Hsu, and Vincent Mougin.

Providing data or critical feedback on data sources

Yohannes Habtegiorgis Abate, Cristiana Abbafati, Hedayat Abbastabar, Samar Abd ElHafeez, Michael Abdelmasseh, Ahmed Abdelwahab, Meriem Abdoun, Auwal Abdullahi, Samrawit Shawel Abebe, Aidin Abedi, E S Abhilash, Hassan Abidi, Richard Gyan Aboagye, Hassan Abolhassani, Lucas Guimarães Abreu, Dariush Abtahi, Niveen ME Abu-Rmeileh, Salahdein Aburuz, Ahmed Abu-Zaid, Akindele Olupelumi Adebiyi, Victor Adekanmbi, Abiola Victor Adepoju, Temitayo Esther Adeyeoluwa, Qorinah Estiningtyas Sakilah Adnani, Muhammad Sohail Afzal, Saira Afzal, Feleke Doyore Agide, Antonella Agodi, Bright Opoku Ahinkorah, Danish Ahmad, Muayyad M Ahmad, Sajjad Ahmad, Ali Ahmed, Ayman Ahmed, Haroon Ahmed, Muktar Beshir Ahmed, Budi Aji, Hossein Akbarialiabad, Hanadi Al Hamad, Fares Alahdab, Tariq A Alalwan, Fahad Mashhour Alanezi, Turki M Alanzi, Almaza Albakri, Mohammed Albashtawy, Mohammad T AlBataineh, Jacqueline Elizabeth Alcalde-Rabanal, Robert W Aldridge, Mulubirhan Assefa Alemayohu, Yihun Mulugeta Alemu, Khalid F Alhabib, Abid Ali, Amjad Ali, Liaqat Ali, Syed Shujait Shujait Ali, Reyhaneh Alikhani, Mohammad A M Aljasir, Syed Mohamed Aljunid, François Alla, Peter Allebeck, Joseph Uy Almazan, Louay Almidani, Omar Almidani, Mahmoud A Alomari, Awais Altaf, Nelson Alvis-Guzman, Hassan Alwafi, Hany Aly, Azmeraw T Amare, Edward Kwabena Ameyaw, Hubert Amu, Ganiyu Adeniyi Amusa, Deanna Anderlini, David B Anderson, Pedro Prata Andrade, Hossein Ansari, Davood Anvari, Saeid Anvari, Saleha Anwar, Sumadi Lukman Anwar, Razique Anwer, Muhammad Aqeel, Jalal Arabloo, Olatunde Aremu, Hany Ariffin, Benedetta Armocida, Johan Ärnlöv, Anton A Artamonov, Judie Arulappan, Mohsen Asadi-Lari, Akeza Awealom Asgedom, Seyyed Shamsadin Athari, Bantalem Tilaye Tilaye Atinafu, Prince Atorkey, Alok Atreya, Beatriz Paulina Ayala Quintanilla, Ahmed Y Azzam, Ashish D Badiye, Soroush Baghdadi, Sara Bagherieh, Atif Amin Baig, Abdulaziz T Bako, Senthilkumar Balakrishnan, Ovidiu Constantin Baltatu, Maciej Banach, Palash Chandra Banik, Martina Barchitta, Mainak Bardhan, Till Winfried Bärnighausen, Hiba Jawdat Barqawi, Lope H Barrero, Amadou Barrow, Sandra Barteit, Zarrin Basharat, Sanjay Basu, Nebiyou Simegnew Bayileyegn, Emad Behboudi, Priyamadhaba Behera, Masoud Behzadifar, Diana Fernanda Bejarano Ramirez, Yannick Béjot, Sefealem Assefa Belay, Chalie Mulu Belete, Michelle L Bell, Olorunjuwon Omolaja Bello, Apostolos Beloukas, Azizullah Beran, Betyna N Berice, Akshaya Srikanth Bhagavathula, Dinesh Bhandari, Kayleigh Bhangdia, Sonu Bhaskar, Vivek Bhat, Gurjit Kaur Bhatti, Jasvinder Singh Bhatti, Zulfiqar A Bhutta, Boris Bikbov, Jessica Devin Bishai, Anca Vasilica Bobirca, Virginia Bodolica, Obasanjo Afolabi Bolarinwa, Archith Boloor,

Berrak Bora Basara, Hamed Borhany, Souad Bouaoud, Antoine Boustany, Christopher Boxe, Dejana Braithwaite, Luisa C Brant, Michael Brauer, Traolach Brugha, Lemma N Bulto, Danilo Buonsenso, Florentino Luciano Caetano dos Santos, Luciana Aparecida Campos, Chao Cao, Juan J Carrero, Márcia Carvalho, Joao Mauricio Castaldelli-Maia, Carlos A Castañeda-Orjuela, Ferrán Catalá-López, Alberico L Catapano, Christopher R Cederroth, Francieli Cembranel, Muthia Cenderadewi, Joshua Chadwick, Gashaw Sisay Chanie, Periklis Charalampous, Vijay Kumar Chattu, Akhilanand Chaurasia, Angela W Chen, Ching-Yu Cheng, Abdulaal Chitheer, William C S Cho, Bryan Chong, Hitesh Chopra, Rajiv Chowdhury, Eric Chung, Eunice Chung, Zinhle Cindi, Rafael M Claro, Alyssa Columbus, Haley Comfort, Joao Conde, Samuele Cortese, Paolo Angelo Cortesi, Ewerton Cousin, Rosa A S Couto, Michael H Criqui, Natália Cruz-Martins, Matthew Cunningham, Siyu Dai, Xiaochen Dai, Giovanni Damiani, Saswati Das, Ana Maria Dascalu, Claudio Alberto Dávila-Cervantes, Aklilu Tamire Debele, Shayom Debopadhaya, Louisa Degenhardt, Lee Deitesfeld, Ivan Delgado-Enciso, Berecha Hundessa Demessa, Andreas K Demetriades, Niloofar Deravi, Nebiyu Dereje, Nikolaos Dervenis, Emina Dervišević, Hardik Dineshbhai Desai, Rupak Desai, Vinoth Gnana Chellaiyan Devanbu, Meghnath Dhimal, Sameer Dhingra, Vishal R Dhulipala, Diana Dias da Silva, Daniel Diaz, Michael J Diaz, Huanghe Ding, Ricardo Jorge Dinis-Oliveira, M Ashworth Dirac, Thao Huynh Phuong Do, Masoud Dodangeh, Milad Dodangeh, Klara Georgieva Dokova, Christiane Dolecek, Regina-Mae Villanueva Dominguez, Wendel Mombaque dos Santos, Rajkumar Doshi, Leila Doshmangir, Robert Kokou Dowou, Tim Robert Driscoll, Haneil Larson Dsouza, Viola Dsouza, Bruce B Duncan, Andre Rodrigues Duraes, Senbagam Duraisamy, Paulina Agnieszka Dzianach, Chidiebere Peter Echieh, Diyan Ermawan Effendi, Ebrahim Eini, Michael Ekholuenetale, Temitope Cyrus Ekundayo, Teshome Bekele Elema, Legesse Tesfaye Elilo, Ryenchindorj Erkhembayar, Sharareh Eskandarieh, Habtamu Esubalew, Adewale Oluwaseun Fadaka, Adeniyi Francis Fagbamigbe, Mohammad Fareed, Carla Sofia e Sá Farinha, Andre Faro, Abidemi Omolara Fasanmi, Ali Fatehizadeh, Pooria Fazeli, Alireza Feizkhah, Seyed-Mohammad Fereshtehnejad, David Flood, Artem Alekseevich Fomenkov, Richard Charles Franklin, Joseph Friedman, Takeshi Fukumoto, John E Fuller, Peter Andras Gaal, Muktar A Gadanya, Santosh Gaihre, Yaseen Galali, Silvano Gallus, Mandukhai Ganbat, William M Gardner, Tilaye Gebru Gebi, Teferi Gebru Gebremeskel, Simona Roxana Georgescu, Peter W Gething, Molla Getie, Ahmad Ghashghaee, Ajnish Ghimire, Sama Ghoba, Alem Girmay, Laszlo Göbölös, Ali Golchin, Mahaveer Golechha, Pouya Goleij, Nelson G M Gomes, Houman Goudarzi, Bárbara Niegia Garcia Goulart, Ayman Grada, Giuseppe Grosso, Avirup Guha, Rafael Alves Guimarães, Snigdha Gulati, Anish Kumar Gupta, Manoj Kumar Gupta, Rajat Das Gupta, Rajeev Gupta, Sapna Gupta, Vijai Kumar Gupta, Najah R Hadi, Nils Haep, Nima Hafezi-Nejad, Demewoz Haile, Alemayehu Hailu, Arvin Haj-Mirzaian, Brian J Hall, Rabih Halwani, Erin B Hamilton, Chieh Han, Arief Hargono, Josep Maria Haro, M Tasdik Hasan, Soheil Hassanipour, Hadi Hassankhani, Johannes Haubold, Simon I Hay, Jeffrey J Hebert, Mahsa Heidari-Foroozan, Claudiu Herteliu, Nobuyuki Horita, Md Mahbub Hossain, Mehdi Hosseinzadeh, Chengxi Hu, Michael Hultström, Javid Hussain, Nawfal R Hussein, Hong-Han Huynh, Segun Emmanuel Ibitoye, Desta Ijo, Kevin S Ikuta, Olayinka Stephen Ilesanmi, Arnaud Iradukunda, Sheikh Mohammed Shariful Islam, Nahlah Elkudssiah Ismail, Gaetano Isola, Mahalaxmi Iyer, Linda Merin J, Jalil Jaafari, Kathryn H Jacobsen, Morteza Jafarinia, Khushleen Jaggi, Nader Jahanmehr, Haitham Jahrami, Nityanand Jain, Tahereh Javaheri, Sathish Kumar Jayapal, Shubha Jayaram, Yingzhao Jin, Jost B Jonas, Tamas Joo, Abel Joseph, Charity Ehimwenma Joshua, Jacek Jerzy Jozwiak, Mikk Jürisson, Vaishali K, Billingsley Kaambwa, Zubair Kabir, Vidya Kadashetti, Dler Hussein Kadir, Leila R Kalankesh, Sivesh Kathir Kamarajah, Naser Kamyari, Himal Kandel, Arun R Kanmanthareddy, Rami S Kantar, Neeti Kapoor, André Karch, Salah Eddin Karimi, Faizan Zaffar Kashoo, Nicholas J Kassebaum, Norito Kawakami, Gbenga A Kayode, Tahseen Haider Kazmi, Fassikaw Kebede, Peter Njenga Keiyoro, Cathleen Keller, Emmanuelle Kesse-Guyot, Himanshu Khajuria,

Nauman Khalid, Faham Khamesipour, Ikramullah Khan, M Nuruzzaman Khan, Maseer Khan, Moien AB Khan, Mahammed Ziauddin Khan suheb, Khaled Khatab, Fatemeh Khatami, Feriha Fatima Khidri, Mohammad Khorgamphar, Grace Kim, Jihee Kim, Min Seo Kim, Yun Jin Kim, Adnan Kisa, Sezer Kisa, Desmond Klu, Ann Kristin Skrindo Knudsen, Jonathan M Kocarnik, Sonali Kochhar, Timea Kocsis, Gerbrand Koren, Soewarta Kosen, Sindhura Lakshmi Koulmane Laxminarayana, Kewal Krishan, Hare Krishna, Vijay Krishnamoorthy, Yuvaraj Krishnamoorthy, Barthelemy Kuate Defo, Burcu Kucuk Bicer, Akshay Kumar, Ashish Kumar, Dian Kusuma, Alexander Kwarteng, Hmwe Hmwe Kyu, Anton C J Lager, Dharmesh Kumar Lal, Savita Lasrado, Kamaluddin Latief, Kaveh Latifinaibin, Nhi Huu Hanh Le, Thao Thi Thu Le, Trang Diep Thanh Le, Caterina Ledda, Munjae Lee, Seung Won Lee, Shaun Wen Huey Lee, James Leigh, Yichong Li, Andrew Tiyamike Makhiringa Likaka, Lee-Ling Lim, Stephen S Lim, Megan Lindstrom, Gang Liu, Jue Liu, Shiwei Liu, Xuefeng Liu, Erand Llanaj, Michael J Loftus, Rubén López-Bueno, Stefan Lorkowski, Paulo A Lotufo, Rafael Lozano, Jailos Lubinda, Zheng Feei Ma, Christian Madsen, Azzam A Maghazachi, Razzagh Mahmoudi, Azeem Majeed, Elaheh Malakan Rad, Venkatesh Maled, Kashish Malhotra, Deborah Carvalho Malta, Abdullah A Mamun, Mohammad Ali Mansournia, Sajid Maqsood, Joemer C Maravilla, Agustina M Marconi, Parham Mardi, Abdoljalal Marjani, Bernardo Alfonso Martinez-Guerra, Francisco Rogerlândio Martins-Melo, Sharmeen Maryam, Roy Rillera Marzo, Awoke Masrie, Alexander G Mathioudakis, Jishanth Mattumpuram, Richard Matzopoulos, Andrea Maugeri, Mohsen Mazidi, Anna Laura W McKowen, Steven M McPhail, Enkeleint A Mechili, John Robert Carabeo Medina, Jitendra Kumar Meena, Entezar Mehrabi Nasab, Tesfahun Mekene Meto, Gebrekiros Gebremichael Meles, Walter Mendoza, Ritesh G Menezes, Haftu Asmerom Meresa, Atte Meretoja, Kukulege Chamila Dinushi Mettananda, Sachith Mettananda, Georgia Micha, Irmina Maria Michalek, Ted R Miller, Edward J Mills, Le Huu Nhat Minh, Erkin M Mirrakhimov, Awoke Misganaw, Mohammadreza Mobayen, Madeline E Moberg, Soheil Mohammadi, Abdollah Mohammadian-Hafshejani, Mustapha Mohammed, Salahuddin Mohammed, Shafiu Mohammed, Viswanathan Mohan, Ali H Mokdad, Mariam Molokhia, Sara Momtazmanesh, Lorenzo Monasta, Mohammad Ali Moni, Yousef Moradi, Maziar Moradi-Lakeh, Shane Douglas Morrison, Jakub Morze, Jonathan F Mosser, Vincent Mougin, Emmanuel A Mpolya, Sumaira Mubarik, Ulrich Otto Mueller, Faraz Mughal, Francesk Mulita, Christopher J L Murray, Fungai Musaigwa, Ghulam Mustafa, Saravanan Muthupandian, Raman Muthusamy, Ahamarshan Jayaraman Nagarajan, Gabriele Nagel, Mohsen Naghavi, Ganesh R Naik, Sanjeev Nair, Sreenivas Narasimha Swamy, Aparna Ichalangod Narayana, Shumaila Nargus, Bruno Ramos Nascimento, Zuhair S Natto, Biswa Prakash Nayak, Vinod C Nayak, Ionut Negoi, Ruxandra Irina Negoi, Haruna Asura Nggada, Josephine W Ngunjiri, Anh Hoang Nguyen, Dang H Nguyen, Hau Thi Hien Nguyen, Phat Tuan Nguyen, Van Thanh Nguyen, Robina Khan Niazi, Taxiarchis Konstantinos Nikolouzakis, Chukwudi A Nnaji, Lawrence Achilles Nnyanzi, Shuhei Nomura, Bo Norrving, Jean Jacques Noubiap, Mpiko Ntsekhe, Dieta Nurrika, Jerry John Nutor, Bogdan Oancea, Kehinde O Obamiro, Mary Aigbiremo Oboh, Ismail A Odetokun, Nkechi Martina Odogwu, Ayodipupo Sikiru Oguntade, Osaretin Christabel Okonji, Andrew T Olagunju, Matthew Idowu Olatubi, Bolajoko Olubukunola Olusanya, Jacob Olusegun Olusanya, Yinka Doris Oluwafemi, Hany A Omar, Ahmed Omar Bali, Maureene Auma Ondayo, Kanyin Liane Ong, Sokking Ong, Obinna E Onwujekwe, Kenneth Ikenna Onyedibe, Alberto Ortiz, Samuel M Ostroff, Adrian Otoiu, Stanislav S Otstavnov, Amel Ouyahia, Mayowa O Owolabi, Yaz Ozten, Mahesh Padukudru P A, Jagadish Rao Padubidri, Raffaele Palladino, Adrian Pana, Songhomitra Panda-Jonas, Seithikurippu R Pandi-Perumal, Helena Ullyartha Pangaribuan, Leonidas D Panos, Anca Mihaela Pantea Stoian, Paraskevi Papadopoulou, Romil R Parikh, Seoyeon Park, Ashwaghosha Parthasarathi, Maja Pasovic, Hemal M Patel, Jay Patel, Sangram Kishor Patel, Shankargouda Patil, Uttam Paudel, Spencer A Pease, Veincent Christian Filipino Pepito, Prince Peprah, Mario F P Peres, Arokiasamy Perianayagam, Konrad Pesudovs, William A Petri, Hoang Tran

Pham, Anil K Philip, Daniela Pierannunzio, David M Pigott, Zahra Zahid Piracha, Vivek Podder, Kevan R Polkinghorne, Ramesh Poluru, Maarten J Postma, Naeimeh Pourtaheri, Sergio I Prada, Elton Junio Sady Prates, TINA PRISCILLA, Natalie Pritchett, Bharathi M Purohit, Jagadeesh Puvvula, Asma Saleem Qazi, Raghu Anekal Radhakrishnan, Venkatraman Radhakrishnan, Hadi Raeisi Shahraki, Quinn Rafferty, Nasiru Raheem, Fakher Rahim, Md Jillur Rahim, Vafa Rahimi-Movaghar, Mohammad Hifz Ur Rahman, Amir Masoud Rahmani, Shakthi Kumaran Ramasamy, Sheena Ramazanu, Chhabi Lal Ranabhat, Nemanja Rancic, Chythra R Rao, Mithun Rao, Sowmya J Rao, Sina Rashedi, Vahid Rashedi, Santosh Kumar Rauniyar, Ramin Ravangard, Nakul Ravikumar, Salman Rawaf, Lal Rawal, Reza Rawassizadeh, Christian Razo, Elrashdy Moustafa Mohamed Redwan, Lennart Reifels, Luis Felipe Reyes, Maryam Rezaei, Taeho Gregory Rhee, Mavra A Riaz, Antonio Luiz P Ribeiro, Hannah R Riva, Hannah Elizabeth Robinson-Oden, Célia Fortuna Rodrigues, Mónica Rodrigues, Leonardo Roever, Emma Lynn Best Rogowski, Peter Rohloff, Debby Syahru Romadlon, Michele Romoli, Luca Ronfani, Gholamreza Roshandel, Himanshu Sekhar Rout, Enrico Rubagotti, Susan Fred Rumisha, Tilleye Runghien, Godfrey M Rwegerera, Andrzej Rynkiewicz, Aly M A Saad, Zahra Saadatian, Siamak Sabour, Basema Saddik, Adam Saddler, Bashdar Abuzed Sadee, Ehsan Sadeghi, Umar Saeed, Sher Zaman Safi, Rajesh Sagar, Narjes Saheb Sharif-Askari, Zahra Saif, Mirza Rizwan Sajid, Afeez Abolarinwa Salami, Marwa Rashad Salem, Sara Samadzadeh, Abdallah M Samy, Juan Sanabria, Francesca Sanna, Damian Francesco Santomauro, Itamar S Santos, Milena M Santric-Milicevic, Babak Saravi, Sachin C Sarode, Brijesh Sathian, Maheswar Satpathy, Md Abu Sayeed, Mete Saylan, Mehdi Sayyah, Markus P Schlaich, Maria Inês Schmidt, David C Schwebel, Mario Šekerija, Subramanian Senthilkumaran, Dragos Serban, Yashendra Sethi, Amir Shafaat, Pritik A Shah, Syed Mahboob Shah, Masood Ali Shaikh, Sunder Sham, Muhammad Aaqib Shamim, Mehran Shams-Beyranvand, Abhishek Shankar, Javad Sharifi-Rad, Vishal Sharma, Rajesh P Shastry, Maryam Shayan, Jiabin Shen, Adithi Shetty, B Suresh Kumar Shetty, Peilin Shi, Kenji Shibuya, Aminu Shittu, K M Shivakumar, Farhad Shokraneh, Sina Shool, Luís Manuel Lopes Rodrigues Silva, Abhinav Singh, Narinder Pal Singh, Paramdeep Singh, Valentin Yurievich Skryabin, Anna Aleksandrovna Skryabina, Sameh S M Soliman, Reed J D Sorensen, Joan B Soriano, Ireneous N Soyiri, Michael Spartalis, Chandrashekhar T Sreeramareddy, Jeffrey D Stanaway, Muhammad Haroon Stanikzai, Benjamin A Stark, Caroline Stein, Fridolin Steinbeis, Caitlyn Steiner, Jaimie D Steinmetz, Aleksandar Stevanović, Mark A Stokes, Vetriselvan Subramaniyan, Muhammad Suleman, Rizwan Suliankatchi Abdulkader, Abida Sultana, Haitong Zhe Sun, Johan Sundström, Chandan Kumar Swain, Mindy D Szeto, Miklós Szócska, Rafael Tabarés-Seisdedos, Seyyed Mohammad Tabatabaei, Shima Tabatabai, Mircea Tampa, Ker-Kan Tan, Elvis Enowbeyang Tarkang, Nuno Taveira, Yibekal Manaye Tefera, Pugazhenthan Thangaraju, Nihal Thomas, Nikhil Kenny Thomas, Azalea M Thomson, Chern Choong Chern Thum, Tala Tillawi, Roman Topor-Madry, Mathilde Touvier, Marcos Roberto Tovani-Palone, Jasmine T Tran, Domenico Trico, Guesh Mebrahtom Tsegay, Munkhtuya Tumurkhuu, Irfan Ullah, Sana Ullah, Srikanth Umakanthan, Chukwuma David Umeokonkwo, Bhaskaran Unnikrishnan, Era Upadhyay, Jibrin Sammani Usman, Jef Van den Eynde, Shoban Babu Varthya, Tommi Juhani Vasankari, Balachandar Vellingiri, Narayanaswamy Venketasubramanian, Nicholas Alexander Verghese, Georgios-Ioannis Verras, Maria Viskadourou, Sergey Konstantinovitch Vladimirov, Vasily Vlassov, Bay Vo, Avina Vongpradith, Theo Vos, Yasir Waheed, Shu Wang, Stefanie Watson, Daniel J Weiss, Ronny Westerman, Joanna L Whisnant, Taweewat Wiangkham, Dakshitha Praneeth Wickramasinghe, Angga Wilandika, Peter Willeit, Charles D A Wolfe, Yen Jun Wong, Utoomporn Wongsin, Eve E Wool, Juan Xia, Suowen Xu, Yvonne Yiru Xu, Ali Yadollahpour, Kazumasa Yamagishi, Yao Yao, Habib Yaribeygi, Pengpeng Ye, Sisay Shewasinad Yehualashet, Siyan Yi, Amanuel Yigezu, Dong Keon Yon, Naohiro Yonemoto, Mustafa Z Younis, Chuanhua Yu, Yong Yu, Faith H Yuh, Siddhesh Zadey, Vesna Zadnik, Iman Zare, Mohammed G M

Zeariya, Zhi-Jiang Zhang, Magdalena Zielińska, Mohammad Zoladl, Zhiyong Zou, Liesl J Zuhlke, Alimuddin Zumla, Elric Zweck.

Developing methods or computational machinery

Cristiana Abbafati, Samrawit Shawel Abebe, Dariush Abtahi, Qorinah Estiningtyas Sakilah Adnani, Saira Afzal, Muktar Beshir Ahmed, Hossein Akbarialiabad, Mohammed Albashtawy, Yihun Mulugeta Alemu, Hubert Amu, Hossein Ansari, Davood Anvari, Aleksandr Y Aravkin, Michael Benjamin Arndt, Judie Arulappan, Ahmed Y Azzam, Gregory J Bertolacci, Akshaya Srikanth Bhagavathula, Jessica Devin Bishai, Somayeh Bohlouli, Milad Bonakdar Hashemi, Hamed Borhany, Kelly M Cercy, Catherine S Chen, Kaleb Coberly, Rebecca M Cogen, Haley Comfort, Rosa A S Couto, Garland T Culbreth, Matthew Cunningham, Xiaochen Dai, Hardik Dineshbhai Desai, Paulina Agnieszka Dzianach, Michael Ekholuenetale, Ali Fatehizadeh, Natalie C Galles, MA Garcia-Gordillo, William M Gardner, Tilaye Gebru Gebi, Peter W Gething, Sama Ghoba, Alem Girmay, Shi-Yang Guan, Rafael Alves Guimarães, Manoj Kumar Gupta, Demewoz Haile, Chieh Han, Mohammad Hasanian, Simon I Hay, Hamed Hesami, Mehdi Hosseinzadeh, Rebecca L Hsu, Hong-Han Huynh, Kevin S Ikuta, Arnaud Iradukunda, Gaetano Isola, Linda Merin J, Morteza Jafarinia, Khushleen Jaggi, Haitham Jahrami, Abubakar Ibrahim Jatau, Tahereh Javaheri, Sathish Kumar Jayapal, Olatunji Johnson, Charity Ehimwenma Joshua, Dler Hussein Kadir, Sivesh Kathir Kamarajah, Faizan Zaffar Kashoo, Nicholas J Kassebaum, Peter Njenga Keiyoro, Cathleen Keller, Alireza Khalilian, M Nuruzzaman Khan, Zahra Khorrami, Adnan Kisa, Sezer Kisa, Jonathan M Kocarnik, Alexander Kwarteng, Nhi Huu Hanh Le, Thao Thi Thu Le, James Leigh, Megan Lindstrom, Runben Liu, Erand Llanaj, Kelsey Lynn Maass, Razzagh Mahmoudi, Awoke Masrie, Le Huu Nhat Minh, Madeline E Moberg, Abdollah Mohammadian-Hafshejani, Hoda Mojiri-Forushani, Ali H Mokdad, Mohammad Ali Moni, Yousef Moradi, Vincent Mougin, Francesk Mulita, Christopher J L Murray, Mohsen Naghavi, Pirouz Naghavi, Josephine W Ngunjiri, Phat Tuan Nguyen, Van Thanh Nguyen, Osaretin Christabel Okonji, Bolajoko Olubukunola Olusanya, Jacob Olusegun Olusanya, Kanyin Liane Ong, Michal Ordak, Adrian Pana, Maja Pasovic, Spencer A Pease, Hoang Tran Pham, Saeed Pirouzpanah, Hadi Raeisi Shahraki, Quinn Rafferty, Mohammad Hifz Ur Rahman, Amir Masoud Rahmani, Chhabi Lal Ranabhat, Giridhara Rathnaiah Babu, Ramin Ravangard, Reza Rawassizadeh, Christian Razo, Robert C Reiner Jr, Maryam Rezaei, Mónica Rodrigues, Emma Lynn Best Rogowski, Gregory A Roth, Himanshu Sekhar Rout, Enrico Rubagotti, Susan Fred Rumisha, Adam Saddler, Umar Saeed, Zahra Saif, Abdallah M Samy, Francesca Sanna, Maheswar Satpathy, Mehdi Sayyah, Austin E Schumacher, Javad Sharifi-Rad, Kenji Shibuya, Youn Ho Shin, Sarah Brooke Sirota, Reed J D Sorensen, Michael Spartalis, Sandra Spearman, Jeffrey D Stanaway, Caitlyn Steiner, Jaimie D Steinmetz, Muhammad Suleman, Chandan Kumar Swain, Yibekal Manaye Tefera, Pugazhenthan Thangaraju, Chern Choong Chern Thum, Tala Tillawi, Era Upadhyay, Bay Vo, Theo Vos, Daniel J Weiss, Ronny Westerman, Joanna L Whisnant, Shadrach Wilson, Yen Jun Wong, Eve E Wool, Zenghong Wu, Wang-Dong Xu, Yvonne Yiru Xu, Amanuel Yigezu, Mohammed G M Zeariya, Jingya Zhang.

Providing critical feedback on methods or results

Amirali Aali, Hazim S Ababneh, Yohannes Habtegiorgis Abate, Cristiana Abbafati, Rouzbeh Abbasgholizadeh, Hedayat Abbastabar, Samar Abd ElHafeez, Michael Abdelmasseh, Sherief Abd-Elsalam, Mohammad Abdollahi, Meriem Abdoun, Deldar Morad Abdulah, Auwal Abdullahi, Mesfin Abebe, Samrawit Shawel Abebe, Kedir Hussein Abegaz, E S Abhilash, Hassan Abidi, Richard Gyan Aboagye, Hassan Abolhassani, Meysam Abolmaali, Mohamed Abouzid, Girma Beressa Aboye, Lucas Guimarães Abreu, Woldu Aberhe Abrha, Dariush Abtahi, Samir Abu Rumeileh, Hasan Abualruz, Bilyaminu Abubakar, Eman Abu-Gharbieh, Salahdein Aburuz, Ahmed Abu-Zaid, Manfred Mario Kokou Accrombessi, Tadele Girum Adal, Abdu A Adamu, Isaac Yeboah Addo, Akindele Olupelumi Adebiyi, Victor Adekanmbi, Abiola

Victor Adepoju, Charles Oluwaseun Adetunji, Juliana Bunmi Adetunji, Temitayo Esther Adeyeoluwa, Daniel Adedayo Adeyinka, Olorunsola Israel Adeyomoye, Biruk Adie Admass, Qorinah Estiningtyas Sakilah Adnani, Saryia Adra, Aanuoluwapo Adeyimika Afolabi, Muhammad Sohail Afzal, Saira Afzal, Suneth Buddhika Agampodi, Pradyumna Agasthi, Manik Aggarwal, Shahin Aghamiri, Feleke Doyore Agide, Antonella Agodi, Anurag Agrawal, Williams Agyemang-Duah, Bright Opoku Ahinkorah, Aqeel Ahmad, Danish Ahmad, Firdos Ahmad, Muayyad M Ahmad, Sajjad Ahmad, Shahzaib Ahmad, Tauseef Ahmad, Keivan Ahmadi, Amir Mahmoud Ahmadzade, Ali Ahmed, Ayman Ahmed, Haroon Ahmed, Luai A Ahmed, Mehrunnisha Sharif Ahmed, Meqdad Saleh Ahmed, Muktar Beshir Ahmed, Syed Anees Ahmed, Budi Aji, Essona Matatom Akara, Hossein Akbarialiabad, Karolina Akinosoglou, Tomi Akinyemiju, Samuel Akyirem, Hanadi Al Hamad, Syed Mahfuz Al Hasan, Fares Alahdab, Samer O Alalalmeh, Ziyad Al-Aly, Khurshid Alam, Manjurul Alam, Noore Alam, Rasmieh Mustafa Al-amer, Fahad Mashhour Alanezi, Turki M Alanzi, Sayer Al-Azzam, Almaza Albakri, Mohammed Albashtawy, Mohammad T AlBataineh, Jacqueline Elizabeth Alcalde-Rabanal, Khalifah A Aldawsari, Robert W Aldridge, Haileselasie Berhane Alema, Mulubirhan Assefa Alemayohu, Sharifullah Alemi, Yihun Mulugeta Alemu, Adel Ali Saeed Al-Gheethi, Khalid F Alhabib, Mohammed Khaled Al-Hanawi, Abid Ali, Amjad Ali, Liaqat Ali, Mohammed Usman Ali, Rafat Ali, Shahid Ali, Syed Shujait Shujait Ali, Gianfranco Alicandro, Sheikh Mohammad Alif, Reyhaneh Alikhani, Yousef Alimohamadi, Ahmednur Adem Aliyi, Mohammad A M Aljasir, Syed Mohamed Aljunid, Peter Allebeck, Sabah Al-Marwani, Sadeq Ali Ali Al-Maweri, Joseph Uy Almazan, Hesham M Al-Mekhlafi, Louay Almidani, Omar Almidani, Mahmoud A Alomari, Basem Al-Omari, Jaber S Alqahtani, Shehabaldin Alqalyoobi, Ahmed Yaseen Alqutaibi, Salman Khalifah Al-Sabah, Zaid Altaany, Awais Altaf, Jaffar A Al-Tawfiq, Khalid A Altirkawi, Nelson Alvis-Guzman, Hassan Alwafi, Yaser Mohammed Al-Worafi, Hany Aly, Safwat Aly, Karem H Alzoubi, Azmeraw T Amare, Prince M Amegbor, Edward Kwabena Ameyaw, Tarek Tawfik Amin, Alireza Amindarolzarbi, Sohrab Amiri, Hubert Amu, Dickson A Amugsi, Ganiyu Adeniyi Amusa, Robert Ancuceanu, Deanna Anderlini, David B Anderson, Pedro Prata Andrade, Catalina Liliana Andrei, Tudorel Andrei, Colin Angus, Amir Anoushiravani, Ansariadi Ansariadi, Alireza Ansari-Moghaddam, Ernoiz Antriyandarti, Davood Anvari, Saeid Anvari, Saleha Anwar, Sumadi Lukman Anwar, Razique Anwer, Anayochukwu Edward Anyasodor, Juan Pablo Arab, Jalal Arabloo, Mosab Arafat, Demelash Areda, Abdulfatai Aremu, Olatunde Aremu, Hany Ariffin, Mesay Arkew, Benedetta Armocida, Mahwish Arooj, Anton A Artamonov, Judie Arulappan, Raphael Taiwo Aruleba, Ashokan Arumugam, Akeza Awealom Asgedom, Mohammad Asghari-Jafarabadi, Muhammad Ashraf, Armin Aslani, Thomas Astell-Burt, Mohammad Athar, Seyyed Shamsadin Athari, Bantalem Tilaye Tilaye Atinafu, Habtamu Wondmagegn Atlaw, Prince Atorkey, Maha Moh'd Wahbi Atout, Alok Atreya, Avinash Aujayeb, Marcel Ausloos, Abolfazl Avan, Atalel Fentahun Awedew, Amlaku Mulat Aweke, Beatriz Paulina Ayala Quintanilla, Haleh Ayatollahi, Jose L Ayuso-Mateos, Seyed Mohammad Ayyoubzadeh, Sina Azadnajafabad, Rui M S Azevedo, Ahmed Y Azzam, Darshan B B, Abraham Samuel Babu, Muhammad Badar, Ashish D Badiye, Soroush Baghdadi, Nasser Bagheri, Sara Bagherieh, Sulaiman Bah, Saeed Bahadorikhalili, Najmeh Bahmanziari, Ruhai Bai, Atif Amin Baig, Jennifer L Baker, Abdulaziz T Bako, Ravleen Kaur Bakshi, Senthilkumar Balakrishnan, Ovidiu Constantin Baltatu, Maciej Banach, Palash Chandra Banik, Hansi Bansal, Kannu Bansal, Franca Barbic, Martina Barchitta, Mainak Bardhan, Erfan Bardideh, Till Winfried Bärnighausen, Hiba Jawdat Barqawi, Lope H Barrero, Amadou Barrow, Sandra Barteit, Lingkan Barua, Zarrin Basharat, Pritish Baskaran, Buddha Basnyat, Quique Bassat, João Diogo Basso, Ann V L Basting, Sanjay Basu, Kavita Batra, Bernhard T Baune, Mohsen Bayati, Nebiyou Simegnew Bayileyegn, Thomas Beaney, Massimiliano Beghi, Emad Behboudi, Priyamadhaba Behera, Amir Hossein Behnoush, Masoud Behzadifar, Sefealem Assefa Belay, Chalie Mulu Belete, Michelle L Bell, Olorunjuwon Omolaja Bello, Luis Belo, Apostolos Beloukas, Rose Grace Bender, Isabela M Bensenor, Alemshet Yirga

Berhie, Betyna N Berice, Robert S Bernstein, Gregory J Bertolacci, Devidas S Bhagat, Akshaya Srikanth Bhagavathula, Neeraj Bhala, Ashish Bhalla, Dinesh Bhandari, Nikha Bhardwaj, Pankaj Bhardwaj, Prarthna V Bhardwaj, Ashish Bhargava, Sonu Bhaskar, Vivek Bhat, Gurjit Kaur Bhatti, Jasvinder Singh Bhatti, Manpreet S Bhatti, Rajbir Bhatti, Zulfiqar A Bhutta, Boris Bikbov, Jessica Devin Bishai, Francesca Bisulli, Atanu Biswas, Bijit Biswas, Bikes Destaw Bitew, Veera R Bitra, Micheal Kofi Boachie, Virginia Bodolica, Aadam Olalekan Bodunrin, Eyob Ketema Bogale, Kassawmar Angaw Bogale, Archith Boloor, Milad Bonakdar Hashemi, Aime Bonny, Kaustubh Bora, Hamed Borhany, Arturo Borzutzky, Souad Bouaoud, Antoine Boustany, Christopher Boxe, Edward J Boyko, Oliver J Brady, Dejana Braithwaite, Luisa C Brant, Alexandra Brazinova, Javier Brazo-Sayavera, Nicholas J K Breitborde, Hermann Brenner, Gabrielle Britton, Julie Brown, Traolach Brugha, Norma B Bulamu, Lemma N Bulto, Danilo Buonsenso, Richard A Burns, Reinhard Busse, Yasser Bustanji, Nadeem Shafique Butt, Zahid A Butt, Florentino Luciano Caetano dos Santos, Luis Alberto Cámera, Luciana Aparecida Campos, Ismael R Campos-Nonato, Chao Cao, Angelo Capodici, Rosario Cárdenas, Cristina G Carvalheiro, Márcia Carvalho, Joao Mauricio Castaldelli-Maia, Carlos A Castañeda-Orjuela, Giulio Castelpietra, Ferrán Catalá-López, Alberico L Catapano, Christopher R Cederroth, Luca Cegolon, Francieli Cembranel, Muthia Cenderadewi, Ester Cerin, Muge Cevik, Joshua Chadwick, Yaacoub Chahine, Chiranjib Chakraborty, Promit Ananyo Chakraborty, Raymond N C Chan, Eeshwar K Chandrasekar, Chin-Kuo Chang, Jung-Chen Chang, Periklis Charalampous, Vijay Kumar Chattu, Pankaj Chaturvedi, Victoria Chatzimavridou-Grigoriadou, Akhilanand Chaurasia, An-Tian Chen, Haowei Chen, Meng Xuan Chen, Ching-Yu Cheng, Esther T W Cheng, Nicolas Cherbuin, Wondimye Ashenafi Cheru, Odgerel Chimed-Ochir, Ritesh Chimoriya, Jesus Lorenzo Chirinos-Caceres, William C S Cho, Bryan Chong, Hitesh Chopra, Sonali Gajanan Choudhari, Rajiv Chowdhury, Devasahayam J Christopher, Isaac Sunday Chukwu, Eric Chung, Erin Chung, Eunice Chung, Sheng-Chia Chung, Muhammad Chutiyami, Zinhle Cindi, Iolanda Cioffi, Mareli M Claassens, Alyssa Columbus, Joao Conde, Samuele Cortese, Paolo Angelo Cortesi, Vera Marisa Costa, Simona Costanzo, Ewerton Cousin, Rosa A S Couto, Richard G Cowden, Kenneth Michael Cramer, Michael H Criqui, Natália Cruz-Martins, Silvia Magali Cuadra-Hernández, Garland T Culbreth, Patricia Cullen, Matthew Cunningham, Omid Dadras, Siyu Dai, Xiaochen Dai, Zhaoli Dai, Lachlan L Dalli, Giovanni Damiani, Jiregna Darega Gela, Jai K Das, Saswati Das, Subasish Das, Ana Maria Dascalu, Nihar Ranjan Dash, Mohsen Dashti, Anna Dastiridou, Gail Davey, Claudio Alberto Dávila-Cervantes, Nicole Davis Weaver, Kairat Davletov, Diego De Leo, Katie de Luca, Aklilu Tamire Debele, Shayom Debopadhaya, Louisa Degenhardt, Azizallah Dehghan, Cristian Del Bo', Ivan Delgado-Enciso, Berecha Hundessa Demessa, Andreas K Demetriades, Ke Deng, Xinlei Deng, Niloofar Deravi, Nebiyu Dereje, Nikolaos Dervenis, Emina Dervišević, Don C Des Jarlais, Hardik Dineshbhai Desai, Rupak Desai, Vinoth Gnana Chellaiyan Devanbu, Syed Masudur Rahman Dewan, Arkadeep Dhali, Kuldeep Dhama, Meghnath Dhimal, Sameer Dhingra, Vishal R Dhulipala, Diana Dias da Silva, Daniel Diaz, Michael J Diaz, Adriana Dima, Delaney D Ding, Huanghe Ding, Ricardo Jorge Dinis-Oliveira, M Ashworth Dirac, Shirin Djalalinia, Thao Huynh Phuong Do, Camila Bruneli do Prado, Saeid Doaei, Masoud Dodangeh, Milad Dodangeh, Klara Georgieva Dokova, Christiane Dolecek, Deepa Dongarwar, Mario D'Oria, Fariba Dorostkar, E Ray Dorsey, Wendel Mombaque dos Santos, Rajkumar Doshi, Leila Doshmangir, Robert Kokou Dowou, Haneil Larson Dsouza, Viola Dsouza, Mi Du, John Dube, Senbagam Duraisamy, Oyewole Christopher Durojaiye, Laura Dwyer-Lindgren, Paulina Agnieszka Dzianach, Arkadiusz Marian Dziedzic, Abdel Rahman E’mar, Ejemai Eboreime, Alireza Ebrahimi, Chidiebere Peter Echieh, Hisham Atan Edinur, David Edvardsson, Kristina Edvardsson, Defi Efendi, Ferry Efendi, Diyan Ermawan Effendi, Terje Andreas Eikemo, Ebrahim Eini, Michael Ekholuenetale, Temitope Cyrus Ekundayo, Iman El Sayed, Noha Mousaad Elemam, Frank J Elgar, Islam Y Elgendy, Ghada Metwally Tawfik ElGohary, Hala Rashad Elhabashy, Muhammed Elhadi, Waseem El-Huneidi, Legesse Tesfaye Elilo,

Omar Abdelsadek Abdou Elmeligy, Mohamed A Elmonem, Ibrahim Elsohaby, Theophilus I Emeto, Luchuo Engelbert Bain, Babak Eshrati, Sharareh Eskandarieh, Juan Espinosa-Montero, Habtamu Esubalew, Farshid Etaee, Natalia Fabin, Adewale Oluwaseun Fadaka, Adeniyi Francis Fagbamigbe, Ayesha Fahim, Saman Fahimi, Aliasghar Fakhri-Demeshghieh, Luca Falzone, Mohammad Fareed, Carla Sofia e Sá Farinha, Pawan Sirwan Faris, Andre Faro, Abidemi Omolara Fasanmi, Ali Fatehizadeh, Hamed Fattahi, Pooria Fazeli, Valery L Feigin, Ginenus Fekadu, Xiaoru Feng, Seyed-Mohammad Fereshtehnejad, Daniela Ferrante, Alize J Ferrari, Getahun Fetensa, Bikila Regassa Feyisa, Irina Filip, Florian Fischer, Joanne Flavel, David Flood, Bobirca Teodor Florin, Nataliya A Foigt, Morenike Oluwatoyin Folayan, Artem Alekseevich Fomenkov, Masoud Foroutan, Ingeborg Forthun, Daniela Fortuna, Matteo Foschi, Kayode Raphael Fowobaje, Kate Louise Francis, Richard Charles Franklin, Alberto Freitas, Joseph Friedman, Sara D Friedman, Takeshi Fukumoto, Blima Fux, Peter Andras Gaal, Muktar A Gadanya, Abhay Motiramji Gaidhane, Santosh Gaihre, Emmanuela Gakidou, Yaseen Galali, Natalie C Galles, Mandukhai Ganbat, Aravind P Gandhi, Balasankar Ganesan, Mohammad Arfat Ganiyani, William M Gardner, Jalaj Garg, Naval Garg, Rupesh K Gautam, Semiu Olatunde Gbadamosi, Tilaye Gebru Gebi, Miglas W Gebregergis, Mesfin Gebrehiwot, Teferi Gebru Gebremeskel, Simona Roxana Georgescu, Tamirat Getachew, Molla Getie, Keyghobad Ghadiri, Sulmaz Ghahramani, Khalid Yaser Ghailan, Mohammad-Reza Ghasemi, Ghazal Ghasempour Dabaghi, Afsaneh Ghasemzadeh, Ahmad Ghashghaee, Fariba Ghassemi, Ramy Mohamed Ghazy, Ajnish Ghimire, Sama Ghoba, Maryam Gholamalizadeh, Asadollah Gholamian, Ali Gholamrezanezhad, Pooyan Ghorbani Vajargah, Aloke Gopal Ghoshal, Paramjit Singh Gill, Tiffany K Gill, Themba G Ginindza, Alem Girmay, James C Glasbey, Elena V Gnedovskaya, Laszlo Göbölös, Myron Anthony Godinho, Amit Goel, Mohamad Goldust, Mahaveer Golechha, Nelson G M Gomes, Philimon N Gona, Sameer Vali Gopalani, Alessandra C Goulart, Bárbara Niegia Garcia Goulart, Anmol Goyal, Ayman Grada, Simon Matthew Graham, Michal Grivna, Shi-Yang Guan, Mohammed Ibrahim Mohialdeen Gubari, Mesay Dechasa Gudeta, Avirup Guha, Stefano Guicciardi, Rafael Alves Guimarães, Snigdha Gulati, Damitha Asanga Gunawardane, Sasidhar Gunturu, Cui Guo, Anish Kumar Gupta, Bhawna Gupta, Manoj Kumar Gupta, Mohak Gupta, Rajat Das Gupta, Rajeev Gupta, Sapna Gupta, Veer Bala Gupta, Vijai Kumar Gupta, Vivek Kumar Gupta, Lami Gurmessa, Reyna Alma Gutiérrez, Farrokh Habibzadeh, Parham Habibzadeh, Rasool Haddadi, Mostafa Hadei, Najah R Hadi, Nils Haep, Nima Hafezi-Nejad, Alemayehu Hailu, Arvin Haj-Mirzaian, Esam S Halboub, Brian J Hall, Sebastian Haller, Rabih Halwani, Randah R Hamadeh, Samer Hamidi, Erin B Hamilton, Chieh Han, Qiuxia Han, Asif Hanif, Fahad Hanna, Md Nuruzzaman Haque, Harapan Harapan, Ahmed I Hasaballah, Ikramul Hasan, M Tasdik Hasan, Hamidreza Hasani, Abdiwahab Hashi, Md Saquib Hasnain, Ikrama Hassan, Soheil Hassanipour, Hadi Hassankhani, Johannes Haubold, Rasmus J Havmoeller, Simon I Hay, Jiawei He, Omar E Hegazi, Golnaz Heidari, Mohammad Heidari, Mahsa Heidari-Foroozan, Bartosz Helfer, Delia Hendrie, Brenda Yuliana Herrera-Serna, Claudiu Herteliu, Hamed Hesami, Kamal Hezam, Catherine L Hill, Yuta Hiraike, Ramesh Holla, Nobuyuki Horita, Md Mahbub Hossain, Sahadat Hossain, Mohammad-Salar Hosseini, Hassan Hosseinzadeh, Mehdi Hosseinzadeh, Ahmad Hosseinzadeh Adli, Mihaela Hostiuc, Mohamed Hsairi, Vivian Chia-rong Hsieh, Rebecca L Hsu, Chengxi Hu, Michael Hultström, Ayesha Humayun, Tsegaye Gebreyes Hundie, Javid Hussain, M Azhar Hussain, Nawfal R Hussein, Foziya Mohammed Hussien, Hong-Han Huynh, Bing-Fang Hwang, Segun Emmanuel Ibitoye, Khalid S Ibrahim, Pulwasha Maria Iftikhar, Desta Ijo, Kevin S Ikuta, Paul Chukwudi Ikwegbue, Olayinka Stephen Ilesanmi, Irena M Ilic, Milena D Ilic, Mohammad Tarique Imam, Mustapha Immurana, Sumant Inamdar, Muhammad Iqhrammullah, Arnaud Iradukunda, Kenneth Chukwuemeka Iregbu, Md Rabiul Islam, Sheikh Mohammed Shariful Islam, Farhad Islami, Faisal Ismail, Nahlah Elkudssiah Ismail, Gaetano Isola, Masao Iwagami, Chidozie C D Iwu, Mahalaxmi Iyer, Linda Merin J, Jalil Jaafari, Louis Jacob, Kathryn H Jacobsen, Farhad Jadidi-Niaragh,

Morteza Jafarinia, Abdollah Jafarzadeh, Khushleen Jaggi, Nader Jahanmehr, Haitham Jahrami, Nityanand Jain, Ammar Abdulrahman Jairoun, Abhishek Jaiswal, Elham Jamshidi, Mark M Janko, Abubakar Ibrahim Jatau, Sabzali Javadov, Tahereh Javaheri, Sathish Kumar Jayapal, Shubha Jayaram, Rime Jebai, Sun Ha Jee, Jayakumar Jeganathan, Anil K Jha, Ravi Prakash Jha, Heng Jiang, Yingzhao Jin, Olatunji Johnson, Mohammad Jokar, Jost B Jonas, Tamas Joo, Abel Joseph, Nitin Joseph, Charity Ehimwenma Joshua, Grace Joshy, Jacek Jerzy Jozwiak, Mikk Jürisson, Vaishali K, Billingsley Kaambwa, Ali Kabir, Zubair Kabir, Vidya Kadashetti, Dler Hussein Kadir, Rizwan Kalani, Laleh R Kalankesh, Leila R Kalankesh, Feroze Kaliyadan, Sanjay Kalra, Vineet Kumar Kamal, Sivesh Kathir Kamarajah, Rajesh Kamath, Zahra Kamiab, Naser Kamyari, Thanigaivelan Kanagasabai, Tanuj Kanchan, Himal Kandel, Arun R Kanmanthareddy, Edmund Wedam Kanmiki, Kehinde Kazeem Kanmodi, Suthanthira Kannan S, Sushil Kumar Kansal, Rami S Kantar, Neeti Kapoor, Mehrdad Karajizadeh, Ibraheem M Karaye, André Karch, Asima Karim, Salah Eddin Karimi, Arman Karimi Behnagh, Faizan Zaffar Kashoo, Qalandar Hussein Abdulkarim Kasnazani, Nicholas J Kassebaum, Joonas H Kauppila, Navjot Kaur, Gbenga A Kayode, Foad Kazemi, Sina Kazemian, Tahseen Haider Kazmi, Getu Mosisa Kebebew, Adera Debella Kebede, Fassikaw Kebede, Peter Njenga Keiyoro, Jaimon Terence Kelly, John H Kempen, Jessica A Kerr, Himanshu Khajuria, Amirmohammad Khalaji, Nauman Khalid, Anees Ahmed Khalil, Alireza Khalilian, Faham Khamesipour, Ajmal Khan, Asaduzzaman Khan, Ikramullah Khan, Imteyaz A Khan, M Nuruzzaman Khan, Maseer Khan, Mohammad Jobair Khan, Moien AB Khan, Zeeshan Ali Khan, Mahammed Ziauddin Khan suheb, Shaghayegh Khanmohammadi, Khaled Khatab, Haitham Khatatbeh, Moawiah Mohammad Khatatbeh, Armin Khavandegar, Feriha Fatima Khidri, Mohammad Khorgamphar, Moein Khormali, Zahra Khorrami, Ahmad Khosravi, Mohammad Ali Khosravi, Zemene Demelash Kifle, Grace Kim, Jihee Kim, Kwanghyun Kim, Min Seo Kim, Yun Jin Kim, Ruth W Kimokoti, Adnan Kisa, Sezer Kisa, Desmond Klu, Ann Kristin Skrindo Knudsen, Jonathan M Kocarnik, Sonali Kochhar, Timea Kocsis, David S Q Koh, Ali-Asghar Kolahi, Kairi Kolves, Farzad Kompani, Gerbrand Koren, Karel Kostev, Parvaiz A Koul, Sindhura Lakshmi Koulmane Laxminarayana, Kewal Krishan, Hare Krishna, Varun Krishna, Vijay Krishnamoorthy, Yuvaraj Krishnamoorthy, Barthelemy Kuate Defo, Md Abdul Kuddus, Mohammed Kuddus, Ilari Kuitunen, Vishnutheertha Kulkarni, Akshay Kumar, Ashish Kumar, Harish Kumar, Manasi Kumar, Fantahun Tarekegn Kumie, Satyajit Kundu, Om P Kurmi, Asep Kusnali, Dian Kusuma, Alexander Kwarteng, Ilias Kyriopoulos, Hmwe Hmwe Kyu, Carlo La Vecchia, Muhammad Awwal Ladan, Abraham K Lagat, Daphne Teck Ching Lai, Dharmesh Kumar Lal, Tea Lallukka, Hilton Lam, Judit Lám, Francesco Lanfranchi, Justin J Lang, Van Charles Lansingh, Savita Lasrado, Zohra S Lassi, Kamaluddin Latief, Kaveh Latifinaibin, Paolo Lauriola, Nhi Huu Hanh Le, Thao Thi Thu Le, Trang Diep Thanh Le, Caterina Ledda, Jorge R Ledesma, Munjae Lee, Seung Won Lee, Shaun Wen Huey Lee, Wei-Chen Lee, Yo Han Lee, Kate E LeGrand, James Leigh, Elvynna Leong, Temesgen L Lerango, Ming-Chieh Li, Wei Li, Xiaopan Li, Zhihui Li, Virendra S Ligade, Andrew Tiyamike Makhiringa Likaka, Lee-Ling Lim, Stephen S Lim, Chaojie Liu, Gang Liu, Jue Liu, Runben Liu, Shiwei Liu, Xiaofeng Liu, Xuefeng Liu, Erand Llanaj, Rubén López-Bueno, Arianna Maever Loreche, Stefan Lorkowski, Paulo A Lotufo, Jailos Lubinda, Giancarlo Lucchetti, Alessandra Lugo, Zheng Feei Ma, Monika Machoy, Farzan Madadizadeh, Christian Madsen, Áurea M Madureira-Carvalho, Azzam A Maghazachi, Sandeep B Maharaj, Soleiman Mahjoub, Mansour Adam Mahmoud, Alireza Mahmoudi, Elham Mahmoudi, Razzagh Mahmoudi, Azeem Majeed, Elaheh Malakan Rad, Venkatesh Maled, Reza Malekzadeh, Armaan K Malhotra, Kashish Malhotra, Ahmad Azam Malik, Iram Malik, Deborah Carvalho Malta, Abdullah A Mamun, Pejman Mansouri, Mohammad Ali Mansournia, Lorenzo Giovanni Mantovani, Sajid Maqsood, Hamid Reza Marateb, Joemer C Maravilla, Agustina M Marconi, Parham Mardi, Mirko Marino, Abdoljalal Marjani, Gabriel Martinez, Bernardo Alfonso Martinez-Guerra, Ramon Martinez-Piedra, Daniela Martini, Santi Martini, Francisco Rogerlândio Martins-Melo, Miquel Martorell, Wolfgang Marx, Sharmeen Maryam,

Roy Rillera Marzo, Anthony Masaka, Awoke Masrie, Alexander G Mathioudakis, Manu Raj Mathur, Jishanth Mattumpuram, Richard Matzopoulos, Richard James Maude, Andrea Maugeri, Mahsa Mayeli, Maryam Mazaheri, Mohsen Mazidi, John J McGrath, Martin McKee, Anna Laura W McKowen, Steven M McPhail, Enkeleint A Mechili, John Robert Carabeo Medina, Rishi P Mediratta, Jitendra Kumar Meena, Rahul Mehra, Kamran Mehrabani-Zeinabad, Entezar Mehrabi Nasab, Tesfahun Mekene Meto, Gebrekiros Gebremichael Meles, Max Alberto Mendez Mendez-Lopez, Walter Mendoza, Ritesh G Menezes, Belayneh Mengist, Alexios-Fotios A Mentis, Sultan Ayoub Meo, Haftu Asmerom Meresa, Atte Meretoja, Tuomo J Meretoja, Abera M Mersha, Bezawit Afework Mesfin, Tomislav Mestrovic, Kukulege Chamila Dinushi Mettananda, Sachith Mettananda, Peter Meylakhs, Adquate Mhlanga, Laurette Mhlanga, Tianyue Mi, Tomasz Miazgowski, Georgia Micha, Irmina Maria Michalek, Ted R Miller, Edward J Mills, Le Huu Nhat Minh, GK Mini, Andreea Mirica, Erkin M Mirrakhimov, Mizan Kiros Mirutse, Maryam Mirzaei, Awoke Misganaw, Sanjeev Misra, Prasanna Mithra, Chaitanya Mittal, Madeline E Moberg, Ashraf Mohamadkhani, Jama Mohamed, Mouhand F H Mohamed, Nouh Saad Mohamed, Sakineh Mohammad-Alizadeh-Charandabi, Soheil Mohammadi, Abdollah Mohammadian-Hafshejani, Noushin Mohammadifard, Hassen Mohammed, Mustapha Mohammed, Salahuddin Mohammed, Shafiu Mohammed, Hoda Mojiri-Forushani, Amin Mokari, Ali H Mokdad, Sabrina Molinaro, Mariam Molokhia, Sara Momtazmanesh, Lorenzo Monasta, Stefania Mondello, Mohammad Ali Moni, AmirAli Moodi Ghalibaf, Maryam Moradi, Yousef Moradi, Maziar Moradi-Lakeh, Maliheh Moradzadeh, Paula Moraga, Lidia Morawska, Rafael Silveira Moreira, Negar Morovatdar, Jonathan F Mosser, Rohith Motappa, Vincent Mougin, Simin Mouodi, Seyed Ehsan Mousavi, Amin Mousavi Khaneghah, Emmanuel A Mpolya, Sumaira Mubarik, Lorenzo Muccioli, Ulrich Otto Mueller, Faraz Mughal, Sumoni Mukherjee, Francesk Mulita, Kavita Munjal, Efrén Murillo-Zamora, Christopher J L Murray, Fungai Musaigwa, Khaled M Musallam, Ghulam Mustafa, Saravanan Muthupandian, Raman Muthusamy, Muhammad Muzaffar, Woojae Myung, Ahamarshan Jayaraman Nagarajan, Mohsen Naghavi, Pirouz Naghavi, Aliya Naheed, Ganesh R Naik, Gurudatta Naik, Firzan Nainu, Sanjeev Nair, Hastyar Hama Rashid Najmuldeen, Vinay Nangia, Atta Abbas Naqvi, Sreenivas Narasimha Swamy, Aparna Ichalangod Narayana, Shumaila Nargus, Bruno Ramos Nascimento, Abdulqadir J Nashwan, Zuhair S Natto, Javaid Nauman, Muhammad Naveed, Biswa Prakash Nayak, Vinod C Nayak, Athare Nazri-Panjaki, Rawlance Ndejjo, Hadush Negash, Ionut Negoi, Ruxandra Irina Negoi, Seyed Aria Nejadghaderi, Chakib Nejjari, Evangelia Nena, Samata Nepal, Marie Ng, Haruna Asura Nggada, Georges Nguefack-Tsague, Josephine W Ngunjiri, Anh Hoang Nguyen, Dang H Nguyen, Phat Tuan Nguyen, Van Thanh Nguyen, Robina Khan Niazi, Katie R Nielsen, Yeshambel T Nigatu, Ali Nikoobar, Amin Reza Nikpoor, Dina Nur Anggraini Ningrum, Chukwudi A Nnaji, Lawrence Achilles Nnyanzi, Efaq Ali Noman, Shuhei Nomura, Mamoona Noreen, Nafise Noroozi, Bo Norrving, Jean Jacques Noubiap, Chisom Adaobi Nri-Ezedi, George Ntaios, Mpiko Ntsekhe, Dieta Nurrika, Jerry John Nutor, Bogdan Oancea, Kehinde O Obamiro, Mary Aigbiremo Oboh, Ismail A Odetokun, Nkechi Martina Odogwu, Martin James O'Donnell, Michael Safo Oduro, Akinyemi O D Ofakunrin, Ayodipupo Sikiru Oguntade, Hassan Okati-Aliabad, Akinkunmi Paul Okekunle, Osaretin Christabel Okonji, Andrew T Olagunju, Muideen Tunbosun Olaiya, Matthew Idowu Olatubi, Gláucia Maria Moraes Oliveira, Isaac Iyinoluwa Olufadewa, Bolajoko Olubukunola Olusanya, Jacob Olusegun Olusanya, Yinka Doris Oluwafemi, Hany A Omar, Ahmed Omar Bali, Goran Latif Omer, Maureene Auma Ondayo, Kanyin Liane Ong, Obinna E Onwujekwe, Kenneth Ikenna Onyedibe, Michal Ordak, Doris V Ortega-Altamirano, Alberto Ortiz, Wael M S Osman, Samuel M Ostroff, Uchechukwu Levi Osuagwu, Adrian Otoiu, Nikita Otstavnov, Stanislav S Otstavnov, Amel Ouyahia, Mayowa O Owolabi, Mahesh Padukudru P A, Jagadish Rao Padubidri, Pramod Kumar Pal, Tamás Palicz, Claudia Palladino, Raffaele Palladino, Feng Pan, Hai-Feng Pan, Adrian Pana, Paramjot Panda, Songhomitra Panda-Jonas, Seithikurippu R Pandi-Perumal, Helena

Ullyartha Pangaribuan, Georgios D Panos, Leonidas D Panos, Ioannis Pantazopoulos, Anca Mihaela Pantea Stoian, Paraskevi Papadopoulou, Romil R Parikh, Seoyeon Park, Ashwaghosha Parthasarathi, Ava Pashaei, Maja Pasovic, Roberto Passera, Deepak Kumar Pasupula, Hemal M Patel, Jay Patel, Sangram Kishor Patel, Shankargouda Patil, Dimitrios Patoulias, Uttam Paudel, Amy E Peden, Paolo Pedersini, Veincent Christian Filipino Pepito, Emmanuel K Peprah, Prince Peprah, João Perdigão, Marcos Pereira, Mario F P Peres, Arokiasamy Perianayagam, Richard G Pestell, Konrad Pesudovs, Fanny Emily Petermann-Rocha, William A Petri, Hoang Tran Pham, Anil K Philip, Michael R Phillips, Daniela Pierannunzio, Manon Pigeolet, David M Pigott, Zahra Zahid Piracha, Michael A Piradov, Saeed Pirouzpanah, Nishad Plakkal, Evgenii Plotnikov, Vivek Podder, Dimitri Poddighe, Kevan R Polkinghorne, Ramesh Poluru, Ville T Ponkilainen, Fabio Porru, Maarten J Postma, Govinda Raj Poudel, Akram Pourshams, Naeimeh Pourtaheri, Sergio I Prada, Pranil Man Singh Pradhan, Manya Prasad, Akila Prashant, Elton Junio Sady Prates, TINA PRISCILLA, Natalie Pritchett, Bharathi M Purohit, Jagadeesh Puvvula, Nameer Hashim Qasim, Ibrahim Qattea, Asma Saleem Qazi, Gangzhen Qian, Suli Qiu, Maryam Faiz Qureshi, Amir Radfar, Raghu Anekal Radhakrishnan, Venkatraman Radhakrishnan, Hadi Raeisi Shahraki, Quinn Rafferty, Alberto Raggi, Pankaja Raghav Raghav, Fakher Rahim, Md Jillur Rahim, Vafa Rahimi-Movaghar, Md Mosfequr Rahman, Mohammad Hifz Ur Rahman, Mosiur Rahman, Muhammad Aziz Rahman, Amir Masoud Rahmani, Shayan Rahmani, Vahid Rahmanian, Sathish Rajaa, Prashant Rajput, Ivo Rakovac, Shakthi Kumaran Ramasamy, Kritika Rana, Chhabi Lal Ranabhat, Nemanja Rancic, Amey Rane, Chythra R Rao, Indu Ramachandra Rao, Mithun Rao, Sowmya J Rao, Drona Prakash Rasali, Sina Rashedi, Vahid Rashedi, Mohammad-Mahdi Rashidi, Azad Rasul, Giridhara Rathnaiah Babu, Santosh Kumar Rauniyar, Ramin Ravangard, Nakul Ravikumar, David Laith Rawaf, Salman Rawaf, Lal Rawal, Reza Rawassizadeh, Bharat Rawlley, Rabail Zehra Raza, Christian Razo, Elrashdy Moustafa Mohamed Redwan, Faizan Ur Rehman, Lennart Reifels, Robert C Reiner Jr, Luis Felipe Reyes, Maryam Rezaei, Nazila Rezaei, Negar Rezaei, Mohsen Rezaeian, Taeho Gregory Rhee, Mavra A Riaz, Antonio Luiz P Ribeiro, Jennifer Rickard, Hannah R Riva, Célia Fortuna Rodrigues, Mónica Rodrigues, Leonardo Roever, Emma Lynn Best Rogowski, Peter Rohloff, Debby Syahru Romadlon, Esperanza Romero-Rodríguez, Gholamreza Roshandel, Himanshu Sekhar Rout, Nitai Roy, Priyanka Roy, Enrico Rubagotti, Godfrey M Rwegerera, Andrzej Rynkiewicz, Chandan S N, Aly M A Saad, Zahra Saadatian, Korosh Saber, Maha Mohamed Saber-Ayad, Morteza SaberiKamarposhti, Siamak Sabour, Perminder S Sachdev, Basema Saddik, Bashdar Abuzed Sadee, Ehsan Sadeghi, Erfan Sadeghi, Mohammad Reza Saeb, Umar Saeed, Sher Zaman Safi, Rajesh Sagar, Amene Saghazadeh, Fatemeh Saheb Sharif-Askari, Narjes Saheb Sharif-Askari, Soumya Swaroop Sahoo, Umakanta Sahoo, Monalisha Sahu, Zahra Saif, Mirza Rizwan Sajid, Joseph W Sakshaug, Payman Salamati, Afeez Abolarinwa Salami, Mohamed A Saleh, Sana Salehi, Marwa Rashad Salem, Hossein Samadi Kafil, Sara Samadzadeh, Saad Samargandy, Yoseph Leonardo Samodra, Abdallah M Samy, Juan Sanabria, Itamar S Santos, Milena M Santric-Milicevic, Bruno Piassi Sao Jose, Sivan Yegnanarayana Iyer Saraswathy, Babak Saravi, Yaser Sarikhani, Tanmay Sarkar, Rodrigo Sarmiento-Suárez, Gargi Sachin Sarode, Sachin C Sarode, Arash Sarveazad, Brijesh Sathian, Thirunavukkarasu Sathish, Maheswar Satpathy, Abu Sayeed, Md Abu Sayeed, Mete Saylan, Mehdi Sayyah, Nikolaos Scarmeas, Benedikt Michael Schaarschmidt, Markus P Schlaich, Winfried Schlee, Ione Jayce Ceola Schneider, Art Schuermans, Austin E Schumacher, Aletta Elisabeth Schutte, Michaël Schwarzinger, David C Schwebel, Falk Schwendicke, Mario Šekerija, Sabyasachi Senapati, Subramanian Senthilkumaran, Sadaf G Sepanlou, Dragos Serban, Yashendra Sethi, Feng Sha, Amir Shafaat, Mahan Shafie, Nilay S Shah, Pritik A Shah, Syed Mahboob Shah, Saeed Shahabi, Ataollah Shahbandi, Izza Shahid, Samiah Shahid, Wajeehah Shahid, Hamid R Shahsavari, Moyad Jamal Shahwan, Masood Ali Shaikh, Alireza Shakeri, Ali S Shalash, Muhammad Aaqib Shamim, Mehran Shams-Beyranvand, Hina Shamshad, Mohammad Anas Shamsi,

Mohd Shanawaz, Abhishek Shankar, Sadaf Sharfaei, Amin Sharifan, Javad Sharifi-Rad, Rajesh Sharma, Saurab Sharma, Vishal Sharma, Rajesh P Shastry, Amin Shavandi, Maryam Shayan, Aziz Sheikh, Rahim Ali Sheikhi, Jiabin Shen, Pavanchand H Shetty, Kenji Shibuya, Desalegn Shiferaw, Mika Shigematsu, Min-Jeong Shin, Youn Ho Shin, Rahman Shiri, Nebiyu Aniley Shitaye, Aminu Shittu, Ivy Shiue, K M Shivakumar, Velizar Shivarov, Farhad Shokraneh, Azad Shokri, Sina Shool, Seyed Afshin Shorofi, Sunil Shrestha, Kerem Shuval, Emmanuel Edwar Siddig, João Pedro Silva, Luís Manuel Lopes Rodrigues Silva, Soraia Silva, Anjali Singal, Abhinav Singh, Balbir Bagicha Singh, Garima Singh, Jasbir Singh, Narinder Pal Singh, Paramdeep Singh, Dhirendra Narain Sinha, Robert Sinto, Md Shahjahan Siraj, Freddy Sitas, Shravan Sivakumar, Valentin Yurievich Skryabin, Anna Aleksandrovna Skryabina, David A Sleet, Hamidreza Soleimani, Sameh S M Soliman, Suhang Song, Yimeng Song, Reed J D Sorensen, Ireneous N Soyiri, Michael Spartalis, Chandrashekhar T Sreeramareddy, Jeffrey D Stanaway, Muhammad Haroon Stanikzai, Benjamin A Stark, Joseph R Starnes, Antonina V Starodubova, Fridolin Steinbeis, Caitlyn Steiner, Jaimie D Steinmetz, Paschalis Steiropoulos, Aleksandar Stevanović, Leo Stockfelt, Mark A Stokes, Stefan Stortecky, Vetriselvan Subramaniyan, Muhammad Suleman, Rizwan Suliankatchi Abdulkader, Abida Sultana, Haitong Zhe Sun, Jing Sun, David Sunkersing, Chandan Kumar Swain, Lukasz Szarpak, Mindy D Szeto, Miklós Szócska, Payam Tabaee Damavandi, Rafael Tabarés-Seisdedos, Shima Tabatabai, Mohammad Tabish, JYOTHI TADAKAMADLA, Santosh Kumar Tadakamadla, Yasaman Taheri Abkenar, Moslem Taheri Soodejani, Jabeen Taiba, Ken Takahashi, Iman M Talaat, Ashis Talukder, Mircea Tampa, Jacques Lukenze Tamuzi, Ker-Kan Tan, Sarmila Tandukar, Haosu Tang, Hong K Tang, Ingan Ukur Tarigan, Mengistie Kassahun Tariku, Md Tariqujjaman, Elvis Enowbeyang Tarkang, Razieh Tavakoli Oliaee, Seyed Mohammad Tavangar, Yibekal Manaye Tefera, Mohamad-Hani Temsah, Reem Mohamad Hani Temsah, Masayuki Teramoto, Enoch Teye-Kwadjo, Rishu Thakur, Pugazhenthan Thangaraju, Kavumpurathu Raman Thankappan, Rasiah Thayakaran, Nihal Thomas, Nikhil Kenny Thomas, Azalea M Thomson, Amanda G Thrift, Chern Choong Chern Thum, Lau Caspar Thygesen, Jing Tian, Ales Tichopad, Jansje Henny Vera Ticoalu, Tala Tillawi, Tenaw Yimer Tiruye, Marcello Tonelli, Roman Topor-Madry, Adetunji T Toriola, Mathilde Touvier, Marcos Roberto Tovani-Palone, Jasmine T Tran, Nghia Minh Tran, Domenico Trico, Samuel Joseph Tromans, Guesh Mebrahtom Tsegay, Evangelia Eirini Tsermpini, Munkhtuya Tumurkhuu, Kang Tung, Stefanos Tyrovolas, Sayed Mohammad Nazim Uddin, Aniefiok John Udoakang, Arit Udoh, Atta Ullah, Irfan Ullah, Saeed Ullah, Sana Ullah, Srikanth Umakanthan, Chukwuma David Umeokonkwo, Bhaskaran Unnikrishnan, Carolyn Anne Unsworth, Era Upadhyay, Daniele Urso, Jibrin Sammani Usman, Seyed Mohammad Vahabi, Asokan Govindaraj Vaithinathan, Rohollah Valizadeh, Sarah M Van de Velde, Jef Van den Eynde, Orsolya Varga, Priya Vart, Shoban Babu Varthya, Milena Vasic, Siavash Vaziri, Balachandar Vellingiri, Narayanaswamy Venketasubramanian, Madhur Verma, Massimiliano Veroux, Georgios-Ioannis Verras, Dominique Vervoort, Jorge Hugo Villafañe, Gabriela Ines Villanueva, Manish Vinayak, Francesco S Violante, Maria Viskadourou, Bay Vo, Stein Emil Vollset, Theo Vos, Isidora S Vujcic, Hatem A Wafa, Yasir Waheed, Richard G Wamai, Cong Wang, Shu Wang, Song Wang, Yanzhong Wang, Yuan-Pang Wang, Muhammad Waqas, Paul Ward, Emebet Gashaw Wassie, Stephanie Louise Watson Watson, Kosala Gayan Weerakoon, Melissa Y Wei, Ronny Westerman, Joanna L Whisnant, Taweewat Wiangkham, Dakshitha Praneeth Wickramasinghe, Nuwan Darshana Wickramasinghe, Angga Wilandika, Caroline Wilkerson, Peter Willeit, Shadrach Wilson, Axel Walter Wolf, Yohannes Addisu Wondimagegene, Yen Jun Wong, Utoomporn Wongsin, Eve E Wool, Ai-Min Wu, Chenkai Wu, Felicia Wu, Zenghong Wu, Juan Xia, Hong Xiao, Yang Xie, Wang-Dong Xu, Xiaoyue Xu, Yvonne Yiru Xu, Ali Yadollahpour, Kazumasa Yamagishi, Danting Yang, Lin Yang, Yuichiro Yano, Yao Yao, Habib Yaribeygi, Pengpeng Ye, Sisay Shewasinad Yehualashet, Metin Yesiltepe, Subah Abderehim Yesuf, Saber Yezli, Siyan Yi, Amanuel Yigezu, Arzu Yiğit, Vahit Yiğit, Paul Yip, Malede Berihun Yismaw, Yazachew

Yismaw, Dong Keon Yon, Naohiro Yonemoto, Seok-Jun Yoon, Yuyi You, Mustafa Z Younis, Zabihollah Yousefi, Chuanhua Yu, Yong Yu, Faith H Yuh, Siddhesh Zadey, Vesna Zadnik, Nima Zafari, Fathiah Zakham, Nazar Zaki, Sojib Bin Zaman, Nelson Zamora, Moein Zangiabadian, Armin Zarrintan, Mohammed G M Zeariya, Haijun Zhang, Jianrong Zhang, Jingya Zhang, Liqun Zhang, Yunquan Zhang, Zhi-Jiang Zhang, Hanqing Zhao, Chenwen Zhong, Juexiao Zhou, Bin Zhu, Lei Zhu, Magdalena Zielińska, Osama A Zitoun, Mohammad Zoladl, Liesl J Zuhlke, Alimuddin Zumla, Elric Zweck, Samer H Zyoud.

Drafting the work or revising it critically for important intellectual content

Amirali Aali, Hazim S Ababneh, Yohannes Habtegiorgis Abate, Cristiana Abbafati, Mohammadreza Abbasian, Mohsen Abbasi-Kangevari, Samar Abd ElHafeez, Michael Abdelmasseh, Sherief Abd-Elsalam, Ahmed Abdelwahab, Mohammad-Amin Abdollahifar, Auwal Abdullahi, Mesfin Abebe, Samrawit Shawel Abebe, Aidin Abedi, Kedir Hussein Abegaz, Hassan Abidi, Olumide Abiodun, Hassan Abolhassani, Meysam Abolmaali, Mohamed Abouzid, Girma Beressa Aboye, Lucas Guimarães Abreu, Dariush Abtahi, Samir Abu Rumeileh, Hasan Abualruz, Bilyaminu Abubakar, Eman Abu-Gharbieh, Niveen ME Abu-Rmeileh, Salahdein Aburuz, Ahmed Abu-Zaid, Tadele Girum Adal, Abdu A Adamu, Isaac Yeboah Addo, Giovanni Addolorato, Akindele Olupelumi Adebiyi, Victor Adekanmbi, Abiola Victor Adepoju, Charles Oluwaseun Adetunji, Juliana Bunmi Adetunji, Daniel Adedayo Adeyinka, Olorunsola Israel Adeyomoye, Biruk Adie Admass, Qorinah Estiningtyas Sakilah Adnani, Saryia Adra, Aanuoluwapo Adeyimika Afolabi, Muhammad Sohail Afzal, Saira Afzal, Pradyumna Agasthi, Manik Aggarwal, Feleke Doyore Agide, Antonella Agodi, Bright Opoku Ahinkorah, Danish Ahmad, Firdos Ahmad, Muayyad M Ahmad, Ali Ahmed, Ayman Ahmed, Haroon Ahmed, Luai A Ahmed, Muktar Beshir Ahmed, Syed Anees Ahmed, Marjan Ajami, Essona Matatom Akara, Hossein Akbarialiabad, Mohammed Ahmed Akkaif, Samuel Akyirem, Fares Alahdab, Samer O Alalalmeh, Tariq A Alalwan, Khurshid Alam, Rasmieh Mustafa Al-amer, Sayer Al-Azzam, Almaza Albakri, Mohammed Albashtawy, Mohammad T AlBataineh, Khalifah A Aldawsari, Wafa A Aldhaleei, Mulubirhan Assefa Alemayohu, Yihun Mulugeta Alemu, Khalid F Alhabib, Fadwa Alhalaiqa Naji Alhalaiqa, Mohammed Khaled Al-Hanawi, Abid Ali, Amjad Ali, Liaqat Ali, Mohammed Usman Ali, Rafat Ali, Shahid Ali, Syed Shujait Shujait Ali, Gianfranco Alicandro, Ahmednur Adem Aliyi, Peter Allebeck, Sadeq Ali Ali Al-Maweri, Louay Almidani, Mahmoud A Alomari, Jordi Alonso, Jaber S Alqahtani, Shehabaldin Alqalyoobi, Ahmed Yaseen Alqutaibi, Awais Altaf, Jaffar A Al-Tawfiq, Deborah Oyine Aluh, Nelson Alvis-Guzman, Hassan Alwafi, Yaser Mohammed Al-Worafi, Hany Aly, Safwat Aly, Karem H Alzoubi, Reza Amani, Azmeraw T Amare, Prince M Amegbor, Tarek Tawfik Amin, Alireza Amindarolzarbi, Sohrab Amiri, Mohammad Hosein Amirzade-Iranaq, Hubert Amu, Dickson A Amugsi, Ganiyu Adeniyi Amusa, Robert Ancuceanu, Deanna Anderlini, David B Anderson, Pedro Prata Andrade, Catalina Liliana Andrei, Tudorel Andrei, Colin Angus, Abhishek Anil, Sneha Anil, Amir Anoushiravani, Hossein Ansari, Catherine M Antony, Ernoiz Antriyandarti, Saeid Anvari, Saleha Anwar, Anayochukwu Edward Anyasodor, Muhammad Aqeel, Juan Pablo Arab, Jalal Arabloo, Mosab Arafat, Abdulfatai Aremu, Olatunde Aremu, Hany Ariffin, Mesay Arkew, Benedetta Armocida, Johan Ärnlöv, Mahwish Arooj, Judie Arulappan, Raphael Taiwo Aruleba, Ashokan Arumugam, Malke Asaad, Akeza Awealom Asgedom, Mona Asghariahmadabad, Muhammad Ashraf, Armin Aslani, Seyyed Shamsadin Athari, Bantalem Tilaye Tilaye Atinafu, Habtamu Wondmagegn Atlaw, Prince Atorkey, Maha Moh'd Wahbi Atout, Alok Atreya, Avinash Aujayeb, Marcel Ausloos, Abolfazl Avan, Amlaku Mulat Aweke, Beatriz Paulina Ayala Quintanilla, Sina Azadnajafabad, Rui M S Azevedo, Ahmed Y Azzam, Abraham Samuel Babu, Muhammad Badar, Ashish D Badiye, Soroush Baghdadi, Sara Bagherieh, Sulaiman Bah, Ruhai Bai, Atif Amin Baig, Jennifer L Baker, Abdulaziz T Bako, Senthilkumar Balakrishnan, Madhan Balasubramanian, Ovidiu Constantin Baltatu, Kiran Bam, Maciej Banach, Soham Bandyopadhyay, Palash

Chandra Banik, Hansi Bansal, Kannu Bansal, Martina Barchitta, Mainak Bardhan, Erfan Bardideh, Suzanne Lyn Barker-Collo, Till Winfried Bärnighausen, Francesco Barone-Adesi, Hiba Jawdat Barqawi, Amadou Barrow, Azadeh Bashiri, Afisu Basiru, Pritish Baskaran, Buddha Basnyat, Quique Bassat, João Diogo Basso, Sanjay Basu, Bernhard T Baune, Thomas Beaney, Neeraj Bedi, Massimiliano Beghi, Priyamadhaba Behera, Amir Hossein Behnoush, Masoud Behzadifar, Maryam Beiranvand, Diana Fernanda Bejarano Ramirez, Yannick Béjot, Sefealem Assefa Belay, Chalie Mulu Belete, Michelle L Bell, Muhammad Bashir Bello, Olorunjuwon Omolaja Bello, Luis Belo, Apostolos Beloukas, Isabela M Bensenor, Zombor Berezvai, Gregory J Bertolacci, Paulo J G Bettencourt, Kebede A Beyene, Akshaya Srikanth Bhagavathula, Neeraj Bhala, Dinesh Bhandari, Prarthna V Bhardwaj, Ashish Bhargava, Sonu Bhaskar, Vivek Bhat, Gurjit Kaur Bhatti, Jasvinder Singh Bhatti, Manpreet S Bhatti, Rajbir Bhatti, Boris Bikbov, Catherine Bisignano, Atanu Biswas, Saeid Bitaraf, Veera R Bitra, Tone Bjørge, Mary Sefa Boampong, Anca Vasilica Bobirca, Virginia Bodolica, Aadam Olalekan Bodunrin, Milad Bonakdar Hashemi, Aime Bonny, Kaustubh Bora, Hamed Borhany, Arturo Borzutzky, Souad Bouaoud, Christopher Boxe, Edward J Boyko, Oliver J Brady, Dejana Braithwaite, Luisa C Brant, Javier Brazo-Sayavera, Nicholas J K Breitborde, Susanne Breitner, Hermann Brenner, Andrey Nikolaevich Briko, Nikolay Ivanovich Briko, Gabrielle Britton, Julie Brown, Traolach Brugha, Norma B Bulamu, Danilo Buonsenso, Yasser Bustanji, Florentino Luciano Caetano dos Santos, Daniela Calina, Luciana Aparecida Campos, Ismael R Campos-Nonato, Yin Cao, Angelo Capodici, Sinclair Carr, Giulia Carreras, Juan J Carrero, Andrea Carugno, Felix Carvalho, Joao Mauricio Castaldelli-Maia, Carlos A Castañeda-Orjuela, Giulio Castelpietra, Ferrán Catalá-López, Alberico L Catapano, Maria Sofia Cattaruzza, Christopher R Cederroth, Luca Cegolon, Francieli Cembranel, Muthia Cenderadewi, Ester Cerin, Muge Cevik, Joshua Chadwick, Yaacoub Chahine, Chiranjib Chakraborty, Jeffrey Shi Kai Chan, Rama Mohan Chandika, Eeshwar K Chandrasekar, Chin-Kuo Chang, Vijay Kumar Chattu, Pankaj Chaturvedi, Victoria Chatzimavridou-Grigoriadou, Akhilanand Chaurasia, An-Tian Chen, Haowei Chen, Meng Xuan Chen, Simiao Chen, Nicolas Cherbuin, Wondimye Ashenafi Cheru, Ju-Huei Chien, Ritesh Chimoriya, Patrick R Ching, Jesus Lorenzo Chirinos-Caceres, William C S Cho, Bryan Chong, Hitesh Chopra, Rajiv Chowdhury, Devasahayam J Christopher, Eric Chung, Muhammad Chutiyami, Iolanda Cioffi, Mareli M Claassens, Rafael M Claro, Alyssa Columbus, Joao Conde, Samuele Cortese, Paolo Angelo Cortesi, Vera Marisa Costa, Ewerton Cousin, Rosa A S Couto, Richard G Cowden, Michael H Criqui, Natália Cruz-Martins, Silvia Magali Cuadra-Hernández, Patricia Cullen, Matthew Cunningham, Sriharsha Dadana, Siyu Dai, Zhaoli Dai, Giovanni Damiani, Saswati Das, Subasish Das, Ana Maria Dascalu, Nihar Ranjan Dash, Mohsen Dashti, Gail Davey, Claudio Alberto Dávila-Cervantes, Nicole Davis Weaver, Diego De Leo, Katie de Luca, Shayom Debopadhaya, Louisa Degenhardt, Cristian Del Bo', Ivan Delgado-Enciso, Andreas K Demetriades, Edgar Denova-Gutiérrez, Niloofar Deravi, Nebiyu Dereje, Nikolaos Dervenis, Don C Des Jarlais, Hardik Dineshbhai Desai, Rupak Desai, Syed Masudur Rahman Dewan, Arkadeep Dhali, Meghnath Dhimal, Sameer Dhingra, Vishal R Dhulipala, Diana Dias da Silva, Daniel Diaz, Michael J Diaz, Adriana Dima, Delaney D Ding, M Ashworth Dirac, Camila Bruneli do Prado, Masoud Dodangeh, Milad Dodangeh, Sushil Dohare, Wanyue Dong, Deepa Dongarwar, Mario D'Oria, E Ray Dorsey, Wendel Mombaque dos Santos, Rajkumar Doshi, Leila Doshmangir, Robert Kokou Dowou, Tim Robert Driscoll, Haneil Larson Dsouza, Mi Du, John Dube, Bruce B Duncan, Senbagam Duraisamy, Oyewole Christopher Durojaiye, Arkadiusz Marian Dziedzic, Abdel Rahman E'mar, Ejemai Eboreime, Chidiebere Peter Echieh, David Edvardsson, Diyan Ermawan Effendi, Ebrahim Eini, Michael Ekholuenetale, Iman El Sayed, Iffat Elbarazi, Teshome Bekele Elema, Noha Mousaad Elemam, Frank J Elgar, Islam Y Elgendy, Ghada Metwally Tawfik ElGohary, Muhammed Elhadi, Omar Abdelsadek Abdou Elmeligy, Mohamed A Elmonem, Mohammed Elshaer, Ibrahim Elsohaby, Theophilus I Emeto, Ryenchindorj Erkhembayar, Christopher Imokhuede Esezobor, Sharareh Eskandarieh, Juan Espinosa-

Montero, Farshid Etaee, Natalia Fabin, Adeniyi Francis Fagbamigbe, Ayesha Fahim, Saman Fahimi, Aliasghar Fakhri-Demeshghieh, Luca Falzone, Mohammad Fareed, MoezAlIslam Ezzat Mahmoud Faris, Andre Faro, Ali Fatehizadeh, Nelsensius Klau Fauk, Pooria Fazeli, Valery L Feigin, Seyed-Mohammad Fereshtehnejad, Abdullah Hamid Feroze, Nuno Ferreira, Getahun Fetensa, Irina Filip, Florian Fischer, Joanne Flavel, David Flood, Nataliya A Foigt, Morenike Oluwatoyin Folayan, Behzad Foroutan, Masoud Foroutan, Ingeborg Forthun, Matteo Foschi, Kate Louise Francis, Richard Charles Franklin, Alberto Freitas, Joseph Friedman, Takeshi Fukumoto, Peter Andras Gaal, Muktar A Gadanya, Santosh Gaihre, Yaseen Galali, Silvano Gallus, Aravind P Gandhi, Balasankar Ganesan, Mohammad Arfat Ganiyani, MA Garcia-Gordillo, Jalaj Garg, Semiu Olatunde Gbadamosi, Tilaye Gebru Gebi, Miglas W Gebregergis, Simona Roxana Georgescu, Tamirat Getachew, Sulmaz Ghahramani, Mohammad-Reza Ghasemi, Ghazal Ghasempour Dabaghi, Afsaneh Ghasemzadeh, Fariba Ghassemi, Ramy Mohamed Ghazy, Ajnish Ghimire, Sama Ghoba, Nasim Gholizadeh, Mahsa Ghorbani, Paramjit Singh Gill, Tiffany K Gill, Richard F Gillum, Alem Girmay, James C Glasbey, Elena V Gnedovskaya, Laszlo Göbölös, Myron Anthony Godinho, Mohamad Goldust, Nelson G M Gomes, Philimon N Gona, Sameer Vali Gopalani, Giuseppe Gorini, Alessandra C Goulart, Bárbara Niegia Garcia Goulart, Anmol Goyal, Ayman Grada, Michal Grivna, Shi-Yang Guan, Giovanni Guarducci, Mesay Dechasa Gudeta, Avirup Guha, Stefano Guicciardi, Snigdha Gulati, Damitha Asanga Gunawardane, Sasidhar Gunturu, Cui Guo, Bhawna Gupta, Manoj Kumar Gupta, Mohak Gupta, Rajat Das Gupta, Rajeev Gupta, Sapna Gupta, Veer Bala Gupta, Vivek Kumar Gupta, Lami Gurmessa, Reyna Alma Gutiérrez, Farrokh Habibzadeh, Parham Habibzadeh, Mostafa Hadei, Najah R Hadi, Nils Haep, Nima Hafezi-Nejad, Alemayehu Hailu, Arvin Haj-Mirzaian, Esam S Halboub, Brian J Hall, Rabih Halwani, Randah R Hamadeh, Sajid Hameed, Nasrin Hanifi, Graeme J Hankey, Fahad Hanna, Md Abdul Hannan, Harapan Harapan, Josep Maria Haro, Ahmed I Hasaballah, M Tasdik Hasan, Hamidreza Hasani, Abdiwahab Hashi, Md Saquib Hasnain, Johannes Haubold, Rasmus J Havmoeller, Simon I Hay, Jeffrey J Hebert, Omar E Hegazi, Golnaz Heidari, Mohammad Heidari, Bartosz Helfer, Claudiu Herteliu, Hamed Hesami, Kamal Hezam, Yuta Hiraike, Ramesh Holla, Nobuyuki Horita, Md Mahbub Hossain, Sahadat Hossain, Mohammad-Salar Hosseini, Ahmad Hosseinzadeh Adli, Sorin Hostiuc, Vivian Chia-rong Hsieh, Rebecca L Hsu, Junjie Huang, Michael Hultström, Tsegaye Gebreyes Hundie, Javid Hussain, M Azhar Hussain, Foziya Mohammed Hussien, Hong-Han Huynh, Segun Emmanuel Ibitoye, Pulwasha Maria Iftikhar, Desta Ijo, Adalia I Ikiroma, Paul Chukwudi Ikwegbue, Olayinka Stephen Ilesanmi, Irena M Ilic, Milena D Ilic, Mustapha Immurana, Sumant Inamdar, Endang Indriasih, Muhammad Iqhrammullah, Arnaud Iradukunda, Kenneth Chukwuemeka Iregbu, Md Rabiul Islam, Sheikh Mohammed Shariful Islam, Farhad Islami, Faisal Ismail, Nahlah Elkudssiah Ismail, Hiroyasu Iso, Gaetano Isola, Chidozie C D Iwu, Ihoghosa Osamuyi Iyamu, Mahalaxmi Iyer, Linda Merin J, Louis Jacob, Kathryn H Jacobsen, Morteza Jafarinia, Abdollah Jafarzadeh, Khushleen Jaggi, Kasra Jahankhani, Nader Jahanmehr, Haitham Jahrami, Abhishek Jaiswal, Mark M Janko, Sathish Kumar Jayapal, Shubha Jayaram, Rime Jebai, Ravi Prakash Jha, Jost B Jonas, Tamas Joo, Abel Joseph, Nitin Joseph, Charity Ehimwenma Joshua, Jacek Jerzy Jozwiak, Mikk Jürisson, Ali Kabir, Vidya Kadashetti, Rizwan Kalani, Laleh R Kalankesh, Feroze Kaliyadan, Sanjay Kalra, Sivesh Kathir Kamarajah, Rajesh Kamath, Thanigaivelan Kanagasabai, Himal Kandel, Arun R Kanmanthareddy, Edmund Wedam Kanmiki, Kehinde Kazeem Kanmodi, Suthanthira Kannan S, Rami S Kantar, Neeti Kapoor, Shama D Karanth, Reema A Karasneh, André Karch, Asima Karim, Faizan Zaffar Kashoo, Hengameh Kasraei, Nicholas J Kassebaum, Joonas H Kauppila, Navjot Kaur, Norito Kawakami, Gbenga A Kayode, Foad Kazemi, Tahseen Haider Kazmi, Getu Mosisa Kebebew, Fassikaw Kebede, Tibebeselassie S Keflie, Peter Njenga Keiyoro, Jaimon Terence Kelly, John H Kempen, Jessica A Kerr, Emmanuelle Kesse-Guyot, Himanshu Khajuria, Amirmohammad Khalaji, Nauman Khalid, Anees Ahmed Khalil, Ajmal Khan, Gulfaraz Khan, Imteyaz A Khan, M Nuruzzaman Khan, Maseer Khan, Mohammad

Jobair Khan, Moien AB Khan, Zeeshan Ali Khan, Mahammed Ziauddin Khan suheb, Shaghayegh Khanmohammadi, Khaled Khatab, Haitham Khatatbeh, Moawiah Mohammad Khatatbeh, Armin Khavandegar, Hamid Reza Khayat Kashani, Feriha Fatima Khidri, Elaheh Khodadoust, Mohammad Khorgamphar, Ahmad Khosravi, Grace Kim, Jihee Kim, Kwanghyun Kim, Min Seo Kim, Yun Jin Kim, Kasey E Kinzel, Adnan Kisa, Desmond Klu, Ann Kristin Skrindo Knudsen, Jonathan M Kocarnik, Sonali Kochhar, Timea Kocsis, Kairi Kolves, Farzad Kompani, Parvaiz A Koul, Sindhura Lakshmi Koulmane Laxminarayana, Kewal Krishan, Varun Krishna, Kris J Krohn, Barthelemy Kuate Defo, Burcu Kucuk Bicer, Md Abdul Kuddus, Mohammed Kuddus, Ilari Kuitunen, Mukhtar Kulimbet, Vishnutheertha Kulkarni, Akshay Kumar, Manasi Kumar, Rakesh Kumar, Madhulata Kumari, Fantahun Tarekegn Kumie, Om P Kurmi, Asep Kusnali, Dian Kusuma, Alexander Kwarteng, Hmwe Hmwe Kyu, Carlo La Vecchia, Ben Lacey, Muhammad Awwal Ladan, Lucie Laflamme, Anton C J Lager, Abdelilah Lahmar, Ratilal Lalloo, Tea Lallukka, Hilton Lam, Judit Lám, Kelsey R Landrum, Justin J Lang, Berthold Langguth, Van Charles Lansingh, Ariane Laplante-Lévesque, Bagher Larijani, Anders O Larsson, Savita Lasrado, Kamaluddin Latief, Kaveh Latifinaibin, Nhi Huu Hanh Le, Thao Thi Thu Le, Caterina Ledda, Munjae Lee, Paul H Lee, Kate E LeGrand, Elvynna Leong, Wei Li, Zhihui Li, Lee-Ling Lim, Christine Linehan, Chaojie Liu, Jue Liu, Runben Liu, Xiaofeng Liu, Erand Llanaj, Michael J Loftus, Rubén López-Bueno, Platon D Lopukhov, Paulo A Lotufo, Giancarlo Lucchetti, Alessandra Lugo, Raimundas Lunevicius, Zheng Feei Ma, Nikolaos Machairas, Monika Machoy, Farzan Madadizadeh, Áurea M Madureira-Carvalho, Soleiman Mahjoub, Mansour Adam Mahmoud, Elham Mahmoudi, Razzagh Mahmoudi, Irsa Fatima Makhdoom, Elaheh Malakan Rad, Venkatesh Maled, Reza Malekzadeh, Armaan K Malhotra, Kashish Malhotra, Ahmad Azam Malik, Deborah Carvalho Malta, Abdullah A Mamun, Lorenzo Giovanni Mantovani, Sajid Maqsood, Bishnu P Marasini, Hamid Reza Marateb, Joemer C Maravilla, Agustina M Marconi, Parham Mardi, Mirko Marino, Bernardo Alfonso Martinez-Guerra, Ramon Martinez-Piedra, Daniela Martini, Francisco Rogerlândio Martins-Melo, Miquel Martorell, Wolfgang Marx, Sharmeen Maryam, Roy Rillera Marzo, Awoke Masrie, Stephanie Mathieson, Alexander G Mathioudakis, Jishanth Mattumpuram, Andrea Maugeri, Pallab K Maulik, Mahsa Mayeli, Anna Laura W McKowen, Susan A McLaughlin, Steven M McPhail, Enkeleint A Mechili, Rishi P Mediratta, Jitendra Kumar Meena, Kamran Mehrabani-Zeinabad, Entezar Mehrabi Nasab, Max Alberto Mendez Mendez-Lopez, Walter Mendoza, Ritesh G Menezes, Alexios-Fotios A Mentis, Sultan Ayoub Meo, Haftu Asmerom Meresa, Atte Meretoja, Tuomo J Meretoja, Tomislav Mestrovic, Kukulege Chamila Dinushi Mettananda, Sachith Mettananda, Tomasz Miazgowski, Georgia Micha, Irmina Maria Michalek, Ted R Miller, Edward J Mills, Le Huu Nhat Minh, GK Mini, Pouya Mir Mohammad Sadeghi, Antonio Mirijello, Awoke Misganaw, Ashim Mishra, Philip B Mitchell, Prasanna Mithra, Chaitanya Mittal, Mouhand F H Mohamed, Nouh Saad Mohamed, Sakineh Mohammad-Alizadeh-Charandabi, Soheil Mohammadi, Abdollah Mohammadian-Hafshejani, Hussen Mohammed, Mustapha Mohammed, Salahuddin Mohammed, Shafiu Mohammed, Viswanathan Mohan, Amin Mokari, Ali H Mokdad, Sabrina Molinaro, Mariam Molokhia, Sara Momtazmanesh, Lorenzo Monasta, Stefania Mondello, Mohammad Ali Moni, AmirAli Moodi Ghalibaf, Maryam Moradi, Yousef Moradi, Maziar Moradi-Lakeh, Maliheh Moradzadeh, Paula Moraga, Rafael Silveira Moreira, Shane Douglas Morrison, Jakub Morze, Jonathan F Mosser, Rohith Motappa, Vincent Mougin, Simin Mouodi, Parsa Mousavi, Seyed Ehsan Mousavi, Amin Mousavi Khaneghah, Emmanuel A Mpolya, Matías Mrejen, Ulrich Otto Mueller, Faraz Mughal, Francesk Mulita, Efrén Murillo-Zamora, Christopher J L Murray, Fungai Musaigwa, Khaled M Musallam, Ahmad Mustafa, Ghulam Mustafa, Saravanan Muthupandian, Muhammad Muzaffar, Ahamarshan Jayaraman Nagarajan, Gabriele Nagel, Mohsen Naghavi, Sanjeev Nair, Noureddin Nakhostin Ansari, Sreenivas Narasimha Swamy, Shumaila Nargus, Bruno Ramos Nascimento, Gustavo G Nascimento, Samar Nasehi, Abdulqadir J Nashwan, Zuhair S Natto, Javaid Nauman, Biswa Prakash Nayak, Vinod C Nayak, Sabina Onyinye

Nduaguba, Hadush Negash, Ionut Negoi, Ruxandra Irina Negoi, Serban Mircea Negru, Seyed Aria Nejadghaderi, Samata Nepal, Georges Nguefack-Tsague, Josephine W Ngunjiri, Dang H Nguyen, Phat Tuan Nguyen, Van Thanh Nguyen, Robina Khan Niazi, Katie R Nielsen, Yeshambel T Nigatu, Taxiarchis Konstantinos Nikolouzakis, Fatemeh Nikoomanesh, Amin Reza Nikpoor, Lawrence Achilles Nnyanzi, Mamoona Noreen, Bo Norrving, Jean Jacques Noubiap, Chisom Adaobi Nri-Ezedi, George Ntaios, Mpiko Ntsekhe, Virginia Nuñez-Samudio, Dieta Nurrika, Bogdan Oancea, Kehinde O Obamiro, Ismail A Odetokun, Nkechi Martina Odogwu, Martin James O'Donnell, Akinyemi O D Ofakunrin, Abiola Ogunkoya, Ayodipupo Sikiru Oguntade, In-Hwan Oh, Sylvester Reuben Okeke, Akinkunmi Paul Okekunle, Osaretin Christabel Okonji, Andrew T Olagunju, Matthew Idowu Olatubi, Bolajoko Olubukunola Olusanya, Jacob Olusegun Olusanya, Hany A Omar, Maureene Auma Ondayo, Obinna E Onwujekwe, Kenneth Ikenna Onyedibe, Michal Ordak, Orish Ebere Orisakwe, Verner N Orish, Doris V Ortega-Altamirano, Alberto Ortiz, Wael M S Osman, Samuel M Ostroff, Uchechukwu Levi Osuagwu, Adrian Otoiu, Nikita Otstavnov, Stanislav S Otstavnov, Amel Ouyahia, Guoqing Ouyang, Mayowa O Owolabi, Mahesh Padukudru P A, Alicia Padron-Monedero, Jagadish Rao Padubidri, Tamás Palicz, Raul Felipe Palma-Alvarez, Feng Pan, Paramjot Panda, Songhomitra Panda-Jonas, Helena Ullyartha Pangaribuan, Leonidas D Panos, Ioannis Pantazopoulos, Anca Mihaela Pantea Stoian, Romil R Parikh, Ashwaghosha Parthasarathi, Maja Pasovic, Roberto Passera, Jay Patel, Shankargouda Patil, Dimitrios Patoulias, Venkata Suresh Patthipati, Uttam Paudel, Hamidreza Pazoki Toroudi, Amy E Peden, Paolo Pedersini, Umberto Pensato, Veincent Christian Filipino Pepito, João Perdigão, Marcos Pereira, Mario F P Peres, Norberto Perico, Richard G Pestell, Konrad Pesudovs, Fanny Emily Petermann-Rocha, Hoang Tran Pham, Anil K Philip, Michael R Phillips, Daniela Pierannunzio, Manon Pigeolet, Thomas Pilgrim, Zahra Zahid Piracha, Michael A Piradov, Saeed Pirouzpanah, Nishad Plakkal, Vivek Podder, Dimitri Poddighe, Suzanne Polinder, Ville T Ponkilainen, Fabio Porru, Maarten J Postma, Govinda Raj Poudel, Sergio I Prada, Pranil Man Singh Pradhan, Thejeswar N Prakasham, Manya Prasad, Elton Junio Sady Prates, TINA PRISCILLA, Nameer Hashim Qasim, Ibrahim Qattea, Asma Saleem Qazi, Suli Qiu, Mehrdad Rabiee Rad, Amir Radfar, Raghu Anekal Radhakrishnan, Venkatraman Radhakrishnan, Hadi Raeisi Shahraki, Alberto Raggi, Pankaja Raghav Raghav, Fakher Rahim, Vafa Rahimi-Movaghar, Mohammad Hifz Ur Rahman, Shayan Rahmani, Sathish Rajaa, Prashant Rajput, Ivo Rakovac, Shakthi Kumaran Ramasamy, Kritika Rana, Chhabi Lal Ranabhat, Nemanja Rancic, Chythra R Rao, Indu Ramachandra Rao, Mithun Rao, Sowmya J Rao, Davide Rasella, Vahid Rashedi, Ashkan Rasouli-Saravani, Giridhara Rathnaiah Babu, Nakul Ravikumar, David Laith Rawaf, Salman Rawaf, Lal Rawal, Bharat Rawlley, Rabail Zehra Raza, Christian Razo, Elrashdy Moustafa Mohamed Redwan, Faizan Ur Rehman, Lennart Reifels, Giuseppe Remuzzi, Luis Felipe Reyes, Maryam Rezaei, Nazila Rezaei, Taeho Gregory Rhee, Mavra A Riaz, Antonio Luiz P Ribeiro, Jennifer Rickard, Hannah R Riva, Mónica Rodrigues, Leonardo Roever, Emma Lynn Best Rogowski, Peter Rohloff, Debby Syahru Romadlon, Esperanza Romero-Rodríguez, Michele Romoli, Luca Ronfani, Himanshu Sekhar Rout, Nitai Roy, Priyanka Roy, Enrico Rubagotti, Guilherme de Andrade Ruela, Susan Fred Rumisha, Godfrey M Rwegerera, Chandan S N, Aly M A Saad, Zahra Saadatian, Korosh Saber, Maha Mohamed Saber-Ayad, Morteza SaberiKamarposhti, Siamak Sabour, Simona Sacco, Perminder S Sachdev, Rajesh Sachdeva, Basema Saddik, Bashdar Abuzed Sadee, Farideh Sadeghian, Umar Saeed, Fahimeh Safaeinejad, Rajesh Sagar, Dominic Sagoe, Fatemeh Saheb Sharif-Askari, Narjes Saheb Sharif-Askari, Amirhossein Sahebkar, Soumya Swaroop Sahoo, Monalisha Sahu, Zahra Saif, Mirza Rizwan Sajid, Joseph W Sakshaug, Nasir Salam, Afeez Abolarinwa Salami, Luciane B Salaroli, Marwa Rashad Salem, Mohammed Z Y Salem, Sohrab Salimi, Hossein Samadi Kafil, Sara Samadzadeh, Saad Samargandy, Abdallah M Samy, Juan Sanabria, Itamar S Santos, Milena M Santric-Milicevic, Bruno Piassi Sao Jose, Made Ary Sarasmita, Aswini Saravanan, Babak Saravi, Yaser Sarikhani, Tanmay Sarkar, Rodrigo Sarmiento-Suárez, Gargi Sachin

Sarode, Sachin C Sarode, Arash Sarveazad, Thirunavukkarasu Sathish, Maheswar Satpathy, Abu Sayeed, Md Abu Sayeed, Mete Saylan, Mehdi Sayyah, Nikolaos Scarmeas, Benedikt Michael Schaarschmidt, Maria Inês Schmidt, Ione Jayce Ceola Schneider, Art Schuermans, Aletta Elisabeth Schutte, David C Schwebel, Falk Schwendicke, Mario Šekerija, Siddharthan Selvaraj, Sabyasachi Senapati, Sadaf G Sepanlou, Dragos Serban, Yashendra Sethi, Maryam Shabany, Mahan Shafie, Nilay S Shah, Pritik A Shah, Syed Mahboob Shah, Saeed Shahabi, Izza Shahid, Samiah Shahid, Moyad Jamal Shahwan, Ahmed Shaikh, Alireza Shakeri, Ali S Shalash, Muhammad Aaqib Shamim, Mehran Shams-Beyranvand, Mohammad Anas Shamsi, Mohd Shanawaz, Abhishek Shankar, Amin Sharifan, Javad Sharifi-Rad, Rajesh Sharma, Saurab Sharma, Ujjawal Sharma, Vishal Sharma, Rajesh P Shastry, Amr Mohamed Elsayed Shehabeldine, Jiabin Shen, Pavanchand H Shetty, Kenji Shibuya, Mika Shigematsu, Youn Ho Shin, Reza Shirkoohi, Nebiyu Aniley Shitaye, Aminu Shittu, K M Shivakumar, Sina Shool, Seyed Afshin Shorofi, Sunil Shrestha, Kerem Shuval, Emmanuel Edwar Siddig, João Pedro Silva, Luís Manuel Lopes Rodrigues Silva, Soraia Silva, Colin R Simpson, Abhinav Singh, Balbir Bagicha Singh, Garima Singh, Jasbir Singh, Narinder Pal Singh, Paramdeep Singh, Surjit Singh, Robert Sinto, Shravan Sivakumar, Valentin Yurievich Skryabin, Anna Aleksandrovna Skryabina, Bogdan Socea, Anton Sokhan, Ranjan Solanki, Shipra Solanki, Hamidreza Soleimani, Sameh S M Soliman, Suhang Song, Joan B Soriano, Ireneous N Soyiri, Michael Spartalis, Chandrashekhar T Sreeramareddy, Jeffrey D Stanaway, Muhammad Haroon Stanikzai, Joseph R Starnes, Antonina V Starodubova, Dan J Stein, Fridolin Steinbeis, Paschalis Steiropoulos, Leo Stockfelt, Stefan Stortecky, Vetriselvan Subramaniyan, Muhammad Suleman, Abida Sultana, Haitong Zhe Sun, Johan Sundström, David Sunkersing, Katharina S Sunnerhagen, Chandan Kumar Swain, Lukasz Szarpak, Miklós Szócska, Payam Tabaee Damavandi, Rafael Tabarés-Seisdedos, Ozra Tabatabaei Malazy, Seyed-Amir Tabatabaeizadeh, Shima Tabatabai, Mohammad Tabish, JYOTHI TADAKAMADLA, Santosh Kumar Tadakamadla, Yasaman Taheri Abkenar, Iman M Talaat, Mircea Tampa, Jacques Lukenze Tamuzi, Ker-Kan Tan, Elvis Enowbeyang Tarkang, Razieh Tavakoli Oliaee, Seyed Mohammad Tavangar, Nuno Taveira, Yibekal Manaye Tefera, Mohamad-Hani Temsah, Reem Mohamad Hani Temsah, Masayuki Teramoto, Riki Tesler, Pugazhenthan Thangaraju, Samar Tharwat, Nihal Thomas, Amanda G Thrift, Chern Choong Chern Thum, Jing Tian, Ales Tichopad, Tala Tillawi, Tenaw Yimer Tiruye, Mariya Vladimirovna Titova, Marcello Tonelli, Roman Topor-Madry, Adetunji T Toriola, Mathilde Touvier, Marcos Roberto Tovani-Palone, Jasmine T Tran, Nghia Minh Tran, Domenico Trico, Samuel Joseph Tromans, Thien Tan Tri Tai Truyen, Aristidis Tsatsakis, Evangelia Eirini Tsermpini, Kang Tung, Stefanos Tyrovolas, Aniefiok John Udoakang, Arit Udoh, Atta Ullah, Irfan Ullah, Saeed Ullah, Srikanth Umakanthan, Brigid Unim, Bhaskaran Unnikrishnan, Carolyn Anne Unsworth, Era Upadhyay, Daniele Urso, Jibrin Sammani Usman, Asokan Govindaraj Vaithinathan, Jef Van den Eynde, Orsolya Varga, Shoban Babu Varthya, Tommi Juhani Vasankari, Balachandar Vellingiri, Narayanaswamy Venketasubramanian, Madhur Verma, Massimiliano Veroux, Georgios-Ioannis Verras, Dominique Vervoort, Jorge Hugo Villafañe, Gabriela Ines Villanueva, Francesco S Violante, Maria Viskadourou, Vasily Vlassov, Stein Emil Vollset, Rade Vukovic, Hatem A Wafa, Yasir Waheed, Richard G Wamai, Cong Wang, Ning Wang, Shu Wang, Song Wang, Yanzhong Wang, Yuan-Pang Wang, Paul Ward, Emebet Gashaw Wassie, Stephanie Louise Watson Watson, Melissa Y Wei, Robert G Weintraub, Ronny Westerman, Taweewat Wiangkham, Dakshitha Praneeth Wickramasinghe, Nuwan Darshana Wickramasinghe, Caroline Wilkerson, Peter Willeit, Shadrach Wilson, Marcin W Wojewodzic, Axel Walter Wolf, Yen Jun Wong, Eve E Wool, Ai-Min Wu, Xinsheng Wu, Juan Xia, Hong Xiao, Kazumasa Yamagishi, Lin Yang, Yao Yao, Sisay Shewasinad Yehualashet, Metin Yesiltepe, Saber Yezli, Amanuel Yigezu, Arzu Yiğit, Vahit Yiğit, Dong Keon Yon, Naohiro Yonemoto, Nima Zafari, Sojib Bin Zaman, Ramin Zand, Heather J Zar, Iman Zare, Armin Zarrintan, Mohammed G M Zeariya, Zahra

Zeinali, Haijun Zhang, Jianrong Zhang, Chenwen Zhong, Makan Ziafati, Magdalena Zielińska, Osama A Zitoun, Mohammad Zoladl, Liesl J Zuhlke, Alimuddin Zumla, Samer H Zyoud.

Managing the estimation or publications process

Robert W Aldridge, Catherine M Antony, Catherine S Chen, Matthew Cunningham, Nicole Davis Weaver, Alize J Ferrari, Erin B Hamilton, Simon I Hay, Nicholas J Kassebaum, Molly B Kassel, Jonathan M Kocarnik, Kris J Krohn, Kate E LeGrand, Anna Laura W McKowen, Madeline E Moberg, Ali H Mokdad, Christopher J L Murray, Mohsen Naghavi, Amanda Novotney, Kanyin Liane Ong, Maja Pasovic, Caitlyn Steiner, Jaimie D Steinmetz, Anna E Torre, Eve E Wool.
