# Supplementary material for: Global burden of 288 causes of death and life expectancy decomposition in 204 countries and territories and 811 subnational locations, 1990–2021: a systematic analysis for the Global Burden of Disease Study 2021
Source: Lancet. 2024 May 18;403(10440):2100–32. doi: 10.1016/S0140-6736(24)00367-2 (PMC11126520; doi:10.1016/S0140-6736(24)00367-2)

THE LANCET

Supplementary appendix 2

This appendix formed part of the original submission and has been peer reviewed. We post it as supplied by the authors.

Supplement to: GBD 2021 Causes of Death Collaborators. Global burden of 288 causes of death and life expectancy decomposition in 204 countries and territories and 811 subnational locations, 1990–2021: a systematic analysis for the Global Burden of Disease Study 2021. *Lancet* 2024; published online April 3. https://doi.org/10.1016/S0140-6736(24)00367-2.

Appendix 2: supplementary results appendix to "Global burden of 288 causes of death and life expectancy decomposition in 204 countries and territories and 811 subnational locations, 1990–2021: a systematic analysis for the Global Burden of Disease Study 2021"

This appendix provides additional figures containing more detailed results for "Global burden of 288 causes of death and life expectancy decomposition in 204 countries and territories and 811 subnational locations, 1990–2021: a systematic analysis for the Global Burden of Disease Study 2021."

Figures

Figure S1 Percentage change in age-standardised mortality rate and number of deaths from previous year 4

Figure S2 Leading Level 3 causes of global age-standardised YLL rate per 100 000 for 1990, 2019, and 2021 for males and females combined 5

Figure S3 Age-standardised YLL rate per 100 000 by location for COVID-19 in 2020 (A), for other pandemic-related mortality in 2020 (B), for COVID-19 in 2021 (C), and for other pandemic-related mortality in 2021 (D)......6

Figure S4 Age-standardised mortality rate per 100 000 by location for COVID-19 in 2020 (A), for other pandemic-related mortality in 2020 (B), for COVID-19 in 2021 (C), and for other pandemic-related mortality in 2021 (D)......10

Figure S5 Sex difference in global mortality for Level 2 causes plus COVID-19 and other COVID-19 pandemic-related outcomes by age group, 2021......14

Figure S6 Number of years of life expectancy gained or lost due to different causes by countries in the central Europe, eastern Europe, and central Asia super-region, 1990–2021 15

Figure S7 Number of years of life expectancy gained or lost due to different causes by countries in the high-income super-region, 1990–2021 16

Figure S8 Number of years of life expectancy gained or lost due to different causes by countries in the Latin America and the Caribbean super-region, 1990–2021......17

Figure S9 Number of years of life expectancy gained or lost due to different causes by countries in the north Africa and the Middle East super-region, 1990–2021 18

Figure S10 Number of years of life expectancy gained or lost due to different causes by countries in the south Asia super-region, 1990–2021......19

Figure S11 Number of years of life expectancy gained or lost due to different causes by countries in the southeast Asia, east Asia, and Oceania super-region, 1990–2021 20

Figure S12 Number of years of life expectancy gained or lost due to different causes by countries in the sub-Saharan Africa super-region, 1990–2021 21

Figure S13 Impact of malaria on life expectancy by GBD region, over four periods of time 22

Figure S14 Impact of tuberculosis on life expectancy by GBD region, over four periods of time 23

Figure S15 Impact of measles on life expectancy by GBD region, over four periods of time......24

Figure S16 Impact of neonatal disorders on life expectancy by GBD region, over four periods of time 25

Figure S17 Impact of ischaemic heart disease on life expectancy by GBD region, over four periods of time 26

Figure S18 Impact of nutritional deficiencies on life expectancy by GBD region, over four periods of time.....27

Figure S19 Impact of neoplasms on life expectancy by GBD region, over four periods of time 28
Figure S20 Impact of chronic respiratory diseases on life expectancy by GBD region, over four periods of time……………..29
Figure S21 Impact of digestive diseases on life expectancy by GBD region, over four periods of time 30
Figure S22 Impact of diabetes and kidney diseases on life expectancy by GBD region, over four periods of time……………..31
Figure S23 Impact of stroke on life expectancy by GBD region, over four periods of time................................32
Figure S24 Impact of transport injuries on life expectancy by GBD region, over four periods of time 33
Figure S25 Impact of self-harm and interpersonal violence (except of war and terrorism) on life expectancy by GBD region, over four periods of time..34
Figure S26 Impact of unintentional injuries (except of natural disaster) on life expectancy by GBD region, over four periods of time 35
Figure S27 Impact of HIV/AIDS on life expectancy by GBD region, over four periods of time........................36
Figure S28 Under-5 mortality rate per 100 000 due to enteric infections by location, in 1990 (A) and 2021 (B)……………..37
Figure S29 Under-5 mortality rate per 100 000 due to lower respiratory infections by location, in 1990 (A) and 2021 (B)………...38
Figure S30 Age-standardised mortality rate per 100 000 due to HIV/AIDS by location, in 1990 (A) and 2021 (B)……………..39
Figure S31 Under-5 mortality rate per 100 000 due to malaria by location, in 1990 (A) and 2021 (B) 40
Figure S32 Age-standardised mortality rate per 100 000 due to tuberculosis by location, in 1990 (A) and 2021 (B)……………..41
Figure S33 Under-5 mortality rate per 100 000 due to measles by location, in 1990 (A) and 2021 (B)..............42
Figure S34 Neonatal mortality rate per 100 000 by location, in 1990 (A) and 2021 (B) 43
Figure S35 Under-5 mortality rate per 100 000 due to nutritional deficiencies by location, in 1990 (A) and 2021 (B)……………..44
Figure S36 Age-standardised mortality rate per 100 000 due to stroke by location, in 1990 (A) and 2021 (B)..45
Figure S37 Age-standardised mortality rate per 100 000 due to ischaemic heart disease by location, in 1990 (A) and 2021 (B)…..46
Figure S38 Age-standardised mortality rate per 100 000 due to neoplasms by location, in 1990 (A) and 2021 (B)……………..47
Figure S39 Age-standardised mortality rate per 100 000 due to chronic respiratory diseases by location, in 1990 (A) and 2021 (B) 48
Figure S40 Age-standardised mortality rate per 100 000 due to digestive diseases by location, in 1990 (A) and 2021 (B)………...49
Figure S41 Age-standardised mortality rate per 100 000 due to diabetes and kidney diseases by location, in 1990 (A) and 2021 (B)..50
Figure S42 Age-standardised mortality rate per 100 000 due to non-communicable diseases by location, in 1990 (A) and 2021 (B) 51
Figure S43 Age-standardised mortality rate per 100 000 due to transport injuries by location, in 1990 (A) and 2021 (B)………...52

Figure S44 Age-standardised mortality rate per 100 000 due to non-disaster unintentional injuries by location, in 1990 (A) and 2021 (B) 53
Figure S45 Age-standardised mortality rate per 100 000 due to intentional injuries (excluding war) by location, in 1990 (A) and 2021 (B) 54
Figure S46 Under-5 mortality rate per 100 000 due to diarrhoeal diseases by location, in 1990 (A) and 2021 (B)……………..55

Figure S1 Percentage change in age-standardised mortality rate and number of deaths from previous year

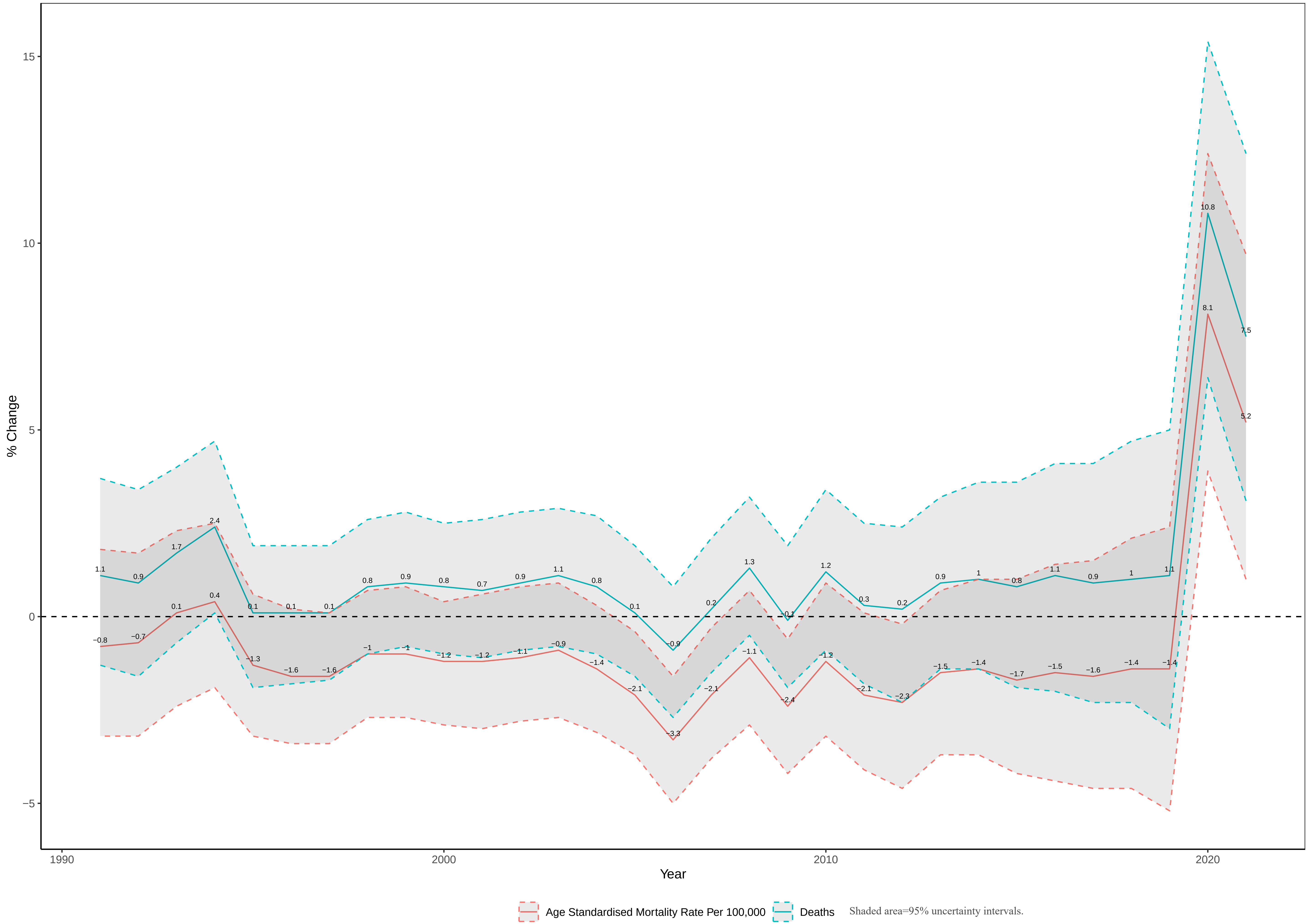

Shaded area=95% uncertainty intervals.

Figure S2 **Leading Level 3 causes of global age-standardised YLL rate per 100 000 for 1990, 2019, and 2021 for males and females combined**

Leading causes 1990	Age-standardized rate of YLLs (per 100,000) 1990	Leading causes 2019	Age-standardized rate of YLLs (per 100,000) 2019	Leading causes 2021	Age-standardized rate of YLLs (per 100,000) 2021
1 Neonatal disorders	4140.9 (3913.3 to 4394.2)	1 Neonatal disorders	2765.0 (2413.7 to 3172.9)	1 Neonatal disorders	2658.6 (2277.2 to 3090.3)
2 Lower respiratory infect	3463.8 (3088.8 to 3871.1)	2 Ischemic heart disease	2203.1 (2080.4 to 2308.6)	2 COVID-19	2324.5 (2202.2 to 2482.8)
3 Diarrheal diseases	3219.0 (2602.2 to 3878.6)	3 Stroke	1747.4 (1619.0 to 1865.3)	3 Ischemic heart disease	2164.5 (2026.6 to 2288.3)
4 Ischemic heart disease	3058.9 (2920.3 to 3172.7)	4 Lower respiratory infect	1463.3 (1286.9 to 1654.1)	4 Stroke	1707.3 (1573.0 to 1842.4)
5 Stroke	2885.5 (2719.9 to 3035.0)	5 Diarrheal diseases	834.1 (658.0 to 1042.3)	5 Lower respiratory infect	1162.6 (1009.9 to 1327.9)
6 Tuberculosis	1556.3 (1357.5 to 1713.4)	6 COPD	783.6 (722.4 to 841.3)	6 Other COVID Outcomes	951.3 (730.6 to 1264.9)
7 COPD	1323.7 (1185.6 to 1431.3)	7 Congenital defects	768.0 (656.2 to 946.1)	7 Malaria	774.8 (294.1 to 1541.5)
8 Congenital defects	1322.8 (825.6 to 1718.8)	8 Road injuries	741.3 (697.1 to 790.8)	8 COPD	766.2 (699.2 to 838.4)
9 Road injuries	1139.1 (1081.3 to 1201.4)	9 Malaria	683.2 (280.8 to 1301.8)	9 Diarrheal diseases	736.4 (569.6 to 923.6)
10 Measles	957.2 (342.4 to 1955.1)	10 Tuberculosis	564.7 (510.2 to 628.5)	10 Road injuries	711.3 (661.7 to 761.7)
11 Malaria	929.4 (465.6 to 1855.6)	11 Cirrhosis liver	551.5 (515.7 to 596.3)	11 Congenital defects	706.4 (593.9 to 871.5)
12 Cirrhosis liver	790.9 (725.6 to 884.9)	12 Lung cancer	534.0 (488.7 to 581.9)	12 Cirrhosis liver	537.6 (497.4 to 587.8)
13 Lung cancer	684.2 (649.3 to 721.1)	13 HIV/AIDS	505.4 (455.5 to 581.0)	13 Lung cancer	526.6 (473.5 to 581.2)
14 Self-harm	655.7 (566.1 to 695.5)	14 Diabetes	441.6 (417.7 to 462.9)	14 Tuberculosis	526.1 (468.7 to 595.8)
15 Drowning	644.7 (582.3 to 707.8)	15 Chronic kidney disease	425.8 (395.7 to 454.6)	15 HIV/AIDS	446.0 (408.4 to 502.5)
16 Protein-energy malnutrition	641.9 (531.7 to 797.2)	16 Self-harm	407.3 (382.1 to 430.4)	16 Diabetes	439.2 (410.6 to 465.8)
17 Stomach cancer	553.5 (497.1 to 602.6)	17 Alzheimer's disease	306.6 (76.2 to 811.2)	17 Chronic kidney disease	424.8 (391.4 to 456.2)
18 Meningitis	535.1 (465.7 to 618.2)	18 Hypertensive heart disease	299.2 (248.4 to 327.1)	18 Self-harm	399.5 (370.6 to 427.3)
19 Diabetes	413.4 (393.1 to 434.6)	19 Interpersonal violence	281.3 (268.2 to 297.2)	19 Alzheimer's disease	309.5 (77.2 to 820.6)
20 Hypertensive heart disease	396.2 (315.4 to 443.3)	20 Colorectal cancer	275.3 (256.2 to 292.2)	20 Hypertensive heart disease	289.6 (242.1 to 319.6)
21 Interpersonal violence	379.6 (367.1 to 391.8)	21 Stomach cancer	267.4 (232.2 to 301.1)	21 Colorectal cancer	270.7 (249.3 to 292.0)
22 Chronic kidney disease	376.3 (348.7 to 409.6)	23 Drowning	233.7 (210.9 to 259.4)	22 Interpersonal violence	272.5 (256.6 to 289.6)
25 Colorectal cancer	346.3 (326.8 to 362.4)	25 Meningitis	221.8 (177.3 to 282.3)		
26 HIV/AIDS	328.9 (254.0 to 432.9)	27 Protein-energy malnutrition	160.9 (133.7 to 190.5)		
29 Alzheimer's disease	308.1 (73.2 to 826.3)	37 Measles	121.5 (46.3 to 263.3)		

Legend:
Communicable, maternal, neonatal and nutritional
Non-communicable
Injuries

Global age–standardized mortality rate for (A) COVID 19 and (B) Other Pandemic Related Mortality per 100,000 population in 2020

(A) Age–standardized mortality rate for COVID 19, 2020

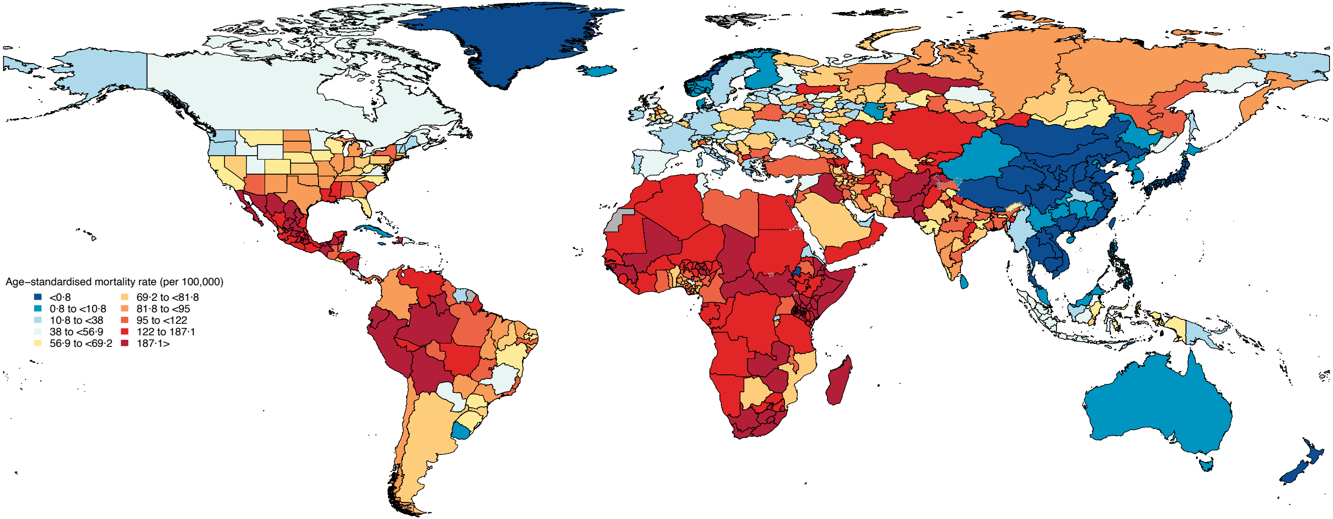

(B) Age–standardized mortality rate for Other Pandemic Related Mortality, 2020

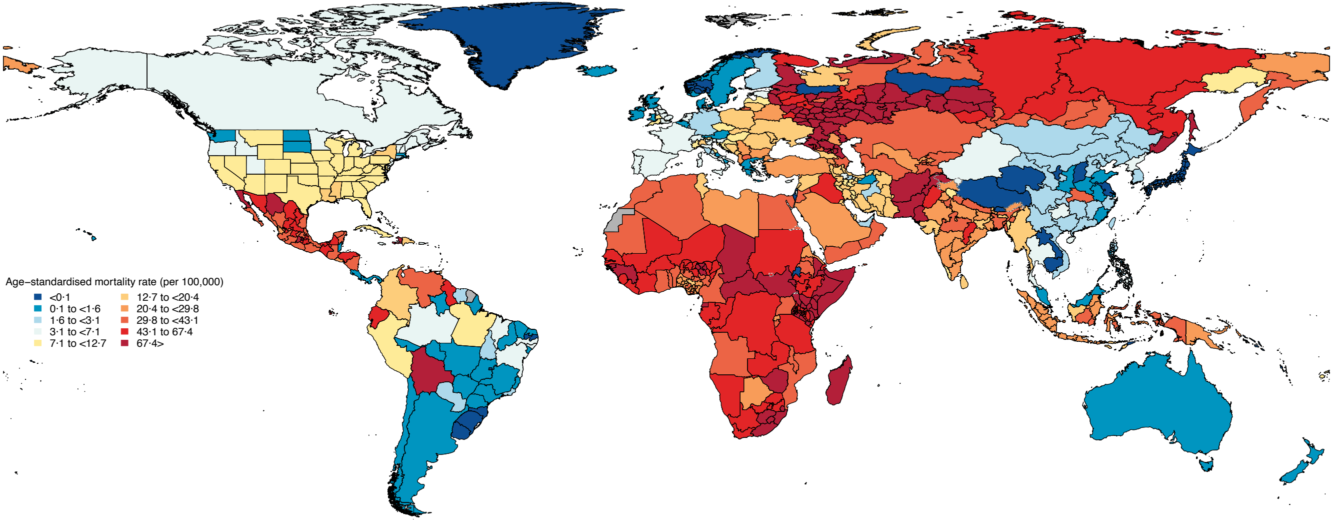

Figure S4 Age-standardised mortality rate per 100 000 by location for COVID-19 in 2020 (A), for other pandemic-related mortality in 2020 (B), for COVID-19 in 2021 (C), and for other pandemic-related mortality in 2021 (D)

(A) COVID-19 in 2020

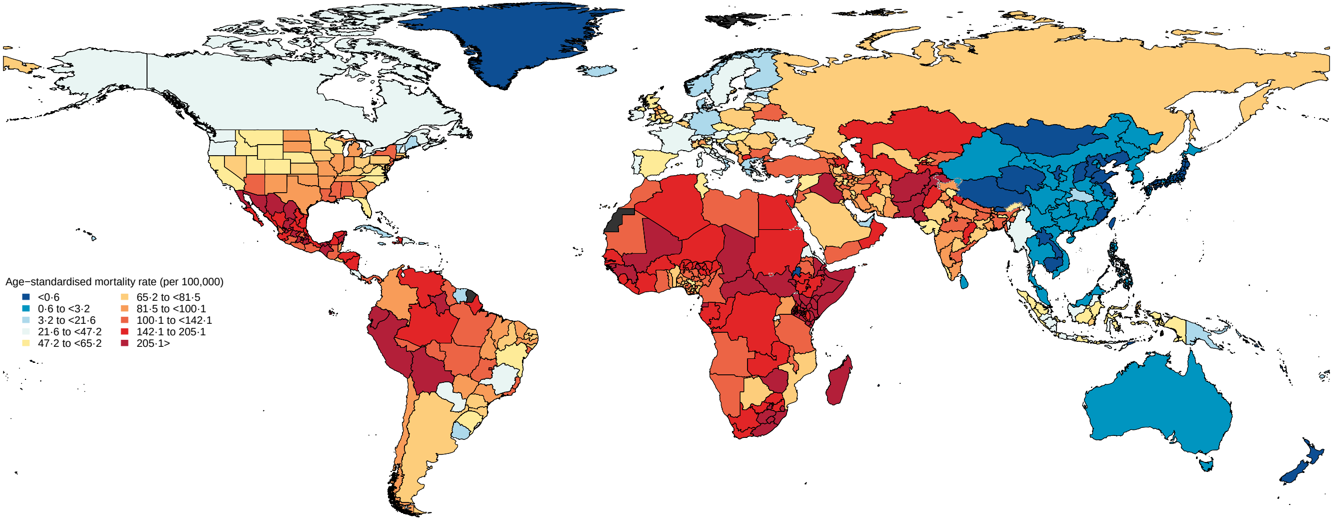

(B) Other pandemic-related mortality in 2020

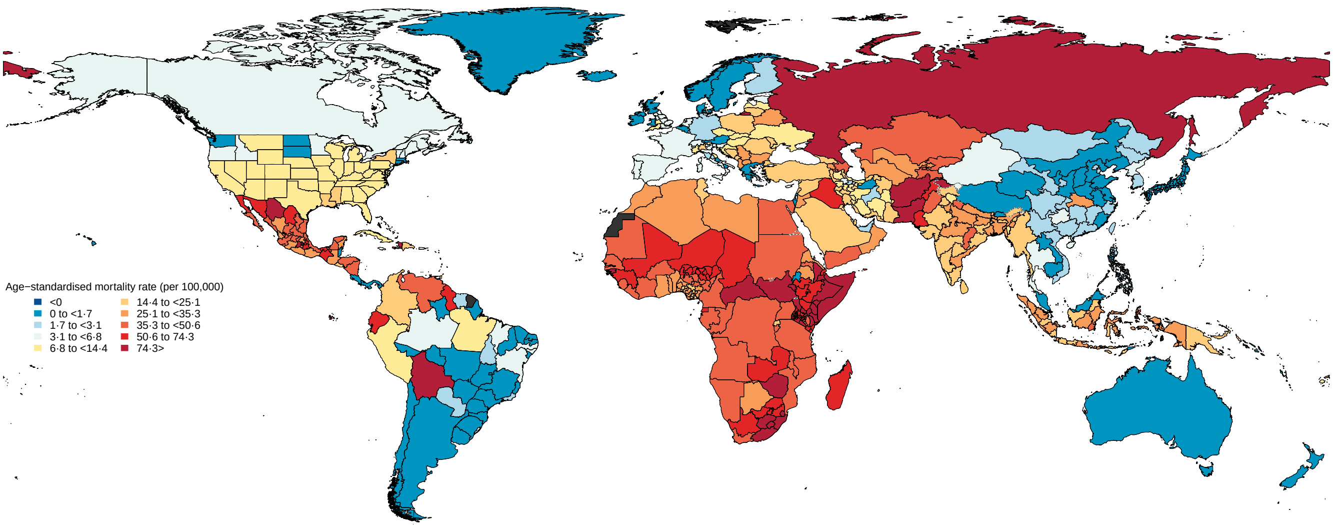

(C) COVID-19 in 2021

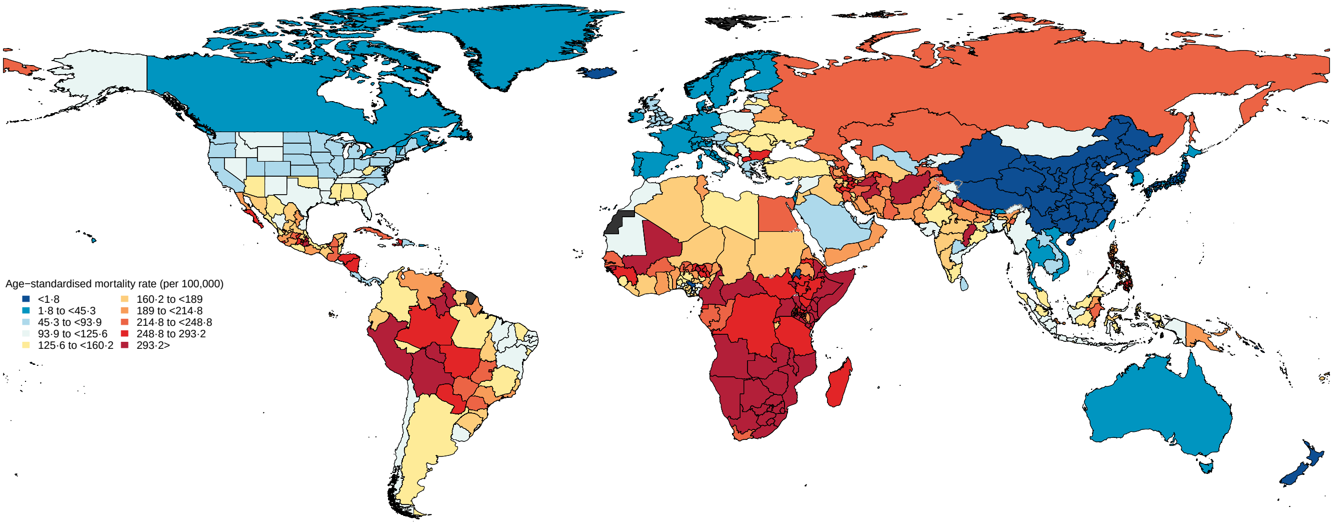

(D) Other pandemic-related mortality in 2021

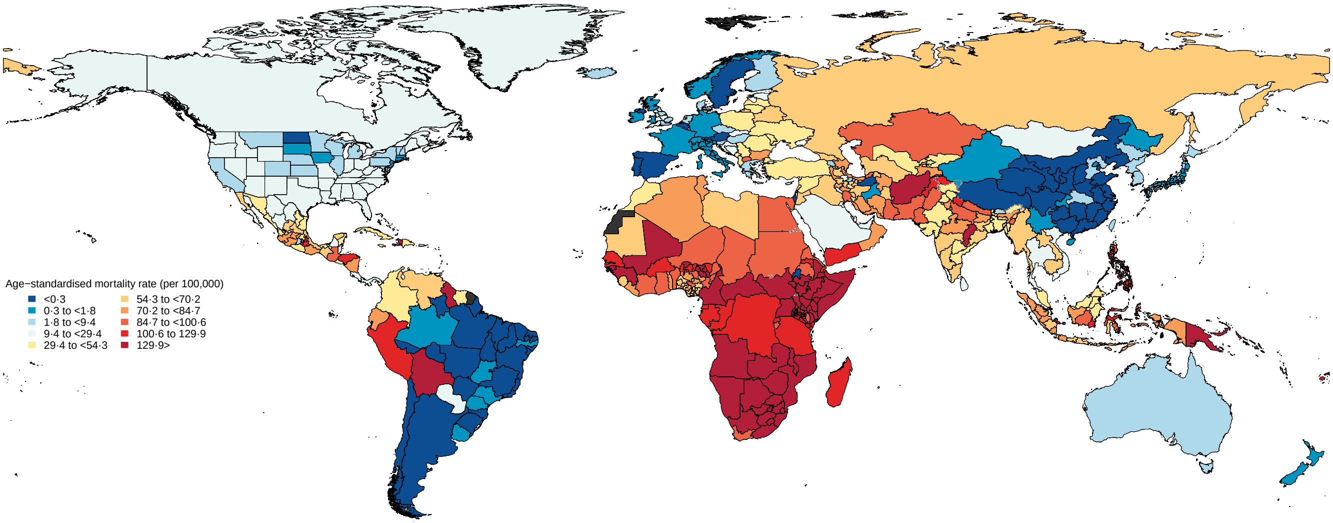

Figure S5 **Sex difference in global mortality for Level 2 causes plus COVID-19 and other COVID-19 pandemic-related outcomes by age group, 2021**

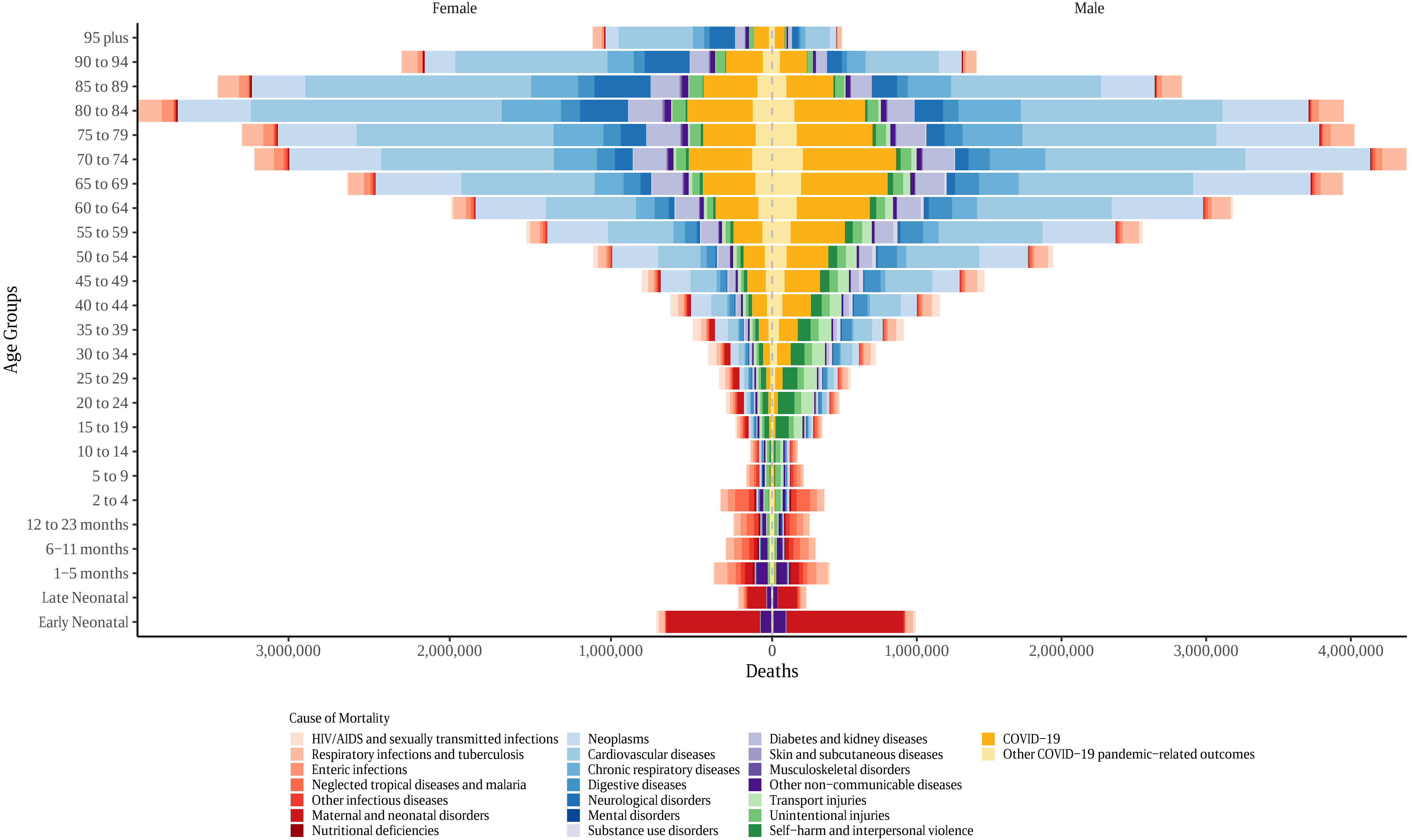

Figure S6 **Number of years of life expectancy gained or lost due to different causes by countries in the central Europe, eastern Europe, and central Asia super-region, 1990–2021**

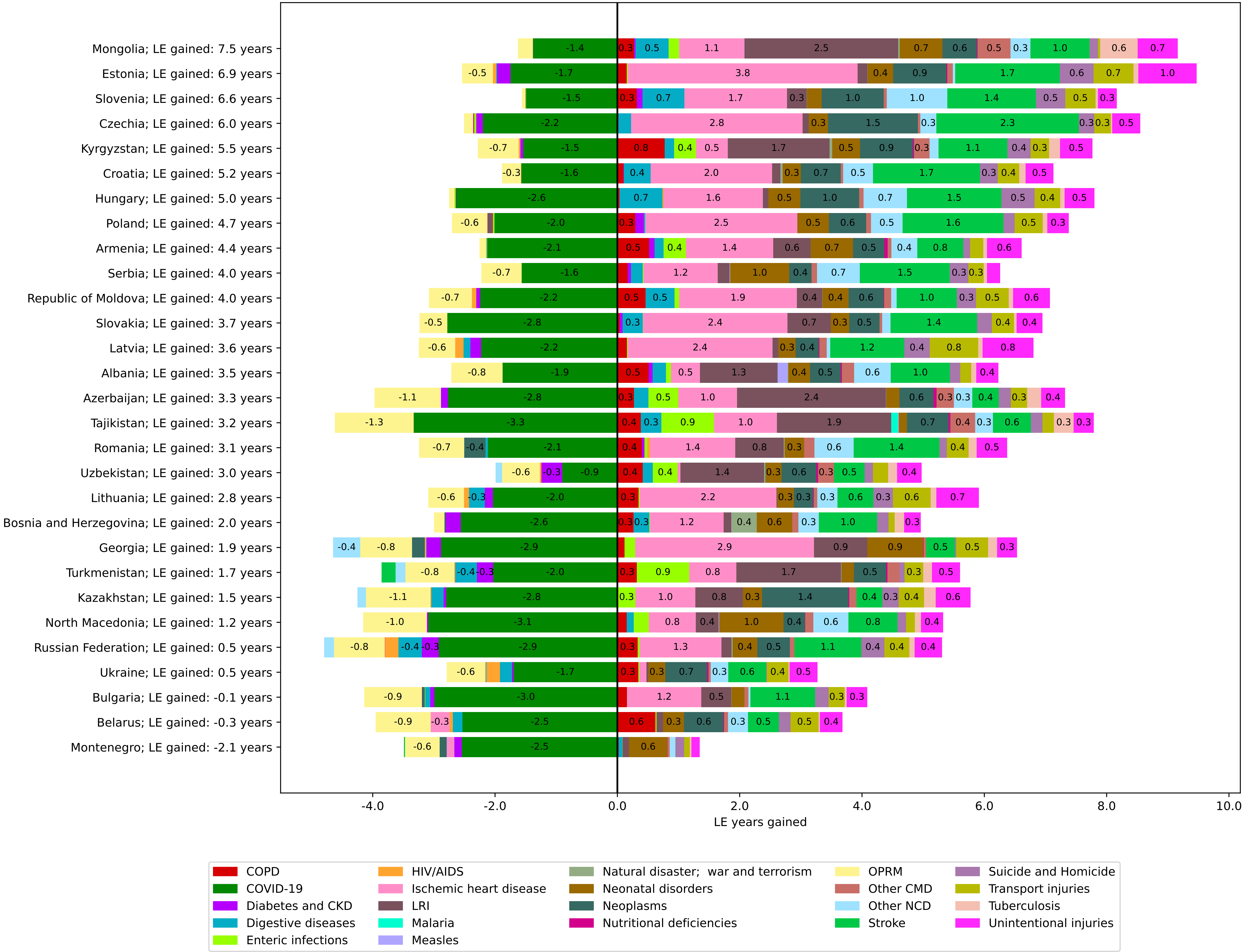

Figure S7 **Number of years of life expectancy gained or lost due to different causes by countries in the high-income super-region, 1990–2021**

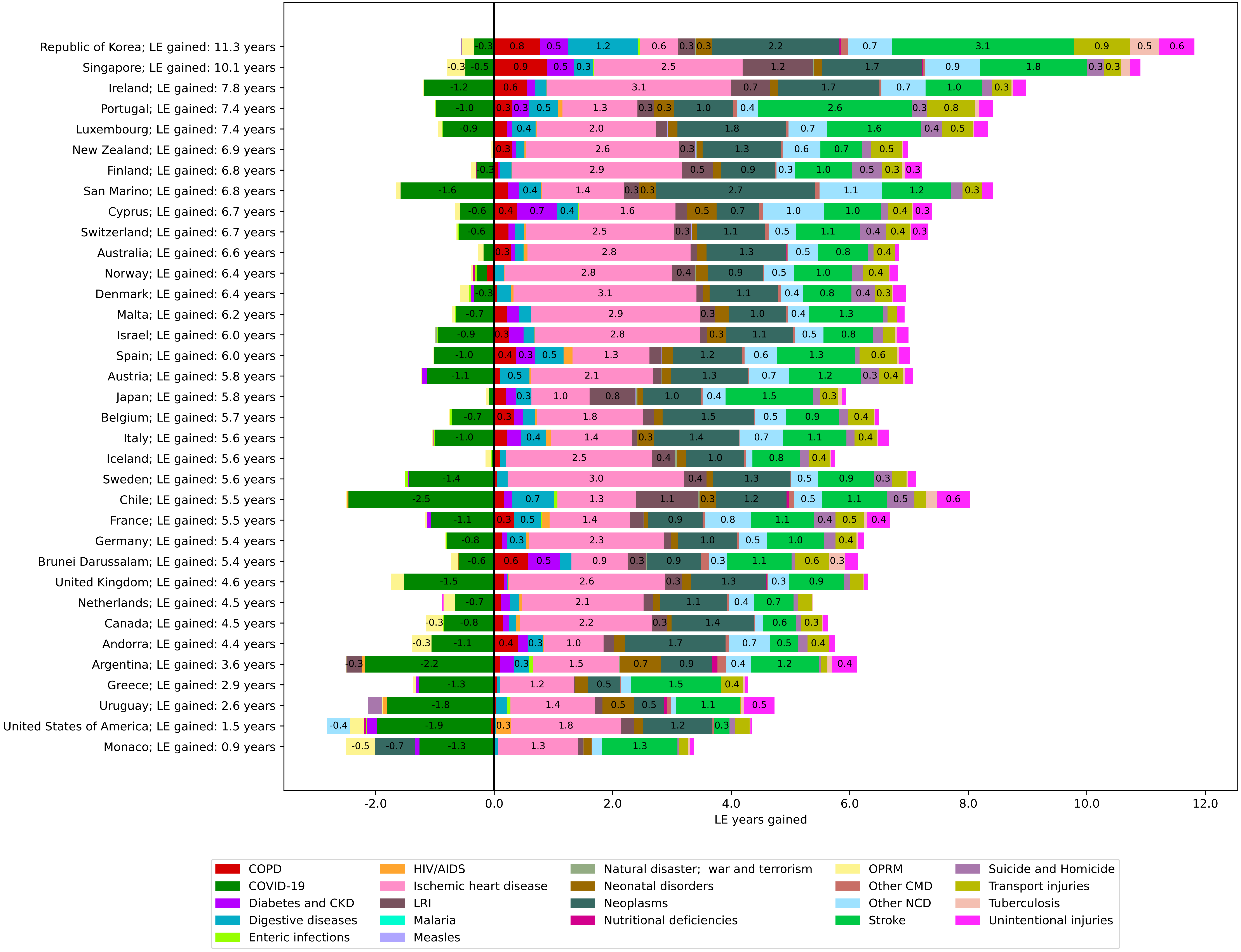

Figure S8 **Number of years of life expectancy gained or lost due to different causes by countries in the Latin America and the Caribbean super-region, 1990–2021**

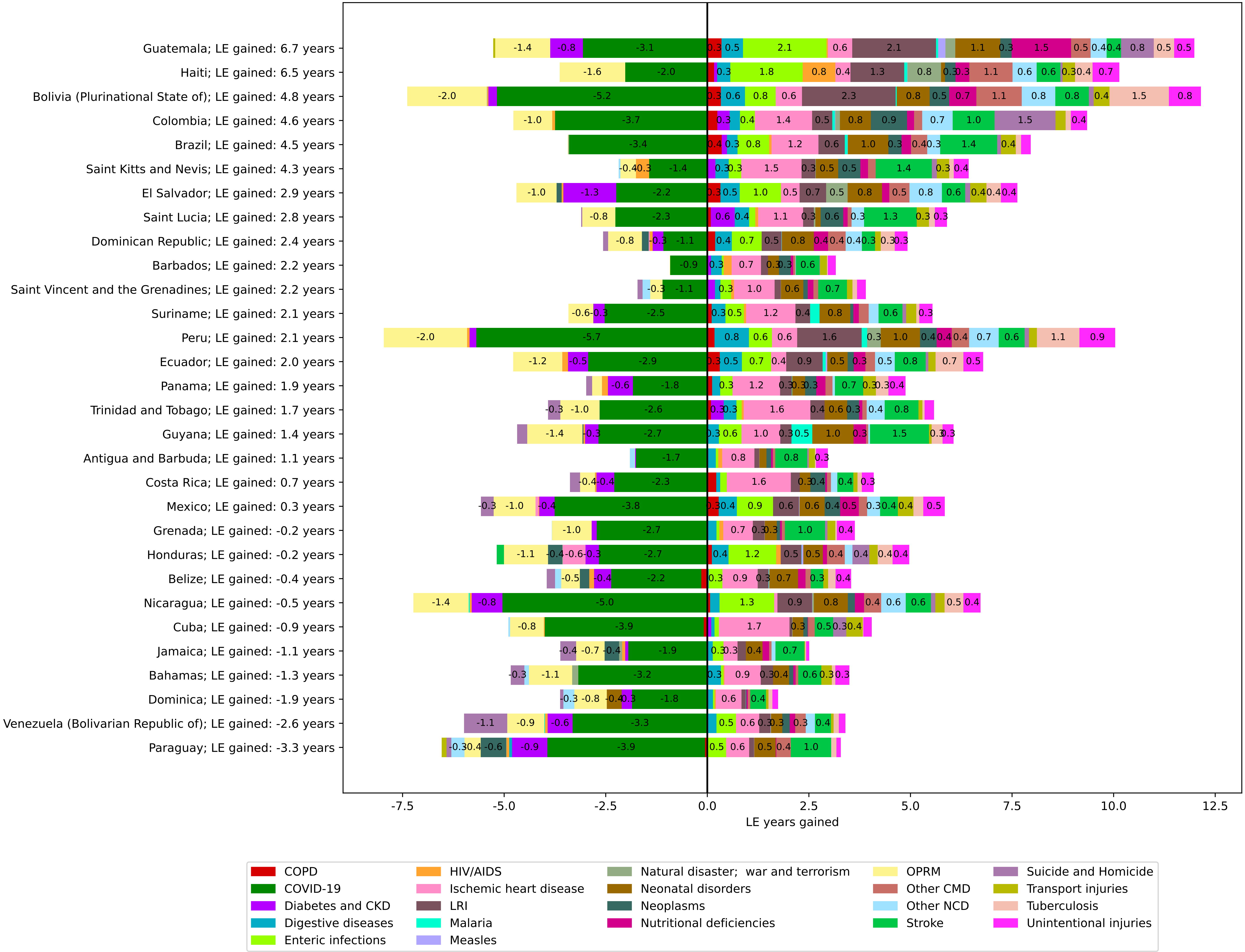

Figure S9 **Number of years of life expectancy gained or lost due to different causes by countries in the north Africa and the Middle East super-region, 1990–2021**

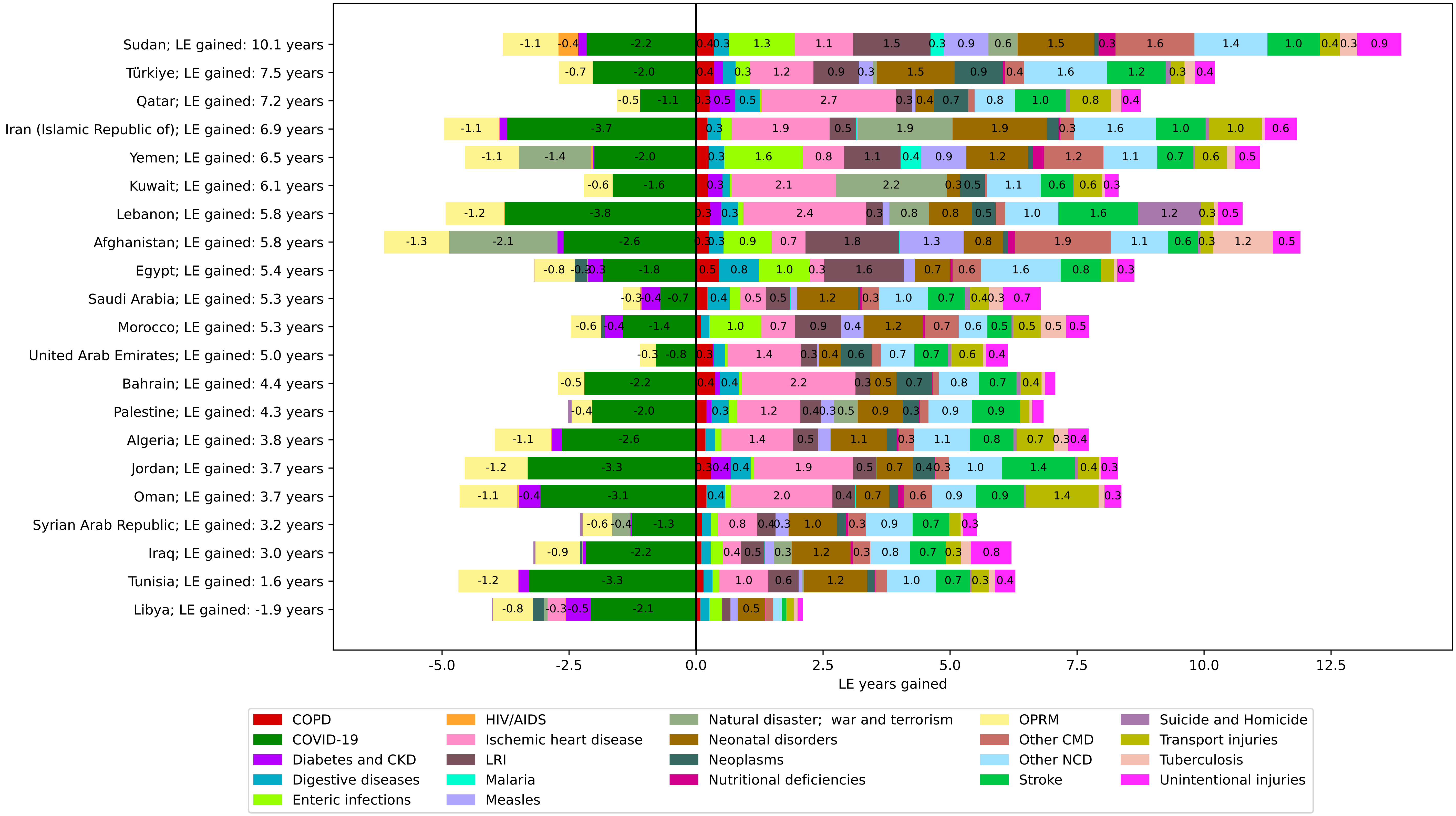

Figure S10 Number of years of life expectancy gained or lost due to different causes by countries in the south Asia super-region, 1990–2021

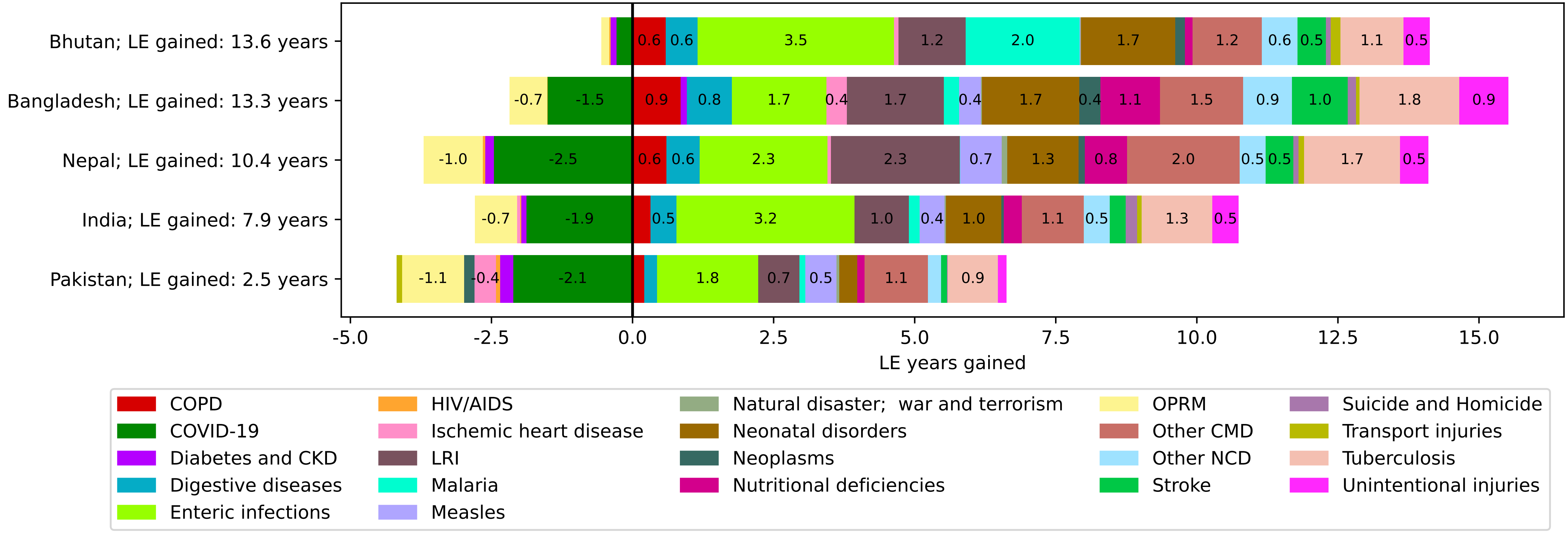

Figure S11 Number of years of life expectancy gained or lost due to different causes by countries in the southeast Asia, east Asia, and Oceania super-region, 1990–2021

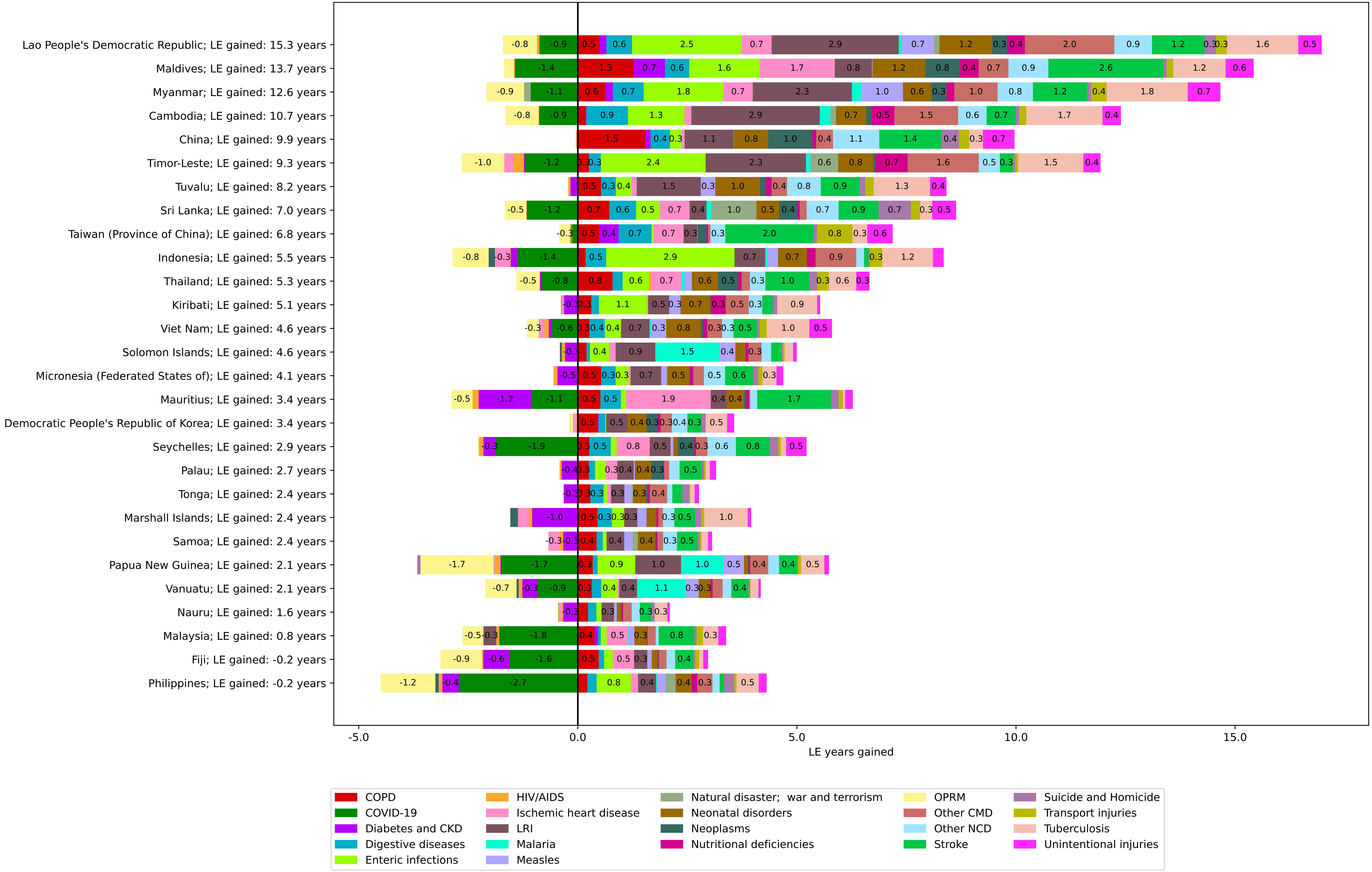

Figure S12 Number of years of life expectancy gained or lost due to different causes by countries in the sub-Saharan Africa super-region, 1990–2021

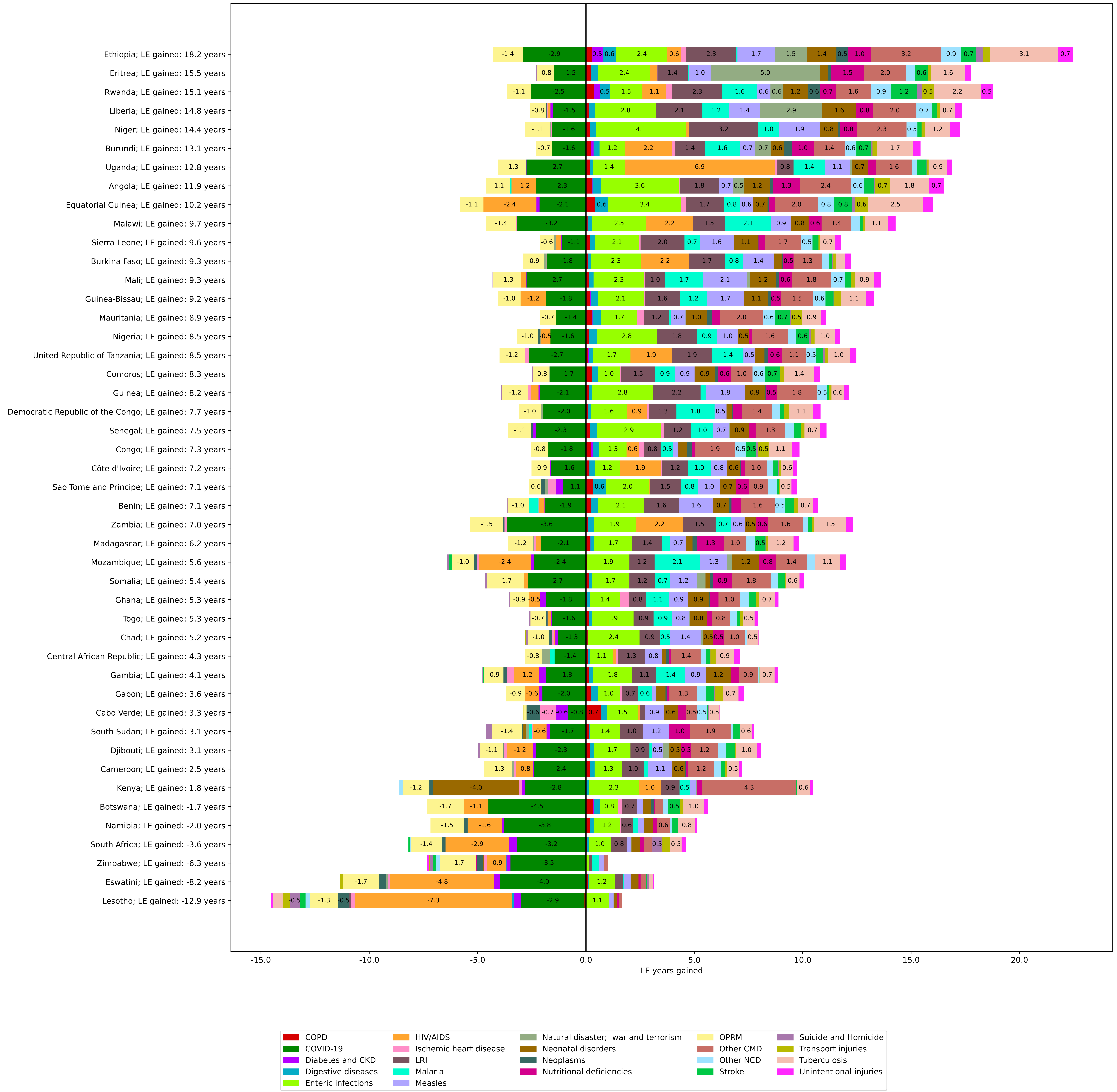

Figure S13 **Impact of malaria on life expectancy by GBD region, over four periods of time**

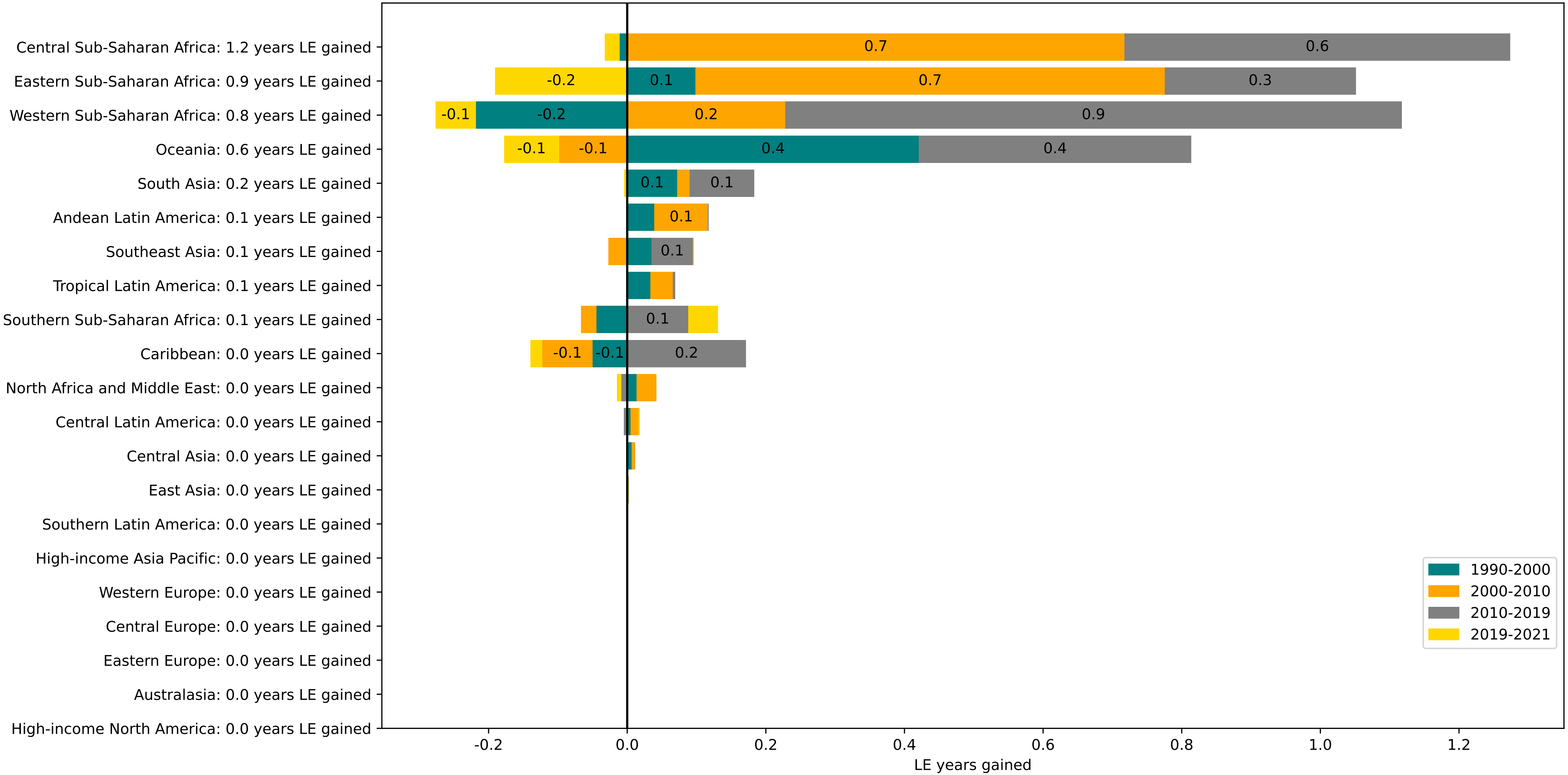

Figure S14 Impact of tuberculosis on life expectancy by GBD region, over four periods of time

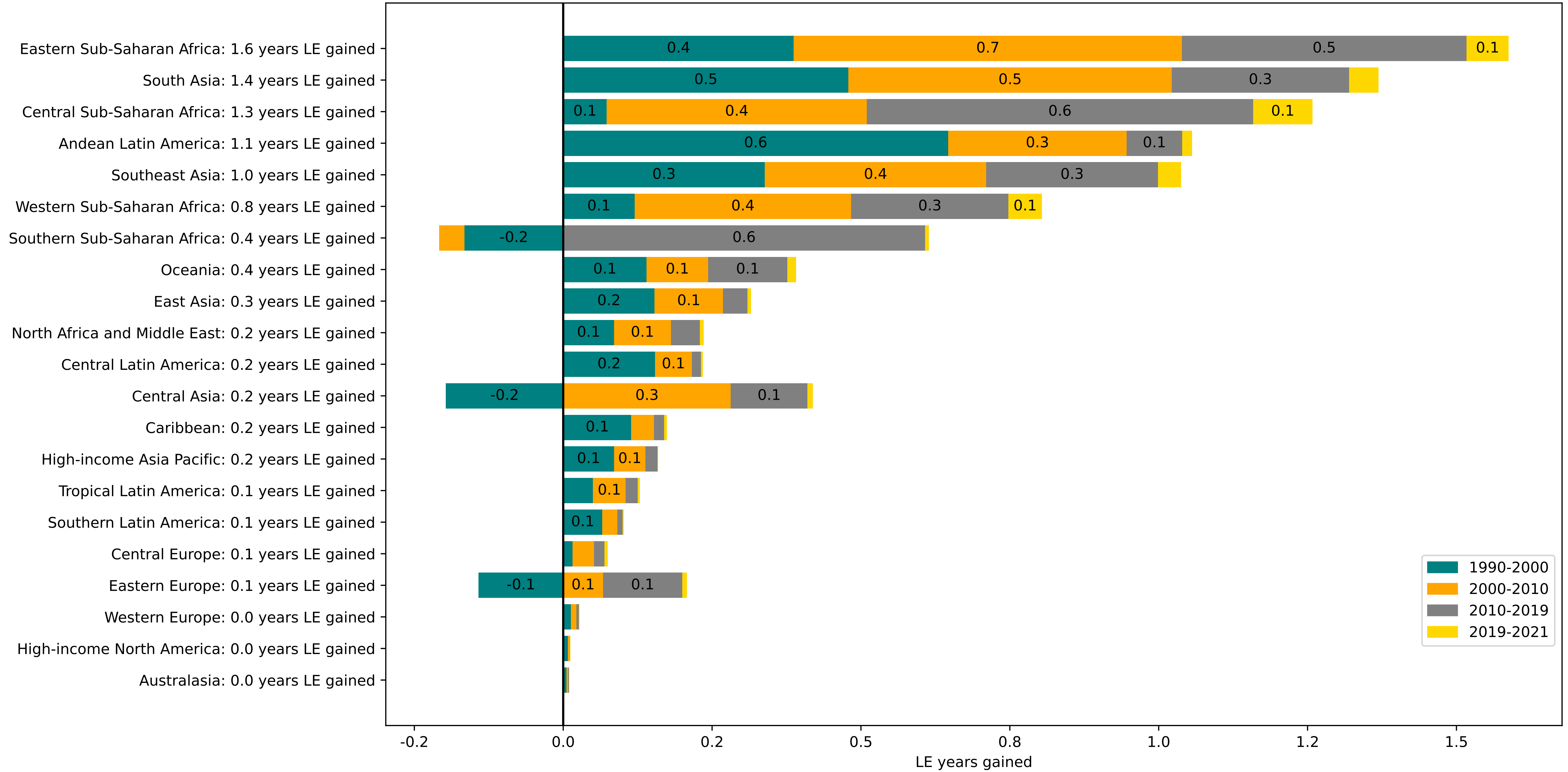

Figure S15 Impact of measles on life expectancy by GBD region, over four periods of time

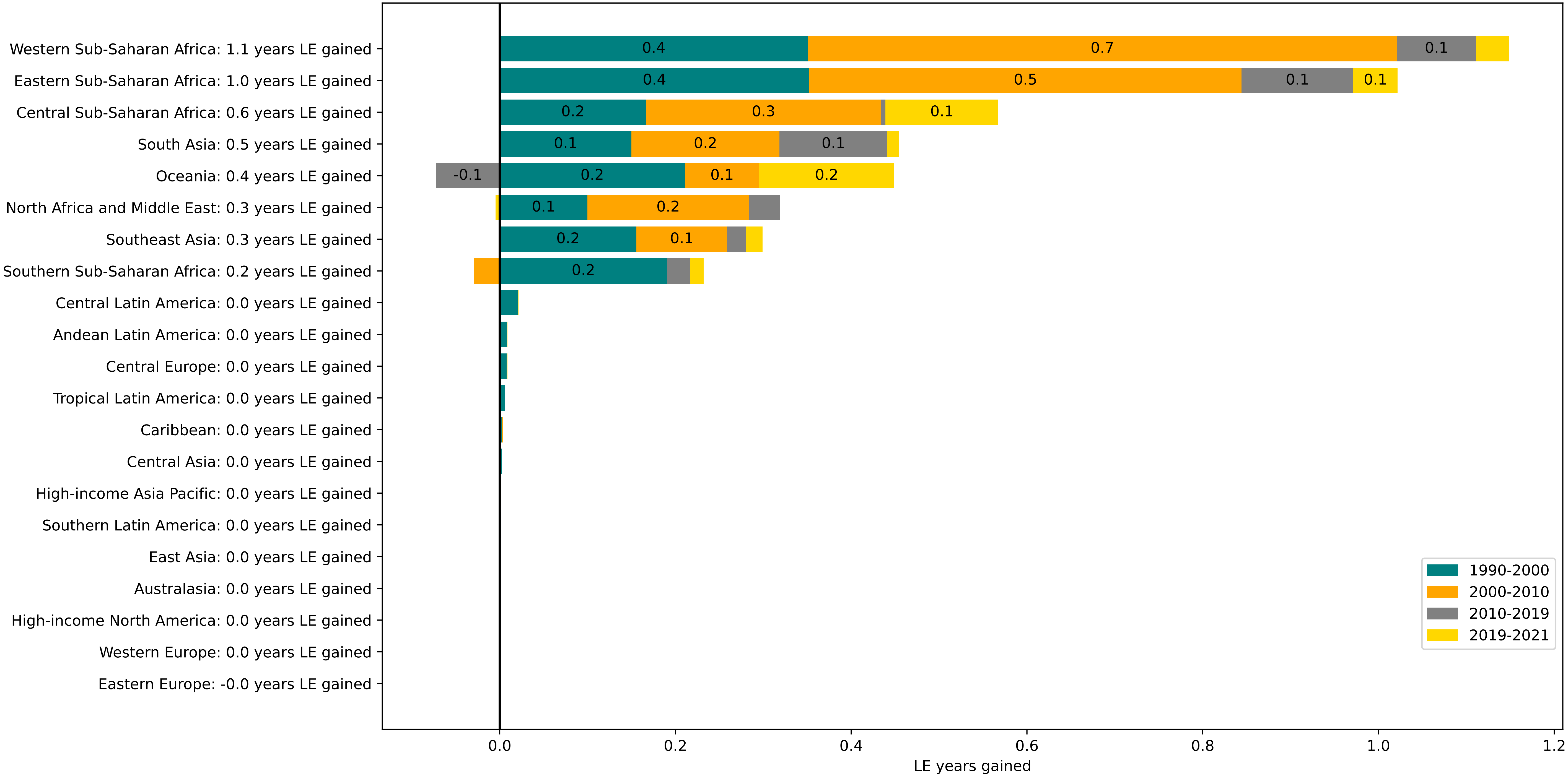

Figure S16 **Impact of neonatal disorders on life expectancy by GBD region, over four periods of time**

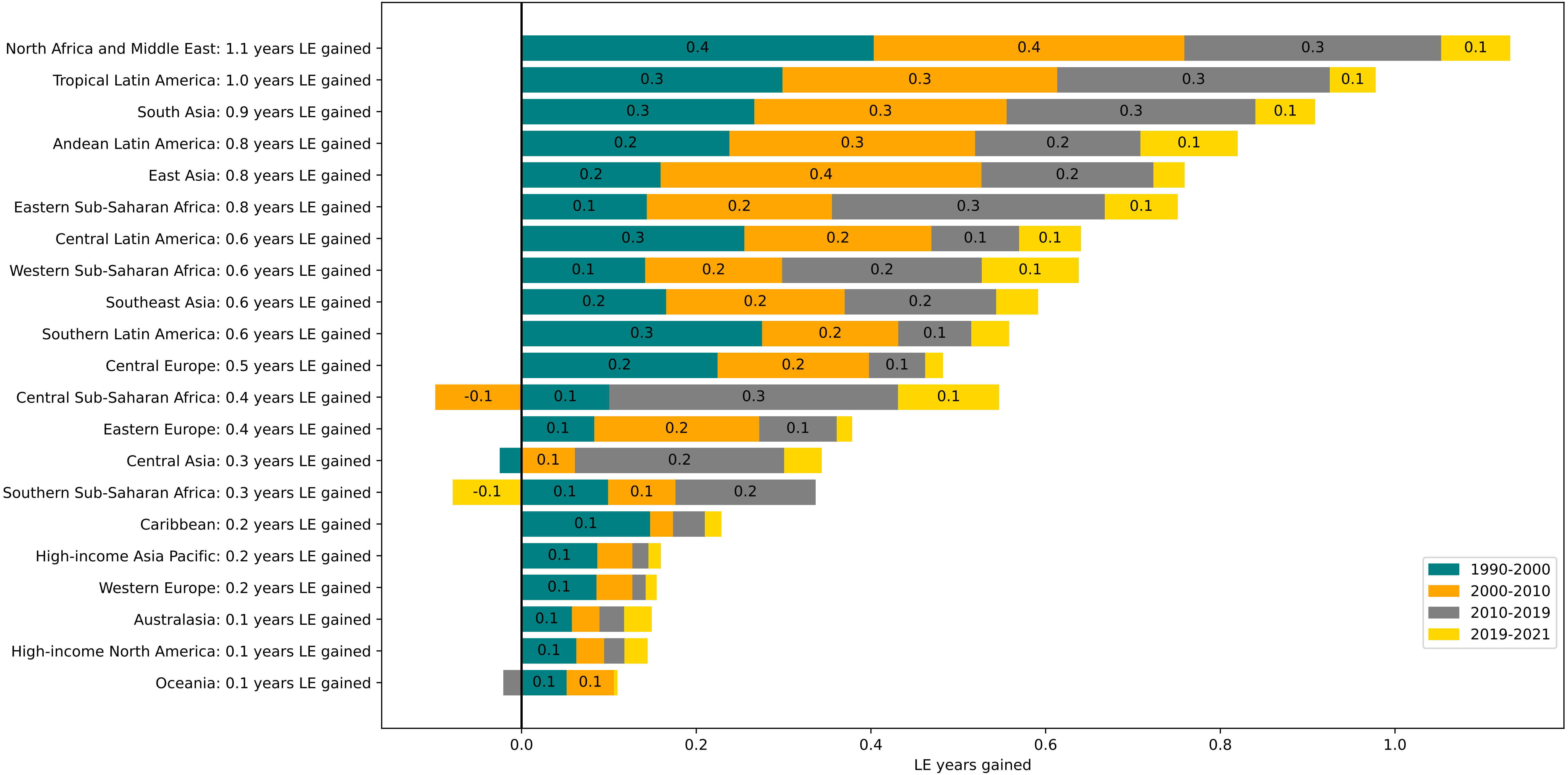

Figure S17 Impact of ischaemic heart disease on life expectancy by GBD region, over four periods of time

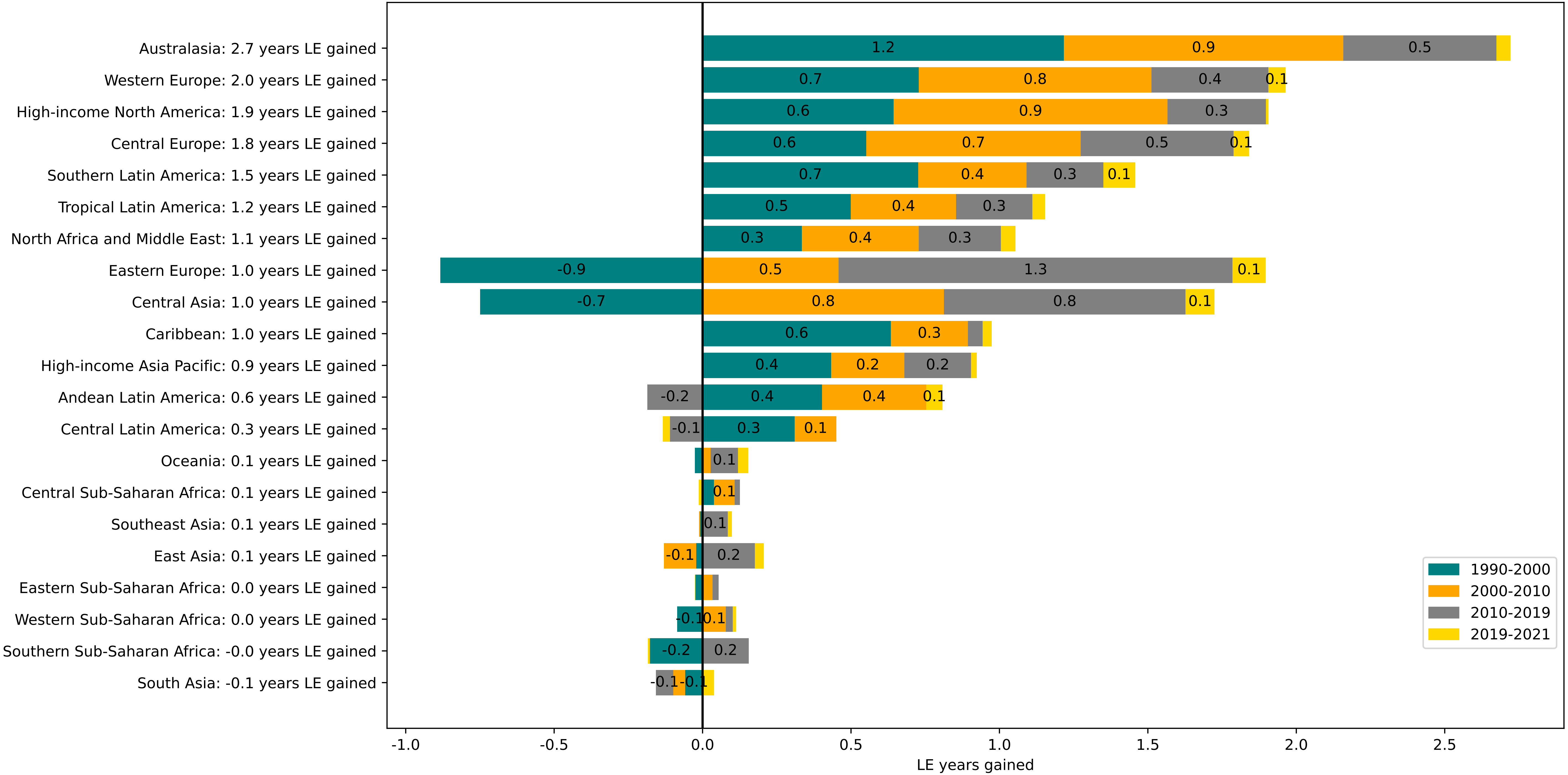

Figure S18 Impact of nutritional deficiencies on life expectancy by GBD region, over four periods of time

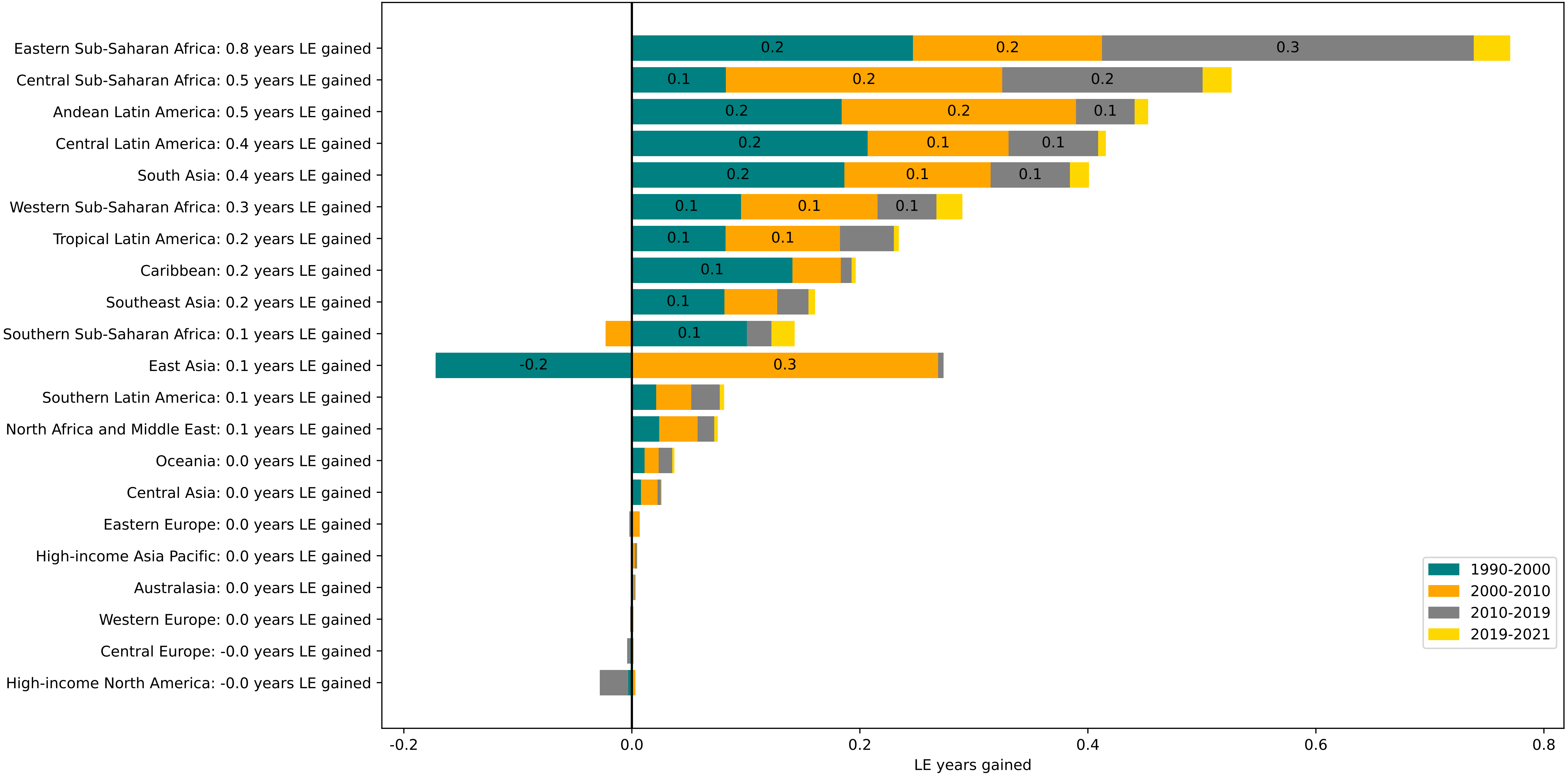

Figure S19 Impact of neoplasms on life expectancy by GBD region, over four periods of time

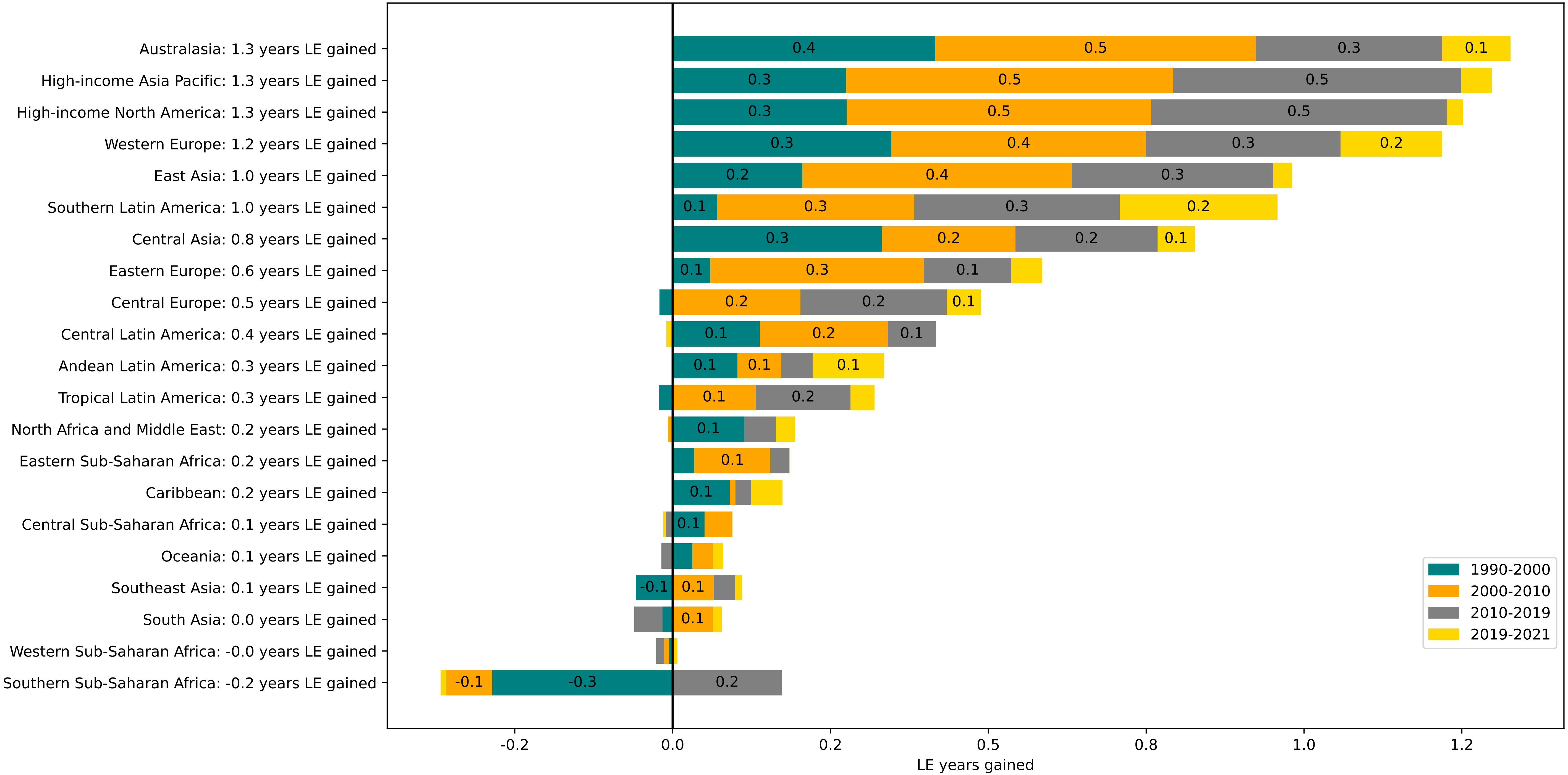

Figure S20 Impact of chronic respiratory diseases on life expectancy by GBD region, over four periods of time

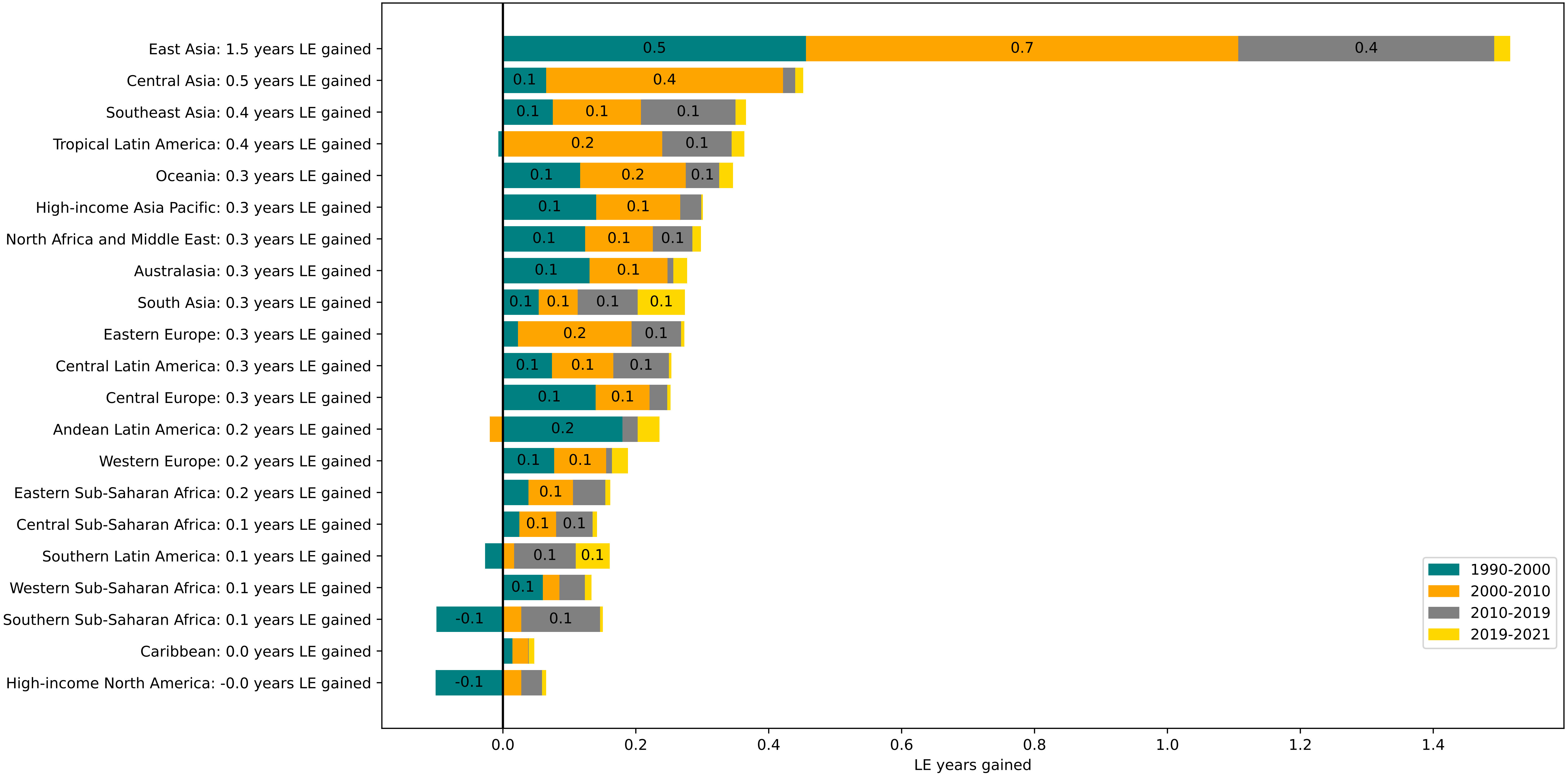

Figure S21 Impact of digestive diseases on life expectancy by GBD region, over four periods of time

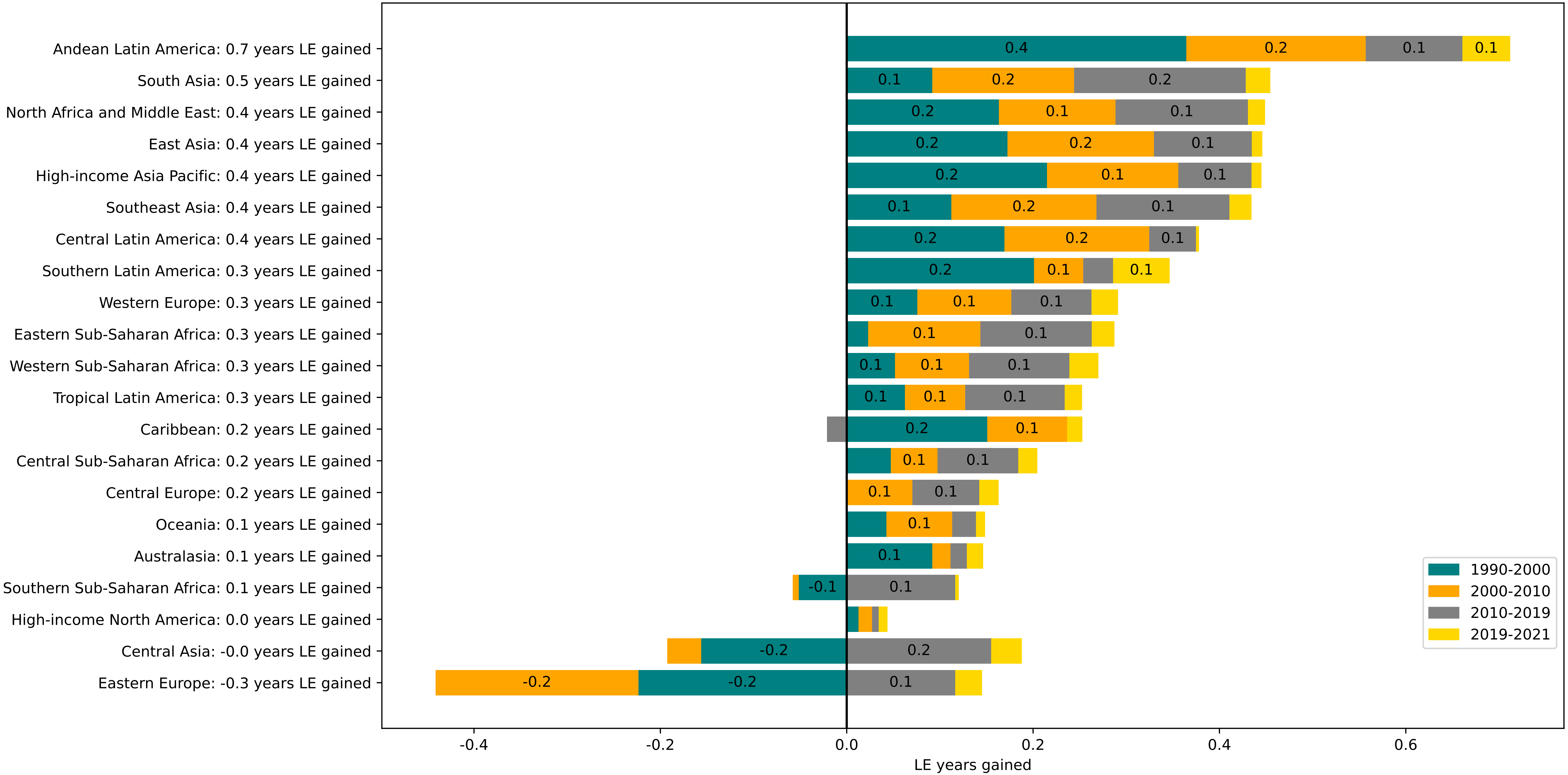

Figure S22 Impact of diabetes and kidney diseases on life expectancy by GBD region, over four periods

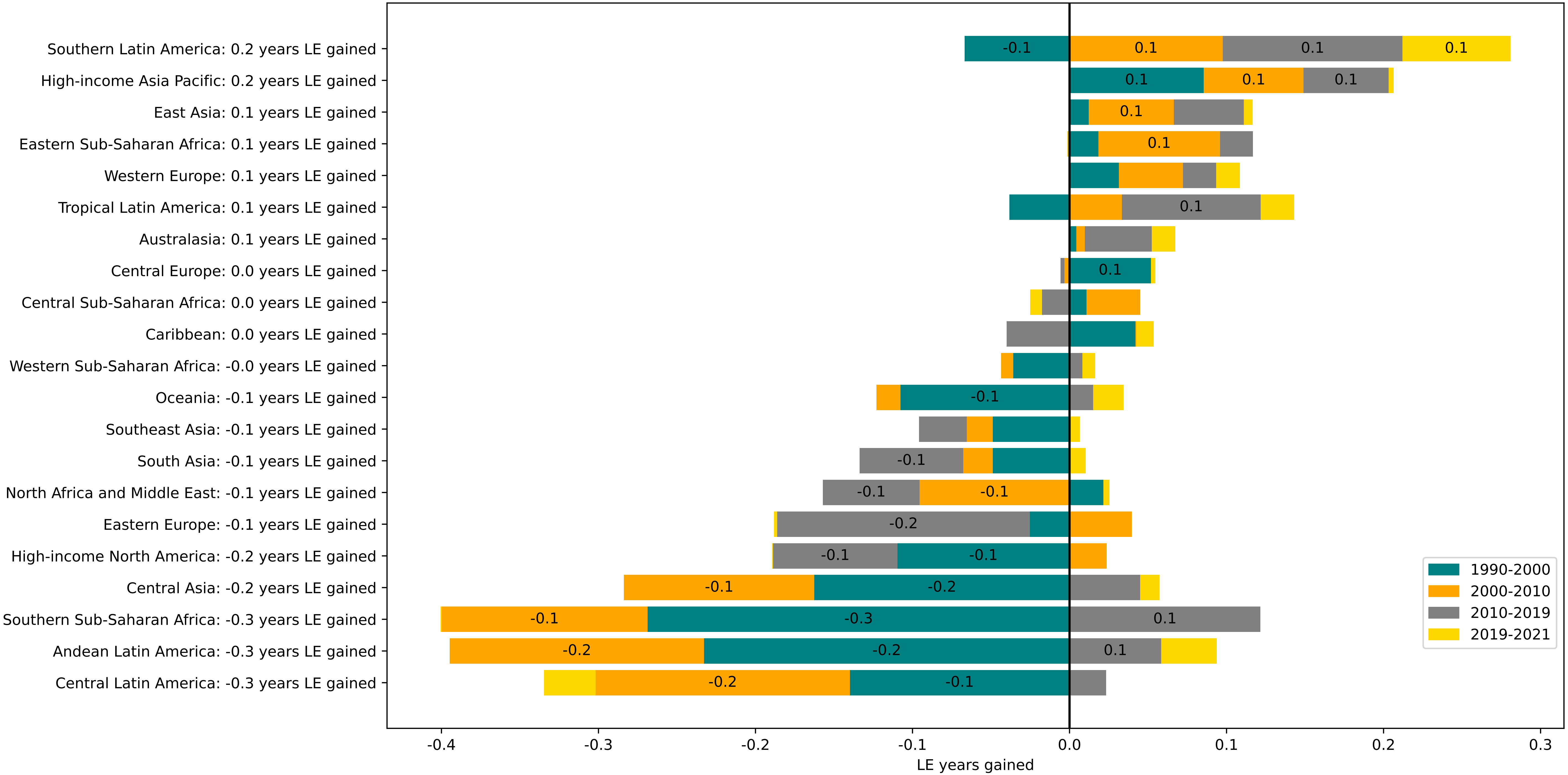

Figure S23 **Impact of stroke on life expectancy by GBD region, over four periods of time**

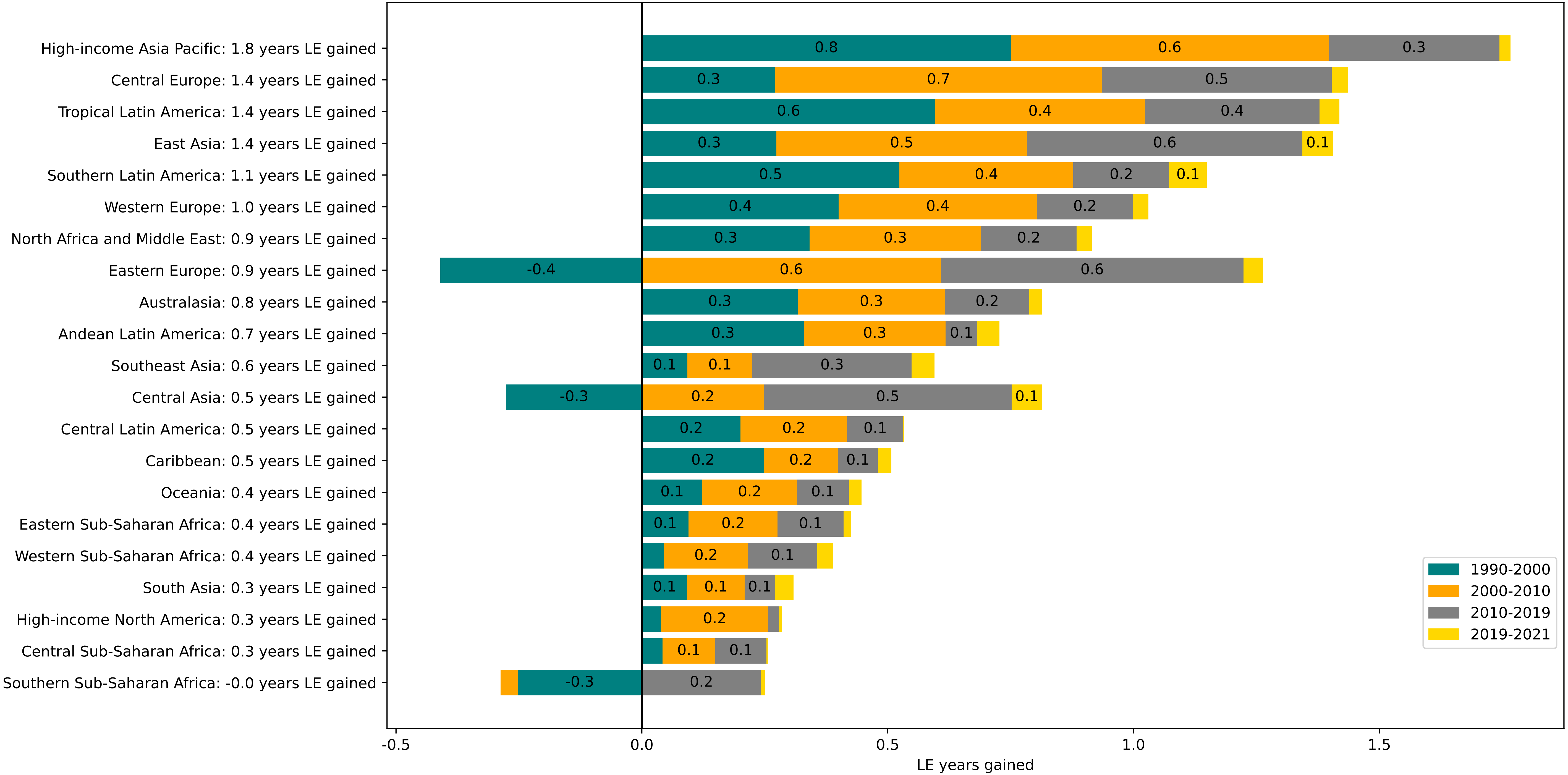

Figure S24 Impact of transport injuries on life expectancy by GBD region, over four periods of time

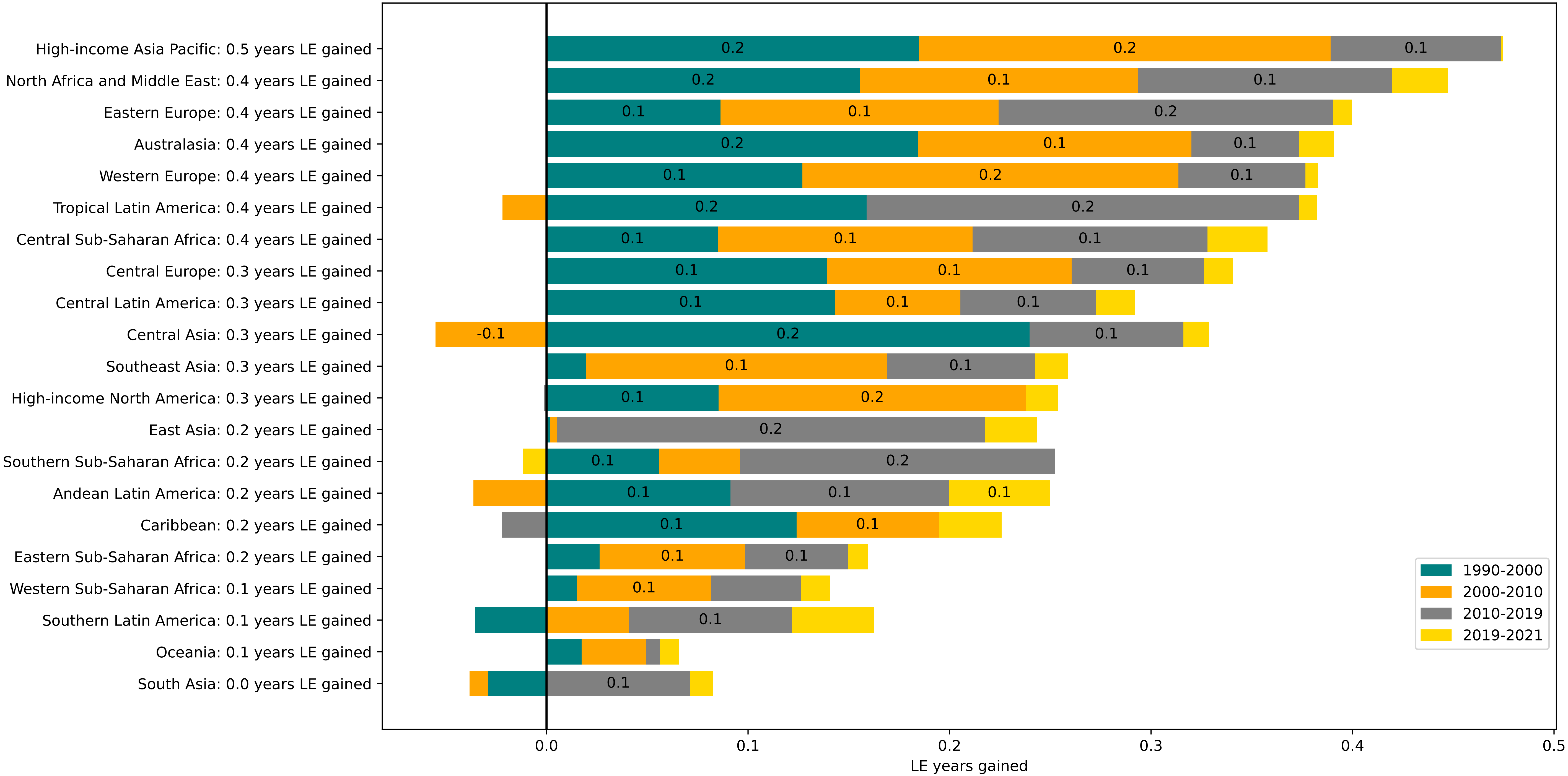

Figure S25 Impact of self-harm and interpersonal violence (except of war and terrorism) on life expectancy by GBD region, over four periods of time

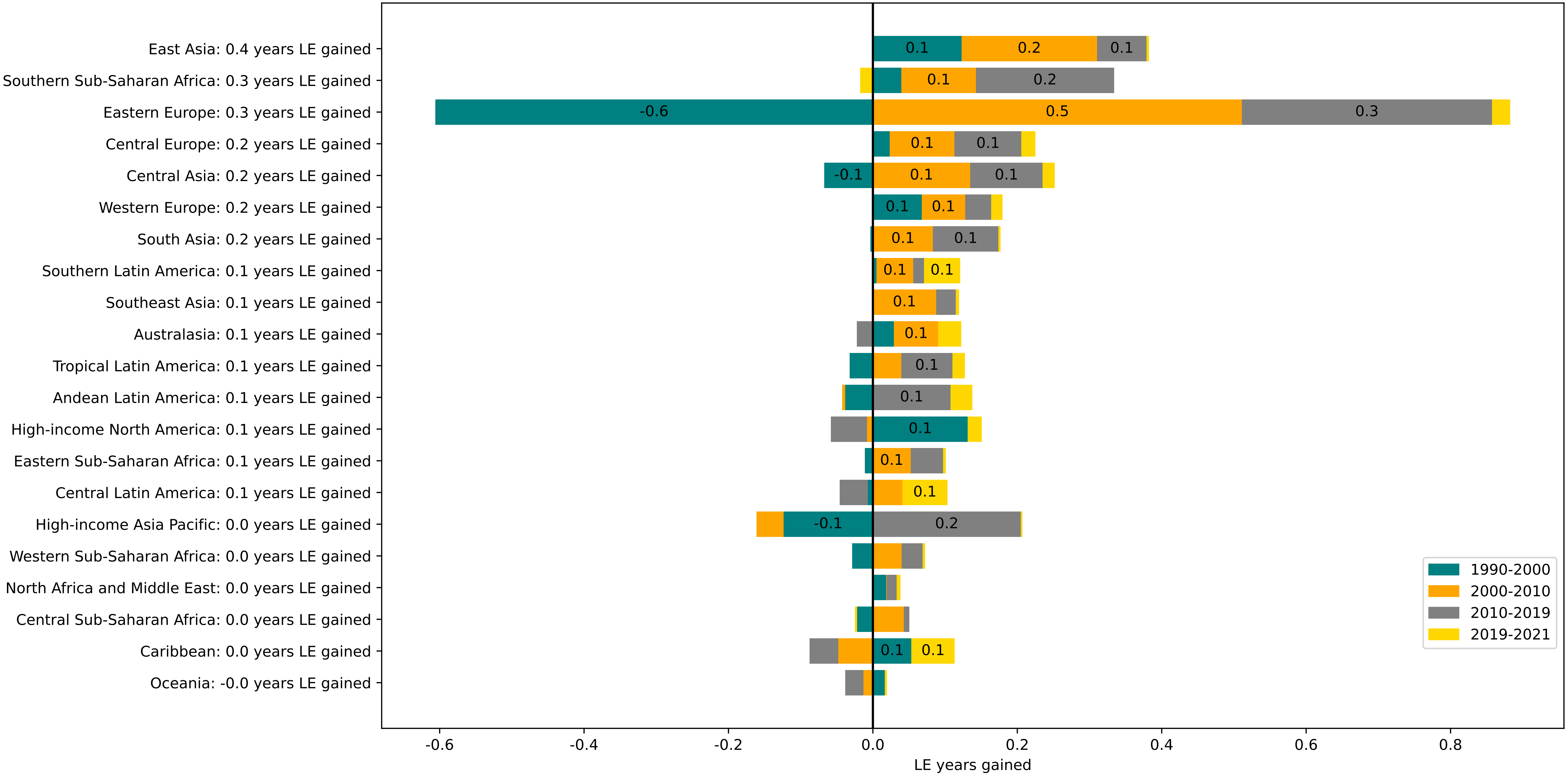

Figure S26 **Impact of unintentional injuries (except of natural disaster) on life expectancy by GBD region, over four periods of time**

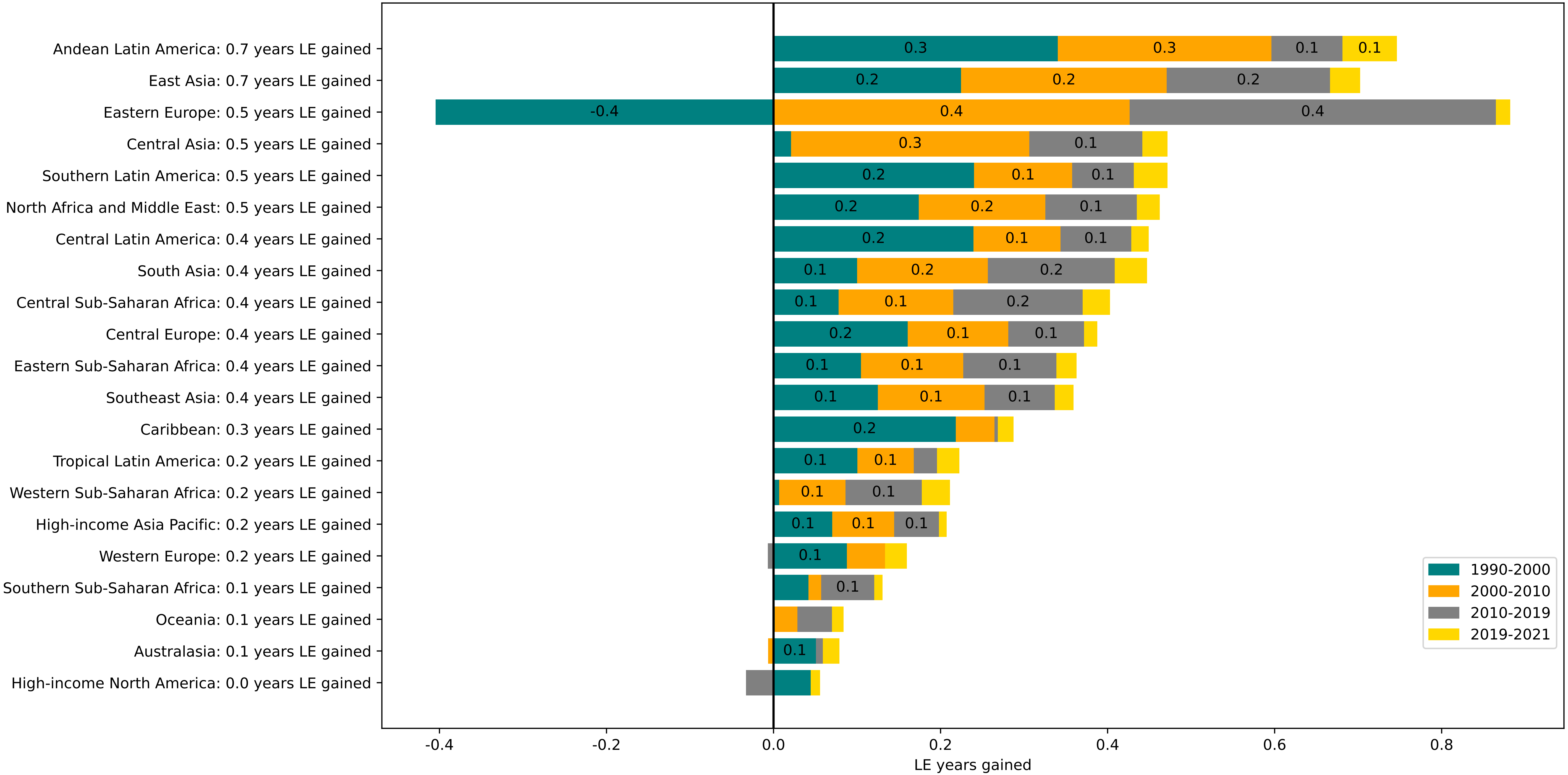

Figure S27 Impact of HIV/AIDS on life expectancy by GBD region, over four periods of time

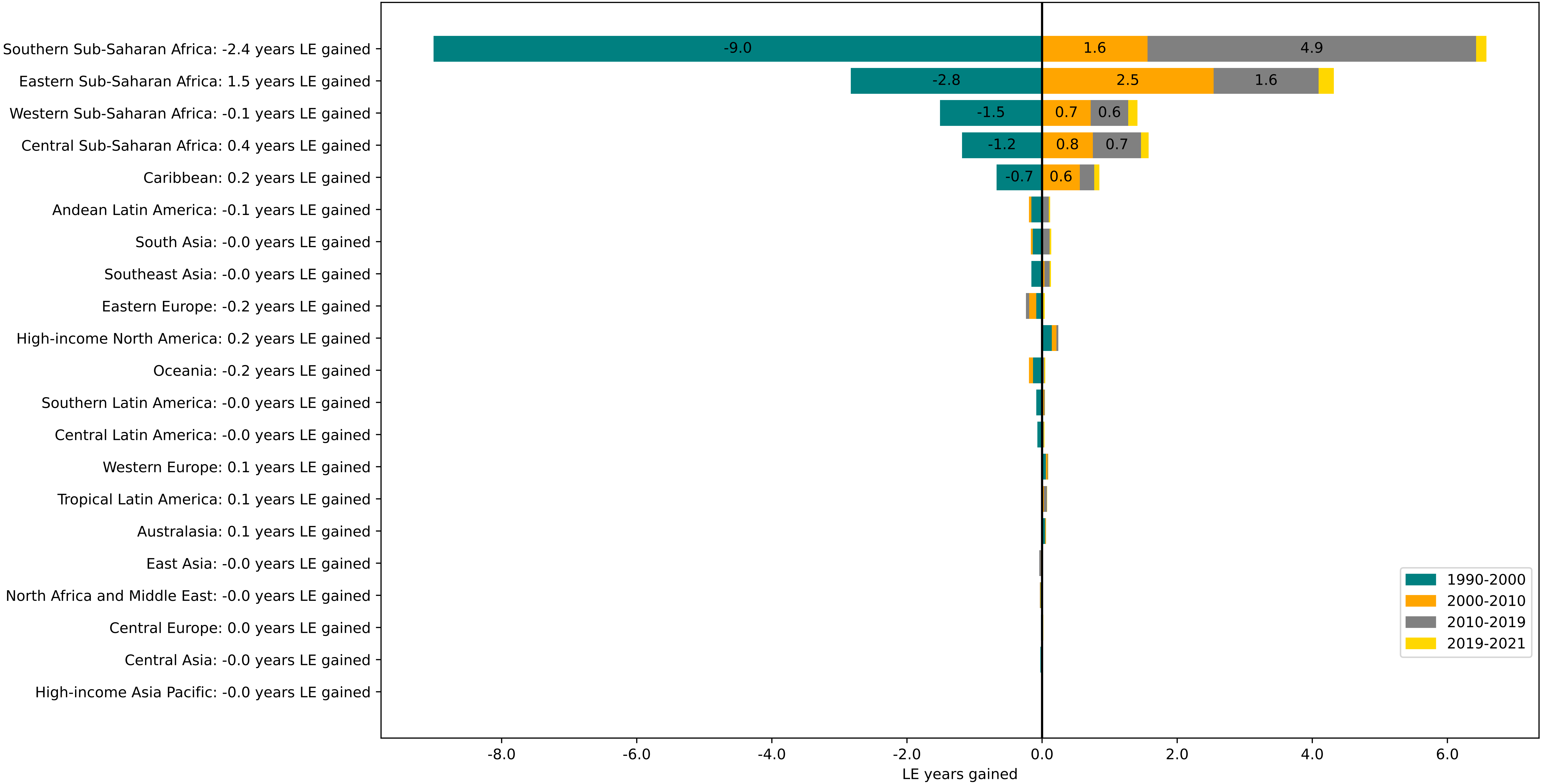

Figure S28 Under−5 mortality rate per 100 000 due to enteric infections by location, in 1990 (A) and 2021 (B)

(A) 1990

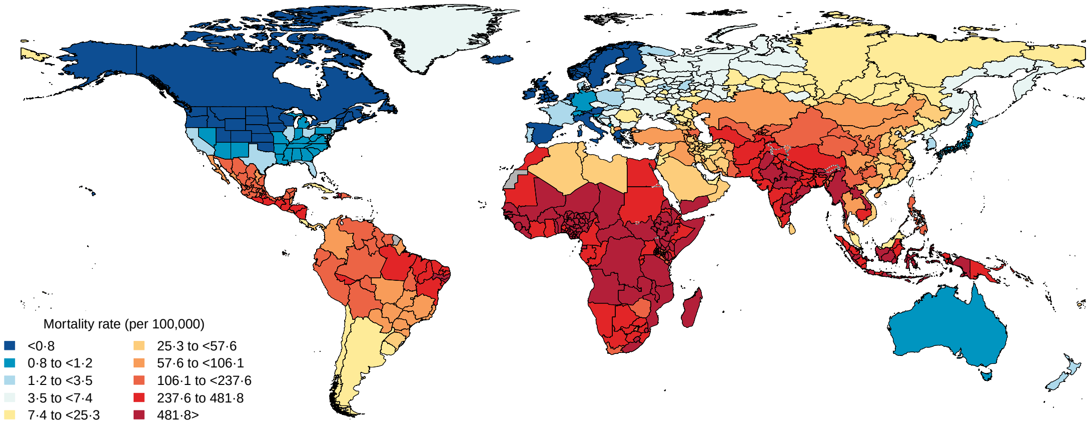

(B) 2021

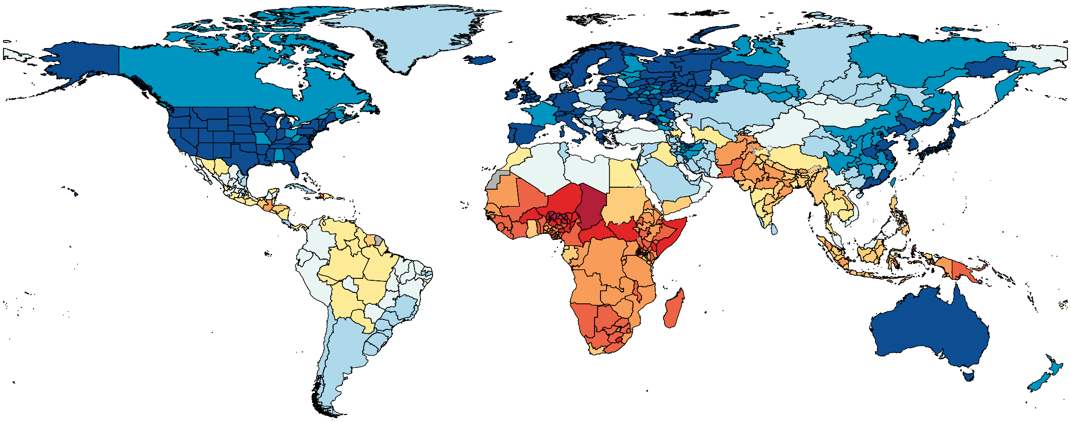

Figure S29 Under−5 mortality rate per 100 000 due to lower respiratory infections by location, in 1990 (A) and 2021 (B)

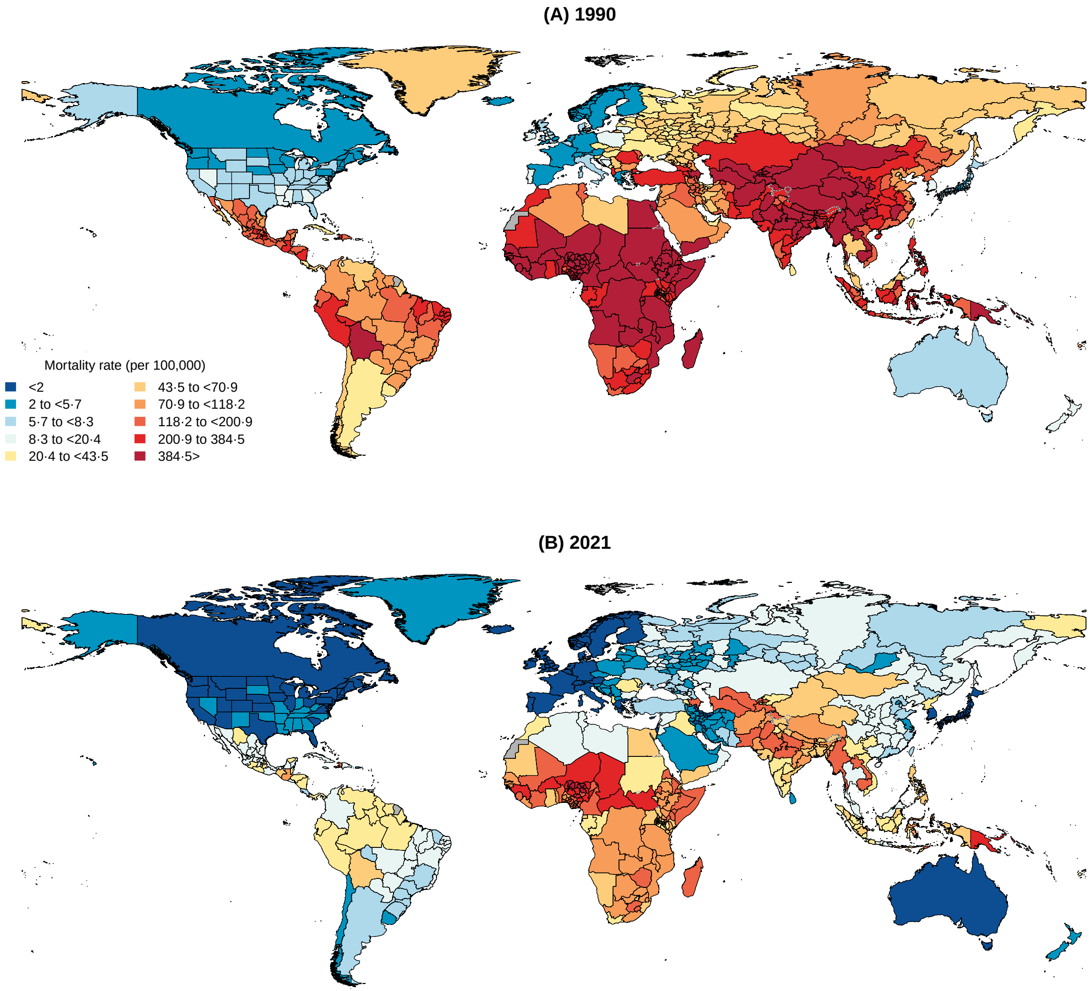

Figure S30 Age−standardised mortality rate per 100 000 due to HIV/AIDS by location, in 1990 (A) and 2021 (B)

(A) 1990

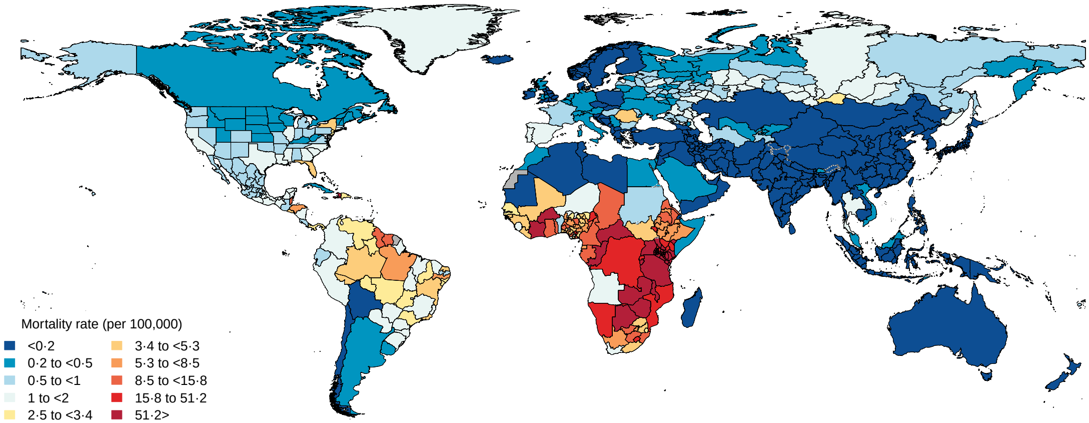

(B) 2021

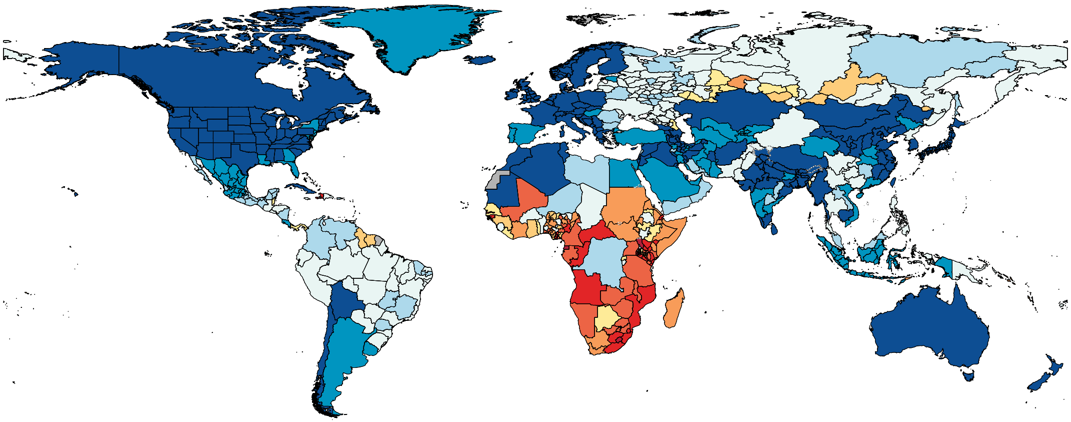

Figure S31 Under−5 mortality rate per 100 000 due to malaria infections by location, in 1990 (A) and 2021 (B)

(A) 1990

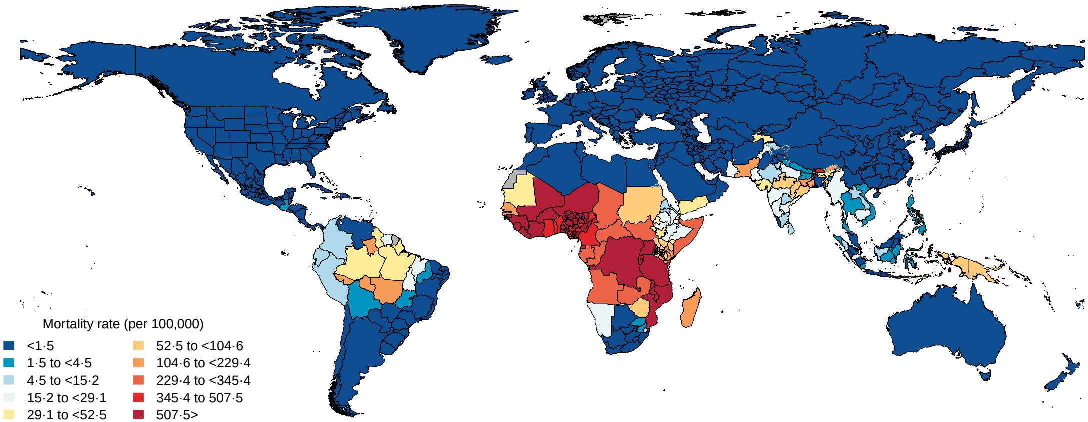

(B) 2021

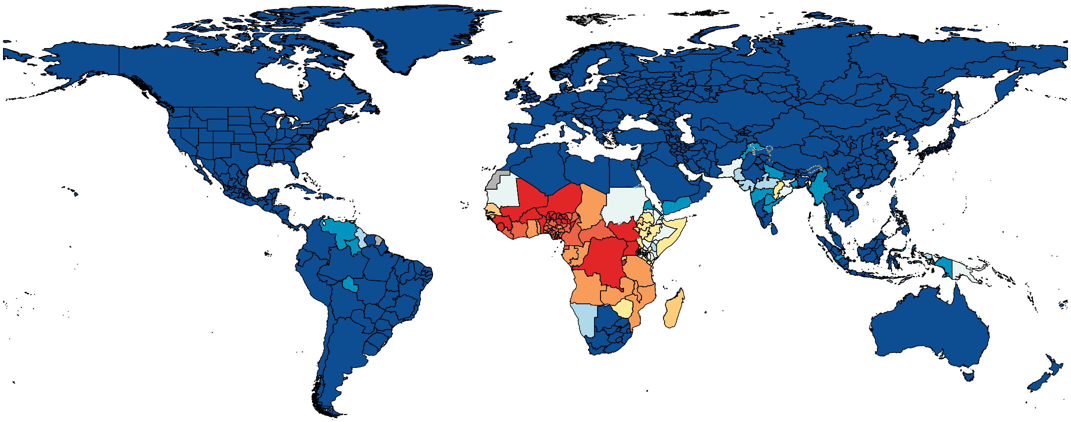

Figure S32 Age−standardised mortality rate per 100 000 due to tuberculosis by location, in 1990 (A) and 2021 (B)

(A) 1990

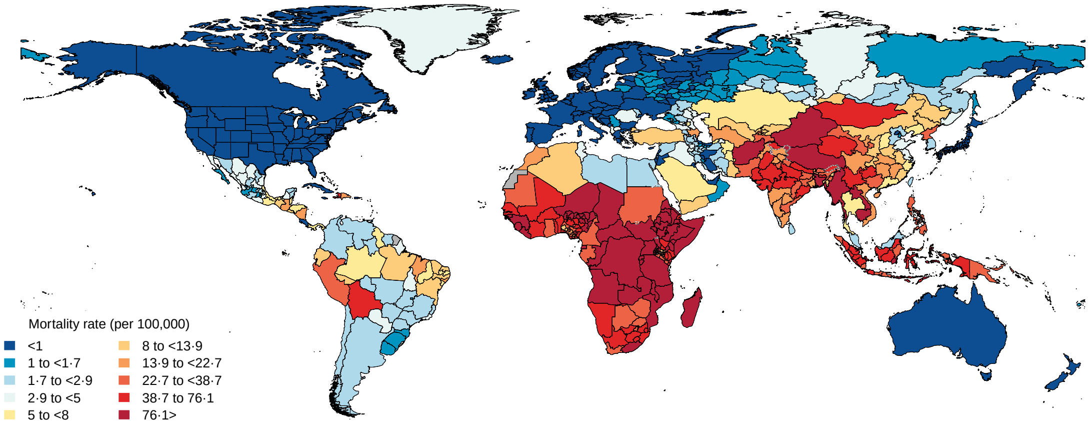

Figure S32 Age−standardised mortality rate per 100 000 due to tuberculosis by location, in 1990 (A) and 2021 (B)

(A) 1990

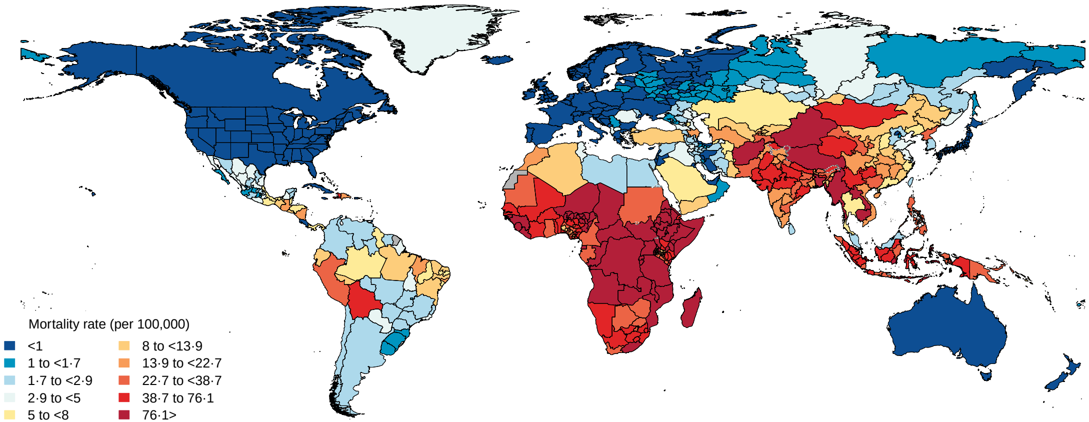

(B) 2021

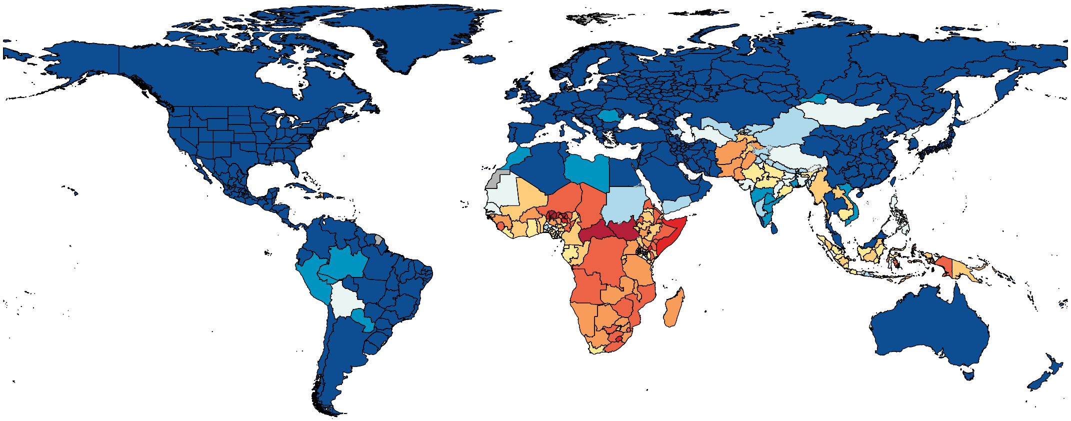

Figure S33 Under−5 mortality rate per 100 000 due to measles infections by location, in 1990 (A) and 2021 (B)

(A) 1990

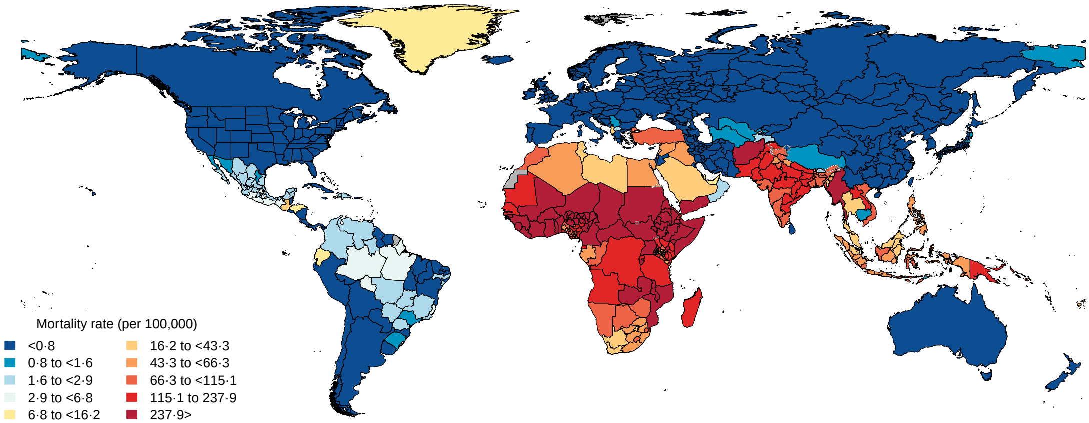

(B) 2021

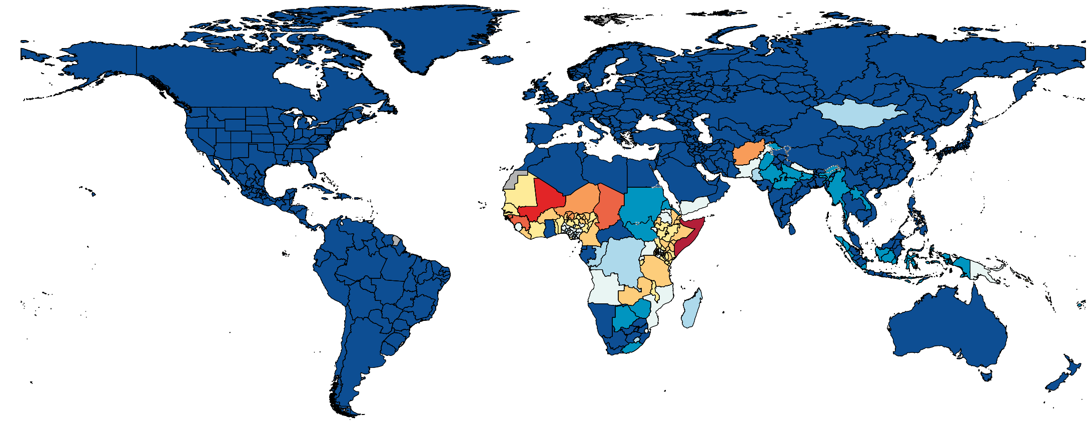

Figure S34 Neonatal mortality rate per 100 000 by location, in 1990 (A) and 2021 (B)

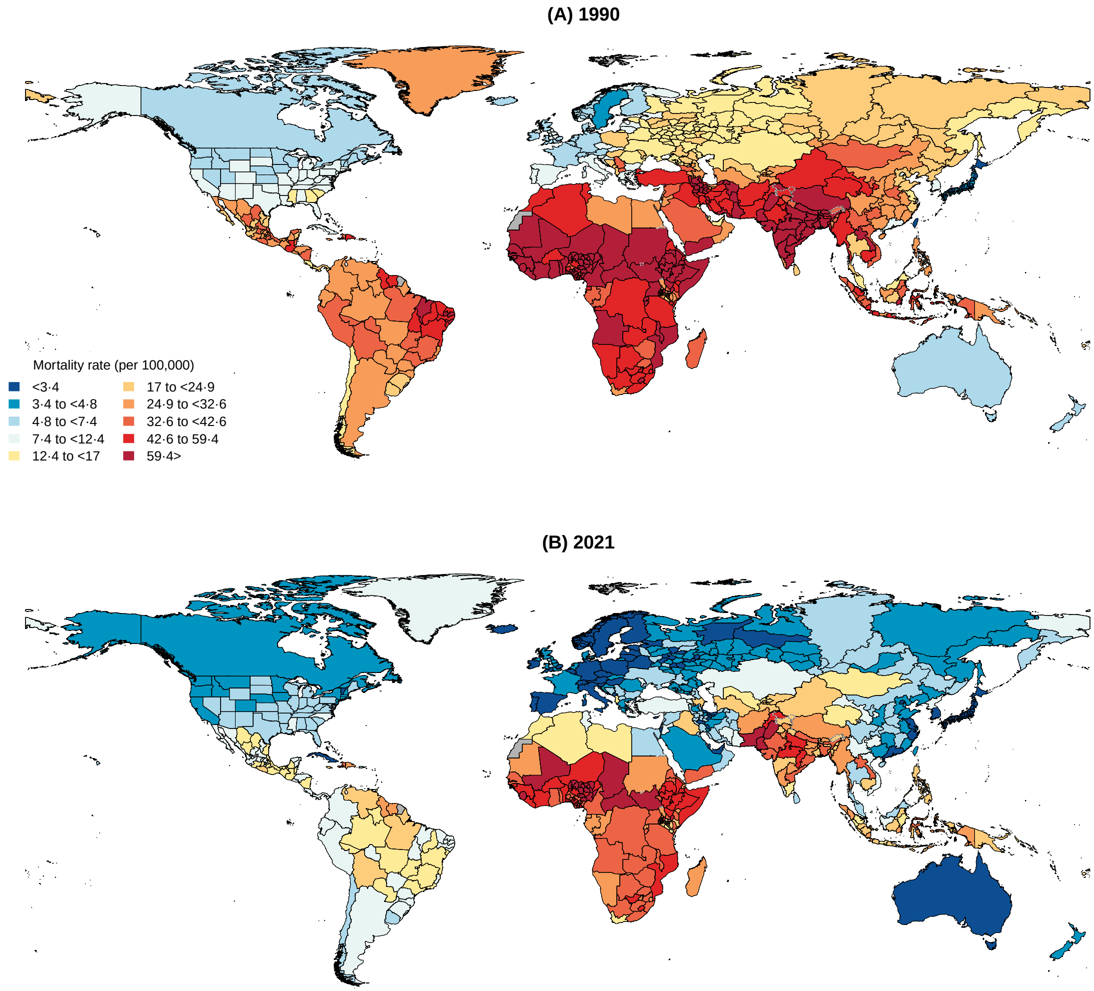

Figure S35 Under−5 mortality rate per 100 000 due to nutritional deficiencies by location, in 1990 (A) and 2021 (B)

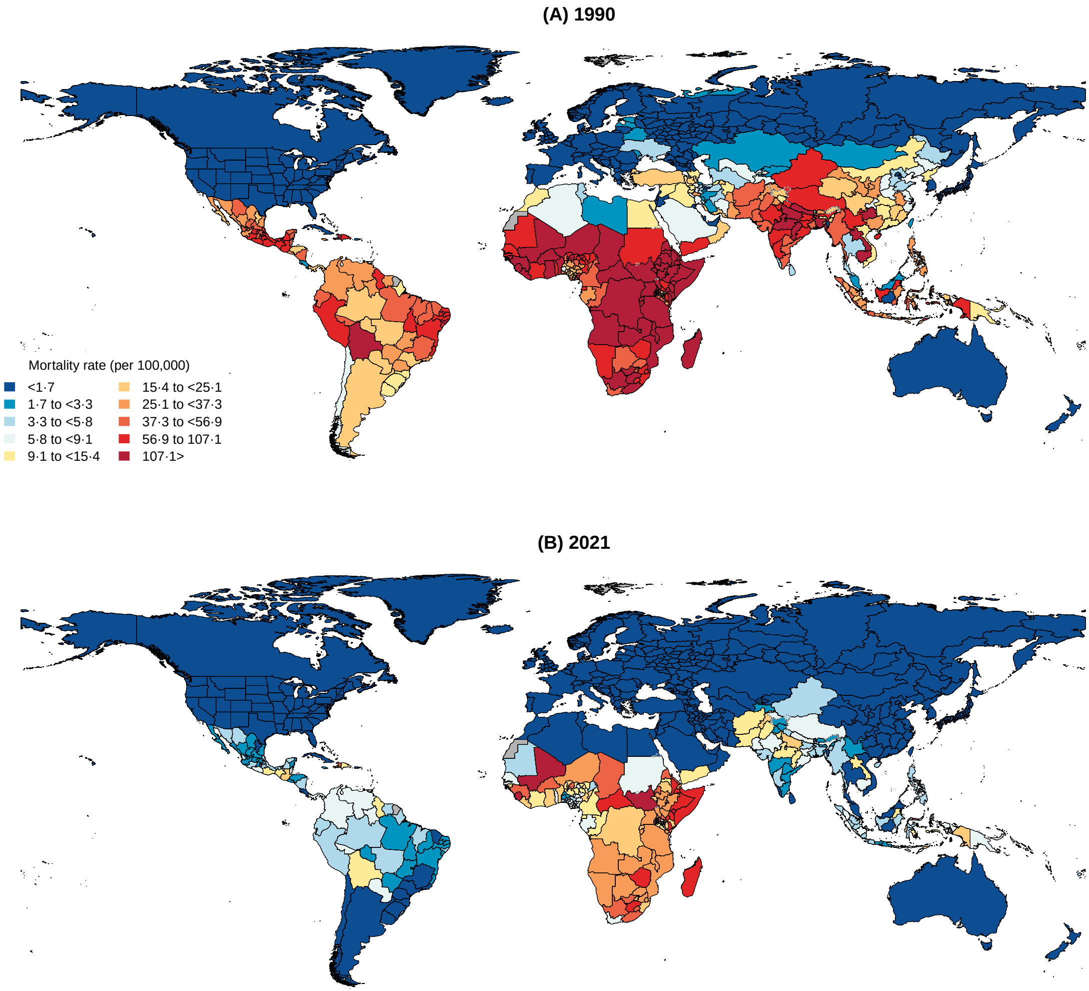

Figure S36 Age−standardised mortality rate per 100 000 due to stroke by location, in 1990 (A) and 2021 (B)

(A) 1990

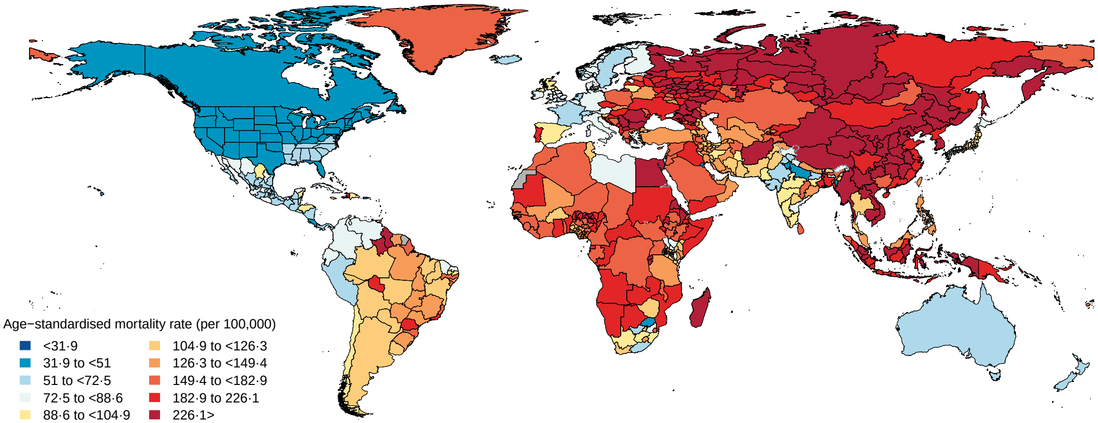

(B) 2021

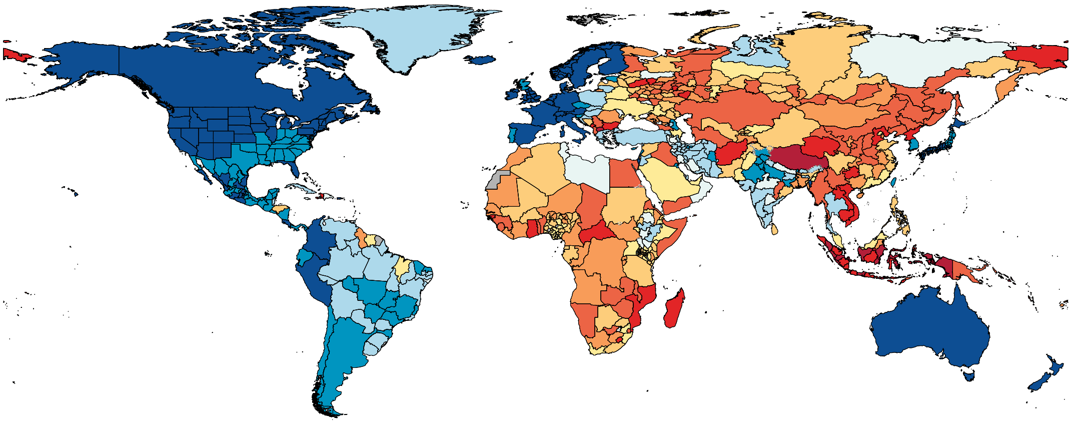

Figure S37 Age−standardised mortality rate per 100 000 due to ischemic heart disease by location, in 1990 (A) and 2021 (B)

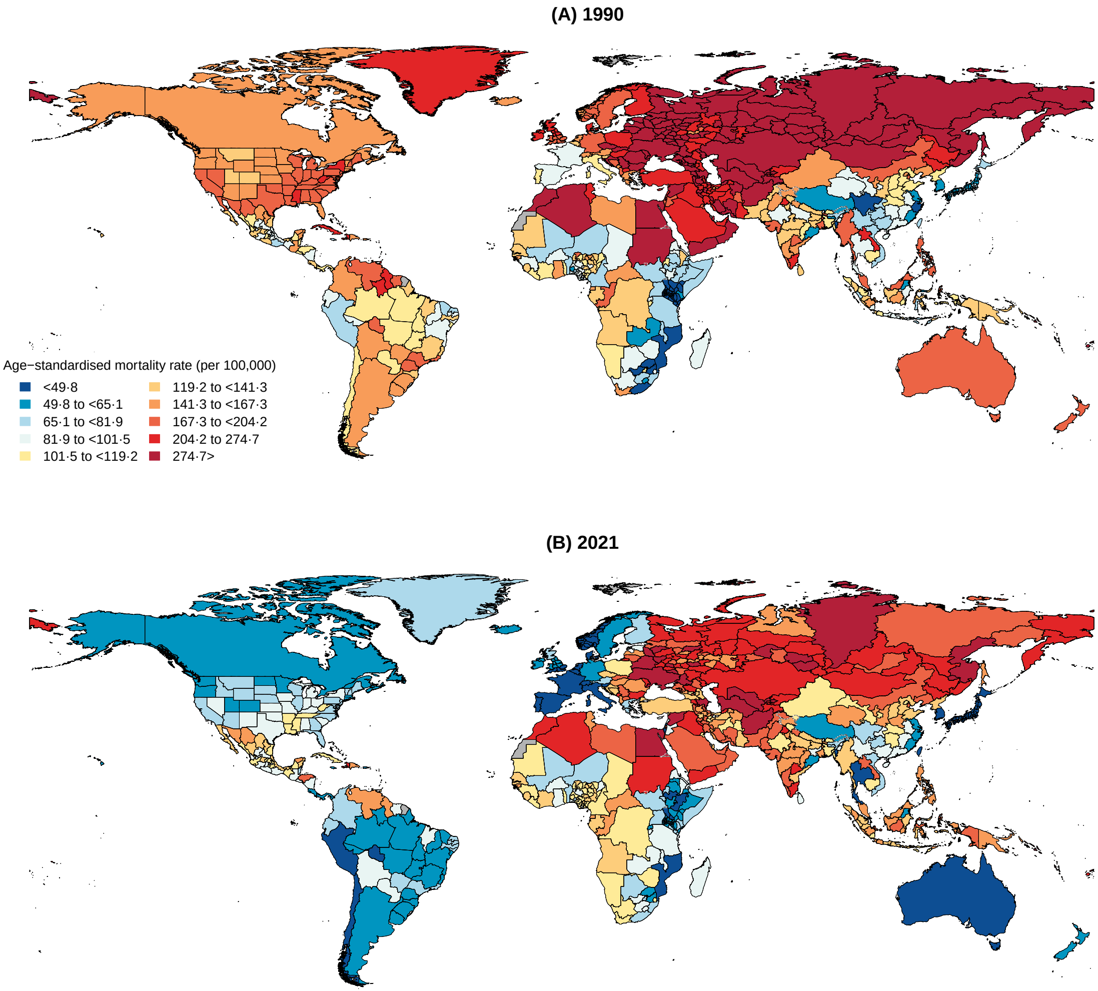

Figure S38 Age−standardised mortality rate per 100 000 due to neoplasms by location, in 1990 (A) and 2021 (B)

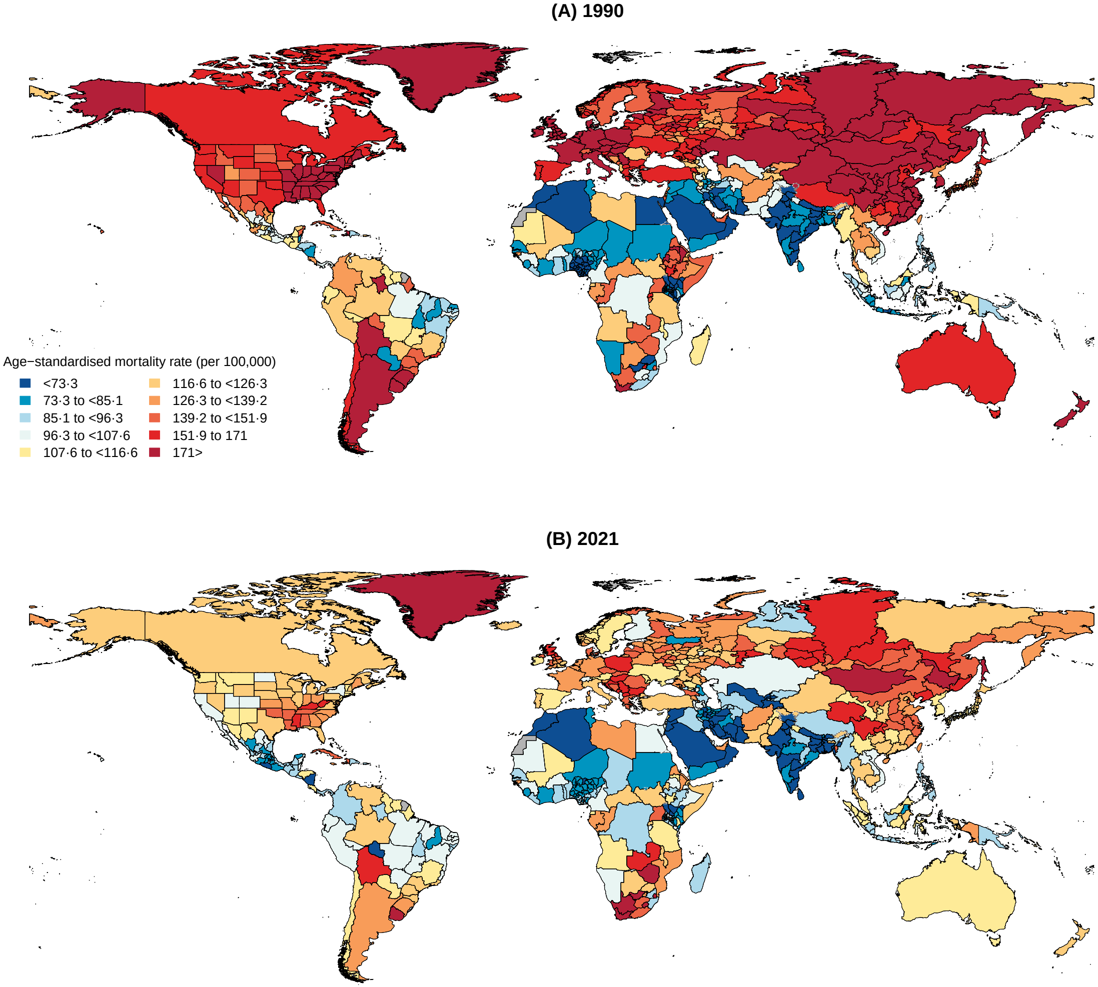

Figure S39 Age−standardised mortality rate per 100 000 due to chronic respiratory disease by location, in 1990 (A) and 2021 (B)

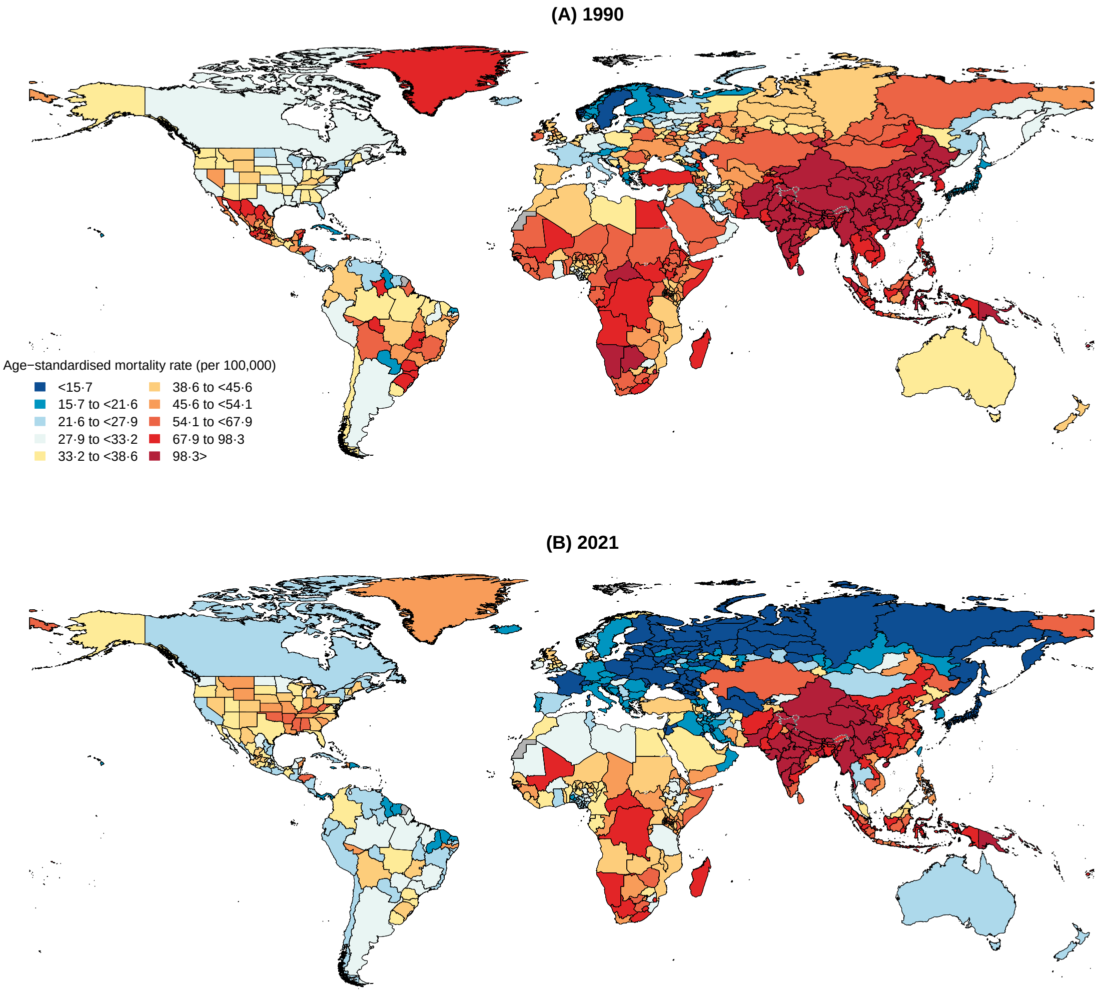

Figure S40 Age−standardised mortality rate per 100 000 due to digestive diseases by location, in 1990 (A) and 2021 (B)

(A) 1990

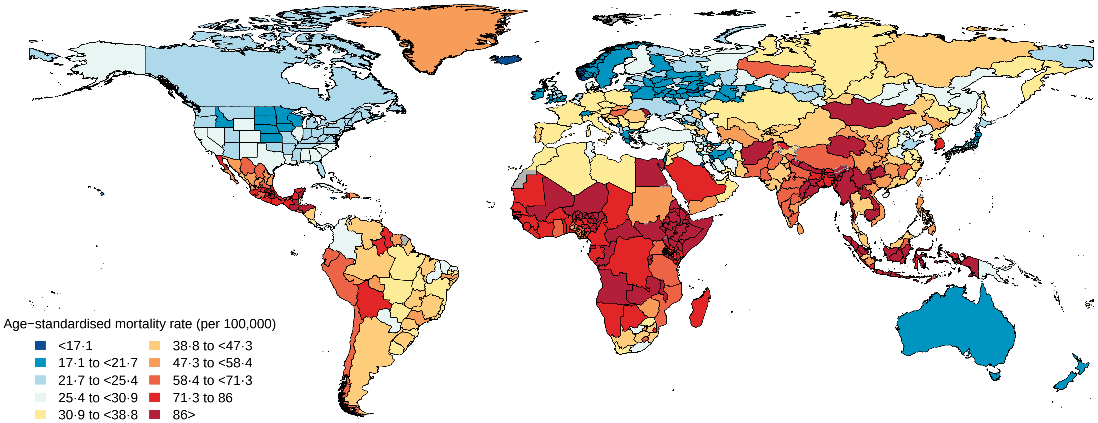

(B) 2021

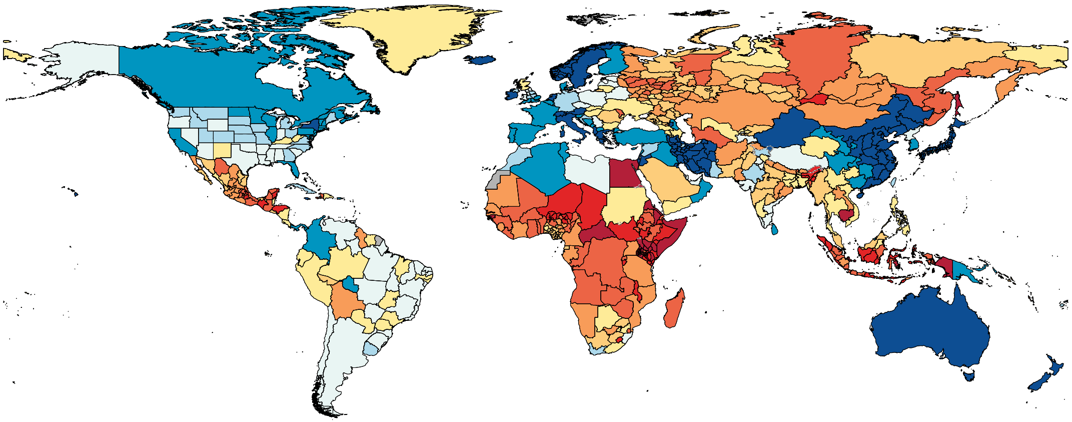

Figure S41 Age−standardised mortality rate per 100 000 due to diabetes and kidney diseases by location, in 1990 (A) and 2021 (B)

(A) 1990

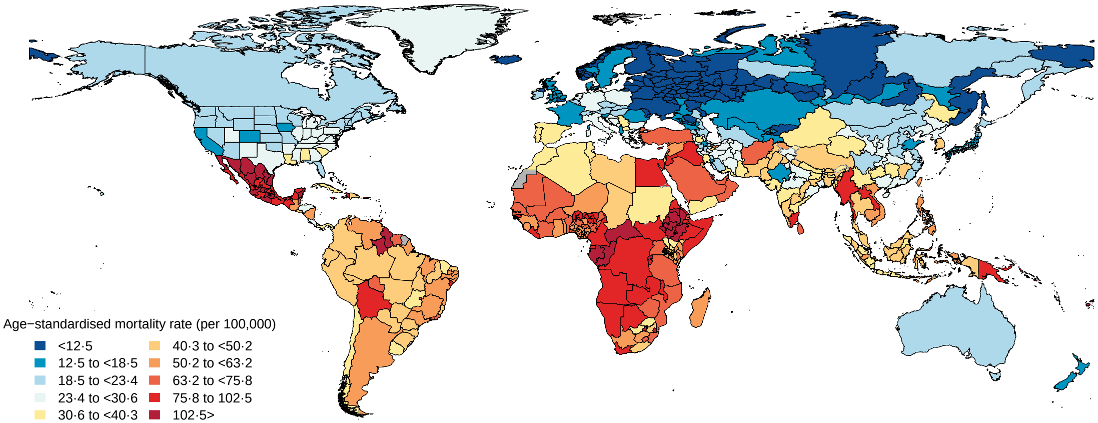

(B) 2021

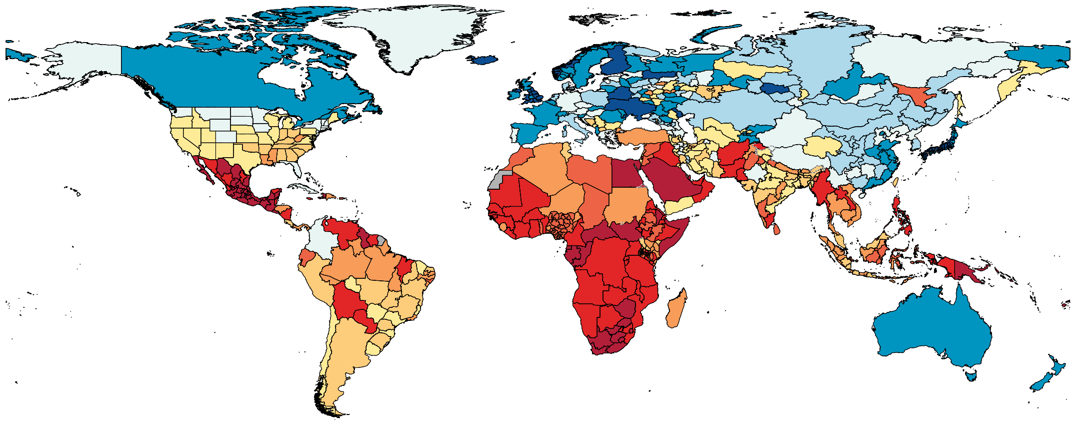

Figure S42 Under−5 mortality rate per 100 000 due to diarrhoeal diseases by location, in 1990 (A) and 2021 (B))

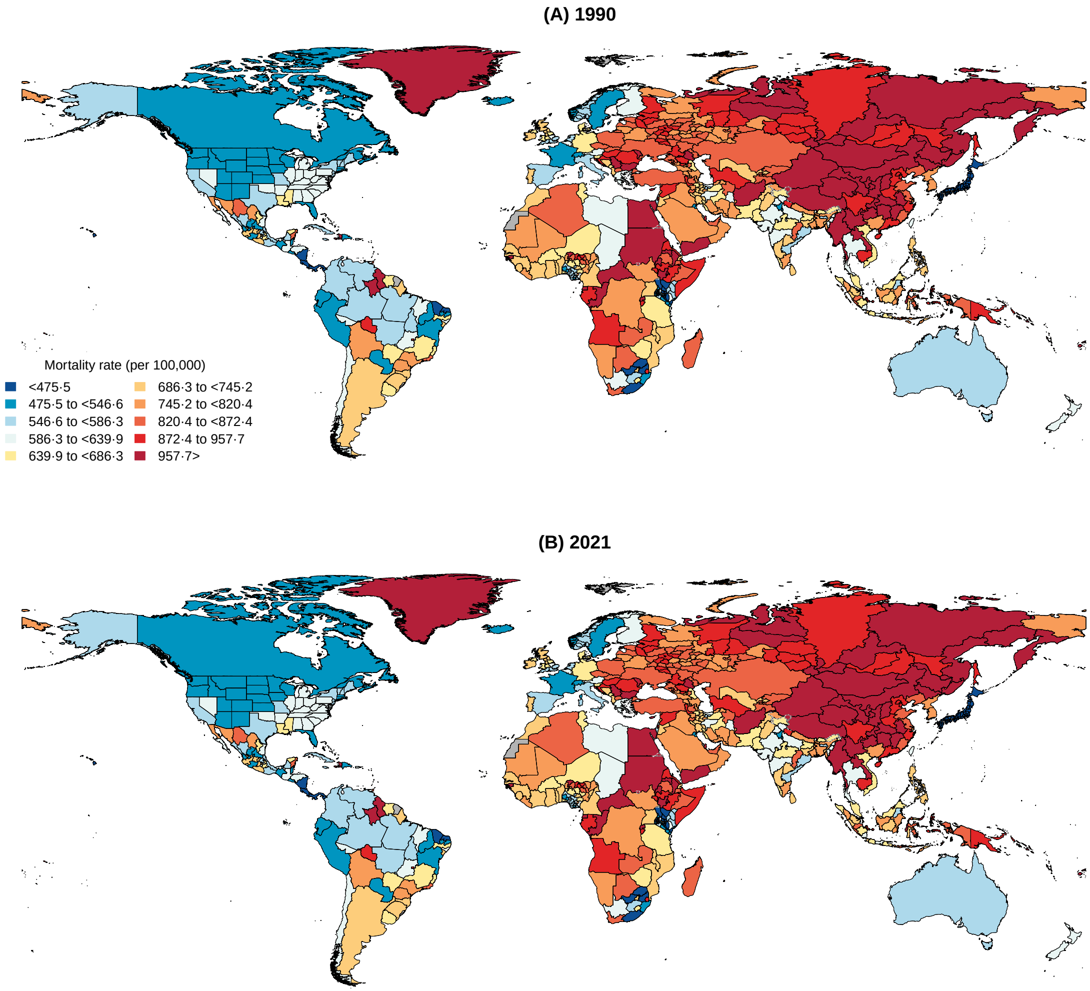

Figure S43 Age−standardised mortality rate per 100 000 due to transport injuries by location, in 1990 (A) and 2021 (B)

(A) 1990

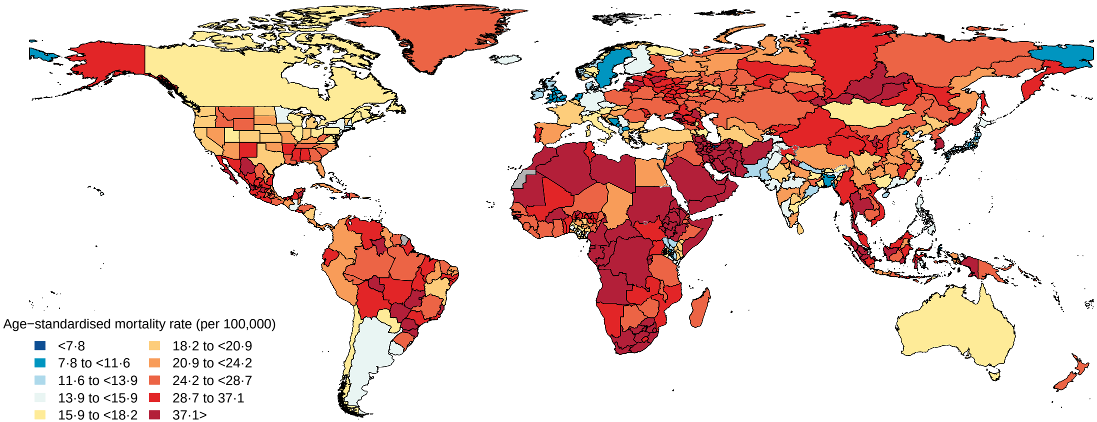

(B) 2021

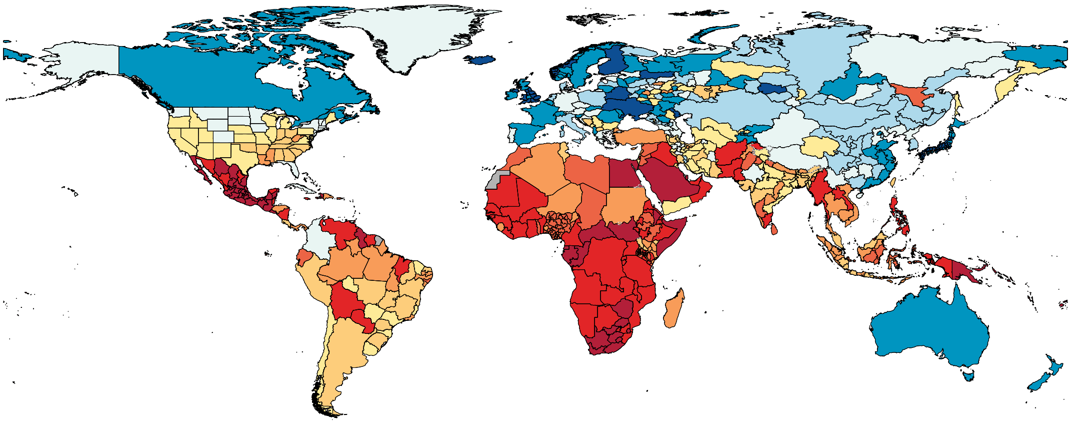

Figure S44 Age−standardised mortality rate per 100 000 due to unintentional injuries by location, in 1990 (A) and 2021 (B)

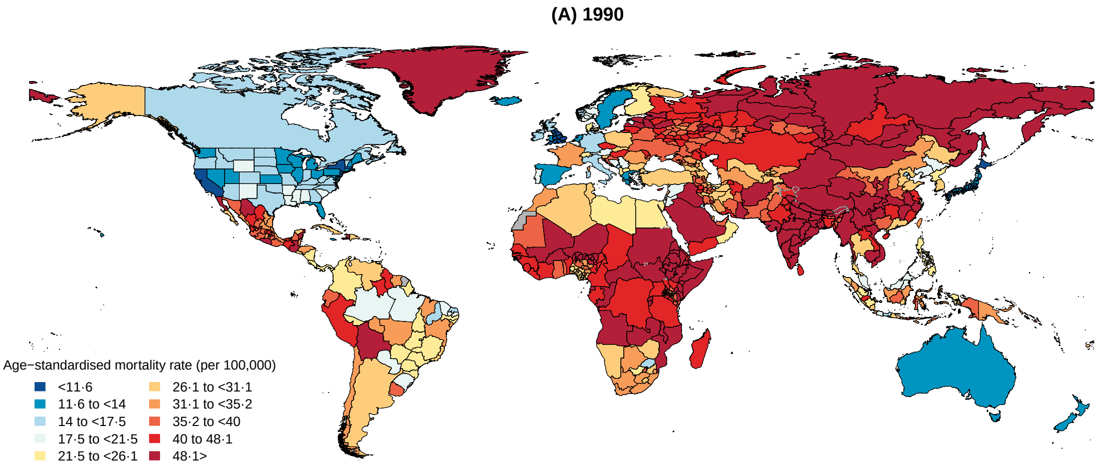

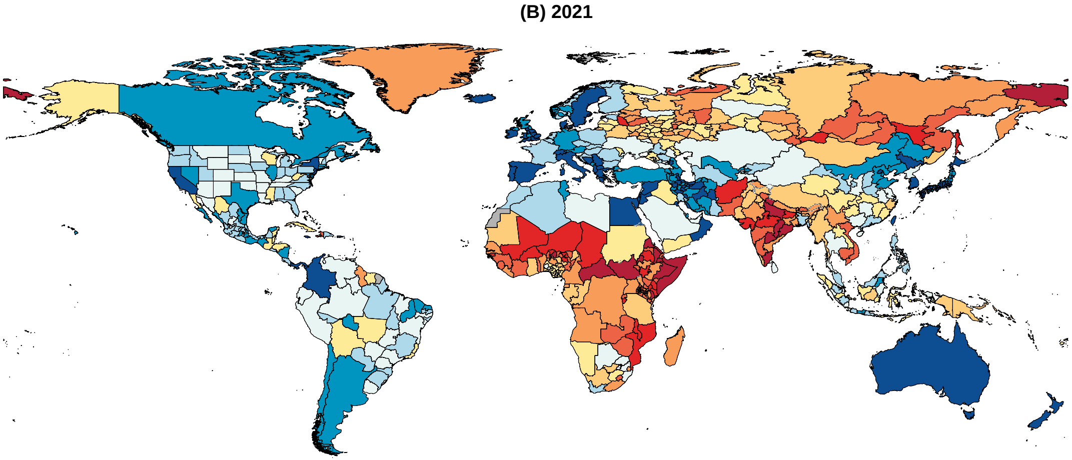

Figure S45 Age−standardised mortality rate per 100 000 due to intentional injuries (excluding war) by location, in 1990 (A) and 2021 (B)

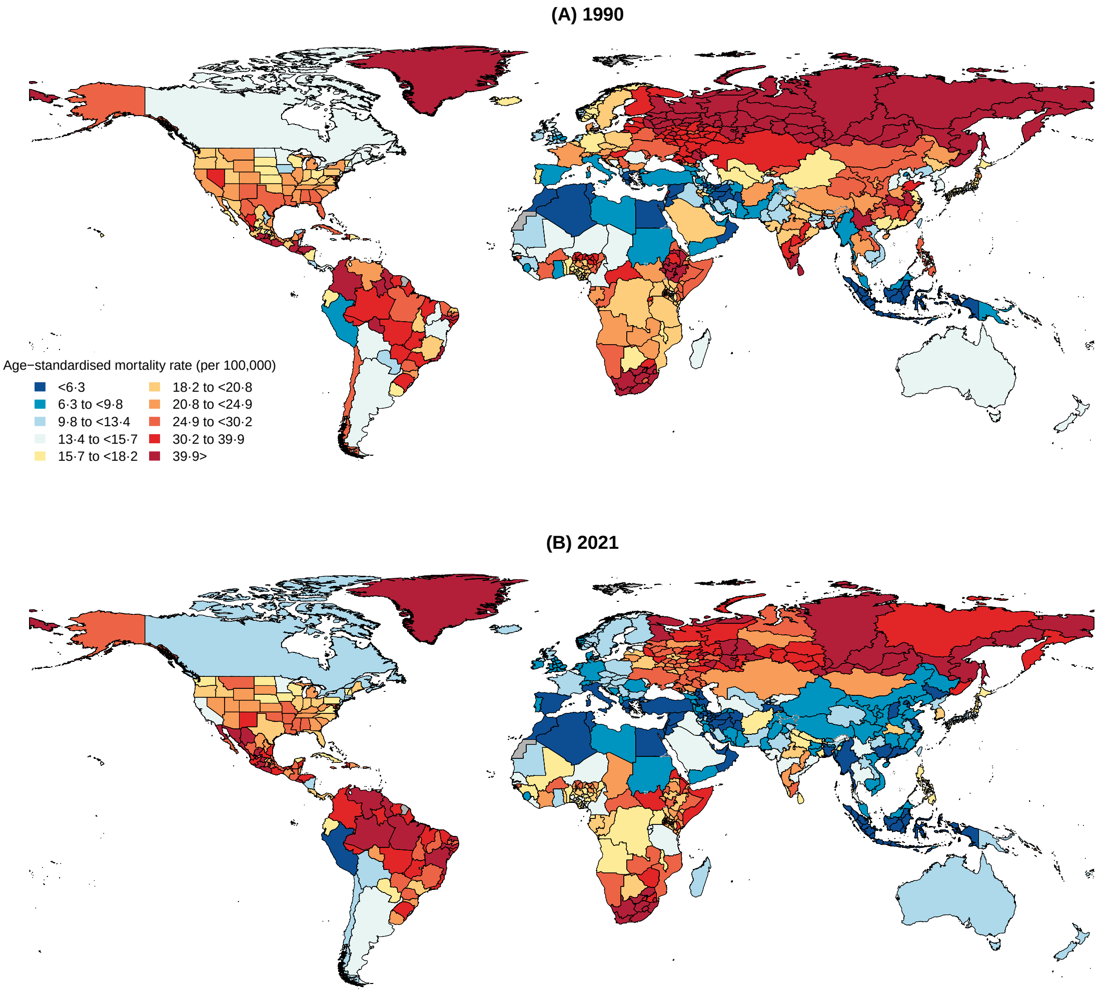

Figure S46 Under−5 mortality rate per 100 000 due to diarrhoeal diseases by location, in 1990 (A) and 2021 (B))

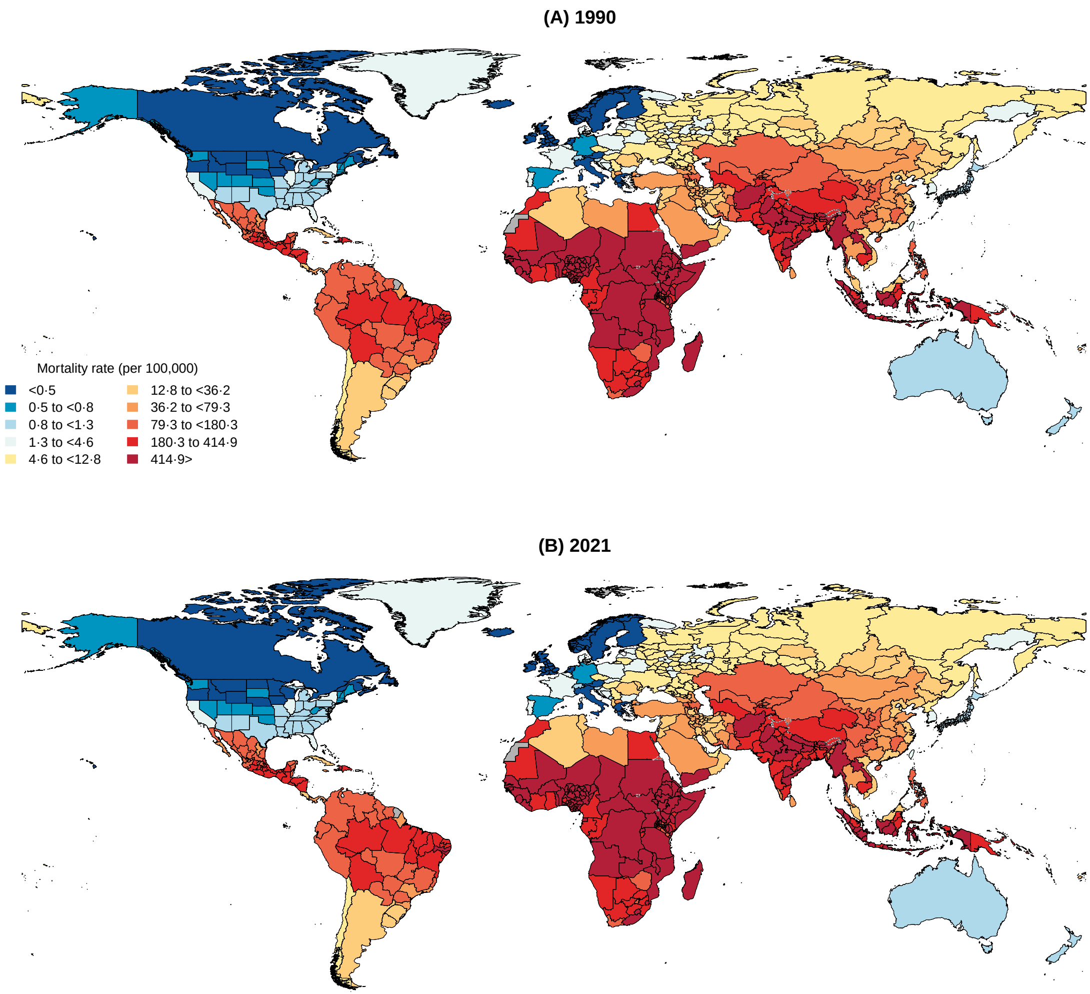

Supplement: Supplementary appendix 2 [file mmc2.pdf]
